# [NiFe]-hydrogenase is essential for cyanobacterium *Synechocystis* sp. PCC 6803 aerobic growth in the dark

**DOI:** 10.1038/srep12424

**Published:** 2015-07-28

**Authors:** Edith De Rosa, Vanessa Checchetto, Cinzia Franchin, Elisabetta Bergantino, Paola Berto, Ildikò Szabò, Giorgio M. Giacometti, Giorgio Arrigoni, Paola Costantini

**Affiliations:** 1 Department of Biology University of Padova, Viale G. Colombo 3, 35131 Padova, Italy; 2 Department of Biomedical Sciences, University of Padova, Viale G. Colombo 3, 35131 Padova, Italy; 3 Proteomics Center of Padova University, Via G. Orus 2/B, 35129 Padova, Italy

## Abstract

The cyanobacterium *Synechocystis* sp. PCC 6803 has a bidirectional [NiFe]-hydrogenase (Hox hydrogenase) which reversibly reduces protons to H_2_. This enzyme is composed of a hydrogenase domain and a diaphorase moiety, which is distinctly homologous to the NADH input module of mitochondrial respiratory Complex I. Hox hydrogenase physiological function is still unclear, since it is not required for *Synechocystis* fitness under standard growth conditions. We analyzed the phenotype under prolonged darkness of three *Synechocystis* knock-out strains, lacking either Hox hydrogenase (ΔHoxE-H) or one of the proteins responsible for the assembly of its NiFe active site (ΔHypA1 and ΔHypB1). We found that Hox hydrogenase is required for *Synechocystis* growth under this condition, regardless of the functional status of its catalytic site, suggesting an additional role beside hydrogen metabolism. Moreover, quantitative proteomic analyses revealed that the expression levels of several subunits of the respiratory NADPH/plastoquinone oxidoreductase (NDH-1) are reduced when *Synechocystis* is grown in the dark. Our findings suggest that the Hox hydrogenase could contribute to electron transport regulation when both photosynthetic and respiratory pathways are down-regulated, and provide a possible explanation for the close evolutionary relationship between mitochondrial respiratory Complex I and cyanobacterial [NiFe]-hydrogenases.

Hydrogen metabolism is one of the most ancient and crucial processes of life. Today, it is at the center of increasing attention in the context of bioenergy production technologies, that will be of critical importance in the near future. In a variety of prokaryotic and eukaryotic microorganisms, hydrogen metabolism is mediated by the hydrogenases, metalloproteins which catalyze the reversible reduction of protons to molecular hydrogen (H_2_) in conditions of strict anaerobiosis^1^. Hydrogenases can be divided in two major classes based on the metal composition of their active sites: i) the [FeFe]-hydrogenases, found in strict anaerobes, fungi, protists, and in some unicellular green algae and ii) the [NiFe]-hydrogenases, widespread among all bacteria families, including cyanobacteria^1^,^2^. Cyanobacteria can have either one or both of two functionally distinct [NiFe]-hydrogenases: an uptake enzyme, which oxidizes H_2_, and a bidirectional hydrogenase, which oxidizes H_2_ but can also generate it from solar energy and water. In particular, the cyanobacterium strain *Synechocystis* sp. PCC 6803 (hereafter referred to as *Synechocystis*) is a commonly used model for both functional and biotechnological applications. *Synechocystis* can grow in both sunlight (phototrophic growth), through oxygenic photosynthesis, and darkness (heterotrophic growth), through glycolysis and oxidative phosphorylation. Moreover, *Synechocystis* genome has been completely sequenced^3^,^4^ and can be easily manipulated exploiting the natural aptitude of this microorganism to be transformed with exogenous DNA.

*Synechocystis* has only a bidirectional [NiFe]-hydrogenase, the Hox hydrogenase; this heteropentameric enzyme is encoded by the *hox* operon, which includes the genes *hoxE, hoxF, hoxU, hoxY*, and *hoxH*, and three additional open reading frames (ORFs)^5^,^6^. The subunits HoxH and HoxY form the catalytic core: HoxH includes the NiFe active site, catalyzing the oxidation of H_2_ or the reduction of protons, and HoxY contains a [4Fe-4S] cluster, mediating the electron transfer to and from the active site. The subunits HoxF, HoxU, and HoxE also contain FeS clusters and form the diaphorase moiety, a flavoprotein interacting with NAD(P)^+^/NAD(P)H. As in all [NiFe]-hydrogenases, the assembly of the NiFe active site and the activation of the Hox hydrogenase require a highly coordinated network of accessory proteins, encoded by the *hypABCDEF* operon and extensively described in *Escherichia coli*^7^.

The precise physiological function of the Hox bidirectional hydrogenase in *Synechocystis* (and in cyanobacteria in general) is still under debate^8^. In particular, it is unknown what role this protein can have in oxygen-rich environments; in fact, the enzyme is active only under anoxic or micro-oxic conditions as it is temporary inactivated by molecular oxygen (O_2_)^1^. It has been proposed that Hox could be involved in both phototrophic and heterotrophic reactions^5^,^9^,^10^. Moreover, the homology of HoxE, HoxF, and HoxU to the respiratory Complex I proteins NuoE, NuoF and NuoG respectively, which are found in other bacteria and in mitochondria, suggests that these Hox proteins could be involved in respiration^5^. However, cyanobacteria lacking the Hox hydrogenase show normal respiration and growth, at least under physiological conditions (see below in Results and Discussion)^11^. Several Hox mutants have been generated to gain new insights into the role of the Hox hydrogenase, and an increasing number of reports suggests that the Hox hydrogenase may play a role only under transient or specific growth conditions^10^,^12^,^13^,^14^,^15^,^16^.

Interestingly, the *hox* and *hyp* genes are constitutively expressed in *Synechocystis* under both anaerobic and aerobic conditions^17^,^18^, even if the Hox hydrogenase is active only under strict anaerobiosis. This supports the hypothesis that this protein could have an additional function in oxygen-rich environments. In this work we analyzed the phenotype of three *Synechocystis* mutant strains, lacking *hypA1* or *hypB1*, or all *hox* genes. Our results indicate that the Hox hydrogenase could be involved in the adaptive response of *Synechocystis* to prolonged darkness, a harsh growth condition frequently occurring in natural environments.

## Methods

All chemicals were of the highest commercially available purity.

### Bacteria strains and standard growth conditions

For photoheterotrophic growth, all *Synechocystis* strains were grown in standard BG11 medium^19^ (supplemented with 5 mM glucose and buffered with HEPES at pH 8.0) at 30 °C under continuous white light (50 μE m^−2^ s^−1^), unless otherwise stated. ΔHypA1 and ΔHypB1 *Synechocystis* strains (see below) were grown in the same medium in the presence of 5 to 50 μg/ml kanamycin. The *hox*^*−*^ strain (ΔHoxE-H, kanamycin resistant) was kindly provided by Jens Appel (Botanical Institute of the Christian-Albrechts-University, Kiel, Germany). Liquid cultures were kept under continuous shaking. When grown under anaerobiosis in the dark, cultures were kept in sealed flasks wrapped with aluminium foil. For cultures grown on plates, BG11 medium was supplemented with 1.5% agar and 0.3% sodium thiosulfate. Cell growth was monitored by measuring the culture optical density at 730 nm (O.D._730_). Chlorophyll concentration was determined spectrophotometrically as described in^20^. The *E. coli* strain XL1-Blue was used for DNA manipulation. *E. coli* cultures were grown in LB medium (with 50 μg/ml kanamycin when requested). Solid medium was obtained by adding 1.5% agar.

### Plasmids construction and generation of ΔHypA1 and ΔHypB1 *Synechocystis* deletion mutants

**Figure 1:**
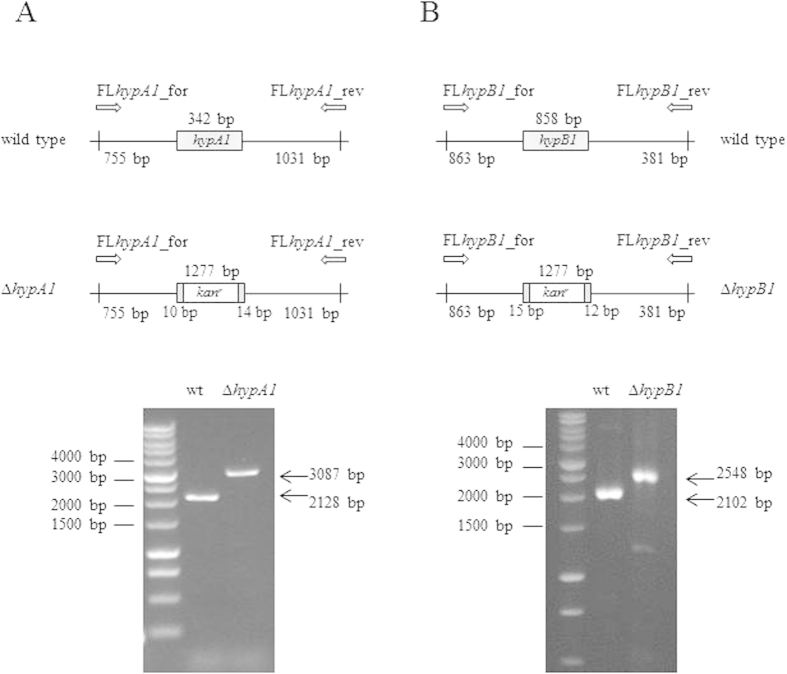
Generation of ΔHypA1 and ΔHypB1 *Synechocystis* strains. *Upper panels*, schematic diagram of the construction of the ΔHypA1 (*panel A*) and ΔHypB1 (*panel B*) knock-out strains. The *hypA1* (*slr1675*) and *hypB1* (*sll1432*) genes were deleted and replaced by a kanamycin-resistance cassette (*kan*^*r*^). The positions of the PCR primers used to verify *kan*^*r*^ correct insertion are indicated (*FLhypA1_for/FLhypA1_rev* and *FLhypB1_for/FLhypB1_rev*). *Lower panels*, agarose gel electrophoresis of PCR products obtained from wild type, ΔHypA1 and ΔHypB1 genomic DNA. *Lane 1*, molecular markers; *lane 2*, DNA from wild type; *lane 3*, DNA from knock-out. The gels are full-length and have been cropped on both sides in order to show only the three relevant lanes.

The *hypA1* (*slr1675* gene) and *hypB1* (*sll1432* gene) knock-out strains (ΔHypA1 and ΔHypB1, respectively) were obtained from wild type *Synechocystis* by homologous double recombination. A part of each gene was replaced with a kanamycin-resistance cassette (*kan*^r^), derived by BamHI digestion from the plasmid pUC4K (Pharmacia). The plasmids used to generate these deletion mutants were based on the pBSKII vector (Agilent Technologies) and contained the *Synechocystis* chromosome regions flanking the *slr1675* and *sll1432* genes, respectively. The regions upstream and downstream *slr1675* and *sll1432* were amplified by a three-steps PCR strategy, using primers designed on the basis of the *Synechocystis* genome sequence accessible through CyanoBase and containing sequences with restriction sites for cloning purposes (listed in Table 1), and the high-fidelity Phusion DNA polymerase (Finnzymes). Each mutant was generated as follows. In the first step, a PCR-up and a PCR-down were separately performed using as template the *Synechocystis* genomic DNA, purified following standard protocols^21^, and the primers couples *i)* SacI*hypA1*_for/BamHI*hypA1*_rev and BamHI*hypA1*_for/EcoRV*hypA1*_rev (for *hypA1*^*−*^) or *ii)* SacI*hypB1*_for/BamHI*hypB1*_rev and BamHI*hypB1*_for/EcoRV*hypB1*_rev (for *hypB1*^*−*^). In a second step, the products of these PCR-up and PCR-down were amplified using the primers couples *i)* SacI*hypA1*_for/EcoRV*hypA1*_rev (for *hypA1*^*−*^) or *ii)* SacI*hypB1*_for/EcoRV*hypB1*_rev (for *hypB1*^*−*^), thus obtaining an overlapping band. In the last step, the overlapping bands were digested by the restriction enzymes SacI and EcoRV and individually cloned in a pBSKII vector, which was finally digested with BamHI to introduce the *kan*^r^ cassette. The correctness of the resulting recombinant plasmids pBSKII_*hypA1*KO_ *kan*^*r*^ and pBSKII_*hypB1*KO_ *kan*^*r*^ was confirmed by DNA sequencing (BMR Genomics, University of Padova). To transform wild type *Synechocystis*^22^, culture was grown until it reached the mid-exponential phase (O.D._730_ : 0.5) and then centrifuged at 3000 × *g* for 5 minutes at room temperature. The cell pellet was suspended in fresh BG11 medium without glucose at a density of 1 × 10^10^ cells/ml and mixed with 0.5 μg/ml of exogenous DNA containing pBSKII_*hypA1*KO_ *kan*^*r*^ and pBSKII_*hypB1*KO_ *kan*^*r*^. The mixture of cells and DNA was incubated for 5 h, and then spread onto a membrane filter resting on a BG11 agar plates. After 24 h, the membrane filter was moved on a BG11 agar plate containing 5 μg/ml kanamycin. Several single recombinant colonies appeared after 10–15 days. Since *Synechocystis* has multiple genome copies, to obtain the complete segregation of the recombinant chromosomes the colonies were subcloned on plates containing increasing antibiotic concentrations up to 50 μg/ml. The complete segregation was verified by PCR using the primers pairs FL*hypA1*_for/FL*hypA1_*rev and FL*hypB1*_for/FL*hypB1*_rev for the ΔHypA1 and ΔHypB1 strains respectively (Fig. 1 and Table 1).

### Hydrogen evolution assay

Hydrogenase activity of whole cell extracts was measured *in vitro*, as previously described^23^. Briefly, H_2_ production from reduced methyl viologen was measured in nitrogen-flushed 13.5 ml sealed vials, using a Clarus 500 gas chromatographer equipped with an Elite-Molesieve column (0.53 mm i.d., 30 m length) (Perkin Elmer). All steps were performed under anaerobic conditions in a glove-box (MBRAUN MB 200B), using oxygen-free solutions.

### Growth experiments

Cultures of wild type and mutant *Synechocystis* strains were grown until they reached the exponential phase (O.D._730_ : 2.5); then, they were diluted 1:10 in fresh medium (O.D._730_ at day 0: 0.2). Each experiment was performed in triplicate i) under continuous white light (50 μE m^−2^ s^−1^), ii) with 12 h light/12 h dark cycles or iii) complete darkness. After five days, we assessed the cultures capabilities to generate colonies on plates: each liquid culture was diluted to an O.D._730_ between 0.3 and 0.4 and quantified using the cell counter Cellometer Auto X4 with a Cellometer SD100 counting chamber (Nexcelom Bioscience LLC.). The cell densities of the diluted cultures ranged between 1 × 10^6^ and 1.5 × 10^6^ cells/ml. Each diluted culture was then plated in three serial dilutions (1:2500, 1:5000 and 1:10000) and incubated at 30 °C in a 20 μE m^−2^ s^−1^ light regimen. Colonies, usually appeared after about ten days, were finally counted. Experiments were made in triplicate.

### Electron microscopy

*Synechocystis* pellets were fixed in 3% glutaraldehyde (0.1 M sodium cacodylate buffer, pH 6.9) overnight at 4 °C and then processed for electron microscopy as described in^24^. Ultrathin sections, cut with an ultramicrotome (LKB Ultratome V), were post-stained with 1% osmium tetroxide/1% potassium ferrycyanide, embedded in a EPON 812 resin and examined under a transmission electron microscope (FEI Tecnai^TM^ F12) operating at 100 kV (at the electron microscopy facility of the Department of Biology, University of Padova).

### Sample collection and protein extraction

Three biological replicates of *Synechocystis* wild type and ΔHoxE-H strains were grown for 5 days under continuous light or darkness as described above. Cells were collected by 5000 × *g* centrifugation for 10 minutes at 4 °C (Allegra^®^ 25R Centrifuge, Beckman Coulter), frozen in liquid nitrogen, and stored at −20 °C. For protein extraction, the cells were washed twice with 50 mM HEPES-KOH, pH 7.5 and then suspended in the presence of 1 mM PMSF. The cells were centrifuged and the pellet was suspended in equal volumes of extraction buffer (50 mM HEPES-KOH, 0.1% SDS, 0.1% Triton X-100, 1 mM PMSF, pH 7.5) and glass beads (150–212 μm diameter). Then the cells were homogenized at 3500 rpm in a Mini Bead Beater (Biospec Products). Intact cells and glass beads were removed by 1000 × *g* centrifugation for 5 minutes (Mikro 22R, Hettich) and the supernatant was incubated in 2% SDS for 30 minutes to facilitate the extraction of membrane proteins. Samples were centrifuged again at 12 000 × *g* for 20 minutes at 4 °C, and the supernatant was stored at −20 °C until further use. Protein concentration was determined by Bradford method (Bradford Reagent, Sigma-Aldrich). The proteins were precipitated in ice-cold acetone (1:4, v/v) at −20 °C overnight and stored at −80 °C.

### Protein digestion and isobaric Tags for Relative and Absolute Quantitation (iTRAQ) labeling

One hundred μg of protein pellets from each of the 12 preparations of *Synechocystis* (3 wild type and 3 ΔHoxE-H strains grown either under light or in the dark) were suspended in 40 μl Laemmli buffer and loaded into a NuPage 12% precast SDS-PAGE (Invitrogen). The electrophoretic process was carried out for few minutes, just enough to allow all proteins to enter the separating gel and focus in single narrow bands. Proteins were stained using SimplyBlue Safestain (Invitrogen) and each band was manually excised from the gel. The staining was then removed and the proteins were reduced with 10 mM dithiotreitol (DTT) in 25 mM triethylammonium bicarbonate (TEAB) at 55 °C for 1 h. Then, cysteines were alkylated using 55 mM iodoacetamide in 25 mM TEAB for 45 minutes in the darkness. Gel slices were thoroughly washed with 50 mM TEAB and acetonitrile (ACN) and vacuum dried in a SpeedVac system. Samples were incubated with 130 μl of sequencing grade modified trypsin (Promega, 12.5 ng/μl in 50 mM TEAB), overnight at 37 °C. Peptides were extracted from the gel slices by two consecutive treatments with 50% ACN (30 minutes, under constant agitation) and finally vacuum dried. Each digested sample was dissolved in iTRAQ dissolution buffer and labeled with iTRAQ tags following the manufacturer’s instructions (AB Sciex). To reduce any potential variability introduced by the labeling reaction, samples from the 3 replicates were labeled with a tag-swapping strategy. To verify the labeling efficiency, each sample was analyzed by liquid chromatography-tandem mass spectrometry (LC-MS/MS) as described below. Acquired data were searched with Mascot search engine, setting iTRAQ labeling as variable modification. No unmodified peptides were identified from the search and all the peptides were correctly modified at the N-terminus and at each lysine residue. Finally, the iTRAQ labeled samples were combined in a 1:1 (wild type:ΔHox) ratio and vacuum dried.

### Strong Cation Exchange Fractionation

Strong cation exchange (SCX) was performed using a SCX cartridge (AB Sciex) and following the manufacturer’s protocol. Each iTRAQ labeled sample was suspended in equilibration buffer (5 mM KH_2_PO_4_, 25% ACN, pH 2.9) and loaded into a SCX cartridge with a syringe-pump system at a 50 μl/min flow rate. Peptides were eluted in an eight-step mode by adding increasing KCl concentrations in equilibration buffer (30, 50, 80, 110, 140, 170, 200, and 350 mM KCl respectively). Each SCX fraction was vacuum dried, suspended in 0.1% formic acid (FA), and desalted using C18 cartridges (Sep-Pack, C18, Waters). Samples were vacuum dried and stored at −20 °C.

### LC-MS/MS analyses

Samples were dissolved in 0.1% FA and analyzed with a LTQ-Orbitrap XL mass spectrometer (ThermoFisher Scientific) coupled online with a Ultimate 3000 nano-HPLC (Dionex - Thermo Fisher Scientific), as previously described^25^. Peptides from each fraction were loaded onto a 10 cm chromatographic column packed into a pico-frit (75 μm I.D., 15 μm tip, New Objective) with C18 material (Aeris Peptide 3.6 μm XB-C18, Phenomenex). Peptides were eluted in 90 minutes using a 3% to 50% linear gradient of ACN/0.1% FA at a 250 nl/min flow rate. The instrument performed spectrometer performed a full scan at high resolution (60000) on the Orbitrap, followed by MS/MS scans on the three most intense ions with both CID and HCD fragmentation. Data were searched with Proteome Discoverer software (Thermo Fisher Scientific) as described below; all peptides identified with high (99%) or medium (95%) confidence were used to create a static exclusion list that was then included in the instrument method. All samples were re-analyzed under identical instrumental and chromatographic conditions except for the application of the exclusion list. All data obtained were merged into a MudPIT protocol and analyzed as described below.

### Data analysis

Raw MS/MS files were analyzed using Proteome Discoverer 1.4 (Thermo Fisher Scientific) connected to a Mascot Search Engine server version 2.2.4 (Matrix Science), using a MudPIT protocol. Data were filtered to exclude MS/MS spectra containing less than 5 peaks and with a total ion count lower than 50. Spectra were searched against the Uniprot *Synechocystis* database (last updated March 2014, 3622 sequences), concatenated with a database of the most common contaminants found in proteomics experiments. Peptide and fragment tolerances were set to 10 ppm and 0.6 Da respectively, enzyme specificity was set to Trypsin with up to 2 missed cleavages. Carbamidomethylcysteine and 4-plex iTRAQ labels at N-terminus and Lys residues were set as fixed modifications; oxidation of methionine was set as variable modification. Percolator and a search against the corresponding randomized database were used to calculate False Discovery Rates (FDR). Proteins were grouped into families according to the principle of maximum parsimony. Data were first filtered by considering as positive hits the peptides that were identified with medium (95%) or high (99%) confidence, based on the q-value calculated by the Percolator algorithm. To obtain the final list of proteins, data were further filtered by considering as positive hits only the proteins that were identified with at least two unique peptides and quantified with at least two independent peptides. Protein expression levels of wild type and mutant strains, under light or darkness growth conditions, were compared; a fold change of 1.5 or greater in the expression level was considered relevant. Statistical significance was determined with a two-tailed Z Test.

## Results and Discussion

Under standard growth conditions, all *Synechocystis* mutant strains lacking one or more of the *hox* genes from the *hoxE-H* operon (coding the cyanobacterial bidirectional [NiFe]-hydrogenase) show no significant differences from the wild type strain. This observation raises the question of what exactly is the role of the Hox hydrogenase. Furthermore, this enzyme is constitutively expressed under both aerobic and anaerobic conditions, suggesting that it likely provides an evolutionary advantage that would manifest under non-standard growth conditions, and that it could have an additional role besides H_2_ metabolism. Most cyanobacteria are aerobic photoautotrophs, and sunlight-driven oxygenic photosynthesis is their main energy metabolism pathway. However, it is well known that some cyanobacterial communities can be exposed and survive to natural environments where regular prolonged periods of complete darkness occur, for example in lake sediments, soil water or in dense aquatic accumulations produced at the surface. Under these conditions cyanobacteria are believed to shift to an anaerobic fermentation metabolism^26^. Interestingly, the *hox* genes are absent in cyanobacterial strains found in open oceans^1^,^27^, where anaerobiosis and darkness are both unlikely, suggesting that [NiFe]-hydrogenase may be especially important under these specific conditions. To explore the potential role played by the Hox hydrogenase in *Synechocystis* growth in the darkness, we analyzed the phenotype of ΔHoxE-H, a *Synechocystis* knock-out strain where the entire *hox* operon has been deleted.

### Physiological characterization of wild type and ΔHoxE-H *Synechocystis* strains under conditions of light or prolonged darkness

**Figure 2:**
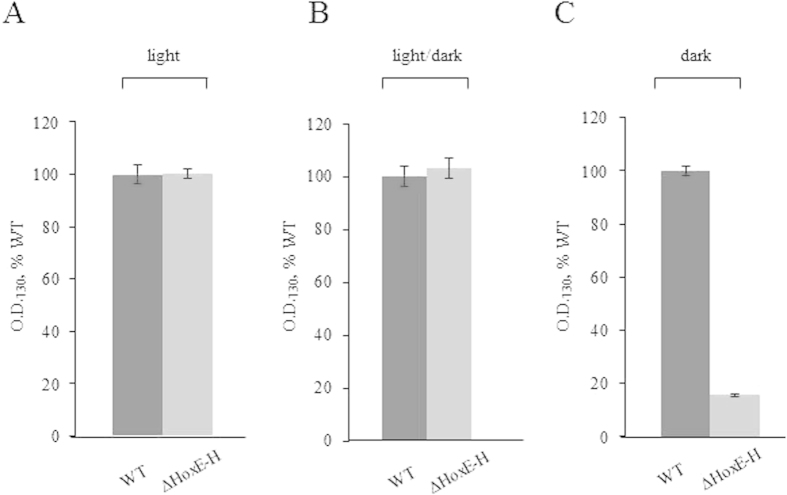
Wild type *Synechocystis* is more resistant to prolonged darkness than ΔHoxE-H strain. *Panel A*, continuous white light (50 μE m^−2^ s^−1^); *panel B*, 12 h cycles of light/dark; *panel C*, continuous darkness. Optical densities at 730 nm (O.D._730_) of wild type (WT, dark grey) and ΔHoxE-H (light grey) cultures were measured after five days of growth, and for each growth condition the values were normalized to the O.D._730_ value of the corresponding WT (*panel A*, 5.2 ± 0.72; *panel B*, 4.03 ± 0.15; *panel C*, 1.8 ± 0.55). Reported data result from the mean of three independent experiments ± Standard Deviation.

**Figure 3:**
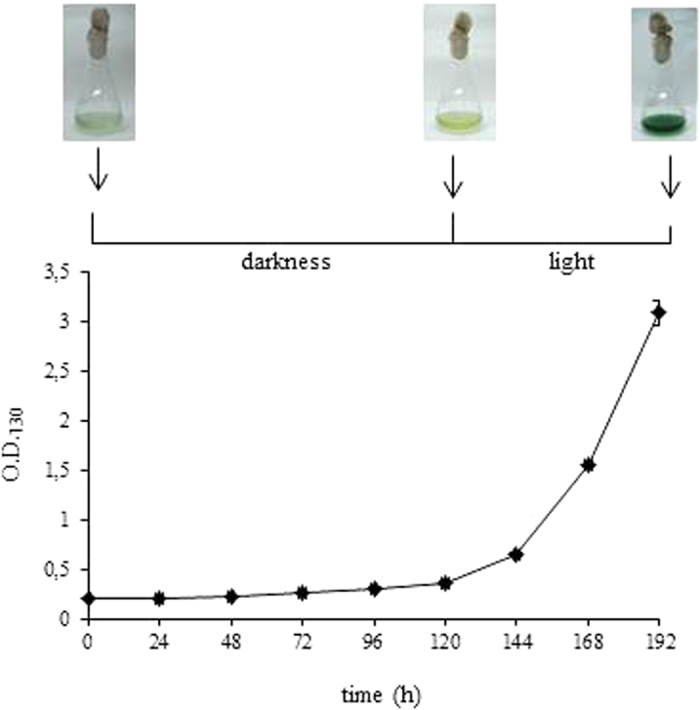
ΔHoxE-H *Synechocystis* is able to resume its growth when exposed to light after five days under complete darkness. Growth curve of ΔHoxE-H *Synechocystis* cultured for five days in the dark and additional three days under normal light intensity. Reported data result from the mean of three independent experiments ± Standard Deviation.

**Figure 4:**
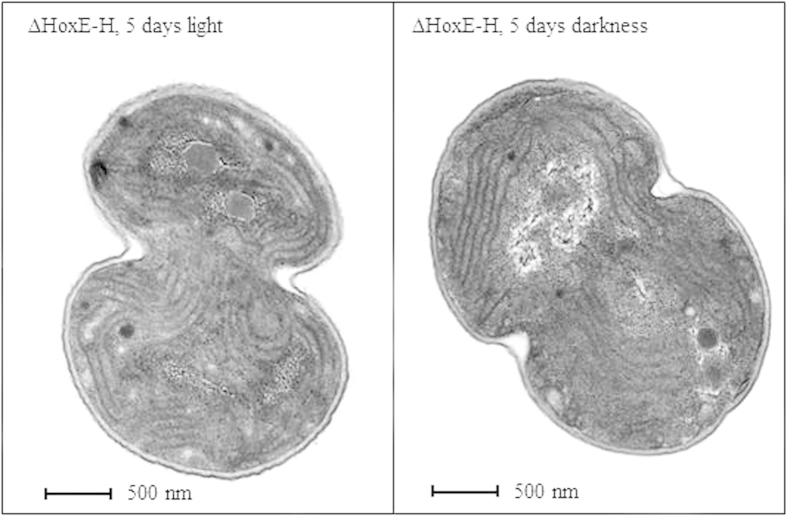
ΔHoxE-H *Synechocystis* cells grown under continuous light or darkness display the same ultrastructural organization. Representative TEM images of ΔHoxE-H cells grown for five days under continuous light (*left panel*) or complete darkness (*right panel*).

To evaluate the effects of the deletion of the entire *hox* operon on *Synechocystis* fitness during prolonged dark periods (up to five days) in the presence of glucose as carbon source, the growth of wild type and ΔHoxE-H strains were compared under three different light conditions: i) continuous white light (50 μE m^−2^ s^−1^), ii) complete darkness or iii) 12 h light/12 h dark cycles. This last condition was added as an additional control, since *Synechocystis* is known to possess a circadian clock system. Figure 2 shows no significant difference between wild type and ΔHoxE-H when grown in continuous light (panel A) or under circadian, 12 h cycles of light/darkness (panel B). This result is consistent with previous data obtained from other *hox* knock-out mutants^11^,^13^,^14^,^15^,^16^,^17^,^18^,^19^. However, under complete darkness ΔHoxE-H showed a severely impaired growth compared to the wild type (panel C). It was previously reported that, despite the presence of glucose in the medium, *Synechocystis* cannot grow under complete darkness unless it is given a daily light pulse (*i.e.* 40 μmol m^−2^ s^−1^ for 5 minutes)^28^. However, our wild type *Synechocystis* strain can grow in the dark without this pulse (supplementary figure S1), which was therefore omitted from our protocols. In the case of the wild type strain, as expected, glucose was able to support heterotrophic growth, even though its growth rate was reduced by 60–70% compared to the growth under photoheterotrophic conditions. In the case of the ΔHoxE-H mutant strain, growth displayed a severe defect as compared to wild type strain cultured in the same conditions (Fig. 2, panel C). Measurements of chlorophyll content further confirmed these results (supplementary figure S2), indicating that the *hox* operon gives *Synechocystis* an adaptive advantage by enabling it to grow in the absence of photosynthetic activity. Interestingly, when after five days in the dark ΔHoxE-H cultures were allowed to grow under normal light conditions, bacteria were able to resume their normal growth rate (Fig. 3). Moreover, they could form colonies on agar plates (see Methods for experimental details). These data were also confirmed by morphological examination of ΔHoxE-H cells using transmission electron microscopy (TEM). TEM analysis showed no gross ultra-structural difference between cells grown under light or darkness conditions (representative images are shown in Fig. 4), further supporting the hypothesis that ΔHoxE-H remains viable in the dark. Altogether, these data suggest that under prolonged darkness ΔHoxE-H enters a reversible quiescent but viable state that allows it to survive in this environment despite lacking the Hox hydrogenase.

It is worth noting that similar results were obtained by incubating wild type and ΔHoxE-H strains in the dark under anaerobic (supplementary figure S3) or in aerobic conditions, which rapidly inhibit the hydrogenase function of the Hox protein^29^. This suggests that the phenotype of wild type *Synechocystis* under prolonged darkness does not depend on H_2_ metabolism. To further investigate this point, we generated two additional knock-out mutant strains, i) ΔHypA1 and ii) ΔHypB1, lacking two accessory proteins required for the assembly of the NiFe active site of the Hox hydrogenase. In both ΔHypA1 and ΔHypB1 the HoxEFU diaphorase activity is preserved whereas the hydrogenase catalytic core is inactivated.

### Physiological characterization of ΔHypA1 and ΔHypB1 *Synechocystis* strains under conditions of light or prolonged darkness

**Figure 5:**
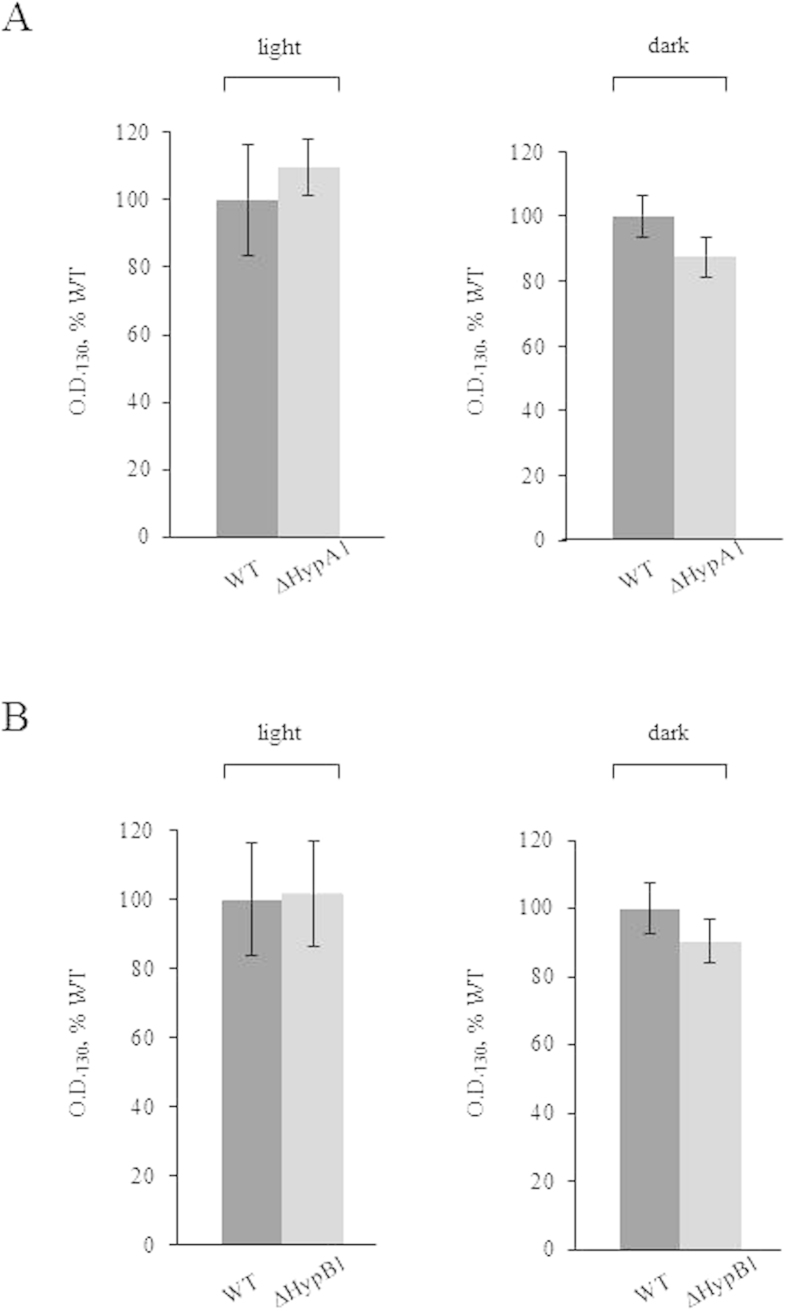
ΔHypA1 and ΔHypB1 *Synechocystis* strains have the same growth phenotype of the wild type strain when cultured in the dark. *Panel A*, ΔHypA1; *panel B*, ΔHypB1. Strains were grown either under continuous white light (50 μE m^−2^ s^−1^) or darkness. O.D._730_ of WT (dark grey) and mutants (light grey) cultures were measured after five days, and for each growth condition the values were normalized to the O.D._730_ of the corresponding WT (5.8 ± 0.9 and 1.6 ± 0.1 for light and darkness, respectively). Reported data result from the mean of three independent experiments ± Standard Deviation.

The biosynthesis of [NiFe]-hydrogenases is a complex, highly conserved process which requires a number of auxiliary proteins^30^,^31^. In particular, in cyanobacteria HypA and HypB are responsible for inserting Ni into the catalytic site, before it can be activated^32^. *Synechocystis* has two homologues *hypA* and *hypB* genes (coding for HypA1 and HypA2, and HypB1 and HypB2, respectively); however, only HypA1 and HypB1 are required for the assembly of the Hox hydrogenase, whereas HypA2 and HypB2 are likely to have a role in the maturation of other metalloproteins^33^. Therefore, we selectively replaced *hypA1* and *hypB1* genes with a kanamycin-resistance cassette (*kan*^*r*^) (Fig. 1) and thus generated the knock-out strains ΔHypA1 and ΔHypB1. The lack of hydrogenase activity, measured by gas chromatography (supplementary table S1), confirmed the lack of an active enzyme in the two mutants. ΔHypA1 and ΔHypB1 were grown under continuous light or prolonged darkness. We measured the growth rate by OD_730_ monitoring (Fig. 5) and chlorophyll quantification (supplementary figure S4), and found no difference between either one of the mutant strains and the wild type under the two growth conditions. This result indicates that H_2_ metabolism is not required for *Synechocystis* growth in the dark.

Taken together, these results suggest that the Hox hydrogenase does play a key role in the adaptive response of *Synechocystis* to prolonged darkness, regardless of whether its NiFe active site module is folded in a functional state or not, and open a new perspective to gain clues on the role of this protein under stress conditions.

Several proteomic studies recently conducted on *Synechocystis* and other cyanobacteria showed that when exposed to different conditions of environmental stress (*e.g.* UV light, high salt concentrations, cold temperature or limited CO_2_ availability), they responded with different survival mechanisms^34^,^35^,^36^,^37^,^38^,^39^. Thus, to gain more insight into the role of the Hox hydrogenase in *Synechocystis* adaptive metabolic response to a prolonged darkness we analyzed and compared the proteomes of both wild type and ΔHoxE-H strains grown either under continuous light or in the dark.

### Quantitative proteomics analysis and comparison of the proteomes of wild type *Synechocystis* under continuous light or in darkness unravel new clues on possible additional functions of the Hox hydrogenase

We compared the expression levels of proteins extracted from cultures of wild type *Synechocystis* that were grown under continuous light or prolonged darkness, using isobaric tag for relative and absolute quantification (iTRAQ) technology and liquid chromatography-tandem mass spectrometry (LC-MS/MS). The analysis was conducted on three independent biological replicates. Across all replicates a total of 780 distinct protein families were identified and quantified. All relevant information relative to proteins and peptides identification and quantification are reported in supplementary tables S2-S5. Of these, 602 proteins (16.4% of the *Synechocystis* theoretical proteome^3^) could be quantified in at least two out the three experiments, and were therefore included in the analysis. Unfortunately, hydrophobic proteins, including membrane-embedded subunits of thylakoid complexes (which are expected to be highly abundant in *Synechocystis*) are underestimated in our analysis, probably due to the intrinsic limitations in the applied methodology. Nevertheless, we determined that 112 proteins were differentially regulated between light and darkness grown organism (p value < 0.05), among which 58 showed an increased abundance (*i.e.* dark/light ratio ≥ 1.50) and 54 a decreased abundance (*i.e.* dark/light ratio ≤ 0.67). We grouped these proteins into different categories according to their known or predicted functions (Table 2). Among them, we identified 39 proteins involved in transcription and translation; almost all of them (38) were up-regulated when the cultures were grown under prolonged darkness. Changes in protein synthesis were also previously observed when *Synechocystis* was exposed to various other stress conditions^14^,^34^,^35^,^36^,^37^,^38^,^39^. More specifically, as expected, several proteins/cofactors belonging to photosystems I and II and/or functionally correlated to photosynthesis, including the electron carrier flavodoxin, show a lower abundance when *Synechocystis* is grown in the absence of light, *i.e.* when glycolysis and oxidative pentose-phosphate metabolic pathways become prevalent. It is worth mentioning that several subunits of the respiratory chain NADPH/plastoquinone oxidoreductase (NDH-1) complex were also found to have a lower expression level under darkness. *Synechocystis* contains a respiratory electron transport chain on both the cytoplasmic and thylakoid membranes, working in an interwoven way with the photosynthetic electron transport chain^40^. The two pathways share some components (plastoquinone (PQ), cytocrome *b*_*6*_*f* and plastocyanin), whereas other complexes are specific for the respiratory electron flow, including NDH-1, succinate dehydrogenase (SDH), and the terminal oxidase. NDH-1 is a proton-pumping NADPH/plastoquinone oxidoreductase and it is analogous to the mitochondrial Complex I; in *Synechocystis*, it is composed of 11 subunits encoded by the *ndh* genes^3^,^41^. NDH-1 has a hydrophobic membrane domain and a hydrophilic domain, and moves the electrons from NADPH to the PQ pool. In *Synechocystis*, respiratory electrons also enter the PQ pool via SDH, an enzyme composed by three subunits (a flavoprotein subunit, a FeS protein subunit, and a membrane subunit) which oxidizes succinate to fumarate, in a reaction similar to that occurring in mitochondria. We identified six NDH-1 subunits, all belonging to the hydrophilic catalytic domain; we found that when wild type *Synechocystis* was grown under prolonged darkness four subunits (Ndh-J, Ndh-K, Ndh-M and Ndh-O, encoded by *ndhJ*/*slr1281*, *ndhK*/*slr1280*, *ndhM*/*slr1623*, and *ndhO*/*sll1690* respectively) were down-regulated in their expression. Furthermore, our data suggest that the remaining two NDH-1 subunits, Ndh-I (*ndhI*/*sll0520*) and Ndh-H (*ndhH*/*slr0261*) are affected in the same way, but they have been excluded from the final list of differentially expressed proteins either because the value is slightly higher than the established cutoff (as in the case of Ndh-I, average value 0.68) or due to the biological variability among the three replicates (as in the case of Ndh-H). Moreover, collected data indicate that also the expression of the SDH flavoprotein subunit (*sdhA*/*slr1233*) is reduced, although it was not included in the final list because it did not completely meet the chosen criteria (altered expression of at least 50% and p < 0.05). Our data also indicate that two antioxidants, the iron superoxide dismutase (*sodB*/*slr1516*) and catalase-peroxidase (*katG*/*sll1987*), show a trend of increased abundance (see supplementary tables S2-S5) indicating that they are likely to have a higher expression level. These results are consistent with a more reductive intracellular environment when *Synechocystis* is grown in the dark, a stress condition that increases the concentration of toxic oxygen species.

The altered expression of all these proteins, in particular NDH-1 hydrophilic subunits, suggests a scenario where the overall respiration electron flow is somehow affected when *Synechocystis* is grown in the dark. Interestingly, a *ndhB*-deficient *Synechocystis* strain, which is completely devoid of the NDH-1 complex, has been shown to display a sustained H_2_ photoevolution^42^, further supporting the hypothesis that the bidirectional Hox hydrogenase can be functionally related to the photosynthetic and respiratory electron transport pathways. However, its specific function(s) is (are) still unclear. Based on our proteomic data, we hypothesize that it could be essential when the photosynthetic and respiratory electron transport chains are temporarily over-reduced during growth under prolonged darkness^2^,^29^,^32^. To further investigate this hypothesis, we performed the same proteomic analysis on the ΔHoxE-H strain grown up to five days either under normal light or in the dark. A comparison between the expression levels of the proteins identified in wild type and the ΔHoxE-H strains under light or darkness conditions is reported in supplementary tables 6-7. Similarly to the wild type strain, the expression of a large number of proteins associated with photosynthesis was reduced when ΔHoxE-H was incubated in the dark. Interestingly, the expression of several NDH-1 hydrophilic subunits, catalase-peroxidase and iron superoxide dismutase were affected in the same way in both strains under prolonged darkness (*i.e.* the expression level in the dark is not significantly different between wild type and mutant strains). However, based on our growth experiments, wild type *Synechocystis* can adjust better than ΔHoxE-H to a light-deprived environment. Thus, the presence of the Hox hydrogenase seems to give *Synechocystis* an adaptive advantage under such a stress condition, by contributing to mantaining a proper redox balance of the electron transport chains when both photosynthetic and respiratory machineries are down-regulated.

Further analyses of overall proteome changes and a closer examination of the metabolic pathways that are altered when *Synechocystis* grows in the dark require additional investigations to be interpretated, and are currently under way in our laboratory.

## Conclusions

The highly evolutionary conserved [NiFe]-hydrogenases are no longer considered simply primordial enzymes whose only function is H_2_ metabolism. Increasing experimental evidence shows that they could have a key role in regulating electron flow pathways under stress conditions.

In this work we show that in *Synechocystis* Hox bidirectional hydrogenase is essential to grow under prolonged darkness (either in anaerobic or aerobic conditions). Interestingly, we found that under this stress condition the expression levels of several hydrophilic NDH-1 subunits in the respiratory complex are reduced. Given the homology between the NAD(P)H oxidoreductase portion of the Hox hydrogenase and the NADH input module of mitochondrial Complex I, we hypothesize that the Hox hydrogenase can compensate for the reduction on the NDH-1 activity and thus allow *Synechocystis* to grow in the dark.

Taken together, our data point toward an additional role for bidirectional Hox hydrogenase beside H_2_ metabolism, and contribute to explain the close evolutionary relationship between [NiFe]-hydrogenases and Complex I in the aerobic respiratory chain.

## Additional Information

**How to cite this article**: De Rosa, E. *et al.* [NiFe]-hydrogenase is essential for cyanobacterium *Synechocystis* sp. PCC 6803 aerobic growth in the dark. *Sci. Rep.* **5**, 12424; doi: 10.1038/srep12424 (2015).

## Supplementary Material

Supplementary Information

## Figures and Tables

**Table 1:** List of the primers used in this study.

Primer name	Primer sequence
SacI*hypA1*_for	5′-TACGTTCAAGAGCTCAGCCCCGAAACC-3′
BamHI*hypA1*_rev	5′-CATATGCACGAAGGGATCCGGTGTGT-3′
BamHI*hypA1*_for	5′-GGATCCGGTGTGTTAGAACTGAGTTGA-3′
EcoRV*hypA1*_rev	5′-ACTGGGGTGGATATCGTCCAAAGAATTATC-3′
SacI*hypB1*_for	5′-ATGAGTCTGGGGAGCTCCGCTTCCG-3′
BamHI*hypB1*_rev	5′-TGTAGTGCGGTGGGAtCCGTTGCCC-3′
BamHI*hypB1*_for	5′-GGATCCGTTGCCCGCATTGGCCTAA-3′
EcoRV*hypB1*_rev	5′-CCGGGCCATAACGGTGGCGATATC-3′
FL*hypA1*_for	5′-GGGCTCAGCGCTCCCGTGCTGG-3′
FL*hypA1_*rev	5′- ATGCCCGCTGGCCCCGGTGGA-3′
FL*hypB1*_for	5′- CCAGCAACCGGGCGGAGCCGT-3′
FL*hypB1*_rev	5′- TGGATGGGGTCCAGGCTTTGGCCCA-3′

**Table 2:** Distribution by functional groups of proteins differentially regulated between control and darkness *Synechocystis* growth.

Functional category	Up-regulated (dark/light ratio ≥ 1.5)	Down-regulated (dark/light ratio ≤ 0.67)
***Nucleotides metabolism, Transcription, Translation***	38	1
***Metabolism***	8	12
Aminoacids	6	5
Lipids	1	2
Carbohydrates	1	5
***Photosynthesis***	0	7
***Respiration***	0	4
***Other functions***	4	17
***Unknown***	8	13
