# Supplementary material for: [NiFe]-hydrogenase is essential for cyanobacterium Synechocystis sp. PCC 6803 aerobic growth in the dark
Source: Sci Rep. 2015 Jul 28;5:12424. doi: 10.1038/srep12424 (PMC4517062; doi:10.1038/srep12424)
Supplement: Supplementary Information [file srep12424-s1.pdf]

[NiFe]-hydrogenase is essential for cyanobacterium *Synechocystis* sp. PCC 6803 aerobic growth in the dark

Edith De Rosa, Vanessa Checchetto, Cinzia Franchin, Elisabetta Bergantino, Paola Berto, Ildikò Szabò, Giorgio M. Giacometti, Giorgio Arrigoni, and Paola Costantini

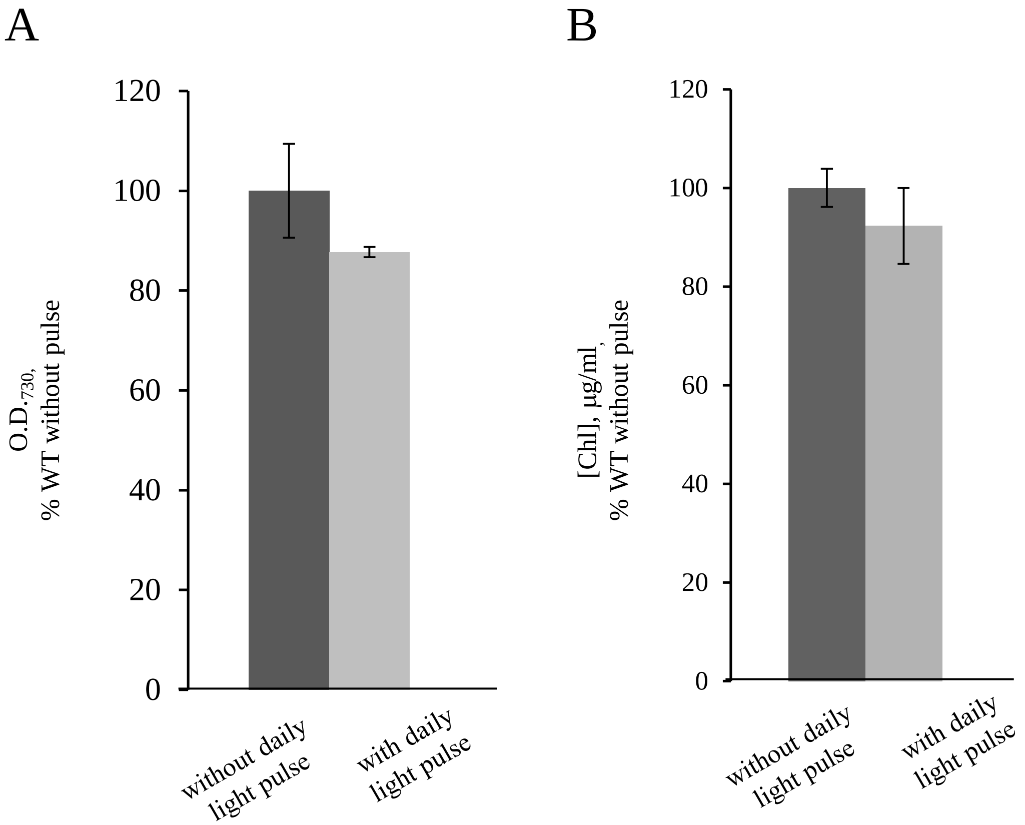

Supplementary figure 1. Heterotrophic growth of wild type *Synechocystis* in the dark, with or without a daily light pulse. Optical densities at 730 nm (O.D.$_{730}$) (panel A) and chlorophyll concentration (panel B) of wild type cultures were measured after five days of growth in the dark, either without (dark grey) or with (light grey) a daily 5 minutes pulse of white light (50 mE m^{-2} s^{-1}). The values were normalized to those of the wild type grown without pulse of light (panel A, 1.7 ± 0.16; panel B, 5.2 µg/ml ± 0.2). Reported data result from the mean of three independent experiments ± Standard Deviation.

[NiFe]-hydrogenase is essential for cyanobacterium *Synechocystis* sp. PCC 6803 aerobic growth in the dark

Edith De Rosa, Vanessa Checchetto, Cinzia Franchin, Elisabetta Bergantino, Paola Berto, Ildikò Szabò, Giorgio M. Giacometti, Giorgio Arrigoni, and Paola Costantini

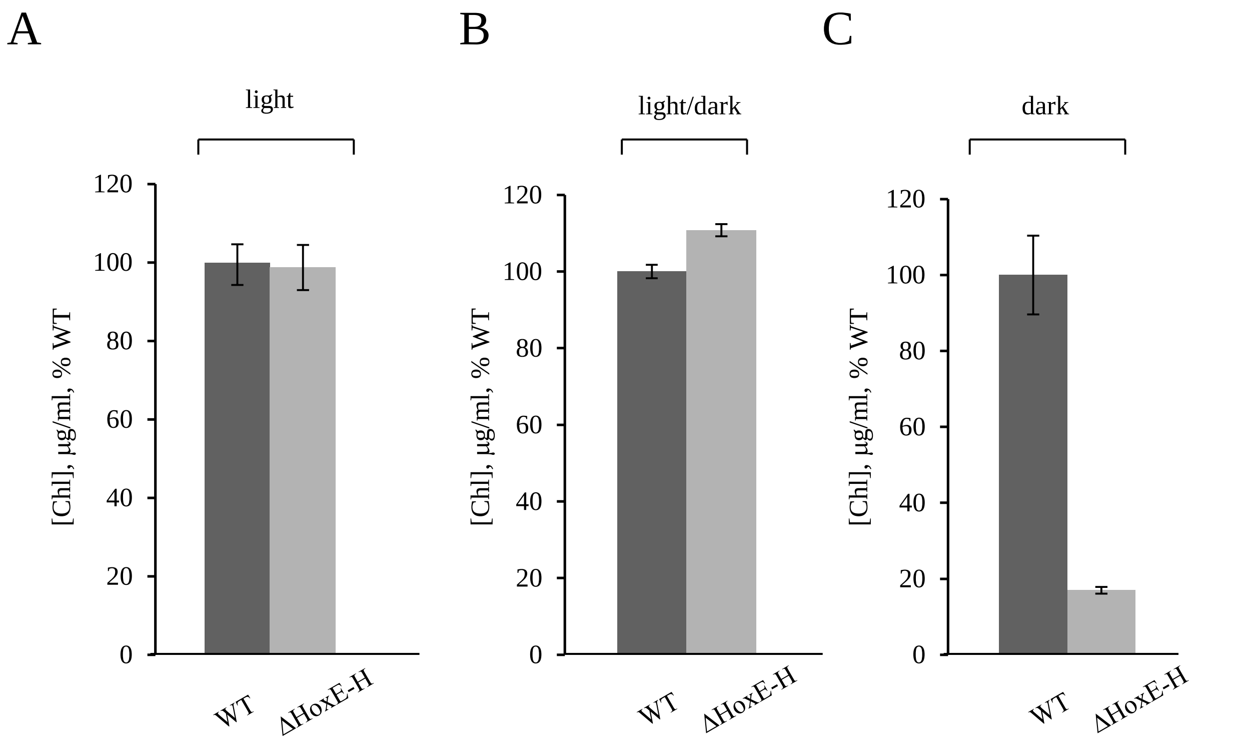

Supplementary figure 2. Wild type *Synechocystis* is more resistant to prolonged darkness than ΔHoxE-H mutant strain. *Panel A*, continuous white light (50 mE m^{-2} s^{-1}); *panel B*, 12 h cycles of light/dark; *panel C*, continuous darkness. Chlorophyll concentration (µg/ml) of wild type (WT, dark grey) and ΔHoxE-H (light grey) cultures was measured after five days of growth, and for each growth condition the values were normalized to the value of the corresponding WT (*panel A*, 15.6 ± 0.73; *panel B*, 13 ± 0.23; *panel C*, 5.3 ± 0.54). Reported data result from the mean of three independent experiments ± Standard Deviation.

[NiFe]-hydrogenase is essential for cyanobacterium *Synechocystis* sp. PCC 6803 aerobic growth in the dark

Edith De Rosa, Vanessa Checchetto, Cinzia Franchin, Elisabetta Bergantino, Paola Berto, Ildikò Szabò, Giorgio M. Giacometti, Giorgio Arrigoni, and Paola Costantini

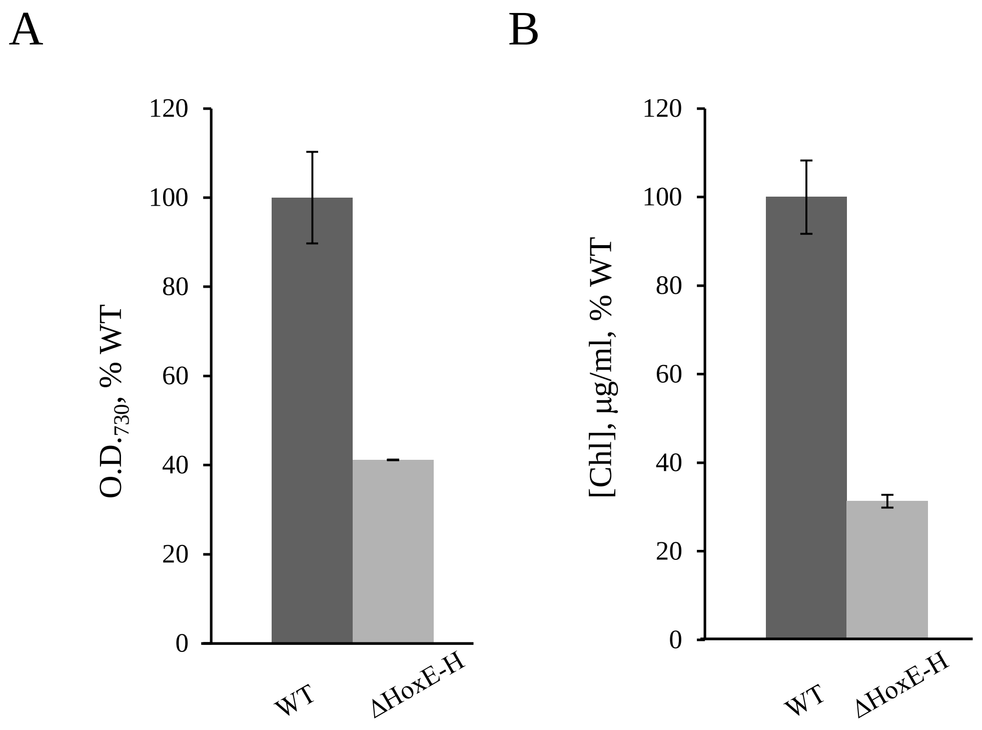

Supplementary figure 3. Wild type *Synechocystis* is more resistant to prolonged darkness in anaerobiosis than ΔHoxE-H strain. Optical densities at 730 nm (O.D.$_{730}$) (panel A) and chlorophyll concentration (panel B) of wild type (dark grey) and ΔHoxE-H (light grey) cultures were measured after five days of anaerobic growth in the dark.The values were normalized to the O.D.$_{730}$ or chlorophyll concentration values of the wild type (panel A, 1.9 ± 0.19; panel B, 4.95 µg/ml ± 0.41). Reported data result from the mean of three independent experiments ± Standard Deviation.

[NiFe]-hydrogenase is essential for cyanobacterium *Synechocystis* sp. PCC 6803 aerobic growth in the dark

Edith De Rosa, Vanessa Checchetto, Cinzia Franchin, Elisabetta Bergantino, Paola Berto, Ildikò Szabò, Giorgio M. Giacometti, Giorgio Arrigoni, and Paola Costantini

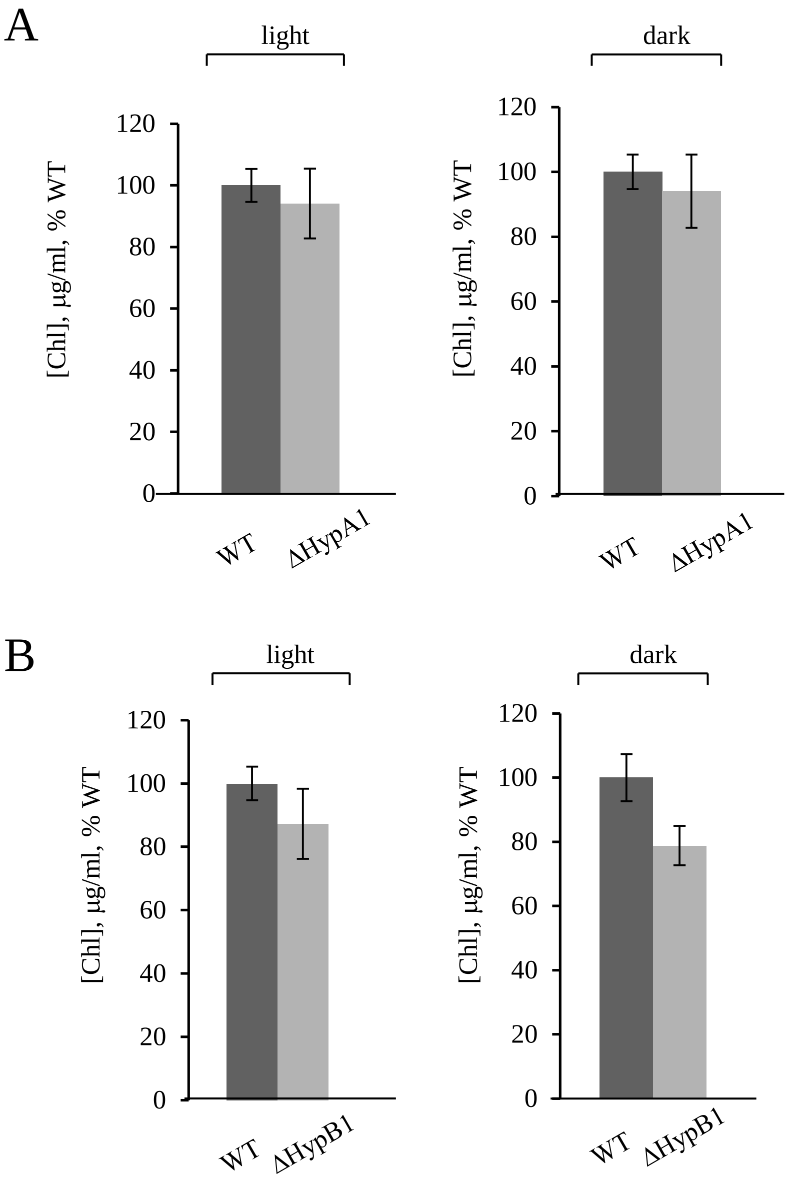

Supplementary figure 4. ΔHypA1 and ΔHypB1 *Synechocystis* strains have the same growth phenotype of the wild type strain when cultured in the dark. *Panel A*, ΔHypA1; *panel B*, ΔHypB1. Strains were grown either under continuous white light (50 mE m^{-2} s^{-1}) or darkness. Chlorophyll concentration of WT (dark grey) and mutants (light grey) cultures were measured after five days, and for each growth condition the values were normalized to the chlrophyll concentration of the WT (20.97 µg/ml ± 1.12 and 4.1 ± 0.3 for light and darkness, respectively). Reported data result from the mean of three independent experiments ± Standard Deviation.

Supplementary table S1. H_2 evolution activities of wild type, ΔHypA1 and ΔHypB1 *Synechocystis* strains

Strain	nmol $H_2 \cdot mg\ Chl^{-1} \cdot min^{-1} \pm SD$[(a)]
wild type	30 ± 3.1
ΔHypA1	N.A.[(b)]
ΔHypB1	N.A.[(b)]

[(a)] Reported values represent the mean of three independent experiments ± Standard Deviation

[(b)] N.A.: not applicable

Supplementary Table S2

[NiFe]-hydrogenase is essential for cyanobacterium *Synechocystis* sp. PCC 6803 aerobic growth in the dark

Edith De Rosa, Vanessa Checchetto, Cinzia Franchin, Elisabetta Bergantino, Paola Berto, Ildikò Szabò, Giorgio M. Giacometti, Giorgio Arrigoni, and Paola Costantini

Accession	Description	Score	Coverage	# Proteins	# Unique Peptides	# Peptides	# PSMs	Dark/Light (116/117)	116/117 Count	116/117 Variability [%]	# AAs	MW [kDa]	calc. pI							
Q54714	C-phycocyanin beta chain OS=Synechocystis sp. (strain PCC 6803 / Kazusa) GN=cpcB PE=1 SV=2 - [	4761,02	87,21	1	18	18	165	0,557	133	26,1	172	18,1	5,14							
	A3	Sequence	# PSMs	# Proteins	# Protein Groups	Protein Group Accessions	Modifications	ΔCn	116/117	116/117 Count	116/117 Variability [%]	q-Value	PEP	IonScore	Exp Value	Charge	MH+ [Da]	ΔM [ppm]	RT [min]	# Missed Cleavages
	High	gEYLSGSQLDALSATVAEGNkR	15	1	1	Q54714	N-Term(iTRAQ4plex); K21(iTRAQ4plex)	0,0000	0,501	9	8,4	0	9,662E-13	114	3,89018E-12	4	2554,33657	0,53	38,57	1
	High	iTGNASAIVSNAAR	47	1	1	Q54714	N-Term(iTRAQ4plex)	0,0000	0,560	42	25,8	0	1,842E-08	90	1,21874E-09	2	1488,82952	2,94	29,65	0
	High	aLFAEQPQLIQPGGNAYTSR	6	1	1	Q54714	N-Term(iTRAQ4plex)	0,0000	0,201	4	4,1	0	4,532E-11	82	6,38841E-09	2	2305,20830	1,09	40,16	0
	High	eTYVALGVPGASVAAGVQk	3	1	1	Q54714	N-Term(iTRAQ4plex); K19(iTRAQ4plex)	0,0000	0,472	2	2,6	0	1,286E-11	80	9,22508E-09	2	2105,18511	0,26	37,99	0
	High	mkEAALDIVNDPNGITR	12	1	1	Q54714	N-Term(iTRAQ4plex); M1(Oxidation); K2(iTRAQ4plex)	0,0000	0,541	8	7,3	0	1,913E-08	79	1,30008E-08	2	2161,15605	1,59	30,86	1
	High	gEYLSGSQLDALSATVAEGNk	4	1	1	Q54714	N-Term(iTRAQ4plex); K21(iTRAQ4plex)	0,0000	0,463	2	0,9	0	2,237E-10	77	1,79048E-08	3	2398,23648	1,00	41,29	0
	High	eAALDIVNDPNGITR	4	1	1	Q54714	N-Term(iTRAQ4plex)	0,0000	0,468	2	2,2	0	4,518E-08	75	2,89048E-08	2	1741,92266	1,42	34,70	0
	High	mkEAALDIVNDPNGITR	7	1	1	Q54714	N-Term(iTRAQ4plex); K2(iTRAQ4plex)	0,0000	0,877	5	4,5	0	1,845E-10	73	5,188E-08	3	2145,15927	0,73	36,42	1
	High	vVSQADAR	6	1	1	Q54714	N-Term(iTRAQ4plex)	0,0000	0,643	5	47,7	0	0,00001867	51	8,01623E-06	2	989,54991	0,25	14,67	0
	High	yVTYATFTGDASVLEDR	2	1	1	Q54714	N-Term(iTRAQ4plex)	0,0000	0,191	1		0	3,598E-07	50	9,07758E-06	3	2052,00565	0,67	43,83	0
	High	rIDSVNR	11	1	1	Q54714	N-Term(iTRAQ4plex)	0,0000	0,612	10	30,5	0	0,0001478	49	1,33343E-05	3	1003,57697	0,42	11,24	1
	High	mFDVFTR	9	1	1	Q54714	N-Term(iTRAQ4plex)	0,0000	0,855	9	10,2	0	0,00008167	48	1,63294E-05	2	1059,54155	0,13	43,61	0
	High	dMEIILR	4	1	1	Q54714	N-Term(iTRAQ4plex)	0,0000	0,587	4	7,0	0	0,002696	36	0,000267898	2	1033,58367	0,37	32,97	0
	High	fDVFTR	4	1	1	Q54714	N-Term(iTRAQ4plex)	0,0000	0,221	4	19,8	0	0,002169	35	0,000309008	2	928,50090	-0,01	31,95	0
	High	rmAAcLR	2	1	1	Q54714	N-Term(iTRAQ4plex); M2(Oxidation); C5(Carbamidomethyl)	0,0000	1,096	2	18,6	0	0,002133	27	0,002103633	3	1037,54660	0,08	10,36	1
	High	mFDVFTR	10	1	1	Q54714	N-Term(iTRAQ4plex); M1(Oxidation)	0,0000	0,593	6	19,4	0	0,002628	19	0,013426722	2	1075,53667	0,32	31,88	0
	High	mAAcLRDMEIILR	1	1	1	Q54714	N-Term(iTRAQ4plex); M1(Oxidation); C4(Carbamidomethyl)	0,0000	1,046	1		0	0,00257	15	0,032134386	3	1751,90970	0,52	39,14	1
	High	iDSVNR	4	1	1	Q54714	N-Term(iTRAQ4plex)	0,0000	0,504	4	5,1	0,001	0,0047	24	0,004206976	2	847,47588	0,53	13,64	0
	High	dmEIILR	3	1	1	Q54714	N-Term(iTRAQ4plex); M2(Oxidation)	0,0000	0,480	2	21,8	0,001	0,00603	15	0,028442647	2	1049,57952	1,25	23,69	0
	High	cLNGLR	3	1	1	Q54714	N-Term(iTRAQ4plex); C1(Carbamidomethyl)	0,0000	0,523	3	2,6	0,004	0,05337	39	0,000118024	2	876,48491	0,79	17,25	0
	High	mAAcLR	6	1	1	Q54714	N-Term(iTRAQ4plex); M1(Oxidation); C4(Carbamidomethyl)	0,0000	0,548	6	11,7	0,006	0,06967	13	0,048191451	2	881,44499	-0,46	12,41	0
	High	mAAcLR	2	1	1	Q54714	N-Term(iTRAQ4plex); C4(Carbamidomethyl)	0,0000	0,737	2	0,6	0,008	0,1058	31	0,000833624	2	865,45098	0,56	17,18	0
Q54715	C-phycocyanin alpha chain OS=Synechocystis sp. (strain PCC 6803 / Kazusa) GN=cpcA PE=1 SV=1 -	4118,13	79,01	1	12	12	148	0,629	119	17,8	162	17,6	5,57							
	A3	Sequence	# PSMs	# Proteins	# Protein Groups	Protein Group Accessions	Modifications	ΔCn	116/117	116/117 Count	116/117 Variability [%]	q-Value	PEP	IonScore	Exp Value	Charge	MH+ [Da]	ΔM [ppm]	RT [min]	# Missed Cleavages
	High	fPYTTQTQGNNFAADQR	9	1	1	Q54715	N-Term(iTRAQ4plex)	0,0000	0,573	9	4,6	0	6,007E-12	114	4,27533E-12	2	2103,01909	8,48	30,84	0
	High	mkTPLTEAVSTADSQGR	20	1	1	Q54715	N-Term(iTRAQ4plex); M1(Oxidation); K2(iTRAQ4plex)	0,0000	0,665	15	16,7	0	3,353E-08	94	4,75534E-10	2	2096,09160	0,92	27,23	1
	High	fLSSTELQIAFGR	22	1	1	Q54715	N-Term(iTRAQ4plex)	0,0000	0,602	19	16,0	0	1,455E-08	85	3,06881E-09	2	1612,88298	0,87	47,23	0
	High	qANAGLQAAk	19	1	1	Q54715	N-Term(iTRAQ4plex); K10(iTRAQ4plex)	0,0000	0,628	11	3,7	0	4,156E-08	82	5,662E-09	2	1259,73235	1,03	15,99	0
	High	tPLTEAVSTADSQGR	6	1	1	Q54715	N-Term(iTRAQ4plex)	0,0000	0,729	5	14,5	0	0,000008233	82	6,20826E-09	2	1676,86443	4,30	30,04	0
	High	aLTDNAQSLVNGAAQAVYNk	10	1	1	Q54715	N-Term(iTRAQ4plex); K20(iTRAQ4plex)	0,0000	0,627	6	5,4	0	6,855E-12	80	9,31044E-09	3	2336,24961	2,01	44,40	0
	High	mkTPLTEAVSTADSQGR	3	1	1	Q54715	N-Term(iTRAQ4plex); K2(iTRAQ4plex)	0,0000	1,179	3	22,7	0	1,36E-10	77	1,80302E-08	3	2080,09751	1,32	30,83	1
	High	tFDLSPSWYVEALk	19	1	1	Q54715	N-Term(iTRAQ4plex); K14(iTRAQ4plex)	0,0000	0,569	17	30,1	0	1,611E-11	73	4,58142E-08	2	1944,03655	0,42	50,24	0
	High	dIGYYLR	12	1	1	Q54715	N-Term(iTRAQ4plex)	0,0000	0,620	9	20,5	0	0,00114	38	0,000160683	2	1043,56450	0,26	34,13	0
	High	aNHGLSGDAR	22	1	1	Q54715	N-Term(iTRAQ4plex)	0,0000	0,659	21	25,1	0	0,00004594	35	0,000308298	3	1141,58310	0,01	7,99	0
	High	lRQANAGLQAAk	2	1	1	Q54715	N-Term(iTRAQ4plex); K12(iTRAQ4plex)	0,0000	1,157	1		0	0,00004114	32	0,000594251	3	1528,92588	6,30	17,02	1
	High	yIkANHGLSGDAR	2	1	1	Q54715	N-Term(iTRAQ4plex); K3(iTRAQ4plex)	0,0000	0,738	2	2,3	0	0,0007687	23	0,00544465	4	1689,92641	-0,65	16,17	1
	High	dEANSYLDYAINALS	2	1	1	Q54715	N-Term(iTRAQ4plex)	0,0000	0,562	1		0,001	0,0103	35	0,000322827	2	1802,86040	2,14	55,02	0
P73282	Transketolase OS=Synechocystis sp. (strain PCC 6803 / Kazusa) GN=tktA PE=3 SV=1 - [P73282_SYN	3301,59	38,51	1	23	23	126	0,533	102	20,2	670	71,7	5,57							
	A3	Sequence	# PSMs	# Proteins	# Protein Groups	Protein Group Accessions	Modifications	ΔCn	116/117	116/117 Count	116/117 Variability [%]	q-Value	PEP	IonScore	Exp Value	Charge	MH+ [Da]	ΔM [ppm]	RT [min]	# Missed Cleavages
	High	vVATQSLDELSINAIR	3	1	1	P73282	N-Term(iTRAQ4plex)	0,0000	0,489	3	11,7	0	3,358E-13	100	9,31044E-11	2	1873,05388	1,44	42,88	0
	High	qAVPNLPGTSIDAVAk	4	1	1	P73282	N-Term(iTRAQ4plex); K16(iTRAQ4plex)	0,0000	0,496	3	16,8	0	3,514E-12	98	1,68644E-10	2	1869,07060	1,15	34,77	0
	High	vTTIIGYGAPNk	9	1	1	P73282	N-Term(iTRAQ4plex); K12(iTRAQ4plex)	0,0000	0,546	7	25,4	0	0,00000007	75	3,33403E-08	2	1521,88957	1,08	31,70	0
	High	fGFSVDNVLAk	8	1	1	P73282	N-Term(iTRAQ4plex); K11(iTRAQ4plex)	0,0000	0,472	7	4,0	0	3,009E-09	74	3,77546E-08	2	1484,83745	1,52	42,79	0
	High	akENAPTLLSLTR	7	1	1	P73282	N-Term(iTRAQ4plex); K2(iTRAQ4plex)	0,0000	0,504	4	0,7	0	1,551E-08	73	4,87495E-08	2	1702,01457	2,57	29,87	1
	High	tLASFTPDQk	4	1	1	P73282	N-Term(iTRAQ4plex); K10(iTRAQ4plex)	0,0000	0,592	4	12,4	0	2,628E-08	70	1,08136E-07	2	1395,77300	0,54	27,98	0
	High	lSVEAATNFGWHk	6	1	1	P73282	N-Term(iTRAQ4plex); K13(iTRAQ4plex)	0,0000	0,594	4	19,6	0	3,057E-11	69	1,36135E-07	3	1747,93888	1,06	36,66	0
	High	aIPNLTVIRPADGNETSGAYk	9	1	1	P73282	N-Term(iTRAQ4plex); K21(iTRAQ4plex)	0,0000	0,509	9	6,1	0	1,578E-09	66	2,74405E-07	3	2475,34769	1,23	35,78	0
	High	sDTAGIHGAALGTDEVAATR	3	1	1	P73282	N-Term(iTRAQ4plex)	0,0000	0,525	2	40,4	0	6,401E-12	64	3,6557E-07	3	2057,03904	0,47	25,56	0
	High	qLSGALPEGWDk	2	1	1	P73282	N-Term(iTRAQ4plex); K12(iTRAQ4plex)	0,0000	0,610	2	20,4	0	3,465E-08	62	6,30957E-07	2	1588,86003	1,67	33,82	0
	High	fLAVDAIEk	3	1	1	P73282	N-Term(iTRAQ4plex); K9(iTRAQ4plex)	0,0000	0,513	3	6,8	0	6,561E-07	59	1,1587E-06	2	1293,76653	0,63	36,69	0
	High	sGHPGLPmGAAPMAFTLWNk	6	1	1	P73282	N-Term(iTRAQ4plex); M8(Oxidation); K20(iTRAQ4plex)	0,0000	0,883	5	23,7	0	1,827E-09	57	1,85768E-06	3	2387,22947	2,14	42,70	0
	High	gASYEAEWNQAFAQYk	3	1	1	P73282	N-Term(iTRAQ4plex); K16(iTRAQ4plex)	0,0000	0,665	2	32,2	0	5,237E-13	56	2,59998E-06	3	2151,04154	1,37	41,66	0
	High	tkYPTEAAAFER	4	1	1	P73282	N-Term(iTRAQ4plex); K2(iTRAQ4plex)	0,0000	0,505	4	3,0	0	0,000000218	55	3,52347E-06	3	1671,89554	0,63	23,52	1
	High	eNAPTLLSLTR	4	1	1	P73282	N-Term(iTRAQ4plex)	0,0000	0,471	4	8,3	0	0,000008321	54	4,01763E-06	2	1358,77764	1,17	35,70	0
	High	aVTDkPSMIk	8	1	1	P73282	N-Term(iTRAQ4plex); K5(iTRAQ4plex); K10(iTRAQ4plex)	0,0000	0,714	6	4,2	0	6,542E-07	50	9,26766E-06	2	1521,90984	4,16	20,27	0
	High	rLSVEAATNFGWHk	2	1	1	P73282	N-Term(iTRAQ4plex); K14(iTRAQ4plex)	0,0000	0,620	2	17,3	0	7,48E-09	50	9,31044E-06	3	1904,04013	1,04	31,23	1
	High	sGHPGLPmGAAPmAFTLWNk	8	1	1	P73282	N-Term(iTRAQ4plex); M8(Oxidation); M13(Oxidation); K20(iTR	0,0000	0,870	6	71,5	0	6,661E-11	47	1,97783E-05	3	2403,22318	1,62	38,26	0
	High	aVTDkPSmIk	12	1	1	P73282	N-Term(iTRAQ4plex); K5(iTRAQ4plex); M8(Oxidation); K10(iTR	0,0000	0,462	8	10,0	0	0,000008206	43	4,5811E-05	2	1537,90166	2,10	16,15	0
	High	fEAYGWHVLHVEDGNTDLAAIAk	1	1	1	P73282	N-Term(iTRAQ4plex); K23(iTRAQ4plex)	0,0000	0,637	1		0	3,532E-07	27	0,001990536	4	2844,45888	1,01	44,24	0
	High	yPTEAAAFER	1	1	1	P73282	N-Term(iTRAQ4plex)	0,0000	0,479	1		0	0,002005	24	0,004149254	2	1298,64995	0,14	28,12	0
	High	sGHPGLPMGAAPMAFTLWNk	1	1	1	P73282	N-Term(iTRAQ4plex); K20(iTRAQ4plex)	0,0000	1,681	1		0	0,00002791	24	0,004425578	4	2371,23305	1,52	46,54	0
	High	nVHFGVR	6	1	1	P73282	N-Term(iTRAQ4plex)	0,0000	0,547	6	16,9	0,001	0,01031	34	0,000422375	2	972,55022	0,64	16,61	0
	High	kAIER	1	1	1	P73282	N-Term(iTRAQ4plex); K1(iTRAQ4plex)	0,0000	0,771	1		0,002	0,01853	13	0,050721174	3	904,57962	-2,46	8,76	1
	High	lAAQGk	6	1	1	P73282	N-Term(iTRAQ4plex); K6(iTRAQ4plex)	0,0000	0,561	5	2,4	0,006	0,07102	28	0,001559445	2	875,55626	1,10	14,68	0
	Medium	vAIAk	5	1	1	P73282	N-Term(iTRAQ4plex); K5(iTRAQ4plex)	0,0000	0,531	2	1,8	0,016	0,2132	23	0,012710601	2	789,54070	-3,76	17,32	0
P77961	Glutamine synthetase OS=Synechocystis sp. (strain PCC 6803 / Kazusa) GN=glnA PE=1 SV=2 - [GLN	3263,85	48,20	1	24	24	130	0,589	105	35,9	473	53,0	5,11							
	A3	Sequence	# PSMs	# Proteins	# Protein Groups	Protein Group Accessions	Modifications	ΔCn	116/117	116/117 Count	116/117 Variability [%]	q-Value	PEP	IonScore	Exp Value	Charge	MH+ [Da]	ΔM [ppm]	RT [min]	# Missed Cleavages
	High	fGQTENSSYYFADSVEGR	3	1	1	P77961	N-Term(iTRAQ4plex)	0,0000	0,514	2	6,0	0	4,847E-11	102	6,82292E-11	2	2200,99468	1,94	36,51	0
	High	lVPGFEAPVNLAYSQGNR	9	1	1	P77961	N-Term(iTRAQ4plex)	0,0000	0,481	7	12,9	0	2,416E-08	90	9,35341E-10	2	2076,10259	1,47	44,00	0
	High	hAPALLAFTNPTTNSYk	15	1	1	P77961	N-Term(iTRAQ4plex); K17(iTRAQ4plex)	0,0000	0,615	10	16,8	0	7,717E-11	88	1,77905E-09	2	2134,15898	2,54	35,16	0
	High	lVNSADNLMIYk	8	1	1	P77961	N-Term(iTRAQ4plex); K12(iTRAQ4plex)	0,0000	1,006	7	17,1	0	1,177E-08	78	1,61582E-08	2	1668,92851	3,10	38,86	0
	High	hHHEVASGGQNELGIk	6	1	1	P77961	N-Term(iTRAQ4plex); K16(iTRAQ4plex)	0,0000	0,456	4	8,1	0	1,152E-10	77	2,21294E-08	2	2001,05461	2,06	13,29	0
	High	rLVPGFEAPVNLAYSQGNR	6	1	1	P77961	N-Term(iTRAQ4plex)	0,0000	0,503	3	3,6	0	3,09E-11	69	1,19399E-07	3	2232,20176	0,49	38,31	1
	High	qGYFPVAPTDTAQDIR	4	1	1	P77961	N-Term(iTRAQ4plex)	0,0000	0,496	2	9,3	0	3,892E-12	69	1,21891E-07	2	1922,97649	1,85	34,90	0
	High	fDkLVNSADNLmIYk	2	1	1	P77961	N-Term(iTRAQ4plex); K3(iTRAQ4plex); M12(Oxidation); K15(iT	0,0000	0,747	2	11,9	0	6,492E-12	64	3,61385E-07	3	2219,21720	2,94	40,55	1
	High	lVNSADNLmIYk	6	1	1	P77961	N-Term(iTRAQ4plex); M9(Oxidation); K12(iTRAQ4plex)	0,0000	0,454	5	9,9	0	5,209E-08	63	4,70099E-07	2	1684,92058	1,38	34,65	0
	High	fDkLVNSADNLMIYk	1	1	1	P77961	N-Term(iTRAQ4plex); K3(iTRAQ4plex); K15(iTRAQ4plex)	0,0000	1,054	1		0	7,845E-12	61	7,72627E-07	3	2203,22220	2,93	44,94	1
	High	iPLSGGNPk	7	1	1	P77961	N-Term(iTRAQ4plex); K9(iTRAQ4plex)	0,0000	0,319	6	6,6	0	0,00002243	57	1,98139E-06	2	1170,71025	1,48	22,76	0
	High	lDNEVNPmR	4	1	1	P77961	N-Term(iTRAQ4plex); M8(Oxidation)	0,0000	0,483	2	24,7	0	0,00006275	56	2,39867E-06	2	1247,61846	1,08	18,85	0
	High	lRPHPYEFSLYYDc	8	1	1	P77961	N-Term(iTRAQ4plex); C14(Carbamidomethyl)	0,0000	0,426	6	21,1	0	4,174E-09	56	2,54665E-06	2	2003,94744	1,60	37,52	0
	High	tPQEVLk	3	1	1	P77961	N-Term(iTRAQ4plex); K7(iTRAQ4plex)	0,0000	0,548	3	3,7	0	0,00003056	53	5,44465E-06	2	1102,67290	1,67	20,39	0
	High	yAGFSQmGLWYIGGILk	4	1	1	P77961	N-Term(iTRAQ4plex); M7(Oxidation); K17(iTRAQ4plex)	0,0000	0,574	4	32,7	0	1,125E-07	51	8,53041E-06	3	2208,18198	2,43	53,74	0
	High	ePTLSMIcSIk	3	1	1	P77961	N-Term(iTRAQ4plex); C8(Carbamidomethyl); K11(iTRAQ4plex)	0,0000	0,668	3	25,9	0	0,000003364	48	1,41244E-05	2	1566,85063	2,06	39,45	0
	High	lDNEVNPMR	2	1	1	P77961	N-Term(iTRAQ4plex)	0,0000	0,997	2	18,1	0	9,382E-07	46	2,4042E-05	2	1231,62370	1,23	25,08	0
	High	ePTLSMIcSIkEPR	4	1	1	P77961	N-Term(iTRAQ4plex); C8(Carbamidomethyl); K11(iTRAQ4plex)	0,0000	0,789	4	5,7	0	1,365E-07	43	7,96834E-05	3	1949,04782	2,01	36,29	1
	High	wIQDENIk	2	1	1	P77961	N-Term(iTRAQ4plex); K8(iTRAQ4plex)	0,0000	0,574	2	20,5	0	0,0007738	41	7,14447E-05	2	1333,73784	1,79	29,71	0
	High	wNTGREEEGGNLGYkPGYk	2	1	1	P77961	N-Term(iTRAQ4plex); K15(iTRAQ4plex); K19(iTRAQ4plex)	0,0000	0,576	2	1,6	0	1,189E-11	40	0,000103507	3	2587,33243	2,26	24,55	1
	High	eEEGGNLGYkPGYk	8	1	1	P77961	N-Term(iTRAQ4plex); K10(iTRAQ4plex); K14(iTRAQ4plex)	0,0000	0,481	6	25,3	0	1,729E-07	39	0,000147973	3	1973,03720	1,59	20,21	0
	High	dGQPLFAGDk	2	1	1	P77961	N-Term(iTRAQ4plex); K10(iTRAQ4plex)	0,0000	0,581	2	21,6	0	0,0002849	38	0,000157387	2	1335,71672	1,49	24,29	0
	High	ePTLSmIcSIk	1	1	1	P77961	N-Term(iTRAQ4plex); M6(Oxidation); C8(Carbamidomethyl); K	0,0000	0,646	1		0	0,000001102	34	0,000416841	3	1582,84330	0,61	33,48	0
	High	ePTLSmIcSIkEPR	2	1	1	P77961	N-Term(iTRAQ4plex); M6(Oxidation); C8(Carbamidomethyl); K	0,0000	0,828	2	56,7	0	0,000001744	32	0,00059016	4	1965,04091	1,07	30,69	1
	High	hAPALLAFTNPTTNSYkR	1	1	1	P77961	N-Term(iTRAQ4plex); K17(iTRAQ4plex)	0,0000	0,956	1		0	0,0001763	31	0,000851138	4	2290,25698	1,00	31,37	1
	High	tGEWYNR	4	1	1	P77961	N-Term(iTRAQ4plex)	0,0000	0,677	4	18,3	0	0,0008322	27	0,00196775	2	1069,51897	0,58	19,48	0
	High	tGEWYNRDPR	4	1	1	P77961	N-Term(iTRAQ4plex)	0,0000	0,704	4	4,5	0,001	0,003827	15	0,034195582	2	1437,70232	2,18	18,56	1
	High	tIAAk	8	1	1	P77961	N-Term(iTRAQ4plex); K5(iTRAQ4plex)	0,0000	0,605	7	4,0	0,004	0,04564	29	0,001396272	2	791,52556	3,33	14,81	0
	High	iIDLk	1	1	1	P77961	N-Term(iTRAQ4plex); K5(iTRAQ4plex)	0,0000	0,479	1		0,005	0,06467	16	0,025880342	2	889,59746	1,53	27,91	0
Q01951	Allophycocyanin alpha chain OS=Synechocystis sp. (strain PCC 6803 / Kazusa) GN=apcA PE=1 SV=3	2535,86	71,43	1	13	14	80	0,646	53	14,4	161	17,4	4,96							
	A3	Sequence	# PSMs	# Proteins	# Protein Groups	Protein Group Accessions	Modifications	ΔCn	116/117	116/117 Count	116/117 Variability [%]	q-Value	PEP	IonScore	Exp Value	Charge	MH+ [Da]	ΔM [ppm]	RT [min]	# Missed Cleavages
	High	rPDIVSPGGNAYGEEMTATcLR	9	1	1	Q01951	N-Term(iTRAQ4plex); C20(Carbamidomethyl)	0,0000	1,004	7	16,9	0	2,35E-16	106	2,73508E-11	3	2538,22171	0,74	33,01	0
	High	sLGTPIEAVAQSVR	12	1	1	Q01951	N-Term(iTRAQ4plex)	0,0000	0,605	10	10,2	0	7,414E-11	96	2,72879E-10	2	1571,88860	0,76	38,68	0
	High	rPDIVSPGGNAYGEEmTATcLR	11	1	1	Q01951	N-Term(iTRAQ4plex); M16(Oxidation); C20(Carbamidomethyl)	0,0000	0,492	6	8,3	0	2,2E-13	90	1,09388E-09	3	2554,21671	0,77	28,71	0
	High	sIVNADAEAR	6	1	1	Q01951	N-Term(iTRAQ4plex)	0,0000	0,635	3	0,0	0	1,042E-07	60	1,01151E-06	2	1189,63103	1,38	18,08	0
	High	yLSPGELDRIk	1	1	1	Q01951	N-Term(iTRAQ4plex); K11(iTRAQ4plex)	0,0000	0,727	1		0	0,000000423	57	2,55934E-06	3	1578,91074	0,84	32,84	1
	High	lVTYGVVSGDVTPIEEIGLVGVR	3	1	1	Q01951	N-Term(iTRAQ4plex)	0,0000	0,652	2	3,7	0	9,75E-10	51	1,06704E-05	3	2516,40818	-0,46	54,05	0
	High	yLSPGELDR	3	1	1	Q01951	N-Term(iTRAQ4plex)	0,0000	0,570	3	2,9	0	0,002697	50	1,05674E-05	2	1193,62871	0,34	29,16	0
	High	iAETLTGSR	6	1	1	Q01951	N-Term(iTRAQ4plex)	0,0000	0,639	3	1,7	0	0,0001218	47	1,79461E-05	2	1091,61821	0,43	23,94	0
	High	aFVTGGAAR	4	1	1	Q01951	N-Term(iTRAQ4plex)	0,0000	0,609	4	5,6	0	0,000003979	47	2,06049E-05	2	993,55974	-0,10	17,29	0
	High	ikAFVTGGAAR	2	1	1	Q01951	N-Term(iTRAQ4plex); K2(iTRAQ4plex)	0,0000	1,005	2	20,5	0	4,403E-07	40	0,000101435	3	1378,84046	-0,36	25,24	1
	High	iAETLTGSRETIVk	1	1	1	Q01951	N-Term(iTRAQ4plex); K14(iTRAQ4plex)	0,0000	0,695	1		0	0,00001626	28	0,001678688	3	1806,05893	0,77	29,06	1
	High	sIVTkSIVNADAEAR	1	1	1	Q01951	N-Term(iTRAQ4plex); K5(iTRAQ4plex)	0,0000	1,029	1		0	1,207E-07	26	0,002243727	3	1862,05735	-0,68	26,57	1
	High	dmDYYLR	4	2	2	P74551;Q01951	N-Term(iTRAQ4plex); M2(Oxidation)	0,0000				0	0,001955	12	0,059016032	2	1135,52141	0,32	22,09	0
	High	dMDYYLR	1	2	2	P74551;Q01951	N-Term(iTRAQ4plex)	0,0000				0,001	0,003346	49	1,1587E-05	2	1119,52886	2,43	31,04	0
	High	eTIVk	4	1	1	Q01951	N-Term(iTRAQ4plex); K5(iTRAQ4plex)	0,0000	0,633	4	5,3	0,001	0,01619	35	0,000339151	2	877,55950	-0,24	15,06	0
	High	sIVTk	12	1	1	Q01951	N-Term(iTRAQ4plex); K5(iTRAQ4plex)	0,0000	0,704	6	5,4	0,003	0,03123	18	0,02362293	2	835,54894	-0,24	15,15	0
Q01952	Allophycocyanin beta chain OS=Synechocystis sp. (strain PCC 6803 / Kazusa) GN=apcB PE=1 SV=1 -	2480,04	98,76	1	17	17	105	0,885	83	27,6	161	17,2	5,60							
	A3	Sequence	# PSMs	# Proteins	# Protein Groups	Protein Group Accessions	Modifications	ΔCn	116/117	116/117 Count	116/117 Variability [%]	q-Value	PEP	IonScore	Exp Value	Charge	MH+ [Da]	ΔM [ppm]	RT [min]	# Missed Cleavages
	High	aASVISANAATIVk	23	1	1	Q01952	N-Term(iTRAQ4plex); K14(iTRAQ4plex)	0,0000	0,864	16	36,8	0	1,472E-10	115	3,08298E-12	2	1603,96355	0,85	32,09	0
	High	mQDAITAVINSADVQGk	6	1	1	Q01952	N-Term(iTRAQ4plex); M1(Oxidation); K17(iTRAQ4plex)	0,0000	0,941	3	5,6	0	9,92E-16	107	1,94971E-11	2	2065,08550	0,79	38,72	0
	High	eVTASLVGADAGk	4	1	1	Q01952	N-Term(iTRAQ4plex); K13(iTRAQ4plex)	0,0000	0,871	4	7,2	0	3,212E-09	81	7,63783E-09	2	1505,84014	-0,84	26,59	0
	High	mQDAITAVINSADVQGk	2	1	1	Q01952	N-Term(iTRAQ4plex); K17(iTRAQ4plex)	0,0000	1,507	1		0	1,096E-12	73	4,80806E-08	3	2049,09153	1,26	45,98	0
	High	yATYAMLAGDASILDER	2	1	1	Q01952	N-Term(iTRAQ4plex)	0,0000	1,293	2	18,7	0	2,833E-10	64	3,78416E-07	3	2003,98771	0,58	47,34	0
	High	aASVISANAATIVkEAVAk	5	1	1	Q01952	N-Term(iTRAQ4plex); K14(iTRAQ4plex); K19(iTRAQ4plex)	0,0000	1,162	4	12,0	0	1,626E-10	64	3,80163E-07	3	2246,34574	0,56	44,83	1
	High	yATYAmLAGDASILDER	2	1	1	Q01952	N-Term(iTRAQ4plex); M6(Oxidation)	0,0000	1,212	2	22,5	0	4,191E-10	56	2,64223E-06	3	2019,98270	0,62	43,71	0
	High	sYFASGELR	5	1	1	Q01952	N-Term(iTRAQ4plex)	0,0000	0,859	4	6,0	0	0,0000127	55	2,95101E-06	2	1173,60222	0,12	27,00	0
	High	eTYNSLGVPISSTVQAIQAIk	7	1	1	Q01952	N-Term(iTRAQ4plex); K21(iTRAQ4plex)	0,0000	0,833	6	23,9	0	1,126E-08	54	4,35482E-06	3	2507,39957	1,42	50,18	0
	High	yLDGAAMDk	2	1	1	Q01952	N-Term(iTRAQ4plex); K9(iTRAQ4plex)	0,0000	1,234	2	18,7	0	0,000004441	52	5,80724E-06	2	1271,65386	-0,46	26,63	0
	High	dLDYYLR	5	1	1	Q01952	N-Term(iTRAQ4plex)	0,0000	0,959	5	21,2	0	0,00004572	50	9,63762E-06	2	1101,56938	-0,29	35,65	0
	High	yLDGAAMDkLk	3	1	1	Q01952	N-Term(iTRAQ4plex); K9(iTRAQ4plex); K11(iTRAQ4plex)	0,0000	2,252	3	0,2	0	1,197E-07	42	7,11524E-05	3	1656,93564	0,06	31,62	1
	High	lkSYFASGELR	2	1	1	Q01952	N-Term(iTRAQ4plex); K2(iTRAQ4plex)	0,0000	0,992	2	37,0	0	0,00008295	40	9,95337E-05	3	1558,88230	-0,57	30,96	1
	High	yLDGAAmDk	3	1	1	Q01952	N-Term(iTRAQ4plex); M7(Oxidation); K9(iTRAQ4plex)	0,0000	0,701	2	2,8	0	0,0003039	39	0,000134887	2	1287,64946	0,08	21,67	0
	High	yLDGAAmDkLk	5	1	1	Q01952	N-Term(iTRAQ4plex); M7(Oxidation); K9(iTRAQ4plex); K11(iTR	0,0000	1,243	5	43,4	0	0,0003936	37	0,000190095	3	1672,92954	-0,55	25,52	1
	High	emGVYLDYIcSGLS	1	1	1	Q01952	N-Term(iTRAQ4plex); M2(Oxidation); C10(Carbamidomethyl)	0,0000	0,855	1		0	0,000005643	30	0,00101151	2	1766,81145	0,94	46,55	0
	High	sLLYSDVTRPGGNmYTTR	6	1	1	Q01952	N-Term(iTRAQ4plex); M14(Oxidation)	0,0000	0,254	4	5,1	0	0,00001313	27	0,002084347	3	2191,09439	0,43	30,10	0
	High	sLLYSDVTRPGGNMYTTR	3	1	1	Q01952	N-Term(iTRAQ4plex)	0,0000	0,542	2	23,4	0	0,00004391	15	0,028838323	3	2175,10031	0,82	33,41	0
	High	eAVAk	7	1	1	Q01952	N-Term(iTRAQ4plex); K5(iTRAQ4plex)	0,0000	0,845	5	5,7	0,003	0,03514	24	0,013979091	2	805,50249	0,36	12,60	0
	Medium	vLNGLk	6	1	1	Q01952	N-Term(iTRAQ4plex); K6(iTRAQ4plex)	0,0000	1,014	4	1,5	0,01	0,1316	27	0,001940752	2	931,61894	1,12	23,71	0
	Medium	yAAcIR	4	1	1	Q01952	N-Term(iTRAQ4plex); C4(Carbamidomethyl)	0,0000	0,786	4	1,6	0,013	0,1733	21	0,007761935	2	897,47325	-0,06	17,56	0
	Medium	rYAAcIR	2	1	1	Q01952	N-Term(iTRAQ4plex); C5(Carbamidomethyl)	0,0000	0,990	2	19,3	0,014	0,1903	14	0,041397108	3	1053,57456	0,12	13,12	1
P73606	Slr1855 protein OS=Synechocystis sp. (strain PCC 6803 / Kazusa) GN=slr1855 PE=4 SV=1 - [P73606	2442,13	46,13	1	32	32	123	0,415	105	28,7	607	70,0	5,25							
	A3	Sequence	# PSMs	# Proteins	# Protein Groups	Protein Group Accessions	Modifications	ΔCn	116/117	116/117 Count	116/117 Variability [%]	q-Value	PEP	IonScore	Exp Value	Charge	MH+ [Da]	ΔM [ppm]	RT [min]	# Missed Cleavages
	High	vIQNFDEGYPDATGTMR	2	1	1	P73606	N-Term(iTRAQ4plex)	0,0000	0,991	2	132,3	0	2,323E-10	96	2,67898E-10	2	2057,97417	1,08	35,65	0
	High	iLHDAEQTIk	11	1	1	P73606	N-Term(iTRAQ4plex); K10(iTRAQ4plex)	0,0000	0,370	9	7,0	0	3,991E-08	83	4,89745E-09	2	1455,84245	1,01	26,53	0
	High	iADLmPAVGSDQQR	1	1	1	P73606	N-Term(iTRAQ4plex); M5(Oxidation)	0,0000	0,330	1		0	2,45E-11	79	1,31513E-08	2	1660,84429	-0,17	27,65	0
	High	vIQNFDEGYPDATGTmR	2	1	1	P73606	N-Term(iTRAQ4plex); M16(Oxidation)	0,0000	0,302	2	7,0	0	1,97E-10	67	1,87919E-07	3	2073,96878	0,92	32,58	0
	High	aQFGDGEIDYR	2	1	1	P73606	N-Term(iTRAQ4plex)	0,0000	0,355	2	7,7	0	0,00001053	67	2,13781E-07	2	1414,67314	0,83	28,21	0
	High	kPAEYVFEk	7	1	1	P73606	N-Term(iTRAQ4plex); K1(iTRAQ4plex); K9(iTRAQ4plex)	0,0000	0,391	7	18,1	0	3,735E-07	63	5,23564E-07	2	1542,89067	0,94	25,14	0
	High	tSDGQEFQVk	1	1	1	P73606	N-Term(iTRAQ4plex); K10(iTRAQ4plex)	0,0000	0,356	1		0	3,662E-07	61	7,81574E-07	2	1426,74187	0,14	19,83	0
	High	kIADLmPAVGSDQQR	7	1	1	P73606	N-Term(iTRAQ4plex); K1(iTRAQ4plex); M6(Oxidation)	0,0000	0,429	4	32,0	0	6,539E-08	57	1,87068E-06	3	1933,04166	0,03	23,41	1
	High	aVVGHNLk	7	1	1	P73606	N-Term(iTRAQ4plex); K8(iTRAQ4plex)	0,0000	0,407	4	21,8	0	0,00002715	57	1,91412E-06	2	1125,69853	0,23	14,19	0
	High	lSPMAYAk	4	1	1	P73606	N-Term(iTRAQ4plex); K8(iTRAQ4plex)	0,0000	0,568	4	5,7	0	0,0003076	52	6,57612E-06	2	1168,66521	1,15	27,60	0
	High	fVDkDEDIIYWYHAIDVQGEk	1	1	1	P73606	N-Term(iTRAQ4plex); K4(iTRAQ4plex); K21(iTRAQ4plex)	0,0000	0,336	1		0	1,562E-11	51	8,60994E-06	4	3015,54848	0,66	46,29	1
	High	iAWNLMR	2	1	1	P73606	N-Term(iTRAQ4plex)	0,0000	0,720	2	3,2	0	0,001599	50	9,26766E-06	2	1047,58977	0,70	37,61	0
	High	lSPmAYAk	6	1	1	P73606	N-Term(iTRAQ4plex); M4(Oxidation); K8(iTRAQ4plex)	0,0000	0,467	4	42,4	0	0,0003263	50	1,03985E-05	2	1184,66045	1,41	21,44	0
	High	gGWYDVVER	4	1	1	P73606	N-Term(iTRAQ4plex)	0,0000	0,556	4	10,7	0	0,000001702	48	1,56304E-05	2	1224,61333	0,27	33,20	0
	High	cHQFVWHDR	3	1	1	P73606	N-Term(iTRAQ4plex); C1(Carbamidomethyl)	0,0000	0,371	3	11,7	0	0,000008258	46	2,71625E-05	2	1428,67156	0,26	16,99	0
	High	tDLSMSGER	1	1	1	P73606	N-Term(iTRAQ4plex)	0,0000	0,691	1		0	0,002709	42	5,70125E-05	2	1139,55083	2,17	17,69	0
	High	qVVFAGk	6	1	1	P73606	N-Term(iTRAQ4plex); K7(iTRAQ4plex)	0,0000	0,384	3	1,6	0	0,0005173	40	0,000108635	2	1036,63896	-0,39	21,37	0
	High	rILHDAEQTIk	2	1	1	P73606	N-Term(iTRAQ4plex); K11(iTRAQ4plex)	0,0000	0,715	2	42,1	0	0,000003347	39	0,000138736	3	1611,94388	1,10	21,18	1
	High	iADLMPAVGSDQQR	1	1	1	P73606	N-Term(iTRAQ4plex)	0,0000	1,002	1		0	0,000005721	38	0,000151695	3	1644,85123	0,96	36,45	0
	High	lLDNHSR	4	1	1	P73606	N-Term(iTRAQ4plex)	0,0000	0,612	4	46,8	0	0,001197	36	0,000226971	2	998,55016	0,17	14,22	0
	High	qVNSLGk	4	1	1	P73606	N-Term(iTRAQ4plex); K7(iTRAQ4plex)	0,0000	0,469	3	15,4	0	0,0007885	35	0,000365926	2	1033,62358	-0,83	15,30	0
	High	sESLGPNk	3	1	1	P73606	N-Term(iTRAQ4plex); K8(iTRAQ4plex)	0,0000	0,453	2	23,0	0	0,0001613	35	0,000421609	2	1119,62627	1,28	14,87	0
	High	sEYGGYFSHLDPLmLDPR	2	1	1	P73606	N-Term(iTRAQ4plex); M14(Oxidation)	0,0000	0,422	1		0	0,000001312	31	0,000887095	3	2257,07260	0,42	45,10	0
	High	tWLTPGR	4	1	1	P73606	N-Term(iTRAQ4plex)	0,0000	0,398	3	3,3	0	0,001025	30	0,00097949	2	974,55504	1,05	25,21	0
	High	kIADLMPAVGSDQQR	1	1	1	P73606	N-Term(iTRAQ4plex); K1(iTRAQ4plex)	0,0000	0,623	1		0	0,00001848	30	0,001083852	3	1917,04923	1,33	31,70	1
	High	ekYVELAk	3	1	1	P73606	N-Term(iTRAQ4plex); K2(iTRAQ4plex); K8(iTRAQ4plex)	0,0000	0,509	3	33,3	0	0,000006345	26	0,002376676	3	1411,85447	1,68	21,93	1
	High	yVELAk	4	1	1	P73606	N-Term(iTRAQ4plex); K6(iTRAQ4plex)	0,0000	0,375	3	9,3	0	0,00249	26	0,00239315	2	1010,61266	0,19	25,03	0
	High	qETDTISR	3	1	1	P73606	N-Term(iTRAQ4plex)	0,0000	0,743	3	2,4	0	0,003058	25	0,003220846	2	1093,56169	0,99	13,80	0
	High	yFPDYDHSPFVQER	3	1	1	P73606	N-Term(iTRAQ4plex)	0,0000	0,393	3	12,2	0	0,000009677	23	0,00478597	3	1943,90586	0,71	36,58	0
	High	lFDkFFLDk	2	1	1	P73606	N-Term(iTRAQ4plex); K4(iTRAQ4plex); K9(iTRAQ4plex)	0,0000	0,421	2	2,3	0	0,0002927	22	0,006341461	3	1604,94211	0,52	43,70	1
	High	hPmDFYFkPLPNGFPDGILR	2	1	1	P73606	N-Term(iTRAQ4plex); M3(Oxidation); K8(iTRAQ4plex)	0,0000	0,354	2	4,6	0	0,000008984	14	0,040548053	4	2665,38491	0,33	45,76	0
	High	mNSLk	1	1	1	P73606	N-Term(iTRAQ4plex); K5(iTRAQ4plex)	0,0000	0,612	1		0,002	0,02377	31	0,001071297	2	880,51506	-1,60	15,82	0
	High	fFLDk	4	1	1	P73606	N-Term(iTRAQ4plex); K5(iTRAQ4plex)	0,0000	0,333	4	0,7	0,003	0,03965	25	0,003265653	2	957,56493	0,14	32,10	0
	High	qDWWIk	3	1	1	P73606	N-Term(iTRAQ4plex); K6(iTRAQ4plex)	0,0000	0,416	3	10,0	0,008	0,1132	28	0,001530982	2	1163,64665	1,27	33,73	0
	High	gTEYLR	4	1	1	P73606	N-Term(iTRAQ4plex)	0,0000	0,539	4	9,7	0,008	0,1082	23	0,004476824	2	882,48082	0,74	16,58	0
	High	iAWNLmR	4	1	1	P73606	N-Term(iTRAQ4plex); M6(Oxidation)	0,0000	0,355	4	5,8	0,008	0,102	21	0,008649082	2	1063,58513	1,11	31,47	0
	Medium	sTVNFR	1	1	1	P73606	N-Term(iTRAQ4plex)	0,0000	0,415	1		0,024	0,3281	13	0,054071698	2	867,48076	0,30	15,81	0
	Medium	tDLSmSGER	1	1	1	P73606	N-Term(iTRAQ4plex); M5(Oxidation)	0,0000	0,415	1		0,044	0,564	29	0,001321204	2	1155,53728	-5,18	12,19	0
Q05972	60 kDa chaperonin 1 OS=Synechocystis sp. (strain PCC 6803 / Kazusa) GN=groL1 PE=1 SV=4 - [CH6	2331,30	49,35	1	29	30	99	0,948	75	11,9	541	57,6	5,10							
	A3	Sequence	# PSMs	# Proteins	# Protein Groups	Protein Group Accessions	Modifications	ΔCn	116/117	116/117 Count	116/117 Variability [%]	q-Value	PEP	IonScore	Exp Value	Charge	MH+ [Da]	ΔM [ppm]	RT [min]	# Missed Cleavages
	High	fGSPQIINDGITIAk	4	1	1	Q05972	N-Term(iTRAQ4plex); K15(iTRAQ4plex)	0,0000	0,862	4	21,0	0	1,92E-14	95	3,17665E-10	2	1862,06462	1,07	40,66	0
	High	vGAATETEmkDR	5	1	1	Q05972	N-Term(iTRAQ4plex); M9(Oxidation); K10(iTRAQ4plex)	0,0000	0,853	4	1,5	0	2,629E-08	95	3,32637E-10	2	1611,82829	1,97	13,04	1
	High	nVAAGANPISLk	10	1	1	Q05972	N-Term(iTRAQ4plex); K12(iTRAQ4plex)	0,0000	0,924	6	4,7	0	8,646E-07	77	2,06524E-08	2	1442,85747	0,33	29,08	0
	High	gVLNVAAVk	8	1	1	Q05972	N-Term(iTRAQ4plex); K9(iTRAQ4plex)	0,0000	1,006	5	4,8	0	4,429E-07	77	2,1576E-08	2	1158,74480	-0,09	28,22	0
	High	vGQEGVISLEEGk	2	1	1	Q05972	N-Term(iTRAQ4plex); K13(iTRAQ4plex)	0,0000	0,883	2	4,6	0	5,083E-08	76	2,23857E-08	2	1632,90801	2,00	30,26	0
	High	ikEHAQPVGDSk	7	1	1	Q05972	N-Term(iTRAQ4plex); K2(iTRAQ4plex); K12(iTRAQ4plex)	0,0000	1,053	5	3,7	0	9,044E-10	70	1,01151E-07	2	1740,99993	1,74	12,50	1
	High	aPAGAPGGDFDY	1	1	1	Q05972	N-Term(iTRAQ4plex)	0,0000	0,616	1		0	6,384E-07	65	2,83772E-07	2	1281,58769	0,66	31,76	0
	High	eIELEDHVENTGVSLIR	1	1	1	Q05972	N-Term(iTRAQ4plex)	0,0000	0,883	1		0	4,73E-11	60	9,24634E-07	3	2097,09684	1,11	38,42	0
	High	tNDVAGDGTTTATVLAHAIVk	2	1	1	Q05972	N-Term(iTRAQ4plex); K21(iTRAQ4plex)	0,0000	1,137	1		0	1,293E-10	59	1,15337E-06	3	2343,27884	1,26	38,32	0
	High	nVAAGANPISLkR	4	1	1	Q05972	N-Term(iTRAQ4plex); K12(iTRAQ4plex)	0,0000	1,279	2	5,8	0	3,015E-08	58	1,7297E-06	3	1598,95841	0,19	23,57	1
	High	lAGGVAVIk	7	2	2	Q05972;P22034	N-Term(iTRAQ4plex); K9(iTRAQ4plex)	0,0000				0	0,00008185	55	3,52347E-06	2	1115,73845	-0,58	27,97	0
	High	iLITDkk	4	1	1	Q05972	N-Term(iTRAQ4plex); K6(iTRAQ4plex); K7(iTRAQ4plex)	0,0000	1,011	4	4,6	0	0,0001115	53	5,34527E-06	2	1262,84233	1,21	20,67	1
	High	qGkPLLIIAEDIEk	7	1	1	Q05972	N-Term(iTRAQ4plex); K3(iTRAQ4plex); K14(iTRAQ4plex)	0,0000	0,947	7	7,6	0	1,403E-08	50	1,03746E-05	3	1999,21762	0,61	43,51	0
	High	aLPAPLkR	5	1	1	Q05972	N-Term(iTRAQ4plex); K7(iTRAQ4plex)	0,0000	0,942	4	5,6	0	0,0004253	48	1,53805E-05	3	1153,76584	-0,12	20,56	1
	High	aTDFLVAR	3	1	1	Q05972	N-Term(iTRAQ4plex)	0,0000	0,967	3	1,4	0	0,00006493	48	1,54159E-05	2	1036,59062	-0,17	30,49	0
	High	lESATVDSLGSAR	1	1	1	Q05972	N-Term(iTRAQ4plex)	0,0000	0,571	1		0	0,00001167	48	1,70204E-05	2	1449,76702	0,28	28,05	0
	High	eALATLVVNR	2	1	1	Q05972	N-Term(iTRAQ4plex)	0,0000	0,866	2	10,4	0	0,00002546	47	1,80289E-05	2	1229,73454	0,89	32,34	0
	High	vGAATETEMkDR	2	1	1	Q05972	N-Term(iTRAQ4plex); K10(iTRAQ4plex)	0,0000	1,634	2	4,5	0	9,131E-07	47	2,05575E-05	3	1595,82950	-0,44	17,08	1
	High	gYISPYFVTDAER	1	1	1	Q05972	N-Term(iTRAQ4plex)	0,0000	0,930	1		0	2,84E-08	44	3,54789E-05	2	1661,83025	0,63	38,12	0
	High	aLPAPLk	1	1	1	Q05972	N-Term(iTRAQ4plex); K7(iTRAQ4plex)	0,0000	0,538	1		0	0,001362	40	0,000108885	2	997,66356	-1,30	25,63	0
	High	sIIYNDEAR	1	1	1	Q05972	N-Term(iTRAQ4plex)	0,0000	0,813	1		0	0,0003319	33	0,000561009	2	1224,63542	1,07	20,85	0
	High	qIEETDSSYDkEk	1	1	1	Q05972	N-Term(iTRAQ4plex); K11(iTRAQ4plex); K13(iTRAQ4plex)	0,0000	0,972	1		0	0,000007909	30	0,001023222	3	2004,02652	6,56	17,00	1
	High	rQIEETDSSYDkEk	1	1	1	Q05972	N-Term(iTRAQ4plex); K12(iTRAQ4plex); K14(iTRAQ4plex)	0,0000	1,042	1		0	2,879E-07	27	0,001883649	4	2160,11464	0,07	15,27	2
	High	vGAATETEmkDRk	3	1	1	Q05972	N-Term(iTRAQ4plex); M9(Oxidation); K10(iTRAQ4plex); K13(iT	0,0000	1,225	2	1,3	0	0,002598	27	0,002027543	3	1884,02176	-0,20	11,72	2
	High	gmDILAEAVAVTLGPk	2	1	1	Q05972	N-Term(iTRAQ4plex); M2(Oxidation); K16(iTRAQ4plex)	0,0000	0,955	2	39,3	0	0,000032	25	0,003299079	3	1889,06632	0,33	51,51	0
	High	eHAQPVGDSk	5	1	1	Q05972	N-Term(iTRAQ4plex); K10(iTRAQ4plex)	0,0000	0,890	5	9,4	0	0,0001966	24	0,003766778	3	1355,71570	-0,06	8,57	0
	High	mEAVLEDPR	2	1	1	Q05972	N-Term(iTRAQ4plex); M1(Oxidation)	0,0000	0,686	1		0	0,002652	20	0,010374568	2	1219,61174	0,63	19,90	0
	High	nVVLEk	4	1	1	Q05972	N-Term(iTRAQ4plex); K6(iTRAQ4plex)	0,0000	0,961	3	3,3	0,001	0,004389	42	6,32368E-05	2	989,62285	-0,53	19,47	0
	High	rINITk	1	1	1	Q05972	N-Term(iTRAQ4plex); K6(iTRAQ4plex)	0,0000	1,039	1		0,007	0,09584	17	0,024679353	3	1032,67792	1,08	15,84	1
	Medium	iLITDk	1	1	1	Q05972	N-Term(iTRAQ4plex); K6(iTRAQ4plex)	0,0000	1,059	1		0,013	0,1713	29	0,001321204	2	990,64250	-1,29	25,41	0
	Medium	iNITk	1	1	1	Q05972	N-Term(iTRAQ4plex); K5(iTRAQ4plex)	0,0000	0,910	1		0,014	0,186	27	0,002979142	2	876,57421	-1,69	22,10	0
P80505	Glyceraldehyde-3-phosphate dehydrogenase 2 OS=Synechocystis sp. (strain PCC 6803 / Kazusa) GN=	2328,93	64,99	1	21	21	86	2,004	71	57,6	337	36,5	6,44							
	A3	Sequence	# PSMs	# Proteins	# Protein Groups	Protein Group Accessions	Modifications	ΔCn	116/117	116/117 Count	116/117 Variability [%]	q-Value	PEP	IonScore	Exp Value	Charge	MH+ [Da]	ΔM [ppm]	RT [min]	# Missed Cleavages
	High	tDSQLEVVGINDTSDPR	2	1	1	P80505	N-Term(iTRAQ4plex)	0,0000	1,882	2	24,8	0	7,502E-10	105	3,42744E-11	2	1989,98686	1,13	31,44	0
	High	aAAVNIVPTSTGAAk	11	1	1	P80505	N-Term(iTRAQ4plex); K15(iTRAQ4plex)	0,0000	2,756	6	4,7	0	1,078E-09	101	7,29407E-11	2	1658,96977	1,07	26,61	0
	High	gPNIGTYVVGVNAHEYk	4	1	1	P80505	N-Term(iTRAQ4plex); K17(iTRAQ4plex)	0,0000	1,478	3	71,2	0	1,403E-16	86	2,31739E-09	3	2106,12339	0,54	33,19	0
	High	vVDLAEIVAk	5	1	1	P80505	N-Term(iTRAQ4plex); K10(iTRAQ4plex)	0,0000	1,377	4	154,0	0	2,064E-08	80	9,01509E-09	2	1344,83586	1,29	42,09	0
	High	gTMTTTHSYTGDQR	6	1	1	P80505	N-Term(iTRAQ4plex)	0,0000	2,869	6	53,5	0	8,798E-08	74	3,95339E-08	2	1699,79021	4,44	13,55	0
	High	vINDNFGIIk	4	1	1	P80505	N-Term(iTRAQ4plex); K10(iTRAQ4plex)	0,0000	2,766	4	16,9	0	0,00001029	66	2,25928E-07	2	1420,84221	1,37	36,78	0
	High	gVLEYTDLELVSSDFR	2	1	1	P80505	N-Term(iTRAQ4plex)	0,0000	1,393	2	23,6	0	7,347E-10	62	5,94251E-07	2	1987,01775	1,82	51,33	0
	High	nTIAEQVNGVLk	2	1	1	P80505	N-Term(iTRAQ4plex); K12(iTRAQ4plex)	0,0000	0,596	2	13,4	0	4,771E-07	61	7,83376E-07	3	1573,91709	1,18	33,89	0
	High	vPTPNVSVVDLVVQVEk	2	1	1	P80505	N-Term(iTRAQ4plex); K17(iTRAQ4plex)	0,0000	1,993	2	14,3	0	1,204E-08	60	1,11679E-06	3	2110,23832	0,98	50,50	0
	High	aVALVIPELQGk	6	1	1	P80505	N-Term(iTRAQ4plex); K12(iTRAQ4plex)	0,0000	2,697	4	31,0	0	0,000004956	58	1,49958E-06	2	1525,95745	1,18	42,29	0
	High	vIAWYDNEWGYSQR	1	1	1	P80505	N-Term(iTRAQ4plex)	0,0000	0,871	1		0	5,075E-08	56	2,66667E-06	3	1930,92258	1,09	43,74	0
	High	vLITAPGk	8	1	1	P80505	N-Term(iTRAQ4plex); K8(iTRAQ4plex)	0,0000	2,372	5	12,4	0	0,00006422	56	2,67282E-06	2	1086,71233	-0,19	26,02	0
	High	gTmTTTHSYTGDQR	8	1	1	P80505	N-Term(iTRAQ4plex); M3(Oxidation)	0,0000	1,703	8	28,0	0	0,0002251	53	4,63415E-06	2	1715,78240	2,81	11,31	0
	High	eAANTSLk	4	1	1	P80505	N-Term(iTRAQ4plex); K8(iTRAQ4plex)	0,0000	1,243	3	17,3	0	0,00001546	51	8,25981E-06	2	1121,64153	0,93	14,66	0
	High	kVLITAPGk	5	1	1	P80505	N-Term(iTRAQ4plex); K1(iTRAQ4plex); K9(iTRAQ4plex)	0,0000	2,824	4	13,4	0	0,00002073	48	1,60313E-05	2	1358,90947	-0,07	19,95	1
	High	hVQAGAk	1	1	1	P80505	N-Term(iTRAQ4plex); K7(iTRAQ4plex)	0,0000	2,713	1		0	0,00003272	43	5,29627E-05	3	998,59751	-1,05	7,60	0
	High	tNAHLLR	6	1	1	P80505	N-Term(iTRAQ4plex)	0,0000	1,683	5	11,5	0	0,0007683	40	0,000111936	2	968,57518	-0,64	13,64	0
	High	vAINGFGR	2	1	1	P80505	N-Term(iTRAQ4plex)	0,0000	3,044	2	0,6	0	0,0001848	38	0,000212903	2	977,56548	0,57	26,97	0
	High	iLDASHR	4	1	1	P80505	N-Term(iTRAQ4plex)	0,0000	1,039	4	23,0	0	0,001019	22	0,006052991	3	955,54465	0,50	14,75	0
	High	yDSmLGk	1	1	1	P80505	N-Term(iTRAQ4plex); M4(Oxidation); K7(iTRAQ4plex)	0,0000	2,131	1		0	0,0008773	21	0,008850545	2	1117,58061	0,38	19,03	0
	High	lNGIALR	1	1	1	P80505	N-Term(iTRAQ4plex)	0,0000	1,924	1		0,002	0,02029	41	7,58525E-05	2	900,57585	1,22	27,28	0
	Medium	cWLGR	1	1	1	P80505	N-Term(iTRAQ4plex); C1(Carbamidomethyl)	0,0000	1,354	1		0,02	0,2669	14	0,036725693	2	835,43688	0,40	22,00	0
P26527	ATP synthase subunit beta OS=Synechocystis sp. (strain PCC 6803 / Kazusa) GN=atpD PE=3 SV=1 -	2292,34	51,35	1	18	18	74	1,502	64	20,7	483	51,7	5,02							
	A3	Sequence	# PSMs	# Proteins	# Protein Groups	Protein Group Accessions	Modifications	ΔCn	116/117	116/117 Count	116/117 Variability [%]	q-Value	PEP	IonScore	Exp Value	Charge	MH+ [Da]	ΔM [ppm]	RT [min]	# Missed Cleavages
	High	fVQAGSEVSALLGR	6	1	1	P26527	N-Term(iTRAQ4plex)	0,0000	1,515	5	16,0	0	2,131E-12	91	8,81049E-10	2	1577,87724	0,25	44,81	0
	High	vGLTALTMAEYFR	8	1	1	P26527	N-Term(iTRAQ4plex)	0,0000	2,174	8	13,0	0	1,177E-09	83	5,74367E-09	2	1615,86565	1,33	50,88	0
	High	mPSAVGYQPTLGTDVGDLQER	1	1	1	P26527	N-Term(iTRAQ4plex); M1(Oxidation)	0,0000	3,368	1		0	6,43E-11	79	1,36449E-08	3	2394,17447	0,67	35,94	0
	High	vGLTALTmAEYFR	7	1	1	P26527	N-Term(iTRAQ4plex); M8(Oxidation)	0,0000	1,370	7	60,9	0	8,607E-08	75	2,97146E-08	2	1631,85918	0,47	43,94	0
	High	gmDVVDTGAPISVPVGTGTLGR	2	1	1	P26527	N-Term(iTRAQ4plex); M2(Oxidation)	0,0000	1,137	1		0	2,098E-11	72	5,94251E-08	3	2259,17868	0,64	38,90	0
	High	yVSLADTIk	3	1	1	P26527	N-Term(iTRAQ4plex); K9(iTRAQ4plex)	0,0000	1,280	3	20,0	0	6,908E-07	68	1,42551E-07	2	1297,76103	0,34	33,76	0
	High	iALVYGQmNEPPGAR	3	1	1	P26527	N-Term(iTRAQ4plex); M8(Oxidation)	0,0000	1,371	3	8,6	0	4,583E-10	67	1,94075E-07	3	1775,92417	0,57	31,02	0
	High	aVAMSSTDGLVR	2	1	1	P26527	N-Term(iTRAQ4plex)	0,0000	1,982	1		0	0,0001523	64	3,77546E-07	2	1350,71782	0,73	26,53	0
	High	aVAmSSTDGLVR	4	1	1	P26527	N-Term(iTRAQ4plex); M4(Oxidation)	0,0000	1,343	2	0,4	0	1,197E-07	62	5,62302E-07	2	1366,71282	0,78	20,32	0
	High	iGLFGGAGVGk	8	1	1	P26527	N-Term(iTRAQ4plex); K11(iTRAQ4plex)	0,0000	1,501	7	13,0	0	1,662E-08	60	9,99931E-07	2	1263,76579	-0,47	34,99	0
	High	iTQVIGPVIDAQFPSGk	3	1	1	P26527	N-Term(iTRAQ4plex); K17(iTRAQ4plex)	0,0000	1,620	2	2,8	0	2,011E-08	60	1,05189E-06	3	2058,18546	0,79	45,81	0
	High	vIDLLTPYR	6	1	1	P26527	N-Term(iTRAQ4plex)	0,0000	1,438	6	6,8	0	0,000002944	54	3,90841E-06	2	1233,73308	0,57	42,75	0
	High	lVDLETkPQVFETGIk	4	1	1	P26527	N-Term(iTRAQ4plex); K7(iTRAQ4plex); K16(iTRAQ4plex)	0,0000	1,545	3	0,4	0	1,559E-10	46	2,71E-05	3	2249,31498	1,42	40,58	0
	High	iYNALk	4	1	1	P26527	N-Term(iTRAQ4plex); K6(iTRAQ4plex)	0,0000	1,708	4	4,6	0	0,002237	45	3,20605E-05	2	1009,62804	-0,41	24,28	0
	High	gPVPAGETFPIHRPAPk	4	1	1	P26527	N-Term(iTRAQ4plex); K17(iTRAQ4plex)	0,0000	1,387	2	14,9	0	9,419E-09	42	6,08093E-05	4	2059,16982	0,31	25,53	0
	High	nSAGNEVAVTcEVQQLLGDNQVR	1	1	1	P26527	N-Term(iTRAQ4plex); C11(Carbamidomethyl)	0,0000	2,161	1		0	5,568E-07	41	7,42968E-05	3	2645,31443	2,78	48,04	0
	High	eVQSTLQR	2	1	1	P26527	N-Term(iTRAQ4plex)	0,0000	1,043	2	14,1	0	0,0002243	40	0,000105189	2	1104,61284	-0,13	15,95	0
	High	iALVYGQMNEPPGAR	1	1	1	P26527	N-Term(iTRAQ4plex)	0,0000	1,504	1		0	0,000002547	35	0,000315479	3	1759,92899	0,43	36,49	0
	High	fLSQPFFVAEVFTGAPGk	1	1	1	P26527	N-Term(iTRAQ4plex); K18(iTRAQ4plex)	0,0000	3,618	1		0	0,001278	32	0,000741157	3	2230,21708	0,87	56,97	0

	High	iFNVLGEPVDNkGPVPAGETFPIHI	1	1	1	P26527	N-Term(iTRAQ4plex); K12(iTRAQ4plex); K29(iTRAQ4plex)	0,0000	1,514	1		0	0,00001333	15	0,031620593	6	3528,97534	1,72	39,89	1
	High	iTSTk	3	1	1	P26527	N-Term(iTRAQ4plex); K5(iTRAQ4plex)	0,0000	1,023	3	0,6	0,008	0,1039	20	0,009161572	2	837,52770	-0,84	12,73	0
P22034	60 kDa chaperonin 2 OS=Synechocystis sp. (strain PCC 6803 / Kazusa) GN=groL2 PE=1 SV=4 - [CH	2280,47	60,14	1	34	35	90	0,768	70	21,2	552	57,7	4,97							
	A3	Sequence	# PSMs	# Proteins	# Protein Groups	Protein Group Accessions	Modifications	ΔCn	116/117	116/117 Count	116/117 Variability [%]	q-Value	PEP	IonScore	Exp Value	Charge	MH+ [Da]	ΔM [ppm]	RT [min]	# Missed Cleavages
	High	sLEAGINALADAVR	2	1	1	P22034	N-Term(iTRAQ4plex)	0,0000	0,622	2	5,6	0	6,613E-12	95	3,04768E-10	2	1543,85674	0,40	48,00	0
	High	tkEIAGDGTTTATIIAQALVR	2	1	1	P22034	N-Term(iTRAQ4plex); K2(iTRAQ4plex)	0,0000	0,742	2	43,0	0	5,452E-12	88	1,7099E-09	3	2418,38285	0,88	44,23	1
	High	rSLEAGINALADAVR	3	1	1	P22034	N-Term(iTRAQ4plex)	0,0000	0,845	2	7,1	0	1,315E-08	79	1,97284E-08	3	1699,95774	0,30	40,45	1
	High	gVLNVAAIk	9	1	1	P22034	N-Term(iTRAQ4plex); K9(iTRAQ4plex)	0,0000	0,751	6	10,2	0	0,000001593	73	4,54957E-08	2	1172,76067	0,09	32,41	0
	High	qLVEFDNPLILITDkk	7	1	1	P22034	N-Term(iTRAQ4plex); K15(iTRAQ4plex); K16(iTRAQ4plex)	0,0000	0,714	5	5,7	0	2,937E-10	69	1,27342E-07	4	2318,37270	1,33	49,75	1
	High	aAVEEGIVPGGGTTLIR	3	1	1	P22034	N-Term(iTRAQ4plex)	0,0000	0,969	3	64,4	0	1,122E-07	64	3,79289E-07	2	1784,00603	1,40	36,27	0
	High	lRIEDALNATk	6	1	1	P22034	N-Term(iTRAQ4plex); K11(iTRAQ4plex)	0,0000	1,198	5	19,6	0	0,000001073	64	4,37492E-07	2	1531,90666	1,30	30,51	1
	High	qYGAPQIVNDGITVAk	2	1	1	P22034	N-Term(iTRAQ4plex); K16(iTRAQ4plex)	0,0000	0,684	2	3,9	0	7,515E-10	62	5,7544E-07	3	1962,09024	0,17	35,06	0
	High	nVAAGANPVALR	6	1	1	P22034	N-Term(iTRAQ4plex)	0,0000	0,727	3	7,5	0	6,911E-07	59	1,28816E-06	2	1296,75249	1,52	24,30	0
	High	iEDALNATk	1	1	1	P22034	N-Term(iTRAQ4plex); K9(iTRAQ4plex)	0,0000	0,945	1		0	8,794E-07	58	2,00421E-06	2	1262,71941	-0,05	26,48	0
	High	vkEATGNQGYNVITGk	3	1	1	P22034	N-Term(iTRAQ4plex); K2(iTRAQ4plex); K16(iTRAQ4plex)	0,0000	0,861	2	7,9	0	9,229E-12	56	2,67898E-06	3	2111,18369	0,74	23,66	1
	High	sLNTELEVVEGmQIDR	1	1	1	P22034	N-Term(iTRAQ4plex); M12(Oxidation)	0,0000	1,200	1		0	0,000000125	55	2,99206E-06	3	1993,00193	-0,50	39,20	0
	High	lAGGVAVIk	7	2	2	Q05972;P22034	N-Term(iTRAQ4plex); K9(iTRAQ4plex)	0,0000				0	0,00008185	55	3,52347E-06	2	1115,73845	-0,58	27,97	0
	High	iEDLIAAGIIDPAk	2	1	1	P22034	N-Term(iTRAQ4plex); K14(iTRAQ4plex)	0,0000	0,689	2	20,6	0	0,000000306	53	5,3576E-06	3	1727,02054	0,67	48,90	0
	High	lIQEVASk	2	1	1	P22034	N-Term(iTRAQ4plex); K8(iTRAQ4plex)	0,0000	0,760	2	9,5	0	0,001065	53	5,54587E-06	2	1175,72185	-1,68	27,45	0
	High	gYISPYFITDSDR	2	1	1	P22034	N-Term(iTRAQ4plex)	0,0000	0,784	2	3,4	0	4,286E-07	52	6,79157E-06	2	1677,82585	1,04	39,61	0
	High	dNTILVAGADkR	3	1	1	P22034	N-Term(iTRAQ4plex); K11(iTRAQ4plex)	0,0000	0,732	2	1,0	0	1,01E-09	50	9,12011E-06	3	1560,89499	0,10	20,73	1
	High	vAADIIAk	2	1	1	P22034	N-Term(iTRAQ4plex); K8(iTRAQ4plex)	0,0000	0,747	1		0	0,000006517	50	1,1828E-05	2	1088,69170	-0,10	26,79	0
	High	nVAAGANPVALRR	1	1	1	P22034	N-Term(iTRAQ4plex)	0,0000	1,077	1		0	0,000007002	50	9,63762E-06	3	1452,85288	0,86	20,13	1
	High	iSAIAELVPVLEAVAR	2	1	1	P22034	N-Term(iTRAQ4plex)	0,0000	0,810	2	58,8	0	0,000004945	48	1,62544E-05	2	1795,08599	2,75	59,92	0
	High	aQLSNDEER	1	1	1	P22034	N-Term(iTRAQ4plex)	0,0000	0,705	1		0	0,00001049	45	2,87058E-05	2	1205,58891	0,84	12,17	0
	High	vGAATETELkDR	2	1	1	P22034	N-Term(iTRAQ4plex); K10(iTRAQ4plex)	0,0000	0,810	2	14,7	0	2,203E-08	39	0,000130008	3	1577,87436	0,38	19,52	1
	High	kEYAASDSDYDkEk	1	1	1	P22034	N-Term(iTRAQ4plex); K1(iTRAQ4plex); K12(iTRAQ4plex); K14(	0,0000	0,764	1		0	4,524E-09	36	0,000241529	4	2225,14345	0,66	15,17	2
	High	eYAASDSDYDkEk	1	1	1	P22034	N-Term(iTRAQ4plex); K11(iTRAQ4plex); K13(iTRAQ4plex)	0,0000	0,683	1		0	1,035E-07	34	0,000435482	3	1952,94468	-0,15	16,98	1
	High	aTLEkDNTILVAGADkR	2	1	1	P22034	N-Term(iTRAQ4plex); K5(iTRAQ4plex); K16(iTRAQ4plex)	0,0000	1,002	2	18,7	0	2,766E-07	32	0,000680722	4	2247,30654	1,42	25,67	2
	High	eATGNQGYNVITGk	1	1	1	P22034	N-Term(iTRAQ4plex); K14(iTRAQ4plex)	0,0000	0,629	1		0	0,000004748	31	0,000799779	3	1739,91794	0,73	24,10	0
	High	aPAFGDRR	1	1	1	P22034	N-Term(iTRAQ4plex)	0,0000	1,232	1		0	0,001419	31	0,000866902	3	1033,56626	0,25	13,39	1
	High	vGAATETELkDRk	1	1	1	P22034	N-Term(iTRAQ4plex); K10(iTRAQ4plex); K13(iTRAQ4plex)	0,0000	1,267	1		0	7,613E-08	24	0,003801631	4	1850,07338	1,40	19,97	2
	High	aLEAPLHQLASNAGVEGSVIVEk	1	1	1	P22034	N-Term(iTRAQ4plex); K23(iTRAQ4plex)	0,0000	0,228	1		0	2,714E-07	15	0,034038468	4	2620,45424	-0,27	42,69	0
	High	lAGkIESFk	1	1	1	P22034	N-Term(iTRAQ4plex); K4(iTRAQ4plex); K9(iTRAQ4plex)	0,0000	1,364	1		0	0,002805	15	0,035397289	3	1424,88343	-0,23	30,36	1
	High	nVLLEk	2	1	1	P22034	N-Term(iTRAQ4plex); K6(iTRAQ4plex)	0,0000	0,714	2	5,6	0,001	0,009191	33	0,000502308	2	1003,63890	-0,12	23,74	0
	High	lISFkDESR	3	1	1	P22034	N-Term(iTRAQ4plex); K5(iTRAQ4plex)	0,0000	0,888	3	10,9	0,001	0,004648	18	0,015522798	3	1382,78839	0,12	25,88	1
	High	iTLGPk	4	1	1	P22034	N-Term(iTRAQ4plex); K6(iTRAQ4plex)	0,0000	0,699	4	5,0	0,003	0,03149	27	0,001985958	2	916,60570	-1,42	22,78	0
	High	iESFk	2	1	1	P22034	N-Term(iTRAQ4plex); K5(iTRAQ4plex)	0,0000	0,927	2	10,9	0,007	0,09847	15	0,034116935	2	911,54302	-1,14	21,85	0
	High	lISFk	1	1	1	P22034	N-Term(iTRAQ4plex); K5(iTRAQ4plex)	0,0000	0,682	1		0,008	0,1064	10	0,095273036	2	895,58348	-2,28	29,92	0
P74390	Negative aliphatic amidase regulator OS=Synechocystis sp. (strain PCC 6803 / Kazusa) GN=amiC PE=	2266,96	40,81	1	16	16	92	1,286	70	17,8	446	48,3	4,89							
	A3	Sequence	# PSMs	# Proteins	# Protein Groups	Protein Group Accessions	Modifications	ΔCn	116/117	116/117 Count	116/117 Variability [%]	q-Value	PEP	IonScore	Exp Value	Charge	MH+ [Da]	ΔM [ppm]	RT [min]	# Missed Cleavages
	High	gGETLGEDYLPLGNTEVTPIITk	1	1	1	P74390	N-Term(iTRAQ4plex); K23(iTRAQ4plex)	0,0000	1,138	1		0	1,467E-13	77	2,39852E-08	3	2705,45359	1,75	46,83	0
	High	aAVEAAGDVGETPEGLEk	3	1	1	P74390	N-Term(iTRAQ4plex); K18(iTRAQ4plex)	0,0000	1,210	2	27,8	0	3,531E-10	73	4,64483E-08	3	2031,05088	1,15	29,22	0
	High	vPVVFGcWTSASR	6	1	1	P74390	N-Term(iTRAQ4plex); C7(Carbamidomethyl)	0,0000	1,081	6	21,5	0	2,365E-07	72	6,7293E-08	2	1609,82878	0,62	43,07	0
	High	nHmLWYPVQYEGQEcSk	5	1	1	P74390	N-Term(iTRAQ4plex); M3(Oxidation); C15(Carbamidomethyl);	0,0000	1,307	4	28,0	0	1,865E-11	69	1,39949E-07	3	2473,15665	1,91	31,87	0
	High	nHMLWYPVQYEGQEcSk	6	1	1	P74390	N-Term(iTRAQ4plex); C15(Carbamidomethyl); K17(iTRAQ4plex	0,0000	2,147	4	17,3	0	7,581E-13	64	4,06415E-07	3	2457,16111	1,67	35,36	0
	High	tFDAPEGmVTmQPNHHISk	6	1	1	P74390	N-Term(iTRAQ4plex); M8(Oxidation); M11(Oxidation); K19(iTR	0,0000	1,007	4	17,9	0	6,341E-11	63	5,14008E-07	4	2460,19448	2,20	21,77	0
	High	fFLVGSDYVFPR	15	1	1	P74390	N-Term(iTRAQ4plex)	0,0000	1,370	12	11,4	0	0,000003588	62	6,38219E-07	2	1590,84587	1,34	50,30	0
	High	qIQAAGLTPDkYPVMSVSVAEEEV	1	1	1	P74390	N-Term(iTRAQ4plex); K11(iTRAQ4plex)	0,0000	1,335	1		0	2,643E-11	61	7,79776E-07	4	3005,58852	0,93	41,83	1
	High	qIQAAGLTPDkYPVmSVSVAEEEV	1	1	1	P74390	N-Term(iTRAQ4plex); K11(iTRAQ4plex); M15(Oxidation)	0,0000	1,594	1		0	7,665E-10	56	3,06689E-06	4	3021,58559	1,64	38,01	1
	High	lIDQDkVPVVFGcWTSASR	5	1	1	P74390	N-Term(iTRAQ4plex); K6(iTRAQ4plex); C13(Carbamidomethyl)	0,0000	1,474	5	6,2	0	9,301E-10	54	3,82798E-06	3	2466,30887	1,40	45,54	1
	High	tFDAPEGMVTMQPNHHISk	3	1	1	P74390	N-Term(iTRAQ4plex); K19(iTRAQ4plex)	0,0000	1,073	2	166,7	0	1,71E-09	53	4,87495E-06	4	2428,20498	2,36	29,74	0
	High	kAVLPVFEAk	8	1	1	P74390	N-Term(iTRAQ4plex); K1(iTRAQ4plex); K10(iTRAQ4plex)	0,0000	1,323	6	12,2	0	6,752E-07	53	5,3576E-06	3	1533,97712	2,74	30,26	1
	High	fVAAFk	8	1	1	P74390	N-Term(iTRAQ4plex); K6(iTRAQ4plex)	0,0000	1,308	5	1,7	0	0,001448	47	2,21804E-05	2	970,59862	2,25	30,04	0
	High	tFDAPEGmVTMQPNHHISk	3	1	1	P74390	N-Term(iTRAQ4plex); M8(Oxidation); K19(iTRAQ4plex)	0,0000	1,100	2	22,5	0	2,876E-11	46	2,73508E-05	4	2444,20205	3,23	24,55	0
	High	aVLPVFEAk	6	1	1	P74390	N-Term(iTRAQ4plex); K9(iTRAQ4plex)	0,0000	1,161	5	7,4	0	0,00002306	45	3,09164E-05	2	1261,77580	-0,06	36,62	0
	High	tDVENPGk	2	1	1	P74390	N-Term(iTRAQ4plex); K8(iTRAQ4plex)	0,0000	1,197	1		0	0,0005275	40	9,63762E-05	2	1147,62163	1,63	13,75	0
	High	tANTIIk	6	1	1	P74390	N-Term(iTRAQ4plex); K7(iTRAQ4plex)	0,0000	1,443	3	1,6	0	0,00231	37	0,000186196	2	1048,66118	0,66	18,02	0
	High	tANTIIkEQLk	2	1	1	P74390	N-Term(iTRAQ4plex); K7(iTRAQ4plex); K11(iTRAQ4plex)	0,0000	2,429	1		0	0,00000107	32	0,000688605	3	1691,04251	-0,15	25,01	1
	High	gFTcDWTR	2	1	1	P74390	N-Term(iTRAQ4plex); C4(Carbamidomethyl)	0,0000	0,950	2	58,6	0	0,001429	29	0,001145434	2	1186,54399	0,67	28,16	0
	High	aAAIGk	3	1	1	P74390	N-Term(iTRAQ4plex); K6(iTRAQ4plex)	0,0000	0,798	3	13,6	0,003	0,0356	23	0,004832694	2	818,53221	-1,98	14,66	0
P74227	Elongation factor Tu OS=Synechocystis sp. (strain PCC 6803 / Kazusa) GN=tuf PE=1 SV=1 - [EFTU_S	2090,06	59,15	1	25	25	101	1,763	81	23,7	399	43,7	5,30							
	A3	Sequence	# PSMs	# Proteins	# Protein Groups	Protein Group Accessions	Modifications	ΔCn	116/117	116/117 Count	116/117 Variability [%]	q-Value	PEP	IonScore	Exp Value	Charge	MH+ [Da]	ΔM [ppm]	RT [min]	# Missed Cleavages
	High	tLEEGmAGDNVGLLLR	2	1	1	P74227	N-Term(iTRAQ4plex); M6(Oxidation)	0,0000	1,602	2	15,9	0	5,424E-13	102	5,72756E-11	2	1847,96758	1,16	38,60	0
	High	tIGAGVVSk	9	1	1	P74227	N-Term(iTRAQ4plex); K9(iTRAQ4plex)	0,0000	1,673	6	25,9	0	0,0000641	70	1,02322E-07	2	1119,69890	1,15	21,32	0
	High	vGEEISIVGIk	3	1	1	P74227	N-Term(iTRAQ4plex); K11(iTRAQ4plex)	0,0000	1,335	2	55,1	0	8,282E-08	69	1,12712E-07	2	1431,86760	1,01	37,15	0
	High	aVDDYIDTPER	2	1	1	P74227	N-Term(iTRAQ4plex)	0,0000	1,763	1		0	0,000007621	62	6,23692E-07	2	1437,69866	0,56	27,37	0
	High	mTVELINPIAIEQGmR	3	1	1	P74227	N-Term(iTRAQ4plex); M15(Oxidation)	0,0000	1,183	3	32,9	0	3,824E-11	62	7,01407E-07	2	1975,04912	0,88	46,86	0
	High	mTVELINPIAIEQGmR	2	1	1	P74227	N-Term(iTRAQ4plex); M1(Oxidation); M15(Oxidation)	0,0000	1,507	2	21,5	0	4,047E-11	61	8,51079E-07	2	1991,04302	0,36	42,14	0
	High	mTVELINPIAIEQGMR	2	1	1	P74227	N-Term(iTRAQ4plex)	0,0000	3,455	2	49,6	0	1,021E-08	61	8,56979E-07	2	1959,05522	1,40	51,33	0
	High	lVVFLNkk	2	1	1	P74227	N-Term(iTRAQ4plex); K7(iTRAQ4plex); K8(iTRAQ4plex)	0,0000	3,011	2	5,2	0	0,0002392	58	1,50998E-06	2	1392,93108	0,56	31,89	1
	High	lVVFLNk	10	1	1	P74227	N-Term(iTRAQ4plex); K7(iTRAQ4plex)	0,0000	1,935	7	4,9	0	0,00143	56	2,42086E-06	2	1120,73479	1,36	37,77	0
	High	tTDVTGTIk	3	1	1	P74227	N-Term(iTRAQ4plex); K9(iTRAQ4plex)	0,0000	1,888	2	4,1	0	0,000002743	56	2,4602E-06	2	1223,70928	0,58	19,63	0
	High	kATVTGVEMFQk	6	1	1	P74227	N-Term(iTRAQ4plex); K1(iTRAQ4plex); K12(iTRAQ4plex)	0,0000	1,124	4	224,0	0	7,579E-09	54	3,81944E-06	3	1771,01474	-0,07	27,55	1
	High	dHVNIGTIGHVDHGk	3	1	1	P74227	N-Term(iTRAQ4plex); K15(iTRAQ4plex)	0,0000	1,608	2	21,8	0	2,68E-08	52	5,82063E-06	4	1887,00625	-0,70	19,65	0
	High	tLEEGMAGDNVGLLLR	1	1	1	P74227	N-Term(iTRAQ4plex)	0,0000	0,858	1		0	5,013E-11	51	7,36156E-06	3	1831,97165	0,62	43,78	0
	High	eHILLAk	6	1	1	P74227	N-Term(iTRAQ4plex); K7(iTRAQ4plex)	0,0000	1,768	5	12,4	0	0,00007586	51	8,37471E-06	2	1111,70757	-0,19	21,06	0
	High	dAILELMk	1	1	1	P74227	N-Term(iTRAQ4plex); K8(iTRAQ4plex)	0,0000	2,360	1		0	0,00000883	51	8,60994E-06	2	1220,71745	0,93	41,09	0
	High	kYEDIDAAPEEk	5	1	1	P74227	N-Term(iTRAQ4plex); K1(iTRAQ4plex); K12(iTRAQ4plex)	0,0000	2,046	3	0,8	0	2,754E-08	47	2,27085E-05	3	1839,96988	-0,10	21,29	1
	High	gITINTAHVEYETDSR	8	1	1	P74227	N-Term(iTRAQ4plex)	0,0000	1,647	5	7,6	0	4,783E-08	47	2,17255E-05	3	1949,96885	0,15	27,55	0
	High	kATVTGVEmFQk	5	1	1	P74227	N-Term(iTRAQ4plex); K1(iTRAQ4plex); M9(Oxidation); K12(iTR	0,0000	2,055	4	5,7	0	4,987E-09	46	3,65876E-05	2	1787,01225	1,39	22,39	1
	High	tkDHVNIGTIGHVDHGk	4	1	1	P74227	N-Term(iTRAQ4plex); K2(iTRAQ4plex); K17(iTRAQ4plex)	0,0000	1,704	3	22,0	0	5,433E-09	43	4,7203E-05	4	2260,25039	-0,84	18,57	1
	High	eYkDAILELmk	2	1	1	P74227	N-Term(iTRAQ4plex); K3(iTRAQ4plex); M10(Oxidation); K11(iT	0,0000	1,350	2	32,1	0	0,000003195	40	0,000106162	3	1801,01383	-0,20	33,94	1
	High	dAILELmk	1	1	1	P74227	N-Term(iTRAQ4plex); M7(Oxidation); K8(iTRAQ4plex)	0,0000	1,063	1		0	0,00001905	38	0,000149958	2	1236,71135	0,10	31,28	0
	High	eLLSDYDFPGDDIPIVAGSALk	1	1	1	P74227	N-Term(iTRAQ4plex); K22(iTRAQ4plex)	0,0000	3,334	1		0	1,048E-07	37	0,000196775	3	2623,37546	0,32	53,17	0
	High	eYkDAILELMk	1	1	1	P74227	N-Term(iTRAQ4plex); K3(iTRAQ4plex); K11(iTRAQ4plex)	0,0000	2,726	1		0	3,827E-08	35	0,000334942	3	1785,01957	0,17	42,05	1
	High	ikmTVELINPIAIEQGmR	1	1	1	P74227	N-Term(iTRAQ4plex); K2(iTRAQ4plex); M3(Oxidation); M17(Ox	0,0000	1,709	1		0	7,159E-07	32	0,000663697	3	2376,32517	0,74	41,60	1
	High	hYAHVDcPGHADYVk	1	1	1	P74227	N-Term(iTRAQ4plex); C7(Carbamidomethyl); K15(iTRAQ4plex)	0,0000	1,473	1		0	3,909E-09	32	0,000665227	4	2056,99062	0,21	16,83	0
	High	aIEGEkEYkDAILELmk	4	1	1	P74227	N-Term(iTRAQ4plex); K6(iTRAQ4plex); K9(iTRAQ4plex); M16(	0,0000	2,018	4	5,2	0	1,089E-10	31	0,000810905	3	2572,44010	0,40	36,48	2
	High	hTPFFANYRPQFYVR	1	1	1	P74227	N-Term(iTRAQ4plex)	0,0000	2,034	1		0	0,00000179	30	0,001018521	4	2087,07485	0,78	37,01	0
	High	gIQkEDIER	3	1	1	P74227	N-Term(iTRAQ4plex); K4(iTRAQ4plex)	0,0000	1,916	3	5,9	0	0,002285	23	0,006236535	3	1375,78022	1,32	16,92	1
	High	aIEGEkEYk	3	1	1	P74227	N-Term(iTRAQ4plex); K6(iTRAQ4plex); K9(iTRAQ4plex)	0,0000	1,727	3	3,4	0	0,000128	21	0,009051027	3	1498,84632	-0,96	15,91	1
	High	kEEGGR	3	1	1	P74227	N-Term(iTRAQ4plex); K1(iTRAQ4plex)	0,0000	1,381	3	30,5	0,002	0,02475	19	0,012536875	3	963,54489	-1,35	7,32	1
	High	qVGVPk	3	1	1	P74227	N-Term(iTRAQ4plex); K6(iTRAQ4plex)	0,0000	0,956	3	0,0	0,003	0,03911	21	0,008317063	2	915,58495	-1,80	15,15	0
P74281	Soluble hydrogenase 42 kD subunit OS=Synechocystis sp. (strain PCC 6803 / Kazusa) GN=sll1559 PE	1920,34	44,79	1	20	20	79	0,481	67	45,2	384	40,7	7,28							
	A3	Sequence	# PSMs	# Proteins	# Protein Groups	Protein Group Accessions	Modifications	ΔCn	116/117	116/117 Count	116/117 Variability [%]	q-Value	PEP	IonScore	Exp Value	Charge	MH+ [Da]	ΔM [ppm]	RT [min]	# Missed Cleavages
	High	aWQAYETATIPR	4	1	1	P74281	N-Term(iTRAQ4plex)	0,0000	0,382	4	3,0	0	0,000001192	77	1,8534E-08	2	1550,80998	1,02	34,74	0
	High	kFDIAMAGGQDHLk	6	1	1	P74281	N-Term(iTRAQ4plex); K1(iTRAQ4plex); K14(iTRAQ4plex)	0,0000	0,736	4	11,2	0	6,463E-11	65	3,45939E-07	4	1963,08217	1,31	28,06	1
	High	tFGLAVEEIk	2	1	1	P74281	N-Term(iTRAQ4plex); K10(iTRAQ4plex)	0,0000	0,541	2	24,8	0	0,000001169	63	6,40764E-07	2	1394,81548	1,50	37,84	0
	High	gYmIPPGLGFVSVSAk	4	1	1	P74281	N-Term(iTRAQ4plex); M3(Oxidation); K16(iTRAQ4plex)	0,0000	0,599	4	77,9	0	1,716E-08	61	7,56781E-07	2	1927,06291	1,41	43,58	0
	High	iIAELTANLk	7	1	1	P74281	N-Term(iTRAQ4plex); K10(iTRAQ4plex)	0,0000	0,418	6	11,4	0	6,243E-08	61	8,05323E-07	2	1373,86260	1,40	45,51	0
	High	aEGLDAIFTR	1	1	1	P74281	N-Term(iTRAQ4plex)	0,0000	0,404	1		0	5,408E-08	60	1,3706E-06	2	1236,67241	1,53	38,58	0
	High	kFDIAmAGGQDHLk	7	1	1	P74281	N-Term(iTRAQ4plex); K1(iTRAQ4plex); M6(Oxidation); K14(iTF	0,0000	0,377	5	8,9	0	3,302E-12	59	1,35737E-06	3	1979,07566	0,58	23,73	1
	High	vLLAMAk	4	1	1	P74281	N-Term(iTRAQ4plex); K7(iTRAQ4plex)	0,0000	0,608	3	5,7	0	0,0002348	58	1,75951E-06	2	1033,67058	2,27	31,29	0
	High	fDIAmAGGQDHLk	2	1	1	P74281	N-Term(iTRAQ4plex); M5(Oxidation); K13(iTRAQ4plex)	0,0000	0,367	1		0	1,022E-10	57	2,07477E-06	3	1706,87656	-0,54	27,32	0
	High	tLLEADSDk	2	1	1	P74281	N-Term(iTRAQ4plex); K9(iTRAQ4plex)	0,0000	0,373	1		0	0,000006048	53	5,48277E-06	2	1279,69963	0,96	22,69	0
	High	fDIAMAGGQDHLk	1	1	1	P74281	N-Term(iTRAQ4plex); K13(iTRAQ4plex)	0,0000	0,647	1		0	6,116E-07	48	1,55586E-05	3	1690,88614	2,11	31,80	0
	High	qMLMIPGPTPVPEk	1	1	1	P74281	N-Term(iTRAQ4plex); K14(iTRAQ4plex)	0,0000	1,107	1		0	0,000001335	46	2,69755E-05	3	1826,01816	1,23	38,53	0
	High	iGHLGFVcDR	5	1	1	P74281	N-Term(iTRAQ4plex); C8(Carbamidomethyl)	0,0000	0,311	4	11,4	0	0,000003061	45	3,22085E-05	2	1317,68596	0,38	27,54	0
	High	aLIITHSETSTGVLNDLAAINAAAK	1	1	1	P74281	N-Term(iTRAQ4plex); K25(iTRAQ4plex)	0,0000	0,359	1		0	1,326E-07	43	4,50786E-05	4	2782,55800	0,94	46,17	0
	High	gYMIPPGLGFVSVSAk	3	1	1	P74281	N-Term(iTRAQ4plex); K16(iTRAQ4plex)	0,0000	0,633	3	14,9	0	0,000001801	43	5,50808E-05	3	1911,06717	0,99	47,03	0
	High	vLVGNNGk	2	1	1	P74281	N-Term(iTRAQ4plex); K8(iTRAQ4plex)	0,0000	0,556	2	1,9	0	0,0003078	41	7,29407E-05	2	1088,66606	-0,54	16,53	0
	High	qmLMIPGPTPVPEk	1	1	1	P74281	N-Term(iTRAQ4plex); M2(Oxidation); K14(iTRAQ4plex)	0,0000	0,937	1		0	0,000001648	41	7,41259E-05	3	1842,01169	0,47	36,31	0
	High	fYLDLk	4	1	1	P74281	N-Term(iTRAQ4plex); K6(iTRAQ4plex)	0,0000	0,325	4	1,6	0	0,0009882	39	0,000127635	2	1086,64482	0,97	37,21	0
	High	kkFDIAMAGGQDHLk	1	1	1	P74281	N-Term(iTRAQ4plex); K1(iTRAQ4plex); K2(iTRAQ4plex); K15(i	0,0000	0,749	1		0	0,00000464	38	0,000152044	4	2235,28872	5,41	25,29	2
	High	kkFDIAmAGGQDHLk	3	1	1	P74281	K1(iTRAQ4plex); N-Term(iTRAQ4plex); K2(iTRAQ4plex); M7(O	0,0000	0,700	2	56,9	0	0,000002484	37	0,000222828	4	2251,27236	0,36	21,05	2
	High	tFGLAVEEIkAEWGk	2	1	1	P74281	N-Term(iTRAQ4plex); K10(iTRAQ4plex); K15(iTRAQ4plex)	0,0000	0,689	2	7,1	0	7,521E-08	35	0,000328829	3	2110,19931	3,97	49,27	1
	High	qmLmIPGPTPVPEk	1	1	1	P74281	N-Term(iTRAQ4plex); M2(Oxidation); M4(Oxidation); K14(iTRA	0,0000	1,552	1		0	2,508E-08	33	0,000515193	3	1858,00705	0,71	32,40	0
	High	fYLDLkk	4	1	1	P74281	N-Term(iTRAQ4plex); K6(iTRAQ4plex); K7(iTRAQ4plex)	0,0000	0,483	4	0,7	0	0,0007955	26	0,002754038	3	1358,84221	1,04	31,80	1
	High	vLLAmAk	7	1	1	P74281	N-Term(iTRAQ4plex); M5(Oxidation); K7(iTRAQ4plex)	0,0000	0,315	5	23,8	0,001	0,007538	29	0,00138666	3	1049,66214	-0,96	22,77	0
	High	aLDPNDFk	2	1	1	P74281	N-Term(iTRAQ4plex); K8(iTRAQ4plex)	0,0000	0,487	2	7,5	0,001	0,01629	20	0,010446481	2	1207,65373	-2,00	27,69	0
	High	aEWGk	1	1	1	P74281	N-Term(iTRAQ4plex); K5(iTRAQ4plex)	0,0000	0,944	1		0,006	0,06882	27	0,002089296	2	878,49565	-2,04	16,11	0
	High	sGDFSk	1	1	1	P74281	N-Term(iTRAQ4plex); K6(iTRAQ4plex)	0,0000	0,485	1		0,007	0,09153	14	0,035889715	2	928,49651	-1,43	13,72	0
P77972	Enolase OS=Synechocystis sp. (strain PCC 6803 / Kazusa) GN=eno PE=3 SV=1 - [ENO_SYNY3]	1906,84	41,44	1	14	14	61	0,403	47	21,6	432	46,5	5,15							
	A3	Sequence	# PSMs	# Proteins	# Protein Groups	Protein Group Accessions	Modifications	ΔCn	116/117	116/117 Count	116/117 Variability [%]	q-Value	PEP	IonScore	Exp Value	Charge	MH+ [Da]	ΔM [ppm]	RT [min]	# Missed Cleavages
	High	sGETEDTTIADLAVATR	3	1	1	P77972	N-Term(iTRAQ4plex)	0,0000	0,509	2	56,8	0	3,301E-08	100	9,86211E-11	2	1893,95513	1,51	35,49	0
	High	lESGAHGIAQVPSGASTGSFEAHE	8	1	1	P77972	N-Term(iTRAQ4plex)	0,0000	0,325	4	3,7	0	6,133E-16	85	3,12586E-09	3	2752,37857	0,50	31,30	0
	High	tQLVGDDLMVTNPVR	2	1	1	P77972	N-Term(iTRAQ4plex)	0,0000	0,711	2	7,6	0	3,461E-10	75	3,28073E-08	2	1801,96269	1,53	37,71	0
	High	sIDLGVANAILIk	6	1	1	P77972	N-Term(iTRAQ4plex); K13(iTRAQ4plex)	0,0000	0,382	5	40,9	0	8,326E-10	75	3,28829E-08	2	1615,00652	1,98	45,94	0
	High	aAAAELAIPLYR	9	1	1	P77972	N-Term(iTRAQ4plex)	0,0000	0,401	6	11,4	0	1,335E-07	69	1,21331E-07	2	1402,81926	1,24	39,11	0
	High	eLGANAILGVSLATAk	2	1	1	P77972	N-Term(iTRAQ4plex); K16(iTRAQ4plex)	0,0000	0,538	2	6,6	0	1,502E-10	67	2,13304E-07	3	1816,08084	1,40	43,71	0
	High	tQLVGDDLmVTNPVR	2	1	1	P77972	N-Term(iTRAQ4plex); M9(Oxidation)	0,0000	0,535	2	101,0	0	3,838E-08	66	2,51171E-07	2	1817,95696	1,16	34,55	0
	High	wGAEVFAVLGk	8	1	1	P77972	N-Term(iTRAQ4plex); K11(iTRAQ4plex)	0,0000	0,411	8	15,5	0	2,349E-09	59	1,16942E-06	2	1464,84746	1,43	50,31	0
	High	vPATIEEIAAR	1	1	1	P77972	N-Term(iTRAQ4plex)	0,0000	0,386	1		0	0,00001138	57	1,89657E-06	2	1313,75603	1,09	35,61	0
	High	lNQIGTLSETLETISLATR	2	1	1	P77972	N-Term(iTRAQ4plex)	0,0000	0,346	2	43,3	0	1,438E-08	57	2,25109E-06	2	2204,22392	-0,73	56,91	0
	High	gLTTSLGTk	6	1	1	P77972	N-Term(iTRAQ4plex); K9(iTRAQ4plex)	0,0000	0,403	3	1,1	0	0,0001479	51	8,83019E-06	2	1165,70464	1,33	22,15	0
	High	gRPTIEAEVR	6	1	1	P77972	N-Term(iTRAQ4plex)	0,0000	0,390	4	4,4	0	0,0006178	40	0,000106407	3	1271,72101	1,68	16,29	0
	High	iEDELGDR	1	1	1	P77972	N-Term(iTRAQ4plex)	0,0000	0,513	1		0	0,00004022	36	0,000260597	2	1090,55058	0,78	22,83	0
	High	aVYAPk	3	1	1	P77972	N-Term(iTRAQ4plex); K6(iTRAQ4plex)	0,0000	0,939	3	3,9	0,002	0,02737	34	0,000393523	2	936,57604	0,37	16,15	0
	Medium	iGLGPk	2	1	1	P77972	N-Term(iTRAQ4plex); K6(iTRAQ4plex)	0,0000	0,396	2	9,0	0,021	0,2806	28	0,001465447	2	872,58183	1,20	20,48	0
Q55544	Phycobiliprotein ApcE OS=Synechocystis sp. (strain PCC 6803 / Kazusa) GN=apcE PE=1 SV=1 - [APC	1808,80	39,51	1	36	36	89	3,499	83	40,2	896	100,2	9,23							
	A3	Sequence	# PSMs	# Proteins	# Protein Groups	Protein Group Accessions	Modifications	ΔCn	116/117	116/117 Count	116/117 Variability [%]	q-Value	PEP	IonScore	Exp Value	Charge	MH+ [Da]	ΔM [ppm]	RT [min]	# Missed Cleavages
	High	aYSQSISYLESQVR	3	1	1	Q55544	N-Term(iTRAQ4plex)	0,0000	0,770	3	21,1	0	1,473E-08	88	1,41896E-09	2	1774,91094	0,96	40,06	0
	High	fVELGQVSAIR	3	1	1	Q55544	N-Term(iTRAQ4plex)	0,0000	3,603	3	51,1	0	0,000001855	67	2,02302E-07	2	1362,78740	0,86	38,16	0
	High	lEIAETLTQNADLIVSR	2	1	1	Q55544	N-Term(iTRAQ4plex)	0,0000	1,572	2	53,2	0	0,000005642	63	5,00253E-07	3	2030,14029	7,49	51,82	0
	High	yLTPGGLQmR	4	1	1	Q55544	N-Term(iTRAQ4plex); M9(Oxidation)	0,0000	3,504	4	22,9	0	0,000008731	63	4,80806E-07	3	1295,68943	-0,34	27,17	0
	High	qPALVGASSDSR	1	1	1	Q55544	N-Term(iTRAQ4plex)	0,0000	3,557	1		0	0,00003591	59	1,33958E-06	2	1331,70745	2,88	17,85	0
	High	fGESSTQALIR	2	1	1	Q55544	N-Term(iTRAQ4plex)	0,0000	4,178	2	9,2	0	0,000005661	57	1,94971E-06	2	1352,72905	-0,03	29,97	0
	High	lSVAEIQLENGDISVR	1	1	1	Q55544	N-Term(iTRAQ4plex)	0,0000	7,816	1		0	8,938E-10	57	2,03222E-06	3	1887,03128	0,44	41,83	0
	High	lSNNEINVk	2	1	1	Q55544	N-Term(iTRAQ4plex); K9(iTRAQ4plex)	0,0000	3,657	2	21,3	0	0,000003912	57	2,08915E-06	2	1318,75640	-0,40	22,92	0
	High	iFTGGSPLSYLEkPVER	12	1	1	Q55544	N-Term(iTRAQ4plex); K13(iTRAQ4plex)	0,0000	4,337	10	14,1	0	9,344E-11	55	3,35714E-06	3	2181,21677	0,42	45,11	1
	High	eIQQYNQILASQGLk	1	1	1	Q55544	N-Term(iTRAQ4plex); K15(iTRAQ4plex)	0,0000	1,638	1		0	2,819E-09	53	5,38233E-06	3	2021,12967	1,32	37,39	0
	High	lYWAPHYVck	1	1	1	Q55544	N-Term(iTRAQ4plex); C9(Carbamidomethyl); K10(iTRAQ4plex)	0,0000	4,662	1		0	1,218E-07	47	1,98139E-05	3	1624,85465	-0,12	33,26	0
	High	aASADYFR	2	1	1	Q55544	N-Term(iTRAQ4plex)	0,0000	1,958	2	11,1	0	0,00004603	47	2,00433E-05	2	1044,52190	-1,15	21,12	0
	High	qFFEPFINSR	3	1	1	Q55544	N-Term(iTRAQ4plex)	0,0000	4,149	3	36,2	0	0,00002371	47	2,07477E-05	2	1428,73967	0,29	41,83	0
	High	sELFLk	2	1	1	Q55544	N-Term(iTRAQ4plex); K6(iTRAQ4plex)	0,0000	4,068	2	16,9	0	0,0008333	45	3,2434E-05	2	1024,62639	-1,70	28,46	0
	High	kQFFEPFINSR	4	1	1	Q55544	N-Term(iTRAQ4plex); K1(iTRAQ4plex)	0,0000	3,664	4	12,5	0	3,962E-08	44	3,83875E-05	3	1700,93515	-0,67	36,11	1
	High	aLELAFR	3	1	1	Q55544	N-Term(iTRAQ4plex)	0,0000	4,766	3	3,6	0	0,0003459	42	9,01454E-05	2	963,57414	-0,29	34,31	0
	High	yAMkPGLSALEk	1	1	1	Q55544	N-Term(iTRAQ4plex); K4(iTRAQ4plex); K12(iTRAQ4plex)	0,0000	12,736	1		0	2,435E-09	40	9,46172E-05	3	1740,00675	-1,32	30,24	0
	High	dMSWFLR	2	1	1	Q55544	N-Term(iTRAQ4plex)	0,0000	7,150	2	5,2	0	0,00009669	38	0,000146208	2	1098,55168	-0,57	42,35	0
	High	lVSTYDk	1	1	1	Q55544	N-Term(iTRAQ4plex); K7(iTRAQ4plex)	0,0000	3,399	1		0	0,0003259	38	0,000147221	2	1113,63896	-0,40	21,22	0
	High	yGPSNMQk	1	1	1	Q55544	N-Term(iTRAQ4plex); K8(iTRAQ4plex)	0,0000	2,224	1		0	0,001174	35	0,000334942	2	1212,62749	-0,86	17,90	0
	High	aSGGSSLARPQLYQTVPVSAISQA	1	1	1	Q55544	N-Term(iTRAQ4plex)	0,0000	0,328	1		0	9,38E-09	34	0,000457279	4	3188,64199	0,07	38,32	0
	High	rFPTLPAANFPNTER	3	1	1	Q55544	N-Term(iTRAQ4plex)	0,0000	1,386	3	131,4	0	0,00002763	34	0,000442558	3	1874,99814	-0,68	34,52	1
	High	yAmkPGLSALEk	2	1	1	Q55544	N-Term(iTRAQ4plex); M3(Oxidation); K4(iTRAQ4plex); K12(iTR	0,0000	4,190	2	15,4	0	0,000001819	31	0,000762026	3	1756,00357	-0,22	26,72	0
	High	qDkELVVPSFTPVVk	1	1	1	Q55544	N-Term(iTRAQ4plex); K3(iTRAQ4plex); K15(iTRAQ4plex)	0,0000	0,821	1		0	0,000009937	24	0,003989973	3	2118,25357	0,03	37,23	1
	High	fPTLPAANFPNTER	2	1	1	Q55544	N-Term(iTRAQ4plex)	0,0000	2,112	2	116,1	0	0,0001064	24	0,004054805	3	1718,89817	-0,08	41,21	0
	High	sVFSSTGPIPPGFRPINIAR	3	1	1	Q55544	N-Term(iTRAQ4plex)	0,0000	3,677	3	1,6	0	3,194E-07	23	0,005046264	3	2257,25992	1,09	43,15	0
	High	rPAPFNk	2	1	1	Q55544	N-Term(iTRAQ4plex); K7(iTRAQ4plex)	0,0000	2,927	2	26,9	0	0,0005926	21	0,008609343	3	1117,67218	0,10	15,09	0
	High	lLGRPTYGR	4	1	1	Q55544	N-Term(iTRAQ4plex)	0,0000	4,469	2	0,3	0	0,002116	16	0,022488993	3	1176,69678	-0,18	20,51	0
	High	sLRDmSWFLR	1	1	1	Q55544	N-Term(iTRAQ4plex); M5(Oxidation)	0,0000	3,443	1		0	0,00241	15	0,030127979	3	1470,76407	-0,26	34,58	1
	High	vDkEVTPR	3	1	1	Q55544	N-Term(iTRAQ4plex); K3(iTRAQ4plex)	0,0000	3,198	3	20,1	0,001	0,004573	28	0,001610534	2	1231,72600	0,90	13,30	1
	High	aPTPANk	7	1	1	Q55544	N-Term(iTRAQ4plex); K7(iTRAQ4plex)	0,0000	2,688	5	7,3	0,001	0,009763	14	0,041301897	2	986,58684	-0,49	11,61	0
	High	yGPSNmQk	1	1	1	Q55544	N-Term(iTRAQ4plex); M6(Oxidation); K8(iTRAQ4plex)	0,0000	2,957	1		0,002	0,02295	13	0,046448319	2	1228,62285	-0,49	12,34	0
	High	aPLNQk	1	1	1	Q55544	N-Term(iTRAQ4plex); K6(iTRAQ4plex)	0,0000	2,032	1		0,003	0,04285	22	0,006760363	2	958,59313	0,75	14,26	0
	Medium	vGSLR	1	1	1	Q55544	N-Term(iTRAQ4plex)	0,0000	3,722	1		0,012	0,1582	13	0,047311858	2	675,42693	-0,13	16,51	0
	Medium	iLIHR	1	1	1	Q55544	N-Term(iTRAQ4plex)	0,0000	1,935	1		0,013	0,1778	15	0,029510054	2	795,53093	-1,52	16,76	0
	Medium	aIEYmHR	2	1	1	Q55544	N-Term(iTRAQ4plex); M5(Oxidation)	0,0000	0,615	2	12,7	0,014	0,1855	11	0,070952876	3	1079,54248	0,02	12,90	0
	Medium	sPLYR	2	1	1	Q55544	N-Term(iTRAQ4plex)	0,0000	1,283	2	43,8	0,016	0,2062	12	0,092754239	2	779,45268	-0,68	17,11	0
	Medium	nAVIk	1	1	1	Q55544	N-Term(iTRAQ4plex); K5(iTRAQ4plex)	0,0000	1,705	1		0,041	0,5316	14	0,038368074	2	832,54772	-2,12	13,76	0
P73317	50S ribosomal protein L2 OS=Synechocystis sp. (strain PCC 6803 / Kazusa) GN=rplB PE=3 SV=1 - [R	1771,43	59,42	1	21	21	77	1,889	68	105,2	276	30,4	11,21							
	A3	Sequence	# PSMs	# Proteins	# Protein Groups	Protein Group Accessions	Modifications	ΔCn	116/117	116/117 Count	116/117 Variability [%]	q-Value	PEP	IonScore	Exp Value	Charge	MH+ [Da]	ΔM [ppm]	RT [min]	# Missed Cleavages
	High	iPLGSEIHNVELVAGR	9	1	1	P73317	N-Term(iTRAQ4plex)	0,0000	2,063	9	106,0	0	6,931E-11	93	4,67735E-10	2	1848,04888	1,53	40,79	0
	High	sAGAFAQVVAk	9	1	1	P73317	N-Term(iTRAQ4plex); K11(iTRAQ4plex)	0,0000	1,339	6	192,2	0	1,612E-07	89	1,46954E-09	2	1336,78533	1,92	29,28	0
	High	iALLFYTDGEkR	8	1	1	P73317	N-Term(iTRAQ4plex); K11(iTRAQ4plex)	0,0000	4,247	8	34,2	0	0,000001782	75	3,41956E-08	2	1713,98223	2,57	39,00	1
	High	qASVSDFTEITk	1	1	1	P73317	N-Term(iTRAQ4plex); K12(iTRAQ4plex)	0,0000	1,314	1		0	4,605E-09	72	6,19398E-08	2	1613,86479	1,41	31,41	0
	High	gSVMNPcDHPHGGGEGR	5	1	1	P73317	N-Term(iTRAQ4plex); C7(Carbamidomethyl)	0,0000	0,750	4	9,7	0	2,507E-09	49	1,12194E-05	3	1907,83719	0,72	12,16	0
	High	sGPVSPWGkPALGAk	4	1	1	P73317	N-Term(iTRAQ4plex); K9(iTRAQ4plex); K15(iTRAQ4plex)	0,0000	2,455	4	68,1	0	2,138E-09	46	2,71625E-05	3	1884,11332	3,46	28,26	0
	High	iALLFYTDGEk	1	1	1	P73317	N-Term(iTRAQ4plex); K11(iTRAQ4plex)	0,0000	1,876	1		0	3,331E-07	45	3,22085E-05	2	1557,87833	1,04	44,44	0
	High	sSALIVR	3	1	1	P73317	N-Term(iTRAQ4plex)	0,0000	2,984	3	51,6	0	0,0005724	43	5,55866E-05	2	889,55998	1,37	18,15	0
	High	vAAIEYDPNR	1	1	1	P73317	N-Term(iTRAQ4plex)	0,0000	1,148	1		0	0,0008813	42	6,88605E-05	2	1291,67851	1,70	25,23	0
	High	gSVmNPcDHPHGGGEGR	8	1	1	P73317	N-Term(iTRAQ4plex); M4(Oxidation); C7(Carbamidomethyl)	0,0000	1,691	5	43,1	0	0,00006152	38	0,00016217	3	1923,83310	1,23	8,59	0
	High	kEcVATLGR	6	1	1	P73317	N-Term(iTRAQ4plex); K1(iTRAQ4plex); C3(Carbamidomethyl)	0,0000	4,026	4	51,3	0	0,0003333	37	0,000190533	3	1321,75481	3,58	15,63	1
	High	nkQNIPAR	3	1	1	P73317	N-Term(iTRAQ4plex); K2(iTRAQ4plex)	0,0000	2,327	3	32,6	0	0,0009053	19	0,013644889	3	1228,74121	3,87	8,88	1
	High	vSNAEFR	3	1	1	P73317	N-Term(iTRAQ4plex)	0,0000	1,338	3	12,2	0,001	0,01048	37	0,000211821	2	966,51402	1,53	16,91	0
	High	eGDYVTIk	1	1	1	P73317	N-Term(iTRAQ4plex); K8(iTRAQ4plex)	0,0000	0,556	1		0,001	0,003486	34	0,000396251	2	1212,67632	4,02	23,82	0
	High	gVITSR	4	1	1	P73317	N-Term(iTRAQ4plex)	0,0000	0,804	4	0,5	0,003	0,04177	30	0,000897367	2	776,47710	3,10	15,08	0
	High	nYRPMTPGTR	2	1	1	P73317	N-Term(iTRAQ4plex)	0,0000	1,474	2	47,9	0,003	0,04018	18	0,017257187	3	1336,69342	1,62	15,15	0
	High	qNIPAR	1	1	1	P73317	N-Term(iTRAQ4plex)	0,0000	1,398	1		0,006	0,07392	24	0,004178015	2	842,49834	2,19	12,64	0
	High	skPEk	1	1	1	P73317	N-Term(iTRAQ4plex); K2(iTRAQ4plex); K5(iTRAQ4plex)	0,0000	5,848	1		0,007	0,08857	11	0,087981768	3	1020,64615	4,69	8,29	1
	Medium	nYRPmTPGTR	2	1	1	P73317	N-Term(iTRAQ4plex); M5(Oxidation)	0,0000	1,033	2	81,7	0,01	0,1337	11	0,085500766	3	1352,68659	0,30	13,11	0
	Medium	ecVATLGR	1	1	1	P73317	N-Term(iTRAQ4plex); C2(Carbamidomethyl)	0,0000	1,409	1		0,013	0,1678	18	0,015594448	2	1049,55535	2,20	20,10	0
	Medium	iIDFk	1	1	1	P73317	N-Term(iTRAQ4plex); K5(iTRAQ4plex)	0,0000	2,367	1		0,026	0,3524	29	0,002024734	2	923,58708	7,19	29,82	0
	Medium	iIDFkR	2	1	1	P73317	N-Term(iTRAQ4plex); K5(iTRAQ4plex)	0,0000	9,357	2	5,6	0,027	0,3636	17	0,018237697	3	1079,68924	7,11	24,31	1
	Medium	aPIGR	1	1	1	P73317	N-Term(iTRAQ4plex)	0,0000	0,948	1		0,037	0,4716	12	0,062225731	2	657,42186	8,22	12,78	0
P73093	Phycobilisome rod-core linker polypeptide CpcG OS=Synechocystis sp. (strain PCC 6803 / Kazusa) GN	1618,46	57,02	1	18	18	66	2,676	58	27,4	235	27,4	9,23							
	A3	Sequence	# PSMs	# Proteins	# Protein Groups	Protein Group Accessions	Modifications	ΔCn	116/117	116/117 Count	116/117 Variability [%]	q-Value	PEP	IonScore	Exp Value	Charge	MH+ [Da]	ΔM [ppm]	RT [min]	# Missed Cleavages
	High	vSAQNINIEASVPR	4	1	1	P73093	N-Term(iTRAQ4plex)	0,0000	1,585	4	95,2	0	4,931E-13	105	3,0267E-11	2	1641,90544	0,81	32,87	0
	High	kLTAGNPmNFLGMAR	3	1	1	P73093	N-Term(iTRAQ4plex); K1(iTRAQ4plex); M8(Oxidation)	0,0000	4,063	3	64,5	0	5,572E-09	61	8,51079E-07	3	1925,03611	1,08	33,81	1
	High	sINPAANTIPk	9	1	1	P73093	N-Term(iTRAQ4plex); K11(iTRAQ4plex)	0,0000	0,898	7	316,1	0	5,815E-07	57	1,82377E-06	2	1413,82952	-0,65	24,94	0
	High	kLTAGNPMNFLGMAR	1	1	1	P73093	N-Term(iTRAQ4plex); K1(iTRAQ4plex)	0,0000	6,519	1		0	1,777E-08	56	2,29599E-06	3	1909,03965	0,28	40,92	1
	High	gLLLSNTFR	6	1	1	P73093	N-Term(iTRAQ4plex)	0,0000	2,865	3	0,1	0	0,000001424	53	4,66627E-06	2	1164,68596	0,17	36,64	0
	High	lTAGNPmNFLGMAR	1	1	1	P73093	N-Term(iTRAQ4plex); M7(Oxidation)	0,0000	6,164	1		0	0,000003432	53	4,74209E-06	2	1652,83708	0,06	39,34	0
	High	qIFFHAFk	4	1	1	P73093	N-Term(iTRAQ4plex); K8(iTRAQ4plex)	0,0000	3,151	3	8,6	0	0,0001014	49	1,17211E-05	2	1325,76201	0,85	33,50	0
	High	lTAGNPMNFLGMAR	2	1	1	P73093	N-Term(iTRAQ4plex)	0,0000	14,433	2	328,1	0	2,585E-07	49	1,21891E-05	2	1636,84331	0,75	46,80	0
	High	kLTAGNPmNFLGmAR	4	1	1	P73093	K1(iTRAQ4plex); N-Term(iTRAQ4plex); M8(Oxidation); M13(Ox	0,0000	3,034	2	1,4	0	5,476E-07	48	1,57025E-05	3	1941,03055	0,83	29,41	1
	High	lTAGNPmNFLGmAR	4	1	1	P73093	N-Term(iTRAQ4plex); M7(Oxidation); M12(Oxidation)	0,0000	2,276	4	31,8	0	0,000002056	47	1,9542E-05	2	1668,83257	0,40	34,39	0
	High	rQLGFPQIVWQNEVR	3	1	1	P73093	N-Term(iTRAQ4plex)	0,0000	2,507	3	40,9	0	2,031E-10	44	4,46373E-05	3	2014,11087	0,25	41,29	1
	High	qLGFPQIVWQNEVR	5	1	1	P73093	N-Term(iTRAQ4plex)	0,0000	3,061	5	43,9	0	1,201E-07	44	4,01763E-05	3	1858,00925	0,00	46,69	0
	High	fVEHcVQk	4	1	1	P73093	N-Term(iTRAQ4plex); C5(Carbamidomethyl); K8(iTRAQ4plex)	0,0000	2,651	4	9,4	0	0,0008477	44	4,05481E-05	2	1334,71294	0,00	17,21	0
	High	iAWSIVVATk	2	1	1	P73093	N-Term(iTRAQ4plex); K10(iTRAQ4plex)	0,0000	2,460	2	20,8	0	0,00008823	35	0,000300587	3	1375,85501	-0,13	42,01	0
	High	eAGELPFNIk	1	1	1	P73093	N-Term(iTRAQ4plex); K10(iTRAQ4plex)	0,0000	0,928	1		0	0,00006131	31	0,000818408	3	1405,79248	-0,36	36,22	0
	High	rFIPQEk	1	1	1	P73093	N-Term(iTRAQ4plex); K7(iTRAQ4plex)	0,0000	2,514	1		0	0,00006917	24	0,005092644	3	1205,72275	-1,45	17,68	1
	High	ekVLESQLR	1	1	1	P73093	N-Term(iTRAQ4plex); K2(iTRAQ4plex)	0,0000	0,663	1		0	0,0007584	23	0,004863736	3	1389,83255	1,53	19,37	1
	High	vLESQLR	3	1	1	P73093	N-Term(iTRAQ4plex)	0,0000	1,951	3	35,4	0,001	0,005475	39	0,000129411	2	988,59075	-0,05	22,44	0
	High	nGQITVR	3	1	1	P73093	N-Term(iTRAQ4plex)	0,0000	1,946	3	39,3	0,001	0,01258	38	0,000165185	2	931,54436	0,20	14,77	0
	High	nSFYEk	1	1	1	P73093	N-Term(iTRAQ4plex); K6(iTRAQ4plex)	0,0000	2,476	1		0,001	0,005604	28	0,001566643	2	1075,56914	2,70	18,46	0
	High	fIPQEk	2	1	1	P73093	N-Term(iTRAQ4plex); K6(iTRAQ4plex)	0,0000	1,579	2	61,1	0,006	0,07713	17	0,020463033	2	1049,62346	0,08	23,19	0
	Medium	nLPGR	2	1	1	P73093	N-Term(iTRAQ4plex)	0,0000	2,689	2	0,7	0,017	0,2241	13	0,056100923	2	700,42174	-0,75	12,70	0
Q55199	Phosphate-binding protein OS=Synechocystis sp. (strain PCC 6803 / Kazusa) GN=pstS PE=3 SV=1 -	1507,42	31,33	1	12	12	59	0,358	48	24,7	383	40,0	4,69							
	A3	Sequence	# PSMs	# Proteins	# Protein Groups	Protein Group Accessions	Modifications	ΔCn	116/117	116/117 Count	116/117 Variability [%]	q-Value	PEP	IonScore	Exp Value	Charge	MH+ [Da]	ΔM [ppm]	RT [min]	# Missed Cleavages
	High	lSQEALAGIMLGNITk	8	1	1	Q55199	N-Term(iTRAQ4plex); K16(iTRAQ4plex)	0,0000	0,376	6	6,4	0	1,679E-11	106	2,76039E-11	2	1947,11980	0,52	49,51	0
	High	sDGSGTTAVFTmNLAAmSPEFk	1	1	1	Q55199	N-Term(iTRAQ4plex); M12(Oxidation); M17(Oxidation); K22(iT	0,0000	0,324	1		0	2,032E-13	91	7,53304E-10	3	2582,23478	-0,39	39,31	0
	High	lSQEALAGImLGNITk	6	1	1	Q55199	N-Term(iTRAQ4plex); M10(Oxidation); K16(iTRAQ4plex)	0,0000	0,303	5	26,1	0	1,693E-11	79	1,87658E-08	2	1963,11553	0,93	43,37	0
	High	tVDFGASDVAmDDEEIAk	1	1	1	Q55199	N-Term(iTRAQ4plex); M11(Oxidation); K18(iTRAQ4plex)	0,0000	0,416	1		0	4,669E-12	69	1,29411E-07	3	2217,04776	0,24	34,24	0
	High	iSPDYTITLk	5	1	1	Q55199	N-Term(iTRAQ4plex); K10(iTRAQ4plex)	0,0000	0,269	5	13,3	0	0,000003159	66	2,49442E-07	2	1438,83904	-0,38	36,32	0
	High	tVDFGASDVAMDDEEIAk	1	1	1	Q55199	N-Term(iTRAQ4plex); K18(iTRAQ4plex)	0,0000	0,371	1		0	2,175E-11	62	6,44125E-07	3	2201,05405	0,79	37,33	0
	High	vAAAADkISPDYTITLk	2	1	1	Q55199	N-Term(iTRAQ4plex); K7(iTRAQ4plex); K17(iTRAQ4plex)	0,0000	0,803	2	77,5	0	3,761E-10	60	1,07145E-06	3	2209,28061	0,07	35,86	1
	High	lVADNPDLTLPDRPITVVHR	19	1	1	Q55199	N-Term(iTRAQ4plex)	0,0000	0,309	13	13,3	0	1,34E-08	50	9,63762E-06	3	2385,33780	0,27	36,99	0
	High	tVEWPTSk	2	1	1	Q55199	N-Term(iTRAQ4plex); K8(iTRAQ4plex)	0,0000	0,358	1		0	0,000004909	48	1,52044E-05	2	1235,68645	-0,80	26,18	0
	High	vAAAADk	5	1	1	Q55199	N-Term(iTRAQ4plex); K7(iTRAQ4plex)	0,0000	0,363	4	9,6	0	0,001761	45	3,38821E-05	2	933,55992	-0,93	12,78	0
	High	ekVAAAADk	3	1	1	Q55199	N-Term(iTRAQ4plex); K2(iTRAQ4plex); K9(iTRAQ4plex)	0,0000	0,379	3	4,8	0	0,00003028	41	8,6896E-05	2	1334,80071	0,21	11,58	1
	High	eTIGDGk	1	1	1	Q55199	N-Term(iTRAQ4plex); K7(iTRAQ4plex)	0,0000	0,408	1		0,001	0,004747	32	0,000639691	2	1007,56133	0,15	14,54	0
	Medium	wNDPk	2	1	1	Q55199	N-Term(iTRAQ4plex); K5(iTRAQ4plex)	0,0000	0,394	2	24,8	0,014	0,1794	13	0,058908945	2	947,51940	0,52	18,18	0
	Medium	fIGGk	3	1	1	Q55199	N-Term(iTRAQ4plex); K5(iTRAQ4plex)	0,0000	0,323	3	9,9	0,015	0,197	16	0,022644879	2	809,51109	-1,57	19,25	0
P74689	Dihydroxy-acid dehydratase OS=Synechocystis sp. (strain PCC 6803 / Kazusa) GN=ilvD PE=3 SV=1 -	1488,56	30,48	1	21	21	73	0,600	62	25,1	561	58,6	5,25							
	A3	Sequence	# PSMs	# Proteins	# Protein Groups	Protein Group Accessions	Modifications	ΔCn	116/117	116/117 Count	116/117 Variability [%]	q-Value	PEP	IonScore	Exp Value	Charge	MH+ [Da]	ΔM [ppm]	RT [min]	# Missed Cleavages
	High	eVIADSIETVcNGQR	2	1	1	P74689	N-Term(iTRAQ4plex); C11(Carbamidomethyl)	0,0000	0,646	2	52,4	0	5,095E-12	77	1,92296E-08	2	1834,90886	0,13	33,80	0
	High	gNLATEGSVAk	4	1	1	P74689	N-Term(iTRAQ4plex); K11(iTRAQ4plex)	0,0000	0,602	2	10,1	0	1,301E-08	76	3,07396E-08	2	1334,75566	2,86	18,23	0
	High	rLLQLNISEEELAQR	1	1	1	P74689	N-Term(iTRAQ4plex)	0,0000	0,712	1		0	5,684E-10	75	3,31872E-08	3	1956,10306	1,80	37,26	1
	High	iQAGDVVVVR	3	1	1	P74689	N-Term(iTRAQ4plex)	0,0000	0,560	2	4,8	0	0,000001737	69	1,25884E-07	2	1199,72368	0,66	28,43	0
	High	iDEETLYGIER	2	1	1	P74689	N-Term(iTRAQ4plex)	0,0000	0,719	2	35,7	0	0,000005094	63	5,3576E-07	2	1481,76189	0,97	35,12	0
	High	vLVEAIkk	5	1	1	P74689	N-Term(iTRAQ4plex); K7(iTRAQ4plex); K8(iTRAQ4plex)	0,0000	0,718	5	2,6	0	0,00007064	62	5,80724E-07	2	1331,90264	2,98	23,83	1
	High	lLQLNISEEELAQR	1	1	1	P74689	N-Term(iTRAQ4plex)	0,0000	0,624	1		0	1,868E-10	58	1,88878E-06	3	1800,00034	1,07	42,92	0
	High	vLVEAIk	2	1	1	P74689	N-Term(iTRAQ4plex); K7(iTRAQ4plex)	0,0000	0,497	2	5,5	0	0,0004484	53	5,01153E-06	2	1059,70232	0,64	29,05	0
	High	yVTTNLHAAGGIPQVMk	6	1	1	P74689	N-Term(iTRAQ4plex); K17(iTRAQ4plex)	0,0000	0,624	4	9,6	0	2,913E-13	52	6,98184E-06	3	2088,15470	1,55	34,71	0
	High	nmPGAMIAMAR	2	1	1	P74689	N-Term(iTRAQ4plex); M2(Oxidation)	0,0000	0,838	2	18,9	0	0,0000293	50	1,04706E-05	2	1322,65178	1,32	26,84	0
	High	nMPGAMIAMAR	1	1	1	P74689	N-Term(iTRAQ4plex)	0,0000	1,101	1		0	3,369E-07	46	2,26449E-05	2	1306,65630	0,90	33,92	0
	High	qILPSQILTR	4	1	1	P74689	N-Term(iTRAQ4plex)	0,0000	0,517	4	9,9	0	0,0006322	44	3,93523E-05	2	1312,80889	1,48	36,25	0
	High	sQVITQGTQR	5	1	1	P74689	N-Term(iTRAQ4plex)	0,0000	0,676	5	22,7	0	0,000001668	43	4,81915E-05	2	1261,69963	1,20	14,09	0
	High	kQILPSQILTR	2	1	1	P74689	N-Term(iTRAQ4plex); K1(iTRAQ4plex)	0,0000	0,516	2	7,3	0	1,462E-07	40	9,20386E-05	3	1585,00284	-0,72	30,22	1
	High	kPVITGPAk	7	1	1	P74689	N-Term(iTRAQ4plex); K1(iTRAQ4plex); K9(iTRAQ4plex)	0,0000	0,568	4	2,3	0	0,000002852	40	9,93047E-05	3	1342,88074	1,84	16,44	0

	High	yVTTNLHAAGGIPQVmk	3	1	1	P74689	N-Term(iTRAQ4plex); M16(Oxidation); K17(iTRAQ4plex)	0,0000	0,160	3	212,7	0	0,000001807	37	0,000192739	3	2104,14914	1,31	31,64	0
	High	aDSTEESAk	1	1	1	P74689	N-Term(iTRAQ4plex); K9(iTRAQ4plex)	0,0000	0,728	1		0	0,001209	33	0,000474209	2	1225,61553	0,37	10,88	0
	High	hkVPVLcDLkPSGk	3	1	1	P74689	N-Term(iTRAQ4plex); K2(iTRAQ4plex); C7(Carbamidomethyl);	0,0000	0,695	3	18,0	0	2,478E-07	32	0,000606694	3	2154,29569	1,93	23,27	1
	High	nmPGAmIAmAR	2	1	1	P74689	N-Term(iTRAQ4plex); M2(Oxidation); M6(Oxidation); M9(Oxida	0,0000	0,437	2	0,7	0	0,00004746	31	0,000866902	2	1354,64092	0,77	15,77	0
	High	nMPGAmIAmAR	4	1	1	P74689	N-Term(iTRAQ4plex); M6(Oxidation); M9(Oxidation)	0,0000	0,634	3	6,5	0	0,000002004	30	0,001020869	2	1338,64690	1,45	21,73	0
	High	rAQWTPPQPR	1	1	1	P74689	N-Term(iTRAQ4plex)	0,0000	0,778	1		0	0,002229	27	0,002027543	3	1380,76334	1,16	18,41	1
	High	vPVLcDLkPSGk	4	1	1	P74689	N-Term(iTRAQ4plex); C5(Carbamidomethyl); K8(iTRAQ4plex);	0,0000	0,551	4	24,9	0	5,463E-07	27	0,002027543	2	1745,03647	0,50	31,85	0
	High	aQWTPPQPR	2	1	1	P74689	N-Term(iTRAQ4plex)	0,0000	0,603	2	0,5	0	0,001204	19	0,013488697	2	1224,66289	1,86	23,67	0
	High	ySLVSR	4	1	1	P74689	N-Term(iTRAQ4plex)	0,0000	0,502	3	0,0	0,001	0,003178	38	0,00016633	2	868,50182	1,06	20,86	0
	Medium	gILAk	2	1	1	P74689	N-Term(iTRAQ4plex); K5(iTRAQ4plex)	0,0000	0,993	1		0,026	0,3524	11	0,195059041	2	789,54125	-3,07	16,82	0
P73203	Phycobilisome 32.1 kDa linker polypeptide, phycocyanin-associated, rod 1 OS=Synechocystis sp. (stra	1434,97	66,32	1	19	19	61	2,098	53	41,2	291	32,5	9,35							
	A3	Sequence	# PSMs	# Proteins	# Protein Groups	Protein Group Accessions	Modifications	ΔCn	116/117	116/117 Count	116/117 Variability [%]	q-Value	PEP	IonScore	Exp Value	Charge	MH+ [Da]	ΔM [ppm]	RT [min]	# Missed Cleavages
	High	lATELGQNTVSAIVGPSGSNAGW/	3	1	1	P73203	N-Term(iTRAQ4plex)	0,0000	1,306	2	178,1	0	2,11E-08	90	8,91189E-10	3	3103,60837	1,33	44,68	0
	High	aLGGTVPFGQASk	8	1	1	P73203	N-Term(iTRAQ4plex); K13(iTRAQ4plex)	0,0000	1,863	6	38,7	0	1,694E-08	89	1,64038E-09	2	1520,86724	-0,21	29,96	0
	High	lkGAESLLTNGSISVR	3	1	1	P73203	N-Term(iTRAQ4plex); K2(iTRAQ4plex)	0,0000	2,246	2	52,9	0	7,487E-09	77	2,23342E-08	3	1933,13468	1,32	36,19	1
	High	gAESLLTNGSISVR	2	1	1	P73203	N-Term(iTRAQ4plex)	0,0000	2,366	1		0	3,79E-08	75	3,96002E-08	2	1547,85259	1,02	34,41	0
	High	vEITAISAPGYPk	4	1	1	P73203	N-Term(iTRAQ4plex); K13(iTRAQ4plex)	0,0000	1,808	4	33,8	0	6,167E-10	71	7,9794E-08	2	1633,94170	0,81	35,06	0
	High	qVLGNDYImDSER	1	1	1	P73203	N-Term(iTRAQ4plex); M9(Oxidation)	0,0000	2,339	1		0	4,692E-10	70	9,70443E-08	2	1699,80852	0,41	28,09	0
	High	qVLGNDYIMDSER	1	1	1	P73203	N-Term(iTRAQ4plex)	0,0000	2,225	1		0	3,443E-09	60	1,04947E-06	2	1683,81645	2,10	32,44	0
	High	aVIVPFEQLNQTLQQINR	3	1	1	P73203	N-Term(iTRAQ4plex)	0,0000	2,442	2	100,6	0	2,763E-10	56	2,618E-06	3	2255,26444	0,68	53,61	0
	High	kFLYNNFQTR	3	1	1	P73203	N-Term(iTRAQ4plex); K1(iTRAQ4plex)	0,0000	2,699	3	5,9	0	3,846E-07	48	1,46545E-05	3	1618,89695	1,57	27,09	1
	High	fNNQVGDR	3	1	1	P73203	N-Term(iTRAQ4plex)	0,0000	2,389	3	2,2	0	0,0001428	45	2,91723E-05	2	1093,55132	0,54	15,26	0
	High	vIELNYk	4	1	1	P73203	N-Term(iTRAQ4plex); K7(iTRAQ4plex)	0,0000	1,675	4	27,6	0	0,002122	44	3,93523E-05	2	1166,70415	1,54	29,19	0
	High	fLYNNFQTR	6	1	1	P73203	N-Term(iTRAQ4plex)	0,0000	2,169	6	27,9	0	0,000001482	39	0,000133958	3	1346,69794	0,42	33,18	0
	High	lGVAPYNESRPVELRPDFSLDDAk	1	1	1	P73203	N-Term(iTRAQ4plex); K24(iTRAQ4plex)	0,0000	2,411	1		0	0,000001008	15	0,040103405	4	2976,56850	0,50	39,70	0
	High	vIELNYkHLLGR	1	1	1	P73203	N-Term(iTRAQ4plex); K7(iTRAQ4plex)	0,0000	6,264	1		0	0,0003328	15	0,034434993	4	1743,05141	-0,33	36,51	1
	High	aITTAASR	3	1	1	P73203	N-Term(iTRAQ4plex)	0,0000	0,789	3	37,9	0,001	0,008372	45	4,48878E-05	2	934,54357	-0,30	15,33	0
	High	sELYkk	1	1	1	P73203	N-Term(iTRAQ4plex); K5(iTRAQ4plex); K6(iTRAQ4plex)	0,0000	1,324	1		0,001	0,005544	28	0,001442016	2	1199,73686	0,73	13,16	1
	High	aGNTPAk	6	1	1	P73203	N-Term(iTRAQ4plex); K7(iTRAQ4plex)	0,0000	1,919	6	46,5	0,002	0,02156	18	0,015738742	3	946,55637	0,36	11,89	0
	High	tVGFTR	5	1	1	P73203	N-Term(iTRAQ4plex)	0,0000	1,827	3	28,4	0,005	0,06284	15	0,033960182	2	824,47490	0,25	18,16	0
	Medium	vASITPASLS	2	1	1	P73203	N-Term(iTRAQ4plex)	0,0000	1,833	2	35,6	0,013	0,1768	24	0,003784164	2	1089,62749	0,22	33,31	0
	Medium	sELYk	1	1	1	P73203	N-Term(iTRAQ4plex); K5(iTRAQ4plex)	0,0000	2,098	1		0,04	0,5138	15	0,03082975	2	927,53929	0,36	15,62	0
P73603	Slr1852 protein OS=Synechocystis sp. (strain PCC 6803 / Kazusa) GN=slr1852 PE=4 SV=1 - [P73603	1383,15	52,66	1	13	13	62	0,373	56	19,0	188	21,8	5,17							
	A3	Sequence	# PSMs	# Proteins	# Protein Groups	Protein Group Accessions	Modifications	ΔCn	116/117	116/117 Count	116/117 Variability [%]	q-Value	PEP	IonScore	Exp Value	Charge	MH+ [Da]	ΔM [ppm]	RT [min]	# Missed Cleavages
	High	aDYSQPGLVPDTLk	4	1	1	P73603	N-Term(iTRAQ4plex); K14(iTRAQ4plex)	0,0000	0,446	4	37,1	0	3,162E-11	88	1,51346E-09	2	1791,97588	1,53	33,61	0
	High	lIAEGIAYVAQk	11	1	1	P73603	N-Term(iTRAQ4plex); K12(iTRAQ4plex)	0,0000	0,347	9	8,3	0	7,593E-11	88	1,76999E-09	2	1563,93767	1,77	44,26	0
	High	cVDDETHETIAk	3	1	1	P73603	N-Term(iTRAQ4plex); C1(Carbamidomethyl); K12(iTRAQ4plex)	0,0000	0,335	2	5,4	0	2,246E-09	81	8,60934E-09	2	1705,83196	0,81	17,97	0
	High	ekLIAEGIAYVAQk	5	1	1	P73603	N-Term(iTRAQ4plex); K2(iTRAQ4plex); K14(iTRAQ4plex)	0,0000	0,520	5	7,2	0	3,026E-11	64	4,17802E-07	3	1965,17466	0,06	41,59	1
	High	gDILTGEYR	2	1	1	P73603	N-Term(iTRAQ4plex)	0,0000	0,431	2	21,8	0	0,000007385	46	2,24373E-05	2	1167,61284	0,16	26,03	0
	High	dWWPTHcEALR	3	1	1	P73603	N-Term(iTRAQ4plex); C7(Carbamidomethyl)	0,0000	0,310	3	12,3	0	6,363E-07	43	4,84139E-05	3	1614,76114	0,45	33,51	0
	High	tEQLTFYR	5	1	1	P73603	N-Term(iTRAQ4plex)	0,0000	0,372	5	12,4	0	0,0000634	40	9,03587E-05	2	1201,63457	0,99	28,66	0
	High	gNVTWVR	5	1	1	P73603	N-Term(iTRAQ4plex)	0,0000	0,365	3	7,9	0	0,0001998	40	9,16157E-05	2	975,54979	0,54	20,47	0
	High	dVFAPDSLLk	6	1	1	P73603	N-Term(iTRAQ4plex); K10(iTRAQ4plex)	0,0000	0,431	5	27,1	0	0,0002277	38	0,00017457	2	1392,79985	1,52	39,75	0
	High	aDYSQPGLVPDTLkEk	1	1	1	P73603	N-Term(iTRAQ4plex); K14(iTRAQ4plex); K16(iTRAQ4plex)	0,0000	0,625	1		0	9,492E-07	34	0,000413421	3	2193,21494	0,99	30,28	1
	High	kNYYLGLk	6	1	1	P73603	N-Term(iTRAQ4plex); K1(iTRAQ4plex); K8(iTRAQ4plex)	0,0000	0,404	6	13,3	0	0,0004822	30	0,000950539	2	1430,87407	0,64	24,67	1
	High	nYYLGLk	6	1	1	P73603	N-Term(iTRAQ4plex); K7(iTRAQ4plex)	0,0000	0,358	6	8,0	0	0,002345	30	0,001044648	2	1158,67632	0,17	30,84	0
	High	gGcYLk	5	1	1	P73603	N-Term(iTRAQ4plex); C3(Carbamidomethyl); K6(iTRAQ4plex)	0,0000	0,373	5	7,5	0	0,003028	22	0,00687021	2	985,53758	-0,34	15,56	0
P73728	Putative peroxiredoxin sll1621 OS=Synechocystis sp. (strain PCC 6803 / Kazusa) GN=sll1621 PE=1 S\	1375,51	60,32	1	16	16	72	0,491	57	28,1	189	21,2	5,05							
	A3	Sequence	# PSMs	# Proteins	# Protein Groups	Protein Group Accessions	Modifications	ΔCn	116/117	116/117 Count	116/117 Variability [%]	q-Value	PEP	IonScore	Exp Value	Charge	MH+ [Da]	ΔM [ppm]	RT [min]	# Missed Cleavages
	High	tTEQIFGGk	4	1	1	P73728	N-Term(iTRAQ4plex); K9(iTRAQ4plex)	0,0000	0,468	3	16,9	0	4,606E-07	71	7,46397E-08	2	1268,71001	0,87	25,11	0
	High	vRDESVPGPNPYR	16	1	1	P73728	N-Term(iTRAQ4plex)	0,0000	0,514	9	13,7	0	2,461E-09	70	1,01618E-07	3	1629,84757	0,60	19,98	1
	High	gAEAPGVSEPVk	3	1	1	P73728	N-Term(iTRAQ4plex); K12(iTRAQ4plex)	0,0000	0,442	2	12,1	0	4,605E-07	68	1,65185E-07	2	1428,79424	0,36	19,79	0
	High	vPSVVFk	4	1	1	P73728	N-Term(iTRAQ4plex); K7(iTRAQ4plex)	0,0000	0,456	4	1,5	0	0,0003692	58	1,51705E-06	2	1063,67595	0,50	30,93	0
	High	lLPDGNGEFTR	2	1	1	P73728	N-Term(iTRAQ4plex)	0,0000	0,490	2	0,8	0	0,00001404	50	9,16157E-06	2	1362,71428	0,61	31,86	0
	High	ySMFVNDGk	1	1	1	P73728	N-Term(iTRAQ4plex); K9(iTRAQ4plex)	0,0000	0,535	1		0	0,000004054	47	1,90095E-05	2	1348,68230	0,98	29,47	0
	High	kVVLFSLPGAFTPTcSSNHLPR	3	1	1	P73728	N-Term(iTRAQ4plex); K1(iTRAQ4plex); C15(Carbamidomethyl)	0,0000	0,239	2	15,2	0	1,681E-12	46	2,4042E-05	4	2716,48574	0,35	42,23	1
	High	vVLFSLPGAFTPTcSSNHLPR	3	1	1	P73728	N-Term(iTRAQ4plex); C14(Carbamidomethyl)	0,0000	0,213	3	24,0	0	7,874E-07	45	3,41956E-05	3	2444,28751	-0,10	47,22	0
	High	ySmFVNDGk	4	1	1	P73728	N-Term(iTRAQ4plex); M3(Oxidation); K9(iTRAQ4plex)	0,0000	0,377	3	1,9	0	0,000009194	43	5,1996E-05	2	1364,67607	0,13	24,12	0
	High	sNLGFGMR	3	1	1	P73728	N-Term(iTRAQ4plex)	0,0000	0,731	3	14,5	0	0,0005774	43	5,43213E-05	2	1025,53252	0,59	25,70	0
	High	kMGMLVEk	2	1	1	P73728	N-Term(iTRAQ4plex); K1(iTRAQ4plex); K8(iTRAQ4plex)	0,0000	0,901	2	15,0	0	0,000002974	38	0,000144867	3	1367,81559	2,97	22,87	1
	High	wEDkTTEQIFGGk	5	1	1	P73728	N-Term(iTRAQ4plex); K4(iTRAQ4plex); K13(iTRAQ4plex)	0,0000	0,511	4	3,9	0	4,138E-08	33	0,000464483	3	1971,05875	2,00	33,32	1
	High	ySMFVNDGkIEk	1	1	1	P73728	N-Term(iTRAQ4plex); K9(iTRAQ4plex); K12(iTRAQ4plex)	0,0000	0,855	1		0	0,000001394	31	0,000883019	3	1863,00840	1,99	30,73	1
	High	ySmFVNDGkIEk	3	1	1	P73728	N-Term(iTRAQ4plex); M3(Oxidation); K9(iTRAQ4plex); K12(iTF	0,0000	0,587	3	0,7	0	2,104E-08	30	0,001231382	3	1879,00064	0,56	27,24	1
	High	kmGMLVEk	2	1	1	P73728	N-Term(iTRAQ4plex); K1(iTRAQ4plex); M2(Oxidation); K8(iTRA	0,0000	0,755	2	6,3	0	0,0001175	29	0,001250173	3	1383,80655	0,08	17,14	1
	High	sNLGFGmR	5	1	1	P73728	N-Term(iTRAQ4plex); M7(Oxidation)	0,0000	0,392	3	2,6	0	0,0007485	24	0,003689521	2	1041,52751	0,66	19,85	0
	High	qIGADkVk	2	1	1	P73728	N-Term(iTRAQ4plex); K6(iTRAQ4plex); K8(iTRAQ4plex)	0,0000	0,740	2	3,0	0	0,001512	22	0,006352871	3	1290,81369	2,41	13,83	1
	High	kmGmLVEk	4	1	1	P73728	N-Term(iTRAQ4plex); K1(iTRAQ4plex); M2(Oxidation); M4(Oxi	0,0000	0,439	3	7,6	0	0,0009306	19	0,011245273	3	1399,80045	-0,65	12,96	1
	High	wEDkTTEQIFGGkk	1	1	1	P73728	N-Term(iTRAQ4plex); K4(iTRAQ4plex); K13(iTRAQ4plex); K14(	0,0000	0,796	1		0	0,00002193	19	0,012330197	4	2243,26015	3,70	29,91	2
	High	mGmLVEk	1	1	1	P73728	N-Term(iTRAQ4plex); M3(Oxidation); K7(iTRAQ4plex)	0,0000	0,415	1		0,001	0,003551	18	0,016480486	2	1111,61089	1,33	22,00	0
	High	mGmLVEk	2	1	1	P73728	N-Term(iTRAQ4plex); M1(Oxidation); M3(Oxidation); K7(iTRAC	0,0000	0,661	2	24,4	0,001	0,009181	11	0,079793957	2	1127,60283	-1,33	15,82	0
	Medium	qIGADk	1	1	1	P73728	N-Term(iTRAQ4plex); K6(iTRAQ4plex)	0,0000	0,618	1		0,013	0,1777	18	0,017296969	2	919,54332	-1,96	13,88	0
P73452	Nitrate transport protein NrtA OS=Synechocystis sp. (strain PCC 6803 / Kazusa) GN=nrtA PE=1 SV=1	1337,54	35,87	1	17	18	52	1,134	44	23,7	446	48,9	5,34							
	A3	Sequence	# PSMs	# Proteins	# Protein Groups	Protein Group Accessions	Modifications	ΔCn	116/117	116/117 Count	116/117 Variability [%]	q-Value	PEP	IonScore	Exp Value	Charge	MH+ [Da]	ΔM [ppm]	RT [min]	# Missed Cleavages
	High	eAAQALEVPADQIPSSPSR	2	1	1	P73452	N-Term(iTRAQ4plex)	0,0000	1,158	2	37,8	0	1,008E-09	95	3,23571E-10	2	2110,09282	1,44	32,78	0
	High	lNVNGQGIQLGNNYk	4	1	1	P73452	N-Term(iTRAQ4plex); K15(iTRAQ4plex)	0,0000	0,947	4	20,7	0	1,275E-11	85	3,15479E-09	2	1920,05803	2,01	33,43	0
	High	vAMTFPGGTHDmWIR	5	1	1	P73452	N-Term(iTRAQ4plex); M12(Oxidation)	0,0000	1,634	5	24,7	0	1,017E-09	73	5,25981E-08	3	1878,91312	1,01	36,96	0
	High	sHDQWFLTENIR	8	1	1	P73452	N-Term(iTRAQ4plex)	0,0000	1,099	7	9,8	0	5,626E-08	73	5,41963E-08	3	1689,84844	1,10	34,93	0
	High	vAMTFPGGTHDMWIR	3	1	1	P73452	N-Term(iTRAQ4plex)	0,0000	2,048	3	3,8	0	6,943E-09	71	7,92447E-08	3	1862,91849	1,17	40,62	0
	High	yWVDNASYPYk	2	1	1	P73452	N-Term(iTRAQ4plex); K11(iTRAQ4plex)	0,0000	0,933	2	22,6	0	2,421E-08	62	6,29463E-07	2	1693,84709	0,39	34,16	0
	High	vGTDAAPLk	2	1	1	P73452	N-Term(iTRAQ4plex); K9(iTRAQ4plex)	0,0000	0,874	1		0	0,00003329	54	3,83681E-06	2	1159,69231	-0,20	20,51	0
	High	vAmTFPGGTHDmWIR	4	1	1	P73452	N-Term(iTRAQ4plex); M3(Oxidation); M12(Oxidation)	0,0000	0,803	4	17,2	0	5,815E-08	47	1,96322E-05	3	1894,90775	0,85	31,34	0
	High	lGFIALTDAAPLIIAk	4	1	1	P73452	N-Term(iTRAQ4plex); K16(iTRAQ4plex)	0,0000	1,026	3	20,1	0	4,86E-10	44	3,56427E-05	3	1915,19034	1,68	56,01	0
	High	vGTDAAPLkEAFAk	2	1	1	P73452	N-Term(iTRAQ4plex); K9(iTRAQ4plex); K14(iTRAQ4plex)	0,0000	1,327	2	14,3	0	1,054E-07	38	0,000159577	3	1850,07859	2,03	31,67	1
	High	wGYLPASTDTk	1	1	1	P73452	N-Term(iTRAQ4plex); K11(iTRAQ4plex)	0,0000	1,003	1		0	0,00003224	37	0,000189221	2	1526,81096	1,06	31,59	0
	High	vPFEDIIDR	2	1	1	P73452	N-Term(iTRAQ4plex)	0,0000	1,193	2	8,6	0	0,0002641	34	0,000395339	2	1247,67558	0,25	41,69	0
	High	vNREDLWR	4	2	2	Q55460;P73452	N-Term(iTRAQ4plex)	0,0000				0	0,002106	20	0,010279453	2	1231,66765	0,99	22,83	1
	High	ekGFYAk	2	1	1	P73452	N-Term(iTRAQ4plex); K2(iTRAQ4plex); K7(iTRAQ4plex)	0,0000	1,157	2	2,0	0,001	0,01043	19	0,015176836	3	1274,74573	-0,92	15,59	1
	High	gFYAk	2	1	1	P73452	N-Term(iTRAQ4plex); K5(iTRAQ4plex)	0,0000	1,096	2	0,2	0,002	0,02824	32	0,000682292	2	873,50707	-0,23	17,86	0
	Medium	qASWGTTR	1	1	1	P73452	N-Term(iTRAQ4plex)	0,0000	0,634	1		0,014	0,1826	12	0,059016032	2	1050,54546	0,53	14,84	0
	Medium	aFGMR	1	1	1	P73452	N-Term(iTRAQ4plex)	0,0000	1,316	1		0,02	0,274	10	0,099076351	2	725,38988	1,85	18,04	0
	Medium	vTDPk	1	1	1	P73452	N-Term(iTRAQ4plex); K5(iTRAQ4plex)	0,0000				0,041	0,5383	19	0,012223323	2	847,51085	-2,25	13,86	0
	Medium	eAFAk	1	1	1	P73452	N-Term(iTRAQ4plex); K5(iTRAQ4plex)	0,0000	1,668	1		0,042	0,547	19	0,013994907	2	853,50109	-1,30	15,10	0
	Medium	rEWFk	1	1	1	P73452	N-Term(iTRAQ4plex); K5(iTRAQ4plex)	0,0000	1,329	1		0,042	0,5466	11	0,079284684	2	1053,60991	1,44	18,89	1
P74551	Allophycocyanin subunit beta-18 OS=Synechocystis sp. (strain PCC 6803 / Kazusa) GN=apcF PE=3 S\	1303,01	63,91	1	14	15	58	0,715	46	28,1	169	18,9	5,07							
	A3	Sequence	# PSMs	# Proteins	# Protein Groups	Protein Group Accessions	Modifications	ΔCn	116/117	116/117 Count	116/117 Variability [%]	q-Value	PEP	IonScore	Exp Value	Charge	MH+ [Da]	ΔM [ppm]	RT [min]	# Missed Cleavages
	High	iAAAAmINANSATIVk	7	1	1	P74551	N-Term(iTRAQ4plex); M6(Oxidation); K16(iTRAQ4plex)	0,0000	0,704	5	10,8	0	4,127E-10	100	9,48353E-11	2	1863,06304	0,95	32,59	0
	High	eTYNSLGVPIGPTVR	4	1	1	P74551	N-Term(iTRAQ4plex)	0,0000	0,649	4	11,8	0	8,742E-12	98	1,77407E-10	2	1746,95220	0,84	35,19	0
	High	dAVTTLIk	1	1	1	P74551	N-Term(iTRAQ4plex); K8(iTRAQ4plex)	0,0000	0,767	1		0	0,00000536	64	4,37492E-07	2	1148,71196	-0,84	28,37	0
	High	yASYALIAADNNVLDER	1	1	1	P74551	N-Term(iTRAQ4plex)	0,0000	0,874	1		0	2,085E-11	61	7,51571E-07	3	2042,03171	0,27	43,48	0
	High	iAAAAMINANSATIVk	2	1	1	P74551	N-Term(iTRAQ4plex); K16(iTRAQ4plex)	0,0000	1,677	2	24,4	0	0,000000625	55	2,91723E-06	2	1847,06925	1,57	37,50	0
	High	rFSAcLR	3	1	1	P74551	N-Term(iTRAQ4plex); C5(Carbamidomethyl)	0,0000	1,271	3	42,8	0	0,00002884	50	9,68211E-06	3	1053,57474	0,29	17,09	1
	High	aYFESGSAR	3	1	1	P74551	N-Term(iTRAQ4plex)	0,0000	0,645	3	4,9	0	6,907E-07	49	1,17482E-05	2	1131,55522	0,08	19,15	0
	High	iAAAAmINANSATIVkR	1	1	1	P74551	N-Term(iTRAQ4plex); M6(Oxidation); K16(iTRAQ4plex)	0,0000	0,597	1		0	0,000006378	42	5,87449E-05	3	2019,16587	1,72	29,02	1
	High	nYDLTGR	4	1	1	P74551	N-Term(iTRAQ4plex)	0,0000	0,636	4	9,3	0	0,0002048	42	6,29463E-05	2	982,50743	-0,01	17,44	0
	High	mRDAVTTLIk	2	1	1	P74551	N-Term(iTRAQ4plex); M1(Oxidation); K10(iTRAQ4plex)	0,0000	1,157	2	21,7	0	0,001666	40	9,1405E-05	2	1451,84990	0,30	22,02	1
	High	mRDAVTTLIk	1	1	1	P74551	N-Term(iTRAQ4plex); K10(iTRAQ4plex)	0,0000	1,044	1		0	0,000001401	35	0,000353973	3	1435,85831	2,62	29,42	1
	High	rDAVTTLIk	2	1	1	P74551	N-Term(iTRAQ4plex); K9(iTRAQ4plex)	0,0000	0,402	1		0	0,00002605	30	0,00093319	3	1304,81403	-0,01	23,14	1
	High	gIQIMk	6	1	1	P74551	N-Term(iTRAQ4plex); K6(iTRAQ4plex)	0,0000	1,066	4	6,6	0	0,002436	28	0,001578299	2	977,60509	-0,55	24,25	0
	High	gIQImk	8	1	1	P74551	N-Term(iTRAQ4plex); M5(Oxidation); K6(iTRAQ4plex)	0,0000	0,682	6	26,6	0	0,001825	23	0,005247712	2	993,59978	-0,77	20,93	0
	High	dmDYYLR	4	2	2	P74551;Q01951	N-Term(iTRAQ4plex); M2(Oxidation)	0,0000				0	0,001955	12	0,059016032	2	1135,52141	0,32	22,09	0
	High	dMDYYLR	1	2	2	P74551;Q01951	N-Term(iTRAQ4plex)	0,0000				0,001	0,003346	49	1,1587E-05	2	1119,52886	2,43	31,04	0
	High	vLQGLR	4	1	1	P74551	N-Term(iTRAQ4plex)	0,0000	0,737	4	2,7	0,002	0,01835	34	0,000385452	2	829,53771	0,09	23,08	0
	High	fSAcLR	3	1	1	P74551	N-Term(iTRAQ4plex); C4(Carbamidomethyl)	0,0000	0,594	3	4,3	0,005	0,06521	19	0,012077304	2	897,47307	-0,28	22,66	0
	Medium	eLSELSV	1	1	1	P74551	N-Term(iTRAQ4plex)	0,0000	0,644	1		0,013	0,1678	32	0,000601132	2	920,50585	0,13	35,54	0
P72871	S-adenosylmethionine synthase OS=Synechocystis sp. (strain PCC 6803 / Kazusa) GN=metK PE=3 S\	1281,87	38,50	1	18	18	59	0,458	51	25,9	426	45,8	5,29							
	A3	Sequence	# PSMs	# Proteins	# Protein Groups	Protein Group Accessions	Modifications	ΔCn	116/117	116/117 Count	116/117 Variability [%]	q-Value	PEP	IonScore	Exp Value	Charge	MH+ [Da]	ΔM [ppm]	RT [min]	# Missed Cleavages
	High	fVVGGPQGDAGLTGR	5	1	1	P72871	N-Term(iTRAQ4plex)	0,0000	0,462	4	27,6	0	2,209E-10	96	2,4042E-10	2	1574,84258	1,13	31,20	0
	High	aAFASSPQAVAV	1	1	1	P72871	N-Term(iTRAQ4plex)	0,0000	0,318	1		0	0,000001435	78	1,75376E-08	2	1262,68743	0,99	33,11	0
	High	fYQDVAAYGHFGR	9	1	1	P72871	N-Term(iTRAQ4plex)	0,0000	0,403	7	10,7	0	1,821E-10	77	2,05102E-08	2	1674,81682	1,36	36,46	0
	High	kIIVDTYGGYSR	4	1	1	P72871	N-Term(iTRAQ4plex); K1(iTRAQ4plex)	0,0000	0,512	4	11,5	0	7,094E-07	55	3,71697E-06	3	1659,93235	0,90	25,32	1
	High	sGQLAYLRPDGk	3	1	1	P72871	N-Term(iTRAQ4plex); K12(iTRAQ4plex)	0,0000	0,432	3	8,3	0	6,627E-07	54	3,8104E-06	2	1592,90129	0,87	23,58	0
	High	fIVNPTGk	6	1	1	P72871	N-Term(iTRAQ4plex); K8(iTRAQ4plex)	0,0000	0,409	3	5,7	0	0,0000221	48	1,72584E-05	2	1163,70439	1,46	27,81	0
	High	kSGQLAYLRPDGk	6	1	1	P72871	N-Term(iTRAQ4plex); K1(iTRAQ4plex); K13(iTRAQ4plex)	0,0000	0,459	5	4,2	0	6,413E-12	45	3,19132E-05	3	1865,09854	0,86	19,98	1
	High	yLFTSESVTEGHPDk	2	1	1	P72871	N-Term(iTRAQ4plex); K15(iTRAQ4plex)	0,0000	0,323	1		0	9,892E-10	45	3,19132E-05	3	1998,00638	0,25	30,25	0
	High	iIVDTYGGYSR	2	1	1	P72871	N-Term(iTRAQ4plex)	0,0000	0,393	2	13,7	0	0,0004018	39	0,000117752	2	1387,73589	1,49	30,78	0
	High	aDLWDVVVGHcFSDIALkPTDk	2	1	1	P72871	N-Term(iTRAQ4plex); C11(Carbamidomethyl); K18(iTRAQ4plex	0,0000	0,385	2	31,5	0	3,602E-07	38	0,000166725	4	2918,54970	1,70	50,25	0
	High	hGGGAFSGk	4	1	1	P72871	N-Term(iTRAQ4plex); K9(iTRAQ4plex)	0,0000	0,808	4	33,8	0	0,001009	37	0,000190095	2	1105,59856	-0,67	11,63	0
	High	sAAYAAR	3	1	1	P72871	N-Term(iTRAQ4plex)	0,0000	0,385	3	16,7	0	0,0001822	37	0,000201823	2	853,46544	0,69	14,34	0
	High	nIVAAGLADk	1	1	1	P72871	N-Term(iTRAQ4plex); K10(iTRAQ4plex)	0,0000	0,567	1		0	0,0001168	23	0,004808061	3	1259,75653	0,26	28,14	0
	High	hALSDDELDk	2	1	1	P72871	N-Term(iTRAQ4plex); K10(iTRAQ4plex)	0,0000	0,478	2	57,4	0	0,00002342	20	0,009225077	3	1430,73474	-1,29	18,11	0
	High	hGGGAFSGkDPTk	4	1	1	P72871	N-Term(iTRAQ4plex); K9(iTRAQ4plex); K13(iTRAQ4plex)	0,0000	0,453	4	8,1	0	0,00003519	16	0,026253626	4	1690,92751	2,24	12,52	1
	High	hGGGAFSGkDPTkVDR	2	1	1	P72871	N-Term(iTRAQ4plex); K9(iTRAQ4plex); K13(iTRAQ4plex)	0,0000	0,682	2	0,7	0	0,001977	11	0,072438593	5	2061,12043	0,10	14,01	2
	High	vDEEk	1	1	1	P72871	N-Term(iTRAQ4plex); K5(iTRAQ4plex)	0,0000	0,820	1		0,006	0,06827	17	0,01827974	2	907,49626	-1,37	11,77	0
	Medium	nLPAER	2	1	1	P72871	N-Term(iTRAQ4plex)	0,0000	0,478	2	4,7	0,011	0,1517	23	0,005247712	2	843,48082	0,36	13,04	0
P74008	Adenosylhomocysteinase OS=Synechocystis sp. (strain PCC 6803 / Kazusa) GN=ahcY PE=3 SV=1 - [I	1268,93	49,88	1	20	20	51	2,547	40	27,8	425	46,2	5,77							
	A3	Sequence	# PSMs	# Proteins	# Protein Groups	Protein Group Accessions	Modifications	ΔCn	116/117	116/117 Count	116/117 Variability [%]	q-Value	PEP	IonScore	Exp Value	Charge	MH+ [Da]	ΔM [ppm]	RT [min]	# Missed Cleavages
	High	qHQLSDIIGTTEETTTGIVR	4	1	1	P74008	N-Term(iTRAQ4plex)	0,0000	1,248	3	7,2	0	1,103E-16	128	1,46218E-13	3	2343,23105	1,60	41,48	0
	High	yGTGQSTLDGIIR	3	1	1	P74008	N-Term(iTRAQ4plex)	0,0000	2,669	3	22,4	0	1,727E-10	86	2,53623E-09	2	1524,81560	1,12	34,10	0
	High	aTNILLAGk	8	1	1	P74008	N-Term(iTRAQ4plex); K9(iTRAQ4plex)	0,0000	2,721	5	5,6	0	0,000004611	81	8,22186E-09	2	1188,75615	0,58	28,45	0
	High	amFNDGVLTFPAmNVNDADTk	1	1	1	P74008	N-Term(iTRAQ4plex); M2(Oxidation); M13(Oxidation); K21(iTR	0,0000	1,816	1		0	3,079E-10	62	6,15134E-07	3	2591,23935	1,25	39,57	0
	High	nkGQLEPGmHSIPVEVDQEIAR	2	1	1	P74008	N-Term(iTRAQ4plex); K2(iTRAQ4plex); M9(Oxidation)	0,0000	2,903	1		0	8,192E-11	59	1,16405E-06	4	2751,43764	1,36	30,02	1
	High	gQLEPGmHSIPVEVDQEIAR	2	1	1	P74008	N-Term(iTRAQ4plex); M7(Oxidation)	0,0000	2,340	1		0	1,228E-08	57	1,79048E-06	3	2365,19474	0,34	32,85	0
	High	tIVVAGYGWcGk	4	1	1	P74008	N-Term(iTRAQ4plex); C10(Carbamidomethyl); K12(iTRAQ4plex	0,0000	2,684	4	25,0	0	8,965E-08	56	2,74138E-06	2	1598,86186	0,95	35,72	0
	High	fAQEkPFAGIR	3	1	1	P74008	N-Term(iTRAQ4plex); K5(iTRAQ4plex)	0,0000	3,024	3	9,6	0	0,00002252	46	3,11302E-05	2	1551,88945	0,53	28,16	0
	High	dISLAPQGR	1	1	1	P74008	N-Term(iTRAQ4plex)	0,0000	2,378	1		0	0,00007603	37	0,000200433	2	1100,61821	0,12	21,37	0
	High	gEDNETYHR	2	1	1	P74008	N-Term(iTRAQ4plex)	0,0000	1,168	2	36,2	0	0,0003511	32	0,000703024	3	1264,56781	0,26	9,58	0
	High	hVIRPEHFAVmk	2	1	1	P74008	N-Term(iTRAQ4plex); M11(Oxidation); K12(iTRAQ4plex)	0,0000	2,456	2	15,6	0	0,00002075	31	0,000767308	4	1767,99453	0,80	19,16	0
	High	yDIkDISLAPQGR	1	1	1	P74008	N-Term(iTRAQ4plex); K4(iTRAQ4plex)	0,0000	1,803	1		0	0,0004662	27	0,002512935	3	1763,99045	0,56	32,94	1
	High	sIIVIGEGR	4	1	1	P74008	N-Term(iTRAQ4plex)	0,0000	2,729	2	12,3	0	0,000273	25	0,003213439	2	1087,65972	0,45	28,89	0
	High	hVQIALDHRPNIIIDDGSDVVATL'	3	1	1	P74008	N-Term(iTRAQ4plex)	0,0000	2,862	2	5,1	0	2,792E-09	24	0,004240262	5	3267,76014	1,04	48,23	0
	High	hFYDNR	3	1	1	P74008	N-Term(iTRAQ4plex)	0,0000	1,630	3	15,6	0	0,002121	24	0,004344802	3	995,48215	0,60	13,60	0
	High	sLkEQAk	4	1	1	P74008	N-Term(iTRAQ4plex); K3(iTRAQ4plex); K7(iTRAQ4plex)	0,0000	2,456	2	2,2	0,001	0,01334	11	0,074984242	3	1235,76642	-1,58	10,14	1
	High	qkYDIk	1	1	1	P74008	N-Term(iTRAQ4plex); K2(iTRAQ4plex); K6(iTRAQ4plex)	0,0000	3,112	1		0,002	0,02369	24	0,003706551	2	1226,74590	-0,81	14,31	1
	High	iEWAAR	1	1	1	P74008	N-Term(iTRAQ4plex)	0,0000	3,424	1		0,007	0,09205	30	0,001104079	2	889,50139	0,15	25,22	0
	Medium	emPVLk	1	1	1	P74008	N-Term(iTRAQ4plex); M2(Oxidation); K6(iTRAQ4plex)	0,0000	1,832	1		0,021	0,2926	15	0,032914911	2	1020,59899	-1,20	15,97	0
	Medium	vATPVk	1	1	1	P74008	N-Term(iTRAQ4plex); K6(iTRAQ4plex)	0,0000	6,275	1		0,024	0,3258	20	0,010739152	2	902,58953	-2,02	15,47	0
P73320	50S ribosomal protein L3 OS=Synechocystis sp. (strain PCC 6803 / Kazusa) GN=rplC PE=3 SV=1 - [R	1263,92	47,89	1	11	11	55	2,033	36	22,0	213	22,7	10,13							
	A3	Sequence	# PSMs	# Proteins	# Protein Groups	Protein Group Accessions	Modifications	ΔCn	116/117	116/117 Count	116/117 Variability [%]	q-Value	PEP	IonScore	Exp Value	Charge	MH+ [Da]	ΔM [ppm]	RT [min]	# Missed Cleavages
	High	mAGQYGASQVTVR	6	1	1	P73320	N-Term(iTRAQ4plex); M1(Oxidation)	0,0000	2,080	4	9,8	0	9,664E-11	94	3,95339E-10	2	1527,77263	1,30	20,08	0
	High	mAGQYGASQVTVR	3	1	1	P73320	N-Term(iTRAQ4plex)	0,0000	2,835	3	58,4	0	2,347E-09	74	3,99917E-08	2	1511,77873	1,98	23,45	0
	High	rmAGQYGASQVTVR	2	1	1	P73320	N-Term(iTRAQ4plex); M2(Oxidation)	0,0000	1,137	1		0	7,328E-08	66	2,36032E-07	3	1683,87394	1,29	15,40	1
	High	lPGSTGAGTTPGR	6	1	1	P73320	N-Term(iTRAQ4plex)	0,0000	1,133	4	123,7	0	0,0002989	58	1,57387E-06	2	1315,70952	0,62	18,72	0
	High	tQDTDGYNAIQVGFLPVk	2	1	1	P73320	N-Term(iTRAQ4plex); K18(iTRAQ4plex)	0,0000	2,230	1		0	2,39E-09	58	1,97105E-06	3	2254,20151	2,52	43,44	0
	High	gFAGYQk	10	1	1	P73320	N-Term(iTRAQ4plex); K7(iTRAQ4plex)	0,0000	2,267	6	10,9	0	0,0001162	49	1,21331E-05	2	1058,59026	2,78	19,43	0
	High	sIGILGTk	8	1	1	P73320	N-Term(iTRAQ4plex); K8(iTRAQ4plex)	0,0000	1,766	4	2,8	0	0,00003601	46	2,76675E-05	2	1076,69475	2,74	27,55	0
	High	gALPGkPGTLLNITPAk	7	1	1	P73320	N-Term(iTRAQ4plex); K6(iTRAQ4plex); K17(iTRAQ4plex)	0,0000	2,033	4	14,8	0	0,000000551	43	5,25981E-05	3	2080,28940	1,88	34,55	0
	High	aLSkPELGHLk	4	1	1	P73320	N-Term(iTRAQ4plex); K4(iTRAQ4plex); K11(iTRAQ4plex)	0,0000	1,981	4	9,0	0	0,00002584	26	0,002624037	3	1625,01908	4,92	23,62	0
	High	aLSkPELGHLkk	1	1	1	P73320	N-Term(iTRAQ4plex); K4(iTRAQ4plex); K11(iTRAQ4plex); K12(	0,0000	2,316	1		0,002	0,02112	15	0,034353422	5	1897,22471	8,75	21,90	1
	High	nLLIIk	5	1	1	P73320	N-Term(iTRAQ4plex); K6(iTRAQ4plex)	0,0000	0,977	3	91,8	0,007	0,0893	41	8,64908E-05	2	1001,70311	6,95	32,13	0
	High	kSNTDPmR	1	1	1	P73320	N-Term(iTRAQ4plex); K1(iTRAQ4plex); M7(Oxidation)	0,0000	1,143	1		0,008	0,1103	11	0,072940713	3	1252,65644	0,48	7,52	1
P73204	Phycobilisome 32.1 kDa linker polypeptide, phycocyanin-associated, rod 2 OS=Synechocystis sp. (stra	1253,64	55,68	1	16	16	56	1,421	48	23,7	273	30,8	9,39							
	A3	Sequence	# PSMs	# Proteins	# Protein Groups	Protein Group Accessions	Modifications	ΔCn	116/117	116/117 Count	116/117 Variability [%]	q-Value	PEP	IonScore	Exp Value	Charge	MH+ [Da]	ΔM [ppm]	RT [min]	# Missed Cleavages
	High	nTASPVYAGSTAESLR	5	1	1	P73204	N-Term(iTRAQ4plex)	0,0000	1,383	4	14,1	0	3,68E-08	86	2,26449E-09	2	1767,89971	0,16	25,78	0
	High	gkAEYLVSYDNLSAk	5	1	1	P73204	N-Term(iTRAQ4plex); K2(iTRAQ4plex); K15(iTRAQ4plex)	0,0000	1,658	3	10,8	0	2,514E-10	70	1,27555E-07	3	2090,14963	0,10	31,89	1
	High	lQVIQGAAPGR	6	1	1	P73204	N-Term(iTRAQ4plex)	0,0000	1,470	4	9,7	0	7,442E-07	61	8,689E-07	2	1253,74455	-0,11	26,49	0
	High	lTSAESLLR	3	1	1	P73204	N-Term(iTRAQ4plex)	0,0000	1,559	3	0,6	0	7,904E-07	59	1,35822E-06	2	1133,66509	0,35	33,74	0
	High	aVALSEVYR	4	1	1	P73204	N-Term(iTRAQ4plex)	0,0000	1,317	3	7,9	0	0,0001325	54	4,12069E-06	2	1151,65410	-0,02	29,66	0
	High	fIELNYk	4	1	1	P73204	N-Term(iTRAQ4plex); K7(iTRAQ4plex)	0,0000	1,649	4	3,6	0	0,0001765	47	1,91854E-05	2	1214,70305	0,58	34,80	0
	High	tSLVSAQR	3	1	1	P73204	N-Term(iTRAQ4plex)	0,0000	1,107	3	30,2	0	0,0002243	46	2,47155E-05	2	1005,58025	-0,70	15,20	0
	High	qVLGNDHLmSQER	5	1	1	P73204	N-Term(iTRAQ4plex); M9(Oxidation)	0,0000	1,190	3	0,1	0	0,000005506	43	5,53312E-05	3	1686,83475	-0,18	17,72	0
	High	lGIVAVDEAIPLELR	3	1	1	P73204	N-Term(iTRAQ4plex)	0,0000	1,007	3	0,9	0	0,000007873	41	7,39554E-05	3	1752,03879	-0,03	51,67	0
	High	sFQVYR	3	1	1	P73204	N-Term(iTRAQ4plex)	0,0000	1,518	3	4,9	0	0,001465	37	0,000220785	2	943,51164	-0,16	21,43	0
	High	fFHSNPQNR	4	1	1	P73204	N-Term(iTRAQ4plex)	0,0000	1,446	4	2,3	0	0,001089	26	0,002552525	3	1290,64771	1,31	15,86	0
	High	qGDTVTmISLA	1	1	1	P73204	N-Term(iTRAQ4plex); M7(Oxidation)	0,0000	1,995	1		0,001	0,01558	37	0,000190095	2	1295,66301	-0,29	32,90	0
	High	eISVR	1	1	1	P73204	N-Term(iTRAQ4plex)	0,0000	1,002	1		0,004	0,04812	19	0,014527178	2	747,44878	0,85	16,11	0
	High	lQQINR	4	1	1	P73204	N-Term(iTRAQ4plex)	0,0000	1,042	4	4,7	0,004	0,04817	12	0,05847497	2	915,54869	-0,61	18,02	0
	High	gFATQR	2	1	1	P73204	N-Term(iTRAQ4plex)	0,0000	3,232	2	82,0	0,005	0,06652	11	0,081652597	2	823,45439	0,12	15,04	0
	Medium	tVGFSR	3	1	1	P73204	N-Term(iTRAQ4plex)	0,0000	1,118	3	10,7	0,027	0,3664	15	0,038542808	2	810,45897	-0,09	16,33	0
P72740	Dihydrolipoyl dehydrogenase OS=Synechocystis sp. (strain PCC 6803 / Kazusa) GN=lpdA PE=1 SV=3	1203,22	48,73	1	20	20	45	0,586	40	42,1	474	50,8	5,55							
	A3	Sequence	# PSMs	# Proteins	# Protein Groups	Protein Group Accessions	Modifications	ΔCn	116/117	116/117 Count	116/117 Variability [%]	q-Value	PEP	IonScore	Exp Value	Charge	MH+ [Da]	ΔM [ppm]	RT [min]	# Missed Cleavages
	High	mmLAHAASGQGVVAVENIcGR	2	1	1	P72740	N-Term(iTRAQ4plex); M1(Oxidation); M2(Oxidation); C19(Carb	0,0000	0,352	1		0	2,115E-16	102	6,29463E-11	3	2347,14566	0,77	29,24	0
	High	vSGPQEVTVIGDNETR	1	1	1	P72740	N-Term(iTRAQ4plex)	0,0000	0,359	1		0	1,976E-09	98	1,76999E-10	2	1844,94597	-0,62	28,85	0
	High	mmLAHAASGQGVVAVENIcGR	2	1	1	P72740	N-Term(iTRAQ4plex); M2(Oxidation); C19(Carbamidomethyl)	0,0000	0,671	2	12,3	0	9,241E-11	86	2,28018E-09	3	2331,15195	1,29	32,59	0
	High	nLGLETVGVETDRR	2	1	1	P72740	N-Term(iTRAQ4plex)	0,0000	0,572	2	5,1	0	0,000002792	82	7,76086E-09	3	1702,92185	0,79	28,29	1
	High	tVFTSDEAVk	1	1	1	P72740	N-Term(iTRAQ4plex); K10(iTRAQ4plex)	0,0000	0,374	1		0	8,162E-07	71	8,85054E-08	2	1384,75932	2,22	25,47	0
	High	nLGLETVGVETDR	1	1	1	P72740	N-Term(iTRAQ4plex)	0,0000	0,600	1		0	2,285E-07	70	9,41825E-08	2	1546,82048	0,71	31,95	0
	High	sRDIETYTGVFATk	2	1	1	P72740	N-Term(iTRAQ4plex); K14(iTRAQ4plex)	0,0000	0,921	2	1,1	0	2,154E-07	64	6,36971E-07	3	1876,01310	4,05	32,02	1
	High	dGkPVPHLWAVGDATGk	3	1	1	P72740	N-Term(iTRAQ4plex); K3(iTRAQ4plex); K17(iTRAQ4plex)	0,0000	0,446	3	31,0	0	5,752E-11	61	8,7694E-07	3	2180,22177	1,33	29,66	0
	High	aIPAAAFTHPEISYVGLTEAQAk	6	1	1	P72740	N-Term(iTRAQ4plex); K23(iTRAQ4plex)	0,0000	0,712	5	34,3	0	1,966E-11	55	2,89734E-06	3	2673,45206	1,10	42,86	0
	High	eAIAAHANDLVSk	4	1	1	P72740	N-Term(iTRAQ4plex); K13(iTRAQ4plex)	0,0000	0,513	2	52,4	0	0,0000006	55	2,97146E-06	3	1626,90781	1,48	23,99	0
	High	aLLAASGR	2	1	1	P72740	N-Term(iTRAQ4plex)	0,0000	0,660	2	19,1	0	0,0006454	51	8,24081E-06	2	902,55443	0,45	18,14	0
	High	tAIIEAk	3	1	1	P72740	N-Term(iTRAQ4plex); K7(iTRAQ4plex)	0,0000	0,426	3	7,9	0	0,0001293	47	1,80289E-05	2	1033,64958	-0,01	19,21	0
	High	iQSDLTNSLTR	1	1	1	P72740	N-Term(iTRAQ4plex)	0,0000	0,386	1		0	0,00003071	47	2,22316E-05	2	1391,76201	0,64	30,98	0
	High	rGFIEVNDQmQVIk	1	1	1	P72740	N-Term(iTRAQ4plex); M10(Oxidation); K14(iTRAQ4plex)	0,0000	0,344	1		0	7,931E-10	46	2,37668E-05	3	1981,08084	1,44	27,95	1
	High	eGFVVSTAk	2	1	1	P72740	N-Term(iTRAQ4plex); K9(iTRAQ4plex)	0,0000	0,346	1		0	0,000008844	46	2,46587E-05	2	1225,70329	0,16	23,21	0
	High	lkVDTIR	4	1	1	P72740	N-Term(iTRAQ4plex); K2(iTRAQ4plex)	0,0000	0,450	4	7,2	0	0,002552	29	0,001361351	2	1132,73198	2,41	19,33	1
	High	kTEVDYR	4	1	1	P72740	N-Term(iTRAQ4plex); K1(iTRAQ4plex)	0,0000	0,731	4	17,9	0	0,002257	20	0,009461718	3	1198,66806	0,86	11,90	1
	High	akEIMLcPGSVPFVPPGIEIDHk	1	1	1	P72740	N-Term(iTRAQ4plex); K2(iTRAQ4plex); C7(Carbamidomethyl);	0,0000	0,903	1		0,001	0,01035	22	0,00654591	4	2966,62807	2,42	44,22	1
	High	eTDGIAk	1	1	1	P72740	N-Term(iTRAQ4plex); K7(iTRAQ4plex)	0,0000	0,750	1		0,003	0,0409	20	0,010255811	2	1021,57469	-2,09	13,84	0
	High	iPATk	1	1	1	P72740	N-Term(iTRAQ4plex); K5(iTRAQ4plex)	0,0000	0,655	1		0,006	0,07371	18	0,015994476	2	817,53630	-2,78	15,03	0
	Medium	gcIPSk	1	1	1	P72740	N-Term(iTRAQ4plex); C2(Carbamidomethyl); K6(iTRAQ4plex)	0,0000	0,721	1		0,043	0,5547	14	0,054617745	2	949,53514	-2,94	15,77	0
P54691	Probable branched-chain-amino-acid aminotransferase OS=Synechocystis sp. (strain PCC 6803 / Kazu	1197,97	56,07	1	19	19	59	0,432	55	19,9	305	33,9	6,33							
	A3	Sequence	# PSMs	# Proteins	# Protein Groups	Protein Group Accessions	Modifications	ΔCn	116/117	116/117 Count	116/117 Variability [%]	q-Value	PEP	IonScore	Exp Value	Charge	MH+ [Da]	ΔM [ppm]	RT [min]	# Missed Cleavages
	High	iSAAYITSALAk	8	1	1	P54691	N-Term(iTRAQ4plex); K12(iTRAQ4plex)	0,0000	0,410	7	20,2	0	4,494E-07	81	8,49122E-09	2	1496,89385	0,77	41,20	0
	High	tEAVESGFDEAILmNSQGk	1	1	1	P54691	N-Term(iTRAQ4plex); M14(Oxidation); K19(iTRAQ4plex)	0,0000	0,144	1		0	6,988E-11	73	5,2236E-08	3	2330,14414	0,70	37,74	0
	High	fLHYDISAEk	6	1	1	P54691	N-Term(iTRAQ4plex); K10(iTRAQ4plex)	0,0000	0,503	6	22,9	0	2,319E-09	72	6,02518E-08	3	1510,81536	0,61	30,06	0
	High	vcEATGMNVFMVR	1	1	1	P54691	N-Term(iTRAQ4plex); C2(Carbamidomethyl)	0,0000	0,717	1		0	1,325E-08	64	3,66412E-07	2	1657,79778	-0,23	39,65	0
	High	sFYIRPLVYSSGLGIAPR	4	1	1	P54691	N-Term(iTRAQ4plex)	0,0000	0,440	3	5,8	0	8,817E-11	57	2,39548E-06	3	2140,20591	1,08	44,39	0
	High	vcEATGmNVFmVR	2	1	1	P54691	N-Term(iTRAQ4plex); C2(Carbamidomethyl); M7(Oxidation); M	0,0000	0,454	2	171,0	0	1,88E-09	54	3,60554E-06	2	1689,78923	0,74	30,25	0
	High	sVLTAVTENREPk	5	1	1	P54691	N-Term(iTRAQ4plex); K13(iTRAQ4plex)	0,0000	0,503	4	2,5	0	0,000000895	53	5,33298E-06	3	1731,98673	1,36	25,77	1
	High	sVLTAVTENR	2	1	1	P54691	N-Term(iTRAQ4plex)	0,0000	0,357	2	4,3	0	0,000001042	45	2,87058E-05	2	1233,69292	0,77	27,11	0
	High	ikEVIVDFVk	3	1	1	P54691	N-Term(iTRAQ4plex); K2(iTRAQ4plex); K10(iTRAQ4plex)	0,0000	0,415	3	12,3	0	4,913E-08	44	3,67257E-05	2	1622,02605	0,44	41,94	1
	High	iENFTLGGDRPITEk	2	1	1	P54691	N-Term(iTRAQ4plex); K15(iTRAQ4plex)	0,0000	0,468	2	10,5	0	0,000000103	44	4,06415E-05	3	1978,08762	1,41	33,28	0
	High	gIPDPEDPGTILLFR	5	1	1	P54691	N-Term(iTRAQ4plex)	0,0000	0,411	4	24,1	0	1,319E-07	43	4,83025E-05	3	1783,97165	0,28	48,55	0
	High	rIENFTLGGDRPITEk	3	1	1	P54691	N-Term(iTRAQ4plex); K16(iTRAQ4plex)	0,0000	0,429	3	3,6	0	3,207E-08	37	0,00018534	3	2134,18851	1,20	28,89	1
	High	fLPIAYFEDk	7	1	1	P54691	N-Term(iTRAQ4plex); K10(iTRAQ4plex)	0,0000	0,398	7	12,3	0	0,000223	32	0,000925482	2	1530,84441	-0,18	48,76	0
	High	yQDWVFk	1	1	1	P54691	N-Term(iTRAQ4plex); K7(iTRAQ4plex)	0,0000	0,435	1		0	0,002353	29	0,00123302	3	1273,68271	0,60	36,36	0
	High	eVIVDFVkk	1	1	1	P54691	N-Term(iTRAQ4plex); K8(iTRAQ4plex); K9(iTRAQ4plex)	0,0000	0,482	1		0,001	0,01084	18	0,016068303	3	1508,95163	6,87	31,35	1
	High	lHNLEk	1	1	1	P54691	N-Term(iTRAQ4plex); K6(iTRAQ4plex)	0,0000	0,415	1		0,001	0,01702	18	0,016367035	3	1041,63013	0,59	15,25	0
	High	kNQPDk	2	1	1	P54691	N-Term(iTRAQ4plex); K1(iTRAQ4plex); K6(iTRAQ4plex)	0,0000	0,393	2	2,9	0,001	0,01371	17	0,027487044	3	1161,68973	-4,71	7,69	1
	High	iSSWYR	2	1	1	P54691	N-Term(iTRAQ4plex)	0,0000	0,502	2	20,1	0,008	0,1128	26	0,002716252	2	955,51225	0,48	26,98	0
	Medium	iTPVk	1	1	1	P54691	N-Term(iTRAQ4plex); K5(iTRAQ4plex)	0,0000	0,401	1		0,035	0,4428	13	0,05	2	845,56676	-3,69	17,31	0
	Medium	sFPLR	2	1	1	P54691	N-Term(iTRAQ4plex)	0,0000	0,450	2	15,5	0,036	0,4677	18	0,036577266	2	763,45903	0,94	20,88	0
P73785	Phosphate-binding protein OS=Synechocystis sp. (strain PCC 6803 / Kazusa) GN=pstS PE=3 SV=1 -	1195,31	47,75	1	17	17	48	0,901	39	14,6	333	35,3	9,92							
	A3	Sequence	# PSMs	# Proteins	# Protein Groups	Protein Group Accessions	Modifications	ΔCn	116/117	116/117 Count	116/117 Variability [%]	q-Value	PEP	IonScore	Exp Value	Charge	MH+ [Da]	ΔM [ppm]	RT [min]	# Missed Cleavages
	High	aTGNTVNYNSVGSGAGIR	5	1	1	P73785	N-Term(iTRAQ4plex)	0,0000	0,773	4	6,8	0	2,733E-14	127	2,0416E-13	2	1881,95537	0,95	25,06	0
	High	gVVmVPTAGGAVAVVYNLPGkk	1	1	1	P73785	N-Term(iTRAQ4plex); M4(Oxidation); K21(iTRAQ4plex); K22(iT	0,0000	1,043	1		0	1,315E-13	95	3,22085E-10	4	2575,50258	0,74	40,64	1
	High	qTPGAIGYVQDTFAR	2	1	1	P73785	N-Term(iTRAQ4plex)	0,0000	0,926	2	4,2	0	1,367E-10	91	8,12774E-10	2	1767,91594	0,72	37,95	0
	High	gVVMVPTAGGAVAVVYNLPGkk	1	1	1	P73785	N-Term(iTRAQ4plex); K21(iTRAQ4plex); K22(iTRAQ4plex)	0,0000	1,191	1		0	5,606E-14	85	2,87058E-09	4	2559,50649	0,28	42,55	1
	High	rNEGPNLQTAk	8	1	1	P73785	N-Term(iTRAQ4plex); K11(iTRAQ4plex)	0,0000	0,856	4	8,6	0	2,382E-07	54	4,23051E-06	2	1515,84929	0,71	13,64	1
	High	kATGNTVNYNSVGSGAGIR	1	1	1	P73785	N-Term(iTRAQ4plex); K1(iTRAQ4plex)	0,0000	0,907	1		0	7,445E-10	52	6,79157E-06	3	2154,15378	1,47	21,46	1
	High	wILTTGQNHNTQLEYTk	2	1	1	P73785	N-Term(iTRAQ4plex); K17(iTRAQ4plex)	0,0000	0,948	2	4,5	0	3,514E-08	50	1,02802E-05	3	2335,23044	0,83	33,62	0
	High	tWNQVDSk	1	1	1	P73785	N-Term(iTRAQ4plex); K8(iTRAQ4plex)	0,0000	0,993	1		0	0,0000288	47	2,22828E-05	2	1265,67241	-0,34	18,88	0
	High	vIAAVNk	4	1	1	P73785	N-Term(iTRAQ4plex); K7(iTRAQ4plex)	0,0000	0,921	3	13,9	0	0,0008062	45	3,52347E-05	2	1002,65453	-0,49	20,18	0
	High	iFTGELk	2	1	1	P73785	N-Term(iTRAQ4plex); K7(iTRAQ4plex)	0,0000	0,906	2	3,0	0	0,002574	42	6,87021E-05	2	1095,66741	1,97	30,53	0
	High	iGASQDPNWGFQVLk	1	1	1	P73785	N-Term(iTRAQ4plex); K15(iTRAQ4plex)	0,0000	0,803	1		0	0,000002233	39	0,00011995	3	1948,05375	0,31	42,89	0
	High	yFAEYkk	2	1	1	P73785	N-Term(iTRAQ4plex); K6(iTRAQ4plex); K7(iTRAQ4plex)	0,0000	1,554	2	8,4	0	0,002335	15	0,029306908	3	1380,78797	-0,57	22,93	1
	High	yFAEYk	1	1	1	P73785	N-Term(iTRAQ4plex); K6(iTRAQ4plex)	0,0000	0,971	1		0,001	0,005268	23	0,004570566	2	1108,59148	-0,22	27,11	0
	High	iPPAVAQR	4	1	1	P73785	N-Term(iTRAQ4plex)	0,0000	0,804	4	6,9	0,002	0,0189	26	0,002269708	2	995,61174	-0,13	19,18	0
	High	nANVAk	4	1	1	P73785	N-Term(iTRAQ4plex); K6(iTRAQ4plex)	0,0000	1,195	4	40,2	0,002	0,02207	26	0,002666674	2	904,54521	-0,27	11,87	0
	High	iALGk	4	1	1	P73785	N-Term(iTRAQ4plex); K5(iTRAQ4plex)	0,0000	0,690	2	47,0	0,003	0,0332	31	0,001923827	2	789,54338	-0,36	20,32	0
	Medium	lPNTPIR	3	1	1	P73785	N-Term(iTRAQ4plex)	0,0000	0,788	3	4,1	0,02	0,26	20	0,010665225	2	954,58513	-0,19	23,87	0
	Medium	aISGAk	1	1	1	P73785	N-Term(iTRAQ4plex); K6(iTRAQ4plex)	0,0000	0,822	1		0,036	0,4647	11	0,073955419	2	834,52733	-1,70	12,48	0
P72848	Oxygen-dependent coproporphyrinogen-III oxidase OS=Synechocystis sp. (strain PCC 6803 / Kazusa)	1176,45	40,88	1	14	14	57	0,660	48	21,2	340	38,9	5,82							
	A3	Sequence	# PSMs	# Proteins	# Protein Groups	Protein Group Accessions	Modifications	ΔCn	116/117	116/117 Count	116/117 Variability [%]	q-Value	PEP	IonScore	Exp Value	Charge	MH+ [Da]	ΔM [ppm]	RT [min]	# Missed Cleavages
	High	tESILMSLPPLVR	4	1	1	P72848	N-Term(iTRAQ4plex)	0,0000	0,982	4	15,0	0	0,00003684	66	2,60597E-07	2	1599,93620	6,31	50,26	0
	High	tESILmSLPPLVR	6	1	1	P72848	N-Term(iTRAQ4plex); M6(Oxidation)	0,0000	0,633	6	36,7	0	2,533E-08	64	3,60554E-07	2	1615,92229	0,79	47,27	0
	High	yVEFNLVYDR	1	1	1	P72848	N-Term(iTRAQ4plex)	0,0000	0,633	1		0	0,000001504	61	8,58954E-07	2	1461,75078	0,89	41,28	0
	High	nPYIPTVHLNYR	5	1	1	P72848	N-Term(iTRAQ4plex)	0,0000	0,584	5	1,4	0	1,673E-08	59	1,3365E-06	3	1630,88303	0,50	33,92	0
	High	gTIFGLQTNGR	3	1	1	P72848	N-Term(iTRAQ4plex)	0,0000	0,660	3	0,1	0	1,252E-08	58	1,69422E-06	2	1307,71941	0,42	30,86	0
	High	qRPEAEGHEFYATGTSMVLHPk	6	1	1	P72848	N-Term(iTRAQ4plex); K22(iTRAQ4plex)	0,0000	0,770	4	1,4	0	1,444E-13	54	3,99945E-06	4	2773,40175	1,68	29,41	0

	High	aFLPAYSPIVEk	6	1	1	P72848	N-Term(iTRAQ4plex); K12(iTRAQ4plex)	0,0000	0,622	5	5,2	0	5,637E-08	52	6,09495E-06	3	1622,94138	1,07	40,13	0
	High	qRPEAEGHEFYATGTSmVLHPk	6	1	1	P72848	N-Term(iTRAQ4plex); M17(Oxidation); K22(iTRAQ4plex)	0,0000	0,506	3	8,9	0	6,445E-11	49	1,37079E-05	4	2789,39297	0,34	26,10	0
	High	nAcDQTHGEFYPVFk	3	1	1	P72848	N-Term(iTRAQ4plex); C3(Carbamidomethyl); K15(iTRAQ4plex)	0,0000	0,776	2	38,6	0	4,978E-12	47	2,15263E-05	3	2101,00833	1,50	32,19	0
	High	wcDEYFYLk	2	1	1	P72848	N-Term(iTRAQ4plex); C2(Carbamidomethyl); K9(iTRAQ4plex)	0,0000	0,614	2	7,8	0	5,673E-08	46	2,76675E-05	2	1611,77776	1,34	42,51	0
	High	sLPPSILk	5	1	1	P72848	N-Term(iTRAQ4plex); K8(iTRAQ4plex)	0,0000	0,624	3	9,0	0	0,002028	36	0,000247725	2	1142,73870	-0,05	29,28	0
	High	gkFQEDSWQR	4	1	1	P72848	N-Term(iTRAQ4plex); K2(iTRAQ4plex)	0,0000	0,859	4	2,4	0	0,000001606	32	0,000580764	3	1568,80680	0,50	20,50	1
	High	fQEDSWQR	1	1	1	P72848	N-Term(iTRAQ4plex)	0,0000	0,666	1		0	0,0003419	24	0,00425569	2	1239,58867	0,94	23,96	0
	High	rWcDEYFYLk	1	1	1	P72848	N-Term(iTRAQ4plex); C3(Carbamidomethyl); K10(iTRAQ4plex)	0,0000	0,621	1		0	0,000002817	21	0,008317063	3	1767,87851	1,02	36,87	1
	High	qFQLYR	3	1	1	P72848	N-Term(iTRAQ4plex)	0,0000	0,613	3	1,8	0,001	0,007658	36	0,000254665	2	998,55284	-1,15	25,80	0
	Medium	fLVPQDWVNS	1	1	1	P72848	N-Term(iTRAQ4plex)	0,0000	1,239	1		0,047	0,6015	13	0,046770284	2	1348,70439	1,91	48,24	0
P73922	D-fructose 1,6-bisphosphatase class 2/sedoheptulose 1,7-bisphosphatase OS=Synechocystis sp. (stra	1162,39	35,65	1	16	16	64	1,467	50	24,4	345	37,1	5,21							
	A3	Sequence	# PSMs	# Proteins	# Protein Groups	Protein Group Accessions	Modifications	ΔCn	116/117	116/117 Count	116/117 Variability [%]	q-Value	PEP	IonScore	Exp Value	Charge	MH+ [Da]	ΔM [ppm]	RT [min]	# Missed Cleavages
	High	tQSLVISSQSSTAR	5	1	1	P73922	N-Term(iTRAQ4plex)	0,0000	1,328	4	24,5	0	8,558E-10	98	1,76592E-10	2	1608,86870	0,82	21,92	0
	High	sIEELVVVVMDRPR	8	1	1	P73922	N-Term(iTRAQ4plex)	0,0000	2,789	6	22,8	0	3,239E-11	65	3,04068E-07	3	1786,00138	-0,02	50,27	0
	High	nTADQVAVEAmR	1	1	1	P73922	N-Term(iTRAQ4plex); M11(Oxidation)	0,0000	1,137	1		0	0,000004033	61	7,9794E-07	2	1464,73198	5,87	19,04	0
	High	sIEELVVVVmDRPR	10	1	1	P73922	N-Term(iTRAQ4plex); M10(Oxidation)	0,0000	1,486	5	39,4	0	7,596E-08	60	1,03985E-06	3	1801,99601	-0,18	41,80	0
	High	kLAAPPAAk	5	1	1	P73922	N-Term(iTRAQ4plex); K1(iTRAQ4plex); K9(iTRAQ4plex)	0,0000	1,701	3	6,3	0	0,00001355	56	2,30659E-06	2	1298,85137	-0,53	14,97	1
	High	gGLFAAPDFYmkk	1	1	1	P73922	N-Term(iTRAQ4plex); M11(Oxidation); K12(iTRAQ4plex); K13(	0,0000	1,746	1		0	0,00003955	38	0,00016367	3	1893,03531	2,53	35,23	1
	High	lASMGIk	1	1	1	P73922	N-Term(iTRAQ4plex); K7(iTRAQ4plex)	0,0000	3,277	1		0	0,0001769	37	0,000218364	2	1007,61681	0,61	26,06	0
	High	lASmGIk	3	1	1	P73922	N-Term(iTRAQ4plex); M4(Oxidation); K7(iTRAQ4plex)	0,0000	1,526	3	0,0	0	0,0001633	34	0,000530774	2	1023,61064	-0,46	17,65	0
	High	lAAPPAAk	6	1	1	P73922	N-Term(iTRAQ4plex); K8(iTRAQ4plex)	0,0000	1,593	3	6,7	0	0,00146	34	0,000423614	2	1026,65471	-0,31	19,08	0
	High	hkELIQEIR	3	1	1	P73922	N-Term(iTRAQ4plex); K2(iTRAQ4plex)	0,0000	1,794	3	6,1	0	0,000003326	32	0,000606694	3	1453,87461	1,13	19,18	1
	High	gGLFAAPDFYmk	1	1	1	P73922	N-Term(iTRAQ4plex); M11(Oxidation); K12(iTRAQ4plex)	0,0000	1,359	1		0	0,000001094	31	0,00072272	3	1620,83255	-0,57	40,54	0
	High	fVDTVHmk	5	1	1	P73922	N-Term(iTRAQ4plex); M7(Oxidation); K8(iTRAQ4plex)	0,0000	1,357	4	5,2	0	0,001373	27	0,001999724	3	1280,69107	-0,06	17,73	0
	High	fVDTVHMk	3	1	1	P73922	N-Term(iTRAQ4plex); K8(iTRAQ4plex)	0,0000	1,696	3	2,9	0	0,002476	23	0,004763981	3	1264,69681	0,45	23,97	0
	High	gGLFAAPDFYMk	1	1	1	P73922	N-Term(iTRAQ4plex); K12(iTRAQ4plex)	0,0000	1,018	1		0	0,00002705	22	0,005688136	3	1604,83774	-0,51	44,33	0
	High	gHVDIDk	2	1	1	P73922	N-Term(iTRAQ4plex); K7(iTRAQ4plex)	0,0000	1,238	2	22,2	0	0,002783	21	0,007177447	3	1071,60395	0,22	12,85	0
	High	fVDTVHmkESPk	1	1	1	P73922	N-Term(iTRAQ4plex); M7(Oxidation); K8(iTRAQ4plex); K12(iTF	0,0000	0,721	1		0	0,00196	15	0,037084233	4	1866,01589	0,16	19,38	1
	High	fFHGGVR	4	1	1	P73922	N-Term(iTRAQ4plex)	0,0000	1,403	4	3,3	0,001	0,007103	18	0,015738742	3	963,52869	0,58	18,67	0
	High	iLSDcLNR	2	1	1	P73922	N-Term(iTRAQ4plex); C5(Carbamidomethyl)	0,0000	1,030	2	9,5	0,001	0,005372	17	0,022129419	2	1134,60564	-0,14	26,76	0
	High	sATENLk	1	1	1	P73922	N-Term(iTRAQ4plex); K7(iTRAQ4plex)	0,0000	1,062	1		0,003	0,04338	22	0,006441248	2	1050,60271	-0,63	13,64	0
	Medium	vIQLH	1	1	1	P73922	N-Term(iTRAQ4plex)	0,0000	1,461	1		0,04	0,5216	13	0,053329806	2	753,47399	0,04	20,92	0
Q55664	Fructose-bisphosphate aldolase class 2 OS=Synechocystis sp. (strain PCC 6803 / Kazusa) GN=fbaA P	1141,75	38,44	1	12	12	41	2,099	35	18,0	359	38,9	5,74							
	A3	Sequence	# PSMs	# Proteins	# Protein Groups	Protein Group Accessions	Modifications	ΔCn	116/117	116/117 Count	116/117 Variability [%]	q-Value	PEP	IonScore	Exp Value	Charge	MH+ [Da]	ΔM [ppm]	RT [min]	# Missed Cleavages
	High	tQVDALAVAIGTSHGAYk	7	1	1	Q55664	N-Term(iTRAQ4plex); K18(iTRAQ4plex)	0,0000	2,099	5	14,7	0	1,307E-14	92	8,5489E-10	3	2090,15293	2,12	36,79	0
	High	yQQFWTAGNASk	4	1	1	Q55664	N-Term(iTRAQ4plex); K12(iTRAQ4plex)	0,0000	2,613	4	3,3	0	1,559E-09	86	2,30659E-09	2	1688,86553	1,20	31,66	0
	High	kPTGEVLAISR	5	1	1	Q55664	N-Term(iTRAQ4plex); K1(iTRAQ4plex)	0,0000	2,166	3	6,1	0	3,249E-08	77	2,02754E-08	3	1458,89014	1,27	22,41	0
	High	qLTLDDYAAk	2	1	1	Q55664	N-Term(iTRAQ4plex); K10(iTRAQ4plex)	0,0000	1,244	2	81,1	0	4,752E-08	54	3,6389E-06	2	1425,78423	1,00	28,94	0
	High	lAITAAFR	4	1	1	Q55664	N-Term(iTRAQ4plex)	0,0000	2,203	4	6,5	0	0,00002606	51	8,58954E-06	2	1006,61815	1,52	35,72	0
	High	kVNIDTDNR	6	1	1	Q55664	N-Term(iTRAQ4plex); K1(iTRAQ4plex)	0,0000	2,113	6	7,6	0	0,00001703	50	1,34558E-05	2	1362,75896	0,71	14,33	1
	High	sYAGENFLR	2	1	1	Q55664	N-Term(iTRAQ4plex)	0,0000	1,418	1		0	0,000005894	34	0,000358897	2	1200,61382	0,69	28,91	0
	High	gELTATSR	1	1	1	Q55664	N-Term(iTRAQ4plex)	0,0000	1,014	1		0	0,0002925	25	0,003288289	2	978,53545	1,81	13,36	0
	High	hFLkPSIk	1	1	1	Q55664	N-Term(iTRAQ4plex); K4(iTRAQ4plex); K8(iTRAQ4plex)	0,0000	2,430	1		0	0,002124	19	0,012105145	3	1401,89389	-0,25	19,05	0
	High	hLVLGAVETYPHIPIAmHQDHGN:	2	1	1	Q55664	N-Term(iTRAQ4plex); M17(Oxidation); C28(Carbamidomethyl)	0,0000	1,399	2	52,5	0	4,063E-08	18	0,014353903	5	3815,89216	1,41	35,75	0
	High	iEEIHR	3	1	1	Q55664	N-Term(iTRAQ4plex)	0,0000	2,408	3	10,7	0,001	0,004775	22	0,00595621	2	940,53398	0,75	14,77	0
	High	aLVPmR	4	1	1	Q55664	N-Term(iTRAQ4plex); M5(Oxidation)	0,0000	1,870	3	2,5	0,002	0,02702	13	0,052236011	2	846,49944	0,73	16,20	0
P54205	Ribulose bisphosphate carboxylase large chain OS=Synechocystis sp. (strain PCC 6803 / Kazusa) GN=	1106,75	44,04	1	22	22	57	3,090	50	70,2	470	52,5	6,15							
	A3	Sequence	# PSMs	# Proteins	# Protein Groups	Protein Group Accessions	Modifications	ΔCn	116/117	116/117 Count	116/117 Variability [%]	q-Value	PEP	IonScore	Exp Value	Charge	MH+ [Da]	ΔM [ppm]	RT [min]	# Missed Cleavages
	High	tFQGPPHGITVER	5	1	1	P54205	N-Term(iTRAQ4plex)	0,0000	3,982	3	13,9	0	4,367E-08	72	5,9016E-08	2	1582,84709	0,78	25,00	0
	High	lTYYTPDYTPk	2	1	1	P54205	N-Term(iTRAQ4plex); K11(iTRAQ4plex)	0,0000	3,415	2	5,9	0	2,271E-07	71	8,70903E-08	2	1649,86785	0,82	31,95	0
	High	fLFVQEAIEk	5	1	1	P54205	N-Term(iTRAQ4plex); K10(iTRAQ4plex)	0,0000	3,933	5	36,7	0	8,452E-07	67	1,78226E-07	2	1511,87151	0,18	43,89	0
	High	gHYLNVTAGTcEEmmk	3	1	1	P54205	N-Term(iTRAQ4plex); C11(Carbamidomethyl); M14(Oxidation);	0,0000	2,215	2	78,7	0	6,164E-12	55	3,18398E-06	3	2161,00138	2,17	21,79	0
	High	aMHAVVDR	2	1	1	P54205	N-Term(iTRAQ4plex)	0,0000	0,981	2	0,0	0	0,00008842	51	8,03471E-06	2	1042,55852	0,05	13,07	0
	High	lSGGDHLHSGTVVGk	6	1	1	P54205	N-Term(iTRAQ4plex); K15(iTRAQ4plex)	0,0000	2,642	4	29,9	0	2,067E-10	47	2,05102E-05	3	1751,96427	-0,02	19,23	0
	High	dTDLLAcFR	1	1	1	P54205	N-Term(iTRAQ4plex); C7(Carbamidomethyl)	0,0000	3,983	1		0	0,00000507	38	0,000155228	2	1254,62676	-0,14	38,57	0
	High	vALEAcVQAR	2	1	1	P54205	N-Term(iTRAQ4plex); C6(Carbamidomethyl)	0,0000	2,656	2	1,4	0	0,0001446	36	0,000237121	2	1260,68584	0,56	25,25	0
	High	aVYEcLR	3	1	1	P54205	N-Term(iTRAQ4plex); C5(Carbamidomethyl)	0,0000	3,312	3	42,1	0	0,001209	34	0,000432484	2	1054,54704	-0,16	22,45	0
	High	gITMGFVDLMR	2	1	1	P54205	N-Term(iTRAQ4plex)	0,0000	1,066	2	121,2	0	0,000348	32	0,000606694	2	1383,72453	-0,03	48,51	0
	High	gGLDFTkDDENINSQPFmR	1	1	1	P54205	N-Term(iTRAQ4plex); K7(iTRAQ4plex); M18(Oxidation)	0,0000	4,101	1		0	1,205E-08	31	0,000774408	3	2488,20353	0,71	33,15	1
	High	lEDIRFPVALIk	3	1	1	P54205	N-Term(iTRAQ4plex); K12(iTRAQ4plex)	0,0000	7,194	3	22,6	0	0,000009829	30	0,000928902	3	1702,05393	1,95	46,59	1
	High	wSPELAAAcELWk	1	1	1	P54205	N-Term(iTRAQ4plex); C9(Carbamidomethyl); K13(iTRAQ4plex)	0,0000	0,943	1		0	0,00003	29	0,001227354	3	1848,95560	-0,07	47,19	0
	High	gHYLNVTAGTcEEmmkR	4	1	1	P54205	N-Term(iTRAQ4plex); C11(Carbamidomethyl); M14(Oxidation);	0,0000	3,096	3	12,7	0	0,00000901	28	0,001496132	4	2317,09926	0,63	19,90	1
	High	amHAVVDR	1	1	1	P54205	N-Term(iTRAQ4plex); M2(Oxidation)	0,0000	1,724	1		0	0,002736	24	0,003588971	3	1058,55347	0,09	10,26	0
	High	dRFLFVQEAIEk	2	1	1	P54205	N-Term(iTRAQ4plex); K12(iTRAQ4plex)	0,0000	1,611	2	6,5	0	0,00002801	22	0,005942511	3	1782,99771	-0,89	38,97	1
	High	yGRPLLGcTIkPk	1	1	1	P54205	N-Term(iTRAQ4plex); C8(Carbamidomethyl); K11(iTRAQ4plex)	0,0000	7,157	1		0	0,000001573	18	0,016068303	4	1935,15676	-0,35	25,53	0
	High	dNGILLHIHR	1	1	1	P54205	N-Term(iTRAQ4plex)	0,0000	1,506	1		0	0,002538	17	0,018321879	4	1331,76650	0,02	24,40	0
	High	fPVALIk	4	1	1	P54205	N-Term(iTRAQ4plex); K7(iTRAQ4plex)	0,0000	4,146	4	7,4	0,001	0,006621	36	0,000242086	2	1075,71160	-0,20	37,78	0
	High	aQAETNEmk	2	1	1	P54205	N-Term(iTRAQ4plex); M8(Oxidation); K9(iTRAQ4plex)	0,0000	1,662	2	39,4	0,001	0,006862	20	0,01002236	2	1325,65935	-1,23	10,81	0
	High	gHYLNVTAGTcEEmMk	1	1	1	P54205	N-Term(iTRAQ4plex); C11(Carbamidomethyl); M14(Oxidation);	0,0000	0,918	1		0,001	0,01204	16	0,023225764	4	2145,00258	0,38	25,71	0
	High	nHGIHFR	1	1	1	P54205	N-Term(iTRAQ4plex)	0,0000	0,502	1		0,001	0,0126	10	0,093534107	3	1024,55857	2,76	12,30	0
	Medium	aEFAk	1	1	1	P54205	N-Term(iTRAQ4plex); K5(iTRAQ4plex)	0,0000	1,806	1		0,018	0,2389	16	0,023012829	2	853,49986	-2,73	15,56	0
	Medium	lGLSAk	3	1	1	P54205	N-Term(iTRAQ4plex); K6(iTRAQ4plex)	0,0000	2,620	2	104,8	0,018	0,2317	12	0,104013871	2	876,57390	-2,05	19,85	0
P29256	Photosystem I reaction center subunit III OS=Synechocystis sp. (strain PCC 6803 / Kazusa) GN=psaF	1075,15	56,36	1	11	11	41	0,725	31	22,3	165	18,2	7,37							
	A3	Sequence	# PSMs	# Proteins	# Protein Groups	Protein Group Accessions	Modifications	ΔCn	116/117	116/117 Count	116/117 Variability [%]	q-Value	PEP	IonScore	Exp Value	Charge	MH+ [Da]	ΔM [ppm]	RT [min]	# Missed Cleavages
	High	nFLNTTNDPNSGk	4	1	1	P29256	N-Term(iTRAQ4plex); K13(iTRAQ4plex)	0,0000	0,703	4	7,9	0	4,435E-09	87	1,86196E-09	2	1709,87261	1,68	22,66	0
	High	skNFLNTTNDPNSGk	3	1	1	P29256	N-Term(iTRAQ4plex); K2(iTRAQ4plex); K15(iTRAQ4plex)	0,0000	0,911	2	5,5	0	1,89E-09	70	9,35341E-08	2	2069,10307	2,07	19,11	1
	High	yASALcGPEGYPHLIVDGR	4	1	1	P29256	N-Term(iTRAQ4plex); C6(Carbamidomethyl)	0,0000	0,696	3	15,8	0	1,402E-13	69	1,19116E-07	3	2219,10459	0,43	39,33	0
	High	nPEmQEVVINVPLAIk	2	1	1	P29256	N-Term(iTRAQ4plex); M4(Oxidation); K16(iTRAQ4plex)	0,0000	0,581	2	10,8	0	9,637E-12	60	1,02087E-06	2	2098,18389	0,84	42,24	0
	High	eSkNPEmQEVVINVPLAIkk	2	1	1	P29256	N-Term(iTRAQ4plex); K3(iTRAQ4plex); M7(Oxidation); K19(iTF	0,0000	1,108	1		0	2,529E-14	56	2,32258E-06	4	2858,65420	1,18	34,61	2
	High	eSkNPEMQEVVINVPLAIk	3	1	1	P29256	N-Term(iTRAQ4plex); K3(iTRAQ4plex); K19(iTRAQ4plex)	0,0000	1,437	2	8,1	0	3,561E-13	55	3,07588E-06	4	2570,45937	0,19	41,98	1
	High	sYLIEIR	7	1	1	P29256	N-Term(iTRAQ4plex)	0,0000	0,573	4	3,3	0	0,001912	47	2,00433E-05	2	1037,61101	-0,17	33,58	0
	High	lVmkDSEIPTSPR	5	1	1	P29256	N-Term(iTRAQ4plex); M3(Oxidation); K4(iTRAQ4plex)	0,0000	0,682	3	0,8	0	0,0000276	42	5,9837E-05	3	1776,97647	-0,22	23,64	1
	High	nPEMQEVVINVPLAIk	2	1	1	P29256	N-Term(iTRAQ4plex); K16(iTRAQ4plex)	0,0000	0,988	2	33,4	0	0,000002177	41	8,47169E-05	3	2082,18711	-0,05	46,28	0
	High	dSEIPTSPR	3	1	1	P29256	N-Term(iTRAQ4plex)	0,0000	0,700	2	7,5	0	0,0001312	38	0,000163294	2	1145,59197	0,04	17,49	0
	High	eSkNPEmQEVVINVPLAIk	3	1	1	P29256	N-Term(iTRAQ4plex); K3(iTRAQ4plex); M7(Oxidation); K19(iTF	0,0000	0,439	3	16,4	0	1,353E-09	37	0,000208435	4	2586,45180	-0,77	38,22	1
	High	nPEMQEVVINVPLAIkk	1	1	1	P29256	N-Term(iTRAQ4plex); K16(iTRAQ4plex); K17(iTRAQ4plex)	0,0000	1,201	1		0	4,12E-09	35	0,000292395	4	2354,38588	0,70	41,64	1
	High	nPEmQEVVINVPLAIkk	1	1	1	P29256	N-Term(iTRAQ4plex); M4(Oxidation); K16(iTRAQ4plex); K17(iT	0,0000	0,826	1		0	1,431E-08	33	0,000524771	3	2370,38303	1,63	37,45	1
	High	kmLGGFLWPLAAVGEYTSGk	1	1	1	P29256	N-Term(iTRAQ4plex); K1(iTRAQ4plex); M2(Oxidation); K20(iTF	0,0000	0,725	1		0	0,000001823	27	0,002218043	4	2573,41396	-0,90	52,58	1
P52983	Glucose-6-phosphate isomerase OS=Synechocystis sp. (strain PCC 6803 / Kazusa) GN=pgi PE=3 SV=	1074,62	35,22	1	18	18	51	0,508	41	24,1	531	58,3	5,76							
	A3	Sequence	# PSMs	# Proteins	# Protein Groups	Protein Group Accessions	Modifications	ΔCn	116/117	116/117 Count	116/117 Variability [%]	q-Value	PEP	IonScore	Exp Value	Charge	MH+ [Da]	ΔM [ppm]	RT [min]	# Missed Cleavages
	High	aVSFYGSLVNVNAYHQPGVEAGk	4	1	1	P52983	N-Term(iTRAQ4plex); K23(iTRAQ4plex)	0,0000	0,374	3	2,1	0	3,704E-14	103	5,48239E-11	3	2695,41391	2,08	40,47	0
	High	aILSTLQNESGPIALEALATk	1	1	1	P52983	N-Term(iTRAQ4plex); K21(iTRAQ4plex)	0,0000	0,493	1		0	8,108E-11	70	1,04947E-07	3	2428,39505	2,00	52,16	0
	High	aAASILELQk	3	1	1	P52983	N-Term(iTRAQ4plex); K10(iTRAQ4plex)	0,0000	1,296	3	165,3	0	0,00001241	67	2,11334E-07	2	1331,81767	2,97	32,40	0
	High	gAIANPDEQR	2	1	1	P52983	N-Term(iTRAQ4plex)	0,0000	0,483	1		0	1,541E-08	64	4,1973E-07	2	1214,62554	0,74	13,87	0
	High	sTLVVTTSk	5	1	1	P52983	N-Term(iTRAQ4plex); K9(iTRAQ4plex)	0,0000	0,455	3	3,5	0	9,036E-07	60	9,39658E-07	2	1223,74761	2,17	19,98	0
	High	qALFENQR	3	1	1	P52983	N-Term(iTRAQ4plex)	0,0000	0,551	3	5,7	0	0,000005841	55	2,91052E-06	2	1149,61418	0,75	19,23	0
	High	kAAASILELQk	3	1	1	P52983	N-Term(iTRAQ4plex); K1(iTRAQ4plex); K11(iTRAQ4plex)	0,0000	0,578	2	11,1	0	1,477E-07	53	5,52039E-06	3	1604,01596	3,25	27,47	1
	High	aFVADVHQGNIkPPTAPk	2	1	1	P52983	N-Term(iTRAQ4plex); K12(iTRAQ4plex); K18(iTRAQ4plex)	0,0000	0,493	1		0	1,424E-07	47	2,03222E-05	4	2322,33437	2,10	27,10	0
	High	vLNcLk	2	1	1	P52983	N-Term(iTRAQ4plex); C4(Carbamidomethyl); K6(iTRAQ4plex)	0,0000	0,428	2	24,0	0	0,0004158	45	5,0157E-05	2	1034,62847	1,34	25,02	0
	High	gSTDQHAYVQQLR	6	1	1	P52983	N-Term(iTRAQ4plex)	0,0000	0,533	4	39,1	0	0,000004668	33	0,000538233	3	1646,83811	0,83	18,73	0
	High	yLQQLVmESLGk	3	1	1	P52983	N-Term(iTRAQ4plex); M7(Oxidation); K12(iTRAQ4plex)	0,0000	0,529	3	84,8	0	0,00002001	31	0,00077085	3	1712,95072	0,67	48,97	0
	High	kDMVILPYkDR	1	1	1	P52983	N-Term(iTRAQ4plex); K1(iTRAQ4plex); K9(iTRAQ4plex)	0,0000	0,805	1		0	0,000042	25	0,003097205	4	1810,06826	3,38	25,46	2
	High	mVGHYWLR	2	1	1	P52983	N-Term(iTRAQ4plex)	0,0000	0,651	1		0	0,00007598	23	0,004753024	3	1205,63788	0,70	28,61	0
	High	mVGHYWLR	4	1	1	P52983	N-Term(iTRAQ4plex); M1(Oxidation)	0,0000	0,544	3	40,6	0	0,0005548	21	0,007361563	3	1221,63251	0,46	23,32	0
	High	kDmVILPYkDR	2	1	1	P52983	N-Term(iTRAQ4plex); K1(iTRAQ4plex); M3(Oxidation); K9(iTRA	0,0000	0,632	2	19,1	0	0,00006769	19	0,013273028	4	1826,06179	2,59	22,18	2
	High	dMVILPYkDR	1	1	1	P52983	N-Term(iTRAQ4plex); K8(iTRAQ4plex)	0,0000	0,596	1		0	0,0001265	18	0,020386082	3	1537,86783	1,77	29,70	1
	High	vQAPEQVETVYk	1	1	1	P52983	N-Term(iTRAQ4plex); K12(iTRAQ4plex)	0,0000	1,589	1		0	0,0003788	12	0,084510372	3	1678,92588	0,26	27,99	0
	High	nPALAPNDGIR	1	1	1	P52983	N-Term(iTRAQ4plex)	0,0000	0,465	1		0,001	0,00355	44	4,04548E-05	2	1281,70500	1,39	21,77	0
	High	lQIQWR	4	1	1	P52983	N-Term(iTRAQ4plex)	0,0000	0,484	4	4,8	0,001	0,006214	38	0,000159577	2	987,58672	1,09	33,74	0
	High	aEITEPLR	1	1	1	P52983	N-Term(iTRAQ4plex)	0,0000	0,608	1		0,002	0,02898	16	0,023821549	2	1072,61370	1,64	22,69	0
Q55517	Sll0529 protein OS=Synechocystis sp. (strain PCC 6803 / Kazusa) GN=sll0529 PE=4 SV=1 - [Q55517	1072,38	31,46	1	15	15	47	0,497	36	44,7	731	78,3	5,60							
	A3	Sequence	# PSMs	# Proteins	# Protein Groups	Protein Group Accessions	Modifications	ΔCn	116/117	116/117 Count	116/117 Variability [%]	q-Value	PEP	IonScore	Exp Value	Charge	MH+ [Da]	ΔM [ppm]	RT [min]	# Missed Cleavages
	High	rGETTASAGQLmAYNGLTAQALGI	2	1	1	Q55517	N-Term(iTRAQ4plex); M12(Oxidation)	0,0000	0,277	1		0	1,778E-13	94	4,22639E-10	3	2712,35135	0,76	34,00	1
	High	rPTPSTITLFTAGALEQGR	10	1	1	Q55517	N-Term(iTRAQ4plex)	0,0000	0,390	6	10,4	0	4,3E-12	82	7,66185E-09	3	2160,19168	1,05	40,55	0
	High	sHGGPASQTVVTESVLPLADLGTL	2	1	1	Q55517	N-Term(iTRAQ4plex); K27(iTRAQ4plex)	0,0000	0,478	2	52,7	0	4,414E-12	76	2,48296E-08	4	2975,66787	0,74	50,83	0
	High	aLSPEAWQLVR	2	1	1	Q55517	N-Term(iTRAQ4plex)	0,0000	0,451	2	14,1	0	0,00003842	54	3,8104E-06	2	1413,79839	0,89	40,25	0
	High	qVATTAMGELVVHVGk	1	1	1	Q55517	N-Term(iTRAQ4plex); K16(iTRAQ4plex)	0,0000	0,817	1		0	3,399E-09	51	8,27885E-06	3	1928,08756	-0,13	36,76	0
	High	qVATTAmGELVVHVGk	2	1	1	Q55517	N-Term(iTRAQ4plex); M7(Oxidation); K16(iTRAQ4plex)	0,0000	0,699	1		0	4,426E-09	50	8,93244E-06	3	1944,08310	0,19	28,59	0
	High	lAFTQAVLEATDk	1	1	1	Q55517	N-Term(iTRAQ4plex); K13(iTRAQ4plex)	0,0000	0,796	1		0	1,179E-07	45	3,04068E-05	3	1694,95768	0,54	43,97	0
	High	gIPITASk	5	1	1	Q55517	N-Term(iTRAQ4plex); K8(iTRAQ4plex)	0,0000	0,472	3	8,0	0	0,0003851	45	3,06175E-05	2	1074,67607	-0,07	22,33	0
	High	sHNLYPGDSLDkDYIVSALQER	2	1	1	Q55517	N-Term(iTRAQ4plex); K12(iTRAQ4plex)	0,0000	0,623	2	21,4	0	6,77E-12	43	4,63415E-05	4	2808,44277	0,73	40,50	1
	High	nVILVNAk	7	1	1	Q55517	N-Term(iTRAQ4plex); K8(iTRAQ4plex)	0,0000	0,473	5	12,3	0	0,0009551	43	4,9314E-05	2	1158,74529	0,34	24,76	0
	High	fSFQQR	3	1	1	Q55517	N-Term(iTRAQ4plex)	0,0000	0,383	3	4,6	0	0,0009384	31	0,000831706	2	956,50780	0,78	23,46	0
	High	nTVTEVGHHAPGFYSTmFLDR	3	1	1	Q55517	N-Term(iTRAQ4plex); M17(Oxidation)	0,0000	0,513	2	94,5	0	9,842E-10	25	0,002998955	4	2539,21596	0,10	33,11	0
	High	fFGENLPDFDR	1	1	1	Q55517	N-Term(iTRAQ4plex)	0,0000	0,550	1		0	0,002069	23	0,004742092	2	1500,72637	1,56	43,20	0
	High	nTVTEVGHHAPGFYSTMFLDR	2	1	1	Q55517	N-Term(iTRAQ4plex)	0,0000	1,108	2	19,7	0	0,000001231	18	0,015811388	4	2523,22255	0,70	37,19	0
	High	aAQAALGGTLTVLIVk	1	1	1	Q55517	N-Term(iTRAQ4plex); K16(iTRAQ4plex)	0,0000	0,365	1		0	0,0003529	18	0,016594723	3	1814,13425	-0,64	46,34	0
	High	nGWTHQRPEIENYFAAmmR	1	1	1	Q55517	N-Term(iTRAQ4plex); M17(Oxidation); M18(Oxidation)	0,0000	0,660	1		0	0,000002201	12	0,065158339	4	2527,17104	-0,70	33,33	0
	High	vVSVINPR	2	1	1	Q55517	N-Term(iTRAQ4plex)	0,0000	0,468	2	12,4	0,001	0,006358	37	0,000190533	2	1027,63786	-0,21	24,22	0
P26287	Apocytochrome f OS=Synechocystis sp. (strain PCC 6803 / Kazusa) GN=petA PE=3 SV=1 - [CYF_SYN	1037,46	42,68	1	14	14	48	0,764	43	25,4	328	35,2	5,16							
	A3	Sequence	# PSMs	# Proteins	# Protein Groups	Protein Group Accessions	Modifications	ΔCn	116/117	116/117 Count	116/117 Variability [%]	q-Value	PEP	IonScore	Exp Value	Charge	MH+ [Da]	ΔM [ppm]	RT [min]	# Missed Cleavages
	High	gQIYPTGLLSNNNAFk	10	1	1	P26287	N-Term(iTRAQ4plex); K16(iTRAQ4plex)	0,0000	0,749	7	5,0	0	2,725E-11	86	2,36576E-09	2	2025,10332	1,25	39,81	0
	High	dTEVVLQNPTR	1	1	1	P26287	N-Term(iTRAQ4plex)	0,0000	0,590	1		0	0,000004915	67	2,19771E-07	2	1415,76238	0,88	23,81	0
	High	iPYDLDSQQVLGDGSk	2	1	1	P26287	N-Term(iTRAQ4plex); K16(iTRAQ4plex)	0,0000	1,181	2	79,5	0	2,224E-10	58	1,61425E-06	3	2023,06089	1,10	37,70	0
	High	gGLNVGAVLmLPEGFk	2	1	1	P26287	N-Term(iTRAQ4plex); M10(Oxidation); K16(iTRAQ4plex)	0,0000	0,804	2	14,8	0	2,827E-07	56	2,64223E-06	2	1906,07768	3,44	50,12	0
	High	vGGTYFQPYR	6	1	1	P26287	N-Term(iTRAQ4plex)	0,0000	0,590	6	4,3	0	0,000003777	49	1,18842E-05	2	1331,68779	0,99	30,94	0
	High	fAVHLGANR	4	1	1	P26287	N-Term(iTRAQ4plex)	0,0000	0,714	4	5,5	0	0,00002215	49	1,38666E-05	2	1128,63970	0,19	22,69	0
	High	aAEVEIPQAVLPDTVFEAVVk	2	1	1	P26287	N-Term(iTRAQ4plex); K21(iTRAQ4plex)	0,0000	0,993	2	13,9	0	0,000009207	43	4,56005E-05	3	2513,41458	1,57	54,50	0
	High	vQAAELNF	1	1	1	P26287	N-Term(iTRAQ4plex)	0,0000	1,023	1		0	0,001692	37	0,000191412	2	1035,55974	0,56	37,25	0
	High	lSEGLkEk	5	1	1	P26287	N-Term(iTRAQ4plex); K6(iTRAQ4plex); K8(iTRAQ4plex)	0,0000	0,900	5	10,1	0	0,0002274	34	0,000413971	2	1335,82146	0,49	17,74	1
	High	iVcANcHLAQk	2	1	1	P26287	N-Term(iTRAQ4plex); C3(Carbamidomethyl); C6(Carbamidome	0,0000	0,595	2	24,5	0	1,99E-08	32	0,000619398	3	1601,85129	1,14	20,07	0
	High	gRGQIYPTGLLSNNNAFk	1	1	1	P26287	N-Term(iTRAQ4plex); K18(iTRAQ4plex)	0,0000	1,828	1		0	7,559E-08	31	0,000736156	4	2238,22500	0,72	34,74	1
	High	gGLNVGAVLMLPEGFk	3	1	1	P26287	N-Term(iTRAQ4plex); K16(iTRAQ4plex)	0,0000	1,315	2	1,4	0	0,0001353	28	0,001530982	3	1890,07999	2,00	52,26	0
	High	sINYGk	5	1	1	P26287	N-Term(iTRAQ4plex); K6(iTRAQ4plex)	0,0000	0,832	4	22,2	0	0,001478	24	0,004285189	2	969,56084	0,08	15,85	0
	High	lSEGLk	2	1	1	P26287	N-Term(iTRAQ4plex); K6(iTRAQ4plex)	0,0000	0,603	2	4,4	0,004	0,04578	28	0,001737681	2	934,58092	-0,28	19,57	0
	Medium	iAPPDR	2	1	1	P26287	N-Term(iTRAQ4plex)	0,0000	0,889	2	12,2	0,021	0,2937	15	0,030267044	2	812,47508	0,47	15,58	0
P73605	Slr1854 protein OS=Synechocystis sp. (strain PCC 6803 / Kazusa) GN=slr1854 PE=4 SV=1 - [P73605	1029,89	50,51	1	9	9	44	0,528	36	24,0	198	22,3	4,84							
	A3	Sequence	# PSMs	# Proteins	# Protein Groups	Protein Group Accessions	Modifications	ΔCn	116/117	116/117 Count	116/117 Variability [%]	q-Value	PEP	IonScore	Exp Value	Charge	MH+ [Da]	ΔM [ppm]	RT [min]	# Missed Cleavages
	High	gIIAIGAYAMDR	5	1	1	P73605	N-Term(iTRAQ4plex)	0,0000	0,761	4	7,3	0	1,266E-08	72	6,78731E-08	2	1394,75994	1,17	41,62	0
	High	gIIAIGAYAmDR	6	1	1	P73605	N-Term(iTRAQ4plex); M10(Oxidation)	0,0000	0,449	4	14,7	0	1,981E-08	61	8,01623E-07	2	1410,75371	0,35	35,25	0
	High	nQAPAVIFLR	7	1	1	P73605	N-Term(iTRAQ4plex)	0,0000	0,556	6	10,6	0	0,000278	54	4,39511E-06	2	1272,75578	0,99	36,08	0
	High	aAATEGLk	2	1	1	P73605	N-Term(iTRAQ4plex); K8(iTRAQ4plex)	0,0000	0,506	1		0	0,00001138	49	1,23586E-05	2	1048,62383	-0,28	15,71	0
	High	hPGmIEEFVk	5	1	1	P73605	N-Term(iTRAQ4plex); M4(Oxidation); K10(iTRAQ4plex)	0,0000	0,452	5	1,5	0	1,726E-07	45	3,08298E-05	3	1490,79224	0,42	23,59	0
	High	kAAATEGLk	6	1	1	P73605	N-Term(iTRAQ4plex); K1(iTRAQ4plex); K9(iTRAQ4plex)	0,0000	0,517	5	5,4	0	0,000001832	45	3,21344E-05	3	1320,82074	-0,31	12,84	1
	High	hPGMIEEFVk	6	1	1	P73605	N-Term(iTRAQ4plex); K10(iTRAQ4plex)	0,0000	0,737	5	14,9	0	0,000005021	35	0,000348095	3	1474,79862	1,30	29,67	0
	High	lGTIcHSLWLFcADPDLLk	1	1	1	P73605	N-Term(iTRAQ4plex); C5(Carbamidomethyl); C12(Carbamidom	0,0000	0,387	1		0	8,84E-08	31	0,000776194	3	2547,33451	0,09	52,94	0
	High	wFNEYFPAQGYEVEYISHLWGQP	1	1	1	P73605	N-Term(iTRAQ4plex); K26(iTRAQ4plex)	0,0000	0,712	1		0	1,796E-07	22	0,006636972	4	3518,73305	0,86	58,97	0
	High	tFVEQIEA	2	1	1	P73605	N-Term(iTRAQ4plex)	0,0000	0,481	1		0,001	0,01138	37	0,000199054	2	1080,56951	0,10	35,57	0
	High	yQATVk	3	1	1	P73605	N-Term(iTRAQ4plex); K6(iTRAQ4plex)	0,0000	0,517	3	3,3	0,001	0,005533	27	0,001802893	2	997,59215	0,09	15,38	0
P72827	Iron uptake protein A1 OS=Synechocystis sp. (strain PCC 6803 / Kazusa) GN=futA1 PE=1 SV=1 - [FL	1015,20	37,22	1	13	13	42	0,184	34	58,3	360	39,3	4,93							
	A3	Sequence	# PSMs	# Proteins	# Protein Groups	Protein Group Accessions	Modifications	ΔCn	116/117	116/117 Count	116/117 Variability [%]	q-Value	PEP	IonScore	Exp Value	Charge	MH+ [Da]	ΔM [ppm]	RT [min]	# Missed Cleavages
	High	aIAENVGVFFPNQEGR	2	1	1	P72827	N-Term(iTRAQ4plex)	0,0000	0,173	2	46,7	0	1,137E-11	90	1,04225E-09	2	1891,97881	0,23	41,24	0
	High	hYNTDNELYAk	7	1	1	P72827	N-Term(iTRAQ4plex); K11(iTRAQ4plex)	0,0000	0,128	5	17,1	0	0,000302	69	1,23018E-07	2	1655,84014	8,07	17,93	0
	High	gTHVNVSGVGVk	7	1	1	P72827	N-Term(iTRAQ4plex); K13(iTRAQ4plex)	0,0000	0,139	4	14,6	0	1,368E-07	69	1,25594E-07	2	1540,90740	1,56	21,42	0
	High	sEGANSPADVLLTVDLAR	1	1	1	P72827	N-Term(iTRAQ4plex)	0,0000	0,194	1		0	1,361E-08	64	3,70655E-07	3	1972,04862	0,90	46,20	0
	High	lGPALAPATk	5	1	1	P72827	N-Term(iTRAQ4plex); K10(iTRAQ4plex)	0,0000	0,242	5	32,2	0	0,000009514	60	9,26766E-07	2	1226,77190	0,63	25,50	0
	High	sVASFGEFk	5	1	1	P72827	N-Term(iTRAQ4plex); K9(iTRAQ4plex)	0,0000	0,152	3	15,8	0	0,00009543	55	3,41169E-06	2	1259,68791	0,37	31,53	0
	High	vkPEELSTYEELADPk	1	1	1	P72827	N-Term(iTRAQ4plex); K2(iTRAQ4plex); K16(iTRAQ4plex)	0,0000	0,130	1		0	7,803E-10	52	5,64898E-06	3	2280,23490	0,58	35,65	0
	High	vNLIEGk	2	1	1	P72827	N-Term(iTRAQ4plex); K7(iTRAQ4plex)	0,0000	0,167	2	9,0	0	0,0003546	49	1,20504E-05	2	1060,66106	0,53	24,85	0
	High	gFVSNFAR	4	1	1	P72827	N-Term(iTRAQ4plex)	0,0000	0,128	3	0,6	0	0,0001959	47	2,14768E-05	2	1041,56059	0,73	27,74	0
	High	vIMYNk	2	1	1	P72827	N-Term(iTRAQ4plex); K6(iTRAQ4plex)	0,0000	0,363	2	48,7	0	0,00065	45	3,14753E-05	2	1055,61675	0,54	22,21	0
	High	vImYNk	3	1	1	P72827	N-Term(iTRAQ4plex); M3(Oxidation); K6(iTRAQ4plex)	0,0000	0,145	3	43,6	0	0,001439	37	0,000193629	2	1071,61138	0,26	19,26	0
	High	sPDGMWFGFTk	1	1	1	P72827	N-Term(iTRAQ4plex); K11(iTRAQ4plex)	0,0000	0,431	1		0	0,0001314	26	0,002311905	3	1560,77707	0,71	43,07	0
	High	ikSEGANSPADVLLTVDLAR	1	1	1	P72827	N-Term(iTRAQ4plex); K2(iTRAQ4plex)	0,0000	1,077	1		0	0,0006241	22	0,006949763	4	2357,33144	1,48	43,35	1
	High	aDELLER	1	1	1	P72827	N-Term(iTRAQ4plex)	0,0000	0,835	1		0,001	0,003386	30	0,000999931	2	989,53783	-0,61	22,97	0
P27181	ATP synthase subunit b OS=Synechocystis sp. (strain PCC 6803 / Kazusa) GN=atpF PE=3 SV=2 - [AT	1008,32	49,16	1	15	15	41	0,782	29	23,8	179	19,8	5,14							
	A3	Sequence	# PSMs	# Proteins	# Protein Groups	Protein Group Accessions	Modifications	ΔCn	116/117	116/117 Count	116/117 Variability [%]	q-Value	PEP	IonScore	Exp Value	Charge	MH+ [Da]	ΔM [ppm]	RT [min]	# Missed Cleavages
	High	mQEAAAQDLGAEQER	2	1	1	P27181	N-Term(iTRAQ4plex); M1(Oxidation)	0,0000	0,887	2	92,6	0	4,863E-12	76	2,39867E-08	3	1806,84171	0,42	18,90	0
	High	rmQEAAAQDLGAEQER	3	1	1	P27181	N-Term(iTRAQ4plex); M2(Oxidation)	0,0000	0,647	2	14,1	0	1,059E-10	73	4,56005E-08	3	1962,94388	0,92	16,01	1
	High	iADAIEEAETR	2	1	1	P27181	N-Term(iTRAQ4plex)	0,0000	0,951	2	45,5	0	4,797E-08	66	2,28544E-07	2	1361,70378	0,61	33,66	0
	High	iAEQAVAk	5	1	1	P27181	N-Term(iTRAQ4plex); K8(iTRAQ4plex)	0,0000	0,650	3	2,8	0	0,000002911	65	2,93745E-07	2	1117,68169	-0,25	18,67	0
	High	qEIATQTEADLR	2	1	1	P27181	N-Term(iTRAQ4plex)	0,0000	0,563	1		0	2,251E-09	59	1,16405E-06	2	1518,78789	-0,12	24,34	0
	High	sIAQLGGR	6	1	1	P27181	N-Term(iTRAQ4plex)	0,0000	0,683	3	3,3	0	0,000007095	59	1,2161E-06	2	945,55956	-0,29	19,98	0
	High	kSAQILAEEEk	4	1	1	P27181	N-Term(iTRAQ4plex); K1(iTRAQ4plex); K11(iTRAQ4plex)	0,0000	0,725	2	1,4	0	1,088E-07	55	3,39602E-06	3	1677,97373	-0,62	19,46	1
	High	rMQEAAAQDLGAEQER	1	1	1	P27181	N-Term(iTRAQ4plex)	0,0000	1,045	1		0	0,000007337	49	1,16405E-05	3	1946,94944	1,17	20,64	1
	High	sAQILAEEEkk	2	1	1	P27181	N-Term(iTRAQ4plex); K10(iTRAQ4plex); K11(iTRAQ4plex)	0,0000	0,782	1		0	1,222E-08	44	3,92618E-05	3	1677,97428	-0,29	20,66	1
	High	kSAQILAEEEkk	2	1	1	P27181	N-Term(iTRAQ4plex); K1(iTRAQ4plex); K11(iTRAQ4plex); K12(	0,0000	0,898	2	14,3	0	2,183E-09	41	7,48118E-05	3	1950,17191	0,06	18,95	2
	High	qEIATQTEADLRR	2	1	1	P27181	N-Term(iTRAQ4plex)	0,0000	0,913	2	8,7	0	0,00006984	33	0,000490874	3	1674,88987	0,40	22,33	1
	High	rIAEQAVAk	4	1	1	P27181	N-Term(iTRAQ4plex); K9(iTRAQ4plex)	0,0000	0,845	2	9,6	0	0,0001702	18	0,014859356	3	1273,78333	0,19	14,10	1
	High	iVQEAGQR	1	1	1	P27181	N-Term(iTRAQ4plex)	0,0000	0,656	1		0,001	0,008609	30	0,001009183	2	1044,59172	-0,14	14,71	0
	High	vIAELkR	2	1	1	P27181	N-Term(iTRAQ4plex); K6(iTRAQ4plex)	0,0000	0,920	2	3,2	0,003	0,03356	24	0,0041973	3	1116,73459	0,23	21,65	1
	High	vIAELk	2	1	1	P27181	N-Term(iTRAQ4plex); K6(iTRAQ4plex)	0,0000	0,800	2	2,2	0,005	0,05599	30	0,000957128	2	960,63317	-0,05	25,90	0
	Medium	lAQAk	1	1	1	P27181	N-Term(iTRAQ4plex); K5(iTRAQ4plex)	0,0000	0,945	1		0,047	0,6141	29	0,001693987	2	818,52965	-5,10	12,60	0
P73418	DNA-binding protein HU OS=Synechocystis sp. (strain PCC 6803 / Kazusa) GN=hup PE=3 SV=2 - [DE	991,93	58,00	1	9	9	53	0,783	40	25,3	100	10,9	8,63							
	A3	Sequence	# PSMs	# Proteins	# Protein Groups	Protein Group Accessions	Modifications	ΔCn	116/117	116/117 Count	116/117 Variability [%]	q-Value	PEP	IonScore	Exp Value	Charge	MH+ [Da]	ΔM [ppm]	RT [min]	# Missed Cleavages
	High	akEQLGESITk	8	1	1	P73418	N-Term(iTRAQ4plex); K2(iTRAQ4plex); K11(iTRAQ4plex)	0,0000	0,738	7	12,1	0	2,356E-07	64	4,28519E-07	2	1635,96550	0,80	19,00	1
	High	vTLVGFGSFEAR	8	1	1	P73418	N-Term(iTRAQ4plex)	0,0000	0,651	8	11,6	0	7,927E-09	61	8,74923E-07	2	1426,78288	1,22	43,01	0
	High	eQLGESITk	2	1	1	P73418	N-Term(iTRAQ4plex); K9(iTRAQ4plex)	0,0000	0,719	2	17,7	0	0,000001727	56	2,56431E-06	2	1292,73039	0,28	22,14	0
	High	gELVHAVTDk	8	1	1	P73418	N-Term(iTRAQ4plex); K10(iTRAQ4plex)	0,0000	0,767	5	13,1	0	4,318E-08	56	2,58208E-06	2	1356,77263	0,04	20,81	0
	High	vPAFSAGk	5	1	1	P73418	N-Term(iTRAQ4plex); K8(iTRAQ4plex)	0,0000	0,757	3	1,6	0	0,0009605	49	1,38347E-05	2	1064,63457	0,26	24,10	0
	High	eQLGESITkk	2	1	1	P73418	N-Term(iTRAQ4plex); K9(iTRAQ4plex); K10(iTRAQ4plex)	0,0000	0,939	2	0,6	0	3,233E-07	43	4,97703E-05	3	1564,93137	2,75	18,81	1
	High	mNkGELVHAVTDk	6	1	1	P73418	N-Term(iTRAQ4plex); M1(Oxidation); K3(iTRAQ4plex); K13(iTF	0,0000	1,003	4	10,2	0	2,624E-07	35	0,000290382	3	1890,04923	0,67	22,07	1
	High	mNkGELVHAVTDk	2	1	1	P73418	N-Term(iTRAQ4plex); K3(iTRAQ4plex); K13(iTRAQ4plex)	0,0000	1,561	2	0,4	0	0,000001746	27	0,002092656	3	1874,05478	0,93	27,99	1
	High	mLIPATk	4	1	1	P73418	N-Term(iTRAQ4plex); K7(iTRAQ4plex)	0,0000	1,298	2	19,9	0	0,003064	21	0,007463972	2	1061,66301	-0,13	26,78	0
	High	mLIPATk	6	1	1	P73418	N-Term(iTRAQ4plex); M1(Oxidation); K7(iTRAQ4plex)	0,0000	0,656	3	2,8	0,001	0,004969	40	0,000128879	2	1077,65788	-0,16	22,08	0
	Medium	vAPEkk	2	1	1	P73418	N-Term(iTRAQ4plex); K5(iTRAQ4plex); K6(iTRAQ4plex)	0,0000	0,844	2	4,2	0,011	0,1382	11	0,083554531	2	1103,71355	-1,21	10,11	1
P73511	Glycogen phosphorylase OS=Synechocystis sp. (strain PCC 6803 / Kazusa) GN=glgP PE=3 SV=1 - [P	961,83	28,62	1	21	21	48	0,386	45	80,1	849	96,6	5,29							
	A3	Sequence	# PSMs	# Proteins	# Protein Groups	Protein Group Accessions	Modifications	ΔCn	116/117	116/117 Count	116/117 Variability [%]	q-Value	PEP	IonScore	Exp Value	Charge	MH+ [Da]	ΔM [ppm]	RT [min]	# Missed Cleavages
	High	aAPGYFTAk	3	1	1	P73511	N-Term(iTRAQ4plex); K9(iTRAQ4plex)	0,0000	0,155	2	10,7	0	0,0000363	57	1,92739E-06	2	1213,68559	3,00	23,48	0
	High	lSNLISSR	2	1	1	P73511	N-Term(iTRAQ4plex)	0,0000	0,396	2	2,5	0	0,001051	52	6,25738E-06	2	1033,61370	1,39	28,85	0
	High	mAILNVAR	3	1	1	P73511	N-Term(iTRAQ4plex)	0,0000	0,488	3	14,5	0	0,001569	51	8,51079E-06	2	1031,61772	2,39	32,46	0
	High	vIFLPDYNVk	2	1	1	P73511	N-Term(iTRAQ4plex); K10(iTRAQ4plex)	0,0000	1,082	2	305,7	0	0,000006486	48	1,47221E-05	2	1495,87993	2,42	41,69	0
	High	wIAGSLVk	3	1	1	P73511	N-Term(iTRAQ4plex); K8(iTRAQ4plex)	0,0000	0,246	3	65,2	0	0,001639	47	1,87055E-05	2	1161,72649	2,63	34,79	0
	High	tFIYGGk	3	1	1	P73511	N-Term(iTRAQ4plex); K7(iTRAQ4plex)	0,0000	0,257	2	53,1	0	0,0003948	44	3,80163E-05	2	1073,62651	2,93	24,44	0
	High	nNPNLDVTPR	1	1	1	P73511	N-Term(iTRAQ4plex)	0,0000	0,345	1		0	0,000005893	44	4,08291E-05	2	1283,68669	3,29	19,75	0
	High	hLEIIYEINQR	3	1	1	P73511	N-Term(iTRAQ4plex)	0,0000	0,265	3	184,7	0	1,133E-08	44	4,23614E-05	3	1571,86850	1,44	33,56	0
	High	tDLVVNPDSLFDVQVk	1	1	1	P73511	N-Term(iTRAQ4plex); K16(iTRAQ4plex)	0,0000	0,236	1		0	1,371E-07	43	5,41243E-05	3	2077,14841	3,08	48,94	0
	High	rWMVLSNPR	2	1	1	P73511	N-Term(iTRAQ4plex)	0,0000	0,400	1		0	0,00003264	43	5,59719E-05	3	1302,72452	1,79	27,32	1
	High	lINSVADVVNNDPTIGDR	1	1	1	P73511	N-Term(iTRAQ4plex)	0,0000	1,508	1		0	2,069E-09	42	6,94976E-05	3	2056,08158	1,15	41,86	0
	High	rWmVLSNPR	2	1	1	P73511	N-Term(iTRAQ4plex); M3(Oxidation)	0,0000	0,484	2	139,6	0	0,00002848	41	8,01623E-05	3	1318,71814	0,79	23,12	1
	High	lLVDEHHYEWQR	4	1	1	P73511	N-Term(iTRAQ4plex)	0,0000	0,207	4	47,0	0	5,346E-07	38	0,000154515	3	1768,89054	1,00	28,56	0
	High	gIPYDTPILGYk	1	1	1	P73511	N-Term(iTRAQ4plex); K12(iTRAQ4plex)	0,0000	0,306	1		0	0,000004892	36	0,000229599	3	1624,92185	1,82	39,60	0
	High	mAYLATVGSHAINGVAALHSQLVI	1	1	1	P73511	N-Term(iTRAQ4plex); K24(iTRAQ4plex)	0,0000	0,387	1		0	7,964E-07	35	0,000283772	4	2739,52773	2,13	48,43	0
	High	mAILNVAR	1	1	1	P73511	N-Term(iTRAQ4plex); M1(Oxidation)	0,0000	0,214	1		0	0,0005013	34	0,000548742	2	1047,61138	1,15	24,49	0
	High	wSLPLFGEmLPR	6	1	1	P73511	N-Term(iTRAQ4plex); M9(Oxidation)	0,0000	0,405	6	40,5	0	5,632E-07	32	0,000698184	3	1605,85965	1,01	51,30	0
	High	lGGHTEPYTDDQGNYR	2	1	1	P73511	N-Term(iTRAQ4plex)	0,0000	0,287	2	73,3	0	6,239E-08	31	0,00075504	3	1966,90464	1,75	20,93	0
	High	vYPAADLSEQISTAGk	1	1	1	P73511	N-Term(iTRAQ4plex); K16(iTRAQ4plex)	0,0000	1,110	1		0	0,000002507	31	0,000812774	3	1938,04612	1,98	35,55	0
	High	aLADNLFYLQGk	1	1	1	P73511	N-Term(iTRAQ4plex); K12(iTRAQ4plex)	0,0000	1,188	1		0	0,00008309	17	0,020699984	3	1640,92691	1,13	42,39	0
	High	mSSENLTk	1	1	1	P73511	N-Term(iTRAQ4plex); K8(iTRAQ4plex)	0,0000	0,571	1		0,001	0,00604	31	0,000820295	2	1197,64775	7,49	17,57	0
	High	vSTANNLR	2	1	1	P73511	N-Term(iTRAQ4plex)	0,0000	0,861	2	30,8	0,006	0,06987	25	0,00306881	2	1018,57756	1,33	14,60	0
	Medium	mkYPNDGDR	2	1	1	P73511	N-Term(iTRAQ4plex); M1(Oxidation); K2(iTRAQ4plex)	0,0000	0,339	2	5,7	0,01	0,13	15	0,029714608	3	1399,69031	1,75	9,04	1
Q55318	Ferredoxin--NADP reductase OS=Synechocystis sp. (strain PCC 6803 / Kazusa) GN=petH PE=1 SV=2	954,49	38,01	1	21	21	66	0,533	59	19,6	413	46,3	6,04							
	A3	Sequence	# PSMs	# Proteins	# Protein Groups	Protein Group Accessions	Modifications	ΔCn	116/117	116/117 Count	116/117 Variability [%]	q-Value	PEP	IonScore	Exp Value	Charge	MH+ [Da]	ΔM [ppm]	RT [min]	# Missed Cleavages
	High	mYSPGYVATSSR	2	1	1	Q55318	N-Term(iTRAQ4plex); M1(Oxidation)	0,0000	0,447	2	7,9	0	0,00001475	59	1,32425E-06	2	1478,70659	-0,03	20,19	0
	High	kADDIPVNIYRPk	8	1	1	Q55318	N-Term(iTRAQ4plex); K1(iTRAQ4plex); K13(iTRAQ4plex)	0,0000	0,569	6	9,5	0	7,532E-13	57	2,0463E-06	3	1961,15732	1,46	26,30	1
	High	lYSIASTR	4	1	1	Q55318	N-Term(iTRAQ4plex)	0,0000	0,577	3	2,3	0	0,0008456	56	2,27494E-06	2	1054,60210	0,72	25,12	0
	High	vLENYPLVR	3	1	1	Q55318	N-Term(iTRAQ4plex)	0,0000	0,460	3	0,5	0	0,00002302	48	1,64426E-05	2	1246,72893	1,05	33,56	0
	High	lTYAISR	4	1	1	Q55318	N-Term(iTRAQ4plex)	0,0000	0,488	3	4,7	0	0,0009905	46	2,53495E-05	2	967,56926	-0,05	25,20	0
	High	gLAWLIFGIPk	2	1	1	Q55318	N-Term(iTRAQ4plex); K11(iTRAQ4plex)	0,0000	0,659	2	85,8	0	0,000003857	41	7,41259E-05	2	1502,94109	4,86	57,91	0
	High	aDDIPVNIYRPk	2	1	1	Q55318	N-Term(iTRAQ4plex); K12(iTRAQ4plex)	0,0000	0,602	1		0	0,00006237	38	0,000203392	3	1688,95969	1,34	29,41	0
	High	hGDFGDDkTVSLcVR	5	1	1	Q55318	N-Term(iTRAQ4plex); K8(iTRAQ4plex); C13(Carbamidomethyl)	0,0000	0,449	5	26,6	0	4,434E-08	37	0,000193629	4	1994,00710	3,33	24,70	1
	High	tMTTTPk	1	1	1	Q55318	N-Term(iTRAQ4plex); K7(iTRAQ4plex)	0,0000	0,829	1		0	0,002803	35	0,000304768	2	1067,60478	3,61	13,61	0
	High	tHTYmcGLk	1	1	1	Q55318	N-Term(iTRAQ4plex); M5(Oxidation); C6(Carbamidomethyl); K	0,0000	0,449	1		0	0,00004451	34	0,000423614	3	1414,70670	0,40	13,77	0
	High	sENILYkDDLEk	3	1	1	Q55318	N-Term(iTRAQ4plex); K7(iTRAQ4plex); K12(iTRAQ4plex)	0,0000	0,497	3	8,2	0	1,278E-07	29	0,001238711	3	1899,04758	2,12	27,55	1
	High	tVSLcVR	3	1	1	Q55318	N-Term(iTRAQ4plex); C5(Carbamidomethyl)	0,0000	0,454	2	14,3	0	0,00214	28	0,001734528	2	978,55266	0,37	22,95	0
	High	sGSTFITVPLkR	2	1	1	Q55318	N-Term(iTRAQ4plex); K11(iTRAQ4plex)	0,0000	0,548	1		0	0,0001149	26	0,002360315	3	1593,95505	-1,03	30,37	1
	High	mFkEQHEDYk	4	1	1	Q55318	N-Term(iTRAQ4plex); M1(Oxidation); K3(iTRAQ4plex); K10(iTF	0,0000	0,526	4	10,4	0	0,0001279	24	0,003580717	4	1802,91274	1,10	14,37	1
	High	tmTTTPk	4	1	1	Q55318	N-Term(iTRAQ4plex); M2(Oxidation); K7(iTRAQ4plex)	0,0000	0,460	4	6,4	0	0,0008189	22	0,006682978	2	1083,59526	-0,54	9,84	0
	High	tHTYMcGLk	2	1	1	Q55318	N-Term(iTRAQ4plex); C6(Carbamidomethyl); K9(iTRAQ4plex)	0,0000	0,887	2	5,5	0	0,0004874	21	0,007673085	3	1398,71701	4,14	18,91	0
	High	eQHEDYkFk	3	1	1	Q55318	N-Term(iTRAQ4plex); K7(iTRAQ4plex); K9(iTRAQ4plex)	0,0000	0,569	3	3,2	0	0,0003828	19	0,011454338	3	1655,88224	4,16	14,10	1
	High	mFkEQHEDYkFk	2	1	1	Q55318	N-Term(iTRAQ4plex); M1(Oxidation); K3(iTRAQ4plex); K10(iTF	0,0000	0,827	2	3,0	0	0,000001633	18	0,016709752	5	2222,18214	2,66	19,34	2
	High	mYIQHR	3	1	1	Q55318	N-Term(iTRAQ4plex)	0,0000	0,906	3	20,3	0,001	0,01627	12	0,069979366	3	991,52835	1,97	13,91	0
	High	aFLWR	3	1	1	Q55318	N-Term(iTRAQ4plex)	0,0000	0,524	3	1,8	0,004	0,05362	29	0,001315134	2	836,49108	1,34	32,39	0
	High	eWTTFQR	1	1	1	Q55318	N-Term(iTRAQ4plex)	0,0000	0,625	1		0,005	0,06547	24	0,00362218	2	1111,56682	1,36	26,07	0
	Medium	wHVETY	2	1	1	Q55318	N-Term(iTRAQ4plex)	0,0000	0,560	2	7,1	0,012	0,1643	13	0,049541597	2	978,48149	1,37	27,18	0
	Medium	tPYIGk	2	1	1	Q55318	N-Term(iTRAQ4plex); K6(iTRAQ4plex)	0,0000	0,499	2	3,1	0,02	0,2599	18	0,022342308	2	966,58806	1,88	19,59	0
P42352	50S ribosomal protein L9 OS=Synechocystis sp. (strain PCC 6803 / Kazusa) GN=rplI PE=3 SV=2 - [R	953,40	53,95	1	11	11	45	2,062	33	35,7	152	16,6	9,20							
	A3	Sequence	# PSMs	# Proteins	# Protein Groups	Protein Group Accessions	Modifications	ΔCn	116/117	116/117 Count	116/117 Variability [%]	q-Value	PEP	IonScore	Exp Value	Charge	MH+ [Da]	ΔM [ppm]	RT [min]	# Missed Cleavages
	High	vVLNETINk	6	1	1	P42352	N-Term(iTRAQ4plex); K9(iTRAQ4plex)	0,0000	1,168	6	101,2	0	0,000002248	73	5,38233E-08	2	1317,80120	2,38	27,09	0
	High	tGFYQAQIk	7	1	1	P42352	N-Term(iTRAQ4plex); K9(iTRAQ4plex)	0,0000	2,062	5	41,4	0	9,159E-07	66	2,46587E-07	2	1343,75823	1,52	26,12	0
	High	lGFTGDLVEVAPGYAR	3	1	1	P42352	N-Term(iTRAQ4plex)	0,0000	1,302	2	123,2	0	6,149E-09	64	4,3448E-07	3	1808,96848	1,15	44,04	0

	High	gISLPDIHk	7	1	1	P42352	N-Term(iTRAQ4plex); K9(iTRAQ4plex)	0,0000	1,598	4	13,8	0	0,00001946	57	2,21294E-06	2	1267,76225	0,77	30,33	0
	High	gLGVVATPGILR	9	1	1	P42352	N-Term(iTRAQ4plex)	0,0000	2,266	5	29,6	0	0,00001142	56	2,66054E-06	2	1296,81377	1,32	37,49	0
	High	kVALETIGR	6	1	1	P42352	N-Term(iTRAQ4plex); K1(iTRAQ4plex)	0,0000	2,400	3	0,1	0	0,00005183	49	1,39306E-05	3	1274,80652	2,38	22,11	1
	High	vALETIGR	1	1	1	P42352	N-Term(iTRAQ4plex)	0,0000	3,795	1		0	0,00005407	33	0,000593173	2	1002,60710	0,65	26,53	0
	High	lkELER	1	1	1	P42352	N-Term(iTRAQ4plex); K2(iTRAQ4plex)	0,0000	1,780	1		0,002	0,02782	15	0,028840315	3	1075,67484	3,20	17,57	1
	High	nYLIPk	4	1	1	P42352	N-Term(iTRAQ4plex); K6(iTRAQ4plex)	0,0000	2,398	4	5,9	0,006	0,07035	31	0,000820352	2	1035,64641	2,23	26,04	0
	Medium	rGISLPDIHk	1	1	1	P42352	N-Term(iTRAQ4plex); K10(iTRAQ4plex)	0,0000	2,676	1		0,016	0,218	16	0,024041967	4	1423,86574	2,35	25,92	1
	Medium	fVIkk	1	1	1	P42352	N-Term(iTRAQ4plex); K4(iTRAQ4plex); K5(iTRAQ4plex)	0,0000	2,951	1		0,016	0,2088	11	0,084133703	3	1066,74350	8,08	20,26	1
P22358	Chaperone protein dnaK2 OS=Synechocystis sp. (strain PCC 6803 / Kazusa) GN=dnaK2 PE=2 SV=1 -	916,78	31,13	1	19	19	40	0,997	36	17,0	636	67,6	4,86							
	A3	Sequence	# PSMs	# Proteins	# Protein Groups	Protein Group Accessions	Modifications	ΔCn	116/117	116/117 Count	116/117 Variability [%]	q-Value	PEP	IonScore	Exp Value	Charge	MH+ [Da]	ΔM [ppm]	RT [min]	# Missed Cleavages
	High	qFAPEEISAQVLR	2	1	1	P22358	N-Term(iTRAQ4plex)	0,0000	1,003	2	8,7	0	1,512E-09	75	2,93069E-08	2	1631,88811	0,43	40,32	0
	High	tTPSVVGYAk	6	1	1	P22358	N-Term(iTRAQ4plex); K10(iTRAQ4plex)	0,0000	0,993	4	17,7	0	4,007E-08	68	1,71779E-07	2	1310,75615	0,23	23,13	0
	High	iINEPTAASLAYGLDkk	4	1	1	P22358	N-Term(iTRAQ4plex); K16(iTRAQ4plex); K17(iTRAQ4plex)	0,0000	1,069	3	8,0	0	2,572E-11	66	2,63615E-07	3	2236,29325	0,84	37,66	1
	High	sALDEIVLVGGSTR	1	1	1	P22358	N-Term(iTRAQ4plex)	0,0000	0,765	1		0	2,745E-07	63	5,85731E-07	2	1560,87053	-0,57	41,49	0
	High	kNQADSLVYQAEk	1	1	1	P22358	N-Term(iTRAQ4plex); K1(iTRAQ4plex); K13(iTRAQ4plex)	0,0000	0,741	1		0	0,000000129	58	1,48926E-06	3	1926,06791	1,15	22,31	1
	High	rQAVmNPGNTFYSVk	2	1	1	P22358	N-Term(iTRAQ4plex); M5(Oxidation); K15(iTRAQ4plex)	0,0000	1,260	1		0	1,919E-09	50	9,68211E-06	3	2016,06003	1,22	24,16	1
	High	kLVDDASk	3	1	1	P22358	N-Term(iTRAQ4plex); K1(iTRAQ4plex); K8(iTRAQ4plex)	0,0000	1,038	3	4,5	0	0,0001337	42	5,83405E-05	3	1307,78858	-0,71	14,25	1
	High	kIVDFLAGEFQk	1	1	1	P22358	N-Term(iTRAQ4plex); K1(iTRAQ4plex); K12(iTRAQ4plex)	0,0000	0,976	1		0	2,234E-07	40	9,22508E-05	3	1827,06992	-2,28	43,46	1
	High	iAGIEVLR	3	1	1	P22358	N-Term(iTRAQ4plex)	0,0000	1,030	3	2,3	0	0,0009254	40	9,8168E-05	2	1014,64263	-0,22	33,99	0
	High	qAVmNPGNTFYSVk	2	1	1	P22358	N-Term(iTRAQ4plex); M4(Oxidation); K14(iTRAQ4plex)	0,0000	0,419	2	92,2	0	0,00000449	40	0,000106652	3	1859,95676	0,17	28,23	0
	High	eAESNAAADkER	2	1	1	P22358	N-Term(iTRAQ4plex); K10(iTRAQ4plex)	0,0000	0,718	2	0,0	0	0,000001309	38	0,000145201	3	1578,79703	0,50	9,01	1
	High	akFEEIcSDLIDR	2	1	1	P22358	N-Term(iTRAQ4plex); K2(iTRAQ4plex); C7(Carbamidomethyl)	0,0000	1,106	2	1,7	0	9,641E-09	36	0,000258208	3	1883,97928	0,90	37,51	1
	High	nTTIPTk	3	1	1	P22358	N-Term(iTRAQ4plex); K7(iTRAQ4plex)	0,0000	0,785	3	6,3	0	0,0005666	34	0,000463866	2	1062,64018	0,41	16,65	0
	High	hLDTTLSR	3	1	1	P22358	N-Term(iTRAQ4plex)	0,0000	1,098	3	3,1	0	0,00005039	32	0,000594251	3	1086,60266	0,23	15,16	0
	High	ikAEGLIk	1	1	1	P22358	N-Term(iTRAQ4plex); K2(iTRAQ4plex); K8(iTRAQ4plex)	0,0000	0,873	1		0	0,00009839	30	0,000965984	3	1303,86563	-1,33	23,82	1
	High	qITELGDkVPAADkIk	1	1	1	P22358	N-Term(iTRAQ4plex); K8(iTRAQ4plex); K14(iTRAQ4plex); K16(	0,0000	0,948	1		0	5,308E-09	28	0,001629184	4	2302,38222	-0,28	30,53	2
	High	eQSISITGASTLPDTEVDR	1	1	1	P22358	N-Term(iTRAQ4plex)	0,0000	0,653	1		0	0,001349	17	0,022129419	3	2163,09061	0,36	34,70	0
	High	nTTIPTkk	1	1	1	P22358	N-Term(iTRAQ4plex); K7(iTRAQ4plex); K8(iTRAQ4plex)	0,0000	0,870	1		0,008	0,1033	12	0,068702099	3	1334,83298	-2,85	13,27	1
	Medium	sLGTFR	1	1	1	P22358	N-Term(iTRAQ4plex)	0,0000	1,089	1		0,018	0,2362	14	0,042753336	2	824,47490	0,25	20,37	0
P19569	Photosystem I reaction center subunit II OS=Synechocystis sp. (strain PCC 6803 / Kazusa) GN=psaD	916,40	48,94	1	7	7	35	0,635	30	12,3	141	15,6	8,90							
	A3	Sequence	# PSMs	# Proteins	# Protein Groups	Protein Group Accessions	Modifications	ΔCn	116/117	116/117 Count	116/117 Variability [%]	q-Value	PEP	IonScore	Exp Value	Charge	MH+ [Da]	ΔM [ppm]	RT [min]	# Missed Cleavages
	High	fGGSTGGLLSk	7	1	1	P19569	N-Term(iTRAQ4plex); K11(iTRAQ4plex)	0,0000	0,637	6	4,4	0	5,273E-08	92	6,17974E-10	2	1311,75127	0,12	30,06	0
	High	kEQcLALGTQLR	5	1	1	P19569	N-Term(iTRAQ4plex); K1(iTRAQ4plex); C4(Carbamidomethyl)	0,0000	0,632	5	11,0	0	1,301E-08	77	2,11821E-08	2	1704,96794	0,58	24,44	1
	High	iGQNPEPVTIk	5	1	1	P19569	N-Term(iTRAQ4plex); K11(iTRAQ4plex)	0,0000	0,559	5	26,9	0	9,926E-08	71	9,20851E-08	2	1483,87273	0,29	26,41	0
	High	tELSGQPPk	6	1	1	P19569	N-Term(iTRAQ4plex); K9(iTRAQ4plex)	0,0000	0,683	4	7,2	0	0,00004353	54	4,40555E-06	2	1244,70854	-0,29	16,22	0
	High	vYPSGEVQYLHPADGVFPEk	4	1	1	P19569	N-Term(iTRAQ4plex); K20(iTRAQ4plex)	0,0000	0,620	4	16,2	0	1,876E-09	46	2,59998E-05	3	2520,29990	-0,58	38,56	0
	High	rIGQNPEPVTIk	5	1	1	P19569	N-Term(iTRAQ4plex); K12(iTRAQ4plex)	0,0000	0,623	3	4,1	0	0,000004102	44	3,91715E-05	2	1639,97441	0,61	21,35	1
	High	iQDYk	3	1	1	P19569	N-Term(iTRAQ4plex); K5(iTRAQ4plex)	0,0000	0,753	3	1,5	0,005	0,06426	23	0,004549566	2	954,55162	1,85	18,47	0
	>sp|TRYP_PIG|	900,20	28,57	1	5	5	33	0,437	25	21,0	231	24,4	7,18							
	A3	Sequence	# PSMs	# Proteins	# Protein Groups	Protein Group Accessions	Modifications	ΔCn	116/117	116/117 Count	116/117 Variability [%]	q-Value	PEP	IonScore	Exp Value	Charge	MH+ [Da]	ΔM [ppm]	RT [min]	# Missed Cleavages
	High	lGEHNIDVLEGNEQFINAAk	7	1	1		N-Term(iTRAQ4plex); K20(iTRAQ4plex)	0,0000	0,428	4	3,5	0	5,519E-17	119	1,31945E-12	3	2499,31070	0,97	39,46	0
	High	iITHPNFNGNTLDNDImLIk	5	1	1		N-Term(iTRAQ4plex); M17(Oxidation); K20(iTRAQ4plex)	0,0000	0,439	5	22,8	0	1,569E-11	71	9,89966E-08	3	2587,38407	1,83	43,38	0
	High	iITHPNFNGNTLDNDIMLIk	4	1	1		N-Term(iTRAQ4plex); K20(iTRAQ4plex)	0,0000	0,484	4	18,5	0	3,949E-11	54	4,37492E-06	3	2571,38742	1,17	45,44	0
	High	lSSPATLNSR	7	1	1		N-Term(iTRAQ4plex)	0,0000	0,410	5	12,2	0	0,00009999	49	1,18024E-05	2	1189,66619	0,36	22,02	0
	High	vATVSLPR	9	1	1		N-Term(iTRAQ4plex)	0,0000	0,413	6	4,4	0	0,000009433	49	1,39627E-05	2	986,61205	0,52	26,06	0
	High	nkPGVYTk	1	1	1		N-Term(iTRAQ4plex); K2(iTRAQ4plex); K8(iTRAQ4plex)	0,0000	0,510	1		0	0,0001657	26	0,002716252	3	1338,80738	-2,38	12,49	0
P73294	50S ribosomal protein L13 OS=Synechocystis sp. (strain PCC 6803 / Kazusa) GN=rplM PE=3 SV=1 -	871,27	75,50	1	9	9	37	1,388	30	22,2	151	17,0	9,99							
	A3	Sequence	# PSMs	# Proteins	# Protein Groups	Protein Group Accessions	Modifications	ΔCn	116/117	116/117 Count	116/117 Variability [%]	q-Value	PEP	IonScore	Exp Value	Charge	MH+ [Da]	ΔM [ppm]	RT [min]	# Missed Cleavages
	High	tVLPTIDNLDHk	6	1	1	P73294	N-Term(iTRAQ4plex); K12(iTRAQ4plex)	0,0000	1,455	4	27,8	0	2,622E-08	77	1,98596E-08	3	1653,94443	1,81	32,93	0
	High	vYAGPSHPHAAQQPETLVINTIPA	7	1	1	P73294	N-Term(iTRAQ4plex)	0,0000	1,071	5	46,7	0	2,509E-07	64	3,58072E-07	3	2897,50797	1,81	35,31	0
	High	lATEVATILR	10	1	1	P73294	N-Term(iTRAQ4plex)	0,0000	1,492	7	6,7	0	0,000003356	64	4,44601E-07	2	1230,75603	1,77	42,75	0
	High	iVESAVR	4	1	1	P73294	N-Term(iTRAQ4plex)	0,0000	1,462	4	11,2	0	0,001004	43	5,49503E-05	2	917,55443	0,81	18,05	0
	High	wYVIDAEGQR	4	1	1	P73294	N-Term(iTRAQ4plex)	0,0000	1,170	4	21,0	0	0,0007701	39	0,000134887	2	1380,70647	2,60	34,52	0
	High	nkPTFTPHMDTGDFVIIINAEk	2	1	1	P73294	N-Term(iTRAQ4plex); K2(iTRAQ4plex); K22(iTRAQ4plex)	0,0000	1,199	2	54,7	0	0,0002987	15	0,035050792	4	2920,56777	2,53	42,76	0
	High	hSGRPGGmkEETFEk	1	1	1	P73294	N-Term(iTRAQ4plex); M8(Oxidation); K9(iTRAQ4plex); K15(iTF	0,0000	1,417	1		0,001	0,01596	18	0,017138389	3	2138,10733	2,25	11,45	1
	Medium	kLFTk	1	1	1	P73294	N-Term(iTRAQ4plex); K1(iTRAQ4plex); K5(iTRAQ4plex)	0,0000	1,721	1		0,019	0,2507	17	0,020089541	3	1068,71830	3,89	17,07	1
	Medium	iEVTGR	2	1	1	P73294	N-Term(iTRAQ4plex)	0,0000	1,195	2	10,8	0,021	0,2851	19	0,017780475	2	818,48668	1,73	17,14	0
P80507	Inorganic pyrophosphatase OS=Synechocystis sp. (strain PCC 6803 / Kazusa) GN=ppa PE=1 SV=2 -	869,27	55,03	1	11	11	50	0,378	42	18,0	169	19,1	4,88							
	A3	Sequence	# PSMs	# Proteins	# Protein Groups	Protein Group Accessions	Modifications	ΔCn	116/117	116/117 Count	116/117 Variability [%]	q-Value	PEP	IonScore	Exp Value	Charge	MH+ [Da]	ΔM [ppm]	RT [min]	# Missed Cleavages
	High	aGLINVLIEIPAGSk	6	1	1	P80507	N-Term(iTRAQ4plex); K15(iTRAQ4plex)	0,0000	0,379	4	9,3	0	1,698E-09	99	1,30608E-10	2	1783,09746	2,38	50,67	0
	High	vTEILGWk	5	1	1	P80507	N-Term(iTRAQ4plex); K8(iTRAQ4plex)	0,0000	0,379	4	0,8	0	0,000007117	54	3,8815E-06	2	1233,74504	0,39	39,32	0
	High	sINDLAGHR	6	1	1	P80507	N-Term(iTRAQ4plex)	0,0000	0,382	4	4,2	0	0,00004493	50	1,11422E-05	3	1126,60947	0,80	16,43	0
	High	lDEIAEFFR	4	1	1	P80507	N-Term(iTRAQ4plex)	0,0000	0,314	4	5,3	0	0,000001543	48	1,47901E-05	2	1283,67583	0,43	50,52	0
	High	kVTEILGWk	6	1	1	P80507	N-Term(iTRAQ4plex); K1(iTRAQ4plex); K9(iTRAQ4plex)	0,0000	0,370	4	1,9	0	0,0003513	44	3,79289E-05	2	1505,94328	1,12	34,54	1
	High	dVDAVLPLVEEcVk	2	1	1	P80507	N-Term(iTRAQ4plex); C12(Carbamidomethyl); K14(iTRAQ4plex	0,0000	0,583	2	10,7	0	2,113E-08	30	0,001021863	3	1874,02146	1,63	45,61	0
	High	nkYEFDkDmNcFALDR	5	1	1	P80507	N-Term(iTRAQ4plex); K2(iTRAQ4plex); K7(iTRAQ4plex); M9(O:	0,0000	0,304	4	1,6	0	0,000001846	25	0,003111501	3	2514,21366	0,73	28,35	2
	High	nkYEFDkDMNcFALDR	3	1	1	P80507	N-Term(iTRAQ4plex); K2(iTRAQ4plex); K7(iTRAQ4plex); C11(C	0,0000	0,621	3	5,7	0	5,692E-08	25	0,003183978	4	2498,22085	1,58	33,67	2
	High	iLcVPAkDPR	4	1	1	P80507	N-Term(iTRAQ4plex); C3(Carbamidomethyl); K7(iTRAQ4plex)	0,0000	0,417	4	10,3	0,001	0,01177	32	0,000693378	2	1456,85576	0,60	25,08	1
	High	iLcVPAk	1	1	1	P80507	N-Term(iTRAQ4plex); C3(Carbamidomethyl); K7(iTRAQ4plex)	0,0000	0,307	1		0,001	0,01355	27	0,00194971	2	1088,67375	-0,26	27,89	0
	High	yTYVk	3	1	1	P80507	N-Term(iTRAQ4plex); K5(iTRAQ4plex)	0,0000	0,375	3	1,5	0,001	0,00416	24	0,004153294	2	961,55968	0,00	18,84	0
	Medium	iPAQPk	5	1	1	P80507	N-Term(iTRAQ4plex); K6(iTRAQ4plex)	0,0000	0,396	5	8,2	0,015	0,1948	15	0,029374468	2	941,60185	-0,42	16,51	0
P27179	ATP synthase subunit alpha OS=Synechocystis sp. (strain PCC 6803 / Kazusa) GN=atpA PE=3 SV=1 -	850,32	30,42	1	14	14	46	2,026	37	25,4	503	53,9	5,08							
	A3	Sequence	# PSMs	# Proteins	# Protein Groups	Protein Group Accessions	Modifications	ΔCn	116/117	116/117 Count	116/117 Variability [%]	q-Value	PEP	IonScore	Exp Value	Charge	MH+ [Da]	ΔM [ppm]	RT [min]	# Missed Cleavages
	High	tTGQIAQIPIGDAmVGR	4	1	1	P27179	N-Term(iTRAQ4plex); M14(Oxidation)	0,0000	2,088	3	3,9	0	2,548E-08	66	2,64832E-07	2	1888,01140	1,82	34,07	0
	High	yVEIINSSk	2	1	1	P27179	N-Term(iTRAQ4plex); K9(iTRAQ4plex)	0,0000	1,903	2	42,6	0	0,000007611	60	1,1091E-06	2	1340,76653	0,09	29,55	0
	High	eAYPGDVFYIHSR	5	1	1	P27179	N-Term(iTRAQ4plex)	0,0000	2,433	3	7,0	0	1,903E-10	55	3,04768E-06	3	1697,84031	-0,07	33,04	0
	High	vTEFAQGLR	3	1	1	P27179	N-Term(iTRAQ4plex)	0,0000	2,164	3	5,4	0	0,000003408	54	4,23643E-06	2	1164,65227	2,48	29,52	0
	High	aSTVAQIIDTLTEk	3	1	1	P27179	N-Term(iTRAQ4plex); K14(iTRAQ4plex)	0,0000	2,820	3	66,5	0	0,000003147	50	9,01509E-06	3	1778,01743	1,36	53,82	0
	High	vSIRPDEISSIIR	10	1	1	P27179	N-Term(iTRAQ4plex)	0,0000	1,874	6	5,5	0	3,739E-07	50	1,11165E-05	3	1628,94504	-0,13	39,24	0
	High	vGSAAQTk	7	1	1	P27179	N-Term(iTRAQ4plex); K8(iTRAQ4plex)	0,0000	2,158	6	44,7	0	0,000352	44	3,79289E-05	2	1049,61931	-0,04	12,29	0
	High	tAIAIDTIINQk	1	1	1	P27179	N-Term(iTRAQ4plex); K12(iTRAQ4plex)	0,0000	1,462	1		0	0,0000217	35	0,000351878	3	1588,95151	0,15	38,96	0
	High	eLIIGDRk	2	1	1	P27179	N-Term(iTRAQ4plex); K8(iTRAQ4plex)	0,0000	2,211	2	39,8	0	0,002218	23	0,005345274	3	1231,76236	0,87	20,52	1
	High	vVDSLGRPIDGk	3	1	1	P27179	N-Term(iTRAQ4plex); K12(iTRAQ4plex)	0,0000	1,371	2	61,0	0	0,0004583	18	0,017296969	3	1543,90555	0,57	25,81	0
	High	kSVcEPmQTGITAIDAmIPIGR	1	1	1	P27179	K1(iTRAQ4plex); N-Term(iTRAQ4plex); C4(Carbamidomethyl);	0,0000	1,678	1		0	4,818E-08	16	0,026119809	4	2708,40542	1,08	36,60	1
	High	gPISSTATR	2	1	1	P27179	N-Term(iTRAQ4plex)	0,0000	2,050	2	2,7	0,001	0,005278	51	8,78962E-06	2	1033,57549	-0,37	13,17	0
	High	qMSLLmR	1	1	1	P27179	N-Term(iTRAQ4plex); M6(Oxidation)	0,0000	3,480	1		0,003	0,03518	16	0,026422263	2	1038,55583	0,14	24,89	0
	High	eLIIGDR	1	1	1	P27179	N-Term(iTRAQ4plex)	0,0000	1,928	1		0,005	0,06371	23	0,00490874	2	959,56493	0,70	24,30	0
	Medium	qMSLLMR	1	1	1	P27179	N-Term(iTRAQ4plex)	0,0000	3,989	1		0,025	0,3426	10	0,089324379	2	1022,56072	-0,06	29,39	0
P73409	Slr1841 protein OS=Synechocystis sp. (strain PCC 6803 / Kazusa) GN=slr1841 PE=4 SV=1 - [P73409	846,36	15,08	1	5	9	29	1,594	17	22,5	630	67,6	4,65							
	A3	Sequence	# PSMs	# Proteins	# Protein Groups	Protein Group Accessions	Modifications	ΔCn	116/117	116/117 Count	116/117 Variability [%]	q-Value	PEP	IonScore	Exp Value	Charge	MH+ [Da]	ΔM [ppm]	RT [min]	# Missed Cleavages
	High	gGTQGIVGTTAANLNR	8	1	1	P73409	N-Term(iTRAQ4plex)	0,0000	1,740	4	2,7	0	2,247E-10	114	3,58072E-12	2	1673,90593	0,45	27,78	0
	High	tLNNQTDNNQAVFQNR	3	1	1	P73409	N-Term(iTRAQ4plex)	0,0000	1,096	2	59,8	0	2,243E-10	74	3,88124E-08	3	2020,99198	0,11	22,90	0
	High	kTLNNQTDNNQAVFQNR	6	1	1	P73409	N-Term(iTRAQ4plex); K1(iTRAQ4plex)	0,0000	1,587	3	11,5	0	4,617E-11	62	5,9837E-07	3	2293,18961	0,36	19,74	1
	High	rLmQEFEAELAALGAR	1	2	2	P73409;P73103	N-Term(iTRAQ4plex); M3(Oxidation)	0,0000				0	0,000002579	49	1,18024E-05	3	1965,03531	0,40	48,01	1
	High	lTGEVIFAPTAIFGTk	7	1	1	P73409	N-Term(iTRAQ4plex); K16(iTRAQ4plex)	0,0000	1,907	7	59,4	0	5,606E-07	41	8,43277E-05	3	1953,12979	-0,10	52,19	0
	High	lmQEFEAELAALGAR	1	2	2	P73409;P73103	N-Term(iTRAQ4plex); M2(Oxidation)	0,0000				0	0,000001108	35	0,000349921	3	1808,93277	-0,35	51,97	0
	High	yGcIVGYPDR	1	2	2	P73409;P73103	N-Term(iTRAQ4plex); C3(Carbamidomethyl)	0,0000				0	0,00009834	33	0,000486374	2	1343,65203	-1,07	28,81	0
	High	dVQPTAWAYEALk	1	2	2	P73409;P73103	N-Term(iTRAQ4plex); K13(iTRAQ4plex)	0,0000				0	0,000001707	33	0,000562586	3	1779,95212	0,06	41,35	0
	High	vDNLEAR	1	1	1	P73409	N-Term(iTRAQ4plex)	0,0000	1,177	1		0,008	0,1125	16	0,023986672	2	960,52306	-0,06	16,00	0
Q55460	Bicarbonate-binding protein CmpA OS=Synechocystis sp. (strain PCC 6803 / Kazusa) GN=cmpA PE=1	838,98	40,71	1	16	17	38	0,196	27	129,2	452	49,4	5,77							
	A3	Sequence	# PSMs	# Proteins	# Protein Groups	Protein Group Accessions	Modifications	ΔCn	116/117	116/117 Count	116/117 Variability [%]	q-Value	PEP	IonScore	Exp Value	Charge	MH+ [Da]	ΔM [ppm]	RT [min]	# Missed Cleavages
	High	aAHTFPNVNQDFWIR	3	1	1	Q55460	N-Term(iTRAQ4plex)	0,0000	0,229	3	37,3	0	1,24E-11	74	3,66412E-08	3	1959,99662	1,00	39,76	0
	High	yGmTGVEVSk	1	1	1	Q55460	N-Term(iTRAQ4plex); M3(Oxidation); K10(iTRAQ4plex)	0,0000	0,196	1		0	0,000004206	61	7,5504E-07	2	1374,71892	0,84	20,85	0
	High	nRPEVVQIVSGR	6	1	1	Q55460	N-Term(iTRAQ4plex)	0,0000	0,113	4	80,0	0	6,232E-09	61	7,85181E-07	3	1497,86313	0,85	24,42	0
	High	gVNLDITk	2	1	1	Q55460	N-Term(iTRAQ4plex); K8(iTRAQ4plex)	0,0000	0,074	1		0	0,00005618	58	1,65947E-06	2	1147,69499	2,15	27,23	0
	High	sHDLWFLTESIR	5	1	1	Q55460	N-Term(iTRAQ4plex)	0,0000	0,130	3	87,1	0	2,338E-07	55	3,12586E-06	2	1647,86235	0,71	44,08	0
	High	lGYIPIVEAAPLIIAQEk	2	1	1	Q55460	N-Term(iTRAQ4plex); K18(iTRAQ4plex)	0,0000	0,512	1		0	9,953E-11	51	7,72627E-06	3	2226,33780	1,15	56,12	0
	High	vPmYVLAQLITQGNGIAVAPmHE	1	1	1	Q55460	N-Term(iTRAQ4plex); M3(Oxidation); M21(Oxidation); K25(iTR	0,0000	0,126	1		0	0,000002181	51	8,60934E-06	4	2957,58608	0,97	49,51	0
	High	eAATEAGFTADIPSSTSR	1	1	1	Q55460	N-Term(iTRAQ4plex)	0,0000	0,650	1		0	0,000001673	51	8,64908E-06	3	1954,94895	0,73	31,13	0
	High	nYFNVPTTILESPFk	2	1	1	Q55460	N-Term(iTRAQ4plex); K15(iTRAQ4plex)	0,0000	0,108	1		0	1,143E-07	47	1,82797E-05	3	2058,11789	1,38	51,64	0
	High	qANWASAR	2	1	1	Q55460	N-Term(iTRAQ4plex)	0,0000	0,376	2	2,6	0	0,00001503	47	2,14274E-05	2	1047,54631	1,02	16,40	0
	High	gPLYWk	2	1	1	Q55460	N-Term(iTRAQ4plex); K6(iTRAQ4plex)	0,0000	0,414	2	2844,2	0	0,002277	30	0,000995337	2	1051,62004	2,05	30,94	0
	High	vNREDLWR	4	2	2	Q55460;P73452	N-Term(iTRAQ4plex)	0,0000				0	0,002106	20	0,010279453	2	1231,66765	0,99	22,83	1
	High	aDWVDkNPk	3	1	1	Q55460	N-Term(iTRAQ4plex); K6(iTRAQ4plex); K9(iTRAQ4plex)	0,0000	0,242	3	41,3	0	0,0004019	18	0,014486718	3	1504,85282	2,92	20,84	1
	High	iIDkVNR	1	1	1	Q55460	N-Term(iTRAQ4plex); K4(iTRAQ4plex)	0,0000	0,263	1		0,001	0,003612	18	0,014353903	3	1145,72843	3,43	17,71	1
	Medium	gFFAk	1	1	1	Q55460	N-Term(iTRAQ4plex); K5(iTRAQ4plex)	0,0000	0,115	1		0,01	0,1258	18	0,015030382	2	857,51311	0,87	23,90	0
	Medium	wGFHk	1	1	1	Q55460	N-Term(iTRAQ4plex); K5(iTRAQ4plex)	0,0000	0,079	1		0,026	0,3506	12	0,063123242	3	962,55283	8,07	22,17	0
	Medium	aADYIk	1	1	1	Q55460	N-Term(iTRAQ4plex); K6(iTRAQ4plex)	0,0000	0,562	1		0,045	0,581	11	0,083596732	2	968,56755	2,10	18,11	0
P72870	Allophycocyanin subunit alpha-B OS=Synechocystis sp. (strain PCC 6803 / Kazusa) GN=apcD PE=3 S	836,10	73,91	1	11	11	40	1,120	33	9,9	161	17,9	5,58							
	A3	Sequence	# PSMs	# Proteins	# Protein Groups	Protein Group Accessions	Modifications	ΔCn	116/117	116/117 Count	116/117 Variability [%]	q-Value	PEP	IonScore	Exp Value	Charge	MH+ [Da]	ΔM [ppm]	RT [min]	# Missed Cleavages
	High	sVVSQVILQADDQLR	3	1	1	P72870	N-Term(iTRAQ4plex)	0,0000	1,120	3	39,7	0	1,197E-09	69	1,17211E-07	2	1815,00994	0,34	45,37	0
	High	gIQAFLTTGAQR	9	1	1	P72870	N-Term(iTRAQ4plex)	0,0000	1,127	5	7,3	0	2,102E-07	64	4,37218E-07	2	1406,78777	0,34	36,48	0
	High	lVTYGVLAGNkEPIETTGLIGVk	7	1	1	P72870	N-Term(iTRAQ4plex); K11(iTRAQ4plex); K23(iTRAQ4plex)	0,0000	1,143	4	1,8	0	9,981E-11	61	8,18408E-07	3	2804,65281	1,06	44,55	1
	High	aPGGNAYGQR	5	1	1	P72870	N-Term(iTRAQ4plex)	0,0000	1,130	5	19,9	0	0,000004392	61	8,4522E-07	2	1134,57768	0,36	13,53	0
	High	kIVDQAQk	4	1	1	P72870	N-Term(iTRAQ4plex); K1(iTRAQ4plex); K8(iTRAQ4plex)	0,0000	1,144	4	5,5	0	0,00002967	47	2,05102E-05	2	1361,85137	2,70	12,63	1
	High	iAETLAENEkk	2	1	1	P72870	N-Term(iTRAQ4plex); K10(iTRAQ4plex); K11(iTRAQ4plex)	0,0000	1,153	2	10,1	0	4,535E-08	40	0,000109893	3	1677,97483	0,04	22,46	1
	High	emYNSLNVPVPGmVDAVTVLk	2	1	1	P72870	N-Term(iTRAQ4plex); M2(Oxidation); M13(Oxidation); K21(iTR	0,0000	1,145	2	0,2	0	0,000001296	38	0,00015921	3	2596,36283	0,87	44,14	0
	High	dYGWYLR	2	1	1	P72870	N-Term(iTRAQ4plex)	0,0000	1,013	2	0,6	0	0,002289	35	0,000312586	2	1116,55889	-0,53	34,05	0
	High	lVTYGVLAGNk	2	1	1	P72870	N-Term(iTRAQ4plex); K11(iTRAQ4plex)	0,0000	0,875	2	11,5	0	0,002145	34	0,000382798	3	1422,85498	-0,65	33,90	0
	High	qYNQcLR	3	1	1	P72870	N-Term(iTRAQ4plex); C5(Carbamidomethyl)	0,0000	0,871	3	13,8	0,001	0,01051	23	0,005610092	2	1125,55974	0,52	14,92	0
	High	kHPEYR	1	1	1	P72870	N-Term(iTRAQ4plex); K1(iTRAQ4plex)	0,0000	1,432	1		0,003	0,03268	17	0,018662508	3	1117,63565	0,00	7,98	1
P73308	50S ribosomal protein L5 OS=Synechocystis sp. (strain PCC 6803 / Kazusa) GN=rplE PE=3 SV=2 - [R	822,55	52,22	1	12	12	41	2,494	35	46,6	180	20,2	9,69							
	A3	Sequence	# PSMs	# Proteins	# Protein Groups	Protein Group Accessions	Modifications	ΔCn	116/117	116/117 Count	116/117 Variability [%]	q-Value	PEP	IonScore	Exp Value	Charge	MH+ [Da]	ΔM [ppm]	RT [min]	# Missed Cleavages
	High	lINLALPR	9	1	1	P73308	N-Term(iTRAQ4plex)	0,0000	3,002	6	4,2	0	0,00001398	65	3,22827E-07	2	1053,69145	1,26	40,50	0
	High	aLESSLTELATITGQkPVVTR	4	1	1	P73308	N-Term(iTRAQ4plex); K16(iTRAQ4plex)	0,0000	2,173	4	29,9	0	2,931E-08	55	3,35714E-06	3	2502,44474	2,61	47,27	0
	High	gNYSLGIR	5	1	1	P73308	N-Term(iTRAQ4plex)	0,0000	3,021	3	2,9	0	0,0001483	54	4,06415E-06	2	1023,57195	1,53	23,78	0
	High	tLYQETILPk	5	1	1	P73308	N-Term(iTRAQ4plex); K10(iTRAQ4plex)	0,0000	0,345	5	43,4	0	2,013E-07	53	4,87495E-06	2	1493,88591	2,77	34,27	0
	High	lQEEFGYk	1	1	1	P73308	N-Term(iTRAQ4plex); K8(iTRAQ4plex)	0,0000	2,494	1		0	0,0002275	46	2,29071E-05	2	1301,70244	3,41	29,01	0
	High	kAIAGFk	2	1	1	P73308	N-Term(iTRAQ4plex); K1(iTRAQ4plex); K7(iTRAQ4plex)	0,0000	3,167	2	1,4	0	0,001841	31	0,000843277	3	1166,76715	4,27	17,90	1
	High	nIHQVPk	2	1	1	P73308	N-Term(iTRAQ4plex); K7(iTRAQ4plex)	0,0000	1,262	2	13,1	0	0,002828	30	0,000959334	2	1123,68743	4,28	12,04	0
	High	gISPNSFDGR	1	1	1	P73308	N-Term(iTRAQ4plex)	0,0000	2,715	1		0	0,001866	30	0,001073915	2	1193,60539	1,87	23,59	0
	High	aLGMPFR	3	1	1	P73308	N-Term(iTRAQ4plex)	0,0000	1,521	3	42,8	0,001	0,005071	34	0,000364729	2	935,52800	2,81	31,26	0
	High	aLGmPFR	3	1	1	P73308	N-Term(iTRAQ4plex); M4(Oxidation)	0,0000	1,760	3	0,1	0,001	0,01343	21	0,00744732	2	951,52141	1,18	22,85	0
	High	aIAGFk	4	1	1	P73308	N-Term(iTRAQ4plex); K6(iTRAQ4plex)	0,0000	2,669	3	29,9	0,008	0,1106	35	0,000330347	2	894,56938	4,75	22,39	0
	Medium	aLGmPFRS	1	1	1	P73308	N-Term(iTRAQ4plex); M4(Oxidation)	0,0000	0,826	1		0,017	0,2212	11	0,086890041	2	1038,55461	2,21	24,27	1
	Medium	mYAFLDR	1	1	1	P73308	N-Term(iTRAQ4plex)	0,0000	2,671	1		0,018	0,2294	24	0,003971641	2	1059,54338	1,87	34,66	0
P52415	Glucose-1-phosphate adenylyltransferase OS=Synechocystis sp. (strain PCC 6803 / Kazusa) GN=glgC	809,38	20,27	1	9	9	25	3,291	25	37,2	439	49,3	6,47							
	A3	Sequence	# PSMs	# Proteins	# Protein Groups	Protein Group Accessions	Modifications	ΔCn	116/117	116/117 Count	116/117 Variability [%]	q-Value	PEP	IonScore	Exp Value	Charge	MH+ [Da]	ΔM [ppm]	RT [min]	# Missed Cleavages
	High	iYVLTQFNSASLNR	4	1	1	P52415	N-Term(iTRAQ4plex)	0,0000	3,308	4	23,6	0	1,076E-10	105	3,18398E-11	2	1769,96758	0,50	43,17	0
	High	vLAIILGGGAGTR	8	1	1	P52415	N-Term(iTRAQ4plex)	0,0000	3,102	8	105,4	0	0,000000048	76	2,29599E-08	2	1341,83428	0,56	46,41	0
	High	lNPYIASmGIYVFk	1	1	1	P52415	N-Term(iTRAQ4plex); M8(Oxidation); K14(iTRAQ4plex)	0,0000	2,988	1		0	4,345E-09	46	2,47725E-05	3	1920,05710	1,43	46,31	0
	High	qYLWLFR	3	1	1	P52415	N-Term(iTRAQ4plex)	0,0000	4,161	3	4,1	0	0,00002139	43	4,76398E-05	2	1169,65874	-0,06	46,93	0
	High	kAPELGLmk	2	1	1	P52415	N-Term(iTRAQ4plex); K1(iTRAQ4plex); M8(Oxidation); K9(iTR/	0,0000	3,385	2	4,2	0	0,000001012	41	0,000152067	3	1434,86954	-1,36	20,88	1
	High	nVmIVNk	2	1	1	P52415	N-Term(iTRAQ4plex); M3(Oxidation); K7(iTRAQ4plex)	0,0000	2,004	2	17,5	0	0,00009434	36	0,00025408	2	1121,65837	-0,67	18,41	0
	High	kAPELGLMk	1	1	1	P52415	N-Term(iTRAQ4plex); K1(iTRAQ4plex); K9(iTRAQ4plex)	0,0000	3,776	1		0	0,000002141	33	0,000536995	3	1418,87482	-1,23	25,05	1
	High	akPAVPLAGk	1	1	1	P52415	N-Term(iTRAQ4plex); K2(iTRAQ4plex); K10(iTRAQ4plex)	0,0000	3,618	1		0	0,000005029	27	0,002137814	3	1383,90479	-0,03	19,51	0
	High	eVLHNLLEk	1	1	1	P52415	N-Term(iTRAQ4plex); K9(iTRAQ4plex)	0,0000	1,534	1		0	0,000009638	21	0,00816526	3	1382,82428	-0,23	30,44	0
	High	lYPLTk	2	1	1	P52415	N-Term(iTRAQ4plex); K6(iTRAQ4plex)	0,0000	1,739	2	29,1	0,009	0,1165	23	0,004487144	2	1022,64855	-0,29	27,25	0
P74690	Probable phosphoketolase OS=Synechocystis sp. (strain PCC 6803 / Kazusa) GN=slr0453 PE=3 SV=2	793,55	12,92	1	11	11	27	1,393	27	32,3	805	90,8	6,21							
	A3	Sequence	# PSMs	# Proteins	# Protein Groups	Protein Group Accessions	Modifications	ΔCn	116/117	116/117 Count	116/117 Variability [%]	q-Value	PEP	IonScore	Exp Value	Charge	MH+ [Da]	ΔM [ppm]	RT [min]	# Missed Cleavages
	High	fVSVIDLLk	8	1	1	P74690	N-Term(iTRAQ4plex); K9(iTRAQ4plex)	0,0000	1,533	8	36,6	0	4,788E-09	74	3,96251E-08	2	1321,83623	2,15	51,12	0
	High	vTSPFSLSPFGQAR	4	1	1	P74690	N-Term(iTRAQ4plex)	0,0000	1,894	4	10,7	0	1,963E-07	70	9,24634E-08	2	1637,87920	1,44	44,21	0
	High	aANYLAVGMIYLR	2	1	1	P74690	N-Term(iTRAQ4plex)	0,0000	1,028	2	13,6	0	0,000005462	54	3,76678E-06	2	1598,88786	2,07	48,86	0
	High	lHAVYEVSk	1	1	1	P74690	N-Term(iTRAQ4plex); K9(iTRAQ4plex)	0,0000	0,764	1		0	0,000007189	54	4,22078E-06	2	1333,77703	3,90	21,74	0
	High	qAHLQYQDmTSAIR	2	1	1	P74690	N-Term(iTRAQ4plex); M9(Oxidation)	0,0000	1,288	2	20,8	0	2,797E-10	51	8,85054E-06	3	1821,90451	0,58	20,35	0
	High	aANYLAVGmIYLR	1	1	1	P74690	N-Term(iTRAQ4plex); M9(Oxidation)	0,0000	1,393	1		0	2,604E-08	49	1,18842E-05	2	1614,88254	1,90	43,06	0
	High	qFFPNLR	1	1	1	P74690	N-Term(iTRAQ4plex)	0,0000	1,392	1		0	0,0001103	36	0,000263009	2	1065,59636	0,14	34,18	0
	High	iNNPSVLSR	2	1	1	P74690	N-Term(iTRAQ4plex)	0,0000	1,192	2	2,3	0	0,00211	33	0,000451825	2	1143,66265	2,07	23,03	0
	High	qAHLQYQDMTSAIR	1	1	1	P74690	N-Term(iTRAQ4plex)	0,0000	0,803	1		0	0,000001214	31	0,000760274	3	1805,91153	1,66	28,23	0
	High	wPMVVMR	1	1	1	P74690	N-Term(iTRAQ4plex)	0,0000	1,715	1		0,006	0,06843	19	0,012734151	2	1062,57475	3,58	38,16	0
	High	iSHEELk	1	1	1	P74690	N-Term(iTRAQ4plex); K7(iTRAQ4plex)	0,0000	1,431	1		0,009	0,1176	21	0,007761935	3	1143,66474	3,08	14,63	0
	Medium	fINPIR	2	1	1	P74690	N-Term(iTRAQ4plex)	0,0000	1,362	2	14,0	0,011	0,1453	34	0,000402689	2	903,55412	0,93	28,37	0
	Medium	aIAPEGDkR	1	1	1	P74690	N-Term(iTRAQ4plex); K8(iTRAQ4plex)	0,0000	0,972	1		0,019	0,2507	11	0,08016227	3	1244,72211	1,57	11,67	1
P73128	Sulfolipid biosynthesis protein SqdB OS=Synechocystis sp. (strain PCC 6803 / Kazusa) GN=sqdB PE=	792,34	35,51	1	15	15	40	0,606	31	18,2	383	43,2	6,01							
	A3	Sequence	# PSMs	# Proteins	# Protein Groups	Protein Group Accessions	Modifications	ΔCn	116/117	116/117 Count	116/117 Variability [%]	q-Value	PEP	IonScore	Exp Value	Charge	MH+ [Da]	ΔM [ppm]	RT [min]	# Missed Cleavages
	High	rYWDAQLGAETLTPIAPIR	6	1	1	P73128	N-Term(iTRAQ4plex)	0,0000	0,669	3	12,6	0	7,473E-10	80	1,00686E-08	3	2315,26480	0,81	43,06	1
	High	lDYDGVFGTALNR	1	1	1	P73128	N-Term(iTRAQ4plex)	0,0000	0,560	1		0	4,534E-08	77	1,98139E-08	2	1584,81523	0,84	41,86	0
	High	fcIQAAIGHPLTVYGk	6	1	1	P73128	N-Term(iTRAQ4plex); C2(Carbamidomethyl); K16(iTRAQ4plex)	0,0000	0,524	4	4,8	0	2,271E-11	61	1,0566E-06	3	2063,13901	1,90	41,64	0
	High	gYEVGILDSLVR	3	1	1	P73128	N-Term(iTRAQ4plex)	0,0000	0,617	3	24,8	0	9,247E-07	58	1,74582E-06	2	1464,81926	0,92	47,00	0
	High	qFQPDAVVHFGEQR	5	1	1	P73128	N-Term(iTRAQ4plex)	0,0000	0,518	3	3,2	0	4,253E-08	52	6,80722E-06	3	1801,91178	0,84	33,30	0
	High	dTLPYPk	1	1	1	P73128	N-Term(iTRAQ4plex); K7(iTRAQ4plex)	0,0000	0,644	1		0	0,00134	39	0,000121891	2	1121,64385	-0,58	23,27	0
	High	lDRWYELTGk	3	1	1	P73128	N-Term(iTRAQ4plex); K10(iTRAQ4plex)	0,0000	0,884	3	1,7	0	0,00001493	39	0,000131817	2	1568,86980	1,44	34,96	1
	High	wYELTGk	2	1	1	P73128	N-Term(iTRAQ4plex); K7(iTRAQ4plex)	0,0000	0,584	2	0,2	0	0,0002974	36	0,000226449	2	1184,65654	0,97	32,45	0
	High	kDTLPYPk	2	1	1	P73128	N-Term(iTRAQ4plex); K1(iTRAQ4plex); K8(iTRAQ4plex)	0,0000	0,598	2	6,0	0	0,000003782	31	0,000753304	3	1393,83973	-1,29	19,55	1
	High	vEIDHLENPR	2	1	1	P73128	N-Term(iTRAQ4plex)	0,0000	0,711	1		0	0,000001515	29	0,00135822	3	1365,72498	0,46	23,91	0
	High	qPGSFYHLSk	1	1	1	P73128	N-Term(iTRAQ4plex); K10(iTRAQ4plex)	0,0000	0,557	1		0	0,0001306	24	0,003881236	3	1451,78940	0,60	23,85	0
	High	sAPFSmIDR	1	1	1	P73128	N-Term(iTRAQ4plex); M6(Oxidation)	0,0000	0,601	1		0	0,00164	23	0,004977027	2	1183,58928	-0,46	24,45	0
	High	gLLDIR	3	1	1	P73128	N-Term(iTRAQ4plex)	0,0000	0,606	2	18,0	0,002	0,02521	22	0,006729302	2	830,52147	-0,22	29,53	0
	High	iWGLR	3	1	1	P73128	N-Term(iTRAQ4plex)	0,0000	0,606	3	4,8	0,009	0,1152	16	0,025291233	2	788,49022	0,34	30,20	0
	Medium	gLLDIRDTVR	1	1	1	P73128	N-Term(iTRAQ4plex)	0,0000	0,991	1		0,031	0,3996	13	0,048974499	3	1301,77109	4,05	31,40	1
P73348	Rehydrin OS=Synechocystis sp. (strain PCC 6803 / Kazusa) GN=slr1198 PE=1 SV=1 - [P73348_SYNY	774,11	46,45	1	11	11	43	0,694	34	16,6	211	23,5	5,30							
	A3	Sequence	# PSMs	# Proteins	# Protein Groups	Protein Group Accessions	Modifications	ΔCn	116/117	116/117 Count	116/117 Variability [%]	q-Value	PEP	IonScore	Exp Value	Charge	MH+ [Da]	ΔM [ppm]	RT [min]	# Missed Cleavages
	High	vIALSVDDVESHk	4	1	1	P73348	N-Term(iTRAQ4plex); K13(iTRAQ4plex)	0,0000	0,664	2	0,6	0	7,312E-10	72	7,60911E-08	3	1699,94748	0,33	33,30	0
	High	vSDLYGMIHPNALNNLTVR	4	1	1	P73348	N-Term(iTRAQ4plex)	0,0000	0,902	3	5,7	0	3,918E-09	56	3,08447E-06	3	2271,20542	0,76	46,28	0
	High	sVFIIDPAk	2	1	1	P73348	N-Term(iTRAQ4plex); K9(iTRAQ4plex)	0,0000	0,621	2	2,0	0	0,000008945	55	3,35714E-06	2	1277,77141	0,49	35,88	0
	High	gVEEIkPYLR	7	1	1	P73348	N-Term(iTRAQ4plex); K6(iTRAQ4plex)	0,0000	0,725	7	3,0	0	0,00003458	52	6,83864E-06	2	1491,87688	-0,33	28,95	0
	High	vSDLYGmIHPNALNNLTVR	7	1	1	P73348	N-Term(iTRAQ4plex); M7(Oxidation)	0,0000	0,737	5	11,8	0	4,404E-09	50	1,20058E-05	3	2287,19986	0,55	40,68	0
	High	lTFTYPASTGR	3	1	1	P73348	N-Term(iTRAQ4plex)	0,0000	0,637	2	1,8	0	0,000007794	46	2,74138E-05	2	1357,72453	0,94	32,56	0
	High	nFDEILR	1	1	1	P73348	N-Term(iTRAQ4plex)	0,0000	0,532	1		0	0,0001183	45	3,13307E-05	2	1050,57121	1,09	32,37	0
	High	cVVVPSISTEDAk	1	1	1	P73348	N-Term(iTRAQ4plex); C1(Carbamidomethyl); K13(iTRAQ4plex)	0,0000	0,978	1		0	0,000003747	35	0,000344661	3	1692,90744	-0,39	30,50	0
	High	sVFIIDPAkk	4	1	1	P73348	N-Term(iTRAQ4plex); K9(iTRAQ4plex); K10(iTRAQ4plex)	0,0000	0,847	3	2,3	0	0,00001815	25	0,003090082	3	1549,96964	1,17	30,48	1
	High	lkPEFDk	3	1	1	P73348	N-Term(iTRAQ4plex); K2(iTRAQ4plex); K7(iTRAQ4plex)	0,0000	0,567	3	1,7	0	0,001819	19	0,012330197	3	1308,78586	-2,23	21,08	0
	High	lkPEFDkR	2	1	1	P73348	N-Term(iTRAQ4plex); K2(iTRAQ4plex); K7(iTRAQ4plex)	0,0000	0,726	2	1,5	0	0,0009187	16	0,023065879	3	1464,89252	1,79	18,33	1
	High	lTPQPNk	5	1	1	P73348	N-Term(iTRAQ4plex); K7(iTRAQ4plex)	0,0000	0,679	3	0,2	0,002	0,01791	11	0,076027376	2	1085,65557	-0,16	16,06	0
P72776	Pyridoxine 5'-phosphate synthase OS=Synechocystis sp. (strain PCC 6803 / Kazusa) GN=pdxJ PE=3 S	767,94	28,93	1	6	6	28	0,810	23	24,2	242	26,5	5,62							
	A3	Sequence	# PSMs	# Proteins	# Protein Groups	Protein Group Accessions	Modifications	ΔCn	116/117	116/117 Count	116/117 Variability [%]	q-Value	PEP	IonScore	Exp Value	Charge	MH+ [Da]	ΔM [ppm]	RT [min]	# Missed Cleavages
	High	mLTLGVNIDHVATIR	6	1	1	P72776	N-Term(iTRAQ4plex); M1(Oxidation)	0,0000	0,775	5	21,3	0	1,899E-11	81	7,51951E-09	3	1813,01364	0,74	38,19	0
	High	hIPVSLFIDPDVAQLk	9	1	1	P72776	N-Term(iTRAQ4plex); K16(iTRAQ4plex)	0,0000	0,833	8	20,4	0	1,06E-09	68	1,5065E-07	3	2080,20774	1,53	46,44	0
	High	yANAPTADDQAR	2	1	1	P72776	N-Term(iTRAQ4plex)	0,0000	0,739	1		0	5,107E-07	64	3,6389E-07	2	1436,69060	1,33	16,15	0
	High	mLTLGVNIDHVATIR	1	1	1	P72776	N-Term(iTRAQ4plex)	0,0000	1,625	1		0	2,454E-07	53	5,59368E-06	3	1797,01902	0,91	42,10	0
	High	tHLNLEMAATk	1	1	1	P72776	N-Term(iTRAQ4plex); K11(iTRAQ4plex)	0,0000	0,439	1		0	7,447E-07	46	2,42086E-05	3	1516,84018	0,37	24,64	0
	High	fIELHTGk	5	1	1	P72776	N-Term(iTRAQ4plex); K8(iTRAQ4plex)	0,0000	0,810	3	4,4	0	0,00001551	46	2,5408E-05	3	1232,72441	0,21	24,54	0
	High	tHLNLEmAATk	2	1	1	P72776	N-Term(iTRAQ4plex); M7(Oxidation); K11(iTRAQ4plex)	0,0000	0,887	2	11,8	0	0,000004447	36	0,000229071	3	1532,83609	1,02	19,94	0
	High	aILVGmER	2	1	1	P72776	N-Term(iTRAQ4plex); M6(Oxidation)	0,0000	0,316	2	123,7	0,001	0,00826	34	0,000583617	2	1048,59502	0,79	22,22	0
P73306	50S ribosomal protein L6 OS=Synechocystis sp. (strain PCC 6803 / Kazusa) GN=rplF PE=3 SV=1 - [R	763,55	43,02	1	7	7	31	1,766	25	48,8	179	19,7	10,18							
	A3	Sequence	# PSMs	# Proteins	# Protein Groups	Protein Group Accessions	Modifications	ΔCn	116/117	116/117 Count	116/117 Variability [%]	q-Value	PEP	IonScore	Exp Value	Charge	MH+ [Da]	ΔM [ppm]	RT [min]	# Missed Cleavages
	High	vSVDIQGSHLSVk	5	1	1	P73306	N-Term(iTRAQ4plex); K13(iTRAQ4plex)	0,0000	2,158	3	46,0	0	7,242E-09	80	8,91189E-09	2	1656,95574	2,05	30,18	0
	High	vIVAQEGETITVTR	1	1	1	P73306	N-Term(iTRAQ4plex)	0,0000	2,371	1		0	8,573E-09	77	2,37236E-08	2	1659,94243	1,56	31,48	0
	High	tLVANmVDGVAQGFER	2	1	1	P73306	N-Term(iTRAQ4plex); M6(Oxidation)	0,0000	2,702	1		0	3,672E-11	75	3,39602E-08	2	1866,95256	1,31	37,33	0
	High	tLVANMVDGVAQGFER	2	1	1	P73306	N-Term(iTRAQ4plex)	0,0000	0,596	2	28,9	0	1,388E-10	70	1,104E-07	2	1850,96062	2,93	51,36	0
	High	rLEIQGVGYR	7	1	1	P73306	N-Term(iTRAQ4plex)	0,0000	1,293	5	19,4	0	0,0002601	52	6,74822E-06	2	1334,76787	1,29	24,60	1
	High	aVRPPEPYk	5	1	1	P73306	N-Term(iTRAQ4plex); K9(iTRAQ4plex)	0,0000	1,775	5	20,5	0	0,002146	42	5,9837E-05	2	1344,78777	-0,04	18,16	0
	High	yQGEYVR	4	1	1	P73306	N-Term(iTRAQ4plex)	0,0000	1,540	4	24,9	0	0,0004563	40	9,86211E-05	2	1058,54009	1,28	17,92	0
	High	rPIPLPAk	5	1	1	P73306	N-Term(iTRAQ4plex); K8(iTRAQ4plex)	0,0000	3,556	4	32,5	0	0,003005	30	0,00090985	3	1179,78540	3,20	20,34	0
P73911	Catalase-peroxidase OS=Synechocystis sp. (strain PCC 6803 / Kazusa) GN=katG PE=1 SV=1 - [KATG	746,67	22,55	1	18	18	47	0,667	47	24,4	754	84,4	5,74							
	A3	Sequence	# PSMs	# Proteins	# Protein Groups	Protein Group Accessions	Modifications	ΔCn	116/117	116/117 Count	116/117 Variability [%]	q-Value	PEP	IonScore	Exp Value	Charge	MH+ [Da]	ΔM [ppm]	RT [min]	# Missed Cleavages
	High	vLGTNHGGTk	2	1	1	P73911	N-Term(iTRAQ4plex); K10(iTRAQ4plex)	0,0000	0,618	2	5,3	0	1,431E-07	85	2,83772E-09	2	1271,73259	1,22	11,12	0
	High	vDLVFGSNSILR	5	1	1	P73911	N-Term(iTRAQ4plex)	0,0000	0,560	5	45,8	0	0,000001992	60	1,64242E-06	2	1463,83513	0,84	44,71	0
	High	qDYAVSPEELLLER	3	1	1	P73911	N-Term(iTRAQ4plex)	0,0000	0,673	3	18,6	0	8,844E-08	57	2,11349E-06	3	1805,94175	0,84	43,91	0
	High	tQLmGLTAPEmTVLIGGmR	1	1	1	P73911	N-Term(iTRAQ4plex); M4(Oxidation); M11(Oxidation); M18(Ox	0,0000	0,494	1		0	3,735E-09	46	2,74138E-05	3	2211,13309	1,18	39,19	0
	High	fYREPDYFAEVFAk	4	1	1	P73911	N-Term(iTRAQ4plex); K14(iTRAQ4plex)	0,0000	0,744	4	15,7	0	1,308E-08	44	4,17802E-05	3	2070,06040	1,38	44,64	1
	High	sYAEVYAQDDNREk	1	1	1	P73911	N-Term(iTRAQ4plex); K14(iTRAQ4plex)	0,0000	0,853	1		0	0,000002329	42	5,9975E-05	3	1975,96305	1,54	19,58	1
	High	vYGFAGGR	3	1	1	P73911	N-Term(iTRAQ4plex)	0,0000	0,580	3	5,9	0	0,001045	39	0,000139627	2	970,52324	0,54	22,77	0
	High	lLWPIk	6	1	1	P73911	N-Term(iTRAQ4plex); K6(iTRAQ4plex)	0,0000	0,609	6	4,6	0	0,002492	31	0,000785181	2	1057,70244	1,14	41,23	0
	High	hNPImTDADmAmIkDPIYR	3	1	1	P73911	N-Term(iTRAQ4plex); M5(Oxidation); M10(Oxidation); M12(Ox	0,0000	0,659	3	116,8	0	3,962E-08	27	0,001840645	5	2568,25330	1,29	28,56	1
	High	tQLmGLTAPEMTVLIGGmR	1	1	1	P73911	N-Term(iTRAQ4plex); M4(Oxidation); M18(Oxidation)	0,0000	0,562	1		0	0,0002612	27	0,002051021	3	2195,13608	0,23	46,23	0
	High	hNPImTDADMAmIkDPIYR	1	1	1	P73911	N-Term(iTRAQ4plex); M5(Oxidation); M12(Oxidation); K14(iTR	0,0000	1,281	1		0	4,839E-08	27	0,002228281	4	2552,25796	1,13	32,58	1
	High	lLWPIkk	1	1	1	P73911	N-Term(iTRAQ4plex); K6(iTRAQ4plex); K7(iTRAQ4plex)	0,0000	0,927	1		0	0,001387	23	0,005419635	3	1329,89930	0,77	34,13	1
	High	vYGFAGGREDIWHPEk	2	1	1	P73911	N-Term(iTRAQ4plex); K16(iTRAQ4plex)	0,0000	0,704	2	1,5	0	0,00002204	22	0,006251295	4	2149,10756	0,28	33,44	1
	High	vMNADRFDLPR	2	1	1	P73911	N-Term(iTRAQ4plex)	0,0000	1,342	2	11,0	0	0,0006491	20	0,009953367	3	1477,77298	1,83	30,32	1
	High	hNPImTDADMAMIkDPIYR	2	1	1	P73911	N-Term(iTRAQ4plex); M5(Oxidation); K14(iTRAQ4plex)	0,0000	1,164	2	21,5	0	0,0005262	20	0,010255811	4	2536,26552	2,12	35,76	1
	High	hGVFTDR	2	1	1	P73911	N-Term(iTRAQ4plex)	0,0000	0,613	2	8,1	0	0,001071	13	0,05482391	3	975,51413	1,29	14,00	0
	High	vmNADRFDLPR	2	1	1	P73911	N-Term(iTRAQ4plex); M2(Oxidation)	0,0000	0,721	2	53,3	0,001	0,01091	20	0,01030315	3	1493,76661	0,95	25,78	1
	High	rLLWPIk	1	1	1	P73911	N-Term(iTRAQ4plex); K7(iTRAQ4plex)	0,0000	0,784	1		0,001	0,004518	14	0,037841645	3	1213,80558	2,65	33,48	1
	High	iRLEPQk	1	1	1	P73911	N-Term(iTRAQ4plex); K7(iTRAQ4plex)	0,0000	1,033	1		0,002	0,02861	20	0,009225077	3	1171,74552	4,58	18,73	1
	High	wTATk	2	1	1	P73911	N-Term(iTRAQ4plex); K5(iTRAQ4plex)	0,0000	0,667	2	0,1	0,008	0,1077	27	0,002356032	2	894,53008	1,50	17,72	0
	Medium	nWPGNEPTR	1	1	1	P73911	N-Term(iTRAQ4plex)	0,0000	0,742	1		0,012	0,1543	17	0,019497099	2	1214,60393	0,36	18,52	0
	Medium	tTFAR	1	1	1	P73911	N-Term(iTRAQ4plex)	0,0000	1,053	1		0,034	0,4411	12	0,065459096	2	739,42290	1,32	15,37	0
Q55247	Nitrogen regulatory protein P-II OS=Synechocystis sp. (strain PCC 6803 / Kazusa) GN=glnB PE=1 SV	734,00	68,75	1	9	9	25	0,791	23	15,5	112	12,4	6,81							
	A3	Sequence	# PSMs	# Proteins	# Protein Groups	Protein Group Accessions	Modifications	ΔCn	116/117	116/117 Count	116/117 Variability [%]	q-Value	PEP	IonScore	Exp Value	Charge	MH+ [Da]	ΔM [ppm]	RT [min]	# Missed Cleavages
	High	iALVNAGIVGmTVSEVR	6	1	1	Q55247	N-Term(iTRAQ4plex); M11(Oxidation)	0,0000	0,670	5	5,4	0	3,31E-14	113	4,81948E-12	2	1889,06816	1,81	44,83	0
	High	iALVNAGIVGMTVSEVR	4	1	1	Q55247	N-Term(iTRAQ4plex)	0,0000	0,792	3	84,8	0	4,43E-11	63	5,83973E-07	3	1873,07224	1,28	48,80	0
	High	gSEYTVEFLQk	1	1	1	Q55247	N-Term(iTRAQ4plex); K11(iTRAQ4plex)	0,0000	0,716	1		0	5,505E-07	63	5,44465E-07	2	1588,84758	0,92	34,63	0
	High	iFISPVDSVVR	4	1	1	Q55247	N-Term(iTRAQ4plex)	0,0000	0,810	4	6,5	0	0,0003407	54	3,69803E-06	2	1375,80705	0,31	40,41	0
	High	vEAIIRPFk	3	1	1	Q55247	N-Term(iTRAQ4plex); K9(iTRAQ4plex)	0,0000	0,784	3	1,0	0	0,000004957	52	5,86098E-06	2	1360,85637	0,62	32,85	0
	High	kVEAIIRPFk	2	1	1	Q55247	N-Term(iTRAQ4plex); K1(iTRAQ4plex); K10(iTRAQ4plex)	0,0000	0,914	2	6,0	0	0,000002014	36	0,000256431	3	1633,05429	1,06	28,53	1
	High	vEAIIRPFkLDEVk	1	1	1	Q55247	N-Term(iTRAQ4plex); K9(iTRAQ4plex); K14(iTRAQ4plex)	0,0000	0,826	1		0	0,000005691	14	0,038456522	3	2089,27549	0,42	38,17	1
	High	tGEIGDGk	1	1	1	Q55247	N-Term(iTRAQ4plex); K8(iTRAQ4plex)	0,0000	0,749	1		0,001	0,0104	26	0,002716252	2	1064,58245	-0,19	14,44	0
	High	tGEkDTEAI	1	1	1	Q55247	N-Term(iTRAQ4plex); K4(iTRAQ4plex)	0,0000	0,791	1		0,007	0,08768	24	0,004101758	2	1251,66765	0,43	15,37	1
	Medium	lVSAAR	2	1	1	Q55247	N-Term(iTRAQ4plex)	0,0000	1,068	2	11,1	0,012	0,1549	23	0,013551062	2	760,47978	0,00	16,07	0

P73287	Bacterioferritin OS=Synechocystis sp. (strain PCC 6803 / Kazusa) GN=bfr PE=3 SV=1 - [P73287_SYN	733,39	36,36	1	5	5	24	0,525	21	12,1	176	20,2	4,61							
	A3	Sequence	# PSMs	# Proteins	# Protein Groups	Protein Group Accessions	Modifications	ΔCn	116/117	116/117 Count	116/117 Variability [%]	q-Value	PEP	IonScore	Exp Value	Charge	MH+ [Da]	ΔM [ppm]	RT [min]	# Missed Cleavages
	High	dGLVNSIAFFETQR	2	1	1	P73287	N-Term(iTRAQ4plex)	0,0000	0,494	2	3,3	0	2,063E-11	81	7,92447E-09	2	1740,90544	0,95	47,21	0
	High	lQLTAINQYFLHAR	13	1	1	P73287	N-Term(iTRAQ4plex)	0,0000	0,524	10	10,0	0	3,909E-08	68	1,61053E-07	2	1832,03252	1,39	46,50	0
	High	nWGLNALNQYEYk	3	1	1	P73287	N-Term(iTRAQ4plex); K13(iTRAQ4plex)	0,0000	0,517	3	7,7	0	1,936E-11	66	2,24373E-07	2	1900,98271	1,64	40,84	0
	High	vLFLEGLPNLQNLEk	2	1	1	P73287	N-Term(iTRAQ4plex); K15(iTRAQ4plex)	0,0000	0,619	2	32,5	0	1,306E-09	45	2,81819E-05	3	2015,18113	1,55	54,68	0
	High	qHLNQALk	4	1	1	P73287	N-Term(iTRAQ4plex); K8(iTRAQ4plex)	0,0000	0,754	4	22,8	0	0,0004374	45	2,91723E-05	2	1239,74394	2,22	12,98	0
P72938	Extracellular nuclease OS=Synechocystis sp. (strain PCC 6803 / Kazusa) GN=nucH PE=4 SV=1 - [P72	727,87	6,55	1	8	8	23	0,158	19	21,4	1879	196,4	3,99							
	A3	Sequence	# PSMs	# Proteins	# Protein Groups	Protein Group Accessions	Modifications	ΔCn	116/117	116/117 Count	116/117 Variability [%]	q-Value	PEP	IonScore	Exp Value	Charge	MH+ [Da]	ΔM [ppm]	RT [min]	# Missed Cleavages
	High	qANLFTYAAGGTLSR	5	1	1	P72938	N-Term(iTRAQ4plex)	0,0000	0,170	4	24,8	0	9,108E-12	83	4,62349E-09	2	1713,90691	1,64	38,43	0
	High	iIGGLSQNLGR	4	1	1	P72938	N-Term(iTRAQ4plex)	0,0000	0,118	3	3,0	0	2,003E-08	68	1,45201E-07	2	1271,75603	0,61	32,50	0
	High	vTGFNVLNYFk	5	1	1	P72938	N-Term(iTRAQ4plex); K11(iTRAQ4plex)	0,0000	0,158	5	8,9	0	7,051E-08	65	3,05471E-07	2	1589,89495	1,21	49,90	0
	High	lSQGGRPQQFTQFNDANVAGYGA	1	1	1	P72938	N-Term(iTRAQ4plex)	0,0000				0	1,411E-09	62	6,93378E-07	3	2889,42240	2,57	35,07	0
	High	dLVTGETFTAVVNHFk	1	1	1	P72938	N-Term(iTRAQ4plex); K16(iTRAQ4plex)	0,0000	0,185	1		0	6,831E-15	58	1,72659E-06	3	2066,11631	0,09	45,84	0
	High	vNDRPVTPENVGGSLk	2	1	1	P72938	N-Term(iTRAQ4plex); K16(iTRAQ4plex)	0,0000	0,146	2	6,0	0	1,211E-09	55	2,89048E-06	3	1970,09177	0,40	25,33	0
	High	sNLIITGIIDGPLPGGLPk	1	1	1	P72938	N-Term(iTRAQ4plex); K19(iTRAQ4plex)	0,0000	0,173	1		0	1,755E-08	45	3,30347E-05	3	2163,29935	0,07	52,11	0
	High	nRPTVAQTFR	4	1	1	P72938	N-Term(iTRAQ4plex)	0,0000	0,236	3	47,6	0	0,00001078	35	0,000383171	3	1333,74598	0,17	16,61	0
P73596	Sll1307 protein OS=Synechocystis sp. (strain PCC 6803 / Kazusa) GN=sll1307 PE=4 SV=1 - [P73596_	726,16	41,71	1	5	5	23	0,393	16	13,2	175	17,8	6,52							
	A3	Sequence	# PSMs	# Proteins	# Protein Groups	Protein Group Accessions	Modifications	ΔCn	116/117	116/117 Count	116/117 Variability [%]	q-Value	PEP	IonScore	Exp Value	Charge	MH+ [Da]	ΔM [ppm]	RT [min]	# Missed Cleavages
	High	vVGGLGVSGTPIGmVDEGcAIk	1	1	1	P73596	N-Term(iTRAQ4plex); M14(Oxidation); C19(Carbamidomethyl);	0,0000	0,386	1		0	5,32E-13	86	2,69135E-09	3	2420,28055	1,52	39,64	0
	High	aYSAVTLATNHNLDR	4	1	1	P73596	N-Term(iTRAQ4plex)	0,0000	0,398	3	2,4	0	1,993E-09	82	6,561E-09	2	1789,93096	-0,23	27,73	0
	High	aAQSAIAAcR	4	1	1	P73596	N-Term(iTRAQ4plex); C9(Carbamidomethyl)	0,0000	0,392	2	4,5	0	0,000001964	76	2,78593E-08	2	1162,61260	0,55	15,44	0
	High	tSGILASmQAk	5	1	1	P73596	N-Term(iTRAQ4plex); M8(Oxidation); K11(iTRAQ4plex)	0,0000	0,523	3	10,8	0	0,000002037	69	1,19391E-07	2	1410,79448	5,64	19,50	0
	High	sDGALVHTVQTSFNk	8	1	1	P73596	N-Term(iTRAQ4plex); K15(iTRAQ4plex)	0,0000	0,377	6	15,0	0	1,24E-09	54	4,92418E-06	3	1892,01120	-0,23	30,96	0
	High	tSGILASMQAk	1	1	1	P73596	N-Term(iTRAQ4plex); K11(iTRAQ4plex)	0,0000	0,265	1		0	0,000004323	30	0,001023739	3	1394,79197	0,26	29,32	0
P73722	SOS function regulatory protein OS=Synechocystis sp. (strain PCC 6803 / Kazusa) GN=lexA PE=3 SV	721,40	50,25	1	14	14	36	1,201	32	14,4	203	22,7	6,11							
	A3	Sequence	# PSMs	# Proteins	# Protein Groups	Protein Group Accessions	Modifications	ΔCn	116/117	116/117 Count	116/117 Variability [%]	q-Value	PEP	IonScore	Exp Value	Charge	MH+ [Da]	ΔM [ppm]	RT [min]	# Missed Cleavages
	High	gGELVEADAEEVEk	1	1	1	P73722	N-Term(iTRAQ4plex); K14(iTRAQ4plex)	0,0000	0,713	1		0	5,596E-09	62	6,23692E-07	3	1762,89530	0,18	28,86	0
	High	gVSVIGELk	9	1	1	P73722	N-Term(iTRAQ4plex); K9(iTRAQ4plex)	0,0000	1,194	6	1,4	0	0,00005672	60	1,00224E-06	2	1189,74065	0,98	32,04	0
	High	gYVDWTDGk	1	1	1	P73722	N-Term(iTRAQ4plex); K9(iTRAQ4plex)	0,0000	1,236	1		0	0,000006239	49	1,28224E-05	2	1328,67327	0,56	28,20	0
	High	iDFAPLMk	1	1	1	P73722	N-Term(iTRAQ4plex); K8(iTRAQ4plex)	0,0000	1,868	1		0	0,0005049	49	1,33958E-05	2	1222,71184	0,82	40,94	0
	High	sVTGEEEIEDGELVAASIk	3	1	1	P73722	N-Term(iTRAQ4plex); K19(iTRAQ4plex)	0,0000	1,367	3	14,1	0	3,757E-08	46	2,28018E-05	3	2264,17783	1,31	43,54	0
	High	sSVFALR	4	1	1	P73722	N-Term(iTRAQ4plex)	0,0000	1,127	4	2,2	0	0,0006044	44	4,385E-05	2	923,54338	0,29	27,99	0
	High	gPGQELk	1	1	1	P73722	N-Term(iTRAQ4plex); K7(iTRAQ4plex)	0,0000	1,065	1		0	0,0003131	41	8,33624E-05	2	1016,59770	-0,19	15,41	0
	High	kSSVFALR	3	1	1	P73722	N-Term(iTRAQ4plex); K1(iTRAQ4plex)	0,0000	1,438	3	1,1	0	0,0001453	37	0,000201359	3	1195,73993	-0,18	21,65	1
	High	iLHQkPk	3	1	1	P73722	N-Term(iTRAQ4plex); K5(iTRAQ4plex); K7(iTRAQ4plex)	0,0000	1,305	3	2,2	0	0,0001384	34	0,000408291	2	1295,85271	0,26	10,22	0
	High	rYYQDGTk	1	1	1	P73722	N-Term(iTRAQ4plex); K8(iTRAQ4plex)	0,0000	1,070	1		0	0,0001618	34	0,00042459	3	1318,69919	-0,14	11,45	1
	High	aSNNkGPGQELk	3	1	1	P73722	N-Term(iTRAQ4plex); K5(iTRAQ4plex); K12(iTRAQ4plex)	0,0000	1,519	2	2,1	0	0,000002334	29	0,001230184	3	1674,95194	1,19	12,81	1
	High	iDFAPLmkk	1	1	1	P73722	N-Term(iTRAQ4plex); M7(Oxidation); K8(iTRAQ4plex); K9(iTRA	0,0000	1,360	1		0	0,002446	21	0,00810905	3	1510,90317	0,26	30,84	1
	High	yYQDGTk	1	1	1	P73722	N-Term(iTRAQ4plex); K7(iTRAQ4plex)	0,0000	0,764	1		0,001	0,007913	12	0,058340481	2	1162,59917	0,78	15,73	0
	High	iDFAPLmk	2	1	1	P73722	N-Term(iTRAQ4plex); M7(Oxidation); K8(iTRAQ4plex)	0,0000	0,892	2	9,7	0,005	0,05579	19	0,013181657	2	1238,70610	0,28	35,76	0
	High	sPAPIQSR	2	1	1	P73722	N-Term(iTRAQ4plex)	0,0000	1,129	2	3,7	0,005	0,05585	15	0,030058687	2	999,56975	-0,64	12,82	0
Q55511	Trigger factor OS=Synechocystis sp. (strain PCC 6803 / Kazusa) GN=tig PE=3 SV=1 - [TIG_SYNY3]	710,04	26,54	1	14	14	30	0,649	29	15,9	471	52,6	4,41							
	A3	Sequence	# PSMs	# Proteins	# Protein Groups	Protein Group Accessions	Modifications	ΔCn	116/117	116/117 Count	116/117 Variability [%]	q-Value	PEP	IonScore	Exp Value	Charge	MH+ [Da]	ΔM [ppm]	RT [min]	# Missed Cleavages
	High	gSDFEVTLEDGR	1	1	1	Q55511	N-Term(iTRAQ4plex)	0,0000	0,627	1		0	1,617E-08	75	3,13307E-08	2	1468,70464	0,65	30,73	0
	High	lVAQTAMELER	1	1	1	Q55511	N-Term(iTRAQ4plex)	0,0000	0,769	1		0	3,849E-08	66	2,82611E-07	2	1404,76445	0,48	34,67	0
	High	lVNFVESSLTESk	3	1	1	Q55511	N-Term(iTRAQ4plex); K13(iTRAQ4plex)	0,0000	0,542	3	18,3	0	1,465E-07	53	4,75302E-06	2	1740,96135	-0,51	46,05	0
	High	lVAQTAmELER	1	1	1	Q55511	N-Term(iTRAQ4plex); M7(Oxidation)	0,0000	0,513	1		0	0,0001645	46	2,46037E-05	2	1420,75908	0,27	31,81	0
	High	vYENVVk	1	1	1	Q55511	N-Term(iTRAQ4plex); K7(iTRAQ4plex)	0,0000	0,578	1		0	0,000919	42	6,2513E-05	2	1138,66911	-1,70	21,30	0
	High	dNSRPEAIANLk	2	1	1	Q55511	N-Term(iTRAQ4plex); K12(iTRAQ4plex)	0,0000	0,584	2	0,0	0	2,393E-07	41	9,43938E-05	3	1615,90134	0,43	21,84	0
	High	kVYENVVk	3	1	1	Q55511	N-Term(iTRAQ4plex); K1(iTRAQ4plex); K8(iTRAQ4plex)	0,0000	0,699	3	2,2	0	3,877E-07	40	0,000106407	3	1410,86652	-1,11	18,37	1
	High	qEELPIIGNFSLR	2	1	1	Q55511	N-Term(iTRAQ4plex)	0,0000	0,706	2	23,7	0	8,761E-07	39	0,000120495	3	1659,91843	-0,17	49,04	0
	High	qFQEQAk	2	1	1	Q55511	N-Term(iTRAQ4plex); K7(iTRAQ4plex)	0,0000	0,555	2	4,2	0	0,0001731	37	0,000180705	2	1166,63933	-1,28	13,44	0
	High	mGLDVSQLFR	1	1	1	Q55511	N-Term(iTRAQ4plex)	0,0000	0,760	1		0	0,001938	31	0,000753304	2	1309,70610	0,43	46,91	0
	High	aTAIEELIDDSIk	1	1	1	Q55511	N-Term(iTRAQ4plex); K13(iTRAQ4plex)	0,0000	0,560	1		0	0,00007202	30	0,001061622	3	1705,94931	1,79	51,27	0
	High	tVNIPGFR	4	1	1	Q55511	N-Term(iTRAQ4plex)	0,0000	0,725	4	4,6	0,001	0,007519	36	0,000271132	2	1047,60723	0,42	32,13	0
	High	mGLDVSQLFR	1	1	1	Q55511	N-Term(iTRAQ4plex); M1(Oxidation)	0,0000	0,681	1		0,001	0,004201	36	0,00024042	2	1325,70122	0,58	41,76	0
	High	lGQSYIk	2	1	1	Q55511	N-Term(iTRAQ4plex); K7(iTRAQ4plex)	0,0000	0,616	2	7,8	0,001	0,004431	28	0,001541594	2	1096,66081	0,31	23,36	0
	High	aIVIQR	4	1	1	Q55511	N-Term(iTRAQ4plex)	0,0000	0,642	3	1,2	0,001	0,01186	13	0,047859704	2	843,55260	-0,81	18,40	0
	Medium	lkEIk	1	1	1	Q55511	N-Term(iTRAQ4plex); K2(iTRAQ4plex); K5(iTRAQ4plex)	0,0000	0,658	1		0,016	0,205	17	0,020891518	3	1062,72260	-1,99	16,03	1
P52231	Thioredoxin OS=Synechocystis sp. (strain PCC 6803 / Kazusa) GN=trxA PE=1 SV=3 - [THIO_SYNY3]	708,67	46,73	1	4	4	28	0,607	23	37,0	107	11,7	4,93							
	A3	Sequence	# PSMs	# Proteins	# Protein Groups	Protein Group Accessions	Modifications	ΔCn	116/117	116/117 Count	116/117 Variability [%]	q-Value	PEP	IonScore	Exp Value	Charge	MH+ [Da]	ΔM [ppm]	RT [min]	# Missed Cleavages
	High	lNTDENPNTASQYGIR	1	1	1	P52231	N-Term(iTRAQ4plex)	0,0000	0,558	1		0	2,213E-12	115	3,15479E-12	2	1936,94927	0,57	27,14	0
	High	sIPTLMIFk	10	1	1	P52231	N-Term(iTRAQ4plex); K9(iTRAQ4plex)	0,0000	0,781	7	5,0	0	0,00000407	71	7,88806E-08	2	1337,81059	0,04	46,18	0
	High	mVAPVVDEISQQYEGk	1	1	1	P52231	N-Term(iTRAQ4plex); M1(Oxidation); K16(iTRAQ4plex)	0,0000	0,538	1		0	2,707E-11	67	1,81539E-07	3	2097,08017	1,18	37,06	0
	High	tTLASTLEk	7	1	1	P52231	N-Term(iTRAQ4plex); K9(iTRAQ4plex)	0,0000	0,607	5	6,4	0	0,000029	60	1,04947E-06	2	1251,74065	0,62	25,82	0
	High	sIPTLmIFk	9	1	1	P52231	N-Term(iTRAQ4plex); M6(Oxidation); K9(iTRAQ4plex)	0,0000	0,557	9	27,4	0	0,0002384	48	1,67098E-05	2	1353,80632	0,64	41,94	0
P74102	Orange carotenoid-binding protein OS=Synechocystis sp. (strain PCC 6803 / Kazusa) GN=slr1963 PE	698,23	46,06	1	12	12	28	1,929	25	20,6	317	34,6	5,05							
	A3	Sequence	# PSMs	# Proteins	# Protein Groups	Protein Group Accessions	Modifications	ΔCn	116/117	116/117 Count	116/117 Variability [%]	q-Value	PEP	IonScore	Exp Value	Charge	MH+ [Da]	ΔM [ppm]	RT [min]	# Missed Cleavages
	High	eIQAmGPLQQTQAmcDLANR	1	1	1	P74102	N-Term(iTRAQ4plex); M5(Oxidation); M14(Oxidation); C15(Car	0,0000	1,829	1		0	3,636E-14	86	2,79235E-09	3	2451,15671	0,78	30,63	0
	High	gIFPNTLAADVVPATIAR	3	1	1	P74102	N-Term(iTRAQ4plex)	0,0000	2,855	3	44,3	0	1,1E-11	83	5,43213E-09	2	1970,12126	1,04	48,20	0
	High	tYASWSPNIk	2	1	1	P74102	N-Term(iTRAQ4plex); K10(iTRAQ4plex)	0,0000	1,391	2	64,6	0	0,000002227	67	2,31578E-07	2	1454,78362	-3,16	30,36	0
	High	tLTIAAPGAASmQLAENALk	3	1	1	P74102	N-Term(iTRAQ4plex); M12(Oxidation); K20(iTRAQ4plex)	0,0000	1,773	2	7,9	0	1,763E-13	66	2,5527E-07	3	2275,25919	0,92	40,62	0
	High	iAEPVVPPQDTASR	1	1	1	P74102	N-Term(iTRAQ4plex)	0,0000	2,368	1		0	5,891E-07	65	3,54318E-07	2	1623,88408	1,08	26,58	0
	High	vQTPWFGGNVGmNIAWR	1	1	1	P74102	N-Term(iTRAQ4plex); M12(Oxidation)	0,0000	1,469	1		0	1,26E-08	59	1,19942E-06	3	2093,05222	0,66	46,24	0
	High	tLTIAAPGAASMQLAENALk	1	1	1	P74102	N-Term(iTRAQ4plex); K20(iTRAQ4plex)	0,0000	1,749	1		0	3,053E-08	53	6,57932E-06	3	2259,26273	0,24	46,96	0
	High	fLLNPEGk	4	1	1	P74102	N-Term(iTRAQ4plex); K8(iTRAQ4plex)	0,0000	1,697	2	1,0	0	0,00002779	41	8,79023E-05	2	1205,71306	-0,17	31,72	0
	High	fFREEcQNLk	3	1	1	P74102	N-Term(iTRAQ4plex); C6(Carbamidomethyl); K10(iTRAQ4plex)	0,0000	1,943	3	1,1	0	0,000001275	39	0,000123586	2	1658,85893	1,57	25,21	1
	High	eLLNFAR	3	1	1	P74102	N-Term(iTRAQ4plex)	0,0000	1,984	3	8,8	0	0,001042	39	0,000127644	2	1006,58019	-0,05	32,75	0
	High	nAVVDmGFTAGkDGkR	1	1	1	P74102	N-Term(iTRAQ4plex); M6(Oxidation); K12(iTRAQ4plex); K15(iT	0,0000	0,988	1		0	0,000101	31	0,00080904	4	2114,14004	0,53	19,14	2
	High	rIAEPVVPPQDTASR	1	1	1	P74102	N-Term(iTRAQ4plex)	0,0000	1,050	1		0	0,00005534	30	0,001176787	3	1779,98618	1,54	21,89	1
	High	lGFWYR	4	1	1	P74102	N-Term(iTRAQ4plex)	0,0000	2,354	4	19,3	0,001	0,006032	22	0,006917832	2	985,53734	-0,29	37,86	0
P72691	Glycogen operon protein GlgX OS=Synechocystis sp. (strain PCC 6803 / Kazusa) GN=glgX PE=4 SV=	691,02	20,11	1	13	14	30	0,404	25	26,6	746	83,4	5,06							
	A3	Sequence	# PSMs	# Proteins	# Protein Groups	Protein Group Accessions	Modifications	ΔCn	116/117	116/117 Count	116/117 Variability [%]	q-Value	PEP	IonScore	Exp Value	Charge	MH+ [Da]	ΔM [ppm]	RT [min]	# Missed Cleavages
	High	aNHPVVGGLILDSLR	4	1	1	P72691	N-Term(iTRAQ4plex)	0,0000	0,331	3	7,1	0	8,775E-10	76	2,63265E-08	3	1704,98948	0,99	39,93	0
	High	yWVSEMHVDGFR	3	1	1	P72691	N-Term(iTRAQ4plex)	0,0000	0,594	3	31,8	0	1,23E-10	62	6,82292E-07	3	1669,79148	0,06	39,22	0
	High	fDLASVLVR	2	1	1	P72691	N-Term(iTRAQ4plex)	0,0000	0,294	2	2,2	0	0,000009973	56	2,63141E-06	2	1163,69109	0,48	45,54	0
	High	dLPGScVVWHGVk	3	1	1	P72691	N-Term(iTRAQ4plex); C6(Carbamidomethyl); K13(iTRAQ4plex)	0,0000	0,406	2	13,3	0	3,288E-09	47	2,07E-05	3	1741,93210	1,30	32,27	0
	High	aGQVYAYR	3	1	1	P72691	N-Term(iTRAQ4plex)	0,0000	0,404	3	1,8	0	0,0000392	39	0,000123302	2	1071,57097	0,56	18,68	0
	High	lLGSPDIYYR	2	1	1	P72691	N-Term(iTRAQ4plex)	0,0000	0,564	2	29,2	0	0,0000943	39	0,000140595	2	1340,73381	0,54	36,05	0
	High	kAAmALGDNcAQALR	1	1	1	P72691	K1(iTRAQ4plex); N-Term(iTRAQ4plex); M4(Oxidation); C10(Ca	0,0000	0,722	1		0	2,494E-08	38	0,000154871	3	1893,98911	0,69	20,47	1
	High	sSVVLmAk	4	1	1	P72691	N-Term(iTRAQ4plex); M6(Oxidation); K8(iTRAQ4plex)	0,0000	0,388	2	14,9	0	0,0006648	37	0,000245866	2	1138,67827	3,36	19,17	0
	High	yWVSEmHVDGFR	1	1	1	P72691	N-Term(iTRAQ4plex); M6(Oxidation)	0,0000	0,524	1		0	1,358E-07	34	0,000395339	3	1685,78666	0,21	32,00	0
	High	nPNSGLSENkR	1	1	1	P72691	N-Term(iTRAQ4plex); K10(iTRAQ4plex)	0,0000	0,347	1		0	0,0001135	32	0,000756307	3	1503,81406	1,49	10,20	1
	High	vTLLLFDRPNDPAPAR	2	1	1	P72691	N-Term(iTRAQ4plex)	0,0000	0,330	2	9,6	0	0,001863	27	0,002108628	3	1939,09201	1,94	40,95	0
	High	rGTYAGLIEk	1	1	1	P72691	N-Term(iTRAQ4plex); K10(iTRAQ4plex)	0,0000	1,624	1		0	0,001015	23	0,005520393	3	1395,82001	0,11	22,68	1
	High	tIELHR	1	1	1	P72691	N-Term(iTRAQ4plex)	0,0000	0,368	1		0,001	0,00418	24	0,003827983	3	912,53876	0,45	15,37	0
	High	vLLDPYAk	1	2	2	P73608;P72691	N-Term(iTRAQ4plex); K8(iTRAQ4plex)	0,0000				0,001	0,008689	20	0,013675504	2	1206,73870	4,17	31,38	0
	High	tFYYWHVFVk	1	1	1	P72691	N-Term(iTRAQ4plex); K10(iTRAQ4plex)	0,0000	0,736	1		0,001	0,007894	13	0,052236011	3	1677,90543	1,35	43,17	0
P77968	Superoxide dismutase [Fe] OS=Synechocystis sp. (strain PCC 6803 / Kazusa) GN=sodB PE=1 SV=3 -	690,35	39,20	1	5	5	30	0,530	29	13,6	199	21,7	5,14							
	A3	Sequence	# PSMs	# Proteins	# Protein Groups	Protein Group Accessions	Modifications	ΔCn	116/117	116/117 Count	116/117 Variability [%]	q-Value	PEP	IonScore	Exp Value	Charge	MH+ [Da]	ΔM [ppm]	RT [min]	# Missed Cleavages
	High	rPDYIADFLGk	17	1	1	P77968	N-Term(iTRAQ4plex); K11(iTRAQ4plex)	0,0000	0,529	16	3,9	0	1,736E-07	59	1,40919E-06	3	1582,88504	1,17	39,56	0
	High	qAGATQFGSGWAWLVLDNGTLk	2	1	1	P77968	N-Term(iTRAQ4plex); K22(iTRAQ4plex)	0,0000	0,608	2	14,6	0	0,000004697	58	1,73768E-06	3	2608,38736	4,24	55,25	0
	High	sTLEFHHDk	5	1	1	P77968	N-Term(iTRAQ4plex); K9(iTRAQ4plex)	0,0000	0,423	5	9,6	0	0,00006326	34	0,000404548	2	1401,73516	-0,96	13,49	0
	High	aVAGDASk	5	1	1	P77968	N-Term(iTRAQ4plex); K8(iTRAQ4plex)	0,0000	0,647	5	8,4	0	0,001093	33	0,000561009	2	1006,57665	-0,52	12,38	0
	High	hHAAYVNNFNNAVAGTDLDNQSI	1	1	1	P77968	N-Term(iTRAQ4plex); K28(iTRAQ4plex)	0,0000	0,465	1		0	4,062E-08	30	0,001081359	4	3357,67422	1,16	39,59	0
P72939	Alkaline phosphatase OS=Synechocystis sp. (strain PCC 6803 / Kazusa) GN=sll0654 PE=4 SV=1 - [P7	688,53	9,58	1	11	11	29	0,287	27	28,3	1409	149,3	4,15							
	A3	Sequence	# PSMs	# Proteins	# Protein Groups	Protein Group Accessions	Modifications	ΔCn	116/117	116/117 Count	116/117 Variability [%]	q-Value	PEP	IonScore	Exp Value	Charge	MH+ [Da]	ΔM [ppm]	RT [min]	# Missed Cleavages
	High	tINSGVSLISk	4	1	1	P72939	N-Term(iTRAQ4plex); K11(iTRAQ4plex)	0,0000	0,535	4	14,7	0	4,966E-07	73	4,47682E-08	2	1406,85124	3,91	30,07	0
	High	hVDIIVAGGSNTR	2	1	1	P72939	N-Term(iTRAQ4plex)	0,0000	0,288	2	61,6	0	0,000001265	70	9,8168E-08	2	1482,81511	0,37	20,45	0
	High	vSVGFLPDmVTFSPDGTk	2	1	1	P72939	N-Term(iTRAQ4plex); M9(Oxidation); K18(iTRAQ4plex)	0,0000	0,316	2	56,6	0	2,29E-09	68	1,47221E-07	3	2201,14139	0,49	45,07	0
	High	vVLLAHmQQIAIEQELAVR	3	1	1	P72939	N-Term(iTRAQ4plex); M7(Oxidation)	0,0000	0,256	2	30,7	0	4,712E-11	63	4,76398E-07	3	2321,31571	1,05	44,34	0
	High	tYAFVGLER	3	1	1	P72939	N-Term(iTRAQ4plex)	0,0000	0,266	3	7,3	0	3,206E-07	52	6,71382E-06	2	1199,65447	0,29	33,21	0
	High	aELQSAGVR	4	1	1	P72939	N-Term(iTRAQ4plex)	0,0000	0,314	3	18,9	0	0,00007152	47	2,09021E-05	2	1074,60307	0,60	16,39	0
	High	fLGQIFDADGNVVR	3	1	1	P72939	N-Term(iTRAQ4plex)	0,0000	0,254	3	18,0	0	0,00001838	43	4,66627E-05	2	1694,89995	0,97	46,41	0
	High	iQNLAFR	3	1	1	P72939	N-Term(iTRAQ4plex)	0,0000	0,238	3	0,9	0	0,0008504	30	0,001370598	2	1005,59551	-0,70	29,00	0
	High	eSNVFGVSNVYLEGSRPAIR	2	1	1	P72939	N-Term(iTRAQ4plex)	0,0000	0,292	2	14,1	0	0,00007631	25	0,003250648	3	2338,22824	0,42	39,34	0
	High	lLDENDRLR	1	1	1	P72939	N-Term(iTRAQ4plex)	0,0000	0,346	1		0,001	0,006454	17	0,020795531	3	1287,71253	-0,98	23,03	1
	High	lTVTTk	2	1	1	P72939	N-Term(iTRAQ4plex); K6(iTRAQ4plex)	0,0000	0,338	2	8,4	0,009	0,1153	42	6,33826E-05	2	950,61308	0,65	18,60	0
P48939	30S ribosomal protein S4 OS=Synechocystis sp. (strain PCC 6803 / Kazusa) GN=rpsD PE=3 SV=1 - [	669,53	28,22	1	8	8	30	2,028	25	84,5	202	23,2	10,48							
	A3	Sequence	# PSMs	# Proteins	# Protein Groups	Protein Group Accessions	Modifications	ΔCn	116/117	116/117 Count	116/117 Variability [%]	q-Value	PEP	IonScore	Exp Value	Charge	MH+ [Da]	ΔM [ppm]	RT [min]	# Missed Cleavages
	High	rATGSTGQALLELLEMR	1	1	1	P48939	N-Term(iTRAQ4plex)	0,0000	2,039	1		0	3,707E-10	66	2,25928E-07	3	1990,09146	2,11	49,31	1
	High	lGmAGTIPGAR	3	1	1	P48939	N-Term(iTRAQ4plex); M3(Oxidation)	0,0000	3,924	2	29,1	0	0,000002823	62	6,12308E-07	2	1203,66619	2,08	23,02	0
	High	rAYPPGQHGQAR	3	1	1	P48939	N-Term(iTRAQ4plex)	0,0000	1,486	3	1,2	0	3,389E-08	55	3,16206E-06	3	1481,78620	1,31	7,70	1
	High	lGMAGTIPGAR	2	1	1	P48939	N-Term(iTRAQ4plex)	0,0000	5,113	1		0	0,00003381	54	4,8661E-06	2	1187,67229	2,97	28,60	0
	High	aYPPGQHGQAR	4	1	1	P48939	N-Term(iTRAQ4plex)	0,0000	1,727	4	22,7	0	0,000000172	52	5,92884E-06	3	1325,68631	2,40	10,47	0
	High	rATGSTGQALLELLEmR	7	1	1	P48939	N-Term(iTRAQ4plex); M16(Oxidation)	0,0000	1,066	6	128,5	0	5,718E-08	50	1,09144E-05	3	2006,08628	2,04	45,32	1
	High	rLGELPGLSR	5	1	1	P48939	N-Term(iTRAQ4plex)	0,0000	3,086	3	120,1	0	0,00009377	40	9,24634E-05	3	1241,74710	1,94	27,44	1
	High	aTGSTGQALLELLEMR	1	1	1	P48939	N-Term(iTRAQ4plex)	0,0000	0,555	1		0	0,00001028	33	0,000501153	3	1833,98709	0,51	54,38	0
	High	lGELPGLSR	3	1	1	P48939	N-Term(iTRAQ4plex)	0,0000	3,613	3	21,7	0	0,002646	31	0,000824081	2	1085,64580	2,05	33,82	0
	High	rSEYAIR	1	1	1	P48939	N-Term(iTRAQ4plex)	0,0000	1,236	1		0,002	0,01756	21	0,007797763	3	1038,58490	3,48	13,59	1
P74267	50S ribosomal protein L27 OS=Synechocystis sp. (strain PCC 6803 / Kazusa) GN=rpmA PE=3 SV=1 -	664,56	59,77	1	6	6	26	1,746	20	62,3	87	9,4	11,24							
	A3	Sequence	# PSMs	# Proteins	# Protein Groups	Protein Group Accessions	Modifications	ΔCn	116/117	116/117 Count	116/117 Variability [%]	q-Value	PEP	IonScore	Exp Value	Charge	MH+ [Da]	ΔM [ppm]	RT [min]	# Missed Cleavages
	High	yGGQTVTAGSIIVR	7	1	1	P74267	N-Term(iTRAQ4plex)	0,0000	2,291	5	61,9	0	1,088E-11	92	7,41633E-10	2	1565,87920	1,52	30,76	0
	High	gkDDTLFALIDGVVk	5	1	1	P74267	N-Term(iTRAQ4plex); K2(iTRAQ4plex); K15(iTRAQ4plex)	0,0000	1,779	4	85,4	0	3,938E-08	58	1,43539E-06	3	2023,18686	3,38	50,73	1
	High	kVSVYPATAE	4	1	1	P74267	N-Term(iTRAQ4plex); K1(iTRAQ4plex)	0,0000	2,115	3	0,9	0	0,0001905	53	5,52039E-06	2	1352,77056	3,06	21,89	1
	High	rYGGQTVTAGSIIVR	2	1	1	P74267	N-Term(iTRAQ4plex)	0,0000	0,892	2	33,9	0	4,428E-07	46	2,25408E-05	3	1721,98001	1,20	25,73	1
	High	gTQVHPGNNVGR	7	1	1	P74267	N-Term(iTRAQ4plex)	0,0000	1,726	5	55,4	0	0,0001922	46	2,42086E-05	3	1379,72797	1,37	11,06	0
	High	vSVYPATAE	1	1	1	P74267	N-Term(iTRAQ4plex)	0,0000	1,118	1		0,004	0,0461	35	0,000345915	2	1080,57121	1,69	27,27	0
P29254	Photosystem I P700 chlorophyll a apoprotein A1 OS=Synechocystis sp. (strain PCC 6803 / Kazusa) GI	661,62	12,52	1	12	12	39	2,238	32	13,0	751	82,9	7,77							
	A3	Sequence	# PSMs	# Proteins	# Protein Groups	Protein Group Accessions	Modifications	ΔCn	116/117	116/117 Count	116/117 Variability [%]	q-Value	PEP	IonScore	Exp Value	Charge	MH+ [Da]	ΔM [ppm]	RT [min]	# Missed Cleavages
	High	nVNNLLDR	3	1	1	P29254	N-Term(iTRAQ4plex)	0,0000	2,372	3	0,8	0	0,000009303	54	3,54789E-06	2	1101,61357	0,23	24,66	0
	High	dIPLPHEFILEPSk	5	1	1	P29254	N-Term(iTRAQ4plex); K14(iTRAQ4plex)	0,0000	2,230	3	5,1	0	1,868E-10	54	3,70655E-06	3	1923,08402	0,51	41,58	0
	High	gPFTGEGHk	3	1	1	P29254	N-Term(iTRAQ4plex); K9(iTRAQ4plex)	0,0000	2,083	3	8,8	0	0,000001412	50	9,0567E-06	3	1217,65097	-0,62	13,59	0
	High	aLSIIQGR	6	1	1	P29254	N-Term(iTRAQ4plex)	0,0000	2,536	3	9,6	0	0,00007696	50	1,11936E-05	2	1001,62218	-0,25	27,98	0
	High	lNVAPAIQPR	4	1	1	P29254	N-Term(iTRAQ4plex)	0,0000	1,747	4	42,1	0	0,00001804	44	4,00867E-05	2	1222,73943	0,45	28,57	0
	High	eILEAHk	3	1	1	P29254	N-Term(iTRAQ4plex); K7(iTRAQ4plex)	0,0000	2,086	3	16,6	0	0,00091	38	0,00014288	2	1127,66899	2,38	15,95	0
	High	tNWGIGHSMk	1	1	1	P29254	N-Term(iTRAQ4plex); K10(iTRAQ4plex)	0,0000	3,358	1		0	0,00002628	29	0,001312109	3	1418,74536	0,03	23,06	0
	High	lVPDkANLGFR	2	1	1	P29254	N-Term(iTRAQ4plex); K5(iTRAQ4plex)	0,0000	2,367	2	84,2	0	0,0009381	21	0,008297935	3	1517,90357	-0,47	32,22	1
	High	tNWGIGHSmk	3	1	1	P29254	N-Term(iTRAQ4plex); M9(Oxidation); K10(iTRAQ4plex)	0,0000	1,615	2	40,9	0	0,0003121	20	0,010446481	3	1434,73981	-0,29	17,41	0
	High	eILEAHkGPFTGEGHk	2	1	1	P29254	N-Term(iTRAQ4plex); K7(iTRAQ4plex); K16(iTRAQ4plex)	0,0000	2,429	1		0	0,000002115	20	0,010592537	5	2182,19862	0,22	21,72	1
	High	wGkPGHFDR	2	1	1	P29254	N-Term(iTRAQ4plex); K3(iTRAQ4plex)	0,0000	2,507	2	20,1	0	0,0001895	17	0,019632247	3	1387,74726	-0,08	18,92	0
	High	aNLGFR	4	1	1	P29254	N-Term(iTRAQ4plex)	0,0000	2,479	4	3,4	0,001	0,006789	32	0,00060256	2	821,47502	-0,01	20,47	0
	High	fPcDGPGR	1	1	1	P29254	N-Term(iTRAQ4plex); C3(Carbamidomethyl)	0,0000	1,860	1		0,008	0,11	17	0,021328976	2	1049,49541	-0,11	20,20	0
P72761	Carbon dioxide-concentrating mechanism protein CcmK homolog 2 OS=Synechocystis sp. (strain PCC	645,05	70,87	1	4	6	30	0,672	13	40,9	103	11,1	5,41							
	A3	Sequence	# PSMs	# Proteins	# Protein Groups	Protein Group Accessions	Modifications	ΔCn	116/117	116/117 Count	116/117 Variability [%]	q-Value	PEP	IonScore	Exp Value	Charge	MH+ [Da]	ΔM [ppm]	RT [min]	# Missed Cleavages
	High	sIAVGMIETR	4	1	1	P72761	N-Term(iTRAQ4plex)	0,0000	0,938	3	6,6	0	7,652E-08	62	5,9975E-07	2	1220,68059	1,32	30,83	0
	High	yTEEVEQFR	2	1	1	P72761	N-Term(iTRAQ4plex)	0,0000	0,649	2	33,3	0	0,000007506	54	4,32484E-06	2	1344,65886	2,69	29,86	0
	High	vTLVGYEk	10	2	2	P72760;P72761	N-Term(iTRAQ4plex); K8(iTRAQ4plex)	0,0000				0	0,0002113	49	1,18842E-05	2	1196,71489	1,65	27,58	0
	High	sIAVGmIETR	5	1	1	P72761	N-Term(iTRAQ4plex); M6(Oxidation)	0,0000	0,592	4	19,8	0	0,000005325	48	1,74253E-05	2	1236,67534	1,17	25,30	0
	High	gFPAVVEAADSmVk	1	1	1	P72761	N-Term(iTRAQ4plex); M12(Oxidation); K14(iTRAQ4plex)	0,0000	0,672	1		0	2,248E-07	42	6,23692E-05	3	1724,91428	0,62	37,49	0
	High	gFPAVVEAADSMVk	2	1	1	P72761	N-Term(iTRAQ4plex); K14(iTRAQ4plex)	0,0000	0,395	2	12,2	0	0,000004475	39	0,00013551	3	1708,92185	2,08	46,29	0
	High	vNGGEVLSTHIIARPHENLEYVLP	1	1	1	P72761	N-Term(iTRAQ4plex)	0,0000	0,814	1		0	6,976E-07	21	0,008609343	5	3070,69562	1,20	41,09	0
	High	vTVIVR	5	2	2	P72760;P72761	N-Term(iTRAQ4plex)	0,0000				0,003	0,04246	28	0,001573874	2	830,55919	1,39	22,25	0
P74229	30S ribosomal protein S7 OS=Synechocystis sp. (strain PCC 6803 / Kazusa) GN=rpsG PE=3 SV=1 - [	644,27	53,85	1	10	10	30	1,593	26	91,8	156	17,4	10,45							
	A3	Sequence	# PSMs	# Proteins	# Protein Groups	Protein Group Accessions	Modifications	ΔCn	116/117	116/117 Count	116/117 Variability [%]	q-Value	PEP	IonScore	Exp Value	Charge	MH+ [Da]	ΔM [ppm]	RT [min]	# Missed Cleavages
	High	rPVPPDPVYNSTLLSMTIR	2	1	1	P74229	N-Term(iTRAQ4plex)	0,0000	1,392	1		0	8,507E-13	74	3,6557E-08	3	2300,25821	1,23	42,40	0
	High	rPVPPDPVYNSTLLSmTIR	4	1	1	P74229	N-Term(iTRAQ4plex); M16(Oxidation)	0,0000	1,266	2	47,9	0	8,508E-13	71	8,59791E-08	3	2316,25248	0,94	37,95	0
	High	kSLASSIVYNALASVGEk	2	1	1	P74229	N-Term(iTRAQ4plex); K1(iTRAQ4plex); K18(iTRAQ4plex)	0,0000	2,456	2	56,8	0	7,291E-10	64	3,95367E-07	3	2269,31961	2,99	49,14	1
	High	vGGATYQVPmEVRPAR	3	1	1	P74229	N-Term(iTRAQ4plex); M10(Oxidation)	0,0000	2,763	3	19,5	0	0,000009791	59	1,14017E-06	3	1890,99918	0,77	25,27	0
	High	nLTPLVEVk	1	1	1	P74229	N-Term(iTRAQ4plex); K9(iTRAQ4plex)	0,0000	1,611	1		0	0,00008969	51	7,92447E-06	2	1300,81035	1,89	33,22	0
	High	vGGATYQVPMEVRPAR	2	1	1	P74229	N-Term(iTRAQ4plex)	0,0000	3,114	2	20,4	0	1,879E-07	50	1,02322E-05	3	1875,00547	1,42	28,67	0
	High	wLVHFSR	1	1	1	P74229	N-Term(iTRAQ4plex)	0,0000	2,617	1		0	0,00007292	48	1,5065E-05	3	1088,61289	0,64	32,38	0
	High	sLASSIVYNALASVGEk	2	1	1	P74229	N-Term(iTRAQ4plex); K17(iTRAQ4plex)	0,0000	2,058	2	77,2	0	0,00001393	45	2,98518E-05	3	1997,12217	3,19	54,09	0
	High	kRPVPPDPVYNSTLLSmTIR	3	1	1	P74229	N-Term(iTRAQ4plex); K1(iTRAQ4plex); M17(Oxidation)	0,0000	1,343	3	219,9	0	2,128E-09	43	4,76398E-05	3	2588,44791	0,22	33,84	1
	High	gTALALR	4	1	1	P74229	N-Term(iTRAQ4plex)	0,0000	1,575	3	21,8	0	0,001915	39	0,00013551	2	845,53368	1,33	19,02	0
	High	rVGGATYQVPMEVRPAR	2	1	1	P74229	N-Term(iTRAQ4plex)	0,0000	1,064	2	17,5	0	0,0008081	26	0,002535129	4	2031,10573	0,89	24,30	1
	High	rVGGATYQVPmEVRPAR	1	1	1	P74229	N-Term(iTRAQ4plex); M11(Oxidation)	0,0000	0,354	1		0	0,000987	16	0,025001727	4	2047,10000	0,57	21,77	1
	High	kRPVPPDPVYNSTLLSMTIR	1	1	1	P74229	N-Term(iTRAQ4plex); K1(iTRAQ4plex)	0,0000	2,231	1		0	6,155E-08	13	0,051400815	4	2572,45839	2,32	38,29	1
	High	aFAHYR	2	1	1	P74229	N-Term(iTRAQ4plex)	0,0000	1,162	2	33,7	0,002	0,02396	14	0,038991506	3	908,48828	2,61	13,94	0
	>sp|K2C1_HUMAN|	627,91	16,17	1	11	12	28	0,595	19	18,8	643	65,8	8,12							
	A3	Sequence	# PSMs	# Proteins	# Protein Groups	Protein Group Accessions	Modifications	ΔCn	116/117	116/117 Count	116/117 Variability [%]	q-Value	PEP	IonScore	Exp Value	Charge	MH+ [Da]	ΔM [ppm]	RT [min]	# Missed Cleavages
	High	tNAENEFVTIkk	4	1	1		N-Term(iTRAQ4plex); K11(iTRAQ4plex); K12(iTRAQ4plex)	0,0000	0,656	2	7,8	0	9,593E-11	67	1,81958E-07	3	1826,03977	0,74	24,83	1
	High	sLNNQFASFIDk	1	1	1		N-Term(iTRAQ4plex); K12(iTRAQ4plex)	0,0000	0,413	1		0	1,346E-08	60	1,10146E-06	2	1671,89385	-0,38	40,19	0
	High	skAEAESLYQSk	6	1	1		N-Term(iTRAQ4plex); K2(iTRAQ4plex); K12(iTRAQ4plex)	0,0000	0,525	3	11,5	0	9,629E-09	54	3,8104E-06	3	1772,97452	-0,54	22,68	1
	High	sLVNLGGSk	5	1	1		N-Term(iTRAQ4plex); K9(iTRAQ4plex)	0,0000	0,571	3	3,5	0	0,000001241	51	8,35545E-06	2	1162,70329	-0,11	24,83	0
	High	sLNNQFASFIDkVR	2	1	1		N-Term(iTRAQ4plex); K12(iTRAQ4plex)	0,0000	0,872	2	3,3	0	7,36E-10	47	1,89657E-05	3	1927,06638	1,22	43,03	1
	High	sISISVAR	3	1	1		N-Term(iTRAQ4plex)	0,0000	0,571	2	6,0	0	0,001497	46	2,31723E-05	2	976,59056	-0,24	24,81	0
	High	fLEQQNQVLQTk	1	2	2		N-Term(iTRAQ4plex); K12(iTRAQ4plex)	0,0000				0	1,775E-07	38	0,000189539	3	1763,98917	-0,16	31,16	0
	High	nkYEDEINkR	2	1	1		N-Term(iTRAQ4plex); K2(iTRAQ4plex); K9(iTRAQ4plex)	0,0000	0,419	2	6,7	0	0,00001201	33	0,000514008	3	1740,96036	-0,07	16,44	2
	High	rVDQLkSDQSR	1	1	1		N-Term(iTRAQ4plex); K6(iTRAQ4plex)	0,0000	0,845	1		0	0,0001176	20	0,012828659	4	1619,90664	-0,09	12,14	2
	High	lRSEIDNVkk	1	1	1		N-Term(iTRAQ4plex); K9(iTRAQ4plex); K10(iTRAQ4plex)	0,0000	0,566	1		0	0,001045	14	0,040735214	4	1633,99563	-0,33	19,43	2
	Medium	rVDQLk	1	1	1		N-Term(iTRAQ4plex); K6(iTRAQ4plex)	0,0000	0,595	1		0,017	0,2239	22	0,010424088	3	1046,65427	-1,73	13,49	1
	Medium	eREQIk	1	1	1		N-Term(iTRAQ4plex); K6(iTRAQ4plex)	0,0000	0,960	1		0,031	0,3999	11	0,131926396	3	1090,64536	-0,50	10,40	1
P55038	Ferredoxin-dependent glutamate synthase 2 OS=Synechocystis sp. (strain PCC 6803 / Kazusa) GN=g	612,07	13,56	1	16	17	27	1,000	25	11,1	1556	169,4	5,76							
	A3	Sequence	# PSMs	# Proteins	# Protein Groups	Protein Group Accessions	Modifications	ΔCn	116/117	116/117 Count	116/117 Variability [%]	q-Value	PEP	IonScore	Exp Value	Charge	MH+ [Da]	ΔM [ppm]	RT [min]	# Missed Cleavages
	High	nPGYTSNFAVYHR	1	1	1	P55038	N-Term(iTRAQ4plex)	0,0000	1,059	1		0	0,000000211	59	1,3551E-06	3	1669,81986	-0,29	25,56	0
	High	vcHTNNcPVGVATQQER	2	1	1	P55038	N-Term(iTRAQ4plex); C2(Carbamidomethyl); C7(Carbamidome	0,0000	0,794	2	26,2	0	1,576E-11	57	2,18258E-06	3	2113,99912	0,13	17,51	0
	High	eLLAQWFNTR	3	1	1	P55038	N-Term(iTRAQ4plex)	0,0000	1,061	3	8,9	0	0,00001059	54	3,89915E-06	2	1421,76555	-0,19	46,77	0
	High	lTVLGWR	2	1	1	P55038	N-Term(iTRAQ4plex)	0,0000	1,090	2	3,2	0	0,0002	52	7,03024E-06	2	988,60686	0,81	38,98	0
	High	hAGSPWELGVTEVHR	2	1	1	P55038	N-Term(iTRAQ4plex)	0,0000	1,104	2	8,7	0	8,658E-08	48	1,44534E-05	4	1818,93825	0,79	28,69	0
	High	lLYDYFk	3	1	1	P55038	N-Term(iTRAQ4plex); K7(iTRAQ4plex)	0,0000	0,980	3	6,3	0	0,0004011	46	2,74909E-05	2	1249,70842	1,07	42,38	0
	High	sLITAHVEHTGSPk	3	1	1	P55038	N-Term(iTRAQ4plex); K14(iTRAQ4plex)	0,0000	0,928	3	21,2	0	3,675E-07	45	5,10612E-05	3	1764,98581	0,63	18,89	0
	High	ePTFcmGDDTPLAVLSHkPR	1	1	1	P55038	N-Term(iTRAQ4plex); C5(Carbamidomethyl); M6(Oxidation); K	0,0000	0,943	1		0	3,595E-07	43	5,27193E-05	4	2575,29360	1,86	36,66	0
	High	sLLAHLGYR	1	1	1	P55038	N-Term(iTRAQ4plex)	0,0000	1,060	1		0	0,00001507	40	0,000110146	3	1173,68634	0,22	31,01	0
	High	kVSEYIAmLR	1	1	1	P55038	N-Term(iTRAQ4plex); K1(iTRAQ4plex); M8(Oxidation)	0,0000	0,682	1		0	0,00006427	28	0,001937911	3	1513,86023	-3,22	32,55	1
	High	lVAEIGIGTIAAGVAk	1	1	1	P55038	N-Term(iTRAQ4plex); K16(iTRAQ4plex)	0,0000	1,666	1		0	0,000386	24	0,004245902	3	1771,09531	1,18	46,49	0
	High	gkPDHTLVEQALk	2	1	1	P55038	N-Term(iTRAQ4plex); K2(iTRAQ4plex); K13(iTRAQ4plex)	0,0000	0,906	1		0	0,00001059	23	0,005092957	4	1868,10024	1,94	22,77	0
	High	mAQGAkPGEGGQLPGkk	1	2	2	P55037;P55038	N-Term(iTRAQ4plex); M1(Oxidation); K6(iTRAQ4plex); K16(iTF	0,0000				0	0,001184	18	0,014420158	4	2246,27724	-0,06	14,92	1
	High	lDRIDLPTALk	1	1	1	P55038	N-Term(iTRAQ4plex); K11(iTRAQ4plex)	0,0000	0,987	1		0	0,0002002	18	0,014453399	3	1542,94736	1,01	36,69	1
	High	iNPEIITLQR	1	1	1	P55038	N-Term(iTRAQ4plex)	0,0000	0,609	1		0	0,002285	18	0,016179683	2	1340,80364	1,32	36,52	0
	High	dRPVTALR	1	1	1	P55038	N-Term(iTRAQ4plex)	0,0000	1,134	1		0	0,0006048	15	0,030547101	3	1071,63819	-0,90	13,94	0
	High	lGVGMVFLPQEPSAR	1	1	1	P55038	N-Term(iTRAQ4plex)	0,0000	0,862	1		0,001	0,01189	24	0,003698026	3	1744,95426	0,30	45,17	0
Q55648	Sll0314 protein OS=Synechocystis sp. (strain PCC 6803 / Kazusa) GN=sll0314 PE=4 SV=1 - [Q55648	606,38	26,43	1	10	10	29	0,603	21	11,4	314	35,4	5,97							
	A3	Sequence	# PSMs	# Proteins	# Protein Groups	Protein Group Accessions	Modifications	ΔCn	116/117	116/117 Count	116/117 Variability [%]	q-Value	PEP	IonScore	Exp Value	Charge	MH+ [Da]	ΔM [ppm]	RT [min]	# Missed Cleavages
	High	yTPFSQSEQVISR	2	1	1	Q55648	N-Term(iTRAQ4plex)	0,0000	1,046	2	89,2	0	0,000001439	67	2,49533E-07	2	1685,86247	0,55	33,98	0
	High	yAQQTLESAQR	1	1	1	Q55648	N-Term(iTRAQ4plex)	0,0000	0,551	1		0	0,00001624	58	1,69422E-06	2	1438,74175	0,72	21,40	0
	High	gSYLQAVGk	1	1	1	Q55648	N-Term(iTRAQ4plex); K9(iTRAQ4plex)	0,0000	0,552	1		0	0,000004415	57	2,08304E-06	2	1210,70220	-1,01	25,20	0
	High	kYAQQTLESAQR	2	1	1	Q55648	N-Term(iTRAQ4plex); K1(iTRAQ4plex)	0,0000	0,681	1		0	1,906E-07	52	6,53085E-06	3	1710,93692	-0,48	16,95	1
	High	gYLDLFLSR	6	1	1	Q55648	N-Term(iTRAQ4plex)	0,0000	0,563	4	1,6	0	0,0000359	51	7,60274E-06	2	1227,68620	0,64	47,53	0
	High	lQQAQQYYAmALQk	2	1	1	Q55648	N-Term(iTRAQ4plex); M10(Oxidation); K14(iTRAQ4plex)	0,0000	0,674	2	62,0	0	5,432E-12	50	9,7268E-06	3	1988,05094	-0,23	36,24	0
	High	lAQNPSLk	6	1	1	Q55648	N-Term(iTRAQ4plex); K8(iTRAQ4plex)	0,0000	0,603	3	0,1	0	0,0003763	48	1,44534E-05	2	1158,70879	0,24	20,34	0
	High	aLATTYR	3	1	1	Q55648	N-Term(iTRAQ4plex)	0,0000	0,551	2	14,4	0	0,001969	31	0,000707897	2	939,53789	-0,13	17,56	0
	High	kGSYLQAVGk	2	1	1	Q55648	N-Term(iTRAQ4plex); K1(iTRAQ4plex); K10(iTRAQ4plex)	0,0000	0,927	2	63,1	0	0,00006226	31	0,000756781	3	1482,89948	-0,66	22,06	1
	High	tGTVFEHLDk	3	1	1	Q55648	N-Term(iTRAQ4plex); K10(iTRAQ4plex)	0,0000	0,604	2	7,7	0	0,00004989	23	0,005187642	3	1434,78321	0,05	25,40	0
	High	lQQAQQYYAMALQk	1	1	1	Q55648	N-Term(iTRAQ4plex); K14(iTRAQ4plex)	0,0000	0,661	1		0	0,00007031	19	0,016021303	3	1972,05814	0,84	39,46	0
P73303	50S ribosomal protein L15 OS=Synechocystis sp. (strain PCC 6803 / Kazusa) GN=rplO PE=3 SV=1 - [	604,25	45,58	1	7	7	19	1,730	15	141,7	147	15,2	10,37							
	A3	Sequence	# PSMs	# Proteins	# Protein Groups	Protein Group Accessions	Modifications	ΔCn	116/117	116/117 Count	116/117 Variability [%]	q-Value	PEP	IonScore	Exp Value	Charge	MH+ [Da]	ΔM [ppm]	RT [min]	# Missed Cleavages
	High	gIAAGQGAScGFGmR	6	1	1	P73303	N-Term(iTRAQ4plex); C10(Carbamidomethyl); M14(Oxidation)	0,0000	1,680	4	169,5	0	1,916E-11	87	1,80289E-09	3	1599,75034	0,90	22,06	0
	High	gIAAGQGAScGFGMR	4	1	1	P73303	N-Term(iTRAQ4plex); C10(Carbamidomethyl)	0,0000	2,743	2	1,0	0	2,816E-07	83	5,23564E-09	2	1583,75688	1,83	26,98	0
	High	iEAAGGSVVAQG	1	1	1	P73303	N-Term(iTRAQ4plex)	0,0000	0,852	1		0	0,000004776	78	1,67483E-08	2	1202,65105	1,04	25,68	0
	High	aGFEGGQMPLYR	1	1	1	P73303	N-Term(iTRAQ4plex)	0,0000	1,715	1		0	3,126E-07	55	3,26565E-06	2	1469,73235	-0,32	32,06	0
	High	mNLSELSPk	1	1	1	P73303	N-Term(iTRAQ4plex); K9(iTRAQ4plex)	0,0000	1,730	1		0	0,00001035	45	2,82468E-05	2	1306,73113	2,44	31,04	0
	High	aGFEGGQmPLYR	1	1	1	P73303	N-Term(iTRAQ4plex); M8(Oxidation)	0,0000	3,464	1		0	0,00005891	37	0,000181958	2	1485,72893	0,81	25,69	0
	High	hFTVVNVGk	2	1	1	P73303	N-Term(iTRAQ4plex); K9(iTRAQ4plex)	0,0000	1,173	2	480,8	0	0,00002895	26	0,002409739	3	1288,76282	0,94	22,44	0
	High	hFPLYNPk	2	1	1	P73303	N-Term(iTRAQ4plex); K8(iTRAQ4plex)	0,0000	1,281	2	170,4	0	0,0003101	17	0,020604876	3	1303,74231	1,68	24,77	0
	High	lkHFPLYNPk	1	1	1	P73303	N-Term(iTRAQ4plex); K2(iTRAQ4plex); K10(iTRAQ4plex)	0,0000	0,537	1		0	0,0002943	13	0,045600542	4	1689,02859	4,35	27,26	1
P72797	Transaldolase OS=Synechocystis sp. (strain PCC 6803 / Kazusa) GN=tal PE=3 SV=1 - [TAL_SYNY3]	591,20	26,85	1	13	13	29	0,932	23	14,0	391	43,1	5,06							
	A3	Sequence	# PSMs	# Proteins	# Protein Groups	Protein Group Accessions	Modifications	ΔCn	116/117	116/117 Count	116/117 Variability [%]	q-Value	PEP	IonScore	Exp Value	Charge	MH+ [Da]	ΔM [ppm]	RT [min]	# Missed Cleavages
	High	akDEAGENASIk	3	1	1	P72797	N-Term(iTRAQ4plex); K2(iTRAQ4plex); K12(iTRAQ4plex)	0,0000	0,853	2	0,8	0	7,889E-11	61	8,83465E-07	3	1664,91886	0,53	13,42	1
	High	iLQIIPGR	7	1	1	P72797	N-Term(iTRAQ4plex)	0,0000	0,938	4	1,4	0	0,00004157	58	1,69032E-06	2	1053,69048	0,33	36,86	0
	High	vTLISPFVGR	5	1	1	P72797	N-Term(iTRAQ4plex)	0,0000	0,916	3	3,5	0	0,00005212	49	1,14543E-05	2	1232,75017	1,46	41,51	0
	High	iASTWEGIk	1	1	1	P72797	N-Term(iTRAQ4plex); K9(iTRAQ4plex)	0,0000	0,735	1		0	0,000001115	49	1,2473E-05	2	1292,74602	0,56	32,10	0
	High	iLDWYk	1	1	1	P72797	N-Term(iTRAQ4plex); K6(iTRAQ4plex)	0,0000	0,930	1		0	0,0002824	45	3,14029E-05	2	1125,65569	0,91	37,90	0
	High	lAVAFGLk	1	1	1	P72797	N-Term(iTRAQ4plex); K8(iTRAQ4plex)	0,0000	1,071	1		0	0,00001091	45	3,42744E-05	2	1106,71855	0,84	37,80	0
	High	nLLEQLR	1	1	1	P72797	N-Term(iTRAQ4plex)	0,0000	1,130	1		0	0,001502	45	3,49921E-05	2	1029,61821	0,84	32,03	0
	High	kLDPATVPQDIEk	2	1	1	P72797	N-Term(iTRAQ4plex); K1(iTRAQ4plex); K13(iTRAQ4plex)	0,0000	1,003	2	31,8	0	7,709E-09	42	6,7293E-05	3	1886,09781	0,99	27,84	1
	High	vLDGQEHIk	1	1	1	P72797	N-Term(iTRAQ4plex); K9(iTRAQ4plex)	0,0000	1,726	1		0	0,002888	30	0,001056745	3	1326,76325	0,94	18,83	0
	High	gkNLLEQLR	1	1	1	P72797	N-Term(iTRAQ4plex); K2(iTRAQ4plex)	0,0000	1,033	1		0	0,0009034	29	0,001124527	3	1358,83985	2,94	28,19	1
	High	tEVmGASFR	2	1	1	P72797	N-Term(iTRAQ4plex); M4(Oxidation)	0,0000	0,770	2	9,6	0	0,0007948	19	0,012444287	2	1157,57903	4,19	18,11	0
	High	mHAEDPmASEk	2	1	1	P72797	N-Term(iTRAQ4plex); M1(Oxidation); M7(Oxidation); K11(iTRA	0,0000	0,913	2	9,4	0	0,0002595	18	0,015274606	3	1565,71762	-0,16	8,71	0
	High	iLDWYkk	2	1	1	P72797	N-Term(iTRAQ4plex); K6(iTRAQ4plex); K7(iTRAQ4plex)	0,0000	1,866	2	15,8	0,001	0,0151	16	0,02415294	3	1397,85852	4,88	31,21	1
P0DJF8	S-methyl-5'-thioadenosine phosphorylase OS=Synechocystis sp. (strain PCC 6803 / Kazusa) GN=mtnl	588,62	24,85	1	8	8	29	0,611	22	14,2	326	35,3	6,86							
	A3	Sequence	# PSMs	# Proteins	# Protein Groups	Protein Group Accessions	Modifications	ΔCn	116/117	116/117 Count	116/117 Variability [%]	q-Value	PEP	IonScore	Exp Value	Charge	MH+ [Da]	ΔM [ppm]	RT [min]	# Missed Cleavages
	High	qLAANPFESIAHR	4	1	1	P0DJF8	N-Term(iTRAQ4plex)	0,0000	0,644	2	6,1	0	1,726E-12	72	6,20826E-08	3	1597,85794	0,74	33,54	0
	High	nAVNAQQVILETVk	3	1	1	P0DJF8	N-Term(iTRAQ4plex); K14(iTRAQ4plex)	0,0000	1,141	3	29,9	0	6,731E-08	51	8,03471E-06	3	1815,06058	1,49	38,99	0
	High	sWGGTVIGMTNLPEAk	2	1	1	P0DJF8	N-Term(iTRAQ4plex); K16(iTRAQ4plex)	0,0000	0,766	2	32,6	0	5,478E-07	50	1,34925E-05	3	1949,04471	2,14	41,48	0
	High	lSLLLGk	8	1	1	P0DJF8	N-Term(iTRAQ4plex); K7(iTRAQ4plex)	0,0000	0,574	5	3,9	0	0,0004962	48	1,70204E-05	2	1031,70745	0,71	38,70	0
	High	gHHLLPSEIPFR	4	1	1	P0DJF8	N-Term(iTRAQ4plex)	0,0000	0,560	3	3,5	0	0,00009143	32	0,00064561	3	1546,86197	0,55	30,41	0

	High	vAFLAR	4	1	1	P0DJF8	N-Term(iTRAQ4plex)	0,0000	0,542	3	0,7	0,001	0,01152	32	0,001199604	2	820,51744	1,55	27,45	0
	Medium	aNIHGMk	1	1	1	P0DJF8	N-Term(iTRAQ4plex); K7(iTRAQ4plex)	0,0000	0,953	1		0,016	0,2042	15	0,034432615	3	1058,60611	3,94	12,37	0
	Medium	aNIHGmk	2	1	1	P0DJF8	N-Term(iTRAQ4plex); M6(Oxidation); K7(iTRAQ4plex)	0,0000	0,611	2	0,6	0,019	0,2433	19	0,012530546	2	1074,59697	0,11	9,80	0
	Medium	yYPPAP	1	1	1	P0DJF8	N-Term(iTRAQ4plex)	0,0000	0,466	1		0,034	0,4365	16	0,022697081	2	851,44298	1,18	28,82	0
P73202	Phycobilisome 8.9 kDa linker polypeptide, phycocyanin-associated, rod OS=Synechocystis sp. (strain	588,25	81,93	1	6	6	20	1,048	17	12,1	83	9,3	9,41							
	A3	Sequence	# PSMs	# Proteins	# Protein Groups	Protein Group Accessions	Modifications	ΔCn	116/117	116/117 Count	116/117 Variability [%]	q-Value	PEP	IonScore	Exp Value	Charge	MH+ [Da]	ΔM [ppm]	RT [min]	# Missed Cleavages
	High	mLGQSSLVGYSNTQAANR	4	1	1	P73202	N-Term(iTRAQ4plex); M1(Oxidation)	0,0000	1,131	3	9,2	0	2,374E-11	91	8,41337E-10	2	2057,02324	1,43	28,35	0
	High	mLGQSSLVGYSNTQAANR	1	1	1	P73202	N-Term(iTRAQ4plex)	0,0000	0,704	1		0	5,628E-09	61	8,33624E-07	3	2041,02701	0,80	32,56	0
	High	lGGTIVNIRPYQADSNEQN	2	1	1	P73202	N-Term(iTRAQ4plex)	0,0000	0,982	2	7,3	0	1,126E-09	53	4,88619E-06	3	2233,13413	0,50	35,91	0
	High	vFVYEVSGLR	4	1	1	P73202	N-Term(iTRAQ4plex)	0,0000	1,001	3	10,6	0	0,0001982	43	5,29627E-05	2	1312,73882	0,47	39,12	0
	High	sGSVFIk	5	1	1	P73202	N-Term(iTRAQ4plex); K7(iTRAQ4plex)	0,0000	1,049	4	2,1	0	0,001935	41	7,44681E-05	2	1025,62248	-0,87	21,55	0
	High	qTDANENSAHDIR	2	1	1	P73202	N-Term(iTRAQ4plex)	0,0000	0,886	2	12,2	0	0,0000025	33	0,000477496	3	1614,75840	-0,31	10,00	0
	High	qTDANENSAHDIRR	2	1	1	P73202	N-Term(iTRAQ4plex)	0,0000	1,424	2	40,3	0	0,00009517	25	0,003507276	4	1770,85915	-0,49	8,62	1
P73297	DNA-directed RNA polymerase subunit alpha OS=Synechocystis sp. (strain PCC 6803 / Kazusa) GN=r	577,69	19,11	1	8	8	22	1,304	17	10,9	314	35,0	4,79							
	A3	Sequence	# PSMs	# Proteins	# Protein Groups	Protein Group Accessions	Modifications	ΔCn	116/117	116/117 Count	116/117 Variability [%]	q-Value	PEP	IonScore	Exp Value	Charge	MH+ [Da]	ΔM [ppm]	RT [min]	# Missed Cleavages
	High	vLLSNLPGAAVTAIR	5	1	1	P73297	N-Term(iTRAQ4plex)	0,0000	1,356	4	5,4	0	2,406E-11	86	2,47725E-09	2	1639,00420	1,11	45,11	0
	High	gQGTTVGNALR	4	1	1	P73297	N-Term(iTRAQ4plex)	0,0000	1,354	2	5,9	0	0,00000125	69	1,15072E-07	2	1217,67217	0,21	16,81	0
	High	iAGVNHEFATILGVR	4	1	1	P73297	N-Term(iTRAQ4plex)	0,0000	1,203	4	8,9	0	1,052E-10	68	1,45871E-07	2	1740,98906	0,73	43,05	0
	High	rVLLSNLPGAAVTAIR	1	1	1	P73297	N-Term(iTRAQ4plex)	0,0000	1,225	1		0	8,079E-07	38	0,000156304	3	1795,10373	0,13	39,41	1
	High	lGITLPHEk	4	1	1	P73297	N-Term(iTRAQ4plex); K9(iTRAQ4plex)	0,0000	1,325	2	6,2	0	0,0001831	31	0,00079794	3	1295,79233	-0,19	28,46	0
	High	kNQQQYSk	2	1	1	P73297	N-Term(iTRAQ4plex); K1(iTRAQ4plex); K8(iTRAQ4plex)	0,0000	1,472	2	1,1	0	0,00001243	23	0,005235643	3	1455,82679	-0,83	8,47	1
	High	rLGITLPHEk	1	1	1	P73297	N-Term(iTRAQ4plex); K10(iTRAQ4plex)	0,0000	1,416	1		0	0,002095	17	0,018534036	4	1451,89406	0,25	24,16	1
	High	nQQQYSk	1	1	1	P73297	N-Term(iTRAQ4plex); K7(iTRAQ4plex)	0,0000	0,997	1		0,001	0,005025	17	0,018662508	2	1183,63188	0,78	12,45	0
P73243	CheY subfamily OS=Synechocystis sp. (strain PCC 6803 / Kazusa) GN=slr2024 PE=4 SV=1 - [P73243	576,69	46,11	1	7	7	17	1,044	16	10,6	180	20,2	6,81							
	A3	Sequence	# PSMs	# Proteins	# Protein Groups	Protein Group Accessions	Modifications	ΔCn	116/117	116/117 Count	116/117 Variability [%]	q-Value	PEP	IonScore	Exp Value	Charge	MH+ [Da]	ΔM [ppm]	RT [min]	# Missed Cleavages
	High	hAPVAAVASGAAPAGDLAAEVQQ	3	1	1	P73243	N-Term(iTRAQ4plex); K25(iTRAQ4plex)	0,0000	0,971	3	8,3	0	1,894E-18	118	1,86719E-12	3	2630,45359	1,16	41,52	0
	High	vkDmLPQGNFEVIEAk	1	1	1	P73243	N-Term(iTRAQ4plex); K2(iTRAQ4plex); M4(Oxidation); K16(iTF	0,0000	0,830	1		0	7,997E-12	68	1,89631E-07	3	2266,24710	-0,31	33,91	1
	High	qLGQLVTFIk	3	1	1	P73243	N-Term(iTRAQ4plex); K10(iTRAQ4plex)	0,0000	1,050	3	8,1	0	0,000000034	67	1,8534E-07	2	1434,89324	0,65	43,48	0
	High	aIPLVLMSGR	2	1	1	P73243	N-Term(iTRAQ4plex)	0,0000	1,940	2	6,0	0	0,000002364	53	5,5077E-06	2	1200,72624	0,58	40,85	0
	High	kEEVLEk	3	1	1	P73243	N-Term(iTRAQ4plex); K1(iTRAQ4plex); K7(iTRAQ4plex)	0,0000	1,038	3	2,5	0	0,0001797	42	5,91521E-05	3	1306,79303	-0,94	15,94	1
	High	qQVAQmHGEIDLLkk	1	1	1	P73243	N-Term(iTRAQ4plex); M6(Oxidation); K14(iTRAQ4plex); K15(iT	0,0000	1,162	1		0	2,91E-08	35	0,00034609	4	2186,23305	0,11	24,50	1
	High	kQLGQLVTFIk	2	1	1	P73243	N-Term(iTRAQ4plex); K1(iTRAQ4plex); K11(iTRAQ4plex)	0,0000	1,108	1		0	0,000005345	32	0,000647098	3	1707,08951	0,11	37,55	1
	High	qQVAQMHGEIDLLkk	1	1	1	P73243	N-Term(iTRAQ4plex); K14(iTRAQ4plex); K15(iTRAQ4plex)	0,0000	1,167	1		0	0,000001619	21	0,007327739	4	2170,23525	-1,22	28,84	1
	High	aIPLVLmSGR	1	1	1	P73243	N-Term(iTRAQ4plex); M7(Oxidation)	0,0000	0,973	1		0	0,00006936	21	0,007620264	2	1216,72087	0,34	33,72	0
P77969	Delta-aminolevulinic acid dehydratase OS=Synechocystis sp. (strain PCC 6803 / Kazusa) GN=hemB P	569,45	34,86	1	13	13	28	0,635	23	17,3	327	36,1	5,08							
	A3	Sequence	# PSMs	# Proteins	# Protein Groups	Protein Group Accessions	Modifications	ΔCn	116/117	116/117 Count	116/117 Variability [%]	q-Value	PEP	IonScore	Exp Value	Charge	MH+ [Da]	ΔM [ppm]	RT [min]	# Missed Cleavages
	High	dAADSSPQFGDR	2	1	1	P77969	N-Term(iTRAQ4plex)	0,0000	0,569	1		0	0,00005281	60	9,28902E-07	2	1409,64604	3,28	17,78	0
	High	eVVSmPGVYQLSVDk	1	1	1	P77969	N-Term(iTRAQ4plex); M5(Oxidation); K15(iTRAQ4plex)	0,0000	0,653	1		0	9,332E-10	57	1,99054E-06	3	1955,03885	-0,51	33,53	0
	High	vLNDPTLELLkk	6	1	1	P77969	N-Term(iTRAQ4plex); K11(iTRAQ4plex); K12(iTRAQ4plex)	0,0000	0,804	5	4,8	0	1,994E-07	50	1,06652E-05	3	1815,13236	0,42	37,30	1
	High	yASAYYGPFR	4	1	1	P77969	N-Term(iTRAQ4plex)	0,0000	0,623	3	0,3	0	0,00008236	46	2,43204E-05	2	1338,66081	0,67	32,91	0
	High	aGADLILTYHAk	4	1	1	P77969	N-Term(iTRAQ4plex); K12(iTRAQ4plex)	0,0000	0,623	2	0,1	0	0,000003202	45	3,22827E-05	3	1560,89994	0,70	30,30	0
	High	vTLETLTSFkR	1	1	1	P77969	N-Term(iTRAQ4plex); K10(iTRAQ4plex)	0,0000	1,029	1		0	3,244E-07	40	9,59334E-05	3	1582,94199	0,81	38,19	1
	High	vLNDPTLELLk	2	1	1	P77969	N-Term(iTRAQ4plex); K11(iTRAQ4plex)	0,0000	0,565	2	18,9	0	0,000003053	40	9,88485E-05	3	1542,93363	-0,61	42,04	0
	High	vTLETLTSFk	2	1	1	P77969	N-Term(iTRAQ4plex); K10(iTRAQ4plex)	0,0000	0,503	2	8,5	0	0,00004734	39	0,000116942	3	1426,83908	-0,35	42,80	0
	High	kTAVSQANAGADVIAPSGmmDG	1	1	1	P77969	K1(iTRAQ4plex); N-Term(iTRAQ4plex); M19(Oxidation); M20(C	0,0000	0,975	1		0	0,00003114	35	0,00034512	4	3125,59804	0,55	34,89	1
	High	dAADSSPQFGDRR	1	1	1	P77969	N-Term(iTRAQ4plex)	0,0000	0,376	1		0	0,003072	25	0,003388208	3	1565,74326	0,46	15,12	1
	High	rAGADLILTYHAk	1	1	1	P77969	N-Term(iTRAQ4plex); K13(iTRAQ4plex)	0,0000	0,617	1		0	0,0002514	23	0,004797003	4	1717,00051	0,32	25,88	1
	High	mFPTIRPR	1	1	1	P77969	N-Term(iTRAQ4plex)	0,0000	1,108	1		0,003	0,04053	16	0,025597014	3	1161,66748	-0,75	26,24	0
	High	aTEAVk	1	1	1	P77969	N-Term(iTRAQ4plex); K6(iTRAQ4plex)	0,0000	0,713	1		0,008	0,1093	26	0,002728978	2	906,54808	-1,98	13,38	0
	Medium	mFPTIRPR	1	1	1	P77969	N-Term(iTRAQ4plex); M1(Oxidation)	0,0000	0,593	1		0,012	0,16	10	0,096376246	3	1177,66330	0,03	21,16	0
P54206	Ribulose bisphosphate carboxylase small chain OS=Synechocystis sp. (strain PCC 6803 / Kazusa) GN	563,84	61,95	1	6	6	19	0,976	16	7,8	113	13,2	5,48							
	A3	Sequence	# PSMs	# Proteins	# Protein Groups	Protein Group Accessions	Modifications	ΔCn	116/117	116/117 Count	116/117 Variability [%]	q-Value	PEP	IonScore	Exp Value	Charge	MH+ [Da]	ΔM [ppm]	RT [min]	# Missed Cleavages
	High	rYETLSYLPPLTDQQIAk	9	1	1	P54206	N-Term(iTRAQ4plex); K18(iTRAQ4plex)	0,0000	0,963	7	5,2	0	2,39E-11	73	4,87495E-08	3	2424,34018	1,01	39,58	1
	High	lPFFGGATANEVLAEVR	2	1	1	P54206	N-Term(iTRAQ4plex)	0,0000	0,946	2	20,7	0	0,000007982	59	1,12972E-06	2	1935,04961	2,01	53,36	0
	High	qcQTVSFIVHkPNQNQGR	3	1	1	P54206	N-Term(iTRAQ4plex); C2(Carbamidomethyl); K11(iTRAQ4plex)	0,0000	1,030	2	5,7	0	2,171E-09	47	1,80705E-05	4	2429,27285	0,73	23,46	0
	High	sENPNcYIR	3	1	1	P54206	N-Term(iTRAQ4plex); C6(Carbamidomethyl)	0,0000	0,953	3	4,8	0	0,000003948	43	5,2236E-05	2	1296,61308	0,58	20,29	0
	High	vIGFDNIk	1	1	1	P54206	N-Term(iTRAQ4plex); K8(iTRAQ4plex)	0,0000	0,986	1		0	0,00002186	42	6,74481E-05	2	1193,71147	-1,50	33,14	0
	High	yETLSYLPPLTDQQIAk	1	1	1	P54206	N-Term(iTRAQ4plex); K17(iTRAQ4plex)	0,0000	1,724	1		0	0,000008305	29	0,001210515	3	2268,23856	0,86	44,11	0
P74061	Ribulose-phosphate 3-epimerase OS=Synechocystis sp. (strain PCC 6803 / Kazusa) GN=rpe PE=1 SV	555,56	43,04	1	9	9	24	0,302	21	37,5	230	25,0	5,43							
	A3	Sequence	# PSMs	# Proteins	# Protein Groups	Protein Group Accessions	Modifications	ΔCn	116/117	116/117 Count	116/117 Variability [%]	q-Value	PEP	IonScore	Exp Value	Charge	MH+ [Da]	ΔM [ppm]	RT [min]	# Missed Cleavages
	High	nIVVAPSILSADFSR	5	1	1	P74061	N-Term(iTRAQ4plex)	0,0000	0,239	4	8,0	0	3,6E-09	94	3,80163E-10	2	1732,97380	1,34	45,33	0
	High	skNIVVAPSILSADFSR	2	1	1	P74061	N-Term(iTRAQ4plex); K2(iTRAQ4plex)	0,0000	0,620	2	41,3	0	3,08E-10	66	2,35489E-07	3	2092,20133	0,38	39,98	1
	High	kTLDVHLmIVEPEk	3	1	1	P74061	N-Term(iTRAQ4plex); K1(iTRAQ4plex); M8(Oxidation); K14(iTF	0,0000	0,273	2	15,2	0	0,000001063	38	0,000143209	4	2100,20986	-0,04	31,51	1
	High	rPEPQLATV	3	1	1	P74061	N-Term(iTRAQ4plex)	0,0000	0,267	3	10,2	0	0,00203	35	0,000284426	2	1154,66570	0,57	22,91	0
	High	yVEDFAk	1	1	1	P74061	N-Term(iTRAQ4plex); K7(iTRAQ4plex)	0,0000	0,266	1		0	0,0001971	35	0,000313307	2	1159,62419	0,37	27,36	0
	High	aGADIISVHVEHNASPHLHR	4	1	1	P74061	N-Term(iTRAQ4plex)	0,0000	0,322	3	68,3	0	1,717E-07	32	0,000699794	5	2304,20881	0,41	23,74	0
	High	fVPNITIGPLIVDAIRPLTk	1	1	1	P74061	N-Term(iTRAQ4plex); K20(iTRAQ4plex)	0,0000	0,182	1		0	0,00003111	25	0,003250648	3	2465,51438	1,83	58,67	0
	High	kTLDVHLMIVEPEk	1	1	1	P74061	N-Term(iTRAQ4plex); K1(iTRAQ4plex); K14(iTRAQ4plex)	0,0000	1,365	1		0	0,000001139	20	0,009549266	4	2084,21572	0,33	34,39	1
	High	lGEEIk	1	1	1	P74061	N-Term(iTRAQ4plex); K6(iTRAQ4plex)	0,0000	0,341	1		0,002	0,03036	22	0,005767266	2	976,59203	0,29	20,62	0
	High	tLcQIR	3	1	1	P74061	N-Term(iTRAQ4plex); C3(Carbamidomethyl)	0,0000	0,420	3	36,1	0,002	0,02863	20	0,009549266	2	934,52654	0,49	18,37	0
Q55662	ATP-dependent Clp protease regulatory subunit OS=Synechocystis sp. (strain PCC 6803 / Kazusa) GN	555,36	17,78	1	12	14	29	2,719	24	64,8	821	91,1	5,50							
	A3	Sequence	# PSMs	# Proteins	# Protein Groups	Protein Group Accessions	Modifications	ΔCn	116/117	116/117 Count	116/117 Variability [%]	q-Value	PEP	IonScore	Exp Value	Charge	MH+ [Da]	ΔM [ppm]	RT [min]	# Missed Cleavages
	High	nPNRPIASFIFSGPTGVGk	2	1	1	Q55662	N-Term(iTRAQ4plex); K19(iTRAQ4plex)	0,0000	3,500	2	34,2	0	1,002E-13	69	1,23934E-07	3	2247,25119	1,02	40,35	0
	High	lDEIIVFR	1	1	1	Q55662	N-Term(iTRAQ4plex)	0,0000	4,228	1		0	0,000009216	60	1,10454E-06	2	1148,67937	-0,22	43,49	0
	High	lLHmEDTLHQR	4	1	1	Q55662	N-Term(iTRAQ4plex); M4(Oxidation)	0,0000	1,350	4	78,5	0	2,373E-07	57	1,86196E-06	3	1552,80246	0,12	17,36	0
	High	nTLLImTSNIGSk	1	1	1	Q55662	N-Term(iTRAQ4plex); M6(Oxidation); K13(iTRAQ4plex)	0,0000	0,590	1		0	0,00001298	50	1,11679E-05	3	1695,95542	0,02	31,76	0
	High	tAIAEGLAQR	2	3	3	P74459;P74361;Q55662	N-Term(iTRAQ4plex)	0,0000				0	5,338E-07	46	3,02268E-05	2	1173,67082	-0,03	23,31	0
	High	lLHMEDTLHQR	1	1	1	Q55662	N-Term(iTRAQ4plex)	0,0000	2,921	1		0	0,000001227	44	4,23614E-05	4	1536,80764	0,18	24,91	0
	High	rPYTVVLFDEIEk	2	1	1	Q55662	N-Term(iTRAQ4plex); K13(iTRAQ4plex)	0,0000	2,736	2	27,2	0	5,537E-11	40	8,97367E-05	3	1897,06839	0,54	41,49	1
	High	rVLELSLEEAR	2	1	1	Q55662	N-Term(iTRAQ4plex)	0,0000	1,824	2	3,5	0	0,00001566	37	0,000199972	3	1458,83878	-0,65	30,79	1
	High	gSGFVAVEIPFTPR	1	1	1	Q55662	N-Term(iTRAQ4plex)	0,0000	1,310	1		0	0,00002266	33	0,00067129	3	1620,88694	0,16	44,73	0
	High	eISEILLR	1	1	1	Q55662	N-Term(iTRAQ4plex)	0,0000	2,644	1		0	0,00007773	24	0,003672569	2	1116,67449	-0,04	37,11	0
	High	nYFRPEFLNR	2	1	1	Q55662	N-Term(iTRAQ4plex)	0,0000	3,415	2	12,4	0	0,0001095	23	0,004920056	3	1499,78757	-0,02	33,80	0
	High	yRGEFEER	2	3	3	P74459;P74361;Q55662	N-Term(iTRAQ4plex)	0,0000				0	0,002186	16	0,027731286	3	1229,60511	1,59	15,30	1
	High	vIQILGR	4	1	1	Q55662	N-Term(iTRAQ4plex)	0,0000	5,119	3	2,2	0	0,002812	15	0,03258142	2	942,62175	0,05	31,92	0
	High	qLNkEEVk	2	1	1	Q55662	N-Term(iTRAQ4plex); K4(iTRAQ4plex); K8(iTRAQ4plex)	0,0000	2,920	2	33,2	0,001	0,005332	13	0,054196346	3	1419,85086	-1,62	14,38	1
	Medium	aIASSk	2	1	1	Q55662	N-Term(iTRAQ4plex); K6(iTRAQ4plex)	0,0000	1,440	2	3,1	0,025	0,347	15	0,03813588	2	864,53600	-3,83	12,31	0
P73309	50S ribosomal protein L24 OS=Synechocystis sp. (strain PCC 6803 / Kazusa) GN=rplX PE=3 SV=1 - [	554,23	52,17	1	8	8	33	1,247	25	30,8	115	12,8	10,08							
	A3	Sequence	# PSMs	# Proteins	# Protein Groups	Protein Group Accessions	Modifications	ΔCn	116/117	116/117 Count	116/117 Variability [%]	q-Value	PEP	IonScore	Exp Value	Charge	MH+ [Da]	ΔM [ppm]	RT [min]	# Missed Cleavages
	High	tIPSHSQVVVk	5	1	1	P73309	N-Term(iTRAQ4plex); K11(iTRAQ4plex)	0,0000	1,244	4	3,0	0	2,252E-07	77	2,05102E-08	2	1482,88970	0,96	19,10	0
	High	kGDTIQVISGk	6	1	1	P73309	N-Term(iTRAQ4plex); K1(iTRAQ4plex); K11(iTRAQ4plex)	0,0000	1,247	3	6,0	0	0,000001639	59	1,30909E-06	2	1577,96099	1,44	20,69	1
	High	icYTVTDDGRk	2	1	1	P73309	N-Term(iTRAQ4plex); C2(Carbamidomethyl); K11(iTRAQ4plex)	0,0000	1,126	2	0,7	0	6,379E-07	48	1,71779E-05	3	1615,83835	1,92	20,66	1
	High	vMLYSTk	4	1	1	P73309	N-Term(iTRAQ4plex); K7(iTRAQ4plex)	0,0000	2,042	3	0,3	0	0,0001067	48	1,7578E-05	2	1129,65703	3,61	24,62	0
	High	vmLYSTk	6	1	1	P73309	N-Term(iTRAQ4plex); M2(Oxidation); K7(iTRAQ4plex)	0,0000	1,020	3	2,5	0	0,002131	44	4,40555E-05	2	1145,64983	1,71	22,04	0
	High	kGDTIQVISGkDk	1	1	1	P73309	N-Term(iTRAQ4plex); K1(iTRAQ4plex); K11(iTRAQ4plex); K13(	0,0000	2,132	1		0	0,000007996	41	7,94273E-05	4	1965,19362	5,56	20,05	2
	Medium	tkPAPHR	2	1	1	P73309	N-Term(iTRAQ4plex); K2(iTRAQ4plex)	0,0000	1,579	2	1,1	0,015	0,1944	11	0,088301891	3	1094,67276	4,98	7,54	0
	Medium	vGEVLR	3	1	1	P73309	N-Term(iTRAQ4plex)	0,0000	1,004	3	2,1	0,034	0,4375	21	0,009713879	2	816,50701	1,23	19,20	0
	Medium	gVNIR	4	1	1	P73309	N-Term(iTRAQ4plex)	0,0000	1,526	4	22,2	0,034	0,4391	10	0,095273036	2	702,43913	1,74	14,76	0
P72659	Polyribonucleotide nucleotidyltransferase OS=Synechocystis sp. (strain PCC 6803 / Kazusa) GN=pnp	548,97	19,36	1	13	13	27	2,581	24	48,8	718	77,8	5,27							
	A3	Sequence	# PSMs	# Proteins	# Protein Groups	Protein Group Accessions	Modifications	ΔCn	116/117	116/117 Count	116/117 Variability [%]	q-Value	PEP	IonScore	Exp Value	Charge	MH+ [Da]	ΔM [ppm]	RT [min]	# Missed Cleavages
	High	kIITVLGQFDLGk	2	1	1	P72659	N-Term(iTRAQ4plex); K1(iTRAQ4plex); K13(iTRAQ4plex)	0,0000	2,989	2	7,8	0	3,05E-12	72	5,83405E-08	3	1864,16397	0,39	44,46	1
	High	aImQALPAR	3	1	1	P72659	N-Term(iTRAQ4plex); M3(Oxidation)	0,0000	2,492	2	10,3	0	0,000003208	55	3,22085E-06	2	1130,64690	-0,35	20,50	0
	High	kLNEGEVYLGR	4	1	1	P72659	N-Term(iTRAQ4plex); K1(iTRAQ4plex)	0,0000	1,795	3	120,9	0	0,000002305	50	9,73171E-06	3	1565,88974	0,47	24,15	1
	High	kPVSGAAmGLIk	1	1	1	P72659	N-Term(iTRAQ4plex); K1(iTRAQ4plex); M8(Oxidation); K12(iTF	0,0000	3,651	1		0	1,168E-08	45	3,17665E-05	3	1619,98990	1,22	21,33	0
	High	kQIVDDGVR	1	1	1	P72659	N-Term(iTRAQ4plex); K1(iTRAQ4plex)	0,0000	0,369	1		0	0,0005359	43	6,82197E-05	3	1317,77140	-1,15	15,31	1
	High	lGIHPDEAAEAR	1	1	1	P72659	N-Term(iTRAQ4plex)	0,0000	0,963	1		0	0,0001058	41	7,83376E-05	3	1422,74558	-0,17	20,95	0
	High	ePRPELSPFAPR	2	1	1	P72659	N-Term(iTRAQ4plex)	0,0000	1,955	2	61,0	0	0,000001491	38	0,000158478	3	1539,83823	-1,19	27,73	0
	High	eIGHGALAER	2	1	1	P72659	N-Term(iTRAQ4plex)	0,0000	1,803	2	105,8	0	0,001409	32	0,000586098	2	1196,64934	-0,93	15,18	0
	High	kLEQVRPIScEVGFLPR	3	1	1	P72659	N-Term(iTRAQ4plex); K1(iTRAQ4plex); C10(Carbamidomethyl)	0,0000	2,822	2	8,2	0	2,08E-09	29	0,001250173	4	2316,31093	0,35	35,24	1
	High	iIPIGAFVEVLPGk	3	1	1	P72659	N-Term(iTRAQ4plex); K14(iTRAQ4plex)	0,0000	0,927	3	93,0	0	0,0001721	17	0,02018227	3	1741,08744	0,44	53,16	0
	High	lVGNLYk	1	1	1	P72659	N-Term(iTRAQ4plex); K7(iTRAQ4plex)	0,0000	2,496	1		0,001	0,003811	32	0,00059016	2	1094,67974	-1,35	27,10	0
	High	lHILDk	2	1	1	P72659	N-Term(iTRAQ4plex); K6(iTRAQ4plex)	0,0000	1,342	2	301,8	0,001	0,006566	29	0,001238711	3	1026,65421	-0,78	24,52	0
	High	eGRPPEk	2	1	1	P72659	N-Term(iTRAQ4plex); K7(iTRAQ4plex)	0,0000	2,271	2	44,7	0,007	0,09388	11	0,083554531	3	1100,63010	-0,15	8,42	0
P16033	Photosystem Q(B) protein 2 OS=Synechocystis sp. (strain PCC 6803 / Kazusa) GN=psbA2 PE=1 SV=	545,15	16,67	1	5	5	18	0,824	17	17,6	360	39,7	5,64							
	A3	Sequence	# PSMs	# Proteins	# Protein Groups	Protein Group Accessions	Modifications	ΔCn	116/117	116/117 Count	116/117 Variability [%]	q-Value	PEP	IonScore	Exp Value	Charge	MH+ [Da]	ΔM [ppm]	RT [min]	# Missed Cleavages
	High	lIFQYASFNNSR	6	1	1	P16033	N-Term(iTRAQ4plex)	0,0000	0,827	6	0,8	0	3,868E-07	64	3,69803E-07	2	1603,83599	0,65	42,33	0
	High	vIGTWADVLNR	4	1	1	P16033	N-Term(iTRAQ4plex)	0,0000	0,782	4	10,6	0	0,000001421	61	7,85181E-07	2	1387,78203	0,40	43,62	0
	High	aNIGFEVmHER	4	1	1	P16033	N-Term(iTRAQ4plex); M8(Oxidation)	0,0000	0,755	3	4,4	0	4,302E-08	54	4,31489E-06	3	1462,72318	0,14	22,66	0
	High	fGQEEETYNIVAAHGYFGR	1	1	1	P16033	N-Term(iTRAQ4plex)	0,0000	0,938	1		0	4,886E-08	44	4,22639E-05	3	2332,11362	0,89	40,56	0
	High	aNIGFEVMHER	2	1	1	P16033	N-Term(iTRAQ4plex)	0,0000	1,150	2	7,3	0	3,698E-07	39	0,000127635	3	1446,72800	-0,04	29,02	0
	High	qWELSYR	1	1	1	P16033	N-Term(iTRAQ4plex)	0,0000	0,660	1		0	0,0001199	30	0,000946172	2	1125,58049	-0,40	29,01	0
Q55665	Glutamate-1-semialdehyde 2,1-aminomutase OS=Synechocystis sp. (strain PCC 6803 / Kazusa) GN=	541,87	21,71	1	7	7	18	0,757	16	31,7	433	45,9	5,27							
	A3	Sequence	# PSMs	# Proteins	# Protein Groups	Protein Group Accessions	Modifications	ΔCn	116/117	116/117 Count	116/117 Variability [%]	q-Value	PEP	IonScore	Exp Value	Charge	MH+ [Da]	ΔM [ppm]	RT [min]	# Missed Cleavages
	High	vNATPFITTk	3	1	1	Q55665	N-Term(iTRAQ4plex); K10(iTRAQ4plex)	0,0000	1,452	3	104,2	0	2,025E-07	75	2,87058E-08	2	1379,81291	-0,58	29,62	0
	High	vIGGGLPVGAYGGR	5	1	1	Q55665	N-Term(iTRAQ4plex)	0,0000	0,706	4	22,3	0	1,524E-09	72	5,67505E-08	2	1416,80840	0,26	33,27	0
	High	vSYGGAQAR	3	1	1	Q55665	N-Term(iTRAQ4plex)	0,0000	0,678	3	0,8	0	0,000005452	57	1,82377E-06	2	1052,56072	0,15	14,01	0
	High	fVNSGTEAcMSVLR	1	1	1	Q55665	N-Term(iTRAQ4plex); C9(Carbamidomethyl)	0,0000	1,450	1		0	0,000006868	56	2,63009E-06	2	1714,84123	2,25	35,68	0
	High	sEEIFAAAQHLmPGGVSSPVR	2	1	1	Q55665	N-Term(iTRAQ4plex); M12(Oxidation)	0,0000	0,521	1		0	8,565E-11	52	5,74077E-06	3	2343,19187	1,45	39,51	0
	High	sEEIFAAAQHLMPGGVSSPVR	1	1	1	Q55665	N-Term(iTRAQ4plex)	0,0000	1,147	1		0	2,537E-07	49	1,40919E-05	3	2327,19669	1,35	45,54	0
	High	sVGGQPIVFDR	1	1	1	Q55665	N-Term(iTRAQ4plex)	0,0000	0,659	1		0	0,0001817	44	4,27533E-05	2	1318,72405	0,32	28,26	0
	High	fEGcYHGHADmFLVk	2	1	1	Q55665	N-Term(iTRAQ4plex); C4(Carbamidomethyl); M11(Oxidation);	0,0000	0,794	2	20,8	0	4,537E-08	21	0,007078969	4	2115,00283	-0,12	29,01	0
P23349	50S ribosomal protein L7/L12 OS=Synechocystis sp. (strain PCC 6803 / Kazusa) GN=rplL PE=1 SV=3	538,16	52,34	1	9	9	30	0,665	22	13,5	128	13,3	4,77							
	A3	Sequence	# PSMs	# Proteins	# Protein Groups	Protein Group Accessions	Modifications	ΔCn	116/117	116/117 Count	116/117 Variability [%]	q-Value	PEP	IonScore	Exp Value	Charge	MH+ [Da]	ΔM [ppm]	RT [min]	# Missed Cleavages
	High	sLSLLEASELVk	7	1	1	P23349	N-Term(iTRAQ4plex); K12(iTRAQ4plex)	0,0000	0,598	6	19,7	0	6,927E-10	65	2,99206E-07	2	1576,94023	0,12	43,52	0
	High	tITGLGLk	7	1	1	P23349	N-Term(iTRAQ4plex); K8(iTRAQ4plex)	0,0000	0,660	4	1,3	0	0,00002145	53	4,47682E-06	2	1090,70842	0,89	29,31	0
	High	qIEEAGGk	2	1	1	P23349	N-Term(iTRAQ4plex); K8(iTRAQ4plex)	0,0000	0,567	1		0	0,000008604	47	2,13781E-05	2	1119,62370	-1,02	12,87	0
	High	kQIEEAGGk	2	1	1	P23349	N-Term(iTRAQ4plex); K1(iTRAQ4plex); K9(iTRAQ4plex)	0,0000	0,813	2	15,7	0	0,0001182	41	7,90624E-05	3	1391,81696	-3,54	11,43	1
	High	eLVESTPk	1	1	1	P23349	N-Term(iTRAQ4plex); K8(iTRAQ4plex)	0,0000	0,611	1		0	0,001894	39	0,000112453	2	1190,68743	0,27	18,89	0
	High	sAATDQILEQLk	1	1	1	P23349	N-Term(iTRAQ4plex); K12(iTRAQ4plex)	0,0000	0,471	1		0	0,0001213	34	0,000372366	3	1604,91007	0,16	37,05	0
	High	eATGkDDAEAIkk	1	1	1	P23349	N-Term(iTRAQ4plex); K5(iTRAQ4plex); K12(iTRAQ4plex); K13(	0,0000	0,733	1		0	9,468E-08	19	0,013644889	4	1952,11440	-0,14	15,53	2
	High	iAVLk	7	1	1	P23349	N-Term(iTRAQ4plex); K5(iTRAQ4plex)	0,0000	0,656	4	3,8	0,002	0,02524	14	0,044357801	2	831,58983	-0,95	23,72	0
	High	kIAVLk	2	1	1	P23349	N-Term(iTRAQ4plex); K1(iTRAQ4plex); K6(iTRAQ4plex)	0,0000	0,760	2	7,4	0,005	0,05623	25	0,00349921	3	1103,78601	-1,48	18,99	1
P28371	Elongation factor G 1 OS=Synechocystis sp. (strain PCC 6803 / Kazusa) GN=fusA PE=1 SV=2 - [EFG1	533,73	21,58	1	16	16	30	2,027	27	18,4	695	76,7	5,02							
	A3	Sequence	# PSMs	# Proteins	# Protein Groups	Protein Group Accessions	Modifications	ΔCn	116/117	116/117 Count	116/117 Variability [%]	q-Value	PEP	IonScore	Exp Value	Charge	MH+ [Da]	ΔM [ppm]	RT [min]	# Missed Cleavages
	High	gVLAGYPVVDLk	2	1	1	P28371	N-Term(iTRAQ4plex); K12(iTRAQ4plex)	0,0000	1,306	1		0	5,279E-08	64	3,62243E-07	2	1518,91448	0,68	40,97	0
	High	kLDFFPTYGGSSFk	5	1	1	P28371	N-Term(iTRAQ4plex); K1(iTRAQ4plex); K14(iTRAQ4plex)	0,0000	1,754	4	25,7	0	1,273E-08	51	8,03471E-06	3	2026,10282	0,89	39,30	1
	High	iYSGTLSk	2	1	1	P28371	N-Term(iTRAQ4plex); K8(iTRAQ4plex)	0,0000	1,461	2	68,4	0	0,0006102	45	3,44326E-05	2	1156,68132	-0,25	24,03	0
	High	nIGIFAHVDAGk	3	1	1	P28371	N-Term(iTRAQ4plex); K12(iTRAQ4plex)	0,0000	2,209	2	11,0	0	9,621E-08	44	4,21667E-05	3	1529,86914	0,82	31,03	0
	High	tHGVEVEmGkPQVAYR	2	1	1	P28371	N-Term(iTRAQ4plex); M8(Oxidation); K10(iTRAQ4plex)	0,0000	1,983	2	15,2	0	7,179E-08	36	0,000265442	3	2105,10990	2,21	20,25	1
	High	vTGGNVPR	3	1	1	P28371	N-Term(iTRAQ4plex)	0,0000	2,556	3	23,4	0	0,003051	35	0,000293069	2	943,54436	0,19	14,03	0
	High	lIYINkLDR	1	1	1	P28371	N-Term(iTRAQ4plex); K6(iTRAQ4plex)	0,0000	3,347	1		0	0,000008576	35	0,000331872	3	1435,88926	1,20	32,87	1
	High	nVQTGHTLcDPk	1	1	1	P28371	N-Term(iTRAQ4plex); C9(Carbamidomethyl); K12(iTRAQ4plex)	0,0000	2,279	1		0	0,000005883	34	0,0004385	3	1657,85794	0,54	16,58	0
	High	fGALTFTR	3	1	1	P28371	N-Term(iTRAQ4plex)	0,0000	2,099	3	8,9	0	0,0009151	29	0,001148074	2	1056,59685	0,91	35,10	0
	High	gmGELHLDIk	1	1	1	P28371	N-Term(iTRAQ4plex); M2(Oxidation); K10(iTRAQ4plex)	0,0000	0,774	1		0	0,001507	26	0,002710004	3	1416,77423	-1,22	26,43	0
	High	kQSGGSGQYAk	1	1	1	P28371	N-Term(iTRAQ4plex); K1(iTRAQ4plex); K11(iTRAQ4plex)	0,0000	2,117	1		0,002	0,02094	15	0,031839776	3	1542,86313	2,00	8,96	1
	High	lIYINk	2	1	1	P28371	N-Term(iTRAQ4plex); K6(iTRAQ4plex)	0,0000	1,745	2	42,1	0,003	0,0376	30	0,001111655	2	1051,67461	-0,75	29,94	0
	High	gFDQSVVk	1	1	1	P28371	N-Term(iTRAQ4plex); K8(iTRAQ4plex)	0,0000	0,589	1		0,008	0,1114	17	0,025963785	2	1167,66081	-0,35	21,62	0
	High	sQETGPmGVR	1	1	1	P28371	N-Term(iTRAQ4plex); M7(Oxidation)	0,0000	1,796	1		0,008	0,108	12	0,065158339	2	1221,60283	1,12	11,69	0
	Medium	lGmALSk	1	1	1	P28371	N-Term(iTRAQ4plex); M3(Oxidation); K7(iTRAQ4plex)	0,0000	1,540	1		0,032	0,4128	22	0,007597975	2	1023,60759	-3,44	20,13	0
	Medium	aLAFk	1	1	1	P28371	N-Term(iTRAQ4plex); K5(iTRAQ4plex)	0,0000	1,795	1		0,038	0,49	22	0,007062688	2	837,54131	-2,82	22,39	0
P10549	Photosystem II manganese-stabilizing polypeptide OS=Synechocystis sp. (strain PCC 6803 / Kazusa)	532,43	27,01	1	8	8	35	0,434	28	12,3	274	29,9	4,94							
	A3	Sequence	# PSMs	# Proteins	# Protein Groups	Protein Group Accessions	Modifications	ΔCn	116/117	116/117 Count	116/117 Variability [%]	q-Value	PEP	IonScore	Exp Value	Charge	MH+ [Da]	ΔM [ppm]	RT [min]	# Missed Cleavages
	High	gLYTGYDNAVALPSAADkFR	4	1	1	P10549	N-Term(iTRAQ4plex); K18(iTRAQ4plex)	0,0000	0,432	2	1,7	0	2,328E-11	71	7,26106E-08	3	2417,27121	0,33	39,76	1
	High	gTIEVEPNTk	3	1	1	P10549	N-Term(iTRAQ4plex); K10(iTRAQ4plex)	0,0000	0,401	2	0,8	0	7,218E-08	71	7,4131E-08	2	1375,77202	3,53	19,11	0
	High	qTTSLEQIR	4	1	1	P10549	N-Term(iTRAQ4plex)	0,0000	0,394	4	8,9	0	0,00000546	58	1,41896E-06	2	1219,67583	-0,40	20,53	0
	High	kETPLGk	3	1	1	P10549	N-Term(iTRAQ4plex); K1(iTRAQ4plex); K7(iTRAQ4plex)	0,0000	0,571	2	26,6	0	0,001714	32	0,000595621	2	1204,75932	-2,69	13,01	1
	High	qkAEYVk	2	1	1	P10549	N-Term(iTRAQ4plex); K2(iTRAQ4plex); K7(iTRAQ4plex)	0,0000	0,467	2	1,7	0	0,0003169	17	0,018028932	3	1297,78168	-1,79	11,76	1
	High	gIFYGR	5	1	1	P10549	N-Term(iTRAQ4plex)	0,0000	0,398	5	12,8	0,001	0,00584	23	0,005482391	2	856,47972	-0,06	23,34	0
	High	eEPVNkR	7	1	1	P10549	N-Term(iTRAQ4plex); K6(iTRAQ4plex)	0,0000	0,449	7	8,7	0,001	0,01116	15	0,031944249	3	1159,66578	-1,38	8,35	1
	High	gAGFLDPk	7	1	1	P10549	N-Term(iTRAQ4plex); K8(iTRAQ4plex)	0,0000	0,441	4	12,2	0,001	0,01215	13	0,045705662	2	1092,62761	-1,46	24,41	0
P52208	6-phosphogluconate dehydrogenase, decarboxylating OS=Synechocystis sp. (strain PCC 6803 / Kazus	531,43	24,90	1	10	10	26	1,455	24	39,3	482	52,8	5,22							
	A3	Sequence	# PSMs	# Proteins	# Protein Groups	Protein Group Accessions	Modifications	ΔCn	116/117	116/117 Count	116/117 Variability [%]	q-Value	PEP	IonScore	Exp Value	Charge	MH+ [Da]	ΔM [ppm]	RT [min]	# Missed Cleavages
	High	vAASGQLSGPSk	5	1	1	P52208	N-Term(iTRAQ4plex); K12(iTRAQ4plex)	0,0000	0,934	4	31,2	0	2,911E-09	96	2,34407E-10	2	1389,79692	2,07	18,91	0
	High	aVLPQNLTQAQR	3	1	1	P52208	N-Term(iTRAQ4plex)	0,0000	1,571	3	19,5	0	6,724E-08	71	7,98343E-08	2	1482,85149	0,37	27,57	0
	High	gFPIAVFNR	6	1	1	P52208	N-Term(iTRAQ4plex)	0,0000	1,355	6	34,5	0	0,000002466	46	2,64223E-05	2	1164,66545	0,69	41,01	0
	High	aAYTVEEFVQLLERPR	1	1	1	P52208	N-Term(iTRAQ4plex)	0,0000	1,912	1		0	0,00000581	33	0,000610815	3	2065,12168	0,84	55,03	0
	High	kILVMVk	1	1	1	P52208	N-Term(iTRAQ4plex); K1(iTRAQ4plex); K7(iTRAQ4plex)	0,0000	3,379	1		0	0,00008521	28	0,001682558	3	1262,85770	-1,39	25,67	1
	High	kDPETGGHLIDYILDAAGQk	1	1	1	P52208	N-Term(iTRAQ4plex); K1(iTRAQ4plex); K20(iTRAQ4plex)	0,0000	1,366	1		0	2,853E-08	26	0,002798788	4	2573,39589	0,89	45,00	1
	High	dYFGAHTYER	1	1	1	P52208	N-Term(iTRAQ4plex)	0,0000	1,047	1		0	0,00004309	25	0,003435342	3	1402,65039	-0,30	22,89	0
	High	kILVmVk	2	1	1	P52208	N-Term(iTRAQ4plex); K1(iTRAQ4plex); M5(Oxidation); K7(iTRA	0,0000	1,821	1		0	0,001578	23	0,005116465	3	1278,85251	-1,45	21,58	1
	High	aFkDNPQLPNLLLAPEFk	1	1	1	P52208	N-Term(iTRAQ4plex); K3(iTRAQ4plex); K18(iTRAQ4plex)	0,0000	1,589	1		0	0,000001247	19	0,011271196	4	2487,43545	0,73	47,00	1
	High	vMSSLkEER	2	1	1	P52208	N-Term(iTRAQ4plex); K6(iTRAQ4plex)	0,0000	1,348	2	63,1	0,001	0,008474	15	0,029510054	3	1366,76044	0,10	19,48	1
	Medium	sPNkTEk	1	1	1	P52208	N-Term(iTRAQ4plex); K4(iTRAQ4plex); K7(iTRAQ4plex)	0,0000	1,978	1		0,017	0,2253	13	0,057436676	3	1235,72898	-2,43	7,93	1
	Medium	vmSSLkEER	2	1	1	P52208	N-Term(iTRAQ4plex); M2(Oxidation); K6(iTRAQ4plex)	0,0000	1,343	2	3,9	0,026	0,3528	11	0,081090505	3	1382,75580	0,42	13,68	1
P74070	Elongation factor Ts OS=Synechocystis sp. (strain PCC 6803 / Kazusa) GN=tsf PE=1 SV=3 - [EFTS_S	526,22	52,75	1	11	11	32	0,957	27	9,0	218	24,2	5,45							
	A3	Sequence	# PSMs	# Proteins	# Protein Groups	Protein Group Accessions	Modifications	ΔCn	116/117	116/117 Count	116/117 Variability [%]	q-Value	PEP	IonScore	Exp Value	Charge	MH+ [Da]	ΔM [ppm]	RT [min]	# Missed Cleavages
	High	gIASADkk	4	1	1	P74070	N-Term(iTRAQ4plex); K7(iTRAQ4plex); K8(iTRAQ4plex)	0,0000	1,167	4	8,4	0	0,0001174	56	2,5304E-06	2	1221,74944	-2,70	10,88	1
	High	tAAEGLVHSYIHFGGR	5	1	1	P74070	N-Term(iTRAQ4plex)	0,0000	0,901	3	9,1	0	8,39E-09	52	6,98184E-06	3	1858,96909	0,53	35,91	0
	High	fNLGEGIEk	2	1	1	P74070	N-Term(iTRAQ4plex); K9(iTRAQ4plex)	0,0000	0,916	2	1,8	0	0,000001962	45	3,01974E-05	2	1294,72429	-0,21	31,77	0
	High	lkELSLLDQPYIk	9	1	1	P74070	N-Term(iTRAQ4plex); K2(iTRAQ4plex); K13(iTRAQ4plex)	0,0000	0,943	6	1,9	0	3,202E-09	44	3,73224E-05	3	1992,21054	-0,01	41,73	1
	High	aETNFAEEVAAAAk	1	1	1	P74070	N-Term(iTRAQ4plex); K14(iTRAQ4plex)	0,0000	0,951	1		0	0,000004949	40	8,97367E-05	3	1709,89634	0,84	37,08	0
	High	aLkENEGDLEk	2	1	1	P74070	N-Term(iTRAQ4plex); K3(iTRAQ4plex); K11(iTRAQ4plex)	0,0000	1,293	2	27,4	0	0,000005396	36	0,000225944	3	1677,93784	-0,32	18,64	1
	High	aEITAQLVk	1	1	1	P74070	N-Term(iTRAQ4plex); K9(iTRAQ4plex)	0,0000	0,458	1		0	0,001972	26	0,002678983	3	1260,77524	-1,08	26,98	0
	High	ekEIEmGRDDLGk	2	1	1	P74070	N-Term(iTRAQ4plex); K2(iTRAQ4plex); M6(Oxidation); K13(iTF	0,0000	1,052	2	18,7	0	0,000007206	25	0,003467129	3	1968,04428	0,51	18,71	2
	High	tGAGmmDckk	2	1	1	P74070	N-Term(iTRAQ4plex); M5(Oxidation); M6(Oxidation); C8(Carba	0,0000	1,332	2	9,5	0	0,0003235	11	0,075330353	3	1562,77127	0,72	7,96	1
	High	sIEWLR	2	1	1	P74070	N-Term(iTRAQ4plex)	0,0000	0,988	2	4,2	0,001	0,008086	27	0,002055749	2	947,54265	-0,49	31,97	0
	High	kPANIk	2	1	1	P74070	N-Term(iTRAQ4plex); K1(iTRAQ4plex); K6(iTRAQ4plex)	0,0000	0,849	2	9,4	0,007	0,08902	16	0,024432618	3	1102,72748	-3,06	10,92	0
Q55361	Sll0887 protein OS=Synechocystis sp. (strain PCC 6803 / Kazusa) GN=sll0887 PE=4 SV=1 - [Q55361	525,48	23,77	1	10	10	25	0,569	23	15,2	446	47,6	4,94							
	A3	Sequence	# PSMs	# Proteins	# Protein Groups	Protein Group Accessions	Modifications	ΔCn	116/117	116/117 Count	116/117 Variability [%]	q-Value	PEP	IonScore	Exp Value	Charge	MH+ [Da]	ΔM [ppm]	RT [min]	# Missed Cleavages
	High	fYLNSAGAk	2	1	1	Q55361	N-Term(iTRAQ4plex); K9(iTRAQ4plex)	0,0000	0,586	2	2,5	0	3,365E-07	61	7,19399E-07	2	1258,70561	1,75	26,46	0
	High	fDTQPTGHANIGSk	2	1	1	Q55361	N-Term(iTRAQ4plex); K14(iTRAQ4plex)	0,0000	0,644	2	23,9	0	3,919E-09	61	7,24386E-07	3	1760,91941	1,36	21,10	0
	High	sYASIYLYSR	3	1	1	Q55361	N-Term(iTRAQ4plex)	0,0000	0,757	3	52,1	0	0,00004944	61	7,65544E-07	2	1366,71379	1,07	33,76	0
	High	rFDTQPTGHANIGSk	2	1	1	Q55361	N-Term(iTRAQ4plex); K15(iTRAQ4plex)	0,0000	0,499	2	13,3	0	1,993E-07	45	3,34942E-05	4	1917,02138	1,69	17,64	1
	High	yTVVFSAR	3	1	1	Q55361	N-Term(iTRAQ4plex)	0,0000	0,564	3	4,3	0	0,000142	45	3,40385E-05	2	1086,61101	4,21	30,38	0
	High	mLDIQAIADHAHQGAAALGIk	2	1	1	Q55361	N-Term(iTRAQ4plex); M1(Oxidation); K21(iTRAQ4plex)	0,0000	0,610	2	49,3	0	3,799E-07	36	0,000263009	4	2448,33144	1,72	43,89	0
	High	mLDIQAIADHAHQGAAALGIk	1	1	1	Q55361	N-Term(iTRAQ4plex); K21(iTRAQ4plex)	0,0000	0,684	1		0	2,449E-09	33	0,000494526	4	2432,33608	1,54	47,24	0
	High	iTVGAHFYHVYSEk	2	1	1	Q55361	N-Term(iTRAQ4plex); K14(iTRAQ4plex)	0,0000	0,573	2	16,9	0	4,036E-10	33	0,00054572	3	1939,03342	0,92	30,25	0
	High	tLSHLDYRPIPTGk	5	1	1	Q55361	N-Term(iTRAQ4plex); K14(iTRAQ4plex)	0,0000	0,569	3	12,9	0	0,000001716	27	0,00198139	3	1886,07456	0,38	26,55	0
	High	rVDIIk	2	1	1	Q55361	N-Term(iTRAQ4plex); K6(iTRAQ4plex)	0,0000	0,555	2	2,6	0,001	0,009782	25	0,003243172	3	1031,68375	2,12	17,79	1
	High	fDGEGTPTR	1	1	1	Q55361	N-Term(iTRAQ4plex)	0,0000	1,306	1		0,002	0,02483	19	0,01216102	2	1123,55046	0,35	16,50	0
Q55707	Uncharacterized protein sll0617 OS=Synechocystis sp. (strain PCC 6803 / Kazusa) GN=sll0617 PE=3	523,78	29,96	1	9	9	19	0,544	15	26,2	267	28,9	5,03							
	A3	Sequence	# PSMs	# Proteins	# Protein Groups	Protein Group Accessions	Modifications	ΔCn	116/117	116/117 Count	116/117 Variability [%]	q-Value	PEP	IonScore	Exp Value	Charge	MH+ [Da]	ΔM [ppm]	RT [min]	# Missed Cleavages
	High	sLTDTAAAYQTQLAQQR	2	1	1	Q55707	N-Term(iTRAQ4plex)	0,0000	0,495	2	23,7	0	7,341E-13	112	6,2513E-12	2	2010,03948	1,08	34,17	0
	High	aNAELQQTLGGLGTSSATSAFER	1	1	1	Q55707	N-Term(iTRAQ4plex)	0,0000	0,579	1		0	4,183E-12	83	5,06956E-09	3	2453,24155	1,05	42,35	0
	High	kSLTDTAAAYQTQLAQQR	1	1	1	Q55707	N-Term(iTRAQ4plex); K1(iTRAQ4plex)	0,0000	0,531	1		0	1,051E-09	69	1,79037E-07	3	2282,23459	0,11	31,17	1
	High	nLAALEAk	8	1	1	Q55707	N-Term(iTRAQ4plex); K8(iTRAQ4plex)	0,0000	0,544	5	19,0	0	0,000001491	64	4,23614E-07	2	1117,68144	-0,47	25,94	0
	High	lNQDTQEAk	1	1	1	Q55707	N-Term(iTRAQ4plex); K9(iTRAQ4plex)	0,0000	0,458	1		0	2,023E-07	58	1,65566E-06	2	1334,71562	0,13	13,64	0
	High	aNLNDLVSk	1	1	1	Q55707	N-Term(iTRAQ4plex); K9(iTRAQ4plex)	0,0000	0,376	1		0	0,0000235	31	0,00079794	2	1261,73393	-1,21	28,79	0
	High	rNLAALEAk	3	1	1	Q55707	N-Term(iTRAQ4plex); K9(iTRAQ4plex)	0,0000	0,765	2	37,1	0	0,0000211	30	0,001050492	3	1273,78177	-1,03	18,77	1
	High	tmSENLR	1	1	1	Q55707	N-Term(iTRAQ4plex); M2(Oxidation)	0,0000	0,951	1		0,003	0,03312	12	0,067759471	2	1010,50487	-0,87	12,73	0
	Medium	rLNNL	1	1	1	Q55707	N-Term(iTRAQ4plex)	0,0000	0,451	1		0,019	0,2566	11	0,087090344	2	773,47429	-0,95	17,38	1
P80046	Isocitrate dehydrogenase [NADP] OS=Synechocystis sp. (strain PCC 6803 / Kazusa) GN=icd PE=1 SV	523,61	26,53	1	13	13	29	0,284	24	17,9	475	52,2	5,58							
	A3	Sequence	# PSMs	# Proteins	# Protein Groups	Protein Group Accessions	Modifications	ΔCn	116/117	116/117 Count	116/117 Variability [%]	q-Value	PEP	IonScore	Exp Value	Charge	MH+ [Da]	ΔM [ppm]	RT [min]	# Missed Cleavages
	High	gIGAAIANR	3	1	1	P80046	N-Term(iTRAQ4plex)	0,0000	0,261	2	16,5	0	0,00001085	63	5,3576E-07	2	986,58684	0,45	21,67	0
	High	kGIGAAIANR	2	1	1	P80046	N-Term(iTRAQ4plex); K1(iTRAQ4plex)	0,0000	0,276	1		0	1,835E-07	53	4,50786E-06	3	1258,78489	1,16	17,21	1
	High	iTFVAGkPVVPNDPIIPYIR	2	1	1	P80046	N-Term(iTRAQ4plex); K7(iTRAQ4plex)	0,0000	0,284	2	17,2	0	5,519E-11	48	1,6367E-05	3	2497,48203	1,39	47,26	0
	High	yYPGTPSPHk	1	1	1	P80046	N-Term(iTRAQ4plex); K10(iTRAQ4plex)	0,0000	0,594	1		0	0,00005505	42	6,98184E-05	3	1434,76398	1,40	19,36	0
	High	lQPPSVGSk	2	1	1	P80046	N-Term(iTRAQ4plex); K9(iTRAQ4plex)	0,0000	0,249	2	3,7	0	0,0001867	41	8,39402E-05	2	1200,72099	1,60	23,14	0
	High	gPLTTPVGGGIR	4	1	1	P80046	N-Term(iTRAQ4plex)	0,0000	0,361	4	49,4	0	0,0003606	40	0,000111165	2	1268,74541	0,82	27,95	0
	High	eVTYDLAR	1	1	1	P80046	N-Term(iTRAQ4plex)	0,0000	0,333	1		0	0,00003268	37	0,000202754	2	1110,59233	1,03	23,61	0
	High	lDSGIGIkPISk	3	1	1	P80046	N-Term(iTRAQ4plex); K8(iTRAQ4plex); K12(iTRAQ4plex)	0,0000	0,288	2	3,0	0	3,769E-07	36	0,000255841	3	1660,03965	1,61	29,49	1
	High	qMVTLVHk	2	1	1	P80046	N-Term(iTRAQ4plex); K8(iTRAQ4plex)	0,0000	0,388	1		0	0,001846	28	0,00155228	3	1243,74634	2,27	21,61	0
	High	tPEkLDIIVYR	1	1	1	P80046	N-Term(iTRAQ4plex); K4(iTRAQ4plex)	0,0000	0,460	1		0	0,0003797	22	0,00582063	3	1634,97562	2,21	33,77	1
	High	qIFDLYTcVRPcR	2	1	1	P80046	N-Term(iTRAQ4plex); C8(Carbamidomethyl); C12(Carbamidom	0,0000	0,253	2	8,0	0	0,00001081	21	0,007924466	3	1871,93937	0,87	37,92	0
	High	sLNVALR	3	1	1	P80046	N-Term(iTRAQ4plex)	0,0000	0,286	3	16,1	0,001	0,009942	30	0,001047056	2	916,57048	0,89	24,44	0
	High	qmVTLVHk	2	1	1	P80046	N-Term(iTRAQ4plex); M2(Oxidation); K8(iTRAQ4plex)	0,0000	0,251	1		0,001	0,004471	11	0,086890041	3	1259,74134	2,31	16,87	0
	High	vDkPLk	1	1	1	P80046	N-Term(iTRAQ4plex); K3(iTRAQ4plex); K6(iTRAQ4plex)	0,0000	0,344	1		0,007	0,08726	23	0,005332981	2	1131,74248	-3,27	14,12	0
Q55499	Single-stranded DNA-binding protein 1 OS=Synechocystis sp. (strain PCC 6803 / Kazusa) GN=ssb PE	514,73	69,42	1	11	11	28	0,534	24	39,7	121	13,6	7,34							
	A3	Sequence	# PSMs	# Proteins	# Protein Groups	Protein Group Accessions	Modifications	ΔCn	116/117	116/117 Count	116/117 Variability [%]	q-Value	PEP	IonScore	Exp Value	Charge	MH+ [Da]	ΔM [ppm]	RT [min]	# Missed Cleavages
	High	sVNSIHLVGR	5	1	1	Q55499	N-Term(iTRAQ4plex)	0,0000	0,404	3	45,5	0	3,549E-07	80	1,04225E-08	2	1225,71538	1,64	22,36	0
	High	vNNLDLLGSk	3	1	1	Q55499	N-Term(iTRAQ4plex); K10(iTRAQ4plex)	0,0000	0,391	2	1,2	0	0,000001001	69	1,25139E-07	2	1360,80693	2,25	33,43	0
	High	tAEIAGNYVk	4	1	1	Q55499	N-Term(iTRAQ4plex); K10(iTRAQ4plex)	0,0000	0,935	4	110,2	0	0,000008684	54	6,19624E-06	2	1353,76457	2,15	24,15	0
	High	gSLIGIQGSLk	3	1	1	Q55499	N-Term(iTRAQ4plex); K11(iTRAQ4plex)	0,0000	0,714	3	13,0	0	0,000003116	48	1,59577E-05	2	1360,84233	1,52	33,44	0
	High	kGSLIGIQGSLk	4	1	1	Q55499	N-Term(iTRAQ4plex); K1(iTRAQ4plex); K12(iTRAQ4plex)	0,0000	0,668	3	64,2	0	4,24E-09	42	6,76036E-05	3	1633,03653	-0,47	27,72	1
	High	dNAEATmNNYPEEF	1	1	1	Q55499	N-Term(iTRAQ4plex); M7(Oxidation)	0,0000	0,571	1		0	0,000575	31	0,000853041	2	1804,74822	1,62	31,48	0
	High	vNNLDLLGSkR	1	1	1	Q55499	N-Term(iTRAQ4plex); K10(iTRAQ4plex)	0,0000	0,804	1		0	0,0008062	24	0,003638899	3	1516,91187	4,53	28,19	1
	High	fDHWEDR	3	1	1	Q55499	N-Term(iTRAQ4plex)	0,0000	0,507	3	16,0	0	0,001147	16	0,026544222	3	1148,52582	1,43	22,19	0
	High	fDHWEDRNSGTPR	1	1	1	Q55499	N-Term(iTRAQ4plex)	0,0000	0,969	1		0	0,001566	16	0,027413848	4	1760,82485	1,52	20,54	1
	High	skPVIR	1	1	1	Q55499	N-Term(iTRAQ4plex); K2(iTRAQ4plex)	0,0000	0,526	1		0,001	0,003329	18	0,01541594	3	987,65393	-1,43	10,56	0
	Medium	aGRDPEVk	2	1	1	Q55499	N-Term(iTRAQ4plex); K8(iTRAQ4plex)	0,0000	0,470	2	2,0	0,019	0,255	12	0,066281554	3	1159,66596	-1,23	10,92	1
P74384	Adenylosuccinate lyase OS=Synechocystis sp. (strain PCC 6803 / Kazusa) GN=purB PE=3 SV=1 - [PU	513,53	32,02	1	11	11	24	0,876	20	9,8	431	48,4	5,92							
	A3	Sequence	# PSMs	# Proteins	# Protein Groups	Protein Group Accessions	Modifications	ΔCn	116/117	116/117 Count	116/117 Variability [%]	q-Value	PEP	IonScore	Exp Value	Charge	MH+ [Da]	ΔM [ppm]	RT [min]	# Missed Cleavages
	High	vVQGSAHQAWNTEGGNFEELVR	1	1	1	P74384	N-Term(iTRAQ4plex)	0,0000	0,582	1		0	3,749E-11	66	2,4042E-07	3	2572,26633	0,06	37,18	0
	High	vAQSIAVGk	2	1	1	P74384	N-Term(iTRAQ4plex); K9(iTRAQ4plex)	0,0000	0,911	1		0	2,887E-07	63	5,47511E-07	2	1160,72490	0,63	20,87	0
	High	vLLALVEk	4	1	1	P74384	N-Term(iTRAQ4plex); K8(iTRAQ4plex)	0,0000	0,823	3	1,2	0	0,0003108	56	2,63009E-06	2	1172,78923	3,01	41,78	0
	High	hAEYVQQLALLAASLER	2	1	1	P74384	N-Term(iTRAQ4plex)	0,0000	0,925	1		0	1,21E-08	55	3,47512E-06	3	2056,13504	2,05	51,58	0
	High	gHAVAALENVALWHER	3	1	1	P74384	N-Term(iTRAQ4plex)	0,0000	1,009	2	30,3	0	1,077E-11	53	4,78597E-06	3	1917,02341	1,14	35,39	0
	High	vAFPDccILTHFMLk	2	1	1	P74384	N-Term(iTRAQ4plex); C6(Carbamidomethyl); C7(Carbamidome	0,0000	1,121	2	4,6	0	7,399E-07	36	0,000248869	3	2140,10355	1,82	51,01	0
	High	vAFPDccILTHFmLk	2	1	1	P74384	N-Term(iTRAQ4plex); C6(Carbamidomethyl); C7(Carbamidome	0,0000	0,929	2	10,9	0	0,00002047	32	0,000636752	3	2156,09818	1,68	47,35	0

	High	nmNVYGGVIFSQk	1	1	1	P74384	N-Term(iTRAQ4plex); M2(Oxidation); K13(iTRAQ4plex)	0,0000	0,889	1		0	0,00006295	22	0,00687021	3	1760,92691	1,42	32,30	0
	High	sHGIHAEPITFGFk	1	1	1	P74384	N-Term(iTRAQ4plex); K14(iTRAQ4plex)	0,0000	4,013	1		0	0,001127	10	0,093534107	4	1828,99599	0,61	29,86	0
	High	nLLVYPENmkR	1	1	1	P74384	N-Term(iTRAQ4plex); M9(Oxidation); K10(iTRAQ4plex)	0,0000	1,525	1		0,001	0,004057	22	0,006251295	3	1680,93821	2,16	24,76	1
	High	yTVmVGR	3	1	1	P74384	N-Term(iTRAQ4plex); M4(Oxidation)	0,0000	0,827	3	5,8	0,002	0,02064	20	0,009119479	2	985,52587	0,12	21,33	0
	Medium	fAVEIR	2	1	1	P74384	N-Term(iTRAQ4plex)	0,0000	0,899	2	10,9	0,02	0,2659	35	0,000507131	2	878,52257	1,04	27,83	0
Q59994	Triosephosphate isomerase OS=Synechocystis sp. (strain PCC 6803 / Kazusa) GN=tpiA PE=3 SV=1 -	507,38	28,10	1	9	9	29	0,515	23	25,0	242	26,1	5,16							
	A3	Sequence	# PSMs	# Proteins	# Protein Groups	Protein Group Accessions	Modifications	ΔCn	116/117	116/117 Count	116/117 Variability [%]	q-Value	PEP	IonScore	Exp Value	Charge	MH+ [Da]	ΔM [ppm]	RT [min]	# Missed Cleavages
	High	rQYFGETDETANLR	2	1	1	Q59994	N-Term(iTRAQ4plex)	0,0000	0,489	2	19,9	0	1,494E-09	62	6,2513E-07	3	1843,90702	0,79	24,54	1
	High	kIIIAGNWk	1	1	1	Q59994	N-Term(iTRAQ4plex); K1(iTRAQ4plex); K9(iTRAQ4plex)	0,0000	0,680	1		0	1,136E-07	52	6,02518E-06	3	1474,95023	2,18	29,24	1
	High	qYFGETDETANLR	2	1	1	Q59994	N-Term(iTRAQ4plex)	0,0000	0,774	2	25,4	0	1,054E-07	48	1,52395E-05	3	1687,80796	2,08	29,38	0
	High	dAGETEQVIVDQVk	1	1	1	Q59994	N-Term(iTRAQ4plex); K14(iTRAQ4plex)	0,0000	0,990	1		0	1,443E-09	45	2,86398E-05	3	1818,97007	0,70	32,65	0
	High	iIIAGNWk	7	1	1	Q59994	N-Term(iTRAQ4plex); K8(iTRAQ4plex)	0,0000	0,542	6	8,4	0	0,0002689	41	8,85054E-05	2	1202,75212	1,78	35,38	0
	High	aGLIPILcVGESk	5	1	1	Q59994	N-Term(iTRAQ4plex); C8(Carbamidomethyl); K13(iTRAQ4plex)	0,0000	0,420	4	24,1	0	7,545E-07	33	0,000447682	3	1644,96000	0,15	44,49	0
	High	vLAAQk	2	1	1	Q59994	N-Term(iTRAQ4plex); K6(iTRAQ4plex)	0,0000	1,309	2	11,2	0,001	0,01357	29	0,001250173	2	917,60192	-0,36	16,20	0
	High	vIGLIR	8	1	1	Q59994	N-Term(iTRAQ4plex)	0,0000	0,451	4	2,8	0,003	0,0365	36	0,000244889	2	814,56359	0,57	29,86	0
	Medium	iVNFQP	1	1	1	Q59994	N-Term(iTRAQ4plex)	0,0000	0,435	1		0,021	0,2816	13	0,052356427	2	861,49547	0,44	35,80	0
P73318	50S ribosomal protein L23 OS=Synechocystis sp. (strain PCC 6803 / Kazusa) GN=rplW PE=3 SV=1 -	506,10	73,27	1	9	9	36	1,723	29	21,5	101	11,5	10,05							
	A3	Sequence	# PSMs	# Proteins	# Protein Groups	Protein Group Accessions	Modifications	ΔCn	116/117	116/117 Count	116/117 Variability [%]	q-Value	PEP	IonScore	Exp Value	Charge	MH+ [Da]	ΔM [ppm]	RT [min]	# Missed Cleavages
	High	aAIELLFDVk	4	1	1	P73318	N-Term(iTRAQ4plex); K10(iTRAQ4plex)	0,0000	1,368	4	40,5	0	7,594E-08	48	1,72572E-05	2	1406,85356	2,69	49,56	0
	High	aTLQLEDNk	2	1	1	P73318	N-Term(iTRAQ4plex); K9(iTRAQ4plex)	0,0000	0,690	2	37,6	0	0,00004418	41	8,77001E-05	2	1319,74407	2,37	23,29	0
	High	rLADLIIkPIVTEk	2	1	1	P73318	N-Term(iTRAQ4plex); K8(iTRAQ4plex); K14(iTRAQ4plex)	0,0000	2,103	1		0	2,751E-07	37	0,000211821	4	2041,31765	3,27	38,92	1
	High	lADLIIkPIVTEk	14	1	1	P73318	N-Term(iTRAQ4plex); K7(iTRAQ4plex); K13(iTRAQ4plex)	0,0000	1,934	10	4,3	0	0,000004276	33	0,000480806	3	1885,21579	3,15	43,72	0
	High	eGDSIQLFPDV	1	1	1	P73318	N-Term(iTRAQ4plex)	0,0000	1,723	1		0	0,002756	31	0,000807179	2	1363,68767	1,05	47,57	0
	High	yVFDVRPEATkPEIk	3	1	1	P73318	N-Term(iTRAQ4plex); K11(iTRAQ4plex); K15(iTRAQ4plex)	0,0000	1,515	3	10,5	0	1,361E-07	21	0,008355453	3	2224,27164	0,63	32,07	0
	High	vTGVNTAR	4	1	1	P73318	N-Term(iTRAQ4plex)	0,0000	1,765	4	19,2	0,001	0,01743	40	0,000103507	2	961,55638	1,70	14,52	0
	High	rAVVTLk	1	1	1	P73318	N-Term(iTRAQ4plex); K7(iTRAQ4plex)	0,0000	1,213	1		0,001	0,005204	36	0,000235489	3	1074,72882	4,71	16,28	1
	High	aVVTLk	5	1	1	P73318	N-Term(iTRAQ4plex); K6(iTRAQ4plex)	0,0000	1,831	3	8,0	0,001	0,009584	34	0,000416092	2	918,62572	3,34	20,75	0
P73319	50S ribosomal protein L4 OS=Synechocystis sp. (strain PCC 6803 / Kazusa) GN=rplD PE=3 SV=1 - [R	504,43	33,33	1	9	9	28	1,915	24	56,0	210	23,3	10,20							
	A3	Sequence	# PSMs	# Proteins	# Protein Groups	Protein Group Accessions	Modifications	ΔCn	116/117	116/117 Count	116/117 Variability [%]	q-Value	PEP	IonScore	Exp Value	Charge	MH+ [Da]	ΔM [ppm]	RT [min]	# Missed Cleavages
	High	aDNMVVVEAFGDQFSQPk	1	1	1	P73319	N-Term(iTRAQ4plex); K18(iTRAQ4plex)	0,0000	1,108	1		0	6,805E-09	74	3,75811E-08	3	2270,14231	2,50	45,08	0
	High	vAkEENAAHIVHR	4	1	1	P73319	N-Term(iTRAQ4plex); K3(iTRAQ4plex)	0,0000	2,949	3	11,0	0	2,079E-09	55	2,97831E-06	3	1762,00248	3,52	15,62	1
	High	gGGVIFGPkPR	4	1	1	P73319	N-Term(iTRAQ4plex); K9(iTRAQ4plex)	0,0000	2,358	4	12,6	0	0,00003114	50	1,08885E-05	2	1372,83513	3,46	20,25	0
	High	aDNmVVVEAFGDQFSQPk	1	1	1	P73319	N-Term(iTRAQ4plex); M4(Oxidation); K18(iTRAQ4plex)	0,0000	1,774	1		0	8,536E-07	48	1,4288E-05	3	2286,13639	2,11	39,53	0
	High	eENAAHIVHR	7	1	1	P73319	N-Term(iTRAQ4plex)	0,0000	1,619	5	32,1	0	2,586E-07	46	2,50017E-05	3	1319,69501	0,99	11,98	0
	High	eLATALTR	3	1	1	P73319	N-Term(iTRAQ4plex)	0,0000	1,354	3	1,3	0	0,00008487	38	0,000168256	2	1018,60228	0,90	23,95	0
	High	tKELATALTR	4	1	1	P73319	N-Term(iTRAQ4plex); K2(iTRAQ4plex)	0,0000	2,927	3	13,9	0	0,00002809	37	0,00020416	2	1391,84929	2,31	22,18	1
	High	iQEVYGE	1	1	1	P73319	N-Term(iTRAQ4plex)	0,0000	0,562	1		0,002	0,02082	21	0,008034706	2	981,50334	2,42	27,24	0
	Medium	nIPYLk	2	1	1	P73319	N-Term(iTRAQ4plex); K6(iTRAQ4plex)	0,0000	2,267	2	14,5	0,018	0,2352	27	0,002331835	2	1035,64885	4,59	29,14	0
	Medium	sPLWR	1	1	1	P73319	N-Term(iTRAQ4plex)	0,0000	1,145	1		0,025	0,3389	11	0,088505448	2	802,47106	2,29	22,73	0
P73307	30S ribosomal protein S8 OS=Synechocystis sp. (strain PCC 6803 / Kazusa) GN=rpsH PE=3 SV=1 - [I	503,06	51,88	1	8	8	27	2,811	20	45,3	133	14,7	9,99							
	A3	Sequence	# PSMs	# Proteins	# Protein Groups	Protein Group Accessions	Modifications	ΔCn	116/117	116/117 Count	116/117 Variability [%]	q-Value	PEP	IonScore	Exp Value	Charge	MH+ [Da]	ΔM [ppm]	RT [min]	# Missed Cleavages
	High	vLGGIGIAIVSTSHGImTDR	7	1	1	P73307	N-Term(iTRAQ4plex); M17(Oxidation)	0,0000	3,721	5	10,5	0	1,168E-11	70	9,2683E-08	3	2157,18436	1,14	44,37	0
	High	qPLINTLQR	3	1	1	P73307	N-Term(iTRAQ4plex)	0,0000	3,366	3	18,3	0	0,001223	48	1,45201E-05	2	1226,73467	0,73	26,96	0
	High	mLVLTLk	4	1	1	P73307	N-Term(iTRAQ4plex); K7(iTRAQ4plex)	0,0000	7,955	2	55,2	0	0,001326	45	4,92415E-05	2	1105,73137	5,09	39,25	0
	High	tRQPLINTLQR	2	1	1	P73307	N-Term(iTRAQ4plex)	0,0000	2,432	2	7,1	0	0,000005982	44	4,42558E-05	3	1483,88449	1,29	22,46	1
	High	mLVLTLk	4	1	1	P73307	N-Term(iTRAQ4plex); M1(Oxidation); K7(iTRAQ4plex)	0,0000	2,828	2	17,7	0	0,0001798	41	8,64908E-05	2	1121,72356	2,58	33,12	0
	High	mTLSIAk	3	1	1	P73307	N-Term(iTRAQ4plex); M1(Oxidation); K7(iTRAQ4plex)	0,0000	1,663	2	42,8	0	0,0003626	31	0,00084522	2	1067,63835	0,97	21,25	0
	High	hSTTQVPTTk	1	1	1	P73307	N-Term(iTRAQ4plex); K10(iTRAQ4plex)	0,0000	0,785	1		0,001	0,003315	15	0,029038221	3	1387,78214	2,72	11,03	0
	High	vYSPSkk	1	1	1	P73307	N-Term(iTRAQ4plex); K6(iTRAQ4plex); K7(iTRAQ4plex)	0,0000	2,467	1		0,005	0,06771	10	0,096376246	3	1240,77463	9,74	12,75	1
	Medium	vSkPGLR	2	1	1	P73307	N-Term(iTRAQ4plex); K3(iTRAQ4plex)	0,0000	2,184	2	55,5	0,044	0,5594	10	0,096598416	2	1044,68010	3,14	15,05	0
P73264	3-amino-5-hydroxybenzoic acid synthase OS=Synechocystis sp. (strain PCC 6803 / Kazusa) GN=slr11	501,68	34,13	1	10	10	21	0,451	19	58,7	378	41,5	5,64							
	A3	Sequence	# PSMs	# Proteins	# Protein Groups	Protein Group Accessions	Modifications	ΔCn	116/117	116/117 Count	116/117 Variability [%]	q-Value	PEP	IonScore	Exp Value	Charge	MH+ [Da]	ΔM [ppm]	RT [min]	# Missed Cleavages
	High	qAENTTVLVPTNTNFATVASVLR	1	1	1	P73264	N-Term(iTRAQ4plex)	0,0000	0,693	1		0	3,496E-10	67	2,01925E-07	3	2590,39853	1,06	48,71	0
	High	sGTLILGNHTQEFER	2	1	1	P73264	N-Term(iTRAQ4plex)	0,0000	0,332	2	17,7	0	1,087E-08	62	5,67505E-07	3	1845,95908	0,80	28,59	0
	High	mTEIHALLGQIHLAk	4	1	1	P73264	N-Term(iTRAQ4plex); M1(Oxidation); K15(iTRAQ4plex)	0,0000	0,383	3	3,7	0	3,362E-12	54	3,69803E-06	3	1979,13883	1,89	39,78	0
	High	qAGNLADGASFSFFPTk	1	1	1	P73264	N-Term(iTRAQ4plex); K17(iTRAQ4plex)	0,0000	0,671	1		0	4,552E-10	51	7,72627E-06	3	2046,05619	1,29	43,51	0
	High	aYELITTPLR	3	1	1	P73264	N-Term(iTRAQ4plex)	0,0000	0,386	3	23,4	0	0,00008905	50	1,07887E-05	2	1320,76543	0,78	37,66	0
	High	hIcPPLTSGTTPEEATR	2	1	1	P73264	N-Term(iTRAQ4plex); C3(Carbamidomethyl)	0,0000	0,783	2	36,8	0	2,621E-08	43	4,81915E-05	3	2011,00559	1,00	23,70	0
	High	mTEIHALLGQIHLAk	2	1	1	P73264	N-Term(iTRAQ4plex); K15(iTRAQ4plex)	0,0000	0,589	1		0	1,326E-07	36	0,000242644	4	1963,14504	2,48	44,93	0
	High	tFAPSLAmVk	1	1	1	P73264	N-Term(iTRAQ4plex); M8(Oxidation); K10(iTRAQ4plex)	0,0000	0,723	1		0	0,00003029	33	0,000456005	3	1368,77954	-0,32	31,50	0
	High	gmNFGGLHTDFGNSTR	2	1	1	P73264	N-Term(iTRAQ4plex); M2(Oxidation)	0,0000	0,634	2	59,8	0	0,000007807	28	0,001682558	3	1870,86289	0,29	27,14	0
	High	rGmNFGGLHTDFGNSTR	1	1	1	P73264	N-Term(iTRAQ4plex); M3(Oxidation)	0,0000	0,680	1		0	0,0004225	13	0,05023079	4	2026,96291	-0,27	23,77	1
	High	gMNFGGLHTDFGNSTR	1	1	1	P73264	N-Term(iTRAQ4plex)	0,0000	1,839	1		0,002	0,01964	20	0,010863506	3	1854,86972	1,24	33,66	0
	Medium	aSQYk	1	1	1	P73264	N-Term(iTRAQ4plex); K5(iTRAQ4plex)	0,0000	0,177	1		0,01	0,1327	16	0,024601977	2	884,50755	-0,49	13,21	0
P72854	Sulfite reductase [ferredoxin] OS=Synechocystis sp. (strain PCC 6803 / Kazusa) GN=sir PE=1 SV=1 -	501,49	21,89	1	14	14	25	0,815	24	16,6	635	71,4	8,47							
	A3	Sequence	# PSMs	# Proteins	# Protein Groups	Protein Group Accessions	Modifications	ΔCn	116/117	116/117 Count	116/117 Variability [%]	q-Value	PEP	IonScore	Exp Value	Charge	MH+ [Da]	ΔM [ppm]	RT [min]	# Missed Cleavages
	High	gFNVLAGGGLGR	3	1	1	P72854	N-Term(iTRAQ4plex)	0,0000	0,880	3	17,4	0	2,357E-07	70	1,03507E-07	2	1261,71416	0,60	35,83	0
	High	lTANHNILLYDINAQDk	2	1	1	P72854	N-Term(iTRAQ4plex); K17(iTRAQ4plex)	0,0000	0,544	2	54,5	0	1,538E-11	68	1,73368E-07	3	2244,22684	1,84	37,14	0
	High	kVVDQFQLPLR	2	1	1	P72854	N-Term(iTRAQ4plex); K1(iTRAQ4plex)	0,0000	0,759	2	12,0	0	7,058E-10	52	6,12308E-06	3	1630,98960	0,77	35,02	1
	High	qGLQIHGIVk	3	1	1	P72854	N-Term(iTRAQ4plex); K10(iTRAQ4plex)	0,0000	0,808	2	2,4	0	0,00005322	41	7,41259E-05	3	1380,85755	0,70	24,57	0
	High	lAAAYTEk	1	1	1	P72854	N-Term(iTRAQ4plex); K8(iTRAQ4plex)	0,0000	1,102	1		0	0,002041	37	0,000191854	2	1154,66289	-2,67	21,03	0
	High	aIVATQR	2	1	1	P72854	N-Term(iTRAQ4plex)	0,0000	0,849	2	7,2	0	0,002788	35	0,000311867	2	902,55449	0,53	14,10	0
	High	vTTPTAAPR	1	1	1	P72854	N-Term(iTRAQ4plex)	0,0000	1,076	1		0	0,001244	34	0,000395339	2	1057,61308	0,78	16,32	0
	High	eFSHGYTAPAk	2	1	1	P72854	N-Term(iTRAQ4plex); K11(iTRAQ4plex)	0,0000	0,759	2	4,0	0	0,00002238	33	0,000524771	3	1495,77875	0,25	18,73	0
	High	nVmAPAAPFR	2	1	1	P72854	N-Term(iTRAQ4plex); M3(Oxidation)	0,0000	0,605	2	14,5	0	0,003058	29	0,001221715	2	1233,65434	1,00	26,26	0
	High	ikDEGDFQLk	1	1	1	P72854	N-Term(iTRAQ4plex); K2(iTRAQ4plex); K10(iTRAQ4plex)	0,0000	1,243	1		0	0,0008386	17	0,020463033	3	1624,92789	0,50	26,81	1
	High	eSFGDFcHR	1	1	1	P72854	N-Term(iTRAQ4plex); C7(Carbamidomethyl)	0,0000	0,764	1		0,001	0,005004	15	0,031839776	3	1298,57251	1,57	21,48	0
	High	vGFTALR	2	1	1	P72854	N-Term(iTRAQ4plex)	0,0000	1,945	2	124,4	0,004	0,04983	22	0,008776751	2	907,54930	1,21	27,50	0
	High	imPSVNAR	2	1	1	P72854	N-Term(iTRAQ4plex); M2(Oxidation)	0,0000	0,791	2	14,0	0,004	0,05443	18	0,017578022	2	1047,57256	-1,17	18,61	0
	High	tHNkEETFAR	1	1	1	P72854	N-Term(iTRAQ4plex); K4(iTRAQ4plex)	0,0000	1,091	1		0,006	0,0789	25	0,003273181	3	1520,80808	1,37	9,56	1
P54123	Ribonuclease J OS=Synechocystis sp. (strain PCC 6803 / Kazusa) GN=slr0551 PE=3 SV=1 - [RNJ_SY	500,86	13,91	1	10	10	18	4,516	17	31,8	640	70,4	7,74							
	A3	Sequence	# PSMs	# Proteins	# Protein Groups	Protein Group Accessions	Modifications	ΔCn	116/117	116/117 Count	116/117 Variability [%]	q-Value	PEP	IonScore	Exp Value	Charge	MH+ [Da]	ΔM [ppm]	RT [min]	# Missed Cleavages
	High	iLPLGGLHEIGk	3	1	1	P54123	N-Term(iTRAQ4plex); K12(iTRAQ4plex)	0,0000	5,055	3	34,0	0	2,948E-09	56	2,37668E-06	3	1534,95572	-0,17	37,71	0
	High	lLEELIVR	2	1	1	P54123	N-Term(iTRAQ4plex)	0,0000	4,579	2	10,1	0	0,00002895	54	3,64729E-06	2	1128,71074	-0,16	45,41	0
	High	vNIILSLAQk	2	1	1	P54123	N-Term(iTRAQ4plex); K10(iTRAQ4plex)	0,0000	4,029	2	14,2	0	0,00001284	47	1,90095E-05	3	1386,89261	0,23	44,03	0
	High	mLLALTRPk	1	1	1	P54123	N-Term(iTRAQ4plex); M1(Oxidation); K9(iTRAQ4plex)	0,0000	1,476	1		0	0,00002504	47	2,14274E-05	3	1346,84131	-1,44	25,03	0
	High	tNLQTVSPR	2	1	1	P54123	N-Term(iTRAQ4plex)	0,0000	3,045	2	8,8	0	0,00003673	47	2,1677E-05	2	1159,65618	0,85	17,29	0
	High	sFVVEFIR	3	1	1	P54123	N-Term(iTRAQ4plex)	0,0000	4,816	3	22,5	0	0,0007182	43	4,94277E-05	2	1140,65373	0,29	42,15	0
	High	sMLNVIAHAR	2	1	1	P54123	N-Term(iTRAQ4plex)	0,0000	4,588	1		0	0,000007604	33	0,000538233	3	1255,70606	-0,11	29,26	0
	High	fFVPVHGEHR	1	1	1	P54123	N-Term(iTRAQ4plex)	0,0000	4,516	1		0	0,00007312	31	0,000878962	4	1368,72915	-0,16	22,93	0
	High	kLGYIk	1	1	1	P54123	N-Term(iTRAQ4plex); K1(iTRAQ4plex); K6(iTRAQ4plex)	0,0000	7,103	1		0	0,0023	21	0,007310886	3	1153,76364	-2,82	18,54	1
	High	lAMALLR	1	1	1	P54123	N-Term(iTRAQ4plex)	0,0000	10,324	1		0,004	0,05096	31	0,000807179	2	931,58806	0,09	37,32	0
P27724	NAD(P)H-quinone oxidoreductase subunit H OS=Synechocystis sp. (strain PCC 6803 / Kazusa) GN=n	495,11	20,56	1	9	9	20	1,468	18	16,3	394	45,5	6,06							
	A3	Sequence	# PSMs	# Proteins	# Protein Groups	Protein Group Accessions	Modifications	ΔCn	116/117	116/117 Count	116/117 Variability [%]	q-Value	PEP	IonScore	Exp Value	Charge	MH+ [Da]	ΔM [ppm]	RT [min]	# Missed Cleavages
	High	iPAGEHYVR	2	1	1	P27724	N-Term(iTRAQ4plex)	0,0000	1,308	2	13,9	0	0,000001303	73	5,08124E-08	3	1185,65009	0,32	18,35	0
	High	tNImYVPYVSR	3	1	1	P27724	N-Term(iTRAQ4plex); M4(Oxidation)	0,0000	1,391	2	9,3	0	0,00002477	60	9,03587E-07	2	1502,77959	0,13	32,38	0
	High	sADFNNLQILPHILk	5	1	1	P27724	N-Term(iTRAQ4plex); K15(iTRAQ4plex)	0,0000	1,510	4	11,0	0	7,959E-10	53	4,89745E-06	3	2011,15958	0,82	45,27	0
	High	lITNNPIFR	3	1	1	P27724	N-Term(iTRAQ4plex)	0,0000	1,623	3	5,0	0	0,000001235	50	1,03985E-05	2	1231,72820	0,19	37,60	0
	High	kSEWNDFQYQYIAk	1	1	1	P27724	N-Term(iTRAQ4plex); K1(iTRAQ4plex); K14(iTRAQ4plex)	0,0000	1,815	1		0	7,439E-10	46	2,594E-05	3	2252,17142	0,09	35,32	1
	High	tNIMYVPYVSR	1	1	1	P27724	N-Term(iTRAQ4plex)	0,0000	1,528	1		0	0,000004135	43	4,7203E-05	2	1486,78508	0,40	37,05	0
	High	vImLELNR	1	1	1	P27724	N-Term(iTRAQ4plex); M3(Oxidation)	0,0000	1,324	1		0	0,001473	26	0,004699353	2	1147,66240	-0,17	32,52	0
	High	lINNNYFR	2	1	1	P27724	N-Term(iTRAQ4plex)	0,0000	1,474	2	10,2	0	0,001875	21	0,007128038	2	1197,64910	-0,50	32,71	0
	High	kVAPTFk	1	1	1	P27724	N-Term(iTRAQ4plex); K1(iTRAQ4plex); K7(iTRAQ4plex)	0,0000	3,125	1		0,001	0,01609	19	0,013365032	3	1222,78379	-3,76	17,41	1
	High	vAPTFk	1	1	1	P27724	N-Term(iTRAQ4plex); K6(iTRAQ4plex)	0,0000	0,763	1		0,006	0,07682	27	0,001827974	2	950,58916	-2,30	21,07	0
P73304	30S ribosomal protein S5 OS=Synechocystis sp. (strain PCC 6803 / Kazusa) GN=rpsE PE=3 SV=1 - [I	494,02	49,13	1	8	8	18	2,317	16	170,3	173	18,2	10,59							
	A3	Sequence	# PSMs	# Proteins	# Protein Groups	Protein Group Accessions	Modifications	ΔCn	116/117	116/117 Count	116/117 Variability [%]	q-Value	PEP	IonScore	Exp Value	Charge	MH+ [Da]	ΔM [ppm]	RT [min]	# Missed Cleavages
	High	qLGSNNPLNNAR	4	1	1	P73304	N-Term(iTRAQ4plex)	0,0000	2,174	4	357,5	0	3,416E-09	92	5,662E-10	2	1441,76360	0,52	22,48	0
	High	aAINALETLR	4	1	1	P73304	N-Term(iTRAQ4plex)	0,0000	4,927	3	2,7	0	0,00000234	63	5,98661E-07	2	1215,71929	1,22	35,32	0
	High	vVVRPAAPGTGVIAGGAVR	4	1	1	P73304	N-Term(iTRAQ4plex)	0,0000	1,040	3	83,4	0	9,243E-09	57	1,78226E-06	3	1891,13797	1,10	29,11	0
	High	tVLELAGVk	1	1	1	P73304	N-Term(iTRAQ4plex); K9(iTRAQ4plex)	0,0000	3,263	1		0	0,00001554	52	5,94251E-06	2	1217,77239	1,31	35,25	0
	High	kQLIEVPLTk	2	1	1	P73304	N-Term(iTRAQ4plex); K1(iTRAQ4plex); K10(iTRAQ4plex)	0,0000	1,460	2	63,8	0	0,000001408	23	0,004655539	3	1601,03867	1,52	29,94	1
	High	tFSEVAEER	1	1	1	P73304	N-Term(iTRAQ4plex)	0,0000	0,395	1		0	0,0001451	20	0,009077578	2	1211,60259	0,08	22,32	0
	High	ekkEDTNWQER	1	1	1	P73304	N-Term(iTRAQ4plex); K2(iTRAQ4plex); K3(iTRAQ4plex)	0,0000	2,217	1		0	0,0001698	11	0,076554373	4	1895,00124	1,53	12,27	2
	Medium	vIQIR	1	1	1	P73304	N-Term(iTRAQ4plex)	0,0000	2,422	1		0,041	0,5306	18	0,014255091	2	772,51726	1,42	22,29	0
Q55141	Acetolactate synthase small subunit OS=Synechocystis sp. (strain PCC 6803 / Kazusa) GN=ilvH PE=3	490,68	48,26	1	9	9	23	1,003	21	19,9	172	18,9	5,43							
	A3	Sequence	# PSMs	# Proteins	# Protein Groups	Protein Group Accessions	Modifications	ΔCn	116/117	116/117 Count	116/117 Variability [%]	q-Value	PEP	IonScore	Exp Value	Charge	MH+ [Da]	ΔM [ppm]	RT [min]	# Missed Cleavages
	High	rGFNIESLAVGSAEQGDVSR	2	1	1	Q55141	N-Term(iTRAQ4plex)	0,0000	0,883	1		0	1,753E-11	82	6,83864E-09	3	2236,14438	0,20	36,70	1
	High	aEVIELAQVFR	3	1	1	Q55141	N-Term(iTRAQ4plex)	0,0000	1,022	3	22,8	0	5,927E-07	64	4,23614E-07	2	1418,81328	0,59	47,01	0
	High	vSANAPNRAEVIELAQVFR	1	1	1	Q55141	N-Term(iTRAQ4plex)	0,0000	1,051	1		0	4,768E-08	56	2,40974E-06	3	2228,22892	0,91	43,94	1
	High	hTLSVLVEDEAGVLTR	1	1	1	Q55141	N-Term(iTRAQ4plex)	0,0000	0,657	1		0	0,000001014	51	7,58525E-06	3	1883,03482	-0,38	42,38	0
	High	mVAILQmLAk	4	1	1	Q55141	N-Term(iTRAQ4plex); M7(Oxidation); K10(iTRAQ4plex)	0,0000	1,169	3	9,5	0	0,00000215	34	0,000382798	3	1421,84635	0,03	46,60	0
	High	mVAILQmLAk	1	1	1	Q55141	N-Term(iTRAQ4plex); M1(Oxidation); M7(Oxidation); K10(iTRA	0,0000	0,879	1		0	0,000425	33	0,00051996	3	1437,84043	-0,55	36,99	0
	High	iAGLFAR	2	1	1	Q55141	N-Term(iTRAQ4plex)	0,0000	0,974	2	4,4	0,001	0,005456	41	8,18408E-05	2	891,55345	0,18	33,69	0
	High	mVAILQMLAk	1	1	1	Q55141	N-Term(iTRAQ4plex); K10(iTRAQ4plex)	0,0000	8,212	1		0,001	0,008864	31	0,000824081	3	1405,85254	0,82	52,90	0
	High	vSANAPNR	5	1	1	Q55141	N-Term(iTRAQ4plex)	0,0000	1,068	5	26,0	0,001	0,01342	31	0,000837471	2	972,53392	-0,43	13,02	0
	High	lVNVIk	1	1	1	Q55141	N-Term(iTRAQ4plex); K6(iTRAQ4plex)	0,0000	0,647	1		0,002	0,02798	22	0,006208262	2	973,66417	-0,69	27,27	0
	Medium	iALVR	2	1	1	Q55141	N-Term(iTRAQ4plex)	0,0000	0,846	2	3,7	0,034	0,4398	15	0,031547867	2	715,49407	-0,89	22,51	0
P73597	Sll1306 protein OS=Synechocystis sp. (strain PCC 6803 / Kazusa) GN=sll1306 PE=4 SV=1 - [P73597	483,56	37,31	1	11	11	23	0,547	20	24,5	335	38,2	8,18							
	A3	Sequence	# PSMs	# Proteins	# Protein Groups	Protein Group Accessions	Modifications	ΔCn	116/117	116/117 Count	116/117 Variability [%]	q-Value	PEP	IonScore	Exp Value	Charge	MH+ [Da]	ΔM [ppm]	RT [min]	# Missed Cleavages
	High	fFSSGQYLTELk	4	1	1	P73597	N-Term(iTRAQ4plex); K12(iTRAQ4plex)	0,0000	0,572	3	3,2	0	7,193E-11	75	3,22085E-08	2	1707,92192	1,34	43,10	0
	High	aVGYNAPGLR	4	1	1	P73597	N-Term(iTRAQ4plex)	0,0000	0,463	3	6,6	0	0,000002899	58	1,73768E-06	2	1161,65032	0,52	23,00	0
	High	kIDIANFALNDPNTIR	1	1	1	P73597	N-Term(iTRAQ4plex); K1(iTRAQ4plex)	0,0000	0,560	1		0	4,326E-12	53	5,58432E-06	3	2103,18180	0,79	39,52	1
	High	yGYTEGVPR	2	1	1	P73597	N-Term(iTRAQ4plex)	0,0000	0,442	2	6,3	0	0,0001343	43	5,03466E-05	2	1185,60307	0,84	22,82	0
	High	lQRPEHVYVFEDFLk	2	1	1	P73597	N-Term(iTRAQ4plex); K15(iTRAQ4plex)	0,0000	0,478	2	0,6	0	5,962E-11	42	6,30914E-05	3	2208,20670	0,49	40,59	0
	High	iLDLLDkYk	3	1	1	P73597	N-Term(iTRAQ4plex); K7(iTRAQ4plex); K9(iTRAQ4plex)	0,0000	0,537	2	0,1	0	0,000001164	35	0,00028772	3	1552,96799	0,34	44,23	1
	High	gHEAAAHGWDWDNEFNmTAPQ	2	1	1	P73597	N-Term(iTRAQ4plex); M17(Oxidation)	0,0000	1,038	2	71,0	0	1,211E-08	30	0,00097717	4	2829,22182	0,88	31,83	0
	High	mmAVTmHDR	1	1	1	P73597	N-Term(iTRAQ4plex); M1(Oxidation); M2(Oxidation); M6(Oxida	0,0000	0,317	1		0	0,000008259	15	0,034116935	3	1283,56702	0,52	8,57	0
	High	mmAVTMHDR	1	1	1	P73597	N-Term(iTRAQ4plex); M1(Oxidation); M2(Oxidation)	0,0000	0,557	1		0	0,0000589	12	0,070302376	3	1267,57148	0,03	11,29	0
	High	sIMVVPYAVYLNDIR	1	1	1	P73597	N-Term(iTRAQ4plex)	0,0000	0,726	1		0,001	0,003347	32	0,000656431	3	1897,03818	0,39	52,71	0
	High	fWPDNIR	1	1	1	P73597	N-Term(iTRAQ4plex)	0,0000	0,642	1		0,001	0,01023	30	0,001088855	2	1091,57549	0,00	34,42	0
	High	iLDLLDk	1	1	1	P73597	N-Term(iTRAQ4plex); K7(iTRAQ4plex)	0,0000	1,262	1		0,001	0,01003	23	0,005248075	2	1117,70745	0,29	42,84	0
P29255	Photosystem I P700 chlorophyll a apoprotein A2 OS=Synechocystis sp. (strain PCC 6803 / Kazusa) Gl	479,78	14,36	1	10	10	25	2,373	20	18,2	731	81,2	6,95							
	A3	Sequence	# PSMs	# Proteins	# Protein Groups	Protein Group Accessions	Modifications	ΔCn	116/117	116/117 Count	116/117 Variability [%]	q-Value	PEP	IonScore	Exp Value	Charge	MH+ [Da]	ΔM [ppm]	RT [min]	# Missed Cleavages
	High	rIWYGIATAHDFETHDGmTEENL'	1	1	1	P29255	N-Term(iTRAQ4plex); M18(Oxidation); K26(iTRAQ4plex)	0,0000	2,761	1		0	1,261E-12	63	4,95416E-07	4	3429,64419	0,84	36,01	1
	High	fSQDLAQDPTTR	2	1	1	P29255	N-Term(iTRAQ4plex)	0,0000	2,165	2	27,0	0	6,169E-08	61	8,14648E-07	2	1522,76299	0,74	25,35	0
	High	dFGYSFPcDGPGR	1	1	1	P29255	N-Term(iTRAQ4plex); C8(Carbamidomethyl)	0,0000	2,375	1		0	1,502E-09	58	1,52746E-06	2	1618,70769	-0,02	33,77	0
	High	wkDkPVALSIVQAR	3	1	1	P29255	N-Term(iTRAQ4plex); K2(iTRAQ4plex); K4(iTRAQ4plex)	0,0000	3,006	2	11,8	0	1,843E-09	52	6,01132E-06	4	2043,24441	0,22	31,97	1
	High	dkPVALSIVQAR	5	1	1	P29255	N-Term(iTRAQ4plex); K2(iTRAQ4plex)	0,0000	2,522	3	4,6	0	8,823E-08	51	8,7694E-06	3	1584,96787	0,16	28,60	0
	High	eILNAHk	4	1	1	P29255	N-Term(iTRAQ4plex); K7(iTRAQ4plex)	0,0000	2,219	3	9,9	0	0,0001092	41	8,41337E-05	2	1112,66582	-0,74	14,87	0
	High	tNWGIGHSIk	3	1	1	P29255	N-Term(iTRAQ4plex); K10(iTRAQ4plex)	0,0000	2,189	2	24,7	0	0,00001268	32	0,000688605	3	1400,78839	-0,35	24,17	0
	High	fRPSLAWFk	1	1	1	P29255	N-Term(iTRAQ4plex); K9(iTRAQ4plex)	0,0000	1,536	1		0	0,0001004	15	0,033187154	3	1439,83829	-1,33	40,02	0
	High	tPLANLVR	3	1	1	P29255	N-Term(iTRAQ4plex)	0,0000	2,224	3	39,1	0,001	0,01038	30	0,001035071	2	1027,63823	0,14	31,75	0
	Medium	lmPDkk	1	1	1	P29255	N-Term(iTRAQ4plex); M2(Oxidation); K5(iTRAQ4plex); K6(iTRA	0,0000	1,509	1		0,014	0,1818	22	0,006713825	2	1179,71062	-2,17	11,48	1
	Medium	lMPDkk	1	1	1	P29255	N-Term(iTRAQ4plex); K5(iTRAQ4plex); K6(iTRAQ4plex)	0,0000	2,407	1		0,038	0,4888	12	0,062230029	3	1163,71158	-5,74	16,06	1
P74720	Sll1106 protein OS=Synechocystis sp. (strain PCC 6803 / Kazusa) GN=sll1106 PE=4 SV=1 - [P74720	477,87	46,20	1	7	7	20	0,717	16	12,0	171	18,0	4,82							
	A3	Sequence	# PSMs	# Proteins	# Protein Groups	Protein Group Accessions	Modifications	ΔCn	116/117	116/117 Count	116/117 Variability [%]	q-Value	PEP	IonScore	Exp Value	Charge	MH+ [Da]	ΔM [ppm]	RT [min]	# Missed Cleavages
	High	tSLSQDEESQLQAVLDR	3	1	1	P74720	N-Term(iTRAQ4plex)	0,0000	0,614	2	27,5	0	9,04E-12	80	9,79422E-09	2	2063,03887	0,72	41,99	0
	High	lQVEHLIEmEDAAVVVkDEk	5	1	1	P74720	N-Term(iTRAQ4plex); M9(Oxidation); K17(iTRAQ4plex); K20(iT	0,0000	0,612	3	2,8	0	4,18E-13	62	5,70125E-07	3	2743,49723	2,16	39,52	1
	High	vLADVSk	2	1	1	P74720	N-Term(iTRAQ4plex); K7(iTRAQ4plex)	0,0000	0,672	2	1,8	0	0,0006666	58	2,59019E-06	2	1019,63365	-0,29	22,59	0
	High	eLGETmcPGSSALFVLVk	1	1	1	P74720	N-Term(iTRAQ4plex); M6(Oxidation); C7(Carbamidomethyl); K	0,0000	0,551	1		0	1,433E-07	49	1,36678E-05	3	2242,17167	0,63	44,97	0
	High	lQVEHLIEMEDAAVVVkDEk	1	1	1	P74720	N-Term(iTRAQ4plex); K17(iTRAQ4plex); K20(iTRAQ4plex)	0,0000	0,929	1		0	6,318E-09	48	2,30645E-05	3	2727,50187	2,01	43,81	1
	High	hGLLDGVPAAE	2	1	1	P74720	N-Term(iTRAQ4plex)	0,0000	0,748	2	6,3	0	0,002477	32	0,000650085	2	1222,66130	5,26	30,92	0
	Medium	kVTPDk	5	1	1	P74720	N-Term(iTRAQ4plex); K1(iTRAQ4plex); K6(iTRAQ4plex)	0,0000	0,756	5	7,5	0,011	0,1392	22	0,005874488	3	1119,70539	-3,93	9,13	1
	Medium	vTPDk	1	1	1	P74720	N-Term(iTRAQ4plex); K5(iTRAQ4plex)	0,0000				0,039	0,5012	10	0,097991702	2	847,51085	-2,25	13,87	0
Q55585	Probable succinate-semialdehyde dehydrogenase [NADP(+)] OS=Synechocystis sp. (strain PCC 6803	472,05	15,42	1	7	7	18	0,494	16	44,4	454	48,7	5,14							
	A3	Sequence	# PSMs	# Proteins	# Protein Groups	Protein Group Accessions	Modifications	ΔCn	116/117	116/117 Count	116/117 Variability [%]	q-Value	PEP	IonScore	Exp Value	Charge	MH+ [Da]	ΔM [ppm]	RT [min]	# Missed Cleavages
	High	fAAPALmAGNVGLLk	4	1	1	Q55585	N-Term(iTRAQ4plex); M7(Oxidation); K15(iTRAQ4plex)	0,0000	0,355	3	4,7	0	3,75E-13	80	1,05674E-08	2	1777,03044	1,08	41,65	0
	High	fAAPALMAGNVGLLk	3	1	1	Q55585	N-Term(iTRAQ4plex); K15(iTRAQ4plex)	0,0000	0,602	2	13,6	0	2,529E-07	66	2,75404E-07	2	1761,03238	-0,70	46,79	0
	High	kAADILEQR	4	1	1	Q55585	N-Term(iTRAQ4plex); K1(iTRAQ4plex)	0,0000	0,596	4	20,2	0	0,00002712	49	1,29113E-05	3	1331,78775	-0,61	20,01	1
	High	hLPFAQR	2	1	1	Q55585	N-Term(iTRAQ4plex)	0,0000	0,491	2	28,5	0	0,0004859	24	0,003605537	3	1012,58036	-0,53	19,55	0
	High	rDEWAALMTLEMGk	1	1	1	Q55585	N-Term(iTRAQ4plex); K14(iTRAQ4plex)	0,0000	0,952	1		0	0,00006476	15	0,033648833	3	1939,00064	-0,73	44,83	1
	High	lDLAQETFQSFR	1	1	1	Q55585	N-Term(iTRAQ4plex)	0,0000	1,283	1		0,001	0,009601	13	0,04709448	2	1598,83025	0,44	44,26	0
	High	aFQALk	1	1	1	Q55585	N-Term(iTRAQ4plex); K6(iTRAQ4plex)	0,0000	0,432	1		0,006	0,06928	34	0,00042459	2	965,60106	-1,23	22,91	0
	High	iPFGGIk	2	1	1	Q55585	N-Term(iTRAQ4plex); K7(iTRAQ4plex)	0,0000	0,368	2	1,8	0,006	0,0831	22	0,005767266	2	1019,64812	-1,07	32,50	0
P74510	Dihydrolipoamide acetyltransferase component (E2) of pyruvate dehydrogenase complex OS=Synech	470,07	33,26	1	15	15	23	1,382	21	29,0	433	44,9	6,19							
	A3	Sequence	# PSMs	# Proteins	# Protein Groups	Protein Group Accessions	Modifications	ΔCn	116/117	116/117 Count	116/117 Variability [%]	q-Value	PEP	IonScore	Exp Value	Charge	MH+ [Da]	ΔM [ppm]	RT [min]	# Missed Cleavages
	High	vDLATIAGTGPHGR	1	1	1	P74510	N-Term(iTRAQ4plex)	0,0000	0,598	1		0	2,26E-10	65	2,91052E-07	3	1508,82959	-0,42	29,32	0
	High	vTSTPSVPVGQTVPLTTFQk	3	1	1	P74510	N-Term(iTRAQ4plex); K20(iTRAQ4plex)	0,0000	0,873	3	2,1	0	1,195E-13	62	6,91783E-07	3	2375,34519	1,13	39,29	0
	High	kHPIVNASYTDQGIIYHk	1	1	1	P74510	N-Term(iTRAQ4plex); K1(iTRAQ4plex); K18(iTRAQ4plex)	0,0000	1,283	1		0	7,822E-11	58	1,81844E-06	4	2516,39809	-0,20	24,56	1
	High	qmAVNVTcDHR	1	1	1	P74510	N-Term(iTRAQ4plex); M2(Oxidation); C8(Carbamidomethyl)	0,0000	1,425	1		0	5,91E-09	49	1,25594E-05	3	1490,69638	0,19	13,01	0
	High	aLVQNmVAAmAAPTFR	1	1	1	P74510	N-Term(iTRAQ4plex); M6(Oxidation); M10(Oxidation)	0,0000	0,828	1		0	2,655E-09	48	1,7297E-05	3	1866,96952	0,41	32,45	0
	High	gVTmTALLAk	2	1	1	P74510	N-Term(iTRAQ4plex); M4(Oxidation); K10(iTRAQ4plex)	0,0000	1,070	1		0	0,0000375	43	4,62349E-05	2	1308,78118	0,91	26,55	0
	High	iVSWTk	1	1	1	P74510	N-Term(iTRAQ4plex); K6(iTRAQ4plex)	0,0000	1,408	1		0	0,002212	38	0,000169032	2	1021,62749	-0,95	27,33	0
	High	vIYGAHAAAFLk	1	1	1	P74510	N-Term(iTRAQ4plex); K12(iTRAQ4plex)	0,0000	2,006	1		0	0,00000265	34	0,00044157	3	1548,91532	0,79	32,29	0
	High	aLVQNMVAAMAAPTFR	2	1	1	P74510	N-Term(iTRAQ4plex)	0,0000	1,239	2	30,3	0	0,00004646	31	0,000774408	2	1834,98296	2,20	50,19	0
	High	aVALALkk	1	1	1	P74510	N-Term(iTRAQ4plex); K7(iTRAQ4plex); K8(iTRAQ4plex)	0,0000	2,095	1		0	0,000007055	26	0,002349471	3	1245,86094	-0,77	23,27	1
	High	aVALALk	2	1	1	P74510	N-Term(iTRAQ4plex); K7(iTRAQ4plex)	0,0000	1,382	1		0,001	0,007028	25	0,003213439	2	973,66460	-0,27	27,16	0
	High	rQmAVNVTcDHR	1	1	1	P74510	N-Term(iTRAQ4plex); M3(Oxidation); C9(Carbamidomethyl)	0,0000	0,899	1		0,002	0,01923	24	0,004374919	4	1646,79714	-0,05	9,62	1
	High	iIASPR	2	1	1	P74510	N-Term(iTRAQ4plex)	0,0000	1,531	2	8,4	0,002	0,02001	18	0,015274606	2	800,51122	0,17	19,01	0
	High	wkELVER	1	1	1	P74510	N-Term(iTRAQ4plex); K2(iTRAQ4plex)	0,0000	2,125	1		0,002	0,02042	17	0,020652375	3	1247,73648	1,14	27,26	1
	Medium	tSPAPTPR	1	1	1	P74510	N-Term(iTRAQ4plex)	0,0000	1,419	1		0,011	0,1479	22	0,006854409	2	970,54393	0,09	11,99	0
	Medium	sPGDkVEk	2	1	1	P74510	N-Term(iTRAQ4plex); K5(iTRAQ4plex); K8(iTRAQ4plex)	0,0000	1,219	2	9,9	0,016	0,2173	17	0,018406449	3	1291,75687	-1,04	11,40	1
P73608	Glycogen operon protein GlgX OS=Synechocystis sp. (strain PCC 6803 / Kazusa) GN=glgX PE=4 SV=	468,00	12,87	1	9	10	24	0,562	19	20,1	707	79,8	5,86							
	A3	Sequence	# PSMs	# Proteins	# Protein Groups	Protein Group Accessions	Modifications	ΔCn	116/117	116/117 Count	116/117 Variability [%]	q-Value	PEP	IonScore	Exp Value	Charge	MH+ [Da]	ΔM [ppm]	RT [min]	# Missed Cleavages
	High	sVVILLAk	4	1	1	P73608	N-Term(iTRAQ4plex); K8(iTRAQ4plex)	0,0000	0,654	2	2,6	0	0,00006284	61	8,62919E-07	2	1130,77739	1,99	36,15	0
	High	fDLASILGR	3	1	1	P73608	N-Term(iTRAQ4plex)	0,0000	0,554	3	2,1	0	0,000003136	51	8,18408E-06	2	1135,65972	0,43	48,41	0
	High	gTFAGILSk	3	1	1	P73608	N-Term(iTRAQ4plex); K9(iTRAQ4plex)	0,0000	0,556	2	5,4	0	0,00005291	50	9,95337E-06	2	1181,71367	0,34	33,90	0
	High	vLAFMLcGR	2	1	1	P73608	N-Term(iTRAQ4plex); C7(Carbamidomethyl)	0,0000	0,846	2	3,9	0	0,00001982	45	3,31872E-05	2	1210,65727	1,26	45,14	0
	High	gPTISFR	4	1	1	P73608	N-Term(iTRAQ4plex)	0,0000	0,496	4	37,2	0	0,001777	42	7,37751E-05	2	921,52837	0,98	25,54	0
	High	rAPQPFVEIPFPESFR	2	1	1	P73608	N-Term(iTRAQ4plex)	0,0000	0,571	2	11,1	0	0,000000178	42	7,06269E-05	3	2061,10544	0,75	45,94	1
	High	yWTAEFHIDGFR	1	1	1	P73608	N-Term(iTRAQ4plex)	0,0000	0,457	1		0	5,418E-07	38	0,000173368	3	1685,82145	1,27	43,99	0
	High	vLLDPYAk	1	2	2	P73608;P72691	N-Term(iTRAQ4plex); K8(iTRAQ4plex)	0,0000				0,001	0,008689	20	0,013675504	2	1206,73870	4,17	31,38	0
	High	lAHPVLR	3	1	1	P73608	N-Term(iTRAQ4plex)	0,0000	0,539	2	5,1	0,001	0,01152	15	0,03451199	3	949,60718	0,84	16,68	0
	High	hcIAFR	1	1	1	P73608	N-Term(iTRAQ4plex); C2(Carbamidomethyl)	0,0000	0,944	1		0,004	0,04556	11	0,074296782	3	947,50193	1,82	15,13	0
P37101	Phosphoribulokinase OS=Synechocystis sp. (strain PCC 6803 / Kazusa) GN=prk PE=2 SV=1 - [KPPR_	467,10	39,16	1	11	11	26	1,632	22	17,4	332	37,9	5,43							
	A3	Sequence	# PSMs	# Proteins	# Protein Groups	Protein Group Accessions	Modifications	ΔCn	116/117	116/117 Count	116/117 Variability [%]	q-Value	PEP	IonScore	Exp Value	Charge	MH+ [Da]	ΔM [ppm]	RT [min]	# Missed Cleavages
	High	gHTYEDILASINAR	5	1	1	P37101	N-Term(iTRAQ4plex)	0,0000	1,733	3	2,9	0	7,184E-09	63	6,07203E-07	3	1703,88462	0,74	38,30	0
	High	kAAGVTALDPR	2	1	1	P37101	N-Term(iTRAQ4plex); K1(iTRAQ4plex)	0,0000	1,094	2	44,4	0	0,0002357	54	3,79289E-06	2	1386,83293	1,55	18,51	1
	High	vEPNkVVVIEGLHPLYDER	2	1	1	P37101	N-Term(iTRAQ4plex); K5(iTRAQ4plex)	0,0000	1,617	2	16,3	0	2,321E-09	53	5,03466E-06	4	2494,39248	0,65	38,68	1
	High	lTcTYPGIk	1	1	1	P37101	N-Term(iTRAQ4plex); C3(Carbamidomethyl); K9(iTRAQ4plex)	0,0000	1,384	1		0	5,365E-07	45	2,98518E-05	2	1340,74797	-0,51	26,89	0
	High	kPDFTAYIEPQk	3	1	1	P37101	N-Term(iTRAQ4plex); K1(iTRAQ4plex); K12(iTRAQ4plex)	0,0000	1,137	3	2,2	0	1,402E-10	44	4,25004E-05	3	1869,04978	0,82	32,49	1
	High	vVVIEGLHPLYDER	4	1	1	P37101	N-Term(iTRAQ4plex)	0,0000	1,620	3	1,1	0	0,000000243	36	0,000235489	3	1782,98801	0,50	38,93	0
	High	qYADVVIQVLPTR	1	1	1	P37101	N-Term(iTRAQ4plex)	0,0000	0,470	1		0	0,00001817	34	0,000456227	3	1645,93961	0,11	41,89	0
	High	kLTcTYPGIk	3	1	1	P37101	N-Term(iTRAQ4plex); K1(iTRAQ4plex); C4(Carbamidomethyl);	0,0000	1,509	2	6,2	0	0,00007997	21	0,007961044	3	1612,94602	0,21	23,31	1
	High	hkDYPGTDNGTGLFQVLVGLk	1	1	1	P37101	N-Term(iTRAQ4plex); K2(iTRAQ4plex); K21(iTRAQ4plex)	0,0000	2,138	1		0	3,003E-08	19	0,01140171	4	2691,48501	0,71	46,32	1
	High	sGQSImkPIYNHETGLLDPPEkVE	1	1	1	P37101	N-Term(iTRAQ4plex); M6(Oxidation); K7(iTRAQ4plex); K22(iTF	0,0000	1,978	1		0	0,00002239	13	0,05482391	5	3613,93415	-1,15	29,73	1
	High	lIEDkESk	3	1	1	P37101	N-Term(iTRAQ4plex); K5(iTRAQ4plex); K8(iTRAQ4plex)	0,0000	1,714	3	6,1	0,001	0,008156	14	0,043148927	3	1393,82361	-1,92	16,87	1
P73704	General secretion pathway protein G OS=Synechocystis sp. (strain PCC 6803 / Kazusa) GN=hofG PE=	461,56	17,26	1	3	3	16	1,288	12	15,9	168	17,6	4,78							
	A3	Sequence	# PSMs	# Proteins	# Protein Groups	Protein Group Accessions	Modifications	ΔCn	116/117	116/117 Count	116/117 Variability [%]	q-Value	PEP	IonScore	Exp Value	Charge	MH+ [Da]	ΔM [ppm]	RT [min]	# Missed Cleavages
	High	sMSGGTFYDSGTR	2	1	1	P73704	N-Term(iTRAQ4plex)	0,0000	1,821	2	3,2	0	2,308E-09	72	6,62171E-08	2	1509,67876	1,78	24,21	0
	High	smSGGTFYDSGTR	5	1	1	P73704	N-Term(iTRAQ4plex); M2(Oxidation)	0,0000	1,174	4	10,7	0	3,304E-07	57	2,1231E-06	2	1525,67180	0,53	17,74	0
	High	sTIGALNR	6	1	1	P73704	N-Term(iTRAQ4plex)	0,0000	1,521	3	0,8	0	0,0008167	48	1,65185E-05	2	975,57097	0,59	17,41	0
	High	aFSTVVcR	3	1	1	P73704	N-Term(iTRAQ4plex); C7(Carbamidomethyl)	0,0000	1,222	3	1,0	0	0,001439	44	4,04548E-05	2	1083,57475	0,91	23,24	0
P73679	Isopenicillin N epimerase OS=Synechocystis sp. (strain PCC 6803 / Kazusa) GN=cefD PE=3 SV=1 - [I	460,65	28,68	1	10	10	22	0,410	19	30,9	401	44,0	5,87							
	A3	Sequence	# PSMs	# Proteins	# Protein Groups	Protein Group Accessions	Modifications	ΔCn	116/117	116/117 Count	116/117 Variability [%]	q-Value	PEP	IonScore	Exp Value	Charge	MH+ [Da]	ΔM [ppm]	RT [min]	# Missed Cleavages
	High	accHYITNEEEIEHLLSR	2	1	1	P73679	N-Term(iTRAQ4plex); C2(Carbamidomethyl); C3(Carbamidome	0,0000	0,351	2	3,9	0	1,158E-14	74	4,10176E-08	3	2418,13163	0,71	38,16	0
	High	vLVDGAQSAGSLPLDFSR	1	1	1	P73679	N-Term(iTRAQ4plex)	0,0000	0,562	1		0	2,09E-08	67	2,36261E-07	3	1976,05863	0,82	43,51	0
	High	lEVDYYAFTGHk	5	1	1	P73679	N-Term(iTRAQ4plex); K12(iTRAQ4plex)	0,0000	0,381	3	2,5	0	4,209E-11	62	6,109E-07	3	1730,89963	0,24	36,40	0
	High	wFAGPAGVGGLYIHGDAk	4	1	1	P73679	N-Term(iTRAQ4plex); K18(iTRAQ4plex)	0,0000	0,840	4	53,2	0	1,125E-08	51	1,03731E-05	3	2104,12845	3,12	41,48	0
	High	lGEINPTYVGWR	2	1	1	P73679	N-Term(iTRAQ4plex)	0,0000	0,360	2	10,8	0	0,0000109	41	7,63783E-05	2	1548,83183	1,74	40,15	0
	High	qQFPGLANk	2	1	1	P73679	N-Term(iTRAQ4plex); K9(iTRAQ4plex)	0,0000	0,349	1		0	0,0001274	33	0,000466627	2	1290,74346	2,00	24,89	0
	High	yQAIcQR	3	1	1	P73679	N-Term(iTRAQ4plex); C5(Carbamidomethyl)	0,0000	0,331	3	6,9	0	0,002683	32	0,000628015	2	1082,55400	0,60	16,62	0
	High	sITYGSk	1	1	1	P73679	N-Term(iTRAQ4plex); K7(iTRAQ4plex)	0,0000	0,478	1		0,001	0,00386	37	0,000200895	2	1043,59636	-1,13	16,58	0
	High	hQGDYPVR	1	1	1	P73679	N-Term(iTRAQ4plex)	0,0000	1,123	1		0,003	0,04148	16	0,023494705	3	1115,57187	0,38	11,43	0
	Medium	fGVTYR	1	1	1	P73679	N-Term(iTRAQ4plex)	0,0000	0,592	1		0,048	0,6276	10	0,094617181	2	886,49108	0,84	22,67	0
P29107	Ketol-acid reductoisomerase OS=Synechocystis sp. (strain PCC 6803 / Kazusa) GN=ilvC PE=1 SV=3 -	459,89	30,51	1	7	7	15	2,286	14	69,3	331	35,8	5,01							
	A3	Sequence	# PSMs	# Proteins	# Protein Groups	Protein Group Accessions	Modifications	ΔCn	116/117	116/117 Count	116/117 Variability [%]	q-Value	PEP	IonScore	Exp Value	Charge	MH+ [Da]	ΔM [ppm]	RT [min]	# Missed Cleavages
	High	rTYEQGQGVPALFAVYQDASGQA	1	1	1	P29107	N-Term(iTRAQ4plex)	0,0000	2,203	1		0	7,572E-13	79	1,34577E-08	3	2756,39298	2,04	43,10	1
	High	qILDEIQSGQFAR	2	1	1	P29107	N-Term(iTRAQ4plex)	0,0000	1,494	2	74,2	0	1,757E-08	69	1,60023E-07	2	1648,87932	1,06	39,77	0
	High	aMFSWLk	3	1	1	P29107	N-Term(iTRAQ4plex); K7(iTRAQ4plex)	0,0000	3,965	3	5,5	0	0,0000225	61	7,67308E-07	2	1170,65862	0,19	42,96	0
	High	tVAIIGYGSQGHAHALNLk	3	1	1	P29107	N-Term(iTRAQ4plex); K19(iTRAQ4plex)	0,0000	1,603	2	37,7	0	1,14E-10	60	1,00686E-06	4	2238,26113	0,61	31,36	0
	High	eSEELIEEVGkDLR	1	1	1	P29107	N-Term(iTRAQ4plex); K11(iTRAQ4plex)	0,0000	3,097	1		0	0,000003841	38	0,000159577	3	1934,03189	-0,13	40,72	1
	High	iVTEETk	1	1	1	P29107	N-Term(iTRAQ4plex); K7(iTRAQ4plex)	0,0000	1,280	1		0	0,0002515	37	0,000223342	2	1107,64873	-1,14	17,47	0
	High	eFVLENQAGkPGFTAmR	2	1	1	P29107	N-Term(iTRAQ4plex); K10(iTRAQ4plex); M16(Oxidation)	0,0000	1,881	2	26,2	0	1,131E-07	34	0,000354789	3	2199,14719	0,02	31,02	0
	High	amFSWLk	2	1	1	P29107	N-Term(iTRAQ4plex); M2(Oxidation); K7(iTRAQ4plex)	0,0000	3,351	2	20,3	0,001	0,01338	29	0,001137549	2	1186,65349	0,15	35,82	0
P73488	Sll1130 protein OS=Synechocystis sp. (strain PCC 6803 / Kazusa) GN=sll1130 PE=4 SV=1 - [P73488	455,86	46,96	1	5	5	30	2,274	20	16,0	115	12,9	8,81							
	A3	Sequence	# PSMs	# Proteins	# Protein Groups	Protein Group Accessions	Modifications	ΔCn	116/117	116/117 Count	116/117 Variability [%]	q-Value	PEP	IonScore	Exp Value	Charge	MH+ [Da]	ΔM [ppm]	RT [min]	# Missed Cleavages
	High	sAGLFTPSVVR	8	1	1	P73488	N-Term(iTRAQ4plex)	0,0000	2,242	5	6,1	0	0,00002696	56	2,48296E-06	2	1277,73369	0,18	32,80	0
	High	rPALILSDAPAFNNR	6	1	1	P73488	N-Term(iTRAQ4plex)	0,0000	1,489	4	75,5	0	7,635E-08	52	6,71382E-06	3	1799,00626	0,97	34,35	0
	High	iGHSVmAmITSAk	6	1	1	P73488	N-Term(iTRAQ4plex); M6(Oxidation); M8(Oxidation); K13(iTRA	0,0000	2,063	3	10,9	0	6,205E-08	47	2,06049E-05	3	1665,89096	0,17	23,02	0
	High	lFTLEHk	3	1	1	P73488	N-Term(iTRAQ4plex); K7(iTRAQ4plex)	0,0000	2,547	3	8,4	0	0,0003444	41	0,000103501	2	1175,70232	-0,31	26,38	0
	High	iGHSVMAmITSAk	2	1	1	P73488	N-Term(iTRAQ4plex); M8(Oxidation); K13(iTRAQ4plex)	0,0000	3,453	1		0	3,661E-07	37	0,000207	3	1649,89597	0,12	30,42	0
	High	sAFPHVFk	4	1	1	P73488	N-Term(iTRAQ4plex); K8(iTRAQ4plex)	0,0000	2,373	3	9,8	0	0,0008967	23	0,004874948	3	1220,70307	0,03	26,93	0
	High	iGHSVMAMITSAk	1	1	1	P73488	N-Term(iTRAQ4plex); K13(iTRAQ4plex)	0,0000	1,083	1		0,001	0,003189	14	0,046151014	3	1633,90024	-0,38	37,69	0
P09193	Photosystem II 44 kDa reaction center protein OS=Synechocystis sp. (strain PCC 6803 / Kazusa) GN=	451,74	12,83	1	6	6	16	1,108	13	11,8	460	50,3	6,60							
	A3	Sequence	# PSMs	# Proteins	# Protein Groups	Protein Group Accessions	Modifications	ΔCn	116/117	116/117 Count	116/117 Variability [%]	q-Value	PEP	IonScore	Exp Value	Charge	MH+ [Da]	ΔM [ppm]	RT [min]	# Missed Cleavages
	High	lGANIASAQGPTGLGk	5	1	1	P09193	N-Term(iTRAQ4plex); K16(iTRAQ4plex)	0,0000	0,801	4	44,1	0	1,408E-09	88	1,49613E-09	2	1742,99993	-0,26	30,61	0
	High	dLPSTGFAWWSGNAR	2	1	1	P09193	N-Term(iTRAQ4plex)	0,0000	1,717	2	81,5	0	7,527E-07	45	3,14029E-05	2	1808,88457	0,47	45,20	0
	High	aAAAGFEk	3	1	1	P09193	N-Term(iTRAQ4plex); K8(iTRAQ4plex)	0,0000	1,074	2	3,4	0	0,0006335	43	4,92006E-05	2	1052,60796	9,55	17,77	0

	High	lINLSGk	3	1	1	P09193	N-Term(iTRAQ4plex); K7(iTRAQ4plex)	0,0000	1,259	2	11,7	0	0,002612	40	9,57128E-05	2	1032,66387	-1,66	28,37	0
	High	gPWLEPLR	1	1	1	P09193	N-Term(iTRAQ4plex)	0,0000	1,246	1		0	0,0001966	37	0,000199972	2	1111,63750	-0,54	35,43	0
	Medium	fWDFR	2	1	1	P09193	N-Term(iTRAQ4plex)	0,0000	1,171	2	3,5	0,029	0,3885	16	0,024886854	2	914,46434	0,23	38,07	0
P73311	30S ribosomal protein S17 OS=Synechocystis sp. (strain PCC 6803 / Kazusa) GN=rpsQ PE=3 SV=1 -	450,44	64,20	1	5	5	16	2,654	15	32,6	81	9,3	9,94							
	A3	Sequence	# PSMs	# Proteins	# Protein Groups	Protein Group Accessions	Modifications	ΔCn	116/117	116/117 Count	116/117 Variability [%]	q-Value	PEP	IonScore	Exp Value	Charge	MH+ [Da]	ΔM [ppm]	RT [min]	# Missed Cleavages
	High	tVVVAVESR	3	1	1	P73311	N-Term(iTRAQ4plex)	0,0000	2,651	2	0,2	0	0,00008194	63	5,89089E-07	2	1103,65654	2,18	22,50	0
	High	vGIVVSNk	4	1	1	P73311	N-Term(iTRAQ4plex); K8(iTRAQ4plex)	0,0000	1,345	4	104,7	0	0,000003041	63	5,49503E-07	2	1103,70781	4,63	22,19	0
	High	wQVINIMSHSS	2	1	1	P73311	N-Term(iTRAQ4plex)	0,0000	5,481	2	7,2	0	0,00003807	52	6,41165E-06	2	1445,73638	2,48	41,86	0
	High	aHDEENQcQEGDkVR	2	1	1	P73311	N-Term(iTRAQ4plex); C8(Carbamidomethyl); K13(iTRAQ4plex)	0,0000	2,826	2	22,8	0	8,397E-07	47	1,87919E-05	4	2102,98061	1,99	8,88	1
	High	iQETRPLSk	4	1	1	P73311	N-Term(iTRAQ4plex); K9(iTRAQ4plex)	0,0000	3,338	4	17,1	0	0,00201	47	2,17756E-05	2	1359,83147	8,54	17,05	0
	High	wQVINImSHSS	1	1	1	P73311	N-Term(iTRAQ4plex); M7(Oxidation)	0,0000	2,144	1		0	0,001239	37	0,000183641	2	1461,72966	1,34	34,62	0
Q55727	Slr0645 protein OS=Synechocystis sp. (strain PCC 6803 / Kazusa) GN=slr0645 PE=4 SV=1 - [Q55727	446,54	49,51	1	9	9	18	0,275	15	38,9	206	22,8	4,74							
	A3	Sequence	# PSMs	# Proteins	# Protein Groups	Protein Group Accessions	Modifications	ΔCn	116/117	116/117 Count	116/117 Variability [%]	q-Value	PEP	IonScore	Exp Value	Charge	MH+ [Da]	ΔM [ppm]	RT [min]	# Missed Cleavages
	High	iDRDEELAISLIQVGR	3	1	1	Q55727	N-Term(iTRAQ4plex)	0,0000	0,390	2	73,0	0	1,588E-10	62	6,96578E-07	3	1971,10160	1,21	45,73	1
	High	vAYIYANNEPmGR	3	1	1	Q55727	N-Term(iTRAQ4plex); M11(Oxidation)	0,0000	0,275	3	35,4	0	2,452E-07	61	7,86991E-07	2	1657,81462	1,27	26,33	0
	High	sAEAVIENR	1	1	1	Q55727	N-Term(iTRAQ4plex)	0,0000	0,469	1		0	3,893E-07	48	1,77407E-05	2	1132,60869	0,69	20,14	0
	High	rYDNVTAEk	5	1	1	Q55727	N-Term(iTRAQ4plex); K9(iTRAQ4plex)	0,0000	0,282	4	17,3	0	0,00001121	41	8,01623E-05	3	1383,74860	1,10	14,40	1
	High	wEIAQASTIALAk	2	1	1	Q55727	N-Term(iTRAQ4plex); K13(iTRAQ4plex)	0,0000	0,326	2	61,7	0	0,00009174	38	0,00016367	3	1689,98014	1,36	40,98	0
	High	kATAFFQALDDQLQAAGAk	1	1	1	Q55727	N-Term(iTRAQ4plex); K1(iTRAQ4plex); K19(iTRAQ4plex)	0,0000				0	0,0001729	31	0,000731089	3	2426,34262	0,89	48,01	1
	High	aTAFFQALDDQLQAAGAk	1	1	1	Q55727	N-Term(iTRAQ4plex); K18(iTRAQ4plex)	0,0000	0,097	1		0	6,181E-07	29	0,001273415	3	2154,14756	1,92	53,30	0
	High	sSSmATADDPNGPTR	1	1	1	Q55727	N-Term(iTRAQ4plex); M4(Oxidation)	0,0000	0,525	1		0	0,0002651	26	0,002703772	3	1666,74588	-0,05	11,30	0
	High	lILEASQk	1	1	1	Q55727	N-Term(iTRAQ4plex); K8(iTRAQ4plex)	0,0000	0,214	1		0,001	0,004021	31	0,000744681	2	1189,73979	0,27	26,30	0
P73314	30S ribosomal protein S3 OS=Synechocystis sp. (strain PCC 6803 / Kazusa) GN=rpsC PE=1 SV=3 - [I	445,08	34,17	1	8	8	20	3,695	18	40,1	240	27,1	9,98							
	A3	Sequence	# PSMs	# Proteins	# Protein Groups	Protein Group Accessions	Modifications	ΔCn	116/117	116/117 Count	116/117 Variability [%]	q-Value	PEP	IonScore	Exp Value	Charge	MH+ [Da]	ΔM [ppm]	RT [min]	# Missed Cleavages
	High	tALTTYGILGIk	5	1	1	P73314	N-Term(iTRAQ4plex); K12(iTRAQ4plex)	0,0000	3,897	5	71,0	0	3,506E-10	74	4,00839E-08	2	1538,94145	1,18	42,76	0
	High	tLNNAGISDIR	1	1	1	P73314	N-Term(iTRAQ4plex)	0,0000	3,504	1		0	0,000004464	69	1,25017E-07	2	1317,72502	0,52	25,71	0
	High	kAEQIELGIHTARPGVVVGR	3	1	1	P73314	N-Term(iTRAQ4plex); K1(iTRAQ4plex)	0,0000	4,661	2	13,3	0	8,961E-07	50	1,07887E-05	5	2418,42161	1,31	28,26	1
	High	gGSGIEQLR	2	1	1	P73314	N-Term(iTRAQ4plex)	0,0000	1,454	2	10,8	0	0,002249	38	0,000158114	2	1060,58733	0,51	18,50	0
	High	rYPELLQEDHk	3	1	1	P73314	N-Term(iTRAQ4plex); K11(iTRAQ4plex)	0,0000	2,684	3	21,4	0	0,000005688	30	0,001506924	3	1715,93003	-1,11	21,42	1
	High	vPLHTLR	2	1	1	P73314	N-Term(iTRAQ4plex)	0,0000	3,852	2	14,8	0	0,001334	24	0,004139711	3	979,61762	0,69	19,49	0
	High	vWIFk	3	1	1	P73314	N-Term(iTRAQ4plex); K5(iTRAQ4plex)	0,0000	4,659	2	14,0	0,002	0,03076	28	0,001541594	2	980,61858	1,44	37,72	0
	Medium	iHPVGFR	1	1	1	P73314	N-Term(iTRAQ4plex)	0,0000	4,519	1		0,015	0,1929	21	0,008709034	3	969,57593	0,87	19,92	0
P73853	IMP dehydrogenase subunit OS=Synechocystis sp. (strain PCC 6803 / Kazusa) GN=guaB PE=3 SV=1	438,53	32,82	1	11	11	20	1,102	16	59,5	387	40,2	5,34							
	A3	Sequence	# PSMs	# Proteins	# Protein Groups	Protein Group Accessions	Modifications	ΔCn	116/117	116/117 Count	116/117 Variability [%]	q-Value	PEP	IonScore	Exp Value	Charge	MH+ [Da]	ΔM [ppm]	RT [min]	# Missed Cleavages
	High	gFHWGmATPSPVLPR	2	1	1	P73853	N-Term(iTRAQ4plex); M6(Oxidation)	0,0000	0,694	2	39,0	0	2,324E-10	61	7,74408E-07	3	1812,93656	1,60	34,90	0
	High	gVLGVGVPQPTAIADcAAAR	1	1	1	P73853	N-Term(iTRAQ4plex); C16(Carbamidomethyl)	0,0000	0,627	1		0	7,781E-07	54	4,23643E-06	3	2067,11716	1,61	41,36	0
	High	iNVGTTGTIR	3	1	1	P73853	N-Term(iTRAQ4plex)	0,0000	1,815	2	9,2	0	0,000007645	51	7,50221E-06	2	1175,68901	2,14	24,40	0
	High	eILVGPAk	2	1	1	P73853	N-Term(iTRAQ4plex); K8(iTRAQ4plex)	0,0000	1,545	1		0	0,0001242	48	1,6217E-05	2	1114,71367	5,57	23,60	0
	High	gFHWGMATPSPVLPR	1	1	1	P73853	N-Term(iTRAQ4plex)	0,0000	0,491	1		0	2,131E-09	45	3,10435E-05	3	1796,94175	1,67	39,08	0
	High	rAYGIDEIALVPGVR	2	1	1	P73853	N-Term(iTRAQ4plex)	0,0000	1,298	2	41,1	0	0,000004819	40	0,000107887	3	1773,01700	1,69	39,21	1
	High	tSmGTLGAk	2	1	1	P73853	N-Term(iTRAQ4plex); M3(Oxidation); K9(iTRAQ4plex)	0,0000	1,844	1		0	0,0009509	35	0,000291052	2	1169,64763	3,21	14,41	0
	High	aYGIDEIALVPGVR	1	1	1	P73853	N-Term(iTRAQ4plex)	0,0000	0,892	1		0	0,00007665	34	0,000430497	3	1616,91263	-0,16	44,25	0
	High	lDDGTHNLLGAIk	1	1	1	P73853	N-Term(iTRAQ4plex); K13(iTRAQ4plex)	0,0000	1,429	1		0	0,00006921	33	0,000521159	3	1654,94236	3,42	34,64	0
	High	aGAAAVLVGIGPGAAcTSR	1	1	1	P73853	N-Term(iTRAQ4plex); C16(Carbamidomethyl)	0,0000	1,652	1		0	0,0002317	24	0,00374973	3	1842,99881	0,58	36,08	0
	High	tLDPALADTR	1	1	1	P73853	N-Term(iTRAQ4plex)	0,0000	0,865	1		0	0,002564	20	0,011116549	2	1216,66753	1,72	25,09	0
	High	aQQLGmGk	2	1	1	P73853	N-Term(iTRAQ4plex); M6(Oxidation); K8(iTRAQ4plex)	0,0000	1,221	1		0,001	0,009697	20	0,011727064	2	1136,63921	4,90	13,96	0
	High	aQQLGMGk	1	1	1	P73853	N-Term(iTRAQ4plex); K8(iTRAQ4plex)	0,0000	0,960	1		0,001	0,01381	18	0,017059646	2	1120,64897	9,14	16,81	0
Q55835	Iron uptake protein A2 OS=Synechocystis sp. (strain PCC 6803 / Kazusa) GN=futA2 PE=1 SV=1 - [FU	437,17	29,48	1	9	9	19	0,381	16	25,5	346	38,1	6,18							
	A3	Sequence	# PSMs	# Proteins	# Protein Groups	Protein Group Accessions	Modifications	ΔCn	116/117	116/117 Count	116/117 Variability [%]	q-Value	PEP	IonScore	Exp Value	Charge	MH+ [Da]	ΔM [ppm]	RT [min]	# Missed Cleavages
	High	gTHVNVSGAGVLk	4	1	1	Q55835	N-Term(iTRAQ4plex); K13(iTRAQ4plex)	0,0000	0,404	2	24,9	0	1,221E-07	65	2,97146E-07	3	1526,89020	0,56	20,85	0
	High	hPDGLWYGFTQR	4	1	1	Q55835	N-Term(iTRAQ4plex)	0,0000	0,325	3	9,6	0	6,25E-09	58	1,5065E-06	3	1620,80490	0,57	34,84	0
	High	iLVRPSSNVYNLSLTASR	2	1	1	Q55835	N-Term(iTRAQ4plex)	0,0000	0,315	2	2,8	0	7,384E-07	50	9,65984E-06	3	2134,21231	1,02	37,30	0
	High	aEQAGLFQPVR	1	1	1	Q55835	N-Term(iTRAQ4plex)	0,0000	0,378	1		0	0,000000204	48	1,6633E-05	2	1359,75090	0,54	28,16	0
	High	wLQGLVGNFAR	2	1	1	Q55835	N-Term(iTRAQ4plex)	0,0000	0,405	2	16,2	0	0,00001163	40	8,95303E-05	2	1404,78923	1,66	50,05	0
	High	vSLFFPNTGSGER	1	1	1	Q55835	N-Term(iTRAQ4plex)	0,0000	0,562	1		0	0,0002933	35	0,000294422	2	1554,80681	2,24	39,00	0
	High	tINLYSSR	1	1	1	Q55835	N-Term(iTRAQ4plex)	0,0000	0,385	1		0	0,00005659	30	0,000941825	2	1097,60735	0,18	23,42	0
	High	iAIHGEPETR	1	1	1	Q55835	N-Term(iTRAQ4plex)	0,0000	0,521	1		0	0,00008254	29	0,001250173	3	1266,69339	0,85	18,08	0
	High	vLYYSR	3	1	1	Q55835	N-Term(iTRAQ4plex)	0,0000	0,454	3	10,5	0,001	0,006908	31	0,00088914	2	944,53380	1,71	21,66	0
P73463	Slr1220 protein OS=Synechocystis sp. (strain PCC 6803 / Kazusa) GN=slr1220 PE=4 SV=1 - [P73463	435,96	13,25	1	4	4	14	0,538	12	8,3	317	36,0	4,32							
	A3	Sequence	# PSMs	# Proteins	# Protein Groups	Protein Group Accessions	Modifications	ΔCn	116/117	116/117 Count	116/117 Variability [%]	q-Value	PEP	IonScore	Exp Value	Charge	MH+ [Da]	ΔM [ppm]	RT [min]	# Missed Cleavages
	High	lRNEFINTEVITR	4	1	1	P73463	N-Term(iTRAQ4plex)	0,0000	0,540	3	5,0	0	6,287E-09	74	4,28519E-08	3	1748,97873	0,64	33,70	1
	High	lNQLSVGLLER	5	1	1	P73463	N-Term(iTRAQ4plex)	0,0000	0,415	5	9,5	0	0,000004731	61	8,81417E-07	2	1385,82427	0,67	41,03	0
	High	vRDFQFDLATGk	3	1	1	P73463	N-Term(iTRAQ4plex); K12(iTRAQ4plex)	0,0000	0,547	2	2,8	0	1,312E-07	57	1,92393E-06	3	1684,92716	0,61	34,03	1
	High	kLGVVk	2	1	1	P73463	N-Term(iTRAQ4plex); K1(iTRAQ4plex); K6(iTRAQ4plex)	0,0000	0,593	2	10,2	0,003	0,03177	20	0,009182692	3	1075,75302	-3,09	15,42	1
P74071	30S ribosomal protein S2 OS=Synechocystis sp. (strain PCC 6803 / Kazusa) GN=rpsB PE=3 SV=1 - [I	434,47	30,11	1	10	10	19	1,950	19	42,3	269	30,1	5,27							
	A3	Sequence	# PSMs	# Proteins	# Protein Groups	Protein Group Accessions	Modifications	ΔCn	116/117	116/117 Count	116/117 Variability [%]	q-Value	PEP	IonScore	Exp Value	Charge	MH+ [Da]	ΔM [ppm]	RT [min]	# Missed Cleavages
	High	lkELEALEASGGIDR	2	1	1	P74071	N-Term(iTRAQ4plex); K2(iTRAQ4plex)	0,0000	1,826	2	34,3	0	5,953E-10	66	2,5668E-07	3	1889,05899	0,37	38,32	1
	High	rQAAGIISQEAQR	1	1	1	P74071	N-Term(iTRAQ4plex)	0,0000	0,890	1		0	0,000003589	54	4,16841E-06	3	1571,88242	5,68	17,24	1
	High	kLPDVVVIVDQR	3	1	1	P74071	N-Term(iTRAQ4plex); K1(iTRAQ4plex)	0,0000	1,906	3	0,2	0	2,326E-07	46	2,50017E-05	3	1669,02427	-0,52	35,72	1
	High	lADAIYEGR	1	1	1	P74071	N-Term(iTRAQ4plex)	0,0000	1,169	1		0	0,00002461	43	4,5391E-05	2	1151,61724	-0,44	28,03	0
	High	lkELEALEASGGIDRR	3	1	1	P74071	N-Term(iTRAQ4plex); K2(iTRAQ4plex)	0,0000	3,532	3	9,5	0	7,682E-07	39	0,000123018	4	2045,16250	1,51	35,21	2
	High	kFLFVGTk	1	1	1	P74071	N-Term(iTRAQ4plex); K1(iTRAQ4plex); K8(iTRAQ4plex)	0,0000	2,661	1		0	0,0002325	27	0,001811215	3	1371,87265	0,15	29,33	1
	High	eHNAIHEcQk	2	1	1	P74071	N-Term(iTRAQ4plex); C8(Carbamidomethyl); K10(iTRAQ4plex)	0,0000	0,833	2	69,0	0	0,000004773	26	0,002306588	4	1553,77248	-0,53	8,10	0
	High	fLFVGTk	2	1	1	P74071	N-Term(iTRAQ4plex); K7(iTRAQ4plex)	0,0000	2,196	2	15,7	0	0,001943	22	0,006323682	2	1099,67522	-0,18	35,63	0
	High	sSTINGk	1	1	1	P74071	N-Term(iTRAQ4plex); K7(iTRAQ4plex)	0,0000	1,483	1		0,001	0,005133	23	0,004875285	2	994,57695	-0,20	11,69	0
	High	yLGGIk	3	1	1	P74071	N-Term(iTRAQ4plex); K6(iTRAQ4plex)	0,0000	1,950	3	1,9	0,009	0,1147	28	0,001757802	2	938,59007	-1,35	24,96	0
P27320	Ferredoxin-1 OS=Synechocystis sp. (strain PCC 6803 / Kazusa) GN=petF PE=1 SV=2 - [FER_SYNY3]	428,14	17,53	1	3	3	21	0,377	19	24,0	97	10,4	3,95							
	A3	Sequence	# PSMs	# Proteins	# Protein Groups	Protein Group Accessions	Modifications	ΔCn	116/117	116/117 Count	116/117 Variability [%]	q-Value	PEP	IonScore	Exp Value	Charge	MH+ [Da]	ΔM [ppm]	RT [min]	# Missed Cleavages
	High	aGAcSTcAGk	13	1	1	P27320	N-Term(iTRAQ4plex); C4(Carbamidomethyl); C7(Carbamidome	0,0000	0,350	11	23,5	0	1,983E-08	68	1,69032E-07	2	1270,61223	-0,02	8,51	0
	High	mASYTVk	1	1	1	P27320	N-Term(iTRAQ4plex); K7(iTRAQ4plex)	0,0000	0,674	1		0	0,0001117	49	1,21051E-05	2	1087,60649	0,45	20,43	0
	High	mASYTVk	2	1	1	P27320	N-Term(iTRAQ4plex); M1(Oxidation); K7(iTRAQ4plex)	0,0000	0,478	2	7,2	0	0,001919	37	0,000223342	2	1103,60002	-0,81	15,10	0
	High	aSYTVk	5	1	1	P27320	N-Term(iTRAQ4plex); K6(iTRAQ4plex)	0,0000	0,421	5	7,0	0,001	0,004245	41	7,72627E-05	2	956,56517	-0,35	15,26	0
P73196	DNA ligase OS=Synechocystis sp. (strain PCC 6803 / Kazusa) GN=lig PE=4 SV=1 - [P73196_SYNY3]	425,10	22,95	1	10	10	15	0,893	15	10,2	562	61,4	5,08							
	A3	Sequence	# PSMs	# Proteins	# Protein Groups	Protein Group Accessions	Modifications	ΔCn	116/117	116/117 Count	116/117 Variability [%]	q-Value	PEP	IonScore	Exp Value	Charge	MH+ [Da]	ΔM [ppm]	RT [min]	# Missed Cleavages
	High	qAVNEVAETRPk	2	1	1	P73196	N-Term(iTRAQ4plex); K12(iTRAQ4plex)	0,0000	0,834	2	10,2	0	8,955E-10	71	8,04253E-08	3	1629,91605	-0,15	17,55	0
	High	dQVQQELQTAR	1	1	1	P73196	N-Term(iTRAQ4plex)	0,0000	0,766	1		0	0,000003709	65	3,32637E-07	2	1459,76103	-0,79	22,41	0
	High	lVQLNSLVTR	3	1	1	P73196	N-Term(iTRAQ4plex)	0,0000	0,904	3	0,1	0	5,009E-07	64	3,8634E-07	2	1286,79228	0,77	37,63	0
	High	nQLTENYQAQEk	1	1	1	P73196	N-Term(iTRAQ4plex); K12(iTRAQ4plex)	0,0000	0,839	1		0	5,849E-09	50	9,57128E-06	3	1753,89695	0,58	19,37	0
	High	kVVILGTLNAmNR	1	1	1	P73196	N-Term(iTRAQ4plex); K1(iTRAQ4plex); M11(Oxidation)	0,0000	1,356	1		0	1,804E-07	38	0,000177407	3	1733,03263	-1,17	28,89	1
	High	kLAQSQPSDQPPTLIAGSGTPAVk	1	1	1	P73196	N-Term(iTRAQ4plex); K1(iTRAQ4plex); K24(iTRAQ4plex)	0,0000	1,073	1		0	1,635E-09	35	0,000336488	4	2823,59243	-0,60	29,79	1
	High	vkLETTQAEGDRLk	1	1	1	P73196	N-Term(iTRAQ4plex); K2(iTRAQ4plex); K14(iTRAQ4plex)	0,0000	0,835	1		0	0,00009882	26	0,00232793	4	2020,17434	-0,99	21,16	2
	High	mVAALQHQLAHYDDIQSEk	1	1	1	P73196	N-Term(iTRAQ4plex); K19(iTRAQ4plex)	0,0000	1,505	1		0	3,827E-07	25	0,002818188	4	2485,27920	1,75	39,71	0
	High	fVGLQk	2	1	1	P73196	N-Term(iTRAQ4plex); K6(iTRAQ4plex)	0,0000	0,893	2	10,2	0,001	0,009317	28	0,001674827	2	979,61595	-1,98	25,67	0
	High	tEPPRENk	2	1	1	P73196	N-Term(iTRAQ4plex); K8(iTRAQ4plex)	0,0000	0,870	2	7,4	0,002	0,02572	16	0,02517503	3	1258,69800	-1,12	8,20	1
P73530	30S ribosomal protein S1 homolog A OS=Synechocystis sp. (strain PCC 6803 / Kazusa) GN=rps1A PE	420,41	30,49	1	10	10	20	1,042	18	19,6	328	36,5	4,67							
	A3	Sequence	# PSMs	# Proteins	# Protein Groups	Protein Group Accessions	Modifications	ΔCn	116/117	116/117 Count	116/117 Variability [%]	q-Value	PEP	IonScore	Exp Value	Charge	MH+ [Da]	ΔM [ppm]	RT [min]	# Missed Cleavages
	High	kmNGLEVAQVVVGSVR	3	1	1	P73530	N-Term(iTRAQ4plex); K1(iTRAQ4plex); M2(Oxidation)	0,0000	0,987	2	27,4	0	3,018E-09	58	1,62918E-06	3	1990,13687	0,52	36,62	1
	High	tAAYIPIQEmSINR	3	1	1	P73530	N-Term(iTRAQ4plex); M10(Oxidation)	0,0000	0,930	2	9,4	0	4,693E-07	51	8,8914E-06	3	1766,92325	0,26	31,94	0
	High	vMIIDLDAER	1	1	1	P73530	N-Term(iTRAQ4plex)	0,0000	1,052	1		0	0,000009482	47	2,17756E-05	2	1318,71733	1,18	39,10	0
	High	gFIPGSHISAR	2	1	1	P73530	N-Term(iTRAQ4plex)	0,0000	0,731	2	7,5	0	0,0001763	42	6,32368E-05	3	1285,71466	1,00	22,28	0
	High	gALIDIGAk	1	1	1	P73530	N-Term(iTRAQ4plex); K9(iTRAQ4plex)	0,0000	1,079	1		0	0,000004124	40	9,57128E-05	2	1145,71269	-0,51	29,32	0
	High	eAkEDLVGEDLPLk	1	1	1	P73530	N-Term(iTRAQ4plex); K3(iTRAQ4plex); K14(iTRAQ4plex)	0,0000	1,180	1		0	3,467E-08	40	0,000141295	3	1988,13004	1,20	32,49	1
	High	vmIIDLDAER	1	1	1	P73530	N-Term(iTRAQ4plex); M2(Oxidation)	0,0000	0,856	1		0	0,00003831	36	0,000269755	2	1334,71099	0,22	35,03	0
	High	sNVFATNR	3	1	1	P73530	N-Term(iTRAQ4plex)	0,0000	0,973	3	3,9	0	0,0007998	32	0,000567505	2	1052,56047	-0,08	17,66	0
	High	kMNGLEVAQVVVGSVR	1	1	1	P73530	N-Term(iTRAQ4plex); K1(iTRAQ4plex)	0,0000	1,478	1		0	0,00005976	18	0,015388252	4	1974,14480	1,96	41,87	1
	High	iSLSTk	2	1	1	P73530	N-Term(iTRAQ4plex); K6(iTRAQ4plex)	0,0000	1,183	2	1,0	0,001	0,009886	42	6,01132E-05	2	936,59642	-0,42	21,29	0
	High	rIEYMR	1	1	1	P73530	N-Term(iTRAQ4plex)	0,0000	1,591	1		0,004	0,04822	22	0,005793887	3	1011,55268	0,04	15,68	1
	Medium	lVLSHR	1	1	1	P73530	N-Term(iTRAQ4plex)	0,0000	1,281	1		0,041	0,5384	25	0,002897143	2	868,54930	0,90	16,33	0
P72760	Carbon dioxide-concentrating mechanism protein CcmK homolog 1 OS=Synechocystis sp. (strain PCC	415,66	45,95	1	4	6	24	0,580	7	7,1	111	12,1	6,55							
	A3	Sequence	# PSMs	# Proteins	# Protein Groups	Protein Group Accessions	Modifications	ΔCn	116/117	116/117 Count	116/117 Variability [%]	q-Value	PEP	IonScore	Exp Value	Charge	MH+ [Da]	ΔM [ppm]	RT [min]	# Missed Cleavages
	High	vTLVGYEk	10	2	2	P72760;P72761	N-Term(iTRAQ4plex); K8(iTRAQ4plex)	0,0000				0	0,0002113	49	1,18842E-05	2	1196,71489	1,65	27,58	0
	High	eIVNPSIIR	4	1	1	P72760	N-Term(iTRAQ4plex)	0,0000	0,567	2	3,7	0	0,00163	41	8,66902E-05	2	1184,71684	4,10	29,93	0
	High	yTEAVEQFR	3	1	1	P72760	N-Term(iTRAQ4plex)	0,0000	0,559	3	3,8	0	0,00005327	40	0,0001104	2	1286,65361	2,99	29,88	0
	High	gDVSEVQASVTAGIENIRR	1	1	1	P72760	N-Term(iTRAQ4plex)	0,0000	0,943	1		0	0,0008503	16	0,025715164	3	2145,13987	0,81	40,69	1
	High	gDVSEVQASVTAGIENIR	1	1	1	P72760	N-Term(iTRAQ4plex)	0,0000	1,776	1		0,001	0,00319	37	0,000199054	3	1989,04062	1,82	43,30	0
	High	vTVIVR	5	2	2	P72760;P72761	N-Term(iTRAQ4plex)	0,0000				0,003	0,04246	28	0,001573874	2	830,55919	1,39	22,25	0
P73817	Hemolysin OS=Synechocystis sp. (strain PCC 6803 / Kazusa) GN=sll1951 PE=4 SV=1 - [P73817_SYN	408,86	3,50	1	5	5	13	0,262	10	18,9	1741	178,2	3,75							
	A3	Sequence	# PSMs	# Proteins	# Protein Groups	Protein Group Accessions	Modifications	ΔCn	116/117	116/117 Count	116/117 Variability [%]	q-Value	PEP	IonScore	Exp Value	Charge	MH+ [Da]	ΔM [ppm]	RT [min]	# Missed Cleavages
	High	aVGGDDELVGGR	2	1	1	P73817	N-Term(iTRAQ4plex)	0,0000	0,264	1		0	0,000000141	78	1,67483E-08	2	1288,66191	0,37	21,13	0
	High	gVAAISGAHTVR	4	1	1	P73817	N-Term(iTRAQ4plex)	0,0000	0,345	2	70,6	0	4,69E-08	74	4,43578E-08	3	1282,73508	0,18	17,07	0
	High	lEALTNk	1	1	1	P73817	N-Term(iTRAQ4plex); K7(iTRAQ4plex)	0,0000	0,259	1		0	0,0001633	52	7,20606E-06	2	1076,65508	-0,31	24,56	0
	High	aLSPNVIAALQIMYTGR	1	1	1	P73817	N-Term(iTRAQ4plex)	0,0000	0,302	1		0	3,204E-08	45	2,90382E-05	3	1962,09555	-0,41	54,79	0
	High	yPFFQAPQTADkR	5	1	1	P73817	N-Term(iTRAQ4plex); K12(iTRAQ4plex)	0,0000	0,258	5	14,1	0	0,000001834	36	0,000303664	3	1856,99173	1,05	31,52	1
P77965	DNA-directed RNA polymerase subunit beta OS=Synechocystis sp. (strain PCC 6803 / Kazusa) GN=rp	405,29	13,61	1	12	12	21	3,426	18	30,3	1102	123,3	5,30							
	A3	Sequence	# PSMs	# Proteins	# Protein Groups	Protein Group Accessions	Modifications	ΔCn	116/117	116/117 Count	116/117 Variability [%]	q-Value	PEP	IonScore	Exp Value	Charge	MH+ [Da]	ΔM [ppm]	RT [min]	# Missed Cleavages
	High	lRPGEPPTVSGGQQLLESR	1	1	1	P77965	N-Term(iTRAQ4plex)	0,0000	3,102	1		0	3,922E-13	94	4,23614E-10	3	2165,18039	0,37	31,66	0
	High	eLQSLGLDIAAHk	2	1	1	P77965	N-Term(iTRAQ4plex); K13(iTRAQ4plex)	0,0000	3,475	1		0	1,799E-09	54	3,57248E-06	3	1682,96628	-1,02	36,88	0
	High	tHGIVTYVDATEIR	1	1	1	P77965	N-Term(iTRAQ4plex)	0,0000	3,378	1		0	5,941E-08	52	6,42643E-06	3	1718,91996	0,32	31,27	0
	High	lELHFLGk	6	1	1	P77965	N-Term(iTRAQ4plex); K8(iTRAQ4plex)	0,0000	4,372	4	5,6	0	0,0003985	43	5,38233E-05	2	1244,75945	-0,88	36,47	0
	High	dTVHGLLEEASQRPNk	2	1	1	P77965	N-Term(iTRAQ4plex); K16(iTRAQ4plex)	0,0000	5,350	2	49,7	0	1,31E-08	39	0,00013213	4	2082,11977	0,73	29,20	0
	High	vIVNQIVR	2	1	1	P77965	N-Term(iTRAQ4plex)	0,0000	2,867	2	6,6	0	0,00008216	39	0,000132425	2	1084,69609	0,15	29,41	0
	High	tYSASLIPNR	1	1	1	P77965	N-Term(iTRAQ4plex)	0,0000	2,022	1		0	0,000244	38	0,000235033	2	1265,69719	0,12	28,25	0
	High	kLSAQVLLk	1	1	1	P77965	N-Term(iTRAQ4plex); K1(iTRAQ4plex); K9(iTRAQ4plex)	0,0000	3,278	1		0	0,00001854	36	0,000279235	3	1431,96164	-0,48	27,23	1
	High	iSALGPGGLTR	1	1	1	P77965	N-Term(iTRAQ4plex)	0,0000	1,087	1		0	0,001782	30	0,000901509	2	1185,71391	5,63	29,79	0
	High	qAVPLLRPERPLVGTGLEAQAAR	1	1	1	P77965	N-Term(iTRAQ4plex)	0,0000	4,089	1		0	2,622E-07	22	0,006165524	4	2586,49819	0,79	34,35	0
	High	sIPRPGTPESFk	1	1	1	P77965	N-Term(iTRAQ4plex); K12(iTRAQ4plex)	0,0000	2,676	1		0,001	0,009537	19	0,011613684	3	1603,90519	0,32	24,90	0
	Medium	kIQVGDk	2	1	1	P77965	N-Term(iTRAQ4plex); K1(iTRAQ4plex); K7(iTRAQ4plex)	0,0000	3,650	2	31,3	0,018	0,2421	14	0,044357801	3	1219,77088	-2,12	13,76	1
P73954	Membrane-associated protein slr1513 OS=Synechocystis sp. (strain PCC 6803 / Kazusa) GN=slr1513	398,21	40,91	1	6	6	22	0,164	16	48,3	110	12,0	7,08							
	A3	Sequence	# PSMs	# Proteins	# Protein Groups	Protein Group Accessions	Modifications	ΔCn	116/117	116/117 Count	116/117 Variability [%]	q-Value	PEP	IonScore	Exp Value	Charge	MH+ [Da]	ΔM [ppm]	RT [min]	# Missed Cleavages
	High	gYTVMNTGGk	2	1	1	P73954	N-Term(iTRAQ4plex); K10(iTRAQ4plex)	0,0000	0,164	1		0	1,914E-08	69	1,2161E-07	2	1315,69316	0,98	19,56	0
	High	fEILTETR	3	1	1	P73954	N-Term(iTRAQ4plex)	0,0000	0,166	3	6,9	0	0,00006257	60	9,37497E-07	2	1152,63713	-0,88	33,87	0
	High	gYTVmNTGGk	8	1	1	P73954	N-Term(iTRAQ4plex); M5(Oxidation); K10(iTRAQ4plex)	0,0000	0,245	5	164,1	0	5,215E-08	56	2,26971E-06	2	1331,68657	-0,17	14,83	0
	High	emAEEIADR	2	1	1	P73954	N-Term(iTRAQ4plex); M2(Oxidation)	0,0000	0,193	1		0	0,0002191	47	1,91854E-05	2	1223,56902	-0,39	15,95	0
	High	eMAEEIADR	1	1	1	P73954	N-Term(iTRAQ4plex)	0,0000	0,451	1		0	0,0001715	34	0,000430497	2	1207,57427	-0,26	22,59	0
	High	lVIVTEk	1	1	1	P73954	N-Term(iTRAQ4plex); K7(iTRAQ4plex)	0,0000	0,164	1		0	0,0004577	30	0,001071445	2	1089,71147	-0,66	28,06	0
	Medium	iLLkk	2	1	1	P73954	N-Term(iTRAQ4plex); K4(iTRAQ4plex); K5(iTRAQ4plex)	0,0000	0,114	2	17,9	0,015	0,1925	12	0,063382593	3	1046,76276	-3,27	19,29	1
	Medium	akPANk	3	1	1	P73954	N-Term(iTRAQ4plex); K2(iTRAQ4plex); K6(iTRAQ4plex)	0,0000	0,087	2	30,2	0,017	0,2275	20	0,013487869	3	1060,67667	-6,83	7,67	0
P73296	50S ribosomal protein L17 OS=Synechocystis sp. (strain PCC 6803 / Kazusa) GN=rplQ PE=3 SV=1 - [	396,80	51,72	1	7	7	39	1,749	31	75,7	116	13,2	11,06							
	A3	Sequence	# PSMs	# Proteins	# Protein Groups	Protein Group Accessions	Modifications	ΔCn	116/117	116/117 Count	116/117 Variability [%]	q-Value	PEP	IonScore	Exp Value	Charge	MH+ [Da]	ΔM [ppm]	RT [min]	# Missed Cleavages
	High	aLGYmYDkPTVHALFADAPSR	6	1	1	P73296	N-Term(iTRAQ4plex); M5(Oxidation); K8(iTRAQ4plex)	0,0000	2,256	4	15,7	0	7,135E-11	45	3,01974E-05	3	2627,35672	1,38	36,49	0
	High	rGDNAEMAVIELV	5	1	1	P73296	N-Term(iTRAQ4plex)	0,0000	0,762	4	77,9	0	0,002515	44	4,10176E-05	2	1560,81999	1,73	44,66	1
	High	vPQLGkPADQR	4	1	1	P73296	N-Term(iTRAQ4plex); K6(iTRAQ4plex)	0,0000	1,915	4	28,2	0	0,00005563	38	0,000151695	2	1496,88140	1,74	19,96	0
	High	aLTTELIR	4	1	1	P73296	N-Term(iTRAQ4plex)	0,0000	2,093	3	42,4	0	0,002661	36	0,00028178	2	1060,65361	4,99	30,24	0
	High	aLGYMYDkPTVHALFADAPSR	5	1	1	P73296	N-Term(iTRAQ4plex); K8(iTRAQ4plex)	0,0000	4,878	3	11,8	0	1,964E-07	33	0,000530848	3	2611,36631	3,11	39,55	0
	High	mITLAk	4	1	1	P73296	N-Term(iTRAQ4plex); M1(Oxidation); K6(iTRAQ4plex)	0,0000	1,396	3	34,3	0,001	0,005387	25	0,003892075	2	980,60643	1,16	20,70	0
	High	gDNAEmAVIELV	2	1	1	P73296	N-Term(iTRAQ4plex); M6(Oxidation)	0,0000	0,562	2	0,0	0,001	0,01144	22	0,006323682	2	1420,71160	0,36	46,26	0
	High	mITLAk	4	1	1	P73296	N-Term(iTRAQ4plex); K6(iTRAQ4plex)	0,0000	3,763	4	76,2	0,006	0,08188	28	0,001663298	2	964,61693	6,79	26,84	0
	High	rGDNAEmAVIELV	2	1	1	P73296	N-Term(iTRAQ4plex); M7(Oxidation)	0,0000	1,089	2	40,3	0,008	0,1046	26	0,002360315	2	1576,81474	1,61	39,62	1
	High	gDNAEMAVIELV	1	1	1	P73296	N-Term(iTRAQ4plex)	0,0000				0,009	0,1231	12	0,068860473	2	1404,72038	3,00	50,88	0
	High	rRGDNAEMAVIELV	1	1	1	P73296	N-Term(iTRAQ4plex)	0,0000	0,682	1		0,009	0,1203	11	0,083940201	3	1716,92118	1,61	39,24	2
	Medium	rRGDNAEmAVIELV	1	1	1	P73296	N-Term(iTRAQ4plex); M8(Oxidation)	0,0000	0,949	1		0,022	0,2979	12	0,070626877	3	1732,91599	1,53	34,96	2
Q55087	Geranylgeranyl diphosphate reductase OS=Synechocystis sp. (strain PCC 6803 / Kazusa) GN=chlP PE	390,87	23,83	1	10	10	20	1,708	17	36,2	407	44,9	8,00							
	A3	Sequence	# PSMs	# Proteins	# Protein Groups	Protein Group Accessions	Modifications	ΔCn	116/117	116/117 Count	116/117 Variability [%]	q-Value	PEP	IonScore	Exp Value	Charge	MH+ [Da]	ΔM [ppm]	RT [min]	# Missed Cleavages
	High	lTFDSYLYk	1	1	1	Q55087	N-Term(iTRAQ4plex); K9(iTRAQ4plex)	0,0000	1,838	1		0	0,000003882	74	3,68104E-08	2	1437,78826	1,01	40,26	0
	High	aGIETYLFER	1	1	1	Q55087	N-Term(iTRAQ4plex)	0,0000	1,708	1		0	0,000001775	57	1,95871E-06	2	1342,71233	-0,03	40,02	0
	High	tVVPANPLVQmk	1	1	1	Q55087	N-Term(iTRAQ4plex); M11(Oxidation); K12(iTRAQ4plex)	0,0000	0,790	1		0	0,000004058	50	8,91189E-06	3	1600,92990	-2,27	30,60	0
	High	tIGSLLR	3	1	1	Q55087	N-Term(iTRAQ4plex)	0,0000	2,162	3	1,1	0	0,0001589	48	1,56304E-05	2	903,57659	2,40	30,99	0
	High	rEVLDGFLR	3	1	1	Q55087	N-Term(iTRAQ4plex)	0,0000	1,505	2	7,2	0	0,000005052	45	3,26565E-05	3	1248,71720	-0,76	32,08	1
	High	kLEGGEIIk	2	1	1	Q55087	N-Term(iTRAQ4plex); K1(iTRAQ4plex); K9(iTRAQ4plex)	0,0000	1,420	2	29,2	0	0,00001012	43	4,92006E-05	3	1418,89112	-2,26	21,16	1
	High	yDHVAVGTGTMk	1	1	1	Q55087	N-Term(iTRAQ4plex); K12(iTRAQ4plex)	0,0000	0,979	1		0	2,221E-08	37	0,000187919	3	1566,81845	-0,27	21,97	0
	High	yDHVAVGTGTmk	2	1	1	Q55087	N-Term(iTRAQ4plex); M11(Oxidation); K12(iTRAQ4plex)	0,0000	1,339	1		0	1,374E-08	37	0,00020463	3	1582,81363	-0,10	18,43	0
	High	ikDLQAGIR	2	1	1	Q55087	N-Term(iTRAQ4plex); K2(iTRAQ4plex)	0,0000	2,133	1		0	0,0004714	33	0,000546978	3	1301,81247	-1,47	22,92	1
	High	vEAHPIPEHPRPR	2	1	1	Q55087	N-Term(iTRAQ4plex)	0,0000	0,778	2	50,7	0	0,00004217	16	0,029642171	5	1678,92591	0,03	14,34	0
	High	iRLPEDk	2	1	1	Q55087	N-Term(iTRAQ4plex); K7(iTRAQ4plex)	0,0000	2,391	2	14,9	0	0,002568	13	0,048191451	3	1158,70896	0,38	22,25	1
P27183	ATP synthase subunit b' OS=Synechocystis sp. (strain PCC 6803 / Kazusa) GN=atpG PE=3 SV=1 - [A	389,67	29,37	1	4	4	13	1,016	11	5,1	143	16,2	5,22							
	A3	Sequence	# PSMs	# Proteins	# Protein Groups	Protein Group Accessions	Modifications	ΔCn	116/117	116/117 Count	116/117 Variability [%]	q-Value	PEP	IonScore	Exp Value	Charge	MH+ [Da]	ΔM [ppm]	RT [min]	# Missed Cleavages
	High	qkETAAQEIEAQR	7	1	1	P27183	N-Term(iTRAQ4plex); K2(iTRAQ4plex)	0,0000	1,013	5	2,8	0	0,000002114	77	2,18867E-08	2	1789,97637	6,51	18,25	1
	High	rQSQAVIADAQAEAR	2	1	1	P27183	N-Term(iTRAQ4plex)	0,0000	0,976	2	7,1	0	8,167E-09	65	3,16206E-07	3	1757,93863	0,61	20,71	1
	High	lLGPELIk	2	1	1	P27183	N-Term(iTRAQ4plex); K8(iTRAQ4plex)	0,0000	1,041	2	3,5	0	0,00002853	56	2,32793E-06	2	1170,76982	-0,20	36,82	0
	High	rLAAEk	2	1	1	P27183	N-Term(iTRAQ4plex); K6(iTRAQ4plex)	0,0000	0,809	2	41,8	0,001	0,01006	16	0,023931505	3	975,61841	-0,57	10,18	1
P73133	Acetylornithine aminotransferase OS=Synechocystis sp. (strain PCC 6803 / Kazusa) GN=argD PE=3 S	384,86	22,14	1	9	9	19	0,606	15	11,8	429	46,5	5,24							
	A3	Sequence	# PSMs	# Proteins	# Protein Groups	Protein Group Accessions	Modifications	ΔCn	116/117	116/117 Count	116/117 Variability [%]	q-Value	PEP	IonScore	Exp Value	Charge	MH+ [Da]	ΔM [ppm]	RT [min]	# Missed Cleavages
	High	gLAGGVPIGAmmck	2	1	1	P73133	N-Term(iTRAQ4plex); M11(Oxidation); M12(Oxidation); C13(Ca	0,0000	0,790	2	11,1	0	3,465E-07	51	8,60934E-06	3	1681,86838	0,30	26,80	0
	High	nQYPTLFTEVR	3	1	1	P73133	N-Term(iTRAQ4plex)	0,0000	0,611	3	12,1	0	0,000004252	49	1,23871E-05	2	1511,79863	0,75	36,93	0
	High	tLATITATGQPk	2	1	1	P73133	N-Term(iTRAQ4plex); K12(iTRAQ4plex)	0,0000	0,301	2	260,1	0	1,954E-07	48	1,73768E-05	2	1489,88432	0,99	25,59	0
	High	aVSDQIQk	2	1	1	P73133	N-Term(iTRAQ4plex); K8(iTRAQ4plex)	0,0000	0,600	1		0	0,000007983	43	5,81007E-05	2	1176,68352	0,71	16,67	0
	High	lHHVSNLYYIPEQGELAk	1	1	1	P73133	N-Term(iTRAQ4plex); K18(iTRAQ4plex)	0,0000	1,063	1		0	0,000004857	42	6,86591E-05	4	2399,29824	0,85	32,69	0
	High	fPIAIAR	6	1	1	P73133	N-Term(iTRAQ4plex)	0,0000	0,594	3	3,0	0	0,0003772	35	0,000294422	2	931,58501	0,44	33,03	0
	High	wIVEHScADR	1	1	1	P73133	N-Term(iTRAQ4plex); C7(Carbamidomethyl)	0,0000	0,656	1		0	0,000006497	28	0,001741687	3	1416,68259	1,05	21,59	0
	High	lLDNVQAR	1	1	1	P73133	N-Term(iTRAQ4plex)	0,0000	0,567	1		0	0,0004316	24	0,003836807	2	1072,62285	-0,29	25,29	0
	Medium	qAIATLV	1	1	1	P73133	N-Term(iTRAQ4plex)	0,0000	0,577	1		0,015	0,1911	16	0,024715534	2	859,53691	-0,06	34,69	0
P73289	50S ribosomal protein L25 OS=Synechocystis sp. (strain PCC 6803 / Kazusa) GN=rplY PE=3 SV=2 - [	375,62	37,76	1	4	4	21	1,052	16	111,8	98	11,1	8,94							
	A3	Sequence	# PSMs	# Proteins	# Protein Groups	Protein Group Accessions	Modifications	ΔCn	116/117	116/117 Count	116/117 Variability [%]	q-Value	PEP	IonScore	Exp Value	Charge	MH+ [Da]	ΔM [ppm]	RT [min]	# Missed Cleavages
	High	aLSIEcQQRPEk	8	1	1	P73289	N-Term(iTRAQ4plex); C6(Carbamidomethyl); K12(iTRAQ4plex)	0,0000	1,594	6	85,8	0	1,378E-07	63	6,20437E-07	2	1746,94170	0,32	20,12	0
	High	nLYHLAFFAGk	3	1	1	P73289	N-Term(iTRAQ4plex); K11(iTRAQ4plex)	0,0000	0,556	3	370,8	0	4,963E-08	61	7,99779E-07	2	1568,88677	2,54	38,49	0
	High	tAITMLR	4	1	1	P73289	N-Term(iTRAQ4plex)	0,0000	2,782	3	64,1	0	0,001629	34	0,000430497	2	949,56517	3,19	26,37	0
	High	eTPIEVk	1	1	1	P73289	N-Term(iTRAQ4plex); K7(iTRAQ4plex)	0,0000	0,995	1		0,001	0,01135	35	0,000400112	2	1103,65849	3,10	20,65	0
	High	tAITmLR	5	1	1	P73289	N-Term(iTRAQ4plex); M5(Oxidation)	0,0000	0,875	3	11,0	0,001	0,01746	14	0,040308133	2	965,55785	0,82	20,26	0
P72777	Ycf54-like protein OS=Synechocystis sp. (strain PCC 6803 / Kazusa) GN=slr1780 PE=3 SV=1 - [YC54	372,77	23,31	1	3	3	17	0,846	11	3,5	133	15,4	4,91							
	A3	Sequence	# PSMs	# Proteins	# Protein Groups	Protein Group Accessions	Modifications	ΔCn	116/117	116/117 Count	116/117 Variability [%]	q-Value	PEP	IonScore	Exp Value	Charge	MH+ [Da]	ΔM [ppm]	RT [min]	# Missed Cleavages
	High	nVAIVSTNk	8	1	1	P72777	N-Term(iTRAQ4plex); K9(iTRAQ4plex)	0,0000	0,836	4	2,8	0	5,561E-07	68	1,85329E-07	2	1233,74101	0,39	21,14	0
	High	fLLEEEPFEEVLkER	1	1	1	P72777	N-Term(iTRAQ4plex); K13(iTRAQ4plex)	0,0000	1,015	1		0	2,716E-08	38	0,000171277	3	2195,18406	0,07	49,14	1
	High	sFIVWVk	8	1	1	P72777	N-Term(iTRAQ4plex); K7(iTRAQ4plex)	0,0000	0,856	6	4,4	0,001	0,005429	44	3,6557E-05	2	1166,71819	0,49	40,23	0
P74450	Slr0151 protein OS=Synechocystis sp. (strain PCC 6803 / Kazusa) GN=slr0151 PE=4 SV=1 - [P74450	369,62	12,81	1	5	5	16	2,408	11	43,8	320	34,9	5,07							
	A3	Sequence	# PSMs	# Proteins	# Protein Groups	Protein Group Accessions	Modifications	ΔCn	116/117	116/117 Count	116/117 Variability [%]	q-Value	PEP	IonScore	Exp Value	Charge	MH+ [Da]	ΔM [ppm]	RT [min]	# Missed Cleavages
	High	hQDLLAVVTELR	8	1	1	P74450	N-Term(iTRAQ4plex)	0,0000	3,195	5	2,8	0	5,838E-09	67	2,25267E-07	3	1537,88211	0,13	42,89	0
	High	qLEEHLLANQR	2	1	1	P74450	N-Term(iTRAQ4plex)	0,0000	0,981	2	120,0	0	0,000002344	47	1,94089E-05	3	1494,81406	-0,33	23,46	0
	High	sLALQGk	3	1	1	P74450	N-Term(iTRAQ4plex); K7(iTRAQ4plex)	0,0000	1,587	2	32,7	0	0,000405	40	8,91189E-05	2	1004,63353	-0,74	19,53	0
	High	kQLEEHLLANQR	2	1	1	P74450	N-Term(iTRAQ4plex); K1(iTRAQ4plex)	0,0000	2,776	1		0	3,029E-07	36	0,000256431	4	1767,01235	0,43	20,68	1
	High	gDLAGAFHHR	1	1	1	P74450	N-Term(iTRAQ4plex)	0,0000	1,745	1		0	0,000004125	30	0,001059181	3	1224,63572	0,20	16,77	0
P74002	Uncharacterized protein slr1322 OS=Synechocystis sp. (strain PCC 6803 / Kazusa) GN=slr1322 PE=3	368,53	23,72	1	11	11	18	0,605	17	20,9	489	53,1	5,64							
	A3	Sequence	# PSMs	# Proteins	# Protein Groups	Protein Group Accessions	Modifications	ΔCn	116/117	116/117 Count	116/117 Variability [%]	q-Value	PEP	IonScore	Exp Value	Charge	MH+ [Da]	ΔM [ppm]	RT [min]	# Missed Cleavages
	High	eSLSTLLGLGR	5	1	1	P74002	N-Term(iTRAQ4plex)	0,0000	0,544	4	6,9	0	0,000005719	59	1,67623E-06	2	1289,75640	1,40	45,59	0
	High	lTQSVGFSLFcADGEHR	1	1	1	P74002	N-Term(iTRAQ4plex); C11(Carbamidomethyl)	0,0000	0,704	1		0	6,446E-09	55	3,09008E-06	3	2068,00595	0,98	41,38	0
	High	qSYAYAAASR	2	1	1	P74002	N-Term(iTRAQ4plex)	0,0000	0,497	2	13,9	0	0,00001363	54	4,17802E-06	2	1231,62004	1,02	17,49	0
	High	gkGDcYVSTNDISFNGLR	1	1	1	P74002	N-Term(iTRAQ4plex); K2(iTRAQ4plex); C5(Carbamidomethyl)	0,0000	2,622	1		0	6,545E-08	40	9,20386E-05	3	2291,13901	2,66	33,57	1
	High	gATLIGTATEIMNk	1	1	1	P74002	N-Term(iTRAQ4plex); K14(iTRAQ4plex)	0,0000	0,605	1		0	4,552E-07	32	0,00059837	3	1707,95853	1,83	44,18	0
	High	lDHYASHAQSR	2	1	1	P74002	N-Term(iTRAQ4plex)	0,0000	0,728	2	18,2	0	0,0001072	30	0,001096402	3	1428,71088	0,57	12,06	0
	High	aAGADFVEFFLEk	1	1	1	P74002	N-Term(iTRAQ4plex); K13(iTRAQ4plex)	0,0000	0,665	1		0	0,0003886	29	0,001547739	3	1731,92704	4,25	54,14	0
	High	rQSYAYAAASR	1	1	1	P74002	N-Term(iTRAQ4plex)	0,0000	1,032	1		0	0,0008475	27	0,001887861	3	1387,72254	1,90	13,20	1
	High	ekDQWLHR	2	1	1	P74002	N-Term(iTRAQ4plex); K2(iTRAQ4plex)	0,0000	0,653	2	21,7	0	0,0004927	17	0,021036331	3	1399,77143	2,10	16,39	1
	High	tTPFLDkk	1	1	1	P74002	N-Term(iTRAQ4plex); K7(iTRAQ4plex); K8(iTRAQ4plex)	0,0000	0,599	1		0,001	0,003825	17	0,020276827	3	1381,84339	1,34	20,64	1
	High	aAYFR	1	1	1	P74002	N-Term(iTRAQ4plex)	0,0000	0,521	1		0,006	0,07153	13	0,048191451	2	771,42742	0,53	17,81	0
P74266	50S ribosomal protein L21 OS=Synechocystis sp. (strain PCC 6803 / Kazusa) GN=rplU PE=3 SV=1 - [	368,35	65,32	1	6	6	11	1,049	8	20,6	124	13,7	9,52							
	A3	Sequence	# PSMs	# Proteins	# Protein Groups	Protein Group Accessions	Modifications	ΔCn	116/117	116/117 Count	116/117 Variability [%]	q-Value	PEP	IonScore	Exp Value	Charge	MH+ [Da]	ΔM [ppm]	RT [min]	# Missed Cleavages
	High	dGDNVTIGQPYVAGATVEGEILS	1	1	1	P74266	N-Term(iTRAQ4plex)	0,0000	1,167	1		0	3,561E-11	81	8,74923E-09	3	2742,38651	1,78	43,92	0
	High	sYAIIEIGGTQIR	3	1	1	P74266	N-Term(iTRAQ4plex)	0,0000				0	8,952E-11	75	3,08298E-08	2	1564,88481	2,07	39,01	0
	High	sISVNGTAIAEALEVDAk	2	1	1	P74266	N-Term(iTRAQ4plex); K18(iTRAQ4plex)	0,0000	0,884	2	114,0	0	9,228E-08	62	6,23692E-07	3	2076,14682	1,96	46,24	0
	High	fYEINHLDAAPEDSYVVDk	1	1	1	P74266	N-Term(iTRAQ4plex); K19(iTRAQ4plex)	0,0000	0,927	1		0	4,8E-10	37	0,000184064	4	2513,25014	2,48	38,45	0
	High	kVIVYk	2	1	1	P74266	N-Term(iTRAQ4plex); K1(iTRAQ4plex); K6(iTRAQ4plex)	0,0000	1,179	2	5,1	0	0,001869	25	0,002957808	3	1181,80286	3,94	17,47	1
	High	vIVYk	2	1	1	P74266	N-Term(iTRAQ4plex); K5(iTRAQ4plex)	0,0000	0,864	2	16,8	0,002	0,02459	27	0,002051021	2	909,60747	6,93	21,33	0
P73600	Sll1785 protein OS=Synechocystis sp. (strain PCC 6803 / Kazusa) GN=sll1785 PE=1 SV=1 - [P73600	367,44	19,40	1	5	5	19	0,746	18	21,9	268	30,0	5,30							
	A3	Sequence	# PSMs	# Proteins	# Protein Groups	Protein Group Accessions	Modifications	ΔCn	116/117	116/117 Count	116/117 Variability [%]	q-Value	PEP	IonScore	Exp Value	Charge	MH+ [Da]	ΔM [ppm]	RT [min]	# Missed Cleavages
	High	lTDLNNLPELTNAQR	1	1	1	P73600	N-Term(iTRAQ4plex)	0,0000	1,251	1		0	2,2E-11	64	3,86367E-07	3	1856,00022	0,40	39,83	0
	High	tLPIVFVWMR	4	1	1	P73600	N-Term(iTRAQ4plex)	0,0000	0,874	4	46,6	0	0,00001345	54	4,32514E-06	2	1405,81536	0,47	55,56	0
	High	aAFASEAPk	1	1	1	P73600	N-Term(iTRAQ4plex); K9(iTRAQ4plex)	0,0000	0,685	1		0	0,000332	44	3,89915E-05	2	1179,66045	-0,67	18,72	0
	High	eYFQAVGPR	2	1	1	P73600	N-Term(iTRAQ4plex)	0,0000	0,798	2	12,3	0	0,00001316	44	4,30497E-05	2	1210,63432	0,50	26,89	0
	High	tLPIVFVWmR	9	1	1	P73600	N-Term(iTRAQ4plex); M9(Oxidation)	0,0000	0,730	9	16,2	0	0,0001673	35	0,000329587	2	1421,81028	0,47	50,23	0
	High	lkNDkDLER	1	1	1	P73600	N-Term(iTRAQ4plex); K2(iTRAQ4plex); K5(iTRAQ4plex)	0,0000	0,860	1		0	0,000784	16	0,027667505	3	1562,92398	0,85	13,07	2
P73458	Carboxyl-terminal protease OS=Synechocystis sp. (strain PCC 6803 / Kazusa) GN=prc PE=3 SV=1 - [	363,47	19,39	1	10	10	20	0,728	16	19,6	423	46,8	5,21							
	A3	Sequence	# PSMs	# Proteins	# Protein Groups	Protein Group Accessions	Modifications	ΔCn	116/117	116/117 Count	116/117 Variability [%]	q-Value	PEP	IonScore	Exp Value	Charge	MH+ [Da]	ΔM [ppm]	RT [min]	# Missed Cleavages
	High	gkPGTSVVLTIER	4	1	1	P73458	N-Term(iTRAQ4plex); K2(iTRAQ4plex)	0,0000	0,686	2	4,4	0	5,428E-09	63	4,78597E-07	3	1644,98948	0,45	26,80	1
	High	iVVVAPIEDTPAYNAGILSk	1	1	1	P73458	N-Term(iTRAQ4plex); K20(iTRAQ4plex)	0,0000	0,506	1		0	3,339E-13	59	1,18842E-06	3	2358,35447	0,89	46,79	0
	High	eNVVGYIFDLR	1	1	1	P73458	N-Term(iTRAQ4plex)	0,0000	0,568	1		0	0,000007521	45	3,51536E-05	2	1468,79216	0,32	50,42	0
	High	rGEVEQQSANk	2	1	1	P73458	N-Term(iTRAQ4plex); K11(iTRAQ4plex)	0,0000	0,662	2	11,7	0	0,000000343	41	8,85054E-05	3	1533,82074	-1,07	8,22	1
	High	tLIEIHPVR	4	1	1	P73458	N-Term(iTRAQ4plex)	0,0000	0,735	2	3,1	0	0,00001202	36	0,000235489	3	1221,74430	0,56	27,17	0
	High	rGEVEQQSANkR	2	1	1	P73458	N-Term(iTRAQ4plex); K11(iTRAQ4plex)	0,0000	0,887	2	1,3	0	8,826E-07	29	0,001121941	3	1689,92612	1,55	7,92	2
	High	gEVEQQSANkR	2	1	1	P73458	N-Term(iTRAQ4plex); K10(iTRAQ4plex)	0,0000	0,668	2	14,4	0	0,0001167	27	0,00207825	3	1533,82459	1,44	8,29	1
	High	lEkENVVGYIFDLR	1	1	1	P73458	N-Term(iTRAQ4plex); K3(iTRAQ4plex)	0,0000	1,121	1		0	0,00002433	24	0,004365158	3	1983,11698	0,80	48,56	1
	High	kELQQNR	1	1	1	P73458	N-Term(iTRAQ4plex); K1(iTRAQ4plex)	0,0000	0,954	1		0	0,001114	15	0,03889944	3	1203,70475	-0,06	9,26	1
	High	gLVQSVR	2	1	1	P73458	N-Term(iTRAQ4plex)	0,0000	0,763	2	2,6	0,001	0,003972	28	0,001489258	2	902,55370	-0,35	19,21	0
P36236	50S ribosomal protein L1 OS=Synechocystis sp. (strain PCC 6803 / Kazusa) GN=rplA PE=3 SV=1 - [R	363,26	36,55	1	10	10	30	1,477	26	59,3	238	25,8	8,73							
	A3	Sequence	# PSMs	# Proteins	# Protein Groups	Protein Group Accessions	Modifications	ΔCn	116/117	116/117 Count	116/117 Variability [%]	q-Value	PEP	IonScore	Exp Value	Charge	MH+ [Da]	ΔM [ppm]	RT [min]	# Missed Cleavages
	High	gGTVTADLAAAVNEFk	2	1	1	P36236	N-Term(iTRAQ4plex); K16(iTRAQ4plex)	0,0000	1,365	2	59,0	0	0,0001929	42	6,50085E-05	3	1852,00284	-1,45	48,64	0
	High	tGIVHVmFGk	5	1	1	P36236	N-Term(iTRAQ4plex); M7(Oxidation); K10(iTRAQ4plex)	0,0000	1,823	3	48,1	0	0,000117	39	0,000131211	3	1392,79181	0,43	26,75	0
	High	lYSPLEAMELLk	7	1	1	P36236	N-Term(iTRAQ4plex); K12(iTRAQ4plex)	0,0000	1,046	7	133,5	0	0,00000339	38	0,000148583	2	1694,96282	-0,79	53,85	0
	High	aSFSADDLLANLk	1	1	1	P36236	N-Term(iTRAQ4plex); K13(iTRAQ4plex)	0,0000	3,126	1		0	0,00006242	32	0,00059975	3	1652,91153	1,04	46,50	0
	High	tGIVHVMFGk	2	1	1	P36236	N-Term(iTRAQ4plex); K10(iTRAQ4plex)	0,0000	2,234	2	33,5	0	0,0002035	23	0,004518247	3	1376,79709	0,58	32,06	0

	A3	Sequence	# PSMs	# Proteins	# Protein Groups	Protein Group Accessions	Modifications	ΔCn	116/117	116/117 Count	116/117 Variability [%]	q-Value	PEP	IonScore	Exp Value	Charge	MH+ [Da]	ΔM [ppm]	RT [min]	# Missed Cleavages
	High	IYSPLEAmELLk	2	1	1	P36236	N-Term(iTRAQ4plex); M8(Oxidation); K12(iTRAQ4plex)	0,0000	1,299	2	18,8	0	0,000006705	19	0,011748164	3	1710,96128	1,29	43,11	0
	High	rMQAAIAk	1	1	1	P36236	N-Term(iTRAQ4plex); K8(iTRAQ4plex)	0,0000	1,317	1		0	0,001164	15	0,033882075	3	1176,71170	-0,73	14,11	1
	High	mQAAIAk	1	1	1	P36236	N-Term(iTRAQ4plex); M1(Oxidation); K7(iTRAQ4plex)	0,0000	1,562	1		0,001	0,01191	39	0,000138357	2	1036,60466	-1,64	13,00	0
	High	aLQETIDR	1	1	1	P36236	N-Term(iTRAQ4plex)	0,0000	1,239	1		0,001	0,003499	32	0,000642643	2	1089,60271	0,56	19,93	0
	High	vAVLAR	5	1	1	P36236	N-Term(iTRAQ4plex)	0,0000	2,194	3	20,5	0,001	0,007064	32	0,000653085	2	772,51610	-0,10	19,62	0
	High	tTVSLPk	2	1	1	P36236	N-Term(iTRAQ4plex); K7(iTRAQ4plex)	0,0000	0,655	2	53,3	0,003	0,03265	39	0,000112712	2	1033,64763	-1,89	21,50	0
	Medium	nRPSGAk	1	1	1	P36236	N-Term(iTRAQ4plex); K7(iTRAQ4plex)	0,0000	1,513	1		0,048	0,6258	12	0,062946271	3	1017,60230	-2,04	7,52	0
Q55557	Virginiamycin B hydrolase Vgb OS=Synechocystis sp. (strain PCC 6803 / Kazusa) GN=sll0173 PE=4 S	360,70	25,57	1	8	8	16	0,878	16	16,7	352	38,5	6,80							
	A3	Sequence	# PSMs	# Proteins	# Protein Groups	Protein Group Accessions	Modifications	ΔCn	116/117	116/117 Count	116/117 Variability [%]	q-Value	PEP	IonScore	Exp Value	Charge	MH+ [Da]	ΔM [ppm]	RT [min]	# Missed Cleavages
	High	vTIYPTVPGIGTR	4	1	1	Q55557	N-Term(iTRAQ4plex)	0,0000	0,846	4	16,4	0	0,000002545	53	4,52866E-06	2	1517,88298	1,41	37,93	0
	High	iFPAPQGFGPYGMTATPk	1	1	1	Q55557	N-Term(iTRAQ4plex); K18(iTRAQ4plex)	0,0000	1,591	1		0	1,856E-08	51	7,76194E-06	3	2168,15110	2,66	43,58	0
	High	iFPAPQGFGPYGmTATPk	2	1	1	Q55557	N-Term(iTRAQ4plex); M13(Oxidation); K18(iTRAQ4plex)	0,0000	0,862	2	12,6	0	2,87E-08	50	1,00918E-05	3	2184,14389	1,67	39,90	0
	High	ykVTIYPTVPGIGTR	2	1	1	Q55557	N-Term(iTRAQ4plex); K2(iTRAQ4plex)	0,0000	1,123	2	7,8	0	2,206E-07	42	6,4859E-05	3	1953,14408	1,47	36,59	1
	High	iWAGQQGVDQIVLFER	1	1	1	Q55557	N-Term(iTRAQ4plex)	0,0000	0,866	1		0	0,000007533	39	0,000183387	3	2003,08646	1,65	48,39	0
	High	gEVWYTSLAGNYIAR	1	1	1	Q55557	N-Term(iTRAQ4plex)	0,0000	0,973	1		0	6,786E-07	38	0,00014756	3	1843,94913	1,72	43,85	0
	High	tYLPEVFPIPDDk	1	1	1	Q55557	N-Term(iTRAQ4plex); K13(iTRAQ4plex)	0,0000	0,704	1		0	0,00001855	26	0,002703772	3	1821,98874	0,56	45,58	0
	High	fNPQNk	2	1	1	Q55557	N-Term(iTRAQ4plex); K6(iTRAQ4plex)	0,0000	0,547	2	23,5	0,003	0,03503	24	0,004344802	2	1035,58452	1,89	15,01	0
	Medium	lPGLGPR	2	1	1	Q55557	N-Term(iTRAQ4plex)	0,0000	0,864	2	4,3	0,016	0,2079	17	0,021575954	2	853,53807	0,50	25,49	0
P32422	Photosystem I iron-sulfur center OS=Synechocystis sp. (strain PCC 6803 / Kazusa) GN=psaC PE=1 S	360,25	65,43	1	5	5	13	0,443	11	7,6	81	8,8	5,95							
	A3	Sequence	# PSMs	# Proteins	# Protein Groups	Protein Group Accessions	Modifications	ΔCn	116/117	116/117 Count	116/117 Variability [%]	q-Value	PEP	IonScore	Exp Value	Charge	MH+ [Da]	ΔM [ppm]	RT [min]	# Missed Cleavages
	High	iYDTcIGcTQcVR	1	1	1	P32422	N-Term(iTRAQ4plex); C5(Carbamidomethyl); C8(Carbamidome	0,0000	0,507	1		0	7,563E-09	73	4,5811E-08	2	1789,81633	0,62	27,92	0
	High	aAQIASSPR	5	1	1	P32422	N-Term(iTRAQ4plex)	0,0000	0,448	4	3,4	0	0,0009554	47	2,01359E-05	2	1044,59148	-0,37	15,74	0
	High	vYLGAETTR	3	1	1	P32422	N-Term(iTRAQ4plex)	0,0000	0,402	3	1,5	0	0,0003027	46	2,63615E-05	2	1153,63408	0,60	22,30	0
	High	rcETAcPTDFLSIR	3	1	1	P32422	N-Term(iTRAQ4plex); C2(Carbamidomethyl); C6(Carbamidome	0,0000	0,442	2	0,5	0	9,3E-09	45	3,28073E-05	3	1869,90836	0,81	34,18	1
	High	tEDcVGck	1	1	1	P32422	N-Term(iTRAQ4plex); C4(Carbamidomethyl); C7(Carbamidome	0,0000	0,412	1		0	0,00003336	39	0,000125884	2	1256,58562	0,20	12,21	0
P73902	Sll0236 protein OS=Synechocystis sp. (strain PCC 6803 / Kazusa) GN=sll0236 PE=4 SV=1 - [P73902	358,84	17,92	1	11	11	23	0,489	20	30,9	558	63,9	5,25							
	A3	Sequence	# PSMs	# Proteins	# Protein Groups	Protein Group Accessions	Modifications	ΔCn	116/117	116/117 Count	116/117 Variability [%]	q-Value	PEP	IonScore	Exp Value	Charge	MH+ [Da]	ΔM [ppm]	RT [min]	# Missed Cleavages
	High	vEIGGWSFk	3	1	1	P73902	N-Term(iTRAQ4plex); K9(iTRAQ4plex)	0,0000	0,560	3	30,1	0	3,613E-07	57	1,95884E-06	2	1310,73613	1,06	40,46	0
	High	kTDSVTLITGk	1	1	1	P73902	N-Term(iTRAQ4plex); K1(iTRAQ4plex); K11(iTRAQ4plex)	0,0000	0,415	1		0	0,00002474	48	1,4756E-05	3	1594,97641	1,50	23,33	1
	High	fAITHALmAPR	1	1	1	P73902	N-Term(iTRAQ4plex); M8(Oxidation)	0,0000	1,364	1		0	0,0004153	38	0,000170596	3	1387,76548	1,27	29,95	0
	High	aEAEFWQQHR	3	1	1	P73902	N-Term(iTRAQ4plex)	0,0000	0,565	3	28,1	0	0,000007616	37	0,000182797	3	1445,70426	-0,01	24,02	0
	High	iHQELPHLEGR	2	1	1	P73902	N-Term(iTRAQ4plex)	0,0000	0,458	2	16,0	0	0,000006132	35	0,000335714	4	1472,80922	0,10	22,46	0
	High	fAITHALMAPR	2	1	1	P73902	N-Term(iTRAQ4plex)	0,0000	0,513	1		0	0,0008681	34	0,000377546	3	1371,77039	1,16	32,95	0
	High	yLTTPYLLR	3	1	1	P73902	N-Term(iTRAQ4plex)	0,0000	0,321	3	0,2	0	0,000325	31	0,000765544	2	1283,74809	0,07	41,05	0
	High	sSVRPYPYPEER	2	1	1	P73902	N-Term(iTRAQ4plex)	0,0000	0,323	1		0	0,00005241	24	0,003732244	3	1623,82614	0,84	19,53	0
	High	aVRPIQVTLk	1	1	1	P73902	N-Term(iTRAQ4plex); K10(iTRAQ4plex)	0,0000	0,316	1		0	0,000213	23	0,004742092	3	1412,92151	1,65	25,70	0
	High	hLIWVIR	2	1	1	P73902	N-Term(iTRAQ4plex)	0,0000	0,336	1		0	0,0009995	22	0,006222573	3	1080,68103	1,06	33,57	0
	High	kAVRPIQVTLk	2	1	1	P73902	N-Term(iTRAQ4plex); K1(iTRAQ4plex); K11(iTRAQ4plex)	0,0000	0,536	2	21,4	0,001	0,009102	19	0,012358621	4	1685,11904	1,67	22,73	1
	High	lTITAQAER	1	1	1	P73902	N-Term(iTRAQ4plex)	0,0000	0,866	1		0,002	0,02202	20	0,009035871	2	1146,66106	0,97	22,88	0
P21697	Plastocyanin OS=Synechocystis sp. (strain PCC 6803 / Kazusa) GN=petE PE=1 SV=1 - [PLAS_SYNY3	357,68	39,68	1	4	4	14	0,610	10	17,8	126	13,1	6,77							
	A3	Sequence	# PSMs	# Proteins	# Protein Groups	Protein Group Accessions	Modifications	ΔCn	116/117	116/117 Count	116/117 Variability [%]	q-Value	PEP	IonScore	Exp Value	Charge	MH+ [Da]	ΔM [ppm]	RT [min]	# Missed Cleavages
	High	lSPHNIVFAADGVDADTAAk	5	1	1	P21697	N-Term(iTRAQ4plex); K20(iTRAQ4plex)	0,0000	0,588	3	15,7	0	3,603E-15	127	2,08915E-13	3	2300,21646	1,69	36,02	0
	High	mGSDSGALVFEPSTVTIk	2	1	1	P21697	N-Term(iTRAQ4plex); M1(Oxidation); K18(iTRAQ4plex)	0,0000	0,569	1		0	1,249E-07	53	6,0699E-06	3	2143,12186	1,06	38,58	0
	High	gAGMVGk	2	1	1	P21697	N-Term(iTRAQ4plex); K7(iTRAQ4plex)	0,0000	0,996	2	15,7	0,002	0,02436	32	0,00067293	2	907,52947	2,29	14,68	0
	High	gAGmVGk	2	1	1	P21697	N-Term(iTRAQ4plex); M4(Oxidation); K7(iTRAQ4plex)	0,0000	0,561	3	2,1	0,003	0,04215	32	0,000665273	2	923,52135	-1,03	13,33	0
	Medium	wVNNk	2	1	1	P21697	N-Term(iTRAQ4plex); K5(iTRAQ4plex)	0,0000	0,736	1		0,02	0,2702	18	0,014620762	2	948,54997	-0,59	17,15	0
Q55013	Cytochrome c-550 OS=Synechocystis sp. (strain PCC 6803 / Kazusa) GN=psbV PE=1 SV=2 - [CY550_	355,92	34,38	1	4	4	8	0,591	8	51,1	160	17,9	4,93							
	A3	Sequence	# PSMs	# Proteins	# Protein Groups	Protein Group Accessions	Modifications	ΔCn	116/117	116/117 Count	116/117 Variability [%]	q-Value	PEP	IonScore	Exp Value	Charge	MH+ [Da]	ΔM [ppm]	RT [min]	# Missed Cleavages
	High	tNNNVSLGLADLAGAEPR	2	1	1	Q55013	N-Term(iTRAQ4plex)	0,0000	0,786	2	126,7	0	4,186E-16	117	2,0463E-12	2	1956,02861	0,94	39,74	0
	High	tIPLDEAGGTTTLTAR	2	1	1	Q55013	N-Term(iTRAQ4plex)	0,0000	0,617	2	45,0	0	3,083E-09	93	4,61286E-10	2	1760,95329	1,23	32,29	0
	High	iFVDTcTQcHLQGk	2	1	1	Q55013	N-Term(iTRAQ4plex); C6(Carbamidomethyl); C9(Carbamidome	0,0000	0,571	2	43,6	0	1,639E-10	53	4,9885E-06	3	1995,00400	0,46	27,61	0
	Medium	wGGTIYF	2	1	1	Q55013	N-Term(iTRAQ4plex)	0,0000	0,549	2	62,4	0,029	0,3854	13	0,051757108	2	987,50585	0,19	46,75	0
P73546	Phosphorylase OS=Synechocystis sp. (strain PCC 6803 / Kazusa) GN=glgP PE=3 SV=1 - [P73546_SY	354,58	9,71	1	8	8	19	4,439	16	52,8	855	98,2	6,14							
	A3	Sequence	# PSMs	# Proteins	# Protein Groups	Protein Group Accessions	Modifications	ΔCn	116/117	116/117 Count	116/117 Variability [%]	q-Value	PEP	IonScore	Exp Value	Charge	MH+ [Da]	ΔM [ppm]	RT [min]	# Missed Cleavages
	High	hLGNNLINLGLYDk	2	1	1	P73546	N-Term(iTRAQ4plex); K14(iTRAQ4plex)	0,0000	5,012	1		0	1,746E-11	59	1,25305E-06	3	1872,05985	0,88	38,29	0
	High	nLAAYLLk	6	1	1	P73546	N-Term(iTRAQ4plex); K8(iTRAQ4plex)	0,0000	5,175	5	11,8	0	0,000006932	56	2,80524E-06	2	1193,74968	0,04	40,34	0
	High	hLEIIYEINHR	3	1	1	P73546	N-Term(iTRAQ4plex)	0,0000	3,069	2	80,2	0	0,000001343	46	2,61198E-05	3	1580,86521	-0,86	29,59	0
	High	qNPHAEIVPR	1	1	1	P73546	N-Term(iTRAQ4plex)	0,0000	2,834	1		0	0,000003076	37	0,00018534	3	1304,71845	-0,57	14,83	0
	High	lGGHTEIIHNEk	2	1	1	P73546	N-Term(iTRAQ4plex); K12(iTRAQ4plex)	0,0000	2,664	2	56,0	0	4,053E-07	27	0,002779137	3	1635,90616	0,27	17,13	0
	High	rWILLSNPELSALVTEk	2	1	1	P73546	N-Term(iTRAQ4plex); K17(iTRAQ4plex)	0,0000	3,310	2	35,5	0	0,0003237	26	0,002716252	3	2257,32071	2,13	49,49	1
	High	ikQNPHAEIVPR	1	1	1	P73546	N-Term(iTRAQ4plex); K2(iTRAQ4plex)	0,0000	2,735	1		0	0,0004184	16	0,025466544	4	1689,99965	-0,38	17,73	1
	High	yEFGIFHQR	2	1	1	P73546	N-Term(iTRAQ4plex)	0,0000	6,096	2	317,0	0,001	0,01282	18	0,015134567	3	1340,68659	-0,17	32,78	0
P05429	Photosystem II CP47 chlorophyll apoprotein OS=Synechocystis sp. (strain PCC 6803 / Kazusa) GN=ps	348,86	16,37	1	9	9	23	1,069	21	36,3	507	55,9	5,85							
	A3	Sequence	# PSMs	# Proteins	# Protein Groups	Protein Group Accessions	Modifications	ΔCn	116/117	116/117 Count	116/117 Variability [%]	q-Value	PEP	IonScore	Exp Value	Charge	MH+ [Da]	ΔM [ppm]	RT [min]	# Missed Cleavages
	High	tGAmNSGDGIAQEWIGHPIFk	3	1	1	P05429	N-Term(iTRAQ4plex); M4(Oxidation); K21(iTRAQ4plex)	0,0000	1,009	2	8,6	0	1,693E-11	79	1,15603E-08	3	2533,27658	0,68	41,91	0
	High	lAFYDYVGNSPAk	1	1	1	P05429	N-Term(iTRAQ4plex); K13(iTRAQ4plex)	0,0000	0,736	1		0	0,000000229	56	2,7477E-06	3	1732,91489	0,01	38,40	0
	High	gYFQEEIQR	1	1	1	P05429	N-Term(iTRAQ4plex)	0,0000	0,518	1		0	0,0001729	49	1,14543E-05	2	1313,65911	-1,19	27,20	0
	High	vHTVVLNDPGR	3	1	1	P05429	N-Term(iTRAQ4plex)	0,0000	1,078	3	9,7	0	0,00006133	41	7,9794E-05	3	1350,76059	-0,35	19,95	0
	High	qGMFVLPFMAR	2	1	1	P05429	N-Term(iTRAQ4plex)	0,0000	2,499	2	105,9	0	0,0001192	36	0,000243204	2	1440,76348	1,51	50,62	0
	High	qGmFVLPFmAR	3	1	1	P05429	N-Term(iTRAQ4plex); M3(Oxidation); M9(Oxidation)	0,0000	0,780	3	3,9	0	0,0001768	18	0,016556556	2	1472,75127	0,10	39,67	0
	High	qGmFVLPFMAR	3	1	1	P05429	N-Term(iTRAQ4plex); M3(Oxidation)	0,0000	1,295	3	31,4	0,001	0,003216	24	0,003689521	2	1456,75640	0,13	45,76	0
	High	aDIPFRR	3	1	1	P05429	N-Term(iTRAQ4plex)	0,0000	1,163	3	2,1	0,001	0,006791	14	0,041972999	3	1018,59143	-0,04	20,44	1
	High	aDIPFR	2	1	1	P05429	N-Term(iTRAQ4plex)	0,0000	0,732	1		0,003	0,04066	13	0,0468781	2	862,48967	-0,79	24,71	0
	High	gLPWYR	1	1	1	P05429	N-Term(iTRAQ4plex)	0,0000	1,538	1		0,006	0,07692	22	0,006240068	2	935,52141	-0,60	31,46	0
	Medium	gGLFR	1	1	1	P05429	N-Term(iTRAQ4plex)	0,0000	1,355	1		0,04	0,5131	17	0,028450589	2	693,41612	-0,48	22,94	0
P48957	50S ribosomal protein L20 OS=Synechocystis sp. (strain PCC 6803 / Kazusa) GN=rplT PE=3 SV=1 - [	343,17	57,26	1	11	11	29	1,595	26	21,0	117	13,5	12,02							
	A3	Sequence	# PSMs	# Proteins	# Protein Groups	Protein Group Accessions	Modifications	ΔCn	116/117	116/117 Count	116/117 Variability [%]	q-Value	PEP	IonScore	Exp Value	Charge	MH+ [Da]	ΔM [ppm]	RT [min]	# Missed Cleavages
	High	mLAQLAVLDPAAFSEVVk	1	1	1	P48957	N-Term(iTRAQ4plex); M1(Oxidation); K18(iTRAQ4plex)	0,0000	1,908	1		0	1,216E-08	70	1,28332E-07	3	2206,24344	1,72	52,04	0
	High	tANQQVMk	3	1	1	P48957	N-Term(iTRAQ4plex); K8(iTRAQ4plex)	0,0000	1,522	2	4,2	0	0,00006472	46	2,78593E-05	2	1207,67705	5,22	12,81	0
	High	kmLAQLAVLDPAAFSEVVk	2	1	1	P48957	N-Term(iTRAQ4plex); K1(iTRAQ4plex); M2(Oxidation); K19(iTF	0,0000	1,847	2	19,8	0	5,576E-07	43	5,52039E-05	4	2478,44765	4,43	46,98	1
	High	tANQQVmk	7	1	1	P48957	N-Term(iTRAQ4plex); M7(Oxidation); K8(iTRAQ4plex)	0,0000	1,616	6	21,4	0	0,00002496	35	0,000356377	2	1223,66716	1,23	12,08	0
	High	kANIEINR	2	1	1	P48957	N-Term(iTRAQ4plex); K1(iTRAQ4plex)	0,0000	1,853	2	4,2	0	0,0002593	29	0,001276351	3	1245,75373	1,56	15,34	1
	High	qEGmSYSk	3	1	1	P48957	N-Term(iTRAQ4plex); M4(Oxidation); K8(iTRAQ4plex)	0,0000	1,246	3	5,3	0,001	0,01652	17	0,020463033	2	1233,60820	4,72	12,49	0
	High	vAATAk	2	1	1	P48957	N-Term(iTRAQ4plex); K6(iTRAQ4plex)	0,0000	1,403	1		0,002	0,0226	18	0,015630397	2	848,54314	-1,48	12,78	0
	High	kANIEINRk	2	1	1	P48957	N-Term(iTRAQ4plex); K1(iTRAQ4plex); K9(iTRAQ4plex)	0,0000	1,459	2	35,3	0,002	0,02446	13	0,046662715	4	1517,95852	6,39	14,01	2
	High	rLWITR	1	1	1	P48957	N-Term(iTRAQ4plex)	0,0000	1,713	1		0,005	0,06538	31	0,000827885	3	988,61771	0,43	22,66	1
	Medium	iNAAAR	4	1	1	P48957	N-Term(iTRAQ4plex)	0,0000	1,551	4	8,2	0,022	0,3037	21	0,00814648	2	759,46105	2,19	14,00	0
	Medium	lWITR	1	1	1	P48957	N-Term(iTRAQ4plex)	0,0000	1,714	1		0,024	0,3304	20	0,009505391	2	832,51769	1,83	29,26	0
	Medium	lTGQLkk	1	1	1	P48957	N-Term(iTRAQ4plex); K6(iTRAQ4plex); K7(iTRAQ4plex)	0,0000	2,808	1		0,045	0,5795	15	0,033187154	3	1219,81977	8,14	17,12	1
P73510	Sll1358 protein OS=Synechocystis sp. (strain PCC 6803 / Kazusa) GN=sll1358 PE=1 SV=1 - [P73510_	342,75	29,19	1	8	8	15	0,583	13	55,6	394	43,1	6,23							
	A3	Sequence	# PSMs	# Proteins	# Protein Groups	Protein Group Accessions	Modifications	ΔCn	116/117	116/117 Count	116/117 Variability [%]	q-Value	PEP	IonScore	Exp Value	Charge	MH+ [Da]	ΔM [ppm]	RT [min]	# Missed Cleavages
	High	kQVYISSYGPASGPLASATPQGQT	2	1	1	P73510	N-Term(iTRAQ4plex); K1(iTRAQ4plex); K26(iTRAQ4plex)	0,0000	0,481	1		0	1,811E-10	83	4,61286E-09	4	3039,65053	0,97	31,61	1
	High	qVGTYNFPVSk	4	1	1	P73510	N-Term(iTRAQ4plex); K11(iTRAQ4plex)	0,0000	1,083	3	9,8	0	3,339E-09	78	2,02276E-08	2	1527,84758	4,32	30,06	0
	High	gWGHSIEGIGPDTAk	2	1	1	P73510	N-Term(iTRAQ4plex); K15(iTRAQ4plex)	0,0000	0,489	2	26,4	0	1,332E-09	54	3,67257E-06	3	1812,94992	0,88	29,20	0
	High	iEVPHTHNLLGQQPLVSLGGNELR	2	1	1	P73510	N-Term(iTRAQ4plex)	0,0000	0,511	2	36,7	0	2,935E-09	36	0,000268534	4	2765,51919	0,43	41,73	0
	High	gGLWYFPR	2	1	1	P73510	N-Term(iTRAQ4plex)	0,0000	0,445	2	3,1	0	0,001806	32	0,000636752	2	1139,61223	0,32	40,44	0
	High	kLPVQDTIFSLPTQP	1	1	1	P73510	N-Term(iTRAQ4plex); K1(iTRAQ4plex)	0,0000	0,848	1		0	0,0001285	27	0,001901907	3	1972,13742	0,82	46,11	1
	High	sLSNVVWGk	1	1	1	P73510	N-Term(iTRAQ4plex); K9(iTRAQ4plex)	0,0000	8,306	1		0,001	0,007535	18	0,014420158	3	1277,74619	0,44	32,13	0
	Medium	gYGHAIR	1	1	1	P73510	N-Term(iTRAQ4plex)	0,0000	0,976	1		0,018	0,2388	11	0,075368401	3	917,50879	1,54	12,01	0
Q55176	Slr0483 protein OS=Synechocystis sp. (strain PCC 6803 / Kazusa) GN=slr0483 PE=4 SV=1 - [Q5517€	341,67	25,50	1	5	5	16	0,847	13	17,4	149	16,9	7,24							
	A3	Sequence	# PSMs	# Proteins	# Protein Groups	Protein Group Accessions	Modifications	ΔCn	116/117	116/117 Count	116/117 Variability [%]	q-Value	PEP	IonScore	Exp Value	Charge	MH+ [Da]	ΔM [ppm]	RT [min]	# Missed Cleavages
	High	rQELASEFGALk	8	1	1	Q55176	N-Term(iTRAQ4plex); K12(iTRAQ4plex)	0,0000	0,807	5	10,1	0	8,225E-08	66	2,74138E-07	3	1636,92624	0,06	30,38	1
	High	tDVGPITTPNPQk	2	1	1	Q55176	N-Term(iTRAQ4plex); K13(iTRAQ4plex)	0,0000	0,997	2	8,9	0	3,462E-11	63	4,92254E-07	2	1655,92034	-0,22	24,63	0
	High	rQELASEFGALkEQIFGG	1	1	1	Q55176	N-Term(iTRAQ4plex); K12(iTRAQ4plex)	0,0000	1,386	1		0	0,000001248	55	2,98538E-06	3	2268,22446	0,76	50,81	2
	High	qELASEFGALk	1	1	1	Q55176	N-Term(iTRAQ4plex); K11(iTRAQ4plex)	0,0000	0,311	1		0	0,0004798	26	0,003068386	3	1480,82480	-0,15	35,50	0
	Medium	tATAGIk	4	1	1	Q55176	N-Term(iTRAQ4plex); K7(iTRAQ4plex)	0,0000	0,777	4	15,6	0,013	0,1733	23	0,008531749	2	949,59130	-0,82	13,23	0
P24602	Bacterioferritin OS=Synechocystis sp. (strain PCC 6803 / Kazusa) GN=bfr PE=1 SV=2 - [BFR_SYNY3]	340,51	60,26	1	8	8	18	0,437	15	19,8	156	18,3	4,82							
	A3	Sequence	# PSMs	# Proteins	# Protein Groups	Protein Group Accessions	Modifications	ΔCn	116/117	116/117 Count	116/117 Variability [%]	q-Value	PEP	IonScore	Exp Value	Charge	MH+ [Da]	ΔM [ppm]	RT [min]	# Missed Cleavages
	High	eAmAVcEQEQDYQSR	1	1	1	P24602	N-Term(iTRAQ4plex); M3(Oxidation); C6(Carbamidomethyl)	0,0000	0,592	1		0	1,191E-08	62	6,85441E-07	3	2003,85807	1,23	20,71	0
	High	iGLQNYLQSQmS	2	1	1	P24602	N-Term(iTRAQ4plex); M11(Oxidation)	0,0000	0,281	1		0	9,475E-07	51	7,60274E-06	2	1541,77666	1,04	38,67	0
	High	iGLQNYLQSQMS	1	1	1	P24602	N-Term(iTRAQ4plex)	0,0000	0,540	1		0	0,000001054	41	8,55008E-05	2	1525,78227	1,40	44,08	0
	High	dQYFIHSR	6	1	1	P24602	N-Term(iTRAQ4plex)	0,0000	0,437	5	19,8	0	0,00002215	40	0,000108885	2	1209,61479	1,23	19,26	0
	High	gkPAVLAQLHk	4	1	1	P24602	N-Term(iTRAQ4plex); K2(iTRAQ4plex); K11(iTRAQ4plex)	0,0000	0,471	3	7,7	0	2,246E-07	37	0,000182797	4	1594,02065	2,60	24,06	1
	High	iLFLEETPDLSQQDPIR	1	1	1	P24602	N-Term(iTRAQ4plex)	0,0000	0,421	1		0	2,205E-07	37	0,000191854	3	2158,15281	0,70	49,33	0
	High	iLADTEEDHAYWLEk	1	1	1	P24602	N-Term(iTRAQ4plex); K15(iTRAQ4plex)	0,0000	0,331	1		0	4,933E-09	28	0,001774067	3	2121,07486	0,26	38,09	0
	High	mYQDWGLEk	1	1	1	P24602	N-Term(iTRAQ4plex); M1(Oxidation); K9(iTRAQ4plex)	0,0000	0,450	1		0	0,0002171	24	0,00432484	2	1473,73540	4,57	29,86	0
	High	qLGLIEk	1	1	1	P24602	N-Term(iTRAQ4plex); K7(iTRAQ4plex)	0,0000	0,359	1		0,002	0,02442	38	0,000270053	2	1088,69231	0,47	26,98	0
P73479	Succinate dehydrogenase flavoprotein subunit OS=Synechocystis sp. (strain PCC 6803 / Kazusa) GN=	340,40	13,74	1	7	7	15	0,760	13	53,4	575	63,7	5,67							
	A3	Sequence	# PSMs	# Proteins	# Protein Groups	Protein Group Accessions	Modifications	ΔCn	116/117	116/117 Count	116/117 Variability [%]	q-Value	PEP	IonScore	Exp Value	Charge	MH+ [Da]	ΔM [ppm]	RT [min]	# Missed Cleavages
	High	qAFQDcmTSHcGVFR	1	1	1	P73479	N-Term(iTRAQ4plex); C6(Carbamidomethyl); M7(Oxidation); C	0,0000	0,539	1		0	8,284E-09	52	5,64898E-06	3	2003,86612	0,90	23,95	0
	High	lGSNSLLEcVVYGR	4	1	1	P73479	N-Term(iTRAQ4plex); C9(Carbamidomethyl)	0,0000	0,760	3	55,7	0	5,241E-08	45	3,27318E-05	2	1710,89910	1,47	45,25	0
	High	aVmVATGGYGR	2	1	1	P73479	N-Term(iTRAQ4plex); M3(Oxidation)	0,0000	0,522	2	11,7	0	0,00007501	45	3,28829E-05	2	1241,64397	0,84	19,73	0
	High	iPFcWEEAHR	1	1	1	P73479	N-Term(iTRAQ4plex); C4(Carbamidomethyl)	0,0000	1,128	1		0	1,962E-07	42	6,74481E-05	3	1488,71848	0,67	33,76	0
	High	aVMVATGGYGR	2	1	1	P73479	N-Term(iTRAQ4plex)	0,0000	0,644	1		0	0,0004408	34	0,000415882	2	1225,65251	3,67	23,20	0
	High	qAFQDcMTSHcGVFR	2	1	1	P73479	N-Term(iTRAQ4plex); C6(Carbamidomethyl); C11(Carbamidom	0,0000	0,969	2	14,3	0	0,000008339	31	0,000736156	3	1987,87131	0,96	29,07	0
	High	rFmEDYAPSR	1	1	1	P73479	N-Term(iTRAQ4plex); M3(Oxidation)	0,0000	0,754	1		0	0,0007736	30	0,000935341	3	1431,68241	1,14	17,37	1
	High	hTLASFDGEQIk	1	1	1	P73479	N-Term(iTRAQ4plex); K12(iTRAQ4plex)	0,0000	1,346	1		0	0,000005465	28	0,001599448	3	1633,88248	2,23	26,17	0
	High	aITLEIR	1	1	1	P73479	N-Term(iTRAQ4plex)	0,0000	0,995	1		0,001	0,007999	32	0,000663697	2	959,60228	1,72	29,52	0
P72704	Probable peptidyl-prolyl cis-trans isomerase sll0227 OS=Synechocystis sp. (strain PCC 6803 / Kazusa)	336,00	24,80	1	6	6	17	0,819	14	24,6	246	26,6	5,14							
	A3	Sequence	# PSMs	# Proteins	# Protein Groups	Protein Group Accessions	Modifications	ΔCn	116/117	116/117 Count	116/117 Variability [%]	q-Value	PEP	IonScore	Exp Value	Charge	MH+ [Da]	ΔM [ppm]	RT [min]	# Missed Cleavages
	High	vITGQNNLEk	2	1	1	P72704	N-Term(iTRAQ4plex); K10(iTRAQ4plex)	0,0000	0,739	2	0,9	0	5,36E-08	73	4,68781E-08	2	1403,81182	1,52	21,10	0
	High	aLGNQAGFPVMLPHk	6	1	1	P72704	N-Term(iTRAQ4plex); K15(iTRAQ4plex)	0,0000	0,984	4	15,8	0	1,686E-09	47	1,80717E-05	3	1868,04837	1,51	36,73	0
	High	aLGNQAGFPVmLPHk	3	1	1	P72704	N-Term(iTRAQ4plex); M11(Oxidation); K15(iTRAQ4plex)	0,0000	0,667	2	27,8	0	1,521E-09	42	7,18094E-05	3	1884,04227	0,95	34,39	0
	High	gDGTGGYVDkNTQRPR	2	1	1	P72704	N-Term(iTRAQ4plex); K10(iTRAQ4plex)	0,0000	0,982	2	3,0	0	0,000002145	19	0,011829598	4	2009,04299	1,32	13,98	1
	High	nIPLEIk	2	1	1	P72704	N-Term(iTRAQ4plex); K7(iTRAQ4plex)	0,0000	0,672	2	5,6	0,001	0,01523	32	0,00064561	2	1114,71172	3,83	30,44	0
	High	tGAVAMAR	1	1	1	P72704	N-Term(iTRAQ4plex)	0,0000	1,007	1		0,002	0,02523	20	0,009461718	2	920,51103	0,63	14,24	0
	Medium	iQSAk	1	1	1	P72704	N-Term(iTRAQ4plex); K5(iTRAQ4plex)	0,0000	0,828	1		0,036	0,4669	14	0,041112132	2	834,52873	0,00	13,48	0
P49433	Glyceraldehyde-3-phosphate dehydrogenase 1 OS=Synechocystis sp. (strain PCC 6803 / Kazusa) GN=	334,85	19,76	1	7	7	17	1,293	15	20,8	339	36,1	6,24							
	A3	Sequence	# PSMs	# Proteins	# Protein Groups	Protein Group Accessions	Modifications	ΔCn	116/117	116/117 Count	116/117 Variability [%]	q-Value	PEP	IonScore	Exp Value	Charge	MH+ [Da]	ΔM [ppm]	RT [min]	# Missed Cleavages
	High	gAAQNIIPSSTGAAk	4	1	1	P49433	N-Term(iTRAQ4plex); K15(iTRAQ4plex)	0,0000	1,104	3	31,1	0	1,321E-11	97	2,1329E-10	2	1673,94365	0,68	24,24	0
	High	vPTPNVSVVDLTFk	2	1	1	P49433	N-Term(iTRAQ4plex); K14(iTRAQ4plex)	0,0000	1,242	2	24,5	0	5,567E-08	58	1,67483E-06	3	1804,04715	0,69	45,11	0
	High	iGINGFGR	1	1	1	P49433	N-Term(iTRAQ4plex)	0,0000	1,259	1		0	0,000217	45	3,64067E-05	2	977,56481	-0,12	29,61	0
	High	aAALVLPQLk	3	1	1	P49433	N-Term(iTRAQ4plex); K10(iTRAQ4plex)	0,0000	1,467	2	28,8	0	0,000297	41	7,34463E-05	3	1311,86020	-0,06	38,50	0
	High	rVIISAPSkDPEk	1	1	1	P49433	N-Term(iTRAQ4plex); K9(iTRAQ4plex); K13(iTRAQ4plex)	0,0000	1,485	1		0	0,0001142	26	0,002741385	4	1872,12588	-1,09	20,18	2
	High	vIISAPSkDPEk	2	1	1	P49433	N-Term(iTRAQ4plex); K8(iTRAQ4plex); K12(iTRAQ4plex)	0,0000	1,238	2	6,4	0	0,001317	18	0,016942208	3	1716,02561	-0,69	22,83	1
	High	lTGMAFR	2	1	1	P49433	N-Term(iTRAQ4plex)	0,0000	2,407	2	16,6	0,001	0,003205	36	0,000251171	2	939,52031	0,03	29,17	0
	Medium	lTGmAFR	2	1	1	P49433	N-Term(iTRAQ4plex); M4(Oxidation)	0,0000	1,398	2	20,2	0,015	0,2037	10	0,091615721	2	955,51494	-0,27	22,21	0
	>sp|K22E_HUMAN|	330,63	15,81	1	8	9	13	0,450	11	18,4	645	65,8	8,00							
	A3	Sequence	# PSMs	# Proteins	# Protein Groups	Protein Group Accessions	Modifications	ΔCn	116/117	116/117 Count	116/117 Variability [%]	q-Value	PEP	IonScore	Exp Value	Charge	MH+ [Da]	ΔM [ppm]	RT [min]	# Missed Cleavages
	High	gFSSGSAVVSGGSR	2	1	1		N-Term(iTRAQ4plex)	0,0000	0,444	2	1,9	0	7,402E-09	82	6,66761E-09	2	1398,71037	0,67	20,22	0
	High	sLVGLGGTk	2	1	1		N-Term(iTRAQ4plex); K9(iTRAQ4plex)	0,0000	0,509	1		0	0,000002515	43	5,09296E-05	2	1119,69707	-0,49	24,20	0
	High	hGGGGGGFGGGGFGSR	1	1	1		N-Term(iTRAQ4plex)	0,0000	0,817	1		0	0,00000108	41	7,49842E-05	3	1464,68470	-0,19	16,22	0
	High	fLEQQNQVLQTk	1	2	2		N-Term(iTRAQ4plex); K12(iTRAQ4plex)	0,0000				0	1,775E-07	38	0,000189539	3	1763,98917	-0,16	31,16	0
	High	lLRDYQELMNVk	1	1	1		N-Term(iTRAQ4plex); K12(iTRAQ4plex)	0,0000	0,436	1		0	0,000001051	37	0,00020416	3	1810,01364	0,04	40,28	1
	High	fASFIDkVR	1	1	1		N-Term(iTRAQ4plex); K7(iTRAQ4plex)	0,0000	0,626	1		0	0,0001705	36	0,000324435	3	1370,80332	-0,12	35,49	1
	High	lQGEIAHVk	1	1	1		N-Term(iTRAQ4plex); K9(iTRAQ4plex)	0,0000	0,399	1		0	0,00002972	33	0,000480806	3	1282,77179	-0,30	22,10	0
	High	skEEAEALYHSk	3	1	1		N-Term(iTRAQ4plex); K2(iTRAQ4plex); K12(iTRAQ4plex)	0,0000	0,479	3	34,6	0	1,646E-07	29	0,001196658	3	1823,98404	-1,28	21,06	1
	High	kYEDEINkR	1	1	1		N-Term(iTRAQ4plex); K1(iTRAQ4plex); K8(iTRAQ4plex)	0,0000	0,397	1		0	0,00001263	19	0,01213305	4	1626,91677	-0,49	15,09	2
P72703	Agmatinase 1 OS=Synechocystis sp. (strain PCC 6803 / Kazusa) GN=speB1 PE=3 SV=1 - [SPEB1_SY	330,54	13,73	1	5	5	17	0,494	15	23,1	306	33,4	5,01							
	A3	Sequence	# PSMs	# Proteins	# Protein Groups	Protein Group Accessions	Modifications	ΔCn	116/117	116/117 Count	116/117 Variability [%]	q-Value	PEP	IonScore	Exp Value	Charge	MH+ [Da]	ΔM [ppm]	RT [min]	# Missed Cleavages
	High	fVVAIGGEHAITTGVVR	5	1	1	P72703	N-Term(iTRAQ4plex)	0,0000	0,445	4	34,3	0	2,746E-11	70	1,03507E-07	3	1870,06803	0,66	37,39	0
	High	rVLELGLPTLPIAIR	3	1	1	P72703	N-Term(iTRAQ4plex)	0,0000	0,494	3	15,4	0	0,00000456	33	0,000447682	3	1805,15055	0,64	49,32	1
	High	ekNIPVFWAR	1	1	1	P72703	N-Term(iTRAQ4plex); K2(iTRAQ4plex)	0,0000	0,664	1		0	0,00001048	28	0,001662256	3	1547,89615	1,58	33,71	1
	High	nIPVFWAR	6	1	1	P72703	N-Term(iTRAQ4plex)	0,0000	0,502	5	13,9	0	0,0007995	28	0,001584784	2	1146,65508	0,87	39,86	0
	High	vLELGLPTLPIAIR	2	1	1	P72703	N-Term(iTRAQ4plex)	0,0000	0,375	2	9,2	0	0,002295	26	0,002477251	2	1649,05315	2,96	55,43	0
P74162	Sll1380 protein OS=Synechocystis sp. (strain PCC 6803 / Kazusa) GN=sll1380 PE=4 SV=1 - [P74162_	330,03	38,32	1	7	7	11	0,549	11	16,0	214	23,9	8,91							
	A3	Sequence	# PSMs	# Proteins	# Protein Groups	Protein Group Accessions	Modifications	ΔCn	116/117	116/117 Count	116/117 Variability [%]	q-Value	PEP	IonScore	Exp Value	Charge	MH+ [Da]	ΔM [ppm]	RT [min]	# Missed Cleavages
	High	nLDFTLNAPAGHLPSPR	1	1	1	P74162	N-Term(iTRAQ4plex)	0,0000	0,705	1		0	4,729E-08	56	2,68159E-06	3	1964,04618	-0,47	37,45	0
	High	tIDQLWLQHSNNR	2	1	1	P74162	N-Term(iTRAQ4plex)	0,0000	0,522	2	11,2	0	4,143E-07	53	5,21159E-06	3	1768,91764	-1,98	32,34	0
	High	lLLAATNR	2	1	1	P74162	N-Term(iTRAQ4plex)	0,0000	0,493	2	7,2	0	0,001529	53	5,38233E-06	2	1015,63713	-0,94	28,72	0
	High	fQVcQSGQVPTAPVR	2	1	1	P74162	N-Term(iTRAQ4plex); C4(Carbamidomethyl)	0,0000	0,527	2	10,0	0	2,807E-09	51	7,79776E-06	3	1817,94541	0,26	29,73	0
	High	rLLLAATNR	1	1	1	P74162	N-Term(iTRAQ4plex)	0,0000	0,897	1		0	0,00005475	27	0,002022879	3	1171,73911	-0,08	20,96	1
	High	fGFTPQLEAFLATGNRPGR	1	1	1	P74162	N-Term(iTRAQ4plex)	0,0000	0,436	1		0	0,000008306	26	0,002322576	3	2223,18125	0,92	49,29	0
	High	kENQWVIFk	2	1	1	P74162	N-Term(iTRAQ4plex); K1(iTRAQ4plex); K9(iTRAQ4plex)	0,0000	0,577	2	7,6	0	0,00009255	25	0,003097205	3	1623,95761	-0,43	31,99	1
Q55118	Putative thylakoid lumen peptidyl-prolyl cis-trans isomerase sll0408 OS=Synechocystis sp. (strain PCC	329,53	17,68	1	8	8	16	0,563	15	12,9	379	41,4	4,84							
	A3	Sequence	# PSMs	# Proteins	# Protein Groups	Protein Group Accessions	Modifications	ΔCn	116/117	116/117 Count	116/117 Variability [%]	q-Value	PEP	IonScore	Exp Value	Charge	MH+ [Da]	ΔM [ppm]	RT [min]	# Missed Cleavages
	High	aIPLEILVk	3	1	1	Q55118	N-Term(iTRAQ4plex); K9(iTRAQ4plex)	0,0000	0,622	3	23,2	0	0,00000383	63	5,41963E-07	2	1283,85552	1,08	44,46	0
	High	lQDSLEDISNHIR	1	1	1	Q55118	N-Term(iTRAQ4plex)	0,0000	0,658	1		0	3,835E-08	47	2,17255E-05	3	1683,87723	-0,63	38,39	0
	High	rLQDSLEDISNHIR	3	1	1	Q55118	N-Term(iTRAQ4plex)	0,0000	0,565	2	9,8	0	8,011E-07	43	5,0579E-05	3	1839,97977	0,20	35,59	1
	High	tDLTALTEAVEAk	1	1	1	Q55118	N-Term(iTRAQ4plex); K13(iTRAQ4plex)	0,0000	0,650	1		0	0,000002278	40	9,18269E-05	3	1649,91977	-0,17	41,44	0
	High	kFYDGLPFIR	1	1	1	Q55118	N-Term(iTRAQ4plex); K1(iTRAQ4plex)	0,0000	0,534	1		0	3,706E-07	39	0,000127057	3	1543,88852	0,63	39,33	1
	High	aANLTITLkEDk	1	1	1	Q55118	N-Term(iTRAQ4plex); K9(iTRAQ4plex); K12(iTRAQ4plex)	0,0000	0,749	1		0	3,295E-07	34	0,000406415	3	1749,04831	0,03	27,98	1
	High	dkEQVISFR	2	1	1	Q55118	N-Term(iTRAQ4plex); K2(iTRAQ4plex)	0,0000	0,638	2	18,6	0	0,00008085	33	0,00045811	3	1409,79709	-1,44	23,38	1
	High	fYDGLPFIR	4	1	1	Q55118	N-Term(iTRAQ4plex)	0,0000	0,517	4	1,2	0	0,002001	31	0,000707897	2	1271,69097	0,35	45,68	0
P74135	Sll1873 protein OS=Synechocystis sp. (strain PCC 6803 / Kazusa) GN=sll1873 PE=4 SV=1 - [P74135_	329,40	56,20	1	6	6	17	0,573	14	22,0	121	12,9	9,99							
	A3	Sequence	# PSMs	# Proteins	# Protein Groups	Protein Group Accessions	Modifications	ΔCn	116/117	116/117 Count	116/117 Variability [%]	q-Value	PEP	IonScore	Exp Value	Charge	MH+ [Da]	ΔM [ppm]	RT [min]	# Missed Cleavages
	High	vDPSQVAFASGDPIPQNVAR	1	1	1	P74135	N-Term(iTRAQ4plex)	0,0000	0,398	1		0	3,24E-10	63	5,33298E-07	3	2212,14859	0,28	38,29	0
	High	aAAPVETPASAPVAPAPk	3	1	1	P74135	N-Term(iTRAQ4plex); K18(iTRAQ4plex)	0,0000	0,473	2	7,5	0	5,926E-11	56	2,47155E-06	3	1933,10044	0,34	25,85	0
	High	sEVAPVEkPAPTTSk	5	1	1	P74135	N-Term(iTRAQ4plex); K8(iTRAQ4plex); K15(iTRAQ4plex)	0,0000	0,429	3	9,9	0	3,792E-08	47	2,0463E-05	3	1973,12656	-0,72	18,69	0
	High	rTPGPSLNR	3	1	1	P74135	N-Term(iTRAQ4plex)	0,0000	0,667	3	32,3	0	0,00005713	41	8,35545E-05	3	1141,65589	0,02	12,34	1
	High	kLFGAk	1	1	1	P74135	N-Term(iTRAQ4plex); K1(iTRAQ4plex); K6(iTRAQ4plex)	0,0000	0,500	1		0,002	0,02433	12	0,058340481	3	1095,72333	-1,57	17,15	1
	Medium	lFGAk	4	1	1	P74135	N-Term(iTRAQ4plex); K5(iTRAQ4plex)	0,0000	0,650	4	3,4	0,024	0,3228	18	0,020378924	2	823,52696	-1,28	21,80	0
P77973	Argininosuccinate synthase OS=Synechocystis sp. (strain PCC 6803 / Kazusa) GN=argG PE=1 SV=1 -	327,48	18,75	1	7	7	17	1,858	13	42,4	400	44,5	5,14							
	A3	Sequence	# PSMs	# Proteins	# Protein Groups	Protein Group Accessions	Modifications	ΔCn	116/117	116/117 Count	116/117 Variability [%]	q-Value	PEP	IonScore	Exp Value	Charge	MH+ [Da]	ΔM [ppm]	RT [min]	# Missed Cleavages
	High	lNEIAGNHGVGR	3	1	1	P77973	N-Term(iTRAQ4plex)	0,0000	1,835	2	10,2	0	0,000000474	62	5,79389E-07	3	1380,74686	0,27	20,50	0
	High	sIQANALYENR	1	1	1	P77973	N-Term(iTRAQ4plex)	0,0000	2,367	1		0	0,000001182	59	1,30008E-06	2	1422,74700	0,84	26,37	0
	High	eIYEAPALLVLIDAHR	5	1	1	P77973	N-Term(iTRAQ4plex)	0,0000	1,403	4	43,8	0	3,565E-09	54	3,92618E-06	3	1967,11576	3,79	51,34	0
	High	gLWYSPLk	3	1	1	P77973	N-Term(iTRAQ4plex); K8(iTRAQ4plex)	0,0000	2,676	2	13,5	0	0,0000138	39	0,000136449	2	1251,73503	0,84	37,49	0
	High	yGADAVAHGcTGk	2	1	1	P77973	N-Term(iTRAQ4plex); C10(Carbamidomethyl); K13(iTRAQ4plex	0,0000	2,632	1		0	0,000002066	37	0,00020416	3	1594,78781	-0,52	17,28	0
	High	yGVESPVkk	1	1	1	P77973	N-Term(iTRAQ4plex); K8(iTRAQ4plex); K9(iTRAQ4plex)	0,0000	1,858	1		0	0,0007193	18	0,017059646	3	1438,86353	0,37	19,06	1
	Medium	vLAPAR	2	1	1	P77973	N-Term(iTRAQ4plex)	0,0000	1,164	2	2,4	0,02	0,2626	17	0,021775594	2	770,50084	0,41	16,86	0
P74375	Slr0442 protein OS=Synechocystis sp. (strain PCC 6803 / Kazusa) GN=slr0442 PE=4 SV=1 - [P74375	327,01	11,62	1	7	7	14	0,780	14	22,8	611	62,9	5,21							
	A3	Sequence	# PSMs	# Proteins	# Protein Groups	Protein Group Accessions	Modifications	ΔCn	116/117	116/117 Count	116/117 Variability [%]	q-Value	PEP	IonScore	Exp Value	Charge	MH+ [Da]	ΔM [ppm]	RT [min]	# Missed Cleavages
	High	aSTPmPSIPLSALASAR	2	1	1	P74375	N-Term(iTRAQ4plex); M5(Oxidation)	0,0000	0,623	2	11,1	0	3,578E-08	61	9,44915E-07	3	1829,99155	0,17	39,15	0
	High	kFELNFDNTGGk	3	1	1	P74375	N-Term(iTRAQ4plex); K1(iTRAQ4plex); K12(iTRAQ4plex)	0,0000	0,853	3	3,8	0	1,037E-07	57	1,89221E-06	3	1801,98465	2,08	29,73	1
	High	aSTPMPSIPLSALASAR	4	1	1	P74375	N-Term(iTRAQ4plex)	0,0000	1,276	4	5,4	0	0,000006225	54	3,9443E-06	3	1813,99820	1,04	43,85	0
	High	lVVEGVSSk	1	1	1	P74375	N-Term(iTRAQ4plex); K9(iTRAQ4plex)	0,0000	0,808	1		0	0,0002508	51	7,67308E-06	2	1205,73613	1,44	24,74	0
	High	vGVEEENkGk	1	1	1	P74375	N-Term(iTRAQ4plex); K8(iTRAQ4plex); K10(iTRAQ4plex)	0,0000	0,754	1		0	0,0007567	25	0,003950916	3	1520,86750	1,98	13,45	1
	High	iPQGQVIGR	1	1	1	P74375	N-Term(iTRAQ4plex)	0,0000	0,669	1		0,001	0,003297	36	0,000264223	2	1111,67204	1,43	22,99	0
	High	vQGLLASSk	1	1	1	P74375	N-Term(iTRAQ4plex); K9(iTRAQ4plex)	0,0000	0,677	1		0,002	0,02665	18	0,015630397	3	1190,73529	0,48	25,91	0
	Medium	iVSYk	1	1	1	P74375	N-Term(iTRAQ4plex); K5(iTRAQ4plex)	0,0000	0,664	1		0,023	0,3067	27	0,002127992	2	897,56200	-3,10	18,95	0
P72758	Carbon dioxide concentrating mechanism protein CcmM OS=Synechocystis sp. (strain PCC 6803 / Kaz	326,82	18,34	1	9	9	14	1,950	11	49,3	687	73,1	8,59							
	A3	Sequence	# PSMs	# Proteins	# Protein Groups	Protein Group Accessions	Modifications	ΔCn	116/117	116/117 Count	116/117 Variability [%]	q-Value	PEP	IonScore	Exp Value	Charge	MH+ [Da]	ΔM [ppm]	RT [min]	# Missed Cleavages
	High	sLLAQGYGIGAEHANER	2	1	1	P72758	N-Term(iTRAQ4plex)	0,0000	2,974	1		0	1,312E-10	80	9,07821E-09	3	1929,99204	1,07	30,23	0
	High	sAVAHQTGNLAGDSANQLR	1	1	1	P72758	N-Term(iTRAQ4plex)	0,0000	1,752	1		0	1,693E-11	67	2,13781E-07	3	2054,05130	0,82	21,52	0
	High	rVLETIIQRP	1	1	1	P72758	N-Term(iTRAQ4plex)	0,0000	2,288	1		0	2,991E-08	56	2,47155E-06	3	1368,84418	-0,16	29,61	1
	High	rVLEQIIQRPGDNVVAGR	3	1	1	P72758	N-Term(iTRAQ4plex)	0,0000	2,263	2	7,2	0	7,465E-08	43	5,12826E-05	4	2164,24209	-0,52	31,16	1
	High	rVVEVIIQRPGDVPGSPSR	2	1	1	P72758	N-Term(iTRAQ4plex)	0,0000	0,932	1		0	1,531E-07	41	0,000101236	4	2205,25869	0,07	29,03	1
	High	fLADHTNEYVR	1	1	1	P72758	N-Term(iTRAQ4plex)	0,0000	1,091	1		0	0,00004908	31	0,000748118	3	1508,76084	-0,40	25,95	0
	High	sAPVSSAGGSSAGGLTPEVIATVR	1	1	1	P72758	N-Term(iTRAQ4plex)	0,0000	1,424	1		0	0,0007001	31	0,000762026	3	2315,23716	2,04	37,35	0
	High	vLEQIIQRPGDNVVAGR	2	1	1	P72758	N-Term(iTRAQ4plex)	0,0000	2,564	2	42,3	0	4,058E-07	29	0,001253688	3	2008,14292	0,41	36,08	0
	High	aLLHQGYk	1	1	1	P72758	N-Term(iTRAQ4plex); K8(iTRAQ4plex)	0,0000	1,233	1		0,001	0,003337	22	0,007164423	3	1217,72275	-1,42	16,75	0
P72700	Slr0244 protein OS=Synechocystis sp. (strain PCC 6803 / Kazusa) GN=slr0244 PE=4 SV=1 - [P72700	326,33	30,63	1	9	9	15	1,974	15	8,9	284	31,2	5,25							
	A3	Sequence	# PSMs	# Proteins	# Protein Groups	Protein Group Accessions	Modifications	ΔCn	116/117	116/117 Count	116/117 Variability [%]	q-Value	PEP	IonScore	Exp Value	Charge	MH+ [Da]	ΔM [ppm]	RT [min]	# Missed Cleavages
	High	lLGTSLSDYIR	3	1	1	P72700	N-Term(iTRAQ4plex)	0,0000	2,336	3	10,5	0	3,218E-07	66	2,62404E-07	2	1381,78130	0,37	44,42	0
	High	vNAPcPVLLTR	2	1	1	P72700	N-Term(iTRAQ4plex); C5(Carbamidomethyl)	0,0000	1,966	2	1,5	0	0,00007227	56	2,25928E-06	2	1383,79045	0,38	32,27	0
	High	vNPDLkPDLLPLSR	2	1	1	P72700	N-Term(iTRAQ4plex); K6(iTRAQ4plex)	0,0000	2,115	2	1,3	0	3,552E-08	31	0,000762026	3	1865,11008	0,08	37,55	0
	High	rLGIAYR	1	1	1	P72700	N-Term(iTRAQ4plex)	0,0000	2,408	1		0	0,0006827	31	0,000885054	3	992,61225	0,05	18,38	1
	High	cTVTGGkPGEk	1	1	1	P72700	N-Term(iTRAQ4plex); C1(Carbamidomethyl); K7(iTRAQ4plex);	0,0000	1,980	1		0	0,000228	27	0,001922959	3	1565,86649	-1,08	11,76	0
	High	aSITILHVVPPQITTEAFTEk	1	1	1	P72700	N-Term(iTRAQ4plex); K21(iTRAQ4plex)	0,0000	1,963	1		0	0,0000387	25	0,003427441	4	2583,46694	1,26	47,24	0
	High	rPSIAk	2	1	1	P72700	N-Term(iTRAQ4plex); K6(iTRAQ4plex)	0,0000	1,663	2	2,2	0,001	0,007939	15	0,029038221	3	959,62305	-1,04	11,39	0
	High	lGIAYR	2	1	1	P72700	N-Term(iTRAQ4plex)	0,0000	1,885	2	1,0	0,006	0,06905	24	0,004101758	2	836,51134	0,32	24,22	0
	Medium	vSTVLR	1	1	1	P72700	N-Term(iTRAQ4plex)	0,0000	1,800	1		0,045	0,5796	17	0,020463033	2	818,52074	-1,11	19,98	0
P36239	50S ribosomal protein L19 OS=Synechocystis sp. (strain PCC 6803 / Kazusa) GN=rplS PE=3 SV=1 - [	326,17	38,52	1	6	6	13	3,969	11	62,9	122	13,8	10,64							
	A3	Sequence	# PSMs	# Proteins	# Protein Groups	Protein Group Accessions	Modifications	ΔCn	116/117	116/117 Count	116/117 Variability [%]	q-Value	PEP	IonScore	Exp Value	Charge	MH+ [Da]	ΔM [ppm]	RT [min]	# Missed Cleavages
	High	kIFQGVGVER	3	1	1	P36239	N-Term(iTRAQ4plex); K1(iTRAQ4plex)	0,0000	5,595	3	19,1	0	9,901E-07	58	1,62544E-06	3	1420,85337	1,31	23,89	1
	High	vFLLHSPR	6	1	1	P36239	N-Term(iTRAQ4plex)	0,0000	4,823	4	30,1	0	0,00003065	55	3,34942E-06	2	1112,67082	1,00	26,45	0
	High	iQPYEGTVIAk	1	1	1	P36239	N-Term(iTRAQ4plex); K11(iTRAQ4plex)	0,0000	2,582	1		0	0,00006159	44	3,69803E-05	2	1506,87908	1,35	29,80	0
	High	rNGGISETITVR	1	1	1	P36239	N-Term(iTRAQ4plex)	0,0000	1,165	1		0	0,0001481	41	9,60269E-05	3	1446,81736	1,93	19,13	1
	High	iFQGVGVER	1	1	1	P36239	N-Term(iTRAQ4plex)	0,0000	3,858	1		0	0,0001838	33	0,000536995	2	1148,65495	0,42	28,89	0
	Medium	iVEGGk	1	1	1	P36239	N-Term(iTRAQ4plex); K6(iTRAQ4plex)	0,0000	1,361	1		0,046	0,5922	25	0,003206048	2	890,55193	-3,41	15,14	0
P72851	50S ribosomal protein L28 OS=Synechocystis sp. (strain PCC 6803 / Kazusa) GN=rpmB PE=3 SV=1 -	326,12	53,85	1	7	7	17	2,103	15	67,6	78	9,0	11,81							
	A3	Sequence	# PSMs	# Proteins	# Protein Groups	Protein Group Accessions	Modifications	ΔCn	116/117	116/117 Count	116/117 Variability [%]	q-Value	PEP	IonScore	Exp Value	Charge	MH+ [Da]	ΔM [ppm]	RT [min]	# Missed Cleavages
	High	lSTTAIk	3	1	1	P72851	N-Term(iTRAQ4plex); K7(iTRAQ4plex)	0,0000	3,130	3	5,4	0	0,0001359	57	1,82797E-06	2	1021,65422	4,54	20,80	0
	High	lQQANLQWk	2	1	1	P72851	N-Term(iTRAQ4plex); K9(iTRAQ4plex)	0,0000	1,945	2	144,6	0	0,001098	54	4,02717E-06	2	1416,83049	7,27	30,64	0
	High	eAGINLNkF	3	1	1	P72851	N-Term(iTRAQ4plex); K8(iTRAQ4plex)	0,0000	2,103	3	8,1	0	0,0005946	48	1,82469E-05	2	1293,74553	3,85	33,10	1
	High	vWWPEGNR	2	1	1	P72851	N-Term(iTRAQ4plex)	0,0000	1,555	2	1,5	0	0,0002623	33	0,000541963	2	1187,60881	0,81	33,41	0
	High	kLQQANLQWk	1	1	1	P72851	N-Term(iTRAQ4plex); K1(iTRAQ4plex); K10(iTRAQ4plex)	0,0000	5,331	1		0	0,0000634	31	0,000822186	3	1689,02066	2,04	25,55	1
	High	gINAmAk	4	1	1	P72851	N-Term(iTRAQ4plex); M5(Oxidation); K7(iTRAQ4plex)	0,0000	1,081	2	24,7	0,001	0,009039	24	0,004017631	2	1008,57872	3,63	13,94	0
	High	rVWWPEGNR	2	1	1	P72851	N-Term(iTRAQ4plex)	0,0000	2,956	2	41,5	0,001	0,005335	15	0,036702685	3	1343,71067	1,26	26,94	1
P74177	DNA-directed RNA polymerase subunit gamma OS=Synechocystis sp. (strain PCC 6803 / Kazusa) GN=	324,71	12,30	1	7	7	19	2,214	17	91,7	626	70,9	7,49							
	A3	Sequence	# PSMs	# Proteins	# Protein Groups	Protein Group Accessions	Modifications	ΔCn	116/117	116/117 Count	116/117 Variability [%]	q-Value	PEP	IonScore	Exp Value	Charge	MH+ [Da]	ΔM [ppm]	RT [min]	# Missed Cleavages
	High	lAAPVTHVWYLk	4	1	1	P74177	N-Term(iTRAQ4plex); K12(iTRAQ4plex)	0,0000	5,278	2	24,4	0	1,662E-08	55	3,03368E-06	3	1685,99887	0,43	38,70	0
	High	lQEILAPEIVR	2	1	1	P74177	N-Term(iTRAQ4plex)	0,0000	2,582	2	43,0	0	0,00000907	53	4,48714E-06	2	1537,94414	0,44	46,62	0
	High	lGIQAFEPILVEGR	3	1	1	P74177	N-Term(iTRAQ4plex)	0,0000	2,727	3	145,8	0	0,000003466	43	5,61009E-05	2	1685,97173	0,58	49,84	0
	High	lREEILASk	2	1	1	P74177	N-Term(iTRAQ4plex); K9(iTRAQ4plex)	0,0000	3,675	2	87,1	0	0,000001158	37	0,00021231	3	1346,82456	-0,04	25,37	1
	High	emAIELFQPFVIHR	4	1	1	P74177	N-Term(iTRAQ4plex); M2(Oxidation)	0,0000	2,861	4	88,6	0	0,000001724	36	0,000244889	3	1890,00437	-1,12	46,89	0
	High	eMAIELFQPFVIHR	2	1	1	P74177	N-Term(iTRAQ4plex)	0,0000	1,561	2	15,7	0	0,0009048	14	0,043972509	3	1874,01432	1,46	51,19	0

	High	tVVGANNR	1	1	1	P74177	N-Term(iTRAQ4plex)	0,0000	0,813	1		0,002	0,01825	18	0,016556556	2	974,54973	-0,27	12,63	0
	Medium	tIAEALVF	1	1	1	P74177	N-Term(iTRAQ4plex)	0,0000	1,247	1		0,018	0,2322	11	0,071116439	2	1007,58953	0,12	49,41	0
P37277	Photosystem I reaction center subunit XI OS=Synechocystis sp. (strain PCC 6803 / Kazusa) GN=psaL	322,11	11,46	1	3	3	15	0,911	11	14,6	157	16,6	4,83							
	A3	Sequence	# PSMs	# Proteins	# Protein Groups	Protein Group Accessions	Modifications	ΔCn	116/117	116/117 Count	116/117 Variability [%]	q-Value	PEP	IonScore	Exp Value	Charge	MH+ [Da]	ΔM [ppm]	RT [min]	# Missed Cleavages
	High	tFIGNLPAYR	8	1	1	P37277	N-Term(iTRAQ4plex)	0,0000	0,808	6	24,8	0	1,926E-07	50	1,10146E-05	3	1295,72330	0,33	36,87	0
	High	gLSPILR	3	1	1	P37277	N-Term(iTRAQ4plex)	0,0000	1,129	3	10,4	0	0,001294	48	1,69032E-05	2	899,58013	0,69	27,43	0
	High	kGLSPILR	4	1	1	P37277	N-Term(iTRAQ4plex); K1(iTRAQ4plex)	0,0000	0,896	2	1,5	0	0,0001805	35	0,00034512	3	1171,77738	0,72	21,91	1
P73315	50S ribosomal protein L22 OS=Synechocystis sp. (strain PCC 6803 / Kazusa) GN=rplV PE=3 SV=1 - [	320,65	43,80	1	6	6	19	1,418	16	58,1	121	13,5	9,99							
	A3	Sequence	# PSMs	# Proteins	# Protein Groups	Protein Group Accessions	Modifications	ΔCn	116/117	116/117 Count	116/117 Variability [%]	q-Value	PEP	IonScore	Exp Value	Charge	MH+ [Da]	ΔM [ppm]	RT [min]	# Missed Cleavages
	High	kPTcHITVAVAPALADN	2	1	1	P73315	N-Term(iTRAQ4plex); K1(iTRAQ4plex); C4(Carbamidomethyl)	0,0000	2,090	2	5,3	0	0,0000118	62	5,92884E-07	3	2066,13608	2,58	29,66	0
	High	tkLDTTAEVk	7	1	1	P73315	N-Term(iTRAQ4plex); K2(iTRAQ4plex); K10(iTRAQ4plex)	0,0000	2,170	4	21,5	0	0,000002937	48	1,6367E-05	3	1537,92436	5,33	17,81	1
	High	rVLDQIR	4	1	1	P73315	N-Term(iTRAQ4plex)	0,0000	1,344	4	21,6	0	0,0007322	37	0,000190095	3	1043,64640	2,07	17,56	1
	High	eALIILEFMPYk	3	1	1	P73315	N-Term(iTRAQ4plex); K12(iTRAQ4plex)	0,0000	0,527	3	1,5	0	0,0003002	21	0,007761935	3	1755,00309	1,45	56,32	0
	High	acEPVLk	2	1	1	P73315	N-Term(iTRAQ4plex); C2(Carbamidomethyl); K7(iTRAQ4plex)	0,0000	1,244	2	42,8	0,001	0,007825	33	0,000446653	2	1104,63664	3,69	18,87	0
	High	vLDQIR	1	1	1	P73315	N-Term(iTRAQ4plex)	0,0000	0,826	1		0,002	0,01965	35	0,000287058	2	887,54460	1,68	22,86	0
P74456	Ribosome-recycling factor OS=Synechocystis sp. (strain PCC 6803 / Kazusa) GN=frr PE=3 SV=1 - [RI	318,86	32,42	1	6	6	19	0,479	18	31,4	182	20,2	5,81							
	A3	Sequence	# PSMs	# Proteins	# Protein Groups	Protein Group Accessions	Modifications	ΔCn	116/117	116/117 Count	116/117 Variability [%]	q-Value	PEP	IonScore	Exp Value	Charge	MH+ [Da]	ΔM [ppm]	RT [min]	# Missed Cleavages
	High	rIDELLAAk	6	1	1	P74456	N-Term(iTRAQ4plex); K9(iTRAQ4plex)	0,0000	0,328	5	8,3	0	0,00002496	50	9,86211E-06	3	1316,81430	0,19	30,26	1
	High	aNASLLDR	3	1	1	P74456	N-Term(iTRAQ4plex)	0,0000	0,532	3	8,5	0	0,0005643	43	5,0579E-05	2	1003,56584	0,53	20,67	0
	High	aISLSDLGLTPNNDGk	1	1	1	P74456	N-Term(iTRAQ4plex); K16(iTRAQ4plex)	0,0000	0,579	1		0	2,357E-08	36	0,000273508	3	1903,03836	0,43	41,31	0
	High	lAELkDHMQk	2	1	1	P74456	N-Term(iTRAQ4plex); K5(iTRAQ4plex); K10(iTRAQ4plex)	0,0000	0,829	2	3,7	0	0,00005105	15	0,036890115	3	1644,94828	0,92	24,26	1
	High	lAELkDHmQk	1	1	1	P74456	N-Term(iTRAQ4plex); K5(iTRAQ4plex); M8(Oxidation); K10(iTR	0,0000	0,391	1		0	0,0004126	14	0,060184401	3	1660,94016	-0,92	17,64	1
	High	lNIPPLTAER	2	1	1	P74456	N-Term(iTRAQ4plex)	0,0000	0,417	2	10,8	0,001	0,00453	40	9,39658E-05	2	1267,74956	0,36	35,80	0
	High	sFNTIR	4	1	1	P74456	N-Term(iTRAQ4plex)	0,0000	0,479	4	1,9	0,001	0,01321	32	0,000687068	2	881,49553	-0,71	19,29	0
P73821	D-3-phosphoglycerate dehydrogenase OS=Synechocystis sp. (strain PCC 6803 / Kazusa) GN=serA PE	317,75	13,00	1	10	10	16	2,795	12	41,3	554	59,2	5,69							
	A3	Sequence	# PSMs	# Proteins	# Protein Groups	Protein Group Accessions	Modifications	ΔCn	116/117	116/117 Count	116/117 Variability [%]	q-Value	PEP	IonScore	Exp Value	Charge	MH+ [Da]	ΔM [ppm]	RT [min]	# Missed Cleavages
	High	tLGVVGLGk	2	1	1	P73821	N-Term(iTRAQ4plex); K9(iTRAQ4plex)	0,0000	2,851	1		0	2,866E-07	67	2,1576E-07	2	1131,73699	2,63	29,70	0
	High	iGSHVAGVAk	4	1	1	P73821	N-Term(iTRAQ4plex); K10(iTRAQ4plex)	0,0000	2,389	2	65,5	0	0,000004735	55	3,11867E-06	2	1226,74858	2,13	16,13	0
	High	kQFIGTEVYk	2	1	1	P73821	N-Term(iTRAQ4plex); K1(iTRAQ4plex); K10(iTRAQ4plex)	0,0000	2,946	2	10,5	0	1,513E-07	43	5,1047E-05	3	1644,96952	0,60	24,42	1
	High	kTLGVVGLGk	1	1	1	P73821	N-Term(iTRAQ4plex); K1(iTRAQ4plex); K10(iTRAQ4plex)	0,0000	0,809	1		0	8,221E-07	36	0,000264832	3	1403,92911	-1,37	24,72	1
	High	dYSGSLHLk	1	1	1	P73821	N-Term(iTRAQ4plex); K9(iTRAQ4plex)	0,0000	0,938	1		0	0,0002265	30	0,000963762	3	1307,71955	-0,18	22,87	0
	High	hIPDANk	1	1	1	P73821	N-Term(iTRAQ4plex); K7(iTRAQ4plex)	0,0000	0,252	1		0	0,001499	22	0,005727565	3	1082,61792	-1,63	12,43	0
	High	kQFIGTEVYkk	1	1	1	P73821	N-Term(iTRAQ4plex); K1(iTRAQ4plex); K10(iTRAQ4plex); K11(	0,0000	3,732	1		0	0,000005876	20	0,009098504	4	1917,16506	-0,26	22,41	2
	High	gLLSQALR	2	1	1	P73821	N-Term(iTRAQ4plex)	0,0000	2,845	1		0,001	0,008956	37	0,000202288	2	1001,62963	7,18	30,75	0
	High	lLAYDPFISQER	1	1	1	P73821	N-Term(iTRAQ4plex)	0,0000	1,816	1		0,001	0,003176	28	0,00143539	2	1595,85613	0,69	45,78	0
	High	lRPYLk	1	1	1	P73821	N-Term(iTRAQ4plex); K6(iTRAQ4plex)	0,0000	3,211	1		0,005	0,06177	12	0,061089983	3	1077,70227	-0,01	20,77	0
P12975	Photosystem I reaction center subunit IV OS=Synechocystis sp. (strain PCC 6803 / Kazusa) GN=psaE	315,24	17,57	1	2	2	17	0,288	9	4,2	74	8,1	8,50							
	A3	Sequence	# PSMs	# Proteins	# Protein Groups	Protein Group Accessions	Modifications	ΔCn	116/117	116/117 Count	116/117 Variability [%]	q-Value	PEP	IonScore	Exp Value	Charge	MH+ [Da]	ΔM [ppm]	RT [min]	# Missed Cleavages
	High	sGILYPVIVR	16	1	1	P12975	N-Term(iTRAQ4plex)	0,0000	0,287	8	2,5	0	0,000347	56	2,58803E-06	2	1260,78081	0,91	37,81	0
	High	sGILYPVIVRFDR	1	1	1	P12975	N-Term(iTRAQ4plex)	0,0000	0,331	1		0,001	0,007709	19	0,012217153	3	1678,97660	0,26	43,92	1
P74344	Molybdopterin biosynthesis MoeB protein OS=Synechocystis sp. (strain PCC 6803 / Kazusa) GN=moe	312,21	22,96	1	9	9	18	1,763	17	51,2	392	42,7	5,05							
	A3	Sequence	# PSMs	# Proteins	# Protein Groups	Protein Group Accessions	Modifications	ΔCn	116/117	116/117 Count	116/117 Variability [%]	q-Value	PEP	IonScore	Exp Value	Charge	MH+ [Da]	ΔM [ppm]	RT [min]	# Missed Cleavages
	High	iILGAPNTLSGR	2	1	1	P74344	N-Term(iTRAQ4plex)	0,0000	2,331	2	6,8	0	0,00001273	63	4,96558E-07	2	1355,81304	0,19	33,70	0
	High	eLLDGHEGILIAHck	3	1	1	P74344	N-Term(iTRAQ4plex); C14(Carbamidomethyl); K15(iTRAQ4plex	0,0000	1,247	3	5,0	0	1,28E-08	40	0,000105049	4	1993,07741	-0,28	31,60	0
	High	hIILPEVGLDGQk	2	1	1	P74344	N-Term(iTRAQ4plex); K13(iTRAQ4plex)	0,0000	1,219	2	49,2	0	3,867E-08	39	0,000121331	3	1707,00565	0,74	33,98	0
	High	lLLFNAWDmk	3	1	1	P74344	N-Term(iTRAQ4plex); M9(Oxidation); K10(iTRAQ4plex)	0,0000	2,405	3	25,8	0	0,00002347	39	0,000136763	2	1554,85979	0,31	48,72	0
	High	qIIHGTSWVGkPk	1	1	1	P74344	N-Term(iTRAQ4plex); K11(iTRAQ4plex); K13(iTRAQ4plex)	0,0000	2,391	1		0	1,605E-07	36	0,000237121	4	1883,12148	-0,68	23,26	0
	High	lRPNPVRPVIEk	2	1	1	P74344	N-Term(iTRAQ4plex); K12(iTRAQ4plex)	0,0000	2,053	2	22,9	0	0,000116	26	0,002558409	3	1706,06797	-0,02	22,04	0
	High	hIILPEVGLDGQkR	1	1	1	P74344	N-Term(iTRAQ4plex); K13(iTRAQ4plex)	0,0000	0,667	1		0	0,002769	21	0,008016227	4	1863,10573	0,12	29,76	1
	High	lLLFNAWDMk	1	1	1	P74344	N-Term(iTRAQ4plex); K10(iTRAQ4plex)	0,0000	0,543	1		0	0,0001772	16	0,028229153	3	1538,86484	0,30	51,66	0
	High	gGITAWSR	1	1	1	P74344	N-Term(iTRAQ4plex)	0,0000	0,428	1		0,001	0,005013	14	0,03638899	2	991,54448	0,30	22,59	0
	High	aLSLLk	2	1	1	P74344	N-Term(iTRAQ4plex); K6(iTRAQ4plex)	0,0000	2,513	1		0,002	0,03029	37	0,000190972	2	932,63671	-1,71	29,76	0
P74233	Slr1160 protein OS=Synechocystis sp. (strain PCC 6803 / Kazusa) GN=slr1160 PE=4 SV=1 - [P74233	311,20	29,90	1	5	5	13	0,390	10	25,7	204	22,2	5,55							
	A3	Sequence	# PSMs	# Proteins	# Protein Groups	Protein Group Accessions	Modifications	ΔCn	116/117	116/117 Count	116/117 Variability [%]	q-Value	PEP	IonScore	Exp Value	Charge	MH+ [Da]	ΔM [ppm]	RT [min]	# Missed Cleavages
	High	aNNEPALAVPLLLQIIR	2	1	1	P74233	N-Term(iTRAQ4plex)	0,0000	0,364	2	59,0	0	1,887E-07	60	1,03746E-06	3	1989,19956	0,88	59,87	0
	High	eFRPLNQEGSILSVGAGR	7	1	1	P74233	N-Term(iTRAQ4plex)	0,0000	0,349	4	4,7	0	1,783E-13	59	1,30017E-06	3	2074,11856	1,11	36,25	0
	High	sQNPTTDLGR	1	1	1	P74233	N-Term(iTRAQ4plex)	0,0000	0,418	1		0	0,00002916	39	0,000133036	2	1232,63469	-0,40	16,24	0
	High	iAALETGQLR	1	1	1	P74233	N-Term(iTRAQ4plex)	0,0000	0,477	1		0	0,00001915	35	0,000394183	2	1215,71843	0,52	30,57	0
	High	iAALETGQLRDEATYR	2	1	1	P74233	N-Term(iTRAQ4plex)	0,0000	0,489	2	13,0	0	0,0000236	28	0,001584784	3	1951,03727	0,34	32,88	1
P74251	Sll1089 protein OS=Synechocystis sp. (strain PCC 6803 / Kazusa) GN=sll1089 PE=4 SV=1 - [P74251	306,53	15,49	1	3	3	11	0,461	9	27,6	213	23,5	4,97							
	A3	Sequence	# PSMs	# Proteins	# Protein Groups	Protein Group Accessions	Modifications	ΔCn	116/117	116/117 Count	116/117 Variability [%]	q-Value	PEP	IonScore	Exp Value	Charge	MH+ [Da]	ΔM [ppm]	RT [min]	# Missed Cleavages
	High	iYAmGVIQPLTSTR	4	1	1	P74251	N-Term(iTRAQ4plex); M4(Oxidation)	0,0000	0,409	4	26,0	0	4,109E-09	74	4,43578E-08	2	1709,93877	0,61	36,20	0
	High	iYAMGVIQPLTSTR	4	1	1	P74251	N-Term(iTRAQ4plex)	0,0000	0,553	3	0,1	0	1,314E-07	58	1,69898E-06	2	1693,94463	1,08	42,68	0
	High	aYLVGNk	1	1	1	P74251	N-Term(iTRAQ4plex); K7(iTRAQ4plex)	0,0000	0,461	1		0	0,00006513	49	1,23586E-05	2	1052,63396	-0,29	19,99	0
	High	fQRPPSGVPPIR	2	1	1	P74251	N-Term(iTRAQ4plex)	0,0000	0,458	1		0	0,00004683	28	0,001682558	3	1494,86652	0,20	25,89	0
Q59978	Protein GrpE OS=Synechocystis sp. (strain PCC 6803 / Kazusa) GN=grpE PE=3 SV=1 - [GRPE_SYNY3	301,27	30,92	1	7	7	14	0,544	13	10,1	249	27,6	4,68							
	A3	Sequence	# PSMs	# Proteins	# Protein Groups	Protein Group Accessions	Modifications	ΔCn	116/117	116/117 Count	116/117 Variability [%]	q-Value	PEP	IonScore	Exp Value	Charge	MH+ [Da]	ΔM [ppm]	RT [min]	# Missed Cleavages
	High	gYLLDDIVLR	3	1	1	Q59978	N-Term(iTRAQ4plex)	0,0000	0,511	3	8,9	0	0,000001234	63	4,48714E-07	2	1320,76433	-0,06	46,34	0
	High	tITELLPVVDNFER	2	1	1	Q59978	N-Term(iTRAQ4plex)	0,0000	0,483	2	10,3	0	0,00004565	53	5,86017E-06	2	1789,98357	1,04	51,44	0
	High	tQIkPNSDGENQIHk	2	1	1	Q59978	N-Term(iTRAQ4plex); K4(iTRAQ4plex); K15(iTRAQ4plex)	0,0000	0,552	2	11,1	0	7,466E-11	51	7,70903E-06	3	2141,16794	0,19	16,41	0
	High	sSEEIIAILQk	1	1	1	Q59978	N-Term(iTRAQ4plex); K11(iTRAQ4plex)	0,0000	0,466	1		0	0,000002366	41	7,58525E-05	3	1518,89737	-0,53	41,42	0
	High	yVALAAEFDNFRk	1	1	1	Q59978	N-Term(iTRAQ4plex); K13(iTRAQ4plex)	0,0000	0,661	1		0	0,001147	22	0,00687021	3	1831,99088	-2,00	42,21	1
	High	ekEEQAk	2	1	1	Q59978	N-Term(iTRAQ4plex); K2(iTRAQ4plex); K7(iTRAQ4plex)	0,0000	0,553	2	2,5	0	0,001694	15	0,03467129	3	1293,73587	-1,23	7,81	1
	High	sYQGVYk	3	1	1	Q59978	N-Term(iTRAQ4plex); K7(iTRAQ4plex)	0,0000	0,569	2	3,3	0,001	0,01255	28	0,00172175	2	1132,62370	-0,33	17,37	0
P36237	50S ribosomal protein L11 OS=Synechocystis sp. (strain PCC 6803 / Kazusa) GN=rplK PE=3 SV=1 - [	297,13	45,39	1	7	7	17	1,923	14	25,7	141	15,0	9,45							
	A3	Sequence	# PSMs	# Proteins	# Protein Groups	Protein Group Accessions	Modifications	ΔCn	116/117	116/117 Count	116/117 Variability [%]	q-Value	PEP	IonScore	Exp Value	Charge	MH+ [Da]	ΔM [ppm]	RT [min]	# Missed Cleavages
	High	sFTFILk	4	1	1	P36237	N-Term(iTRAQ4plex); K7(iTRAQ4plex)	0,0000	2,175	4	2,0	0	0,00002154	61	7,2773E-07	2	1143,70110	-0,46	40,16	0
	High	tPPASVLIR	4	1	1	P36237	N-Term(iTRAQ4plex)	0,0000	2,145	2	11,3	0	0,0001582	46	2,2489E-05	2	1097,68035	0,36	27,20	0
	High	lALPAGk	4	1	1	P36237	N-Term(iTRAQ4plex); K7(iTRAQ4plex)	0,0000	1,791	3	5,6	0,001	0,009225	36	0,000274138	2	957,63365	0,10	25,39	0
	High	eIAQTk	1	1	1	P36237	N-Term(iTRAQ4plex); K6(iTRAQ4plex)	0,0000	1,394	1		0,001	0,005439	20	0,013656323	2	977,58702	0,04	12,64	0
	High	tADkPGmIIPVEISVFEDR	1	1	1	P36237	N-Term(iTRAQ4plex); K4(iTRAQ4plex); M7(Oxidation)	0,0000	1,626	1		0,001	0,003588	15	0,030267044	4	2421,29360	-0,12	44,62	0
	High	gSSEPNkNk	1	1	1	P36237	N-Term(iTRAQ4plex); K7(iTRAQ4plex); K9(iTRAQ4plex)	0,0000	3,351	1		0,001	0,007162	12	0,059425111	3	1392,77716	-2,56	7,80	1
	Medium	kAAGVEk	2	1	1	P36237	N-Term(iTRAQ4plex); K1(iTRAQ4plex); K7(iTRAQ4plex)	0,0000	1,398	2	6,3	0,012	0,1591	26	0,002605974	3	1134,71482	-5,18	9,16	1
Q59987	Light-dependent protochlorophyllide reductase OS=Synechocystis sp. (strain PCC 6803 / Kazusa) GN	295,14	24,22	1	11	11	21	2,544	21	65,1	322	36,0	8,38							
	A3	Sequence	# PSMs	# Proteins	# Protein Groups	Protein Group Accessions	Modifications	ΔCn	116/117	116/117 Count	116/117 Variability [%]	q-Value	PEP	IonScore	Exp Value	Charge	MH+ [Da]	ΔM [ppm]	RT [min]	# Missed Cleavages
	High	gYVSQELAGER	1	1	1	Q59987	N-Term(iTRAQ4plex)	0,0000	2,514	1		0	0,00001076	62	6,99794E-07	2	1352,69255	-0,11	23,95	0
	High	tIFPWFQk	6	1	1	Q59987	N-Term(iTRAQ4plex); K8(iTRAQ4plex)	0,0000	4,144	6	12,7	0	0,000003995	49	1,18296E-05	2	1354,77715	0,70	46,97	0
	High	lIILGTVTANSk	1	1	1	Q59987	N-Term(iTRAQ4plex); K12(iTRAQ4plex)	0,0000	1,845	1		0	0,00000145	37	0,000197683	3	1517,95136	0,53	39,00	0
	High	kPIAmINNk	2	1	1	Q59987	N-Term(iTRAQ4plex); K1(iTRAQ4plex); M5(Oxidation); K9(iTRA	0,0000	1,682	2	67,6	0	0,0000242	33	0,000449749	3	1476,89399	0,48	15,61	1
	High	rFVAQFR	1	1	1	Q59987	N-Term(iTRAQ4plex)	0,0000	2,390	1		0	0,000467	29	0,001383471	3	1067,62369	0,55	20,89	1
	High	lcNmLTTR	1	1	1	Q59987	N-Term(iTRAQ4plex); C2(Carbamidomethyl); M4(Oxidation)	0,0000	2,659	1		0	0,0004251	24	0,003917148	2	1168,59392	0,34	20,59	0
	High	kPIAmINNkk	1	1	1	Q59987	K1(iTRAQ4plex); N-Term(iTRAQ4plex); M5(Oxidation); K9(iTRA	0,0000	3,798	1		0	0,00007147	15	0,030127979	4	1749,08779	-1,44	14,87	1
	High	kPIAMINNk	1	1	1	Q59987	N-Term(iTRAQ4plex); K1(iTRAQ4plex); K9(iTRAQ4plex)	0,0000	1,378	1		0	0,0007852	13	0,055076965	3	1460,89899	0,43	20,64	1
	High	nHYSLFR	1	1	1	Q59987	N-Term(iTRAQ4plex)	0,0000	3,192	1		0,001	0,01253	12	0,064264333	3	1080,57080	0,09	21,55	0
	High	eLGRPLk	2	1	1	Q59987	N-Term(iTRAQ4plex); K7(iTRAQ4plex)	0,0000	1,874	2	43,8	0,001	0,006277	11	0,073615625	3	1100,70243	-0,55	15,81	0
	High	fVAQFR	3	1	1	Q59987	N-Term(iTRAQ4plex)	0,0000	2,065	3	211,4	0,003	0,03709	29	0,00119116	2	911,52172	-0,30	27,31	0
	Medium	acPDADkR	1	1	1	Q59987	N-Term(iTRAQ4plex); C2(Carbamidomethyl); K7(iTRAQ4plex)	0,0000	1,587	1		0,015	0,1996	15	0,03467129	3	1220,63001	0,32	8,89	1
P74421	Phosphoglycerate kinase OS=Synechocystis sp. (strain PCC 6803 / Kazusa) GN=pgk PE=3 SV=2 - [P	294,74	36,91	1	13	13	20	1,381	17	49,3	401	41,8	5,19							
	A3	Sequence	# PSMs	# Proteins	# Protein Groups	Protein Group Accessions	Modifications	ΔCn	116/117	116/117 Count	116/117 Variability [%]	q-Value	PEP	IonScore	Exp Value	Charge	MH+ [Da]	ΔM [ppm]	RT [min]	# Missed Cleavages
	High	fHAGEEGNDAEFAk	1	1	1	P74421	N-Term(iTRAQ4plex); K14(iTRAQ4plex)	0,0000	1,724	1		0	5,423E-09	50	9,41825E-06	3	1809,86515	0,26	22,62	0
	High	iASLPNGGVALLENLR	1	1	1	P74421	N-Term(iTRAQ4plex)	0,0000	1,727	1		0	0,000005488	41	7,74408E-05	3	1781,04068	0,25	49,61	0
	High	aQGLNTGk	3	1	1	P74421	N-Term(iTRAQ4plex); K8(iTRAQ4plex)	0,0000	1,091	2	36,0	0	0,0001249	40	0,000126095	2	1076,62981	-0,42	14,22	0
	High	eLQFLQGAIEAPk	1	1	1	P74421	N-Term(iTRAQ4plex); K13(iTRAQ4plex)	0,0000	1,008	1		0	0,000002578	39	0,000134577	3	1731,98783	-0,33	43,34	0
	High	rPLVAIVGGSk	3	1	1	P74421	N-Term(iTRAQ4plex); K11(iTRAQ4plex)	0,0000	1,210	2	41,6	0	0,000006188	34	0,000416841	3	1384,88843	0,39	24,35	0
	High	iGVIETLLDk	2	1	1	P74421	N-Term(iTRAQ4plex); K10(iTRAQ4plex)	0,0000	1,077	2	141,5	0	6,621E-07	33	0,000454957	2	1388,86113	0,57	48,03	0
	High	vILGSHFGRPk	3	1	1	P74421	N-Term(iTRAQ4plex); K11(iTRAQ4plex)	0,0000	1,075	2	123,4	0	0,00009355	31	0,000731089	4	1498,90957	-0,08	22,61	0
	High	lGELLGQPVVk	1	1	1	P74421	N-Term(iTRAQ4plex); K11(iTRAQ4plex)	0,0000	1,534	1		0	0,00001679	31	0,000774408	3	1440,90314	0,18	38,60	0
	High	aHASTEGVTHFLSPNVAGYLIEk	1	1	1	P74421	N-Term(iTRAQ4plex); K23(iTRAQ4plex)	0,0000	1,055	1		0	4,656E-08	31	0,000874923	4	2729,45229	0,78	42,08	0
	High	sLVEEDkLDLAk	1	1	1	P74421	N-Term(iTRAQ4plex); K7(iTRAQ4plex); K12(iTRAQ4plex)	0,0000	1,677	1		0	0,000008839	19	0,0125603	3	1792,04148	-0,77	30,73	1
	High	aALPTIk	1	1	1	P74421	N-Term(iTRAQ4plex); K7(iTRAQ4plex)	0,0000	2,389	1		0,001	0,003813	33	0,000557147	2	1001,65984	0,07	25,97	0
	Medium	sLmAk	1	1	1	P74421	N-Term(iTRAQ4plex); M3(Oxidation); K5(iTRAQ4plex)	0,0000	1,244	1		0,024	0,3315	12	0,069979366	2	853,50426	-1,54	12,74	0
	Medium	lTPVGDR	1	1	1	P74421	N-Term(iTRAQ4plex)	0,0000	0,546	1		0,029	0,3887	12	0,064560964	2	901,52355	1,29	16,84	0
P73334	DNA-directed RNA polymerase subunit beta' OS=Synechocystis sp. (strain PCC 6803 / Kazusa) GN=rp	293,79	7,14	1	9	9	13	2,432	12	70,3	1317	144,7	4,84							
	A3	Sequence	# PSMs	# Proteins	# Protein Groups	Protein Group Accessions	Modifications	ΔCn	116/117	116/117 Count	116/117 Variability [%]	q-Value	PEP	IonScore	Exp Value	Charge	MH+ [Da]	ΔM [ppm]	RT [min]	# Missed Cleavages
	High	rLVDVSQDVIVR	2	1	1	P73334	N-Term(iTRAQ4plex)	0,0000	1,413	2	3,4	0	5,33E-10	63	5,16381E-07	3	1542,90927	0,52	30,79	1
	High	tFHTGGVFTGEVAR	2	1	1	P73334	N-Term(iTRAQ4plex)	0,0000	3,397	1		0	3,481E-08	63	5,5335E-07	3	1622,84140	0,38	26,91	0
	High	hIEVIVR	1	1	1	P73334	N-Term(iTRAQ4plex)	0,0000	0,688	1		0	0,00003265	40	0,000108635	3	1009,62851	0,99	18,54	0
	High	vQNHSIVAELIDDRYR	2	1	1	P73334	N-Term(iTRAQ4plex)	0,0000	3,648	2	25,9	0	0,00001523	36	0,000232258	4	2072,10146	0,42	36,81	1
	High	gLkENVIIGR	1	1	1	P73334	N-Term(iTRAQ4plex); K3(iTRAQ4plex)	0,0000	1,836	1		0	0,00000704	30	0,001088855	3	1386,86762	0,33	23,24	1
	High	kQGYEVTk	1	1	1	P73334	N-Term(iTRAQ4plex); K1(iTRAQ4plex); K8(iTRAQ4plex)	0,0000	2,127	1		0	0,00003496	29	0,001216102	3	1384,81556	-0,35	13,34	1
	High	kLIALAYR	1	1	1	P73334	N-Term(iTRAQ4plex); K1(iTRAQ4plex)	0,0000	4,405	1		0	0,00006484	29	0,00132425	3	1235,80680	-0,83	28,29	1
	High	gDNLVLLVFER	2	1	1	P73334	N-Term(iTRAQ4plex)	0,0000	3,736	2	45,9	0,001	0,003998	29	0,001199499	2	1418,81877	4,46	51,38	0
	Medium	tTTGGmIR	1	1	1	P73334	N-Term(iTRAQ4plex); M6(Oxidation)	0,0000	1,971	1		0,018	0,2305	11	0,079610436	2	996,52696	0,48	12,06	0
P72866	30S ribosomal protein S15 OS=Synechocystis sp. (strain PCC 6803 / Kazusa) GN=rpsO PE=3 SV=1 -	292,07	26,97	1	4	4	12	2,277	10	13,3	89	10,4	10,90							
	A3	Sequence	# PSMs	# Proteins	# Protein Groups	Protein Group Accessions	Modifications	ΔCn	116/117	116/117 Count	116/117 Variability [%]	q-Value	PEP	IonScore	Exp Value	Charge	MH+ [Da]	ΔM [ppm]	RT [min]	# Missed Cleavages
	High	lLSFINAR	4	1	1	P72866	N-Term(iTRAQ4plex)	0,0000	2,457	3	0,0	0	0,00004722	59	1,42462E-06	2	1077,65483	1,02	42,06	0
	High	iTQLTGHLk	4	1	1	P72866	N-Term(iTRAQ4plex); K9(iTRAQ4plex)	0,0000	2,201	3	10,0	0	7,448E-07	50	9,95337E-06	2	1298,80571	1,73	25,14	0
	High	rLLSFINAR	1	1	1	P72866	N-Term(iTRAQ4plex)	0,0000	2,080	1		0	0,00001026	47	2,05102E-05	3	1233,75528	0,35	33,22	1
	High	yQALIk	3	1	1	P72866	N-Term(iTRAQ4plex); K6(iTRAQ4plex)	0,0000	1,941	3	10,2	0	0,001069	30	0,001061622	2	1023,64507	0,95	25,60	0
P74500	Slr1940 protein OS=Synechocystis sp. (strain PCC 6803 / Kazusa) GN=slr1940 PE=4 SV=1 - [P74500	290,40	11,20	1	5	5	10	0,328	9	40,6	482	51,2	4,89							
	A3	Sequence	# PSMs	# Proteins	# Protein Groups	Protein Group Accessions	Modifications	ΔCn	116/117	116/117 Count	116/117 Variability [%]	q-Value	PEP	IonScore	Exp Value	Charge	MH+ [Da]	ΔM [ppm]	RT [min]	# Missed Cleavages
	High	lTLSGSGNNLk	3	1	1	P74500	N-Term(iTRAQ4plex); K11(iTRAQ4plex)	0,0000	0,560	3	20,2	0	5,531E-07	72	6,65227E-08	2	1391,81072	0,74	26,41	0
	High	nVGSIGAPGAR	3	1	1	P74500	N-Term(iTRAQ4plex)	0,0000	0,275	2	12,8	0	0,000006793	62	6,88605E-07	2	1142,64006	0,15	18,45	0
	High	nQGFNLSk	2	1	1	P74500	N-Term(iTRAQ4plex); K8(iTRAQ4plex)	0,0000	0,294	2	4,8	0	0,0003444	42	5,71439E-05	2	1195,66826	0,75	22,42	0
	High	fRPPSTYTLR	1	1	1	P74500	N-Term(iTRAQ4plex)	0,0000	0,685	1		0	0,0008133	23	0,004774963	3	1381,77207	0,86	27,12	0
	High	sFLVVWNAPQPFER	1	1	1	P74500	N-Term(iTRAQ4plex)	0,0000	0,434	1		0	0,002893	21	0,00827885	3	1833,97867	0,97	49,13	0
P72699	UPF0045 protein sll0230 OS=Synechocystis sp. (strain PCC 6803 / Kazusa) GN=sll0230 PE=3 SV=1 -	288,18	44,63	1	4	4	11	0,403	9	15,6	121	13,3	7,43							
	A3	Sequence	# PSMs	# Proteins	# Protein Groups	Protein Group Accessions	Modifications	ΔCn	116/117	116/117 Count	116/117 Variability [%]	q-Value	PEP	IonScore	Exp Value	Charge	MH+ [Da]	ΔM [ppm]	RT [min]	# Missed Cleavages
	High	acHEAVHALGAPR	7	1	1	P72699	N-Term(iTRAQ4plex); C2(Carbamidomethyl)	0,0000	0,425	5	21,5	0	2,165E-09	84	3,77546E-09	2	1532,78691	-0,26	14,33	0
	High	hTmHAYGTNIEGDWDEVFAAVk	1	1	1	P72699	N-Term(iTRAQ4plex); M3(Oxidation); K22(iTRAQ4plex)	0,0000	0,366	1		0	1,598E-07	30	0,001076391	4	2795,33657	0,98	44,99	0
	High	tDRPQTmDEk	2	1	1	P72699	N-Term(iTRAQ4plex); M7(Oxidation); K10(iTRAQ4plex)	0,0000	0,362	2	0,5	0	0,002423	19	0,013457674	3	1524,75757	0,58	8,23	0
	High	sVETWLENS	1	1	1	P72699	N-Term(iTRAQ4plex)	0,0000	0,782	1		0,001	0,006996	37	0,000202754	2	1208,59245	0,72	40,05	0
Q55458	Uncharacterized protein slr0039 OS=Synechocystis sp. (strain PCC 6803 / Kazusa) GN=slr0039 PE=4	284,21	11,20	1	4	4	10	1,271	9	24,0	357	38,4	4,91							
	A3	Sequence	# PSMs	# Proteins	# Protein Groups	Protein Group Accessions	Modifications	ΔCn	116/117	116/117 Count	116/117 Variability [%]	q-Value	PEP	IonScore	Exp Value	Charge	MH+ [Da]	ΔM [ppm]	RT [min]	# Missed Cleavages
	High	iNAAVLLVGR	4	1	1	Q55458	N-Term(iTRAQ4plex)	0,0000	1,271	3	5,5	0	0,000000737	76	2,57614E-08	2	1169,74809	-0,53	39,92	0
	High	yHSPLVVDGLLk	2	1	1	Q55458	N-Term(iTRAQ4plex); K12(iTRAQ4plex)	0,0000	0,938	2	26,0	0	0,00003312	39	0,000132187	3	1628,96024	-0,74	36,44	0
	High	kPLVFLDEk	3	1	1	Q55458	N-Term(iTRAQ4plex); K1(iTRAQ4plex); K9(iTRAQ4plex)	0,0000	1,491	3	13,9	0	6,476E-08	32	0,000571439	3	1520,93918	-1,37	31,63	0
	High	eLAHQLNAk	1	1	1	Q55458	N-Term(iTRAQ4plex); K9(iTRAQ4plex)	0,0000	1,627	1		0,001	0,008109	14	0,038191789	3	1311,76691	3,49	15,75	0
P73103	Slr1908 protein OS=Synechocystis sp. (strain PCC 6803 / Kazusa) GN=slr1908 PE=4 SV=1 - [P73103	282,91	14,55	1	4	8	12	0,673	8	20,5	591	64,5	5,26							
	A3	Sequence	# PSMs	# Proteins	# Protein Groups	Protein Group Accessions	Modifications	ΔCn	116/117	116/117 Count	116/117 Variability [%]	q-Value	PEP	IonScore	Exp Value	Charge	MH+ [Da]	ΔM [ppm]	RT [min]	# Missed Cleavages
	High	lQAGSVPNLSGPTGTNMAR	1	1	1	P73103	N-Term(iTRAQ4plex)	0,0000	0,778	1		0	2,191E-10	70	1,14391E-07	3	2015,04801	0,93	33,91	0
	High	lQAGSVPNLSGPTGTNmAR	2	1	1	P73103	N-Term(iTRAQ4plex); M17(Oxidation)	0,0000	0,609	2	15,4	0	1,247E-07	63	5,5077E-07	3	2031,04392	1,41	30,49	0
	High	nSPYIIEAQYQFPVNk	1	1	1	P73103	N-Term(iTRAQ4plex); K16(iTRAQ4plex)	0,0000	0,765	1		0	1,111E-10	51	8,74923E-06	3	2199,17044	0,72	40,56	0
	High	rLmQEFEAELAALGAR	1	2	2	P73409;P73103	N-Term(iTRAQ4plex); M3(Oxidation)	0,0000				0	0,000002579	49	1,18024E-05	3	1965,03531	0,40	48,01	1
	High	lmQEFEAELAALGAR	1	2	2	P73409;P73103	N-Term(iTRAQ4plex); M2(Oxidation)	0,0000				0	0,000001108	35	0,000349921	3	1808,93277	-0,35	51,97	0
	High	yGcIVGYPDR	1	2	2	P73409;P73103	N-Term(iTRAQ4plex); C3(Carbamidomethyl)	0,0000				0	0,00009834	33	0,000486374	2	1343,65203	-1,07	28,81	0
	High	dVQPTAWAYEALk	1	2	2	P73409;P73103	N-Term(iTRAQ4plex); K13(iTRAQ4plex)	0,0000				0	0,000001707	33	0,000562586	3	1779,95212	0,06	41,35	0
	High	ySPFVNR	3	1	1	P73103	N-Term(iTRAQ4plex)	0,0000	0,571	3	1,9	0,001	0,01688	12	0,057938868	2	1026,54741	-1,46	24,95	0
	Medium	tTFkF	1	1	1	P73103	N-Term(iTRAQ4plex); K4(iTRAQ4plex)	0,0000	0,749	1		0,045	0,5823	14	0,042851892	2	931,54802	-1,20	27,42	1
Q55387	Periplasmic binding protein of ABC transporter for natural amino acids OS=Synechocystis sp. (strain F	280,19	13,66	1	5	5	11	0,759	11	17,1	454	46,6	4,56							
	A3	Sequence	# PSMs	# Proteins	# Protein Groups	Protein Group Accessions	Modifications	ΔCn	116/117	116/117 Count	116/117 Variability [%]	q-Value	PEP	IonScore	Exp Value	Charge	MH+ [Da]	ΔM [ppm]	RT [min]	# Missed Cleavages
	High	sLEAFTAQWk	3	1	1	Q55387	N-Term(iTRAQ4plex); K10(iTRAQ4plex)	0,0000	0,783	3	3,6	0	4,758E-09	75	3,31108E-08	2	1468,80535	1,00	40,53	0
	High	tAPPDTYQAQALAALAk	1	1	1	Q55387	N-Term(iTRAQ4plex); K17(iTRAQ4plex)	0,0000	1,708	1		0	8,715E-11	66	2,9779E-07	3	2018,11649	0,18	38,84	0
	High	nNImmISPGSTSPVFTDQAk	1	1	1	Q55387	N-Term(iTRAQ4plex); M4(Oxidation); M5(Oxidation); K20(iTRA	0,0000	0,638	1		0	1,219E-13	58	1,46208E-06	3	2458,22202	0,94	32,48	0
	High	sNTGAGIQSk	5	1	1	Q55387	N-Term(iTRAQ4plex); K10(iTRAQ4plex)	0,0000	0,759	5	11,8	0	0,00005705	58	1,69587E-06	2	1250,69560	1,03	13,01	0
	Medium	gFWAR	1	1	1	Q55387	N-Term(iTRAQ4plex)	0,0000	0,637	1		0,016	0,2149	19	0,012617404	2	780,42790	0,70	24,22	0
P73313	50S ribosomal protein L16 OS=Synechocystis sp. (strain PCC 6803 / Kazusa) GN=rplP PE=3 SV=1 - [	279,40	35,25	1	4	4	12	1,314	12	86,5	139	16,0	11,06							
	A3	Sequence	# PSMs	# Proteins	# Protein Groups	Protein Group Accessions	Modifications	ΔCn	116/117	116/117 Count	116/117 Variability [%]	q-Value	PEP	IonScore	Exp Value	Charge	MH+ [Da]	ΔM [ppm]	RT [min]	# Missed Cleavages
	High	vmFELAGVSEEVAR	2	1	1	P73313	N-Term(iTRAQ4plex); M2(Oxidation)	0,0000	2,886	2	210,0	0	4,767E-09	76	2,50594E-08	2	1696,87163	1,12	40,47	0
	High	vMFELAGVSEEVAR	1	1	1	P73313	N-Term(iTRAQ4plex)	0,0000	0,496	1		0	6,019E-07	54	4,40524E-06	2	1680,87700	1,30	46,02	0
	High	gSPEYWVAVVkPGR	5	1	1	P73313	N-Term(iTRAQ4plex); K11(iTRAQ4plex)	0,0000	1,480	5	82,8	0	2,973E-07	39	0,000160953	4	1833,03029	2,24	35,79	0
	High	iFPDkPVTmRPAETR	1	1	1	P73313	N-Term(iTRAQ4plex); K5(iTRAQ4plex); M9(Oxidation)	0,0000	36,134	1		0	0,0006596	11	0,114903635	4	2062,13735	0,73	23,35	0
	High	qIEAAR	3	1	1	P73313	N-Term(iTRAQ4plex)	0,0000	0,834	3	1,9	0,003	0,0363	28	0,001908525	2	831,48180	1,54	13,26	0
Q55113	Slr0431 protein OS=Synechocystis sp. (strain PCC 6803 / Kazusa) GN=slr0431 PE=4 SV=1 - [Q5511	276,03	8,40	1	2	2	6	0,428	6	10,0	250	27,0	8,37							
	A3	Sequence	# PSMs	# Proteins	# Protein Groups	Protein Group Accessions	Modifications	ΔCn	116/117	116/117 Count	116/117 Variability [%]	q-Value	PEP	IonScore	Exp Value	Charge	MH+ [Da]	ΔM [ppm]	RT [min]	# Missed Cleavages
	High	vVLSSLNLTPQNIVR	4	1	1	Q55113	N-Term(iTRAQ4plex)	0,0000	0,455	4	13,5	0	1,251E-10	96	2,52912E-10	2	1797,07390	1,33	45,18	0
	High	lVAYLR	2	1	1	Q55113	N-Term(iTRAQ4plex)	0,0000	0,411	2	3,9	0,006	0,07715	31	0,001114064	2	878,56072	3,06	31,01	0
P52981	1,4-alpha-glucan branching enzyme GlgB OS=Synechocystis sp. (strain PCC 6803 / Kazusa) GN=glgB	274,35	15,84	1	11	11	17	1,555	16	22,2	770	89,5	5,62							
	A3	Sequence	# PSMs	# Proteins	# Protein Groups	Protein Group Accessions	Modifications	ΔCn	116/117	116/117 Count	116/117 Variability [%]	q-Value	PEP	IonScore	Exp Value	Charge	MH+ [Da]	ΔM [ppm]	RT [min]	# Missed Cleavages
	High	gVYFAVWAPNAR	3	1	1	P52981	N-Term(iTRAQ4plex)	0,0000	1,564	3	30,9	0	0,000001132	66	2,31206E-07	2	1494,79949	1,36	42,10	0
	High	lGAHLmTVDGVk	1	1	1	P52981	N-Term(iTRAQ4plex); M6(Oxidation); K12(iTRAQ4plex)	0,0000	1,546	1		0	8,591E-07	52	6,63697E-06	3	1544,87180	0,56	25,52	0
	High	aLFTYMFTHPGk	1	1	1	P52981	N-Term(iTRAQ4plex); K12(iTRAQ4plex)	0,0000	3,113	1		0	1,693E-07	38	0,000167869	3	1700,91080	2,06	42,07	0
	High	tDPYGFYQEVRPk	1	1	1	P52981	N-Term(iTRAQ4plex); K13(iTRAQ4plex)	0,0000	1,450	1		0	1,907E-08	36	0,00030757	3	1887,98490	0,29	31,53	0
	High	aLFTYmFTHPGk	2	1	1	P52981	N-Term(iTRAQ4plex); M6(Oxidation); K12(iTRAQ4plex)	0,0000	1,827	2	1,5	0	5,423E-09	29	0,00128289	3	1716,90305	0,49	34,84	0
	High	aYLPTAEAVTVLLPTDRR	2	1	1	P52981	N-Term(iTRAQ4plex)	0,0000	1,510	2	3,0	0	0,0002444	26	0,003001876	3	2130,20523	0,58	45,64	1
	High	qFFTELNHLYk	1	1	1	P52981	N-Term(iTRAQ4plex); K11(iTRAQ4plex)	0,0000	2,250	1		0	0,000003349	25	0,0028772	3	1727,93662	0,39	39,99	0
	High	hSVVSFIR	1	1	1	P52981	N-Term(iTRAQ4plex)	0,0000	2,257	1		0	0,001279	25	0,003491162	3	1088,63468	1,24	24,31	0
	High	aLFTYmFTHPGkk	1	1	1	P52981	N-Term(iTRAQ4plex); M6(Oxidation); K12(iTRAQ4plex); K13(iT	0,0000	1,545	1		0	0,0009228	20	0,011116549	4	1989,10451	2,65	29,98	1
	High	nWEGHIYEk	1	1	1	P52981	N-Term(iTRAQ4plex); K9(iTRAQ4plex)	0,0000	1,796	1		0	0,002824	19	0,013030768	3	1463,75122	-0,64	22,59	0
	High	dGHGLAFFDGTHLYEHGDPR	2	1	1	P52981	N-Term(iTRAQ4plex)	0,0000	1,449	1		0	0,00006766	15	0,028705823	5	2385,11396	0,42	33,20	0
	Medium	sNmLGk	1	1	1	P52981	N-Term(iTRAQ4plex); M3(Oxidation); K6(iTRAQ4plex)	0,0000	1,031	1		0,027	0,3654	10	0,095273036	2	953,53118	-1,76	12,27	0
P73298	30S ribosomal protein S11 OS=Synechocystis sp. (strain PCC 6803 / Kazusa) GN=rpsK PE=3 SV=1 -	269,02	22,31	1	2	2	9	2,279	6	132,0	130	13,8	11,36							
	A3	Sequence	# PSMs	# Proteins	# Protein Groups	Protein Group Accessions	Modifications	ΔCn	116/117	116/117 Count	116/117 Variability [%]	q-Value	PEP	IonScore	Exp Value	Charge	MH+ [Da]	ΔM [ppm]	RT [min]	# Missed Cleavages
	High	kGTPYAAQTAADSAAR	4	1	1	P73298	N-Term(iTRAQ4plex); K1(iTRAQ4plex)	0,0000	4,287	2	12,6	0	5,388E-09	84	3,8815E-09	3	1866,99295	0,91	17,35	1
	High	aLQGAGLEITLIR	5	1	1	P73298	N-Term(iTRAQ4plex)	0,0000	1,168	4	28,9	0	0,000006429	63	5,06956E-07	2	1498,90923	1,21	42,82	0
Q55120	Biotin carboxyl carrier protein of acetyl-CoA carboxylase OS=Synechocystis sp. (strain PCC 6803 / Ka	267,19	14,29	1	2	2	9	0,471	9	38,6	154	16,3	4,32							
	A3	Sequence	# PSMs	# Proteins	# Protein Groups	Protein Group Accessions	Modifications	ΔCn	116/117	116/117 Count	116/117 Variability [%]	q-Value	PEP	IonScore	Exp Value	Charge	MH+ [Da]	ΔM [ppm]	RT [min]	# Missed Cleavages
	High	aINFTELR	3	1	1	Q55120	N-Term(iTRAQ4plex)	0,0000	0,471	3	5,6	0	0,00002894	56	2,38765E-06	2	1107,62773	-0,16	34,11	0
	High	wTAIVSPmVGTFYR	4	1	1	Q55120	N-Term(iTRAQ4plex); M8(Oxidation)	0,0000	0,407	4	17,2	0	0,00002778	53	4,84139E-06	3	1787,92765	0,27	44,35	0
	High	wTAIVSPMVGTFYR	2	1	1	Q55120	N-Term(iTRAQ4plex)	0,0000	0,784	2	3,6	0	0,000246	31	0,000860934	2	1771,93535	1,75	50,87	0
P74769	Ssr1528 protein OS=Synechocystis sp. (strain PCC 6803 / Kazusa) GN=ssr1528 PE=4 SV=1 - [P7476	266,84	47,87	1	4	4	9	0,417	8	13,2	94	10,1	7,42							
	A3	Sequence	# PSMs	# Proteins	# Protein Groups	Protein Group Accessions	Modifications	ΔCn	116/117	116/117 Count	116/117 Variability [%]	q-Value	PEP	IonScore	Exp Value	Charge	MH+ [Da]	ΔM [ppm]	RT [min]	# Missed Cleavages
	High	hIVQAELDQk	3	1	1	P74769	N-Term(iTRAQ4plex); K10(iTRAQ4plex)	0,0000	0,388	3	4,8	0	0,000001037	61	8,14648E-07	2	1468,83660	0,24	19,03	0
	High	lELYHQVMGLEAGR	2	1	1	P74769	N-Term(iTRAQ4plex)	0,0000	0,656	2	6,2	0	8,995E-09	56	2,26971E-06	3	1759,92826	0,02	39,16	0
	High	lELYHQVmGLEAGR	1	1	1	P74769	N-Term(iTRAQ4plex); M8(Oxidation)	0,0000	0,393	1		0	1,595E-09	38	0,000145201	3	1775,92234	-0,45	34,79	0
	High	dkEIAFFYGAk	1	1	1	P74769	N-Term(iTRAQ4plex); K2(iTRAQ4plex); K11(iTRAQ4plex)	0,0000	0,436	1		0	4,126E-08	35	0,00034512	3	1720,95993	-2,05	33,58	1
	High	lIDAGFAPLkDk	2	1	1	P74769	N-Term(iTRAQ4plex); K10(iTRAQ4plex); K12(iTRAQ4plex)	0,0000	0,399	1		0	0,00007144	19	0,011994165	3	1720,03470	-1,32	35,89	1
Q55119	Elongation factor P OS=Synechocystis sp. (strain PCC 6803 / Kazusa) GN=efp PE=3 SV=1 - [EFP_SY	266,41	37,97	1	5	5	12	0,559	10	39,9	187	20,4	4,89							
	A3	Sequence	# PSMs	# Proteins	# Protein Groups	Protein Group Accessions	Modifications	ΔCn	116/117	116/117 Count	116/117 Variability [%]	q-Value	PEP	IonScore	Exp Value	Charge	MH+ [Da]	ΔM [ppm]	RT [min]	# Missed Cleavages
	High	sVQTGNVVEk	4	1	1	Q55119	N-Term(iTRAQ4plex); K10(iTRAQ4plex)	0,0000	0,559	2	2,1	0	2,041E-08	68	1,69032E-07	2	1348,76726	-0,17	17,46	0
	High	aGETVPQANIEk	3	1	1	Q55119	N-Term(iTRAQ4plex); K12(iTRAQ4plex)	0,0000	1,413	3	102,4	0	7,995E-08	56	2,31723E-06	2	1544,85344	0,74	22,16	0
	High	gDTATGGTkPAILETGAQVmVPLF	1	1	1	Q55119	N-Term(iTRAQ4plex); K9(iTRAQ4plex); M20(Oxidation)	0,0000	0,371	1		0	1,031E-09	52	6,85441E-06	4	3332,77871	0,69	46,13	0
	High	vVEFLHVkPGk	2	1	1	Q55119	N-Term(iTRAQ4plex); K8(iTRAQ4plex); K11(iTRAQ4plex)	0,0000	0,400	2	5,6	0	3,068E-08	30	0,000891189	3	1685,04764	0,10	29,24	1
	High	sVmQHTYk	1	1	1	Q55119	N-Term(iTRAQ4plex); M3(Oxidation); K8(iTRAQ4plex)	0,0000	0,484	1		0	0,0003974	24	0,004456255	3	1297,67987	-1,09	9,69	0
	High	sVMQHTYk	1	1	1	Q55119	N-Term(iTRAQ4plex); K8(iTRAQ4plex)	0,0000	0,698	1		0,005	0,06818	12	0,069178319	3	1281,68772	1,05	13,83	0
P74467	Probable ATP-dependent Clp protease proteolytic subunit 3 OS=Synechocystis sp. (strain PCC 6803 /	265,96	29,21	1	6	6	13	1,504	11	23,9	202	22,4	6,04							
	A3	Sequence	# PSMs	# Proteins	# Protein Groups	Protein Group Accessions	Modifications	ΔCn	116/117	116/117 Count	116/117 Variability [%]	q-Value	PEP	IonScore	Exp Value	Charge	MH+ [Da]	ΔM [ppm]	RT [min]	# Missed Cleavages
	High	iIFLGQEVNDSIANR	2	1	1	P74467	N-Term(iTRAQ4plex)	0,0000	1,442	2	1,4	0	3,718E-11	79	1,21891E-08	2	1833,00005	0,71	44,35	0
	High	wIDIYTR	2	1	1	P74467	N-Term(iTRAQ4plex)	0,0000	1,644	2	10,4	0	0,0001269	45	2,97831E-05	2	1110,60686	0,38	36,97	0
	High	lALPHAR	4	1	1	P74467	N-Term(iTRAQ4plex)	0,0000	1,728	2	3,5	0	0,001686	32	0,000580724	3	921,57529	0,22	20,75	0
	High	imIHQPmGGTGR	1	1	1	P74467	N-Term(iTRAQ4plex); M2(Oxidation); M7(Oxidation)	0,0000	0,701	1		0	0,0003708	26	0,002471553	3	1473,74128	-0,73	17,57	0
	High	iMIHQPmGGTGR	1	1	1	P74467	N-Term(iTRAQ4plex); M7(Oxidation)	0,0000	0,903	1		0,001	0,007285	12	0,068072234	3	1457,74775	0,22	18,85	0
	High	pIGVPSVPFR	2	1	1	P74467	N-Term(iTRAQ4plex)	0,0000	1,792	2	26,4	0,003	0,03984	25	0,003005869	2	1212,72331	0,95	36,84	0
	Medium	lPGSQYER	1	1	1	P74467	N-Term(iTRAQ4plex)	0,0000	1,153	1		0,013	0,1659	28	0,001472211	2	1093,57500	-0,79	20,88	0
P73299	30S ribosomal protein S13 OS=Synechocystis sp. (strain PCC 6803 / Kazusa) GN=rpsM PE=3 SV=1 -	265,45	54,33	1	9	9	17	1,161	17	30,6	127	14,6	10,59							
	A3	Sequence	# PSMs	# Proteins	# Protein Groups	Protein Group Accessions	Modifications	ΔCn	116/117	116/117 Count	116/117 Variability [%]	q-Value	PEP	IonScore	Exp Value	Charge	MH+ [Da]	ΔM [ppm]	RT [min]	# Missed Cleavages
	High	sHEILDATGVSPDVR	1	1	1	P73299	N-Term(iTRAQ4plex)	0,0000	1,161	1		0	2,602E-08	61	8,51079E-07	3	1739,90366	-0,49	27,36	0
	High	vkDLSDEDALk	1	1	1	P73299	N-Term(iTRAQ4plex); K2(iTRAQ4plex); K11(iTRAQ4plex)	0,0000	1,930	1		0	9,079E-09	56	2,36576E-06	3	1664,94468	0,93	23,77	1
	High	iAGVDLPR	2	1	1	P73299	N-Term(iTRAQ4plex)	0,0000	1,567	2	24,6	0	0,0008038	51	8,14648E-06	2	984,59526	-0,64	29,80	0
	High	rLGDIGTYR	1	1	1	P73299	N-Term(iTRAQ4plex)	0,0000	1,726	1		0	0,00008726	46	2,89674E-05	3	1194,67197	0,67	21,85	1
	High	lGDIGTYR	2	1	1	P73299	N-Term(iTRAQ4plex)	0,0000	0,917	2	41,4	0	0,0004537	31	0,000864908	2	1038,57024	0,18	26,78	0
	High	rWEAmNIk	2	1	1	P73299	N-Term(iTRAQ4plex); M5(Oxidation); K8(iTRAQ4plex)	0,0000	1,121	2	7,1	0	0,0002909	17	0,017987467	3	1351,73957	0,04	18,93	1
	High	rLTVAGk	3	1	1	P73299	N-Term(iTRAQ4plex); K7(iTRAQ4plex)	0,0000	1,105	3	31,3	0,001	0,01444	23	0,008877128	3	1032,67499	-1,77	13,66	1
	Medium	qGLPVR	4	1	1	P73299	N-Term(iTRAQ4plex)	0,0000	0,899	4	10,9	0,01	0,1319	23	0,004677028	2	813,50542	-1,13	16,41	0
	Medium	kTPAk	1	1	1	P73299	N-Term(iTRAQ4plex); K1(iTRAQ4plex); K5(iTRAQ4plex)	0,0000	1,558	1		0,044	0,5682	11	0,087896181	3	976,64618	-5,49	8,04	1
Q55196	Phosphate import ATP-binding protein PstB 1 OS=Synechocystis sp. (strain PCC 6803 / Kazusa) GN=	264,71	17,47	1	4	4	8	0,554	8	14,7	269	30,2	6,02							
	A3	Sequence	# PSMs	# Proteins	# Protein Groups	Protein Group Accessions	Modifications	ΔCn	116/117	116/117 Count	116/117 Variability [%]	q-Value	PEP	IonScore	Exp Value	Charge	MH+ [Da]	ΔM [ppm]	RT [min]	# Missed Cleavages
	High	aSGFSLSGGQQQR	3	1	1	Q55196	N-Term(iTRAQ4plex)	0,0000	0,499	3	1,7	0	4,43E-10	77	2,06524E-08	2	1466,74700	0,08	23,02	0
	High	lNDLIESFR	1	1	1	Q55196	N-Term(iTRAQ4plex)	0,0000	0,531	1		0	0,00001999	43	5,41624E-05	2	1250,68535	-0,65	45,00	0
	High	aALWDEVkDk	2	1	1	Q55196	N-Term(iTRAQ4plex); K8(iTRAQ4plex); K10(iTRAQ4plex)	0,0000	0,588	2	2,3	0	2,118E-07	31	0,00084522	3	1606,91397	-1,58	31,23	1
	High	kYIGMVFQkPNPFPk	1	1	1	Q55196	N-Term(iTRAQ4plex); K1(iTRAQ4plex); K9(iTRAQ4plex); K15(iT	0,0000	0,989	1		0	6,514E-07	23	0,004539103	4	2370,38540	-0,02	35,70	1
	High	kYIGmVFQkPNPFPk	1	1	1	Q55196	K1(iTRAQ4plex); N-Term(iTRAQ4plex); M5(Oxidation); K9(iTRA	0,0000	0,929	1		0	0,00006113	13	0,050578973	4	2386,37685	-1,47	32,48	1
P73672	4-hydroxy-3-methylbut-2-en-1-yl diphosphate synthase OS=Synechocystis sp. (strain PCC 6803 / Kaz	264,14	20,84	1	6	6	12	1,266	10	11,2	403	44,1	5,96							
	A3	Sequence	# PSMs	# Proteins	# Protein Groups	Protein Group Accessions	Modifications	ΔCn	116/117	116/117 Count	116/117 Variability [%]	q-Value	PEP	IonScore	Exp Value	Charge	MH+ [Da]	ΔM [ppm]	RT [min]	# Missed Cleavages
	High	tLFNLEDVLHEVR	2	1	1	P73672	N-Term(iTRAQ4plex)	0,0000	1,385	2	10,5	0	2,17E-11	76	2,49442E-08	3	1728,94077	0,35	50,03	0
	High	rVPETDGVQELINLIk	3	1	1	P73672	N-Term(iTRAQ4plex); K16(iTRAQ4plex)	0,0000	1,170	3	17,8	0	4,431E-09	54	3,9443E-06	3	2112,22867	0,91	49,58	1
	High	iRETLEPLVISLR	4	1	1	P73672	N-Term(iTRAQ4plex)	0,0000	1,210	2	3,8	0	5,919E-07	42	5,94251E-05	3	1683,02964	0,62	42,66	1
	High	sTAGIATLLADGIGDTIR	1	1	1	P73672	N-Term(iTRAQ4plex)	0,0000	2,209	1		0	0,00003086	37	0,000256555	3	1889,04526	-0,45	53,57	0
	High	iNPGLYVFEkPDAQR	1	1	1	P73672	N-Term(iTRAQ4plex); K10(iTRAQ4plex)	0,0000	1,292	1		0	0,000001081	23	0,00540717	3	2035,12278	0,60	36,85	0
	High	vPVmLAAYR	1	1	1	P73672	N-Term(iTRAQ4plex); M4(Oxidation)	0,0000	1,017	1		0,005	0,05555	28	0,001566643	2	1179,66789	0,18	30,89	0
P72929	Uncharacterized protein sll1021 OS=Synechocystis sp. (strain PCC 6803 / Kazusa) GN=sll1021 PE=4	261,54	9,96	1	6	6	10	1,159	8	36,5	673	74,4	5,12							
	A3	Sequence	# PSMs	# Proteins	# Protein Groups	Protein Group Accessions	Modifications	ΔCn	116/117	116/117 Count	116/117 Variability [%]	q-Value	PEP	IonScore	Exp Value	Charge	MH+ [Da]	ΔM [ppm]	RT [min]	# Missed Cleavages
	High	lAVIVAQQDAQQHR	2	1	1	P72929	N-Term(iTRAQ4plex)	0,0000	1,272	1		0	8,443E-09	68	1,4157E-07	3	1720,95609	-0,85	26,60	0
	High	kELESQEAQQQR	2	1	1	P72929	N-Term(iTRAQ4plex); K1(iTRAQ4plex)	0,0000	0,960	2	18,3	0	1,179E-08	58	1,44877E-06	3	1761,93253	-0,49	11,95	1
	High	kTIEASQLQAk	1	1	1	P72929	N-Term(iTRAQ4plex); K1(iTRAQ4plex); K11(iTRAQ4plex)	0,0000	1,238	1		0	2,398E-08	46	2,58803E-05	3	1648,99546	-0,22	18,48	1
	High	tQITEQTAAIAIANk	1	1	1	P72929	N-Term(iTRAQ4plex); K15(iTRAQ4plex)	0,0000	0,905	1		0	0,000003549	41	8,7694E-05	3	1861,06290	-0,25	33,33	0

	High	kGQISEADIk	2	1	1	P72929	N-Term(iTRAQ4plex); K1(iTRAQ4plex); K10(iTRAQ4plex)	0,0000	1,019	2	57,0	0	0,0007633	38	0,000174169	3	1520,89908	-1,17	18,30	1
	High	kGQISEADIkDALEk	2	1	1	P72929	N-Term(iTRAQ4plex); K1(iTRAQ4plex); K10(iTRAQ4plex); K15(	0,0000	2,216	1		0	5,776E-07	34	0,00037151	4	2221,28701	-0,73	31,74	2
	>sp|K1C10_HUMAN|	261,25	14,84	1	9	10	15	0,528	14	22,2	593	59,5	5,21							
	A3	Sequence	# PSMs	# Proteins	# Protein Groups	Protein Group Accessions	Modifications	ΔCn	116/117	116/117 Count	116/117 Variability [%]	q-Value	PEP	IonScore	Exp Value	Charge	MH+ [Da]	ΔM [ppm]	RT [min]	# Missed Cleavages
	High	sQYEQLAEQNRk	2	1	1		N-Term(iTRAQ4plex); K12(iTRAQ4plex)	0,0000	0,511	2	5,7	0	8,855E-09	46	2,29071E-05	3	1781,94138	1,63	18,58	1
	High	lkYENEVALR	2	1	1		N-Term(iTRAQ4plex); K2(iTRAQ4plex)	0,0000	0,421	2	9,5	0	0,000003979	40	9,54993E-05	3	1522,88376	0,38	25,65	1
	High	lASYLDkVR	2	1	1		N-Term(iTRAQ4plex); K7(iTRAQ4plex)	0,0000	0,603	2	4,5	0	0,00001702	39	0,000112194	3	1352,81357	-0,34	32,36	1
	High	qSVEADINGLRR	1	1	1		N-Term(iTRAQ4plex)	0,0000	0,554	1		0	0,00003112	34	0,000416841	3	1501,82096	0,38	26,10	1
	High	iRLENEIQTYR	1	1	1		N-Term(iTRAQ4plex)	0,0000	0,492	1		0	0,00001974	32	0,000654591	3	1578,87357	0,95	29,23	1
	High	rVLDELTLTk	1	1	1		N-Term(iTRAQ4plex); K10(iTRAQ4plex)	0,0000	1,034	1		0	0,00001489	29	0,001279293	3	1475,90320	-0,27	31,94	1
	High	ikEWYEk	3	1	1		N-Term(iTRAQ4plex); K2(iTRAQ4plex); K7(iTRAQ4plex)	0,0000	0,462	3	10,8	0	0,0001409	16	0,028196117	3	1427,82309	-1,95	23,89	1
	High	lASYLDk	1	2	2		N-Term(iTRAQ4plex); K7(iTRAQ4plex)	0,0000				0,001	0,00638	28	0,001415696	2	1097,64348	-0,93	29,68	0
	High	sEITELRR	1	1	1		N-Term(iTRAQ4plex)	0,0000	0,823	1		0,002	0,02333	18	0,025970717	3	1147,65900	3,32	15,93	1
	High	nHEEEMkDLR	1	1	1		N-Term(iTRAQ4plex); K7(iTRAQ4plex)	0,0000	0,733	1		0,006	0,08143	12	0,068860473	3	1588,80063	0,89	14,09	1
P74426	Sll0359 protein OS=Synechocystis sp. (strain PCC 6803 / Kazusa) GN=sll0359 PE=1 SV=1 - [P74426_	260,34	18,06	1	4	4	12	1,492	9	16,5	155	17,2	9,11							
	A3	Sequence	# PSMs	# Proteins	# Protein Groups	Protein Group Accessions	Modifications	ΔCn	116/117	116/117 Count	116/117 Variability [%]	q-Value	PEP	IonScore	Exp Value	Charge	MH+ [Da]	ΔM [ppm]	RT [min]	# Missed Cleavages
	High	acGYYSTSk	4	1	1	P74426	N-Term(iTRAQ4plex); C2(Carbamidomethyl); K9(iTRAQ4plex)	0,0000	1,336	3	16,5	0	0,000001187	63	5,46978E-07	2	1324,64519	0,47	16,69	0
	High	gVPLDPSGTk	1	1	1	P74426	N-Term(iTRAQ4plex); K10(iTRAQ4plex)	0,0000	1,248	1		0	0,000003866	57	2,16257E-06	2	1258,72392	-0,51	21,52	0
	High	vkELSHLPR	2	1	1	P74426	N-Term(iTRAQ4plex); K2(iTRAQ4plex)	0,0000	1,933	2	5,1	0	0,00001661	34	0,000404752	3	1366,84183	0,66	20,29	1
	High	eLSHLPR	4	1	1	P74426	N-Term(iTRAQ4plex)	0,0000	1,566	3	2,5	0	0,00003702	26	0,002360315	3	995,57535	-0,12	17,36	0
P72655	Uncharacterized protein slr1128 OS=Synechocystis sp. (strain PCC 6803 / Kazusa) GN=slr1128 PE=3	259,28	19,31	1	6	6	12	2,033	12	41,9	321	35,7	5,68							
	A3	Sequence	# PSMs	# Proteins	# Protein Groups	Protein Group Accessions	Modifications	ΔCn	116/117	116/117 Count	116/117 Variability [%]	q-Value	PEP	IonScore	Exp Value	Charge	MH+ [Da]	ΔM [ppm]	RT [min]	# Missed Cleavages
	High	nILSTLEGVR	3	1	1	P72655	N-Term(iTRAQ4plex)	0,0000	1,989	3	6,5	0	0,000004012	54	4,44601E-06	2	1245,72881	0,36	42,63	0
	High	lTPGLNFTVPILDR	2	1	1	P72655	N-Term(iTRAQ4plex)	0,0000	2,360	2	31,5	0	0,00001426	47	2,37783E-05	2	1699,98760	0,71	51,03	0
	High	kLTPGLNFTVPILDR	3	1	1	P72655	N-Term(iTRAQ4plex); K1(iTRAQ4plex)	0,0000	2,783	3	2,5	0	7,129E-09	44	4,44601E-05	3	1972,18412	0,35	45,52	1
	High	aTAEALSILTEk	1	1	1	P72655	N-Term(iTRAQ4plex); K12(iTRAQ4plex)	0,0000	1,159	1		0	0,0003332	26	0,002875397	3	1534,89328	0,12	39,84	0
	High	iVNEkNEYLVER	2	1	1	P72655	N-Term(iTRAQ4plex); K5(iTRAQ4plex)	0,0000	1,848	2	22,4	0	0,000003013	25	0,002857393	3	1794,00144	0,79	25,88	1
	High	aAILNAEAEQQkk	1	1	1	P72655	N-Term(iTRAQ4plex); K12(iTRAQ4plex); K13(iTRAQ4plex)	0,0000	0,984	1		0	0,0001939	24	0,004385004	3	1846,07975	2,09	21,27	1
Q55385	Probable 30S ribosomal protein PSRP-3 OS=Synechocystis sp. (strain PCC 6803 / Kazusa) GN=slr092	258,29	40,18	1	4	4	10	0,985	10	6,4	112	12,6	5,12							
	A3	Sequence	# PSMs	# Proteins	# Protein Groups	Protein Group Accessions	Modifications	ΔCn	116/117	116/117 Count	116/117 Variability [%]	q-Value	PEP	IonScore	Exp Value	Charge	MH+ [Da]	ΔM [ppm]	RT [min]	# Missed Cleavages
	High	gTSPLTSYFFWPR	4	1	1	Q55385	N-Term(iTRAQ4plex)	0,0000	1,000	4	70,3	0	1,177E-07	57	2,13781E-06	3	1702,87131	0,18	51,67	0
	High	aDAWQQLkDELEAk	2	1	1	Q55385	N-Term(iTRAQ4plex); K8(iTRAQ4plex); K14(iTRAQ4plex)	0,0000	1,016	2	1,2	0	3,676E-09	43	4,47682E-05	3	2077,13028	0,59	43,67	1
	High	fPEVVFSGSN	1	1	1	Q55385	N-Term(iTRAQ4plex)	0,0000	0,950	1		0	0,0006355	42	5,78056E-05	2	1226,61858	0,95	42,53	0
	High	hWIAEADR	3	1	1	Q55385	N-Term(iTRAQ4plex)	0,0000	0,971	3	4,1	0	0,0002657	31	0,000858954	3	1141,58731	0,18	17,99	0
Q05971	10 kDa chaperonin OS=Synechocystis sp. (strain PCC 6803 / Kazusa) GN=groS PE=1 SV=3 - [CH10_	257,98	61,17	1	6	6	11	0,526	10	14,1	103	10,9	5,22							
	A3	Sequence	# PSMs	# Proteins	# Protein Groups	Protein Group Accessions	Modifications	ΔCn	116/117	116/117 Count	116/117 Variability [%]	q-Value	PEP	IonScore	Exp Value	Charge	MH+ [Da]	ΔM [ppm]	RT [min]	# Missed Cleavages
	High	ekPQIGEVVQVGPGk	3	1	1	Q05971	N-Term(iTRAQ4plex); K2(iTRAQ4plex); K15(iTRAQ4plex)	0,0000	0,502	3	4,4	0	8,758E-10	64	4,14925E-07	3	1997,17453	-0,54	27,52	0
	High	aAISINVSTVkPLGDR	4	1	1	Q05971	N-Term(iTRAQ4plex); K11(iTRAQ4plex)	0,0000	0,549	3	41,7	0	1,741E-10	54	4,32484E-06	3	1929,13840	0,62	34,07	0
	High	ekPQIGEVVQVGPGkR	1	1	1	Q05971	N-Term(iTRAQ4plex); K2(iTRAQ4plex); K15(iTRAQ4plex)	0,0000	0,514	1		0	2,926E-09	31	0,000737853	4	2153,27700	0,12	24,97	1
	High	rNDDGTYSPVEVk	1	1	1	Q05971	N-Term(iTRAQ4plex); K13(iTRAQ4plex)	0,0000	0,777	1		0	0,0001221	24	0,003908139	3	1767,91147	-0,07	19,69	1
	High	vSPAEEk	1	1	1	Q05971	N-Term(iTRAQ4plex); K7(iTRAQ4plex)	0,0000	0,471	1		0,001	0,01494	21	0,007961044	2	1047,59209	-0,37	14,47	0
	High	tAGGILLPDNAk	1	1	1	Q05971	N-Term(iTRAQ4plex); K12(iTRAQ4plex)	0,0000	0,746	1		0,004	0,04416	11	0,083940201	3	1457,85614	-0,35	28,56	0
Q55660	Slr0001 protein OS=Synechocystis sp. (strain PCC 6803 / Kazusa) GN=slr0001 PE=4 SV=1 - [Q55660	257,84	19,38	1	4	4	14	0,422	13	9,4	160	17,4	5,15							
	A3	Sequence	# PSMs	# Proteins	# Protein Groups	Protein Group Accessions	Modifications	ΔCn	116/117	116/117 Count	116/117 Variability [%]	q-Value	PEP	IonScore	Exp Value	Charge	MH+ [Da]	ΔM [ppm]	RT [min]	# Missed Cleavages
	High	dVAAQFFkPQR	7	1	1	Q55660	N-Term(iTRAQ4plex); K8(iTRAQ4plex)	0,0000	0,422	7	3,2	0	0,000005383	63	5,04626E-07	2	1594,89641	1,24	28,73	0
	High	kIPHGLYVcGVk	4	1	1	Q55660	N-Term(iTRAQ4plex); K1(iTRAQ4plex); C9(Carbamidomethyl);	0,0000	0,413	3	4,7	0	2,058E-08	45	2,81819E-05	3	1803,06845	0,50	25,50	1
	High	iPHGLYVcGVk	1	1	1	Q55660	N-Term(iTRAQ4plex); C8(Carbamidomethyl); K11(iTRAQ4plex)	0,0000	0,396	1		0	0,000001964	30	0,00950539	3	1530,87293	1,58	29,58	0
	High	mLDEQAkk	2	1	1	Q55660	N-Term(iTRAQ4plex); M1(Oxidation); K7(iTRAQ4plex); K8(iTRA	0,0000	1,026	2	12,0	0,002	0,02282	22	0,006265706	3	1410,80912	7,38	11,66	1
P54224	Uroporphyrinogen decarboxylase OS=Synechocystis sp. (strain PCC 6803 / Kazusa) GN=hemE PE=3	257,43	18,00	1	6	6	14	0,850	13	14,6	350	39,1	5,96							
	A3	Sequence	# PSMs	# Proteins	# Protein Groups	Protein Group Accessions	Modifications	ΔCn	116/117	116/117 Count	116/117 Variability [%]	q-Value	PEP	IonScore	Exp Value	Charge	MH+ [Da]	ΔM [ppm]	RT [min]	# Missed Cleavages
	High	kAGQGGHIFNLGHGVLVGTPEDN	3	1	1	P54224	N-Term(iTRAQ4plex); K1(iTRAQ4plex)	0,0000	0,669	2	21,6	0	2,054E-10	50	1,13161E-05	5	2860,54334	0,42	32,06	1
	High	aGQGGHIFNLGHGVLVGTPEDNV	1	1	1	P54224	N-Term(iTRAQ4plex)	0,0000	0,810	1		0	2,356E-11	46	2,68516E-05	4	2588,34511	0,00	35,20	0
	High	qmAFSEPAILHSFLDk	3	1	1	P54224	N-Term(iTRAQ4plex); M2(Oxidation); K16(iTRAQ4plex)	0,0000	0,975	3	13,8	0	5,968E-09	38	0,000171384	3	2138,12033	0,38	46,89	0
	High	rPPVWMMR	1	1	1	P54224	N-Term(iTRAQ4plex)	0,0000	1,426	1		0	0,000008663	35	0,000349116	3	1216,65653	0,09	27,10	0
	High	fFFETAk	2	1	1	P54224	N-Term(iTRAQ4plex); K7(iTRAQ4plex)	0,0000	0,836	2	2,3	0	0,0003975	30	0,000939658	2	1177,64946	-0,12	35,78	0
	High	qMAFSEPAILHSFLDk	1	1	1	P54224	N-Term(iTRAQ4plex); K16(iTRAQ4plex)	0,0000	1,179	1		0	0,00002603	17	0,020747702	3	2122,12461	0,00	49,60	0
	High	tILGTLR	3	1	1	P54224	N-Term(iTRAQ4plex)	0,0000	0,887	3	8,1	0,003	0,03123	30	0,000924634	2	917,58977	-0,32	29,45	0
P73607	Anti-sigma factor antagonist OS=Synechocystis sp. (strain PCC 6803 / Kazusa) GN=slr1856 PE=3 SV	255,53	19,27	1	2	2	9	0,693	9	57,6	109	11,9	4,41							
	A3	Sequence	# PSMs	# Proteins	# Protein Groups	Protein Group Accessions	Modifications	ΔCn	116/117	116/117 Count	116/117 Variability [%]	q-Value	PEP	IonScore	Exp Value	Charge	MH+ [Da]	ΔM [ppm]	RT [min]	# Missed Cleavages
	High	iHEVILPLAEPGSk	5	1	1	P73607	N-Term(iTRAQ4plex); K14(iTRAQ4plex)	0,0000	0,995	5	57,6	0	4,094E-09	52	5,83405E-06	3	1791,06260	0,38	34,91	0
	High	lLLYLYR	4	1	1	P73607	N-Term(iTRAQ4plex)	0,0000	0,525	4	18,7	0	0,0002819	40	9,70443E-05	2	1097,68413	0,17	45,21	0
P26290	Cytochrome b6-f complex iron-sulfur subunit 2 OS=Synechocystis sp. (strain PCC 6803 / Kazusa) GN	252,19	28,89	1	4	4	10	0,792	8	0,7	180	19,0	5,11							
	A3	Sequence	# PSMs	# Proteins	# Protein Groups	Protein Group Accessions	Modifications	ΔCn	116/117	116/117 Count	116/117 Variability [%]	q-Value	PEP	IonScore	Exp Value	Charge	MH+ [Da]	ΔM [ppm]	RT [min]	# Missed Cleavages
	High	vTEFLASHNAGDR	3	1	1	P26290	N-Term(iTRAQ4plex)	0,0000	0,786	3	10,8	0	1,752E-09	82	5,95621E-09	2	1560,78923	0,31	26,36	0
	High	vLAQGLk	5	1	1	P26290	N-Term(iTRAQ4plex); K7(iTRAQ4plex)	0,0000	0,792	3	0,1	0	0,00175	38	0,000169032	2	1016,66973	-0,93	22,61	0
	High	yLIPPSSGGSGGGVTAk	1	1	1	P26290	N-Term(iTRAQ4plex); K17(iTRAQ4plex)	0,0000	0,794	1		0	3,156E-09	36	0,000253495	3	1836,01059	0,00	29,18	0
	Medium	mTQISGSPDVPDLGR	1	1	1	P26290	N-Term(iTRAQ4plex); M1(Oxidation)	0,0000	0,729	1		0,049	0,631	13	0,054572017	4	1732,86818	1,43	11,47	0
P73609	Anti-sigma factor antagonist OS=Synechocystis sp. (strain PCC 6803 / Kazusa) GN=slr1859 PE=3 SV	251,17	45,37	1	4	4	10	0,641	9	12,8	108	12,0	4,74							
	A3	Sequence	# PSMs	# Proteins	# Protein Groups	Protein Group Accessions	Modifications	ΔCn	116/117	116/117 Count	116/117 Variability [%]	q-Value	PEP	IonScore	Exp Value	Charge	MH+ [Da]	ΔM [ppm]	RT [min]	# Missed Cleavages
	High	ekIEEVAQAEISk	1	1	1	P73609	N-Term(iTRAQ4plex); K2(iTRAQ4plex); K13(iTRAQ4plex)	0,0000	0,744	1		0	6,742E-09	65	3,10435E-07	3	1906,08396	-0,96	30,24	1
	High	lTLIGELDGGTAPLFk	2	1	1	P73609	N-Term(iTRAQ4plex); K16(iTRAQ4plex)	0,0000	0,567	2	18,3	0	0,000005356	44	4,47621E-05	3	1933,12223	-1,39	52,76	0
	High	aFNIESEIINNAAk	1	1	1	P73609	N-Term(iTRAQ4plex); K14(iTRAQ4plex)	0,0000	0,805	1		0	4,282E-07	39	0,000127929	3	1821,99662	0,92	41,71	0
	High	iLVFAk	6	1	1	P73609	N-Term(iTRAQ4plex); K6(iTRAQ4plex)	0,0000	0,611	5	5,7	0,001	0,01432	39	0,000120773	2	978,65874	-0,30	34,28	0
P73008	Methyl-accepting chemotaxis protein OS=Synechocystis sp. (strain PCC 6803 / Kazusa) GN=mcpA PE	248,27	3,22	1	3	3	8	1,733	7	33,0	869	93,2	4,40							
	A3	Sequence	# PSMs	# Proteins	# Protein Groups	Protein Group Accessions	Modifications	ΔCn	116/117	116/117 Count	116/117 Variability [%]	q-Value	PEP	IonScore	Exp Value	Charge	MH+ [Da]	ΔM [ppm]	RT [min]	# Missed Cleavages
	High	iVAVISQIASR	5	1	1	P73008	N-Term(iTRAQ4plex)	0,0000	1,893	4	12,9	0	0,000007284	76	2,66054E-08	2	1300,80828	1,02	45,18	0
	High	tVGGILQIR	2	1	1	P73008	N-Term(iTRAQ4plex)	0,0000	1,183	2	24,9	0	0,0002891	34	0,000443578	2	1100,69109	0,21	33,92	0
	Medium	sITADTVk	1	1	1	P73008	N-Term(iTRAQ4plex); K8(iTRAQ4plex)	0,0000	0,815	1		0,016	0,2137	24	0,004092324	2	1122,65996	-0,83	17,71	0
P73335	Uncharacterized deoxyribonuclease sll1786 OS=Synechocystis sp. (strain PCC 6803 / Kazusa) GN=sll	247,61	24,90	1	5	5	9	0,561	9	27,4	261	29,2	5,87							
	A3	Sequence	# PSMs	# Proteins	# Protein Groups	Protein Group Accessions	Modifications	ΔCn	116/117	116/117 Count	116/117 Variability [%]	q-Value	PEP	IonScore	Exp Value	Charge	MH+ [Da]	ΔM [ppm]	RT [min]	# Missed Cleavages
	High	hVPLETLAQQTTTNAR	1	1	1	P73335	N-Term(iTRAQ4plex)	0,0000	0,562	1		0	3,309E-10	73	5,28409E-08	3	1924,03855	0,85	28,77	0
	High	lLVETDcPFLAPVPQR	2	1	1	P73335	N-Term(iTRAQ4plex); C7(Carbamidomethyl)	0,0000	0,480	2	23,5	0	1,657E-08	57	1,79875E-06	2	1999,08013	-0,13	45,68	0
	High	hVAEAIAALR	1	1	1	P73335	N-Term(iTRAQ4plex)	0,0000	0,494	1		0	0,00001573	54	3,9808E-06	2	1194,70757	-0,01	26,39	0
	High	eLNkPVIIHcR	2	1	1	P73335	N-Term(iTRAQ4plex); K4(iTRAQ4plex); C10(Carbamidomethyl)	0,0000	0,412	2	5,3	0	5,193E-07	38	0,000148242	4	1666,96755	0,60	21,73	0
	High	aDNRDHQIEVFR	3	1	1	P73335	N-Term(iTRAQ4plex)	0,0000	0,672	3	23,4	0	0,00001924	27	0,00198139	3	1643,83756	0,28	19,13	1
Q55602	NifS protein OS=Synechocystis sp. (strain PCC 6803 / Kazusa) GN=nifS PE=3 SV=1 - [Q55602_SYNY	246,79	17,36	1	6	6	9	0,496	9	20,4	386	41,7	6,46							
	A3	Sequence	# PSMs	# Proteins	# Protein Groups	Protein Group Accessions	Modifications	ΔCn	116/117	116/117 Count	116/117 Variability [%]	q-Value	PEP	IonScore	Exp Value	Charge	MH+ [Da]	ΔM [ppm]	RT [min]	# Missed Cleavages
	High	tEASHVLYALGR	2	1	1	Q55602	N-Term(iTRAQ4plex)	0,0000	0,436	2	34,4	0	1,411E-08	66	2,32258E-07	2	1460,79912	0,88	29,17	0
	High	sGTLPVPLIVGLAk	2	1	1	Q55602	N-Term(iTRAQ4plex); K14(iTRAQ4plex)	0,0000	0,334	2	3,7	0	0,000003659	49	1,20495E-05	3	1653,05747	1,27	48,16	0
	High	lAPQLLGGGQEGNFR	1	1	1	Q55602	N-Term(iTRAQ4plex)	0,0000	0,783	1		0	0,000002627	49	1,22744E-05	3	1700,92179	0,98	37,45	0
	High	aLLTTLQPR	1	1	1	Q55602	N-Term(iTRAQ4plex)	0,0000	0,526	1		0	0,00004983	48	1,49613E-05	2	1156,71843	1,18	31,52	0
	High	dkTSAQASLR	2	1	1	Q55602	N-Term(iTRAQ4plex); K2(iTRAQ4plex)	0,0000	0,484	2	16,8	0	0,00008211	31	0,00101137	3	1364,77530	1,22	12,64	1
	Medium	gIGGLYR	1	1	1	Q55602	N-Term(iTRAQ4plex)	0,0000	0,496	1		0,032	0,4122	25	0,003380415	2	879,51818	1,46	22,53	0
	>sp|K1C9_HUMAN|	243,20	10,75	1	4	6	7	0,551	5	10,5	623	62,1	5,30							
	A3	Sequence	# PSMs	# Proteins	# Protein Groups	Protein Group Accessions	Modifications	ΔCn	116/117	116/117 Count	116/117 Variability [%]	q-Value	PEP	IonScore	Exp Value	Charge	MH+ [Da]	ΔM [ppm]	RT [min]	# Missed Cleavages
	High	gGSGGSYGGGGSGGGYGGGSGS	1	1	1		N-Term(iTRAQ4plex)	0,0000	0,592	1		0	1,05E-16	118	1,45201E-12	3	1935,83091	0,54	12,93	0
	High	fSSSSGYGGGSSR	2	1	1		N-Term(iTRAQ4plex)	0,0000	0,530	2	5,3	0	0,000006022	62	5,754E-07	2	1379,63078	-0,03	15,98	0
	High	kGPAAIQk	1	1	1		N-Term(iTRAQ4plex); K1(iTRAQ4plex); K8(iTRAQ4plex)	0,0000	0,551	1		0	0,002214	30	0,000941825	3	1244,80075	-3,49	12,96	1
	High	ikFEMEQNLR	1	1	1		N-Term(iTRAQ4plex); K2(iTRAQ4plex)	0,0000	0,737	1		0	8,282E-07	25	0,002957808	3	1595,88187	0,03	31,13	1
	High	lASYLDk	1	2	2		N-Term(iTRAQ4plex); K7(iTRAQ4plex)	0,0000				0,001	0,00638	28	0,001415696	2	1097,64348	-0,93	29,68	0
	High	kAALEk	1	2	2	;P72675	N-Term(iTRAQ4plex); K1(iTRAQ4plex); K6(iTRAQ4plex)	0,0000				0,002	0,02083	20	0,010739152	3	1091,70865	-5,70	11,37	1
P17253	ATP synthase gamma chain OS=Synechocystis sp. (strain PCC 6803 / Kazusa) GN=atpG PE=3 SV=1	241,37	20,06	1	7	7	12	2,334	10	37,2	314	34,6	6,89							
	A3	Sequence	# PSMs	# Proteins	# Protein Groups	Protein Group Accessions	Modifications	ΔCn	116/117	116/117 Count	116/117 Variability [%]	q-Value	PEP	IonScore	Exp Value	Charge	MH+ [Da]	ΔM [ppm]	RT [min]	# Missed Cleavages
	High	aLQESAASELAAR	1	1	1	P17253	N-Term(iTRAQ4plex)	0,0000	2,635	1		0	1,042E-08	76	2,70377E-08	2	1460,78240	-0,14	27,07	0
	High	lSFAETELPLFEQR	2	1	1	P17253	N-Term(iTRAQ4plex)	0,0000	2,661	2	23,4	0	0,000008197	54	3,8104E-06	2	1823,96965	1,97	50,33	0
	High	aVALLVVTGDR	2	1	1	P17253	N-Term(iTRAQ4plex)	0,0000	1,302	2	38,2	0	0,00008305	41	8,7694E-05	2	1257,76579	0,82	37,85	0
	High	lVLVGSk	2	1	1	P17253	N-Term(iTRAQ4plex); K7(iTRAQ4plex)	0,0000	3,285	1		0,001	0,01015	34	0,000389915	2	1003,67528	-0,14	27,21	0
	High	lVAAAk	3	1	1	P17253	N-Term(iTRAQ4plex); K6(iTRAQ4plex)	0,0000	2,384	2	26,9	0,003	0,04277	19	0,012189054	2	860,58006	-0,84	16,61	0
	Medium	kITEAmR	1	1	1	P17253	N-Term(iTRAQ4plex); K1(iTRAQ4plex); M6(Oxidation)	0,0000	2,392	1		0,021	0,2935	10	0,089736681	3	1152,66428	-0,57	10,73	1
	Medium	iQSVk	1	1	1	P17253	N-Term(iTRAQ4plex); K5(iTRAQ4plex)	0,0000	1,331	1		0,03	0,3908	14	0,042851892	2	862,55950	-0,63	16,09	0
P73321	Uncharacterized protein slr1894 OS=Synechocystis sp. (strain PCC 6803 / Kazusa) GN=slr1894 PE=3	241,07	33,33	1	7	7	15	0,374	14	56,3	156	17,7	5,57							
	A3	Sequence	# PSMs	# Proteins	# Protein Groups	Protein Group Accessions	Modifications	ΔCn	116/117	116/117 Count	116/117 Variability [%]	q-Value	PEP	IonScore	Exp Value	Charge	MH+ [Da]	ΔM [ppm]	RT [min]	# Missed Cleavages
	High	aTINIGIPEADR	2	1	1	P73321	N-Term(iTRAQ4plex)	0,0000	0,255	1		0	0,0000758	63	4,797E-07	2	1413,78240	0,37	33,67	0
	High	aTINIGIPEADRIk	1	1	1	P73321	N-Term(iTRAQ4plex); K14(iTRAQ4plex)	0,0000	0,604	1		0	8,898E-08	48	1,41244E-05	3	1799,06284	-0,08	34,62	1
	High	gHETIVQScR	3	1	1	P73321	N-Term(iTRAQ4plex); C9(Carbamidomethyl)	0,0000	0,554	3	41,8	0	0,000004148	38	0,000169813	3	1330,66550	0,05	11,40	0
	High	iAESLkk	4	1	1	P73321	N-Term(iTRAQ4plex); K6(iTRAQ4plex); K7(iTRAQ4plex)	0,0000	0,332	4	1,1	0,001	0,009533	31	0,00072773	3	1220,79033	-2,89	18,08	1
	High	sLDVFAPGTYkEFAk	1	1	1	P73321	N-Term(iTRAQ4plex); K11(iTRAQ4plex); K15(iTRAQ4plex)	0,0000	0,518	1		0,001	0,008719	19	0,014467473	3	2105,16117	-1,52	40,48	1
	High	tAWmLR	2	1	1	P73321	N-Term(iTRAQ4plex); M4(Oxidation)	0,0000	0,513	2	63,1	0,002	0,0236	14	0,036641227	2	937,50463	0,00	24,15	0
	High	tAWMLR	1	1	1	P73321	N-Term(iTRAQ4plex)	0,0000	0,391	1		0,003	0,04258	23	0,00512826	2	921,50920	-0,55	29,22	0
	Medium	iAESLk	1	1	1	P73321	N-Term(iTRAQ4plex); K6(iTRAQ4plex)	0,0000	0,885	1		0,011	0,1439	40	8,93305E-05	2	948,59490	-2,04	20,91	0
P77966	DNA gyrase subunit B OS=Synechocystis sp. (strain PCC 6803 / Kazusa) GN=gyrB PE=3 SV=1 - [GYF	240,87	5,10	1	6	6	9	2,818	8	49,3	1078	122,7	6,64							
	A3	Sequence	# PSMs	# Proteins	# Protein Groups	Protein Group Accessions	Modifications	ΔCn	116/117	116/117 Count	116/117 Variability [%]	q-Value	PEP	IonScore	Exp Value	Charge	MH+ [Da]	ΔM [ppm]	RT [min]	# Missed Cleavages
	High	sALETVLTVLHAGGk	2	1	1	P77966	N-Term(iTRAQ4plex); K15(iTRAQ4plex)	0,0000	3,996	1		0	1,907E-09	64	4,18765E-07	3	1784,05570	2,04	51,33	0
	High	fQAILPLR	3	1	1	P77966	N-Term(iTRAQ4plex)	0,0000	3,013	3	18,9	0	0,00003043	41	7,65544E-05	2	1101,69048	0,32	40,71	0
	High	lkELAYLNAGVR	1	1	1	P77966	N-Term(iTRAQ4plex); K2(iTRAQ4plex)	0,0000	2,018	1		0	4,898E-09	38	0,000174973	3	1634,98349	0,15	35,39	1
	High	rEFIETYGTk	1	1	1	P77966	N-Term(iTRAQ4plex); K10(iTRAQ4plex)	0,0000	2,098	1		0	0,00003369	37	0,000184489	3	1531,83634	0,29	23,17	0
	High	rFQAILPLR	1	1	1	P77966	N-Term(iTRAQ4plex)	0,0000	1,472	1		0	0,00007232	34	0,000413971	3	1257,79090	-0,28	32,45	1
	High	tLLLTFFYR	1	1	1	P77966	N-Term(iTRAQ4plex)	0,0000	7,327	1		0	0,0006294	33	0,000609791	2	1317,76885	0,07	54,88	0
P74326	Ssr1600 protein OS=Synechocystis sp. (strain PCC 6803 / Kazusa) GN=ssr1600 PE=4 SV=1 - [P7432	239,75	50,00	1	3	3	7	0,844	7	18,5	70	7,5	7,42							
	A3	Sequence	# PSMs	# Proteins	# Protein Groups	Protein Group Accessions	Modifications	ΔCn	116/117	116/117 Count	116/117 Variability [%]	q-Value	PEP	IonScore	Exp Value	Charge	MH+ [Da]	ΔM [ppm]	RT [min]	# Missed Cleavages
	High	qGSVQIVTNPR	4	1	1	P74326	N-Term(iTRAQ4plex)	0,0000	0,827	4	9,2	0	5,551E-07	71	7,56781E-08	2	1342,75652	0,40	20,08	0
	High	fLHLHNSLAEAIAATTEG	1	1	1	P74326	N-Term(iTRAQ4plex)	0,0000	0,315	1		0	0,00001628	43	4,66627E-05	3	2039,06943	0,75	45,63	0
	Medium	vTQTVk	2	1	1	P74326	N-Term(iTRAQ4plex); K6(iTRAQ4plex)	0,0000	0,954	2	3,4	0,011	0,1444	14	0,043648568	2	963,60784	0,12	13,18	0
Q55734	Sll0395 protein OS=Synechocystis sp. (strain PCC 6803 / Kazusa) GN=sll0395 PE=4 SV=1 - [Q55734	239,56	27,36	1	5	5	9	0,998	8	16,4	212	23,7	6,52							
	A3	Sequence	# PSMs	# Proteins	# Protein Groups	Protein Group Accessions	Modifications	ΔCn	116/117	116/117 Count	116/117 Variability [%]	q-Value	PEP	IonScore	Exp Value	Charge	MH+ [Da]	ΔM [ppm]	RT [min]	# Missed Cleavages
	High	tLNLYFLR	3	1	1	Q55734	N-Term(iTRAQ4plex)	0,0000	1,120	3	3,0	0	0,00008044	55	3,41169E-06	2	1183,69646	0,74	44,27	0
	High	tFTTGNVLVVSHk	2	1	1	Q55734	N-Term(iTRAQ4plex); K13(iTRAQ4plex)	0,0000	0,978	1		0	1,224E-09	52	7,08241E-06	3	1690,97458	0,90	29,12	0
	High	lINLPIQk	1	1	1	Q55734	N-Term(iTRAQ4plex); K8(iTRAQ4plex)	0,0000	0,900	1		0	0,0004015	43	4,77496E-05	2	1226,81059	2,52	37,68	0
	High	hGETTSSQTGTFcGR	1	1	1	Q55734	N-Term(iTRAQ4plex); C13(Carbamidomethyl)	0,0000	1,018	1		0	2,423E-09	38	0,000174973	3	1769,80057	0,67	12,84	0
	High	dVSWTAIFASPLHR	2	1	1	Q55734	N-Term(iTRAQ4plex)	0,0000	0,538	2	77,8	0	0,000002334	35	0,000294422	3	1743,93143	0,86	46,65	0
P74729	HrEpiB OS=Synechocystis sp. (strain PCC 6803 / Kazusa) GN=sll0576 PE=4 SV=1 - [P74729_SYNY3]	236,92	37,50	1	9	9	14	2,165	12	21,3	312	34,9	7,62							
	A3	Sequence	# PSMs	# Proteins	# Protein Groups	Protein Group Accessions	Modifications	ΔCn	116/117	116/117 Count	116/117 Variability [%]	q-Value	PEP	IonScore	Exp Value	Charge	MH+ [Da]	ΔM [ppm]	RT [min]	# Missed Cleavages
	High	aVVIFEGHFk	3	1	1	P74729	N-Term(iTRAQ4plex); K10(iTRAQ4plex)	0,0000	2,299	2	17,4	0	1,961E-07	49	1,1508E-05	3	1434,83484	0,04	33,63	0
	High	gkPYNVGLEDANLSk	2	1	1	P74729	N-Term(iTRAQ4plex); K2(iTRAQ4plex); K15(iTRAQ4plex)	0,0000	3,694	2	121,5	0	0,000001343	45	2,89714E-05	3	2037,13126	-1,40	28,15	0
	High	vFLHGLENFESMk	1	1	1	P74729	N-Term(iTRAQ4plex); K13(iTRAQ4plex)	0,0000	2,200	1		0	1,216E-09	33	0,000553312	3	1838,97141	0,02	41,49	0
	High	vFLHGLENFESmk	1	1	1	P74729	N-Term(iTRAQ4plex); M12(Oxidation); K13(iTRAQ4plex)	0,0000	2,130	1		0	3,653E-09	32	0,00064561	3	1854,96622	-0,04	37,89	0
	High	fcTEESPLRPISLYGVTk	1	1	1	P74729	N-Term(iTRAQ4plex); C2(Carbamidomethyl); K18(iTRAQ4plex)	0,0000	2,762	1		0	6,958E-08	29	0,00128289	3	2385,27390	0,50	40,76	0
	High	kYLPNFVYLEAPIGEDPDkR	1	1	1	P74729	N-Term(iTRAQ4plex); K1(iTRAQ4plex); K19(iTRAQ4plex)	0,0000	0,966	1		0	0,000001931	24	0,0044154	5	2796,53455	1,73	41,70	2
	High	lATVFGmAPR	1	1	1	P74729	N-Term(iTRAQ4plex); M7(Oxidation)	0,0000	2,336	1		0	0,00004722	23	0,00544465	2	1222,67363	0,10	34,21	0
	High	gYTILR	2	1	1	P74729	N-Term(iTRAQ4plex)	0,0000	1,966	2	19,1	0,001	0,01093	35	0,000315479	2	866,52104	-0,68	23,14	0
	High	nYIHIR	1	1	1	P74729	N-Term(iTRAQ4plex)	0,0000	1,052	1		0,003	0,04227	15	0,03467129	3	959,55429	-0,03	18,52	0
	High	tADVIIPLAALVGAPLcSR	1	1	1	P74729	N-Term(iTRAQ4plex); C17(Carbamidomethyl)	0,0000				0,005	0,05974	15	0,030058687	3	2081,19083	-0,09	57,78	0
P73048	Sll1638 protein OS=Synechocystis sp. (strain PCC 6803 / Kazusa) GN=sll1638 PE=1 SV=1 - [P73048_	236,34	24,16	1	4	4	12	0,562	12	18,2	149	16,5	7,34							
	A3	Sequence	# PSMs	# Proteins	# Protein Groups	Protein Group Accessions	Modifications	ΔCn	116/117	116/117 Count	116/117 Variability [%]	q-Value	PEP	IonScore	Exp Value	Charge	MH+ [Da]	ΔM [ppm]	RT [min]	# Missed Cleavages
	High	iAQLQVYVNPIAVAR	3	1	1	P73048	N-Term(iTRAQ4plex)	0,0000	0,531	3	2,7	0	7,266E-11	70	1,10146E-07	2	1799,06792	1,05	44,80	0
	High	eVFGHLER	4	1	1	P73048	N-Term(iTRAQ4plex)	0,0000	0,585	4	3,1	0	0,0003562	47	1,81121E-05	2	1130,60674	-0,69	20,32	0
	High	rDmLGLASSLLPk	2	1	1	P73048	N-Term(iTRAQ4plex); M3(Oxidation); K13(iTRAQ4plex)	0,0000	0,986	2	20,5	0	5,923E-07	30	0,000984011	3	1704,99222	0,06	36,36	1
	High	dMLGLASSLLPk	2	1	1	P73048	N-Term(iTRAQ4plex); K12(iTRAQ4plex)	0,0000	0,579	2	38,8	0	0,002517	17	0,019141237	3	1532,89588	-0,14	50,13	0
	High	rDMLGLASSLLPk	1	1	1	P73048	N-Term(iTRAQ4plex); K13(iTRAQ4plex)	0,0000	0,501	1		0	0,002751	15	0,034274411	3	1688,99448	-1,61	43,49	1
P36265	Transcription termination/antitermination protein NusG OS=Synechocystis sp. (strain PCC 6803 / Kaz	236,09	15,12	1	3	3	8	1,330	6	1,2	205	23,4	6,35							
	A3	Sequence	# PSMs	# Proteins	# Protein Groups	Protein Group Accessions	Modifications	ΔCn	116/117	116/117 Count	116/117 Variability [%]	q-Value	PEP	IonScore	Exp Value	Charge	MH+ [Da]	ΔM [ppm]	RT [min]	# Missed Cleavages
	High	nTPHVINFVGSEQk	1	1	1	P36265	N-Term(iTRAQ4plex); K14(iTRAQ4plex)	0,0000	1,170	1		0	3,543E-10	64	4,385E-07	3	1858,00595	-0,11	27,08	0
	High	aLLSIFGR	4	1	1	P36265	N-Term(iTRAQ4plex)	0,0000	1,298	2	3,9	0	0,000327	51	8,24884E-06	2	1020,63231	0,04	44,28	0
	High	iFPGYVLIR	3	1	1	P36265	N-Term(iTRAQ4plex)	0,0000	1,337	3	1,0	0	0,0001976	46	2,87203E-05	2	1221,74773	0,08	44,44	0
P73244	Slr2025 protein OS=Synechocystis sp. (strain PCC 6803 / Kazusa) GN=slr2025 PE=4 SV=1 - [P73244	234,81	25,49	1	3	3	13	0,472	8	13,2	153	16,9	4,44							
	A3	Sequence	# PSMs	# Proteins	# Protein Groups	Protein Group Accessions	Modifications	ΔCn	116/117	116/117 Count	116/117 Variability [%]	q-Value	PEP	IonScore	Exp Value	Charge	MH+ [Da]	ΔM [ppm]	RT [min]	# Missed Cleavages
	High	eNHLVALHQR	7	1	1	P73244	N-Term(iTRAQ4plex)	0,0000	0,499	5	23,1	0	3,837E-07	62	7,03024E-07	2	1360,75749	0,63	13,90	0
	High	tVADLSPSEISR	2	1	1	P73244	N-Term(iTRAQ4plex)	0,0000	0,382	1		0	0,000007298	57	2,12799E-06	2	1418,76018	-0,43	28,55	0
	High	aITLVATLADDHDDRLk	4	1	1	P73244	N-Term(iTRAQ4plex); K17(iTRAQ4plex)	0,0000	0,472	2	3,0	0	3,274E-10	46	3,32591E-05	4	2155,19741	0,57	38,17	1
P73302	Adenylate kinase 1 OS=Synechocystis sp. (strain PCC 6803 / Kazusa) GN=adk1 PE=3 SV=2 - [KAD1_	231,81	38,38	1	5	5	10	0,849	9	27,7	185	20,2	5,59							
	A3	Sequence	# PSMs	# Proteins	# Protein Groups	Protein Group Accessions	Modifications	ΔCn	116/117	116/117 Count	116/117 Variability [%]	q-Value	PEP	IonScore	Exp Value	Charge	MH+ [Da]	ΔM [ppm]	RT [min]	# Missed Cleavages
	High	nVNQAIFLDELLVNIGHR	3	1	1	P73302	N-Term(iTRAQ4plex)	0,0000	0,990	2	65,5	0	1,062E-08	49	1,16673E-05	3	2209,22477	1,68	54,45	0
	High	aGWILDGFPR	2	1	1	P73302	N-Term(iTRAQ4plex)	0,0000	0,750	2	8,9	0	0,0000269	41	7,26056E-05	2	1275,69756	0,68	45,28	0
	High	gTQAVGLAETLGIPHISTGDmLR	1	1	1	P73302	N-Term(iTRAQ4plex); M21(Oxidation)	0,0000	0,485	1		0	0,000000206	39	0,000149071	3	2497,32712	2,77	43,74	0
	High	gLIFLGAPGSGk	2	1	1	P73302	N-Term(iTRAQ4plex); K12(iTRAQ4plex)	0,0000	0,950	2	12,0	0	0,000006788	33	0,000485255	3	1404,84546	0,07	38,10	0
	High	tHWVINLk	2	1	1	P73302	N-Term(iTRAQ4plex); K8(iTRAQ4plex)	0,0000	0,857	2	1,5	0	0,000008287	31	0,000858954	3	1298,78336	0,79	31,27	0
Q55522	Valine--tRNA ligase OS=Synechocystis sp. (strain PCC 6803 / Kazusa) GN=valS PE=1 SV=1 - [SYV_S	230,58	10,00	1	10	10	16	3,060	14	29,8	910	102,7	5,17							
	A3	Sequence	# PSMs	# Proteins	# Protein Groups	Protein Group Accessions	Modifications	ΔCn	116/117	116/117 Count	116/117 Variability [%]	q-Value	PEP	IonScore	Exp Value	Charge	MH+ [Da]	ΔM [ppm]	RT [min]	# Missed Cleavages
	High	lNQVIQQTR	1	1	1	Q55522	N-Term(iTRAQ4plex)	0,0000	3,276	1		0	0,000003312	56	2,80524E-06	2	1243,72429	0,29	24,41	0
	High	kNVVQALEAQGFLVk	2	1	1	Q55522	N-Term(iTRAQ4plex); K1(iTRAQ4plex); K15(iTRAQ4plex)	0,0000	5,301	2	104,5	0	1,258E-10	50	1,04706E-05	3	2076,25559	0,67	44,43	1
	High	nVVQALEAQGFLVk	2	1	1	Q55522	N-Term(iTRAQ4plex); K14(iTRAQ4plex)	0,0000	3,109	2		0	3,258E-08	50	1,11686E-05	3	1804,05613	-0,57	48,32	0
	High	qVLAHTLDSIIk	3	1	1	Q55522	N-Term(iTRAQ4plex); K12(iTRAQ4plex)	0,0000	3,272	2	12,3	0	4,367E-08	38	0,00016633	3	1625,98294	0,02	38,13	0
	High	wQTAWEESHAFk	2	1	1	Q55522	N-Term(iTRAQ4plex); K12(iTRAQ4plex)	0,0000	2,258	2	114,4	0	2,741E-09	34	0,000412069	3	1807,90061	-0,02	34,08	0
	High	hNLPFINLLNk	1	1	1	Q55522	N-Term(iTRAQ4plex); K11(iTRAQ4plex)	0,0000	3,501	1		0	0,000007688	28	0,001765916	3	1610,96134	-0,48	42,91	0
	High	vPVEPLLSTQWFVk	1	1	1	Q55522	N-Term(iTRAQ4plex); K14(iTRAQ4plex)	0,0000	1,812	1		0	0,00009249	21	0,007361563	3	1931,12235	-1,12	51,90	0
	High	gkVPVEPLLSTQWFVk	2	1	1	Q55522	N-Term(iTRAQ4plex); K2(iTRAQ4plex); K16(iTRAQ4plex)	0,0000	2,156	2	42,7	0	2,825E-07	18	0,015775023	4	2260,34243	-0,26	45,88	1
	High	vYRDWLVk	1	1	1	Q55522	N-Term(iTRAQ4plex); K8(iTRAQ4plex)	0,0000	5,935	1		0	0,0009176	13	0,045705662	3	1366,80823	-0,24	30,38	1
	High	lkDWcISR	1	1	1	Q55522	N-Term(iTRAQ4plex); K2(iTRAQ4plex); C5(Carbamidomethyl)	0,0000	2,397	1		0,001	0,003929	12	0,063969065	3	1365,75373	-1,04	25,95	1
P72839	Slr1301 protein OS=Synechocystis sp. (strain PCC 6803 / Kazusa) GN=slr1301 PE=4 SV=1 - [P72839	230,57	14,21	1	8	8	12	0,462	11	17,2	535	61,7	4,84							
	A3	Sequence	# PSMs	# Proteins	# Protein Groups	Protein Group Accessions	Modifications	ΔCn	116/117	116/117 Count	116/117 Variability [%]	q-Value	PEP	IonScore	Exp Value	Charge	MH+ [Da]	ΔM [ppm]	RT [min]	# Missed Cleavages
	High	eGTLWILNLSNSR	3	1	1	P72839	N-Term(iTRAQ4plex)	0,0000	0,481	3	2,1	0	0,000000482	55	3,06881E-06	2	1646,89983	0,93	48,53	0
	High	eVLQGILHR	1	1	1	P72839	N-Term(iTRAQ4plex)	0,0000	0,398	1		0	0,000001367	45	3,49116E-05	3	1208,72486	1,36	29,83	0
	High	nkLEQNLQSTR	2	1	1	P72839	N-Term(iTRAQ4plex); K2(iTRAQ4plex)	0,0000	0,428	1		0	3,366E-07	43	4,94277E-05	3	1618,91288	0,83	18,57	1
	High	nFLGGYk	1	1	1	P72839	N-Term(iTRAQ4plex); K7(iTRAQ4plex)	0,0000	0,462	1		0	0,0001893	41	7,9794E-05	2	1086,61882	0,19	27,46	0
	High	kTNFEQNR	1	1	1	P72839	N-Term(iTRAQ4plex); K1(iTRAQ4plex)	0,0000	0,567	1		0	0,000007325	29	0,00118296	3	1324,72153	0,25	10,89	1
	High	lkEQQSEIER	2	1	1	P72839	N-Term(iTRAQ4plex); K2(iTRAQ4plex)	0,0000	0,538	2	31,8	0	0,000005679	29	0,001651746	3	1547,86374	0,36	15,92	1
	High	qEVLLQHLk	1	1	1	P72839	N-Term(iTRAQ4plex); K9(iTRAQ4plex)	0,0000	0,411	1		0	0,00001421	24	0,003881236	3	1395,85691	0,48	27,32	0
	High	mLYLAEIkk	1	1	1	P72839	N-Term(iTRAQ4plex); K8(iTRAQ4plex); K9(iTRAQ4plex)	0,0000	0,658	1		0,005	0,06333	11	0,077085023	3	1540,96048	6,98	34,48	1
Q55756	Carbamoyl-phosphate synthase large chain OS=Synechocystis sp. (strain PCC 6803 / Kazusa) GN=ca	230,05	6,66	1	6	6	9	2,774	8	44,0	1081	119,0	4,98							
	A3	Sequence	# PSMs	# Proteins	# Protein Groups	Protein Group Accessions	Modifications	ΔCn	116/117	116/117 Count	116/117 Variability [%]	q-Value	PEP	IonScore	Exp Value	Charge	MH+ [Da]	ΔM [ppm]	RT [min]	# Missed Cleavages
	High	sLGITPVYk	1	1	1	Q55756	N-Term(iTRAQ4plex); K9(iTRAQ4plex)	0,0000	2,470	1		0	0,000001773	74	3,9443E-08	2	1265,77080	0,03	29,14	0
	High	lRDYSIAIIR	3	1	1	Q55756	N-Term(iTRAQ4plex)	0,0000	3,033	2	44,0	0	0,00000396	57	2,14783E-06	3	1363,81784	0,00	33,50	1
	High	vmILGGGPNR	2	1	1	Q55756	N-Term(iTRAQ4plex); M2(Oxidation)	0,0000	3,196	2	3,8	0	0,00003428	36	0,000277952	2	1173,65312	0,01	24,42	0
	High	kTPASFEPTIDYVVTk	1	1	1	Q55756	N-Term(iTRAQ4plex); K1(iTRAQ4plex); K16(iTRAQ4plex)	0,0000	0,886	1		0	1,475E-08	29	0,001202264	4	2228,25380	-0,04	36,66	1
	High	hETLPTLSHLR	1	1	1	Q55756	N-Term(iTRAQ4plex)	0,0000	1,626	1		0	1,175E-07	26	0,002942374	4	1447,81399	0,12	23,89	0
	High	iSYPVVVRPSYVLGGR	1	1	1	Q55756	N-Term(iTRAQ4plex)	0,0000	4,551	1		0	0,000104	18	0,017578022	3	1906,10373	0,31	39,67	0
P54416	ATP-dependent Clp protease proteolytic subunit 1 OS=Synechocystis sp. (strain PCC 6803 / Kazusa) (	229,35	29,80	1	5	5	11	0,759	8	8,0	198	21,7	5,03							
	A3	Sequence	# PSMs	# Proteins	# Protein Groups	Protein Group Accessions	Modifications	ΔCn	116/117	116/117 Count	116/117 Variability [%]	q-Value	PEP	IonScore	Exp Value	Charge	MH+ [Da]	ΔM [ppm]	RT [min]	# Missed Cleavages
	High	imIHQPLGGAQGQATDIEIQAk	1	1	1	P54416	N-Term(iTRAQ4plex); M2(Oxidation); K22(iTRAQ4plex)	0,0000	1,084	1		0	6,602E-14	55	3,33403E-06	4	2623,41347	0,67	34,73	0
	High	aFDIYSR	1	1	1	P54416	N-Term(iTRAQ4plex)	0,0000	0,783	1		0	0,0001356	44	4,10176E-05	2	1015,53313	0,19	27,55	0
	High	iVFLGQEVR	2	1	1	P54416	N-Term(iTRAQ4plex)	0,0000	0,744	2	9,5	0	0,0002971	44	5,25027E-05	2	1204,71733	0,21	38,06	0
	High	aLLNQHLANHTGk	6	1	1	P54416	N-Term(iTRAQ4plex); K13(iTRAQ4plex)	0,0000	0,737	3	5,0	0	0,00001007	42	6,70177E-05	4	1704,97439	-0,24	18,65	0
	Medium	rPSASDPI	1	1	1	P54416	N-Term(iTRAQ4plex)	0,0000	0,467	1		0,025	0,3475	18	0,015666429	2	986,53972	0,97	17,29	0
P27180	ATP synthase subunit delta OS=Synechocystis sp. (strain PCC 6803 / Kazusa) GN=atpH PE=3 SV=1 -	227,18	28,11	1	6	6	11	1,115	8	16,6	185	20,1	6,79							
	A3	Sequence	# PSMs	# Proteins	# Protein Groups	Protein Group Accessions	Modifications	ΔCn	116/117	116/117 Count	116/117 Variability [%]	q-Value	PEP	IonScore	Exp Value	Charge	MH+ [Da]	ΔM [ppm]	RT [min]	# Missed Cleavages
	High	vGSQVFDSSLR	2	1	1	P27180	N-Term(iTRAQ4plex)	0,0000	0,976	2	13,3	0	0,000001036	62	6,51583E-07	2	1338,71831	3,63	30,04	0
	High	vGLSLGTAL	2	1	1	P27180	N-Term(iTRAQ4plex)	0,0000	1,119	1		0	0,002557	45	3,47512E-05	2	974,60051	0,21	43,18	0
	High	vDGDILGGIVIk	1	1	1	P27180	N-Term(iTRAQ4plex); K12(iTRAQ4plex)	0,0000	0,933	1		0	0,00003069	40	0,00010964	3	1486,90775	-0,42	43,77	0
	High	mkGSLYSSk	1	1	1	P27180	N-Term(iTRAQ4plex); M1(Oxidation); K2(iTRAQ4plex); K9(iTRA	0,0000	1,740	1		0	0,0007107	25	0,003539729	3	1448,81577	0,99	13,24	1
	High	rVGLSLGTAL	4	1	1	P27180	N-Term(iTRAQ4plex)	0,0000	1,132	2	2,8	0,001	0,01041	16	0,02465869	2	1130,70183	0,36	35,82	1
	High	lTDAQkDQVk	1	1	1	P27180	N-Term(iTRAQ4plex); K6(iTRAQ4plex); K10(iTRAQ4plex)	0,0000	1,552	1		0,001	0,01503	14	0,040644333	3	1577,92856	3,96	15,76	1
P72805	Sll1665 protein OS=Synechocystis sp. (strain PCC 6803 / Kazusa) GN=sll1665 PE=4 SV=1 - [P72805_	224,70	5,94	1	4	4	14	0,649	13	16,7	589	63,6	3,57							
	A3	Sequence	# PSMs	# Proteins	# Protein Groups	Protein Group Accessions	Modifications	ΔCn	116/117	116/117 Count	116/117 Variability [%]	q-Value	PEP	IonScore	Exp Value	Charge	MH+ [Da]	ΔM [ppm]	RT [min]	# Missed Cleavages
	High	tALEDELQSLR	2	1	1	P72805	N-Term(iTRAQ4plex)	0,0000	0,581	2	0,3	0	0,000002099	63	4,76398E-07	2	1418,76250	1,20	39,93	0
	High	lESLESSHR	5	1	1	P72805	N-Term(iTRAQ4plex)	0,0000	0,737	4	30,4	0	0,0002379	51	7,19399E-06	2	1201,63591	5,45	17,15	0
	High	aETQALQAR	3	1	1	P72805	N-Term(iTRAQ4plex)	0,0000	0,647	3	0,5	0	0,00004472	43	5,55866E-05	2	1131,62444	0,48	14,96	0
	High	kLDSTk	4	1	1	P72805	N-Term(iTRAQ4plex); K1(iTRAQ4plex); K6(iTRAQ4plex)	0,0000	0,805	4	16,1	0,001	0,008342	18	0,014756046	3	1123,70313	-1,40	12,06	1
P74667	Diaminopimelate epimerase OS=Synechocystis sp. (strain PCC 6803 / Kazusa) GN=dapF PE=1 SV=1	223,11	15,05	1	3	3	7	1,306	7	34,7	279	30,1	4,91							
	A3	Sequence	# PSMs	# Proteins	# Protein Groups	Protein Group Accessions	Modifications	ΔCn	116/117	116/117 Count	116/117 Variability [%]	q-Value	PEP	IonScore	Exp Value	Charge	MH+ [Da]	ΔM [ppm]	RT [min]	# Missed Cleavages
	High	iHTLAGVITPQLLADGQVk	4	1	1	P74667	N-Term(iTRAQ4plex); K19(iTRAQ4plex)	0,0000	1,370	4	12,5	0	8,527E-13	76	2,594E-08	4	2262,34365	0,53	43,08	0
	High	lYmTGPAQR	1	1	1	P74667	N-Term(iTRAQ4plex); M3(Oxidation)	0,0000	1,040	1		0	0,002234	29	0,001367634	2	1196,62297	1,27	17,46	0
	High	lYMTGPAQR	1	1	1	P74667	N-Term(iTRAQ4plex)	0,0000	1,643	1		0	0,0004393	25	0,002917226	2	1180,62663	0,08	24,00	0
	High	yHGLGNDFILVDNR	1	1	1	P74667	N-Term(iTRAQ4plex)	0,0000	0,732	1		0	0,0005168	15	0,032357131	3	1776,91513	0,07	36,63	0
P74296	Phenylalanine--tRNA ligase beta subunit OS=Synechocystis sp. (strain PCC 6803 / Kazusa) GN=pheT	222,25	5,68	1	5	5	9	2,778	7	103,9	810	87,8	4,97							
	A3	Sequence	# PSMs	# Proteins	# Protein Groups	Protein Group Accessions	Modifications	ΔCn	116/117	116/117 Count	116/117 Variability [%]	q-Value	PEP	IonScore	Exp Value	Charge	MH+ [Da]	ΔM [ppm]	RT [min]	# Missed Cleavages
	High	sWAAGVVLGR	1	1	1	P74296	N-Term(iTRAQ4plex)	0,0000	1,453	1		0	6,419E-07	64	3,8832E-07	2	1159,66973	-0,64	33,67	0
	High	gLLEAIFQR	5	1	1	P74296	N-Term(iTRAQ4plex)	0,0000	3,832	3	14,9	0	0,000000339	54	4,23614E-06	2	1190,70122	-0,17	49,35	0
	High	sLAFSLAYR	1	1	1	P74296	N-Term(iTRAQ4plex)	0,0000	1,259	1		0	0,0001551	45	4,59138E-05	2	1171,65972	0,44	37,30	0
	High	fGQLHPQLR	1	1	1	P74296	N-Term(iTRAQ4plex)	0,0000	2,778	1		0	0,001037	32	0,000657612	3	1239,70594	-1,58	23,81	0
	Medium	gQSVPAGQR	1	1	1	P74296	N-Term(iTRAQ4plex)	0,0000	1,050	1		0,038	0,4887	11	0,087291108	2	1043,57060	-0,82	9,81	0
Q55432	Sll0822 protein OS=Synechocystis sp. (strain PCC 6803 / Kazusa) GN=sll0822 PE=1 SV=1 - [Q55432	221,14	27,91	1	4	4	13	2,096	10	14,5	129	14,0	8,59							
	A3	Sequence	# PSMs	# Proteins	# Protein Groups	Protein Group Accessions	Modifications	ΔCn	116/117	116/117 Count	116/117 Variability [%]	q-Value	PEP	IonScore	Exp Value	Charge	MH+ [Da]	ΔM [ppm]	RT [min]	# Missed Cleavages
	High	icGYVTSTk	2	1	1	Q55432	N-Term(iTRAQ4plex); C2(Carbamidomethyl); K9(iTRAQ4plex)	0,0000	1,932	2	1,4	0	0,00002302	60	9,59334E-07	2	1316,71172	-0,41	23,47	0
	High	sNATkPLTGEALLEk	7	1	1	Q55432	N-Term(iTRAQ4plex); K5(iTRAQ4plex); K15(iTRAQ4plex)	0,0000	2,264	4	2,4	0	6,563E-10	45	3,28073E-05	3	2004,17063	0,22	28,73	0
	High	vkELGDLPk	1	1	1	Q55432	N-Term(iTRAQ4plex); K2(iTRAQ4plex); K9(iTRAQ4plex)	0,0000	2,328	1		0	0,00001429	25	0,002824685	3	1430,89157	-1,92	24,82	1
	High	vkELGDLPkEEk	3	1	1	Q55432	N-Term(iTRAQ4plex); K2(iTRAQ4plex); K9(iTRAQ4plex); K12(iT	0,0000	1,935	3	24,8	0	0,00003244	13	0,046022479	4	1961,17495	-0,82	22,67	2
Q55953	Sll0781 protein OS=Synechocystis sp. (strain PCC 6803 / Kazusa) GN=sll0781 PE=4 SV=1 - [Q55953	220,89	38,06	1	6	6	13	0,480	11	21,8	155	18,6	6,24							
	A3	Sequence	# PSMs	# Proteins	# Protein Groups	Protein Group Accessions	Modifications	ΔCn	116/117	116/117 Count	116/117 Variability [%]	q-Value	PEP	IonScore	Exp Value	Charge	MH+ [Da]	ΔM [ppm]	RT [min]	# Missed Cleavages
	High	tTPLVPPFTk	6	1	1	Q55953	N-Term(iTRAQ4plex); K10(iTRAQ4plex)	0,0000	0,480	5	21,8	0	0,000001307	59	1,25305E-06	2	1388,84148	1,64	34,27	0
	High	rEASINDkPIAESER	2	1	1	Q55953	N-Term(iTRAQ4plex); K8(iTRAQ4plex)	0,0000	0,365	1		0	0,0000012	35	0,000341979	4	2003,07766	0,79	16,26	1
	High	rFFWDAPGDRPLDHPGLINTPE	2	1	1	Q55953	N-Term(iTRAQ4plex)	0,0000	0,512	2	27,4	0	0,0001822	33	0,000536995	4	2694,35634	0,64	41,84	1
	High	eASINDkPIAESER	1	1	1	Q55953	N-Term(iTRAQ4plex); K7(iTRAQ4plex)	0,0000	0,422	1		0	3,715E-07	29	0,001355096	3	1846,97678	0,99	19,68	0
	High	eIAIAk	1	1	1	Q55953	N-Term(iTRAQ4plex); K6(iTRAQ4plex)	0,0000	0,561	1		0,009	0,114	34	0,000416841	2	932,60259	0,72	21,01	0
	Medium	iREFLR	1	1	1	Q55953	N-Term(iTRAQ4plex)	0,0000	0,496	1		0,016	0,2063	18	0,016710906	3	977,60291	1,64	24,20	1
P73604	Slr1853 protein OS=Synechocystis sp. (strain PCC 6803 / Kazusa) GN=slr1853 PE=4 SV=1 - [P73604	220,32	20,35	1	2	2	9	0,374	6	6,8	113	12,1	4,84							

	A3	Sequence	# PSMs	# Proteins	# Protein Groups	Protein Group Accessions	Modifications	ΔCn	116/117	116/117 Count	116/117 Variability [%]	q-Value	PEP	IonScore	Exp Value	Charge	MH+ [Da]	ΔM [ppm]	RT [min]	# Missed Cleavages
	High	aAGAFAALk	8	1	1	P73604	N-Term(iTRAQ4plex); K9(iTRAQ4plex)	0,0000	0,374	5	4,0	0	0,00004562	62	6,36752E-07	2	1107,67656	0,06	28,59	0
	High	vAGEAWGLPLIDQk	1	1	1	P73604	N-Term(iTRAQ4plex); K14(iTRAQ4plex)	0,0000	0,350	1		0	0,000001653	33	0,000486374	3	1785,01664	0,93	45,04	0
P73270	Probable agmatinase 2 OS=Synechocystis sp. (strain PCC 6803 / Kazusa) GN=speB2 PE=3 SV=1 - [S	220,31	15,38	1	5	5	8	0,552	6	8,5	390	42,9	5,24							
	A3	Sequence	# PSMs	# Proteins	# Protein Groups	Protein Group Accessions	Modifications	ΔCn	116/117	116/117 Count	116/117 Variability [%]	q-Value	PEP	IonScore	Exp Value	Charge	MH+ [Da]	ΔM [ppm]	RT [min]	# Missed Cleavages
	High	nLVQLGIGGWQVPR	1	1	1	P73270	N-Term(iTRAQ4plex)	0,0000	0,509	1		0	6,309E-14	90	1,03985E-09	3	1680,97025	2,13	46,33	0
	High	vGIIHFDR	4	1	1	P73270	N-Term(iTRAQ4plex)	0,0000	0,517	2	9,1	0	0,00001283	56	2,4602E-06	3	1100,63568	2,14	27,76	0
	High	vIcDTMAHLVVSGQLPR	1	1	1	P73270	N-Term(iTRAQ4plex); C3(Carbamidomethyl)	0,0000	0,571	1		0	1,521E-09	51	8,16526E-06	3	2040,08731	1,06	44,03	0
	High	gELPHYAGINTFMk	1	1	1	P73270	N-Term(iTRAQ4plex); K14(iTRAQ4plex)	0,0000	1,072	1		0	0,000003163	26	0,002311905	3	1865,98862	3,40	36,15	0
	Medium	sIPTFSR	1	1	1	P73270	N-Term(iTRAQ4plex)	0,0000	0,554	1		0,036	0,4667	20	0,010543141	2	951,53996	2,04	24,93	0
P73654	Ssl3364 protein OS=Synechocystis sp. (strain PCC 6803 / Kazusa) GN=ssl3364 PE=4 SV=1 - [P73654	218,61	27,03	1	2	2	5	0,411	5	5,0	74	8,3	4,22							
	A3	Sequence	# PSMs	# Proteins	# Protein Groups	Protein Group Accessions	Modifications	ΔCn	116/117	116/117 Count	116/117 Variability [%]	q-Value	PEP	IonScore	Exp Value	Charge	MH+ [Da]	ΔM [ppm]	RT [min]	# Missed Cleavages
	High	qQHPTQTTLEk	3	1	1	P73654	N-Term(iTRAQ4plex); K11(iTRAQ4plex)	0,0000	0,406	3	1,7	0	3,086E-07	63	5,01153E-07	2	1598,87969	3,51	13,70	0
	High	iEQELANAR	2	1	1	P73654	N-Term(iTRAQ4plex)	0,0000	0,480	2	0,9	0	0,000004521	56	2,70377E-06	2	1187,65007	-0,04	25,30	0
P73410	Cysteine synthase OS=Synechocystis sp. (strain PCC 6803 / Kazusa) GN=cysK PE=3 SV=2 - [CYSK_S	218,29	16,03	1	4	4	8	0,514	7	8,8	312	33,2	5,80							
	A3	Sequence	# PSMs	# Proteins	# Protein Groups	Protein Group Accessions	Modifications	ΔCn	116/117	116/117 Count	116/117 Variability [%]	q-Value	PEP	IonScore	Exp Value	Charge	MH+ [Da]	ΔM [ppm]	RT [min]	# Missed Cleavages
	High	iASNITELIGR	4	1	1	P73410	N-Term(iTRAQ4plex)	0,0000	0,514	3	6,3	0	9,445E-07	72	6,109E-08	2	1330,78252	1,04	41,84	0
	High	eEGILSGISTGAALAAAIk	1	1	1	P73410	N-Term(iTRAQ4plex); K19(iTRAQ4plex)	0,0000	0,462	1		0	0,0001052	43	4,76398E-05	3	2060,19669	6,04	51,97	0
	High	lIVmIQPSFGER	1	1	1	P73410	N-Term(iTRAQ4plex); M4(Oxidation)	0,0000	0,546	1		0,001	0,008478	23	0,005069557	2	1549,84770	-3,38	40,36	0
	High	lIVMIQPSFGER	1	1	1	P73410	N-Term(iTRAQ4plex)	0,0000	0,243	1		0,001	0,01654	15	0,045012566	3	1533,85956	1,00	44,76	0
	High	iGINmINR	1	1	1	P73410	N-Term(iTRAQ4plex); M5(Oxidation)	0,0000	0,517	1		0,007	0,09228	32	0,000587449	2	1090,61662	0,59	24,22	0
Q6ZEQ0	Non-heme chloroperoxidase OS=Synechocystis sp. (strain PCC 6803 / Kazusa) GN=sll5080 PE=4 SV=	217,46	25,91	1	5	5	12	0,248	11	192,2	274	30,1	5,60							
	A3	Sequence	# PSMs	# Proteins	# Protein Groups	Protein Group Accessions	Modifications	ΔCn	116/117	116/117 Count	116/117 Variability [%]	q-Value	PEP	IonScore	Exp Value	Charge	MH+ [Da]	ΔM [ppm]	RT [min]	# Missed Cleavages
	High	tEANPGGLPIEVFDGFR	2	1	1	Q6ZEQ0	N-Term(iTRAQ4plex)	0,0000	0,256	2	206,3	0	1,142E-08	70	9,59334E-08	2	1963,00578	0,78	49,79	0
	High	iYPGGSHSLGDTSk	3	1	1	Q6ZEQ0	N-Term(iTRAQ4plex); K14(iTRAQ4plex)	0,0000	0,128	2	127,1	0	0,000009359	39	0,000137711	3	1706,89762	1,41	20,76	0
	High	aVLMGAVPPLMLk	3	1	1	Q6ZEQ0	N-Term(iTRAQ4plex); K13(iTRAQ4plex)	0,0000	0,212	3	10,1	0	0,001448	27	0,001940752	3	1627,99344	3,20	48,10	0
	High	sQFFLDIASGPFFGFNRPGAk	1	1	1	Q6ZEQ0	N-Term(iTRAQ4plex); K21(iTRAQ4plex)	0,0000	0,209	1		0	0,000006667	25	0,002998955	3	2591,37430	3,59	53,96	0
	High	sTITTk	1	1	1	Q6ZEQ0	N-Term(iTRAQ4plex); K6(iTRAQ4plex)	0,0000	0,492	1		0,001	0,00694	40	0,000105674	2	938,57762	1,65	12,47	0
	High	aVLmGAVPPLmLk	2	1	1	Q6ZEQ0	N-Term(iTRAQ4plex); M4(Oxidation); M11(Oxidation); K13(iTR	0,0000	1,564	2	163,1	0,001	0,01081	14	0,038368074	3	1659,97959	0,92	39,36	0
Q55386	Slr0924 protein OS=Synechocystis sp. (strain PCC 6803 / Kazusa) GN=slr0924 PE=4 SV=1 - [Q55386	217,36	14,34	1	3	3	6	0,502	6	12,1	286	31,3	5,72							
	A3	Sequence	# PSMs	# Proteins	# Protein Groups	Protein Group Accessions	Modifications	ΔCn	116/117	116/117 Count	116/117 Variability [%]	q-Value	PEP	IonScore	Exp Value	Charge	MH+ [Da]	ΔM [ppm]	RT [min]	# Missed Cleavages
	High	iVPTQEAIQFIR	3	1	1	Q55386	N-Term(iTRAQ4plex)	0,0000	0,512	3	6,3	0	2,601E-08	80	1,03031E-08	2	1558,90801	0,39	42,83	0
	High	qkPDVGSQVSVQPVSLGEVVk	1	1	1	Q55386	N-Term(iTRAQ4plex); K2(iTRAQ4plex); K21(iTRAQ4plex)	0,0000	0,731	1		0	1,214E-09	38	0,000150998	4	2612,49868	0,10	35,16	0
	High	sLSAQQPk	2	1	1	Q55386	N-Term(iTRAQ4plex); K8(iTRAQ4plex)	0,0000	0,462	2	8,2	0	0,0008573	35	0,000318398	2	1146,66973	-2,09	14,74	0
Q57038	Cytochrome b6 OS=Synechocystis sp. (strain PCC 6803 / Kazusa) GN=petB PE=3 SV=1 - [CYB6_SYN	215,58	15,32	1	4	4	6	2,605	6	28,1	222	25,0	9,01							
	A3	Sequence	# PSMs	# Proteins	# Protein Groups	Protein Group Accessions	Modifications	ΔCn	116/117	116/117 Count	116/117 Variability [%]	q-Value	PEP	IonScore	Exp Value	Charge	MH+ [Da]	ΔM [ppm]	RT [min]	# Missed Cleavages
	High	gSESVGQATLTR	2	1	1	Q57038	N-Term(iTRAQ4plex)	0,0000	2,399	2	31,2	0	0,000000228	85	3,45915E-09	2	1349,71501	0,62	17,99	0
	High	vYLTGGFk	1	1	1	Q57038	N-Term(iTRAQ4plex); K8(iTRAQ4plex)	0,0000	2,574	1		0	0,00002333	40	0,000103269	2	1172,69072	-0,91	31,44	0
	High	vFQWFNDR	2	1	1	Q57038	N-Term(iTRAQ4plex)	0,0000	2,968	2	17,7	0	0,000005517	39	0,000112972	2	1255,63408	0,01	41,93	0
	High	eVTESk	1	1	1	Q57038	N-Term(iTRAQ4plex); K6(iTRAQ4plex)	0,0000	1,094	1		0,002	0,03102	14	0,041397108	2	980,54936	-0,92	12,80	0
P73312	50S ribosomal protein L29 OS=Synechocystis sp. (strain PCC 6803 / Kazusa) GN=rpmC PE=3 SV=1 -	213,30	53,42	1	5	5	11	0,889	10	11,5	73	8,5	7,53							
	A3	Sequence	# PSMs	# Proteins	# Protein Groups	Protein Group Accessions	Modifications	ΔCn	116/117	116/117 Count	116/117 Variability [%]	q-Value	PEP	IonScore	Exp Value	Charge	MH+ [Da]	ΔM [ppm]	RT [min]	# Missed Cleavages
	High	lAQLLTVER	4	1	1	P73312	N-Term(iTRAQ4plex)	0,0000	0,891	3	0,6	0	0,000008882	62	6,39691E-07	2	1186,72869	0,89	38,23	0
	High	rPENPHEFk	2	1	1	P73312	N-Term(iTRAQ4plex); K9(iTRAQ4plex)	0,0000	1,021	2	0,4	0	4,258E-07	52	6,36752E-06	3	1441,77976	0,49	12,30	0
	High	qLENSPSEEA	1	1	1	P73312	N-Term(iTRAQ4plex)	0,0000	0,396	1		0	0,0003047	41	8,7694E-05	2	1247,58733	0,08	19,12	0
	High	fQQATR	2	1	1	P73312	N-Term(iTRAQ4plex)	0,0000	0,797	2	0,5	0,002	0,02936	11	0,07128038	2	894,49150	0,11	14,58	0
	Medium	lFQLR	2	1	1	P73312	N-Term(iTRAQ4plex)	0,0000	0,887	2	6,9	0,018	0,2415	19	0,021480187	2	820,51616	0,00	31,50	0
Q55828	LL-diaminopimelate aminotransferase OS=Synechocystis sp. (strain PCC 6803 / Kazusa) GN=dapL PE	212,03	21,60	1	6	6	10	2,169	9	33,0	412	45,1	5,62							
	A3	Sequence	# PSMs	# Proteins	# Protein Groups	Protein Group Accessions	Modifications	ΔCn	116/117	116/117 Count	116/117 Variability [%]	q-Value	PEP	IonScore	Exp Value	Charge	MH+ [Da]	ΔM [ppm]	RT [min]	# Missed Cleavages
	High	rVNAFTTANPNAQVIk	2	1	1	Q55828	N-Term(iTRAQ4plex); K16(iTRAQ4plex)	0,0000	1,047	2	210,2	0	1,544E-10	61	7,76194E-07	3	2032,15482	0,28	25,96	1
	High	tPHGLSSWDFFDk	2	1	1	Q55828	N-Term(iTRAQ4plex); K13(iTRAQ4plex)	0,0000	2,633	2	29,3	0	3,152E-11	51	7,12804E-06	3	1824,91623	0,15	41,28	0
	High	lLHTVNVVGTPGSGFGAAGEGYFF	1	1	1	Q55828	N-Term(iTRAQ4plex)	0,0000	1,238	1		0	0,000004382	47	2,4344E-05	3	2550,32352	0,50	43,27	0
	High	kVDLIYLcFPNNPTGATATk	1	1	1	Q55828	N-Term(iTRAQ4plex); K1(iTRAQ4plex); C8(Carbamidomethyl);	0,0000	0,727	1		0	0,0000174	36	0,000302268	4	2655,45498	0,33	42,21	1
	High	iAQHDFQAR	3	1	1	Q55828	N-Term(iTRAQ4plex)	0,0000	2,551	2	4,4	0	0,0001165	28	0,001713839	3	1229,65061	-0,14	16,44	0
	Medium	iSAFNSR	1	1	1	Q55828	N-Term(iTRAQ4plex)	0,0000	1,746	1		0,012	0,1558	14	0,038456522	2	938,51836	0,78	20,24	0
Q55847	Sll0470 protein OS=Synechocystis sp. (strain PCC 6803 / Kazusa) GN=sll0470 PE=4 SV=1 - [Q55847	211,78	19,35	1	5	5	10	0,466	9	45,6	186	20,7	8,78							
	A3	Sequence	# PSMs	# Proteins	# Protein Groups	Protein Group Accessions	Modifications	ΔCn	116/117	116/117 Count	116/117 Variability [%]	q-Value	PEP	IonScore	Exp Value	Charge	MH+ [Da]	ΔM [ppm]	RT [min]	# Missed Cleavages
	High	vYLGTWIVSINR	5	1	1	Q55847	N-Term(iTRAQ4plex)	0,0000	0,421	5	14,8	0	5,285E-08	80	1,09893E-08	2	1564,89763	0,52	49,21	0
	High	rQGNIEIEDVVWDk	1	1	1	Q55847	N-Term(iTRAQ4plex); K14(iTRAQ4plex)	0,0000	0,688	1		0	0,000001584	39	0,000134577	3	1989,06552	0,55	35,89	1
	High	rQGNIEIEDVVWDkESR	2	1	1	Q55847	N-Term(iTRAQ4plex); K14(iTRAQ4plex)	0,0000	0,819	1		0	3,433E-08	39	0,000137395	4	2361,24038	0,08	32,62	2
	High	lYIPAkk	1	1	1	Q55847	N-Term(iTRAQ4plex); K6(iTRAQ4plex); K7(iTRAQ4plex)	0,0000	0,643	1		0	0,0002923	29	0,001336503	3	1264,83442	-0,72	24,05	1
	High	lYIPAk	1	1	1	Q55847	N-Term(iTRAQ4plex); K6(iTRAQ4plex)	0,0000	0,830	1		0,002	0,01781	29	0,001137549	2	992,63725	-1,04	28,70	0
P73598	Sll1305 protein OS=Synechocystis sp. (strain PCC 6803 / Kazusa) GN=sll1305 PE=4 SV=1 - [P73598	211,45	5,42	1	2	2	5	1,082	5	6,2	295	32,9	4,97							
	A3	Sequence	# PSMs	# Proteins	# Protein Groups	Protein Group Accessions	Modifications	ΔCn	116/117	116/117 Count	116/117 Variability [%]	q-Value	PEP	IonScore	Exp Value	Charge	MH+ [Da]	ΔM [ppm]	RT [min]	# Missed Cleavages
	High	lLLEQLGEHFR	4	1	1	P73598	N-Term(iTRAQ4plex)	0,0000	1,082	4	3,1	0	0,000001177	69	1,19726E-07	2	1498,84868	-0,80	43,38	0
	High	aFLAk	1	1	1	P73598	N-Term(iTRAQ4plex); K5(iTRAQ4plex)	0,0000	1,185	1		0,007	0,09376	19	0,011586973	2	837,54241	-1,51	20,19	0
P72785	4-alpha-glucanotransferase OS=Synechocystis sp. (strain PCC 6803 / Kazusa) GN=malQ PE=3 SV=1	211,15	5,74	1	3	3	10	0,719	9	21,2	505	57,1	4,88							
	A3	Sequence	# PSMs	# Proteins	# Protein Groups	Protein Group Accessions	Modifications	ΔCn	116/117	116/117 Count	116/117 Variability [%]	q-Value	PEP	IonScore	Exp Value	Charge	MH+ [Da]	ΔM [ppm]	RT [min]	# Missed Cleavages
	High	aTGFAWWIk	5	1	1	P72785	N-Term(iTRAQ4plex); K9(iTRAQ4plex)	0,0000	0,719	5	26,5	0	5,266E-07	58	1,53098E-06	2	1367,77727	4,25	44,99	0
	High	vLHFAFDSDR	4	1	1	P72785	N-Term(iTRAQ4plex)	0,0000	0,731	3	0,8	0	0,000002421	44	4,36486E-05	3	1350,69431	1,48	33,58	0
	Medium	aNLQYLDIVR	1	1	1	P72785	N-Term(iTRAQ4plex)	0,0000	0,675	1		0,031	0,4039	10	0,090985043	2	1348,77397	2,53	38,94	0
P73405	Pyruvate dehydrogenase E1 beta subunit OS=Synechocystis sp. (strain PCC 6803 / Kazusa) GN=pdhE	210,63	6,79	1	2	2	8	3,008	6	9,2	324	35,7	5,54							
	A3	Sequence	# PSMs	# Proteins	# Protein Groups	Protein Group Accessions	Modifications	ΔCn	116/117	116/117 Count	116/117 Variability [%]	q-Value	PEP	IonScore	Exp Value	Charge	MH+ [Da]	ΔM [ppm]	RT [min]	# Missed Cleavages
	High	aETLLFAALR	4	1	1	P73405	N-Term(iTRAQ4plex)	0,0000	3,182	4	9,2	0	7,985E-07	60	9,86211E-07	2	1248,74358	0,23	46,72	0
	High	lEAYFHAVPGLk	4	1	1	P73405	N-Term(iTRAQ4plex); K12(iTRAQ4plex)	0,0000	2,679	2	15,0	0	0,0001967	36	0,000252331	3	1632,93583	0,37	40,70	0
P23353	Chorismate synthase OS=Synechocystis sp. (strain PCC 6803 / Kazusa) GN=aroC PE=3 SV=2 - [ARO	210,45	10,50	1	3	3	7	1,423	7	32,3	362	39,3	5,99							
	A3	Sequence	# PSMs	# Proteins	# Protein Groups	Protein Group Accessions	Modifications	ΔCn	116/117	116/117 Count	116/117 Variability [%]	q-Value	PEP	IonScore	Exp Value	Charge	MH+ [Da]	ΔM [ppm]	RT [min]	# Missed Cleavages
	High	iAFkPTATIGQEQk	2	1	1	P23353	N-Term(iTRAQ4plex); K4(iTRAQ4plex); K14(iTRAQ4plex)	0,0000	1,950	2	15,3	0	1,666E-10	54	3,8634E-06	3	1964,15421	0,04	28,02	0
	High	yRPSHADATYEAk	2	1	1	P23353	N-Term(iTRAQ4plex); K13(iTRAQ4plex)	0,0000	0,842	2	91,2	0	1,269E-08	50	9,01571E-06	3	1796,91703	0,02	14,37	0
	High	tLGTPIAILVR	3	1	1	P23353	N-Term(iTRAQ4plex)	0,0000	1,349	3	15,8	0	0,00001361	48	1,57025E-05	3	1297,83213	-0,24	43,40	0
P74410	30S ribosomal protein S16 OS=Synechocystis sp. (strain PCC 6803 / Kazusa) GN=rpsP PE=3 SV=1 -	209,66	60,98	1	6	6	12	1,456	12	52,3	82	9,6	10,52							
	A3	Sequence	# PSMs	# Proteins	# Protein Groups	Protein Group Accessions	Modifications	ΔCn	116/117	116/117 Count	116/117 Variability [%]	q-Value	PEP	IonScore	Exp Value	Charge	MH+ [Da]	ΔM [ppm]	RT [min]	# Missed Cleavages
	High	lkEGAQPTDTVR	4	1	1	P74410	N-Term(iTRAQ4plex); K2(iTRAQ4plex)	0,0000	1,522	4	96,0	0	0,0001483	42	6,54591E-05	2	1602,91130	3,68	16,49	1
	High	dGRPLEELGFYNPR	2	1	1	P74410	N-Term(iTRAQ4plex)	0,0000	1,257	2	11,1	0	1,769E-08	35	0,000322085	3	1806,92649	0,50	35,97	0
	High	rDGRPLEELGFYNPR	2	1	1	P74410	N-Term(iTRAQ4plex)	0,0000	1,884	2	12,7	0	0,0009554	27	0,001945226	3	1963,02921	1,28	31,91	1
	High	aQVFEQLkA	1	1	1	P74410	N-Term(iTRAQ4plex); K8(iTRAQ4plex)	0,0000	0,979	1		0	0,0008981	23	0,004698617	2	1321,77715	4,01	30,54	1
	High	sILTk	1	1	1	P74410	N-Term(iTRAQ4plex); K5(iTRAQ4plex)	0,0000	1,807	1		0,004	0,04804	28	0,001725719	2	849,56584	1,24	18,49	0
	High	iVAMHSTTR	1	1	1	P74410	N-Term(iTRAQ4plex)	0,0000	1,057	1		0,005	0,05603	16	0,02432036	3	1159,63968	1,94	15,08	0
	Medium	iVAmHSTTR	1	1	1	P74410	N-Term(iTRAQ4plex); M4(Oxidation)	0,0000	1,565	1		0,02	0,2761	27	0,001790482	2	1175,63323	0,75	11,28	0
P74367	Photosystem II lipoprotein Psb27 OS=Synechocystis sp. (strain PCC 6803 / Kazusa) GN=psb27 PE=1	209,13	35,82	1	5	5	10	0,994	10	22,1	134	14,8	9,33							
	A3	Sequence	# PSMs	# Proteins	# Protein Groups	Protein Group Accessions	Modifications	ΔCn	116/117	116/117 Count	116/117 Variability [%]	q-Value	PEP	IonScore	Exp Value	Charge	MH+ [Da]	ΔM [ppm]	RT [min]	# Missed Cleavages
	High	rLQLEFTQAER	3	1	1	P74367	N-Term(iTRAQ4plex)	0,0000	1,206	3	4,1	0	0,000005574	52	6,89275E-06	2	1534,84770	1,20	27,69	1
	High	gQINDYISR	3	1	1	P74367	N-Term(iTRAQ4plex)	0,0000	0,918	3	22,2	0	0,000001025	46	2,23857E-05	2	1209,63518	0,61	24,31	0
	High	kGDAGGLk	2	1	1	P74367	N-Term(iTRAQ4plex); K1(iTRAQ4plex); K8(iTRAQ4plex)	0,0000	0,925	2	17,0	0	0,001113	26	0,002243727	3	1177,72510	-1,20	10,47	1
	High	qEVQDTAR	1	1	1	P74367	N-Term(iTRAQ4plex)	0,0000	0,874	1		0	0,0004613	25	0,003451199	2	1090,56084	-0,11	11,50	0
	High	eAIDLPQDAPNRQEVQDTAR	1	1	1	P74367	N-Term(iTRAQ4plex)	0,0000	0,954	1		0	0,0004654	23	0,005419635	3	2410,20700	-0,41	27,76	1
Q01950	Phycobilisome 7.8 kDa linker polypeptide, allophycocyanin-associated, core OS=Synechocystis sp. (st	208,64	50,75	1	3	3	8	2,433	7	20,2	67	7,7	10,93							
	A3	Sequence	# PSMs	# Proteins	# Protein Groups	Protein Group Accessions	Modifications	ΔCn	116/117	116/117 Count	116/117 Variability [%]	q-Value	PEP	IonScore	Exp Value	Charge	MH+ [Da]	ΔM [ppm]	RT [min]	# Missed Cleavages
	High	vELATGRPGTNAGLA	3	1	1	Q01950	N-Term(iTRAQ4plex)	0,0000	3,063	2	20,0	0	0,000000694	63	5,29894E-07	2	1570,86797	0,61	28,03	0
	High	eLQNTYFTk	1	1	1	Q01950	N-Term(iTRAQ4plex); K9(iTRAQ4plex)	0,0000	2,785	1		0	0,00001722	53	5,59368E-06	2	1431,77239	0,12	28,74	0
	High	iTAcVPSQTR	4	1	1	Q01950	N-Term(iTRAQ4plex); C4(Carbamidomethyl)	0,0000	1,641	4	61,4	0	0,00001197	43	4,7203E-05	2	1276,68096	0,73	21,20	0
P74193	Threonine synthase OS=Synechocystis sp. (strain PCC 6803 / Kazusa) GN=thrC PE=3 SV=1 - [THRC	208,19	19,37	1	4	4	6	1,881	6	12,0	382	40,4	7,24							
	A3	Sequence	# PSMs	# Proteins	# Protein Groups	Protein Group Accessions	Modifications	ΔCn	116/117	116/117 Count	116/117 Variability [%]	q-Value	PEP	IonScore	Exp Value	Charge	MH+ [Da]	ΔM [ppm]	RT [min]	# Missed Cleavages
	High	mmGFQAAGSAPFIQGQPISHPET	2	1	1	P74193	N-Term(iTRAQ4plex); M1(Oxidation); M2(Oxidation)	0,0000	1,881	2	2,6	0	1,483E-09	74	4,29507E-08	3	3203,61399	1,26	41,72	0
	High	aVIcASTGNTSAAAAAYAR	1	1	1	P74193	N-Term(iTRAQ4plex); C4(Carbamidomethyl)	0,0000	0,834	1		0	4,689E-09	57	1,85768E-06	3	1969,99015	0,95	28,29	0
	High	aGLkPDLEIVAk	2	1	1	P74193	N-Term(iTRAQ4plex); K4(iTRAQ4plex); K12(iTRAQ4plex)	0,0000	2,039	2	6,5	0	0,00000161	36	0,000237121	3	1686,05417	0,91	31,52	0
	High	aFVIIPDGYVALGk	1	1	1	P74193	N-Term(iTRAQ4plex); K14(iTRAQ4plex)	0,0000	0,440	1		0,001	0,004327	28	0,001509976	3	1751,03666	1,16	47,75	0
P73316	30S ribosomal protein S19 OS=Synechocystis sp. (strain PCC 6803 / Kazusa) GN=rpsS PE=3 SV=1 -	207,69	36,96	1	4	4	9	2,292	9	11,7	92	10,3	11,14							
	A3	Sequence	# PSMs	# Proteins	# Protein Groups	Protein Group Accessions	Modifications	ΔCn	116/117	116/117 Count	116/117 Variability [%]	q-Value	PEP	IonScore	Exp Value	Charge	MH+ [Da]	ΔM [ppm]	RT [min]	# Missed Cleavages
	High	kGPFVAASLLR	2	1	1	P73316	N-Term(iTRAQ4plex); K1(iTRAQ4plex)	0,0000	2,464	2	0,9	0	3,468E-08	56	2,594E-06	3	1446,90497	0,98	33,32	1
	High	gPFVAASLLR	3	1	1	P73316	N-Term(iTRAQ4plex)	0,0000	2,300	3	0,5	0	4,914E-07	45	3,42529E-05	2	1174,70720	0,58	39,96	0
	High	qHVPVFVSEQMVGHk	2	1	1	P73316	N-Term(iTRAQ4plex); K15(iTRAQ4plex)	0,0000	1,536	2	30,1	0	3,122E-08	28	0,001516946	4	2010,08413	0,37	28,92	0
	High	qHVPVFVSEQmVGHk	1	1	1	P73316	N-Term(iTRAQ4plex); M11(Oxidation); K15(iTRAQ4plex)	0,0000	1,910	1		0	6,121E-08	27	0,001914124	4	2026,07876	0,22	24,09	0
	High	lGEFAPTR	1	1	1	P73316	N-Term(iTRAQ4plex)	0,0000	1,885	1		0,004	0,04459	18	0,015847837	2	1034,57439	-0,74	26,23	0
P74447	Ferredoxin OS=Synechocystis sp. (strain PCC 6803 / Kazusa) GN=slr0148 PE=4 SV=1 - [P74447_SYN	207,50	10,75	1	2	2	6	0,816	5	12,8	186	20,6	5,06							
	A3	Sequence	# PSMs	# Proteins	# Protein Groups	Protein Group Accessions	Modifications	ΔCn	116/117	116/117 Count	116/117 Variability [%]	q-Value	PEP	IonScore	Exp Value	Charge	MH+ [Da]	ΔM [ppm]	RT [min]	# Missed Cleavages
	High	tLEVITTHNR	3	1	1	P74447	N-Term(iTRAQ4plex)	0,0000	0,792	2	4,3	0	0,000001	58	1,41896E-06	2	1327,74748	1,82	20,37	0
	High	rAEENILNPR	3	1	1	P74447	N-Term(iTRAQ4plex)	0,0000	0,889	3	36,5	0	0,00008325	57	1,80289E-06	2	1355,75920	5,88	17,67	1
P73328	Slr1900 protein OS=Synechocystis sp. (strain PCC 6803 / Kazusa) GN=slr1900 PE=4 SV=1 - [P73328	204,83	14,57	1	5	5	10	0,501	10	29,6	247	27,4	4,87							
	A3	Sequence	# PSMs	# Proteins	# Protein Groups	Protein Group Accessions	Modifications	ΔCn	116/117	116/117 Count	116/117 Variability [%]	q-Value	PEP	IonScore	Exp Value	Charge	MH+ [Da]	ΔM [ppm]	RT [min]	# Missed Cleavages
	High	aHLTVTQGDER	2	1	1	P73328	N-Term(iTRAQ4plex)	0,0000	0,588	2	9,5	0	7,73E-08	52	5,8475E-06	3	1370,71387	-0,46	14,42	0
	High	gTEIcQVSR	2	1	1	P73328	N-Term(iTRAQ4plex); C5(Carbamidomethyl)	0,0000	0,430	2	7,2	0	0,0000022	47	2,19265E-05	2	1193,60588	-0,54	17,36	0
	High	yNAIFkDPk	2	1	1	P73328	N-Term(iTRAQ4plex); K6(iTRAQ4plex); K9(iTRAQ4plex)	0,0000	0,493	2	30,8	0	0,0006089	18	0,017934854	4	1527,89101	0,96	26,22	1
	High	yNAIFk	2	1	1	P73328	N-Term(iTRAQ4plex); K6(iTRAQ4plex)	0,0000	0,352	2	39,3	0,001	0,01521	25	0,003133069	2	1043,61260	-0,19	28,03	0
	High	kQGNFEk	2	1	1	P73328	N-Term(iTRAQ4plex); K1(iTRAQ4plex); K7(iTRAQ4plex)	0,0000	0,668	2	14,7	0,001	0,005331	18	0,016496533	3	1282,74634	-1,27	11,12	1
P48946	30S ribosomal protein S18 OS=Synechocystis sp. (strain PCC 6803 / Kazusa) GN=rpsR PE=3 SV=1 -	203,22	25,35	1	2	2	11	2,015	10	16,6	71	8,4	10,76							
	A3	Sequence	# PSMs	# Proteins	# Protein Groups	Protein Group Accessions	Modifications	ΔCn	116/117	116/117 Count	116/117 Variability [%]	q-Value	PEP	IonScore	Exp Value	Charge	MH+ [Da]	ΔM [ppm]	RT [min]	# Missed Cleavages
	High	lVALLPFVNk	10	1	1	P48946	N-Term(iTRAQ4plex); K10(iTRAQ4plex)	0,0000	2,025	9	12,7	0	0,000001189	60	1,00686E-06	2	1401,91081	2,56	49,80	0
	High	rITGLTAk	1	1	1	P48946	N-Term(iTRAQ4plex); K8(iTRAQ4plex)	0,0000	1,765	1		0,001	0,006526	17	0,020369014	3	1147,74167	1,33	16,76	1
P74448	Slr0149 protein OS=Synechocystis sp. (strain PCC 6803 / Kazusa) GN=slr0149 PE=3 SV=1 - [P74448	203,16	20,51	1	3	3	7	2,318	6	36,5	156	18,4	5,26							
	A3	Sequence	# PSMs	# Proteins	# Protein Groups	Protein Group Accessions	Modifications	ΔCn	116/117	116/117 Count	116/117 Variability [%]	q-Value	PEP	IonScore	Exp Value	Charge	MH+ [Da]	ΔM [ppm]	RT [min]	# Missed Cleavages
	High	aYNYTEYAk	1	1	1	P74448	N-Term(iTRAQ4plex); K9(iTRAQ4plex)	0,0000	2,615	1		0	2,528E-07	66	2,78593E-07	2	1410,71404	-0,22	23,86	0
	High	iRDSEEQIIHR	5	1	1	P74448	N-Term(iTRAQ4plex)	0,0000	2,409	4	30,3	0	1,507E-07	47	2,13781E-05	3	1539,83640	0,24	19,88	1
	High	gLPQDTFHmGNR	1	1	1	P74448	N-Term(iTRAQ4plex); M9(Oxidation)	0,0000	0,959	1		0	0,000006163	37	0,000193629	3	1532,74024	0,36	20,09	0
P74643	Phosphoglucomutase OS=Synechocystis sp. (strain PCC 6803 / Kazusa) GN=pgm PE=1 SV=1 - [P746	202,25	7,41	1	3	3	7	1,519	7	6,8	567	61,1	6,11							
	A3	Sequence	# PSMs	# Proteins	# Protein Groups	Protein Group Accessions	Modifications	ΔCn	116/117	116/117 Count	116/117 Variability [%]	q-Value	PEP	IonScore	Exp Value	Charge	MH+ [Da]	ΔM [ppm]	RT [min]	# Missed Cleavages
	High	fTPTPAVSYAILTHNQGR	2	1	1	P74643	N-Term(iTRAQ4plex)	0,0000	1,482	2	3,0	0	9,969E-13	94	4,31699E-10	3	2117,12748	0,67	38,11	0
	High	rLDYEQALk	2	1	1	P74643	N-Term(iTRAQ4plex); K9(iTRAQ4plex)	0,0000	1,309	2	22,4	0	0,000002461	32	0,000622257	3	1423,81519	0,30	24,14	1
	High	tGkDPGLHYQDLTAk	3	1	1	P74643	N-Term(iTRAQ4plex); K3(iTRAQ4plex); K15(iTRAQ4plex)	0,0000	1,597	3	6,5	0	0,000002561	29	0,00118296	4	2076,14638	0,66	23,79	1
P72940	Probable adenylyl-sulfate kinase OS=Synechocystis sp. (strain PCC 6803 / Kazusa) GN=cysC PE=3 S\	201,63	28,25	1	4	4	9	1,394	6	5,9	177	19,7	5,97							
	A3	Sequence	# PSMs	# Proteins	# Protein Groups	Protein Group Accessions	Modifications	ΔCn	116/117	116/117 Count	116/117 Variability [%]	q-Value	PEP	IonScore	Exp Value	Charge	MH+ [Da]	ΔM [ppm]	RT [min]	# Missed Cleavages
	High	iGFVSHLLTR	2	1	1	P72940	N-Term(iTRAQ4plex)	0,0000	1,403	1		0	0,000003377	58	1,41244E-06	3	1286,77054	0,29	38,43	0
	High	gVTIWLTGLSGAGk	4	1	1	P72940	N-Term(iTRAQ4plex); K14(iTRAQ4plex)	0,0000	1,360	3	11,9	0	1,014E-07	55	3,49921E-06	3	1647,96805	0,47	46,62	0
	High	nGVIVLVSAISPYAAIR	1	1	1	P72940	N-Term(iTRAQ4plex)	0,0000	1,892	1		0	0,000001027	32	0,000691783	3	1887,12101	1,34	54,28	0
	High	tTITHALEk	2	1	1	P72940	N-Term(iTRAQ4plex); K9(iTRAQ4plex)	0,0000	1,384	1		0	0,002576	18	0,01779888	3	1301,76624	-0,39	18,90	0
P72941	Putative biopolymer transport protein ExbB-like 2 OS=Synechocystis sp. (strain PCC 6803 / Kazusa) G	199,13	7,93	1	2	2	5	1,424	5	3,5	227	25,1	5,24							
	A3	Sequence	# PSMs	# Proteins	# Protein Groups	Protein Group Accessions	Modifications	ΔCn	116/117	116/117 Count	116/117 Variability [%]	q-Value	PEP	IonScore	Exp Value	Charge	MH+ [Da]	ΔM [ppm]	RT [min]	# Missed Cleavages
	High	kVGSELEVLYR	2	1	1	P72941	N-Term(iTRAQ4plex); K1(iTRAQ4plex)	0,0000	1,397	2	0,7	0	7,179E-08	71	7,07897E-08	3	1580,92600	0,60	31,79	1
	High	yLLAPLR	3	1	1	P72941	N-Term(iTRAQ4plex)	0,0000	1,525	3	0,3	0	0,002686	42	8,03421E-05	2	989,62639	-0,05	35,43	0
P72817	Universal stress protein Sll1654 OS=Synechocystis sp. (strain PCC 6803 / Kazusa) GN=sll1654 PE=3	198,57	45,86	1	5	5	11	0,928	10	30,6	157	16,8	5,52							
	A3	Sequence	# PSMs	# Proteins	# Protein Groups	Protein Group Accessions	Modifications	ΔCn	116/117	116/117 Count	116/117 Variability [%]	q-Value	PEP	IonScore	Exp Value	Charge	MH+ [Da]	ΔM [ppm]	RT [min]	# Missed Cleavages
	High	lLEAAQAVFSQQGIATk	1	1	1	P72817	N-Term(iTRAQ4plex); K17(iTRAQ4plex)	0,0000	1,028	1		0	1,225E-12	67	2,17866E-07	3	2063,17527	0,62	48,64	0
	High	iHQSQLILLSVVEk	3	1	1	P72817	N-Term(iTRAQ4plex); K14(iTRAQ4plex)	0,0000	0,913	2	45,3	0	4,899E-07	40	0,000108885	3	1895,15915	1,21	42,40	0
	High	nPPGQDHEAHGmDSPEAVAk	1	1	1	P72817	N-Term(iTRAQ4plex); M12(Oxidation); K20(iTRAQ4plex)	0,0000	0,758	1		0	0,000001008	20	0,009418245	4	2391,12148	-0,99	14,50	0
	High	tILFPLDR	5	1	1	P72817	N-Term(iTRAQ4plex)	0,0000	0,974	5	14,4	0,001	0,004241	37	0,000215774	2	1118,66911	0,05	40,51	0
	Medium	vINLSPcPVLVVP	1	1	1	P72817	N-Term(iTRAQ4plex); C7(Carbamidomethyl)	0,0000	0,502	1		0,043	0,5514	12	0,067448144	2	1550,91118	0,94	52,27	0
P73111	Sll1835 protein OS=Synechocystis sp. (strain PCC 6803 / Kazusa) GN=sll1835 PE=4 SV=1 - [P73111	198,26	6,79	1	2	2	6	1,975	4	12,4	265	28,8	4,93							
	A3	Sequence	# PSMs	# Proteins	# Protein Groups	Protein Group Accessions	Modifications	ΔCn	116/117	116/117 Count	116/117 Variability [%]	q-Value	PEP	IonScore	Exp Value	Charge	MH+ [Da]	ΔM [ppm]	RT [min]	# Missed Cleavages
	High	gQLTGAQYIVLGR	5	1	1	P73111	N-Term(iTRAQ4plex)	0,0000	2,065	3	11,7	0	7,359E-09	78	1,73768E-08	2	1519,87297	1,07	35,88	0
	Medium	aPVGk	1	1	1	P73111	N-Term(iTRAQ4plex); K5(iTRAQ4plex)	0,0000	1,890	1		0,013	0,1781	10	0,09749223	2	759,49608	-0,84	13,19	0
P74518	Light-repressed protein A homolog OS=Synechocystis sp. (strain PCC 6803 / Kazusa) GN=lrtA PE=3 S	197,26	33,51	1	6	6	11	1,628	8	28,9	191	21,9	6,54							
	A3	Sequence	# PSMs	# Proteins	# Protein Groups	Protein Group Accessions	Modifications	ΔCn	116/117	116/117 Count	116/117 Variability [%]	q-Value	PEP	IonScore	Exp Value	Charge	MH+ [Da]	ΔM [ppm]	RT [min]	# Missed Cleavages
	High	nHGGYGVIQPHQAS	4	1	1	P74518	N-Term(iTRAQ4plex)	0,0000	1,932	2	1,7	0	0,000004373	51	8,20295E-06	2	1608,80083	0,54	16,31	0
	High	nkDTDEINVIYIR	3	1	1	P74518	N-Term(iTRAQ4plex); K2(iTRAQ4plex)	0,0000	1,628	2	0,2	0	2,239E-07	51	8,8914E-06	3	1881,03293	0,47	31,59	1
	High	vDVHLSVAR	1	1	1	P74518	N-Term(iTRAQ4plex)	0,0000	0,873	1		0	0,0002675	26	0,002660541	3	1139,66370	-1,46	23,41	0
	High	hFQNLTTk	1	1	1	P74518	N-Term(iTRAQ4plex); K8(iTRAQ4plex)	0,0000	0,541	1		0	0,00004179	24	0,006340029	3	1276,72483	-0,30	17,05	0
	High	iQDkQHGNVk	1	1	1	P74518	N-Term(iTRAQ4plex); K4(iTRAQ4plex); K10(iTRAQ4plex)	0,0000	2,653	1		0	0,00005831	19	0,014059504	3	1598,93424	0,22	9,31	1
	High	aPELPSEVLR	1	1	1	P74518	N-Term(iTRAQ4plex)	0,0000	1,333	1		0,001	0,006881	35	0,000345915	2	1254,71782	0,28	32,93	0
P74360	Uncharacterized protein sll1526 OS=Synechocystis sp. (strain PCC 6803 / Kazusa) GN=sll1526 PE=4	197,19	12,45	1	5	5	10	2,597	8	43,4	506	57,7	7,50							
	A3	Sequence	# PSMs	# Proteins	# Protein Groups	Protein Group Accessions	Modifications	ΔCn	116/117	116/117 Count	116/117 Variability [%]	q-Value	PEP	IonScore	Exp Value	Charge	MH+ [Da]	ΔM [ppm]	RT [min]	# Missed Cleavages
	High	lEQLLLAk	1	1	1	P74360	N-Term(iTRAQ4plex); K9(iTRAQ4plex)	0,0000	3,944	1		0	0,000002855	54	4,02689E-06	2	1328,87627	0,51	48,91	0
	High	lALLPQEHLQALGNLR	5	1	1	P74360	N-Term(iTRAQ4plex)	0,0000	3,010	3	18,4	0	5,529E-10	48	1,72572E-05	3	1930,13614	0,32	43,18	0
	High	aNYFTQNPILkPR	2	1	1	P74360	N-Term(iTRAQ4plex); K11(iTRAQ4plex)	0,0000	1,733	2	95,3	0	4,104E-08	41	8,31706E-05	3	1850,05405	0,72	33,00	0
	High	kQDGPQFVWQVLSSQGk	1	1	1	P74360	N-Term(iTRAQ4plex); K1(iTRAQ4plex); K17(iTRAQ4plex)	0,0000	2,264	1		0	0,00001399	15	0,033960182	4	2364,30703	1,42	43,85	1
	High	qAIVNFIR	1	1	1	P74360	N-Term(iTRAQ4plex)	0,0000	1,943	1		0,001	0,004863	24	0,005449213	2	1104,66435	-0,25	33,72	0
P73527	6,7-dimethyl-8-ribityllumazine synthase OS=Synechocystis sp. (strain PCC 6803 / Kazusa) GN=ribH P	196,55	29,27	1	4	4	11	0,370	8	24,4	164	17,6	6,29							
	A3	Sequence	# PSMs	# Proteins	# Protein Groups	Protein Group Accessions	Modifications	ΔCn	116/117	116/117 Count	116/117 Variability [%]	q-Value	PEP	IonScore	Exp Value	Charge	MH+ [Da]	ΔM [ppm]	RT [min]	# Missed Cleavages
	High	gQTPHFDFVAGEAAk	2	1	1	P73527	N-Term(iTRAQ4plex); K15(iTRAQ4plex)	0,0000	0,489	2	39,2	0	1,596E-09	59	1,37711E-06	3	1862,96500	0,54	30,13	0
	High	tVYEGSFTPPARPFR	2	1	1	P73527	N-Term(iTRAQ4plex)	0,0000	0,379	2	22,5	0	0,0001254	30	0,001076935	3	1868,97855	0,50	32,36	0
	High	lLSGcQDcLkR	1	1	1	P73527	N-Term(iTRAQ4plex); C5(Carbamidomethyl); C8(Carbamidome	0,0000	0,532	1		0	0,0001729	22	0,005675054	3	1637,87235	1,06	23,38	1
	High	fALVIAR	6	1	1	P73527	N-Term(iTRAQ4plex)	0,0000	0,318	3	15,6	0,001	0,006663	44	6,19966E-05	2	933,60045	0,22	35,09	0
P74746	Slr0600 protein OS=Synechocystis sp. (strain PCC 6803 / Kazusa) GN=slr0600 PE=4 SV=1 - [P74746	195,92	10,12	1	4	4	9	0,583	8	11,0	326	36,0	6,05							
	A3	Sequence	# PSMs	# Proteins	# Protein Groups	Protein Group Accessions	Modifications	ΔCn	116/117	116/117 Count	116/117 Variability [%]	q-Value	PEP	IonScore	Exp Value	Charge	MH+ [Da]	ΔM [ppm]	RT [min]	# Missed Cleavages
	High	iFALGDLkk	3	1	1	P74746	N-Term(iTRAQ4plex); K8(iTRAQ4plex); K9(iTRAQ4plex)	0,0000	0,587	3	2,1	0	0,00002195	53	5,55866E-06	2	1436,92546	3,70	34,40	1
	High	iFALGDLk	1	1	1	P74746	N-Term(iTRAQ4plex); K8(iTRAQ4plex)	0,0000	0,416	1		0	0,0001743	42	6,2513E-05	2	1164,72307	-0,03	40,78	0
	High	lADHGYPLHEAAITk	3	1	1	P74746	N-Term(iTRAQ4plex); K15(iTRAQ4plex)	0,0000	0,476	2	15,5	0	0,000003323	37	0,000207955	4	1924,05520	1,08	25,97	0
	High	kDSLYPIFR	2	1	1	P74746	N-Term(iTRAQ4plex); K1(iTRAQ4plex)	0,0000	0,629	2	8,2	0	3,351E-07	35	0,000291052	3	1426,83048	0,55	32,17	1
P73757	Neopullulanase OS=Synechocystis sp. (strain PCC 6803 / Kazusa) GN=nplT PE=4 SV=1 - [P73757_SY	195,21	10,43	1	4	4	8	0,381	6	20,8	508	58,0	5,25							
	A3	Sequence	# PSMs	# Proteins	# Protein Groups	Protein Group Accessions	Modifications	ΔCn	116/117	116/117 Count	116/117 Variability [%]	q-Value	PEP	IonScore	Exp Value	Charge	MH+ [Da]	ΔM [ppm]	RT [min]	# Missed Cleavages
	High	iIATAGmGYVFQR	1	1	1	P73757	N-Term(iTRAQ4plex); M7(Oxidation)	0,0000	0,325	1		0	5,436E-09	57	1,92739E-06	2	1586,84929	0,70	35,25	0
	High	fAIGQQQAIAVAQQPk	2	1	1	P73757	N-Term(iTRAQ4plex); K16(iTRAQ4plex)	0,0000	0,529	2	41,4	0	4,894E-10	55	3,04068E-06	3	1986,14005	1,26	33,95	0
	High	aLPQFNHDHPDVR	4	1	1	P73757	N-Term(iTRAQ4plex)	0,0000	0,344	2	6,1	0	5,872E-08	39	0,000116405	4	1689,85915	0,79	23,21	0
	High	kAVFYQIFPDR	1	1	1	P73757	N-Term(iTRAQ4plex); K1(iTRAQ4plex)	0,0000	0,436	1		0	0,0001566	21	0,007655437	3	1671,94754	0,85	37,24	1
Q55552	IMP dehydrogenase OS=Synechocystis sp. (strain PCC 6803 / Kazusa) GN=guaB PE=4 SV=1 - [Q555	193,93	20,65	1	3	3	7	0,904	7	18,3	155	17,3	5,34							
	A3	Sequence	# PSMs	# Proteins	# Protein Groups	Protein Group Accessions	Modifications	ΔCn	116/117	116/117 Count	116/117 Variability [%]	q-Value	PEP	IonScore	Exp Value	Charge	MH+ [Da]	ΔM [ppm]	RT [min]	# Missed Cleavages
	High	qLIGILTQGDIIR	4	1	1	Q55552	N-Term(iTRAQ4plex)	0,0000	1,010	4	5,8	0	3,578E-07	68	1,74973E-07	2	1583,96050	0,20	48,06	0
	High	rLPVLNVESR	1	1	1	Q55552	N-Term(iTRAQ4plex)	0,0000	0,810	1		0	1,112E-07	50	9,95337E-06	3	1326,79676	-0,53	26,75	1
	High	eAAHLmNEk	2	1	1	Q55552	N-Term(iTRAQ4plex); M6(Oxidation); K9(iTRAQ4plex)	0,0000	0,755	2	7,1	0	0,0004234	14	0,041876464	3	1346,69712	-0,43	11,42	0
P72585	GDP-L-fucose synthase OS=Synechocystis sp. (strain PCC 6803 / Kazusa) GN=fcl PE=3 SV=1 - [P725	192,95	15,71	1	5	5	6	1,068	6	47,6	312	34,7	5,90							
	A3	Sequence	# PSMs	# Proteins	# Protein Groups	Protein Group Accessions	Modifications	ΔCn	116/117	116/117 Count	116/117 Variability [%]	q-Value	PEP	IonScore	Exp Value	Charge	MH+ [Da]	ΔM [ppm]	RT [min]	# Missed Cleavages
	High	qVVAQLIAAGGDRPk	1	1	1	P72585	N-Term(iTRAQ4plex); K15(iTRAQ4plex)	0,0000	1,414	1		0	4,665E-12	72	6,7293E-08	3	1811,07419	-0,01	32,51	0
	High	kALLVQLESYR	1	1	1	P72585	N-Term(iTRAQ4plex); K1(iTRAQ4plex)	0,0000	1,698	1		0	5,497E-08	49	1,2161E-05	3	1607,97342	0,68	37,09	1
	High	aLLVQLESYR	2	1	1	P72585	N-Term(iTRAQ4plex)	0,0000	0,967	2	41,1	0	0,0009995	40	0,000100224	2	1335,77617	0,65	42,02	0
	High	sSHVIPALIHk	1	1	1	P72585	N-Term(iTRAQ4plex); K11(iTRAQ4plex)	0,0000	0,768	1		0	0,000003027	29	0,001180239	4	1489,90908	-0,17	23,36	0
	High	iLVTGGAGFLGk	1	1	1	P72585	N-Term(iTRAQ4plex); K12(iTRAQ4plex)	0,0000	0,904	1		0,001	0,003362	30	0,001016179	3	1420,87616	-0,35	39,41	0
P72673	Thylakoid-associated protein slr0729 OS=Synechocystis sp. (strain PCC 6803 / Kazusa) GN=slr0729 P	192,17	46,53	1	4	4	7	0,579	7	22,0	101	10,9	4,70							
	A3	Sequence	# PSMs	# Proteins	# Protein Groups	Protein Group Accessions	Modifications	ΔCn	116/117	116/117 Count	116/117 Variability [%]	q-Value	PEP	IonScore	Exp Value	Charge	MH+ [Da]	ΔM [ppm]	RT [min]	# Missed Cleavages
	High	vNISLLDALSSTmVNR	2	1	1	P72673	N-Term(iTRAQ4plex); M13(Oxidation)	0,0000	0,458	2	13,2	0	3,979E-10	54	4,20698E-06	2	1893,02300	-0,14	48,05	0
	High	vYPLLEkDELGR	1	1	1	P72673	N-Term(iTRAQ4plex); K7(iTRAQ4plex)	0,0000	0,579	1		0	0,000001938	42	6,09537E-05	3	1719,99002	0,95	34,18	1
	High	vNISLLDALSSTMVNR	1	1	1	P72673	N-Term(iTRAQ4plex)	0,0000	0,873	1		0	0,001534	34	0,00042459	3	1877,02677	-0,84	54,18	0
	High	lLTLLEEYQSk	2	1	1	P72673	N-Term(iTRAQ4plex); K11(iTRAQ4plex)	0,0000	0,589	2	13,0	0	0,00009998	31	0,000812774	3	1624,93998	-0,03	49,17	0
	High	aGVEAALk	1	1	1	P72673	N-Term(iTRAQ4plex); K8(iTRAQ4plex)	0,0000	0,701	1		0,001	0,005335	40	0,000115128	2	1046,64397	-0,85	19,44	0
Q55436	Slr0848 protein OS=Synechocystis sp. (strain PCC 6803 / Kazusa) GN=slr0848 PE=1 SV=1 - [Q55436	188,89	18,05	1	5	5	8	0,645	8	11,7	277	31,8	4,89							
	A3	Sequence	# PSMs	# Proteins	# Protein Groups	Protein Group Accessions	Modifications	ΔCn	116/117	116/117 Count	116/117 Variability [%]	q-Value	PEP	IonScore	Exp Value	Charge	MH+ [Da]	ΔM [ppm]	RT [min]	# Missed Cleavages
	High	qVTHQEIQQFR	2	1	1	Q55436	N-Term(iTRAQ4plex)	0,0000	0,663	2	7,0	0	1,067E-07	56	2,55252E-06	2	1557,82512	-0,21	19,37	0
	High	aIAILER	2	1	1	Q55436	N-Term(iTRAQ4plex)	0,0000	0,625	2	7,4	0	0,0007229	41	8,85054E-05	2	929,58940	-0,72	28,79	0
	High	ikDESGIIQk	2	1	1	Q55436	N-Term(iTRAQ4plex); K2(iTRAQ4plex); K10(iTRAQ4plex)	0,0000	0,477	2	39,8	0	0,000001641	37	0,000192296	3	1562,94614	-1,07	22,41	1
	High	mIEVAHQEAQR	1	1	1	Q55436	N-Term(iTRAQ4plex); M1(Oxidation)	0,0000	0,705	1		0	0,00003105	26	0,002443262	3	1471,74433	-0,08	14,06	0
	High	qTQQEcDTLRR	1	1	1	Q55436	N-Term(iTRAQ4plex); C6(Carbamidomethyl)	0,0000	1,019	1		0,001	0,009568	10	0,095933437	3	1578,77726	-0,17	11,89	1
Q55690	Glycine--tRNA ligase beta subunit OS=Synechocystis sp. (strain PCC 6803 / Kazusa) GN=glyS PE=3 S	187,12	5,54	1	5	5	9	3,229	9	31,2	722	80,0	4,86							
	A3	Sequence	# PSMs	# Proteins	# Protein Groups	Protein Group Accessions	Modifications	ΔCn	116/117	116/117 Count	116/117 Variability [%]	q-Value	PEP	IonScore	Exp Value	Charge	MH+ [Da]	ΔM [ppm]	RT [min]	# Missed Cleavages
	High	lNLLGLIR	3	1	1	Q55690	N-Term(iTRAQ4plex)	0,0000	2,987	3	11,9	0	0,0001823	49	1,33958E-05	2	1055,70610	0,31	48,88	0
	High	lALSWLk	3	1	1	Q55690	N-Term(iTRAQ4plex); K7(iTRAQ4plex)	0,0000	3,403	3	7,8	0	0,0003655	48	1,58844E-05	2	1118,71721	-0,36	43,10	0
	High	aVYQALLAIYPk	1	1	1	Q55690	N-Term(iTRAQ4plex); K12(iTRAQ4plex)	0,0000	0,524	1		0	0,00002573	23	0,005457202	3	1637,98789	0,59	48,13	0
	High	rLAVLIk	1	1	1	Q55690	N-Term(iTRAQ4plex); K7(iTRAQ4plex)	0,0000	4,893	1		0,001	0,01369	19	0,012245316	3	1100,77576	-0,04	24,21	1
	Medium	fPRPIR	1	1	1	Q55690	N-Term(iTRAQ4plex)	0,0000	1,986	1		0,019	0,248	11	0,079610436	3	929,58039	0,23	18,98	0
Q55900	Septum site-determining protein MinD OS=Synechocystis sp. (strain PCC 6803 / Kazusa) GN=minD P	182,54	23,31	1	5	5	10	1,217	10	22,7	266	29,0	5,41							
	A3	Sequence	# PSMs	# Proteins	# Protein Groups	Protein Group Accessions	Modifications	ΔCn	116/117	116/117 Count	116/117 Variability [%]	q-Value	PEP	IonScore	Exp Value	Charge	MH+ [Da]	ΔM [ppm]	RT [min]	# Missed Cleavages
	High	lSVPGLAFQNIAR	2	1	1	Q55900	N-Term(iTRAQ4plex)	0,0000	1,511	2	36,2	0	0,0004799	35	0,000318398	2	1529,89299	0,59	44,91	0
	High	lPNLVLLPAAQNR	4	1	1	Q55900	N-Term(iTRAQ4plex)	0,0000	0,819	4	77,8	0	0,00009272	35	0,000340385	3	1562,94980	-0,09	44,80	0
	High	rLEGQDIPFLDFmAAHNTLLNR	2	1	1	Q55900	N-Term(iTRAQ4plex); M13(Oxidation)	0,0000	1,244	2	0,4	0	0,00003097	33	0,000559642	4	2731,41396	1,17	46,37	1
	High	rLPNLVLLPAAQNR	1	1	1	Q55900	N-Term(iTRAQ4plex)	0,0000	1,001	1		0	0,00007623	30	0,000950539	3	1719,05143	0,21	37,95	1
	High	tTTTANLGAALAR	1	1	1	Q55900	N-Term(iTRAQ4plex)	0,0000	0,586	1		0,001	0,008698	42	5,62302E-05	2	1404,79021	-1,82	27,26	0
P73643	Sll1762 protein OS=Synechocystis sp. (strain PCC 6803 / Kazusa) GN=sll1762 PE=4 SV=1 - [P73643	182,35	24,35	1	8	8	9	0,541	9	32,6	382	42,0	4,94							
	A3	Sequence	# PSMs	# Proteins	# Protein Groups	Protein Group Accessions	Modifications	ΔCn	116/117	116/117 Count	116/117 Variability [%]	q-Value	PEP	IonScore	Exp Value	Charge	MH+ [Da]	ΔM [ppm]	RT [min]	# Missed Cleavages
	High	kGTVDAVAGDGIQLAGLR	1	1	1	P73643	N-Term(iTRAQ4plex); K1(iTRAQ4plex)	0,0000	0,541	1		0	5,331E-10	65	2,92395E-07	3	2029,16581	0,64	36,24	1
	High	qFFNYLVITHEQVmEPk	1	1	1	P73643	N-Term(iTRAQ4plex); M14(Oxidation); K17(iTRAQ4plex)	0,0000	1,260	1		0	5,57E-10	44	3,76868E-05	4	2427,26162	-0,22	42,79	0
	High	rVAmVPNTIVEDAVk	1	1	1	P73643	N-Term(iTRAQ4plex); M4(Oxidation); K15(iTRAQ4plex)	0,0000	0,807	1		0	3,564E-08	35	0,000404295	3	1946,09836	-0,02	30,85	1
	High	iREEVGk	2	1	1	P73643	N-Term(iTRAQ4plex); K7(iTRAQ4plex)	0,0000	0,435	2	12,0	0	0,0002558	26	0,003026519	3	1118,67743	0,19	14,50	1
	High	iREEVGkELGR	1	1	1	P73643	N-Term(iTRAQ4plex); K7(iTRAQ4plex)	0,0000	0,522	1		0	0,0003971	25	0,003228271	3	1573,92752	0,67	21,36	2
	High	iVQNEIEVVPVTSVR	1	1	1	P73643	N-Term(iTRAQ4plex)	0,0000	1,051	1		0	0,0001246	23	0,005116465	3	1826,05350	1,67	38,46	0
	High	yGVGcMVR	1	1	1	P73643	N-Term(iTRAQ4plex); C5(Carbamidomethyl)	0,0000	0,671	1		0,002	0,02792	30	0,000965984	2	1085,53520	-0,07	24,59	0
	High	vIPQPAETR	1	1	1	P73643	N-Term(iTRAQ4plex)	0,0000	0,477	1		0,007	0,09366	23	0,004753024	2	1154,66509	0,05	20,78	0
P73471	Phosphoribosylaminoimidazole-succinocarboxamide synthase OS=Synechocystis sp. (strain PCC 6803	182,04	9,47	1	2	2	4	1,115	4	26,5	264	29,7	5,29							
	A3	Sequence	# PSMs	# Proteins	# Protein Groups	Protein Group Accessions	Modifications	ΔCn	116/117	116/117 Count	116/117 Variability [%]	q-Value	PEP	IonScore	Exp Value	Charge	MH+ [Da]	ΔM [ppm]	RT [min]	# Missed Cleavages
	High	gAINcAISAALFR	2	1	1	P73471	N-Term(iTRAQ4plex); C5(Carbamidomethyl)	0,0000	1,031	2	26,5	0	9,586E-08	73	5,06956E-08	2	1507,81792	0,46	44,45	0
	High	aVNIIPLEVVVR	2	1	1	P73471	N-Term(iTRAQ4plex)	0,0000	1,207	2	26,7	0	0,00008038	49	1,27635E-05	2	1465,92351	0,80	46,97	0
P74494	Nucleoside diphosphate kinase OS=Synechocystis sp. (strain PCC 6803 / Kazusa) GN=ndk PE=1 SV=	180,78	34,23	1	4	4	12	0,780	10	14,6	149	16,7	5,55							
	A3	Sequence	# PSMs	# Proteins	# Protein Groups	Protein Group Accessions	Modifications	ΔCn	116/117	116/117 Count	116/117 Variability [%]	q-Value	PEP	IonScore	Exp Value	Charge	MH+ [Da]	ΔM [ppm]	RT [min]	# Missed Cleavages
	High	nIIHGSDAIETAk	1	1	1	P74494	N-Term(iTRAQ4plex); K13(iTRAQ4plex)	0,0000	0,688	1		0	3,574E-09	54	4,40739E-06	3	1656,91770	1,05	22,49	0
	High	qmIGATDPHAAAPGTIR	2	1	1	P74494	N-Term(iTRAQ4plex); M2(Oxidation)	0,0000	0,728	2	6,4	0	0,00002988	41	8,51079E-05	3	1866,96292	0,85	20,43	0
	High	qLIGEIVGR	2	1	1	P74494	N-Term(iTRAQ4plex)	0,0000	0,778	1		0	0,0002293	38	0,000144202	2	1128,68657	0,71	34,02	0
	High	tFImIkPDGVQR	3	1	1	P74494	N-Term(iTRAQ4plex); M4(Oxidation); K6(iTRAQ4plex)	0,0000	0,804	3	4,2	0	0,000004832	29	0,00118842	3	1708,96817	1,33	28,55	0

	High	qMIGATDPHAAAPGTIR	1	1	1	P74494	N-Term(iTRAQ4plex)	0,0000	0,717	1		0	0,0007461	27	0,001874865	3	1850,97013	2,00	24,59	0
	High	tFIMIkPDGVQR	3	1	1	P74494	N-Term(iTRAQ4plex); K6(iTRAQ4plex)	0,0000	1,498	2	25,5	0	0,00056	22	0,006763777	3	1692,97629	3,13	31,64	0
P72656	Ribonuclease E/G-like protein OS=Synechocystis sp. (strain PCC 6803 / Kazusa) GN=rne PE=3 SV=1	180,04	9,79	1	5	5	8	2,885	7	64,8	674	74,9	6,16							
	A3	Sequence	# PSMs	# Proteins	# Protein Groups	Protein Group Accessions	Modifications	ΔCn	116/117	116/117 Count	116/117 Variability [%]	q-Value	PEP	IonScore	Exp Value	Charge	MH+ [Da]	ΔM [ppm]	RT [min]	# Missed Cleavages
	High	aRPQIAQLTELGLVELTR	3	1	1	P72656	N-Term(iTRAQ4plex)	0,0000	2,885	3	54,7	0	1,885E-08	72	6,94976E-08	3	2152,25791	0,37	48,41	0
	High	aLAVLIkPPGmGLLVR	2	1	1	P72656	N-Term(iTRAQ4plex); K7(iTRAQ4plex); M11(Oxidation)	0,0000	4,302	1		0	2,362E-08	33	0,000476398	3	1952,23514	0,90	43,20	0
	High	lVEkPILSPPVAk	1	1	1	P72656	N-Term(iTRAQ4plex); K4(iTRAQ4plex); K13(iTRAQ4plex)	0,0000	4,185	1		0	1,406E-07	26	0,002529123	3	1823,17197	-0,61	32,27	0
	High	hQVAAVFWk	1	1	1	P72656	N-Term(iTRAQ4plex); K9(iTRAQ4plex)	0,0000	0,491	1		0	0,00006381	18	0,016518477	3	1373,79334	0,07	28,31	0
	High	lTGNISmPGR	1	1	1	P72656	N-Term(iTRAQ4plex); M7(Oxidation)	0,0000	1,308	1		0,007	0,08688	11	0,075678062	2	1205,64421	1,06	20,97	0
P72807	Sll1663 protein OS=Synechocystis sp. (strain PCC 6803 / Kazusa) GN=sll1663 PE=4 SV=1 - [P72807_	178,04	10,91	1	2	2	5	0,609	4	8,4	220	24,1	4,65							
	A3	Sequence	# PSMs	# Proteins	# Protein Groups	Protein Group Accessions	Modifications	ΔCn	116/117	116/117 Count	116/117 Variability [%]	q-Value	PEP	IonScore	Exp Value	Charge	MH+ [Da]	ΔM [ppm]	RT [min]	# Missed Cleavages
	High	aDLEQSLQLLR	1	1	1	P72807	N-Term(iTRAQ4plex)	0,0000	0,667	1		0	0,000003411	66	2,80524E-07	2	1429,81291	-0,18	42,40	0
	High	lALAVGHLEHPDR	4	1	1	P72807	N-Term(iTRAQ4plex)	0,0000	0,595	3	6,9	0	4,074E-07	46	2,76675E-05	4	1571,87685	-0,42	28,95	0
Q55865	Sll0585 protein OS=Synechocystis sp. (strain PCC 6803 / Kazusa) GN=sll0585 PE=4 SV=1 - [Q55865	177,66	11,11	1	3	3	7	0,325	7	17,3	342	37,6	7,03							
	A3	Sequence	# PSMs	# Proteins	# Protein Groups	Protein Group Accessions	Modifications	ΔCn	116/117	116/117 Count	116/117 Variability [%]	q-Value	PEP	IonScore	Exp Value	Charge	MH+ [Da]	ΔM [ppm]	RT [min]	# Missed Cleavages
	High	sSLkPFQALAVTSTGTmER	2	1	1	Q55865	N-Term(iTRAQ4plex); K4(iTRAQ4plex); M17(Oxidation)	0,0000	0,289	2	10,5	0	1,178E-08	61	1,00539E-06	3	2328,24881	0,67	34,46	0
	High	rLEVIGELAmL	3	1	1	Q55865	N-Term(iTRAQ4plex); M10(Oxidation)	0,0000	0,365	3	36,4	0	0,001217	48	1,71384E-05	2	1403,80583	0,64	47,42	1
	High	qVFNILWR	2	1	1	Q55865	N-Term(iTRAQ4plex)	0,0000	0,336	2	4,7	0	0,000009406	36	0,000234947	2	1219,70745	0,50	44,77	0
P74468	Aminopeptidase P OS=Synechocystis sp. (strain PCC 6803 / Kazusa) GN=pepP PE=3 SV=1 - [P74468	177,31	12,47	1	5	5	9	0,525	9	21,4	441	49,5	5,63							
	A3	Sequence	# PSMs	# Proteins	# Protein Groups	Protein Group Accessions	Modifications	ΔCn	116/117	116/117 Count	116/117 Variability [%]	q-Value	PEP	IonScore	Exp Value	Charge	MH+ [Da]	ΔM [ppm]	RT [min]	# Missed Cleavages
	High	acDLSAIAHQR	1	1	1	P74468	N-Term(iTRAQ4plex); C2(Carbamidomethyl)	0,0000	0,535	1		0	7,216E-08	56	2,66054E-06	3	1385,70847	0,59	22,20	0
	High	tGHWLGLDVHDAGNYk	2	1	1	P74468	N-Term(iTRAQ4plex); K16(iTRAQ4plex)	0,0000	0,434	2	11,9	0	1,195E-08	46	2,32793E-05	4	2071,05922	-0,37	31,10	0
	High	qGYGPQALVNSHPLVHPLR	2	1	1	P74468	N-Term(iTRAQ4plex)	0,0000	0,422	2	22,9	0	1,265E-07	37	0,000181958	4	2227,22158	-0,06	33,16	0
	High	rAcDLSAIAHQR	2	1	1	P74468	N-Term(iTRAQ4plex); C3(Carbamidomethyl)	0,0000	1,311	2	13,0	0	0,00001779	32	0,0005662	4	1541,80959	0,53	17,63	1
	High	ykPFYmHR	2	1	1	P74468	N-Term(iTRAQ4plex); K2(iTRAQ4plex); M6(Oxidation)	0,0000	0,564	2	10,6	0	0,002247	23	0,005057897	4	1445,75954	-0,46	16,62	0
P72864	Carboxysome formation protein OS=Synechocystis sp. (strain PCC 6803 / Kazusa) GN=ccmA PE=4 S\	175,95	26,78	1	9	9	16	1,492	14	21,4	351	37,6	6,37							
	A3	Sequence	# PSMs	# Proteins	# Protein Groups	Protein Group Accessions	Modifications	ΔCn	116/117	116/117 Count	116/117 Variability [%]	q-Value	PEP	IonScore	Exp Value	Charge	MH+ [Da]	ΔM [ppm]	RT [min]	# Missed Cleavages
	High	ncLDLSVLPVLR	2	1	1	P72864	N-Term(iTRAQ4plex); C2(Carbamidomethyl)	0,0000	1,486	2	23,0	0	0,000001631	61	7,48118E-07	2	1542,88152	1,32	47,82	0
	High	qLAVIGQTVGR	1	1	1	P72864	N-Term(iTRAQ4plex)	0,0000	1,492	1		0	0,0001509	39	0,000139949	2	1285,77251	1,25	29,25	0
	High	vGAQDkPVLLk	2	1	1	P72864	N-Term(iTRAQ4plex); K6(iTRAQ4plex); K11(iTRAQ4plex)	0,0000	1,652	2	6,4	0	0,000004602	36	0,000280524	3	1600,01511	-0,46	25,30	0
	High	kVGAQDkPVLLk	2	1	1	P72864	N-Term(iTRAQ4plex); K1(iTRAQ4plex); K7(iTRAQ4plex); K12(i	0,0000	1,926	1		0	0,000001826	26	0,002703772	4	1872,21242	-0,24	21,76	1
	High	sLTHLPImIDPSHGTGk	2	1	1	P72864	N-Term(iTRAQ4plex); M8(Oxidation); K17(iTRAQ4plex)	0,0000	0,938	2	71,3	0	0,0001076	25	0,003329605	4	2108,14101	-0,14	31,26	0
	High	tSPYAFQGHGESALGLLAAAk	2	1	1	P72864	N-Term(iTRAQ4plex); K21(iTRAQ4plex)	0,0000	1,297	1		0	0,00001	12	0,059016032	4	2377,27822	1,14	41,85	0
	High	tFDGHYTR	1	1	1	P72864	N-Term(iTRAQ4plex)	0,0000	0,809	1		0,001	0,01566	13	0,050812435	3	1140,55497	-0,43	14,25	0
	High	aSLTFR	2	1	1	P72864	N-Term(iTRAQ4plex)	0,0000	1,557	2	6,3	0,002	0,02236	18	0,016825578	2	838,49040	0,07	21,46	0
	High	vEQPFkR	2	1	1	P72864	N-Term(iTRAQ4plex); K6(iTRAQ4plex)	0,0000	1,645	2	21,3	0,005	0,05959	15	0,038101612	3	1191,71006	1,01	16,39	1
Q55514	Slr0552 protein OS=Synechocystis sp. (strain PCC 6803 / Kazusa) GN=slr0552 PE=4 SV=1 - [Q55514	173,69	19,26	1	4	4	7	0,951	5	9,2	244	26,7	4,98							
	A3	Sequence	# PSMs	# Proteins	# Protein Groups	Protein Group Accessions	Modifications	ΔCn	116/117	116/117 Count	116/117 Variability [%]	q-Value	PEP	IonScore	Exp Value	Charge	MH+ [Da]	ΔM [ppm]	RT [min]	# Missed Cleavages
	High	aGSQLVFk	4	1	1	Q55514	N-Term(iTRAQ4plex); K8(iTRAQ4plex)	0,0000	0,936	2	2,4	0	0,00002868	53	4,85255E-06	2	1137,68657	-0,42	25,22	0
	High	vASAFVEk	1	1	1	Q55514	N-Term(iTRAQ4plex); K8(iTRAQ4plex)	0,0000	1,012	1		0	0,000006145	50	1,04706E-05	2	1138,67046	-0,54	25,26	0
	High	eLDPTLVNEQFLk	1	1	1	Q55514	N-Term(iTRAQ4plex); K13(iTRAQ4plex)	0,0000	0,812	1		0	0,000000611	44	3,68952E-05	3	1834,02390	2,07	41,48	0
	High	kFITGTLLASGAWQDLGk	1	1	1	Q55514	N-Term(iTRAQ4plex); K1(iTRAQ4plex); K18(iTRAQ4plex)	0,0000	1,323	1		0	0,00001053	19	0,012273545	4	2338,34853	-0,44	45,84	1
Q55497	Ornithine carbamoyltransferase OS=Synechocystis sp. (strain PCC 6803 / Kazusa) GN=argF PE=3 SV	173,34	8,77	1	4	4	8	2,043	8	26,6	308	33,6	5,64							
	A3	Sequence	# PSMs	# Proteins	# Protein Groups	Protein Group Accessions	Modifications	ΔCn	116/117	116/117 Count	116/117 Variability [%]	q-Value	PEP	IonScore	Exp Value	Charge	MH+ [Da]	ΔM [ppm]	RT [min]	# Missed Cleavages
	High	sLLQLAADLk	3	1	1	Q55497	N-Term(iTRAQ4plex); K10(iTRAQ4plex)	0,0000	1,875	3	0,1	0	3,335E-07	76	2,71625E-08	2	1359,84551	0,36	48,07	0
	High	yIDILAVR	1	1	1	Q55497	N-Term(iTRAQ4plex)	0,0000	1,432	1		0	0,002774	35	0,000333403	2	1106,66960	0,51	41,81	0
	High	iLGLLFYk	3	1	1	Q55497	N-Term(iTRAQ4plex); K8(iTRAQ4plex)	0,0000	2,309	3	16,2	0	0,0001497	33	0,000457057	2	1254,81194	4,39	50,97	0
	High	kILGLLFYk	1	1	1	Q55497	N-Term(iTRAQ4plex); K1(iTRAQ4plex); K9(iTRAQ4plex)	0,0000	2,226	1		0	0,0005179	16	0,025940002	3	1527,00153	-1,26	43,61	1
P73407	Carbon dioxide-concentrating mechanism protein CcmK homolog 4 OS=Synechocystis sp. (strain PCC	172,60	12,50	1	2	2	7	0,734	7	2,9	112	11,9	6,06							
	A3	Sequence	# PSMs	# Proteins	# Protein Groups	Protein Group Accessions	Modifications	ΔCn	116/117	116/117 Count	116/117 Variability [%]	q-Value	PEP	IonScore	Exp Value	Charge	MH+ [Da]	ΔM [ppm]	RT [min]	# Missed Cleavages
	High	iTIVGYIR	4	1	1	P73407	N-Term(iTRAQ4plex)	0,0000	0,714	4	3,0	0	0,00004291	41	7,81574E-05	2	1078,67497	0,79	37,30	0
	High	fTLNIR	3	1	1	P73407	N-Term(iTRAQ4plex)	0,0000	0,747	3	1,7	0,001	0,003777	36	0,000302924	2	907,54833	0,15	32,06	0
P73393	Sll1734 protein OS=Synechocystis sp. (strain PCC 6803 / Kazusa) GN=sll1734 PE=4 SV=1 - [P73393_	172,09	8,82	1	4	4	8	0,575	8	26,4	431	50,0	5,88							
	A3	Sequence	# PSMs	# Proteins	# Protein Groups	Protein Group Accessions	Modifications	ΔCn	116/117	116/117 Count	116/117 Variability [%]	q-Value	PEP	IonScore	Exp Value	Charge	MH+ [Da]	ΔM [ppm]	RT [min]	# Missed Cleavages
	High	smYNVTSAVIQALR	2	1	1	P73393	N-Term(iTRAQ4plex); M2(Oxidation)	0,0000	0,800	2	3,9	0	1,027E-08	51	7,31089E-06	3	1712,91208	-0,09	44,52	0
	High	lkDIQSDSYR	2	1	1	P73393	N-Term(iTRAQ4plex); K2(iTRAQ4plex)	0,0000	0,356	2	51,2	0	0,0003065	37	0,000206049	3	1512,82450	-1,03	20,81	1
	High	vFLNPLR	2	1	1	P73393	N-Term(iTRAQ4plex)	0,0000	0,558	2	7,9	0,001	0,007111	36	0,000244326	2	1002,62163	-0,07	34,50	0
	High	sYNAQAk	2	1	1	P73393	N-Term(iTRAQ4plex); K7(iTRAQ4plex)	0,0000	0,626	2	16,4	0,001	0,00884	19	0,012105145	2	1069,58379	-3,97	11,43	0
P73826	3-oxoacyl-[acyl-carrier-protein] reductase 2 OS=Synechocystis sp. (strain PCC 6803 / Kazusa) GN=fa	171,89	22,92	1	5	5	7	0,849	6	19,8	240	25,3	6,60							
	A3	Sequence	# PSMs	# Proteins	# Protein Groups	Protein Group Accessions	Modifications	ΔCn	116/117	116/117 Count	116/117 Variability [%]	q-Value	PEP	IonScore	Exp Value	Charge	MH+ [Da]	ΔM [ppm]	RT [min]	# Missed Cleavages
	High	kAGSIVAISSISGER	2	1	1	P73826	N-Term(iTRAQ4plex); K1(iTRAQ4plex)	0,0000	0,932	1		0	1,317E-08	51	7,69077E-06	3	1763,02842	1,04	27,93	1
	High	vIVVTGGNR	1	1	1	P73826	N-Term(iTRAQ4plex)	0,0000	0,866	1		0	0,00001204	48	1,61425E-05	2	1058,64421	0,29	20,13	0
	High	lGPVYGVVANAGITk	1	1	1	P73826	N-Term(iTRAQ4plex); K15(iTRAQ4plex)	0,0000	0,715	1		0	4,763E-07	43	5,4978E-05	3	1747,03781	1,22	40,28	0
	High	aGSIVAISSISGER	1	1	1	P73826	N-Term(iTRAQ4plex)	0,0000	0,645	1		0	0,000002858	40	0,000100455	2	1490,82976	0,14	33,23	0
	High	gVAYSIkPFIEGmYER	1	1	1	P73826	N-Term(iTRAQ4plex); K7(iTRAQ4plex); M13(Oxidation)	0,0000	0,833	1		0	0,00005746	27	0,002499827	3	2164,13651	0,63	41,08	0
	High	gVAYSIkPFIEGMYER	1	1	1	P73826	N-Term(iTRAQ4plex); K7(iTRAQ4plex)	0,0000	1,713	1		0	0,00007052	15	0,033263658	3	2148,14115	0,42	46,91	0
Q55332	Photosystem II 12 kDa extrinsic protein OS=Synechocystis sp. (strain PCC 6803 / Kazusa) GN=psbU	171,85	25,19	1	2	2	5	0,316	5	10,7	131	14,2	4,60							
	A3	Sequence	# PSMs	# Proteins	# Protein Groups	Protein Group Accessions	Modifications	ΔCn	116/117	116/117 Count	116/117 Variability [%]	q-Value	PEP	IonScore	Exp Value	Charge	MH+ [Da]	ΔM [ppm]	RT [min]	# Missed Cleavages
	High	gFYPNLASEIIk	4	1	1	Q55332	N-Term(iTRAQ4plex); K12(iTRAQ4plex)	0,0000	0,311	4	7,4	0	0,00001819	56	2,51171E-06	2	1639,93010	0,18	43,78	0
	High	nAPYDTVEEVLDIPGLSETQk	1	1	1	Q55332	N-Term(iTRAQ4plex); K21(iTRAQ4plex)	0,0000	0,381	1		0	2,869E-07	42	8,47117E-05	3	2606,35215	3,11	52,64	0
P73807	Histidinol-phosphate aminotransferase OS=Synechocystis sp. (strain PCC 6803 / Kazusa) GN=hisC PE	171,65	18,91	1	5	5	7	0,396	7	47,6	349	38,7	5,12							
	A3	Sequence	# PSMs	# Proteins	# Protein Groups	Protein Group Accessions	Modifications	ΔCn	116/117	116/117 Count	116/117 Variability [%]	q-Value	PEP	IonScore	Exp Value	Charge	MH+ [Da]	ΔM [ppm]	RT [min]	# Missed Cleavages
	High	vGLAIAAPALIEEMDkVR	2	1	1	P73807	N-Term(iTRAQ4plex); K16(iTRAQ4plex)	0,0000	0,273	2	59,3	0	3,188E-13	72	6,99794E-08	3	2184,26914	1,19	49,90	1
	High	hTPAYVPGEQPQTNDFIk	2	1	1	P73807	N-Term(iTRAQ4plex); K18(iTRAQ4plex)	0,0000	0,499	2	48,9	0	2,613E-09	47	1,81539E-05	3	2330,20676	2,05	30,00	0
	High	lIFLASPNPPk	1	1	1	P73807	N-Term(iTRAQ4plex); K11(iTRAQ4plex)	0,0000	0,391	1		0	0,00002656	38	0,000167098	3	1484,90906	0,74	39,88	0
	High	lAQVLGTAALR	1	1	1	P73807	N-Term(iTRAQ4plex)	0,0000	1,244	1		0,001	0,01336	26	0,002624037	2	1256,78227	1,22	38,35	0
	High	vSIRPSVR	1	1	1	P73807	N-Term(iTRAQ4plex)	0,0000	0,536	1		0,006	0,06878	13	0,05116465	3	1057,66028	0,38	16,91	0
P74582	Aconitate hydratase 2 OS=Synechocystis sp. (strain PCC 6803 / Kazusa) GN=acnB PE=3 SV=1 - [AC(	171,64	5,65	1	4	4	6	0,912	4	11,7	868	93,5	5,31							
	A3	Sequence	# PSMs	# Proteins	# Protein Groups	Protein Group Accessions	Modifications	ΔCn	116/117	116/117 Count	116/117 Variability [%]	q-Value	PEP	IonScore	Exp Value	Charge	MH+ [Da]	ΔM [ppm]	RT [min]	# Missed Cleavages
	High	gHPVAYVGDVVGTGSSR	1	1	1	P74582	N-Term(iTRAQ4plex)	0,0000	0,360	1		0	4,45E-12	99	1,26756E-10	3	1801,93174	0,19	25,88	0
	High	gGVALRPGDGIIHSWLNR	2	1	1	P74582	N-Term(iTRAQ4plex)	0,0000	0,938	1		0	0,000002768	41	0,000105726	4	2062,14394	0,58	38,56	0
	High	iTVTVFk	1	1	1	P74582	N-Term(iTRAQ4plex); K7(iTRAQ4plex)	0,0000	0,886	1		0,002	0,02123	34	0,000358072	2	1095,70195	0,30	33,24	0
	High	iPLLIGR	2	1	1	P74582	N-Term(iTRAQ4plex)	0,0000	1,036	1		0,003	0,03174	20	0,009999309	2	925,63140	-0,16	38,63	0
P74446	Slr0147 protein OS=Synechocystis sp. (strain PCC 6803 / Kazusa) GN=slr0147 PE=4 SV=1 - [P74446	167,55	10,27	1	3	3	9	3,577	7	47,8	292	32,3	8,44							
	A3	Sequence	# PSMs	# Proteins	# Protein Groups	Protein Group Accessions	Modifications	ΔCn	116/117	116/117 Count	116/117 Variability [%]	q-Value	PEP	IonScore	Exp Value	Charge	MH+ [Da]	ΔM [ppm]	RT [min]	# Missed Cleavages
	High	nLFILGLk	5	1	1	P74446	N-Term(iTRAQ4plex); K8(iTRAQ4plex)	0,0000	3,925	4	18,0	0	0,000004674	56	2,62404E-06	2	1205,78667	0,52	44,88	0
	High	sHGYGLVDIDLk	2	1	1	P74446	N-Term(iTRAQ4plex); K12(iTRAQ4plex)	0,0000	0,537	1		0	0,00003193	26	0,002285441	3	1604,89048	1,13	34,45	0
	High	lIALPDTLIk	2	1	1	P74446	N-Term(iTRAQ4plex); K10(iTRAQ4plex)	0,0000	2,151	2	87,5	0	0,001271	22	0,00654591	3	1384,90271	0,65	45,40	0
P74188	Slr1276 protein OS=Synechocystis sp. (strain PCC 6803 / Kazusa) GN=slr1276 PE=4 SV=1 - [P74188	167,18	13,45	1	3	3	6	0,463	6	29,9	275	30,0	4,34							
	A3	Sequence	# PSMs	# Proteins	# Protein Groups	Protein Group Accessions	Modifications	ΔCn	116/117	116/117 Count	116/117 Variability [%]	q-Value	PEP	IonScore	Exp Value	Charge	MH+ [Da]	ΔM [ppm]	RT [min]	# Missed Cleavages
	High	rLANAEVQLQEAQQR	1	1	1	P74188	N-Term(iTRAQ4plex)	0,0000	0,943	1		0	5,687E-11	59	1,3212E-06	3	1898,03281	0,16	23,26	1
	High	tSLVNPEFPVNVVR	2	1	1	P74188	N-Term(iTRAQ4plex)	0,0000	0,334	2	21,7	0	2,313E-08	51	7,94273E-06	2	1714,96135	0,26	40,89	0
	High	lQPLILMk	3	1	1	P74188	N-Term(iTRAQ4plex); K8(iTRAQ4plex)	0,0000	0,554	3	1,3	0	0,0001749	46	2,5408E-05	2	1243,80596	0,71	40,89	0
P73732	Extracellular solute-binding protein OS=Synechocystis sp. (strain PCC 6803 / Kazusa) GN=slr1740 PE	166,88	7,22	1	3	3	7	0,729	7	5,5	582	63,6	5,19							
	A3	Sequence	# PSMs	# Proteins	# Protein Groups	Protein Group Accessions	Modifications	ΔCn	116/117	116/117 Count	116/117 Variability [%]	q-Value	PEP	IonScore	Exp Value	Charge	MH+ [Da]	ΔM [ppm]	RT [min]	# Missed Cleavages
	High	vTSFkPGDVVLYEVNPHYR	2	1	1	P73732	N-Term(iTRAQ4plex); K5(iTRAQ4plex)	0,0000	0,720	2	1,7	0	4,952E-09	52	7,11857E-06	4	2508,35097	0,80	38,44	0
	High	kNIGFQQVEIk	2	1	1	P73732	N-Term(iTRAQ4plex); K1(iTRAQ4plex); K11(iTRAQ4plex)	0,0000	0,947	2	7,7	0	1,467E-08	48	1,64048E-05	3	1736,04489	1,03	27,31	1
	High	fPHPFLQDPLVR	3	1	1	P73732	N-Term(iTRAQ4plex)	0,0000	0,729	3	5,5	0	0,00001258	36	0,000247155	3	1609,89804	0,54	40,80	0
P72631	Sll1064 protein OS=Synechocystis sp. (strain PCC 6803 / Kazusa) GN=sll1064 PE=4 SV=1 - [P72631_	164,62	9,68	1	6	6	9	0,343	9	12,0	568	64,7	5,77							
	A3	Sequence	# PSMs	# Proteins	# Protein Groups	Protein Group Accessions	Modifications	ΔCn	116/117	116/117 Count	116/117 Variability [%]	q-Value	PEP	IonScore	Exp Value	Charge	MH+ [Da]	ΔM [ppm]	RT [min]	# Missed Cleavages
	High	sFFPVLQGR	2	1	1	P72631	N-Term(iTRAQ4plex)	0,0000	0,334	2	4,0	0	0,000004495	52	8,74802E-06	2	1194,67632	0,93	39,99	0
	High	qYYAVTFGNIR	3	1	1	P72631	N-Term(iTRAQ4plex)	0,0000	0,372	3	15,1	0	0,000013	44	4,15882E-05	2	1475,77825	1,28	36,61	0
	High	hQAVITGLR	1	1	1	P72631	N-Term(iTRAQ4plex)	0,0000	0,347	1		0	0,000001193	43	6,98088E-05	3	1138,68143	0,08	16,78	0
	High	lSLRPIWR	1	1	1	P72631	N-Term(iTRAQ4plex)	0,0000	0,269	1		0	0,001525	19	0,011480743	3	1184,73905	0,48	35,66	0
	High	aLHLQFk	1	1	1	P72631	N-Term(iTRAQ4plex); K7(iTRAQ4plex)	0,0000	0,275	1		0,001	0,003296	17	0,019722865	3	1144,71000	1,65	26,84	0
	Medium	tPQQDSSTAGR	1	1	1	P72631	N-Term(iTRAQ4plex)	0,0000	0,571	1		0,026	0,3544	16	0,025643069	2	1291,63713	0,94	9,97	0
P72799	Slr1794 protein OS=Synechocystis sp. (strain PCC 6803 / Kazusa) GN=slr1794 PE=4 SV=1 - [P72799	163,71	7,73	1	3	3	8	1,856	7	18,1	362	39,1	6,13							
	A3	Sequence	# PSMs	# Proteins	# Protein Groups	Protein Group Accessions	Modifications	ΔCn	116/117	116/117 Count	116/117 Variability [%]	q-Value	PEP	IonScore	Exp Value	Charge	MH+ [Da]	ΔM [ppm]	RT [min]	# Missed Cleavages
	High	aLVLTFLGk	5	1	1	P72799	N-Term(iTRAQ4plex); K9(iTRAQ4plex)	0,0000	1,844	4	10,0	0	0,00003962	47	1,79461E-05	2	1249,81230	0,04	47,62	0
	High	lkEVEAQYLR	2	1	1	P72799	N-Term(iTRAQ4plex); K2(iTRAQ4plex)	0,0000	2,168	2	28,0	0	6,593E-07	36	0,000228544	3	1536,89939	0,36	28,91	1
	High	vFLPGFDkR	1	1	1	P72799	N-Term(iTRAQ4plex); K8(iTRAQ4plex)	0,0000	2,617	1		0	0,00006033	26	0,002460198	3	1366,80823	-0,25	33,60	1
Q55131	Slr0049 protein OS=Synechocystis sp. (strain PCC 6803 / Kazusa) GN=slr0049 PE=4 SV=1 - [Q55131	161,48	3,52	1	2	2	7	1,763	5	6,3	398	44,0	5,06							
	A3	Sequence	# PSMs	# Proteins	# Protein Groups	Protein Group Accessions	Modifications	ΔCn	116/117	116/117 Count	116/117 Variability [%]	q-Value	PEP	IonScore	Exp Value	Charge	MH+ [Da]	ΔM [ppm]	RT [min]	# Missed Cleavages
	High	iVPLQFLk	6	1	1	Q55131	N-Term(iTRAQ4plex); K8(iTRAQ4plex)	0,0000	1,795	4	5,8	0	0,000006011	56	2,25408E-06	2	1245,81853	0,96	45,17	0
	High	nIPTLk	1	1	1	Q55131	N-Term(iTRAQ4plex); K6(iTRAQ4plex)	0,0000	1,012	1		0,007	0,09769	25	0,003357144	2	973,62737	-1,12	22,92	0
P20804	Acyl carrier protein OS=Synechocystis sp. (strain PCC 6803 / Kazusa) GN=acpP PE=1 SV=2 - [ACP_S	159,90	20,78	1	2	2	8	0,543	7	10,9	77	8,6	4,07							
	A3	Sequence	# PSMs	# Proteins	# Protein Groups	Protein Group Accessions	Modifications	ΔCn	116/117	116/117 Count	116/117 Variability [%]	q-Value	PEP	IonScore	Exp Value	Charge	MH+ [Da]	ΔM [ppm]	RT [min]	# Missed Cleavages
	High	aVEHIESk	5	1	1	P20804	N-Term(iTRAQ4plex); K8(iTRAQ4plex)	0,0000	0,543	5	10,9	0	0,00004645	55	3,52371E-06	2	1200,68181	-0,73	13,53	0
	High	mNQEIFEk	1	1	1	P20804	N-Term(iTRAQ4plex); K8(iTRAQ4plex)	0,0000	0,777	1		0	0,0007501	39	0,000126465	2	1326,69646	-0,13	27,50	0
	High	mNQEIFEk	2	1	1	P20804	N-Term(iTRAQ4plex); M1(Oxidation); K8(iTRAQ4plex)	0,0000	0,517	1		0	0,0008869	38	0,000142551	2	1342,69133	-0,16	21,55	0
P74490	Pyruvate dehydrogenase E1 component, alpha subunit OS=Synechocystis sp. (strain PCC 6803 / Kaz	159,33	12,57	1	4	4	7	1,913	6	59,0	342	38,1	5,19							
	A3	Sequence	# PSMs	# Proteins	# Protein Groups	Protein Group Accessions	Modifications	ΔCn	116/117	116/117 Count	116/117 Variability [%]	q-Value	PEP	IonScore	Exp Value	Charge	MH+ [Da]	ΔM [ppm]	RT [min]	# Missed Cleavages
	High	lPILFVVENNk	2	1	1	P74490	N-Term(iTRAQ4plex); K11(iTRAQ4plex)	0,0000	2,767	2	16,5	0	9,707E-07	54	3,72366E-06	2	1573,95903	2,16	49,21	0
	High	aTSQPEIYkk	2	1	1	P74490	N-Term(iTRAQ4plex); K9(iTRAQ4plex); K10(iTRAQ4plex)	0,0000	2,410	1		0	0,00001135	44	6,18146E-05	3	1596,93308	0,59	16,31	1
	High	dHVHALSAGVPAR	2	1	1	P74490	N-Term(iTRAQ4plex)	0,0000	1,046	2	59,9	0	0,000003147	44	4,38531E-05	3	1473,80463	0,20	17,00	0
	Medium	wAIGMAHER	1	1	1	P74490	N-Term(iTRAQ4plex)	0,0000	1,020	1		0,012	0,1581	24	0,004364857	3	1214,62040	-1,43	23,85	0
P74079	Slr1342 protein OS=Synechocystis sp. (strain PCC 6803 / Kazusa) GN=slr1342 PE=4 SV=1 - [P74079	159,02	14,16	1	4	4	8	2,362	7	27,1	445	48,5	5,30							
	A3	Sequence	# PSMs	# Proteins	# Protein Groups	Protein Group Accessions	Modifications	ΔCn	116/117	116/117 Count	116/117 Variability [%]	q-Value	PEP	IonScore	Exp Value	Charge	MH+ [Da]	ΔM [ppm]	RT [min]	# Missed Cleavages
	High	lLGQIIPGcSLVR	2	1	1	P74079	N-Term(iTRAQ4plex); C9(Carbamidomethyl)	0,0000	2,008	2	16,8	0	0,0009939	51	7,24386E-06	2	1569,92705	0,18	45,27	0
	High	qGLRDESPIFFILSNTR	2	1	1	P74079	N-Term(iTRAQ4plex)	0,0000	2,840	2	25,8	0	8,837E-07	47	1,84064E-05	3	2137,15610	1,78	49,97	1
	High	lALATEAVDHYLVVSR	1	1	1	P74079	N-Term(iTRAQ4plex)	0,0000	2,826	1		0	0,00001858	34	0,000397365	3	1901,05991	-0,75	46,28	0
	High	fPLLPVVLFPGNVGNAR	3	1	1	P74079	N-Term(iTRAQ4plex)	0,0000	1,507	2	74,7	0,001	0,006527	25	0,003250648	2	1954,14800	4,32	57,84	0
P72950	Sll0647 protein OS=Synechocystis sp. (strain PCC 6803 / Kazusa) GN=sll0647 PE=4 SV=1 - [P72950_	156,97	9,77	1	3	3	8	0,440	6	23,0	256	28,7	5,52							
	A3	Sequence	# PSMs	# Proteins	# Protein Groups	Protein Group Accessions	Modifications	ΔCn	116/117	116/117 Count	116/117 Variability [%]	q-Value	PEP	IonScore	Exp Value	Charge	MH+ [Da]	ΔM [ppm]	RT [min]	# Missed Cleavages
	High	fSLYALGLSHR	4	1	1	P72950	N-Term(iTRAQ4plex)	0,0000	0,375	2	5,3	0	1,728E-09	54	5,73236E-06	3	1407,78751	0,69	39,12	0
	High	kLPAAIk	2	1	1	P72950	N-Term(iTRAQ4plex); K1(iTRAQ4plex); K7(iTRAQ4plex)	0,0000	0,531	2	0,8	0	0,00003363	34	0,000375811	3	1172,80671	-2,06	19,35	1
	High	lQEYFHR	2	1	1	P72950	N-Term(iTRAQ4plex)	0,0000	0,440	2	10,1	0	0,0003228	33	0,000561009	3	1136,59787	0,84	25,81	0
P73790	Putative pterin-4-alpha-carbinolamine dehydratase OS=Synechocystis sp. (strain PCC 6803 / Kazusa)	155,79	34,38	1	2	2	5	1,093	5	49,8	96	10,7	6,52							
	A3	Sequence	# PSMs	# Proteins	# Protein Groups	Protein Group Accessions	Modifications	ΔCn	116/117	116/117 Count	116/117 Variability [%]	q-Value	PEP	IonScore	Exp Value	Charge	MH+ [Da]	ΔM [ppm]	RT [min]	# Missed Cleavages
	High	fANFNQAFGFmTR	1	1	1	P73790	N-Term(iTRAQ4plex); M11(Oxidation)	0,0000	0,764	1		0	1,367E-07	60	1,07392E-06	3	1710,81882	0,51	42,90	0
	High	lALVAETLNHHPEWSNVYNR	2	1	1	P73790	N-Term(iTRAQ4plex)	0,0000	1,029	2	9,0	0	4,049E-10	46	2,26986E-05	4	2507,29213	0,36	42,84	0
	High	fANFNQAFGFMTR	2	1	1	P73790	N-Term(iTRAQ4plex)	0,0000	2,045	2	48,3	0	0,0001417	39	0,000114017	2	1694,82402	0,58	48,68	0
P73914	Squalene-hopene-cyclase OS=Synechocystis sp. (strain PCC 6803 / Kazusa) GN=shc PE=4 SV=1 - [P	154,91	6,80	1	5	5	8	0,307	8	39,6	647	72,0	4,96							
	A3	Sequence	# PSMs	# Proteins	# Protein Groups	Protein Group Accessions	Modifications	ΔCn	116/117	116/117 Count	116/117 Variability [%]	q-Value	PEP	IonScore	Exp Value	Charge	MH+ [Da]	ΔM [ppm]	RT [min]	# Missed Cleavages
	High	iWGTAAQRPLEk	1	1	1	P73914	N-Term(iTRAQ4plex); K12(iTRAQ4plex)	0,0000	0,291	1		0	0,00000139	45	3,0267E-05	3	1657,96579	1,77	26,99	0
	High	nYLLQQQR	2	1	1	P73914	N-Term(iTRAQ4plex)	0,0000	0,335	2	4,9	0	0,00007829	43	5,1996E-05	2	1206,67229	0,94	22,26	0
	High	qYFPLIALAR	3	1	1	P73914	N-Term(iTRAQ4plex)	0,0000	0,218	3	12,6	0,001	0,0103	30	0,00103269	2	1335,79241	1,38	47,79	0
	High	fAPQIk	1	1	1	P73914	N-Term(iTRAQ4plex); K6(iTRAQ4plex)	0,0000	0,397	1		0,001	0,003598	27	0,002232019	2	991,62016	2,29	23,34	0
	High	aGQWLLDk	1	1	1	P73914	N-Term(iTRAQ4plex); K8(iTRAQ4plex)	0,0000	0,587	1		0,001	0,007817	12	0,070825443	2	1218,71062	1,72	32,69	0
P74756	47 kD protein OS=Synechocystis sp. (strain PCC 6803 / Kazusa) GN=slr0609 PE=4 SV=1 - [P74756_	152,65	12,53	1	3	3	5	2,770	5	39,7	383	43,2	5,91							
	A3	Sequence	# PSMs	# Proteins	# Protein Groups	Protein Group Accessions	Modifications	ΔCn	116/117	116/117 Count	116/117 Variability [%]	q-Value	PEP	IonScore	Exp Value	Charge	MH+ [Da]	ΔM [ppm]	RT [min]	# Missed Cleavages
	High	tTLLNYILSNQQGLk	1	1	1	P74756	N-Term(iTRAQ4plex); K15(iTRAQ4plex)	0,0000	2,900	1		0	8,683E-11	64	3,74947E-07	3	1994,15366	0,59	51,09	0
	High	qGLPVTIITGFLGSGk	3	1	1	P74756	N-Term(iTRAQ4plex); K16(iTRAQ4plex)	0,0000	2,770	3	67,4	0	0,00002704	43	4,48714E-05	3	1876,12040	3,05	55,46	0
	High	sNQLVLIGQDLDHDkIR	1	1	1	P74756	N-Term(iTRAQ4plex); K15(iTRAQ4plex)	0,0000	2,140	1		0	5,767E-07	37	0,000194075	4	2252,25991	-0,12	36,28	1
P72623	Probable glycogen synthase 2 OS=Synechocystis sp. (strain PCC 6803 / Kazusa) GN=glgA2 PE=3 SV	151,77	12,02	1	6	6	7	1,509	7	41,2	491	56,2	6,48							
	A3	Sequence	# PSMs	# Proteins	# Protein Groups	Protein Group Accessions	Modifications	ΔCn	116/117	116/117 Count	116/117 Variability [%]	q-Value	PEP	IonScore	Exp Value	Charge	MH+ [Da]	ΔM [ppm]	RT [min]	# Missed Cleavages
	High	aGGLGDVIYGLSR	1	1	1	P72623	N-Term(iTRAQ4plex)	0,0000	1,509	1		0	6,131E-07	61	8,14648E-07	2	1421,78679	-0,12	42,56	0
	High	gHYYGALDDHmR	1	1	1	P72623	N-Term(iTRAQ4plex); M11(Oxidation)	0,0000	0,906	1		0	0,00001792	38	0,000144202	4	1594,71853	-0,25	16,23	0
	High	yGAVPVVR	1	1	1	P72623	N-Term(iTRAQ4plex)	0,0000	1,361	1		0	0,000199	29	0,002394818	2	1004,60082	-0,15	25,56	0
	High	aAMEFLLR	1	1	1	P72623	N-Term(iTRAQ4plex)	0,0000	1,971	1		0	0,0005194	27	0,00182377	2	1094,61504	0,10	39,99	0
	High	yDHIWGLHDAYR	1	1	1	P72623	N-Term(iTRAQ4plex)	0,0000	1,481	1		0	0,00001993	21	0,007797763	4	1689,82570	0,16	31,54	0
	High	fAFFSk	2	1	1	P72623	N-Term(iTRAQ4plex); K6(iTRAQ4plex)	0,0000	2,106	2	2,2	0,001	0,0134	28	0,001570363	2	1034,59148	0,13	36,81	0
Q55788	Sensory transduction histidine kinase OS=Synechocystis sp. (strain PCC 6803 / Kazusa) GN=slr0073 I	150,72	14,60	1	5	5	9	0,870	7	16,5	315	35,0	5,86							
	A3	Sequence	# PSMs	# Proteins	# Protein Groups	Protein Group Accessions	Modifications	ΔCn	116/117	116/117 Count	116/117 Variability [%]	q-Value	PEP	IonScore	Exp Value	Charge	MH+ [Da]	ΔM [ppm]	RT [min]	# Missed Cleavages
	High	tLAPVVLk	2	1	1	Q55788	N-Term(iTRAQ4plex); K8(iTRAQ4plex)	0,0000	0,987	2	23,5	0	0,0001495	52	5,86098E-06	2	1128,76213	2,34	29,90	0
	High	qENWQHLLSLSk	2	1	1	Q55788	N-Term(iTRAQ4plex); K12(iTRAQ4plex)	0,0000	0,933	1		0	2,194E-08	36	0,000255252	3	1770,97428	0,08	38,36	0
	High	qAIANPLHSYR	3	1	1	Q55788	N-Term(iTRAQ4plex)	0,0000	0,861	2	18,1	0	0,00005161	35	0,000292395	3	1413,77222	0,20	23,02	0
	High	eHQLPVDQk	1	1	1	Q55788	N-Term(iTRAQ4plex); K9(iTRAQ4plex)	0,0000	0,870	1		0	0,0002583	19	0,011775246	3	1381,76612	-1,23	15,46	0
	Medium	mLQLFk	1	1	1	Q55788	N-Term(iTRAQ4plex); K6(iTRAQ4plex)	0,0000	0,771	1		0,03	0,3921	23	0,005320715	2	1067,64910	-3,26	38,20	0
P73305	50S ribosomal protein L18 OS=Synechocystis sp. (strain PCC 6803 / Kazusa) GN=rplR PE=3 SV=1 - [	150,36	20,00	1	3	3	9	1,739	6	76,9	120	13,2	10,92							
	A3	Sequence	# PSMs	# Proteins	# Protein Groups	Protein Group Accessions	Modifications	ΔCn	116/117	116/117 Count	116/117 Variability [%]	q-Value	PEP	IonScore	Exp Value	Charge	MH+ [Da]	ΔM [ppm]	RT [min]	# Missed Cleavages
	High	gINQVVFDR	5	1	1	P73305	N-Term(iTRAQ4plex)	0,0000	2,514	3	27,4	0	2,945E-07	60	9,18269E-07	2	1191,66106	0,65	28,93	0
	High	hLSGTSERPR	1	1	1	P73305	N-Term(iTRAQ4plex)	0,0000	0,948	1		0,007	0,09737	16	0,029356065	4	1283,69521	1,17	8,00	0
	Medium	iAVFR	3	1	1	P73305	N-Term(iTRAQ4plex)	0,0000	0,995	2	64,9	0,019	0,2484	12	0,084900454	2	749,47930	0,32	26,83	0
P74707	Peptide chain release factor 1 OS=Synechocystis sp. (strain PCC 6803 / Kazusa) GN=prfA PE=3 SV=1	149,05	6,58	1	3	3	9	1,005	8	14,0	365	41,1	5,00							
	A3	Sequence	# PSMs	# Proteins	# Protein Groups	Protein Group Accessions	Modifications	ΔCn	116/117	116/117 Count	116/117 Variability [%]	q-Value	PEP	IonScore	Exp Value	Charge	MH+ [Da]	ΔM [ppm]	RT [min]	# Missed Cleavages
	High	lTILLLPk	5	1	1	P74707	N-Term(iTRAQ4plex); K8(iTRAQ4plex)	0,0000	0,933	5	12,0	0	0,0005498	40	0,000108136	2	1198,83745	-0,24	45,55	0
	High	vETAVDLFHkPTGIR	3	1	1	P74707	N-Term(iTRAQ4plex); K10(iTRAQ4plex)	0,0000	1,016	2	2,6	0	0,000003673	35	0,000337264	4	1971,12551	-0,57	33,34	0
	High	rLTILLLPk	1	1	1	P74707	N-Term(iTRAQ4plex); K9(iTRAQ4plex)	0,0000	1,634	1		0,001	0,004312	25	0,00292395	3	1354,93787	-0,73	38,23	1
P73411	Glucose-6-phosphate 1-dehydrogenase OS=Synechocystis sp. (strain PCC 6803 / Kazusa) GN=zwf PE	145,94	13,75	1	7	7	8	1,497	8	49,9	509	57,8	7,17							
	A3	Sequence	# PSMs	# Proteins	# Protein Groups	Protein Group Accessions	Modifications	ΔCn	116/117	116/117 Count	116/117 Variability [%]	q-Value	PEP	IonScore	Exp Value	Charge	MH+ [Da]	ΔM [ppm]	RT [min]	# Missed Cleavages
	High	kVSEIAIQFR	1	1	1	P73411	N-Term(iTRAQ4plex); K1(iTRAQ4plex)	0,0000	1,924	1		0	0,000001062	52	5,67505E-06	3	1478,89304	-0,22	30,54	1
	High	kLVPAIYQmk	1	1	1	P73411	N-Term(iTRAQ4plex); K1(iTRAQ4plex); M9(Oxidation); K10(iTR	0,0000	1,132	1		0	6,409E-07	37	0,000177816	3	1638,99881	0,66	25,60	1
	High	wQGVPFYLR	1	1	1	P73411	N-Term(iTRAQ4plex)	0,0000	1,983	1		0	0,002608	35	0,000341169	2	1309,71709	-0,23	42,94	0
	High	rLPPELTVVGFAR	1	1	1	P73411	N-Term(iTRAQ4plex)	0,0000	1,165	1		0	0,0002087	30	0,000993047	3	1598,95017	0,13	38,67	1
	High	fANAIFEPLWNR	1	1	1	P73411	N-Term(iTRAQ4plex)	0,0000	9,912	1		0	0,0004116	24	0,004120691	2	1621,86113	0,21	50,10	0
	High	iVIEkPFGR	2	1	1	P73411	N-Term(iTRAQ4plex); K5(iTRAQ4plex)	0,0000	1,282	2	87,9	0,003	0,03143	23	0,004476824	3	1346,83820	-1,25	30,88	0
	Medium	iDHYLGk	1	1	1	P73411	N-Term(iTRAQ4plex); K7(iTRAQ4plex)	0,0000	0,565	1		0,011	0,1471	11	0,109129798	3	1133,65473	-0,88	22,64	0
Q55596	3-ketoacyl-acyl carrier protein reductase OS=Synechocystis sp. (strain PCC 6803 / Kazusa) GN=fabG	145,43	21,24	1	3	3	6	0,799	6	27,5	259	28,3	10,18							
	A3	Sequence	# PSMs	# Proteins	# Protein Groups	Protein Group Accessions	Modifications	ΔCn	116/117	116/117 Count	116/117 Variability [%]	q-Value	PEP	IonScore	Exp Value	Charge	MH+ [Da]	ΔM [ppm]	RT [min]	# Missed Cleavages
	High	hPGLQAIALPLDLTQPVAVNTAIAI	1	1	1	Q55596	N-Term(iTRAQ4plex)	0,0000	0,355	1		0	1,115E-09	53	5,32072E-06	4	2723,56777	-0,44	49,61	0
	High	fAILGFSQALR	4	1	1	Q55596	N-Term(iTRAQ4plex)	0,0000	0,799	4	19,0	0	0,00003459	42	7,13498E-05	2	1366,79521	-0,86	48,52	0
	High	hNIAVVTLLPTLTDTDmAk	1	1	1	Q55596	N-Term(iTRAQ4plex); M17(Oxidation); K19(iTRAQ4plex)	0,0000	1,216	1		0	0,001379	14	0,03964508	4	2357,29482	-1,75	42,93	0
Q55709	Protein translocase subunit SecA OS=Synechocystis sp. (strain PCC 6803 / Kazusa) GN=secA PE=3 S	144,97	6,01	1	5	5	5	1,973	5	47,5	932	106,9	5,12							
	A3	Sequence	# PSMs	# Proteins	# Protein Groups	Protein Group Accessions	Modifications	ΔCn	116/117	116/117 Count	116/117 Variability [%]	q-Value	PEP	IonScore	Exp Value	Charge	MH+ [Da]	ΔM [ppm]	RT [min]	# Missed Cleavages
	High	gVHVVTVNDYLAR	1	1	1	Q55709	N-Term(iTRAQ4plex)	0,0000	0,820	1		0	2,671E-08	51	8,10905E-06	3	1586,87784	0,43	31,07	0
	High	tPLIISGQVERPTEk	1	1	1	Q55709	N-Term(iTRAQ4plex); K15(iTRAQ4plex)	0,0000	2,675	1		0	0,000001052	48	1,65566E-05	3	1956,13620	-0,34	28,50	0
	High	tFFHEEVR	1	1	1	Q55709	N-Term(iTRAQ4plex)	0,0000	1,817	1		0	0,00003681	36	0,000269135	3	1208,61838	0,25	22,03	0
	High	kVETYYYDIR	1	1	1	Q55709	N-Term(iTRAQ4plex); K1(iTRAQ4plex)	0,0000	1,973	1		0	0,00002648	32	0,000680769	3	1637,87674	-0,61	26,88	1
	High	eHLQSmEALR	1	1	1	Q55709	N-Term(iTRAQ4plex); M6(Oxidation)	0,0000	6,538	1		0	0,000002015	31	0,000807179	3	1373,69556	-0,63	15,03	0
P74438	Dihydroorotase OS=Synechocystis sp. (strain PCC 6803 / Kazusa) GN=pyrC PE=3 SV=1 - [PYRC_SYN	143,92	15,50	1	4	5	10	0,695	4	29,8	342	38,1	6,80							
	A3	Sequence	# PSMs	# Proteins	# Protein Groups	Protein Group Accessions	Modifications	ΔCn	116/117	116/117 Count	116/117 Variability [%]	q-Value	PEP	IonScore	Exp Value	Charge	MH+ [Da]	ΔM [ppm]	RT [min]	# Missed Cleavages
	High	fFLGTDSAPHAR	1	1	1	P74438	N-Term(iTRAQ4plex)	0,0000	1,228	1		0	0,00001055	40	9,01509E-05	3	1462,75641	0,29	28,58	0
	High	gGIcPHFYcLPILk	1	1	1	P74438	N-Term(iTRAQ4plex); C4(Carbamidomethyl); C9(Carbamidome	0,0000	0,470	1		0	0,00001071	30	0,00102658	3	1963,05759	2,00	43,13	0
	High	lSLLHAATSGNPk	1	1	1	P74438	N-Term(iTRAQ4plex); K13(iTRAQ4plex)	0,0000	0,693	1		0	0,00009684	26	0,003733286	3	1596,93106	-0,09	27,73	0
	High	yLIPLR	6	2	2	P74438;Q55641	N-Term(iTRAQ4plex)	0,0000				0,001	0,007505	27	0,002552368	2	918,58965	0,35	35,48	0
	High	aVLPHTVR	1	1	1	P74438	N-Term(iTRAQ4plex)	0,0000	0,697	1		0,005	0,05869	26	0,00229599	3	1036,64069	2,19	16,10	0
P19050	NAD(P)H-quinone oxidoreductase subunit K 1 OS=Synechocystis sp. (strain PCC 6803 / Kazusa) GN=	143,86	18,15	1	6	6	13	0,841	12	15,2	248	27,3	8,13							
	A3	Sequence	# PSMs	# Proteins	# Protein Groups	Protein Group Accessions	Modifications	ΔCn	116/117	116/117 Count	116/117 Variability [%]	q-Value	PEP	IonScore	Exp Value	Charge	MH+ [Da]	ΔM [ppm]	RT [min]	# Missed Cleavages
	High	kVANESIQER	1	1	1	P19050	N-Term(iTRAQ4plex); K1(iTRAQ4plex)	0,0000	0,892	1		0	9,434E-08	47	2,24749E-05	3	1461,82633	-0,05	12,73	1
	High	aITQQTHR	1	1	1	P19050	N-Term(iTRAQ4plex)	0,0000	0,769	1		0	0,00009056	18	0,014255091	3	1098,61411	0,42	10,04	0
	High	fGLVPR	5	1	1	P19050	N-Term(iTRAQ4plex)	0,0000	0,841	4	1,1	0,001	0,00377	27	0,001922959	2	832,51634	0,20	27,67	0
	High	iLNPASR	3	1	1	P19050	N-Term(iTRAQ4plex)	0,0000	1,205	3	2,9	0,001	0,009371	26	0,002254084	2	914,55449	0,52	18,96	0
	High	yLQQGTR	2	1	1	P19050	N-Term(iTRAQ4plex)	0,0000	0,709	2	8,2	0,001	0,006223	23	0,005350479	2	1009,55425	-0,47	15,91	0
	Medium	mAPALVR	1	1	1	P19050	N-Term(iTRAQ4plex); M1(Oxidation)	0,0000	1,263	1		0,025	0,3422	10	0,09749223	2	917,53667	0,79	17,14	0
P73456	Cell division protein FtsZ OS=Synechocystis sp. (strain PCC 6803 / Kazusa) GN=ftsZ PE=1 SV=1 - [F1	143,80	7,67	1	3	3	5	1,225	5	10,5	430	44,7	4,73							
	A3	Sequence	# PSMs	# Proteins	# Protein Groups	Protein Group Accessions	Modifications	ΔCn	116/117	116/117 Count	116/117 Variability [%]	q-Value	PEP	IonScore	Exp Value	Charge	MH+ [Da]	ΔM [ppm]	RT [min]	# Missed Cleavages
	High	vIGVGGGGcNAVNR	2	1	1	P73456	N-Term(iTRAQ4plex); C9(Carbamidomethyl)	0,0000	0,922	2	13,0	0	2,113E-10	76	2,76675E-08	2	1473,77153	0,13	22,02	0
	High	gLGAGGNPAIGQk	2	1	1	P73456	N-Term(iTRAQ4plex); K13(iTRAQ4plex)	0,0000	1,269	2	5,2	0	0,0001415	26	0,002747704	3	1427,82126	0,22	20,95	0
	Medium	ekPQAk	1	1	1	P73456	N-Term(iTRAQ4plex); K2(iTRAQ4plex); K6(iTRAQ4plex)	0,0000	1,309	1		0,038	0,4807	11	0,083502185	3	1132,70395	-0,96	8,13	0
P73037	Peptidyl-prolyl cis-trans isomerase OS=Synechocystis sp. (strain PCC 6803 / Kazusa) GN=ytfC PE=4 S	143,41	17,91	1	4	4	8	0,718	7	13,4	201	21,5	8,94							
	A3	Sequence	# PSMs	# Proteins	# Protein Groups	Protein Group Accessions	Modifications	ΔCn	116/117	116/117 Count	116/117 Variability [%]	q-Value	PEP	IonScore	Exp Value	Charge	MH+ [Da]	ΔM [ppm]	RT [min]	# Missed Cleavages
	High	nkPFTFTIGVGQVIk	3	1	1	P73037	N-Term(iTRAQ4plex); K2(iTRAQ4plex); K15(iTRAQ4plex)	0,0000	0,758	3	3,5	0	7,071E-10	46	2,26971E-05	3	2081,25107	1,30	41,06	0
	High	vEVHYTGR	2	1	1	P73037	N-Term(iTRAQ4plex)	0,0000	0,686	2	6,7	0	0,00006626	40	9,99931E-05	2	1104,59270	0,77	15,25	0
	High	lIIPPDLAYGSR	1	1	1	P73037	N-Term(iTRAQ4plex)	0,0000	0,630	1		0	0,001739	32	0,000639691	2	1458,84563	1,30	41,66	0
	High	kLIIPPDLAYGSR	2	1	1	P73037	N-Term(iTRAQ4plex); K1(iTRAQ4plex)	0,0000	0,572	1		0	0,000007245	32	0,000688605	3	1731,04062	-0,08	34,94	1
P73636	30S ribosomal protein S6 OS=Synechocystis sp. (strain PCC 6803 / Kazusa) GN=rpsF PE=3 SV=1 - [F	143,30	38,05	1	5	5	13	1,556	12	15,2	113	13,2	8,37							
	A3	Sequence	# PSMs	# Proteins	# Protein Groups	Protein Group Accessions	Modifications	ΔCn	116/117	116/117 Count	116/117 Variability [%]	q-Value	PEP	IonScore	Exp Value	Charge	MH+ [Da]	ΔM [ppm]	RT [min]	# Missed Cleavages
	High	vSQEVTk	3	1	1	P73636	N-Term(iTRAQ4plex); K7(iTRAQ4plex)	0,0000	1,319	2	7,0	0	0,0004881	37	0,00021576	2	1078,63664	1,83	15,12	0
	High	yQEFLTNNAAEEVSVk	1	1	1	P73636	N-Term(iTRAQ4plex); K16(iTRAQ4plex)	0,0000	0,900	1		0	7,141E-08	27	0,001844888	3	2130,09977	1,87	38,34	0
	High	lTPEkPEk	3	1	1	P73636	N-Term(iTRAQ4plex); K5(iTRAQ4plex); K8(iTRAQ4plex)	0,0000	1,592	3	9,7	0	0,0005227	21	0,008116947	3	1373,83747	0,73	17,30	0
	High	rLAYQIR	2	1	1	P73636	N-Term(iTRAQ4plex)	0,0000	2,846	2	18,0	0	0,0007303	16	0,023067472	3	1063,65021	0,85	18,59	1
	High	fLSIk	4	1	1	P73636	N-Term(iTRAQ4plex); K5(iTRAQ4plex)	0,0000	1,558	4	6,3	0,004	0,04756	31	0,00077085	2	895,58769	2,42	29,58	0
P74142	30S ribosomal protein S1 homolog B OS=Synechocystis sp. (strain PCC 6803 / Kazusa) GN=rps1b PE	143,03	9,18	1	3	3	7	1,127	7	19,2	305	33,8	5,25							
	A3	Sequence	# PSMs	# Proteins	# Protein Groups	Protein Group Accessions	Modifications	ΔCn	116/117	116/117 Count	116/117 Variability [%]	q-Value	PEP	IonScore	Exp Value	Charge	MH+ [Da]	ΔM [ppm]	RT [min]	# Missed Cleavages
	High	aHILEANQDNNk	2	1	1	P74142	N-Term(iTRAQ4plex); K12(iTRAQ4plex)	0,0000	1,371	2	10,1	0	3,998E-08	53	4,69862E-06	3	1654,87333	-1,10	15,00	0
	High	rIQQAESmGk	2	1	1	P74142	N-Term(iTRAQ4plex); M8(Oxidation); K10(iTRAQ4plex)	0,0000	1,007	2	4,2	0	0,00009101	23	0,005320715	3	1451,78785	-0,05	10,11	1
	High	rIQQAESMGk	1	1	1	P74142	N-Term(iTRAQ4plex); K10(iTRAQ4plex)	0,0000	0,834	1		0	0,00004286	20	0,009771697	3	1435,79139	-1,13	14,95	1
	High	lVLTQR	2	1	1	P74142	N-Term(iTRAQ4plex)	0,0000	1,172	2	5,8	0,005	0,05695	32	0,000612308	2	873,56401	0,19	21,37	0
P73293	30S ribosomal protein S9 OS=Synechocystis sp. (strain PCC 6803 / Kazusa) GN=rpsI PE=3 SV=1 - [R	142,47	29,93	1	5	5	9	2,849	8	24,7	137	15,1	10,13							
	A3	Sequence	# PSMs	# Proteins	# Protein Groups	Protein Group Accessions	Modifications	ΔCn	116/117	116/117 Count	116/117 Variability [%]	q-Value	PEP	IonScore	Exp Value	Charge	MH+ [Da]	ΔM [ppm]	RT [min]	# Missed Cleavages
	High	iANYIQSLk	3	1	1	P73293	N-Term(iTRAQ4plex); K9(iTRAQ4plex)	0,0000	3,511	2	12,6	0	0,00002228	53	5,49503E-06	2	1337,80437	0,93	38,61	0
	High	vVYWGTGR	2	1	1	P73293	N-Term(iTRAQ4plex)	0,0000	2,600	2	29,5	0	0,0001324	35	0,000314029	2	1081,59148	0,33	27,59	0
	High	qPLkAEGYLTR	1	1	1	P73293	N-Term(iTRAQ4plex); K4(iTRAQ4plex)	0,0000	2,568	1		0	0,0005989	14	0,043548179	3	1563,91056	0,52	24,32	1
	High	kAPQYSk	2	1	1	P73293	N-Term(iTRAQ4plex); K1(iTRAQ4plex); K7(iTRAQ4plex)	0,0000	2,727	2	24,2	0,001	0,008017	21	0,008317063	3	1253,75479	-2,39	10,67	1
	Medium	kAAIAR	1	1	1	P73293	N-Term(iTRAQ4plex); K1(iTRAQ4plex)	0,0000	0,869	1		0,034	0,4383	17	0,018705529	3	917,61225	-1,35	10,06	1
P73812	Slr2060 protein OS=Synechocystis sp. (strain PCC 6803 / Kazusa) GN=slr2060 PE=4 SV=1 - [P73812	140,90	4,09	1	2	2	8	0,614	5	2,2	465	53,0	6,02							
	A3	Sequence	# PSMs	# Proteins	# Protein Groups	Protein Group Accessions	Modifications	ΔCn	116/117	116/117 Count	116/117 Variability [%]	q-Value	PEP	IonScore	Exp Value	Charge	MH+ [Da]	ΔM [ppm]	RT [min]	# Missed Cleavages
	High	lVENFVAVTGHYVk	1	1	1	P73812	N-Term(iTRAQ4plex); K14(iTRAQ4plex)	0,0000	2,557	1		0	2,841E-09	42	6,29506E-05	3	1864,05814	0,54	41,96	0
	High	iAIAk	7	1	1	P73812	N-Term(iTRAQ4plex); K5(iTRAQ4plex)	0,0000	0,613	4	1,1	0,003	0,0363	20	0,010863506	2	803,55681	-3,12	20,81	0
P72707	Sll0224 protein OS=Synechocystis sp. (strain PCC 6803 / Kazusa) GN=sll0224 PE=4 SV=1 - [P72707_	140,27	15,44	1	4	4	6	0,465	5	9,4	298	32,8	4,42							
	A3	Sequence	# PSMs	# Proteins	# Protein Groups	Protein Group Accessions	Modifications	ΔCn	116/117	116/117 Count	116/117 Variability [%]	q-Value	PEP	IonScore	Exp Value	Charge	MH+ [Da]	ΔM [ppm]	RT [min]	# Missed Cleavages
	High	iEQTGVINAGTR	1	1	1	P72707	N-Term(iTRAQ4plex)	0,0000	0,338	1		0	1,749E-08	66	2,67282E-07	2	1402,77812	0,72	23,17	0

	High	nLVNYSLVk	3	1	1	P72707	N-Term(iTRAQ4plex); K9(iTRAQ4plex)	0,0000	0,425	2	22,8	0	0,000006254	50	9,93047E-06	2	1337,80474	1,20	32,25	0
	High	aILDFQPGATIVEVk	1	1	1	P72707	N-Term(iTRAQ4plex); K15(iTRAQ4plex)	0,0000	0,465	1		0	0,00008896	35	0,000327318	3	1889,10202	1,76	45,91	0
	High	kTSDNPQQWk	1	1	1	P72707	N-Term(iTRAQ4plex); K1(iTRAQ4plex); K10(iTRAQ4plex)	0,0000	0,480	1		0	0,00008584	24	0,003564265	3	1663,91123	-0,95	17,74	1
P73120	Sll1830 protein OS=Synechocystis sp. (strain PCC 6803 / Kazusa) GN=sll1830 PE=4 SV=1 - [P73120	139,75	12,29	1	4	4	10	0,878	10	17,1	358	39,6	4,87							
	A3	Sequence	# PSMs	# Proteins	# Protein Groups	Protein Group Accessions	Modifications	ΔCn	116/117	116/117 Count	116/117 Variability [%]	q-Value	PEP	IonScore	Exp Value	Charge	MH+ [Da]	ΔM [ppm]	RT [min]	# Missed Cleavages
	High	dLVYGLHR	1	1	1	P73120	N-Term(iTRAQ4plex)	0,0000	0,599	1		0	0,0001738	37	0,000221294	3	1116,62848	0,22	23,16	0
	High	qLQSLTPVPALSILLPTHR	2	1	1	P73120	N-Term(iTRAQ4plex)	0,0000	1,055	2	16,3	0	0,00006507	33	0,000486374	3	2228,32505	0,12	52,11	0
	High	lYYLPFPVQAR	6	1	1	P73120	N-Term(iTRAQ4plex)	0,0000	0,878	6	17,1	0	0,00009842	29	0,001255943	2	1510,85539	1,00	47,37	0
	High	iALVLR	1	1	1	P73120	N-Term(iTRAQ4plex)	0,0000	0,801	1		0,002	0,02032	35	0,000346713	2	828,57891	0,16	31,92	0
Q55484	Diaminopimelate decarboxylase OS=Synechocystis sp. (strain PCC 6803 / Kazusa) GN=lysA PE=3 SV	139,71	9,59	1	5	5	7	2,605	7	39,6	469	50,8	5,45							
	A3	Sequence	# PSMs	# Proteins	# Protein Groups	Protein Group Accessions	Modifications	ΔCn	116/117	116/117 Count	116/117 Variability [%]	q-Value	PEP	IonScore	Exp Value	Charge	MH+ [Da]	ΔM [ppm]	RT [min]	# Missed Cleavages
	High	tPASPSPNQNLLPLTAVINk	3	1	1	Q55484	N-Term(iTRAQ4plex); K20(iTRAQ4plex)	0,0000	2,339	3	23,3	0	4,621E-08	56	2,57022E-06	3	2363,35709	1,41	44,91	0
	High	kVVPNIR	1	1	1	Q55484	N-Term(iTRAQ4plex); K1(iTRAQ4plex)	0,0000	6,074	1		0	0,0004136	24	0,003881236	3	1113,73532	0,59	17,44	1
	High	iYFHGNNk	1	1	1	Q55484	N-Term(iTRAQ4plex); K8(iTRAQ4plex)	0,0000	2,605	1		0	0,0001522	18	0,017659158	3	1280,69876	-0,17	18,06	0
	Medium	hcESGDILVk	1	1	1	Q55484	N-Term(iTRAQ4plex); C2(Carbamidomethyl); K10(iTRAQ4plex)	0,0000	0,578	1		0,02	0,2782	18	0,015775023	3	1445,77329	4,96	17,64	0
	Medium	vVPNIR	1	1	1	Q55484	N-Term(iTRAQ4plex)	0,0000	2,911	1		0,02	0,2682	12	0,056620018	2	841,53856	1,10	21,59	0
P28463	Glutamyl-tRNA reductase OS=Synechocystis sp. (strain PCC 6803 / Kazusa) GN=hemA PE=3 SV=1 -	139,06	4,92	1	2	2	7	4,644	5	65,5	427	47,5	6,76							
	A3	Sequence	# PSMs	# Proteins	# Protein Groups	Protein Group Accessions	Modifications	ΔCn	116/117	116/117 Count	116/117 Variability [%]	q-Value	PEP	IonScore	Exp Value	Charge	MH+ [Da]	ΔM [ppm]	RT [min]	# Missed Cleavages
	High	lEEALTHLR	5	1	1	P28463	N-Term(iTRAQ4plex)	0,0000	3,600	3	39,1	0	0,00003306	47	2,13781E-05	3	1225,70285	0,58	32,01	0
	High	yLFTLLHEDAVR	2	1	1	P28463	N-Term(iTRAQ4plex)	0,0000	10,098	2	60,0	0	3,427E-08	45	3,99862E-05	3	1620,88639	-0,17	45,15	0
P26525	NAD(P)H-quinone oxidoreductase subunit I OS=Synechocystis sp. (strain PCC 6803 / Kazusa) GN=nd	137,75	29,53	1	6	6	13	0,801	11	14,0	193	22,1	6,76							
	A3	Sequence	# PSMs	# Proteins	# Protein Groups	Protein Group Accessions	Modifications	ΔCn	116/117	116/117 Count	116/117 Variability [%]	q-Value	PEP	IonScore	Exp Value	Charge	MH+ [Da]	ΔM [ppm]	RT [min]	# Missed Cleavages
	High	gVIEPHNLPk	4	1	1	P26525	N-Term(iTRAQ4plex); K10(iTRAQ4plex)	0,0000	0,902	2	17,6	0	0,000002031	37	0,000188352	3	1391,82483	-0,08	21,72	0
	High	hDLNYDNVALGR	2	1	1	P26525	N-Term(iTRAQ4plex)	0,0000	0,703	2	34,0	0	0,000007855	30	0,00097268	3	1530,78247	2,82	24,39	0
	High	iHFEFDk	4	1	1	P26525	N-Term(iTRAQ4plex); K7(iTRAQ4plex)	0,0000	0,752	4	7,1	0	0,002077	24	0,004999693	2	1223,66521	-0,90	30,24	0
	High	rPITVQYPYEk	1	1	1	P26525	N-Term(iTRAQ4plex); K11(iTRAQ4plex)	0,0000	0,854	1		0	2,555E-07	23	0,00489745	3	1681,95206	0,28	25,04	0
	High	eLGYLPk	1	1	1	P26525	N-Term(iTRAQ4plex); K7(iTRAQ4plex)	0,0000	0,723	1		0,001	0,003405	29	0,001321204	2	1107,66338	-1,68	27,94	0
	High	aGQHPEDLVk	1	1	1	P26525	N-Term(iTRAQ4plex); K10(iTRAQ4plex)	0,0000	0,855	1		0,001	0,01619	20	0,010863506	3	1381,76484	-2,17	16,78	0
P73061	Urease subunit alpha OS=Synechocystis sp. (strain PCC 6803 / Kazusa) GN=ureC PE=3 SV=1 - [URE	136,77	15,47	1	7	7	14	0,549	11	53,6	569	61,0	6,21							
	A3	Sequence	# PSMs	# Proteins	# Protein Groups	Protein Group Accessions	Modifications	ΔCn	116/117	116/117 Count	116/117 Variability [%]	q-Value	PEP	IonScore	Exp Value	Charge	MH+ [Da]	ΔM [ppm]	RT [min]	# Missed Cleavages
	High	aTTcTPGAWNIHR	2	1	1	P73061	N-Term(iTRAQ4plex); C4(Carbamidomethyl)	0,0000	0,323	1		0	9,106E-08	62	6,28015E-07	3	1628,80936	0,57	23,23	0
	High	mLQAADGFPINLGFLGk	1	1	1	P73061	N-Term(iTRAQ4plex); M1(Oxidation); K17(iTRAQ4plex)	0,0000	0,537	1		0	0,00001161	28	0,001690324	3	2096,15073	2,57	52,40	0
	High	vIHTYHTEGAGGGHAPDIIk	3	1	1	P73061	N-Term(iTRAQ4plex); K20(iTRAQ4plex)	0,0000	0,446	2	75,5	0	0,000003344	26	0,003093091	4	2361,25893	1,49	22,02	0
	High	lGLSkPLVAVk	3	1	1	P73061	N-Term(iTRAQ4plex); K5(iTRAQ4plex); K11(iTRAQ4plex)	0,0000	0,401	2	3,9	0	0,00002606	23	0,004487144	3	1557,04886	1,57	31,69	0
	High	tWQTAHk	1	1	1	P73061	N-Term(iTRAQ4plex); K7(iTRAQ4plex)	0,0000	0,854	1		0	0,001321	22	0,006933779	3	1159,64600	-0,20	12,75	0
	High	hSYAHTFGPTVGDk	2	1	1	P73061	N-Term(iTRAQ4plex); K14(iTRAQ4plex)	0,0000	1,632	2	143,9	0	0,001709	15	0,029510054	4	1804,92434	1,25	18,51	0
	Medium	iYAIGk	2	1	1	P73061	N-Term(iTRAQ4plex); K6(iTRAQ4plex)	0,0000	0,584	2	9,4	0,024	0,3309	11	0,085150911	2	952,60661	-0,39	24,49	0
P73810	DNA topoisomerase 1 OS=Synechocystis sp. (strain PCC 6803 / Kazusa) GN=topA PE=3 SV=1 - [TOF	136,07	5,79	1	5	5	6	1,489	6	60,8	898	99,3	8,82							
	A3	Sequence	# PSMs	# Proteins	# Protein Groups	Protein Group Accessions	Modifications	ΔCn	116/117	116/117 Count	116/117 Variability [%]	q-Value	PEP	IonScore	Exp Value	Charge	MH+ [Da]	ΔM [ppm]	RT [min]	# Missed Cleavages
	High	rIDFPGYFR	1	1	1	P73810	N-Term(iTRAQ4plex)	0,0000	2,188	1		0	0,000001291	46	2,31191E-05	3	1314,70813	0,43	34,25	1
	High	kASATGTAk	1	1	1	P73810	N-Term(iTRAQ4plex); K1(iTRAQ4plex); K9(iTRAQ4plex)	0,0000	2,158	1		0	0,001018	33	0,000475302	3	1266,77167	-1,99	7,84	1
	High	iTQLSVLLk	2	1	1	P73810	N-Term(iTRAQ4plex); K9(iTRAQ4plex)	0,0000	0,771	2	9,4	0	6,278E-07	32	0,000630914	2	1302,85991	-0,01	42,47	0
	High	gAQEAHEAIRPAGTEFR	1	1	1	P73810	N-Term(iTRAQ4plex)	0,0000	1,875	1		0	0,000001905	31	0,000714447	4	1984,01198	0,10	19,38	0
	High	lmTLGGTk	1	1	1	P73810	N-Term(iTRAQ4plex); M2(Oxidation); K8(iTRAQ4plex)	0,0000	1,182	1		0,001	0,006342	34	0,000496489	2	1124,65727	-1,35	20,19	0
P74463	Twitching motility protein OS=Synechocystis sp. (strain PCC 6803 / Kazusa) GN=pilT PE=4 SV=1 - [P	135,70	21,95	1	8	8	10	1,962	10	56,2	369	40,6	7,03							
	A3	Sequence	# PSMs	# Proteins	# Protein Groups	Protein Group Accessions	Modifications	ΔCn	116/117	116/117 Count	116/117 Variability [%]	q-Value	PEP	IonScore	Exp Value	Charge	MH+ [Da]	ΔM [ppm]	RT [min]	# Missed Cleavages
	High	lIFSmLNNSQR	2	1	1	P74463	N-Term(iTRAQ4plex); M5(Oxidation)	0,0000	1,962	2	15,0	0	0,00002006	42	6,93378E-05	2	1482,78667	0,74	34,91	0
	High	iPNFEQLGLPNIVR	2	1	1	P74463	N-Term(iTRAQ4plex)	0,0000	3,981	2	46,5	0	0,00000957	40	0,000104947	3	1754,01175	2,03	51,47	0
	High	aAQIYSAIQTGAk	1	1	1	P74463	N-Term(iTRAQ4plex); K13(iTRAQ4plex)	0,0000	0,634	1		0	0,0001871	25	0,002883832	3	1609,91471	-0,32	30,93	0
	High	sPkPGEFGR	1	1	1	P74463	N-Term(iTRAQ4plex); K3(iTRAQ4plex)	0,0000	2,512	1		0	0,0004705	18	0,016083949	3	1262,70948	-0,09	14,60	0
	High	kSPkPGEFGR	1	1	1	P74463	N-Term(iTRAQ4plex); K1(iTRAQ4plex); K4(iTRAQ4plex)	0,0000	2,765	1		0	0,001226	11	0,073615625	4	1534,90676	0,09	13,08	1
	High	sGkPDELQR	1	1	1	P74463	N-Term(iTRAQ4plex); K3(iTRAQ4plex)	0,0000	1,364	1		0,001	0,004559	23	0,004666594	3	1317,73633	-0,15	12,16	0
	High	amLSNSLLAVFAQNLVk	1	1	1	P74463	N-Term(iTRAQ4plex); M2(Oxidation); K17(iTRAQ4plex)	0,0000	1,524	1		0,001	0,003208	18	0,016404765	3	2123,20841	-2,52	56,78	0
	Medium	sFSNALR	1	1	1	P74463	N-Term(iTRAQ4plex)	0,0000	1,239	1		0,035	0,4444	10	0,094182454	2	938,51824	0,65	19,50	0
P74618	6-phosphogluconolactonase OS=Synechocystis sp. (strain PCC 6803 / Kazusa) GN=pgl PE=1 SV=3 -	135,64	15,42	1	4	4	7	1,195	7	4,4	240	26,5	5,27							
	A3	Sequence	# PSMs	# Proteins	# Protein Groups	Protein Group Accessions	Modifications	ΔCn	116/117	116/117 Count	116/117 Variability [%]	q-Value	PEP	IonScore	Exp Value	Charge	MH+ [Da]	ΔM [ppm]	RT [min]	# Missed Cleavages
	High	sVVFLVAGASk	1	1	1	P74618	N-Term(iTRAQ4plex); K11(iTRAQ4plex)	0,0000	0,568	1		0	0,000884	39	0,000126174	3	1365,83438	-0,05	37,03	0
	High	lITVGNk	1	1	1	P74618	N-Term(iTRAQ4plex); K7(iTRAQ4plex)	0,0000	1,196	1		0	0,000678	37	0,000222316	2	1032,66557	0,00	23,07	0
	High	lTFTIPLINR	3	1	1	P74618	N-Term(iTRAQ4plex)	0,0000	1,126	3	10,6	0	0,001169	31	0,000737853	2	1331,81841	1,22	49,07	0
	High	iHVFWGDER	2	1	1	P74618	N-Term(iTRAQ4plex)	0,0000	1,213	2	2,2	0	0,0008012	28	0,001599448	3	1302,67115	-0,03	32,31	0
P74186	Membrane protein OS=Synechocystis sp. (strain PCC 6803 / Kazusa) GN=pilM PE=4 SV=1 - [P74186	135,60	9,81	1	4	4	6	0,752	6	7,9	367	40,9	4,60							
	A3	Sequence	# PSMs	# Proteins	# Protein Groups	Protein Group Accessions	Modifications	ΔCn	116/117	116/117 Count	116/117 Variability [%]	q-Value	PEP	IonScore	Exp Value	Charge	MH+ [Da]	ΔM [ppm]	RT [min]	# Missed Cleavages
	High	kSSGVGLEITPER	1	1	1	P74186	N-Term(iTRAQ4plex); K1(iTRAQ4plex)	0,0000	0,716	1		0	3,576E-09	65	3,14029E-07	3	1660,94712	-0,09	21,89	1
	High	vQVLLVATR	2	1	1	P74186	N-Term(iTRAQ4plex)	0,0000	0,822	2	6,5	0	0,000817	34	0,000431489	2	1142,73808	0,25	35,10	0
	High	fTSLFGR	2	1	1	P74186	N-Term(iTRAQ4plex)	0,0000	0,756	2	7,8	0,001	0,01413	34	0,000402689	2	971,54296	-0,16	36,62	0
	High	lSIPTVk	1	1	1	P74186	N-Term(iTRAQ4plex); K7(iTRAQ4plex)	0,0000	0,655	1		0,001	0,003783	26	0,002437642	2	1045,68572	-0,25	31,07	0
P72646	Sll1054 protein OS=Synechocystis sp. (strain PCC 6803 / Kazusa) GN=sll1054 PE=3 SV=1 - [P72646_	135,12	9,09	1	2	2	8	0,261	7	13,6	187	21,0	4,79							
	A3	Sequence	# PSMs	# Proteins	# Protein Groups	Protein Group Accessions	Modifications	ΔCn	116/117	116/117 Count	116/117 Variability [%]	q-Value	PEP	IonScore	Exp Value	Charge	MH+ [Da]	ΔM [ppm]	RT [min]	# Missed Cleavages
	High	sIASYFWmR	4	1	1	P72646	N-Term(iTRAQ4plex); M8(Oxidation)	0,0000	0,239	3	21,3	0	0,00209	41	8,25981E-05	2	1320,65349	0,56	42,68	0
	High	sIASYFWMR	3	1	1	P72646	N-Term(iTRAQ4plex)	0,0000	0,276	3	8,0	0	0,00001584	39	0,00012473	2	1304,65947	1,25	46,61	0
	High	kFNFDVSR	1	1	1	P72646	N-Term(iTRAQ4plex); K1(iTRAQ4plex)	0,0000	0,196	1		0	0,001456	32	0,000705	3	1300,72684	1,24	25,00	1
Q55383	Malate dehydrogenase OS=Synechocystis sp. (strain PCC 6803 / Kazusa) GN=mdh PE=3 SV=1 - [MD	134,89	5,56	1	2	2	3	1,890	3	0,4	324	34,3	5,03							
	A3	Sequence	# PSMs	# Proteins	# Protein Groups	Protein Group Accessions	Modifications	ΔCn	116/117	116/117 Count	116/117 Variability [%]	q-Value	PEP	IonScore	Exp Value	Charge	MH+ [Da]	ΔM [ppm]	RT [min]	# Missed Cleavages
	High	nANIVAQGAR	2	1	1	Q55383	N-Term(iTRAQ4plex)	0,0000	1,757	2	10,8	0	0,000005317	66	2,50594E-07	2	1157,65178	0,86	16,83	0
	High	vTGLPSQR	1	1	1	Q55383	N-Term(iTRAQ4plex)	0,0000	1,895	1		0,001	0,009055	36	0,000281642	2	1001,58653	0,49	18,45	0
P72688	Transcription termination/antitermination protein NusA OS=Synechocystis sp. (strain PCC 6803 / Kazu	134,79	11,14	1	5	5	7	1,077	7	30,7	458	51,3	4,61							
	A3	Sequence	# PSMs	# Proteins	# Protein Groups	Protein Group Accessions	Modifications	ΔCn	116/117	116/117 Count	116/117 Variability [%]	q-Value	PEP	IonScore	Exp Value	Charge	MH+ [Da]	ΔM [ppm]	RT [min]	# Missed Cleavages
	High	rEQLPNDNYR	1	1	1	P72688	N-Term(iTRAQ4plex)	0,0000	0,867	1		0	3,565E-08	43	4,75302E-05	3	1448,73630	0,00	15,23	1
	High	gPQLVVSR	2	1	1	P72688	N-Term(iTRAQ4plex)	0,0000	1,394	2	8,3	0	0,0000747	41	8,87095E-05	2	999,60747	0,69	21,33	0
	High	eAkPPSPSVGPR	2	1	1	P72688	N-Term(iTRAQ4plex); K3(iTRAQ4plex)	0,0000	1,206	2	16,9	0	0,000008607	28	0,001573874	3	1509,86185	-0,63	15,95	0
	High	dkEAIEQSILER	1	1	1	P72688	N-Term(iTRAQ4plex); K2(iTRAQ4plex)	0,0000	1,042	1		0	3,502E-07	28	0,001737681	3	1718,95237	-0,22	31,31	1
	High	iHAVVNELR	1	1	1	P72688	N-Term(iTRAQ4plex)	0,0000	1,017	1		0,001	0,007055	23	0,005545874	3	1194,70667	-0,75	24,41	0
P77962	Serine hydroxymethyltransferase OS=Synechocystis sp. (strain PCC 6803 / Kazusa) GN=glyA PE=3 S\	134,46	17,56	1	7	7	13	2,599	12	99,9	427	46,2	6,32							
	A3	Sequence	# PSMs	# Proteins	# Protein Groups	Protein Group Accessions	Modifications	ΔCn	116/117	116/117 Count	116/117 Variability [%]	q-Value	PEP	IonScore	Exp Value	Charge	MH+ [Da]	ΔM [ppm]	RT [min]	# Missed Cleavages
	High	sVFPGTQGGPLEHVITAk	2	1	1	P77962	N-Term(iTRAQ4plex); K18(iTRAQ4plex)	0,0000	4,946	1		0	5,105E-09	50	1,03031E-05	3	2126,18698	0,99	38,39	0
	High	aVAFGEALkPEFk	4	1	1	P77962	N-Term(iTRAQ4plex); K9(iTRAQ4plex); K13(iTRAQ4plex)	0,0000	1,466	4	132,5	0	3,158E-07	36	0,000225408	3	1839,07468	0,32	35,92	0
	High	qIEFDkFR	2	1	1	P77962	N-Term(iTRAQ4plex); K6(iTRAQ4plex)	0,0000	2,496	2	203,6	0	0,00004686	26	0,002529123	3	1370,76697	-0,09	28,81	1
	High	wFEVAHYGVEk	2	1	1	P77962	N-Term(iTRAQ4plex); K11(iTRAQ4plex)	0,0000	3,547	2	37,6	0	0,000002673	24	0,004101758	3	1652,86832	0,47	36,40	0
	High	qQALEVkPk	1	1	1	P77962	N-Term(iTRAQ4plex); K7(iTRAQ4plex); K9(iTRAQ4plex)	0,0000	3,926	1		0	0,0001809	24	0,0044154	3	1472,91574	-0,25	16,05	0
	High	lDYDkIR	1	1	1	P77962	N-Term(iTRAQ4plex); K5(iTRAQ4plex)	0,0000	2,383	1		0	0,002948	13	0,061788836	3	1210,70340	-0,01	21,95	1
	High	yAEGLPGkR	1	1	1	P77962	N-Term(iTRAQ4plex); K8(iTRAQ4plex)	0,0000	2,253	1		0,002	0,02812	20	0,010237391	3	1278,74091	0,03	18,49	1
P73426	2-C-methyl-D-erythritol 2,4-cyclodiphosphate synthase OS=Synechocystis sp. (strain PCC 6803 / Kazu	134,38	13,04	1	2	2	5	0,315	4	12,9	161	17,4	6,52							
	A3	Sequence	# PSMs	# Proteins	# Protein Groups	Protein Group Accessions	Modifications	ΔCn	116/117	116/117 Count	116/117 Variability [%]	q-Value	PEP	IonScore	Exp Value	Charge	MH+ [Da]	ΔM [ppm]	RT [min]	# Missed Cleavages
	High	lLAQVHQLILER	3	1	1	P73426	N-Term(iTRAQ4plex)	0,0000	0,289	2	12,4	0	3,696E-11	75	3,19132E-08	3	1576,96524	-0,22	44,26	0
	High	lkPHIQAMk	1	1	1	P73426	N-Term(iTRAQ4plex); K2(iTRAQ4plex); K9(iTRAQ4plex)	0,0000	0,789	1		0	0,0001122	22	0,00654591	4	1497,92653	-2,31	19,77	0
	High	lkPHIQAmk	1	1	1	P73426	N-Term(iTRAQ4plex); K2(iTRAQ4plex); M8(Oxidation); K9(iTRA	0,0000	0,316	1		0	0,0002432	10	0,091826917	4	1513,92409	-0,54	16,30	0
P74592	ATP phosphoribosyltransferase regulatory subunit OS=Synechocystis sp. (strain PCC 6803 / Kazusa) (	133,31	5,74	1	2	2	5	1,919	5	56,1	401	44,3	5,07							
	A3	Sequence	# PSMs	# Proteins	# Protein Groups	Protein Group Accessions	Modifications	ΔCn	116/117	116/117 Count	116/117 Variability [%]	q-Value	PEP	IonScore	Exp Value	Charge	MH+ [Da]	ΔM [ppm]	RT [min]	# Missed Cleavages
	High	nLALQLFDLR	3	1	1	P74592	N-Term(iTRAQ4plex)	0,0000	2,506	3	145,2	0	0,00009408	53	5,34527E-06	2	1346,79265	1,00	50,79	0
	High	lGLRPELTASIAR	2	1	1	P74592	N-Term(iTRAQ4plex)	0,0000	1,608	2	26,6	0	1,807E-07	47	2,14768E-05	3	1540,92972	0,33	33,10	0
P74338	NAD(P)H-quinone oxidoreductase subunit M OS=Synechocystis sp. (strain PCC 6803 / Kazusa) GN=n	133,09	16,53	1	2	2	4	0,288	4	7,9	121	14,1	4,70							
	A3	Sequence	# PSMs	# Proteins	# Protein Groups	Protein Group Accessions	Modifications	ΔCn	116/117	116/117 Count	116/117 Variability [%]	q-Value	PEP	IonScore	Exp Value	Charge	MH+ [Da]	ΔM [ppm]	RT [min]	# Missed Cleavages
	High	vLNYSmGLPk	2	1	1	P74338	N-Term(iTRAQ4plex); M6(Oxidation); K10(iTRAQ4plex)	0,0000	0,283	2	7,9	0	0,0001156	49	1,25017E-05	2	1425,80351	1,46	30,17	0
	High	iGSDLEHFIR	2	1	1	P74338	N-Term(iTRAQ4plex)	0,0000	0,301	2	11,9	0	0,00001596	42	6,76036E-05	2	1330,72295	-0,50	36,75	0
P73437	ATP-dependent zinc metalloprotease FtsH 4 OS=Synechocystis sp. (strain PCC 6803 / Kazusa) GN=fts	131,91	11,94	1	4	7	9	2,353	4	24,4	628	68,2	5,41							
	A3	Sequence	# PSMs	# Proteins	# Protein Groups	Protein Group Accessions	Modifications	ΔCn	116/117	116/117 Count	116/117 Variability [%]	q-Value	PEP	IonScore	Exp Value	Charge	MH+ [Da]	ΔM [ppm]	RT [min]	# Missed Cleavages
	High	eIVEQGHNQALAILEHNR	2	1	1	P73437	N-Term(iTRAQ4plex)	0,0000	1,474	1		0	2,742E-11	48	1,71779E-05	4	2215,17080	0,33	33,16	0
	High	gVLLVGPPGTGk	1	3	3	Q55700;P72991;P73437	N-Term(iTRAQ4plex); K12(iTRAQ4plex)	0,0000				0	0,001499	31	0,000749842	3	1382,85981	-0,88	31,27	0
	High	yTALGAk	1	1	1	P73437	N-Term(iTRAQ4plex); K7(iTRAQ4plex)	0,0000	2,528	1		0	0,001761	25	0,003213439	2	1011,60649	-1,21	19,12	0
	High	vRDLFEQAk	2	2	2	P72991;P73437	N-Term(iTRAQ4plex); K9(iTRAQ4plex)	0,0000				0	0,0002062	23	0,004581102	3	1393,80448	0,19	26,88	1
	High	aIkPQPQWQR	1	1	1	P73437	N-Term(iTRAQ4plex); K3(iTRAQ4plex)	0,0000	2,190	1		0	0,00002342	21	0,007361563	3	1539,90030	0,30	19,67	0
	High	yQLkPEAEDEGkEk	1	1	1	P73437	N-Term(iTRAQ4plex); K4(iTRAQ4plex); K12(iTRAQ4plex); K14(	0,0000	3,030	1		0	0,000002406	16	0,026253626	4	2240,22695	0,57	19,59	1
	Medium	tLLAk	1	3	3	Q55700;P72991;P73437	N-Term(iTRAQ4plex); K5(iTRAQ4plex)	0,0000				0,029	0,3849	29	0,001121941	2	833,56822	-1,98	18,81	0
P73145	Thylakoid-associated single-stranded DNA-binding protein slr1034 OS=Synechocystis sp. (strain PCC (	130,12	13,95	1	2	2	6	0,713	6	15,3	129	14,4	4,74							
	A3	Sequence	# PSMs	# Proteins	# Protein Groups	Protein Group Accessions	Modifications	ΔCn	116/117	116/117 Count	116/117 Variability [%]	q-Value	PEP	IonScore	Exp Value	Charge	MH+ [Da]	ΔM [ppm]	RT [min]	# Missed Cleavages
	High	vVGWGNLANTIk	3	1	1	P73145	N-Term(iTRAQ4plex); K12(iTRAQ4plex)	0,0000	0,900	3	25,6	0	0,0004829	42	8,01512E-05	3	1559,91794	1,99	40,62	0
	High	lIIEGR	3	1	1	P73145	N-Term(iTRAQ4plex)	0,0000	0,673	3	4,1	0,001	0,007004	37	0,000189657	2	844,53716	-0,17	23,37	0
P73107	Sll1837 protein OS=Synechocystis sp. (strain PCC 6803 / Kazusa) GN=sll1837 PE=4 SV=1 - [P73107_	129,14	15,17	1	2	2	5	0,632	4	8,3	145	15,5	4,72							
	A3	Sequence	# PSMs	# Proteins	# Protein Groups	Protein Group Accessions	Modifications	ΔCn	116/117	116/117 Count	116/117 Variability [%]	q-Value	PEP	IonScore	Exp Value	Charge	MH+ [Da]	ΔM [ppm]	RT [min]	# Missed Cleavages
	High	kAIAEmAGSESIk	2	1	1	P73107	N-Term(iTRAQ4plex); K1(iTRAQ4plex); M6(Oxidation); K13(iTF	0,0000	0,576	1		0	2,07E-08	67	1,93629E-07	3	1783,00266	1,71	18,46	1
	High	iSDGVAPNRk	3	1	1	P73107	N-Term(iTRAQ4plex); K10(iTRAQ4plex)	0,0000	0,645	3	6,1	0	0,0002572	31	0,000776194	3	1344,78550	1,25	14,54	1
P73971	Probable cytosol aminopeptidase OS=Synechocystis sp. (strain PCC 6803 / Kazusa) GN=pepA PE=3 S	129,08	4,27	1	3	3	3	3,079	3	27,5	492	52,1	5,11							
	A3	Sequence	# PSMs	# Proteins	# Protein Groups	Protein Group Accessions	Modifications	ΔCn	116/117	116/117 Count	116/117 Variability [%]	q-Value	PEP	IonScore	Exp Value	Charge	MH+ [Da]	ΔM [ppm]	RT [min]	# Missed Cleavages
	High	kLILVGLGEIEk	1	1	1	P73971	N-Term(iTRAQ4plex); K1(iTRAQ4plex); K12(iTRAQ4plex)	0,0000	2,485	1		0	5,995E-11	94	4,20698E-10	3	1744,13797	4,07	40,81	1
	High	lILVGLGEIEk	1	1	1	P73971	N-Term(iTRAQ4plex); K11(iTRAQ4plex)	0,0000	3,693	1		0	0,000246	33	0,000451825	3	1471,93723	2,30	46,84	0
	High	iVSGVILAR	1	1	1	P73971	N-Term(iTRAQ4plex)	0,0000	3,079	1		0	0,0000206	28	0,001621698	2	1071,70171	0,95	33,78	0
P74561	1-(5-phosphoribosyl)-5-[(5-phosphoribosylamino)methylideneamino] imidazole-4-carboxamide isome	129,03	13,28	1	3	3	5	0,862	4	34,4	256	27,3	5,02							
	A3	Sequence	# PSMs	# Proteins	# Protein Groups	Protein Group Accessions	Modifications	ΔCn	116/117	116/117 Count	116/117 Variability [%]	q-Value	PEP	IonScore	Exp Value	Charge	MH+ [Da]	ΔM [ppm]	RT [min]	# Missed Cleavages
	High	lGAAAIIYTDIHR	2	1	1	P74561	N-Term(iTRAQ4plex)	0,0000	0,697	1		0	2,495E-08	59	1,58744E-06	3	1557,88712	0,08	38,16	0
	High	lHLVDLDGAk	2	1	1	P74561	N-Term(iTRAQ4plex); K10(iTRAQ4plex)	0,0000	1,081	2	1,9	0	3,854E-07	45	3,23571E-05	3	1368,80967	0,51	31,06	0
	High	iLPAIDLLGGk	1	1	1	P74561	N-Term(iTRAQ4plex); K11(iTRAQ4plex)	0,0000	0,639	1		0	0,00001039	19	0,011939056	2	1397,89726	0,14	47,72	0
Q55410	Sll0540 protein OS=Synechocystis sp. (strain PCC 6803 / Kazusa) GN=sll0540 PE=4 SV=1 - [Q55410	128,94	5,21	1	2	2	5	0,409	4	16,7	307	32,5	4,93							
	A3	Sequence	# PSMs	# Proteins	# Protein Groups	Protein Group Accessions	Modifications	ΔCn	116/117	116/117 Count	116/117 Variability [%]	q-Value	PEP	IonScore	Exp Value	Charge	MH+ [Da]	ΔM [ppm]	RT [min]	# Missed Cleavages
	High	qLFYFYk	2	1	1	Q55410	N-Term(iTRAQ4plex); K7(iTRAQ4plex)	0,0000	0,375	2	16,7	0	0,00007695	54	3,54789E-06	2	1296,72173	-1,03	40,82	0
	High	gVVNLILGk	3	1	1	Q55410	N-Term(iTRAQ4plex); K9(iTRAQ4plex)	0,0000	0,475	2	26,4	0	0,00000097	43	5,30848E-05	2	1200,79155	-0,25	41,80	0
Q55848	Ribose-phosphate pyrophosphokinase OS=Synechocystis sp. (strain PCC 6803 / Kazusa) GN=prs PE=	128,25	7,81	1	3	3	4	1,984	4	90,9	333	36,4	7,20							
	A3	Sequence	# PSMs	# Proteins	# Protein Groups	Protein Group Accessions	Modifications	ΔCn	116/117	116/117 Count	116/117 Variability [%]	q-Value	PEP	IonScore	Exp Value	Charge	MH+ [Da]	ΔM [ppm]	RT [min]	# Missed Cleavages
	High	lVANLITGAGAQR	2	1	1	Q55848	N-Term(iTRAQ4plex)	0,0000	1,206	2	137,8	0	1,569E-08	54	4,14665E-06	2	1427,84538	0,17	37,19	0
	High	kLNDAPLAIIDkR	1	1	1	Q55848	N-Term(iTRAQ4plex); K1(iTRAQ4plex); K12(iTRAQ4plex)	0,0000	4,638	1		0	9,316E-07	30	0,000891189	4	1899,17434	-0,46	29,95	2
	High	kLNDAPLAIIDk	1	1	1	Q55848	N-Term(iTRAQ4plex); K1(iTRAQ4plex); K12(iTRAQ4plex)	0,0000	1,628	1		0	0,000001096	30	0,000995337	3	1743,07376	-0,19	33,79	1
P73886	Ribosome-binding ATPase YchF OS=Synechocystis sp. (strain PCC 6803 / Kazusa) GN=ychF PE=3 SV	128,15	7,16	1	2	2	5	1,932	4	37,9	363	39,3	4,84							
	A3	Sequence	# PSMs	# Proteins	# Protein Groups	Protein Group Accessions	Modifications	ΔCn	116/117	116/117 Count	116/117 Variability [%]	q-Value	PEP	IonScore	Exp Value	Charge	MH+ [Da]	ΔM [ppm]	RT [min]	# Missed Cleavages
	High	sTLFNALVANAk	2	1	1	P73886	N-Term(iTRAQ4plex); K12(iTRAQ4plex)	0,0000	1,672	2	37,9	0	9,639E-08	64	3,68104E-07	3	1536,89811	-0,48	42,08	0
	High	gNkDLLEEVAILER	3	1	1	P73886	N-Term(iTRAQ4plex); K3(iTRAQ4plex)	0,0000	2,238	2	38,3	0	6,866E-11	54	4,18013E-06	3	1887,08146	1,29	43,33	1
P73392	Uncharacterized protein sll1735 OS=Synechocystis sp. (strain PCC 6803 / Kazusa) GN=sll1735 PE=4	126,85	13,53	1	2	2	4	0,193	3	4,7	133	14,1	4,48							
	A3	Sequence	# PSMs	# Proteins	# Protein Groups	Protein Group Accessions	Modifications	ΔCn	116/117	116/117 Count	116/117 Variability [%]	q-Value	PEP	IonScore	Exp Value	Charge	MH+ [Da]	ΔM [ppm]	RT [min]	# Missed Cleavages
	High	iLTYHVVAGk	3	1	1	P73392	N-Term(iTRAQ4plex); K10(iTRAQ4plex)	0,0000	0,156	2	37,6	0	1,018E-07	77	2,21294E-08	2	1388,85185	1,03	26,71	0
	High	fTQADLcR	1	1	1	P73392	N-Term(iTRAQ4plex); C7(Carbamidomethyl)	0,0000	0,193	1		0	0,00167	39	0,000133343	2	1154,57488	0,33	23,58	0
Q55770	Sll0185 protein OS=Synechocystis sp. (strain PCC 6803 / Kazusa) GN=sll0185 PE=4 SV=1 - [Q55770	126,46	9,79	1	5	5	7	1,063	7	6,7	419	47,0	5,12							
	A3	Sequence	# PSMs	# Proteins	# Protein Groups	Protein Group Accessions	Modifications	ΔCn	116/117	116/117 Count	116/117 Variability [%]	q-Value	PEP	IonScore	Exp Value	Charge	MH+ [Da]	ΔM [ppm]	RT [min]	# Missed Cleavages
	High	sQGGQQLALR	2	1	1	Q55770	N-Term(iTRAQ4plex)	0,0000	1,007	2	14,9	0	1,827E-07	56	2,55841E-06	2	1201,67766	0,55	17,35	0
	High	kSQLLAEVEk	1	1	1	Q55770	N-Term(iTRAQ4plex); K1(iTRAQ4plex); K10(iTRAQ4plex)	0,0000	0,950	1		0	0,00007419	23	0,004560054	3	1576,96323	-0,15	26,13	1
	High	akEDRPSLk	1	1	1	Q55770	N-Term(iTRAQ4plex); K2(iTRAQ4plex); K9(iTRAQ4plex)	0,0000	1,103	1		0	0,000457	22	0,005793887	3	1475,88938	-0,85	10,81	1
	High	iTLmPR	1	1	1	Q55770	N-Term(iTRAQ4plex); M4(Oxidation)	0,0000	1,049	1		0,002	0,02418	20	0,01002236	2	890,52519	0,19	21,70	0
	Medium	iTLMPR	1	1	1	Q55770	N-Term(iTRAQ4plex)	0,0000	1,521	1		0,027	0,3603	28	0,001682674	2	874,52922	-1,02	27,47	0
	Medium	wLTLAR	1	1	1	Q55770	N-Term(iTRAQ4plex)	0,0000	1,063	1		0,034	0,4348	24	0,004295068	2	903,55364	0,39	34,78	0
P54384	3-isopropylmalate dehydratase large subunit OS=Synechocystis sp. (strain PCC 6803 / Kazusa) GN=le	126,37	2,99	1	2	2	7	0,769	5	5,0	468	50,4	5,69							
	A3	Sequence	# PSMs	# Proteins	# Protein Groups	Protein Group Accessions	Modifications	ΔCn	116/117	116/117 Count	116/117 Variability [%]	q-Value	PEP	IonScore	Exp Value	Charge	MH+ [Da]	ΔM [ppm]	RT [min]	# Missed Cleavages
	High	dVILHIIR	6	1	1	P54384	N-Term(iTRAQ4plex)	0,0000	0,769	4	2,5	0	0,00003293	43	5,58432E-05	2	1122,70989	-1,51	32,21	0
	High	aVQWWR	1	1	1	P54384	N-Term(iTRAQ4plex)	0,0000	0,471	1		0,001	0,01034	30	0,00097717	2	989,54344	-0,34	30,87	0
Q55366	Ferredoxin--nitrite reductase OS=Synechocystis sp. (strain PCC 6803 / Kazusa) GN=nirA PE=4 SV=1	125,58	9,16	1	5	5	9	3,253	8	76,0	502	55,9	6,61							
	A3	Sequence	# PSMs	# Proteins	# Protein Groups	Protein Group Accessions	Modifications	ΔCn	116/117	116/117 Count	116/117 Variability [%]	q-Value	PEP	IonScore	Exp Value	Charge	MH+ [Da]	ΔM [ppm]	RT [min]	# Missed Cleavages
	High	kFNIAIEGSR	2	1	1	Q55366	N-Term(iTRAQ4plex); K1(iTRAQ4plex)	0,0000	4,181	1		0	1,347E-08	48	1,93723E-05	3	1422,83182	0,74	25,78	1
	High	eLTVPkPVR	1	1	1	Q55366	N-Term(iTRAQ4plex); K6(iTRAQ4plex)	0,0000	1,093	1		0	0,00001004	45	3,05471E-05	3	1326,83365	-0,86	22,48	0
	High	fREEVAAk	1	1	1	Q55366	N-Term(iTRAQ4plex); K8(iTRAQ4plex)	0,0000	2,708	1		0	0,0001731	30	0,001093881	3	1237,71347	-0,70	16,81	1
	High	lPFPLLTAAPk	4	1	1	Q55366	N-Term(iTRAQ4plex); K11(iTRAQ4plex)	0,0000	3,576	4	130,8	0	0,00008868	23	0,004497488	3	1455,91834	0,37	47,93	0
	High	aNkFETVk	1	1	1	Q55366	N-Term(iTRAQ4plex); K3(iTRAQ4plex); K8(iTRAQ4plex)	0,0000	3,909	1		0	0,0006046	13	0,048974499	3	1368,82074	-0,29	17,91	1
P74361	Chaperone protein ClpB 2 OS=Synechocystis sp. (strain PCC 6803 / Kazusa) GN=clpB2 PE=3 SV=1 -	125,08	5,85	1	3	5	9	2,064	4	16,9	872	98,1	5,26							
	A3	Sequence	# PSMs	# Proteins	# Protein Groups	Protein Group Accessions	Modifications	ΔCn	116/117	116/117 Count	116/117 Variability [%]	q-Value	PEP	IonScore	Exp Value	Charge	MH+ [Da]	ΔM [ppm]	RT [min]	# Missed Cleavages
	High	tAIAEGLAQR	2	3	3	P74459;P74361;Q55662	N-Term(iTRAQ4plex)	0,0000				0	5,338E-07	46	3,02268E-05	2	1173,67082	-0,03	23,31	0
	High	lLHLEDELHSR	3	1	1	P74361	N-Term(iTRAQ4plex)	0,0000	2,123	2	16,9	0	0,000001163	42	6,38263E-05	4	1505,81875	-0,37	30,49	0
	High	vDETIIFHGLQk	1	1	1	P74361	N-Term(iTRAQ4plex); K12(iTRAQ4plex)	0,0000	1,650	1		0	0,000002843	26	0,00232793	3	1687,96268	0,30	37,29	0
	High	eNFRPEFLNR	1	1	1	P74361	N-Term(iTRAQ4plex)	0,0000	2,246	1		0	0,0004757	25	0,003280726	3	1465,76657	-0,21	29,47	0
	High	yRGEFEER	2	3	3	P74459;P74361;Q55662	N-Term(iTRAQ4plex)	0,0000				0	0,002186	16	0,027731286	3	1229,60511	1,59	15,30	1
Q6ZEI6	Slr7012 protein OS=Synechocystis sp. (strain PCC 6803 / Kazusa) GN=slr7012 PE=4 SV=1 - [Q6ZEI6	124,96	17,02	1	5	5	6	0,236	5	21,6	329	36,5	6,35							
	A3	Sequence	# PSMs	# Proteins	# Protein Groups	Protein Group Accessions	Modifications	ΔCn	116/117	116/117 Count	116/117 Variability [%]	q-Value	PEP	IonScore	Exp Value	Charge	MH+ [Da]	ΔM [ppm]	RT [min]	# Missed Cleavages
	High	hSQSFPVFQTDGVLNTTR	2	1	1	Q6ZEI6	N-Term(iTRAQ4plex)	0,0000	0,205	1		0	3,565E-08	74	4,31489E-08	3	2178,10782	0,81	36,60	0
	High	sQFQPSFPR	1	1	1	Q6ZEI6	N-Term(iTRAQ4plex)	0,0000	0,213	1		0	0,0005147	36	0,000227494	2	1237,64519	0,46	27,87	0
	High	nLGLTSADk	1	1	1	Q6ZEI6	N-Term(iTRAQ4plex); K9(iTRAQ4plex)	0,0000	0,416	1		0	0,0004066	29	0,001273415	2	1206,69792	3,87	20,47	0
	High	lASGHYVHFLmLR	1	1	1	Q6ZEI6	N-Term(iTRAQ4plex); M11(Oxidation)	0,0000	0,236	1		0	0,000006296	21	0,007395542	4	1703,91738	0,08	34,75	0
	Medium	yGAQESR	1	1	1	Q6ZEI6	N-Term(iTRAQ4plex)	0,0000	0,712	1		0,044	0,5679	12	0,062082615	2	954,47795	1,89	11,94	0
P73960	3-isopropylmalate dehydrogenase OS=Synechocystis sp. (strain PCC 6803 / Kazusa) GN=leuB PE=3 S	124,23	10,77	1	3	3	6	0,924	5	32,5	362	38,6	4,84							
	A3	Sequence	# PSMs	# Proteins	# Protein Groups	Protein Group Accessions	Modifications	ΔCn	116/117	116/117 Count	116/117 Variability [%]	q-Value	PEP	IonScore	Exp Value	Charge	MH+ [Da]	ΔM [ppm]	RT [min]	# Missed Cleavages
	High	aNPLAQVLSAAmmLR	3	1	1	P73960	N-Term(iTRAQ4plex); M12(Oxidation); M13(Oxidation)	0,0000	0,422	2	169,1	0	0,00006207	43	5,61009E-05	3	1761,94736	0,05	55,85	0
	High	sQRPETGLLAIR	2	1	1	P73960	N-Term(iTRAQ4plex)	0,0000	1,045	2	15,0	0	3,855E-07	41	7,63783E-05	3	1484,86713	0,35	26,26	0
	High	eLTGGIYFGkPk	1	1	1	P73960	N-Term(iTRAQ4plex); K10(iTRAQ4plex); K12(iTRAQ4plex)	0,0000	0,746	1		0	0,00002269	20	0,008953029	4	1742,01943	-1,08	30,31	0
Q55894	Phosphomethylpyrimidine synthase OS=Synechocystis sp. (strain PCC 6803 / Kazusa) GN=thiC PE=3	124,02	11,33	1	5	5	7	0,830	7	43,1	459	51,1	5,74							
	A3	Sequence	# PSMs	# Proteins	# Protein Groups	Protein Group Accessions	Modifications	ΔCn	116/117	116/117 Count	116/117 Variability [%]	q-Value	PEP	IonScore	Exp Value	Charge	MH+ [Da]	ΔM [ppm]	RT [min]	# Missed Cleavages
	High	iAAHAADIAR	2	1	1	Q55894	N-Term(iTRAQ4plex)	0,0000	0,857	2	37,7	0	3,395E-07	57	2,21804E-06	2	1152,66118	0,48	17,95	0
	High	qGQTNVSQMHYAR	1	1	1	Q55894	N-Term(iTRAQ4plex)	0,0000	0,830	1		0	0,0001314	29	0,001164046	3	1663,80796	-0,71	15,74	0
	High	qGQTNVSQmHYAR	1	1	1	Q55894	N-Term(iTRAQ4plex); M9(Oxidation)	0,0000	1,193	1		0	0,00001178	23	0,005332981	3	1679,80497	0,54	10,66	0
	High	rENLPVELIkDEVAR	1	1	1	Q55894	N-Term(iTRAQ4plex); K10(iTRAQ4plex)	0,0000	0,581	1		0	7,728E-08	23	0,005457202	4	2069,19668	0,42	37,40	2
	Medium	dkEmVSQR	1	1	1	Q55894	N-Term(iTRAQ4plex); K2(iTRAQ4plex); M4(Oxidation)	0,0000	0,732	1		0,014	0,1864	13	0,054321281	3	1296,68213	0,06	8,99	1
	Medium	lLLSVk	1	1	1	Q55894	N-Term(iTRAQ4plex); K6(iTRAQ4plex)	0,0000	1,363	1		0,016	0,2116	28	0,001737681	2	960,66771	-1,96	31,35	0
P73736	N-acetylmuramoyl-L-alanine amidase OS=Synechocystis sp. (strain PCC 6803 / Kazusa) GN=amiA PE	123,58	5,86	1	4	4	5	0,663	5	8,2	649	70,0	9,83							
	A3	Sequence	# PSMs	# Proteins	# Protein Groups	Protein Group Accessions	Modifications	ΔCn	116/117	116/117 Count	116/117 Variability [%]	q-Value	PEP	IonScore	Exp Value	Charge	MH+ [Da]	ΔM [ppm]	RT [min]	# Missed Cleavages
	High	vQPTAQLIPNPTR	1	1	1	P73736	N-Term(iTRAQ4plex)	0,0000	0,627	1		0	0,000001363	63	6,92035E-07	2	1578,90947	0,64	32,50	0
	High	iVVDLPGTTLR	2	1	1	P73736	N-Term(iTRAQ4plex)	0,0000	0,747	2	17,8	0	0,001474	44	3,89018E-05	2	1327,80669	0,05	39,58	0
	High	nVVNSLNVR	1	1	1	P73736	N-Term(iTRAQ4plex)	0,0000	0,641	1		0,005	0,05872	23	0,004487144	2	1158,66997	-1,04	25,09	0
	Medium	fYVLR	1	1	1	P73736	N-Term(iTRAQ4plex)	0,0000	0,758	1		0,013	0,172	17	0,021133431	2	841,50743	2,59	29,93	0
P73668	UDP-N-acetylmuramoylalanine--D-glutamate ligase OS=Synechocystis sp. (strain PCC 6803 / Kazusa)	123,06	6,19	1	2	2	4	2,892	4	7,5	452	49,0	5,76							
	A3	Sequence	# PSMs	# Proteins	# Protein Groups	Protein Group Accessions	Modifications	ΔCn	116/117	116/117 Count	116/117 Variability [%]	q-Value	PEP	IonScore	Exp Value	Charge	MH+ [Da]	ΔM [ppm]	RT [min]	# Missed Cleavages
	High	aITETLLTFTGVPHR	3	1	1	P73668	N-Term(iTRAQ4plex)	0,0000	2,962	3	7,1	0	0,00000615	57	2,18368E-06	3	1800,01316	-0,28	45,14	0
	High	tTTTSLVQAIFQk	1	1	1	P73668	N-Term(iTRAQ4plex); K13(iTRAQ4plex)	0,0000	2,677	1		0	2,666E-07	45	2,93745E-05	3	1725,99991	0,56	46,21	0
P72741	Slr1097 protein OS=Synechocystis sp. (strain PCC 6803 / Kazusa) GN=slr1097 PE=4 SV=1 - [P72741	122,58	26,38	1	5	5	7	1,504	7	32,2	163	19,0	4,70							
	A3	Sequence	# PSMs	# Proteins	# Protein Groups	Protein Group Accessions	Modifications	ΔCn	116/117	116/117 Count	116/117 Variability [%]	q-Value	PEP	IonScore	Exp Value	Charge	MH+ [Da]	ΔM [ppm]	RT [min]	# Missed Cleavages
	High	vFVLADWLk	2	1	1	P72741	N-Term(iTRAQ4plex); K9(iTRAQ4plex)	0,0000	1,828	2	2,5	0	0,000001306	49	1,52299E-05	2	1378,83391	0,13	52,15	0
	High	yAAmLVPHEFHR	1	1	1	P72741	N-Term(iTRAQ4plex); M4(Oxidation)	0,0000	0,919	1		0	0,00003725	33	0,000461286	4	1630,82802	-0,05	28,52	0
	High	kYQLPAQFR	1	1	1	P72741	N-Term(iTRAQ4plex); K1(iTRAQ4plex)	0,0000	0,717	1		0	0,001119	24	0,004187646	3	1438,84146	0,38	26,56	1
	Medium	aISLNLNR	1	1	1	P72741	N-Term(iTRAQ4plex)	0,0000	0,773	1		0,011	0,1397	21	0,007797763	2	1044,62761	-0,61	27,83	0
	Medium	lWFIR	2	1	1	P72741	N-Term(iTRAQ4plex)	0,0000	1,513	2	0,9	0,012	0,1648	23	0,005523181	2	878,53667	-0,27	42,93	0
P73848	3-hydroxyacyl-[acyl-carrier-protein] dehydratase FabZ OS=Synechocystis sp. (strain PCC 6803 / Kazu	122,57	24,39	1	4	4	6	1,581	5	27,1	164	17,9	5,53							
	A3	Sequence	# PSMs	# Proteins	# Protein Groups	Protein Group Accessions	Modifications	ΔCn	116/117	116/117 Count	116/117 Variability [%]	q-Value	PEP	IonScore	Exp Value	Charge	MH+ [Da]	ΔM [ppm]	RT [min]	# Missed Cleavages
	High	gkFFAFAGIDGVR	1	1	1	P73848	N-Term(iTRAQ4plex); K2(iTRAQ4plex)	0,0000	1,831	1		0	0,000004095	52	5,87449E-06	3	1672,94510	2,21	40,29	1
	High	fFAFAGIDGVR	3	1	1	P73848	N-Term(iTRAQ4plex)	0,0000	1,429	2	15,1	0	0,00004642	46	2,57614E-05	2	1343,72429	1,03	46,81	0
	High	rPVVPGDQLImTVELQSFk	1	1	1	P73848	N-Term(iTRAQ4plex); M11(Oxidation); K19(iTRAQ4plex)	0,0000	1,953	1		0	5,861E-07	25	0,003040675	4	2461,37417	0,56	45,85	0
	High	yPFALVDR	1	1	1	P73848	N-Term(iTRAQ4plex)	0,0000	1,321	1		0,001	0,005541	21	0,00763783	2	1124,62248	0,35	37,32	0
P73804	Sll1961 protein OS=Synechocystis sp. (strain PCC 6803 / Kazusa) GN=sll1961 PE=4 SV=1 - [P73804_	122,51	3,61	1	2	2	5	0,897	4	35,0	388	42,9	5,95							
	A3	Sequence	# PSMs	# Proteins	# Protein Groups	Protein Group Accessions	Modifications	ΔCn	116/117	116/117 Count	116/117 Variability [%]	q-Value	PEP	IonScore	Exp Value	Charge	MH+ [Da]	ΔM [ppm]	RT [min]	# Missed Cleavages
	High	vLVTVPQR	3	1	1	P73804	N-Term(iTRAQ4plex)	0,0000	0,750	3	15,0	0	0,00199	36	0,000237668	2	1055,66850	-0,83	25,50	0
	High	sIAILk	2	1	1	P73804	N-Term(iTRAQ4plex); K6(iTRAQ4plex)	0,0000	1,457	1		0,002	0,02469	42	6,79157E-05	2	932,63689	-1,51	26,39	0
Q55367	Cyanate hydratase OS=Synechocystis sp. (strain PCC 6803 / Kazusa) GN=cynS PE=1 SV=1 - [CYNS_	122,30	26,17	1	5	5	7	0,474	7	12,1	149	16,5	5,21							
	A3	Sequence	# PSMs	# Proteins	# Protein Groups	Protein Group Accessions	Modifications	ΔCn	116/117	116/117 Count	116/117 Variability [%]	q-Value	PEP	IonScore	Exp Value	Charge	MH+ [Da]	ΔM [ppm]	RT [min]	# Missed Cleavages
	High	gLGPVVPTDPLIYR	1	1	1	Q55367	N-Term(iTRAQ4plex)	0,0000	0,437	1		0	0,000002541	60	1,11165E-06	2	1640,95098	1,04	42,62	0
	High	fYEImQVYGmPmk	1	1	1	Q55367	N-Term(iTRAQ4plex); M5(Oxidation); M10(Oxidation); M12(Ox	0,0000	0,383	1		0	6,343E-09	30	0,001059181	3	1972,94638	0,12	35,35	0
	High	eVIHEk	1	1	1	Q55367	N-Term(iTRAQ4plex); K6(iTRAQ4plex)	0,0000	0,493	1		0,001	0,003087	12	0,056489796	3	1042,61350	-0,04	11,91	0
	Medium	fLPYk	3	1	1	Q55367	N-Term(iTRAQ4plex); K5(iTRAQ4plex)	0,0000	0,474	3	2,1	0,012	0,1647	29	0,001380289	2	955,58617	0,68	28,46	0
	Medium	fLPYkk	1	1	1	Q55367	N-Term(iTRAQ4plex); K5(iTRAQ4plex); K6(iTRAQ4plex)	0,0000	0,637	1		0,029	0,3878	11	0,077085023	3	1227,78522	2,18	23,49	1
P73588	Sll1315 protein OS=Synechocystis sp. (strain PCC 6803 / Kazusa) GN=sll1315 PE=4 SV=1 - [P73588_	122,03	4,83	1	2	2	3	0,720	3	13,6	352	38,9	7,23							
	A3	Sequence	# PSMs	# Proteins	# Protein Groups	Protein Group Accessions	Modifications	ΔCn	116/117	116/117 Count	116/117 Variability [%]	q-Value	PEP	IonScore	Exp Value	Charge	MH+ [Da]	ΔM [ppm]	RT [min]	# Missed Cleavages
	High	lYQSIGVAR	2	1	1	P73588	N-Term(iTRAQ4plex)	0,0000	0,688	2	6,8	0	5,583E-07	61	8,53041E-07	2	1150,66997	-0,12	27,40	0
	High	iISLTTGk	1	1	1	P73588	N-Term(iTRAQ4plex); K8(iTRAQ4plex)	0,0000	0,796	1		0,001	0,005943	39	0,000138666	2	1120,71892	0,81	28,53	0
P73066	Ycf23 protein OS=Synechocystis sp. (strain PCC 6803 / Kazusa) GN=ycf23 PE=4 SV=1 - [P73066_SY	121,96	12,90	1	3	3	5	0,429	5	21,5	248	25,4	5,33							
	A3	Sequence	# PSMs	# Proteins	# Protein Groups	Protein Group Accessions	Modifications	ΔCn	116/117	116/117 Count	116/117 Variability [%]	q-Value	PEP	IonScore	Exp Value	Charge	MH+ [Da]	ΔM [ppm]	RT [min]	# Missed Cleavages
	High	rFEGPEVLALTk	3	1	1	P73066	N-Term(iTRAQ4plex); K12(iTRAQ4plex)	0,0000	0,381	3	3,7	0	2,517E-08	46	2,54665E-05	3	1647,96805	0,47	34,35	1
	High	aAPTLAAAHAISR	1	1	1	P73066	N-Term(iTRAQ4plex)	0,0000	0,535	1		0	0,000002765	44	3,58072E-05	3	1393,80347	0,14	22,76	0
	Medium	qLIQQVR	1	1	1	P73066	N-Term(iTRAQ4plex)	0,0000	1,113	1		0,017	0,2198	33	0,000524771	2	1028,63420	0,85	19,91	0
P73913	Acetohydroxy acid synthase OS=Synechocystis sp. (strain PCC 6803 / Kazusa) GN=ilvG PE=3 SV=1 -	120,83	3,54	1	2	2	5	5,080	3	7,2	621	67,7	6,18							
	A3	Sequence	# PSMs	# Proteins	# Protein Groups	Protein Group Accessions	Modifications	ΔCn	116/117	116/117 Count	116/117 Variability [%]	q-Value	PEP	IonScore	Exp Value	Charge	MH+ [Da]	ΔM [ppm]	RT [min]	# Missed Cleavages
	High	fEAAGEIEHILVR	3	1	1	P73913	N-Term(iTRAQ4plex)	0,0000	4,958	2	3,6	0	4,911E-10	57	2,17756E-06	3	1627,89273	0,15	44,05	0
	High	hVLEQLLQR	2	1	1	P73913	N-Term(iTRAQ4plex)	0,0000	5,931	1		0	2,064E-07	49	1,30308E-05	3	1279,76053	0,16	31,62	0
Q55558	Sll0172 protein OS=Synechocystis sp. (strain PCC 6803 / Kazusa) GN=sll0172 PE=4 SV=1 - [Q55558	120,60	21,05	1	3	3	5	1,085	5	2,3	152	16,3	5,38							
	A3	Sequence	# PSMs	# Proteins	# Protein Groups	Protein Group Accessions	Modifications	ΔCn	116/117	116/117 Count	116/117 Variability [%]	q-Value	PEP	IonScore	Exp Value	Charge	MH+ [Da]	ΔM [ppm]	RT [min]	# Missed Cleavages
	High	vLDVFNVk	2	1	1	Q55558	N-Term(iTRAQ4plex); K8(iTRAQ4plex)	0,0000	1,088	2	1,9	0	0,000005804	53	5,21159E-06	2	1221,74553	0,79	39,99	0
	High	lTPVTTDGASYPTQLIAR	1	1	1	Q55558	N-Term(iTRAQ4plex)	0,0000	0,624	1		0	0,00000237	33	0,000446653	3	2048,11722	1,33	39,43	0
	Medium	lYPQSk	2	1	1	Q55558	N-Term(iTRAQ4plex); K6(iTRAQ4plex)	0,0000	1,300	2	27,3	0,021	0,2832	24	0,004233483	2	1023,60796	0,24	16,92	0
P52986	Homoserine dehydrogenase OS=Synechocystis sp. (strain PCC 6803 / Kazusa) GN=hom PE=3 SV=1	119,41	5,08	1	2	2	4	2,303	3	7,1	433	45,5	5,01							
	A3	Sequence	# PSMs	# Proteins	# Protein Groups	Protein Group Accessions	Modifications	ΔCn	116/117	116/117 Count	116/117 Variability [%]	q-Value	PEP	IonScore	Exp Value	Charge	MH+ [Da]	ΔM [ppm]	RT [min]	# Missed Cleavages
	High	iAILASLGFGGR	2	1	1	P52986	N-Term(iTRAQ4plex)	0,0000	2,365	2	11,1	0	0,00002448	59	1,19666E-06	2	1318,79985	2,61	51,44	0
	High	sLILQAIAHk	2	1	1	P52986	N-Term(iTRAQ4plex); K10(iTRAQ4plex)	0,0000	2,303	1		0	0,000001269	42	6,57612E-05	3	1381,87717	0,14	35,27	0
P74226	30S ribosomal protein S10 OS=Synechocystis sp. (strain PCC 6803 / Kazusa) GN=rpsJ PE=3 SV=1 - [	117,68	40,00	1	6	6	9	1,835	8	29,6	105	12,0	9,77							
	A3	Sequence	# PSMs	# Proteins	# Protein Groups	Protein Group Accessions	Modifications	ΔCn	116/117	116/117 Count	116/117 Variability [%]	q-Value	PEP	IonScore	Exp Value	Charge	MH+ [Da]	ΔM [ppm]	RT [min]	# Missed Cleavages

	High	tNAAAVGPIPLPTkR	2	1	1	P74226	N-Term(iTRAQ4plex); K14(iTRAQ4plex)	0,0000	2,228	1		0	1,73E-10	43	4,63415E-05	3	1794,08677	1,51	29,45	1
	High	kIYcVLR	2	1	1	P74226	N-Term(iTRAQ4plex); K1(iTRAQ4plex); C4(Carbamidomethyl)	0,0000	2,187	2	3,1	0	0,00002799	31	0,000731089	3	1239,74778	-0,66	23,64	1
	High	rIIDIYQPSSk	1	1	1	P74226	N-Term(iTRAQ4plex); K11(iTRAQ4plex)	0,0000	1,973	1		0	0,00008276	29	0,001345767	3	1607,93717	0,76	26,32	1
	High	tNAAAVGPIPLPTk	2	1	1	P74226	N-Term(iTRAQ4plex); K14(iTRAQ4plex)	0,0000	0,990	2	21,8	0	0,0000979	25	0,003183978	3	1637,98203	-0,55	34,30	0
	High	sPHVDkDSR	1	1	1	P74226	N-Term(iTRAQ4plex); K6(iTRAQ4plex)	0,0000	1,707	1		0,001	0,0123	16	0,027859287	3	1328,71689	0,58	8,15	1
	High	iYcVLR	1	1	1	P74226	N-Term(iTRAQ4plex); C3(Carbamidomethyl)	0,0000	1,216	1		0,006	0,07631	18	0,016709752	2	967,55156	0,00	28,55	0
P73655	Tryptophan--tRNA ligase OS=Synechocystis sp. (strain PCC 6803 / Kazusa) GN=trpS PE=3 SV=2 - [S	117,11	6,53	1	2	2	4	0,846	4	1,1	337	37,7	6,02							
	A3	Sequence	# PSMs	# Proteins	# Protein Groups	Protein Group Accessions	Modifications	ΔCn	116/117	116/117 Count	116/117 Variability [%]	q-Value	PEP	IonScore	Exp Value	Charge	MH+ [Da]	ΔM [ppm]	RT [min]	# Missed Cleavages
	High	aSQTAQQTLAR	2	1	1	P73655	N-Term(iTRAQ4plex)	0,0000	0,846	2	1,0	0	2,366E-08	64	3,63053E-07	2	1318,72026	0,51	15,16	0
	High	iNLLDPPEmIk	2	1	1	P73655	N-Term(iTRAQ4plex); M9(Oxidation); K11(iTRAQ4plex)	0,0000	0,692	2	31,6	0,001	0,006242	11	0,084912183	3	1586,90293	-2,35	57,49	0
P72675	Slr0731 protein OS=Synechocystis sp. (strain PCC 6803 / Kazusa) GN=slr0731 PE=4 SV=1 - [P72675	117,10	9,95	1	3	4	5	1,710	4	27,2	402	44,7	4,50							
	A3	Sequence	# PSMs	# Proteins	# Protein Groups	Protein Group Accessions	Modifications	ΔCn	116/117	116/117 Count	116/117 Variability [%]	q-Value	PEP	IonScore	Exp Value	Charge	MH+ [Da]	ΔM [ppm]	RT [min]	# Missed Cleavages
	High	tRPDYYGPPWQLR	1	1	1	P72675	N-Term(iTRAQ4plex)	0,0000	1,071	1		0	1,357E-10	54	3,56427E-06	3	1792,92624	0,60	33,83	0
	High	iENWFFTLGGR	2	1	1	P72675	N-Term(iTRAQ4plex)	0,0000	2,048	2	16,8	0	0,000001556	40	0,000102558	2	1483,78227	0,54	51,77	0
	High	qIQALLEQYR	1	1	1	P72675	N-Term(iTRAQ4plex)	0,0000	1,598	1		0	0,0000493	37	0,000219265	2	1405,79228	0,19	40,40	0
	High	kAALEk	1	2	2	;P72675	N-Term(iTRAQ4plex); K1(iTRAQ4plex); K6(iTRAQ4plex)	0,0000				0,002	0,02083	20	0,010739152	3	1091,70865	-5,70	11,37	1
P73681	Slr2144 protein OS=Synechocystis sp. (strain PCC 6803 / Kazusa) GN=slr2144 PE=4 SV=1 - [P73681	116,89	7,31	1	2	2	5	0,713	5	4,1	301	33,5	5,67							
	A3	Sequence	# PSMs	# Proteins	# Protein Groups	Protein Group Accessions	Modifications	ΔCn	116/117	116/117 Count	116/117 Variability [%]	q-Value	PEP	IonScore	Exp Value	Charge	MH+ [Da]	ΔM [ppm]	RT [min]	# Missed Cleavages
	High	lELLQAGGLFk	4	1	1	P73681	N-Term(iTRAQ4plex); K11(iTRAQ4plex)	0,0000	0,722	4	3,4	0	3,758E-07	60	1,11936E-06	2	1476,90459	1,16	47,94	0
	High	tAQLNGQITSR	1	1	1	P73681	N-Term(iTRAQ4plex)	0,0000	0,573	1		0,001	0,003214	20	0,010336956	2	1332,73992	3,51	18,88	0
P74565	RNA polymerase sigma factor SigA OS=Synechocystis sp. (strain PCC 6803 / Kazusa) GN=sigA PE=3	116,12	6,59	1	3	3	5	3,362	5	42,3	425	49,9	6,27							
	A3	Sequence	# PSMs	# Proteins	# Protein Groups	Protein Group Accessions	Modifications	ΔCn	116/117	116/117 Count	116/117 Variability [%]	q-Value	PEP	IonScore	Exp Value	Charge	MH+ [Da]	ΔM [ppm]	RT [min]	# Missed Cleavages
	High	lPVHLYETISR	2	1	1	P74565	N-Term(iTRAQ4plex)	0,0000	2,698	2	84,2	0	0,000002249	45	3,08298E-05	3	1471,83936	0,27	34,86	0
	High	lVVSIAk	1	1	1	P74565	N-Term(iTRAQ4plex); K7(iTRAQ4plex)	0,0000	3,397	1		0	0,0009602	39	0,000127635	2	1017,68968	-1,36	29,82	0
	High	kkPYTEDSIR	2	1	1	P74565	N-Term(iTRAQ4plex); K1(iTRAQ4plex); K2(iTRAQ4plex)	0,0000	2,802	2	27,5	0	0,00001543	31	0,000837471	4	1668,96682	1,38	14,04	1
P74607	UPF0296 protein ssl2874 OS=Synechocystis sp. (strain PCC 6803 / Kazusa) GN=ssl2874 PE=3 SV=1	115,94	40,45	1	4	4	6	0,894	5	6,9	89	9,9	9,60							
	A3	Sequence	# PSMs	# Proteins	# Protein Groups	Protein Group Accessions	Modifications	ΔCn	116/117	116/117 Count	116/117 Variability [%]	q-Value	PEP	IonScore	Exp Value	Charge	MH+ [Da]	ΔM [ppm]	RT [min]	# Missed Cleavages
	High	sQLIDATYGR	1	1	1	P74607	N-Term(iTRAQ4plex)	0,0000	0,689	1		0	0,000001979	60	9,61546E-07	2	1267,67827	1,54	25,65	0
	High	vVAIVSPESAPIkR	2	1	1	P74607	N-Term(iTRAQ4plex); K13(iTRAQ4plex)	0,0000	0,854	1		0	2,912E-09	52	5,74077E-06	3	1754,08060	1,54	29,43	1
	High	fVVDkDFk	2	1	1	P74607	N-Term(iTRAQ4plex); K5(iTRAQ4plex); K8(iTRAQ4plex)	0,0000	0,910	2	2,7	0	0,0008919	17	0,02018227	3	1429,84082	-0,51	27,50	1
	High	fVVDkDFkENAk	1	1	1	P74607	N-Term(iTRAQ4plex); K5(iTRAQ4plex); K8(iTRAQ4plex); K12(i	0,0000	1,075	1		0	0,000003577	10	0,090775783	4	2016,16091	-0,16	25,22	2
P74521	Glycogen synthase 1 OS=Synechocystis sp. (strain PCC 6803 / Kazusa) GN=glgA1 PE=3 SV=1 - [GLG	115,81	7,13	1	3	3	6	2,659	5	58,5	477	54,3	6,09							
	A3	Sequence	# PSMs	# Proteins	# Protein Groups	Protein Group Accessions	Modifications	ΔCn	116/117	116/117 Count	116/117 Variability [%]	q-Value	PEP	IonScore	Exp Value	Charge	MH+ [Da]	ΔM [ppm]	RT [min]	# Missed Cleavages
	High	iPLYLFGHPAFDSR	2	1	1	P74521	N-Term(iTRAQ4plex)	0,0000	3,499	2	13,6	0	0,000003539	43	4,97703E-05	3	1776,95707	0,93	46,56	0
	High	nAmVVGIVAR	1	1	1	P74521	N-Term(iTRAQ4plex); M3(Oxidation)	0,0000	0,923	1		0	0,00000885	37	0,00020369	2	1189,68340	-0,85	24,08	0
	High	vLHQLGHDVR	3	1	1	P74521	N-Term(iTRAQ4plex)	0,0000	2,012	2	43,2	0	0,000006075	28	0,00156304	3	1317,74997	-0,65	16,92	0
P73921	Sll1979 protein OS=Synechocystis sp. (strain PCC 6803 / Kazusa) GN=sll1979 PE=4 SV=1 - [P73921	114,52	14,79	1	3	3	6	1,146	6	36,0	142	15,9	5,06							
	A3	Sequence	# PSMs	# Proteins	# Protein Groups	Protein Group Accessions	Modifications	ΔCn	116/117	116/117 Count	116/117 Variability [%]	q-Value	PEP	IonScore	Exp Value	Charge	MH+ [Da]	ΔM [ppm]	RT [min]	# Missed Cleavages
	High	iNYALAMLANkk	1	1	1	P73921	N-Term(iTRAQ4plex); K11(iTRAQ4plex); K12(iTRAQ4plex)	0,0000	2,300	1		0	1,892E-08	44	4,27533E-05	3	1782,06974	1,41	40,26	1
	High	iNYALAmLANk	1	1	1	P73921	N-Term(iTRAQ4plex); M7(Oxidation); K11(iTRAQ4plex)	0,0000	0,817	1		0	0,000008444	33	0,000558432	3	1525,86481	-0,19	38,33	0
	High	iNYALAMLANk	2	1	1	P73921	N-Term(iTRAQ4plex); K11(iTRAQ4plex)	0,0000	1,146	2	9,3	0	0,00005854	27	0,002022879	3	1509,86936	-0,55	44,79	0
	High	iDGYHPQAR	1	1	1	P73921	N-Term(iTRAQ4plex)	0,0000	0,860	1		0	0,001705	26	0,002254084	3	1200,62345	-0,64	15,35	0
	High	iNYALAmLANkk	1	1	1	P73921	N-Term(iTRAQ4plex); M7(Oxidation); K11(iTRAQ4plex); K12(i	0,0000	1,377	1		0	0,000132	25	0,002850821	3	1798,05942	-1,51	35,18	1
Q55200	Protein SphX OS=Synechocystis sp. (strain PCC 6803 / Kazusa) GN=sphX PE=3 SV=1 - [SPHX_SYNY	113,97	15,48	1	5	5	8	0,375	6	16,0	336	36,8	4,77							
	A3	Sequence	# PSMs	# Proteins	# Protein Groups	Protein Group Accessions	Modifications	ΔCn	116/117	116/117 Count	116/117 Variability [%]	q-Value	PEP	IonScore	Exp Value	Charge	MH+ [Da]	ΔM [ppm]	RT [min]	# Missed Cleavages
	High	sLTVEELkR	4	1	1	Q55200	N-Term(iTRAQ4plex); K8(iTRAQ4plex)	0,0000	0,341	2	12,1	0	0,0001103	37	0,000213781	3	1362,81882	-0,52	23,74	1
	High	tDIADASRPINk	1	1	1	Q55200	N-Term(iTRAQ4plex); K12(iTRAQ4plex)	0,0000	0,389	1		0	0,00000717	36	0,000228034	3	1588,89090	0,73	19,82	0
	High	rIWEPAAEk	1	1	1	Q55200	N-Term(iTRAQ4plex); K9(iTRAQ4plex)	0,0000	0,458	1		0	0,001728	26	0,00225928	3	1387,79267	-0,71	22,32	1
	High	yVELPIAFDAITVVSNPk	1	1	1	Q55200	N-Term(iTRAQ4plex); K18(iTRAQ4plex)	0,0000	0,136	1		0,001	0,005928	11	0,087694025	3	2264,27542	-1,19	55,58	0
	Medium	iSFNk	1	1	1	Q55200	N-Term(iTRAQ4plex); K5(iTRAQ4plex)	0,0000	0,380	1		0,019	0,2533	27	0,001874865	2	896,54393	-0,51	23,49	0
P77970	Glutamate--ammonia ligase OS=Synechocystis sp. (strain PCC 6803 / Kazusa) GN=glnN PE=3 SV=1	112,86	9,94	1	6	6	8	7,128	7	56,0	724	79,2	5,47							
	A3	Sequence	# PSMs	# Proteins	# Protein Groups	Protein Group Accessions	Modifications	ΔCn	116/117	116/117 Count	116/117 Variability [%]	q-Value	PEP	IonScore	Exp Value	Charge	MH+ [Da]	ΔM [ppm]	RT [min]	# Missed Cleavages
	High	tSPFAFTGNR	2	1	1	P77970	N-Term(iTRAQ4plex)	0,0000	6,627	2	53,3	0	0,00001023	41	8,29793E-05	2	1241,64079	1,01	28,78	0
	High	tVIYPAAVEYLSk	1	1	1	P77970	N-Term(iTRAQ4plex); K13(iTRAQ4plex)	0,0000	3,714	1		0	0,00003547	36	0,000255252	3	1741,99967	1,03	42,30	0
	High	tQSVHQIINRPLSSGk	2	1	1	P77970	N-Term(iTRAQ4plex); K16(iTRAQ4plex)	0,0000	7,735	1		0	8,933E-07	24	0,003863403	4	2053,17739	0,82	22,59	0
	High	lQYcAQTLRPLMDEVR	1	1	1	P77970	N-Term(iTRAQ4plex); C4(Carbamidomethyl)	0,0000	7,128	1		0	0,000621	23	0,005259809	3	2137,10410	1,20	40,33	0
	High	kVPLLR	1	1	1	P77970	N-Term(iTRAQ4plex); K1(iTRAQ4plex)	0,0000	10,137	1		0,001	0,006654	22	0,006729302	3	1013,70719	-0,20	20,82	1
	High	aAIATASNDHR	1	1	1	P77970	N-Term(iTRAQ4plex)	0,0000	1,069	1		0,002	0,02047	20	0,011040024	3	1270,66275	0,53	10,62	0
P23350	50S ribosomal protein L10 OS=Synechocystis sp. (strain PCC 6803 / Kazusa) GN=rplJ PE=3 SV=1 - [	112,85	18,50	1	4	4	12	1,690	9	39,6	173	18,7	8,60							
	A3	Sequence	# PSMs	# Proteins	# Protein Groups	Protein Group Accessions	Modifications	ΔCn	116/117	116/117 Count	116/117 Variability [%]	q-Value	PEP	IonScore	Exp Value	Charge	MH+ [Da]	ΔM [ppm]	RT [min]	# Missed Cleavages
	High	eVPASVAR	2	1	1	P23350	N-Term(iTRAQ4plex)	0,0000	0,890	2	13,3	0	0,001887	31	0,000812774	2	972,55980	0,31	15,74	0
	High	gLQAHVDkE	4	1	1	P23350	N-Term(iTRAQ4plex); K8(iTRAQ4plex)	0,0000	1,687	2	39,8	0	0,0004666	24	0,00400839	3	1284,71436	-0,54	14,81	1
	High	lRPLGGTck	3	1	1	P23350	N-Term(iTRAQ4plex); C8(Carbamidomethyl); K9(iTRAQ4plex)	0,0000	1,690	3	20,9	0	0,0004799	23	0,00594169	3	1289,76075	0,39	16,89	0
	High	iALGIk	3	1	1	P23350	N-Term(iTRAQ4plex); K6(iTRAQ4plex)	0,0000	2,351	2	11,4	0,005	0,06594	27	0,001819575	2	902,62737	-0,41	28,30	0
P74250	Hydroperoxy fatty acid reductase gpx1 OS=Synechocystis sp. (strain PCC 6803 / Kazusa) GN=gpx1 P	112,61	16,57	1	3	3	6	0,208	5	10,0	169	18,4	7,09							
	A3	Sequence	# PSMs	# Proteins	# Protein Groups	Protein Group Accessions	Modifications	ΔCn	116/117	116/117 Count	116/117 Variability [%]	q-Value	PEP	IonScore	Exp Value	Charge	MH+ [Da]	ΔM [ppm]	RT [min]	# Missed Cleavages
	High	yGVTFPLFEk	3	1	1	P74250	N-Term(iTRAQ4plex); K10(iTRAQ4plex)	0,0000	0,200	3	4,6	0	1,054E-07	45	3,61335E-05	2	1488,83403	-0,05	45,89	0
	High	vEVNGPNAHPLFk	2	1	1	P74250	N-Term(iTRAQ4plex); K13(iTRAQ4plex)	0,0000	0,223	1		0	0,000007954	18	0,014722108	3	1709,95841	0,37	28,31	0
	High	wNFTk	1	1	1	P74250	N-Term(iTRAQ4plex); K5(iTRAQ4plex)	0,0000	0,208	1		0,001	0,01587	28	0,001541594	2	983,55315	-2,17	27,02	0
P73833	Cyanophycin synthetase OS=Synechocystis sp. (strain PCC 6803 / Kazusa) GN=cphA PE=3 SV=1 - [C	112,37	7,56	1	6	6	7	1,787	6	29,2	873	94,6	5,58							
	A3	Sequence	# PSMs	# Proteins	# Protein Groups	Protein Group Accessions	Modifications	ΔCn	116/117	116/117 Count	116/117 Variability [%]	q-Value	PEP	IonScore	Exp Value	Charge	MH+ [Da]	ΔM [ppm]	RT [min]	# Missed Cleavages
	High	amVQLGYGVHQQR	2	1	1	P73833	N-Term(iTRAQ4plex); M2(Oxidation)	0,0000	1,744	1		0	1,102E-07	57	1,82377E-06	3	1646,85733	1,19	20,56	0
	High	kIPWmLLSAR	1	1	1	P73833	N-Term(iTRAQ4plex); K1(iTRAQ4plex); M5(Oxidation)	0,0000	3,270	1		0	0,00002251	38	0,000157467	3	1518,90607	-0,56	37,61	1
	High	tNQDPNRGDGHANILTk	1	1	1	P73833	N-Term(iTRAQ4plex); K17(iTRAQ4plex)	0,0000	1,540	1		0	0,00005017	21	0,007310886	4	2139,11464	0,04	16,16	1
	High	mHVAPSQGLPR	1	1	1	P73833	N-Term(iTRAQ4plex); M1(Oxidation)	0,0000	1,831	1		0,001	0,004848	26	0,002415294	3	1352,72312	0,40	15,32	0
	High	mNLFNLGR	1	1	1	P73833	N-Term(iTRAQ4plex); M1(Oxidation)	0,0000	1,408	1		0,008	0,1133	11	0,082979345	2	1124,60076	0,38	32,19	0
	Medium	lLAHIYR	1	1	1	P73833	N-Term(iTRAQ4plex)	0,0000	4,098	1		0,011	0,1349	21	0,007193993	3	1029,63297	0,37	26,28	0
Q6ZEP2	Slr5088 protein OS=Synechocystis sp. (strain PCC 6803 / Kazusa) GN=slr5088 PE=4 SV=1 - [Q6ZEP2	111,41	16,81	1	3	3	4	0,324	3	14,1	238	26,2	5,11							
	A3	Sequence	# PSMs	# Proteins	# Protein Groups	Protein Group Accessions	Modifications	ΔCn	116/117	116/117 Count	116/117 Variability [%]	q-Value	PEP	IonScore	Exp Value	Charge	MH+ [Da]	ΔM [ppm]	RT [min]	# Missed Cleavages
	High	qSGRPEVTFGASk	2	1	1	Q6ZEP2	N-Term(iTRAQ4plex); K13(iTRAQ4plex)	0,0000	0,324	1		0	3,39E-08	54	4,4401E-06	3	1651,90555	2,97	19,28	0
	High	gNGELIPLHDVINLVk	1	1	1	Q6ZEP2	N-Term(iTRAQ4plex); K16(iTRAQ4plex)	0,0000	0,294	1		0	0,000001944	42	6,53085E-05	3	2019,19205	3,91	47,59	0
	High	lILTGSTSGLR	1	1	1	Q6ZEP2	N-Term(iTRAQ4plex)	0,0000	0,443	1		0,007	0,08512	42	6,74059E-05	2	1261,76262	2,34	32,20	0
P72991	ATP-dependent zinc metalloprotease FtsH 3 OS=Synechocystis sp. (strain PCC 6803 / Kazusa) GN=fts	111,07	7,47	1	2	5	7	1,616	3	4,0	616	67,2	5,24							
	A3	Sequence	# PSMs	# Proteins	# Protein Groups	Protein Group Accessions	Modifications	ΔCn	116/117	116/117 Count	116/117 Variability [%]	q-Value	PEP	IonScore	Exp Value	Charge	MH+ [Da]	ΔM [ppm]	RT [min]	# Missed Cleavages
	High	gVLLVGPPGTGk	1	3	3	Q55700;P72991;P73437	N-Term(iTRAQ4plex); K12(iTRAQ4plex)	0,0000				0	0,001499	31	0,000749842	3	1382,85981	-0,88	31,27	0
	High	qVVVDRPDYAGR	2	1	1	P72991	N-Term(iTRAQ4plex)	0,0000	1,328	2	29,7	0	0,0007251	29	0,001238711	3	1518,81580	0,81	19,54	0
	High	vRDLFEQAk	2	2	2	P72991;P73437	N-Term(iTRAQ4plex); K9(iTRAQ4plex)	0,0000				0	0,0002062	23	0,004581102	3	1393,80448	0,19	26,88	1
	High	eILNVHAR	1	1	1	P72991	N-Term(iTRAQ4plex)	0,0000	1,660	1		0,001	0,006564	38	0,00017457	3	1095,64569	5,98	17,89	0
	Medium	tLLAk	1	3	3	Q55700;P72991;P73437	N-Term(iTRAQ4plex); K5(iTRAQ4plex)	0,0000				0,029	0,3849	29	0,001121941	2	833,56822	-1,98	18,81	0
Q55621	Amidophosphoribosyltransferase OS=Synechocystis sp. (strain PCC 6803 / Kazusa) GN=purF PE=3 S\	110,99	12,12	1	4	4	5	3,584	4	31,8	495	53,6	5,76							
	A3	Sequence	# PSMs	# Proteins	# Protein Groups	Protein Group Accessions	Modifications	ΔCn	116/117	116/117 Count	116/117 Variability [%]	q-Value	PEP	IonScore	Exp Value	Charge	MH+ [Da]	ΔM [ppm]	RT [min]	# Missed Cleavages
	High	lTYFGLYALQHR	1	1	1	Q55621	N-Term(iTRAQ4plex)	0,0000	4,713	1		0	1,151E-09	51	7,11164E-06	3	1625,89267	0,38	42,06	0
	High	tVEAGELVHITESGLVSHR	2	1	1	Q55621	N-Term(iTRAQ4plex)	0,0000	3,101	1		0	7,411E-09	49	1,48833E-05	4	2178,16372	0,06	37,99	0
	High	dPHGIRPLVIGVLEEETPR	1	1	1	Q55621	N-Term(iTRAQ4plex)	0,0000	4,142	1		0	0,000007093	13	0,050721174	4	2271,26137	1,55	42,08	0
	High	tFIQPTQHmR	1	1	1	Q55621	N-Term(iTRAQ4plex); M9(Oxidation)	0,0000	0,517	1		0,001	0,004347	33	0,000476398	3	1418,73539	1,59	16,17	0
P73746	Sll0854 protein OS=Synechocystis sp. (strain PCC 6803 / Kazusa) GN=sll0854 PE=4 SV=1 - [P73746	110,62	14,94	1	4	4	5	0,276	4	6,4	308	34,5	5,12							
	A3	Sequence	# PSMs	# Proteins	# Protein Groups	Protein Group Accessions	Modifications	ΔCn	116/117	116/117 Count	116/117 Variability [%]	q-Value	PEP	IonScore	Exp Value	Charge	MH+ [Da]	ΔM [ppm]	RT [min]	# Missed Cleavages
	High	lLHSIIGGVAVSSTR	1	1	1	P73746	N-Term(iTRAQ4plex)	0,0000	0,189	1		0	8,215E-11	68	1,53451E-07	3	1653,97721	0,20	37,49	0
	High	iYPASVVk	1	1	1	P73746	N-Term(iTRAQ4plex); K8(iTRAQ4plex)	0,0000				0	0,000889	29	0,001129718	2	1164,72539	1,98	26,42	0
	High	nmLTTNAVAR	2	1	1	P73746	N-Term(iTRAQ4plex); M2(Oxidation)	0,0000	0,288	2	3,9	0	0,0001224	29	0,001185687	2	1250,66533	0,75	17,10	0
	High	lLPFISQAFANAH	1	1	1	P73746	N-Term(iTRAQ4plex)	0,0000	0,271	1		0,001	0,01308	18	0,016404765	3	1572,86606	0,34	54,59	0
P74466	Putative ATP-dependent Clp protease proteolytic subunit-like OS=Synechocystis sp. (strain PCC 6803	110,21	10,67	1	2	2	3	1,024	3	3,5	225	24,9	5,00							
	A3	Sequence	# PSMs	# Proteins	# Protein Groups	Protein Group Accessions	Modifications	ΔCn	116/117	116/117 Count	116/117 Variability [%]	q-Value	PEP	IonScore	Exp Value	Charge	MH+ [Da]	ΔM [ppm]	RT [min]	# Missed Cleavages
	High	tFYLTPAQAk	2	1	1	P74466	N-Term(iTRAQ4plex); K10(iTRAQ4plex)	0,0000	1,077	2	7,5	0	0,000005583	54	4,385E-06	2	1427,81462	0,65	29,45	0
	High	aSLPHATIVLNQNR	1	1	1	P74466	N-Term(iTRAQ4plex)	0,0000	1,000	1		0	4,885E-09	47	1,97683E-05	3	1677,95267	0,56	28,40	0
P73274	Leucine--tRNA ligase OS=Synechocystis sp. (strain PCC 6803 / Kazusa) GN=leuS PE=3 SV=1 - [SYL_	110,04	4,03	1	4	4	6	3,712	6	47,2	869	98,2	5,29							
	A3	Sequence	# PSMs	# Proteins	# Protein Groups	Protein Group Accessions	Modifications	ΔCn	116/117	116/117 Count	116/117 Variability [%]	q-Value	PEP	IonScore	Exp Value	Charge	MH+ [Da]	ΔM [ppm]	RT [min]	# Missed Cleavages
	High	lRDWLISR	1	1	1	P73274	N-Term(iTRAQ4plex)	0,0000	3,307	1		0	0,00005931	40	0,000117001	3	1202,71140	-1,04	33,89	1
	High	fAVRPETLVTNEPLTk	2	1	1	P73274	N-Term(iTRAQ4plex); K16(iTRAQ4plex)	0,0000	2,488	2	66,6	0	2,867E-09	36	0,000262404	3	2103,20981	2,15	35,92	0
	High	mFILFk	2	1	1	P73274	N-Term(iTRAQ4plex); K6(iTRAQ4plex)	0,0000	6,663	2	14,3	0,001	0,009451	13	0,049770271	2	1086,66203	-0,35	45,35	0
	High	qWFLk	1	1	1	P73274	N-Term(iTRAQ4plex); K5(iTRAQ4plex)	0,0000	3,681	1		0,007	0,0854	22	0,006822916	2	1009,60533	-1,98	32,59	0
P54899	N-acetyl-gamma-glutamyl-phosphate reductase OS=Synechocystis sp. (strain PCC 6803 / Kazusa) GN	110,01	9,69	1	3	3	5	1,702	5	15,5	351	38,2	6,71							
	A3	Sequence	# PSMs	# Proteins	# Protein Groups	Protein Group Accessions	Modifications	ΔCn	116/117	116/117 Count	116/117 Variability [%]	q-Value	PEP	IonScore	Exp Value	Charge	MH+ [Da]	ΔM [ppm]	RT [min]	# Missed Cleavages
	High	gILATVYATLR	2	1	1	P54899	N-Term(iTRAQ4plex)	0,0000	1,616	2	7,7	0	0,000002525	47	2,04644E-05	2	1321,79643	0,30	50,74	0
	High	vIVLSAIDNLVk	2	1	1	P54899	N-Term(iTRAQ4plex); K12(iTRAQ4plex)	0,0000	2,009	2	26,0	0	0,00005083	33	0,000480806	3	1571,99741	-0,06	51,07	0
	High	iLPHGVYPQTk	1	1	1	P54899	N-Term(iTRAQ4plex); K11(iTRAQ4plex)	0,0000	2,247	1		0	0,0003461	19	0,011297179	3	1540,90775	-0,81	26,02	0
P74122	Arginine biosynthesis bifunctional protein ArgJ OS=Synechocystis sp. (strain PCC 6803 / Kazusa) GN=	109,98	8,96	1	3	3	4	0,987	4	33,9	413	43,3	5,47							
	A3	Sequence	# PSMs	# Proteins	# Protein Groups	Protein Group Accessions	Modifications	ΔCn	116/117	116/117 Count	116/117 Variability [%]	q-Value	PEP	IonScore	Exp Value	Charge	MH+ [Da]	ΔM [ppm]	RT [min]	# Missed Cleavages
	High	tIAGSSLVk	1	1	1	P74122	N-Term(iTRAQ4plex); K9(iTRAQ4plex)	0,0000	0,795	1		0	0,000004938	48	1,61797E-05	2	1163,72405	0,19	22,88	0
	High	lEAmLTAVcQHLAk	1	1	1	P74122	N-Term(iTRAQ4plex); M4(Oxidation); C9(Carbamidomethyl); K	0,0000	0,785	1		0	0,000008576	36	0,000239436	3	1889,02164	-0,60	37,48	0
	High	lEAMLTAVcQHLAk	1	1	1	P74122	N-Term(iTRAQ4plex); C9(Carbamidomethyl); K14(iTRAQ4plex)	0,0000	1,225	1		0	0,000002721	36	0,000255252	3	1873,02829	0,24	48,55	0
	High	tIALETEIDGRPVR	1	1	1	P74122	N-Term(iTRAQ4plex)	0,0000	1,489	1		0	0,0001454	30	0,001343325	3	1713,96054	-0,64	30,29	0
P72647	Sll1053 protein OS=Synechocystis sp. (strain PCC 6803 / Kazusa) GN=sll1053 PE=4 SV=1 - [P72647	109,63	7,31	1	3	3	4	0,189	4	22,5	520	56,2	4,97							
	A3	Sequence	# PSMs	# Proteins	# Protein Groups	Protein Group Accessions	Modifications	ΔCn	116/117	116/117 Count	116/117 Variability [%]	q-Value	PEP	IonScore	Exp Value	Charge	MH+ [Da]	ΔM [ppm]	RT [min]	# Missed Cleavages
	High	vGQALASVk	1	1	1	P72647	N-Term(iTRAQ4plex); K9(iTRAQ4plex)	0,0000	0,284	1		0	0,000002912	46	2,39332E-05	2	1160,72270	-1,26	25,10	0
	High	lRPGLVVEILDGQEk	2	1	1	P72647	N-Term(iTRAQ4plex); K15(iTRAQ4plex)	0,0000	0,162	2	11,1	0	2,192E-10	40	0,000101852	3	1954,15866	0,54	44,96	0
	High	lQAGERPETIAQAR	1	1	1	P72647	N-Term(iTRAQ4plex)	0,0000	0,203	1		0	0,00006751	29	0,00118842	3	1683,92594	0,01	20,87	0
P74576	Biosynthetic arginine decarboxylase 1 OS=Synechocystis sp. (strain PCC 6803 / Kazusa) GN=speA1 P	109,35	4,03	1	2	2	5	2,369	5	2,5	695	78,2	5,22							
	A3	Sequence	# PSMs	# Proteins	# Protein Groups	Protein Group Accessions	Modifications	ΔCn	116/117	116/117 Count	116/117 Variability [%]	q-Value	PEP	IonScore	Exp Value	Charge	MH+ [Da]	ΔM [ppm]	RT [min]	# Missed Cleavages
	High	nIGLPLLLR	4	1	1	P74576	N-Term(iTRAQ4plex)	0,0000	2,375	4	1,7	0	0,00001521	43	4,69862E-05	2	1152,75847	-0,06	45,84	0
	High	sVLELHPLIEVHQPGTPPR	1	1	1	P74576	N-Term(iTRAQ4plex)	0,0000	1,929	1		0	0,00001266	22	0,006251295	4	2263,26723	-0,34	36,48	0
P73720	Putative OxPP cycle protein OpcA OS=Synechocystis sp. (strain PCC 6803 / Kazusa) GN=opcA PE=4 S	108,63	6,95	1	3	3	3	0,740	2	60,6	475	52,0	5,11							
	A3	Sequence	# PSMs	# Proteins	# Protein Groups	Protein Group Accessions	Modifications	ΔCn	116/117	116/117 Count	116/117 Variability [%]	q-Value	PEP	IonScore	Exp Value	Charge	MH+ [Da]	ΔM [ppm]	RT [min]	# Missed Cleavages
	High	gNHAQALmYLGWVASR	1	1	1	P73720	N-Term(iTRAQ4plex); M8(Oxidation)	0,0000				0	2,637E-09	61	7,29407E-07	3	1933,98502	1,36	39,57	0
	High	qALQTAFETAHR	1	1	1	P73720	N-Term(iTRAQ4plex)	0,0000	0,507	1		0	1,064E-07	49	1,25884E-05	3	1516,79853	-0,25	25,21	0
	High	yVWWk	1	1	1	P73720	N-Term(iTRAQ4plex); K5(iTRAQ4plex)	0,0000	1,079	1		0,001	0,003135	24	0,00372366	2	1069,60735	0,03	36,99	0
P74737	Protein RecA OS=Synechocystis sp. (strain PCC 6803 / Kazusa) GN=recA PE=3 SV=1 - [RECA_SYNY3	108,35	7,91	1	3	3	5	1,500	5	81,5	354	37,8	5,22							
	A3	Sequence	# PSMs	# Proteins	# Protein Groups	Protein Group Accessions	Modifications	ΔCn	116/117	116/117 Count	116/117 Variability [%]	q-Value	PEP	IonScore	Exp Value	Charge	MH+ [Da]	ΔM [ppm]	RT [min]	# Missed Cleavages
	High	sGcLVIFLNQLR	3	1	1	P74737	N-Term(iTRAQ4plex); C3(Carbamidomethyl)	0,0000	2,545	3	91,9	0	0,000002134	61	7,36156E-07	2	1563,88103	0,78	50,08	0
	High	kGTEGEYGIR	1	1	1	P74737	N-Term(iTRAQ4plex); K1(iTRAQ4plex)	0,0000	0,927	1		0	0,0002725	24	0,003935229	3	1397,76130	-1,03	14,51	1
	Medium	fYASVR	1	1	1	P74737	N-Term(iTRAQ4plex)	0,0000	1,253	1		0,019	0,2555	23	0,005469782	2	886,48943	-1,02	21,00	0
Q55393	Diflavin flavoprotein A 1 OS=Synechocystis sp. (strain PCC 6803 / Kazusa) GN=dfa1 PE=1 SV=1 - [D	108,25	5,58	1	3	3	5	2,069	5	6,4	573	63,5	5,90							
	A3	Sequence	# PSMs	# Proteins	# Protein Groups	Protein Group Accessions	Modifications	ΔCn	116/117	116/117 Count	116/117 Variability [%]	q-Value	PEP	IonScore	Exp Value	Charge	MH+ [Da]	ΔM [ppm]	RT [min]	# Missed Cleavages
	High	hNVGELLHR	2	1	1	Q55393	N-Term(iTRAQ4plex)	0,0000	1,471	2	54,5	0	0,000002844	43	5,1996E-05	3	1218,68268	0,23	17,46	0
	High	fLDLGLR	2	1	1	Q55393	N-Term(iTRAQ4plex)	0,0000	1,679	2	38,6	0	0,0005826	35	0,000519126	2	977,59044	0,37	40,30	0
	High	vALQFLDNFVHQPFER	1	1	1	Q55393	N-Term(iTRAQ4plex)	0,0000	2,069	1		0	1,566E-07	32	0,000874802	3	2104,11307	1,60	54,25	0
P73173	PilJ protein OS=Synechocystis sp. (strain PCC 6803 / Kazusa) GN=pilJ PE=4 SV=1 - [P73173_SYNY3	106,68	5,98	1	5	5	5	0,842	5	32,0	953	103,1	4,61							
	A3	Sequence	# PSMs	# Proteins	# Protein Groups	Protein Group Accessions	Modifications	ΔCn	116/117	116/117 Count	116/117 Variability [%]	q-Value	PEP	IonScore	Exp Value	Charge	MH+ [Da]	ΔM [ppm]	RT [min]	# Missed Cleavages
	High	dIQGQPLALLVR	1	1	1	P73173	N-Term(iTRAQ4plex)	0,0000	1,039	1		0	7,22E-08	48	1,50998E-05	2	1466,88176	0,39	42,31	0
	High	sQLVSQSLQSLAk	1	1	1	P73173	N-Term(iTRAQ4plex); K13(iTRAQ4plex)	0,0000	0,842	1		0	0,0001404	41	8,55008E-05	3	1676,97763	-0,54	35,59	0
	High	qPIPAPLAAQVR	1	1	1	P73173	N-Term(iTRAQ4plex)	0,0000	3,528	1		0	0,00005582	35	0,000305471	3	1404,84455	0,09	32,12	0
	High	rLAESSLEISk	1	1	1	P73173	N-Term(iTRAQ4plex); K11(iTRAQ4plex)	0,0000	0,706	1		0,002	0,0241	13	0,057331418	3	1520,88544	-2,13	23,77	1
	High	iIASANAQR	1	1	1	P73173	N-Term(iTRAQ4plex)	0,0000	0,630	1		0,004	0,05381	22	0,00687368	2	1087,63481	0,69	16,69	0
Q55233	Protein DrgA OS=Synechocystis sp. (strain PCC 6803 / Kazusa) GN=drgA PE=1 SV=1 - [DRGA_SYNY	105,06	18,57	1	4	4	7	0,876	7	40,4	210	23,7	9,19							
	A3	Sequence	# PSMs	# Proteins	# Protein Groups	Protein Group Accessions	Modifications	ΔCn	116/117	116/117 Count	116/117 Variability [%]	q-Value	PEP	IonScore	Exp Value	Charge	MH+ [Da]	ΔM [ppm]	RT [min]	# Missed Cleavages
	High	lPADYAIGPmVAIGk	1	1	1	Q55233	N-Term(iTRAQ4plex); M10(Oxidation); K15(iTRAQ4plex)	0,0000	0,570	1		0	7,948E-09	45	2,95121E-05	3	1820,02585	1,52	39,24	0
	High	sIGMAMQNLMLAAk	2	1	1	Q55233	N-Term(iTRAQ4plex); K14(iTRAQ4plex)	0,0000	0,907	2	46,4	0	0,00001067	34	0,000377546	3	1766,96006	1,72	48,07	0
	High	sIGmAMQNLMLAAk	1	1	1	Q55233	N-Term(iTRAQ4plex); M4(Oxidation); K14(iTRAQ4plex)	0,0000	1,095	1		0	0,00007238	26	0,002365756	3	1782,95725	2,98	46,44	0
	High	fLIIRDPQLR	2	1	1	Q55233	N-Term(iTRAQ4plex)	0,0000	0,922	2	7,6	0	0,0002196	16	0,02415294	3	1414,86591	0,54	35,76	1
	Medium	fLIIR	1	1	1	Q55233	N-Term(iTRAQ4plex)	0,0000	0,504	1		0,026	0,3542	25	0,007644758	2	805,54247	1,01	34,30	0
P74211	Pyridoxine/pyridoxamine 5'-phosphate oxidase OS=Synechocystis sp. (strain PCC 6803 / Kazusa) GN	104,18	18,22	1	3	3	4	1,110	4	24,9	214	24,6	6,34							
	A3	Sequence	# PSMs	# Proteins	# Protein Groups	Protein Group Accessions	Modifications	ΔCn	116/117	116/117 Count	116/117 Variability [%]	q-Value	PEP	IonScore	Exp Value	Charge	MH+ [Da]	ΔM [ppm]	RT [min]	# Missed Cleavages
	High	lQFNLLDGQWHR	2	1	1	P74211	N-Term(iTRAQ4plex)	0,0000	1,309	2	4,9	0	1,827E-08	55	3,14029E-06	3	1670,88883	0,26	43,48	0
	High	qYENQSIPRPPHWGGFR	1	1	1	P74211	N-Term(iTRAQ4plex)	0,0000	0,789	1		0	0,000001095	26	0,00273508	4	2213,11245	0,13	31,52	0
	High	iEFWQGRPSR	1	1	1	P74211	N-Term(iTRAQ4plex)	0,0000	0,973	1		0,001	0,01439	20	0,008994355	3	1419,76279	0,98	29,46	0
Q55466	Sll0518 protein OS=Synechocystis sp. (strain PCC 6803 / Kazusa) GN=sll0518 PE=4 SV=1 - [Q55466	104,09	6,49	1	3	3	4	2,487	4	35,6	385	43,1	8,38							
	A3	Sequence	# PSMs	# Proteins	# Protein Groups	Protein Group Accessions	Modifications	ΔCn	116/117	116/117 Count	116/117 Variability [%]	q-Value	PEP	IonScore	Exp Value	Charge	MH+ [Da]	ΔM [ppm]	RT [min]	# Missed Cleavages
	High	fYQILAR	1	1	1	Q55466	N-Term(iTRAQ4plex)	0,0000	3,670	1		0	0,0003335	42	6,20826E-05	2	1054,61675	0,14	35,36	0
	High	rPVFSVYR	2	1	1	Q55466	N-Term(iTRAQ4plex)	0,0000	2,487	2	11,4	0	0,000001257	41	8,4522E-05	3	1167,67517	-0,30	23,60	0
	High	nYYQAIGWLR	1	1	1	Q55466	N-Term(iTRAQ4plex)	0,0000	1,055	1		0,002	0,01878	22	0,00734003	2	1427,75176	-2,43	42,76	0
P74175	HlyD family of secretion proteins OS=Synechocystis sp. (strain PCC 6803 / Kazusa) GN=hlyD PE=4 S\	103,75	2,92	1	2	2	4	1,619	3	9,9	583	64,7	5,45							
	A3	Sequence	# PSMs	# Proteins	# Protein Groups	Protein Group Accessions	Modifications	ΔCn	116/117	116/117 Count	116/117 Variability [%]	q-Value	PEP	IonScore	Exp Value	Charge	MH+ [Da]	ΔM [ppm]	RT [min]	# Missed Cleavages
	High	iLAGLLGR	3	1	1	P74175	N-Term(iTRAQ4plex)	0,0000	1,674	2	4,9	0	0,0003365	53	5,34527E-06	2	956,63725	-0,11	41,87	0
	High	kIESLETVR	1	1	1	P74175	N-Term(iTRAQ4plex); K1(iTRAQ4plex)	0,0000	0,962	1		0	0,00003186	34	0,000372366	3	1362,81974	0,15	21,10	1
Q55759	GTP cyclohydrolase 1 OS=Synechocystis sp. (strain PCC 6803 / Kazusa) GN=folE PE=3 SV=1 - [GCH1	103,17	13,68	1	3	3	6	1,432	5	9,4	234	26,6	6,62							
	A3	Sequence	# PSMs	# Proteins	# Protein Groups	Protein Group Accessions	Modifications	ΔCn	116/117	116/117 Count	116/117 Variability [%]	q-Value	PEP	IonScore	Exp Value	Charge	MH+ [Da]	ΔM [ppm]	RT [min]	# Missed Cleavages
	High	tREEFLNLIR	4	1	1	Q55759	N-Term(iTRAQ4plex)	0,0000	1,432	3	9,4	0	5,039E-07	52	6,4121E-06	3	1434,81809	-0,35	35,88	1
	High	yHPENVELNkEk	1	1	1	Q55759	N-Term(iTRAQ4plex); K10(iTRAQ4plex); K12(iTRAQ4plex)	0,0000	1,515	1		0	0,00001666	19	0,016206821	4	1932,05556	0,23	20,01	1
	High	aHLAYIPNQk	1	1	1	Q55759	N-Term(iTRAQ4plex); K10(iTRAQ4plex)	0,0000	0,673	1		0	0,003044	12	0,0608093	3	1442,83600	0,12	21,43	0
P74228	Elongation factor G 2 OS=Synechocystis sp. (strain PCC 6803 / Kazusa) GN=fusB PE=3 SV=1 - [EFG2	103,04	6,80	1	5	5	6	2,491	6	55,9	691	75,4	5,05							
	A3	Sequence	# PSMs	# Proteins	# Protein Groups	Protein Group Accessions	Modifications	ΔCn	116/117	116/117 Count	116/117 Variability [%]	q-Value	PEP	IonScore	Exp Value	Charge	MH+ [Da]	ΔM [ppm]	RT [min]	# Missed Cleavages
	High	gSYVLNSTk	1	1	1	P74228	N-Term(iTRAQ4plex); K9(iTRAQ4plex)	0,0000	2,442	1		0	0,000001681	54	3,91715E-06	2	1256,70793	-0,76	18,94	0
	High	kAVQAEGk	1	1	1	P74228	N-Term(iTRAQ4plex); K1(iTRAQ4plex); K8(iTRAQ4plex)	0,0000	3,384	1		0	0,0007149	35	0,000339602	3	1262,77393	-4,24	8,69	1
	High	iLFYSGVVHk	2	1	1	P74228	N-Term(iTRAQ4plex); K10(iTRAQ4plex)	0,0000	3,068	2	28,5	0	0,000001516	33	0,000503466	3	1450,86563	-0,30	34,55	0
	High	rGHIEGQETEQGIAk	1	1	1	P74228	N-Term(iTRAQ4plex); K15(iTRAQ4plex)	0,0000	0,511	1		0,001	0,01248	11	0,079983426	4	1941,04152	1,16	13,33	1
	Medium	lTFVR	1	1	1	P74228	N-Term(iTRAQ4plex)	0,0000	1,309	1		0,025	0,3448	13	0,057834808	2	779,48998	0,47	26,44	0
Q55843	Phosphoribosylformylglycinamidine synthase 1 OS=Synechocystis sp. (strain PCC 6803 / Kazusa) GN	102,62	15,18	1	2	2	5	1,516	4	42,2	224	24,4	5,97							
	A3	Sequence	# PSMs	# Proteins	# Protein Groups	Protein Group Accessions	Modifications	ΔCn	116/117	116/117 Count	116/117 Variability [%]	q-Value	PEP	IonScore	Exp Value	Charge	MH+ [Da]	ΔM [ppm]	RT [min]	# Missed Cleavages
	High	gYQSQQVTTLPIAHGEGR	2	1	1	Q55843	N-Term(iTRAQ4plex)	0,0000	0,898	1		0	2,542E-09	54	4,45625E-06	3	2098,11692	0,33	32,50	0
	High	fSPIMTAIIDHANAGk	3	1	1	Q55843	N-Term(iTRAQ4plex); K16(iTRAQ4plex)	0,0000	1,551	3	6,7	0	1,472E-08	42	7,4676E-05	3	1974,07468	1,27	45,65	0
P74506	Slr1944 protein OS=Synechocystis sp. (strain PCC 6803 / Kazusa) GN=slr1944 PE=4 SV=1 - [P74506	102,60	5,20	1	3	3	5	1,865	4	8,7	538	58,6	4,77							
	A3	Sequence	# PSMs	# Proteins	# Protein Groups	Protein Group Accessions	Modifications	ΔCn	116/117	116/117 Count	116/117 Variability [%]	q-Value	PEP	IonScore	Exp Value	Charge	MH+ [Da]	ΔM [ppm]	RT [min]	# Missed Cleavages
	High	iFTWLGk	3	1	1	P74506	N-Term(iTRAQ4plex); K7(iTRAQ4plex)	0,0000	1,977	2	3,2	0	0,0002602	44	3,66412E-05	2	1152,70268	0,62	44,00	0
	High	fYTQHLkPEELEk	1	1	1	P74506	N-Term(iTRAQ4plex); K7(iTRAQ4plex); K13(iTRAQ4plex)	0,0000	1,797	1		0	6,377E-08	27	0,002066281	4	2094,16054	0,46	28,42	0
	High	gLLNHSFk	1	1	1	P74506	N-Term(iTRAQ4plex); K8(iTRAQ4plex)	0,0000	1,559	1		0	0,0002044	21	0,010431188	3	1203,70905	0,18	23,89	0
Q55698	Slr0226 protein OS=Synechocystis sp. (strain PCC 6803 / Kazusa) GN=slr0226 PE=4 SV=1 - [Q55698	102,56	2,90	1	2	2	6	0,450	5	0,7	482	50,8	4,98							
	A3	Sequence	# PSMs	# Proteins	# Protein Groups	Protein Group Accessions	Modifications	ΔCn	116/117	116/117 Count	116/117 Variability [%]	q-Value	PEP	IonScore	Exp Value	Charge	MH+ [Da]	ΔM [ppm]	RT [min]	# Missed Cleavages
	High	lIIFSR	5	1	1	Q55698	N-Term(iTRAQ4plex)	0,0000	0,449	4	0,3	0	0,00247	38	0,000164323	2	892,57518	1,68	38,91	0
	High	fLVLMHVk	1	1	1	Q55698	N-Term(iTRAQ4plex); K8(iTRAQ4plex)	0,0000	2,970	1		0	0,0007459	30	0,00090567	3	1274,79068	0,73	38,02	0
P74588	Slr1557 protein OS=Synechocystis sp. (strain PCC 6803 / Kazusa) GN=slr1557 PE=4 SV=1 - [P74588	102,41	7,59	1	3	3	4	0,942	4	23,7	369	40,2	4,78							
	A3	Sequence	# PSMs	# Proteins	# Protein Groups	Protein Group Accessions	Modifications	ΔCn	116/117	116/117 Count	116/117 Variability [%]	q-Value	PEP	IonScore	Exp Value	Charge	MH+ [Da]	ΔM [ppm]	RT [min]	# Missed Cleavages
	High	fGVSISTVSR	2	1	1	P74588	N-Term(iTRAQ4plex)	0,0000	1,023	2	23,7	0	0,000001316	61	8,72911E-07	2	1196,67534	-0,21	31,98	0
	High	rPQIVSIk	1	1	1	P74588	N-Term(iTRAQ4plex); K8(iTRAQ4plex)	0,0000	1,015	1		0	0,002852	27	0,001849141	3	1228,79651	-1,21	20,93	0
	High	rGELIVRPLk	1	1	1	P74588	N-Term(iTRAQ4plex); K10(iTRAQ4plex)	0,0000	0,511	1		0	0,00004738	18	0,016105344	4	1468,95656	-0,06	21,50	1
P74453	Sll0148 protein OS=Synechocystis sp. (strain PCC 6803 / Kazusa) GN=sll0148 PE=4 SV=1 - [P74453	102,40	3,67	1	2	2	7	2,611	5	17,8	735	78,5	5,91							
	A3	Sequence	# PSMs	# Proteins	# Protein Groups	Protein Group Accessions	Modifications	ΔCn	116/117	116/117 Count	116/117 Variability [%]	q-Value	PEP	IonScore	Exp Value	Charge	MH+ [Da]	ΔM [ppm]	RT [min]	# Missed Cleavages
	High	gLSVAAILRPDLk	4	1	1	P74453	N-Term(iTRAQ4plex); K13(iTRAQ4plex)	0,0000	2,771	2	8,8	0	5,936E-11	46	2,23857E-05	3	1641,03055	0,20	41,25	0
	High	nLALTVALAHDLER	3	1	1	P74453	N-Term(iTRAQ4plex)	0,0000	2,432	3	83,7	0	0,00000361	30	0,000935341	3	1679,95584	-0,18	43,34	0
Q55673	UDP-N-acetylglucosamine 1-carboxyvinyltransferase OS=Synechocystis sp. (strain PCC 6803 / Kazusa	102,04	4,79	1	2	2	6	0,158	6	69,5	438	46,6	5,97							
	A3	Sequence	# PSMs	# Proteins	# Protein Groups	Protein Group Accessions	Modifications	ΔCn	116/117	116/117 Count	116/117 Variability [%]	q-Value	PEP	IonScore	Exp Value	Charge	MH+ [Da]	ΔM [ppm]	RT [min]	# Missed Cleavages
	High	aSFFVIGPLLSR	5	1	1	Q55673	N-Term(iTRAQ4plex)	0,0000	0,133	5	51,2	0	0,00001991	45	3,25065E-05	2	1450,85515	0,85	52,33	0
	High	lQHVAELQR	1	1	1	Q55673	N-Term(iTRAQ4plex)	0,0000	1,124	1		0,004	0,05224	14	0,03872309	3	1237,71357	0,15	19,65	0
Q55404	Acetyl-coenzyme A synthetase OS=Synechocystis sp. (strain PCC 6803 / Kazusa) GN=acsA PE=3 SV=	101,56	5,21	1	3	3	4	3,279	4	131,9	653	73,0	5,76							
	A3	Sequence	# PSMs	# Proteins	# Protein Groups	Protein Group Accessions	Modifications	ΔCn	116/117	116/117 Count	116/117 Variability [%]	q-Value	PEP	IonScore	Exp Value	Charge	MH+ [Da]	ΔM [ppm]	RT [min]	# Missed Cleavages
	High	iGAPHSVVFGGFSAEALR	1	1	1	Q55404	N-Term(iTRAQ4plex)	0,0000	1,685	1		0	2,046E-09	57	2,02754E-06	3	1959,05857	0,82	40,66	0
	High	wIFDLk	2	1	1	Q55404	N-Term(iTRAQ4plex); K6(iTRAQ4plex)	0,0000	6,455	2	1,7	0,001	0,004164	37	0,00018534	2	1109,65825	-1,37	44,75	0
	High	hTYWEHIQPk	1	1	1	Q55404	N-Term(iTRAQ4plex); K10(iTRAQ4plex)	0,0000	1,479	1		0,001	0,004187	11	0,083940201	4	1626,86220	-0,55	20,88	0
P72891	Slr1613 protein OS=Synechocystis sp. (strain PCC 6803 / Kazusa) GN=slr1613 PE=4 SV=1 - [P72891	101,50	12,76	1	2	2	4	0,273	4	42,7	196	22,3	4,86							
	A3	Sequence	# PSMs	# Proteins	# Protein Groups	Protein Group Accessions	Modifications	ΔCn	116/117	116/117 Count	116/117 Variability [%]	q-Value	PEP	IonScore	Exp Value	Charge	MH+ [Da]	ΔM [ppm]	RT [min]	# Missed Cleavages
	High	kEPDESYNIHIVk	1	1	1	P72891	N-Term(iTRAQ4plex); K1(iTRAQ4plex); K13(iTRAQ4plex)	0,0000	0,139	1		0	2,832E-11	51	1,0887E-05	4	2004,11587	1,61	24,60	1
	High	sTIGLLLEAYmR	3	1	1	P72891	N-Term(iTRAQ4plex); M11(Oxidation)	0,0000	0,285	3	12,5	0	2,989E-07	46	2,29599E-05	2	1526,84014	2,09	47,66	0
Q55542	Slr0334 protein OS=Synechocystis sp. (strain PCC 6803 / Kazusa) GN=slr0334 PE=4 SV=1 - [Q55542	101,14	13,64	1	3	3	5	4,472	4	32,9	110	11,6	7,85							
	A3	Sequence	# PSMs	# Proteins	# Protein Groups	Protein Group Accessions	Modifications	ΔCn	116/117	116/117 Count	116/117 Variability [%]	q-Value	PEP	IonScore	Exp Value	Charge	MH+ [Da]	ΔM [ppm]	RT [min]	# Missed Cleavages
	High	kPAYYEAk	2	1	1	Q55542	N-Term(iTRAQ4plex); K1(iTRAQ4plex); K8(iTRAQ4plex)	0,0000	2,176	2	236,4	0	0,000002976	41	8,18408E-05	3	1401,81058	0,26	15,80	0
	High	kAQLIVk	2	1	1	Q55542	N-Term(iTRAQ4plex); K1(iTRAQ4plex); K7(iTRAQ4plex)	0,0000	5,610	1		0	0,00004577	27	0,002027543	3	1231,84613	-0,08	17,86	1
	High	aQLIVk	1	1	1	Q55542	N-Term(iTRAQ4plex); K6(iTRAQ4plex)	0,0000	3,640	1		0,005	0,0661	34	0,000358897	2	959,64995	0,78	21,69	0
P74741	Bifunctional purine biosynthesis protein PurH OS=Synechocystis sp. (strain PCC 6803 / Kazusa) GN=|	101,03	8,81	1	3	3	3	1,303	3	142,5	511	54,5	5,36							
	A3	Sequence	# PSMs	# Proteins	# Protein Groups	Protein Group Accessions	Modifications	ΔCn	116/117	116/117 Count	116/117 Variability [%]	q-Value	PEP	IonScore	Exp Value	Charge	MH+ [Da]	ΔM [ppm]	RT [min]	# Missed Cleavages
	High	hTNPcGVALAPTLVEAYQk	1	1	1	P74741	N-Term(iTRAQ4plex); C5(Carbamidomethyl); K19(iTRAQ4plex)	0,0000	1,303	1		0	1,898E-10	48	1,59221E-05	4	2357,25405	0,60	35,25	0
	High	tAAAAGITTIIQPGGSmR	1	1	1	P74741	N-Term(iTRAQ4plex); M17(Oxidation)	0,0000	0,291	1		0	1,897E-07	40	0,00016	3	1876,00614	-0,97	34,52	0
	High	iHGGILAR	1	1	1	P74741	N-Term(iTRAQ4plex)	0,0000	2,651	1		0,001	0,005004	41	9,34099E-05	3	980,61234	0,14	18,38	0
P45450	UDP-N-acetylmuramoyl-tripeptide--D-alanyl-D-alanine ligase OS=Synechocystis sp. (strain PCC 6803	100,18	7,93	1	3	3	4	1,662	3	51,5	454	48,3	5,29							
	A3	Sequence	# PSMs	# Proteins	# Protein Groups	Protein Group Accessions	Modifications	ΔCn	116/117	116/117 Count	116/117 Variability [%]	q-Value	PEP	IonScore	Exp Value	Charge	MH+ [Da]	ΔM [ppm]	RT [min]	# Missed Cleavages
	High	eLIAAVLSQFGNVHk	2	1	1	P45450	N-Term(iTRAQ4plex); K15(iTRAQ4plex)	0,0000	2,306	1		0	3,754E-10	71	7,11164E-08	3	1914,10196	-1,67	51,87	0
	High	fTIPIIGVTGSVGk	1	1	1	P45450	N-Term(iTRAQ4plex); K14(iTRAQ4plex)	0,0000	0,960	1		0,001	0,007924	12	0,059425111	3	1677,01920	0,13	47,69	0
	High	lSLFDIR	1	1	1	P45450	N-Term(iTRAQ4plex)	0,0000	1,662	1		0,008	0,1132	30	0,000937497	2	1007,60082	0,18	43,02	0
Q55469	UDP-N-acetylmuramoyl-L-alanyl-D-glutamate--2,6-diaminopimelate ligase OS=Synechocystis sp. (stra	100,09	10,30	1	3	3	3	1,675	3	14,0	505	54,6	5,41							
	A3	Sequence	# PSMs	# Proteins	# Protein Groups	Protein Group Accessions	Modifications	ΔCn	116/117	116/117 Count	116/117 Variability [%]	q-Value	PEP	IonScore	Exp Value	Charge	MH+ [Da]	ΔM [ppm]	RT [min]	# Missed Cleavages
	High	tTTSHLIEYFLNQQQR	1	1	1	Q55469	N-Term(iTRAQ4plex)	0,0000	1,525	1		0	2,086E-09	60	9,57128E-07	3	2123,09671	-1,65	44,10	0
	High	sSALLGTLYTR	1	1	1	Q55469	N-Term(iTRAQ4plex)	0,0000	2,407	1		0	0,0009954	53	5,1047E-06	2	1325,75481	0,18	35,26	0
	High	tkRPLmGNIAAQLADLAVVTSDNF	1	1	1	Q55469	N-Term(iTRAQ4plex); K2(iTRAQ4plex); M6(Oxidation)	0,0000	1,675	1		0	0,00001248	11	0,074125904	4	2955,63173	0,95	42,88	1
P51634	Magnesium-chelatase subunit ChlI OS=Synechocystis sp. (strain PCC 6803 / Kazusa) GN=chlI PE=3 S	99,88	9,80	1	3	3	4	1,772	4	22,6	357	39,4	5,08							
	A3	Sequence	# PSMs	# Proteins	# Protein Groups	Protein Group Accessions	Modifications	ΔCn	116/117	116/117 Count	116/117 Variability [%]	q-Value	PEP	IonScore	Exp Value	Charge	MH+ [Da]	ΔM [ppm]	RT [min]	# Missed Cleavages
	High	rVVFPFTAIVGQDEMk	1	1	1	P51634	N-Term(iTRAQ4plex); K16(iTRAQ4plex)	0,0000	1,817	1		0	1,074E-09	46	2,68516E-05	3	2125,17350	0,75	44,66	1
	High	lALLLNVIDPk	1	1	1	P51634	N-Term(iTRAQ4plex); K11(iTRAQ4plex)	0,0000	2,781	1		0	0,000009619	34	0,000388124	3	1496,96610	0,42	53,74	0
	High	fGMHAEIR	1	1	1	P51634	N-Term(iTRAQ4plex)	0,0000	1,345	1		0	0,0008404	31	0,000818408	3	1104,57190	-2,00	20,38	0
	High	rVVFPFTAIVGQDEmk	1	1	1	P51634	N-Term(iTRAQ4plex); M15(Oxidation); K16(iTRAQ4plex)	0,0000	1,728	1		0	8,814E-07	28	0,001503142	4	2141,16689	0,04	42,60	1
P48576	2-isopropylmalate synthase OS=Synechocystis sp. (strain PCC 6803 / Kazusa) GN=leuA PE=3 SV=1 -	99,84	4,50	1	2	2	4	1,399	3	18,4	533	57,4	5,49							
	A3	Sequence	# PSMs	# Proteins	# Protein Groups	Protein Group Accessions	Modifications	ΔCn	116/117	116/117 Count	116/117 Variability [%]	q-Value	PEP	IonScore	Exp Value	Charge	MH+ [Da]	ΔM [ppm]	RT [min]	# Missed Cleavages
	High	aGNAALEELVmALHVR	2	1	1	P48576	N-Term(iTRAQ4plex); M11(Oxidation)	0,0000	2,529	1		0	2,729E-10	64	5,65371E-07	3	1854,00309	0,33	51,62	0
	High	aGNAALEELVMALHVR	1	1	1	P48576	N-Term(iTRAQ4plex)	0,0000	1,399	1		0	7,854E-07	39	0,000118032	3	1838,00937	0,98	52,46	0
	High	aYLGALNR	1	1	1	P48576	N-Term(iTRAQ4plex)	0,0000	1,237	1		0,003	0,03912	24	0,004622852	2	1021,58983	-1,27	26,10	0

P73601	Sll1784 protein OS=Synechocystis sp. (strain PCC 6803 / Kazusa) GN=sll1784 PE=4 SV=1 - [P73601_	99,16	7,49	1	2	2	4	0,458	3	1,4	267	29,7	5,11							
	A3	Sequence	# PSMs	# Proteins	# Protein Groups	Protein Group Accessions	Modifications	ΔCn	116/117	116/117 Count	116/117 Variability [%]	q-Value	PEP	IonScore	Exp Value	Charge	MH+ [Da]	ΔM [ppm]	RT [min]	# Missed Cleavages
	High	gGLQLFQSVk	3	1	1	P73601	N-Term(iTRAQ4plex); K10(iTRAQ4plex)	0,0000	0,498	2	13,7	0	2,524E-07	70	1,05189E-07	2	1364,81438	0,24	36,38	0
	High	hLPAITEESk	1	1	1	P73601	N-Term(iTRAQ4plex); K10(iTRAQ4plex)	0,0000	0,458	1		0	0,00003256	28	0,002197405	3	1412,79856	-0,16	20,82	0
P74459	Chaperone protein ClpB 1 OS=Synechocystis sp. (strain PCC 6803 / Kazusa) GN=clpB1 PE=3 SV=1 -	98,60	4,68	1	2	4	6	2,089	2	71,3	898	101,3	5,34							
	A3	Sequence	# PSMs	# Proteins	# Protein Groups	Protein Group Accessions	Modifications	ΔCn	116/117	116/117 Count	116/117 Variability [%]	q-Value	PEP	IonScore	Exp Value	Charge	MH+ [Da]	ΔM [ppm]	RT [min]	# Missed Cleavages
	High	tAIAEGLAQR	2	3	3	P74459;P74361;Q55662	N-Term(iTRAQ4plex)	0,0000				0	5,338E-07	46	3,02268E-05	2	1173,67082	-0,03	23,31	0
	High	rPYSVVLLDEVEk	1	1	1	P74459	N-Term(iTRAQ4plex); K13(iTRAQ4plex)	0,0000	1,356	1		0	4,768E-08	40	9,09913E-05	3	1835,05552	2,06	36,54	0
	High	iDDLIIFHTLk	1	1	1	P74459	N-Term(iTRAQ4plex); K11(iTRAQ4plex)	0,0000	3,218	1		0	0,00005616	29	0,001330363	3	1615,96579	-0,25	46,69	0
	High	yRGEFEER	2	3	3	P74459;P74361;Q55662	N-Term(iTRAQ4plex)	0,0000				0	0,002186	16	0,027731286	3	1229,60511	1,59	15,30	1
P74598	Uncharacterized WD repeat-containing protein sll1491 OS=Synechocystis sp. (strain PCC 6803 / Kazu	98,52	7,18	1	3	3	6	0,561	6	15,8	348	37,3	5,52							
	A3	Sequence	# PSMs	# Proteins	# Protein Groups	Protein Group Accessions	Modifications	ΔCn	116/117	116/117 Count	116/117 Variability [%]	q-Value	PEP	IonScore	Exp Value	Charge	MH+ [Da]	ΔM [ppm]	RT [min]	# Missed Cleavages
	High	iWDLSALAR	4	1	1	P74598	N-Term(iTRAQ4plex)	0,0000	0,550	4	12,8	0	0,000003196	45	3,14029E-05	2	1188,68608	0,26	44,59	0
	High	vETLVGHTDk	1	1	1	P74598	N-Term(iTRAQ4plex); K10(iTRAQ4plex)	0,0000	0,817	1		0	0,0001293	24	0,004111213	3	1386,78376	0,45	20,23	0
	High	eLFSVR	1	1	1	P74598	N-Term(iTRAQ4plex)	0,0000	0,562	1		0,004	0,04784	33	0,000502308	2	894,51531	-1,41	27,68	0
P72895	Slr1617 protein OS=Synechocystis sp. (strain PCC 6803 / Kazusa) GN=slr1617 PE=4 SV=1 - [P72895	98,11	8,52	1	3	3	4	2,297	4	64,9	411	45,4	8,15							
	A3	Sequence	# PSMs	# Proteins	# Protein Groups	Protein Group Accessions	Modifications	ΔCn	116/117	116/117 Count	116/117 Variability [%]	q-Value	PEP	IonScore	Exp Value	Charge	MH+ [Da]	ΔM [ppm]	RT [min]	# Missed Cleavages
	High	yLAEILIDHcR	2	1	1	P72895	N-Term(iTRAQ4plex); C10(Carbamidomethyl)	0,0000	2,297	2	44,1	0	4,804E-07	54	4,09232E-06	3	1546,81638	-0,31	41,46	0
	High	gFIGTALQGALINSEFR	1	1	1	P72895	N-Term(iTRAQ4plex)	0,0000	0,682	1		0	0,0001327	21	0,008713433	3	1938,05582	-0,41	52,50	0
	High	fIFNFFk	1	1	1	P72895	N-Term(iTRAQ4plex); K7(iTRAQ4plex)	0,0000	3,847	1		0,001	0,01273	33	0,000481915	2	1250,71550	-1,69	51,98	0
P73553	Large-conductance mechanosensitive channel OS=Synechocystis sp. (strain PCC 6803 / Kazusa) GN=	97,77	14,48	1	2	2	4	1,113	4	7,3	145	15,8	4,84							
	A3	Sequence	# PSMs	# Proteins	# Protein Groups	Protein Group Accessions	Modifications	ΔCn	116/117	116/117 Count	116/117 Variability [%]	q-Value	PEP	IonScore	Exp Value	Charge	MH+ [Da]	ΔM [ppm]	RT [min]	# Missed Cleavages
	High	lITTLENQQSSSQ	1	1	1	P73553	N-Term(iTRAQ4plex)	0,0000	1,224	1		0	5,516E-09	95	2,83772E-10	2	1592,82561	0,49	33,84	0
	High	dFkDFILR	3	1	1	P73553	N-Term(iTRAQ4plex); K3(iTRAQ4plex)	0,0000	1,088	3	6,7	0	0,0007398	15	0,035315878	3	1341,77625	-0,51	37,47	1
P33779	Global nitrogen regulator OS=Synechocystis sp. (strain PCC 6803 / Kazusa) GN=ntcA PE=4 SV=1 - [I	97,52	15,56	1	3	3	5	1,524	5	38,0	225	25,0	7,05							
	A3	Sequence	# PSMs	# Proteins	# Protein Groups	Protein Group Accessions	Modifications	ΔCn	116/117	116/117 Count	116/117 Variability [%]	q-Value	PEP	IonScore	Exp Value	Charge	MH+ [Da]	ΔM [ppm]	RT [min]	# Missed Cleavages
	High	lSHQAIAEAIGSTR	2	1	1	P33779	N-Term(iTRAQ4plex)	0,0000	1,452	2	7,2	0	2,369E-09	53	5,59719E-06	3	1597,87845	0,35	28,19	0
	High	eNSVFGVLSLVTGQR	1	1	1	P33779	N-Term(iTRAQ4plex)	0,0000	1,104	1		0,005	0,06263	18	0,017257187	3	1749,95725	-2,51	55,10	0
	Medium	vYFLLk	2	1	1	P33779	N-Term(iTRAQ4plex); K6(iTRAQ4plex)	0,0000	2,123	2	12,4	0,012	0,1565	19	0,011586973	2	1070,68535	0,11	40,02	0
P74395	N utilization substance protein B homolog OS=Synechocystis sp. (strain PCC 6803 / Kazusa) GN=nusI	97,08	15,84	1	2	2	5	1,693	4	2,6	221	24,7	6,02							
	A3	Sequence	# PSMs	# Proteins	# Protein Groups	Protein Group Accessions	Modifications	ΔCn	116/117	116/117 Count	116/117 Variability [%]	q-Value	PEP	IonScore	Exp Value	Charge	MH+ [Da]	ΔM [ppm]	RT [min]	# Missed Cleavages
	High	kTDGAPVTPGEPR	3	1	1	P74395	N-Term(iTRAQ4plex); K1(iTRAQ4plex)	0,0000	1,545	2	13,8	0	0,0002033	52	6,83864E-06	3	1612,88687	-1,79	14,93	1
	High	kDTkPETLEQGDLEALLLAATR	2	1	1	P74395	N-Term(iTRAQ4plex); K1(iTRAQ4plex); K4(iTRAQ4plex)	0,0000	1,722	2	2,6	0	2,751E-07	27	0,001832188	4	2844,60756	1,13	47,42	1
P73824	Hydroperoxy fatty acid reductase gpx2 OS=Synechocystis sp. (strain PCC 6803 / Kazusa) GN=gpx2 P	96,49	25,32	1	3	3	5	0,221	4	22,3	154	16,6	4,81							
	A3	Sequence	# PSMs	# Proteins	# Protein Groups	Protein Group Accessions	Modifications	ΔCn	116/117	116/117 Count	116/117 Variability [%]	q-Value	PEP	IonScore	Exp Value	Charge	MH+ [Da]	ΔM [ppm]	RT [min]	# Missed Cleavages
	High	tkYDVDFTLLEk	1	1	1	P73824	N-Term(iTRAQ4plex); K2(iTRAQ4plex); K12(iTRAQ4plex)	0,0000	0,247	1		0	8,086E-10	60	1,01904E-06	3	1904,07437	0,13	38,23	1
	High	pLPTSLTTLDGTPLAPEVIADk	1	1	1	P73824	N-Term(iTRAQ4plex); K22(iTRAQ4plex)	0,0000	0,176	1		0	0,00003165	40	9,43996E-05	3	2537,43692	2,04	49,28	0
	Medium	fLIGR	3	1	1	P73824	N-Term(iTRAQ4plex)	0,0000	0,228	2	22,3	0,011	0,1431	12	0,073267271	2	749,47948	0,57	27,08	0
Q55390	Sll0553 protein OS=Synechocystis sp. (strain PCC 6803 / Kazusa) GN=sll0553 PE=4 SV=1 - [Q55390	96,12	13,62	1	5	5	5	1,780	4	8,6	345	38,2	5,38							
	A3	Sequence	# PSMs	# Proteins	# Protein Groups	Protein Group Accessions	Modifications	ΔCn	116/117	116/117 Count	116/117 Variability [%]	q-Value	PEP	IonScore	Exp Value	Charge	MH+ [Da]	ΔM [ppm]	RT [min]	# Missed Cleavages
	High	rIYPEIIDAYLESATQPR	1	1	1	Q55390	N-Term(iTRAQ4plex)	0,0000	1,757	1		0	6,85E-08	51	7,12804E-06	3	2279,21592	0,28	48,30	1
	High	vLAPDLLGWGR	1	1	1	Q55390	N-Term(iTRAQ4plex)	0,0000	1,802	1		0	0,00007325	38	0,000167869	2	1340,78166	0,68	46,71	0
	High	rLAALNPNAIk	1	1	1	Q55390	N-Term(iTRAQ4plex); K11(iTRAQ4plex)	0,0000	1,606	1		0	0,00002295	25	0,003435342	3	1468,91864	-1,10	25,61	1
	High	rFLALLQ	1	1	1	Q55390	N-Term(iTRAQ4plex)	0,0000	2,040	1		0,001	0,003217	22	0,007592638	2	1004,63744	0,08	40,38	1
	Medium	fLALLQ	1	1	1	Q55390	N-Term(iTRAQ4plex)	0,0000				0,04	0,5148	11	0,078337554	2	848,53642	0,22	49,97	0
P73942	Proline--tRNA ligase OS=Synechocystis sp. (strain PCC 6803 / Kazusa) GN=proS PE=3 SV=2 - [SYP_S	95,82	6,80	1	3	3	6	2,657	4	31,3	603	66,9	5,47							
	A3	Sequence	# PSMs	# Proteins	# Protein Groups	Protein Group Accessions	Modifications	ΔCn	116/117	116/117 Count	116/117 Variability [%]	q-Value	PEP	IonScore	Exp Value	Charge	MH+ [Da]	ΔM [ppm]	RT [min]	# Missed Cleavages
	High	fkDSELIGIPYR	1	1	1	P73942	N-Term(iTRAQ4plex); K2(iTRAQ4plex)	0,0000	2,240	1		0	0,00002141	36	0,000244326	3	1725,97702	-0,47	36,16	1
	High	gIEVGHIFQLGTk	4	1	1	P73942	N-Term(iTRAQ4plex); K13(iTRAQ4plex)	0,0000	3,265	2	5,2	0	0,0000088	35	0,000310435	3	1686,97702	-0,68	38,73	0
	High	aLAGDRPVHDPTGELk	1	1	1	P73942	N-Term(iTRAQ4plex); K16(iTRAQ4plex)	0,0000	2,072	1		0	0,000001239	16	0,026361493	4	1964,08071	0,14	22,07	0
P73222	Slr2005 protein OS=Synechocystis sp. (strain PCC 6803 / Kazusa) GN=slr2005 PE=4 SV=1 - [P73222	93,18	13,03	1	3	3	3	0,869	3	17,9	261	28,0	9,64							
	A3	Sequence	# PSMs	# Proteins	# Protein Groups	Protein Group Accessions	Modifications	ΔCn	116/117	116/117 Count	116/117 Variability [%]	q-Value	PEP	IonScore	Exp Value	Charge	MH+ [Da]	ΔM [ppm]	RT [min]	# Missed Cleavages
	High	aAmAILNPR	1	1	1	P73222	N-Term(iTRAQ4plex); M3(Oxidation)	0,0000	0,869	1		0	0,00002068	49	1,23586E-05	2	1116,63115	-0,44	21,14	0
	High	iLLGSPDLALIPVGGGPk	1	1	1	P73222	N-Term(iTRAQ4plex); K18(iTRAQ4plex)	0,0000	0,680	1		0	0,000006583	31	0,000874923	3	2005,23099	0,46	50,30	0
	High	iLANPFR	1	1	1	P73222	N-Term(iTRAQ4plex)	0,0000	0,980	1		0,001	0,003198	40	0,00011091	2	974,59038	-0,03	33,98	0
P74416	Glycine dehydrogenase (decarboxylating) OS=Synechocystis sp. (strain PCC 6803 / Kazusa) GN=gcvF	92,98	5,70	1	4	4	4	0,422	4	39,5	983	107,3	5,39							
	A3	Sequence	# PSMs	# Proteins	# Protein Groups	Protein Group Accessions	Modifications	ΔCn	116/117	116/117 Count	116/117 Variability [%]	q-Value	PEP	IonScore	Exp Value	Charge	MH+ [Da]	ΔM [ppm]	RT [min]	# Missed Cleavages
	High	fGIPLGYGGPHAAYFATk	1	1	1	P74416	N-Term(iTRAQ4plex); K18(iTRAQ4plex)	0,0000	0,268	1		0	1,683E-11	50	9,26766E-06	3	2155,16013	1,02	41,40	0
	High	gNNELVAHEcILDLRPLk	1	1	1	P74416	N-Term(iTRAQ4plex); C10(Carbamidomethyl); K18(iTRAQ4plex	0,0000	0,448	1		0	6,092E-08	35	0,000408235	4	2379,30752	0,74	37,85	0
	High	sHLQAFLPR	1	1	1	P74416	N-Term(iTRAQ4plex)	0,0000	0,747	1		0	0,00212	27	0,002093968	3	1212,69779	0,65	27,26	0
	High	rLENYYPILFR	1	1	1	P74416	N-Term(iTRAQ4plex)	0,0000	0,398	1		0	0,000003304	21	0,008495507	3	1627,90848	0,47	40,87	1
Q55512	Aspartate-semialdehyde dehydrogenase OS=Synechocystis sp. (strain PCC 6803 / Kazusa) GN=asd P	92,82	7,10	1	2	2	2	1,876	2	42,8	338	36,6	6,04							
	A3	Sequence	# PSMs	# Proteins	# Protein Groups	Protein Group Accessions	Modifications	ΔCn	116/117	116/117 Count	116/117 Variability [%]	q-Value	PEP	IonScore	Exp Value	Charge	MH+ [Da]	ΔM [ppm]	RT [min]	# Missed Cleavages
	High	kGAALNAVQIAELLVER	1	1	1	Q55512	N-Term(iTRAQ4plex); K1(iTRAQ4plex)	0,0000	2,474	1		0	4,542E-09	75	2,95101E-08	3	2083,24857	0,35	50,61	1
	High	iTATcVR	1	1	1	Q55512	N-Term(iTRAQ4plex); C5(Carbamidomethyl)	0,0000	1,423	1		0,001	0,009466	31	0,000883019	2	964,53581	-0,86	16,75	0
P73043	Glutamate decarboxylase OS=Synechocystis sp. (strain PCC 6803 / Kazusa) GN=gad PE=3 SV=1 - [P	92,50	8,35	1	4	4	4	2,179	4	76,6	467	53,0	5,41							
	A3	Sequence	# PSMs	# Proteins	# Protein Groups	Protein Group Accessions	Modifications	ΔCn	116/117	116/117 Count	116/117 Variability [%]	q-Value	PEP	IonScore	Exp Value	Charge	MH+ [Da]	ΔM [ppm]	RT [min]	# Missed Cleavages
	High	gLANSVSk	1	1	1	P73043	N-Term(iTRAQ4plex); K8(iTRAQ4plex)	0,0000	1,103	1		0	0,0007076	39	0,000135266	2	1063,63188	-2,94	17,16	0
	High	gWLVPAYR	1	1	1	P73043	N-Term(iTRAQ4plex)	0,0000	3,139	1		0	0,0001568	39	0,000134887	2	1105,62712	-0,35	34,56	0
	High	aIAYFASQPDHkPk	1	1	1	P73043	N-Term(iTRAQ4plex); K12(iTRAQ4plex); K14(iTRAQ4plex)	0,0000	3,783	1		0	5,212E-07	31	0,000765544	3	2005,12351	0,18	23,43	0
	High	nREDLVVQR	1	1	1	P73043	N-Term(iTRAQ4plex)	0,0000	1,512	1		0	0,0003329	23	0,005081243	3	1272,71399	-0,10	15,19	1
Q54735	Carbonic anhydrase OS=Synechocystis sp. (strain PCC 6803 / Kazusa) GN=icfA PE=1 SV=1 - [CYNT_	91,66	12,04	1	3	3	4	1,621	3	90,5	274	30,7	6,23							
	A3	Sequence	# PSMs	# Proteins	# Protein Groups	Protein Group Accessions	Modifications	ΔCn	116/117	116/117 Count	116/117 Variability [%]	q-Value	PEP	IonScore	Exp Value	Charge	MH+ [Da]	ΔM [ppm]	RT [min]	# Missed Cleavages
	High	lPLVYDWLk	1	1	1	Q54735	N-Term(iTRAQ4plex); K9(iTRAQ4plex)	0,0000	2,732	1		0	0,00007687	48	3,42791E-05	2	1434,86138	1,01	51,56	0
	High	gDLSLHGWIYR	2	1	1	Q54735	N-Term(iTRAQ4plex)	0,0000	1,621	1		0	4,855E-07	39	0,000126174	3	1460,77585	-0,58	36,12	0
	High	dLFEQLSHGQHPR	1	1	1	Q54735	N-Term(iTRAQ4plex)	0,0000	0,955	1		0	0,0005567	33	0,000496558	4	1707,86941	0,60	28,47	0
Q55641	Ribonuclease D OS=Synechocystis sp. (strain PCC 6803 / Kazusa) GN=rnd PE=4 SV=1 - [Q55641_SY	91,28	8,29	1	2	3	8	1,106	2	30,4	217	24,4	6,21							
	A3	Sequence	# PSMs	# Proteins	# Protein Groups	Protein Group Accessions	Modifications	ΔCn	116/117	116/117 Count	116/117 Variability [%]	q-Value	PEP	IonScore	Exp Value	Charge	MH+ [Da]	ΔM [ppm]	RT [min]	# Missed Cleavages
	High	iFHFAR	1	1	1	Q55641	N-Term(iTRAQ4plex)	0,0000	0,905	1		0	0,001985	31	0,001251319	3	934,53797	0,00	25,19	0
	High	yLIPLR	6	2	2	P74438;Q55641	N-Term(iTRAQ4plex)	0,0000				0,001	0,007505	27	0,002552368	2	918,58965	0,35	35,48	0
	High	hTFDIk	1	1	1	Q55641	N-Term(iTRAQ4plex); K6(iTRAQ4plex)	0,0000	1,351	1		0,001	0,004335	12	0,091699718	3	1048,60343	0,44	18,19	0
P74241	Sulfate adenylyltransferase OS=Synechocystis sp. (strain PCC 6803 / Kazusa) GN=sat PE=3 SV=1 - [	90,59	10,00	1	4	4	6	1,593	6	25,2	390	43,7	6,25							
	A3	Sequence	# PSMs	# Proteins	# Protein Groups	Protein Group Accessions	Modifications	ΔCn	116/117	116/117 Count	116/117 Variability [%]	q-Value	PEP	IonScore	Exp Value	Charge	MH+ [Da]	ΔM [ppm]	RT [min]	# Missed Cleavages
	High	vILGINPSAmR	1	1	1	P74241	N-Term(iTRAQ4plex); M10(Oxidation)	0,0000	1,599	1		0	0,0006437	33	0,000536995	2	1330,76372	0,25	32,91	0
	High	eAIFHALIR	3	1	1	P74241	N-Term(iTRAQ4plex)	0,0000	1,586	3	16,0	0	0,001215	31	0,000767308	3	1213,71723	-0,15	34,15	0
	High	ePHPLFPk	1	1	1	P74241	N-Term(iTRAQ4plex); K8(iTRAQ4plex)	0,0000	2,366	1		0	0,00000211	30	0,000943996	3	1252,72959	0,27	21,87	0
	High	nYGcTHFIVGR	1	1	1	P74241	N-Term(iTRAQ4plex); C4(Carbamidomethyl)	0,0000	1,274	1		0	0,00003347	26	0,002691349	3	1467,72699	-0,94	24,72	0
P73016	Enoyl-[acyl-carrier-protein] reductase [NADH] FabI OS=Synechocystis sp. (strain PCC 6803 / Kazusa)	90,31	9,69	1	2	2	3	3,104	3	12,2	258	27,6	6,43							
	A3	Sequence	# PSMs	# Proteins	# Protein Groups	Protein Group Accessions	Modifications	ΔCn	116/117	116/117 Count	116/117 Variability [%]	q-Value	PEP	IonScore	Exp Value	Charge	MH+ [Da]	ΔM [ppm]	RT [min]	# Missed Cleavages
	High	lDILIHcLAFADk	2	1	1	P73016	N-Term(iTRAQ4plex); C7(Carbamidomethyl); K13(iTRAQ4plex)	0,0000	3,234	2	6,1	0	1,942E-07	44	3,61385E-05	3	1817,02274	-0,37	50,30	0
	High	vIPNYNLmGVAk	1	1	1	P73016	N-Term(iTRAQ4plex); M8(Oxidation); K12(iTRAQ4plex)	0,0000	0,965	1		0	0,000000627	36	0,000229071	3	1622,91721	-0,41	34,00	0
P74230	30S ribosomal protein S12 OS=Synechocystis sp. (strain PCC 6803 / Kazusa) GN=rpsL PE=3 SV=1 - [	90,28	11,90	1	2	2	4	3,748	4	31,0	126	14,2	11,22							
	A3	Sequence	# PSMs	# Proteins	# Protein Groups	Protein Group Accessions	Modifications	ΔCn	116/117	116/117 Count	116/117 Variability [%]	q-Value	PEP	IonScore	Exp Value	Charge	MH+ [Da]	ΔM [ppm]	RT [min]	# Missed Cleavages
	High	pTIQQLIR	2	1	1	P74230	N-Term(iTRAQ4plex)	0,0000	4,598	2	12,9	0	0,000567	41	7,77983E-05	2	1112,69219	1,21	32,30	0
	High	vYTTTPk	2	1	1	P74230	N-Term(iTRAQ4plex); K7(iTRAQ4plex)	0,0000	3,002	2	15,6	0	0,002696	34	0,000404548	2	1097,65080	5,76	16,73	0
P74073	Sll1258 protein OS=Synechocystis sp. (strain PCC 6803 / Kazusa) GN=sll1258 PE=4 SV=1 - [P74073_	90,15	14,51	1	2	2	3	1,111	3	2,6	193	21,4	6,38							
	A3	Sequence	# PSMs	# Proteins	# Protein Groups	Protein Group Accessions	Modifications	ΔCn	116/117	116/117 Count	116/117 Variability [%]	q-Value	PEP	IonScore	Exp Value	Charge	MH+ [Da]	ΔM [ppm]	RT [min]	# Missed Cleavages
	High	lAIPANVTVLcIGk	2	1	1	P74073	N-Term(iTRAQ4plex); C11(Carbamidomethyl); K14(iTRAQ4plex	0,0000	1,121	2	1,3	0	0,000002768	43	4,77496E-05	3	1757,06137	0,90	45,89	0
	High	nFNPHNLEATQLHR	1	1	1	P74073	N-Term(iTRAQ4plex)	0,0000	0,963	1		0	1,294E-09	35	0,000305471	4	1834,94680	2,10	24,44	0
P74535	Sll1336 protein OS=Synechocystis sp. (strain PCC 6803 / Kazusa) GN=sll1336 PE=4 SV=1 - [P74535_	89,92	6,10	1	4	4	5	3,464	5	195,0	705	78,3	5,62							
	A3	Sequence	# PSMs	# Proteins	# Protein Groups	Protein Group Accessions	Modifications	ΔCn	116/117	116/117 Count	116/117 Variability [%]	q-Value	PEP	IonScore	Exp Value	Charge	MH+ [Da]	ΔM [ppm]	RT [min]	# Missed Cleavages
	High	rVELLVEQIAWEmR	1	1	1	P74535	N-Term(iTRAQ4plex); M13(Oxidation)	0,0000	1,377	1		0	1,994E-07	41	0,000105528	3	1932,04978	0,18	44,41	1
	High	vLNFNLGVER	1	1	1	P74535	N-Term(iTRAQ4plex)	0,0000	3,464	1		0	0,000005018	31	0,000822601	2	1304,74529	0,71	39,70	0
	High	wLWAGYGFR	2	1	1	P74535	N-Term(iTRAQ4plex)	0,0000	8,165	2	1,9	0	0,0005291	30	0,00090567	2	1299,67546	-0,06	47,74	0
	High	wLDTEVVSLR	1	1	1	P74535	N-Term(iTRAQ4plex)	0,0000	2,537	1		0	0,00161	19	0,01315134	2	1361,75530	0,52	42,20	0
Q55421	Elongation factor G-like protein OS=Synechocystis sp. (strain PCC 6803 / Kazusa) GN=sll0830 PE=3 S	89,46	3,29	1	2	2	5	2,122	5	18,7	669	73,3	5,00							
	A3	Sequence	# PSMs	# Proteins	# Protein Groups	Protein Group Accessions	Modifications	ΔCn	116/117	116/117 Count	116/117 Variability [%]	q-Value	PEP	IonScore	Exp Value	Charge	MH+ [Da]	ΔM [ppm]	RT [min]	# Missed Cleavages
	High	vLTLAPLFk	4	1	1	Q55421	N-Term(iTRAQ4plex); K9(iTRAQ4plex)	0,0000	2,259	4	48,5	0	0,000001463	52	6,88605E-06	2	1289,84355	0,00	47,03	0
	High	akQPFGEVLQALk	1	1	1	Q55421	N-Term(iTRAQ4plex); K2(iTRAQ4plex); K13(iTRAQ4plex)	0,0000	1,878	1		0,003	0,03806	18	0,016105344	3	1861,13047	1,76	41,24	1
P73104	NarL subfamily OS=Synechocystis sp. (strain PCC 6803 / Kazusa) GN=slr1909 PE=4 SV=1 - [P73104	89,41	12,21	1	2	2	5	0,827	4	27,7	213	24,0	5,58							
	A3	Sequence	# PSMs	# Proteins	# Protein Groups	Protein Group Accessions	Modifications	ΔCn	116/117	116/117 Count	116/117 Variability [%]	q-Value	PEP	IonScore	Exp Value	Charge	MH+ [Da]	ΔM [ppm]	RT [min]	# Missed Cleavages
	High	lLQHNNHPLEDNAR	4	1	1	P73104	N-Term(iTRAQ4plex)	0,0000	0,847	3	7,3	0	5,292E-08	41	7,24386E-05	4	1814,93764	-0,12	17,81	0
	High	tVESHVSNmLNk	1	1	1	P73104	N-Term(iTRAQ4plex); M9(Oxidation); K12(iTRAQ4plex)	0,0000	0,567	1		0	3,934E-08	40	0,000101618	3	1662,87192	-0,27	18,60	0
P74534	Sll1338 protein OS=Synechocystis sp. (strain PCC 6803 / Kazusa) GN=sll1338 PE=4 SV=1 - [P74534_	89,36	17,11	1	2	2	3	0,684	3	2,2	187	20,2	4,70							
	A3	Sequence	# PSMs	# Proteins	# Protein Groups	Protein Group Accessions	Modifications	ΔCn	116/117	116/117 Count	116/117 Variability [%]	q-Value	PEP	IonScore	Exp Value	Charge	MH+ [Da]	ΔM [ppm]	RT [min]	# Missed Cleavages
	High	tDFYSAAHPTWLTR	2	1	1	P74534	N-Term(iTRAQ4plex)	0,0000	0,621	2	14,4	0	6,373E-07	39	0,000120218	3	1809,90250	-0,88	35,45	0
	High	sAPAPVAPAPAPAPmPIR	1	1	1	P74534	N-Term(iTRAQ4plex); M15(Oxidation)	0,0000	0,694	1		0	0,00008383	39	0,000125017	3	1871,03189	-0,63	27,37	0
P73866	Potassium-transporting ATPase A chain OS=Synechocystis sp. (strain PCC 6803 / Kazusa) GN=kdpA F	89,15	4,84	1	2	2	4	0,542	4	13,3	558	59,3	5,41							
	A3	Sequence	# PSMs	# Proteins	# Protein Groups	Protein Group Accessions	Modifications	ΔCn	116/117	116/117 Count	116/117 Variability [%]	q-Value	PEP	IonScore	Exp Value	Charge	MH+ [Da]	ΔM [ppm]	RT [min]	# Missed Cleavages
	High	iARPLESLIFAGSGITk	2	1	1	P73866	N-Term(iTRAQ4plex); K17(iTRAQ4plex)	0,0000	0,583	2	22,3	0	0,000001036	50	1,12901E-05	3	2061,23288	0,86	47,10	0
	High	gPVAHFEIIk	2	1	1	P73866	N-Term(iTRAQ4plex); K10(iTRAQ4plex)	0,0000	0,536	2	13,3	0	0,000005514	39	0,00013551	3	1398,83448	-0,22	30,27	0
P72814	Slr1800 protein OS=Synechocystis sp. (strain PCC 6803 / Kazusa) GN=slr1800 PE=4 SV=1 - [P72814	88,69	6,20	1	3	3	4	0,654	4	2,0	355	39,4	4,84							
	A3	Sequence	# PSMs	# Proteins	# Protein Groups	Protein Group Accessions	Modifications	ΔCn	116/117	116/117 Count	116/117 Variability [%]	q-Value	PEP	IonScore	Exp Value	Charge	MH+ [Da]	ΔM [ppm]	RT [min]	# Missed Cleavages
	High	gTGFLNWLk	2	1	1	P72814	N-Term(iTRAQ4plex); K9(iTRAQ4plex)	0,0000	0,645	2	0,8	0	0,000008819	49	1,4578E-05	2	1323,76628	-0,06	45,61	0
	High	lFTTWk	1	1	1	P72814	N-Term(iTRAQ4plex); K6(iTRAQ4plex)	0,0000	0,659	1		0	0,002305	38	0,00016217	2	1083,64299	-1,03	35,62	0
	High	hLAPLVk	1	1	1	P72814	N-Term(iTRAQ4plex); K7(iTRAQ4plex)	0,0000	1,860	1		0	0,002275	11	0,088098802	3	1065,70154	-0,71	19,41	0
Q55168	Phytochrome-like protein cph1 OS=Synechocystis sp. (strain PCC 6803 / Kazusa) GN=cph1 PE=1 SV=	88,35	3,07	1	3	3	5	23,724	4	121,9	748	84,2	5,12							
	A3	Sequence	# PSMs	# Proteins	# Protein Groups	Protein Group Accessions	Modifications	ΔCn	116/117	116/117 Count	116/117 Variability [%]	q-Value	PEP	IonScore	Exp Value	Charge	MH+ [Da]	ΔM [ppm]	RT [min]	# Missed Cleavages
	High	aIVNLILR	2	1	1	Q55168	N-Term(iTRAQ4plex)	0,0000	34,283	1		0	0,001949	40	9,0567E-05	2	1055,70635	0,54	41,95	0
	High	lFIHNPIR	1	1	1	Q55168	N-Term(iTRAQ4plex)	0,0000	100,000	1		0	0,0001243	15	0,029374468	3	1153,69718	0,79	29,22	0
	High	iFVIFQR	2	1	1	Q55168	N-Term(iTRAQ4plex)	0,0000	10,760	2	69,3	0,001	0,00455	39	0,000127342	2	1066,65386	0,80	43,42	0
P74771	NAD(P)H-quinone oxidoreductase subunit O OS=Synechocystis sp. (strain PCC 6803 / Kazusa) GN=n	87,76	40,28	1	4	4	8	0,457	8	12,9	72	8,3	7,25							
	A3	Sequence	# PSMs	# Proteins	# Protein Groups	Protein Group Accessions	Modifications	ΔCn	116/117	116/117 Count	116/117 Variability [%]	q-Value	PEP	IonScore	Exp Value	Charge	MH+ [Da]	ΔM [ppm]	RT [min]	# Missed Cleavages
	High	fYVPTPNVWLR	4	1	1	P74771	N-Term(iTRAQ4plex)	0,0000	0,456	4	7,6	0	0,0003753	35	0,000322827	3	1535,84946	0,21	48,58	0
	High	lPDYLFHSk	2	1	1	P74771	N-Term(iTRAQ4plex); K9(iTRAQ4plex)	0,0000	0,466	2	17,8	0	0,0002031	25	0,004406848	3	1407,78586	-1,15	35,09	0
	High	rLPDYLFHSk	1	1	1	P74771	N-Term(iTRAQ4plex); K10(iTRAQ4plex)	0,0000	0,445	1		0	0,0007004	19	0,017503626	4	1563,88857	-0,02	29,85	1
	Medium	lDQIEALA	1	1	1	P74771	N-Term(iTRAQ4plex)	0,0000	0,574	1		0,028	0,3705	18	0,016709752	2	1016,57421	-0,27	41,29	0
P73503	Slr1437 protein OS=Synechocystis sp. (strain PCC 6803 / Kazusa) GN=slr1437 PE=4 SV=1 - [P73503	87,45	32,14	1	4	4	5	1,179	5	26,3	112	12,7	5,22							
	A3	Sequence	# PSMs	# Proteins	# Protein Groups	Protein Group Accessions	Modifications	ΔCn	116/117	116/117 Count	116/117 Variability [%]	q-Value	PEP	IonScore	Exp Value	Charge	MH+ [Da]	ΔM [ppm]	RT [min]	# Missed Cleavages
	High	cAYVAIGEFkPGR	1	1	1	P73503	N-Term(iTRAQ4plex); C1(Carbamidomethyl); K10(iTRAQ4plex)	0,0000	1,179	1		0	1,771E-09	49	1,28816E-05	3	1755,94553	0,02	32,07	0
	High	sAEFFDcLQHk	1	1	1	P73503	N-Term(iTRAQ4plex); C7(Carbamidomethyl); K11(iTRAQ4plex)	0,0000	1,404	1		0	0,0000019	36	0,000253495	3	1669,82553	0,51	31,04	0
	High	aISTYk	2	1	1	P73503	N-Term(iTRAQ4plex); K6(iTRAQ4plex)	0,0000	0,841	2	3,7	0,001	0,007851	19	0,012077304	2	970,58165	0,51	17,44	0
	Medium	gAFLLR	1	1	1	P73503	N-Term(iTRAQ4plex)	0,0000	1,227	1		0,016	0,2103	22	0,005688529	2	820,51549	-0,83	27,67	0
Q55445	Putative methyl-accepting chemotaxis protein sll0041 OS=Synechocystis sp. (strain PCC 6803 / Kazus	87,33	6,20	1	5	5	5	2,788	5	29,5	1000	108,3	4,79							
	A3	Sequence	# PSMs	# Proteins	# Protein Groups	Protein Group Accessions	Modifications	ΔCn	116/117	116/117 Count	116/117 Variability [%]	q-Value	PEP	IonScore	Exp Value	Charge	MH+ [Da]	ΔM [ppm]	RT [min]	# Missed Cleavages
	High	gNLLGLLIAHQcSGPR	1	1	1	Q55445	N-Term(iTRAQ4plex); C12(Carbamidomethyl)	0,0000	2,788	1		0	6,546E-08	53	5,57418E-06	3	1850,01944	0,36	43,58	0
	High	lVFYYHPR	1	1	1	Q55445	N-Term(iTRAQ4plex)	0,0000	3,388	1		0	0,002738	34	0,000401763	3	1238,68018	-0,06	30,30	0
	High	dIYNAGLTPcHIGQLkPFEVk	1	1	1	Q55445	N-Term(iTRAQ4plex); C10(Carbamidomethyl); K16(iTRAQ4plex	0,0000	3,326	1		0	1,557E-09	16	0,023986672	4	2832,54336	-0,34	39,86	0
	High	aSLTVPLHR	1	1	1	Q55445	N-Term(iTRAQ4plex)	0,0000	1,020	1		0,001	0,007274	22	0,006760363	3	1137,68937	2,88	22,75	0
	Medium	qLAQQANR	1	1	1	Q55445	N-Term(iTRAQ4plex)	0,0000	1,972	1		0,01	0,1333	15	0,032958695	2	1072,59819	0,18	11,51	0
P74060	Putative sulfur carrier protein slr0821 OS=Synechocystis sp. (strain PCC 6803 / Kazusa) GN=slr0821	87,32	20,18	1	2	2	3	0,826	3	0,7	109	11,7	4,67							
	A3	Sequence	# PSMs	# Proteins	# Protein Groups	Protein Group Accessions	Modifications	ΔCn	116/117	116/117 Count	116/117 Variability [%]	q-Value	PEP	IonScore	Exp Value	Charge	MH+ [Da]	ΔM [ppm]	RT [min]	# Missed Cleavages
	High	rFLNFYVNEEDIR	2	1	1	P74060	N-Term(iTRAQ4plex)	0,0000	0,921	2	16,2	0	2,82E-08	47	2,0463E-05	3	1858,95829	0,76	39,24	1
	High	lcDEEGkPR	1	1	1	P74060	N-Term(iTRAQ4plex); C2(Carbamidomethyl); K7(iTRAQ4plex)	0,0000	0,823	1		0	0,0005263	20	0,010161785	3	1391,71854	-0,45	13,48	0
P73057	Nucleoid-associated protein slr1847 OS=Synechocystis sp. (strain PCC 6803 / Kazusa) GN=slr1847 PE	87,24	23,68	1	3	3	6	0,455	5	3,1	114	12,1	4,60							
	A3	Sequence	# PSMs	# Proteins	# Protein Groups	Protein Group Accessions	Modifications	ΔCn	116/117	116/117 Count	116/117 Variability [%]	q-Value	PEP	IonScore	Exp Value	Charge	MH+ [Da]	ΔM [ppm]	RT [min]	# Missed Cleavages
	High	gFGFGLGk	1	1	1	P73057	N-Term(iTRAQ4plex); K8(iTRAQ4plex)	0,0000	0,445	1		0	0,0006755	35	0,000325065	2	1070,62187	-1,73	33,21	0
	High	aQQVQEGAk	2	1	1	P73057	N-Term(iTRAQ4plex); K9(iTRAQ4plex)	0,0000	0,376	2	31,2	0	0,000009352	34	0,000480505	2	1246,69866	-0,60	10,16	0
	High	ikELQEAFQk	3	1	1	P73057	N-Term(iTRAQ4plex); K2(iTRAQ4plex); K10(iTRAQ4plex)	0,0000	0,460	2	1,5	0	0,000002299	30	0,00104947	3	1665,98801	-1,20	31,56	1
P73424	Slr1540 protein OS=Synechocystis sp. (strain PCC 6803 / Kazusa) GN=slr1540 PE=4 SV=1 - [P73424	85,59	14,47	1	5	5	6	2,048	6	22,7	311	35,1	6,61							
	A3	Sequence	# PSMs	# Proteins	# Protein Groups	Protein Group Accessions	Modifications	ΔCn	116/117	116/117 Count	116/117 Variability [%]	q-Value	PEP	IonScore	Exp Value	Charge	MH+ [Da]	ΔM [ppm]	RT [min]	# Missed Cleavages
	High	fIGIHLcR	2	1	1	P73424	N-Term(iTRAQ4plex); C7(Carbamidomethyl)	0,0000	2,489	2	7,0	0	0,00001639	37	0,000194075	3	1159,65268	0,00	31,11	0
	High	qQHFFADIQk	1	1	1	P73424	N-Term(iTRAQ4plex); K10(iTRAQ4plex)	0,0000	1,803	1		0	0,000000192	36	0,000223857	3	1549,83579	-0,50	26,90	0
	High	vLVAQGHEVVLFNR	1	1	1	P73424	N-Term(iTRAQ4plex)	0,0000	1,348	1		0	0,001508	16	0,024208618	3	1724,99448	0,93	35,43	0
	High	lVHYDPk	1	1	1	P73424	N-Term(iTRAQ4plex); K7(iTRAQ4plex)	0,0000	1,756	1		0,001	0,005868	17	0,021414002	3	1159,67145	0,06	18,51	0
	High	kAFPLR	1	1	1	P73424	N-Term(iTRAQ4plex); K1(iTRAQ4plex)	0,0000	2,325	1		0,002	0,02539	16	0,023119051	3	1019,65940	-1,02	17,78	1
Q55782	Slr0209 protein OS=Synechocystis sp. (strain PCC 6803 / Kazusa) GN=slr0209 PE=4 SV=1 - [Q55782	84,81	7,18	1	2	2	6	2,827	4	13,0	348	40,7	5,63							
	A3	Sequence	# PSMs	# Proteins	# Protein Groups	Protein Group Accessions	Modifications	ΔCn	116/117	116/117 Count	116/117 Variability [%]	q-Value	PEP	IonScore	Exp Value	Charge	MH+ [Da]	ΔM [ppm]	RT [min]	# Missed Cleavages
	High	nVNDLAILTHLVNk	3	1	1	Q55782	N-Term(iTRAQ4plex); K14(iTRAQ4plex)	0,0000	3,016	2	13,0	0	1,46E-10	38	0,00016367	3	1852,09073	0,66	45,00	0
	High	gGLIEGIIHSk	3	1	1	Q55782	N-Term(iTRAQ4plex); K11(iTRAQ4plex)	0,0000	1,975	2	61,3	0	0,00001813	26	0,00237121	3	1411,84952	-1,17	34,55	0
Q55426	Slr0841 protein OS=Synechocystis sp. (strain PCC 6803 / Kazusa) GN=slr0841 PE=4 SV=1 - [Q55426	84,53	7,56	1	2	2	4	0,800	4	7,0	291	31,5	4,92							
	A3	Sequence	# PSMs	# Proteins	# Protein Groups	Protein Group Accessions	Modifications	ΔCn	116/117	116/117 Count	116/117 Variability [%]	q-Value	PEP	IonScore	Exp Value	Charge	MH+ [Da]	ΔM [ppm]	RT [min]	# Missed Cleavages
	High	kAcTAEVmAQEQR	2	1	1	Q55426	K1(iTRAQ4plex); N-Term(iTRAQ4plex); C3(Carbamidomethyl);	0,0000	0,535	2	66,3	0	1,569E-07	48	1,4157E-05	3	1825,91471	0,42	13,55	1
	High	kAcTAEVMAQEQR	1	1	1	Q55426	N-Term(iTRAQ4plex); K1(iTRAQ4plex); C3(Carbamidomethyl)	0,0000	0,797	1		0	0,000000352	45	2,99206E-05	3	1809,92118	1,19	17,51	1
	High	gAPHSGSLR	1	1	1	Q55426	N-Term(iTRAQ4plex)	0,0000	0,877	1		0,009	0,1233	12	0,067603628	3	1025,56034	-0,54	9,32	0
P73832	Cyanophycinase OS=Synechocystis sp. (strain PCC 6803 / Kazusa) GN=cphB PE=1 SV=1 - [CPHB_SY	83,78	8,49	1	2	2	4	1,352	4	12,7	271	29,4	6,52							
	A3	Sequence	# PSMs	# Proteins	# Protein Groups	Protein Group Accessions	Modifications	ΔCn	116/117	116/117 Count	116/117 Variability [%]	q-Value	PEP	IonScore	Exp Value	Charge	MH+ [Da]	ΔM [ppm]	RT [min]	# Missed Cleavages
	High	lNILVHGEVYHQVk	2	1	1	P73832	N-Term(iTRAQ4plex); K14(iTRAQ4plex)	0,0000	1,371	2	12,7	0	3,485E-13	44	3,77546E-05	3	1937,12271	0,82	30,10	0
	High	eILQTFWSR	2	1	1	P73832	N-Term(iTRAQ4plex)	0,0000	1,032	2	54,0	0	0,00002831	37	0,000217756	2	1323,72002	1,68	43,44	0
P72977	16.6 kDa small heat shock protein, molecular chaperon OS=Synechocystis sp. (strain PCC 6803 / Kaz	83,33	25,34	1	3	3	3	0,573	3	33,5	146	16,6	5,10							
	A3	Sequence	# PSMs	# Proteins	# Protein Groups	Protein Group Accessions	Modifications	ΔCn	116/117	116/117 Count	116/117 Variability [%]	q-Value	PEP	IonScore	Exp Value	Charge	MH+ [Da]	ΔM [ppm]	RT [min]	# Missed Cleavages
	High	vIPVPGAIQNTEVk	1	1	1	P72977	N-Term(iTRAQ4plex); K14(iTRAQ4plex)	0,0000	0,573	1		0	3,989E-08	44	3,8104E-05	3	1753,04825	1,14	35,22	0
	High	sLILYNPLR	1	1	1	P72977	N-Term(iTRAQ4plex)	0,0000	0,412	1		0	0,0008528	43	4,52866E-05	2	1232,75017	1,47	41,61	0
	High	aNYDAGILTLTLPk	1	1	1	P72977	N-Term(iTRAQ4plex); K14(iTRAQ4plex)	0,0000	0,714	1		0	0,001507	22	0,006713825	3	1778,03354	1,85	45,45	0
Q55589	Cell division cycle protein OS=Synechocystis sp. (strain PCC 6803 / Kazusa) GN=slr0374 PE=4 SV=1	83,29	4,19	1	2	2	4	1,554	4	63,6	501	56,4	6,27							
	A3	Sequence	# PSMs	# Proteins	# Protein Groups	Protein Group Accessions	Modifications	ΔCn	116/117	116/117 Count	116/117 Variability [%]	q-Value	PEP	IonScore	Exp Value	Charge	MH+ [Da]	ΔM [ppm]	RT [min]	# Missed Cleavages
	High	aRPASASVAEYQR	2	1	1	Q55589	N-Term(iTRAQ4plex)	0,0000	1,049	2	20,7	0	9,449E-08	46	2,43204E-05	3	1549,82022	-0,10	15,53	0
	High	lWGLPLLR	2	1	1	Q55589	N-Term(iTRAQ4plex)	0,0000	2,615	2	41,0	0,001	0,01186	25	0,003435342	2	1111,71050	-0,33	50,09	0
Q55541	Slr0333 protein OS=Synechocystis sp. (strain PCC 6803 / Kazusa) GN=slr0333 PE=4 SV=1 - [Q55541	83,26	37,74	1	4	4	5	1,940	5	25,2	106	11,4	8,48							
	A3	Sequence	# PSMs	# Proteins	# Protein Groups	Protein Group Accessions	Modifications	ΔCn	116/117	116/117 Count	116/117 Variability [%]	q-Value	PEP	IonScore	Exp Value	Charge	MH+ [Da]	ΔM [ppm]	RT [min]	# Missed Cleavages
	High	sSAPQYGLPGDkVEVIk	2	1	1	Q55541	N-Term(iTRAQ4plex); K12(iTRAQ4plex); K17(iTRAQ4plex)	0,0000	2,065	2	26,4	0	0,000001815	41	8,8914E-05	3	2220,26346	1,53	31,82	1
	High	sDFIIFADGGE	1	1	1	Q55541	N-Term(iTRAQ4plex)	0,0000	1,940	1		0,002	0,02298	22	0,007062688	2	1314,63469	0,93	46,70	0
	High	tQPTIk	1	1	1	Q55541	N-Term(iTRAQ4plex); K6(iTRAQ4plex)	0,0000	1,445	1		0,002	0,02226	17	0,021686492	2	975,60820	0,50	13,37	0
	Medium	aVGWIR	1	1	1	Q55541	N-Term(iTRAQ4plex)	0,0000	2,294	1		0,013	0,1786	30	0,001165179	2	845,51158	0,19	25,10	0
Q55564	Sll0162 protein OS=Synechocystis sp. (strain PCC 6803 / Kazusa) GN=sll0162 PE=4 SV=1 - [Q55564	83,19	10,31	1	2	2	2	1,046	2	8,5	194	22,7	5,81							
	A3	Sequence	# PSMs	# Proteins	# Protein Groups	Protein Group Accessions	Modifications	ΔCn	116/117	116/117 Count	116/117 Variability [%]	q-Value	PEP	IonScore	Exp Value	Charge	MH+ [Da]	ΔM [ppm]	RT [min]	# Missed Cleavages
	High	iSQAQSLLQEIQQR	1	1	1	Q55564	N-Term(iTRAQ4plex)	0,0000	0,987	1		0	6,323E-10	80	1,05674E-08	3	1785,99479	0,45	43,03	0
	High	kWELEk	1	1	1	Q55564	N-Term(iTRAQ4plex); K1(iTRAQ4plex); K6(iTRAQ4plex)	0,0000	1,108	1		0	0,001572	17	0,031356797	3	1264,76163	-0,73	21,29	1
Q55776	Sll0180 protein OS=Synechocystis sp. (strain PCC 6803 / Kazusa) GN=sll0180 PE=4 SV=1 - [Q55776	82,95	6,79	1	3	3	3	0,717	3	18,6	501	53,9	5,78							
	A3	Sequence	# PSMs	# Proteins	# Protein Groups	Protein Group Accessions	Modifications	ΔCn	116/117	116/117 Count	116/117 Variability [%]	q-Value	PEP	IonScore	Exp Value	Charge	MH+ [Da]	ΔM [ppm]	RT [min]	# Missed Cleavages
	High	aTVQANQSSVSASR	1	1	1	Q55776	N-Term(iTRAQ4plex)	0,0000	0,717	1		0	1,416E-08	61	7,42968E-07	2	1549,80657	0,94	15,07	0
	High	aQANLNLAQINFk	1	1	1	Q55776	N-Term(iTRAQ4plex); K13(iTRAQ4plex)	0,0000	0,632	1		0	5,366E-08	32	0,000647098	3	1732,99491	0,02	38,37	0
	Medium	sAVQQAk	1	1	1	Q55776	N-Term(iTRAQ4plex); K7(iTRAQ4plex)	0,0000	0,812	1		0,01	0,1239	16	0,026741718	2	1019,60863	-0,16	11,08	0
P74507	2,3-bisphosphoglycerate-independent phosphoglycerate mutase OS=Synechocystis sp. (strain PCC 68	82,48	4,89	1	2	2	3	0,803	3	5,1	532	57,9	5,83							
	A3	Sequence	# PSMs	# Proteins	# Protein Groups	Protein Group Accessions	Modifications	ΔCn	116/117	116/117 Count	116/117 Variability [%]	q-Value	PEP	IonScore	Exp Value	Charge	MH+ [Da]	ΔM [ppm]	RT [min]	# Missed Cleavages
	High	lQGISQLcIHAITDGR	1	1	1	P74507	N-Term(iTRAQ4plex); C8(Carbamidomethyl)	0,0000	0,803	1		0	0,000004919	39	0,000121891	3	1926,03659	0,92	39,22	0
	High	kIPGHGGEVk	2	1	1	P74507	N-Term(iTRAQ4plex); K1(iTRAQ4plex); K10(iTRAQ4plex)	0,0000	0,782	2	9,0	0	0,00007514	36	0,000251171	3	1453,88523	0,05	10,96	1
P73762	Sll0837 protein OS=Synechocystis sp. (strain PCC 6803 / Kazusa) GN=sll0837 PE=4 SV=1 - [P73762_	82,36	11,56	1	3	3	3	1,024	3	21,2	294	32,9	4,81							
	A3	Sequence	# PSMs	# Proteins	# Protein Groups	Protein Group Accessions	Modifications	ΔCn	116/117	116/117 Count	116/117 Variability [%]	q-Value	PEP	IonScore	Exp Value	Charge	MH+ [Da]	ΔM [ppm]	RT [min]	# Missed Cleavages
	High	fTLGNAYFQk	1	1	1	P73762	N-Term(iTRAQ4plex); K10(iTRAQ4plex)	0,0000	0,889	1		0	0,000001619	49	1,16405E-05	2	1476,81059	1,12	37,35	0
	High	kAQDALNLDSR	1	1	1	P73762	N-Term(iTRAQ4plex); K1(iTRAQ4plex)	0,0000	1,024	1		0	0,00003297	43	4,59166E-05	3	1518,84858	0,46	19,60	1
	Medium	aLFATPTMVEVLR	1	1	1	P73762	N-Term(iTRAQ4plex)	0,0000	2,338	1		0,011	0,1479	16	0,027100045	2	1591,90068	0,50	47,90	0
P74341	Sll1537 protein OS=Synechocystis sp. (strain PCC 6803 / Kazusa) GN=sll1537 PE=3 SV=1 - [P74341_	81,67	12,95	1	2	2	7	1,050	6	20,5	139	15,9	4,70							
	A3	Sequence	# PSMs	# Proteins	# Protein Groups	Protein Group Accessions	Modifications	ΔCn	116/117	116/117 Count	116/117 Variability [%]	q-Value	PEP	IonScore	Exp Value	Charge	MH+ [Da]	ΔM [ppm]	RT [min]	# Missed Cleavages
	High	vEWGETLEAALkR	1	1	1	P74341	N-Term(iTRAQ4plex); K12(iTRAQ4plex)	0,0000	1,208	1		0	0,00004014	13	0,044768238	3	1790,00827	1,76	42,70	1
	High	vLIVk	6	1	1	P74341	N-Term(iTRAQ4plex); K5(iTRAQ4plex)	0,0000	1,037	5	18,1	0,008	0,1056	32	0,000665227	2	859,62657	5,42	25,59	0
P74275	Delta-1-pyrroline-5-carboxylate dehydrogenase OS=Synechocystis sp. (strain PCC 6803 / Kazusa) GN	80,08	3,74	1	4	4	7	3,352	7	174,5	990	110,0	5,67							
	A3	Sequence	# PSMs	# Proteins	# Protein Groups	Protein Group Accessions	Modifications	ΔCn	116/117	116/117 Count	116/117 Variability [%]	q-Value	PEP	IonScore	Exp Value	Charge	MH+ [Da]	ΔM [ppm]	RT [min]	# Missed Cleavages
	High	lLPGMAYLIR	4	1	1	P74275	N-Term(iTRAQ4plex)	0,0000	4,994	4	186,7	0	0,000628	33	0,000539473	2	1290,77275	0,21	48,59	0
	High	lLDWTMGHPGLR	1	1	1	P74275	N-Term(iTRAQ4plex)	0,0000	3,182	1		0	0,0001338	27	0,002192653	3	1539,82047	-1,19	40,58	0
	High	dLILNILk	1	1	1	P74275	N-Term(iTRAQ4plex); K8(iTRAQ4plex)	0,0000	5,409	1		0,002	0,02991	33	0,000448714	2	1229,80901	1,50	46,61	0
	Medium	tPDHINR	1	1	1	P74275	N-Term(iTRAQ4plex)	0,0000	1,425	1		0,023	0,3191	14	0,035724816	3	996,53702	2,70	10,80	0
Q55190	High-affinity Na(+)/H(+) antiporter NhaS3 OS=Synechocystis sp. (strain PCC 6803 / Kazusa) GN=nha	80,03	5,42	1	2	2	4	0,944	4	13,1	461	47,8	4,61							
	A3	Sequence	# PSMs	# Proteins	# Protein Groups	Protein Group Accessions	Modifications	ΔCn	116/117	116/117 Count	116/117 Variability [%]	q-Value	PEP	IonScore	Exp Value	Charge	MH+ [Da]	ΔM [ppm]	RT [min]	# Missed Cleavages
	High	lLSPFYVSLVNR	3	1	1	Q55190	N-Term(iTRAQ4plex)	0,0000	1,009	3	9,3	0	0,00001315	39	0,000131211	2	1551,90593	2,81	49,83	0
	High	kEEAPEkPVPTPD	1	1	1	Q55190	N-Term(iTRAQ4plex); K1(iTRAQ4plex); K7(iTRAQ4plex)	0,0000	0,883	1		0	0,00003055	16	0,022907094	3	1869,03293	-0,05	18,40	1
P72802	Mitochondrial outer membrane 72K protein OS=Synechocystis sp. (strain PCC 6803 / Kazusa) GN=m	80,01	7,89	1	2	2	4	0,625	3	14,9	266	29,9	5,30							
	A3	Sequence	# PSMs	# Proteins	# Protein Groups	Protein Group Accessions	Modifications	ΔCn	116/117	116/117 Count	116/117 Variability [%]	q-Value	PEP	IonScore	Exp Value	Charge	MH+ [Da]	ΔM [ppm]	RT [min]	# Missed Cleavages
	High	aEAILTQIR	2	1	1	P72802	N-Term(iTRAQ4plex)	0,0000	0,565	1		0	0,000001723	56	2,97435E-06	2	1158,69731	0,84	32,55	0
	High	[illegible]	[illegible]	1	1	P72802	N-Term(iTRAQ4plex)	0,0000	[illegible]	2	[illegible]	0	[illegible]	[illegible]	[illegible]	3	[illegible]	[illegible]	[illegible]	0
P73163	Putative carboxymethylenebutenolidase OS=Synechocystis sp. (strain PCC 6803 / Kazusa) GN=[illegible]	79,22	18,29	1	2	2	2	[illegible]	2	[illegible]	[illegible]	[illegible]	[illegible]							
	A3	Sequence	# PSMs	# Proteins	# Protein Groups	Protein Group Accessions	Modifications	ΔCn	116/117	116/117 Count	116/117 Variability [%]	q-Value	PEP	IonScore	Exp Value	Charge	MH+ [Da]	ΔM [ppm]	RT [min]	# Missed Cleavages
	High	[illegible]	1	1	1	P73163	N-Term(iTRAQ4plex)	0,0000	[illegible]	1		0	[illegible]	[illegible]	[illegible]	3	[illegible]	[illegible]	[illegible]	0
	High	[illegible]	1	1	1	P73163	N-Term(iTRAQ4plex)	0,0000	[illegible]	1		0	[illegible]	[illegible]	[illegible]	[illegible]	[illegible]	[illegible]	[illegible]	0
P72689	Translation initiation factor IF-2 OS=Synechocystis sp. (strain PCC 6803 / Kazusa) GN=infB PE=3 SV=1 -	79,06	9,29	1	9	9	10	[illegible]	10	[illegible]	[illegible]	[illegible]	[illegible]							
	A3	Sequence	# PSMs	# Proteins	# Protein Groups	Protein Group Accessions	Modifications	ΔCn	116/117	116/117 Count	116/117 Variability [%]	q-Value	PEP	IonScore	Exp Value	Charge	MH+ [Da]	ΔM [ppm]	RT [min]	# Missed Cleavages
	High	[illegible]	[illegible]	1	1	P72689	[illegible]	0,0000	[illegible]	[illegible]	[illegible]	[illegible]	[illegible]	[illegible]	[illegible]	[illegible]	[illegible]	[illegible]	[illegible]	[illegible]
	High	[illegible]	[illegible]	1	1	P72689	[illegible]	0,0000	[illegible]	[illegible]	[illegible]	[illegible]	[illegible]	[illegible]	[illegible]	[illegible]	[illegible]	[illegible]	[illegible]	[illegible]
	High	[illegible]	[illegible]	1	1	P72689	[illegible]	0,0000	[illegible]	[illegible]	[illegible]	[illegible]	[illegible]	[illegible]	[illegible]	[illegible]	[illegible]	[illegible]	[illegible]	[illegible]
	High	[illegible]	[illegible]	1	1	P72689	[illegible]	0,0000	[illegible]	[illegible]	[illegible]	[illegible]	[illegible]	[illegible]	[illegible]	[illegible]	[illegible]	[illegible]	[illegible]	[illegible]
	High	[illegible]	[illegible]	1	1	P72689	[illegible]	0,0000	[illegible]	[illegible]	[illegible]	[illegible]	[illegible]	[illegible]	[illegible]	[illegible]	[illegible]	[illegible]	[illegible]	[illegible]
	High	[illegible]	[illegible]	1	1	P72689	[illegible]	0,0000	[illegible]	[illegible]	[illegible]	[illegible]	[illegible]	[illegible]	[illegible]	[illegible]	[illegible]	[illegible]	[illegible]	[illegible]
	High	[illegible]	[illegible]	1	1	P72689	[illegible]	0,0000	[illegible]	[illegible]	[illegible]	[illegible]	[illegible]	[illegible]	[illegible]	[illegible]	[illegible]	[illegible]	[illegible]	[illegible]
	Medium	[illegible]	[illegible]	1	1	P72689	[illegible]	0,0000	[illegible]	[illegible]	[illegible]	[illegible]	[illegible]	[illegible]	[illegible]	[illegible]	[illegible]	[illegible]	[illegible]	[illegible]
	Medium	[illegible]	[illegible]	1	1	P72689	[illegible]	0,0000	[illegible]	[illegible]	[illegible]	[illegible]	[illegible]	[illegible]	[illegible]	[illegible]	[illegible]	[illegible]	[illegible]	[illegible]
P73283	3-oxoacyl-[acyl-carrier-protein] synthase 2 OS=Synechocystis sp. (strain PCC 6803 / Kazusa) GN=[illegible]	78,96	5,53	1	2	2	4	[illegible]	3	[illegible]	[illegible]	[illegible]	[illegible]							
	A3	Sequence	# PSMs	# Proteins	# Protein Groups	Protein Group Accessions	Modifications	ΔCn	116/117	116/117 Count	116/117 Variability [%]	q-Value	PEP	IonScore	Exp Value	Charge	MH+ [Da]	ΔM [ppm]	RT [min]	# Missed Cleavages
	High	[illegible]	[illegible]	1	1	P73283	[illegible]	0,0000	[illegible]	[illegible]	[illegible]	0	[illegible]	[illegible]	[illegible]	[illegible]	[illegible]	[illegible]	[illegible]	[illegible]
	High	[illegible]	[illegible]	1	1	P73283	[illegible]	0,0000	[illegible]	[illegible]	[illegible]	0	[illegible]	[illegible]	[illegible]	[illegible]	[illegible]	[illegible]	[illegible]	[illegible]
Q55738	DNA gyrase subunit A OS=Synechocystis sp. (strain PCC 6803 / Kazusa) GN=gyrA PE=3 SV=1 - [GYR	78,91	7,21	1	5	5	5	[illegible]	5	[illegible]	[illegible]	[illegible]	[illegible]							

	A3	Sequence	# PSMs	# Proteins	# Protein Groups	Protein Group Accessions	Modifications	ΔCn	116/117	116/117 Count	116/117 Variability [%]	q-Value	PEP	IonScore	Exp Value	Charge	MH+ [Da]	ΔM [ppm]	RT [min]	# Missed Cleavages
	High	lRPLSTNSLLR	1	1	1	Q55738	N-Term(iTRAQ4plex)	0,0000	2,654	1		0	0,000007642	39	0,000127929	3	1413,86524	-0,44	30,82	0
	High	rmPASTFGTQNR	1	1	1	Q55738	N-Term(iTRAQ4plex); M2(Oxidation)	0,0000	1,107	1		0	0,0003675	26	0,002382155	3	1525,76438	-1,22	12,21	1
	High	gVVYSLNAYQIPIASR	1	1	1	Q55738	N-Term(iTRAQ4plex)	0,0000	0,995	1		0	0,001835	25	0,002870582	3	1895,05204	0,67	43,44	0
	High	gVASIETmEHPGRPDR	1	1	1	Q55738	N-Term(iTRAQ4plex); M8(Oxidation)	0,0000	0,692	1		0,001	0,01513	15	0,034831326	4	1911,94668	0,13	17,72	0
	High	iVIELkR	1	1	1	Q55738	N-Term(iTRAQ4plex); K6(iTRAQ4plex)	0,0000	3,429	1		0,003	0,03764	26	0,002630086	3	1158,78238	0,95	26,84	1
Q55360	Sll0888 protein OS=Synechocystis sp. (strain PCC 6803 / Kazusa) GN=sll0888 PE=4 SV=1 - [Q55360	78,75	12,11	1	2	2	4	0,449	3	29,1	190	21,9	4,94							
	A3	Sequence	# PSMs	# Proteins	# Protein Groups	Protein Group Accessions	Modifications	ΔCn	116/117	116/117 Count	116/117 Variability [%]	q-Value	PEP	IonScore	Exp Value	Charge	MH+ [Da]	ΔM [ppm]	RT [min]	# Missed Cleavages
	High	nRFPDLTIIR	2	1	1	Q55360	N-Term(iTRAQ4plex)	0,0000	0,497	2	45,7	0	0,00004903	42	6,69838E-05	3	1388,81375	0,47	35,37	1
	High	gHGLELEVHGEPk	2	1	1	Q55360	N-Term(iTRAQ4plex); K13(iTRAQ4plex)	0,0000	0,449	1		0	0,000002634	36	0,00264832	4	1689,91775	0,86	20,92	0
P73851	Aspartate--tRNA ligase OS=Synechocystis sp. (strain PCC 6803 / Kazusa) GN=aspS PE=3 SV=1 - [SY	78,34	5,68	1	4	4	6	2,109	6	38,7	599	67,2	5,31							
	A3	Sequence	# PSMs	# Proteins	# Protein Groups	Protein Group Accessions	Modifications	ΔCn	116/117	116/117 Count	116/117 Variability [%]	q-Value	PEP	IonScore	Exp Value	Charge	MH+ [Da]	ΔM [ppm]	RT [min]	# Missed Cleavages
	High	ikPGGDLFk	3	1	1	P73851	N-Term(iTRAQ4plex); K2(iTRAQ4plex); K9(iTRAQ4plex)	0,0000	2,157	3	6,7	0	0,00001653	41	8,31706E-05	3	1406,87171	-1,05	26,27	0
	High	lVmLLAk	1	1	1	P73851	N-Term(iTRAQ4plex); M3(Oxidation); K7(iTRAQ4plex)	0,0000	0,748	1		0	0,0002184	27	0,002036901	2	1091,71013	0,03	34,38	0
	High	lSQNLQLR	1	1	1	P73851	N-Term(iTRAQ4plex)	0,0000	1,620	1		0	0,0005523	25	0,003349423	2	1115,66570	0,31	26,93	0
	High	nIDLPRPFPR	1	1	1	P73851	N-Term(iTRAQ4plex)	0,0000	2,918	1		0	0,0004445	17	0,021476821	3	1368,78742	0,38	32,13	0
P74721	Zinc-containing alcohol dehydrogenase family OS=Synechocystis sp. (strain PCC 6803 / Kazusa) GN=	78,08	5,65	1	3	3	4	0,313	4	35,0	336	35,8	6,10							
	A3	Sequence	# PSMs	# Proteins	# Protein Groups	Protein Group Accessions	Modifications	ΔCn	116/117	116/117 Count	116/117 Variability [%]	q-Value	PEP	IonScore	Exp Value	Charge	MH+ [Da]	ΔM [ppm]	RT [min]	# Missed Cleavages
	High	fDYIISTVNLk	1	1	1	P74721	N-Term(iTRAQ4plex); K11(iTRAQ4plex)	0,0000	0,225	1		0	0,00005658	35	0,000298518	3	1600,91800	-0,56	45,49	0
	High	vVLSHSk	2	1	1	P74721	N-Term(iTRAQ4plex); K7(iTRAQ4plex)	0,0000	0,313	2	26,0	0,001	0,006569	41	8,85054E-05	2	1057,66033	-0,46	13,02	0
	Medium	vVLSHSkN	1	1	1	P74721	N-Term(iTRAQ4plex); K7(iTRAQ4plex)	0,0000	0,416	1		0,019	0,2493	11	0,086890041	3	1171,70340	-0,29	12,36	1
P72937	Slr0670 protein OS=Synechocystis sp. (strain PCC 6803 / Kazusa) GN=slr0670 PE=4 SV=1 - [P72937	77,52	19,24	1	4	4	6	1,564	5	18,1	291	32,7	5,54							
	A3	Sequence	# PSMs	# Proteins	# Protein Groups	Protein Group Accessions	Modifications	ΔCn	116/117	116/117 Count	116/117 Variability [%]	q-Value	PEP	IonScore	Exp Value	Charge	MH+ [Da]	ΔM [ppm]	RT [min]	# Missed Cleavages
	High	rPELLEIR	2	1	1	P72937	N-Term(iTRAQ4plex)	0,0000	1,485	2	10,3	0	0,0001111	38	0,000148583	3	1169,71204	-0,24	26,36	0
	High	qQFSPLVPNIITEVR	1	1	1	P72937	N-Term(iTRAQ4plex)	0,0000	1,008	1		0	0,0001896	35	0,000293069	3	1885,06839	1,04	47,35	0
	High	lFGSHTLSLAk	2	1	1	P72937	N-Term(iTRAQ4plex); K11(iTRAQ4plex)	0,0000	1,564	1		0	0,0001901	12	0,082258587	3	1461,86643	-0,26	33,56	0
	High	vPVmILRPQLVSTYTVEEmALR	1	1	1	P72937	N-Term(iTRAQ4plex); M4(Oxidation); M19(Oxidation)	0,0000	1,931	1		0	0,0001218	11	0,086093429	3	2721,47989	-0,07	45,81	0
P73280	Sll1072 protein OS=Synechocystis sp. (strain PCC 6803 / Kazusa) GN=sll1072 PE=4 SV=1 - [P73280_	77,23	10,31	1	2	2	3	0,505	3	13,1	223	25,2	5,20							
	A3	Sequence	# PSMs	# Proteins	# Protein Groups	Protein Group Accessions	Modifications	ΔCn	116/117	116/117 Count	116/117 Variability [%]	q-Value	PEP	IonScore	Exp Value	Charge	MH+ [Da]	ΔM [ppm]	RT [min]	# Missed Cleavages
	High	aELIEGIVHVASPLR	2	1	1	P73280	N-Term(iTRAQ4plex)	0,0000	0,483	2	6,5	0	0,000001423	49	1,31513E-05	3	1748,02018	0,81	45,44	0
	High	vYENHGVk	1	1	1	P73280	N-Term(iTRAQ4plex); K8(iTRAQ4plex)	0,0000	0,557	1		0,006	0,08331	16	0,023603152	3	1233,68653	2,85	12,59	0
P73618	Deoxyribose-phosphate aldolase OS=Synechocystis sp. (strain PCC 6803 / Kazusa) GN=deoC PE=3 S	76,87	10,22	1	2	2	3	0,372	3	24,6	225	23,9	5,62							
	A3	Sequence	# PSMs	# Proteins	# Protein Groups	Protein Group Accessions	Modifications	ΔCn	116/117	116/117 Count	116/117 Variability [%]	q-Value	PEP	IonScore	Exp Value	Charge	MH+ [Da]	ΔM [ppm]	RT [min]	# Missed Cleavages
	High	eAIATccAQALHHk	2	1	1	P73618	N-Term(iTRAQ4plex); C6(Carbamidomethyl); C7(Carbamidome	0,0000	0,343	2	12,1	0	2,513E-12	60	1,00918E-06	3	1897,96451	1,56	19,05	0
	High	lLHQISQGR	1	1	1	P73618	N-Term(iTRAQ4plex)	0,0000	0,663	1		0	0,001416	16	0,024944224	3	1195,70313	0,27	19,43	0
P72584	Magnesium-protoporphyrin IX monomethyl ester [oxidative] cyclase 1 OS=Synechocystis sp. (strain F	76,11	10,06	1	3	3	5	2,283	5	15,5	358	42,1	7,12							
	A3	Sequence	# PSMs	# Proteins	# Protein Groups	Protein Group Accessions	Modifications	ΔCn	116/117	116/117 Count	116/117 Variability [%]	q-Value	PEP	IonScore	Exp Value	Charge	MH+ [Da]	ΔM [ppm]	RT [min]	# Missed Cleavages
	High	lYLmkPIAVDQLAGAVR	1	1	1	P72584	N-Term(iTRAQ4plex); M4(Oxidation); K5(iTRAQ4plex)	0,0000	2,108	1		0	1,235E-09	49	1,18842E-05	3	2162,25949	-0,72	42,49	0
	High	qLFVEFLER	3	1	1	P72584	N-Term(iTRAQ4plex)	0,0000	2,533	3	15,5	0	0,000004891	38	0,00015921	2	1324,73943	0,92	47,89	0
	High	aQPHTLNDWk	1	1	1	P72584	N-Term(iTRAQ4plex); K10(iTRAQ4plex)	0,0000	1,574	1		0,001	0,003894	12	0,065761242	3	1497,80436	-0,61	20,93	0
Q55700	ATP-dependent zinc metalloprotease FtsH 2 OS=Synechocystis sp. (strain PCC 6803 / Kazusa) GN=fts	75,77	6,86	1	3	5	5	1,474	3	41,9	627	68,5	5,41							
	A3	Sequence	# PSMs	# Proteins	# Protein Groups	Protein Group Accessions	Modifications	ΔCn	116/117	116/117 Count	116/117 Variability [%]	q-Value	PEP	IonScore	Exp Value	Charge	MH+ [Da]	ΔM [ppm]	RT [min]	# Missed Cleavages
	High	gVLLVGPPGTGk	1	3	3	Q55700;P72991;P73437	N-Term(iTRAQ4plex); K12(iTRAQ4plex)	0,0000				0	0,001499	31	0,000749842	3	1382,85981	-0,88	31,27	0
	High	kEILEVHAR	1	1	1	Q55700	N-Term(iTRAQ4plex); K1(iTRAQ4plex)	0,0000	0,784	1		0,002	0,02532	19	0,011640456	4	1382,83486	-0,71	17,82	1
	High	qLAEQGHQmAR	1	1	1	Q55700	N-Term(iTRAQ4plex); M9(Oxidation)	0,0000	1,474	1		0,002	0,01983	18	0,015702543	3	1428,71445	0,69	8,99	0
	Medium	vTLIPR	1	1	1	Q55700	N-Term(iTRAQ4plex)	0,0000	1,933	1		0,014	0,1815	30	0,001073915	2	842,55803	0,00	25,74	0
	Medium	tLLAk	1	3	3	Q55700;P72991;P73437	N-Term(iTRAQ4plex); K5(iTRAQ4plex)	0,0000				0,029	0,3849	29	0,001121941	2	833,56822	-1,98	18,81	0
P74527	Aminopeptidase OS=Synechocystis sp. (strain PCC 6803 / Kazusa) GN=ape2 PE=4 SV=1 - [P74527_	75,56	1,96	1	2	2	2	3,355	2	65,3	869	97,9	5,77							
	A3	Sequence	# PSMs	# Proteins	# Protein Groups	Protein Group Accessions	Modifications	ΔCn	116/117	116/117 Count	116/117 Variability [%]	q-Value	PEP	IonScore	Exp Value	Charge	MH+ [Da]	ΔM [ppm]	RT [min]	# Missed Cleavages
	High	iAWVLIk	1	1	1	P74527	N-Term(iTRAQ4plex); K7(iTRAQ4plex)	0,0000	5,015	1		0	0,00008431	55	3,06881E-06	2	1130,75334	-0,59	43,44	0
	High	aVLGDELFFR	1	1	1	P74527	N-Term(iTRAQ4plex)	0,0000	2,244	1		0,001	0,003559	33	0,000451825	2	1310,72014	-1,85	44,65	0
P73493	Glutathione synthetase OS=Synechocystis sp. (strain PCC 6803 / Kazusa) GN=gshB PE=3 SV=1 - [GS	75,54	9,69	1	2	2	3	1,425	2	25,9	320	35,0	5,55							
	A3	Sequence	# PSMs	# Proteins	# Protein Groups	Protein Group Accessions	Modifications	ΔCn	116/117	116/117 Count	116/117 Variability [%]	q-Value	PEP	IonScore	Exp Value	Charge	MH+ [Da]	ΔM [ppm]	RT [min]	# Missed Cleavages
	High	iILLDGDPIGAVNR	1	1	1	P73493	N-Term(iTRAQ4plex)	0,0000	1,200	1		0	5,999E-07	54	4,16841E-06	2	1609,93950	0,03	45,30	0
	High	qFLEEHGAAVLkPLGGk	2	1	1	P73493	N-Term(iTRAQ4plex); K12(iTRAQ4plex); K17(iTRAQ4plex)	0,0000	1,692	1		0	1,442E-08	29	0,001279293	4	2226,29702	-0,05	33,57	0
P74145	Sll1390 protein OS=Synechocystis sp. (strain PCC 6803 / Kazusa) GN=sll1390 PE=4 SV=1 - [P74145_	75,39	10,44	1	3	3	4	1,191	4	3,8	249	27,4	4,74							
	A3	Sequence	# PSMs	# Proteins	# Protein Groups	Protein Group Accessions	Modifications	ΔCn	116/117	116/117 Count	116/117 Variability [%]	q-Value	PEP	IonScore	Exp Value	Charge	MH+ [Da]	ΔM [ppm]	RT [min]	# Missed Cleavages
	High	yNQALIEADkR	2	1	1	P74145	N-Term(iTRAQ4plex); K10(iTRAQ4plex)	0,0000	1,213	2	3,8	0	0,000002566	36	0,000241529	3	1608,89536	0,34	22,55	1
	High	lAQSSGQEAR	1	1	1	P74145	N-Term(iTRAQ4plex)	0,0000	0,901	1		0,001	0,007623	21	0,007833755	2	1190,62505	0,36	12,08	0
	Medium	fVVIR	1	1	1	P74145	N-Term(iTRAQ4plex)	0,0000	1,199	1		0,041	0,5351	24	0,008038439	2	777,50926	-1,40	26,65	0
Q57310	Lipid-A-disaccharide synthase OS=Synechocystis sp. (strain PCC 6803 / Kazusa) GN=lpxB PE=3 SV=1	75,21	8,63	1	3	3	5	2,237	5	17,3	394	43,4	6,62							
	A3	Sequence	# PSMs	# Proteins	# Protein Groups	Protein Group Accessions	Modifications	ΔCn	116/117	116/117 Count	116/117 Variability [%]	q-Value	PEP	IonScore	Exp Value	Charge	MH+ [Da]	ΔM [ppm]	RT [min]	# Missed Cleavages
	High	fIIPTWQIQQR	2	1	1	Q57310	N-Term(iTRAQ4plex)	0,0000	2,823	2	4,6	0	0,0006556	33	0,000469862	2	1573,89897	1,15	45,31	0
	High	fNLPFVSPTNIVLNR	2	1	1	Q57310	N-Term(iTRAQ4plex)	0,0000	1,994	2	0,1	0	0,0007453	28	0,001610534	2	1875,06682	3,13	51,84	0
	High	lLLPVSLk	1	1	1	Q57310	N-Term(iTRAQ4plex); K8(iTRAQ4plex)	0,0000	2,237	1		0,001	0,009456	29	0,001409191	2	1170,80693	0,42	42,97	0
P09192	Photosystem II D2 protein OS=Synechocystis sp. (strain PCC 6803 / Kazusa) GN=psbD PE=3 SV=2 -	74,65	4,26	1	2	2	4	0,754	4	23,6	352	39,5	5,55							
	A3	Sequence	# PSMs	# Proteins	# Protein Groups	Protein Group Accessions	Modifications	ΔCn	116/117	116/117 Count	116/117 Variability [%]	q-Value	PEP	IonScore	Exp Value	Charge	MH+ [Da]	ΔM [ppm]	RT [min]	# Missed Cleavages
	High	nILLNEGmR	2	1	1	P09192	N-Term(iTRAQ4plex); M8(Oxidation)	0,0000	0,888	2	8,3	0	0,0002035	46	2,81171E-05	2	1219,65923	0,53	24,73	0
	High	nILLNEGMR	1	1	1	P09192	N-Term(iTRAQ4plex)	0,0000	0,613	1		0,001	0,01356	20	0,010567445	2	1203,66313	-0,44	29,59	0
	High	qFEISR	1	1	1	P09192	N-Term(iTRAQ4plex)	0,0000	0,677	1		0,001	0,005284	14	0,037065512	2	923,50701	0,30	20,07	0
Q55074	Bifunctional pantoate ligase/cytidylate kinase OS=Synechocystis sp. (strain PCC 6803 / Kazusa) GN=	74,63	4,48	1	2	2	2	1,430	2	47,0	513	55,4	5,68							
	A3	Sequence	# PSMs	# Proteins	# Protein Groups	Protein Group Accessions	Modifications	ΔCn	116/117	116/117 Count	116/117 Variability [%]	q-Value	PEP	IonScore	Exp Value	Charge	MH+ [Da]	ΔM [ppm]	RT [min]	# Missed Cleavages
	High	kYGDQGGIVAEGR	1	1	1	Q55074	N-Term(iTRAQ4plex); K1(iTRAQ4plex)	0,0000	1,058	1		0	0,00002721	46	2,76039E-05	3	1637,89469	5,92	16,81	1
	High	lIDNLVLNHR	1	1	1	Q55074	N-Term(iTRAQ4plex)	0,0000	1,932	1		0	0,0000321	42	6,22257E-05	3	1350,79786	0,30	35,02	0
Q55374	Slr0907 protein OS=Synechocystis sp. (strain PCC 6803 / Kazusa) GN=slr0907 PE=4 SV=1 - [Q55374	73,83	3,16	1	3	3	4	6,134	4	151,2	1014	115,3	5,25							
	A3	Sequence	# PSMs	# Proteins	# Protein Groups	Protein Group Accessions	Modifications	ΔCn	116/117	116/117 Count	116/117 Variability [%]	q-Value	PEP	IonScore	Exp Value	Charge	MH+ [Da]	ΔM [ppm]	RT [min]	# Missed Cleavages
	High	fWILPAVVPHNLIk	1	1	1	Q55374	N-Term(iTRAQ4plex); K14(iTRAQ4plex)	0,0000	13,364	1		0	0,000001755	31	0,000814648	3	1935,18216	-0,07	51,39	0
	High	fLIDPLINR	2	1	1	Q55374	N-Term(iTRAQ4plex)	0,0000	6,134	2	136,7	0	0,00052	30	0,000943996	2	1357,83281	0,27	50,51	0
	High	fATHPELR	1	1	1	Q55374	N-Term(iTRAQ4plex)	0,0000	1,563	1		0,007	0,09761	29	0,001258838	3	1114,61872	5,49	18,29	0
P73599	Uncharacterized protein sll1304 OS=Synechocystis sp. (strain PCC 6803 / Kazusa) GN=sll1304 PE=3	72,56	8,36	1	2	2	3	0,488	3	5,0	287	32,8	5,99							
	A3	Sequence	# PSMs	# Proteins	# Protein Groups	Protein Group Accessions	Modifications	ΔCn	116/117	116/117 Count	116/117 Variability [%]	q-Value	PEP	IonScore	Exp Value	Charge	MH+ [Da]	ΔM [ppm]	RT [min]	# Missed Cleavages
	High	gTPGSDSFAWGHWFk	1	1	1	P73599	N-Term(iTRAQ4plex); K15(iTRAQ4plex)	0,0000	0,579	1		0	2,063E-07	50	1,08885E-05	3	1967,96537	0,53	42,61	0
	High	fGVHTFIWk	2	1	1	P73599	N-Term(iTRAQ4plex); K9(iTRAQ4plex)	0,0000	0,480	2	2,5	0	0,0001143	26	0,002296149	3	1422,81433	0,48	38,10	0
P48944	30S ribosomal protein S14 OS=Synechocystis sp. (strain PCC 6803 / Kazusa) GN=rpsN PE=3 SV=1 -	72,24	43,00	1	4	4	8	1,712	6	18,8	100	11,8	10,74							
	A3	Sequence	# PSMs	# Proteins	# Protein Groups	Protein Group Accessions	Modifications	ΔCn	116/117	116/117 Count	116/117 Variability [%]	q-Value	PEP	IonScore	Exp Value	Charge	MH+ [Da]	ΔM [ppm]	RT [min]	# Missed Cleavages
	High	eWAHQGLLPGVTk	5	1	1	P48944	N-Term(iTRAQ4plex); K13(iTRAQ4plex)	0,0000	1,660	3	9,3	0	1,533E-07	36	0,00024042	3	1723,97403	0,36	31,97	0
	High	qAETLEDkLAVHQk	1	1	1	P48944	N-Term(iTRAQ4plex); K8(iTRAQ4plex); K14(iTRAQ4plex)	0,0000	2,222	1		0	4,189E-08	23	0,005057897	4	2042,16054	-0,06	26,18	1
	High	eALkEEFR	1	1	1	P48944	N-Term(iTRAQ4plex); K4(iTRAQ4plex)	0,0000	1,968	1		0	0,001746	12	0,059974965	3	1309,73590	0,33	21,00	1
	High	cQVTGRPR	1	1	1	P48944	N-Term(iTRAQ4plex); C1(Carbamidomethyl)	0,0000	1,530	1		0,005	0,06804	10	0,094399567	3	1117,60151	-0,19	8,88	0
P73946	Slr1506 protein OS=Synechocystis sp. (strain PCC 6803 / Kazusa) GN=slr1506 PE=4 SV=1 - [P73946	72,21	8,20	1	6	6	7	0,978	7	21,1	622	68,8	5,30							
	A3	Sequence	# PSMs	# Proteins	# Protein Groups	Protein Group Accessions	Modifications	ΔCn	116/117	116/117 Count	116/117 Variability [%]	q-Value	PEP	IonScore	Exp Value	Charge	MH+ [Da]	ΔM [ppm]	RT [min]	# Missed Cleavages
	High	qGYYPTEFIDRPk	1	1	1	P73946	N-Term(iTRAQ4plex); K13(iTRAQ4plex)	0,0000	0,810	1		0	0,00001577	37	0,000211334	3	1902,00162	0,85	31,81	0
	High	fYVDVYRPQR	2	1	1	P73946	N-Term(iTRAQ4plex)	0,0000	1,093	2	38,4	0	0,0002309	25	0,003326366	3	1486,79413	1,20	30,07	0
	High	fYLISQk	1	1	1	P73946	N-Term(iTRAQ4plex); K7(iTRAQ4plex)	0,0000	0,850	1		0	0,0002424	22	0,00654591	2	1186,71062	2,69	33,27	0
	High	rFYVDVYRPQR	1	1	1	P73946	N-Term(iTRAQ4plex)	0,0000	1,109	1		0	0,0000981	13	0,045391027	4	1642,89431	0,51	24,88	1
	High	lREIMGLR	1	1	1	P73946	N-Term(iTRAQ4plex)	0,0000	1,275	1		0,001	0,003887	24	0,003953393	3	1131,68177	2,51	29,45	1
	Medium	dISYVIDELERR	1	1	1	P73946	N-Term(iTRAQ4plex)	0,0000	0,978	1		0,012	0,1547	12	0,066787908	3	1651,88193	2,85	47,70	1
P74302	Slr0937 protein OS=Synechocystis sp. (strain PCC 6803 / Kazusa) GN=slr0937 PE=4 SV=1 - [P74302	71,96	7,19	1	2	2	2	0,885	2	2,6	417	46,1	4,37							
	A3	Sequence	# PSMs	# Proteins	# Protein Groups	Protein Group Accessions	Modifications	ΔCn	116/117	116/117 Count	116/117 Variability [%]	q-Value	PEP	IonScore	Exp Value	Charge	MH+ [Da]	ΔM [ppm]	RT [min]	# Missed Cleavages
	High	eVkPWASVGDVLASFTR	1	1	1	P74302	N-Term(iTRAQ4plex); K3(iTRAQ4plex)	0,0000	0,901	1		0	0,000001834	45	2,94422E-05	3	2150,18314	-0,82	52,36	0
	High	lASTEQELLGHQR	1	1	1	P74302	N-Term(iTRAQ4plex)	0,0000	0,870	1		0	4,291E-08	40	0,000107887	3	1625,87253	-0,17	29,29	0
Q55758	Bifunctional protein PyrR OS=Synechocystis sp. (strain PCC 6803 / Kazusa) GN=pyrR PE=3 SV=1 - [F	70,76	14,61	1	2	2	3	1,258	3	7,2	178	19,9	9,17							
	A3	Sequence	# PSMs	# Proteins	# Protein Groups	Protein Group Accessions	Modifications	ΔCn	116/117	116/117 Count	116/117 Variability [%]	q-Value	PEP	IonScore	Exp Value	Charge	MH+ [Da]	ΔM [ppm]	RT [min]	# Missed Cleavages
	High	tKIPLSLTGk	1	1	1	Q55758	N-Term(iTRAQ4plex); K2(iTRAQ4plex); K10(iTRAQ4plex)	0,0000	1,258	1		0	0,00001229	36	0,000238215	3	1489,96762	-0,12	27,76	1
	High	aALNAVTEYGRPQVIR	2	1	1	Q55758	N-Term(iTRAQ4plex)	0,0000	1,137	2	22,4	0	1,926E-07	32	0,000768596	3	1902,06864	0,42	32,32	0
P54386	NADP-specific glutamate dehydrogenase OS=Synechocystis sp. (strain PCC 6803 / Kazusa) GN=gdhA	70,58	6,07	1	3	3	3	1,059	2	35,8	428	47,3	5,16							
	A3	Sequence	# PSMs	# Proteins	# Protein Groups	Protein Group Accessions	Modifications	ΔCn	116/117	116/117 Count	116/117 Variability [%]	q-Value	PEP	IonScore	Exp Value	Charge	MH+ [Da]	ΔM [ppm]	RT [min]	# Missed Cleavages
	High	kISPAVVTGkPVTmGGSQGR	1	1	1	P54386	N-Term(iTRAQ4plex); K1(iTRAQ4plex); K10(iTRAQ4plex); M14	0,0000				0	0,000000535	43	5,32072E-05	4	2418,38759	0,50	21,73	1
	High	iSPAVVTGkPVTmGGSQGR	1	1	1	P54386	N-Term(iTRAQ4plex); K9(iTRAQ4plex); M13(Oxidation)	0,0000	0,838	1		0	0,00001172	35	0,000341956	3	2146,18998	0,30	24,78	0
	High	iFPGYR	1	1	1	P54386	N-Term(iTRAQ4plex)	0,0000	1,339	1		0,002	0,02965	19	0,011994165	2	896,51109	0,02	26,86	0
P73355	Ferredoxin component OS=Synechocystis sp. (strain PCC 6803 / Kazusa) GN=slr1205 PE=4 SV=1 - [	69,30	31,10	1	4	4	7	0,878	5	13,3	164	17,5	8,31							
	A3	Sequence	# PSMs	# Proteins	# Protein Groups	Protein Group Accessions	Modifications	ΔCn	116/117	116/117 Count	116/117 Variability [%]	q-Value	PEP	IonScore	Exp Value	Charge	MH+ [Da]	ΔM [ppm]	RT [min]	# Missed Cleavages
	High	ikDGAIVcPFHR	3	1	1	P73355	N-Term(iTRAQ4plex); K2(iTRAQ4plex); C8(Carbamidomethyl)	0,0000	0,850	2	8,4	0	0,000003284	28	0,001516946	4	1700,95009	-0,48	26,67	1
	High	mSAEkPLPVFPVR	2	1	1	P73355	N-Term(iTRAQ4plex); M1(Oxidation); K5(iTRAQ4plex)	0,0000	0,720	1		0	0,00007707	27	0,002019246	3	1775,01340	0,30	30,93	0
	High	dGAIVcPFHR	1	1	1	P73355	N-Term(iTRAQ4plex); C6(Carbamidomethyl)	0,0000	0,966	1		0	0,000511	27	0,002172551	3	1315,66806	-1,33	24,39	0
	High	vNQQAILLLNEGGTIHAVSNQcPH	1	1	1	P73355	N-Term(iTRAQ4plex); C22(Carbamidomethyl); K26(iTRAQ4plex	0,0000	0,878	1		0	7,809E-07	22	0,006917832	5	3142,69422	-2,94	39,74	0
P73152	Uncharacterized thylakoid-associated protein sll0982 OS=Synechocystis sp. (strain PCC 6803 / Kazusa	69,22	17,97	1	2	2	2	0,456	2	3,8	128	14,3	4,92							
	A3	Sequence	# PSMs	# Proteins	# Protein Groups	Protein Group Accessions	Modifications	ΔCn	116/117	116/117 Count	116/117 Variability [%]	q-Value	PEP	IonScore	Exp Value	Charge	MH+ [Da]	ΔM [ppm]	RT [min]	# Missed Cleavages
	High	tTNNSTLLDTIQQGFR	1	1	1	P73152	N-Term(iTRAQ4plex)	0,0000	0,468	1		0	0,000008754	50	1,00224E-05	3	1953,01755	0,87	44,60	0
	High	aSQWAQk	1	1	1	P73152	N-Term(iTRAQ4plex); K7(iTRAQ4plex)	0,0000	0,445	1		0	0,0005735	32	0,000595662	2	1106,62004	0,32	15,90	0
P74511	Thylakoid membrane protein slr1949 OS=Synechocystis sp. (strain PCC 6803 / Kazusa) GN=slr1949 F	69,21	12,74	1	3	3	5	1,638	4	17,5	212	23,9	9,13							
	A3	Sequence	# PSMs	# Proteins	# Protein Groups	Protein Group Accessions	Modifications	ΔCn	116/117	116/117 Count	116/117 Variability [%]	q-Value	PEP	IonScore	Exp Value	Charge	MH+ [Da]	ΔM [ppm]	RT [min]	# Missed Cleavages
	High	nLLFWHEVAR	3	1	1	P74511	N-Term(iTRAQ4plex)	0,0000	1,740	2	17,5	0	0,000001114	41	7,2272E-05	3	1428,78687	-0,01	37,46	0
	High	tLIEQTPNRR	1	1	1	P74511	N-Term(iTRAQ4plex)	0,0000	1,733	1		0	0,001129	24	0,004245902	3	1371,78293	0,30	16,24	1
	Medium	aTTLATR	1	1	1	P74511	N-Term(iTRAQ4plex)	0,0000	1,002	1		0,046	0,5959	11	0,079977901	2	889,55974	1,10	21,94	0
P74445	Slr0146 protein OS=Synechocystis sp. (strain PCC 6803 / Kazusa) GN=slr0146 PE=4 SV=1 - [P74445	68,94	12,94	1	2	2	3	2,068	3	3,8	170	19,7	4,83							
	A3	Sequence	# PSMs	# Proteins	# Protein Groups	Protein Group Accessions	Modifications	ΔCn	116/117	116/117 Count	116/117 Variability [%]	q-Value	PEP	IonScore	Exp Value	Charge	MH+ [Da]	ΔM [ppm]	RT [min]	# Missed Cleavages
	High	aEDHYLQPPEIISFHk	2	1	1	P74445	N-Term(iTRAQ4plex); K16(iTRAQ4plex)	0,0000	1,757	2	28,6	0	0,000003039	34	0,000392816	4	2212,16274	-0,63	36,89	0
	High	lSALLk	1	1	1	P74445	N-Term(iTRAQ4plex); K6(iTRAQ4plex)	0,0000	2,068	1		0,001	0,01414	32	0,000629463	2	932,63780	-0,53	31,62	0
P74474	D-alanyl-D-alanine carboxypeptidase OS=Synechocystis sp. (strain PCC 6803 / Kazusa) GN=slr1924 F	68,72	8,50	1	3	3	3	0,303	3	34,8	400	44,3	4,75							
	A3	Sequence	# PSMs	# Proteins	# Protein Groups	Protein Group Accessions	Modifications	ΔCn	116/117	116/117 Count	116/117 Variability [%]	q-Value	PEP	IonScore	Exp Value	Charge	MH+ [Da]	ΔM [ppm]	RT [min]	# Missed Cleavages
	High	rEPEFFSAIQLSTk	1	1	1	P74474	N-Term(iTRAQ4plex); K14(iTRAQ4plex)	0,0000	0,242	1		0	1,236E-07	43	5,25981E-05	3	1941,07138	1,51	39,53	1
	High	sHAPNSPPIDAk	1	1	1	P74474	N-Term(iTRAQ4plex); K12(iTRAQ4plex)	0,0000	0,403	1		0	0,000005684	33	0,000482158	3	1521,82752	0,73	15,43	0
	High	lVSLITGk	1	1	1	P74474	N-Term(iTRAQ4plex); K8(iTRAQ4plex)	0,0000	0,303	1		0,001	0,008787	19	0,013426722	2	1118,73808	-0,59	37,19	0
P72967	Slr0695 protein OS=Synechocystis sp. (strain PCC 6803 / Kazusa) GN=slr0695 PE=4 SV=1 - [P72967	68,60	8,09	1	2	2	4	1,069	4	18,3	173	19,0	9,04							
	A3	Sequence	# PSMs	# Proteins	# Protein Groups	Protein Group Accessions	Modifications	ΔCn	116/117	116/117 Count	116/117 Variability [%]	q-Value	PEP	IonScore	Exp Value	Charge	MH+ [Da]	ΔM [ppm]	RT [min]	# Missed Cleavages
	High	nNPTAANk	3	1	1	P72967	N-Term(iTRAQ4plex); K8(iTRAQ4plex)	0,0000	1,160	3	18,0	0	0,0002498	43	4,84139E-05	2	1117,62175	1,19	10,95	0
	High	vFAQEk	1	1	1	P72967	N-Term(iTRAQ4plex); K6(iTRAQ4plex)	0,0000	0,909	1		0	0,001811	26	0,002741385	2	1009,59368	1,58	16,73	0
P27589	Cytochrome b6-f complex subunit 4 OS=Synechocystis sp. (strain PCC 6803 / Kazusa) GN=petD PE=	68,50	17,50	1	4	4	5	2,312	5	25,1	160	17,4	6,04							
	A3	Sequence	# PSMs	# Proteins	# Protein Groups	Protein Group Accessions	Modifications	ΔCn	116/117	116/117 Count	116/117 Variability [%]	q-Value	PEP	IonScore	Exp Value	Charge	MH+ [Da]	ΔM [ppm]	RT [min]	# Missed Cleavages
	High	kPDLSDPDLR	1	1	1	P27589	N-Term(iTRAQ4plex); K1(iTRAQ4plex)	0,0000	1,957	1		0	0,00003535	34	0,0004385	3	1443,80463	0,01	22,33	0
	High	fQNPFR	2	1	1	P27589	N-Term(iTRAQ4plex)	0,0000	2,431	2	7,4	0,001	0,01642	26	0,002354887	2	952,51329	1,21	30,28	0
	Medium	sLTLGLF	1	1	1	P27589	N-Term(iTRAQ4plex)	0,0000	0,903	1		0,014	0,1852	20	0,009161572	2	894,54167	-0,05	50,99	0
	Medium	iLPNk	1	1	1	P27589	N-Term(iTRAQ4plex); K5(iTRAQ4plex)	0,0000	3,164	1		0,038	0,49	11	0,081652597	2	872,57945	-1,52	19,88	0
P73370	Slr2070 protein OS=Synechocystis sp. (strain PCC 6803 / Kazusa) GN=slr2070 PE=4 SV=1 - [P73370	67,48	9,51	1	2	2	2	1,552	2	23,8	284	31,9	5,71							
	A3	Sequence	# PSMs	# Proteins	# Protein Groups	Protein Group Accessions	Modifications	ΔCn	116/117	116/117 Count	116/117 Variability [%]	q-Value	PEP	IonScore	Exp Value	Charge	MH+ [Da]	ΔM [ppm]	RT [min]	# Missed Cleavages
	High	sLGVVLVPQR	1	1	1	P73370	N-Term(iTRAQ4plex)	0,0000	1,818	1		0	0,00004288	54	3,68952E-06	2	1211,75945	0,14	34,01	0
	High	wIGHDPEILDIHSIELR	1	1	1	P73370	N-Term(iTRAQ4plex)	0,0000	1,324	1		0	0,000003617	26	0,002415294	4	2187,16713	-0,37	43,73	0
P73719	Sll1628 protein OS=Synechocystis sp. (strain PCC 6803 / Kazusa) GN=sll1628 PE=4 SV=1 - [P73719_	65,90	6,77	1	2	2	2	0,906	2	30,9	384	43,1	4,46							
	A3	Sequence	# PSMs	# Proteins	# Protein Groups	Protein Group Accessions	Modifications	ΔCn	116/117	116/117 Count	116/117 Variability [%]	q-Value	PEP	IonScore	Exp Value	Charge	MH+ [Da]	ΔM [ppm]	RT [min]	# Missed Cleavages
	High	fQTTVLASPSPNLVLAAR	1	1	1	P73719	N-Term(iTRAQ4plex)	0,0000	0,739	1		0	5,009E-12	55	3,43534E-06	3	2029,15720	0,43	43,91	0
	High	fFQQLDHR	1	1	1	P73719	N-Term(iTRAQ4plex)	0,0000	1,110	1		0	0,000479	24	0,003732244	3	1234,64518	0,18	27,38	0
P73867	Potassium-transporting ATPase B chain OS=Synechocystis sp. (strain PCC 6803 / Kazusa) GN=kdpB F	65,65	3,62	1	2	2	2	0,425	2	16,1	690	72,9	6,14							
	A3	Sequence	# PSMs	# Proteins	# Protein Groups	Protein Group Accessions	Modifications	ΔCn	116/117	116/117 Count	116/117 Variability [%]	q-Value	PEP	IonScore	Exp Value	Charge	MH+ [Da]	ΔM [ppm]	RT [min]	# Missed Cleavages
	High	fRPLSADQLLQR	1	1	1	P73867	N-Term(iTRAQ4plex)	0,0000	0,382	1		0	2,689E-07	44	3,76678E-05	3	1587,91056	1,10	35,08	0
	High	vSHLGGTPLAVcR	1	1	1	P73867	N-Term(iTRAQ4plex); C12(Carbamidomethyl)	0,0000	0,474	1		0	0,000000146	34	0,000360554	3	1510,82709	-0,67	24,71	0
P74645	Circadian clock protein KaiB OS=Synechocystis sp. (strain PCC 6803 / Kazusa) GN=kaiB PE=1 SV=1 -	65,09	21,90	1	2	2	2	1,022	2	5,3	105	11,9	6,79							
	A3	Sequence	# PSMs	# Proteins	# Protein Groups	Protein Group Accessions	Modifications	ΔCn	116/117	116/117 Count	116/117 Variability [%]	q-Value	PEP	IonScore	Exp Value	Charge	MH+ [Da]	ΔM [ppm]	RT [min]	# Missed Cleavages
	High	lYVAGNTPNSVR	1	1	1	P74645	N-Term(iTRAQ4plex)	0,0000	0,986	1		0	0,000006389	49	1,15072E-05	2	1434,78313	0,66	26,29	0
	High	kIIGDLSDREk	1	1	1	P74645	N-Term(iTRAQ4plex); K1(iTRAQ4plex); K11(iTRAQ4plex)	0,0000	1,059	1		0	0,0009647	29	0,001342672	4	1706,01589	-0,83	20,90	2
Q55171	Slr0476 protein OS=Synechocystis sp. (strain PCC 6803 / Kazusa) GN=slr0476 PE=4 SV=1 - [Q55171	64,56	27,94	1	3	3	6	0,735	5	34,2	136	14,4	7,59							
	A3	Sequence	# PSMs	# Proteins	# Protein Groups	Protein Group Accessions	Modifications	ΔCn	116/117	116/117 Count	116/117 Variability [%]	q-Value	PEP	IonScore	Exp Value	Charge	MH+ [Da]	ΔM [ppm]	RT [min]	# Missed Cleavages
	High	hIGGLDLLPTPk	2	1	1	Q55171	N-Term(iTRAQ4plex); K12(iTRAQ4plex)	0,0000	1,047	2	19,3	0	0,00001467	35	0,000293745	3	1548,93399	-0,80	35,62	0
	High	aGSSkDESALVkPk	1	1	1	Q55171	N-Term(iTRAQ4plex); K5(iTRAQ4plex); K12(iTRAQ4plex); K14(	0,0000	0,735	1		0	6,244E-07	27	0,002157595	4	1993,17666	-0,48	16,39	1
	High	gmPTNRPIEASk	3	1	1	Q55171	N-Term(iTRAQ4plex); M2(Oxidation); K12(iTRAQ4plex)	0,0000	0,586	2	15,0	0	0,0003629	20	0,013776155	3	1604,86539	-0,95	13,21	0
P26522	NAD(P)H-quinone oxidoreductase subunit 1 OS=Synechocystis sp. (strain PCC 6803 / Kazusa) GN=nd	64,11	5,11	1	2	2	4	1,230	4	11,6	372	40,5	5,06							
	A3	Sequence	# PSMs	# Proteins	# Protein Groups	Protein Group Accessions	Modifications	ΔCn	116/117	116/117 Count	116/117 Variability [%]	q-Value	PEP	IonScore	Exp Value	Charge	MH+ [Da]	ΔM [ppm]	RT [min]	# Missed Cleavages
	High	ySLLGGLR	2	1	1	P26522	N-Term(iTRAQ4plex)	0,0000	1,133	2	4,6	0	0,0004948	32	0,000635287	2	1022,61150	-0,03	35,98	0
	High	lVFkEDVVPAk	2	1	1	P26522	N-Term(iTRAQ4plex); K4(iTRAQ4plex); K11(iTRAQ4plex)	0,0000	1,330	2	3,9	0	2,157E-07	23	0,00540717	3	1677,02982	-0,80	32,32	1
P73058	Histidinol dehydrogenase 1 OS=Synechocystis sp. (strain PCC 6803 / Kazusa) GN=hisD1 PE=3 SV=1	63,77	6,45	1	2	2	2	0,889	2	24,3	434	46,5	5,53							
	A3	Sequence	# PSMs	# Proteins	# Protein Groups	Protein Group Accessions	Modifications	ΔCn	116/117	116/117 Count	116/117 Variability [%]	q-Value	PEP	IonScore	Exp Value	Charge	MH+ [Da]	ΔM [ppm]	RT [min]	# Missed Cleavages
	High	lGGVQALAAIAYGTk	1	1	1	P73058	N-Term(iTRAQ4plex); K15(iTRAQ4plex)	0,0000	0,757	1		0	0,0001364	39	0,00011587	3	1721,02036	0,19	46,82	0
	High	iTPLPTVGLYVPR	1	1	1	P73058	N-Term(iTRAQ4plex)	0,0000	1,045	1		0	0,0001857	37	0,000180705	3	1569,94864	0,07	44,70	0
P74113	Slr1970 protein OS=Synechocystis sp. (strain PCC 6803 / Kazusa) GN=slr1970 PE=4 SV=1 - [P74113	63,77	10,40	1	2	2	3	0,574	3	29,7	173	19,4	4,58							
	A3	Sequence	# PSMs	# Proteins	# Protein Groups	Protein Group Accessions	Modifications	ΔCn	116/117	116/117 Count	116/117 Variability [%]	q-Value	PEP	IonScore	Exp Value	Charge	MH+ [Da]	ΔM [ppm]	RT [min]	# Missed Cleavages
	High	kPLEPLVk	2	1	1	P74113	N-Term(iTRAQ4plex); K1(iTRAQ4plex); K8(iTRAQ4plex)	0,0000	0,648	2	49,8	0	0,00001092	36	0,000272251	3	1355,89753	-0,84	22,62	0
	High	yFDRPQIADR	1	1	1	P74113	N-Term(iTRAQ4plex)	0,0000	0,574	1		0	0,001652	20	0,010964025	3	1424,74070	0,26	25,49	0
P72756	Sll1033 protein OS=Synechocystis sp. (strain PCC 6803 / Kazusa) GN=sll1033 PE=4 SV=1 - [P72756_	63,19	2,54	1	2	2	3	2,278	3	25,8	668	73,7	4,46							
	A3	Sequence	# PSMs	# Proteins	# Protein Groups	Protein Group Accessions	Modifications	ΔCn	116/117	116/117 Count	116/117 Variability [%]	q-Value	PEP	IonScore	Exp Value	Charge	MH+ [Da]	ΔM [ppm]	RT [min]	# Missed Cleavages
	High	vRPQIPLDVW	1	1	1	P72756	N-Term(iTRAQ4plex)	0,0000	2,720	1		0	0,0001506	42	6,91783E-05	2	1366,79729	0,65	44,23	0
	High	aIIPYFR	2	1	1	P72756	N-Term(iTRAQ4plex)	0,0000	2,091	2	12,7	0,005	0,06579	27	0,002575787	2	1023,61028	-0,51	37,27	0
P73557	RNA-binding protein OS=Synechocystis sp. (strain PCC 6803 / Kazusa) GN=ssr1480 PE=4 SV=1 - [P7	62,82	14,46	2	4	4	9	0,831	9	43,1	83	9,4	5,01							
	A3	Sequence	# PSMs	# Proteins	# Protein Groups	Protein Group Accessions	Modifications	ΔCn	116/117	116/117 Count	116/117 Variability [%]	q-Value	PEP	IonScore	Exp Value	Charge	MH+ [Da]	ΔM [ppm]	RT [min]	# Missed Cleavages
	High	rVQLPTDR	2	2	1	P73557	N-Term(iTRAQ4plex)	0,0000	0,871	2	40,6	0	0,0002911	33	0,000521159	3	1128,66034	-0,24	17,33	1
	High	rVQLPTDRETGR	2	2	1	P73557	N-Term(iTRAQ4plex)	0,0000	1,326	2	3,2	0	0,002394	22	0,005807243	4	1571,87343	-0,03	17,11	2
	High	vQLPTDR	4	2	1	P73557	N-Term(iTRAQ4plex)	0,0000	0,768	4	7,7	0	0,001439	16	0,023548866	2	972,55974	0,25	21,78	0
	Medium	vQLPTDRETGR	1	2	1	P73557	N-Term(iTRAQ4plex)	0,0000	1,330	1		0,018	0,2345	17	0,022231563	3	1415,77320	0,59	20,28	1
Q55160	Biotin carboxylase OS=Synechocystis sp. (strain PCC 6803 / Kazusa) GN=accC PE=4 SV=1 - [Q55160	62,75	6,25	1	2	2	2	4,012	2	28,0	448	49,1	7,28							
	A3	Sequence	# PSMs	# Proteins	# Protein Groups	Protein Group Accessions	Modifications	ΔCn	116/117	116/117 Count	116/117 Variability [%]	q-Value	PEP	IonScore	Exp Value	Charge	MH+ [Da]	ΔM [ppm]	RT [min]	# Missed Cleavages
	High	lLEEAPSPFLTPHLR	1	1	1	Q55160	N-Term(iTRAQ4plex)	0,0000	3,333	1		0	1,289E-07	43	4,59166E-05	3	1864,04514	0,09	42,38	0
	High	lIVWAGDRPSAIk	1	1	1	Q55160	N-Term(iTRAQ4plex); K13(iTRAQ4plex)	0,0000	4,830	1		0	0,000002358	32	0,000576727	3	1714,02500	-0,27	34,95	0
Q55626	Bifunctional protein FolD OS=Synechocystis sp. (strain PCC 6803 / Kazusa) GN=folD PE=3 SV=1 - [F(	62,68	8,84	1	2	2	2	0,668	2	30,7	294	30,9	6,01							
	A3	Sequence	# PSMs	# Proteins	# Protein Groups	Protein Group Accessions	Modifications	ΔCn	116/117	116/117 Count	116/117 Variability [%]	q-Value	PEP	IonScore	Exp Value	Charge	MH+ [Da]	ΔM [ppm]	RT [min]	# Missed Cleavages
	High	dIAQLVAQGHR	1	1	1	Q55626	N-Term(iTRAQ4plex)	0,0000	0,546	1		0	0,00001014	41	7,11164E-05	3	1351,75559	-0,54	26,63	0
	High	sILVGkPLALmLLEk	1	1	1	Q55626	N-Term(iTRAQ4plex); K6(iTRAQ4plex); M11(Oxidation); K15(iT	0,0000	0,818	1		0	7,159E-09	34	0,000378416	3	2073,30869	0,23	47,63	0
Q55513	4-hydroxy-tetrahydrodipicolinate synthase OS=Synechocystis sp. (strain PCC 6803 / Kazusa) GN=dap	62,49	8,31	1	2	2	2	0,602	2	21,4	301	31,8	5,08							
	A3	Sequence	# PSMs	# Proteins	# Protein Groups	Protein Group Accessions	Modifications	ΔCn	116/117	116/117 Count	116/117 Variability [%]	q-Value	PEP	IonScore	Exp Value	Charge	MH+ [Da]	ΔM [ppm]	RT [min]	# Missed Cleavages
	High	gSVIAGTGSNcTR	1	1	1	Q55513	N-Term(iTRAQ4plex); C11(Carbamidomethyl)	0,0000	0,522	1		0	0,00000374	54	3,8104E-06	2	1423,71147	2,41	14,99	0
	High	iLFcATNPIPVk	1	1	1	Q55513	N-Term(iTRAQ4plex); C4(Carbamidomethyl); K12(iTRAQ4plex)	0,0000	0,694	1		0	0,0001874	21	0,007378533	3	1660,97458	2,81	41,62	0
P74074	Slr1338 protein OS=Synechocystis sp. (strain PCC 6803 / Kazusa) GN=slr1338 PE=4 SV=1 - [P74074	62,44	12,00	1	2	2	3	0,869	2	23,6	200	22,4	4,51							
	A3	Sequence	# PSMs	# Proteins	# Protein Groups	Protein Group Accessions	Modifications	ΔCn	116/117	116/117 Count	116/117 Variability [%]	q-Value	PEP	IonScore	Exp Value	Charge	MH+ [Da]	ΔM [ppm]	RT [min]	# Missed Cleavages
	High	sRWELVQESTLALAR	2	1	1	P74074	N-Term(iTRAQ4plex)	0,0000	0,742	1		0	8,716E-10	55	2,91723E-06	3	1903,05429	1,28	40,03	1
	High	rYDNVNASk	1	1	1	P74074	N-Term(iTRAQ4plex); K9(iTRAQ4plex)	0,0000	1,017	1		0	0,002445	20	0,009749223	3	1354,73297	0,90	11,99	1
P72709	Slr0250 protein OS=Synechocystis sp. (strain PCC 6803 / Kazusa) GN=slr0250 PE=4 SV=1 - [P72709	61,05	14,61	1	2	2	3	0,567	2	42,0	178	19,2	5,87							
	A3	Sequence	# PSMs	# Proteins	# Protein Groups	Protein Group Accessions	Modifications	ΔCn	116/117	116/117 Count	116/117 Variability [%]	q-Value	PEP	IonScore	Exp Value	Charge	MH+ [Da]	ΔM [ppm]	RT [min]	# Missed Cleavages
	High	nLQATAAQAQYFSQER	1	1	1	P72709	N-Term(iTRAQ4plex)	0,0000	0,744	1		0	0,000000108	40	0,000109136	3	1969,98447	-0,21	32,93	0
	High	gNVQVYYPAR	2	1	1	P72709	N-Term(iTRAQ4plex)	0,0000	0,432	1		0	0,0002092	34	0,000389018	2	1310,69756	0,14	23,87	0
P49057	GMP synthase [glutamine-hydrolyzing] OS=Synechocystis sp. (strain PCC 6803 / Kazusa) GN=guaA P	60,99	10,89	1	4	4	5	1,220	5	93,4	542	61,0	6,24							
	A3	Sequence	# PSMs	# Proteins	# Protein Groups	Protein Group Accessions	Modifications	ΔCn	116/117	116/117 Count	116/117 Variability [%]	q-Value	PEP	IonScore	Exp Value	Charge	MH+ [Da]	ΔM [ppm]	RT [min]	# Missed Cleavages
	High	lIGHEFIQVFEEESNR	1	1	1	P49057	N-Term(iTRAQ4plex)	0,0000	4,923	1		0	4,238E-09	42	5,8475E-05	3	2091,06351	0,33	47,17	0
	High	rHPFPGPGLAIR	2	1	1	P49057	N-Term(iTRAQ4plex)	0,0000	1,099	2	15,7	0	0,0008424	25	0,003902947	3	1461,85636	0,26	23,59	1
	High	aLFGVQFHPEVVHSVGGIALIR	1	1	1	P49057	N-Term(iTRAQ4plex)	0,0000	4,365	1		0	0,000665	20	0,010964025	4	2490,41152	0,50	47,55	0
	High	tYAHPVVLR	1	1	1	P49057	N-Term(iTRAQ4plex)	0,0000	0,715	1		0,006	0,07939	12	0,057805612	3	1199,70188	0,12	19,33	0
P72586	GDP-mannose 4,6-dehydratase OS=Synechocystis sp. (strain PCC 6803 / Kazusa) GN=rfbD PE=1 SV	60,69	14,36	1	4	4	5	1,376	5	62,2	362	41,3	6,47							
	A3	Sequence	# PSMs	# Proteins	# Protein Groups	Protein Group Accessions	Modifications	ΔCn	116/117	116/117 Count	116/117 Variability [%]	q-Value	PEP	IonScore	Exp Value	Charge	MH+ [Da]	ΔM [ppm]	RT [min]	# Missed Cleavages
	High	iLEDVkPTEIYNLGAQSHVR	2	1	1	P72586	N-Term(iTRAQ4plex); K6(iTRAQ4plex)	0,0000	2,101	2	5,7	0	0,000000406	34	0,000379289	4	2570,41421	-1,53	39,44	0
	High	lHYGDLTDGTTLR	1	1	1	P72586	N-Term(iTRAQ4plex)	0,0000	1,251	1		0	0,00003503	27	0,00198139	3	1605,83457	-0,48	30,13	0
	High	vYGHWQTVNYR	1	1	1	P72586	N-Term(iTRAQ4plex)	0,0000	1,376	1		0	0,002686	10	0,096376246	3	1566,79337	-0,02	26,30	0
	High	rGETFVTR	1	1	1	P72586	N-Term(iTRAQ4plex)	0,0000	0,913	1		0,006	0,07824	16	0,028117066	3	1109,61875	0,30	13,27	1
P74396	Slr0280 protein OS=Synechocystis sp. (strain PCC 6803 / Kazusa) GN=slr0280 PE=4 SV=1 - [P74396	60,66	7,87	1	3	3	3	1,039	3	115,8	610	65,4	9,76							
	A3	Sequence	# PSMs	# Proteins	# Protein Groups	Protein Group Accessions	Modifications	ΔCn	116/117	116/117 Count	116/117 Variability [%]	q-Value	PEP	IonScore	Exp Value	Charge	MH+ [Da]	ΔM [ppm]	RT [min]	# Missed Cleavages
	High	lQVSTLTSPFR	1	1	1	P74396	N-Term(iTRAQ4plex)	0,0000	1,039	1		0	0,0007372	41	8,72911E-05	2	1392,79680	0,02	39,84	0
	High	sPGISLRPLmANPTmAQGTAPLV'	1	1	1	P74396	N-Term(iTRAQ4plex); M10(Oxidation); M15(Oxidation)	0,0000	0,433	1		0	0,000006599	29	0,001267564	4	2939,59755	1,60	41,08	0
	High	vHFQLPVGYk	1	1	1	P74396	N-Term(iTRAQ4plex); K10(iTRAQ4plex)	0,0000	1,935	1		0	0,00007581	17	0,019362882	3	1475,86255	0,84	35,05	0
P73918	Acetolactate synthase OS=Synechocystis sp. (strain PCC 6803 / Kazusa) GN=ilvB PE=3 SV=1 - [P739	60,25	6,00	1	3	3	4	1,003	4	49,4	550	60,1	5,41							
	A3	Sequence	# PSMs	# Proteins	# Protein Groups	Protein Group Accessions	Modifications	ΔCn	116/117	116/117 Count	116/117 Variability [%]	q-Value	PEP	IonScore	Exp Value	Charge	MH+ [Da]	ΔM [ppm]	RT [min]	# Missed Cleavages
	High	qGkPTPYGASLR	1	1	1	P73918	N-Term(iTRAQ4plex); K3(iTRAQ4plex)	0,0000	1,284	1		0	0,00009139	30	0,001037457	3	1562,89103	1,08	17,85	0
	High	qIVRPSITPEVVR	2	1	1	P73918	N-Term(iTRAQ4plex)	0,0000	1,074	2	49,4	0	8,692E-08	30	0,001083852	3	1637,98331	0,82	28,61	0
	Medium	fGNPDFVk	1	1	1	P73918	N-Term(iTRAQ4plex); K8(iTRAQ4plex)	0,0000	0,470	1		0,029	0,3856	14	0,043148927	3	1211,67597	7,96	20,58	0
Q55766	Ribose-5-phosphate isomerase A OS=Synechocystis sp. (strain PCC 6803 / Kazusa) GN=rpiA PE=3 SV	60,14	9,36	1	2	2	4	0,670	3	14,6	235	24,7	5,03							
	A3	Sequence	# PSMs	# Proteins	# Protein Groups	Protein Group Accessions	Modifications	ΔCn	116/117	116/117 Count	116/117 Variability [%]	q-Value	PEP	IonScore	Exp Value	Charge	MH+ [Da]	ΔM [ppm]	RT [min]	# Missed Cleavages
	High	kYGIPLTTLDVADR	2	1	1	Q55766	N-Term(iTRAQ4plex); K1(iTRAQ4plex)	0,0000	0,601	1		0	1,983E-08	42	5,86098E-05	3	1850,06101	-0,88	36,85	1
	High	lGGkPELR	2	1	1	Q55766	N-Term(iTRAQ4plex); K4(iTRAQ4plex)	0,0000	0,703	2	7,3	0,003	0,03649	29	0,001156032	2	1157,72966	4,45	17,69	0
P73849	Probable 2-phosphosulfolactate phosphatase OS=Synechocystis sp. (strain PCC 6803 / Kazusa) GN=c	60,07	9,54	1	2	2	2	1,222	2	39,0	241	26,0	5,10							
	A3	Sequence	# PSMs	# Proteins	# Protein Groups	Protein Group Accessions	Modifications	ΔCn	116/117	116/117 Count	116/117 Variability [%]	q-Value	PEP	IonScore	Exp Value	Charge	MH+ [Da]	ΔM [ppm]	RT [min]	# Missed Cleavages
	High	sDLLNLFk	1	1	1	P73849	N-Term(iTRAQ4plex); K8(iTRAQ4plex)	0,0000	1,575	1		0	0,000004798	53	5,4572E-06	2	1237,74016	0,55	44,49	0
	High	vkDcPQLVTASLVNR	1	1	1	P73849	N-Term(iTRAQ4plex); K2(iTRAQ4plex); C4(Carbamidomethyl)	0,0000	0,949	1		0	0,0002088	21	0,011202932	3	1988,12052	0,17	31,18	1
P74571	Oligopeptidase A OS=Synechocystis sp. (strain PCC 6803 / Kazusa) GN=prlC PE=3 SV=1 - [P74571_	60,06	3,51	1	2	2	2	1,853	2	103,8	713	80,2	5,11							
	A3	Sequence	# PSMs	# Proteins	# Protein Groups	Protein Group Accessions	Modifications	ΔCn	116/117	116/117 Count	116/117 Variability [%]	q-Value	PEP	IonScore	Exp Value	Charge	MH+ [Da]	ΔM [ppm]	RT [min]	# Missed Cleavages
	High	qINFSLLDLELHHR	1	1	1	P74571	N-Term(iTRAQ4plex)	0,0000	3,298	1		0	0,000001305	42	6,90192E-05	4	1879,03151	0,43	42,60	0
	High	kmAPNVAAVEk	1	1	1	P74571	N-Term(iTRAQ4plex); K1(iTRAQ4plex); M2(Oxidation); K11(iTF	0,0000	1,041	1		0	0,001599	31	0,000712804	3	1605,94425	5,21	17,75	1
P73290	Adenylosuccinate synthetase OS=Synechocystis sp. (strain PCC 6803 / Kazusa) GN=purA PE=3 SV=2	59,91	9,46	1	3	3	3	2,703	3	29,1	444	48,8	5,45							
	A3	Sequence	# PSMs	# Proteins	# Protein Groups	Protein Group Accessions	Modifications	ΔCn	116/117	116/117 Count	116/117 Variability [%]	q-Value	PEP	IonScore	Exp Value	Charge	MH+ [Da]	ΔM [ppm]	RT [min]	# Missed Cleavages
	High	lLWTVEYk	1	1	1	P73290	N-Term(iTRAQ4plex); K8(iTRAQ4plex)	0,0000	2,703	1		0	0,00004492	49	1,40919E-05	2	1339,78716	0,55	40,97	0
	High	iYDAIHQR	1	1	1	P73290	N-Term(iTRAQ4plex)	0,0000	1,440	1		0	0,0003677	22	0,005793887	3	1159,63355	-0,43	19,99	0
	High	sADVVVRPQGGVNAGHTIVVNDC	1	1	1	P73290	N-Term(iTRAQ4plex); K26(iTRAQ4plex)	0,0000	3,276	1		0	0,00003201	15	0,031188896	4	2996,61782	0,70	31,24	0
P72845	Sll1188 protein OS=Synechocystis sp. (strain PCC 6803 / Kazusa) GN=sll1188 PE=4 SV=1 - [P72845_	59,08	15,24	1	2	2	3	0,652	3	7,5	164	18,8	5,00							
	A3	Sequence	# PSMs	# Proteins	# Protein Groups	Protein Group Accessions	Modifications	ΔCn	116/117	116/117 Count	116/117 Variability [%]	q-Value	PEP	IonScore	Exp Value	Charge	MH+ [Da]	ΔM [ppm]	RT [min]	# Missed Cleavages
	High	nVVTGIYFLmTk	1	1	1	P72845	N-Term(iTRAQ4plex); M10(Oxidation); K12(iTRAQ4plex)	0,0000	0,619	1		0	0,000004476	34	0,000407352	3	1689,94663	-1,30	42,78	0

	High	lELLPHPEGGFYk	2	1	1	P72845	N-Term(iTRAQ4plex); K13(iTRAQ4plex)	0,0000	0,751	2	21,2	0	6,164E-07	32	0,000594251	3	1787,99338	-0,05	38,76	0
P73602	Uncharacterized protein sll1783 OS=Synechocystis sp. (strain PCC 6803 / Kazusa) GN=sll1783 PE=3	59,06	23,13	1	2	2	3	0,619	3	27,4	147	16,8	4,96							
	A3	Sequence	# PSMs	# Proteins	# Protein Groups	Protein Group Accessions	Modifications	ΔCn	116/117	116/117 Count	116/117 Variability [%]	q-Value	PEP	IonScore	Exp Value	Charge	MH+ [Da]	ΔM [ppm]	RT [min]	# Missed Cleavages
	High	kEEFIALSQTFIEPSR	1	1	1	P73602	N-Term(iTRAQ4plex); K1(iTRAQ4plex)	0,0000	0,619	1		0	2,325E-07	49	1,44675E-05	3	2183,19962	2,06	43,27	1
	High	aALDYHFQTPYFHEFVEk	2	1	1	P73602	N-Term(iTRAQ4plex); K18(iTRAQ4plex)	0,0000	0,725	2	53,7	0	2,035E-07	23	0,004497488	4	2530,26504	0,18	42,11	0
P72719	Bacterioferritin comigratory protein OS=Synechocystis sp. (strain PCC 6803 / Kazusa) GN=bcp PE=4	58,94	15,22	1	2	2	2	0,887	2	2,2	184	20,4	4,86							
	A3	Sequence	# PSMs	# Proteins	# Protein Groups	Protein Group Accessions	Modifications	ΔCn	116/117	116/117 Count	116/117 Variability [%]	q-Value	PEP	IonScore	Exp Value	Charge	MH+ [Da]	ΔM [ppm]	RT [min]	# Missed Cleavages
	High	hTYVIDPEGILR	1	1	1	P72719	N-Term(iTRAQ4plex)	0,0000	0,900	1		0	3,031E-08	37	0,000193629	3	1556,85477	-0,37	36,52	0
	High	fLGVRPATHSEEVLAR	1	1	1	P72719	N-Term(iTRAQ4plex)	0,0000	0,874	1		0	0,000002081	35	0,000328829	4	1926,06765	-0,10	27,78	0
P74299	Phosphoenolpyruvate carboxylase OS=Synechocystis sp. (strain PCC 6803 / Kazusa) GN=ppc PE=3 S	58,81	5,51	1	6	6	6	1,614	6	71,8	1034	118,9	5,78							
	A3	Sequence	# PSMs	# Proteins	# Protein Groups	Protein Group Accessions	Modifications	ΔCn	116/117	116/117 Count	116/117 Variability [%]	q-Value	PEP	IonScore	Exp Value	Charge	MH+ [Da]	ΔM [ppm]	RT [min]	# Missed Cleavages
	High	kLDQLQGSVTGR	1	1	1	P74299	N-Term(iTRAQ4plex); K1(iTRAQ4plex)	0,0000	2,107	1		0	0,0008537	28	0,001625436	3	1589,92051	-0,55	23,10	1
	High	eTFPHLR	1	1	1	P74299	N-Term(iTRAQ4plex)	0,0000	2,950	1		0	0,0001034	25	0,00306881	3	1043,57544	-0,03	22,35	0
	High	sLQAVAQSHR	1	1	1	P74299	N-Term(iTRAQ4plex)	0,0000	1,659	1		0	0,0002264	22	0,006621708	3	1240,68747	-0,33	16,03	0
	High	rPLIPQEmPFSER	1	1	1	P74299	N-Term(iTRAQ4plex); M8(Oxidation)	0,0000	0,723	1		0	0,002183	11	0,077798282	3	1759,92954	0,74	28,83	0
	High	yFYFk	1	1	1	P74299	N-Term(iTRAQ4plex); K5(iTRAQ4plex)	0,0000	0,562	1		0,001	0,01712	23	0,00496558	2	1055,57866	-1,66	34,42	0
	High	wPFFNmVISk	1	1	1	P74299	N-Term(iTRAQ4plex); M6(Oxidation); K10(iTRAQ4plex)	0,0000	1,571	1		0,002	0,02113	12	0,061939829	3	1572,85160	1,82	47,01	0
Q55501	Slr0929 protein OS=Synechocystis sp. (strain PCC 6803 / Kazusa) GN=slr0929 PE=4 SV=1 - [Q55501	58,74	13,33	1	2	2	2	1,501	2	6,8	195	21,7	8,88							
	A3	Sequence	# PSMs	# Proteins	# Protein Groups	Protein Group Accessions	Modifications	ΔCn	116/117	116/117 Count	116/117 Variability [%]	q-Value	PEP	IonScore	Exp Value	Charge	MH+ [Da]	ΔM [ppm]	RT [min]	# Missed Cleavages
	High	lPFYVASQAGSTALIR	1	1	1	Q55501	N-Term(iTRAQ4plex)	0,0000	1,434	1		0	1,892E-09	59	1,33343E-06	3	1838,02970	0,21	46,03	0
	Medium	kYPHIVVDTR	1	1	1	Q55501	N-Term(iTRAQ4plex); K1(iTRAQ4plex)	0,0000	1,571	1		0,018	0,2359	10	0,099304746	4	1515,88711	-0,98	19,57	1
P48959	50S ribosomal protein L35 OS=Synechocystis sp. (strain PCC 6803 / Kazusa) GN=rpmI PE=3 SV=1 -	58,59	32,84	1	2	2	3	1,215	3	53,4	67	7,9	11,36							
	A3	Sequence	# PSMs	# Proteins	# Protein Groups	Protein Group Accessions	Modifications	ΔCn	116/117	116/117 Count	116/117 Variability [%]	q-Value	PEP	IonScore	Exp Value	Charge	MH+ [Da]	ΔM [ppm]	RT [min]	# Missed Cleavages
	High	rLSNLALVHEADEk	2	1	1	P48959	N-Term(iTRAQ4plex); K14(iTRAQ4plex)	0,0000	1,281	2	63,2	0	4,082E-07	39	0,000134267	3	1883,05716	-0,95	24,71	1
	High	fRPTGSGk	1	1	1	P48959	N-Term(iTRAQ4plex); K8(iTRAQ4plex)	0,0000	1,215	1		0	0,002157	16	0,026975531	3	1137,66181	-0,08	11,84	0
P73067	1-deoxy-D-xylulose-5-phosphate synthase OS=Synechocystis sp. (strain PCC 6803 / Kazusa) GN=dxs	58,33	4,22	1	2	2	3	9,357	3	144,4	640	69,3	6,28							
	A3	Sequence	# PSMs	# Proteins	# Protein Groups	Protein Group Accessions	Modifications	ΔCn	116/117	116/117 Count	116/117 Variability [%]	q-Value	PEP	IonScore	Exp Value	Charge	MH+ [Da]	ΔM [ppm]	RT [min]	# Missed Cleavages
	High	fVkPLDTELILPLAER	2	1	1	P73067	N-Term(iTRAQ4plex); K3(iTRAQ4plex)	0,0000	16,243	2	97,6	0	0,00001234	38	0,000157025	3	2142,27976	0,94	47,72	0
	High	vFAHTLTTLAk	1	1	1	P73067	N-Term(iTRAQ4plex); K11(iTRAQ4plex)	0,0000	4,573	1		0	0,0001948	21	0,008259809	3	1489,89786	-0,17	31,98	0
P72897	Slr1619 protein OS=Synechocystis sp. (strain PCC 6803 / Kazusa) GN=slr1619 PE=4 SV=1 - [P72897	58,17	7,23	1	2	2	3	0,954	2	22,3	249	27,7	5,14							
	A3	Sequence	# PSMs	# Proteins	# Protein Groups	Protein Group Accessions	Modifications	ΔCn	116/117	116/117 Count	116/117 Variability [%]	q-Value	PEP	IonScore	Exp Value	Charge	MH+ [Da]	ΔM [ppm]	RT [min]	# Missed Cleavages
	High	tAVWEYLk	2	1	1	P72897	N-Term(iTRAQ4plex); K8(iTRAQ4plex)	0,0000	1,107	1		0	0,000007666	51	7,31089E-06	2	1297,74004	0,44	38,76	0
	High	eAIEAHPmVk	1	1	1	P72897	N-Term(iTRAQ4plex); M8(Oxidation); K10(iTRAQ4plex)	0,0000	0,823	1		0	0,001076	20	0,010398483	3	1428,77771	1,23	16,66	0
P73390	Poly(3-hydroxyalkanoate) synthase OS=Synechocystis sp. (strain PCC 6803 / Kazusa) GN=phbC PE=	58,11	5,03	1	2	2	4	1,620	4	4,4	378	42,9	4,77							
	A3	Sequence	# PSMs	# Proteins	# Protein Groups	Protein Group Accessions	Modifications	ΔCn	116/117	116/117 Count	116/117 Variability [%]	q-Value	PEP	IonScore	Exp Value	Charge	MH+ [Da]	ΔM [ppm]	RT [min]	# Missed Cleavages
	High	dLPPAIAHWLSER	2	1	1	P73390	N-Term(iTRAQ4plex)	0,0000	1,593	2	4,4	0	0,0007281	28	0,001479006	3	1648,89402	0,72	46,92	0
	High	lLNFLR	2	1	1	P73390	N-Term(iTRAQ4plex)	0,0000	1,651	2	4,8	0,001	0,003412	27	0,003091207	2	919,58483	0,26	42,69	0
P54902	Probable gamma-glutamyl phosphate reductase OS=Synechocystis sp. (strain PCC 6803 / Kazusa) GN	57,56	6,70	1	2	2	2	1,502	2	39,4	433	46,3	5,63							
	A3	Sequence	# PSMs	# Proteins	# Protein Groups	Protein Group Accessions	Modifications	ΔCn	116/117	116/117 Count	116/117 Variability [%]	q-Value	PEP	IonScore	Exp Value	Charge	MH+ [Da]	ΔM [ppm]	RT [min]	# Missed Cleavages
	High	qAALHLGSLNTAER	1	1	1	P54902	N-Term(iTRAQ4plex)	0,0000	1,162	1		0	0,0001063	36	0,000279235	3	1624,88980	0,62	26,04	0
	High	scQVLTEVIQTALAk	1	1	1	P54902	N-Term(iTRAQ4plex); C2(Carbamidomethyl); K15(iTRAQ4plex)	0,0000	1,941	1		0	0,0004537	35	0,000343701	3	1949,09891	0,45	53,40	0
Q59992	Tryptophan synthase beta chain OS=Synechocystis sp. (strain PCC 6803 / Kazusa) GN=trpB PE=3 SV	57,37	12,14	1	5	5	5	1,351	5	1,7	412	45,0	6,71							
	A3	Sequence	# PSMs	# Proteins	# Protein Groups	Protein Group Accessions	Modifications	ΔCn	116/117	116/117 Count	116/117 Variability [%]	q-Value	PEP	IonScore	Exp Value	Charge	MH+ [Da]	ΔM [ppm]	RT [min]	# Missed Cleavages
	High	iIAETGAGQHGVATATVcAR	1	1	1	Q59992	N-Term(iTRAQ4plex); C18(Carbamidomethyl)	0,0000	1,100	1		0	8,058E-08	32	0,000676036	3	2127,11258	1,31	25,65	0
	High	gDkDVQTVAk	1	1	1	Q59992	N-Term(iTRAQ4plex); K3(iTRAQ4plex); K10(iTRAQ4plex)	0,0000	1,336	1		0	0,0002746	21	0,007446805	3	1492,87195	1,60	13,22	1
	High	iVINcSGR	1	1	1	Q59992	N-Term(iTRAQ4plex); C5(Carbamidomethyl)	0,0000	1,391	1		0,001	0,01552	16	0,024944224	2	1062,58513	0,44	20,52	0
	High	mNLLGAR	1	1	1	Q59992	N-Term(iTRAQ4plex)	0,0000	1,358	1		0,002	0,03106	21	0,007979947	2	918,53179	0,65	26,57	0
	Medium	lNVFR	1	1	1	Q59992	N-Term(iTRAQ4plex)	0,0000	1,351	1		0,043	0,5515	20	0,011835354	2	792,48442	-0,56	26,11	0
P73489	Ssl2245 protein OS=Synechocystis sp. (strain PCC 6803 / Kazusa) GN=ssl2245 PE=4 SV=1 - [P73489	57,30	15,91	1	2	2	5	0,655	5	20,3	88	9,2	4,35							
	A3	Sequence	# PSMs	# Proteins	# Protein Groups	Protein Group Accessions	Modifications	ΔCn	116/117	116/117 Count	116/117 Variability [%]	q-Value	PEP	IonScore	Exp Value	Charge	MH+ [Da]	ΔM [ppm]	RT [min]	# Missed Cleavages
	High	aQGPAPGk	4	1	1	P73489	N-Term(iTRAQ4plex); K8(iTRAQ4plex)	0,0000	0,612	4	10,4	0,001	0,003182	33	0,000502308	2	1013,59783	-0,41	11,65	0
	High	sINAYk	1	1	1	P73489	N-Term(iTRAQ4plex); K6(iTRAQ4plex)	0,0000	0,893	1		0,001	0,007863	25	0,002837723	2	983,57683	0,43	18,01	0
Q55780	Slr0208 protein OS=Synechocystis sp. (strain PCC 6803 / Kazusa) GN=slr0208 PE=4 SV=1 - [Q55780	56,96	8,49	1	2	2	2	0,923	2	22,6	259	29,9	5,07							
	A3	Sequence	# PSMs	# Proteins	# Protein Groups	Protein Group Accessions	Modifications	ΔCn	116/117	116/117 Count	116/117 Variability [%]	q-Value	PEP	IonScore	Exp Value	Charge	MH+ [Da]	ΔM [ppm]	RT [min]	# Missed Cleavages
	High	iQFALPSIPk	1	1	1	Q55780	N-Term(iTRAQ4plex); K10(iTRAQ4plex)	0,0000	0,794	1		0	0,00001187	37	0,000189657	3	1401,87055	-0,21	44,65	0
	High	dkLDQVIYSLIR	1	1	1	Q55780	N-Term(iTRAQ4plex); K2(iTRAQ4plex)	0,0000	1,073	1		0	0,00001065	33	0,000529627	3	1751,03061	0,01	51,69	1
Q55584	Cation or drug efflux system protein OS=Synechocystis sp. (strain PCC 6803 / Kazusa) GN=slr0369 P	56,66	2,03	1	3	3	3	1,759	3	18,8	1083	117,5	4,88							
	A3	Sequence	# PSMs	# Proteins	# Protein Groups	Protein Group Accessions	Modifications	ΔCn	116/117	116/117 Count	116/117 Variability [%]	q-Value	PEP	IonScore	Exp Value	Charge	MH+ [Da]	ΔM [ppm]	RT [min]	# Missed Cleavages
	High	sLFFVk	1	1	1	Q55584	N-Term(iTRAQ4plex); K6(iTRAQ4plex)	0,0000	1,995	1		0	0,001381	43	4,47682E-05	2	1028,63689	-1,37	36,18	0
	High	yGNIITAILk	1	1	1	Q55584	N-Term(iTRAQ4plex); K10(iTRAQ4plex)	0,0000	1,759	1		0	0,0004479	26	0,002404197	3	1393,86527	-0,34	48,63	0
	Medium	fQINAR	1	1	1	Q55584	N-Term(iTRAQ4plex)	0,0000	1,544	1		0,019	0,2571	12	0,06123081	2	892,51244	0,33	20,81	0
P74308	Aldehyde reductase OS=Synechocystis sp. (strain PCC 6803 / Kazusa) GN=slr0942 PE=4 SV=1 - [P74	56,16	16,51	1	4	4	5	1,229	5	11,8	327	36,0	5,15							
	A3	Sequence	# PSMs	# Proteins	# Protein Groups	Protein Group Accessions	Modifications	ΔCn	116/117	116/117 Count	116/117 Variability [%]	q-Value	PEP	IonScore	Exp Value	Charge	MH+ [Da]	ΔM [ppm]	RT [min]	# Missed Cleavages
	High	kLEmVLSmAR	1	1	1	P74308	K1(iTRAQ4plex); N-Term(iTRAQ4plex); M4(Oxidation); M8(Oxi	0,0000	1,302	1		0	0,001275	28	0,001523947	3	1497,83887	1,12	27,06	1
	High	lWSNAHHPDAVLPALEk	1	1	1	P74308	N-Term(iTRAQ4plex); K17(iTRAQ4plex)	0,0000	1,135	1		0	0,0009452	24	0,004446313	4	2186,20083	2,15	35,57	0
	High	aVDLGLcHHIGVSNFSLk	1	1	1	P74308	N-Term(iTRAQ4plex); C7(Carbamidomethyl); K18(iTRAQ4plex)	0,0000	2,007	1		0	0,000006308	20	0,010432603	4	2255,22573	2,12	38,26	0
	High	rEELWITSk	1	1	1	P74308	N-Term(iTRAQ4plex); K9(iTRAQ4plex)	0,0000	0,817	1		0,001	0,003934	24	0,004385307	3	1449,83338	2,03	27,39	1
	High	kLEMVLSMAR	1	1	1	P74308	N-Term(iTRAQ4plex); K1(iTRAQ4plex)	0,0000	1,229	1		0,002	0,0248	12	0,059425111	3	1465,85547	5,53	36,78	1
P72772	Magnesium-chelatase subunit ChlD OS=Synechocystis sp. (strain PCC 6803 / Kazusa) GN=chlD PE=3	56,07	3,25	1	2	2	3	2,478	3	101,6	676	73,6	5,19							
	A3	Sequence	# PSMs	# Proteins	# Protein Groups	Protein Group Accessions	Modifications	ΔCn	116/117	116/117 Count	116/117 Variability [%]	q-Value	PEP	IonScore	Exp Value	Charge	MH+ [Da]	ΔM [ppm]	RT [min]	# Missed Cleavages
	High	aIHTLLPPIEIIk	2	1	1	P72772	N-Term(iTRAQ4plex); K13(iTRAQ4plex)	0,0000	3,292	2	44,1	0	4,06E-08	37	0,000198596	3	1746,11313	-0,04	43,92	0
	High	kFVSTGFGk	1	1	1	P72772	N-Term(iTRAQ4plex); K1(iTRAQ4plex); K9(iTRAQ4plex)	0,0000	0,773	1		0	0,002102	18	0,014255091	3	1402,84018	-1,21	22,62	1
P72861	Uncharacterized protein sll0936 OS=Synechocystis sp. (strain PCC 6803 / Kazusa) GN=sll0936 PE=3	55,96	3,19	1	2	2	2	1,231	2	156,4	501	55,2	5,35							
	A3	Sequence	# PSMs	# Proteins	# Protein Groups	Protein Group Accessions	Modifications	ΔCn	116/117	116/117 Count	116/117 Variability [%]	q-Value	PEP	IonScore	Exp Value	Charge	MH+ [Da]	ΔM [ppm]	RT [min]	# Missed Cleavages
	High	vLFDVLk	1	1	1	P72861	N-Term(iTRAQ4plex); K7(iTRAQ4plex)	0,0000	2,606	1		0	0,0009034	40	0,000109388	2	1121,71599	-1,16	42,15	0
	High	gFIQLLETR	1	1	1	P72861	N-Term(iTRAQ4plex)	0,0000	0,581	1		0	0,0007662	30	0,001214486	2	1220,71147	-0,42	44,76	0
Q55498	N5-carboxyaminoimidazole ribonucleotide mutase OS=Synechocystis sp. (strain PCC 6803 / Kazusa) (	55,88	17,61	1	2	2	3	0,739	3	1,6	176	18,6	6,15							
	A3	Sequence	# PSMs	# Proteins	# Protein Groups	Protein Group Accessions	Modifications	ΔCn	116/117	116/117 Count	116/117 Variability [%]	q-Value	PEP	IonScore	Exp Value	Charge	MH+ [Da]	ΔM [ppm]	RT [min]	# Missed Cleavages
	High	mVEYAQTAHQR	1	1	1	Q55498	N-Term(iTRAQ4plex); M1(Oxidation)	0,0000	0,747	1		0	0,000004348	36	0,000226971	3	1493,72889	0,08	12,28	0
	High	nAGLLAVQILASHNPVLLEk	2	1	1	Q55498	N-Term(iTRAQ4plex); K20(iTRAQ4plex)	0,0000	0,648	2	19,6	0	0,00003953	31	0,000729407	3	2388,42350	0,72	53,40	0
P72751	Sll1036 protein OS=Synechocystis sp. (strain PCC 6803 / Kazusa) GN=sll1036 PE=4 SV=1 - [P72751	55,73	10,27	1	3	3	3	0,480	3	10,3	292	32,6	5,33							
	A3	Sequence	# PSMs	# Proteins	# Protein Groups	Protein Group Accessions	Modifications	ΔCn	116/117	116/117 Count	116/117 Variability [%]	q-Value	PEP	IonScore	Exp Value	Charge	MH+ [Da]	ΔM [ppm]	RT [min]	# Missed Cleavages
	High	vGWGHSTWQEAVk	1	1	1	P72751	N-Term(iTRAQ4plex); K13(iTRAQ4plex)	0,0000	0,480	1		0	6,789E-08	38	0,000172572	3	1772,93332	0,59	30,19	0
	High	gSIPcPGPTTVR	1	1	1	P72751	N-Term(iTRAQ4plex); C5(Carbamidomethyl)	0,0000	0,941	1		0,001	0,004473	28	0,001749726	2	1385,73442	1,16	22,70	0
	Medium	fWGVR	1	1	1	P72751	N-Term(iTRAQ4plex)	0,0000	0,448	1		0,04	0,5211	17	0,022078522	2	808,45983	1,45	27,99	0
P74328	Uncharacterized tRNA/rRNA methyltransferase slr0955 OS=Synechocystis sp. (strain PCC 6803 / Kazu	55,20	8,59	1	3	3	3	1,331	3	378,0	384	42,8	9,64							
	A3	Sequence	# PSMs	# Proteins	# Protein Groups	Protein Group Accessions	Modifications	ΔCn	116/117	116/117 Count	116/117 Variability [%]	q-Value	PEP	IonScore	Exp Value	Charge	MH+ [Da]	ΔM [ppm]	RT [min]	# Missed Cleavages
	High	iWITSHLR	1	1	1	P74328	N-Term(iTRAQ4plex)	0,0000	6,027	1		0	0,000001273	48	1,62544E-05	3	1169,69153	0,31	30,35	0
	High	qTALHQADLR	1	1	1	P74328	N-Term(iTRAQ4plex)	0,0000	1,331	1		0,001	0,01631	20	0,009267658	3	1296,71308	-0,79	15,55	0
	High	gFDRPTLSHTASDLR	1	1	1	P74328	N-Term(iTRAQ4plex)	0,0000	0,437	1		0,003	0,04036	12	0,065912837	4	1816,94155	-0,41	23,23	0
P73547	Precorrin decarboxylase OS=Synechocystis sp. (strain PCC 6803 / Kazusa) GN=cbiT PE=4 SV=1 - [P7	54,86	11,01	1	2	2	2	0,866	2	5,7	227	24,8	6,21							
	A3	Sequence	# PSMs	# Proteins	# Protein Groups	Protein Group Accessions	Modifications	ΔCn	116/117	116/117 Count	116/117 Variability [%]	q-Value	PEP	IonScore	Exp Value	Charge	MH+ [Da]	ΔM [ppm]	RT [min]	# Missed Cleavages
	High	qILQETWAHLAPGGR	1	1	1	P73547	N-Term(iTRAQ4plex)	0,0000	0,900	1		0	4,938E-08	42	6,77595E-05	3	1820,98844	-0,23	40,45	0
	High	lLIISALQLk	1	1	1	P73547	N-Term(iTRAQ4plex); K10(iTRAQ4plex)	0,0000	0,834	1		0	0,000003418	26	0,002404197	3	1399,94703	-1,47	51,31	0
Q55799	General secretion pathway protein E OS=Synechocystis sp. (strain PCC 6803 / Kazusa) GN=gspE PE=	54,16	1,60	1	2	2	3	3,174	3	57,4	813	90,1	5,52							
	A3	Sequence	# PSMs	# Proteins	# Protein Groups	Protein Group Accessions	Modifications	ΔCn	116/117	116/117 Count	116/117 Variability [%]	q-Value	PEP	IonScore	Exp Value	Charge	MH+ [Da]	ΔM [ppm]	RT [min]	# Missed Cleavages
	High	iLILGLR	2	1	1	Q55799	N-Term(iTRAQ4plex)	0,0000	3,801	2	27,2	0	0,002633	41	7,53304E-05	2	941,66289	0,05	42,37	0
	Medium	kFNPFk	1	1	1	Q55799	N-Term(iTRAQ4plex); K1(iTRAQ4plex); K6(iTRAQ4plex)	0,0000	2,048	1		0,013	0,1716	17	0,020415969	3	1212,74100	-4,54	22,45	1
P73545	Uncharacterized thylakoid-associated protein ssl2501 OS=Synechocystis sp. (strain PCC 6803 / Kazus	53,34	24,72	1	3	3	3	0,544	3	6,0	89	10,6	4,67							
	A3	Sequence	# PSMs	# Proteins	# Protein Groups	Protein Group Accessions	Modifications	ΔCn	116/117	116/117 Count	116/117 Variability [%]	q-Value	PEP	IonScore	Exp Value	Charge	MH+ [Da]	ΔM [ppm]	RT [min]	# Missed Cleavages
	High	lTDTFETSLk	1	1	1	P73545	N-Term(iTRAQ4plex); K10(iTRAQ4plex)	0,0000	0,609	1		0	0,0001494	48	1,48242E-05	2	1442,79912	0,69	34,92	0
	High	fFSTWmESLPk	1	1	1	P73545	N-Term(iTRAQ4plex); M6(Oxidation); K11(iTRAQ4plex)	0,0000	0,544	1		0	0,0009298	18	0,015594448	3	1676,86136	1,00	43,62	0
	High	kFFSTWmESLPk	1	1	1	P73545	N-Term(iTRAQ4plex); K1(iTRAQ4plex); M7(Oxidation); K12(iTF	0,0000	0,523	1		0	0,000007179	14	0,058950018	3	1949,05863	0,98	37,81	1
P72749	GTP-binding protein TypA/BipA homolog OS=Synechocystis sp. (strain PCC 6803 / Kazusa) GN=typA	52,87	3,52	1	2	2	2	1,207	2	25,1	597	66,0	5,10							
	A3	Sequence	# PSMs	# Proteins	# Protein Groups	Protein Group Accessions	Modifications	ΔCn	116/117	116/117 Count	116/117 Variability [%]	q-Value	PEP	IonScore	Exp Value	Charge	MH+ [Da]	ΔM [ppm]	RT [min]	# Missed Cleavages
	High	nVAIIAHVDHGk	1	1	1	P72749	N-Term(iTRAQ4plex); K12(iTRAQ4plex)	0,0000	1,425	1		0	6,815E-07	35	0,000350728	4	1561,90420	-0,72	21,15	0
	High	aGQQAALVk	1	1	1	P72749	N-Term(iTRAQ4plex); K9(iTRAQ4plex)	0,0000	1,022	1		0	0,001334	31	0,000734463	2	1173,71831	-0,94	16,03	0
P73697	Slr1812 protein OS=Synechocystis sp. (strain PCC 6803 / Kazusa) GN=slr1812 PE=4 SV=1 - [P73697	52,65	12,03	1	2	2	2	0,868	2	70,1	158	19,1	5,07							
	A3	Sequence	# PSMs	# Proteins	# Protein Groups	Protein Group Accessions	Modifications	ΔCn	116/117	116/117 Count	116/117 Variability [%]	q-Value	PEP	IonScore	Exp Value	Charge	MH+ [Da]	ΔM [ppm]	RT [min]	# Missed Cleavages
	High	nHWEAEIINFR	1	1	1	P73697	N-Term(iTRAQ4plex)	0,0000	1,329	1		0	4,858E-08	49	1,20218E-05	3	1572,80326	-0,47	36,95	0
	High	lLFEHLLk	1	1	1	P73697	N-Term(iTRAQ4plex); K8(iTRAQ4plex)	0,0000	0,567	1		0,001	0,005597	16	0,022540835	3	1300,82270	-0,35	40,80	0
P73862	Rubisco operon transcriptional regulator OS=Synechocystis sp. (strain PCC 6803 / Kazusa) GN=rbcR	52,21	4,43	1	2	2	4	0,490	3	1,0	316	35,7	8,50							
	A3	Sequence	# PSMs	# Proteins	# Protein Groups	Protein Group Accessions	Modifications	ΔCn	116/117	116/117 Count	116/117 Variability [%]	q-Value	PEP	IonScore	Exp Value	Charge	MH+ [Da]	ΔM [ppm]	RT [min]	# Missed Cleavages
	High	lAVQNLFHR	3	1	1	P73862	N-Term(iTRAQ4plex)	0,0000	0,491	2	0,5	0	0,00001877	36	0,000276675	3	1241,72376	0,17	34,75	0
	Medium	yFIPR	1	1	1	P73862	N-Term(iTRAQ4plex)	0,0000	0,482	1		0,034	0,4325	20	0,009161572	2	839,48900	-0,72	26,78	0
P74371	Chromophore lyase CpcT/CpeT OS=Synechocystis sp. (strain PCC 6803 / Kazusa) GN=cpcT PE=3 SV=	51,38	15,31	1	3	3	5	1,042	5	19,3	196	22,6	5,33							
	A3	Sequence	# PSMs	# Proteins	# Protein Groups	Protein Group Accessions	Modifications	ΔCn	116/117	116/117 Count	116/117 Variability [%]	q-Value	PEP	IonScore	Exp Value	Charge	MH+ [Da]	ΔM [ppm]	RT [min]	# Missed Cleavages
	High	lkVVEDHIEIENFk	2	1	1	P74371	N-Term(iTRAQ4plex); K2(iTRAQ4plex); K14(iTRAQ4plex)	0,0000	1,185	2	11,8	0	5,576E-07	30	0,001068981	4	2145,23208	1,89	35,87	1
	High	vAIRPLDQAk	2	1	1	P74371	N-Term(iTRAQ4plex); K10(iTRAQ4plex)	0,0000	0,950	2	13,8	0	0,00003475	19	0,012763507	3	1398,86744	0,20	22,56	0
	Medium	vkDEEk	1	1	1	P74371	N-Term(iTRAQ4plex); K2(iTRAQ4plex); K6(iTRAQ4plex)	0,0000	0,916	1		0,01	0,1319	13	0,046341491	3	1179,69065	-3,30	8,90	1
P73201	Serine--tRNA ligase OS=Synechocystis sp. (strain PCC 6803 / Kazusa) GN=serS PE=3 SV=1 - [SYS_S	51,13	4,42	1	2	2	3	3,484	3	44,3	430	48,0	5,59							
	A3	Sequence	# PSMs	# Proteins	# Protein Groups	Protein Group Accessions	Modifications	ΔCn	116/117	116/117 Count	116/117 Variability [%]	q-Value	PEP	IonScore	Exp Value	Charge	MH+ [Da]	ΔM [ppm]	RT [min]	# Missed Cleavages
	High	vPVVLQDFLk	1	1	1	P73201	N-Term(iTRAQ4plex); K10(iTRAQ4plex)	0,0000	3,484	1		0	0,00001553	31	0,00077085	3	1445,89762	0,39	50,21	0
	High	aLINFMLER	2	1	1	P73201	N-Term(iTRAQ4plex)	0,0000	2,801	2	86,4	0	0,0001006	23	0,00512826	2	1250,70513	0,26	45,89	0
P74155	Membrane protein insertase YidC OS=Synechocystis sp. (strain PCC 6803 / Kazusa) GN=yidC PE=1 S	50,72	5,21	1	2	2	2	2,377	2	76,9	384	42,6	6,62							
	A3	Sequence	# PSMs	# Proteins	# Protein Groups	Protein Group Accessions	Modifications	ΔCn	116/117	116/117 Count	116/117 Variability [%]	q-Value	PEP	IonScore	Exp Value	Charge	MH+ [Da]	ΔM [ppm]	RT [min]	# Missed Cleavages
	High	tGFLFIk	1	1	1	P74155	N-Term(iTRAQ4plex); K7(iTRAQ4plex)	0,0000	3,765	1		0,001	0,005552	40	0,000111165	2	1113,69023	-0,75	38,96	0
	High	nSQILTPTYSVTk	1	1	1	P74155	N-Term(iTRAQ4plex); K13(iTRAQ4plex)	0,0000	1,501	1		0,001	0,01237	25	0,004080354	3	1739,98825	5,78	29,82	0
P73592	Slr1406 protein OS=Synechocystis sp. (strain PCC 6803 / Kazusa) GN=slr1406 PE=4 SV=1 - [P73592	49,92	13,26	1	2	2	2	0,592	2	13,5	181	18,7	7,84							
	A3	Sequence	# PSMs	# Proteins	# Protein Groups	Protein Group Accessions	Modifications	ΔCn	116/117	116/117 Count	116/117 Variability [%]	q-Value	PEP	IonScore	Exp Value	Charge	MH+ [Da]	ΔM [ppm]	RT [min]	# Missed Cleavages
	High	gDDATPHTIENSFYk	1	1	1	P73592	N-Term(iTRAQ4plex); K15(iTRAQ4plex)	0,0000	0,540	1		0	9,654E-08	38	0,000148242	3	1982,97287	1,53	27,51	0
	High	eGLVQAVIR	1	1	1	P73592	N-Term(iTRAQ4plex)	0,0000	0,648	1		0,001	0,004023	25	0,003427441	2	1128,68608	0,27	31,62	0
P19125	NAD(P)H-quinone oxidoreductase subunit J OS=Synechocystis sp. (strain PCC 6803 / Kazusa) GN=nd	49,46	16,20	1	3	3	5	0,641	4	29,9	179	20,6	4,53							
	A3	Sequence	# PSMs	# Proteins	# Protein Groups	Protein Group Accessions	Modifications	ΔCn	116/117	116/117 Count	116/117 Variability [%]	q-Value	PEP	IonScore	Exp Value	Charge	MH+ [Da]	ΔM [ppm]	RT [min]	# Missed Cleavages
	High	sLVSFYHLVk	2	1	1	P19125	N-Term(iTRAQ4plex); K10(iTRAQ4plex)	0,0000	0,482	1		0	2,077E-07	37	0,000214274	3	1480,87882	1,48	38,90	0
	High	iLmPEDWVGWPLRk	2	1	1	P19125	N-Term(iTRAQ4plex); M3(Oxidation); K14(iTRAQ4plex)	0,0000	0,780	2	8,8	0	0,0008006	11	0,072105768	3	2044,13138	1,02	48,12	1
	Medium	vFLPR	1	1	1	P19125	N-Term(iTRAQ4plex)	0,0000	0,558	1		0,023	0,3178	12	0,061797372	2	775,49443	-0,35	25,64	0
P26533	ATP synthase epsilon chain OS=Synechocystis sp. (strain PCC 6803 / Kazusa) GN=atpC PE=1 SV=3 -	49,46	17,65	1	2	2	4	0,824	3	14,0	136	14,6	5,26							
	A3	Sequence	# PSMs	# Proteins	# Protein Groups	Protein Group Accessions	Modifications	ΔCn	116/117	116/117 Count	116/117 Variability [%]	q-Value	PEP	IonScore	Exp Value	Charge	MH+ [Da]	ΔM [ppm]	RT [min]	# Missed Cleavages
	High	qAYTAAQGALEEANRGEDkPNQL	2	1	1	P26533	N-Term(iTRAQ4plex); K19(iTRAQ4plex); K24(iTRAQ4plex)	0,0000	0,905	1		0	2,407E-08	46	2,99497E-05	4	3034,59170	-0,06	30,13	1
	High	gEDkPNQLk	2	1	1	P26533	N-Term(iTRAQ4plex); K4(iTRAQ4plex); K9(iTRAQ4plex)	0,0000	0,706	2	23,2	0,001	0,006233	11	0,083170633	3	1460,84452	0,81	13,17	0
P73695	Thylakoid-associated protein sll1697 OS=Synechocystis sp. (strain PCC 6803 / Kazusa) GN=sll1697 P	49,06	8,72	1	2	2	2	0,686	2	1,1	321	35,0	6,96							
	A3	Sequence	# PSMs	# Proteins	# Protein Groups	Protein Group Accessions	Modifications	ΔCn	116/117	116/117 Count	116/117 Variability [%]	q-Value	PEP	IonScore	Exp Value	Charge	MH+ [Da]	ΔM [ppm]	RT [min]	# Missed Cleavages
	High	lGLNPTAAELTFPLSHPR	1	1	1	P73695	N-Term(iTRAQ4plex)	0,0000	0,691	1		0	1,76E-09	49	1,23871E-05	3	2078,15219	0,30	44,22	0
	Medium	fGEmLVETAk	1	1	1	P73695	N-Term(iTRAQ4plex); M4(Oxidation); K10(iTRAQ4plex)	0,0000	0,681	1		0,034	0,4307	11	0,087694025	3	1428,76828	2,49	13,50	0
P74219	Slr1533 protein OS=Synechocystis sp. (strain PCC 6803 / Kazusa) GN=slr1533 PE=4 SV=1 - [P74219	49,03	29,57	1	3	3	4	1,562	3	10,8	115	12,8	5,12							
	A3	Sequence	# PSMs	# Proteins	# Protein Groups	Protein Group Accessions	Modifications	ΔCn	116/117	116/117 Count	116/117 Variability [%]	q-Value	PEP	IonScore	Exp Value	Charge	MH+ [Da]	ΔM [ppm]	RT [min]	# Missed Cleavages
	High	aIGAQVNQGGRPEk	2	1	1	P74219	N-Term(iTRAQ4plex); K14(iTRAQ4plex)	0,0000	1,680	1		0	0,00000255	35	0,000321344	3	1712,96500	0,20	16,08	0
	High	tAPLTLNLLQGSVR	1	1	1	P74219	N-Term(iTRAQ4plex)	0,0000	0,538	1		0	0,0002292	26	0,002630086	3	1626,96640	0,26	43,65	0
	Medium	vLLTLR	1	1	1	P74219	N-Term(iTRAQ4plex)	0,0000	1,562	1		0,016	0,2102	11	0,070952876	2	858,58898	-0,42	31,24	0
P73796	Urease subunit gamma OS=Synechocystis sp. (strain PCC 6803 / Kazusa) GN=ureA PE=3 SV=1 - [UR	48,93	19,00	1	2	2	4	0,384	3	2,0	100	11,0	5,21							
	A3	Sequence	# PSMs	# Proteins	# Protein Groups	Protein Group Accessions	Modifications	ΔCn	116/117	116/117 Count	116/117 Variability [%]	q-Value	PEP	IonScore	Exp Value	Charge	MH+ [Da]	ΔM [ppm]	RT [min]	# Missed Cleavages
	High	lVTVHEPIR	3	1	1	P73796	N-Term(iTRAQ4plex)	0,0000	0,381	2	1,0	0	0,0002849	28	0,001729697	3	1207,72712	-0,70	23,49	0
	Medium	mQLSPQEkDk	1	1	1	P73796	N-Term(iTRAQ4plex); M1(Oxidation); K8(iTRAQ4plex); K10(iTF	0,0000	0,590	1		0,03	0,3933	20	0,009183326	3	1651,91324	5,01	12,85	1
P74751	Elongation factor 4 OS=Synechocystis sp. (strain PCC 6803 / Kazusa) GN=lepA PE=3 SV=1 - [LEPA_S	48,49	4,64	1	3	3	3	4,299	3	36,6	603	66,9	5,22							
	A3	Sequence	# PSMs	# Proteins	# Protein Groups	Protein Group Accessions	Modifications	ΔCn	116/117	116/117 Count	116/117 Variability [%]	q-Value	PEP	IonScore	Exp Value	Charge	MH+ [Da]	ΔM [ppm]	RT [min]	# Missed Cleavages
	High	lkVEEPFIk	1	1	1	P74751	N-Term(iTRAQ4plex); K2(iTRAQ4plex); K9(iTRAQ4plex)	0,0000	5,460	1		0	0,00001281	31	0,000777983	3	1534,95462	-1,50	31,75	1
	High	nFSIIAHIDHGk	1	1	1	P74751	N-Term(iTRAQ4plex); K12(iTRAQ4plex)	0,0000	2,153	1		0	0,0004091	28	0,001555858	4	1639,91359	-1,40	28,83	0
	High	lkELIPR	1	1	1	P74751	N-Term(iTRAQ4plex); K2(iTRAQ4plex)	0,0000	4,299	1		0,009	0,1233	15	0,029172255	3	1156,76438	-1,09	27,69	1
P74379	Slr0443 protein OS=Synechocystis sp. (strain PCC 6803 / Kazusa) GN=slr0443 PE=4 SV=1 - [P74379	47,70	11,20	1	3	3	4	0,735	3	11,2	125	14,6	4,58							
	A3	Sequence	# PSMs	# Proteins	# Protein Groups	Protein Group Accessions	Modifications	ΔCn	116/117	116/117 Count	116/117 Variability [%]	q-Value	PEP	IonScore	Exp Value	Charge	MH+ [Da]	ΔM [ppm]	RT [min]	# Missed Cleavages
	High	rGLYGLLR	1	1	1	P74379	N-Term(iTRAQ4plex)	0,0000	0,923	1		0	0,0006239	23	0,004634149	3	1091,68009	-0,48	29,27	1
	High	gLYGLLR	2	1	1	P74379	N-Term(iTRAQ4plex)	0,0000	0,681	1		0,001	0,01599	32	0,000570125	2	935,57945	-0,04	36,15	0
	High	lSHFIk	1	1	1	P74379	N-Term(iTRAQ4plex); K6(iTRAQ4plex)	0,0000	0,735	1		0,006	0,07398	11	0,146230528	3	1032,64405	-0,38	22,51	0
P72662	Cysteine synthase OS=Synechocystis sp. (strain PCC 6803 / Kazusa) GN=cysM PE=4 SV=1 - [P72662	47,03	6,46	1	2	2	2	0,851	2	25,7	325	34,7	5,40							
	A3	Sequence	# PSMs	# Proteins	# Protein Groups	Protein Group Accessions	Modifications	ΔCn	116/117	116/117 Count	116/117 Variability [%]	q-Value	PEP	IonScore	Exp Value	Charge	MH+ [Da]	ΔM [ppm]	RT [min]	# Missed Cleavages
	High	vVYQLLR	1	1	1	P72662	N-Term(iTRAQ4plex)	0,0000	0,718	1		0	0,001282	45	3,08298E-05	2	1034,64812	0,21	34,76	0
	High	hGFVDSIGHTPLIR	1	1	1	P72662	N-Term(iTRAQ4plex)	0,0000	1,010	1		0	0,0002098	15	0,032060479	4	1692,92959	-0,40	31,65	0
Q55905	Phosphoenolpyruvate synthase OS=Synechocystis sp. (strain PCC 6803 / Kazusa) GN=ppsA PE=3 SV	46,70	3,42	1	2	2	2	5,519	2	43,2	818	89,9	5,47							
	A3	Sequence	# PSMs	# Proteins	# Protein Groups	Protein Group Accessions	Modifications	ΔCn	116/117	116/117 Count	116/117 Variability [%]	q-Value	PEP	IonScore	Exp Value	Charge	MH+ [Da]	ΔM [ppm]	RT [min]	# Missed Cleavages
	High	lEFIIANHIk	1	1	1	Q55905	N-Term(iTRAQ4plex); K10(iTRAQ4plex)	0,0000	7,294	1		0	0,00000932	36	0,000250017	3	1485,90424	0,70	38,58	0
	High	nVSQINQFRPGEVLITNR	1	1	1	Q55905	N-Term(iTRAQ4plex)	0,0000	4,177	1		0	2,776E-07	24	0,004679304	3	2229,22354	0,64	35,88	0
Q55508	Indole-3-glycerol phosphate synthase OS=Synechocystis sp. (strain PCC 6803 / Kazusa) GN=trpC PE	46,68	7,46	1	2	2	2	0,824	2	53,0	295	32,9	5,99							
	A3	Sequence	# PSMs	# Proteins	# Protein Groups	Protein Group Accessions	Modifications	ΔCn	116/117	116/117 Count	116/117 Variability [%]	q-Value	PEP	IonScore	Exp Value	Charge	MH+ [Da]	ΔM [ppm]	RT [min]	# Missed Cleavages
	High	vLALDGVQLIGVNNR	1	1	1	Q55508	N-Term(iTRAQ4plex)	0,0000	0,589	1		0	0,00003517	28	0,001667132	3	1725,01535	0,78	45,85	0
	Medium	lQQAGAR	1	1	1	Q55508	N-Term(iTRAQ4plex)	0,0000	1,152	1		0,012	0,1564	32	0,000642643	2	887,51903	1,20	12,70	0
P73007	Slr1043 protein OS=Synechocystis sp. (strain PCC 6803 / Kazusa) GN=slr1043 PE=4 SV=1 - [P73007	45,93	14,44	1	2	2	3	0,355	3	14,4	187	20,7	4,50							
	A3	Sequence	# PSMs	# Proteins	# Protein Groups	Protein Group Accessions	Modifications	ΔCn	116/117	116/117 Count	116/117 Variability [%]	q-Value	PEP	IonScore	Exp Value	Charge	MH+ [Da]	ΔM [ppm]	RT [min]	# Missed Cleavages
	High	lLDHVALLR	1	1	1	P73007	N-Term(iTRAQ4plex)	0,0000	0,312	1		0	0,000001162	40	0,000107145	3	1193,74884	0,12	36,74	0
	High	iTPIPNASPLLLGTINLR	2	1	1	P73007	N-Term(iTRAQ4plex)	0,0000	0,373	2	7,2	0	0,002531	19	0,012301838	3	2047,24308	1,67	52,38	0
P77964	Protein translocase subunit SecY OS=Synechocystis sp. (strain PCC 6803 / Kazusa) GN=secY PE=3 S	45,84	4,52	1	2	2	3	3,379	2	31,8	442	48,0	9,70							
	A3	Sequence	# PSMs	# Proteins	# Protein Groups	Protein Group Accessions	Modifications	ΔCn	116/117	116/117 Count	116/117 Variability [%]	q-Value	PEP	IonScore	Exp Value	Charge	MH+ [Da]	ΔM [ppm]	RT [min]	# Missed Cleavages
	High	rIPIISAR	2	1	1	P77964	N-Term(iTRAQ4plex)	0,0000	4,166	1		0	0,000009142	39	0,000125017	3	1069,69590	-0,35	24,17	1
	High	mGSSIPGIRPGk	1	1	1	P77964	N-Term(iTRAQ4plex); M1(Oxidation); K12(iTRAQ4plex)	0,0000	2,741	1		0	0,002072	20	0,010398483	3	1503,85434	-0,85	19,70	0
P72914	Ssr1766 protein OS=Synechocystis sp. (strain PCC 6803 / Kazusa) GN=ssr1766 PE=4 SV=1 - [P7291	45,56	10,53	1	2	2	3	0,840	3	3,0	76	8,7	9,99							
	A3	Sequence	# PSMs	# Proteins	# Protein Groups	Protein Group Accessions	Modifications	ΔCn	116/117	116/117 Count	116/117 Variability [%]	q-Value	PEP	IonScore	Exp Value	Charge	MH+ [Da]	ΔM [ppm]	RT [min]	# Missed Cleavages
	High	kLGFFLER	1	1	1	P72914	N-Term(iTRAQ4plex); K1(iTRAQ4plex)	0,0000	1,175	1		0	0,0002075	37	0,000215263	3	1297,78507	-1,57	33,67	1
	High	lGFFLER	2	1	1	P72914	N-Term(iTRAQ4plex)	0,0000	0,832	2	1,5	0,009	0,1218	22	0,009233535	2	1025,59026	0,18	41,74	0
P73637	5-oxo-1,2,5-tricarboxilic-3-penten acid decarboxilase/isomer OS=Synechocystis sp. (strain PCC 6803	45,17	11,96	1	2	2	3	0,351	3	18,3	276	30,0	4,49							
	A3	Sequence	# PSMs	# Proteins	# Protein Groups	Protein Group Accessions	Modifications	ΔCn	116/117	116/117 Count	116/117 Variability [%]	q-Value	PEP	IonScore	Exp Value	Charge	MH+ [Da]	ΔM [ppm]	RT [min]	# Missed Cleavages
	High	gFDGFcPLGPWVVR	2	1	1	P73637	N-Term(iTRAQ4plex); C6(Carbamidomethyl)	0,0000	0,330	2	9,1	0	0,00006698	24	0,00400839	2	1750,88689	0,71	51,28	0
	High	hIDPEAHLETTVNDEETPR	1	1	1	P73637	N-Term(iTRAQ4plex)	0,0000	0,566	1		0	4,913E-07	23	0,004874948	4	2347,12710	-0,53	25,49	0
P72587	Biosynthetic arginine decarboxylase 2 OS=Synechocystis sp. (strain PCC 6803 / Kazusa) GN=speA2 P	44,87	2,43	1	3	3	3	4,834	3	13,8	659	74,4	5,48							
	A3	Sequence	# PSMs	# Proteins	# Protein Groups	Protein Group Accessions	Modifications	ΔCn	116/117	116/117 Count	116/117 Variability [%]	q-Value	PEP	IonScore	Exp Value	Charge	MH+ [Da]	ΔM [ppm]	RT [min]	# Missed Cleavages
	High	lGLPLLIR	1	1	1	P72587	N-Term(iTRAQ4plex)	0,0000	5,302	1		0	0,00197	29	0,001294106	2	1038,71550	-0,11	46,12	0
	High	kLGLPLLIR	1	1	1	P72587	N-Term(iTRAQ4plex); K1(iTRAQ4plex)	0,0000	4,834	1		0	0,000007069	27	0,002192653	3	1310,91217	-0,36	38,76	1
	High	kIFEITR	1	1	1	P72587	N-Term(iTRAQ4plex); K1(iTRAQ4plex)	0,0000	3,542	1		0,001	0,006078	15	0,028639802	3	1194,74338	-1,27	24,84	1
P73050	Ferripyochelin binding protein OS=Synechocystis sp. (strain PCC 6803 / Kazusa) GN=fbp PE=4 SV=1	44,68	13,16	1	2	2	2	0,472	1		190	19,5	7,62							
	A3	Sequence	# PSMs	# Proteins	# Protein Groups	Protein Group Accessions	Modifications	ΔCn	116/117	116/117 Count	116/117 Variability [%]	q-Value	PEP	IonScore	Exp Value	Charge	MH+ [Da]	ΔM [ppm]	RT [min]	# Missed Cleavages
	High	aVVHAAHIER	1	1	1	P73050	N-Term(iTRAQ4plex)	0,0000	0,472	1		0	0,000001719	35	0,000309721	3	1246,71411	0,32	11,62	0
	High	gSLIGIGATILDNVR	1	1	1	P73050	N-Term(iTRAQ4plex)	0,0000				0,001	0,003397	23	0,005482391	3	1642,96213	0,74	54,16	0
P72747	Slr1103 protein OS=Synechocystis sp. (strain PCC 6803 / Kazusa) GN=slr1103 PE=4 SV=1 - [P72747	43,48	3,12	1	2	2	2	0,830	2	65,9	706	79,8	5,39							
	A3	Sequence	# PSMs	# Proteins	# Protein Groups	Protein Group Accessions	Modifications	ΔCn	116/117	116/117 Count	116/117 Variability [%]	q-Value	PEP	IonScore	Exp Value	Charge	MH+ [Da]	ΔM [ppm]	RT [min]	# Missed Cleavages
	High	ikPTSQNEGYSYR	1	1	1	P72747	N-Term(iTRAQ4plex); K2(iTRAQ4plex)	0,0000	1,245	1		0	0,000007019	38	0,000142223	3	1830,95835	-0,28	18,66	0
	High	hIFVIEDQk	1	1	1	P72747	N-Term(iTRAQ4plex); K9(iTRAQ4plex)	0,0000	0,554	1		0	0,00001421	18	0,015738742	3	1416,80939	0,31	25,60	0
Q55966	Sll0676 protein OS=Synechocystis sp. (strain PCC 6803 / Kazusa) GN=sll0676 PE=4 SV=1 - [Q55966	43,44	12,22	1	2	2	2	1,760	2	57,5	180	20,7	6,57							
	A3	Sequence	# PSMs	# Proteins	# Protein Groups	Protein Group Accessions	Modifications	ΔCn	116/117	116/117 Count	116/117 Variability [%]	q-Value	PEP	IonScore	Exp Value	Charge	MH+ [Da]	ΔM [ppm]	RT [min]	# Missed Cleavages
	High	wFTSFFk	1	1	1	Q55966	N-Term(iTRAQ4plex); K7(iTRAQ4plex)	0,0000	2,524	1		0	0,00008032	34	0,000358897	2	1250,68035	-0,71	48,44	0
	High	lRPDPTVmALSLNFR	1	1	1	Q55966	N-Term(iTRAQ4plex); M8(Oxidation)	0,0000	1,227	1		0	0,000002734	22	0,006294627	3	1890,03916	0,16	39,65	0
Q55187	Phenylalanine--tRNA ligase alpha subunit OS=Synechocystis sp. (strain PCC 6803 / Kazusa) GN=pheS	43,32	5,14	1	2	2	3	2,480	2	2,3	331	37,1	6,15							
	A3	Sequence	# PSMs	# Proteins	# Protein Groups	Protein Group Accessions	Modifications	ΔCn	116/117	116/117 Count	116/117 Variability [%]	q-Value	PEP	IonScore	Exp Value	Charge	MH+ [Da]	ΔM [ppm]	RT [min]	# Missed Cleavages
	High	kGELSLILk	1	1	1	Q55187	N-Term(iTRAQ4plex); K1(iTRAQ4plex); K9(iTRAQ4plex)	0,0000	2,441	1		0	0,0001829	36	0,000244889	3	1432,94336	-2,08	31,77	1
	High	gLAFTHLk	2	1	1	Q55187	N-Term(iTRAQ4plex); K8(iTRAQ4plex)	0,0000	2,519	1		0	0,002738	16	0,02552525	3	1174,71860	-0,06	26,74	0
P73031	Ssl1918 protein OS=Synechocystis sp. (strain PCC 6803 / Kazusa) GN=ssl1918 PE=4 SV=1 - [P73031	43,07	18,56	1	2	2	2	0,605	2	11,1	97	11,3	5,01							
	A3	Sequence	# PSMs	# Proteins	# Protein Groups	Protein Group Accessions	Modifications	ΔCn	116/117	116/117 Count	116/117 Variability [%]	q-Value	PEP	IonScore	Exp Value	Charge	MH+ [Da]	ΔM [ppm]	RT [min]	# Missed Cleavages
	High	rEGNQILWQLER	1	1	1	P73031	N-Term(iTRAQ4plex)	0,0000	0,562	1		0	0,000006902	30	0,00090985	3	1685,92051	0,04	33,68	1
	Medium	nWVVSR	1	1	1	P73031	N-Term(iTRAQ4plex)	0,0000	0,652	1		0,016	0,205	26	0,002703772	2	904,51134	-0,89	20,17	0
P74569	Aspartokinase OS=Synechocystis sp. (strain PCC 6803 / Kazusa) GN=lysC PE=1 SV=1 - [P74569_SYN	43,06	3,17	1	2	2	4	1,198	4	26,3	600	63,5	5,38							
	A3	Sequence	# PSMs	# Proteins	# Protein Groups	Protein Group Accessions	Modifications	ΔCn	116/117	116/117 Count	116/117 Variability [%]	q-Value	PEP	IonScore	Exp Value	Charge	MH+ [Da]	ΔM [ppm]	RT [min]	# Missed Cleavages
	High	vVAPPVQNR	2	1	1	P74569	N-Term(iTRAQ4plex)	0,0000	0,890	2	49,6	0,002	0,01982	26	0,00239315	2	1123,67131	0,76	20,23	0
	High	vPDRPGVASk	2	1	1	P74569	N-Term(iTRAQ4plex); K10(iTRAQ4plex)	0,0000	1,400	2	26,3	0,004	0,04972	13	0,053575965	3	1313,77988	1,43	15,63	0
P72968	Slr1590 protein OS=Synechocystis sp. (strain PCC 6803 / Kazusa) GN=slr1590 PE=4 SV=1 - [P72968	42,57	6,10	1	2	2	3	1,819	3	14,8	295	32,6	5,00							
	A3	Sequence	# PSMs	# Proteins	# Protein Groups	Protein Group Accessions	Modifications	ΔCn	116/117	116/117 Count	116/117 Variability [%]	q-Value	PEP	IonScore	Exp Value	Charge	MH+ [Da]	ΔM [ppm]	RT [min]	# Missed Cleavages
	High	kVTGETDPALVk	1	1	1	P72968	N-Term(iTRAQ4plex); K1(iTRAQ4plex); K12(iTRAQ4plex)	0,0000	1,326	1		0	4,319E-07	42	5,72756E-05	3	1690,00888	-1,34	19,45	1
	High	skPIFk	2	1	1	P72968	N-Term(iTRAQ4plex); K2(iTRAQ4plex); K6(iTRAQ4plex)	0,0000	1,912	2	7,4	0,001	0,01516	13	0,048191451	3	1151,74775	-3,05	16,42	0
P72622	Peptide methionine sulfoxide reductase MsrA 1 OS=Synechocystis sp. (strain PCC 6803 / Kazusa) GN	42,28	9,46	1	2	2	5	0,656	5	14,2	222	24,2	5,26							
	A3	Sequence	# PSMs	# Proteins	# Protein Groups	Protein Group Accessions	Modifications	ΔCn	116/117	116/117 Count	116/117 Variability [%]	q-Value	PEP	IonScore	Exp Value	Charge	MH+ [Da]	ΔM [ppm]	RT [min]	# Missed Cleavages
	High	vFWESHNPTQGMR	1	1	1	P72622	N-Term(iTRAQ4plex)	0,0000	0,722	1		0	0,00009013	26	0,002517503	3	1732,83945	2,77	30,00	0
	High	gFFDLFGk	3	1	1	P72622	N-Term(iTRAQ4plex); K8(iTRAQ4plex)	0,0000	0,610	3	4,7	0	0,001574	19	0,011323222	2	1218,67717	0,83	49,35	0
	High	vFWESHNPTQGmR	1	1	1	P72622	N-Term(iTRAQ4plex); M12(Oxidation)	0,0000	0,811	1		0	0,0000576	19	0,012734151	3	1748,83115	0,90	26,08	0
P74591	Shikimate dehydrogenase OS=Synechocystis sp. (strain PCC 6803 / Kazusa) GN=aroE PE=3 SV=1 - [	41,85	9,31	1	2	2	2	0,698	2	36,1	290	31,1	7,25							
	A3	Sequence	# PSMs	# Proteins	# Protein Groups	Protein Group Accessions	Modifications	ΔCn	116/117	116/117 Count	116/117 Variability [%]	q-Value	PEP	IonScore	Exp Value	Charge	MH+ [Da]	ΔM [ppm]	RT [min]	# Missed Cleavages
	High	lPPTAIAYDLIYTPRPTR	1	1	1	P74591	N-Term(iTRAQ4plex)	0,0000	0,551	1		0	0,00001771	29	0,00119116	3	2202,24295	1,17	46,19	0
	High	qALLHHLEk	1	1	1	P74591	N-Term(iTRAQ4plex); K9(iTRAQ4plex)	0,0000	0,884	1		0	0,000006192	26	0,00273508	4	1376,82656	0,94	19,71	0
P73439	Sll1461 protein OS=Synechocystis sp. (strain PCC 6803 / Kazusa) GN=sll1461 PE=4 SV=1 - [P73439	41,66	9,09	1	2	2	2	0,562	2	100,2	220	25,5	4,69							
	A3	Sequence	# PSMs	# Proteins	# Protein Groups	Protein Group Accessions	Modifications	ΔCn	116/117	116/117 Count	116/117 Variability [%]	q-Value	PEP	IonScore	Exp Value	Charge	MH+ [Da]	ΔM [ppm]	RT [min]	# Missed Cleavages
	High	vAPDAMVALGRPk	1	1	1	P73439	N-Term(iTRAQ4plex); K13(iTRAQ4plex)	0,0000	0,986	1		0	6,062E-07	35	0,000306881	3	1612,94712	1,44	29,19	0
	High	kLQFYER	1	1	1	P73439	N-Term(iTRAQ4plex); K1(iTRAQ4plex)	0,0000	0,320	1		0	0,002734	20	0,011090982	3	1271,73584	0,63	22,01	1
P73443	Lysine--tRNA ligase OS=Synechocystis sp. (strain PCC 6803 / Kazusa) GN=lysS PE=3 SV=1 - [SYK_S	41,24	4,90	1	3	3	3	2,235	3	18,5	510	57,3	5,66							
	A3	Sequence	# PSMs	# Proteins	# Protein Groups	Protein Group Accessions	Modifications	ΔCn	116/117	116/117 Count	116/117 Variability [%]	q-Value	PEP	IonScore	Exp Value	Charge	MH+ [Da]	ΔM [ppm]	RT [min]	# Missed Cleavages
	High	iATELHLk	1	1	1	P73443	N-Term(iTRAQ4plex); K8(iTRAQ4plex)	0,0000	2,235	1		0	0,0001594	31	0,000729407	3	1212,75373	-1,43	27,52	0
	High	skPGLVER	1	1	1	P73443	N-Term(iTRAQ4plex); K2(iTRAQ4plex)	0,0000	1,303	1		0,001	0,0162	16	0,022488993	3	1173,71738	-1,74	13,05	0
	High	dVIAFPLLk	1	1	1	P73443	N-Term(iTRAQ4plex); K9(iTRAQ4plex)	0,0000	2,529	1		0,003	0,03574	19	0,011427994	2	1303,82024	-1,99	46,15	0
P73336	30S ribosomal protein S20 OS=Synechocystis sp. (strain PCC 6803 / Kazusa) GN=rpsT PE=3 SV=1 -	41,08	16,49	1	2	2	3	1,083	3	3,8	97	10,7	10,29							
	A3	Sequence	# PSMs	# Proteins	# Protein Groups	Protein Group Accessions	Modifications	ΔCn	116/117	116/117 Count	116/117 Variability [%]	q-Value	PEP	IonScore	Exp Value	Charge	MH+ [Da]	ΔM [ppm]	RT [min]	# Missed Cleavages
	High	rIEIAER	2	1	1	P73336	N-Term(iTRAQ4plex)	0,0000	1,097	2	1,9	0,001	0,005754	31	0,000753304	3	1030,61368	1,05	15,25	1
	Medium	aLQSALPAA	1	1	1	P73336	N-Term(iTRAQ4plex)	0,0000	0,773	1		0,043	0,5508	13	0,053575965	2	985,57994	0,04	31,07	0
P72720	Glutamine--fructose-6-phosphate aminotransferase [isomerizing] OS=Synechocystis sp. (strain PCC 6	40,85	6,02	1	3	3	4	2,657	3	53,9	631	69,6	5,85							

	A3	Sequence	# PSMs	# Proteins	# Protein Groups	Protein Group Accessions	Modifications	ΔCn	116/117	116/117 Count	116/117 Variability [%]	q-Value	PEP	IonScore	Exp Value	Charge	MH+ [Da]	ΔM [ppm]	RT [min]	# Missed Cleavages
	High	gINFPIALEGALk	1	1	1	P72720	N-Term(iTRAQ4plex); K13(iTRAQ4plex)	0,0000	1,506	1		0	0,0001847	38	0,000170596	3	1630,97733	0,13	48,67	0
	High	hGPIALLDAk	1	1	1	P72720	N-Term(iTRAQ4plex); K10(iTRAQ4plex)	0,0000				0	0,0002543	16	0,024043628	3	1322,80359	0,08	28,94	0
	High	lTPLGVEVYDFNLkR	2	1	1	P72720	N-Term(iTRAQ4plex); K14(iTRAQ4plex)	0,0000	3,151	2	25,7	0,001	0,005998	11	0,087291108	3	2052,17466	0,69	44,30	1
P74305	Protease OS=Synechocystis sp. (strain PCC 6803 / Kazusa) GN=pqqE PE=3 SV=1 - [P74305_SYNY3]	40,72	4,96	1	2	2	2	0,667	2	25,7	524	59,6	5,15							
	A3	Sequence	# PSMs	# Proteins	# Protein Groups	Protein Group Accessions	Modifications	ΔCn	116/117	116/117 Count	116/117 Variability [%]	q-Value	PEP	IonScore	Exp Value	Charge	MH+ [Da]	ΔM [ppm]	RT [min]	# Missed Cleavages
	High	rPVIGYDEDIR	1	1	1	P74305	N-Term(iTRAQ4plex)	0,0000	0,562	1		0	0,00002168	35	0,000334942	3	1476,79465	1,28	24,20	0
	High	vAQETFRPENSTIGR	1	1	1	P74305	N-Term(iTRAQ4plex)	0,0000	0,791	1		0	0,002608	19	0,013255395	3	1848,97062	1,14	23,63	0
Q55862	Sll0588 protein OS=Synechocystis sp. (strain PCC 6803 / Kazusa) GN=sll0588 PE=4 SV=1 - [Q55862	40,69	11,11	1	2	2	2	1,490	2	38,6	153	16,5	5,45							
	A3	Sequence	# PSMs	# Proteins	# Protein Groups	Protein Group Accessions	Modifications	ΔCn	116/117	116/117 Count	116/117 Variability [%]	q-Value	PEP	IonScore	Exp Value	Charge	MH+ [Da]	ΔM [ppm]	RT [min]	# Missed Cleavages
	High	fYQDALTk	1	1	1	Q55862	N-Term(iTRAQ4plex); K8(iTRAQ4plex)	0,0000	1,159	1		0	0,00002647	40	9,41825E-05	2	1273,70476	1,32	28,27	0
	High	lPRPQGSPk	1	1	1	Q55862	N-Term(iTRAQ4plex); K9(iTRAQ4plex)	0,0000	1,917	1		0,004	0,04389	13	0,045182474	3	1267,77491	1,88	15,70	0
P73448	Nitrate reductase OS=Synechocystis sp. (strain PCC 6803 / Kazusa) GN=narB PE=3 SV=1 - [NARB_S	40,63	3,08	1	2	2	2	0,922	2	19,7	714	79,6	7,23							
	A3	Sequence	# PSMs	# Proteins	# Protein Groups	Protein Group Accessions	Modifications	ΔCn	116/117	116/117 Count	116/117 Variability [%]	q-Value	PEP	IonScore	Exp Value	Charge	MH+ [Da]	ΔM [ppm]	RT [min]	# Missed Cleavages
	High	rVTLSPAFR	1	1	1	P73448	N-Term(iTRAQ4plex)	0,0000	1,052	1		0	0,002367	29	0,00118842	3	1190,71314	0,41	24,07	1
	High	eAGGLSHLLPGYR	1	1	1	P73448	N-Term(iTRAQ4plex)	0,0000	0,809	1		0	0,000307	24	0,00363053	3	1513,82499	0,39	33,63	0
P74358	Sll1528 protein OS=Synechocystis sp. (strain PCC 6803 / Kazusa) GN=sll1528 PE=4 SV=1 - [P74358_	40,42	2,21	1	2	2	2	1,982	2	82,8	996	114,5	6,42							
	A3	Sequence	# PSMs	# Proteins	# Protein Groups	Protein Group Accessions	Modifications	ΔCn	116/117	116/117 Count	116/117 Variability [%]	q-Value	PEP	IonScore	Exp Value	Charge	MH+ [Da]	ΔM [ppm]	RT [min]	# Missed Cleavages
	High	vTERPDNAHGVPFQEk	1	1	1	P74358	N-Term(iTRAQ4plex); K16(iTRAQ4plex)	0,0000	1,217	1		0	0,00001019	20	0,009844401	4	2112,10879	0,52	21,04	0
	High	lPLFLk	1	1	1	P74358	N-Term(iTRAQ4plex); K6(iTRAQ4plex)	0,0000	3,227	1		0,009	0,1223	33	0,00047203	2	1018,68956	-0,76	41,47	0
P09190	Cytochrome b559 subunit alpha OS=Synechocystis sp. (strain PCC 6803 / Kazusa) GN=psbE PE=1 S\	39,96	32,10	1	2	2	2	0,471	2	4,2	81	9,4	4,59							
	A3	Sequence	# PSMs	# Proteins	# Protein Groups	Protein Group Accessions	Modifications	ΔCn	116/117	116/117 Count	116/117 Variability [%]	q-Value	PEP	IonScore	Exp Value	Charge	MH+ [Da]	ΔM [ppm]	RT [min]	# Missed Cleavages
	High	sGTTGERPFSDIVTSIR	1	1	1	P09190	N-Term(iTRAQ4plex)	0,0000	0,457	1		0	0,0000106	24	0,003784164	3	1967,03281	0,66	35,28	0
	High	qELPILQER	1	1	1	P09190	N-Term(iTRAQ4plex)	0,0000	0,484	1		0,001	0,008717	29	0,001330363	2	1269,72710	-1,00	31,91	0
Q55614	High-affinity branched-chain amino acid transport ATP-binding protein OS=Synechocystis sp. (strain F	39,90	6,43	1	2	2	2	2,023	2	82,8	373	41,2	5,02							
	A3	Sequence	# PSMs	# Proteins	# Protein Groups	Protein Group Accessions	Modifications	ΔCn	116/117	116/117 Count	116/117 Variability [%]	q-Value	PEP	IonScore	Exp Value	Charge	MH+ [Da]	ΔM [ppm]	RT [min]	# Missed Cleavages
	High	tTFLDVITGk	1	1	1	Q55614	N-Term(iTRAQ4plex); K10(iTRAQ4plex)	0,0000	1,243	1		0	0,001011	30	0,000935341	3	1382,81284	-0,39	46,58	0
	High	sVGGLLETIGLTPk	1	1	1	Q55614	N-Term(iTRAQ4plex); K14(iTRAQ4plex)	0,0000	3,294	1		0	0,0004973	23	0,005457202	3	1673,00974	0,56	49,07	0
Q55658	Sll0023 protein OS=Synechocystis sp. (strain PCC 6803 / Kazusa) GN=sll0023 PE=4 SV=1 - [Q55658	39,75	5,37	1	3	3	3	1,809	3	67,0	447	51,6	4,63							
	A3	Sequence	# PSMs	# Proteins	# Protein Groups	Protein Group Accessions	Modifications	ΔCn	116/117	116/117 Count	116/117 Variability [%]	q-Value	PEP	IonScore	Exp Value	Charge	MH+ [Da]	ΔM [ppm]	RT [min]	# Missed Cleavages
	High	lLLEGLHVR	1	1	1	Q55658	N-Term(iTRAQ4plex)	0,0000	2,727	1		0	0,00001812	37	0,000203222	3	1193,74903	0,27	35,99	0
	High	iRPEYWPALk	1	1	1	Q55658	N-Term(iTRAQ4plex); K10(iTRAQ4plex)	0,0000	1,809	1		0	0,0008437	16	0,0259998	3	1560,91367	-0,27	34,00	0
	Medium	mQPIk	1	1	1	Q55658	N-Term(iTRAQ4plex); K5(iTRAQ4plex)	0,0000	1,040	1		0,035	0,4439	11	0,077798282	2	904,55889	6,66	17,39	0
P72653	Sll1049 protein OS=Synechocystis sp. (strain PCC 6803 / Kazusa) GN=sll1049 PE=4 SV=1 - [P72653_	39,71	3,37	1	2	2	2	2,420	2	47,2	564	63,9	5,06							
	A3	Sequence	# PSMs	# Proteins	# Protein Groups	Protein Group Accessions	Modifications	ΔCn	116/117	116/117 Count	116/117 Variability [%]	q-Value	PEP	IonScore	Exp Value	Charge	MH+ [Da]	ΔM [ppm]	RT [min]	# Missed Cleavages
	High	kIESVGLPFSR	1	1	1	P72653	N-Term(iTRAQ4plex); K1(iTRAQ4plex)	0,0000	3,275	1		0	0,00003599	40	0,000106652	3	1520,90137	-1,69	32,39	1
	High	gHIWVYGR	1	1	1	P72653	N-Term(iTRAQ4plex)	0,0000	1,788	1		0,002	0,01898	11	0,072940713	3	1131,61859	0,52	22,58	0
P74236	Slr1161 protein OS=Synechocystis sp. (strain PCC 6803 / Kazusa) GN=slr1161 PE=4 SV=1 - [P74236	39,55	8,54	1	2	2	3	1,188	3	22,3	199	22,9	5,85							
	A3	Sequence	# PSMs	# Proteins	# Protein Groups	Protein Group Accessions	Modifications	ΔCn	116/117	116/117 Count	116/117 Variability [%]	q-Value	PEP	IonScore	Exp Value	Charge	MH+ [Da]	ΔM [ppm]	RT [min]	# Missed Cleavages
	High	kLYELPR	2	1	1	P74236	N-Term(iTRAQ4plex); K1(iTRAQ4plex)	0,0000	1,098	2	11,7	0	0,0007666	24	0,00591686	3	1206,74457	-0,26	23,05	1
	High	sFLDRIENLk	1	1	1	P74236	N-Term(iTRAQ4plex); K10(iTRAQ4plex)	0,0000	1,378	1		0,001	0,00343	18	0,017699867	3	1522,88413	0,61	40,77	1
P73344	Slr1196 protein OS=Synechocystis sp. (strain PCC 6803 / Kazusa) GN=slr1196 PE=4 SV=1 - [P73344	38,97	3,96	1	2	2	2	0,529	2	3,8	429	48,0	5,38							
	A3	Sequence	# PSMs	# Proteins	# Protein Groups	Protein Group Accessions	Modifications	ΔCn	116/117	116/117 Count	116/117 Variability [%]	q-Value	PEP	IonScore	Exp Value	Charge	MH+ [Da]	ΔM [ppm]	RT [min]	# Missed Cleavages
	High	ikEQLVQR	1	1	1	P73344	N-Term(iTRAQ4plex); K2(iTRAQ4plex)	0,0000	0,516	1		0	0,0003576	28	0,001705965	3	1301,81449	0,08	18,75	1
	High	aYTLAWSIk	1	1	1	P73344	N-Term(iTRAQ4plex); K9(iTRAQ4plex)	0,0000	0,543	1		0	0,001614	24	0,003706551	2	1340,78191	0,18	38,78	0
P72852	Slr0962 protein OS=Synechocystis sp. (strain PCC 6803 / Kazusa) GN=slr0962 PE=4 SV=1 - [P72852	38,93	5,66	1	2	2	2	1,348	2	12,9	477	51,8	4,12							
	A3	Sequence	# PSMs	# Proteins	# Protein Groups	Protein Group Accessions	Modifications	ΔCn	116/117	116/117 Count	116/117 Variability [%]	q-Value	PEP	IonScore	Exp Value	Charge	MH+ [Da]	ΔM [ppm]	RT [min]	# Missed Cleavages
	High	tPVAVDDkk	1	1	1	P72852	N-Term(iTRAQ4plex); K8(iTRAQ4plex); K9(iTRAQ4plex)	0,0000	1,470	1		0	0,0002213	26	0,002264488	3	1404,84034	-1,38	15,17	1
	High	eEkPVNPAPEKEEEVLLk	1	1	1	P72852	N-Term(iTRAQ4plex); K3(iTRAQ4plex); K11(iTRAQ4plex); K18(	0,0000	1,236	1		0	1,682E-08	25	0,002818188	4	2654,51333	1,27	28,24	1
P72804	Thylakoid membrane protein slr1796 OS=Synechocystis sp. (strain PCC 6803 / Kazusa) GN=slr1796 F	37,88	8,96	1	2	2	2	0,757	2	1,7	201	23,0	5,48							
	A3	Sequence	# PSMs	# Proteins	# Protein Groups	Protein Group Accessions	Modifications	ΔCn	116/117	116/117 Count	116/117 Variability [%]	q-Value	PEP	IonScore	Exp Value	Charge	MH+ [Da]	ΔM [ppm]	RT [min]	# Missed Cleavages
	High	kIFDAQGALk	1	1	1	P72804	N-Term(iTRAQ4plex); K1(iTRAQ4plex); K10(iTRAQ4plex)	0,0000	0,765	1		0	0,00003855	38	0,000162544	3	1522,92981	-1,29	25,23	1
	Medium	dLFDLLPR	1	1	1	P72804	N-Term(iTRAQ4plex)	0,0000	0,748	1		0,013	0,171	12	0,070140685	2	1132,65288	4,02	49,72	0
P73068	Rubredoxin OS=Synechocystis sp. (strain PCC 6803 / Kazusa) GN=rub PE=3 SV=1 - [RUBR_SYNY3]	37,77	20,87	1	2	2	5	1,626	4	16,9	115	12,6	7,03							
	A3	Sequence	# PSMs	# Proteins	# Protein Groups	Protein Group Accessions	Modifications	ΔCn	116/117	116/117 Count	116/117 Variability [%]	q-Value	PEP	IonScore	Exp Value	Charge	MH+ [Da]	ΔM [ppm]	RT [min]	# Missed Cleavages
	High	tSVSPGTPFEALPLNWk	1	1	1	P73068	N-Term(iTRAQ4plex); K17(iTRAQ4plex)	0,0000	1,918	1		0	0,000006319	35	0,000290382	3	2132,16684	1,76	48,73	0
	High	sERPPEk	4	1	1	P73068	N-Term(iTRAQ4plex); K7(iTRAQ4plex)	0,0000	1,507	3	11,0	0	0,001211	12	0,068544088	3	1130,64026	-0,50	11,12	0
Q6ZED7	Uncharacterized protein OS=Synechocystis sp. (strain PCC 6803 / Kazusa) GN=slr7061 PE=4 SV=1 -	37,30	4,27	1	2	2	2	1,359	2	32,2	422	47,9	5,20							
	A3	Sequence	# PSMs	# Proteins	# Protein Groups	Protein Group Accessions	Modifications	ΔCn	116/117	116/117 Count	116/117 Variability [%]	q-Value	PEP	IonScore	Exp Value	Charge	MH+ [Da]	ΔM [ppm]	RT [min]	# Missed Cleavages
	High	vIFDFTHGYR	1	1	1	Q6ZED7	N-Term(iTRAQ4plex)	0,0000	1,680	1		0	0,000003193	36	0,000263009	3	1398,72882	0,10	38,27	0
	High	akPFNLIk	1	1	1	Q6ZED7	N-Term(iTRAQ4plex); K2(iTRAQ4plex); K8(iTRAQ4plex)	0,0000	1,100	1		0	0,002399	15	0,035315878	3	1362,87934	-2,94	27,01	0
Q55798	6-carboxy-5,6,7,8-tetrahydropterin synthase OS=Synechocystis sp. (strain PCC 6803 / Kazusa) GN=q	36,99	23,26	1	3	3	3	0,402	3	21,3	129	14,5	7,01							
	A3	Sequence	# PSMs	# Proteins	# Protein Groups	Protein Group Accessions	Modifications	ΔCn	116/117	116/117 Count	116/117 Variability [%]	q-Value	PEP	IonScore	Exp Value	Charge	MH+ [Da]	ΔM [ppm]	RT [min]	# Missed Cleavages
	High	eFSFEAAHQLPHHEGk	1	1	1	Q55798	N-Term(iTRAQ4plex); K16(iTRAQ4plex)	0,0000	0,464	1		0	0,00002593	25	0,00439634	4	2152,08388	1,12	24,87	0
	High	mWIIYk	1	1	1	Q55798	N-Term(iTRAQ4plex); K6(iTRAQ4plex)	0,0000	0,273	1		0,002	0,02513	20	0,009505391	2	1141,66997	1,53	38,93	0
	High	aHLDPLVk	1	1	1	Q55798	N-Term(iTRAQ4plex); K8(iTRAQ4plex)	0,0000	0,402	1		0,002	0,0178	19	0,013676344	3	1180,72785	-1,19	22,45	0
Q55444	Methyl-accepting chemotaxis protein II OS=Synechocystis sp. (strain PCC 6803 / Kazusa) GN=tar PE	36,75	11,04	1	2	2	3	0,608	2	11,2	163	17,7	5,26							
	A3	Sequence	# PSMs	# Proteins	# Protein Groups	Protein Group Accessions	Modifications	ΔCn	116/117	116/117 Count	116/117 Variability [%]	q-Value	PEP	IonScore	Exp Value	Charge	MH+ [Da]	ΔM [ppm]	RT [min]	# Missed Cleavages
	High	qFAAQTHLLALk	2	1	1	Q55444	N-Term(iTRAQ4plex); K12(iTRAQ4plex)	0,0000	0,564	1		0	3,248E-07	31	0,000707897	3	1628,97177	-0,55	33,38	0
	Medium	aINLIR	1	1	1	Q55444	N-Term(iTRAQ4plex)	0,0000	0,655	1		0,019	0,2477	18	0,014892582	2	843,55242	-1,03	25,36	0
P73589	C4-dicarboxylase binding protein OS=Synechocystis sp. (strain PCC 6803 / Kazusa) GN=dctP PE=4 S\	36,74	5,42	1	2	2	2	1,746	2	28,5	369	41,1	7,62							
	A3	Sequence	# PSMs	# Proteins	# Protein Groups	Protein Group Accessions	Modifications	ΔCn	116/117	116/117 Count	116/117 Variability [%]	q-Value	PEP	IonScore	Exp Value	Charge	MH+ [Da]	ΔM [ppm]	RT [min]	# Missed Cleavages
	High	iVTSIASkPIYk	1	1	1	P73589	N-Term(iTRAQ4plex); K8(iTRAQ4plex); K12(iTRAQ4plex)	0,0000	2,108	1		0	0,000002559	20	0,009999309	3	1752,09775	-1,03	29,81	0
	High	iTmNQVFR	1	1	1	P73589	N-Term(iTRAQ4plex); M3(Oxidation)	0,0000	1,447	1		0,001	0,006175	30	0,001056745	2	1168,62651	-0,02	24,18	0
P55037	Ferredoxin-dependent glutamate synthase 1 OS=Synechocystis sp. (strain PCC 6803 / Kazusa) GN=g	36,05	4,19	1	4	5	5	0,776	4	26,9	1550	169,0	5,92							
	A3	Sequence	# PSMs	# Proteins	# Protein Groups	Protein Group Accessions	Modifications	ΔCn	116/117	116/117 Count	116/117 Variability [%]	q-Value	PEP	IonScore	Exp Value	Charge	MH+ [Da]	ΔM [ppm]	RT [min]	# Missed Cleavages
	High	mAQGAkPGEGGQLPGkk	1	2	2	P55037;P55038	N-Term(iTRAQ4plex); M1(Oxidation); K6(iTRAQ4plex); K16(iTF	0,0000				0	0,001184	18	0,014420158	4	2246,27724	-0,06	14,92	1
	High	hAGLPWELGLAETHQTLVLNNLR	1	1	1	P55037	N-Term(iTRAQ4plex)	0,0000	0,751	1		0	0,00006964	16	0,025820818	4	2726,47939	-2,41	46,36	0
	High	kVYPWIAk	1	1	1	P55037	N-Term(iTRAQ4plex); K1(iTRAQ4plex); K8(iTRAQ4plex)	0,0000	0,803	1		0,001	0,004993	22	0,005874488	3	1436,89661	-1,66	27,37	1
	High	vTATLPITNINR	1	1	1	P55037	N-Term(iTRAQ4plex)	0,0000	0,562	1		0,002	0,01936	18	0,01415696	2	1456,86150	0,72	35,23	0
	High	kLYVIR	1	1	1	P55037	N-Term(iTRAQ4plex); K1(iTRAQ4plex)	0,0000	4,169	1		0,003	0,04312	12	0,057407681	3	1079,71680	-1,05	21,03	1
P73699	Slr1814 protein OS=Synechocystis sp. (strain PCC 6803 / Kazusa) GN=slr1814 PE=4 SV=1 - [P73699	35,89	12,34	1	2	2	2	0,659	2	5,8	154	18,4	5,58							
	A3	Sequence	# PSMs	# Proteins	# Protein Groups	Protein Group Accessions	Modifications	ΔCn	116/117	116/117 Count	116/117 Variability [%]	q-Value	PEP	IonScore	Exp Value	Charge	MH+ [Da]	ΔM [ppm]	RT [min]	# Missed Cleavages
	High	lQLLQVLEDNPSLR	1	1	1	P73699	N-Term(iTRAQ4plex)	0,0000	0,633	1		0	0,0001697	32	0,000682624	3	1782,02433	0,05	52,60	0
	Medium	iLNFR	1	1	1	P73699	N-Term(iTRAQ4plex)	0,0000	0,685	1		0,015	0,1922	17	0,020652375	2	806,49999	-0,65	29,09	0
Q55177	Sensory transduction histidine kinase OS=Synechocystis sp. (strain PCC 6803 / Kazusa) GN=slr0484 I	35,88	3,85	1	2	2	2	1,597	2	139,5	675	75,8	5,60							
	A3	Sequence	# PSMs	# Proteins	# Protein Groups	Protein Group Accessions	Modifications	ΔCn	116/117	116/117 Count	116/117 Variability [%]	q-Value	PEP	IonScore	Exp Value	Charge	MH+ [Da]	ΔM [ppm]	RT [min]	# Missed Cleavages
	High	kLVQDFLTLSQLESGR	1	1	1	Q55177	N-Term(iTRAQ4plex); K1(iTRAQ4plex)	0,0000	3,220	1		0	0,00161	28	0,00141896	3	2122,21415	1,44	47,93	1
	High	rFEGVWTFDR	1	1	1	Q55177	N-Term(iTRAQ4plex)	0,0000	0,793	1		0	0,00002145	20	0,009077578	3	1456,74671	0,88	34,59	1
P73803	Slr1263 protein OS=Synechocystis sp. (strain PCC 6803 / Kazusa) GN=slr1263 PE=4 SV=1 - [P73803	35,20	5,74	1	2	2	3	0,729	2	14,4	470	51,7	9,14							
	A3	Sequence	# PSMs	# Proteins	# Protein Groups	Protein Group Accessions	Modifications	ΔCn	116/117	116/117 Count	116/117 Variability [%]	q-Value	PEP	IonScore	Exp Value	Charge	MH+ [Da]	ΔM [ppm]	RT [min]	# Missed Cleavages
	High	gRFDVFAHHVVQASPEGk	2	1	1	P73803	N-Term(iTRAQ4plex); K18(iTRAQ4plex)	0,0000	0,802	1		0	5,033E-09	31	0,000746397	4	2269,20595	-0,94	27,08	1
	Medium	fVIPAGQSR	1	1	1	P73803	N-Term(iTRAQ4plex)	0,0000	0,662	1		0,023	0,3069	13	0,044665274	2	1118,64531	1,26	28,14	0
P74478	Slr1926 protein OS=Synechocystis sp. (strain PCC 6803 / Kazusa) GN=slr1926 PE=4 SV=1 - [P74478	35,00	14,35	1	3	3	3	0,787	2	4,0	223	24,6	9,28							
	A3	Sequence	# PSMs	# Proteins	# Protein Groups	Protein Group Accessions	Modifications	ΔCn	116/117	116/117 Count	116/117 Variability [%]	q-Value	PEP	IonScore	Exp Value	Charge	MH+ [Da]	ΔM [ppm]	RT [min]	# Missed Cleavages
	High	kLVTLFYFD	1	1	1	P74478	N-Term(iTRAQ4plex); K1(iTRAQ4plex)	0,0000	0,766	1		0,002	0,02452	23	0,004886524	2	1433,82903	0,52	49,43	1
	High	iALLGSR	1	1	1	P74478	N-Term(iTRAQ4plex)	0,0000	0,808	1		0,007	0,08671	23	0,005211587	2	873,56322	-0,73	28,84	0
	Medium	aDPNLLTVILPQSLDR	1	1	1	P74478	N-Term(iTRAQ4plex)	0,0000				0,043	0,5574	15	0,030972054	2	1909,07842	-4,80	52,56	0
P74719	Sll1107 protein OS=Synechocystis sp. (strain PCC 6803 / Kazusa) GN=sll1107 PE=4 SV=1 - [P74719_	34,92	8,91	1	2	2	2	3,049	2	8,6	258	28,4	9,58							
	A3	Sequence	# PSMs	# Proteins	# Protein Groups	Protein Group Accessions	Modifications	ΔCn	116/117	116/117 Count	116/117 Variability [%]	q-Value	PEP	IonScore	Exp Value	Charge	MH+ [Da]	ΔM [ppm]	RT [min]	# Missed Cleavages
	High	yLYLVLPNLER	1	1	1	P74719	N-Term(iTRAQ4plex)	0,0000	3,230	1		0	0,0006168	32	0,000642643	2	1536,88909	-1,03	52,04	0
	High	tVLVLIPkPLSR	1	1	1	P74719	N-Term(iTRAQ4plex); K8(iTRAQ4plex)	0,0000	2,878	1		0	0,002602	16	0,025001727	3	1624,07553	-0,55	40,20	0
P72753	Uracil phosphoribosyltransferase OS=Synechocystis sp. (strain PCC 6803 / Kazusa) GN=upp PE=3 SV	34,76	17,59	1	3	3	3	0,932	3	86,7	216	23,6	6,39							
	A3	Sequence	# PSMs	# Proteins	# Protein Groups	Protein Group Accessions	Modifications	ΔCn	116/117	116/117 Count	116/117 Variability [%]	q-Value	PEP	IonScore	Exp Value	Charge	MH+ [Da]	ΔM [ppm]	RT [min]	# Missed Cleavages
	High	lVSVVAAPTALQk	1	1	1	P72753	N-Term(iTRAQ4plex); K13(iTRAQ4plex)	0,0000	0,932	1		0	0,0003741	25	0,002951005	3	1584,99204	-0,45	36,49	0
	High	iYHLGLVR	1	1	1	P72753	N-Term(iTRAQ4plex)	0,0000	2,089	1		0	0,001047	22	0,006137196	3	1114,68500	-0,32	28,48	0
	Medium	aSLIDPQTPFVIVPILR	1	1	1	P72753	N-Term(iTRAQ4plex)	0,0000	0,562	1		0,012	0,1639	13	0,046022479	3	2023,21122	1,93	56,62	0
P52276	Asparagine--tRNA ligase OS=Synechocystis sp. (strain PCC 6803 / Kazusa) GN=asnS PE=3 SV=2 - [S	34,65	4,76	1	2	2	3	1,562	3	15,9	462	52,7	5,43							
	A3	Sequence	# PSMs	# Proteins	# Protein Groups	Protein Group Accessions	Modifications	ΔCn	116/117	116/117 Count	116/117 Variability [%]	q-Value	PEP	IonScore	Exp Value	Charge	MH+ [Da]	ΔM [ppm]	RT [min]	# Missed Cleavages
	High	yGSVPHAGFGLGFER	2	1	1	P52276	N-Term(iTRAQ4plex)	0,0000	1,481	2	7,9	0	0,00003858	26	0,002494422	3	1737,88401	0,59	35,36	0
	High	hSFEFLR	1	1	1	P52276	N-Term(iTRAQ4plex)	0,0000	3,225	1		0	0,002464	20	0,010374568	3	1079,57489	-0,54	26,97	0
P74473	Slr1923 protein OS=Synechocystis sp. (strain PCC 6803 / Kazusa) GN=slr1923 PE=4 SV=1 - [P74473	34,63	4,02	1	2	2	2	0,429	2	52,1	398	44,5	7,27							
	A3	Sequence	# PSMs	# Proteins	# Protein Groups	Protein Group Accessions	Modifications	ΔCn	116/117	116/117 Count	116/117 Variability [%]	q-Value	PEP	IonScore	Exp Value	Charge	MH+ [Da]	ΔM [ppm]	RT [min]	# Missed Cleavages
	High	fSIDSHFTR	1	1	1	P74473	N-Term(iTRAQ4plex)	0,0000	0,309	1		0	0,00001291	35	0,000343534	3	1253,63834	-0,95	27,38	0
	Medium	kIVEQYk	1	1	1	P74473	N-Term(iTRAQ4plex); K1(iTRAQ4plex); K7(iTRAQ4plex)	0,0000	0,598	1		0,013	0,1734	11	0,076202638	3	1339,82709	-2,89	16,48	1
P74457	Uridylate kinase OS=Synechocystis sp. (strain PCC 6803 / Kazusa) GN=pyrH PE=3 SV=1 - [PYRH_SY	34,33	6,15	1	2	2	2	1,309	2	24,7	260	27,6	5,33							
	A3	Sequence	# PSMs	# Proteins	# Protein Groups	Protein Group Accessions	Modifications	ΔCn	116/117	116/117 Count	116/117 Variability [%]	q-Value	PEP	IonScore	Exp Value	Charge	MH+ [Da]	ΔM [ppm]	RT [min]	# Missed Cleavages
	High	rFTTLTYSHVLAEDLk	1	1	1	P74457	N-Term(iTRAQ4plex); K16(iTRAQ4plex)	0,0000	1,543	1		0	4,763E-08	30	0,001009253	4	2182,21059	-0,22	36,36	1
	High	fTTLTYSHVLAEDLk	1	1	1	P74457	N-Term(iTRAQ4plex); K15(iTRAQ4plex)	0,0000	1,111	1		0	0,00002409	17	0,018237697	3	2026,10831	-0,81	41,39	0
P73623	Putative quercetin 2,3-dioxygenase sll1773 OS=Synechocystis sp. (strain PCC 6803 / Kazusa) GN=sll1	33,16	11,21	1	2	2	2	0,835	2	38,8	232	25,7	5,39							
	A3	Sequence	# PSMs	# Proteins	# Protein Groups	Protein Group Accessions	Modifications	ΔCn	116/117	116/117 Count	116/117 Variability [%]	q-Value	PEP	IonScore	Exp Value	Charge	MH+ [Da]	ΔM [ppm]	RT [min]	# Missed Cleavages
	High	aWLQIIR	1	1	1	P73623	N-Term(iTRAQ4plex)	0,0000	1,075	1		0,001	0,0172	33	0,000481915	2	1043,64910	0,81	40,77	0
	High	vINEDYIAPGQGFATHGHk	1	1	1	P73623	N-Term(iTRAQ4plex); K19(iTRAQ4plex)	0,0000	0,649	1		0,001	0,005599	12	0,068768996	4	2342,21596	1,17	29,15	0
P74357	Sll1530 protein OS=Synechocystis sp. (strain PCC 6803 / Kazusa) GN=sll1530 PE=4 SV=1 - [P74357_	32,97	6,22	1	2	2	2	2,439	2	74,4	386	43,7	6,18							
	A3	Sequence	# PSMs	# Proteins	# Protein Groups	Protein Group Accessions	Modifications	ΔCn	116/117	116/117 Count	116/117 Variability [%]	q-Value	PEP	IonScore	Exp Value	Charge	MH+ [Da]	ΔM [ppm]	RT [min]	# Missed Cleavages
	High	tLVSAkPHLVLETQR	1	1	1	P74357	N-Term(iTRAQ4plex); K6(iTRAQ4plex)	0,0000	1,559	1		0	0,0001463	21	0,007690773	4	1980,18545	0,49	28,58	0
	High	vLAPEIDkk	1	1	1	P74357	N-Term(iTRAQ4plex); K8(iTRAQ4plex); K9(iTRAQ4plex)	0,0000	3,817	1		0,001	0,005589	25	0,003273181	3	1444,90693	-2,11	22,88	1
P72874	Translation initiation factor IF-3 OS=Synechocystis sp. (strain PCC 6803 / Kazusa) GN=infC PE=3 SV=	32,73	19,21	1	4	4	5	1,479	5	20,5	177	20,5	9,33							
	A3	Sequence	# PSMs	# Proteins	# Protein Groups	Protein Group Accessions	Modifications	ΔCn	116/117	116/117 Count	116/117 Variability [%]	q-Value	PEP	IonScore	Exp Value	Charge	MH+ [Da]	ΔM [ppm]	RT [min]	# Missed Cleavages
	High	iRFPEIR	1	1	1	P72874	N-Term(iTRAQ4plex)	0,0000	1,696	1		0	0,002448	21	0,007567806	3	1074,65366	-0,39	30,97	1
	High	ykIDEHDYQVR	1	1	1	P72874	N-Term(iTRAQ4plex); K2(iTRAQ4plex)	0,0000	1,479	1		0	0,00003002	17	0,01811215	4	1753,91213	0,54	22,03	1
	High	eIQHAHLAk	1	1	1	P72874	N-Term(iTRAQ4plex); K9(iTRAQ4plex)	0,0000	1,079	1		0	0,0004605	17	0,020276827	4	1334,77822	-0,07	13,21	0
	High	ykFEQEk	2	1	1	P72874	N-Term(iTRAQ4plex); K2(iTRAQ4plex); K7(iTRAQ4plex)	0,0000	1,368	2	14,6	0	0,0001909	16	0,023931505	3	1403,78812	-0,98	17,69	1
Q6ZEU6	Sll5034 protein OS=Synechocystis sp. (strain PCC 6803 / Kazusa) GN=sll5034 PE=4 SV=1 - [Q6ZEU6	32,28	11,11	1	2	2	2	0,521	2	13,8	207	23,2	7,34							
	A3	Sequence	# PSMs	# Proteins	# Protein Groups	Protein Group Accessions	Modifications	ΔCn	116/117	116/117 Count	116/117 Variability [%]	q-Value	PEP	IonScore	Exp Value	Charge	MH+ [Da]	ΔM [ppm]	RT [min]	# Missed Cleavages
	High	nPDHkPNNLHDQIALAk	1	1	1	Q6ZEU6	N-Term(iTRAQ4plex); K5(iTRAQ4plex); K17(iTRAQ4plex)	0,0000	0,475	1		0	2,337E-07	23	0,004497799	4	2357,30459	-0,20	20,08	0
	High	tEAPTk	1	1	1	Q6ZEU6	N-Term(iTRAQ4plex); K6(iTRAQ4plex)	0,0000	0,572	1		0,003	0,04016	23	0,00778266	2	934,54314	-1,76	12,14	0
Q55449	Slr0031 protein OS=Synechocystis sp. (strain PCC 6803 / Kazusa) GN=slr0031 PE=4 SV=1 - [Q55449	32,27	6,34	1	4	4	5	0,349	5	54,9	584	66,9	5,58							
	A3	Sequence	# PSMs	# Proteins	# Protein Groups	Protein Group Accessions	Modifications	ΔCn	116/117	116/117 Count	116/117 Variability [%]	q-Value	PEP	IonScore	Exp Value	Charge	MH+ [Da]	ΔM [ppm]	RT [min]	# Missed Cleavages
	High	iRPIALEk	1	1	1	Q55449	N-Term(iTRAQ4plex); K8(iTRAQ4plex)	0,0000	0,333	1		0	0,00006727	25	0,00289048	3	1227,80436	1,30	25,10	0
	High	vkSEAGQEk	2	1	1	Q55449	N-Term(iTRAQ4plex); K2(iTRAQ4plex); K9(iTRAQ4plex)	0,0000	0,579	2	23,8	0	0,0007608	13	0,045811025	3	1407,82212	3,78	8,81	1
	High	fGFRDPEk	1	1	1	Q55449	N-Term(iTRAQ4plex); K8(iTRAQ4plex)	0,0000	0,349	1		0,005	0,06775	10	0,092044957	3	1283,70032	1,27	23,77	1
	Medium	fPVWTPGSYLVR	1	1	1	Q55449	N-Term(iTRAQ4plex)	0,0000	0,057	1		0,01	0,1266	19	0,011829598	2	1565,86321	2,23	47,63	0
P27319	Flavodoxin OS=Synechocystis sp. (strain PCC 6803 / Kazusa) GN=isiB PE=1 SV=2 - [FLAV_SYNY3]	32,17	11,18	1	2	2	3	0,153	3	40,3	170	18,8	4,30							
	A3	Sequence	# PSMs	# Proteins	# Protein Groups	Protein Group Accessions	Modifications	ΔCn	116/117	116/117 Count	116/117 Variability [%]	q-Value	PEP	IonScore	Exp Value	Charge	MH+ [Da]	ΔM [ppm]	RT [min]	# Missed Cleavages
	High	tWVSEIkPILQS	2	1	1	P27319	N-Term(iTRAQ4plex); K7(iTRAQ4plex)	0,0000	0,134	2	19,6	0	0,0006094	18	0,019641798	3	1688,98368	0,65	45,71	0
	High	iSGLGGk	1	1	1	P27319	N-Term(iTRAQ4plex); K7(iTRAQ4plex)	0,0000	0,340	1		0,002	0,01934	26	0,002460198	2	919,58202	0,54	19,94	0
P74309	Fructose-bisphosphate aldolase class 1 OS=Synechocystis sp. (strain PCC 6803 / Kazusa) GN=fda PE	32,11	4,67	1	2	2	2	0,897	2	118,9	300	33,1	5,83							
	A3	Sequence	# PSMs	# Proteins	# Protein Groups	Protein Group Accessions	Modifications	ΔCn	116/117	116/117 Count	116/117 Variability [%]	q-Value	PEP	IonScore	Exp Value	Charge	MH+ [Da]	ΔM [ppm]	RT [min]	# Missed Cleavages
	High	qIVPILk	1	1	1	P74309	N-Term(iTRAQ4plex); K7(iTRAQ4plex)	0,0000	0,476	1		0	0,00301	29	0,00118296	2	1098,75029	1,25	31,72	0
	High	hLNQLPk	1	1	1	P74309	N-Term(iTRAQ4plex); K7(iTRAQ4plex)	0,0000	1,689	1		0,001	0,006253	16	0,025940002	3	1137,70008	1,59	16,02	0
Q55663	1-deoxy-D-xylulose 5-phosphate reductoisomerase OS=Synechocystis sp. (strain PCC 6803 / Kazusa)	31,76	5,84	1	2	2	2	1,524	2	51,0	394	42,5	5,02							
	A3	Sequence	# PSMs	# Proteins	# Protein Groups	Protein Group Accessions	Modifications	ΔCn	116/117	116/117 Count	116/117 Variability [%]	q-Value	PEP	IonScore	Exp Value	Charge	MH+ [Da]	ΔM [ppm]	RT [min]	# Missed Cleavages
	High	lPLLYALSWPER	1	1	1	Q55663	N-Term(iTRAQ4plex)	0,0000	2,108	1		0	0,00005913	27	0,002069998	2	1601,91582	-0,88	56,33	0
	High	lPFVTVQDALk	1	1	1	Q55663	N-Term(iTRAQ4plex); K11(iTRAQ4plex)	0,0000	1,101	1		0	0,00245	18	0,016069413	3	1518,91486	0,94	44,99	0
P73069	Ycf48-like protein OS=Synechocystis sp. (strain PCC 6803 / Kazusa) GN=slr2034 PE=3 SV=1 - [YC48	31,52	4,68	1	2	2	2	1,240	2	10,3	342	37,3	4,89							
	A3	Sequence	# PSMs	# Proteins	# Protein Groups	Protein Group Accessions	Modifications	ΔCn	116/117	116/117 Count	116/117 Variability [%]	q-Value	PEP	IonScore	Exp Value	Charge	MH+ [Da]	ΔM [ppm]	RT [min]	# Missed Cleavages
	High	yVAVSAR	1	1	1	P73069	N-Term(iTRAQ4plex)	0,0000	1,329	1		0,001	0,01308	25	0,003097205	2	909,52880	1,47	17,72	0
	High	rLQTmGYGk	1	1	1	P73069	N-Term(iTRAQ4plex); M5(Oxidation); K9(iTRAQ4plex)	0,0000	1,158	1		0,001	0,00764	19	0,011349324	3	1357,74955	-0,38	14,96	1
Q55737	Slr0416 protein OS=Synechocystis sp. (strain PCC 6803 / Kazusa) GN=slr0416 PE=4 SV=1 - [Q55737	31,17	14,38	1	2	2	3	0,395	2	25,6	146	16,3	5,12							
	A3	Sequence	# PSMs	# Proteins	# Protein Groups	Protein Group Accessions	Modifications	ΔCn	116/117	116/117 Count	116/117 Variability [%]	q-Value	PEP	IonScore	Exp Value	Charge	MH+ [Da]	ΔM [ppm]	RT [min]	# Missed Cleavages
	High	kAGGTYLSSEELR	1	1	1	Q55737	N-Term(iTRAQ4plex); K1(iTRAQ4plex)	0,0000	0,469	1		0	0,00003435	23	0,004720304	3	1698,92575	-0,45	21,33	1
	High	iLLLHLIk	2	1	1	Q55737	N-Term(iTRAQ4plex); K8(iTRAQ4plex)	0,0000	0,334	1		0	0,001424	21	0,008071793	3	1250,88050	0,18	43,27	0
Q55139	Glutathione S-transferase OS=Synechocystis sp. (strain PCC 6803 / Kazusa) GN=gst PE=4 SV=1 - [Q	30,23	14,67	1	3	3	5	0,974	5	3,8	184	20,4	6,57							
	A3	Sequence	# PSMs	# Proteins	# Protein Groups	Protein Group Accessions	Modifications	ΔCn	116/117	116/117 Count	116/117 Variability [%]	q-Value	PEP	IonScore	Exp Value	Charge	MH+ [Da]	ΔM [ppm]	RT [min]	# Missed Cleavages
	High	qAPYLAINPFGk	1	1	1	Q55139	N-Term(iTRAQ4plex); K12(iTRAQ4plex)	0,0000	0,843	1		0	0,00002241	14	0,040550854	3	1606,91947	-0,07	41,93	0
	High	iLQSLEk	1	1	1	Q55139	N-Term(iTRAQ4plex); K7(iTRAQ4plex)	0,0000	1,301	1		0,001	0,005491	19	0,012530546	2	1118,70110	-1,13	31,07	0
	High	lYGAPQSR	3	1	1	Q55139	N-Term(iTRAQ4plex)	0,0000	0,974	3	1,2	0,004	0,04747	21	0,007708502	2	1035,56987	-0,49	16,97	0
Q55716	Glycine--tRNA ligase alpha subunit OS=Synechocystis sp. (strain PCC 6803 / Kazusa) GN=glyQ PE=3	30,17	7,36	1	2	2	2	1,130	2	51,8	299	34,3	4,87							
	A3	Sequence	# PSMs	# Proteins	# Protein Groups	Protein Group Accessions	Modifications	ΔCn	116/117	116/117 Count	116/117 Variability [%]	q-Value	PEP	IonScore	Exp Value	Charge	MH+ [Da]	ΔM [ppm]	RT [min]	# Missed Cleavages
	High	gAGTmNPHTFLR	1	1	1	Q55716	N-Term(iTRAQ4plex); M5(Oxidation)	0,0000	1,570	1		0	0,0003569	25	0,003483133	3	1461,73871	-0,17	19,04	0
	High	eVAHIYLQQR	1	1	1	Q55716	N-Term(iTRAQ4plex)	0,0000	0,813	1		0	0,00008912	19	0,013771144	3	1400,77686	0,12	22,52	0
P74630	Sll0735 protein OS=Synechocystis sp. (strain PCC 6803 / Kazusa) GN=sll0735 PE=4 SV=1 - [P74630_	29,98	7,94	1	3	3	3	1,418	3	7,7	529	62,0	6,25							
	A3	Sequence	# PSMs	# Proteins	# Protein Groups	Protein Group Accessions	Modifications	ΔCn	116/117	116/117 Count	116/117 Variability [%]	q-Value	PEP	IonScore	Exp Value	Charge	MH+ [Da]	ΔM [ppm]	RT [min]	# Missed Cleavages
	High	gNPHQQVcRPSQSSWGYk	1	1	1	P74630	N-Term(iTRAQ4plex); C8(Carbamidomethyl); K18(iTRAQ4plex)	0,0000	1,494	1		0	0,000008989	14	0,038901828	4	2404,18349	0,65	19,17	0
	High	tGTmVPYAVR	1	1	1	P74630	N-Term(iTRAQ4plex); M4(Oxidation)	0,0000	1,418	1		0,001	0,01621	23	0,0049885	2	1254,66435	0,82	21,90	0
	Medium	yEAHLSLLQELLAk	1	1	1	P74630	N-Term(iTRAQ4plex); K14(iTRAQ4plex)	0,0000	0,546	1		0,023	0,3167	19	0,012911301	4	1916,11025	0,36	50,10	0
P74625	Phycobilisome rod-core linker polypeptide CpcG OS=Synechocystis sp. (strain PCC 6803 / Kazusa) GN	29,94	7,23	1	2	2	2	0,162	2	47,0	249	28,5	9,10							
	A3	Sequence	# PSMs	# Proteins	# Protein Groups	Protein Group Accessions	Modifications	ΔCn	116/117	116/117 Count	116/117 Variability [%]	q-Value	PEP	IonScore	Exp Value	Charge	MH+ [Da]	ΔM [ppm]	RT [min]	# Missed Cleavages
	High	fVQmcIQR	1	1	1	P74625	N-Term(iTRAQ4plex); M4(Oxidation); C5(Carbamidomethyl)	0,0000	0,120	1		0	0,001271	19	0,013676344	2	1241,62456	-0,49	23,46	0
	High	ekLEAIGYFR	1	1	1	P74625	N-Term(iTRAQ4plex); K2(iTRAQ4plex)	0,0000	0,219	1		0,002	0,02291	25	0,004376511	3	1513,85923	-1,65	32,28	1
P74752	Slr0605 protein OS=Synechocystis sp. (strain PCC 6803 / Kazusa) GN=slr0605 PE=4 SV=1 - [P74752	27,75	5,33	1	2	2	3	1,793	3	10,1	319	36,5	6,58							
	A3	Sequence	# PSMs	# Proteins	# Protein Groups	Protein Group Accessions	Modifications	ΔCn	116/117	116/117 Count	116/117 Variability [%]	q-Value	PEP	IonScore	Exp Value	Charge	MH+ [Da]	ΔM [ppm]	RT [min]	# Missed Cleavages
	High	fDVVYVGHFk	1	1	1	P74752	N-Term(iTRAQ4plex); K10(iTRAQ4plex)	0,0000	0,693	1		0	0,00003391	24	0,003854517	3	1498,83496	3,52	35,87	0
	Medium	lFTTLIR	2	1	1	P74752	N-Term(iTRAQ4plex)	0,0000	1,855	2	5,0	0,015	0,1917	14	0,039261782	2	1007,63793	0,90	40,19	0
P74124	Renin-binding protein OS=Synechocystis sp. (strain PCC 6803 / Kazusa) GN=slr1975 PE=4 SV=1 - [P	26,75	5,37	1	2	2	2	2,141	2	21,8	391	45,6	5,91							
	A3	Sequence	# PSMs	# Proteins	# Protein Groups	Protein Group Accessions	Modifications	ΔCn	116/117	116/117 Count	116/117 Variability [%]	q-Value	PEP	IonScore	Exp Value	Charge	MH+ [Da]	ΔM [ppm]	RT [min]	# Missed Cleavages
	High	rGEVLLNLk	1	1	1	P74124	N-Term(iTRAQ4plex); K9(iTRAQ4plex)	0,0000	2,475	1		0	0,0008882	23	0,004602248	3	1329,84519	-0,38	27,47	1
	High	lEPkPQWLEIAR	1	1	1	P74124	N-Term(iTRAQ4plex); K4(iTRAQ4plex)	0,0000	1,852	1		0	0,00007481	17	0,024065378	3	1768,03635	0,18	37,65	0
P73286	Ssl3451 protein OS=Synechocystis sp. (strain PCC 6803 / Kazusa) GN=ssl3451 PE=4 SV=1 - [P73286	26,27	22,22	1	2	2	2	1,093	2	88,3	81	9,1	5,11							
	A3	Sequence	# PSMs	# Proteins	# Protein Groups	Protein Group Accessions	Modifications	ΔCn	116/117	116/117 Count	116/117 Variability [%]	q-Value	PEP	IonScore	Exp Value	Charge	MH+ [Da]	ΔM [ppm]	RT [min]	# Missed Cleavages
	High	lIARPPYLk	1	1	1	P73286	N-Term(iTRAQ4plex); K9(iTRAQ4plex)	0,0000	0,655	1		0	0,0002758	25	0,003075884	3	1358,87592	-0,24	30,14	0
	High	rPAGYWGVk	1	1	1	P73286	N-Term(iTRAQ4plex); K9(iTRAQ4plex)	0,0000	1,825	1		0,001	0,007987	14	0,041086376	3	1321,76062	-1,00	21,07	0
P73660	Porphobilinogen deaminase OS=Synechocystis sp. (strain PCC 6803 / Kazusa) GN=hemC PE=3 SV=1	24,69	6,88	1	2	2	3	1,238	2	31,6	320	34,9	5,73							
	A3	Sequence	# PSMs	# Proteins	# Protein Groups	Protein Group Accessions	Modifications	ΔCn	116/117	116/117 Count	116/117 Variability [%]	q-Value	PEP	IonScore	Exp Value	Charge	MH+ [Da]	ΔM [ppm]	RT [min]	# Missed Cleavages
	High	rVNPADALVLNAk	1	1	1	P73660	N-Term(iTRAQ4plex); K13(iTRAQ4plex)	0,0000	1,005	1		0	0,001218	24	0,004364857	3	1669,00046	0,29	29,42	1
	High	tDLAVHSLk	2	1	1	P73660	N-Term(iTRAQ4plex); K9(iTRAQ4plex)	0,0000	1,525	1		0	0,0002979	11	0,077440831	3	1271,75470	-1,17	20,45	0
P73092	Chromophore lyase CpcS/CpeS 2 OS=Synechocystis sp. (strain PCC 6803 / Kazusa) GN=cpcS2 PE=3	24,01	8,33	1	2	2	2	0,957	2	30,9	192	21,9	4,72							
	A3	Sequence	# PSMs	# Proteins	# Protein Groups	Protein Group Accessions	Modifications	ΔCn	116/117	116/117 Count	116/117 Variability [%]	q-Value	PEP	IonScore	Exp Value	Charge	MH+ [Da]	ΔM [ppm]	RT [min]	# Missed Cleavages
	High	aVGGAFVR	1	1	1	P73092	N-Term(iTRAQ4plex)	0,0000	0,781	1		0,004	0,05262	23	0,007657575	2	920,54314	-0,35	20,44	0
	Medium	tTHHLAFR	1	1	1	P73092	N-Term(iTRAQ4plex)	0,0000	1,173	1		0,014	0,1865	15	0,031693486	3	1126,62384	0,03	12,53	0
Q55625	Ribosome-binding factor A OS=Synechocystis sp. (strain PCC 6803 / Kazusa) GN=rbfA PE=3 SV=1 - [	23,19	21,80	1	2	2	2	1,359	2	79,7	133	14,7	5,91							
	A3	Sequence	# PSMs	# Proteins	# Protein Groups	Protein Group Accessions	Modifications	ΔCn	116/117	116/117 Count	116/117 Variability [%]	q-Value	PEP	IonScore	Exp Value	Charge	MH+ [Da]	ΔM [ppm]	RT [min]	# Missed Cleavages
	High	aSTmAGLHSAAPFVR	1	1	1	Q55625	N-Term(iTRAQ4plex); M4(Oxidation)	0,0000	0,847	1		0	0,0006776	23	0,00478597	3	1675,86985	-0,52	24,66	0
	High	eVSQmLLHEIkDDR	1	1	1	Q55625	N-Term(iTRAQ4plex); M5(Oxidation); K11(iTRAQ4plex)	0,0000	2,182	1		0	0,00002142	14	0,057987418	4	2017,06350	0,38	31,77	1
Q55184	Sll0456 protein OS=Synechocystis sp. (strain PCC 6803 / Kazusa) GN=sll0456 PE=4 SV=1 - [Q55184	21,90	5,41	1	2	2	2	1,336	2	226,8	333	37,4	7,15							
	A3	Sequence	# PSMs	# Proteins	# Protein Groups	Protein Group Accessions	Modifications	ΔCn	116/117	116/117 Count	116/117 Variability [%]	q-Value	PEP	IonScore	Exp Value	Charge	MH+ [Da]	ΔM [ppm]	RT [min]	# Missed Cleavages
	High	wLPLIR	1	1	1	Q55184	N-Term(iTRAQ4plex)	0,0000	3,315	1		0,001	0,006856	22	0,006441248	2	941,60472	-0,64	42,65	0
	High	tLTLINLLNPER	1	1	1	Q55184	N-Term(iTRAQ4plex)	0,0000	0,538	1		0,004	0,04971	13	0,053452744	2	1540,91960	1,06	51,44	0
P73377	Mannose-6-phosphate isomerase OS=Synechocystis sp. (strain PCC 6803 / Kazusa) GN=manA PE=4	20,97	18,60	1	2	2	2	0,745	2	9,7	129	14,6	5,94							
	A3	Sequence	# PSMs	# Proteins	# Protein Groups	Protein Group Accessions	Modifications	ΔCn	116/117	116/117 Count	116/117 Variability [%]	q-Value	PEP	IonScore	Exp Value	Charge	MH+ [Da]	ΔM [ppm]	RT [min]	# Missed Cleavages
	High	sEHWVVVSGTAk	1	1	1	P73377	N-Term(iTRAQ4plex); K12(iTRAQ4plex)	0,0000	0,795	1		0	0,0002985	21	0,007979396	3	1587,87632	1,86	22,51	0
	Medium	eSTYVPmNTPHR	1	1	1	P73377	N-Term(iTRAQ4plex); M7(Oxidation)	0,0000	0,698	1		0,018	0,2323	11	0,077798282	3	1591,76749	1,22	16,46	0

Supplementary Table S3

[NiFe]-hydrogenase is essential for cyanobacterium *Synechocystis* sp. PCC 6803 aerobic growth in the dark

Edith De Rosa, Vanessa Checchetto, Cinzia Franchin, Elisabetta Bergantino, Paola Berto, Ildikò Szabò, Giorgio M. Giacometti, Giorgio Arrigoni, and Paola Costantini

Accession	Description	Score	Coverage	# Proteins	# Unique Peptides	# Peptides	# PSMs	Dark/Light (117/114)	117/114 Count	117/114 Variability [%]	# AAs	MW [kDa]	calc. pI							
Q54715	C-phycocyanin alpha chain OS=Synechocystis sp. (strain PCC 6803 / Kaz	3557,15	79,01	1	13	13	119	1,005	99	31,8	162	17,6	5,57							
	A3	Sequence	# PSMs	# Proteins	# Protein Groups	Protein Group Accessions	Modifications	ΔCn	117/114	117/114 Count	117/114 Variability [%]	q-Value	PEP	IonScore	Exp Value	Charge	MH+ [Da]	ΔM [ppm]	RT [min]	# Missed Cleavages
	High	tPLTEAVSTADSQGR	2	1	1	Q54715	N-Term(iTRAQ4plex)	0,0000	1,000	2	3,2	0	1,971E-10	100	9,22508E-11	2	1676,85906	1,09	29,16	0
	High	fPYTTQTQGNNFAADQR	11	1	1	Q54715	N-Term(iTRAQ4plex)	0,0000	0,927	11	12,0	0	2,517E-14	100	9,59334E-11	2	2103,00469	1,63	29,93	0
	High	tFDLSPSWYVEALk	11	1	1	Q54715	N-Term(iTRAQ4plex); K14(iTRAQ4plex)	0,0000	1,014	10	17,8	0	9,041E-11	98	1,41896E-10	2	1944,03826	1,30	49,35	0
	High	mkTPLTEAVSTADSQGR	15	1	1	Q54715	N-Term(iTRAQ4plex); M1(Oxidation); K2(iTRAQ4plex)	0,0000	1,245	12	28,1	0	1,268E-10	89	1,61503E-09	3	2096,09214	1,18	25,59	1
	High	mkTPLTEAVSTADSQGR	3	1	1	Q54715	N-Term(iTRAQ4plex); K2(iTRAQ4plex)	0,0000	1,673	3	4,8	0	2,424E-09	87	2,11349E-09	3	2080,09659	0,88	29,68	1
	High	fLSSTELQIAFGR	14	1	1	Q54715	N-Term(iTRAQ4plex)	0,0000	1,238	12	12,5	0	2,337E-09	82	5,8475E-09	2	1612,88420	1,62	45,89	0
	High	qANAGLQAAk	18	1	1	Q54715	N-Term(iTRAQ4plex); K10(iTRAQ4plex)	0,0000	1,092	10	15,0	0	1,291E-07	81	7,12804E-09	2	1259,73406	2,39	13,88	0
	High	kTPLTEAVSTADSQGR	3	1	1	Q54715	N-Term(iTRAQ4plex); K1(iTRAQ4plex)	0,0000	1,082	2	9,0	0	4,86E-08	65	4,55114E-07	3	1949,05716	1,49	25,98	1
	High	aLTDNAQSLVNGAAQAVYNk	2	1	1	Q54715	N-Term(iTRAQ4plex); K20(iTRAQ4plex)	0,0000	1,083	1		0	1,085E-13	64	4,37522E-07	3	2336,24869	1,62	42,90	0
	High	aNHGLSGDAR	24	1	1	Q54715	N-Term(iTRAQ4plex)	0,0000	0,718	24	28,2	0	0,000000691	58	1,7578E-06	2	1141,58476	1,46	8,80	0
	High	dIGYYLR	12	1	1	Q54715	N-Term(iTRAQ4plex)	0,0000	1,011	9	8,1	0	0,00266	44	4,11121E-05	2	1043,56621	1,90	32,21	0
	High	dEANSYLDYAINALS	1	1	1	Q54715	N-Term(iTRAQ4plex)	0,0000				0	0,01089	42	5,72756E-05	2	1802,85991	1,87	53,71	0
	High	lRQANAGLQAAk	1	1	1	Q54715	N-Term(iTRAQ4plex); K12(iTRAQ4plex)	0,0000	1,095	1		0	0,0007514	37	0,000179461	3	1528,92102	3,13	16,40	1
	High	yIkANHGLSGDAR	2	1	1	Q54715	N-Term(iTRAQ4plex); K3(iTRAQ4plex)	0,0000	1,066	2	13,6	0	0,00009444	17	0,021926535	3	1689,92905	0,91	15,32	1
Q54714	C-phycocyanin beta chain OS=Synechocystis sp. (strain PCC 6803 / Kaz	3483,65	66,28	1	15	15	125	0,899	104	20,0	172	18,1	5,14							
	A3	Sequence	# PSMs	# Proteins	# Protein Groups	Protein Group Accessions	Modifications	ΔCn	117/114	117/114 Count	117/114 Variability [%]	q-Value	PEP	IonScore	Exp Value	Charge	MH+ [Da]	ΔM [ppm]	RT [min]	# Missed Cleavages
	High	gEYLSGSQLDALSATVAEGNkR	14	1	1	Q54714	N-Term(iTRAQ4plex); K21(iTRAQ4plex)	0,0000	1,084	9	13,8	0	1,958E-15	106	2,48296E-11	4	2554,33755	0,92	37,33	1
	High	iTGNASAIVSNAAR	23	1	1	Q54714	N-Term(iTRAQ4plex)	0,0000	0,845	22	23,3	0	1,373E-07	93	6,17888E-10	2	1488,82903	2,61	28,09	0
	High	aLFAEQPQLIQPGGNAYTSR	6	1	1	Q54714	N-Term(iTRAQ4plex)	0,0000	0,705	4	25,7	0	2,041E-08	77	2,34095E-08	2	2305,21025	1,93	39,20	0
	High	gEYLSGSQLDALSATVAEGNk	4	1	1	Q54714	N-Term(iTRAQ4plex); K21(iTRAQ4plex)	0,0000	0,815	3	0,1	0	1,21E-11	77	2,13781E-08	3	2398,23685	1,15	40,03	0
	High	mkEAALDIVNDPNGITR	5	1	1	Q54714	N-Term(iTRAQ4plex); K2(iTRAQ4plex)	0,0000	1,202	3	40,6	0	9,795E-14	77	2,22331E-08	3	2145,15891	0,56	35,38	1
	High	eAALDIVNDPNGITR	5	1	1	Q54714	N-Term(iTRAQ4plex)	0,0000	0,849	3	2,7	0	2,385E-09	76	2,40974E-08	2	1741,92375	2,05	33,83	0
	High	mkEAALDIVNDPNGITR	10	1	1	Q54714	N-Term(iTRAQ4plex); M1(Oxidation); K2(iTRAQ4plex)	0,0000	1,240	6	12,3	0	1,546E-12	69	1,39949E-07	3	2161,15683	1,95	29,88	1
	High	mFDVFTR	9	1	1	Q54714	N-Term(iTRAQ4plex)	0,0000	1,035	9	5,2	0	0,003181	53	5,2236E-06	2	1059,54265	1,17	37,75	0
	High	vVSQADAR	7	1	1	Q54714	N-Term(iTRAQ4plex)	0,0000	0,875	7	19,2	0	0,0006739	44	4,33481E-05	2	989,55065	0,99	11,23	0
	High	dMEIILR	3	1	1	Q54714	N-Term(iTRAQ4plex)	0,0000	0,928	3	9,0	0	0,0005926	42	7,06269E-05	2	1033,58440	1,08	31,51	0
	High	rIDSVNR	9	1	1	Q54714	N-Term(iTRAQ4plex)	0,0000	0,925	9	11,0	0	0,001776	37	0,000182797	3	1003,57853	1,97	11,61	1
	High	mFDVFTR	9	1	1	Q54714	N-Term(iTRAQ4plex); M1(Oxidation)	0,0000	0,947	6	5,4	0	0,007357	33	0,000540717	2	1075,53777	1,34	30,88	0
	High	rmAAcLR	3	1	1	Q54714	N-Term(iTRAQ4plex); M2(Oxidation); C5(Carbamidometh	0,0000	1,097	3	10,5	0	0,005123	26	0,002716252	3	1037,54651	-0,01	11,26	1
	High	iDSVNR	3	1	1	Q54714	N-Term(iTRAQ4plex)	0,0000	0,785	3	0,9	0	0,009696	18	0,016903242	2	847,47637	1,11	13,10	0
	High	fDVFTR	1	1	1	Q54714	N-Term(iTRAQ4plex)	0,0000	0,585	1		0,001	0,0189	28	0,001588437	2	928,50237	1,56	30,50	0
	High	cLNGLR	3	1	1	Q54714	N-Term(iTRAQ4plex); C1(Carbamidomethyl)	0,0000	0,997	3	2,9	0,006	0,06866	39	0,000127929	2	876,48491	0,79	15,99	0
	High	dmEIILR	3	1	1	Q54714	N-Term(iTRAQ4plex); M2(Oxidation)	0,0000	0,689	3	6,6	0,006	0,07989	16	0,0259998	2	1049,57891	0,67	22,56	0
	Medium	mAAcLR	5	1	1	Q54714	N-Term(iTRAQ4plex); M1(Oxidation); C4(Carbamidometh	0,0000	0,717	4	14,3	0,013	0,1665	15	0,031330693	2	881,44744	2,31	12,35	0
	Medium	mAAcLR	3	1	1	Q54714	N-Term(iTRAQ4plex); C4(Carbamidomethyl)	0,0000	0,841	3	1,3	0,025	0,3069	18	0,015274606	2	865,45183	1,55	16,98	0
P73282	Transketolase OS=Synechocystis sp. (strain PCC 6803 / Kazusa) GN=tkt	3401,34	32,69	1	21	21	127	1,301	100	20,9	670	71,7	5,57							
	A3	Sequence	# PSMs	# Proteins	# Protein Groups	Protein Group Accessions	Modifications	ΔCn	117/114	117/114 Count	117/114 Variability [%]	q-Value	PEP	IonScore	Exp Value	Charge	MH+ [Da]	ΔM [ppm]	RT [min]	# Missed Cleavages
	High	vVATQSLDELSINAIR	3	1	1	P73282	N-Term(iTRAQ4plex)	0,0000	1,268	3	18,0	0	1,02E-11	119	1,14807E-12	2	1873,05278	0,85	41,94	0
	High	qAVPNLPGTSIDAVAk	3	1	1	P73282	N-Term(iTRAQ4plex); K16(iTRAQ4plex)	0,0000	1,104	3	8,4	0	5,144E-12	94	3,62218E-10	2	1869,06999	0,82	33,94	0
	High	sDTAGIHGAALGTDEVAATR	1	1	1	P73282	N-Term(iTRAQ4plex)	0,0000	1,152	1		0	1,879E-13	85	3,46713E-09	3	2057,03867	0,29	24,11	0
	High	tLASFTPDQk	3	1	1	P73282	N-Term(iTRAQ4plex); K10(iTRAQ4plex)	0,0000	2,060	2	68,1	0	1,668E-08	74	4,25569E-08	2	1395,77153	-0,51	26,92	0
	High	vTTIIGYGAPNk	9	1	1	P73282	N-Term(iTRAQ4plex); K12(iTRAQ4plex)	0,0000	1,352	7	14,9	0	1,202E-09	73	4,92006E-08	2	1521,88860	0,43	30,24	0
	High	akENAPTLLSLTR	10	1	1	P73282	N-Term(iTRAQ4plex); K2(iTRAQ4plex)	0,0000	1,512	6	7,4	0	0,000000709	71	7,24386E-08	2	1702,01140	0,71	29,29	1
	High	sGHPGLPMGAAPmAFTLWNk	6	1	1	P73282	N-Term(iTRAQ4plex); M13(Oxidation); K20(iTRAQ4plex)	0,0000	1,344	6	26,0	0	4,064E-10	68	1,67098E-07	3	2387,22434	-0,01	41,45	0
	High	lSVEAATNFGWHk	8	1	1	P73282	N-Term(iTRAQ4plex); K13(iTRAQ4plex)	0,0000	1,330	5	12,8	0	3,864E-11	66	2,5408E-07	3	1747,93814	0,64	35,10	0
	High	eNAPTLLSLTR	4	1	1	P73282	N-Term(iTRAQ4plex)	0,0000	0,945	3	4,9	0	0,00003429	65	3,07588E-07	2	1358,77446	-1,16	35,25	0
	High	fGFSVDNVLAk	8	1	1	P73282	N-Term(iTRAQ4plex); K11(iTRAQ4plex)	0,0000	1,161	6	20,6	0	4,462E-09	63	4,86374E-07	2	1484,83647	0,87	41,54	0
	High	tkYPTEAAAFER	8	1	1	P73282	N-Term(iTRAQ4plex); K2(iTRAQ4plex)	0,0000	1,351	6	5,9	0	5,833E-07	63	4,94277E-07	3	1671,89408	-0,25	23,95	1
	High	aIPNLTVIRPADGNETSGAYk	9	1	1	P73282	N-Term(iTRAQ4plex); K21(iTRAQ4plex)	0,0000	1,323	8	19,5	0	3,157E-09	62	7,03024E-07	4	2475,34463	-0,01	34,14	0
	High	qLSGALPEGWDk	1	1	1	P73282	N-Term(iTRAQ4plex); K12(iTRAQ4plex)	0,0000	1,219	1		0	0,000007523	59	1,37781E-06	2	1588,85820	0,51	32,73	0
	High	sGHPGLPmGAAPmAFTLWNk	10	1	1	P73282	N-Term(iTRAQ4plex); M8(Oxidation); M13(Oxidation); K2	0,0000	1,308	8	14,8	0	1,333E-09	59	1,39316E-06	4	2403,21938	0,04	37,40	0
	High	aVTDkPSmIk	12	1	1	P73282	N-Term(iTRAQ4plex); K5(iTRAQ4plex); M8(Oxidation); K	0,0000	1,184	8	31,2	0	0,000001136	56	2,45454E-06	2	1537,89739	-0,68	15,64	0
	High	sGHPGLPMGAAPMAFTLWNk	4	1	1	P73282	N-Term(iTRAQ4plex); K20(iTRAQ4plex)	0,0000	1,353	4	12,2	0	3,544E-11	56	2,52912E-06	3	2371,22971	0,11	45,16	0
	High	rLSVEAATNFGWHk	2	1	1	P73282	N-Term(iTRAQ4plex); K14(iTRAQ4plex)	0,0000	1,479	2	7,6	0	3,425E-11	54	4,36486E-06	4	1904,03762	-0,28	30,81	1
	High	fLAVDAIEk	2	1	1	P73282	N-Term(iTRAQ4plex); K9(iTRAQ4plex)	0,0000	1,212	2	18,8	0	0,000000264	53	4,797E-06	2	1293,76616	0,35	35,06	0
	High	aVTDkPSMIk	6	1	1	P73282	N-Term(iTRAQ4plex); K5(iTRAQ4plex); K10(iTRAQ4plex)	0,0000	1,479	4	21,2	0	0,000003345	40	0,00011091	2	1521,89983	-2,42	19,25	0
	High	nVHFGVR	6	1	1	P73282	N-Term(iTRAQ4plex)	0,0000	1,098	6	15,6	0	0,005892	22	0,006802959	3	972,54984	0,25	17,54	0
	High	yPTEAAAFER	1	1	1	P73282	N-Term(iTRAQ4plex)	0,0000	0,964	1		0,001	0,01211	25	0,003258142	2	1298,64812	-1,27	27,14	0
	High	kAIER	3	1	1	P73282	N-Term(iTRAQ4plex); K1(iTRAQ4plex)	0,0000	1,623	3	4,9	0,001	0,0119	13	0,058774548	3	904,58008	-1,95	10,17	1
	High	lAAQGk	4	1	1	P73282	N-Term(iTRAQ4plex); K6(iTRAQ4plex)	0,0000	1,181	3	1,2	0,006	0,07241	28	0,00171779	2	875,55358	-1,97	11,12	0
	Medium	vAIAk	4	1	1	P73282	N-Term(iTRAQ4plex); K5(iTRAQ4plex)	0,0000	1,332	2	9,9	0,011	0,1422	23	0,012887423	2	789,54131	-2,99	16,38	0
P77961	Glutamine synthetase OS=Synechocystis sp. (strain PCC 6803 / Kazusa)	3034,31	43,55	1	22	22	124	1,308	96	23,6	473	53,0	5,11							
	A3	Sequence	# PSMs	# Proteins	# Protein Groups	Protein Group Accessions	Modifications	ΔCn	117/114	117/114 Count	117/114 Variability [%]	q-Value	PEP	IonScore	Exp Value	Charge	MH+ [Da]	ΔM [ppm]	RT [min]	# Missed Cleavages
	High	lVPGFEAPVNLAYSQGNR	8	1	1	P77961	N-Term(iTRAQ4plex)	0,0000	1,055	7	41,7	0	1,106E-09	111	7,36156E-12	2	2076,10137	0,88	43,06	0
	High	fGQTENSSYYFADSVEGR	3	1	1	P77961	N-Term(iTRAQ4plex)	0,0000	1,607	2	0,6	0	2,205E-13	106	2,71625E-11	2	2200,99224	0,83	35,58	0
	High	rLVPGFEAPVNLAYSQGNR	5	1	1	P77961	N-Term(iTRAQ4plex)	0,0000	1,204	3	1,2	0	2,326E-13	95	3,35111E-10	3	2232,20377	1,40	37,42	1
	High	hAPALLAFTNPTTNSYk	18	1	1	P77961	N-Term(iTRAQ4plex); K17(iTRAQ4plex)	0,0000	1,461	13	31,8	0	9,42E-10	94	4,40524E-10	2	2134,15215	-0,66	33,72	0
	High	qGYFPVAPTDTAQDIR	3	1	1	P77961	N-Term(iTRAQ4plex)	0,0000	1,476	2	0,8	0	2,372E-10	79	1,35198E-08	2	1922,97368	0,39	34,02	0
	High	lVNSADNLMIYk	5	1	1	P77961	N-Term(iTRAQ4plex); K12(iTRAQ4plex)	0,0000	1,482	5	32,9	0	5,591E-11	78	1,52746E-08	2	1668,92229	-0,63	37,46	0
	High	lVNSADNLmIYk	7	1	1	P77961	N-Term(iTRAQ4plex); M9(Oxidation); K12(iTRAQ4plex)	0,0000	1,169	6	17,5	0	2,693E-08	77	1,93281E-08	2	1684,91765	-0,36	33,45	0
	High	hHHEVASGGQNELGIk	5	1	1	P77961	N-Term(iTRAQ4plex); K16(iTRAQ4plex)	0,0000	1,346	3	0,6	0	1,717E-11	67	1,96322E-07	3	2001,05362	1,56	12,83	0
	High	iPLSGGNPk	9	1	1	P77961	N-Term(iTRAQ4plex); K9(iTRAQ4plex)	0,0000	1,194	6	5,5	0	0,000008069	62	5,78056E-07	2	1170,70732	-1,02	21,32	0
	High	lRPHPYEFSLYYDc	12	1	1	P77961	N-Term(iTRAQ4plex); C14(Carbamidomethyl)	0,0000	1,490	7	9,8	0	4,534E-08	60	1,00918E-06	2	2003,94499	0,38	36,73	0
	High	ePTLSMIcSIk	3	1	1	P77961	N-Term(iTRAQ4plex); C8(Carbamidomethyl); K11(iTRAQ	0,0000	1,054	3	10,6	0	0,0000198	58	1,64805E-06	2	1566,84514	-1,45	38,14	0
	High	lDNEVNPmR	4	1	1	P77961	N-Term(iTRAQ4plex); M8(Oxidation)	0,0000	1,392	2	3,1	0	0,006064	55	3,17665E-06	2	1247,61736	0,20	17,09	0
	High	dGQPLFAGDk	1	1	1	P77961	N-Term(iTRAQ4plex); K10(iTRAQ4plex)	0,0000	2,178	1		0	0,00004626	49	1,33958E-05	2	1335,71416	-0,43	22,64	0
	High	wIQDENIk	2	1	1	P77961	N-Term(iTRAQ4plex); K8(iTRAQ4plex)	0,0000	1,247	2	32,8	0	0,0002408	46	2,77313E-05	2	1333,73540	-0,04	28,24	0
	High	eEEGGNLGYkPGYk	10	1	1	P77961	N-Term(iTRAQ4plex); K10(iTRAQ4plex); K14(iTRAQ4plex	0,0000	1,212	6	19,6	0	4,501E-08	45	3,74267E-05	3	1973,03427	0,11	19,76	0
	High	wNTGREEEGGNLGYkPGYk	6	1	1	P77961	N-Term(iTRAQ4plex); K15(iTRAQ4plex); K19(iTRAQ4plex	0,0000	1,804	6	22,9	0	1,945E-12	43	5,49503E-05	3	2587,32804	0,56	23,61	1
	High	tPQEVLk	3	1	1	P77961	N-Term(iTRAQ4plex); K7(iTRAQ4plex)	0,0000	1,540	3	0,7	0	0,0005253	42	6,50085E-05	2	1102,66911	-1,76	19,48	0
	High	lDNEVNPMR	2	1	1	P77961	N-Term(iTRAQ4plex)	0,0000	1,326	2	18,1	0	8,229E-07	39	0,000114017	2	1231,62175	-0,36	23,48	0
	High	hAPALLAFTNPTTNSYkR	2	1	1	P77961	N-Term(iTRAQ4plex); K17(iTRAQ4plex)	0,0000	1,586	2	38,7	0	4,545E-08	39	0,000140595	4	2290,25210	-1,13	31,37	1
	High	ePTLSMIcSIkEPR	2	1	1	P77961	N-Term(iTRAQ4plex); C8(Carbamidomethyl); K11(iTRAQ	0,0000	1,136	2	117,8	0	4,384E-07	31	0,001060152	3	1949,04142	-1,28	35,30	1
	High	tGEWYNR	4	1	1	P77961	N-Term(iTRAQ4plex)	0,0000	0,868	4	9,6	0	0,0003371	27	0,001940752	2	1069,51811	-0,22	17,64	0
	High	fDkLVNSADNLmIYk	1	1	1	P77961	N-Term(iTRAQ4plex); K3(iTRAQ4plex); M12(Oxidation); I	0,0000	1,308	1		0	0,00001697	18	0,014553586	3	2219,20951	-0,52	39,84	1
	High	tGEWYNRDPR	2	1	1	P77961	N-Term(iTRAQ4plex)	0,0000	1,633	2	46,0	0	0,003633	14	0,038545173	2	1437,69951	0,23	17,64	1
	High	ePTLSmIcSIkEPR	1	1	1	P77961	N-Term(iTRAQ4plex); M6(Oxidation); C8(Carbamidometh	0,0000	1,125	1		0	0,000506	10	0,089530293	3	1965,03934	0,27	30,02	1
	High	tIAAk	6	1	1	P77961	N-Term(iTRAQ4plex); K5(iTRAQ4plex)	0,0000	1,154	5	1,7	0,007	0,09613	29	0,001364489	2	791,52208	-1,07	11,88	0
P74390	Negative aliphatic amidase regulator OS=Synechocystis sp. (strain PCC	2500,93	30,72	1	15	15	97	2,613	72	65,6	446	48,3	4,89							
	A3	Sequence	# PSMs	# Proteins	# Protein Groups	Protein Group Accessions	Modifications	ΔCn	117/114	117/114 Count	117/114 Variability [%]	q-Value	PEP	IonScore	Exp Value	Charge	MH+ [Da]	ΔM [ppm]	RT [min]	# Missed Cleavages
	High	aAVEAAGDVGETPEGLEk	4	1	1	P74390	N-Term(iTRAQ4plex); K18(iTRAQ4plex)	0,0000	3,102	3	55,0	0	8,08E-16	124	4,20698E-13	2	2031,04729	-0,61	28,35	0
	High	nHMLWYPVQYEGQEcSk	6	1	1	P74390	N-Term(iTRAQ4plex); C15(Carbamidomethyl); K17(iTRAC	0,0000	2,439	4	89,0	0	2,731E-10	80	1,09893E-08	3	2457,15891	0,77	34,16	0
	High	vPVVFGcWTSASR	6	1	1	P74390	N-Term(iTRAQ4plex); C7(Carbamidomethyl)	0,0000	1,487	6	46,9	0	1,07E-08	75	3,49921E-08	2	1609,82829	0,32	41,97	0
	High	fFLVGSDYVFPR	12	1	1	P74390	N-Term(iTRAQ4plex)	0,0000	2,275	11	45,5	0	2,013E-09	71	7,19399E-08	2	1590,84441	0,42	49,42	0
	High	nHmLWYPVQYEGQEcSk	7	1	1	P74390	N-Term(iTRAQ4plex); M3(Oxidation); C15(Carbamidomet	0,0000	2,235	5	47,3	0	6,548E-11	69	1,24157E-07	3	2473,15445	1,02	30,33	0
	High	tFDAPEGMVTMQPNHHISk	3	1	1	P74390	N-Term(iTRAQ4plex); K19(iTRAQ4plex)	0,0000	2,675	2	93,9	0	7,213E-13	68	1,4756E-07	4	2428,19765	-0,66	28,45	0
	High	lIDQDkVPVVFGcWTSASR	4	1	1	P74390	N-Term(iTRAQ4plex); K6(iTRAQ4plex); C13(Carbamidom	0,0000	2,545	4	92,3	0	2,021E-11	66	2,58208E-07	3	2466,30594	0,21	44,41	1
	High	aVLPVFEAk	6	1	1	P74390	N-Term(iTRAQ4plex); K9(iTRAQ4plex)	0,0000	2,819	5	42,0	0	0,00001208	61	8,28303E-07	2	1261,77617	0,23	34,73	0
	High	tFDAPEGmVTmQPNHHISk	10	1	1	P74390	N-Term(iTRAQ4plex); M8(Oxidation); M11(Oxidation); K1	0,0000	2,875	6	18,2	0	8,165E-10	59	1,36763E-06	4	2460,18935	0,11	21,48	0
	High	kAVLPVFEAk	9	1	1	P74390	N-Term(iTRAQ4plex); K1(iTRAQ4plex); K10(iTRAQ4plex)	0,0000	4,977	6	27,9	0	2,599E-07	58	1,69813E-06	2	1533,97063	-1,49	29,17	1
	High	fVAAFk	9	1	1	P74390	N-Term(iTRAQ4plex); K6(iTRAQ4plex)	0,0000	3,903	5	5,9	0	0,002288	47	2,13781E-05	2	970,59459	-1,90	28,56	0
	High	tANTIIk	6	1	1	P74390	N-Term(iTRAQ4plex); K7(iTRAQ4plex)	0,0000	3,386	3	12,5	0	0,008472	44	3,61385E-05	2	1048,65935	-1,09	16,22	0
	High	tFDAPEGmVTMQPNHHISk	5	1	1	P74390	N-Term(iTRAQ4plex); M8(Oxidation); K19(iTRAQ4plex)	0,0000	1,609	4	30,0	0	6,882E-07	39	0,000121331	4	2444,19155	-1,07	23,72	0
	High	gNkFFLVGSDYVFPR	1	1	1	P74390	N-Term(iTRAQ4plex); K3(iTRAQ4plex)	0,0000	1,858	1		0	1,014E-07	38	0,000170596	3	2034,10141	-1,85	43,37	1
	High	tDVENPGk	2	1	1	P74390	N-Term(iTRAQ4plex); K8(iTRAQ4plex)	0,0000	2,509	1		0	0,001502	36	0,000264832	2	1147,61797	-1,56	11,07	0
	High	tANTIIkEQLk	3	1	1	P74390	N-Term(iTRAQ4plex); K7(iTRAQ4plex); K11(iTRAQ4plex)	0,0000	4,390	2	22,2	0	0,00004977	32	0,000696578	3	1691,04068	-1,23	24,39	1
	High	gFTcDWTR	2	1	1	P74390	N-Term(iTRAQ4plex); C4(Carbamidomethyl)	0,0000	1,989	2	36,7	0	0,0004288	26	0,002285441	2	1186,54265	-0,46	26,80	0
	Medium	aAAIGk	2	1	1	P74390	N-Term(iTRAQ4plex); K6(iTRAQ4plex)	0,0000	1,619	2	10,9	0,017	0,2089	18	0,015819374	2	818,53063	-3,92	11,90	0
P26527	ATP synthase subunit beta OS=Synechocystis sp. (strain PCC 6803 / Ka	2225,58	46,58	1	17	17	69	1,427	59	16,0	483	51,7	5,02							
	A3	Sequence	# PSMs	# Proteins	# Protein Groups	Protein Group Accessions	Modifications	ΔCn	117/114	117/114 Count	117/114 Variability [%]	q-Value	PEP	IonScore	Exp Value	Charge	MH+ [Da]	ΔM [ppm]	RT [min]	# Missed Cleavages
	High	fVQAGSEVSALLGR	5	1	1	P26527	N-Term(iTRAQ4plex)	0,0000	1,408	5	3,4	0	1,288E-10	88	1,51771E-09	2	1577,87859	1,10	43,24	0
	High	vGLTALTMAEYFR	7	1	1	P26527	N-Term(iTRAQ4plex)	0,0000	1,434	7	7,3	0	3,696E-08	87	2,14877E-09	2	1615,86589	1,48	49,28	0
	High	vGLTALTmAEYFR	5	1	1	P26527	N-Term(iTRAQ4plex); M8(Oxidation)	0,0000	1,472	5	0,6	0	9,988E-08	75	2,93069E-08	2	1631,86077	1,44	43,01	0
	High	mPSAVGYQPTLGTDVGDLQER	1	1	1	P26527	N-Term(iTRAQ4plex); M1(Oxidation)	0,0000	0,826	1		0	2,866E-12	70	1,04947E-07	3	2394,17575	1,21	34,84	0
	High	iALVYGQmNEPPGAR	4	1	1	P26527	N-Term(iTRAQ4plex); M8(Oxidation)	0,0000	1,178	4	7,8	0	7,177E-08	69	1,28224E-07	2	1775,92620	1,72	30,09	0
	High	aVAmSSTDGLVR	3	1	1	P26527	N-Term(iTRAQ4plex); M4(Oxidation)	0,0000	1,492	2	2,8	0	2,289E-08	69	1,39949E-07	2	1366,71257	0,60	19,34	0
	High	iGLFGGAGVGk	9	1	1	P26527	N-Term(iTRAQ4plex); K11(iTRAQ4plex)	0,0000	1,384	6	31,6	0	5,665E-08	66	2,67282E-07	2	1263,76738	0,79	33,06	0
	High	gmDVVDTGAPISVPVGTGTLGR	1	1	1	P26527	N-Term(iTRAQ4plex); M2(Oxidation)	0,0000	1,430	1		0	3,247E-10	64	4,30497E-07	3	2259,17905	0,80	37,40	0
	High	yVSLADTIk	5	1	1	P26527	N-Term(iTRAQ4plex); K9(iTRAQ4plex)	0,0000	1,277	4	6,2	0	5,307E-07	59	1,39627E-06	2	1297,76140	0,62	32,64	0
	High	fLSQPFFVAEVFTGAPGk	1	1	1	P26527	N-Term(iTRAQ4plex); K18(iTRAQ4plex)	0,0000	1,576	1		0	0,000002728	49	1,58456E-05	3	2230,21836	1,44	54,56	0
	High	aVAMSSTDGLVR	1	1	1	P26527	N-Term(iTRAQ4plex)	0,0000	0,915	1		0	0,002142	45	2,83772E-05	2	1350,71709	0,19	25,71	0
	High	iYNALk	4	1	1	P26527	N-Term(iTRAQ4plex); K6(iTRAQ4plex)	0,0000	1,482	4	21,4	0	0,005108	45	2,95781E-05	2	1009,62871	0,26	22,88	0
	High	iFNVLGEPVDNkGPVPAGETFPIHI	7	1	1	P26527	N-Term(iTRAQ4plex); K12(iTRAQ4plex); K29(iTRAQ4plex	0,0000	1,787	4	6,9	0	0,000002501	43	4,85255E-05	4	3528,97475	1,55	38,94	1
	High	lVDLETkPQVFETGIk	3	1	1	P26527	N-Term(iTRAQ4plex); K7(iTRAQ4plex); K16(iTRAQ4plex)	0,0000	1,681	3	6,6	0	6,748E-11	40	9,03587E-05	3	2249,31186	0,04	39,26	0
	High	eVQSTLQR	2	1	1	P26527	N-Term(iTRAQ4plex)	0,0000	1,309	2	1,8	0	0,00006353	40	9,12011E-05	2	1104,61370	0,64	14,41	0
	High	iALVYGQMNEPPGAR	1	1	1	P26527	N-Term(iTRAQ4plex)	0,0000	1,356	1		0	0,001308	39	0,000113232	3	1759,92826	0,01	34,99	0
	High	vIDLLTPYR	2	1	1	P26527	N-Term(iTRAQ4plex)	0,0000	1,361	2	8,9	0	0,0004697	39	0,000139637	2	1233,73296	0,47	41,47	0
	High	gPVPAGETFPIHRPAPk	3	1	1	P26527	N-Term(iTRAQ4plex); K17(iTRAQ4plex)	0,0000	0,886	2	3,1	0	3,552E-07	37	0,000187055	4	2059,17226	1,49	24,05	0
	High	iTQVIGPVIDAQFPSGk	1	1	1	P26527	N-Term(iTRAQ4plex); K17(iTRAQ4plex)	0,0000	1,390	1		0	0,000001596	37	0,000207	3	2058,18601	1,06	44,27	0
	High	iTSTk	4	1	1	P26527	N-Term(iTRAQ4plex); K5(iTRAQ4plex)	0,0000	1,135	3	8,2	0,005	0,06046	17	0,020043336	2	837,52855	0,18	12,34	0
Q05972	60 kDa chaperonin 1 OS=Synechocystis sp. (strain PCC 6803 / Kazusa) (	1936,42	45,29	1	26	27	85	1,290	59	25,0	541	57,6	5,10							
	A3	Sequence	# PSMs	# Proteins	# Protein Groups	Protein Group Accessions	Modifications	ΔCn	117/114	117/114 Count	117/114 Variability [%]	q-Value	PEP	IonScore	Exp Value	Charge	MH+ [Da]	ΔM [ppm]	RT [min]	# Missed Cleavages
	High	nVAAGANPISLk	9	1	1	Q05972	N-Term(iTRAQ4plex); K12(iTRAQ4plex)	0,0000	1,117	6	22,0	0	1,453E-08	80	1,07392E-08	2	1442,85820	0,84	26,70	0
	High	fGSPQIINDGITIAk	2	1	1	Q05972	N-Term(iTRAQ4plex); K15(iTRAQ4plex)	0,0000	0,938	2	47,9	0	3,722E-07	77	2,16257E-08	2	1862,06572	1,66	39,61	0
	High	gVLNVAAVk	7	1	1	Q05972	N-Term(iTRAQ4plex); K9(iTRAQ4plex)	0,0000	1,526	4	7,0	0	2,518E-07	77	2,20785E-08	2	1158,74529	0,33	26,98	0
	High	lESATVDSLGSAR	1	1	1	Q05972	N-Term(iTRAQ4plex)	0,0000	0,673	1		0	4,051E-07	69	1,35822E-07	2	1449,76714	0,36	27,26	0
	High	aPAGAPGGDFDY	1	1	1	Q05972	N-Term(iTRAQ4plex)	0,0000	1,096	1		0	0,0000135	67	2,0369E-07	2	1281,58769	0,66	30,81	0
	High	aLPAPLkR	4	1	1	Q05972	N-Term(iTRAQ4plex); K7(iTRAQ4plex)	0,0000	1,402	3	8,2	0	0,001195	61	7,51571E-07	3	1153,76484	-0,99	19,50	1
	High	tNDVAGDGTTTATVLAHAIVk	3	1	1	Q05972	N-Term(iTRAQ4plex); K21(iTRAQ4plex)	0,0000	1,214	2	7,1	0	1,631E-11	60	1,06407E-06	3	2343,27665	0,32	37,66	0
	High	qGkPLLIIAEDIEk	4	1	1	Q05972	N-Term(iTRAQ4plex); K3(iTRAQ4plex); K14(iTRAQ4plex)	0,0000	0,912	4	14,2	0	2,577E-08	59	1,3551E-06	3	1999,21909	1,34	42,03	0
	High	lAGGVAVIk	11	2	2	Q05972;P22034	N-Term(iTRAQ4plex); K9(iTRAQ4plex)	0,0000				0	0,00004804	58	1,67483E-06	2	1115,73857	-0,47	27,62	0
	High	ikEHAQPVGDSk	8	1	1	Q05972	N-Term(iTRAQ4plex); K2(iTRAQ4plex); K12(iTRAQ4plex)	0,0000	1,336	6	10,6	0	4,56E-09	55	3,26565E-06	3	1740,99687	-0,01	11,97	1
	High	nVAAGANPISLkR	2	1	1	Q05972	N-Term(iTRAQ4plex); K12(iTRAQ4plex)	0,0000	2,254	1		0	3,889E-10	55	3,31108E-06	3	1598,95713	-0,61	22,44	1
	High	eALATLVVNR	1	1	1	Q05972	N-Term(iTRAQ4plex)	0,0000	0,687	1		0	0,0000857	53	5,18764E-06	2	1229,73454	0,89	31,45	0
	High	gYISPYFVTDAER	1	1	1	Q05972	N-Term(iTRAQ4plex)	0,0000	1,293	1		0	5,293E-08	47	1,82377E-05	2	1661,83122	1,22	37,26	0
	High	vGAATETEmkDR	3	1	1	Q05972	N-Term(iTRAQ4plex); M9(Oxidation); K10(iTRAQ4plex)	0,0000	1,505	2	2,6	0	1,178E-07	47	2,02288E-05	3	1611,82523	0,07	11,07	1
	High	kFGSPQIINDGITIAk	2	1	1	Q05972	N-Term(iTRAQ4plex); K1(iTRAQ4plex); K16(iTRAQ4plex)	0,0000	1,495	1		0	0,000003725	45	3,45915E-05	3	2134,26175	0,98	34,64	1
	High	eIELEDHVENTGVSLIR	1	1	1	Q05972	N-Term(iTRAQ4plex)	0,0000	1,242	1		0	6,186E-11	43	4,96558E-05	3	2097,09629	0,85	37,11	0
	High	vGQEGVISLEEGk	1	1	1	Q05972	N-Term(iTRAQ4plex); K13(iTRAQ4plex)	0,0000	0,747	1		0	5,349E-08	42	6,99794E-05	3	1632,90580	0,65	29,42	0
	High	aLPAPLk	1	1	1	Q05972	N-Term(iTRAQ4plex); K7(iTRAQ4plex)	0,0000	0,817	1		0	0,003567	39	0,000114017	2	997,66411	-0,75	24,04	0
	High	iLITDkk	6	1	1	Q05972	N-Term(iTRAQ4plex); K6(iTRAQ4plex); K7(iTRAQ4plex)	0,0000	1,580	6	24,5	0	0,003908	39	0,000127635	2	1262,83977	-0,82	20,13	1
	High	sIIYNDEAR	1	1	1	Q05972	N-Term(iTRAQ4plex)	0,0000	1,117	1		0	0,0006487	39	0,00013551	2	1224,63555	1,17	19,79	0
	High	vGAATETEMkDR	2	1	1	Q05972	N-Term(iTRAQ4plex); K10(iTRAQ4plex)	0,0000	1,279	2	14,8	0	8,109E-09	38	0,000152044	3	1595,83023	0,02	16,28	1
	High	vGAATETEmkDRk	4	1	1	Q05972	N-Term(iTRAQ4plex); M9(Oxidation); K10(iTRAQ4plex); I	0,0000	1,834	4	0,8	0	0,002131	18	0,015380484	3	1884,02323	0,57	10,88	2
	High	eHAQPVGDSk	1	1	1	Q05972	N-Term(iTRAQ4plex); K10(iTRAQ4plex)	0,0000	1,105	1		0	0,0003383	17	0,01874865	3	1355,71607	0,21	8,26	0
	High	nVVLEk	4	1	1	Q05972	N-Term(iTRAQ4plex); K6(iTRAQ4plex)	0,0000	1,149	3	17,3	0	0,00351	15	0,028181883	2	989,62395	0,58	19,26	0
	High	iLITDk	1	1	1	Q05972	N-Term(iTRAQ4plex); K6(iTRAQ4plex)	0,0000				0,005	0,06704	31	0,000716094	2	990,64336	-0,42	23,90	0
	High	gmDILAEAVAVTLGPk	2	1	1	Q05972	N-Term(iTRAQ4plex); M2(Oxidation); K16(iTRAQ4plex)	0,0000	1,693	2	46,8	0,005	0,06631	12	0,062805836	3	1889,06815	1,30	49,90	0
	Medium	rINITk	1	1	1	Q05972	N-Term(iTRAQ4plex); K6(iTRAQ4plex)	0,0000	1,808	1		0,01	0,1303	14	0,059474907	3	1032,67746	0,64	14,92	1
	Medium	aPGFGDRR	1	1	1	Q05972	N-Term(iTRAQ4plex)	0,0000	1,476	1		0,045	0,5492	11	0,079793957	3	1019,54981	-0,53	11,50	1
P73317	50S ribosomal protein L2 OS=Synechocystis sp. (strain PCC 6803 / Kazu	1916,21	59,42	1	20	20	93	1,582	77	99,5	276	30,4	11,21							
	A3	Sequence	# PSMs	# Proteins	# Protein Groups	Protein Group Accessions	Modifications	ΔCn	117/114	117/114 Count	117/114 Variability [%]	q-Value	PEP	IonScore	Exp Value	Charge	MH+ [Da]	ΔM [ppm]	RT [min]	# Missed Cleavages
	High	qASVSDFTEITk	1	1	1	P73317	N-Term(iTRAQ4plex); K12(iTRAQ4plex)	0,0000	2,889	1		0	1,025E-10	101	7,53304E-11	2	1613,86174	-0,48	30,43	0
	High	iPLGSEIHNVELVAGR	9	1	1	P73317	N-Term(iTRAQ4plex)	0,0000	1,469	9	92,3	0	9,819E-12	97	2,12814E-10	2	1848,04643	0,21	39,73	0
	High	sAGAFAQVVAk	9	1	1	P73317	N-Term(iTRAQ4plex); K11(iTRAQ4plex)	0,0000	1,384	6	111,1	0	6,322E-07	82	8,5686E-09	2	1336,78093	-1,37	27,15	0
	High	sGPVSPWGkPALGAk	6	1	1	P73317	N-Term(iTRAQ4plex); K9(iTRAQ4plex); K15(iTRAQ4plex)	0,0000	3,513	4	119,6	0	2,355E-10	62	6,32368E-07	3	1884,10398	-1,50	27,57	0
	High	iALLFYTDGEk	3	1	1	P73317	N-Term(iTRAQ4plex); K11(iTRAQ4plex)	0,0000	0,629	3	18,9	0	1,235E-09	60	1,07324E-06	2	1557,87578	-0,60	43,50	0
	High	iALLFYTDGEkR	7	1	1	P73317	N-Term(iTRAQ4plex); K11(iTRAQ4plex)	0,0000	3,339	7	58,2	0	0,000003035	59	1,26756E-06	3	1713,97576	-1,21	38,27	1
	High	gSVmNPcDHPHGGGEGR	12	1	1	P73317	N-Term(iTRAQ4plex); M4(Oxidation); C7(Carbamidometh	0,0000	2,111	7	126,6	0	0,000000394	44	3,75811E-05	3	1923,82926	-0,77	8,20	0
	High	sSALIVR	3	1	1	P73317	N-Term(iTRAQ4plex)	0,0000	0,967	2	16,8	0	0,001066	43	4,9885E-05	2	889,55718	-1,78	17,39	0
	High	gSVMNPcDHPHGGGEGR	3	1	1	P73317	N-Term(iTRAQ4plex); C7(Carbamidomethyl)	0,0000	1,007	2	15,3	0	0,0002914	40	9,31044E-05	4	1907,83449	-0,69	11,05	0
	High	kEcVATLGR	9	1	1	P73317	N-Term(iTRAQ4plex); K1(iTRAQ4plex); C3(Carbamidome	0,0000	1,748	5	41,4	0	0,00007763	37	0,000179048	2	1321,74968	-0,29	14,41	1
	High	vAAIEYDPNR	1	1	1	P73317	N-Term(iTRAQ4plex)	0,0000	1,959	1		0	0,0004656	36	0,00025408	2	1291,67485	-1,14	24,19	0
	High	vSNAEFR	3	1	1	P73317	N-Term(iTRAQ4plex)	0,0000	1,251	3	1,2	0	0,004496	33	0,000466627	2	966,51164	-0,93	15,96	0
	High	nkQNIPAR	3	1	1	P73317	N-Term(iTRAQ4plex); K2(iTRAQ4plex)	0,0000	2,505	3	52,4	0	0,0004667	33	0,000503466	2	1228,73442	-1,66	8,53	1
	High	eGDYVTIk	1	1	1	P73317	N-Term(iTRAQ4plex); K8(iTRAQ4plex)	0,0000	0,379	1		0	0,006722	21	0,008127744	2	1212,66899	-2,02	22,27	0
	High	nYRPMTPGTR	3	1	1	P73317	N-Term(iTRAQ4plex)	0,0000	1,011	3	7,4	0	0,006114	14	0,044153995	3	1336,68985	-1,06	13,64	0
	High	gVITSR	4	1	1	P73317	N-Term(iTRAQ4plex)	0,0000	0,980	4	14,0	0,004	0,0549	16	0,024829616	2	776,47374	-1,23	13,17	0
	High	nYRPmTPGTR	2	1	1	P73317	N-Term(iTRAQ4plex); M5(Oxidation)	0,0000	2,144	2	27,6	0,005	0,06424	16	0,025117129	3	1352,68549	-0,51	11,63	0
	High	iIDFkR	3	1	1	P73317	N-Term(iTRAQ4plex); K5(iTRAQ4plex)	0,0000	15,050	3	17,6	0,006	0,08289	18	0,014824157	3	1079,67945	-1,96	23,58	1
	High	skPEk	4	1	1	P73317	N-Term(iTRAQ4plex); K2(iTRAQ4plex); K5(iTRAQ4plex)	0,0000	4,433	4	45,6	0,006	0,078	12	0,066983834	3	1020,63782	-3,48	7,76	0
	Medium	qNIPAR	2	1	1	P73317	N-Term(iTRAQ4plex)	0,0000	2,278	2	81,9	0,011	0,1425	22	0,006981842	2	842,49553	-1,14	9,58	0
	Medium	iIDFk	2	1	1	P73317	N-Term(iTRAQ4plex); K5(iTRAQ4plex)	0,0000	1,911	2	91,7	0,023	0,2942	19	0,012911301	2	923,57787	-2,79	28,82	0
	Medium	aPIGR	3	1	1	P73317	N-Term(iTRAQ4plex)	0,0000	1,585	3	35,7	0,025	0,3151	17	0,021977081	2	657,41564	-1,24	11,97	0
P80505	Glyceraldehyde-3-phosphate dehydrogenase 2 OS=Synechocystis sp. (st	1896,60	48,96	1	17	17	80	1,061	61	18,4	337	36,5	6,44							
	A3	Sequence	# PSMs	# Proteins	# Protein Groups	Protein Group Accessions	Modifications	ΔCn	117/114	117/114 Count	117/114 Variability [%]	q-Value	PEP	IonScore	Exp Value	Charge	MH+ [Da]	ΔM [ppm]	RT [min]	# Missed Cleavages
	High	vVDLAEIVAk	4	1	1	P80505	N-Term(iTRAQ4plex); K10(iTRAQ4plex)	0,0000	0,970	3	12,0	0	4,535E-08	86	2,3333E-09	2	1344,83403	-0,07	40,79	0
	High	aAAVNIVPTSTGAAk	9	1	1	P80505	N-Term(iTRAQ4plex); K15(iTRAQ4plex)	0,0000	1,287	5	9,1	0	7,046E-13	85	2,93069E-09	2	1658,96794	-0,04	25,73	0
	High	gPNIGTYVVGVNAHEYk	4	1	1	P80505	N-Term(iTRAQ4plex); K17(iTRAQ4plex)	0,0000	1,001	3	2,2	0	5,034E-12	85	3,40557E-09	3	2106,12302	0,36	31,76	0
	High	gTMTTTHSYTGDQR	8	1	1	P80505	N-Term(iTRAQ4plex)	0,0000	1,147	6	3,4	0	3,088E-10	74	4,32484E-08	2	1699,78154	-0,65	12,23	0
	High	vINDNFGIIk	3	1	1	P80505	N-Term(iTRAQ4plex); K10(iTRAQ4plex)	0,0000	0,963	3	47,1	0	0,000009912	72	5,72756E-08	2	1420,83989	-0,26	35,22	0
	High	gTmTTTHSYTGDQR	11	1	1	P80505	N-Term(iTRAQ4plex); M3(Oxidation)	0,0000	1,036	8	7,5	0	3,188E-07	70	1,11936E-07	2	1715,77995	1,39	8,46	0
	High	aVALVIPELQGk	7	1	1	P80505	N-Term(iTRAQ4plex); K12(iTRAQ4plex)	0,0000	0,930	6	44,1	0	0,000002509	61	8,35545E-07	2	1525,95378	-1,22	40,89	0
	High	tDSQLEVVGINDTSDPR	1	1	1	P80505	N-Term(iTRAQ4plex)	0,0000	1,383	1		0	8,064E-09	57	1,8534E-06	3	1989,98453	-0,05	30,52	0
	High	vLITAPGk	8	1	1	P80505	N-Term(iTRAQ4plex); K8(iTRAQ4plex)	0,0000	1,061	5	13,0	0	0,0001249	57	2,08915E-06	2	1086,71050	-1,88	24,95	0
	High	kVLITAPGk	4	1	1	P80505	N-Term(iTRAQ4plex); K1(iTRAQ4plex); K9(iTRAQ4plex)	0,0000	1,284	3	69,7	0	0,00001955	56	2,31723E-06	2	1358,90825	-0,97	19,35	1
	High	eAANTSLk	2	1	1	P80505	N-Term(iTRAQ4plex); K8(iTRAQ4plex)	0,0000	1,276	1		0	0,00000824	49	1,38666E-05	2	1121,63884	-1,47	12,41	0
	High	nTIAEQVNGVLk	1	1	1	P80505	N-Term(iTRAQ4plex); K12(iTRAQ4plex)	0,0000	0,565	1		0	0,00005627	40	9,61546E-05	3	1573,91526	0,02	32,95	0
	High	vAINGFGR	3	1	1	P80505	N-Term(iTRAQ4plex)	0,0000	1,139	3	14,1	0	0,000988	39	0,000168233	2	977,56407	-0,87	25,16	0
	High	hVQAGAk	1	1	1	P80505	N-Term(iTRAQ4plex); K7(iTRAQ4plex)	0,0000	0,939	1		0	0,0006529	31	0,000724386	3	998,59385	-4,72	7,59	0
	High	tNAHLLR	6	1	1	P80505	N-Term(iTRAQ4plex)	0,0000	0,806	4	8,3	0	0,0002756	28	0,001636703	2	968,57585	0,05	12,26	0
	High	iLDASHR	4	1	1	P80505	N-Term(iTRAQ4plex)	0,0000	0,892	4	18,7	0	0,000573	25	0,003388208	3	955,54401	-0,17	13,68	0
	High	cWLGR	3	1	1	P80505	N-Term(iTRAQ4plex); C1(Carbamidomethyl)	0,0000	1,505	3	1,6	0,004	0,05175	15	0,03061752	2	835,43523	-1,57	20,28	0
	High	lNGIALR	1	1	1	P80505	N-Term(iTRAQ4plex)	0,0000	0,965	1		0,006	0,07244	29	0,001282242	2	900,57427	-0,54	25,82	0
Q01951	Allophycocyanin alpha chain OS=Synechocystis sp. (strain PCC 6803 / K	1769,58	62,73	1	11	12	60	1,087	38	13,2	161	17,4	4,96							
	A3	Sequence	# PSMs	# Proteins	# Protein Groups	Protein Group Accessions	Modifications	ΔCn	117/114	117/114 Count	117/114 Variability [%]	q-Value	PEP	IonScore	Exp Value	Charge	MH+ [Da]	ΔM [ppm]	RT [min]	# Missed Cleavages
	High	rPDIVSPGGNAYGEEMTATcLR	5	1	1	Q01951	N-Term(iTRAQ4plex); C20(Carbamidomethyl)	0,0000	1,082	3	3,4	0	1,384E-16	99	1,15603E-10	3	2538,22281	1,17	31,94	0
	High	sLGTPIEAVAQSVR	9	1	1	Q01951	N-Term(iTRAQ4plex)	0,0000	1,068	7	15,2	0	2,188E-10	95	2,97831E-10	2	1571,88982	1,54	37,76	0
	High	rPDIVSPGGNAYGEEmTATcLR	10	1	1	Q01951	N-Term(iTRAQ4plex); M16(Oxidation); C20(Carbamidom	0,0000	0,965	6	8,8	0	1,162E-10	85	3,01974E-09	3	2554,21817	1,34	27,63	0
	High	sIVNADAEAR	5	1	1	Q01951	N-Term(iTRAQ4plex)	0,0000	1,170	3	10,0	0	2,166E-07	65	2,93745E-07	2	1189,63115	1,49	16,36	0
	High	aFVTGGAAR	4	1	1	Q01951	N-Term(iTRAQ4plex)	0,0000	1,160	3	3,8	0	0,000003436	52	6,30914E-06	2	993,56029	0,45	15,53	0
	High	ikAFVTGGAAR	2	1	1	Q01951	N-Term(iTRAQ4plex); K2(iTRAQ4plex)	0,0000	1,857	2	40,9	0	5,954E-09	41	8,13185E-05	3	1378,84174	0,57	24,22	1
	High	iAETLTGSR	6	1	1	Q01951	N-Term(iTRAQ4plex)	0,0000	1,186	3	3,1	0	0,0003662	40	9,41825E-05	2	1091,61882	0,99	22,89	0
	High	dmDYYLR	3	2	2	P74551;Q01951	N-Term(iTRAQ4plex); M2(Oxidation)	0,0000				0	0,003725	36	0,00025408	2	1135,52165	0,53	20,78	0
	High	dMDYYLR	1	2	2	P74551;Q01951	N-Term(iTRAQ4plex)	0,0000				0	0,00333	32	0,000668298	2	1119,52739	1,12	29,51	0
	High	qAGDRLFQk	1	1	1	Q01951	N-Term(iTRAQ4plex); K9(iTRAQ4plex)	0,0000	3,595	1		0	0,002258	22	0,005997497	3	1350,77323	-0,01	17,81	1
	High	iAETLTGSRETIVk	1	1	1	Q01951	N-Term(iTRAQ4plex); K14(iTRAQ4plex)	0,0000	2,472	1		0	0,001834	20	0,009816801	3	1806,05692	-0,35	28,39	1
	High	yLSPGELDR	2	1	1	Q01951	N-Term(iTRAQ4plex)	0,0000	0,983	2	2,8	0,001	0,02128	34	0,000367257	2	1193,62871	0,34	28,17	0
	High	eTIVk	3	1	1	Q01951	N-Term(iTRAQ4plex); K5(iTRAQ4plex)	0,0000	0,880	3	8,2	0,004	0,0514	30	0,001014829	2	877,55931	-0,45	13,61	0
	High	sIVTk	8	1	1	Q01951	N-Term(iTRAQ4plex); K5(iTRAQ4plex)	0,0000	1,085	4	2,8	0,005	0,06552	17	0,028862488	2	835,54906	-0,09	15,41	0
P73606	Slr1855 protein OS=Synechocystis sp. (strain PCC 6803 / Kazusa) GN=s	1767,08	45,47	1	32	32	101	1,028	84	23,4	607	70,0	5,25							
	A3	Sequence	# PSMs	# Proteins	# Protein Groups	Protein Group Accessions	Modifications	ΔCn	117/114	117/114 Count	117/114 Variability [%]	q-Value	PEP	IonScore	Exp Value	Charge	MH+ [Da]	ΔM [ppm]	RT [min]	# Missed Cleavages
	High	vIQNFDEGYPDATGTmR	2	1	1	P73606	N-Term(iTRAQ4plex); M16(Oxidation)	0,0000	0,801	2	3,5	0	2,958E-09	84	4,11121E-09	2	2073,97002	1,52	31,73	0
	High	iADLmPAVGSDQQR	1	1	1	P73606	N-Term(iTRAQ4plex); M5(Oxidation)	0,0000	0,755	1		0	3,254E-09	78	1,51695E-08	2	1660,84563	0,64	26,60	0
	High	iLHDAEQTIk	7	1	1	P73606	N-Term(iTRAQ4plex); K10(iTRAQ4plex)	0,0000	0,900	6	17,7	0	7,702E-08	65	3,01974E-07	2	1455,84294	1,34	26,05	0
	High	aQFGDGEIDYR	1	1	1	P73606	N-Term(iTRAQ4plex)	0,0000	0,851	1		0	0,000003415	54	3,57248E-06	2	1414,67119	-0,55	26,47	0
	High	aVVGHNLk	4	1	1	P73606	N-Term(iTRAQ4plex); K8(iTRAQ4plex)	0,0000	1,152	2	0,6	0	0,00002109	53	5,3576E-06	2	1125,70012	1,64	12,91	0
	High	tSDGQEFQVk	2	1	1	P73606	N-Term(iTRAQ4plex); K10(iTRAQ4plex)	0,0000	1,029	1		0	5,771E-07	52	5,68814E-06	2	1426,74358	1,34	19,04	0
	High	lSPMAYAk	3	1	1	P73606	N-Term(iTRAQ4plex); K8(iTRAQ4plex)	0,0000	1,111	2	11,7	0	0,0003574	50	1,09388E-05	2	1168,66435	0,42	26,24	0
	High	lSPmAYAk	5	1	1	P73606	N-Term(iTRAQ4plex); M4(Oxidation); K8(iTRAQ4plex)	0,0000	0,950	3	0,9	0	0,0002869	50	1,10146E-05	2	1184,66155	2,33	20,48	0
	High	iAWNLMR	3	1	1	P73606	N-Term(iTRAQ4plex)	0,0000	1,128	3	19,5	0	0,005488	45	2,94422E-05	2	1047,58940	0,35	36,26	0
	High	lFDkFFLDk	4	1	1	P73606	N-Term(iTRAQ4plex); K4(iTRAQ4plex); K9(iTRAQ4plex)	0,0000	1,158	4	9,2	0	5,391E-07	45	3,35268E-05	2	1604,94255	0,80	43,32	1
	High	kPAEYVFEk	8	1	1	P73606	N-Term(iTRAQ4plex); K1(iTRAQ4plex); K9(iTRAQ4plex)	0,0000	1,345	8	8,7	0	6,896E-07	45	3,12586E-05	2	1542,89104	1,18	23,65	0
	High	yVELAk	4	1	1	P73606	N-Term(iTRAQ4plex); K6(iTRAQ4plex)	0,0000	0,884	3	14,3	0	0,004357	40	9,24634E-05	2	1010,61467	2,18	23,32	0

	High	qVVFAGk	6	1	1	P73606	N-Term(iTRAQ4plex); K7(iTRAQ4plex)	0,0000	1,201	3	12,5	0	0,0005447	40	9,31044E-05	2	1036,64043	1,02	19,66	0
	High	gGWYDVVER	1	1	1	P73606	N-Term(iTRAQ4plex)	0,0000	0,900	1		0	0,000004974	39	0,000125305	2	1224,61394	0,77	31,64	0
	High	kIADLmPAVGSDQQR	3	1	1	P73606	N-Term(iTRAQ4plex); K1(iTRAQ4plex); M6(Oxidation)	0,0000	0,916	2	32,7	0	3,889E-07	35	0,00032434	3	1933,04331	0,88	22,41	1
	High	tDLSmSGER	1	1	1	P73606	N-Term(iTRAQ4plex); M5(Oxidation)	0,0000	1,161	1		0	0,002707	32	0,000587449	2	1155,53826	-4,34	11,15	0
	High	sESLGPNk	2	1	1	P73606	N-Term(iTRAQ4plex); K8(iTRAQ4plex)	0,0000	0,995	1		0	0,0006414	32	0,000756676	2	1119,62627	1,28	12,68	0
	High	yFPDYDHSPFVQER	3	1	1	P73606	N-Term(iTRAQ4plex)	0,0000	0,835	3	10,0	0	0,00003101	32	0,000639691	3	1943,90750	1,55	35,81	0
	High	tWLTPGR	4	1	1	P73606	N-Term(iTRAQ4plex)	0,0000	0,910	3	8,6	0	0,004889	32	0,000654636	2	974,55474	0,74	24,79	0
	High	qVNSLGk	5	1	1	P73606	N-Term(iTRAQ4plex); K7(iTRAQ4plex)	0,0000	1,091	3	0,6	0	0,003048	31	0,001388302	2	1033,62334	-1,06	13,08	0
	High	cHQFVWHDR	3	1	1	P73606	N-Term(iTRAQ4plex); C1(Carbamidomethyl)	0,0000	1,110	3	15,2	0	5,015E-08	30	0,000959334	3	1428,67188	0,48	16,53	0
	High	kIADLMPAVGSDQQR	1	1	1	P73606	N-Term(iTRAQ4plex); K1(iTRAQ4plex)	0,0000	0,981	1		0	0,00003404	30	0,00098168	3	1917,04813	0,75	30,84	1
	High	rILHDAEQTIk	2	1	1	P73606	N-Term(iTRAQ4plex); K11(iTRAQ4plex)	0,0000	1,611	1		0	0,00001257	30	0,00110202	3	1611,94260	0,31	19,88	1
	High	fYEDWSHDTTWGWQQNR	1	1	1	P73606	N-Term(iTRAQ4plex)	0,0000	0,901	1		0	0,0006799	25	0,003005869	3	2400,05655	0,61	40,95	0
	High	ekYVELAk	3	1	1	P73606	N-Term(iTRAQ4plex); K2(iTRAQ4plex); K8(iTRAQ4plex)	0,0000	1,345	3	15,8	0	0,00001199	24	0,0041973	3	1411,85181	-0,21	20,89	1
	High	sEYGGYFSHLDPLmLDPR	1	1	1	P73606	N-Term(iTRAQ4plex); M14(Oxidation)	0,0000	0,786	1		0	0,0005389	23	0,004742092	3	2257,07425	1,15	44,39	0
	High	lLDNHSR	6	1	1	P73606	N-Term(iTRAQ4plex)	0,0000	1,115	6	19,9	0	0,005807	19	0,011297179	3	998,55109	1,11	12,91	0
	High	hPmDFYFkPLPNGFPDGILR	1	1	1	P73606	N-Term(iTRAQ4plex); M3(Oxidation); K8(iTRAQ4plex)	0,0000	1,206	1		0	0,0001022	16	0,027731286	4	2665,38955	2,07	44,88	0
	High	sTVNFRQETDTISR	1	1	1	P73606	N-Term(iTRAQ4plex)	0,0000	1,076	1		0	0,0008135	15	0,031620593	3	1797,92081	-0,22	22,95	1
	High	iAWNLmR	4	1	1	P73606	N-Term(iTRAQ4plex); M6(Oxidation)	0,0000	0,811	4	1,6	0,001	0,02105	35	0,000306175	2	1063,58464	0,65	30,30	0
	High	qDWWIk	2	1	1	P73606	N-Term(iTRAQ4plex); K6(iTRAQ4plex)	0,0000	1,080	2	2,1	0,002	0,03214	26	0,002505936	2	1163,64678	1,37	32,73	0
	High	qETDTISR	1	1	1	P73606	N-Term(iTRAQ4plex)	0,0000	1,393	1		0,002	0,02235	16	0,023821549	2	1093,56182	1,10	11,51	0
	High	gTEYLR	3	1	1	P73606	N-Term(iTRAQ4plex)	0,0000	0,977	3	19,6	0,006	0,07279	27	0,001870553	2	882,48070	0,61	15,05	0
	High	fFLDk	2	1	1	P73606	N-Term(iTRAQ4plex); K5(iTRAQ4plex)	0,0000	0,754	2	34,5	0,006	0,08535	25	0,003213439	2	957,56560	0,84	30,82	0
	High	mNSLk	1	1	1	P73606	N-Term(iTRAQ4plex); K5(iTRAQ4plex)	0,0000	1,109	1		0,009	0,1158	30	0,001147916	2	880,51506	-1,60	14,83	0
P74227	Elongation factor Tu OS=Synechocystis sp. (strain PCC 6803 / Kazusa) G	1755,86	58,15	1	24	24	86	1,301	64	30,9	399	43,7	5,30							
	A3	Sequence	# PSMs	# Proteins	# Protein Groups	Protein Group Accessions	Modifications	ΔCn	117/114	117/114 Count	117/114 Variability [%]	q-Value	PEP	IonScore	Exp Value	Charge	MH+ [Da]	ΔM [ppm]	RT [min]	# Missed Cleavages
	High	aVDDYIDTPER	1	1	1	P74227	N-Term(iTRAQ4plex)	0,0000	1,078	1		0	4,316E-08	86	2,70377E-09	2	1437,69841	0,39	26,50	0
	High	tLEEGmAGDNVGLLLR	2	1	1	P74227	N-Term(iTRAQ4plex); M6(Oxidation)	0,0000	1,066	2	10,3	0	1,383E-11	83	4,83025E-09	2	1847,96697	0,83	37,75	0
	High	vGEEISIVGIk	3	1	1	P74227	N-Term(iTRAQ4plex); K11(iTRAQ4plex)	0,0000	1,069	2	13,1	0	6,263E-09	77	1,81121E-08	2	1431,86724	0,75	35,68	0
	High	tIGAGVVSk	8	1	1	P74227	N-Term(iTRAQ4plex); K9(iTRAQ4plex)	0,0000	1,316	5	23,3	0	0,0000109	69	1,29709E-07	2	1119,69805	0,38	20,16	0
	High	mTVELINPIAIEQGmR	3	1	1	P74227	N-Term(iTRAQ4plex); M1(Oxidation); M15(Oxidation)	0,0000	1,045	2	6,1	0	2,934E-09	66	2,24373E-07	2	1991,04411	0,91	41,33	0
	High	gITINTAHVEYETDSR	6	1	1	P74227	N-Term(iTRAQ4plex)	0,0000	1,153	3	9,3	0	4,328E-08	55	3,47512E-06	3	1949,96500	-1,82	26,80	0
	High	vkVGEEISIVGIk	1	1	1	P74227	N-Term(iTRAQ4plex); K2(iTRAQ4plex); K13(iTRAQ4plex)	0,0000	0,837	1		0	5,323E-10	54	3,78416E-06	3	1803,13108	-0,30	33,58	1
	High	kATVTGVEMFQk	5	1	1	P74227	N-Term(iTRAQ4plex); K1(iTRAQ4plex); K12(iTRAQ4plex)	0,0000	1,304	4	28,9	0	7,4E-10	53	4,7863E-06	3	1771,01419	-0,38	25,86	1
	High	eHILLAk	8	1	1	P74227	N-Term(iTRAQ4plex); K7(iTRAQ4plex)	0,0000	1,345	4	0,8	0	0,0001141	51	8,01623E-06	2	1111,70696	-0,74	18,91	0
	High	tTDVTGTIk	3	1	1	P74227	N-Term(iTRAQ4plex); K9(iTRAQ4plex)	0,0000	1,173	2	26,1	0	0,000003828	50	1,03507E-05	2	1223,70781	-0,62	18,15	0
	High	kYEDIDAAPEEk	2	1	1	P74227	N-Term(iTRAQ4plex); K1(iTRAQ4plex); K12(iTRAQ4plex)	0,0000	1,541	1		0	8,384E-08	49	1,27994E-05	3	1839,97080	0,40	19,49	1
	High	mTVELINPIAIEQGmR	3	1	1	P74227	N-Term(iTRAQ4plex); M15(Oxidation)	0,0000	1,482	3	23,3	0	1,054E-07	48	1,48242E-05	3	1975,04789	0,25	46,00	0
	High	dHVNIGTIGHVDHGk	3	1	1	P74227	N-Term(iTRAQ4plex); K15(iTRAQ4plex)	0,0000	0,946	2	8,9	0	3,156E-10	48	1,56664E-05	4	1887,00796	0,21	18,53	0
	High	lVVFLNkk	3	1	1	P74227	N-Term(iTRAQ4plex); K7(iTRAQ4plex); K8(iTRAQ4plex)	0,0000	2,003	3	15,6	0	0,002045	45	2,85739E-05	2	1392,92900	-0,93	31,28	1
	High	kATVTGVEmFQk	7	1	1	P74227	N-Term(iTRAQ4plex); K1(iTRAQ4plex); M9(Oxidation); K	0,0000	1,305	5	27,9	0	3,18E-08	42	9,16925E-05	3	1787,00882	-0,53	22,41	1
	High	lVVFLNk	10	1	1	P74227	N-Term(iTRAQ4plex); K7(iTRAQ4plex)	0,0000	1,579	6	7,8	0	0,0006633	41	7,51571E-05	2	1120,73393	0,60	36,42	0
	High	eYkDAILELMk	1	1	1	P74227	N-Term(iTRAQ4plex); K3(iTRAQ4plex); K11(iTRAQ4plex)	0,0000	1,346	1		0	6,975E-08	39	0,00013365	3	1785,01883	-0,24	41,79	1
	High	hTPFFANYRPQFYVR	3	1	1	P74227	N-Term(iTRAQ4plex)	0,0000	1,457	3	10,3	0	5,995E-07	37	0,000191854	4	2087,07363	0,19	35,83	0
	High	hYAHVDcPGHADYVk	1	1	1	P74227	N-Term(iTRAQ4plex); C7(Carbamidomethyl); K15(iTRAQ4	0,0000	1,170	1		0	6,67E-09	35	0,000331108	4	2056,98964	-0,26	15,68	0
	High	aTVTGVEmFQk	2	1	1	P74227	N-Term(iTRAQ4plex); M8(Oxidation); K11(iTRAQ4plex)	0,0000	0,889	2	3,7	0	0,00000404	28	0,001492691	3	1514,81321	0,31	25,74	0
	High	aIEGEkEYk	3	1	1	P74227	N-Term(iTRAQ4plex); K6(iTRAQ4plex); K9(iTRAQ4plex)	0,0000	1,422	3	0,9	0	0,00001287	27	0,002156244	3	1498,84504	-1,81	14,65	1
	High	tkDHVNIGTIGHVDHGk	1	1	1	P74227	N-Term(iTRAQ4plex); K2(iTRAQ4plex); K17(iTRAQ4plex)	0,0000	0,978	1		0	4,592E-07	24	0,0044154	5	2260,25632	1,79	17,40	1
	High	aIEGEkEYkDAILELmk	1	1	1	P74227	N-Term(iTRAQ4plex); K6(iTRAQ4plex); K9(iTRAQ4plex);	0,0000	1,639	1		0	0,0001423	23	0,004476824	3	2572,44120	0,82	36,40	2
	High	gIQkEDIER	2	1	1	P74227	N-Term(iTRAQ4plex); K4(iTRAQ4plex)	0,0000	1,621	2	0,0	0	0,002663	20	0,011290101	3	1375,77793	-0,34	15,66	1
	High	gmVLAkPGSITPHTEFEGEVYVLk	1	1	1	P74227	N-Term(iTRAQ4plex); M2(Oxidation); K6(iTRAQ4plex); K	0,0000	1,390	1		0	6,738E-09	16	0,022488993	4	3050,66054	0,25	36,77	0
	High	qVGVPk	3	1	1	P74227	N-Term(iTRAQ4plex); K6(iTRAQ4plex)	0,0000	0,750	3	14,5	0,002	0,0261	24	0,004158819	2	915,58544	-1,27	13,79	0
P74281	Soluble hydrogenase 42 kD subunit OS=Synechocystis sp. (strain PCC 6	1740,37	38,28	1	19	19	74	0,949	59	37,4	384	40,7	7,28							
	A3	Sequence	# PSMs	# Proteins	# Protein Groups	Protein Group Accessions	Modifications	ΔCn	117/114	117/114 Count	117/114 Variability [%]	q-Value	PEP	IonScore	Exp Value	Charge	MH+ [Da]	ΔM [ppm]	RT [min]	# Missed Cleavages
	High	aWQAYETATIPR	5	1	1	P74281	N-Term(iTRAQ4plex)	0,0000	0,696	5	48,1	0	1,613E-07	90	9,11948E-10	2	1550,80937	0,63	33,76	0
	High	iIAELTANLk	7	1	1	P74281	N-Term(iTRAQ4plex); K10(iTRAQ4plex)	0,0000	1,172	5	18,3	0	2,322E-09	72	6,26571E-08	2	1373,86052	-0,11	44,36	0
	High	kFDIAMAGGQDHLk	8	1	1	P74281	N-Term(iTRAQ4plex); K1(iTRAQ4plex); K14(iTRAQ4plex)	0,0000	1,281	6	25,3	0	1,091E-11	61	7,12804E-07	3	1963,07792	-0,86	27,42	1
	High	vLLAMAk	4	1	1	P74281	N-Term(iTRAQ4plex); K7(iTRAQ4plex)	0,0000	0,966	2	26,1	0	0,0001988	57	2,34095E-06	2	1033,66667	-1,51	30,06	0
	High	kFDIAmAGGQDHLk	8	1	1	P74281	N-Term(iTRAQ4plex); K1(iTRAQ4plex); M6(Oxidation); K	0,0000	0,964	4	4,8	0	5,826E-12	57	2,56861E-06	3	1979,07566	0,58	23,28	1
	High	aEGLDAIFTR	1	1	1	P74281	N-Term(iTRAQ4plex)	0,0000	0,832	1		0	0,00001404	56	3,20561E-06	2	1236,67095	0,34	37,26	0
	High	tFGLAVEEIk	3	1	1	P74281	N-Term(iTRAQ4plex); K10(iTRAQ4plex)	0,0000	0,525	2	30,5	0	0,000002169	52	6,41165E-06	2	1394,81304	-0,25	36,46	0
	High	iGHLGFVcDR	5	1	1	P74281	N-Term(iTRAQ4plex); C8(Carbamidomethyl)	0,0000	0,761	5	27,4	0	0,000002173	49	1,21619E-05	2	1317,68620	0,57	26,78	0
	High	fDIAmAGGQDHLk	1	1	1	P74281	N-Term(iTRAQ4plex); M5(Oxidation); K13(iTRAQ4plex)	0,0000	0,644	1		0	0,000000524	48	1,71384E-05	3	1706,87564	-1,08	26,60	0
	High	tLLEADSDk	2	1	1	P74281	N-Term(iTRAQ4plex); K9(iTRAQ4plex)	0,0000	1,019	1		0	4,323E-07	47	1,90546E-05	2	1279,69805	-0,28	21,03	0
	High	aLDPNDFk	1	1	1	P74281	N-Term(iTRAQ4plex); K8(iTRAQ4plex)	0,0000	0,985	1		0	0,0005539	47	2,20785E-05	2	1207,65532	-0,68	26,69	0
	High	gYmIPPGLGFVSVSAk	3	1	1	P74281	N-Term(iTRAQ4plex); M3(Oxidation); K16(iTRAQ4plex)	0,0000	0,944	2	2,2	0	1,397E-07	46	2,25928E-05	3	1927,06107	0,45	42,64	0
	High	kkFDIAMAGGQDHLk	1	1	1	P74281	N-Term(iTRAQ4plex); K1(iTRAQ4plex); K2(iTRAQ4plex);	0,0000	1,693	1		0	2,058E-08	42	6,29463E-05	4	2235,27309	-1,58	24,11	2
	High	vLVGNNGk	2	1	1	P74281	N-Term(iTRAQ4plex); K8(iTRAQ4plex)	0,0000	0,869	2	21,4	0	0,00001558	39	0,000113493	2	1088,66582	-0,76	15,10	0
	High	fYLDLk	4	1	1	P74281	N-Term(iTRAQ4plex); K6(iTRAQ4plex)	0,0000	0,666	4	18,2	0	0,0006841	39	0,000137711	2	1086,64336	-0,38	36,17	0
	High	qmLmIPGPTPVPEk	2	1	1	P74281	N-Term(iTRAQ4plex); M2(Oxidation); M4(Oxidation); K14	0,0000	0,654	2	82,8	0	1,197E-08	38	0,000157387	3	1858,00705	0,71	31,52	0
	High	tFGLAVEEIkAEWGk	1	1	1	P74281	N-Term(iTRAQ4plex); K10(iTRAQ4plex); K15(iTRAQ4plex	0,0000	3,893	1		0	9,048E-09	38	0,00016217	3	2110,19071	-0,11	47,96	1
	High	fYLDLkk	8	1	1	P74281	N-Term(iTRAQ4plex); K6(iTRAQ4plex); K7(iTRAQ4plex)	0,0000	1,561	8	32,0	0	0,001138	37	0,000186196	2	1358,83782	-2,20	31,13	1
	High	gYMIPPGLGFVSVSAk	1	1	1	P74281	N-Term(iTRAQ4plex); K16(iTRAQ4plex)	0,0000	0,855	1		0	4,241E-08	36	0,000271762	3	1911,06461	-0,35	45,75	0
	High	qMLmIPGPTPVPEk	1	1	1	P74281	N-Term(iTRAQ4plex); M4(Oxidation); K14(iTRAQ4plex)	0,0000	1,144	1		0	0,000005085	35	0,000341169	3	1842,00931	-0,82	35,48	0
	High	vLLAmAk	4	1	1	P74281	N-Term(iTRAQ4plex); M5(Oxidation); K7(iTRAQ4plex)	0,0000	0,883	2	10,8	0,002	0,0325	13	0,046128571	2	1049,66362	0,45	21,45	0
	High	sGDFSk	1	1	1	P74281	N-Term(iTRAQ4plex); K6(iTRAQ4plex)	0,0000	0,662	1		0,003	0,03586	17	0,019141237	2	928,49706	-0,84	12,87	0
	Medium	aEWGk	1	1	1	P74281	N-Term(iTRAQ4plex); K5(iTRAQ4plex)	0,0000	0,917	1		0,012	0,1459	12	0,05834451	2	878,49504	-2,74	15,13	0
Q01952	Allophycocyanin beta chain OS=Synechocystis sp. (strain PCC 6803 / Ka	1714,50	88,20	1	16	16	72	1,271	53	26,5	161	17,2	5,60							
	A3	Sequence	# PSMs	# Proteins	# Protein Groups	Protein Group Accessions	Modifications	ΔCn	117/114	117/114 Count	117/114 Variability [%]	q-Value	PEP	IonScore	Exp Value	Charge	MH+ [Da]	ΔM [ppm]	RT [min]	# Missed Cleavages
	High	aASVISANAATIVk	17	1	1	Q01952	N-Term(iTRAQ4plex); K14(iTRAQ4plex)	0,0000	1,366	10	17,0	0	1,663E-12	113	5,08124E-12	2	1603,96428	1,31	30,82	0
	High	eVTASLVGADAGk	3	1	1	Q01952	N-Term(iTRAQ4plex); K13(iTRAQ4plex)	0,0000	1,231	3	79,6	0	3,469E-09	97	2,13796E-10	2	1505,84172	0,21	25,10	0
	High	aASVISANAATIVkEAVAk	6	1	1	Q01952	N-Term(iTRAQ4plex); K14(iTRAQ4plex); K19(iTRAQ4plex	0,0000	1,758	4	5,7	0	3,488E-10	80	9,50539E-09	3	2246,34684	1,05	43,49	1
	High	mQDAITAVINSADVQGk	4	1	1	Q01952	N-Term(iTRAQ4plex); M1(Oxidation); K17(iTRAQ4plex)	0,0000	1,308	2	3,7	0	3,322E-11	75	2,86398E-08	3	2065,08872	2,35	37,70	0
	High	sYFASGELR	5	1	1	Q01952	N-Term(iTRAQ4plex)	0,0000	1,270	4	0,8	0	0,000001396	57	2,0463E-06	2	1173,60344	1,16	26,64	0
	High	mQDAITAVINSADVQGk	1	1	1	Q01952	N-Term(iTRAQ4plex); K17(iTRAQ4plex)	0,0000	1,231	1		0	1,672E-09	57	2,09879E-06	3	2049,09134	1,17	44,44	0
	High	yLDGAAMDk	1	1	1	Q01952	N-Term(iTRAQ4plex); K9(iTRAQ4plex)	0,0000	1,340	1		0	0,00000106	50	9,31044E-06	2	1271,65422	-0,17	25,18	0
	High	dLDYYLR	4	1	1	Q01952	N-Term(iTRAQ4plex)	0,0000	1,185	4	1,4	0	0,00008516	42	6,80722E-05	2	1101,56999	0,26	34,27	0
	High	yLDGAAMDkLk	2	1	1	Q01952	N-Term(iTRAQ4plex); K9(iTRAQ4plex); K11(iTRAQ4plex)	0,0000	2,942	2	2,9	0	0,00001248	40	0,000113029	3	1656,93656	0,61	30,82	1
	High	eTYNSLGVPISSTVQAIQAIk	2	1	1	Q01952	N-Term(iTRAQ4plex); K21(iTRAQ4plex)	0,0000	1,552	2	23,4	0	0,000003999	39	0,000114543	3	2507,39957	1,42	49,24	0
	High	sLLYSDVTRPGGNmYTTR	5	1	1	Q01952	N-Term(iTRAQ4plex); M14(Oxidation)	0,0000	0,703	3	3,0	0	0,003395	38	0,000165947	3	2191,09604	1,18	28,63	0
	High	yLDGAAmDk	3	1	1	Q01952	N-Term(iTRAQ4plex); M7(Oxidation); K9(iTRAQ4plex)	0,0000	1,051	2	23,5	0	0,0001978	37	0,000184914	2	1287,65178	1,89	20,00	0
	High	rYAAcIR	1	1	1	Q01952	N-Term(iTRAQ4plex); C5(Carbamidomethyl)	0,0000	1,329	1		0	0,0002872	35	0,000319867	3	1053,57557	1,08	11,91	1
	High	lkSYFASGELR	1	1	1	Q01952	N-Term(iTRAQ4plex); K2(iTRAQ4plex)	0,0000	1,552	1		0	0,00007188	33	0,000473119	3	1558,88376	0,37	30,19	1
	High	yLDGAAmDkLk	2	1	1	Q01952	N-Term(iTRAQ4plex); M7(Oxidation); K9(iTRAQ4plex); K	0,0000	1,825	2	2,1	0	0,005951	33	0,000496558	3	1672,93100	0,33	24,79	1
	High	sLLYSDVTRPGGNMYTTR	3	1	1	Q01952	N-Term(iTRAQ4plex)	0,0000	0,766	3	2,4	0	0,00000821	30	0,000941825	3	2175,10013	0,73	32,12	0
	High	emGVYLDYIcSGLS	1	1	1	Q01952	N-Term(iTRAQ4plex); M2(Oxidation); C10(Carbamidomet	0,0000				0,001	0,01479	29	0,001393061	2	1766,81255	1,56	45,59	0
	High	vLNGLk	6	1	1	Q01952	N-Term(iTRAQ4plex); K6(iTRAQ4plex)	0,0000	1,664	3	0,9	0,009	0,1207	19	0,011748164	2	931,61931	1,52	22,23	0
	Medium	eAVAk	2	1	1	Q01952	N-Term(iTRAQ4plex); K5(iTRAQ4plex)	0,0000	1,170	2	7,4	0,027	0,3353	28	0,005122877	2	805,50041	-2,21	10,83	0
	Medium	yAAcIR	3	1	1	Q01952	N-Term(iTRAQ4plex); C4(Carbamidomethyl)	0,0000	1,174	3	2,6	0,028	0,3397	21	0,008709034	2	897,47392	0,69	17,34	0
P22034	60 kDa chaperonin 2 OS=Synechocystis sp. (strain PCC 6803 / Kazusa)	1667,30	49,82	1	28	29	66	1,072	45	36,1	552	57,7	4,97							
	A3	Sequence	# PSMs	# Proteins	# Protein Groups	Protein Group Accessions	Modifications	ΔCn	117/114	117/114 Count	117/114 Variability [%]	q-Value	PEP	IonScore	Exp Value	Charge	MH+ [Da]	ΔM [ppm]	RT [min]	# Missed Cleavages
	High	rSLEAGINALADAVR	3	1	1	P22034	N-Term(iTRAQ4plex)	0,0000	1,334	3	6,1	0	1,943E-10	81	1,11967E-08	3	1699,95792	0,40	39,80	1
	High	gVLNVAAIk	7	1	1	P22034	N-Term(iTRAQ4plex); K9(iTRAQ4plex)	0,0000	1,289	4	15,8	0	3,134E-07	70	8,91189E-08	2	1172,76018	-0,32	30,41	0
	High	nVAAGANPVALR	6	1	1	P22034	N-Term(iTRAQ4plex)	0,0000	1,074	3	7,5	0	0,000007795	66	2,77952E-07	2	1296,75163	0,86	23,23	0
	High	vAADIIAk	2	1	1	P22034	N-Term(iTRAQ4plex); K8(iTRAQ4plex)	0,0000	0,993	1		0	0,000007363	63	6,52995E-07	2	1088,69170	-0,10	25,28	0
	High	vkEATGNQGYNVITGk	2	1	1	P22034	N-Term(iTRAQ4plex); K2(iTRAQ4plex); K16(iTRAQ4plex)	0,0000	1,434	1		0	4,027E-11	62	6,32368E-07	3	2111,18204	-0,04	22,66	1
	High	aAVEEGIVPGGGTTLIR	2	1	1	P22034	N-Term(iTRAQ4plex)	0,0000	1,038	2	4,1	0	1,856E-08	61	7,26056E-07	2	1784,00456	0,58	35,31	0
	High	sLEAGINALADAVR	2	1	1	P22034	N-Term(iTRAQ4plex)	0,0000	0,764	2	4,4	0	1,077E-08	59	1,1587E-06	2	1543,85173	-2,84	46,29	0
	High	lAGGVAVIk	11	2	2	Q05972;P22034	N-Term(iTRAQ4plex); K9(iTRAQ4plex)	0,0000				0	0,00004804	58	1,67483E-06	2	1115,73857	-0,47	27,62	0
	High	iEDALNATk	1	1	1	P22034	N-Term(iTRAQ4plex); K9(iTRAQ4plex)	0,0000	0,899	1		0	5,958E-07	57	2,3875E-06	2	1262,72063	0,91	25,04	0
	High	lIQEVASk	1	1	1	P22034	N-Term(iTRAQ4plex); K8(iTRAQ4plex)	0,0000	1,001	1		0	0,00002939	52	5,76727E-06	2	1175,72295	-0,75	26,27	0
	High	qYGAPQIVNDGITVAk	1	1	1	P22034	N-Term(iTRAQ4plex); K16(iTRAQ4plex)	0,0000	0,697	1		0	1,365E-08	51	7,74462E-06	3	1962,09098	0,54	34,21	0
	High	dNTILVAGADkR	2	1	1	P22034	N-Term(iTRAQ4plex); K11(iTRAQ4plex)	0,0000	0,904	2	22,2	0	4,453E-09	49	1,1722E-05	3	1560,89463	-0,14	19,92	1
	High	vGAATETELkDR	1	1	1	P22034	N-Term(iTRAQ4plex); K10(iTRAQ4plex)	0,0000	1,014	1		0	0,000000218	44	3,88124E-05	3	1577,87271	-0,67	18,55	1
	High	qLVEFDNPLILITDkk	3	1	1	P22034	N-Term(iTRAQ4plex); K15(iTRAQ4plex); K16(iTRAQ4plex	0,0000	1,116	3	15,2	0	0,000003704	43	4,9314E-05	3	2318,37302	1,47	48,80	1
	High	gYISPYFITDSDR	1	1	1	P22034	N-Term(iTRAQ4plex)	0,0000	0,884	1		0	0,000000332	41	7,17745E-05	2	1677,82488	0,46	38,62	0
	High	nVAAGANPVALRR	2	1	1	P22034	N-Term(iTRAQ4plex)	0,0000	2,240	1		0	0,0000847	40	9,48353E-05	3	1452,85205	0,29	19,00	1
	High	eYAASDSDYDkEk	1	1	1	P22034	N-Term(iTRAQ4plex); K11(iTRAQ4plex); K13(iTRAQ4plex	0,0000	1,038	1		0	8,413E-10	40	9,65984E-05	3	1952,94321	-0,90	15,99	1
	High	iEDLIAAGIIDPAk	2	1	1	P22034	N-Term(iTRAQ4plex); K14(iTRAQ4plex)	0,0000	0,587	2	8,0	0	0,000004888	40	0,000106162	3	1727,02036	0,56	47,60	0
	High	lRIEDALNATk	3	1	1	P22034	N-Term(iTRAQ4plex); K11(iTRAQ4plex)	0,0000	1,867	3	19,8	0	0,000004301	37	0,00020369	3	1531,90409	-0,38	29,55	1
	High	nVLLEk	1	1	1	P22034	N-Term(iTRAQ4plex); K6(iTRAQ4plex)	0,0000	0,661	1		0	0,006075	37	0,00021576	2	1003,63848	-0,55	22,20	0
	High	aTLEkDNTILVAGADkR	2	1	1	P22034	N-Term(iTRAQ4plex); K5(iTRAQ4plex); K16(iTRAQ4plex)	0,0000	2,341	1		0	3,554E-07	35	0,000293069	4	2247,30239	-0,42	24,69	2
	High	tkEIAGDGTTTATIIAQALVR	1	1	1	P22034	N-Term(iTRAQ4plex); K2(iTRAQ4plex)	0,0000	2,995	1		0	0,00000184	30	0,000911948	3	2418,38266	0,81	43,45	1
	High	aQLSNDEER	1	1	1	P22034	N-Term(iTRAQ4plex)	0,0000	1,072	1		0	0,0001609	30	0,001018521	2	1205,58806	0,13	11,56	0
	High	eATGNQGYNVITGk	1	1	1	P22034	N-Term(iTRAQ4plex); K14(iTRAQ4plex)	0,0000	0,914	1		0	0,00002493	27	0,002084347	3	1739,91574	-0,54	23,32	0
	High	lAGkIESFk	1	1	1	P22034	N-Term(iTRAQ4plex); K4(iTRAQ4plex); K9(iTRAQ4plex)	0,0000	1,702	1		0	0,004343	10	0,094399567	3	1424,88233	-1,00	30,40	1
	High	aPAFGDRR	1	1	1	P22034	N-Term(iTRAQ4plex)	0,0000	9,685	1		0,001	0,01921	21	0,008016227	3	1033,56525	-0,72	12,14	1
	High	lISFkDESR	2	1	1	P22034	N-Term(iTRAQ4plex); K5(iTRAQ4plex)	0,0000	1,166	2	34,2	0,001	0,01143	15	0,031186742	3	1382,78785	-0,28	24,82	1
	Medium	iTLGPk	2	1	1	P22034	N-Term(iTRAQ4plex); K6(iTRAQ4plex)	0,0000	0,925	2	48,2	0,012	0,156	30	0,000897367	2	916,60594	-1,15	21,39	0
	Medium	lISFk	1	1	1	P22034	N-Term(iTRAQ4plex); K5(iTRAQ4plex)	0,0000	0,805	1		0,048	0,586	16	0,026975531	2	895,58306	-2,76	28,73	0
P73093	Phycobilisome rod-core linker polypeptide CpcG OS=Synechocystis sp. (	1645,32	59,57	1	19	19	65	0,963	53	16,4	235	27,4	9,23							
	A3	Sequence	# PSMs	# Proteins	# Protein Groups	Protein Group Accessions	Modifications	ΔCn	117/114	117/114 Count	117/114 Variability [%]	q-Value	PEP	IonScore	Exp Value	Charge	MH+ [Da]	ΔM [ppm]	RT [min]	# Missed Cleavages
	High	vSAQNINIEASVPR	4	1	1	P73093	N-Term(iTRAQ4plex)	0,0000	0,972	4	6,8	0	7,367E-12	109	1,24157E-11	2	1641,90691	1,70	32,24	0
	High	kLTAGNPmNFLGMAR	2	1	1	P73093	N-Term(iTRAQ4plex); K1(iTRAQ4plex); M8(Oxidation)	0,0000	0,933	2	9,7	0	2,144E-10	74	4,30497E-08	3	1925,03702	1,56	32,24	1
	High	kLTAGNPmNFLGmAR	5	1	1	P73093	K1(iTRAQ4plex); N-Term(iTRAQ4plex); M8(Oxidation); M	0,0000	1,069	3	11,0	0	6,782E-12	69	1,34577E-07	3	1941,03238	1,78	28,05	1
	High	sINPAANTIPk	8	1	1	P73093	N-Term(iTRAQ4plex); K11(iTRAQ4plex)	0,0000	0,928	6	44,0	0	0,0000017	66	2,69135E-07	2	1413,83415	2,64	23,28	0
	High	qLGFPQIVWQNEVR	4	1	1	P73093	N-Term(iTRAQ4plex)	0,0000	0,970	3	10,5	0	2,981E-11	57	1,90533E-06	2	1858,01189	1,42	45,58	0
	High	lTAGNPmNFLGmAR	4	1	1	P73093	N-Term(iTRAQ4plex); M7(Oxidation); M12(Oxidation)	0,0000	0,906	4	7,4	0	0,00000558	55	3,19867E-06	2	1668,83440	1,49	33,79	0
	High	gLLLSNTFR	6	1	1	P73093	N-Term(iTRAQ4plex)	0,0000	1,072	3	1,6	0	0,00001793	53	4,68781E-06	2	1164,68694	1,01	35,76	0
	High	rQLGFPQIVWQNEVR	4	1	1	P73093	N-Term(iTRAQ4plex)	0,0000	1,066	2	2,9	0	4,875E-10	53	4,7863E-06	3	2014,11271	1,16	39,74	1
	High	kLTAGNPMNFLGMAR	1	1	1	P73093	N-Term(iTRAQ4plex); K1(iTRAQ4plex)	0,0000	1,196	1		0	0,000000262	52	6,109E-06	3	1909,04203	1,53	40,18	1
	High	eAGELPFNIk	2	1	1	P73093	N-Term(iTRAQ4plex); K10(iTRAQ4plex)	0,0000	0,933	2	12,0	0	0,00001121	44	4,13971E-05	2	1405,79509	1,50	34,99	0
	High	qIFFHAFk	6	1	1	P73093	N-Term(iTRAQ4plex); K8(iTRAQ4plex)	0,0000	0,934	4	12,1	0	0,0004684	38	0,000161797	2	1325,76311	1,68	32,53	0
	High	fVEHcVQk	1	1	1	P73093	N-Term(iTRAQ4plex); C5(Carbamidomethyl); K8(iTRAQ4p	0,0000	0,871	1		0	0,004046	35	0,000287058	2	1334,71391	0,73	16,18	0
	High	iAWSIVVATk	2	1	1	P73093	N-Term(iTRAQ4plex); K10(iTRAQ4plex)	0,0000	0,795	2	25,4	0	0,000178	35	0,00028772	3	1375,85620	0,74	40,93	0
	High	lTAGNPMNFLGMAR	1	1	1	P73093	N-Term(iTRAQ4plex)	0,0000	1,161	1		0	0,009654	32	0,000693378	2	1636,84514	1,87	45,45	0
	High	ekVLESQLR	2	1	1	P73093	N-Term(iTRAQ4plex); K2(iTRAQ4plex)	0,0000	1,356	2	2,4	0	0,0001877	28	0,001611348	3	1389,82926	-0,84	19,15	1
	High	nSFYEk	1	1	1	P73093	N-Term(iTRAQ4plex); K6(iTRAQ4plex)	0,0000	0,963	1		0	0,003665	24	0,003698026	2	1075,56792	1,56	16,53	0
	High	rFIPQEk	1	1	1	P73093	N-Term(iTRAQ4plex); K7(iTRAQ4plex)	0,0000	1,288	1		0	0,001917	23	0,00474242	3	1205,72550	0,83	16,52	1
	High	nGQITVR	2	1	1	P73093	N-Term(iTRAQ4plex)	0,0000	0,852	2	7,8	0,001	0,01863	50	1,07887E-05	2	931,54405	-0,13	13,15	0
	High	vLESQLR	3	1	1	P73093	N-Term(iTRAQ4plex)	0,0000	0,682	3	30,2	0,002	0,02226	43	5,39473E-05	2	988,59172	0,94	20,66	0
	High	yDAYHR	2	1	1	P73093	N-Term(iTRAQ4plex)	0,0000	0,761	2	0,4	0,007	0,0911	13	0,048080614	3	968,47245	1,86	11,22	0
	Medium	nLPGR	2	1	1	P73093	N-Term(iTRAQ4plex)	0,0000	0,884	2	3,6	0,015	0,1883	11	0,075852518	2	700,42357	1,86	11,59	0
	Medium	fIPQEk	2	1	1	P73093	N-Term(iTRAQ4plex); K6(iTRAQ4plex)	0,0000	0,649	2	87,0	0,016	0,1981	17	0,019407518	2	1049,62541	1,94	22,18	0
Q55460	Bicarbonate-binding protein CmpA OS=Synechocystis sp. (strain PCC 68	1603,03	43,81	1	19	21	68	0,739	51	28,2	452	49,4	5,77							
	A3	Sequence	# PSMs	# Proteins	# Protein Groups	Protein Group Accessions	Modifications	ΔCn	117/114	117/114 Count	117/114 Variability [%]	q-Value	PEP	IonScore	Exp Value	Charge	MH+ [Da]	ΔM [ppm]	RT [min]	# Missed Cleavages
	High	nRPEVVQIVSGR	7	1	1	Q55460	N-Term(iTRAQ4plex)	0,0000	0,754	4	12,3	0	1,321E-07	73	5,16381E-08	3	1497,86130	-0,37	23,62	0
	High	dGIGNVSYPYk	3	1	1	Q55460	N-Term(iTRAQ4plex); K11(iTRAQ4plex)	0,0000	0,683	2	22,4	0	5,982E-10	71	8,03471E-08	2	1500,79289	-0,53	27,63	0
	High	sHDLWFLTESIR	15	1	1	Q55460	N-Term(iTRAQ4plex)	0,0000	0,736	10	26,9	0	1,852E-09	68	1,64426E-07	2	1647,86199	0,49	42,99	0
	High	aAHTFPNVNQDFWIR	6	1	1	Q55460	N-Term(iTRAQ4plex)	0,0000	0,718	6	17,7	0	4,239E-11	66	2,56431E-07	3	1959,99479	0,06	37,89	0
	High	gVNLDITk	2	1	1	Q55460	N-Term(iTRAQ4plex); K8(iTRAQ4plex)	0,0000	0,553	1		0	0,000006544	58	1,65566E-06	2	1147,69133	-1,04	25,76	0
	High	yGMTGVEVSk	2	1	1	Q55460	N-Term(iTRAQ4plex); K10(iTRAQ4plex)	0,0000	0,806	2	72,7	0	2,814E-07	58	1,70204E-06	2	1358,72246	-0,29	25,48	0
	High	yGmTGVEVSk	3	1	1	Q55460	N-Term(iTRAQ4plex); M3(Oxidation); K10(iTRAQ4plex)	0,0000	0,531	3	18,0	0	6,651E-07	57	1,98596E-06	2	1374,71660	-0,85	19,59	0
	High	qANWASAR	3	1	1	Q55460	N-Term(iTRAQ4plex)	0,0000	0,815	3	4,7	0	0,0000376	54	4,14925E-06	2	1047,54485	-0,38	14,55	0
	High	gPLYWk	2	1	1	Q55460	N-Term(iTRAQ4plex); K6(iTRAQ4plex)	0,0000	0,440	2	66,6	0	0,0004365	51	8,53041E-06	2	1051,61797	0,08	29,87	0
	High	gQYTmGDGQPAIDDFQk	1	1	1	Q55460	N-Term(iTRAQ4plex); M5(Oxidation); K17(iTRAQ4plex)	0,0000	0,658	1		0	1,372E-10	50	9,3319E-06	3	2175,02579	-0,43	28,53	0
	High	nYFNVPTTILESPFk	1	1	1	Q55460	N-Term(iTRAQ4plex); K15(iTRAQ4plex)	0,0000	0,110	1		0	8,979E-07	41	7,29407E-05	3	2058,11588	0,40	50,17	0
	High	lGYIPIVEAAPLIIAQEk	1	1	1	Q55460	N-Term(iTRAQ4plex); K18(iTRAQ4plex)	0,0000	0,772	1		0	1,488E-07	32	0,000588803	3	2226,33487	-0,17	54,89	0
	High	nAIPDLDTAQk	1	1	1	Q55460	N-Term(iTRAQ4plex); K11(iTRAQ4plex)	0,0000	0,592	1		0	0,009174	30	0,00101202	3	1473,81241	-1,88	25,75	0
	High	eAATEAGFTADIPSSTSR	1	1	1	Q55460	N-Term(iTRAQ4plex)	0,0000	1,860	1		0	0,00002587	29	0,001156032	3	1954,94748	-0,02	30,25	0
	High	iIDkVNR	4	1	1	Q55460	N-Term(iTRAQ4plex); K4(iTRAQ4plex)	0,0000	0,875	4	3,8	0	0,004617	25	0,003901048	2	1145,72246	-1,78	16,67	1
	High	vNREDLWR	6	2	2	Q55460;P73452	N-Term(iTRAQ4plex)	0,0000				0	0,006727	20	0,009267658	3	1231,66605	-0,31	22,06	1
	High	aDWVDkNPk	3	1	1	Q55460	N-Term(iTRAQ4plex); K6(iTRAQ4plex); K9(iTRAQ4plex)	0,0000	0,430	3	62,0	0	0,000243	20	0,009289022	3	1504,84604	-1,59	18,99	1
	High	iIDkVNREDLWR	1	1	1	Q55460	N-Term(iTRAQ4plex); K4(iTRAQ4plex)	0,0000	1,309	1		0	0,0000798	12	0,072727363	4	1845,05666	-1,03	31,67	2
	High	aADYIk	1	1	1	Q55460	N-Term(iTRAQ4plex); K6(iTRAQ4plex)	0,0000	0,316	1		0,001	0,0119	38	0,000170204	2	968,56389	-1,68	16,68	0
	High	wGFHk	2	1	1	Q55460	N-Term(iTRAQ4plex); K5(iTRAQ4plex)	0,0000	0,658	2	9,2	0,009	0,1215	12	0,060529907	2	962,54363	-1,49	21,18	0
	Medium	eDLWR	1	2	2	Q55460;P73452	N-Term(iTRAQ4plex)	0,0000				0,025	0,309	13	0,053084778	2	862,45232	-1,91	22,24	0
	Medium	gFFAk	2	1	1	Q55460	N-Term(iTRAQ4plex); K5(iTRAQ4plex)	0,0000	0,832	2	0,7	0,028	0,345	16	0,022854409	2	857,50951	-3,33	22,69	0
P74689	Dihydroxy-acid dehydratase OS=Synechocystis sp. (strain PCC 6803 / Ka	1474,67	28,52	1	20	20	74	1,448	57	35,7	561	58,6	5,25							
	A3	Sequence	# PSMs	# Proteins	# Protein Groups	Protein Group Accessions	Modifications	ΔCn	117/114	117/114 Count	117/114 Variability [%]	q-Value	PEP	IonScore	Exp Value	Charge	MH+ [Da]	ΔM [ppm]	RT [min]	# Missed Cleavages
	High	gNLATEGSVAk	4	1	1	P74689	N-Term(iTRAQ4plex); K11(iTRAQ4plex)	0,0000	1,382	3	8,4	0	1,102E-08	70	1,41561E-07	2	1334,75041	-1,07	16,59	0
	High	vLVEAIkk	5	1	1	P74689	N-Term(iTRAQ4plex); K7(iTRAQ4plex); K8(iTRAQ4plex)	0,0000	2,823	5	31,2	0	0,00005552	67	1,80289E-07	2	1331,89726	-1,06	23,26	1
	High	kQILPSQILTR	3	1	1	P74689	N-Term(iTRAQ4plex); K1(iTRAQ4plex)	0,0000	1,602	2	30,3	0	3,308E-10	66	2,58803E-07	3	1585,00357	-0,25	29,07	1
	High	iQAGDVVVVR	2	1	1	P74689	N-Term(iTRAQ4plex)	0,0000	0,710	2	86,4	0	0,000000174	64	4,25569E-07	2	1199,72295	0,04	27,56	0
	High	rLLQLNISEEELAQR	2	1	1	P74689	N-Term(iTRAQ4plex)	0,0000	2,086	2	13,2	0	3,017E-11	64	4,25569E-07	3	1956,09885	-0,35	35,83	1
	High	nmPGAMIAMAR	2	1	1	P74689	N-Term(iTRAQ4plex); M2(Oxidation)	0,0000	1,434	2	15,2	0	0,00000402	61	7,11164E-07	2	1322,64971	-0,25	25,85	0
	High	yVTTNLHAAGGIPQVMk	8	1	1	P74689	N-Term(iTRAQ4plex); K17(iTRAQ4plex)	0,0000	1,378	5	35,4	0	9,416E-12	59	1,4494E-06	3	2088,15158	0,06	32,68	0
	High	lLQLNISEEELAQR	1	1	1	P74689	N-Term(iTRAQ4plex)	0,0000	1,117	1		0	6,5E-09	56	3,2502E-06	3	1799,99906	0,36	41,98	0
	High	sQVITQGTQR	5	1	1	P74689	N-Term(iTRAQ4plex)	0,0000	1,204	4	41,5	0	0,00001178	51	7,90624E-06	2	1261,69853	0,33	12,97	0
	High	kPVITGPAk	6	1	1	P74689	N-Term(iTRAQ4plex); K1(iTRAQ4plex); K9(iTRAQ4plex)	0,0000	1,877	3	35,2	0	6,935E-07	47	1,86196E-05	3	1342,87635	-1,43	15,56	0
	High	aDSTEESAk	1	1	1	P74689	N-Term(iTRAQ4plex); K9(iTRAQ4plex)	0,0000	1,829	1		0	0,00003287	47	1,9542E-05	2	1225,61431	-0,62	8,33	0
	High	yVTTNLHAAGGIPQVmk	5	1	1	P74689	N-Term(iTRAQ4plex); M16(Oxidation); K17(iTRAQ4plex)	0,0000	1,416	2	1,8	0	1,432E-07	46	2,60597E-05	3	2104,14695	0,27	30,27	0
	High	vLVEAIk	2	1	1	P74689	N-Term(iTRAQ4plex); K7(iTRAQ4plex)	0,0000	1,047	1		0	0,001206	45	2,97831E-05	2	1059,70134	-0,28	27,57	0
	High	qILPSQILTR	6	1	1	P74689	N-Term(iTRAQ4plex)	0,0000	1,617	5	4,3	0	1,046E-07	44	3,9443E-05	3	1312,80622	-0,55	35,47	0
	High	ySLVSR	3	1	1	P74689	N-Term(iTRAQ4plex)	0,0000	1,507	3	12,4	0	0,003081	38	0,000145871	2	868,50066	-0,27	19,95	0
	High	eVIADSIETVcNGQR	1	1	1	P74689	N-Term(iTRAQ4plex); C11(Carbamidomethyl)	0,0000	1,146	1		0	1,551E-07	34	0,000382798	3	1834,90830	-0,17	32,89	0
	High	nmPGAmIAmAR	3	1	1	P74689	N-Term(iTRAQ4plex); M2(Oxidation); M6(Oxidation); M9	0,0000	1,203	2	7,7	0	0,0003512	32	0,000662171	2	1354,64104	0,86	14,80	0
	High	nMPGAmIAmAR	3	1	1	P74689	N-Term(iTRAQ4plex); M6(Oxidation); M9(Oxidation)	0,0000	1,247	2	22,4	0	0,00000653	30	0,000916157	2	1338,64592	0,72	20,69	0
	High	aQWTPPQPR	3	1	1	P74689	N-Term(iTRAQ4plex)	0,0000	1,568	2	8,9	0	0,001286	30	0,000979422	2	1224,66008	-0,43	21,79	0
	High	vPVLcDLkPSGk	4	1	1	P74689	N-Term(iTRAQ4plex); C5(Carbamidomethyl); K8(iTRAQ4p	0,0000	1,146	4	17,5	0	0,00001987	27	0,001832188	3	1745,03482	-0,44	31,01	0
	High	hkVPVLcDLkPSGk	3	1	1	P74689	N-Term(iTRAQ4plex); K2(iTRAQ4plex); C7(Carbamidome	0,0000	2,006	3	9,6	0	1,506E-07	23	0,005495029	3	2154,28800	-1,64	22,61	1
	High	rAQWTPPQPR	1	1	1	P74689	N-Term(iTRAQ4plex)	0,0000	0,943	1		0	0,0008535	20	0,01018521	3	1380,76142	-0,23	17,33	1
	Medium	gILAk	1	1	1	P74689	N-Term(iTRAQ4plex); K5(iTRAQ4plex)	0,0000	1,402	1		0,039	0,4849	11	0,216354576	2	789,54045	-4,07	17,87	0
P72740	Dihydrolipoyl dehydrogenase OS=Synechocystis sp. (strain PCC 6803 / K	1443,92	42,19	1	18	18	57	1,392	45	42,5	474	50,8	5,55							
	A3	Sequence	# PSMs	# Proteins	# Protein Groups	Protein Group Accessions	Modifications	ΔCn	117/114	117/114 Count	117/114 Variability [%]	q-Value	PEP	IonScore	Exp Value	Charge	MH+ [Da]	ΔM [ppm]	RT [min]	# Missed Cleavages
	High	mmLAHAASGQGVVAVENIcGR	5	1	1	P72740	N-Term(iTRAQ4plex); M1(Oxidation); M2(Oxidation); C19	0,0000	1,901	3	34,0	0	1,051E-13	106	2,4602E-11	3	2347,14548	0,70	27,85	0
	High	mmLAHAASGQGVVAVENIcGR	3	1	1	P72740	N-Term(iTRAQ4plex); M2(Oxidation); C19(Carbamidomet	0,0000	1,375	2	22,6	0	1,454E-14	99	1,35198E-10	3	2331,14829	-0,28	32,57	0
	High	mMLAHAASGQGVVAVENIcGR	1	1	1	P72740	N-Term(iTRAQ4plex); C19(Carbamidomethyl)	0,0000	0,896	1		0	7,645E-16	83	5,1047E-09	3	2315,15439	0,16	34,35	0
	High	nLGLETVGVETDR	1	1	1	P72740	N-Term(iTRAQ4plex)	0,0000	0,801	1		0	0,000001205	70	9,93047E-08	2	1546,81975	0,24	31,04	0
	High	dGkPVPHLWAVGDATGk	11	1	1	P72740	N-Term(iTRAQ4plex); K3(iTRAQ4plex); K17(iTRAQ4plex)	0,0000	1,994	7	20,2	0	3,785E-12	66	2,44326E-07	3	2180,21793	-0,44	29,32	0
	High	tVFTSDEAVk	1	1	1	P72740	N-Term(iTRAQ4plex); K10(iTRAQ4plex)	0,0000	1,059	1		0	0,000002096	66	2,61198E-07	2	1384,75566	-0,43	23,94	0
	High	eAIAAHANDLVSk	4	1	1	P72740	N-Term(iTRAQ4plex); K13(iTRAQ4plex)	0,0000	1,261	2	40,5	0	9,645E-11	64	4,04548E-07	3	1626,90488	-0,32	23,02	0
	High	eGFVVSTAk	2	1	1	P72740	N-Term(iTRAQ4plex); K9(iTRAQ4plex)	0,0000	1,222	1		0	0,000004995	56	2,55841E-06	2	1225,70171	-1,13	22,47	0
	High	sRDIETYTGVFATk	2	1	1	P72740	N-Term(iTRAQ4plex); K14(iTRAQ4plex)	0,0000	1,855	2	44,6	0	3,006E-07	52	1,01887E-05	3	1876,00559	0,05	30,65	1
	High	rGFIEVNDQmQVIk	3	1	1	P72740	N-Term(iTRAQ4plex); M10(Oxidation); K14(iTRAQ4plex)	0,0000	1,640	3	0,8	0	3,865E-10	51	7,29407E-06	3	1981,07718	-0,40	26,94	1
	High	rGFIEVNDQMQVIk	2	1	1	P72740	N-Term(iTRAQ4plex); K14(iTRAQ4plex)	0,0000	1,434	2	6,3	0	2,565E-08	50	9,83943E-06	3	1965,08164	-0,73	32,66	1
	High	nLGLETVGVETDRR	1	1	1	P72740	N-Term(iTRAQ4plex)	0,0000	1,982	1		0	0,00001756	49	1,59923E-05	3	1702,92038	-0,07	27,26	1
	High	aLLAASGR	3	1	1	P72740	N-Term(iTRAQ4plex)	0,0000	1,285	3	14,2	0	0,004961	45	3,34497E-05	2	902,55278	-1,38	17,15	0
	High	tAIIEAk	3	1	1	P72740	N-Term(iTRAQ4plex); K7(iTRAQ4plex)	0,0000	1,023	2	46,1	0	0,0007529	45	3,13307E-05	2	1033,64714	-2,38	17,90	0
	High	aIPAAAFTHPEISYVGLTEAQAk	3	1	1	P72740	N-Term(iTRAQ4plex); K23(iTRAQ4plex)	0,0000	1,186	2	44,7	0	6,25E-09	40	0,000110408	4	2673,45058	0,55	41,29	0
	High	lkVDTIR	5	1	1	P72740	N-Term(iTRAQ4plex); K2(iTRAQ4plex)	0,0000	1,828	5	31,5	0	0,001264	28	0,001617968	2	1132,72710	-1,90	18,64	1
	High	sQDFDYDLVIIGAGVGGHGAALH/	1	1	1	P72740	N-Term(iTRAQ4plex); K26(iTRAQ4plex)	0,0000	1,653	1		0	7,276E-07	22	0,006251295	4	2898,53725	0,66	43,44	0
	High	kTEVDYR	2	1	1	P72740	N-Term(iTRAQ4plex); K1(iTRAQ4plex)	0,0000	1,062	2	0,7	0	0,00306	16	0,026789833	3	1198,66605	-0,82	10,57	1
	High	dIETYTGVFATk	1	1	1	P72740	N-Term(iTRAQ4plex); K12(iTRAQ4plex)	0,0000	0,786	1		0	0,01034	12	0,057540019	3	1632,87137	-0,60	35,86	0
	High	iPATk	2	1	1	P72740	N-Term(iTRAQ4plex); K5(iTRAQ4plex)	0,0000	1,106	2	0,7	0,007	0,09071	19	0,012472974	2	817,53655	-2,48	14,03	0
	High	gcIPSk	1	1	1	P72740	N-Term(iTRAQ4plex); C2(Carbamidomethyl); K6(iTRAQ4p	0,0000	0,931	1		0,007	0,09129	10	0,106095714	2	949,53691	-1,07	13,54	0
Q55544	Phycobiliprotein ApcE OS=Synechocystis sp. (strain PCC 6803 / Kazusa)	1419,46	35,83	1	32	32	72	0,909	64	14,0	896	100,2	9,23							
	A3	Sequence	# PSMs	# Proteins	# Protein Groups	Protein Group Accessions	Modifications	ΔCn	117/114	117/114 Count	117/114 Variability [%]	q-Value	PEP	IonScore	Exp Value	Charge	MH+ [Da]	ΔM [ppm]	RT [min]	# Missed Cleavages
	High	gPAVNNQVGNPSAVGEFPGSLGA	1	1	1	Q55544	N-Term(iTRAQ4plex); K24(iTRAQ4plex)	0,0000	0,630	1		0	1,526E-13	67	2,18258E-07	3	2555,34995	1,65	34,84	0
	High	eIQQYNQILASQGLk	1	1	1	Q55544	N-Term(iTRAQ4plex); K15(iTRAQ4plex)	0,0000	0,571	1		0	8,43E-11	66	2,66667E-07	3	2021,12967	1,32	36,68	0
	High	fGESSTQALIR	2	1	1	Q55544	N-Term(iTRAQ4plex)	0,0000	0,822	2	6,4	0	0,000004754	65	3,09008E-07	2	1352,73039	0,96	27,73	0
	High	fVELGQVSAIR	3	1	1	Q55544	N-Term(iTRAQ4plex)	0,0000	0,841	3	12,4	0	0,000003	63	4,48745E-07	2	1362,78728	0,77	37,20	0
	High	iFTGGSPLSYLEkPVER	7	1	1	Q55544	N-Term(iTRAQ4plex); K13(iTRAQ4plex)	0,0000	0,901	5	2,0	0	1,762E-10	62	6,12308E-07	3	2181,21878	1,35	43,92	0
	High	lSNNEINVk	2	1	1	Q55544	N-Term(iTRAQ4plex); K9(iTRAQ4plex)	0,0000	0,772	2	13,1	0	8,279E-07	61	7,53304E-07	2	1318,75884	1,46	21,86	0
	High	lNGGLPGAk	2	1	1	Q55544	N-Term(iTRAQ4plex); K9(iTRAQ4plex)	0,0000	0,897	1		0	0,00005228	57	1,86625E-06	2	1114,68315	0,76	21,05	0
	High	aYSQSISYLESQVR	1	1	1	Q55544	N-Term(iTRAQ4plex)	0,0000	0,362	1		0	4,328E-08	56	2,42086E-06	3	1774,91123	1,13	39,07	0
	High	qFFEPFINSR	2	1	1	Q55544	N-Term(iTRAQ4plex)	0,0000	0,879	2	4,9	0	0,0003851	52	6,17974E-06	2	1428,74126	1,40	40,62	0
	High	qPALVGASSDSR	1	1	1	Q55544	N-Term(iTRAQ4plex)	0,0000	0,760	1		0	0,0008145	50	9,39658E-06	2	1331,70610	1,87	16,78	0
	High	yLTPGGLQmR	4	1	1	Q55544	N-Term(iTRAQ4plex); M9(Oxidation)	0,0000	0,813	4	6,9	0	0,00008079	46	2,44326E-05	3	1295,68998	0,08	26,38	0
	High	sELFLk	2	1	1	Q55544	N-Term(iTRAQ4plex); K6(iTRAQ4plex)	0,0000	0,686	2	62,3	0	0,00595	45	3,56774E-05	2	1024,62920	1,04	27,03	0
	High	aASADYFR	1	1	1	Q55544	N-Term(iTRAQ4plex)	0,0000	0,834	1		0	0,00005097	44	3,56427E-05	2	1044,52422	1,07	19,78	0
	High	rFPTLPAANFPNTER	2	1	1	Q55544	N-Term(iTRAQ4plex)	0,0000	0,975	2	5,4	0	4,751E-07	43	5,17571E-05	3	1875,00052	0,59	33,07	1

	High	yAmkPGLSALEk	3	1	1	Q55544	N-Term(iTRAQ4plex); M3(Oxidation); K4(iTRAQ4plex); K	0,0000	0,990	2	2,3	0	0,000001477	41	7,79776E-05	3	1756,00522	0,72	25,33	0
	High	lYWAPHYVck	2	1	1	Q55544	N-Term(iTRAQ4plex); C9(Carbamidomethyl); K10(iTRAQ	0,0000	0,843	2	21,4	0	0,00000026	41	8,51079E-05	3	1624,85556	0,44	32,09	0
	High	kQFFEPFINSR	4	1	1	Q55544	N-Term(iTRAQ4plex); K1(iTRAQ4plex)	0,0000	1,052	4	15,3	0	3,773E-08	38	0,00015921	3	1700,93845	1,27	35,21	1
	High	aLELAFR	4	1	1	Q55544	N-Term(iTRAQ4plex)	0,0000	0,916	4	12,0	0	0,007192	35	0,000359072	2	963,57567	1,29	32,58	0
	High	aIEYmHR	3	1	1	Q55544	N-Term(iTRAQ4plex); M5(Oxidation)	0,0000	0,943	3	22,5	0	0,0001459	34	0,000444601	2	1079,54167	-0,73	12,11	0
	High	qDkELVVPSFTPVVk	1	1	1	Q55544	N-Term(iTRAQ4plex); K3(iTRAQ4plex); K15(iTRAQ4plex)	0,0000	0,554	1		0	0,000001148	32	0,000563599	3	2118,25760	1,93	35,89	1
	High	lVSTYDk	1	1	1	Q55544	N-Term(iTRAQ4plex); K7(iTRAQ4plex)	0,0000	0,913	1		0	0,004075	30	0,000901509	2	1113,64092	1,36	19,46	0
	High	sVFSSTGPIPPGFRPINIAR	5	1	1	Q55544	N-Term(iTRAQ4plex)	0,0000	1,000	5	2,8	0	0,000006915	29	0,001177525	3	2257,26029	1,26	42,05	0
	High	sLRDmSWFLR	1	1	1	Q55544	N-Term(iTRAQ4plex); M5(Oxidation)	0,0000	2,086	1		0	0,001743	25	0,002930691	3	1470,76453	0,05	33,88	1
	High	eYSDAFGEDTVPYER	1	1	1	Q55544	N-Term(iTRAQ4plex)	0,0000	1,160	1		0	2,393E-08	22	0,006530854	2	1921,85918	0,99	32,68	0
	High	yAMkPGLSALEk	1	1	1	Q55544	N-Term(iTRAQ4plex); K4(iTRAQ4plex); K12(iTRAQ4plex)	0,0000	0,846	1		0	0,000001579	21	0,007128038	3	1740,01316	2,37	29,16	0
	High	lLGRPTYGR	4	1	1	Q55544	N-Term(iTRAQ4plex)	0,0000	0,969	2	9,6	0	0,01016	19	0,013365032	3	1176,69779	0,67	19,45	0
	High	rPAPFNk	1	1	1	Q55544	N-Term(iTRAQ4plex); K7(iTRAQ4plex)	0,0000	0,970	1		0	0,008447	13	0,048191451	3	1117,67282	0,68	13,75	0
	High	dMSWFLR	1	1	1	Q55544	N-Term(iTRAQ4plex)	0,0000	0,854	1		0,001	0,01693	17	0,021328976	2	1098,55266	0,31	41,17	0
	High	aPTPANk	4	1	1	Q55544	N-Term(iTRAQ4plex); K7(iTRAQ4plex)	0,0000	0,994	2	18,6	0,001	0,01645	11	0,07463972	2	986,58714	-0,19	9,76	0
	High	vDkEVTPR	2	1	1	Q55544	N-Term(iTRAQ4plex); K3(iTRAQ4plex)	0,0000	0,943	2	4,2	0,001	0,02049	10	0,096827786	3	1231,72684	1,58	12,18	1
	High	rLLGRPTYGR	1	1	1	Q55544	N-Term(iTRAQ4plex)	0,0000	0,916	1		0,006	0,07268	11	0,081652597	4	1332,79946	1,01	14,68	1
	Medium	yGPSNmQk	1	1	1	Q55544	N-Term(iTRAQ4plex); M6(Oxidation); K8(iTRAQ4plex)	0,0000	1,029	1		0,012	0,1478	21	0,008317063	2	1228,62444	0,80	11,30	0
	Medium	aPLNQk	1	1	1	Q55544	N-Term(iTRAQ4plex); K6(iTRAQ4plex)	0,0000	0,829	1		0,022	0,2676	21	0,008452205	2	958,59282	0,43	11,90	0
P72871	S-adenosylmethionine synthase OS=Synechocystis sp. (strain PCC 6803	1390,43	25,59	1	13	13	53	1,266	42	20,0	426	45,8	5,29							
	A3	Sequence	# PSMs	# Proteins	# Protein Groups	Protein Group Accessions	Modifications	ΔCn	117/114	117/114 Count	117/114 Variability [%]	q-Value	PEP	IonScore	Exp Value	Charge	MH+ [Da]	ΔM [ppm]	RT [min]	# Missed Cleavages
	High	fVVGGPQGDAGLTGR	2	1	1	P72871	N-Term(iTRAQ4plex)	0,0000	1,239	2	3,3	0	2,772E-08	85	2,96463E-09	2	1574,84258	1,13	30,30	0
	High	fYQDVAAYGHFGR	13	1	1	P72871	N-Term(iTRAQ4plex)	0,0000	1,184	10	8,0	0	2,618E-10	82	5,95621E-09	2	1674,81511	0,34	34,96	0
	High	kIIVDTYGGYSR	7	1	1	P72871	N-Term(iTRAQ4plex); K1(iTRAQ4plex)	0,0000	1,467	5	9,2	0	6,93E-08	72	7,76571E-08	3	1659,93088	0,01	24,09	1
	High	aAFASSPQAVAV	1	1	1	P72871	N-Term(iTRAQ4plex)	0,0000	1,631	1		0	0,00002672	56	2,49442E-06	2	1262,68608	-0,07	32,18	0
	High	fIVNPTGk	6	1	1	P72871	N-Term(iTRAQ4plex); K8(iTRAQ4plex)	0,0000	1,522	3	0,8	0	0,00002497	52	6,93728E-06	2	1163,70293	0,20	26,89	0
	High	sGQLAYLRPDGk	4	1	1	P72871	N-Term(iTRAQ4plex); K12(iTRAQ4plex)	0,0000	1,392	3	22,8	0	6,098E-10	47	2,19771E-05	3	1592,90165	1,09	22,83	0
	High	kSGQLAYLRPDGk	6	1	1	P72871	N-Term(iTRAQ4plex); K1(iTRAQ4plex); K13(iTRAQ4plex)	0,0000	1,630	4	21,6	0	1,462E-10	38	0,00017578	3	1865,09598	-0,51	19,69	1
	High	hGGGAFSGk	2	1	1	P72871	N-Term(iTRAQ4plex); K9(iTRAQ4plex)	0,0000	1,492	2	12,9	0	0,0001497	33	0,000488619	2	1105,59770	-1,44	10,85	0
	High	sAAYAAR	3	1	1	P72871	N-Term(iTRAQ4plex)	0,0000	1,035	3	0,1	0	0,003742	33	0,000509296	2	853,46495	0,11	10,92	0
	High	iIVDTYGGYSR	2	1	1	P72871	N-Term(iTRAQ4plex)	0,0000	0,982	2	7,0	0	0,0004809	27	0,001972287	2	1387,73479	0,70	30,24	0
	High	hGGGAFSGkDPTk	3	1	1	P72871	N-Term(iTRAQ4plex); K9(iTRAQ4plex); K13(iTRAQ4plex)	0,0000	1,577	3	6,3	0	0,0006678	19	0,014467473	4	1690,92360	-0,07	11,66	1
	Medium	nLPAER	3	1	1	P72871	N-Term(iTRAQ4plex)	0,0000	1,265	3	6,2	0,012	0,1542	23	0,00468781	2	843,48052	0,00	12,13	0
	Medium	nIVAAGLADk	1	1	1	P72871	N-Term(iTRAQ4plex); K10(iTRAQ4plex)	0,0000	0,757	1		0,016	0,1964	27	0,001936288	3	1259,75580	-0,32	27,38	0
P77972	Enolase OS=Synechocystis sp. (strain PCC 6803 / Kazusa) GN=eno PE=	1356,22	34,26	1	13	13	53	1,011	38	19,1	432	46,5	5,15							
	A3	Sequence	# PSMs	# Proteins	# Protein Groups	Protein Group Accessions	Modifications	ΔCn	117/114	117/114 Count	117/114 Variability [%]	q-Value	PEP	IonScore	Exp Value	Charge	MH+ [Da]	ΔM [ppm]	RT [min]	# Missed Cleavages
	High	sGETEDTTIADLAVATR	2	1	1	P77972	N-Term(iTRAQ4plex)	0,0000	0,984	2	2,0	0	3,026E-10	102	6,35287E-11	2	1893,95268	0,22	34,54	0
	High	wGAEVFAVLGk	5	1	1	P77972	N-Term(iTRAQ4plex); K11(iTRAQ4plex)	0,0000	0,897	5	26,8	0	6,314E-09	76	2,38215E-08	2	1464,84648	0,76	48,87	0
	High	lESGAHGIAQVPSGASTGSFEAHE	7	1	1	P77972	N-Term(iTRAQ4plex)	0,0000	0,836	4	19,6	0	9,644E-08	69	1,12972E-07	4	2752,37807	0,32	30,75	0
	High	aAAAELAIPLYR	10	1	1	P77972	N-Term(iTRAQ4plex)	0,0000	1,129	6	12,5	0	1,714E-09	69	1,23871E-07	2	1402,81865	0,80	38,29	0
	High	tQLVGDDLmVTNPVR	1	1	1	P77972	N-Term(iTRAQ4plex); M9(Oxidation)	0,0000	0,867	1		0	5,599E-09	66	2,31723E-07	2	1817,95659	0,96	33,52	0
	High	vPATIEEIAAR	1	1	1	P77972	N-Term(iTRAQ4plex)	0,0000	0,974	1		0	0,00001382	57	2,07955E-06	2	1313,75530	0,53	34,75	0
	High	gLTTSLGTk	6	1	1	P77972	N-Term(iTRAQ4plex); K9(iTRAQ4plex)	0,0000	1,330	3	1,5	0	0,0002551	51	8,39402E-06	2	1165,70342	0,28	20,85	0
	High	tQLVGDDLMVTNPVR	1	1	1	P77972	N-Term(iTRAQ4plex)	0,0000	1,170	1		0	0,000007094	46	2,32258E-05	3	1801,95957	-0,20	36,84	0
	High	iEDELGDR	1	1	1	P77972	N-Term(iTRAQ4plex)	0,0000	1,097	1		0	0,000009274	44	3,72366E-05	2	1090,54997	0,22	22,10	0
	High	gRPTIEAEVR	12	1	1	P77972	N-Term(iTRAQ4plex)	0,0000	1,056	8	9,1	0	0,00003013	44	4,33481E-05	3	1271,71872	-0,12	15,20	0
	High	kELGANAILGVSLATAk	1	1	1	P77972	N-Term(iTRAQ4plex); K1(iTRAQ4plex); K17(iTRAQ4plex)	0,0000	0,804	1		0	4,901E-07	38	0,000143209	3	2088,27683	0,72	36,61	1
	High	eLGANAILGVSLATAk	1	1	1	P77972	N-Term(iTRAQ4plex); K16(iTRAQ4plex)	0,0000	0,768	1		0	0,00001882	34	0,000370681	3	1816,07846	0,09	42,18	0
	Medium	iGLGPk	2	1	1	P77972	N-Term(iTRAQ4plex); K6(iTRAQ4plex)	0,0000	1,176	1		0,015	0,1923	10	0,0920386	2	872,58153	0,85	19,00	0
	Medium	aVYAPk	3	1	1	P77972	N-Term(iTRAQ4plex); K6(iTRAQ4plex)	0,0000	0,908	3	2,4	0,016	0,1996	29	0,001285198	2	936,57414	-1,65	14,48	0
P73320	50S ribosomal protein L3 OS=Synechocystis sp. (strain PCC 6803 / Kazu	1277,04	46,95	1	11	11	53	2,467	34	70,0	213	22,7	10,13							
	A3	Sequence	# PSMs	# Proteins	# Protein Groups	Protein Group Accessions	Modifications	ΔCn	117/114	117/114 Count	117/114 Variability [%]	q-Value	PEP	IonScore	Exp Value	Charge	MH+ [Da]	ΔM [ppm]	RT [min]	# Missed Cleavages
	High	mAGQYGASQVTVR	4	1	1	P73320	N-Term(iTRAQ4plex)	0,0000	2,112	4	150,4	0	2,192E-10	88	1,41579E-09	2	1511,77593	0,13	22,98	0
	High	mAGQYGASQVTVR	6	1	1	P73320	N-Term(iTRAQ4plex); M1(Oxidation)	0,0000	2,293	4	71,0	0	4,198E-10	84	4,01763E-09	2	1527,77056	-0,06	19,15	0
	High	tQDTDGYNAIQVGFLPVk	2	1	1	P73320	N-Term(iTRAQ4plex); K18(iTRAQ4plex)	0,0000	2,222	1		0	2,211E-11	69	1,41641E-07	3	2254,19547	-0,16	42,50	0
	High	lPGSTGAGTTPGR	6	1	1	P73320	N-Term(iTRAQ4plex)	0,0000	1,910	4	107,4	0	0,000001461	67	2,20785E-07	2	1315,70915	0,34	17,73	0
	High	sIGILGTk	6	1	1	P73320	N-Term(iTRAQ4plex); K8(iTRAQ4plex)	0,0000	2,816	3	11,4	0	0,0001133	54	3,9443E-06	2	1076,68889	-2,70	26,20	0
	High	gFAGYQk	8	1	1	P73320	N-Term(iTRAQ4plex); K7(iTRAQ4plex)	0,0000	2,332	5	165,8	0	0,0005	47	1,98596E-05	2	1058,58501	-2,18	18,00	0
	High	rmAGQYGASQVTVR	2	1	1	P73320	N-Term(iTRAQ4plex); M2(Oxidation)	0,0000	1,188	1		0	0,000001299	44	4,13019E-05	3	1683,87229	0,31	14,58	1
	High	gALPGkPGTLLNITPAk	7	1	1	P73320	N-Term(iTRAQ4plex); K6(iTRAQ4plex); K17(iTRAQ4plex)	0,0000	2,808	4	45,1	0	2,929E-07	38	0,000163294	3	2080,28263	-1,38	33,20	0
	High	aLSkPELGHLk	4	1	1	P73320	N-Term(iTRAQ4plex); K4(iTRAQ4plex); K11(iTRAQ4plex)	0,0000	2,887	4	6,4	0	3,248E-07	24	0,003588971	3	1625,00736	-2,29	23,66	0
	High	aLSkPELGHLkk	2	1	1	P73320	N-Term(iTRAQ4plex); K4(iTRAQ4plex); K11(iTRAQ4plex)	0,0000	3,802	2	121,3	0	0,000004858	18	0,014222306	3	1897,20005	-4,25	21,11	1
	High	nLLIIk	5	1	1	P73320	N-Term(iTRAQ4plex); K6(iTRAQ4plex)	0,0000	1,080	1		0,004	0,04882	31	0,000717745	2	1001,69383	-2,31	31,18	0
	High	lTVVR	1	1	1	P73320	N-Term(iTRAQ4plex)	0,0000	0,951	1		0,008	0,1079	13	0,045182474	2	731,48815	-2,01	17,73	0
Q55664	Fructose-bisphosphate aldolase class 2 OS=Synechocystis sp. (strain PC	1253,22	38,44	1	12	12	48	1,088	40	18,6	359	38,9	5,74							
	A3	Sequence	# PSMs	# Proteins	# Protein Groups	Protein Group Accessions	Modifications	ΔCn	117/114	117/114 Count	117/114 Variability [%]	q-Value	PEP	IonScore	Exp Value	Charge	MH+ [Da]	ΔM [ppm]	RT [min]	# Missed Cleavages
	High	tQVDALAVAIGTSHGAYk	9	1	1	Q55664	N-Term(iTRAQ4plex); K18(iTRAQ4plex)	0,0000	1,068	7	8,9	0	4,074E-15	88	1,87461E-09	3	2090,15274	2,04	35,73	0
	High	yQQFWTAGNASk	5	1	1	Q55664	N-Term(iTRAQ4plex); K12(iTRAQ4plex)	0,0000	1,144	5	13,9	0	1,267E-10	81	7,88806E-09	2	1688,86589	1,41	30,84	0
	High	kPTGEVLAISR	7	1	1	Q55664	N-Term(iTRAQ4plex); K1(iTRAQ4plex)	0,0000	1,391	5	4,6	0	5,204E-09	74	4,17802E-08	3	1458,89060	1,58	21,78	0
	High	sYAGENFLR	3	1	1	Q55664	N-Term(iTRAQ4plex)	0,0000	1,099	3	4,5	0	0,00000118	53	4,61286E-06	2	1200,61382	0,69	27,42	0
	High	lAITAAFR	7	1	1	Q55664	N-Term(iTRAQ4plex)	0,0000	1,418	4	4,8	0	0,00006073	50	9,0985E-06	2	1006,61730	0,67	33,68	0
	High	kVNIDTDNR	5	1	1	Q55664	N-Term(iTRAQ4plex); K1(iTRAQ4plex)	0,0000	1,148	5	13,4	0	0,000006646	46	2,82429E-05	3	1362,75895	0,70	12,89	1
	High	qLTLDDYAAk	1	1	1	Q55664	N-Term(iTRAQ4plex); K10(iTRAQ4plex)	0,0000	0,965	1		0	0,000007277	45	2,97146E-05	2	1425,78374	0,66	28,26	0
	High	hFLkPSIk	1	1	1	Q55664	N-Term(iTRAQ4plex); K4(iTRAQ4plex); K8(iTRAQ4plex)	0,0000	0,746	1		0	0,0001202	20	0,010470562	3	1401,89618	1,39	18,69	0
	High	hLVLGAVETYPHIPIAmHQDHGN	2	1	1	Q55664	N-Term(iTRAQ4plex); M17(Oxidation); C28(Carbamidom	0,0000	0,891	2	10,5	0	0,00000491	19	0,011323222	5	3815,88880	0,53	35,76	0
	High	hLVLGAVETYPHIPIAMHQDHGN	2	1	1	Q55664	N-Term(iTRAQ4plex); C28(Carbamidomethyl)	0,0000	0,991	2	13,8	0	9,248E-07	17	0,018965749	6	3799,89050	-0,36	37,52	0
	High	iEEIHR	2	1	1	Q55664	N-Term(iTRAQ4plex)	0,0000	0,706	2	73,9	0	0,0076	15	0,032882892	3	940,53412	0,90	13,29	0
	High	gELTATSR	1	1	1	Q55664	N-Term(iTRAQ4plex)	0,0000	1,385	1		0,001	0,01541	17	0,018364115	2	978,53264	-1,06	12,24	0
	High	aLVPmR	3	1	1	Q55664	N-Term(iTRAQ4plex); M5(Oxidation)	0,0000	1,025	2	1,5	0,005	0,06179	12	0,066676072	2	846,49919	0,44	15,21	0
P74008	Adenosylhomocysteinase OS=Synechocystis sp. (strain PCC 6803 / Kazu	1177,57	38,35	1	15	15	44	2,889	35	40,3	425	46,2	5,77							
	A3	Sequence	# PSMs	# Proteins	# Protein Groups	Protein Group Accessions	Modifications	ΔCn	117/114	117/114 Count	117/114 Variability [%]	q-Value	PEP	IonScore	Exp Value	Charge	MH+ [Da]	ΔM [ppm]	RT [min]	# Missed Cleavages
	High	qHQLSDIIGTTEETTGIVR	4	1	1	P74008	N-Term(iTRAQ4plex)	0,0000	1,809	3	97,1	0	3,031E-17	129	1,30317E-13	3	2343,23014	1,20	40,12	0
	High	tIVVAGYGWcGk	4	1	1	P74008	N-Term(iTRAQ4plex); C10(Carbamidomethyl); K12(iTRAC	0,0000	3,455	4	24,7	0	1,118E-10	83	4,83025E-09	2	1598,86199	1,02	34,25	0
	High	yGTGQSTLDGIIR	3	1	1	P74008	N-Term(iTRAQ4plex)	0,0000	2,889	3	28,6	0	3,798E-08	80	1,01202E-08	2	1524,81511	0,80	33,22	0
	High	amFNDGVLTFPAmNVNDADTk	1	1	1	P74008	N-Term(iTRAQ4plex); M2(Oxidation); M13(Oxidation); K2	0,0000	2,501	1		0	2,405E-13	77	2,0369E-08	3	2591,23789	0,68	38,56	0
	High	nkGQLEPGMHSIPVEVDQEIAR	1	1	1	P74008	N-Term(iTRAQ4plex); K2(iTRAQ4plex)	0,0000	1,949	1		0	2,159E-10	63	4,87741E-07	4	2735,43667	-0,85	31,76	1
	High	aTNILLAGk	7	1	1	P74008	N-Term(iTRAQ4plex); K9(iTRAQ4plex)	0,0000	3,256	4	19,6	0	0,000002801	63	5,0579E-07	2	1188,75688	1,19	27,51	0
	High	nkGQLEPGmHSIPVEVDQEIAR	1	1	1	P74008	N-Term(iTRAQ4plex); K2(iTRAQ4plex); M9(Oxidation)	0,0000	1,612	1		0	7,197E-12	53	4,66627E-06	4	2751,43447	0,21	28,95	1
	High	dISLAPQGR	1	1	1	P74008	N-Term(iTRAQ4plex)	0,0000	1,724	1		0	0,0001474	43	4,7203E-05	2	1100,61833	0,23	20,45	0
	High	fAQEkPFAGIR	4	1	1	P74008	N-Term(iTRAQ4plex); K5(iTRAQ4plex)	0,0000	3,728	4	2,5	0	6,809E-08	42	8,16923E-05	2	1551,88982	0,77	27,10	0
	High	gQLEPGmHSIPVEVDQEIAR	1	1	1	P74008	N-Term(iTRAQ4plex); M7(Oxidation)	0,0000	2,264	1		0	6,855E-10	38	0,00014157	3	2365,19565	0,72	31,91	0
	High	sIIVIGEGR	4	1	1	P74008	N-Term(iTRAQ4plex)	0,0000	3,117	2	3,4	0	0,001406	36	0,000266667	2	1087,65972	0,45	28,01	0
	High	hFYDNR	4	1	1	P74008	N-Term(iTRAQ4plex)	0,0000	1,842	3	61,6	0	0,0002305	27	0,002041597	3	995,48151	-0,04	14,28	0
	High	hVIRPEHFAVmk	2	1	1	P74008	N-Term(iTRAQ4plex); M11(Oxidation); K12(iTRAQ4plex)	0,0000	1,975	2	57,0	0	0,00000949	25	0,003396018	4	1767,99428	0,66	17,91	0
	High	sLkEQAk	4	1	1	P74008	N-Term(iTRAQ4plex); K3(iTRAQ4plex); K7(iTRAQ4plex)	0,0000	3,563	2	1,3	0	0,005774	20	0,011040024	3	1235,76780	-0,47	9,45	1
	High	hVIRPEHFAVMk	1	1	1	P74008	N-Term(iTRAQ4plex); K12(iTRAQ4plex)	0,0000	3,841	1		0	0,0002962	13	0,053084778	4	1751,99941	0,69	20,38	0
	High	iEWAAR	1	1	1	P74008	N-Term(iTRAQ4plex)	0,0000	1,399	1		0,003	0,04004	23	0,004988845	2	889,50084	-0,46	23,61	0
	Medium	vATPVk	1	1	1	P74008	N-Term(iTRAQ4plex); K6(iTRAQ4plex)	0,0000	2,248	1		0,012	0,1511	27	0,002027543	2	902,59093	-0,46	14,60	0
P74551	Allophycocyanin subunit beta-18 OS=Synechocystis sp. (strain PCC 680	1136,28	68,05	1	15	16	50	1,127	36	24,1	169	18,9	5,07							
	A3	Sequence	# PSMs	# Proteins	# Protein Groups	Protein Group Accessions	Modifications	ΔCn	117/114	117/114 Count	117/114 Variability [%]	q-Value	PEP	IonScore	Exp Value	Charge	MH+ [Da]	ΔM [ppm]	RT [min]	# Missed Cleavages
	High	iAAAmINANSATIVk	6	1	1	P74551	N-Term(iTRAQ4plex); M6(Oxidation); K16(iTRAQ4plex)	0,0000	0,888	5	19,7	0	1,56E-11	109	1,21331E-11	2	1863,06450	1,73	31,60	0
	High	eTYNSLGVPIGPTVR	6	1	1	P74551	N-Term(iTRAQ4plex)	0,0000	1,165	4	7,9	0	3,279E-10	105	3,30347E-11	2	1746,95317	1,40	34,61	0
	High	dAVTTLIk	1	1	1	P74551	N-Term(iTRAQ4plex); K8(iTRAQ4plex)	0,0000	0,771	1		0	0,000003353	58	1,67869E-06	2	1148,71306	0,12	26,86	0
	High	rFSAcLR	2	1	1	P74551	N-Term(iTRAQ4plex); C5(Carbamidomethyl)	0,0000	1,336	2	3,4	0	0,0006868	49	1,25017E-05	3	1053,57566	1,16	15,83	1
	High	aYFESGSAR	4	1	1	P74551	N-Term(iTRAQ4plex)	0,0000	1,231	3	3,1	0	0,00001464	43	4,48714E-05	2	1131,55559	0,40	17,24	0
	High	mRDAVTTLIk	3	1	1	P74551	N-Term(iTRAQ4plex); M1(Oxidation); K10(iTRAQ4plex)	0,0000	1,677	3	19,7	0	0,00000995	37	0,00020416	3	1451,85303	2,46	21,49	1
	High	dmDYYLR	3	2	2	P74551;Q01951	N-Term(iTRAQ4plex); M2(Oxidation)	0,0000				0	0,003725	36	0,00025408	2	1135,52165	0,53	20,78	0
	High	nYDLTGR	2	1	1	P74551	N-Term(iTRAQ4plex)	0,0000	0,939	2	20,9	0	0,004578	33	0,000508124	2	982,50823	0,80	16,75	0
	High	dMDYYLR	1	2	2	P74551;Q01951	N-Term(iTRAQ4plex)	0,0000				0	0,00333	32	0,000668298	2	1119,52739	1,12	29,51	0
	High	iAAAmINANSATIVkR	2	1	1	P74551	N-Term(iTRAQ4plex); M6(Oxidation); K16(iTRAQ4plex)	0,0000	1,290	2	61,0	0	0,001175	31	0,00088914	3	2019,16385	0,72	28,22	1
	High	yASYALIAADNNVLDER	1	1	1	P74551	N-Term(iTRAQ4plex)	0,0000	0,948	1		0	9,325E-07	28	0,00154871	3	2042,03318	0,99	42,48	0
	High	rDAVTTLIk	1	1	1	P74551	N-Term(iTRAQ4plex); K9(iTRAQ4plex)	0,0000	1,123	1		0	0,000001967	27	0,002046303	3	1304,81513	0,83	22,07	1
	High	gIQIMk	3	1	1	P74551	N-Term(iTRAQ4plex); K6(iTRAQ4plex)	0,0000	1,127	2	26,5	0	0,006362	26	0,002524581	2	977,60588	0,26	23,03	0
	High	gIQImk	6	1	1	P74551	N-Term(iTRAQ4plex); M5(Oxidation); K6(iTRAQ4plex)	0,0000	1,139	3	11,5	0	0,005306	25	0,003380415	2	993,60124	0,71	19,88	0
	High	vLQGLR	5	1	1	P74551	N-Term(iTRAQ4plex)	0,0000	1,180	3	5,2	0,001	0,01501	34	0,000398997	2	829,53887	1,48	21,69	0
	High	nAMDELk	1	1	1	P74551	N-Term(iTRAQ4plex); K7(iTRAQ4plex)	0,0000	1,354	1		0,004	0,05354	29	0,001294106	2	1108,59307	1,77	20,01	0
	Medium	eLSELSV	1	1	1	P74551	N-Term(iTRAQ4plex)	0,0000	0,917	1		0,012	0,1516	36	0,000252912	2	920,50585	0,13	34,67	0
	Medium	fSAcLR	2	1	1	P74551	N-Term(iTRAQ4plex); C4(Carbamidomethyl)	0,0000	0,921	2	12,9	0,017	0,2154	16	0,027350798	2	897,47429	1,08	20,94	0
P52983	Glucose-6-phosphate isomerase OS=Synechocystis sp. (strain PCC 6803	1131,17	29,19	1	16	16	49	0,691	38	29,0	531	58,3	5,76							
	A3	Sequence	# PSMs	# Proteins	# Protein Groups	Protein Group Accessions	Modifications	ΔCn	117/114	117/114 Count	117/114 Variability [%]	q-Value	PEP	IonScore	Exp Value	Charge	MH+ [Da]	ΔM [ppm]	RT [min]	# Missed Cleavages
	High	aVSFYGSLVNVNAYHQPGVEAGk	4	1	1	P52983	N-Term(iTRAQ4plex); K23(iTRAQ4plex); K24(iTRAQ4plex	0,0000	1,306	2	10,0	0	5,663E-16	83	4,92006E-09	4	2967,60708	0,59	36,06	1
	High	aAASILELQk	2	1	1	P52983	N-Term(iTRAQ4plex); K10(iTRAQ4plex)	0,0000	0,862	2	28,2	0	0,00000152	68	1,73368E-07	2	1331,81279	-0,69	31,48	0
	High	aVSFYGSLVNVNAYHQPGVEAGk	2	1	1	P52983	N-Term(iTRAQ4plex); K23(iTRAQ4plex)	0,0000	0,988	2	45,8	0	9,68E-14	67	1,94971E-07	4	2695,40932	0,38	39,05	0
	High	mNNQQLWQR	2	1	1	P52983	N-Term(iTRAQ4plex); M1(Oxidation)	0,0000	0,694	2	42,7	0	0,000001466	54	4,29507E-06	2	1377,68181	0,28	19,28	0
	High	sTLVVTTSk	5	1	1	P52983	N-Term(iTRAQ4plex); K9(iTRAQ4plex)	0,0000	0,647	3	1,0	0	0,000001206	54	4,45625E-06	2	1223,74394	-0,82	18,96	0
	High	kAAASILELQk	3	1	1	P52983	N-Term(iTRAQ4plex); K1(iTRAQ4plex); K11(iTRAQ4plex)	0,0000	0,412	2	47,4	0	0,00002054	50	1,08885E-05	3	1604,00864	-1,32	26,72	1
	High	nPALAPNDGIR	1	1	1	P52983	N-Term(iTRAQ4plex)	0,0000	0,569	1		0	0,0003451	49	1,26174E-05	2	1281,70329	0,05	20,82	0
	High	gAIANPDEQR	1	1	1	P52983	N-Term(iTRAQ4plex)	0,0000	0,571	1		0	6,678E-08	45	2,81819E-05	2	1214,62468	0,04	12,86	0
	High	vLNcLk	3	1	1	P52983	N-Term(iTRAQ4plex); C4(Carbamidomethyl); K6(iTRAQ4	0,0000	0,454	3	5,2	0	0,0008098	45	3,71458E-05	2	1034,62651	-0,55	23,34	0
	High	qALFENQR	3	1	1	P52983	N-Term(iTRAQ4plex)	0,0000	0,725	3	24,7	0	0,00007658	42	5,70125E-05	2	1149,61284	-0,42	19,36	0
	High	gSTDQHAYVQQLR	6	1	1	P52983	N-Term(iTRAQ4plex)	0,0000	0,795	4	14,1	0	0,00001104	36	0,000255841	3	1646,83646	-0,17	16,86	0
	High	aFVADVHQGNIkPPTAPk	4	1	1	P52983	N-Term(iTRAQ4plex); K12(iTRAQ4plex); K18(iTRAQ4plex	0,0000	0,734	2	19,8	0	0,000006547	34	0,000369803	4	2322,33144	0,84	26,41	0
	High	mVGHYWLR	3	1	1	P52983	N-Term(iTRAQ4plex)	0,0000	0,581	2	4,9	0	0,0000241	32	0,000654591	3	1205,63642	-0,51	27,60	0
	High	mVGHYWLR	4	1	1	P52983	N-Term(iTRAQ4plex); M1(Oxidation)	0,0000	0,661	3	14,9	0	0,00001893	30	0,000891189	3	1221,63214	0,16	22,98	0
	High	kDmVILPYkDR	2	1	1	P52983	N-Term(iTRAQ4plex); K1(iTRAQ4plex); M3(Oxidation); K	0,0000	0,820	2	27,7	0	0,00001352	28	0,001617968	3	1826,05387	-1,75	21,60	2
	High	yLQQLVmESLGk	1	1	1	P52983	N-Term(iTRAQ4plex); M7(Oxidation); K12(iTRAQ4plex)	0,0000	0,731	1		0	0,000006346	26	0,002333297	3	1712,94779	-1,04	47,09	0
	High	lQIQWR	3	1	1	P52983	N-Term(iTRAQ4plex)	0,0000	0,649	3	8,0	0,001	0,01258	27	0,001866251	2	987,58562	-0,03	31,70	0
P73204	Phycobilisome 32.1 kDa linker polypeptide, phycocyanin-associated, rod	1128,58	54,58	1	16	16	47	0,462	38	15,6	273	30,8	9,39							
	A3	Sequence	# PSMs	# Proteins	# Protein Groups	Protein Group Accessions	Modifications	ΔCn	117/114	117/114 Count	117/114 Variability [%]	q-Value	PEP	IonScore	Exp Value	Charge	MH+ [Da]	ΔM [ppm]	RT [min]	# Missed Cleavages
	High	nTASPVYAGSTAESLR	4	1	1	P73204	N-Term(iTRAQ4plex)	0,0000	0,487	4	31,2	0	1,401E-10	87	1,82797E-09	2	1767,90300	2,03	25,38	0
	High	gkAEYLVSYDNLSAk	4	1	1	P73204	N-Term(iTRAQ4plex); K2(iTRAQ4plex); K15(iTRAQ4plex)	0,0000	0,570	2	9,9	0	1,22E-08	84	4,69009E-09	3	2090,15586	3,08	30,36	1
	High	lTSAESLLR	5	1	1	P73204	N-Term(iTRAQ4plex)	0,0000	0,451	3	4,9	0	0,000001025	64	5,61477E-07	2	1133,66557	0,78	32,02	0
	High	lQVIQGAAPGR	7	1	1	P73204	N-Term(iTRAQ4plex)	0,0000	0,441	4	8,4	0	0,000001456	61	8,66902E-07	2	1253,74651	1,45	25,56	0
	High	aVALSEVYR	3	1	1	P73204	N-Term(iTRAQ4plex)	0,0000	0,515	3	24,9	0	0,0007892	50	1,05918E-05	2	1151,65434	0,19	27,39	0
	High	qVLGNDHLmSQER	3	1	1	P73204	N-Term(iTRAQ4plex); M9(Oxidation)	0,0000	0,498	2	2,3	0	4,893E-08	49	1,19942E-05	3	1686,83622	0,69	16,48	0
	High	fIELNYk	3	1	1	P73204	N-Term(iTRAQ4plex); K7(iTRAQ4plex)	0,0000	0,347	3	15,2	0	0,0007862	48	1,72175E-05	2	1214,70525	2,39	33,18	0
	High	fFHSNPQNR	4	1	1	P73204	N-Term(iTRAQ4plex)	0,0000	0,426	4	2,5	0	0,008302	44	4,32484E-05	2	1290,64995	3,05	14,72	0
	High	tSLVSAQR	3	1	1	P73204	N-Term(iTRAQ4plex)	0,0000	0,479	3	7,8	0	0,00002621	39	0,000127635	2	1005,58275	1,79	14,15	0
	High	sFQVYR	3	1	1	P73204	N-Term(iTRAQ4plex)	0,0000	0,389	3	4,0	0	0,007414	37	0,000208435	2	943,51348	1,78	21,34	0
	High	lGIVAVDEAIPLELR	1	1	1	P73204	N-Term(iTRAQ4plex)	0,0000				0	0,003292	25	0,002844265	3	1752,04117	1,33	50,08	0
	High	lQQINR	3	1	1	P73204	N-Term(iTRAQ4plex)	0,0000	0,546	3	23,9	0	0,009576	22	0,006949763	2	915,55089	1,79	14,86	0
	High	qGDTVTmISLA	1	1	1	P73204	N-Term(iTRAQ4plex); M7(Oxidation)	0,0000	0,480	1		0,003	0,03935	30	0,001061622	2	1295,66545	1,59	32,04	0
	Medium	gFATQR	1	1	1	P73204	N-Term(iTRAQ4plex)	0,0000	0,850	1		0,013	0,1707	10	0,095933437	2	823,45427	-0,03	12,78	0
	Medium	qkFFHSNPQNR	1	1	1	P73204	N-Term(iTRAQ4plex); K2(iTRAQ4plex)	0,0000	0,816	1		0,015	0,1934	10	0,098898408	4	1690,90236	0,44	13,21	1
	Medium	tVGFSR	1	1	1	P73204	N-Term(iTRAQ4plex)	0,0000	0,455	1		0,036	0,4483	17	0,021912798	2	810,46062	1,94	15,18	0
P73785	Phosphate-binding protein OS=Synechocystis sp. (strain PCC 6803 / Kaz	1125,25	43,54	1	15	15	36	0,642	29	8,6	333	35,3	9,92							
	A3	Sequence	# PSMs	# Proteins	# Protein Groups	Protein Group Accessions	Modifications	ΔCn	117/114	117/114 Count	117/114 Variability [%]	q-Value	PEP	IonScore	Exp Value	Charge	MH+ [Da]	ΔM [ppm]	RT [min]	# Missed Cleavages
	High	aTGNTVNYNSVGSGAGIR	5	1	1	P73785	N-Term(iTRAQ4plex)	0,0000	0,637	4	3,1	0	1,17E-15	129	1,25884E-13	2	1881,95757	2,12	24,06	0
	High	gVVmVPTAGGAVAVYNLPGkk	1	1	1	P73785	N-Term(iTRAQ4plex); M4(Oxidation); K21(iTRAQ4plex); I	0,0000	0,944	1		0	4,285E-12	101	8,41337E-11	4	2575,50918	3,30	39,63	1
	High	gVVMVPTAGGAVAVYNLPGkk	1	1	1	P73785	N-Term(iTRAQ4plex); K21(iTRAQ4plex); K22(iTRAQ4plex	0,0000	0,717	1		0	1,102E-09	82	5,71439E-09	4	2559,51162	2,28	41,55	1
	High	kATGNTVNYNSVGSGAGIR	4	1	1	P73785	N-Term(iTRAQ4plex); K1(iTRAQ4plex)	0,0000	0,614	2	16,9	0	5,483E-09	68	1,56304E-07	3	2154,15891	3,85	20,46	1
	High	rNEGPNLQTAk	5	1	1	P73785	N-Term(iTRAQ4plex); K11(iTRAQ4plex)	0,0000	0,760	3	18,4	0	6,817E-08	61	7,11164E-07	2	1515,85307	3,21	12,57	1
	High	qTPGAIGYVQDTFAR	1	1	1	P73785	N-Term(iTRAQ4plex)	0,0000	0,655	1		0	7,33E-08	58	1,59945E-06	3	1767,91697	1,30	37,06	0
	High	vIAAVNk	4	1	1	P73785	N-Term(iTRAQ4plex); K7(iTRAQ4plex)	0,0000	0,606	3	34,1	0	0,007177	41	8,24081E-05	2	1002,65929	4,26	18,38	0
	High	wILTTGQNHNTQLEYTk	2	1	1	P73785	N-Term(iTRAQ4plex); K17(iTRAQ4plex)	0,0000	0,636	2	4,6	0	0,00000014	37	0,000218872	3	2335,23484	2,71	32,17	0
	High	iGASQDPNWGFQVLk	1	1	1	P73785	N-Term(iTRAQ4plex); K15(iTRAQ4plex)	0,0000	0,663	1		0	0,002018	30	0,001304271	3	1948,05942	3,23	41,42	0
	High	yFAEYkk	1	1	1	P73785	N-Term(iTRAQ4plex); K6(iTRAQ4plex); K7(iTRAQ4plex)	0,0000	2,404	1		0	0,002972	18	0,017296969	3	1380,79090	1,55	21,90	1
	High	yFAEYk	1	1	1	P73785	N-Term(iTRAQ4plex); K6(iTRAQ4plex)	0,0000	0,433	1		0,003	0,04033	24	0,004092324	2	1108,59661	4,41	25,61	0
	High	iPPAVAQR	3	1	1	P73785	N-Term(iTRAQ4plex)	0,0000	0,639	3	3,8	0,006	0,07607	40	0,000109388	2	995,61351	1,64	18,13	0
	Medium	lPNTPIR	1	1	1	P73785	N-Term(iTRAQ4plex)	0,0000	0,641	1		0,011	0,1454	26	0,002488685	2	954,58739	2,17	23,12	0
	Medium	iFTGELk	3	1	1	P73785	N-Term(iTRAQ4plex); K7(iTRAQ4plex)	0,0000	0,549	2	4,6	0,021	0,2638	38	0,000146208	2	1095,67009	4,42	29,02	0
	Medium	nANVAk	1	1	1	P73785	N-Term(iTRAQ4plex); K6(iTRAQ4plex)	0,0000	0,673	1		0,031	0,3798	33	0,000508124	2	904,55101	6,14	9,24	0
	Medium	iALGk	2	1	1	P73785	N-Term(iTRAQ4plex); K5(iTRAQ4plex)	0,0000	0,630	2	2,8	0,031	0,3841	19	0,034211025	2	789,54186	-2,29	18,45	0
P73452	Nitrate transport protein NrtA OS=Synechocystis sp. (strain PCC 6803 /	1085,98	34,53	1	16	18	52	1,758	39	28,3	446	48,9	5,34							
	A3	Sequence	# PSMs	# Proteins	# Protein Groups	Protein Group Accessions	Modifications	ΔCn	117/114	117/114 Count	117/114 Variability [%]	q-Value	PEP	IonScore	Exp Value	Charge	MH+ [Da]	ΔM [ppm]	RT [min]	# Missed Cleavages
	High	vAMTFPGGTHDMWIR	2	1	1	P73452	N-Term(iTRAQ4plex)	0,0000	1,908	2	22,1	0	7,287E-09	70	1,08635E-07	3	1862,91629	-0,01	39,11	0
	High	vGTDAAPLk	2	1	1	P73452	N-Term(iTRAQ4plex); K9(iTRAQ4plex)	0,0000	1,823	1		0	0,00002559	68	1,5775E-07	2	1159,69084	-1,46	18,79	0
	High	lGFIALTDAAPLIIAk	4	1	1	P73452	N-Term(iTRAQ4plex); K16(iTRAQ4plex)	0,0000	2,040	4	33,3	0	7,534E-07	67	2,07955E-07	2	1915,18767	0,28	53,95	0
	High	eAAQALEVPADQIPSSPSR	2	1	1	P73452	N-Term(iTRAQ4plex)	0,0000	1,957	1		0	7,604E-11	61	7,12804E-07	3	2110,09092	0,54	31,80	0
	High	yGMPDVEVLk	1	1	1	P73452	N-Term(iTRAQ4plex); K10(iTRAQ4plex)	0,0000	1,676	1		0	0,00001824	57	2,06524E-06	2	1438,78423	-0,85	36,69	0
	High	vGTDAAPLkEAFAk	2	1	1	P73452	N-Term(iTRAQ4plex); K9(iTRAQ4plex); K14(iTRAQ4plex)	0,0000	1,194	2	123,8	0	4,086E-08	52	6,19398E-06	3	1850,07639	0,84	30,65	1
	High	lNVNGQGIQLGNNYk	3	1	1	P73452	N-Term(iTRAQ4plex); K15(iTRAQ4plex)	0,0000	1,164	3	192,1	0	3,99E-10	52	6,41165E-06	3	1920,05564	0,76	32,44	0
	High	vAMTFPGGTHDmWIR	3	1	1	P73452	N-Term(iTRAQ4plex); M12(Oxidation)	0,0000	1,758	3	21,4	0	0,000001897	52	6,94976E-06	3	1878,91166	0,23	35,62	0
	High	sHDQWFLTENIR	9	1	1	P73452	N-Term(iTRAQ4plex)	0,0000	1,870	6	8,9	0	6,361E-08	50	9,31044E-06	2	1689,84648	-0,06	32,92	0
	High	vPFEDIIDR	2	1	1	P73452	N-Term(iTRAQ4plex)	0,0000	1,788	2	8,1	0	0,0006334	40	0,00010964	2	1247,67522	-0,04	40,15	0
	High	vAmTFPGGTHDmWIR	3	1	1	P73452	N-Term(iTRAQ4plex); M3(Oxidation); M12(Oxidation)	0,0000	1,178	3	36,1	0	3,081E-07	33	0,000456005	3	1894,90830	1,14	30,33	0
	High	wGYLPASTDTk	2	1	1	P73452	N-Term(iTRAQ4plex); K11(iTRAQ4plex)	0,0000	1,453	2	7,6	0	0,000001405	31	0,000855008	2	1526,80913	-0,14	31,14	0
	High	vNREDLWR	6	2	2	Q55460;P73452	N-Term(iTRAQ4plex)	0,0000				0	0,006727	20	0,009267658	3	1231,66605	-0,31	22,06	1
	High	ekGFYAk	2	1	1	P73452	N-Term(iTRAQ4plex); K2(iTRAQ4plex); K7(iTRAQ4plex)	0,0000	2,663	2	21,4	0	0,001374	19	0,014627875	3	1274,74692	0,02	14,22	1
	High	gFYAk	2	1	1	P73452	N-Term(iTRAQ4plex); K5(iTRAQ4plex)	0,0000	1,625	2	1,0	0,001	0,01682	40	0,000105189	2	873,50646	-0,93	15,93	0
	High	qASWGTTR	2	1	1	P73452	N-Term(iTRAQ4plex)	0,0000	1,285	2	7,2	0,002	0,02507	36	0,000257022	2	1050,54558	0,64	13,86	0
	High	rEWFk	2	1	1	P73452	N-Term(iTRAQ4plex); K5(iTRAQ4plex)	0,0000	1,959	2	6,3	0,004	0,05425	11	0,083362361	3	1053,60724	-1,10	18,37	1
	Medium	eDLWR	1	2	2	Q55460;P73452	N-Term(iTRAQ4plex)	0,0000				0,025	0,309	13	0,053084778	2	862,45232	-1,91	22,24	0
	Medium	vTDPk	1	1	1	P73452	N-Term(iTRAQ4plex); K5(iTRAQ4plex)	0,0000				0,036	0,4508	19	0,011945087	2	847,51109	-1,96	11,80	0
	Medium	eAFAk	1	1	1	P73452	N-Term(iTRAQ4plex); K5(iTRAQ4plex)	0,0000	1,300	1		0,041	0,5025	18	0,014620762	2	853,49932	-3,38	14,38	0
Q55199	Phosphate-binding protein OS=Synechocystis sp. (strain PCC 6803 / Kaz	1064,20	31,33	1	12	12	44	0,553	36	24,5	383	40,0	4,69							
	A3	Sequence	# PSMs	# Proteins	# Protein Groups	Protein Group Accessions	Modifications	ΔCn	117/114	117/114 Count	117/114 Variability [%]	q-Value	PEP	IonScore	Exp Value	Charge	MH+ [Da]	ΔM [ppm]	RT [min]	# Missed Cleavages
	High	tVDFGASDVAmDDEEIAk	1	1	1	Q55199	N-Term(iTRAQ4plex); M11(Oxidation); K18(iTRAQ4plex)	0,0000	0,665	1		0	1,495E-10	76	2,79879E-08	3	2217,05198	2,14	33,33	0
	High	sDGSGTTAVFTmNLAAmSPEFk	1	1	1	Q55199	N-Term(iTRAQ4plex); M12(Oxidation); M17(Oxidation); K	0,0000	0,525	1		0	0,000001475	63	5,48239E-07	3	2582,24521	3,65	38,45	0
	High	iSPDYTITLk	5	1	1	Q55199	N-Term(iTRAQ4plex); K10(iTRAQ4plex)	0,0000	0,489	5	22,8	0	7,37E-08	59	1,33343E-06	2	1438,84245	2,00	34,31	0
	High	lSQEALAGImLGNITk	5	1	1	Q55199	N-Term(iTRAQ4plex); M10(Oxidation); K16(iTRAQ4plex)	0,0000	0,508	5	58,8	0	9,894E-11	56	2,87019E-06	3	1963,11930	2,85	42,40	0
	High	lVADNPDLTLPDRPITVVHR	17	1	1	Q55199	N-Term(iTRAQ4plex)	0,0000	0,602	11	14,8	0	0,000002745	47	1,94971E-05	4	2385,33877	0,67	36,34	0
	High	tVEWPTSk	4	1	1	Q55199	N-Term(iTRAQ4plex); K8(iTRAQ4plex)	0,0000	0,468	2	6,0	0	0,0008976	47	2,15263E-05	2	1235,69194	3,65	25,02	0
	High	lSQEALAGIMLGNITk	3	1	1	Q55199	N-Term(iTRAQ4plex); K16(iTRAQ4plex)	0,0000	0,676	3	8,0	0	0,0001214	45	2,95781E-05	3	1947,12760	4,52	48,25	0
	High	ekVAAAADk	2	1	1	Q55199	N-Term(iTRAQ4plex); K2(iTRAQ4plex); K9(iTRAQ4plex)	0,0000	1,126	2	10,3	0	0,002126	36	0,000263009	2	1334,80400	2,68	10,37	1
	High	ekVAAAADkISPDYTITLk	1	1	1	Q55199	N-Term(iTRAQ4plex); K2(iTRAQ4plex); K9(iTRAQ4plex);	0,0000	1,646	1		0	2,124E-08	27	0,002118215	4	2610,52065	0,21	32,83	2
	High	vAAAADk	2	1	1	Q55199	N-Term(iTRAQ4plex); K7(iTRAQ4plex)	0,0000	0,471	2	27,4	0,004	0,05078	48	1,69587E-05	2	933,56529	4,83	11,04	0
	High	eTIGDGk	1	1	1	Q55199	N-Term(iTRAQ4plex); K7(iTRAQ4plex)	0,0000	0,552	1		0,007	0,09321	28	0,001640476	2	1007,56517	3,96	12,43	0
	Medium	fIGGk	1	1	1	Q55199	N-Term(iTRAQ4plex); K5(iTRAQ4plex)	0,0000	0,535	1		0,036	0,4519	15	0,029853826	2	809,51634	4,91	18,15	0
	Medium	wNDPk	1	1	1	Q55199	N-Term(iTRAQ4plex); K5(iTRAQ4plex)	0,0000	0,503	1		0,043	0,5294	15	0,037444901	2	947,52190	3,16	16,67	0
P72827	Iron uptake protein A1 OS=Synechocystis sp. (strain PCC 6803 / Kazusa	1045,58	34,44	1	11	11	43	0,244	33	23,3	360	39,3	4,93							
	A3	Sequence	# PSMs	# Proteins	# Protein Groups	Protein Group Accessions	Modifications	ΔCn	117/114	117/114 Count	117/114 Variability [%]	q-Value	PEP	IonScore	Exp Value	Charge	MH+ [Da]	ΔM [ppm]	RT [min]	# Missed Cleavages
	High	hYNTDNELYAk	7	1	1	P72827	N-Term(iTRAQ4plex); K11(iTRAQ4plex)	0,0000	0,237	6	29,2	0	5,314E-09	76	2,63009E-08	2	1655,83318	3,87	17,33	0
	High	aIAENVGVFFPNQEGR	2	1	1	P72827	N-Term(iTRAQ4plex)	0,0000	0,322	2	38,3	0	1,32E-10	74	4,46684E-08	2	1891,98259	2,23	40,39	0
	High	gTHVNVSGVGVVk	6	1	1	P72827	N-Term(iTRAQ4plex); K13(iTRAQ4plex)	0,0000	0,231	3	8,6	0	0,000000234	72	6,19398E-08	2	1540,91033	3,46	20,51	0
	High	lGPALAPATk	7	1	1	P72827	N-Term(iTRAQ4plex); K10(iTRAQ4plex)	0,0000	0,298	6	45,1	0	0,0004555	65	2,87058E-07	2	1226,77507	3,22	24,40	0
	High	sVASFGEFk	5	1	1	P72827	N-Term(iTRAQ4plex); K9(iTRAQ4plex)	0,0000	0,364	4	79,4	0	0,0003364	60	1,08136E-06	2	1259,69206	3,67	30,10	0
	High	vkPEELSTYEELADPk	1	1	1	P72827	N-Term(iTRAQ4plex); K2(iTRAQ4plex); K16(iTRAQ4plex)	0,0000	0,244	1		0	7,436E-10	48	1,49958E-05	3	2280,24167	3,55	34,07	0
	High	gFVSNFAR	5	1	1	P72827	N-Term(iTRAQ4plex)	0,0000	0,228	3	15,1	0	0,01044	47	2,00895E-05	2	1041,56182	1,90	26,29	0
	High	ikSEGANSPADVLLTVDLAR	1	1	1	P72827	N-Term(iTRAQ4plex); K2(iTRAQ4plex)	0,0000	0,352	1		0	0,000008705	36	0,000254665	4	2357,33071	1,17	41,73	1
	High	imNEAGWk	1	1	1	P72827	N-Term(iTRAQ4plex); M2(Oxidation); K8(iTRAQ4plex)	0,0000	0,319	1		0	0,006994	34	0,000379289	2	1252,66387	3,20	21,47	0
	High	vIMYNk	3	1	1	P72827	N-Term(iTRAQ4plex); K6(iTRAQ4plex)	0,0000	0,230	2	15,9	0	0,006058	34	0,000413019	2	1055,61943	3,08	21,14	0
	High	vImYNk	3	1	1	P72827	N-Term(iTRAQ4plex); M3(Oxidation); K6(iTRAQ4plex)	0,0000	0,209	3	9,7	0,002	0,02991	39	0,000126465	2	1071,61467	3,34	17,64	0
	High	vNLIEGk	2	1	1	P72827	N-Term(iTRAQ4plex); K7(iTRAQ4plex)	0,0000	0,205	1		0,003	0,04168	45	5,87858E-05	2	1060,66545	4,67	23,79	0
P42352	50S ribosomal protein L9 OS=Synechocystis sp. (strain PCC 6803 / Kazu	1039,67	55,26	1	11	11	53	2,770	39	61,2	152	16,6	9,20							
	A3	Sequence	# PSMs	# Proteins	# Protein Groups	Protein Group Accessions	Modifications	ΔCn	117/114	117/114 Count	117/114 Variability [%]	q-Value	PEP	IonScore	Exp Value	Charge	MH+ [Da]	ΔM [ppm]	RT [min]	# Missed Cleavages
	High	vVLNETINk	6	1	1	P42352	N-Term(iTRAQ4plex); K9(iTRAQ4plex)	0,0000	1,751	5	131,9	0	6,868E-07	73	5,5077E-08	2	1317,79619	-1,42	26,02	0
	High	lGFTGDLVEVAPGYAR	2	1	1	P42352	N-Term(iTRAQ4plex)	0,0000	1,969	2	6,1	0	2,097E-09	70	9,57128E-08	2	1808,96660	0,11	43,14	0
	High	gISLPDIHk	7	1	1	P42352	N-Term(iTRAQ4plex); K9(iTRAQ4plex)	0,0000	2,855	4	20,6	0	0,00001726	68	1,55228E-07	2	1267,75859	-2,12	29,09	0
	High	vkVVLNETINk	1	1	1	P42352	N-Term(iTRAQ4plex); K2(iTRAQ4plex); K11(iTRAQ4plex)	0,0000	2,316	1		0	2,678E-08	54	3,81918E-06	3	1689,06040	-1,84	27,51	1
	High	tGFYQAQIk	8	1	1	P42352	N-Term(iTRAQ4plex); K9(iTRAQ4plex)	0,0000	2,679	6	50,7	0	6,432E-07	52	6,39691E-06	2	1343,75481	-1,02	24,51	0
	High	gLGVVATPGILR	9	1	1	P42352	N-Term(iTRAQ4plex)	0,0000	4,152	5	13,2	0	0,000006932	52	6,62171E-06	2	1296,81194	-0,09	36,69	0
	High	kVALETIGR	7	1	1	P42352	N-Term(iTRAQ4plex); K1(iTRAQ4plex)	0,0000	3,539	4	44,9	0	0,00005373	49	1,25884E-05	3	1274,80240	-0,85	21,09	1
	High	nYLIPk	7	1	1	P42352	N-Term(iTRAQ4plex); K6(iTRAQ4plex)	0,0000	2,655	6	51,7	0,002	0,03321	11	0,088715601	3	1035,64222	-1,82	24,47	0
	High	rGISLPDIHk	1	1	1	P42352	N-Term(iTRAQ4plex); K10(iTRAQ4plex)	0,0000	2,770	1		0,005	0,06728	13	0,054321281	4	1423,86074	-1,17	24,82	1
	High	lkELER	3	1	1	P42352	N-Term(iTRAQ4plex); K2(iTRAQ4plex)	0,0000	2,001	3	48,9	0,008	0,1065	10	0,109693589	3	1075,66806	-3,10	15,77	1
	Medium	fVIkk	2	1	1	P42352	N-Term(iTRAQ4plex); K4(iTRAQ4plex); K5(iTRAQ4plex)	0,0000	4,618	2	46,1	0,013	0,1629	21	0,008870947	2	1066,73100	-3,63	19,14	1
P54691	Probable branched-chain-amino-acid aminotransferase OS=Synechocyst	1038,19	51,80	1	17	17	52	1,158	45	27,7	305	33,9	6,33							
	A3	Sequence	# PSMs	# Proteins	# Protein Groups	Protein Group Accessions	Modifications	ΔCn	117/114	117/114 Count	117/114 Variability [%]	q-Value	PEP	IonScore	Exp Value	Charge	MH+ [Da]	ΔM [ppm]	RT [min]	# Missed Cleavages
	High	iSAAYITSALAk	9	1	1	P54691	N-Term(iTRAQ4plex); K12(iTRAQ4plex)	0,0000	1,117	6	24,7	0	1,614E-08	91	8,35545E-10	2	1496,89336	0,45	40,16	0

	High	sVLTAVTENREPk	4	1	1	P54691	N-Term(iTRAQ4plex); K13(iTRAQ4plex)	0,0000	1,543	3	15,0	0	4,551E-08	66	2,78612E-07	3	1731,98417	-0,12	24,61	1
	High	sFYIRPLVYSSGLGIAPR	4	1	1	P54691	N-Term(iTRAQ4plex)	0,0000	1,371	3	6,7	0	1,532E-10	61	8,65345E-07	3	2140,20444	0,40	43,28	0
	High	ikEVIVDFVk	5	1	1	P54691	N-Term(iTRAQ4plex); K2(iTRAQ4plex); K10(iTRAQ4plex)	0,0000	1,168	5	30,2	0	1,284E-08	55	2,97146E-06	2	1622,02446	-0,54	40,83	1
	High	fLHYDISAEk	5	1	1	P54691	N-Term(iTRAQ4plex); K10(iTRAQ4plex)	0,0000	0,982	5	11,7	0	1,692E-07	54	4,39511E-06	2	1510,81401	-0,27	29,26	0
	High	iSVATHALHYGTAAFGGLR	2	1	1	P54691	N-Term(iTRAQ4plex)	0,0000	1,426	1		0	8,144E-10	52	6,28015E-06	3	2086,13303	0,74	37,09	0
	High	fLPIAYFEDk	4	1	1	P54691	N-Term(iTRAQ4plex); K10(iTRAQ4plex)	0,0000	0,445	4	3,4	0	0,00001052	52	1,01477E-05	2	1530,84429	-0,26	47,92	0
	High	iENFTLGGDRPITEk	1	1	1	P54691	N-Term(iTRAQ4plex); K15(iTRAQ4plex)	0,0000	1,253	1		0	9,856E-09	50	9,35341E-06	3	1978,08451	-0,17	32,12	0
	High	sVLTAVTENR	2	1	1	P54691	N-Term(iTRAQ4plex)	0,0000	0,822	2	9,9	0	7,103E-08	40	9,90764E-05	2	1233,69170	-0,22	26,35	0
	High	rIENFTLGGDRPITEk	5	1	1	P54691	N-Term(iTRAQ4plex); K16(iTRAQ4plex)	0,0000	1,301	5	2,1	0	1,751E-13	36	0,000256431	3	2134,18558	-0,18	28,30	1
	High	yQDWVFk	1	1	1	P54691	N-Term(iTRAQ4plex); K7(iTRAQ4plex)	0,0000	0,897	1		0	0,0001365	27	0,002207852	2	1273,68096	-0,78	35,45	0
	High	lHNLEk	1	1	1	P54691	N-Term(iTRAQ4plex); K6(iTRAQ4plex)	0,0000	0,601	1		0	0,007523	19	0,011994165	3	1041,62885	-0,64	14,02	0
	High	kNQPDk	2	1	1	P54691	N-Term(iTRAQ4plex); K1(iTRAQ4plex); K6(iTRAQ4plex)	0,0000	1,488	2	10,1	0	0,001329	18	0,015559656	3	1161,69495	-0,22	7,54	1
	High	gIPDPEDPGTILLFR	1	1	1	P54691	N-Term(iTRAQ4plex)	0,0000	0,891	1		0,002	0,02415	20	0,009246343	2	1783,97136	0,12	46,85	0
	High	iSSWYR	2	1	1	P54691	N-Term(iTRAQ4plex)	0,0000	1,171	2	16,0	0,003	0,04456	29	0,00132425	2	955,51177	-0,03	25,92	0
	High	iTPVk	2	1	1	P54691	N-Term(iTRAQ4plex); K5(iTRAQ4plex)	0,0000	0,836	1		0,007	0,09614	11	0,085500766	2	845,56743	-2,89	15,30	0
	Medium	sFPLR	2	1	1	P54691	N-Term(iTRAQ4plex)	0,0000	1,173	2	2,0	0,016	0,2039	14	0,070637944	2	763,45769	-0,82	20,59	0
P72848	Oxygen-dependent coproporphyrinogen-III oxidase OS=Synechocystis s	1027,97	35,00	1	11	11	46	1,502	36	19,7	340	38,9	5,82							
	A3	Sequence	# PSMs	# Proteins	# Protein Groups	Protein Group Accessions	Modifications	ΔCn	117/114	117/114 Count	117/114 Variability [%]	q-Value	PEP	IonScore	Exp Value	Charge	MH+ [Da]	ΔM [ppm]	RT [min]	# Missed Cleavages
	High	tESILmSLPPLVR	4	1	1	P72848	N-Term(iTRAQ4plex); M6(Oxidation)	0,0000	1,265	4	5,9	0	6,696E-10	66	2,73508E-07	2	1615,92278	1,09	45,61	0
	High	gTIFGLQTNGR	3	1	1	P72848	N-Term(iTRAQ4plex)	0,0000	1,790	2	7,6	0	4,292E-07	63	4,97703E-07	2	1307,71733	-1,17	29,84	0
	High	qRPEAEGHEFYATGTSmVLHPk	6	1	1	P72848	N-Term(iTRAQ4plex); M17(Oxidation); K22(iTRAQ4plex)	0,0000	1,510	4	10,1	0	1,299E-12	61	8,12774E-07	4	2789,39321	0,43	25,39	0
	High	nPYIPTVHLNYR	5	1	1	P72848	N-Term(iTRAQ4plex)	0,0000	1,606	4	14,3	0	2,553E-08	58	1,70596E-06	3	1630,88230	0,05	32,31	0
	High	aFLPAYSPIVEk	6	1	1	P72848	N-Term(iTRAQ4plex); K12(iTRAQ4plex)	0,0000	1,576	5	27,2	0	0,000001397	56	2,59998E-06	2	1622,94060	0,59	38,96	0
	High	tESILMSLPPLVR	3	1	1	P72848	N-Term(iTRAQ4plex)	0,0000	0,915	3	73,7	0	0,000001104	55	3,21657E-06	2	1599,92729	0,74	48,66	0
	High	wcDEYFYLk	1	1	1	P72848	N-Term(iTRAQ4plex); C2(Carbamidomethyl); K9(iTRAQ4p	0,0000	1,258	1		0	2,756E-08	51	7,94273E-06	2	1611,77605	0,28	41,42	0
	High	qRPEAEGHEFYATGTSMVLHPk	6	1	1	P72848	N-Term(iTRAQ4plex); K22(iTRAQ4plex)	0,0000	1,748	4	11,4	0	1,216E-15	45	2,82488E-05	4	2773,39712	0,01	29,50	0
	High	nAcDQTHGEFYPVFk	2	1	1	P72848	N-Term(iTRAQ4plex); C3(Carbamidomethyl); K15(iTRAQ	0,0000	1,561	1		0	1,522E-13	45	3,25065E-05	3	2101,00486	-0,15	31,19	0
	High	sLPPSILk	4	1	1	P72848	N-Term(iTRAQ4plex); K8(iTRAQ4plex)	0,0000	1,488	2	5,7	0	0,00002649	37	0,000197229	2	1142,73821	-0,47	27,63	0
	High	qFQLYR	3	1	1	P72848	N-Term(iTRAQ4plex)	0,0000	1,514	3	7,2	0	0,004661	37	0,000209397	2	998,55321	-0,79	24,58	0
	High	gkFQEDSWQR	2	1	1	P72848	N-Term(iTRAQ4plex); K2(iTRAQ4plex)	0,0000	1,529	2	52,4	0	8,974E-07	32	0,000681066	3	1568,80588	-0,08	19,48	1
	High	rWcDEYFYLk	1	1	1	P72848	N-Term(iTRAQ4plex); C3(Carbamidomethyl); K10(iTRAQ	0,0000	1,461	1		0	4,509E-08	23	0,004476824	3	1767,87723	0,29	35,85	1
P73203	Phycobilisome 32.1 kDa linker polypeptide, phycocyanin-associated, rod	1013,70	50,17	1	15	15	43	0,818	38	18,4	291	32,5	9,35							
	A3	Sequence	# PSMs	# Proteins	# Protein Groups	Protein Group Accessions	Modifications	ΔCn	117/114	117/114 Count	117/114 Variability [%]	q-Value	PEP	IonScore	Exp Value	Charge	MH+ [Da]	ΔM [ppm]	RT [min]	# Missed Cleavages
	High	aLGGTVPFGQASk	8	1	1	P73203	N-Term(iTRAQ4plex); K13(iTRAQ4plex)	0,0000	0,819	6	20,3	0	5,015E-09	89	1,45445E-09	2	1520,86968	1,39	28,47	0
	High	lkGAESLLTNGSISVR	3	1	1	P73203	N-Term(iTRAQ4plex); K2(iTRAQ4plex)	0,0000	1,212	3	6,1	0	1,646E-10	71	8,80988E-08	3	1933,13358	0,76	35,24	1
	High	fLYNNFQTR	5	1	1	P73203	N-Term(iTRAQ4plex)	0,0000	0,775	5	9,2	0	7,638E-07	49	1,13232E-05	3	1346,69712	-0,19	32,55	0
	High	kFLYNNFQTR	5	1	1	P73203	N-Term(iTRAQ4plex); K1(iTRAQ4plex)	0,0000	0,844	5	13,4	0	9,323E-07	45	2,94422E-05	3	1618,89603	1,00	26,50	1
	High	qVLGNDYImDSER	1	1	1	P73203	N-Term(iTRAQ4plex); M9(Oxidation)	0,0000	1,108	1		0	2,707E-08	44	3,74085E-05	2	1699,80864	0,48	27,28	0
	High	vIELNYk	3	1	1	P73203	N-Term(iTRAQ4plex); K7(iTRAQ4plex)	0,0000	0,795	2	8,0	0	0,004828	44	3,89942E-05	2	1166,70305	0,60	28,29	0
	High	fNNQVGDR	2	1	1	P73203	N-Term(iTRAQ4plex)	0,0000	0,773	2	7,2	0	0,0001232	42	6,01132E-05	2	1093,55193	1,10	14,23	0
	High	aITTAASR	2	1	1	P73203	N-Term(iTRAQ4plex)	0,0000	0,604	2	10,3	0	0,009404	39	0,000156796	2	934,54527	1,53	11,91	0
	High	vEITAISAPGYPk	2	1	1	P73203	N-Term(iTRAQ4plex); K13(iTRAQ4plex)	0,0000	0,487	2	21,5	0	0,00002095	36	0,000250017	3	1633,94309	1,66	34,18	0
	High	vIELNYkHLLGR	1	1	1	P73203	N-Term(iTRAQ4plex); K7(iTRAQ4plex)	0,0000	0,317	1		0	0,000005	21	0,007888601	4	1743,05251	0,30	36,14	1
	High	aGNTPAk	5	1	1	P73203	N-Term(iTRAQ4plex); K7(iTRAQ4plex)	0,0000	0,846	4	6,4	0,001	0,02191	15	0,033588399	2	946,55809	2,18	8,08	0
	High	sELYkk	1	1	1	P73203	N-Term(iTRAQ4plex); K5(iTRAQ4plex); K6(iTRAQ4plex)	0,0000	1,236	1		0,001	0,02166	15	0,033960182	3	1199,73008	-4,93	12,61	1
	High	lGVAPYNESRPVELRPDFSLDDAk	1	1	1	P73203	N-Term(iTRAQ4plex); K24(iTRAQ4plex)	0,0000	0,831	1		0,002	0,02699	21	0,009118219	3	2976,57639	3,15	38,27	0
	Medium	tVGFTR	3	1	1	P73203	N-Term(iTRAQ4plex)	0,0000	0,726	2	5,2	0,017	0,2159	25	0,003026704	2	824,47557	1,07	15,55	0
	Medium	vASITPASLS	1	1	1	P73203	N-Term(iTRAQ4plex)	0,0000	0,864	1		0,044	0,5467	31	0,00088914	2	1089,62761	0,34	32,42	0
P54205	Ribulose bisphosphate carboxylase large chain OS=Synechocystis sp. (s	971,70	33,83	1	17	17	50	1,158	42	23,5	470	52,5	6,15							
	A3	Sequence	# PSMs	# Proteins	# Protein Groups	Protein Group Accessions	Modifications	ΔCn	117/114	117/114 Count	117/114 Variability [%]	q-Value	PEP	IonScore	Exp Value	Charge	MH+ [Da]	ΔM [ppm]	RT [min]	# Missed Cleavages
	High	tFQGPPHGITVER	5	1	1	P54205	N-Term(iTRAQ4plex)	0,0000	1,025	3	2,4	0	0,000000031	95	3,31108E-10	2	1582,84673	0,55	24,04	0
	High	lTYYTPDYTPk	1	1	1	P54205	N-Term(iTRAQ4plex); K11(iTRAQ4plex)	0,0000	1,260	1		0	4,355E-09	65	3,03368E-07	2	1649,86772	0,74	30,98	0
	High	fLFVQEAIEk	5	1	1	P54205	N-Term(iTRAQ4plex); K10(iTRAQ4plex)	0,0000	1,038	5	68,7	0	0,000002705	65	3,47512E-07	2	1511,87261	0,91	42,80	0
	High	gHYLNVTAGTcEEmmk	3	1	1	P54205	N-Term(iTRAQ4plex); C11(Carbamidomethyl); M14(Oxida	0,0000	0,919	2	9,7	0	3,238E-12	59	1,18842E-06	3	2160,99881	0,98	20,91	0
	High	lSGGDHLHSGTVVGk	4	1	1	P54205	N-Term(iTRAQ4plex); K15(iTRAQ4plex)	0,0000	1,267	3	7,2	0	1,082E-07	58	1,42551E-06	3	1751,96610	1,02	18,15	0
	High	gHYLNVTAGTcEEmmkR	3	1	1	P54205	N-Term(iTRAQ4plex); C11(Carbamidomethyl); M14(Oxida	0,0000	1,297	2	33,1	0	1,13E-08	41	8,33624E-05	4	2317,09780	-0,01	18,82	1
	High	fEFEAmDTL	1	1	1	P54205	N-Term(iTRAQ4plex); M6(Oxidation)	0,0000	0,775	1		0	0,001092	39	0,000122735	2	1262,57353	0,28	41,24	0
	High	dTDLLAcFR	1	1	1	P54205	N-Term(iTRAQ4plex); C7(Carbamidomethyl)	0,0000	1,397	1		0	0,000004656	39	0,000140595	2	1254,62639	-0,44	37,62	0
	High	fPVALIk	4	1	1	P54205	N-Term(iTRAQ4plex); K7(iTRAQ4plex)	0,0000	1,300	4	6,3	0	0,005584	38	0,000164048	2	1075,71135	-0,42	36,76	0
	High	aVYEcLR	3	1	1	P54205	N-Term(iTRAQ4plex); C5(Carbamidomethyl)	0,0000	1,109	3	31,0	0	0,0008048	33	0,000462349	2	1054,54680	-0,39	21,39	0
	High	amHAVVDR	2	1	1	P54205	N-Term(iTRAQ4plex); M2(Oxidation)	0,0000	0,778	2	46,0	0	0,00007848	33	0,000497703	2	1058,55400	0,59	9,21	0
	High	aMHAVVDR	1	1	1	P54205	N-Term(iTRAQ4plex)	0,0000	0,535	1		0	0,0004857	33	0,000501153	3	1042,55884	0,36	12,12	0
	High	vALEAcVQAR	1	1	1	P54205	N-Term(iTRAQ4plex); C6(Carbamidomethyl)	0,0000	1,185	1		0	0,0001889	31	0,000788806	2	1260,68498	-0,11	24,12	0
	High	lEDIRFPVALIk	4	1	1	P54205	N-Term(iTRAQ4plex); K12(iTRAQ4plex)	0,0000	2,440	4	8,8	0	0,000002862	27	0,001905329	3	1702,05027	-0,21	45,48	1
	High	dNGILLHIHR	3	1	1	P54205	N-Term(iTRAQ4plex)	0,0000	1,251	2	29,4	0	0,001281	26	0,002792351	3	1331,76725	0,58	23,02	0
	High	aQAETNEmk	2	1	1	P54205	N-Term(iTRAQ4plex); M8(Oxidation); K9(iTRAQ4plex)	0,0000	0,975	2	6,9	0	0,002681	25	0,003250648	3	1325,65976	-0,92	8,81	0
	High	yGRPLLGcTIkPk	2	1	1	P54205	N-Term(iTRAQ4plex); C8(Carbamidomethyl); K11(iTRAQ	0,0000	1,679	2	6,2	0	7,402E-07	21	0,008569787	3	1935,15653	-0,47	25,04	0
	High	lGLSAk	5	1	1	P54205	N-Term(iTRAQ4plex); K6(iTRAQ4plex)	0,0000	1,276	3	3,0	0,005	0,06204	11	0,117309658	2	876,57482	-1,01	18,76	0
P73409	Slr1841 protein OS=Synechocystis sp. (strain PCC 6803 / Kazusa) GN=s	953,30	16,67	1	4	9	28	1,926	15	10,2	630	67,6	4,65							
	A3	Sequence	# PSMs	# Proteins	# Protein Groups	Protein Group Accessions	Modifications	ΔCn	117/114	117/114 Count	117/114 Variability [%]	q-Value	PEP	IonScore	Exp Value	Charge	MH+ [Da]	ΔM [ppm]	RT [min]	# Missed Cleavages
	High	gGTQGIVGTTAANLNR	8	1	1	P73409	N-Term(iTRAQ4plex)	0,0000	1,910	4	1,6	0	4,129E-10	112	6,35287E-12	2	1673,90788	1,61	26,15	0
	High	kTLNNQTDNNQAVFQNR	7	1	1	P73409	N-Term(iTRAQ4plex); K1(iTRAQ4plex)	0,0000	1,988	5	3,5	0	6,269E-13	88	1,6633E-09	3	2293,18998	0,52	18,83	1
	High	tLNNQTDNNQAVFQNR	2	1	1	P73409	N-Term(iTRAQ4plex)	0,0000	1,636	1		0	2,089E-10	82	6,15134E-09	3	2020,99417	1,20	21,87	0
	High	lTGEVIFAPTAIFGTk	5	1	1	P73409	N-Term(iTRAQ4plex); K16(iTRAQ4plex)	0,0000	1,780	5	18,1	0	5,563E-07	64	3,57248E-07	2	1953,13664	3,40	50,92	0
	High	dVQPTAWAYEALk	1	2	2	P73409;P73103	N-Term(iTRAQ4plex); K13(iTRAQ4plex)	0,0000				0	4,474E-09	41	9,69154E-05	3	1779,95450	1,40	40,25	0
	High	yGcIVGYPDR	2	2	2	P73409;P73103	N-Term(iTRAQ4plex); C3(Carbamidomethyl)	0,0000				0	0,00001184	37	0,00021576	2	1343,65495	1,11	27,69	0
	High	lIQENVAVLREDIDk	1	2	2	P73409;P73103	N-Term(iTRAQ4plex); K15(iTRAQ4plex)	0,0000				0	1,237E-09	36	0,000276675	3	2043,17026	0,66	39,66	1
	High	rLmQEFEAELAALGAR	1	2	2	P73409;P73103	N-Term(iTRAQ4plex); M3(Oxidation)	0,0000				0	0,0001955	28	0,001644258	3	1965,03641	0,96	46,80	1
	High	eDIDkLk	1	2	2	P73409;P73103	N-Term(iTRAQ4plex); K5(iTRAQ4plex); K7(iTRAQ4plex)	0,0000				0	0,005364	12	0,069818418	3	1292,77814	-0,36	18,21	1
P73603	Slr1852 protein OS=Synechocystis sp. (strain PCC 6803 / Kazusa) GN=s	952,21	47,87	1	12	12	48	0,973	42	19,5	188	21,8	5,17							
	A3	Sequence	# PSMs	# Proteins	# Protein Groups	Protein Group Accessions	Modifications	ΔCn	117/114	117/114 Count	117/114 Variability [%]	q-Value	PEP	IonScore	Exp Value	Charge	MH+ [Da]	ΔM [ppm]	RT [min]	# Missed Cleavages
	High	lIAEGIAYVAQk	10	1	1	P73603	N-Term(iTRAQ4plex); K12(iTRAQ4plex)	0,0000	0,994	8	39,7	0	3,469E-10	71	8,47169E-08	2	1563,93755	1,69	43,02	0
	High	aDYSQPGLVPDTLk	3	1	1	P73603	N-Term(iTRAQ4plex); K14(iTRAQ4plex)	0,0000	0,973	3	13,1	0	2,692E-10	68	1,43539E-07	2	1791,97576	1,46	31,98	0
	High	ekLIAEGIAYVAQk	3	1	1	P73603	N-Term(iTRAQ4plex); K2(iTRAQ4plex); K14(iTRAQ4plex)	0,0000	1,102	3	2,3	0	2,035E-09	55	3,35714E-06	3	1965,17649	1,00	40,81	1
	High	dVFAPDSLLk	3	1	1	P73603	N-Term(iTRAQ4plex); K10(iTRAQ4plex)	0,0000	1,097	2	2,4	0	0,00004891	48	1,45536E-05	2	1392,80058	2,04	38,40	0
	High	kNYYLGLk	5	1	1	P73603	N-Term(iTRAQ4plex); K1(iTRAQ4plex); K8(iTRAQ4plex)	0,0000	1,068	5	32,8	0	0,001345	45	2,8312E-05	2	1430,87541	1,58	24,11	1
	High	gNVTWVR	5	1	1	P73603	N-Term(iTRAQ4plex)	0,0000	0,939	3	0,4	0	0,00132	40	8,93244E-05	2	975,55028	1,04	19,37	0
	High	tEQLTFYR	4	1	1	P73603	N-Term(iTRAQ4plex)	0,0000	0,851	4	8,1	0	0,0009421	38	0,00014157	2	1201,63542	1,71	27,05	0
	High	dWWPTHcEALR	1	1	1	P73603	N-Term(iTRAQ4plex); C7(Carbamidomethyl)	0,0000	0,572	1		0	5,962E-07	38	0,000161797	3	1614,76169	0,79	32,00	0
	High	aDYSQPGLVPDTLkEk	1	1	1	P73603	N-Term(iTRAQ4plex); K14(iTRAQ4plex); K16(iTRAQ4plex	0,0000	1,235	1		0	8,116E-07	35	0,000309877	3	2193,21622	1,57	29,68	1
	High	nYYLGLk	6	1	1	P73603	N-Term(iTRAQ4plex); K7(iTRAQ4plex)	0,0000	0,992	6	11,4	0	0,006196	31	0,000751571	2	1158,67803	1,65	29,90	0
	High	cVDDETHETIAk	2	1	1	P73603	N-Term(iTRAQ4plex); C1(Carbamidomethyl); K12(iTRAQ	0,0000	0,779	1		0	6,423E-07	31	0,000870903	3	1705,83323	1,56	16,79	0
	High	gGcYLk	5	1	1	P73603	N-Term(iTRAQ4plex); C3(Carbamidomethyl); K6(iTRAQ4p	0,0000	0,760	5	6,0	0	0,005154	24	0,003999171	2	985,53838	0,46	14,58	0
	>sp|K2C1_HUMAN|	945,29	23,48	1	13	14	36	1,835	25	48,3	643	65,8	8,12							
	A3	Sequence	# PSMs	# Proteins	# Protein Groups	Protein Group Accessions	Modifications	ΔCn	117/114	117/114 Count	117/114 Variability [%]	q-Value	PEP	IonScore	Exp Value	Charge	MH+ [Da]	ΔM [ppm]	RT [min]	# Missed Cleavages
	High	gGGGGGYGSGGSSYGSGGGSYG	2	1	1		N-Term(iTRAQ4plex)	0,0000	1,373	2	56,0	0	7,47743E-20	132	6,7293E-14	3	2528,05570	0,66	15,94	0
	High	skAEAESLYQSkYEELQITAGR	1	1	1		N-Term(iTRAQ4plex); K2(iTRAQ4plex); K12(iTRAQ4plex)	0,0000	5,084	1		0	5,034E-13	69	1,30909E-07	4	2933,55898	0,30	35,65	2
	High	sLNNQFASFIDkVR	7	1	1		N-Term(iTRAQ4plex); K12(iTRAQ4plex)	0,0000	3,051	4	8,9	0	2,728E-12	57	2,07E-06	3	1927,06546	0,75	42,18	1
	High	sLVNLGGSk	5	1	1		N-Term(iTRAQ4plex); K9(iTRAQ4plex)	0,0000	1,662	3	8,4	0	5,101E-07	54	3,63053E-06	2	1162,70391	0,41	23,67	0
	High	skAEAESLYQSk	5	1	1		N-Term(iTRAQ4plex); K2(iTRAQ4plex); K12(iTRAQ4plex)	0,0000	1,835	3	18,4	0	5,931E-09	52	6,65227E-06	3	1772,97617	0,39	21,37	1
	High	sLNNQFASFIDk	1	1	1		N-Term(iTRAQ4plex); K12(iTRAQ4plex)	0,0000	1,347	1		0	0,00001413	49	1,12972E-05	2	1671,89629	1,08	39,09	0
	High	sISISVAR	3	1	1		N-Term(iTRAQ4plex)	0,0000	1,303	2	39,9	0	0,00002047	48	1,59945E-05	2	976,59050	-0,30	23,80	0
	High	tNAENEFVTIkk	4	1	1		N-Term(iTRAQ4plex); K11(iTRAQ4plex); K12(iTRAQ4plex	0,0000	2,791	2	11,3	0	9,358E-11	48	1,6217E-05	3	1826,03922	0,44	24,66	1
	High	fLEQQNQVLQTk	1	2	2		N-Term(iTRAQ4plex); K12(iTRAQ4plex)	0,0000				0	4,062E-09	45	3,26361E-05	3	1763,98899	-0,26	30,29	0
	High	wELLQQVDTSTR	1	1	1		N-Term(iTRAQ4plex)	0,0000	1,826	1		0	0,00002988	35	0,000295101	2	1619,85491	2,41	39,58	0
	High	nkYEDEINkR	2	1	1		N-Term(iTRAQ4plex); K2(iTRAQ4plex); K9(iTRAQ4plex)	0,0000	1,596	2	1,6	0	0,00001111	25	0,0028772	3	1740,96054	0,03	15,32	2
	High	lRSEIDNVkk	2	1	1		N-Term(iTRAQ4plex); K9(iTRAQ4plex); K10(iTRAQ4plex)	0,0000	3,272	2	14,0	0	0,00005045	18	0,015630397	4	1633,99563	-0,33	18,29	2
	High	rVDQLkSDQSR	1	1	1		N-Term(iTRAQ4plex); K6(iTRAQ4plex)	0,0000	2,240	1		0,001	0,02191	14	0,048773199	4	1619,90578	-0,62	10,76	2
	High	rVDQLk	1	1	1		N-Term(iTRAQ4plex); K6(iTRAQ4plex)	0,0000	1,068	1		0,003	0,04548	21	0,007344631	3	1046,65555	-0,50	12,16	1
P22358	Chaperone protein dnaK2 OS=Synechocystis sp. (strain PCC 6803 / Kazu	924,10	25,00	1	16	16	36	1,169	31	31,3	636	67,6	4,86							
	A3	Sequence	# PSMs	# Proteins	# Protein Groups	Protein Group Accessions	Modifications	ΔCn	117/114	117/114 Count	117/114 Variability [%]	q-Value	PEP	IonScore	Exp Value	Charge	MH+ [Da]	ΔM [ppm]	RT [min]	# Missed Cleavages
	High	tTPSVVGYAk	7	1	1	P22358	N-Term(iTRAQ4plex); K10(iTRAQ4plex)	0,0000	1,169	5	17,7	0	0,000004818	81	8,10905E-09	2	1310,75749	1,26	22,16	0
	High	iINEPTAASLAYGLDkk	2	1	1	P22358	N-Term(iTRAQ4plex); K16(iTRAQ4plex); K17(iTRAQ4plex	0,0000	1,134	2	44,1	0	1,841E-10	68	1,41896E-07	3	2236,29215	0,35	36,18	1
	High	kNQADSLVYQAEk	3	1	1	P22358	N-Term(iTRAQ4plex); K1(iTRAQ4plex); K13(iTRAQ4plex)	0,0000	1,159	2	52,5	0	1,222E-08	63	5,24771E-07	3	1926,06626	0,29	21,62	1
	High	iAGIEVLR	3	1	1	P22358	N-Term(iTRAQ4plex)	0,0000	1,302	3	1,4	0	0,0005948	60	1,11936E-06	2	1014,64330	0,45	32,38	0
	Medium	qAVmNPGNTFYSVk	1	1	1	P22358	N-Term(iTRAQ4plex); M4(Oxidation); K14(iTRAQ4plex)	0,0000	0,740	1		0	6,692E-08	57	1,80289E-06	3	1859,95823	0,96	28,06	0
	High	iDkSALDEIVLVGGSTR	1	1	1	P22358	N-Term(iTRAQ4plex); K3(iTRAQ4plex)	0,0000	1,336	1		0	4,812E-11	53	5,18764E-06	3	2061,18033	0,41	41,26	1
	High	qFAPEEISAQVLR	2	1	1	P22358	N-Term(iTRAQ4plex)	0,0000	1,057	2	18,9	0	4,019E-08	52	6,2513E-06	2	1631,88762	0,13	39,30	0
	High	eAESNAAADkER	1	1	1	P22358	N-Term(iTRAQ4plex); K10(iTRAQ4plex)	0,0000	1,371	1		0	2,969E-08	47	1,97229E-05	3	1578,79465	-1,01	8,39	1
	High	rQAVmNPGNTFYSVk	2	1	1	P22358	N-Term(iTRAQ4plex); M5(Oxidation); K15(iTRAQ4plex)	0,0000	1,951	1		0	4,765E-12	46	2,76039E-05	3	2016,05765	0,04	23,38	1
	High	hLDTTLSR	3	1	1	P22358	N-Term(iTRAQ4plex)	0,0000	1,177	3	39,6	0	0,000008189	43	4,75302E-05	3	1086,60266	0,23	14,25	0
	High	kLVDDASk	3	1	1	P22358	N-Term(iTRAQ4plex); K1(iTRAQ4plex); K8(iTRAQ4plex)	0,0000	0,888	3	13,1	0	0,00052	39	0,000122453	3	1307,78583	-2,81	12,66	1
	High	nTTIPTk	3	1	1	P22358	N-Term(iTRAQ4plex); K7(iTRAQ4plex)	0,0000	0,951	3	8,7	0	0,0003097	35	0,000368462	2	1062,63945	-0,28	14,62	0
	High	ikAEGLIk	2	1	1	P22358	N-Term(iTRAQ4plex); K2(iTRAQ4plex); K8(iTRAQ4plex)	0,0000	1,646	1		0	0,000004949	26	0,002280185	3	1303,86554	-1,40	22,73	1
	High	iPAVQEVVkk	1	1	1	P22358	N-Term(iTRAQ4plex); K9(iTRAQ4plex); K10(iTRAQ4plex)	0,0000	3,558	1		0	0,001216	20	0,009862114	3	1542,99240	-1,27	26,09	1
	Medium	aEGIDLR	1	1	1	P22358	N-Term(iTRAQ4plex)	0,0000	0,731	1		0,03	0,3778	18	0,016786881	2	917,51787	0,62	19,25	0
	Medium	sLGTFR	1	1	1	P22358	N-Term(iTRAQ4plex)	0,0000	1,068	1		0,036	0,4563	10	0,095712796	2	824,47399	-0,86	19,78	0
P73728	Putative peroxiredoxin sll1621 OS=Synechocystis sp. (strain PCC 6803 /	919,43	48,15	1	13	13	48	0,877	38	23,2	189	21,2	5,05							
	A3	Sequence	# PSMs	# Proteins	# Protein Groups	Protein Group Accessions	Modifications	ΔCn	117/114	117/114 Count	117/114 Variability [%]	q-Value	PEP	IonScore	Exp Value	Charge	MH+ [Da]	ΔM [ppm]	RT [min]	# Missed Cleavages
	High	vRDESVPGPNPYR	10	1	1	P73728	N-Term(iTRAQ4plex)	0,0000	0,956	5	1,4	0	0,00000084	68	1,5775E-07	3	1629,84702	0,26	18,76	1
	High	vVLFSLPGAFTPTcSSNHLPR	2	1	1	P73728	N-Term(iTRAQ4plex); C14(Carbamidomethyl)	0,0000	0,667	2	26,6	0	1,308E-10	62	6,35287E-07	3	2444,28861	0,35	46,67	0
	High	tTEQIFGGk	2	1	1	P73728	N-Term(iTRAQ4plex); K9(iTRAQ4plex)	0,0000	0,573	1		0	5,693E-07	62	6,87021E-07	2	1268,70976	0,68	23,44	0
	High	vPSVVFk	3	1	1	P73728	N-Term(iTRAQ4plex); K7(iTRAQ4plex)	0,0000	0,933	3	10,5	0	0,007597	58	1,50314E-06	2	1063,67522	-0,19	30,67	0
	High	kVVLFSLPGAFTPTcSSNHLPR	2	1	1	P73728	N-Term(iTRAQ4plex); K1(iTRAQ4plex); C15(Carbamidom	0,0000	0,933	1		0	6,861E-13	53	4,92006E-06	4	2716,48574	0,35	42,05	1
	High	sNLGFGMR	2	1	1	P73728	N-Term(iTRAQ4plex)	0,0000	1,201	2	57,9	0	0,001732	43	4,47682E-05	2	1025,53227	0,35	24,10	0
	High	ySMFVNDGk	1	1	1	P73728	N-Term(iTRAQ4plex); K9(iTRAQ4plex)	0,0000	0,612	1		0	0,000001862	43	4,60225E-05	2	1348,68132	0,26	28,06	0
	High	wEDkTTEQIFGGk	2	1	1	P73728	N-Term(iTRAQ4plex); K4(iTRAQ4plex); K13(iTRAQ4plex)	0,0000	0,758	2	0,5	0	2,123E-09	41	7,29407E-05	3	1971,05564	0,42	32,65	1
	High	kmGMLVEk	2	1	1	P73728	N-Term(iTRAQ4plex); K1(iTRAQ4plex); M2(Oxidation); K	0,0000	0,782	2	6,9	0	0,0004534	38	0,000165185	3	1383,80399	-1,77	15,46	1
	High	ySmFVNDGk	4	1	1	P73728	N-Term(iTRAQ4plex); M3(Oxidation); K9(iTRAQ4plex)	0,0000	0,680	3	7,4	0	0,00005473	38	0,000177407	2	1364,67607	0,13	22,88	0
	High	kMGMLVEk	1	1	1	P73728	N-Term(iTRAQ4plex); K1(iTRAQ4plex); K8(iTRAQ4plex)	0,0000	1,098	1		0	0,00001581	33	0,000451825	3	1367,80863	-2,12	21,73	1
	High	sNLGFGmR	5	1	1	P73728	N-Term(iTRAQ4plex); M7(Oxidation)	0,0000	0,849	4	5,4	0	0,00231	28	0,001705965	2	1041,52776	0,89	18,95	0
	High	kmGmLVEk	4	1	1	P73728	N-Term(iTRAQ4plex); K1(iTRAQ4plex); M2(Oxidation); M	0,0000	1,121	3	1,0	0	0,001026	27	0,001985958	3	1399,79953	-1,30	12,02	1
	High	ySmFVNDGkIEk	2	1	1	P73728	N-Term(iTRAQ4plex); M3(Oxidation); K9(iTRAQ4plex); K	0,0000	1,191	2	14,7	0	4,178E-07	27	0,002324838	3	1879,00010	0,27	26,19	1
	High	qIGADkVk	3	1	1	P73728	N-Term(iTRAQ4plex); K6(iTRAQ4plex); K8(iTRAQ4plex)	0,0000	1,609	3	4,0	0	0,0003025	24	0,004038214	3	1290,81021	-0,28	12,53	1
	High	ySMFVNDGkIEk	1	1	1	P73728	N-Term(iTRAQ4plex); K9(iTRAQ4plex); K12(iTRAQ4plex)	0,0000	1,347	1		0	3,471E-07	24	0,004790631	3	1863,00272	-1,05	29,57	1
	Medium	mGmLVEk	1	1	1	P73728	N-Term(iTRAQ4plex); M1(Oxidation); M3(Oxidation); K7(	0,0000	0,793	1		0,011	0,1455	11	0,076907732	2	1127,60356	-0,68	14,46	0
	Medium	qIGADk	1	1	1	P73728	N-Term(iTRAQ4plex); K6(iTRAQ4plex)	0,0000	0,833	1		0,028	0,3422	19	0,013930606	2	919,54430	-0,90	11,59	0
P27181	ATP synthase subunit b OS=Synechocystis sp. (strain PCC 6803 / Kazus	916,09	49,16	1	16	16	41	1,519	31	39,5	179	19,8	5,14							
	A3	Sequence	# PSMs	# Proteins	# Protein Groups	Protein Group Accessions	Modifications	ΔCn	117/114	117/114 Count	117/114 Variability [%]	q-Value	PEP	IonScore	Exp Value	Charge	MH+ [Da]	ΔM [ppm]	RT [min]	# Missed Cleavages
	High	rmQEAAAQDLGAEQER	2	1	1	P27181	N-Term(iTRAQ4plex); M2(Oxidation)	0,0000	1,176	1		0	1,069E-09	77	2,1329E-08	3	1962,94443	1,20	14,84	1
	High	sIAQLGGR	5	1	1	P27181	N-Term(iTRAQ4plex)	0,0000	1,242	3	12,5	0	0,000009168	65	3,18398E-07	2	945,56035	0,55	19,24	0
	High	mQEAAAQDLGAEQER	1	1	1	P27181	N-Term(iTRAQ4plex); M1(Oxidation)	0,0000	1,005	1		0	4,23E-10	63	5,34527E-07	3	1806,84262	0,93	17,81	0
	High	iADAIEEAETR	1	1	1	P27181	N-Term(iTRAQ4plex)	0,0000	1,534	1		0	7,79E-08	59	1,31817E-06	2	1361,70354	0,43	31,94	0
	High	sAQILAEEEk	1	1	1	P27181	N-Term(iTRAQ4plex); K10(iTRAQ4plex)	0,0000	0,847	1		0	7,305E-08	57	2,01359E-06	2	1405,77922	1,06	21,83	0
	High	qEIATQTEADLR	1	1	1	P27181	N-Term(iTRAQ4plex)	0,0000	0,858	1		0	1,182E-09	55	3,19867E-06	2	1518,78972	1,08	23,33	0
	High	kSAQILAEEEk	5	1	1	P27181	N-Term(iTRAQ4plex); K1(iTRAQ4plex); K11(iTRAQ4plex)	0,0000	1,347	3	48,2	0	0,000001908	51	8,39402E-06	3	1677,97629	0,91	18,41	1
	High	iAEQAVAk	5	1	1	P27181	N-Term(iTRAQ4plex); K8(iTRAQ4plex)	0,0000	1,186	3	13,9	0	0,00001542	51	9,80376E-06	2	1117,68352	1,39	17,05	0
	High	rIAEQAVAk	5	1	1	P27181	N-Term(iTRAQ4plex); K9(iTRAQ4plex)	0,0000	2,134	3	12,3	0	0,00001711	43	5,61048E-05	3	1273,78301	-0,06	12,87	1
	High	kSAQILAEEEkk	3	1	1	P27181	N-Term(iTRAQ4plex); K1(iTRAQ4plex); K11(iTRAQ4plex)	0,0000	2,183	3	7,8	0	6,199E-11	42	6,74481E-05	3	1950,17154	-0,13	17,67	2
	High	qEIATQTEADLRR	2	1	1	P27181	N-Term(iTRAQ4plex)	0,0000	2,019	2	35,3	0	0,0002064	33	0,000485255	3	1674,88858	-0,36	21,60	1
	High	vIAELkR	4	1	1	P27181	N-Term(iTRAQ4plex); K6(iTRAQ4plex)	0,0000	2,248	3	21,4	0	0,004899	24	0,003749471	2	1116,73516	0,73	20,59	1
	High	iVQEAGQR	1	1	1	P27181	N-Term(iTRAQ4plex)	0,0000	0,725	1		0,001	0,01739	19	0,011271196	2	1044,59172	-0,14	13,50	0
	High	vIAELk	2	1	1	P27181	N-Term(iTRAQ4plex); K6(iTRAQ4plex)	0,0000	1,437	2	7,6	0,005	0,06095	28	0,001745702	2	960,63188	-1,39	24,34	0
	Medium	lAQAk	2	1	1	P27181	N-Term(iTRAQ4plex); K5(iTRAQ4plex)	0,0000	1,440	2	16,1	0,018	0,2329	30	0,00101202	2	818,53142	-2,94	11,74	0
	Medium	kLAQAk	1	1	1	P27181	N-Term(iTRAQ4plex); K1(iTRAQ4plex); K6(iTRAQ4plex)	0,0000	1,901	1		0,027	0,3333	23	0,00512826	2	1090,72942	-1,32	9,62	1
Q55318	Ferredoxin--NADP reductase OS=Synechocystis sp. (strain PCC 6803 / K	907,66	34,87	1	18	18	54	0,750	47	27,9	413	46,3	6,04							
	A3	Sequence	# PSMs	# Proteins	# Protein Groups	Protein Group Accessions	Modifications	ΔCn	117/114	117/114 Count	117/114 Variability [%]	q-Value	PEP	IonScore	Exp Value	Charge	MH+ [Da]	ΔM [ppm]	RT [min]	# Missed Cleavages
	High	mYSPGYVATSSR	3	1	1	Q55318	N-Term(iTRAQ4plex); M1(Oxidation)	0,0000	0,583	2	15,5	0	0,000009622	76	2,41529E-08	2	1478,70891	1,54	19,22	0
	High	gLAWLIFGIPk	3	1	1	Q55318	N-Term(iTRAQ4plex); K11(iTRAQ4plex)	0,0000	0,372	2	164,7	0	2,416E-09	71	8,14648E-08	2	1502,93169	-1,40	56,26	0
	High	kADDIPVNIYRPk	7	1	1	Q55318	N-Term(iTRAQ4plex); K1(iTRAQ4plex); K13(iTRAQ4plex)	0,0000	0,897	6	14,9	0	3,11E-14	54	4,11121E-06	3	1961,14945	-2,56	25,64	1
	High	sGSTFITVPLkR	4	1	1	Q55318	N-Term(iTRAQ4plex); K11(iTRAQ4plex)	0,0000	0,943	3	13,9	0	2,625E-07	50	9,03587E-06	3	1593,95853	1,15	29,25	1
	High	lYSIASTR	3	1	1	Q55318	N-Term(iTRAQ4plex)	0,0000	0,682	2	15,0	0	0,002548	50	1,03507E-05	2	1054,60112	-0,21	24,93	0
	High	vLENYPLVR	3	1	1	Q55318	N-Term(iTRAQ4plex)	0,0000	0,652	3	7,9	0	0,00004297	47	1,80705E-05	2	1246,72759	-0,03	32,47	0
	High	lTYAISR	5	1	1	Q55318	N-Term(iTRAQ4plex)	0,0000	0,732	3	0,7	0	0,008387	46	2,77313E-05	2	967,56828	-1,06	23,57	0
	High	hGDFGDDkTVSLcVR	2	1	1	Q55318	N-Term(iTRAQ4plex); K8(iTRAQ4plex); C13(Carbamidom	0,0000	1,094	2	36,5	0	2,655E-08	45	3,05471E-05	4	1993,99758	-1,44	24,24	1
	High	tmTTTPk	3	1	1	Q55318	N-Term(iTRAQ4plex); M2(Oxidation); K7(iTRAQ4plex)	0,0000	0,707	3	0,9	0	0,002839	37	0,000202754	2	1083,59441	-1,32	8,87	0
	High	tHTYmcGLk	2	1	1	Q55318	N-Term(iTRAQ4plex); M5(Oxidation); C6(Carbamidometh	0,0000	0,539	2	13,2	0	0,007147	37	0,000222316	2	1414,70427	-1,32	12,60	0
	High	sENILYkDDLEk	1	1	1	Q55318	N-Term(iTRAQ4plex); K7(iTRAQ4plex); K12(iTRAQ4plex)	0,0000	0,815	1		0	6,165E-08	30	0,000963762	3	1899,03971	-2,03	26,50	1
	High	tVSLcVR	2	1	1	Q55318	N-Term(iTRAQ4plex); C5(Carbamidomethyl)	0,0000	0,585	2	9,5	0	0,001007	30	0,001435302	2	978,55162	-0,69	21,18	0
	High	aDDIPVNIYRPk	1	1	1	Q55318	N-Term(iTRAQ4plex); K12(iTRAQ4plex)	0,0000	0,789	1		0	0,0001506	29	0,001403399	3	1688,95603	-0,83	28,35	0
	High	mYSPGYVATSSR	1	1	1	Q55318	N-Term(iTRAQ4plex)	0,0000	0,753	1		0	0,000007214	28	0,001765916	2	1462,71294	0,84	23,56	0
	High	tHTYMcGLk	1	1	1	Q55318	N-Term(iTRAQ4plex); C6(Carbamidomethyl); K9(iTRAQ4p	0,0000	0,614	1		0	0,0003674	25	0,00289048	3	1398,70823	-2,14	17,83	0
	High	tMTTTPk	2	1	1	Q55318	N-Term(iTRAQ4plex); K7(iTRAQ4plex)	0,0000	1,275	2	7,2	0	0,00511	19	0,013395842	2	1067,59783	-2,91	12,32	0
	High	mFkEQHEDYk	3	1	1	Q55318	N-Term(iTRAQ4plex); M1(Oxidation); K3(iTRAQ4plex); K	0,0000	0,647	3	13,3	0	0,0001018	18	0,016179683	4	1802,90713	-2,02	13,43	1
	High	mFkEQHEDYk	1	1	1	Q55318	N-Term(iTRAQ4plex); K3(iTRAQ4plex); K10(iTRAQ4plex)	0,0000	0,548	1		0	0,0007264	13	0,048861861	4	1786,91067	-2,90	16,87	1
	High	eQHEDYkFk	2	1	1	Q55318	N-Term(iTRAQ4plex); K7(iTRAQ4plex); K9(iTRAQ4plex)	0,0000	1,135	2	13,6	0,001	0,01718	15	0,028639802	4	1655,86916	-3,74	14,18	1
	High	aFLWR	2	1	1	Q55318	N-Term(iTRAQ4plex)	0,0000	0,805	2	4,4	0,003	0,0384	25	0,002964627	2	836,48973	-0,26	31,13	0
	Medium	tPYIGk	2	1	1	Q55318	N-Term(iTRAQ4plex); K6(iTRAQ4plex)	0,0000	0,632	2	2,4	0,019	0,2384	19	0,011856869	2	966,58385	-2,48	17,73	0
	Medium	mYIQHR	1	1	1	Q55318	N-Term(iTRAQ4plex)	0,0000	1,282	1		0,033	0,4153	10	0,089736681	3	991,52524	-1,17	12,68	0
P19569	Photosystem I reaction center subunit II OS=Synechocystis sp. (strain F	854,74	48,94	1	7	7	34	0,682	25	17,9	141	15,6	8,90							
	A3	Sequence	# PSMs	# Proteins	# Protein Groups	Protein Group Accessions	Modifications	ΔCn	117/114	117/114 Count	117/114 Variability [%]	q-Value	PEP	IonScore	Exp Value	Charge	MH+ [Da]	ΔM [ppm]	RT [min]	# Missed Cleavages
	High	fGGSTGGLLSk	10	1	1	P19569	N-Term(iTRAQ4plex); K11(iTRAQ4plex)	0,0000	0,757	6	9,7	0	8,956E-08	82	6,94976E-09	2	1311,75237	0,95	28,54	0
	High	kEQcLALGTQLR	5	1	1	P19569	N-Term(iTRAQ4plex); K1(iTRAQ4plex); C4(Carbamidome	0,0000	0,657	4	7,6	0	4,223E-07	71	8,22186E-08	2	1704,97099	2,37	23,72	1
	High	iGQNPEPVTIk	4	1	1	P19569	N-Term(iTRAQ4plex); K11(iTRAQ4plex)	0,0000	0,777	4	41,0	0	0,000001055	71	8,64908E-08	2	1483,87737	3,42	25,33	0
	High	vYPSGEVQYLHPADGVFPEk	3	1	1	P19569	N-Term(iTRAQ4plex); K20(iTRAQ4plex)	0,0000	0,605	3	29,2	0	1,532E-09	51	7,14447E-06	3	2520,30411	1,09	37,59	0
	High	tELSGQPPk	4	1	1	P19569	N-Term(iTRAQ4plex); K9(iTRAQ4plex)	0,0000	0,615	2	4,2	0	0,0003003	48	1,43219E-05	2	1244,71062	1,38	15,01	0
	High	rIGQNPEPVTIk	5	1	1	P19569	N-Term(iTRAQ4plex); K12(iTRAQ4plex)	0,0000	0,804	3	8,5	0	5,004E-07	38	0,000154515	3	1639,97568	1,38	20,17	1
	High	iQDYk	3	1	1	P19569	N-Term(iTRAQ4plex); K5(iTRAQ4plex)	0,0000	0,616	3	9,1	0,005	0,06497	21	0,00799779	2	954,55022	0,38	16,81	0
P73308	50S ribosomal protein L5 OS=Synechocystis sp. (strain PCC 6803 / Kazu	854,41	60,00	1	13	13	38	1,666	33	39,8	180	20,2	9,69							
	A3	Sequence	# PSMs	# Proteins	# Protein Groups	Protein Group Accessions	Modifications	ΔCn	117/114	117/114 Count	117/114 Variability [%]	q-Value	PEP	IonScore	Exp Value	Charge	MH+ [Da]	ΔM [ppm]	RT [min]	# Missed Cleavages
	High	tLYQETILPk	4	1	1	P73308	N-Term(iTRAQ4plex); K10(iTRAQ4plex)	0,0000	1,630	4	15,7	0	8,507E-09	69	1,21891E-07	2	1493,87981	-1,32	33,88	0
	High	lINLALPR	7	1	1	P73308	N-Term(iTRAQ4plex)	0,0000	2,406	5	15,6	0	0,00006823	63	5,40717E-07	2	1053,68913	-0,94	39,25	0
	High	lQEEFGYk	1	1	1	P73308	N-Term(iTRAQ4plex); K8(iTRAQ4plex)	0,0000	2,135	1		0	0,0004024	56	2,55252E-06	2	1301,69560	-1,84	27,57	0
	High	gNYSLGIR	4	1	1	P73308	N-Term(iTRAQ4plex)	0,0000	1,905	3	33,1	0	0,00002301	54	4,03618E-06	2	1023,56999	-0,38	22,24	0
	High	aLESSLTELATITGQkPVVTR	3	1	1	P73308	N-Term(iTRAQ4plex); K16(iTRAQ4plex)	0,0000	2,015	3	4,9	0	1,329E-09	54	4,13019E-06	3	2502,43155	-2,66	45,87	0
	High	gLGEASQNAk	2	1	1	P73308	N-Term(iTRAQ4plex); K10(iTRAQ4plex)	0,0000	2,153	1		0	0,00000034	51	7,62026E-06	2	1262,69280	-1,21	12,76	0
	High	iREGMPVGVMVTLR	1	1	1	P73308	N-Term(iTRAQ4plex)	0,0000	0,655	1		0	2,174E-07	43	5,53312E-05	3	1701,96000	-1,49	35,92	1
	High	aLGMPFR	3	1	1	P73308	N-Term(iTRAQ4plex)	0,0000	1,287	3	31,0	0	0,002567	39	0,000143943	2	935,52336	-2,15	29,91	0
	High	nIHQVPk	2	1	1	P73308	N-Term(iTRAQ4plex); K7(iTRAQ4plex)	0,0000	1,187	2	22,9	0	0,005786	33	0,000539473	2	1123,67790	-4,19	10,51	0
	High	kAIAGFk	2	1	1	P73308	N-Term(iTRAQ4plex); K1(iTRAQ4plex); K7(iTRAQ4plex)	0,0000	3,569	2	9,3	0	0,005166	26	0,002415294	3	1166,75708	-4,36	17,72	1
	High	aLGmPFR	3	1	1	P73308	N-Term(iTRAQ4plex); M4(Oxidation)	0,0000	1,509	3	2,9	0	0,01007	21	0,007429678	2	951,51860	-1,77	21,82	0
	High	mYAFLDR	1	1	1	P73308	N-Term(iTRAQ4plex); M1(Oxidation)	0,0000	1,351	1		0,002	0,02688	15	0,031913174	2	1075,53533	-0,92	27,80	0
	Medium	aIAGFk	4	1	1	P73308	N-Term(iTRAQ4plex); K6(iTRAQ4plex)	0,0000	0,978	3	11,1	0,018	0,2309	12	0,068386441	2	894,56157	-3,99	20,64	0
	Medium	aLGmPFRS	1	1	1	P73308	N-Term(iTRAQ4plex); M4(Oxidation)	0,0000	1,117	1		0,028	0,3477	11	0,085304119	2	1038,55156	-0,73	21,73	1
P73605	Slr1854 protein OS=Synechocystis sp. (strain PCC 6803 / Kazusa) GN=s	847,04	27,78	1	7	7	40	0,972	31	17,5	198	22,3	4,84							
	A3	Sequence	# PSMs	# Proteins	# Protein Groups	Protein Group Accessions	Modifications	ΔCn	117/114	117/114 Count	117/114 Variability [%]	q-Value	PEP	IonScore	Exp Value	Charge	MH+ [Da]	ΔM [ppm]	RT [min]	# Missed Cleavages
	High	aAATEGLk	2	1	1	P73605	N-Term(iTRAQ4plex); K8(iTRAQ4plex)	0,0000	0,765	1		0	0,00003695	64	4,03081E-07	2	1048,62578	1,59	13,63	0
	High	gIIAIGAYAmDR	4	1	1	P73605	N-Term(iTRAQ4plex); M10(Oxidation)	0,0000	1,047	3	25,1	0	1,621E-08	59	1,36763E-06	2	1410,75542	1,56	34,37	0
	High	kAAATEGLk	6	1	1	P73605	N-Term(iTRAQ4plex); K1(iTRAQ4plex); K9(iTRAQ4plex)	0,0000	1,109	4	4,4	0	0,00001953	57	1,83219E-06	3	1320,82523	3,09	11,60	1
	High	nQAPAVIFLR	8	1	1	P73605	N-Term(iTRAQ4plex)	0,0000	0,946	6	5,0	0	0,00002694	53	4,9314E-06	3	1272,75629	1,39	35,73	0
	High	gIIAIGAYAMDR	3	1	1	P73605	N-Term(iTRAQ4plex)	0,0000	1,088	2	6,3	0	0,000001279	45	3,57024E-05	2	1394,76042	1,52	40,19	0
	High	hPGMIEEFVk	4	1	1	P73605	N-Term(iTRAQ4plex); K10(iTRAQ4plex)	0,0000	1,096	4	1,3	0	1,886E-07	41	9,13477E-05	3	1474,79844	1,18	28,64	0
	High	hPGmIEEFVk	9	1	1	P73605	N-Term(iTRAQ4plex); M4(Oxidation); K10(iTRAQ4plex)	0,0000	0,879	7	13,5	0	0,000001821	39	0,0001277	2	1490,79546	2,58	22,61	0
	High	tFVEQIEA	1	1	1	P73605	N-Term(iTRAQ4plex)	0,0000	1,028	1		0	0,009062	31	0,000866902	2	1080,57073	1,23	34,71	0
	High	yQATVk	3	1	1	P73605	N-Term(iTRAQ4plex); K6(iTRAQ4plex)	0,0000	0,765	3	4,4	0,003	0,04065	28	0,001632939	2	997,59429	2,23	14,49	0
Q55517	Sll0529 protein OS=Synechocystis sp. (strain PCC 6803 / Kazusa) GN=s	835,83	25,72	1	13	13	40	0,474	32	22,5	731	78,3	5,60							
	A3	Sequence	# PSMs	# Proteins	# Protein Groups	Protein Group Accessions	Modifications	ΔCn	117/114	117/114 Count	117/114 Variability [%]	q-Value	PEP	IonScore	Exp Value	Charge	MH+ [Da]	ΔM [ppm]	RT [min]	# Missed Cleavages
	High	rPTPSTITLFTAGALEQGR	9	1	1	Q55517	N-Term(iTRAQ4plex)	0,0000	0,448	5	4,0	0	1,294E-09	78	2,27685E-08	3	2160,19168	1,05	39,44	0
	High	aLSPEAWQLVR	2	1	1	Q55517	N-Term(iTRAQ4plex)	0,0000	0,547	2	44,9	0	0,000008372	54	3,77546E-06	2	1413,79814	0,72	38,89	0
	High	sHGGPASQTVVTESVLPLADLGTL	1	1	1	Q55517	N-Term(iTRAQ4plex); K27(iTRAQ4plex)	0,0000	0,262	1		0	0,0004079	50	9,57128E-06	4	2975,67129	1,88	49,66	0
	High	nTVTEVGHHAPGFYSTmFLDR	4	1	1	Q55517	N-Term(iTRAQ4plex); M17(Oxidation)	0,0000	0,538	3	33,1	0	6,461E-07	49	1,2161E-05	4	2539,22133	2,22	31,93	0
	High	gIPITASk	5	1	1	Q55517	N-Term(iTRAQ4plex); K8(iTRAQ4plex)	0,0000	0,463	3	8,2	0	0,001309	47	2,46259E-05	2	1074,67766	1,41	20,65	0
	High	nVILVNAk	6	1	1	Q55517	N-Term(iTRAQ4plex); K8(iTRAQ4plex)	0,0000	0,568	5	41,3	0	0,00165	45	3,11867E-05	2	1158,74651	1,39	23,59	0
	High	lAFTQAVLEATDk	1	1	1	Q55517	N-Term(iTRAQ4plex); K13(iTRAQ4plex)	0,0000	0,484	1		0	0,000005913	38	0,000155586	3	1694,95877	1,18	42,39	0
	High	qVATTAmGELVVHVGk	1	1	1	Q55517	N-Term(iTRAQ4plex); M7(Oxidation); K16(iTRAQ4plex)	0,0000	1,763	1		0	2,024E-07	38	0,000161053	3	1944,08493	1,13	27,89	0
	High	sHNLYPGDSLDkDYIVSALQER	2	1	1	Q55517	N-Term(iTRAQ4plex); K12(iTRAQ4plex)	0,0000	0,602	2	64,9	0	1,73E-10	38	0,000171779	4	2808,44643	2,03	40,11	1
	High	vVSVINPR	2	1	1	Q55517	N-Term(iTRAQ4plex)	0,0000	0,379	2	14,7	0	0,001214	37	0,000194971	2	1027,63982	1,69	23,74	0
	High	fSFQQR	3	1	1	Q55517	N-Term(iTRAQ4plex)	0,0000	0,414	3	0,6	0	0,007633	32	0,000588803	2	956,50859	1,61	21,95	0
	High	nTVTEVGHHAPGFYSTMFLDR	2	1	1	Q55517	N-Term(iTRAQ4plex)	0,0000	0,589	2	20,4	0	2,064E-08	23	0,005545874	4	2523,22207	0,50	36,42	0
	High	nGWTHQRPEIENYFAAmmR	1	1	1	Q55517	N-Term(iTRAQ4plex); M17(Oxidation); M18(Oxidation)	0,0000	0,714	1		0	0,00006331	11	0,073955419	4	2527,17348	0,26	32,43	0

	Medium	yLTLQTTASk	1	1	1	Q55517	N-Term(iTRAQ4plex); K10(iTRAQ4plex)	0,0000	0,552	1		0,03	0,3692	12	0,058609768	2	1413,82134	1,53	27,43	0
P73596	Sll1307 protein OS=Synechocystis sp. (strain PCC 6803 / Kazusa) GN=s	821,28	29,14	1	4	4	26	0,824	18	7,3	175	17,8	6,52							
	A3	Sequence	# PSMs	# Proteins	# Protein Groups	Protein Group Accessions	Modifications	ΔCn	117/114	117/114 Count	117/114 Variability [%]	q-Value	PEP	IonScore	Exp Value	Charge	MH+ [Da]	ΔM [ppm]	RT [min]	# Missed Cleavages
	High	aYSAVTLATNHNLDR	8	1	1	P73596	N-Term(iTRAQ4plex)	0,0000	0,807	6	9,6	0	1,315E-12	100	1,01384E-10	2	1789,93376	1,33	26,96	0
	High	aAQSAIAAcR	5	1	1	P73596	N-Term(iTRAQ4plex); C9(Carbamidomethyl)	0,0000	0,822	3	6,6	0	0,00002425	78	1,57387E-08	2	1162,61492	2,54	13,76	0
	High	tSGILASmQAk	7	1	1	P73596	N-Term(iTRAQ4plex); M8(Oxidation); K11(iTRAQ4plex)	0,0000	0,810	5	7,7	0	0,00000102	63	5,06956E-07	2	1410,78972	2,27	18,21	0
	High	sDGALVHTVQTSFNk	6	1	1	P73596	N-Term(iTRAQ4plex); K15(iTRAQ4plex)	0,0000	0,853	4	8,8	0	2,777E-07	46	3,63185E-05	3	1892,01688	2,77	29,85	0
Q55648	Sll0314 protein OS=Synechocystis sp. (strain PCC 6803 / Kazusa) GN=s	817,34	31,21	1	11	11	32	1,269	24	20,3	314	35,4	5,97							
	A3	Sequence	# PSMs	# Proteins	# Protein Groups	Protein Group Accessions	Modifications	ΔCn	117/114	117/114 Count	117/114 Variability [%]	q-Value	PEP	IonScore	Exp Value	Charge	MH+ [Da]	ΔM [ppm]	RT [min]	# Missed Cleavages
	High	yTPFSQSEQVISR	3	1	1	Q55648	N-Term(iTRAQ4plex)	0,0000	1,094	3	22,8	0	1,114E-09	80	1,31561E-08	2	1685,86211	0,33	32,21	0
	High	yAQQTLESAQR	2	1	1	Q55648	N-Term(iTRAQ4plex)	0,0000	1,186	2	7,4	0	0,000003052	65	2,91052E-07	2	1438,74077	0,05	20,25	0
	High	lQQAQQYYAmALQk	1	1	1	Q55648	N-Term(iTRAQ4plex); M10(Oxidation); K14(iTRAQ4plex)	0,0000	1,612	1		0	1,012E-12	60	1,07887E-06	3	1988,05259	0,60	35,24	0
	High	kYAQQTLESAQR	3	1	1	Q55648	N-Term(iTRAQ4plex); K1(iTRAQ4plex)	0,0000	1,384	2	13,5	0	3,593E-08	56	2,29071E-06	3	1710,93729	-0,26	15,93	1
	High	gSYLQAVGk	4	1	1	Q55648	N-Term(iTRAQ4plex); K9(iTRAQ4plex)	0,0000	1,215	2	1,2	0	3,443E-07	52	7,2262E-06	2	1210,70354	0,10	24,47	0
	High	aLATTYR	2	1	1	Q55648	N-Term(iTRAQ4plex)	0,0000	1,252	2	2,6	0	0,0004918	50	9,0985E-06	2	939,53832	0,32	15,73	0
	High	aSAINPNDPELNLLR	1	1	1	Q55648	N-Term(iTRAQ4plex)	0,0000	1,412	1		0	9,061E-08	49	1,16942E-05	2	1780,96831	0,48	37,55	0
	High	lAQNPSLk	5	1	1	Q55648	N-Term(iTRAQ4plex); K8(iTRAQ4plex)	0,0000	1,564	3	18,8	0	0,0002962	46	2,67898E-05	2	1158,70867	0,14	18,85	0
	High	gYLDLFLSR	6	1	1	Q55648	N-Term(iTRAQ4plex)	0,0000	1,439	4	9,5	0	0,00009046	42	5,662E-05	2	1227,68559	0,14	46,03	0
	High	lQQAQQYYAMALQk	1	1	1	Q55648	N-Term(iTRAQ4plex); K14(iTRAQ4plex)	0,0000	0,916	1		0	2,425E-08	40	0,00010464	3	1972,05796	0,75	38,32	0
	High	kGSYLQAVGk	2	1	1	Q55648	N-Term(iTRAQ4plex); K1(iTRAQ4plex); K10(iTRAQ4plex)	0,0000	1,490	1		0	0,00001993	34	0,000380163	3	1482,90085	0,27	20,93	1
	High	tGTVFEHLDk	2	1	1	Q55648	N-Term(iTRAQ4plex); K10(iTRAQ4plex)	0,0000	1,194	2	9,9	0	0,00005902	28	0,001523947	3	1434,78348	0,24	24,93	0
P74229	30S ribosomal protein S7 OS=Synechocystis sp. (strain PCC 6803 / Kazu	793,15	53,21	1	9	9	31	1,519	26	37,6	156	17,4	10,45							
	A3	Sequence	# PSMs	# Proteins	# Protein Groups	Protein Group Accessions	Modifications	ΔCn	117/114	117/114 Count	117/114 Variability [%]	q-Value	PEP	IonScore	Exp Value	Charge	MH+ [Da]	ΔM [ppm]	RT [min]	# Missed Cleavages
	High	rPVPPDPVYNSTLLSMTIR	2	1	1	P74229	N-Term(iTRAQ4plex)	0,0000	1,702	1		0	8,656E-14	88	1,61425E-09	3	2300,25675	0,59	41,37	0
	High	rPVPPDPVYNSTLLSmTIR	5	1	1	P74229	N-Term(iTRAQ4plex); M16(Oxidation)	0,0000	1,514	3	19,9	0	7,027E-12	73	5,36281E-08	3	2316,25211	0,78	37,03	0
	High	nLTPLVEVk	3	1	1	P74229	N-Term(iTRAQ4plex); K9(iTRAQ4plex)	0,0000	1,182	3	7,2	0	0,00006999	64	4,14925E-07	2	1300,80620	-1,30	30,91	0
	High	vGGATYQVPMEVRPAR	1	1	1	P74229	N-Term(iTRAQ4plex)	0,0000	2,331	1		0	1,868E-07	54	4,44601E-06	3	1875,00455	0,93	27,86	0
	High	kRPVPPDPVYNSTLLSmTIR	5	1	1	P74229	N-Term(iTRAQ4plex); K1(iTRAQ4plex); M17(Oxidation)	0,0000	1,932	4	8,0	0	9,595E-07	51	7,41259E-06	3	2588,44809	0,29	33,41	1
	High	sLASSIVYNALASVGEk	1	1	1	P74229	N-Term(iTRAQ4plex); K17(iTRAQ4plex)	0,0000	1,160	1		0	6,188E-08	51	8,81846E-06	3	1997,11246	-1,67	51,97	0
	High	vGGATYQVPmEVRPAR	4	1	1	P74229	N-Term(iTRAQ4plex); M10(Oxidation)	0,0000	1,624	4	22,1	0	0,0001231	44	3,78416E-05	3	1890,99771	0,00	24,12	0
	High	wLVHFSR	1	1	1	P74229	N-Term(iTRAQ4plex)	0,0000	2,100	1		0	0,0002028	41	8,78962E-05	3	1088,61115	-0,96	31,28	0
	High	gTALALR	4	1	1	P74229	N-Term(iTRAQ4plex)	0,0000	1,510	3	17,4	0	0,003244	37	0,000205102	2	845,53185	-0,84	17,90	0
	High	aFAHYR	2	1	1	P74229	N-Term(iTRAQ4plex)	0,0000	1,483	2	36,9	0	0,008875	31	0,000803471	3	908,48517	-0,82	13,08	0
	High	rVGGATYQVPMEVRPAR	1	1	1	P74229	N-Term(iTRAQ4plex)	0,0000	1,031	1		0	0,000006691	29	0,001418071	4	2031,10256	-0,67	23,95	1
	High	kRPVPPDPVYNSTLLSMTIR	1	1	1	P74229	N-Term(iTRAQ4plex); K1(iTRAQ4plex)	0,0000	2,155	1		0	2,499E-09	27	0,001879187	4	2572,45180	-0,24	37,75	1
	High	rVGGATYQVPmEVRPAR	1	1	1	P74229	N-Term(iTRAQ4plex); M11(Oxidation)	0,0000	1,520	1		0	0,009082	21	0,00790624	4	2047,09926	0,21	20,75	1
P73922	D-fructose 1,6-bisphosphatase class 2/sedoheptulose 1,7-bisphosphatas	783,49	30,72	1	15	15	43	1,227	34	24,0	345	37,1	5,21							
	A3	Sequence	# PSMs	# Proteins	# Protein Groups	Protein Group Accessions	Modifications	ΔCn	117/114	117/114 Count	117/114 Variability [%]	q-Value	PEP	IonScore	Exp Value	Charge	MH+ [Da]	ΔM [ppm]	RT [min]	# Missed Cleavages
	High	tQSLVISSQSSTAR	6	1	1	P73922	N-Term(iTRAQ4plex)	0,0000	1,028	4	28,5	0	1,6E-09	99	1,16137E-10	2	1608,86992	1,58	20,08	0
	High	sIEELVVVmDRPR	3	1	1	P73922	N-Term(iTRAQ4plex); M10(Oxidation)	0,0000	1,320	2	8,5	0	2,863E-07	56	2,48296E-06	3	1801,99729	0,53	40,38	0
	High	kLAAPPAAk	4	1	1	P73922	N-Term(iTRAQ4plex); K1(iTRAQ4plex); K9(iTRAQ4plex)	0,0000	1,481	2	26,5	0	0,0001317	54	3,58072E-06	2	1298,85198	-0,06	14,12	1
	High	lASMGIk	1	1	1	P73922	N-Term(iTRAQ4plex); K7(iTRAQ4plex)	0,0000	1,270	1		0	0,0008748	42	7,81954E-05	2	1007,61510	-1,09	24,59	0
	High	gGLFAAPDFYmk	1	1	1	P73922	N-Term(iTRAQ4plex); M11(Oxidation); K12(iTRAQ4plex)	0,0000	0,975	1		0	1,718E-07	42	6,82292E-05	3	1620,83365	0,10	39,18	0
	High	gGLFAAPDFYmkk	1	1	1	P73922	N-Term(iTRAQ4plex); M11(Oxidation); K12(iTRAQ4plex);	0,0000	5,243	1		0	1,297E-07	40	0,000103985	3	1893,02817	-1,24	33,51	1
	High	gHVDIDkSATENLk	1	1	1	P73922	N-Term(iTRAQ4plex); K7(iTRAQ4plex); K14(iTRAQ4plex)	0,0000	2,301	1		0	4,337E-09	39	0,000206106	4	1959,08645	-0,37	20,62	1
	High	lAAPPAAk	4	1	1	P73922	N-Term(iTRAQ4plex); K8(iTRAQ4plex)	0,0000	0,952	2	28,0	0	0,003116	34	0,000374085	2	1026,65544	0,41	17,10	0
	High	lASmGIk	2	1	1	P73922	N-Term(iTRAQ4plex); M4(Oxidation); K7(iTRAQ4plex)	0,0000	0,953	2	11,9	0	0,000536	32	0,00075842	2	1023,61040	-0,70	16,68	0
	High	sIEELVVVMDRPR	1	1	1	P73922	N-Term(iTRAQ4plex)	0,0000	1,679	1		0	0,000001389	32	0,000680722	3	1786,00156	0,08	49,24	0
	High	hkELIQEIR	3	1	1	P73922	N-Term(iTRAQ4plex); K2(iTRAQ4plex)	0,0000	1,879	3	10,1	0	0,00004904	29	0,001342672	3	1453,87397	0,69	17,86	1
	High	fVDTVHMk	2	1	1	P73922	N-Term(iTRAQ4plex); K8(iTRAQ4plex)	0,0000	1,227	2	1,7	0	0,0001763	25	0,00310456	3	1264,69754	1,03	23,35	0
	High	iLSDcLNR	1	1	1	P73922	N-Term(iTRAQ4plex); C5(Carbamidomethyl)	0,0000	1,399	1		0	0,0047	25	0,003147531	2	1134,60576	-0,03	25,60	0
	High	fVDTVHmk	4	1	1	P73922	N-Term(iTRAQ4plex); M7(Oxidation); K8(iTRAQ4plex)	0,0000	1,059	3	17,8	0	0,001989	23	0,005011526	3	1280,69226	0,87	16,92	0
	High	fFHGGVR	4	1	1	P73922	N-Term(iTRAQ4plex)	0,0000	1,149	4	13,7	0	0,006154	22	0,006222573	3	963,52915	1,05	17,47	0
	High	fVDTVHmkESPk	2	1	1	P73922	N-Term(iTRAQ4plex); M7(Oxidation); K8(iTRAQ4plex); K	0,0000	1,995	1		0	0,00003046	14	0,037341288	4	1866,01565	0,03	18,29	1
	High	gGLFAAPDFYMk	1	1	1	P73922	N-Term(iTRAQ4plex); K12(iTRAQ4plex)	0,0000	1,172	1		0	0,0005385	14	0,03749471	3	1604,83390	-2,91	43,38	0
	High	gHVDIDk	1	1	1	P73922	N-Term(iTRAQ4plex); K7(iTRAQ4plex)	0,0000	0,778	1		0	0,01061	13	0,052356427	3	1071,60358	-0,12	11,73	0
	High	sATENLk	1	1	1	P73922	N-Term(iTRAQ4plex); K7(iTRAQ4plex)	0,0000	1,070	1		0,002	0,02848	28	0,001648049	2	1050,60283	-0,51	12,74	0
P72776	Pyridoxine 5'-phosphate synthase OS=Synechocystis sp. (strain PCC 68(	777,74	32,23	1	7	7	29	1,459	25	32,8	242	26,5	5,62							
	A3	Sequence	# PSMs	# Proteins	# Protein Groups	Protein Group Accessions	Modifications	ΔCn	117/114	117/114 Count	117/114 Variability [%]	q-Value	PEP	IonScore	Exp Value	Charge	MH+ [Da]	ΔM [ppm]	RT [min]	# Missed Cleavages
	High	mLTLGVNIDHVATIR	4	1	1	P72776	N-Term(iTRAQ4plex); M1(Oxidation)	0,0000	1,573	4	19,3	0	2,222E-10	83	4,51825E-09	3	1813,01163	-0,37	37,32	0
	High	mLTLGVNIDHVATIR	3	1	1	P72776	N-Term(iTRAQ4plex)	0,0000	1,459	3	5,4	0	3,94E-11	65	3,57024E-07	3	1797,01737	-0,01	40,52	0
	High	hIPVSLFIDPDVAQLk	9	1	1	P72776	N-Term(iTRAQ4plex); K16(iTRAQ4plex)	0,0000	1,540	7	28,4	0	2,713E-11	65	3,45915E-07	3	2080,20536	0,38	45,01	0
	High	yANAPTADDQAR	1	1	1	P72776	N-Term(iTRAQ4plex)	0,0000	1,263	1		0	0,00004118	49	1,26174E-05	2	1436,68974	0,74	15,11	0
	High	fIELHTGk	6	1	1	P72776	N-Term(iTRAQ4plex); K8(iTRAQ4plex)	0,0000	1,800	4	29,8	0	0,00001433	48	1,55228E-05	2	1232,72209	-1,67	23,92	0
	High	tHLNLEMAATk	1	1	1	P72776	N-Term(iTRAQ4plex); K11(iTRAQ4plex)	0,0000	1,159	1		0	0,000001834	38	0,000146545	3	1516,83716	-1,62	23,45	0
	High	tHLNLEmAATk	2	1	1	P72776	N-Term(iTRAQ4plex); M7(Oxidation); K11(iTRAQ4plex)	0,0000	1,162	2	44,7	0	0,000002362	38	0,000173368	3	1532,83426	-0,18	18,85	0
	High	aILVGmER	2	1	1	P72776	N-Term(iTRAQ4plex); M6(Oxidation)	0,0000	1,068	2	14,3	0,001	0,01458	31	0,001081753	2	1048,59368	-0,49	19,51	0
	Medium	lAmLGLPF	1	1	1	P72776	N-Term(iTRAQ4plex); M3(Oxidation)	0,0000	1,690	1		0,018	0,2365	23	0,006150966	2	1021,58702	-0,28	51,16	0
P26287	Apocytochrome f OS=Synechocystis sp. (strain PCC 6803 / Kazusa) GN=	762,51	35,67	1	12	12	31	1,246	28	29,4	328	35,2	5,16							
	A3	Sequence	# PSMs	# Proteins	# Protein Groups	Protein Group Accessions	Modifications	ΔCn	117/114	117/114 Count	117/114 Variability [%]	q-Value	PEP	IonScore	Exp Value	Charge	MH+ [Da]	ΔM [ppm]	RT [min]	# Missed Cleavages
	High	gQIYPTGLLSNNNAFk	8	1	1	P26287	N-Term(iTRAQ4plex); K16(iTRAQ4plex)	0,0000	1,368	6	29,1	0	8,713E-13	106	2,37668E-11	2	2025,10271	0,94	38,99	0
	High	iPYDLDSQQVLGDGSk	1	1	1	P26287	N-Term(iTRAQ4plex); K16(iTRAQ4plex)	0,0000	0,633	1		0	4,409E-11	61	7,94273E-07	3	2023,05960	0,46	36,87	0
	High	dTEVVLQNPTR	1	1	1	P26287	N-Term(iTRAQ4plex)	0,0000	1,419	1		0	0,000005389	60	1,11679E-06	2	1415,76152	0,28	22,73	0
	High	vGGTYFQPYR	4	1	1	P26287	N-Term(iTRAQ4plex)	0,0000	1,051	4	5,5	0	0,00000201	46	2,59998E-05	2	1331,68682	0,26	29,51	0
	High	lSEGLkEk	4	1	1	P26287	N-Term(iTRAQ4plex); K6(iTRAQ4plex); K8(iTRAQ4plex)	0,0000	1,684	4	17,4	0	0,00004794	42	6,47098E-05	2	1335,81890	-1,43	16,62	1
	High	sINYGk	4	1	1	P26287	N-Term(iTRAQ4plex); K6(iTRAQ4plex)	0,0000	1,149	4	12,2	0	0,002709	40	9,26766E-05	2	969,56059	-0,17	14,21	0
	High	fAVHLGANR	3	1	1	P26287	N-Term(iTRAQ4plex)	0,0000	1,437	3	8,0	0	0,0000291	39	0,000131817	2	1128,63970	0,19	21,55	0
	High	vQAAELNF	1	1	1	P26287	N-Term(iTRAQ4plex)	0,0000	1,247	1		0	0,0002169	38	0,00014756	2	1035,55901	-0,15	36,39	0
	High	iVcANcHLAQk	1	1	1	P26287	N-Term(iTRAQ4plex); C3(Carbamidomethyl); C6(Carbam	0,0000	1,078	1		0	1,04E-08	35	0,000324317	3	1601,85074	0,80	18,92	0
	High	gGLNVGAVLmLPEGFk	2	1	1	P26287	N-Term(iTRAQ4plex); M10(Oxidation); K16(iTRAQ4plex)	0,0000	1,591	1		0	0,00004726	32	0,000754567	3	1906,06894	-1,14	48,63	0
	High	lSEGLk	1	1	1	P26287	N-Term(iTRAQ4plex); K6(iTRAQ4plex)	0,0000	0,768	1		0,005	0,06699	27	0,001786364	2	934,58104	-0,15	17,75	0
	Medium	iAPPDR	1	1	1	P26287	N-Term(iTRAQ4plex)	0,0000	1,509	1		0,037	0,4586	17	0,021775594	2	812,47417	-0,66	14,36	0
P73418	DNA-binding protein HU OS=Synechocystis sp. (strain PCC 6803 / Kazus	761,23	60,00	1	11	11	46	1,321	36	22,2	100	10,9	8,63							
	A3	Sequence	# PSMs	# Proteins	# Protein Groups	Protein Group Accessions	Modifications	ΔCn	117/114	117/114 Count	117/114 Variability [%]	q-Value	PEP	IonScore	Exp Value	Charge	MH+ [Da]	ΔM [ppm]	RT [min]	# Missed Cleavages
	High	vTLVGFGSFEAR	4	1	1	P73418	N-Term(iTRAQ4plex)	0,0000	1,204	4	5,9	0	0,000002264	66	2,58208E-07	2	1426,78325	1,48	42,10	0
	High	vPAFSAGk	5	1	1	P73418	N-Term(iTRAQ4plex); K8(iTRAQ4plex)	0,0000	1,329	3	1,7	0	0,0002637	51	7,56781E-06	2	1064,63347	-0,77	22,50	0
	High	eQLGESITk	2	1	1	P73418	N-Term(iTRAQ4plex); K9(iTRAQ4plex)	0,0000	1,025	2	2,3	0	0,000002389	48	1,51346E-05	2	1292,73149	1,13	20,37	0
	High	mNkGELVHAVTDk	9	1	1	P73418	N-Term(iTRAQ4plex); M1(Oxidation); K3(iTRAQ4plex); K	0,0000	1,692	5	19,2	0	3,551E-10	42	6,01174E-05	3	1890,04996	1,06	21,26	1
	High	gELVHAVTDk	4	1	1	P73418	N-Term(iTRAQ4plex); K10(iTRAQ4plex)	0,0000	0,961	4	26,0	0	0,00007748	42	6,79157E-05	2	1356,77312	0,40	20,28	0
	High	akEQLGESITkk	1	1	1	P73418	N-Term(iTRAQ4plex); K2(iTRAQ4plex); K11(iTRAQ4plex)	0,0000	3,796	1		0	6,913E-09	38	0,000148583	3	1908,15982	-0,73	18,16	2
	High	akEQLGESITk	2	1	1	P73418	N-Term(iTRAQ4plex); K2(iTRAQ4plex); K11(iTRAQ4plex)	0,0000	1,238	2	21,0	0	1,773E-07	37	0,000188352	3	1635,96805	2,36	18,40	1
	High	eQLGESITkk	2	1	1	P73418	N-Term(iTRAQ4plex); K9(iTRAQ4plex); K10(iTRAQ4plex)	0,0000	4,090	2	38,0	0	0,000002539	36	0,000239315	3	1564,92679	-0,18	17,72	1
	High	mNkGELVHAVTDk	4	1	1	P73418	N-Term(iTRAQ4plex); K3(iTRAQ4plex); K13(iTRAQ4plex)	0,0000	1,316	4	9,9	0	6,279E-08	32	0,000709887	3	1874,05295	-0,05	27,07	1
	High	vAPEkk	4	1	1	P73418	N-Term(iTRAQ4plex); K5(iTRAQ4plex); K6(iTRAQ4plex)	0,0000	1,435	4	22,6	0	0,002351	21	0,008432765	3	1103,71460	-0,25	8,46	1
	High	mLIPATk	2	1	1	P73418	N-Term(iTRAQ4plex); K7(iTRAQ4plex)	0,0000	1,263	1		0	0,005782	18	0,017416866	2	1061,66326	0,10	25,32	0
	High	mLIPATk	6	1	1	P73418	N-Term(iTRAQ4plex); M1(Oxidation); K7(iTRAQ4plex)	0,0000	1,348	3	3,8	0,001	0,01288	28	0,001738526	2	1077,65874	0,63	21,15	0
	High	dkVAPEkk	1	1	1	P73418	N-Term(iTRAQ4plex); K2(iTRAQ4plex); K7(iTRAQ4plex);	0,0000	1,526	1		0,001	0,01622	13	0,054950292	4	1490,93850	-0,25	8,46	2
P73128	Sulfolipid biosynthesis protein SqdB OS=Synechocystis sp. (strain PCC 6	753,83	36,29	1	15	15	32	1,048	25	17,8	383	43,2	6,01							
	A3	Sequence	# PSMs	# Proteins	# Protein Groups	Protein Group Accessions	Modifications	ΔCn	117/114	117/114 Count	117/114 Variability [%]	q-Value	PEP	IonScore	Exp Value	Charge	MH+ [Da]	ΔM [ppm]	RT [min]	# Missed Cleavages
	High	fcIQAAIGHPLTVYGk	7	1	1	P73128	N-Term(iTRAQ4plex); C2(Carbamidomethyl); K16(iTRAQ4	0,0000	1,224	4	5,9	0	3,35E-15	103	5,91439E-11	3	2063,13736	1,11	40,52	0
	High	rYWDAQLGAETLTPIAPIR	6	1	1	P73128	N-Term(iTRAQ4plex)	0,0000	0,976	4	4,1	0	2,196E-08	80	9,46172E-09	3	2315,26499	0,89	41,70	1
	High	gYEVGILDSLVR	2	1	1	P73128	N-Term(iTRAQ4plex)	0,0000	1,086	2	9,5	0	1,416E-08	65	2,97852E-07	2	1464,81902	0,75	45,40	0
	High	qFQPDAVVHFGEQR	2	1	1	P73128	N-Term(iTRAQ4plex)	0,0000	0,636	2	31,6	0	2,009E-09	61	7,19399E-07	3	1801,91141	0,64	32,21	0
	High	lDYDGVFGTALNR	1	1	1	P73128	N-Term(iTRAQ4plex)	0,0000	0,992	1		0	0,00001125	55	2,90382E-06	2	1584,81340	-0,31	40,75	0
	High	qPGSFYHLSk	1	1	1	P73128	N-Term(iTRAQ4plex); K10(iTRAQ4plex)	0,0000	1,199	1		0	0,000001642	35	0,000321344	3	1451,78812	-0,29	22,68	0
	High	wYELTGk	1	1	1	P73128	N-Term(iTRAQ4plex); K7(iTRAQ4plex)	0,0000	1,048	1		0	0,0002584	34	0,000414925	2	1184,65581	0,35	31,11	0
	High	vEIDHLENPR	1	1	1	P73128	N-Term(iTRAQ4plex)	0,0000	0,548	1		0	0,00005631	29	0,00126174	3	1365,72361	-0,54	22,80	0
	High	kDTLPYPk	2	1	1	P73128	N-Term(iTRAQ4plex); K1(iTRAQ4plex); K8(iTRAQ4plex)	0,0000	0,791	2	63,4	0	0,00003214	29	0,00129113	3	1393,84018	-0,97	18,48	1
	High	vHDSHNIHFAck	1	1	1	P73128	N-Term(iTRAQ4plex); C11(Carbamidomethyl); K12(iTRAC	0,0000	0,510	1		0	0,00002211	29	0,001330363	4	1752,88552	0,72	14,55	0
	High	lDRWYELTGk	1	1	1	P73128	N-Term(iTRAQ4plex); K10(iTRAQ4plex)	0,0000	1,176	1		0	0,000001748	18	0,016594723	3	1568,86649	-0,67	34,63	1
	High	dTLPYPk	1	1	1	P73128	N-Term(iTRAQ4plex); K7(iTRAQ4plex)	0,0000	0,949	1		0,003	0,04538	40	0,000106706	2	1121,64495	0,40	22,51	0
	High	gLLDIR	2	1	1	P73128	N-Term(iTRAQ4plex)	0,0000	1,088	1		0,003	0,03957	22	0,006456096	2	830,52110	-0,66	28,69	0
	High	gLLDIRDTVR	2	1	1	P73128	N-Term(iTRAQ4plex)	0,0000	2,177	1		0,003	0,04483	13	0,05615555	3	1301,76752	1,30	30,42	1
	Medium	iWGLR	2	1	1	P73128	N-Term(iTRAQ4plex)	0,0000	1,199	2	1,2	0,023	0,2943	16	0,024886854	2	788,48949	-0,59	29,35	0
P27179	ATP synthase subunit alpha OS=Synechocystis sp. (strain PCC 6803 / Ka	735,26	28,23	1	14	14	33	1,168	27	20,1	503	53,9	5,08							
	A3	Sequence	# PSMs	# Proteins	# Protein Groups	Protein Group Accessions	Modifications	ΔCn	117/114	117/114 Count	117/114 Variability [%]	q-Value	PEP	IonScore	Exp Value	Charge	MH+ [Da]	ΔM [ppm]	RT [min]	# Missed Cleavages
	High	sTLVIYDDLSk	2	1	1	P27179	N-Term(iTRAQ4plex); K11(iTRAQ4plex)	0,0000	1,123	1		0	5,446E-10	82	6,12308E-09	2	1541,86821	1,09	35,36	0
	High	tAIAIDTIINQk	2	1	1	P27179	N-Term(iTRAQ4plex); K12(iTRAQ4plex)	0,0000	0,840	2	23,8	0	8,555E-09	70	1,18411E-07	2	1588,95159	0,19	37,66	0
	High	aSTVAQIIDTLTEk	2	1	1	P27179	N-Term(iTRAQ4plex); K14(iTRAQ4plex)	0,0000	1,311	2	18,9	0	2,295E-09	65	3,08298E-07	2	1778,01787	1,61	51,70	0
	High	yVEIINSSk	1	1	1	P27179	N-Term(iTRAQ4plex); K9(iTRAQ4plex)	0,0000	1,179	1		0	0,0001113	55	3,19132E-06	2	1340,76787	1,09	28,03	0
	High	eAYPGDVFYIHSR	6	1	1	P27179	N-Term(iTRAQ4plex)	0,0000	1,299	4	7,2	0	2,826E-11	48	1,54159E-05	3	1697,84159	0,68	31,97	0
	High	vTEFAQGLR	1	1	1	P27179	N-Term(iTRAQ4plex)	0,0000	0,977	1		0	0,00001732	48	1,63001E-05	2	1164,65105	1,43	28,69	0
	High	vSIRPDEISSIIR	5	1	1	P27179	N-Term(iTRAQ4plex)	0,0000	1,277	3	6,7	0	2,532E-07	46	2,29071E-05	3	1628,94596	0,43	37,98	0
	High	tTGQIAQIPIGDAmVGR	1	1	1	P27179	N-Term(iTRAQ4plex); M14(Oxidation)	0,0000	0,888	1		0	8,145E-07	43	4,91122E-05	3	1888,00962	0,88	33,16	0
	High	vGSAAQTk	4	1	1	P27179	N-Term(iTRAQ4plex); K8(iTRAQ4plex)	0,0000	1,303	4	7,5	0	0,001087	42	5,754E-05	2	1049,61992	0,54	9,49	0
	High	gPISSTATR	2	1	1	P27179	N-Term(iTRAQ4plex)	0,0000	1,023	2	6,8	0	0,005402	41	8,43277E-05	2	1033,57695	1,04	12,70	0
	High	vVDSLGRPIDGk	2	1	1	P27179	N-Term(iTRAQ4plex); K12(iTRAQ4plex)	0,0000	1,182	1		0	0,0008216	27	0,001940752	3	1543,90647	1,16	24,69	0
	High	eLIIGDRk	1	1	1	P27179	N-Term(iTRAQ4plex); K8(iTRAQ4plex)	0,0000	1,876	1		0	0,004385	23	0,004863736	3	1231,76181	0,43	19,04	1
	High	qMSLLMR	2	1	1	P27179	N-Term(iTRAQ4plex)	0,0000	1,011	2	15,4	0,003	0,03661	19	0,011427994	2	1022,56114	0,36	28,69	0
	High	qMSLLmR	1	1	1	P27179	N-Term(iTRAQ4plex); M6(Oxidation)	0,0000	0,836	1		0,007	0,08998	18	0,014926913	2	1038,55571	0,02	23,91	0
	Medium	eLIIGDR	1	1	1	P27179	N-Term(iTRAQ4plex)	0,0000	1,468	1		0,013	0,17	21	0,00827885	2	959,56456	0,32	23,69	0
P0DJF8	S-methyl-5'-thioadenosine phosphorylase OS=Synechocystis sp. (strain	721,86	24,23	1	7	7	31	1,437	21	21,4	326	35,3	6,86							
	A3	Sequence	# PSMs	# Proteins	# Protein Groups	Protein Group Accessions	Modifications	ΔCn	117/114	117/114 Count	117/114 Variability [%]	q-Value	PEP	IonScore	Exp Value	Charge	MH+ [Da]	ΔM [ppm]	RT [min]	# Missed Cleavages
	High	qLAANPFESIAHR	7	1	1	P0DJF8	N-Term(iTRAQ4plex)	0,0000	1,430	3	8,6	0	9,743E-09	76	2,79235E-08	3	1597,85666	-0,06	32,06	0
	High	aLQYAVLTPPDkFPAATYEk	4	1	1	P0DJF8	N-Term(iTRAQ4plex); K12(iTRAQ4plex); K20(iTRAQ4plex	0,0000	2,105	3	11,0	0	8,844E-09	59	1,40272E-06	3	2655,47477	-0,42	39,77	1
	High	lSLLLGk	7	1	1	P0DJF8	N-Term(iTRAQ4plex); K7(iTRAQ4plex)	0,0000	1,442	5	13,7	0	0,0001759	50	1,02795E-05	2	1031,70635	-0,36	37,55	0
	High	gHHLLPSEIPFR	4	1	1	P0DJF8	N-Term(iTRAQ4plex)	0,0000	1,534	2	11,5	0	0,00000284	43	4,90874E-05	3	1546,86069	-0,27	29,34	0
	High	nAVNAQQVILETVk	3	1	1	P0DJF8	N-Term(iTRAQ4plex); K14(iTRAQ4plex)	0,0000	0,965	3	11,5	0	2,241E-09	42	6,1372E-05	3	1815,05838	0,28	37,88	0
	High	vAFLAR	4	1	1	P0DJF8	N-Term(iTRAQ4plex)	0,0000	1,638	3	2,8	0,001	0,01477	33	0,000530884	2	820,51579	-0,46	26,30	0
	High	aNIHGmk	2	1	1	P0DJF8	N-Term(iTRAQ4plex); M6(Oxidation); K7(iTRAQ4plex)	0,0000	1,410	2	2,8	0,001	0,02036	18	0,015702543	2	1074,59856	1,59	8,87	0
P29254	Photosystem I P700 chlorophyll a apoprotein A1 OS=Synechocystis sp. (	709,85	12,65	1	13	13	41	0,649	33	10,3	751	82,9	7,77							
	A3	Sequence	# PSMs	# Proteins	# Protein Groups	Protein Group Accessions	Modifications	ΔCn	117/114	117/114 Count	117/114 Variability [%]	q-Value	PEP	IonScore	Exp Value	Charge	MH+ [Da]	ΔM [ppm]	RT [min]	# Missed Cleavages
	High	dIPLPHEFILEPSk	3	1	1	P29254	N-Term(iTRAQ4plex); K14(iTRAQ4plex)	0,0000	0,618	2	9,5	0	3,103E-11	63	5,36995E-07	3	1923,08457	0,80	40,63	0
	High	nVNNLLDR	3	1	1	P29254	N-Term(iTRAQ4plex)	0,0000	0,615	3	0,4	0	0,000004548	62	6,88605E-07	2	1101,61455	1,11	24,15	0
	High	lLDAGVAPk	3	1	1	P29254	N-Term(iTRAQ4plex); K9(iTRAQ4plex)	0,0000	0,777	2	25,7	0	5,752E-07	58	1,70204E-06	2	1171,72942	0,42	25,99	0
	High	aLSIIQGR	5	1	1	P29254	N-Term(iTRAQ4plex)	0,0000	0,668	3	1,0	0	0,0004454	43	4,88619E-05	2	1001,62370	1,27	27,04	0
	High	lNVAPAIQPR	6	1	1	P29254	N-Term(iTRAQ4plex)	0,0000	0,622	4	3,2	0	4,535E-07	39	0,000134887	2	1222,73992	0,85	27,59	0
	High	gPFTGEGHk	5	1	1	P29254	N-Term(iTRAQ4plex); K9(iTRAQ4plex)	0,0000	0,594	5	14,7	0	0,0002589	39	0,000137079	2	1217,65251	0,65	12,49	0
	High	tNWGIGHSMk	3	1	1	P29254	N-Term(iTRAQ4plex); K10(iTRAQ4plex)	0,0000	0,550	2	14,1	0	0,000006256	33	0,000521159	3	1418,74637	0,74	21,17	0
	High	tNWGIGHSmk	2	1	1	P29254	N-Term(iTRAQ4plex); M9(Oxidation); K10(iTRAQ4plex)	0,0000	0,657	1		0	0,00008565	32	0,000654591	3	1434,74237	1,49	16,32	0
	High	lVPDkANLGFR	2	1	1	P29254	N-Term(iTRAQ4plex); K5(iTRAQ4plex)	0,0000	0,747	2	20,2	0	0,0004752	26	0,002722513	3	1517,90366	-0,41	31,38	1
	High	eILEAHk	2	1	1	P29254	N-Term(iTRAQ4plex); K7(iTRAQ4plex)	0,0000	0,625	2	15,4	0	0,0006084	20	0,00948353	3	1127,66773	1,26	15,35	0
	High	tNWGIGHSmkEILEAHk	1	1	1	P29254	N-Term(iTRAQ4plex); M9(Oxidation); K10(iTRAQ4plex); I	0,0000	0,935	1		0	0,000002206	18	0,019784896	5	2399,28907	1,01	26,04	1
	High	wGkPGHFDR	2	1	1	P29254	N-Term(iTRAQ4plex); K3(iTRAQ4plex)	0,0000	0,646	2	9,6	0	0,00005197	17	0,01883519	3	1387,74909	1,24	17,66	0
	High	eILEAHkGPFTGEGHk	1	1	1	P29254	N-Term(iTRAQ4plex); K7(iTRAQ4plex); K16(iTRAQ4plex)	0,0000	1,267	1		0	0,000005544	13	0,053951062	5	2182,19907	0,43	20,38	1
	High	aNLGFR	3	1	1	P29254	N-Term(iTRAQ4plex)	0,0000	0,656	3	5,5	0,001	0,01412	36	0,000250017	2	821,47588	1,03	18,93	0
P73511	Glycogen phosphorylase OS=Synechocystis sp. (strain PCC 6803 / Kazu:	708,69	16,73	1	15	15	35	0,426	33	68,1	849	96,6	5,29							
	A3	Sequence	# PSMs	# Proteins	# Protein Groups	Protein Group Accessions	Modifications	ΔCn	117/114	117/114 Count	117/114 Variability [%]	q-Value	PEP	IonScore	Exp Value	Charge	MH+ [Da]	ΔM [ppm]	RT [min]	# Missed Cleavages
	High	vIFLPDYNVk	2	1	1	P73511	N-Term(iTRAQ4plex); K10(iTRAQ4plex)	0,0000	0,301	2	68,5	0	6,16E-09	67	2,06049E-07	2	1495,87554	-0,52	40,54	0
	High	hLEIIYEINQR	2	1	1	P73511	N-Term(iTRAQ4plex)	0,0000	0,664	2	62,8	0	4,102E-10	64	4,2655E-07	3	1571,86612	-0,08	32,09	0
	High	aAPGYFTAk	2	1	1	P73511	N-Term(iTRAQ4plex); K9(iTRAQ4plex)	0,0000	0,369	2	40,2	0	0,000005113	52	6,32368E-06	2	1213,67925	-2,23	22,53	0
	High	mAILNVAR	3	1	1	P73511	N-Term(iTRAQ4plex)	0,0000	0,790	3	2,9	0	0,00002542	52	6,63697E-06	2	1031,61443	-0,80	31,08	0
	High	fPAIATk	1	1	1	P73511	N-Term(iTRAQ4plex); K7(iTRAQ4plex)	0,0000	0,207	1		0	0,00222	45	2,88383E-05	2	1035,64031	-3,68	26,50	0
	High	tFIYGGk	4	1	1	P73511	N-Term(iTRAQ4plex); K7(iTRAQ4plex)	0,0000	0,402	3	20,2	0	0,001118	45	3,06175E-05	2	1073,62102	-2,19	23,09	0
	High	wIAGSLVk	3	1	1	P73511	N-Term(iTRAQ4plex); K8(iTRAQ4plex)	0,0000	0,255	3	30,3	0	0,001023	40	9,41825E-05	2	1161,72087	-2,21	32,82	0
	High	rWmVLSNPR	3	1	1	P73511	N-Term(iTRAQ4plex); M3(Oxidation)	0,0000	0,661	2	87,7	0	0,00001297	40	9,88485E-05	3	1318,71686	-0,18	21,98	1
	High	rWMVLSNPR	1	1	1	P73511	N-Term(iTRAQ4plex)	0,0000	0,472	1		0	0,0001023	36	0,000227494	3	1302,72086	-1,02	26,67	1
	High	lLVDEHHYEWQR	4	1	1	P73511	N-Term(iTRAQ4plex)	0,0000	0,402	4	32,7	0	0,000003632	35	0,000314029	3	1768,88926	0,27	28,15	0
	High	lSNLISSR	3	1	1	P73511	N-Term(iTRAQ4plex)	0,0000	0,331	3	4,1	0	0,0001928	33	0,000587735	2	1033,61199	-0,26	28,19	0
	High	vSTANNLR	2	1	1	P73511	N-Term(iTRAQ4plex)	0,0000	0,886	2	25,0	0	0,007065	29	0,001213305	2	1018,57573	-0,46	13,58	0
	High	aLADNLFYLQGk	1	1	1	P73511	N-Term(iTRAQ4plex); K12(iTRAQ4plex)	0,0000	0,828	1		0	0,0001129	20	0,0097268	3	1640,92344	-0,99	40,92	0
	High	wSLPLFGEmLPR	2	1	1	P73511	N-Term(iTRAQ4plex); M9(Oxidation)	0,0000	0,493	2	153,9	0	0,00177	11	0,081464802	2	1605,86174	2,31	49,51	0
	High	gIPYDTPILGYk	1	1	1	P73511	N-Term(iTRAQ4plex); K12(iTRAQ4plex)	0,0000	0,595	1		0,006	0,07873	19	0,011856869	2	1624,91887	-0,02	38,67	0
	Medium	mkYPNDGDR	1	1	1	P73511	N-Term(iTRAQ4plex); M1(Oxidation); K2(iTRAQ4plex)	0,0000	0,771	1		0,019	0,2471	11	0,081277438	3	1399,68637	-1,06	8,53	1
P72691	Glycogen operon protein GlgX OS=Synechocystis sp. (strain PCC 6803 /	704,35	20,51	1	14	15	32	0,928	27	20,5	746	83,4	5,06							
	A3	Sequence	# PSMs	# Proteins	# Protein Groups	Protein Group Accessions	Modifications	ΔCn	117/114	117/114 Count	117/114 Variability [%]	q-Value	PEP	IonScore	Exp Value	Charge	MH+ [Da]	ΔM [ppm]	RT [min]	# Missed Cleavages
	High	aNHPVVGGLILDSLR	5	1	1	P72691	N-Term(iTRAQ4plex)	0,0000	0,981	4	9,0	0	3,781E-09	79	1,27466E-08	3	1704,98929	0,88	38,94	0
	High	gTYAGLIEk	2	1	1	P72691	N-Term(iTRAQ4plex); K9(iTRAQ4plex)	0,0000	0,905	2	13,9	0	6,712E-07	57	2,00895E-06	2	1239,71819	-0,44	26,63	0
	High	dLPGScVVWHGVk	3	1	1	P72691	N-Term(iTRAQ4plex); C6(Carbamidomethyl); K13(iTRAQ	0,0000	1,032	2	3,6	0	1,33E-09	52	6,47098E-06	3	1741,93064	0,46	31,24	0
	High	lLGSPDIYYR	1	1	1	P72691	N-Term(iTRAQ4plex)	0,0000	0,757	1		0	0,00008296	50	1,1091E-05	2	1340,73393	0,63	34,87	0
	High	aGQVYAYR	2	1	1	P72691	N-Term(iTRAQ4plex)	0,0000	0,895	2	14,8	0	0,00002745	49	1,27929E-05	2	1071,57097	0,56	17,00	0
	High	fDLASVLVR	3	1	1	P72691	N-Term(iTRAQ4plex)	0,0000	0,791	3	2,0	0	0,00008072	47	1,84914E-05	2	1163,69097	0,38	44,44	0
	High	sSVVLmAk	4	1	1	P72691	N-Term(iTRAQ4plex); M6(Oxidation); K8(iTRAQ4plex)	0,0000	0,965	2	14,5	0	0,00006968	39	0,000130735	2	1138,67314	-1,14	17,84	0
	High	yWVSEMHVDGFR	1	1	1	P72691	N-Term(iTRAQ4plex)	0,0000	1,209	1		0	0,000009592	35	0,000313307	3	1669,79056	-0,49	38,31	0
	High	nPNSGLSENkR	3	1	1	P72691	N-Term(iTRAQ4plex); K10(iTRAQ4plex)	0,0000	1,068	3	0,5	0	0,01025	33	0,000551306	2	1503,81145	-0,24	9,32	1
	High	vTLLLFDRPNDPAPAR	2	1	1	P72691	N-Term(iTRAQ4plex)	0,0000	0,707	2	12,9	0	0,000005038	32	0,000633826	3	1939,08927	0,52	39,55	0
	High	rGTYAGLIEk	1	1	1	P72691	N-Term(iTRAQ4plex); K10(iTRAQ4plex)	0,0000	1,119	1		0	0,0001508	32	0,000662171	3	1395,82065	0,57	22,19	1
	High	tIELHR	2	1	1	P72691	N-Term(iTRAQ4plex)	0,0000	0,793	2	22,3	0	0,00696	22	0,00687021	3	912,53940	1,15	14,46	0
	High	tPYAASIIYELHVGGFTR	1	1	1	P72691	N-Term(iTRAQ4plex)	0,0000	0,393	1		0	0,0001586	20	0,009161572	3	2139,13565	0,04	47,75	0
	High	tFYYWHVFVk	1	1	1	P72691	N-Term(iTRAQ4plex); K10(iTRAQ4plex)	0,0000	1,166	1		0	0,00001078	14	0,039899734	3	1677,90268	-0,29	41,93	0
	Medium	vLLDPYAk	1	2	2	P73608;P72691	N-Term(iTRAQ4plex); K8(iTRAQ4plex)	0,0000				0,007	0,08816	16	0,029644447	2	1206,73760	3,26	30,68	0
	>sp|TRYP_PIG|	701,80	28,57	1	5	5	29	1,049	21	11,9	231	24,4	7,18							
	A3	Sequence	# PSMs	# Proteins	# Protein Groups	Protein Group Accessions	Modifications	ΔCn	117/114	117/114 Count	117/114 Variability [%]	q-Value	PEP	IonScore	Exp Value	Charge	MH+ [Da]	ΔM [ppm]	RT [min]	# Missed Cleavages
	High	lGEHNIDVLEGNEQFINAAk	4	1	1		N-Term(iTRAQ4plex); K20(iTRAQ4plex)	0,0000	1,076	2	1,9	0	4,243E-15	106	2,68652E-11	3	2499,31180	1,41	38,42	0
	High	iITHPNFNGNTLDNDIMLIk	3	1	1		N-Term(iTRAQ4plex); K20(iTRAQ4plex)	0,0000	1,204	3	37,9	0	1,301E-13	76	2,39867E-08	3	2571,38633	0,75	43,89	0
	High	iITHPNFNGNTLDNDImLIk	5	1	1		N-Term(iTRAQ4plex); M17(Oxidation); K20(iTRAQ4plex)	0,0000	1,086	4	16,2	0	8,801E-14	66	3,24475E-07	3	2587,38297	1,41	42,13	0
	High	vATVSLPR	9	1	1		N-Term(iTRAQ4plex)	0,0000	1,046	7	3,0	0	0,0001995	47	2,14768E-05	2	986,61205	0,52	24,29	0
	High	lSSPATLNSR	6	1	1		N-Term(iTRAQ4plex)	0,0000	1,070	3	3,0	0	0,003907	44	3,6557E-05	2	1189,66667	0,77	21,04	0
	High	nkPGVYTk	2	1	1		N-Term(iTRAQ4plex); K2(iTRAQ4plex); K8(iTRAQ4plex)	0,0000	0,603	2	36,0	0	0,0005098	31	0,000814648	3	1338,80902	-1,15	11,54	0
P73287	Bacterioferritin OS=Synechocystis sp. (strain PCC 6803 / Kazusa) GN=b	688,78	36,36	1	5	5	22	1,111	19	23,7	176	20,2	4,61							
	A3	Sequence	# PSMs	# Proteins	# Protein Groups	Protein Group Accessions	Modifications	ΔCn	117/114	117/114 Count	117/114 Variability [%]	q-Value	PEP	IonScore	Exp Value	Charge	MH+ [Da]	ΔM [ppm]	RT [min]	# Missed Cleavages
	High	lQLTAINQYFLHAR	13	1	1	P73287	N-Term(iTRAQ4plex)	0,0000	1,121	10	24,7	0	6,798E-08	75	3,19132E-08	2	1832,03215	1,19	45,08	0
	High	dGLVNSIAFFETQR	2	1	1	P73287	N-Term(iTRAQ4plex)	0,0000	1,555	2	57,3	0	2,434E-09	63	5,08124E-07	2	1740,90434	0,32	45,88	0
	High	nWGLNALNQYEYk	3	1	1	P73287	N-Term(iTRAQ4plex); K13(iTRAQ4plex)	0,0000	1,111	3	5,3	0	1,68E-10	60	1,05918E-06	2	1900,98186	1,19	39,73	0
	High	qHLNQALk	3	1	1	P73287	N-Term(iTRAQ4plex); K8(iTRAQ4plex)	0,0000	1,289	3	3,8	0	0,0009414	39	0,000136763	2	1239,74016	-0,84	11,87	0
	High	vLFLEGLPNLQNLEk	1	1	1	P73287	N-Term(iTRAQ4plex); K15(iTRAQ4plex)	0,0000	0,563	1		0	0,0001356	36	0,000240974	3	2015,17563	-1,18	53,32	0
P73297	DNA-directed RNA polymerase subunit alpha OS=Synechocystis sp. (stra	681,89	21,66	1	9	9	26	1,231	20	10,2	314	35,0	4,79							
	A3	Sequence	# PSMs	# Proteins	# Protein Groups	Protein Group Accessions	Modifications	ΔCn	117/114	117/114 Count	117/114 Variability [%]	q-Value	PEP	IonScore	Exp Value	Charge	MH+ [Da]	ΔM [ppm]	RT [min]	# Missed Cleavages
	High	iAGVNHEFATILGVR	8	1	1	P73297	N-Term(iTRAQ4plex)	0,0000	1,149	6	6,4	0	5,475E-12	91	7,48118E-10	2	1740,98992	1,22	42,09	0
	High	vLLSNLPGAAVTAIR	5	1	1	P73297	N-Term(iTRAQ4plex)	0,0000	1,223	4	14,7	0	1,208E-09	83	4,78597E-09	2	1639,00322	0,51	44,16	0
	High	gQGTTVGNALR	3	1	1	P73297	N-Term(iTRAQ4plex)	0,0000	1,501	2	11,4	0	2,373E-07	81	8,49122E-09	2	1217,67253	0,51	15,86	0
	High	rVLLSNLPGAAVTAIR	1	1	1	P73297	N-Term(iTRAQ4plex)	0,0000	1,483	1		0	3,955E-07	48	1,69422E-05	3	1795,10318	-0,18	37,83	1
	High	lGITLPHEk	5	1	1	P73297	N-Term(iTRAQ4plex); K9(iTRAQ4plex)	0,0000	1,287	3	11,4	0	0,001847	38	0,000168644	3	1295,79334	0,59	27,49	0
	High	fSLEPLDR	1	1	1	P73297	N-Term(iTRAQ4plex)	0,0000	1,235	1		0	0,005365	36	0,000254665	2	1120,61223	0,26	33,40	0
	High	nQQQYSk	1	1	1	P73297	N-Term(iTRAQ4plex); K7(iTRAQ4plex)	0,0000	1,170	1		0	0,0005051	25	0,003154787	2	1183,62956	-1,18	9,59	0
	High	kNQQQYSk	1	1	1	P73297	N-Term(iTRAQ4plex); K1(iTRAQ4plex); K8(iTRAQ4plex)	0,0000	1,651	1		0	0,00002979	21	0,007567806	3	1455,82752	-0,32	7,99	1
	High	rLGITLPHEk	1	1	1	P73297	N-Term(iTRAQ4plex); K10(iTRAQ4plex)	0,0000	1,295	1		0,001	0,01128	14	0,041112132	4	1451,89272	-0,67	22,92	1
P73294	50S ribosomal protein L13 OS=Synechocystis sp. (strain PCC 6803 / Ka	681,33	78,81	1	10	10	32	1,566	26	81,1	151	17,0	9,99							
	A3	Sequence	# PSMs	# Proteins	# Protein Groups	Protein Group Accessions	Modifications	ΔCn	117/114	117/114 Count	117/114 Variability [%]	q-Value	PEP	IonScore	Exp Value	Charge	MH+ [Da]	ΔM [ppm]	RT [min]	# Missed Cleavages
	High	tVLPTIDNLDHk	3	1	1	P73294	N-Term(iTRAQ4plex); K12(iTRAQ4plex)	0,0000	2,046	2	50,1	0	6,763E-08	87	2,18258E-09	3	1653,93967	-1,07	31,72	0
	High	lATEVATILR	7	1	1	P73294	N-Term(iTRAQ4plex)	0,0000	1,775	5	2,6	0	5,72E-08	78	1,48242E-08	2	1230,75371	-0,12	41,88	0
	High	vYAGPSHPHAAQQPETLVINTIPA	7	1	1	P73294	N-Term(iTRAQ4plex)	0,0000	1,817	4	82,7	0	1,126E-07	67	1,83641E-07	3	2897,50321	0,17	33,37	0
	High	wYVIDAEGQR	1	1	1	P73294	N-Term(iTRAQ4plex)	0,0000	1,649	1		0	0,001919	30	0,000911948	2	1380,70317	0,22	33,64	0
	High	iVESAVR	3	1	1	P73294	N-Term(iTRAQ4plex)	0,0000	0,709	3	3,2	0	0,007035	30	0,000970443	2	917,55351	-0,19	17,03	0
	High	nkPTFTPHmDTGDFVIIINAEk	3	1	1	P73294	N-Term(iTRAQ4plex); K2(iTRAQ4plex); M9(Oxidation); K	0,0000	1,255	3	81,7	0	0,00000262	30	0,001009183	4	2936,55337	-0,66	37,42	0
	High	hSGRPGGmkEETFEk	1	1	1	P73294	N-Term(iTRAQ4plex); M8(Oxidation); K9(iTRAQ4plex); K	0,0000	2,639	1		0	0,00006147	20	0,010989299	3	2138,10257	0,02	10,98	1
	High	hSGRPGGMkEETFEk	1	1	1	P73294	N-Term(iTRAQ4plex); K9(iTRAQ4plex); K15(iTRAQ4plex)	0,0000	1,848	1		0	0,001004	14	0,039352289	3	2122,10648	-0,53	14,49	1
	Medium	iEVTGR	1	1	1	P73294	N-Term(iTRAQ4plex)	0,0000	1,132	1		0,018	0,2313	23	0,006066572	2	818,48442	-1,03	15,41	0
	Medium	kLFTk	3	1	1	P73294	N-Term(iTRAQ4plex); K1(iTRAQ4plex); K5(iTRAQ4plex)	0,0000	2,427	3	31,0	0,022	0,273	14	0,038456522	3	1068,71216	-1,85	16,18	1
	Medium	gmLPk	2	1	1	P73294	N-Term(iTRAQ4plex); M2(Oxidation); K5(iTRAQ4plex)	0,0000	0,892	2	1,7	0,026	0,3222	10	0,116665743	2	849,50945	-1,43	12,38	0
P72939	Alkaline phosphatase OS=Synechocystis sp. (strain PCC 6803 / Kazusa)	678,80	9,51	1	11	11	24	0,514	22	13,5	1409	149,3	4,15							
	A3	Sequence	# PSMs	# Proteins	# Protein Groups	Protein Group Accessions	Modifications	ΔCn	117/114	117/114 Count	117/114 Variability [%]	q-Value	PEP	IonScore	Exp Value	Charge	MH+ [Da]	ΔM [ppm]	RT [min]	# Missed Cleavages
	High	tINSGVSLISk	3	1	1	P72939	N-Term(iTRAQ4plex); K11(iTRAQ4plex)	0,0000	0,750	3	22,9	0	0,000006246	79	1,33036E-08	2	1406,85137	4,00	29,23	0
	High	vVLLAHMQQIAIEQELAVR	2	1	1	P72939	N-Term(iTRAQ4plex)	0,0000	0,521	1		0	4,083E-15	77	1,86625E-08	3	2305,32254	1,82	47,93	0
	High	hVDIIVAGGSNTR	4	1	1	P72939	N-Term(iTRAQ4plex)	0,0000	0,560	4	21,0	0	2,659E-07	65	3,14753E-07	2	1482,81828	2,51	19,68	0
	High	sDNkGPEPEGVTVGTIDGR	2	1	1	P72939	N-Term(iTRAQ4plex); K4(iTRAQ4plex)	0,0000	0,573	1		0	1,41E-08	60	1,07639E-06	3	2216,14597	2,78	24,18	1
	High	aELQSAGVR	2	1	1	P72939	N-Term(iTRAQ4plex)	0,0000	0,533	2	6,7	0	0,0001564	53	4,53942E-06	2	1074,60466	2,07	15,43	0
	High	fLGQIFDADGNVVR	2	1	1	P72939	N-Term(iTRAQ4plex)	0,0000	0,449	2	35,8	0	9,506E-08	51	8,66902E-06	2	1694,90154	1,90	45,07	0
	High	tYAFVGLER	2	1	1	P72939	N-Term(iTRAQ4plex)	0,0000	0,465	2	3,9	0	0,00002936	49	1,39949E-05	2	1199,65666	2,12	32,29	0

	High	iQNLAFR	3	1	1	P72939	N-Term(iTRAQ4plex)	0,0000	0,492	3	2,5	0	0,00964	29	0,001816903	2	1005,59911	2,88	28,22	0
	High	vSVGFLPDmVTFSPDGTk	1	1	1	P72939	N-Term(iTRAQ4plex); M9(Oxidation); K18(iTRAQ4plex)	0,0000	0,452	1		0	0,003183	16	0,026666745	3	2201,14469	1,98	44,05	0
	High	lLDENDRLR	1	1	1	P72939	N-Term(iTRAQ4plex)	0,0000	0,672	1		0	0,004168	14	0,039171482	3	1287,71765	3,01	22,00	1
	Medium	lTVTTk	2	1	1	P72939	N-Term(iTRAQ4plex); K6(iTRAQ4plex)	0,0000	0,431	2	20,3	0,037	0,4572	41	7,37853E-05	2	950,61742	5,21	16,97	0
P52415	Glucose-1-phosphate adenylyltransferase OS=Synechocystis sp. (strain	673,15	21,18	1	9	9	24	0,897	20	20,4	439	49,3	6,47							
	A3	Sequence	# PSMs	# Proteins	# Protein Groups	Protein Group Accessions	Modifications	ΔCn	117/114	117/114 Count	117/114 Variability [%]	q-Value	PEP	IonScore	Exp Value	Charge	MH+ [Da]	ΔM [ppm]	RT [min]	# Missed Cleavages
	High	vLAIILGGGAGTR	5	1	1	P52415	N-Term(iTRAQ4plex)	0,0000	1,006	4	8,3	0	0,000001211	83	4,90874E-09	2	1341,83440	0,65	45,48	0
	High	iYVLTQFNSASLNR	4	1	1	P52415	N-Term(iTRAQ4plex)	0,0000	0,889	3	2,7	0	8,518E-08	72	6,54591E-08	2	1769,96904	1,33	42,25	0
	High	lNPYIASmGIYVFk	1	1	1	P52415	N-Term(iTRAQ4plex); M8(Oxidation); K14(iTRAQ4plex)	0,0000	0,652	1		0	1,467E-08	51	8,39402E-06	3	1920,05600	0,85	44,82	0
	High	qYLWLFR	3	1	1	P52415	N-Term(iTRAQ4plex)	0,0000	0,974	3	15,1	0	0,00006974	45	3,43534E-05	2	1169,66057	1,51	45,41	0
	High	kAPELGLmk	4	1	1	P52415	N-Term(iTRAQ4plex); K1(iTRAQ4plex); M8(Oxidation); K	0,0000	0,806	3	5,2	0	6,442E-07	42	0,000107441	3	1434,87082	-0,46	20,56	1
	High	kAPELGLMk	1	1	1	P52415	N-Term(iTRAQ4plex); K1(iTRAQ4plex); K9(iTRAQ4plex)	0,0000	0,288	1		0	0,000005364	38	0,000174169	3	1418,87812	1,09	23,97	1
	High	nVmIVNk	3	1	1	P52415	N-Term(iTRAQ4plex); M3(Oxidation); K7(iTRAQ4plex)	0,0000	0,862	2	7,3	0	0,001923	34	0,000403618	2	1121,65996	0,75	17,92	0
	High	akPAVPLAGk	1	1	1	P52415	N-Term(iTRAQ4plex); K2(iTRAQ4plex); K10(iTRAQ4plex)	0,0000	1,003	1		0	0,0001852	27	0,001972287	3	1383,90799	2,29	18,45	0
	High	eVLHNLLEk	1	1	1	P52415	N-Term(iTRAQ4plex); K9(iTRAQ4plex)	0,0000	0,691	1		0	0,00007282	18	0,014386992	3	1382,82712	1,82	29,54	0
	Medium	nVTIADGTVI	1	1	1	P52415	N-Term(iTRAQ4plex)	0,0000	1,382	1		0,015	0,1932	41	8,29793E-05	2	1146,64946	0,65	35,31	0
P52208	6-phosphogluconate dehydrogenase, decarboxylating OS=Synechocystis	670,73	15,56	1	7	7	27	0,750	22	22,6	482	52,8	5,22							
	A3	Sequence	# PSMs	# Proteins	# Protein Groups	Protein Group Accessions	Modifications	ΔCn	117/114	117/114 Count	117/114 Variability [%]	q-Value	PEP	IonScore	Exp Value	Charge	MH+ [Da]	ΔM [ppm]	RT [min]	# Missed Cleavages
	High	vAASGQLSGPSk	8	1	1	P52208	N-Term(iTRAQ4plex); K12(iTRAQ4plex)	0,0000	0,888	6	50,9	0	3,631E-09	86	2,44326E-09	2	1389,79656	1,81	17,63	0
	High	aVLPQNLTQAQR	3	1	1	P52208	N-Term(iTRAQ4plex)	0,0000	0,661	3	1,0	0	6,643E-07	71	7,7983E-08	2	1482,85320	1,52	26,61	0
	High	gFPIAVFNR	7	1	1	P52208	N-Term(iTRAQ4plex)	0,0000	0,733	6	7,6	0	0,002081	51	7,39554E-06	2	1164,66606	1,22	40,15	0
	High	kILVMVk	2	1	1	P52208	N-Term(iTRAQ4plex); K1(iTRAQ4plex); K7(iTRAQ4plex)	0,0000	0,971	2	35,2	0	0,00007203	39	0,000116405	3	1262,85642	-2,40	24,97	1
	High	aAYTVEEFVQLLERPR	1	1	1	P52208	N-Term(iTRAQ4plex)	0,0000	0,791	1		0	0,0002747	30	0,001235088	3	2065,12442	2,17	53,46	0
	High	kILVmVk	4	1	1	P52208	N-Term(iTRAQ4plex); K1(iTRAQ4plex); M5(Oxidation); K	0,0000	0,958	2	20,2	0	0,000392	27	0,002207852	3	1278,85865	3,35	19,50	1
	High	dYFGAHTYER	1	1	1	P52208	N-Term(iTRAQ4plex)	0,0000	1,470	1		0	0,000119	21	0,008240812	3	1402,65186	0,74	21,74	0
	Medium	vMSSLkEER	1	1	1	P52208	N-Term(iTRAQ4plex); K6(iTRAQ4plex)	0,0000	0,621	1		0,014	0,1791	14	0,03749471	3	1366,76154	0,90	18,85	1
P74690	Probable phosphoketolase OS=Synechocystis sp. (strain PCC 6803 / Kaz	670,44	14,29	1	11	11	27	0,463	27	33,2	805	90,8	6,21							
	A3	Sequence	# PSMs	# Proteins	# Protein Groups	Protein Group Accessions	Modifications	ΔCn	117/114	117/114 Count	117/114 Variability [%]	q-Value	PEP	IonScore	Exp Value	Charge	MH+ [Da]	ΔM [ppm]	RT [min]	# Missed Cleavages
	High	vTSPFSLSPFGQAR	7	1	1	P74690	N-Term(iTRAQ4plex)	0,0000	0,406	7	12,3	0	4,67E-08	83	4,47682E-09	2	1637,87859	1,07	43,21	0
	High	fVSVIDLLk	6	1	1	P74690	N-Term(iTRAQ4plex); K9(iTRAQ4plex)	0,0000	0,440	6	12,4	0	5,732E-08	65	3,35714E-07	2	1321,83269	-0,53	50,07	0
	High	qAHLQYQDmTSAIR	2	1	1	P74690	N-Term(iTRAQ4plex); M9(Oxidation)	0,0000	0,786	2	6,0	0	2,68E-10	56	2,48296E-06	3	1821,90470	0,68	19,22	0
	High	aANYLAVGMIYLR	1	1	1	P74690	N-Term(iTRAQ4plex)	0,0000	0,372	1		0	1,846E-08	56	2,73508E-06	2	1598,88469	0,09	47,67	0
	High	iNNPSVLSR	1	1	1	P74690	N-Term(iTRAQ4plex)	0,0000	0,456	1		0	0,0005386	37	0,000220785	2	1143,66106	0,69	21,91	0
	High	aANYLAVGmIYLR	2	1	1	P74690	N-Term(iTRAQ4plex); M9(Oxidation)	0,0000	0,665	2	13,4	0	0,00001214	30	0,00097268	3	1614,87925	-0,14	42,18	0
	High	qFFPNLR	1	1	1	P74690	N-Term(iTRAQ4plex)	0,0000	0,459	1		0	0,006015	29	0,001288161	2	1065,59538	-0,78	32,40	0
	High	qAHLQYQDMTSAIR	1	1	1	P74690	N-Term(iTRAQ4plex)	0,0000	1,004	1		0	0,000001986	24	0,003732244	3	1805,90677	-0,98	27,44	0
	High	sYkPEELFDEQGTLkPGFk	1	1	1	P74690	N-Term(iTRAQ4plex); K3(iTRAQ4plex); K15(iTRAQ4plex)	0,0000	0,668	1		0	6,923E-07	21	0,009889657	4	2789,51699	-1,37	36,08	0
	High	iSHEELk	1	1	1	P74690	N-Term(iTRAQ4plex); K7(iTRAQ4plex)	0,0000	0,573	1		0	0,002887	17	0,021675544	3	1143,65860	-2,29	13,46	0
	High	fINPIR	2	1	1	P74690	N-Term(iTRAQ4plex)	0,0000	0,529	2	20,0	0,001	0,01432	29	0,001145434	2	903,55278	-0,56	27,66	0
	High	lHAVYEVSkk	1	1	1	P74690	N-Term(iTRAQ4plex); K9(iTRAQ4plex); K10(iTRAQ4plex)	0,0000	0,923	1		0,005	0,06353	13	0,046234909	4	1605,96120	-4,77	18,87	1
	Medium	vAGAHIk	1	1	1	P74690	N-Term(iTRAQ4plex); K7(iTRAQ4plex)	0,0000	0,859	1		0,026	0,3189	19	0,011856869	3	983,61774	-6,41	11,82	0
P73853	IMP dehydrogenase subunit OS=Synechocystis sp. (strain PCC 6803 / K	669,24	30,49	1	10	10	23	0,868	18	25,4	387	40,2	5,34							
	A3	Sequence	# PSMs	# Proteins	# Protein Groups	Protein Group Accessions	Modifications	ΔCn	117/114	117/114 Count	117/114 Variability [%]	q-Value	PEP	IonScore	Exp Value	Charge	MH+ [Da]	ΔM [ppm]	RT [min]	# Missed Cleavages
	High	rIQEIQAAGGIAAVSLTPVGASk	2	1	1	P73853	N-Term(iTRAQ4plex); K23(iTRAQ4plex)	0,0000	0,873	2	12,5	0	2,265E-09	92	6,19398E-10	3	2525,46769	0,88	37,27	1
	High	tSMGTLGAk	2	1	1	P73853	N-Term(iTRAQ4plex); K9(iTRAQ4plex)	0,0000	0,781	1		0	0,000002097	64	4,28519E-07	2	1153,64458	-3,80	17,44	0
	High	aGAAAVLVGIGPGAAcTSR	2	1	1	P73853	N-Term(iTRAQ4plex); C16(Carbamidomethyl)	0,0000	0,913	2	18,8	0	1,091E-08	62	6,3387E-07	2	1842,99968	1,05	35,07	0
	High	iNVGTTGTIR	3	1	1	P73853	N-Term(iTRAQ4plex)	0,0000	0,908	2	0,0	0	5,107E-08	57	2,10848E-06	2	1175,68645	-0,04	23,37	0
	High	gFHWGmATPSPVLPR	2	1	1	P73853	N-Term(iTRAQ4plex); M6(Oxidation)	0,0000	1,489	2	259,0	0	2,281E-08	54	3,91715E-06	3	1812,93363	-0,02	33,33	0
	High	iQEIQAAGGIAAVSLTPVGASk	1	1	1	P73853	N-Term(iTRAQ4plex); K22(iTRAQ4plex)	0,0000	0,603	1		0	0,00009869	47	1,86196E-05	3	2369,36148	-1,20	42,25	0
	High	rAYGIDEIALVPGVR	4	1	1	P73853	N-Term(iTRAQ4plex)	0,0000	1,040	3	17,3	0	6,732E-08	45	3,41169E-05	3	1773,01444	0,24	37,71	1
	High	tSmGTLGAk	2	1	1	P73853	N-Term(iTRAQ4plex); M3(Oxidation); K9(iTRAQ4plex)	0,0000	0,826	1		0	0,00218	38	0,000167483	2	1169,64238	-1,27	12,18	0
	High	gFHWGMATPSPVLPR	1	1	1	P73853	N-Term(iTRAQ4plex)	0,0000	1,338	1		0	0,000000423	33	0,000602811	3	1796,93698	-0,98	37,55	0
	High	aQQLGmGk	2	1	1	P73853	N-Term(iTRAQ4plex); M6(Oxidation); K8(iTRAQ4plex)	0,0000	1,237	1		0	0,00185	31	0,000966925	2	1136,63176	-1,65	11,56	0
	High	lDDGTHNLLGAIk	1	1	1	P73853	N-Term(iTRAQ4plex); K13(iTRAQ4plex)	0,0000	0,694	1		0	0,0001852	22	0,005874488	3	1654,93448	-1,34	33,28	0
	Medium	iASVGk	1	1	1	P73853	N-Term(iTRAQ4plex); K6(iTRAQ4plex)	0,0000	0,829	1		0,037	0,463	30	0,000950539	2	862,55449	-6,44	15,13	0
	>sp|K1C10_HUMAN|	650,72	17,88	1	11	11	24	1,734	22	52,4	593	59,5	5,21							
	A3	Sequence	# PSMs	# Proteins	# Protein Groups	Protein Group Accessions	Modifications	ΔCn	117/114	117/114 Count	117/114 Variability [%]	q-Value	PEP	IonScore	Exp Value	Charge	MH+ [Da]	ΔM [ppm]	RT [min]	# Missed Cleavages
	High	gSLGGGFSSGGFSGGSFSR	4	1	1		N-Term(iTRAQ4plex)	0,0000	1,554	3	17,9	0	1,001E-16	117	1,84914E-12	2	1851,87639	1,13	33,94	0
	High	vLDELTLTk	1	1	1		N-Term(iTRAQ4plex); K9(iTRAQ4plex)	0,0000	1,029	1		0	0,00003799	65	2,89048E-07	2	1319,80510	1,99	36,59	0
	High	lkYENEVALR	3	1	1		N-Term(iTRAQ4plex); K2(iTRAQ4plex)	0,0000	2,204	3	14,0	0	2,287E-07	48	1,77419E-05	3	1522,88395	0,50	24,92	1
	High	sQYEQLAEQNRk	3	1	1		N-Term(iTRAQ4plex); K12(iTRAQ4plex)	0,0000	3,285	2	3,2	0	1,381E-09	42	5,80724E-05	3	1781,93918	0,40	17,96	1
	High	iRLENEIQTYR	2	1	1		N-Term(iTRAQ4plex)	0,0000	1,789	2	46,6	0	0,000005808	41	8,7694E-05	3	1578,87302	0,61	28,03	1
	High	lASYLDkVR	3	1	1		N-Term(iTRAQ4plex); K7(iTRAQ4plex)	0,0000	1,929	3	1,0	0	0,000001519	38	0,00016633	3	1352,81467	0,47	31,74	1
	High	rVLDELTLTk	1	1	1		N-Term(iTRAQ4plex); K10(iTRAQ4plex)	0,0000	0,561	1		0	0,00005829	28	0,001506503	3	1475,90512	1,03	31,19	1
	High	qSVEADINGLRR	2	1	1		N-Term(iTRAQ4plex)	0,0000	1,338	2	54,2	0	0,0009742	25	0,003357144	3	1501,82032	-0,05	24,88	1
	High	vTmQNLNDRLASYLDkVR	1	1	1		N-Term(iTRAQ4plex); M3(Oxidation); K16(iTRAQ4plex)	0,0000	1,716	1		0	0,001744	23	0,005357597	4	2440,32314	0,41	38,77	2
	High	ikEWYEk	3	1	1		N-Term(iTRAQ4plex); K2(iTRAQ4plex); K7(iTRAQ4plex)	0,0000	1,151	3	5,4	0	0,00001165	17	0,021154009	3	1427,82566	-0,16	23,59	1
	High	sEITELRR	1	1	1		N-Term(iTRAQ4plex)	0,0000	1,852	1		0,001	0,01316	15	0,044292865	3	1147,65470	-0,43	15,21	1
P52231	Thioredoxin OS=Synechocystis sp. (strain PCC 6803 / Kazusa) GN=trxA	650,40	46,73	1	4	4	22	1,157	18	18,6	107	11,7	4,93							
	A3	Sequence	# PSMs	# Proteins	# Protein Groups	Protein Group Accessions	Modifications	ΔCn	117/114	117/114 Count	117/114 Variability [%]	q-Value	PEP	IonScore	Exp Value	Charge	MH+ [Da]	ΔM [ppm]	RT [min]	# Missed Cleavages
	High	lNTDENPNTASQYGIR	1	1	1	P52231	N-Term(iTRAQ4plex)	0,0000	1,242	1		0	3,916E-12	100	1,03269E-10	2	1936,95085	1,39	26,26	0
	High	sIPTLMIFk	7	1	1	P52231	N-Term(iTRAQ4plex); K9(iTRAQ4plex)	0,0000	1,344	6	31,8	0	0,0000595	66	2,30128E-07	2	1337,81182	0,95	44,92	0
	High	mVAPVVDEISQQYEGk	1	1	1	P52231	N-Term(iTRAQ4plex); M1(Oxidation); K16(iTRAQ4plex)	0,0000	1,113	1		0	5,279E-13	66	2,67898E-07	3	2097,07999	1,09	36,25	0
	High	tTLASTLEk	6	1	1	P52231	N-Term(iTRAQ4plex); K9(iTRAQ4plex)	0,0000	0,956	4	33,0	0	0,00004673	56	2,44326E-06	2	1251,74211	1,79	24,22	0
	High	sIPTLmIFk	7	1	1	P52231	N-Term(iTRAQ4plex); M6(Oxidation); K9(iTRAQ4plex)	0,0000	1,150	6	14,9	0	0,0002047	46	2,48869E-05	2	1353,80718	1,27	41,00	0
P73348	Rehydrin OS=Synechocystis sp. (strain PCC 6803 / Kazusa) GN=slr1198	645,04	48,82	1	12	12	36	1,275	28	20,1	211	23,5	5,30							
	A3	Sequence	# PSMs	# Proteins	# Protein Groups	Protein Group Accessions	Modifications	ΔCn	117/114	117/114 Count	117/114 Variability [%]	q-Value	PEP	IonScore	Exp Value	Charge	MH+ [Da]	ΔM [ppm]	RT [min]	# Missed Cleavages
	High	vIALSVDDVESHk	3	1	1	P73348	N-Term(iTRAQ4plex); K13(iTRAQ4plex)	0,0000	1,009	2	17,5	0	2,203E-13	71	1,04312E-07	3	1699,94730	0,22	32,32	0
	High	sVFIIDPAk	3	1	1	P73348	N-Term(iTRAQ4plex); K9(iTRAQ4plex)	0,0000	1,139	2	28,9	0	0,000001126	56	2,36576E-06	2	1277,77117	0,30	33,88	0
	High	gVEEIkPYLR	7	1	1	P73348	N-Term(iTRAQ4plex); K6(iTRAQ4plex)	0,0000	1,360	6	17,5	0	0,000107	47	1,92739E-05	2	1491,87834	0,65	27,81	0
	High	lTFTYPASTGR	4	1	1	P73348	N-Term(iTRAQ4plex)	0,0000	1,335	3	2,0	0	0,000138	47	2,08915E-05	2	1357,72429	0,76	32,08	0
	High	vSDLYGmIHPNALNNLTVR	4	1	1	P73348	N-Term(iTRAQ4plex); M7(Oxidation)	0,0000	1,229	3	0,9	0	1,883E-10	46	2,63265E-05	3	2287,20114	1,11	39,58	0
	High	nFDEILR	1	1	1	P73348	N-Term(iTRAQ4plex)	0,0000	1,082	1		0	0,0005894	37	0,000194523	2	1050,57134	1,21	30,87	0
	High	vSDLYGMIHPNALNNLTVR	1	1	1	P73348	N-Term(iTRAQ4plex)	0,0000	1,269	1		0	1,758E-07	36	0,000318553	3	2271,20395	0,12	45,06	0
	High	sVFIIDPAkk	2	1	1	P73348	N-Term(iTRAQ4plex); K9(iTRAQ4plex); K10(iTRAQ4plex)	0,0000	1,280	2	11,2	0	0,00009369	27	0,002069998	3	1549,96671	-0,72	30,00	1
	High	lkPEFDk	2	1	1	P73348	N-Term(iTRAQ4plex); K2(iTRAQ4plex); K7(iTRAQ4plex)	0,0000	0,593	2	20,7	0	0,00001632	22	0,005636377	3	1308,78843	-0,27	20,80	0
	High	cVVVPSISTEDAk	1	1	1	P73348	N-Term(iTRAQ4plex); C1(Carbamidomethyl); K13(iTRAQ	0,0000	0,891	1		0	0,0009824	19	0,015149304	3	1692,90781	-0,17	29,75	0
	High	lkPEFDkR	1	1	1	P73348	N-Term(iTRAQ4plex); K2(iTRAQ4plex); K7(iTRAQ4plex)	0,0000	1,712	1		0	0,001615	16	0,025584092	3	1464,89087	0,66	17,10	1
	High	lTPQPNk	6	1	1	P73348	N-Term(iTRAQ4plex); K7(iTRAQ4plex)	0,0000	1,471	3	13,7	0	0,0104	11	0,081090505	2	1085,65605	0,29	15,14	0
	High	vkFPk	1	1	1	P73348	N-Term(iTRAQ4plex); K2(iTRAQ4plex); K5(iTRAQ4plex)	0,0000	1,622	1		0,005	0,05966	11	0,072271989	3	1050,70172	-1,77	17,24	1
P72938	Extracellular nuclease OS=Synechocystis sp. (strain PCC 6803 / Kazusa)	644,55	3,83	1	5	5	16	0,326	14	12,2	1879	196,4	3,99							
	A3	Sequence	# PSMs	# Proteins	# Protein Groups	Protein Group Accessions	Modifications	ΔCn	117/114	117/114 Count	117/114 Variability [%]	q-Value	PEP	IonScore	Exp Value	Charge	MH+ [Da]	ΔM [ppm]	RT [min]	# Missed Cleavages
	High	qANLFTYAAGGTLSR	4	1	1	P72938	N-Term(iTRAQ4plex)	0,0000	0,458	4	18,6	0	3,315E-11	89	1,16137E-09	2	1713,90935	3,06	37,66	0
	High	vTGFNVLNYFk	8	1	1	P72938	N-Term(iTRAQ4plex); K11(iTRAQ4plex)	0,0000	0,317	6	10,3	0	3,328E-09	85	2,97146E-09	2	1589,90007	4,44	48,28	0
	High	iIGGLSQNLGR	2	1	1	P72938	N-Term(iTRAQ4plex)	0,0000	0,317	2	0,2	0	0,000001187	66	2,69755E-07	2	1271,75859	2,62	32,11	0
	High	lSQGGRPQQFTQFNDANVAGYG	1	1	1	P72938	N-Term(iTRAQ4plex)	0,0000	0,214	1		0	4,34E-14	62	6,47098E-07	3	2889,42185	2,38	33,88	0
	High	nRPTVAQTFR	1	1	1	P72938	N-Term(iTRAQ4plex)	0,0000	0,323	1		0	0,0006483	30	0,000897367	3	1333,74900	2,44	15,48	0
P48939	30S ribosomal protein S4 OS=Synechocystis sp. (strain PCC 6803 / Kazu	643,12	28,22	1	7	7	32	1,543	24	27,0	202	23,2	10,48							
	A3	Sequence	# PSMs	# Proteins	# Protein Groups	Protein Group Accessions	Modifications	ΔCn	117/114	117/114 Count	117/114 Variability [%]	q-Value	PEP	IonScore	Exp Value	Charge	MH+ [Da]	ΔM [ppm]	RT [min]	# Missed Cleavages
	High	lGMAGTIPGAR	2	1	1	P48939	N-Term(iTRAQ4plex)	0,0000	2,556	1		0	0,00002427	55	3,64067E-06	2	1187,66692	-1,55	27,73	0
	High	lGmAGTIPGAR	4	1	1	P48939	N-Term(iTRAQ4plex); M3(Oxidation)	0,0000	1,614	2	6,8	0	0,000009696	55	3,17665E-06	2	1203,66240	-1,06	22,00	0
	High	rAYPPGQHGQAR	3	1	1	P48939	N-Term(iTRAQ4plex)	0,0000	1,422	3	5,4	0	2,06E-09	55	3,38821E-06	3	1481,78290	-0,91	7,69	1
	High	rATGSTGQALLELLEMR	1	1	1	P48939	N-Term(iTRAQ4plex)	0,0000	1,991	1		0	1,588E-08	51	7,07897E-06	3	1990,08615	-0,56	48,04	1
	High	rLGELPGLSR	5	1	1	P48939	N-Term(iTRAQ4plex)	0,0000	1,637	3	55,4	0	0,000000547	51	7,44681E-06	3	1241,74353	-0,93	26,51	1
	High	rATGSTGQALLELLEmR	7	1	1	P48939	N-Term(iTRAQ4plex); M16(Oxidation)	0,0000	1,666	6	18,3	0	7,497E-09	49	1,18842E-05	3	2006,08078	-0,70	44,24	1
	High	aYPPGQHGQAR	4	1	1	P48939	N-Term(iTRAQ4plex)	0,0000	1,543	3	10,0	0	0,00003199	37	0,000199972	3	1325,68109	-1,54	8,47	0
	High	rSEYAIR	1	1	1	P48939	N-Term(iTRAQ4plex)	0,0000	0,999	1		0	0,00731	33	0,000503466	3	1038,57959	-1,63	12,12	1
	High	lGELPGLSR	5	1	1	P48939	N-Term(iTRAQ4plex)	0,0000	1,272	4	2,0	0,001	0,02105	22	0,00564898	2	1085,64165	-1,77	32,88	0
P29256	Photosystem I reaction center subunit III OS=Synechocystis sp. (strain	628,48	44,85	1	9	9	30	0,995	20	24,8	165	18,2	7,37							
	A3	Sequence	# PSMs	# Proteins	# Protein Groups	Protein Group Accessions	Modifications	ΔCn	117/114	117/114 Count	117/114 Variability [%]	q-Value	PEP	IonScore	Exp Value	Charge	MH+ [Da]	ΔM [ppm]	RT [min]	# Missed Cleavages
	High	nFLNTTNDPNSGk	3	1	1	P29256	N-Term(iTRAQ4plex); K13(iTRAQ4plex)	0,0000	0,859	2	4,9	0	2,85E-08	75	3,52347E-08	2	1709,87151	1,04	21,80	0
	High	skNFLNTTNDPNSGk	4	1	1	P29256	N-Term(iTRAQ4plex); K2(iTRAQ4plex); K15(iTRAQ4plex)	0,0000	1,409	3	2,7	0	5,862E-13	66	2,59418E-07	3	2069,09928	0,24	18,80	1
	High	eSkNPEMQEVVINVPLAIk	1	1	1	P29256	N-Term(iTRAQ4plex); K3(iTRAQ4plex); K19(iTRAQ4plex)	0,0000	1,406	1		0	9,652E-11	56	2,77313E-06	4	2570,45839	-0,19	41,01	1
	High	yASALcGPEGYPHLIVDGR	4	1	1	P29256	N-Term(iTRAQ4plex); C6(Carbamidomethyl)	0,0000	0,687	3	15,0	0	3,149E-11	50	9,01509E-06	3	2219,10532	0,76	37,80	0
	High	sYLIEIR	6	1	1	P29256	N-Term(iTRAQ4plex)	0,0000	0,885	4	3,4	0	0,005114	47	1,99512E-05	2	1037,61186	0,66	32,11	0
	High	nPEmQEVVINVPLAIk	2	1	1	P29256	N-Term(iTRAQ4plex); M4(Oxidation); K16(iTRAQ4plex)	0,0000	0,633	1		0	1,068E-08	46	2,64223E-05	3	2098,18375	0,78	41,30	0
	High	lVmkDSEIPTSPR	5	1	1	P29256	N-Term(iTRAQ4plex); M3(Oxidation); K4(iTRAQ4plex)	0,0000	1,127	3	10,4	0	0,00000354	43	5,51048E-05	3	1776,97794	0,61	22,60	1
	High	dSEIPTSPR	2	1	1	P29256	N-Term(iTRAQ4plex)	0,0000	0,950	1		0	0,0003388	41	7,5504E-05	2	1145,59221	0,25	16,57	0
	High	nPEMQEVVINVPLAIkk	1	1	1	P29256	N-Term(iTRAQ4plex); K16(iTRAQ4plex); K17(iTRAQ4plex	0,0000	1,148	1		0	0,000003386	34	0,000357248	4	2354,38540	0,49	40,70	1
	High	eSkNPEmQEVVINVPLAIk	2	1	1	P29256	N-Term(iTRAQ4plex); K3(iTRAQ4plex); M7(Oxidation); K	0,0000	1,459	1		0	0,000003029	27	0,001954204	4	2586,45498	0,46	37,40	1
P73303	50S ribosomal protein L15 OS=Synechocystis sp. (strain PCC 6803 / Kaz	624,74	44,22	1	6	6	20	1,921	15	47,6	147	15,2	10,37							
	A3	Sequence	# PSMs	# Proteins	# Protein Groups	Protein Group Accessions	Modifications	ΔCn	117/114	117/114 Count	117/114 Variability [%]	q-Value	PEP	IonScore	Exp Value	Charge	MH+ [Da]	ΔM [ppm]	RT [min]	# Missed Cleavages
	High	gIAAGQGASc GFGMR	3	1	1	P73303	N-Term(iTRAQ4plex); C10(Carbamidomethyl)	0,0000	1,215	2	462,8	0	3,942E-08	82	6,4561E-09	2	1583,75371	-0,18	26,09	0
	High	gIAAGQGAScGFGmR	7	1	1	P73303	N-Term(iTRAQ4plex); C10(Carbamidomethyl); M14(Oxida	0,0000	1,388	4	113,4	0	8,244E-12	80	1,06407E-08	2	1599,74858	-0,20	21,64	0
	High	mNLSELSPk	2	1	1	P73303	N-Term(iTRAQ4plex); M1(Oxidation); K9(iTRAQ4plex)	0,0000	2,061	1		0	0,000001426	54	3,62243E-06	2	1322,72270	-0,11	22,23	0
	High	hFTVVNVGk	3	1	1	P73303	N-Term(iTRAQ4plex); K9(iTRAQ4plex)	0,0000	1,416	3	50,0	0	0,0000395	54	4,13971E-06	2	1288,75957	-1,58	21,45	0
	High	iEAAGGSVVAQG	1	1	1	P73303	N-Term(iTRAQ4plex)	0,0000	1,428	1		0	0,00004825	49	1,31513E-05	2	1202,64934	-0,38	24,73	0
	High	aGFEGGQmPLYR	2	1	1	P73303	N-Term(iTRAQ4plex); M8(Oxidation)	0,0000	1,963	2	3,2	0	0,0005371	33	0,000485255	2	1485,72783	0,07	25,18	0
	High	hFPLYNPk	2	1	1	P73303	N-Term(iTRAQ4plex); K8(iTRAQ4plex)	0,0000	2,181	2	24,1	0	0,005145	30	0,000963762	2	1303,73735	-2,13	23,99	0
P77968	Superoxide dismutase [Fe] OS=Synechocystis sp. (strain PCC 6803 / Ka	619,96	28,14	1	4	4	27	1,654	24	25,3	199	21,7	5,14							
	A3	Sequence	# PSMs	# Proteins	# Protein Groups	Protein Group Accessions	Modifications	ΔCn	117/114	117/114 Count	117/114 Variability [%]	q-Value	PEP	IonScore	Exp Value	Charge	MH+ [Da]	ΔM [ppm]	RT [min]	# Missed Cleavages
	High	hHAAYVNNFNNAVAGTDLDNQSI	2	1	1	P77968	N-Term(iTRAQ4plex); K28(iTRAQ4plex)	0,0000	1,802	1		0	1,19E-12	72	6,99794E-08	4	3357,67495	1,38	38,42	0
	High	rPDYIADFLGk	15	1	1	P77968	N-Term(iTRAQ4plex); K11(iTRAQ4plex)	0,0000	1,889	14	19,2	0	1,366E-10	60	1,104E-06	2	1582,88384	0,40	37,80	0
	High	sTLEFHHDk	6	1	1	P77968	N-Term(iTRAQ4plex); K9(iTRAQ4plex)	0,0000	1,289	5	32,7	0	0,00002628	39	0,000115072	2	1401,73516	-0,96	13,24	0
	High	aVAGDASk	4	1	1	P77968	N-Term(iTRAQ4plex); K8(iTRAQ4plex)	0,0000	1,514	4	11,8	0	0,0001259	30	0,000937497	2	1006,57585	-1,31	8,94	0
P74267	50S ribosomal protein L27 OS=Synechocystis sp. (strain PCC 6803 / Kaz	609,63	59,77	1	6	6	25	1,514	18	95,2	87	9,4	11,24							
	A3	Sequence	# PSMs	# Proteins	# Protein Groups	Protein Group Accessions	Modifications	ΔCn	117/114	117/114 Count	117/114 Variability [%]	q-Value	PEP	IonScore	Exp Value	Charge	MH+ [Da]	ΔM [ppm]	RT [min]	# Missed Cleavages
	High	yGGQTVTAGSIIVR	7	1	1	P74267	N-Term(iTRAQ4plex)	0,0000	3,144	4	4,0	0	2,006E-10	83	5,35048E-09	2	1565,87761	0,50	29,87	0
	High	gTQVHPGNNVGR	6	1	1	P74267	N-Term(iTRAQ4plex)	0,0000	1,714	4	55,5	0	0,000003957	49	1,24157E-05	2	1379,72588	-0,14	8,43	0
	High	rYGGQTVTAGSIIVR	2	1	1	P74267	N-Term(iTRAQ4plex)	0,0000	1,116	2	33,7	0	8,344E-08	45	3,13307E-05	3	1721,97745	-0,29	24,48	1
	High	kVSVYPATAE	7	1	1	P74267	N-Term(iTRAQ4plex); K1(iTRAQ4plex)	0,0000	1,073	5	103,7	0	0,000167	40	9,84011E-05	2	1352,76567	-0,55	20,55	1
	High	gkDDTLFALIDGVVk	2	1	1	P74267	N-Term(iTRAQ4plex); K2(iTRAQ4plex); K15(iTRAQ4plex)	0,0000	1,190	2	51,1	0	0,0001819	39	0,000117211	3	2023,17716	-1,42	49,30	1
	High	vSVYPATAE	1	1	1	P74267	N-Term(iTRAQ4plex)	0,0000	0,742	1		0,008	0,1069	37	0,000217756	2	1080,56890	-0,45	26,33	0
P28371	Elongation factor G 1 OS=Synechocystis sp. (strain PCC 6803 / Kazusa)	608,25	19,42	1	15	15	31	0,952	26	13,5	695	76,7	5,02							
	A3	Sequence	# PSMs	# Proteins	# Protein Groups	Protein Group Accessions	Modifications	ΔCn	117/114	117/114 Count	117/114 Variability [%]	q-Value	PEP	IonScore	Exp Value	Charge	MH+ [Da]	ΔM [ppm]	RT [min]	# Missed Cleavages
	High	kLDFFPTYGGSSFk	6	1	1	P28371	N-Term(iTRAQ4plex); K1(iTRAQ4plex); K14(iTRAQ4plex)	0,0000	1,036	4	31,1	0	3,712E-10	64	3,74085E-07	3	2026,10282	0,89	38,78	1
	High	iYSGTLSk	1	1	1	P28371	N-Term(iTRAQ4plex); K8(iTRAQ4plex)	0,0000	1,143	1		0	0,0001652	61	8,74923E-07	2	1156,68205	0,39	22,34	0
	High	gVLAGYPVVDLk	2	1	1	P28371	N-Term(iTRAQ4plex); K12(iTRAQ4plex)	0,0000	0,805	2	35,3	0	1,376E-08	60	1,08143E-06	2	1518,91472	0,84	39,85	0
	High	nIGIFAHVDAGk	3	1	1	P28371	N-Term(iTRAQ4plex); K12(iTRAQ4plex)	0,0000	0,786	2	34,4	0	3,119E-08	50	1,00686E-05	3	1529,86905	0,76	29,71	0
	High	vTGGNVPR	2	1	1	P28371	N-Term(iTRAQ4plex)	0,0000	0,844	2	3,5	0	0,006821	39	0,000136449	2	943,54338	-0,85	12,93	0
	High	tHGVEVEmGkPQVAYR	3	1	1	P28371	N-Term(iTRAQ4plex); M8(Oxidation); K10(iTRAQ4plex)	0,0000	0,934	2	3,7	0	1,086E-07	36	0,000232793	4	2105,10805	1,33	19,62	0
	High	kQSGGSGQYAk	1	1	1	P28371	N-Term(iTRAQ4plex); K1(iTRAQ4plex); K11(iTRAQ4plex)	0,0000	0,886	1		0	0,000000543	36	0,000269135	3	1542,86020	0,11	8,05	1
	High	gFDQSVVk	1	1	1	P28371	N-Term(iTRAQ4plex); K8(iTRAQ4plex)	0,0000	1,085	1		0	0,002197	30	0,001314952	2	1167,66069	-0,46	20,75	0
	High	nVQTGHTLcDPk	2	1	1	P28371	N-Term(iTRAQ4plex); C9(Carbamidomethyl); K12(iTRAQ	0,0000	0,828	2	12,0	0	4,608E-07	29	0,001121941	3	1657,85978	1,64	15,40	0
	High	lIYINkLDR	1	1	1	P28371	N-Term(iTRAQ4plex); K6(iTRAQ4plex)	0,0000	1,636	1		0	0,00005621	26	0,002465869	3	1435,88779	0,18	31,81	1
	High	fGALTFTR	3	1	1	P28371	N-Term(iTRAQ4plex)	0,0000	0,947	3	9,5	0	0,002459	26	0,002494422	2	1056,59709	1,14	34,05	0
	High	tHGVEVEMGkPQVAYR	1	1	1	P28371	N-Term(iTRAQ4plex); K10(iTRAQ4plex)	0,0000	0,983	1		0	0,00001052	20	0,010964025	4	2089,10830	-0,98	23,78	0
	High	lGmALSk	2	1	1	P28371	N-Term(iTRAQ4plex); M3(Oxidation); K7(iTRAQ4plex)	0,0000	1,370	1		0,001	0,01189	11	0,111406367	2	1023,61089	-0,22	19,41	0
	Medium	lIYINk	1	1	1	P28371	N-Term(iTRAQ4plex); K6(iTRAQ4plex)	0,0000	0,654	1		0,013	0,172	36	0,000225928	2	1051,67607	0,64	28,42	0
	Medium	qSLPk	1	1	1	P28371	N-Term(iTRAQ4plex); K5(iTRAQ4plex)	0,0000	1,018	1		0,015	0,1884	15	0,031839776	2	860,54332	-1,24	12,72	0
	Medium	aLAFk	1	1	1	P28371	N-Term(iTRAQ4plex); K5(iTRAQ4plex)	0,0000	0,998	1		0,027	0,3328	22	0,006137196	2	837,54222	-1,72	21,07	0
P73319	50S ribosomal protein L4 OS=Synechocystis sp. (strain PCC 6803 / Kazu	604,09	30,95	1	10	10	35	2,103	26	89,2	210	23,3	10,20							
	A3	Sequence	# PSMs	# Proteins	# Protein Groups	Protein Group Accessions	Modifications	ΔCn	117/114	117/114 Count	117/114 Variability [%]	q-Value	PEP	IonScore	Exp Value	Charge	MH+ [Da]	ΔM [ppm]	RT [min]	# Missed Cleavages
	High	gGGVIFGPkPR	5	1	1	P73319	N-Term(iTRAQ4plex); K9(iTRAQ4plex)	0,0000	2,342	4	54,2	0	0,00002321	62	6,79157E-07	2	1372,82707	-2,41	19,67	0
	High	eENAAHIVHR	9	1	1	P73319	N-Term(iTRAQ4plex)	0,0000	1,268	5	4,2	0	2,178E-07	54	3,98997E-06	2	1319,69438	0,51	11,01	0
	High	vAkEENAAHIVHR	4	1	1	P73319	N-Term(iTRAQ4plex); K3(iTRAQ4plex)	0,0000	4,687	3	45,6	0	5,856E-10	53	5,66486E-06	3	1761,99442	-1,05	14,59	1
	High	tkELATALTR	7	1	1	P73319	N-Term(iTRAQ4plex); K2(iTRAQ4plex)	0,0000	3,845	4	44,6	0	0,000012	45	3,25814E-05	2	1391,84441	-1,20	21,04	1
	High	eLATALTR	2	1	1	P73319	N-Term(iTRAQ4plex)	0,0000	1,022	2	12,5	0	0,0001299	37	0,000211821	2	1018,59990	-1,44	23,00	0
	High	qGNASAk	1	1	1	P73319	N-Term(iTRAQ4plex); K7(iTRAQ4plex)	0,0000	2,232	1		0	0,004873	33	0,000569768	2	963,54320	-3,10	7,45	0
	High	wGAkPEk	2	1	1	P73319	N-Term(iTRAQ4plex); K4(iTRAQ4plex); K7(iTRAQ4plex)	0,0000	2,867	2	49,5	0	0,00291	27	0,002177559	2	1247,74358	-2,94	15,61	0
	High	vDcIVk	1	1	1	P73319	N-Term(iTRAQ4plex); C3(Carbamidomethyl); K6(iTRAQ4	0,0000	0,740	1		0,003	0,04112	29	0,001670873	2	1021,59307	-2,34	18,95	0
	High	sPLWR	2	1	1	P73319	N-Term(iTRAQ4plex)	0,0000	1,460	2	42,8	0,006	0,07749	20	0,010714453	2	802,46825	-1,20	21,46	0
	High	nIPYLk	2	1	1	P73319	N-Term(iTRAQ4plex); K6(iTRAQ4plex)	0,0000	2,348	2	30,2	0,007	0,08564	29	0,001318166	2	1035,64128	-2,72	27,42	0
P72758	Carbon dioxide concentrating mechanism protein CcmM OS=Synechocys	603,96	25,04	1	14	14	23	1,145	20	19,8	687	73,1	8,59							
	A3	Sequence	# PSMs	# Proteins	# Protein Groups	Protein Group Accessions	Modifications	ΔCn	117/114	117/114 Count	117/114 Variability [%]	q-Value	PEP	IonScore	Exp Value	Charge	MH+ [Da]	ΔM [ppm]	RT [min]	# Missed Cleavages
	High	sAVAHQTGNLAGDSANQLR	1	1	1	P72758	N-Term(iTRAQ4plex)	0,0000	0,816	1		0	3,565E-12	81	8,39402E-09	3	2054,05075	0,55	19,84	0
	High	sLLAQGYGIGAEHANER	1	1	1	P72758	N-Term(iTRAQ4plex)	0,0000	1,008	1		0	1,531E-11	77	1,88365E-08	3	1929,99039	0,22	28,95	0
	High	iQPGVSVAPGSSIR	1	1	1	P72758	N-Term(iTRAQ4plex)	0,0000	1,188	1		0	1,348E-08	72	5,9837E-08	2	1511,86638	0,07	26,85	0
	High	rVVEVIIQRPGDVPGSPSR	3	1	1	P72758	N-Term(iTRAQ4plex)	0,0000	1,259	2	3,0	0	3,474E-07	54	5,51963E-06	4	2205,26113	1,18	27,91	1
	High	sAPVSSAGGSSAGGLTPEVIATVR	1	1	1	P72758	N-Term(iTRAQ4plex)	0,0000	0,585	1		0	0,0003074	52	5,82103E-06	3	2315,23276	0,14	35,96	0
	High	rVLEQIIQRPGDNVVAGR	3	1	1	P72758	N-Term(iTRAQ4plex)	0,0000	1,395	2	7,3	0	1,48E-08	50	1,06407E-05	4	2164,24331	0,05	30,04	1
	High	rVLETIIQRP	2	1	1	P72758	N-Term(iTRAQ4plex)	0,0000	1,241	2	43,8	0	1,152E-07	49	1,37079E-05	3	1368,84436	-0,03	27,91	1
	High	sLLAQGYk	3	1	1	P72758	N-Term(iTRAQ4plex); K8(iTRAQ4plex)	0,0000	1,221	2	11,2	0	0,000003435	48	1,46882E-05	2	1167,69768	0,07	23,68	0
	High	tGSWLTGGTIGSHR	2	1	1	P72758	N-Term(iTRAQ4plex)	0,0000	0,885	2	59,0	0	0,00001172	34	0,000360554	3	1573,82077	0,25	26,46	0
	High	vLEQIIQRPGDNVVAGR	2	1	1	P72758	N-Term(iTRAQ4plex)	0,0000	1,075	2	11,0	0	0,000005134	30	0,001122508	3	2008,14182	-0,13	34,59	0
	High	fLADHTNEYVR	1	1	1	P72758	N-Term(iTRAQ4plex)	0,0000	0,740	1		0	0,002095	26	0,002415294	3	1508,76194	0,33	25,26	0
	High	tALASRPWSk	1	1	1	P72758	N-Term(iTRAQ4plex); K10(iTRAQ4plex)	0,0000	1,103	1		0	0,0003451	24	0,003917148	3	1404,81919	-0,72	20,60	0
	High	vVEVIIQRPGDVPGSPSR	1	1	1	P72758	N-Term(iTRAQ4plex)	0,0000	1,180	1		0	0,00007109	23	0,005610092	3	2049,15763	0,11	32,04	0
	High	aLLHQGYk	1	1	1	P72758	N-Term(iTRAQ4plex); K8(iTRAQ4plex)	0,0000	1,063	1		0	0,003618	16	0,031345936	3	1217,72321	-1,04	15,65	0
P73202	Phycobilisome 8.9 kDa linker polypeptide, phycocyanin-associated, rod (	600,26	81,93	1	6	6	19	0,909	16	14,4	83	9,3	9,41							
	A3	Sequence	# PSMs	# Proteins	# Protein Groups	Protein Group Accessions	Modifications	ΔCn	117/114	117/114 Count	117/114 Variability [%]	q-Value	PEP	IonScore	Exp Value	Charge	MH+ [Da]	ΔM [ppm]	RT [min]	# Missed Cleavages
	High	mLGQSSLVGYSNTQAANR	4	1	1	P73202	N-Term(iTRAQ4plex); M1(Oxidation)	0,0000	0,882	4	17,9	0	1,192E-14	103	4,48714E-11	2	2057,02349	1,55	26,30	0
	High	lGGTIVNIRPYQADSNEQN	3	1	1	P73202	N-Term(iTRAQ4plex)	0,0000	0,902	3	10,9	0	1,294E-08	51	8,80988E-06	2	2233,13725	1,90	35,04	0
	High	vFVYEVSGLR	5	1	1	P73202	N-Term(iTRAQ4plex)	0,0000	0,916	3	7,9	0	0,0001545	45	2,81819E-05	2	1312,74028	1,59	37,70	0
	High	sGSVFIk	4	1	1	P73202	N-Term(iTRAQ4plex); K7(iTRAQ4plex)	0,0000	0,938	3	19,8	0	0,00251	43	4,75302E-05	2	1025,62676	3,29	20,21	0
	High	qTDANENSAHDIR	1	1	1	P73202	N-Term(iTRAQ4plex)	0,0000	0,656	1		0	4,635E-09	43	5,1047E-05	3	1614,76279	2,41	9,02	0
	High	qTDANENSAHDIRR	2	1	1	P73202	N-Term(iTRAQ4plex)	0,0000	0,885	2	18,9	0	0,001093	17	0,020652375	4	1770,86098	0,55	8,29	1
Q59994	Triosephosphate isomerase OS=Synechocystis sp. (strain PCC 6803 / Ka	590,68	31,40	1	9	9	30	1,268	25	21,2	242	26,1	5,16							
	A3	Sequence	# PSMs	# Proteins	# Protein Groups	Protein Group Accessions	Modifications	ΔCn	117/114	117/114 Count	117/114 Variability [%]	q-Value	PEP	IonScore	Exp Value	Charge	MH+ [Da]	ΔM [ppm]	RT [min]	# Missed Cleavages
	High	qYFGETDETANLR	2	1	1	Q59994	N-Term(iTRAQ4plex)	0,0000	1,081	2	24,0	0	2,107E-10	75	2,93745E-08	2	1687,80559	0,68	28,39	0
	High	rQYFGETDETANLR	1	1	1	Q59994	N-Term(iTRAQ4plex)	0,0000	1,381	1		0	3,751E-11	61	7,37853E-07	3	1843,90500	-0,30	23,80	1
	High	aGLIPILcVGESk	7	1	1	Q59994	N-Term(iTRAQ4plex); C8(Carbamidomethyl); K13(iTRAQ	0,0000	1,256	6	29,0	0	3,176E-10	58	1,65566E-06	2	1644,95989	0,08	43,06	0
	High	iIIAGNWk	6	1	1	Q59994	N-Term(iTRAQ4plex); K8(iTRAQ4plex)	0,0000	1,441	5	16,6	0	0,000478	45	3,45915E-05	2	1202,74956	-0,36	33,60	0
	High	kIIIAGNWk	2	1	1	Q59994	N-Term(iTRAQ4plex); K1(iTRAQ4plex); K9(iTRAQ4plex)	0,0000	0,921	2	10,8	0	0,00002045	43	4,60225E-05	3	1474,94391	-2,11	28,58	1
	High	eVVLcVPFTDLSGmSQQLHGGR	1	1	1	Q59994	N-Term(iTRAQ4plex); C5(Carbamidomethyl); M14(Oxidat	0,0000	2,234	1		0	0,000001263	39	0,000121051	3	2590,28995	0,94	42,42	0
	High	vLAAQk	3	1	1	Q59994	N-Term(iTRAQ4plex); K6(iTRAQ4plex)	0,0000	1,101	3	7,3	0,001	0,01869	26	0,002666674	2	917,60033	-2,09	14,47	0
	High	vIGLIR	7	1	1	Q59994	N-Term(iTRAQ4plex)	0,0000	1,352	4	8,4	0,006	0,08262	15	0,030972054	2	814,56273	-0,48	29,44	0
	Medium	iVNFQP	1	1	1	Q59994	N-Term(iTRAQ4plex)	0,0000	1,313	1		0,025	0,3051	14	0,043049688	2	861,49431	-0,91	34,77	0
Q55361	Sll0887 protein OS=Synechocystis sp. (strain PCC 6803 / Kazusa) GN=sl	586,98	27,13	1	11	11	23	0,896	22	14,8	446	47,6	4,94							
	A3	Sequence	# PSMs	# Proteins	# Protein Groups	Protein Group Accessions	Modifications	ΔCn	117/114	117/114 Count	117/114 Variability [%]	q-Value	PEP	IonScore	Exp Value	Charge	MH+ [Da]	ΔM [ppm]	RT [min]	# Missed Cleavages
	High	fYLNSAGAk	3	1	1	Q55361	N-Term(iTRAQ4plex); K9(iTRAQ4plex)	0,0000	0,842	3	6,8	0	0,000003128	63	5,15193E-07	2	1258,70195	-1,16	25,67	0
	High	sYASIYLYSR	3	1	1	Q55361	N-Term(iTRAQ4plex)	0,0000	0,880	3	1,4	0	0,00001943	56	2,48296E-06	2	1366,71208	-0,18	32,26	0
	High	nGVLTGLLHSAGTAk	1	1	1	Q55361	N-Term(iTRAQ4plex); K15(iTRAQ4plex)	0,0000	0,910	1		0	3,454E-07	56	2,62404E-06	3	1727,00498	-0,27	33,34	0
	High	rFDTQPTGHANIGSk	3	1	1	Q55361	N-Term(iTRAQ4plex); K15(iTRAQ4plex)	0,0000	1,028	3	21,5	0	1,931E-08	48	1,42551E-05	4	1917,01870	0,29	16,73	1
	High	yTVVFSAR	3	1	1	Q55361	N-Term(iTRAQ4plex)	0,0000	0,988	3	4,0	0	0,00004977	48	1,64805E-05	2	1086,60625	-0,17	28,83	0
	High	fDTQPTGHANIGSk	1	1	1	Q55361	N-Term(iTRAQ4plex); K14(iTRAQ4plex)	0,0000	0,987	1		0	7,611E-08	42	6,88605E-05	3	1760,91703	0,01	19,45	0
	High	iTVGAHFYHVYSEk	2	1	1	Q55361	N-Term(iTRAQ4plex); K14(iTRAQ4plex)	0,0000	0,943	2	13,8	0	1,311E-11	34	0,00036557	3	1939,03122	-0,21	29,16	0
	High	tLSHLDYRPIPTGk	4	1	1	Q55361	N-Term(iTRAQ4plex); K14(iTRAQ4plex)	0,0000	0,795	3	21,7	0	3,535E-10	31	0,000716094	3	1886,07181	-1,08	25,96	0
	High	mLDIQAIADHAHQGAAALGIk	1	1	1	Q55361	N-Term(iTRAQ4plex); M1(Oxidation); K21(iTRAQ4plex)	0,0000	0,810	1		0	4,976E-08	19	0,011640456	4	2448,32583	-0,58	42,98	0
	High	rVDIIk	1	1	1	Q55361	N-Term(iTRAQ4plex); K6(iTRAQ4plex)	0,0000	0,855	1		0,002	0,02528	25	0,003435342	3	1031,68018	-1,35	16,45	1
	Medium	fDGEGTPTR	1	1	1	Q55361	N-Term(iTRAQ4plex)	0,0000	1,076	1		0,024	0,3025	10	0,093104357	2	1123,55058	0,46	15,43	0
P73306	50S ribosomal protein L6 OS=Synechocystis sp. (strain PCC 6803 / Kazu	580,36	48,60	1	10	10	29	1,660	25	49,0	179	19,7	10,18							
	A3	Sequence	# PSMs	# Proteins	# Protein Groups	Protein Group Accessions	Modifications	ΔCn	117/114	117/114 Count	117/114 Variability [%]	q-Value	PEP	IonScore	Exp Value	Charge	MH+ [Da]	ΔM [ppm]	RT [min]	# Missed Cleavages
	High	vIVAQEGETITVTR	1	1	1	P73306	N-Term(iTRAQ4plex)	0,0000	1,083	1		0	2,185E-09	95	3,91004E-10	2	1659,93999	0,09	30,63	0
	High	tLVANmVDGVAQGFER	1	1	1	P73306	N-Term(iTRAQ4plex); M6(Oxidation)	0,0000	0,588	1		0	3,922E-10	71	8,0904E-08	3	1866,95029	0,10	36,46	0
	High	vSVDIQGSHLSVk	5	1	1	P73306	N-Term(iTRAQ4plex); K13(iTRAQ4plex)	0,0000	1,660	3	46,1	0	2,003E-07	59	1,37079E-06	3	1656,95029	-1,24	29,11	0
	High	tLVANMVDGVAQGFER	2	1	1	P73306	N-Term(iTRAQ4plex)	0,0000	0,968	2	2,2	0	2,065E-07	58	1,7099E-06	2	1850,95867	1,88	49,54	0
	High	rLEIQGVGYR	5	1	1	P73306	N-Term(iTRAQ4plex)	0,0000	1,822	4	40,4	0	2,934E-07	46	2,73967E-05	3	1334,76468	-1,10	24,03	1
	High	yQGEYVR	4	1	1	P73306	N-Term(iTRAQ4plex)	0,0000	2,035	4	6,2	0	0,0001434	38	0,000153098	2	1058,53752	-1,14	16,95	0
	High	rPIPLPAk	4	1	1	P73306	N-Term(iTRAQ4plex); K8(iTRAQ4plex)	0,0000	3,785	3	37,2	0	0,003188	22	0,006382194	3	1179,77954	-1,77	19,24	1
	High	aVRPPEPYk	4	1	1	P73306	N-Term(iTRAQ4plex); K9(iTRAQ4plex)	0,0000	1,693	4	19,9	0	0,009047	16	0,022801846	3	1344,78577	-1,53	16,36	0
	High	vSVDIQGSHLSVkGPk	1	1	1	P73306	N-Term(iTRAQ4plex); K13(iTRAQ4plex); K16(iTRAQ4plex	0,0000	1,214	1		0	0,00002246	11	0,074125904	4	2083,22329	-0,16	27,62	1
	High	aVRPPEPYkGk	1	1	1	P73306	N-Term(iTRAQ4plex); K9(iTRAQ4plex); K11(iTRAQ4plex)	0,0000	1,253	1		0,001	0,01748	13	0,049452619	4	1674,00210	-2,53	15,94	1
	Medium	qLPEk	1	1	1	P73306	N-Term(iTRAQ4plex); K5(iTRAQ4plex)	0,0000	1,867	1		0,025	0,3143	23	0,005533119	2	902,55358	-1,53	13,98	0
P80507	Inorganic pyrophosphatase OS=Synechocystis sp. (strain PCC 6803 / Ka	578,45	46,75	1	10	10	40	0,858	35	29,7	169	19,1	4,88							
	A3	Sequence	# PSMs	# Proteins	# Protein Groups	Protein Group Accessions	Modifications	ΔCn	117/114	117/114 Count	117/114 Variability [%]	q-Value	PEP	IonScore	Exp Value	Charge	MH+ [Da]	ΔM [ppm]	RT [min]	# Missed Cleavages
	High	vTEILGWk	1	1	1	P80507	N-Term(iTRAQ4plex); K8(iTRAQ4plex)	0,0000	0,728	1		0	0,000001941	54	3,91194E-06	2	1233,74248	-1,69	38,39	0
	High	kVTEILGWk	8	1	1	P80507	N-Term(iTRAQ4plex); K1(iTRAQ4plex); K9(iTRAQ4plex)	0,0000	0,967	6	34,8	0	0,00009854	51	8,07179E-06	3	1505,94248	0,58	33,42	1

	High	lDEIAEFFR	3	1	1	P80507	N-Term(iTRAQ4plex)	0,0000	0,612	3	11,7	0	0,00001258	48	1,53805E-05	2	1283,67766	1,86	49,48	0
	High	iLcVPAk	2	1	1	P80507	N-Term(iTRAQ4plex); C3(Carbamidomethyl); K7(iTRAQ4	0,0000	0,702	2	10,2	0	0,00432	45	2,85739E-05	2	1088,67217	-1,72	26,41	0
	High	aGLINVLIEIPAGSk	3	1	1	P80507	N-Term(iTRAQ4plex); K15(iTRAQ4plex)	0,0000	0,826	2	45,8	0	0,0000158	42	6,53085E-05	2	1783,09648	1,83	49,05	0
	High	sINDLAGHR	7	1	1	P80507	N-Term(iTRAQ4plex)	0,0000	0,858	5	9,8	0	0,00007718	40	0,000100918	3	1126,60855	-0,02	15,43	0
	High	nkYEFDkDmNcFALDR	3	1	1	P80507	N-Term(iTRAQ4plex); K2(iTRAQ4plex); K7(iTRAQ4plex);	0,0000	0,831	3	11,5	0	0,00001187	26	0,002636149	4	2514,21401	0,87	28,32	2
	High	nkYEFDkDMNcFALDR	1	1	1	P80507	N-Term(iTRAQ4plex); K2(iTRAQ4plex); K7(iTRAQ4plex);	0,0000	0,879	1		0	0,000000243	24	0,003989973	4	2498,21547	-0,57	32,86	2
	High	iPAQPk	7	1	1	P80507	N-Term(iTRAQ4plex); K6(iTRAQ4plex)	0,0000	0,967	7	12,9	0,002	0,0264	17	0,021378144	3	941,60089	-1,44	14,81	0
	High	iLcVPAkDPR	2	1	1	P80507	N-Term(iTRAQ4plex); C3(Carbamidomethyl); K7(iTRAQ4	0,0000	0,724	2	10,3	0,003	0,03818	20	0,010813593	2	1456,85527	0,27	24,21	1
	High	yTYVk	3	1	1	P80507	N-Term(iTRAQ4plex); K5(iTRAQ4plex)	0,0000	0,898	3	22,5	0,006	0,07002	28	0,001894055	2	961,55931	-0,38	16,94	0
P80046	Isocitrate dehydrogenase [NADP] OS=Synechocystis sp. (strain PCC 680	565,96	22,74	1	11	11	32	0,485	22	14,7	475	52,2	5,58							
	A3	Sequence	# PSMs	# Proteins	# Protein Groups	Protein Group Accessions	Modifications	ΔCn	117/114	117/114 Count	117/114 Variability [%]	q-Value	PEP	IonScore	Exp Value	Charge	MH+ [Da]	ΔM [ppm]	RT [min]	# Missed Cleavages
	High	gIGAAIANR	4	1	1	P80046	N-Term(iTRAQ4plex)	0,0000	0,479	2	1,2	0	0,0003729	62	5,662E-07	2	986,58586	-0,54	19,68	0
	High	kGIGAAIANR	3	1	1	P80046	N-Term(iTRAQ4plex); K1(iTRAQ4plex)	0,0000	0,460	2	44,8	0	9,403E-08	56	2,40974E-06	3	1258,78470	1,01	16,10	1
	High	lQPPSVGSk	4	1	1	P80046	N-Term(iTRAQ4plex); K9(iTRAQ4plex)	0,0000	0,466	2	9,5	0	0,0003099	44	4,14925E-05	2	1200,71977	0,58	22,13	0
	High	gPLTTPVGGGIR	4	1	1	P80046	N-Term(iTRAQ4plex)	0,0000	0,526	3	6,2	0	0,0000427	43	5,16381E-05	2	1268,74553	0,92	26,42	0
	High	iTFVAGkPVVPNDPIIPYIR	2	1	1	P80046	N-Term(iTRAQ4plex); K7(iTRAQ4plex)	0,0000	0,381	2	29,2	0	2,556E-08	41	7,67308E-05	3	2497,48075	0,87	46,29	0
	High	qMVTLVHk	2	1	1	P80046	N-Term(iTRAQ4plex); K8(iTRAQ4plex)	0,0000	0,487	1		0	0,00001456	39	0,000125884	3	1243,74359	0,06	20,59	0
	High	lDIIVYR	1	1	1	P80046	N-Term(iTRAQ4plex)	0,0000	0,443	1		0	0,0002864	34	0,000436486	2	1035,63188	-0,04	34,62	0
	High	lDSGIGIkPISk	4	1	1	P80046	N-Term(iTRAQ4plex); K8(iTRAQ4plex); K12(iTRAQ4plex)	0,0000	0,556	2	8,9	0	0,000007816	33	0,00049885	3	1660,03983	1,72	28,49	0
	High	qIFDLYTcVRPcR	3	1	1	P80046	N-Term(iTRAQ4plex); C8(Carbamidomethyl); C12(Carbar	0,0000	0,882	3	5,0	0	0,000009339	28	0,001455359	3	1871,93808	0,19	36,91	0
	High	yYPGTPSPHk	1	1	1	P80046	N-Term(iTRAQ4plex); K10(iTRAQ4plex)	0,0000	0,541	1		0	0,00676	14	0,040548053	3	1434,76325	0,89	18,04	0
	High	sLNVALR	4	1	1	P80046	N-Term(iTRAQ4plex)	0,0000	0,446	3	1,7	0,001	0,01369	29	0,001303077	2	916,57054	0,96	22,98	0
P74162	Sll1380 protein OS=Synechocystis sp. (strain PCC 6803 / Kazusa) GN=s	564,80	38,79	1	8	8	20	0,764	17	32,8	214	23,9	8,91							
	A3	Sequence	# PSMs	# Proteins	# Protein Groups	Protein Group Accessions	Modifications	ΔCn	117/114	117/114 Count	117/114 Variability [%]	q-Value	PEP	IonScore	Exp Value	Charge	MH+ [Da]	ΔM [ppm]	RT [min]	# Missed Cleavages
	High	nLDFTLNAPAGHLPSPR	3	1	1	P74162	N-Term(iTRAQ4plex)	0,0000	0,757	2	11,8	0	2,006E-10	85	3,96002E-09	3	1964,04691	-0,10	35,97	0
	High	fQVcQSGQVPTAPVR	4	1	1	P74162	N-Term(iTRAQ4plex); C4(Carbamidomethyl)	0,0000	0,580	4	11,3	0	5,852E-08	81	8,43277E-09	2	1817,94792	1,64	28,76	0
	High	fGFTPQLEAFLATGNRPGR	4	1	1	P74162	N-Term(iTRAQ4plex)	0,0000	0,635	3	18,1	0	0,000005828	54	3,60554E-06	4	2223,18032	0,50	47,74	0
	High	lLLAATNR	2	1	1	P74162	N-Term(iTRAQ4plex)	0,0000	0,655	2	1,1	0	0,00004448	46	2,72879E-05	2	1015,63841	0,32	26,47	0
	High	kNLDFTLNAPAGHLPSPR	2	1	1	P74162	N-Term(iTRAQ4plex); K1(iTRAQ4plex)	0,0000	0,877	1		0	4,917E-12	43	5,47254E-05	4	2236,24575	0,72	32,31	1
	High	rLLLAATNR	1	1	1	P74162	N-Term(iTRAQ4plex)	0,0000	1,156	1		0	0,00004488	41	8,33624E-05	3	1171,73948	0,23	19,83	1
	High	tIDQLWLQHSNNR	2	1	1	P74162	N-Term(iTRAQ4plex)	0,0000	0,897	2	24,1	0	9,909E-10	36	0,000229071	3	1768,92331	1,23	31,70	0
	High	kENQWVIFk	2	1	1	P74162	N-Term(iTRAQ4plex); K1(iTRAQ4plex); K9(iTRAQ4plex)	0,0000	0,904	2	15,9	0	0,0004114	23	0,004763981	3	1623,95890	0,36	31,30	1
P74061	Ribulose-phosphate 3-epimerase OS=Synechocystis sp. (strain PCC 680	558,83	31,30	1	7	7	19	0,644	15	14,4	230	25,0	5,43							
	A3	Sequence	# PSMs	# Proteins	# Protein Groups	Protein Group Accessions	Modifications	ΔCn	117/114	117/114 Count	117/114 Variability [%]	q-Value	PEP	IonScore	Exp Value	Charge	MH+ [Da]	ΔM [ppm]	RT [min]	# Missed Cleavages
	High	nIVVAPSILSADFSR	5	1	1	P74061	N-Term(iTRAQ4plex)	0,0000	0,550	4	22,8	0	2,532E-10	82	6,96578E-09	2	1732,97356	1,20	44,44	0
	High	skNIVVAPSILSADFSR	2	1	1	P74061	N-Term(iTRAQ4plex); K2(iTRAQ4plex)	0,0000	1,344	2	11,1	0	4,364E-12	80	9,57128E-09	3	2092,20133	0,38	38,41	1
	High	aGADIISVHVEHNASPHLHR	4	1	1	P74061	N-Term(iTRAQ4plex)	0,0000	0,654	2	2,3	0	3,607E-10	51	8,31706E-06	5	2304,20927	0,61	22,64	0
	High	rPEPQLATV	3	1	1	P74061	N-Term(iTRAQ4plex)	0,0000	0,681	3	6,2	0	0,005328	49	1,18842E-05	2	1154,66606	0,89	21,93	0
	High	kTLDVHLmIVEPEk	2	1	1	P74061	N-Term(iTRAQ4plex); K1(iTRAQ4plex); M8(Oxidation); K	0,0000	0,930	1		0	1,259E-07	43	5,21159E-05	4	2100,21108	0,54	30,95	1
	High	tLcQIR	2	1	1	P74061	N-Term(iTRAQ4plex); C3(Carbamidomethyl)	0,0000	0,564	2	10,5	0,001	0,01606	32	0,000629463	2	934,52605	-0,03	16,53	0
	High	lGEEIk	1	1	1	P74061	N-Term(iTRAQ4plex); K6(iTRAQ4plex)	0,0000	0,459	1		0,001	0,01808	19	0,011721144	2	976,59154	-0,21	18,88	0
P73530	30S ribosomal protein S1 homolog A OS=Synechocystis sp. (strain PCC	555,34	29,27	1	10	10	27	1,385	23	29,4	328	36,5	4,67							
	A3	Sequence	# PSMs	# Proteins	# Protein Groups	Protein Group Accessions	Modifications	ΔCn	117/114	117/114 Count	117/114 Variability [%]	q-Value	PEP	IonScore	Exp Value	Charge	MH+ [Da]	ΔM [ppm]	RT [min]	# Missed Cleavages
	High	kMNGLEVAQVVVGSVR	3	1	1	P73530	N-Term(iTRAQ4plex); K1(iTRAQ4plex)	0,0000	1,197	3	25,5	0	1,035E-11	80	1,04759E-08	3	1974,14151	0,30	40,09	1
	High	kmNGLEVAQVVVGSVR	2	1	1	P73530	N-Term(iTRAQ4plex); K1(iTRAQ4plex); M2(Oxidation)	0,0000	1,248	2	72,3	0	1,123E-08	59	1,25017E-06	3	1990,13578	-0,03	35,13	1
	High	vmIIDLDAER	1	1	1	P73530	N-Term(iTRAQ4plex); M2(Oxidation)	0,0000	1,317	1		0	2,479E-07	54	4,18765E-06	2	1334,71111	0,31	34,07	0
	High	tAAYIPIQEmSINR	3	1	1	P73530	N-Term(iTRAQ4plex); M10(Oxidation)	0,0000	1,001	2	0,0	0	0,00001744	44	3,88124E-05	3	1766,92362	0,46	30,86	0
	High	gFIPGSHISAR	3	1	1	P73530	N-Term(iTRAQ4plex)	0,0000	1,601	2	5,8	0	0,00000152	42	6,99794E-05	3	1285,71329	-0,07	21,21	0
	High	qLQAEDATVR	1	1	1	P73530	N-Term(iTRAQ4plex)	0,0000	1,419	1		0	0,00002205	40	9,3319E-05	2	1274,68254	0,31	16,90	0
	High	gALIDIGAk	2	1	1	P73530	N-Term(iTRAQ4plex); K9(iTRAQ4plex)	0,0000	1,607	1		0	0,000001254	39	0,000134577	2	1145,71257	-0,62	28,63	0
	High	sNVFATNR	2	1	1	P73530	N-Term(iTRAQ4plex)	0,0000	0,987	2	34,9	0	0,0001657	32	0,000629463	2	1052,56072	0,15	16,04	0
	High	rIEYMR	2	1	1	P73530	N-Term(iTRAQ4plex)	0,0000	1,175	2	24,7	0,001	0,01253	16	0,02415294	3	1011,55240	-0,23	14,96	1
	High	iSLSTk	4	1	1	P73530	N-Term(iTRAQ4plex); K6(iTRAQ4plex)	0,0000	1,808	3	26,4	0,002	0,03252	46	2,40974E-05	2	936,59606	-0,81	19,58	0
	High	lVLSHR	2	1	1	P73530	N-Term(iTRAQ4plex)	0,0000	1,494	2	5,6	0,005	0,05793	27	0,002207852	2	868,54790	-0,72	15,00	0
	High	rIEYmR	2	1	1	P73530	N-Term(iTRAQ4plex); M5(Oxidation)	0,0000	1,193	2	54,3	0,006	0,06996	12	0,063091377	3	1027,54740	-0,15	11,05	1
Q55499	Single-stranded DNA-binding protein 1 OS=Synechocystis sp. (strain PC	551,00	64,46	1	10	10	22	1,061	18	25,7	121	13,6	7,34							
	A3	Sequence	# PSMs	# Proteins	# Protein Groups	Protein Group Accessions	Modifications	ΔCn	117/114	117/114 Count	117/114 Variability [%]	q-Value	PEP	IonScore	Exp Value	Charge	MH+ [Da]	ΔM [ppm]	RT [min]	# Missed Cleavages
	High	vNNLDLLGSk	2	1	1	Q55499	N-Term(iTRAQ4plex); K10(iTRAQ4plex)	0,0000	0,932	2	2,3	0	0,000006241	67	2,80507E-07	2	1360,80229	-1,16	32,25	0
	High	gSLIGIQGSLk	4	1	1	Q55499	N-Term(iTRAQ4plex); K11(iTRAQ4plex)	0,0000	0,922	4	31,8	0	5,033E-08	60	1,02795E-06	2	1360,83891	-0,99	31,40	0
	High	tAEIAGNYVk	1	1	1	Q55499	N-Term(iTRAQ4plex); K10(iTRAQ4plex)	0,0000	0,946	1		0	0,000003636	59	1,29927E-06	2	1353,76091	-0,56	22,58	0
	High	dNAEATmNNYPEEF	1	1	1	Q55499	N-Term(iTRAQ4plex); M7(Oxidation)	0,0000	1,910	1		0	2,023E-07	58	1,52044E-06	2	1804,74516	-0,07	29,84	0
	High	sVNSIHLVGR	4	1	1	Q55499	N-Term(iTRAQ4plex)	0,0000	1,196	2	10,1	0	0,000007437	55	3,53159E-06	3	1225,71320	-0,14	21,35	0
	High	kGSLIGIQGSLk	5	1	1	Q55499	N-Term(iTRAQ4plex); K1(iTRAQ4plex); K12(iTRAQ4plex)	0,0000	1,450	3	38,6	0	9,268E-09	47	2,02288E-05	3	1633,03690	-0,25	26,91	1
	High	vNNLDLLGSkR	1	1	1	Q55499	N-Term(iTRAQ4plex); K10(iTRAQ4plex)	0,0000	1,616	1		0	0,00001733	33	0,000527193	3	1516,90354	-0,96	26,96	1
	High	fDHWEDR	1	1	1	Q55499	N-Term(iTRAQ4plex)	0,0000	0,865	1		0	0,003311	16	0,022437269	3	1148,52408	-0,08	21,19	0
	High	skPVIR	2	1	1	Q55499	N-Term(iTRAQ4plex); K2(iTRAQ4plex)	0,0000	0,891	2	3,2	0	0,001693	16	0,022801846	3	987,65366	-1,71	9,66	0
	High	aGRDPEVk	1	1	1	Q55499	N-Term(iTRAQ4plex); K8(iTRAQ4plex)	0,0000	1,046	1		0,008	0,1053	25	0,003535027	2	1159,66533	-1,77	9,26	1
P73318	50S ribosomal protein L23 OS=Synechocystis sp. (strain PCC 6803 / Kaz	543,81	78,22	1	10	10	37	2,175	28	63,1	101	11,5	10,05							
	A3	Sequence	# PSMs	# Proteins	# Protein Groups	Protein Group Accessions	Modifications	ΔCn	117/114	117/114 Count	117/114 Variability [%]	q-Value	PEP	IonScore	Exp Value	Charge	MH+ [Da]	ΔM [ppm]	RT [min]	# Missed Cleavages
	High	aAIELLFDVk	4	1	1	P73318	N-Term(iTRAQ4plex); K10(iTRAQ4plex)	0,0000	1,643	4	127,0	0	5,588E-08	57	1,79875E-06	2	1406,84990	0,09	47,93	0
	High	aTLQLEDNk	2	1	1	P73318	N-Term(iTRAQ4plex); K9(iTRAQ4plex)	0,0000	1,997	1		0	0,00004198	48	1,70608E-05	2	1319,73894	-1,51	21,54	0
	High	eGDSIQLFPDV	1	1	1	P73318	N-Term(iTRAQ4plex)	0,0000	3,860	1		0	0,0003847	48	1,75376E-05	2	1363,68596	-0,20	46,74	0
	High	lADLIIkPIVTEk	12	1	1	P73318	N-Term(iTRAQ4plex); K7(iTRAQ4plex); K13(iTRAQ4plex)	0,0000	2,535	8	25,4	0	4,708E-07	40	0,000109893	4	1885,20644	-1,81	42,96	0
	High	rLADLIIkPIVTEk	5	1	1	P73318	N-Term(iTRAQ4plex); K8(iTRAQ4plex); K14(iTRAQ4plex)	0,0000	4,011	3	33,8	0	2,605E-10	39	0,00013273	4	2041,30984	-0,56	37,79	1
	High	aVVTLk	4	1	1	P73318	N-Term(iTRAQ4plex); K6(iTRAQ4plex)	0,0000	1,294	3	9,3	0	0,00797	36	0,000270377	2	918,61992	-2,97	20,81	0
	High	vTGVNTAR	3	1	1	P73318	N-Term(iTRAQ4plex)	0,0000	0,941	3	4,4	0	0,008178	21	0,008530412	2	961,55486	0,12	11,81	0
	High	yVFDVRPEATkPEIk	2	1	1	P73318	N-Term(iTRAQ4plex); K11(iTRAQ4plex); K15(iTRAQ4plex	0,0000	1,985	2	26,7	0	0,0001697	19	0,013426722	3	2224,26999	-0,11	31,35	0
	High	rAVVTLk	1	1	1	P73318	N-Term(iTRAQ4plex); K7(iTRAQ4plex)	0,0000	1,471	1		0,004	0,05408	24	0,004216674	3	1074,72242	-1,26	15,10	1
	High	fmGFk	3	1	1	P73318	N-Term(iTRAQ4plex); M2(Oxidation); K5(iTRAQ4plex)	0,0000	1,581	2	6,9	0,009	0,1195	18	0,017457016	2	933,50859	-2,22	22,64	0
P54206	Ribulose bisphosphate carboxylase small chain OS=Synechocystis sp. (s	539,62	46,90	1	4	4	21	1,545	16	13,6	113	13,2	5,48							
	A3	Sequence	# PSMs	# Proteins	# Protein Groups	Protein Group Accessions	Modifications	ΔCn	117/114	117/114 Count	117/114 Variability [%]	q-Value	PEP	IonScore	Exp Value	Charge	MH+ [Da]	ΔM [ppm]	RT [min]	# Missed Cleavages
	High	rYETLSYLPPLTDQQIAk	10	1	1	P54206	N-Term(iTRAQ4plex); K18(iTRAQ4plex)	0,0000	1,545	8	17,3	0	1,259E-10	58	1,78316E-06	3	2424,34293	2,14	38,66	1
	High	qcQTVSFIVHkPNQNQGR	5	1	1	P54206	N-Term(iTRAQ4plex); C2(Carbamidomethyl); K11(iTRAQ	0,0000	1,638	3	2,1	0	2,919E-11	57	1,97229E-06	4	2429,27334	0,93	22,82	0
	High	vIGFDNIk	3	1	1	P54206	N-Term(iTRAQ4plex); K8(iTRAQ4plex)	0,0000	1,330	2	8,4	0	0,00001427	55	3,47512E-06	2	1193,71233	-0,79	31,97	0
	High	sENPNcYIR	3	1	1	P54206	N-Term(iTRAQ4plex); C6(Carbamidomethyl)	0,0000	1,515	3	14,5	0	0,00001333	49	1,29411E-05	2	1296,61308	0,58	19,08	0
	>sp|K22E_HUMAN|	538,28	17,83	1	10	11	22	1,463	17	27,1	645	65,8	8,00							
	A3	Sequence	# PSMs	# Proteins	# Protein Groups	Protein Group Accessions	Modifications	ΔCn	117/114	117/114 Count	117/114 Variability [%]	q-Value	PEP	IonScore	Exp Value	Charge	MH+ [Da]	ΔM [ppm]	RT [min]	# Missed Cleavages
	High	gFSSGSAVVSGGSR	3	1	1		N-Term(iTRAQ4plex)	0,0000	1,755	2	0,3	0	8,403E-11	93	5,02308E-10	2	1398,71013	0,50	19,71	0
	High	hGGGGGGFGGGGFGSR	1	1	1		N-Term(iTRAQ4plex)	0,0000	1,340	1		0	3,079E-09	57	2,11334E-06	3	1464,68406	-0,63	15,05	0
	High	sLVGLGGTk	4	1	1		N-Term(iTRAQ4plex); K9(iTRAQ4plex)	0,0000	1,364	2	9,5	0	0,000002654	50	1,06162E-05	2	1119,69768	0,06	23,53	0
	High	fLEQQNQVLQTk	1	2	2		N-Term(iTRAQ4plex); K12(iTRAQ4plex)	0,0000				0	4,062E-09	45	3,26361E-05	3	1763,98899	-0,26	30,29	0
	High	fASFIDkVR	2	1	1		N-Term(iTRAQ4plex); K7(iTRAQ4plex)	0,0000	2,979	2	4,1	0	0,00006868	44	5,53513E-05	3	1370,80323	-0,19	34,42	1
	High	tAAENDFVTLkk	2	1	1		N-Term(iTRAQ4plex); K11(iTRAQ4plex); K12(iTRAQ4plex	0,0000	1,518	2	8,9	0	6,353E-08	42	6,91783E-05	3	1769,01798	0,58	25,57	1
	High	skEEAEALYHSk	2	1	1		N-Term(iTRAQ4plex); K2(iTRAQ4plex); K12(iTRAQ4plex)	0,0000	1,127	2	0,9	0	4,484E-11	39	0,000114543	3	1823,98569	-0,37	20,40	1
	High	lLRDYQELMNVk	1	1	1		N-Term(iTRAQ4plex); K12(iTRAQ4plex)	0,0000	2,836	1		0	0,000008919	35	0,000326565	3	1810,01529	0,95	39,90	1
	High	lQGEIAHVk	2	1	1		N-Term(iTRAQ4plex); K9(iTRAQ4plex)	0,0000	1,085	1		0	0,0001158	29	0,001342672	3	1282,77243	0,20	21,44	0
	High	kYEDEINkR	3	1	1		N-Term(iTRAQ4plex); K1(iTRAQ4plex); K8(iTRAQ4plex)	0,0000	1,463	3	1,3	0	0,00004755	28	0,001527461	3	1626,91825	0,42	14,09	2
	High	lQGEIAHVkk	1	1	1		N-Term(iTRAQ4plex); K9(iTRAQ4plex); K10(iTRAQ4plex)	0,0000	1,870	1		0	0,006571	14	0,040548053	4	1554,96694	-1,46	19,35	1
P73243	CheY subfamily OS=Synechocystis sp. (strain PCC 6803 / Kazusa) GN=s	530,23	46,11	1	7	7	18	1,064	15	9,5	180	20,2	6,81							
	A3	Sequence	# PSMs	# Proteins	# Protein Groups	Protein Group Accessions	Modifications	ΔCn	117/114	117/114 Count	117/114 Variability [%]	q-Value	PEP	IonScore	Exp Value	Charge	MH+ [Da]	ΔM [ppm]	RT [min]	# Missed Cleavages
	High	qLGQLVTFIk	4	1	1	P73243	N-Term(iTRAQ4plex); K10(iTRAQ4plex)	0,0000	1,090	4	8,8	0	1,878E-08	77	2,22316E-08	2	1434,89531	2,10	42,28	0
	High	hAPVAAVASGAAPAGDLAAEVQQ	2	1	1	P73243	N-Term(iTRAQ4plex); K25(iTRAQ4plex)	0,0000	0,953	2	16,4	0	8,321E-09	74	4,47908E-08	3	2630,45285	0,88	40,55	0
	High	vkDmLPQGNFEVIEAk	1	1	1	P73243	N-Term(iTRAQ4plex); K2(iTRAQ4plex); M4(Oxidation); K	0,0000	0,999	1		0	1,597E-13	69	1,54138E-07	3	2266,24912	0,58	33,07	1
	High	aIPLVLMSGR	3	1	1	P73243	N-Term(iTRAQ4plex)	0,0000	1,098	2	2,9	0	0,00009812	44	4,15882E-05	2	1200,72649	0,79	39,69	0
	High	qQVAQmHGEIDLLkk	2	1	1	P73243	N-Term(iTRAQ4plex); M6(Oxidation); K14(iTRAQ4plex); I	0,0000	1,503	1		0	5,522E-09	39	0,000145274	4	2186,23208	-0,33	23,35	1
	High	kEEVLEk	1	1	1	P73243	N-Term(iTRAQ4plex); K1(iTRAQ4plex); K7(iTRAQ4plex)	0,0000	0,911	1		0	0,00185	33	0,000459166	3	1306,79477	0,39	14,87	1
	High	kQLGQLVTFIk	1	1	1	P73243	N-Term(iTRAQ4plex); K1(iTRAQ4plex); K11(iTRAQ4plex)	0,0000	1,076	1		0	0,00002681	32	0,000683864	3	1707,09208	1,61	37,07	1
	High	aIPLVLmSGR	3	1	1	P73243	N-Term(iTRAQ4plex); M7(Oxidation)	0,0000	0,934	2	9,8	0	0,000005005	31	0,000734463	2	1216,72136	0,74	32,74	0
	High	qQVAQMHGEIDLLkk	1	1	1	P73243	N-Term(iTRAQ4plex); K14(iTRAQ4plex); K15(iTRAQ4plex	0,0000	1,296	1		0	1,228E-07	24	0,004036175	4	2170,23867	0,36	28,49	1
P72797	Transaldolase OS=Synechocystis sp. (strain PCC 6803 / Kazusa) GN=tal	526,45	23,53	1	12	12	25	0,832	19	15,9	391	43,1	5,06							
	A3	Sequence	# PSMs	# Proteins	# Protein Groups	Protein Group Accessions	Modifications	ΔCn	117/114	117/114 Count	117/114 Variability [%]	q-Value	PEP	IonScore	Exp Value	Charge	MH+ [Da]	ΔM [ppm]	RT [min]	# Missed Cleavages
	High	iASTWEGIk	1	1	1	P72797	N-Term(iTRAQ4plex); K9(iTRAQ4plex)	0,0000	0,537	1		0	4,821E-07	54	3,70655E-06	2	1292,74504	-0,20	31,28	0
	High	iLQIIPGR	6	1	1	P72797	N-Term(iTRAQ4plex)	0,0000	0,811	4	5,8	0	0,0002107	54	4,44601E-06	2	1053,69109	0,91	35,88	0
	High	nLLEQLR	1	1	1	P72797	N-Term(iTRAQ4plex)	0,0000	0,961	1		0	0,0001347	45	3,28073E-05	2	1029,61748	0,13	31,28	0
	High	vTLISPFVGR	4	1	1	P72797	N-Term(iTRAQ4plex)	0,0000	0,807	3	5,2	0	0,000879	43	4,59166E-05	2	1232,74944	0,86	40,06	0
	High	akDEAGENASIkDIVR	4	1	1	P72797	N-Term(iTRAQ4plex); K2(iTRAQ4plex); K12(iTRAQ4plex)	0,0000	1,321	2	6,2	0	8,425E-09	43	6,19313E-05	4	2148,19692	-0,75	22,76	2
	High	akDEAGENASIk	1	1	1	P72797	N-Term(iTRAQ4plex); K2(iTRAQ4plex); K12(iTRAQ4plex)	0,0000	0,768	1		0	5,511E-08	43	5,06991E-05	3	1664,91721	-0,46	12,16	1
	High	iLDWYk	1	1	1	P72797	N-Term(iTRAQ4plex); K6(iTRAQ4plex)	0,0000	0,604	1		0	0,001159	41	8,62919E-05	2	1125,65618	1,34	36,83	0
	High	lAVAFGLk	3	1	1	P72797	N-Term(iTRAQ4plex); K8(iTRAQ4plex)	0,0000	0,876	2	4,3	0	0,00003524	39	0,000126465	2	1106,71880	1,06	36,60	0
	High	kLDPATVPQDIEk	1	1	1	P72797	N-Term(iTRAQ4plex); K1(iTRAQ4plex); K13(iTRAQ4plex)	0,0000	0,858	1		0	6,321E-07	32	0,000594251	3	1886,09543	-0,27	26,88	1
	High	aLEVLEHLLEER	1	1	1	P72797	N-Term(iTRAQ4plex)	0,0000	0,701	1		0	0,0001358	23	0,004909079	3	1594,88962	-1,59	53,46	0
	High	gkNLLEQLR	1	1	1	P72797	N-Term(iTRAQ4plex); K2(iTRAQ4plex)	0,0000	0,644	1		0	0,002007	14	0,036641227	3	1358,83280	-2,24	27,01	1
	High	iLDWYkk	1	1	1	P72797	N-Term(iTRAQ4plex); K6(iTRAQ4plex); K7(iTRAQ4plex)	0,0000	2,805	1		0	0,002789	13	0,045078557	3	1397,84800	-2,65	30,87	1
P74102	Orange carotenoid-binding protein OS=Synechocystis sp. (strain PCC 68	523,92	35,33	1	9	9	21	1,200	19	22,4	317	34,6	5,05							
	A3	Sequence	# PSMs	# Proteins	# Protein Groups	Protein Group Accessions	Modifications	ΔCn	117/114	117/114 Count	117/114 Variability [%]	q-Value	PEP	IonScore	Exp Value	Charge	MH+ [Da]	ΔM [ppm]	RT [min]	# Missed Cleavages
	High	gIFPNTLAADVVPATIAR	3	1	1	P74102	N-Term(iTRAQ4plex)	0,0000	1,363	2	47,0	0	1,415E-09	77	1,99054E-08	2	1970,12297	1,90	47,32	0
	High	tYASWSPNIk	5	1	1	P74102	N-Term(iTRAQ4plex); K10(iTRAQ4plex)	0,0000	1,071	5	27,0	0	1,921E-08	64	3,58072E-07	2	1454,78838	0,12	29,88	0
	High	tLTIAAPGAASmQLAENALk	1	1	1	P74102	N-Term(iTRAQ4plex); M12(Oxidation); K20(iTRAQ4plex)	0,0000	1,200	1		0	3,631E-10	64	4,18794E-07	3	2275,26010	1,32	39,77	0
	High	eLLNFAR	2	1	1	P74102	N-Term(iTRAQ4plex)	0,0000	1,304	2	10,6	0	0,0003787	47	1,8197E-05	2	1006,58061	0,38	31,34	0
	High	fLLNPEGk	4	1	1	P74102	N-Term(iTRAQ4plex); K8(iTRAQ4plex)	0,0000	1,138	3	0,5	0	0,00001936	45	3,22849E-05	2	1205,71257	-0,58	30,26	0
	High	nAVVDmGFTAGkDGkR	1	1	1	P74102	N-Term(iTRAQ4plex); M6(Oxidation); K12(iTRAQ4plex); I	0,0000	1,756	1		0	4,362E-07	35	0,000319132	4	2114,13686	-0,98	18,44	2
	High	fFREEcQNLk	1	1	1	P74102	N-Term(iTRAQ4plex); C6(Carbamidomethyl); K10(iTRAQ	0,0000	1,869	1		0	0,0007051	17	0,018406449	3	1658,85733	0,60	24,42	1
	High	vQTPWFGGNVGmNIAWR	1	1	1	P74102	N-Term(iTRAQ4plex); M12(Oxidation)	0,0000	1,061	1		0	0,0001308	13	0,054697818	3	2093,05094	0,05	45,17	0
	High	lGFWYR	3	1	1	P74102	N-Term(iTRAQ4plex)	0,0000	1,410	3	8,2	0,002	0,02258	22	0,006094948	2	985,53819	0,58	37,10	0
Q55835	Iron uptake protein A2 OS=Synechocystis sp. (strain PCC 6803 / Kazusa	523,29	29,48	1	9	9	24	0,377	21	25,8	346	38,1	6,18							
	A3	Sequence	# PSMs	# Proteins	# Protein Groups	Protein Group Accessions	Modifications	ΔCn	117/114	117/114 Count	117/114 Variability [%]	q-Value	PEP	IonScore	Exp Value	Charge	MH+ [Da]	ΔM [ppm]	RT [min]	# Missed Cleavages
	High	gTHVNVSGAGVLk	4	1	1	Q55835	N-Term(iTRAQ4plex); K13(iTRAQ4plex)	0,0000	0,419	2	16,6	0	2,75E-08	69	1,1587E-07	3	1526,89167	1,52	19,77	0
	High	hPDGLWYGFTQR	5	1	1	Q55835	N-Term(iTRAQ4plex)	0,0000	0,408	5	15,1	0	1,191E-08	69	1,23586E-07	3	1620,80564	1,02	33,74	0
	High	iLVRPSSNVYNLSLTASR	3	1	1	Q55835	N-Term(iTRAQ4plex)	0,0000	0,383	2	2,3	0	9,414E-08	44	4,28519E-05	3	2134,21286	1,28	36,16	0
	High	tINLYSSR	2	1	1	Q55835	N-Term(iTRAQ4plex)	0,0000	0,319	2	8,9	0	0,00002602	39	0,000122735	2	1097,60771	0,51	22,39	0
	High	vSLFFPNTGSGER	3	1	1	Q55835	N-Term(iTRAQ4plex)	0,0000	0,302	3	5,7	0	0,00001426	39	0,000139627	2	1554,80535	1,29	38,01	0
	High	wLQGLVGNFAR	2	1	1	Q55835	N-Term(iTRAQ4plex)	0,0000	0,792	2	17,5	0	0,000005423	34	0,000379289	2	1404,78874	1,32	48,91	0
	High	iAIHGEPETR	1	1	1	Q55835	N-Term(iTRAQ4plex)	0,0000	0,313	1		0	0,0002227	26	0,002594	3	1266,69294	0,49	16,94	0
	High	vLYYSR	3	1	1	Q55835	N-Term(iTRAQ4plex)	0,0000	0,337	3	8,8	0	0,00651	25	0,003515362	2	944,53398	1,90	21,93	0
	High	aEQAGLFQPVR	1	1	1	Q55835	N-Term(iTRAQ4plex)	0,0000	0,796	1		0	0,001803	21	0,008749233	2	1359,75163	1,07	27,47	0
P29255	Photosystem I P700 chlorophyll a apoprotein A2 OS=Synechocystis sp. (	522,37	15,18	1	11	11	33	0,654	26	14,4	731	81,2	6,95							
	A3	Sequence	# PSMs	# Proteins	# Protein Groups	Protein Group Accessions	Modifications	ΔCn	117/114	117/114 Count	117/114 Variability [%]	q-Value	PEP	IonScore	Exp Value	Charge	MH+ [Da]	ΔM [ppm]	RT [min]	# Missed Cleavages
	High	dFGYSFPcDGPGR	1	1	1	P29255	N-Term(iTRAQ4plex); C8(Carbamidomethyl)	0,0000	0,828	1		0	9,704E-10	71	8,33624E-08	2	1618,70915	0,89	32,72	0
	High	tNWGIGHSIk	5	1	1	P29255	N-Term(iTRAQ4plex); K10(iTRAQ4plex)	0,0000	0,606	3	2,1	0	0,007766	62	6,87021E-07	2	1400,80168	9,13	22,96	0
	High	dkPVALSIVQAR	4	1	1	P29255	N-Term(iTRAQ4plex); K2(iTRAQ4plex)	0,0000	0,696	3	16,0	0	2,29E-09	60	8,99435E-07	3	1584,96988	1,44	27,91	0
	High	fSQDLAQDPTTR	1	1	1	P29255	N-Term(iTRAQ4plex)	0,0000	0,568	1		0	8,408E-08	59	1,40595E-06	2	1522,76335	0,98	24,45	0
	High	eILNAHk	8	1	1	P29255	N-Term(iTRAQ4plex); K7(iTRAQ4plex)	0,0000	0,657	5	7,6	0	0,000207	43	4,59166E-05	2	1112,66850	1,68	14,40	0
	High	tPLANLVR	3	1	1	P29255	N-Term(iTRAQ4plex)	0,0000	0,590	3	8,3	0	0,002853	42	6,60648E-05	2	1027,63921	1,09	30,32	0
	High	rIWYGIATAHDFETHDGmTEENL'	1	1	1	P29255	N-Term(iTRAQ4plex); M18(Oxidation); K26(iTRAQ4plex)	0,0000	0,650	1		0	6,114E-10	36	0,000229599	4	3429,64663	1,55	35,56	1
	High	wkDkPVALSIVQAR	3	1	1	P29255	N-Term(iTRAQ4plex); K2(iTRAQ4plex); K4(iTRAQ4plex)	0,0000	0,749	2	17,5	0	1,787E-07	33	0,000495416	4	2043,24685	1,41	31,03	1
	High	fRPSLAWFk	2	1	1	P29255	N-Term(iTRAQ4plex); K9(iTRAQ4plex)	0,0000	0,554	2	1,7	0	0,0000451	20	0,011167861	3	1439,84278	1,78	38,73	0
	High	lmPDkk	3	1	1	P29255	N-Term(iTRAQ4plex); M2(Oxidation); K5(iTRAQ4plex); K	0,0000	0,829	3	9,7	0,002	0,03394	14	0,04425578	3	1179,71354	0,31	10,36	1
	Medium	lMPDkk	1	1	1	P29255	N-Term(iTRAQ4plex); K5(iTRAQ4plex); K6(iTRAQ4plex)	0,0000	0,642	1		0,014	0,1822	15	0,028904802	3	1163,71781	-0,39	14,71	1
	Medium	aTkFPk	1	1	1	P29255	N-Term(iTRAQ4plex); K3(iTRAQ4plex); K6(iTRAQ4plex)	0,0000	0,861	1		0,021	0,2571	12	0,070140685	3	1123,71567	-3,82	12,91	1
P73304	30S ribosomal protein S5 OS=Synechocystis sp. (strain PCC 6803 / Kazu	518,16	45,09	1	9	9	19	1,641	15	18,7	173	18,2	10,59							
	A3	Sequence	# PSMs	# Proteins	# Protein Groups	Protein Group Accessions	Modifications	ΔCn	117/114	117/114 Count	117/114 Variability [%]	q-Value	PEP	IonScore	Exp Value	Charge	MH+ [Da]	ΔM [ppm]	RT [min]	# Missed Cleavages
	High	qLGSNNPLNNAR	5	1	1	P73304	N-Term(iTRAQ4plex)	0,0000	1,257	4	52,1	0	2,96E-10	82	5,68814E-09	2	1441,76384	0,68	21,52	0
	High	aAINALETLR	4	1	1	P73304	N-Term(iTRAQ4plex)	0,0000	1,461	3	0,7	0	2,564E-07	71	9,69835E-08	2	1215,71733	-0,39	34,17	0
	High	tVLELAGVk	2	1	1	P73304	N-Term(iTRAQ4plex); K9(iTRAQ4plex)	0,0000	1,641	1		0	0,0001747	55	3,20605E-06	2	1217,76836	-1,99	33,70	0
	High	qLIEVPLTk	1	1	1	P73304	N-Term(iTRAQ4plex); K9(iTRAQ4plex)	0,0000	1,448	1		0	0,000006711	50	1,06652E-05	2	1328,83794	-0,95	34,01	0
	High	vVVRPAAPGTGVIAGGAVR	3	1	1	P73304	N-Term(iTRAQ4plex)	0,0000	1,290	2	59,3	0	5,589E-08	48	1,5921E-05	3	1891,13669	0,42	27,61	0
	High	kQLIEVPLTk	1	1	1	P73304	N-Term(iTRAQ4plex); K1(iTRAQ4plex); K10(iTRAQ4plex)	0,0000	3,737	1		0	0,00001825	24	0,003801631	3	1601,03116	-3,17	29,12	1
	High	ekkEDTNWQER	1	1	1	P73304	N-Term(iTRAQ4plex); K2(iTRAQ4plex); K3(iTRAQ4plex)	0,0000	2,146	1		0	0,00001388	16	0,026913489	4	1894,99404	-2,27	12,27	2
	High	kGVADGk	1	1	1	P73304	N-Term(iTRAQ4plex); K1(iTRAQ4plex); K7(iTRAQ4plex)	0,0000	1,687	1		0,009	0,113	13	0,046555394	3	1106,68512	-3,86	8,17	1
	Medium	kGVADGkk	1	1	1	P73304	N-Term(iTRAQ4plex); K1(iTRAQ4plex); K7(iTRAQ4plex);	0,0000	1,765	1		0,014	0,1793	10	0,097716973	4	1378,88320	-2,35	7,84	2
P72761	Carbon dioxide-concentrating mechanism protein CcmK homolog 2 OS=	511,09	70,87	1	4	6	24	1,867	8	32,4	103	11,1	5,41							
	A3	Sequence	# PSMs	# Proteins	# Protein Groups	Protein Group Accessions	Modifications	ΔCn	117/114	117/114 Count	117/114 Variability [%]	q-Value	PEP	IonScore	Exp Value	Charge	MH+ [Da]	ΔM [ppm]	RT [min]	# Missed Cleavages
	High	sIAVGMIETR	3	1	1	P72761	N-Term(iTRAQ4plex)	0,0000	2,122	2	0,9	0	1,413E-07	70	9,88485E-08	2	1220,67851	-0,38	29,91	0
	High	yTEEVEQFR	1	1	1	P72761	N-Term(iTRAQ4plex)	0,0000	1,884	1		0	0,000002164	54	4,45625E-06	2	1344,65557	0,24	28,92	0
	High	vTLVGYEk	6	2	2	P72760;P72761	N-Term(iTRAQ4plex); K8(iTRAQ4plex)	0,0000				0	0,0003198	49	1,13493E-05	2	1196,71233	-0,49	26,17	0
	High	gFPAVVEAADSMVk	2	1	1	P72761	N-Term(iTRAQ4plex); K14(iTRAQ4plex)	0,0000	0,891	2	2,3	0	0,0005061	45	2,89714E-05	3	1708,91581	-1,46	45,27	0
	High	sIAVGmIETR	4	1	1	P72761	N-Term(iTRAQ4plex); M6(Oxidation)	0,0000	1,606	2	21,2	0	0,000003214	45	3,39932E-05	2	1236,67327	-0,51	23,71	0
	High	vNGGEVLSTHIIARPHENLEYVLP	2	1	1	P72761	N-Term(iTRAQ4plex)	0,0000	3,319	1		0	5,565E-08	37	0,000213781	4	3070,69253	0,19	40,07	0
	High	vTVIVR	6	2	2	P72760;P72761	N-Term(iTRAQ4plex)	0,0000				0,002	0,03444	24	0,003872309	2	830,55742	-0,74	20,16	0
Q55511	Trigger factor OS=Synechocystis sp. (strain PCC 6803 / Kazusa) GN=tig	498,63	27,39	1	14	14	25	1,328	24	27,8	471	52,6	4,41							
	A3	Sequence	# PSMs	# Proteins	# Protein Groups	Protein Group Accessions	Modifications	ΔCn	117/114	117/114 Count	117/114 Variability [%]	q-Value	PEP	IonScore	Exp Value	Charge	MH+ [Da]	ΔM [ppm]	RT [min]	# Missed Cleavages
	High	lVNFVESSLTESk	2	1	1	Q55511	N-Term(iTRAQ4plex); K13(iTRAQ4plex)	0,0000	1,016	2	17,5	0	2,691E-09	60	9,20386E-07	2	1740,95989	-1,35	45,09	0
	High	iQPTEAEVk	1	1	1	Q55511	N-Term(iTRAQ4plex); K9(iTRAQ4plex)	0,0000	1,285	1		0	2,545E-07	52	6,39691E-06	2	1302,75041	-0,28	19,49	0
	High	kVYENVVk	2	1	1	Q55511	N-Term(iTRAQ4plex); K1(iTRAQ4plex); K8(iTRAQ4plex)	0,0000	1,197	2	100,7	0	4,616E-07	49	1,40595E-05	3	1410,86744	-0,46	17,63	1
	High	mGLDVSQLFR	1	1	1	Q55511	N-Term(iTRAQ4plex); M1(Oxidation)	0,0000	0,996	1		0	0,001301	44	4,05481E-05	2	1325,70098	0,40	40,65	0
	High	lVAQTAmELER	1	1	1	Q55511	N-Term(iTRAQ4plex); M7(Oxidation)	0,0000	1,309	1		0	0,00001004	43	5,44503E-05	2	1420,76018	1,04	30,96	0
	High	qFQEQAk	2	1	1	Q55511	N-Term(iTRAQ4plex); K7(iTRAQ4plex)	0,0000	1,110	2	7,5	0	0,000225	40	9,8168E-05	2	1166,64043	-0,33	12,67	0
	High	dNSRPEAIANLk	2	1	1	Q55511	N-Term(iTRAQ4plex); K12(iTRAQ4plex)	0,0000	1,535	2	5,3	0	8,953E-08	38	0,000198127	3	1615,90097	0,20	21,08	0
	High	tVNIPGFR	3	1	1	Q55511	N-Term(iTRAQ4plex)	0,0000	1,203	3	13,2	0	0,003058	37	0,000221912	2	1047,60747	0,65	30,78	0
	High	aTAIEELIDDSIk	1	1	1	Q55511	N-Term(iTRAQ4plex); K13(iTRAQ4plex)	0,0000	1,592	1		0	0,00001568	28	0,001513457	2	1705,94730	0,61	49,59	0
	High	lGQSYIk	2	1	1	Q55511	N-Term(iTRAQ4plex); K7(iTRAQ4plex)	0,0000	1,429	2	33,7	0	0,004214	24	0,003801631	2	1096,66106	0,53	21,97	0
	High	qEELPIIGNFSLR	1	1	1	Q55511	N-Term(iTRAQ4plex)	0,0000	1,888	1		0	0,0002865	23	0,004549881	2	1659,92204	2,01	47,22	0
	High	mGLDVSQLFR	1	1	1	Q55511	N-Term(iTRAQ4plex)	0,0000	1,054	1		0	0,0002635	23	0,004874948	2	1309,70635	0,62	45,91	0
	High	aIVIQR	3	1	1	Q55511	N-Term(iTRAQ4plex)	0,0000	1,369	2	2,5	0,001	0,01337	13	0,05163807	2	843,55345	0,20	17,39	0
	High	lkEIk	2	1	1	Q55511	N-Term(iTRAQ4plex); K2(iTRAQ4plex); K5(iTRAQ4plex)	0,0000	1,813	2	8,4	0,002	0,02979	12	0,066522721	3	1062,72177	-2,76	14,73	1
	Medium	mkVTQEk	1	1	1	Q55511	N-Term(iTRAQ4plex); M1(Oxidation); K2(iTRAQ4plex); K	0,0000	2,268	1		0,035	0,4357	14	0,040454795	3	1311,76627	-0,30	8,16	1
P74071	30S ribosomal protein S2 OS=Synechocystis sp. (strain PCC 6803 / Kazu	490,22	31,97	1	12	12	22	1,777	20	23,4	269	30,1	5,27							
	A3	Sequence	# PSMs	# Proteins	# Protein Groups	Protein Group Accessions	Modifications	ΔCn	117/114	117/114 Count	117/114 Variability [%]	q-Value	PEP	IonScore	Exp Value	Charge	MH+ [Da]	ΔM [ppm]	RT [min]	# Missed Cleavages
	High	qAAGIISQEAQR	1	1	1	P74071	N-Term(iTRAQ4plex)	0,0000	1,749	1		0	9,523E-07	72	5,754E-08	2	1415,77300	0,45	21,17	0
	High	lkELEALEASGGIDR	1	1	1	P74071	N-Term(iTRAQ4plex); K2(iTRAQ4plex)	0,0000	1,054	1		0	1,174E-09	65	3,76177E-07	3	1889,05698	-0,70	36,80	1
	High	kLPDVVVIVDQR	3	1	1	P74071	N-Term(iTRAQ4plex); K1(iTRAQ4plex)	0,0000	1,806	3	0,7	0	7,692E-10	55	3,26565E-06	3	1669,02518	0,03	35,20	1
	High	rQAAGIISQEAQR	1	1	1	P74071	N-Term(iTRAQ4plex)	0,0000	1,360	1		0	0,000001865	51	7,83376E-06	3	1571,87455	0,68	16,53	1
	High	lkELEALEASGGIDRR	4	1	1	P74071	N-Term(iTRAQ4plex); K2(iTRAQ4plex)	0,0000	3,689	4	10,3	0	1,864E-08	46	2,48313E-05	4	2045,15786	-0,76	34,43	2
	High	eHNAIHEcQk	1	1	1	P74071	N-Term(iTRAQ4plex); C8(Carbamidomethyl); K10(iTRAQ	0,0000	1,627	1		0	0,000003485	27	0,002013585	4	1553,77358	0,17	7,98	0
	High	kFLFVGTk	2	1	1	P74071	N-Term(iTRAQ4plex); K1(iTRAQ4plex); K8(iTRAQ4plex)	0,0000	1,893	2	33,7	0	0,0005438	26	0,002660541	3	1371,87073	-1,25	28,65	1
	High	fLFVGTk	3	1	1	P74071	N-Term(iTRAQ4plex); K7(iTRAQ4plex)	0,0000	1,898	2	13,1	0	0,001215	23	0,004644832	2	1099,67363	-1,62	34,87	0
	High	sSTINGk	1	1	1	P74071	N-Term(iTRAQ4plex); K7(iTRAQ4plex)	0,0000	1,573	1		0,001	0,01867	32	0,000656739	2	994,57530	-1,85	10,16	0
	High	yLGGIk	3	1	1	P74071	N-Term(iTRAQ4plex); K6(iTRAQ4plex)	0,0000	1,132	2	82,9	0,001	0,01785	15	0,0349921	2	938,58953	-1,93	23,74	0
	High	cGAHYVNQR	1	1	1	P74071	N-Term(iTRAQ4plex); C1(Carbamidomethyl)	0,0000	1,512	1		0,007	0,09575	11	0,087896181	3	1248,60147	-0,76	8,83	0
	Medium	lIVGk	1	1	1	P74071	N-Term(iTRAQ4plex); K5(iTRAQ4plex)	0,0000	2,045	1		0,016	0,1989	25	0,00306881	2	817,57274	-2,72	19,70	0
P72659	Polyribonucleotide nucleotidyltransferase OS=Synechocystis sp. (strain F	488,58	16,71	1	11	11	22	0,997	19	11,7	718	77,8	5,27							
	A3	Sequence	# PSMs	# Proteins	# Protein Groups	Protein Group Accessions	Modifications	ΔCn	117/114	117/114 Count	117/114 Variability [%]	q-Value	PEP	IonScore	Exp Value	Charge	MH+ [Da]	ΔM [ppm]	RT [min]	# Missed Cleavages
	High	kIITVLGQFDLGk	2	1	1	P72659	N-Term(iTRAQ4plex); K1(iTRAQ4plex); K13(iTRAQ4plex)	0,0000	1,134	2	5,7	0	1,149E-09	62	6,02518E-07	3	1864,16562	1,28	43,48	1
	High	aImQALPAR	3	1	1	P72659	N-Term(iTRAQ4plex); M3(Oxidation)	0,0000	0,944	2	1,9	0	0,000003082	58	1,63294E-06	2	1130,64763	0,30	19,52	0
	High	iIPIGAFVEVLPGk	4	1	1	P72659	N-Term(iTRAQ4plex); K14(iTRAQ4plex)	0,0000	0,759	4	14,0	0	0,000004259	50	9,63762E-06	2	1741,08879	1,21	51,49	0
	High	kLNEGEVYLGR	3	1	1	P72659	N-Term(iTRAQ4plex); K1(iTRAQ4plex)	0,0000	1,064	2	9,7	0	0,000001155	46	2,74909E-05	3	1565,88938	0,24	22,98	1
	High	ePRPELSPFAPR	1	1	1	P72659	N-Term(iTRAQ4plex)	0,0000	1,008	1		0	1,653E-09	43	4,63415E-05	3	1539,84024	0,12	26,83	0
	High	kPVSGAAmGLIk	1	1	1	P72659	N-Term(iTRAQ4plex); K1(iTRAQ4plex); M8(Oxidation); K	0,0000	1,007	1		0	1,872E-07	36	0,000239867	3	1619,98679	-0,70	20,29	1
	High	eIGHGALAER	1	1	1	P72659	N-Term(iTRAQ4plex)	0,0000	0,934	1		0	0,0001131	35	0,000301974	3	1196,65024	-0,18	13,88	0
	High	kLEQVRPIScEVGFLPR	2	1	1	P72659	N-Term(iTRAQ4plex); K1(iTRAQ4plex); C10(Carbamidom	0,0000	1,387	1		0	5,607E-08	34	0,00036389	4	2316,30898	-0,49	34,55	1
	High	lVGNLYk	1	1	1	P72659	N-Term(iTRAQ4plex); K7(iTRAQ4plex)	0,0000	0,814	1		0	0,003727	30	0,001020869	2	1094,68157	0,32	26,33	0
	High	lHILDk	2	1	1	P72659	N-Term(iTRAQ4plex); K6(iTRAQ4plex)	0,0000	0,977	2	8,7	0	0,004006	26	0,002488685	3	1026,65467	-0,33	23,86	0
	High	kQIVDDGVR	2	1	1	P72659	N-Term(iTRAQ4plex); K1(iTRAQ4plex)	0,0000	1,128	2	8,8	0	0,0007204	24	0,005418886	3	1317,77332	0,30	14,16	1
Q55665	Glutamate-1-semialdehyde 2,1-aminomutase OS=Synechocystis sp. (str	486,13	24,71	1	7	7	19	1,117	17	16,4	433	45,9	5,27							
	A3	Sequence	# PSMs	# Proteins	# Protein Groups	Protein Group Accessions	Modifications	ΔCn	117/114	117/114 Count	117/114 Variability [%]	q-Value	PEP	IonScore	Exp Value	Charge	MH+ [Da]	ΔM [ppm]	RT [min]	# Missed Cleavages
	High	vIGGGLPVGAYGGR	5	1	1	Q55665	N-Term(iTRAQ4plex)	0,0000	1,002	4	14,9	0	7,542E-08	72	6,91783E-08	2	1416,80852	0,34	32,71	0
	High	vNATPFITTk	4	1	1	Q55665	N-Term(iTRAQ4plex); K10(iTRAQ4plex)	0,0000	1,696	4	62,5	0	7,601E-07	62	6,2513E-07	2	1379,81316	-0,40	28,77	0
	High	vSYGGAQAR	3	1	1	Q55665	N-Term(iTRAQ4plex)	0,0000	1,142	3	13,1	0	0,000003914	51	7,29407E-06	2	1052,56096	0,39	12,96	0
	High	aGSGVATLGLPDSPGVPSNTTk	1	1	1	Q55665	N-Term(iTRAQ4plex); K22(iTRAQ4plex)	0,0000	0,912	1		0	2,191E-10	47	2,95348E-05	3	2314,25113	0,77	33,72	0
	High	sEEIFAAAQHLmPGGVSSPVR	2	1	1	Q55665	N-Term(iTRAQ4plex); M12(Oxidation)	0,0000	1,008	1		0	3,329E-10	46	2,50017E-05	3	2343,19004	0,67	38,03	0
	High	tLEILQkPGSYEYLDk	1	1	1	Q55665	N-Term(iTRAQ4plex); K7(iTRAQ4plex); K16(iTRAQ4plex)	0,0000	1,056	1		0	2,241E-09	25	0,003090082	3	2329,30167	0,04	37,40	0
	High	fEGcYHGHADMFLVk	1	1	1	Q55665	N-Term(iTRAQ4plex); C4(Carbamidomethyl); K15(iTRAQ	0,0000	1,277	1		0	0,000519	18	0,016254365	4	2099,00673	-0,68	31,70	0
	High	fEGcYHGHADmFLVk	2	1	1	Q55665	N-Term(iTRAQ4plex); C4(Carbamidomethyl); M11(Oxidat	0,0000	1,151	2	19,2	0	9,035E-09	15	0,035153616	4	2115,00332	0,11	28,12	0
P73911	Catalase-peroxidase OS=Synechocystis sp. (strain PCC 6803 / Kazusa) (	483,32	20,69	1	17	17	35	1,332	31	28,6	754	84,4	5,74							
	A3	Sequence	# PSMs	# Proteins	# Protein Groups	Protein Group Accessions	Modifications	ΔCn	117/114	117/114 Count	117/114 Variability [%]	q-Value	PEP	IonScore	Exp Value	Charge	MH+ [Da]	ΔM [ppm]	RT [min]	# Missed Cleavages
	High	vDLVFGSNSILR	3	1	1	P73911	N-Term(iTRAQ4plex)	0,0000	1,512	3	6,6	0	0,00002085	70	1,31634E-07	2	1463,83354	-0,25	43,27	0
	High	vLGTNHGGTk	3	1	1	P73911	N-Term(iTRAQ4plex); K10(iTRAQ4plex)	0,0000	1,673	2	9,2	0	0,000001437	70	9,83943E-08	2	1271,73149	0,36	10,20	0
	High	fYREPDYFAEVFAk	3	1	1	P73911	N-Term(iTRAQ4plex); K14(iTRAQ4plex)	0,0000	1,290	3	12,9	0	2,285E-08	52	6,06694E-06	3	2070,05948	0,94	43,28	1

	High	qDYAVSPEELLLER	1	1	1	P73911	N-Term(iTRAQ4plex)	0,0000	1,359	1		0	0,000002508	48	1,74181E-05	2	1805,94182	0,88	42,84	0
	High	tQLmGLTAPEmTVLIGGmR	2	1	1	P73911	N-Term(iTRAQ4plex); M4(Oxidation); M11(Oxidation); M	0,0000	1,317	2	37,2	0	1,915E-09	47	2,12799E-05	3	2211,13034	-0,07	38,05	0
	High	vYGFAGGR	2	1	1	P73911	N-Term(iTRAQ4plex)	0,0000	1,320	2	1,3	0	0,001511	35	0,000324317	2	970,52275	0,04	20,99	0
	High	lLWPIk	6	1	1	P73911	N-Term(iTRAQ4plex); K6(iTRAQ4plex)	0,0000	1,519	4	7,6	0	0,009691	31	0,00075504	2	1057,70098	-0,25	40,35	0
	High	vYGFAGGREDIWHPEk	1	1	1	P73911	N-Term(iTRAQ4plex); K16(iTRAQ4plex)	0,0000	1,947	1		0	0,004632	22	0,006426433	4	2149,10586	-0,52	32,40	1
	High	hNPImTDADmAmIkDPIYR	1	1	1	P73911	N-Term(iTRAQ4plex); M5(Oxidation); M10(Oxidation); M	0,0000	1,157	1		0	2,431E-07	20	0,011065474	4	2568,25112	0,44	27,34	1
	High	lLWPIkk	1	1	1	P73911	N-Term(iTRAQ4plex); K6(iTRAQ4plex); K7(iTRAQ4plex)	0,0000	1,076	1		0	0,001392	16	0,025059362	3	1329,89490	-2,53	33,14	1
	High	hGVFTDR	2	1	1	P73911	N-Term(iTRAQ4plex)	0,0000	1,134	2	16,9	0	0,00166	11	0,075504008	3	975,51322	0,35	13,06	0
	High	rLLWPIk	2	1	1	P73911	N-Term(iTRAQ4plex); K7(iTRAQ4plex)	0,0000	3,156	1		0	0,001451	11	0,076378303	3	1213,80283	0,39	32,44	1
	High	tQLmGLTAPEMTVLIGGmR	1	1	1	P73911	N-Term(iTRAQ4plex); M4(Oxidation); M18(Oxidation)	0,0000	0,959	1		0,001	0,01986	15	0,028573932	3	2195,13187	-1,69	45,20	0
	High	iRLEPQk	1	1	1	P73911	N-Term(iTRAQ4plex); K7(iTRAQ4plex)	0,0000	6,151	1		0,003	0,03952	21	0,008471689	3	1171,73929	-0,73	17,69	1
	High	nWPGNEPTR	2	1	1	P73911	N-Term(iTRAQ4plex)	0,0000	1,130	2	20,0	0,003	0,03991	14	0,043348094	2	1214,60344	-0,04	17,98	0
	High	vMNADRFDLPR	1	1	1	P73911	N-Term(iTRAQ4plex)	0,0000	0,956	1		0,006	0,0714	10	0,094835296	3	1477,76969	-0,40	28,91	1
	Medium	wTATk	2	1	1	P73911	N-Term(iTRAQ4plex); K5(iTRAQ4plex)	0,0000	0,934	2	1,2	0,028	0,3417	14	0,050954348	2	894,52605	-3,01	16,06	0
	Medium	tTFAR	1	1	1	P73911	N-Term(iTRAQ4plex)	0,0000	0,970	1		0,049	0,6068	11	0,082218586	2	739,41997	-2,64	13,20	0
P77969	Delta-aminolevulinic acid dehydratase OS=Synechocystis sp. (strain PCC	481,25	24,77	1	9	9	24	1,190	21	27,6	327	36,1	5,08							
	A3	Sequence	# PSMs	# Proteins	# Protein Groups	Protein Group Accessions	Modifications	ΔCn	117/114	117/114 Count	117/114 Variability [%]	q-Value	PEP	IonScore	Exp Value	Charge	MH+ [Da]	ΔM [ppm]	RT [min]	# Missed Cleavages
	High	vTLETLTSFk	2	1	1	P77969	N-Term(iTRAQ4plex); K10(iTRAQ4plex)	0,0000	0,998	2	15,3	0	6,275E-07	67	2,08435E-07	2	1426,84111	1,07	41,76	0
	High	ikEmTNLPVAAYNVSGEYSmVk	1	1	1	P77969	N-Term(iTRAQ4plex); K2(iTRAQ4plex); M4(Oxidation); M	0,0000	0,673	1		0	7,912E-13	51	8,74923E-06	4	2908,51992	1,31	34,71	1
	High	vLNDPTLELLkk	3	1	1	P77969	N-Term(iTRAQ4plex); K11(iTRAQ4plex); K12(iTRAQ4plex	0,0000	1,439	3	3,5	0	3,575E-09	49	1,19391E-05	3	1815,13327	0,92	36,13	1
	High	aGADLILTYHAk	7	1	1	P77969	N-Term(iTRAQ4plex); K12(iTRAQ4plex)	0,0000	1,429	5	52,7	0	0,000004514	43	5,61009E-05	3	1560,89957	0,47	29,21	0
	High	yASAYYGPFR	3	1	1	P77969	N-Term(iTRAQ4plex)	0,0000	1,045	3	4,2	0	0,000001006	42	5,9975E-05	2	1338,66142	1,13	31,35	0
	High	vTLETLTSFkR	1	1	1	P77969	N-Term(iTRAQ4plex); K10(iTRAQ4plex)	0,0000	2,142	1		0	3,515E-08	38	0,000152395	3	1582,94089	0,12	36,63	1
	High	rAGADLILTYHAk	3	1	1	P77969	N-Term(iTRAQ4plex); K13(iTRAQ4plex)	0,0000	1,254	2	12,0	0	0,000009711	34	0,000358072	4	1717,00088	0,53	24,80	1
	High	mFPTIRPR	2	1	1	P77969	N-Term(iTRAQ4plex)	0,0000	1,348	2	8,9	0	0,01116	18	0,016564918	3	1161,66794	-0,36	25,62	0
	High	mFPTIRPR	1	1	1	P77969	N-Term(iTRAQ4plex); M1(Oxidation)	0,0000	1,112	1		0	0,009844	16	0,023065879	3	1177,66413	0,73	20,07	0
	High	aTEAVk	1	1	1	P77969	N-Term(iTRAQ4plex); K6(iTRAQ4plex)	0,0000	0,555	1		0,002	0,03248	32	0,000591521	2	906,54961	-0,30	10,91	0
P23349	50S ribosomal protein L7/L12 OS=Synechocystis sp. (strain PCC 6803 /	479,20	52,34	1	8	8	26	1,481	19	20,0	128	13,3	4,77							
	A3	Sequence	# PSMs	# Proteins	# Protein Groups	Protein Group Accessions	Modifications	ΔCn	117/114	117/114 Count	117/114 Variability [%]	q-Value	PEP	IonScore	Exp Value	Charge	MH+ [Da]	ΔM [ppm]	RT [min]	# Missed Cleavages
	High	sLSLLEASELVk	4	1	1	P23349	N-Term(iTRAQ4plex); K12(iTRAQ4plex)	0,0000	1,452	3	15,5	0	5,151E-11	71	7,96104E-08	2	1576,94048	0,27	42,43	0
	High	sAATDQILEQLk	2	1	1	P23349	N-Term(iTRAQ4plex); K12(iTRAQ4plex)	0,0000	0,954	2	36,2	0	6,819E-11	69	1,25594E-07	2	1604,90935	-0,29	35,76	0
	High	tITGLGLk	6	1	1	P23349	N-Term(iTRAQ4plex); K8(iTRAQ4plex)	0,0000	1,635	3	1,3	0	0,00001776	53	4,60225E-06	2	1090,70830	0,78	27,88	0
	High	kQIEEAGGk	2	1	1	P23349	N-Term(iTRAQ4plex); K1(iTRAQ4plex); K9(iTRAQ4plex)	0,0000	1,261	2	8,7	0	0,00001765	47	1,89657E-05	3	1391,81980	-1,50	10,36	1
	High	eLVESTPk	1	1	1	P23349	N-Term(iTRAQ4plex); K8(iTRAQ4plex)	0,0000	1,944	1		0	0,0001584	35	0,000341169	2	1190,68682	-0,25	17,07	0
	High	kIAVLk	2	1	1	P23349	N-Term(iTRAQ4plex); K1(iTRAQ4plex); K6(iTRAQ4plex)	0,0000	1,067	2	1,8	0	0,009718	23	0,005023079	3	1103,78611	-1,40	17,72	1
	High	eATGkDDAEAIkk	2	1	1	P23349	N-Term(iTRAQ4plex); K5(iTRAQ4plex); K12(iTRAQ4plex)	0,0000	2,222	2	51,8	0	3,958E-08	20	0,009035871	4	1952,11184	-1,46	14,40	2
	High	iAVLk	7	1	1	P23349	N-Term(iTRAQ4plex); K5(iTRAQ4plex)	0,0000	1,506	4	5,6	0,003	0,03793	16	0,026483172	2	831,58965	-1,17	22,62	0
P73817	Hemolysin OS=Synechocystis sp. (strain PCC 6803 / Kazusa) GN=sll195	476,91	3,33	1	5	5	14	0,710	12	35,1	1741	178,2	3,75							
	A3	Sequence	# PSMs	# Proteins	# Protein Groups	Protein Group Accessions	Modifications	ΔCn	117/114	117/114 Count	117/114 Variability [%]	q-Value	PEP	IonScore	Exp Value	Charge	MH+ [Da]	ΔM [ppm]	RT [min]	# Missed Cleavages
	High	aQAAQAAINLAQLR	3	1	1	P73817	N-Term(iTRAQ4plex)	0,0000	0,479	3	2,2	0	5,237E-11	107	2,07014E-11	2	1582,91838	2,37	36,11	0
	High	gVAAISGAHTVR	2	1	1	P73817	N-Term(iTRAQ4plex)	0,0000	0,736	1		0	1,385E-07	67	1,98153E-07	2	1282,73711	1,76	15,95	0
	High	aVGGDDELVGGR	2	1	1	P73817	N-Term(iTRAQ4plex)	0,0000	0,684	1		0	0,000002137	65	3,49921E-07	2	1288,66448	2,36	20,16	0
	High	lEALTNk	1	1	1	P73817	N-Term(iTRAQ4plex); K7(iTRAQ4plex)	0,0000	0,512	1		0	0,003866	49	2,07535E-05	2	1076,65984	4,11	22,93	0
	High	yPFFQAPQTADkR	6	1	1	P73817	N-Term(iTRAQ4plex); K12(iTRAQ4plex)	0,0000	0,893	6	16,9	0	0,000006732	26	0,002547941	3	1856,99485	2,72	30,22	1
P73307	30S ribosomal protein S8 OS=Synechocystis sp. (strain PCC 6803 / Kazu	475,17	51,88	1	8	8	22	1,500	18	19,4	133	14,7	9,99							
	A3	Sequence	# PSMs	# Proteins	# Protein Groups	Protein Group Accessions	Modifications	ΔCn	117/114	117/114 Count	117/114 Variability [%]	q-Value	PEP	IonScore	Exp Value	Charge	MH+ [Da]	ΔM [ppm]	RT [min]	# Missed Cleavages
	High	vLGGIGIAIVSTSHGImTDR	4	1	1	P73307	N-Term(iTRAQ4plex); M17(Oxidation)	0,0000	1,661	3	17,3	0	3,757E-10	67	2,24075E-07	3	2157,18308	0,54	43,25	0
	High	hSTTQVPTTk	3	1	1	P73307	N-Term(iTRAQ4plex); K10(iTRAQ4plex)	0,0000	1,279	2	8,6	0	0,0001713	61	8,70903E-07	2	1387,77776	-0,44	9,94	0
	High	qPLINTLQR	2	1	1	P73307	N-Term(iTRAQ4plex)	0,0000	1,579	2	1,5	0	0,0001971	49	1,34267E-05	2	1226,73381	0,03	26,00	0
	High	mLVLTLk	2	1	1	P73307	N-Term(iTRAQ4plex); K7(iTRAQ4plex)	0,0000	1,448	2	17,2	0	0,0003916	43	4,96558E-05	2	1105,72344	-2,09	38,21	0
	High	tRQPLINTLQR	2	1	1	P73307	N-Term(iTRAQ4plex)	0,0000	1,211	2	7,4	0	0,00003767	40	0,000100455	3	1483,88294	0,25	21,06	1
	High	mTLSIAk	2	1	1	P73307	N-Term(iTRAQ4plex); M1(Oxidation); K7(iTRAQ4plex)	0,0000	1,096	2	9,5	0	0,004213	33	0,000492006	2	1067,63384	-3,26	19,78	0
	High	mLVLTLk	5	1	1	P73307	N-Term(iTRAQ4plex); M1(Oxidation); K7(iTRAQ4plex)	0,0000	1,655	3	4,6	0	0,001653	32	0,000623692	2	1121,71782	-2,53	32,52	0
	High	vYSPSkk	1	1	1	P73307	N-Term(iTRAQ4plex); K6(iTRAQ4plex); K7(iTRAQ4plex)	0,0000	2,128	1		0,007	0,08649	11	0,085500766	3	1240,75769	-3,91	11,45	1
	High	vSkPGLR	1	1	1	P73307	N-Term(iTRAQ4plex); K3(iTRAQ4plex)	0,0000	1,439	1		0,008	0,1013	15	0,028705823	2	1044,67485	-1,88	13,69	0
P73902	Sll0236 protein OS=Synechocystis sp. (strain PCC 6803 / Kazusa) GN=sl	463,11	20,97	1	12	12	25	1,037	23	17,6	558	63,9	5,25							
	A3	Sequence	# PSMs	# Proteins	# Protein Groups	Protein Group Accessions	Modifications	ΔCn	117/114	117/114 Count	117/114 Variability [%]	q-Value	PEP	IonScore	Exp Value	Charge	MH+ [Da]	ΔM [ppm]	RT [min]	# Missed Cleavages
	High	fAITHALMAPR	2	1	1	P73902	N-Term(iTRAQ4plex)	0,0000	0,954	2	0,7	0	1,231E-07	58	1,46208E-06	2	1371,77080	1,45	31,50	0
	High	vEIGGWSFk	1	1	1	P73902	N-Term(iTRAQ4plex); K9(iTRAQ4plex)	0,0000	0,743	1		0	0,000001883	54	4,00119E-06	2	1310,73393	-0,61	39,55	0
	High	fAITHALmAPR	2	1	1	P73902	N-Term(iTRAQ4plex); M8(Oxidation)	0,0000	1,720	2	67,6	0	2,634E-07	48	1,68256E-05	3	1387,76282	-0,65	28,46	0
	High	yLTTPYLLR	3	1	1	P73902	N-Term(iTRAQ4plex)	0,0000	1,037	3	17,6	0	0,001187	43	4,74209E-05	2	1283,74675	-0,98	39,67	0
	High	iHQELPHLEGR	3	1	1	P73902	N-Term(iTRAQ4plex)	0,0000	0,967	3	58,1	0	0,00000384	41	7,44681E-05	3	1472,80863	-0,30	21,96	0
	High	sSVRPYPYPEER	3	1	1	P73902	N-Term(iTRAQ4plex)	0,0000	0,886	2	25,5	0	0,0001914	36	0,000226449	3	1623,82431	-0,28	18,42	0
	High	kTDSVTLITGk	1	1	1	P73902	N-Term(iTRAQ4plex); K1(iTRAQ4plex); K11(iTRAQ4plex)	0,0000	1,090	1		0	0,0000091	34	0,000397164	3	1594,97165	-1,48	22,21	1
	High	gkELTGYNcISAYHDFR	1	1	1	P73902	N-Term(iTRAQ4plex); K2(iTRAQ4plex); C9(Carbamidome	0,0000	1,448	1		0	1,173E-08	31	0,000786991	4	2319,14126	-0,78	29,63	1
	High	aEAEFWQQHR	1	1	1	P73902	N-Term(iTRAQ4plex)	0,0000	0,942	1		0	0,00003425	30	0,001027945	3	1445,70417	-0,07	23,37	0
	High	hLIWVIR	3	1	1	P73902	N-Term(iTRAQ4plex)	0,0000	0,916	2	26,4	0	0,0005903	29	0,001244429	2	1080,67949	-0,36	32,56	0
	High	aVRPIQVTLk	1	1	1	P73902	N-Term(iTRAQ4plex); K10(iTRAQ4plex)	0,0000	1,220	1		0	0,0002938	24	0,0044154	3	1412,91767	-1,08	24,45	0
	High	kAVRPIQVTLk	3	1	1	P73902	N-Term(iTRAQ4plex); K1(iTRAQ4plex); K11(iTRAQ4plex)	0,0000	1,158	3	26,2	0	0,003252	15	0,035072765	3	1685,11301	-1,90	21,96	1
	Medium	lTITAQAER	1	1	1	P73902	N-Term(iTRAQ4plex)	0,0000	0,594	1		0,015	0,1878	14	0,040176306	2	1146,65996	0,01	21,84	0
P73103	Slr1908 protein OS=Synechocystis sp. (strain PCC 6803 / Kazusa) GN=s	462,24	16,58	1	3	8	15	0,505	9	7,7	591	64,5	5,26							
	A3	Sequence	# PSMs	# Proteins	# Protein Groups	Protein Group Accessions	Modifications	ΔCn	117/114	117/114 Count	117/114 Variability [%]	q-Value	PEP	IonScore	Exp Value	Charge	MH+ [Da]	ΔM [ppm]	RT [min]	# Missed Cleavages
	High	lQAGSVPNLSGPTGTNMAR	3	1	1	P73103	N-Term(iTRAQ4plex)	0,0000	0,572	3	18,7	0	3,67E-08	90	1,01391E-09	2	2015,04888	1,36	32,14	0
	High	lQAGSVPNLSGPTGTNmAR	2	1	1	P73103	N-Term(iTRAQ4plex); M17(Oxidation)	0,0000	0,541	2	2,7	0	0,00001052	58	1,61797E-06	2	2031,04387	1,39	29,32	0
	High	nSPYIIEAQYQFPVNk	1	1	1	P73103	N-Term(iTRAQ4plex); K16(iTRAQ4plex)	0,0000	0,473	1		0	2,135E-09	49	1,16942E-05	3	2199,17447	2,55	39,59	0
	High	dVQPTAWAYEALk	1	2	2	P73409;P73103	N-Term(iTRAQ4plex); K13(iTRAQ4plex)	0,0000				0	4,474E-09	41	9,69154E-05	3	1779,95450	1,40	40,25	0
	High	yGcIVGYPDR	2	2	2	P73409;P73103	N-Term(iTRAQ4plex); C3(Carbamidomethyl)	0,0000				0	0,00001184	37	0,00021576	2	1343,65495	1,11	27,69	0
	High	lIQENVAVLREDIDk	1	2	2	P73409;P73103	N-Term(iTRAQ4plex); K15(iTRAQ4plex)	0,0000				0	1,237E-09	36	0,000276675	3	2043,17026	0,66	39,66	1
	High	rLmQEFEAELAALGAR	1	2	2	P73409;P73103	N-Term(iTRAQ4plex); M3(Oxidation)	0,0000				0	0,0001955	28	0,001644258	3	1965,03641	0,96	46,80	1
	High	eDIDkLk	1	2	2	P73409;P73103	N-Term(iTRAQ4plex); K5(iTRAQ4plex); K7(iTRAQ4plex)	0,0000				0	0,005364	12	0,069818418	3	1292,77814	-0,36	18,21	1
	High	ySPFVNR	3	1	1	P73103	N-Term(iTRAQ4plex)	0,0000	0,483	3	0,2	0,003	0,03759	26	0,002301283	2	1026,55022	1,27	23,17	0
P74510	Dihydrolipoamide acetyltransferase component (E2) of pyruvate dehydro	455,92	31,18	1	15	15	27	1,083	21	29,9	433	44,9	6,19							
	A3	Sequence	# PSMs	# Proteins	# Protein Groups	Protein Group Accessions	Modifications	ΔCn	117/114	117/114 Count	117/114 Variability [%]	q-Value	PEP	IonScore	Exp Value	Charge	MH+ [Da]	ΔM [ppm]	RT [min]	# Missed Cleavages
	High	vDLATIAGTGPHGR	1	1	1	P74510	N-Term(iTRAQ4plex)	0,0000	0,907	1		0	9,962E-09	67	1,94523E-07	3	1508,83088	0,43	28,31	0
	High	eLkVDLATIAGTGPHGR	2	1	1	P74510	N-Term(iTRAQ4plex); K3(iTRAQ4plex)	0,0000	2,194	2	20,9	0	1,597E-08	48	1,73368E-05	4	2023,15359	-0,17	33,06	1
	High	vIYGAHAAAFLk	4	1	1	P74510	N-Term(iTRAQ4plex); K12(iTRAQ4plex)	0,0000	0,978	2	13,4	0	8,289E-09	47	1,92752E-05	3	1548,91532	0,79	30,55	0
	High	vTSTPSVPVGQTVPLTTFQk	3	1	1	P74510	N-Term(iTRAQ4plex); K20(iTRAQ4plex)	0,0000	1,396	2	8,3	0	3,673E-11	47	2,10363E-05	3	2375,34427	0,74	38,38	0
	High	gVTmTALLAk	2	1	1	P74510	N-Term(iTRAQ4plex); M4(Oxidation); K10(iTRAQ4plex)	0,0000	1,258	1		0	4,337E-07	47	2,1576E-05	2	1308,78142	1,10	25,61	0
	High	kHPIVNASYTDQGIIYHk	1	1	1	P74510	N-Term(iTRAQ4plex); K1(iTRAQ4plex); K18(iTRAQ4plex)	0,0000	1,074	1		0	2,563E-11	46	3,06689E-05	4	2516,40005	0,58	24,22	1
	High	aLVQNmVAAmAAPTFR	2	1	1	P74510	N-Term(iTRAQ4plex); M6(Oxidation); M10(Oxidation)	0,0000	0,769	2	7,8	0	2,886E-08	46	2,78593E-05	3	1866,97007	0,71	30,82	0
	High	iVSWTk	1	1	1	P74510	N-Term(iTRAQ4plex); K6(iTRAQ4plex)	0,0000	0,872	1		0	0,003656	38	0,000168256	2	1021,62737	-1,07	26,25	0
	High	aVALALkk	2	1	1	P74510	N-Term(iTRAQ4plex); K7(iTRAQ4plex); K8(iTRAQ4plex)	0,0000	2,248	1		0	0,00002072	33	0,000487495	3	1245,86011	-1,43	22,13	1
	High	hPIVNASYTDQGIIYHk	1	1	1	P74510	N-Term(iTRAQ4plex); K17(iTRAQ4plex)	0,0000	1,467	1		0	2,754E-07	32	0,000647098	4	2244,20180	0,11	27,05	0
	High	iIASPR	2	1	1	P74510	N-Term(iTRAQ4plex)	0,0000	1,051	2	4,4	0,001	0,01548	19	0,013303625	2	800,51195	1,08	16,88	0
	High	aVALALk	2	1	1	P74510	N-Term(iTRAQ4plex); K7(iTRAQ4plex)	0,0000	0,814	1		0,002	0,03355	41	7,83376E-05	2	973,66582	0,99	25,66	0
	High	wkELVER	1	1	1	P74510	N-Term(iTRAQ4plex); K2(iTRAQ4plex)	0,0000	1,564	1		0,005	0,06549	12	0,057543994	3	1247,73383	-0,99	26,64	1
	Medium	sPGDkVEk	1	1	1	P74510	N-Term(iTRAQ4plex); K5(iTRAQ4plex); K8(iTRAQ4plex)	0,0000	1,106	1		0,013	0,1636	13	0,054572017	3	1291,75779	-0,33	10,09	1
	Medium	tSPAPTPR	2	1	1	P74510	N-Term(iTRAQ4plex)	0,0000	1,109	2	4,0	0,031	0,3792	14	0,039533931	2	970,54430	0,47	11,04	0
P73309	50S ribosomal protein L24 OS=Synechocystis sp. (strain PCC 6803 / Kaz	453,04	52,17	1	8	8	29	2,519	21	44,3	115	12,8	10,08							
	A3	Sequence	# PSMs	# Proteins	# Protein Groups	Protein Group Accessions	Modifications	ΔCn	117/114	117/114 Count	117/114 Variability [%]	q-Value	PEP	IonScore	Exp Value	Charge	MH+ [Da]	ΔM [ppm]	RT [min]	# Missed Cleavages
	High	kGDTIQVISGk	6	1	1	P73309	N-Term(iTRAQ4plex); K1(iTRAQ4plex); K11(iTRAQ4plex)	0,0000	3,247	4	24,2	0	2,929E-07	59	1,23871E-06	3	1577,95621	-1,58	20,05	1
	High	icYTVTDDGRk	2	1	1	P73309	N-Term(iTRAQ4plex); C2(Carbamidomethyl); K11(iTRAQ	0,0000	3,066	2	13,2	0	0,000002043	45	3,00587E-05	3	1615,83268	-1,59	19,82	1
	High	vmLYSTk	6	1	1	P73309	N-Term(iTRAQ4plex); M2(Oxidation); K7(iTRAQ4plex)	0,0000	2,176	3	22,4	0	0,001123	44	4,42558E-05	2	1145,64617	-1,49	21,37	0
	High	vMLYSTk	5	1	1	P73309	N-Term(iTRAQ4plex); K7(iTRAQ4plex)	0,0000	2,870	3	0,2	0	0,0009498	43	4,50786E-05	2	1129,64983	-2,77	23,56	0
	High	tIPSHSQVVVk	3	1	1	P73309	N-Term(iTRAQ4plex); K11(iTRAQ4plex)	0,0000	1,693	2	64,4	0	0,0006142	34	0,000357248	3	1482,88639	-1,27	18,07	0
	High	kGDTIQVISGkDk	1	1	1	P73309	N-Term(iTRAQ4plex); K1(iTRAQ4plex); K11(iTRAQ4plex)	0,0000	2,996	1		0	0,000003222	23	0,005432128	4	1965,17702	-2,89	18,80	2
	High	tkPAPHR	3	1	1	P73309	N-Term(iTRAQ4plex); K2(iTRAQ4plex)	0,0000	2,113	3	23,2	0,001	0,01478	18	0,016216981	2	1094,66582	-1,36	7,57	0
	Medium	vGEVLR	1	1	1	P73309	N-Term(iTRAQ4plex)	0,0000	1,194	1		0,022	0,2806	19	0,014975892	2	816,50481	-1,46	18,26	0
	Medium	gVNIR	2	1	1	P73309	N-Term(iTRAQ4plex)	0,0000	0,989	2	20,9	0,031	0,3821	17	0,01811215	2	702,43724	-0,96	13,68	0
P73488	Sll1130 protein OS=Synechocystis sp. (strain PCC 6803 / Kazusa) GN=sl	445,48	46,96	1	5	5	26	1,097	18	14,3	115	12,9	8,81							
	A3	Sequence	# PSMs	# Proteins	# Protein Groups	Protein Group Accessions	Modifications	ΔCn	117/114	117/114 Count	117/114 Variability [%]	q-Value	PEP	IonScore	Exp Value	Charge	MH+ [Da]	ΔM [ppm]	RT [min]	# Missed Cleavages
	High	rPALILSDAPAFNNR	4	1	1	P73488	N-Term(iTRAQ4plex)	0,0000	1,155	3	13,7	0	2,262E-09	55	3,09008E-06	3	1799,00589	0,77	33,04	0
	High	sAGLFTPSVVR	7	1	1	P73488	N-Term(iTRAQ4plex)	0,0000	1,202	5	7,6	0	0,0001982	54	4,2459E-06	2	1277,73516	1,33	31,87	0
	High	iGHSVMAMITSAk	1	1	1	P73488	N-Term(iTRAQ4plex); K13(iTRAQ4plex)	0,0000	0,976	1		0	2,467E-08	49	1,31734E-05	3	1633,90281	1,19	37,33	0
	High	iGHSVmAmITSAk	4	1	1	P73488	N-Term(iTRAQ4plex); M6(Oxidation); M8(Oxidation); K13	0,0000	1,019	2	15,4	0	2,487E-07	47	1,95871E-05	3	1665,89371	1,82	21,81	0
	High	iGHSVMAmITSAk	2	1	1	P73488	N-Term(iTRAQ4plex); M8(Oxidation); K13(iTRAQ4plex)	0,0000	1,030	1		0	0,00000331	43	4,64483E-05	3	1649,89762	1,12	29,18	0
	High	sAFPHVFk	4	1	1	P73488	N-Term(iTRAQ4plex); K8(iTRAQ4plex)	0,0000	1,063	2	19,7	0	0,002581	34	0,000405481	3	1220,70600	2,43	25,84	0
	High	lFTLEHk	4	1	1	P73488	N-Term(iTRAQ4plex); K7(iTRAQ4plex)	0,0000	1,082	4	2,0	0	0,005076	25	0,004235552	2	1175,70513	2,07	26,57	0
P74070	Elongation factor Ts OS=Synechocystis sp. (strain PCC 6803 / Kazusa) G	441,83	41,74	1	9	9	28	1,194	23	26,1	218	24,2	5,45							
	A3	Sequence	# PSMs	# Proteins	# Protein Groups	Protein Group Accessions	Modifications	ΔCn	117/114	117/114 Count	117/114 Variability [%]	q-Value	PEP	IonScore	Exp Value	Charge	MH+ [Da]	ΔM [ppm]	RT [min]	# Missed Cleavages
	High	tAAEGLVHSYIHFGGR	6	1	1	P74070	N-Term(iTRAQ4plex)	0,0000	1,118	3	9,3	0	1,15E-09	60	1,05189E-06	4	1858,96975	0,88	34,80	0
	High	aEITAQLVk	1	1	1	P74070	N-Term(iTRAQ4plex); K9(iTRAQ4plex)	0,0000	1,002	1		0	2,179E-07	52	6,54636E-06	2	1260,77715	0,44	26,57	0
	High	gIASADkk	7	1	1	P74070	N-Term(iTRAQ4plex); K7(iTRAQ4plex); K8(iTRAQ4plex)	0,0000	1,311	7	8,8	0	0,00001473	49	1,46281E-05	2	1221,75054	-1,80	9,40	1
	High	lkELSLLDQPYIk	6	1	1	P74070	N-Term(iTRAQ4plex); K2(iTRAQ4plex); K13(iTRAQ4plex)	0,0000	1,194	5	21,9	0	5,255E-10	45	3,30347E-05	3	1992,21164	0,54	40,42	1
	High	fNLGEGIEk	1	1	1	P74070	N-Term(iTRAQ4plex); K9(iTRAQ4plex)	0,0000	0,975	1		0	0,000001988	44	4,15882E-05	2	1294,72478	0,16	30,36	0
	High	aETNFAEEVAAAAk	1	1	1	P74070	N-Term(iTRAQ4plex); K14(iTRAQ4plex)	0,0000	0,825	1		0	0,0001073	33	0,000514008	3	1709,89634	0,84	35,75	0
	High	kPANIk	2	1	1	P74070	N-Term(iTRAQ4plex); K1(iTRAQ4plex); K6(iTRAQ4plex)	0,0000	1,454	2	0,4	0	0,0007431	22	0,005901603	3	1102,73059	-0,24	9,74	0
	High	tGAGmmDckk	3	1	1	P74070	N-Term(iTRAQ4plex); M5(Oxidation); M6(Oxidation); C8(	0,0000	1,990	2	21,6	0	0,0005231	13	0,050812435	3	1562,76816	-1,27	7,79	1
	High	sIEWLR	1	1	1	P74070	N-Term(iTRAQ4plex)	0,0000	0,930	1		0,002	0,0302	15	0,030058687	2	947,54381	0,73	30,81	0
P73296	50S ribosomal protein L17 OS=Synechocystis sp. (strain PCC 6803 / Kaz	438,53	51,72	1	6	6	36	2,097	27	41,3	116	13,2	11,06							
	A3	Sequence	# PSMs	# Proteins	# Protein Groups	Protein Group Accessions	Modifications	ΔCn	117/114	117/114 Count	117/114 Variability [%]	q-Value	PEP	IonScore	Exp Value	Charge	MH+ [Da]	ΔM [ppm]	RT [min]	# Missed Cleavages
	High	aLTTELIR	4	1	1	P73296	N-Term(iTRAQ4plex)	0,0000	2,128	3	15,9	0	0,003121	52	6,17974E-06	2	1060,64702	-1,22	29,31	0
	High	aLGYmYDkPTVHALFADAPSR	10	1	1	P73296	N-Term(iTRAQ4plex); M5(Oxidation); K8(iTRAQ4plex)	0,0000	2,632	7	12,2	0	2,732E-12	46	2,70377E-05	3	2627,35196	-0,43	35,68	0
	High	aLGYMYDkPTVHALFADAPSR	6	1	1	P73296	N-Term(iTRAQ4plex); K8(iTRAQ4plex)	0,0000	2,418	5	70,4	0	1,662E-08	29	0,00132425	3	2611,35880	0,23	39,32	0
	High	vPQLGkPADQR	3	1	1	P73296	N-Term(iTRAQ4plex); K6(iTRAQ4plex)	0,0000	1,318	3	0,5	0	0,005577	15	0,033187154	3	1496,87674	-1,37	18,92	0
	High	rRGDNAEmAVIELV	2	1	1	P73296	N-Term(iTRAQ4plex); M8(Oxidation)	0,0000	1,011	2	53,9	0,001	0,01211	21	0,007744083	3	1732,91233	-0,58	33,82	2
	High	mITLAk	6	1	1	P73296	N-Term(iTRAQ4plex); M1(Oxidation); K6(iTRAQ4plex)	0,0000	1,656	3	13,9	0,005	0,06189	27	0,00226559	2	980,60350	-1,83	19,27	0
	Medium	mITLAk	3	1	1	P73296	N-Term(iTRAQ4plex); K6(iTRAQ4plex)	0,0000	2,769	2	202,6	0,013	0,1744	31	0,000851079	2	964,60784	-2,63	25,40	0
	Medium	rGDNAEMAVIELV	2	1	1	P73296	N-Term(iTRAQ4plex)	0,0000	1,285	2	54,1	0,015	0,1863	28	0,001698126	2	1560,81633	-0,62	43,00	1
P73510	Sll1358 protein OS=Synechocystis sp. (strain PCC 6803 / Kazusa) GN=sl	437,87	22,59	1	7	7	19	1,089	18	18,5	394	43,1	6,23							
	A3	Sequence	# PSMs	# Proteins	# Protein Groups	Protein Group Accessions	Modifications	ΔCn	117/114	117/114 Count	117/114 Variability [%]	q-Value	PEP	IonScore	Exp Value	Charge	MH+ [Da]	ΔM [ppm]	RT [min]	# Missed Cleavages
	High	qVGTYNFPVSk	5	1	1	P73510	N-Term(iTRAQ4plex); K11(iTRAQ4plex)	0,0000	0,964	5	17,8	0	9,882E-10	75	3,85827E-08	2	1527,84429	2,17	29,09	0
	High	iEVPHTHNLLGQQPLVSLGGNELR	3	1	1	P73510	N-Term(iTRAQ4plex)	0,0000	0,672	3	98,2	0	7,656E-11	72	6,60648E-08	3	2765,52353	2,01	40,09	0
	High	gWGHSIEGIGPDTAk	2	1	1	P73510	N-Term(iTRAQ4plex); K15(iTRAQ4plex)	0,0000	0,982	2	35,1	0	4,866E-10	61	8,10905E-07	3	1812,95139	1,68	28,14	0
	High	kLPVQDTIFSLPTQP	4	1	1	P73510	N-Term(iTRAQ4plex); K1(iTRAQ4plex)	0,0000	1,272	3	14,1	0	0,006081	40	9,59819E-05	2	1972,13823	1,23	44,69	1
	High	iTLTSPEGk	1	1	1	P73510	N-Term(iTRAQ4plex); K9(iTRAQ4plex)	0,0000	1,229	1		0	0,0002472	36	0,000274789	2	1233,73223	2,37	24,17	0
	High	gGLWYFPR	3	1	1	P73510	N-Term(iTRAQ4plex)	0,0000	1,153	3	0,8	0	0,005228	31	0,000862919	2	1139,61382	1,71	38,97	0
	Medium	gYGHAIR	1	1	1	P73510	N-Term(iTRAQ4plex)	0,0000	1,063	1		0,037	0,4575	15	0,035496808	3	917,50879	1,54	11,17	0
P16033	Photosystem Q(B) protein 2 OS=Synechocystis sp. (strain PCC 6803 / K	436,27	16,67	1	5	5	12	0,370	12	23,4	360	39,7	5,64							
	A3	Sequence	# PSMs	# Proteins	# Protein Groups	Protein Group Accessions	Modifications	ΔCn	117/114	117/114 Count	117/114 Variability [%]	q-Value	PEP	IonScore	Exp Value	Charge	MH+ [Da]	ΔM [ppm]	RT [min]	# Missed Cleavages
	High	lIFQYASFNNSR	5	1	1	P16033	N-Term(iTRAQ4plex)	0,0000	0,341	5	21,1	0	0,000001239	64	3,55607E-07	2	1603,83794	1,87	41,06	0
	High	vIGTWADVLNR	2	1	1	P16033	N-Term(iTRAQ4plex)	0,0000	0,347	2	6,6	0	0,00004322	59	1,2161E-06	2	1387,78386	1,72	42,50	0
	High	aNIGFEVmHER	2	1	1	P16033	N-Term(iTRAQ4plex); M8(Oxidation)	0,0000	0,484	2	62,0	0	3,808E-08	58	1,5775E-06	3	1462,72455	1,08	21,42	0
	High	fGQEEETYNIVAAHGYFGR	1	1	1	P16033	N-Term(iTRAQ4plex)	0,0000	0,450	1		0	1,176E-11	50	9,97631E-06	3	2332,11399	1,05	39,57	0
	High	qWELSYR	1	1	1	P16033	N-Term(iTRAQ4plex)	0,0000	0,505	1		0	0,00311	38	0,00014756	2	1125,58257	1,44	28,18	0
	High	aNIGFEVMHER	1	1	1	P16033	N-Term(iTRAQ4plex)	0,0000	0,591	1		0	0,000004526	22	0,005727565	3	1446,72937	0,91	27,89	0
P27320	Ferredoxin-1 OS=Synechocystis sp. (strain PCC 6803 / Kazusa) GN=petl	430,76	17,53	1	3	3	20	0,869	18	18,3	97	10,4	3,95							
	A3	Sequence	# PSMs	# Proteins	# Protein Groups	Protein Group Accessions	Modifications	ΔCn	117/114	117/114 Count	117/114 Variability [%]	q-Value	PEP	IonScore	Exp Value	Charge	MH+ [Da]	ΔM [ppm]	RT [min]	# Missed Cleavages
	High	aGAcSTcAGk	12	1	1	P27320	N-Term(iTRAQ4plex); C4(Carbamidomethyl); C7(Carbam	0,0000	0,889	10	12,8	0	0,000001105	64	3,69803E-07	2	1270,61235	0,08	8,21	0
	High	mASYTVk	1	1	1	P27320	N-Term(iTRAQ4plex); K7(iTRAQ4plex)	0,0000	1,025	1		0	0,000276	53	5,09296E-06	2	1087,60674	0,67	18,57	0
	High	aSYTVk	5	1	1	P27320	N-Term(iTRAQ4plex); K6(iTRAQ4plex)	0,0000	0,709	5	9,7	0	0,007046	33	0,000483025	2	956,56487	-0,67	14,03	0
	High	mASYTVk	2	1	1	P27320	N-Term(iTRAQ4plex); M1(Oxidation); K7(iTRAQ4plex)	0,0000	0,943	2	18,0	0	0,0006924	33	0,00052236	2	1103,60185	0,84	13,70	0
P55038	Ferredoxin-dependent glutamate synthase 2 OS=Synechocystis sp. (stra	427,33	9,70	1	12	12	17	0,786	15	15,5	1556	169,4	5,76							
	A3	Sequence	# PSMs	# Proteins	# Protein Groups	Protein Group Accessions	Modifications	ΔCn	117/114	117/114 Count	117/114 Variability [%]	q-Value	PEP	IonScore	Exp Value	Charge	MH+ [Da]	ΔM [ppm]	RT [min]	# Missed Cleavages
	High	vcHTNNcPVGVATQQER	2	1	1	P55038	N-Term(iTRAQ4plex); C2(Carbamidomethyl); C7(Carbam	0,0000	0,802	2	18,6	0	6,091E-09	57	2,21804E-06	3	2114,00003	0,56	16,15	0
	High	eLLAQWFNTR	1	1	1	P55038	N-Term(iTRAQ4plex)	0,0000	0,540	1		0	0,0000463	53	5,46978E-06	2	1421,76445	-0,96	45,77	0
	High	lTVLGWR	3	1	1	P55038	N-Term(iTRAQ4plex)	0,0000	0,786	3	7,4	0	0,0007568	52	6,7293E-06	2	988,60619	0,13	37,97	0
	High	nPGYTSNFAVYHR	1	1	1	P55038	N-Term(iTRAQ4plex)	0,0000	0,737	1		0	8,386E-10	48	1,7578E-05	3	1669,82114	0,48	24,44	0
	High	hAGSPWELGVTEVHR	1	1	1	P55038	N-Term(iTRAQ4plex)	0,0000	0,879	1		0	1,649E-07	45	2,98518E-05	4	1818,93679	-0,01	28,10	0
	High	fcTGGmSLGALSR	1	1	1	P55038	N-Term(iTRAQ4plex); C2(Carbamidomethyl); M6(Oxidatic	0,0000	0,890	1		0	9,131E-09	45	3,39602E-05	2	1516,73943	1,65	29,54	0
	High	kLENFGFVNYRPGGEYHmNSPEn	2	1	1	P55038	N-Term(iTRAQ4plex); K1(iTRAQ4plex); M18(Oxidation); I	0,0000	0,846	1		0	3,855E-11	41	8,49122E-05	5	3395,65260	0,35	30,76	1
	High	sLLAHLGYR	1	1	1	P55038	N-Term(iTRAQ4plex)	0,0000	0,715	1		0	0,00001199	39	0,00011587	3	1173,68634	0,22	30,43	0
	High	lLYDYFk	1	1	1	P55038	N-Term(iTRAQ4plex); K7(iTRAQ4plex)	0,0000	0,778	1		0	0,0001862	38	0,000177088	2	1249,70574	-1,08	41,14	0
	High	gkPDHTLVEQALk	2	1	1	P55038	N-Term(iTRAQ4plex); K2(iTRAQ4plex); K13(iTRAQ4plex)	0,0000	0,663	2	42,5	0	0,000002837	36	0,000267898	3	1868,09525	-0,73	22,03	0
	High	sLITAHVEHTGSPk	1	1	1	P55038	N-Term(iTRAQ4plex); K14(iTRAQ4plex)	0,0000				0	0,00005419	26	0,003811782	4	1764,98452	-0,10	18,20	0
	High	dRPVTALR	1	1	1	P55038	N-Term(iTRAQ4plex)	0,0000	1,343	1		0,005	0,06851	16	0,023119051	3	1071,63874	-0,39	12,78	0
P29107	Ketol-acid reductoisomerase OS=Synechocystis sp. (strain PCC 6803 / K	421,32	24,17	1	5	5	17	1,353	14	30,8	331	35,8	5,01							
	A3	Sequence	# PSMs	# Proteins	# Protein Groups	Protein Group Accessions	Modifications	ΔCn	117/114	117/114 Count	117/114 Variability [%]	q-Value	PEP	IonScore	Exp Value	Charge	MH+ [Da]	ΔM [ppm]	RT [min]	# Missed Cleavages
	High	rTYEQGQGVPALFAVYQDASGQA	1	1	1	P29107	N-Term(iTRAQ4plex)	0,0000	1,718	1		0	9,812E-14	99	1,36135E-10	3	2756,38913	0,65	42,18	1
	High	qILDEIQSGQFAR	1	1	1	P29107	N-Term(iTRAQ4plex)	0,0000	1,289	1		0	8,805E-10	62	8,00168E-07	2	1648,87932	1,06	38,83	0
	High	aMFSWLk	3	1	1	P29107	N-Term(iTRAQ4plex); K7(iTRAQ4plex)	0,0000	1,820	3	5,7	0	0,00008221	56	2,32258E-06	2	1170,65996	1,33	41,79	0
	High	tVAIIGYGSQGHAHALNLk	5	1	1	P29107	N-Term(iTRAQ4plex); K19(iTRAQ4plex)	0,0000	1,336	3	29,4	0	2,597E-12	51	7,5504E-06	4	2238,26064	0,39	30,24	0
	High	eFVLENQAGkPGFTAmR	3	1	1	P29107	N-Term(iTRAQ4plex); K10(iTRAQ4plex); M16(Oxidation)	0,0000	1,255	2	41,0	0	2,951E-07	30	0,000891189	3	2199,14811	0,44	29,95	0
	High	amFSWLk	4	1	1	P29107	N-Term(iTRAQ4plex); M2(Oxidation); K7(iTRAQ4plex)	0,0000	1,334	4	11,8	0,005	0,06301	17	0,02018227	2	1186,65337	0,05	34,24	0
P72854	Sulfite reductase [ferredoxin] OS=Synechocystis sp. (strain PCC 6803 /	419,96	21,57	1	12	12	19	0,804	18	19,3	635	71,4	8,47							
	A3	Sequence	# PSMs	# Proteins	# Protein Groups	Protein Group Accessions	Modifications	ΔCn	117/114	117/114 Count	117/114 Variability [%]	q-Value	PEP	IonScore	Exp Value	Charge	MH+ [Da]	ΔM [ppm]	RT [min]	# Missed Cleavages
	High	gFNVLAGGGLGR	1	1	1	P72854	N-Term(iTRAQ4plex)	0,0000	0,708	1		0	2,418E-09	83	4,54957E-09	2	1261,71465	0,99	34,96	0
	High	lTANHNILLYDINAQDk	2	1	1	P72854	N-Term(iTRAQ4plex); K17(iTRAQ4plex)	0,0000	0,689	2	24,8	0	7,27E-12	70	1,10655E-07	3	2244,22428	0,70	36,40	0
	High	kVVDQFQLPLR	2	1	1	P72854	N-Term(iTRAQ4plex); K1(iTRAQ4plex)	0,0000	0,607	2	9,6	0	2,035E-08	57	2,09397E-06	3	1630,98795	-0,24	33,39	1
	High	rVSLSDEmYAQLk	1	1	1	P72854	N-Term(iTRAQ4plex); M8(Oxidation); K13(iTRAQ4plex)	0,0000	1,005	1		0	0,000001008	43	4,90874E-05	3	1843,98319	0,29	26,06	1
	High	eFSHGYTAPAk	1	1	1	P72854	N-Term(iTRAQ4plex); K11(iTRAQ4plex)	0,0000	0,922	1		0	0,000006864	32	0,000591521	3	1495,77802	-0,24	17,52	0
	High	qGLQIHGIVk	3	1	1	P72854	N-Term(iTRAQ4plex); K10(iTRAQ4plex)	0,0000	0,855	2	10,4	0	0,00104	27	0,001782256	3	1380,85535	-0,89	23,44	0
	High	nVmAPAAPFR	2	1	1	P72854	N-Term(iTRAQ4plex); M3(Oxidation)	0,0000	0,819	2	14,5	0	0,00007061	26	0,002546654	2	1233,65337	0,21	24,69	0
	High	tHNkEETFAR	1	1	1	P72854	N-Term(iTRAQ4plex); K4(iTRAQ4plex)	0,0000	0,851	1		0	0,01043	17	0,021526331	3	1520,80579	-0,14	9,28	1
	High	qVETYmGkPFQSFkPLPAWR	1	1	1	P72854	N-Term(iTRAQ4plex); M6(Oxidation); K8(iTRAQ4plex); K	0,0000	0,978	1		0	8,597E-07	14	0,049307163	4	2858,54213	1,15	37,61	0
	High	aIVATQR	2	1	1	P72854	N-Term(iTRAQ4plex)	0,0000	0,855	2	24,8	0,001	0,01552	26	0,002500173	2	902,55400	-0,01	13,10	0
	High	vGFTALR	1	1	1	P72854	N-Term(iTRAQ4plex)	0,0000	0,565	1		0,006	0,07167	27	0,002525421	2	907,54851	0,34	26,26	0
	High	vTTPTAAPR	2	1	1	P72854	N-Term(iTRAQ4plex)	0,0000	0,800	2	3,9	0,007	0,09236	24	0,003917148	2	1057,61284	0,54	15,97	0
P73546	Phosphorylase OS=Synechocystis sp. (strain PCC 6803 / Kazusa) GN=gl	419,18	10,18	1	9	9	19	0,800	16	27,0	855	98,2	6,14							
	A3	Sequence	# PSMs	# Proteins	# Protein Groups	Protein Group Accessions	Modifications	ΔCn	117/114	117/114 Count	117/114 Variability [%]	q-Value	PEP	IonScore	Exp Value	Charge	MH+ [Da]	ΔM [ppm]	RT [min]	# Missed Cleavages
	High	hLGNNLINLGLYDk	2	1	1	P73546	N-Term(iTRAQ4plex); K14(iTRAQ4plex)	0,0000	0,621	2	9,8	0	5,077E-11	59	1,12972E-06	3	1872,06095	1,47	37,12	0
	High	nLAAYLLk	6	1	1	P73546	N-Term(iTRAQ4plex); K8(iTRAQ4plex)	0,0000	0,860	4	25,7	0	0,00007872	53	5,55866E-06	2	1193,75261	2,49	39,26	0
	High	hLEIIYEINHR	3	1	1	P73546	N-Term(iTRAQ4plex)	0,0000	0,855	2	15,4	0	1,179E-08	50	1,104E-05	4	1580,86733	0,48	29,25	0
	High	tFIFGGk	1	1	1	P73546	N-Term(iTRAQ4plex); K7(iTRAQ4plex)	0,0000	0,520	1		0	0,005843	45	3,16935E-05	2	1057,62981	1,27	30,26	0
	High	qNPHAEIVPR	1	1	1	P73546	N-Term(iTRAQ4plex)	0,0000	0,797	1		0	8,154E-08	41	7,72627E-05	3	1304,72000	0,62	13,53	0
	High	qHLAALEIIHLYNR	1	1	1	P73546	N-Term(iTRAQ4plex)	0,0000	0,636	1		0	9,065E-10	41	8,63759E-05	4	1835,04201	0,63	37,69	0
	High	yEFGIFHQR	2	1	1	P73546	N-Term(iTRAQ4plex)	0,0000	0,902	2	12,9	0	0,007252	25	0,003396018	3	1340,68851	1,27	32,24	0
	High	lGGHTEIIHNEk	2	1	1	P73546	N-Term(iTRAQ4plex); K12(iTRAQ4plex)	0,0000	0,830	2	15,2	0	0,00007039	24	0,004463729	4	1635,90847	1,68	16,83	0
	High	ikQNPHAEIVPR	1	1	1	P73546	N-Term(iTRAQ4plex); K2(iTRAQ4plex)	0,0000	1,142	1		0	0,0000226	22	0,006251295	4	1690,00234	1,21	16,85	1
P73608	Glycogen operon protein GlgX OS=Synechocystis sp. (strain PCC 6803 /	416,56	11,46	1	8	9	20	0,528	15	25,4	707	79,8	5,86							
	A3	Sequence	# PSMs	# Proteins	# Protein Groups	Protein Group Accessions	Modifications	ΔCn	117/114	117/114 Count	117/114 Variability [%]	q-Value	PEP	IonScore	Exp Value	Charge	MH+ [Da]	ΔM [ppm]	RT [min]	# Missed Cleavages
	High	sVVILLAk	3	1	1	P73608	N-Term(iTRAQ4plex); K8(iTRAQ4plex)	0,0000	0,568	2	15,1	0	0,0005536	61	7,99779E-07	2	1130,77703	1,67	34,15	0
	High	gPTISFR	3	1	1	P73608	N-Term(iTRAQ4plex)	0,0000	0,528	3	33,4	0	0,00008254	56	2,90342E-06	2	921,52861	1,25	24,04	0
	High	gTFAGILSk	2	1	1	P73608	N-Term(iTRAQ4plex); K9(iTRAQ4plex)	0,0000	0,380	1		0	0,0000833	55	3,11867E-06	2	1181,71575	2,10	32,22	0
	High	rAPQPFVEIPFPESFR	3	1	1	P73608	N-Term(iTRAQ4plex)	0,0000	0,482	2	15,0	0	3,646E-09	51	8,20295E-06	3	2061,10544	0,75	44,43	1
	High	fDLASILGR	2	1	1	P73608	N-Term(iTRAQ4plex)	0,0000	0,425	2	7,3	0	0,000005044	43	4,68781E-05	2	1135,66008	0,76	47,36	0
	High	vLAFMLcGR	2	1	1	P73608	N-Term(iTRAQ4plex); C7(Carbamidomethyl)	0,0000	0,620	2	5,8	0	5,775E-07	42	6,63697E-05	2	1210,65569	-0,05	44,06	0
	High	aGYAATGk	1	1	1	P73608	N-Term(iTRAQ4plex); K8(iTRAQ4plex)	0,0000	0,625	1		0	0,0024	24	0,003953393	2	1026,58330	1,03	11,47	0
	High	lAHPVLR	3	1	1	P73608	N-Term(iTRAQ4plex)	0,0000	0,549	2	15,7	0	0,003621	20	0,010115096	3	949,60691	0,55	16,18	0
	High	vLLDPYAk	1	2	2	P73608;P72691	N-Term(iTRAQ4plex); K8(iTRAQ4plex)	0,0000				0,007	0,08816	16	0,029644447	2	1206,73760	3,26	30,68	0
P72870	Allophycocyanin subunit alpha-B OS=Synechocystis sp. (strain PCC 6803	415,19	56,52	1	10	10	22	1,622	19	29,6	161	17,9	5,58							
	A3	Sequence	# PSMs	# Proteins	# Protein Groups	Protein Group Accessions	Modifications	ΔCn	117/114	117/114 Count	117/114 Variability [%]	q-Value	PEP	IonScore	Exp Value	Charge	MH+ [Da]	ΔM [ppm]	RT [min]	# Missed Cleavages
	High	gIQAFLTTGAQR	7	1	1	P72870	N-Term(iTRAQ4plex)	0,0000	1,983	4	2,4	0	0,000000224	67	2,46259E-07	2	1406,78813	0,60	36,20	0
	High	aPGGNAYGQR	3	1	1	P72870	N-Term(iTRAQ4plex)	0,0000	1,527	3	4,8	0	0,0001356	39	0,000136763	2	1134,57768	0,36	10,87	0
	High	iAETLAENEkk	1	1	1	P72870	N-Term(iTRAQ4plex); K10(iTRAQ4plex); K11(iTRAQ4plex	0,0000	1,858	1		0	1,716E-08	37	0,000180289	3	1677,97519	0,26	21,32	1
	High	kIVDQAQk	4	1	1	P72870	N-Term(iTRAQ4plex); K1(iTRAQ4plex); K8(iTRAQ4plex)	0,0000	1,878	4	12,3	0	0,00002836	35	0,000329587	2	1361,84697	-0,53	11,55	1
	High	lVTYGVLAGNkEPIETTGLIGVk	1	1	1	P72870	N-Term(iTRAQ4plex); K11(iTRAQ4plex); K23(iTRAQ4plex	0,0000	1,482	1		0	4,395E-07	29	0,001129718	4	2804,65175	0,68	43,54	1
	High	sVVSQVILQADDQLR	1	1	1	P72870	N-Term(iTRAQ4plex)	0,0000	1,037	1		0	0,002645	20	0,010161785	3	1815,00986	0,30	44,10	0
	High	lVTYGVLAGNk	1	1	1	P72870	N-Term(iTRAQ4plex); K11(iTRAQ4plex)	0,0000	0,815	1		0	0,001983	19	0,012021814	3	1422,85782	1,35	32,98	0
	High	iAETLAENEk	1	1	1	P72870	N-Term(iTRAQ4plex); K10(iTRAQ4plex)	0,0000	0,616	1		0	0,002588	14	0,037667778	3	1405,77994	1,57	24,73	0
	High	qYNQcLR	2	1	1	P72870	N-Term(iTRAQ4plex); C5(Carbamidomethyl)	0,0000	1,348	2	2,3	0,002	0,02268	32	0,000703024	2	1125,56059	1,28	13,97	0
	High	kHPEYR	1	1	1	P72870	N-Term(iTRAQ4plex); K1(iTRAQ4plex)	0,0000	1,723	1		0,008	0,09723	17	0,018534036	3	1117,63657	0,82	7,91	1
Q55247	Nitrogen regulatory protein P-II OS=Synechocystis sp. (strain PCC 6803	407,38	58,93	1	7	7	18	1,187	18	21,5	112	12,4	6,81							
	A3	Sequence	# PSMs	# Proteins	# Protein Groups	Protein Group Accessions	Modifications	ΔCn	117/114	117/114 Count	117/114 Variability [%]	q-Value	PEP	IonScore	Exp Value	Charge	MH+ [Da]	ΔM [ppm]	RT [min]	# Missed Cleavages
	High	iALVNAGIVGmTVSEVR	4	1	1	Q55247	N-Term(iTRAQ4plex); M11(Oxidation)	0,0000	0,914	4	22,0	0	2,072E-08	74	4,37918E-08	3	1889,06687	1,12	43,72	0
	High	iFISPVDSVVR	3	1	1	Q55247	N-Term(iTRAQ4plex)	0,0000	1,113	3	6,9	0	0,0008982	47	1,79048E-05	2	1375,80791	0,93	39,84	0
	High	vEAIIRPFk	5	1	1	Q55247	N-Term(iTRAQ4plex); K9(iTRAQ4plex)	0,0000	1,215	5	8,8	0	0,00009145	47	1,82797E-05	2	1360,85661	0,80	32,06	0
	High	iALVNAGIVGMTVSEVR	1	1	1	Q55247	N-Term(iTRAQ4plex)	0,0000	0,910	1		0	0,000007444	39	0,00015045	3	1873,07206	1,19	47,19	0

	High	kVEAIIRPFk	2	1	1	Q55247	N-Term(iTRAQ4plex); K1(iTRAQ4plex); K10(iTRAQ4plex)	0,0000	1,544	2	2,0	0	0,000001738	25	0,003364883	3	1633,05338	0,50	27,41	1
	High	yRGSEYTVEFLQk	1	1	1	Q55247	N-Term(iTRAQ4plex); K13(iTRAQ4plex)	0,0000	1,213	1		0	0,00000018	23	0,005770179	3	1908,01187	0,69	31,60	1
	Medium	tGEkDTEAI	1	1	1	Q55247	N-Term(iTRAQ4plex); K4(iTRAQ4plex)	0,0000	1,331	1		0,016	0,2039	26	0,002779521	2	1251,67009	2,38	14,59	1
	Medium	lVSAAR	1	1	1	Q55247	N-Term(iTRAQ4plex)	0,0000	0,832	1		0,035	0,4339	25	0,008709101	2	760,48064	1,12	13,92	0
P74384	Adenylosuccinate lyase OS=Synechocystis sp. (strain PCC 6803 / Kazusa	402,33	22,97	1	8	8	17	1,186	12	38,2	431	48,4	5,92							
	A3	Sequence	# PSMs	# Proteins	# Protein Groups	Protein Group Accessions	Modifications	ΔCn	117/114	117/114 Count	117/114 Variability [%]	q-Value	PEP	IonScore	Exp Value	Charge	MH+ [Da]	ΔM [ppm]	RT [min]	# Missed Cleavages
	High	gHAVAALENVALWHER	4	1	1	P74384	N-Term(iTRAQ4plex)	0,0000	1,563	2	12,6	0	5,833E-12	68	1,74169E-07	3	1917,02359	1,24	34,29	0
	High	vAQSIAVGk	4	1	1	P74384	N-Term(iTRAQ4plex); K9(iTRAQ4plex)	0,0000	1,186	2	7,2	0	4,215E-07	67	1,80302E-07	2	1160,72319	-0,84	20,11	0
	High	hAEYVQQLALLAASLER	1	1	1	P74384	N-Term(iTRAQ4plex)	0,0000	0,731	1		0	3,805E-08	44	4,07352E-05	3	2056,13047	-0,17	50,29	0
	High	vLLALVEk	2	1	1	P74384	N-Term(iTRAQ4plex); K8(iTRAQ4plex)	0,0000	1,563	1		0	0,00001272	44	4,2655E-05	2	1172,78545	-0,22	40,53	0
	High	nmNVYGGVIFSQk	1	1	1	P74384	N-Term(iTRAQ4plex); M2(Oxidation); K13(iTRAQ4plex)	0,0000	0,950	1		0	0,000005466	42	6,88605E-05	3	1760,92142	-1,70	31,99	0
	High	vAFPDccILTHFmLk	1	1	1	P74384	N-Term(iTRAQ4plex); C6(Carbamidomethyl); C7(Carbam	0,0000	1,659	1		0	0,000004019	37	0,000199054	3	2156,09415	-0,19	45,94	0
	High	vAFPDccILTHFMLk	1	1	1	P74384	N-Term(iTRAQ4plex); C6(Carbamidomethyl); C7(Carbam	0,0000	2,284	1		0	0,0001435	23	0,004774963	3	2140,10154	0,88	49,74	0
	High	yTVmVGR	2	1	1	P74384	N-Term(iTRAQ4plex); M4(Oxidation)	0,0000	1,071	2	6,3	0	0,009766	20	0,011014632	2	985,52519	-0,56	18,78	0
	High	sHGIHAEPITFGFk	1	1	1	P74384	N-Term(iTRAQ4plex); K14(iTRAQ4plex)	0,0000	0,485	1		0	0,0001598	15	0,034038468	4	1828,99550	0,34	29,61	0
P73867	Potassium-transporting ATPase B chain OS=Synechocystis sp. (strain PC	401,99	13,04	1	9	9	16	0,104	15	39,9	690	72,9	6,14							
	A3	Sequence	# PSMs	# Proteins	# Protein Groups	Protein Group Accessions	Modifications	ΔCn	117/114	117/114 Count	117/114 Variability [%]	q-Value	PEP	IonScore	Exp Value	Charge	MH+ [Da]	ΔM [ppm]	RT [min]	# Missed Cleavages
	High	vAQFNVVATSGR	2	1	1	P73867	N-Term(iTRAQ4plex)	0,0000	0,081	2	2,1	0	0,000006019	64	4,25569E-07	2	1392,77580	2,99	27,59	0
	High	lIDLVTIGk	1	1	1	P73867	N-Term(iTRAQ4plex); K9(iTRAQ4plex)	0,0000	0,082	1		0	0,000006559	63	4,5391E-07	2	1259,82463	5,47	42,49	0
	High	fRPLSADQLLQR	3	1	1	P73867	N-Term(iTRAQ4plex)	0,0000	0,083	3	6,3	0	7,404E-08	49	1,26465E-05	3	1587,91275	2,49	34,08	0
	High	vSHLGGTPLAVcR	1	1	1	P73867	N-Term(iTRAQ4plex); C12(Carbamidomethyl)	0,0000	0,138	1		0	0,000003242	43	0,00005	3	1510,83075	1,75	23,78	0
	High	iTASVIAk	3	1	1	P73867	N-Term(iTRAQ4plex); K8(iTRAQ4plex)	0,0000	0,193	2	40,5	0	0,005614	38	0,000164805	2	1090,71489	6,82	24,50	0
	High	iISDELIIR	1	1	1	P73867	N-Term(iTRAQ4plex)	0,0000	0,099	1		0	0,0008617	33	0,000457057	2	1215,74687	3,22	38,13	0
	High	tGTITLGNR	2	1	1	P73867	N-Term(iTRAQ4plex)	0,0000	0,133	2	13,1	0,002	0,03291	41	0,000107377	2	1076,62163	3,30	18,21	0
	High	sTQTQTYAQR	2	1	1	P73867	N-Term(iTRAQ4plex)	0,0000	0,092	2	18,3	0,003	0,03624	34	0,000357248	2	1327,67620	2,95	9,31	0
	Medium	kGAVDAIR	1	1	1	P73867	N-Term(iTRAQ4plex); K1(iTRAQ4plex)	0,0000	0,350	1		0,024	0,302	14	0,051663193	3	1117,70111	7,07	12,21	1
P73704	General secretion pathway protein G OS=Synechocystis sp. (strain PCC	401,18	17,26	1	3	3	13	1,239	10	12,9	168	17,6	4,78							
	A3	Sequence	# PSMs	# Proteins	# Protein Groups	Protein Group Accessions	Modifications	ΔCn	117/114	117/114 Count	117/114 Variability [%]	q-Value	PEP	IonScore	Exp Value	Charge	MH+ [Da]	ΔM [ppm]	RT [min]	# Missed Cleavages
	High	sMSGGTFYDSGTR	1	1	1	P73704	N-Term(iTRAQ4plex)	0,0000	1,314	1		0	1,37E-10	87	1,83641E-09	2	1509,67790	1,21	22,02	0
	High	smSGGTFYDSGTR	4	1	1	P73704	N-Term(iTRAQ4plex); M2(Oxidation)	0,0000	1,336	3	3,6	0	2,92E-08	59	1,3365E-06	2	1525,67339	1,57	16,71	0
	High	aFSTVVcR	3	1	1	P73704	N-Term(iTRAQ4plex); C7(Carbamidomethyl)	0,0000	1,078	3	2,0	0	0,001407	46	2,57614E-05	2	1083,57524	1,36	22,19	0
	High	sTIGALNR	5	1	1	P73704	N-Term(iTRAQ4plex)	0,0000	1,205	3	9,0	0	0,00236	45	3,0267E-05	2	975,57213	1,78	17,17	0
P73597	Sll1306 protein OS=Synechocystis sp. (strain PCC 6803 / Kazusa) GN=sl	400,67	32,24	1	9	9	15	1,020	15	5,8	335	38,2	8,18							
	A3	Sequence	# PSMs	# Proteins	# Protein Groups	Protein Group Accessions	Modifications	ΔCn	117/114	117/114 Count	117/114 Variability [%]	q-Value	PEP	IonScore	Exp Value	Charge	MH+ [Da]	ΔM [ppm]	RT [min]	# Missed Cleavages
	High	fFSSGQYLTELk	2	1	1	P73597	N-Term(iTRAQ4plex); K12(iTRAQ4plex)	0,0000	0,889	2	7,5	0	7,465E-12	90	9,48353E-10	2	1707,92400	2,56	41,87	0
	High	kIDIANFALNDPNTIR	2	1	1	P73597	N-Term(iTRAQ4plex); K1(iTRAQ4plex)	0,0000	0,587	2	44,2	0	3,356E-11	62	6,32368E-07	3	2103,18509	2,36	37,94	1
	High	aVGYNAPGLR	2	1	1	P73597	N-Term(iTRAQ4plex)	0,0000	1,049	2	1,7	0	4,363E-07	56	2,78593E-06	2	1161,65154	1,57	22,69	0
	High	lQRPEHVYVFEDFLk	2	1	1	P73597	N-Term(iTRAQ4plex); K15(iTRAQ4plex)	0,0000	1,048	2	0,8	0	1,844E-11	51	7,7085E-06	3	2208,21091	2,40	40,16	0
	High	nVDIILk	1	1	1	P73597	N-Term(iTRAQ4plex); K7(iTRAQ4plex)	0,0000	0,990	1		0	0,0004081	50	1,06407E-05	2	1102,70989	2,21	29,05	0
	High	iLDLLDkYk	2	1	1	P73597	N-Term(iTRAQ4plex); K7(iTRAQ4plex); K9(iTRAQ4plex)	0,0000	1,103	2	11,6	0	0,0002167	29	0,001156032	3	1552,97293	3,52	43,26	1
	High	gHEAAAHGWDWDNEFNmTAPQ	1	1	1	P73597	N-Term(iTRAQ4plex); M17(Oxidation)	0,0000	1,055	1		0	6,004E-07	22	0,006052991	4	2829,22378	1,57	30,71	0
	High	mmAVTmHDR	1	1	1	P73597	N-Term(iTRAQ4plex); M1(Oxidation); M2(Oxidation); M6	0,0000	0,969	1		0	0,0001942	11	0,080717928	3	1283,56784	1,16	7,98	0
	High	mmAVTMHDR	1	1	1	P73597	N-Term(iTRAQ4plex); M1(Oxidation); M2(Oxidation)	0,0000	1,046	1		0	0,0004702	11	0,083940201	3	1267,57203	0,47	10,12	0
	High	fWPDNIR	1	1	1	P73597	N-Term(iTRAQ4plex)	0,0000	0,932	1		0,001	0,01588	27	0,002127992	2	1091,57720	1,57	33,52	0
Q55087	Geranylgeranyl diphosphate reductase OS=Synechocystis sp. (strain PC	399,79	24,57	1	10	10	22	1,178	19	25,9	407	44,9	8,00							
	A3	Sequence	# PSMs	# Proteins	# Protein Groups	Protein Group Accessions	Modifications	ΔCn	117/114	117/114 Count	117/114 Variability [%]	q-Value	PEP	IonScore	Exp Value	Charge	MH+ [Da]	ΔM [ppm]	RT [min]	# Missed Cleavages
	High	lTFDSYLYk	1	1	1	Q55087	N-Term(iTRAQ4plex); K9(iTRAQ4plex)	0,0000	1,236	1		0	0,000007175	69	1,1587E-07	2	1437,78899	1,52	39,36	0
	High	yDHVAVGTGTmk	2	1	1	Q55087	N-Term(iTRAQ4plex); M11(Oxidation); K12(iTRAQ4plex)	0,0000	0,960	2	31,2	0	1,119E-08	46	2,42086E-05	3	1582,81400	0,13	17,28	0
	High	tIGSLLR	3	1	1	Q55087	N-Term(iTRAQ4plex)	0,0000	1,178	3	23,2	0	0,0008459	46	2,42644E-05	2	903,57506	0,71	29,45	0
	High	rEVLDGFLR	3	1	1	Q55087	N-Term(iTRAQ4plex)	0,0000	1,223	2	5,0	0	0,00004384	40	9,24634E-05	3	1248,71811	-0,03	31,49	1
	High	kLEGGEIIk	3	1	1	Q55087	N-Term(iTRAQ4plex); K1(iTRAQ4plex); K9(iTRAQ4plex)	0,0000	0,897	3	15,1	0	3,547E-07	39	0,000121331	3	1418,89396	-0,26	19,13	1
	High	tVVPANPLVQmk	1	1	1	Q55087	N-Term(iTRAQ4plex); M11(Oxidation); K12(iTRAQ4plex)	0,0000	0,962	1		0	0,00001896	37	0,000223342	3	1600,93448	0,59	29,81	0
	High	yGATYLVLDILQR	1	1	1	Q55087	N-Term(iTRAQ4plex)	0,0000	1,375	1		0	0,000009075	33	0,000481915	3	1668,94736	1,92	53,41	0
	High	ikDLQAGIR	4	1	1	Q55087	N-Term(iTRAQ4plex); K2(iTRAQ4plex)	0,0000	1,564	2	6,0	0	0,0003027	31	0,000812774	3	1301,81348	-0,70	22,33	1
	High	vEAHPIPEHPRPR	3	1	1	Q55087	N-Term(iTRAQ4plex)	0,0000	0,915	3	18,0	0	0,0001408	28	0,002233281	3	1678,92331	-1,51	13,35	0
	Medium	iRLPEDk	1	1	1	Q55087	N-Term(iTRAQ4plex); K7(iTRAQ4plex)	0,0000	2,077	1		0,023	0,2831	13	0,047311858	3	1158,71051	1,72	21,11	1
P73334	DNA-directed RNA polymerase subunit beta' OS=Synechocystis sp. (stra	399,08	7,67	1	10	10	19	1,175	17	39,7	1317	144,7	4,84							
	A3	Sequence	# PSMs	# Proteins	# Protein Groups	Protein Group Accessions	Modifications	ΔCn	117/114	117/114 Count	117/114 Variability [%]	q-Value	PEP	IonScore	Exp Value	Charge	MH+ [Da]	ΔM [ppm]	RT [min]	# Missed Cleavages
	High	tFHTGGVFTGEVAR	2	1	1	P73334	N-Term(iTRAQ4plex)	0,0000	1,153	1		0	9,058E-09	58	1,6788E-06	3	1622,84177	0,61	25,94	0
	High	vQNHSIVAELIDDRYR	4	1	1	P73334	N-Term(iTRAQ4plex)	0,0000	1,527	4	2,3	0	0,00000308	43	5,49503E-05	3	2072,10239	0,87	35,76	1
	High	kGLVDTALR	3	1	1	P73334	N-Term(iTRAQ4plex); K1(iTRAQ4plex)	0,0000	1,212	2	25,9	0	0,00004549	42	6,109E-05	3	1260,78724	-0,48	20,83	1
	High	yTPVLLGITk	3	1	1	P73334	N-Term(iTRAQ4plex); K10(iTRAQ4plex)	0,0000	0,784	3	58,1	0	0,000005394	41	8,64908E-05	2	1392,87029	-0,14	38,34	0
	High	gLkENVIIGR	1	1	1	P73334	N-Term(iTRAQ4plex); K3(iTRAQ4plex)	0,0000	1,565	1		0	0,0001735	38	0,000158114	3	1386,86716	0,00	22,73	1
	High	kQGYEVTk	2	1	1	P73334	N-Term(iTRAQ4plex); K1(iTRAQ4plex); K8(iTRAQ4plex)	0,0000	1,117	2	7,5	0	0,00003923	37	0,000187919	3	1384,81491	-0,81	11,90	1
	High	gDNLVLLVFER	1	1	1	P73334	N-Term(iTRAQ4plex)	0,0000	1,550	1		0	0,00001652	35	0,000319132	2	1418,81328	0,59	49,77	0
	High	hIEVIVR	1	1	1	P73334	N-Term(iTRAQ4plex)	0,0000	1,072	1		0	0,0002927	32	0,000657612	3	1009,62824	0,72	17,85	0
	High	kLIALAYR	1	1	1	P73334	N-Term(iTRAQ4plex); K1(iTRAQ4plex)	0,0000	1,573	1		0	0,0001549	31	0,000841337	3	1235,80707	-0,61	27,65	1
	Medium	tTTGGmIR	1	1	1	P73334	N-Term(iTRAQ4plex); M6(Oxidation)	0,0000	1,600	1		0,043	0,5323	11	0,085500766	2	996,52617	-0,32	10,65	0
P73289	50S ribosomal protein L25 OS=Synechocystis sp. (strain PCC 6803 / Kaz	397,72	37,76	1	4	4	20	1,930	14	68,3	98	11,1	8,94							
	A3	Sequence	# PSMs	# Proteins	# Protein Groups	Protein Group Accessions	Modifications	ΔCn	117/114	117/114 Count	117/114 Variability [%]	q-Value	PEP	IonScore	Exp Value	Charge	MH+ [Da]	ΔM [ppm]	RT [min]	# Missed Cleavages
	High	nLYHLAFFAGk	6	1	1	P73289	N-Term(iTRAQ4plex); K11(iTRAQ4plex)	0,0000	2,459	4	24,3	0	2,259E-09	70	9,88485E-08	2	1568,88030	-1,59	38,37	0
	High	tAITMLR	3	1	1	P73289	N-Term(iTRAQ4plex)	0,0000	1,120	3	25,8	0	0,0007627	43	8,14776E-05	2	949,56084	-1,38	25,73	0
	High	aLSIEcQQRPEk	7	1	1	P73289	N-Term(iTRAQ4plex); C6(Carbamidomethyl); K12(iTRAQ	0,0000	2,609	4	63,1	0	3,088E-08	39	0,000153955	3	1746,94004	-0,63	19,10	0
	High	eTPIEVk	1	1	1	P73289	N-Term(iTRAQ4plex); K7(iTRAQ4plex)	0,0000	1,781	1		0	0,00339	33	0,00055077	2	1103,64897	-5,53	18,86	0
	High	tAITmLR	3	1	1	P73289	N-Term(iTRAQ4plex); M5(Oxidation)	0,0000	1,059	2	20,2	0	0,00424	23	0,005168835	2	965,55583	-1,27	19,31	0
P73722	SOS function regulatory protein OS=Synechocystis sp. (strain PCC 6803	393,99	25,12	1	8	8	21	1,229	19	21,6	203	22,7	6,11							
	A3	Sequence	# PSMs	# Proteins	# Protein Groups	Protein Group Accessions	Modifications	ΔCn	117/114	117/114 Count	117/114 Variability [%]	q-Value	PEP	IonScore	Exp Value	Charge	MH+ [Da]	ΔM [ppm]	RT [min]	# Missed Cleavages
	High	gVSVIGELk	6	1	1	P73722	N-Term(iTRAQ4plex); K9(iTRAQ4plex)	0,0000	1,240	4	4,1	0	0,0005757	66	2,66054E-07	2	1189,74162	1,80	30,60	0
	High	sSVFALR	3	1	1	P73722	N-Term(iTRAQ4plex)	0,0000	1,075	3	15,5	0	0,002314	42	5,76727E-05	2	923,54369	0,62	25,81	0
	High	kSSVFALR	3	1	1	P73722	N-Term(iTRAQ4plex); K1(iTRAQ4plex)	0,0000	1,229	3	20,5	0	0,0000412	42	6,90192E-05	3	1195,74167	1,28	21,75	1
	High	iLHQkPk	2	1	1	P73722	N-Term(iTRAQ4plex); K5(iTRAQ4plex); K7(iTRAQ4plex)	0,0000	1,124	2	0,4	0	0,0004973	36	0,000243764	2	1295,85466	1,77	9,84	0
	High	aSNNkGPGQELk	4	1	1	P73722	N-Term(iTRAQ4plex); K5(iTRAQ4plex); K12(iTRAQ4plex)	0,0000	2,003	4	20,3	0	0,000001602	24	0,004139711	3	1674,95011	0,10	11,52	1
	High	gPGQELk	1	1	1	P73722	N-Term(iTRAQ4plex); K7(iTRAQ4plex)	0,0000	0,975	1		0,001	0,01184	27	0,001857676	2	1016,59819	0,29	13,55	0
	High	yYQDGTk	1	1	1	P73722	N-Term(iTRAQ4plex); K7(iTRAQ4plex)	0,0000	1,039	1		0,005	0,06446	18	0,016254365	2	1162,59856	0,26	13,48	0
	Medium	sPAPIQSR	1	1	1	P73722	N-Term(iTRAQ4plex)	0,0000	1,456	1		0,013	0,1705	10	0,096598416	2	999,57091	0,52	11,74	0
Q55662	ATP-dependent Clp protease regulatory subunit OS=Synechocystis sp. (	392,90	11,57	1	9	9	24	1,635	21	23,3	821	91,1	5,50							
	A3	Sequence	# PSMs	# Proteins	# Protein Groups	Protein Group Accessions	Modifications	ΔCn	117/114	117/114 Count	117/114 Variability [%]	q-Value	PEP	IonScore	Exp Value	Charge	MH+ [Da]	ΔM [ppm]	RT [min]	# Missed Cleavages
	High	lLHmEDTLHQR	2	1	1	Q55662	N-Term(iTRAQ4plex); M4(Oxidation)	0,0000	1,039	2	3,6	0	1,025E-09	61	7,41259E-07	3	1552,80210	-0,12	16,80	0
	High	nPNRPIASFIFSGPTGVGk	3	1	1	Q55662	N-Term(iTRAQ4plex); K19(iTRAQ4plex)	0,0000	1,920	3	46,0	0	2,588E-09	60	1,01625E-06	3	2247,24991	0,45	38,69	0
	High	rPYTVVLFDEIEk	4	1	1	Q55662	N-Term(iTRAQ4plex); K13(iTRAQ4plex)	0,0000	1,761	4	26,8	0	1,021E-10	58	1,61797E-06	3	1897,06766	0,15	40,48	0
	High	nTLLImTSNIGSk	1	1	1	Q55662	N-Term(iTRAQ4plex); M6(Oxidation); K13(iTRAQ4plex)	0,0000	0,780	1		0	0,00003516	39	0,000125884	3	1695,95578	0,24	30,93	0
	High	vIQILGR	5	1	1	Q55662	N-Term(iTRAQ4plex)	0,0000	1,518	3	2,8	0	0,01088	32	0,000701407	2	942,62163	-0,08	31,06	0
	High	nYFRPEFLNR	3	1	1	Q55662	N-Term(iTRAQ4plex)	0,0000	1,828	3	2,7	0	0,00002532	28	0,001644258	3	1499,78830	0,47	31,96	0
	High	yRGEFEER	2	1	1	Q55662	N-Term(iTRAQ4plex)	0,0000	2,245	2	24,3	0	0,003285	13	0,053575965	3	1229,60337	0,17	14,41	1
	High	aIASSk	3	1	1	Q55662	N-Term(iTRAQ4plex); K6(iTRAQ4plex)	0,0000	1,358	2	7,8	0,003	0,03832	16	0,033754662	2	864,53612	-3,69	11,34	0
	Medium	qLNkEEVk	1	1	1	Q55662	N-Term(iTRAQ4plex); K4(iTRAQ4plex); K8(iTRAQ4plex)	0,0000	1,714	1		0,015	0,1872	11	0,088098802	3	1419,85205	-0,79	13,34	1
Q55585	Probable succinate-semialdehyde dehydrogenase [NADP(+)] OS=Synec	387,93	9,69	1	5	5	13	0,693	12	23,5	454	48,7	5,14							
	A3	Sequence	# PSMs	# Proteins	# Protein Groups	Protein Group Accessions	Modifications	ΔCn	117/114	117/114 Count	117/114 Variability [%]	q-Value	PEP	IonScore	Exp Value	Charge	MH+ [Da]	ΔM [ppm]	RT [min]	# Missed Cleavages
	High	fAAPALmAGNVGLLk	4	1	1	Q55585	N-Term(iTRAQ4plex); M7(Oxidation); K15(iTRAQ4plex)	0,0000	0,557	3	1,2	0	5,135E-09	77	2,22828E-08	3	1777,03086	1,31	40,75	0
	High	fAAPALMAGNVGLLk	2	1	1	Q55585	N-Term(iTRAQ4plex); K15(iTRAQ4plex)	0,0000	0,637	2	32,1	0	3,49E-08	72	6,66761E-08	3	1761,03440	0,45	45,27	0
	High	kAADILEQR	2	1	1	Q55585	N-Term(iTRAQ4plex); K1(iTRAQ4plex)	0,0000	0,925	2	20,1	0	0,00007748	41	8,8914E-05	3	1331,78922	0,49	19,16	1
	High	hLPFAQR	1	1	1	Q55585	N-Term(iTRAQ4plex)	0,0000	0,778	1		0	0,0001163	26	0,002460198	3	1012,58200	1,10	18,40	0
	High	iPFGGIk	3	1	1	Q55585	N-Term(iTRAQ4plex); K7(iTRAQ4plex)	0,0000	0,592	3	7,9	0,002	0,02568	31	0,00072773	2	1019,64903	-0,17	31,43	0
	Medium	aFQALk	1	1	1	Q55585	N-Term(iTRAQ4plex); K6(iTRAQ4plex)	0,0000	0,738	1		0,012	0,1538	34	0,000409232	2	965,60222	-0,03	21,20	0
P73133	Acetylornithine aminotransferase OS=Synechocystis sp. (strain PCC 680	387,32	18,65	1	7	7	18	1,215	14	13,1	429	46,5	5,24							
	A3	Sequence	# PSMs	# Proteins	# Protein Groups	Protein Group Accessions	Modifications	ΔCn	117/114	117/114 Count	117/114 Variability [%]	q-Value	PEP	IonScore	Exp Value	Charge	MH+ [Da]	ΔM [ppm]	RT [min]	# Missed Cleavages
	High	tLATITATGQPk	2	1	1	P73133	N-Term(iTRAQ4plex); K12(iTRAQ4plex)	0,0000	1,101	2	11,5	0	1,785E-08	57	2,20785E-06	2	1489,88579	1,97	24,60	0
	High	nQYPTLFTEVR	2	1	1	P73133	N-Term(iTRAQ4plex)	0,0000	1,177	2	6,8	0	0,000005152	53	4,84139E-06	2	1511,79912	1,08	36,04	0
	High	gLAGGVPIGAmmck	3	1	1	P73133	N-Term(iTRAQ4plex); M11(Oxidation); M12(Oxidation); C	0,0000	0,982	2	24,6	0	6,643E-08	43	4,56005E-05	3	1681,86911	0,74	25,93	0
	High	aVSDQIQk	1	1	1	P73133	N-Term(iTRAQ4plex); K8(iTRAQ4plex)	0,0000	1,266	1		0	0,000009814	43	5,47896E-05	2	1176,68376	0,91	15,79	0
	High	lHHVSNLYYIPEQGELAk	3	1	1	P73133	N-Term(iTRAQ4plex); K18(iTRAQ4plex)	0,0000	1,245	3	26,3	0	8,333E-07	43	6,33787E-05	4	2399,29995	1,56	31,86	0
	High	fPIAIAR	6	1	1	P73133	N-Term(iTRAQ4plex)	0,0000	1,426	3	4,6	0	0,008732	30	0,00095273	2	931,58513	0,58	31,70	0
	High	wIVEHScADR	1	1	1	P73133	N-Term(iTRAQ4plex); C7(Carbamidomethyl)	0,0000	1,198	1		0	0,000002164	28	0,001592099	3	1416,68213	0,73	20,26	0
P74251	Sll1089 protein OS=Synechocystis sp. (strain PCC 6803 / Kazusa) GN=sl	385,17	15,49	1	3	3	11	1,040	10	17,3	213	23,5	4,97							
	A3	Sequence	# PSMs	# Proteins	# Protein Groups	Protein Group Accessions	Modifications	ΔCn	117/114	117/114 Count	117/114 Variability [%]	q-Value	PEP	IonScore	Exp Value	Charge	MH+ [Da]	ΔM [ppm]	RT [min]	# Missed Cleavages
	High	iYAMGVIQPLTSTR	4	1	1	P74251	N-Term(iTRAQ4plex)	0,0000	1,135	4	6,1	0	2,938E-11	77	2,22941E-08	2	1693,94487	1,22	41,71	0
	High	iYAmGVIQPLTSTR	3	1	1	P74251	N-Term(iTRAQ4plex); M4(Oxidation)	0,0000	0,880	3	15,9	0	8,095E-10	72	6,98184E-08	2	1709,93950	1,04	35,61	0
	High	aYLVGNk	2	1	1	P74251	N-Term(iTRAQ4plex); K7(iTRAQ4plex)	0,0000	0,924	2	10,1	0	0,0001874	44	3,55607E-05	2	1052,63420	-0,06	18,85	0
	High	fQRPPSGVPPIR	2	1	1	P74251	N-Term(iTRAQ4plex)	0,0000	1,169	1		0	0,0001501	26	0,002269708	3	1494,86615	-0,04	24,68	0
P27724	NAD(P)H-quinone oxidoreductase subunit H OS=Synechocystis sp. (stra	384,49	18,53	1	8	8	16	0,622	15	19,0	394	45,5	6,06							
	A3	Sequence	# PSMs	# Proteins	# Protein Groups	Protein Group Accessions	Modifications	ΔCn	117/114	117/114 Count	117/114 Variability [%]	q-Value	PEP	IonScore	Exp Value	Charge	MH+ [Da]	ΔM [ppm]	RT [min]	# Missed Cleavages
	High	iPAGEHYVR	3	1	1	P27724	N-Term(iTRAQ4plex)	0,0000	0,622	3	27,1	0	3,362E-07	54	3,7151E-06	3	1185,64991	0,17	17,31	0
	High	tNImYVPYVSR	2	1	1	P27724	N-Term(iTRAQ4plex); M4(Oxidation)	0,0000	0,543	2	10,9	0	0,000003943	50	9,11948E-06	2	1502,78069	0,86	31,03	0
	High	sADFNNLQILPHILk	2	1	1	P27724	N-Term(iTRAQ4plex); K15(iTRAQ4plex)	0,0000	0,707	1		0	6,256E-08	46	2,39315E-05	3	2011,16068	1,37	43,83	0
	High	lITNNPIFR	2	1	1	P27724	N-Term(iTRAQ4plex)	0,0000	0,518	2	1,3	0	0,000007021	44	4,21667E-05	2	1231,72893	0,78	36,10	0
	High	kSEWNDFQYQYIAk	1	1	1	P27724	N-Term(iTRAQ4plex); K1(iTRAQ4plex); K14(iTRAQ4plex)	0,0000	0,653	1		0	3,457E-08	42	6,2513E-05	3	2252,17142	0,09	34,10	1
	High	tNIMYVPYVSR	1	1	1	P27724	N-Term(iTRAQ4plex)	0,0000	0,546	1		0	0,000003328	38	0,000173368	2	1486,78581	0,89	36,16	0
	High	lINNNYFR	3	1	1	P27724	N-Term(iTRAQ4plex)	0,0000	0,645	3	3,1	0	0,000004009	35	0,000297146	2	1197,64983	0,11	32,06	0
	High	kVAPTFk	1	1	1	P27724	N-Term(iTRAQ4plex); K1(iTRAQ4plex); K7(iTRAQ4plex)	0,0000	1,049	1		0	0,002252	14	0,041301897	3	1222,78690	-1,21	16,23	1
	High	vAPTFk	1	1	1	P27724	N-Term(iTRAQ4plex); K6(iTRAQ4plex)	0,0000	0,632	1		0,005	0,05895	27	0,002187611	2	950,59203	0,72	19,39	0
P10549	Photosystem II manganese-stabilizing polypeptide OS=Synechocystis sp	382,80	27,01	1	9	9	23	1,001	19	26,7	274	29,9	4,94							
	A3	Sequence	# PSMs	# Proteins	# Protein Groups	Protein Group Accessions	Modifications	ΔCn	117/114	117/114 Count	117/114 Variability [%]	q-Value	PEP	IonScore	Exp Value	Charge	MH+ [Da]	ΔM [ppm]	RT [min]	# Missed Cleavages
	High	gLYTGYDNAVALPSAADkFR	4	1	1	P10549	N-Term(iTRAQ4plex); K18(iTRAQ4plex)	0,0000	1,182	2	6,6	0	1,403E-11	82	6,60648E-09	3	2417,27524	2,00	38,78	1
	High	qTTSLEQIR	2	1	1	P10549	N-Term(iTRAQ4plex)	0,0000	0,891	2	12,0	0	0,000001359	59	1,18024E-06	2	1219,67778	1,20	19,46	0
	High	gTIEVEPNTk	2	1	1	P10549	N-Term(iTRAQ4plex); K10(iTRAQ4plex)	0,0000	1,073	1		0	3,408E-08	58	1,73659E-06	2	1375,77031	2,29	17,81	0
	High	qkAEYVk	4	1	1	P10549	N-Term(iTRAQ4plex); K2(iTRAQ4plex); K7(iTRAQ4plex)	0,0000	1,483	4	7,2	0	0,0003495	34	0,000356427	2	1297,78618	1,67	10,60	1
	High	gLYTGYDNAVALPSAADk	1	1	1	P10549	N-Term(iTRAQ4plex); K18(iTRAQ4plex)	0,0000	1,791	1		0	5,799E-08	29	0,001161368	3	2114,10550	2,19	35,74	0
	High	kETPLGk	1	1	1	P10549	N-Term(iTRAQ4plex); K1(iTRAQ4plex); K7(iTRAQ4plex)	0,0000	1,045	1		0	0,002844	18	0,016254365	3	1204,76154	-0,85	12,33	1
	High	eEPVNkR	3	1	1	P10549	N-Term(iTRAQ4plex); K6(iTRAQ4plex)	0,0000	0,963	3	5,7	0	0,007188	13	0,055614662	3	1159,66989	2,17	8,15	1
	High	gIFYGR	3	1	1	P10549	N-Term(iTRAQ4plex)	0,0000	0,867	3	4,6	0,002	0,02661	26	0,00232793	2	856,48161	2,15	22,37	0
	High	gAGFLDPk	3	1	1	P10549	N-Term(iTRAQ4plex); K8(iTRAQ4plex)	0,0000	0,818	2	18,5	0,004	0,04854	30	0,00090985	2	1092,63262	3,12	23,27	0
P73264	3-amino-5-hydroxybenzoic acid synthase OS=Synechocystis sp. (strain F	382,65	24,60	1	7	7	18	0,877	17	24,2	378	41,5	5,64							
	A3	Sequence	# PSMs	# Proteins	# Protein Groups	Protein Group Accessions	Modifications	ΔCn	117/114	117/114 Count	117/114 Variability [%]	q-Value	PEP	IonScore	Exp Value	Charge	MH+ [Da]	ΔM [ppm]	RT [min]	# Missed Cleavages
	High	mTEIHALLGQIHLAk	5	1	1	P73264	N-Term(iTRAQ4plex); M1(Oxidation); K15(iTRAQ4plex)	0,0000	0,808	4	17,4	0	8,188E-13	59	1,22453E-06	3	1979,13553	0,22	38,66	0
	High	hIcPPLTSGTTPEEATR	2	1	1	P73264	N-Term(iTRAQ4plex); C3(Carbamidomethyl)	0,0000	0,881	2	26,2	0	1,109E-09	54	3,62218E-06	3	2011,00412	0,27	23,16	0
	High	sGTLILGNHTQEFER	2	1	1	P73264	N-Term(iTRAQ4plex)	0,0000	0,733	2	14,8	0	8,775E-10	51	8,55008E-06	3	1845,95871	0,60	27,84	0
	High	aYELITTPLR	2	1	1	P73264	N-Term(iTRAQ4plex)	0,0000	0,801	2	0,1	0	0,000008336	50	9,50539E-06	2	1320,76506	0,50	36,30	0
	High	mTEIHALLGQIHLAk	2	1	1	P73264	N-Term(iTRAQ4plex); K15(iTRAQ4plex)	0,0000	0,950	2	12,0	0	5,293E-09	45	3,07036E-05	4	1963,13967	-0,26	43,83	0
	High	rGmNFGGLHTDFGNSTR	2	1	1	P73264	N-Term(iTRAQ4plex); M3(Oxidation)	0,0000	1,042	2	10,4	0	0,0001508	33	0,000553312	4	2026,96389	0,21	23,14	1
	High	gLYVWEDTAHAHGSQLQGk	1	1	1	P73264	N-Term(iTRAQ4plex); K19(iTRAQ4plex)	0,0000	1,182	1		0	0,0005977	27	0,001972287	4	2385,21865	-0,16	29,71	0
	High	gmNFGGLHTDFGNSTR	2	1	1	P73264	N-Term(iTRAQ4plex); M2(Oxidation)	0,0000	1,041	2	19,2	0	0,000008485	24	0,004073521	3	1870,86216	-0,10	26,53	0
P73954	Membrane-associated protein slr1513 OS=Synechocystis sp. (strain PCC	381,57	48,18	1	7	7	18	0,348	13	91,0	110	12,0	7,08							
	A3	Sequence	# PSMs	# Proteins	# Protein Groups	Protein Group Accessions	Modifications	ΔCn	117/114	117/114 Count	117/114 Variability [%]	q-Value	PEP	IonScore	Exp Value	Charge	MH+ [Da]	ΔM [ppm]	RT [min]	# Missed Cleavages
	High	gYTVMNTGGk	2	1	1	P73954	N-Term(iTRAQ4plex); K10(iTRAQ4plex)	0,0000	0,348	1		0	4,671E-08	71	8,03471E-08	2	1315,68901	-2,18	18,79	0
	High	gYTVmNTGGk	7	1	1	P73954	N-Term(iTRAQ4plex); M5(Oxidation); K10(iTRAQ4plex)	0,0000	0,439	4	89,3	0	1,392E-08	66	2,38765E-07	2	1331,68535	-1,08	13,73	0
	High	emAEEIADR	1	1	1	P73954	N-Term(iTRAQ4plex); M2(Oxidation)	0,0000	0,361	1		0	0,00006072	49	1,3365E-05	2	1223,56853	-0,79	14,71	0
	High	fEILTETR	1	1	1	P73954	N-Term(iTRAQ4plex)	0,0000	0,588	1		0	0,00002791	46	2,38215E-05	2	1152,63750	-0,56	32,31	0
	High	iIDESGAk	1	1	1	P73954	N-Term(iTRAQ4plex); K8(iTRAQ4plex)	0,0000	0,704	1		0	0,00007373	44	4,03618E-05	2	1120,64324	-1,79	17,18	0
	High	lVIVTEk	2	1	1	P73954	N-Term(iTRAQ4plex); K7(iTRAQ4plex)	0,0000	0,359	1		0	0,0007105	29	0,001247297	2	1089,71001	-2,01	26,61	0
	High	akPANk	2	1	1	P73954	N-Term(iTRAQ4plex); K2(iTRAQ4plex); K6(iTRAQ4plex)	0,0000	0,209	2	11,9	0	0,007703	13	0,052598094	3	1060,68454	0,60	7,63	0
	High	iLLkk	2	1	1	P73954	N-Term(iTRAQ4plex); K4(iTRAQ4plex); K5(iTRAQ4plex)	0,0000	0,232	2	51,8	0,006	0,08379	13	0,048525498	3	1046,76294	-3,09	19,12	1
	>sp|K1C9_HUMAN|	379,47	13,16	1	7	7	12	1,440	12	20,3	623	62,1	5,30							
	A3	Sequence	# PSMs	# Proteins	# Protein Groups	Protein Group Accessions	Modifications	ΔCn	117/114	117/114 Count	117/114 Variability [%]	q-Value	PEP	IonScore	Exp Value	Charge	MH+ [Da]	ΔM [ppm]	RT [min]	# Missed Cleavages
	High	gGSGGSYGGGGSGGGYGGGSGS	3	1	1		N-Term(iTRAQ4plex)	0,0000	1,380	3	18,4	0	4,341E-17	98	1,69813E-10	2	1935,83037	0,26	11,67	0
	High	fSSSSGYGGGSSR	2	1	1		N-Term(iTRAQ4plex)	0,0000	1,618	2	10,0	0	2,934E-07	67	1,82377E-07	2	1379,63188	0,77	14,97	0
	High	hGVQELEIELQSQLSkk	1	1	1		N-Term(iTRAQ4plex); K16(iTRAQ4plex); K17(iTRAQ4plex	0,0000	1,643	1		0	4,034E-11	42	6,2513E-05	4	2398,36513	-0,63	39,34	1
	High	ikFEMEQNLR	1	1	1		N-Term(iTRAQ4plex); K2(iTRAQ4plex)	0,0000	1,503	1		0	8,221E-07	23	0,005345274	3	1595,88132	-0,32	29,63	1
	High	kGPAAIQk	1	1	1		N-Term(iTRAQ4plex); K1(iTRAQ4plex); K8(iTRAQ4plex)	0,0000	1,899	1		0,007	0,09419	21	0,008297935	3	1244,80158	-2,83	12,08	1
	Medium	kAALEk	2	1	1		N-Term(iTRAQ4plex); K1(iTRAQ4plex); K6(iTRAQ4plex)	0,0000	1,196	2	9,5	0,013	0,1595	16	0,027162517	3	1091,70920	-5,20	11,28	1
	Medium	iGLGGR	2	1	1		N-Term(iTRAQ4plex)	0,0000	1,134	2	15,7	0,026	0,3182	11	0,07851814	2	716,45336	-0,31	16,60	0
P74426	Sll0359 protein OS=Synechocystis sp. (strain PCC 6803 / Kazusa) GN=sl	377,72	30,32	1	6	6	16	1,054	11	18,7	155	17,2	9,11							
	A3	Sequence	# PSMs	# Proteins	# Protein Groups	Protein Group Accessions	Modifications	ΔCn	117/114	117/114 Count	117/114 Variability [%]	q-Value	PEP	IonScore	Exp Value	Charge	MH+ [Da]	ΔM [ppm]	RT [min]	# Missed Cleavages
	High	acGYYSTSk	5	1	1	P74426	N-Term(iTRAQ4plex); C2(Carbamidomethyl); K9(iTRAQ4p	0,0000	1,054	3	13,3	0	4,562E-07	66	2,76039E-07	2	1324,64678	1,67	14,65	0
	High	qITDSDDEEEV	1	1	1	P74426	N-Term(iTRAQ4plex)	0,0000	1,053	1		0	0,00002405	60	1,03031E-06	2	1423,62126	1,36	22,51	0
	High	gVPLDPSGTk	2	1	1	P74426	N-Term(iTRAQ4plex); K10(iTRAQ4plex)	0,0000	1,116	1		0	0,000007641	57	2,16755E-06	2	1258,72478	0,17	20,68	0
	High	sGDEFEIk	2	1	1	P74426	N-Term(iTRAQ4plex); K8(iTRAQ4plex)	0,0000	1,215	1		0	0,000002108	52	6,7293E-06	2	1212,63591	0,69	22,98	0
	High	eLSHLPR	4	1	1	P74426	N-Term(iTRAQ4plex)	0,0000	0,835	3	16,0	0	0,008891	40	0,000106162	2	995,57585	0,38	15,87	0
	High	vkELSHLPR	2	1	1	P74426	N-Term(iTRAQ4plex); K2(iTRAQ4plex)	0,0000	1,241	2	18,8	0	0,00001824	37	0,000210955	3	1366,84229	1,00	19,08	1
P72851	50S ribosomal protein L28 OS=Synechocystis sp. (strain PCC 6803 / Kaz	375,45	61,54	1	7	7	17	1,315	16	16,0	78	9,0	11,81							
	A3	Sequence	# PSMs	# Proteins	# Protein Groups	Protein Group Accessions	Modifications	ΔCn	117/114	117/114 Count	117/114 Variability [%]	q-Value	PEP	IonScore	Exp Value	Charge	MH+ [Da]	ΔM [ppm]	RT [min]	# Missed Cleavages
	High	lSTTAIk	3	1	1	P72851	N-Term(iTRAQ4plex); K7(iTRAQ4plex)	0,0000	1,403	3	9,9	0	0,0001023	63	5,48239E-07	2	1021,64769	-1,86	20,71	0
	High	lQQANLQWk	4	1	1	P72851	N-Term(iTRAQ4plex); K9(iTRAQ4plex)	0,0000	1,253	4	56,9	0	0,00001706	52	6,65227E-06	2	1416,81719	-2,12	29,52	0
	High	kLQQANLQWk	2	1	1	P72851	N-Term(iTRAQ4plex); K1(iTRAQ4plex); K10(iTRAQ4plex)	0,0000	1,793	2	67,6	0	4,034E-07	42	7,01407E-05	3	1689,01627	-0,56	24,48	1
	High	eAGINLNkF	2	1	1	P72851	N-Term(iTRAQ4plex); K8(iTRAQ4plex)	0,0000	1,690	2	33,5	0	0,0008587	41	9,27234E-05	2	1293,73857	-1,53	32,17	1
	High	cQLTGk	1	1	1	P72851	N-Term(iTRAQ4plex); C1(Carbamidomethyl); K6(iTRAQ4p	0,0000	0,877	1		0	0,006632	33	0,000494277	2	994,55742	-1,98	12,93	0
	High	rVWWPEGNR	2	1	1	P72851	N-Term(iTRAQ4plex)	0,0000	1,417	2	26,1	0	0,0005957	26	0,00277332	3	1343,70746	-1,13	25,85	1
	High	gINAmAk	3	1	1	P72851	N-Term(iTRAQ4plex); M5(Oxidation); K7(iTRAQ4plex)	0,0000	1,311	2	2,0	0	0,00987	15	0,03467129	2	1008,57421	-0,85	11,33	0
P48957	50S ribosomal protein L20 OS=Synechocystis sp. (strain PCC 6803 / Kaz	373,94	57,26	1	11	11	30	1,831	27	39,9	117	13,5	12,02							
	A3	Sequence	# PSMs	# Proteins	# Protein Groups	Protein Group Accessions	Modifications	ΔCn	117/114	117/114 Count	117/114 Variability [%]	q-Value	PEP	IonScore	Exp Value	Charge	MH+ [Da]	ΔM [ppm]	RT [min]	# Missed Cleavages
	High	kmLAQLAVLDPAAFSEVVk	3	1	1	P48957	N-Term(iTRAQ4plex); K1(iTRAQ4plex); M2(Oxidation); K	0,0000	1,659	3	12,6	0	3,202E-10	79	1,36763E-08	3	2478,43521	-0,59	45,73	1
	High	tANQQVMk	4	1	1	P48957	N-Term(iTRAQ4plex); K8(iTRAQ4plex)	0,0000	1,866	2	2,8	0	0,00002226	46	2,79235E-05	2	1207,66826	-2,06	12,06	0
	High	kANIEINR	3	1	1	P48957	N-Term(iTRAQ4plex); K1(iTRAQ4plex)	0,0000	2,373	3	17,7	0	0,00183	37	0,000202288	3	1245,74906	-2,18	14,11	1
	High	tANQQVmk	5	1	1	P48957	N-Term(iTRAQ4plex); M7(Oxidation); K8(iTRAQ4plex)	0,0000	1,752	4	52,9	0	0,00001956	37	0,00023426	2	1223,66411	-1,27	9,39	0
	High	kANIEINRk	2	1	1	P48957	N-Term(iTRAQ4plex); K1(iTRAQ4plex); K9(iTRAQ4plex)	0,0000	2,685	2	52,1	0	0,001521	12	0,064116529	3	1517,94568	-2,06	12,75	2
	High	vAATAk	3	1	1	P48957	N-Term(iTRAQ4plex); K6(iTRAQ4plex)	0,0000	1,444	3	13,4	0,006	0,07829	27	0,002177559	2	848,54155	-3,35	10,40	0
	High	lWITR	3	1	1	P48957	N-Term(iTRAQ4plex)	0,0000	2,394	2	0,8	0,008	0,105	19	0,011271196	2	832,51573	-0,52	28,20	0
	Medium	rLWITR	2	1	1	P48957	N-Term(iTRAQ4plex)	0,0000	1,467	2	71,7	0,014	0,1749	21	0,008127744	3	988,61652	-0,77	21,41	1
	Medium	iNAAAR	3	1	1	P48957	N-Term(iTRAQ4plex)	0,0000	1,522	3	15,6	0,021	0,2616	21	0,008830189	2	759,45848	-1,19	11,83	0
	Medium	qEGmSYSk	1	1	1	P48957	N-Term(iTRAQ4plex); M4(Oxidation); K8(iTRAQ4plex)	0,0000	1,968	1		0,029	0,3553	14	0,040361752	2	1233,60002	-1,91	9,63	0
	Medium	lTGQLkk	1	1	1	P48957	N-Term(iTRAQ4plex); K6(iTRAQ4plex); K7(iTRAQ4plex)	0,0000	7,972	1		0,037	0,4584	14	0,044052444	3	1219,80640	-2,82	15,98	1
	Medium	aNIEINRk	1	1	1	P48957	N-Term(iTRAQ4plex); K8(iTRAQ4plex)	0,0000	2,727	1		0,046	0,5629	18	0,015487096	3	1245,74952	-1,82	14,63	1
P74450	Slr0151 protein OS=Synechocystis sp. (strain PCC 6803 / Kazusa) GN=s	371,12	12,81	1	4	4	13	2,366	10	45,2	320	34,9	5,07							
	A3	Sequence	# PSMs	# Proteins	# Protein Groups	Protein Group Accessions	Modifications	ΔCn	117/114	117/114 Count	117/114 Variability [%]	q-Value	PEP	IonScore	Exp Value	Charge	MH+ [Da]	ΔM [ppm]	RT [min]	# Missed Cleavages
	High	hQDLLAVVTELR	6	1	1	P74450	N-Term(iTRAQ4plex)	0,0000	2,314	5	37,2	0	4,843E-08	77	2,53921E-08	3	1537,88266	0,49	41,82	0
	High	kQLEEHLLANQR	4	1	1	P74450	N-Term(iTRAQ4plex); K1(iTRAQ4plex)	0,0000	3,166	2	0,2	0	1,061E-09	50	9,16157E-06	3	1767,01224	0,37	19,84	1
	High	gDLAGAFHHR	1	1	1	P74450	N-Term(iTRAQ4plex)	0,0000	1,145	1		0	0,00001545	42	6,05299E-05	3	1224,63590	0,35	15,55	0
	High	sLALQGk	2	1	1	P74450	N-Term(iTRAQ4plex); K7(iTRAQ4plex)	0,0000	1,711	2	55,0	0	0,0005713	34	0,000427533	2	1004,63378	-0,50	17,60	0
P74135	Sll1873 protein OS=Synechocystis sp. (strain PCC 6803 / Kazusa) GN=sl	367,31	57,85	1	7	7	14	0,826	12	35,9	121	12,9	9,99							
	A3	Sequence	# PSMs	# Proteins	# Protein Groups	Protein Group Accessions	Modifications	ΔCn	117/114	117/114 Count	117/114 Variability [%]	q-Value	PEP	IonScore	Exp Value	Charge	MH+ [Da]	ΔM [ppm]	RT [min]	# Missed Cleavages
	High	vDPSQVAFASGDPIPQNVAR	1	1	1	P74135	N-Term(iTRAQ4plex)	0,0000	0,513	1		0	1,927E-11	62	6,48634E-07	3	2212,15226	1,94	37,31	0
	High	akVDPSQVAFASGDPIPQNVAR	2	1	1	P74135	N-Term(iTRAQ4plex); K2(iTRAQ4plex)	0,0000	1,685	2	0,3	0	8,049E-09	61	7,36156E-07	3	2555,39005	3,10	33,78	1
	High	aAAPVETPASAPVAPAPk	1	1	1	P74135	N-Term(iTRAQ4plex); K18(iTRAQ4plex)	0,0000	0,831	1		0	1,097E-07	54	4,44631E-06	3	1933,10392	2,14	24,92	0
	High	rTPGPSLNR	3	1	1	P74135	N-Term(iTRAQ4plex)	0,0000	0,821	3	6,3	0	0,0002432	44	3,68104E-05	3	1141,65845	2,27	11,09	1
	High	sEVAPVEkPAPTTSk	4	1	1	P74135	N-Term(iTRAQ4plex); K8(iTRAQ4plex); K15(iTRAQ4plex)	0,0000	0,653	2	0,0	0	1,885E-07	44	4,21667E-05	3	1973,13645	4,29	17,91	0
	Medium	kLFGAk	1	1	1	P74135	N-Term(iTRAQ4plex); K1(iTRAQ4plex); K6(iTRAQ4plex)	0,0000	0,808	1		0,013	0,1653	14	0,03827983	3	1095,73166	6,03	16,20	1
	Medium	lFGAk	2	1	1	P74135	N-Term(iTRAQ4plex); K5(iTRAQ4plex)	0,0000	1,056	2	3,8	0,026	0,3263	18	0,019732461	2	823,52941	1,69	21,51	0
Q55141	Acetolactate synthase small subunit OS=Synechocystis sp. (strain PCC 6	366,84	48,26	1	8	8	16	1,215	15	28,5	172	18,9	5,43							
	A3	Sequence	# PSMs	# Proteins	# Protein Groups	Protein Group Accessions	Modifications	ΔCn	117/114	117/114 Count	117/114 Variability [%]	q-Value	PEP	IonScore	Exp Value	Charge	MH+ [Da]	ΔM [ppm]	RT [min]	# Missed Cleavages
	High	rGFNIESLAVGSAEQGDVSR	1	1	1	Q55141	N-Term(iTRAQ4plex)	0,0000	1,519	1		0	1,248E-12	88	1,48583E-09	3	2236,14383	-0,05	35,82	1
	High	hTLSVLVEDEAGVLTR	1	1	1	Q55141	N-Term(iTRAQ4plex)	0,0000	1,085	1		0	5,173E-08	81	8,41337E-09	3	1883,03666	0,60	40,72	0
	High	mVAILQmLAk	2	1	1	Q55141	N-Term(iTRAQ4plex); M7(Oxidation); K10(iTRAQ4plex)	0,0000	1,259	2	34,2	0	0,000009272	47	2,06049E-05	3	1421,84681	0,35	45,98	0
	High	mVAILQMLAk	1	1	1	Q55141	N-Term(iTRAQ4plex); K10(iTRAQ4plex)	0,0000	1,253	1		0	0,000002993	44	3,73224E-05	2	1405,84905	-1,66	51,40	0
	High	aEVIELAQVFR	2	1	1	Q55141	N-Term(iTRAQ4plex)	0,0000	1,023	2	5,1	0	0,00004373	41	8,29793E-05	2	1418,81279	0,24	45,64	0
	High	iAGLFAR	3	1	1	Q55141	N-Term(iTRAQ4plex)	0,0000	1,834	2	13,1	0	0,0009613	40	0,000108385	2	891,55327	-0,03	32,08	0
	High	mVAILQmLAk	1	1	1	Q55141	N-Term(iTRAQ4plex); M1(Oxidation); M7(Oxidation); K1(	0,0000	0,553	1		0	0,000007995	34	0,000414925	3	1437,84088	-0,23	35,98	0
	High	vSANAPNR	2	1	1	Q55141	N-Term(iTRAQ4plex)	0,0000	1,350	2	0,6	0,001	0,01158	20	0,009439957	2	972,53392	-0,43	9,93	0
	High	lVNVIk	1	1	1	Q55141	N-Term(iTRAQ4plex); K6(iTRAQ4plex)	0,0000	1,215	1		0,007	0,08749	29	0,001158697	2	973,66332	-1,57	26,37	0
	Medium	iALVR	2	1	1	Q55141	N-Term(iTRAQ4plex)	0,0000	0,993	2	26,2	0,01	0,1297	16	0,025643069	2	715,49419	-0,72	21,27	0
P24602	Bacterioferritin OS=Synechocystis sp. (strain PCC 6803 / Kazusa) GN=bf	359,66	55,77	1	8	8	17	0,902	15	15,4	156	18,3	4,82							
	A3	Sequence	# PSMs	# Proteins	# Protein Groups	Protein Group Accessions	Modifications	ΔCn	117/114	117/114 Count	117/114 Variability [%]	q-Value	PEP	IonScore	Exp Value	Charge	MH+ [Da]	ΔM [ppm]	RT [min]	# Missed Cleavages
	High	iGLQNYLQSQmS	1	1	1	P24602	N-Term(iTRAQ4plex); M11(Oxidation)	0,0000	0,774	1		0	4,767E-07	64	3,63053E-07	2	1541,77568	0,41	37,80	0
	High	iLFLEETPDLSQQDPIR	1	1	1	P24602	N-Term(iTRAQ4plex)	0,0000	0,641	1		0	8,484E-07	49	1,14017E-05	3	2158,15354	1,03	48,53	0
	High	iLADTEEDHAYWLEk	1	1	1	P24602	N-Term(iTRAQ4plex); K15(iTRAQ4plex)	0,0000	0,565	1		0	2,559E-10	43	4,85255E-05	3	2121,07486	0,26	36,83	0
	High	eAmAVcEQEQDYQSR	1	1	1	P24602	N-Term(iTRAQ4plex); M3(Oxidation); C6(Carbamidometh	0,0000	0,952	1		0	0,00001227	38	0,000150304	3	2003,85752	0,96	19,58	0
	High	dQYFIHSR	6	1	1	P24602	N-Term(iTRAQ4plex)	0,0000	0,940	5	13,2	0	0,00002548	38	0,00015775	2	1209,61333	0,02	18,07	0
	High	gkPAVLAQLHk	4	1	1	P24602	N-Term(iTRAQ4plex); K2(iTRAQ4plex); K11(iTRAQ4plex)	0,0000	1,118	3	26,5	0	5,336E-08	37	0,000179461	3	1594,01456	-1,22	23,35	0
	High	mkGkPAVLAQLHk	1	1	1	P24602	N-Term(iTRAQ4plex); M1(Oxidation); K2(iTRAQ4plex); K	0,0000	0,813	1		0	0,003533	15	0,034116935	5	2013,24171	-3,60	20,77	1

	High	qLGLIEk	2	1	1	P24602	N-Term(iTRAQ4plex); K7(iTRAQ4plex)	0,0000	0,843	2	2,1	0,005	0,06032	31	0,000935284	2	1088,68938	-2,22	26,53	0
P09193	Photosystem II 44 kDa reaction center protein OS=Synechocystis sp. (st	358,91	10,87	1	5	5	11	0,477	8	44,1	460	50,3	6,60							
	A3	Sequence	# PSMs	# Proteins	# Protein Groups	Protein Group Accessions	Modifications	ΔCn	117/114	117/114 Count	117/114 Variability [%]	q-Value	PEP	IonScore	Exp Value	Charge	MH+ [Da]	ΔM [ppm]	RT [min]	# Missed Cleavages
	High	IGANIASAQGPTGLGk	6	1	1	P09193	N-Term(iTRAQ4plex); K16(iTRAQ4plex)	0,0000	0,552	4	62,8	0	7,584E-14	101	8,51079E-11	2	1743,00347	1,77	29,70	0
	High	lINLSGk	1	1	1	P09193	N-Term(iTRAQ4plex); K7(iTRAQ4plex)	0,0000	0,387	1		0	0,004532	42	0,000113285	2	1032,66802	2,36	27,15	0
	High	gPWLEPLR	1	1	1	P09193	N-Term(iTRAQ4plex)	0,0000	0,381	1		0	0,00000841	42	6,77595E-05	2	1111,63933	1,11	34,05	0
	High	aAAAGFEk	2	1	1	P09193	N-Term(iTRAQ4plex); K8(iTRAQ4plex)	0,0000	0,549	1		0	0,00004999	40	9,86211E-05	2	1052,59673	-1,12	15,78	0
	High	lRNDIQPWQVR	1	1	1	P09193	N-Term(iTRAQ4plex)	0,0000	1,201	1		0	0,00009026	29	0,001164046	3	1568,87876	0,59	27,88	1
P32422	Photosystem I iron-sulfur center OS=Synechocystis sp. (strain PCC 6803	358,32	65,43	1	5	5	11	0,592	11	8,4	81	8,8	5,95							
	A3	Sequence	# PSMs	# Proteins	# Protein Groups	Protein Group Accessions	Modifications	ΔCn	117/114	117/114 Count	117/114 Variability [%]	q-Value	PEP	IonScore	Exp Value	Charge	MH+ [Da]	ΔM [ppm]	RT [min]	# Missed Cleavages
	High	iYDTcIGcTQcVR	1	1	1	P32422	N-Term(iTRAQ4plex); C5(Carbamidomethyl); C8(Carbam	0,0000	0,609	1		0	2,285E-08	86	2,72879E-09	2	1789,81633	0,62	27,18	0
	High	rcETAcPTDFLSIR	2	1	1	P32422	N-Term(iTRAQ4plex); C2(Carbamidomethyl); C6(Carbam	0,0000	0,689	2	13,7	0	2,298E-10	59	1,27929E-06	3	1869,90836	0,81	32,38	1
	High	vYLGAETTR	4	1	1	P32422	N-Term(iTRAQ4plex)	0,0000	0,584	4	3,8	0	0,00007535	49	1,13755E-05	2	1153,63432	0,82	21,29	0
	High	aAQIASSPR	3	1	1	P32422	N-Term(iTRAQ4plex)	0,0000	0,584	3	7,2	0	0,003233	47	1,91412E-05	2	1044,59355	1,62	12,08	0
	High	tEDcVGck	1	1	1	P32422	N-Term(iTRAQ4plex); C4(Carbamidomethyl); C7(Carbam	0,0000	0,559	1		0,001	0,01625	33	0,000518764	2	1256,58904	2,92	11,66	0
P72866	30S ribosomal protein S15 OS=Synechocystis sp. (strain PCC 6803 / Kaz	355,63	33,71	1	5	5	16	2,085	13	29,0	89	10,4	10,90							
	A3	Sequence	# PSMs	# Proteins	# Protein Groups	Protein Group Accessions	Modifications	ΔCn	117/114	117/114 Count	117/114 Variability [%]	q-Value	PEP	IonScore	Exp Value	Charge	MH+ [Da]	ΔM [ppm]	RT [min]	# Missed Cleavages
	High	iTQLTGHLk	4	1	1	P72866	N-Term(iTRAQ4plex); K9(iTRAQ4plex)	0,0000	1,600	3	29,4	0	4,085E-07	60	1,11679E-06	2	1298,80217	-1,00	24,38	0
	High	lLSFINAR	6	1	1	P72866	N-Term(iTRAQ4plex)	0,0000	2,454	5	7,3	0	0,00001069	55	3,18559E-06	2	1077,65361	-0,11	40,33	0
	High	rLLSFINAR	2	1	1	P72866	N-Term(iTRAQ4plex)	0,0000	2,555	1		0	0,000007348	39	0,000133343	3	1233,75501	0,12	32,20	1
	High	yQALIk	3	1	1	P72866	N-Term(iTRAQ4plex); K6(iTRAQ4plex)	0,0000	1,868	3	19,5	0	0,003801	32	0,000623692	2	1023,64311	-0,96	24,41	0
	Medium	sLTQIR	1	1	1	P72866	N-Term(iTRAQ4plex)	0,0000	1,660	1		0,033	0,4128	16	0,023766761	2	861,52678	-0,78	17,71	0
P27183	ATP synthase subunit b' OS=Synechocystis sp. (strain PCC 6803 / Kazus	353,51	39,16	1	5	5	11	1,674	8	19,1	143	16,2	5,22							
	A3	Sequence	# PSMs	# Proteins	# Protein Groups	Protein Group Accessions	Modifications	ΔCn	117/114	117/114 Count	117/114 Variability [%]	q-Value	PEP	IonScore	Exp Value	Charge	MH+ [Da]	ΔM [ppm]	RT [min]	# Missed Cleavages
	High	aITQEYEQQITDAR	1	1	1	P27183	N-Term(iTRAQ4plex)	0,0000	1,606	1		0	9,267E-11	73	5,04626E-08	2	1809,91191	1,07	30,15	0
	High	rQSQAVIADAQAEAR	3	1	1	P27183	N-Term(iTRAQ4plex)	0,0000	1,222	2	38,5	0	1,414E-08	67	1,92739E-07	3	1757,93882	0,72	19,14	1
	High	qkETAAQEIEAQR	4	1	1	P27183	N-Term(iTRAQ4plex); K2(iTRAQ4plex)	0,0000	1,991	2	2,7	0	5,676E-09	62	7,65064E-07	3	1789,96689	1,22	17,75	1
	High	lLGPELIk	2	1	1	P27183	N-Term(iTRAQ4plex); K8(iTRAQ4plex)	0,0000	1,798	2	4,4	0	0,000003215	56	2,23857E-06	2	1170,77068	0,53	35,02	0
	High	rLAAEk	1	1	1	P27183	N-Term(iTRAQ4plex); K6(iTRAQ4plex)	0,0000	1,227	1		0,001	0,01995	13	0,04591663	3	975,61832	-0,66	9,49	1
P72760	Carbon dioxide-concentrating mechanism protein CcmK homolog 1 OS=	351,71	52,25	1	3	5	18	1,800	4	1,8	111	12,1	6,55							
	A3	Sequence	# PSMs	# Proteins	# Protein Groups	Protein Group Accessions	Modifications	ΔCn	117/114	117/114 Count	117/114 Variability [%]	q-Value	PEP	IonScore	Exp Value	Charge	MH+ [Da]	ΔM [ppm]	RT [min]	# Missed Cleavages
	High	vTLVGYEk	6	2	2	P72760;P72761	N-Term(iTRAQ4plex); K8(iTRAQ4plex)	0,0000				0	0,0003198	49	1,13493E-05	2	1196,71233	-0,49	26,17	0
	High	yTEAVEQFR	1	1	1	P72760	N-Term(iTRAQ4plex)	0,0000	1,809	1		0	0,000009106	48	1,42223E-05	2	1286,64995	0,14	28,94	0
	High	eIVNPSIIR	4	1	1	P72760	N-Term(iTRAQ4plex)	0,0000	1,778	2	1,1	0	0,000247	40	0,000103507	2	1184,71172	-0,23	29,04	0
	High	vNGGEVLSNHIIARPHENLEYVLP	1	1	1	P72760	N-Term(iTRAQ4plex)	0,0000	2,849	1		0	4,14E-10	34	0,00042655	4	3083,68960	0,78	39,25	0
	High	vTVIVR	6	2	2	P72760;P72761	N-Term(iTRAQ4plex)	0,0000				0,002	0,03444	24	0,003872309	2	830,55742	-0,74	20,16	0
P73335	Uncharacterized deoxyribonuclease sll1786 OS=Synechocystis sp. (strai	349,24	24,90	1	5	5	12	0,925	9	16,7	261	29,2	5,87							
	A3	Sequence	# PSMs	# Proteins	# Protein Groups	Protein Group Accessions	Modifications	ΔCn	117/114	117/114 Count	117/114 Variability [%]	q-Value	PEP	IonScore	Exp Value	Charge	MH+ [Da]	ΔM [ppm]	RT [min]	# Missed Cleavages
	High	hVPLETLAQQTTTNAR	2	1	1	P73335	N-Term(iTRAQ4plex)	0,0000	0,826	2	19,8	0	7,482E-11	70	1,07887E-07	3	1924,03873	0,94	28,00	0
	High	hVAEAIAALR	5	1	1	P73335	N-Term(iTRAQ4plex)	0,0000	0,823	4	15,5	0	0,00002616	54	3,82798E-06	2	1194,70732	-0,21	25,29	0
	High	lLVETDcPFLAPVPQR	1	1	1	P73335	N-Term(iTRAQ4plex); C7(Carbamidomethyl)	0,0000				0	0,00003451	51	7,39554E-06	2	1999,08537	2,49	44,55	0
	High	eLNkPVIIHcR	3	1	1	P73335	N-Term(iTRAQ4plex); K4(iTRAQ4plex); C10(Carbamidom	0,0000	1,190	2	21,1	0	7,821E-08	44	4,27533E-05	4	1666,96670	0,09	20,90	0
	High	aDNRDHQIEVFR	1	1	1	P73335	N-Term(iTRAQ4plex)	0,0000	0,945	1		0	0,0001842	17	0,022027743	4	1643,83620	-0,54	18,99	1
P73311	30S ribosomal protein S17 OS=Synechocystis sp. (strain PCC 6803 / Kaz	345,67	66,67	1	5	5	19	1,597	13	28,4	81	9,3	9,94							
	A3	Sequence	# PSMs	# Proteins	# Protein Groups	Protein Group Accessions	Modifications	ΔCn	117/114	117/114 Count	117/114 Variability [%]	q-Value	PEP	IonScore	Exp Value	Charge	MH+ [Da]	ΔM [ppm]	RT [min]	# Missed Cleavages
	High	tVVVAVESR	2	1	1	P73311	N-Term(iTRAQ4plex)	0,0000	1,516	1		0	0,0002233	58	1,52395E-06	2	1103,65300	-1,03	20,79	0
	High	vGIVVSNk	6	1	1	P73311	N-Term(iTRAQ4plex); K8(iTRAQ4plex)	0,0000	1,845	4	23,7	0	0,00004654	50	1,08385E-05	2	1103,70012	-2,33	21,15	0
	High	wQVINIMSHSS	2	1	1	P73311	N-Term(iTRAQ4plex)	0,0000	1,345	2	10,7	0	0,000002325	47	2,0369E-05	2	1445,73174	-0,73	40,92	0
	High	iQETRPLSk	7	1	1	P73311	N-Term(iTRAQ4plex); K9(iTRAQ4plex)	0,0000	1,837	4	25,3	0	0,00002144	47	2,33986E-05	2	1359,81731	-1,87	16,71	0
	High	wQVINImSHSS	1	1	1	P73311	N-Term(iTRAQ4plex); M7(Oxidation)	0,0000	1,533	1		0	0,000006135	33	0,00052236	2	1461,72734	-0,25	33,61	0
	High	fkAHDEENQcQEGDkVR	1	1	1	P73311	N-Term(iTRAQ4plex); K2(iTRAQ4plex); C10(Carbamidom	0,0000	2,348	1		0	1,098E-08	33	0,000557147	4	2522,23623	-2,24	12,92	2
P37277	Photosystem I reaction center subunit XI OS=Synechocystis sp. (strain I	343,57	11,46	1	3	3	17	0,709	13	14,7	157	16,6	4,83							
	A3	Sequence	# PSMs	# Proteins	# Protein Groups	Protein Group Accessions	Modifications	ΔCn	117/114	117/114 Count	117/114 Variability [%]	q-Value	PEP	IonScore	Exp Value	Charge	MH+ [Da]	ΔM [ppm]	RT [min]	# Missed Cleavages
	High	tFIGNLPAYR	8	1	1	P37277	N-Term(iTRAQ4plex)	0,0000	0,645	7	15,3	0	0,00003335	51	7,83376E-06	2	1295,72527	1,85	35,01	0
	High	gLSPILR	4	1	1	P37277	N-Term(iTRAQ4plex)	0,0000	0,770	3	1,1	0	0,001784	46	2,74138E-05	2	899,58049	1,10	26,91	0
	High	kGLSPILR	5	1	1	P37277	N-Term(iTRAQ4plex); K1(iTRAQ4plex)	0,0000	0,862	3	27,5	0	0,00191	28	0,001617968	3	1171,77848	1,65	21,05	1
P54123	Ribonuclease J OS=Synechocystis sp. (strain PCC 6803 / Kazusa) GN=sl	343,48	12,34	1	9	9	18	1,432	14	14,7	640	70,4	7,74							
	A3	Sequence	# PSMs	# Proteins	# Protein Groups	Protein Group Accessions	Modifications	ΔCn	117/114	117/114 Count	117/114 Variability [%]	q-Value	PEP	IonScore	Exp Value	Charge	MH+ [Da]	ΔM [ppm]	RT [min]	# Missed Cleavages
	High	lLEELIVR	1	1	1	P54123	N-Term(iTRAQ4plex)	0,0000	1,474	1		0	0,00000861	57	1,87055E-06	2	1128,71135	0,38	43,71	0
	High	tNLQTVSPR	3	1	1	P54123	N-Term(iTRAQ4plex)	0,0000	1,361	2	21,8	0	0,00005964	48	1,58478E-05	2	1159,65544	0,22	16,22	0
	High	iLPLGGLHEIGk	2	1	1	P54123	N-Term(iTRAQ4plex); K12(iTRAQ4plex)	0,0000	1,048	2	28,9	0	9,968E-07	48	1,64048E-05	3	1534,95627	0,19	36,53	0
	High	mLLALTRPk	1	1	1	P54123	N-Term(iTRAQ4plex); M1(Oxidation); K9(iTRAQ4plex)	0,0000	1,036	1		0	0,00005519	40	9,95337E-05	3	1346,84333	0,06	24,35	0
	High	sFVVEFIR	2	1	1	P54123	N-Term(iTRAQ4plex)	0,0000	1,453	2	1,5	0	0,001352	34	0,000435482	2	1140,65361	0,18	40,95	0
	High	sMLNVIAHAR	4	1	1	P54123	N-Term(iTRAQ4plex)	0,0000	1,681	2	24,8	0	0,00000406	31	0,000818408	3	1255,70615	-0,04	28,30	0
	High	kLGYIk	1	1	1	P54123	N-Term(iTRAQ4plex); K1(iTRAQ4plex); K6(iTRAQ4plex)	0,0000	1,797	1		0	0,000322	31	0,000855008	3	1153,76374	-2,74	17,61	1
	High	fFVPVHGEHR	1	1	1	P54123	N-Term(iTRAQ4plex)	0,0000	1,317	1		0	0,00339	28	0,001774067	4	1368,72927	-0,07	21,71	0
	High	smLNVIAHAR	2	1	1	P54123	N-Term(iTRAQ4plex); M2(Oxidation)	0,0000	1,292	1		0	0,000002293	22	0,006886047	3	1271,70124	0,10	18,32	0
	Medium	lAMALLR	1	1	1	P54123	N-Term(iTRAQ4plex)	0,0000	1,571	1		0,015	0,1862	23	0,004863736	2	931,58763	-0,37	36,22	0
P73463	Slr1220 protein OS=Synechocystis sp. (strain PCC 6803 / Kazusa) GN=s	339,92	16,40	1	5	5	13	1,273	10	23,7	317	36,0	4,32							
	A3	Sequence	# PSMs	# Proteins	# Protein Groups	Protein Group Accessions	Modifications	ΔCn	117/114	117/114 Count	117/114 Variability [%]	q-Value	PEP	IonScore	Exp Value	Charge	MH+ [Da]	ΔM [ppm]	RT [min]	# Missed Cleavages
	High	lRNEFINTEVITR	6	1	1	P73463	N-Term(iTRAQ4plex)	0,0000	1,653	3	0,7	0	2,264E-07	59	1,23302E-06	3	1748,97855	0,53	32,07	1
	High	lNQLSVGLLER	3	1	1	P73463	N-Term(iTRAQ4plex)	0,0000	1,108	3	5,7	0	0,00003581	56	2,91864E-06	2	1385,82427	0,67	39,87	0
	High	vRDFQFDLATGk	2	1	1	P73463	N-Term(iTRAQ4plex); K12(iTRAQ4plex)	0,0000	1,417	2	27,2	0	3,962E-10	53	5,52318E-06	3	1684,92643	0,17	32,90	1
	High	kLGVVk	1	1	1	P73463	N-Term(iTRAQ4plex); K1(iTRAQ4plex); K6(iTRAQ4plex)	0,0000	1,251	1		0,002	0,02388	28	0,00172175	3	1075,75275	-3,35	14,04	1
	High	lGIGRPSWER	1	1	1	P73463	N-Term(iTRAQ4plex)	0,0000	1,660	1		0,002	0,02377	12	0,063969065	3	1314,73999	0,04	25,92	0
P77973	Argininosuccinate synthase OS=Synechocystis sp. (strain PCC 6803 / Ka	334,86	21,25	1	9	9	16	1,104	13	21,7	400	44,5	5,14							
	A3	Sequence	# PSMs	# Proteins	# Protein Groups	Protein Group Accessions	Modifications	ΔCn	117/114	117/114 Count	117/114 Variability [%]	q-Value	PEP	IonScore	Exp Value	Charge	MH+ [Da]	ΔM [ppm]	RT [min]	# Missed Cleavages
	High	sIQANALYENR	1	1	1	P77973	N-Term(iTRAQ4plex)	0,0000	0,955	1		0	0,000003264	65	2,81819E-07	2	1422,74724	1,02	24,08	0
	High	gLWYSPLk	4	1	1	P77973	N-Term(iTRAQ4plex); K8(iTRAQ4plex)	0,0000	1,109	3	3,9	0	0,00001564	50	9,01509E-06	2	1251,73516	0,93	36,48	0
	High	lNEIAGNHGVGR	2	1	1	P77973	N-Term(iTRAQ4plex)	0,0000	1,083	1		0	6,701E-08	50	1,11422E-05	3	1380,74759	0,80	19,35	0
	High	eIYEAPALLVLIDAHR	1	1	1	P77973	N-Term(iTRAQ4plex)	0,0000	2,078	1		0	0,000001294	47	1,9542E-05	3	1967,11191	1,83	49,94	0
	High	sREIYEAPALLVLIDAHR	1	1	1	P77973	N-Term(iTRAQ4plex)	0,0000	2,355	1		0	0,00001252	43	4,80806E-05	4	2210,24135	-0,05	45,44	1
	High	yGADAVAHGcTGk	2	1	1	P77973	N-Term(iTRAQ4plex); C10(Carbamidomethyl); K13(iTRAC	0,0000	1,104	1		0	7,817E-08	37	0,000180289	3	1594,78781	-0,52	16,16	0
	High	yGVESPVkk	2	1	1	P77973	N-Term(iTRAQ4plex); K8(iTRAQ4plex); K9(iTRAQ4plex)	0,0000	1,412	2	32,6	0	0,0002634	29	0,001315134	3	1438,86271	-0,21	18,32	1
	High	vLAPAR	2	1	1	P77973	N-Term(iTRAQ4plex)	0,0000	0,860	2	21,2	0,007	0,09088	16	0,023225764	2	770,50182	1,68	14,32	0
	Medium	kSSPYSIDR	1	1	1	P77973	N-Term(iTRAQ4plex); K1(iTRAQ4plex)	0,0000	0,812	1		0,029	0,3619	18	0,015274606	3	1340,74125	-0,01	14,20	1
P12975	Photosystem I reaction center subunit IV OS=Synechocystis sp. (strain I	333,30	41,89	1	3	3	15	0,745	10	10,4	74	8,1	8,50							
	A3	Sequence	# PSMs	# Proteins	# Protein Groups	Protein Group Accessions	Modifications	ΔCn	117/114	117/114 Count	117/114 Variability [%]	q-Value	PEP	IonScore	Exp Value	Charge	MH+ [Da]	ΔM [ppm]	RT [min]	# Missed Cleavages
	High	sGILYPVIVR	13	1	1	P12975	N-Term(iTRAQ4plex)	0,0000	0,745	8	6,6	0	0,00003211	58	1,65947E-06	2	1260,78301	2,65	36,94	0
	High	rTESYWYGDVGTVASVEk	1	1	1	P12975	N-Term(iTRAQ4plex); K18(iTRAQ4plex)	0,0000	0,609	1		0	1,678E-08	57	2,02288E-06	3	2335,18613	2,24	33,31	1
	High	sGILYPVIVRFDR	1	1	1	P12975	N-Term(iTRAQ4plex)	0,0000	0,848	1		0	0,002755	13	0,046131757	3	1678,97568	-0,28	42,99	1
P21697	Plastocyanin OS=Synechocystis sp. (strain PCC 6803 / Kazusa) GN=petE	332,97	39,68	1	4	4	10	1,082	9	16,5	126	13,1	6,77							
	A3	Sequence	# PSMs	# Proteins	# Protein Groups	Protein Group Accessions	Modifications	ΔCn	117/114	117/114 Count	117/114 Variability [%]	q-Value	PEP	IonScore	Exp Value	Charge	MH+ [Da]	ΔM [ppm]	RT [min]	# Missed Cleavages
	High	lSPHNIVFAADGVDADTAAk	2	1	1	P21697	N-Term(iTRAQ4plex); K20(iTRAQ4plex)	0,0000	1,242	2	9,4	0	4,542E-18	127	2,07477E-13	3	2300,21134	-0,54	34,74	0
	High	mGSDSGALVFEPSTVTIk	1	1	1	P21697	N-Term(iTRAQ4plex); M1(Oxidation); K18(iTRAQ4plex)	0,0000	0,916	1		0	0,000004533	41	9,75397E-05	3	2143,12149	0,89	37,75	0
	High	gAGmVGk	3	1	1	P21697	N-Term(iTRAQ4plex); M4(Oxidation); K7(iTRAQ4plex)	0,0000	1,175	2	4,2	0	0,01051	17	0,020941125	2	923,52068	-1,76	9,02	0
	High	gAGMVGk	2	1	1	P21697	N-Term(iTRAQ4plex); K7(iTRAQ4plex)	0,0000	0,971	2	16,2	0,007	0,09186	37	0,000207	2	907,52312	-4,70	12,45	0
	Medium	wVNNk	2	1	1	P21697	N-Term(iTRAQ4plex); K5(iTRAQ4plex)	0,0000	0,979	2	2,2	0,022	0,2772	26	0,002254084	2	948,54869	-1,94	15,70	0
P72777	Ycf54-like protein OS=Synechocystis sp. (strain PCC 6803 / Kazusa) GN	329,87	16,54	1	3	3	16	1,694	11	35,3	133	15,4	4,91							
	A3	Sequence	# PSMs	# Proteins	# Protein Groups	Protein Group Accessions	Modifications	ΔCn	117/114	117/114 Count	117/114 Variability [%]	q-Value	PEP	IonScore	Exp Value	Charge	MH+ [Da]	ΔM [ppm]	RT [min]	# Missed Cleavages
	High	nVAIVSTNk	7	1	1	P72777	N-Term(iTRAQ4plex); K9(iTRAQ4plex)	0,0000	1,344	5	48,6	0	0,000002401	68	1,8834E-07	2	1233,74053	-0,01	18,32	0
	High	sFIVWVk	8	1	1	P72777	N-Term(iTRAQ4plex); K7(iTRAQ4plex)	0,0000	2,125	5	4,7	0	0,003119	17	0,018922129	2	1166,71709	-0,45	38,82	0
	Medium	akAPEk	1	1	1	P72777	N-Term(iTRAQ4plex); K2(iTRAQ4plex); K6(iTRAQ4plex)	0,0000	2,977	1		0,014	0,1756	11	0,097431153	3	1075,68106	-2,34	8,07	1
Q55828	LL-diaminopimelate aminotransferase OS=Synechocystis sp. (strain PCC	329,21	16,75	1	6	6	13	0,992	11	11,6	412	45,1	5,62							
	A3	Sequence	# PSMs	# Proteins	# Protein Groups	Protein Group Accessions	Modifications	ΔCn	117/114	117/114 Count	117/114 Variability [%]	q-Value	PEP	IonScore	Exp Value	Charge	MH+ [Da]	ΔM [ppm]	RT [min]	# Missed Cleavages
	High	lLHTVNVVGTPGSGFGAAGEGYFF	2	1	1	Q55828	N-Term(iTRAQ4plex)	0,0000	1,477	1		0	4,626E-13	96	2,86677E-10	3	2550,32279	0,21	41,70	0
	High	rVNAFTTANPNAQVIk	2	1	1	Q55828	N-Term(iTRAQ4plex); K16(iTRAQ4plex)	0,0000	1,284	2	6,4	0	7,105E-10	54	4,27533E-06	3	2032,15757	1,63	24,81	1
	High	tPHGLSSWDFFDk	3	1	1	Q55828	N-Term(iTRAQ4plex); K13(iTRAQ4plex)	0,0000	0,971	3	4,5	0	4,236E-08	48	1,58844E-05	3	1824,91953	1,96	40,46	0
	High	iAQHDFQAR	3	1	1	Q55828	N-Term(iTRAQ4plex)	0,0000	1,031	2	5,8	0	0,00003004	40	0,000101852	3	1229,65290	1,73	14,94	0
	High	vNAFTTANPNAQVIk	1	1	1	Q55828	N-Term(iTRAQ4plex); K15(iTRAQ4plex)	0,0000	0,941	1		0	7,603E-09	37	0,000281273	3	1876,05576	1,40	30,02	0
	High	iSAFNSR	2	1	1	Q55828	N-Term(iTRAQ4plex)	0,0000	0,642	2	36,8	0	0,01039	14	0,039533931	2	938,51805	0,46	19,15	0
P77965	DNA-directed RNA polymerase subunit beta OS=Synechocystis sp. (strai	328,15	11,07	1	11	11	18	1,164	16	18,3	1102	123,3	5,30							
	A3	Sequence	# PSMs	# Proteins	# Protein Groups	Protein Group Accessions	Modifications	ΔCn	117/114	117/114 Count	117/114 Variability [%]	q-Value	PEP	IonScore	Exp Value	Charge	MH+ [Da]	ΔM [ppm]	RT [min]	# Missed Cleavages
	High	lRPGEPPTVSGGQQLLESR	1	1	1	P77965	N-Term(iTRAQ4plex)	0,0000	0,988	1		0	1,448E-13	93	5,17571E-10	3	2165,18131	0,79	30,76	0
	High	tHGIVTYVDATEIR	1	1	1	P77965	N-Term(iTRAQ4plex)	0,0000	1,930	1		0	9,17E-09	51	7,96104E-06	3	1718,91886	-0,32	30,52	0
	High	tYSASLIPNR	1	1	1	P77965	N-Term(iTRAQ4plex)	0,0000	1,114	1		0	0,0001594	42	8,26282E-05	2	1265,69756	0,41	26,04	0
	High	kLSAQVLLk	1	1	1	P77965	N-Term(iTRAQ4plex); K1(iTRAQ4plex); K9(iTRAQ4plex)	0,0000	1,243	1		0	0,00001549	40	0,000108136	3	1431,96183	-0,35	26,26	1
	High	lELHFLGk	6	1	1	P77965	N-Term(iTRAQ4plex); K8(iTRAQ4plex)	0,0000	1,297	4	5,1	0	0,003487	37	0,000205102	2	1244,76189	1,08	36,42	0
	High	iSALGPGGLTR	1	1	1	P77965	N-Term(iTRAQ4plex)	0,0000	1,147	1		0	0,00005556	37	0,000207477	2	1185,70818	0,79	28,88	0
	High	vIVNQIVR	2	1	1	P77965	N-Term(iTRAQ4plex)	0,0000	0,960	2	9,9	0	0,0007063	32	0,000680722	2	1084,69621	0,26	27,59	0
	High	kLRPGEPPTVSGGQQLLESR	1	1	1	P77965	N-Term(iTRAQ4plex); K1(iTRAQ4plex)	0,0000	0,993	1		0	1,004E-07	31	0,00077085	4	2437,37832	0,69	27,43	1
	High	qAVPLLRPERPLVGTGLEAQAAR	2	1	1	P77965	N-Term(iTRAQ4plex)	0,0000	1,337	2	3,1	0	1,376E-09	27	0,002152633	4	2586,49795	0,69	32,88	0
	High	sIPRPGTPESFk	1	1	1	P77965	N-Term(iTRAQ4plex); K12(iTRAQ4plex)	0,0000	1,175	1		0	0,0001655	21	0,007869914	3	1603,90519	0,32	23,94	0
	High	kIQVGDk	1	1	1	P77965	N-Term(iTRAQ4plex); K1(iTRAQ4plex); K7(iTRAQ4plex)	0,0000	1,154	1		0,006	0,08071	18	0,017138389	3	1219,76996	-2,87	12,99	1
P72940	Probable adenylyl-sulfate kinase OS=Synechocystis sp. (strain PCC 6803	326,30	22,03	1	4	4	14	0,989	10	15,4	177	19,7	5,97							
	A3	Sequence	# PSMs	# Proteins	# Protein Groups	Protein Group Accessions	Modifications	ΔCn	117/114	117/114 Count	117/114 Variability [%]	q-Value	PEP	IonScore	Exp Value	Charge	MH+ [Da]	ΔM [ppm]	RT [min]	# Missed Cleavages
	High	iGFVSHLLTR	3	1	1	P72940	N-Term(iTRAQ4plex)	0,0000	0,975	2	12,7	0	1,331E-07	56	2,594E-06	3	1286,77118	0,79	37,31	0
	High	tTITHALEk	5	1	1	P72940	N-Term(iTRAQ4plex); K9(iTRAQ4plex)	0,0000	0,888	3	14,8	0	6,633E-07	55	3,16514E-06	2	1301,76702	0,21	18,23	0
	High	gVTIWLTGLSGAGk	5	1	1	P72940	N-Term(iTRAQ4plex); K14(iTRAQ4plex)	0,0000	1,121	4	17,4	0	7,642E-07	55	3,20605E-06	2	1647,97002	1,66	45,09	0
	High	gLGFSk	1	1	1	P72940	N-Term(iTRAQ4plex); K6(iTRAQ4plex)	0,0000	0,671	1		0	0,009608	42	6,68298E-05	2	896,54399	-0,45	19,87	0
P74500	Slr1940 protein OS=Synechocystis sp. (strain PCC 6803 / Kazusa) GN=s	322,66	11,83	1	6	6	11	0,751	10	9,9	482	51,2	4,89							
	A3	Sequence	# PSMs	# Proteins	# Protein Groups	Protein Group Accessions	Modifications	ΔCn	117/114	117/114 Count	117/114 Variability [%]	q-Value	PEP	IonScore	Exp Value	Charge	MH+ [Da]	ΔM [ppm]	RT [min]	# Missed Cleavages
	High	nVGSIGAPGAR	3	1	1	P74500	N-Term(iTRAQ4plex)	0,0000	0,751	2	2,1	0	0,00001183	62	7,04644E-07	2	1142,64018	0,25	17,41	0
	High	nQGFNLSk	2	1	1	P74500	N-Term(iTRAQ4plex); K8(iTRAQ4plex)	0,0000	0,708	2	3,0	0	0,0001269	54	3,82798E-06	2	1195,66765	0,24	20,97	0
	High	fRPPSTYTLR	1	1	1	P74500	N-Term(iTRAQ4plex)	0,0000	0,712	1		0	0,000006704	49	1,31817E-05	3	1381,77198	0,79	25,81	0
	High	lTLSGSGNNLk	3	1	1	P74500	N-Term(iTRAQ4plex); K11(iTRAQ4plex)	0,0000	0,831	3	20,6	0	0,00003189	41	7,48118E-05	2	1391,81133	1,18	25,36	0
	High	rNVGSIGAPGAR	1	1	1	P74500	N-Term(iTRAQ4plex)	0,0000	0,889	1		0	0,00003211	40	9,57128E-05	3	1298,74207	0,81	12,80	1
	High	nLDSLNQIFVGAAGGk	1	1	1	P74500	N-Term(iTRAQ4plex); K16(iTRAQ4plex)	0,0000	1,137	1		0	0,0008871	28	0,001879072	3	1892,04929	0,65	43,38	0
P48946	30S ribosomal protein S18 OS=Synechocystis sp. (strain PCC 6803 / Kaz	321,40	64,79	1	6	6	16	1,655	13	37,3	71	8,4	10,76							
	A3	Sequence	# PSMs	# Proteins	# Protein Groups	Protein Group Accessions	Modifications	ΔCn	117/114	117/114 Count	117/114 Variability [%]	q-Value	PEP	IonScore	Exp Value	Charge	MH+ [Da]	ΔM [ppm]	RT [min]	# Missed Cleavages
	High	lVALLPFVNk	9	1	1	P48946	N-Term(iTRAQ4plex); K10(iTRAQ4plex)	0,0000	1,575	7	35,7	0	2,714E-08	73	4,83025E-08	2	1401,90813	0,64	48,38	0
	High	iTGLTAk	1	1	1	P48946	N-Term(iTRAQ4plex); K7(iTRAQ4plex)	0,0000	1,101	1		0	0,002427	46	2,42086E-05	2	991,63823	-0,80	20,88	0
	High	lVALLPFVNkEI	2	1	1	P48946	N-Term(iTRAQ4plex); K10(iTRAQ4plex)	0,0000	4,280	2	62,6	0	0,0003071	37	0,000208915	2	1644,03288	-0,62	53,04	1
	High	rLSPLPPNQPIDYkDTELLR	1	1	1	P48946	N-Term(iTRAQ4plex); K14(iTRAQ4plex)	0,0000	1,420	1		0	0,00002894	21	0,007344631	4	2653,48964	-0,75	35,59	2
	High	rITGLTAk	2	1	1	P48946	N-Term(iTRAQ4plex); K8(iTRAQ4plex)	0,0000	2,112	1		0	0,001599	20	0,00948353	3	1147,74012	-0,03	15,64	1
	Medium	kFITER	1	1	1	P48946	N-Term(iTRAQ4plex); K1(iTRAQ4plex)	0,0000	2,055	1		0,016	0,1976	13	0,051996008	3	1081,65973	-1,01	13,92	1
P73313	50S ribosomal protein L16 OS=Synechocystis sp. (strain PCC 6803 / Kaz	319,78	24,46	1	3	3	12	1,199	10	57,9	139	16,0	11,06							
	A3	Sequence	# PSMs	# Proteins	# Protein Groups	Protein Group Accessions	Modifications	ΔCn	117/114	117/114 Count	117/114 Variability [%]	q-Value	PEP	IonScore	Exp Value	Charge	MH+ [Da]	ΔM [ppm]	RT [min]	# Missed Cleavages
	High	gSPEYWVAVVkPGR	9	1	1	P73313	N-Term(iTRAQ4plex); K11(iTRAQ4plex)	0,0000	1,606	7	68,7	0	3,678E-10	74	3,95724E-08	2	1833,02532	-0,48	34,38	0
	High	vmFELAGVSEEVAR	1	1	1	P73313	N-Term(iTRAQ4plex); M2(Oxidation)	0,0000	1,143	1		0	2,843E-10	73	4,59166E-08	2	1696,86907	-0,39	39,41	0
	High	qIEAAR	2	1	1	P73313	N-Term(iTRAQ4plex)	0,0000	1,128	2	0,7	0,002	0,03243	25	0,003347325	2	831,48064	0,15	11,95	0
Q55176	Slr0483 protein OS=Synechocystis sp. (strain PCC 6803 / Kazusa) GN=s	314,31	21,48	1	3	3	12	0,988	9	15,0	149	16,9	7,24							
	A3	Sequence	# PSMs	# Proteins	# Protein Groups	Protein Group Accessions	Modifications	ΔCn	117/114	117/114 Count	117/114 Variability [%]	q-Value	PEP	IonScore	Exp Value	Charge	MH+ [Da]	ΔM [ppm]	RT [min]	# Missed Cleavages
	High	rQELASEFGALk	8	1	1	Q55176	N-Term(iTRAQ4plex); K12(iTRAQ4plex)	0,0000	1,131	5	9,2	0	8,632E-08	68	1,61053E-07	2	1636,92790	1,08	29,03	1
	High	tDVGPITTPNPQk	1	1	1	Q55176	N-Term(iTRAQ4plex); K13(iTRAQ4plex)	0,0000	0,894	1		0	0,000002244	43	6,17153E-05	3	1655,92307	1,43	23,43	0
	High	tATAGIk	3	1	1	Q55176	N-Term(iTRAQ4plex); K7(iTRAQ4plex)	0,0000	0,939	3	9,1	0,008	0,1035	23	0,009226523	2	949,59245	0,40	13,40	0
Q55013	Cytochrome c-550 OS=Synechocystis sp. (strain PCC 6803 / Kazusa) GN	313,83	41,88	1	5	5	9	0,989	8	24,3	160	17,9	4,93							
	A3	Sequence	# PSMs	# Proteins	# Protein Groups	Protein Group Accessions	Modifications	ΔCn	117/114	117/114 Count	117/114 Variability [%]	q-Value	PEP	IonScore	Exp Value	Charge	MH+ [Da]	ΔM [ppm]	RT [min]	# Missed Cleavages
	High	tIPLDEAGGTTTLTAR	2	1	1	Q55013	N-Term(iTRAQ4plex)	0,0000	1,121	2	26,6	0	2,683E-09	103	5,08124E-11	2	1760,95391	1,57	31,33	0
	High	tNNNVSLGLADLAGAEPR	1	1	1	Q55013	N-Term(iTRAQ4plex)	0,0000	1,025	1		0	3,932E-11	58	1,73368E-06	3	1956,02854	0,90	38,72	0
	High	rDNVLALVEFLk	1	1	1	Q55013	N-Term(iTRAQ4plex); K12(iTRAQ4plex)	0,0000	0,805	1		0	0,000002891	45	2,89714E-05	3	1705,02591	0,46	49,35	1
	High	iFVDTcTQcHLQGk	3	1	1	Q55013	N-Term(iTRAQ4plex); C6(Carbamidomethyl); C9(Carbam	0,0000	0,897	2	9,1	0	8,206E-10	44	4,09232E-05	3	1995,00235	-0,36	26,77	0
	High	qFTNGQk	2	1	1	Q55013	N-Term(iTRAQ4plex); K7(iTRAQ4plex)	0,0000	1,264	2	12,1	0	0,002484	40	0,000103031	2	1110,61431	-0,26	11,73	0
P77966	DNA gyrase subunit B OS=Synechocystis sp. (strain PCC 6803 / Kazusa)	313,55	4,27	1	5	5	14	0,987	11	21,7	1078	122,7	6,64							
	A3	Sequence	# PSMs	# Proteins	# Protein Groups	Protein Group Accessions	Modifications	ΔCn	117/114	117/114 Count	117/114 Variability [%]	q-Value	PEP	IonScore	Exp Value	Charge	MH+ [Da]	ΔM [ppm]	RT [min]	# Missed Cleavages
	High	sALETVLTVLHAGGk	4	1	1	P77966	N-Term(iTRAQ4plex); K15(iTRAQ4plex)	0,0000	1,139	3	2,3	0	5,514E-10	73	5,18764E-08	3	1784,05295	0,50	50,02	0
	High	fQAILPLR	3	1	1	P77966	N-Term(iTRAQ4plex)	0,0000	0,854	3	1,8	0	0,00001264	49	1,35198E-05	2	1101,69084	0,65	39,62	0
	High	lkELAYLNAGVR	3	1	1	P77966	N-Term(iTRAQ4plex); K2(iTRAQ4plex)	0,0000	1,124	2	26,3	0	4,697E-08	43	4,52866E-05	3	1634,98331	0,04	34,22	1
	High	rFQAILPLR	3	1	1	P77966	N-Term(iTRAQ4plex)	0,0000	1,064	2	13,4	0	0,00001361	31	0,000714447	3	1257,79090	-0,28	32,16	1
	High	rEFIETYGTk	1	1	1	P77966	N-Term(iTRAQ4plex); K10(iTRAQ4plex)	0,0000	0,602	1		0	0,0004239	15	0,029038221	3	1531,83634	0,29	22,08	1
Q59987	Light-dependent protochlorophyllide reductase OS=Synechocystis sp. (s	311,63	27,33	1	11	11	22	0,831	20	16,4	322	36,0	8,38							
	A3	Sequence	# PSMs	# Proteins	# Protein Groups	Protein Group Accessions	Modifications	ΔCn	117/114	117/114 Count	117/114 Variability [%]	q-Value	PEP	IonScore	Exp Value	Charge	MH+ [Da]	ΔM [ppm]	RT [min]	# Missed Cleavages
	High	gYVSQELAGER	1	1	1	Q59987	N-Term(iTRAQ4plex)	0,0000	0,871	1		0	0,000005561	58	1,72572E-06	2	1352,69402	0,97	22,97	0
	High	tIFPWFQk	8	1	1	Q59987	N-Term(iTRAQ4plex); K8(iTRAQ4plex)	0,0000	0,823	7	19,6	0	0,0001646	42	5,662E-05	2	1354,77898	2,05	45,47	0
	High	lDLGYLDSVR	1	1	1	Q59987	N-Term(iTRAQ4plex)	0,0000	0,877	1		0	0,0001017	41	7,2272E-05	2	1294,71367	1,00	40,47	0
	High	rFVAQFR	1	1	1	Q59987	N-Term(iTRAQ4plex)	0,0000	0,845	1		0	0,000849	35	0,000325814	3	1067,62497	1,75	20,03	1
	High	kPIAmINNk	1	1	1	Q59987	N-Term(iTRAQ4plex); K1(iTRAQ4plex); M5(Oxidation); K	0,0000	1,173	1		0	2,509E-07	31	0,000709529	3	1476,89426	0,67	14,47	0
	High	lIILGTVTANSk	2	1	1	Q59987	N-Term(iTRAQ4plex); K12(iTRAQ4plex)	0,0000	0,671	2	13,8	0	0,0008069	21	0,007533035	3	1517,95154	0,66	38,22	0
	High	lcNmLTTR	2	1	1	Q59987	N-Term(iTRAQ4plex); C2(Carbamidomethyl); M4(Oxidatic	0,0000	0,918	1		0	0,007679	19	0,011829598	2	1168,59416	0,55	19,48	0
	High	eLGRPLk	1	1	1	Q59987	N-Term(iTRAQ4plex); K7(iTRAQ4plex)	0,0000	0,830	1		0	0,006336	14	0,037408475	3	1100,70407	0,94	14,70	0
	High	gWHVIMAcR	1	1	1	Q59987	N-Term(iTRAQ4plex); C8(Carbamidomethyl)	0,0000	0,880	1		0,001	0,0144	20	0,009267658	3	1273,64270	0,95	26,28	0
	High	fVAQFR	3	1	1	Q59987	N-Term(iTRAQ4plex)	0,0000	0,651	3	0,9	0,003	0,04586	28	0,001595769	2	911,52245	0,51	26,33	0
	High	nHYSLFR	1	1	1	Q59987	N-Term(iTRAQ4plex)	0,0000	1,040	1		0,003	0,04658	18	0,014722108	3	1080,57209	1,28	20,71	0
P36237	50S ribosomal protein L11 OS=Synechocystis sp. (strain PCC 6803 / Kaz	307,04	27,66	1	5	5	16	2,238	12	33,0	141	15,0	9,45							
	A3	Sequence	# PSMs	# Proteins	# Protein Groups	Protein Group Accessions	Modifications	ΔCn	117/114	117/114 Count	117/114 Variability [%]	q-Value	PEP	IonScore	Exp Value	Charge	MH+ [Da]	ΔM [ppm]	RT [min]	# Missed Cleavages
	High	sFTFILk	5	1	1	P36237	N-Term(iTRAQ4plex); K7(iTRAQ4plex)	0,0000	2,499	4	5,5	0	0,00004979	62	6,87021E-07	2	1143,70207	0,39	39,14	0
	High	kAAGVEk	1	1	1	P36237	N-Term(iTRAQ4plex); K1(iTRAQ4plex); K7(iTRAQ4plex)	0,0000	1,563	1		0	0,0004098	35	0,00034512	3	1134,71857	-1,88	8,21	1
	High	lALPAGk	5	1	1	P36237	N-Term(iTRAQ4plex); K7(iTRAQ4plex)	0,0000	1,404	3	22,0	0	0,001948	30	0,000968211	2	957,63274	-0,86	24,31	0
	High	gSSEPNkNk	2	1	1	P36237	N-Term(iTRAQ4plex); K7(iTRAQ4plex); K9(iTRAQ4plex)	0,0000	2,675	2	14,3	0	0,00001765	22	0,005834048	3	1392,77808	-1,90	7,69	1
	High	tPPASVLIR	3	1	1	P36237	N-Term(iTRAQ4plex)	0,0000	1,751	2	27,0	0,001	0,01965	33	0,000457057	2	1097,68035	0,36	26,22	0
P05429	Photosystem II CP47 chlorophyll apoprotein OS=Synechocystis sp. (stra	300,59	14,60	1	8	8	24	0,340	19	29,4	507	55,9	5,85							
	A3	Sequence	# PSMs	# Proteins	# Protein Groups	Protein Group Accessions	Modifications	ΔCn	117/114	117/114 Count	117/114 Variability [%]	q-Value	PEP	IonScore	Exp Value	Charge	MH+ [Da]	ΔM [ppm]	RT [min]	# Missed Cleavages
	High	tGAmNSGDGIAQEWIGHPIFk	3	1	1	P05429	N-Term(iTRAQ4plex); M4(Oxidation); K21(iTRAQ4plex)	0,0000	0,317	3	90,6	0	2,043E-11	57	2,05575E-06	4	2533,27920	1,71	41,04	0
	High	vHTVVLNDPGR	4	1	1	P05429	N-Term(iTRAQ4plex)	0,0000	0,280	3	13,4	0	0,00002764	56	2,5408E-06	3	1350,76224	0,87	18,78	0
	High	lAFYDYVGNSPAk	2	1	1	P05429	N-Term(iTRAQ4plex); K13(iTRAQ4plex)	0,0000	0,350	1		0	7,191E-07	35	0,000315479	3	1732,91800	1,80	37,36	0
	High	qGmFVLPFmAR	5	1	1	P05429	N-Term(iTRAQ4plex); M3(Oxidation); M9(Oxidation)	0,0000	0,273	4	5,2	0	0,0018	30	0,001059181	2	1472,75322	1,42	38,59	0
	High	qGmFVLPFMAR	2	1	1	P05429	N-Term(iTRAQ4plex); M3(Oxidation)	0,0000	0,544	2	53,7	0	0,0005828	25	0,003176655	2	1456,75749	0,88	44,74	0
	High	qGMFVLPFMAR	1	1	1	P05429	N-Term(iTRAQ4plex)	0,0000	0,309	1		0,001	0,01741	23	0,005320715	2	1440,76103	-0,18	49,46	0
	High	aDIPFRR	3	1	1	P05429	N-Term(iTRAQ4plex)	0,0000	0,378	2	3,4	0,005	0,05886	13	0,046770284	3	1018,59244	0,95	19,28	1
	High	gLPWYR	1	1	1	P05429	N-Term(iTRAQ4plex)	0,0000	1,603	1		0,008	0,1042	18	0,017425663	2	935,52233	0,38	30,22	0
	Medium	aDIPFR	2	1	1	P05429	N-Term(iTRAQ4plex)	0,0000	0,350	1		0,012	0,146	13	0,050699071	2	862,49199	1,90	23,98	0
	Medium	gGLFR	1	1	1	P05429	N-Term(iTRAQ4plex)	0,0000	0,340	1		0,049	0,6101	22	0,008873425	2	693,41692	0,67	19,05	0
P73302	Adenylate kinase 1 OS=Synechocystis sp. (strain PCC 6803 / Kazusa) GI	298,47	25,95	1	4	4	11	1,163	9	8,2	185	20,2	5,59							
	A3	Sequence	# PSMs	# Proteins	# Protein Groups	Protein Group Accessions	Modifications	ΔCn	117/114	117/114 Count	117/114 Variability [%]	q-Value	PEP	IonScore	Exp Value	Charge	MH+ [Da]	ΔM [ppm]	RT [min]	# Missed Cleavages
	High	nVNQAIFLDELLVNIGHR	3	1	1	P73302	N-Term(iTRAQ4plex)	0,0000	0,990	2	15,9	0	7,953E-13	72	6,66807E-08	3	2209,22184	0,35	52,77	0
	High	gLIFLGAPGSGk	4	1	1	P73302	N-Term(iTRAQ4plex); K12(iTRAQ4plex)	0,0000	1,181	3	32,0	0	0,000001591	61	8,27885E-07	2	1404,84648	0,79	36,85	0
	High	aGWILDGFPR	2	1	1	P73302	N-Term(iTRAQ4plex)	0,0000	1,186	2	3,4	0	0,00004352	40	0,000100455	2	1275,69756	0,68	43,99	0
	High	tHWVINLk	2	1	1	P73302	N-Term(iTRAQ4plex); K8(iTRAQ4plex)	0,0000	1,306	2	17,3	0	0,00003435	34	0,000416841	3	1298,78226	-0,06	30,67	0
P73314	30S ribosomal protein S3 OS=Synechocystis sp. (strain PCC 6803 / Kazu	297,13	28,33	1	7	7	19	1,511	15	16,4	240	27,1	9,98							
	A3	Sequence	# PSMs	# Proteins	# Protein Groups	Protein Group Accessions	Modifications	ΔCn	117/114	117/114 Count	117/114 Variability [%]	q-Value	PEP	IonScore	Exp Value	Charge	MH+ [Da]	ΔM [ppm]	RT [min]	# Missed Cleavages
	High	tALTTYGILGIk	4	1	1	P73314	N-Term(iTRAQ4plex); K12(iTRAQ4plex)	0,0000	1,354	3	43,7	0	7,201E-10	91	7,69077E-10	2	1538,94011	0,31	41,25	0
	High	kAEQIELGIHTARPGVVVGR	3	1	1	P73314	N-Term(iTRAQ4plex); K1(iTRAQ4plex)	0,0000	1,832	2	17,0	0	2,539E-08	44	3,70655E-05	5	2418,41856	0,05	27,16	1
	High	rYPELLQEDHk	4	1	1	P73314	N-Term(iTRAQ4plex); K11(iTRAQ4plex)	0,0000	1,411	4	13,9	0	2,038E-08	33	0,00067467	3	1715,93204	0,07	21,47	1
	High	vWIFk	5	1	1	P73314	N-Term(iTRAQ4plex); K5(iTRAQ4plex)	0,0000	1,550	3	3,8	0,001	0,01527	22	0,006165524	2	980,61571	-1,49	37,01	0
	High	vPLHTLR	1	1	1	P73314	N-Term(iTRAQ4plex)	0,0000	1,511	1		0,003	0,03891	18	0,017497258	3	979,61716	0,22	17,76	0
	High	iHPVGFR	1	1	1	P73314	N-Term(iTRAQ4plex)	0,0000	1,502	1		0,007	0,09008	14	0,040083903	3	969,57456	-0,54	18,68	0
	Medium	iQVSGR	1	1	1	P73314	N-Term(iTRAQ4plex)	0,0000	0,805	1		0,037	0,4578	22	0,006591284	2	803,48631	0,90	13,78	0
P73196	DNA ligase OS=Synechocystis sp. (strain PCC 6803 / Kazusa) GN=lig PE	295,38	9,96	1	7	7	14	1,376	13	16,6	562	61,4	5,08							
	A3	Sequence	# PSMs	# Proteins	# Protein Groups	Protein Group Accessions	Modifications	ΔCn	117/114	117/114 Count	117/114 Variability [%]	q-Value	PEP	IonScore	Exp Value	Charge	MH+ [Da]	ΔM [ppm]	RT [min]	# Missed Cleavages
	High	qAVNEVAETRPk	3	1	1	P73196	N-Term(iTRAQ4plex); K12(iTRAQ4plex)	0,0000	1,406	2	25,2	0	4,117E-10	56	2,52576E-06	3	1629,91587	-0,26	15,76	0
	High	kVVILGTLNAmNR	1	1	1	P73196	N-Term(iTRAQ4plex); K1(iTRAQ4plex); M11(Oxidation)	0,0000	0,973	1		0	3,691E-09	55	3,28829E-06	3	1733,03592	0,73	28,08	1
	High	lVQLNSLVTR	3	1	1	P73196	N-Term(iTRAQ4plex)	0,0000	1,404	3	3,0	0	0,000002255	54	3,83681E-06	2	1286,79167	0,29	36,76	0
	High	vVILGTLNAmNR	2	1	1	P73196	N-Term(iTRAQ4plex); M10(Oxidation)	0,0000	1,194	2	4,6	0	0,007632	31	0,000716094	2	1460,83904	0,97	34,56	0
	High	fVGLQk	2	1	1	P73196	N-Term(iTRAQ4plex); K6(iTRAQ4plex)	0,0000	1,468	2	7,3	0	0,07402	28	0,001741687	2	979,61778	-0,12	24,08	0
	High	tEPPRENk	2	1	1	P73196	N-Term(iTRAQ4plex); K8(iTRAQ4plex)	0,0000	1,414	2	11,5	0	0,008199	11	0,077798282	3	1258,69983	0,34	8,03	1
	Medium	aPASPAk	1	1	1	P73196	N-Term(iTRAQ4plex); K7(iTRAQ4plex)	0,0000	1,282	1		0,042	0,5181	14	0,039991713	2	929,56529	-0,62	9,83	0
P73008	Methyl-accepting chemotaxis protein OS=Synechocystis sp. (strain PCC	294,77	3,80	1	3	3	8	0,774	7	9,3	869	93,2	4,40							
	A3	Sequence	# PSMs	# Proteins	# Protein Groups	Protein Group Accessions	Modifications	ΔCn	117/114	117/114 Count	117/114 Variability [%]	q-Value	PEP	IonScore	Exp Value	Charge	MH+ [Da]	ΔM [ppm]	RT [min]	# Missed Cleavages
	High	iVAVISQIASR	5	1	1	P73008	N-Term(iTRAQ4plex)	0,0000	0,783	4	3,5	0	0,000003885	76	2,74138E-08	2	1300,80876	1,39	43,65	0
	High	vAGSLQTLVSISR	1	1	1	P73008	N-Term(iTRAQ4plex)	0,0000	0,838	1		0	1,734E-08	61	9,89313E-07	2	1474,87151	0,33	39,82	0
	High	tVGGILQIR	2	1	1	P73008	N-Term(iTRAQ4plex)	0,0000	0,667	2	3,4	0	0,0002356	48	1,62544E-05	2	1100,69194	0,99	32,94	0
P73679	Isopenicillin N epimerase OS=Synechocystis sp. (strain PCC 6803 / Kazu	293,51	25,94	1	9	9	14	1,042	13	13,4	401	44,0	5,87							
	A3	Sequence	# PSMs	# Proteins	# Protein Groups	Protein Group Accessions	Modifications	ΔCn	117/114	117/114 Count	117/114 Variability [%]	q-Value	PEP	IonScore	Exp Value	Charge	MH+ [Da]	ΔM [ppm]	RT [min]	# Missed Cleavages
	High	wFAGPAGVGGLYIHGDAk	3	1	1	P73679	N-Term(iTRAQ4plex); K18(iTRAQ4plex)	0,0000	1,051	2	0,7	0	6,566E-13	57	2,7235E-06	3	2104,12625	2,07	40,45	0
	High	accHYITNEEEIEHLLSR	2	1	1	P73679	N-Term(iTRAQ4plex); C2(Carbamidomethyl); C3(Carbam	0,0000	1,089	2	6,7	0	6,268E-13	50	9,07758E-06	4	2418,13051	0,25	38,27	0

	High	yQAIcQR	2	1	1	P73679	N-Term(iTRAQ4plex); C5(Carbamidomethyl)	0,0000	1,138	2	4,5	0	0,0003349	39	0,000122172	2	1082,55058	-2,55	15,62	0
	High	vLVDGAQSAGSLPLDFSR	1	1	1	P73679	N-Term(iTRAQ4plex)	0,0000	0,668	1		0	0,00003078	39	0,000153601	3	1976,05771	0,35	42,57	0
	High	lEVDYYAFTGHk	1	1	1	P73679	N-Term(iTRAQ4plex); K12(iTRAQ4plex)	0,0000	0,965	1		0	9,622E-09	38	0,000158114	3	1730,89652	-1,56	35,19	0
	High	sITYGSk	1	1	1	P73679	N-Term(iTRAQ4plex); K7(iTRAQ4plex)	0,0000	0,894	1		0	0,000669	37	0,000198139	2	1043,59673	-0,78	14,55	0
	High	qQFPGLANk	1	1	1	P73679	N-Term(iTRAQ4plex); K9(iTRAQ4plex)	0,0000	0,913	1		0	0,0009075	32	0,000695024	2	1290,74016	-0,56	24,21	0
	Medium	fGVTYR	2	1	1	P73679	N-Term(iTRAQ4plex)	0,0000	0,752	2	5,3	0,02	0,2541	14	0,036810355	2	886,48998	-0,40	21,25	0
	Medium	rHQGDYPVR	1	1	1	P73679	N-Term(iTRAQ4plex)	0,0000	1,096	1		0,036	0,4495	20	0,010591806	3	1271,67231	-0,20	8,85	1
P72700	Slr0244 protein OS=Synechocystis sp. (strain PCC 6803 / Kazusa) GN=s	286,04	26,06	1	9	9	15	1,183	15	11,7	284	31,2	5,25							
	A3	Sequence	# PSMs	# Proteins	# Protein Groups	Protein Group Accessions	Modifications	ΔCn	117/114	117/114 Count	117/114 Variability [%]	q-Value	PEP	IonScore	Exp Value	Charge	MH+ [Da]	ΔM [ppm]	RT [min]	# Missed Cleavages
	High	lLGTSLSDYIR	2	1	1	P72700	N-Term(iTRAQ4plex)	0,0000	1,221	2	4,6	0	1,72E-08	66	2,61198E-07	2	1381,78191	0,81	43,14	0
	High	vNAPcPVLLTR	2	1	1	P72700	N-Term(iTRAQ4plex); C5(Carbamidomethyl)	0,0000	1,336	2	10,9	0	0,001002	55	3,53159E-06	2	1383,79057	0,47	31,35	0
	High	rLGIAYR	1	1	1	P72700	N-Term(iTRAQ4plex)	0,0000	1,160	1		0	0,005318	33	0,000465554	3	992,61206	-0,13	17,51	1
	High	vNPDLkPDLLPLSR	2	1	1	P72700	N-Term(iTRAQ4plex); K6(iTRAQ4plex)	0,0000	1,137	2	4,2	0	0,000005064	27	0,002027543	3	1865,11136	0,77	36,27	0
	High	cTVTGGkPGEk	2	1	1	P72700	N-Term(iTRAQ4plex); C1(Carbamidomethyl); K7(iTRAQ4p	0,0000	1,397	2	7,1	0	0,0001905	25	0,003318715	3	1565,86631	-1,20	10,45	0
	High	rVmVALDk	1	1	1	P72700	N-Term(iTRAQ4plex); M3(Oxidation); K8(iTRAQ4plex)	0,0000	1,026	1		0	0,0002451	22	0,006011322	3	1235,73841	-0,04	17,51	1
	High	rPSIAk	1	1	1	P72700	N-Term(iTRAQ4plex); K6(iTRAQ4plex)	0,0000	1,020	1		0,003	0,04532	18	0,014255091	3	959,62177	-2,37	9,97	0
	High	lGIAYR	2	1	1	P72700	N-Term(iTRAQ4plex)	0,0000	1,301	2	3,1	0,007	0,09164	25	0,00305471	2	836,51140	0,39	22,67	0
	Medium	vSTVLR	2	1	1	P72700	N-Term(iTRAQ4plex)	0,0000	1,090	2	0,6	0,021	0,2617	16	0,024944224	2	818,52172	0,09	19,08	0
P36265	Transcription termination/antitermination protein NusG OS=Synechocyst	284,66	15,12	1	3	3	12	1,627	9	6,8	205	23,4	6,35							
	A3	Sequence	# PSMs	# Proteins	# Protein Groups	Protein Group Accessions	Modifications	ΔCn	117/114	117/114 Count	117/114 Variability [%]	q-Value	PEP	IonScore	Exp Value	Charge	MH+ [Da]	ΔM [ppm]	RT [min]	# Missed Cleavages
	High	iFPGYVLIR	4	1	1	P36265	N-Term(iTRAQ4plex)	0,0000	1,685	4	6,8	0	0,000003253	48	1,51074E-05	2	1221,74785	0,18	43,11	0
	High	aLLSIFGR	7	1	1	P36265	N-Term(iTRAQ4plex)	0,0000	1,659	4	4,0	0	0,0008733	48	1,58998E-05	2	1020,63262	0,33	42,81	0
	High	nTPHVINFVGSEQk	1	1	1	P36265	N-Term(iTRAQ4plex); K14(iTRAQ4plex)	0,0000	1,230	1		0	7,256E-12	40	9,57128E-05	3	1858,00797	0,97	26,10	0
Q55385	Probable 30S ribosomal protein PSRP-3 OS=Synechocystis sp. (strain PC	284,24	36,61	1	4	4	12	1,939	12	44,2	112	12,6	5,12							
	A3	Sequence	# PSMs	# Proteins	# Protein Groups	Protein Group Accessions	Modifications	ΔCn	117/114	117/114 Count	117/114 Variability [%]	q-Value	PEP	IonScore	Exp Value	Charge	MH+ [Da]	ΔM [ppm]	RT [min]	# Missed Cleavages
	High	gTSPLTSYFFWPR	7	1	1	Q55385	N-Term(iTRAQ4plex)	0,0000	2,212	7	57,4	0	1,542E-08	66	2,29599E-07	2	1702,87285	1,08	49,84	0
	High	qISmAEAQGk	1	1	1	Q55385	N-Term(iTRAQ4plex); M4(Oxidation); K10(iTRAQ4plex)	0,0000	1,895	1		0	0,0001204	50	1,09136E-05	2	1366,72515	0,89	13,07	0
	High	fPEVVFSGSN	1	1	1	Q55385	N-Term(iTRAQ4plex)	0,0000	1,460	1		0	0,006033	49	1,21331E-05	2	1226,61736	-0,05	41,08	0
	High	hWIAEADR	3	1	1	Q55385	N-Term(iTRAQ4plex)	0,0000	1,985	3	88,7	0	0,00005068	27	0,001827974	3	1141,58731	0,18	16,34	0
P72704	Probable peptidyl-prolyl cis-trans isomerase sll0227 OS=Synechocystis s	283,34	26,42	1	7	7	14	0,944	12	30,6	246	26,6	5,14							
	A3	Sequence	# PSMs	# Proteins	# Protein Groups	Protein Group Accessions	Modifications	ΔCn	117/114	117/114 Count	117/114 Variability [%]	q-Value	PEP	IonScore	Exp Value	Charge	MH+ [Da]	ΔM [ppm]	RT [min]	# Missed Cleavages
	High	vITGQNNLEk	2	1	1	P72704	N-Term(iTRAQ4plex); K10(iTRAQ4plex)	0,0000	1,150	2	10,8	0	9,166E-08	69	1,14543E-07	2	1403,81047	0,56	20,24	0
	High	aLGNQAGFPVmLPHk	3	1	1	P72704	N-Term(iTRAQ4plex); M11(Oxidation); K15(iTRAQ4plex)	0,0000	0,742	2	41,7	0	2,928E-13	50	1,11096E-05	3	1884,03989	-0,31	33,21	0
	High	aLGNQAGFPVMLPHk	2	1	1	P72704	N-Term(iTRAQ4plex); K15(iTRAQ4plex)	0,0000	1,107	2	28,3	0	6,479E-11	47	1,99526E-05	3	1868,04599	0,23	35,67	0
	High	nIPLEIk	1	1	1	P72704	N-Term(iTRAQ4plex); K7(iTRAQ4plex)	0,0000	0,892	1		0	0,002562	36	0,0002594	2	1114,70732	-0,11	28,96	0
	High	gDGTGGYVDkNTQRPR	3	1	1	P72704	N-Term(iTRAQ4plex); K10(iTRAQ4plex)	0,0000	1,663	2	18,6	0	0,000001061	20	0,010591806	4	2009,04006	-0,14	12,64	1
	High	qGDkIQSAk	1	1	1	P72704	N-Term(iTRAQ4plex); K4(iTRAQ4plex); K9(iTRAQ4plex)	0,0000	0,908	1		0	0,0006516	17	0,021182148	3	1406,83197	-0,57	9,76	1
	Medium	iQSAk	1	1	1	P72704	N-Term(iTRAQ4plex); K5(iTRAQ4plex)	0,0000	0,757	1		0,021	0,2615	14	0,041206906	2	834,53093	2,63	11,11	0
	Medium	tGAVAmAR	1	1	1	P72704	N-Term(iTRAQ4plex); M6(Oxidation)	0,0000	0,787	1		0,03	0,3729	24	0,004111213	2	936,50426	-1,19	9,38	0
P73826	3-oxoacyl-[acyl-carrier-protein] reductase 2 OS=Synechocystis sp. (strai	280,53	25,00	1	5	5	11	0,754	9	17,6	240	25,3	6,60							
	A3	Sequence	# PSMs	# Proteins	# Protein Groups	Protein Group Accessions	Modifications	ΔCn	117/114	117/114 Count	117/114 Variability [%]	q-Value	PEP	IonScore	Exp Value	Charge	MH+ [Da]	ΔM [ppm]	RT [min]	# Missed Cleavages
	High	gNVGQTNYSATk	3	1	1	P73826	N-Term(iTRAQ4plex); K12(iTRAQ4plex)	0,0000	0,711	2	8,8	0	6,471E-10	69	1,33036E-07	2	1527,80351	1,93	14,05	0
	High	kAGSIVAISSISGER	3	1	1	P73826	N-Term(iTRAQ4plex); K1(iTRAQ4plex)	0,0000	0,857	2	27,7	0	1,694E-08	57	2,0463E-06	3	1763,03061	2,28	27,28	1
	High	vIVVTGGNR	2	1	1	P73826	N-Term(iTRAQ4plex)	0,0000	0,657	2	10,4	0	0,00003226	52	6,01132E-06	2	1058,64458	0,64	19,05	0
	High	lLQEmGAk	1	1	1	P73826	N-Term(iTRAQ4plex); M5(Oxidation); K8(iTRAQ4plex)	0,0000	0,785	1		0	0,007476	29	0,001279293	3	1193,68450	3,55	18,14	0
	High	gVAYSIkPFIEGmYER	2	1	1	P73826	N-Term(iTRAQ4plex); K7(iTRAQ4plex); M13(Oxidation)	0,0000	0,892	2	2,6	0	0,00001665	18	0,02084059	3	2164,13761	1,13	39,53	0
P17253	ATP synthase gamma chain OS=Synechocystis sp. (strain PCC 6803 / Ka	277,64	26,11	1	9	9	12	0,953	9	9,0	314	34,6	6,89							
	A3	Sequence	# PSMs	# Proteins	# Protein Groups	Protein Group Accessions	Modifications	ΔCn	117/114	117/114 Count	117/114 Variability [%]	q-Value	PEP	IonScore	Exp Value	Charge	MH+ [Da]	ΔM [ppm]	RT [min]	# Missed Cleavages
	High	aLQESAASELAAR	1	1	1	P17253	N-Term(iTRAQ4plex)	0,0000	1,086	1		0	6,683E-08	78	1,71779E-08	2	1460,78435	1,19	26,19	0
	High	gLcGGYNVNAIk	2	1	1	P17253	N-Term(iTRAQ4plex); C3(Carbamidomethyl); K12(iTRAQ	0,0000	1,017	2	12,8	0	1,457E-09	67	2,05575E-07	2	1553,83757	1,74	28,16	0
	High	aVALLVVTGDR	1	1	1	P17253	N-Term(iTRAQ4plex)	0,0000	0,762	1		0	0,000003983	42	6,76036E-05	2	1257,76494	0,14	36,03	0
	High	lVLVGSk	2	1	1	P17253	N-Term(iTRAQ4plex); K7(iTRAQ4plex)	0,0000	0,910	1		0	0,006543	41	8,51079E-05	2	1003,67644	1,02	25,79	0
	High	nQGIAVk	1	1	1	P17253	N-Term(iTRAQ4plex); K7(iTRAQ4plex)	0,0000	0,903	1		0	0,0003751	34	0,000411121	2	1017,63054	1,00	14,10	0
	High	lSFAETELPLFEQR	1	1	1	P17253	N-Term(iTRAQ4plex)	0,0000				0,001	0,01786	13	0,047203044	3	1823,96775	0,93	48,95	0
	High	kITEAmR	1	1	1	P17253	N-Term(iTRAQ4plex); K1(iTRAQ4plex); M6(Oxidation)	0,0000	1,203	1		0,003	0,03671	13	0,050695569	3	1152,66565	0,62	9,40	1
	High	lVAAAk	2	1	1	P17253	N-Term(iTRAQ4plex); K6(iTRAQ4plex)	0,0000	1,013	1		0,005	0,05713	20	0,009077578	2	860,58196	1,36	15,56	0
	Medium	iQSVk	1	1	1	P17253	N-Term(iTRAQ4plex); K5(iTRAQ4plex)	0,0000	0,953	1		0,036	0,449	17	0,020795531	2	862,55846	-1,83	13,85	0
Q55734	Sll0395 protein OS=Synechocystis sp. (strain PCC 6803 / Kazusa) GN=s	277,60	27,36	1	5	5	12	0,643	11	19,1	212	23,7	6,52							
	A3	Sequence	# PSMs	# Proteins	# Protein Groups	Protein Group Accessions	Modifications	ΔCn	117/114	117/114 Count	117/114 Variability [%]	q-Value	PEP	IonScore	Exp Value	Charge	MH+ [Da]	ΔM [ppm]	RT [min]	# Missed Cleavages
	High	dVSWTAIFASPLHR	3	1	1	Q55734	N-Term(iTRAQ4plex)	0,0000	0,566	3	4,3	0	1,534E-09	47	1,9542E-05	3	1743,93198	1,17	45,35	0
	High	hGETTSSQTGTFcGR	1	1	1	Q55734	N-Term(iTRAQ4plex); C13(Carbamidomethyl)	0,0000	0,875	1		0	2,282E-08	44	3,72366E-05	3	1769,79966	0,16	11,19	0
	High	tLNLYFLR	3	1	1	Q55734	N-Term(iTRAQ4plex)	0,0000	0,779	3	10,9	0	0,0003859	44	4,13019E-05	2	1183,69621	0,53	43,04	0
	High	lINLPIQk	3	1	1	Q55734	N-Term(iTRAQ4plex); K8(iTRAQ4plex)	0,0000	0,643	3	2,8	0	0,000511	43	4,61286E-05	2	1226,80852	0,83	36,19	0
	High	tFTTGNVLVVSHk	2	1	1	Q55734	N-Term(iTRAQ4plex); K13(iTRAQ4plex)	0,0000	0,620	1		0	1,172E-09	43	5,70402E-05	3	1690,97440	0,79	28,04	0
Q55788	Sensory transduction histidine kinase OS=Synechocystis sp. (strain PCC	276,67	18,73	1	6	6	14	0,928	10	11,0	315	35,0	5,86							
	A3	Sequence	# PSMs	# Proteins	# Protein Groups	Protein Group Accessions	Modifications	ΔCn	117/114	117/114 Count	117/114 Variability [%]	q-Value	PEP	IonScore	Exp Value	Charge	MH+ [Da]	ΔM [ppm]	RT [min]	# Missed Cleavages
	High	qAIANPLHSYR	3	1	1	Q55788	N-Term(iTRAQ4plex)	0,0000	1,026	2	4,2	0	4,647E-07	55	2,97831E-06	3	1413,77130	-0,45	22,29	0
	High	gGAAmLGYTSIQk	1	1	1	Q55788	N-Term(iTRAQ4plex); M5(Oxidation); K13(iTRAQ4plex)	0,0000	0,950	1		0	3,014E-07	53	4,95416E-06	3	1600,86069	-0,04	25,51	0
	High	tLAPVVLk	5	1	1	Q55788	N-Term(iTRAQ4plex); K8(iTRAQ4plex)	0,0000	0,872	3	6,3	0	0,0004202	50	1,05189E-05	2	1128,76079	1,15	29,24	0
	High	qENWQHLLSLSk	3	1	1	Q55788	N-Term(iTRAQ4plex); K12(iTRAQ4plex)	0,0000	0,897	2	16,5	0	0,000009816	39	0,000130008	3	1770,97629	1,22	36,68	0
	High	ekLQEYcTR	1	1	1	Q55788	N-Term(iTRAQ4plex); K2(iTRAQ4plex); C7(Carbamidome	0,0000	0,823	1		0	0,004735	16	0,023876464	3	1514,78830	0,49	14,94	1
	High	mLQLFk	1	1	1	Q55788	N-Term(iTRAQ4plex); K6(iTRAQ4plex)	0,0000	0,947	1		0,005	0,05959	28	0,001648049	2	1067,65215	-0,40	36,76	0
P73298	30S ribosomal protein S11 OS=Synechocystis sp. (strain PCC 6803 / Kaz	274,59	22,31	1	2	2	6	1,536	5	22,5	130	13,8	11,36							
	A3	Sequence	# PSMs	# Proteins	# Protein Groups	Protein Group Accessions	Modifications	ΔCn	117/114	117/114 Count	117/114 Variability [%]	q-Value	PEP	IonScore	Exp Value	Charge	MH+ [Da]	ΔM [ppm]	RT [min]	# Missed Cleavages
	High	kGTPYAAQTAADSAAR	3	1	1	P73298	N-Term(iTRAQ4plex); K1(iTRAQ4plex)	0,0000	1,708	2	23,9	0	7,249E-08	89	1,38986E-09	3	1866,99039	-0,46	15,95	1
	High	aLQGAGLEITLIR	3	1	1	P73298	N-Term(iTRAQ4plex)	0,0000	1,536	3	22,5	0	4,146E-09	52	6,38219E-06	2	1498,90874	0,88	41,83	0
P74002	Uncharacterized protein slr1322 OS=Synechocystis sp. (strain PCC 6803	272,83	12,68	1	8	8	13	0,926	13	12,1	489	53,1	5,64							
	A3	Sequence	# PSMs	# Proteins	# Protein Groups	Protein Group Accessions	Modifications	ΔCn	117/114	117/114 Count	117/114 Variability [%]	q-Value	PEP	IonScore	Exp Value	Charge	MH+ [Da]	ΔM [ppm]	RT [min]	# Missed Cleavages
	High	eSLSTLLGLGR	3	1	1	P74002	N-Term(iTRAQ4plex)	0,0000	0,932	3	3,5	0	0,00001062	62	7,14795E-07	2	1289,75481	0,17	44,03	0
	High	qSYAYAAASR	2	1	1	P74002	N-Term(iTRAQ4plex)	0,0000	0,971	2	0,6	0	0,00004724	47	1,85768E-05	2	1231,61821	-0,46	15,68	0
	High	lDHYASHAQSR	2	1	1	P74002	N-Term(iTRAQ4plex)	0,0000	0,889	2	6,1	0	0,00001275	33	0,000496558	3	1428,70996	-0,07	10,98	0
	High	ekDQWLHR	2	1	1	P74002	N-Term(iTRAQ4plex); K2(iTRAQ4plex)	0,0000	0,827	2	4,8	0	0,00408	28	0,001632939	4	1399,76711	-0,98	16,05	1
	High	rQSYAYAAASR	1	1	1	P74002	N-Term(iTRAQ4plex)	0,0000	1,870	1		0	0,0000502	21	0,007961044	3	1387,71924	-0,48	11,99	1
	High	tTPFLDkk	1	1	1	P74002	N-Term(iTRAQ4plex); K7(iTRAQ4plex); K8(iTRAQ4plex)	0,0000	1,139	1		0,007	0,08612	21	0,008709034	3	1381,83835	-2,31	20,07	1
	Medium	aAYFR	1	1	1	P74002	N-Term(iTRAQ4plex)	0,0000	0,431	1		0,015	0,1888	14	0,039899734	2	771,42601	-1,29	17,00	0
	Medium	lSSGcGVR	1	1	1	P74002	N-Term(iTRAQ4plex); C5(Carbamidomethyl)	0,0000	0,914	1		0,032	0,3947	21	0,007602738	2	979,51012	-1,07	13,16	0
P73315	50S ribosomal protein L22 OS=Synechocystis sp. (strain PCC 6803 / Kaz	270,48	33,88	1	5	5	16	2,281	12	106,9	121	13,5	9,99							
	A3	Sequence	# PSMs	# Proteins	# Protein Groups	Protein Group Accessions	Modifications	ΔCn	117/114	117/114 Count	117/114 Variability [%]	q-Value	PEP	IonScore	Exp Value	Charge	MH+ [Da]	ΔM [ppm]	RT [min]	# Missed Cleavages
	High	kPTcHITVAVAPALADN	4	1	1	P73315	N-Term(iTRAQ4plex); K1(iTRAQ4plex); C4(Carbamidome	0,0000	4,062	4	76,1	0	8,548E-07	52	6,79157E-06	3	2066,12894	-0,87	28,51	0
	High	tkLDTTAEVk	4	1	1	P73315	N-Term(iTRAQ4plex); K2(iTRAQ4plex); K10(iTRAQ4plex)	0,0000	5,318	2	6,6	0	4,786E-07	40	9,70443E-05	3	1537,91288	-2,14	16,43	1
	High	rVLDQIR	3	1	1	P73315	N-Term(iTRAQ4plex)	0,0000	1,480	3	21,0	0	0,0002629	38	0,000149269	3	1043,64347	-0,74	16,47	1
	High	acEPVLk	4	1	1	P73315	N-Term(iTRAQ4plex); C2(Carbamidomethyl); K7(iTRAQ4p	0,0000	1,968	2	72,7	0	0,005944	21	0,008629189	2	1104,63005	-2,28	17,32	0
	High	vLDQIR	1	1	1	P73315	N-Term(iTRAQ4plex)	0,0000	0,394	1		0,002	0,02827	34	0,000390814	2	887,54180	-1,49	22,06	0
P74421	Phosphoglycerate kinase OS=Synechocystis sp. (strain PCC 6803 / Kazu	269,50	21,70	1	8	8	14	0,984	11	18,5	401	41,8	5,19							
	A3	Sequence	# PSMs	# Proteins	# Protein Groups	Protein Group Accessions	Modifications	ΔCn	117/114	117/114 Count	117/114 Variability [%]	q-Value	PEP	IonScore	Exp Value	Charge	MH+ [Da]	ΔM [ppm]	RT [min]	# Missed Cleavages
	High	aQGLNTGk	2	1	1	P74421	N-Term(iTRAQ4plex); K8(iTRAQ4plex)	0,0000	1,091	1		0	0,00001103	48	1,74655E-05	2	1076,63152	1,17	11,75	0
	High	vILGSHFGRPk	3	1	1	P74421	N-Term(iTRAQ4plex); K11(iTRAQ4plex)	0,0000	0,860	2	35,2	0	0,000001119	46	2,25928E-05	3	1498,91297	2,19	21,48	0
	High	fHAGEEGNDAEFAk	1	1	1	P74421	N-Term(iTRAQ4plex); K14(iTRAQ4plex)	0,0000	0,655	1		0	6,088E-09	43	4,62349E-05	3	1809,86771	1,68	21,44	0
	High	rPLVAIVGGSk	3	1	1	P74421	N-Term(iTRAQ4plex); K11(iTRAQ4plex)	0,0000	1,061	2	7,2	0	4,542E-07	39	0,000133036	3	1384,89127	2,43	23,23	0
	High	lGELLGQPVVk	1	1	1	P74421	N-Term(iTRAQ4plex); K11(iTRAQ4plex)	0,0000	0,580	1		0	0,00001549	36	0,000224373	3	1440,90305	0,12	37,58	0
	High	aALPTIk	2	1	1	P74421	N-Term(iTRAQ4plex); K7(iTRAQ4plex)	0,0000	0,944	2	6,2	0	0,00526	32	0,000660648	2	1001,66014	0,37	24,31	0
	High	sLVEEDkLDLAk	1	1	1	P74421	N-Term(iTRAQ4plex); K7(iTRAQ4plex); K12(iTRAQ4plex)	0,0000	0,844	1		0	0,0005222	17	0,020417379	3	1792,04624	1,89	29,84	1
	High	eLQFLQGAIEAPk	1	1	1	P74421	N-Term(iTRAQ4plex); K13(iTRAQ4plex)	0,0000	1,174	1		0	0,001094	13	0,049655802	3	1731,98801	-0,23	41,90	0
Q55118	Putative thylakoid lumen peptidyl-prolyl cis-trans isomerase sll0408 OS=	268,77	11,87	1	5	5	9	1,119	9	17,1	379	41,4	4,84							
	A3	Sequence	# PSMs	# Proteins	# Protein Groups	Protein Group Accessions	Modifications	ΔCn	117/114	117/114 Count	117/114 Variability [%]	q-Value	PEP	IonScore	Exp Value	Charge	MH+ [Da]	ΔM [ppm]	RT [min]	# Missed Cleavages
	High	aIPLEILVk	2	1	1	Q55118	N-Term(iTRAQ4plex); K9(iTRAQ4plex)	0,0000	1,184	2	23,8	0	9,438E-08	61	8,74923E-07	2	1283,85442	0,23	43,21	0
	High	kFYDGLPFIR	1	1	1	Q55118	N-Term(iTRAQ4plex); K1(iTRAQ4plex)	0,0000	1,169	1		0	2,837E-07	47	2,26307E-05	3	1543,88944	1,22	38,31	1
	High	rLQDSLEDISNHIR	2	1	1	Q55118	N-Term(iTRAQ4plex)	0,0000	0,789	2	26,2	0	5,003E-07	44	3,68104E-05	4	1839,98000	0,32	34,52	1
	High	aANLTITLkEDk	2	1	1	Q55118	N-Term(iTRAQ4plex); K9(iTRAQ4plex); K12(iTRAQ4plex)	0,0000	1,382	2	14,4	0	5,721E-07	44	3,8634E-05	3	1749,04776	-0,28	26,81	1
	High	fYDGLPFIR	2	1	1	Q55118	N-Term(iTRAQ4plex)	0,0000	1,062	2	7,8	0	0,0004613	43	5,40717E-05	2	1271,69097	0,35	44,73	0
P52981	1,4-alpha-glucan branching enzyme GlgB OS=Synechocystis sp. (strain	268,33	8,83	1	7	7	12	0,771	11	7,6	770	89,5	5,62							
	A3	Sequence	# PSMs	# Proteins	# Protein Groups	Protein Group Accessions	Modifications	ΔCn	117/114	117/114 Count	117/114 Variability [%]	q-Value	PEP	IonScore	Exp Value	Charge	MH+ [Da]	ΔM [ppm]	RT [min]	# Missed Cleavages
	High	gVYFAVWAPNAR	2	1	1	P52981	N-Term(iTRAQ4plex)	0,0000	0,800	2	4,5	0	0,000001118	64	3,96251E-07	2	1494,79790	0,30	40,95	0
	High	vNQWVIR	1	1	1	P52981	N-Term(iTRAQ4plex)	0,0000	0,714	1		0	0,0002045	41	8,49551E-05	2	1058,62273	-0,03	28,51	0
	High	hSVVSFIR	1	1	1	P52981	N-Term(iTRAQ4plex)	0,0000	0,655	1		0	0,00000364	37	0,000188786	3	1088,63376	0,40	23,53	0
	High	aLFTYmFTHPGk	1	1	1	P52981	N-Term(iTRAQ4plex); M6(Oxidation); K12(iTRAQ4plex)	0,0000	0,733	1		0	0,000003088	36	0,000258208	3	1716,90360	0,81	33,23	0
	High	aLFTYMFTHPGk	1	1	1	P52981	N-Term(iTRAQ4plex); K12(iTRAQ4plex)	0,0000	0,874	1		0	6,579E-07	29	0,001161449	3	1700,90549	-1,06	40,90	0
	High	qFFTELNHLYk	4	1	1	P52981	N-Term(iTRAQ4plex); K11(iTRAQ4plex)	0,0000	0,806	3	0,8	0	0,000001501	29	0,001166729	3	1727,93698	0,61	39,25	0
	High	lGAHLmTVDGVk	1	1	1	P52981	N-Term(iTRAQ4plex); M6(Oxidation); K12(iTRAQ4plex)	0,0000	0,637	1		0	0,000006641	20	0,010518892	3	1544,87070	-0,15	24,46	0
	High	lIPYVk	1	1	1	P52981	N-Term(iTRAQ4plex); K6(iTRAQ4plex)	0,0000	0,751	1		0,001	0,01852	26	0,002728789	2	1020,66857	-1,00	31,01	0
P74446	Slr0147 protein OS=Synechocystis sp. (strain PCC 6803 / Kazusa) GN=s	268,07	5,48	1	2	2	8	2,331	6	25,1	292	32,3	8,44							
	A3	Sequence	# PSMs	# Proteins	# Protein Groups	Protein Group Accessions	Modifications	ΔCn	117/114	117/114 Count	117/114 Variability [%]	q-Value	PEP	IonScore	Exp Value	Charge	MH+ [Da]	ΔM [ppm]	RT [min]	# Missed Cleavages
	High	nLFILGLk	7	1	1	P74446	N-Term(iTRAQ4plex); K8(iTRAQ4plex)	0,0000	2,391	5	18,5	0	9,845E-07	59	1,16137E-06	2	1205,78642	0,32	43,80	0
	High	tWGSAFYk	1	1	1	P74446	N-Term(iTRAQ4plex); K8(iTRAQ4plex)	0,0000	1,713	1		0	0,00000328	54	3,68952E-06	2	1247,66680	0,40	30,61	0
P36239	50S ribosomal protein L19 OS=Synechocystis sp. (strain PCC 6803 / Kaz	265,24	24,59	1	4	4	10	2,261	9	24,5	122	13,8	10,64							
	A3	Sequence	# PSMs	# Proteins	# Protein Groups	Protein Group Accessions	Modifications	ΔCn	117/114	117/114 Count	117/114 Variability [%]	q-Value	PEP	IonScore	Exp Value	Charge	MH+ [Da]	ΔM [ppm]	RT [min]	# Missed Cleavages
	High	kIFQGVGVER	3	1	1	P36239	N-Term(iTRAQ4plex); K1(iTRAQ4plex)	0,0000	2,660	3	14,9	0	5,815E-07	61	8,689E-07	2	1420,84990	-1,13	22,87	1
	High	vFLLHSPR	5	1	1	P36239	N-Term(iTRAQ4plex)	0,0000	2,309	4	8,3	0	0,000006703	51	7,14447E-06	3	1112,66936	-0,32	26,11	0
	High	rNGGISETITVR	1	1	1	P36239	N-Term(iTRAQ4plex)	0,0000	1,410	1		0	5,521E-07	50	1,07992E-05	3	1446,81479	0,16	18,33	1
	High	iFQGVGVER	1	1	1	P36239	N-Term(iTRAQ4plex)	0,0000	1,737	1		0,001	0,01814	26	0,002792351	2	1148,65361	-0,75	28,22	0
Q55690	Glycine--tRNA ligase beta subunit OS=Synechocystis sp. (strain PCC 68	264,62	6,37	1	6	6	12	1,046	11	16,7	722	80,0	4,86							
	A3	Sequence	# PSMs	# Proteins	# Protein Groups	Protein Group Accessions	Modifications	ΔCn	117/114	117/114 Count	117/114 Variability [%]	q-Value	PEP	IonScore	Exp Value	Charge	MH+ [Da]	ΔM [ppm]	RT [min]	# Missed Cleavages
	High	lNLLGLIR	5	1	1	Q55690	N-Term(iTRAQ4plex)	0,0000	1,124	5	10,7	0	0,00002612	52	5,79389E-06	2	1055,70622	0,43	47,36	0
	High	lALSWLk	2	1	1	Q55690	N-Term(iTRAQ4plex); K7(iTRAQ4plex)	0,0000	0,985	2	8,1	0	0,00008157	52	6,98184E-06	2	1118,71916	1,38	41,60	0
	High	aVYQALLAIYPk	1	1	1	Q55690	N-Term(iTRAQ4plex); K12(iTRAQ4plex)	0,0000	1,470	1		0	0,00008371	32	0,0005662	3	1637,98844	0,93	46,97	0
	High	rLAVLIk	1	1	1	Q55690	N-Term(iTRAQ4plex); K7(iTRAQ4plex)	0,0000	1,194	1		0,001	0,01187	32	0,000568814	3	1100,77622	0,37	24,31	1
	High	tFFLQR	1	1	1	Q55690	N-Term(iTRAQ4plex)	0,0000	0,907	1		0,001	0,01804	24	0,003810395	2	955,54863	0,46	29,67	0
	High	fPRPIR	2	1	1	Q55690	N-Term(iTRAQ4plex)	0,0000	0,936	1		0,003	0,04157	11	0,076554373	3	929,58084	0,72	18,34	0
P72839	Slr1301 protein OS=Synechocystis sp. (strain PCC 6803 / Kazusa) GN=s	264,09	15,70	1	9	9	12	0,871	12	27,0	535	61,7	4,84							
	A3	Sequence	# PSMs	# Proteins	# Protein Groups	Protein Group Accessions	Modifications	ΔCn	117/114	117/114 Count	117/114 Variability [%]	q-Value	PEP	IonScore	Exp Value	Charge	MH+ [Da]	ΔM [ppm]	RT [min]	# Missed Cleavages
	High	eVLQGILHR	2	1	1	P72839	N-Term(iTRAQ4plex)	0,0000	0,753	2	3,3	0	8,023E-07	55	2,93745E-06	3	1208,72367	0,37	28,65	0
	High	nkLEQNLQSTR	2	1	1	P72839	N-Term(iTRAQ4plex); K2(iTRAQ4plex)	0,0000	1,268	2	20,4	0	8,968E-07	43	5,44465E-05	3	1618,91233	0,49	17,48	1
	High	nFLGGYk	2	1	1	P72839	N-Term(iTRAQ4plex); K7(iTRAQ4plex)	0,0000	0,869	2	7,7	0	0,000985	38	0,000158114	2	1086,61748	-1,05	25,96	0
	High	kTNFEQNR	1	1	1	P72839	N-Term(iTRAQ4plex); K1(iTRAQ4plex)	0,0000	1,021	1		0	0,000007421	33	0,00052236	3	1324,72272	1,15	9,57	1
	High	nQALIQQk	1	1	1	P72839	N-Term(iTRAQ4plex); K8(iTRAQ4plex)	0,0000	0,720	1		0	0,00008392	33	0,000528409	2	1230,74114	0,22	15,69	0
	High	eGTLWILNLSNSR	1	1	1	P72839	N-Term(iTRAQ4plex)	0,0000	0,250	1		0	0,001302	32	0,000586098	3	1646,90036	1,26	47,01	0
	High	mLYLAEIkk	1	1	1	P72839	N-Term(iTRAQ4plex); M1(Oxidation); K8(iTRAQ4plex); K'	0,0000	1,122	1		0	0,000145	26	0,002601293	3	1556,94339	-0,80	27,84	1
	High	qEVLLQHLk	1	1	1	P72839	N-Term(iTRAQ4plex); K9(iTRAQ4plex)	0,0000	0,828	1		0	0,000009043	23	0,005271934	3	1395,85627	0,02	26,02	0
	High	lkEQQSEIER	1	1	1	P72839	N-Term(iTRAQ4plex); K2(iTRAQ4plex)	0,0000	1,107	1		0	0,004049	16	0,030898924	3	1547,86484	1,07	14,50	1
P36236	50S ribosomal protein L1 OS=Synechocystis sp. (strain PCC 6803 / Kazu	263,13	23,53	1	7	7	21	1,440	17	22,5	238	25,8	8,73							
	A3	Sequence	# PSMs	# Proteins	# Protein Groups	Protein Group Accessions	Modifications	ΔCn	117/114	117/114 Count	117/114 Variability [%]	q-Value	PEP	IonScore	Exp Value	Charge	MH+ [Da]	ΔM [ppm]	RT [min]	# Missed Cleavages
	High	aSFSADDLLANLk	2	1	1	P36236	N-Term(iTRAQ4plex); K13(iTRAQ4plex)	0,0000	1,516	2	6,1	0	0,00004221	38	0,000173768	3	1652,90824	-0,96	45,47	0
	High	mQAAIAk	2	1	1	P36236	N-Term(iTRAQ4plex); M1(Oxidation); K7(iTRAQ4plex)	0,0000	1,209	2	3,7	0	0,0002112	34	0,000379289	2	1036,60576	-0,58	11,99	0
	High	tGIVHVmFGk	6	1	1	P36236	N-Term(iTRAQ4plex); M7(Oxidation); K10(iTRAQ4plex)	0,0000	1,616	3	5,0	0	0,0001095	31	0,000758578	3	1392,79218	0,69	25,76	0
	High	lYSPLEAmELLk	1	1	1	P36236	N-Term(iTRAQ4plex); M8(Oxidation); K12(iTRAQ4plex)	0,0000	1,038	1		0	0,00003398	21	0,008891397	3	1710,96018	0,65	42,83	0
	High	tGIVHVMFGk	2	1	1	P36236	N-Term(iTRAQ4plex); K10(iTRAQ4plex)	0,0000	2,313	1		0	0,00002224	20	0,009749223	3	1376,79590	-0,29	31,40	0
	High	lYSPLEAMELLk	1	1	1	P36236	N-Term(iTRAQ4plex); K12(iTRAQ4plex)	0,0000	0,457	1		0	0,0008719	15	0,033882075	3	1694,96518	0,61	52,31	0
	High	rmQAAIAk	1	1	1	P36236	N-Term(iTRAQ4plex); M2(Oxidation); K8(iTRAQ4plex)	0,0000	1,105	1		0	0,005919	12	0,062000142	3	1192,70688	-0,50	8,89	1
	High	tTVSLPk	2	1	1	P36236	N-Term(iTRAQ4plex); K7(iTRAQ4plex)	0,0000	1,148	2	9,2	0,001	0,0159	37	0,000209397	2	1033,64897	-0,59	20,42	0
	High	vAVLAR	3	1	1	P36236	N-Term(iTRAQ4plex)	0,0000	1,440	3	9,4	0,002	0,03296	27	0,001849141	2	772,51549	-0,89	18,52	0
	High	rMQAAIAk	1	1	1	P36236	N-Term(iTRAQ4plex); K8(iTRAQ4plex)	0,0000	1,654	1		0,006	0,07422	15	0,035072765	3	1176,71042	-1,82	13,36	1
P73821	D-3-phosphoglycerate dehydrogenase OS=Synechocystis sp. (strain PCC	253,10	9,75	1	7	7	11	0,938	9	20,2	554	59,2	5,69							
	A3	Sequence	# PSMs	# Proteins	# Protein Groups	Protein Group Accessions	Modifications	ΔCn	117/114	117/114 Count	117/114 Variability [%]	q-Value	PEP	IonScore	Exp Value	Charge	MH+ [Da]	ΔM [ppm]	RT [min]	# Missed Cleavages
	High	tLGVVGLGk	4	1	1	P73821	N-Term(iTRAQ4plex); K9(iTRAQ4plex)	0,0000	0,974	2	5,6	0	2,004E-07	73	4,73119E-08	2	1131,73528	1,12	28,99	0
	High	kQFIGTEVYk	1	1	1	P73821	N-Term(iTRAQ4plex); K1(iTRAQ4plex); K10(iTRAQ4plex)	0,0000	1,038	1		0	2,684E-08	48	1,41579E-05	3	1644,96823	-0,18	23,57	1
	High	iIQAGSQLk	1	1	1	P73821	N-Term(iTRAQ4plex); K9(iTRAQ4plex)	0,0000	0,819	1		0	0,002846	43	5,236E-05	2	1245,77788	0,76	24,01	0
	High	lLAYDPFISQER	1	1	1	P73821	N-Term(iTRAQ4plex)	0,0000	0,730	1		0	0,0001438	35	0,000325065	2	1595,85820	1,99	44,40	0
	High	hIPDANk	2	1	1	P73821	N-Term(iTRAQ4plex); K7(iTRAQ4plex)	0,0000	0,678	2	6,8	0	0,008033	24	0,003638899	3	1082,62104	1,25	10,89	0
	High	kQFIGTEVYkk	1	1	1	P73821	N-Term(iTRAQ4plex); K1(iTRAQ4plex); K10(iTRAQ4plex)	0,0000	0,942	1		0	3,463E-07	21	0,008769403	4	1917,16506	-0,26	21,23	2
	High	lRPYLk	1	1	1	P73821	N-Term(iTRAQ4plex); K6(iTRAQ4plex)	0,0000	1,159	1		0,001	0,01881	21	0,007571629	3	1077,70383	1,43	19,91	0
P37101	Phosphoribulokinase OS=Synechocystis sp. (strain PCC 6803 / Kazusa) (	252,48	28,61	1	8	8	16	0,931	13	22,2	332	37,9	5,43							
	A3	Sequence	# PSMs	# Proteins	# Protein Groups	Protein Group Accessions	Modifications	ΔCn	117/114	117/114 Count	117/114 Variability [%]	q-Value	PEP	IonScore	Exp Value	Charge	MH+ [Da]	ΔM [ppm]	RT [min]	# Missed Cleavages
	High	gHTYEDILASINAR	2	1	1	P37101	N-Term(iTRAQ4plex)	0,0000	1,122	2	10,2	0	2,538E-07	66	3,60026E-07	3	1703,88517	1,06	36,92	0
	High	kPDFTAYIEPQk	3	1	1	P37101	N-Term(iTRAQ4plex); K1(iTRAQ4plex); K12(iTRAQ4plex)	0,0000	0,829	2	9,7	0	8,813E-11	56	2,93354E-06	3	1869,04978	0,82	31,61	0
	High	vVVIEGLHPLYDER	1	1	1	P37101	N-Term(iTRAQ4plex)	0,0000	0,928	1		0	0,000001728	33	0,00051047	3	1782,98838	0,71	38,00	0
	High	kLTcTYPGIk	3	1	1	P37101	N-Term(iTRAQ4plex); K1(iTRAQ4plex); C4(Carbamidome	0,0000	1,224	2	18,9	0	0,00006037	27	0,002233418	3	1612,94565	-0,02	22,25	1
	High	lIEDkESk	2	1	1	P37101	N-Term(iTRAQ4plex); K5(iTRAQ4plex); K8(iTRAQ4plex)	0,0000	0,749	2	12,2	0	0,0002753	14	0,036472876	3	1393,82535	-0,67	15,85	1
	High	hkDYPGTDNGTGLFQVLVGLk	1	1	1	P37101	N-Term(iTRAQ4plex); K2(iTRAQ4plex); K21(iTRAQ4plex)	0,0000	1,140	1		0	7,978E-09	14	0,036810355	4	2691,48867	2,07	45,97	1
	High	kAAGVTALDPR	2	1	1	P37101	N-Term(iTRAQ4plex); K1(iTRAQ4plex)	0,0000	0,889	2	6,8	0,003	0,04142	23	0,0049885	3	1386,83237	1,15	18,08	1
	Medium	sTFLR	1	1	1	P37101	N-Term(iTRAQ4plex)	0,0000	1,262	1		0,031	0,3898	13	0,049313974	2	767,45384	0,81	17,72	0
P73411	Glucose-6-phosphate 1-dehydrogenase OS=Synechocystis sp. (strain PC	251,07	15,72	1	7	7	11	0,458	11	10,2	509	57,8	7,17							
	A3	Sequence	# PSMs	# Proteins	# Protein Groups	Protein Group Accessions	Modifications	ΔCn	117/114	117/114 Count	117/114 Variability [%]	q-Value	PEP	IonScore	Exp Value	Charge	MH+ [Da]	ΔM [ppm]	RT [min]	# Missed Cleavages
	High	kVSEIAIQFR	2	1	1	P73411	N-Term(iTRAQ4plex); K1(iTRAQ4plex)	0,0000	0,518	2	25,5	0	8,997E-08	57	2,07955E-06	3	1478,89716	2,56	29,52	1
	High	rLPPELTVVGFAR	1	1	1	P73411	N-Term(iTRAQ4plex)	0,0000	0,643	1		0	1,457E-07	52	5,94251E-06	3	1598,95127	0,82	37,49	1
	High	qVPLLIFQSVAHQANPNVLSLR	1	1	1	P73411	N-Term(iTRAQ4plex)	0,0000	0,343	1		0	2,03E-08	42	5,78056E-05	3	2588,48288	1,34	51,68	0
	High	wQGVPFYLR	2	1	1	P73411	N-Term(iTRAQ4plex)	0,0000	0,465	2	2,4	0	0,0006056	35	0,000334172	2	1309,71904	1,26	41,89	0
	High	kLVPAIYQmk	1	1	1	P73411	N-Term(iTRAQ4plex); K1(iTRAQ4plex); M9(Oxidation); K	0,0000	0,628	1		0	0,00002768	32	0,000612308	3	1639,00101	2,00	24,81	1
	High	iVIEkPFGR	2	1	1	P73411	N-Term(iTRAQ4plex); K5(iTRAQ4plex)	0,0000	0,414	2	4,6	0	0,00004215	30	0,000899435	3	1346,84113	0,93	29,52	0
	High	iDHYLGk	2	1	1	P73411	N-Term(iTRAQ4plex); K7(iTRAQ4plex)	0,0000	0,464	2	2,0	0,001	0,01567	15	0,04710763	3	1133,65747	1,54	20,88	0
P73598	Sll1305 protein OS=Synechocystis sp. (strain PCC 6803 / Kazusa) GN=sl	248,71	6,78	1	2	2	6	0,702	6	4,6	295	32,9	4,97							
	A3	Sequence	# PSMs	# Proteins	# Protein Groups	Protein Group Accessions	Modifications	ΔCn	117/114	117/114 Count	117/114 Variability [%]	q-Value	PEP	IonScore	Exp Value	Charge	MH+ [Da]	ΔM [ppm]	RT [min]	# Missed Cleavages
	High	lLLEQLGEHFR	5	1	1	P73598	N-Term(iTRAQ4plex)	0,0000	0,691	5	4,6	0	2,563E-08	71	8,51908E-08	3	1498,85172	1,22	42,96	0
	Medium	yYSRPGAmk	1	1	1	P73598	N-Term(iTRAQ4plex); M8(Oxidation); K9(iTRAQ4plex)	0,0000	0,795	1		0,037	0,4611	18	0,014420158	3	1376,72669	2,31	12,37	0
P73316	30S ribosomal protein S19 OS=Synechocystis sp. (strain PCC 6803 / Kaz	243,69	28,26	1	3	3	9	1,695	8	21,6	92	10,3	11,14							
	A3	Sequence	# PSMs	# Proteins	# Protein Groups	Protein Group Accessions	Modifications	ΔCn	117/114	117/114 Count	117/114 Variability [%]	q-Value	PEP	IonScore	Exp Value	Charge	MH+ [Da]	ΔM [ppm]	RT [min]	# Missed Cleavages
	High	gPFVAASLLR	3	1	1	P73316	N-Term(iTRAQ4plex)	0,0000	1,757	3	10,7	0	2,479E-07	57	2,0239E-06	2	1174,70671	0,17	38,91	0
	High	kGPFVAASLLR	3	1	1	P73316	N-Term(iTRAQ4plex); K1(iTRAQ4plex)	0,0000	1,509	3	12,4	0	0,000002236	45	3,42744E-05	3	1446,90287	-0,47	32,36	1
	High	qHVPVFVSEQMVGHk	1	1	1	P73316	N-Term(iTRAQ4plex); K15(iTRAQ4plex)	0,0000	2,034	1		0	1,992E-09	43	5,08159E-05	4	2010,08144	-0,97	27,81	0
	High	qHVPVFVSEQmVGHk	2	1	1	P73316	N-Term(iTRAQ4plex); M11(Oxidation); K15(iTRAQ4plex)	0,0000	1,377	2		0	4,061E-07	31	0,000853041	4	2026,08022	0,95	23,05	0
P72655	Uncharacterized protein slr1128 OS=Synechocystis sp. (strain PCC 6803	243,46	15,26	1	4	4	9	0,602	9	18,9	321	35,7	5,68							
	A3	Sequence	# PSMs	# Proteins	# Protein Groups	Protein Group Accessions	Modifications	ΔCn	117/114	117/114 Count	117/114 Variability [%]	q-Value	PEP	IonScore	Exp Value	Charge	MH+ [Da]	ΔM [ppm]	RT [min]	# Missed Cleavages
	High	kLTPGLNFTVPILDR	4	1	1	P72655	N-Term(iTRAQ4plex); K1(iTRAQ4plex)	0,0000	0,637	4	22,8	0	3,664E-08	56	2,72879E-06	3	1972,18302	-0,21	44,44	1
	High	nILSTLEGVR	3	1	1	P72655	N-Term(iTRAQ4plex)	0,0000	0,554	3	21,3	0	0,000001536	55	2,90382E-06	2	1245,73027	1,53	41,19	0
	High	iVNEkNEYLVER	1	1	1	P72655	N-Term(iTRAQ4plex); K5(iTRAQ4plex)	0,0000	0,856	1		0	0,000006708	37	0,000219265	3	1794,00125	0,69	25,54	1
	High	aTAEALSILTEk	1	1	1	P72655	N-Term(iTRAQ4plex); K12(iTRAQ4plex)	0,0000	0,602	1		0	0,007808	31	0,000863759	3	1534,89511	1,31	38,69	0
P73321	Uncharacterized protein slr1894 OS=Synechocystis sp. (strain PCC 6803	239,74	29,49	1	5	5	13	0,611	11	35,2	156	17,7	5,57							
	A3	Sequence	# PSMs	# Proteins	# Protein Groups	Protein Group Accessions	Modifications	ΔCn	117/114	117/114 Count	117/114 Variability [%]	q-Value	PEP	IonScore	Exp Value	Charge	MH+ [Da]	ΔM [ppm]	RT [min]	# Missed Cleavages
	High	aTINIGIPEADR	2	1	1	P73321	N-Term(iTRAQ4plex)	0,0000	0,485	1		0	0,00002745	66	2,59998E-07	2	1413,78264	0,54	32,77	0
	High	sLDVFAPGTYk	3	1	1	P73321	N-Term(iTRAQ4plex); K11(iTRAQ4plex)	0,0000	0,579	2	8,0	0	1,066E-08	60	9,0985E-07	2	1485,82024	0,71	35,17	0
	High	gHETIVQScR	2	1	1	P73321	N-Term(iTRAQ4plex); C9(Carbamidomethyl)	0,0000	0,771	2	54,3	0	0,000004238	42	6,19398E-05	3	1330,66660	0,87	10,65	0
	High	iAESLkk	3	1	1	P73321	N-Term(iTRAQ4plex); K6(iTRAQ4plex); K7(iTRAQ4plex)	0,0000	0,937	3	21,1	0	0,001171	40	0,000107145	2	1220,79277	-0,89	16,98	1
	High	tAWMLR	1	1	1	P73321	N-Term(iTRAQ4plex)	0,0000	0,435	1		0,001	0,01172	22	0,005635987	2	921,50957	-0,16	28,16	0
	High	tAWmLR	2	1	1	P73321	N-Term(iTRAQ4plex); M4(Oxidation)	0,0000	0,568	2	15,7	0,003	0,03764	13	0,047970032	2	937,50597	1,43	23,41	0
P73458	Carboxyl-terminal protease OS=Synechocystis sp. (strain PCC 6803 / Ka	238,72	9,69	1	5	5	13	2,062	9	22,4	423	46,8	5,21							
	A3	Sequence	# PSMs	# Proteins	# Protein Groups	Protein Group Accessions	Modifications	ΔCn	117/114	117/114 Count	117/114 Variability [%]	q-Value	PEP	IonScore	Exp Value	Charge	MH+ [Da]	ΔM [ppm]	RT [min]	# Missed Cleavages
	High	gkPGTSVVLTIER	4	1	1	P73458	N-Term(iTRAQ4plex); K2(iTRAQ4plex)	0,0000	2,606	2	12,7	0	1,724E-11	64	4,3448E-07	3	1644,98911	0,23	25,43	0
	High	rGEVEQQSANk	4	1	1	P73458	N-Term(iTRAQ4plex); K11(iTRAQ4plex)	0,0000	1,877	3	7,5	0	0,000001389	44	3,81918E-05	3	1533,82312	0,48	7,99	1
	High	tLIEIHPVR	3	1	1	P73458	N-Term(iTRAQ4plex)	0,0000	1,328	2	72,7	0	0,00001547	36	0,000237668	3	1221,74366	0,03	26,32	0
	High	rGEVEQQSANkR	1	1	1	P73458	N-Term(iTRAQ4plex); K11(iTRAQ4plex)	0,0000	4,139	1		0	0,00000193	26	0,002546654	4	1689,92238	-0,66	7,69	2
	High	kELQQNR	1	1	1	P73458	N-Term(iTRAQ4plex); K1(iTRAQ4plex)	0,0000	1,734	1		0	0,006261	12	0,082396737	3	1203,70584	0,85	8,56	1
Q55458	Uncharacterized protein slr0039 OS=Synechocystis sp. (strain PCC 6803	237,34	11,20	1	4	4	6	0,937	6	23,1	357	38,4	4,91							
	A3	Sequence	# PSMs	# Proteins	# Protein Groups	Protein Group Accessions	Modifications	ΔCn	117/114	117/114 Count	117/114 Variability [%]	q-Value	PEP	IonScore	Exp Value	Charge	MH+ [Da]	ΔM [ppm]	RT [min]	# Missed Cleavages
	High	iNAAVLLVGR	3	1	1	Q55458	N-Term(iTRAQ4plex)	0,0000	1,063	3	3,1	0	2,143E-07	76	2,76039E-08	2	1169,74846	-0,22	38,42	0
	High	yHSPLVVDGLLk	1	1	1	Q55458	N-Term(iTRAQ4plex); K12(iTRAQ4plex)	0,0000	0,682	1		0	0,0004903	37	0,000188352	3	1628,96939	4,88	35,07	0
	High	kPLVFLDEk	1	1	1	Q55458	N-Term(iTRAQ4plex); K1(iTRAQ4plex); K9(iTRAQ4plex)	0,0000	0,781	1		0	0,000001448	34	0,000368104	3	1520,94092	-0,23	30,53	0
	High	eLAHQLNAk	1	1	1	Q55458	N-Term(iTRAQ4plex); K9(iTRAQ4plex)	0,0000	0,844	1		0	0,001004	14	0,041017577	3	1311,76142	-0,70	15,29	0
P74746	Slr0600 protein OS=Synechocystis sp. (strain PCC 6803 / Kazusa) GN=s	237,27	10,12	1	4	4	10	0,818	10	45,1	326	36,0	6,05							
	A3	Sequence	# PSMs	# Proteins	# Protein Groups	Protein Group Accessions	Modifications	ΔCn	117/114	117/114 Count	117/114 Variability [%]	q-Value	PEP	IonScore	Exp Value	Charge	MH+ [Da]	ΔM [ppm]	RT [min]	# Missed Cleavages
	High	lADHGYPLHEAAITk	2	1	1	P74746	N-Term(iTRAQ4plex); K15(iTRAQ4plex)	0,0000	0,698	2	24,2	0	1,733E-08	52	5,62302E-06	4	1924,05556	1,27	24,96	0
	High	iFALGDLkk	5	1	1	P74746	N-Term(iTRAQ4plex); K8(iTRAQ4plex); K9(iTRAQ4plex)	0,0000	1,137	5	44,5	0	0,000001009	52	6,20826E-06	2	1436,91936	-0,54	34,40	1
	High	kDSLYPIFR	2	1	1	P74746	N-Term(iTRAQ4plex); K1(iTRAQ4plex)	0,0000	0,697	2	15,1	0	1,024E-07	41	7,76194E-05	3	1426,82984	0,11	31,41	1
	High	iFALGDLk	1	1	1	P74746	N-Term(iTRAQ4plex); K8(iTRAQ4plex)	0,0000	0,489	1		0	0,001196	29	0,001402717	2	1164,72490	1,54	39,21	0
P72785	4-alpha-glucanotransferase OS=Synechocystis sp. (strain PCC 6803 / Ka	235,39	8,51	1	4	4	10	0,694	9	50,6	505	57,1	4,88							
	A3	Sequence	# PSMs	# Proteins	# Protein Groups	Protein Group Accessions	Modifications	ΔCn	117/114	117/114 Count	117/114 Variability [%]	q-Value	PEP	IonScore	Exp Value	Charge	MH+ [Da]	ΔM [ppm]	RT [min]	# Missed Cleavages
	High	aTGFAWWIk	6	1	1	P72785	N-Term(iTRAQ4plex); K9(iTRAQ4plex)	0,0000	0,694	5	50,6	0	0,000002696	58	1,5775E-06	2	1367,76860	-2,09	44,06	0

	High	aNLQYLDIVR	1	1	1	P72785	N-Term(iTRAQ4plex)	0,0000	0,729	1		0	3,661E-07	48	1,63376E-05	2	1348,76933	-0,90	37,48	0
	High	vLHFAFDSDR	2	1	1	P72785	N-Term(iTRAQ4plex)	0,0000	0,508	2	37,4	0	0,00005926	39	0,000112194	3	1350,69083	-1,10	32,44	0
	High	cSGILLHPTSLPSR	1	1	1	P72785	N-Term(iTRAQ4plex); C1(Carbamidomethyl)	0,0000	1,116	1		0	0,000006619	31	0,000860934	3	1681,91635	-0,77	28,66	0
Q55900	Septum site-determining protein MinD OS=Synechocystis sp. (strain PCC	229,58	17,29	1	4	4	7	0,855	7	32,5	266	29,0	5,41							
	A3	Sequence	# PSMs	# Proteins	# Protein Groups	Protein Group Accessions	Modifications	ΔCn	117/114	117/114 Count	117/114 Variability [%]	q-Value	PEP	IonScore	Exp Value	Charge	MH+ [Da]	ΔM [ppm]	RT [min]	# Missed Cleavages
	High	lSVPGLAFQNIAR	2	1	1	Q55900	N-Term(iTRAQ4plex)	0,0000	0,849	2	0,9	0	0,00000376	61	8,74923E-07	2	1529,89372	1,07	43,68	0
	High	tTTTANLGAALAR	2	1	1	Q55900	N-Term(iTRAQ4plex)	0,0000	0,763	2	51,6	0	0,000001952	53	5,5335E-06	2	1404,79363	0,61	26,33	0
	High	lPNLVLLPAAQNR	2	1	1	Q55900	N-Term(iTRAQ4plex)	0,0000	1,652	2	118,5	0	0,0001798	44	4,01763E-05	2	1562,95110	0,74	43,99	0
	High	iSLIVNR	1	1	1	Q55900	N-Term(iTRAQ4plex)	0,0000	0,550	1		0,001	0,02062	32	0,00064561	2	958,61730	0,71	28,83	0
P74667	Diaminopimelate epimerase OS=Synechocystis sp. (strain PCC 6803 / Ka	228,91	17,20	1	4	4	8	0,957	8	7,1	279	30,1	4,91							
	A3	Sequence	# PSMs	# Proteins	# Protein Groups	Protein Group Accessions	Modifications	ΔCn	117/114	117/114 Count	117/114 Variability [%]	q-Value	PEP	IonScore	Exp Value	Charge	MH+ [Da]	ΔM [ppm]	RT [min]	# Missed Cleavages
	High	iHTLAGVITPQLLADGQVk	3	1	1	P74667	N-Term(iTRAQ4plex); K19(iTRAQ4plex)	0,0000	1,250	3	10,5	0	4,685E-10	64	4,36486E-07	3	2262,34476	1,02	42,04	0
	High	yHGLGNDFILVDNR	2	1	1	P74667	N-Term(iTRAQ4plex)	0,0000	0,954	2	10,1	0	7,077E-08	43	4,49749E-05	3	1776,91605	0,59	35,79	0
	High	lYmTGPAQR	2	1	1	P74667	N-Term(iTRAQ4plex); M3(Oxidation)	0,0000	0,946	2	2,6	0	0,003321	31	0,000887095	2	1196,62200	0,46	16,95	0
	High	aLSFSk	1	1	1	P74667	N-Term(iTRAQ4plex); K6(iTRAQ4plex)	0,0000	0,952	1		0,001	0,01977	38	0,000159577	2	940,56951	-1,17	22,13	0
P49433	Glyceraldehyde-3-phosphate dehydrogenase 1 OS=Synechocystis sp. (st	228,77	15,63	1	5	5	11	0,831	9	16,6	339	36,1	6,24							
	A3	Sequence	# PSMs	# Proteins	# Protein Groups	Protein Group Accessions	Modifications	ΔCn	117/114	117/114 Count	117/114 Variability [%]	q-Value	PEP	IonScore	Exp Value	Charge	MH+ [Da]	ΔM [ppm]	RT [min]	# Missed Cleavages
	High	gAAQNIIPSSTGAAk	4	1	1	P49433	N-Term(iTRAQ4plex); K15(iTRAQ4plex)	0,0000	0,831	3	3,8	0	3,603E-09	93	4,57057E-10	2	1673,94524	1,63	23,18	0
	High	aAALVLPQLk	4	1	1	P49433	N-Term(iTRAQ4plex); K10(iTRAQ4plex)	0,0000	0,891	3	12,9	0	0,0003711	54	4,35482E-06	2	1311,86272	1,86	37,53	0
	High	iGINGFGR	1	1	1	P49433	N-Term(iTRAQ4plex)	0,0000	0,827	1		0	0,001799	33	0,0006197	2	977,56609	1,19	28,19	0
	High	rVIISAPSkDPEk	1	1	1	P49433	N-Term(iTRAQ4plex); K9(iTRAQ4plex); K13(iTRAQ4plex)	0,0000	0,928	1		0	0,000008354	19	0,013802889	4	1872,12868	0,41	19,35	2
	High	lTGMAFR	1	1	1	P49433	N-Term(iTRAQ4plex)	0,0000	0,634	1		0,009	0,1213	19	0,013771144	2	939,52129	1,07	27,77	0
P73048	Sll1638 protein OS=Synechocystis sp. (strain PCC 6803 / Kazusa) GN=s	227,55	24,16	1	4	4	10	0,848	10	22,3	149	16,5	7,34							
	A3	Sequence	# PSMs	# Proteins	# Protein Groups	Protein Group Accessions	Modifications	ΔCn	117/114	117/114 Count	117/114 Variability [%]	q-Value	PEP	IonScore	Exp Value	Charge	MH+ [Da]	ΔM [ppm]	RT [min]	# Missed Cleavages
	High	iAQLQVYVNPIAVAR	4	1	1	P73048	N-Term(iTRAQ4plex)	0,0000	1,020	4	10,8	0	2,005E-09	72	5,74077E-08	2	1799,06804	1,12	43,71	0
	High	rDmLGLASSLLPk	2	1	1	P73048	N-Term(iTRAQ4plex); M3(Oxidation); K13(iTRAQ4plex)	0,0000	0,848	2	10,4	0	1,504E-07	34	0,000455186	3	1704,99167	-0,26	35,40	1
	High	rDMLGLASSLLPk	1	1	1	P73048	N-Term(iTRAQ4plex); K13(iTRAQ4plex)	0,0000	0,705	1		0	0,000001344	28	0,00154871	3	1688,99430	-1,72	43,06	1
	High	eVFGHLER	2	1	1	P73048	N-Term(iTRAQ4plex)	0,0000	0,848	2	9,5	0	0,00102	23	0,005495029	3	1130,60776	0,22	19,95	0
	High	dMLGLASSLLPk	1	1	1	P73048	N-Term(iTRAQ4plex); K12(iTRAQ4plex)	0,0000	0,539	1		0,007	0,08823	20	0,009907635	3	1532,89524	-0,55	48,70	0
Q55707	Uncharacterized protein sll0617 OS=Synechocystis sp. (strain PCC 6803	227,53	21,35	1	6	6	10	1,024	7	12,1	267	28,9	5,03							
	A3	Sequence	# PSMs	# Proteins	# Protein Groups	Protein Group Accessions	Modifications	ΔCn	117/114	117/114 Count	117/114 Variability [%]	q-Value	PEP	IonScore	Exp Value	Charge	MH+ [Da]	ΔM [ppm]	RT [min]	# Missed Cleavages
	High	aNAELQQTLGGLGTSSATSAFER	1	1	1	Q55707	N-Term(iTRAQ4plex)	0,0000	0,412	1		0	6,328E-09	69	1,22453E-07	3	2453,24210	1,28	41,25	0
	High	nLAALEAk	4	1	1	Q55707	N-Term(iTRAQ4plex); K8(iTRAQ4plex)	0,0000	1,026	2	11,8	0	0,00002	50	1,20891E-05	2	1117,68328	1,17	24,60	0
	High	aNLNDLVSk	1	1	1	Q55707	N-Term(iTRAQ4plex); K9(iTRAQ4plex)	0,0000	1,024	1		0	0,000004393	43	4,46653E-05	2	1261,73699	1,21	27,26	0
	High	lNQDTQEAk	1	1	1	Q55707	N-Term(iTRAQ4plex); K9(iTRAQ4plex)	0,0000	0,903	1		0	7,756E-07	40	9,48353E-05	2	1334,71648	0,77	12,82	0
	High	rNLAALEAk	2	1	1	Q55707	N-Term(iTRAQ4plex); K9(iTRAQ4plex)	0,0000	1,034	1		0	0,0005439	37	0,000218258	3	1273,78562	1,99	18,23	1
	Medium	kNmLQAR	1	1	1	Q55707	N-Term(iTRAQ4plex); K1(iTRAQ4plex); M3(Oxidation)	0,0000	1,312	1		0,035	0,441	11	0,081840826	3	1164,67682	0,56	8,32	1
P74490	Pyruvate dehydrogenase E1 component, alpha subunit OS=Synechocyst	224,29	16,67	1	5	5	9	1,195	8	18,2	342	38,1	5,19							
	A3	Sequence	# PSMs	# Proteins	# Protein Groups	Protein Group Accessions	Modifications	ΔCn	117/114	117/114 Count	117/114 Variability [%]	q-Value	PEP	IonScore	Exp Value	Charge	MH+ [Da]	ΔM [ppm]	RT [min]	# Missed Cleavages
	High	wAIGmAHER	2	1	1	P74490	N-Term(iTRAQ4plex); M5(Oxidation)	0,0000	0,932	1		0	9,822E-07	61	8,12774E-07	3	1230,61750	0,36	18,16	0
	High	aGEGPTLIEALTYR	1	1	1	P74490	N-Term(iTRAQ4plex)	0,0000	1,203	1		0	1,026E-08	60	1,13289E-06	2	1634,88811	0,64	43,32	0
	High	wAIGMAHER	1	1	1	P74490	N-Term(iTRAQ4plex)	0,0000	1,187	1		0	0,000001763	48	1,58114E-05	3	1214,62241	0,23	23,11	0
	High	dHVHALSAGVPAR	1	1	1	P74490	N-Term(iTRAQ4plex)	0,0000	1,394	1		0	0,000002092	37	0,000215278	3	1473,80454	0,14	15,69	0
	High	lPILFVVENNk	2	1	1	P74490	N-Term(iTRAQ4plex); K11(iTRAQ4plex)	0,0000	1,069	2	30,6	0	0,000001534	31	0,000751571	3	1573,95719	0,98	48,19	0
	High	aTSQPEIYkk	2	1	1	P74490	N-Term(iTRAQ4plex); K9(iTRAQ4plex); K10(iTRAQ4plex)	0,0000	1,439	2	38,8	0	0,00007134	30	0,001503458	3	1596,93161	-0,33	15,23	1
P72585	GDP-L-fucose synthase OS=Synechocystis sp. (strain PCC 6803 / Kazusa	222,15	15,71	1	5	5	7	0,996	7	27,6	312	34,7	5,90							
	A3	Sequence	# PSMs	# Proteins	# Protein Groups	Protein Group Accessions	Modifications	ΔCn	117/114	117/114 Count	117/114 Variability [%]	q-Value	PEP	IonScore	Exp Value	Charge	MH+ [Da]	ΔM [ppm]	RT [min]	# Missed Cleavages
	High	qVVAQLIAAGGDRPk	2	1	1	P72585	N-Term(iTRAQ4plex); K15(iTRAQ4plex)	0,0000	1,034	2	5,6	0	2,705E-11	75	2,83772E-08	3	1811,07602	1,00	31,45	0
	High	kALLVQLESYR	1	1	1	P72585	N-Term(iTRAQ4plex); K1(iTRAQ4plex)	0,0000	1,196	1		0	1,78E-09	41	7,83376E-05	3	1607,97287	0,33	36,64	1
	High	aLLVQLESYR	1	1	1	P72585	N-Term(iTRAQ4plex)	0,0000	0,904	1		0	0,0001279	35	0,000291723	2	1335,77495	-0,27	40,67	0
	High	sSHVIPALIHk	1	1	1	P72585	N-Term(iTRAQ4plex); K11(iTRAQ4plex)	0,0000	0,756	1		0	0,000008594	33	0,000524771	4	1489,91005	0,48	22,97	0
	High	iLVTGGAGFLGk	2	1	1	P72585	N-Term(iTRAQ4plex); K12(iTRAQ4plex)	0,0000	0,721	2	98,8	0	0,004663	25	0,002917226	3	1420,87690	0,16	38,44	0
P19050	NAD(P)H-quinone oxidoreductase subunit K 1 OS=Synechocystis sp. (st	221,77	23,79	1	8	8	15	0,594	15	13,1	248	27,3	8,13							
	A3	Sequence	# PSMs	# Proteins	# Protein Groups	Protein Group Accessions	Modifications	ΔCn	117/114	117/114 Count	117/114 Variability [%]	q-Value	PEP	IonScore	Exp Value	Charge	MH+ [Da]	ΔM [ppm]	RT [min]	# Missed Cleavages
	High	kVANESIQER	2	1	1	P19050	N-Term(iTRAQ4plex); K1(iTRAQ4plex)	0,0000	0,628	2	3,3	0	5,163E-08	53	5,6195E-06	3	1461,82633	-0,05	11,80	1
	High	yLQQGTR	2	1	1	P19050	N-Term(iTRAQ4plex)	0,0000	0,596	2	15,6	0	0,0003831	28	0,001633052	2	1009,55492	0,19	14,91	0
	High	yYSTSHQmk	1	1	1	P19050	N-Term(iTRAQ4plex); M8(Oxidation); K9(iTRAQ4plex)	0,0000	0,373	1		0	0,0005671	15	0,028971435	3	1448,71003	1,24	10,30	0
	High	fGLVPR	3	1	1	P19050	N-Term(iTRAQ4plex)	0,0000	0,594	3	0,3	0,001	0,01972	31	0,000748118	2	832,51683	0,79	26,23	0
	High	iLNPASR	3	1	1	P19050	N-Term(iTRAQ4plex)	0,0000	0,504	3	3,7	0,001	0,0166	27	0,001909721	2	914,55419	0,19	17,75	0
	Medium	mAPALVR	1	1	1	P19050	N-Term(iTRAQ4plex); M1(Oxidation)	0,0000	0,594	1		0,022	0,2795	13	0,054321281	2	917,53630	0,40	16,17	0
	Medium	aITQQTHR	1	1	1	P19050	N-Term(iTRAQ4plex)	0,0000	0,684	1		0,036	0,4462	17	0,020891518	2	1098,61504	1,27	9,64	0
	Medium	vATAk	2	1	1	P19050	N-Term(iTRAQ4plex); K5(iTRAQ4plex)	0,0000	0,637	2	2,7	0,041	0,5	31	0,00188983	2	777,50145	-7,49	10,89	0
P74266	50S ribosomal protein L21 OS=Synechocystis sp. (strain PCC 6803 / Kaz	220,65	15,32	1	3	3	10	1,041	10	44,6	124	13,7	9,52							
	A3	Sequence	# PSMs	# Proteins	# Protein Groups	Protein Group Accessions	Modifications	ΔCn	117/114	117/114 Count	117/114 Variability [%]	q-Value	PEP	IonScore	Exp Value	Charge	MH+ [Da]	ΔM [ppm]	RT [min]	# Missed Cleavages
	High	sYAIIEIGGTQIR	3	1	1	P74266	N-Term(iTRAQ4plex)	0,0000	0,950	3	3,3	0	5,725E-11	75	3,34172E-08	2	1564,88262	0,67	38,31	0
	High	kVIVYk	4	1	1	P74266	N-Term(iTRAQ4plex); K1(iTRAQ4plex); K6(iTRAQ4plex)	0,0000	1,557	4	29,4	0	0,002986	21	0,008709034	3	1181,79370	-3,80	16,62	1
	High	vIVYk	3	1	1	P74266	N-Term(iTRAQ4plex); K5(iTRAQ4plex)	0,0000	1,115	3	54,4	0,006	0,0771	27	0,002127992	2	909,59899	-2,40	21,13	0
P72703	Agmatinase 1 OS=Synechocystis sp. (strain PCC 6803 / Kazusa) GN=sp	219,69	13,73	1	4	4	13	1,002	10	35,3	306	33,4	5,01							
	A3	Sequence	# PSMs	# Proteins	# Protein Groups	Protein Group Accessions	Modifications	ΔCn	117/114	117/114 Count	117/114 Variability [%]	q-Value	PEP	IonScore	Exp Value	Charge	MH+ [Da]	ΔM [ppm]	RT [min]	# Missed Cleavages
	High	fVVAIGGEHAITTGVVR	4	1	1	P72703	N-Term(iTRAQ4plex)	0,0000	0,780	3	11,1	0	4,577E-09	66	2,34407E-07	3	1870,06693	0,08	36,50	0
	High	nIPVFWAR	5	1	1	P72703	N-Term(iTRAQ4plex)	0,0000	1,176	4	11,6	0	0,0006464	32	0,000571439	2	1146,65386	-0,19	39,02	0
	High	rVLELGLPTLPIAIR	3	1	1	P72703	N-Term(iTRAQ4plex)	0,0000	0,650	2	62,9	0	0,00002256	23	0,00468781	3	1805,14908	-0,17	48,46	1
	High	ekNIPVFWAR	1	1	1	P72703	N-Term(iTRAQ4plex); K2(iTRAQ4plex)	0,0000	1,633	1		0	0,009433	11	0,113588344	3	1547,89139	-1,50	33,44	1
Q55131	Slr0049 protein OS=Synechocystis sp. (strain PCC 6803 / Kazusa) GN=s	217,94	9,55	1	3	3	7	0,775	6	13,4	398	44,0	5,06							
	A3	Sequence	# PSMs	# Proteins	# Protein Groups	Protein Group Accessions	Modifications	ΔCn	117/114	117/114 Count	117/114 Variability [%]	q-Value	PEP	IonScore	Exp Value	Charge	MH+ [Da]	ΔM [ppm]	RT [min]	# Missed Cleavages
	High	vmIVGAGGVGSVVAHk	1	1	1	Q55131	N-Term(iTRAQ4plex); M2(Oxidation); K16(iTRAQ4plex)	0,0000	0,690	1		0	1,805E-14	76	2,65442E-08	3	1785,03073	0,65	29,86	0
	High	iVPLQFLk	5	1	1	Q55131	N-Term(iTRAQ4plex); K8(iTRAQ4plex)	0,0000	0,775	4	6,7	0	0,0002346	59	1,30909E-06	2	1245,81975	1,94	43,86	0
	High	fWmTFSEAYINHLR	1	1	1	Q55131	N-Term(iTRAQ4plex); M3(Oxidation)	0,0000	0,883	1		0	2,874E-07	11	0,083170633	3	1974,96384	-0,75	46,09	0
P74494	Nucleoside diphosphate kinase OS=Synechocystis sp. (strain PCC 6803 ,	217,33	34,23	1	4	4	13	0,862	10	17,5	149	16,7	5,55							
	A3	Sequence	# PSMs	# Proteins	# Protein Groups	Protein Group Accessions	Modifications	ΔCn	117/114	117/114 Count	117/114 Variability [%]	q-Value	PEP	IonScore	Exp Value	Charge	MH+ [Da]	ΔM [ppm]	RT [min]	# Missed Cleavages
	High	nIIHGSDAIETAk	2	1	1	P74494	N-Term(iTRAQ4plex); K13(iTRAQ4plex)	0,0000	0,715	1		0	1,607E-08	56	2,9779E-06	3	1656,91458	-0,83	21,29	0
	High	qLIGEIVGR	4	1	1	P74494	N-Term(iTRAQ4plex)	0,0000	0,841	3	7,2	0	0,00006535	48	1,5065E-05	2	1128,68596	0,17	33,07	0
	High	qmIGATDPHAAAPGTIR	2	1	1	P74494	N-Term(iTRAQ4plex); M2(Oxidation)	0,0000	0,807	2	19,4	0	1,217E-08	40	9,3319E-05	3	1866,96183	0,26	19,38	0
	High	tFIMIkPDGVQR	3	1	1	P74494	N-Term(iTRAQ4plex); K6(iTRAQ4plex)	0,0000	0,954	2	10,8	0	0,00005202	28	0,001745702	3	1692,96970	-0,76	30,56	0
	High	tFImIkPDGVQR	2	1	1	P74494	N-Term(iTRAQ4plex); M4(Oxidation); K6(iTRAQ4plex)	0,0000	0,884	2	17,5	0	0,000005662	27	0,002046303	3	1708,96506	-0,49	27,60	0
P72864	Carboxysome formation protein OS=Synechocystis sp. (strain PCC 6803	217,10	25,93	1	9	9	14	0,717	13	24,0	351	37,6	6,37							
	A3	Sequence	# PSMs	# Proteins	# Protein Groups	Protein Group Accessions	Modifications	ΔCn	117/114	117/114 Count	117/114 Variability [%]	q-Value	PEP	IonScore	Exp Value	Charge	MH+ [Da]	ΔM [ppm]	RT [min]	# Missed Cleavages
	High	qLAVIGQTVGR	2	1	1	P72864	N-Term(iTRAQ4plex)	0,0000	0,599	2	6,4	0	0,000001012	50	8,97367E-06	2	1285,77190	0,77	29,23	0
	High	aAGAQFLR	1	1	1	P72864	N-Term(iTRAQ4plex)	0,0000	0,707	1		0	0,00006761	40	0,000113548	2	977,56560	0,69	20,96	0
	High	tSPYAFQGHGESALGLLAAAk	2	1	1	P72864	N-Term(iTRAQ4plex); K21(iTRAQ4plex)	0,0000	0,757	1		0	9,988E-07	38	0,000151695	4	2377,27773	0,94	40,90	0
	High	kVGAQDkPVLLk	1	1	1	P72864	N-Term(iTRAQ4plex); K1(iTRAQ4plex); K7(iTRAQ4plex);	0,0000	0,944	1		0	6,468E-07	33	0,000496558	4	1872,21120	-0,89	20,99	1
	High	sLTHLPImIDPSHGTGk	1	1	1	P72864	N-Term(iTRAQ4plex); M8(Oxidation); K17(iTRAQ4plex)	0,0000	0,612	1		0	3,496E-07	28	0,001425509	4	2108,14443	1,48	30,27	0
	High	vGAQDkPVLLk	2	1	1	P72864	N-Term(iTRAQ4plex); K6(iTRAQ4plex); K11(iTRAQ4plex)	0,0000	0,577	2	5,0	0	0,00002018	27	0,001874865	3	1600,01621	0,23	24,16	0
	High	sLTHLPIMIDPSHGTGk	1	1	1	P72864	N-Term(iTRAQ4plex); K17(iTRAQ4plex)	0,0000	0,748	1		0	2,475E-07	26	0,002605974	4	2092,14663	0,11	33,31	0
	High	nmQNFSmLk	1	1	1	P72864	N-Term(iTRAQ4plex); M2(Oxidation); M7(Oxidation); K9(	0,0000	0,717	1		0	0,0000103	20	0,009098504	2	1432,71782	0,76	19,98	0
	High	aSLTFR	2	1	1	P72864	N-Term(iTRAQ4plex)	0,0000	0,817	2	7,8	0,006	0,06955	13	0,046341491	2	838,49144	1,31	20,60	0
	High	vEQPFkR	1	1	1	P72864	N-Term(iTRAQ4plex); K6(iTRAQ4plex)	0,0000	0,926	1		0,008	0,103	12	0,08451686	3	1191,70896	0,09	15,26	1
P72656	Ribonuclease E/G-like protein OS=Synechocystis sp. (strain PCC 6803 /	215,68	9,79	1	5	5	8	1,089	7	37,0	674	74,9	6,16							
	A3	Sequence	# PSMs	# Proteins	# Protein Groups	Protein Group Accessions	Modifications	ΔCn	117/114	117/114 Count	117/114 Variability [%]	q-Value	PEP	IonScore	Exp Value	Charge	MH+ [Da]	ΔM [ppm]	RT [min]	# Missed Cleavages
	High	aRPQIAQLTELGLVELTR	2	1	1	P72656	N-Term(iTRAQ4plex)	0,0000	1,132	2	43,4	0	4,174E-11	76	2,27494E-08	3	2152,25754	0,20	47,97	0
	High	lVEkPILSPPVAk	1	1	1	P72656	N-Term(iTRAQ4plex); K4(iTRAQ4plex); K13(iTRAQ4plex)	0,0000	0,831	1		0	1,534E-10	52	6,79157E-06	3	1823,17380	0,40	31,58	0
	High	skGPSPSPTk	1	1	1	P72656	N-Term(iTRAQ4plex); K2(iTRAQ4plex); K10(iTRAQ4plex)	0,0000	1,210	1		0	0,001078	36	0,000429865	3	1417,83890	0,97	8,51	1
	High	aLAVLIkPPGmGLLVR	2	1	1	P72656	N-Term(iTRAQ4plex); K7(iTRAQ4plex); M11(Oxidation)	0,0000	1,327	1		0	0,00000192	30	0,000939658	3	1952,23569	1,18	41,79	0
	High	hQVAAVFWk	1	1	1	P72656	N-Term(iTRAQ4plex); K9(iTRAQ4plex)	0,0000	0,571	1		0	0,0001691	22	0,006367515	3	1373,79471	1,07	28,45	0
	High	aLAVLIkPPGMGLLVR	1	1	1	P72656	N-Term(iTRAQ4plex); K7(iTRAQ4plex)	0,0000	1,089	1		0	0,00133	14	0,040083903	3	1936,23978	0,68	47,36	0
P74375	Slr0442 protein OS=Synechocystis sp. (strain PCC 6803 / Kazusa) GN=s	215,25	6,87	1	4	4	12	1,520	11	37,4	611	62,9	5,21							
	A3	Sequence	# PSMs	# Proteins	# Protein Groups	Protein Group Accessions	Modifications	ΔCn	117/114	117/114 Count	117/114 Variability [%]	q-Value	PEP	IonScore	Exp Value	Charge	MH+ [Da]	ΔM [ppm]	RT [min]	# Missed Cleavages
	High	aSTPmPSIPLSALASAR	4	1	1	P74375	N-Term(iTRAQ4plex); M5(Oxidation)	0,0000	1,116	4	45,1	0	0,000008261	58	1,66492E-06	2	1829,99138	0,08	38,24	0
	High	vQGLLASSk	2	1	1	P74375	N-Term(iTRAQ4plex); K9(iTRAQ4plex)	0,0000	1,520	1		0	0,000002416	42	6,7293E-05	2	1190,73406	-0,56	25,10	0
	High	aSTPMPSIPLSALASAR	3	1	1	P74375	N-Term(iTRAQ4plex)	0,0000	1,191	3	34,4	0	0,0003035	39	0,000138986	3	1813,99656	0,13	42,97	0
	High	iPQGQVIGR	2	1	1	P74375	N-Term(iTRAQ4plex)	0,0000	1,728	2	8,2	0	0,001631	37	0,000184489	2	1111,67046	0,00	22,09	0
	Medium	tcTYIPR	1	1	1	P74375	N-Term(iTRAQ4plex); C2(Carbamidomethyl)	0,0000	1,898	1		0,022	0,27	11	0,085304119	2	1054,54631	-0,84	18,88	0
P74193	Threonine synthase OS=Synechocystis sp. (strain PCC 6803 / Kazusa) G	213,76	26,70	1	6	6	8	1,597	8	30,4	382	40,4	7,24							
	A3	Sequence	# PSMs	# Proteins	# Protein Groups	Protein Group Accessions	Modifications	ΔCn	117/114	117/114 Count	117/114 Variability [%]	q-Value	PEP	IonScore	Exp Value	Charge	MH+ [Da]	ΔM [ppm]	RT [min]	# Missed Cleavages
	High	qLSEHYPVTLVNSVNPYR	2	1	1	P74193	N-Term(iTRAQ4plex)	0,0000	1,799	2	9,0	0	1,164E-09	62	6,01132E-07	3	2260,18558	0,57	37,17	0
	High	mmGFQAAGSAPFIQGQPISHPET	1	1	1	P74193	N-Term(iTRAQ4plex); M1(Oxidation); M2(Oxidation)	0,0000	1,505	1		0	1,539E-07	59	1,17482E-06	4	3203,61245	0,78	40,81	0
	High	aGLkPDLEIVAk	2	1	1	P74193	N-Term(iTRAQ4plex); K4(iTRAQ4plex); K12(iTRAQ4plex)	0,0000	1,566	2	32,6	0	2,395E-07	38	0,00016217	3	1686,05051	-1,26	30,58	0
	High	aVIcASTGNTSAAAAAYAR	1	1	1	P74193	N-Term(iTRAQ4plex); C4(Carbamidomethyl)	0,0000	1,235	1		0	0,00005344	24	0,004158819	3	1969,98850	0,12	27,25	0
	High	aFVIIPDGYVALGk	1	1	1	P74193	N-Term(iTRAQ4plex); K14(iTRAQ4plex)	0,0000	1,961	1		0	0,008116	23	0,004602248	3	1751,03153	-1,77	46,25	0
	High	dRGmTmAISk	1	1	1	P74193	N-Term(iTRAQ4plex); M4(Oxidation); M6(Oxidation); K1(	0,0000	1,022	1		0,001	0,01899	13	0,052236011	3	1429,73801	-0,14	9,92	1
Q55366	Ferredoxin--nitrite reductase OS=Synechocystis sp. (strain PCC 6803 / K	210,75	14,14	1	7	7	11	0,833	10	27,4	502	55,9	6,61							
	A3	Sequence	# PSMs	# Proteins	# Protein Groups	Protein Group Accessions	Modifications	ΔCn	117/114	117/114 Count	117/114 Variability [%]	q-Value	PEP	IonScore	Exp Value	Charge	MH+ [Da]	ΔM [ppm]	RT [min]	# Missed Cleavages
	High	qAGLNYVGLHVPVGR	2	1	1	Q55366	N-Term(iTRAQ4plex)	0,0000	1,178	1		0	8,805E-13	56	2,64223E-06	3	1723,97293	0,27	33,15	0
	High	lPFPLLTAAPk	3	1	1	Q55366	N-Term(iTRAQ4plex); K11(iTRAQ4plex)	0,0000	0,851	3	31,6	0	0,0000274	54	3,59724E-06	2	1455,92046	1,83	46,18	0
	High	eLTVPkPVR	1	1	1	Q55366	N-Term(iTRAQ4plex); K6(iTRAQ4plex)	0,0000	0,495	1		0	0,00004429	46	2,56431E-05	3	1326,83585	0,80	21,85	0
	High	kFNIAIEGSR	2	1	1	Q55366	N-Term(iTRAQ4plex); K1(iTRAQ4plex)	0,0000	0,806	2	1,9	0	0,000000186	42	7,50207E-05	3	1422,83265	1,32	24,73	1
	High	fREEVAAk	1	1	1	Q55366	N-Term(iTRAQ4plex); K8(iTRAQ4plex)	0,0000	0,938	1		0	0,0004233	34	0,00039443	3	1237,71375	-0,48	15,58	1
	High	dAHLGTcVHk	1	1	1	Q55366	N-Term(iTRAQ4plex); C7(Carbamidomethyl); K10(iTRAQ4	0,0000	1,503	1		0	0,0007022	12	0,066369723	4	1425,75014	-0,69	13,00	0
	High	aNkFETVk	1	1	1	Q55366	N-Term(iTRAQ4plex); K3(iTRAQ4plex); K8(iTRAQ4plex)	0,0000	0,701	1		0	0,001742	11	0,074984242	3	1368,82010	-0,76	16,62	1
P74729	HrEpiB OS=Synechocystis sp. (strain PCC 6803 / Kazusa) GN=sll0576 PE	210,08	26,60	1	8	8	13	0,741	11	23,0	312	34,9	7,62							
	A3	Sequence	# PSMs	# Proteins	# Protein Groups	Protein Group Accessions	Modifications	ΔCn	117/114	117/114 Count	117/114 Variability [%]	q-Value	PEP	IonScore	Exp Value	Charge	MH+ [Da]	ΔM [ppm]	RT [min]	# Missed Cleavages
	High	aVVIFEGHFk	4	1	1	P74729	N-Term(iTRAQ4plex); K10(iTRAQ4plex)	0,0000	0,743	2	0,3	0	5,759E-07	57	2,12814E-06	3	1434,83704	1,57	32,54	0
	High	lATVFGMAPR	1	1	1	P74729	N-Term(iTRAQ4plex)	0,0000	0,636	1		0	0,000001193	49	1,16942E-05	2	1206,67900	0,34	38,06	0
	High	vFLHGLENFESmk	1	1	1	P74729	N-Term(iTRAQ4plex); M12(Oxidation); K13(iTRAQ4plex)	0,0000	0,533	1		0	1,736E-08	39	0,00013365	3	1854,96439	-1,03	36,21	0
	High	lATVFGmAPR	1	1	1	P74729	N-Term(iTRAQ4plex); M7(Oxidation)	0,0000	0,696	1		0	0,00004042	30	0,001056745	2	1222,67485	1,10	32,45	0
	High	gYTILR	2	1	1	P74729	N-Term(iTRAQ4plex)	0,0000	0,645	2	25,0	0	0,00558	25	0,003357144	2	866,52141	-0,26	21,82	0
	High	nYIHIR	1	1	1	P74729	N-Term(iTRAQ4plex)	0,0000	0,595	1		0	0,003346	16	0,026059736	3	959,55539	1,11	17,33	0
	High	mRVDLLVNDFVYR	1	1	1	P74729	N-Term(iTRAQ4plex); M1(Oxidation)	0,0000	1,440	1		0	0,0001204	14	0,043850041	3	1799,96134	1,00	40,66	1
	High	kEDLIkEQLk	1	1	1	P74729	N-Term(iTRAQ4plex); K1(iTRAQ4plex); K6(iTRAQ4plex);	0,0000	0,773	1		0	0,0002699	11	0,070952876	3	1820,13828	2,38	22,78	2
	High	gkPYNVGLEDANLSk	1	1	1	P74729	N-Term(iTRAQ4plex); K2(iTRAQ4plex); K15(iTRAQ4plex)	0,0000	0,901	1		0	0,00005619	11	0,079793957	3	2037,13657	1,20	27,13	0
Q05971	10 kDa chaperonin OS=Synechocystis sp. (strain PCC 6803 / Kazusa) GI	209,51	41,75	1	4	4	7	0,846	7	21,7	103	10,9	5,22							
	A3	Sequence	# PSMs	# Proteins	# Protein Groups	Protein Group Accessions	Modifications	ΔCn	117/114	117/114 Count	117/114 Variability [%]	q-Value	PEP	IonScore	Exp Value	Charge	MH+ [Da]	ΔM [ppm]	RT [min]	# Missed Cleavages
	High	aAISINVSTVkPLGDR	3	1	1	Q05971	N-Term(iTRAQ4plex); K11(iTRAQ4plex)	0,0000	0,994	3	1,0	0	9,597E-10	67	1,84914E-07	3	1929,14060	1,76	32,90	0
	High	ekPQIGEVVQVGPGk	2	1	1	Q05971	N-Term(iTRAQ4plex); K2(iTRAQ4plex); K15(iTRAQ4plex)	0,0000	0,909	2	10,8	0	1,116E-10	52	6,03907E-06	3	1997,17435	-0,63	26,56	0
	High	vSPAEEk	1	1	1	Q05971	N-Term(iTRAQ4plex); K7(iTRAQ4plex)	0,0000	0,686	1		0	0,006523	28	0,001617968	2	1047,59221	-0,25	12,35	0
	High	vLYSk	1	1	1	Q05971	N-Term(iTRAQ4plex); K5(iTRAQ4plex)	0,0000	0,826	1		0,006	0,08372	27	0,001849141	2	897,56584	1,19	16,88	0
P73244	Slr2025 protein OS=Synechocystis sp. (strain PCC 6803 / Kazusa) GN=s	208,65	25,49	1	3	3	9	0,952	6	9,6	153	16,9	4,44							
	A3	Sequence	# PSMs	# Proteins	# Protein Groups	Protein Group Accessions	Modifications	ΔCn	117/114	117/114 Count	117/114 Variability [%]	q-Value	PEP	IonScore	Exp Value	Charge	MH+ [Da]	ΔM [ppm]	RT [min]	# Missed Cleavages
	High	eNHLVALHQR	6	1	1	P73244	N-Term(iTRAQ4plex)	0,0000	0,915	4	5,8	0	7,093E-08	65	2,89714E-07	2	1360,75652	-0,09	13,59	0
	High	tVADLSPSEISR	1	1	1	P73244	N-Term(iTRAQ4plex)	0,0000	1,004	1		0	0,000003065	52	6,16552E-06	2	1418,76067	-0,09	27,67	0
	High	aITLVATLADDHDDRLk	2	1	1	P73244	N-Term(iTRAQ4plex); K17(iTRAQ4plex)	0,0000	1,154	1		0	3,385E-11	41	9,68682E-05	4	2155,19668	0,23	37,39	1
P74474	D-alanyl-D-alanine carboxypeptidase OS=Synechocystis sp. (strain PCC	208,04	14,25	1	5	5	6	0,340	6	9,6	400	44,3	4,75							
	A3	Sequence	# PSMs	# Proteins	# Protein Groups	Protein Group Accessions	Modifications	ΔCn	117/114	117/114 Count	117/114 Variability [%]	q-Value	PEP	IonScore	Exp Value	Charge	MH+ [Da]	ΔM [ppm]	RT [min]	# Missed Cleavages
	High	sLFQIGSITk	2	1	1	P74474	N-Term(iTRAQ4plex); K10(iTRAQ4plex)	0,0000	0,558	2	95,0	0	1,292E-08	77	2,06524E-08	2	1381,83379	3,21	38,51	0
	High	sHAPNSPPIDAk	1	1	1	P74474	N-Term(iTRAQ4plex); K12(iTRAQ4plex)	0,0000	0,347	1		0	0,000001448	49	1,30975E-05	3	1521,83072	2,84	14,19	0
	High	vYGQSYAELIDkk	1	1	1	P74474	N-Term(iTRAQ4plex); K12(iTRAQ4plex); K13(iTRAQ4plex	0,0000	0,334	1		0	6,998E-09	45	3,13612E-05	3	1946,10056	2,38	31,22	1
	High	rEPEFFSAIQLSTk	1	1	1	P74474	N-Term(iTRAQ4plex); K14(iTRAQ4plex)	0,0000	0,389	1		0	4,455E-09	44	3,58897E-05	3	1941,07358	2,65	38,39	1
	High	lVSLITGk	1	1	1	P74474	N-Term(iTRAQ4plex); K8(iTRAQ4plex)	0,0000	0,313	1		0	0,0009253	30	0,001023222	2	1118,74272	3,55	36,22	0
P73732	Extracellular solute-binding protein OS=Synechocystis sp. (strain PCC 68	207,68	10,48	1	4	4	8	1,283	8	4,9	582	63,6	5,19							
	A3	Sequence	# PSMs	# Proteins	# Protein Groups	Protein Group Accessions	Modifications	ΔCn	117/114	117/114 Count	117/114 Variability [%]	q-Value	PEP	IonScore	Exp Value	Charge	MH+ [Da]	ΔM [ppm]	RT [min]	# Missed Cleavages
	High	vTSFkPGDVVLYEVNPHYR	2	1	1	P73732	N-Term(iTRAQ4plex); K5(iTRAQ4plex)	0,0000	1,423	2	12,6	0	6,425E-10	61	9,42742E-07	4	2508,35073	0,70	38,40	0
	High	kNIGFQQVEIk	1	1	1	P73732	N-Term(iTRAQ4plex); K1(iTRAQ4plex); K11(iTRAQ4plex)	0,0000	1,253	1		0	1,621E-09	55	3,25814E-06	3	1736,04581	1,56	26,56	1
	High	fPHPFLQDPLVR	4	1	1	P73732	N-Term(iTRAQ4plex)	0,0000	1,295	4	7,8	0	2,215E-07	36	0,000231723	3	1609,89731	0,09	39,76	0
	High	iLSWQAPTILNPHLATGFk	1	1	1	P73732	N-Term(iTRAQ4plex); K19(iTRAQ4plex)	0,0000	1,248	1		0	0,0000134	35	0,000353159	3	2395,37455	0,19	47,70	0
P73527	6,7-dimethyl-8-ribityllumazine synthase OS=Synechocystis sp. (strain PC	206,68	29,27	1	4	4	11	0,682	8	15,2	164	17,6	6,29							
	A3	Sequence	# PSMs	# Proteins	# Protein Groups	Protein Group Accessions	Modifications	ΔCn	117/114	117/114 Count	117/114 Variability [%]	q-Value	PEP	IonScore	Exp Value	Charge	MH+ [Da]	ΔM [ppm]	RT [min]	# Missed Cleavages
	High	gQTPHFDFVAGEAAk	2	1	1	P73527	N-Term(iTRAQ4plex); K15(iTRAQ4plex)	0,0000	0,830	2	14,2	0	7,096E-07	48	1,68256E-05	3	1862,96408	0,05	29,07	0
	High	fALVIAR	6	1	1	P73527	N-Term(iTRAQ4plex)	0,0000	0,671	3	4,9	0	0,00757	44	5,46221E-05	2	933,60057	0,35	34,23	0
	High	tVYEGSFTPPARPFR	2	1	1	P73527	N-Term(iTRAQ4plex)	0,0000	0,580	2	8,9	0	0,000002885	28	0,001828897	3	1868,97873	0,60	31,30	0
	High	iLSGcQDcLkR	1	1	1	P73527	N-Term(iTRAQ4plex); C5(Carbamidomethyl); C8(Carbam	0,0000	0,925	1		0	0,001198	19	0,013212044	3	1637,87015	-0,28	22,38	1
P54384	3-isopropylmalate dehydratase large subunit OS=Synechocystis sp. (stra	204,53	4,06	1	3	3	11	1,183	8	27,9	468	50,4	5,69							
	A3	Sequence	# PSMs	# Proteins	# Protein Groups	Protein Group Accessions	Modifications	ΔCn	117/114	117/114 Count	117/114 Variability [%]	q-Value	PEP	IonScore	Exp Value	Charge	MH+ [Da]	ΔM [ppm]	RT [min]	# Missed Cleavages
	High	dVILHIIR	8	1	1	P54384	N-Term(iTRAQ4plex)	0,0000	1,276	5	16,7	0	0,00009137	45	3,28073E-05	3	1122,71143	-0,14	32,01	0
	High	aVQWWR	2	1	1	P54384	N-Term(iTRAQ4plex)	0,0000	1,089	2	17,7	0	0,001966	34	0,000408291	2	989,54448	0,70	30,35	0
	Medium	kLGVk	1	1	1	P54384	N-Term(iTRAQ4plex); K1(iTRAQ4plex); K5(iTRAQ4plex)	0,0000	0,879	1		0,037	0,4637	15	0,029851764	3	976,68619	-1,78	11,86	1
P74518	Light-repressed protein A homolog OS=Synechocystis sp. (strain PCC 68	204,34	42,93	1	7	7	11	1,297	10	24,5	191	21,9	6,54							
	A3	Sequence	# PSMs	# Proteins	# Protein Groups	Protein Group Accessions	Modifications	ΔCn	117/114	117/114 Count	117/114 Variability [%]	q-Value	PEP	IonScore	Exp Value	Charge	MH+ [Da]	ΔM [ppm]	RT [min]	# Missed Cleavages
	High	nHGGYGVIQPHQAS	2	1	1	P74518	N-Term(iTRAQ4plex)	0,0000	1,297	2	2,5	0	0,000001714	52	6,05299E-06	2	1608,80120	0,77	15,15	0
	High	nkDTDEINVIYIR	2	1	1	P74518	N-Term(iTRAQ4plex); K2(iTRAQ4plex)	0,0000	1,423	2	7,5	0	4,258E-07	49	1,28816E-05	3	1881,03238	0,18	30,38	1
	High	hFQNLTTk	2	1	1	P74518	N-Term(iTRAQ4plex); K8(iTRAQ4plex)	0,0000	1,142	2	8,3	0	0,000005548	41	0,000119975	2	1276,72527	0,05	15,34	0
	High	aPELPSEVLR	1	1	1	P74518	N-Term(iTRAQ4plex)	0,0000	0,767	1		0	0,006226	27	0,002218043	2	1254,71709	-0,31	32,04	0
	High	tSEIVEDkPVEENLIGDR	1	1	1	P74518	N-Term(iTRAQ4plex); K8(iTRAQ4plex)	0,0000	1,025	1		0	8,604E-07	25	0,002944422	3	2331,22885	0,24	31,61	0
	High	vDVHLSVAR	1	1	1	P74518	N-Term(iTRAQ4plex)	0,0000				0	0,0004286	22	0,006965784	3	1139,66544	0,07	23,13	0
	High	iQDkQHGNVk	2	1	1	P74518	N-Term(iTRAQ4plex); K4(iTRAQ4plex); K10(iTRAQ4plex)	0,0000	1,585	2	1,9	0	0,00002512	17	0,020322166	3	1598,93058	-2,07	8,99	1
P54416	ATP-dependent Clp protease proteolytic subunit 1 OS=Synechocystis sp	204,00	29,80	1	5	5	11	1,025	8	2,9	198	21,7	5,03							
	A3	Sequence	# PSMs	# Proteins	# Protein Groups	Protein Group Accessions	Modifications	ΔCn	117/114	117/114 Count	117/114 Variability [%]	q-Value	PEP	IonScore	Exp Value	Charge	MH+ [Da]	ΔM [ppm]	RT [min]	# Missed Cleavages
	High	aLLNQHLANHTGk	7	1	1	P54416	N-Term(iTRAQ4plex); K13(iTRAQ4plex)	0,0000	1,026	4	2,1	0	4,655E-07	49	1,23934E-05	3	1704,97666	1,09	17,53	0
	High	iVFLGQEVR	1	1	1	P54416	N-Term(iTRAQ4plex)	0,0000	1,032	1		0	0,0004965	40	0,000127403	2	1204,71843	1,13	36,63	0
	High	imIHQPLGGAQGQATDIEIQAk	1	1	1	P54416	N-Term(iTRAQ4plex); M2(Oxidation); K22(iTRAQ4plex)	0,0000	0,676	1		0	9,169E-10	37	0,000178636	4	2623,41372	0,77	33,02	0
	High	aFDIYSR	1	1	1	P54416	N-Term(iTRAQ4plex)	0,0000	0,654	1		0	0,0001947	33	0,000509296	2	1015,53282	-0,11	26,11	0
	Medium	rPSASDPI	1	1	1	P54416	N-Term(iTRAQ4plex)	0,0000	1,046	1		0,033	0,415	18	0,015274606	2	986,53935	0,60	16,07	0
Q55557	Virginiamycin B hydrolase Vgb OS=Synechocystis sp. (strain PCC 6803 /	201,03	13,07	1	5	5	9	1,818	9	12,3	352	38,5	6,80							
	A3	Sequence	# PSMs	# Proteins	# Protein Groups	Protein Group Accessions	Modifications	ΔCn	117/114	117/114 Count	117/114 Variability [%]	q-Value	PEP	IonScore	Exp Value	Charge	MH+ [Da]	ΔM [ppm]	RT [min]	# Missed Cleavages
	High	vTIYPTVPGIGTR	2	1	1	Q55557	N-Term(iTRAQ4plex)	0,0000	1,844	2	10,2	0	1,319E-07	60	1,00455E-06	2	1517,88201	0,77	37,32	0
	High	iFPAPQGFGPYGmTATPk	2	1	1	Q55557	N-Term(iTRAQ4plex); M13(Oxidation); K18(iTRAQ4plex)	0,0000	2,010	2	14,9	0	1,104E-11	50	1,08136E-05	3	2184,14243	1,00	38,95	0
	High	ykVTIYPTVPGIGTR	1	1	1	Q55557	N-Term(iTRAQ4plex); K2(iTRAQ4plex)	0,0000	2,666	1		0	0,00002149	40	0,000104947	3	1953,14261	0,72	35,55	1
	High	fNPQNk	2	1	1	Q55557	N-Term(iTRAQ4plex); K6(iTRAQ4plex)	0,0000	1,214	2	26,5	0,002	0,03506	22	0,006560999	2	1035,58135	-1,18	14,39	0
	High	lPGLGPR	2	1	1	Q55557	N-Term(iTRAQ4plex)	0,0000	1,840	2	4,3	0,002	0,02507	11	0,07463972	2	853,53771	0,07	24,79	0
P73393	Sll1734 protein OS=Synechocystis sp. (strain PCC 6803 / Kazusa) GN=sl	200,98	10,44	1	5	5	9	0,407	9	155,9	431	50,0	5,88							
	A3	Sequence	# PSMs	# Proteins	# Protein Groups	Protein Group Accessions	Modifications	ΔCn	117/114	117/114 Count	117/114 Variability [%]	q-Value	PEP	IonScore	Exp Value	Charge	MH+ [Da]	ΔM [ppm]	RT [min]	# Missed Cleavages
	High	smYNVTSAVIQALR	3	1	1	P73393	N-Term(iTRAQ4plex); M2(Oxidation)	0,0000	0,903	3	15,8	0	5,585E-09	64	3,62218E-07	3	1712,91245	0,13	43,30	0
	High	vFLNPLR	2	1	1	P73393	N-Term(iTRAQ4plex)	0,0000	0,224	2	23,1	0	0,005594	36	0,000228018	2	1002,62248	0,78	33,02	0
	High	lkDIQSDSYR	1	1	1	P73393	N-Term(iTRAQ4plex); K2(iTRAQ4plex)	0,0000	0,447	1		0	0,00001932	36	0,000273508	3	1512,82605	0,00	19,51	1
	High	sYNAQAk	2	1	1	P73393	N-Term(iTRAQ4plex); K7(iTRAQ4plex)	0,0000	0,339	2	27,6	0,001	0,01145	29	0,001377114	2	1069,58843	0,36	10,10	0
	Medium	fLRPEAR	1	1	1	P73393	N-Term(iTRAQ4plex)	0,0000	0,878	1		0,016	0,2025	10	0,09418896	3	1032,60733	0,20	15,88	0
P74643	Phosphoglucomutase OS=Synechocystis sp. (strain PCC 6803 / Kazusa)	200,72	6,88	1	3	3	7	0,696	6	28,3	567	61,1	6,11							
	A3	Sequence	# PSMs	# Proteins	# Protein Groups	Protein Group Accessions	Modifications	ΔCn	117/114	117/114 Count	117/114 Variability [%]	q-Value	PEP	IonScore	Exp Value	Charge	MH+ [Da]	ΔM [ppm]	RT [min]	# Missed Cleavages
	High	fTPTPAVSYAILTHNQGR	3	1	1	P74643	N-Term(iTRAQ4plex)	0,0000	0,861	2	5,2	0	2,185E-13	93	6,60969E-10	3	2117,12784	0,84	36,71	0
	High	aPGNQAAIGGLk	2	1	1	P74643	N-Term(iTRAQ4plex); K12(iTRAQ4plex)	0,0000	0,568	2	1,0	0	0,0009101	36	0,000229071	3	1384,81757	1,76	21,77	0
	High	rLDYEQALk	2	1	1	P74643	N-Term(iTRAQ4plex); K9(iTRAQ4plex)	0,0000	0,696	2	5,0	0	0,000006335	34	0,000369828	3	1423,81610	0,94	22,51	1
Q55776	Sll0180 protein OS=Synechocystis sp. (strain PCC 6803 / Kazusa) GN=sl	200,35	8,78	1	4	4	7	0,557	7	20,0	501	53,9	5,78							
	A3	Sequence	# PSMs	# Proteins	# Protein Groups	Protein Group Accessions	Modifications	ΔCn	117/114	117/114 Count	117/114 Variability [%]	q-Value	PEP	IonScore	Exp Value	Charge	MH+ [Da]	ΔM [ppm]	RT [min]	# Missed Cleavages
	High	aTVQANQSSVSASR	2	1	1	Q55776	N-Term(iTRAQ4plex)	0,0000	0,417	2	8,3	0	1,71E-10	78	1,45536E-08	2	1549,80644	0,86	13,66	0
	High	aQANLNLAQINFk	1	1	1	Q55776	N-Term(iTRAQ4plex); K13(iTRAQ4plex)	0,0000	0,494	1		0	0,000001324	33	0,000514008	3	1732,99710	1,29	37,45	0
	High	iSFISPTANR	1	1	1	Q55776	N-Term(iTRAQ4plex)	0,0000	0,729	1		0	0,0002985	31	0,000801678	2	1249,70403	1,51	30,66	0
	High	sAVQQAk	3	1	1	Q55776	N-Term(iTRAQ4plex); K7(iTRAQ4plex)	0,0000	0,588	3	8,1	0,001	0,01639	23	0,005060452	2	1019,60887	0,08	9,89	0
P54224	Uroporphyrinogen decarboxylase OS=Synechocystis sp. (strain PCC 680	199,47	20,00	1	7	7	11	0,908	11	10,4	350	39,1	5,96							
	A3	Sequence	# PSMs	# Proteins	# Protein Groups	Protein Group Accessions	Modifications	ΔCn	117/114	117/114 Count	117/114 Variability [%]	q-Value	PEP	IonScore	Exp Value	Charge	MH+ [Da]	ΔM [ppm]	RT [min]	# Missed Cleavages
	High	aGQGGHIFNLGHGVLVGTPEDNV	1	1	1	P54224	N-Term(iTRAQ4plex)	0,0000	0,743	1		0	3,847E-11	62	5,8475E-07	4	2588,34804	1,13	33,55	0
	High	kAGQGGHIFNLGHGVLVGTPEDN	1	1	1	P54224	N-Term(iTRAQ4plex); K1(iTRAQ4plex)	0,0000	0,749	1		0	2,173E-08	52	6,92944E-06	4	2860,54482	0,93	31,07	1
	High	fFFETAk	2	1	1	P54224	N-Term(iTRAQ4plex); K7(iTRAQ4plex)	0,0000	0,862	2	9,6	0	0,0001311	39	0,000119942	2	1177,65141	1,54	34,48	0
	High	rPPVWMMR	1	1	1	P54224	N-Term(iTRAQ4plex)	0,0000	0,962	1		0	0,0001359	29	0,001377114	3	1216,65644	0,01	26,82	0
	High	qmAFSEPAILHSFLDk	1	1	1	P54224	N-Term(iTRAQ4plex); M2(Oxidation); K16(iTRAQ4plex)	0,0000	0,992	1		0	0,000001782	23	0,00512826	3	2138,12510	2,61	45,46	0
	High	rPPVWmmR	1	1	1	P54224	N-Term(iTRAQ4plex); M6(Oxidation); M7(Oxidation)	0,0000	0,953	1		0	0,003168	14	0,037667778	3	1248,64634	0,06	16,09	0
	High	dkYPSFR	2	1	1	P54224	N-Term(iTRAQ4plex); K2(iTRAQ4plex)	0,0000	0,915	2	1,1	0,001	0,01566	11	0,08240812	3	1200,66227	0,60	17,73	1
	High	tILGTLR	2	1	1	P54224	N-Term(iTRAQ4plex)	0,0000	0,840	2	1,3	0,006	0,07098	28	0,001534511	2	917,59081	0,81	27,96	0
Q55848	Ribose-phosphate pyrophosphokinase OS=Synechocystis sp. (strain PCC	198,27	11,71	1	3	3	5	1,033	5	4,1	333	36,4	7,20							
	A3	Sequence	# PSMs	# Proteins	# Protein Groups	Protein Group Accessions	Modifications	ΔCn	117/114	117/114 Count	117/114 Variability [%]	q-Value	PEP	IonScore	Exp Value	Charge	MH+ [Da]	ΔM [ppm]	RT [min]	# Missed Cleavages
	High	lVANLITGAGAQR	3	1	1	Q55848	N-Term(iTRAQ4plex)	0,0000	1,056	3	0,9	0	1,184E-09	71	8,80436E-08	2	1427,84599	0,60	36,20	0
	High	qITAVLPYYGYAR	1	1	1	Q55848	N-Term(iTRAQ4plex)	0,0000	1,033	1		0	0,000001295	41	8,31706E-05	2	1658,90447	1,32	39,48	0
	High	kLNDAPLAIIDkR	1	1	1	Q55848	N-Term(iTRAQ4plex); K1(iTRAQ4plex); K12(iTRAQ4plex)	0,0000	0,890	1		0	0,000003358	40	9,7717E-05	4	1899,17849	1,73	29,62	2
P73120	Sll1830 protein OS=Synechocystis sp. (strain PCC 6803 / Kazusa) GN=sl	197,07	12,29	1	4	4	11	0,765	10	24,6	358	39,6	4,87							
	A3	Sequence	# PSMs	# Proteins	# Protein Groups	Protein Group Accessions	Modifications	ΔCn	117/114	117/114 Count	117/114 Variability [%]	q-Value	PEP	IonScore	Exp Value	Charge	MH+ [Da]	ΔM [ppm]	RT [min]	# Missed Cleavages
	High	lYYLPFPVQAR	4	1	1	P73120	N-Term(iTRAQ4plex)	0,0000	0,688	4	8,5	0	0,000009937	54	3,99917E-06	2	1510,85564	1,16	45,79	0

	High	qLQSLTPVPALSILLPTHR	3	1	1	P73120	N-Term(iTRAQ4plex)	0,0000	0,980	3	95,5	0	2,83E-09	51	7,16094E-06	4	2228,32656	0,80	50,53	0
	High	rFIQEVDR	1	1	1	P73120	N-Term(iTRAQ4plex)	0,0000	1,194	1		0	0,000204	34	0,000416841	3	1206,67206	0,73	17,62	1
	High	iALVLR	3	1	1	P73120	N-Term(iTRAQ4plex)	0,0000	0,739	2	13,5	0,003	0,03786	34	0,0004385	2	828,57988	1,34	31,10	0
P73600	Sll1785 protein OS=Synechocystis sp. (strain PCC 6803 / Kazusa) GN=s	194,68	17,54	1	4	4	15	0,912	15	22,4	268	30,0	5,30							
	A3	Sequence	# PSMs	# Proteins	# Protein Groups	Protein Group Accessions	Modifications	ΔCn	117/114	117/114 Count	117/114 Variability [%]	q-Value	PEP	IonScore	Exp Value	Charge	MH+ [Da]	ΔM [ppm]	RT [min]	# Missed Cleavages
	High	tLPIVFVWMR	2	1	1	P73600	N-Term(iTRAQ4plex)	0,0000	1,111	2	1,1	0	0,000003641	38	0,00017378	2	1405,81548	0,56	54,70	0
	High	tLPIVFVWmR	10	1	1	P73600	N-Term(iTRAQ4plex); M9(Oxidation)	0,0000	0,910	10	19,8	0	0,00003962	35	0,000341979	3	1421,81009	0,34	48,80	0
	High	qLIYSPNHYmHGFVNPTDk	1	1	1	P73600	N-Term(iTRAQ4plex); M10(Oxidation); K19(iTRAQ4plex)	0,0000	0,849	1		0	9,727E-07	16	0,025880342	4	2565,28457	1,82	29,45	0
	High	eYFQAVGPR	1	1	1	P73600	N-Term(iTRAQ4plex)	0,0000	1,142	1		0	0,004833	15	0,029783107	2	1210,63518	1,20	26,01	0
	High	lkNDkDLER	1	1	1	P73600	N-Term(iTRAQ4plex); K2(iTRAQ4plex); K5(iTRAQ4plex)	0,0000	0,698	1		0	0,001365	11	0,087291108	3	1562,92582	2,02	12,67	2
P74326	Ssr1600 protein OS=Synechocystis sp. (strain PCC 6803 / Kazusa) GN=s	193,98	50,00	1	3	3	7	1,283	7	19,1	70	7,5	7,42							
	A3	Sequence	# PSMs	# Proteins	# Protein Groups	Protein Group Accessions	Modifications	ΔCn	117/114	117/114 Count	117/114 Variability [%]	q-Value	PEP	IonScore	Exp Value	Charge	MH+ [Da]	ΔM [ppm]	RT [min]	# Missed Cleavages
	High	qGSVQIVTNPR	4	1	1	P74326	N-Term(iTRAQ4plex)	0,0000	1,206	4	34,3	0	4,568E-09	66	2,31191E-07	2	1342,75664	0,50	19,85	0
	High	fLHLHNSLAEAIAATTEG	1	1	1	P74326	N-Term(iTRAQ4plex)	0,0000	1,184	1		0	0,0001229	27	0,001999724	3	2039,06852	0,30	44,50	0
	High	vTQTVk	2	1	1	P74326	N-Term(iTRAQ4plex); K6(iTRAQ4plex)	0,0000	1,323	2	4,5	0,001	0,02118	24	0,0044154	2	963,60790	0,19	11,82	0
Q55119	Elongation factor P OS=Synechocystis sp. (strain PCC 6803 / Kazusa) Gl	191,98	21,93	1	4	4	8	1,043	7	30,3	187	20,4	4,89							
	A3	Sequence	# PSMs	# Proteins	# Protein Groups	Protein Group Accessions	Modifications	ΔCn	117/114	117/114 Count	117/114 Variability [%]	q-Value	PEP	IonScore	Exp Value	Charge	MH+ [Da]	ΔM [ppm]	RT [min]	# Missed Cleavages
	High	sVQTGNVVEk	2	1	1	Q55119	N-Term(iTRAQ4plex); K10(iTRAQ4plex)	0,0000	0,928	2	25,4	0	9,53E-08	74	3,87231E-08	2	1348,77007	1,91	16,32	0
	High	vVEFLHVkPGk	2	1	1	Q55119	N-Term(iTRAQ4plex); K8(iTRAQ4plex); K11(iTRAQ4plex)	0,0000	0,582	2	14,5	0	9,926E-08	35	0,000315479	3	1685,05423	4,01	28,19	0
	High	aGETVPQANIEk	1	1	1	Q55119	N-Term(iTRAQ4plex); K12(iTRAQ4plex)	0,0000	1,273	1		0	0,00007493	33	0,000525981	2	1544,85515	1,85	21,44	0
	High	sVmQHTYk	2	1	1	Q55119	N-Term(iTRAQ4plex); M3(Oxidation); K8(iTRAQ4plex)	0,0000	1,132	1		0	0,002808	22	0,006456096	3	1297,68390	2,01	8,94	0
	High	sVMQHTYk	1	1	1	Q55119	N-Term(iTRAQ4plex); K8(iTRAQ4plex)	0,0000	1,043	1		0	0,0007028	15	0,031766547	3	1281,68689	0,40	12,96	0
Q55497	Ornithine carbamoyltransferase OS=Synechocystis sp. (strain PCC 6803	191,82	8,77	1	4	4	7	0,939	6	5,8	308	33,6	5,64							
	A3	Sequence	# PSMs	# Proteins	# Protein Groups	Protein Group Accessions	Modifications	ΔCn	117/114	117/114 Count	117/114 Variability [%]	q-Value	PEP	IonScore	Exp Value	Charge	MH+ [Da]	ΔM [ppm]	RT [min]	# Missed Cleavages
	High	sLLQLAADLk	2	1	1	Q55497	N-Term(iTRAQ4plex); K10(iTRAQ4plex)	0,0000	1,055	2	34,8	0	0,000002297	53	4,84139E-06	2	1359,84624	0,90	46,71	0
	High	iLGLLFYk	2	1	1	Q55497	N-Term(iTRAQ4plex); K8(iTRAQ4plex)	0,0000	0,952	2	4,2	0	0,000001599	51	8,64908E-06	2	1254,80620	-0,18	49,82	0
	High	yIDILAVR	1	1	1	Q55497	N-Term(iTRAQ4plex)	0,0000	0,906	1		0	0,001506	34	0,000395339	2	1106,67021	1,06	40,36	0
	High	kILGLLFYk	2	1	1	Q55497	N-Term(iTRAQ4plex); K1(iTRAQ4plex); K9(iTRAQ4plex)	0,0000	0,953	1		0	5,113E-08	31	0,000772627	3	1527,00309	-0,25	42,24	1
P73456	Cell division protein FtsZ OS=Synechocystis sp. (strain PCC 6803 / Kazu	191,33	4,42	1	2	2	5	1,212	3	6,5	430	44,7	4,73							
	A3	Sequence	# PSMs	# Proteins	# Protein Groups	Protein Group Accessions	Modifications	ΔCn	117/114	117/114 Count	117/114 Variability [%]	q-Value	PEP	IonScore	Exp Value	Charge	MH+ [Da]	ΔM [ppm]	RT [min]	# Missed Cleavages
	High	gLGAGGNPAIGQk	4	1	1	P73456	N-Term(iTRAQ4plex); K13(iTRAQ4plex)	0,0000	1,185	2	3,2	0	2,61E-10	89	1,40605E-09	2	1427,82305	1,47	19,88	0
	Medium	ekPQAk	1	1	1	P73456	N-Term(iTRAQ4plex); K2(iTRAQ4plex); K6(iTRAQ4plex)	0,0000	1,642	1		0,027	0,3298	11	0,091347747	3	1132,70221	-2,50	7,86	0
P73672	4-hydroxy-3-methylbut-2-en-1-yl diphosphate synthase OS=Synechocys	191,06	13,90	1	5	5	12	1,233	11	27,0	403	44,1	5,96							
	A3	Sequence	# PSMs	# Proteins	# Protein Groups	Protein Group Accessions	Modifications	ΔCn	117/114	117/114 Count	117/114 Variability [%]	q-Value	PEP	IonScore	Exp Value	Charge	MH+ [Da]	ΔM [ppm]	RT [min]	# Missed Cleavages
	High	iRETLEPLVISLR	6	1	1	P73672	N-Term(iTRAQ4plex)	0,0000	1,475	5	10,4	0	3,896E-08	49	1,32425E-05	3	1683,03019	0,95	41,65	1
	High	vPVmLAAYR	2	1	1	P73672	N-Term(iTRAQ4plex); M4(Oxidation)	0,0000	1,075	2	8,5	0	0,001501	44	4,03618E-05	2	1179,66802	0,29	29,92	0
	High	vTVPSmAHAk	1	1	1	P73672	N-Term(iTRAQ4plex); M6(Oxidation); K10(iTRAQ4plex)	0,0000	0,898	1		0	0,0003395	41	8,22186E-05	3	1344,75684	1,50	15,21	0
	High	iNPGLYVFEkPDAQR	1	1	1	P73672	N-Term(iTRAQ4plex); K10(iTRAQ4plex)	0,0000	1,100	1		0	0,000002282	16	0,025407972	3	2035,12314	0,78	35,90	0
	High	qAGYIALYR	2	1	1	P73672	N-Term(iTRAQ4plex)	0,0000	0,880	2	63,1	0,001	0,01275	16	0,026180022	2	1198,67168	1,32	27,87	0
P72799	Slr1794 protein OS=Synechocystis sp. (strain PCC 6803 / Kazusa) GN=s	189,25	8,84	1	3	4	11	0,972	8	17,1	362	39,1	6,13							
	A3	Sequence	# PSMs	# Proteins	# Protein Groups	Protein Group Accessions	Modifications	ΔCn	117/114	117/114 Count	117/114 Variability [%]	q-Value	PEP	IonScore	Exp Value	Charge	MH+ [Da]	ΔM [ppm]	RT [min]	# Missed Cleavages
	High	aLVLTFLGk	7	1	1	P72799	N-Term(iTRAQ4plex); K9(iTRAQ4plex)	0,0000	0,956	5	7,3	0	6,227E-07	46	2,49442E-05	2	1249,81267	0,33	46,63	0
	High	tTVAIAAAk	1	1	1	P72799	N-Term(iTRAQ4plex); K9(iTRAQ4plex)	0,0000	0,667	1		0	0,000001756	39	0,00011587	2	1133,71208	-1,04	18,91	0
	High	vFLPGFDkR	2	1	1	P72799	N-Term(iTRAQ4plex); K8(iTRAQ4plex)	0,0000	1,215	2	0,2	0	0,000007335	29	0,001199416	3	1366,80750	-0,79	32,84	1
	Medium	sPALk	1	2	2	P74230;P72799	N-Term(iTRAQ4plex); K5(iTRAQ4plex)	0,0000				0,04	0,4947	18	0,017296969	2	803,51995	-3,71	13,10	0
P74233	Slr1160 protein OS=Synechocystis sp. (strain PCC 6803 / Kazusa) GN=s	187,77	13,73	1	2	2	5	1,268	4	8,8	204	22,2	5,55							
	A3	Sequence	# PSMs	# Proteins	# Protein Groups	Protein Group Accessions	Modifications	ΔCn	117/114	117/114 Count	117/114 Variability [%]	q-Value	PEP	IonScore	Exp Value	Charge	MH+ [Da]	ΔM [ppm]	RT [min]	# Missed Cleavages
	High	eFRPLNQEGSILSVGAGR	4	1	1	P74233	N-Term(iTRAQ4plex)	0,0000	1,297	3	6,7	0	1,844E-11	75	3,25837E-08	3	2074,11948	1,55	35,45	0
	High	iAALETGQLR	1	1	1	P74233	N-Term(iTRAQ4plex)	0,0000	0,213	1		0,001	0,01172	18	0,01837305	2	1215,71953	1,42	29,94	0
P72807	Sll1663 protein OS=Synechocystis sp. (strain PCC 6803 / Kazusa) GN=sl	184,75	14,09	1	3	3	8	0,951	6	12,4	220	24,1	4,65							
	A3	Sequence	# PSMs	# Proteins	# Protein Groups	Protein Group Accessions	Modifications	ΔCn	117/114	117/114 Count	117/114 Variability [%]	q-Value	PEP	IonScore	Exp Value	Charge	MH+ [Da]	ΔM [ppm]	RT [min]	# Missed Cleavages
	High	lALAVGHLEHPDR	6	1	1	P72807	N-Term(iTRAQ4plex)	0,0000	0,951	4	8,3	0	1,124E-08	47	1,78226E-05	3	1571,87802	0,33	28,39	0
	High	aDLEQSLQLLR	1	1	1	P72807	N-Term(iTRAQ4plex)	0,0000	1,246	1		0	0,000009096	36	0,000277313	2	1429,81352	0,25	41,25	0
	High	rGFDLLk	1	1	1	P72807	N-Term(iTRAQ4plex); K7(iTRAQ4plex)	0,0000	0,862	1		0,003	0,04347	20	0,008953029	3	1136,70252	-0,46	25,25	1
P73293	30S ribosomal protein S9 OS=Synechocystis sp. (strain PCC 6803 / Kazu	184,19	29,93	1	5	5	10	1,758	10	7,6	137	15,1	10,13							
	A3	Sequence	# PSMs	# Proteins	# Protein Groups	Protein Group Accessions	Modifications	ΔCn	117/114	117/114 Count	117/114 Variability [%]	q-Value	PEP	IonScore	Exp Value	Charge	MH+ [Da]	ΔM [ppm]	RT [min]	# Missed Cleavages
	High	iANYIQSLk	2	1	1	P73293	N-Term(iTRAQ4plex); K9(iTRAQ4plex)	0,0000	1,804	2	2,5	0	3,036E-07	54	3,72366E-06	2	1337,80303	-0,08	37,71	0
	High	vVYWGTGR	3	1	1	P73293	N-Term(iTRAQ4plex)	0,0000	1,750	3	1,5	0	0,00002884	33	0,000502308	2	1081,59087	-0,24	26,36	0
	High	kAPQYSk	2	1	1	P73293	N-Term(iTRAQ4plex); K1(iTRAQ4plex); K7(iTRAQ4plex)	0,0000	1,825	2	3,3	0	0,003645	29	0,001124527	3	1253,75470	-2,46	9,37	1
	High	qPLkAEGYLTR	2	1	1	P73293	N-Term(iTRAQ4plex); K4(iTRAQ4plex)	0,0000	1,194	2	35,6	0	0,0001797	23	0,005610092	3	1563,90781	-1,23	23,86	1
	High	kAAIAR	1	1	1	P73293	N-Term(iTRAQ4plex); K1(iTRAQ4plex)	0,0000	0,784	1		0,005	0,06309	15	0,030267044	3	917,60968	-4,14	9,13	1
Q55196	Phosphate import ATP-binding protein PstB 1 OS=Synechocystis sp. (str	183,91	17,84	1	5	5	6	0,359	5	6,4	269	30,2	6,02							
	A3	Sequence	# PSMs	# Proteins	# Protein Groups	Protein Group Accessions	Modifications	ΔCn	117/114	117/114 Count	117/114 Variability [%]	q-Value	PEP	IonScore	Exp Value	Charge	MH+ [Da]	ΔM [ppm]	RT [min]	# Missed Cleavages
	High	aSGFSLSGGQQQR	2	1	1	Q55196	N-Term(iTRAQ4plex)	0,0000	0,365	2	2,6	0	2,196E-09	83	4,47682E-09	2	1466,75029	2,33	20,53	0
	High	lNDLIESFR	1	1	1	Q55196	N-Term(iTRAQ4plex)	0,0000	0,397	1		0	0,00003044	40	0,000105123	2	1250,68901	2,28	43,93	0
	High	kYIGmVFQkPNPFPk	1	1	1	Q55196	K1(iTRAQ4plex); N-Term(iTRAQ4plex); M5(Oxidation); K	0,0000	0,344	1		0	2,76E-08	25	0,003311083	4	2386,39077	4,36	32,13	1
	High	yIGmVFQkPNPFPk	1	1	1	Q55196	N-Term(iTRAQ4plex); M4(Oxidation); K8(iTRAQ4plex); K	0,0000	0,240	1		0	0,0006935	13	0,046770284	4	2114,19106	3,66	36,10	0
	High	rAALWDEVkDk	1	1	1	Q55196	N-Term(iTRAQ4plex); K9(iTRAQ4plex); K11(iTRAQ4plex)	0,0000				0	0,008595	13	0,051519306	4	1763,02614	4,82	25,46	2
P26525	NAD(P)H-quinone oxidoreductase subunit I OS=Synechocystis sp. (strai	183,77	41,45	1	8	8	14	0,672	11	12,8	193	22,1	6,76							
	A3	Sequence	# PSMs	# Proteins	# Protein Groups	Protein Group Accessions	Modifications	ΔCn	117/114	117/114 Count	117/114 Variability [%]	q-Value	PEP	IonScore	Exp Value	Charge	MH+ [Da]	ΔM [ppm]	RT [min]	# Missed Cleavages
	High	gVIEPHNLPk	4	1	1	P26525	N-Term(iTRAQ4plex); K10(iTRAQ4plex)	0,0000	0,567	2	26,5	0	0,0001781	41	7,9794E-05	3	1391,82730	1,69	20,39	0
	High	hDLNYDNVALGR	1	1	1	P26525	N-Term(iTRAQ4plex)	0,0000	0,950	1		0	1,571E-07	37	0,000211821	3	1530,77918	0,66	24,11	0
	High	yIGQGLAVTFDHMSR	1	1	1	P26525	N-Term(iTRAQ4plex)	0,0000	0,730	1		0	0,0001361	33	0,000462349	3	1838,93259	-0,79	40,02	0
	High	rPITVQYPYEk	3	1	1	P26525	N-Term(iTRAQ4plex); K11(iTRAQ4plex)	0,0000	0,693	2	26,6	0	0,000003027	32	0,000671382	2	1681,95403	1,45	23,81	0
	High	eLGYLPk	1	1	1	P26525	N-Term(iTRAQ4plex); K7(iTRAQ4plex)	0,0000	0,676	1		0	0,00436	31	0,000777983	2	1107,66387	-1,24	26,50	0
	High	mFNNILk	1	1	1	P26525	N-Term(iTRAQ4plex); M1(Oxidation); K7(iTRAQ4plex)	0,0000	0,672	1		0	0,00133	23	0,005187642	2	1183,67632	1,31	26,73	0
	High	iHFEFDk	2	1	1	P26525	N-Term(iTRAQ4plex); K7(iTRAQ4plex)	0,0000	0,617	2	7,8	0	0,0007415	20	0,011889983	3	1223,66755	1,01	30,19	0
	Medium	vTEDPmVTPLR	1	1	1	P26525	N-Term(iTRAQ4plex); M6(Oxidation)	0,0000	0,617	1		0,018	0,236	13	0,055203931	2	1417,74919	0,98	24,94	0
Q55113	Slr0431 protein OS=Synechocystis sp. (strain PCC 6803 / Kazusa) GN=s	183,69	12,00	1	3	3	7	1,047	7	12,0	250	27,0	8,37							
	A3	Sequence	# PSMs	# Proteins	# Protein Groups	Protein Group Accessions	Modifications	ΔCn	117/114	117/114 Count	117/114 Variability [%]	q-Value	PEP	IonScore	Exp Value	Charge	MH+ [Da]	ΔM [ppm]	RT [min]	# Missed Cleavages
	High	vVLSSLNLTPQNIVR	3	1	1	Q55113	N-Term(iTRAQ4plex)	0,0000	1,133	3	24,0	0	3,628E-11	64	4,13971E-07	2	1797,07414	1,46	43,75	0
	High	fIATPETLk	1	1	1	Q55113	N-Term(iTRAQ4plex); K9(iTRAQ4plex)	0,0000	0,702	1		0,002	0,03032	30	0,001086351	2	1307,78435	2,30	31,24	0
	High	lVAYLR	3	1	1	Q55113	N-Term(iTRAQ4plex)	0,0000	1,047	3	5,1	0,007	0,09208	30	0,001124372	2	878,55913	1,26	29,53	0
P74036	dTDP-glucose 4-6-dehydratase OS=Synechocystis sp. (strain PCC 6803 /	183,36	7,93	1	2	2	3	0,823	3	24,7	328	36,9	5,81							
	A3	Sequence	# PSMs	# Proteins	# Protein Groups	Protein Group Accessions	Modifications	ΔCn	117/114	117/114 Count	117/114 Variability [%]	q-Value	PEP	IonScore	Exp Value	Charge	MH+ [Da]	ΔM [ppm]	RT [min]	# Missed Cleavages
	High	iLVTGGAGFIGSHLIDR	2	1	1	P74036	N-Term(iTRAQ4plex)	0,0000	0,758	2	12,2	0	2,666E-13	97	2,03222E-10	3	1870,06858	0,96	41,99	0
	High	gNVNTIGPR	1	1	1	P74036	N-Term(iTRAQ4plex)	0,0000	1,038	1		0	0,000397	34	0,000421667	2	1071,60320	0,40	14,27	0
P74467	Probable ATP-dependent Clp protease proteolytic subunit 3 OS=Synech	181,28	34,16	1	7	7	13	0,970	11	16,7	202	22,4	6,04							
	A3	Sequence	# PSMs	# Proteins	# Protein Groups	Protein Group Accessions	Modifications	ΔCn	117/114	117/114 Count	117/114 Variability [%]	q-Value	PEP	IonScore	Exp Value	Charge	MH+ [Da]	ΔM [ppm]	RT [min]	# Missed Cleavages
	High	lALPHAR	4	1	1	P74467	N-Term(iTRAQ4plex)	0,0000	1,159	2	6,4	0	0,001075	48	1,67098E-05	3	921,57575	0,72	19,72	0
	High	iIFLGQEVNDSIANR	1	1	1	P74467	N-Term(iTRAQ4plex)	0,0000	0,997	1		0	2,178E-10	38	0,000146208	3	1833,00016	0,76	43,19	0
	High	vIENSTmGNN	1	1	1	P74467	N-Term(iTRAQ4plex); M7(Oxidation)	0,0000	1,142	1		0	0,005197	38	0,000161797	2	1238,57805	-1,88	14,81	0
	High	wIDIYTR	1	1	1	P74467	N-Term(iTRAQ4plex)	0,0000	0,932	1		0	0,000169	30	0,001066522	2	1110,60686	0,38	35,83	0
	High	imIHQPmGGTGR	2	1	1	P74467	N-Term(iTRAQ4plex); M2(Oxidation); M7(Oxidation)	0,0000	0,756	2	37,7	0	0,0002253	15	0,031766547	3	1473,74375	0,95	16,29	0
	High	iMIHQPmGGTGR	1	1	1	P74467	N-Term(iTRAQ4plex); M7(Oxidation)	0,0000	0,875	1		0	0,001199	14	0,042556902	3	1457,74848	0,72	17,89	0
	High	pIGVPSVPFR	2	1	1	P74467	N-Term(iTRAQ4plex)	0,0000	0,878	2	14,9	0,001	0,01291	23	0,004602248	2	1212,72344	1,05	35,93	0
	Medium	lPGSQYER	1	1	1	P74467	N-Term(iTRAQ4plex)	0,0000	1,085	1		0,025	0,3167	15	0,033187154	2	1093,57622	0,32	19,78	0
P73328	Slr1900 protein OS=Synechocystis sp. (strain PCC 6803 / Kazusa) GN=s	179,20	20,24	1	8	8	12	0,989	12	60,6	247	27,4	4,87							
	A3	Sequence	# PSMs	# Proteins	# Protein Groups	Protein Group Accessions	Modifications	ΔCn	117/114	117/114 Count	117/114 Variability [%]	q-Value	PEP	IonScore	Exp Value	Charge	MH+ [Da]	ΔM [ppm]	RT [min]	# Missed Cleavages
	High	aHLTVTQGDER	2	1	1	P73328	N-Term(iTRAQ4plex)	0,0000	0,793	2	12,3	0	9,169E-07	55	3,12586E-06	3	1370,71497	0,34	13,15	0
	High	gTEIcQVSR	1	1	1	P73328	N-Term(iTRAQ4plex); C5(Carbamidomethyl)	0,0000	1,258	1		0	9,947E-07	48	1,7457E-05	2	1193,60503	-1,26	14,84	0
	High	yEGEVEVk	1	1	1	P73328	N-Term(iTRAQ4plex); K8(iTRAQ4plex)	0,0000	0,615	1		0	0,000004744	29	0,001235862	2	1240,66765	1,03	20,31	0
	High	yNAIFkDPk	3	1	1	P73328	N-Term(iTRAQ4plex); K6(iTRAQ4plex); K9(iTRAQ4plex)	0,0000	0,880	3	35,8	0	0,0005315	20	0,011592611	4	1527,88881	-0,48	25,53	1
	High	kQGNFEk	2	1	1	P73328	N-Term(iTRAQ4plex); K1(iTRAQ4plex); K7(iTRAQ4plex)	0,0000	1,827	2	5,3	0	0,006023	17	0,02062494	3	1282,74652	-1,13	10,04	1
	High	aHLTVTQGDERYEGEVEVk	1	1	1	P73328	N-Term(iTRAQ4plex); K19(iTRAQ4plex)	0,0000	1,949	1		0	0,0001783	16	0,028470342	4	2448,25991	-0,43	25,24	1
	High	yNAIFk	1	1	1	P73328	N-Term(iTRAQ4plex); K6(iTRAQ4plex)	0,0000	0,630	1		0,001	0,01377	30	0,00103269	2	1043,61052	-2,18	26,90	0
	Medium	dIVTHR	1	1	1	P73328	N-Term(iTRAQ4plex)	0,0000	1,231	1		0,017	0,2285	20	0,009615459	2	884,50566	-1,57	12,41	0
Q55432	Sll0822 protein OS=Synechocystis sp. (strain PCC 6803 / Kazusa) GN=sl	179,04	27,91	1	4	4	11	1,063	10	40,2	129	14,0	8,59							
	A3	Sequence	# PSMs	# Proteins	# Protein Groups	Protein Group Accessions	Modifications	ΔCn	117/114	117/114 Count	117/114 Variability [%]	q-Value	PEP	IonScore	Exp Value	Charge	MH+ [Da]	ΔM [ppm]	RT [min]	# Missed Cleavages
	High	icGYVTSTk	2	1	1	Q55432	N-Term(iTRAQ4plex); C2(Carbamidomethyl); K9(iTRAQ4	0,0000	0,924	2	27,9	0	0,00002675	52	6,22257E-06	2	1316,71269	0,33	22,70	0
	High	sNATkPLTGEALLEk	5	1	1	Q55432	N-Term(iTRAQ4plex); K5(iTRAQ4plex); K15(iTRAQ4plex)	0,0000	1,072	4	38,8	0	9,511E-09	41	8,47169E-05	3	2004,17173	0,77	27,38	0
	High	vkELGDLPk	1	1	1	Q55432	N-Term(iTRAQ4plex); K2(iTRAQ4plex); K9(iTRAQ4plex)	0,0000	0,920	1		0	0,0000432	25	0,003303467	3	1430,89588	1,09	23,79	1
	High	vkELGDLPkEEk	3	1	1	Q55432	N-Term(iTRAQ4plex); K2(iTRAQ4plex); K9(iTRAQ4plex);	0,0000	1,533	3	29,2	0	0,000005074	18	0,016142471	4	1961,17788	0,68	22,87	2
Q55522	Valine--tRNA ligase OS=Synechocystis sp. (strain PCC 6803 / Kazusa) Gl	177,04	9,67	1	8	8	12	1,032	9	20,1	910	102,7	5,17							
	A3	Sequence	# PSMs	# Proteins	# Protein Groups	Protein Group Accessions	Modifications	ΔCn	117/114	117/114 Count	117/114 Variability [%]	q-Value	PEP	IonScore	Exp Value	Charge	MH+ [Da]	ΔM [ppm]	RT [min]	# Missed Cleavages
	High	lNQVIQQTR	2	1	1	Q55522	N-Term(iTRAQ4plex)	0,0000	0,956	2	19,5	0	0,00003385	55	2,89048E-06	2	1243,72453	0,48	23,36	0
	High	qVLAHTLDSIIk	2	1	1	Q55522	N-Term(iTRAQ4plex); K12(iTRAQ4plex)	0,0000	1,180	1		0	8,142E-07	40	0,000104225	3	1625,98404	0,70	36,90	0
	High	hNLPFINLLNk	2	1	1	Q55522	N-Term(iTRAQ4plex); K11(iTRAQ4plex)	0,0000	1,275	1		0	0,00001941	32	0,000605299	3	1610,96354	0,89	41,83	0
	High	kNVVQALEAQGFLVk	2	1	1	Q55522	N-Term(iTRAQ4plex); K1(iTRAQ4plex); K15(iTRAQ4plex)	0,0000	1,096	1		0	0,0042	30	0,001091365	3	2076,25742	1,56	43,67	1
	High	lkDWcISR	1	1	1	Q55522	N-Term(iTRAQ4plex); K2(iTRAQ4plex); C5(Carbamidome	0,0000	0,609	1		0	0,00001466	19	0,011323222	3	1365,75400	-0,84	25,46	1
	High	gkVPVEPLLSTQWFVk	1	1	1	Q55522	N-Term(iTRAQ4plex); K2(iTRAQ4plex); K16(iTRAQ4plex)	0,0000	0,950	1		0	0,0004885	19	0,013771144	4	2260,34463	0,71	45,12	1
	High	tITLPLVNR	1	1	1	Q55522	N-Term(iTRAQ4plex)	0,0000	1,032	1		0,001	0,01786	21	0,008491218	2	1170,73467	1,67	35,96	0
	High	vYRDWLVk	1	1	1	Q55522	N-Term(iTRAQ4plex); K8(iTRAQ4plex)	0,0000	0,670	1		0,002	0,02641	10	0,098394314	3	1366,80979	0,90	30,11	1
P73312	50S ribosomal protein L29 OS=Synechocystis sp. (strain PCC 6803 / Kaz	176,97	46,58	1	4	4	8	1,889	8	34,7	73	8,5	7,53							
	A3	Sequence	# PSMs	# Proteins	# Protein Groups	Protein Group Accessions	Modifications	ΔCn	117/114	117/114 Count	117/114 Variability [%]	q-Value	PEP	IonScore	Exp Value	Charge	MH+ [Da]	ΔM [ppm]	RT [min]	# Missed Cleavages
	High	lAQLLTVER	3	1	1	P73312	N-Term(iTRAQ4plex)	0,0000	2,145	3	2,6	0	0,000001417	58	1,44202E-06	2	1186,72771	0,06	36,73	0
	High	rPENPHEFk	3	1	1	P73312	N-Term(iTRAQ4plex); K9(iTRAQ4plex)	0,0000	2,174	3	25,5	0	4,662E-07	30	0,001047056	3	1441,77893	-0,09	10,84	0
	High	qLENSPSEEA	1	1	1	P73312	N-Term(iTRAQ4plex)	0,0000	1,369	1		0,001	0,02081	33	0,000469862	2	1247,58733	0,08	17,05	0
	Medium	fQQATR	1	1	1	P73312	N-Term(iTRAQ4plex)	0,0000	1,367	1		0,027	0,3314	11	0,088098802	2	894,49053	-0,98	11,99	0
Q55387	Periplasmic binding protein of ABC transporter for natural amino acids C	175,74	14,98	1	6	6	12	1,247	12	48,2	454	46,6	4,56							
	A3	Sequence	# PSMs	# Proteins	# Protein Groups	Protein Group Accessions	Modifications	ΔCn	117/114	117/114 Count	117/114 Variability [%]	q-Value	PEP	IonScore	Exp Value	Charge	MH+ [Da]	ΔM [ppm]	RT [min]	# Missed Cleavages
	High	sNTGAGIQSk	6	1	1	Q55387	N-Term(iTRAQ4plex); K10(iTRAQ4plex)	0,0000	1,513	6	24,9	0	0,00003904	51	8,30213E-06	2	1250,69597	1,32	10,24	0
	High	tAPPDTYQAQALAALAk	1	1	1	Q55387	N-Term(iTRAQ4plex); K17(iTRAQ4plex)	0,0000	0,782	1		0	9,134E-11	43	5,75321E-05	3	2018,11649	0,18	37,88	0
	High	sLEAFTAQWk	2	1	1	Q55387	N-Term(iTRAQ4plex); K10(iTRAQ4plex)	0,0000	0,951	2	41,3	0	0,0001822	29	0,001273415	3	1468,80484	0,66	39,60	0
	Medium	dANGVSLLSGALGTVPGADGk	1	1	1	Q55387	N-Term(iTRAQ4plex); K21(iTRAQ4plex)	0,0000	0,599	1		0,01	0,128	23	0,008576927	3	2187,18821	0,99	43,46	0
	Medium	kGEFk	1	1	1	Q55387	N-Term(iTRAQ4plex); K1(iTRAQ4plex); K5(iTRAQ4plex)	0,0000	1,250	1		0,016	0,2002	12	0,065008479	3	1040,64365	-2,70	9,80	1
	Medium	gFWAR	1	1	1	Q55387	N-Term(iTRAQ4plex)	0,0000	1,642	1		0,04	0,4961	15	0,035315878	2	780,42754	0,24	23,09	0
Q55852	Sll0596 protein OS=Synechocystis sp. (strain PCC 6803 / Kazusa) GN=sl	174,31	22,54	1	4	4	8	0,775	6	15,6	244	26,3	6,10							
	A3	Sequence	# PSMs	# Proteins	# Protein Groups	Protein Group Accessions	Modifications	ΔCn	117/114	117/114 Count	117/114 Variability [%]	q-Value	PEP	IonScore	Exp Value	Charge	MH+ [Da]	ΔM [ppm]	RT [min]	# Missed Cleavages
	High	mGSNPALATPEHGQR	4	1	1	Q55852	N-Term(iTRAQ4plex); M1(Oxidation)	0,0000	0,775	2	11,2	0	6,561E-07	57	2,21294E-06	3	1725,84684	0,51	12,81	0
	High	fYFINGHGGNIATLk	1	1	1	Q55852	N-Term(iTRAQ4plex); K15(iTRAQ4plex)	0,0000	0,849	1		0	5,745E-12	45	3,10435E-05	3	1940,06485	0,81	37,26	0
	High	gVYQLAk	2	1	1	Q55852	N-Term(iTRAQ4plex); K7(iTRAQ4plex)	0,0000	0,660	2	6,2	0	0,0002993	42	5,94251E-05	2	1066,64897	-0,88	21,16	0
	High	aAFSETYHHLGSHGQDVR	1	1	1	Q55852	N-Term(iTRAQ4plex)	0,0000	1,294	1		0	0,001548	16	0,024886854	4	2156,03994	0,42	19,33	0
P73971	Probable cytosol aminopeptidase OS=Synechocystis sp. (strain PCC 680	173,97	9,96	1	5	5	6	0,982	6	35,1	492	52,1	5,11							
	A3	Sequence	# PSMs	# Proteins	# Protein Groups	Protein Group Accessions	Modifications	ΔCn	117/114	117/114 Count	117/114 Variability [%]	q-Value	PEP	IonScore	Exp Value	Charge	MH+ [Da]	ΔM [ppm]	RT [min]	# Missed Cleavages
	High	kLILVGLGEIEk	1	1	1	P73971	N-Term(iTRAQ4plex); K1(iTRAQ4plex); K12(iTRAQ4plex)	0,0000	1,241	1		0	9,98E-11	78	1,4387E-08	3	1744,12241	-4,86	40,11	1
	High	tLGVSLPQR	1	1	1	P73971	N-Term(iTRAQ4plex)	0,0000	0,784	1		0	0,00006472	47	2,15263E-05	2	1114,66948	-0,57	27,69	0
	High	iVSGVILAR	2	1	1	P73971	N-Term(iTRAQ4plex)	0,0000	0,982	2	23,8	0	0,0005994	43	4,74209E-05	2	1071,69975	-0,88	32,31	0
	High	lTLADALVFAEk	1	1	1	P73971	N-Term(iTRAQ4plex); K12(iTRAQ4plex)	0,0000	0,597	1		0	0,001249	34	0,00068129	3	1578,92997	-2,92	49,23	0
	High	kLAIIGk	1	1	1	P73971	N-Term(iTRAQ4plex); K1(iTRAQ4plex); K7(iTRAQ4plex)	0,0000	1,243	1		0	0,002808	25	0,003475122	3	1174,81766	-6,05	22,78	1
P74367	Photosystem II lipoprotein Psb27 OS=Synechocystis sp. (strain PCC 680	173,52	20,90	1	3	3	6	1,598	5	13,1	134	14,8	9,33							
	A3	Sequence	# PSMs	# Proteins	# Protein Groups	Protein Group Accessions	Modifications	ΔCn	117/114	117/114 Count	117/114 Variability [%]	q-Value	PEP	IonScore	Exp Value	Charge	MH+ [Da]	ΔM [ppm]	RT [min]	# Missed Cleavages
	High	rLQLEFTQAER	4	1	1	P74367	N-Term(iTRAQ4plex)	0,0000	1,741	3	2,6	0	0,00007526	54	4,29925E-06	2	1534,84734	0,96	26,89	1
	High	gQINDYISR	1	1	1	P74367	N-Term(iTRAQ4plex)	0,0000	1,464	1		0	2,069E-07	53	4,49749E-06	2	1209,63469	0,20	23,26	0
	High	kGDAGGLk	1	1	1	P74367	N-Term(iTRAQ4plex); K1(iTRAQ4plex); K8(iTRAQ4plex)	0,0000	1,598	1		0	0,007248	28	0,00156304	3	1177,72400	-2,14	9,06	1
Q55426	Slr0841 protein OS=Synechocystis sp. (strain PCC 6803 / Kazusa) GN=s	173,41	11,34	1	3	3	7	1,000	7	28,1	291	31,5	4,92							
	A3	Sequence	# PSMs	# Proteins	# Protein Groups	Protein Group Accessions	Modifications	ΔCn	117/114	117/114 Count	117/114 Variability [%]	q-Value	PEP	IonScore	Exp Value	Charge	MH+ [Da]	ΔM [ppm]	RT [min]	# Missed Cleavages
	High	kAcTAEVmAQEQR	2	1	1	Q55426	K1(iTRAQ4plex); N-Term(iTRAQ4plex); C3(Carbamidome	0,0000	0,768	2	3,2	0	0,000000155	55	2,93745E-06	3	1825,91452	0,32	12,53	1
	High	aIPQLQFLGDR	1	1	1	Q55426	N-Term(iTRAQ4plex)	0,0000	0,728	1		0	0,00000522	45	2,89048E-05	2	1401,79839	0,90	40,91	0
	High	kAcTAEVMAQEQR	2	1	1	Q55426	N-Term(iTRAQ4plex); K1(iTRAQ4plex); C3(Carbamidome	0,0000	1,159	2	5,8	0	0,000000507	42	6,7293E-05	3	1809,91935	0,17	16,79	1
	High	gAPHSGSLR	2	1	1	Q55426	N-Term(iTRAQ4plex)	0,0000	1,060	2	8,7	0,007	0,09572	12	0,066064782	3	1025,56208	1,15	9,03	0
P73866	Potassium-transporting ATPase A chain OS=Synechocystis sp. (strain PC	172,80	4,84	1	2	2	5	0,088	5	23,4	558	59,3	5,41							
	A3	Sequence	# PSMs	# Proteins	# Protein Groups	Protein Group Accessions	Modifications	ΔCn	117/114	117/114 Count	117/114 Variability [%]	q-Value	PEP	IonScore	Exp Value	Charge	MH+ [Da]	ΔM [ppm]	RT [min]	# Missed Cleavages
	High	iARPLESLIFAGSGITk	2	1	1	P73866	N-Term(iTRAQ4plex); K17(iTRAQ4plex)	0,0000	0,259	2	204,9	0	2,328E-09	62	5,95922E-07	3	2061,23984	4,23	45,91	0
	High	gPVAHFEIIk	3	1	1	P73866	N-Term(iTRAQ4plex); K10(iTRAQ4plex)	0,0000	0,085	3	4,3	0	9,105E-07	49	1,12194E-05	3	1398,84180	5,01	28,88	0
P74177	DNA-directed RNA polymerase subunit gamma OS=Synechocystis sp. (s	172,35	11,02	1	6	6	10	1,012	8	22,0	626	70,9	7,49							
	A3	Sequence	# PSMs	# Proteins	# Protein Groups	Protein Group Accessions	Modifications	ΔCn	117/114	117/114 Count	117/114 Variability [%]	q-Value	PEP	IonScore	Exp Value	Charge	MH+ [Da]	ΔM [ppm]	RT [min]	# Missed Cleavages
	High	lQEILAPEIIVR	1	1	1	P74177	N-Term(iTRAQ4plex)	0,0000	1,013	1		0	0,000001129	48	1,45201E-05	2	1537,94414	0,44	45,31	0
	High	lAAPVTHVWYLk	2	1	1	P74177	N-Term(iTRAQ4plex); K12(iTRAQ4plex)	0,0000	1,314	1		0	0,000001296	43	5,27193E-05	3	1685,99997	1,08	37,52	0
	High	emAIELFQPFVIHR	2	1	1	P74177	N-Term(iTRAQ4plex); M2(Oxidation)	0,0000	0,931	2	2,6	0	3,864E-07	39	0,000122453	3	1890,00748	0,52	45,25	0
	High	lGIQAFEPILVEGR	2	1	1	P74177	N-Term(iTRAQ4plex)	0,0000	1,387	1		0	0,000003453	37	0,000211334	3	1685,96903	-1,02	48,42	0
	High	lREEILASk	1	1	1	P74177	N-Term(iTRAQ4plex); K9(iTRAQ4plex)	0,0000	2,309	1		0	0,000007043	31	0,00079794	3	1346,82383	-0,59	24,16	1
	High	tVVGANNR	2	1	1	P74177	N-Term(iTRAQ4plex)	0,0000	0,919	2	14,2	0,004	0,05157	20	0,010351422	2	974,55156	1,61	11,70	0
Q55660	Slr0001 protein OS=Synechocystis sp. (strain PCC 6803 / Kazusa) GN=s	171,35	20,00	1	4	4	11	1,152	10	16,0	160	17,4	5,15							
	A3	Sequence	# PSMs	# Proteins	# Protein Groups	Protein Group Accessions	Modifications	ΔCn	117/114	117/114 Count	117/114 Variability [%]	q-Value	PEP	IonScore	Exp Value	Charge	MH+ [Da]	ΔM [ppm]	RT [min]	# Missed Cleavages
	High	dVAAQFFkPQR	5	1	1	Q55660	N-Term(iTRAQ4plex); K8(iTRAQ4plex)	0,0000	1,249	5	3,1	0	1,004E-07	41	7,36207E-05	3	1594,89383	-0,38	28,29	0
	High	dcLGYVEcR	1	1	1	Q55660	N-Term(iTRAQ4plex); C2(Carbamidomethyl); C8(Carbam	0,0000	0,814	1		0	0,000005098	40	9,16157E-05	2	1315,58953	0,28	23,39	0
	High	iPHGLYVcGVk	1	1	1	Q55660	N-Term(iTRAQ4plex); C8(Carbamidomethyl); K11(iTRAQ	0,0000	0,960	1		0	3,938E-08	36	0,000252331	3	1530,87073	0,14	28,43	0
	High	kIPHGLYVcGVk	4	1	1	Q55660	N-Term(iTRAQ4plex); K1(iTRAQ4plex); C9(Carbamidome	0,0000	1,127	3	6,5	0	3,718E-09	26	0,002785929	3	1803,06589	-0,92	24,30	1
Q55469	UDP-N-acetylmuramoyl-L-alanyl-D-glutamate--2,6-diaminopimelate ligas	171,22	5,35	1	2	2	3	1,139	3	6,6	505	54,6	5,41							
	A3	Sequence	# PSMs	# Proteins	# Protein Groups	Protein Group Accessions	Modifications	ΔCn	117/114	117/114 Count	117/114 Variability [%]	q-Value	PEP	IonScore	Exp Value	Charge	MH+ [Da]	ΔM [ppm]	RT [min]	# Missed Cleavages
	High	tTTSHLIEYFLNQQQR	2	1	1	Q55469	N-Term(iTRAQ4plex)	0,0000	1,252	2	14,1	0	1,149E-12	75	3,07588E-08	3	2123,10111	0,42	42,85	0
	High	sSALLGTLYTR	1	1	1	Q55469	N-Term(iTRAQ4plex)	0,0000	1,089	1		0	0,001772	47	1,88352E-05	2	1325,75652	1,47	34,25	0
P74468	Aminopeptidase P OS=Synechocystis sp. (strain PCC 6803 / Kazusa) GN	170,60	16,55	1	7	7	9	0,953	8	11,5	441	49,5	5,63							
	A3	Sequence	# PSMs	# Proteins	# Protein Groups	Protein Group Accessions	Modifications	ΔCn	117/114	117/114 Count	117/114 Variability [%]	q-Value	PEP	IonScore	Exp Value	Charge	MH+ [Da]	ΔM [ppm]	RT [min]	# Missed Cleavages
	High	mHPVVSSAEYR	1	1	1	P74468	N-Term(iTRAQ4plex); M1(Oxidation)	0,0000	1,016	1		0	6,711E-07	53	5,17571E-06	3	1435,71302	0,67	13,83	0
	High	qGYGPQALVNSHPLVHPLR	3	1	1	P74468	N-Term(iTRAQ4plex)	0,0000	0,953	2	11,5	0	8,036E-10	41	7,5504E-05	4	2227,22182	0,05	32,18	0
	High	acDLSAIAHQR	1	1	1	P74468	N-Term(iTRAQ4plex); C2(Carbamidomethyl)	0,0000	1,023	1		0	0,000009787	40	9,48353E-05	3	1385,70847	0,59	21,02	0
	High	tGHWLGLDVHDAGNYk	1	1	1	P74468	N-Term(iTRAQ4plex); K16(iTRAQ4plex)	0,0000	0,573	1		0	3,567E-10	40	0,000106898	4	2071,06142	0,69	30,92	0
	High	ykPFYmHR	1	1	1	P74468	N-Term(iTRAQ4plex); K2(iTRAQ4plex); M6(Oxidation)	0,0000	0,894	1		0	0,0004922	20	0,010045464	3	1445,76064	0,30	16,24	0
	High	rAcDLSAIAHQR	1	1	1	P74468	N-Term(iTRAQ4plex); C3(Carbamidomethyl)	0,0000	2,070	1		0	0,002089	19	0,012617404	4	1541,80861	-0,10	17,84	1
	High	iHYHLGR	1	1	1	P74468	N-Term(iTRAQ4plex)	0,0000	0,342	1		0,005	0,05978	12	0,065761242	3	1039,59125	-0,50	14,87	0
P74741	Bifunctional purine biosynthesis protein PurH OS=Synechocystis sp. (str	170,35	11,15	1	4	4	5	0,983	5	4,2	511	54,5	5,36							
	A3	Sequence	# PSMs	# Proteins	# Protein Groups	Protein Group Accessions	Modifications	ΔCn	117/114	117/114 Count	117/114 Variability [%]	q-Value	PEP	IonScore	Exp Value	Charge	MH+ [Da]	ΔM [ppm]	RT [min]	# Missed Cleavages
	High	hTNPcGVALAPTLVEAYQk	2	1	1	P74741	N-Term(iTRAQ4plex); C5(Carbamidomethyl); K19(iTRAQ	0,0000	0,989	2	0,8	0	5,224E-11	76	2,80524E-08	3	2357,25290	0,11	34,38	0
	High	iHGGILAR	1	1	1	P74741	N-Term(iTRAQ4plex)	0,0000	0,906	1		0	0,0006732	42	6,98864E-05	3	980,61261	0,42	17,19	0
	High	tAAAAGITTIIQPGGSmR	1	1	1	P74741	N-Term(iTRAQ4plex); M17(Oxidation)	0,0000	0,919	1		0	0,00002156	38	0,000284763	3	1876,00833	0,20	33,12	0
	High	tTLGVGAGQmNR	1	1	1	P74741	N-Term(iTRAQ4plex); M10(Oxidation)	0,0000	1,012	1		0,001	0,01741	34	0,000356427	2	1364,70879	1,07	16,20	0
P73609	Anti-sigma factor antagonist OS=Synechocystis sp. (strain PCC 6803 / K	170,10	30,56	1	3	3	5	0,887	5	10,8	108	12,0	4,74							
	A3	Sequence	# PSMs	# Proteins	# Protein Groups	Protein Group Accessions	Modifications	ΔCn	117/114	117/114 Count	117/114 Variability [%]	q-Value	PEP	IonScore	Exp Value	Charge	MH+ [Da]	ΔM [ppm]	RT [min]	# Missed Cleavages
	High	ekIEEVAQAEISk	1	1	1	P73609	N-Term(iTRAQ4plex); K2(iTRAQ4plex); K13(iTRAQ4plex)	0,0000	0,887	1		0	6,827E-10	63	5,4572E-07	3	1906,08799	1,16	29,27	1
	High	aFNIESEIINNAAk	1	1	1	P73609	N-Term(iTRAQ4plex); K14(iTRAQ4plex)	0,0000	0,865	1		0	2,627E-08	32	0,000668298	3	1821,99698	1,12	40,30	0
	Medium	iLVFAk	3	1	1	P73609	N-Term(iTRAQ4plex); K6(iTRAQ4plex)	0,0000	1,050	3	13,6	0,013	0,1597	42	5,662E-05	2	978,66167	2,69	33,56	0
Q57038	Cytochrome b6 OS=Synechocystis sp. (strain PCC 6803 / Kazusa) GN=p	169,42	15,32	1	4	4	6	1,226	6	19,2	222	25,0	9,01							
	A3	Sequence	# PSMs	# Proteins	# Protein Groups	Protein Group Accessions	Modifications	ΔCn	117/114	117/114 Count	117/114 Variability [%]	q-Value	PEP	IonScore	Exp Value	Charge	MH+ [Da]	ΔM [ppm]	RT [min]	# Missed Cleavages
	High	gSESVGQATLTR	2	1	1	Q57038	N-Term(iTRAQ4plex)	0,0000	1,387	2	9,9	0	3,548E-07	77	1,82797E-08	2	1349,71526	0,80	16,96	0
	High	vYLTGGFk	1	1	1	Q57038	N-Term(iTRAQ4plex); K8(iTRAQ4plex)	0,0000	1,310	1		0	0,00005624	40	9,57128E-05	2	1172,69280	0,86	30,00	0
	High	vFQWFNDR	2	1	1	Q57038	N-Term(iTRAQ4plex)	0,0000	0,858	2	4,1	0	0,00005985	29	0,001218905	2	1255,63481	0,60	40,48	0
	High	eVTESk	1	1	1	Q57038	N-Term(iTRAQ4plex); K6(iTRAQ4plex)	0,0000	1,158	1		0,005	0,05732	16	0,025820818	2	980,55028	0,01	10,41	0
Q55484	Diaminopimelate decarboxylase OS=Synechocystis sp. (strain PCC 6803	169,21	17,27	1	6	6	8	2,567	8	18,5	469	50,8	5,45							
	A3	Sequence	# PSMs	# Proteins	# Protein Groups	Protein Group Accessions	Modifications	ΔCn	117/114	117/114 Count	117/114 Variability [%]	q-Value	PEP	IonScore	Exp Value	Charge	MH+ [Da]	ΔM [ppm]	RT [min]	# Missed Cleavages
	High	lGRPAAVLVNQGQANLILQR	1	1	1	Q55484	N-Term(iTRAQ4plex)	0,0000	2,736	1		0	1,267E-11	78	1,5065E-08	3	2275,34928	0,56	37,61	0
	High	tPASPSPNQNLLPLTAVINk	2	1	1	Q55484	N-Term(iTRAQ4plex); K20(iTRAQ4plex)	0,0000	3,815	2	104,2	0	2,239E-07	54	4,05481E-06	3	2363,35489	0,48	43,44	0
	High	kVVPNIR	1	1	1	Q55484	N-Term(iTRAQ4plex); K1(iTRAQ4plex)	0,0000	2,588	1		0	0,002798	23	0,005057897	3	1113,73413	-0,48	16,32	1
	High	iYFHGNNk	2	1	1	Q55484	N-Term(iTRAQ4plex); K8(iTRAQ4plex)	0,0000	2,295	2	26,7	0	0,001666	21	0,007128038	3	1280,69831	-0,53	16,91	0
	High	sLVGSAcVTAYR	1	1	1	Q55484	N-Term(iTRAQ4plex); C7(Carbamidomethyl)	0,0000	2,546	1		0	0,000007107	20	0,010273013	2	1427,74370	0,24	27,92	0
	High	lTPGIEcHTHEYIk	1	1	1	Q55484	N-Term(iTRAQ4plex); C7(Carbamidomethyl); K14(iTRAQ	0,0000	1,671	1		0	0,00005541	14	0,043850041	4	1986,03554	-0,10	23,99	0
P23353	Chorismate synthase OS=Synechocystis sp. (strain PCC 6803 / Kazusa)	169,14	10,50	1	3	3	6	0,836	6	14,0	362	39,3	5,99							
	A3	Sequence	# PSMs	# Proteins	# Protein Groups	Protein Group Accessions	Modifications	ΔCn	117/114	117/114 Count	117/114 Variability [%]	q-Value	PEP	IonScore	Exp Value	Charge	MH+ [Da]	ΔM [ppm]	RT [min]	# Missed Cleavages
	High	tLGTPIAILVR	4	1	1	P23353	N-Term(iTRAQ4plex)	0,0000	0,910	4	9,5	0	9,329E-07	57	2,1576E-06	2	1297,83391	1,13	42,12	0
	High	iAFkPTATIGQEQk	1	1	1	P23353	N-Term(iTRAQ4plex); K4(iTRAQ4plex); K14(iTRAQ4plex)	0,0000	0,796	1		0	7,703E-11	44	3,62218E-05	3	1964,15421	0,04	27,54	0
	High	yRPSHADATYEAk	1	1	1	P23353	N-Term(iTRAQ4plex); K13(iTRAQ4plex)	0,0000	0,812	1		0	6,174E-09	44	4,37522E-05	3	1796,91831	0,73	14,02	0
Q55466	Sll0518 protein OS=Synechocystis sp. (strain PCC 6803 / Kazusa) GN=sl	168,93	12,21	1	5	5	7	1,170	7	8,7	385	43,1	8,38							
	A3	Sequence	# PSMs	# Proteins	# Protein Groups	Protein Group Accessions	Modifications	ΔCn	117/114	117/114 Count	117/114 Variability [%]	q-Value	PEP	IonScore	Exp Value	Charge	MH+ [Da]	ΔM [ppm]	RT [min]	# Missed Cleavages
	High	gVWQFIPQcR	1	1	1	Q55466	N-Term(iTRAQ4plex); C9(Carbamidomethyl)	0,0000	1,111	1		0	0,00004685	45	3,31872E-05	2	1434,74346	0,10	40,88	0
	High	fYQILAR	2	1	1	Q55466	N-Term(iTRAQ4plex)	0,0000	1,138	2	4,1	0	0,000999	40	9,70443E-05	2	1054,61711	0,48	33,34	0
	High	rPVFSVYR	2	1	1	Q55466	N-Term(iTRAQ4plex)	0,0000	1,089	2	19,5	0	0,00001236	38	0,000157025	3	1167,67563	0,09	22,70	0
	High	rAIAVAAALEGk	1	1	1	Q55466	N-Term(iTRAQ4plex); K12(iTRAQ4plex)	0,0000	1,329	1		0	0,003357	31	0,000786991	3	1457,90412	-0,12	27,26	1
	High	nYYQAIGWLR	1	1	1	Q55466	N-Term(iTRAQ4plex)	0,0000	1,253	1		0,002	0,02523	21	0,009261852	2	1427,74675	-5,93	41,83	0
P73757	Neopullulanase OS=Synechocystis sp. (strain PCC 6803 / Kazusa) GN=n	168,72	10,43	1	5	5	7	0,835	6	8,6	508	58,0	5,25							
	A3	Sequence	# PSMs	# Proteins	# Protein Groups	Protein Group Accessions	Modifications	ΔCn	117/114	117/114 Count	117/114 Variability [%]	q-Value	PEP	IonScore	Exp Value	Charge	MH+ [Da]	ΔM [ppm]	RT [min]	# Missed Cleavages
	High	aVFYQIFPDR	1	1	1	P73757	N-Term(iTRAQ4plex)	0,0000	0,846	1		0	0,000001124	58	1,75376E-06	2	1399,74932	0,16	42,52	0
	High	fAIGQQQAIAVAQQPk	2	1	1	P73757	N-Term(iTRAQ4plex); K16(iTRAQ4plex)	0,0000	0,695	2	26,5	0	4,087E-10	49	1,37711E-05	3	1986,14078	1,63	32,25	0

	High	iIATAGmGYVFQR	1	1	1	P73757	N-Term(iTRAQ4plex); M7(Oxidation)	0,0000	0,698	1		0	4,853E-08	40	0,000106898	2	1586,85063	1,55	34,52	0
	High	aLPQFNHDHPDVR	2	1	1	P73757	N-Term(iTRAQ4plex)	0,0000	0,841	1		0	0,000002623	33	0,000521159	4	1689,85866	0,50	22,23	0
	High	kAVFYQIFPDR	1	1	1	P73757	N-Term(iTRAQ4plex); K1(iTRAQ4plex)	0,0000	0,925	1		0	0,000002836	22	0,006886047	3	1671,94773	0,96	37,38	1
P72646	Sll1054 protein OS=Synechocystis sp. (strain PCC 6803 / Kazusa) GN=sl	168,38	9,09	1	2	2	8	0,607	7	13,1	187	21,0	4,79							
	A3	Sequence	# PSMs	# Proteins	# Protein Groups	Protein Group Accessions	Modifications	ΔCn	117/114	117/114 Count	117/114 Variability [%]	q-Value	PEP	IonScore	Exp Value	Charge	MH+ [Da]	ΔM [ppm]	RT [min]	# Missed Cleavages
	High	sIASYFWmR	5	1	1	P72646	N-Term(iTRAQ4plex); M8(Oxidation)	0,0000	0,558	4	14,8	0	0,0002257	42	5,64898E-05	2	1320,65373	0,75	41,36	0
	High	kFNFDVSR	1	1	1	P72646	N-Term(iTRAQ4plex); K1(iTRAQ4plex)	0,0000	0,456	1		0	0,001224	35	0,000293069	3	1300,72556	0,26	24,17	1
	High	sIASYFWMR	2	1	1	P72646	N-Term(iTRAQ4plex)	0,0000	0,621	2	2,0	0	0,0008222	28	0,001625436	2	1304,65764	-0,15	45,39	0
P72781	NarL subfamily OS=Synechocystis sp. (strain PCC 6803 / Kazusa) GN=sl	167,23	7,45	1	2	2	8	1,465	8	7,8	282	31,4	5,53							
	A3	Sequence	# PSMs	# Proteins	# Protein Groups	Protein Group Accessions	Modifications	ΔCn	117/114	117/114 Count	117/114 Variability [%]	q-Value	PEP	IonScore	Exp Value	Charge	MH+ [Da]	ΔM [ppm]	RT [min]	# Missed Cleavages
	High	fQSLPVVFLTAR	7	1	1	P72781	N-Term(iTRAQ4plex)	0,0000	1,482	7	6,8	0	1,247E-08	55	3,22827E-06	3	1521,89087	-0,10	48,38	0
	High	fALQHGLTE	1	1	1	P72781	N-Term(iTRAQ4plex)	0,0000	1,171	1		0	0,0003467	27	0,001918536	2	1159,62212	-0,61	28,48	0
Q01950	Phycobilisome 7.8 kDa linker polypeptide, allophycocyanin-associated, c	166,53	50,75	1	3	3	5	1,226	5	6,3	67	7,7	10,93							
	A3	Sequence	# PSMs	# Proteins	# Protein Groups	Protein Group Accessions	Modifications	ΔCn	117/114	117/114 Count	117/114 Variability [%]	q-Value	PEP	IonScore	Exp Value	Charge	MH+ [Da]	ΔM [ppm]	RT [min]	# Missed Cleavages
	High	vELATGRPGTNAGLA	2	1	1	Q01950	N-Term(iTRAQ4plex)	0,0000	1,173	2	6,6	0	4,124E-08	60	1,05972E-06	2	1570,86772	0,45	27,26	0
	High	eLQNTYFTk	1	1	1	Q01950	N-Term(iTRAQ4plex); K9(iTRAQ4plex)	0,0000	1,157	1		0	0,0001798	52	7,1151E-06	2	1431,77471	1,74	27,22	0
	High	iTAcVPSQTR	2	1	1	Q01950	N-Term(iTRAQ4plex); C4(Carbamidomethyl)	0,0000	1,274	2	0,6	0	0,0001699	43	4,73119E-05	2	1276,68157	1,20	20,91	0
P74456	Ribosome-recycling factor OS=Synechocystis sp. (strain PCC 6803 / Kaz	166,39	32,42	1	6	6	11	0,846	11	23,3	182	20,2	5,81							
	A3	Sequence	# PSMs	# Proteins	# Protein Groups	Protein Group Accessions	Modifications	ΔCn	117/114	117/114 Count	117/114 Variability [%]	q-Value	PEP	IonScore	Exp Value	Charge	MH+ [Da]	ΔM [ppm]	RT [min]	# Missed Cleavages
	High	rIDELLAAk	4	1	1	P74456	N-Term(iTRAQ4plex); K9(iTRAQ4plex)	0,0000	0,604	4	15,5	0	0,00003649	43	4,81915E-05	3	1316,81375	-0,23	29,70	1
	High	aISLSDLGLTPNNDGk	1	1	1	P74456	N-Term(iTRAQ4plex); K16(iTRAQ4plex)	0,0000	0,921	1		0	0,00001257	29	0,001345767	3	1903,04001	1,30	39,95	0
	High	lNIPPLTAER	1	1	1	P74456	N-Term(iTRAQ4plex)	0,0000	0,729	1		0	0,00207	27	0,002004334	2	1267,75090	1,42	35,12	0
	High	aNASLLDR	1	1	1	P74456	N-Term(iTRAQ4plex)	0,0000	0,846	1		0	0,005371	24	0,003926178	2	1003,56554	0,22	19,12	0
	High	sFNTIR	3	1	1	P74456	N-Term(iTRAQ4plex)	0,0000	0,970	3	2,8	0	0,008803	22	0,006266139	2	881,49700	0,95	16,70	0
	High	lAELkDHmQk	1	1	1	P74456	N-Term(iTRAQ4plex); K5(iTRAQ4plex); M8(Oxidation); K	0,0000	1,190	1		0	0,00299	16	0,031095521	3	1660,94291	0,73	17,45	1
Q55865	Sll0585 protein OS=Synechocystis sp. (strain PCC 6803 / Kazusa) GN=sl	165,15	11,11	1	3	3	8	0,666	7	10,7	342	37,6	7,03							
	A3	Sequence	# PSMs	# Proteins	# Protein Groups	Protein Group Accessions	Modifications	ΔCn	117/114	117/114 Count	117/114 Variability [%]	q-Value	PEP	IonScore	Exp Value	Charge	MH+ [Da]	ΔM [ppm]	RT [min]	# Missed Cleavages
	High	sSLkPFQALAVTSTGTMER	1	1	1	Q55865	N-Term(iTRAQ4plex); K4(iTRAQ4plex)	0,0000	0,666	1		0	2,994E-12	64	5,57113E-07	3	2312,25272	0,17	36,85	0
	High	sSLkPFQALAVTSTGTmER	3	1	1	Q55865	N-Term(iTRAQ4plex); K4(iTRAQ4plex); M17(Oxidation)	0,0000	0,587	2	20,6	0	1,267E-10	48	2,00601E-05	3	2328,25028	1,30	33,26	0
	High	qVFNILWR	3	1	1	Q55865	N-Term(iTRAQ4plex)	0,0000	0,614	3	12,4	0	0,00002213	41	7,96104E-05	2	1219,70769	0,70	43,26	0
	High	rLEVIGELAmL	1	1	1	Q55865	N-Term(iTRAQ4plex); M10(Oxidation)	0,0000	0,716	1		0,003	0,04036	25	0,003012798	2	1403,80596	0,73	46,32	1
P74618	6-phosphogluconolactonase OS=Synechocystis sp. (strain PCC 6803 / Ka	165,04	15,42	1	4	4	8	0,923	8	14,3	240	26,5	5,27							
	A3	Sequence	# PSMs	# Proteins	# Protein Groups	Protein Group Accessions	Modifications	ΔCn	117/114	117/114 Count	117/114 Variability [%]	q-Value	PEP	IonScore	Exp Value	Charge	MH+ [Da]	ΔM [ppm]	RT [min]	# Missed Cleavages
	High	lITVGNk	1	1	1	P74618	N-Term(iTRAQ4plex); K7(iTRAQ4plex)	0,0000	0,826	1		0	0,0001961	53	5,29627E-06	2	1032,66643	0,83	22,35	0
	High	sVVFLVAGASk	1	1	1	P74618	N-Term(iTRAQ4plex); K11(iTRAQ4plex)	0,0000	0,556	1		0	0,0003233	46	2,36576E-05	3	1365,83603	1,15	35,83	0
	High	lTFTIPLINR	3	1	1	P74618	N-Term(iTRAQ4plex)	0,0000	1,074	3	4,0	0	0,00007445	32	0,0006109	2	1331,81828	1,13	47,83	0
	High	iHVFWGDER	3	1	1	P74618	N-Term(iTRAQ4plex)	0,0000	0,922	3	0,1	0	0,0009828	26	0,00227494	3	1302,67234	0,88	31,64	0
P74410	30S ribosomal protein S16 OS=Synechocystis sp. (strain PCC 6803 / Kaz	164,43	70,73	1	7	7	14	1,336	13	24,2	82	9,6	10,52							
	A3	Sequence	# PSMs	# Proteins	# Protein Groups	Protein Group Accessions	Modifications	ΔCn	117/114	117/114 Count	117/114 Variability [%]	q-Value	PEP	IonScore	Exp Value	Charge	MH+ [Da]	ΔM [ppm]	RT [min]	# Missed Cleavages
	High	lkEGAQPTDTVR	3	1	1	P74410	N-Term(iTRAQ4plex); K2(iTRAQ4plex)	0,0000	1,028	3	31,2	0	0,000002313	44	4,385E-05	3	1602,90360	-1,12	15,33	1
	High	aQVFEQLkA	1	1	1	P74410	N-Term(iTRAQ4plex); K8(iTRAQ4plex)	0,0000	1,553	1		0	0,0007543	33	0,000536995	2	1321,76982	-1,53	29,84	1
	High	iVAmHSTTR	2	1	1	P74410	N-Term(iTRAQ4plex); M4(Oxidation)	0,0000	1,063	2	34,8	0	0,0003532	32	0,000567505	2	1175,63176	-0,50	9,65	0
	High	lDVPAIVk	1	1	1	P74410	N-Term(iTRAQ4plex); K8(iTRAQ4plex)	0,0000	1,049	1		0	0,006277	29	0,001177525	2	1142,73589	-2,51	33,16	0
	High	dGRPLEELGFYNPR	1	1	1	P74410	N-Term(iTRAQ4plex)	0,0000	2,637	1		0	2,427E-08	24	0,003881236	3	1806,92374	-1,02	34,79	0
	High	rDGRPLEELGFYNPR	2	1	1	P74410	N-Term(iTRAQ4plex)	0,0000	1,662	2	10,3	0	0,000002121	21	0,007779828	4	1963,02688	0,09	30,78	1
	High	iVAMHSTTR	1	1	1	P74410	N-Term(iTRAQ4plex)	0,0000	1,137	1		0,001	0,01463	12	0,058340481	3	1159,63538	-1,77	13,90	0
	Medium	sILTk	3	1	1	P74410	N-Term(iTRAQ4plex); K5(iTRAQ4plex)	0,0000	1,336	2	14,1	0,019	0,2399	12	0,05847497	2	849,56023	-5,37	17,29	0
Q55729	Methionine--tRNA ligase OS=Synechocystis sp. (strain PCC 6803 / Kazus	164,07	3,20	1	2	2	5	0,601	5	43,3	532	61,4	5,33							
	A3	Sequence	# PSMs	# Proteins	# Protein Groups	Protein Group Accessions	Modifications	ΔCn	117/114	117/114 Count	117/114 Variability [%]	q-Value	PEP	IonScore	Exp Value	Charge	MH+ [Da]	ΔM [ppm]	RT [min]	# Missed Cleavages
	High	lSAYLLSPIVPR	4	1	1	Q55729	N-Term(iTRAQ4plex)	0,0000	0,717	4	27,7	0	7,211E-08	58	1,7578E-06	2	1472,89836	1,76	46,43	0
	High	yYFLk	1	1	1	Q55729	N-Term(iTRAQ4plex); K5(iTRAQ4plex)	0,0000	0,454	1		0,008	0,1033	24	0,004412633	2	1021,59874	2,62	32,05	0
Q55759	GTP cyclohydrolase 1 OS=Synechocystis sp. (strain PCC 6803 / Kazusa)	163,87	13,68	1	3	3	10	1,136	7	6,6	234	26,6	6,62							
	A3	Sequence	# PSMs	# Proteins	# Protein Groups	Protein Group Accessions	Modifications	ΔCn	117/114	117/114 Count	117/114 Variability [%]	q-Value	PEP	IonScore	Exp Value	Charge	MH+ [Da]	ΔM [ppm]	RT [min]	# Missed Cleavages
	High	tREEFLNLIR	8	1	1	Q55759	N-Term(iTRAQ4plex)	0,0000	1,136	5	6,6	0	0,00002361	45	3,07588E-05	3	1434,81855	-0,03	34,95	1
	High	yHPENVELNkEk	1	1	1	Q55759	N-Term(iTRAQ4plex); K10(iTRAQ4plex); K12(iTRAQ4plex	0,0000	0,980	1		0	0,000164	28	0,002024575	4	1932,05434	-0,40	18,98	1
	High	aHLAYIPNQk	1	1	1	Q55759	N-Term(iTRAQ4plex); K10(iTRAQ4plex)	0,0000	1,174	1		0	9,884E-07	28	0,001636703	3	1442,83673	0,63	20,33	0
Q55953	Sll0781 protein OS=Synechocystis sp. (strain PCC 6803 / Kazusa) GN=sl	162,73	21,94	1	4	4	9	0,955	8	33,6	155	18,6	6,24							
	A3	Sequence	# PSMs	# Proteins	# Protein Groups	Protein Group Accessions	Modifications	ΔCn	117/114	117/114 Count	117/114 Variability [%]	q-Value	PEP	IonScore	Exp Value	Charge	MH+ [Da]	ΔM [ppm]	RT [min]	# Missed Cleavages
	High	tTPLVPPFTk	5	1	1	Q55953	N-Term(iTRAQ4plex); K10(iTRAQ4plex)	0,0000	0,800	4	26,8	0	0,00003853	51	8,3946E-06	2	1388,83904	-0,12	33,35	0
	High	iREFLR	2	1	1	Q55953	N-Term(iTRAQ4plex)	0,0000	1,233	2	28,7	0,002	0,02476	12	0,070631755	2	977,60112	-0,19	22,91	1
	High	lVkELWAFGENR	1	1	1	Q55953	N-Term(iTRAQ4plex); K3(iTRAQ4plex)	0,0000	1,101	1		0,002	0,0305	11	0,083021256	3	1749,98691	-1,24	42,45	1
	Medium	eIAIAk	1	1	1	Q55953	N-Term(iTRAQ4plex); K6(iTRAQ4plex)	0,0000	0,618	1		0,015	0,1923	41	7,2272E-05	2	932,59953	-2,55	19,19	0
P73037	Peptidyl-prolyl cis-trans isomerase OS=Synechocystis sp. (strain PCC 68	161,73	17,91	1	3	3	10	1,304	8	20,4	201	21,5	8,94							
	A3	Sequence	# PSMs	# Proteins	# Protein Groups	Protein Group Accessions	Modifications	ΔCn	117/114	117/114 Count	117/114 Variability [%]	q-Value	PEP	IonScore	Exp Value	Charge	MH+ [Da]	ΔM [ppm]	RT [min]	# Missed Cleavages
	High	nkPFTFTIGVGQVIk	5	1	1	P73037	N-Term(iTRAQ4plex); K2(iTRAQ4plex); K15(iTRAQ4plex)	0,0000	1,413	4	10,9	0	1,012E-08	50	9,7268E-06	3	2081,25144	1,47	39,72	0
	High	kLIIPPDLAYGSR	4	1	1	P73037	N-Term(iTRAQ4plex); K1(iTRAQ4plex)	0,0000	1,280	3	8,1	0	4,174E-08	33	0,000492006	3	1731,04209	0,76	33,50	1
	High	vEVHYTGR	1	1	1	P73037	N-Term(iTRAQ4plex)	0,0000	0,887	1		0	0,0007426	19	0,012189054	3	1104,59223	0,35	14,32	0
P73604	Slr1853 protein OS=Synechocystis sp. (strain PCC 6803 / Kazusa) GN=s	161,55	20,35	1	2	2	6	0,775	4	9,3	113	12,1	4,84							
	A3	Sequence	# PSMs	# Proteins	# Protein Groups	Protein Group Accessions	Modifications	ΔCn	117/114	117/114 Count	117/114 Variability [%]	q-Value	PEP	IonScore	Exp Value	Charge	MH+ [Da]	ΔM [ppm]	RT [min]	# Missed Cleavages
	High	aAGAFAALk	5	1	1	P73604	N-Term(iTRAQ4plex); K9(iTRAQ4plex)	0,0000	0,807	3	18,7	0	0,0003403	65	3,26565E-07	2	1107,67815	1,49	27,41	0
	High	vAGEAWGLPLIDQk	1	1	1	P73604	N-Term(iTRAQ4plex); K14(iTRAQ4plex)	0,0000	0,744	1		0	0,0000125	23	0,004753024	3	1785,01902	2,27	43,55	0
Q55756	Carbamoyl-phosphate synthase large chain OS=Synechocystis sp. (strai	157,92	7,49	1	7	7	11	1,141	10	25,6	1081	119,0	4,98							
	A3	Sequence	# PSMs	# Proteins	# Protein Groups	Protein Group Accessions	Modifications	ΔCn	117/114	117/114 Count	117/114 Variability [%]	q-Value	PEP	IonScore	Exp Value	Charge	MH+ [Da]	ΔM [ppm]	RT [min]	# Missed Cleavages
	High	lRDYSIAIIR	3	1	1	Q55756	N-Term(iTRAQ4plex)	0,0000	1,447	2	6,5	0	0,000002211	53	5,4325E-06	3	1363,81821	0,27	33,03	1
	High	vmILGGGPNR	1	1	1	Q55756	N-Term(iTRAQ4plex); M2(Oxidation)	0,0000	1,094	1		0	0,000002283	50	1,02795E-05	2	1173,65386	0,64	23,44	0
	High	hETLPTLSHLR	1	1	1	Q55756	N-Term(iTRAQ4plex)	0,0000	0,587	1		0	0,000001561	22	0,008085332	4	1447,81533	1,05	22,48	0
	High	iSYPVVVRPSYVLGGR	2	1	1	Q55756	N-Term(iTRAQ4plex)	0,0000	1,341	2	9,8	0	0,00001285	20	0,010567445	3	1906,10575	1,37	38,21	0
	High	kTPASFEPTIDYVVTk	2	1	1	Q55756	N-Term(iTRAQ4plex); K1(iTRAQ4plex); K16(iTRAQ4plex)	0,0000	1,141	2	0,6	0	0,000001655	19	0,012359474	4	2228,25576	0,83	35,53	1
	High	tYIEPLIPEIVEk	1	1	1	Q55756	N-Term(iTRAQ4plex); K13(iTRAQ4plex)	0,0000	0,985	1		0	0,004842	14	0,051562477	3	1832,06852	1,39	45,53	0
	High	fAFEk	1	1	1	Q55756	N-Term(iTRAQ4plex); K5(iTRAQ4plex)	0,0000	0,764	1		0,002	0,0338	12	0,069337791	2	929,53349	0,00	25,56	0
P73654	Ssl3364 protein OS=Synechocystis sp. (strain PCC 6803 / Kazusa) GN=s	157,21	27,03	1	2	2	4	0,928	4	3,0	74	8,3	4,22							
	A3	Sequence	# PSMs	# Proteins	# Protein Groups	Protein Group Accessions	Modifications	ΔCn	117/114	117/114 Count	117/114 Variability [%]	q-Value	PEP	IonScore	Exp Value	Charge	MH+ [Da]	ΔM [ppm]	RT [min]	# Missed Cleavages
	High	qQHPTQTTLEk	3	1	1	P73654	N-Term(iTRAQ4plex); K11(iTRAQ4plex)	0,0000	0,911	3	5,4	0	1,905E-09	71	8,85054E-08	2	1598,87554	0,92	12,09	0
	High	iEQELANAR	1	1	1	P73654	N-Term(iTRAQ4plex)	0,0000	0,949	1		0	0,000008343	58	1,59577E-06	2	1187,65105	0,79	24,67	0
P74447	Ferredoxin OS=Synechocystis sp. (strain PCC 6803 / Kazusa) GN=slr014	156,38	10,75	1	2	2	6	1,043	5	6,9	186	20,6	5,06							
	A3	Sequence	# PSMs	# Proteins	# Protein Groups	Protein Group Accessions	Modifications	ΔCn	117/114	117/114 Count	117/114 Variability [%]	q-Value	PEP	IonScore	Exp Value	Charge	MH+ [Da]	ΔM [ppm]	RT [min]	# Missed Cleavages
	High	rAEENILNPR	3	1	1	P74447	N-Term(iTRAQ4plex)	0,0000	1,043	3	21,7	0	0,00002067	51	8,7694E-06	3	1355,74903	-1,63	16,93	1
	High	tLEVITTHNR	3	1	1	P74447	N-Term(iTRAQ4plex)	0,0000	1,067	2	3,5	0	0,00001219	48	1,65185E-05	3	1327,74607	0,76	19,52	0
P74582	Aconitate hydratase 2 OS=Synechocystis sp. (strain PCC 6803 / Kazusa)	155,91	6,80	1	5	5	8	1,088	5	12,4	868	93,5	5,31							
	A3	Sequence	# PSMs	# Proteins	# Protein Groups	Protein Group Accessions	Modifications	ΔCn	117/114	117/114 Count	117/114 Variability [%]	q-Value	PEP	IonScore	Exp Value	Charge	MH+ [Da]	ΔM [ppm]	RT [min]	# Missed Cleavages
	High	skDTNIASAAATALSk	1	1	1	P74582	N-Term(iTRAQ4plex); K2(iTRAQ4plex); K16(iTRAQ4plex)	0,0000	0,807	1		0	1,018E-08	66	2,72879E-07	3	1981,12973	0,35	29,75	1
	High	aGGYILGGk	2	1	1	P74582	N-Term(iTRAQ4plex); K9(iTRAQ4plex)	0,0000	1,244	1		0	0,000001572	55	2,84426E-06	2	1123,67192	0,47	23,35	0
	High	gGVALRPGDGIIHSWLNR	2	1	1	P74582	N-Term(iTRAQ4plex)	0,0000	1,088	1		0	0,00000351	41	9,40116E-05	4	2062,14370	0,46	37,49	0
	High	iPLLIGR	2	1	1	P74582	N-Term(iTRAQ4plex)	0,0000	1,159	1		0	0,01066	25	0,003380415	2	925,63213	0,63	36,98	0
	High	aGFLTAIAk	1	1	1	P74582	N-Term(iTRAQ4plex); K9(iTRAQ4plex)	0,0000	1,001	1		0	0,0007298	11	0,087291108	2	1179,73406	0,04	33,82	0
P72699	UPF0045 protein sll0230 OS=Synechocystis sp. (strain PCC 6803 / Kazu	155,71	19,01	1	2	2	9	0,952	6	10,9	121	13,3	7,43							
	A3	Sequence	# PSMs	# Proteins	# Protein Groups	Protein Group Accessions	Modifications	ΔCn	117/114	117/114 Count	117/114 Variability [%]	q-Value	PEP	IonScore	Exp Value	Charge	MH+ [Da]	ΔM [ppm]	RT [min]	# Missed Cleavages
	High	acHEAVHALGAPR	7	1	1	P72699	N-Term(iTRAQ4plex); C2(Carbamidomethyl)	0,0000	0,900	4	12,6	0	1,284E-10	56	2,47725E-06	3	1532,78739	0,05	13,47	0
	High	tDRPQTmDEk	2	1	1	P72699	N-Term(iTRAQ4plex); M7(Oxidation); K10(iTRAQ4plex)	0,0000	0,996	2	9,5	0	0,00004198	17	0,020939678	3	1524,75867	1,30	8,05	0
P74395	N utilization substance protein B homolog OS=Synechocystis sp. (strain	155,68	14,93	1	3	3	6	1,695	5	23,6	221	24,7	6,02							
	A3	Sequence	# PSMs	# Proteins	# Protein Groups	Protein Group Accessions	Modifications	ΔCn	117/114	117/114 Count	117/114 Variability [%]	q-Value	PEP	IonScore	Exp Value	Charge	MH+ [Da]	ΔM [ppm]	RT [min]	# Missed Cleavages
	High	eLSLLSLSQLSR	1	1	1	P74395	N-Term(iTRAQ4plex)	0,0000	1,983	1		0	4,502E-08	52	6,50718E-06	2	1489,87090	0,15	45,60	0
	High	kTDGAPVTPGEPR	3	1	1	P74395	N-Term(iTRAQ4plex); K1(iTRAQ4plex)	0,0000	1,425	2	33,1	0	0,00001938	47	1,92296E-05	3	1612,88907	-0,42	14,14	1
	High	aSNLNSAk	2	1	1	P74395	N-Term(iTRAQ4plex); K8(iTRAQ4plex)	0,0000	1,382	2	31,0	0	0,0006761	42	6,20826E-05	2	1092,62590	0,67	10,74	0
P73601	Sll1784 protein OS=Synechocystis sp. (strain PCC 6803 / Kazusa) GN=sl	155,23	10,11	1	3	3	7	0,750	6	21,4	267	29,7	5,11							
	A3	Sequence	# PSMs	# Proteins	# Protein Groups	Protein Group Accessions	Modifications	ΔCn	117/114	117/114 Count	117/114 Variability [%]	q-Value	PEP	IonScore	Exp Value	Charge	MH+ [Da]	ΔM [ppm]	RT [min]	# Missed Cleavages
	High	gGLQLFQSVk	4	1	1	P73601	N-Term(iTRAQ4plex); K10(iTRAQ4plex)	0,0000	0,718	3	20,4	0	3,632E-08	68	1,7457E-07	2	1364,81658	1,85	35,25	0
	High	hLPAITEESk	2	1	1	P73601	N-Term(iTRAQ4plex); K10(iTRAQ4plex)	0,0000	0,894	2	45,7	0	0,0001584	30	0,001007371	3	1412,80048	1,20	19,72	0
	Medium	lPTSLTk	1	1	1	P73601	N-Term(iTRAQ4plex); K7(iTRAQ4plex)	0,0000	0,783	1		0,04	0,4906	31	0,000862919	2	1047,66680	1,49	25,24	0
P73405	Pyruvate dehydrogenase E1 beta subunit OS=Synechocystis sp. (strain I	154,75	13,58	1	4	4	12	1,057	9	16,7	324	35,7	5,54							
	A3	Sequence	# PSMs	# Proteins	# Protein Groups	Protein Group Accessions	Modifications	ΔCn	117/114	117/114 Count	117/114 Variability [%]	q-Value	PEP	IonScore	Exp Value	Charge	MH+ [Da]	ΔM [ppm]	RT [min]	# Missed Cleavages
	High	aETLLFAALR	3	1	1	P73405	N-Term(iTRAQ4plex)	0,0000	1,057	3	12,5	0	2,948E-07	53	5,04626E-06	2	1248,74370	0,32	45,06	0
	High	lEAYFHAVPGLk	7	1	1	P73405	N-Term(iTRAQ4plex); K12(iTRAQ4plex)	0,0000	1,110	4	15,9	0	0,00003183	28	0,001741687	3	1632,93637	0,70	39,33	0
	High	yTSGGNYQIPmVIR	1	1	1	P73405	N-Term(iTRAQ4plex); M11(Oxidation)	0,0000	1,092	1		0	0,003053	21	0,008336236	2	1758,90093	2,48	34,57	0
	High	aEVVRPGk	1	1	1	P73405	N-Term(iTRAQ4plex); K8(iTRAQ4plex)	0,0000	0,852	1		0	0,0003063	18	0,014961323	3	1143,70768	-1,03	11,37	0
P73299	30S ribosomal protein S13 OS=Synechocystis sp. (strain PCC 6803 / Kaz	154,46	33,86	1	6	6	9	1,541	8	27,1	127	14,6	10,59							
	A3	Sequence	# PSMs	# Proteins	# Protein Groups	Protein Group Accessions	Modifications	ΔCn	117/114	117/114 Count	117/114 Variability [%]	q-Value	PEP	IonScore	Exp Value	Charge	MH+ [Da]	ΔM [ppm]	RT [min]	# Missed Cleavages
	High	iAGVDLPR	2	1	1	P73299	N-Term(iTRAQ4plex)	0,0000	1,107	1		0	0,0003169	51	7,12804E-06	2	984,59575	-0,15	28,42	0
	High	rLGDIGTYR	1	1	1	P73299	N-Term(iTRAQ4plex)	0,0000	1,809	1		0	0,00001786	50	1,17138E-05	3	1194,67096	-0,18	20,78	1
	High	rLTVAGk	2	1	1	P73299	N-Term(iTRAQ4plex); K7(iTRAQ4plex)	0,0000	1,739	2	11,6	0	0,001379	30	0,001504298	3	1032,67710	0,27	12,82	1
	High	rWEAmNIk	1	1	1	P73299	N-Term(iTRAQ4plex); M5(Oxidation); K8(iTRAQ4plex)	0,0000	0,969	1		0	0,00005139	21	0,007585252	3	1351,73993	0,31	17,92	1
	High	qGLPVR	2	1	1	P73299	N-Term(iTRAQ4plex)	0,0000	1,399	2	7,8	0,002	0,02383	22	0,005942511	2	813,50597	-0,45	14,77	0
	High	kTPAk	1	1	1	P73299	N-Term(iTRAQ4plex); K1(iTRAQ4plex); K5(iTRAQ4plex)	0,0000	2,947	1		0,008	0,09835	12	0,06123081	3	976,64966	-1,92	7,81	1
P73607	Anti-sigma factor antagonist OS=Synechocystis sp. (strain PCC 6803 / K	154,12	19,27	1	2	2	6	1,054	6	21,6	109	11,9	4,41							
	A3	Sequence	# PSMs	# Proteins	# Protein Groups	Protein Group Accessions	Modifications	ΔCn	117/114	117/114 Count	117/114 Variability [%]	q-Value	PEP	IonScore	Exp Value	Charge	MH+ [Da]	ΔM [ppm]	RT [min]	# Missed Cleavages
	High	iHEVILPLAEPGSk	3	1	1	P73607	N-Term(iTRAQ4plex); K14(iTRAQ4plex)	0,0000	0,790	3	23,9	0	7,361E-10	44	4,00839E-05	3	1791,06443	1,41	33,87	0
	High	lLLYLYR	3	1	1	P73607	N-Term(iTRAQ4plex)	0,0000	1,196	3	5,3	0	0,000401	43	5,55866E-05	2	1097,68486	0,84	43,90	0
P74074	Slr1338 protein OS=Synechocystis sp. (strain PCC 6803 / Kazusa) GN=s	152,67	12,00	1	2	2	6	0,991	5	10,1	200	22,4	4,51							
	A3	Sequence	# PSMs	# Proteins	# Protein Groups	Protein Group Accessions	Modifications	ΔCn	117/114	117/114 Count	117/114 Variability [%]	q-Value	PEP	IonScore	Exp Value	Charge	MH+ [Da]	ΔM [ppm]	RT [min]	# Missed Cleavages
	High	sRWELVQESTLALAR	4	1	1	P74074	N-Term(iTRAQ4plex)	0,0000	1,244	3	1,2	0	7,629E-07	52	6,60648E-06	3	1903,05265	0,42	39,28	1
	High	rYDNVNASk	2	1	1	P74074	N-Term(iTRAQ4plex); K9(iTRAQ4plex)	0,0000	0,958	2	5,0	0	0,0006282	27	0,001972287	3	1354,73142	-0,25	10,73	1
P73851	Aspartate--tRNA ligase OS=Synechocystis sp. (strain PCC 6803 / Kazusa	152,03	7,18	1	5	5	9	0,793	8	13,0	599	67,2	5,31							
	A3	Sequence	# PSMs	# Proteins	# Protein Groups	Protein Group Accessions	Modifications	ΔCn	117/114	117/114 Count	117/114 Variability [%]	q-Value	PEP	IonScore	Exp Value	Charge	MH+ [Da]	ΔM [ppm]	RT [min]	# Missed Cleavages
	High	lSQNLQLR	1	1	1	P73851	N-Term(iTRAQ4plex)	0,0000	0,797	1		0	0,00003892	56	2,62404E-06	2	1115,66631	0,85	25,36	0
	High	lVmLLAk	2	1	1	P73851	N-Term(iTRAQ4plex); M3(Oxidation); K7(iTRAQ4plex)	0,0000	0,872	1		0	0,0002708	38	0,000160313	2	1091,71086	0,70	32,98	0
	High	rPPESLNEk	2	1	1	P73851	N-Term(iTRAQ4plex); K9(iTRAQ4plex)	0,0000	0,823	2	6,2	0	0,000007162	31	0,000803471	3	1357,76859	0,56	12,98	0
	High	ikPGGDLFk	2	1	1	P73851	N-Term(iTRAQ4plex); K2(iTRAQ4plex); K9(iTRAQ4plex)	0,0000	0,839	2	23,4	0	0,00002367	28	0,001588437	3	1406,87354	0,25	24,66	0
	High	nIDLPRPFPR	2	1	1	P73851	N-Term(iTRAQ4plex)	0,0000	0,716	2	13,9	0	0,00004295	22	0,007046444	3	1368,78788	0,72	31,22	0
P73305	50S ribosomal protein L18 OS=Synechocystis sp. (strain PCC 6803 / Kaz	151,64	20,00	1	3	3	9	1,889	6	30,3	120	13,2	10,92							
	A3	Sequence	# PSMs	# Proteins	# Protein Groups	Protein Group Accessions	Modifications	ΔCn	117/114	117/114 Count	117/114 Variability [%]	q-Value	PEP	IonScore	Exp Value	Charge	MH+ [Da]	ΔM [ppm]	RT [min]	# Missed Cleavages
	High	gINQVVFDR	5	1	1	P73305	N-Term(iTRAQ4plex)	0,0000	1,932	3	22,4	0	3,362E-07	61	8,56979E-07	2	1191,66008	-0,17	28,00	0
	High	hLSGTSERPR	1	1	1	P73305	N-Term(iTRAQ4plex)	0,0000	1,210	1		0	0,0004038	12	0,064475198	4	1283,69387	0,13	7,81	0
	Medium	lAVFR	3	1	1	P73305	N-Term(iTRAQ4plex)	0,0000	2,094	2	18,7	0,01	0,1266	11	0,109121421	2	749,47844	-0,82	25,73	0
P74344	Molybdopterin biosynthesis MoeB protein OS=Synechocystis sp. (strain I	150,75	14,54	1	6	6	9	1,415	8	19,7	392	42,7	5,05							
	A3	Sequence	# PSMs	# Proteins	# Protein Groups	Protein Group Accessions	Modifications	ΔCn	117/114	117/114 Count	117/114 Variability [%]	q-Value	PEP	IonScore	Exp Value	Charge	MH+ [Da]	ΔM [ppm]	RT [min]	# Missed Cleavages
	High	iILGAPNTLSGR	2	1	1	P74344	N-Term(iTRAQ4plex)	0,0000	1,373	2	8,7	0	0,000005047	43	4,95416E-05	2	1355,81389	0,82	32,80	0
	High	hIILPEVGLDGQk	1	1	1	P74344	N-Term(iTRAQ4plex); K13(iTRAQ4plex)	0,0000	0,822	1		0	4,007E-07	41	8,35545E-05	3	1707,00473	0,20	32,92	0
	High	aLSLLk	1	1	1	P74344	N-Term(iTRAQ4plex); K6(iTRAQ4plex)	0,0000	1,450	1		0	0,00816	35	0,000337264	2	932,63793	-0,40	28,73	0
	High	hIILPEVGLDGQkR	1	1	1	P74344	N-Term(iTRAQ4plex); K13(iTRAQ4plex)	0,0000	0,747	1		0	0,000001957	28	0,001686437	4	1863,10232	-1,71	29,76	1
	High	lRPNPVRPVIEk	3	1	1	P74344	N-Term(iTRAQ4plex); K12(iTRAQ4plex)	0,0000	1,590	2	21,0	0	0,0001767	21	0,007227199	4	1706,06936	0,80	21,81	0
	High	qIIHGTSWVGkPk	1	1	1	P74344	N-Term(iTRAQ4plex); K11(iTRAQ4plex); K13(iTRAQ4plex	0,0000	1,684	1		0	0,00002319	16	0,023657563	4	1883,12380	0,55	22,64	0
P73407	Carbon dioxide-concentrating mechanism protein CcmK homolog 4 OS=	150,54	12,50	1	2	2	6	1,433	6	7,4	112	11,9	6,06							
	A3	Sequence	# PSMs	# Proteins	# Protein Groups	Protein Group Accessions	Modifications	ΔCn	117/114	117/114 Count	117/114 Variability [%]	q-Value	PEP	IonScore	Exp Value	Charge	MH+ [Da]	ΔM [ppm]	RT [min]	# Missed Cleavages
	High	iTIVGYIR	3	1	1	P73407	N-Term(iTRAQ4plex)	0,0000	1,522	3	3,3	0	0,00005083	42	6,41165E-05	2	1078,67473	0,56	35,79	0
	High	fTLNIR	3	1	1	P73407	N-Term(iTRAQ4plex)	0,0000	1,377	3	0,7	0	0,005338	39	0,000181691	2	907,54869	0,55	30,68	0
P74720	Sll1106 protein OS=Synechocystis sp. (strain PCC 6803 / Kazusa) GN=sl	149,07	23,98	1	6	6	9	0,921	8	16,8	171	18,0	4,82							
	A3	Sequence	# PSMs	# Proteins	# Protein Groups	Protein Group Accessions	Modifications	ΔCn	117/114	117/114 Count	117/114 Variability [%]	q-Value	PEP	IonScore	Exp Value	Charge	MH+ [Da]	ΔM [ppm]	RT [min]	# Missed Cleavages
	High	tSLSQDEESQLQAVLDR	1	1	1	P74720	N-Term(iTRAQ4plex)	0,0000	0,911	1		0	5,036E-11	62	6,02518E-07	3	2063,03904	0,80	40,99	0
	High	vLADVSk	1	1	1	P74720	N-Term(iTRAQ4plex); K7(iTRAQ4plex)	0,0000	0,878	1		0	0,0002523	58	2,60215E-06	2	1019,63359	-0,35	20,87	0
	High	kVTPDkVLADVSk	1	1	1	P74720	N-Term(iTRAQ4plex); K1(iTRAQ4plex); K6(iTRAQ4plex);	0,0000	0,963	1		0	0,001125	16	0,026666745	4	1976,22085	-1,51	26,51	2
	High	kVTPDk	3	1	1	P74720	N-Term(iTRAQ4plex); K1(iTRAQ4plex); K6(iTRAQ4plex)	0,0000	1,166	3	34,5	0,001	0,01826	20	0,010091832	3	1119,70667	-2,79	8,39	1
	High	hGLLDGVPAAE	2	1	1	P74720	N-Term(iTRAQ4plex)	0,0000	0,754	2	3,5	0,005	0,06644	32	0,00059837	2	1222,65520	0,27	29,50	0
	Medium	vTPDk	1	1	1	P74720	N-Term(iTRAQ4plex); K5(iTRAQ4plex)	0,0000				0,031	0,3891	11	0,080018296	2	847,51109	-1,96	11,80	0
Q55847	Sll0470 protein OS=Synechocystis sp. (strain PCC 6803 / Kazusa) GN=sl	148,92	19,35	1	4	4	7	1,363	7	38,0	186	20,7	8,78							
	A3	Sequence	# PSMs	# Proteins	# Protein Groups	Protein Group Accessions	Modifications	ΔCn	117/114	117/114 Count	117/114 Variability [%]	q-Value	PEP	IonScore	Exp Value	Charge	MH+ [Da]	ΔM [ppm]	RT [min]	# Missed Cleavages
	High	vYLGTWIVSINR	4	1	1	Q55847	N-Term(iTRAQ4plex)	0,0000	1,204	4	28,1	0	1,633E-07	58	1,41896E-06	2	1564,89836	0,98	47,57	0
	High	lYIPAk	1	1	1	Q55847	N-Term(iTRAQ4plex); K6(iTRAQ4plex)	0,0000	0,725	1		0	0,00254	30	0,001002236	2	992,63896	0,68	27,28	0
	High	lYIPAkk	1	1	1	Q55847	N-Term(iTRAQ4plex); K6(iTRAQ4plex); K7(iTRAQ4plex)	0,0000	1,894	1		0	0,0002552	27	0,002098795	3	1264,83423	-0,86	22,93	1
	High	rQGNIEIEDVVWDkESR	1	1	1	Q55847	N-Term(iTRAQ4plex); K14(iTRAQ4plex)	0,0000	1,370	1		0	0,00009882	15	0,034831326	4	2361,24087	0,29	32,57	2
P27180	ATP synthase subunit delta OS=Synechocystis sp. (strain PCC 6803 / Ka	148,63	24,86	1	5	5	8	1,698	7	65,3	185	20,1	6,79							
	A3	Sequence	# PSMs	# Proteins	# Protein Groups	Protein Group Accessions	Modifications	ΔCn	117/114	117/114 Count	117/114 Variability [%]	q-Value	PEP	IonScore	Exp Value	Charge	MH+ [Da]	ΔM [ppm]	RT [min]	# Missed Cleavages
	High	qFTNTVLAEVTSALk	1	1	1	P27180	N-Term(iTRAQ4plex); K15(iTRAQ4plex)	0,0000	1,136	1		0	0,0000098	42	6,38542E-05	3	1910,08298	-0,41	51,35	0
	High	vDGDILGGIVIk	1	1	1	P27180	N-Term(iTRAQ4plex); K12(iTRAQ4plex)	0,0000	0,761	1		0	0,00001552	36	0,000271	3	1486,91013	1,19	42,32	0
	High	mkGSLYSSk	3	1	1	P27180	N-Term(iTRAQ4plex); M1(Oxidation); K2(iTRAQ4plex); K	0,0000	2,990	2	23,6	0	0,0008666	19	0,013334293	3	1448,81458	0,17	11,98	1
	High	vGLSLGTAL	1	1	1	P27180	N-Term(iTRAQ4plex)	0,0000	1,773	1		0,005	0,0586	43	4,48714E-05	2	974,60100	0,71	42,25	0
	High	rVGLSLGTAL	2	1	1	P27180	N-Term(iTRAQ4plex)	0,0000	1,572	2	11,5	0,006	0,07926	35	0,000348313	2	1130,70232	0,79	33,84	1
P72707	Sll0224 protein OS=Synechocystis sp. (strain PCC 6803 / Kazusa) GN=sl	148,45	11,41	1	3	3	5	0,855	4	3,0	298	32,8	4,42							
	A3	Sequence	# PSMs	# Proteins	# Protein Groups	Protein Group Accessions	Modifications	ΔCn	117/114	117/114 Count	117/114 Variability [%]	q-Value	PEP	IonScore	Exp Value	Charge	MH+ [Da]	ΔM [ppm]	RT [min]	# Missed Cleavages
	High	nLVNYSLVk	3	1	1	P72707	N-Term(iTRAQ4plex); K9(iTRAQ4plex)	0,0000	0,856	2	3,0	0	5,797E-08	64	4,00839E-07	2	1337,80449	1,02	30,76	0
	High	kTSDNPQQWk	1	1	1	P72707	N-Term(iTRAQ4plex); K1(iTRAQ4plex); K10(iTRAQ4plex)	0,0000	0,871	1		0	5,461E-08	31	0,000714447	3	1663,91068	-1,28	16,17	1
	High	aILDFQPGATIVEVk	1	1	1	P72707	N-Term(iTRAQ4plex); K15(iTRAQ4plex)	0,0000	0,820	1		0	0,00004612	31	0,000816582	3	1889,10056	0,99	44,38	0
P73810	DNA topoisomerase 1 OS=Synechocystis sp. (strain PCC 6803 / Kazusa)	146,49	6,35	1	5	5	7	1,070	7	6,2	898	99,3	8,82							
	A3	Sequence	# PSMs	# Proteins	# Protein Groups	Protein Group Accessions	Modifications	ΔCn	117/114	117/114 Count	117/114 Variability [%]	q-Value	PEP	IonScore	Exp Value	Charge	MH+ [Da]	ΔM [ppm]	RT [min]	# Missed Cleavages
	High	lVGYTLSPLLWk	2	1	1	P73810	N-Term(iTRAQ4plex); K12(iTRAQ4plex)	0,0000	0,925	2	21,9	0	0,00001035	53	5,1996E-06	2	1678,02190	2,19	49,72	0
	High	rIDFPGYFR	1	1	1	P73810	N-Term(iTRAQ4plex)	0,0000	1,046	1		0	0,00001492	40	9,35341E-05	3	1314,70795	0,29	33,26	1
	High	kASATGTAk	1	1	1	P73810	N-Term(iTRAQ4plex); K1(iTRAQ4plex); K9(iTRAQ4plex)	0,0000	1,271	1		0	0,004461	33	0,000481915	3	1266,77433	0,11	7,78	1
	High	lTGkPWAVVNTEEkPGVR	1	1	1	P73810	N-Term(iTRAQ4plex); K4(iTRAQ4plex); K14(iTRAQ4plex)	0,0000	0,942	1		0	1,889E-10	29	0,001264649	4	2413,39443	0,66	30,46	0
	High	iTQLSVLLk	2	1	1	P73810	N-Term(iTRAQ4plex); K9(iTRAQ4plex)	0,0000	1,113	2	0,4	0	0,000003263	27	0,00181539	2	1302,86113	0,92	40,98	0
P74241	Sulfate adenylyltransferase OS=Synechocystis sp. (strain PCC 6803 / Ka	146,42	10,00	1	4	4	9	0,982	8	14,4	390	43,7	6,25							
	A3	Sequence	# PSMs	# Proteins	# Protein Groups	Protein Group Accessions	Modifications	ΔCn	117/114	117/114 Count	117/114 Variability [%]	q-Value	PEP	IonScore	Exp Value	Charge	MH+ [Da]	ΔM [ppm]	RT [min]	# Missed Cleavages
	High	nYGcTHFIVGR	1	1	1	P74241	N-Term(iTRAQ4plex); C4(Carbamidomethyl)	0,0000	1,097	1		0	0,000003923	42	5,80724E-05	3	1467,72864	0,19	23,53	0
	High	vILGINPSAmR	2	1	1	P74241	N-Term(iTRAQ4plex); M10(Oxidation)	0,0000	0,916	2	6,2	0	0,00005642	36	0,000274138	2	1330,76470	0,98	30,90	0
	High	eAIFHALIR	5	1	1	P74241	N-Term(iTRAQ4plex)	0,0000	1,030	4	11,1	0	0,0001081	32	0,000633826	2	1213,71807	0,54	33,40	0
	High	ePHPLFPk	1	1	1	P74241	N-Term(iTRAQ4plex); K8(iTRAQ4plex)	0,0000	0,863	1		0	0,000004662	25	0,003380415	3	1252,73087	1,29	21,24	0
P72805	Sll1665 protein OS=Synechocystis sp. (strain PCC 6803 / Kazusa) GN=sl	146,21	5,94	1	4	4	8	1,073	8	42,2	589	63,6	3,57							
	A3	Sequence	# PSMs	# Proteins	# Protein Groups	Protein Group Accessions	Modifications	ΔCn	117/114	117/114 Count	117/114 Variability [%]	q-Value	PEP	IonScore	Exp Value	Charge	MH+ [Da]	ΔM [ppm]	RT [min]	# Missed Cleavages
	High	tALEDELQSLR	1	1	1	P72805	N-Term(iTRAQ4plex)	0,0000	1,190	1		0	6,164E-08	63	4,74209E-07	2	1418,76177	0,69	38,48	0
	High	aETQALQAR	2	1	1	P72805	N-Term(iTRAQ4plex)	0,0000	0,930	2	5,7	0	0,0003808	33	0,000534527	2	1131,62419	0,26	14,30	0
	High	lESLESSHR	3	1	1	P72805	N-Term(iTRAQ4plex)	0,0000	0,672	3	5,1	0	0,002517	23	0,00544465	3	1201,62906	-0,25	16,63	0
	High	kLDSTk	2	1	1	P72805	N-Term(iTRAQ4plex); K1(iTRAQ4plex); K6(iTRAQ4plex)	0,0000	1,409	2	2,4	0,001	0,01129	18	0,016903242	3	1123,70295	-1,56	9,43	1
Q55168	Phytochrome-like protein cph1 OS=Synechocystis sp. (strain PCC 6803 /	145,59	4,81	1	4	4	5	3,318	5	27,4	748	84,2	5,12							
	A3	Sequence	# PSMs	# Proteins	# Protein Groups	Protein Group Accessions	Modifications	ΔCn	117/114	117/114 Count	117/114 Variability [%]	q-Value	PEP	IonScore	Exp Value	Charge	MH+ [Da]	ΔM [ppm]	RT [min]	# Missed Cleavages
	High	sVASGLLAIPIAR	1	1	1	Q55168	N-Term(iTRAQ4plex)	0,0000	3,318	1		0	2,401E-08	86	2,67282E-09	2	1411,87541	0,02	42,73	0
	High	iFVIFQR	2	1	1	Q55168	N-Term(iTRAQ4plex)	0,0000	4,223	2	8,9	0	0,0009917	37	0,000202754	2	1066,65325	0,23	42,32	0
	High	lFIHNPIR	1	1	1	Q55168	N-Term(iTRAQ4plex)	0,0000	3,197	1		0	0,004235	17	0,020228795	3	1153,69645	0,16	29,25	0
	High	aIVNLILR	1	1	1	Q55168	N-Term(iTRAQ4plex)	0,0000	1,548	1		0,001	0,02082	29	0,001204953	2	1055,70549	-0,27	41,16	0
P72688	Transcription termination/antitermination protein NusA OS=Synechocyst	144,42	9,17	1	5	5	7	1,089	7	17,0	458	51,3	4,61							
	A3	Sequence	# PSMs	# Proteins	# Protein Groups	Protein Group Accessions	Modifications	ΔCn	117/114	117/114 Count	117/114 Variability [%]	q-Value	PEP	IonScore	Exp Value	Charge	MH+ [Da]	ΔM [ppm]	RT [min]	# Missed Cleavages
	High	rEQLPNDNYR	1	1	1	P72688	N-Term(iTRAQ4plex)	0,0000	2,093	1		0	8,421E-08	43	4,65554E-05	3	1448,73777	1,02	14,44	1
	High	eAkPPSPSVGPR	2	1	1	P72688	N-Term(iTRAQ4plex); K3(iTRAQ4plex)	0,0000	1,020	2	7,2	0	0,000003913	39	0,000114017	3	1509,86258	-0,15	14,74	0
	High	gPQLVVSR	2	1	1	P72688	N-Term(iTRAQ4plex)	0,0000	1,117	2	3,7	0	0,0001303	37	0,000194523	2	999,60723	0,44	20,22	0
	High	iHAVVNELR	1	1	1	P72688	N-Term(iTRAQ4plex)	0,0000	0,546	1		0	0,00002932	29	0,00138666	3	1194,70703	-0,44	22,53	0
	High	iHAVVNELRGEk	1	1	1	P72688	N-Term(iTRAQ4plex); K12(iTRAQ4plex)	0,0000	1,795	1		0	0,0007215	25	0,003539729	3	1652,96885	0,11	24,68	1
P73426	2-C-methyl-D-erythritol 2,4-cyclodiphosphate synthase OS=Synechocyst	144,41	13,04	1	2	2	6	0,762	5	18,8	161	17,4	6,52							
	A3	Sequence	# PSMs	# Proteins	# Protein Groups	Protein Group Accessions	Modifications	ΔCn	117/114	117/114 Count	117/114 Variability [%]	q-Value	PEP	IonScore	Exp Value	Charge	MH+ [Da]	ΔM [ppm]	RT [min]	# Missed Cleavages
	High	lLAQVHQLILER	4	1	1	P73426	N-Term(iTRAQ4plex)	0,0000	0,762	3	2,4	0	1,762E-09	70	9,16157E-08	3	1576,96616	0,36	43,14	0
	High	lkPHIQAMk	1	1	1	P73426	N-Term(iTRAQ4plex); K2(iTRAQ4plex); K9(iTRAQ4plex)	0,0000	0,865	1		0	0,0002921	12	0,068702099	4	1497,92580	-2,80	18,65	0
	High	lkPHIQAmk	1	1	1	P73426	N-Term(iTRAQ4plex); K2(iTRAQ4plex); M8(Oxidation); K	0,0000	0,565	1		0	0,0004408	10	0,095712796	4	1513,92641	1,00	15,28	0
P74591	Shikimate dehydrogenase OS=Synechocystis sp. (strain PCC 6803 / Kaz	144,38	13,10	1	3	3	5	0,522	4	16,3	290	31,1	7,25							
	A3	Sequence	# PSMs	# Proteins	# Protein Groups	Protein Group Accessions	Modifications	ΔCn	117/114	117/114 Count	117/114 Variability [%]	q-Value	PEP	IonScore	Exp Value	Charge	MH+ [Da]	ΔM [ppm]	RT [min]	# Missed Cleavages
	High	tAVILGYGGAAR	3	1	1	P74591	N-Term(iTRAQ4plex)	0,0000	0,468	2	14,3	0	1,461E-08	74	4,35482E-08	2	1292,74516	0,63	27,60	0
	High	lPPTAIAYDLIYTPRPTR	1	1	1	P74591	N-Term(iTRAQ4plex)	0,0000	0,529	1		0	0,0001359	21	0,007761935	3	2202,24277	1,09	45,29	0
	High	fLQHAQER	1	1	1	P74591	N-Term(iTRAQ4plex)	0,0000	0,823	1		0	0,0004724	16	0,025291233	3	1172,62992	0,52	14,44	0
P74338	NAD(P)H-quinone oxidoreductase subunit M OS=Synechocystis sp. (stra	144,13	23,97	1	3	3	7	0,563	7	36,7	121	14,1	4,70							
	A3	Sequence	# PSMs	# Proteins	# Protein Groups	Protein Group Accessions	Modifications	ΔCn	117/114	117/114 Count	117/114 Variability [%]	q-Value	PEP	IonScore	Exp Value	Charge	MH+ [Da]	ΔM [ppm]	RT [min]	# Missed Cleavages
	High	vLNYSMGLPk	1	1	1	P74338	N-Term(iTRAQ4plex); K10(iTRAQ4plex)	0,0000	0,569	1		0	0,000006125	60	1,14073E-06	2	1409,80791	0,99	34,41	0
	High	vLNYSmGLPk	3	1	1	P74338	N-Term(iTRAQ4plex); M6(Oxidation); K10(iTRAQ4plex)	0,0000	0,563	3	36,7	0	0,00003063	41	8,47169E-05	2	1425,80144	0,00	29,58	0
	High	iGSDLEHFIR	2	1	1	P74338	N-Term(iTRAQ4plex)	0,0000	0,461	2	12,6	0	0,000004031	28	0,00172175	3	1330,72492	0,98	36,37	0
	Medium	vENQETAGk	1	1	1	P74338	N-Term(iTRAQ4plex); K9(iTRAQ4plex)	0,0000	1,167	1		0,038	0,4702	16	0,024545394	2	1263,68254	3,33	10,54	0
P72941	Putative biopolymer transport protein ExbB-like 2 OS=Synechocystis sp.	141,48	10,57	1	3	3	5	0,827	5	4,6	227	25,1	5,24							
	A3	Sequence	# PSMs	# Proteins	# Protein Groups	Protein Group Accessions	Modifications	ΔCn	117/114	117/114 Count	117/114 Variability [%]	q-Value	PEP	IonScore	Exp Value	Charge	MH+ [Da]	ΔM [ppm]	RT [min]	# Missed Cleavages
	High	kVGSELEVLYR	1	1	1	P72941	N-Term(iTRAQ4plex); K1(iTRAQ4plex)	0,0000	0,850	1		0	0,000000303	54	3,85452E-06	3	1580,92673	1,06	30,62	1

	High	yLLAPLR	3	1	1	P72941	N-Term(iTRAQ4plex)	0,0000	0,827	3	4,6	0	0,00123	51	9,59275E-06	2	989,62718	0,75	35,05	0
	High	rFPIAk	1	1	1	P72941	N-Term(iTRAQ4plex); K6(iTRAQ4plex)	0,0000	0,720	1		0,001	0,01146	17	0,020843469	3	1019,66123	0,78	18,25	1
Q55436	Slr0848 protein OS=Synechocystis sp. (strain PCC 6803 / Kazusa) GN=s	141,44	10,11	1	3	3	5	0,900	5	12,0	277	31,8	4,89							
	A3	Sequence	# PSMs	# Proteins	# Protein Groups	Protein Group Accessions	Modifications	ΔCn	117/114	117/114 Count	117/114 Variability [%]	q-Value	PEP	IonScore	Exp Value	Charge	MH+ [Da]	ΔM [ppm]	RT [min]	# Missed Cleavages
	High	qVTHQEIQQFR	2	1	1	Q55436	N-Term(iTRAQ4plex)	0,0000	0,923	2	3,7	0	7,015E-08	49	1,38347E-05	3	1557,82688	0,92	18,24	0
	High	aIAILER	2	1	1	Q55436	N-Term(iTRAQ4plex)	0,0000	0,884	2	14,8	0	0,0003196	39	0,000131211	2	929,59014	0,07	28,08	0
	High	ikDESGIIQk	1	1	1	Q55436	N-Term(iTRAQ4plex); K2(iTRAQ4plex); K10(iTRAQ4plex)	0,0000	0,738	1		0	2,926E-07	30	0,001013841	3	1562,94852	0,45	21,17	1
P73270	Probable agmatinase 2 OS=Synechocystis sp. (strain PCC 6803 / Kazusa	140,04	9,23	1	3	3	8	1,714	5	27,0	390	42,9	5,24							
	A3	Sequence	# PSMs	# Proteins	# Protein Groups	Protein Group Accessions	Modifications	ΔCn	117/114	117/114 Count	117/114 Variability [%]	q-Value	PEP	IonScore	Exp Value	Charge	MH+ [Da]	ΔM [ppm]	RT [min]	# Missed Cleavages
	High	nLVQLGIGGWQVPR	1	1	1	P73270	N-Term(iTRAQ4plex)	0,0000	1,433	1		0	3,791E-10	57	1,90095E-06	3	1680,96549	-0,70	45,38	0
	High	vGIIHFDR	6	1	1	P73270	N-Term(iTRAQ4plex)	0,0000	1,965	3	18,0	0	0,0001425	44	4,15882E-05	3	1100,63211	-1,11	26,97	0
	High	gELPHYAGINTFmk	1	1	1	P73270	N-Term(iTRAQ4plex); M13(Oxidation); K14(iTRAQ4plex)	0,0000	0,956	1		0	2,298E-09	40	0,000103746	3	1881,97409	-1,64	31,01	0
P74296	Phenylalanine--tRNA ligase beta subunit OS=Synechocystis sp. (strain P	139,63	4,44	1	4	4	6	1,107	6	6,4	810	87,8	4,97							
	A3	Sequence	# PSMs	# Proteins	# Protein Groups	Protein Group Accessions	Modifications	ΔCn	117/114	117/114 Count	117/114 Variability [%]	q-Value	PEP	IonScore	Exp Value	Charge	MH+ [Da]	ΔM [ppm]	RT [min]	# Missed Cleavages
	High	sLAFSLAYR	1	1	1	P74296	N-Term(iTRAQ4plex)	0,0000	1,084	1		0	0,0001173	53	6,65186E-06	2	1171,66020	0,85	35,64	0
	High	gLLEAIFQR	3	1	1	P74296	N-Term(iTRAQ4plex)	0,0000	1,131	3	0,1	0	2,495E-07	41	8,18465E-05	2	1190,70232	0,75	48,01	0
	High	fGQLHPQLR	1	1	1	P74296	N-Term(iTRAQ4plex)	0,0000	0,889	1		0	0,00003745	26	0,00239315	3	1239,70923	1,08	23,09	0
	High	gQSVPAGQR	1	1	1	P74296	N-Term(iTRAQ4plex)	0,0000	0,756	1		0,001	0,01856	20	0,009077578	2	1043,57158	0,11	8,90	0
P74308	Aldehyde reductase OS=Synechocystis sp. (strain PCC 6803 / Kazusa) G	138,45	16,51	1	4	4	8	0,652	7	6,2	327	36,0	5,15							
	A3	Sequence	# PSMs	# Proteins	# Protein Groups	Protein Group Accessions	Modifications	ΔCn	117/114	117/114 Count	117/114 Variability [%]	q-Value	PEP	IonScore	Exp Value	Charge	MH+ [Da]	ΔM [ppm]	RT [min]	# Missed Cleavages
	High	kLEmVLSmAR	1	1	1	P74308	K1(iTRAQ4plex); N-Term(iTRAQ4plex); M4(Oxidation); M	0,0000	0,631	1		0	0,000009371	49	1,29709E-05	3	1497,83649	-0,47	25,77	1
	High	kLEMVLSMAR	1	1	1	P74308	N-Term(iTRAQ4plex); K1(iTRAQ4plex)	0,0000	0,652	1		0	0,000002736	34	0,00036389	3	1465,84494	-1,65	36,26	1
	High	lWSNAHHPDAVLPALEk	2	1	1	P74308	N-Term(iTRAQ4plex); K17(iTRAQ4plex)	0,0000	0,717	2	6,2	0	0,000004568	32	0,000594251	3	2186,19651	0,18	35,04	0
	High	aVDLGLcHHIGVSNFSLk	2	1	1	P74308	N-Term(iTRAQ4plex); C7(Carbamidomethyl); K18(iTRAQ	0,0000	0,660	1		0	0,000002116	23	0,004766387	4	2255,21963	-0,59	37,69	0
	High	rEELWITSk	2	1	1	P74308	N-Term(iTRAQ4plex); K9(iTRAQ4plex)	0,0000	0,402	2	73,2	0	0,00145	23	0,00474242	3	1449,82926	-0,81	26,32	1
P73833	Cyanophycin synthetase OS=Synechocystis sp. (strain PCC 6803 / Kazus	137,97	7,67	1	6	6	8	1,038	7	18,2	873	94,6	5,58							
	A3	Sequence	# PSMs	# Proteins	# Protein Groups	Protein Group Accessions	Modifications	ΔCn	117/114	117/114 Count	117/114 Variability [%]	q-Value	PEP	IonScore	Exp Value	Charge	MH+ [Da]	ΔM [ppm]	RT [min]	# Missed Cleavages
	High	amVQLGYGVHQQR	1	1	1	P73833	N-Term(iTRAQ4plex); M2(Oxidation)	0,0000	1,038	1		0	1,662E-10	42	6,82292E-05	3	1646,85605	0,41	19,62	0
	High	kIPWmLLSAR	3	1	1	P73833	N-Term(iTRAQ4plex); K1(iTRAQ4plex); M5(Oxidation)	0,0000	1,252	2	1,5	0	0,000002729	36	0,00022751	3	1518,90689	-0,02	36,87	1
	High	yHALVDYAHNPAGYR	1	1	1	P73833	N-Term(iTRAQ4plex)	0,0000	0,919	1		0	0,000002188	29	0,001185687	4	1890,93764	0,46	27,17	0
	High	fGVVGGPGDRR	1	1	1	P73833	N-Term(iTRAQ4plex)	0,0000	0,729	1		0	0,001356	28	0,001516946	3	1260,69294	-0,05	18,77	1
	High	mHVAPSQGLPR	1	1	1	P73833	N-Term(iTRAQ4plex); M1(Oxidation)	0,0000	0,951	1		0,001	0,01156	29	0,001339584	3	1352,72202	-0,41	14,05	0
	High	lLAHIYR	1	1	1	P73833	N-Term(iTRAQ4plex)	0,0000	1,104	1		0,003	0,0418	17	0,019542045	3	1029,63233	-0,25	25,02	0
P54899	N-acetyl-gamma-glutamyl-phosphate reductase OS=Synechocystis sp. (	137,87	9,69	1	3	3	7	0,686	6	13,0	351	38,2	6,71							
	A3	Sequence	# PSMs	# Proteins	# Protein Groups	Protein Group Accessions	Modifications	ΔCn	117/114	117/114 Count	117/114 Variability [%]	q-Value	PEP	IonScore	Exp Value	Charge	MH+ [Da]	ΔM [ppm]	RT [min]	# Missed Cleavages
	High	gILATVYATLR	4	1	1	P54899	N-Term(iTRAQ4plex)	0,0000	0,714	3	11,9	0	5,574E-07	60	1,05189E-06	2	1321,79729	0,94	49,08	0
	High	iLPHGVYPQTk	2	1	1	P54899	N-Term(iTRAQ4plex); K11(iTRAQ4plex)	0,0000	0,673	2	13,0	0	0,000005527	28	0,001640476	3	1540,91196	1,92	24,85	0
	High	vIVLSAIDNLVk	1	1	1	P54899	N-Term(iTRAQ4plex); K12(iTRAQ4plex)	0,0000	0,493	1		0	0,001561	18	0,016367035	3	1572,00125	2,38	49,35	0
P26290	Cytochrome b6-f complex iron-sulfur subunit 2 OS=Synechocystis sp. (s	137,83	20,56	1	3	3	7	1,058	7	30,8	180	19,0	5,11							
	A3	Sequence	# PSMs	# Proteins	# Protein Groups	Protein Group Accessions	Modifications	ΔCn	117/114	117/114 Count	117/114 Variability [%]	q-Value	PEP	IonScore	Exp Value	Charge	MH+ [Da]	ΔM [ppm]	RT [min]	# Missed Cleavages
	High	vTEFLASHNAGDR	2	1	1	P26290	N-Term(iTRAQ4plex)	0,0000	1,171	2	15,1	0	2,307E-07	43	4,85255E-05	3	1560,78971	0,62	25,70	0
	High	vLAQGLk	3	1	1	P26290	N-Term(iTRAQ4plex); K7(iTRAQ4plex)	0,0000	1,243	3	39,4	0	0,008065	33	0,000601095	2	1016,67174	1,05	21,73	0
	High	yLIPPSSGGSGGGVTAk	2	1	1	P26290	N-Term(iTRAQ4plex); K17(iTRAQ4plex)	0,0000	0,700	2	39,9	0	7,688E-07	32	0,00059975	3	1836,01224	0,90	27,44	0
P74142	30S ribosomal protein S1 homolog B OS=Synechocystis sp. (strain PCC	136,27	12,79	1	4	4	6	1,365	6	16,3	305	33,8	5,25							
	A3	Sequence	# PSMs	# Proteins	# Protein Groups	Protein Group Accessions	Modifications	ΔCn	117/114	117/114 Count	117/114 Variability [%]	q-Value	PEP	IonScore	Exp Value	Charge	MH+ [Da]	ΔM [ppm]	RT [min]	# Missed Cleavages
	High	aHILEANQDNNk	2	1	1	P74142	N-Term(iTRAQ4plex); K12(iTRAQ4plex)	0,0000	1,365	2	5,6	0	4,193E-09	56	2,57022E-06	3	1654,87662	0,89	14,13	0
	High	rIQQAESmGk	2	1	1	P74142	N-Term(iTRAQ4plex); M8(Oxidation); K10(iTRAQ4plex)	0,0000	1,461	2	16,3	0	0,000006089	30	0,00098168	3	1451,78904	0,77	9,09	1
	High	aLDkHDYHAEk	1	1	1	P74142	N-Term(iTRAQ4plex); K4(iTRAQ4plex); K11(iTRAQ4plex)	0,0000	0,995	1		0	0,008367	21	0,007498424	4	1758,95009	0,10	12,38	1
	Medium	lVLTQR	1	1	1	P74142	N-Term(iTRAQ4plex)	0,0000	1,700	1		0,036	0,4551	20	0,010494699	2	873,56438	0,61	21,16	0
P74188	Slr1276 protein OS=Synechocystis sp. (strain PCC 6803 / Kazusa) GN=s	135,78	13,45	1	3	3	3	0,733	3	41,7	275	30,0	4,34							
	A3	Sequence	# PSMs	# Proteins	# Protein Groups	Protein Group Accessions	Modifications	ΔCn	117/114	117/114 Count	117/114 Variability [%]	q-Value	PEP	IonScore	Exp Value	Charge	MH+ [Da]	ΔM [ppm]	RT [min]	# Missed Cleavages
	High	tSLVNPEFPVNVVR	1	1	1	P74188	N-Term(iTRAQ4plex)	0,0000	0,490	1		0	0,000001598	66	2,77313E-07	2	1714,96318	1,33	39,76	0
	High	rLANAEVQLQEAQQR	1	1	1	P74188	N-Term(iTRAQ4plex)	0,0000	0,961	1		0	4,059E-11	55	3,24317E-06	3	1898,03464	1,12	23,09	1
	High	lQPLILMk	1	1	1	P74188	N-Term(iTRAQ4plex); K8(iTRAQ4plex)	0,0000	0,733	1		0	0,00008661	41	7,32774E-05	2	1243,80498	-0,07	40,07	0
P72950	Sll0647 protein OS=Synechocystis sp. (strain PCC 6803 / Kazusa) GN=s	135,37	9,77	1	3	3	5	1,302	4	12,0	256	28,7	5,52							
	A3	Sequence	# PSMs	# Proteins	# Protein Groups	Protein Group Accessions	Modifications	ΔCn	117/114	117/114 Count	117/114 Variability [%]	q-Value	PEP	IonScore	Exp Value	Charge	MH+ [Da]	ΔM [ppm]	RT [min]	# Missed Cleavages
	High	fSLYALGLSHR	3	1	1	P72950	N-Term(iTRAQ4plex)	0,0000	1,219	2	12,0	0	0,000000167	59	1,74634E-06	3	1407,78623	-0,22	38,88	0
	High	kLPAAIk	1	1	1	P72950	N-Term(iTRAQ4plex); K1(iTRAQ4plex); K7(iTRAQ4plex)	0,0000	1,835	1		0	0,0002186	40	0,000109893	3	1172,80725	-1,59	18,13	1
	High	lQEYFHR	1	1	1	P72950	N-Term(iTRAQ4plex)	0,0000	1,283	1		0	0,000001567	32	0,000608093	3	1136,59668	-0,21	24,67	0
Q55972	Slr0708 protein OS=Synechocystis sp. (strain PCC 6803 / Kazusa) GN=s	135,06	8,71	1	2	2	2	1,049	2	0,7	402	44,7	5,26							
	A3	Sequence	# PSMs	# Proteins	# Protein Groups	Protein Group Accessions	Modifications	ΔCn	117/114	117/114 Count	117/114 Variability [%]	q-Value	PEP	IonScore	Exp Value	Charge	MH+ [Da]	ΔM [ppm]	RT [min]	# Missed Cleavages
	High	vPmAASQGSYAVGPAHAR	1	1	1	Q55972	N-Term(iTRAQ4plex); M3(Oxidation)	0,0000	1,045	1		0	3,183E-14	87	2,10848E-09	3	1929,97428	1,06	21,01	0
	High	tTPIQAPPVPVQYQVVk	1	1	1	Q55972	N-Term(iTRAQ4plex); K17(iTRAQ4plex)	0,0000	1,054	1		0	1,12E-12	61	7,37853E-07	3	2153,26059	1,53	36,78	0
Q55738	DNA gyrase subunit A OS=Synechocystis sp. (strain PCC 6803 / Kazusa)	132,82	8,95	1	6	6	7	1,013	7	24,2	860	95,0	5,26							
	A3	Sequence	# PSMs	# Proteins	# Protein Groups	Protein Group Accessions	Modifications	ΔCn	117/114	117/114 Count	117/114 Variability [%]	q-Value	PEP	IonScore	Exp Value	Charge	MH+ [Da]	ΔM [ppm]	RT [min]	# Missed Cleavages
	High	yHPHGDTAVYDALVR	2	1	1	Q55738	N-Term(iTRAQ4plex)	0,0000	0,744	2	14,8	0	3,472E-10	44	3,7151E-05	3	1857,93711	0,35	27,12	0
	High	lRPLSTNSLLR	1	1	1	Q55738	N-Term(iTRAQ4plex)	0,0000	0,989	1		0	3,205E-07	43	5,52039E-05	3	1413,86625	0,27	29,68	0
	High	kTALSAFSNIR	1	1	1	Q55738	N-Term(iTRAQ4plex); K1(iTRAQ4plex)	0,0000	1,039	1		0	0,0003228	26	0,002779521	3	1495,88431	0,52	28,52	1
	High	rmPASTFGTQNR	1	1	1	Q55738	N-Term(iTRAQ4plex); M2(Oxidation)	0,0000	1,190	1		0	0,0001385	24	0,003766778	3	1525,76767	0,94	10,94	1
	High	rVPIGQFR	1	1	1	Q55738	N-Term(iTRAQ4plex)	0,0000	2,809	1		0	0,0001414	20	0,010398483	3	1116,67646	0,52	20,43	1
	High	rLTALEADkITAEHDELQTk	1	1	1	Q55738	N-Term(iTRAQ4plex); K9(iTRAQ4plex); K20(iTRAQ4plex)	0,0000	1,013	1		0	1,383E-07	10	0,089736681	5	2714,50727	0,85	28,72	2
P72631	Sll1064 protein OS=Synechocystis sp. (strain PCC 6803 / Kazusa) GN=s	132,53	12,85	1	6	6	10	0,910	9	8,9	568	64,7	5,77							
	A3	Sequence	# PSMs	# Proteins	# Protein Groups	Protein Group Accessions	Modifications	ΔCn	117/114	117/114 Count	117/114 Variability [%]	q-Value	PEP	IonScore	Exp Value	Charge	MH+ [Da]	ΔM [ppm]	RT [min]	# Missed Cleavages
	High	sFFPVLQGR	3	1	1	P72631	N-Term(iTRAQ4plex)	0,0000	0,866	3	1,9	0	0,00001234	45	4,35422E-05	2	1194,67461	-0,50	38,61	0
	High	hQAVITGLR	1	1	1	P72631	N-Term(iTRAQ4plex)	0,0000	0,910	1		0	0,00002916	35	0,000399667	3	1138,68015	-1,05	15,70	0
	High	qYYAVTFGNIR	1	1	1	P72631	N-Term(iTRAQ4plex)	0,0000	0,857	1		0	0,00003644	32	0,000595621	2	1475,77446	-1,28	35,89	0
	High	lSLRPIWR	2	1	1	P72631	N-Term(iTRAQ4plex)	0,0000	1,047	1		0	0,00005893	25	0,002857393	3	1184,73795	-0,44	34,62	0
	High	aLHLQFk	2	1	1	P72631	N-Term(iTRAQ4plex); K7(iTRAQ4plex)	0,0000	1,093	2	16,9	0	0,0005571	20	0,010279453	3	1144,70606	-1,79	25,74	0
	High	yQESSQQLHkPENWGHGQHIFEF	1	1	1	P72631	N-Term(iTRAQ4plex); K10(iTRAQ4plex)	0,0000	0,935	1		0	0,000008983	12	0,060113222	5	3675,83601	1,74	29,81	0
Q55727	Slr0645 protein OS=Synechocystis sp. (strain PCC 6803 / Kazusa) GN=s	132,29	24,27	1	4	4	6	0,610	6	41,2	206	22,8	4,74							
	A3	Sequence	# PSMs	# Proteins	# Protein Groups	Protein Group Accessions	Modifications	ΔCn	117/114	117/114 Count	117/114 Variability [%]	q-Value	PEP	IonScore	Exp Value	Charge	MH+ [Da]	ΔM [ppm]	RT [min]	# Missed Cleavages
	High	vAYIYANNEPmGR	1	1	1	Q55727	N-Term(iTRAQ4plex); M11(Oxidation)	0,0000	0,387	1		0	0,00001333	46	2,60597E-05	2	1657,81487	1,42	25,35	0
	High	rYDNVTAEk	3	1	1	Q55727	N-Term(iTRAQ4plex); K9(iTRAQ4plex)	0,0000	0,632	3	10,6	0	0,0000109	42	5,74077E-05	3	1383,74594	-0,82	13,50	1
	High	wEIAQASTIALAk	1	1	1	Q55727	N-Term(iTRAQ4plex); K13(iTRAQ4plex)	0,0000	0,932	1		0	0,000564	22	0,006108998	3	1689,97629	-0,91	39,90	0
	High	sSSmATADDPNGPTR	1	1	1	Q55727	N-Term(iTRAQ4plex); M4(Oxidation)	0,0000	0,506	1		0,003	0,04222	32	0,000588803	2	1666,74809	1,28	9,61	0
P73479	Succinate dehydrogenase flavoprotein subunit OS=Synechocystis sp. (st	131,96	6,43	1	3	3	4	0,723	4	7,6	575	63,7	5,67							
	A3	Sequence	# PSMs	# Proteins	# Protein Groups	Protein Group Accessions	Modifications	ΔCn	117/114	117/114 Count	117/114 Variability [%]	q-Value	PEP	IonScore	Exp Value	Charge	MH+ [Da]	ΔM [ppm]	RT [min]	# Missed Cleavages
	High	lGSNSLLEcVVYGR	1	1	1	P73479	N-Term(iTRAQ4plex); C9(Carbamidomethyl)	0,0000	0,617	1		0	6,483E-09	77	1,89657E-08	2	1710,89775	0,69	43,66	0
	High	aVmVATGGYGR	2	1	1	P73479	N-Term(iTRAQ4plex); M3(Oxidation)	0,0000	0,761	2	6,3	0	0,00008139	32	0,000698184	2	1241,64202	-0,74	18,81	0
	High	hTLASFDGEQIk	1	1	1	P73479	N-Term(iTRAQ4plex); K12(iTRAQ4plex)	0,0000	0,716	1		0	0,0007233	30	0,000986211	3	1633,87680	-1,24	25,41	0
Q55778	Glutamate--tRNA ligase OS=Synechocystis sp. (strain PCC 6803 / Kazus	131,64	8,07	1	3	3	4	0,857	4	20,9	483	54,0	5,48							
	A3	Sequence	# PSMs	# Proteins	# Protein Groups	Protein Group Accessions	Modifications	ΔCn	117/114	117/114 Count	117/114 Variability [%]	q-Value	PEP	IonScore	Exp Value	Charge	MH+ [Da]	ΔM [ppm]	RT [min]	# Missed Cleavages
	High	kAIQQLLDQGLAYR	1	1	1	Q55778	N-Term(iTRAQ4plex); K1(iTRAQ4plex)	0,0000	1,279	1		0	6,099E-10	57	2,02754E-06	3	1905,11716	0,58	40,46	1
	High	tAVFNWLFAR	2	1	1	Q55778	N-Term(iTRAQ4plex)	0,0000	0,745	2	4,7	0	0,0001764	48	1,56304E-05	2	1368,75603	1,10	50,47	0
	High	iAPSPTGNLHIGTAR	1	1	1	Q55778	N-Term(iTRAQ4plex)	0,0000	0,954	1		0	4,258E-08	47	2,31159E-05	3	1648,92368	-0,91	25,99	0
P73061	Urease subunit alpha OS=Synechocystis sp. (strain PCC 6803 / Kazusa)	130,79	13,36	1	7	7	14	0,676	11	10,4	569	61,0	6,21							
	A3	Sequence	# PSMs	# Proteins	# Protein Groups	Protein Group Accessions	Modifications	ΔCn	117/114	117/114 Count	117/114 Variability [%]	q-Value	PEP	IonScore	Exp Value	Charge	MH+ [Da]	ΔM [ppm]	RT [min]	# Missed Cleavages
	High	aTTcTPGAWNIHR	2	1	1	P73061	N-Term(iTRAQ4plex); C4(Carbamidomethyl)	0,0000	0,772	1		0	7,45E-08	50	1,01852E-05	3	1628,80844	0,01	22,05	0
	High	vIHTYHTEGAGGGHAPDIIk	4	1	1	P73061	N-Term(iTRAQ4plex); K20(iTRAQ4plex)	0,0000	0,662	3	2,9	0	4,093E-07	33	0,000522869	4	2361,25844	1,28	22,15	0
	High	hSYAHTFGPTVGDk	2	1	1	P73061	N-Term(iTRAQ4plex); K14(iTRAQ4plex)	0,0000	0,603	2	17,0	0	0,000009989	32	0,000691783	4	1804,92397	1,04	18,59	0
	High	lGLSkPLVAVk	3	1	1	P73061	N-Term(iTRAQ4plex); K5(iTRAQ4plex); K11(iTRAQ4plex)	0,0000	0,770	2	15,5	0	8,298E-07	21	0,007797763	3	1557,04648	0,05	30,81	0
	High	tWQTAHk	1	1	1	P73061	N-Term(iTRAQ4plex); K7(iTRAQ4plex)	0,0000	0,799	1		0	0,0005832	20	0,009267658	3	1159,64756	1,15	11,40	0
	High	iYAIGk	1	1	1	P73061	N-Term(iTRAQ4plex); K6(iTRAQ4plex)	0,0000	0,468	1		0,009	0,1218	19	0,01166729	2	952,60784	0,89	22,73	0
	Medium	fGGGk	1	1	1	P73061	N-Term(iTRAQ4plex); K5(iTRAQ4plex)	0,0000	0,631	1		0,034	0,4259	14	0,043348094	2	753,44817	-2,12	12,63	0
Q55602	NifS protein OS=Synechocystis sp. (strain PCC 6803 / Kazusa) GN=nifS	130,69	17,88	1	6	6	9	0,835	9	43,0	386	41,7	6,46							
	A3	Sequence	# PSMs	# Proteins	# Protein Groups	Protein Group Accessions	Modifications	ΔCn	117/114	117/114 Count	117/114 Variability [%]	q-Value	PEP	IonScore	Exp Value	Charge	MH+ [Da]	ΔM [ppm]	RT [min]	# Missed Cleavages
	High	sGTLPVPLIVGLAk	3	1	1	Q55602	N-Term(iTRAQ4plex); K14(iTRAQ4plex)	0,0000	0,504	3	19,6	0	0,00001836	39	0,000140272	3	1653,05307	-1,39	46,70	0
	High	aLLTTLQPR	1	1	1	Q55602	N-Term(iTRAQ4plex)	0,0000	0,663	1		0	0,00005095	32	0,000633826	2	1156,71672	-0,30	30,86	0
	High	dkTSAQASLR	2	1	1	Q55602	N-Term(iTRAQ4plex); K2(iTRAQ4plex)	0,0000	1,039	2	8,8	0	0,00007488	29	0,001158697	3	1364,77127	-1,73	11,00	1
	High	tEASHVLYALGR	1	1	1	Q55602	N-Term(iTRAQ4plex)	0,0000	0,914	1		0	0,000001024	28	0,001737681	3	1460,79636	-1,01	28,38	0
	High	hLVTVETEHQAVLAPcR	1	1	1	Q55602	N-Term(iTRAQ4plex); C16(Carbamidomethyl)	0,0000	0,835	1		0	0,000001898	23	0,005938788	4	2104,10756	-0,71	23,34	0
	Medium	gIGGLYR	1	1	1	Q55602	N-Term(iTRAQ4plex)	0,0000	0,356	1		0,023	0,2936	14	0,043148927	2	879,51518	-1,94	21,79	0
P73681	Slr2144 protein OS=Synechocystis sp. (strain PCC 6803 / Kazusa) GN=s	130,59	9,63	1	3	3	5	1,633	5	30,1	301	33,5	5,67							
	A3	Sequence	# PSMs	# Proteins	# Protein Groups	Protein Group Accessions	Modifications	ΔCn	117/114	117/114 Count	117/114 Variability [%]	q-Value	PEP	IonScore	Exp Value	Charge	MH+ [Da]	ΔM [ppm]	RT [min]	# Missed Cleavages
	High	lELLQAGGLFk	3	1	1	P73681	N-Term(iTRAQ4plex); K11(iTRAQ4plex)	0,0000	1,339	3	51,3	0	2,972E-09	81	8,12774E-09	2	1476,90288	0,00	46,86	0
	High	qTFVQNk	1	1	1	P73681	N-Term(iTRAQ4plex); K7(iTRAQ4plex)	0,0000	1,909	1		0	0,0006332	35	0,000355786	2	1152,66057	-0,85	14,66	0
	High	tAQLNGQITSR	1	1	1	P73681	N-Term(iTRAQ4plex)	0,0000	1,633	1		0,002	0,03366	16	0,026205514	2	1332,73516	-0,06	17,74	0
P74416	Glycine dehydrogenase (decarboxylating) OS=Synechocystis sp. (strain	129,05	3,87	1	3	3	4	0,659	3	25,1	983	107,3	5,39							
	A3	Sequence	# PSMs	# Proteins	# Protein Groups	Protein Group Accessions	Modifications	ΔCn	117/114	117/114 Count	117/114 Variability [%]	q-Value	PEP	IonScore	Exp Value	Charge	MH+ [Da]	ΔM [ppm]	RT [min]	# Missed Cleavages
	High	fGIPLGYGGPHAAYFATk	2	1	1	P74416	N-Term(iTRAQ4plex); K18(iTRAQ4plex)	0,0000	0,557	1		0	9,862E-09	50	9,68211E-06	3	2155,16086	1,36	40,12	0
	High	sHLQAFLPR	1	1	1	P74416	N-Term(iTRAQ4plex)	0,0000	0,659	1		0	0,0005455	46	2,45454E-05	3	1212,69861	1,33	26,16	0
	High	rLENYYPILFR	1	1	1	P74416	N-Term(iTRAQ4plex)	0,0000	0,802	1		0	5,066E-07	27	0,001910686	3	1627,90775	0,02	39,73	1
Q55589	Cell division cycle protein OS=Synechocystis sp. (strain PCC 6803 / Kazu	128,10	4,19	1	2	2	4	0,625	4	19,2	501	56,4	6,27							
	A3	Sequence	# PSMs	# Proteins	# Protein Groups	Protein Group Accessions	Modifications	ΔCn	117/114	117/114 Count	117/114 Variability [%]	q-Value	PEP	IonScore	Exp Value	Charge	MH+ [Da]	ΔM [ppm]	RT [min]	# Missed Cleavages
	High	aRPASASVAEYQR	2	1	1	Q55589	N-Term(iTRAQ4plex)	0,0000	1,027	2	66,5	0	2,226E-10	73	5,39473E-08	3	1549,82077	0,26	14,28	0
	High	lWGLPLLR	2	1	1	Q55589	N-Term(iTRAQ4plex)	0,0000	0,549	2	5,8	0	0,0004822	32	0,000592884	2	1111,71147	0,55	49,00	0
P74769	Ssr1528 protein OS=Synechocystis sp. (strain PCC 6803 / Kazusa) GN=s	127,32	32,98	1	3	3	6	0,805	6	4,2	94	10,1	7,42							
	A3	Sequence	# PSMs	# Proteins	# Protein Groups	Protein Group Accessions	Modifications	ΔCn	117/114	117/114 Count	117/114 Variability [%]	q-Value	PEP	IonScore	Exp Value	Charge	MH+ [Da]	ΔM [ppm]	RT [min]	# Missed Cleavages
	High	hIVQAELDQk	4	1	1	P74769	N-Term(iTRAQ4plex); K10(iTRAQ4plex)	0,0000	0,805	4	2,0	0	7,201E-09	40	9,0985E-05	2	1468,83623	-0,01	18,36	0
	High	dkEIAFFYGAk	1	1	1	P74769	N-Term(iTRAQ4plex); K2(iTRAQ4plex); K11(iTRAQ4plex)	0,0000	0,772	1		0	0,0001415	23	0,005034658	3	1720,96506	0,93	33,16	1
	High	lIDAGFAPLkDk	1	1	1	P74769	N-Term(iTRAQ4plex); K10(iTRAQ4plex); K12(iTRAQ4plex	0,0000	0,857	1		0	0,0002369	21	0,007761935	3	1720,03562	-0,79	34,77	1
P74226	30S ribosomal protein S10 OS=Synechocystis sp. (strain PCC 6803 / Kaz	127,31	40,00	1	6	6	9	1,487	9	37,3	105	12,0	9,77							
	A3	Sequence	# PSMs	# Proteins	# Protein Groups	Protein Group Accessions	Modifications	ΔCn	117/114	117/114 Count	117/114 Variability [%]	q-Value	PEP	IonScore	Exp Value	Charge	MH+ [Da]	ΔM [ppm]	RT [min]	# Missed Cleavages
	High	tNAAAVGPIPLPTk	2	1	1	P74226	N-Term(iTRAQ4plex); K14(iTRAQ4plex)	0,0000	1,176	2	63,9	0	4,563E-07	38	0,000175376	3	1637,98185	-0,66	33,29	0
	High	tNAAAVGPIPLPTkR	1	1	1	P74226	N-Term(iTRAQ4plex); K14(iTRAQ4plex)	0,0000	1,735	1		0	1,009E-09	36	0,000269135	3	1794,08365	-0,22	28,44	1
	High	kIYcVLR	3	1	1	P74226	N-Term(iTRAQ4plex); K1(iTRAQ4plex); C4(Carbamidome	0,0000	1,897	3	26,7	0	0,00007472	27	0,002008954	3	1239,74677	-1,47	22,94	1
	High	rIIDIYQPSSk	1	1	1	P74226	N-Term(iTRAQ4plex); K11(iTRAQ4plex)	0,0000	1,342	1		0	0,001402	20	0,01091365	3	1607,93607	0,08	25,57	1
	High	iYcVLR	1	1	1	P74226	N-Term(iTRAQ4plex); C3(Carbamidomethyl)	0,0000	0,898	1		0,001	0,0121	25	0,003364883	2	967,55083	-0,75	27,00	0
	High	sPHVDkDSR	1	1	1	P74226	N-Term(iTRAQ4plex); K6(iTRAQ4plex)	0,0000	1,100	1		0,001	0,01211	13	0,055843162	3	1328,71634	0,17	8,03	1
Q6ZEI6	Slr7012 protein OS=Synechocystis sp. (strain PCC 6803 / Kazusa) GN=s	127,21	16,41	1	5	5	5	0,592	5	30,7	329	36,5	6,35							
	A3	Sequence	# PSMs	# Proteins	# Protein Groups	Protein Group Accessions	Modifications	ΔCn	117/114	117/114 Count	117/114 Variability [%]	q-Value	PEP	IonScore	Exp Value	Charge	MH+ [Da]	ΔM [ppm]	RT [min]	# Missed Cleavages
	High	hSQSFPVFQTDGVLNTTR	1	1	1	Q6ZEI6	N-Term(iTRAQ4plex)	0,0000	0,571	1		0	3,118E-12	61	8,31706E-07	3	2178,10654	0,22	35,58	0
	High	sQFQPSFPR	1	1	1	Q6ZEI6	N-Term(iTRAQ4plex)	0,0000	0,391	1		0	0,0006292	36	0,000243204	2	1237,64604	1,15	26,92	0
	High	lASGHYVHFLmLR	1	1	1	Q6ZEI6	N-Term(iTRAQ4plex); M11(Oxidation)	0,0000	0,807	1		0	7,304E-07	27	0,001888814	4	1703,91628	-0,57	33,74	0
	High	nLGLTSADk	1	1	1	Q6ZEI6	N-Term(iTRAQ4plex); K9(iTRAQ4plex)	0,0000	0,725	1		0,002	0,02934	28	0,001468825	2	1206,68962	-3,01	19,68	0
	High	lVMFk	1	1	1	Q6ZEI6	N-Term(iTRAQ4plex); K5(iTRAQ4plex)	0,0000	0,592	1		0,006	0,07688	27	0,002132898	2	925,57524	-3,35	30,96	0
P74360	Uncharacterized protein sll1526 OS=Synechocystis sp. (strain PCC 6803	127,15	7,31	1	3	3	5	0,951	4	2,4	506	57,7	7,50							
	A3	Sequence	# PSMs	# Proteins	# Protein Groups	Protein Group Accessions	Modifications	ΔCn	117/114	117/114 Count	117/114 Variability [%]	q-Value	PEP	IonScore	Exp Value	Charge	MH+ [Da]	ΔM [ppm]	RT [min]	# Missed Cleavages
	High	aNYFTQNPILkPR	1	1	1	P74360	N-Term(iTRAQ4plex); K11(iTRAQ4plex)	0,0000	0,962	1		0	3,662E-10	51	8,4522E-06	3	1850,05460	1,02	31,63	0
	High	lALLPQEHLQALGNLR	2	1	1	P74360	N-Term(iTRAQ4plex)	0,0000	0,810	2	27,4	0	1,146E-07	39	0,000128224	3	1930,13651	0,51	42,12	0
	High	fALVVPIk	2	1	1	P74360	N-Term(iTRAQ4plex); K8(iTRAQ4plex)	0,0000	0,940	1		0	0,002432	39	0,000139949	2	1174,78313	2,47	40,23	0
P74466	Putative ATP-dependent Clp protease proteolytic subunit-like OS=Synec	127,13	16,44	1	3	3	4	1,052	4	7,9	225	24,9	5,00							
	A3	Sequence	# PSMs	# Proteins	# Protein Groups	Protein Group Accessions	Modifications	ΔCn	117/114	117/114 Count	117/114 Variability [%]	q-Value	PEP	IonScore	Exp Value	Charge	MH+ [Da]	ΔM [ppm]	RT [min]	# Missed Cleavages
	High	tFYLTPAQAk	2	1	1	P74466	N-Term(iTRAQ4plex); K10(iTRAQ4plex)	0,0000	1,109	2	2,1	0	0,000003233	57	1,98139E-06	2	1427,81462	0,65	28,63	0
	High	tGAQGQATDIQIR	1	1	1	P74466	N-Term(iTRAQ4plex)	0,0000	1,011	1		0	1,685E-07	56	2,29071E-06	2	1502,80547	0,71	20,01	0
	High	aSLPHATIVLNQNR	1	1	1	P74466	N-Term(iTRAQ4plex)	0,0000	0,867	1		0	0,00000562	23	0,004753024	3	1677,95285	0,67	27,18	0
P72929	Uncharacterized protein sll1021 OS=Synechocystis sp. (strain PCC 6803	126,81	4,75	1	3	3	3	0,685	3	6,9	673	74,4	5,12							
	A3	Sequence	# PSMs	# Proteins	# Protein Groups	Protein Group Accessions	Modifications	ΔCn	117/114	117/114 Count	117/114 Variability [%]	q-Value	PEP	IonScore	Exp Value	Charge	MH+ [Da]	ΔM [ppm]	RT [min]	# Missed Cleavages
	High	lAVIVAQQDAQQHR	1	1	1	P72929	N-Term(iTRAQ4plex)	0,0000	0,718	1		0	1,206E-09	66	2,28544E-07	3	1720,95737	-0,10	25,20	0
	High	kTIEASQLQAk	1	1	1	P72929	N-Term(iTRAQ4plex); K1(iTRAQ4plex); K11(iTRAQ4plex)	0,0000	0,625	1		0	7,081E-08	51	7,29407E-06	3	1648,99894	1,89	17,72	1
	High	vAQALQk	1	1	1	P72929	N-Term(iTRAQ4plex); K7(iTRAQ4plex)	0,0000	0,685	1		0,004	0,05269	35	0,000311867	2	1045,66374	2,79	17,14	0
P74521	Glycogen synthase 1 OS=Synechocystis sp. (strain PCC 6803 / Kazusa) (	126,62	8,60	1	4	4	7	0,910	7	11,7	477	54,3	6,09							
	A3	Sequence	# PSMs	# Proteins	# Protein Groups	Protein Group Accessions	Modifications	ΔCn	117/114	117/114 Count	117/114 Variability [%]	q-Value	PEP	IonScore	Exp Value	Charge	MH+ [Da]	ΔM [ppm]	RT [min]	# Missed Cleavages
	High	nAmVVGIVAR	2	1	1	P74521	N-Term(iTRAQ4plex); M3(Oxidation)	0,0000	0,947	2	5,9	0	0,00002181	39	0,000124157	2	1189,68535	0,79	23,30	0
	High	iPLYLFGHPAFDSR	3	1	1	P74521	N-Term(iTRAQ4plex)	0,0000	0,905	3	5,4	0	0,000001072	37	0,000184489	3	1776,95652	0,62	45,78	0
	High	vLHQLGHDVR	1	1	1	P74521	N-Term(iTRAQ4plex)	0,0000	0,757	1		0	0,0001985	19	0,013365955	4	1317,75210	0,96	16,55	0
	High	fkADWQk	1	1	1	P74521	N-Term(iTRAQ4plex); K2(iTRAQ4plex); K7(iTRAQ4plex)	0,0000	0,997	1		0	0,002116	11	0,074984242	3	1354,78223	-1,57	21,35	1
Q55762	Sll0188 protein OS=Synechocystis sp. (strain PCC 6803 / Kazusa) GN=s	124,84	9,38	1	3	3	3	1,215	3	6,2	501	52,4	5,12							
	A3	Sequence	# PSMs	# Proteins	# Protein Groups	Protein Group Accessions	Modifications	ΔCn	117/114	117/114 Count	117/114 Variability [%]	q-Value	PEP	IonScore	Exp Value	Charge	MH+ [Da]	ΔM [ppm]	RT [min]	# Missed Cleavages
	High	lRENTLVSIAQQGQALk	1	1	1	Q55762	N-Term(iTRAQ4plex); K17(iTRAQ4plex)	0,0000	1,080	1		0	2,057E-11	70	9,54927E-08	3	2157,26072	0,59	35,96	1
	High	sRYPIFNQLAEQAIANR	1	1	1	Q55762	N-Term(iTRAQ4plex)	0,0000	1,215	1		0	8,46E-12	55	2,90382E-06	3	2135,14853	0,31	40,76	1
	High	lPITGPPIPSLPR	1	1	1	Q55762	N-Term(iTRAQ4plex)	0,0000	1,267	1		0	0,01016	25	0,002951005	2	1501,92449	1,43	43,27	0
P74625	Phycobilisome rod-core linker polypeptide CpcG OS=Synechocystis sp. (	124,45	20,48	1	5	5	9	0,162	9	53,4	249	28,5	9,10							
	A3	Sequence	# PSMs	# Proteins	# Protein Groups	Protein Group Accessions	Modifications	ΔCn	117/114	117/114 Count	117/114 Variability [%]	q-Value	PEP	IonScore	Exp Value	Charge	MH+ [Da]	ΔM [ppm]	RT [min]	# Missed Cleavages
	High	tLPLIAYAPVSQNQR	1	1	1	P74625	N-Term(iTRAQ4plex)	0,0000	0,121	1		0	0,0000159	38	0,000176185	3	1815,02835	2,09	39,95	0
	High	fVQmcIQR	2	1	1	P74625	N-Term(iTRAQ4plex); M4(Oxidation); C5(Carbamidometh	0,0000	0,107	2	11,0	0	0,0001829	29	0,001255943	2	1241,62871	2,86	21,87	0
	High	rNFEVNNNYR	1	1	1	P74625	N-Term(iTRAQ4plex)	0,0000	1,111	1		0	0,0002533	28	0,001509976	3	1469,74024	2,46	15,96	1
	High	ekLEAIGYFR	2	1	1	P74625	N-Term(iTRAQ4plex); K2(iTRAQ4plex)	0,0000	0,237	2	101,4	0,002	0,02796	27	0,002033243	3	1513,86692	3,43	31,67	1
	Medium	nQAPLTYR	3	1	1	P74625	N-Term(iTRAQ4plex)	0,0000	0,187	3	21,9	0,011	0,1407	21	0,008336236	2	1106,61101	3,18	17,51	0
Q55770	Sll0185 protein OS=Synechocystis sp. (strain PCC 6803 / Kazusa) GN=s	123,79	9,79	1	5	5	6	0,924	6	12,7	419	47,0	5,12							
	A3	Sequence	# PSMs	# Proteins	# Protein Groups	Protein Group Accessions	Modifications	ΔCn	117/114	117/114 Count	117/114 Variability [%]	q-Value	PEP	IonScore	Exp Value	Charge	MH+ [Da]	ΔM [ppm]	RT [min]	# Missed Cleavages
	High	sQGGQQLALR	2	1	1	Q55770	N-Term(iTRAQ4plex)	0,0000	0,998	2	1,7	0	0,000002208	52	6,53085E-06	2	1201,67827	1,06	16,28	0
	High	kSQLLAEVEk	1	1	1	Q55770	N-Term(iTRAQ4plex); K1(iTRAQ4plex); K10(iTRAQ4plex)	0,0000	0,732	1		0	0,0002364	22	0,005688136	3	1576,96469	0,78	25,25	1
	High	wLTLAR	1	1	1	Q55770	N-Term(iTRAQ4plex)	0,0000	1,162	1		0,003	0,04434	37	0,000206049	2	903,55388	0,66	33,50	0
	Medium	iTLmPR	1	1	1	Q55770	N-Term(iTRAQ4plex); M4(Oxidation)	0,0000	0,852	1		0,023	0,2861	19	0,012330197	2	890,52605	1,14	20,60	0
	Medium	akEDRPSLk	1	1	1	Q55770	N-Term(iTRAQ4plex); K2(iTRAQ4plex); K9(iTRAQ4plex)	0,0000	0,866	1		0,041	0,5125	14	0,039991713	3	1475,88691	-2,52	10,28	1
P51634	Magnesium-chelatase subunit ChlI OS=Synechocystis sp. (strain PCC 68	123,63	9,80	1	3	3	5	1,232	5	17,7	357	39,4	5,08							
	A3	Sequence	# PSMs	# Proteins	# Protein Groups	Protein Group Accessions	Modifications	ΔCn	117/114	117/114 Count	117/114 Variability [%]	q-Value	PEP	IonScore	Exp Value	Charge	MH+ [Da]	ΔM [ppm]	RT [min]	# Missed Cleavages
	High	rVVFPFTAIVGQDEmk	1	1	1	P51634	N-Term(iTRAQ4plex); M15(Oxidation); K16(iTRAQ4plex)	0,0000	1,094	1		0	6,263E-09	52	6,95024E-06	4	2141,16787	0,49	41,27	1
	High	fGMHAEIR	1	1	1	P51634	N-Term(iTRAQ4plex)	0,0000	1,038	1		0	0,00001587	34	0,000400839	3	1104,57410	-0,01	19,76	0
	High	lALLLNVIDPk	2	1	1	P51634	N-Term(iTRAQ4plex); K11(iTRAQ4plex)	0,0000	1,394	2	17,1	0	0,0003273	33	0,000477496	2	1496,96428	-0,80	52,00	0
	High	fGmHAEIR	1	1	1	P51634	N-Term(iTRAQ4plex); M3(Oxidation)	0,0000	1,232	1		0,001	0,01375	25	0,003507276	3	1120,56882	-0,19	14,37	0
P73424	Slr1540 protein OS=Synechocystis sp. (strain PCC 6803 / Kazusa) GN=s	122,65	20,26	1	6	6	7	0,957	6	30,4	311	35,1	6,61							
	A3	Sequence	# PSMs	# Proteins	# Protein Groups	Protein Group Accessions	Modifications	ΔCn	117/114	117/114 Count	117/114 Variability [%]	q-Value	PEP	IonScore	Exp Value	Charge	MH+ [Da]	ΔM [ppm]	RT [min]	# Missed Cleavages
	High	vLVAQGHEVVLFNR	1	1	1	P73424	N-Term(iTRAQ4plex)	0,0000	0,978	1		0	8,362E-08	56	2,31723E-06	3	1724,99540	1,46	33,85	0
	High	qQHFFADIQk	1	1	1	P73424	N-Term(iTRAQ4plex); K10(iTRAQ4plex)	0,0000	0,728	1		0	1,052E-07	49	1,25305E-05	3	1549,83707	0,32	25,66	0
	High	fIGIHLcR	2	1	1	P73424	N-Term(iTRAQ4plex); C7(Carbamidomethyl)	0,0000	1,380	1		0	0,0007193	25	0,003303467	3	1159,65287	0,15	30,21	0
	High	gNRPDPVNGVAQIHGDRR	1	1	1	P73424	N-Term(iTRAQ4plex)	0,0000	1,509	1		0	0,00000979	24	0,004036454	5	2102,10722	-0,61	16,40	1
	High	lVHYDPk	1	1	1	P73424	N-Term(iTRAQ4plex); K7(iTRAQ4plex)	0,0000	0,841	1		0	0,0006216	16	0,025923751	3	1159,67200	0,54	17,46	0
	Medium	kAFPLR	1	1	1	P73424	N-Term(iTRAQ4plex); K1(iTRAQ4plex)	0,0000	0,936	1		0,027	0,3302	18	0,017296969	3	1019,65812	-2,28	17,58	1
P74438	Dihydroorotase OS=Synechocystis sp. (strain PCC 6803 / Kazusa) GN=p	122,25	11,70	1	4	4	6	0,996	6	5,2	342	38,1	6,80							
	A3	Sequence	# PSMs	# Proteins	# Protein Groups	Protein Group Accessions	Modifications	ΔCn	117/114	117/114 Count	117/114 Variability [%]	q-Value	PEP	IonScore	Exp Value	Charge	MH+ [Da]	ΔM [ppm]	RT [min]	# Missed Cleavages
	High	fFLGTDSAPHAR	1	1	1	P74438	N-Term(iTRAQ4plex)	0,0000	1,083	1		0	6,069E-07	45	3,15479E-05	3	1462,75641	0,29	27,53	0
	High	yLIPLR	3	1	1	P74438	N-Term(iTRAQ4plex)	0,0000	0,971	3	1,4	0	0,004627	31	0,00112705	2	918,58940	0,09	32,85	0
	High	aVLPHTVR	1	1	1	P74438	N-Term(iTRAQ4plex)	0,0000	1,604	1		0	0,0002239	25	0,003075884	3	1036,63813	-0,28	15,37	0
	High	gGIcPHFYcLPILk	1	1	1	P74438	N-Term(iTRAQ4plex); C4(Carbamidomethyl); C9(Carbam	0,0000	0,961	1		0	4,303E-08	24	0,004003065	3	1963,05429	0,33	42,22	0
P77970	Glutamate--ammonia ligase OS=Synechocystis sp. (strain PCC 6803 / Ka	122,19	4,42	1	3	3	6	2,579	5	19,0	724	79,2	5,47							
	A3	Sequence	# PSMs	# Proteins	# Protein Groups	Protein Group Accessions	Modifications	ΔCn	117/114	117/114 Count	117/114 Variability [%]	q-Value	PEP	IonScore	Exp Value	Charge	MH+ [Da]	ΔM [ppm]	RT [min]	# Missed Cleavages
	High	tQSVHQIINRPLSSGk	3	1	1	P77970	N-Term(iTRAQ4plex); K16(iTRAQ4plex)	0,0000	2,355	2	13,5	0	4,843E-09	47	2,00895E-05	3	2053,17679	0,53	21,45	0
	High	tSPFAFTGNR	2	1	1	P77970	N-Term(iTRAQ4plex)	0,0000	2,621	2	16,6	0	0,000003019	35	0,000327318	2	1241,63945	-0,07	27,91	0
	High	kVPLLR	1	1	1	P77970	N-Term(iTRAQ4plex); K1(iTRAQ4plex)	0,0000	4,167	1		0	0,007844	18	0,014386992	3	1013,70646	-0,92	20,00	1
Q55698	Slr0226 protein OS=Synechocystis sp. (strain PCC 6803 / Kazusa) GN=s	122,04	2,90	1	2	2	8	0,682	6	5,1	482	50,8	4,98							
	A3	Sequence	# PSMs	# Proteins	# Protein Groups	Protein Group Accessions	Modifications	ΔCn	117/114	117/114 Count	117/114 Variability [%]	q-Value	PEP	IonScore	Exp Value	Charge	MH+ [Da]	ΔM [ppm]	RT [min]	# Missed Cleavages
	High	fLVLMHVk	1	1	1	Q55698	N-Term(iTRAQ4plex); K8(iTRAQ4plex)	0,0000	2,878	1		0	0,0002038	38	0,000141896	3	1274,78785	-1,49	36,88	0
	High	lIIFSR	6	1	1	Q55698	N-Term(iTRAQ4plex)	0,0000	0,682	4	4,6	0	0,009613	36	0,00028556	2	892,57317	-0,58	37,18	0
	High	fLVLmHVk	1	1	1	Q55698	N-Term(iTRAQ4plex); M5(Oxidation); K8(iTRAQ4plex)	0,0000	0,663	1		0	0,00008343	19	0,012588385	3	1290,78366	-0,77	29,21	0
P73636	30S ribosomal protein S6 OS=Synechocystis sp. (strain PCC 6803 / Kazu	121,61	23,89	1	4	4	8	1,880	8	32,4	113	13,2	8,37							
	A3	Sequence	# PSMs	# Proteins	# Protein Groups	Protein Group Accessions	Modifications	ΔCn	117/114	117/114 Count	117/114 Variability [%]	q-Value	PEP	IonScore	Exp Value	Charge	MH+ [Da]	ΔM [ppm]	RT [min]	# Missed Cleavages
	High	vSQEVTk	2	1	1	P73636	N-Term(iTRAQ4plex); K7(iTRAQ4plex)	0,0000	1,603	2	6,9	0	0,0001786	34	0,00043448	2	1078,63371	-0,88	13,12	0
	High	lTPEkPEk	2	1	1	P73636	N-Term(iTRAQ4plex); K5(iTRAQ4plex); K8(iTRAQ4plex)	0,0000	1,345	2	98,3	0	0,002026	22	0,007566761	3	1373,83463	-1,33	15,91	0
	High	rLAYQIR	1	1	1	P73636	N-Term(iTRAQ4plex)	0,0000	2,900	1		0	0,005318	17	0,022129419	3	1063,64884	-0,44	17,39	1
	High	fLSIk	3	1	1	P73636	N-Term(iTRAQ4plex); K5(iTRAQ4plex)	0,0000	2,104	3	5,3	0,003	0,03897	35	0,000325814	2	895,58470	-0,92	28,57	0
P73471	Phosphoribosylaminoimidazole-succinocarboxamide synthase OS=Synec	121,55	9,47	1	2	2	3	1,240	3	11,6	264	29,7	5,29							
	A3	Sequence	# PSMs	# Proteins	# Protein Groups	Protein Group Accessions	Modifications	ΔCn	117/114	117/114 Count	117/114 Variability [%]	q-Value	PEP	IonScore	Exp Value	Charge	MH+ [Da]	ΔM [ppm]	RT [min]	# Missed Cleavages
	High	gAINcAISAALFR	1	1	1	P73471	N-Term(iTRAQ4plex); C5(Carbamidomethyl)	0,0000	1,240	1		0	0,000000234	74	4,4154E-08	2	1507,81706	-0,10	43,11	0
	High	aVNIIPLEVVVR	2	1	1	P73471	N-Term(iTRAQ4plex)	0,0000	1,286	2	17,0	0	0,0001369	52	6,63697E-06	2	1465,92229	-0,03	45,71	0
P74506	Slr1944 protein OS=Synechocystis sp. (strain PCC 6803 / Kazusa) GN=s	119,98	5,20	1	3	3	5	0,951	5	20,2	538	58,6	4,77							
	A3	Sequence	# PSMs	# Proteins	# Protein Groups	Protein Group Accessions	Modifications	ΔCn	117/114	117/114 Count	117/114 Variability [%]	q-Value	PEP	IonScore	Exp Value	Charge	MH+ [Da]	ΔM [ppm]	RT [min]	# Missed Cleavages
	High	iFTWLGk	3	1	1	P74506	N-Term(iTRAQ4plex); K7(iTRAQ4plex)	0,0000	1,089	3	22,0	0	0,001501	49	1,34577E-05	2	1152,70452	2,21	43,07	0
	High	fFNTTFGk	1	1	1	P74506	N-Term(iTRAQ4plex); K8(iTRAQ4plex)	0,0000	0,951	1		0	0,00001401	46	2,37121E-05	2	1249,68437	1,93	33,60	0
	High	fYTQHLkPEELEk	1	1	1	P74506	N-Term(iTRAQ4plex); K7(iTRAQ4plex); K13(iTRAQ4plex)	0,0000	0,683	1		0	1,747E-07	19	0,014634999	4	2094,16421	2,20	27,57	0
Q55233	Protein DrgA OS=Synechocystis sp. (strain PCC 6803 / Kazusa) GN=drg	119,32	21,90	1	5	5	9	0,457	9	45,2	210	23,7	9,19							

	A3	Sequence	# PSMs	# Proteins	# Protein Groups	Protein Group Accessions	Modifications	ΔCn	117/114	117/114 Count	117/114 Variability [%]	q-Value	PEP	IonScore	Exp Value	Charge	MH+ [Da]	ΔM [ppm]	RT [min]	# Missed Cleavages
	High	sIGmAmQNLmLAAk	1	1	1	Q55233	N-Term(iTRAQ4plex); M4(Oxidation); M6(Oxidation); M1(	0,0000	0,628	1		0	2,167E-10	43	5,2236E-05	3	1814,94394	1,20	27,21	0
	High	sIGMAMQNLMLAAk	1	1	1	Q55233	N-Term(iTRAQ4plex); K14(iTRAQ4plex)	0,0000	0,437	1		0	0,000003869	35	0,000328829	3	1766,95694	-0,04	46,98	0
	High	lPADYAIGPmVAIGk	1	1	1	Q55233	N-Term(iTRAQ4plex); M10(Oxidation); K15(iTRAQ4plex)	0,0000	0,292	1		0	0,000006268	33	0,000447713	3	1820,02475	0,91	38,25	0
	High	sIGMAmQNLMLAAk	1	1	1	Q55233	N-Term(iTRAQ4plex); M6(Oxidation); K14(iTRAQ4plex)	0,0000	0,342	1		0	0,008831	29	0,00150797	3	1782,95249	0,31	45,39	0
	High	fLIIRDPQLR	2	1	1	Q55233	N-Term(iTRAQ4plex)	0,0000	0,708	2	23,2	0	0,003387	19	0,011560324	3	1414,86554	0,28	34,02	1
	High	hFDPDHR	1	1	1	Q55233	N-Term(iTRAQ4plex)	0,0000	1,084	1		0,001	0,01654	14	0,038812356	3	1067,51731	3,16	9,34	0
	Medium	fLIIR	2	1	1	Q55233	N-Term(iTRAQ4plex)	0,0000	0,436	2	7,2	0,025	0,3085	19	0,036928678	2	805,54253	1,08	34,02	0
Q55393	Diflavin flavoprotein A 1 OS=Synechocystis sp. (strain PCC 6803 / Kazus	119,24	5,76	1	4	4	6	0,561	5	42,3	573	63,5	5,90							
	A3	Sequence	# PSMs	# Proteins	# Protein Groups	Protein Group Accessions	Modifications	ΔCn	117/114	117/114 Count	117/114 Variability [%]	q-Value	PEP	IonScore	Exp Value	Charge	MH+ [Da]	ΔM [ppm]	RT [min]	# Missed Cleavages
	High	hNVGELLHR	3	1	1	Q55393	N-Term(iTRAQ4plex)	0,0000	0,592	2	35,9	0	0,00004259	40	0,000100224	3	1218,68268	0,23	17,18	0
	High	fLDLGLR	1	1	1	Q55393	N-Term(iTRAQ4plex)	0,0000	0,561	1		0	0,003419	37	0,000332689	2	977,59087	0,81	39,37	0
	High	lSQAIAk	1	1	1	Q55393	N-Term(iTRAQ4plex); K7(iTRAQ4plex)	0,0000	0,427	1		0	0,008692	34	0,000385646	2	1018,65196	2,00	18,87	0
	High	aEGLTAVHHR	1	1	1	Q55393	N-Term(iTRAQ4plex)	0,0000	0,840	1		0	0,001089	26	0,00232793	3	1234,67914	1,47	10,43	0
P74230	30S ribosomal protein S12 OS=Synechocystis sp. (strain PCC 6803 / Kaz	119,22	15,87	1	2	3	5	1,345	4	13,2	126	14,2	11,22							
	A3	Sequence	# PSMs	# Proteins	# Protein Groups	Protein Group Accessions	Modifications	ΔCn	117/114	117/114 Count	117/114 Variability [%]	q-Value	PEP	IonScore	Exp Value	Charge	MH+ [Da]	ΔM [ppm]	RT [min]	# Missed Cleavages
	High	pTIQQLIR	2	1	1	P74230	N-Term(iTRAQ4plex)	0,0000	1,284	2	15,9	0	0,00381	40	8,99435E-05	2	1112,68999	-0,77	30,84	0
	High	vYTTTPk	2	1	1	P74230	N-Term(iTRAQ4plex); K7(iTRAQ4plex)	0,0000	1,384	2	13,2	0	0,00305	37	0,000193183	2	1097,64128	-2,92	14,69	0
	Medium	sPALk	1	2	2	P74230;P72799	N-Term(iTRAQ4plex); K5(iTRAQ4plex)	0,0000				0,04	0,4947	18	0,017296969	2	803,51995	-3,71	13,10	0
P73066	Ycf23 protein OS=Synechocystis sp. (strain PCC 6803 / Kazusa) GN=ycf	118,37	12,90	1	3	3	5	0,257	5	23,3	248	25,4	5,33							
	A3	Sequence	# PSMs	# Proteins	# Protein Groups	Protein Group Accessions	Modifications	ΔCn	117/114	117/114 Count	117/114 Variability [%]	q-Value	PEP	IonScore	Exp Value	Charge	MH+ [Da]	ΔM [ppm]	RT [min]	# Missed Cleavages
	High	aAPTLAAAHAISR	1	1	1	P73066	N-Term(iTRAQ4plex)	0,0000	0,503	1		0	0,00006385	37	0,000202288	3	1393,80631	2,18	21,64	0
	High	rFEGPEVLALTk	2	1	1	P73066	N-Term(iTRAQ4plex); K12(iTRAQ4plex)	0,0000	0,237	2	12,2	0	0,0004807	37	0,000209397	3	1647,97318	3,58	32,43	1
	High	qLIQQVR	2	1	1	P73066	N-Term(iTRAQ4plex)	0,0000	0,254	2	21,7	0,009	0,1171	32	0,000694976	2	1028,63579	2,40	19,51	0
P73914	Squalene-hopene-cyclase OS=Synechocystis sp. (strain PCC 6803 / Kazu	117,97	3,86	1	3	3	6	0,428	6	20,3	647	72,0	4,96							
	A3	Sequence	# PSMs	# Proteins	# Protein Groups	Protein Group Accessions	Modifications	ΔCn	117/114	117/114 Count	117/114 Variability [%]	q-Value	PEP	IonScore	Exp Value	Charge	MH+ [Da]	ΔM [ppm]	RT [min]	# Missed Cleavages
	High	nYLLQQQR	2	1	1	P73914	N-Term(iTRAQ4plex)	0,0000	0,426	2	21,5	0	0,000008792	43	5,36658E-05	2	1206,67082	-0,27	21,21	0
	High	qYFPLIALAR	3	1	1	P73914	N-Term(iTRAQ4plex)	0,0000	0,384	3	3,8	0	0,001143	30	0,001018521	2	1335,79094	0,28	46,11	0
	Medium	kQGAINk	1	1	1	P73914	N-Term(iTRAQ4plex); K1(iTRAQ4plex); K7(iTRAQ4plex)	0,0000	0,760	1		0,01	0,1271	17	0,021675544	3	1190,75333	-4,04	8,35	1
P73626	Sll1771 protein OS=Synechocystis sp. (strain PCC 6803 / Kazusa) GN=sl	117,92	8,66	1	2	2	4	0,995	4	20,0	254	28,5	4,56							
	A3	Sequence	# PSMs	# Proteins	# Protein Groups	Protein Group Accessions	Modifications	ΔCn	117/114	117/114 Count	117/114 Variability [%]	q-Value	PEP	IonScore	Exp Value	Charge	MH+ [Da]	ΔM [ppm]	RT [min]	# Missed Cleavages
	High	rDmGTTAVLIAFR	1	1	1	P73626	N-Term(iTRAQ4plex); M3(Oxidation)	0,0000	0,787	1		0	3,083E-09	61	8,44098E-07	3	1610,88114	0,36	31,55	1
	High	hVLFQcLGR	2	1	1	P73626	N-Term(iTRAQ4plex); C6(Carbamidomethyl)	0,0000	0,995	2	10,4	0	3,838E-07	44	4,00839E-05	3	1273,69526	-0,29	26,58	0
	High	rDMGTTAVLIAFR	1	1	1	P73626	N-Term(iTRAQ4plex)	0,0000	1,211	1		0	0,000002073	25	0,003355041	3	1594,88486	-0,49	36,46	1
P73574	3-oxoacyl-[acyl-carrier-protein] reductase 1 OS=Synechocystis sp. (strai	116,95	12,55	1	2	2	3	0,985	3	4,0	247	25,7	6,77							
	A3	Sequence	# PSMs	# Proteins	# Protein Groups	Protein Group Accessions	Modifications	ΔCn	117/114	117/114 Count	117/114 Variability [%]	q-Value	PEP	IonScore	Exp Value	Charge	MH+ [Da]	ΔM [ppm]	RT [min]	# Missed Cleavages
	High	iINITSVAGmmGNPGQANYSAAk	2	1	1	P73574	N-Term(iTRAQ4plex); M10(Oxidation); M11(Oxidation); K	0,0000	0,972	2	2,0	0	4,076E-12	76	2,60597E-08	3	2628,33615	-0,13	35,01	0
	High	aGVIGFTk	1	1	1	P73574	N-Term(iTRAQ4plex); K8(iTRAQ4plex)	0,0000	1,196	1		0	0,0004732	43	5,02308E-05	2	1080,66557	-0,01	25,69	0
P74423	Alanine--tRNA ligase OS=Synechocystis sp. (strain PCC 6803 / Kazusa) (	116,86	4,56	1	3	3	3	1,132	3	8,3	877	96,1	5,27							
	A3	Sequence	# PSMs	# Proteins	# Protein Groups	Protein Group Accessions	Modifications	ΔCn	117/114	117/114 Count	117/114 Variability [%]	q-Value	PEP	IonScore	Exp Value	Charge	MH+ [Da]	ΔM [ppm]	RT [min]	# Missed Cleavages
	High	rIEAVAGPSVLDYLNVR	1	1	1	P74423	N-Term(iTRAQ4plex)	0,0000	1,070	1		0	2,951E-08	74	4,29507E-08	3	2016,13749	0,77	43,23	1
	High	vSLVAAFSPQLVk	1	1	1	P74423	N-Term(iTRAQ4plex); K13(iTRAQ4plex)	0,0000	1,132	1		0	9,144E-07	37	0,000188352	3	1647,01004	0,99	44,34	0
	High	eSGIFIHFGR	1	1	1	P74423	N-Term(iTRAQ4plex)	0,0000	1,364	1		0	0,0002226	32	0,000635287	3	1306,70313	0,49	33,60	0
Q55894	Phosphomethylpyrimidine synthase OS=Synechocystis sp. (strain PCC 6	116,09	11,11	1	5	5	7	0,906	7	17,9	459	51,1	5,74							
	A3	Sequence	# PSMs	# Proteins	# Protein Groups	Protein Group Accessions	Modifications	ΔCn	117/114	117/114 Count	117/114 Variability [%]	q-Value	PEP	IonScore	Exp Value	Charge	MH+ [Da]	ΔM [ppm]	RT [min]	# Missed Cleavages
	High	iAAHAADIAR	1	1	1	Q55894	N-Term(iTRAQ4plex)	0,0000	0,906	1		0	0,000003391	47	2,0146E-05	3	1152,66071	0,07	16,89	0
	High	qGQTNVSQmHYAR	1	1	1	Q55894	N-Term(iTRAQ4plex); M9(Oxidation)	0,0000	1,022	1		0	2,341E-08	40	9,41825E-05	3	1679,80570	0,98	9,39	0
	High	qGQTNVSQMHYAR	1	1	1	Q55894	N-Term(iTRAQ4plex)	0,0000	1,016	1		0	0,0004726	40	0,000106898	3	1663,80942	0,17	14,58	0
	High	rENLPVELIkDEVAR	1	1	1	Q55894	N-Term(iTRAQ4plex); K10(iTRAQ4plex)	0,0000	0,860	1		0	0,00008994	16	0,026361493	4	2069,19692	0,54	36,34	2
	High	tLGQLTR	1	1	1	Q55894	N-Term(iTRAQ4plex)	0,0000	0,695	1		0	0,002676	12	0,069979366	2	932,56383	-0,80	19,11	0
	High	lLLSVk	2	1	1	Q55894	N-Term(iTRAQ4plex); K6(iTRAQ4plex)	0,0000	0,988	2	37,7	0,006	0,08339	25	0,002897143	2	960,66979	0,20	30,65	0
P72741	Slr1097 protein OS=Synechocystis sp. (strain PCC 6803 / Kazusa) GN=s	115,88	20,86	1	5	5	7	1,052	7	18,2	163	19,0	4,70							
	A3	Sequence	# PSMs	# Proteins	# Protein Groups	Protein Group Accessions	Modifications	ΔCn	117/114	117/114 Count	117/114 Variability [%]	q-Value	PEP	IonScore	Exp Value	Charge	MH+ [Da]	ΔM [ppm]	RT [min]	# Missed Cleavages
	High	kYQLPAQFR	1	1	1	P72741	N-Term(iTRAQ4plex); K1(iTRAQ4plex)	0,0000	0,931	1		0	0,00001692	35	0,00028772	3	1438,84302	1,46	26,23	1
	High	yAAmLVPHEFHR	1	1	1	P72741	N-Term(iTRAQ4plex); M4(Oxidation)	0,0000	0,911	1		0	0,000002189	30	0,000901509	4	1630,82900	0,55	27,82	0
	High	yAAMLVPHEFHR	1	1	1	P72741	N-Term(iTRAQ4plex)	0,0000	1,028	1		0	0,0006422	27	0,002187611	4	1614,83486	1,04	31,56	0
	High	yQLPAQFR	1	1	1	P72741	N-Term(iTRAQ4plex)	0,0000	1,058	1		0,002	0,03523	31	0,000847169	2	1166,64556	1,43	31,56	0
	Medium	lWFIR	2	1	1	P72741	N-Term(iTRAQ4plex)	0,0000	1,238	2	2,2	0,029	0,3528	24	0,004397332	2	878,53765	0,84	41,77	0
	Medium	aISLNLNR	1	1	1	P72741	N-Term(iTRAQ4plex)	0,0000	1,052	1		0,029	0,3615	23	0,004560054	2	1044,62883	0,56	22,28	0
P73643	Sll1762 protein OS=Synechocystis sp. (strain PCC 6803 / Kazusa) GN=sl	115,31	15,71	1	6	6	6	0,801	6	12,2	382	42,0	4,94							
	A3	Sequence	# PSMs	# Proteins	# Protein Groups	Protein Group Accessions	Modifications	ΔCn	117/114	117/114 Count	117/114 Variability [%]	q-Value	PEP	IonScore	Exp Value	Charge	MH+ [Da]	ΔM [ppm]	RT [min]	# Missed Cleavages
	High	rVAmVPNTIVEDAVk	1	1	1	P73643	N-Term(iTRAQ4plex); M4(Oxidation); K15(iTRAQ4plex)	0,0000	0,701	1		0	1,468E-08	57	2,05775E-06	3	1946,10093	1,30	29,92	1
	High	qFFNYLVITHEQVmEPk	1	1	1	P73643	N-Term(iTRAQ4plex); M14(Oxidation); K17(iTRAQ4plex)	0,0000	0,765	1		0	1,419E-08	32	0,000628058	3	2427,26346	0,54	41,86	0
	High	iREEVGkELGR	1	1	1	P73643	N-Term(iTRAQ4plex); K7(iTRAQ4plex)	0,0000	1,365	1		0	0,0004712	27	0,002069998	3	1573,92716	0,43	21,00	2
	High	yGVGcMVR	1	1	1	P73643	N-Term(iTRAQ4plex); C5(Carbamidomethyl)	0,0000	0,744	1		0,001	0,02137	29	0,00120773	2	1085,53594	0,61	23,65	0
	High	iREEVGk	1	1	1	P73643	N-Term(iTRAQ4plex); K7(iTRAQ4plex)	0,0000	0,839	1		0,005	0,05653	11	0,101200171	3	1118,67899	1,58	13,31	1
	Medium	vIPQPAETR	1	1	1	P73643	N-Term(iTRAQ4plex)	0,0000	0,876	1		0,012	0,1478	22	0,005793887	2	1154,66533	0,26	19,65	0
P72673	Thylakoid-associated protein slr0729 OS=Synechocystis sp. (strain PCC	115,20	34,65	1	3	3	5	1,157	5	13,6	101	10,9	4,70							
	A3	Sequence	# PSMs	# Proteins	# Protein Groups	Protein Group Accessions	Modifications	ΔCn	117/114	117/114 Count	117/114 Variability [%]	q-Value	PEP	IonScore	Exp Value	Charge	MH+ [Da]	ΔM [ppm]	RT [min]	# Missed Cleavages
	High	aGVEAALk	1	1	1	P72673	N-Term(iTRAQ4plex); K8(iTRAQ4plex)	0,0000	1,106	1		0	0,001131	50	1,13027E-05	2	1046,64409	-0,73	18,66	0
	High	vNISLLDALSSTmVNR	1	1	1	P72673	N-Term(iTRAQ4plex); M13(Oxidation)	0,0000	1,056	1		0	9,567E-07	37	0,000205102	3	1893,02378	0,27	47,06	0
	High	lLTLLEEYQSk	3	1	1	P72673	N-Term(iTRAQ4plex); K11(iTRAQ4plex)	0,0000	1,428	3	9,6	0	7,084E-07	33	0,000557147	3	1624,94107	0,64	47,43	0
P74448	Slr0149 protein OS=Synechocystis sp. (strain PCC 6803 / Kazusa) GN=s	115,12	14,74	1	2	2	5	1,708	5	38,4	156	18,4	5,26							
	A3	Sequence	# PSMs	# Proteins	# Protein Groups	Protein Group Accessions	Modifications	ΔCn	117/114	117/114 Count	117/114 Variability [%]	q-Value	PEP	IonScore	Exp Value	Charge	MH+ [Da]	ΔM [ppm]	RT [min]	# Missed Cleavages
	High	iRDSEEQIIHR	4	1	1	P74448	N-Term(iTRAQ4plex)	0,0000	1,921	4	43,9	0	0,000002974	43	5,38233E-05	3	1539,83603	0,01	19,06	1
	High	gLPQDTFHmGNR	1	1	1	P74448	N-Term(iTRAQ4plex); M9(Oxidation)	0,0000	1,708	1		0	0,01019	22	0,005928844	3	1532,74042	0,48	18,96	0
Q55410	Sll0540 protein OS=Synechocystis sp. (strain PCC 6803 / Kazusa) GN=sl	115,06	5,21	1	2	2	4	0,696	3	0,3	307	32,5	4,93							
	A3	Sequence	# PSMs	# Proteins	# Protein Groups	Protein Group Accessions	Modifications	ΔCn	117/114	117/114 Count	117/114 Variability [%]	q-Value	PEP	IonScore	Exp Value	Charge	MH+ [Da]	ΔM [ppm]	RT [min]	# Missed Cleavages
	High	qLFYFYk	2	1	1	Q55410	N-Term(iTRAQ4plex); K7(iTRAQ4plex)	0,0000	0,667	2	6,7	0	0,00003414	49	1,18296E-05	2	1296,72527	1,70	39,63	0
	High	gVVNLILGk	2	1	1	Q55410	N-Term(iTRAQ4plex); K9(iTRAQ4plex)	0,0000	0,696	1		0	0,000006043	44	4,29507E-05	2	1200,79350	1,37	40,75	0
P73812	Slr2060 protein OS=Synechocystis sp. (strain PCC 6803 / Kazusa) GN=s	114,96	4,09	1	2	2	6	0,799	4	19,3	465	53,0	6,02							
	A3	Sequence	# PSMs	# Proteins	# Protein Groups	Protein Group Accessions	Modifications	ΔCn	117/114	117/114 Count	117/114 Variability [%]	q-Value	PEP	IonScore	Exp Value	Charge	MH+ [Da]	ΔM [ppm]	RT [min]	# Missed Cleavages
	High	lVENFVAVTGHYVk	1	1	1	P73812	N-Term(iTRAQ4plex); K14(iTRAQ4plex)	0,0000	0,606	1		0	7,606E-07	33	0,000516884	3	1864,06034	1,72	41,63	0
	High	lAIAk	5	1	1	P73812	N-Term(iTRAQ4plex); K5(iTRAQ4plex)	0,0000	0,834	3	12,8	0,001	0,01499	28	0,001482416	2	803,55834	-1,22	19,92	0
P74186	Membrane protein OS=Synechocystis sp. (strain PCC 6803 / Kazusa) GN	114,60	13,62	1	5	5	6	0,668	6	35,8	367	40,9	4,60							
	A3	Sequence	# PSMs	# Proteins	# Protein Groups	Protein Group Accessions	Modifications	ΔCn	117/114	117/114 Count	117/114 Variability [%]	q-Value	PEP	IonScore	Exp Value	Charge	MH+ [Da]	ΔM [ppm]	RT [min]	# Missed Cleavages
	High	vNVAQLTSQGQSYk	1	1	1	P74186	N-Term(iTRAQ4plex); K14(iTRAQ4plex)	0,0000	1,294	1		0	7,407E-10	57	1,87068E-06	3	1810,99271	1,40	30,50	0
	High	kSSGVGLEITPER	1	1	1	P74186	N-Term(iTRAQ4plex); K1(iTRAQ4plex)	0,0000	0,712	1		0	0,00000213	40	0,000105431	3	1660,94822	0,57	21,26	1
	High	lSIPTVk	1	1	1	P74186	N-Term(iTRAQ4plex); K7(iTRAQ4plex)	0,0000	0,627	1		0,001	0,01864	15	0,033034672	2	1045,68596	-0,02	30,36	0
	High	fTSLFGR	2	1	1	P74186	N-Term(iTRAQ4plex)	0,0000	0,528	2	1,3	0,003	0,03691	14	0,037322438	2	971,54393	0,84	35,24	0
	Medium	qVATAVPmR	1	1	1	P74186	N-Term(iTRAQ4plex); M8(Oxidation)	0,0000	0,929	1		0,028	0,3418	22	0,005969941	2	1132,62663	0,08	15,66	0
P74707	Peptide chain release factor 1 OS=Synechocystis sp. (strain PCC 6803 /	114,52	6,58	1	3	3	8	1,434	7	28,2	365	41,1	5,00							
	A3	Sequence	# PSMs	# Proteins	# Protein Groups	Protein Group Accessions	Modifications	ΔCn	117/114	117/114 Count	117/114 Variability [%]	q-Value	PEP	IonScore	Exp Value	Charge	MH+ [Da]	ΔM [ppm]	RT [min]	# Missed Cleavages
	High	lTILLLPk	3	1	1	P74707	N-Term(iTRAQ4plex); K8(iTRAQ4plex)	0,0000	1,726	3	0,3	0	0,001407	44	3,69803E-05	2	1198,83794	0,17	44,51	0
	High	vETAVDLFHkPTGIR	4	1	1	P74707	N-Term(iTRAQ4plex); K10(iTRAQ4plex)	0,0000	0,863	3	21,7	0	0,000001679	35	0,000285082	4	1971,12685	0,11	33,46	0
	High	rLTILLLPk	1	1	1	P74707	N-Term(iTRAQ4plex); K9(iTRAQ4plex)	0,0000	1,030	1		0	0,0004512	20	0,009015089	3	1354,93824	-0,46	37,14	1
P73918	Acetolactate synthase OS=Synechocystis sp. (strain PCC 6803 / Kazusa)	114,14	8,18	1	4	4	6	0,873	5	4,2	550	60,1	5,41							
	A3	Sequence	# PSMs	# Proteins	# Protein Groups	Protein Group Accessions	Modifications	ΔCn	117/114	117/114 Count	117/114 Variability [%]	q-Value	PEP	IonScore	Exp Value	Charge	MH+ [Da]	ΔM [ppm]	RT [min]	# Missed Cleavages
	High	qIVRPSITPEVVR	3	1	1	P73918	N-Term(iTRAQ4plex)	0,0000	0,876	2	0,4	0	3,358E-07	46	2,38765E-05	3	1637,98276	0,48	27,09	0
	High	qGkPTPYGASLR	1	1	1	P73918	N-Term(iTRAQ4plex); K3(iTRAQ4plex)	0,0000	0,898	1		0	0,002435	28	0,001570254	3	1562,88901	-0,21	16,77	0
	High	akNPIILAGNGTIR	1	1	1	P73918	N-Term(iTRAQ4plex); K2(iTRAQ4plex)	0,0000	0,826	1		0	0,003169	24	0,00425569	3	1726,05667	-0,67	26,71	1
	Medium	vYAAFR	1	1	1	P73918	N-Term(iTRAQ4plex)	0,0000	0,834	1		0,012	0,157	25	0,00289048	2	870,49486	-0,65	22,41	0
P72749	GTP-binding protein TypA/BipA homolog OS=Synechocystis sp. (strain P	114,04	3,18	1	2	2	3	1,002	2	9,4	597	66,0	5,10							
	A3	Sequence	# PSMs	# Proteins	# Protein Groups	Protein Group Accessions	Modifications	ΔCn	117/114	117/114 Count	117/114 Variability [%]	q-Value	PEP	IonScore	Exp Value	Charge	MH+ [Da]	ΔM [ppm]	RT [min]	# Missed Cleavages
	High	gVFFITPGTk	2	1	1	P72749	N-Term(iTRAQ4plex); K10(iTRAQ4plex)	0,0000	0,941	1		0	3,582E-07	73	5,24807E-08	2	1354,79863	0,96	35,79	0
	High	aGQQAALVk	1	1	1	P72749	N-Term(iTRAQ4plex); K9(iTRAQ4plex)	0,0000	1,068	1		0	0,0004993	50	9,57128E-06	2	1173,71977	0,30	15,14	0
P72623	Probable glycogen synthase 2 OS=Synechocystis sp. (strain PCC 6803 /	113,73	11,61	1	6	6	8	0,618	8	10,7	491	56,2	6,48							
	A3	Sequence	# PSMs	# Proteins	# Protein Groups	Protein Group Accessions	Modifications	ΔCn	117/114	117/114 Count	117/114 Variability [%]	q-Value	PEP	IonScore	Exp Value	Charge	MH+ [Da]	ΔM [ppm]	RT [min]	# Missed Cleavages
	High	gHYYGALDDHmR	1	1	1	P72623	N-Term(iTRAQ4plex); M11(Oxidation)	0,0000	0,853	1		0	5,944E-07	40	0,000109388	4	1594,71901	0,05	15,29	0
	High	aGGLGDVIYGLSR	1	1	1	P72623	N-Term(iTRAQ4plex)	0,0000	0,649	1		0	0,00001686	38	0,000150998	2	1421,78667	-0,20	41,08	0
	High	yGAVPVVR	2	1	1	P72623	N-Term(iTRAQ4plex)	0,0000	0,651	2	20,2	0	0,0001408	32	0,001208572	2	1004,60210	1,13	23,54	0
	High	fAFFSk	2	1	1	P72623	N-Term(iTRAQ4plex); K6(iTRAQ4plex)	0,0000	0,600	2	6,7	0	0,003589	26	0,002443431	2	1034,59270	1,31	35,79	0
	High	yDHIWGLHDAYR	1	1	1	P72623	N-Term(iTRAQ4plex)	0,0000	0,607	1		0	0,0005584	12	0,066217077	4	1689,82619	0,45	30,65	0
	High	wFWHEk	1	1	1	P72623	N-Term(iTRAQ4plex); K6(iTRAQ4plex)	0,0000	0,575	1		0	0,00139	12	0,068386441	3	1220,64896	2,83	29,95	0
P72689	Translation initiation factor IF-2 OS=Synechocystis sp. (strain PCC 6803	113,37	6,89	1	5	5	8	1,152	6	16,2	1001	108,1	5,95							
	A3	Sequence	# PSMs	# Proteins	# Protein Groups	Protein Group Accessions	Modifications	ΔCn	117/114	117/114 Count	117/114 Variability [%]	q-Value	PEP	IonScore	Exp Value	Charge	MH+ [Da]	ΔM [ppm]	RT [min]	# Missed Cleavages
	High	rGDQVLFEGNIDSLkR	3	1	1	P72689	N-Term(iTRAQ4plex); K15(iTRAQ4plex)	0,0000	1,132	2	21,9	0	0,000000185	38	0,000203296	4	2135,18130	0,04	31,33	2
	High	aAGVPLIVAINk	1	1	1	P72689	N-Term(iTRAQ4plex); K12(iTRAQ4plex)	0,0000	1,232	1		0	0,0009728	33	0,000488619	3	1453,93375	-0,53	37,67	0
	High	tAVPAPAkPk	1	1	1	P72689	N-Term(iTRAQ4plex); K8(iTRAQ4plex); K10(iTRAQ4plex)	0,0000	1,563	1		0	0,001259	29	0,001161368	3	1411,90061	0,61	14,82	0
	High	rPPVVTImGHVDHGk	1	1	1	P72689	N-Term(iTRAQ4plex); M8(Oxidation); K15(iTRAQ4plex)	0,0000	1,055	1		0	0,00000785	25	0,003427678	5	1947,08387	0,07	17,13	0
	High	sGGPSPARPTPPPRPk	2	1	1	P72689	N-Term(iTRAQ4plex); K16(iTRAQ4plex)	0,0000	1,077	1		0	0,004551	15	0,036323635	4	1887,08205	0,89	13,33	0
P73503	Slr1437 protein OS=Synechocystis sp. (strain PCC 6803 / Kazusa) GN=s	113,09	22,32	1	3	3	4	1,305	4	10,0	112	12,7	5,22							
	A3	Sequence	# PSMs	# Proteins	# Protein Groups	Protein Group Accessions	Modifications	ΔCn	117/114	117/114 Count	117/114 Variability [%]	q-Value	PEP	IonScore	Exp Value	Charge	MH+ [Da]	ΔM [ppm]	RT [min]	# Missed Cleavages
	High	cAYVAIGEFkPGR	2	1	1	P73503	N-Term(iTRAQ4plex); C1(Carbamidomethyl); K10(iTRAQ	0,0000	1,305	2	6,8	0	2,849E-09	53	5,29627E-06	3	1755,94498	-0,29	31,12	0
	High	aISTYk	1	1	1	P73503	N-Term(iTRAQ4plex); K6(iTRAQ4plex)	0,0000	1,079	1		0	0,005153	27	0,002108483	2	970,58104	-0,12	15,46	0
	Medium	gAFLLR	1	1	1	P73503	N-Term(iTRAQ4plex)	0,0000	1,425	1		0,022	0,2797	21	0,008531001	2	820,51543	-0,91	26,64	0
P74394	Sll0272 protein OS=Synechocystis sp. (strain PCC 6803 / Kazusa) GN=sl	113,07	17,20	1	2	2	3	0,604	3	36,8	157	17,6	8,78							
	A3	Sequence	# PSMs	# Proteins	# Protein Groups	Protein Group Accessions	Modifications	ΔCn	117/114	117/114 Count	117/114 Variability [%]	q-Value	PEP	IonScore	Exp Value	Charge	MH+ [Da]	ΔM [ppm]	RT [min]	# Missed Cleavages
	High	iGLGPVALDFSk	2	1	1	P74394	N-Term(iTRAQ4plex); K12(iTRAQ4plex)	0,0000	0,518	2	23,3	0	5,563E-09	55	3,25814E-06	2	1504,90007	1,51	43,45	0
	High	qFNLYFFQEDIkGPk	1	1	1	P74394	N-Term(iTRAQ4plex); K12(iTRAQ4plex); K15(iTRAQ4plex	0,0000	0,769	1		0	1,04E-09	43	4,47682E-05	4	2306,25527	0,30	43,45	1
Q55386	Slr0924 protein OS=Synechocystis sp. (strain PCC 6803 / Kazusa) GN=s	113,03	6,99	1	2	2	5	1,765	4	14,0	286	31,3	5,72							
	A3	Sequence	# PSMs	# Proteins	# Protein Groups	Protein Group Accessions	Modifications	ΔCn	117/114	117/114 Count	117/114 Variability [%]	q-Value	PEP	IonScore	Exp Value	Charge	MH+ [Da]	ΔM [ppm]	RT [min]	# Missed Cleavages
	High	iVPTQEAIQFIR	2	1	1	Q55386	N-Term(iTRAQ4plex)	0,0000	1,939	2	7,8	0	7,629E-07	58	1,59945E-06	2	1558,90935	1,25	41,49	0
	High	sLSAQQPk	3	1	1	Q55386	N-Term(iTRAQ4plex); K8(iTRAQ4plex)	0,0000	1,519	2	16,3	0	0,002291	37	0,000223559	2	1146,67327	1,00	12,59	0
P77962	Serine hydroxymethyltransferase OS=Synechocystis sp. (strain PCC 680	112,13	15,46	1	6	6	8	1,615	8	22,1	427	46,2	6,32							
	A3	Sequence	# PSMs	# Proteins	# Protein Groups	Protein Group Accessions	Modifications	ΔCn	117/114	117/114 Count	117/114 Variability [%]	q-Value	PEP	IonScore	Exp Value	Charge	MH+ [Da]	ΔM [ppm]	RT [min]	# Missed Cleavages
	High	sVFPGTQGGPLEHVITAk	1	1	1	P77962	N-Term(iTRAQ4plex); K18(iTRAQ4plex)	0,0000	1,626	1		0	1,234E-08	50	9,46172E-06	3	2126,18735	1,16	37,50	0
	High	aVAFGEALkPEFk	1	1	1	P77962	N-Term(iTRAQ4plex); K9(iTRAQ4plex); K13(iTRAQ4plex)	0,0000	1,435	1		0	0,000006845	30	0,000935341	3	1839,07450	0,22	33,59	0
	High	qQALEVkPk	1	1	1	P77962	N-Term(iTRAQ4plex); K7(iTRAQ4plex); K9(iTRAQ4plex)	0,0000	1,925	1		0	0,00001132	27	0,002079553	3	1472,91602	-0,06	14,91	0
	High	yAEGLPGkR	1	1	1	P77962	N-Term(iTRAQ4plex); K8(iTRAQ4plex)	0,0000	2,247	1		0	0,0009043	23	0,004497799	3	1278,74155	0,53	17,44	1
	High	qIEFDkFR	2	1	1	P77962	N-Term(iTRAQ4plex); K6(iTRAQ4plex)	0,0000	1,276	2	69,5	0	0,0008721	21	0,007111644	3	1370,76670	-0,29	27,04	1
	Medium	lGSPAmTTR	2	1	1	P77962	N-Term(iTRAQ4plex); M6(Oxidation)	0,0000	1,574	2	2,7	0,014	0,178	21	0,007942734	2	1093,57805	-1,11	13,41	0
P09192	Photosystem II D2 protein OS=Synechocystis sp. (strain PCC 6803 / Kaz	112,06	5,40	1	2	2	3	0,367	3	9,1	352	39,5	5,55							
	A3	Sequence	# PSMs	# Proteins	# Protein Groups	Protein Group Accessions	Modifications	ΔCn	117/114	117/114 Count	117/114 Variability [%]	q-Value	PEP	IonScore	Exp Value	Charge	MH+ [Da]	ΔM [ppm]	RT [min]	# Missed Cleavages
	High	aYDFVSQELR	1	1	1	P09192	N-Term(iTRAQ4plex)	0,0000	0,478	1		0	0,00001173	66	2,76039E-07	2	1371,70488	1,71	35,10	0
	High	nILLNEGmR	2	1	1	P09192	N-Term(iTRAQ4plex); M8(Oxidation)	0,0000	0,356	2	4,5	0	0,007284	35	0,000315479	2	1219,66020	1,33	23,81	0
Q55383	Malate dehydrogenase OS=Synechocystis sp. (strain PCC 6803 / Kazusa	111,47	5,56	1	2	2	3	0,863	3	10,9	324	34,3	5,03							
	A3	Sequence	# PSMs	# Proteins	# Protein Groups	Protein Group Accessions	Modifications	ΔCn	117/114	117/114 Count	117/114 Variability [%]	q-Value	PEP	IonScore	Exp Value	Charge	MH+ [Da]	ΔM [ppm]	RT [min]	# Missed Cleavages
	High	nANIVAQGAR	2	1	1	Q55383	N-Term(iTRAQ4plex)	0,0000	0,854	2	12,4	0	0,00001057	60	9,03587E-07	2	1157,65141	0,54	14,33	0
	High	vTGLPSQR	1	1	1	Q55383	N-Term(iTRAQ4plex)	0,0000	0,863	1		0,005	0,05785	35	0,000376442	2	1001,58690	0,85	17,36	0
P73410	Cysteine synthase OS=Synechocystis sp. (strain PCC 6803 / Kazusa) GN	111,18	9,94	1	3	3	5	0,953	5	16,7	312	33,2	5,80							
	A3	Sequence	# PSMs	# Proteins	# Protein Groups	Protein Group Accessions	Modifications	ΔCn	117/114	117/114 Count	117/114 Variability [%]	q-Value	PEP	IonScore	Exp Value	Charge	MH+ [Da]	ΔM [ppm]	RT [min]	# Missed Cleavages
	High	iASNITELIGR	2	1	1	P73410	N-Term(iTRAQ4plex)	0,0000	0,977	2	30,0	0	0,00001112	66	2,56431E-07	2	1330,78203	0,67	40,64	0
	High	lIVmIQPSFGER	2	1	1	P73410	N-Term(iTRAQ4plex); M4(Oxidation)	0,0000	1,008	2	8,3	0	0,0001883	30	0,00093319	2	1549,84758	-3,46	39,47	0
	High	iGINmINR	1	1	1	P73410	N-Term(iTRAQ4plex); M5(Oxidation)	0,0000	0,937	1		0	0,008649	27	0,002212942	2	1090,61650	0,48	22,85	0
P73370	Slr2070 protein OS=Synechocystis sp. (strain PCC 6803 / Kazusa) GN=s	111,10	9,15	1	2	2	3	1,067	3	3,2	284	31,9	5,71							
	A3	Sequence	# PSMs	# Proteins	# Protein Groups	Protein Group Accessions	Modifications	ΔCn	117/114	117/114 Count	117/114 Variability [%]	q-Value	PEP	IonScore	Exp Value	Charge	MH+ [Da]	ΔM [ppm]	RT [min]	# Missed Cleavages
	High	sLGVVLVPQR	2	1	1	P73370	N-Term(iTRAQ4plex)	0,0000	1,078	2	1,6	0	0,0001812	57	1,92739E-06	2	1211,76079	1,25	32,13	0
	High	yTAHITLGYFDPAIEk	1	1	1	P73370	N-Term(iTRAQ4plex); K16(iTRAQ4plex)	0,0000	0,943	1		0	6,713E-10	45	2,8772E-05	3	2127,13767	0,55	40,62	0
P73489	Ssl2245 protein OS=Synechocystis sp. (strain PCC 6803 / Kazusa) GN=s	110,41	15,91	1	2	2	8	1,208	7	15,6	88	9,2	4,35							
	A3	Sequence	# PSMs	# Proteins	# Protein Groups	Protein Group Accessions	Modifications	ΔCn	117/114	117/114 Count	117/114 Variability [%]	q-Value	PEP	IonScore	Exp Value	Charge	MH+ [Da]	ΔM [ppm]	RT [min]	# Missed Cleavages
	High	aQGPAPGk	7	1	1	P73489	N-Term(iTRAQ4plex); K8(iTRAQ4plex)	0,0000	1,210	6	10,9	0	0,002294	37	0,000217756	2	1013,59868	0,44	10,72	0
	High	sINAYk	1	1	1	P73489	N-Term(iTRAQ4plex); K6(iTRAQ4plex)	0,0000	0,795	1		0,002	0,03319	33	0,000532072	2	983,57750	1,11	16,00	0
P73807	Histidinol-phosphate aminotransferase OS=Synechocystis sp. (strain PC	109,36	13,75	1	4	4	4	0,552	4	29,0	349	38,7	5,12							
	A3	Sequence	# PSMs	# Proteins	# Protein Groups	Protein Group Accessions	Modifications	ΔCn	117/114	117/114 Count	117/114 Variability [%]	q-Value	PEP	IonScore	Exp Value	Charge	MH+ [Da]	ΔM [ppm]	RT [min]	# Missed Cleavages
	High	lAQVLGTAALR	1	1	1	P73807	N-Term(iTRAQ4plex)	0,0000	0,522	1		0	0,000001514	50	8,91189E-06	2	1256,78032	-0,34	36,79	0
	High	lIFLASPNPPk	1	1	1	P73807	N-Term(iTRAQ4plex); K11(iTRAQ4plex)	0,0000	0,268	1		0	0,00002323	46	2,79254E-05	3	1484,90641	-1,04	38,76	0
	High	hTPAYVPGEQPQTNDFIk	1	1	1	P73807	N-Term(iTRAQ4plex); K18(iTRAQ4plex)	0,0000	0,584	1		0	1,982E-09	39	0,000116137	3	2330,20163	-0,15	28,94	0
	High	vSIRPSVR	1	1	1	P73807	N-Term(iTRAQ4plex)	0,0000	0,766	1		0,003	0,03624	11	0,078337554	3	1057,65863	-1,18	15,76	0
Q55404	Acetyl-coenzyme A synthetase OS=Synechocystis sp. (strain PCC 6803 /	108,40	6,13	1	4	4	4	1,033	4	22,4	653	73,0	5,76							
	A3	Sequence	# PSMs	# Proteins	# Protein Groups	Protein Group Accessions	Modifications	ΔCn	117/114	117/114 Count	117/114 Variability [%]	q-Value	PEP	IonScore	Exp Value	Charge	MH+ [Da]	ΔM [ppm]	RT [min]	# Missed Cleavages
	High	yGVNIFYTAPTAIR	1	1	1	Q55404	N-Term(iTRAQ4plex)	0,0000	1,189	1		0	1,348E-09	65	3,13307E-07	2	1729,94023	0,47	42,77	0
	High	iITYAQLHR	1	1	1	Q55404	N-Term(iTRAQ4plex)	0,0000	0,897	1		0	2,046E-07	51	7,9794E-06	3	1258,73911	0,21	25,58	0
	High	tkADVTmTAGR	1	1	1	Q55404	N-Term(iTRAQ4plex); K2(iTRAQ4plex); M7(Oxidation)	0,0000	0,858	1		0,001	0,01255	16	0,025820818	3	1454,78830	0,49	11,23	1
	Medium	wIFDLk	1	1	1	Q55404	N-Term(iTRAQ4plex); K6(iTRAQ4plex)	0,0000	1,209	1		0,01	0,133	16	0,02792351	2	1109,65886	-0,82	43,58	0
P72647	Sll1053 protein OS=Synechocystis sp. (strain PCC 6803 / Kazusa) GN=sl	107,74	4,62	1	2	2	4	0,387	3	5,1	520	56,2	4,97							
	A3	Sequence	# PSMs	# Proteins	# Protein Groups	Protein Group Accessions	Modifications	ΔCn	117/114	117/114 Count	117/114 Variability [%]	q-Value	PEP	IonScore	Exp Value	Charge	MH+ [Da]	ΔM [ppm]	RT [min]	# Missed Cleavages
	High	vGQALASVk	2	1	1	P72647	N-Term(iTRAQ4plex); K9(iTRAQ4plex)	0,0000	0,401	1		0	7,776E-07	58	1,63682E-06	2	1160,72453	0,32	24,31	0
	High	lRPGLVVEILDGQEk	2	1	1	P72647	N-Term(iTRAQ4plex); K15(iTRAQ4plex)	0,0000	0,354	2	13,2	0	5,515E-10	53	5,59719E-06	3	1954,15921	0,82	43,93	0
P73746	Sll0854 protein OS=Synechocystis sp. (strain PCC 6803 / Kazusa) GN=sl	106,99	15,26	1	4	4	5	0,625	3	27,1	308	34,5	5,12							
	A3	Sequence	# PSMs	# Proteins	# Protein Groups	Protein Group Accessions	Modifications	ΔCn	117/114	117/114 Count	117/114 Variability [%]	q-Value	PEP	IonScore	Exp Value	Charge	MH+ [Da]	ΔM [ppm]	RT [min]	# Missed Cleavages
	High	lLHSIIGGVAVSSTR	2	1	1	P73746	N-Term(iTRAQ4plex)	0,0000	0,523	1		0	2,814E-09	68	1,61797E-07	3	1653,97702	0,08	35,99	0
	High	rLLPFISQAFANAH	1	1	1	P73746	N-Term(iTRAQ4plex)	0,0000	1,600	1		0	0,005023	28	0,001603135	3	1728,96769	0,60	45,05	1
	High	iYPASVVk	1	1	1	P73746	N-Term(iTRAQ4plex); K8(iTRAQ4plex)	0,0000				0,004	0,05059	33	0,000456005	2	1164,72515	1,77	25,82	0
	Medium	nmLTTNAVAR	1	1	1	P73746	N-Term(iTRAQ4plex); M2(Oxidation)	0,0000	0,625	1		0,012	0,1464	15	0,035072765	2	1250,66472	0,26	16,71	0
P74250	Hydroperoxy fatty acid reductase gpx1 OS=Synechocystis sp. (strain PC	106,98	16,57	1	3	3	5	0,401	5	16,3	169	18,4	7,09							
	A3	Sequence	# PSMs	# Proteins	# Protein Groups	Protein Group Accessions	Modifications	ΔCn	117/114	117/114 Count	117/114 Variability [%]	q-Value	PEP	IonScore	Exp Value	Charge	MH+ [Da]	ΔM [ppm]	RT [min]	# Missed Cleavages
	High	yGVTFPLFEk	3	1	1	P74250	N-Term(iTRAQ4plex); K10(iTRAQ4plex)	0,0000	0,401	3	3,2	0	3,326E-07	44	5,36962E-05	2	1488,83708	2,00	44,61	0
	High	vEVNGPNAHPLFk	1	1	1	P74250	N-Term(iTRAQ4plex); K13(iTRAQ4plex)	0,0000	0,472	1		0	0,0003135	26	0,002437642	3	1709,96189	2,41	27,33	0
	Medium	wNFTk	1	1	1	P74250	N-Term(iTRAQ4plex); K5(iTRAQ4plex)	0,0000	0,303	1		0,029	0,3601	28	0,001670975	2	983,55846	3,22	26,42	0
P74511	Thylakoid membrane protein slr1949 OS=Synechocystis sp. (strain PCC	106,71	9,43	1	2	2	5	1,166	4	9,1	212	23,9	9,13							
	A3	Sequence	# PSMs	# Proteins	# Protein Groups	Protein Group Accessions	Modifications	ΔCn	117/114	117/114 Count	117/114 Variability [%]	q-Value	PEP	IonScore	Exp Value	Charge	MH+ [Da]	ΔM [ppm]	RT [min]	# Missed Cleavages
	High	nLLFWHEVAR	4	1	1	P74511	N-Term(iTRAQ4plex)	0,0000	1,097	3	0,3	0	0,000003237	41	8,49122E-05	3	1428,78778	0,63	36,76	0
	High	tLIEQTPNRR	1	1	1	P74511	N-Term(iTRAQ4plex)	0,0000	1,707	1		0,003	0,04369	25	0,003539729	3	1371,78376	0,90	15,11	1
Q55584	Cation or drug efflux system protein OS=Synechocystis sp. (strain PCC 6	106,62	2,49	1	3	3	5	0,676	5	10,6	1083	117,5	4,88							
	A3	Sequence	# PSMs	# Proteins	# Protein Groups	Protein Group Accessions	Modifications	ΔCn	117/114	117/114 Count	117/114 Variability [%]	q-Value	PEP	IonScore	Exp Value	Charge	MH+ [Da]	ΔM [ppm]	RT [min]	# Missed Cleavages
	High	sLFFVk	2	1	1	Q55584	N-Term(iTRAQ4plex); K6(iTRAQ4plex)	0,0000	0,649	2	4,4	0	0,008337	44	3,73224E-05	2	1028,63872	0,41	34,21	0
	High	yGNIITAILk	2	1	1	Q55584	N-Term(iTRAQ4plex); K10(iTRAQ4plex)	0,0000	0,812	2	27,7	0	0,0002411	30	0,000943996	3	1393,86728	1,11	46,99	0
	High	lkNWDERPGQk	1	1	1	Q55584	N-Term(iTRAQ4plex); K2(iTRAQ4plex); K11(iTRAQ4plex)	0,0000	0,822	1		0	0,001666	11	0,075330353	4	1803,02407	0,16	18,16	1
P72586	GDP-mannose 4,6-dehydratase OS=Synechocystis sp. (strain PCC 6803	106,54	21,55	1	7	7	7	0,813	7	40,6	362	41,3	6,47							
	A3	Sequence	# PSMs	# Proteins	# Protein Groups	Protein Group Accessions	Modifications	ΔCn	117/114	117/114 Count	117/114 Variability [%]	q-Value	PEP	IonScore	Exp Value	Charge	MH+ [Da]	ΔM [ppm]	RT [min]	# Missed Cleavages
	High	vYGHWQTVNYR	1	1	1	P72586	N-Term(iTRAQ4plex)	0,0000	0,579	1		0	0,00004193	36	0,00025175	3	1566,79520	1,15	25,05	0
	High	kLYLGNIDSk	1	1	1	P72586	N-Term(iTRAQ4plex); K1(iTRAQ4plex); K10(iTRAQ4plex)	0,0000	0,446	1		0	0,000000944	34	0,000354789	3	1582,95243	-0,29	25,74	1
	High	iLEDVkPTEIYNLGAQSHVR	1	1	1	P72586	N-Term(iTRAQ4plex); K6(iTRAQ4plex)	0,0000	0,739	1		0	0,000001456	29	0,001221715	4	2570,42104	1,13	38,30	0
	High	lHYGDLTDGTTLR	1	1	1	P72586	N-Term(iTRAQ4plex)	0,0000	1,193	1		0	2,366E-07	29	0,00132425	3	1605,83640	0,66	29,36	0
	High	rGETFVTR	1	1	1	P72586	N-Term(iTRAQ4plex)	0,0000	1,058	1		0	0,005667	24	0,003890183	3	1109,61847	0,05	12,11	1
	High	gYQVHGIIR	1	1	1	P72586	N-Term(iTRAQ4plex)	0,0000	0,813	1		0	0,0002055	13	0,048861861	3	1186,67951	-1,54	19,40	0
	Medium	sPYAcAk	1	1	1	P72586	N-Term(iTRAQ4plex); C5(Carbamidomethyl); K7(iTRAQ4p	0,0000	0,969	1		0,024	0,3037	19	0,012682045	2	1084,56719	-2,55	12,99	0
P72814	Slr1800 protein OS=Synechocystis sp. (strain PCC 6803 / Kazusa) GN=s	106,28	6,20	1	3	3	5	1,203	4	8,1	355	39,4	4,84							
	A3	Sequence	# PSMs	# Proteins	# Protein Groups	Protein Group Accessions	Modifications	ΔCn	117/114	117/114 Count	117/114 Variability [%]	q-Value	PEP	IonScore	Exp Value	Charge	MH+ [Da]	ΔM [ppm]	RT [min]	# Missed Cleavages
	High	gTGFLNWLk	3	1	1	P72814	N-Term(iTRAQ4plex); K9(iTRAQ4plex)	0,0000	1,139	2	2,7	0	2,223E-07	50	1,1153E-05	2	1323,76750	0,86	44,13	0
	High	hLAPLVk	1	1	1	P72814	N-Term(iTRAQ4plex); K7(iTRAQ4plex)	0,0000	1,756	1		0	0,0006015	15	0,035234653	3	1065,70255	0,24	18,18	0
	High	lFTTWk	1	1	1	P72814	N-Term(iTRAQ4plex); K6(iTRAQ4plex)	0,0000	1,247	1		0,002	0,03564	41	7,46397E-05	2	1083,64580	1,56	34,04	0
Q55421	Elongation factor G-like protein OS=Synechocystis sp. (strain PCC 6803	105,35	3,29	1	2	2	3	1,053	3	8,6	669	73,3	5,00							
	A3	Sequence	# PSMs	# Proteins	# Protein Groups	Protein Group Accessions	Modifications	ΔCn	117/114	117/114 Count	117/114 Variability [%]	q-Value	PEP	IonScore	Exp Value	Charge	MH+ [Da]	ΔM [ppm]	RT [min]	# Missed Cleavages
	High	akQPFGEVLQALk	2	1	1	Q55421	N-Term(iTRAQ4plex); K2(iTRAQ4plex); K13(iTRAQ4plex)	0,0000	1,084	2	4,3	0	3,987E-08	66	2,79235E-07	3	1861,12699	-0,11	40,28	1
	High	vLTLAPLFk	1	1	1	Q55421	N-Term(iTRAQ4plex); K9(iTRAQ4plex)	0,0000	0,713	1		0	0,000733	22	0,006698383	3	1289,84443	0,67	46,05	0
P74299	Phosphoenolpyruvate carboxylase OS=Synechocystis sp. (strain PCC 68	104,84	7,16	1	7	7	7	0,978	7	6,6	1034	118,9	5,78							
	A3	Sequence	# PSMs	# Proteins	# Protein Groups	Protein Group Accessions	Modifications	ΔCn	117/114	117/114 Count	117/114 Variability [%]	q-Value	PEP	IonScore	Exp Value	Charge	MH+ [Da]	ΔM [ppm]	RT [min]	# Missed Cleavages
	High	kLDQLQGSVTGR	1	1	1	P74299	N-Term(iTRAQ4plex); K1(iTRAQ4plex)	0,0000	0,715	1		0	0,0005357	43	5,34527E-05	3	1589,92105	-0,20	22,12	1
	High	gLVLGk	1	1	1	P74299	N-Term(iTRAQ4plex); K6(iTRAQ4plex)	0,0000	1,081	1		0	0,005806	29	0,001180239	2	874,59636	-0,09	21,47	0
	High	rPLIPQEmPFSER	1	1	1	P74299	N-Term(iTRAQ4plex); M8(Oxidation)	0,0000	1,116	1		0	9,66E-08	28	0,001725719	3	1759,92899	0,43	27,70	0
	High	aIPWVFSWTQTR	1	1	1	P74299	N-Term(iTRAQ4plex)	0,0000	0,963	1		0	0,00003822	26	0,003442786	2	1635,87712	0,42	50,76	0
	High	sLQAVAQSHR	1	1	1	P74299	N-Term(iTRAQ4plex)	0,0000	1,022	1		0	0,002062	24	0,003706551	3	1240,68848	0,48	15,05	0
	High	eTFPHLR	1	1	1	P74299	N-Term(iTRAQ4plex)	0,0000	0,951	1		0	0,004194	20	0,010068621	3	1043,57636	0,85	21,21	0
	High	tIVPLGLLQISLLk	1	1	1	P74299	N-Term(iTRAQ4plex); K14(iTRAQ4plex)	0,0000	0,978	1		0	0,007487	14	0,037754611	3	1796,18442	-1,09	55,78	0
P74598	Uncharacterized WD repeat-containing protein sll1491 OS=Synechocysti	104,33	10,92	1	4	4	4	1,243	4	20,3	348	37,3	5,52							
	A3	Sequence	# PSMs	# Proteins	# Protein Groups	Protein Group Accessions	Modifications	ΔCn	117/114	117/114 Count	117/114 Variability [%]	q-Value	PEP	IonScore	Exp Value	Charge	MH+ [Da]	ΔM [ppm]	RT [min]	# Missed Cleavages
	High	iWDLSALAR	1	1	1	P74598	N-Term(iTRAQ4plex)	0,0000	1,128	1		0	0,000001596	48	1,61797E-05	2	1188,68559	-0,15	43,52	0
	High	wVATANk	1	1	1	P74598	N-Term(iTRAQ4plex); K7(iTRAQ4plex)	0,0000	0,983	1		0	0,00003557	45	4,3143E-05	2	1077,62969	0,15	19,49	0
	High	eLFSVR	1	1	1	P74598	N-Term(iTRAQ4plex)	0,0000	1,369	1		0	0,00574	39	0,000144607	2	894,51677	0,23	26,48	0

	Medium	gPSLEINTIAVSPNNR	1	1	1	P74598	N-Term(iTRAQ4plex)	0,0000	1,480	1		0,017	0,2099	12	0,060534087	3	1825,98886	-0,03	34,38	0
P74228	Elongation factor G 2 OS=Synechocystis sp. (strain PCC 6803 / Kazusa)	104,19	6,80	1	5	5	7	1,229	6	14,5	691	75,4	5,05							
	A3	Sequence	# PSMs	# Proteins	# Protein Groups	Protein Group Accessions	Modifications	ΔCn	117/114	117/114 Count	117/114 Variability [%]	q-Value	PEP	IonScore	Exp Value	Charge	MH+ [Da]	ΔM [ppm]	RT [min]	# Missed Cleavages
	High	gSYVLNSTk	3	1	1	P74228	N-Term(iTRAQ4plex); K9(iTRAQ4plex)	0,0000	1,229	2	3,2	0	4,328E-07	56	2,77952E-06	2	1256,70806	-0,66	18,04	0
	High	iLFYSGVVHk	1	1	1	P74228	N-Term(iTRAQ4plex); K10(iTRAQ4plex)	0,0000	1,367	1		0	0,000000578	33	0,000559719	3	1450,86737	0,90	33,45	0
	High	kAVQAEGk	1	1	1	P74228	N-Term(iTRAQ4plex); K1(iTRAQ4plex); K8(iTRAQ4plex)	0,0000	1,791	1		0	0,0001451	28	0,001492691	3	1262,77530	-3,15	8,23	1
	High	rGHIEGQETEQGIAk	1	1	1	P74228	N-Term(iTRAQ4plex); K15(iTRAQ4plex)	0,0000	0,741	1		0	0,0000263	13	0,055586586	4	1941,03847	-0,41	13,17	1
	Medium	yQVPR	1	1	1	P74228	N-Term(iTRAQ4plex)	0,0000	1,124	1		0,049	0,5979	15	0,031986742	2	806,46489	0,96	16,41	0
P73173	PilJ protein OS=Synechocystis sp. (strain PCC 6803 / Kazusa) GN=pilJ P	103,27	3,88	1	4	4	6	0,611	5	12,9	953	103,1	4,61							
	A3	Sequence	# PSMs	# Proteins	# Protein Groups	Protein Group Accessions	Modifications	ΔCn	117/114	117/114 Count	117/114 Variability [%]	q-Value	PEP	IonScore	Exp Value	Charge	MH+ [Da]	ΔM [ppm]	RT [min]	# Missed Cleavages
	High	sQLVSQSLQSLAk	1	1	1	P73173	N-Term(iTRAQ4plex); K13(iTRAQ4plex)	0,0000	0,667	1		0	0,00001002	45	3,46713E-05	3	1676,98294	2,62	34,73	0
	High	iIASANAQR	1	1	1	P73173	N-Term(iTRAQ4plex)	0,0000	0,692	1		0	0,001432	36	0,000241651	2	1087,63542	1,25	15,56	0
	High	sNLTLQk	3	1	1	P73173	N-Term(iTRAQ4plex); K7(iTRAQ4plex)	0,0000	0,598	2	3,3	0,003	0,04538	11	0,086291895	2	1091,67082	4,15	17,96	0
	High	nLQTTAQk	1	1	1	P73173	N-Term(iTRAQ4plex); K8(iTRAQ4plex)	0,0000	0,528	1		0,006	0,08426	31	0,000941694	2	1191,69841	4,06	12,43	0
P72937	Slr0670 protein OS=Synechocystis sp. (strain PCC 6803 / Kazusa) GN=s	102,35	11,68	1	3	3	4	0,580	4	34,3	291	32,7	5,54							
	A3	Sequence	# PSMs	# Proteins	# Protein Groups	Protein Group Accessions	Modifications	ΔCn	117/114	117/114 Count	117/114 Variability [%]	q-Value	PEP	IonScore	Exp Value	Charge	MH+ [Da]	ΔM [ppm]	RT [min]	# Missed Cleavages
	High	rPELLEIR	2	1	1	P72937	N-Term(iTRAQ4plex)	0,0000	0,580	2	7,2	0	0,0000189	57	1,87487E-06	3	1169,71396	1,40	24,54	0
	High	lFGSHTLSLAk	1	1	1	P72937	N-Term(iTRAQ4plex); K11(iTRAQ4plex)	0,0000	0,277	1		0	0,0004836	25	0,003603279	3	1461,86789	0,74	32,34	0
	High	qQFSPLVPNIITEVR	1	1	1	P72937	N-Term(iTRAQ4plex)	0,0000	0,866	1		0,009	0,1214	22	0,00595621	3	1885,06729	0,46	46,28	0
P73553	Large-conductance mechanosensitive channel OS=Synechocystis sp. (st	101,72	14,48	1	2	2	2	0,793	2	20,8	145	15,8	4,84							
	A3	Sequence	# PSMs	# Proteins	# Protein Groups	Protein Group Accessions	Modifications	ΔCn	117/114	117/114 Count	117/114 Variability [%]	q-Value	PEP	IonScore	Exp Value	Charge	MH+ [Da]	ΔM [ppm]	RT [min]	# Missed Cleavages
	High	lITTLENQQSSSQ	1	1	1	P73553	N-Term(iTRAQ4plex)	0,0000	0,911	1		0	5,155E-08	102	6,82292E-11	2	1592,82536	0,33	32,38	0
	High	dFkDFILR	1	1	1	P73553	N-Term(iTRAQ4plex); K3(iTRAQ4plex)	0,0000	0,691	1		0	0,003245	13	0,049200555	3	1341,77542	-1,13	36,50	1
Q55514	Slr0552 protein OS=Synechocystis sp. (strain PCC 6803 / Kazusa) GN=s	101,05	8,20	1	2	2	5	0,897	3	36,2	244	26,7	4,98							
	A3	Sequence	# PSMs	# Proteins	# Protein Groups	Protein Group Accessions	Modifications	ΔCn	117/114	117/114 Count	117/114 Variability [%]	q-Value	PEP	IonScore	Exp Value	Charge	MH+ [Da]	ΔM [ppm]	RT [min]	# Missed Cleavages
	High	aGSQLVFk	4	1	1	Q55514	N-Term(iTRAQ4plex); K8(iTRAQ4plex)	0,0000	1,024	2	19,8	0	0,00009455	50	1,01151E-05	2	1137,68816	0,98	24,18	0
	High	fLAEQQPQTVFG	1	1	1	Q55514	N-Term(iTRAQ4plex)	0,0000	0,708	1		0,003	0,04325	23	0,004886186	2	1508,78740	0,52	41,39	0
P74453	Sll0148 protein OS=Synechocystis sp. (strain PCC 6803 / Kazusa) GN=sl	100,90	3,67	1	2	2	4	0,957	3	16,8	735	78,5	5,91							
	A3	Sequence	# PSMs	# Proteins	# Protein Groups	Protein Group Accessions	Modifications	ΔCn	117/114	117/114 Count	117/114 Variability [%]	q-Value	PEP	IonScore	Exp Value	Charge	MH+ [Da]	ΔM [ppm]	RT [min]	# Missed Cleavages
	High	gLSVAAILRPDLk	3	1	1	P74453	N-Term(iTRAQ4plex); K13(iTRAQ4plex)	0,0000	1,012	2	8,4	0	1,6E-10	49	1,23586E-05	3	1641,03000	-0,13	40,42	0
	High	nLALTVALAHDLER	1	1	1	P74453	N-Term(iTRAQ4plex)	0,0000	0,795	1		0	0,00001105	43	4,94277E-05	3	1679,95621	0,03	42,04	0
P73668	UDP-N-acetylmuramoylalanine--D-glutamate ligase OS=Synechocystis s	100,76	6,19	1	2	2	3	0,943	3	3,4	452	49,0	5,76							
	A3	Sequence	# PSMs	# Proteins	# Protein Groups	Protein Group Accessions	Modifications	ΔCn	117/114	117/114 Count	117/114 Variability [%]	q-Value	PEP	IonScore	Exp Value	Charge	MH+ [Da]	ΔM [ppm]	RT [min]	# Missed Cleavages
	High	tTTTSLVQAIFQk	1	1	1	P73668	N-Term(iTRAQ4plex); K13(iTRAQ4plex)	0,0000	1,049	1		0	9,536E-07	49	1,19391E-05	3	1726,00010	0,67	44,58	0
	High	aITETLLTFTGVPHR	2	1	1	P73668	N-Term(iTRAQ4plex)	0,0000	0,932	2	1,7	0	2,365E-09	43	5,00035E-05	3	1800,01554	1,04	43,90	0
P73145	Thylakoid-associated single-stranded DNA-binding protein slr1034 OS=S	100,27	18,60	1	2	2	5	1,163	5	10,0	129	14,4	4,74							
	A3	Sequence	# PSMs	# Proteins	# Protein Groups	Protein Group Accessions	Modifications	ΔCn	117/114	117/114 Count	117/114 Variability [%]	q-Value	PEP	IonScore	Exp Value	Charge	MH+ [Da]	ΔM [ppm]	RT [min]	# Missed Cleavages
	High	vVGWGNLANTIk	3	1	1	P73145	N-Term(iTRAQ4plex); K12(iTRAQ4plex)	0,0000	1,163	3	10,0	0	0,0001346	44	4,37705E-05	3	1559,91556	0,46	39,16	0
	High	mNSFVLMATVIR	1	1	1	P73145	N-Term(iTRAQ4plex); M1(Oxidation)	0,0000	1,145	1		0	0,00005729	42	6,7293E-05	3	1541,83098	0,57	43,59	0
	High	mNSFVLMATVIR	1	1	1	P73145	N-Term(iTRAQ4plex)	0,0000	1,496	1		0	0,0004929	25	0,003566065	2	1525,83513	-0,04	48,72	0
P74211	Pyridoxine/pyridoxamine 5'-phosphate oxidase OS=Synechocystis sp. (s	99,62	18,22	1	3	3	5	0,943	5	8,0	214	24,6	6,34							
	A3	Sequence	# PSMs	# Proteins	# Protein Groups	Protein Group Accessions	Modifications	ΔCn	117/114	117/114 Count	117/114 Variability [%]	q-Value	PEP	IonScore	Exp Value	Charge	MH+ [Da]	ΔM [ppm]	RT [min]	# Missed Cleavages
	High	lQFNLLDGQWHR	2	1	1	P74211	N-Term(iTRAQ4plex)	0,0000	0,888	2	0,9	0	6,554E-10	51	8,01623E-06	3	1670,88974	0,81	42,22	0
	High	qYENQSIPRPPHWGGFR	1	1	1	P74211	N-Term(iTRAQ4plex)	0,0000	0,943	1		0	1,429E-08	25	0,003515362	4	2213,11220	0,02	31,30	0
	High	iEFWQGRPSR	2	1	1	P74211	N-Term(iTRAQ4plex)	0,0000	1,111	2	20,0	0	0,0003842	16	0,027540385	3	1419,76197	0,40	28,46	0
P73283	3-oxoacyl-[acyl-carrier-protein] synthase 2 OS=Synechocystis sp. (strain	98,63	5,53	1	2	2	4	1,258	3	2,0	416	44,0	5,73							
	A3	Sequence	# PSMs	# Proteins	# Protein Groups	Protein Group Accessions	Modifications	ΔCn	117/114	117/114 Count	117/114 Variability [%]	q-Value	PEP	IonScore	Exp Value	Charge	MH+ [Da]	ΔM [ppm]	RT [min]	# Missed Cleavages
	High	qALGNHAYNIAVSSTk	2	1	1	P73283	N-Term(iTRAQ4plex); K16(iTRAQ4plex)	0,0000	1,275	1		0	8,369E-10	52	6,83864E-06	3	1962,06662	0,96	24,46	0
	High	aIAWALk	2	1	1	P73283	N-Term(iTRAQ4plex); K7(iTRAQ4plex)	0,0000	1,222	2	4,3	0	0,0003096	37	0,000214873	2	1060,67558	-0,16	33,08	0
P73599	Uncharacterized protein sll1304 OS=Synechocystis sp. (strain PCC 6803	98,52	8,36	1	2	2	4	0,374	4	11,5	287	32,8	5,99							
	A3	Sequence	# PSMs	# Proteins	# Protein Groups	Protein Group Accessions	Modifications	ΔCn	117/114	117/114 Count	117/114 Variability [%]	q-Value	PEP	IonScore	Exp Value	Charge	MH+ [Da]	ΔM [ppm]	RT [min]	# Missed Cleavages
	High	gTPGSDSFAWGHWFk	3	1	1	P73599	N-Term(iTRAQ4plex); K15(iTRAQ4plex)	0,0000	0,365	3	16,3	0	5,074E-07	52	6,06694E-06	3	1967,96665	1,18	41,70	0
	High	fGVHTFIWk	1	1	1	P73599	N-Term(iTRAQ4plex); K9(iTRAQ4plex)	0,0000	0,382	1		0	0,0002267	27	0,001866251	3	1422,81598	1,64	37,05	0
P74607	UPF0296 protein ssl2874 OS=Synechocystis sp. (strain PCC 6803 / Kazu	98,38	26,97	1	2	2	3	0,744	2	21,7	89	9,9	9,60							
	A3	Sequence	# PSMs	# Proteins	# Protein Groups	Protein Group Accessions	Modifications	ΔCn	117/114	117/114 Count	117/114 Variability [%]	q-Value	PEP	IonScore	Exp Value	Charge	MH+ [Da]	ΔM [ppm]	RT [min]	# Missed Cleavages
	High	sQLIDATYGR	1	1	1	P74607	N-Term(iTRAQ4plex)	0,0000	0,644	1		0	0,000001657	55	3,27318E-06	2	1267,67815	1,45	24,63	0
	High	vVAIVSPESAPIkR	2	1	1	P74607	N-Term(iTRAQ4plex); K13(iTRAQ4plex)	0,0000	0,860	1		0	1,079E-07	49	1,15337E-05	3	1754,07932	0,81	28,42	1
P74702	Heat shock protein OS=Synechocystis sp. (strain PCC 6803 / Kazusa) Gl	98,05	3,80	1	2	2	6	0,949	3	1,9	658	75,0	5,03							
	A3	Sequence	# PSMs	# Proteins	# Protein Groups	Protein Group Accessions	Modifications	ΔCn	117/114	117/114 Count	117/114 Variability [%]	q-Value	PEP	IonScore	Exp Value	Charge	MH+ [Da]	ΔM [ppm]	RT [min]	# Missed Cleavages
	High	gNITIHTENIFPIIk	4	1	1	P74702	N-Term(iTRAQ4plex); K15(iTRAQ4plex)	0,0000	0,943	2	0,9	0	1,859E-08	52	6,66761E-06	3	1998,16092	-0,88	40,29	0
	High	aLNNPNINIk	2	1	1	P74702	N-Term(iTRAQ4plex); K10(iTRAQ4plex)	0,0000	1,709	1		0	0,00002939	19	0,011245273	2	1398,82866	-1,50	25,43	0
P73016	Enoyl-[acyl-carrier-protein] reductase [NADH] FabI OS=Synechocystis s	97,91	13,95	1	3	3	4	0,906	4	21,2	258	27,6	6,43							
	A3	Sequence	# PSMs	# Proteins	# Protein Groups	Protein Group Accessions	Modifications	ΔCn	117/114	117/114 Count	117/114 Variability [%]	q-Value	PEP	IonScore	Exp Value	Charge	MH+ [Da]	ΔM [ppm]	RT [min]	# Missed Cleavages
	High	hAFVTGIANNR	2	1	1	P73016	N-Term(iTRAQ4plex)	0,0000	0,906	2	3,8	0	0,000001249	45	3,22085E-05	2	1343,73088	0,59	18,29	0
	High	vIPNYNLmGVAk	1	1	1	P73016	N-Term(iTRAQ4plex); M8(Oxidation); K12(iTRAQ4plex)	0,0000	0,477	1		0	2,949E-07	33	0,000518764	3	1622,91886	0,61	33,10	0
	High	lDILIHcLAFADk	1	1	1	P73016	N-Term(iTRAQ4plex); C7(Carbamidomethyl); K13(iTRAQ	0,0000	1,171	1		0	0,00005494	22	0,00687021	3	1817,02604	1,44	49,20	0
P73921	Sll1979 protein OS=Synechocystis sp. (strain PCC 6803 / Kazusa) GN=sl	96,68	14,79	1	3	3	6	1,261	6	37,9	142	15,9	5,06							
	A3	Sequence	# PSMs	# Proteins	# Protein Groups	Protein Group Accessions	Modifications	ΔCn	117/114	117/114 Count	117/114 Variability [%]	q-Value	PEP	IonScore	Exp Value	Charge	MH+ [Da]	ΔM [ppm]	RT [min]	# Missed Cleavages
	High	iNYALAmLANk	1	1	1	P73921	N-Term(iTRAQ4plex); M7(Oxidation); K11(iTRAQ4plex)	0,0000	1,443	1		0	0,000006162	40	9,50539E-05	3	1525,86444	-0,43	37,56	0
	High	iNYALAmLANkk	1	1	1	P73921	N-Term(iTRAQ4plex); M7(Oxidation); K11(iTRAQ4plex);	0,0000	1,588	1		0	9,773E-07	39	0,000117752	3	1798,05979	-1,31	34,71	1
	High	iNYALAMLANkk	1	1	1	P73921	N-Term(iTRAQ4plex); K11(iTRAQ4plex); K12(iTRAQ4plex	0,0000	3,234	1		0	9,878E-07	31	0,000831706	3	1782,06498	-1,26	39,60	1
	High	iNYALAMLANk	2	1	1	P73921	N-Term(iTRAQ4plex); K11(iTRAQ4plex)	0,0000	0,932	2	5,9	0	0,0005285	22	0,006426433	3	1509,86954	-0,43	43,53	0
	High	iDGYHPQAR	1	1	1	P73921	N-Term(iTRAQ4plex)	0,0000	1,101	1		0	0,00282	16	0,022385665	3	1200,62390	-0,26	14,01	0
P52986	Homoserine dehydrogenase OS=Synechocystis sp. (strain PCC 6803 / K	96,37	5,08	1	2	2	4	0,801	3	1,1	433	45,5	5,01							
	A3	Sequence	# PSMs	# Proteins	# Protein Groups	Protein Group Accessions	Modifications	ΔCn	117/114	117/114 Count	117/114 Variability [%]	q-Value	PEP	IonScore	Exp Value	Charge	MH+ [Da]	ΔM [ppm]	RT [min]	# Missed Cleavages
	High	sLILQAIAHk	3	1	1	P52986	N-Term(iTRAQ4plex); K10(iTRAQ4plex)	0,0000	0,798	2	0,6	0	3,825E-08	55	3,09008E-06	3	1381,87809	0,80	34,61	0
	High	iAILASLGFGGR	1	1	1	P52986	N-Term(iTRAQ4plex)	0,0000	1,080	1		0	0,00002485	34	0,000395339	2	1318,79741	0,76	50,33	0
P74371	Chromophore lyase CpcT/CpeT OS=Synechocystis sp. (strain PCC 6803 /	96,32	11,73	1	2	2	3	1,479	3	5,8	196	22,6	5,33							
	A3	Sequence	# PSMs	# Proteins	# Protein Groups	Protein Group Accessions	Modifications	ΔCn	117/114	117/114 Count	117/114 Variability [%]	q-Value	PEP	IonScore	Exp Value	Charge	MH+ [Da]	ΔM [ppm]	RT [min]	# Missed Cleavages
	High	vWGSVAGAFHFVR	1	1	1	P74371	N-Term(iTRAQ4plex)	0,0000	1,115	1		0	4,937E-10	77	1,99972E-08	3	1576,85044	-0,08	42,90	0
	High	vAIRPLDQAk	2	1	1	P74371	N-Term(iTRAQ4plex); K10(iTRAQ4plex)	0,0000	1,508	2	2,9	0	0,00006496	23	0,004507856	3	1398,86744	0,20	22,01	0
P73862	Rubisco operon transcriptional regulator OS=Synechocystis sp. (strain P	95,81	11,39	1	4	4	7	0,154	6	9,1	316	35,7	8,50							
	A3	Sequence	# PSMs	# Proteins	# Protein Groups	Protein Group Accessions	Modifications	ΔCn	117/114	117/114 Count	117/114 Variability [%]	q-Value	PEP	IonScore	Exp Value	Charge	MH+ [Da]	ΔM [ppm]	RT [min]	# Missed Cleavages
	High	lAVQNLFHR	3	1	1	P73862	N-Term(iTRAQ4plex)	0,0000	0,145	2	2,8	0	0,000005181	48	1,7578E-05	3	1241,72632	2,24	33,55	0
	High	rWYVANLAGk	1	1	1	P73862	N-Term(iTRAQ4plex); K10(iTRAQ4plex)	0,0000	0,340	1		0	0,003034	26	0,002354887	3	1465,85730	3,74	29,28	1
	High	tVGLPLFEQIGk	1	1	1	P73862	N-Term(iTRAQ4plex); K12(iTRAQ4plex)	0,0000	0,158	1		0	0,0001439	13	0,048302544	3	1589,95530	2,98	44,62	0
	Medium	yFIPR	2	1	1	P73862	N-Term(iTRAQ4plex)	0,0000	0,158	2	8,3	0,027	0,3291	19	0,012358621	2	839,49254	3,50	26,40	0
Q59978	Protein GrpE OS=Synechocystis sp. (strain PCC 6803 / Kazusa) GN=grpl	95,80	20,88	1	5	5	6	1,266	6	5,3	249	27,6	4,68							
	A3	Sequence	# PSMs	# Proteins	# Protein Groups	Protein Group Accessions	Modifications	ΔCn	117/114	117/114 Count	117/114 Variability [%]	q-Value	PEP	IonScore	Exp Value	Charge	MH+ [Da]	ΔM [ppm]	RT [min]	# Missed Cleavages
	High	gYLLDDIVLR	1	1	1	Q59978	N-Term(iTRAQ4plex)	0,0000	1,261	1		0	0,00001335	37	0,00018534	2	1320,76579	1,05	45,09	0
	High	yVALAAEFDNFRk	1	1	1	Q59978	N-Term(iTRAQ4plex); K13(iTRAQ4plex)	0,0000	1,834	1		0	0,0001647	30	0,000948353	3	1831,99564	0,60	41,01	1
	High	tQIkPNSDGENQIHk	1	1	1	Q59978	N-Term(iTRAQ4plex); K4(iTRAQ4plex); K15(iTRAQ4plex)	0,0000	0,950	1		0	0,00133	22	0,007247518	3	2141,16959	0,96	15,80	0
	High	ekEEQAk	1	1	1	Q59978	N-Term(iTRAQ4plex); K2(iTRAQ4plex); K7(iTRAQ4plex)	0,0000	1,298	1		0	0,01097	12	0,062369176	3	1293,73468	-2,15	7,71	1
	High	sYQGVYk	2	1	1	Q59978	N-Term(iTRAQ4plex); K7(iTRAQ4plex)	0,0000	1,239	2	3,7	0,001	0,02082	31	0,000753304	2	1132,62505	0,86	16,03	0
P74565	RNA polymerase sigma factor SigA OS=Synechocystis sp. (strain PCC 68	95,62	6,59	1	3	3	4	1,945	4	50,0	425	49,9	6,27							
	A3	Sequence	# PSMs	# Proteins	# Protein Groups	Protein Group Accessions	Modifications	ΔCn	117/114	117/114 Count	117/114 Variability [%]	q-Value	PEP	IonScore	Exp Value	Charge	MH+ [Da]	ΔM [ppm]	RT [min]	# Missed Cleavages
	High	lVVSIAk	1	1	1	P74565	N-Term(iTRAQ4plex); K7(iTRAQ4plex)	0,0000	2,913	1		0	0,0003035	45	3,46713E-05	2	1017,68938	-1,66	28,45	0
	High	lPVHLYETISR	2	1	1	P74565	N-Term(iTRAQ4plex)	0,0000	1,414	2	3,6	0	0,00001037	34	0,000360554	3	1471,83881	-0,10	33,03	0
	High	kkPYTEDSIR	1	1	1	P74565	N-Term(iTRAQ4plex); K1(iTRAQ4plex); K2(iTRAQ4plex)	0,0000	2,612	1		0	6,409E-07	24	0,004139711	4	1668,96377	-0,45	12,84	1
P72662	Cysteine synthase OS=Synechocystis sp. (strain PCC 6803 / Kazusa) GN	95,41	10,15	1	2	2	2	0,594	2	12,0	325	34,7	5,40							
	A3	Sequence	# PSMs	# Proteins	# Protein Groups	Protein Group Accessions	Modifications	ΔCn	117/114	117/114 Count	117/114 Variability [%]	q-Value	PEP	IonScore	Exp Value	Charge	MH+ [Da]	ΔM [ppm]	RT [min]	# Missed Cleavages
	High	lkPGGTVVEGTAGNTGIGLAHIcN	1	1	1	P72662	N-Term(iTRAQ4plex); K2(iTRAQ4plex); C23(Carbamidom	0,0000	0,644	1		0	7,586E-10	56	2,81171E-06	4	2967,64492	1,29	30,86	0
	High	vVYQLLR	1	1	1	P72662	N-Term(iTRAQ4plex)	0,0000	0,548	1		0	0,0042	53	5,1047E-06	2	1034,64861	0,68	32,88	0
P73946	Slr1506 protein OS=Synechocystis sp. (strain PCC 6803 / Kazusa) GN=s	95,24	6,27	1	3	3	5	0,855	4	9,6	622	68,8	5,30							
	A3	Sequence	# PSMs	# Proteins	# Protein Groups	Protein Group Accessions	Modifications	ΔCn	117/114	117/114 Count	117/114 Variability [%]	q-Value	PEP	IonScore	Exp Value	Charge	MH+ [Da]	ΔM [ppm]	RT [min]	# Missed Cleavages
	High	fYLISQk	2	1	1	P73946	N-Term(iTRAQ4plex); K7(iTRAQ4plex)	0,0000	0,901	2	16,9	0	0,0002714	36	0,000275672	2	1186,70928	1,56	32,22	0
	High	vVAVLAANPVNSAIFGVSGLHk	1	1	1	P73946	N-Term(iTRAQ4plex); K22(iTRAQ4plex)	0,0000	0,798	1		0	0,000064	29	0,00135822	4	2451,43593	1,32	48,21	0
	High	fYVDVYRPQR	2	1	1	P73946	N-Term(iTRAQ4plex)	0,0000	0,908	1		0	0,000006272	28	0,001452011	3	1486,79322	0,59	29,22	0
P73355	Ferredoxin component OS=Synechocystis sp. (strain PCC 6803 / Kazusa	95,18	31,10	1	3	3	5	1,201	4	10,5	164	17,5	8,31							
	A3	Sequence	# PSMs	# Proteins	# Protein Groups	Protein Group Accessions	Modifications	ΔCn	117/114	117/114 Count	117/114 Variability [%]	q-Value	PEP	IonScore	Exp Value	Charge	MH+ [Da]	ΔM [ppm]	RT [min]	# Missed Cleavages
	High	vNQQAILLLNEGGTIHAVSNQcPł	1	1	1	P73355	N-Term(iTRAQ4plex); C22(Carbamidomethyl); K26(iTRAC	0,0000	1,159	1		0	4,526E-12	54	3,64729E-06	4	3142,70839	1,57	38,38	0
	High	mSAEkPLPVFPVR	2	1	1	P73355	N-Term(iTRAQ4plex); M1(Oxidation); K5(iTRAQ4plex)	0,0000	1,040	1		0	0,000001806	36	0,000235505	3	1775,01505	1,23	30,01	0
	High	ikDGAIVcPFHR	2	1	1	P73355	N-Term(iTRAQ4plex); K2(iTRAQ4plex); C8(Carbamidome	0,0000	1,289	2	5,2	0	0,000008328	28	0,001774067	4	1700,95302	1,24	25,67	1
P74592	ATP phosphoribosyltransferase regulatory subunit OS=Synechocystis sp.	94,99	7,98	1	3	3	4	0,938	4	7,5	401	44,3	5,07							
	A3	Sequence	# PSMs	# Proteins	# Protein Groups	Protein Group Accessions	Modifications	ΔCn	117/114	117/114 Count	117/114 Variability [%]	q-Value	PEP	IonScore	Exp Value	Charge	MH+ [Da]	ΔM [ppm]	RT [min]	# Missed Cleavages
	High	lGLRPELTASIAR	2	1	1	P74592	N-Term(iTRAQ4plex)	0,0000	0,968	2	14,7	0	0,00001088	43	5,30848E-05	3	1540,93045	0,80	31,70	0
	High	nLALQLFDLR	1	1	1	P74592	N-Term(iTRAQ4plex)	0,0000	0,971	1		0	0,0006316	37	0,000190972	2	1346,79277	1,09	49,62	0
	Medium	nPPEGYHGk	1	1	1	P74592	N-Term(iTRAQ4plex); K9(iTRAQ4plex)	0,0000	0,905	1		0,012	0,1564	11	0,08471689	3	1286,66416	-7,01	10,18	0
Q55988	6-phosphofructokinase 2 OS=Synechocystis sp. (strain PCC 6803 / Kazu	94,65	5,99	1	2	2	3	0,567	3	8,1	384	41,7	5,86							
	A3	Sequence	# PSMs	# Proteins	# Protein Groups	Protein Group Accessions	Modifications	ΔCn	117/114	117/114 Count	117/114 Variability [%]	q-Value	PEP	IonScore	Exp Value	Charge	MH+ [Da]	ΔM [ppm]	RT [min]	# Missed Cleavages
	High	lLATVFGIR	2	1	1	Q55988	N-Term(iTRAQ4plex)	0,0000	0,583	2	4,1	0	0,001452	56	2,4042E-06	2	1133,71782	1,30	47,06	0
	High	vEHADLkPIISIIk	1	1	1	Q55988	N-Term(iTRAQ4plex); K7(iTRAQ4plex); K14(iTRAQ4plex)	0,0000	0,329	1		0	1,245E-07	18	0,017138389	4	2008,25564	1,25	37,07	0
P73720	Putative OxPP cycle protein OpcA OS=Synechocystis sp. (strain PCC 680	93,74	6,95	1	3	3	3	0,607	3	10,5	475	52,0	5,11							
	A3	Sequence	# PSMs	# Proteins	# Protein Groups	Protein Group Accessions	Modifications	ΔCn	117/114	117/114 Count	117/114 Variability [%]	q-Value	PEP	IonScore	Exp Value	Charge	MH+ [Da]	ΔM [ppm]	RT [min]	# Missed Cleavages
	High	gNHAQALmYLGWVASR	1	1	1	P73720	N-Term(iTRAQ4plex); M8(Oxidation)	0,0000	0,956	1		0	7,321E-10	67	1,88352E-07	3	1933,98520	1,45	37,87	0
	High	qALQTAFETAHR	1	1	1	P73720	N-Term(iTRAQ4plex)	0,0000	0,607	1		0	0,00005865	23	0,005558659	3	1516,80155	1,74	24,08	0
	High	yVWWk	1	1	1	P73720	N-Term(iTRAQ4plex); K5(iTRAQ4plex)	0,0000	0,566	1		0,004	0,05043	30	0,001006862	2	1069,60869	1,28	35,94	0
P73390	Poly(3-hydroxyalkanoate) synthase OS=Synechocystis sp. (strain PCC 68	93,60	3,70	1	2	2	3	0,540	3	2,5	378	42,9	4,77							
	A3	Sequence	# PSMs	# Proteins	# Protein Groups	Protein Group Accessions	Modifications	ΔCn	117/114	117/114 Count	117/114 Variability [%]	q-Value	PEP	IonScore	Exp Value	Charge	MH+ [Da]	ΔM [ppm]	RT [min]	# Missed Cleavages
	High	sLVANLLk	1	1	1	P73390	N-Term(iTRAQ4plex); K8(iTRAQ4plex)	0,0000	0,531	1		0	0,000126	58	1,52746E-06	2	1145,75481	4,50	36,69	0
	High	lLNFLR	2	1	1	P73390	N-Term(iTRAQ4plex)	0,0000	0,549	2	2,5	0,002	0,02679	34	0,000576937	2	919,58617	1,72	41,59	0
P73057	Nucleoid-associated protein slr1847 OS=Synechocystis sp. (strain PCC 6	93,46	16,67	1	2	2	4	0,978	4	12,0	114	12,1	4,60							
	A3	Sequence	# PSMs	# Proteins	# Protein Groups	Protein Group Accessions	Modifications	ΔCn	117/114	117/114 Count	117/114 Variability [%]	q-Value	PEP	IonScore	Exp Value	Charge	MH+ [Da]	ΔM [ppm]	RT [min]	# Missed Cleavages
	High	aQQVQEGAk	2	1	1	P73057	N-Term(iTRAQ4plex); K9(iTRAQ4plex)	0,0000	0,978	2	4,7	0	0,000007538	39	0,000135425	2	1246,69829	-0,90	9,29	0
	High	ikELQEAFQk	2	1	1	P73057	N-Term(iTRAQ4plex); K2(iTRAQ4plex); K10(iTRAQ4plex)	0,0000	0,993	2	21,6	0	1,613E-08	37	0,000199054	3	1665,99039	0,23	30,65	1
P74571	Oligopeptidase A OS=Synechocystis sp. (strain PCC 6803 / Kazusa) GN=	92,91	5,75	1	3	3	3	0,749	3	26,1	713	80,2	5,11							
	A3	Sequence	# PSMs	# Proteins	# Protein Groups	Protein Group Accessions	Modifications	ΔCn	117/114	117/114 Count	117/114 Variability [%]	q-Value	PEP	IonScore	Exp Value	Charge	MH+ [Da]	ΔM [ppm]	RT [min]	# Missed Cleavages
	High	qINFSLLDLELHHR	1	1	1	P74571	N-Term(iTRAQ4plex)	0,0000	0,749	1		0	6,998E-07	45	3,24317E-05	4	1879,03261	1,02	41,52	0
	High	kmAPNVAAVEk	1	1	1	P74571	N-Term(iTRAQ4plex); K1(iTRAQ4plex); M2(Oxidation); K	0,0000	1,043	1		0	3,252E-07	38	0,000161425	3	1605,93802	1,33	17,09	1
	High	hGFEQVQPLVVGFISR	1	1	1	P74571	N-Term(iTRAQ4plex)	0,0000	0,630	1		0	0,000001521	37	0,000262972	3	1957,07993	1,15	43,43	0
P74379	Slr0443 protein OS=Synechocystis sp. (strain PCC 6803 / Kazusa) GN=s	92,18	16,80	1	4	4	7	1,239	5	11,3	125	14,6	4,58							
	A3	Sequence	# PSMs	# Proteins	# Protein Groups	Protein Group Accessions	Modifications	ΔCn	117/114	117/114 Count	117/114 Variability [%]	q-Value	PEP	IonScore	Exp Value	Charge	MH+ [Da]	ΔM [ppm]	RT [min]	# Missed Cleavages
	High	gLYGLLR	4	1	1	P74379	N-Term(iTRAQ4plex)	0,0000	1,287	2	5,6	0	0,008602	39	0,000122735	2	935,58049	1,07	35,07	0
	High	rGLYGLLR	1	1	1	P74379	N-Term(iTRAQ4plex)	0,0000	1,220	1		0	0,0004308	32	0,000572756	3	1091,68137	0,69	28,07	1
	High	vLESLHR	1	1	1	P74379	N-Term(iTRAQ4plex)	0,0000	0,926	1		0	0,003456	32	0,000647098	3	997,59208	0,95	18,51	0
	High	lSHFIk	1	1	1	P74379	N-Term(iTRAQ4plex); K6(iTRAQ4plex)	0,0000	1,361	1		0,009	0,1225	13	0,078530443	3	1032,64524	0,77	21,64	0
Q55367	Cyanate hydratase OS=Synechocystis sp. (strain PCC 6803 / Kazusa) GN	91,76	17,45	1	4	4	6	0,734	5	32,5	149	16,5	5,21							
	A3	Sequence	# PSMs	# Proteins	# Protein Groups	Protein Group Accessions	Modifications	ΔCn	117/114	117/114 Count	117/114 Variability [%]	q-Value	PEP	IonScore	Exp Value	Charge	MH+ [Da]	ΔM [ppm]	RT [min]	# Missed Cleavages
	High	gLGPVVPTDPLIYR	1	1	1	Q55367	N-Term(iTRAQ4plex)	0,0000	0,909	1		0	0,000002795	59	1,22172E-06	2	1640,95122	1,19	41,67	0
	High	eVIHEk	1	1	1	Q55367	N-Term(iTRAQ4plex); K6(iTRAQ4plex)	0,0000	0,734	1		0,002	0,02471	11	0,080347063	3	1042,61148	-1,97	10,70	0
	High	fLPYk	3	1	1	Q55367	N-Term(iTRAQ4plex); K5(iTRAQ4plex)	0,0000	0,568	2	23,9	0,006	0,07018	11	0,084522047	2	955,58452	-1,04	27,44	0
	Medium	fLPYkk	1	1	1	Q55367	N-Term(iTRAQ4plex); K5(iTRAQ4plex); K6(iTRAQ4plex)	0,0000	1,392	1		0,015	0,1852	14	0,036472876	3	1227,77872	-3,12	22,73	1
P26522	NAD(P)H-quinone oxidoreductase subunit 1 OS=Synechocystis sp. (strai	91,75	7,53	1	3	3	6	0,412	6	13,0	372	40,5	5,06							
	A3	Sequence	# PSMs	# Proteins	# Protein Groups	Protein Group Accessions	Modifications	ΔCn	117/114	117/114 Count	117/114 Variability [%]	q-Value	PEP	IonScore	Exp Value	Charge	MH+ [Da]	ΔM [ppm]	RT [min]	# Missed Cleavages
	High	ySLLGGLR	3	1	1	P26522	N-Term(iTRAQ4plex)	0,0000	0,397	3	2,3	0	0,00003076	35	0,00030267	2	1022,61260	1,05	34,91	0
	High	kISAAAQQR	1	1	1	P26522	N-Term(iTRAQ4plex); K1(iTRAQ4plex)	0,0000	0,811	1		0	0,0004214	32	0,000659461	3	1260,76187	-0,64	9,20	1
	High	lVFkEDVVPAk	2	1	1	P26522	N-Term(iTRAQ4plex); K4(iTRAQ4plex); K11(iTRAQ4plex)	0,0000	0,397	2	24,2	0	0,00001299	27	0,001936288	3	1677,03202	0,51	31,31	1
P23350	50S ribosomal protein L10 OS=Synechocystis sp. (strain PCC 6803 / Kaz	91,47	18,50	1	4	4	9	1,460	7	25,8	173	18,7	8,60							
	A3	Sequence	# PSMs	# Proteins	# Protein Groups	Protein Group Accessions	Modifications	ΔCn	117/114	117/114 Count	117/114 Variability [%]	q-Value	PEP	IonScore	Exp Value	Charge	MH+ [Da]	ΔM [ppm]	RT [min]	# Missed Cleavages
	High	gLQAHVDkE	4	1	1	P23350	N-Term(iTRAQ4plex); K8(iTRAQ4plex)	0,0000	1,615	2	10,4	0	0,0005431	30	0,001068981	3	1284,71427	-0,61	13,21	1
	High	lRPLGGTck	1	1	1	P23350	N-Term(iTRAQ4plex); C8(Carbamidomethyl); K9(iTRAQ4p	0,0000	1,800	1		0	0,00196	14	0,047087975	3	1289,75782	-1,88	15,72	0
	Medium	eVPASVAR	2	1	1	P23350	N-Term(iTRAQ4plex)	0,0000	1,441	2	2,0	0,013	0,1698	20	0,01091365	2	972,55962	0,12	14,81	0
	Medium	iALGIk	2	1	1	P23350	N-Term(iTRAQ4plex); K6(iTRAQ4plex)	0,0000	0,995	2	27,4	0,015	0,1914	30	0,000926766	2	902,62547	-2,51	26,85	0
Q55758	Bifunctional protein PyrR OS=Synechocystis sp. (strain PCC 6803 / Kazu	90,58	14,61	1	2	2	4	0,922	3	3,0	178	19,9	9,17							
	A3	Sequence	# PSMs	# Proteins	# Protein Groups	Protein Group Accessions	Modifications	ΔCn	117/114	117/114 Count	117/114 Variability [%]	q-Value	PEP	IonScore	Exp Value	Charge	MH+ [Da]	ΔM [ppm]	RT [min]	# Missed Cleavages
	High	aALNAVTEYGRPQVIR	3	1	1	Q55758	N-Term(iTRAQ4plex)	0,0000	0,932	2	1,5	0	7,182E-09	43	5,32964E-05	3	1902,06882	0,52	31,19	0
	High	tkIPLSLTGk	1	1	1	Q55758	N-Term(iTRAQ4plex); K2(iTRAQ4plex); K10(iTRAQ4plex)	0,0000	0,674	1		0	4,088E-07	42	7,01407E-05	3	1489,96909	0,86	27,00	1
Q55673	UDP-N-acetylglucosamine 1-carboxyvinyltransferase OS=Synechocystis	90,40	4,79	1	2	2	4	1,444	4	15,5	438	46,6	5,97							
	A3	Sequence	# PSMs	# Proteins	# Protein Groups	Protein Group Accessions	Modifications	ΔCn	117/114	117/114 Count	117/114 Variability [%]	q-Value	PEP	IonScore	Exp Value	Charge	MH+ [Da]	ΔM [ppm]	RT [min]	# Missed Cleavages
	High	aSFFVIGPLLSR	3	1	1	Q55673	N-Term(iTRAQ4plex)	0,0000	1,359	3	12,8	0	0,0001259	48	1,68256E-05	2	1450,85564	1,19	50,80	0
	High	lQHVAELQR	1	1	1	Q55673	N-Term(iTRAQ4plex)	0,0000	1,535	1		0	0,001045	17	0,021036331	3	1237,71531	1,56	18,69	0
P73274	Leucine--tRNA ligase OS=Synechocystis sp. (strain PCC 6803 / Kazusa) (	90,14	3,45	1	3	3	4	1,015	4	15,6	869	98,2	5,29							
	A3	Sequence	# PSMs	# Proteins	# Protein Groups	Protein Group Accessions	Modifications	ΔCn	117/114	117/114 Count	117/114 Variability [%]	q-Value	PEP	IonScore	Exp Value	Charge	MH+ [Da]	ΔM [ppm]	RT [min]	# Missed Cleavages
	High	fAVRPETLVTNEPLTk	1	1	1	P73274	N-Term(iTRAQ4plex); K16(iTRAQ4plex)	0,0000	0,809	1		0	1,521E-10	45	2,96463E-05	3	2103,20652	0,58	34,88	0
	High	lRDWLISR	1	1	1	P73274	N-Term(iTRAQ4plex)	0,0000	1,085	1		0	0,00001923	29	0,00130674	3	1202,71296	0,25	32,93	1
	High	mFILFk	2	1	1	P73274	N-Term(iTRAQ4plex); K6(iTRAQ4plex)	0,0000	1,055	2	15,6	0,001	0,01729	37	0,000181958	2	1086,66448	1,89	43,42	0
P72683	Geranylgeranyl pyrophosphate synthase OS=Synechocystis sp. (strain P	90,11	7,62	1	2	2	3	1,201	3	10,0	302	32,4	5,11							
	A3	Sequence	# PSMs	# Proteins	# Protein Groups	Protein Group Accessions	Modifications	ΔCn	117/114	117/114 Count	117/114 Variability [%]	q-Value	PEP	IonScore	Exp Value	Charge	MH+ [Da]	ΔM [ppm]	RT [min]	# Missed Cleavages
	High	ySLLAGGk	1	1	1	P72683	N-Term(iTRAQ4plex); K8(iTRAQ4plex)	0,0000	1,201	1		0	0,00001396	57	2,13781E-06	2	1096,66167	1,08	25,65	0
	High	tDITPETLTFIHTHk	2	1	1	P72683	N-Term(iTRAQ4plex); K15(iTRAQ4plex)	0,0000	0,951	2	46,9	0	0,000001569	32	0,0005662	4	2042,11831	1,08	33,02	0
P74675	Sll1542 protein OS=Synechocystis sp. (strain PCC 6803 / Kazusa) GN=sl	89,89	8,67	1	2	2	3	1,089	3	1,7	300	34,8	4,69							
	A3	Sequence	# PSMs	# Proteins	# Protein Groups	Protein Group Accessions	Modifications	ΔCn	117/114	117/114 Count	117/114 Variability [%]	q-Value	PEP	IonScore	Exp Value	Charge	MH+ [Da]	ΔM [ppm]	RT [min]	# Missed Cleavages
	High	rLGELSNEQIGQIR	2	1	1	P74675	N-Term(iTRAQ4plex)	0,0000	1,095	2	0,9	0	1,048E-08	40	0,000107199	3	1756,97843	-0,15	27,83	1
	High	rLSLIETILANk	1	1	1	P74675	N-Term(iTRAQ4plex); K12(iTRAQ4plex)	0,0000	0,742	1		0	0,0001381	36	0,000249442	3	1659,03934	-0,86	45,80	1
P73042	Uncharacterized protein slr1764 OS=Synechocystis sp. (strain PCC 6803	89,71	23,46	1	4	4	5	0,836	4	41,1	179	19,4	4,73							
	A3	Sequence	# PSMs	# Proteins	# Protein Groups	Protein Group Accessions	Modifications	ΔCn	117/114	117/114 Count	117/114 Variability [%]	q-Value	PEP	IonScore	Exp Value	Charge	MH+ [Da]	ΔM [ppm]	RT [min]	# Missed Cleavages
	High	gQSLSLTkPDGSSIQk	1	1	1	P73042	N-Term(iTRAQ4plex); K8(iTRAQ4plex); K16(iTRAQ4plex)	0,0000	0,537	1		0	9,712E-11	37	0,000199154	3	2078,17728	-2,18	25,11	0
	High	kVAFFNDk	1	1	1	P73042	N-Term(iTRAQ4plex); K1(iTRAQ4plex); K8(iTRAQ4plex)	0,0000	0,914	1		0	0,0000282	34	0,000388124	3	1400,82135	-3,48	23,77	1
	High	kQAFNLVNNAk	2	1	1	P73042	N-Term(iTRAQ4plex); K1(iTRAQ4plex); K11(iTRAQ4plex)	0,0000	0,764	1		0	0,0002152	28	0,001737681	3	1678,99161	-2,91	22,66	1
	High	tAISGIk	1	1	1	P73042	N-Term(iTRAQ4plex); K7(iTRAQ4plex)	0,0000	2,511	1		0,002	0,02537	30	0,001004546	2	977,61949	-3,97	17,77	0
Q6ZEQ0	Non-heme chloroperoxidase OS=Synechocystis sp. (strain PCC 6803 / K	89,44	14,60	1	4	4	7	0,815	6	48,8	274	30,1	5,60							
	A3	Sequence	# PSMs	# Proteins	# Protein Groups	Protein Group Accessions	Modifications	ΔCn	117/114	117/114 Count	117/114 Variability [%]	q-Value	PEP	IonScore	Exp Value	Charge	MH+ [Da]	ΔM [ppm]	RT [min]	# Missed Cleavages
	High	iYPGGSHSLGDTSk	1	1	1	Q6ZEQ0	N-Term(iTRAQ4plex); K14(iTRAQ4plex)	0,0000	0,319	1		0	0,00003744	36	0,000247725	3	1706,89396	-0,73	19,84	0
	High	aVLMGAVPPLMLk	2	1	1	Q6ZEQ0	N-Term(iTRAQ4plex); K13(iTRAQ4plex)	0,0000	0,429	2	161,1	0	0,00006769	30	0,001056745	3	1627,98630	-1,19	46,50	0
	High	aVLmGAVPPLmLk	1	1	1	Q6ZEQ0	N-Term(iTRAQ4plex); M4(Oxidation); M11(Oxidation); K1	0,0000	1,349	1		0,006	0,0692	16	0,027037716	3	1659,97629	-1,07	38,41	0
	High	sTITTk	1	1	1	Q6ZEQ0	N-Term(iTRAQ4plex); K6(iTRAQ4plex)	0,0000	0,908	1		0,007	0,08824	23	0,005128614	2	938,57292	-3,36	11,45	0
	Medium	aAFLADR	1	1	1	Q6ZEQ0	N-Term(iTRAQ4plex)	0,0000	0,732	1		0,045	0,5527	16	0,025702183	2	907,51073	-1,21	21,67	0
P73848	3-hydroxyacyl-[acyl-carrier-protein] dehydratase FabZ OS=Synechocysti	89,05	19,51	1	3	3	3	2,302	3	34,8	164	17,9	5,53							
	A3	Sequence	# PSMs	# Proteins	# Protein Groups	Protein Group Accessions	Modifications	ΔCn	117/114	117/114 Count	117/114 Variability [%]	q-Value	PEP	IonScore	Exp Value	Charge	MH+ [Da]	ΔM [ppm]	RT [min]	# Missed Cleavages
	High	fFAFAGIDGVR	1	1	1	P73848	N-Term(iTRAQ4plex)	0,0000	1,753	1		0	0,0002741	45	2,85082E-05	2	1343,72283	-0,06	45,68	0
	High	rPVVPGDQLImTVELQSFk	1	1	1	P73848	N-Term(iTRAQ4plex); M11(Oxidation); K19(iTRAQ4plex)	0,0000	2,302	1		0	6,752E-09	41	7,11164E-05	4	2461,37490	0,86	43,92	0
	High	gkFFAFAGIDGVR	1	1	1	P73848	N-Term(iTRAQ4plex); K2(iTRAQ4plex)	0,0000	2,891	1		0	0,0005189	28	0,001530982	3	1672,93924	-1,29	39,56	1
P73719	Sll1628 protein OS=Synechocystis sp. (strain PCC 6803 / Kazusa) GN=sl	88,87	6,77	1	2	2	3	1,116	3	12,2	384	43,1	4,46							
	A3	Sequence	# PSMs	# Proteins	# Protein Groups	Protein Group Accessions	Modifications	ΔCn	117/114	117/114 Count	117/114 Variability [%]	q-Value	PEP	IonScore	Exp Value	Charge	MH+ [Da]	ΔM [ppm]	RT [min]	# Missed Cleavages
	High	fQTTVLASPSPNLVLAAR	1	1	1	P73719	N-Term(iTRAQ4plex)	0,0000	1,116	1		0	0,00000002	62	5,91521E-07	3	2029,15793	0,79	42,50	0
	High	fFQQLDHR	2	1	1	P73719	N-Term(iTRAQ4plex)	0,0000	0,702	2	96,1	0	0,00004855	27	0,002157595	3	1234,64527	0,25	25,84	0
P74534	Sll1338 protein OS=Synechocystis sp. (strain PCC 6803 / Kazusa) GN=sl	88,72	17,11	1	2	2	3	0,804	3	15,2	187	20,2	4,70							
	A3	Sequence	# PSMs	# Proteins	# Protein Groups	Protein Group Accessions	Modifications	ΔCn	117/114	117/114 Count	117/114 Variability [%]	q-Value	PEP	IonScore	Exp Value	Charge	MH+ [Da]	ΔM [ppm]	RT [min]	# Missed Cleavages
	High	tDFYSAAHPTWLTR	2	1	1	P74534	N-Term(iTRAQ4plex)	0,0000	0,880	2	13,5	0	1,634E-08	48	1,54515E-05	3	1809,90635	1,24	34,59	0
	High	sAPAPVAPAPAPAPmPIR	1	1	1	P74534	N-Term(iTRAQ4plex); M15(Oxidation)	0,0000	0,726	1		0	0,00001607	42	5,63599E-05	3	1871,03373	0,35	26,46	0
Q55445	Putative methyl-accepting chemotaxis protein sll0041 OS=Synechocystis	88,16	3,20	1	3	3	6	0,924	4	17,2	1000	108,3	4,79							
	A3	Sequence	# PSMs	# Proteins	# Protein Groups	Protein Group Accessions	Modifications	ΔCn	117/114	117/114 Count	117/114 Variability [%]	q-Value	PEP	IonScore	Exp Value	Charge	MH+ [Da]	ΔM [ppm]	RT [min]	# Missed Cleavages
	High	gNLLGLLIAHQcSGPR	2	1	1	Q55445	N-Term(iTRAQ4plex); C12(Carbamidomethyl)	0,0000	1,237	1		0	0,000000186	51	8,54472E-06	3	1850,02054	0,95	41,92	0
	High	lVFYYHPR	3	1	1	Q55445	N-Term(iTRAQ4plex)	0,0000	0,924	2	16,7	0	0,0001923	28	0,001702041	3	1238,68082	0,46	30,37	0
	High	qLAQQANR	1	1	1	Q55445	N-Term(iTRAQ4plex)	0,0000	0,820	1		0	0,001309	16	0,025466544	2	1072,59807	0,06	10,37	0
P73588	Sll1315 protein OS=Synechocystis sp. (strain PCC 6803 / Kazusa) GN=sl	86,12	4,83	1	2	2	3	0,824	2	3,5	352	38,9	7,23							
	A3	Sequence	# PSMs	# Proteins	# Protein Groups	Protein Group Accessions	Modifications	ΔCn	117/114	117/114 Count	117/114 Variability [%]	q-Value	PEP	IonScore	Exp Value	Charge	MH+ [Da]	ΔM [ppm]	RT [min]	# Missed Cleavages
	High	lYQSIGVAR	1	1	1	P73588	N-Term(iTRAQ4plex)	0,0000	0,804	1		0	0,00000118	58	1,56664E-06	2	1150,67131	1,05	26,44	0
	High	iISLTTGk	2	1	1	P73588	N-Term(iTRAQ4plex); K8(iTRAQ4plex)	0,0000	0,843	1		0	0,007186	39	0,000125594	2	1120,71965	1,47	27,77	0
P72719	Bacterioferritin comigratory protein OS=Synechocystis sp. (strain PCC 68	85,75	15,22	1	2	2	3	0,705	3	6,0	184	20,4	4,86							
	A3	Sequence	# PSMs	# Proteins	# Protein Groups	Protein Group Accessions	Modifications	ΔCn	117/114	117/114 Count	117/114 Variability [%]	q-Value	PEP	IonScore	Exp Value	Charge	MH+ [Da]	ΔM [ppm]	RT [min]	# Missed Cleavages
	High	fLGVRPATHSEEVLAR	2	1	1	P72719	N-Term(iTRAQ4plex)	0,0000	0,691	2	3,0	0	7,964E-09	41	7,74408E-05	3	1926,06919	0,70	26,86	0
	High	hTYVIDPEGILR	1	1	1	P72719	N-Term(iTRAQ4plex)	0,0000	0,821	1		0	0,000009594	35	0,000314912	3	1556,85953	2,68	34,93	0
Q6ZEQ5	Sll5075 protein OS=Synechocystis sp. (strain PCC 6803 / Kazusa) GN=sl	85,43	15,83	1	2	2	6	0,875	4	9,9	139	15,6	5,15							
	A3	Sequence	# PSMs	# Proteins	# Protein Groups	Protein Group Accessions	Modifications	ΔCn	117/114	117/114 Count	117/114 Variability [%]	q-Value	PEP	IonScore	Exp Value	Charge	MH+ [Da]	ΔM [ppm]	RT [min]	# Missed Cleavages
	High	mkEDFGFTHIALFAk	4	1	1	Q6ZEQ5	N-Term(iTRAQ4plex); M1(Oxidation); K2(iTRAQ4plex); K	0,0000	0,819	2	3,2	0	4,739E-11	48	1,60683E-05	3	2203,19583	0,54	39,42	1
	High	mkEDFGFTHIALFAk	1	1	1	Q6ZEQ5	N-Term(iTRAQ4plex); K2(iTRAQ4plex); K15(iTRAQ4plex)	0,0000	0,982	1		0	2,9E-09	29	0,00214528	4	2187,19912	-0,28	42,16	1
	High	vVWLSDk	1	1	1	Q6ZEQ5	N-Term(iTRAQ4plex); K7(iTRAQ4plex)	0,0000	0,915	1		0,002	0,02292	21	0,008491805	2	1134,67449	-1,46	31,44	0
P73824	Hydroperoxy fatty acid reductase gpx2 OS=Synechocystis sp. (strain PC	84,39	17,53	1	3	3	3	0,697	3	2,3	154	16,6	4,81							
	A3	Sequence	# PSMs	# Proteins	# Protein Groups	Protein Group Accessions	Modifications	ΔCn	117/114	117/114 Count	117/114 Variability [%]	q-Value	PEP	IonScore	Exp Value	Charge	MH+ [Da]	ΔM [ppm]	RT [min]	# Missed Cleavages

	High	tkYDVDFTLLEk	1	1	1	P73824	N-Term(iTRAQ4plex); K2(iTRAQ4plex); K12(iTRAQ4plex)	0,0000	0,657	1		0	1,428E-07	54	4,73579E-06	3	1904,07566	0,80	37,54	1
	High	vVLFVNVASk	1	1	1	P73824	N-Term(iTRAQ4plex); K10(iTRAQ4plex)	0,0000	0,697	1		0	0,00003704	44	4,02689E-05	2	1363,85564	0,33	37,97	0
	High	fLIGR	1	1	1	P73824	N-Term(iTRAQ4plex)	0,0000	0,707	1		0,008	0,1093	12	0,078507295	2	749,47960	0,73	25,52	0
P72762	NADH-glutamate synthase small subunit OS=Synechocystis sp. (strain P	84,30	5,67	1	3	3	5	1,060	4	16,1	494	54,1	5,52							
	A3	Sequence	# PSMs	# Proteins	# Protein Groups	Protein Group Accessions	Modifications	ΔCn	117/114	117/114 Count	117/114 Variability [%]	q-Value	PEP	IonScore	Exp Value	Charge	MH+ [Da]	ΔM [ppm]	RT [min]	# Missed Cleavages
	High	sYLTTATk	3	1	1	P72762	N-Term(iTRAQ4plex); K8(iTRAQ4plex)	0,0000	1,181	2	11,1	0	0,0001874	43	4,66627E-05	2	1172,67668	0,14	18,33	0
	High	aGHWVTVYER	1	1	1	P72762	N-Term(iTRAQ4plex)	0,0000	0,787	1		0	0,002845	20	0,01091365	3	1361,70902	0,54	23,65	0
	High	tPGPNFISAk	1	1	1	P72762	N-Term(iTRAQ4plex); K10(iTRAQ4plex)	0,0000	1,026	1		0,001	0,0169	34	0,000442773	2	1319,75554	-0,50	26,22	0
P73043	Glutamate decarboxylase OS=Synechocystis sp. (strain PCC 6803 / Kazu	83,48	4,71	1	2	2	3	1,138	3	6,6	467	53,0	5,41							
	A3	Sequence	# PSMs	# Proteins	# Protein Groups	Protein Group Accessions	Modifications	ΔCn	117/114	117/114 Count	117/114 Variability [%]	q-Value	PEP	IonScore	Exp Value	Charge	MH+ [Da]	ΔM [ppm]	RT [min]	# Missed Cleavages
	High	aIAYFASQPDHkPk	2	1	1	P73043	N-Term(iTRAQ4plex); K12(iTRAQ4plex); K14(iTRAQ4plex	0,0000	1,097	2	12,0	0	2,169E-07	40	0,000106162	4	2005,12588	1,36	23,55	0
	High	gWLVPAYR	1	1	1	P73043	N-Term(iTRAQ4plex)	0,0000	1,138	1		0,004	0,05451	35	0,000340385	2	1105,62786	0,31	33,24	0
Q55200	Protein SphX OS=Synechocystis sp. (strain PCC 6803 / Kazusa) GN=sph	83,13	5,06	1	2	2	4	0,446	3	17,2	336	36,8	4,77							
	A3	Sequence	# PSMs	# Proteins	# Protein Groups	Protein Group Accessions	Modifications	ΔCn	117/114	117/114 Count	117/114 Variability [%]	q-Value	PEP	IonScore	Exp Value	Charge	MH+ [Da]	ΔM [ppm]	RT [min]	# Missed Cleavages
	High	rIWEPAAEk	1	1	1	Q55200	N-Term(iTRAQ4plex); K9(iTRAQ4plex)	0,0000	0,315	1		0	0,00005818	39	0,000135198	3	1387,79880	3,71	21,25	1
	High	sLTVEELkR	3	1	1	Q55200	N-Term(iTRAQ4plex); K8(iTRAQ4plex)	0,0000	0,472	2	8,6	0	0,001811	37	0,000193642	3	1362,82257	2,24	22,78	1
Q55139	Glutathione S-transferase OS=Synechocystis sp. (strain PCC 6803 / Kazu	82,93	19,02	1	4	4	5	0,667	5	21,2	184	20,4	6,57							
	A3	Sequence	# PSMs	# Proteins	# Protein Groups	Protein Group Accessions	Modifications	ΔCn	117/114	117/114 Count	117/114 Variability [%]	q-Value	PEP	IonScore	Exp Value	Charge	MH+ [Da]	ΔM [ppm]	RT [min]	# Missed Cleavages
	High	lLQSLEk	1	1	1	Q55139	N-Term(iTRAQ4plex); K7(iTRAQ4plex)	0,0000	0,768	1		0	0,0008297	43	5,08124E-05	2	1118,70513	2,47	30,45	0
	High	sPFILGEk	1	1	1	Q55139	N-Term(iTRAQ4plex); K8(iTRAQ4plex)	0,0000	0,667	1		0	0,0003186	37	0,000219771	2	1178,70439	1,72	30,49	0
	High	qAPYLAINPFGk	2	1	1	Q55139	N-Term(iTRAQ4plex); K12(iTRAQ4plex)	0,0000	0,326	2	11,8	0	0,000004997	16	0,025291233	3	1606,92057	0,62	40,82	0
	High	lYGAPQSR	1	1	1	Q55139	N-Term(iTRAQ4plex)	0,0000	0,691	1		0,003	0,03813	26	0,002248899	2	1035,57048	0,09	15,89	0
Q55162	Sll0051 protein OS=Synechocystis sp. (strain PCC 6803 / Kazusa) GN=sl	82,81	11,32	1	2	2	3	0,544	3	44,0	159	17,8	4,54							
	A3	Sequence	# PSMs	# Proteins	# Protein Groups	Protein Group Accessions	Modifications	ΔCn	117/114	117/114 Count	117/114 Variability [%]	q-Value	PEP	IonScore	Exp Value	Charge	MH+ [Da]	ΔM [ppm]	RT [min]	# Missed Cleavages
	High	tGGPFGVVIVk	2	1	1	Q55162	N-Term(iTRAQ4plex); K11(iTRAQ4plex)	0,0000	0,472	2	21,3	0	0,00001403	53	4,90874E-06	2	1361,83806	-1,09	35,43	0
	Medium	qAIAIMR	1	1	1	Q55162	N-Term(iTRAQ4plex)	0,0000	1,001	1		0,017	0,22	24	0,003890183	2	946,56108	-1,48	22,05	0
Q55512	Aspartate-semialdehyde dehydrogenase OS=Synechocystis sp. (strain P	81,24	7,10	1	2	2	2	0,963	2	11,4	338	36,6	6,04							
	A3	Sequence	# PSMs	# Proteins	# Protein Groups	Protein Group Accessions	Modifications	ΔCn	117/114	117/114 Count	117/114 Variability [%]	q-Value	PEP	IonScore	Exp Value	Charge	MH+ [Da]	ΔM [ppm]	RT [min]	# Missed Cleavages
	High	kGAALNAVQIAELLVER	1	1	1	Q55512	N-Term(iTRAQ4plex); K1(iTRAQ4plex)	0,0000	1,039	1		0	4,732E-09	79	1,30008E-08	3	2083,25150	1,76	50,22	1
	High	iTATcVR	1	1	1	Q55512	N-Term(iTRAQ4plex); C5(Carbamidomethyl)	0,0000	0,892	1		0,003	0,04393	16	0,029593021	2	964,53929	2,74	15,71	0
P73058	Histidinol dehydrogenase 1 OS=Synechocystis sp. (strain PCC 6803 / Ka	80,57	6,45	1	2	2	2	1,111	2	2,7	434	46,5	5,53							
	A3	Sequence	# PSMs	# Proteins	# Protein Groups	Protein Group Accessions	Modifications	ΔCn	117/114	117/114 Count	117/114 Variability [%]	q-Value	PEP	IonScore	Exp Value	Charge	MH+ [Da]	ΔM [ppm]	RT [min]	# Missed Cleavages
	High	iTPLPTVGLYVPR	1	1	1	P73058	N-Term(iTRAQ4plex)	0,0000	1,091	1		0	7,626E-07	49	1,38029E-05	2	1569,94914	0,39	43,67	0
	High	lGGVQALAAIAYGTk	1	1	1	P73058	N-Term(iTRAQ4plex); K15(iTRAQ4plex)	0,0000	1,132	1		0	0,0000874	45	3,16935E-05	3	1721,01743	-1,52	45,23	0
P72845	Sll1188 protein OS=Synechocystis sp. (strain PCC 6803 / Kazusa) GN=sl	80,52	15,24	1	2	2	4	0,673	4	23,9	164	18,8	5,00							
	A3	Sequence	# PSMs	# Proteins	# Protein Groups	Protein Group Accessions	Modifications	ΔCn	117/114	117/114 Count	117/114 Variability [%]	q-Value	PEP	IonScore	Exp Value	Charge	MH+ [Da]	ΔM [ppm]	RT [min]	# Missed Cleavages
	High	nVVTGIYFLmTk	2	1	1	P72845	N-Term(iTRAQ4plex); M10(Oxidation); K12(iTRAQ4plex)	0,0000	0,626	2	39,0	0	1,006E-07	43	4,60225E-05	3	1689,94791	-0,55	41,66	0
	High	nVVTGIYFLMTk	1	1	1	P72845	N-Term(iTRAQ4plex); K12(iTRAQ4plex)	0,0000	0,587	1		0	0,0001098	26	0,002552525	3	1673,95310	-0,49	50,22	0
	High	lELLPHPEGGFYk	1	1	1	P72845	N-Term(iTRAQ4plex); K13(iTRAQ4plex)	0,0000	0,772	1		0	0,00003285	25	0,003388208	3	1787,99356	0,05	37,80	0
P27319	Flavodoxin OS=Synechocystis sp. (strain PCC 6803 / Kazusa) GN=isiB P	80,43	11,18	1	2	2	5	0,162	5	53,4	170	18,8	4,30							
	A3	Sequence	# PSMs	# Proteins	# Protein Groups	Protein Group Accessions	Modifications	ΔCn	117/114	117/114 Count	117/114 Variability [%]	q-Value	PEP	IonScore	Exp Value	Charge	MH+ [Da]	ΔM [ppm]	RT [min]	# Missed Cleavages
	High	tWVSEIkPILQS	4	1	1	P27319	N-Term(iTRAQ4plex); K7(iTRAQ4plex)	0,0000	0,137	4	26,1	0	0,009139	36	0,0002594	2	1688,99089	4,92	44,14	0
	Medium	iSGLGGk	1	1	1	P27319	N-Term(iTRAQ4plex); K7(iTRAQ4plex)	0,0000	0,351	1		0,039	0,4849	29	0,001792124	2	919,58824	7,31	17,93	0
P73804	Sll1961 protein OS=Synechocystis sp. (strain PCC 6803 / Kazusa) GN=sl	80,11	3,61	1	2	2	3	0,998	3	30,0	388	42,9	5,95							
	A3	Sequence	# PSMs	# Proteins	# Protein Groups	Protein Group Accessions	Modifications	ΔCn	117/114	117/114 Count	117/114 Variability [%]	q-Value	PEP	IonScore	Exp Value	Charge	MH+ [Da]	ΔM [ppm]	RT [min]	# Missed Cleavages
	High	sIAILk	1	1	1	P73804	N-Term(iTRAQ4plex); K6(iTRAQ4plex)	0,0000	0,819	1		0	0,01062	42	6,41165E-05	2	932,63719	-1,18	25,63	0
	High	vLVTVPQR	2	1	1	P73804	N-Term(iTRAQ4plex)	0,0000	1,103	2	14,8	0	0,0001365	36	0,000258803	2	1055,66924	-0,14	25,15	0
P72765	Slr1772 protein OS=Synechocystis sp. (strain PCC 6803 / Kazusa) GN=s	78,88	7,16	1	2	2	3	0,819	3	20,3	377	41,6	5,74							
	A3	Sequence	# PSMs	# Proteins	# Protein Groups	Protein Group Accessions	Modifications	ΔCn	117/114	117/114 Count	117/114 Variability [%]	q-Value	PEP	IonScore	Exp Value	Charge	MH+ [Da]	ΔM [ppm]	RT [min]	# Missed Cleavages
	High	gLGmHMLYLTATDFGPR	1	1	1	P72765	N-Term(iTRAQ4plex); M4(Oxidation)	0,0000	0,938	1		0	0,000001422	54	3,98997E-06	3	2040,01865	1,10	40,75	0
	High	lVVHHGPEYR	2	1	1	P72765	N-Term(iTRAQ4plex)	0,0000	0,750	2	13,1	0	0,00001278	25	0,002850821	3	1350,73871	-0,89	14,15	0
P52276	Asparagine--tRNA ligase OS=Synechocystis sp. (strain PCC 6803 / Kazus	78,71	7,79	1	3	3	3	0,813	3	3,0	462	52,7	5,43							
	A3	Sequence	# PSMs	# Proteins	# Protein Groups	Protein Group Accessions	Modifications	ΔCn	117/114	117/114 Count	117/114 Variability [%]	q-Value	PEP	IonScore	Exp Value	Charge	MH+ [Da]	ΔM [ppm]	RT [min]	# Missed Cleavages
	High	yGSVPHAGFGLGFER	1	1	1	P52276	N-Term(iTRAQ4plex)	0,0000	0,797	1		0	0,00001053	39	0,000124443	3	1737,88401	0,59	34,48	0
	High	nAcATAIHQFFQER	1	1	1	P52276	N-Term(iTRAQ4plex); C3(Carbamidomethyl)	0,0000	0,873	1		0	2,327E-07	34	0,000442558	3	1836,89481	0,86	32,76	0
	High	hSFEFLR	1	1	1	P52276	N-Term(iTRAQ4plex)	0,0000	0,813	1		0	0,0001522	32	0,000609495	3	1079,57572	0,23	25,75	0
P49057	GMP synthase [glutamine-hydrolyzing] OS=Synechocystis sp. (strain PC	77,58	7,93	1	4	4	5	0,885	5	2,7	542	61,0	6,24							
	A3	Sequence	# PSMs	# Proteins	# Protein Groups	Protein Group Accessions	Modifications	ΔCn	117/114	117/114 Count	117/114 Variability [%]	q-Value	PEP	IonScore	Exp Value	Charge	MH+ [Da]	ΔM [ppm]	RT [min]	# Missed Cleavages
	High	hPFPGPGLAIR	1	1	1	P49057	N-Term(iTRAQ4plex)	0,0000	0,892	1		0	0,00001702	40	9,24634E-05	3	1305,75583	0,74	27,42	0
	High	rHPFPGPGLAIR	2	1	1	P49057	N-Term(iTRAQ4plex)	0,0000	0,856	2	7,6	0	0,001394	29	0,001413815	4	1461,85830	1,58	23,26	1
	High	aLFGVQFHPEVVHSVGGIALIR	1	1	1	P49057	N-Term(iTRAQ4plex)	0,0000	0,885	1		0	0,00004139	21	0,007111644	4	2490,41005	-0,09	46,39	0
	High	tYAHPVVLR	1	1	1	P49057	N-Term(iTRAQ4plex)	0,0000	0,797	1		0,002	0,02212	21	0,008891397	3	1199,70389	1,80	18,47	0
P73545	Uncharacterized thylakoid-associated protein ssl2501 OS=Synechocystis	76,32	24,72	1	2	2	2	0,746	2	15,3	89	10,6	4,67							
	A3	Sequence	# PSMs	# Proteins	# Protein Groups	Protein Group Accessions	Modifications	ΔCn	117/114	117/114 Count	117/114 Variability [%]	q-Value	PEP	IonScore	Exp Value	Charge	MH+ [Da]	ΔM [ppm]	RT [min]	# Missed Cleavages
	High	lTDTFETSLk	1	1	1	P73545	N-Term(iTRAQ4plex); K10(iTRAQ4plex)	0,0000	0,673	1		0	1,241E-07	53	4,61286E-06	2	1442,80144	2,30	33,12	0
	High	kFFSTWmESLPk	1	1	1	P73545	N-Term(iTRAQ4plex); K1(iTRAQ4plex); M7(Oxidation); K	0,0000	0,826	1		0	0,00000475	37	0,000314291	3	1949,05899	1,16	37,21	1
P74328	Uncharacterized tRNA/rRNA methyltransferase slr0955 OS=Synechocyst	75,55	4,43	1	2	2	3	1,648	3	13,8	384	42,8	9,64							
	A3	Sequence	# PSMs	# Proteins	# Protein Groups	Protein Group Accessions	Modifications	ΔCn	117/114	117/114 Count	117/114 Variability [%]	q-Value	PEP	IonScore	Exp Value	Charge	MH+ [Da]	ΔM [ppm]	RT [min]	# Missed Cleavages
	High	iWITSHLR	2	1	1	P74328	N-Term(iTRAQ4plex)	0,0000	1,726	2	6,9	0	0,000002757	47	2,08435E-05	3	1169,69117	-0,01	29,92	0
	High	sGQkPPFGk	1	1	1	P74328	N-Term(iTRAQ4plex); K4(iTRAQ4plex); K9(iTRAQ4plex)	0,0000	1,289	1		0	0,0002751	22	0,006760363	3	1377,81937	-1,53	14,24	0
Q55541	Slr0333 protein OS=Synechocystis sp. (strain PCC 6803 / Kazusa) GN=s	75,52	27,36	1	3	3	4	2,289	4	21,8	106	11,4	8,48							
	A3	Sequence	# PSMs	# Proteins	# Protein Groups	Protein Group Accessions	Modifications	ΔCn	117/114	117/114 Count	117/114 Variability [%]	q-Value	PEP	IonScore	Exp Value	Charge	MH+ [Da]	ΔM [ppm]	RT [min]	# Missed Cleavages
	High	sSAPQYGLPGDkVEVIk	1	1	1	Q55541	N-Term(iTRAQ4plex); K12(iTRAQ4plex); K17(iTRAQ4plex	0,0000	4,387	1		0	2,961E-07	34	0,000377546	3	2220,26200	0,87	30,78	1
	High	aVGWIR	2	1	1	Q55541	N-Term(iTRAQ4plex)	0,0000	2,289	2	16,8	0,002	0,03245	36	0,000310022	2	845,51171	0,33	24,25	0
	High	tQPTIk	1	1	1	Q55541	N-Term(iTRAQ4plex); K6(iTRAQ4plex)	0,0000	1,916	1		0,006	0,07779	22	0,005945513	2	975,60710	-0,63	12,52	0
Q55700	ATP-dependent zinc metalloprotease FtsH 2 OS=Synechocystis sp. (stra	75,04	5,90	1	4	5	6	1,386	5	10,0	627	68,5	5,41							
	A3	Sequence	# PSMs	# Proteins	# Protein Groups	Protein Group Accessions	Modifications	ΔCn	117/114	117/114 Count	117/114 Variability [%]	q-Value	PEP	IonScore	Exp Value	Charge	MH+ [Da]	ΔM [ppm]	RT [min]	# Missed Cleavages
	High	qLAEQGHQmAR	1	1	1	Q55700	N-Term(iTRAQ4plex); M9(Oxidation)	0,0000	1,482	1		0	0,005623	23	0,005558659	3	1428,71518	1,20	8,53	0
	High	kEILEVHAR	1	1	1	Q55700	N-Term(iTRAQ4plex); K1(iTRAQ4plex)	0,0000	0,881	1		0	0,005963	21	0,007567806	4	1382,83547	-0,27	16,56	1
	High	vTLIPR	2	1	1	Q55700	N-Term(iTRAQ4plex)	0,0000	1,357	2	3,1	0,002	0,0318	32	0,000685441	2	842,55834	0,36	24,84	0
	High	vRDLFk	1	1	1	Q55700	N-Term(iTRAQ4plex); K6(iTRAQ4plex)	0,0000	1,811	1		0,003	0,03654	20	0,010715193	3	1065,66465	-1,19	22,61	1
	Medium	tLLAk	1	2	2	Q55700;P73437	N-Term(iTRAQ4plex); K5(iTRAQ4plex)	0,0000				0,046	0,5599	10	0,097268004	2	833,56615	-4,47	18,02	0
P33779	Global nitrogen regulator OS=Synechocystis sp. (strain PCC 6803 / Kazu	74,52	8,89	1	2	2	3	0,994	3	2,4	225	25,0	7,05							
	A3	Sequence	# PSMs	# Proteins	# Protein Groups	Protein Group Accessions	Modifications	ΔCn	117/114	117/114 Count	117/114 Variability [%]	q-Value	PEP	IonScore	Exp Value	Charge	MH+ [Da]	ΔM [ppm]	RT [min]	# Missed Cleavages
	High	lSHQAIAEAIGSTR	1	1	1	P33779	N-Term(iTRAQ4plex)	0,0000	0,613	1		0	3,279E-10	48	1,5775E-05	3	1597,87845	0,35	27,80	0
	High	vYFLLk	2	1	1	P33779	N-Term(iTRAQ4plex); K6(iTRAQ4plex)	0,0000	1,002	2	1,2	0,001	0,0206	30	0,001054314	2	1070,68596	0,68	38,97	0
P73534	Pyruvate kinase 2 OS=Synechocystis sp. (strain PCC 6803 / Kazusa) GN	73,93	7,45	1	4	4	5	0,971	5	11,1	591	62,9	6,64							
	A3	Sequence	# PSMs	# Proteins	# Protein Groups	Protein Group Accessions	Modifications	ΔCn	117/114	117/114 Count	117/114 Variability [%]	q-Value	PEP	IonScore	Exp Value	Charge	MH+ [Da]	ΔM [ppm]	RT [min]	# Missed Cleavages
	High	lGVPVIVGFk	1	1	1	P73534	N-Term(iTRAQ4plex); K10(iTRAQ4plex)	0,0000	1,158	1		0	2,573E-07	50	1,07639E-05	2	1316,85564	0,89	42,83	0
	High	qLIQAGATTFR	1	1	1	P73534	N-Term(iTRAQ4plex)	0,0000	0,766	1		0	0,0002109	22	0,005834048	2	1349,76677	0,71	28,90	0
	High	lNFSHGDHAYHQQSIR	2	1	1	P73534	N-Term(iTRAQ4plex)	0,0000	1,007	2	5,8	0	0,000003503	16	0,023603152	5	2054,00846	0,55	18,77	0
	Medium	sVPVIAk	1	1	1	P73534	N-Term(iTRAQ4plex); K7(iTRAQ4plex)	0,0000	0,971	1		0,017	0,2137	24	0,003681035	2	1001,66283	3,06	21,36	0
Q54735	Carbonic anhydrase OS=Synechocystis sp. (strain PCC 6803 / Kazusa) G	73,42	12,77	1	3	3	3	1,187	3	27,9	274	30,7	6,23							
	A3	Sequence	# PSMs	# Proteins	# Protein Groups	Protein Group Accessions	Modifications	ΔCn	117/114	117/114 Count	117/114 Variability [%]	q-Value	PEP	IonScore	Exp Value	Charge	MH+ [Da]	ΔM [ppm]	RT [min]	# Missed Cleavages
	High	gDLSLHGWIYR	1	1	1	Q54735	N-Term(iTRAQ4plex)	0,0000	1,429	1		0	0,000000288	39	0,000125305	3	1460,77695	0,17	35,23	0
	High	dLFEQLSHGQHPR	1	1	1	Q54735	N-Term(iTRAQ4plex)	0,0000	1,187	1		0	0,00006565	31	0,000792447	4	1707,86941	0,60	28,61	0
	High	nLQTYPAIHSR	1	1	1	Q54735	N-Term(iTRAQ4plex)	0,0000	0,536	1		0	0,00002316	30	0,001181388	3	1443,78229	-0,15	19,03	0
P20804	Acyl carrier protein OS=Synechocystis sp. (strain PCC 6803 / Kazusa) G	72,75	20,78	1	2	2	4	1,074	4	2,0	77	8,6	4,07							
	A3	Sequence	# PSMs	# Proteins	# Protein Groups	Protein Group Accessions	Modifications	ΔCn	117/114	117/114 Count	117/114 Variability [%]	q-Value	PEP	IonScore	Exp Value	Charge	MH+ [Da]	ΔM [ppm]	RT [min]	# Missed Cleavages
	High	aVEHIESk	3	1	1	P20804	N-Term(iTRAQ4plex); K8(iTRAQ4plex)	0,0000	1,075	3	0,0	0	0,0002307	55	3,28073E-06	2	1200,68340	0,59	12,05	0
	High	mNQEIFEk	1	1	1	P20804	N-Term(iTRAQ4plex); M1(Oxidation); K8(iTRAQ4plex)	0,0000	0,930	1		0	0,0003404	28	0,00160683	2	1342,69121	-0,26	20,81	0
P74737	Protein RecA OS=Synechocystis sp. (strain PCC 6803 / Kazusa) GN=rec/	72,46	6,21	1	2	2	2	1,052	2	12,8	354	37,8	5,22							
	A3	Sequence	# PSMs	# Proteins	# Protein Groups	Protein Group Accessions	Modifications	ΔCn	117/114	117/114 Count	117/114 Variability [%]	q-Value	PEP	IonScore	Exp Value	Charge	MH+ [Da]	ΔM [ppm]	RT [min]	# Missed Cleavages
	High	kGTEGEYGIR	1	1	1	P74737	N-Term(iTRAQ4plex); K1(iTRAQ4plex)	0,0000	0,965	1		0	0,000004674	47	1,83219E-05	3	1397,76267	-0,05	13,54	1
	High	sGcLVIFLNQLR	1	1	1	P74737	N-Term(iTRAQ4plex); C3(Carbamidomethyl)	0,0000	1,146	1		0	0,00008178	38	0,000154515	2	1563,88140	1,01	48,88	0
P48944	30S ribosomal protein S14 OS=Synechocystis sp. (strain PCC 6803 / Kaz	72,30	30,00	1	3	3	6	1,237	4	8,3	100	11,8	10,74							
	A3	Sequence	# PSMs	# Proteins	# Protein Groups	Protein Group Accessions	Modifications	ΔCn	117/114	117/114 Count	117/114 Variability [%]	q-Value	PEP	IonScore	Exp Value	Charge	MH+ [Da]	ΔM [ppm]	RT [min]	# Missed Cleavages
	High	eWAHQGLLPGVTk	4	1	1	P48944	N-Term(iTRAQ4plex); K13(iTRAQ4plex)	0,0000	1,402	2	11,9	0	8,678E-07	36	0,000331826	3	1723,97531	1,10	30,62	0
	High	rEALkEEFR	1	1	1	P48944	N-Term(iTRAQ4plex); K5(iTRAQ4plex)	0,0000	1,158	1		0	0,00001326	24	0,004314893	4	1465,83693	0,24	16,25	2
	High	cQVTGRPR	1	1	1	P48944	N-Term(iTRAQ4plex); C1(Carbamidomethyl)	0,0000	1,182	1		0	0,008906	12	0,057407681	3	1117,60196	0,22	8,30	0
Q6ZEC5	Slr7073 protein OS=Synechocystis sp. (strain PCC 6803 / Kazusa) GN=s	71,92	19,30	1	2	2	4	1,338	4	30,1	114	12,4	5,25							
	A3	Sequence	# PSMs	# Proteins	# Protein Groups	Protein Group Accessions	Modifications	ΔCn	117/114	117/114 Count	117/114 Variability [%]	q-Value	PEP	IonScore	Exp Value	Charge	MH+ [Da]	ΔM [ppm]	RT [min]	# Missed Cleavages
	High	rLENQEFLNR	1	1	1	Q6ZEC5	N-Term(iTRAQ4plex)	0,0000	0,583	1		0	3,442E-07	38	0,00014157	3	1462,78864	0,20	20,48	1
	High	lTGQIk	3	1	1	Q6ZEC5	N-Term(iTRAQ4plex); K6(iTRAQ4plex)	0,0000	1,590	3	7,8	0	0,004429	28	0,001761854	2	947,61070	-2,22	18,20	0
Q55542	Slr0334 protein OS=Synechocystis sp. (strain PCC 6803 / Kazusa) GN=s	71,37	13,64	1	3	3	3	7,599	3	53,7	110	11,6	7,85							
	A3	Sequence	# PSMs	# Proteins	# Protein Groups	Protein Group Accessions	Modifications	ΔCn	117/114	117/114 Count	117/114 Variability [%]	q-Value	PEP	IonScore	Exp Value	Charge	MH+ [Da]	ΔM [ppm]	RT [min]	# Missed Cleavages
	High	kPAYYEAk	1	1	1	Q55542	N-Term(iTRAQ4plex); K1(iTRAQ4plex); K8(iTRAQ4plex)	0,0000	10,673	1		0	0,000001395	39	0,000126756	3	1401,80976	-0,33	14,63	0
	High	aQLIVk	1	1	1	Q55542	N-Term(iTRAQ4plex); K6(iTRAQ4plex)	0,0000	4,912	1		0	0,009663	35	0,000296463	2	959,64867	-0,55	19,96	0
	High	kAQLIVk	1	1	1	Q55542	N-Term(iTRAQ4plex); K1(iTRAQ4plex); K7(iTRAQ4plex)	0,0000	7,599	1		0	0,00003624	23	0,004841389	3	1231,84632	0,07	16,70	1
P48959	50S ribosomal protein L35 OS=Synechocystis sp. (strain PCC 6803 / Kaz	70,84	32,84	1	2	2	3	2,024	3	23,4	67	7,9	11,36							
	A3	Sequence	# PSMs	# Proteins	# Protein Groups	Protein Group Accessions	Modifications	ΔCn	117/114	117/114 Count	117/114 Variability [%]	q-Value	PEP	IonScore	Exp Value	Charge	MH+ [Da]	ΔM [ppm]	RT [min]	# Missed Cleavages
	High	rLSNLALVHEADEk	2	1	1	P48959	N-Term(iTRAQ4plex); K14(iTRAQ4plex)	0,0000	2,188	2	11,6	0	0,00001197	41	7,62026E-05	4	1883,05605	-1,54	24,35	1
	High	fRPTGSGk	1	1	1	P48959	N-Term(iTRAQ4plex); K8(iTRAQ4plex)	0,0000	1,703	1		0,001	0,01398	17	0,02018227	3	1137,65906	-2,49	10,34	0
Q55390	Sll0553 protein OS=Synechocystis sp. (strain PCC 6803 / Kazusa) GN=sl	70,49	8,12	1	4	4	4	0,872	4	15,6	345	38,2	5,38							
	A3	Sequence	# PSMs	# Proteins	# Protein Groups	Protein Group Accessions	Modifications	ΔCn	117/114	117/114 Count	117/114 Variability [%]	q-Value	PEP	IonScore	Exp Value	Charge	MH+ [Da]	ΔM [ppm]	RT [min]	# Missed Cleavages
	High	aEYAALSFVR	1	1	1	Q55390	N-Term(iTRAQ4plex)	0,0000	0,795	1		0	0,0003742	35	0,000343534	2	1270,69121	-0,03	36,14	0
	High	vLAPDLLGWGR	1	1	1	Q55390	N-Term(iTRAQ4plex)	0,0000	0,770	1		0	0,000508	24	0,003715096	2	1340,78118	0,31	45,73	0
	High	rFLALLQ	1	1	1	Q55390	N-Term(iTRAQ4plex)	0,0000	0,980	1		0,002	0,02257	32	0,000661291	2	1004,63732	-0,04	38,87	1
	Medium	fLALLQ	1	1	1	Q55390	N-Term(iTRAQ4plex)	0,0000	0,957	1		0,016	0,1999	19	0,013519792	2	848,53679	0,66	49,04	0
Q55737	Slr0416 protein OS=Synechocystis sp. (strain PCC 6803 / Kazusa) GN=s	70,39	14,38	1	2	2	4	0,932	3	24,7	146	16,3	5,12							
	A3	Sequence	# PSMs	# Proteins	# Protein Groups	Protein Group Accessions	Modifications	ΔCn	117/114	117/114 Count	117/114 Variability [%]	q-Value	PEP	IonScore	Exp Value	Charge	MH+ [Da]	ΔM [ppm]	RT [min]	# Missed Cleavages
	High	iLLLHLIk	3	1	1	Q55737	N-Term(iTRAQ4plex); K8(iTRAQ4plex)	0,0000	1,012	2	12,2	0	0,00001482	45	3,28829E-05	2	1250,88078	0,41	42,19	0
	High	kAGGTYLSSEELR	1	1	1	Q55737	N-Term(iTRAQ4plex); K1(iTRAQ4plex)	0,0000	0,690	1		0	0,00001209	27	0,001990536	3	1698,92704	0,31	20,75	1
P74576	Biosynthetic arginine decarboxylase 1 OS=Synechocystis sp. (strain PCC	70,06	4,03	1	2	2	3	0,632	3	27,9	695	78,2	5,22							
	A3	Sequence	# PSMs	# Proteins	# Protein Groups	Protein Group Accessions	Modifications	ΔCn	117/114	117/114 Count	117/114 Variability [%]	q-Value	PEP	IonScore	Exp Value	Charge	MH+ [Da]	ΔM [ppm]	RT [min]	# Missed Cleavages
	High	nIGLPLLLR	2	1	1	P74576	N-Term(iTRAQ4plex)	0,0000	0,576	2	13,8	0	0,000002059	41	7,48118E-05	2	1152,76042	1,63	44,23	0
	High	sVLELHPLIEVHQPGTPPR	1	1	1	P74576	N-Term(iTRAQ4plex)	0,0000	1,064	1		0	0,000000631	32	0,000654636	4	2263,27041	1,06	35,03	0
Q55709	Protein translocase subunit SecA OS=Synechocystis sp. (strain PCC 680	69,47	5,90	1	5	5	5	1,207	5	13,6	932	106,9	5,12							
	A3	Sequence	# PSMs	# Proteins	# Protein Groups	Protein Group Accessions	Modifications	ΔCn	117/114	117/114 Count	117/114 Variability [%]	q-Value	PEP	IonScore	Exp Value	Charge	MH+ [Da]	ΔM [ppm]	RT [min]	# Missed Cleavages
	High	gVHVVTVNDYLAR	1	1	1	Q55709	N-Term(iTRAQ4plex)	0,0000	1,102	1		0	0,000006498	46	2,38215E-05	3	1586,87894	1,13	30,08	0
	High	vYNLQVTITPTNRPSSR	1	1	1	Q55709	N-Term(iTRAQ4plex)	0,0000	1,207	1		0	5,619E-07	37	0,000259526	3	2090,15018	1,27	30,01	0
	High	tFFHEEVRk	1	1	1	Q55709	N-Term(iTRAQ4plex); K9(iTRAQ4plex)	0,0000	2,526	1		0	0,002149	12	0,073272895	4	1480,81594	0,56	19,42	1
	High	akELFLk	1	1	1	Q55709	N-Term(iTRAQ4plex); K2(iTRAQ4plex); K7(iTRAQ4plex)	0,0000	1,677	1		0	0,00112	11	0,074125904	3	1280,82676	-2,73	24,15	1
	High	vAGLMNMFR	1	1	1	Q55709	N-Term(iTRAQ4plex)	0,0000	1,194	1		0	0,001791	11	0,083362361	2	1182,62590	1,22	43,40	0
P72817	Universal stress protein Sll1654 OS=Synechocystis sp. (strain PCC 6803	69,38	14,01	1	2	2	2	1,183	2	18,7	157	16,8	5,52							
	A3	Sequence	# PSMs	# Proteins	# Protein Groups	Protein Group Accessions	Modifications	ΔCn	117/114	117/114 Count	117/114 Variability [%]	q-Value	PEP	IonScore	Exp Value	Charge	MH+ [Da]	ΔM [ppm]	RT [min]	# Missed Cleavages
	High	iHQSQLILLSVVEk	1	1	1	P72817	N-Term(iTRAQ4plex); K14(iTRAQ4plex)	0,0000	1,340	1		0	8,124E-08	49	1,27049E-05	3	1895,15604	-0,43	41,56	0
	High	tILFPLDR	1	1	1	P72817	N-Term(iTRAQ4plex)	0,0000	1,044	1		0	0,0006455	33	0,000452898	2	1118,66911	0,05	39,15	0
Q55558	Sll0172 protein OS=Synechocystis sp. (strain PCC 6803 / Kazusa) GN=sl	69,23	17,11	1	2	2	2	1,509	2	17,3	152	16,3	5,38							
	A3	Sequence	# PSMs	# Proteins	# Protein Groups	Protein Group Accessions	Modifications	ΔCn	117/114	117/114 Count	117/114 Variability [%]	q-Value	PEP	IonScore	Exp Value	Charge	MH+ [Da]	ΔM [ppm]	RT [min]	# Missed Cleavages
	High	vLDVFNVk	1	1	1	Q55558	N-Term(iTRAQ4plex); K8(iTRAQ4plex)	0,0000	1,694	1		0	0,000006099	44	3,69803E-05	2	1221,74541	0,69	39,05	0
	High	lTPVTTDGASYPTQLIAR	1	1	1	Q55558	N-Term(iTRAQ4plex)	0,0000	1,343	1		0	9,006E-10	38	0,000161053	3	2048,11649	0,97	38,49	0
P72584	Magnesium-protoporphyrin IX monomethyl ester [oxidative] cyclase 1 O	69,00	13,41	1	4	4	6	1,233	6	25,9	358	42,1	7,12							
	A3	Sequence	# PSMs	# Proteins	# Protein Groups	Protein Group Accessions	Modifications	ΔCn	117/114	117/114 Count	117/114 Variability [%]	q-Value	PEP	IonScore	Exp Value	Charge	MH+ [Da]	ΔM [ppm]	RT [min]	# Missed Cleavages
	High	kLPIFASNGWQFIk	1	1	1	P72584	N-Term(iTRAQ4plex); K1(iTRAQ4plex); K14(iTRAQ4plex)	0,0000	1,426	1		0	0,00000433	40	9,8168E-05	3	2081,23221	2,39	45,82	1
	High	lYLMkPIAVDQLAGAVR	1	1	1	P72584	N-Term(iTRAQ4plex); K5(iTRAQ4plex)	0,0000	1,372	1		0	0,005346	26	0,002264488	3	2146,26926	1,46	45,49	0
	High	aQPHTLNDWk	1	1	1	P72584	N-Term(iTRAQ4plex); K10(iTRAQ4plex)	0,0000	3,113	1		0	0,0001687	19	0,012167162	3	1497,80729	1,35	19,85	0
	High	lYLmkPIAVDQLAGAVR	2	1	1	P72584	N-Term(iTRAQ4plex); M4(Oxidation); K5(iTRAQ4plex)	0,0000	1,059	2	6,7	0	0,001098	18	0,016329392	3	2162,26444	1,57	41,66	0
	High	yTFFkPk	1	1	1	P72584	N-Term(iTRAQ4plex); K5(iTRAQ4plex); K7(iTRAQ4plex)	0,0000	0,793	1		0	0,0001001	14	0,039807968	3	1362,81433	-0,18	26,35	0
P73942	Proline--tRNA ligase OS=Synechocystis sp. (strain PCC 6803 / Kazusa) G	68,59	5,47	1	3	3	5	0,981	4	2,6	603	66,9	5,47							
	A3	Sequence	# PSMs	# Proteins	# Protein Groups	Protein Group Accessions	Modifications	ΔCn	117/114	117/114 Count	117/114 Variability [%]	q-Value	PEP	IonScore	Exp Value	Charge	MH+ [Da]	ΔM [ppm]	RT [min]	# Missed Cleavages
	High	gIEVGHIFQLGTk	2	1	1	P73942	N-Term(iTRAQ4plex); K13(iTRAQ4plex)	0,0000	1,127	1		0	0,000001034	37	0,000218761	3	1686,97922	0,62	37,36	0
	High	fkDSELIGIPYR	1	1	1	P73942	N-Term(iTRAQ4plex); K2(iTRAQ4plex)	0,0000	0,948	1		0	0,00002319	35	0,000314753	3	1725,97885	0,60	35,41	1
	Medium	fRDEIRPR	2	1	1	P73942	N-Term(iTRAQ4plex)	0,0000	0,981	2	0,1	0,015	0,1868	10	0,129402704	3	1232,69886	0,63	14,77	1
P73825	Acetyl coenzyme A acetyltransferase (Thiolase) OS=Synechocystis sp. (s	68,27	7,33	1	3	3	5	0,889	4	5,1	409	43,3	6,10							
	A3	Sequence	# PSMs	# Proteins	# Protein Groups	Protein Group Accessions	Modifications	ΔCn	117/114	117/114 Count	117/114 Variability [%]	q-Value	PEP	IonScore	Exp Value	Charge	MH+ [Da]	ΔM [ppm]	RT [min]	# Missed Cleavages
	High	kGTQVLASDEGIR	2	1	1	P73825	N-Term(iTRAQ4plex); K1(iTRAQ4plex)	0,0000	0,889	2	4,7	0	0,0000162	42	6,32368E-05	3	1661,94669	2,51	19,15	1
	High	yQLkPLAk	2	1	1	P73825	N-Term(iTRAQ4plex); K4(iTRAQ4plex); K8(iTRAQ4plex)	0,0000	0,807	1		0	0,0001791	20	0,010091832	3	1392,89737	2,49	22,49	0
	High	aGHGQLIPR	1	1	1	P73825	N-Term(iTRAQ4plex)	0,0000	0,923	1		0,002	0,02685	15	0,037471221	3	1092,64295	3,17	13,26	0
Q55360	Sll0888 protein OS=Synechocystis sp. (strain PCC 6803 / Kazusa) GN=sl	68,25	12,11	1	2	2	3	0,620	2	8,4	190	21,9	4,94							
	A3	Sequence	# PSMs	# Proteins	# Protein Groups	Protein Group Accessions	Modifications	ΔCn	117/114	117/114 Count	117/114 Variability [%]	q-Value	PEP	IonScore	Exp Value	Charge	MH+ [Da]	ΔM [ppm]	RT [min]	# Missed Cleavages
	High	nRFPDLTIIR	1	1	1	Q55360	N-Term(iTRAQ4plex)	0,0000	0,586	1		0	0,00002551	40	0,000106414	3	1388,81238	-0,52	34,92	1
	High	gHGLELEVHGEPk	2	1	1	Q55360	N-Term(iTRAQ4plex); K13(iTRAQ4plex)	0,0000	0,656	1		0	0,000009714	39	0,000136449	4	1689,91482	-0,88	20,01	0
P74756	47 kD protein OS=Synechocystis sp. (strain PCC 6803 / Kazusa) GN=slr	66,77	8,09	1	2	2	2	1,591	2	76,9	383	43,2	5,91							
	A3	Sequence	# PSMs	# Proteins	# Protein Groups	Protein Group Accessions	Modifications	ΔCn	117/114	117/114 Count	117/114 Variability [%]	q-Value	PEP	IonScore	Exp Value	Charge	MH+ [Da]	ΔM [ppm]	RT [min]	# Missed Cleavages
	High	qGLPVTIITGFLGSGk	1	1	1	P74756	N-Term(iTRAQ4plex); K16(iTRAQ4plex)	0,0000	2,519	1		0	0,0001021	43	4,94277E-05	3	1876,11710	1,29	54,06	0
	High	tTLLNYILSNQQGLk	1	1	1	P74756	N-Term(iTRAQ4plex); K15(iTRAQ4plex)	0,0000	1,005	1		0	7,303E-07	37	0,00021231	3	1994,15238	-0,05	49,38	0
P74219	Slr1533 protein OS=Synechocystis sp. (strain PCC 6803 / Kazusa) GN=s	66,54	24,35	1	2	2	3	1,562	2	22,1	115	12,8	5,12							
	A3	Sequence	# PSMs	# Proteins	# Protein Groups	Protein Group Accessions	Modifications	ΔCn	117/114	117/114 Count	117/114 Variability [%]	q-Value	PEP	IonScore	Exp Value	Charge	MH+ [Da]	ΔM [ppm]	RT [min]	# Missed Cleavages
	High	tAPLTLNLLQGSVR	1	1	1	P74219	N-Term(iTRAQ4plex)	0,0000	1,348	1		0	0,00003511	41	8,14648E-05	3	1626,96787	1,16	42,35	0
	High	aIGAQVNQGGRPEk	2	1	1	P74219	N-Term(iTRAQ4plex); K14(iTRAQ4plex)	0,0000	1,810	1		0	0,00002518	39	0,000135822	3	1712,96665	1,16	14,96	0
P74189	General secretion pathway protein D OS=Synechocystis sp. (strain PCC	65,50	4,20	1	2	2	2	0,979	2	5,6	785	84,6	4,78							
	A3	Sequence	# PSMs	# Proteins	# Protein Groups	Protein Group Accessions	Modifications	ΔCn	117/114	117/114 Count	117/114 Variability [%]	q-Value	PEP	IonScore	Exp Value	Charge	MH+ [Da]	ΔM [ppm]	RT [min]	# Missed Cleavages
	High	gASPQVGNPSILSPAVPVAPVR	1	1	1	P74189	N-Term(iTRAQ4plex)	0,0000	0,943	1		0	0,000005394	53	4,86374E-06	3	2257,28043	0,81	38,14	0
	High	gQVNVPSLVLR	1	1	1	P74189	N-Term(iTRAQ4plex)	0,0000	1,016	1		0	0,007111	25	0,00289048	2	1325,80388	1,26	35,03	0
P74507	2,3-bisphosphoglycerate-independent phosphoglycerate mutase OS=Sy	64,86	3,20	1	2	2	3	0,952	3	39,9	532	57,9	5,83							
	A3	Sequence	# PSMs	# Proteins	# Protein Groups	Protein Group Accessions	Modifications	ΔCn	117/114	117/114 Count	117/114 Variability [%]	q-Value	PEP	IonScore	Exp Value	Charge	MH+ [Da]	ΔM [ppm]	RT [min]	# Missed Cleavages
	High	lLASAAk	1	1	1	P74507	N-Term(iTRAQ4plex); K7(iTRAQ4plex)	0,0000	0,952	1		0	0,000609	39	0,000124157	2	961,62706	-1,46	18,59	0
	High	kIPGHGGEVk	2	1	1	P74507	N-Term(iTRAQ4plex); K1(iTRAQ4plex); K10(iTRAQ4plex)	0,0000	0,924	2	45,0	0	0,0002944	37	0,000210363	4	1453,88296	-1,51	10,44	1
P74771	NAD(P)H-quinone oxidoreductase subunit O OS=Synechocystis sp. (stra	64,62	29,17	1	2	2	5	0,448	5	19,3	72	8,3	7,25							
	A3	Sequence	# PSMs	# Proteins	# Protein Groups	Protein Group Accessions	Modifications	ΔCn	117/114	117/114 Count	117/114 Variability [%]	q-Value	PEP	IonScore	Exp Value	Charge	MH+ [Da]	ΔM [ppm]	RT [min]	# Missed Cleavages
	High	fYVPTPNVWLR	3	1	1	P74771	N-Term(iTRAQ4plex)	0,0000	0,439	3	2,8	0	0,00004874	36	0,000263009	2	1535,85112	1,29	47,52	0
	High	rLPDYLFHSk	2	1	1	P74771	N-Term(iTRAQ4plex); K10(iTRAQ4plex)	0,0000	0,512	2	0,8	0	0,0002142	20	0,01409105	4	1563,89040	1,15	28,76	1
Q55389	Ferredoxin-thioredoxin reductase, catalytic chain OS=Synechocystis sp.	64,40	19,49	1	2	2	4	1,109	4	23,7	118	13,4	5,00							
	A3	Sequence	# PSMs	# Proteins	# Protein Groups	Protein Group Accessions	Modifications	ΔCn	117/114	117/114 Count	117/114 Variability [%]	q-Value	PEP	IonScore	Exp Value	Charge	MH+ [Da]	ΔM [ppm]	RT [min]	# Missed Cleavages
	High	hkEELGSPLcPcR	2	1	1	Q55389	N-Term(iTRAQ4plex); K2(iTRAQ4plex); C10(Carbamidom	0,0000	0,941	2	11,7	0	5,978E-09	57	2,06049E-06	3	1870,95206	0,75	18,39	1
	High	hYEDkEAEVk	2	1	1	Q55389	N-Term(iTRAQ4plex); K5(iTRAQ4plex); K10(iTRAQ4plex)	0,0000	1,298	2	10,6	0	0,00002571	14	0,039716412	4	1679,89602	-0,28	13,14	1
P55037	Ferredoxin-dependent glutamate synthase 1 OS=Synechocystis sp. (stra	64,02	2,45	1	5	5	6	1,251	5	3,7	1550	169,0	5,92							
	A3	Sequence	# PSMs	# Proteins	# Protein Groups	Protein Group Accessions	Modifications	ΔCn	117/114	117/114 Count	117/114 Variability [%]	q-Value	PEP	IonScore	Exp Value	Charge	MH+ [Da]	ΔM [ppm]	RT [min]	# Missed Cleavages
	High	iGISTIQSYR	1	1	1	P55037	N-Term(iTRAQ4plex)	0,0000	1,029	1		0	0,00005002	37	0,000194971	2	1281,72930	0,74	32,01	0
	High	gNVNWmQAR	2	1	1	P55037	N-Term(iTRAQ4plex); M6(Oxidation)	0,0000	1,263	1		0	0,00008173	33	0,000494277	2	1235,60759	0,31	17,48	0
	High	kVYPWIAk	1	1	1	P55037	N-Term(iTRAQ4plex); K1(iTRAQ4plex); K8(iTRAQ4plex)	0,0000	0,915	1		0	0,006856	14	0,043248396	3	1436,90082	1,27	27,36	1
	High	kLYVIR	1	1	1	P55037	N-Term(iTRAQ4plex); K1(iTRAQ4plex)	0,0000	1,251	1		0	0,00305	12	0,056881364	3	1079,71735	-0,54	20,24	1
	Medium	fFTLR	1	1	1	P55037	N-Term(iTRAQ4plex)	0,0000	1,282	1		0,02	0,2535	19	0,013181657	2	827,48979	0,22	32,53	0
Q55187	Phenylalanine--tRNA ligase alpha subunit OS=Synechocystis sp. (strain	63,86	9,06	1	3	3	4	1,403	4	10,6	331	37,1	6,15							
	A3	Sequence	# PSMs	# Proteins	# Protein Groups	Protein Group Accessions	Modifications	ΔCn	117/114	117/114 Count	117/114 Variability [%]	q-Value	PEP	IonScore	Exp Value	Charge	MH+ [Da]	ΔM [ppm]	RT [min]	# Missed Cleavages
	High	kGELSLILk	1	1	1	Q55187	N-Term(iTRAQ4plex); K1(iTRAQ4plex); K9(iTRAQ4plex)	0,0000	1,501	1		0	0,00002187	35	0,000333403	3	1432,94492	-1,00	31,11	1
	High	gLAFTHLk	1	1	1	Q55187	N-Term(iTRAQ4plex); K8(iTRAQ4plex)	0,0000	1,044	1		0	0,00004181	28	0,001599448	3	1174,71933	0,56	25,61	0
	High	fAmVLHQIDDIRR	2	1	1	Q55187	N-Term(iTRAQ4plex); M3(Oxidation)	0,0000	1,408	2	10,6	0	0,003541	19	0,012704864	3	1773,95597	0,48	35,82	1
Q55799	General secretion pathway protein E OS=Synechocystis sp. (strain PCC	63,70	2,83	1	3	3	5	1,220	4	13,3	813	90,1	5,52							
	A3	Sequence	# PSMs	# Proteins	# Protein Groups	Protein Group Accessions	Modifications	ΔCn	117/114	117/114 Count	117/114 Variability [%]	q-Value	PEP	IonScore	Exp Value	Charge	MH+ [Da]	ΔM [ppm]	RT [min]	# Missed Cleavages
	High	fALSVIHGVk	2	1	1	Q55799	N-Term(iTRAQ4plex); K10(iTRAQ4plex)	0,0000	1,071	1		0	0,0002479	26	0,002618002	3	1358,83985	-0,01	32,93	0
	High	iLILGLR	2	1	1	Q55799	N-Term(iTRAQ4plex)	0,0000	1,347	2	7,5	0,001	0,01573	26	0,002426443	2	941,66258	-0,28	41,15	0
	High	kFNPFk	1	1	1	Q55799	N-Term(iTRAQ4plex); K1(iTRAQ4plex); K6(iTRAQ4plex)	0,0000	1,163	1		0,001	0,01738	21	0,008240812	3	1212,74247	-3,34	22,00	1
Q55726	Sll0602 protein OS=Synechocystis sp. (strain PCC 6803 / Kazusa) GN=sl	63,56	4,20	1	2	2	2	0,862	2	31,1	691	77,7	4,87							
	A3	Sequence	# PSMs	# Proteins	# Protein Groups	Protein Group Accessions	Modifications	ΔCn	117/114	117/114 Count	117/114 Variability [%]	q-Value	PEP	IonScore	Exp Value	Charge	MH+ [Da]	ΔM [ppm]	RT [min]	# Missed Cleavages
	High	sGALTQAVGLGPSAQLHPIIQR	1	1	1	Q55726	N-Term(iTRAQ4plex)	0,0000	1,058	1		0	6,776E-08	59	1,16673E-06	3	2358,34000	1,06	36,90	0
	High	tFIQHLk	1	1	1	Q55726	N-Term(iTRAQ4plex); K7(iTRAQ4plex)	0,0000	0,702	1		0,005	0,05887	17	0,01883519	3	1174,72016	1,27	22,48	0
Q55782	Slr0209 protein OS=Synechocystis sp. (strain PCC 6803 / Kazusa) GN=s	63,48	7,18	1	2	2	4	1,019	4	8,5	348	40,7	5,63							
	A3	Sequence	# PSMs	# Proteins	# Protein Groups	Protein Group Accessions	Modifications	ΔCn	117/114	117/114 Count	117/114 Variability [%]	q-Value	PEP	IonScore	Exp Value	Charge	MH+ [Da]	ΔM [ppm]	RT [min]	# Missed Cleavages
	High	nVNDLAILTHLVNk	2	1	1	Q55782	N-Term(iTRAQ4plex); K14(iTRAQ4plex)	0,0000	1,008	2	8,5	0	0,00000276	30	0,000901509	3	1852,09092	0,76	43,95	0
	High	gGLIEGIIHSk	2	1	1	Q55782	N-Term(iTRAQ4plex); K11(iTRAQ4plex)	0,0000	1,052	2	11,7	0	0,001167	28	0,001530982	3	1411,85199	0,59	33,60	0
Q55780	Slr0208 protein OS=Synechocystis sp. (strain PCC 6803 / Kazusa) GN=s	63,44	8,49	1	2	2	3	1,099	3	15,0	259	29,9	5,07							
	A3	Sequence	# PSMs	# Proteins	# Protein Groups	Protein Group Accessions	Modifications	ΔCn	117/114	117/114 Count	117/114 Variability [%]	q-Value	PEP	IonScore	Exp Value	Charge	MH+ [Da]	ΔM [ppm]	RT [min]	# Missed Cleavages
	High	iQFALPSIPk	2	1	1	Q55780	N-Term(iTRAQ4plex); K10(iTRAQ4plex)	0,0000	0,843	2	40,9	0	0,006868	39	0,000138347	2	1401,87248	1,17	43,42	0
	High	dkLDQVIYSLIR	1	1	1	Q55780	N-Term(iTRAQ4plex); K2(iTRAQ4plex)	0,0000	1,215	1		0	0,0002405	27	0,002113343	3	1751,03189	0,75	50,40	1
P73886	Ribosome-binding ATPase YchF OS=Synechocystis sp. (strain PCC 6803	62,46	5,79	1	2	2	2	0,908	2	16,7	363	39,3	4,84							
	A3	Sequence	# PSMs	# Proteins	# Protein Groups	Protein Group Accessions	Modifications	ΔCn	117/114	117/114 Count	117/114 Variability [%]	q-Value	PEP	IonScore	Exp Value	Charge	MH+ [Da]	ΔM [ppm]	RT [min]	# Missed Cleavages
	High	sTLFNALVANAk	1	1	1	P73886	N-Term(iTRAQ4plex); K12(iTRAQ4plex)	0,0000	0,812	1		0	0,00002779	37	0,000205575	3	1536,89957	0,47	41,02	0

	High	aTYELLGLR	1	1	1	P73886	N-Term(iTRAQ4plex)	0,0000	1,016	1		0,003	0,0399	39	0,000137721	2	1179,68620	0,66	36,17	0
Q59977	Citrate synthase OS=Synechocystis sp. (strain PCC 6803 / Kazusa) GN=	62,46	10,33	1	3	3	3	0,821	3	55,9	397	44,8	6,05							
	A3	Sequence	# PSMs	# Proteins	# Protein Groups	Protein Group Accessions	Modifications	ΔCn	117/114	117/114 Count	117/114 Variability [%]	q-Value	PEP	IonScore	Exp Value	Charge	MH+ [Da]	ΔM [ppm]	RT [min]	# Missed Cleavages
	High	iPTmVAAFHmIR	1	1	1	Q59977	N-Term(iTRAQ4plex); M4(Oxidation); M10(Oxidation)	0,0000	0,577	1		0	7,623E-07	39	0,000125594	3	1562,83207	1,04	30,70	0
	High	iYRPTQIYIGDHNLSYVPmTER	1	1	1	Q59977	N-Term(iTRAQ4plex); M19(Oxidation)	0,0000	0,821	1		0	3,854E-10	33	0,000490874	4	2826,44033	1,32	34,90	0
	High	iMGFGHR	1	1	1	Q59977	N-Term(iTRAQ4plex)	0,0000	1,170	1		0	0,007935	16	0,022959901	3	961,51627	0,42	20,43	0
P73541	Sll1273 protein OS=Synechocystis sp. (strain PCC 6803 / Kazusa) GN=s	62,19	3,80	1	2	2	2	0,798	2	9,2	474	53,9	5,67							
	A3	Sequence	# PSMs	# Proteins	# Protein Groups	Protein Group Accessions	Modifications	ΔCn	117/114	117/114 Count	117/114 Variability [%]	q-Value	PEP	IonScore	Exp Value	Charge	MH+ [Da]	ΔM [ppm]	RT [min]	# Missed Cleavages
	High	sAIFTQLLR	1	1	1	P73541	N-Term(iTRAQ4plex)	0,0000	0,849	1		0	0,0008964	43	4,74209E-05	2	1192,71721	0,12	40,85	0
	High	gIHVITFLR	1	1	1	P73541	N-Term(iTRAQ4plex)	0,0000	0,750	1		0	0,00005258	32	0,000635287	3	1199,73841	0,23	34,22	0
P54386	NADP-specific glutamate dehydrogenase OS=Synechocystis sp. (strain P	62,06	9,81	1	3	3	3	0,572	3	17,6	428	47,3	5,16							
	A3	Sequence	# PSMs	# Proteins	# Protein Groups	Protein Group Accessions	Modifications	ΔCn	117/114	117/114 Count	117/114 Variability [%]	q-Value	PEP	IonScore	Exp Value	Charge	MH+ [Da]	ΔM [ppm]	RT [min]	# Missed Cleavages
	High	tSLSVSIPVR	1	1	1	P54386	N-Term(iTRAQ4plex)	0,0000	0,508	1		0	0,000001824	46	2,63009E-05	2	1202,72258	0,03	31,21	0
	High	kISPAVVTGkPVTmGGSQGR	1	1	1	P54386	N-Term(iTRAQ4plex); K1(iTRAQ4plex); K10(iTRAQ4plex)	0,0000	0,572	1		0	1,981E-08	29	0,001180239	4	2418,38930	1,21	20,68	1
	High	cALLNLPFGGAk	1	1	1	P54386	N-Term(iTRAQ4plex); C1(Carbamidomethyl); K12(iTRAQ4	0,0000	1,159	1		0,004	0,04851	12	0,063382593	2	1548,88152	0,27	42,14	0
P74309	Fructose-bisphosphate aldolase class 1 OS=Synechocystis sp. (strain PC	60,49	7,33	1	3	3	3	0,765	3	4,9	300	33,1	5,83							
	A3	Sequence	# PSMs	# Proteins	# Protein Groups	Protein Group Accessions	Modifications	ΔCn	117/114	117/114 Count	117/114 Variability [%]	q-Value	PEP	IonScore	Exp Value	Charge	MH+ [Da]	ΔM [ppm]	RT [min]	# Missed Cleavages
	High	aQAEALLk	1	1	1	P74309	N-Term(iTRAQ4plex); K8(iTRAQ4plex)	0,0000	0,988	1		0	0,004572	39	0,000113493	2	1131,69609	-1,35	21,96	0
	High	qIVPILk	1	1	1	P74309	N-Term(iTRAQ4plex); K7(iTRAQ4plex)	0,0000	0,765	1		0	0,001009	34	0,000392618	2	1098,74602	-2,64	30,94	0
	High	hLNQLPk	1	1	1	P74309	N-Term(iTRAQ4plex); K7(iTRAQ4plex)	0,0000	0,740	1		0	0,007727	12	0,065761242	3	1137,69687	-1,23	14,46	0
Q55198	Phosphate transport system permease protein PstC OS=Synechocystis s	60,39	5,05	1	2	2	2	0,394	2	20,0	317	33,8	9,61							
	A3	Sequence	# PSMs	# Proteins	# Protein Groups	Protein Group Accessions	Modifications	ΔCn	117/114	117/114 Count	117/114 Variability [%]	q-Value	PEP	IonScore	Exp Value	Charge	MH+ [Da]	ΔM [ppm]	RT [min]	# Missed Cleavages
	High	qASLGLGATR	1	1	1	Q55198	N-Term(iTRAQ4plex)	0,0000	0,450	1		0,002	0,02684	47	1,99972E-05	2	1117,64763	2,68	19,61	0
	High	wETIFR	1	1	1	Q55198	N-Term(iTRAQ4plex)	0,0000	0,345	1		0,009	0,1164	26	0,002280185	2	995,54521	2,11	36,91	0
P72759	Carbon dioxide concentrating mechanism protein CcmL OS=Synechocys	60,04	18,00	1	2	2	2	1,443	2	32,4	100	10,6	9,17							
	A3	Sequence	# PSMs	# Proteins	# Protein Groups	Protein Group Accessions	Modifications	ΔCn	117/114	117/114 Count	117/114 Variability [%]	q-Value	PEP	IonScore	Exp Value	Charge	MH+ [Da]	ΔM [ppm]	RT [min]	# Missed Cleavages
	High	lLLVQFLDTk	1	1	1	P72759	N-Term(iTRAQ4plex); K10(iTRAQ4plex)	0,0000	1,786	1		0	4,264E-07	53	4,65554E-06	2	1477,92351	0,16	51,21	0
	High	tPNLTGVk	1	1	1	P72759	N-Term(iTRAQ4plex); K8(iTRAQ4plex)	0,0000	1,166	1		0,009	0,1184	20	0,010616222	2	1117,68193	-0,02	20,71	0
P73280	Sll1072 protein OS=Synechocystis sp. (strain PCC 6803 / Kazusa) GN=s	59,80	10,31	1	2	2	4	0,733	4	7,6	223	25,2	5,20							
	A3	Sequence	# PSMs	# Proteins	# Protein Groups	Protein Group Accessions	Modifications	ΔCn	117/114	117/114 Count	117/114 Variability [%]	q-Value	PEP	IonScore	Exp Value	Charge	MH+ [Da]	ΔM [ppm]	RT [min]	# Missed Cleavages
	High	aELIEGIVHVASPLR	2	1	1	P73280	N-Term(iTRAQ4plex)	0,0000	0,401	2	107,9	0	0,0000877	38	0,000154159	3	1748,01871	-0,03	44,29	0
	High	vYENHGVk	2	1	1	P73280	N-Term(iTRAQ4plex); K8(iTRAQ4plex)	0,0000	0,771	2	6,1	0,001	0,01596	10	0,099770006	3	1233,67957	-2,79	11,82	0
P72756	Sll1033 protein OS=Synechocystis sp. (strain PCC 6803 / Kazusa) GN=s	57,65	2,54	1	2	2	3	0,960	3	2,2	668	73,7	4,46							
	A3	Sequence	# PSMs	# Proteins	# Protein Groups	Protein Group Accessions	Modifications	ΔCn	117/114	117/114 Count	117/114 Variability [%]	q-Value	PEP	IonScore	Exp Value	Charge	MH+ [Da]	ΔM [ppm]	RT [min]	# Missed Cleavages
	High	vRPQIPLDVW	1	1	1	P72756	N-Term(iTRAQ4plex)	0,0000	0,861	1		0	0,004488	32	0,000691831	2	1366,79827	1,37	43,35	0
	High	aIIPYFR	2	1	1	P72756	N-Term(iTRAQ4plex)	0,0000	0,967	2	1,1	0,001	0,02112	24	0,004548938	2	1023,61150	0,69	36,12	0
P73832	Cyanophycinase OS=Synechocystis sp. (strain PCC 6803 / Kazusa) GN=	57,09	8,49	1	2	2	3	0,953	3	9,4	271	29,4	6,52							
	A3	Sequence	# PSMs	# Proteins	# Protein Groups	Protein Group Accessions	Modifications	ΔCn	117/114	117/114 Count	117/114 Variability [%]	q-Value	PEP	IonScore	Exp Value	Charge	MH+ [Da]	ΔM [ppm]	RT [min]	# Missed Cleavages
	High	eILQTFWSR	2	1	1	P73832	N-Term(iTRAQ4plex)	0,0000	0,865	2	24,2	0	0,0001159	38	0,000149269	2	1323,71977	1,50	42,14	0
	High	lNILVHGEVYHQVk	1	1	1	P73832	N-Term(iTRAQ4plex); K14(iTRAQ4plex)	0,0000	0,953	1		0	2,138E-07	24	0,003749471	4	1937,12136	0,12	30,10	0
Q59992	Tryptophan synthase beta chain OS=Synechocystis sp. (strain PCC 6803	56,37	9,47	1	4	4	4	0,883	4	25,5	412	45,0	6,71							
	A3	Sequence	# PSMs	# Proteins	# Protein Groups	Protein Group Accessions	Modifications	ΔCn	117/114	117/114 Count	117/114 Variability [%]	q-Value	PEP	IonScore	Exp Value	Charge	MH+ [Da]	ΔM [ppm]	RT [min]	# Missed Cleavages
	High	iNNALGQVLLAk	1	1	1	Q59992	N-Term(iTRAQ4plex); K12(iTRAQ4plex)	0,0000	0,856	1		0	0,000008772	39	0,000122735	3	1541,96170	-0,05	40,50	0
	High	gDkDVQTVAk	1	1	1	Q59992	N-Term(iTRAQ4plex); K3(iTRAQ4plex); K10(iTRAQ4plex)	0,0000	0,911	1		0	0,00005439	20	0,009461718	3	1492,86756	-1,34	11,99	1
	High	eDLNHTGAHk	1	1	1	Q59992	N-Term(iTRAQ4plex); K10(iTRAQ4plex)	0,0000	0,649	1		0	0,0057	17	0,021526331	4	1409,73684	-0,53	8,59	0
	High	mNLLGAR	1	1	1	Q59992	N-Term(iTRAQ4plex)	0,0000	2,157	1		0,005	0,0583	19	0,011508004	2	918,53087	-0,34	25,92	0
P72856	DNA polymerase III subunit beta OS=Synechocystis sp. (strain PCC 680	56,07	8,95	1	2	2	3	0,885	2	53,8	391	42,1	4,87							
	A3	Sequence	# PSMs	# Proteins	# Protein Groups	Protein Group Accessions	Modifications	ΔCn	117/114	117/114 Count	117/114 Variability [%]	q-Value	PEP	IonScore	Exp Value	Charge	MH+ [Da]	ΔM [ppm]	RT [min]	# Missed Cleavages
	High	qVLTGVHIk	2	1	1	P72856	N-Term(iTRAQ4plex); K9(iTRAQ4plex)	0,0000	0,630	1		0	3,615E-07	34	0,00037151	3	1282,81089	1,82	22,71	0
	High	aVSSRPTHPVLGNVLLEADADkNY	1	1	1	P72856	N-Term(iTRAQ4plex); K22(iTRAQ4plex)	0,0000	1,244	1		0	2,319E-08	24	0,004045479	5	3123,71534	-0,02	36,84	1
P74685	Sll0443 protein OS=Synechocystis sp. (strain PCC 6803 / Kazusa) GN=s	56,04	5,76	1	2	2	3	0,759	2	3,7	469	51,0	4,77							
	A3	Sequence	# PSMs	# Proteins	# Protein Groups	Protein Group Accessions	Modifications	ΔCn	117/114	117/114 Count	117/114 Variability [%]	q-Value	PEP	IonScore	Exp Value	Charge	MH+ [Da]	ΔM [ppm]	RT [min]	# Missed Cleavages
	High	tQNLLLFVSGLR	2	1	1	P74685	N-Term(iTRAQ4plex)	0,0000	0,778	1		0	0,000007465	32	0,000580724	2	1504,89849	1,10	50,54	0
	High	yGQAIASLHTLLQNk	1	1	1	P74685	N-Term(iTRAQ4plex); K15(iTRAQ4plex)	0,0000	0,740	1		0	0,0003856	31	0,000748118	3	1945,10904	-0,98	43,29	0
P74175	HlyD family of secretion proteins OS=Synechocystis sp. (strain PCC 680	55,53	3,09	1	2	2	4	0,972	3	8,2	583	64,7	5,45							
	A3	Sequence	# PSMs	# Proteins	# Protein Groups	Protein Group Accessions	Modifications	ΔCn	117/114	117/114 Count	117/114 Variability [%]	q-Value	PEP	IonScore	Exp Value	Charge	MH+ [Da]	ΔM [ppm]	RT [min]	# Missed Cleavages
	High	iLAGLLGR	3	1	1	P74175	N-Term(iTRAQ4plex)	0,0000	1,039	2	10,0	0	0,01094	42	6,4561E-05	2	956,63878	1,48	40,85	0
	High	qLQQASLQLR	1	1	1	P74175	N-Term(iTRAQ4plex)	0,0000	0,920	1		0	0,004106	15	0,033960182	2	1328,77849	1,34	25,93	0
P74100	Slr1962 protein OS=Synechocystis sp. (strain PCC 6803 / Kazusa) GN=s	55,10	5,14	1	2	2	2	0,797	2	32,4	370	40,3	6,28							
	A3	Sequence	# PSMs	# Proteins	# Protein Groups	Protein Group Accessions	Modifications	ΔCn	117/114	117/114 Count	117/114 Variability [%]	q-Value	PEP	IonScore	Exp Value	Charge	MH+ [Da]	ΔM [ppm]	RT [min]	# Missed Cleavages
	High	kPVESLADLR	1	1	1	P74100	N-Term(iTRAQ4plex); K1(iTRAQ4plex)	0,0000	0,644	1		0	0,0001967	39	0,000127938	3	1415,84571	-0,27	25,16	0
	High	sANLLmLSR	1	1	1	P74100	N-Term(iTRAQ4plex); M6(Oxidation)	0,0000	0,986	1		0,005	0,0612	29	0,00126588	2	1164,65312	0,31	28,93	0
P72874	Translation initiation factor IF-3 OS=Synechocystis sp. (strain PCC 6803	54,74	19,21	1	4	4	7	1,065	7	33,4	177	20,5	9,33							
	A3	Sequence	# PSMs	# Proteins	# Protein Groups	Protein Group Accessions	Modifications	ΔCn	117/114	117/114 Count	117/114 Variability [%]	q-Value	PEP	IonScore	Exp Value	Charge	MH+ [Da]	ΔM [ppm]	RT [min]	# Missed Cleavages
	High	ykIDEHDYQVR	2	1	1	P72874	N-Term(iTRAQ4plex); K2(iTRAQ4plex)	0,0000	1,163	2	13,2	0	0,000008376	25	0,003523465	4	1753,91372	1,45	21,25	1
	High	iRFPEIR	2	1	1	P72874	N-Term(iTRAQ4plex)	0,0000	1,646	2	5,1	0	0,008942	24	0,0041973	3	1074,65448	0,38	30,24	1
	High	eIQHAHLAk	1	1	1	P72874	N-Term(iTRAQ4plex); K9(iTRAQ4plex)	0,0000	0,878	1		0	0,0002677	16	0,024715534	4	1334,78188	2,67	12,11	0
	High	ykFEQEk	2	1	1	P72874	N-Term(iTRAQ4plex); K2(iTRAQ4plex); K7(iTRAQ4plex)	0,0000	0,812	2	7,7	0	0,0003571	15	0,031258635	3	1403,78885	-0,45	16,64	1
Q55822	CobW protein OS=Synechocystis sp. (strain PCC 6803 / Kazusa) GN=co	54,52	6,53	1	2	2	2	1,929	2	24,0	352	38,9	4,63							
	A3	Sequence	# PSMs	# Proteins	# Protein Groups	Protein Group Accessions	Modifications	ΔCn	117/114	117/114 Count	117/114 Variability [%]	q-Value	PEP	IonScore	Exp Value	Charge	MH+ [Da]	ΔM [ppm]	RT [min]	# Missed Cleavages
	High	lVLQGVGQR	1	1	1	Q55822	N-Term(iTRAQ4plex)	0,0000	1,644	1		0	0,0004332	35	0,000353159	2	1113,68523	-0,79	26,94	0
	High	iPVTVVTGFLGSGk	1	1	1	Q55822	N-Term(iTRAQ4plex); K14(iTRAQ4plex)	0,0000	2,263	1		0	0,00005759	33	0,00049885	3	1663,00412	0,48	46,84	0
	>sp|CAS1_BOVIN|	54,22	12,62	1	2	2	3	1,150	3	0,4	214	24,5	5,02							
	A3	Sequence	# PSMs	# Proteins	# Protein Groups	Protein Group Accessions	Modifications	ΔCn	117/114	117/114 Count	117/114 Variability [%]	q-Value	PEP	IonScore	Exp Value	Charge	MH+ [Da]	ΔM [ppm]	RT [min]	# Missed Cleavages
	High	hQGLPQEVLNENLLR	1	1	1		N-Term(iTRAQ4plex)	0,0000	1,153	1		0	0,0000029	36	0,000264223	3	1904,05002	1,54	36,42	0
	High	fFVAPFPEVFGk	2	1	1		N-Term(iTRAQ4plex); K12(iTRAQ4plex)	0,0000	1,138	2	1,6	0	0,003533	26	0,002338676	2	1672,93938	3,10	51,39	0
Q55663	1-deoxy-D-xylulose 5-phosphate reductoisomerase OS=Synechocystis sp	54,16	5,84	1	2	2	2	0,987	2	7,7	394	42,5	5,02							
	A3	Sequence	# PSMs	# Proteins	# Protein Groups	Protein Group Accessions	Modifications	ΔCn	117/114	117/114 Count	117/114 Variability [%]	q-Value	PEP	IonScore	Exp Value	Charge	MH+ [Da]	ΔM [ppm]	RT [min]	# Missed Cleavages
	High	lPLLYALSWPER	1	1	1	Q55663	N-Term(iTRAQ4plex)	0,0000	1,039	1		0	0,005019	39	0,000133343	2	1601,91997	1,71	54,79	0
	High	iILTASGGAFR	1	1	1	Q55663	N-Term(iTRAQ4plex)	0,0000	0,937	1		0	0,01105	30	0,001872617	2	1249,74004	1,20	34,28	0
P74220	Putative carboxypeptidase slr1534 OS=Synechocystis sp. (strain PCC 68	53,89	8,64	1	2	2	3	1,152	3	0,4	301	32,9	5,47							
	A3	Sequence	# PSMs	# Proteins	# Protein Groups	Protein Group Accessions	Modifications	ΔCn	117/114	117/114 Count	117/114 Variability [%]	q-Value	PEP	IonScore	Exp Value	Charge	MH+ [Da]	ΔM [ppm]	RT [min]	# Missed Cleavages
	High	lTVVSPSGSLR	2	1	1	P74220	N-Term(iTRAQ4plex)	0,0000	1,154	2	0,2	0,001	0,0165	30	0,00091405	2	1259,74346	-0,44	28,56	0
	Medium	sWGYEVIFSQGYHNR	1	1	1	P74220	N-Term(iTRAQ4plex)	0,0000	0,684	1		0,039	0,4839	14	0,039171482	3	1986,95707	-0,43	39,22	0
P72977	16.6 kDa small heat shock protein, molecular chaperon OS=Synechocys	53,13	27,40	1	4	4	4	0,778	4	19,6	146	16,6	5,10							
	A3	Sequence	# PSMs	# Proteins	# Protein Groups	Protein Group Accessions	Modifications	ΔCn	117/114	117/114 Count	117/114 Variability [%]	q-Value	PEP	IonScore	Exp Value	Charge	MH+ [Da]	ΔM [ppm]	RT [min]	# Missed Cleavages
	High	rVIPVPGAIQNTEVk	1	1	1	P72977	N-Term(iTRAQ4plex); K15(iTRAQ4plex)	0,0000	1,081	1		0	0,000000557	45	3,13307E-05	3	1909,14658	-0,42	28,66	1
	High	aNYDAGILTLTLPk	1	1	1	P72977	N-Term(iTRAQ4plex); K14(iTRAQ4plex)	0,0000	0,866	1		0	0,0002161	21	0,007744083	3	1778,02768	-1,45	43,92	0
	Medium	qGFNPk	1	1	1	P72977	N-Term(iTRAQ4plex); K6(iTRAQ4plex)	0,0000	0,666	1		0,025	0,314	12	0,073775135	2	978,55571	-5,51	13,91	0
	Medium	yGSFR	1	1	1	P72977	N-Term(iTRAQ4plex)	0,0000	0,699	1		0,038	0,4738	11	0,088505448	2	773,40550	-0,98	16,12	0
Q55563	Uncharacterized WD repeat-containing protein sll0163 OS=Synechocysti	53,04	0,95	1	2	2	2	0,559	2	10,3	1693	189,8	5,94							
	A3	Sequence	# PSMs	# Proteins	# Protein Groups	Protein Group Accessions	Modifications	ΔCn	117/114	117/114 Count	117/114 Variability [%]	q-Value	PEP	IonScore	Exp Value	Charge	MH+ [Da]	ΔM [ppm]	RT [min]	# Missed Cleavages
	High	sHWLDLR	1	1	1	Q55563	N-Term(iTRAQ4plex)	0,0000	0,522	1		0	0,01035	28	0,001621698	3	1070,58835	1,85	26,57	0
	High	lSLLQGLAR	1	1	1	Q55563	N-Term(iTRAQ4plex)	0,0000	0,599	1		0,002	0,02222	38	0,000152746	2	1114,70757	0,95	40,53	0
P74721	Zinc-containing alcohol dehydrogenase family OS=Synechocystis sp. (st	52,43	2,38	1	2	2	4	0,654	4	68,8	336	35,8	6,10							
	A3	Sequence	# PSMs	# Proteins	# Protein Groups	Protein Group Accessions	Modifications	ΔCn	117/114	117/114 Count	117/114 Variability [%]	q-Value	PEP	IonScore	Exp Value	Charge	MH+ [Da]	ΔM [ppm]	RT [min]	# Missed Cleavages
	High	vVLSHSk	2	1	1	P74721	N-Term(iTRAQ4plex); K7(iTRAQ4plex)	0,0000	0,430	2	8,0	0	0,007165	41	8,18408E-05	2	1057,66142	0,57	11,99	0
	High	vVLSHSkN	2	1	1	P74721	N-Term(iTRAQ4plex); K7(iTRAQ4plex)	0,0000	1,031	2	13,3	0,001	0,01137	24	0,0044154	2	1171,70452	0,66	11,20	1
P73267	Sll1080 protein OS=Synechocystis sp. (strain PCC 6803 / Kazusa) GN=s	52,13	5,97	1	2	2	2	1,139	2	14,9	352	38,2	4,56							
	A3	Sequence	# PSMs	# Proteins	# Protein Groups	Protein Group Accessions	Modifications	ΔCn	117/114	117/114 Count	117/114 Variability [%]	q-Value	PEP	IonScore	Exp Value	Charge	MH+ [Da]	ΔM [ppm]	RT [min]	# Missed Cleavages
	High	rAGVSQEELQLFk	1	1	1	P73267	N-Term(iTRAQ4plex); K13(iTRAQ4plex)	0,0000	1,259	1		0	2,504E-10	45	3,16935E-05	3	1793,01432	-0,95	30,45	1
	High	iLDAQFVk	1	1	1	P73267	N-Term(iTRAQ4plex); K8(iTRAQ4plex)	0,0000	1,030	1		0	0,00005504	20	0,009637625	2	1221,74407	-0,41	32,53	0
P73736	N-acetylmuramoyl-L-alanine amidase OS=Synechocystis sp. (strain PCC	50,63	2,47	1	2	2	2	1,253	2	67,2	649	70,0	9,83							
	A3	Sequence	# PSMs	# Proteins	# Protein Groups	Protein Group Accessions	Modifications	ΔCn	117/114	117/114 Count	117/114 Variability [%]	q-Value	PEP	IonScore	Exp Value	Charge	MH+ [Da]	ΔM [ppm]	RT [min]	# Missed Cleavages
	High	iVVDLPGTTLR	1	1	1	P73736	N-Term(iTRAQ4plex)	0,0000	1,891	1		0	0,005982	40	9,97631E-05	2	1327,80803	1,06	38,52	0
	Medium	fYVLR	1	1	1	P73736	N-Term(iTRAQ4plex)	0,0000	0,830	1		0,017	0,2109	24	0,00432484	2	841,50609	0,99	29,07	0
Q55376	Slr0912 protein OS=Synechocystis sp. (strain PCC 6803 / Kazusa) GN=s	49,67	8,14	1	2	2	2	0,794	2	17,5	258	29,3	5,21							
	A3	Sequence	# PSMs	# Proteins	# Protein Groups	Protein Group Accessions	Modifications	ΔCn	117/114	117/114 Count	117/114 Variability [%]	q-Value	PEP	IonScore	Exp Value	Charge	MH+ [Da]	ΔM [ppm]	RT [min]	# Missed Cleavages
	High	qALAAELHQR	1	1	1	Q55376	N-Term(iTRAQ4plex)	0,0000	0,893	1		0	0,00004771	36	0,000279235	3	1280,71909	-0,08	17,95	0
	High	wLHIDGEHTGR	1	1	1	Q55376	N-Term(iTRAQ4plex)	0,0000	0,706	1		0	0,0004508	27	0,001927392	4	1464,74795	1,00	23,82	0
P73437	ATP-dependent zinc metalloprotease FtsH 4 OS=Synechocystis sp. (stra	49,55	4,94	1	3	4	4	1,162	3	4,0	628	68,2	5,41							
	A3	Sequence	# PSMs	# Proteins	# Protein Groups	Protein Group Accessions	Modifications	ΔCn	117/114	117/114 Count	117/114 Variability [%]	q-Value	PEP	IonScore	Exp Value	Charge	MH+ [Da]	ΔM [ppm]	RT [min]	# Missed Cleavages
	High	yTALGAk	1	1	1	P73437	N-Term(iTRAQ4plex); K7(iTRAQ4plex)	0,0000	1,194	1		0	0,0001221	36	0,000258208	2	1011,60723	-0,49	18,30	0
	High	aIkPQPQWQR	1	1	1	P73437	N-Term(iTRAQ4plex); K3(iTRAQ4plex)	0,0000	1,162	1		0	1,156E-07	21	0,007797763	3	1539,90030	0,30	18,51	0
	High	vRDLFEQAk	1	1	1	P73437	N-Term(iTRAQ4plex); K9(iTRAQ4plex)	0,0000	0,917	1		0	0,00006257	19	0,013739471	3	1393,80301	-0,86	26,27	1
	Medium	tLLAk	1	2	2	Q55700;P73437	N-Term(iTRAQ4plex); K5(iTRAQ4plex)	0,0000				0,046	0,5599	10	0,097268004	2	833,56615	-4,47	18,02	0
P73448	Nitrate reductase OS=Synechocystis sp. (strain PCC 6803 / Kazusa) GN=	49,48	2,94	1	2	2	2	1,384	1		714	79,6	7,23							
	A3	Sequence	# PSMs	# Proteins	# Protein Groups	Protein Group Accessions	Modifications	ΔCn	117/114	117/114 Count	117/114 Variability [%]	q-Value	PEP	IonScore	Exp Value	Charge	MH+ [Da]	ΔM [ppm]	RT [min]	# Missed Cleavages
	High	rGSAQLEVLVTR	1	1	1	P73448	N-Term(iTRAQ4plex)	0,0000				0	0,00002344	32	0,000563599	3	1472,86676	0,11	24,28	1
	High	rVTLSPAFR	1	1	1	P73448	N-Term(iTRAQ4plex)	0,0000	1,384	1		0	0,002298	30	0,000997631	3	1190,71186	-0,67	22,90	1
P74094	Slr1958 protein OS=Synechocystis sp. (strain PCC 6803 / Kazusa) GN=s	49,27	6,50	1	2	2	5	1,147	5	7,9	200	23,0	5,06							
	A3	Sequence	# PSMs	# Proteins	# Protein Groups	Protein Group Accessions	Modifications	ΔCn	117/114	117/114 Count	117/114 Variability [%]	q-Value	PEP	IonScore	Exp Value	Charge	MH+ [Da]	ΔM [ppm]	RT [min]	# Missed Cleavages
	High	mVTFFFIR	3	1	1	P74094	N-Term(iTRAQ4plex); M1(Oxidation)	0,0000	1,147	3	6,7	0	0,00004202	30	0,00095273	2	1220,66252	0,54	44,86	0
	High	mVTFFFIR	1	1	1	P74094	N-Term(iTRAQ4plex)	0,0000	1,305	1		0,002	0,03111	13	0,046341491	2	1204,66802	0,88	50,12	0
	Medium	fGFSR	1	1	1	P74094	N-Term(iTRAQ4plex)	0,0000	1,088	1		0,022	0,2668	11	0,073615625	2	757,41185	0,64	21,34	0
P73449	Nitrate transport protein NrtD OS=Synechocystis sp. (strain PCC 6803 /	49,20	6,93	1	2	2	2	2,745	2	21,4	332	36,5	5,01							
	A3	Sequence	# PSMs	# Proteins	# Protein Groups	Protein Group Accessions	Modifications	ΔCn	117/114	117/114 Count	117/114 Variability [%]	q-Value	PEP	IonScore	Exp Value	Charge	MH+ [Da]	ΔM [ppm]	RT [min]	# Missed Cleavages
	High	nYALDFLFHR	1	1	1	P73449	N-Term(iTRAQ4plex)	0,0000	3,166	1		0	0,00001517	49	1,19942E-05	3	1439,75589	0,46	44,78	0
	High	iGEILDIPFDRPR	1	1	1	P73449	N-Term(iTRAQ4plex)	0,0000	2,379	1		0,005	0,05558	13	0,05202227	3	1684,94986	-0,29	45,00	0
P74357	Sll1530 protein OS=Synechocystis sp. (strain PCC 6803 / Kazusa) GN=s	48,91	8,03	1	3	3	4	0,809	3	37,6	386	43,7	6,18							
	A3	Sequence	# PSMs	# Proteins	# Protein Groups	Protein Group Accessions	Modifications	ΔCn	117/114	117/114 Count	117/114 Variability [%]	q-Value	PEP	IonScore	Exp Value	Charge	MH+ [Da]	ΔM [ppm]	RT [min]	# Missed Cleavages
	High	vLAPEIDkk	1	1	1	P74357	N-Term(iTRAQ4plex); K8(iTRAQ4plex); K9(iTRAQ4plex)	0,0000	1,034	1		0	0,003009	22	0,006621708	3	1444,91260	1,82	21,88	1
	High	tLVSAkPHLVLETQR	1	1	1	P74357	N-Term(iTRAQ4plex); K6(iTRAQ4plex)	0,0000	0,520	1		0	0,001625	12	0,062657059	4	1980,18764	1,60	27,62	0
	High	lVPNLLk	2	1	1	P74357	N-Term(iTRAQ4plex); K7(iTRAQ4plex)	0,0000	0,809	1		0,001	0,02123	10	0,089118938	2	1084,73577	2,30	35,27	0
P74215	Aspartyl/glutamyl-tRNA(Asn/Gln) amidotransferase subunit B OS=Synec	48,28	6,06	1	3	3	3	0,942	3	9,3	495	54,7	5,54							
	A3	Sequence	# PSMs	# Proteins	# Protein Groups	Protein Group Accessions	Modifications	ΔCn	117/114	117/114 Count	117/114 Variability [%]	q-Value	PEP	IonScore	Exp Value	Charge	MH+ [Da]	ΔM [ppm]	RT [min]	# Missed Cleavages
	High	gNmQGFFVGQVmk	1	1	1	P74215	N-Term(iTRAQ4plex); M3(Oxidation); M12(Oxidation); K1	0,0000	0,885	1		0	0,000001741	38	0,000166713	3	1762,88834	1,36	29,38	0
	High	kQYFYPDLPk	1	1	1	P74215	N-Term(iTRAQ4plex); K1(iTRAQ4plex); K10(iTRAQ4plex)	0,0000	0,942	1		0	0,000004501	20	0,009637625	3	1730,98331	-0,50	29,87	1
	High	kTIGITR	1	1	1	P74215	N-Term(iTRAQ4plex); K1(iTRAQ4plex)	0,0000	1,083	1		0	0,0098	16	0,023065879	3	1076,70188	-1,07	13,63	1
P72849	Heme oxygenase 1 OS=Synechocystis sp. (strain PCC 6803 / Kazusa) G	47,56	8,75	1	2	2	2	1,309	2	9,7	240	27,0	6,70							
	A3	Sequence	# PSMs	# Proteins	# Protein Groups	Protein Group Accessions	Modifications	ΔCn	117/114	117/114 Count	117/114 Variability [%]	q-Value	PEP	IonScore	Exp Value	Charge	MH+ [Da]	ΔM [ppm]	RT [min]	# Missed Cleavages
	High	sVNLASQLR	1	1	1	P72849	N-Term(iTRAQ4plex)	0,0000	1,226	1		0,001	0,0141	36	0,000254097	2	1131,66008	-0,17	24,53	0
	High	aIGIMVFNSLTR	1	1	1	P72849	N-Term(iTRAQ4plex)	0,0000	1,397	1		0,005	0,06193	25	0,003443261	2	1465,83513	2,27	48,00	0
Q55862	Sll0588 protein OS=Synechocystis sp. (strain PCC 6803 / Kazusa) GN=s	47,17	11,11	1	2	2	2	1,125	2	6,8	153	16,5	5,45							
	A3	Sequence	# PSMs	# Proteins	# Protein Groups	Protein Group Accessions	Modifications	ΔCn	117/114	117/114 Count	117/114 Variability [%]	q-Value	PEP	IonScore	Exp Value	Charge	MH+ [Da]	ΔM [ppm]	RT [min]	# Missed Cleavages
	High	fYQDALTk	1	1	1	Q55862	N-Term(iTRAQ4plex); K8(iTRAQ4plex)	0,0000	1,075	1		0	0,000001974	42	6,35287E-05	2	1273,70354	0,36	27,54	0
	High	lPRPQGSPk	1	1	1	Q55862	N-Term(iTRAQ4plex); K9(iTRAQ4plex)	0,0000	1,178	1		0	0,001044	18	0,01506503	3	1267,77353	0,80	14,44	0
P74275	Delta-1-pyrroline-5-carboxylate dehydrogenase OS=Synechocystis sp. (s	45,97	1,62	1	2	2	4	0,886	4	13,8	990	110,0	5,67							
	A3	Sequence	# PSMs	# Proteins	# Protein Groups	Protein Group Accessions	Modifications	ΔCn	117/114	117/114 Count	117/114 Variability [%]	q-Value	PEP	IonScore	Exp Value	Charge	MH+ [Da]	ΔM [ppm]	RT [min]	# Missed Cleavages
	High	lLPGMAYLIR	3	1	1	P74275	N-Term(iTRAQ4plex)	0,0000	0,907	3	20,8	0	0,0004232	40	9,07758E-05	2	1290,77361	0,87	47,13	0
	High	yFYQPR	1	1	1	P74275	N-Term(iTRAQ4plex)	0,0000	0,865	1		0,003	0,04279	15	0,032731809	2	1017,52855	1,10	23,88	0
Q55723	Sll0606 protein OS=Synechocystis sp. (strain PCC 6803 / Kazusa) GN=s	45,87	4,41	1	2	2	2	0,676	2	136,4	476	50,6	6,11							
	A3	Sequence	# PSMs	# Proteins	# Protein Groups	Protein Group Accessions	Modifications	ΔCn	117/114	117/114 Count	117/114 Variability [%]	q-Value	PEP	IonScore	Exp Value	Charge	MH+ [Da]	ΔM [ppm]	RT [min]	# Missed Cleavages
	High	kFWGGDVSWR	1	1	1	Q55723	N-Term(iTRAQ4plex); K1(iTRAQ4plex)	0,0000	0,339	1		0	2,822E-07	30	0,001078872	3	1525,81647	0,66	32,19	1
	High	gANRPFAEAAR	1	1	1	Q55723	N-Term(iTRAQ4plex)	0,0000	1,350	1		0	0,0001514	29	0,001196658	3	1303,69818	-0,48	12,39	0
Q55984	Sll0659 protein OS=Synechocystis sp. (strain PCC 6803 / Kazusa) GN=s	45,49	3,26	1	2	2	2	0,671	2	5,0	522	58,4	5,59							
	A3	Sequence	# PSMs	# Proteins	# Protein Groups	Protein Group Accessions	Modifications	ΔCn	117/114	117/114 Count	117/114 Variability [%]	q-Value	PEP	IonScore	Exp Value	Charge	MH+ [Da]	ΔM [ppm]	RT [min]	# Missed Cleavages
	High	fLSGFFPAYk	1	1	1	Q55984	N-Term(iTRAQ4plex); K10(iTRAQ4plex)	0,0000	0,649	1		0	0,001963	29	0,001164046	2	1464,81609	2,12	46,88	0
	High	fLQAGGk	1	1	1	Q55984	N-Term(iTRAQ4plex); K7(iTRAQ4plex)	0,0000	0,694	1		0,001	0,0151	29	0,001210515	2	1008,60857	0,50	19,12	0
P72751	Sll1036 protein OS=Synechocystis sp. (strain PCC 6803 / Kazusa) GN=s	45,41	5,82	1	2	2	3	0,753	3	1,5	292	32,6	5,33							
	A3	Sequence	# PSMs	# Proteins	# Protein Groups	Protein Group Accessions	Modifications	ΔCn	117/114	117/114 Count	117/114 Variability [%]	q-Value	PEP	IonScore	Exp Value	Charge	MH+ [Da]	ΔM [ppm]	RT [min]	# Missed Cleavages
	High	gSIPcPGPTTVR	2	1	1	P72751	N-Term(iTRAQ4plex); C5(Carbamidomethyl)	0,0000	0,749	2	0,7	0	0,00001793	28	0,001538048	2	1385,73247	-0,24	21,56	0
	Medium	fWGVR	1	1	1	P72751	N-Term(iTRAQ4plex)	0,0000	0,828	1		0,048	0,5935	20	0,009098504	2	808,45769	-1,19	27,32	0
P73557	RNA-binding protein OS=Synechocystis sp. (strain PCC 6803 / Kazusa) G	43,95	14,46	2	3	3	6	1,144	5	44,7	83	9,4	5,01							
	A3	Sequence	# PSMs	# Proteins	# Protein Groups	Protein Group Accessions	Modifications	ΔCn	117/114	117/114 Count	117/114 Variability [%]	q-Value	PEP	IonScore	Exp Value	Charge	MH+ [Da]	ΔM [ppm]	RT [min]	# Missed Cleavages
	High	rVQLPTDRETGR	1	2	1	P73557	N-Term(iTRAQ4plex)	0,0000	2,470	1		0	0,0007038	24	0,003706551	4	1571,87356	0,05	15,87	2
	High	rVQLPTDR	4	2	1	P73557	N-Term(iTRAQ4plex)	0,0000	1,144	3	44,7	0	0,0054	12	0,060390692	3	1128,65952	-0,97	15,59	1
	Medium	vQLPTDR	1	2	1	P73557	N-Term(iTRAQ4plex)	0,0000	1,107	1		0,02	0,2517	20	0,010763909	2	972,55998	0,50	21,01	0
P73067	1-deoxy-D-xylulose-5-phosphate synthase OS=Synechocystis sp. (strain	43,83	5,00	1	3	3	4	1,209	4	43,9	640	69,3	6,28							
	A3	Sequence	# PSMs	# Proteins	# Protein Groups	Protein Group Accessions	Modifications	ΔCn	117/114	117/114 Count	117/114 Variability [%]	q-Value	PEP	IonScore	Exp Value	Charge	MH+ [Da]	ΔM [ppm]	RT [min]	# Missed Cleavages
	High	fVkPLDTELILPLAER	2	1	1	P73067	N-Term(iTRAQ4plex); K3(iTRAQ4plex)	0,0000	1,622	2	16,6	0	0,001727	25	0,002831196	3	2142,28196	1,97	46,90	0
	High	vFAHTLTTLAk	1	1	1	P73067	N-Term(iTRAQ4plex); K11(iTRAQ4plex)	0,0000	0,823	1		0	0,0005206	21	0,00714447	3	1489,90006	1,31	30,84	0
	High	lVVPk	1	1	1	P73067	N-Term(iTRAQ4plex); K5(iTRAQ4plex)	0,0000	1,008	1		0,008	0,1054	23	0,005104697	2	843,58922	-1,66	21,24	0
P73468	Cytoplasmic membrane protein for maltose uptake OS=Synechocystis sp	43,07	4,41	1	2	2	2	0,844	2	16,0	408	45,2	8,50							
	A3	Sequence	# PSMs	# Proteins	# Protein Groups	Protein Group Accessions	Modifications	ΔCn	117/114	117/114 Count	117/114 Variability [%]	q-Value	PEP	IonScore	Exp Value	Charge	MH+ [Da]	ΔM [ppm]	RT [min]	# Missed Cleavages
	High	wHGLVAPYVGR	1	1	1	P73468	N-Term(iTRAQ4plex)	0,0000	0,939	1		0	4,221E-07	42	6,29463E-05	3	1398,77698	0,48	32,26	0
	High	iHLFDAk	1	1	1	P73468	N-Term(iTRAQ4plex); K7(iTRAQ4plex)	0,0000	0,758	1		0,002	0,02798	14	0,03908139	3	1131,67783	1,19	27,10	0
P73342	Slr1194 protein OS=Synechocystis sp. (strain PCC 6803 / Kazusa) GN=s	42,14	9,46	1	2	2	2	1,035	2	24,4	222	25,2	4,75							
	A3	Sequence	# PSMs	# Proteins	# Protein Groups	Protein Group Accessions	Modifications	ΔCn	117/114	117/114 Count	117/114 Variability [%]	q-Value	PEP	IonScore	Exp Value	Charge	MH+ [Da]	ΔM [ppm]	RT [min]	# Missed Cleavages
	High	lNPLYEQLIGLR	1	1	1	P73342	N-Term(iTRAQ4plex)	0,0000	0,880	1		0	0,00000851	42	6,09495E-05	2	1572,92388	0,53	48,06	0
	High	iVHIPPLck	1	1	1	P73342	N-Term(iTRAQ4plex); C8(Carbamidomethyl); K9(iTRAQ4p	0,0000	1,217	1		0	0,006586	13	0,05597576	3	1364,83353	0,63	30,31	0
P73288	Aklaviketone reductase OS=Synechocystis sp. (strain PCC 6803 / Kazusa	41,89	8,57	1	2	2	2	0,925	2	6,8	210	22,0	5,85							
	A3	Sequence	# PSMs	# Proteins	# Protein Groups	Protein Group Accessions	Modifications	ΔCn	117/114	117/114 Count	117/114 Variability [%]	q-Value	PEP	IonScore	Exp Value	Charge	MH+ [Da]	ΔM [ppm]	RT [min]	# Missed Cleavages
	High	aIVTSLQGk	1	1	1	P73288	N-Term(iTRAQ4plex); K9(iTRAQ4plex)	0,0000	0,969	1		0,008	0,09687	36	0,000269755	2	1204,75176	1,14	23,66	0
	Medium	lmGQVNLIR	1	1	1	P73288	N-Term(iTRAQ4plex); M2(Oxidation)	0,0000	0,884	1		0,044	0,5448	20	0,013675504	2	1203,70134	1,07	28,33	0
P73336	30S ribosomal protein S20 OS=Synechocystis sp. (strain PCC 6803 / Kaz	41,86	16,49	1	2	2	2	1,300	2	5,9	97	10,7	10,29							
	A3	Sequence	# PSMs	# Proteins	# Protein Groups	Protein Group Accessions	Modifications	ΔCn	117/114	117/114 Count	117/114 Variability [%]	q-Value	PEP	IonScore	Exp Value	Charge	MH+ [Da]	ΔM [ppm]	RT [min]	# Missed Cleavages
	High	rIEIAER	1	1	1	P73336	N-Term(iTRAQ4plex)	0,0000	1,354	1		0	0,0009245	34	0,000420698	3	1030,61222	-0,38	14,07	1
	High	aLQSALPAA	1	1	1	P73336	N-Term(iTRAQ4plex)	0,0000	1,249	1		0,009	0,1237	21	0,007726272	2	985,57994	0,04	30,06	0
Q55513	4-hydroxy-tetrahydrodipicolinate synthase OS=Synechocystis sp. (strain	41,64	8,31	1	2	2	2	0,467	2	25,0	301	31,8	5,08							
	A3	Sequence	# PSMs	# Proteins	# Protein Groups	Protein Group Accessions	Modifications	ΔCn	117/114	117/114 Count	117/114 Variability [%]	q-Value	PEP	IonScore	Exp Value	Charge	MH+ [Da]	ΔM [ppm]	RT [min]	# Missed Cleavages
	High	gSVIAGTGSNcTR	1	1	1	Q55513	N-Term(iTRAQ4plex); C11(Carbamidomethyl)	0,0000	0,396	1		0	0,002774	39	0,000129113	2	1423,70891	0,61	14,25	0
	High	iLFcATNPIPVk	1	1	1	Q55513	N-Term(iTRAQ4plex); C4(Carbamidomethyl); K12(iTRAQ4	0,0000	0,552	1		0,007	0,08955	16	0,026483172	3	1660,97000	0,06	40,70	0
P73618	Deoxyribose-phosphate aldolase OS=Synechocystis sp. (strain PCC 680	41,47	10,22	1	2	2	3	0,440	2	17,9	225	23,9	5,62							
	A3	Sequence	# PSMs	# Proteins	# Protein Groups	Protein Group Accessions	Modifications	ΔCn	117/114	117/114 Count	117/114 Variability [%]	q-Value	PEP	IonScore	Exp Value	Charge	MH+ [Da]	ΔM [ppm]	RT [min]	# Missed Cleavages
	High	eAIATccAQALHHk	2	1	1	P73618	N-Term(iTRAQ4plex); C6(Carbamidomethyl); C7(Carbam	0,0000	0,390	1		0	6,588E-07	28	0,001581139	4	1897,96071	-0,44	18,46	0
	High	lLHQISQGR	1	1	1	P73618	N-Term(iTRAQ4plex)	0,0000	0,496	1		0	0,0000151	23	0,004666272	3	1195,70185	-0,80	18,83	0
P19125	NAD(P)H-quinone oxidoreductase subunit J OS=Synechocystis sp. (strai	41,10	8,38	1	2	2	3	0,621	2	30,8	179	20,6	4,53							
	A3	Sequence	# PSMs	# Proteins	# Protein Groups	Protein Group Accessions	Modifications	ΔCn	117/114	117/114 Count	117/114 Variability [%]	q-Value	PEP	IonScore	Exp Value	Charge	MH+ [Da]	ΔM [ppm]	RT [min]	# Missed Cleavages
	High	sLVSFYHLVk	2	1	1	P19125	N-Term(iTRAQ4plex); K10(iTRAQ4plex)	0,0000	0,507	1		0	6,622E-07	36	0,000238215	3	1480,87818	1,05	37,87	0
	Medium	vFLPR	1	1	1	P19125	N-Term(iTRAQ4plex)	0,0000	0,760	1		0,046	0,5663	10	0,095492663	2	775,49498	0,36	24,55	0
Q46363	Acetazolamide conferring resistance protein zam OS=Synechocystis sp.	40,24	3,32	1	2	2	3	1,044	2	36,1	782	87,5	4,93							
	A3	Sequence	# PSMs	# Proteins	# Protein Groups	Protein Group Accessions	Modifications	ΔCn	117/114	117/114 Count	117/114 Variability [%]	q-Value	PEP	IonScore	Exp Value	Charge	MH+ [Da]	ΔM [ppm]	RT [min]	# Missed Cleavages
	High	aVLNHLLANTLk	2	1	1	Q46363	N-Term(iTRAQ4plex); K12(iTRAQ4plex)	0,0000	1,322	1		0	1,315E-07	38	0,000162544	3	1594,98923	0,57	36,94	0
	High	yPIAQHPPLGEVTk	1	1	1	Q46363	N-Term(iTRAQ4plex); K14(iTRAQ4plex)	0,0000	0,825	1		0	0,0009069	11	0,072105768	3	1838,04728	3,15	28,38	0
P73619	Sll1775 protein OS=Synechocystis sp. (strain PCC 6803 / Kazusa) GN=s	40,22	2,94	1	2	2	2	0,809	2	16,4	817	92,3	4,92							
	A3	Sequence	# PSMs	# Proteins	# Protein Groups	Protein Group Accessions	Modifications	ΔCn	117/114	117/114 Count	117/114 Variability [%]	q-Value	PEP	IonScore	Exp Value	Charge	MH+ [Da]	ΔM [ppm]	RT [min]	# Missed Cleavages
	High	vIGYNLSLAPR	1	1	1	P73619	N-Term(iTRAQ4plex)	0,0000	0,725	1		0	0,001176	33	0,00051047	2	1346,79216	0,64	36,22	0
	High	lYGSFGLLmHWPk	1	1	1	P73619	N-Term(iTRAQ4plex); M9(Oxidation); K13(iTRAQ4plex)	0,0000	0,903	1		0	0,00007079	21	0,009970062	3	1853,00223	-0,02	44,27	0
Q55496	Sll0493 protein OS=Synechocystis sp. (strain PCC 6803 / Kazusa) GN=s	40,17	8,33	1	2	2	3	1,029	3	29,7	228	25,2	6,23							
	A3	Sequence	# PSMs	# Proteins	# Protein Groups	Protein Group Accessions	Modifications	ΔCn	117/114	117/114 Count	117/114 Variability [%]	q-Value	PEP	IonScore	Exp Value	Charge	MH+ [Da]	ΔM [ppm]	RT [min]	# Missed Cleavages
	High	rQFAVIGLGR	1	1	1	Q55496	N-Term(iTRAQ4plex)	0,0000	1,278	1		0	0,002502	28	0,001625436	3	1260,76599	0,18	27,77	1
	High	tSHFLSLIR	2	1	1	Q55496	N-Term(iTRAQ4plex)	0,0000	0,932	2	14,6	0	0,0007074	26	0,003100221	3	1217,71305	0,61	31,41	0
P74569	Aspartokinase OS=Synechocystis sp. (strain PCC 6803 / Kazusa) GN=lys	40,13	3,50	1	2	2	2	1,034	2	23,7	600	63,5	5,38							
	A3	Sequence	# PSMs	# Proteins	# Protein Groups	Protein Group Accessions	Modifications	ΔCn	117/114	117/114 Count	117/114 Variability [%]	q-Value	PEP	IonScore	Exp Value	Charge	MH+ [Da]	ΔM [ppm]	RT [min]	# Missed Cleavages
	High	aAHSAFNLAGTk	1	1	1	P74569	N-Term(iTRAQ4plex); K12(iTRAQ4plex)	0,0000	1,211	1		0	0,0002682	33	0,000552039	3	1475,82254	1,10	19,88	0
	High	vVAPPVQNR	1	1	1	P74569	N-Term(iTRAQ4plex)	0,0000	0,883	1		0	0,007123	21	0,008769403	2	1123,67180	1,20	18,48	0
P74239	Ssl2148 protein OS=Synechocystis sp. (strain PCC 6803 / Kazusa) GN=s	39,98	16,33	1	3	3	6	0,929	6	11,8	98	10,6	4,50							
	A3	Sequence	# PSMs	# Proteins	# Protein Groups	Protein Group Accessions	Modifications	ΔCn	117/114	117/114 Count	117/114 Variability [%]	q-Value	PEP	IonScore	Exp Value	Charge	MH+ [Da]	ΔM [ppm]	RT [min]	# Missed Cleavages
	High	rPDFIPk	3	1	1	P74239	N-Term(iTRAQ4plex); K7(iTRAQ4plex)	0,0000	0,909	3	2,3	0	0,002385	14	0,042530241	3	1160,70224	-0,68	20,91	0
	High	rPVGWER	2	1	1	P74239	N-Term(iTRAQ4plex)	0,0000	1,041	2	16,2	0	0,00498	14	0,038456522	3	1043,58753	0,77	14,05	0
	High	skRPDFIPk	1	1	1	P74239	N-Term(iTRAQ4plex); K2(iTRAQ4plex); K9(iTRAQ4plex)	0,0000	1,065	1		0,002	0,02548	17	0,018028932	4	1519,92763	-2,94	17,37	1
Q55716	Glycine--tRNA ligase alpha subunit OS=Synechocystis sp. (strain PCC 68	39,05	10,37	1	3	3	3	1,031	3	37,5	299	34,3	4,87							
	A3	Sequence	# PSMs	# Proteins	# Protein Groups	Protein Group Accessions	Modifications	ΔCn	117/114	117/114 Count	117/114 Variability [%]	q-Value	PEP	IonScore	Exp Value	Charge	MH+ [Da]	ΔM [ppm]	RT [min]	# Missed Cleavages
	High	gAGTmNPHTFLR	1	1	1	Q55716	N-Term(iTRAQ4plex); M5(Oxidation)	0,0000	1,031	1		0	0,0001159	30	0,00104947	3	1461,74091	1,33	17,77	0
	High	eVAHIYLQQR	1	1	1	Q55716	N-Term(iTRAQ4plex)	0,0000	1,316	1		0	5,828E-07	19	0,012189054	3	1400,77768	0,71	21,23	0
	High	kVDkPISLV	1	1	1	Q55716	N-Term(iTRAQ4plex); K1(iTRAQ4plex); K4(iTRAQ4plex)	0,0000	0,658	1		0,002	0,02812	16	0,024264425	3	1430,93204	0,94	25,80	1
P74587	Carbamoyl-phosphate synthase small chain OS=Synechocystis sp. (strai	38,30	4,17	1	2	2	2	1,102	2	22,8	384	41,8	5,83							

	A3	Sequence	# PSMs	# Proteins	# Protein Groups	Protein Group Accessions	Modifications	ΔCn	117/114	117/114 Count	117/114 Variability [%]	q-Value	PEP	IonScore	Exp Value	Charge	MH+ [Da]	ΔM [ppm]	RT [min]	# Missed Cleavages
	High	iIGIYGIDTR	1	1	1	P74587	N-Term(iTRAQ4plex)	0,0000	0,946	1		0	0,0007517	38	0,00014756	2	1264,73906	0,69	37,79	0
	Medium	tVAGLR	1	1	1	P74587	N-Term(iTRAQ4plex)	0,0000	1,282	1		0,043	0,5336	17	0,050231176	2	760,48076	1,28	13,65	0
P73004	Long-chain-fatty-acid CoA ligase OS=Synechocystis sp. (strain PCC 6803	38,10	4,02	1	2	2	2	0,712	2	3,0	696	77,6	6,67							
	A3	Sequence	# PSMs	# Proteins	# Protein Groups	Protein Group Accessions	Modifications	ΔCn	117/114	117/114 Count	117/114 Variability [%]	q-Value	PEP	IonScore	Exp Value	Charge	MH+ [Da]	ΔM [ppm]	RT [min]	# Missed Cleavages
	High	rPVVTQTYQHLIDEmF	1	1	1	P73004	N-Term(iTRAQ4plex); M15(Oxidation)	0,0000	0,727	1		0	0,000005911	38	0,000154515	3	2137,08945	1,19	37,69	0
	High	dIkPHHIVGVPR	1	1	1	P73004	N-Term(iTRAQ4plex); K3(iTRAQ4plex)	0,0000	0,698	1		0	0,002536	13	0,050578973	4	1655,99624	0,86	16,97	0
Q55171	Slr0476 protein OS=Synechocystis sp. (strain PCC 6803 / Kazusa) GN=s	38,09	17,65	1	2	2	5	1,265	4	59,7	136	14,4	7,59							
	A3	Sequence	# PSMs	# Proteins	# Protein Groups	Protein Group Accessions	Modifications	ΔCn	117/114	117/114 Count	117/114 Variability [%]	q-Value	PEP	IonScore	Exp Value	Charge	MH+ [Da]	ΔM [ppm]	RT [min]	# Missed Cleavages
	High	hIGGLDLLPTPk	2	1	1	Q55171	N-Term(iTRAQ4plex); K12(iTRAQ4plex)	0,0000	0,972	2	288,3	0	0,0002675	22	0,005928844	3	1548,93894	2,39	33,70	0
	High	gMPTNRPIEASk	1	1	1	Q55171	N-Term(iTRAQ4plex); K12(iTRAQ4plex)	0,0000	1,265	1		0	0,00002511	20	0,012328494	3	1588,87387	1,18	16,68	0
	High	gmPTNRPIEASk	2	1	1	Q55171	N-Term(iTRAQ4plex); M2(Oxidation); K12(iTRAQ4plex)	0,0000	1,264	1		0	0,002437	20	0,014666746	3	1604,86850	0,99	11,86	0
Q55374	Slr0907 protein OS=Synechocystis sp. (strain PCC 6803 / Kazusa) GN=s	38,09	3,94	1	3	3	3	0,837	3	20,3	1014	115,3	5,25							
	A3	Sequence	# PSMs	# Proteins	# Protein Groups	Protein Group Accessions	Modifications	ΔCn	117/114	117/114 Count	117/114 Variability [%]	q-Value	PEP	IonScore	Exp Value	Charge	MH+ [Da]	ΔM [ppm]	RT [min]	# Missed Cleavages
	High	fWILPAVVPHNLIk	1	1	1	Q55374	N-Term(iTRAQ4plex); K14(iTRAQ4plex)	0,0000	0,837	1		0	0,0005408	30	0,000954927	3	1935,18418	0,97	50,83	0
	High	kQVFTNITQIFADHEVSR	1	1	1	Q55374	N-Term(iTRAQ4plex); K1(iTRAQ4plex)	0,0000	0,958	1		0	0,000006752	13	0,053452744	4	2421,31606	1,29	41,27	1
	High	fATHPELR	1	1	1	Q55374	N-Term(iTRAQ4plex)	0,0000	0,511	1		0,004	0,05411	21	0,00810905	3	1114,61396	1,22	18,06	0
Q55766	Ribose-5-phosphate isomerase A OS=Synechocystis sp. (strain PCC 680	37,78	9,36	1	2	2	3	0,929	3	1,9	235	24,7	5,03							
	A3	Sequence	# PSMs	# Proteins	# Protein Groups	Protein Group Accessions	Modifications	ΔCn	117/114	117/114 Count	117/114 Variability [%]	q-Value	PEP	IonScore	Exp Value	Charge	MH+ [Da]	ΔM [ppm]	RT [min]	# Missed Cleavages
	High	kYGIPLTTLDVADR	1	1	1	Q55766	N-Term(iTRAQ4plex); K1(iTRAQ4plex)	0,0000	0,467	1		0	0,00008959	32	0,000642643	3	1850,06559	1,60	36,00	1
	High	lGGkPELR	2	1	1	Q55766	N-Term(iTRAQ4plex); K4(iTRAQ4plex)	0,0000	0,935	2	0,9	0,004	0,05499	19	0,012941065	2	1157,72453	0,02	16,95	0
Q55449	Slr0031 protein OS=Synechocystis sp. (strain PCC 6803 / Kazusa) GN=s	37,44	3,42	1	2	2	3	0,859	3	20,5	584	66,9	5,58							
	A3	Sequence	# PSMs	# Proteins	# Protein Groups	Protein Group Accessions	Modifications	ΔCn	117/114	117/114 Count	117/114 Variability [%]	q-Value	PEP	IonScore	Exp Value	Charge	MH+ [Da]	ΔM [ppm]	RT [min]	# Missed Cleavages
	High	iRPIALEk	2	1	1	Q55449	N-Term(iTRAQ4plex); K8(iTRAQ4plex)	0,0000	0,920	2	10,2	0	0,0002227	21	0,00790624	3	1227,79932	-2,80	23,49	0
	Medium	fPVWTPGSYLVR	1	1	1	Q55449	N-Term(iTRAQ4plex)	0,0000	0,638	1		0,03	0,3746	24	0,004456255	2	1565,86284	2,00	46,15	0
P72653	Sll1049 protein OS=Synechocystis sp. (strain PCC 6803 / Kazusa) GN=sl	37,37	4,96	1	2	2	2	1,202	2	16,3	564	63,9	5,06							
	A3	Sequence	# PSMs	# Proteins	# Protein Groups	Protein Group Accessions	Modifications	ΔCn	117/114	117/114 Count	117/114 Variability [%]	q-Value	PEP	IonScore	Exp Value	Charge	MH+ [Da]	ΔM [ppm]	RT [min]	# Missed Cleavages
	High	kIESVGLPFSR	1	1	1	P72653	N-Term(iTRAQ4plex); K1(iTRAQ4plex)	0,0000	1,078	1		0	0,000387	36	0,000265442	3	1520,90357	-0,24	30,89	1
	High	lSYGIkPHIDTPDLALR	1	1	1	P72653	N-Term(iTRAQ4plex); K6(iTRAQ4plex)	0,0000	1,341	1		0	0,00001308	15	0,034432615	4	2197,25747	-0,41	36,57	0
P74207	3-isopropylmalate dehydratase small subunit OS=Synechocystis sp. (stra	36,32	10,00	1	2	2	2	0,898	2	8,7	200	21,6	5,15							
	A3	Sequence	# PSMs	# Proteins	# Protein Groups	Protein Group Accessions	Modifications	ΔCn	117/114	117/114 Count	117/114 Variability [%]	q-Value	PEP	IonScore	Exp Value	Charge	MH+ [Da]	ΔM [ppm]	RT [min]	# Missed Cleavages
	High	eHAPQAIIk	1	1	1	P74207	N-Term(iTRAQ4plex); K9(iTRAQ4plex)	0,0000	0,952	1		0	0,00002288	26	0,00381357	3	1294,77216	-0,01	16,04	0
	High	lPYLHWQTSAA	1	1	1	P74207	N-Term(iTRAQ4plex)	0,0000	0,847	1		0	0,003108	24	0,003801631	2	1430,75578	0,62	39,72	0
P73163	Putative carboxymethylenebutenolidase OS=Synechocystis sp. (strain P(	36,22	9,76	1	2	2	2	0,872	2	15,1	246	26,9	4,96							
	A3	Sequence	# PSMs	# Proteins	# Protein Groups	Protein Group Accessions	Modifications	ΔCn	117/114	117/114 Count	117/114 Variability [%]	q-Value	PEP	IonScore	Exp Value	Charge	MH+ [Da]	ΔM [ppm]	RT [min]	# Missed Cleavages
	High	qAPGFEEGYTPEGIEAGR	1	1	1	P73163	N-Term(iTRAQ4plex)	0,0000	0,788	1		0	0,000002025	29	0,001216102	3	2051,97745	-0,83	32,07	0
	High	yQVNHk	1	1	1	P73163	N-Term(iTRAQ4plex); K6(iTRAQ4plex)	0,0000	0,965	1		0,002	0,03481	20	0,009794223	2	1076,60625	-2,65	9,79	0
P73176	Protein PatA OS=Synechocystis sp. (strain PCC 6803 / Kazusa) GN=sll1	36,10	2,99	1	2	2	2	0,642	2	20,2	401	44,6	6,54							
	A3	Sequence	# PSMs	# Proteins	# Protein Groups	Protein Group Accessions	Modifications	ΔCn	117/114	117/114 Count	117/114 Variability [%]	q-Value	PEP	IonScore	Exp Value	Charge	MH+ [Da]	ΔM [ppm]	RT [min]	# Missed Cleavages
	High	lNYLFQR	1	1	1	P73176	N-Term(iTRAQ4plex)	0,0000	0,561	1		0,004	0,05196	27	0,001931835	2	1097,62444	1,84	34,81	0
	High	sILAk	1	1	1	P73176	N-Term(iTRAQ4plex); K5(iTRAQ4plex)	0,0000	0,735	1		0,009	0,1173	22	0,006338259	2	819,55461	0,47	17,20	0
P74478	Slr1926 protein OS=Synechocystis sp. (strain PCC 6803 / Kazusa) GN=s	35,60	7,17	1	2	2	3	1,071	3	16,2	223	24,6	9,28							
	A3	Sequence	# PSMs	# Proteins	# Protein Groups	Protein Group Accessions	Modifications	ΔCn	117/114	117/114 Count	117/114 Variability [%]	q-Value	PEP	IonScore	Exp Value	Charge	MH+ [Da]	ΔM [ppm]	RT [min]	# Missed Cleavages
	High	kLVTLFYFD	1	1	1	P74478	N-Term(iTRAQ4plex); K1(iTRAQ4plex)	0,0000	1,227	1		0	0,005786	19	0,012416523	2	1433,82927	0,69	48,05	1
	High	iALLGSR	2	1	1	P74478	N-Term(iTRAQ4plex)	0,0000	1,015	2	8,0	0,005	0,05721	26	0,002376676	2	873,56444	0,67	28,09	0
Q55501	Slr0929 protein OS=Synechocystis sp. (strain PCC 6803 / Kazusa) GN=s	35,06	13,33	1	2	2	2	0,620	2	35,5	195	21,7	8,88							
	A3	Sequence	# PSMs	# Proteins	# Protein Groups	Protein Group Accessions	Modifications	ΔCn	117/114	117/114 Count	117/114 Variability [%]	q-Value	PEP	IonScore	Exp Value	Charge	MH+ [Da]	ΔM [ppm]	RT [min]	# Missed Cleavages
	High	lPFYVASQAGSTALIR	1	1	1	Q55501	N-Term(iTRAQ4plex)	0,0000	0,492	1		0	0,000002215	35	0,000311889	3	1838,03171	1,30	44,90	0
	High	kYPHIVVDTR	1	1	1	Q55501	N-Term(iTRAQ4plex); K1(iTRAQ4plex)	0,0000	0,782	1		0	0,007099	11	0,075330353	4	1515,89089	1,51	19,43	1
P73589	C4-dicarboxylase binding protein OS=Synechocystis sp. (strain PCC 680	34,43	9,76	1	3	3	3	0,730	3	18,3	369	41,1	7,62							
	A3	Sequence	# PSMs	# Proteins	# Protein Groups	Protein Group Accessions	Modifications	ΔCn	117/114	117/114 Count	117/114 Variability [%]	q-Value	PEP	IonScore	Exp Value	Charge	MH+ [Da]	ΔM [ppm]	RT [min]	# Missed Cleavages
	High	iVTSIASkPIYk	1	1	1	P73589	N-Term(iTRAQ4plex); K8(iTRAQ4plex); K12(iTRAQ4plex)	0,0000	0,861	1		0	0,000001528	26	0,002238567	3	1752,10324	2,11	28,85	0
	High	iTmNQVFR	1	1	1	P73589	N-Term(iTRAQ4plex); M3(Oxidation)	0,0000	0,646	1		0,001	0,0114	21	0,008034706	2	1168,62920	2,28	22,90	0
	High	lGmVQTkPNLSQAPQk	1	1	1	P73589	N-Term(iTRAQ4plex); M3(Oxidation); K7(iTRAQ4plex); K	0,0000	0,730	1		0,002	0,02675	12	0,062225731	3	2188,25553	3,23	24,04	0
P72985	Slr1600 protein OS=Synechocystis sp. (strain PCC 6803 / Kazusa) GN=s	34,05	8,72	1	2	2	3	1,301	3	3,0	149	17,1	4,98							
	A3	Sequence	# PSMs	# Proteins	# Protein Groups	Protein Group Accessions	Modifications	ΔCn	117/114	117/114 Count	117/114 Variability [%]	q-Value	PEP	IonScore	Exp Value	Charge	MH+ [Da]	ΔM [ppm]	RT [min]	# Missed Cleavages
	High	aIAEIkk	1	1	1	P72985	N-Term(iTRAQ4plex); K6(iTRAQ4plex); K7(iTRAQ4plex)	0,0000	3,263	1		0	0,0001597	24	0,004187646	3	1204,79789	-0,88	15,79	1
	High	vIVTPR	2	1	1	P72985	N-Term(iTRAQ4plex)	0,0000	1,287	2	1,5	0,002	0,03454	23	0,004808061	2	828,54314	0,91	17,33	0
P74358	Sll1528 protein OS=Synechocystis sp. (strain PCC 6803 / Kazusa) GN=sl	33,79	2,21	1	2	2	3	0,571	3	5,1	996	114,5	6,42							
	A3	Sequence	# PSMs	# Proteins	# Protein Groups	Protein Group Accessions	Modifications	ΔCn	117/114	117/114 Count	117/114 Variability [%]	q-Value	PEP	IonScore	Exp Value	Charge	MH+ [Da]	ΔM [ppm]	RT [min]	# Missed Cleavages
	High	fLPPkPQQELmGLFAR	1	1	1	P74358	N-Term(iTRAQ4plex); K5(iTRAQ4plex); M11(Oxidation)	0,0000	0,622	1		0,001	0,01458	12	0,061513439	3	2176,22348	1,98	43,78	0
	Medium	lPLFLk	2	1	1	P74358	N-Term(iTRAQ4plex); K6(iTRAQ4plex)	0,0000	0,561	2	2,6	0,023	0,283	21	0,008850545	2	1018,69414	3,73	40,63	0
P73290	Adenylosuccinate synthetase OS=Synechocystis sp. (strain PCC 6803 / k	33,02	6,31	1	3	3	4	1,423	4	17,0	444	48,8	5,45							
	A3	Sequence	# PSMs	# Proteins	# Protein Groups	Protein Group Accessions	Modifications	ΔCn	117/114	117/114 Count	117/114 Variability [%]	q-Value	PEP	IonScore	Exp Value	Charge	MH+ [Da]	ΔM [ppm]	RT [min]	# Missed Cleavages
	High	lRPHVVDSSLk	1	1	1	P73290	N-Term(iTRAQ4plex); K11(iTRAQ4plex)	0,0000	1,377	1		0	0,0001181	23	0,005211587	3	1538,92435	-0,89	19,17	0
	High	kLLWTVEYk	2	1	1	P73290	N-Term(iTRAQ4plex); K1(iTRAQ4plex); K9(iTRAQ4plex)	0,0000	1,594	2	12,0	0	0,00004793	22	0,005701249	3	1611,98398	0,33	33,35	1
	High	iYDAIHQR	1	1	1	P73290	N-Term(iTRAQ4plex)	0,0000	1,000	1		0	0,004594	14	0,041876464	3	1159,63446	0,36	19,25	0
P73556	Slr0876 protein OS=Synechocystis sp. (strain PCC 6803 / Kazusa) GN=s	32,32	7,58	1	2	2	2	0,820	2	17,0	409	45,2	8,69							
	A3	Sequence	# PSMs	# Proteins	# Protein Groups	Protein Group Accessions	Modifications	ΔCn	117/114	117/114 Count	117/114 Variability [%]	q-Value	PEP	IonScore	Exp Value	Charge	MH+ [Da]	ΔM [ppm]	RT [min]	# Missed Cleavages
	High	gGNALFTFPATPIk	1	1	1	P73556	N-Term(iTRAQ4plex); K14(iTRAQ4plex)	0,0000	0,918	1		0	0,003969	26	0,00232793	3	1721,98569	1,60	42,56	0
	High	kQAPVVATNLLGLLNSk	1	1	1	P73556	N-Term(iTRAQ4plex); K1(iTRAQ4plex); K17(iTRAQ4plex)	0,0000	0,732	1		0	0,0001167	19	0,012559432	4	2198,36147	0,80	45,29	1
P74588	Slr1557 protein OS=Synechocystis sp. (strain PCC 6803 / Kazusa) GN=s	32,32	4,88	1	2	2	2	2,397	2	101,0	369	40,2	4,78							
	A3	Sequence	# PSMs	# Proteins	# Protein Groups	Protein Group Accessions	Modifications	ΔCn	117/114	117/114 Count	117/114 Variability [%]	q-Value	PEP	IonScore	Exp Value	Charge	MH+ [Da]	ΔM [ppm]	RT [min]	# Missed Cleavages
	High	rGELIVRPLk	1	1	1	P74588	N-Term(iTRAQ4plex); K10(iTRAQ4plex)	0,0000	4,220	1		0	0,00008264	29	0,001399491	4	1468,95644	-0,14	20,27	1
	High	rPQIVSIk	1	1	1	P74588	N-Term(iTRAQ4plex); K8(iTRAQ4plex)	0,0000	1,362	1		0	0,002095	17	0,020891518	3	1228,79926	1,03	19,86	0
Q55943	Sll0788 protein OS=Synechocystis sp. (strain PCC 6803 / Kazusa) GN=sl	31,59	12,76	2	2	2	2	5,494	2	103,8	196	22,9	6,96							
	A3	Sequence	# PSMs	# Proteins	# Protein Groups	Protein Group Accessions	Modifications	ΔCn	117/114	117/114 Count	117/114 Variability [%]	q-Value	PEP	IonScore	Exp Value	Charge	MH+ [Da]	ΔM [ppm]	RT [min]	# Missed Cleavages
	High	tWYQQWFk	1	2	1	Q55943	N-Term(iTRAQ4plex); K8(iTRAQ4plex)	0,0000	9,781	1		0	0,00568	32	0,000691783	2	1474,77239	0,16	42,37	0
	High	qmIPHHQmAVmmASNLk	1	2	1	Q55943	N-Term(iTRAQ4plex); M2(Oxidation); M8(Oxidation); M1	0,0000	3,086	1		0	0,000006723	12	0,058206301	4	2319,13540	1,42	16,58	0
Q55798	6-carboxy-5,6,7,8-tetrahydropterin synthase OS=Synechocystis sp. (stra	31,58	17,05	1	2	2	2	0,736	2	12,1	129	14,5	7,01							
	A3	Sequence	# PSMs	# Proteins	# Protein Groups	Protein Group Accessions	Modifications	ΔCn	117/114	117/114 Count	117/114 Variability [%]	q-Value	PEP	IonScore	Exp Value	Charge	MH+ [Da]	ΔM [ppm]	RT [min]	# Missed Cleavages
	High	eFSFEAAHQLPHHEGk	1	1	1	Q55798	N-Term(iTRAQ4plex); K16(iTRAQ4plex)	0,0000	0,798	1		0	0,0001029	15	0,050129256	5	2152,08021	-0,59	23,67	0
	High	mWIIYk	1	1	1	Q55798	N-Term(iTRAQ4plex); K6(iTRAQ4plex)	0,0000	0,678	1		0,002	0,03102	32	0,000693378	2	1141,66667	-1,35	38,01	0
Q55138	Sll0068 protein OS=Synechocystis sp. (strain PCC 6803 / Kazusa) GN=sl	31,36	1,53	1	2	2	2	0,885	2	19,2	1239	137,7	5,96							
	A3	Sequence	# PSMs	# Proteins	# Protein Groups	Protein Group Accessions	Modifications	ΔCn	117/114	117/114 Count	117/114 Variability [%]	q-Value	PEP	IonScore	Exp Value	Charge	MH+ [Da]	ΔM [ppm]	RT [min]	# Missed Cleavages
	High	vLAFWWGR	1	1	1	Q55138	N-Term(iTRAQ4plex)	0,0000	0,778	1		0	0,003016	23	0,004476824	2	1178,65874	-0,36	48,51	0
	High	kLLPTVVYQLR	1	1	1	Q55138	N-Term(iTRAQ4plex); K1(iTRAQ4plex)	0,0000	1,006	1		0	0,0001587	21	0,00814648	3	1618,02903	-0,27	36,94	1
P72720	Glutamine--fructose-6-phosphate aminotransferase [isomerizing] OS=Sy	30,98	3,33	1	2	2	2	0,633	2	6,9	631	69,6	5,85							
	A3	Sequence	# PSMs	# Proteins	# Protein Groups	Protein Group Accessions	Modifications	ΔCn	117/114	117/114 Count	117/114 Variability [%]	q-Value	PEP	IonScore	Exp Value	Charge	MH+ [Da]	ΔM [ppm]	RT [min]	# Missed Cleavages
	High	hGPIALLDAk	1	1	1	P72720	N-Term(iTRAQ4plex); K10(iTRAQ4plex)	0,0000	0,604	1		0	0,0002283	31	0,000796104	3	1322,80606	1,95	28,72	0
	High	ekLENHSNFSR	1	1	1	P72720	N-Term(iTRAQ4plex); K2(iTRAQ4plex)	0,0000	0,663	1		0,001	0,01213	13	0,052236011	4	1648,86684	1,37	12,88	1
P74752	Slr0605 protein OS=Synechocystis sp. (strain PCC 6803 / Kazusa) GN=s	29,83	6,90	1	3	3	5	0,507	5	13,9	319	36,5	6,58							
	A3	Sequence	# PSMs	# Proteins	# Protein Groups	Protein Group Accessions	Modifications	ΔCn	117/114	117/114 Count	117/114 Variability [%]	q-Value	PEP	IonScore	Exp Value	Charge	MH+ [Da]	ΔM [ppm]	RT [min]	# Missed Cleavages
	High	rIQDYPNLWR	1	1	1	P74752	N-Term(iTRAQ4plex)	0,0000	1,026	1		0	0,00002826	14	0,03944573	3	1504,81574	1,05	31,21	1
	High	tLIFR	1	1	1	P74752	N-Term(iTRAQ4plex)	0,0000	0,507	1		0,004	0,05081	28	0,001489258	2	793,50414	-1,42	25,86	0
	High	lFTTLIR	3	1	1	P74752	N-Term(iTRAQ4plex)	0,0000	0,502	3	12,2	0,005	0,06344	14	0,044153995	2	1007,63640	-0,61	39,27	0
P73310	50S ribosomal protein L14 OS=Synechocystis sp. (strain PCC 6803 / Kaz	29,78	18,85	1	3	3	4	1,218	4	15,1	122	13,3	10,08							
	A3	Sequence	# PSMs	# Proteins	# Protein Groups	Protein Group Accessions	Modifications	ΔCn	117/114	117/114 Count	117/114 Variability [%]	q-Value	PEP	IonScore	Exp Value	Charge	MH+ [Da]	ΔM [ppm]	RT [min]	# Missed Cleavages
	High	dALPNmPIkk	1	1	1	P73310	N-Term(iTRAQ4plex); M6(Oxidation); K9(iTRAQ4plex); K	0,0000	0,879	1		0	0,002822	25	0,003396018	3	1574,92691	-2,00	18,75	1
	High	kLMcLR	1	1	1	P73310	N-Term(iTRAQ4plex); K1(iTRAQ4plex); C4(Carbamidome	0,0000	1,474	1		0,004	0,05475	14	0,041780151	3	1108,65451	-2,56	19,23	1
	Medium	vFGPVAR	1	1	1	P73310	N-Term(iTRAQ4plex)	0,0000	1,205	1		0,018	0,2319	17	0,018491409	2	889,53722	-0,48	21,04	0
	Medium	kLmcLR	1	1	1	P73310	K1(iTRAQ4plex); N-Term(iTRAQ4plex); M3(Oxidation); C	0,0000	1,231	1		0,038	0,4799	10	0,097044294	3	1124,64933	-2,61	13,79	1
P73706	Slr1818 protein OS=Synechocystis sp. (strain PCC 6803 / Kazusa) GN=s	29,75	8,46	1	2	2	3	1,032	3	4,9	201	23,3	5,25							
	A3	Sequence	# PSMs	# Proteins	# Protein Groups	Protein Group Accessions	Modifications	ΔCn	117/114	117/114 Count	117/114 Variability [%]	q-Value	PEP	IonScore	Exp Value	Charge	MH+ [Da]	ΔM [ppm]	RT [min]	# Missed Cleavages
	High	gIPVHQVSQR	1	1	1	P73706	N-Term(iTRAQ4plex)	0,0000	1,067	1		0	0,0005682	22	0,00754037	3	1264,72492	0,51	14,62	0
	High	qVAQAQk	2	1	1	P73706	N-Term(iTRAQ4plex); K7(iTRAQ4plex)	0,0000	1,014	2	2,6	0,002	0,03156	20	0,010813593	2	1060,63420	-1,07	9,70	0
P73997	3-dehydroquinate synthase OS=Synechocystis sp. (strain PCC 6803 / Ka	29,66	7,20	1	2	2	3	1,098	2	17,1	361	38,8	5,80							
	A3	Sequence	# PSMs	# Proteins	# Protein Groups	Protein Group Accessions	Modifications	ΔCn	117/114	117/114 Count	117/114 Variability [%]	q-Value	PEP	IonScore	Exp Value	Charge	MH+ [Da]	ΔM [ppm]	RT [min]	# Missed Cleavages
	High	tGVNHPQGk	2	1	1	P73997	N-Term(iTRAQ4plex); K9(iTRAQ4plex)	0,0000	0,979	1		0	0,001719	19	0,011245273	3	1225,69068	1,23	7,93	0
	High	aGYEVFQHLIPAGETHk	1	1	1	P73997	N-Term(iTRAQ4plex); K17(iTRAQ4plex)	0,0000	1,231	1		0	0,00004533	15	0,030058687	4	2185,16738	1,33	37,01	0
Q55687	Long-chain acyl-[acyl-carrier-protein] reductase OS=Synechocystis sp. (	29,55	7,06	1	2	2	2	1,141	2	6,1	340	37,6	6,05							
	A3	Sequence	# PSMs	# Proteins	# Protein Groups	Protein Group Accessions	Modifications	ΔCn	117/114	117/114 Count	117/114 Variability [%]	q-Value	PEP	IonScore	Exp Value	Charge	MH+ [Da]	ΔM [ppm]	RT [min]	# Missed Cleavages
	High	fTTGNTHTAYVIcR	1	1	1	Q55687	N-Term(iTRAQ4plex); C13(Carbamidomethyl)	0,0000	1,095	1		0	1,043E-07	24	0,003638899	3	1784,88626	-0,44	24,80	0
	High	vkADGVHILk	1	1	1	Q55687	N-Term(iTRAQ4plex); K2(iTRAQ4plex); K10(iTRAQ4plex)	0,0000	1,190	1		0,002	0,03523	18	0,015204425	4	1511,96132	-1,37	20,03	1
P73450	Nitrate transport ATP-binding protein NrtC OS=Synechocystis sp. (strain	28,89	2,69	1	2	2	2	1,917	2	6,0	670	75,1	5,77							
	A3	Sequence	# PSMs	# Proteins	# Protein Groups	Protein Group Accessions	Modifications	ΔCn	117/114	117/114 Count	117/114 Variability [%]	q-Value	PEP	IonScore	Exp Value	Charge	MH+ [Da]	ΔM [ppm]	RT [min]	# Missed Cleavages
	High	kFAEAGVk	1	1	1	P73450	N-Term(iTRAQ4plex); K1(iTRAQ4plex); K8(iTRAQ4plex)	0,0000	1,842	1		0	0,002026	28	0,001765916	3	1281,78934	0,17	15,21	1
	High	yPATHLALVk	1	1	1	P73450	N-Term(iTRAQ4plex); K10(iTRAQ4plex)	0,0000	1,996	1		0	0,001199	14	0,036472876	3	1400,85047	0,04	26,74	0
Q55898	Polyphosphate kinase OS=Synechocystis sp. (strain PCC 6803 / Kazusa)	28,78	2,47	1	2	2	2	0,772	2	9,3	728	82,6	5,35							
	A3	Sequence	# PSMs	# Proteins	# Protein Groups	Protein Group Accessions	Modifications	ΔCn	117/114	117/114 Count	117/114 Variability [%]	q-Value	PEP	IonScore	Exp Value	Charge	MH+ [Da]	ΔM [ppm]	RT [min]	# Missed Cleavages
	High	yVHIGTGNYNPk	1	1	1	Q55898	N-Term(iTRAQ4plex); K12(iTRAQ4plex)	0,0000	0,725	1		0	0,000004876	28	0,001682558	3	1650,88712	1,75	23,84	0
	Medium	lLEHSR	1	1	1	Q55898	N-Term(iTRAQ4plex)	0,0000	0,822	1		0,017	0,2141	14	0,039261782	3	898,52597	3,64	13,38	0
P73660	Porphobilinogen deaminase OS=Synechocystis sp. (strain PCC 6803 / Ka	28,68	3,13	1	2	2	3	1,277	2	28,0	320	34,9	5,73							
	A3	Sequence	# PSMs	# Proteins	# Protein Groups	Protein Group Accessions	Modifications	ΔCn	117/114	117/114 Count	117/114 Variability [%]	q-Value	PEP	IonScore	Exp Value	Charge	MH+ [Da]	ΔM [ppm]	RT [min]	# Missed Cleavages
	High	tDLAVHSLk	2	1	1	P73660	N-Term(iTRAQ4plex); K9(iTRAQ4plex)	0,0000	1,060	1		0	0,003948	22	0,006151344	3	1271,75891	2,14	19,79	0
	High	rTDLAVHSLk	1	1	1	P73660	N-Term(iTRAQ4plex); K10(iTRAQ4plex)	0,0000	1,537	1		0,004	0,0498	20	0,010989299	4	1427,85879	1,04	17,03	1
Q55863	Pyruvate kinase 1 OS=Synechocystis sp. (strain PCC 6803 / Kazusa) GN	28,58	5,59	1	2	2	3	0,476	2	3,2	483	53,1	5,86							
	A3	Sequence	# PSMs	# Proteins	# Protein Groups	Protein Group Accessions	Modifications	ΔCn	117/114	117/114 Count	117/114 Variability [%]	q-Value	PEP	IonScore	Exp Value	Charge	MH+ [Da]	ΔM [ppm]	RT [min]	# Missed Cleavages
	High	kGVNLPGLVLTLPSmTTk	2	1	1	Q55863	N-Term(iTRAQ4plex); K1(iTRAQ4plex); M15(Oxidation); I	0,0000	0,487	1		0	0,00002934	27	0,001827974	4	2317,39492	2,56	41,64	1
	Medium	kGEDIHTLk	1	1	1	Q55863	N-Term(iTRAQ4plex); K1(iTRAQ4plex); K9(iTRAQ4plex)	0,0000	0,466	1		0,033	0,4137	14	0,037841645	4	1472,88747	5,26	13,91	1
Q55140	Sll0066 protein OS=Synechocystis sp. (strain PCC 6803 / Kazusa) GN=sl	27,57	2,54	1	2	2	2	0,822	2	42,6	315	34,1	4,87							
	A3	Sequence	# PSMs	# Proteins	# Protein Groups	Protein Group Accessions	Modifications	ΔCn	117/114	117/114 Count	117/114 Variability [%]	q-Value	PEP	IonScore	Exp Value	Charge	MH+ [Da]	ΔM [ppm]	RT [min]	# Missed Cleavages
	High	rLWIYDLk	1	1	1	Q55140	N-Term(iTRAQ4plex); K8(iTRAQ4plex)	0,0000	0,624	1		0	0,0007228	28	0,001745702	3	1394,83957	-0,20	37,72	1
	High	lWIYDLk	1	1	1	Q55140	N-Term(iTRAQ4plex); K7(iTRAQ4plex)	0,0000	1,082	1		0,006	0,07945	11	0,085697865	2	1238,73320	-4,47	44,32	0
P73803	Slr1263 protein OS=Synechocystis sp. (strain PCC 6803 / Kazusa) GN=s	26,93	5,32	1	2	2	2	1,264	2	6,8	470	51,7	9,14							
	A3	Sequence	# PSMs	# Proteins	# Protein Groups	Protein Group Accessions	Modifications	ΔCn	117/114	117/114 Count	117/114 Variability [%]	q-Value	PEP	IonScore	Exp Value	Charge	MH+ [Da]	ΔM [ppm]	RT [min]	# Missed Cleavages
	High	gRFDVFAHHVVQASPEGk	1	1	1	P73803	N-Term(iTRAQ4plex); K18(iTRAQ4plex)	0,0000	1,324	1		0	0,000007149	22	0,006965784	4	2269,20962	0,67	26,91	1
	High	yYHLVQR	1	1	1	P73803	N-Term(iTRAQ4plex)	0,0000	1,208	1		0	0,006872	19	0,015254314	3	1122,61841	0,68	18,82	0
Q55392	Slr0565 protein OS=Synechocystis sp. (strain PCC 6803 / Kazusa) GN=s	26,47	3,69	1	2	2	3	0,811	2	20,0	325	35,4	5,19							
	A3	Sequence	# PSMs	# Proteins	# Protein Groups	Protein Group Accessions	Modifications	ΔCn	117/114	117/114 Count	117/114 Variability [%]	q-Value	PEP	IonScore	Exp Value	Charge	MH+ [Da]	ΔM [ppm]	RT [min]	# Missed Cleavages
	High	sVPWIHR	1	1	1	Q55392	N-Term(iTRAQ4plex)	0,0000	0,710	1		0	0,001045	18	0,0141244	3	1038,59671	0,16	23,41	0
	High	lLFGk	2	1	1	Q55392	N-Term(iTRAQ4plex); K5(iTRAQ4plex)	0,0000	0,927	1		0,003	0,04486	21	0,007961044	2	865,57433	-0,74	29,14	0
P74145	Sll1390 protein OS=Synechocystis sp. (strain PCC 6803 / Kazusa) GN=sl	25,98	9,24	1	3	3	3	1,153	3	31,6	249	27,4	4,74							
	A3	Sequence	# PSMs	# Proteins	# Protein Groups	Protein Group Accessions	Modifications	ΔCn	117/114	117/114 Count	117/114 Variability [%]	q-Value	PEP	IonScore	Exp Value	Charge	MH+ [Da]	ΔM [ppm]	RT [min]	# Missed Cleavages
	High	yNQALIEADkR	1	1	1	P74145	N-Term(iTRAQ4plex); K10(iTRAQ4plex)	0,0000	0,936	1		0	0,0009488	21	0,007815738	3	1608,89444	-0,23	22,33	1
	Medium	lNSDLkk	1	1	1	P74145	N-Term(iTRAQ4plex); K6(iTRAQ4plex); K7(iTRAQ4plex)	0,0000	1,423	1		0,014	0,1799	14	0,043850041	3	1249,78113	-2,31	15,29	1
	Medium	fVVIR	1	1	1	P74145	N-Term(iTRAQ4plex)	0,0000	1,153	1		0,036	0,4438	19	0,022551308	2	777,51024	-0,15	25,87	0
P73049	Slr1768 protein OS=Synechocystis sp. (strain PCC 6803 / Kazusa) GN=s	23,09	4,70	1	2	2	2	1,142	2	13,4	298	32,8	5,62							
	A3	Sequence	# PSMs	# Proteins	# Protein Groups	Protein Group Accessions	Modifications	ΔCn	117/114	117/114 Count	117/114 Variability [%]	q-Value	PEP	IonScore	Exp Value	Charge	MH+ [Da]	ΔM [ppm]	RT [min]	# Missed Cleavages
	High	kQLELISAR	1	1	1	P73049	N-Term(iTRAQ4plex); K1(iTRAQ4plex)	0,0000	1,044	1		0	0,002535	23	0,005151931	3	1345,84092	0,24	21,97	1
	Medium	aIQAk	1	1	1	P73049	N-Term(iTRAQ4plex); K5(iTRAQ4plex)	0,0000	1,250	1		0,024	0,3002	13	0,048774104	2	818,53288	-1,15	11,25	0
P74751	Elongation factor 4 OS=Synechocystis sp. (strain PCC 6803 / Kazusa) Gl	23,00	4,64	1	3	3	3	1,859	3	6,1	603	66,9	5,22							
	A3	Sequence	# PSMs	# Proteins	# Protein Groups	Protein Group Accessions	Modifications	ΔCn	117/114	117/114 Count	117/114 Variability [%]	q-Value	PEP	IonScore	Exp Value	Charge	MH+ [Da]	ΔM [ppm]	RT [min]	# Missed Cleavages
	High	nFSIIAHIDHGk	1	1	1	P74751	N-Term(iTRAQ4plex); K12(iTRAQ4plex)	0,0000	1,232	1		0	0,0000087	21	0,007979396	4	1639,91689	0,61	28,52	0
	High	lkVEEPFIk	1	1	1	P74751	N-Term(iTRAQ4plex); K2(iTRAQ4plex); K9(iTRAQ4plex)	0,0000	1,937	1		0	0,00012	14	0,03749471	3	1534,95920	1,49	30,68	1
	High	lkELIPR	1	1	1	P74751	N-Term(iTRAQ4plex); K2(iTRAQ4plex)	0,0000	1,859	1		0,003	0,03968	14	0,041780151	3	1156,76557	-0,06	27,72	1
P73010	Sec-independent protein translocase protein TatA OS=Synechocystis sp.	22,43	17,46	1	2	2	3	1,137	3	14,5	126	13,7	6,70							
	A3	Sequence	# PSMs	# Proteins	# Protein Groups	Protein Group Accessions	Modifications	ΔCn	117/114	117/114 Count	117/114 Variability [%]	q-Value	PEP	IonScore	Exp Value	Charge	MH+ [Da]	ΔM [ppm]	RT [min]	# Missed Cleavages
	High	gFQEASkEFETELkR	1	1	1	P73010	N-Term(iTRAQ4plex); K7(iTRAQ4plex); K14(iTRAQ4plex)	0,0000	3,068	1		0	0,000004978	22	0,006682978	4	2231,20278	-0,22	33,07	2
	High	kLPEVGR	2	1	1	P73010	N-Term(iTRAQ4plex); K1(iTRAQ4plex)	0,0000	1,083	2	7,2	0	0,001832	12	0,069497632	3	1086,69001	2,41	15,75	1
P73792	Ssr2087 protein OS=Synechocystis sp. (strain PCC 6803 / Kazusa) GN=s	22,04	14,29	1	2	2	2	1,073	2	1,8	84	9,9	7,28							
	A3	Sequence	# PSMs	# Proteins	# Protein Groups	Protein Group Accessions	Modifications	ΔCn	117/114	117/114 Count	117/114 Variability [%]	q-Value	PEP	IonScore	Exp Value	Charge	MH+ [Da]	ΔM [ppm]	RT [min]	# Missed Cleavages
	High	vSTQSPR	1	1	1	P73792	N-Term(iTRAQ4plex)	0,0000	1,060	1		0	0,002805	21	0,007095288	2	918,51348	1,02	10,55	0
	High	kWWIR	1	1	1	P73792	N-Term(iTRAQ4plex); K1(iTRAQ4plex)	0,0000	1,086	1		0,003	0,03673	14	0,060859965	3	1076,66031	-0,42	25,40	1

Supplementary Table S4

[NiFe]-hydrogenase is essential for cyanobacterium *Synechocystis* sp. PCC 6803 aerobic growth in the dark

Edith De Rosa, Vanessa Checchetto, Cinzia Franchin, Elisabetta Bergantino, Paola Berto, Ildikò Szabò, Giorgio M. Giacometti, Giorgio Arrigoni, and Paola Costantini

Accession	Description	Score	Coverage	# Proteins	# Unique Peptides	# Peptides	# PSMs	Dark/Light (114/115)	114/115 Count	114/115 Variability [%]	# AAs	MW [kDa]	calc. pI
Q54714	C-phycocyanin beta chain OS=Synechocystis sp. (strain PCC 6803 / Kazusa) GN=cp	9885,23	86,63	1	20	20	328	0,911	303	13,8	172	18,1	5,14

A3	Sequence	# PSMs	# Proteins	# Protein Groups	Protein Group Accessions	Modifications	ΔCn	114/115	114/115 Count	114/115 Variability [%]	q-Value	PEP	IonScore	Exp Value	Charge	MH+ [Da]	ΔM [ppm]	RT [min]	# Missed Cleavages
High	gEYLSGSQLDALSATVAEGNkR	48	1	1	Q54714	N-Term(iTRAQ4plex); K21(iTRAQ4plex)	0,0000	0,939	43	9,6	0	3,771E-14	133	5,69368E-14	2	2554,33965	1,74	39,57	1
High	iTGNASAIVSNAAR	97	1	1	Q54714	N-Term(iTRAQ4plex)	0,0000	0,885	91	10,3	0	8,044E-10	98	1,82886E-10	2	1488,82622	0,73	57,85	0
High	mkEAALDIVNDPNGITR	14	1	1	Q54714	N-Term(iTRAQ4plex); K2(iTRAQ4plex)	0,0000	0,856	13	25,3	0	9,357E-14	88	1,47571E-09	3	2145,15909	0,65	37,26	1
High	mkEAALDIVNDPNGITR	39	1	1	Q54714	N-Term(iTRAQ4plex); M1(Oxidation); K2(iTRAQ4plex)	0,0000	0,919	34	11,1	0	3,34E-09	84	3,6389E-09	2	2161,15532	1,25	31,73	1
High	aLFAEQPQLIQPGGNAYTSR	6	1	1	Q54714	N-Term(iTRAQ4plex)	0,0000	0,327	5	22,0	0	8,372E-12	84	4,25598E-09	2	2305,20659	0,35	40,94	0
High	eAALDIVNDPNGITR	3	1	1	Q54714	N-Term(iTRAQ4plex)	0,0000	0,871	2	1,6	0	1,281E-09	77	2,09879E-08	2	1741,92217	1,14	35,71	0
High	vVSQADARGEYLSGSQLDALSATVAEGNkR	1	1	1	Q54714	N-Term(iTRAQ4plex); K29(iTRAQ4plex)	0,0000	1,380	1		0	9,231E-12	64	4,13019E-07	4	3380,76943	1,32	39,34	2
High	gEYLSGSQLDALSATVAEGNk	1	1	1	Q54714	N-Term(iTRAQ4plex); K21(iTRAQ4plex)	0,0000	0,991	1		0	8,174E-11	59	1,20218E-06	3	2398,23667	1,08	41,88	0
High	vVSQADAR	7	1	1	Q54714	N-Term(iTRAQ4plex)	0,0000	0,854	6	6,1	0	0,00007902	54	3,60554E-06	2	989,55144	1,79	13,48	0
High	mFDVFTR	33	1	1	Q54714	N-Term(iTRAQ4plex)	0,0000	0,943	33	11,4	0	0,002631	53	5,57147E-06	2	1059,54253	1,05	39,90	0
High	rIDSVNR	25	1	1	Q54714	N-Term(iTRAQ4plex)	0,0000	0,906	25	18,1	0	0,0001187	43	4,77496E-05	3	1003,57697	0,42	12,08	1
High	eTYVALGVPGASVAAGVQk	1	1	1	Q54714	N-Term(iTRAQ4plex); K19(iTRAQ4plex)	0,0000	0,809	1		0	3,624E-10	41	7,26056E-05	3	2105,18479	0,11	38,43	0
High	dMEIILR	3	1	1	Q54714	N-Term(iTRAQ4plex)	0,0000	0,743	3	2,7	0	0,001804	41	8,8914E-05	2	1033,58367	0,37	33,72	0
High	rmAAcLR	3	1	1	Q54714	N-Term(iTRAQ4plex); M2(Oxidation); C5(Carbamidomethyl)	0,0000	0,872	3	8,4	0	0,0001295	38	0,000149613	3	1037,54569	-0,80	11,39	1
High	mAAcLRDmEIILR	2	1	1	Q54714	N-Term(iTRAQ4plex); M1(Oxidation); C4(Carbamidomethyl); M8(Oxidation)	0,0000	0,972	2	30,3	0	0,001038	28	0,001648049	3	1767,90451	0,46	29,45	1
High	mAAcLRDMEIILR	1	1	1	Q54714	N-Term(iTRAQ4plex); M1(Oxidation); C4(Carbamidomethyl)	0,0000	0,706	1		0	0,0007467	21	0,007761935	3	1751,90824	-0,31	40,03	1
High	fDVFTR	3	1	1	Q54714	N-Term(iTRAQ4plex)	0,0000	0,571	3	2,8	0,001	0,01249	35	0,000327318	2	928,50066	-0,28	32,50	0
High	dmEIILR	3	1	1	Q54714	N-Term(iTRAQ4plex); M2(Oxidation)	0,0000	0,898	3	2,1	0,001	0,008161	28	0,001632939	2	1049,57842	0,21	24,59	0
High	mFDVFTR	15	1	1	Q54714	N-Term(iTRAQ4plex); M1(Oxidation)	0,0000	1,043	12	8,9	0,001	0,01239	15	0,029989554	2	1075,53691	0,54	32,32	0
High	rMAAcLR	1	1	1	Q54714	N-Term(iTRAQ4plex); C5(Carbamidomethyl)	0,0000	1,067	1		0,001	0,01415	14	0,044997595	3	1021,54923	-2,33	12,98	1
High	gDcSAIVAEIAGYFDR	1	1	1	Q54714	N-Term(iTRAQ4plex); C3(Carbamidomethyl)	0,0000	1,035	1		0,001	0,01364	10	0,092890223	3	1887,90140	-0,75	50,84	0
High	iDSVNR	6	1	1	Q54714	N-Term(iTRAQ4plex)	0,0000	0,955	6	4,2	0,003	0,03914	15	0,031766547	2	847,47655	1,33	52,11	0
High	mAAcLR	5	1	1	Q54714	N-Term(iTRAQ4plex); M1(Oxidation); C4(Carbamidomethyl)	0,0000	0,905	3	1,7	0,003	0,04285	13	0,046770284	2	881,44664	1,41	13,14	0
High	cLNGLR	5	1	1	Q54714	N-Term(iTRAQ4plex); C1(Carbamidomethyl)	0,0000	0,971	5	8,9	0,005	0,06115	34	0,000375811	2	876,49040	7,06	18,86	0
High	rmAAcLRDmEIILR	1	1	1	Q54714	N-Term(iTRAQ4plex); M2(Oxidation); C5(Carbamidomethyl); M9(Oxidation)	0,0000	0,854	1		0,008	0,09344	12	0,062369176	4	1924,00405	-0,40	24,72	2
Medium	mAAcLR	4	1	1	Q54714	N-Term(iTRAQ4plex); C4(Carbamidomethyl)	0,0000	0,781	4	6,5	0,01	0,117	22	0,006621708	2	865,45104	0,63	16,77	0

Accession	Description	Score	Coverage	# Proteins	# Unique Peptides	# Peptides	# PSMs	Dark/Light (114/115)	114/115 Count	114/115 Variability [%]	# AAs	MW [kDa]	calc. pI
Q54715	C-phycocyanin alpha chain OS=Synechocystis sp. (strain PCC 6803 / Kazusa) GN=c	8184,02	79,01	1	13	13	329	1,041	301	15,2	162	17,6	5,57

A3	Sequence	# PSMs	# Proteins	# Protein Groups	Protein Group Accessions	Modifications	ΔCn	114/115	114/115 Count	114/115 Variability [%]	q-Value	PEP	IonScore	Exp Value	Charge	MH+ [Da]	ΔM [ppm]	RT [min]	# Missed Cleavages
High	fPYTTQTQGNNFAADQR	57	1	1	Q54715	N-Term(iTRAQ4plex)	0,0000	1,055	54	18,8	0	2,271E-16	104	4,13019E-11	2	2103,00151	0,12	31,37	0
High	tFDLSPSWYVEALk	23	1	1	Q54715	N-Term(iTRAQ4plex); K14(iTRAQ4plex)	0,0000	0,954	21	22,5	0	1,957E-13	91	8,18465E-10	2	1944,03752	0,92	51,39	0
High	mkTPLTEAVSTADSQGR	3	1	1	Q54715	N-Term(iTRAQ4plex); K2(iTRAQ4plex)	0,0000	1,263	3	1,0	0	1,725E-10	90	0,000000001	2	2080,09917	2,12	31,19	1
High	mkTPLTEAVSTADSQGR	67	1	1	Q54715	N-Term(iTRAQ4plex); M1(Oxidation); K2(iTRAQ4plex)	0,0000	1,228	62	12,6	0	1,511E-09	90	1,22513E-09	2	2096,09233	1,27	27,10	1
High	tPLTEAVSTADSQGR	4	1	1	Q54715	N-Term(iTRAQ4plex)	0,0000	1,177	4	4,9	0	0,000001211	88	1,61797E-09	2	1676,85796	0,44	30,92	0
High	fLSSTELQIAFGR	14	1	1	Q54715	N-Term(iTRAQ4plex)	0,0000	0,985	14	3,4	0	9,502E-11	83	5,46978E-09	2	1612,88371	1,32	48,82	0
High	qANAGLQAAk	54	1	1	Q54715	N-Term(iTRAQ4plex); K10(iTRAQ4plex)	0,0000	1,044	43	10,2	0	1,299E-07	82	6,88605E-09	2	1259,74040	7,43	14,86	0
High	kTPLTEAVSTADSQGR	4	1	1	Q54715	N-Term(iTRAQ4plex); K1(iTRAQ4plex)	0,0000	0,909	3	6,5	0	3,669E-10	75	4,80972E-08	3	1949,05552	0,65	26,66	1
High	aLTDNAQSLVNGAAQAVYNk	1	1	1	Q54715	N-Term(iTRAQ4plex); K20(iTRAQ4plex)	0,0000	0,644	1		0	3,065E-13	65	2,91072E-07	3	2336,24851	1,54	45,23	0
High	aNHGLSGDAR	37	1	1	Q54715	N-Term(iTRAQ4plex)	0,0000	1,013	35	8,8	0	9,848E-07	51	7,19399E-06	2	1141,58684	3,28	11,11	0
High	lRQANAGLQAAk	2	1	1	Q54715	N-Term(iTRAQ4plex); K12(iTRAQ4plex)	0,0000	0,942	2	45,9	0	1,09E-09	50	1,04706E-05	3	1528,91571	-0,34	17,41	1
High	dIGYYLR	59	1	1	Q54715	N-Term(iTRAQ4plex)	0,0000	0,972	55	6,3	0	0,0005356	47	2,06524E-05	2	1043,56450	0,26	34,59	0
High	aNHGLSGDARDEANSYLDYAINALS	2	1	1	Q54715	N-Term(iTRAQ4plex)	0,0000	0,722	2	23,0	0	0,000007482	31	0,000807179	3	2781,32315	1,23	50,58	1
High	yIkANHGLSGDAR	2	1	1	Q54715	N-Term(iTRAQ4plex); K3(iTRAQ4plex)	0,0000	0,818	2	29,6	0	0,001069	20	0,008911894	3	1689,92539	-1,26	16,01	1

Accession	Description	Score	Coverage	# Proteins	# Unique Peptides	# Peptides	# PSMs	Dark/Light (114/115)	114/115 Count	114/115 Variability [%]	# AAs	MW [kDa]	calc. pI
P77961	Glutamine synthetase OS=Synechocystis sp. (strain PCC 6803 / Kazusa) GN=glnA F	5431,99	44,40	1	23	23	242	1,658	225	36,1	473	53,0	5,11

A3	Sequence	# PSMs	# Proteins	# Protein Groups	Protein Group Accessions	Modifications	ΔCn	114/115	114/115 Count	114/115 Variability [%]	q-Value	PEP	IonScore	Exp Value	Charge	MH+ [Da]	ΔM [ppm]	RT [min]	# Missed Cleavages
High	lVPGFEAPVNLAYSQGNR	8	1	1	P77961	N-Term(iTRAQ4plex)	0,0000	1,966	6	6,0	0	1,69E-11	112	6,77595E-12	2	2076,10234	1,35	45,23	0
High	iPSTPGSLEAALEALEk	2	1	1	P77961	N-Term(iTRAQ4plex); K17(iTRAQ4plex)	0,0000	1,968	2	63,2	0	1,431E-07	103	4,94277E-11	2	2014,13701	2,92	54,36	0
High	lVNSADNLMIYk	5	1	1	P77961	N-Term(iTRAQ4plex); K12(iTRAQ4plex)	0,0000	2,361	5	25,8	0	3,271E-12	94	4,18765E-10	2	1668,92534	1,19	39,75	0
High	hAPALLAFTNPTTNSYk	72	1	1	P77961	N-Term(iTRAQ4plex); K17(iTRAQ4plex)	0,0000	1,690	70	38,9	0	3,528E-11	94	4,21697E-10	2	2134,15776	1,97	36,55	0
High	hHHEVASGGQNELGIk	6	1	1	P77961	N-Term(iTRAQ4plex); K16(iTRAQ4plex)	0,0000	2,365	4	4,2	0	1,221E-12	76	2,26449E-08	2	2001,05376	1,63	13,13	0
High	qGYFPVAPTDTAQDIR	3	1	1	P77961	N-Term(iTRAQ4plex)	0,0000	2,223	3	26,9	0	6,498E-11	75	2,8312E-08	2	1922,97637	1,79	35,56	0
High	lVNSADNLmIYk	8	1	1	P77961	N-Term(iTRAQ4plex); M9(Oxidation); K12(iTRAQ4plex)	0,0000	1,877	7	47,6	0	2,823E-08	74	4,60457E-08	2	1684,92082	1,52	35,26	0
High	eEEGGNLGYkPGYk	14	1	1	P77961	N-Term(iTRAQ4plex); K10(iTRAQ4plex); K14(iTRAQ4plex)	0,0000	2,293	10	55,3	0	3,165E-13	71	9,14495E-08	2	1973,03716	1,57	20,21	0
High	rLVPGFEAPVNLAYSQGNR	5	1	1	P77961	N-Term(iTRAQ4plex)	0,0000	1,221	4	30,4	0	6,874E-11	68	1,58489E-07	3	2232,20249	0,82	39,68	1
High	ePTLSMIcSIk	3	1	1	P77961	N-Term(iTRAQ4plex); C8(Carbamidomethyl); K11(iTRAQ4plex)	0,0000	1,441	3	29,8	0	2,094E-07	68	1,76999E-07	2	1566,84758	0,11	40,50	0
High	fDkLVNSADNLmIYk	6	1	1	P77961	N-Term(iTRAQ4plex); K3(iTRAQ4plex); M12(Oxidation); K15(iTRAQ4plex)	0,0000	1,556	5	9,7	0	9,079E-13	65	3,09721E-07	3	2219,21482	1,87	41,63	1
High	iPLSGGNPk	20	1	1	P77961	N-Term(iTRAQ4plex); K9(iTRAQ4plex)	0,0000	1,398	18	9,9	0	0,000003041	63	5,59719E-07	2	1170,71135	2,42	22,43	0
High	lDNEVNPmR	2	1	1	P77961	N-Term(iTRAQ4plex); M8(Oxidation)	0,0000	1,510	2	4,3	0	0,0002377	55	3,26565E-06	2	1247,61797	0,69	19,02	0
High	lRPHPYEFSLYYDc	19	1	1	P77961	N-Term(iTRAQ4plex); C14(Carbamidomethyl)	0,0000	1,399	17	38,1	0	1,853E-08	51	8,37471E-06	2	2003,94719	1,48	38,70	0
High	tPQEVLk	4	1	1	P77961	N-Term(iTRAQ4plex); K7(iTRAQ4plex)	0,0000	1,940	4	12,8	0	0,002207	50	1,11422E-05	2	1102,67851	6,76	19,34	0
High	wIQDENIk	3	1	1	P77961	N-Term(iTRAQ4plex); K8(iTRAQ4plex)	0,0000	2,146	3	0,3	0	0,0007325	49	1,13755E-05	2	1333,74797	9,38	30,18	0
High	fDkLVNSADNLMIYk	1	1	1	P77961	N-Term(iTRAQ4plex); K3(iTRAQ4plex); K15(iTRAQ4plex)	0,0000	1,658	1		0	4,815E-08	44	3,70655E-05	3	2203,21836	1,18	46,11	1
High	ePTLSMIcSIkEPR	3	1	1	P77961	N-Term(iTRAQ4plex); C8(Carbamidomethyl); K11(iTRAQ4plex)	0,0000	1,909	3	46,8	0	5,507E-08	44	6,29638E-05	3	1949,04563	0,89	37,21	1
High	wNTGREEEGGNLGYkPGYk	19	1	1	P77961	N-Term(iTRAQ4plex); K15(iTRAQ4plex); K19(iTRAQ4plex)	0,0000	2,173	19	11,2	0	2,138E-13	42	5,86098E-05	3	2587,32730	0,28	25,25	1
High	dGQPLFAGDk	1	1	1	P77961	N-Term(iTRAQ4plex); K10(iTRAQ4plex)	0,0000	2,387	1		0	0,0001729	39	0,000130608	2	1335,71489	0,12	23,59	0
High	ePTLSmIcSIk	2	1	1	P77961	N-Term(iTRAQ4plex); M6(Oxidation); C8(Carbamidomethyl); K11(iTRAQ4plex	0,0000	1,033	2	67,7	0	0,00005844	38	0,000168256	2	1582,84429	1,24	34,10	0
High	lDNEVNPMR	3	1	1	P77961	N-Term(iTRAQ4plex)	0,0000	1,246	3	11,3	0	0,00008106	34	0,000378416	2	1231,62212	-0,06	24,90	0
High	tGEWYNR	3	1	1	P77961	N-Term(iTRAQ4plex)	0,0000	1,148	3	5,2	0	0,001367	31	0,000716094	2	1069,52471	5,95	19,34	0
High	ePTLSmIcSIkEPR	3	1	1	P77961	N-Term(iTRAQ4plex); M6(Oxidation); C8(Carbamidomethyl); K11(iTRAQ4plex	0,0000	1,706	3	20,7	0	0,000000213	29	0,001270486	4	1965,03908	0,14	32,07	1
High	hAPALLAFTNPTTNSYkR	2	1	1	P77961	N-Term(iTRAQ4plex); K17(iTRAQ4plex)	0,0000	1,162	2	14,0	0	0,000002533	29	0,001358313	4	2290,25625	0,69	32,28	1
High	tGEWYNRDPR	3	1	1	P77961	N-Term(iTRAQ4plex)	0,0000	1,964	3	2,5	0,001	0,009184	18	0,014255091	2	1437,70232	2,18	18,17	1
High	tIAAk	21	1	1	P77961	N-Term(iTRAQ4plex); K5(iTRAQ4plex)	0,0000	1,598	21	12,4	0,003	0,03604	32	0,000582063	2	791,52281	-0,14	49,26	0
Medium	iIDLk	1	1	1	P77961	N-Term(iTRAQ4plex); K5(iTRAQ4plex)	0,0000	1,888	1		0,02	0,214	26	0,002301283	2	889,59636	0,30	29,09	0

Accession	Description	Score	Coverage	# Proteins	# Unique Peptides	# Peptides	# PSMs	Dark/Light (114/115)	114/115 Count	114/115 Variability [%]	# AAs	MW [kDa]	calc. pI
Q01951	Allophycocyanin alpha chain OS=Synechocystis sp. (strain PCC 6803 / Kazusa) GN=	4709,44	62,73	1	14	15	177	1,097	153	17,1	161	17,4	4,96

A3	Sequence	# PSMs	# Proteins	# Protein Groups	Protein Group Accessions	Modifications	ΔCn	114/115	114/115 Count	114/115 Variability [%]	q-Value	PEP	IonScore	Exp Value	Charge	MH+ [Da]	ΔM [ppm]	RT [min]	# Missed Cleavages
High	sLGTPIEAVAQSVR	52	1	1	Q01951	N-Term(iTRAQ4plex)	0,0000	1,094	50	17,8	0	2,763E-11	102	6,39691E-11	2	1571,88884	0,91	39,88	0
High	rPDIVSPGGNAYGEEMTATcLR	4	1	1	Q01951	N-Term(iTRAQ4plex); C20(Carbamidomethyl)	0,0000	0,946	3	8,1	0	8,725E-16	99	1,30308E-10	3	2538,22062	0,31	33,87	0
High	rPDIVSPGGNAYGEEmTATcLR	14	1	1	Q01951	N-Term(iTRAQ4plex); M16(Oxidation); C20(Carbamidomethyl)	0,0000	0,989	9	8,1	0	9,167E-13	84	4,13971E-09	3	2554,21579	0,41	29,70	0
High	yLSPGELDRIk	2	1	1	Q01951	N-Term(iTRAQ4plex); K11(iTRAQ4plex)	0,0000	0,644	2	0,9	0	3,067E-07	69	1,84981E-07	3	1578,90946	0,03	33,65	1
High	sIVNADAEAR	6	1	1	Q01951	N-Term(iTRAQ4plex)	0,0000	1,116	3	10,0	0	1,089E-07	60	1,104E-06	2	1189,63054	0,97	18,11	0
High	aFVTGGAAR	24	1	1	Q01951	N-Term(iTRAQ4plex)	0,0000	1,061	20	6,2	0	0,000003376	56	2,29599E-06	2	993,56072	0,88	16,16	0
High	iAETLTGSR	14	1	1	Q01951	N-Term(iTRAQ4plex)	0,0000	1,041	13	9,2	0	0,0002371	50	1,02087E-05	2	1091,61772	-0,02	23,30	0
High	ikAFVTGGAAR	3	1	1	Q01951	N-Term(iTRAQ4plex); K2(iTRAQ4plex)	0,0000	0,654	3	17,1	0	8,897E-08	49	1,48316E-05	3	1378,84311	1,57	24,38	1
High	dMDYYLR	4	2	2	P74551;Q01951	N-Term(iTRAQ4plex)	0,0000				0	0,0003235	45	3,42744E-05	2	1119,52605	-0,08	30,39	0
High	yLSPGELDR	8	1	1	Q01951	N-Term(iTRAQ4plex)	0,0000	1,018	8	13,1	0	0,0001356	43	5,1047E-05	2	1193,62908	0,65	28,93	0
High	rPDIVSPGGNAYGEEmTATcLRDmDYYLR	1	1	1	Q01951	N-Term(iTRAQ4plex); M16(Oxidation); C20(Carbamidomethyl); M24(Oxidatio	0,0000	1,191	1		0	8,904E-11	42	6,16552E-05	4	3526,61440	-0,42	36,80	1
High	iAETLTGSRETIVk	2	1	1	Q01951	N-Term(iTRAQ4plex); K14(iTRAQ4plex)	0,0000	0,780	2	12,5	0	9,227E-09	31	0,000818408	3	1806,05765	0,06	29,97	1
High	qAGDRLFQk	2	1	1	Q01951	N-Term(iTRAQ4plex); K9(iTRAQ4plex)	0,0000	0,807	2	6,4	0	0,005829	26	0,002523306	3	1350,77423	0,73	17,99	1
High	eTIVkQAGDR	1	1	1	Q01951	N-Term(iTRAQ4plex); K5(iTRAQ4plex)	0,0000	0,934	1		0	0,0007219	23	0,004644832	3	1404,80371	-0,88	14,25	1
High	dmDYYLR	3	2	2	P74551;Q01951	N-Term(iTRAQ4plex); M2(Oxidation)	0,0000				0,001	0,01054	19	0,011911597	2	1135,52117	0,10	22,56	0
High	eTIVk	9	1	1	Q01951	N-Term(iTRAQ4plex); K5(iTRAQ4plex)	0,0000	1,200	9	6,7	0,004	0,04749	35	0,000360075	2	877,56236	3,03	14,44	0
High	sIVTk	28	1	1	Q01951	N-Term(iTRAQ4plex); K5(iTRAQ4plex)	0,0000	1,282	27	5,0	0,005	0,07106	17	0,026505364	2	835,55077	1,95	53,73	0

Accession	Description	Score	Coverage	# Proteins	# Unique Peptides	# Peptides	# PSMs	Dark/Light (114/115)	114/115 Count	114/115 Variability [%]	# AAs	MW [kDa]	calc. pI
P73282	Transketolase OS=Synechocystis sp. (strain PCC 6803 / Kazusa) GN=tktA PE=3 SV	4057,55	43,58	1	28	28	175	1,395	150	15,8	670	71,7	5,57

A3	Sequence	# PSMs	# Proteins	# Protein Groups	Protein Group Accessions	Modifications	ΔCn	114/115	114/115 Count	114/115 Variability [%]	q-Value	PEP	IonScore	Exp Value	Charge	MH+ [Da]	ΔM [ppm]	RT [min]	# Missed Cleavages
High	vVATQSLDELSINAIR	4	1	1	P73282	N-Term(iTRAQ4plex)	0,0000	1,105	4	41,0	0	5,704E-10	117	2,08435E-12	2	1873,05376	1,37	44,13	0
High	fEAYGWHVLHVEDGNTDLAAIAk	2	1	1	P73282	N-Term(iTRAQ4plex); K23(iTRAQ4plex)	0,0000	0,693	2	189,7	0	1,415E-15	100	1,10146E-10	3	2844,45993	1,38	44,59	0
High	lSVEAATNFGWHk	5	1	1	P73282	N-Term(iTRAQ4plex); K13(iTRAQ4plex)	0,0000	1,362	4	5,6	0	1,97E-13	96	2,48296E-10	2	1747,93901	1,14	36,94	0
High	qAVPNLPGTSIDAVAk	4	1	1	P73282	N-Term(iTRAQ4plex); K16(iTRAQ4plex)	0,0000	1,142	4	70,4	0	1,822E-11	94	4,31489E-10	2	1869,07146	1,61	35,83	0
High	vTTIIGYGAPNk	12	1	1	P73282	N-Term(iTRAQ4plex); K12(iTRAQ4plex)	0,0000	1,310	10	29,1	0	6,506E-10	81	8,47169E-09	2	1521,88921	0,83	32,45	0
High	tLASFTPDQk	3	1	1	P73282	N-Term(iTRAQ4plex); K10(iTRAQ4plex)	0,0000	1,166	3	33,7	0	3,919E-09	74	4,2459E-08	2	1395,77324	0,72	28,66	0
High	fGFSVDNVLAk	4	1	1	P73282	N-Term(iTRAQ4plex); K11(iTRAQ4plex)	0,0000	1,225	4	12,1	0	4,138E-10	73	4,96558E-08	2	1484,83696	1,19	44,19	0
High	sDTAGIHGAALGTDEVAATR	1	1	1	P73282	N-Term(iTRAQ4plex)	0,0000	1,349	1		0	8,709E-12	70	9,95337E-08	3	2057,04911	5,36	25,94	0
High	akENAPTLLSLTR	33	1	1	P73282	N-Term(iTRAQ4plex); K2(iTRAQ4plex)	0,0000	1,288	28	12,6	0	1,625E-07	68	1,72175E-07	2	1702,01250	1,35	30,53	1
High	fLAVDAIEk	3	1	1	P73282	N-Term(iTRAQ4plex); K9(iTRAQ4plex)	0,0000	1,463	3	7,2	0	1,981E-07	66	2,45454E-07	2	1293,76604	0,26	37,64	0
High	aVTDkPSmIk	15	1	1	P73282	N-Term(iTRAQ4plex); K5(iTRAQ4plex); M8(Oxidation); K10(iTRAQ4plex)	0,0000	1,593	9	12,4	0	0,000001992	60	8,95303E-07	2	1537,90483	4,17	15,91	0
High	kNLGWDYAPFEVPQEVLDYTR	1	1	1	P73282	N-Term(iTRAQ4plex); K1(iTRAQ4plex)	0,0000	1,497	1		0	7,565E-10	59	1,19666E-06	3	2828,45652	2,36	52,69	1
High	aVTDkPSMIk	10	1	1	P73282	N-Term(iTRAQ4plex); K5(iTRAQ4plex); K10(iTRAQ4plex)	0,0000	1,525	5	15,0	0	2,755E-08	58	1,43209E-06	2	1521,90605	1,67	20,56	0
High	aIPNLTVIRPADGNETSGAYk	22	1	1	P73282	N-Term(iTRAQ4plex); K21(iTRAQ4plex)	0,0000	1,355	22	14,5	0	3,578E-08	58	1,75547E-06	3	2475,34806	1,38	35,99	0
High	rLSVEAATNFGWHk	3	1	1	P73282	N-Term(iTRAQ4plex); K14(iTRAQ4plex)	0,0000	1,158	2	7,6	0	2,591E-12	57	2,05102E-06	4	1904,03823	0,04	31,13	1
High	tkYPTEAAAFER	8	1	1	P73282	N-Term(iTRAQ4plex); K2(iTRAQ4plex)	0,0000	1,460	8	10,8	0	3,451E-08	56	2,41529E-06	3	1671,89499	0,30	23,86	1
High	eNAPTLLSLTR	2	1	1	P73282	N-Term(iTRAQ4plex)	0,0000	1,493	2	4,8	0	0,00000939	56	2,68516E-06	2	1358,77654	0,36	36,56	0
High	sDTAGIHGAALGTDEVAATRk	2	1	1	P73282	N-Term(iTRAQ4plex); K21(iTRAQ4plex)	0,0000	1,249	1		0	3,381E-10	56	2,75404E-06	4	2329,23501	-0,04	23,50	1
High	gASYEAEWNQAFAQYk	2	1	1	P73282	N-Term(iTRAQ4plex); K16(iTRAQ4plex)	0,0000	0,939	2	33,4	0	3,355E-13	51	8,58954E-06	3	2151,04117	1,20	41,84	0
High	sGHPGLPMGAAPmAFTLWNk	1	1	1	P73282	N-Term(iTRAQ4plex); M13(Oxidation); K20(iTRAQ4plex)	0,0000	0,847	1		0	2,349E-08	44	3,77546E-05	3	2387,22617	0,76	43,65	0
High	sGHPGLPmGAAPmAFTLWNk	2	1	1	P73282	N-Term(iTRAQ4plex); M8(Oxidation); M13(Oxidation); K20(iTRAQ4plex)	0,0000	1,506	2	7,0	0	4,45E-11	42	6,2114E-05	3	2403,22135	0,86	39,16	0
High	yPTEAAAFER	2	1	1	P73282	N-Term(iTRAQ4plex)	0,0000	1,323	2	5,9	0	0,0004173	35	0,000315479	2	1298,64934	-0,33	28,64	0
High	qLSGALPEGWDk	2	1	1	P73282	N-Term(iTRAQ4plex); K12(iTRAQ4plex)	0,0000	0,991	2	22,1	0	0,00003152	28	0,001766808	3	1588,85813	0,47	35,31	0
High	rFEAYGWHVLHVEDGNTDLAAIAk	2	1	1	P73282	N-Term(iTRAQ4plex); K24(iTRAQ4plex)	0,0000	1,301	2	50,8	0	6,906E-07	22	0,007058263	4	3000,56118	1,35	40,51	1
High	nVHFGVR	6	1	1	P73282	N-Term(iTRAQ4plex)	0,0000	1,442	6	18,5	0	0,007039	17	0,020332431	3	972,55341	3,92	15,78	0
High	lAAQGk	4	1	1	P73282	N-Term(iTRAQ4plex); K6(iTRAQ4plex)	0,0000	1,605	3	6,5	0,001	0,008755	15	0,029714608	2	875,55467	-0,71	11,93	0
High	kAIER	3	1	1	P73282	N-Term(iTRAQ4plex); K1(iTRAQ4plex)	0,0000	1,582	3	1,4	0,001	0,01615	10	0,102846448	3	904,58255	0,78	8,42	1
High	vAIAk	11	1	1	P73282	N-Term(iTRAQ4plex); K5(iTRAQ4plex)	0,0000	1,421	9	7,4	0,005	0,06137	23	0,012250846	2	789,54302	-0,82	16,50	0
High	eSVLPk	4	1	1	P73282	N-Term(iTRAQ4plex); K6(iTRAQ4plex)	0,0000	1,486	3	10,7	0,008	0,09857	12	0,06655632	2	960,60369	7,14	18,86	0
Medium	gAYQNR	2	1	1	P73282	N-Term(iTRAQ4plex)	0,0000	1,450	2	4,2	0,012	0,1399	18	0,017699867	2	852,44505	0,72	7,94	0

Accession	Description	Score	Coverage	# Proteins	# Unique Peptides	# Peptides	# PSMs	Dark/Light (114/115)	114/115 Count	114/115 Variability [%]	# AAs	MW [kDa]	calc. pI
Q01952	Allophycocyanin beta chain OS=Synechocystis sp. (strain PCC 6803 / Kazusa) GN=a	3597,28	79,50	1	16	16	165	1,098	152	17,4	161	17,2	5,60

A3	Sequence	# PSMs	# Proteins	# Protein Groups	Protein Group Accessions	Modifications	ΔCn	114/115	114/115 Count	114/115 Variability [%]	q-Value	PEP	IonScore	Exp Value	Charge	MH+ [Da]	ΔM [ppm]	RT [min]	# Missed Cleavages
High	aASVISANAATIVk	65	1	1	Q01952	N-Term(iTRAQ4plex); K14(iTRAQ4plex)	0,0000	1,073	60	24,1	0	3,616E-13	119	1,14543E-12	2	1603,96404	1,15	33,02	0
High	eVTASLVGADAGk	3	1	1	Q01952	N-Term(iTRAQ4plex); K13(iTRAQ4plex)	0,0000	1,337	3	24,3	0	2,471E-10	94	4,35702E-10	2	1505,84282	0,94	26,76	0
High	aASVISANAATIVkEAVAk	2	1	1	Q01952	N-Term(iTRAQ4plex); K14(iTRAQ4plex); K19(iTRAQ4plex)	0,0000	0,573	2	40,1	0	4,337E-14	77	2,06524E-08	3	2246,34537	0,40	45,50	1
High	mQDAITAVINSADVQGk	1	1	1	Q01952	N-Term(iTRAQ4plex); M1(Oxidation); K17(iTRAQ4plex)	0,0000	0,925	1		0	7,354E-10	66	2,70377E-07	3	2065,08634	1,20	39,42	0
High	sYFASGELR	34	1	1	Q01952	N-Term(iTRAQ4plex)	0,0000	1,109	34	11,6	0	0,00008826	63	4,47682E-07	2	1173,60246	0,32	27,38	0
High	yLDGAAmDkLk	6	1	1	Q01952	N-Term(iTRAQ4plex); M7(Oxidation); K9(iTRAQ4plex); K11(iTRAQ4plex)	0,0000	1,118	6	6,5	0	9,056E-09	57	1,87487E-06	2	1672,93511	2,78	25,49	1
High	eTYNSLGVPISSTVQAIQAIk	4	1	1	Q01952	N-Term(iTRAQ4plex); K21(iTRAQ4plex)	0,0000	1,428	4	9,5	0	3,811E-10	52	5,662E-06	3	2507,39847	0,99	51,48	0
High	yLDGAAMDk	1	1	1	Q01952	N-Term(iTRAQ4plex); K9(iTRAQ4plex)	0,0000	1,206	1		0	0,000001158	50	1,03746E-05	2	1271,65727	2,23	26,00	0
High	dLDYYLR	8	1	1	Q01952	N-Term(iTRAQ4plex)	0,0000	1,009	7	9,5	0	0,000487	42	6,08093E-05	2	1101,57012	0,37	36,36	0
High	yLDGAAMDkLk	4	1	1	Q01952	N-Term(iTRAQ4plex); K9(iTRAQ4plex); K11(iTRAQ4plex)	0,0000	0,916	4	3,4	0	0,000004443	37	0,000226148	3	1656,93747	1,17	32,21	1
High	yLDGAAmDk	5	1	1	Q01952	N-Term(iTRAQ4plex); M7(Oxidation); K9(iTRAQ4plex)	0,0000	1,290	5	5,4	0	0,0001958	36	0,000274138	2	1287,65190	1,98	21,93	0
High	lkSYFASGELR	2	1	1	Q01952	N-Term(iTRAQ4plex); K2(iTRAQ4plex)	0,0000	1,098	2	0,7	0	0,000005341	34	0,000428519	3	1558,88083	-1,51	31,87	1
High	sLLYSDVTRPGGNmYTTR	6	1	1	Q01952	N-Term(iTRAQ4plex); M14(Oxidation)	0,0000	0,307	4	11,7	0	0,0003249	33	0,000450786	3	2191,09439	0,43	30,64	0
High	sLLYSDVTRPGGNMYTTR	1	1	1	Q01952	N-Term(iTRAQ4plex)	0,0000	0,235	1		0	0,00001695	26	0,00280524	3	2175,09940	0,40	34,20	0
High	yAAcIRDLDYYLR	1	1	1	Q01952	N-Term(iTRAQ4plex); C4(Carbamidomethyl)	0,0000	0,997	1		0	0,00003294	19	0,013962719	3	1835,92105	-1,12	42,74	1
High	eAVAk	8	1	1	Q01952	N-Term(iTRAQ4plex); K5(iTRAQ4plex)	0,0000	1,214	6	8,7	0,004	0,04939	19	0,042022213	2	805,50218	-0,02	12,07	0
High	vLNGLk	7	1	1	Q01952	N-Term(iTRAQ4plex); K6(iTRAQ4plex)	0,0000	1,129	4	9,4	0,008	0,09772	14	0,039443006	2	931,61876	0,93	23,46	0
Medium	rYAAcIR	1	1	1	Q01952	N-Term(iTRAQ4plex); C5(Carbamidomethyl)	0,0000	0,836	1		0,011	0,1323	10	0,092676581	2	1053,57463	0,19	12,31	1
Medium	yAAcIR	6	1	1	Q01952	N-Term(iTRAQ4plex); C4(Carbamidomethyl)	0,0000	1,232	6	6,3	0,02	0,2218	12	0,061655242	2	897,47368	0,41	55,51	0

Accession	Description	Score	Coverage	# Proteins	# Unique Peptides	# Peptides	# PSMs	Dark/Light (114/115)	114/115 Count	114/115 Variability [%]	# AAs	MW [kDa]	calc. pI
P74390	Negative aliphatic amidase regulator OS=Synechocystis sp. (strain PCC 6803 / Kazu	3063,05	36,32	1	16	16	175	2,508	153	62,3	446	48,3	4,89

A3	Sequence	# PSMs	# Proteins	# Protein Groups	Protein Group Accessions	Modifications	ΔCn	114/115	114/115 Count	114/115 Variability [%]	q-Value	PEP	IonScore	Exp Value	Charge	MH+ [Da]	ΔM [ppm]	RT [min]	# Missed Cleavages
High	nHMLWYPVQYEGQEcSk	2	1	1	P74390	N-Term(iTRAQ4plex); C15(Carbamidomethyl); K17(iTRAQ4plex)	0,0000	0,561	2	15909,4	0	3,529E-14	79	1,39306E-08	3	2457,15891	0,77	36,10	0
High	fFLVGSDYVFPR	16	1	1	P74390	N-Term(iTRAQ4plex)	0,0000	1,915	16	69,5	0	0,00001064	72	6,4859E-08	2	1590,84551	1,11	50,90	0
High	tFDAPEGmVTmQPNHHISk	9	1	1	P74390	N-Term(iTRAQ4plex); M8(Oxidation); M11(Oxidation); K19(iTRAQ4plex)	0,0000	4,496	6	28,3	0	2,123E-10	70	1,10655E-07	4	2460,19497	2,39	22,13	0
High	vPVVFGcWTSASR	5	1	1	P74390	N-Term(iTRAQ4plex); C7(Carbamidomethyl)	0,0000	1,517	5	45,6	0	2,36E-10	69	1,17211E-07	2	1609,82952	1,08	43,91	0
High	nHmLWYPVQYEGQEcSk	4	1	1	P74390	N-Term(iTRAQ4plex); M3(Oxidation); C15(Carbamidomethyl); K17(iTRAQ4ple	0,0000	5,625	3	30,6	0	2,851E-14	69	1,23586E-07	3	2473,15519	1,32	32,52	0
High	kAVLPVFEAk	35	1	1	P74390	N-Term(iTRAQ4plex); K1(iTRAQ4plex); K10(iTRAQ4plex)	0,0000	3,466	30	56,5	0	0,00007342	61	7,48118E-07	2	1533,97551	1,70	30,69	1
High	aVLPVFEAk	7	1	1	P74390	N-Term(iTRAQ4plex); K9(iTRAQ4plex)	0,0000	2,101	6	42,4	0	0,000004769	61	9,08216E-07	2	1261,77703	0,91	37,37	0
High	qIQAAGLTPDkYPVmSVSVAEEEVR	3	1	1	P74390	N-Term(iTRAQ4plex); K11(iTRAQ4plex); M15(Oxidation)	0,0000	3,147	2	93,5	0	3,4E-09	57	2,31046E-06	3	3021,58426	1,20	38,07	1
High	lIDQDkVPVVFGcWTSASR	8	1	1	P74390	N-Term(iTRAQ4plex); K6(iTRAQ4plex); C13(Carbamidomethyl)	0,0000	4,388	7	51,9	0	2,295E-12	55	3,51536E-06	3	2466,30759	0,88	46,48	1
High	tFDAPEGMVTMQPNHHISk	2	1	1	P74390	N-Term(iTRAQ4plex); K19(iTRAQ4plex)	0,0000	3,116	2	12,1	0	1,047E-09	52	6,53085E-06	4	2428,20034	0,45	29,79	0
High	gNkFFLVGSDYVFPR	1	1	1	P74390	N-Term(iTRAQ4plex); K3(iTRAQ4plex)	0,0000	2,940	1		0	3,961E-10	45	3,13307E-05	3	2034,10636	0,59	45,18	1
High	fVAAFk	48	1	1	P74390	N-Term(iTRAQ4plex); K6(iTRAQ4plex)	0,0000	2,416	44	21,6	0	0,006381	43	5,36995E-05	2	970,59923	2,88	30,48	0
High	tDVENPGk	2	1	1	P74390	N-Term(iTRAQ4plex); K8(iTRAQ4plex)	0,0000	5,070	1		0	0,002069	41	8,10905E-05	2	1147,62480	4,40	13,71	0
High	tFDAPEGmVTMQPNHHISk	5	1	1	P74390	N-Term(iTRAQ4plex); M8(Oxidation); K19(iTRAQ4plex)	0,0000	4,575	3	24,1	0	7,557E-11	40	9,86211E-05	4	2444,19716	1,23	24,82	0
High	aAVEAAGDVGETPEGLEk	1	1	1	P74390	N-Term(iTRAQ4plex); K18(iTRAQ4plex)	0,0000	0,619	1		0	0,00000217	34	0,00041973	3	2031,05362	2,50	30,17	0
High	tANTIIkEQLk	2	1	1	P74390	N-Term(iTRAQ4plex); K7(iTRAQ4plex); K11(iTRAQ4plex)	0,0000	3,764	2	0,9	0	0,000004358	33	0,000558432	3	1691,04453	1,04	25,39	1
High	gFTcDWTR	3	1	1	P74390	N-Term(iTRAQ4plex); C4(Carbamidomethyl)	0,0000	2,052	3	14,4	0	0,002189	30	0,000901509	2	1186,54460	1,18	28,18	0
High	tANTIIk	17	1	1	P74390	N-Term(iTRAQ4plex); K7(iTRAQ4plex)	0,0000	1,446	14	9,3	0,001	0,01563	43	5,58432E-05	2	1048,66252	1,94	18,09	0
High	aAAIGk	5	1	1	P74390	N-Term(iTRAQ4plex); K6(iTRAQ4plex)	0,0000	1,987	5	16,6	0,005	0,05849	15	0,030987697	2	818,53313	-0,87	53,17	0

Accession	Description	Score	Coverage	# Proteins	# Unique Peptides	# Peptides	# PSMs	Dark/Light (114/115)	114/115 Count	114/115 Variability [%]	# AAs	MW [kDa]	calc. pI
P74227	Elongation factor Tu OS=Synechocystis sp. (strain PCC 6803 / Kazusa) GN=tuf PE=	2915,58	59,15	1	29	29	130	0,960	112	13,2	399	43,7	5,30

A3	Sequence	# PSMs	# Proteins	# Protein Groups	Protein Group Accessions	Modifications	ΔCn	114/115	114/115 Count	114/115 Variability [%]	q-Value	PEP	IonScore	Exp Value	Charge	MH+ [Da]	ΔM [ppm]	RT [min]	# Missed Cleavages
High	kATVTGVEMFQk	6	1	1	P74227	N-Term(iTRAQ4plex); K1(iTRAQ4plex); K12(iTRAQ4plex)	0,0000	0,898	6	10,4	0	3,007E-11	78	1,58125E-08	2	1771,01714	1,29	27,15	1
High	vGEEISIVGIk	5	1	1	P74227	N-Term(iTRAQ4plex); K11(iTRAQ4plex)	0,0000	0,964	5	5,6	0	6,465E-10	77	2,17756E-08	2	1431,86699	0,58	38,05	0
High	gITINTAHVEYETDSR	7	1	1	P74227	N-Term(iTRAQ4plex)	0,0000	0,844	5	21,1	0	2,466E-08	74	4,43578E-08	2	1949,98271	7,26	28,87	0
High	tTLTAAITmTLAELGGAk	2	1	1	P74227	N-Term(iTRAQ4plex); M9(Oxidation); K18(iTRAQ4plex)	0,0000	1,019	2	26,4	0	1,088E-10	67	1,99054E-07	2	2067,16411	1,48	59,51	0
High	tIGAGVVSk	7	1	1	P74227	N-Term(iTRAQ4plex); K9(iTRAQ4plex)	0,0000	0,928	5	7,9	0	0,00001697	65	2,91723E-07	2	1119,69817	0,49	21,33	0
High	dHVNIGTIGHVDHGk	4	1	1	P74227	N-Term(iTRAQ4plex); K15(iTRAQ4plex)	0,0000	0,798	3	18,5	0	3,628E-11	65	2,97146E-07	3	1887,00858	0,54	19,11	0
High	mTVELINPIAIEQGmR	3	1	1	P74227	N-Term(iTRAQ4plex); M1(Oxidation); M15(Oxidation)	0,0000	1,018	3	4,6	0	1,079E-09	64	4,45625E-07	2	1991,04375	0,73	42,87	0
High	hYAHVDcPGHADYVk	2	1	1	P74227	N-Term(iTRAQ4plex); C7(Carbamidomethyl); K15(iTRAQ4plex)	0,0000	0,841	2	3,7	0	2,898E-11	61	7,2773E-07	3	2056,99051	0,16	16,24	0
High	aTVTGVEmFQk	1	1	1	P74227	N-Term(iTRAQ4plex); M8(Oxidation); K11(iTRAQ4plex)	0,0000	1,137	1		0	1,324E-09	59	1,27929E-06	2	1514,81328	0,35	27,04	0
High	tTDVTGTIk	4	1	1	P74227	N-Term(iTRAQ4plex); K9(iTRAQ4plex)	0,0000	0,967	3	1,6	0	0,000001189	58	1,66713E-06	2	1223,70830	-0,22	19,96	0
High	eVDkPFLmAVEDVFSITGR	2	1	1	P74227	N-Term(iTRAQ4plex); K4(iTRAQ4plex); M8(Oxidation)	0,0000	0,901	2	14,0	0	3,946E-13	55	2,93069E-06	3	2457,29477	0,36	58,08	0
High	aIEGEkEYk	7	1	1	P74227	N-Term(iTRAQ4plex); K6(iTRAQ4plex); K9(iTRAQ4plex)	0,0000	1,108	7	12,3	0	0,00000045	55	3,12744E-06	2	1498,84905	0,86	15,18	1
High	kATVTGVEmFQk	7	1	1	P74227	N-Term(iTRAQ4plex); K1(iTRAQ4plex); M9(Oxidation); K12(iTRAQ4plex)	0,0000	1,039	5	6,4	0	4,883E-10	54	4,99969E-06	2	1787,00932	-0,25	22,36	1
High	kYEDIDAAPEEk	8	1	1	P74227	N-Term(iTRAQ4plex); K1(iTRAQ4plex); K12(iTRAQ4plex)	0,0000	1,101	6	11,5	0	0,000002451	53	5,50038E-06	2	1839,97685	3,69	20,19	1
High	dAILELMk	1	1	1	P74227	N-Term(iTRAQ4plex); K8(iTRAQ4plex)	0,0000	0,873	1		0	0,00001178	53	5,26017E-06	2	1220,71575	-0,47	41,57	0
High	eHILLAk	10	1	1	P74227	N-Term(iTRAQ4plex); K7(iTRAQ4plex)	0,0000	0,855	7	4,9	0	0,0001072	51	7,67308E-06	2	1111,70867	0,80	19,48	0
High	vkVGEEISIVGIk	2	1	1	P74227	N-Term(iTRAQ4plex); K2(iTRAQ4plex); K13(iTRAQ4plex)	0,0000	0,959	2	9,1	0	2,422E-08	50	9,68211E-06	3	1803,13254	0,52	35,49	1
High	tkDHVNIGTIGHVDHGk	2	1	1	P74227	N-Term(iTRAQ4plex); K2(iTRAQ4plex); K17(iTRAQ4plex)	0,0000	0,734	1		0	1,521E-09	49	1,23018E-05	4	2260,25234	0,03	18,13	1
High	lVVFLNkk	4	1	1	P74227	N-Term(iTRAQ4plex); K7(iTRAQ4plex); K8(iTRAQ4plex)	0,0000	0,793	4	14,8	0	0,000164	49	1,29709E-05	3	1392,92942	-0,63	32,13	1
High	ikmTVELINPIAIEQGmR	1	1	1	P74227	N-Term(iTRAQ4plex); K2(iTRAQ4plex); M3(Oxidation); M17(Oxidation)	0,0000	1,098	1		0	6,116E-09	45	2,95101E-05	3	2376,32718	1,59	42,72	1
High	gIQkEDIER	7	1	1	P74227	N-Term(iTRAQ4plex); K4(iTRAQ4plex)	0,0000	0,933	7	4,3	0	0,00002058	43	6,45205E-05	2	1375,77788	-0,38	16,07	1
High	lVVFLNk	15	1	1	P74227	N-Term(iTRAQ4plex); K7(iTRAQ4plex)	0,0000	1,010	11	6,0	0	0,001007	41	7,69077E-05	2	1120,73454	1,14	38,92	0
High	eYkDAILELMk	5	1	1	P74227	N-Term(iTRAQ4plex); K3(iTRAQ4plex); K11(iTRAQ4plex)	0,0000	0,886	5	21,3	0	3,637E-07	39	0,000120773	3	1785,02121	1,09	43,72	1
High	aIEGEkEYkDAILELmk	1	1	1	P74227	N-Term(iTRAQ4plex); K6(iTRAQ4plex); K9(iTRAQ4plex); M16(Oxidation); K17	0,0000	0,999	1		0	7,532E-10	38	0,000162544	3	2572,44211	1,18	36,22	2
High	eYkDAILELmk	5	1	1	P74227	N-Term(iTRAQ4plex); K3(iTRAQ4plex); M10(Oxidation); K11(iTRAQ4plex)	0,0000	1,105	5	9,1	0	6,829E-09	38	0,000167483	3	1801,01419	0,01	33,66	1
High	aIEGEkEYkDAILELMk	1	1	1	P74227	N-Term(iTRAQ4plex); K6(iTRAQ4plex); K9(iTRAQ4plex); K17(iTRAQ4plex)	0,0000	0,934	1		0	1,856E-10	35	0,000295101	3	2556,44748	1,30	43,84	2
High	vGEEISIVGIkDTR	1	1	1	P74227	N-Term(iTRAQ4plex); K11(iTRAQ4plex)	0,0000	0,919	1		0	0,000003251	33	0,00058263	3	1804,04111	-0,44	34,15	1
High	vkVGEEISIVGIkDTR	1	1	1	P74227	N-Term(iTRAQ4plex); K2(iTRAQ4plex); K13(iTRAQ4plex)	0,0000	0,894	1		0	0,00001208	28	0,00171779	4	2175,30703	-0,15	33,72	2
High	hTPFFANYRPQFYVR	3	1	1	P74227	N-Term(iTRAQ4plex)	0,0000	0,823	3	3,6	0	0,000001069	27	0,00182377	4	2087,07583	1,25	35,89	0
High	mTVELINPIAIEQGmR	1	1	1	P74227	N-Term(iTRAQ4plex); M15(Oxidation)	0,0000	1,251	1		0	0,0003685	26	0,002311905	3	1975,05100	1,83	47,67	0
High	qVGVPk	3	1	1	P74227	N-Term(iTRAQ4plex); K6(iTRAQ4plex)	0,0000	0,955	3	2,2	0,001	0,01603	23	0,004591663	2	915,59056	4,33	14,64	0
High	kEEGGR	1	1	1	P74227	N-Term(iTRAQ4plex); K1(iTRAQ4plex)	0,0000	0,951	1		0,006	0,07982	16	0,023669512	3	963,54563	-0,59	7,30	1
Medium	gTVATGR	1	1	1	P74227	N-Term(iTRAQ4plex)	0,0000	0,936	1		0,017	0,1822	21	0,008649082	2	805,46428	-0,72	7,94	0

Accession	Description	Score	Coverage	# Proteins	# Unique Peptides	# Peptides	# PSMs	Dark/Light (114/115)	114/115 Count	114/115 Variability [%]	# AAs	MW [kDa]	calc. pI
Q05972	60 kDa chaperonin 1 OS=Synechocystis sp. (strain PCC 6803 / Kazusa) GN=groL1 F	2663,11	39,00	1	25	26	123	1,093	96	11,3	541	57,6	5,10

A3	Sequence	# PSMs	# Proteins	# Protein Groups	Protein Group Accessions	Modifications	ΔCn	114/115	114/115 Count	114/115 Variability [%]	q-Value	PEP	IonScore	Exp Value	Charge	MH+ [Da]	ΔM [ppm]	RT [min]	# Missed Cleavages
High	fGSPQIINDGITIAk	3	1	1	Q05972	N-Term(iTRAQ4plex); K15(iTRAQ4plex)	0,0000	1,108	3	4,0	0	4,301E-13	84	3,69803E-09	2	1862,06487	1,20	41,36	0
High	vGAATETEmkDR	7	1	1	Q05972	N-Term(iTRAQ4plex); M9(Oxidation); K10(iTRAQ4plex)	0,0000	1,045	6	4,8	0	5,365E-10	83	5,08124E-09	2	1611,82659	0,91	11,57	1
High	ikEHAQPVGDSk	6	1	1	Q05972	N-Term(iTRAQ4plex); K2(iTRAQ4plex); K12(iTRAQ4plex)	0,0000	0,963	3	1,9	0	1,301E-10	79	1,30308E-08	2	1741,00017	1,88	12,62	1
High	nVAAGANPISLk	9	1	1	Q05972	N-Term(iTRAQ4plex); K12(iTRAQ4plex)	0,0000	1,216	6	12,3	0	4,595E-07	77	2,14274E-08	2	1442,86785	7,53	28,78	0
High	gVLNVAAVk	22	1	1	Q05972	N-Term(iTRAQ4plex); K9(iTRAQ4plex)	0,0000	1,096	19	6,2	0	5,005E-07	76	2,32258E-08	2	1158,74602	0,96	29,10	0
High	kINLVQDLVPILEQVAR	3	1	1	Q05972	N-Term(iTRAQ4plex); K1(iTRAQ4plex)	0,0000	0,944	3	1,0	0	1,353E-10	71	8,24081E-08	2	2236,36333	0,06	58,35	1
High	nVAAGANPISLkR	3	1	1	Q05972	N-Term(iTRAQ4plex); K12(iTRAQ4plex)	0,0000	0,934	2	8,9	0	1,685E-09	65	3,43534E-07	2	1598,95976	1,04	23,77	1
High	gYISPYFVTDAER	3	1	1	Q05972	N-Term(iTRAQ4plex)	0,0000	1,136	3	5,2	0	5,54E-09	61	7,39554E-07	2	1661,83037	0,71	39,00	0
High	lAGGVAVIk	13	2	2	Q05972;P22034	N-Term(iTRAQ4plex); K9(iTRAQ4plex)	0,0000				0	0,00006664	59	1,36449E-06	2	1115,74285	3,36	28,43	0
High	eALATLVVNR	2	1	1	Q05972	N-Term(iTRAQ4plex)	0,0000	1,110	2	0,9	0	0,00002207	49	1,14017E-05	2	1229,73369	0,19	33,72	0
High	aLPAPLkR	7	1	1	Q05972	N-Term(iTRAQ4plex); K7(iTRAQ4plex)	0,0000	0,946	5	5,8	0	0,0002619	49	1,40919E-05	3	1153,76767	1,47	19,87	1
High	aLPAPLk	1	1	1	Q05972	N-Term(iTRAQ4plex); K7(iTRAQ4plex)	0,0000	2,133	1		0	0,003095	47	1,87055E-05	2	997,66521	0,35	26,05	0
High	iLITDkk	6	1	1	Q05972	N-Term(iTRAQ4plex); K6(iTRAQ4plex); K7(iTRAQ4plex)	0,0000	1,143	6	14,2	0	0,0003016	43	5,09296E-05	2	1262,84014	-0,53	21,20	1
High	qGkPLLIIAEDIEk	9	1	1	Q05972	N-Term(iTRAQ4plex); K3(iTRAQ4plex); K14(iTRAQ4plex)	0,0000	1,173	9	12,2	0	5,873E-09	42	6,7293E-05	3	1999,21781	0,70	42,83	1
High	aTDFLVAR	3	1	1	Q05972	N-Term(iTRAQ4plex)	0,0000	1,141	3	7,6	0	0,000298	41	0,000102124	2	1036,60063	9,48	30,34	0
High	vGAATETEMkDR	5	1	1	Q05972	N-Term(iTRAQ4plex); K10(iTRAQ4plex)	0,0000	1,048	5	13,2	0	0,0000141	39	0,000113493	3	1595,82914	-0,67	17,08	1
High	kFGSPQIINDGITIAk	4	1	1	Q05972	N-Term(iTRAQ4plex); K1(iTRAQ4plex); K16(iTRAQ4plex)	0,0000	1,338	3	1,3	0	0,0004118	38	0,00015775	3	2134,26249	1,32	36,23	1
High	qIEETDSSYDkEk	1	1	1	Q05972	N-Term(iTRAQ4plex); K11(iTRAQ4plex); K13(iTRAQ4plex)	0,0000	1,108	1		0	2,509E-08	36	0,000252912	3	2004,02561	6,10	16,53	1
High	eHAQPVGDSk	4	1	1	Q05972	N-Term(iTRAQ4plex); K10(iTRAQ4plex)	0,0000	1,097	4	1,3	0	0,00003921	35	0,00028772	3	1355,71524	-0,40	8,59	0
High	rQIEETDSSYDkEk	1	1	1	Q05972	N-Term(iTRAQ4plex); K12(iTRAQ4plex); K14(iTRAQ4plex)	0,0000	1,248	1		0	1,992E-07	34	0,000442781	4	2160,11635	0,86	15,09	2
High	vGQEGVISLEEGk	1	1	1	Q05972	N-Term(iTRAQ4plex); K13(iTRAQ4plex)	0,0000	1,114	1		0	2,21E-08	33	0,000450786	3	1632,90689	1,32	30,54	0
High	vGAATETEmkDRk	2	1	1	Q05972	N-Term(iTRAQ4plex); M9(Oxidation); K10(iTRAQ4plex); K13(iTRAQ4plex)	0,0000	1,052	2	14,0	0	0,0003627	32	0,000639691	3	1884,02304	0,48	11,93	2
High	smTTELEITEGmRFDk	1	1	1	Q05972	N-Term(iTRAQ4plex); M2(Oxidation); M12(Oxidation); K16(iTRAQ4plex)	0,0000	1,271	1		0	2,332E-07	32	0,000653085	3	2208,07871	0,89	30,00	1
High	nVVLEk	3	1	1	Q05972	N-Term(iTRAQ4plex); K6(iTRAQ4plex)	0,0000	1,111	3	4,5	0,001	0,009234	37	0,000216755	2	989,62261	-0,78	20,44	0
High	rINITk	2	1	1	Q05972	N-Term(iTRAQ4plex); K6(iTRAQ4plex)	0,0000	0,710	2	1,9	0,003	0,0396	18	0,030709483	3	1032,67371	-3,00	15,47	1
High	aPGFGDRR	1	1	1	Q05972	N-Term(iTRAQ4plex)	0,0000	0,846	1		0,004	0,04702	26	0,002460198	3	1019,55191	1,54	11,75	1
Medium	iLITDk	1	1	1	Q05972	N-Term(iTRAQ4plex); K6(iTRAQ4plex)	0,0000	1,063	1		0,014	0,1568	23	0,005247712	2	990,64806	4,32	25,75	0

Accession	Description	Score	Coverage	# Proteins	# Unique Peptides	# Peptides	# PSMs	Dark/Light (114/115)	114/115 Count	114/115 Variability [%]	# AAs	MW [kDa]	calc. pI
P22034	60 kDa chaperonin 2 OS=Synechocystis sp. (strain PCC 6803 / Kazusa) GN=groL2 F	2567,98	48,91	1	30	31	97	1,186	70	16,3	552	57,7	4,97

A3	Sequence	# PSMs	# Proteins	# Protein Groups	Protein Group Accessions	Modifications	ΔCn	114/115	114/115 Count	114/115 Variability [%]	q-Value	PEP	IonScore	Exp Value	Charge	MH+ [Da]	ΔM [ppm]	RT [min]	# Missed Cleavages
High	tkEIAGDGTTTATIIAQALVR	2	1	1	P22034	N-Term(iTRAQ4plex); K2(iTRAQ4plex)	0,0000	0,768	2	91,7	0	1,722E-13	105	3,38041E-11	3	2418,38395	1,34	45,37	1
High	eATGNQGYNVITGk	1	1	1	P22034	N-Term(iTRAQ4plex); K14(iTRAQ4plex)	0,0000	1,400	1		0	2,149E-11	79	1,2852E-08	2	1739,91826	0,91	24,67	0
High	qLVEFDNPLILITDkk	6	1	1	P22034	N-Term(iTRAQ4plex); K15(iTRAQ4plex); K16(iTRAQ4plex)	0,0000	1,190	6	9,6	0	5,539E-14	71	7,5504E-08	3	2318,37247	1,23	50,99	1
High	lRIEDALNATk	7	1	1	P22034	N-Term(iTRAQ4plex); K11(iTRAQ4plex)	0,0000	1,060	5	4,8	0	1,223E-07	71	8,59014E-08	2	1531,90569	0,67	30,73	1
High	gVLNVAAIk	8	1	1	P22034	N-Term(iTRAQ4plex); K9(iTRAQ4plex)	0,0000	1,074	5	10,3	0	3,762E-07	71	8,66902E-08	2	1172,76103	0,41	32,53	0
High	nVAAGANPVALR	6	1	1	P22034	N-Term(iTRAQ4plex)	0,0000	0,809	4	46,9	0	0,00000376	69	1,38666E-07	2	1296,75188	1,05	24,65	0
High	rSLEAGINALADAVR	3	1	1	P22034	N-Term(iTRAQ4plex)	0,0000	1,002	2	4,5	0	5,296E-11	67	2,79981E-07	3	1699,95719	-0,03	41,36	1
High	iSAIAELVPVLEAVAR	4	1	1	P22034	N-Term(iTRAQ4plex)	0,0000	1,200	4	14,6	0	1,213E-09	65	3,41956E-07	2	1795,08562	2,55	62,06	0
High	aAVEEGIVPGGGTTLIR	2	1	1	P22034	N-Term(iTRAQ4plex)	0,0000	1,163	2	8,3	0	6,506E-08	62	6,1372E-07	2	1784,00481	0,72	37,34	0
High	vGAATETELkDR	6	1	1	P22034	N-Term(iTRAQ4plex); K10(iTRAQ4plex)	0,0000	1,349	5	13,2	0	1,661E-07	62	6,17974E-07	2	1577,87578	1,28	19,81	1
High	vkEATGNQGYNVITGk	4	1	1	P22034	N-Term(iTRAQ4plex); K2(iTRAQ4plex); K16(iTRAQ4plex)	0,0000	1,350	2	11,9	0	1,036E-10	61	7,56781E-07	3	2111,18406	0,91	23,90	1
High	sLEAGINALADAVR	1	1	1	P22034	N-Term(iTRAQ4plex)	0,0000	1,079	1		0	1,781E-10	60	9,39658E-07	2	1543,85723	0,72	49,35	0
High	lIQEVASk	3	1	1	P22034	N-Term(iTRAQ4plex); K8(iTRAQ4plex)	0,0000	1,199	3	8,3	0	0,00001215	60	1,00918E-06	2	1175,72466	0,71	28,11	0

	High	vAADIIAk	2	1	1	P22034	N-Term(iTRAQ4plex); K8(iTRAQ4plex)	0,0000	1,245	1		0	0,00001669	60	1,79246E-06	2	1088,69231	0,46	27,06	0
	High	lAGGVAVIk	13	2	2	Q05972;P22034	N-Term(iTRAQ4plex); K9(iTRAQ4plex)	0,0000				0	0,00006664	59	1,36449E-06	2	1115,74285	3,36	28,43	0
	High	kISAIAELVPVLEAVAR	1	1	1	P22034	N-Term(iTRAQ4plex); K1(iTRAQ4plex)	0,0000	0,810	1		0	2,096E-09	54	3,99917E-06	3	2067,28049	1,17	56,54	1
	High	iEDALNATk	1	1	1	P22034	N-Term(iTRAQ4plex); K9(iTRAQ4plex)	0,0000	1,141	1		0	0,00000136	45	4,19704E-05	2	1262,71904	-0,34	26,82	0
	High	lISFkDESR	4	1	1	P22034	N-Term(iTRAQ4plex); K5(iTRAQ4plex)	0,0000	1,562	4	6,5	0	0,00003023	45	3,36488E-05	2	1382,79241	3,02	25,97	1
	High	dNTILVAGADkR	2	1	1	P22034	N-Term(iTRAQ4plex); K11(iTRAQ4plex)	0,0000	0,768	2	80,6	0	3,258E-08	43	5,15229E-05	3	1560,89609	0,80	21,05	1
	High	aTLEkDNTILVAGADkR	3	1	1	P22034	N-Term(iTRAQ4plex); K5(iTRAQ4plex); K16(iTRAQ4plex)	0,0000	1,280	2	7,4	0	0,00000117	43	5,29627E-05	4	2247,30190	-0,64	25,99	2
	High	nVAAGANPVALRR	1	1	1	P22034	N-Term(iTRAQ4plex)	0,0000	0,745	1		0	5,219E-08	42	6,76036E-05	3	1452,85178	0,10	20,11	1
	High	qYGAPQIVNDGITVAk	1	1	1	P22034	N-Term(iTRAQ4plex); K16(iTRAQ4plex)	0,0000	0,782	1		0	1,275E-07	39	0,000126174	3	1962,09189	1,01	35,78	0
	High	gYISPYFITDSDR	1	1	1	P22034	N-Term(iTRAQ4plex)	0,0000	1,082	1		0	0,00003383	32	0,000601132	2	1677,82536	0,75	40,38	0
	High	vGAATETELkDRk	2	1	1	P22034	N-Term(iTRAQ4plex); K10(iTRAQ4plex); K13(iTRAQ4plex)	0,0000	1,148	2	7,8	0	0,0000159	31	0,000864908	4	1850,07046	-0,19	19,90	2
	High	lAGkIESFk	1	1	1	P22034	N-Term(iTRAQ4plex); K4(iTRAQ4plex); K9(iTRAQ4plex)	0,0000	1,178	1		0	0,001316	24	0,003917148	3	1424,88324	-0,36	31,36	1
	High	eRIEQLR	1	1	1	P22034	N-Term(iTRAQ4plex)	0,0000	1,771	1		0	0,007858	20	0,00993549	3	1087,63456	0,45	14,63	1
	High	aSAGVk	3	1	1	P22034	N-Term(iTRAQ4plex); K6(iTRAQ4plex)	0,0000	1,368	2	6,6	0	0,003592	19	0,012416523	2	820,51201	-1,32	9,97	0
	High	nVLLEk	2	1	1	P22034	N-Term(iTRAQ4plex); K6(iTRAQ4plex)	0,0000	1,117	2	14,5	0,001	0,0099	34	0,000442558	2	1003,63860	-0,43	23,37	0
	High	aPAFGDRR	2	1	1	P22034	N-Term(iTRAQ4plex)	0,0000	1,309	2	2,1	0,002	0,02148	30	0,000916157	3	1033,57047	4,33	12,32	1
	Medium	iTLGPk	3	1	1	P22034	N-Term(iTRAQ4plex); K6(iTRAQ4plex)	0,0000	1,098	3	3,0	0,015	0,1683	27	0,002233418	2	916,60607	-1,02	23,78	0
	Medium	iEQLRk	1	1	1	P22034	N-Term(iTRAQ4plex); K6(iTRAQ4plex)	0,0000	1,161	1		0,02	0,2171	22	0,006500848	2	1074,68999	2,43	16,31	1
P73317	50S ribosomal protein L2 OS=Synechocystis sp. (strain PCC 6803 / Kazusa) GN=rpl	2522,78	54,35	1	20	20	124	2,150	106	68,0	276	30,4	11,21							
	A3	Sequence	# PSMs	# Proteins	# Protein Groups	Protein Group Accessions	Modifications	ΔCn	114/115	114/115 Count	114/115 Variability [%]	q-Value	PEP	IonScore	Exp Value	Charge	MH+ [Da]	ΔM [ppm]	RT [min]	# Missed Cleavages
	High	iPLGSEIHNVELVAGR	22	1	1	P73317	N-Term(iTRAQ4plex)	0,0000	1,994	22	37,4	0	4,992E-13	105	2,89734E-11	2	1848,04692	0,48	41,12	0
	High	sAGAFAQVVAk	9	1	1	P73317	N-Term(iTRAQ4plex); K11(iTRAQ4plex)	0,0000	2,503	6	73,7	0	3,124E-08	82	8,10793E-09	2	1336,78325	0,37	28,61	0
	High	iALLFYTDGEkR	21	1	1	P73317	N-Term(iTRAQ4plex); K11(iTRAQ4plex)	0,0000	1,957	20	50,5	0	1,028E-07	81	7,62026E-09	2	1713,98027	1,43	39,49	1
	High	sGPVSPWGkPALGAk	8	1	1	P73317	N-Term(iTRAQ4plex); K9(iTRAQ4plex); K15(iTRAQ4plex)	0,0000	4,836	6	139,5	0	1,036E-10	70	9,83943E-08	3	1884,10727	0,25	28,93	0
	High	iALLFYTDGEk	3	1	1	P73317	N-Term(iTRAQ4plex); K11(iTRAQ4plex)	0,0000	2,209	3	11,8	0	1,722E-09	64	4,37918E-07	2	1557,87810	0,89	46,06	0
	High	gSVmNPcDHPHGGGEGR	7	1	1	P73317	N-Term(iTRAQ4plex); M4(Oxidation); C7(Carbamidomethyl)	0,0000	1,475	4	27,6	0	2,904E-08	55	3,34172E-06	2	1923,83306	1,21	8,74	0
	High	kEcVATLGR	9	1	1	P73317	N-Term(iTRAQ4plex); K1(iTRAQ4plex); C3(Carbamidomethyl)	0,0000	6,880	5	393,6	0	2,225E-07	52	6,29463E-06	3	1321,75083	0,57	15,31	1
	High	vAAIEYDPNR	2	1	1	P73317	N-Term(iTRAQ4plex)	0,0000	1,784	2	10,6	0	0,00005946	43	4,59166E-05	2	1291,67693	0,47	25,50	0
	High	sSALIVR	1	1	1	P73317	N-Term(iTRAQ4plex)	0,0000	1,624	1		0	0,00104	42	5,86098E-05	2	889,55901	0,27	19,41	0
	High	vSNAEFR	3	1	1	P73317	N-Term(iTRAQ4plex)	0,0000	1,486	3	4,5	0	0,001729	38	0,00014288	2	966,51329	0,77	17,34	0
	High	gSVMNPcDHPHGGGEGR	3	1	1	P73317	N-Term(iTRAQ4plex); C7(Carbamidomethyl)	0,0000	1,144	2	28,6	0	4,779E-11	36	0,000272251	3	1907,83884	1,58	12,02	0
	High	nkQNIPAR	5	1	1	P73317	N-Term(iTRAQ4plex); K2(iTRAQ4plex)	0,0000	3,463	5	38,2	0	0,0001466	31	0,000726056	2	1228,73674	0,23	9,27	1
	High	nYRPMTPGTR	1	1	1	P73317	N-Term(iTRAQ4plex)	0,0000	1,345	1		0	0,00721	13	0,047311858	3	1336,69123	-0,03	14,77	0
	High	ecVATLGR	3	1	1	P73317	N-Term(iTRAQ4plex); C2(Carbamidomethyl)	0,0000	1,557	3	20,2	0,001	0,01167	23	0,004612857	2	1049,55962	6,27	18,16	0
	High	skPEk	6	1	1	P73317	N-Term(iTRAQ4plex); K2(iTRAQ4plex); K5(iTRAQ4plex)	0,0000	20,890	3	187,8	0,001	0,01232	13	0,053699471	3	1020,64212	0,74	7,96	0
	High	qNIPAR	4	1	1	P73317	N-Term(iTRAQ4plex)	0,0000	2,458	3	10,0	0,001	0,01478	10	0,099533667	2	842,49626	-0,27	10,48	0
	High	sLTTNR	3	1	1	P73317	N-Term(iTRAQ4plex)	0,0000	1,312	3	17,1	0,004	0,04943	25	0,003326366	2	835,47576	0,41	12,46	0
	High	gVITSR	4	1	1	P73317	N-Term(iTRAQ4plex)	0,0000	1,476	4	32,1	0,005	0,06629	25	0,00334172	2	776,47527	0,74	13,88	0
	High	iIDFkR	2	1	1	P73317	N-Term(iTRAQ4plex); K5(iTRAQ4plex)	0,0000	6,512	2	148,8	0,007	0,08922	12	0,066983834	3	1079,68274	1,09	23,74	1
	Medium	nYRPmTPGTR	1	1	1	P73317	N-Term(iTRAQ4plex); M5(Oxidation)	0,0000	1,529	1		0,013	0,1525	15	0,03258142	2	1352,68657	0,29	13,04	0
	Medium	iIDFk	4	1	1	P73317	N-Term(iTRAQ4plex); K5(iTRAQ4plex)	0,0000	3,694	4	34,6	0,014	0,1584	27	0,002993147	2	923,58055	0,11	30,39	0
	Medium	aPIGR	3	1	1	P73317	N-Term(iTRAQ4plex)	0,0000	4,378	3	1,1	0,017	0,1856	18	0,015451477	2	657,41747	1,54	13,34	0
	>sp|TRYP_PIG|	2383,44	28,57	1	5	5	88	1,164	81	12,4	231	24,4	7,18							
	A3	Sequence	# PSMs	# Proteins	# Protein Groups	Protein Group Accessions	Modifications	ΔCn	114/115	114/115 Count	114/115 Variability [%]	q-Value	PEP	IonScore	Exp Value	Charge	MH+ [Da]	ΔM [ppm]	RT [min]	# Missed Cleavages
	High	lGEHNIDVLEGNEQFINAAk	18	1	1		N-Term(iTRAQ4plex); K20(iTRAQ4plex)	0,0000	1,167	14	19,8	0	7,201E-17	114	4,46059E-12	3	2499,31400	2,29	39,93	0
	High	iTTHPNFNGNTLDNDImLIk	9	1	1		N-Term(iTRAQ4plex); M17(Oxidation); K20(iTRAQ4plex)	0,0000	1,140	8	9,5	0	4,055E-14	79	1,4262E-08	3	2587,38297	1,41	43,85	0
	High	iTTHPNFNGNTLDNDIMLIk	5	1	1		N-Term(iTRAQ4plex); K20(iTRAQ4plex)	0,0000	1,130	5	12,1	0	5,12E-15	68	1,4157E-07	3	2571,38504	0,25	46,53	0
	High	vATVSLPR	42	1	1		N-Term(iTRAQ4plex)	0,0000	1,194	41	7,0	0	0,001756	45	3,48313E-05	2	986,61254	1,01	57,53	0
	High	lSSPATLNSR	12	1	1		N-Term(iTRAQ4plex)	0,0000	1,016	11	5,1	0	0,00009889	44	3,78416E-05	2	1189,66667	0,77	21,27	0
	High	nkPGVYTk	2	1	1		N-Term(iTRAQ4plex); K2(iTRAQ4plex); K8(iTRAQ4plex)	0,0000	1,135	2	23,7	0	0,004395	29	0,00130008	3	1338,81415	2,68	11,95	0
P80505	Glyceraldehyde-3-phosphate dehydrogenase 2 OS=Synechocystis sp. (strain PCC 68	2308,07	45,99	1	17	17	90	2,503	72	43,7	337	36,5	6,44							
	A3	Sequence	# PSMs	# Proteins	# Protein Groups	Protein Group Accessions	Modifications	ΔCn	114/115	114/115 Count	114/115 Variability [%]	q-Value	PEP	IonScore	Exp Value	Charge	MH+ [Da]	ΔM [ppm]	RT [min]	# Missed Cleavages
	High	aAAVNIVPTSTGAAk	18	1	1	P80505	N-Term(iTRAQ4plex); K15(iTRAQ4plex)	0,0000	1,210	13	31,7	0	1,268E-10	102	6,7293E-11	2	1658,97197	2,39	27,74	0
	High	vVDLAEIVAk	5	1	1	P80505	N-Term(iTRAQ4plex); K10(iTRAQ4plex)	0,0000	2,821	4	9,3	0	3,388E-09	90	9,65984E-10	2	1344,83696	2,11	42,68	0
	High	nTIAEQVNGVLk	3	1	1	P80505	N-Term(iTRAQ4plex); K12(iTRAQ4plex)	0,0000	1,913	3	93,9	0	8,297E-09	86	2,68516E-09	2	1573,91777	1,62	34,49	0
	High	gPNIGTYVVGVNAHEYk	2	1	1	P80505	N-Term(iTRAQ4plex); K17(iTRAQ4plex)	0,0000	1,809	2	105,7	0	1,031E-14	85	3,25065E-09	3	2106,12467	1,14	33,94	0
	High	gTmTTTHSYTGDQR	11	1	1	P80505	N-Term(iTRAQ4plex); M3(Oxidation)	0,0000	2,897	8	14,1	0	6,884E-10	79	1,27929E-08	2	1715,78008	1,46	11,33	0
	High	aVALVIPELQGk	6	1	1	P80505	N-Term(iTRAQ4plex); K12(iTRAQ4plex)	0,0000	1,435	5	30,4	0	1,087E-08	69	1,25017E-07	2	1525,95769	1,34	43,30	0
	High	vINDNFGIIk	4	1	1	P80505	N-Term(iTRAQ4plex); K10(iTRAQ4plex)	0,0000	3,165	4	4,1	0	7,663E-07	67	1,92296E-07	2	1420,84282	1,80	38,03	0
	High	eAANTSLk	6	1	1	P80505	N-Term(iTRAQ4plex); K8(iTRAQ4plex)	0,0000	2,950	3	11,6	0	0,00001044	63	5,38233E-07	2	1121,64238	1,69	13,14	0
	High	kVLITAPGk	5	1	1	P80505	N-Term(iTRAQ4plex); K1(iTRAQ4plex); K9(iTRAQ4plex)	0,0000	2,673	5	12,7	0	0,000001195	58	1,58844E-06	2	1358,91008	0,38	20,71	1
	High	vLITAPGk	7	1	1	P80505	N-Term(iTRAQ4plex); K8(iTRAQ4plex)	0,0000	3,082	4	16,6	0	0,000147	57	2,08915E-06	2	1086,71453	1,83	26,14	0
	High	hVQAGAk	3	1	1	P80505	N-Term(iTRAQ4plex); K7(iTRAQ4plex)	0,0000	2,945	3	9,3	0	0,0001889	40	0,000106162	3	998,59842	-0,13	7,65	0
	High	gTMTTTHSYTGDQR	5	1	1	P80505	N-Term(iTRAQ4plex)	0,0000	3,313	3	0,7	0	0,000004973	40	0,000111422	3	1699,78250	-0,09	12,96	0
	High	vAINGFGR	3	1	1	P80505	N-Term(iTRAQ4plex)	0,0000	2,575	3	5,6	0	0,001298	33	0,00056909	2	977,56523	0,32	26,95	0
	High	iLDASHR	4	1	1	P80505	N-Term(iTRAQ4plex)	0,0000	1,192	4	25,5	0	0,003412	28	0,001674827	3	955,54529	1,17	13,92	0
	High	tNAHLLR	4	1	1	P80505	N-Term(iTRAQ4plex)	0,0000	1,365	4	13,1	0	0,003347	25	0,003318715	2	968,58092	5,28	12,35	0
	High	yDSmLGk	1	1	1	P80505	N-Term(iTRAQ4plex); M4(Oxidation); K7(iTRAQ4plex)	0,0000	2,662	1		0,001	0,01088	21	0,00814648	2	1117,59014	8,90	19,05	0
	High	lNGIALR	2	1	1	P80505	N-Term(iTRAQ4plex)	0,0000	1,337	2	4,9	0,003	0,03813	36	0,000259998	2	900,57683	2,30	28,34	0
	Medium	cWLGR	1	1	1	P80505	N-Term(iTRAQ4plex); C1(Carbamidomethyl)	0,0000	1,157	1		0,029	0,3161	16	0,0259998	2	835,43694	0,48	21,62	0
Q55199	Phosphate-binding protein OS=Synechocystis sp. (strain PCC 6803 / Kazusa) GN=p	2133,98	19,58	1	9	9	91	0,812	82	14,0	383	40,0	4,69							
	A3	Sequence	# PSMs	# Proteins	# Protein Groups	Protein Group Accessions	Modifications	ΔCn	114/115	114/115 Count	114/115 Variability [%]	q-Value	PEP	IonScore	Exp Value	Charge	MH+ [Da]	ΔM [ppm]	RT [min]	# Missed Cleavages
	High	lSQEALAGIMLGNITk	4	1	1	Q55199	N-Term(iTRAQ4plex); K16(iTRAQ4plex)	0,0000	0,786	4	6,4	0	2,46E-15	115	2,87078E-12	2	1947,12383	2,59	50,91	0
	High	lSQEALAGImLGNITk	4	1	1	Q55199	N-Term(iTRAQ4plex); M10(Oxidation); K16(iTRAQ4plex)	0,0000	0,932	4	10,8	0	1,416E-13	91	1,14904E-09	2	1963,11760	1,99	43,98	0
	High	iSPDYTITLk	7	1	1	Q55199	N-Term(iTRAQ4plex); K10(iTRAQ4plex)	0,0000	0,759	6	12,5	0	8,966E-09	66	2,4602E-07	2	1438,84026	0,47	36,31	0
	High	vAAAADkISPDYTITLk	1	1	1	Q55199	N-Term(iTRAQ4plex); K7(iTRAQ4plex); K17(iTRAQ4plex)	0,0000	0,650	1		0	1,533E-09	59	1,16405E-06	3	2209,28006	-0,18	36,77	1
	High	lVADNPDLTLPDRPITVVHR	59	1	1	Q55199	N-Term(iTRAQ4plex)	0,0000	0,838	53	13,5	0	0,000002453	58	1,7297E-06	4	2385,34072	1,49	37,87	0
	High	tVEWPTSk	3	1	1	Q55199	N-Term(iTRAQ4plex); K8(iTRAQ4plex)	0,0000	0,784	3	1,6	0	0,000007246	55	3,28829E-06	2	1235,68730	-0,11	26,44	0
	High	vAAAADk	4	1	1	Q55199	N-Term(iTRAQ4plex); K7(iTRAQ4plex)	0,0000	0,758	3	0,3	0	0,005879	51	7,94273E-06	2	933,55974	-1,12	13,42	0
	High	ekVAAAADk	6	1	1	Q55199	N-Term(iTRAQ4plex); K2(iTRAQ4plex); K9(iTRAQ4plex)	0,0000	0,744	5	4,8	0	0,0001937	45	2,94422E-05	2	1334,79802	-1,80	11,30	1
	High	eTIGDGk	1	1	1	Q55199	N-Term(iTRAQ4plex); K7(iTRAQ4plex)	0,0000	1,193	1		0,002	0,03137	27	0,001849141	2	1007,56188	0,69	14,22	0
	Medium	fIGGk	2	1	1	Q55199	N-Term(iTRAQ4plex); K5(iTRAQ4plex)	0,0000	0,742	2	0,2	0,029	0,313	18	0,016518477	2	809,51055	-2,25	17,92	0
P73606	Slr1855 protein OS=Synechocystis sp. (strain PCC 6803 / Kazusa) GN=slr1855 PE=	2054,28	37,89	1	27	27	116	1,263	103	17,4	607	70,0	5,25							
	A3	Sequence	# PSMs	# Proteins	# Protein Groups	Protein Group Accessions	Modifications	ΔCn	114/115	114/115 Count	114/115 Variability [%]	q-Value	PEP	IonScore	Exp Value	Charge	MH+ [Da]	ΔM [ppm]	RT [min]	# Missed Cleavages
	High	iLHDAEQTIk	8	1	1	P73606	N-Term(iTRAQ4plex); K10(iTRAQ4plex)	0,0000	1,012	8	2,3	0	4,116E-09	83	5,03466E-09	2	1455,84209	0,76	27,32	0
	High	tSDGQEFQVk	1	1	1	P73606	N-Term(iTRAQ4plex); K10(iTRAQ4plex)	0,0000	1,354	1		0	7,761E-09	67	2,22828E-07	2	1426,74529	2,54	19,66	0
	High	fVDkDEDIIYWYHAIDVQGEk	2	1	1	P73606	N-Term(iTRAQ4plex); K4(iTRAQ4plex); K21(iTRAQ4plex)	0,0000	1,612	2	4,7	0	1,364E-16	61	8,64968E-07	3	3015,55021	1,24	46,71	1
	High	kPAEYVFEk	18	1	1	P73606	N-Term(iTRAQ4plex); K1(iTRAQ4plex); K9(iTRAQ4plex)	0,0000	1,406	18	9,9	0	1,138E-07	58	1,65185E-06	2	1542,88872	-0,32	25,04	0
	High	aVVGHNLk	9	1	1	P73606	N-Term(iTRAQ4plex); K8(iTRAQ4plex)	0,0000	1,247	5	2,6	0	0,00001672	57	1,96775E-06	2	1125,70732	8,04	13,34	0
	High	kIADLMPAVGSDQQR	1	1	1	P73606	N-Term(iTRAQ4plex); K1(iTRAQ4plex)	0,0000	1,086	1		0	3,239E-09	53	5,33298E-06	3	1917,04868	1,04	32,05	1
	High	lSPMAYAk	3	1	1	P73606	N-Term(iTRAQ4plex); K8(iTRAQ4plex)	0,0000	1,321	3	2,4	0	0,0008525	52	6,60648E-06	2	1168,66960	4,91	27,98	0
	High	gGWYDVVER	3	1	1	P73606	N-Term(iTRAQ4plex)	0,0000	0,762	3	11,5	0	0,000005621	51	7,31089E-06	2	1224,61382	0,67	33,87	0
	High	cHQFVWHDR	3	1	1	P73606	N-Term(iTRAQ4plex); C1(Carbamidomethyl)	0,0000	1,248	3	1,7	0	0,00009449	48	1,70596E-05	2	1428,67253	0,94	16,89	0
	High	lFDkFFLDk	2	1	1	P73606	N-Term(iTRAQ4plex); K4(iTRAQ4plex); K9(iTRAQ4plex)	0,0000	1,579	2	0,9	0	0,000132	47	2,15372E-05	2	1604,94621	3,08	43,83	1
	High	iAWNLMR	1	1	1	P73606	N-Term(iTRAQ4plex)	0,0000	1,204	1		0	0,002402	46	2,74138E-05	2	1047,58940	0,35	38,60	0
	High	ekYVELAk	4	1	1	P73606	N-Term(iTRAQ4plex); K2(iTRAQ4plex); K8(iTRAQ4plex)	0,0000	1,488	4	6,5	0	0,00004783	46	2,76039E-05	2	1411,85845	4,49	20,59	1
	High	qVVFAGk	5	1	1	P73606	N-Term(iTRAQ4plex); K7(iTRAQ4plex)	0,0000	1,346	3	3,2	0	0,0009225	44	3,81918E-05	2	1036,63896	-0,39	20,97	0
	High	rILHDAEQTIk	3	1	1	P73606	N-Term(iTRAQ4plex); K11(iTRAQ4plex)	0,0000	1,408	2	42,2	0	1,837E-07	41	8,07587E-05	3	1611,94388	1,10	20,39	1
	High	yVELAk	3	1	1	P73606	N-Term(iTRAQ4plex); K6(iTRAQ4plex)	0,0000	1,223	3	4,0	0	0,002097	40	8,91189E-05	2	1010,61437	1,88	25,23	0
	High	lSPmAYAk	3	1	1	P73606	N-Term(iTRAQ4plex); M4(Oxidation); K8(iTRAQ4plex)	0,0000	1,281	2	14,0	0	0,0007795	38	0,000161053	2	1184,65996	0,99	21,28	0
	High	kIADLmPAVGSDQQR	4	1	1	P73606	N-Term(iTRAQ4plex); K1(iTRAQ4plex); M6(Oxidation)	0,0000	1,072	3	26,3	0	0,000008721	37	0,000181121	3	1933,04074	-0,44	23,63	1
	High	qVNSLGk	5	1	1	P73606	N-Term(iTRAQ4plex); K7(iTRAQ4plex)	0,0000	1,378	3	1,8	0	0,002613	36	0,0002665	2	1033,63030	5,67	14,99	0
	High	sEYGGYFSHLDPLmLDPR	3	1	1	P73606	N-Term(iTRAQ4plex); M14(Oxidation)	0,0000	1,020	3	7,6	0	0,000002236	31	0,000883019	3	2257,07370	0,91	46,85	0
	High	sTVNFRQETDTISR	2	1	1	P73606	N-Term(iTRAQ4plex)	0,0000	1,004	2	3,6	0	0,00002693	30	0,001059181	3	1797,92154	0,18	24,88	1
	High	tWLTPGR	4	1	1	P73606	N-Term(iTRAQ4plex)	0,0000	1,204	3	1,1	0	0,004476	28	0,001911581	2	974,55889	5,00	25,79	0
	High	yFPDYDHSPFVQER	4	1	1	P73606	N-Term(iTRAQ4plex)	0,0000	0,974	4	17,9	0	4,065E-07	26	0,002354887	3	1943,90531	0,42	37,52	0
	High	fFLDkSEYGGYFSHLDPLmLDPR	1	1	1	P73606	N-Term(iTRAQ4plex); K5(iTRAQ4plex); M19(Oxidation)	0,0000	1,031	1		0	0,00371	17	0,018922129	4	3051,51919	0,86	51,84	1
	High	hPmDFYFkPLPNGFPDGILR	2	1	1	P73606	N-Term(iTRAQ4plex); M3(Oxidation); K8(iTRAQ4plex)	0,0000	0,790	2	71,0	0	0,0000258	14	0,040641526	4	2665,38564	0,61	45,51	0
	High	hPMDFYFkPLPNGFPDGILR	1	1	1	P73606	N-Term(iTRAQ4plex); K8(iTRAQ4plex)	0,0000	1,084	1		0	0,00001558	11	0,084327651	4	2649,39370	1,73	48,80	0
	High	iAWNLmR	3	1	1	P73606	N-Term(iTRAQ4plex); M6(Oxidation)	0,0000	1,327	3	4,1	0,001	0,01715	29	0,001396272	2	1063,58464	0,65	32,13	0
	High	lLDNHSR	4	1	1	P73606	N-Term(iTRAQ4plex)	0,0000	1,035	4	9,2	0,001	0,01624	18	0,015487096	3	998,55613	6,15	12,60	0
	High	sTVNFR	2	1	1	P73606	N-Term(iTRAQ4plex)	0,0000	1,168	1		0,002	0,03184	10	0,090985043	2	867,48650	6,91	15,66	0
	High	qDWWIk	4	1	1	P73606	N-Term(iTRAQ4plex); K6(iTRAQ4plex)	0,0000	1,371	4	6,3	0,003	0,04224	28	0,001592099	2	1163,64739	1,90	33,58	0
	High	fFLDk	5	1	1	P73606	N-Term(iTRAQ4plex); K5(iTRAQ4plex)	0,0000	1,364	5	1,9	0,005	0,05887	23	0,004634149	2	957,56542	0,65	32,61	0
	Medium	gTEYLR	3	1	1	P73606	N-Term(iTRAQ4plex)	0,0000	1,210	3	4,3	0,013	0,1522	19	0,013090915	2	882,48046	0,33	16,26	0
P26527	ATP synthase subunit beta OS=Synechocystis sp. (strain PCC 6803 / Kazusa) GN=a	2021,66	37,68	1	15	15	64	1,161	57	16,5	483	51,7	5,02							
	A3	Sequence	# PSMs	# Proteins	# Protein Groups	Protein Group Accessions	Modifications	ΔCn	114/115	114/115 Count	114/115 Variability [%]	q-Value	PEP	IonScore	Exp Value	Charge	MH+ [Da]	ΔM [ppm]	RT [min]	# Missed Cleavages
	High	vGLTALTMAEYFR	9	1	1	P26527	N-Term(iTRAQ4plex)	0,0000	1,060	9	3,6	0	1,464E-09	89	1,23934E-09	2	1615,86516	1,03	51,94	0
	High	fVQAGSEVSALLGR	4	1	1	P26527	N-Term(iTRAQ4plex)	0,0000	1,038	4	11,2	0	2,502E-10	82	6,38542E-09	2	1577,87798	0,72	45,61	0
	High	vGLTALTmAEYFR	6	1	1	P26527	N-Term(iTRAQ4plex); M8(Oxidation)	0,0000	1,155	6	13,2	0	1,595E-08	72	6,1372E-08	2	1631,86040	1,22	44,43	0
	High	iTQVIGPVIDAQFPSGk	1	1	1	P26527	N-Term(iTRAQ4plex); K17(iTRAQ4plex)	0,0000	1,297	1		0	1,549E-07	69	1,20495E-07	3	2058,18692	1,50	46,39	0
	High	iALVYGQmNEPPGAR	2	1	1	P26527	N-Term(iTRAQ4plex); M8(Oxidation)	0,0000	1,171	2	17,1	0	3,182E-09	68	1,56664E-07	2	1775,92546	1,30	32,11	0
	High	aVAmSSTDGLVR	3	1	1	P26527	N-Term(iTRAQ4plex); M4(Oxidation)	0,0000	1,204	2	0,1	0	2,647E-09	66	2,30128E-07	2	1366,71196	0,16	21,30	0
	High	aVAMSSTDGLVR	1	1	1	P26527	N-Term(iTRAQ4plex)	0,0000	0,958	1		0	9,347E-07	63	4,68781E-07	2	1350,71733	0,37	26,84	0
	High	iGLFGGAGVGk	7	1	1	P26527	N-Term(iTRAQ4plex); K11(iTRAQ4plex)	0,0000	1,248	6	15,1	0	2,417E-08	61	8,37471E-07	2	1263,76763	0,98	35,93	0
	High	yVSLADTIk	6	1	1	P26527	N-Term(iTRAQ4plex); K9(iTRAQ4plex)	0,0000	1,305	5	7,5	0	2,311E-07	57	2,22316E-06	2	1297,76128	0,52	34,70	0
	High	eVQSTLQR	3	1	1	P26527	N-Term(iTRAQ4plex)	0,0000	1,256	2	0,9	0	0,00007958	47	1,99526E-05	2	1104,61357	0,53	15,88	0
	High	vIDLLTPYR	5	1	1	P26527	N-Term(iTRAQ4plex)	0,0000	1,210	5	16,1	0	0,00001322	43	4,81948E-05	2	1233,73320	0,67	43,98	0
	High	iALVYGQMNEPPGAR	1	1	1	P26527	N-Term(iTRAQ4plex)	0,0000	0,943	1		0	2,573E-08	43	5,15193E-05	3	1759,93229	2,30	36,45	0
	High	gPVPAGETFPIHRPAPk	4	1	1	P26527	N-Term(iTRAQ4plex); K17(iTRAQ4plex)	0,0000	1,135	3	5,8	0	5,848E-09	42	6,54591E-05	4	2059,17031	0,54	25,25	0
	High	lVDLETkPQVFETGIk	4	1	1	P26527	N-Term(iTRAQ4plex); K7(iTRAQ4plex); K16(iTRAQ4plex)	0,0000	1,254	3	10,2	0	4,409E-10	42	6,85441E-05	3	2249,31369	0,86	41,07	0
	High	iYNALk	4	1	1	P26527	N-Term(iTRAQ4plex); K6(iTRAQ4plex)	0,0000	1,336	4	9,6	0	0,005609	41	7,7085E-05	2	1009,62950	1,05	24,07	0
	High	fLSQPFFVAEVFTGAPGk	1	1	1	P26527	N-Term(iTRAQ4plex); K18(iTRAQ4plex)	0,0000	1,027	1		0	1,976E-09	32	0,000690528	3	2230,21543	0,13	58,22	0
	High	qDVLLFIDNIFR	1	1	1	P26527	N-Term(iTRAQ4plex)	0,0000	1,082	1		0	0,000001466	27	0,002051021	2	1636,92046	1,52	60,35	0
	Medium	iTSTk	2	1	1	P26527	N-Term(iTRAQ4plex); K5(iTRAQ4plex)	0,0000	1,390	1		0,015	0,1707	22	0,006052991	2	837,52904	0,77	13,16	0
P73093	Phycobilisome rod-core linker polypeptide CpcG OS=Synechocystis sp. (strain PCC 6	1923,76	57,45	1	18	18	114	0,871	105	25,0	235	27,4	9,23							
	A3	Sequence	# PSMs	# Proteins	# Protein Groups	Protein Group Accessions	Modifications	ΔCn	114/115	114/115 Count	114/115 Variability [%]	q-Value	PEP	IonScore	Exp Value	Charge	MH+ [Da]	ΔM [ppm]	RT [min]	# Missed Cleavages
	High	vSAQNINIEASVPR	4	1	1	P73093	N-Term(iTRAQ4plex)	0,0000	0,764	4	11,4	0	1,226E-12	110	1,01151E-11	2	1641,90483	0,44	33,62	0
	High	iAWSIVVATk	3	1	1	P73093	N-Term(iTRAQ4plex); K10(iTRAQ4plex)	0,0000	1,078	3	15,1	0	8,352E-09	70	8,99435E-08	2	1375,85564	0,33	42,93	0
	High	sINPAANTIPk	9	1	1	P73093	N-Term(iTRAQ4plex); K11(iTRAQ4plex)	0,0000	0,721	6	7,1	0	3,613E-07	63	4,73119E-07	2	1413,83257	1,51	25,26	0
	High	eAGELPFNIk	2	1	1	P73093	N-Term(iTRAQ4plex); K10(iTRAQ4plex)	0,0000	0,992	2	30,8	0	1,325E-08	62	6,36752E-07	2	1405,79350	0,37	37,54	0
	High	qLGFPQIVWQNEVR	6	1	1	P73093	N-Term(iTRAQ4plex)	0,0000	0,729	6	23,6	0	2,782E-10	60	1,00918E-06	2	1858,01140	1,15	47,14	0
	High	rQLGFPQIVWQNEVR	3	1	1	P73093	N-Term(iTRAQ4plex)	0,0000	0,493	3	0,9	0	5,022E-11	59	1,23364E-06	3	2014,11142	0,52	41,49	1
	High	qIFFHAFk	6	1	1	P73093	N-Term(iTRAQ4plex); K8(iTRAQ4plex)	0,0000	0,649	5	19,7	0	0,0002369	55	3,46713E-06	2	1325,76152	0,49	32,81	0
	High	kLTAGNPmNFLGmAR	4	1	1	P73093	K1(iTRAQ4plex); N-Term(iTRAQ4plex); M8(Oxidation); M13(Oxidation)	0,0000	0,548	3	0,5	0	2,081E-08	49	1,18569E-05	3	1941,02890	-0,02	29,99	1
	High	kLTAGNPMNFLGmAR	1	1	1	P73093	N-Term(iTRAQ4plex); K1(iTRAQ4plex); M13(Oxidation)	0,0000	0,486	1		0	4,781E-11	48	1,50998E-05	3	1925,03409	0,04	37,46	1
	High	gLLLSNTFR	47	1	1	P73093	N-Term(iTRAQ4plex)	0,0000	1,011	43	7,1	0	0,000007399	48	1,7297E-05	2	1164,68547	-0,25	37,60	0
	High	lTAGNPmNFLGmAR	4	1	1	P73093	N-Term(iTRAQ4plex); M7(Oxidation); M12(Oxidation)	0,0000	1,206	4	18,6	0	2,927E-08	46	2,42086E-05	2	1668,83367	1,06	35,32	0
	High	lTAGNPMNFLGmAR	3	1	1	P73093	N-Term(iTRAQ4plex); M12(Oxidation)	0,0000	1,012	3	21,0	0	5,212E-07	46	2,71E-05	2	1652,83794	0,57	43,20	0
	High	lTAGNPMNFLGMAR	2	1	1	P73093	N-Term(iTRAQ4plex)	0,0000	0,883	2	5,2	0	0,0002301	39	0,000123018	2	1636,84441	1,43	48,57	0
	High	fVEHcVQk	3	1	1	P73093	N-Term(iTRAQ4plex); C5(Carbamidomethyl); K8(iTRAQ4plex)	0,0000	0,802	3	1,7	0	0,0005601	37	0,000190095	2	1334,71868	4,30	16,24	0
	High	nSFYEk	2	1	1	P73093	N-Term(iTRAQ4plex); K6(iTRAQ4plex)	0,0000	0,807	2	6,6	0	0,006969	35	0,000306881	2	1075,57158	4,97	18,13	0
	High	ekVLESQLR	3	1	1	P73093	N-Term(iTRAQ4plex); K2(iTRAQ4plex)	0,0000	0,785	3	4,6	0	0,00376	29	0,001300736	3	1389,82972	-0,51	19,62	1
	High	rFIPQEk	2	1	1	P73093	N-Term(iTRAQ4plex); K7(iTRAQ4plex)	0,0000	1,044	2	40,4	0	0,0009254	16	0,029576267	3	1205,72321	-1,07	17,32	1
	High	vLESQLR	3	1	1	P73093	N-Term(iTRAQ4plex)	0,0000	0,771	3	12,0	0,001	0,00817	47	2,03222E-05	2	988,59075	-0,05	21,59	0
	High	nGQITVR	3	1	1	P73093	N-Term(iTRAQ4plex)	0,0000	0,853	3	33,6	0,003	0,04022	34	0,000354789	2	931,54357	-0,65	13,64	0
	High	fIPQEk	3	1	1	P73093	N-Term(iTRAQ4plex); K6(iTRAQ4plex)	0,0000	0,814	3	7,9	0,008	0,09255	20	0,01002236	2	1049,62248	-0,85	24,09	0
	Medium	yDAYHR	1	1	1	P73093	N-Term(iTRAQ4plex)	0,0000	0,798	1		0,034	0,3593	12	0,064412478	2	968,47051	-0,14	11,50	0
Q55544	Phycobiliprotein ApcE OS=Synechocystis sp. (strain PCC 6803 / Kazusa) GN=apcE F	1902,09	40,29	1	36	36	102	0,713	94	20,2	896	100,2	9,23							
	A3	Sequence	# PSMs	# Proteins	# Protein Groups	Protein Group Accessions	Modifications	ΔCn	114/115	114/115 Count	114/115 Variability [%]	q-Value	PEP	IonScore	Exp Value	Charge	MH+ [Da]	ΔM [ppm]	RT [min]	# Missed Cleavages
	High	aYSQSISYLESQVR	3	1	1	Q55544	N-Term(iTRAQ4plex)	0,0000	0,922	3	41,0	0	1,088E-09	83	4,57057E-09	2	1774,91118	1,10	40,88	0
	High	gPAVNNQVGNPSAVGEFPGSLGAk	1	1	1	Q55544	N-Term(iTRAQ4plex); K24(iTRAQ4plex)	0,0000	0,936	1		0	1,279E-12	64	3,6557E-07	3	2555,34848	1,07	36,55	0
	High	fGESSTQALIR	2	1	1	Q55544	N-Term(iTRAQ4plex)	0,0000	0,659	2	4,7	0	5,551E-08	64	4,2459E-07	2	1352,72832	-0,57	29,33	0
	High	fVELGQVSAIR	4	1	1	Q55544	N-Term(iTRAQ4plex)	0,0000	0,630	4	3,7	0	0,000002204	60	1,22009E-06	2	1362,78642	0,15	39,43	0
	High	lYWAPHYVck	1	1	1	Q55544	N-Term(iTRAQ4plex); C9(Carbamidomethyl); K10(iTRAQ4plex)	0,0000	0,525	1		0	3,034E-08	57	1,80289E-06	3	1624,85282	-1,25	33,99	0
	High	eIQQYNQILASQGLk	2	1	1	Q55544	N-Term(iTRAQ4plex); K15(iTRAQ4plex)	0,0000	0,632	2	3,8	0	4,344E-08	55	3,49921E-06	3	2021,13114	2,04	38,35	0
	High	lSNNEINVk	2	1	1	Q55544	N-Term(iTRAQ4plex); K9(iTRAQ4plex)	0,0000	0,691	1		0	0,000001809	54	3,77546E-06	2	1318,76567	6,64	23,72	0
	High	iFTGGSPLSYLEkPVER	10	1	1	Q55544	N-Term(iTRAQ4plex); K13(iTRAQ4plex)	0,0000	0,681	7	15,4	0	2,253E-11	53	5,1047E-06	3	2181,21769	0,84	46,06	0
	High	lNGGLPGAk	3	1	1	Q55544	N-Term(iTRAQ4plex); K9(iTRAQ4plex)	0,0000	0,812	2	10,3	0	0,000005433	52	6,03907E-06	2	1114,68572	3,06	22,94	0
	High	qPALVGASSDSR	2	1	1	Q55544	N-Term(iTRAQ4plex)	0,0000	0,669	2	22,5	0	0,00005515	48	1,52044E-05	2	1331,70354	-0,06	18,03	0
	High	qFFEPFINSR	3	1	1	Q55544	N-Term(iTRAQ4plex)	0,0000	0,683	3	7,9	0	0,00003854	46	2,4042E-05	2	1428,73967	0,29	43,07	0
	High	aASADYFR	3	1	1	Q55544	N-Term(iTRAQ4plex)	0,0000	0,748	3	6,0	0	0,0001103	45	2,88383E-05	2	1044,52251	-0,57	21,55	0
	High	qDkELVVPSFTPVVk	3	1	1	Q55544	N-Term(iTRAQ4plex); K3(iTRAQ4plex); K15(iTRAQ4plex)	0,0000	0,714	3	24,6	0	1,134E-08	45	3,01974E-05	3	2118,25412	0,28	38,13	1
	High	sELFLk	2	1	1	Q55544	N-Term(iTRAQ4plex); K6(iTRAQ4plex)	0,0000	0,739	2	2,2	0	0,001793	45	3,44661E-05	2	1024,63359	5,33	28,64	0
	High	kQFFEPFINSR	3	1	1	Q55544	N-Term(iTRAQ4plex); K1(iTRAQ4plex)	0,0000	0,655	3	7,4	0	0,000004606	43	4,9362E-05	2	1700,93669	0,23	36,74	1
	High	aLELAFR	4	1	1	Q55544	N-Term(iTRAQ4plex)	0,0000	0,705	4	5,5	0	0,001105	41	9,7262E-05	2	963,57384	-0,61	34,96	0
	High	dMSWFLR	3	1	1	Q55544	N-Term(iTRAQ4plex)	0,0000	0,543	3	7,0	0	0,000286	37	0,000181539	2	1098,55083	-1,35	42,74	0
	High	yLTPGGLQmR	4	1	1	Q55544	N-Term(iTRAQ4plex); M9(Oxidation)	0,0000	0,665	4	8,8	0	0,000007857	36	0,000230128	3	1295,69126	1,07	27,58	0
	High	aSGGSSLARPQLYQTVPVSAISQAEQQDR	1	1	1	Q55544	N-Term(iTRAQ4plex)	0,0000				0	1,175E-07	36	0,000282942	4	3188,64663	1,53	38,88	0
	High	rFPTLPAANFPNTER	3	1	1	Q55544	N-Term(iTRAQ4plex)	0,0000	0,787	3	5,5	0	0,000001389	34	0,000396251	3	1875,00034	0,49	35,62	1
	High	yAmkPGLSALEk	2	1	1	Q55544	N-Term(iTRAQ4plex); M3(Oxidation); K4(iTRAQ4plex); K12(iTRAQ4plex)	0,0000	0,803	2	11,6	0	0,000001546	33	0,0004797	3	1756,00302	-0,53	26,96	0
	High	vIEmGTk	1	1	1	Q55544	N-Term(iTRAQ4plex); M4(Oxidation); K7(iTRAQ4plex)	0,0000				0	0,0002583	32	0,000564898	2	1081,61626	-0,31	13,92	0
	High	lVSTYDk	2	1	1	Q55544	N-Term(iTRAQ4plex); K7(iTRAQ4plex)	0,0000	0,736	2	2,7	0	0,003972	32	0,00059975	2	1113,63872	-0,62	20,78	0
	High	gLGVEAQEcR	2	1	1	Q55544	N-Term(iTRAQ4plex); C9(Carbamidomethyl)	0,0000	0,951	2	4,9	0	0,00001488	29	0,001253055	2	1262,62749	-0,41	18,17	0
	High	vDkEVTPR	5	1	1	Q55544	N-Term(iTRAQ4plex); K3(iTRAQ4plex)	0,0000	0,657	5	3,7	0	0,00443	28	0,001534511	2	1231,72405	-0,69	13,14	1
	High	sVFSSTGPIPPGFRPINIAR	8	1	1	Q55544	N-Term(iTRAQ4plex)	0,0000	0,801	8	12,4	0	1,299E-07	23	0,005545874	3	2257,25937	0,85	44,29	0
	High	yGPSNmQk	1	1	1	Q55544	N-Term(iTRAQ4plex); M6(Oxidation); K8(iTRAQ4plex)	0,0000	0,701	1		0	0,005698	22	0,006151344	2	1228,62688	2,79	13,67	0
	High	rPAPFNk	2	1	1	Q55544	N-Term(iTRAQ4plex); K7(iTRAQ4plex)	0,0000	0,742	2	5,7	0	0,005305	21	0,008432765	3	1117,67063	-1,29	14,54	0
	High	sLRDmSWFLR	1	1	1	Q55544	N-Term(iTRAQ4plex); M5(Oxidation)	0,0000	0,691	1		0	0,004808	19	0,01121941	3	1470,76389	-0,38	35,04	1
	High	yAMkPGLSALEk	1	1	1	Q55544	N-Term(iTRAQ4plex); K4(iTRAQ4plex); K12(iTRAQ4plex)	0,0000	0,730	1		0	0,000001876	19	0,013242501	3	1740,00766	-0,79	30,98	0
	High	lLGRPTYGR	5	1	1	Q55544	N-Term(iTRAQ4plex)	0,0000	0,537	4	6,6	0	0,00135	10	0,089736681	3	1176,69678	-0,18	20,58	0
	High	yGPSNMQk	1	1	1	Q55544	N-Term(iTRAQ4plex); K8(iTRAQ4plex)	0,0000	0,584	1		0,001	0,01229	27	0,001945226	2	1212,62517	-2,78	17,40	0
	High	aPTPANk	4	1	1	Q55544	N-Term(iTRAQ4plex); K7(iTRAQ4plex)	0,0000	0,894	4	22,4	0,002	0,01846	13	0,048080614	2	986,58635	-0,99	10,55	0
	High	fPTLPAANFPNTER	1	1	1	Q55544	N-Term(iTRAQ4plex)	0,0000	0,575	1		0,006	0,07212	12	0,066369723	2	1718,89922	0,53	41,78	0
	Medium	qIFER	1	1	1	Q55544	N-Term(iTRAQ4plex)	0,0000	0,822	1		0,01	0,1169	18	0,018832588	2	836,47496	0,32	18,83	0
	Medium	aPLNQk	3	1	1	Q55544	N-Term(iTRAQ4plex); K6(iTRAQ4plex)	0,0000	0,731	3	19,4	0,015	0,1644	23	0,005558659	2	958,59386	1,51	13,73	0
	Medium	nTGTSVk	2	1	1	Q55544	N-Term(iTRAQ4plex); K7(iTRAQ4plex)	0,0000	0,879	2	12,4	0,022	0,2391	19	0,013772095	2	994,57646	-0,69	10,23	0
	Medium	sPLYR	1	1	1	Q55544	N-Term(iTRAQ4plex)	0,0000	1,219	1		0,049	0,5052	11	0,119490607	2	779,45207	-1,46	16,26	0
P74281	Soluble hydrogenase 42 kD subunit OS=Synechocystis sp. (strain PCC 6803 / Kazus	1862,67	34,38	1	17	17	77	1,033	69	16,6	384	40,7	7,28							
	A3	Sequence	# PSMs	# Proteins	# Protein Groups	Protein Group Accessions	Modifications	ΔCn	114/115	114/115 Count	114/115 Variability [%]	q-Value	PEP	IonScore	Exp Value	Charge	MH+ [Da]	ΔM [ppm]	RT [min]	# Missed Cleavages
	High	aWQAYETATIPR	4	1	1	P74281	N-Term(iTRAQ4plex)	0,0000	0,929	3	9,9	0	9,553E-08	77	1,93183E-08	2	1550,80937	0,63	35,51	0
	High	tFGLAVEEIk	5	1	1	P74281	N-Term(iTRAQ4plex); K10(iTRAQ4plex)	0,0000	1,026	5	16,3	0	6,265E-09	74	4,51542E-08	2	1394,81450	0,80	38,89	0
	High	iIAELTANLk	10	1	1	P74281	N-Term(iTRAQ4plex); K10(iTRAQ4plex)	0,0000	0,960	10	17,0	0	7,305E-09	69	1,30909E-07	2	1373,86125	0,43	45,70	0
	High	kFDIAMAGGQDHLk	2	1	1	P74281	N-Term(iTRAQ4plex); K1(iTRAQ4plex); K14(iTRAQ4plex)	0,0000	1,151	2	2,4	0	5,806E-16	67	2,07477E-07	3	1963,08030	0,35	28,51	1
	High	kFDIAmAGGQDHLk	6	1	1	P74281	N-Term(iTRAQ4plex); K1(iTRAQ4plex); M6(Oxidation); K14(iTRAQ4plex)	0,0000	1,334	4	13,0	0	1,252E-13	61	8,09828E-07	3	1979,07804	1,78	24,51	1
	High	aEGLDAIFTR	1	1	1	P74281	N-Term(iTRAQ4plex)	0,0000	0,882	1		0	0,00004918	54	4,66563E-06	2	1236,67107	0,44	39,23	0
	High	vLLAMAk	5	1	1	P74281	N-Term(iTRAQ4plex); K7(iTRAQ4plex)	0,0000	1,006	4	5,9	0	0,0001365	53	5,30143E-06	2	1033,66838	0,14	32,51	0
	High	fDIAmAGGQDHLk	2	1	1	P74281	N-Term(iTRAQ4plex); M5(Oxidation); K13(iTRAQ4plex)	0,0000	0,710	2	13,1	0	3,293E-07	49	1,36135E-05	3	1706,88883	6,65	28,67	0
	High	tFGLAVEEIkAEWGk	4	1	1	P74281	N-Term(iTRAQ4plex); K10(iTRAQ4plex); K15(iTRAQ4plex)	0,0000	0,927	3	7,0	0	5,247E-11	48	1,42223E-05	3	2110,19327	1,11	48,94	1
	High	iGHLGFVcDR	7	1	1	P74281	N-Term(iTRAQ4plex); C8(Carbamidomethyl)	0,0000	1,054	6	16,3	0	8,021E-07	48	1,61436E-05	2	1317,68645	0,75	27,84	0
	High	vLVGNNGk	3	1	1	P74281	N-Term(iTRAQ4plex); K8(iTRAQ4plex)	0,0000	0,643	3	50,1	0	0,00005006	47	1,82377E-05	2	1088,67107	4,06	16,89	0
	High	fDIAMAGGQDHLk	1	1	1	P74281	N-Term(iTRAQ4plex); K13(iTRAQ4plex)	0,0000	1,368	1		0	4,621E-08	47	1,90095E-05	3	1690,88230	-0,16	32,59	0
	High	gYmIPPGLGFVSVSAk	3	1	1	P74281	N-Term(iTRAQ4plex); M3(Oxidation); K16(iTRAQ4plex)	0,0000	0,816	3	17,9	0	0,000001542	45	2,99206E-05	3	1927,06363	1,78	44,79	0
	High	kkFDIAMAGGQDHLk	1	1	1	P74281	N-Term(iTRAQ4plex); K1(iTRAQ4plex); K2(iTRAQ4plex); K15(iTRAQ4plex)	0,0000	1,220	1		0	1,165E-09	45	3,25065E-05	4	2235,27700	0,16	24,65	2
	High	qmLmIPGPTPVPEk	2	1	1	P74281	N-Term(iTRAQ4plex); M2(Oxidation); M4(Oxidation); K14(iTRAQ4plex)	0,0000	0,770	2	26,7	0	6,451E-08	40	9,74922E-05	3	1858,00724	0,81	33,42	0
	High	qMLmIPGPTPVPEk	1	1	1	P74281	N-Term(iTRAQ4plex); M4(Oxidation); K14(iTRAQ4plex)	0,0000	1,343	1		0	3,93E-08	38	0,000165947	3	1842,01096	0,07	37,08	0
	High	aLDPNDFk	1	1	1	P74281	N-Term(iTRAQ4plex); K8(iTRAQ4plex)	0,0000	1,028	1		0	0,00004915	38	0,000168256	2	1207,65666	0,43	27,53	0
	High	fYLDLk	5	1	1	P74281	N-Term(iTRAQ4plex); K6(iTRAQ4plex)	0,0000	1,085	5	7,0	0	0,004676	34	0,000380163	2	1086,64397	0,19	38,33	0
	High	fYLDLkk	5	1	1	P74281	N-Term(iTRAQ4plex); K6(iTRAQ4plex); K7(iTRAQ4plex)	0,0000	1,100	5	12,3	0	0,0002228	32	0,000620826	2	1358,84111	0,23	31,97	1
	High	kkFDIAmAGGQDHLk	2	1	1	P74281	K1(iTRAQ4plex); N-Term(iTRAQ4plex); K2(iTRAQ4plex); M7(Oxidation); K15(	0,0000	1,341	1		0	0,00002115	27	0,001861959	4	2251,27260	0,47	21,74	2
	High	qMLMIPGPTPVPEk	1	1	1	P74281	N-Term(iTRAQ4plex); K14(iTRAQ4plex)	0,0000	1,379	1		0	0,000005382	15	0,032807263	3	1826,01761	0,93	39,48	0
	High	vLLAmAk	5	1	1	P74281	N-Term(iTRAQ4plex); M5(Oxidation); K7(iTRAQ4plex)	0,0000	1,057	4	6,1	0,001	0,01025	22	0,006432691	3	1049,66993	6,45	23,42	0
	Medium	aEWGk	1	1	1	P74281	N-Term(iTRAQ4plex); K5(iTRAQ4plex)	0,0000	1,329	1		0,024	0,2575	26	0,002404363	2	878,49571	-1,97	15,83	0
Q55460	Bicarbonate-binding protein CmpA OS=Synechocystis sp. (strain PCC 6803 / Kazusa	1772,41	36,73	1	15	16	79	0,690	64	23,3	452	49,4	5,77							
	A3	Sequence	# PSMs	# Proteins	# Protein Groups	Protein Group Accessions	Modifications	ΔCn	114/115	114/115 Count	114/115 Variability [%]	q-Value	PEP	IonScore	Exp Value	Charge	MH+ [Da]	ΔM [ppm]	RT [min]	# Missed Cleavages
	High	nYFNVPTTILESPFk	3	1	1	Q55460	N-Term(iTRAQ4plex); K15(iTRAQ4plex)	0,0000	0,697	3	4,7	0	1,626E-11	89	1,21051E-09	2	2058,11675	0,83	52,64	0
	High	aAHTFPNVNQDFWIR	9	1	1	Q55460	N-Term(iTRAQ4plex)	0,0000	0,686	8	18,1	0	3,045E-13	73	5,16381E-08	3	1959,99643	0,90	40,42	0
	High	yGMTGVEVSk	2	1	1	Q55460	N-Term(iTRAQ4plex); K10(iTRAQ4plex)	0,0000	0,686	2	1,8	0	1,159E-08	69	1,19666E-07	2	1358,72527	1,78	26,05	0
	High	sHDLWFLTESIR	16	1	1	Q55460	N-Term(iTRAQ4plex)	0,0000	0,960	15	24,8	0	6,45E-10	64	4,02689E-07	3	1647,86130	0,08	44,95	0
	High	yGmTGVEVSk	5	1	1	Q55460	N-Term(iTRAQ4plex); M3(Oxidation); K10(iTRAQ4plex)	0,0000	0,632	3	5,4	0	7,872E-07	61	7,29407E-07	2	1374,71782	0,04	21,60	0
	High	gVNLDITk	5	1	1	Q55460	N-Term(iTRAQ4plex); K8(iTRAQ4plex)	0,0000	0,629	3	5,2	0	0,000002199	61	9,20851E-07	2	1147,69328	0,66	27,57	0
	High	lGYIPIVEAAPLIIAQEk	2	1	1	Q55460	N-Term(iTRAQ4plex); K18(iTRAQ4plex)	0,0000	0,587	1		0	1,105E-11	58	1,4288E-06	3	2226,33506	-0,09	57,36	0
	High	nRPEVVQIVSGR	9	1	1	Q55460	N-Term(iTRAQ4plex)	0,0000	0,537	5	7,9	0	2,649E-08	57	1,95871E-06	3	1497,86332	0,98	24,82	0
	High	aDWVDkNPk	7	1	1	Q55460	N-Term(iTRAQ4plex); K6(iTRAQ4plex); K9(iTRAQ4plex)	0,0000	0,619	7	15,5	0	0,00003312	56	2,66054E-06	2	1504,85320	3,17	19,60	1
	High	vPMYVLAQLITQGNGIAVAPmHEGk	1	1	1	Q55460	N-Term(iTRAQ4plex); M21(Oxidation); K25(iTRAQ4plex)	0,0000	0,971	1		0	9,124E-11	50	1,02322E-05	4	2941,58828	-0,01	56,75	0
	High	gPLYWk	2	1	1	Q55460	N-Term(iTRAQ4plex); K6(iTRAQ4plex)	0,0000	0,665	2	8,9	0	0,001139	45	2,83772E-05	2	1051,61785	-0,04	31,77	0
	High	qANWASAR	3	1	1	Q55460	N-Term(iTRAQ4plex)	0,0000	0,845	3	8,6	0	0,00003744	44	4,12069E-05	2	1047,54997	4,52	15,81	0
	High	vPmYVLAQLITQGNGIAVAPmHEGk	1	1	1	Q55460	N-Term(iTRAQ4plex); M3(Oxidation); M21(Oxidation); K25(iTRAQ4plex)	0,0000	0,857	1		0	1,956E-09	42	5,90201E-05	4	2957,58242	-0,27	50,10	0
	High	vNREDLWR	4	2	2	Q55460;P73452	N-Term(iTRAQ4plex)	0,0000				0	0,0005999	27	0,001945226	2	1231,66802	1,28	22,94	1
	High	iIDkVNR	5	1	1	Q55460	N-Term(iTRAQ4plex); K4(iTRAQ4plex)	0,0000	0,616	5	11,8	0	0,007502	21	0,007344631	2	1145,73113	5,79	17,01	1
	High	aADYIk	1	1	1	Q55460	N-Term(iTRAQ4plex); K6(iTRAQ4plex)	0,0000	0,812	1		0,002	0,03141	28	0,001528232	2	968,57048	5,13	18,67	0
	Medium	wGFHk	2	1	1	Q55460	N-Term(iTRAQ4plex); K5(iTRAQ4plex)	0,0000	0,612	2	20,0	0,012	0,136	20	0,009659842	2	962,54442	-0,66	22,11	0
	Medium	gFFAk	2	1	1	Q55460	N-Term(iTRAQ4plex); K5(iTRAQ4plex)	0,0000	0,697	2	12,7	0,02	0,2142	11	0,082029489	2	857,51177	-0,70	23,77	0
P74689	Dihydroxy-acid dehydratase OS=Synechocystis sp. (strain PCC 6803 / Kazusa) GN=	1761,92	27,81	1	21	21	100	1,747	83	54,5	561	58,6	5,25							
	A3	Sequence	# PSMs	# Proteins	# Protein Groups	Protein Group Accessions	Modifications	ΔCn	114/115	114/115 Count	114/115 Variability [%]	q-Value	PEP	IonScore	Exp Value	Charge	MH+ [Da]	ΔM [ppm]	RT [min]	# Missed Cleavages
	High	gNLATEGSVAk	6	1	1	P74689	N-Term(iTRAQ4plex); K11(iTRAQ4plex)	0,0000	2,348	3	1,1	0	2,256E-09	76	2,88E-08	2	1334,75835	4,87	17,28	0
	High	vLVEAIkk	5	1	1	P74689	N-Term(iTRAQ4plex); K7(iTRAQ4plex); K8(iTRAQ4plex)	0,0000	2,598	3	6,5	0	0,00008395	67	1,78636E-07	2	1331,90141	2,06	24,02	1
	High	iQAGDVVVVR	3	1	1	P74689	N-Term(iTRAQ4plex)	0,0000	1,889	2	11,6	0	3,845E-08	66	2,77952E-07	2	1199,72319	0,25	29,83	0
	High	kPVITGPAk	10	1	1	P74689	N-Term(iTRAQ4plex); K1(iTRAQ4plex); K9(iTRAQ4plex)	0,0000	2,524	5	10,8	0	2,504E-07	64	3,98997E-07	2	1342,88286	3,42	15,86	0
	High	aDSTEESAk	1	1	1	P74689	N-Term(iTRAQ4plex); K9(iTRAQ4plex)	0,0000	2,730	1		0	0,00001817	57	1,97229E-06	2	1225,61760	2,07	10,47	0
	High	vLVEAIk	4	1	1	P74689	N-Term(iTRAQ4plex); K7(iTRAQ4plex)	0,0000	2,009	4	25,4	0	0,000136	53	4,65554E-06	2	1059,70268	0,99	29,77	0

	High	nMPGAMIAMAR	1	1	1	P74689	N-Term(iTRAQ4plex)	0,0000	1,310	1		0	0,000001731	52	5,80724E-06	2	1306,65544	0,24	34,38	0
	High	rLLQLNISEEELAQR	1	1	1	P74689	N-Term(iTRAQ4plex)	0,0000	1,015	1		0	1,184E-11	51	8,85054E-06	3	1956,09995	0,21	37,65	1
	High	yVTTNLHAAGGIPQVMk	7	1	1	P74689	N-Term(iTRAQ4plex); K17(iTRAQ4plex)	0,0000	1,220	6	28,2	0	9,662E-14	49	1,25893E-05	3	2088,15268	0,58	35,19	0
	High	sQVITQGTQR	6	1	1	P74689	N-Term(iTRAQ4plex)	0,0000	1,371	4	69,1	0	0,000004595	49	1,40595E-05	2	1261,69878	0,53	13,34	0
	High	kQILPSQILTR	5	1	1	P74689	N-Term(iTRAQ4plex); K1(iTRAQ4plex)	0,0000	1,649	4	31,4	0	7,721E-09	46	2,30659E-05	3	1585,00467	0,44	30,43	1
	High	nmPGAmIAMAR	3	1	1	P74689	N-Term(iTRAQ4plex); M2(Oxidation); M6(Oxidation)	0,0000	1,863	3	5,8	0	0,00000279	42	5,88803E-05	2	1338,64592	0,72	19,94	0
	High	qILPSQILTR	13	1	1	P74689	N-Term(iTRAQ4plex)	0,0000	1,029	13	7,9	0	1,791E-07	40	9,18269E-05	3	1312,80750	0,42	36,86	0
	High	nMPGAmIAMAR	2	1	1	P74689	N-Term(iTRAQ4plex); M6(Oxidation)	0,0000	1,686	2	7,0	0	8,777E-07	40	9,18269E-05	2	1322,65080	0,58	26,70	0
	High	yVTTNLHAAGGIPQVmk	2	1	1	P74689	N-Term(iTRAQ4plex); M16(Oxidation); K17(iTRAQ4plex)	0,0000	1,199	2	91,6	0	0,000000113	38	0,000147571	3	2104,14951	1,49	32,02	0
	High	hkVPVLcDLkPSGk	3	1	1	P74689	N-Term(iTRAQ4plex); K2(iTRAQ4plex); C7(Carbamidomethyl); K10(iTRAQ4pl	0,0000	2,074	3	6,3	0	1,56E-11	36	0,000230128	3	2154,29349	0,91	23,43	1
	High	vPVLcDLkPSGk	6	1	1	P74689	N-Term(iTRAQ4plex); C5(Carbamidomethyl); K8(iTRAQ4plex); K12(iTRAQ4pl	0,0000	2,167	6	90,0	0	6,582E-07	34	0,000368104	3	1745,03885	1,87	31,94	0
	High	nmPGAmIAmAR	3	1	1	P74689	N-Term(iTRAQ4plex); M2(Oxidation); M6(Oxidation); M9(Oxidation)	0,0000	2,143	2	7,8	0	0,00001138	32	0,000696578	2	1354,64116	0,95	16,32	0
	High	aQWTPPQPR	2	1	1	P74689	N-Term(iTRAQ4plex)	0,0000	2,113	2	1,2	0	0,004639	27	0,001887861	2	1224,66069	0,06	23,58	0
	High	lLQLNISEEELAQRR	1	1	1	P74689	N-Term(iTRAQ4plex)	0,0000	1,774	1		0	0,00003388	24	0,003999171	3	1956,10233	1,43	39,95	1
	High	rAQWTPPQPR	2	1	1	P74689	N-Term(iTRAQ4plex)	0,0000	0,970	2	10,0	0	0,0003266	23	0,004797003	3	1380,76023	-1,09	18,26	1
	High	ySLVSR	4	1	1	P74689	N-Term(iTRAQ4plex)	0,0000	1,677	4	8,9	0,001	0,01213	31	0,000801623	2	868,50627	6,19	20,47	0
	High	aEAGLR	7	1	1	P74689	N-Term(iTRAQ4plex)	0,0000	1,101	6	9,4	0,003	0,03392	22	0,0136381	2	760,44756	5,46	12,78	0
	High	iSGVk	1	1	1	P74689	N-Term(iTRAQ4plex); K5(iTRAQ4plex)	0,0000	1,904	1		0,008	0,09208	21	0,023111174	2	791,52696	5,10	15,52	0
	Medium	gILAk	2	1	1	P74689	N-Term(iTRAQ4plex); K5(iTRAQ4plex)	0,0000	1,657	2	2,4	0,016	0,174	18	0,037771661	2	789,54680	3,97	18,55	0
P72871	S-adenosylmethionine synthase OS=Synechocystis sp. (strain PCC 6803 / Kazusa) G	1670,85	30,99	1	15	15	82	1,586	71	18,9	426	45,8	5,29							
	A3	Sequence	# PSMs	# Proteins	# Protein Groups	Protein Group Accessions	Modifications	ΔCn	114/115	114/115 Count	114/115 Variability [%]	q-Value	PEP	IonScore	Exp Value	Charge	MH+ [Da]	ΔM [ppm]	RT [min]	# Missed Cleavages
	High	fYQDVAAYGHFGR	29	1	1	P72871	N-Term(iTRAQ4plex)	0,0000	1,426	26	14,1	0	3,365E-10	87	1,89221E-09	2	1674,81670	1,29	37,12	0
	High	fVVGGPQGDAGLTGR	4	1	1	P72871	N-Term(iTRAQ4plex)	0,0000	1,779	3	0,9	0	1,858E-12	87	1,97683E-09	2	1574,84233	0,97	32,27	0
	High	sGQLAYLRPDGk	4	1	1	P72871	N-Term(iTRAQ4plex); K12(iTRAQ4plex)	0,0000	2,058	4	4,3	0	2,826E-09	66	2,47155E-07	2	1592,90386	2,48	23,42	0
	High	kIIVDTYGGYSR	8	1	1	P72871	N-Term(iTRAQ4plex); K1(iTRAQ4plex)	0,0000	1,605	7	5,1	0	7,137E-07	60	9,687E-07	3	1659,93216	0,79	25,00	1
	High	iIVDTYGGYSR	2	1	1	P72871	N-Term(iTRAQ4plex)	0,0000	1,744	2	3,5	0	0,000004304	52	6,83864E-06	2	1387,73454	0,52	31,59	0
	High	fIVNPTGk	8	1	1	P72871	N-Term(iTRAQ4plex); K8(iTRAQ4plex)	0,0000	1,775	5	12,0	0	0,00008639	51	8,64968E-06	2	1163,71160	7,65	28,52	0
	High	kSGQLAYLRPDGk	6	1	1	P72871	N-Term(iTRAQ4plex); K1(iTRAQ4plex); K13(iTRAQ4plex)	0,0000	1,502	4	25,5	0	1,615E-10	46	2,73508E-05	3	1865,09726	0,17	20,63	1
	High	aDLWDVVVGHcFSDIALkPTDk	2	1	1	P72871	N-Term(iTRAQ4plex); C11(Carbamidomethyl); K18(iTRAQ4plex); K22(iTRAQ4	0,0000	1,444	2	30,4	0	1,145E-08	38	0,000173368	4	2918,54897	1,45	50,51	0
	High	hGGGAFSGkDPTk	5	1	1	P72871	N-Term(iTRAQ4plex); K9(iTRAQ4plex); K13(iTRAQ4plex)	0,0000	1,917	5	18,0	0	0,000007286	36	0,000270514	2	1690,92876	2,97	12,03	1
	High	nIVAAGLADk	1	1	1	P72871	N-Term(iTRAQ4plex); K10(iTRAQ4plex)	0,0000	1,426	1		0	0,0001722	33	0,00045811	3	1259,75772	1,20	28,79	0
	High	hALSDDELDk	3	1	1	P72871	N-Term(iTRAQ4plex); K10(iTRAQ4plex)	0,0000	0,946	3	8,6	0	0,000001623	32	0,000665227	3	1430,73713	0,38	18,01	0
	High	sAAYAAR	6	1	1	P72871	N-Term(iTRAQ4plex)	0,0000	1,641	5	5,1	0	0,003799	31	0,000837471	2	853,46599	1,33	11,97	0
	High	hGGGAFSGk	2	1	1	P72871	N-Term(iTRAQ4plex); K9(iTRAQ4plex)	0,0000	1,595	2	1,6	0	0,0005824	15	0,033648833	3	1105,60059	1,17	10,29	0
	High	hGGGAFSGkDPTkVDR	1	1	1	P72871	N-Term(iTRAQ4plex); K9(iTRAQ4plex); K13(iTRAQ4plex)	0,0000	1,466	1		0	0,001991	12	0,063091377	4	2061,12075	0,26	14,10	2
	Medium	nLPAER	1	1	1	P72871	N-Term(iTRAQ4plex)	0,0000	1,526	1		0,043	0,4497	23	0,004931397	2	843,48113	0,73	13,07	0
P73320	50S ribosomal protein L3 OS=Synechocystis sp. (strain PCC 6803 / Kazusa) GN=rpl	1632,70	41,78	1	11	11	81	1,838	58	155,5	213	22,7	10,13							
	A3	Sequence	# PSMs	# Proteins	# Protein Groups	Protein Group Accessions	Modifications	ΔCn	114/115	114/115 Count	114/115 Variability [%]	q-Value	PEP	IonScore	Exp Value	Charge	MH+ [Da]	ΔM [ppm]	RT [min]	# Missed Cleavages
	High	mAGQYGASQVTVR	7	1	1	P73320	N-Term(iTRAQ4plex); M1(Oxidation)	0,0000	1,112	5	119,8	0	1,263E-09	88	1,67869E-09	2	1527,78154	7,13	19,23	0
	High	mAGQYGASQVTVR	4	1	1	P73320	N-Term(iTRAQ4plex)	0,0000	3,555	4	631,0	0	5,804E-10	75	2,86398E-08	2	1511,77690	0,77	24,24	0
	High	gALPGkPGTLLNITPAk	17	1	1	P73320	N-Term(iTRAQ4plex); K6(iTRAQ4plex); K17(iTRAQ4plex)	0,0000	2,081	13	28,5	0	1,153E-12	67	2,00433E-07	2	2080,29009	2,20	35,72	0
	High	rmAGQYGASQVTVR	1	1	1	P73320	N-Term(iTRAQ4plex); M2(Oxidation)	0,0000	1,283	1		0	1,966E-07	59	1,34887E-06	3	1683,87394	1,29	14,97	1
	High	lPGSTGAGTTPGR	4	1	1	P73320	N-Term(iTRAQ4plex)	0,0000	24,051	3	373,2	0	0,00004848	58	1,73768E-06	2	1315,71843	7,39	18,64	0
	High	sIGILGTk	7	1	1	P73320	N-Term(iTRAQ4plex); K8(iTRAQ4plex)	0,0000	20,376	3	22,9	0	0,00004195	51	8,07179E-06	2	1076,69145	-0,32	27,99	0
	High	gFAGYQk	11	1	1	P73320	N-Term(iTRAQ4plex); K7(iTRAQ4plex)	0,0000	1,098	7	30,4	0	0,000254	47	2,00895E-05	2	1058,58830	0,93	20,35	0
	High	aLSkPELGHLk	6	1	1	P73320	N-Term(iTRAQ4plex); K4(iTRAQ4plex); K11(iTRAQ4plex)	0,0000	13,150	4	20,6	0	0,000001384	45	2,96463E-05	2	1625,01323	1,32	24,61	0
	High	aLSkPELGHLkk	3	1	1	P73320	N-Term(iTRAQ4plex); K4(iTRAQ4plex); K11(iTRAQ4plex); K12(iTRAQ4plex)	0,0000	8,225	2	60,0	0	1,031E-08	25	0,003005869	3	1897,21048	1,25	21,46	1
	High	nLLIIk	19	1	1	P73320	N-Term(iTRAQ4plex); K6(iTRAQ4plex)	0,0000	0,846	14	23,0	0	0,003287	23	0,004830254	3	1001,69629	0,15	31,94	0
	High	kSNTDPmR	1	1	1	P73320	N-Term(iTRAQ4plex); K1(iTRAQ4plex); M7(Oxidation)	0,0000	3,834	1		0,001	0,01232	17	0,020987949	3	1252,65589	0,04	7,66	1
	Medium	vYPGk	1	1	1	P73320	N-Term(iTRAQ4plex); K5(iTRAQ4plex)	0,0000	8,016	1		0,014	0,155	32	0,000885302	2	851,52977	8,05	14,80	0
P74008	Adenosylhomocysteinase OS=Synechocystis sp. (strain PCC 6803 / Kazusa) GN=ah	1623,70	51,29	1	20	20	74	0,520	62	31,5	425	46,2	5,77							
	A3	Sequence	# PSMs	# Proteins	# Protein Groups	Protein Group Accessions	Modifications	ΔCn	114/115	114/115 Count	114/115 Variability [%]	q-Value	PEP	IonScore	Exp Value	Charge	MH+ [Da]	ΔM [ppm]	RT [min]	# Missed Cleavages
	High	qHQLSDIIGTTEETTTGIVR	4	1	1	P74008	N-Term(iTRAQ4plex)	0,0000	0,860	3	32,2	0	2,422E-16	100	1,01904E-10	3	2343,22794	0,27	42,64	0
	High	tIVVAGYGWcGk	5	1	1	P74008	N-Term(iTRAQ4plex); C10(Carbamidomethyl); K12(iTRAQ4plex)	0,0000	0,941	5	63,3	0	2,421E-11	81	7,26056E-09	2	1598,86125	0,56	36,59	0
	High	aTNILLAGk	13	1	1	P74008	N-Term(iTRAQ4plex); K9(iTRAQ4plex)	0,0000	0,748	9	64,3	0	2,099E-07	81	8,66902E-09	2	1188,75530	-0,14	29,20	0
	High	yGTGQSTLDGIIR	4	1	1	P74008	N-Term(iTRAQ4plex)	0,0000	0,594	4	40,1	0	9,207E-09	80	9,35341E-09	2	1524,81413	0,16	35,27	0
	High	hVQIALDHRPNIIIDDGSDVVATLVQER	8	1	1	P74008	N-Term(iTRAQ4plex)	0,0000	0,333	6	12,0	0	1,12E-10	62	6,52218E-07	3	3267,75949	0,84	47,90	0
	High	vmPmAEAAHQGDIFITVTGNk	1	1	1	P74008	N-Term(iTRAQ4plex); M2(Oxidation); M4(Oxidation); K21(iTRAQ4plex)	0,0000	0,494	1		0	9,371E-11	62	6,23735E-07	3	2550,29367	0,04	35,05	0
	High	nkGQLEPGmHSIPVEVDQEIAR	1	1	1	P74008	N-Term(iTRAQ4plex); K2(iTRAQ4plex); M9(Oxidation)	0,0000	0,473	1		0	1,641E-09	52	5,94251E-06	4	2751,43300	-0,33	30,78	1
	High	fAQEkPFAGIR	6	1	1	P74008	N-Term(iTRAQ4plex); K5(iTRAQ4plex)	0,0000	0,440	6	4,4	0	0,000003262	48	2,19955E-05	2	1551,88872	0,06	28,07	0
	High	gEDNETYHR	5	1	1	P74008	N-Term(iTRAQ4plex)	0,0000	0,807	4	17,1	0	0,00003456	47	1,86196E-05	2	1264,56708	-0,32	8,93	0
	High	gQLEPGmHSIPVEVDQEIAR	4	1	1	P74008	N-Term(iTRAQ4plex); M7(Oxidation)	0,0000	0,495	2	14,6	0	3,057E-09	42	6,17974E-05	3	2365,19400	0,03	34,25	0
	High	sIIVIGEGR	4	1	1	P74008	N-Term(iTRAQ4plex)	0,0000	0,478	2	2,0	0	0,0006735	39	0,000137395	2	1087,65874	-0,45	30,28	0
	High	dISLAPQGR	2	1	1	P74008	N-Term(iTRAQ4plex)	0,0000	0,519	2	16,8	0	0,00004415	39	0,000140919	2	1100,61797	-0,11	21,75	0
	High	yDIkDISLAPQGR	1	1	1	P74008	N-Term(iTRAQ4plex); K4(iTRAQ4plex)	0,0000	0,436	1		0	0,000007223	33	0,000563246	3	1763,98642	-1,72	33,91	1
	High	hVIRPEHFAVmk	2	1	1	P74008	N-Term(iTRAQ4plex); M11(Oxidation); K12(iTRAQ4plex)	0,0000	0,440	2	2,3	0	8,822E-08	30	0,000965984	3	1767,99204	-0,61	18,48	0
	High	hFYDNR	3	1	1	P74008	N-Term(iTRAQ4plex)	0,0000	1,081	3	27,0	0	0,001488	29	0,001164046	3	995,48197	0,42	14,54	0
	High	nFTEQYILPNGk	1	1	1	P74008	N-Term(iTRAQ4plex); K12(iTRAQ4plex)	0,0000	0,677	1		0	0,002851	16	0,027490412	3	1711,92423	-0,90	34,93	0
	High	hVIRPEHFAVMk	3	1	1	P74008	N-Term(iTRAQ4plex); K12(iTRAQ4plex)	0,0000	0,437	3	4,4	0	0,0001625	16	0,025761432	4	1751,99831	0,07	20,84	0
	High	sLkEQAk	2	1	1	P74008	N-Term(iTRAQ4plex); K3(iTRAQ4plex); K7(iTRAQ4plex)	0,0000	0,495	2	0,6	0,003	0,04185	29	0,001364489	2	1235,76164	-5,44	10,07	1
	Medium	qkYDIk	1	1	1	P74008	N-Term(iTRAQ4plex); K2(iTRAQ4plex); K6(iTRAQ4plex)	0,0000	0,513	1		0,016	0,1735	32	0,000691783	2	1226,73967	-5,88	14,27	1
	Medium	vATPVk	2	1	1	P74008	N-Term(iTRAQ4plex); K6(iTRAQ4plex)	0,0000	0,770	2	2,0	0,022	0,2378	27	0,002157595	2	902,58605	-5,87	14,87	0
	Medium	emPVLk	2	1	1	P74008	N-Term(iTRAQ4plex); M2(Oxidation); K6(iTRAQ4plex)	0,0000	0,625	2	8,4	0,036	0,3805	23	0,007890259	2	1020,60179	1,55	16,65	0
P72740	Dihydrolipoyl dehydrogenase OS=Synechocystis sp. (strain PCC 6803 / Kazusa) GN-	1557,73	52,74	1	18	18	60	1,897	53	33,0	474	50,8	5,55							
	A3	Sequence	# PSMs	# Proteins	# Protein Groups	Protein Group Accessions	Modifications	ΔCn	114/115	114/115 Count	114/115 Variability [%]	q-Value	PEP	IonScore	Exp Value	Charge	MH+ [Da]	ΔM [ppm]	RT [min]	# Missed Cleavages
	High	mMLAHAASGQGVVAVENIcGR	1	1	1	P72740	N-Term(iTRAQ4plex); C19(Carbamidomethyl)	0,0000	1,439	1		0	2,423E-19	100	1,03985E-10	3	2315,15494	0,40	36,91	0
	High	mmLAHAASGQGVVAVENIcGR	2	1	1	P72740	N-Term(iTRAQ4plex); M1(Oxidation); M2(Oxidation); C19(Carbamidomethyl)	0,0000	2,581	1		0	1,945E-14	90	9,90764E-10	3	2347,14621	1,01	29,85	0
	High	mmLAHAASGQGVVAVENIcGR	2	1	1	P72740	N-Term(iTRAQ4plex); M2(Oxidation); C19(Carbamidomethyl)	0,0000	1,166	2	0,7	0	2,146E-15	90	1,06162E-09	3	2331,15122	0,98	33,50	0
	High	nLGLETVGVETDRR	3	1	1	P72740	N-Term(iTRAQ4plex)	0,0000	2,005	3	3,7	0	8,274E-08	72	8,69629E-08	3	1702,92075	0,14	28,80	1
	High	aIPAAAFTHPEISYVGLTEAQAk	5	1	1	P72740	N-Term(iTRAQ4plex); K23(iTRAQ4plex)	0,0000	1,944	4	35,6	0	1,672E-14	72	6,80722E-08	3	2673,45114	0,76	44,03	0
	High	tkEVIDTLEVDAcLVATGR	1	1	1	P72740	N-Term(iTRAQ4plex); K2(iTRAQ4plex); C13(Carbamidomethyl)	0,0000	1,136	1		0	5,309E-10	71	8,16526E-08	3	2378,28971	2,39	50,17	1
	High	eAIAAHANDLVSk	6	1	1	P72740	N-Term(iTRAQ4plex); K13(iTRAQ4plex)	0,0000	1,899	3	27,5	0	1,163E-08	63	5,59719E-07	3	1626,90525	-0,09	25,01	0
	High	sRDIETYTGVFATk	3	1	1	P72740	N-Term(iTRAQ4plex); K14(iTRAQ4plex)	0,0000	2,259	3	16,2	0	8,303E-09	60	1,50776E-06	3	1876,00650	0,54	32,88	1
	High	aLAEkETDGIAk	1	1	1	P72740	N-Term(iTRAQ4plex); K5(iTRAQ4plex); K12(iTRAQ4plex)	0,0000	2,966	1		0	1,226E-08	57	1,84914E-06	2	1677,97881	2,41	20,02	1
	High	rGFIEVNDQmQVIk	3	1	1	P72740	N-Term(iTRAQ4plex); M10(Oxidation); K14(iTRAQ4plex)	0,0000	1,602	3	3,7	0	1,541E-10	55	3,29587E-06	3	1981,07865	0,34	28,29	1
	High	dGkPVPHLWAVGDATGk	6	1	1	P72740	N-Term(iTRAQ4plex); K3(iTRAQ4plex); K17(iTRAQ4plex)	0,0000	2,681	4	23,7	0	5,049E-11	54	4,37492E-06	3	2180,21976	0,40	30,32	0
	High	eGFVVSTAk	2	1	1	P72740	N-Term(iTRAQ4plex); K9(iTRAQ4plex)	0,0000	2,247	2	8,6	0	4,669E-07	53	4,50786E-06	2	1225,70403	0,76	23,89	0
	High	tAIIEAk	3	1	1	P72740	N-Term(iTRAQ4plex); K7(iTRAQ4plex)	0,0000	1,846	3	36,4	0	0,0007621	47	2,73129E-05	2	1033,65715	7,31	19,09	0
	High	iQSDLTNSLTR	2	1	1	P72740	N-Term(iTRAQ4plex)	0,0000	1,715	2	13,7	0	0,00001324	46	2,73508E-05	2	1391,76201	0,64	31,58	0
	High	aLLAASGR	4	1	1	P72740	N-Term(iTRAQ4plex)	0,0000	1,293	4	40,1	0	0,001891	45	3,57024E-05	2	902,55962	6,20	18,75	0
	High	sQDFDYDLVIIGAGVGGHGAALHAVk	1	1	1	P72740	N-Term(iTRAQ4plex); K26(iTRAQ4plex)	0,0000	3,269	1		0	3,353E-08	39	0,000141315	4	2898,54653	3,86	45,52	0
	High	vREmSDQDHLQQLGIQINGVTFTR	1	1	1	P72740	N-Term(iTRAQ4plex); M4(Oxidation)	0,0000	0,369	1		0	0,000003533	30	0,001045665	4	2945,50698	1,66	39,80	1
	High	lkVDTIR	6	1	1	P72740	N-Term(iTRAQ4plex); K2(iTRAQ4plex)	0,0000	2,389	6	11,6	0	0,001978	28	0,001592099	2	1132,73003	0,69	19,09	1
	High	kTEVDYR	6	1	1	P72740	N-Term(iTRAQ4plex); K1(iTRAQ4plex)	0,0000	1,853	6	10,2	0	0,004753	28	0,00171779	2	1198,66924	1,84	10,86	1
	High	iPATk	2	1	1	P72740	N-Term(iTRAQ4plex); K5(iTRAQ4plex)	0,0000	1,538	2	3,5	0,002	0,02749	18	0,015451477	2	817,54210	4,31	14,59	0
P73203	Phycobilisome 32.1 kDa linker polypeptide, phycocyanin-associated, rod 1 OS=Syne	1537,35	56,70	1	16	16	66	0,658	55	12,3	291	32,5	9,35							
	A3	Sequence	# PSMs	# Proteins	# Protein Groups	Protein Group Accessions	Modifications	ΔCn	114/115	114/115 Count	114/115 Variability [%]	q-Value	PEP	IonScore	Exp Value	Charge	MH+ [Da]	ΔM [ppm]	RT [min]	# Missed Cleavages
	High	gAESLLTNGSISVR	3	1	1	P73203	N-Term(iTRAQ4plex)	0,0000	0,761	2	24,0	0	4,472E-11	91	9,31497E-10	2	1547,85161	0,38	35,37	0
	High	lATELGQNTVSAIVGPSGSNAGWAYRPSR	3	1	1	P73203	N-Term(iTRAQ4plex)	0,0000	0,718	2	49,8	0	7,066E-10	88	1,51346E-09	3	3103,60837	1,33	45,96	0
	High	vEITAISAPGYPk	5	1	1	P73203	N-Term(iTRAQ4plex); K13(iTRAQ4plex)	0,0000	0,838	5	3,8	0	4,734E-12	86	2,29071E-09	2	1633,94267	1,41	35,98	0
	High	aLGGTVPFGQASk	8	1	1	P73203	N-Term(iTRAQ4plex); K13(iTRAQ4plex)	0,0000	0,658	5	5,4	0	1,455E-10	85	3,94183E-09	2	1520,86821	0,43	30,60	0
	High	aVIVPFEQLNQTLQQINR	2	1	1	P73203	N-Term(iTRAQ4plex)	0,0000	0,599	2	20,0	0	4,685E-10	60	1,11422E-06	3	2255,26371	0,35	54,26	0
	High	lkGAESLLTNGSISVR	4	1	1	P73203	N-Term(iTRAQ4plex); K2(iTRAQ4plex)	0,0000	0,640	3	6,5	0	1,514E-11	58	1,78727E-06	3	1933,13102	-0,57	36,90	1
	High	aITTAASR	3	1	1	P73203	N-Term(iTRAQ4plex)	0,0000	0,796	3	0,6	0	0,001838	50	1,4097E-05	2	934,54350	-0,36	13,32	0
	High	vIELNYk	6	1	1	P73203	N-Term(iTRAQ4plex); K7(iTRAQ4plex)	0,0000	0,632	5	6,1	0	0,005813	49	1,1749E-05	2	1166,70342	0,92	30,40	0
	High	kFLYNNFQTR	6	1	1	P73203	N-Term(iTRAQ4plex); K1(iTRAQ4plex)	0,0000	0,633	6	5,0	0	2,301E-07	46	2,47725E-05	2	1618,89519	0,48	26,63	1
	High	fNNQVGDR	3	1	1	P73203	N-Term(iTRAQ4plex)	0,0000	0,613	3	10,9	0	0,00002777	43	5,16381E-05	2	1093,55644	5,23	14,78	0
	High	fLYNNFQTR	5	1	1	P73203	N-Term(iTRAQ4plex)	0,0000	0,645	5	7,6	0	0,0006672	40	0,00010964	2	1346,69768	0,23	34,10	0
	High	lGVAPYNESRPVELRPDFSLDDAk	1	1	1	P73203	N-Term(iTRAQ4plex); K24(iTRAQ4plex)	0,0000	0,661	1		0	0,00009822	15	0,040381388	3	2976,57071	1,24	40,77	0
	High	aGNTPAk	6	1	1	P73203	N-Term(iTRAQ4plex); K7(iTRAQ4plex)	0,0000	0,664	5	6,2	0,003	0,03856	17	0,021847044	2	946,55400	-2,14	8,27	0
	High	tVGFTR	9	1	1	P73203	N-Term(iTRAQ4plex)	0,0000	0,616	6	20,8	0,007	0,08482	10	0,099304746	2	824,47490	0,25	16,50	0
	Medium	sELYk	1	1	1	P73203	N-Term(iTRAQ4plex); K5(iTRAQ4plex)	0,0000	0,819	1		0,026	0,2792	28	0,001581139	2	927,54228	3,59	14,71	0
	Medium	sELYkk	1	1	1	P73203	N-Term(iTRAQ4plex); K5(iTRAQ4plex); K6(iTRAQ4plex)	0,0000	0,735	1		0,029	0,3177	10	0,090567005	2	1199,73064	-4,46	12,53	1
P54691	Probable branched-chain-amino-acid aminotransferase OS=Synechocystis sp. (strai	1529,56	51,48	1	19	19	67	1,395	64	24,0	305	33,9	6,33							
	A3	Sequence	# PSMs	# Proteins	# Protein Groups	Protein Group Accessions	Modifications	ΔCn	114/115	114/115 Count	114/115 Variability [%]	q-Value	PEP	IonScore	Exp Value	Charge	MH+ [Da]	ΔM [ppm]	RT [min]	# Missed Cleavages
	High	iENFTLGGDRPITEk	3	1	1	P54691	N-Term(iTRAQ4plex); K15(iTRAQ4plex)	0,0000	1,487	3	6,6	0	3,098E-08	86	2,37121E-09	2	1978,08843	1,81	33,57	0
	High	sVLTAVTENREPk	5	1	1	P54691	N-Term(iTRAQ4plex); K13(iTRAQ4plex)	0,0000	1,352	4	3,1	0	1,513E-09	81	7,69077E-09	2	1731,98577	0,80	25,45	1
	High	iSAAYITSALAk	7	1	1	P54691	N-Term(iTRAQ4plex); K12(iTRAQ4plex)	0,0000	1,264	7	23,0	0	2,633E-08	81	8,39402E-09	2	1496,89507	1,59	42,52	0
	High	fLHYDISAEk	10	1	1	P54691	N-Term(iTRAQ4plex); K10(iTRAQ4plex)	0,0000	1,519	10	14,0	0	9,946E-09	73	5,49503E-08	2	1510,81621	1,18	31,04	0
	High	sFYIRPLVYSSGLGIAPR	4	1	1	P54691	N-Term(iTRAQ4plex)	0,0000	1,193	4	16,6	0	7,255E-12	70	1,15983E-07	3	2140,20572	0,99	44,96	0
	High	fLPIAYFEDk	8	1	1	P54691	N-Term(iTRAQ4plex); K10(iTRAQ4plex)	0,0000	1,311	8	34,1	0	2,727E-08	65	4,20851E-07	2	1530,84563	0,62	49,34	0
	High	vcEATGmNVFmVR	2	1	1	P54691	N-Term(iTRAQ4plex); C2(Carbamidomethyl); M7(Oxidation); M11(Oxidation)	0,0000	1,334	2	0,9	0	4,576E-10	57	1,92739E-06	2	1689,78935	0,81	31,15	0
	High	ikEVIVDFVk	5	1	1	P54691	N-Term(iTRAQ4plex); K2(iTRAQ4plex); K10(iTRAQ4plex)	0,0000	1,378	5	16,6	0	4,57E-08	55	2,88383E-06	2	1622,02690	0,96	41,18	1
	High	sVLTAVTENR	2	1	1	P54691	N-Term(iTRAQ4plex)	0,0000	1,160	2	11,8	0	0,00004469	48	1,7297E-05	2	1233,69243	0,37	27,69	0
	High	iSVATHALHYGTAAFGGLR	1	1	1	P54691	N-Term(iTRAQ4plex)	0,0000	1,777	1		0	7,629E-11	42	6,63697E-05	3	2086,13614	2,23	37,40	0
	High	fLPIAYFEDkFVPFEDAk	2	1	1	P54691	N-Term(iTRAQ4plex); K10(iTRAQ4plex); K18(iTRAQ4plex)	0,0000	1,438	2	50,4	0	0,000003306	39	0,000140919	3	2608,40696	0,21	57,40	1
	High	rIENFTLGGDRPITEk	3	1	1	P54691	N-Term(iTRAQ4plex); K16(iTRAQ4plex)	0,0000	1,390	3	3,7	0	5,109E-12	38	0,00017297	3	2134,18668	0,34	29,34	1
	High	eVIVDFVkk	2	1	1	P54691	N-Term(iTRAQ4plex); K8(iTRAQ4plex); K9(iTRAQ4plex)	0,0000	1,403	2	26,0	0	0,00002602	36	0,000273508	3	1508,94147	0,13	32,09	1
	High	vcEATGMNVFmVR	1	1	1	P54691	N-Term(iTRAQ4plex); C2(Carbamidomethyl); M11(Oxidation)	0,0000	1,470	1		0	0,0007186	34	0,000380163	2	1673,79643	2,01	37,37	0
	High	kNQPDk	3	1	1	P54691	N-Term(iTRAQ4plex); K1(iTRAQ4plex); K6(iTRAQ4plex)	0,0000	2,018	3	4,0	0	0,005909	16	0,031782002	3	1161,69696	1,52	7,80	1
	High	iSSWYR	1	1	1	P54691	N-Term(iTRAQ4plex)	0,0000	1,400	1		0,002	0,02806	25	0,002985176	2	955,51183	0,03	27,08	0
	High	fVPFEDAk	1	1	1	P54691	N-Term(iTRAQ4plex); K8(iTRAQ4plex)	0,0000	1,493	1		0,002	0,02878	25	0,003012798	2	1240,68120	-0,35	34,48	0
	High	iTPVk	4	1	1	P54691	N-Term(iTRAQ4plex); K5(iTRAQ4plex)	0,0000	1,789	2	7,5	0,003	0,04099	22	0,005942511	2	845,57457	5,55	17,08	0
	High	lHNLEk	2	1	1	P54691	N-Term(iTRAQ4plex); K6(iTRAQ4plex)	0,0000	1,182	2	30,7	0,004	0,04532	27	0,002018227	2	1041,62956	0,05	14,40	0
	Medium	sFPLR	1	1	1	P54691	N-Term(iTRAQ4plex)	0,0000	1,426	1		0,036	0,3824	20	0,018838141	2	763,45854	0,30	20,67	0
P73728	Putative peroxiredoxin sll1621 OS=Synechocystis sp. (strain PCC 6803 / Kazusa) GN	1504,84	60,32	1	17	17	76	0,995	63	12,8	189	21,2	5,05							
	A3	Sequence	# PSMs	# Proteins	# Protein Groups	Protein Group Accessions	Modifications	ΔCn	114/115	114/115 Count	114/115 Variability [%]	q-Value	PEP	IonScore	Exp Value	Charge	MH+ [Da]	ΔM [ppm]	RT [min]	# Missed Cleavages
	High	gAEAPGVSEPVk	5	1	1	P73728	N-Term(iTRAQ4plex); K12(iTRAQ4plex)	0,0000	0,966	4	5,6	0	2,696E-08	87	1,84064E-09	2	1428,79570	1,38	19,74	0
	High	vRDESVPGPNPYR	15	1	1	P73728	N-Term(iTRAQ4plex)	0,0000	0,884	8	7,8	0	2,221E-10	69	1,15337E-07	3	1629,84739	0,49	19,92	1
	High	tTEQIFGGk	2	1	1	P73728	N-Term(iTRAQ4plex); K9(iTRAQ4plex)	0,0000	1,159	2	1,2	0	3,216E-07	69	1,31817E-07	2	1268,71184	2,31	25,34	0
	High	qIGADkVk	4	1	1	P73728	N-Term(iTRAQ4plex); K6(iTRAQ4plex); K8(iTRAQ4plex)	0,0000	0,948	4	7,3	0	0,0006496	66	2,29071E-07	2	1290,81877	6,35	12,98	1
	High	vVLFSLPGAFTPTcSSNHLPR	2	1	1	P73728	N-Term(iTRAQ4plex); C14(Carbamidomethyl)	0,0000	1,284	2	11,2	0	3,749E-11	60	9,70443E-07	3	2444,28971	0,80	49,16	0
	High	vPSVVFk	5	1	1	P73728	N-Term(iTRAQ4plex); K7(iTRAQ4plex)	0,0000	1,089	5	2,5	0	0,002326	54	4,21667E-06	2	1063,67644	0,96	31,83	0
	High	ySMFVNDGk	1	1	1	P73728	N-Term(iTRAQ4plex); K9(iTRAQ4plex)	0,0000	0,884	1		0	5,283E-07	50	8,93244E-06	2	1348,68144	0,35	28,91	0
	High	kVVLFSLPGAFTPTcSSNHLPR	2	1	1	P73728	N-Term(iTRAQ4plex); K1(iTRAQ4plex); C15(Carbamidomethyl)	0,0000	1,024	2	7,9	0	5,845E-10	45	2,97146E-05	4	2716,48598	0,44	43,89	1
	High	sNLGFGMR	3	1	1	P73728	N-Term(iTRAQ4plex)	0,0000	0,908	3	16,7	0	0,001131	42	6,08093E-05	2	1025,53203	0,11	25,41	0
	High	ySmFVNDGk	4	1	1	P73728	N-Term(iTRAQ4plex); M3(Oxidation); K9(iTRAQ4plex)	0,0000	1,063	3	2,5	0	0,0001583	40	0,000102795	2	1364,67693	0,76	24,14	0
	High	wEDkTTEQIFGGk	4	1	1	P73728	N-Term(iTRAQ4plex); K4(iTRAQ4plex); K13(iTRAQ4plex)	0,0000	1,002	4	4,3	0	6,396E-08	38	0,000164426	3	1971,05637	0,79	33,90	1
	High	kMGMLVEk	3	1	1	P73728	N-Term(iTRAQ4plex); K1(iTRAQ4plex); K8(iTRAQ4plex)	0,0000	0,966	2	7,0	0	0,000004362	37	0,000186625	3	1367,81174	0,15	22,89	1
	High	lLPDGNGEFTR	1	1	1	P73728	N-Term(iTRAQ4plex)	0,0000	0,824	1		0	0,00155	35	0,000285739	2	1362,71428	0,61	32,44	0
	High	kmGmLVEk	6	1	1	P73728	N-Term(iTRAQ4plex); K1(iTRAQ4plex); M2(Oxidation); M4(Oxidation); K8(iTF	0,0000	1,059	4	14,1	0	0,001128	32	0,000620826	3	1399,80201	0,46	12,71	1
	High	ySMFVNDGkIEk	2	1	1	P73728	N-Term(iTRAQ4plex); K9(iTRAQ4plex); K12(iTRAQ4plex)	0,0000	0,842	2	4,3	0	3,734E-08	31	0,00079688	3	1863,00400	-0,36	31,36	1
	High	ySmFVNDGkIEk	3	1	1	P73728	N-Term(iTRAQ4plex); M3(Oxidation); K9(iTRAQ4plex); K12(iTRAQ4plex)	0,0000	1,014	3	7,5	0	2,206E-07	27	0,002015546	3	1878,99955	-0,03	27,27	1
	High	sNLGFGmR	5	1	1	P73728	N-Term(iTRAQ4plex); M7(Oxidation)	0,0000	0,991	4	2,4	0	0,004625	27	0,002192653	2	1041,53411	6,99	18,76	0
	High	kmGMLVEk	2	1	1	P73728	N-Term(iTRAQ4plex); K1(iTRAQ4plex); M2(Oxidation); K8(iTRAQ4plex)	0,0000	1,162	2	0,6	0	0,001042	24	0,003801631	3	1383,80573	-0,52	16,89	1
	High	tTEQIFGGkk	2	1	1	P73728	N-Term(iTRAQ4plex); K9(iTRAQ4plex); K10(iTRAQ4plex)	0,0000	1,166	2	0,0	0	0,00001091	24	0,003827983	3	1540,90628	0,22	19,30	1
	High	wEDkTTEQIFGGkk	1	1	1	P73728	N-Term(iTRAQ4plex); K4(iTRAQ4plex); K13(iTRAQ4plex); K14(iTRAQ4plex)	0,0000	0,889	1		0	0,00001555	18	0,01541594	4	2243,25234	0,22	29,79	2
	High	mGmLVEk	3	1	1	P73728	N-Term(iTRAQ4plex); M1(Oxidation); M3(Oxidation); K7(iTRAQ4plex)	0,0000	1,008	3	0,1	0,004	0,04631	11	0,075852518	2	1127,60600	1,49	15,40	0
	Medium	qIGADk	1	1	1	P73728	N-Term(iTRAQ4plex); K6(iTRAQ4plex)	0,0000	1,021	1		0,025	0,2748	25	0,003235713	2	919,54473	-0,44	13,26	0
P77968	Superoxide dismutase [Fe] OS=Synechocystis sp. (strain PCC 6803 / Kazusa) GN=s	1457,70	28,14	1	4	4	65	1,840	60	32,0	199	21,7	5,14							
	A3	Sequence	# PSMs	# Proteins	# Protein Groups	Protein Group Accessions	Modifications	ΔCn	114/115	114/115 Count	114/115 Variability [%]	q-Value	PEP	IonScore	Exp Value	Charge	MH+ [Da]	ΔM [ppm]	RT [min]	# Missed Cleavages
	High	hHAAYVNNFNNAVAGTDLDNQSIEDVIk	3	1	1	P77968	N-Term(iTRAQ4plex); K28(iTRAQ4plex)	0,0000	1,805	3	2,8	0	9,242E-11	70	9,18269E-08	4	3357,67470	1,31	40,47	0
	High	rPDYIADFLGk	46	1	1	P77968	N-Term(iTRAQ4plex); K11(iTRAQ4plex)	0,0000	1,906	45	29,1	0	5,263E-08	59	1,33036E-06	3	1582,88541	1,40	39,78	0
	High	sTLEFHHDk	10	1	1	P77968	N-Term(iTRAQ4plex); K9(iTRAQ4plex)	0,0000	2,667	6	15,2	0	0,00005973	53	5,2236E-06	2	1401,73808	1,13	14,16	0
	High	aVAGDASk	6	1	1	P77968	N-Term(iTRAQ4plex); K8(iTRAQ4plex)	0,0000	1,557	6	25,8	0	0,0001716	28	0,001603135	2	1006,57823	1,06	10,95	0
P73204	Phycobilisome 32.1 kDa linker polypeptide, phycocyanin-associated, rod 2 OS=Syne	1431,99	46,52	1	14	14	54	0,411	44	26,9	273	30,8	9,39							
	A3	Sequence	# PSMs	# Proteins	# Protein Groups	Protein Group Accessions	Modifications	ΔCn	114/115	114/115 Count	114/115 Variability [%]	q-Value	PEP	IonScore	Exp Value	Charge	MH+ [Da]	ΔM [ppm]	RT [min]	# Missed Cleavages
	High	nTASPVYAGSTAESLR	6	1	1	P73204	N-Term(iTRAQ4plex)	0,0000	0,457	4	55,8	0	3,059E-12	108	1,64805E-11	2	1767,89922	-0,11	26,69	0
	High	gkAEYLVSYDNLSAk	5	1	1	P73204	N-Term(iTRAQ4plex); K2(iTRAQ4plex); K15(iTRAQ4plex)	0,0000	0,426	4	25,4	0	3,616E-10	75	4,00588E-08	3	2090,14963	0,10	32,54	1
	High	qVLGNDHLmSQER	6	1	1	P73204	N-Term(iTRAQ4plex); M9(Oxidation)	0,0000	0,408	4	11,7	0	1,827E-09	68	1,76592E-07	2	1686,84441	5,55	18,16	0
	High	lQVIQGAAPGR	6	1	1	P73204	N-Term(iTRAQ4plex)	0,0000	0,441	4	44,6	0	5,782E-09	62	5,64898E-07	2	1253,74358	-0,89	26,49	0
	High	lTSAESLLR	4	1	1	P73204	N-Term(iTRAQ4plex)	0,0000	0,354	3	7,1	0	0,00000176	57	2,07E-06	2	1133,66423	-0,41	34,47	0
	High	aVALSEVYR	5	1	1	P73204	N-Term(iTRAQ4plex)	0,0000	0,363	3	8,0	0	0,0004183	50	9,26766E-06	2	1151,65349	-0,55	28,98	0
	High	fIELNYk	3	1	1	P73204	N-Term(iTRAQ4plex); K7(iTRAQ4plex)	0,0000	0,358	3	5,5	0	0,000101	43	4,85255E-05	2	1214,70159	-0,63	36,11	0
	High	tSLVSAQR	3	1	1	P73204	N-Term(iTRAQ4plex)	0,0000	0,558	3	21,1	0	0,00006488	37	0,000210568	2	1005,58568	4,70	14,85	0
	High	sFQVYR	3	1	1	P73204	N-Term(iTRAQ4plex)	0,0000	0,389	3	6,6	0	0,003416	34	0,000374085	2	943,51097	-0,87	22,46	0
	High	fFHSNPQNR	6	1	1	P73204	N-Term(iTRAQ4plex)	0,0000	0,387	6	17,4	0	0,002164	33	0,00052236	2	1290,64751	1,16	16,01	0
	High	lQQINR	3	1	1	P73204	N-Term(iTRAQ4plex)	0,0000	0,635	3	0,1	0,001	0,01213	31	0,000777983	2	915,55345	4,59	16,18	0
	Medium	gYATSDR	1	1	1	P73204	N-Term(iTRAQ4plex)	0,0000	0,904	1		0,016	0,1761	22	0,007014069	2	913,45055	1,04	10,72	0
	Medium	eISVR	2	1	1	P73204	N-Term(iTRAQ4plex)	0,0000	1,388	2	17,6	0,017	0,1849	18	0,018704382	2	747,45384	7,63	13,94	0
	Medium	tVGFSR	1	1	1	P73204	N-Term(iTRAQ4plex)	0,0000	0,440	1		0,041	0,4276	16	0,031113105	2	810,45824	-0,99	16,64	0
P22358	Chaperone protein dnaK2 OS=Synechocystis sp. (strain PCC 6803 / Kazusa) GN=dr	1428,44	34,43	1	21	21	56	1,270	51	10,0	636	67,6	4,86							
	A3	Sequence	# PSMs	# Proteins	# Protein Groups	Protein Group Accessions	Modifications	ΔCn	114/115	114/115 Count	114/115 Variability [%]	q-Value	PEP	IonScore	Exp Value	Charge	MH+ [Da]	ΔM [ppm]	RT [min]	# Missed Cleavages
	High	tTPSVVGYAk	5	1	1	P22358	N-Term(iTRAQ4plex); K10(iTRAQ4plex)	0,0000	1,234	4	2,3	0	0,000001094	72	6,22257E-08	2	1310,76555	7,40	23,89	0
	High	iINEPTAASLAYGLDkk	7	1	1	P22358	N-Term(iTRAQ4plex); K16(iTRAQ4plex); K17(iTRAQ4plex)	0,0000	1,218	5	3,6	0	7,003E-14	69	1,39949E-07	3	2236,29343	0,93	38,10	1
	High	kNQADSLVYQAEk	3	1	1	P22358	N-Term(iTRAQ4plex); K1(iTRAQ4plex); K13(iTRAQ4plex)	0,0000	1,475	2	6,8	0	7,279E-10	68	1,50304E-07	3	1926,06736	0,86	22,53	1
	High	kFDEITNEATEVAYSVVk	2	1	1	P22358	N-Term(iTRAQ4plex); K1(iTRAQ4plex); K18(iTRAQ4plex)	0,0000	1,135	2	23,4	0	1,225E-11	61	8,56979E-07	3	2475,33579	0,58	48,75	1
	High	iDkSALDEIVLVGGSTR	2	1	1	P22358	N-Term(iTRAQ4plex); K3(iTRAQ4plex)	0,0000	1,180	2	20,0	0	1,605E-09	60	9,90764E-07	3	2061,18125	0,86	43,19	1
	High	lTEAAEk	1	1	1	P22358	N-Term(iTRAQ4plex); K7(iTRAQ4plex)	0,0000	1,289	1		0	0,0003042	52	6,16552E-06	2	1049,61272	4,37	16,38	0
	High	kIVDFLAGEFQk	1	1	1	P22358	N-Term(iTRAQ4plex); K1(iTRAQ4plex); K12(iTRAQ4plex)	0,0000	1,305	1		0	6,703E-10	49	1,23871E-05	3	1827,07157	-1,38	43,74	1
	High	iPAVQEVVk	2	1	1	P22358	N-Term(iTRAQ4plex); K9(iTRAQ4plex)	0,0000	1,130	2	23,3	0	0,0006778	48	1,4288E-05	2	1270,79839	0,83	31,96	0
	High	qFAPEEISAQVLR	2	1	1	P22358	N-Term(iTRAQ4plex)	0,0000	1,222	2	2,8	0	0,0000639	48	1,74973E-05	2	1631,88896	0,96	41,19	0
	High	akFEEIcSDLIDR	3	1	1	P22358	N-Term(iTRAQ4plex); K2(iTRAQ4plex); C7(Carbamidomethyl)	0,0000	1,834	3	6,6	0	1,501E-08	44	4,20698E-05	3	1883,97965	1,09	37,45	1
	High	eAESNAAADkER	6	1	1	P22358	N-Term(iTRAQ4plex); K10(iTRAQ4plex)	0,0000	1,293	5	21,2	0	4,623E-09	43	4,51825E-05	3	1578,79758	0,84	11,56	1
	High	kLVDDASk	7	1	1	P22358	N-Term(iTRAQ4plex); K1(iTRAQ4plex); K8(iTRAQ4plex)	0,0000	1,270	7	6,3	0	0,0000296	41	8,7694E-05	2	1307,78972	0,16	13,60	1
	High	qAVmNPGNTFYSVk	1	1	1	P22358	N-Term(iTRAQ4plex); M4(Oxidation); K14(iTRAQ4plex)	0,0000	0,742	1		0	9,845E-08	35	0,000294422	3	1859,95786	0,76	29,16	0
	High	iPAVQEVVkk	2	1	1	P22358	N-Term(iTRAQ4plex); K9(iTRAQ4plex); K10(iTRAQ4plex)	0,0000	1,270	2	10,5	0	0,000006936	35	0,000328073	3	1542,99259	-1,16	26,99	1
	High	hLDTTLSR	4	1	1	P22358	N-Term(iTRAQ4plex)	0,0000	1,158	4	9,4	0	0,0002936	34	0,000357248	3	1086,60312	0,65	14,90	0
	High	nTTIPTk	3	1	1	P22358	N-Term(iTRAQ4plex); K7(iTRAQ4plex)	0,0000	1,358	3	0,5	0	0,0003439	33	0,00055769	2	1062,63982	0,07	16,57	0
	High	qITELGDkVPAADk	1	1	1	P22358	N-Term(iTRAQ4plex); K8(iTRAQ4plex); K14(iTRAQ4plex)	0,0000	1,537	1		0	0,000003662	30	0,000922508	3	1917,10819	3,35	27,61	1
	High	ikAEGLIk	1	1	1	P22358	N-Term(iTRAQ4plex); K2(iTRAQ4plex); K8(iTRAQ4plex)	0,0000	1,357	1		0	0,001553	30	0,001093881	3	1303,86673	-0,49	23,83	1
	High	cGIPVENAIR	1	1	1	P22358	N-Term(iTRAQ4plex); C1(Carbamidomethyl)	0,0000	1,187	1		0	0,000017	27	0,002137814	2	1272,68657	1,13	27,60	0
	High	lDcPAQGk	1	1	1	P22358	N-Term(iTRAQ4plex); C3(Carbamidomethyl); K8(iTRAQ4plex)	0,0000	1,449	1		0	0,0005129	17	0,017904822	2	1176,63042	1,59	15,28	0
	High	aEGLIk	1	1	1	P22358	N-Term(iTRAQ4plex); K6(iTRAQ4plex)	0,0000	1,283	1		0,006	0,0781	21	0,01091298	2	918,59068	4,81	19,07	0
Q55664	Fructose-bisphosphate aldolase class 2 OS=Synechocystis sp. (strain PCC 6803 / Ka	1402,84	26,46	1	10	10	60	1,002	54	10,9	359	38,9	5,74							
	A3	Sequence	# PSMs	# Proteins	# Protein Groups	Protein Group Accessions	Modifications	ΔCn	114/115	114/115 Count	114/115 Variability [%]	q-Value	PEP	IonScore	Exp Value	Charge	MH+ [Da]	ΔM [ppm]	RT [min]	# Missed Cleavages
	High	tQVDALAVAIGTSHGAYk	6	1	1	Q55664	N-Term(iTRAQ4plex); K18(iTRAQ4plex)	0,0000	0,953	4	15,4	0	1,147E-16	90	1,13161E-09	3	2090,15146	1,42	37,89	0
	High	yQQFWTAGNASk	3	1	1	Q55664	N-Term(iTRAQ4plex); K12(iTRAQ4plex)	0,0000	1,220	3	8,7	0	6,496E-11	81	8,60934E-09	2	1688,86650	1,77	31,49	0
	High	kPTGEVLAISR	15	1	1	Q55664	N-Term(iTRAQ4plex); K1(iTRAQ4plex)	0,0000	1,030	13	5,8	0	1,395E-08	69	1,30608E-07	3	1458,89005	1,21	22,72	0
	High	lAITAAFR	13	1	1	Q55664	N-Term(iTRAQ4plex)	0,0000	0,910	11	2,8	0	0,00003034	50	9,7717E-06	2	1006,61669	0,06	36,54	0
	High	kVNIDTDNR	8	1	1	Q55664	N-Term(iTRAQ4plex); K1(iTRAQ4plex)	0,0000	1,036	8	4,4	0	0,00006829	46	2,92543E-05	3	1362,76700	6,61	12,82	1
	High	sYAGENFLR	3	1	1	Q55664	N-Term(iTRAQ4plex)	0,0000	1,061	3	1,1	0	0,000001151	45	3,44326E-05	2	1200,61284	-0,12	29,34	0
	High	hFLkPSIk	2	1	1	Q55664	N-Term(iTRAQ4plex); K4(iTRAQ4plex); K8(iTRAQ4plex)	0,0000	0,999	2	0,9	0	0,0001411	30	0,001047056	2	1401,89446	0,15	19,62	0
	High	gELTATSR	2	1	1	Q55664	N-Term(iTRAQ4plex)	0,0000	0,828	2	42,5	0	0,000266	28	0,001659472	2	978,53349	-0,18	12,90	0
	High	iEEIHR	6	1	1	Q55664	N-Term(iTRAQ4plex)	0,0000	0,915	6	4,2	0	0,003922	23	0,004623491	3	940,53339	0,12	14,37	0
	High	aLVPmR	2	1	1	Q55664	N-Term(iTRAQ4plex); M5(Oxidation)	0,0000	1,063	2	3,1	0,006	0,07524	12	0,065609995	2	846,49956	0,87	16,80	0
P73922	D-fructose 1,6-bisphosphatase class 2/sedoheptulose 1,7-bisphosphatase OS=Syne	1313,27	42,32	1	18	18	63	1,067	55	29,0	345	37,1	5,21							
	A3	Sequence	# PSMs	# Proteins	# Protein Groups	Protein Group Accessions	Modifications	ΔCn	114/115	114/115 Count	114/115 Variability [%]	q-Value	PEP	IonScore	Exp Value	Charge	MH+ [Da]	ΔM [ppm]	RT [min]	# Missed Cleavages
	High	tQSLVISSQSSTAR	6	1	1	P73922	N-Term(iTRAQ4plex)	0,0000	0,934	4	22,0	0	4,981E-10	94	3,98997E-10	2	1608,86736	-0,01	21,10	0
	High	sIEELVVVMDRPR	4	1	1	P73922	N-Term(iTRAQ4plex)	0,0000	0,853	3	0,3	0	5,809E-08	76	2,43204E-08	2	1786,00298	0,88	50,31	0
	High	gGLFAAPDFYMk	1	1	1	P73922	N-Term(iTRAQ4plex); K12(iTRAQ4plex)	0,0000	1,152	1		0	4,47E-08	71	7,44681E-08	2	1604,84148	1,81	44,99	0
	High	sIEELVVVmDRPR	7	1	1	P73922	N-Term(iTRAQ4plex); M10(Oxidation)	0,0000	1,030	6	12,5	0	1,889E-08	65	3,0128E-07	3	1801,99784	0,84	42,21	0
	High	cLGGHFQGQLIYDPEVVk	1	1	1	P73922	N-Term(iTRAQ4plex); C1(Carbamidomethyl); K18(iTRAQ4plex)	0,0000	1,070	1		0	2,072E-10	47	1,97683E-05	3	2348,23551	1,84	37,92	0
	High	kLAAPPAAk	5	1	1	P73922	N-Term(iTRAQ4plex); K1(iTRAQ4plex); K9(iTRAQ4plex)	0,0000	1,054	3	3,4	0	0,000000339	45	2,88383E-05	3	1298,85248	0,32	15,04	1
	High	tGLIGESR	2	1	1	P73922	N-Term(iTRAQ4plex)	0,0000	0,838	2	23,8	0	0,0001567	41	7,14447E-05	2	976,55553	1,14	16,84	0
	High	lASmGIk	2	1	1	P73922	N-Term(iTRAQ4plex); M4(Oxidation); K7(iTRAQ4plex)	0,0000	1,293	2	0,0	0	0,0005048	39	0,000141876	2	1023,61089	-0,22	18,61	0
	High	hkELIQEIR	3	1	1	P73922	N-Term(iTRAQ4plex); K2(iTRAQ4plex)	0,0000	1,083	3	3,1	0	0,000009257	38	0,000176185	2	1453,87322	0,18	18,38	1
	High	lASMGIk	1	1	1	P73922	N-Term(iTRAQ4plex); K7(iTRAQ4plex)	0,0000	1,067	1		0	0,0006876	37	0,00025774	2	1007,61656	0,36	26,48	0
	High	gGLFAAPDFYmkk	2	1	1	P73922	N-Term(iTRAQ4plex); M11(Oxidation); K12(iTRAQ4plex); K13(iTRAQ4plex)	0,0000	1,410	2	4,5	0	1,848E-07	36	0,000242086	3	1893,03055	0,02	35,16	1
	High	fFHGGVR	4	1	1	P73922	N-Term(iTRAQ4plex)	0,0000	0,836	4	1,6	0	0,001494	35	0,000325065	3	963,52869	0,58	18,32	0
	High	gHVDIDkSATENLk	2	1	1	P73922	N-Term(iTRAQ4plex); K7(iTRAQ4plex); K14(iTRAQ4plex)	0,0000	1,012	2	19,4	0	0,00004302	34	0,000491285	3	1959,08933	1,10	21,80	1
	High	lAAPPAAk	4	1	1	P73922	N-Term(iTRAQ4plex); K8(iTRAQ4plex)	0,0000	1,318	3	2,8	0	0,007612	32	0,0005662	2	1026,66167	6,47	18,23	0
	High	fVDTVHMk	3	1	1	P73922	N-Term(iTRAQ4plex); K8(iTRAQ4plex)	0,0000	0,803	3	8,0	0	0,0001378	31	0,000792847	3	1264,69873	1,97	23,65	0
	High	iLSDcLNR	3	1	1	P73922	N-Term(iTRAQ4plex); C5(Carbamidomethyl)	0,0000	1,245	3	7,1	0	0,001672	31	0,00075504	2	1134,60576	-0,03	26,96	0
	High	fVDTVHmk	6	1	1	P73922	N-Term(iTRAQ4plex); M7(Oxidation); K8(iTRAQ4plex)	0,0000	1,131	5	9,7	0	0,00002703	31	0,000843277	2	1280,69304	1,47	18,13	0
	High	nTADQVAVEAmRER	1	1	1	P73922	N-Term(iTRAQ4plex); M11(Oxidation)	0,0000	1,402	1		0	0,001904	18	0,015204425	3	1749,87577	4,95	21,14	1
	High	gHVDIDk	3	1	1	P73922	N-Term(iTRAQ4plex); K7(iTRAQ4plex)	0,0000	1,033	3	6,5	0	0,001293	18	0,016068303	3	1071,60468	0,91	12,34	0
	High	fVDTVHmkESPk	1	1	1	P73922	N-Term(iTRAQ4plex); M7(Oxidation); K8(iTRAQ4plex); K12(iTRAQ4plex)	0,0000	0,860	1		0	0,00005578	17	0,021786592	4	1866,01455	-0,56	19,33	1
	High	sATENLk	2	1	1	P73922	N-Term(iTRAQ4plex); K7(iTRAQ4plex)	0,0000	1,054	2	5,4	0,002	0,02186	37	0,000195519	2	1050,60832	4,72	14,16	0
P72827	Iron uptake protein A1 OS=Synechocystis sp. (strain PCC 6803 / Kazusa) GN=futA1	1305,25	37,50	1	12	12	47	0,383	41	20,6	360	39,3	4,93							
	A3	Sequence	# PSMs	# Proteins	# Protein Groups	Protein Group Accessions	Modifications	ΔCn	114/115	114/115 Count	114/115 Variability [%]	q-Value	PEP	IonScore	Exp Value	Charge	MH+ [Da]	ΔM [ppm]	RT [min]	# Missed Cleavages
	High	hYNTDNELYAk	8	1	1	P72827	N-Term(iTRAQ4plex); K11(iTRAQ4plex)	0,0000	0,338	7	14,2	0	2,086E-10	96	2,63009E-10	2	1655,82329	-2,10	18,29	0
	High	ikSEGANSPADVLLTVDLAR	3	1	1	P72827	N-Term(iTRAQ4plex); K2(iTRAQ4plex)	0,0000	0,384	3	10,2	0	8,704E-14	92	5,95621E-10	3	2357,32926	0,56	43,71	1
	High	gTHVNVSGVGVVk	4	1	1	P72827	N-Term(iTRAQ4plex); K13(iTRAQ4plex)	0,0000	0,368	2	10,0	0	7,536E-09	69	1,13232E-07	2	1540,90447	-0,34	21,82	0
	High	aIAENVGVFFPNQEGR	3	1	1	P72827	N-Term(iTRAQ4plex)	0,0000	0,366	3	108,6	0	1,265E-10	68	1,59945E-07	2	1891,98161	1,72	42,13	0
	High	lGPALAPATk	5	1	1	P72827	N-Term(iTRAQ4plex); K10(iTRAQ4plex)	0,0000	0,418	5	34,4	0	0,0000237	66	2,7477E-07	2	1226,76995	-0,96	25,74	0
	High	sVASFGEFk	4	1	1	P72827	N-Term(iTRAQ4plex); K9(iTRAQ4plex)	0,0000	0,458	4	20,4	0	0,000005012	51	7,37853E-06	2	1259,68584	-1,28	32,12	0
	High	vkPEELSTYEELADPk	3	1	1	P72827	N-Term(iTRAQ4plex); K2(iTRAQ4plex); K16(iTRAQ4plex)	0,0000	0,448	3	7,8	0	1,06E-11	48	1,65566E-05	3	2280,23233	-0,54	36,32	1
	High	vNLIEGk	5	1	1	P72827	N-Term(iTRAQ4plex); K7(iTRAQ4plex)	0,0000	0,338	3	7,3	0	0,007521	44	4,25302E-05	2	1060,65654	-3,73	25,87	0
	High	gFVSNFAR	4	1	1	P72827	N-Term(iTRAQ4plex)	0,0000	0,372	3	3,6	0	0,00375	43	5,5077E-05	2	1041,56316	3,19	28,01	0

	High	sPDGmWFGFTk	1	1	1	P72827	N-Term(iTRAQ4plex); M5(Oxidation); K11(iTRAQ4plex)	0,0000	0,558	1		0	2,883E-08	39	0,000131211	2	1576,76897	-1,21	38,76	0
	High	vImYNk	3	1	1	P72827	N-Term(iTRAQ4plex); M3(Oxidation); K6(iTRAQ4plex)	0,0000	0,383	3	2,3	0	0,005474	38	0,000147221	2	1071,61479	3,45	19,11	0
	High	vIMYNk	2	1	1	P72827	N-Term(iTRAQ4plex); K6(iTRAQ4plex)	0,0000	0,407	2	23,2	0	0,001879	36	0,000233868	2	1055,61479	-1,31	22,69	0
	High	imNEAGWk	1	1	1	P72827	N-Term(iTRAQ4plex); M2(Oxidation); K8(iTRAQ4plex)	0,0000	0,371	1		0	0,004229	32	0,000579389	2	1252,65666	-2,55	22,11	0
	High	sPDGMWFGFTk	1	1	1	P72827	N-Term(iTRAQ4plex); K11(iTRAQ4plex)	0,0000	0,500	1		0	0,000336	31	0,000737853	2	1560,77532	-0,42	43,68	0
P29256	Photosystem I reaction center subunit III OS=Synechocystis sp. (strain PCC 6803 /	1302,58	56,36	1	11	11	45	0,851	38	12,4	165	18,2	7,37							
	A3	Sequence	# PSMs	# Proteins	# Protein Groups	Protein Group Accessions	Modifications	ΔCn	114/115	114/115 Count	114/115 Variability [%]	q-Value	PEP	IonScore	Exp Value	Charge	MH+ [Da]	ΔM [ppm]	RT [min]	# Missed Cleavages
	High	nFLNTTNDPNSGk	4	1	1	P29256	N-Term(iTRAQ4plex); K13(iTRAQ4plex)	0,0000	0,981	4	20,8	0	1,602E-10	98	1,72572E-10	2	1709,87187	1,26	23,03	0
	High	kmLGGFLWPLAAVGEYTSGk	2	1	1	P29256	N-Term(iTRAQ4plex); K1(iTRAQ4plex); M2(Oxidation); K20(iTRAQ4plex)	0,0000	0,935	2	25,2	0	1,708E-11	64	3,70655E-07	3	2573,41971	1,34	53,31	1
	High	yASALcGPEGYPHLIVDGR	5	1	1	P29256	N-Term(iTRAQ4plex); C6(Carbamidomethyl)	0,0000	0,815	3	0,5	0	1,279E-13	61	7,44681E-07	3	2219,10532	0,76	39,54	0
	High	eSkNPEmQEVVINVPLAIk	4	1	1	P29256	N-Term(iTRAQ4plex); K3(iTRAQ4plex); M7(Oxidation); K19(iTRAQ4plex)	0,0000	0,864	3	2,0	0	1,629E-12	55	2,87058E-06	3	2586,45627	0,96	38,61	1
	High	mLGGFLWPLAAVGEYTSGk	2	1	1	P29256	N-Term(iTRAQ4plex); M1(Oxidation); K19(iTRAQ4plex)	0,0000	0,851	2	9,2	0	6,394E-11	54	4,02689E-06	3	2301,22062	0,60	58,41	0
	High	eSkNPEMQEVVINVPLAIk	2	1	1	P29256	N-Term(iTRAQ4plex); K3(iTRAQ4plex); K19(iTRAQ4plex)	0,0000	0,993	2	13,8	0	5,438E-09	53	5,32072E-06	3	2570,46292	1,58	43,05	1
	High	lVmkDSEIPTSPR	8	1	1	P29256	N-Term(iTRAQ4plex); M3(Oxidation); K4(iTRAQ4plex)	0,0000	0,826	6	4,8	0	0,000005062	52	6,83864E-06	2	1776,97649	-0,21	23,71	1
	High	nPEmQEVVINVPLAIkk	2	1	1	P29256	N-Term(iTRAQ4plex); M4(Oxidation); K16(iTRAQ4plex); K17(iTRAQ4plex)	0,0000	0,838	2	0,5	0	4,342E-13	50	8,99435E-06	3	2370,38248	1,40	37,62	1
	High	sYLIEIR	6	1	1	P29256	N-Term(iTRAQ4plex)	0,0000	0,779	5	3,1	0	0,002307	49	1,12453E-05	2	1037,61125	0,07	34,28	0
	High	skNFLNTTNDPNSGk	4	1	1	P29256	N-Term(iTRAQ4plex); K2(iTRAQ4plex); K15(iTRAQ4plex)	0,0000	0,928	3	19,8	0	8,677E-09	49	1,12453E-05	3	2069,10202	1,56	19,73	1
	High	nPEMQEVVINVPLAIkk	1	1	1	P29256	N-Term(iTRAQ4plex); K16(iTRAQ4plex); K17(iTRAQ4plex)	0,0000	1,020	1		0	4,628E-08	42	5,67505E-05	4	2354,38588	0,70	42,26	1
	High	mLGGFLWPLAAVGEYTSGk	1	1	1	P29256	N-Term(iTRAQ4plex); K19(iTRAQ4plex)	0,0000	0,849	1		0	2,238E-09	42	7,01407E-05	3	2285,22965	2,33	60,53	0
	High	nPEmQEVVINVPLAIk	2	1	1	P29256	N-Term(iTRAQ4plex); M4(Oxidation); K16(iTRAQ4plex)	0,0000	1,058	2	7,9	0	3,604E-09	39	0,000120495	3	2098,18320	0,51	43,67	0
	High	dSEIPTSPR	1	1	1	P29256	N-Term(iTRAQ4plex)	0,0000	0,929	1		0	0,00051	37	0,000211821	2	1145,59697	4,41	17,48	0
	High	lVMkDSEIPTSPR	1	1	1	P29256	N-Term(iTRAQ4plex); K4(iTRAQ4plex)	0,0000	0,705	1		0	0,000000904	31	0,000796104	3	1760,98166	-0,16	28,69	1
P74551	Allophycocyanin subunit beta-18 OS=Synechocystis sp. (strain PCC 6803 / Kazusa)	1297,94	53,85	1	13	14	57	1,116	42	22,7	169	18,9	5,07							
	A3	Sequence	# PSMs	# Proteins	# Protein Groups	Protein Group Accessions	Modifications	ΔCn	114/115	114/115 Count	114/115 Variability [%]	q-Value	PEP	IonScore	Exp Value	Charge	MH+ [Da]	ΔM [ppm]	RT [min]	# Missed Cleavages
	High	iAAAAmINANSATIVk	4	1	1	P74551	N-Term(iTRAQ4plex); M6(Oxidation); K16(iTRAQ4plex)	0,0000	1,099	4	34,7	0	1,699E-13	121	8,12774E-13	2	1863,06401	1,47	33,52	0
	High	eTYNSLGVPIGPTVR	6	1	1	P74551	N-Term(iTRAQ4plex)	0,0000	1,069	4	11,1	0	4,263E-12	111	8,689E-12	2	1746,95256	1,05	36,08	0
	High	iAAAAmINANSATIVkR	2	1	1	P74551	N-Term(iTRAQ4plex); M6(Oxidation); K16(iTRAQ4plex)	0,0000	0,757	1		0	6,407E-09	59	1,39627E-06	3	2019,16184	-0,27	29,93	1
	High	dAVTTLIk	1	1	1	P74551	N-Term(iTRAQ4plex); K8(iTRAQ4plex)	0,0000	1,416	1		0	0,0000198	57	1,79048E-06	2	1148,71257	-0,31	27,79	0
	High	aYFESGSAR	5	1	1	P74551	N-Term(iTRAQ4plex)	0,0000	1,187	3	0,8	0	0,000004317	56	2,39315E-06	2	1131,56108	5,26	17,85	0
	High	rFSAcLR	6	1	1	P74551	N-Term(iTRAQ4plex); C5(Carbamidomethyl)	0,0000	1,117	6	23,4	0	0,00003967	50	9,31044E-06	3	1053,57511	0,64	16,65	1
	High	mRDAVTTLIk	1	1	1	P74551	N-Term(iTRAQ4plex); K10(iTRAQ4plex)	0,0000	0,901	1		0	3,535E-07	46	2,27879E-05	3	1435,85483	0,20	29,86	1
	High	dMDYYLR	4	2	2	P74551;Q01951	N-Term(iTRAQ4plex)	0,0000				0	0,0003235	45	3,42744E-05	2	1119,52605	-0,08	30,39	0
	High	nYDLTGR	2	1	1	P74551	N-Term(iTRAQ4plex)	0,0000	1,032	2	11,2	0	0,001539	36	0,000242086	2	982,50719	-0,26	18,41	0
	High	mRDAVTTLIk	5	1	1	P74551	N-Term(iTRAQ4plex); M1(Oxidation); K10(iTRAQ4plex)	0,0000	1,038	4	21,4	0	1,628E-07	36	0,000242644	3	1451,85010	0,44	22,81	1
	High	rDAVTTLIk	1	1	1	P74551	N-Term(iTRAQ4plex); K9(iTRAQ4plex)	0,0000	0,914	1		0	0,00003674	30	0,000928902	3	1304,81266	-1,07	23,16	1
	High	gIQIMk	3	1	1	P74551	N-Term(iTRAQ4plex); K6(iTRAQ4plex)	0,0000	1,169	3	5,6	0	0,007192	26	0,002832626	2	977,60503	-0,61	24,19	0
	High	gIQImk	4	1	1	P74551	N-Term(iTRAQ4plex); M5(Oxidation); K6(iTRAQ4plex)	0,0000	1,294	2	0,1	0	0,007662	24	0,003953393	2	993,60161	1,07	21,06	0
	High	dmDYYLR	3	2	2	P74551;Q01951	N-Term(iTRAQ4plex); M2(Oxidation)	0,0000				0,001	0,01054	19	0,011911597	2	1135,52117	0,10	22,56	0
	High	vLQGLR	6	1	1	P74551	N-Term(iTRAQ4plex)	0,0000	1,039	6	2,9	0,002	0,03229	36	0,000264832	2	829,53942	2,15	21,95	0
	High	nAMDELk	1	1	1	P74551	N-Term(iTRAQ4plex); K7(iTRAQ4plex)	0,0000	1,072	1		0,002	0,02986	24	0,003792888	2	1108,59123	0,11	20,35	0
	Medium	fSAcLR	3	1	1	P74551	N-Term(iTRAQ4plex); C4(Carbamidomethyl)	0,0000	1,471	3	4,3	0,019	0,2094	22	0,006426433	2	897,47386	0,61	22,16	0
P42352	50S ribosomal protein L9 OS=Synechocystis sp. (strain PCC 6803 / Kazusa) GN=rpl	1289,64	61,18	1	13	13	77	2,385	59	100,3	152	16,6	9,20							
	A3	Sequence	# PSMs	# Proteins	# Protein Groups	Protein Group Accessions	Modifications	ΔCn	114/115	114/115 Count	114/115 Variability [%]	q-Value	PEP	IonScore	Exp Value	Charge	MH+ [Da]	ΔM [ppm]	RT [min]	# Missed Cleavages
	High	vVLNETINk	9	1	1	P42352	N-Term(iTRAQ4plex); K9(iTRAQ4plex)	0,0000	2,393	6	147,9	0	7,764E-08	73	5,44465E-08	2	1317,79900	0,71	28,05	0
	High	gISLPDIHk	15	1	1	P42352	N-Term(iTRAQ4plex); K9(iTRAQ4plex)	0,0000	2,069	10	85,6	0	0,000005495	66	2,44889E-07	2	1267,76140	0,10	30,24	0
	High	lGFTGDLVEVAPGYAR	3	1	1	P42352	N-Term(iTRAQ4plex)	0,0000	2,227	3	116,7	0	3,441E-10	65	3,26565E-07	2	1808,96758	0,65	44,85	0
	High	vkVVLNETINk	2	1	1	P42352	N-Term(iTRAQ4plex); K2(iTRAQ4plex); K11(iTRAQ4plex)	0,0000	1,665	2	43,1	0	1,924E-09	59	1,17482E-06	3	1689,06314	-0,22	29,15	1
	High	gLGVVATPGILR	9	1	1	P42352	N-Term(iTRAQ4plex)	0,0000	1,927	5	111,8	0	0,0003002	52	5,8475E-06	2	1296,81328	0,95	38,64	0
	High	tGFYQAQIk	6	1	1	P42352	N-Term(iTRAQ4plex); K9(iTRAQ4plex)	0,0000	1,619	5	256,6	0	4,042E-07	52	5,94251E-06	2	1343,75701	0,62	26,36	0
	High	kVALETIGR	17	1	1	P42352	N-Term(iTRAQ4plex); K1(iTRAQ4plex)	0,0000	2,009	12	53,9	0	0,00004443	52	6,87021E-06	3	1274,80552	1,59	21,52	1
	High	vALETIGR	2	1	1	P42352	N-Term(iTRAQ4plex)	0,0000	3,086	2	18,3	0	0,0001115	44	5,64863E-05	2	1002,60643	-0,02	26,88	0
	High	nYLIPk	7	1	1	P42352	N-Term(iTRAQ4plex); K6(iTRAQ4plex)	0,0000	3,118	7	82,6	0,003	0,0379	12	0,068544088	2	1035,64470	0,58	68,81	0
	High	fVIkk	3	1	1	P42352	N-Term(iTRAQ4plex); K4(iTRAQ4plex); K5(iTRAQ4plex)	0,0000	2,896	3	8,6	0,005	0,05755	21	0,007128038	2	1066,73479	-0,09	20,11	1
	High	lkELER	2	1	1	P42352	N-Term(iTRAQ4plex); K2(iTRAQ4plex)	0,0000	3,813	2	7,0	0,005	0,06073	13	0,056000164	3	1075,67218	0,73	17,07	1
	High	aEkDAAEAR	1	1	1	P42352	N-Term(iTRAQ4plex); K3(iTRAQ4plex)	0,0000	1,543	1		0,007	0,08408	16	0,028719789	3	1248,67920	0,41	10,01	1
	Medium	rGISLPDIHk	1	1	1	P42352	N-Term(iTRAQ4plex); K10(iTRAQ4plex)	0,0000	2,723	1		0,011	0,1267	15	0,032506485	4	1423,86318	0,55	25,49	1
P54205	Ribulose bisphosphate carboxylase large chain OS=Synechocystis sp. (strain PCC 68	1287,63	41,28	1	23	23	65	0,939	61	17,2	470	52,5	6,15							
	A3	Sequence	# PSMs	# Proteins	# Protein Groups	Protein Group Accessions	Modifications	ΔCn	114/115	114/115 Count	114/115 Variability [%]	q-Value	PEP	IonScore	Exp Value	Charge	MH+ [Da]	ΔM [ppm]	RT [min]	# Missed Cleavages
	High	tFQGPPHGITVER	4	1	1	P54205	N-Term(iTRAQ4plex)	0,0000	0,710	4	5,3	0	3,836E-09	85	3,16935E-09	2	1582,84685	0,62	25,03	0
	High	fLFVQEAIEk	7	1	1	P54205	N-Term(iTRAQ4plex); K10(iTRAQ4plex)	0,0000	0,974	7	9,3	0	0,000001236	69	1,27929E-07	2	1511,87297	1,15	45,54	0
	High	gITMGFVDLMR	1	1	1	P54205	N-Term(iTRAQ4plex)	0,0000	0,709	1		0	6,046E-07	58	1,44534E-06	2	1383,72502	0,32	49,70	0
	High	gHYLNVTAGTcEEmmk	1	1	1	P54205	N-Term(iTRAQ4plex); C11(Carbamidomethyl); M14(Oxidation); M15(Oxidatio	0,0000	1,018	1		0	2,25E-10	57	1,87487E-06	3	2161,00064	1,83	22,14	0
	High	lTYYTPDYTPk	2	1	1	P54205	N-Term(iTRAQ4plex); K11(iTRAQ4plex)	0,0000	1,022	2	10,4	0	1,433E-08	53	5,30848E-06	2	1649,86797	0,89	33,36	0
	High	amHAVVDR	6	1	1	P54205	N-Term(iTRAQ4plex); M2(Oxidation)	0,0000	0,835	6	19,3	0	0,00008	52	6,79157E-06	2	1058,55229	-1,03	9,12	0
	High	lSGGDHLHSGTVVGk	4	1	1	P54205	N-Term(iTRAQ4plex); K15(iTRAQ4plex)	0,0000	1,124	3	17,3	0	1,854E-08	50	1,08136E-05	3	1751,96555	0,71	19,18	0
	High	vALEAcVQAR	2	1	1	P54205	N-Term(iTRAQ4plex); C6(Carbamidomethyl)	0,0000	0,914	2	3,5	0	0,000001315	39	0,000133343	2	1260,68535	0,18	25,44	0
	High	aQAETNEmk	5	1	1	P54205	N-Term(iTRAQ4plex); M8(Oxidation); K9(iTRAQ4plex)	0,0000	1,029	5	6,0	0	0,00002229	38	0,000159945	3	1325,66059	-0,30	10,16	0
	High	dRFLFVQEAIEk	2	1	1	P54205	N-Term(iTRAQ4plex); K12(iTRAQ4plex)	0,0000	0,962	2	13,6	0	0,000000121	37	0,000187055	3	1782,99936	0,03	40,28	1
	High	aVYEcLR	3	1	1	P54205	N-Term(iTRAQ4plex); C5(Carbamidomethyl)	0,0000	0,921	3	3,4	0	0,001269	34	0,000368104	2	1054,54778	0,54	22,37	0
	High	dNGILLHIHR	3	1	1	P54205	N-Term(iTRAQ4plex)	0,0000	1,181	2	97,4	0	0,00001843	32	0,000568814	3	1331,76679	0,24	23,53	0
	High	gHYLNVTAGTcEEmmkR	3	1	1	P54205	N-Term(iTRAQ4plex); C11(Carbamidomethyl); M14(Oxidation); M15(Oxidatio	0,0000	0,899	2	5,2	0	0,000009937	31	0,000772627	4	2317,09975	0,84	19,95	1
	High	wSPELAAAcELWk	1	1	1	P54205	N-Term(iTRAQ4plex); C9(Carbamidomethyl); K13(iTRAQ4plex)	0,0000	2,010	1		0	3,833E-07	29	0,00129476	3	1848,95853	1,51	48,57	0
	High	lEDIRFPVALIk	4	1	1	P54205	N-Term(iTRAQ4plex); K12(iTRAQ4plex)	0,0000	0,885	4	1,9	0	9,696E-07	28	0,001530982	3	1702,05082	0,12	47,56	1
	High	yGRPLLGcTIkPk	2	1	1	P54205	N-Term(iTRAQ4plex); C8(Carbamidomethyl); K11(iTRAQ4plex); K13(iTRAQ4p	0,0000	0,289	2	39,9	0	2,073E-09	21	0,00741259	4	1935,15603	-0,73	26,17	0
	High	aMHAVVDR	2	1	1	P54205	N-Term(iTRAQ4plex)	0,0000	1,123	2	9,7	0	0,003811	21	0,008530412	3	1042,55829	-0,17	12,95	0
	High	fPVALIk	4	1	1	P54205	N-Term(iTRAQ4plex); K7(iTRAQ4plex)	0,0000	0,962	4	5,4	0,001	0,01545	38	0,000164805	2	1075,71160	-0,20	38,84	0
	High	nHGIHFR	1	1	1	P54205	N-Term(iTRAQ4plex)	0,0000	0,613	1		0,003	0,03871	12	0,05847497	2	1024,55437	-1,33	12,05	0
	High	lGLSAk	4	1	1	P54205	N-Term(iTRAQ4plex); K6(iTRAQ4plex)	0,0000	1,059	3	3,8	0,006	0,07523	10	0,121712104	2	876,57530	-0,45	20,89	0
	High	aLRLEDIR	1	1	1	P54205	N-Term(iTRAQ4plex)	0,0000	0,845	1		0,007	0,0839	21	0,008550077	3	1129,68152	0,44	25,90	1
	High	rAEFAk	1	1	1	P54205	N-Term(iTRAQ4plex); K6(iTRAQ4plex)	0,0000	0,994	1		0,008	0,0934	12	0,057407681	3	1009,60251	-0,80	11,30	1
	Medium	dkLNk	1	1	1	P54205	N-Term(iTRAQ4plex); K2(iTRAQ4plex); K5(iTRAQ4plex)	0,0000	1,069	1		0,019	0,2035	12	0,061939829	3	1049,66599	-1,84	10,83	1
	Medium	aGVQDYR	1	1	1	P54205	N-Term(iTRAQ4plex)	0,0000	1,094	1		0,035	0,3728	12	0,067759471	2	952,49748	0,63	13,76	0
Q55517	Sll0529 protein OS=Synechocystis sp. (strain PCC 6803 / Kazusa) GN=sll0529 PE=4	1281,53	26,27	1	15	15	44	0,510	37	37,6	731	78,3	5,60							
	A3	Sequence	# PSMs	# Proteins	# Protein Groups	Protein Group Accessions	Modifications	ΔCn	114/115	114/115 Count	114/115 Variability [%]	q-Value	PEP	IonScore	Exp Value	Charge	MH+ [Da]	ΔM [ppm]	RT [min]	# Missed Cleavages
	High	rPTPSTITLFTAGALEQGR	7	1	1	Q55517	N-Term(iTRAQ4plex)	0,0000	0,910	6	47,3	0	3,238E-12	93	6,51543E-10	3	2160,19077	0,62	41,41	0
	High	rGETTASAGQLmAYNGLTAQALGDR	1	1	1	Q55517	N-Term(iTRAQ4plex); M12(Oxidation)	0,0000	0,399	1		0	4,892E-13	88	1,72175E-09	3	2712,35117	0,70	34,26	1
	High	qVATTAMGELVVHVGk	1	1	1	Q55517	N-Term(iTRAQ4plex); K16(iTRAQ4plex)	0,0000	0,434	1		0	1,279E-12	81	8,16526E-09	3	1928,08792	0,06	37,80	0
	High	yGQETAIASGAk	1	1	1	Q55517	N-Term(iTRAQ4plex); K12(iTRAQ4plex)	0,0000	0,506	1		0	0,000000317	72	6,96578E-08	2	1483,79766	-1,26	21,63	0
	High	yLTLQTTASk	3	1	1	Q55517	N-Term(iTRAQ4plex); K10(iTRAQ4plex)	0,0000	0,430	3	9,1	0	3,844E-08	70	9,59334E-08	2	1413,81792	-0,88	28,99	0
	High	aLSPEAWQLVR	3	1	1	Q55517	N-Term(iTRAQ4plex)	0,0000	0,379	3	3,5	0	3,223E-08	56	2,59998E-06	2	1413,79668	-0,32	41,35	0
	High	qVATTAmGELVVHVGk	1	1	1	Q55517	N-Term(iTRAQ4plex); M7(Oxidation); K16(iTRAQ4plex)	0,0000	0,666	1		0	3,072E-10	49	1,34577E-05	3	1944,08384	0,57	28,86	0
	High	nVILVNAk	8	1	1	Q55517	N-Term(iTRAQ4plex); K8(iTRAQ4plex)	0,0000	0,452	5	24,6	0	0,002174	45	3,07588E-05	2	1158,74394	-0,82	25,01	0
	High	lAFTQAVLEATDk	1	1	1	Q55517	N-Term(iTRAQ4plex); K13(iTRAQ4plex)	0,0000	1,517	1		0	4,131E-07	44	3,7151E-05	3	1694,95713	0,21	45,24	0
	High	vVSVINPR	4	1	1	Q55517	N-Term(iTRAQ4plex)	0,0000	0,510	3	10,5	0	0,001172	43	4,80806E-05	2	1027,63762	-0,45	25,32	0
	High	gIPITASk	5	1	1	Q55517	N-Term(iTRAQ4plex); K8(iTRAQ4plex)	0,0000	0,439	3	12,2	0	0,002495	42	6,94976E-05	2	1074,67400	-2,00	21,99	0
	High	sHNLYPGDSLDkDYIVSALQER	2	1	1	Q55517	N-Term(iTRAQ4plex); K12(iTRAQ4plex)	0,0000	0,583	2	50,2	0	7,393E-12	40	0,000106898	4	2808,43886	-0,66	42,02	1
	High	tTLEQNR	1	1	1	Q55517	N-Term(iTRAQ4plex)	0,0000	0,589	1		0	0,004594	36	0,000255252	2	1005,54479	0,22	11,73	0
	High	nTVTEVGHHAPGFYSTmFLDR	1	1	1	Q55517	N-Term(iTRAQ4plex); M17(Oxidation)	0,0000	1,551	1		0	8,091E-08	25	0,003082975	4	2539,21694	0,48	34,00	0
	High	nTVTEVGHHAPGFYSTMFLDR	1	1	1	Q55517	N-Term(iTRAQ4plex)	0,0000	0,997	1		0	6,226E-09	22	0,006456096	4	2523,22109	0,12	38,37	0
	High	fSFQQR	3	1	1	Q55517	N-Term(iTRAQ4plex)	0,0000	0,495	3	26,7	0,001	0,008929	34	0,000377546	2	956,50658	-0,50	23,85	0
	High	dLFAWk	1	1	1	Q55517	N-Term(iTRAQ4plex); K6(iTRAQ4plex)	0,0000	0,677	1		0,008	0,09273	27	0,001972287	2	1067,61003	-2,61	39,18	0
P77972	Enolase OS=Synechocystis sp. (strain PCC 6803 / Kazusa) GN=eno PE=3 SV=1 - [E	1239,18	24,77	1	9	9	49	1,284	38	15,1	432	46,5	5,15							
	A3	Sequence	# PSMs	# Proteins	# Protein Groups	Protein Group Accessions	Modifications	ΔCn	114/115	114/115 Count	114/115 Variability [%]	q-Value	PEP	IonScore	Exp Value	Charge	MH+ [Da]	ΔM [ppm]	RT [min]	# Missed Cleavages
	High	wGAEVFAVLGk	10	1	1	P77972	N-Term(iTRAQ4plex); K11(iTRAQ4plex)	0,0000	1,263	8	22,4	0	1,9E-10	94	3,96251E-10	2	1464,84722	1,26	51,84	0
	High	aAAAELAIPLYR	7	1	1	P77972	N-Term(iTRAQ4plex)	0,0000	1,263	6	5,3	0	1,296E-08	81	7,16094E-09	2	1402,81890	0,98	40,40	0
	High	lESGAHGIAQVPSGASTGSFEAHELR	5	1	1	P77972	N-Term(iTRAQ4plex)	0,0000	1,375	3	6,1	0	1,223E-15	81	7,37853E-09	3	2752,37876	0,56	32,30	0
	High	vPATIEEIAAR	2	1	1	P77972	N-Term(iTRAQ4plex)	0,0000	1,190	2	3,1	0	0,000001269	56	2,74138E-06	2	1313,75615	1,19	36,53	0
	High	gLTTSLGTk	7	1	1	P77972	N-Term(iTRAQ4plex); K9(iTRAQ4plex)	0,0000	1,446	4	6,1	0	0,0001113	50	9,37497E-06	2	1165,70415	0,91	22,19	0
	High	gRPTIEAEVR	9	1	1	P77972	N-Term(iTRAQ4plex)	0,0000	1,270	6	4,7	0	0,00004485	44	4,3448E-05	3	1271,71946	0,46	16,47	0
	High	eLGANAILGVSLATAk	1	1	1	P77972	N-Term(iTRAQ4plex); K16(iTRAQ4plex)	0,0000	0,941	1		0	1,939E-08	41	7,74462E-05	3	1816,08030	1,10	45,05	0
	High	aVYAPk	4	1	1	P77972	N-Term(iTRAQ4plex); K6(iTRAQ4plex)	0,0000	1,254	4	9,6	0,006	0,07811	28	0,001452011	2	936,57573	0,05	15,97	0
	Medium	iGLGPk	4	1	1	P77972	N-Term(iTRAQ4plex); K6(iTRAQ4plex)	0,0000	1,548	4	4,5	0,011	0,1342	32	0,000703024	2	872,58391	3,58	20,35	0
P27179	ATP synthase subunit alpha OS=Synechocystis sp. (strain PCC 6803 / Kazusa) GN=	1220,98	26,84	1	13	13	55	1,021	50	9,8	503	53,9	5,08							
	A3	Sequence	# PSMs	# Proteins	# Protein Groups	Protein Group Accessions	Modifications	ΔCn	114/115	114/115 Count	114/115 Variability [%]	q-Value	PEP	IonScore	Exp Value	Charge	MH+ [Da]	ΔM [ppm]	RT [min]	# Missed Cleavages
	High	aSTVAQIIDTLTEk	5	1	1	P27179	N-Term(iTRAQ4plex); K14(iTRAQ4plex)	0,0000	0,917	5	25,2	0	2,536E-12	82	6,109E-09	2	1778,01641	0,79	55,06	0
	High	tAIAIDTIINQk	3	1	1	P27179	N-Term(iTRAQ4plex); K12(iTRAQ4plex)	0,0000	1,114	3	20,3	0	2,076E-09	66	2,80797E-07	2	1588,95244	0,73	39,59	0
	High	tTGQIAQIPIGDAmVGR	2	1	1	P27179	N-Term(iTRAQ4plex); M14(Oxidation)	0,0000	0,968	2	21,4	0	0,0002129	60	1,0964E-06	2	1888,01140	1,82	35,33	0
	High	yVEIINSSk	5	1	1	P27179	N-Term(iTRAQ4plex); K9(iTRAQ4plex)	0,0000	1,029	5	12,3	0	0,000005089	59	1,29113E-06	2	1340,76592	-0,37	29,26	0
	High	vTEFAQGLR	3	1	1	P27179	N-Term(iTRAQ4plex)	0,0000	0,948	3	5,1	0	0,000002859	58	1,82049E-06	2	1164,65044	0,90	30,23	0
	High	eAYPGDVFYIHSR	5	1	1	P27179	N-Term(iTRAQ4plex)	0,0000	1,013	5	5,8	0	3,71E-10	50	9,86211E-06	3	1697,83994	-0,29	34,06	0
	High	vVDSLGRPIDGk	4	1	1	P27179	N-Term(iTRAQ4plex); K12(iTRAQ4plex)	0,0000	0,997	3	3,5	0	5,273E-08	46	2,58803E-05	2	1543,90508	0,26	25,61	0
	High	vSIRPDEISSIIR	13	1	1	P27179	N-Term(iTRAQ4plex)	0,0000	1,086	10	4,2	0	0,000001841	45	3,38821E-05	2	1628,94805	1,71	40,25	0
	High	vGSAAQTk	5	1	1	P27179	N-Term(iTRAQ4plex); K8(iTRAQ4plex)	0,0000	1,026	4	3,5	0	0,001116	44	3,97164E-05	2	1049,61943	0,07	12,09	0
	High	gPISSTATR	2	1	1	P27179	N-Term(iTRAQ4plex)	0,0000	1,036	2	0,7	0	0,0003758	41	7,49842E-05	2	1033,57598	0,10	12,87	0
	High	eLIIGDRk	4	1	1	P27179	N-Term(iTRAQ4plex); K8(iTRAQ4plex)	0,0000	0,974	4	3,8	0	0,001439	27	0,002228281	3	1231,76172	0,35	20,30	1
	High	vTEFAQGLRDYLk	1	1	1	P27179	N-Term(iTRAQ4plex); K13(iTRAQ4plex)	0,0000	0,654	1		0	0,000002818	11	0,077798282	3	1828,02335	1,42	43,82	1
	High	qMSLLMR	1	1	1	P27179	N-Term(iTRAQ4plex)	0,0000	0,726	1		0,005	0,06194	24	0,003766778	2	1022,56053	-0,24	30,18	0
	Medium	qMSLLmR	1	1	1	P27179	N-Term(iTRAQ4plex); M6(Oxidation)	0,0000	0,934	1		0,011	0,1292	20	0,008953029	2	1038,55522	-0,45	25,03	0
	Medium	qmSLLmR	1	1	1	P27179	N-Term(iTRAQ4plex); M2(Oxidation); M6(Oxidation)	0,0000	0,964	1		0,048	0,4978	13	0,053947336	2	1054,55010	-0,48	18,24	0
P73605	Slr1854 protein OS=Synechocystis sp. (strain PCC 6803 / Kazusa) GN=slr1854 PE=4	1167,54	34,34	1	8	8	41	0,975	34	17,0	198	22,3	4,84							
	A3	Sequence	# PSMs	# Proteins	# Protein Groups	Protein Group Accessions	Modifications	ΔCn	114/115	114/115 Count	114/115 Variability [%]	q-Value	PEP	IonScore	Exp Value	Charge	MH+ [Da]	ΔM [ppm]	RT [min]	# Missed Cleavages
	High	gIIAIGAYAMDR	3	1	1	P73605	N-Term(iTRAQ4plex)	0,0000	0,973	3	0,7	0	1,974E-09	67	1,96874E-07	2	1394,75969	0,99	42,85	0
	High	hPGMIEEFVk	2	1	1	P73605	N-Term(iTRAQ4plex); K10(iTRAQ4plex)	0,0000	0,836	2	2,8	0	1,523E-07	67	2,49861E-07	2	1474,79778	0,73	30,13	0
	High	hPGmIEEFVk	6	1	1	P73605	N-Term(iTRAQ4plex); M4(Oxidation); K10(iTRAQ4plex)	0,0000	0,914	6	6,2	0	3,659E-08	60	1,08885E-06	2	1490,79253	0,61	23,98	0
	High	lGTIcHSLWLFcADPDLLk	2	1	1	P73605	N-Term(iTRAQ4plex); C5(Carbamidomethyl); C12(Carbamidomethyl); K19(iTI	0,0000	0,833	1		0	1,741E-10	59	1,30608E-06	3	2547,33707	1,09	53,76	0
	High	kAAATEGLk	8	1	1	P73605	N-Term(iTRAQ4plex); K1(iTRAQ4plex); K9(iTRAQ4plex)	0,0000	1,343	7	14,9	0	0,000008659	58	1,74973E-06	3	1320,83228	8,43	11,98	1
	High	gIIAIGAYAmDR	6	1	1	P73605	N-Term(iTRAQ4plex); M10(Oxidation)	0,0000	0,944	4	18,3	0	8,936E-09	56	2,38765E-06	2	1410,75469	1,04	36,54	0
	High	nQAPAVIFLR	8	1	1	P73605	N-Term(iTRAQ4plex)	0,0000	0,963	6	7,3	0	5,292E-08	56	2,72879E-06	3	1272,75272	-1,42	36,37	0
	High	aAATEGLk	2	1	1	P73605	N-Term(iTRAQ4plex); K8(iTRAQ4plex)	0,0000	1,073	1		0	0,0000303	44	4,25569E-05	2	1048,62444	0,31	15,14	0
	High	lGTIcHSLWLFcADPDLLkDk	1	1	1	P73605	N-Term(iTRAQ4plex); C5(Carbamidomethyl); C12(Carbamidomethyl); K19(iTI	0,0000	0,839	1		0	0,000000182	30	0,001284393	4	2934,56313	1,66	49,92	1
	Medium	yQATVk	3	1	1	P73605	N-Term(iTRAQ4plex); K6(iTRAQ4plex)	0,0000	1,095	3	1,8	0,01	0,1195	26	0,002685159	2	997,59032	-1,74	15,31	0
P73452	Nitrate transport protein NrtA OS=Synechocystis sp. (strain PCC 6803 / Kazusa) GN	1167,39	30,49	1	15	16	65	2,161	55	42,9	446	48,9	5,34							
	A3	Sequence	# PSMs	# Proteins	# Protein Groups	Protein Group Accessions	Modifications	ΔCn	114/115	114/115 Count	114/115 Variability [%]	q-Value	PEP	IonScore	Exp Value	Charge	MH+ [Da]	ΔM [ppm]	RT [min]	# Missed Cleavages
	High	sHDQWFLTENIR	22	1	1	P73452	N-Term(iTRAQ4plex)	0,0000	1,372	20	24,4	0	3,658E-09	74	4,2655E-08	2	1689,84917	1,53	34,25	0
	High	yWVDNASYPYk	1	1	1	P73452	N-Term(iTRAQ4plex); K11(iTRAQ4plex)	0,0000	2,657	1		0	9,27E-10	67	2,17756E-07	2	1693,85112	2,77	34,66	0
	High	lGFIALTDAAPLIIAk	3	1	1	P73452	N-Term(iTRAQ4plex); K16(iTRAQ4plex)	0,0000	2,790	3	46,9	0	2,694E-07	66	2,44326E-07	2	1915,19548	4,36	56,58	0
	High	vAMTFPGGTHDMWIR	2	1	1	P73452	N-Term(iTRAQ4plex)	0,0000	2,170	2	3,4	0	5,559E-09	59	1,19666E-06	3	1862,91794	0,88	40,93	0
	High	lNVNGQGIQLGNNYk	1	1	1	P73452	N-Term(iTRAQ4plex); K15(iTRAQ4plex)	0,0000	1,424	1		0	2,403E-09	59	1,3365E-06	3	1920,05674	1,33	34,17	0
	High	vGTDAAPLk	4	1	1	P73452	N-Term(iTRAQ4plex); K9(iTRAQ4plex)	0,0000	2,701	3	7,4	0	0,0001008	55	3,14753E-06	2	1159,69206	-0,41	20,03	0
	High	vAMTFPGGTHDmWIR	4	1	1	P73452	N-Term(iTRAQ4plex); M12(Oxidation)	0,0000	2,115	3	25,4	0	6,159E-09	51	7,31089E-06	3	1878,91275	0,82	37,75	0
	High	vGTDAAPLkEAFAk	7	1	1	P73452	N-Term(iTRAQ4plex); K9(iTRAQ4plex); K14(iTRAQ4plex)	0,0000	2,538	6	12,3	0	1,165E-07	41	7,46397E-05	3	1850,07712	1,24	31,62	1
	High	wGYLPASTDTk	2	1	1	P73452	N-Term(iTRAQ4plex); K11(iTRAQ4plex)	0,0000	2,771	2	4,3	0	0,00002139	39	0,000127049	2	1526,81145	1,38	32,64	0
	High	vAmTFPGGTHDmWIR	4	1	1	P73452	N-Term(iTRAQ4plex); M3(Oxidation); M12(Oxidation)	0,0000	1,907	3	21,9	0	1,168E-07	36	0,000231191	3	1894,90830	1,14	32,09	0
	High	vNREDLWR	4	2	2	Q55460;P73452	N-Term(iTRAQ4plex)	0,0000				0	0,0005999	27	0,001945226	2	1231,66802	1,28	22,94	1
	High	ekGFYAk	2	1	1	P73452	N-Term(iTRAQ4plex); K2(iTRAQ4plex); K7(iTRAQ4plex)	0,0000	2,802	2	4,6	0	0,001293	25	0,003476812	2	1274,74895	1,61	15,04	1
	High	qASWGTTR	1	1	1	P73452	N-Term(iTRAQ4plex)	0,0000	1,734	1		0	0,002287	21	0,008336236	2	1050,54570	0,76	15,34	0
	High	gFYAk	2	1	1	P73452	N-Term(iTRAQ4plex); K5(iTRAQ4plex)	0,0000	2,511	2	22,5	0,004	0,04974	29	0,001355096	2	873,50987	2,98	17,43	0
	High	vPFEDIIDR	1	1	1	P73452	N-Term(iTRAQ4plex)	0,0000	1,708	1		0,009	0,1045	13	0,049427655	2	1247,67644	0,93	42,90	0
	Medium	eAFAk	2	1	1	P73452	N-Term(iTRAQ4plex); K5(iTRAQ4plex)	0,0000	1,662	2	52,4	0,012	0,1387	24	0,004314893	2	853,50865	7,56	15,47	0
	Medium	rEWFk	2	1	1	P73452	N-Term(iTRAQ4plex); K5(iTRAQ4plex)	0,0000	3,228	2	0,8	0,012	0,1414	12	0,071976264	3	1053,61072	2,21	18,87	1
	Medium	vTDPk	1	1	1	P73452	N-Term(iTRAQ4plex); K5(iTRAQ4plex)	0,0000	1,451	1		0,039	0,4071	18	0,016594723	2	847,51177	-1,17	12,18	0
P73603	Slr1852 protein OS=Synechocystis sp. (strain PCC 6803 / Kazusa) GN=slr1852 PE=4	1159,83	40,43	1	10	10	72	1,301	67	12,4	188	21,8	5,17							
	A3	Sequence	# PSMs	# Proteins	# Protein Groups	Protein Group Accessions	Modifications	ΔCn	114/115	114/115 Count	114/115 Variability [%]	q-Value	PEP	IonScore	Exp Value	Charge	MH+ [Da]	ΔM [ppm]	RT [min]	# Missed Cleavages
	High	lIAEGIAYVAQk	10	1	1	P73603	N-Term(iTRAQ4plex); K12(iTRAQ4plex)	0,0000	1,323	8	10,5	0	3,55E-11	87	1,77816E-09	2	1563,93755	1,69	45,61	0
	High	ekLIAEGIAYVAQk	4	1	1	P73603	N-Term(iTRAQ4plex); K2(iTRAQ4plex); K14(iTRAQ4plex)	0,0000	1,286	4	8,0	0	5,847E-11	76	2,78593E-08	3	1965,17923	2,39	42,58	1
	High	dVFAPDSLLk	1	1	1	P73603	N-Term(iTRAQ4plex); K10(iTRAQ4plex)	0,0000	1,482	1		0	0,000008113	42	6,44125E-05	2	1392,79997	1,61	40,97	0
	High	gNVTWVR	8	1	1	P73603	N-Term(iTRAQ4plex)	0,0000	1,283	7	8,3	0	0,0006836	39	0,00013365	2	975,55010	0,85	20,58	0
	High	kNYYLGLk	12	1	1	P73603	N-Term(iTRAQ4plex); K1(iTRAQ4plex); K8(iTRAQ4plex)	0,0000	1,596	12	14,2	0	0,0009386	38	0,000172572	2	1430,87517	1,41	24,98	1
	High	tEQLTFYR	6	1	1	P73603	N-Term(iTRAQ4plex)	0,0000	1,286	6	11,8	0	0,001093	37	0,000202754	2	1201,63530	1,60	28,25	0
	High	dWWPTHcEALR	3	1	1	P73603	N-Term(iTRAQ4plex); C7(Carbamidomethyl)	0,0000	1,301	3	28,3	0	1,938E-08	32	0,000691783	3	1614,76114	0,45	33,98	0
	High	cVDDETHETIAk	4	1	1	P73603	N-Term(iTRAQ4plex); C1(Carbamidomethyl); K12(iTRAQ4plex)	0,0000	1,431	2	0,9	0	0,000003154	31	0,000866902	3	1705,82792	-1,56	17,52	0
	High	nYYLGLk	22	1	1	P73603	N-Term(iTRAQ4plex); K7(iTRAQ4plex)	0,0000	1,225	22	6,0	0	0,005951	28	0,001479006	2	1158,67693	0,70	68,24	0
	High	gGcYLk	2	1	1	P73603	N-Term(iTRAQ4plex); C3(Carbamidomethyl); K6(iTRAQ4plex)	0,0000	1,679	2	3,8	0,003	0,03664	17	0,018195752	2	985,54088	3,00	15,65	0
P52983	Glucose-6-phosphate isomerase OS=Synechocystis sp. (strain PCC 6803 / Kazusa) (	1144,82	40,68	1	20	20	54	0,982	46	12,5	531	58,3	5,76							
	A3	Sequence	# PSMs	# Proteins	# Protein Groups	Protein Group Accessions	Modifications	ΔCn	114/115	114/115 Count	114/115 Variability [%]	q-Value	PEP	IonScore	Exp Value	Charge	MH+ [Da]	ΔM [ppm]	RT [min]	# Missed Cleavages
	High	aVSFYGSLVNVNAYHQPGVEAGk	2	1	1	P52983	N-Term(iTRAQ4plex); K23(iTRAQ4plex)	0,0000	1,335	1		0	1,38E-15	78	1,65185E-08	3	2695,41336	1,88	41,66	0
	High	yLQQLVmESLGk	1	1	1	P52983	N-Term(iTRAQ4plex); M7(Oxidation); K12(iTRAQ4plex)	0,0000	0,816	1		0	2,574E-10	74	4,12069E-08	2	1712,95256	1,75	49,50	0
	High	aAASILELQk	3	1	1	P52983	N-Term(iTRAQ4plex); K10(iTRAQ4plex)	0,0000	1,373	3	36,2	0	0,000003529	68	1,76999E-07	2	1331,81487	0,86	33,31	0
	High	sTLVVTTSk	4	1	1	P52983	N-Term(iTRAQ4plex); K9(iTRAQ4plex)	0,0000	0,992	2	4,8	0	0,000001316	60	9,41825E-07	2	1223,74565	0,58	20,01	0
	High	kAAASILELQk	3	1	1	P52983	N-Term(iTRAQ4plex); K1(iTRAQ4plex); K11(iTRAQ4plex)	0,0000	1,058	3	11,3	0	7,842E-07	56	2,69135E-06	3	1604,01010	-0,40	28,46	1
	High	aFVADVHQGNIkPPTAPk	11	1	1	P52983	N-Term(iTRAQ4plex); K12(iTRAQ4plex); K18(iTRAQ4plex)	0,0000	0,995	6	5,4	0	6,624E-10	51	8,80988E-06	3	2322,33158	0,90	27,38	0
	High	qALFENQR	3	1	1	P52983	N-Term(iTRAQ4plex)	0,0000	0,910	3	18,6	0	0,00003061	45	2,94422E-05	2	1149,61382	0,43	20,66	0
	High	vLNcLk	1	1	1	P52983	N-Term(iTRAQ4plex); C4(Carbamidomethyl); K6(iTRAQ4plex)	0,0000	1,054	1		0	0,001298	45	4,94688E-05	2	1034,62895	1,81	25,28	0
	High	nPALAPNDGIR	2	1	1	P52983	N-Term(iTRAQ4plex)	0,0000	0,876	2	6,2	0	0,0004458	44	4,00839E-05	2	1281,70403	0,63	21,92	0
	High	gAIANPDEQR	2	1	1	P52983	N-Term(iTRAQ4plex)	0,0000	0,818	2	28,1	0	2,858E-07	42	7,03024E-05	2	1214,62822	2,95	13,57	0
	High	mNNQQLWQR	1	1	1	P52983	N-Term(iTRAQ4plex); M1(Oxidation)	0,0000	1,016	1		0	0,002197	34	0,000357248	2	1377,68279	0,99	20,90	0
	High	mVGHYWLR	3	1	1	P52983	N-Term(iTRAQ4plex)	0,0000	1,031	3	9,9	0	0,00001653	32	0,000568814	3	1205,63715	0,10	29,22	0
	High	aVFEAQGLHFADYAVAVTmPGSk	1	1	1	P52983	N-Term(iTRAQ4plex); M19(Oxidation); K23(iTRAQ4plex)	0,0000	0,821	1		0	8,729E-07	30	0,001002236	4	2713,39419	1,58	47,44	0
	High	eRDLDGNVVHQGIAVYGNk	1	1	1	P52983	N-Term(iTRAQ4plex); K19(iTRAQ4plex)	0,0000	1,022	1		0	0,00001207	30	0,001059181	3	2372,25528	-0,37	28,94	1
	High	gSTDQHAYVQQLR	3	1	1	P52983	N-Term(iTRAQ4plex)	0,0000	0,931	3	5,0	0	0,0009627	28	0,001725719	3	1646,84726	6,39	18,63	0
	High	kDmVILPYkDR	1	1	1	P52983	N-Term(iTRAQ4plex); K1(iTRAQ4plex); M3(Oxidation); K9(iTRAQ4plex)	0,0000	0,921	1		0	1,72E-08	27	0,001870553	3	1826,05698	-0,04	22,67	2
	High	kDMVILPYkDR	1	1	1	P52983	N-Term(iTRAQ4plex); K1(iTRAQ4plex); K9(iTRAQ4plex)	0,0000	1,123	1		0	0,000001786	21	0,008317063	4	1810,06093	-0,67	26,21	2
	High	dmVILPYkDR	1	1	1	P52983	N-Term(iTRAQ4plex); M2(Oxidation); K8(iTRAQ4plex)	0,0000	0,906	1		0	0,001201	20	0,008932438	3	1553,85990	-0,08	25,30	1
	High	mVGHYWLR	4	1	1	P52983	N-Term(iTRAQ4plex); M1(Oxidation)	0,0000	1,098	4	23,6	0	0,006723	20	0,009418245	2	1221,63213	0,15	24,23	0
	High	vQAPEQVETVYk	1	1	1	P52983	N-Term(iTRAQ4plex); K12(iTRAQ4plex)	0,0000	1,365	1		0	0,002358	16	0,030825492	3	1678,92386	-0,94	28,54	0
	High	dMVILPYkDR	1	1	1	P52983	N-Term(iTRAQ4plex); K8(iTRAQ4plex)	0,0000	0,963	1		0	0,001864	16	0,029833063	3	1537,86582	0,46	30,36	1
	High	nGLAETk	1	1	1	P52983	N-Term(iTRAQ4plex); K7(iTRAQ4plex)	0,0000	0,811	1		0,003	0,03608	24	0,004115216	2	1020,59575	2,88	12,10	0
	High	lQIQWR	2	1	1	P52983	N-Term(iTRAQ4plex)	0,0000	0,766	2	1,7	0,009	0,1048	27	0,002177559	2	987,58599	0,35	32,36	0
	Medium	yLQQLVMESLGk	1	1	1	P52983	N-Term(iTRAQ4plex); K12(iTRAQ4plex)	0,0000	0,946	1		0,03	0,3219	13	0,05023079	3	1696,94547	-5,41	53,83	0
P72848	Oxygen-dependent coproporphyrinogen-III oxidase OS=Synechocystis sp. (strain P	1070,62	35,00	1	11	11	46	1,911	41	29,0	340	38,9	5,82							
	A3	Sequence	# PSMs	# Proteins	# Protein Groups	Protein Group Accessions	Modifications	ΔCn	114/115	114/115 Count	114/115 Variability [%]	q-Value	PEP	IonScore	Exp Value	Charge	MH+ [Da]	ΔM [ppm]	RT [min]	# Missed Cleavages
	High	tESILmSLPPLVR	5	1	1	P72848	N-Term(iTRAQ4plex); M6(Oxidation)	0,0000	1,797	5	52,9	0	4,524E-08	67	2,09411E-07	2	1615,92168	0,41	48,31	0
	High	nPYIPTVHLNYR	6	1	1	P72848	N-Term(iTRAQ4plex)	0,0000	1,977	4	6,2	0	1,16E-08	61	8,0904E-07	3	1630,88230	0,05	33,83	0
	High	gTIFGLQTNGR	3	1	1	P72848	N-Term(iTRAQ4plex)	0,0000	2,021	3	15,4	0	3,823E-08	60	1,04713E-06	2	1307,72002	0,88	32,04	0
	High	aFLPAYSPIVEk	6	1	1	P72848	N-Term(iTRAQ4plex); K12(iTRAQ4plex)	0,0000	1,643	6	44,3	0	0,00004241	59	1,13493E-06	2	1622,94255	1,79	41,52	0
	High	tESILMSLPPLVR	4	1	1	P72848	N-Term(iTRAQ4plex)	0,0000	1,783	4	12,4	0	0,000001144	54	4,41763E-06	2	1599,92815	1,28	51,82	0
	High	sLPPSILk	4	1	1	P72848	N-Term(iTRAQ4plex); K8(iTRAQ4plex)	0,0000	1,524	4	23,1	0	0,0007314	52	6,50085E-06	2	1142,73979	0,92	29,36	0
	High	wcDEYFYLk	2	1	1	P72848	N-Term(iTRAQ4plex); C2(Carbamidomethyl); K9(iTRAQ4plex)	0,0000	2,290	2	29,3	0	8,379E-07	51	7,53304E-06	2	1611,77849	1,80	43,47	0
	High	qRPEAEGHEFYATGTSmVLHPk	4	1	1	P72848	N-Term(iTRAQ4plex); M17(Oxidation); K22(iTRAQ4plex)	0,0000	2,153	2	17,0	0	3,143E-16	49	1,25594E-05	4	2789,39297	0,34	26,20	0
	High	qRPEAEGHEFYATGTSMVLHPk	3	1	1	P72848	N-Term(iTRAQ4plex); K22(iTRAQ4plex)	0,0000	2,315	3	19,3	0	1,582E-18	46	2,26986E-05	4	2773,39834	0,45	30,40	0
	High	nAcDQTHGEFYPVFk	3	1	1	P72848	N-Term(iTRAQ4plex); C3(Carbamidomethyl); K15(iTRAQ4plex)	0,0000	2,538	2	14,1	0	1,412E-11	38	0,000167098	3	2101,00797	1,33	32,93	0
	High	gkFQEDSWQR	2	1	1	P72848	N-Term(iTRAQ4plex); K2(iTRAQ4plex)	0,0000	2,505	2	2,2	0	0,000002732	32	0,000610942	3	1568,80716	0,74	19,88	1
	High	rWcDEYFYLk	1	1	1	P72848	N-Term(iTRAQ4plex); C3(Carbamidomethyl); K10(iTRAQ4plex)	0,0000	2,529	1		0	0,000001311	22	0,006179737	3	1767,87979	1,74	35,96	1
	High	qFQLYR	3	1	1	P72848	N-Term(iTRAQ4plex)	0,0000	1,892	3	14,2	0,001	0,01313	34	0,000444601	2	998,55400	0,01	26,88	0
Q55318	Ferredoxin--NADP reductase OS=Synechocystis sp. (strain PCC 6803 / Kazusa) GN=	1061,62	38,74	1	19	19	69	0,999	61	19,2	413	46,3	6,04							
	A3	Sequence	# PSMs	# Proteins	# Protein Groups	Protein Group Accessions	Modifications	ΔCn	114/115	114/115 Count	114/115 Variability [%]	q-Value	PEP	IonScore	Exp Value	Charge	MH+ [Da]	ΔM [ppm]	RT [min]	# Missed Cleavages
	High	mYSPGYVATSSR	4	1	1	Q55318	N-Term(iTRAQ4plex); M1(Oxidation)	0,0000	1,144	2	1,2	0	1,767E-09	74	3,95339E-08	2	1478,70818	1,05	20,18	0
	High	gLAWLIFGIPk	5	1	1	Q55318	N-Term(iTRAQ4plex); K11(iTRAQ4plex)	0,0000	1,177	4	14,6	0	1,285E-08	69	1,29411E-07	2	1502,93511	0,88	57,82	0
	High	sGSTFITVPLkR	3	1	1	Q55318	N-Term(iTRAQ4plex); K11(iTRAQ4plex)	0,0000	0,887	3	1,4	0	9,842E-07	63	4,75302E-07	2	1593,95781	0,70	30,79	1
	High	lYSIASTR	4	1	1	Q55318	N-Term(iTRAQ4plex)	0,0000	0,894	3	0,4	0	0,0002066	62	5,92884E-07	2	1054,60149	0,14	26,25	0
	High	kADDIPVNIYRPk	10	1	1	Q55318	N-Term(iTRAQ4plex); K1(iTRAQ4plex); K13(iTRAQ4plex)	0,0000	1,118	10	20,5	0	8,315E-15	59	1,36449E-06	3	1961,15403	-0,22	26,66	1
	High	lTYAISR	4	1	1	Q55318	N-Term(iTRAQ4plex)	0,0000	0,909	3	1,0	0	0,001829	46	2,49442E-05	2	967,57219	2,97	25,49	0
	High	vLENYPLVR	4	1	1	Q55318	N-Term(iTRAQ4plex)	0,0000	0,834	3	2,8	0	0,000001337	43	5,12826E-05	2	1246,72795	0,26	34,09	0
	High	hGDFGDDkTVSLcVR	4	1	1	Q55318	N-Term(iTRAQ4plex); K8(iTRAQ4plex); C13(Carbamidomethyl)	0,0000	0,907	4	15,1	0	1,208E-08	37	0,000178636	4	1994,00515	2,36	25,75	1
	High	tMTTTPk	2	1	1	Q55318	N-Term(iTRAQ4plex); K7(iTRAQ4plex)	0,0000	1,278	2	5,8	0	0,003	36	0,000266054	2	1067,60625	4,98	14,11	0
	High	aDDIPVNIYRPk	1	1	1	Q55318	N-Term(iTRAQ4plex); K12(iTRAQ4plex)	0,0000	1,195	1		0	5,887E-07	36	0,00031024	3	1688,95713	-0,18	30,37	0
	High	tmTTTPk	7	1	1	Q55318	N-Term(iTRAQ4plex); M2(Oxidation); K7(iTRAQ4plex)	0,0000	1,045	5	13,8	0	0,0006136	34	0,000400839	2	1083,59599	0,14	8,79	0
	High	sENILYkDDLEk	3	1	1	Q55318	N-Term(iTRAQ4plex); K7(iTRAQ4plex); K12(iTRAQ4plex)	0,0000	0,935	3	9,5	0	8,644E-08	34	0,000432484	3	1899,04337	-0,10	27,50	1
	High	yLEGQSIGIIPPGEDDkGkPHk	1	1	1	Q55318	N-Term(iTRAQ4plex); K17(iTRAQ4plex); K19(iTRAQ4plex); K22(iTRAQ4plex)	0,0000	1,135	1		0	2,087E-10	28	0,001428795	4	2954,64907	1,91	30,54	1
	High	tVSLcVR	2	1	1	Q55318	N-Term(iTRAQ4plex); C5(Carbamidomethyl)	0,0000	0,748	2	4,7	0	0,001723	28	0,001973246	2	978,55235	0,06	22,24	0
	High	eQHEDYkFk	2	1	1	Q55318	N-Term(iTRAQ4plex); K7(iTRAQ4plex); K9(iTRAQ4plex)	0,0000	0,957	2	6,9	0	0,0001412	21	0,008769403	3	1655,87400	-0,82	14,12	1
	High	mFkEQHEDYk	2	1	1	Q55318	N-Term(iTRAQ4plex); M1(Oxidation); K3(iTRAQ4plex); K10(iTRAQ4plex)	0,0000	1,020	2	3,1	0	0,00000266	21	0,008789618	3	1802,91007	-0,39	14,20	1
	High	tHTYmcGLk	2	1	1	Q55318	N-Term(iTRAQ4plex); M5(Oxidation); C6(Carbamidomethyl); K9(iTRAQ4plex)	0,0000	1,147	2	11,7	0	0,0001939	20	0,010068621	3	1414,71558	6,68	12,84	0
	High	mFkEQHEDYkFk	1	1	1	Q55318	N-Term(iTRAQ4plex); M1(Oxidation); K3(iTRAQ4plex); K10(iTRAQ4plex); K1:	0,0000	0,983	1		0	0,00001385	11	0,071116439	4	2222,17544	-0,35	18,77	2
	High	aFLWR	5	1	1	Q55318	N-Term(iTRAQ4plex)	0,0000	0,869	5	5,4	0,007	0,09017	25	0,003459155	2	836,49047	0,61	33,04	0
	Medium	tPYIGk	3	1	1	Q55318	N-Term(iTRAQ4plex); K6(iTRAQ4plex)	0,0000	0,968	3	9,3	0,016	0,1803	20	0,01358714	2	966,59172	5,66	19,00	0
P19569	Photosystem I reaction center subunit II OS=Synechocystis sp. (strain PCC 6803 / I	1048,89	52,48	1	8	8	48	0,671	41	35,2	141	15,6	8,90							
	A3	Sequence	# PSMs	# Proteins	# Protein Groups	Protein Group Accessions	Modifications	ΔCn	114/115	114/115 Count	114/115 Variability [%]	q-Value	PEP	IonScore	Exp Value	Charge	MH+ [Da]	ΔM [ppm]	RT [min]	# Missed Cleavages
	High	fGGSTGGLLSk	9	1	1	P19569	N-Term(iTRAQ4plex); K11(iTRAQ4plex)	0,0000	0,707	7	23,6	0	4,18E-08	83	5,09296E-09	2	1311,75151	0,30	30,25	0
	High	kEQcLALGTQLR	5	1	1	P19569	N-Term(iTRAQ4plex); K1(iTRAQ4plex); C4(Carbamidomethyl)	0,0000	0,665	5	5,2	0	0,000000112	71	7,96104E-08	2	1704,97124	2,51	24,75	1
	High	iGQNPEPVTIk	5	1	1	P19569	N-Term(iTRAQ4plex); K11(iTRAQ4plex)	0,0000	0,590	5	7,4	0	8,749E-08	58	1,74658E-06	2	1483,87309	0,54	27,52	0
	High	tELSGQPPk	8	1	1	P19569	N-Term(iTRAQ4plex); K9(iTRAQ4plex)	0,0000	0,867	6	7,6	0	0,00006341	56	2,5846E-06	2	1244,71477	4,71	16,77	0
	High	vYPSGEVQYLHPADGVFPEk	4	1	1	P19569	N-Term(iTRAQ4plex); K20(iTRAQ4plex)	0,0000	0,486	4	19,1	0	3,514E-11	49	1,28224E-05	3	2520,30246	0,43	38,93	0
	High	rIGQNPEPVTIk	13	1	1	P19569	N-Term(iTRAQ4plex); K12(iTRAQ4plex)	0,0000	0,600	10	34,1	0	0,000007962	44	4,2655E-05	2	1639,97356	0,09	21,64	1
	High	vYPSGEVQYLHPADGVFPEkVNEGR	1	1	1	P19569	N-Term(iTRAQ4plex); K20(iTRAQ4plex)	0,0000	0,533	1		0	3,08E-11	39	0,000130308	4	3075,58047	0,83	38,08	1
	High	iQDYk	3	1	1	P19569	N-Term(iTRAQ4plex); K5(iTRAQ4plex)	0,0000	0,671	3	4,6	0,004	0,05301	21	0,007128038	2	954,55144	1,66	17,49	0
P72797	Transaldolase OS=Synechocystis sp. (strain PCC 6803 / Kazusa) GN=tal PE=3 SV=1	1045,40	49,10	1	19	19	43	0,938	35	14,4	391	43,1	5,06							
	A3	Sequence	# PSMs	# Proteins	# Protein Groups	Protein Group Accessions	Modifications	ΔCn	114/115	114/115 Count	114/115 Variability [%]	q-Value	PEP	IonScore	Exp Value	Charge	MH+ [Da]	ΔM [ppm]	RT [min]	# Missed Cleavages
	High	hGAEEIFHAYDLDGDGFITR	2	1	1	P72797	N-Term(iTRAQ4plex)	0,0000	0,874	2	12,7	0	3,661E-12	76	2,4042E-08	3	2407,14402	0,18	41,22	0
	High	lAEGIAGFTk	1	1	1	P72797	N-Term(iTRAQ4plex); K10(iTRAQ4plex)	0,0000	1,034	1		0	9,949E-07	75	4,37856E-08	2	1294,76409	2,42	34,59	0

	High	iTAAEmSAGLGAAFR	1	1	1	P72797	N-Term(iTRAQ4plex); M6(Oxidation)	0,0000	1,016	1		0	1,915E-11	69	1,22453E-07	2	1625,84599	1,32	35,67	0
	High	akDEAGENASIk	6	1	1	P72797	N-Term(iTRAQ4plex); K2(iTRAQ4plex); K12(iTRAQ4plex)	0,0000	1,062	3	1,5	0	7,691E-09	60	8,93305E-07	3	1664,91703	-0,57	13,59	1
	High	akDEAGENASIkDIVR	2	1	1	P72797	N-Term(iTRAQ4plex); K2(iTRAQ4plex); K12(iTRAQ4plex)	0,0000	0,980	1		0	4,287E-11	60	9,51019E-07	3	2148,19956	0,47	24,04	2
	High	iASTWEGIk	1	1	1	P72797	N-Term(iTRAQ4plex); K9(iTRAQ4plex)	0,0000	0,919	1		0	4,223E-07	54	3,7151E-06	2	1292,74565	0,28	33,00	0
	High	iLQIIPGR	5	1	1	P72797	N-Term(iTRAQ4plex)	0,0000	0,860	4	2,1	0	0,00007028	53	4,46653E-06	2	1053,69084	0,68	37,82	0
	High	aAEVLEQEGIHcNLTLLFGLHQAIAcAEAk	1	1	1	P72797	N-Term(iTRAQ4plex); C12(Carbamidomethyl); C26(Carbamidomethyl); K30(i	0,0000	1,486	1		0	5,313E-10	53	5,95248E-06	4	3594,86880	0,99	55,96	0
	High	yLIGEYAk	2	1	1	P72797	N-Term(iTRAQ4plex); K8(iTRAQ4plex)	0,0000	0,943	2	4,1	0	0,00001067	51	8,6575E-06	2	1244,71257	-0,27	31,79	0
	High	nLLEQLR	3	1	1	P72797	N-Term(iTRAQ4plex)	0,0000	1,244	3	32,2	0	0,0001348	49	1,18569E-05	2	1029,61809	0,72	32,38	0
	High	kLDPATVPQDIEk	1	1	1	P72797	N-Term(iTRAQ4plex); K1(iTRAQ4plex); K13(iTRAQ4plex)	0,0000	0,687	1		0	1,651E-09	43	5,34527E-05	3	1886,09653	0,31	28,45	1
	High	lAVAFGLk	4	1	1	P72797	N-Term(iTRAQ4plex); K8(iTRAQ4plex)	0,0000	0,913	3	4,6	0	0,00004982	43	5,4572E-05	2	1106,71697	-0,60	38,86	0
	High	iLDWYk	3	1	1	P72797	N-Term(iTRAQ4plex); K6(iTRAQ4plex)	0,0000	0,938	3	8,9	0	0,004026	41	8,22186E-05	2	1125,65361	-0,94	37,58	0
	High	vTLISPFVGR	3	1	1	P72797	N-Term(iTRAQ4plex)	0,0000	0,872	2	3,6	0	0,00000102	41	8,8914E-05	2	1232,74883	0,37	42,56	0
	High	mHAEDPmASEk	3	1	1	P72797	N-Term(iTRAQ4plex); M1(Oxidation); M7(Oxidation); K11(iTRAQ4plex)	0,0000	0,856	2	1,9	0	4,331E-07	37	0,000189221	3	1565,71817	0,20	8,77	0
	High	vLDGQEHIk	1	1	1	P72797	N-Term(iTRAQ4plex); K9(iTRAQ4plex)	0,0000	0,940	1		0	0,0004005	33	0,000465554	3	1326,76178	-0,17	19,45	0
	High	tEVmGASFR	2	1	1	P72797	N-Term(iTRAQ4plex); M4(Oxidation)	0,0000	0,892	2	9,1	0	0,00008196	32	0,000638219	2	1157,57414	-0,03	18,84	0
	High	gkNLLEQLR	1	1	1	P72797	N-Term(iTRAQ4plex); K2(iTRAQ4plex)	0,0000	1,364	1		0	0,000152	24	0,003672569	3	1358,83500	-0,63	28,15	1
	High	iLDWYkk	1	1	1	P72797	N-Term(iTRAQ4plex); K6(iTRAQ4plex); K7(iTRAQ4plex)	0,0000	0,857	1		0	0,004042	23	0,005151931	3	1397,84955	-1,54	31,29	1
P73418	DNA-binding protein HU OS=Synechocystis sp. (strain PCC 6803 / Kazusa) GN=hup	1024,91	58,00	1	11	11	57	1,738	44	21,6	100	10,9	8,63							
	A3	Sequence	# PSMs	# Proteins	# Protein Groups	Protein Group Accessions	Modifications	ΔCn	114/115	114/115 Count	114/115 Variability [%]	q-Value	PEP	IonScore	Exp Value	Charge	MH+ [Da]	ΔM [ppm]	RT [min]	# Missed Cleavages
	High	eQLGESITk	2	1	1	P73418	N-Term(iTRAQ4plex); K9(iTRAQ4plex)	0,0000	1,737	2	0,2	0	2,383E-07	58	1,45536E-06	2	1292,73210	1,60	21,69	0
	High	eQLGESITkk	5	1	1	P73418	N-Term(iTRAQ4plex); K9(iTRAQ4plex); K10(iTRAQ4plex)	0,0000	1,601	5	14,2	0	0,00002129	58	1,46208E-06	2	1564,93425	4,59	18,12	1
	High	gELVHAVTDk	9	1	1	P73418	N-Term(iTRAQ4plex); K10(iTRAQ4plex)	0,0000	1,855	6	3,7	0	7,017E-08	56	2,69135E-06	2	1356,77629	2,74	22,50	0
	High	vTLVGFGSFEAR	6	1	1	P73418	N-Term(iTRAQ4plex)	0,0000	1,306	6	13,1	0	1,825E-08	55	3,09721E-06	2	1426,78337	1,56	44,04	0
	High	vPAFSAGk	3	1	1	P73418	N-Term(iTRAQ4plex); K8(iTRAQ4plex)	0,0000	1,468	3	1,3	0	0,001082	48	1,72861E-05	2	1064,63994	5,31	24,44	0
	High	akEQLGESITkk	1	1	1	P73418	N-Term(iTRAQ4plex); K2(iTRAQ4plex); K11(iTRAQ4plex); K12(iTRAQ4plex)	0,0000	1,480	1		0	9,478E-12	46	2,45454E-05	3	1908,16294	0,90	18,89	2
	High	akEQLGESITk	2	1	1	P73418	N-Term(iTRAQ4plex); K2(iTRAQ4plex); K11(iTRAQ4plex)	0,0000	1,950	2	2,3	0	6,266E-07	42	6,80722E-05	3	1635,96787	2,25	19,56	1
	High	mNkGELVHAVTDk	6	1	1	P73418	N-Term(iTRAQ4plex); M1(Oxidation); K3(iTRAQ4plex); K13(iTRAQ4plex)	0,0000	1,990	4	15,2	0	4,824E-09	32	0,0005662	3	1890,05179	2,03	22,32	1
	High	mNkGELVHAVTDk	4	1	1	P73418	N-Term(iTRAQ4plex); K3(iTRAQ4plex); K13(iTRAQ4plex)	0,0000	2,153	4	7,4	0	7,641E-07	24	0,004588786	3	1874,05497	1,03	27,50	1
	High	mLIPATk	6	1	1	P73418	N-Term(iTRAQ4plex); M1(Oxidation); K7(iTRAQ4plex)	0,0000	1,731	3	3,5	0	0,005893	23	0,0054098	2	1077,65911	0,97	21,25	0
	High	mLIPATk	3	1	1	P73418	N-Term(iTRAQ4plex); K7(iTRAQ4plex)	0,0000	1,640	2	6,0	0,001	0,01431	17	0,019997237	2	1061,66667	3,32	27,16	0
	High	vAPEkk	8	1	1	P73418	N-Term(iTRAQ4plex); K5(iTRAQ4plex); K6(iTRAQ4plex)	0,0000	2,136	5	2,6	0,002	0,02758	16	0,02409739	2	1103,71269	-1,98	9,12	1
	High	vAPEk	2	1	1	P73418	N-Term(iTRAQ4plex); K5(iTRAQ4plex)	0,0000	2,058	1		0,007	0,08591	17	0,019283653	2	831,52007	2,67	13,33	0
P73308	50S ribosomal protein L5 OS=Synechocystis sp. (strain PCC 6803 / Kazusa) GN=rpl	1005,21	61,11	1	13	13	45	1,372	41	51,3	180	20,2	9,69							
	A3	Sequence	# PSMs	# Proteins	# Protein Groups	Protein Group Accessions	Modifications	ΔCn	114/115	114/115 Count	114/115 Variability [%]	q-Value	PEP	IonScore	Exp Value	Charge	MH+ [Da]	ΔM [ppm]	RT [min]	# Missed Cleavages
	High	aLESSLTELATITGQkPVVTR	5	1	1	P73308	N-Term(iTRAQ4plex); K16(iTRAQ4plex)	0,0000	0,890	5	47,0	0	2,233E-13	69	1,40595E-07	3	2502,44016	0,78	47,96	0
	High	gLGEASQNAk	2	1	1	P73308	N-Term(iTRAQ4plex); K10(iTRAQ4plex)	0,0000	2,558	1		0	1,742E-07	60	1,02322E-06	2	1262,70256	6,52	14,60	0
	High	tLYQETILPk	5	1	1	P73308	N-Term(iTRAQ4plex); K10(iTRAQ4plex)	0,0000	1,740	5	44,8	0	4,186E-08	59	1,13232E-06	2	1493,88323	0,97	35,43	0
	High	lINLALPR	9	1	1	P73308	N-Term(iTRAQ4plex)	0,0000	1,309	8	49,2	0	0,00005725	59	1,15603E-06	2	1053,69097	0,80	41,62	0
	High	lQEEFGYk	2	1	1	P73308	N-Term(iTRAQ4plex); K8(iTRAQ4plex)	0,0000	1,385	2	95,4	0	0,00002441	53	5,41963E-06	2	1301,69963	1,26	28,81	0
	High	gNYSLGIR	3	1	1	P73308	N-Term(iTRAQ4plex)	0,0000	1,784	3	7,9	0	0,000201	51	9,35813E-06	2	1023,57842	7,85	23,93	0
	High	nIHQVPk	4	1	1	P73308	N-Term(iTRAQ4plex); K7(iTRAQ4plex)	0,0000	1,090	4	55,3	0	0,0002319	46	2,51171E-05	2	1123,68205	-0,50	11,48	0
	High	gISPNSFDGR	1	1	1	P73308	N-Term(iTRAQ4plex)	0,0000	1,529	1		0	0,00106	39	0,000119942	2	1193,60405	0,74	23,86	0
	High	aLGMPFR	2	1	1	P73308	N-Term(iTRAQ4plex)	0,0000	1,048	2	18,7	0	0,004012	35	0,000364074	2	935,52599	0,66	31,63	0
	High	kAIAGFk	5	1	1	P73308	N-Term(iTRAQ4plex); K1(iTRAQ4plex); K7(iTRAQ4plex)	0,0000	1,798	3	20,0	0	0,0006965	32	0,00064561	3	1166,76175	-0,36	18,53	1
	High	eGmPVGVmVTLR	1	1	1	P73308	N-Term(iTRAQ4plex); M3(Oxidation); M8(Oxidation)	0,0000	2,160	1		0	0,005401	17	0,01844888	2	1464,76970	1,72	28,84	0
	High	iREGmPVGVmVTLR	1	1	1	P73308	N-Term(iTRAQ4plex); M5(Oxidation); M10(Oxidation)	0,0000	1,023	1		0,003	0,03348	21	0,00886539	3	1733,94303	-5,38	28,53	1
	High	aLGmPFR	3	1	1	P73308	N-Term(iTRAQ4plex); M4(Oxidation)	0,0000	1,143	3	2,2	0,004	0,04822	15	0,033342641	2	951,52062	0,34	23,15	0
	High	aIAGFk	2	1	1	P73308	N-Term(iTRAQ4plex); K6(iTRAQ4plex)	0,0000	1,666	2	33,6	0,008	0,09424	35	0,000353973	2	894,56462	-0,58	21,17	0
P26287	Apocytochrome f OS=Synechocystis sp. (strain PCC 6803 / Kazusa) GN=petA PE=3	1003,73	28,96	1	11	11	47	1,461	46	25,0	328	35,2	5,16							
	A3	Sequence	# PSMs	# Proteins	# Protein Groups	Protein Group Accessions	Modifications	ΔCn	114/115	114/115 Count	114/115 Variability [%]	q-Value	PEP	IonScore	Exp Value	Charge	MH+ [Da]	ΔM [ppm]	RT [min]	# Missed Cleavages
	High	gQIYPTGLLSNNNAFk	12	1	1	P26287	N-Term(iTRAQ4plex); K16(iTRAQ4plex)	0,0000	1,335	11	53,5	0	2,438E-14	90	8,99435E-10	2	2025,10454	1,85	41,04	0
	High	vGGTYFQPYR	4	1	1	P26287	N-Term(iTRAQ4plex)	0,0000	1,924	4	11,2	0	0,00001657	58	1,58478E-06	2	1331,68706	0,44	31,06	0
	High	fAVHLGANR	11	1	1	P26287	N-Term(iTRAQ4plex)	0,0000	1,345	11	13,3	0	0,000003951	54	3,73224E-06	2	1128,63982	0,30	23,12	0
	High	gGLNVGAVLmLPEGFk	2	1	1	P26287	N-Term(iTRAQ4plex); M10(Oxidation); K16(iTRAQ4plex)	0,0000	1,168	2	4,2	0	3,202E-07	52	6,223E-06	3	1906,07352	1,26	50,71	0
	High	lSEGLkEk	5	1	1	P26287	N-Term(iTRAQ4plex); K6(iTRAQ4plex); K8(iTRAQ4plex)	0,0000	1,743	5	0,6	0	0,0001404	42	6,44125E-05	2	1335,83000	6,88	16,83	1
	High	iVcANcHLAQk	1	1	1	P26287	N-Term(iTRAQ4plex); C3(Carbamidomethyl); C6(Carbamidomethyl); K11(iTR	0,0000	1,732	1		0	1,938E-09	37	0,000217756	3	1601,85074	0,80	19,96	0
	High	vQAAELNF	1	1	1	P26287	N-Term(iTRAQ4plex)	0,0000	0,914	1		0	0,0003488	31	0,000765544	2	1035,55889	-0,27	38,16	0
	High	sINYGk	3	1	1	P26287	N-Term(iTRAQ4plex); K6(iTRAQ4plex)	0,0000	1,627	3	0,6	0	0,007853	25	0,002850821	2	969,56639	5,81	15,56	0
	High	gGLNVGAVLMLPEGFk	1	1	1	P26287	N-Term(iTRAQ4plex); K16(iTRAQ4plex)	0,0000	0,905	1		0	0,0002412	22	0,006537214	3	1890,07669	0,26	53,54	0
	Medium	lSEGLk	4	1	1	P26287	N-Term(iTRAQ4plex); K6(iTRAQ4plex)	0,0000	1,534	4	4,1	0,017	0,182	24	0,004690091	2	934,58330	2,27	18,15	0
	Medium	iAPPDR	2	1	1	P26287	N-Term(iTRAQ4plex)	0,0000	1,461	2	5,0	0,029	0,3132	16	0,023931505	2	812,47502	0,39	16,00	0
	Medium	kQIEk	1	1	1	P26287	N-Term(iTRAQ4plex); K1(iTRAQ4plex); K5(iTRAQ4plex)	0,0000	1,245	1		0,036	0,3756	10	0,098848482	3	1077,69834	-0,82	9,58	1
P52208	6-phosphogluconate dehydrogenase, decarboxylating OS=Synechocystis sp. (strain	995,79	23,44	1	10	10	36	0,510	31	26,9	482	52,8	5,22							
	A3	Sequence	# PSMs	# Proteins	# Protein Groups	Protein Group Accessions	Modifications	ΔCn	114/115	114/115 Count	114/115 Variability [%]	q-Value	PEP	IonScore	Exp Value	Charge	MH+ [Da]	ΔM [ppm]	RT [min]	# Missed Cleavages
	High	vAASGQLSGPSk	9	1	1	P52208	N-Term(iTRAQ4plex); K12(iTRAQ4plex)	0,0000	0,711	6	35,0	0	3,736E-10	86	2,66667E-09	2	1389,80181	5,58	19,47	0
	High	aAYTVEEFVQLLERPR	2	1	1	P52208	N-Term(iTRAQ4plex)	0,0000	0,420	2	1,1	0	2,537E-08	81	1,01604E-08	2	2065,12163	0,82	55,04	0
	High	aVLPQNLTQAQR	3	1	1	P52208	N-Term(iTRAQ4plex)	0,0000	0,459	3	1,2	0	2,323E-07	71	7,7124E-08	2	1482,85039	-0,37	28,07	0
	High	gFPIAVFNR	3	1	1	P52208	N-Term(iTRAQ4plex)	0,0000	0,427	3	0,4	0	5,794E-07	62	7,09887E-07	2	1164,66350	-0,98	42,19	0
	High	aFkDNPQLPNLLLAPEFk	4	1	1	P52208	N-Term(iTRAQ4plex); K3(iTRAQ4plex); K18(iTRAQ4plex)	0,0000	0,633	4	19,3	0	7,107E-09	48	1,60683E-05	3	2487,43521	0,64	47,89	1
	High	vmSSLkEER	2	1	1	P52208	N-Term(iTRAQ4plex); M2(Oxidation); K6(iTRAQ4plex)	0,0000	0,506	2	12,3	0	0,002641	43	4,64483E-05	2	1382,76238	5,17	12,77	1
	High	kDPETGGHLIDYILDAAGQk	2	1	1	P52208	N-Term(iTRAQ4plex); K1(iTRAQ4plex); K20(iTRAQ4plex)	0,0000	0,647	2	13,4	0	9,205E-09	42	5,94251E-05	3	2573,39481	0,47	45,20	1
	High	kILVMVk	3	1	1	P52208	N-Term(iTRAQ4plex); K1(iTRAQ4plex); K7(iTRAQ4plex)	0,0000	0,390	2	27,6	0	0,005265	34	0,00039808	3	1262,85477	-3,71	26,03	1
	High	dYFGAHTYER	1	1	1	P52208	N-Term(iTRAQ4plex)	0,0000	0,634	1		0	0,00001009	33	0,000509296	3	1402,65021	-0,43	22,70	0
	High	kILVmVk	4	1	1	P52208	N-Term(iTRAQ4plex); K1(iTRAQ4plex); M5(Oxidation); K7(iTRAQ4plex)	0,0000	0,534	3	2,9	0	0,003025	27	0,002218043	3	1278,84748	-5,39	20,69	1
	High	iLVMVk	1	1	1	P52208	N-Term(iTRAQ4plex); K6(iTRAQ4plex)	0,0000	1,270	1		0,002	0,01937	38	0,000169032	2	990,65892	-3,53	32,41	0
	High	vMSSLkEER	2	1	1	P52208	N-Term(iTRAQ4plex); K6(iTRAQ4plex)	0,0000	0,453	2	2,9	0,004	0,04517	22	0,006367515	3	1366,75769	-1,91	19,98	1
P29254	Photosystem I P700 chlorophyll a apoprotein A1 OS=Synechocystis sp. (strain PCC	989,65	13,72	1	13	13	68	0,507	59	24,9	751	82,9	7,77							
	A3	Sequence	# PSMs	# Proteins	# Protein Groups	Protein Group Accessions	Modifications	ΔCn	114/115	114/115 Count	114/115 Variability [%]	q-Value	PEP	IonScore	Exp Value	Charge	MH+ [Da]	ΔM [ppm]	RT [min]	# Missed Cleavages
	High	nVNNLLDR	4	1	1	P29254	N-Term(iTRAQ4plex)	0,0000	0,448	3	5,3	0	0,000005921	65	3,22827E-07	2	1101,61272	-0,55	24,74	0
	High	dIPLPHEFILEPSk	5	1	1	P29254	N-Term(iTRAQ4plex); K14(iTRAQ4plex)	0,0000	0,517	4	14,4	0	1,117E-12	65	3,43534E-07	3	1923,08182	-0,63	42,59	0
	High	lLDAGVAPk	5	1	1	P29254	N-Term(iTRAQ4plex); K9(iTRAQ4plex)	0,0000	0,817	4	9,6	0	0,000001725	58	1,68256E-06	2	1171,72820	-0,62	27,85	0
	High	tNWGIGHSmk	5	1	1	P29254	N-Term(iTRAQ4plex); M9(Oxidation); K10(iTRAQ4plex)	0,0000	0,506	4	11,1	0	4,376E-07	53	5,33298E-06	2	1434,74748	5,05	16,67	0
	High	aLSIIQGR	7	1	1	P29254	N-Term(iTRAQ4plex)	0,0000	0,444	5	3,7	0	0,0001203	49	1,13232E-05	2	1001,62114	-1,29	29,25	0
	High	lNVAPAIQPR	9	1	1	P29254	N-Term(iTRAQ4plex)	0,0000	0,645	9	32,4	0	0,000004568	44	4,31489E-05	2	1222,73796	-0,75	28,60	0
	High	gPFTGEGHk	7	1	1	P29254	N-Term(iTRAQ4plex); K9(iTRAQ4plex)	0,0000	0,465	7	10,8	0	0,000005537	42	6,62171E-05	2	1217,65740	4,66	12,69	0
	High	eILEAHk	8	1	1	P29254	N-Term(iTRAQ4plex); K7(iTRAQ4plex)	0,0000	0,570	7	36,6	0	0,0009318	40	0,000106652	2	1127,66472	-1,41	16,36	0
	High	lVPDkANLGFR	2	1	1	P29254	N-Term(iTRAQ4plex); K5(iTRAQ4plex)	0,0000	0,440	2	1,8	0	0,0002859	30	0,001091365	3	1517,90421	-0,05	32,23	1
	High	tNWGIGHSMk	2	1	1	P29254	N-Term(iTRAQ4plex); K10(iTRAQ4plex)	0,0000	0,524	2	11,7	0	6,598E-07	29	0,00135822	3	1418,74427	-0,74	22,52	0
	High	eILEAHkGPFTGEGHk	4	1	1	P29254	N-Term(iTRAQ4plex); K7(iTRAQ4plex); K16(iTRAQ4plex)	0,0000	0,400	2	1,2	0	1,949E-10	28	0,001702159	4	2182,19765	-0,22	21,59	1
	High	wGkPGHFDR	3	1	1	P29254	N-Term(iTRAQ4plex); K3(iTRAQ4plex)	0,0000	0,415	3	1,3	0	0,001787	17	0,020557486	2	1387,74663	-0,53	18,11	0
	High	aNLGFR	3	1	1	P29254	N-Term(iTRAQ4plex)	0,0000	0,584	3	21,2	0,001	0,01055	30	0,00097717	2	821,47960	5,56	20,29	0
	High	fPcDGPGR	4	1	1	P29254	N-Term(iTRAQ4plex); C3(Carbamidomethyl)	0,0000	1,047	4	13,9	0,002	0,01904	13	0,051046974	2	1049,50200	6,17	18,26	0
P73785	Phosphate-binding protein OS=Synechocystis sp. (strain PCC 6803 / Kazusa) GN=p	966,12	46,55	1	14	14	30	1,741	27	13,7	333	35,3	9,92							
	A3	Sequence	# PSMs	# Proteins	# Protein Groups	Protein Group Accessions	Modifications	ΔCn	114/115	114/115 Count	114/115 Variability [%]	q-Value	PEP	IonScore	Exp Value	Charge	MH+ [Da]	ΔM [ppm]	RT [min]	# Missed Cleavages
	High	aTGNTVNYNSVGSGAGIR	4	1	1	P73785	N-Term(iTRAQ4plex)	0,0000	1,468	4	42,4	0	2,959E-13	112	6,66761E-12	2	1881,95708	1,86	25,98	0
	High	gVVmVPTAGGAVAVYNLPGkk	4	1	1	P73785	N-Term(iTRAQ4plex); M4(Oxidation); K21(iTRAQ4plex); K22(iTRAQ4plex)	0,0000	0,835	1		0	3,324E-10	87	1,89221E-09	4	2575,50625	2,16	41,82	1
	High	qTPGAIGYVQDTFAR	2	1	1	P73785	N-Term(iTRAQ4plex)	0,0000	1,727	2	6,8	0	1,596E-11	77	1,81121E-08	2	1767,91728	1,48	38,75	0
	High	aDGSGTTDIFTSHLSAISSTFR	1	1	1	P73785	N-Term(iTRAQ4plex)	0,0000	1,813	1		0	7,765E-10	73	4,86374E-08	3	2415,19608	2,13	46,90	0
	High	rNEGPNLQTAk	8	1	1	P73785	N-Term(iTRAQ4plex); K11(iTRAQ4plex)	0,0000	1,675	5	5,8	0	1,628E-07	61	8,51079E-07	2	1515,85466	4,26	13,63	1
	High	yFAEYkk	2	1	1	P73785	N-Term(iTRAQ4plex); K6(iTRAQ4plex); K7(iTRAQ4plex)	0,0000	1,903	2	0,4	0	0,0006991	49	1,18842E-05	2	1380,79949	7,78	23,05	1
	High	tWNQVDSk	1	1	1	P73785	N-Term(iTRAQ4plex); K8(iTRAQ4plex)	0,0000	1,400	1		0	0,0003365	44	4,45625E-05	2	1265,68437	9,11	18,50	0
	High	wILTTGQNHNTQLEYTk	1	1	1	P73785	N-Term(iTRAQ4plex); K17(iTRAQ4plex)	0,0000	1,918	1		0	3,039E-07	40	9,73171E-05	3	2335,23355	2,16	34,44	0
	High	vIAAVNk	2	1	1	P73785	N-Term(iTRAQ4plex); K7(iTRAQ4plex)	0,0000	1,743	2	4,5	0	0,002926	31	0,000776194	2	1002,65990	4,87	19,66	0
	High	yFAEYk	1	1	1	P73785	N-Term(iTRAQ4plex); K6(iTRAQ4plex)	0,0000	1,930	1		0,001	0,01338	20	0,00948353	2	1108,59990	7,38	27,20	0
	High	iFTGELk	3	1	1	P73785	N-Term(iTRAQ4plex); K7(iTRAQ4plex)	0,0000	1,744	3	1,0	0,002	0,02369	34	0,000358072	2	1095,67082	5,09	30,56	0
	Medium	nANVAk	1	1	1	P73785	N-Term(iTRAQ4plex); K6(iTRAQ4plex)	0,0000	2,087	1		0,019	0,2003	26	0,00232793	2	904,55235	7,63	11,15	0
	Medium	lPNTPIR	2	1	1	P73785	N-Term(iTRAQ4plex)	0,0000	1,121	2	3,3	0,039	0,4054	20	0,010161785	2	954,58678	1,53	24,70	0
	Medium	iPPAVAQR	1	1	1	P73785	N-Term(iTRAQ4plex)	0,0000	1,073	1		0,043	0,445	20	0,009615459	2	995,61315	1,28	20,21	0
P73294	50S ribosomal protein L13 OS=Synechocystis sp. (strain PCC 6803 / Kazusa) GN=rp	962,74	75,50	1	9	9	49	2,644	46	93,1	151	17,0	9,99							
	A3	Sequence	# PSMs	# Proteins	# Protein Groups	Protein Group Accessions	Modifications	ΔCn	114/115	114/115 Count	114/115 Variability [%]	q-Value	PEP	IonScore	Exp Value	Charge	MH+ [Da]	ΔM [ppm]	RT [min]	# Missed Cleavages
	High	vYAGPSHPHAAQQPETLVINTIPAGAN	20	1	1	P73294	N-Term(iTRAQ4plex)	0,0000	2,644	19	88,9	0	6,648E-07	78	1,48926E-08	3	2897,50467	0,67	35,44	0
	High	tVLPTIDNLDHk	2	1	1	P73294	N-Term(iTRAQ4plex); K12(iTRAQ4plex)	0,0000	3,898	2	25,7	0	6,788E-10	78	1,56304E-08	3	1653,94205	0,37	33,69	0
	High	lATEVATILR	8	1	1	P73294	N-Term(iTRAQ4plex)	0,0000	1,549	7	33,4	0	7,701E-09	76	2,47725E-08	2	1230,75493	0,87	44,08	0
	High	wYVIDAEGQR	3	1	1	P73294	N-Term(iTRAQ4plex)	0,0000	1,784	3	82,2	0	0,001061	32	0,000698184	2	1380,70488	1,45	35,70	0
	High	iVESAVR	3	1	1	P73294	N-Term(iTRAQ4plex)	0,0000	2,675	3	14,0	0	0,003356	30	0,001030315	2	917,55358	-0,12	18,91	0
	High	hSGRPGGmkEETFEk	3	1	1	P73294	N-Term(iTRAQ4plex); M8(Oxidation); K9(iTRAQ4plex); K15(iTRAQ4plex)	0,0000	10,379	3	5,4	0	0,0001035	24	0,003999171	2	2138,10576	1,51	11,50	1
	High	hSGRPGGMkEETFEk	1	1	1	P73294	N-Term(iTRAQ4plex); K9(iTRAQ4plex); K15(iTRAQ4plex)	0,0000	63,510	1		0	0,001033	20	0,010374568	3	2122,10978	1,02	14,75	1
	High	nkPTFTPHmDTGDFVIIINAEk	1	1	1	P73294	N-Term(iTRAQ4plex); K2(iTRAQ4plex); M9(Oxidation); K22(iTRAQ4plex)	0,0000	1,177	1		0	4,739E-07	12	0,065609995	5	2936,55775	0,83	39,03	0
	High	iEVTGR	5	1	1	P73294	N-Term(iTRAQ4plex)	0,0000	2,390	4	4,7	0,001	0,01477	19	0,016253367	2	818,48528	0,02	16,74	0
	High	kLFTk	3	1	1	P73294	N-Term(iTRAQ4plex); K1(iTRAQ4plex); K5(iTRAQ4plex)	0,0000	11,177	3	227,9	0,001	0,01077	13	0,04709448	3	1068,71445	0,29	16,74	1
P27181	ATP synthase subunit b OS=Synechocystis sp. (strain PCC 6803 / Kazusa) GN=atpF	937,15	41,34	1	14	14	43	1,861	32	19,3	179	19,8	5,14							
	A3	Sequence	# PSMs	# Proteins	# Protein Groups	Protein Group Accessions	Modifications	ΔCn	114/115	114/115 Count	114/115 Variability [%]	q-Value	PEP	IonScore	Exp Value	Charge	MH+ [Da]	ΔM [ppm]	RT [min]	# Missed Cleavages
	High	sIAQLGGR	5	1	1	P27181	N-Term(iTRAQ4plex)	0,0000	1,717	3	0,1	0	0,00001082	70	9,53212E-08	2	945,56597	6,48	19,93	0
	High	rMQEAAAQDLGAEQER	2	1	1	P27181	N-Term(iTRAQ4plex)	0,0000	1,023	2	0,6	0	2,115E-11	66	2,41529E-07	3	1946,94852	0,70	20,30	1
	High	sAQILAEEEk	1	1	1	P27181	N-Term(iTRAQ4plex); K10(iTRAQ4plex)	0,0000	1,783	1		0	1,667E-08	66	2,48869E-07	2	1405,78032	1,85	22,65	0
	High	rmQEAAAQDLGAEQER	3	1	1	P27181	N-Term(iTRAQ4plex); M2(Oxidation)	0,0000	1,575	2	43,8	0	6,164E-10	64	3,70655E-07	3	1962,94352	0,74	15,73	1
	High	iAEQAVAk	6	1	1	P27181	N-Term(iTRAQ4plex); K8(iTRAQ4plex)	0,0000	2,112	3	9,5	0	0,00001774	55	3,38212E-06	2	1117,68999	7,18	18,58	0
	High	kSAQILAEEEk	5	1	1	P27181	N-Term(iTRAQ4plex); K1(iTRAQ4plex); K11(iTRAQ4plex)	0,0000	2,676	3	5,5	0	0,00000794	52	6,23692E-06	3	1677,97739	1,57	19,59	1
	High	sAQILAEEEkk	2	1	1	P27181	N-Term(iTRAQ4plex); K10(iTRAQ4plex); K11(iTRAQ4plex)	0,0000	2,521	1		0	0,000001008	43	4,70945E-05	3	1677,97885	2,44	20,51	1
	High	rIAEQAVAk	6	1	1	P27181	N-Term(iTRAQ4plex); K9(iTRAQ4plex)	0,0000	1,618	5	13,8	0	0,00002172	42	5,88803E-05	2	1273,79375	8,37	13,31	1
	High	kSAQILAEEEkk	3	1	1	P27181	N-Term(iTRAQ4plex); K1(iTRAQ4plex); K11(iTRAQ4plex); K12(iTRAQ4plex)	0,0000	3,215	3	6,9	0	1,083E-08	38	0,000145201	3	1950,18161	5,04	19,06	2
	High	qEIATQTEADLRR	2	1	1	P27181	N-Term(iTRAQ4plex)	0,0000	1,810	2	5,1	0	0,0006446	29	0,001285198	3	1674,89023	0,62	22,57	1
	High	vIAELk	1	1	1	P27181	N-Term(iTRAQ4plex); K6(iTRAQ4plex)	0,0000	1,871	1		0,002	0,02295	30	0,001025581	2	960,63408	0,90	26,19	0
	High	vIAELkR	2	1	1	P27181	N-Term(iTRAQ4plex); K6(iTRAQ4plex)	0,0000	2,020	2	1,7	0,002	0,02	19	0,013994907	3	1116,73688	2,28	21,54	1
	Medium	kLAQAk	2	1	1	P27181	N-Term(iTRAQ4plex); K1(iTRAQ4plex); K6(iTRAQ4plex)	0,0000	1,403	2	17,8	0,013	0,1486	23	0,005140081	2	1090,73027	-0,54	10,05	1
	Medium	aEVAk	1	1	1	P27181	N-Term(iTRAQ4plex); K5(iTRAQ4plex)	0,0000				0,018	0,1927	11	0,265753454	2	805,50261	0,51	11,88	0
	Medium	lAQAk	2	1	1	P27181	N-Term(iTRAQ4plex); K5(iTRAQ4plex)	0,0000	1,755	2	2,5	0,04	0,4163	15	0,030212683	2	818,53368	-0,18	12,64	0
	>sp|K2C1_HUMAN|	905,62	26,44	1	16	17	33	1,512	27	17,0	643	65,8	8,12							
	A3	Sequence	# PSMs	# Proteins	# Protein Groups	Protein Group Accessions	Modifications	ΔCn	114/115	114/115 Count	114/115 Variability [%]	q-Value	PEP	IonScore	Exp Value	Charge	MH+ [Da]	ΔM [ppm]	RT [min]	# Missed Cleavages
	High	fSScGGGGGSFGAGGGFGSR	1	1	1		N-Term(iTRAQ4plex); C4(Carbamidomethyl)	0,0000	2,058	1		0	4,027E-19	131	7,26056E-14	2	1909,83794	0,55	30,17	0
	High	tHNLEPYFESFINNLR	3	1	1		N-Term(iTRAQ4plex)	0,0000	1,793	3	23,3	0	7,086E-12	79	1,51842E-08	3	2138,08170	1,37	51,45	0
	High	sLNNQFASFIDkVR	2	1	1		N-Term(iTRAQ4plex); K12(iTRAQ4plex)	0,0000	1,345	1		0	3,219E-12	67	2,1231E-07	3	1927,06583	0,94	43,95	1
	High	gSGGGSSGGSIGGR	3	1	1		N-Term(iTRAQ4plex)	0,0000	1,379	3	27,7	0	0,000002396	60	1,05189E-06	2	1236,60576	0,64	9,38	0
	High	fLEQQNQVLQTk	1	2	2		N-Term(iTRAQ4plex); K12(iTRAQ4plex)	0,0000				0	2,846E-08	56	2,93561E-06	2	1763,99321	2,13	31,91	0
	High	tNAENEFVTIkk	3	1	1		N-Term(iTRAQ4plex); K11(iTRAQ4plex); K12(iTRAQ4plex)	0,0000	1,585	2	9,9	0	2,528E-11	56	2,75404E-06	3	1826,04068	1,24	25,64	1
	High	sLNNQFASFIDk	1	1	1		N-Term(iTRAQ4plex); K12(iTRAQ4plex)	0,0000	2,156	1		0	4,233E-08	55	2,91052E-06	2	1671,89604	0,93	41,58	0
	High	skAEAESLYQSk	4	1	1		N-Term(iTRAQ4plex); K2(iTRAQ4plex); K12(iTRAQ4plex)	0,0000	1,674	3	18,9	0	2,111E-09	53	5,17571E-06	3	1772,97818	1,53	22,33	1
	High	skAEAESLYQSkYEELQITAGR	1	1	1		N-Term(iTRAQ4plex); K2(iTRAQ4plex); K12(iTRAQ4plex)	0,0000	1,452	1		0	1,288E-11	52	6,29463E-06	4	2933,55630	-0,62	37,57	2
	High	sLVNLGGSk	4	1	1		N-Term(iTRAQ4plex); K9(iTRAQ4plex)	0,0000	1,517	2	0,5	0	0,00000432	52	6,74481E-06	2	1162,70952	5,24	25,63	0
	High	sISISVAR	2	1	1		N-Term(iTRAQ4plex)	0,0000	1,273	2	6,8	0	0,00005357	39	0,000138029	2	976,59087	0,08	24,70	0
	High	lLRDYQELMNTk	1	1	1		N-Term(iTRAQ4plex); K12(iTRAQ4plex)	0,0000	1,213	1		0	3,566E-08	36	0,000275423	3	1811,99283	0,00	37,51	1
	High	lRSEIDNVk	1	1	1		N-Term(iTRAQ4plex); K9(iTRAQ4plex)	0,0000	1,615	1		0	0,00001046	34	0,00037151	3	1361,79993	0,59	21,46	1
	High	rVDQLk	2	1	1		N-Term(iTRAQ4plex); K6(iTRAQ4plex)	0,0000	1,559	2	0,0	0	0,003282	25	0,003295869	3	1046,65711	0,98	12,65	1
	High	nkYEDEINkR	1	1	1		N-Term(iTRAQ4plex); K2(iTRAQ4plex); K9(iTRAQ4plex)	0,0000	1,869	1		0	0,0001817	25	0,003539729	3	1740,96091	0,24	15,75	2
	High	rVDQLkSDQSR	1	1	1		N-Term(iTRAQ4plex); K6(iTRAQ4plex)	0,0000	1,410	1		0	0,001229	18	0,01986924	4	1619,90810	0,81	11,20	2
	High	lRSEIDNVkk	2	1	1		N-Term(iTRAQ4plex); K9(iTRAQ4plex); K10(iTRAQ4plex)	0,0000	1,016	2	3,5	0	0,000583	16	0,024944224	4	1633,99746	0,79	18,84	2
P73128	Sulfolipid biosynthesis protein SqdB OS=Synechocystis sp. (strain PCC 6803 / Kazus	895,98	48,04	1	20	20	41	1,219	34	18,2	383	43,2	6,01							
	A3	Sequence	# PSMs	# Proteins	# Protein Groups	Protein Group Accessions	Modifications	ΔCn	114/115	114/115 Count	114/115 Variability [%]	q-Value	PEP	IonScore	Exp Value	Charge	MH+ [Da]	ΔM [ppm]	RT [min]	# Missed Cleavages
	High	kIDLFIGDINDYPFLTNALR	3	1	1	P73128	N-Term(iTRAQ4plex); K1(iTRAQ4plex)	0,0000	1,205	3	7,6	0	2,163E-14	86	2,54665E-09	3	2626,45108	1,03	56,35	1
	High	rYWDAQLGAETLTPIAPIR	5	1	1	P73128	N-Term(iTRAQ4plex)	0,0000	1,039	3	11,3	0	5,503E-11	78	1,6367E-08	3	2315,26426	0,57	44,18	1
	High	fcIQAAIGHPLTVYGk	3	1	1	P73128	N-Term(iTRAQ4plex); C2(Carbamidomethyl); K16(iTRAQ4plex)	0,0000	1,370	2	7,0	0	9,286E-14	73	6,29424E-08	3	2063,13938	2,08	42,58	0
	High	gYEVGILDSLVR	3	1	1	P73128	N-Term(iTRAQ4plex)	0,0000	1,109	3	8,9	0	3,13E-08	59	1,37088E-06	2	1464,81902	0,75	48,43	0
	High	aGADLGLk	1	1	1	P73128	N-Term(iTRAQ4plex); K8(iTRAQ4plex)	0,0000	1,512	1		0	0,00002265	56	4,78668E-06	2	1032,63249	3,18	22,13	0
	High	qFQPDAVVHFGEQR	5	1	1	P73128	N-Term(iTRAQ4plex)	0,0000	0,963	4	33,1	0	8,831E-09	54	3,61385E-06	3	1801,90995	-0,18	33,98	0
	High	lDRWYELTGk	2	1	1	P73128	N-Term(iTRAQ4plex); K10(iTRAQ4plex)	0,0000	1,408	2	1,8	0	1,521E-07	43	4,62349E-05	2	1568,86992	1,52	36,20	1
	High	cIELAIANPADkGQFR	1	1	1	P73128	N-Term(iTRAQ4plex); C1(Carbamidomethyl); K12(iTRAQ4plex)	0,0000	1,444	1		0	3,252E-09	42	5,72756E-05	3	2091,12577	-0,11	35,42	1
	High	wYELTGk	1	1	1	P73128	N-Term(iTRAQ4plex); K7(iTRAQ4plex)	0,0000	1,516	1		0	0,0003399	36	0,000242644	2	1184,65557	0,14	32,98	0
	High	aGADLGLkVEIDHLENPR	1	1	1	P73128	N-Term(iTRAQ4plex); K8(iTRAQ4plex)	0,0000	1,169	1		0	1,28E-08	34	0,000427563	4	2235,23672	1,37	37,85	1
	High	vEIDHLENPR	1	1	1	P73128	N-Term(iTRAQ4plex)	0,0000	1,001	1		0	0,00000331	34	0,000433481	3	1365,72489	0,40	23,91	0
	High	kDTLPYPk	2	1	1	P73128	N-Term(iTRAQ4plex); K1(iTRAQ4plex); K8(iTRAQ4plex)	0,0000	1,398	2	3,8	0	4,673E-07	32	0,0006109	3	1393,84211	0,41	20,10	1
	High	qPGSFYHLSk	1	1	1	P73128	N-Term(iTRAQ4plex); K10(iTRAQ4plex)	0,0000	1,225	1		0	0,000002803	31	0,000760274	3	1451,79004	1,04	24,14	0
	High	vHDSHNIHFAck	1	1	1	P73128	N-Term(iTRAQ4plex); C11(Carbamidomethyl); K12(iTRAQ4plex)	0,0000	1,462	1		0	7,948E-08	26	0,002773129	4	1752,88698	1,55	15,26	0
	High	gLLDIRDTVR	6	1	1	P73128	N-Term(iTRAQ4plex)	0,0000	1,191	3	3,2	0	0,0001727	19	0,011967405	3	1301,76761	1,37	31,82	1
	High	sAPFSmIDR	1	1	1	P73128	N-Term(iTRAQ4plex); M6(Oxidation)	0,0000	1,121	1		0,001	0,01281	21	0,008336236	2	1183,58953	-0,26	24,54	0
	High	gLLDIR	1	1	1	P73128	N-Term(iTRAQ4plex)	0,0000	1,501	1		0,005	0,07111	15	0,029646266	2	830,52214	0,59	30,30	0
	Medium	hILPk	1	1	1	P73128	N-Term(iTRAQ4plex); K5(iTRAQ4plex)	0,0000	1,278	1		0,011	0,1267	27	0,002108483	2	895,59618	-0,65	14,59	0
	Medium	dTLPYPk	1	1	1	P73128	N-Term(iTRAQ4plex); K7(iTRAQ4plex)	0,0000	1,440	1		0,017	0,19	23	0,004797003	2	1121,65337	7,91	24,31	0
	Medium	iWGLR	1	1	1	P73128	N-Term(iTRAQ4plex)	0,0000	1,607	1		0,032	0,3431	16	0,027667505	2	788,48961	-0,44	30,99	0
P74070	Elongation factor Ts OS=Synechocystis sp. (strain PCC 6803 / Kazusa) GN=tsf PE=:	879,76	53,21	1	13	13	48	1,397	43	29,6	218	24,2	5,45							
	A3	Sequence	# PSMs	# Proteins	# Protein Groups	Protein Group Accessions	Modifications	ΔCn	114/115	114/115 Count	114/115 Variability [%]	q-Value	PEP	IonScore	Exp Value	Charge	MH+ [Da]	ΔM [ppm]	RT [min]	# Missed Cleavages
	High	tAAEGLVHSYIHFGGR	6	1	1	P74070	N-Term(iTRAQ4plex)	0,0000	0,795	4	5,5	0	8,454E-10	88	1,64048E-09	3	1858,97063	1,36	35,14	0
	High	qAIAELGENIQVR	1	1	1	P74070	N-Term(iTRAQ4plex)	0,0000	1,024	1		0	3,283E-08	70	1,29701E-07	2	1584,88408	0,90	35,78	0
	High	aEITAQLVk	1	1	1	P74070	N-Term(iTRAQ4plex); K9(iTRAQ4plex)	0,0000	1,509	1		0	7,831E-07	59	1,30317E-06	2	1260,77641	-0,14	27,88	0
	High	gIASADkk	6	1	1	P74070	N-Term(iTRAQ4plex); K7(iTRAQ4plex); K8(iTRAQ4plex)	0,0000	1,671	6	8,1	0	0,00001102	58	1,601E-06	2	1221,75200	-0,60	10,18	1
	High	fNLGEGIEk	3	1	1	P74070	N-Term(iTRAQ4plex); K9(iTRAQ4plex)	0,0000	1,158	3	4,4	0	0,00000046	49	1,18024E-05	2	1294,72527	0,54	32,89	0
	High	lkELSLLDQPYIk	6	1	1	P74070	N-Term(iTRAQ4plex); K2(iTRAQ4plex); K13(iTRAQ4plex)	0,0000	1,326	6	20,8	0	3,84E-09	45	3,46713E-05	3	1992,21183	0,63	42,87	1
	High	aLkENEGDLEk	3	1	1	P74070	N-Term(iTRAQ4plex); K3(iTRAQ4plex); K11(iTRAQ4plex)	0,0000	1,669	3	10,4	0	2,864E-07	37	0,000196967	3	1677,94187	2,08	17,92	1
	High	ekEIEmGRDDLGk	5	1	1	P74070	N-Term(iTRAQ4plex); K2(iTRAQ4plex); M6(Oxidation); K13(iTRAQ4plex)	0,0000	1,571	4	3,0	0	0,00007543	33	0,000452866	3	1968,04557	1,16	19,14	2
	High	tGAGmmDck	6	1	1	P74070	N-Term(iTRAQ4plex); M5(Oxidation); M6(Oxidation); C8(Carbamidomethyl);	0,0000	1,964	4	5,2	0	0,0005506	26	0,002582082	4	1562,77346	2,12	8,04	1
	High	ekTGAGmmDck	1	1	1	P74070	N-Term(iTRAQ4plex); K2(iTRAQ4plex); M7(Oxidation); M8(Oxidation); C10(C	0,0000	2,077	1		0	0,00001538	24	0,003766778	3	1691,81467	1,14	8,28	1
	High	kPANIk	2	1	1	P74070	N-Term(iTRAQ4plex); K1(iTRAQ4plex); K6(iTRAQ4plex)	0,0000	1,605	2	0,0	0	0,004498	19	0,012358621	3	1102,72913	-1,57	10,35	0
	High	ekEIEMGRDDLGk	1	1	1	P74070	N-Term(iTRAQ4plex); K2(iTRAQ4plex); K13(iTRAQ4plex)	0,0000	1,214	1		0	0,000174	14	0,046471779	4	1952,04946	0,56	22,73	2
	High	sIEWLR	4	1	1	P74070	N-Term(iTRAQ4plex)	0,0000	1,077	4	4,6	0,001	0,01089	27	0,002093968	2	947,54296	-0,17	32,47	0
	High	ekEIEmGR	3	1	1	P74070	N-Term(iTRAQ4plex); K2(iTRAQ4plex); M6(Oxidation)	0,0000	1,100	3	18,6	0,003	0,03695	18	0,016367035	3	1295,68631	-0,39	10,27	1
P73409	Slr1841 protein OS=Synechocystis sp. (strain PCC 6803 / Kazusa) GN=slr1841 PE=	867,83	12,70	1	4	7	20	1,723	9	10,4	630	67,6	4,65							
	A3	Sequence	# PSMs	# Proteins	# Protein Groups	Protein Group Accessions	Modifications	ΔCn	114/115	114/115 Count	114/115 Variability [%]	q-Value	PEP	IonScore	Exp Value	Charge	MH+ [Da]	ΔM [ppm]	RT [min]	# Missed Cleavages
	High	gGTQGIVGTTAANLNR	7	1	1	P73409	N-Term(iTRAQ4plex)	0,0000	1,760	4	5,1	0	6,826E-10	96	2,76039E-10	2	1673,90679	0,96	27,55	0
	High	lmQEFEAELAALGAR	4	2	2	P73409;P73103	N-Term(iTRAQ4plex); M2(Oxidation)	0,0000				0	2,752E-11	80	1,11422E-08	2	1808,93474	0,74	54,13	0
	High	rLmQEFEAELAALGAR	1	2	2	P73409;P73103	N-Term(iTRAQ4plex); M3(Oxidation)	0,0000				0	9,957E-10	71	7,86991E-08	3	1965,03623	0,87	47,64	1
	High	lTGEVIFAPTAIFGTk	3	1	1	P73409	N-Term(iTRAQ4plex); K16(iTRAQ4plex)	0,0000	1,138	3	5,0	0	2,179E-11	70	9,74922E-08	2	1953,13384	1,97	52,90	0
	High	lMQEFEAELAALGAR	2	2	2	P73409;P73103	N-Term(iTRAQ4plex)	0,0000				0	1,571E-09	69	1,38347E-07	2	1792,93596	-1,41	59,09	0
	High	tLNNQTDNNQAVFQNR	1	1	1	P73409	N-Term(iTRAQ4plex)	0,0000	1,499	1		0	2,265E-10	65	2,96463E-07	2	2020,99308	0,65	23,26	0
	High	kTLNNQTDNNQAVFQNR	1	1	1	P73409	N-Term(iTRAQ4plex); K1(iTRAQ4plex)	0,0000	1,723	1		0	7,738E-11	60	1,05918E-06	3	2293,19107	1,00	19,88	1
	High	lIQENVAVLREDIDk	1	2	2	P73409;P73103	N-Term(iTRAQ4plex); K15(iTRAQ4plex)	0,0000				0	2,089E-09	29	0,001279293	3	2043,16916	0,12	41,73	1
P73318	50S ribosomal protein L23 OS=Synechocystis sp. (strain PCC 6803 / Kazusa) GN=rp	857,89	78,22	1	10	10	53	2,583	46	58,3	101	11,5	10,05							
	A3	Sequence	# PSMs	# Proteins	# Protein Groups	Protein Group Accessions	Modifications	ΔCn	114/115	114/115 Count	114/115 Variability [%]	q-Value	PEP	IonScore	Exp Value	Charge	MH+ [Da]	ΔM [ppm]	RT [min]	# Missed Cleavages
	High	lADLIIkPIVTEk	27	1	1	P73318	N-Term(iTRAQ4plex); K7(iTRAQ4plex); K13(iTRAQ4plex)	0,0000	2,588	24	39,2	0	5,975E-11	75	3,33403E-08	2	1885,21391	2,15	44,78	0
	High	aAIELLFDVk	6	1	1	P73318	N-Term(iTRAQ4plex); K10(iTRAQ4plex)	0,0000	1,525	6	79,4	0	1,53E-08	69	1,22172E-07	2	1406,85137	1,13	50,70	0
	High	aTLQLEDNk	2	1	1	P73318	N-Term(iTRAQ4plex); K9(iTRAQ4plex)	0,0000	2,414	2	17,8	0	0,0000298	54	4,25569E-06	2	1319,74175	0,61	22,88	0
	High	rLADLIIkPIVTEk	4	1	1	P73318	N-Term(iTRAQ4plex); K8(iTRAQ4plex); K14(iTRAQ4plex)	0,0000	10,989	2	10,1	0	8,805E-09	38	0,000154515	4	2041,31362	1,29	37,86	1
	High	aVVTLk	5	1	1	P73318	N-Term(iTRAQ4plex); K6(iTRAQ4plex)	0,0000	5,875	3	27,1	0	0,006198	35	0,000334942	2	918,62346	0,89	21,94	0
	High	yVFDVRPEATkPEIk	2	1	1	P73318	N-Term(iTRAQ4plex); K11(iTRAQ4plex); K15(iTRAQ4plex)	0,0000	6,655	2	97,4	0	0,00002746	25	0,003213439	3	2224,27274	1,13	32,73	0
	High	rAVVTLk	2	1	1	P73318	N-Term(iTRAQ4plex); K7(iTRAQ4plex)	0,0000	2,547	2	28,5	0	0,001277	24	0,004139711	3	1074,72498	1,13	15,84	1
	High	vTGVNTAR	3	1	1	P73318	N-Term(iTRAQ4plex)	0,0000	2,115	3	6,6	0,001	0,01032	31	0,00085584	2	961,55980	5,26	14,08	0
	Medium	fmGFk	1	1	1	P73318	N-Term(iTRAQ4plex); M2(Oxidation); K5(iTRAQ4plex)	0,0000	1,309	1		0,042	0,4356	13	0,046448319	2	933,51116	0,53	23,34	0
	Medium	eGDSIQLFPDV	1	1	1	P73318	N-Term(iTRAQ4plex)	0,0000	1,091	1		0,042	0,4402	10	0,091615721	2	1363,68779	1,14	48,40	0
P73319	50S ribosomal protein L4 OS=Synechocystis sp. (strain PCC 6803 / Kazusa) GN=rpl	840,56	43,81	1	14	14	59	1,761	50	118,4	210	23,3	10,20							
	A3	Sequence	# PSMs	# Proteins	# Protein Groups	Protein Group Accessions	Modifications	ΔCn	114/115	114/115 Count	114/115 Variability [%]	q-Value	PEP	IonScore	Exp Value	Charge	MH+ [Da]	ΔM [ppm]	RT [min]	# Missed Cleavages
	High	eENAAHIVHR	6	1	1	P73319	N-Term(iTRAQ4plex)	0,0000	10,432	4	512,8	0	4,482E-07	74	4,39511E-08	2	1319,69707	2,55	11,54	0
	High	vAkEENAAHIVHR	6	1	1	P73319	N-Term(iTRAQ4plex); K3(iTRAQ4plex)	0,0000	13,720	4	23,4	0	2,859E-09	64	4,29536E-07	2	1761,99895	1,52	14,91	1
	High	tkELATALTR	9	1	1	P73319	N-Term(iTRAQ4plex); K2(iTRAQ4plex)	0,0000	9,265	5	60,8	0	0,000005806	63	4,49749E-07	2	1391,84868	1,87	21,53	1
	High	qGNASAk	2	1	1	P73319	N-Term(iTRAQ4plex); K7(iTRAQ4plex)	0,0000	100,000	1		0	0,002038	50	1,04518E-05	2	963,54637	0,19	7,61	0
	High	gGGVIFGPkPR	12	1	1	P73319	N-Term(iTRAQ4plex); K9(iTRAQ4plex)	0,0000	1,012	12	45,4	0	0,00003369	49	1,27929E-05	2	1372,83550	3,72	19,95	0
	High	eLATALTR	2	1	1	P73319	N-Term(iTRAQ4plex)	0,0000	0,842	2	3,3	0	0,000151	33	0,000544465	2	1018,60167	0,30	24,02	0
	High	rVLLILDEIPENVFLSGR	1	1	1	P73319	N-Term(iTRAQ4plex)	0,0000	1,688	1		0	0,00005488	27	0,002008954	3	2227,29837	2,33	56,13	1
	High	wGAkPEk	3	1	1	P73319	N-Term(iTRAQ4plex); K4(iTRAQ4plex); K7(iTRAQ4plex)	0,0000	12,198	3	193,9	0	0,000393	27	0,002127992	2	1247,74797	0,58	16,29	0
	High	nIPYLkILR	1	1	1	P73319	N-Term(iTRAQ4plex); K6(iTRAQ4plex)	0,0000	1,427	1		0	0,0000927	15	0,029783107	3	1417,91214	-0,86	38,42	1
	High	nIPYLk	11	1	1	P73319	N-Term(iTRAQ4plex); K6(iTRAQ4plex)	0,0000	1,191	11	5,5	0,002	0,02977	29	0,001276439	2	1035,64507	0,94	28,78	0
	High	tAIASR	2	1	1	P73319	N-Term(iTRAQ4plex)	0,0000	1,727	2	9,3	0,002	0,02563	23	0,005140081	2	762,45915	0,14	11,54	0
	High	wGAkPEkR	1	1	1	P73319	N-Term(iTRAQ4plex); K4(iTRAQ4plex); K7(iTRAQ4plex)	0,0000	2,179	1		0,002	0,03225	12	0,062801498	3	1403,84836	-0,01	12,71	1
	High	vDcIVk	1	1	1	P73319	N-Term(iTRAQ4plex); C3(Carbamidomethyl); K6(iTRAQ4plex)	0,0000	4,748	1		0,005	0,06363	30	0,00103992	2	1021,60167	6,09	20,73	0
	Medium	sPLWR	2	1	1	P73319	N-Term(iTRAQ4plex)	0,0000	2,259	2	14,5	0,021	0,2291	28	0,001503038	2	802,47112	2,37	21,97	0
P73287	Bacterioferritin OS=Synechocystis sp. (strain PCC 6803 / Kazusa) GN=bfr PE=3 SV=	830,88	36,36	1	5	5	31	1,592	28	26,0	176	20,2	4,61							
	A3	Sequence	# PSMs	# Proteins	# Protein Groups	Protein Group Accessions	Modifications	ΔCn	114/115	114/115 Count	114/115 Variability [%]	q-Value	PEP	IonScore	Exp Value	Charge	MH+ [Da]	ΔM [ppm]	RT [min]	# Missed Cleavages
	High	nWGLNALNQYEYk	4	1	1	P73287	N-Term(iTRAQ4plex); K13(iTRAQ4plex)	0,0000	1,030	4	58,2	0	6,055E-12	86	2,72251E-09	2	1900,98308	1,83	42,33	0
	High	lQLTAINQYFLHAR	19	1	1	P73287	N-Term(iTRAQ4plex)	0,0000	1,403	16	32,6	0	1,806E-09	77	2,22828E-08	2	1832,03288	1,59	47,34	0
	High	dGLVNSIAFFETQR	1	1	1	P73287	N-Term(iTRAQ4plex)	0,0000	1,631	1		0	1,577E-11	76	2,76675E-08	2	1740,90532	0,88	47,89	0
	High	vLFLEGLPNLQNLEk	3	1	1	P73287	N-Term(iTRAQ4plex); K15(iTRAQ4plex)	0,0000	1,595	3	15,6	0	5,525E-08	54	3,78416E-06	2	2015,17729	-0,35	55,86	0
	High	qHLNQALk	4	1	1	P73287	N-Term(iTRAQ4plex); K8(iTRAQ4plex)	0,0000	1,845	4	0,9	0	0,001027	41	7,48118E-05	2	1239,74748	5,07	12,20	0
P74102	Orange carotenoid-binding protein OS=Synechocystis sp. (strain PCC 6803 / Kazusa	829,63	35,65	1	11	11	30	1,073	30	11,7	317	34,6	5,05							
	A3	Sequence	# PSMs	# Proteins	# Protein Groups	Protein Group Accessions	Modifications	ΔCn	114/115	114/115 Count	114/115 Variability [%]	q-Value	PEP	IonScore	Exp Value	Charge	MH+ [Da]	ΔM [ppm]	RT [min]	# Missed Cleavages
	High	nAVVDmGFTAGk	3	1	1	P74102	N-Term(iTRAQ4plex); M6(Oxidation); K12(iTRAQ4plex)	0,0000	1,135	3	3,4	0	7,605E-11	75	2,90382E-08	2	1513,79338	0,68	24,89	0
	High	gIFPNTLAADVVPATIAR	4	1	1	P74102	N-Term(iTRAQ4plex)	0,0000	1,013	4	11,3	0	6,565E-10	70	9,1405E-08	2	1970,12029	0,54	49,60	0
	High	tYASWSPNIk	3	1	1	P74102	N-Term(iTRAQ4plex); K10(iTRAQ4plex)	0,0000	1,080	3	1,7	0	1,857E-08	70	1,02322E-07	2	1454,79021	1,38	31,65	0
	High	fLLNPEGk	4	1	1	P74102	N-Term(iTRAQ4plex); K8(iTRAQ4plex)	0,0000	1,063	4	13,3	0	0,000006528	58	1,6217E-06	2	1205,71428	0,84	32,51	0
	High	tLTIAAPGAASmQLAENALk	1	1	1	P74102	N-Term(iTRAQ4plex); M12(Oxidation); K20(iTRAQ4plex)	0,0000	1,092	1		0	2,623E-10	56	2,35505E-06	3	2275,25882	0,76	41,86	0
	High	eLLNFAR	3	1	1	P74102	N-Term(iTRAQ4plex)	0,0000	0,997	3	12,9	0	0,005381	39	0,00012106	2	1006,58043	0,20	33,43	0
	High	fFREEcQNLk	4	1	1	P74102	N-Term(iTRAQ4plex); C6(Carbamidomethyl); K10(iTRAQ4plex)	0,0000	1,154	4	7,8	0	0,0001253	38	0,000146545	2	1658,85808	1,05	26,13	1
	High	iFFVAIDLLASPk	1	1	1	P74102	N-Term(iTRAQ4plex); K13(iTRAQ4plex)	0,0000	0,957	1		0	0,00003577	28	0,001485833	3	1722,04685	1,38	59,42	0

	High	nAVVDmGFTAGkDGkR	1	1	1	P74102	N-Term(iTRAQ4plex); M6(Oxidation); K12(iTRAQ4plex); K15(iTRAQ4plex)	0,0000	1,129	1		0	3,078E-07	24	0,004354818	4	2114,14004	0,53	19,52	2
	High	lGFWYR	5	1	1	P74102	N-Term(iTRAQ4plex)	0,0000	0,942	5	8,8	0,001	0,01717	25	0,003235713	2	985,53801	0,39	37,83	0
	Medium	lIPER	1	1	1	P74102	N-Term(iTRAQ4plex)	0,0000	0,886	1		0,049	0,5083	18	0,014520113	2	771,48912	5,95	20,68	0
P72776	Pyridoxine 5'-phosphate synthase OS=Synechocystis sp. (strain PCC 6803 / Kazusa)	828,01	40,91	1	7	7	30	1,447	27	46,8	242	26,5	5,62							
	A3	Sequence	# PSMs	# Proteins	# Protein Groups	Protein Group Accessions	Modifications	ΔCn	114/115	114/115 Count	114/115 Variability [%]	q-Value	PEP	IonScore	Exp Value	Charge	MH+ [Da]	ΔM [ppm]	RT [min]	# Missed Cleavages
	High	mLTLGVNIDHVATIR	2	1	1	P72776	N-Term(iTRAQ4plex)	0,0000	1,324	2	35,0	0	3,773E-14	77	2,09748E-08	3	1797,01865	0,70	43,12	0
	High	mLTLGVNIDHVATIR	3	1	1	P72776	N-Term(iTRAQ4plex); M1(Oxidation)	0,0000	1,269	3	19,4	0	1,112E-11	73	5,28676E-08	3	1813,01474	1,35	38,56	0
	High	hIPVSLFIDPDVAQLk	6	1	1	P72776	N-Term(iTRAQ4plex); K16(iTRAQ4plex)	0,0000	1,289	6	13,3	0	9,199E-10	57	2,11821E-06	3	2080,20755	1,44	46,96	0
	High	aSAQTGAk	4	1	1	P72776	N-Term(iTRAQ4plex); K8(iTRAQ4plex)	0,0000	2,939	3	10,4	0	0,0002236	53	5,54857E-06	2	1021,58806	0,00	8,48	0
	High	fIELHTGk	6	1	1	P72776	N-Term(iTRAQ4plex); K8(iTRAQ4plex)	0,0000	2,360	4	6,8	0	0,00001055	51	7,92447E-06	2	1232,72637	1,79	24,65	0
	High	aILVGMER	1	1	1	P72776	N-Term(iTRAQ4plex)	0,0000	1,715	1		0	0,0000427	47	2,58425E-05	2	1032,59941	0,13	28,45	0
	High	tHLNLEMAATk	2	1	1	P72776	N-Term(iTRAQ4plex); K11(iTRAQ4plex)	0,0000	1,170	2	13,0	0	0,00005423	43	5,30848E-05	3	1516,84687	4,78	25,01	0
	High	tHLNLEmAATk	3	1	1	P72776	N-Term(iTRAQ4plex); M7(Oxidation); K11(iTRAQ4plex)	0,0000	0,956	3	4,8	0	0,00001452	39	0,000118024	3	1532,83829	2,45	20,07	0
	High	iNAGHGLTYWNVRPVAELPGMEELNIGHSIMSR	1	1	1	P72776	N-Term(iTRAQ4plex)	0,0000	1,359	1		0	1,374E-07	31	0,000741259	5	3805,94373	1,28	49,72	0
	High	aILVGmER	2	1	1	P72776	N-Term(iTRAQ4plex); M6(Oxidation)	0,0000	1,204	2	103,9	0,001	0,009736	30	0,001380791	2	1048,59490	0,67	20,49	0
P80507	Inorganic pyrophosphatase OS=Synechocystis sp. (strain PCC 6803 / Kazusa) GN=p	823,13	55,03	1	12	12	49	1,243	44	23,2	169	19,1	4,88							
	A3	Sequence	# PSMs	# Proteins	# Protein Groups	Protein Group Accessions	Modifications	ΔCn	114/115	114/115 Count	114/115 Variability [%]	q-Value	PEP	IonScore	Exp Value	Charge	MH+ [Da]	ΔM [ppm]	RT [min]	# Missed Cleavages
	High	aGLINVLIEIPAGSk	3	1	1	P80507	N-Term(iTRAQ4plex); K15(iTRAQ4plex)	0,0000	1,048	3	31,3	0	1,038E-09	67	1,9542E-07	2	1783,09441	0,67	52,22	0
	High	sINDLAGHR	8	1	1	P80507	N-Term(iTRAQ4plex)	0,0000	1,005	5	11,2	0	0,00003408	58	1,62544E-06	2	1126,60954	0,86	16,42	0
	High	vTEILGWk	2	1	1	P80507	N-Term(iTRAQ4plex); K8(iTRAQ4plex)	0,0000	1,110	2	6,8	0	0,00000565	50	9,88553E-06	2	1233,74565	0,88	40,25	0
	High	lDEIAEFFR	4	1	1	P80507	N-Term(iTRAQ4plex)	0,0000	1,212	4	2,4	0	0,00001767	48	1,7099E-05	2	1283,67632	0,81	51,84	0
	High	kVTEILGWk	7	1	1	P80507	N-Term(iTRAQ4plex); K1(iTRAQ4plex); K9(iTRAQ4plex)	0,0000	1,456	6	23,6	0	0,00004072	44	3,7151E-05	2	1505,94341	1,20	35,13	1
	High	nkYEFDkDmNcFALDR	4	1	1	P80507	N-Term(iTRAQ4plex); K2(iTRAQ4plex); K7(iTRAQ4plex); M9(Oxidation); C11(	0,0000	1,456	4	10,5	0	8,778E-07	36	0,0002594	3	2514,21494	1,24	29,23	2
	High	nkYEFDkDMNcFALDR	4	1	1	P80507	N-Term(iTRAQ4plex); K2(iTRAQ4plex); K7(iTRAQ4plex); C11(Carbamidometh	0,0000	1,445	4	11,9	0	1,472E-09	36	0,000279879	4	2498,21865	0,70	34,19	2
	High	kVTEILGWkDVDAVLPLVEEcVk	1	1	1	P80507	K1(iTRAQ4plex); N-Term(iTRAQ4plex); K9(iTRAQ4plex); C21(Carbamidometh	0,0000	0,909	1		0	0,000001247	27	0,001857676	4	3216,84414	1,26	53,24	2
	High	nkYEFDk	1	1	1	P80507	N-Term(iTRAQ4plex); K2(iTRAQ4plex); K7(iTRAQ4plex)	0,0000	1,089	1		0	0,003588	10	0,093534107	3	1375,75632	-1,36	17,58	1
	High	iLcVPAk	2	1	1	P80507	N-Term(iTRAQ4plex); C3(Carbamidomethyl); K7(iTRAQ4plex)	0,0000	1,646	1		0,002	0,02725	11	0,077085023	2	1088,67558	1,42	28,33	0
	High	iLcVPAkDPR	4	1	1	P80507	N-Term(iTRAQ4plex); C3(Carbamidomethyl); K7(iTRAQ4plex)	0,0000	1,108	4	8,8	0,002	0,02316	10	0,099304746	2	1456,85625	0,94	25,34	1
	High	yTYVk	4	1	1	P80507	N-Term(iTRAQ4plex); K5(iTRAQ4plex)	0,0000	1,264	4	11,4	0,003	0,04141	25	0,004563875	2	961,56395	4,44	18,25	0
	High	iPAQPk	5	1	1	P80507	N-Term(iTRAQ4plex); K6(iTRAQ4plex)	0,0000	1,359	5	7,4	0,004	0,04546	19	0,013676344	2	941,60185	-0,42	16,52	0
P73303	50S ribosomal protein L15 OS=Synechocystis sp. (strain PCC 6803 / Kazusa) GN=r	805,03	59,18	1	9	9	35	2,244	28	78,7	147	15,2	10,37							
	A3	Sequence	# PSMs	# Proteins	# Protein Groups	Protein Group Accessions	Modifications	ΔCn	114/115	114/115 Count	114/115 Variability [%]	q-Value	PEP	IonScore	Exp Value	Charge	MH+ [Da]	ΔM [ppm]	RT [min]	# Missed Cleavages
	High	gIAAGQGAScGFGMR	5	1	1	P73303	N-Term(iTRAQ4plex); C10(Carbamidomethyl)	0,0000	2,699	3	79,7	0	9,813E-11	102	6,41165E-11	2	1583,75444	0,29	27,70	0
	High	gIAAGQGAScGFGmR	5	1	1	P73303	N-Term(iTRAQ4plex); C10(Carbamidomethyl); M14(Oxidation)	0,0000	4,710	3	4,0	0	6,479E-12	83	5,04626E-09	2	1599,74968	0,49	22,14	0
	High	mNLSELSPk	1	1	1	P73303	N-Term(iTRAQ4plex); M1(Oxidation); K9(iTRAQ4plex)	0,0000	4,172	1		0	1,634E-07	64	4,42781E-07	2	1322,72405	0,90	23,32	0
	High	vLGNGELAVALTVHAPcSk	2	1	1	P73303	N-Term(iTRAQ4plex); C17(Carbamidomethyl); K19(iTRAQ4plex)	0,0000	2,348	1		0	2,422E-13	60	1,02322E-06	3	2224,23722	0,42	40,58	0
	High	aGFEGGQMPLYR	1	1	1	P73303	N-Term(iTRAQ4plex)	0,0000	2,358	1		0	2,028E-09	55	3,27318E-06	2	1469,73296	0,10	32,96	0
	High	mNLSELSPk	1	1	1	P73303	N-Term(iTRAQ4plex); K9(iTRAQ4plex)	0,0000	1,771	1		0	5,183E-07	50	9,86211E-06	2	1306,72954	1,23	31,34	0
	High	hFTVVNVGk	5	1	1	P73303	N-Term(iTRAQ4plex); K9(iTRAQ4plex)	0,0000	3,115	4	27,8	0	0,00003622	48	1,54159E-05	2	1288,76213	0,41	22,08	0
	High	aGFEGGQmPLYR	2	1	1	P73303	N-Term(iTRAQ4plex); M8(Oxidation)	0,0000	3,245	2	61,9	0	3,681E-07	42	5,9975E-05	2	1485,72856	0,56	25,97	0
	High	lkHFPLYNPk	1	1	1	P73303	N-Term(iTRAQ4plex); K2(iTRAQ4plex); K10(iTRAQ4plex)	0,0000	1,101	1		0	0,000004087	18	0,016632978	3	1689,02121	-0,01	26,70	1
	High	hFPLYNPk	9	1	1	P73303	N-Term(iTRAQ4plex); K8(iTRAQ4plex)	0,0000	1,378	9	29,0	0	0,00005696	15	0,028442647	3	1303,74048	0,27	24,83	0
	Medium	sRSGTGTk	1	1	1	P73303	N-Term(iTRAQ4plex); K8(iTRAQ4plex)	0,0000				0,039	0,4051	12	0,063528705	2	1081,61626	-3,84	13,93	1
	Medium	aAQAk	2	1	1	P73303	N-Term(iTRAQ4plex); K5(iTRAQ4plex)	0,0000	2,197	2	28,0	0,043	0,4534	15	0,04001117	2	776,48625	-0,80	7,56	0
Q55662	ATP-dependent Clp protease regulatory subunit OS=Synechocystis sp. (strain PCC 6	802,00	21,80	1	13	15	37	1,116	31	15,2	821	91,1	5,50							
	A3	Sequence	# PSMs	# Proteins	# Protein Groups	Protein Group Accessions	Modifications	ΔCn	114/115	114/115 Count	114/115 Variability [%]	q-Value	PEP	IonScore	Exp Value	Charge	MH+ [Da]	ΔM [ppm]	RT [min]	# Missed Cleavages
	High	gSGFVAVEIPFTPR	5	1	1	Q55662	N-Term(iTRAQ4plex)	0,0000	1,116	5	11,6	0	1,305E-08	65	3,84002E-07	2	1620,88884	1,33	46,05	0
	High	lGHNFVGTEQILLGLIGEGTGVAAk	1	1	1	Q55662	N-Term(iTRAQ4plex); K25(iTRAQ4plex)	0,0000	0,747	1		0	9,159E-09	64	3,80163E-07	4	2782,57485	1,51	57,54	0
	High	nPNRPIASFIFSGPTGVGk	5	1	1	Q55662	N-Term(iTRAQ4plex); K19(iTRAQ4plex)	0,0000	1,153	5	8,9	0	1,871E-13	61	8,66962E-07	3	2247,25083	0,86	41,29	0
	High	lLHmEDTLHQR	3	1	1	Q55662	N-Term(iTRAQ4plex); M4(Oxidation)	0,0000	0,930	3	0,7	0	4,881E-09	50	1,04465E-05	3	1552,80136	-0,59	18,01	0
	High	tAIAEGLAQR	3	2	2	P74361;Q55662	N-Term(iTRAQ4plex)	0,0000				0	2,898E-07	45	3,07588E-05	2	1173,66985	-0,86	23,74	0
	High	lDEIIVFR	2	1	1	Q55662	N-Term(iTRAQ4plex)	0,0000	1,148	2	12,2	0	0,00001146	45	3,89671E-05	2	1148,67986	0,20	44,11	0
	High	rVLELSLEEAR	2	1	1	Q55662	N-Term(iTRAQ4plex)	0,0000	0,988	2	8,6	0	0,00001092	44	4,36486E-05	3	1458,83988	0,11	31,48	1
	High	eISEILLR	2	1	1	Q55662	N-Term(iTRAQ4plex)	0,0000	1,117	2	10,2	0	0,00002789	42	5,70125E-05	2	1116,67436	-0,15	38,60	0
	High	rPYTVVLFDEIEk	2	1	1	Q55662	N-Term(iTRAQ4plex); K13(iTRAQ4plex)	0,0000	1,002	2	17,1	0	8,292E-10	40	9,93047E-05	3	1897,06913	0,92	40,79	0
	High	nYFRPEFLNR	2	1	1	Q55662	N-Term(iTRAQ4plex)	0,0000	1,197	2	7,4	0	0,00002804	30	0,001030315	3	1499,78849	0,59	33,30	0
	High	eISLQVTDkFk	1	1	1	Q55662	N-Term(iTRAQ4plex); K9(iTRAQ4plex); K11(iTRAQ4plex)	0,0000	1,144	1		0	8,312E-07	29	0,001402717	3	1740,02579	-0,57	34,01	1
	High	yRGEFEER	3	2	2	P74361;Q55662	N-Term(iTRAQ4plex)	0,0000				0	0,003701	26	0,002306588	3	1229,60428	0,92	14,57	1
	High	qLGHNYIGTEHLLLGLIR	1	1	1	Q55662	N-Term(iTRAQ4plex)	0,0000	0,969	1		0	3,143E-07	23	0,005495029	4	2191,25014	1,50	45,32	0
	High	vIQILGR	3	1	1	Q55662	N-Term(iTRAQ4plex)	0,0000	0,964	3	3,5	0,001	0,0122	34	0,0004385	2	942,62163	-0,08	33,40	0
	Medium	aIASSk	2	1	1	Q55662	N-Term(iTRAQ4plex); K6(iTRAQ4plex)	0,0000	1,346	2	12,2	0,013	0,1464	14	0,048120903	2	864,53722	-2,42	12,68	0
Q55648	Sll0314 protein OS=Synechocystis sp. (strain PCC 6803 / Kazusa) GN=sll0314 PE=4	797,02	26,43	1	10	10	34	1,670	27	12,1	314	35,4	5,97							
	A3	Sequence	# PSMs	# Proteins	# Protein Groups	Protein Group Accessions	Modifications	ΔCn	114/115	114/115 Count	114/115 Variability [%]	q-Value	PEP	IonScore	Exp Value	Charge	MH+ [Da]	ΔM [ppm]	RT [min]	# Missed Cleavages
	High	yTPFSQSEQVISR	1	1	1	Q55648	N-Term(iTRAQ4plex)	0,0000	1,817	1		0	2,468E-09	93	6,79404E-10	2	1685,86284	0,76	34,22	0
	High	yAQQTLESAQR	2	1	1	Q55648	N-Term(iTRAQ4plex)	0,0000	1,734	2	7,7	0	6,138E-07	65	2,8312E-07	2	1438,74114	0,30	22,14	0
	High	gSYLQAVGk	3	1	1	Q55648	N-Term(iTRAQ4plex); K9(iTRAQ4plex)	0,0000	2,056	2	3,5	0	3,271E-07	60	1,26386E-06	2	1210,70476	1,11	25,17	0
	High	kYAQQTLESAQR	4	1	1	Q55648	N-Term(iTRAQ4plex); K1(iTRAQ4plex)	0,0000	1,545	2	0,0	0	5,42E-09	55	2,98518E-06	3	1710,93857	0,49	16,93	1
	High	gYLDLFLSR	8	1	1	Q55648	N-Term(iTRAQ4plex)	0,0000	1,379	7	29,0	0	0,0001081	49	1,16673E-05	2	1227,68657	0,93	48,67	0
	High	lAQNPSLk	5	1	1	Q55648	N-Term(iTRAQ4plex); K8(iTRAQ4plex)	0,0000	1,978	3	8,4	0	0,000301	44	4,15882E-05	2	1158,71037	1,61	19,80	0
	High	lQQAQQYYAmALQk	1	1	1	Q55648	N-Term(iTRAQ4plex); M10(Oxidation); K14(iTRAQ4plex)	0,0000	1,267	1		0	9,136E-11	43	5,57147E-05	3	1988,05185	0,23	37,09	0
	High	lQQAQQYYAMALQk	1	1	1	Q55648	N-Term(iTRAQ4plex); K14(iTRAQ4plex)	0,0000	0,904	1		0	8,022E-10	40	0,000120697	3	1972,05979	1,68	40,10	0
	High	aLATTYR	4	1	1	Q55648	N-Term(iTRAQ4plex)	0,0000	1,729	4	1,5	0	0,001725	36	0,000249442	2	939,53819	0,19	16,36	0
	High	tGTVFEHLDk	4	1	1	Q55648	N-Term(iTRAQ4plex); K10(iTRAQ4plex)	0,0000	1,592	3	0,0	0	0,0000631	30	0,00097268	3	1434,78376	0,43	25,09	0
	High	kGSYLQAVGk	1	1	1	Q55648	N-Term(iTRAQ4plex); K1(iTRAQ4plex); K10(iTRAQ4plex)	0,0000	1,812	1		0	0,00003284	29	0,001306081	3	1482,90131	0,58	21,98	1
P73348	Rehydrin OS=Synechocystis sp. (strain PCC 6803 / Kazusa) GN=slr1198 PE=1 SV=1	786,52	40,28	1	10	10	51	1,203	45	14,6	211	23,5	5,30							
	A3	Sequence	# PSMs	# Proteins	# Protein Groups	Protein Group Accessions	Modifications	ΔCn	114/115	114/115 Count	114/115 Variability [%]	q-Value	PEP	IonScore	Exp Value	Charge	MH+ [Da]	ΔM [ppm]	RT [min]	# Missed Cleavages
	High	vIALSVDDVESHk	1	1	1	P73348	N-Term(iTRAQ4plex); K13(iTRAQ4plex)	0,0000	1,311	1		0	3,322E-12	63	6,68858E-07	3	1699,94748	0,33	34,26	0
	High	vSDLYGmIHPNALNNLTVR	5	1	1	P73348	N-Term(iTRAQ4plex); M7(Oxidation)	0,0000	1,026	4	6,5	0	1,901E-10	60	1,1545E-06	3	2287,20114	1,11	42,19	0
	High	vSDLYGMIHPNALNNLTVR	2	1	1	P73348	N-Term(iTRAQ4plex)	0,0000	1,032	1		0	8,674E-12	59	1,38733E-06	3	2271,20487	0,52	47,21	0
	High	sVFIIDPAkk	5	1	1	P73348	N-Term(iTRAQ4plex); K9(iTRAQ4plex); K10(iTRAQ4plex)	0,0000	1,343	4	14,0	0	4,594E-07	59	1,22172E-06	2	1549,96831	0,32	30,83	1
	High	sVFIIDPAk	2	1	1	P73348	N-Term(iTRAQ4plex); K9(iTRAQ4plex)	0,0000	1,239	2	4,7	0	6,372E-07	56	2,71E-06	2	1277,77104	0,20	36,73	0
	High	gVEEIkPYLR	16	1	1	P73348	N-Term(iTRAQ4plex); K6(iTRAQ4plex)	0,0000	1,227	16	20,2	0	0,000002282	48	1,72572E-05	2	1491,87724	-0,09	28,80	0
	High	lTFTYPASTGR	10	1	1	P73348	N-Term(iTRAQ4plex)	0,0000	1,118	9	17,3	0	0,000004473	47	2,11821E-05	2	1357,72405	0,58	33,79	0
	High	nFDEILR	2	1	1	P73348	N-Term(iTRAQ4plex)	0,0000	1,078	2	20,4	0	0,0003861	43	4,95416E-05	2	1050,57121	1,09	32,88	0
	High	lkPEFDk	1	1	1	P73348	N-Term(iTRAQ4plex); K2(iTRAQ4plex); K7(iTRAQ4plex)	0,0000	4,358	1		0,001	0,008293	20	0,010068621	3	1308,79465	4,49	21,89	0
	High	lkPEFDkR	2	1	1	P73348	N-Term(iTRAQ4plex); K2(iTRAQ4plex); K7(iTRAQ4plex)	0,0000	1,375	2	9,4	0,002	0,02277	11	0,07128038	4	1464,89052	0,43	17,52	1
	High	lTPQPNk	5	1	1	P73348	N-Term(iTRAQ4plex); K7(iTRAQ4plex)	0,0000	1,254	3	5,1	0,003	0,03738	10	0,096598416	2	1085,65666	0,85	16,16	0
P73309	50S ribosomal protein L24 OS=Synechocystis sp. (strain PCC 6803 / Kazusa) GN=r	781,89	68,70	1	9	9	41	4,855	29	125,6	115	12,8	10,08							
	A3	Sequence	# PSMs	# Proteins	# Protein Groups	Protein Group Accessions	Modifications	ΔCn	114/115	114/115 Count	114/115 Variability [%]	q-Value	PEP	IonScore	Exp Value	Charge	MH+ [Da]	ΔM [ppm]	RT [min]	# Missed Cleavages
	High	qEGESGQISSYEAPIHSSk	2	1	1	P73309	N-Term(iTRAQ4plex); K19(iTRAQ4plex)	0,0000	3,189	1		0	3,323E-16	78	1,74973E-08	3	2322,14829	1,31	24,06	0
	High	tIPSHSQVVVk	3	1	1	P73309	N-Term(iTRAQ4plex); K11(iTRAQ4plex)	0,0000	8,279	2	12,7	0	6,882E-08	70	9,79422E-08	2	1482,89055	1,54	19,36	0
	High	kGDTIQVISGk	12	1	1	P73309	N-Term(iTRAQ4plex); K1(iTRAQ4plex); K11(iTRAQ4plex)	0,0000	8,562	8	259,7	0	0,000002185	62	6,98184E-07	2	1577,96111	1,52	21,46	1
	High	icYTVTDDGRk	4	1	1	P73309	N-Term(iTRAQ4plex); C2(Carbamidomethyl); K11(iTRAQ4plex)	0,0000	5,032	4	7,9	0	1,278E-08	59	1,32425E-06	3	1615,83762	1,47	20,92	1
	High	vMLYSTk	5	1	1	P73309	N-Term(iTRAQ4plex); K7(iTRAQ4plex)	0,0000	4,048	3	4,4	0	0,00009649	52	7,03024E-06	2	1129,65544	2,20	25,31	0
	High	vmLYSTk	6	1	1	P73309	N-Term(iTRAQ4plex); M2(Oxidation); K7(iTRAQ4plex)	0,0000	8,185	3	1,2	0	0,0001347	44	4,42588E-05	2	1145,64934	1,28	21,47	0
	High	kGDTIQVISGkDk	1	1	1	P73309	N-Term(iTRAQ4plex); K1(iTRAQ4plex); K11(iTRAQ4plex); K13(iTRAQ4plex)	0,0000	1,526	1		0	0,00002569	17	0,01874865	4	1965,18398	0,66	20,12	2
	High	tkPAPHR	3	1	1	P73309	N-Term(iTRAQ4plex); K2(iTRAQ4plex)	0,0000	15,113	2	2,2	0,002	0,02217	12	0,059837027	3	1094,66819	0,80	7,63	0
	Medium	gVNIR	3	1	1	P73309	N-Term(iTRAQ4plex)	0,0000	1,458	3	8,9	0,019	0,2052	14	0,089368099	2	702,44243	6,43	13,62	0
	Medium	vGEVLR	2	1	1	P73309	N-Term(iTRAQ4plex)	0,0000	2,492	2	5,1	0,039	0,4048	19	0,014301865	2	816,50640	0,48	18,73	0
P23349	50S ribosomal protein L7/L12 OS=Synechocystis sp. (strain PCC 6803 / Kazusa) GN	761,88	52,34	1	9	9	35	1,614	29	24,2	128	13,3	4,77							
	A3	Sequence	# PSMs	# Proteins	# Protein Groups	Protein Group Accessions	Modifications	ΔCn	114/115	114/115 Count	114/115 Variability [%]	q-Value	PEP	IonScore	Exp Value	Charge	MH+ [Da]	ΔM [ppm]	RT [min]	# Missed Cleavages
	High	sLSLLEASELVk	7	1	1	P23349	N-Term(iTRAQ4plex); K12(iTRAQ4plex)	0,0000	1,354	7	26,8	0	2,664E-11	79	1,23302E-08	2	1576,94231	1,43	45,15	0
	High	eATGkDDAEAIkk	2	1	1	P23349	N-Term(iTRAQ4plex); K5(iTRAQ4plex); K12(iTRAQ4plex); K13(iTRAQ4plex)	0,0000	2,054	2	8,9	0	4,362E-11	59	1,39306E-06	3	1952,11759	1,49	15,07	2
	High	tITGLGLk	9	1	1	P23349	N-Term(iTRAQ4plex); K8(iTRAQ4plex)	0,0000	1,473	6	36,4	0	0,0000116	55	3,31108E-06	2	1090,70757	0,11	29,93	0
	High	qIEEAGGk	4	1	1	P23349	N-Term(iTRAQ4plex); K8(iTRAQ4plex)	0,0000	1,541	2	7,0	0	0,000008716	55	4,17744E-06	2	1119,62615	1,16	12,59	0
	High	eLVESTPk	3	1	1	P23349	N-Term(iTRAQ4plex); K8(iTRAQ4plex)	0,0000	1,733	3	13,1	0	0,002008	43	5,83973E-05	2	1190,69499	6,62	18,81	0
	High	kQIEEAGGk	3	1	1	P23349	N-Term(iTRAQ4plex); K1(iTRAQ4plex); K9(iTRAQ4plex)	0,0000	1,341	3	3,0	0	0,00003121	35	0,000314753	3	1391,82273	0,61	11,37	1
	High	sAATDQILEQLk	1	1	1	P23349	N-Term(iTRAQ4plex); K12(iTRAQ4plex)	0,0000	1,064	1		0	0,0003521	24	0,004111213	3	1604,91007	0,16	37,12	0
	High	kIAVLk	2	1	1	P23349	N-Term(iTRAQ4plex); K1(iTRAQ4plex); K6(iTRAQ4plex)	0,0000	1,513	2	23,6	0	0,001984	23	0,005081243	3	1103,78666	-0,90	18,70	1
	High	iAVLk	4	1	1	P23349	N-Term(iTRAQ4plex); K5(iTRAQ4plex)	0,0000	1,830	3	0,1	0,004	0,04669	20	0,01002236	2	831,58989	-0,87	24,62	0
P73511	Glycogen phosphorylase OS=Synechocystis sp. (strain PCC 6803 / Kazusa) GN=glgP	753,96	19,32	1	17	17	38	0,752	37	26,4	849	96,6	5,29							
	A3	Sequence	# PSMs	# Proteins	# Protein Groups	Protein Group Accessions	Modifications	ΔCn	114/115	114/115 Count	114/115 Variability [%]	q-Value	PEP	IonScore	Exp Value	Charge	MH+ [Da]	ΔM [ppm]	RT [min]	# Missed Cleavages
	High	hLEIIYEINQR	4	1	1	P73511	N-Term(iTRAQ4plex)	0,0000	0,547	4	3,7	0	4,886E-09	69	1,2473E-07	2	1571,86601	-0,15	33,43	0
	High	aAPGYFTAk	4	1	1	P73511	N-Term(iTRAQ4plex); K9(iTRAQ4plex)	0,0000	0,761	3	2,1	0	0,000005421	50	1,10655E-05	2	1213,68144	-0,42	23,57	0
	High	vIFLPDYNVk	1	1	1	P73511	N-Term(iTRAQ4plex); K10(iTRAQ4plex)	0,0000	0,708	1		0	1,482E-07	46	2,65442E-05	2	1495,87590	-0,27	43,25	0
	High	tFIYGGk	3	1	1	P73511	N-Term(iTRAQ4plex); K7(iTRAQ4plex)	0,0000	0,636	3	10,2	0	0,0004828	45	2,85739E-05	2	1073,62297	-0,37	25,05	0
	High	wIAGSLVk	3	1	1	P73511	N-Term(iTRAQ4plex); K8(iTRAQ4plex)	0,0000	0,684	3	15,6	0	0,000255	41	7,69077E-05	2	1161,72331	-0,11	35,57	0
	High	rWMVLSNPR	2	1	1	P73511	N-Term(iTRAQ4plex)	0,0000	0,811	2	31,0	0	0,000009099	38	0,000141896	3	1302,72159	-0,45	27,73	1
	High	mSSENLTk	1	1	1	P73511	N-Term(iTRAQ4plex); M1(Oxidation); K8(iTRAQ4plex)	0,0000	0,759	1		0	0,0006043	38	0,00017099	2	1213,63762	3,24	13,85	0
	High	lSNLISSR	3	1	1	P73511	N-Term(iTRAQ4plex)	0,0000	0,456	3	10,6	0	0,002398	38	0,000211925	2	1033,61235	0,09	29,03	0
	High	lLVDEHHYEWQR	4	1	1	P73511	N-Term(iTRAQ4plex)	0,0000	0,653	4	10,4	0	3,069E-08	36	0,000281171	4	1768,88942	0,37	29,65	0
	High	rWmVLSNPR	1	1	1	P73511	N-Term(iTRAQ4plex); M3(Oxidation)	0,0000	0,529	1		0	0,00009206	33	0,000463415	3	1318,71677	-0,25	23,12	1
	High	mAILNVAR	3	1	1	P73511	N-Term(iTRAQ4plex)	0,0000	1,234	3	7,8	0	0,000041	33	0,000561009	2	1031,61565	0,38	32,98	0
	High	fPAIATk	1	1	1	P73511	N-Term(iTRAQ4plex); K7(iTRAQ4plex)	0,0000	0,752	1		0	0,002513	30	0,000895365	2	1035,64543	1,27	27,83	0
	High	aLADNLFYLQGk	1	1	1	P73511	N-Term(iTRAQ4plex); K12(iTRAQ4plex)	0,0000	0,793	1		0	0,0000129	29	0,001218905	3	1640,92563	0,35	42,53	0
	High	mAILNVAR	1	1	1	P73511	N-Term(iTRAQ4plex); M1(Oxidation)	0,0000	0,777	1		0	0,0002918	27	0,002656646	2	1047,61028	0,10	24,22	0
	High	eTILkDFYELWPEk	1	1	1	P73511	N-Term(iTRAQ4plex); K5(iTRAQ4plex); K14(iTRAQ4plex)	0,0000	0,792	1		0	0,00002734	17	0,021562437	3	2243,23752	2,27	52,37	1
	High	wSLPLFGEmLPR	1	1	1	P73511	N-Term(iTRAQ4plex); M9(Oxidation)	0,0000	0,838	1		0	0,0004857	12	0,058880299	2	1605,85918	0,72	51,87	0
	High	qLEPFADLAGFR	1	1	1	P73511	N-Term(iTRAQ4plex)	0,0000	0,824	1		0,002	0,0181	24	0,004178015	2	1507,80266	0,03	47,61	0
	High	vSTANNLR	2	1	1	P73511	N-Term(iTRAQ4plex)	0,0000	1,146	2	17,7	0,005	0,05812	20	0,009527304	2	1018,57884	2,59	14,08	0
	Medium	mkYPNDGDR	1	1	1	P73511	N-Term(iTRAQ4plex); M1(Oxidation); K2(iTRAQ4plex)	0,0000	0,372	1		0,033	0,3534	11	0,084133703	3	1399,68784	-0,02	9,11	1
P29255	Photosystem I P700 chlorophyll a apoprotein A2 OS=Synechocystis sp. (strain PCC	740,07	11,63	1	9	9	47	0,507	36	22,7	731	81,2	6,95							
	A3	Sequence	# PSMs	# Proteins	# Protein Groups	Protein Group Accessions	Modifications	ΔCn	114/115	114/115 Count	114/115 Variability [%]	q-Value	PEP	IonScore	Exp Value	Charge	MH+ [Da]	ΔM [ppm]	RT [min]	# Missed Cleavages
	High	tNWGIGHSIk	7	1	1	P29255	N-Term(iTRAQ4plex); K10(iTRAQ4plex)	0,0000	0,444	5	8,2	0	0,000001522	69	1,25594E-07	2	1400,78789	-0,71	23,78	0
	High	dkPVALSIVQAR	7	1	1	P29255	N-Term(iTRAQ4plex); K2(iTRAQ4plex)	0,0000	0,534	5	0,7	0	7,972E-10	60	9,20386E-07	3	1584,96622	-0,87	28,79	0
	High	qILIEPVFAQWIQATSGk	1	1	1	P29255	N-Term(iTRAQ4plex); K18(iTRAQ4plex)	0,0000	0,461	1		0	1,911E-11	57	2,18761E-06	3	2317,31906	1,36	59,48	0
	High	eILNAHk	10	1	1	P29255	N-Term(iTRAQ4plex); K7(iTRAQ4plex)	0,0000	0,440	6	9,6	0	0,0001559	48	1,54871E-05	2	1112,66448	-1,94	14,29	0
	High	wkDkPVALSIVQAR	4	1	1	P29255	N-Term(iTRAQ4plex); K2(iTRAQ4plex); K4(iTRAQ4plex)	0,0000	0,174	4	15,4	0	1,208E-08	35	0,000348313	4	2043,24282	-0,56	32,37	1
	High	fRPSLAWFk	3	1	1	P29255	N-Term(iTRAQ4plex); K9(iTRAQ4plex)	0,0000	0,480	3	3,0	0	0,000003125	28	0,00155228	3	1439,84021	0,00	38,27	0
	High	tPLANLVR	4	1	1	P29255	N-Term(iTRAQ4plex)	0,0000	0,494	4	25,3	0,001	0,009879	24	0,004374919	2	1027,63799	-0,09	32,38	0
	High	lmPDkk	8	1	1	P29255	N-Term(iTRAQ4plex); M2(Oxidation); K5(iTRAQ4plex); K6(iTRAQ4plex)	0,0000	0,613	5	7,5	0,001	0,01705	17	0,021328976	3	1179,71196	-1,03	10,51	1
	High	lMPDkk	2	1	1	P29255	N-Term(iTRAQ4plex); K5(iTRAQ4plex); K6(iTRAQ4plex)	0,0000	0,531	2	12,3	0,002	0,02682	11	0,071116439	3	1163,71680	-1,25	15,66	1
	Medium	fSQDLAQDPTTRR	1	1	1	P29255	N-Term(iTRAQ4plex)	0,0000	0,881	1		0,021	0,2283	11	0,074468054	3	1678,87369	6,38	23,69	1
P73306	50S ribosomal protein L6 OS=Synechocystis sp. (strain PCC 6803 / Kazusa) GN=rpl	738,84	75,98	1	14	14	41	2,521	34	117,9	179	19,7	10,18							
	A3	Sequence	# PSMs	# Proteins	# Protein Groups	Protein Group Accessions	Modifications	ΔCn	114/115	114/115 Count	114/115 Variability [%]	q-Value	PEP	IonScore	Exp Value	Charge	MH+ [Da]	ΔM [ppm]	RT [min]	# Missed Cleavages
	High	vENNTQVIVSGIDkELLGNTAAk	1	1	1	P73306	N-Term(iTRAQ4plex); K14(iTRAQ4plex); K23(iTRAQ4plex)	0,0000	6,132	1		0	1,323E-12	89	1,24157E-09	3	2845,60233	0,96	47,03	1
	High	vIVAQEGETITVTR	1	1	1	P73306	N-Term(iTRAQ4plex)	0,0000	3,396	1		0	4,596E-10	85	3,2561E-09	2	1659,94121	0,83	32,62	0
	High	vSVDIQGSHLSVk	7	1	1	P73306	N-Term(iTRAQ4plex); K13(iTRAQ4plex)	0,0000	2,668	4	56,2	0	1,026E-09	85	2,8772E-09	2	1656,95220	-0,09	30,85	0
	High	rLEIQGVGYR	3	1	1	P73306	N-Term(iTRAQ4plex)	0,0000	2,886	2	6,5	0	0,0001251	52	8,04818E-06	2	1334,77080	3,49	24,92	1
	High	tLVANmVDGVAQGFER	1	1	1	P73306	N-Term(iTRAQ4plex); M6(Oxidation)	0,0000	0,432	1		0	1,482E-11	51	7,48118E-06	3	1866,94883	-0,69	38,21	0
	High	lEIQGVGYR	1	1	1	P73306	N-Term(iTRAQ4plex)	0,0000	1,205	1		0	0,00008879	44	4,27533E-05	2	1178,66545	0,36	31,12	0
	High	aVRPPEPYk	10	1	1	P73306	N-Term(iTRAQ4plex); K9(iTRAQ4plex)	0,0000	2,544	8	107,1	0	0,00007922	41	8,12774E-05	2	1344,78850	0,50	17,95	0
	High	yQGEYVR	3	1	1	P73306	N-Term(iTRAQ4plex)	0,0000	3,200	3	32,4	0	0,0004889	38	0,000159945	2	1058,53911	0,36	16,70	0
	High	eLLGNTAAk	1	1	1	P73306	N-Term(iTRAQ4plex); K9(iTRAQ4plex)	0,0000	1,044	1		0	0,00001081	35	0,000421873	2	1204,71501	0,84	19,69	0
	High	lTLNVGYSkPVEmTmPQGIEVk	1	1	1	P73306	N-Term(iTRAQ4plex); K9(iTRAQ4plex); M13(Oxidation); M15(Oxidation); K22	0,0000	0,783	1		0	9,499E-09	34	0,000446591	4	2898,57241	1,46	37,32	0
	High	rPIPLPAk	6	1	1	P73306	N-Term(iTRAQ4plex); K8(iTRAQ4plex)	0,0000	5,068	5	68,9	0	0,003019	30	0,000946172	2	1179,78215	0,44	20,52	0
	High	aQAQGNk	2	1	1	P73306	N-Term(iTRAQ4plex); K7(iTRAQ4plex)	0,0000	5,994	2	24,3	0	0,0004041	29	0,001315134	2	1004,57335	0,61	7,81	0
	High	lTLNVGYSkPVEmTMPQGIEVk	1	1	1	P73306	N-Term(iTRAQ4plex); K9(iTRAQ4plex); M13(Oxidation); K22(iTRAQ4plex)	0,0000	0,823	1		0	9,045E-09	12	0,067915672	4	2882,57583	0,89	40,78	0
	High	aVRPPEPYkGk	1	1	1	P73306	N-Term(iTRAQ4plex); K9(iTRAQ4plex); K11(iTRAQ4plex)	0,0000	0,763	1		0	0,005429	10	0,091615721	4	1674,00710	0,46	16,70	1
	Medium	qLPEk	2	1	1	P73306	N-Term(iTRAQ4plex); K5(iTRAQ4plex)	0,0000	1,988	2	15,5	0,019	0,2112	21	0,008408099	2	902,56017	5,77	14,25	0
Q55665	Glutamate-1-semialdehyde 2,1-aminomutase OS=Synechocystis sp. (strain PCC 680	731,04	28,64	1	9	9	21	0,941	17	24,6	433	45,9	5,27							
	A3	Sequence	# PSMs	# Proteins	# Protein Groups	Protein Group Accessions	Modifications	ΔCn	114/115	114/115 Count	114/115 Variability [%]	q-Value	PEP	IonScore	Exp Value	Charge	MH+ [Da]	ΔM [ppm]	RT [min]	# Missed Cleavages
	High	vIGGGLPVGAYGGR	5	1	1	Q55665	N-Term(iTRAQ4plex)	0,0000	0,871	4	20,6	0	1,79E-11	72	6,71382E-08	2	1416,80937	0,95	34,20	0
	High	vNATPFITTk	3	1	1	Q55665	N-Term(iTRAQ4plex); K10(iTRAQ4plex)	0,0000	1,203	3	2,6	0	4,695E-07	70	9,97631E-08	2	1379,81377	0,04	30,48	0
	High	aTLTAPYNDLEAVk	1	1	1	Q55665	N-Term(iTRAQ4plex); K14(iTRAQ4plex)	0,0000	1,108	1		0	2,161E-07	64	4,33481E-07	2	1793,99040	0,90	37,64	0
	High	vSYGGAQAR	3	1	1	Q55665	N-Term(iTRAQ4plex)	0,0000	0,986	2	6,9	0	0,000001511	62	6,68298E-07	2	1052,56035	-0,19	13,60	0
	High	sVGGQPIVFDR	2	1	1	Q55665	N-Term(iTRAQ4plex)	0,0000	0,909	2	0,8	0	0,000002095	53	5,5077E-06	2	1318,72417	0,41	29,79	0
	High	sEEIFAAAQHLmPGGVSSPVR	3	1	1	Q55665	N-Term(iTRAQ4plex); M12(Oxidation)	0,0000	0,758	1		0	9,31E-10	48	1,6217E-05	3	2343,19205	1,53	40,07	0
	High	fVNSGTEAcmSVLR	1	1	1	Q55665	N-Term(iTRAQ4plex); C9(Carbamidomethyl); M10(Oxidation)	0,0000	0,766	1		0	1,614E-07	47	2,11821E-05	2	1730,83440	1,22	30,35	0
	High	sEEIFAAAQHLMPGGVSSPVR	1	1	1	Q55665	N-Term(iTRAQ4plex)	0,0000	0,868	1		0	1,016E-08	41	7,11164E-05	3	2327,19486	0,56	46,96	0
	High	tLEILQkPGSYEYLDk	1	1	1	Q55665	N-Term(iTRAQ4plex); K7(iTRAQ4plex); K16(iTRAQ4plex)	0,0000	1,279	1		0	1,163E-11	36	0,000267898	3	2329,30478	1,38	39,92	0
	High	fEGcYHGHADmFLVk	1	1	1	Q55665	N-Term(iTRAQ4plex); C4(Carbamidomethyl); M11(Oxidation); K15(iTRAQ4ple	0,0000	0,723	1		0	0,0001625	14	0,036725693	4	2115,00332	0,11	29,26	0
P77969	Delta-aminolevulinic acid dehydratase OS=Synechocystis sp. (strain PCC 6803 / Kaz	723,23	35,78	1	13	13	33	1,505	31	10,5	327	36,1	5,08							
	A3	Sequence	# PSMs	# Proteins	# Protein Groups	Protein Group Accessions	Modifications	ΔCn	114/115	114/115 Count	114/115 Variability [%]	q-Value	PEP	IonScore	Exp Value	Charge	MH+ [Da]	ΔM [ppm]	RT [min]	# Missed Cleavages
	High	vTLETLTSFk	3	1	1	P77969	N-Term(iTRAQ4plex); K10(iTRAQ4plex)	0,0000	1,563	3	3,7	0	2,04E-09	86	2,43764E-09	2	1426,84136	1,24	44,21	0
	High	ikEmTNLPVAAYNVSGEYSMVk	1	1	1	P77969	N-Term(iTRAQ4plex); K2(iTRAQ4plex); M4(Oxidation); K22(iTRAQ4plex)	0,0000	1,053	1		0	1,093E-12	68	1,73768E-07	4	2892,52480	1,24	38,33	1
	High	ikEmTNLPVAAYNVSGEYSmVk	1	1	1	P77969	N-Term(iTRAQ4plex); K2(iTRAQ4plex); M4(Oxidation); M20(Oxidation); K22(	0,0000	0,964	1		0	4,844E-12	50	9,31044E-06	4	2908,52041	1,47	35,78	1
	High	aAALNGWIDEQkVTLETLTSFk	1	1	1	P77969	N-Term(iTRAQ4plex); K12(iTRAQ4plex); K22(iTRAQ4plex)	0,0000	1,416	1		0	5,314E-08	50	9,37497E-06	3	2867,59275	1,67	55,66	1
	High	vLNDPTLELLkk	5	1	1	P77969	N-Term(iTRAQ4plex); K11(iTRAQ4plex); K12(iTRAQ4plex)	0,0000	1,510	5	5,6	0	7,919E-08	49	1,20773E-05	3	1815,13309	0,82	38,37	1
	High	vTLETLTSFkR	2	1	1	P77969	N-Term(iTRAQ4plex); K10(iTRAQ4plex)	0,0000	1,392	2	16,2	0	4,274E-08	47	2,21294E-05	3	1582,94199	0,81	38,94	1
	High	aTEAVkk	2	1	1	P77969	N-Term(iTRAQ4plex); K6(iTRAQ4plex); K7(iTRAQ4plex)	0,0000	1,481	2	5,0	0	0,001121	46	2,7477E-05	2	1178,74455	-2,00	9,15	1
	High	aGADLILTYHAk	6	1	1	P77969	N-Term(iTRAQ4plex); K12(iTRAQ4plex)	0,0000	1,633	4	4,5	0	0,00004871	44	4,00839E-05	3	1560,90049	1,06	30,73	0
	High	rAGADLILTYHAk	1	1	1	P77969	N-Term(iTRAQ4plex); K13(iTRAQ4plex)	0,0000	1,420	1		0	6,964E-08	43	5,02308E-05	4	1717,00100	0,61	25,40	1
	High	yASAYYGPFR	3	1	1	P77969	N-Term(iTRAQ4plex)	0,0000	1,548	3	2,9	0	0,000001006	42	6,03907E-05	2	1338,66142	1,13	33,55	0
	High	dAADSSPQFGDRR	1	1	1	P77969	N-Term(iTRAQ4plex)	0,0000	0,997	1		0	0,005358	24	0,00372366	3	1565,74161	-0,59	14,43	1
	High	mFPTIRPR	1	1	1	P77969	N-Term(iTRAQ4plex)	0,0000	1,783	1		0	0,006674	19	0,01349551	3	1161,66840	0,04	26,68	0
	High	mFPTIRPR	3	1	1	P77969	N-Term(iTRAQ4plex); M1(Oxidation)	0,0000	1,465	3	4,0	0,001	0,009722	15	0,028311964	3	1177,66953	5,32	20,66	0
	High	tYQmDPGNSR	1	1	1	P77969	N-Term(iTRAQ4plex); M4(Oxidation)	0,0000	1,786	1		0,002	0,0194	15	0,032357131	3	1328,60355	1,04	8,24	0
	High	aTEAVk	2	1	1	P77969	N-Term(iTRAQ4plex); K6(iTRAQ4plex)	0,0000	1,334	2	5,0	0,004	0,05366	34	0,00042462	2	906,54875	-1,24	12,51	0
P74267	50S ribosomal protein L27 OS=Synechocystis sp. (strain PCC 6803 / Kazusa) GN=r	714,12	59,77	1	5	5	23	2,058	19	56,5	87	9,4	11,24							
	A3	Sequence	# PSMs	# Proteins	# Protein Groups	Protein Group Accessions	Modifications	ΔCn	114/115	114/115 Count	114/115 Variability [%]	q-Value	PEP	IonScore	Exp Value	Charge	MH+ [Da]	ΔM [ppm]	RT [min]	# Missed Cleavages
	High	yGGQTVTAGSIIVR	4	1	1	P74267	N-Term(iTRAQ4plex)	0,0000	1,776	4	72,8	0	3,576E-12	93	5,08619E-10	2	1565,87834	0,97	31,70	0
	High	gkDDTLFALIDGVVk	10	1	1	P74267	N-Term(iTRAQ4plex); K2(iTRAQ4plex); K15(iTRAQ4plex)	0,0000	1,915	8	74,0	0	5,576E-10	73	4,797E-08	2	2023,17668	-1,65	50,65	1
	High	gTQVHPGNNVGR	5	1	1	P74267	N-Term(iTRAQ4plex)	0,0000	2,294	3	28,1	0	0,000007709	52	5,754E-06	2	1379,72795	1,36	9,40	0
	High	kVSVYPATAE	3	1	1	P74267	N-Term(iTRAQ4plex); K1(iTRAQ4plex)	0,0000	2,196	3	1,7	0	0,001938	46	2,56448E-05	2	1352,76799	1,16	21,94	1
	High	rYGGQTVTAGSIIVR	1	1	1	P74267	N-Term(iTRAQ4plex)	0,0000	1,221	1		0	0,00001458	41	8,12774E-05	3	1721,97800	0,03	26,39	1
Q55499	Single-stranded DNA-binding protein 1 OS=Synechocystis sp. (strain PCC 6803 / Ka	708,70	57,85	1	10	10	32	1,560	27	33,5	121	13,6	7,34							
	A3	Sequence	# PSMs	# Proteins	# Protein Groups	Protein Group Accessions	Modifications	ΔCn	114/115	114/115 Count	114/115 Variability [%]	q-Value	PEP	IonScore	Exp Value	Charge	MH+ [Da]	ΔM [ppm]	RT [min]	# Missed Cleavages
	High	sVNSIHLVGR	7	1	1	Q55499	N-Term(iTRAQ4plex)	0,0000	1,792	4	2,8	0	1,961E-09	86	2,39332E-09	2	1225,71391	0,44	22,16	0
	High	tAEIAGNYVk	5	1	1	Q55499	N-Term(iTRAQ4plex); K10(iTRAQ4plex)	0,0000	1,306	4	106,5	0	0,000001394	66	3,35996E-07	2	1353,76274	0,79	23,81	0
	High	vNNLDLLGSk	2	1	1	Q55499	N-Term(iTRAQ4plex); K10(iTRAQ4plex)	0,0000	1,874	2	5,4	0	0,000002162	64	5,43179E-07	2	1360,80608	1,62	34,18	0
	High	gSLIGIQGSLk	2	1	1	Q55499	N-Term(iTRAQ4plex); K11(iTRAQ4plex)	0,0000	1,385	2	71,2	0	1,388E-08	58	1,68256E-06	2	1360,84197	1,25	33,30	0
	High	kGSLIGIQGSLk	6	1	1	Q55499	N-Term(iTRAQ4plex); K1(iTRAQ4plex); K12(iTRAQ4plex)	0,0000	1,723	5	4,9	0	2,016E-07	52	5,71439E-06	3	1633,03873	0,88	28,47	1
	High	vNNLDLLGSkR	2	1	1	Q55499	N-Term(iTRAQ4plex); K10(iTRAQ4plex)	0,0000	1,241	2	7,0	0	5,675E-07	41	8,83879E-05	3	1516,90610	0,73	28,09	1
	High	fDHWEDR	4	1	1	Q55499	N-Term(iTRAQ4plex)	0,0000	1,322	4	11,4	0	0,0006263	32	0,00064561	2	1148,52446	0,25	21,77	0
	High	fDHWEDRNSGTPR	2	1	1	Q55499	N-Term(iTRAQ4plex)	0,0000	1,051	2	4,1	0	0,0003012	22	0,005915208	3	1760,82309	0,52	20,47	1
	High	skPVIR	1	1	1	Q55499	N-Term(iTRAQ4plex); K2(iTRAQ4plex)	0,0000	1,891	1		0,002	0,0209	16	0,025702183	3	987,65650	1,16	10,33	0
	Medium	aGRDPEVk	1	1	1	Q55499	N-Term(iTRAQ4plex); K8(iTRAQ4plex)	0,0000	1,372	1		0,048	0,4978	11	0,078886012	3	1159,66770	0,27	8,94	1
P74071	30S ribosomal protein S2 OS=Synechocystis sp. (strain PCC 6803 / Kazusa) GN=rps	706,16	25,65	1	9	9	28	1,657	26	11,5	269	30,1	5,27							
	A3	Sequence	# PSMs	# Proteins	# Protein Groups	Protein Group Accessions	Modifications	ΔCn	114/115	114/115 Count	114/115 Variability [%]	q-Value	PEP	IonScore	Exp Value	Charge	MH+ [Da]	ΔM [ppm]	RT [min]	# Missed Cleavages
	High	lkELEALEASGGIDR	4	1	1	P74071	N-Term(iTRAQ4plex); K2(iTRAQ4plex)	0,0000	1,542	4	17,3	0	1,405E-09	84	4,04011E-09	2	1889,05901	0,38	39,21	1
	High	qAAGIISQEAQR	2	1	1	P74071	N-Term(iTRAQ4plex)	0,0000	1,665	2	12,2	0	4,258E-07	66	2,35489E-07	2	1415,77153	-0,59	22,29	0
	High	kLPDVVVIVDQR	3	1	1	P74071	N-Term(iTRAQ4plex); K1(iTRAQ4plex)	0,0000	1,533	3	3,6	0	4,743E-10	58	1,62544E-06	3	1669,02756	1,46	35,95	1
	High	lkELEALEASGGIDRR	4	1	1	P74071	N-Term(iTRAQ4plex); K2(iTRAQ4plex)	0,0000	1,643	4	2,2	0	0,00000029	39	0,000116405	4	2045,15981	0,19	35,91	2
	High	eHNAIHEcQk	3	1	1	P74071	N-Term(iTRAQ4plex); C8(Carbamidomethyl); K10(iTRAQ4plex)	0,0000	1,616	2	2,3	0	0,000000631	31	0,000777983	4	1553,77407	0,49	8,27	0
	High	kFLFVGTk	2	1	1	P74071	N-Term(iTRAQ4plex); K1(iTRAQ4plex); K8(iTRAQ4plex)	0,0000	1,832	2	15,1	0	0,003286	30	0,001051889	2	1371,87334	0,65	29,80	1
	High	fLFVGTk	5	1	1	P74071	N-Term(iTRAQ4plex); K7(iTRAQ4plex)	0,0000	1,926	4	2,1	0	0,002214	27	0,002233418	2	1099,67620	0,71	36,64	0
	High	yLGGIk	3	1	1	P74071	N-Term(iTRAQ4plex); K6(iTRAQ4plex)	0,0000	1,652	3	0,6	0,008	0,09256	28	0,00141896	2	938,59197	0,67	25,19	0
	Medium	lIVGk	2	1	1	P74071	N-Term(iTRAQ4plex); K5(iTRAQ4plex)	0,0000	1,985	2	9,2	0,019	0,2059	27	0,001958709	2	817,57469	-0,33	21,52	0
P72691	Glycogen operon protein GlgX OS=Synechocystis sp. (strain PCC 6803 / Kazusa) GN	695,59	18,90	1	15	15	32	1,204	28	11,7	746	83,4	5,06							
	A3	Sequence	# PSMs	# Proteins	# Protein Groups	Protein Group Accessions	Modifications	ΔCn	114/115	114/115 Count	114/115 Variability [%]	q-Value	PEP	IonScore	Exp Value	Charge	MH+ [Da]	ΔM [ppm]	RT [min]	# Missed Cleavages
	High	gTYAGLIEk	2	1	1	P72691	N-Term(iTRAQ4plex); K9(iTRAQ4plex)	0,0000	1,290	1		0	3,657E-07	69	1,24157E-07	2	1239,71636	-1,92	27,06	0
	High	aNHPVVGGLILDSLR	5	1	1	P72691	N-Term(iTRAQ4plex)	0,0000	1,207	5	6,1	0	1,531E-09	64	4,1754E-07	3	1704,98783	0,02	40,84	0
	High	nPNSGLSENkR	2	1	1	P72691	N-Term(iTRAQ4plex); K10(iTRAQ4plex)	0,0000	1,305	2	1,3	0	4,368E-07	52	6,32412E-06	2	1503,81072	-0,73	9,89	1
	High	yWVSEMHVDGFR	1	1	1	P72691	N-Term(iTRAQ4plex)	0,0000	1,410	1		0	1,454E-08	48	1,5921E-05	3	1669,79331	1,15	40,38	0
	High	dLPGScVVWHGVk	2	1	1	P72691	N-Term(iTRAQ4plex); C6(Carbamidomethyl); K13(iTRAQ4plex)	0,0000	1,696	1		0	4,565E-09	45	2,92395E-05	3	1741,93155	0,98	33,08	0
	High	fDLASVLVR	4	1	1	P72691	N-Term(iTRAQ4plex)	0,0000	1,198	4	3,0	0	0,0002122	43	4,73119E-05	2	1163,68987	-0,56	47,03	0
	High	aGQVYAYR	2	1	1	P72691	N-Term(iTRAQ4plex)	0,0000	1,167	2	3,1	0	0,00001956	40	9,90764E-05	2	1071,57073	0,33	18,81	0
	High	sSVVLmAk	5	1	1	P72691	N-Term(iTRAQ4plex); M6(Oxidation); K8(iTRAQ4plex)	0,0000	1,281	3	9,7	0	0,00005645	39	0,000142134	2	1138,67974	4,65	18,32	0
	High	vTLLLFDRPNDPAPAR	3	1	1	P72691	N-Term(iTRAQ4plex)	0,0000	1,099	3	4,3	0	0,00007387	37	0,000214768	3	1939,08908	0,43	41,57	0
	High	tFYYWHVFVk	1	1	1	P72691	N-Term(iTRAQ4plex); K10(iTRAQ4plex)	0,0000	1,607	1		0	0,000004289	34	0,00039443	3	1677,90396	0,48	42,33	0
	High	rGTYAGLIEk	1	1	1	P72691	N-Term(iTRAQ4plex); K10(iTRAQ4plex)	0,0000	0,954	1		0	0,000429	27	0,002098795	3	1395,81983	-0,02	23,12	1
	High	lLGSPDIYYR	1	1	1	P72691	N-Term(iTRAQ4plex)	0,0000	0,746	1		0	0,006628	11	0,088505448	2	1340,74187	6,54	37,07	0
	High	tIELHR	1	1	1	P72691	N-Term(iTRAQ4plex)	0,0000	1,202	1		0,001	0,01593	20	0,009246343	3	912,53894	0,65	15,05	0
	High	vDGPHEPEk	1	1	1	P72691	N-Term(iTRAQ4plex); K9(iTRAQ4plex)	0,0000	1,175	1		0,002	0,0276	19	0,012734151	3	1295,68375	0,25	13,57	0
	Medium	iPYLk	1	1	1	P72691	N-Term(iTRAQ4plex); K5(iTRAQ4plex)	0,0000	1,522	1		0,041	0,4325	21	0,008336236	2	921,60961	9,16	30,37	0
P72761	Carbon dioxide-concentrating mechanism protein CcmK homolog 2 OS=Synechocys	693,86	75,73	1	4	7	39	1,792	9	33,6	103	11,1	5,41							
	A3	Sequence	# PSMs	# Proteins	# Protein Groups	Protein Group Accessions	Modifications	ΔCn	114/115	114/115 Count	114/115 Variability [%]	q-Value	PEP	IonScore	Exp Value	Charge	MH+ [Da]	ΔM [ppm]	RT [min]	# Missed Cleavages
	High	sIAVGMIETR	3	1	1	P72761	N-Term(iTRAQ4plex)	0,0000	2,251	2	1,1	0	2,226E-07	60	9,20386E-07	2	1220,67949	0,42	31,73	0
	High	yTEEVEQFR	2	1	1	P72761	N-Term(iTRAQ4plex)	0,0000	1,691	2	8,6	0	0,00000146	54	4,36486E-06	2	1344,65630	0,78	30,53	0
	High	vTLVGYEk	19	2	2	P72760;P72761	N-Term(iTRAQ4plex); K8(iTRAQ4plex)	0,0000				0	0,0008475	49	1,13755E-05	2	1196,71440	1,24	27,93	0
	High	gFPAVVEAADSmVk	1	1	1	P72761	N-Term(iTRAQ4plex); M12(Oxidation); K14(iTRAQ4plex)	0,0000	0,749	1		0	2,412E-08	46	2,43764E-05	3	1724,91501	1,04	38,28	0
	High	vNGGEVLSTHIIARPHENLEYVLPIR	1	1	1	P72761	N-Term(iTRAQ4plex)	0,0000	1,393	1		0	4,21E-12	44	4,33481E-05	4	3070,69350	0,51	40,45	0
	High	gFPAVVEAADSMVk	1	1	1	P72761	N-Term(iTRAQ4plex); K14(iTRAQ4plex)	0,0000	1,065	1		0	0,00001098	42	6,12308E-05	3	1708,91874	0,26	47,93	0
	High	sIAVGmIETR	3	1	1	P72761	N-Term(iTRAQ4plex); M6(Oxidation)	0,0000	1,990	2	5,5	0	2,837E-07	42	8,05715E-05	2	1236,67473	0,67	24,93	0
	High	vTVIVR	7	2	2	P72760;P72761	N-Term(iTRAQ4plex)	0,0000				0,001	0,01412	37	0,000187055	2	830,55889	1,02	22,67	0
	Medium	iGSGR	2	2	2	P72760;P72761	N-Term(iTRAQ4plex)	0,0000				0,023	0,2444	16	0,0477829	2	633,38042	0,56	7,96	0
Q55511	Trigger factor OS=Synechocystis sp. (strain PCC 6803 / Kazusa) GN=tig PE=3 SV=1	693,26	24,42	1	14	14	37	1,672	33	19,4	471	52,6	4,41							
	A3	Sequence	# PSMs	# Proteins	# Protein Groups	Protein Group Accessions	Modifications	ΔCn	114/115	114/115 Count	114/115 Variability [%]	q-Value	PEP	IonScore	Exp Value	Charge	MH+ [Da]	ΔM [ppm]	RT [min]	# Missed Cleavages

	High	lVNFVESSLTESk	3	1	1	Q55511	N-Term(iTRAQ4plex); K13(iTRAQ4plex)	0,0000	1,303	3	21,7	0	4,035E-08	92	6,3387E-10	2	1740,96636	2,37	46,69	0
	High	lVAQTAMELER	1	1	1	Q55511	N-Term(iTRAQ4plex)	0,0000	1,646	1		0	1,561E-08	65	2,97296E-07	2	1404,76384	0,04	35,43	0
	High	lVAQTAmELER	1	1	1	Q55511	N-Term(iTRAQ4plex); M7(Oxidation)	0,0000	1,694	1		0	0,00000485	45	2,95801E-05	2	1420,75957	0,61	32,70	0
	High	dNSRPEAIANLk	5	1	1	Q55511	N-Term(iTRAQ4plex); K12(iTRAQ4plex)	0,0000	1,705	4	5,4	0	4,968E-07	42	8,89085E-05	3	1615,90207	0,88	22,14	0
	High	kVYENVVk	5	1	1	Q55511	N-Term(iTRAQ4plex); K1(iTRAQ4plex); K8(iTRAQ4plex)	0,0000	2,111	4	8,1	0	0,00001625	42	7,04644E-05	3	1410,87531	5,12	18,21	1
	High	iQPTEAEVkER	1	1	1	Q55511	N-Term(iTRAQ4plex); K9(iTRAQ4plex)	0,0000	0,948	1		0	0,000003191	39	0,000115337	3	1587,89499	0,31	18,94	1
	High	tVNIPGFR	3	1	1	Q55511	N-Term(iTRAQ4plex)	0,0000	1,650	3	10,4	0	0,004159	37	0,000247846	2	1047,60674	-0,05	33,22	0
	High	qFQEQAk	2	1	1	Q55511	N-Term(iTRAQ4plex); K7(iTRAQ4plex)	0,0000	1,589	2	30,3	0	0,0005349	36	0,000252912	2	1166,64787	6,05	14,44	0
	High	iQPTEAEVk	2	1	1	Q55511	N-Term(iTRAQ4plex); K9(iTRAQ4plex)	0,0000	1,847	2	29,5	0	0,0002943	36	0,000271625	2	1302,75810	5,62	21,40	0
	High	vYENVVk	2	1	1	Q55511	N-Term(iTRAQ4plex); K7(iTRAQ4plex)	0,0000	1,651	2	7,2	0	0,001603	28	0,001645859	2	1138,67253	1,30	22,39	0
	High	aAVkQEELPIIGNFSLR	1	1	1	Q55511	N-Term(iTRAQ4plex); K4(iTRAQ4plex)	0,0000	1,474	1		0	0,000000259	23	0,00485255	3	2173,25901	0,28	45,69	1
	High	lGQSYIk	2	1	1	Q55511	N-Term(iTRAQ4plex); K7(iTRAQ4plex)	0,0000	1,825	2	11,5	0,001	0,008211	30	0,000997631	2	1096,66142	0,86	22,94	0
	High	mGLDVSQLFR	3	1	1	Q55511	N-Term(iTRAQ4plex); M1(Oxidation)	0,0000	1,225	3	3,1	0,001	0,01282	29	0,001177525	2	1325,70159	0,86	42,96	0
	High	aIVIQR	4	1	1	Q55511	N-Term(iTRAQ4plex)	0,0000	1,638	2	3,1	0,001	0,01735	14	0,036221798	2	843,55400	0,85	19,39	0
	High	lkEIk	2	1	1	Q55511	N-Term(iTRAQ4plex); K2(iTRAQ4plex); K5(iTRAQ4plex)	0,0000	1,865	2	20,5	0,002	0,01854	19	0,012970897	3	1062,72434	-0,35	15,90	1
P72659	Polyribonucleotide nucleotidyltransferase OS=Synechocystis sp. (strain PCC 6803 /	693,22	21,03	1	15	15	35	0,694	31	16,4	718	77,8	5,27							
	A3	Sequence	# PSMs	# Proteins	# Protein Groups	Protein Group Accessions	Modifications	ΔCn	114/115	114/115 Count	114/115 Variability [%]	q-Value	PEP	IonScore	Exp Value	Charge	MH+ [Da]	ΔM [ppm]	RT [min]	# Missed Cleavages
	High	kIITVLGQFDLGk	1	1	1	P72659	N-Term(iTRAQ4plex); K1(iTRAQ4plex); K13(iTRAQ4plex)	0,0000	0,752	1		0	9,922E-12	57	1,84914E-06	3	1864,16269	-0,29	45,43	1
	High	aImQALPAR	3	1	1	P72659	N-Term(iTRAQ4plex); M3(Oxidation)	0,0000	0,712	2	3,7	0	0,000001901	55	3,26565E-06	2	1130,64958	2,03	20,43	0
	High	lGIHPDEAAEAR	2	1	1	P72659	N-Term(iTRAQ4plex)	0,0000	0,964	2	24,4	0	0,000007599	54	3,68952E-06	3	1422,74457	-0,88	20,76	0
	High	kLNEGEVYLGR	6	1	1	P72659	N-Term(iTRAQ4plex); K1(iTRAQ4plex)	0,0000	0,598	4	1,5	0	0,000006772	51	7,62411E-06	2	1565,88970	0,44	24,55	1
	High	eIGHGALAER	2	1	1	P72659	N-Term(iTRAQ4plex)	0,0000	0,724	2	10,4	0	0,000005463	48	1,53805E-05	3	1196,65006	-0,33	14,78	0
	High	kPVSGAAmGLIk	2	1	1	P72659	N-Term(iTRAQ4plex); K1(iTRAQ4plex); M8(Oxidation); K12(iTRAQ4plex)	0,0000	0,747	2	5,4	0	3,676E-08	45	3,13307E-05	3	1619,99082	1,79	21,80	0
	High	iIPIGAFVEVLPGk	4	1	1	P72659	N-Term(iTRAQ4plex); K14(iTRAQ4plex)	0,0000	0,773	4	20,4	0	0,00005202	44	4,31489E-05	2	1741,09014	1,99	54,68	0
	High	kQIVDDGVR	1	1	1	P72659	N-Term(iTRAQ4plex); K1(iTRAQ4plex)	0,0000	0,671	1		0	0,00003629	41	9,24578E-05	3	1317,77222	-0,53	14,44	1
	High	lVGNLYk	1	1	1	P72659	N-Term(iTRAQ4plex); K7(iTRAQ4plex)	0,0000	0,641	1		0	0,004577	32	0,000636752	2	1094,68572	4,11	28,06	0
	High	ePRPELSPFAPR	2	1	1	P72659	N-Term(iTRAQ4plex)	0,0000	0,730	2	11,8	0	6,095E-07	31	0,000765544	3	1539,84024	0,12	28,38	0
	High	kPVSGAAMGLIk	1	1	1	P72659	N-Term(iTRAQ4plex); K1(iTRAQ4plex); K12(iTRAQ4plex)	0,0000	0,663	1		0	0,001386	29	0,001142799	3	1603,98813	-3,04	26,40	0
	High	kLEQVRPIScEVGFLPR	3	1	1	P72659	N-Term(iTRAQ4plex); K1(iTRAQ4plex); C10(Carbamidomethyl)	0,0000	0,638	2	6,2	0	7,474E-08	26	0,002360315	3	2316,31144	0,57	36,03	1
	High	lHILDk	3	1	1	P72659	N-Term(iTRAQ4plex); K6(iTRAQ4plex)	0,0000	0,554	3	25,6	0	0,000642	26	0,002426443	3	1026,65329	-1,67	24,68	0
	High	eGRPPEk	2	1	1	P72659	N-Term(iTRAQ4plex); K7(iTRAQ4plex)	0,0000	0,742	2	7,2	0	0,005812	16	0,022854409	3	1100,63074	0,43	8,48	0
	High	aTLISR	1	1	1	P72659	N-Term(iTRAQ4plex)	0,0000	0,694	1		0,009	0,1093	12	0,06382194	2	804,50572	-0,34	16,70	0
	High	mLATIR	1	1	1	P72659	N-Term(iTRAQ4plex)	0,0000	0,761	1		0,009	0,1104	11	0,154536117	2	848,51457	0,13	22,93	0
P73289	50S ribosomal protein L25 OS=Synechocystis sp. (strain PCC 6803 / Kazusa) GN=rp	689,91	65,31	1	7	7	37	1,560	33	37,7	98	11,1	8,94							
	A3	Sequence	# PSMs	# Proteins	# Protein Groups	Protein Group Accessions	Modifications	ΔCn	114/115	114/115 Count	114/115 Variability [%]	q-Value	PEP	IonScore	Exp Value	Charge	MH+ [Da]	ΔM [ppm]	RT [min]	# Missed Cleavages
	High	aLSIEcQQRPEk	8	1	1	P73289	N-Term(iTRAQ4plex); C6(Carbamidomethyl); K12(iTRAQ4plex)	0,0000	4,913	5	583,2	0	1,805E-09	79	1,49414E-08	2	1746,93974	-0,79	20,41	0
	High	nLYHLAFFAGk	18	1	1	P73289	N-Term(iTRAQ4plex); K11(iTRAQ4plex)	0,0000	1,440	17	26,6	0	1,03E-09	68	1,54159E-07	2	1568,88310	0,20	40,01	0
	High	iPHLSWEGEAVVQEIQcHPWR	2	1	1	P73289	N-Term(iTRAQ4plex); C17(Carbamidomethyl)	0,0000	1,370	2	45,9	0	5,819E-12	54	4,00839E-06	4	2715,35732	-0,36	47,23	0
	High	eTPIEVk	2	1	1	P73289	N-Term(iTRAQ4plex); K7(iTRAQ4plex)	0,0000	1,935	2	11,2	0	0,003868	48	1,90852E-05	2	1103,66033	4,76	20,78	0
	High	tAITMLR	3	1	1	P73289	N-Term(iTRAQ4plex)	0,0000	1,237	3	5,7	0	0,0003995	40	0,000193273	2	949,56334	1,26	25,90	0
	High	tAITmLR	2	1	1	P73289	N-Term(iTRAQ4plex); M5(Oxidation)	0,0000	1,324	2	29,2	0	0,007066	25	0,004033647	2	965,55687	-0,19	20,47	0
	High	sVTVkETPIEVk	1	1	1	P73289	N-Term(iTRAQ4plex); K5(iTRAQ4plex); K12(iTRAQ4plex)	0,0000	1,995	1		0	0,00003176	23	0,004720304	3	1762,06913	0,26	25,74	1
	High	nLYHLAFFAGkk	1	1	1	P73289	N-Term(iTRAQ4plex); K11(iTRAQ4plex); K12(iTRAQ4plex)	0,0000	1,560	1		0	0,00002172	21	0,00714447	4	1841,07851	-0,71	33,56	1
P54206	Ribulose bisphosphate carboxylase small chain OS=Synechocystis sp. (strain PCC 6	688,02	61,95	1	5	5	26	1,265	24	18,6	113	13,2	5,48							
	A3	Sequence	# PSMs	# Proteins	# Protein Groups	Protein Group Accessions	Modifications	ΔCn	114/115	114/115 Count	114/115 Variability [%]	q-Value	PEP	IonScore	Exp Value	Charge	MH+ [Da]	ΔM [ppm]	RT [min]	# Missed Cleavages
	High	rYETLSYLPPLTDQQIAk	14	1	1	P54206	N-Term(iTRAQ4plex); K18(iTRAQ4plex)	0,0000	1,141	13	18,1	0	2,797E-12	68	1,69118E-07	3	2424,34311	2,22	40,40	1
	High	qcQTVSFIVHkPNQNQGR	5	1	1	P54206	N-Term(iTRAQ4plex); C2(Carbamidomethyl); K11(iTRAQ4plex)	0,0000	1,350	4	9,7	0	3,406E-12	62	5,662E-07	4	2429,27163	0,22	22,99	0
	High	vIGFDNIk	3	1	1	P54206	N-Term(iTRAQ4plex); K8(iTRAQ4plex)	0,0000	1,381	3	8,8	0	0,000004804	57	2,18258E-06	2	1193,71440	0,95	33,73	0
	High	sENPNcYIR	3	1	1	P54206	N-Term(iTRAQ4plex); C6(Carbamidomethyl)	0,0000	1,550	3	4,6	0	0,00001353	48	1,70204E-05	2	1296,61333	0,77	21,15	0
	High	lPFFGGATANEVLAEVR	1	1	1	P54206	N-Term(iTRAQ4plex)	0,0000	0,959	1		0	0,00003699	19	0,011427994	3	1935,04850	1,43	54,59	0
P72870	Allophycocyanin subunit alpha-B OS=Synechocystis sp. (strain PCC 6803 / Kazusa)	675,80	47,83	1	8	8	28	1,211	25	10,6	161	17,9	5,58							
	A3	Sequence	# PSMs	# Proteins	# Protein Groups	Protein Group Accessions	Modifications	ΔCn	114/115	114/115 Count	114/115 Variability [%]	q-Value	PEP	IonScore	Exp Value	Charge	MH+ [Da]	ΔM [ppm]	RT [min]	# Missed Cleavages
	High	gIQAFLTTGAQR	7	1	1	P72870	N-Term(iTRAQ4plex)	0,0000	1,169	5	19,5	0	3,267E-09	66	2,97644E-07	2	1406,78862	0,95	37,65	0
	High	lVTYGVLAGNkEPIETTGLIGVk	2	1	1	P72870	N-Term(iTRAQ4plex); K11(iTRAQ4plex); K23(iTRAQ4plex)	0,0000	1,325	1		0	1,217E-10	65	2,94422E-07	3	2804,65244	0,93	45,11	1
	High	aPGGNAYGQR	4	1	1	P72870	N-Term(iTRAQ4plex)	0,0000	1,199	4	1,0	0	1,898E-07	59	1,22453E-06	2	1134,57964	2,08	13,44	0
	High	kIVDQAQk	6	1	1	P72870	N-Term(iTRAQ4plex); K1(iTRAQ4plex); K8(iTRAQ4plex)	0,0000	1,468	6	15,1	0	0,00001622	47	2,14768E-05	2	1361,84905	1,00	12,52	1
	High	iVDQAQk	1	1	1	P72870	N-Term(iTRAQ4plex); K7(iTRAQ4plex)	0,0000	1,378	1		0	0,0002824	39	0,000129709	2	1089,65679	5,62	15,87	0
	High	iAETLAENEkk	3	1	1	P72870	N-Term(iTRAQ4plex); K10(iTRAQ4plex); K11(iTRAQ4plex)	0,0000	1,251	3	0,8	0	0,000000106	38	0,000154515	3	1677,97574	0,59	21,96	1
	High	dYGWYLR	2	1	1	P72870	N-Term(iTRAQ4plex)	0,0000	1,171	2	5,0	0	0,007874	27	0,002157595	2	1116,55925	-0,20	35,08	0
	High	qYNQcLR	3	1	1	P72870	N-Term(iTRAQ4plex); C5(Carbamidomethyl)	0,0000	1,064	3	9,6	0,001	0,01785	31	0,000714447	2	1125,55950	0,30	14,67	0
P73243	CheY subfamily OS=Synechocystis sp. (strain PCC 6803 / Kazusa) GN=slr2024 PE=	672,58	69,44	1	9	9	20	0,977	19	9,0	180	20,2	6,81							
	A3	Sequence	# PSMs	# Proteins	# Protein Groups	Protein Group Accessions	Modifications	ΔCn	114/115	114/115 Count	114/115 Variability [%]	q-Value	PEP	IonScore	Exp Value	Charge	MH+ [Da]	ΔM [ppm]	RT [min]	# Missed Cleavages
	High	hAPVAAVASGAAPAGDLAAEVQQLk	3	1	1	P73243	N-Term(iTRAQ4plex); K25(iTRAQ4plex)	0,0000	0,920	3	7,0	0	6,001E-15	102	6,70177E-11	3	2630,45395	1,29	42,15	0
	High	qLGQLVTFIk	4	1	1	P73243	N-Term(iTRAQ4plex); K10(iTRAQ4plex)	0,0000	0,998	4	6,5	0	1,836E-09	83	5,4572E-09	2	1434,89324	0,65	45,17	0
	High	vkDmLPQGNFEVIEAk	1	1	1	P73243	N-Term(iTRAQ4plex); K2(iTRAQ4plex); M4(Oxidation); K16(iTRAQ4plex)	0,0000	1,144	1		0	1,004E-12	65	4,61257E-07	3	2266,25132	1,55	34,49	1
	High	mSGWEVFQSVQERPELk	1	1	1	P73243	N-Term(iTRAQ4plex); M1(Oxidation); K17(iTRAQ4plex)	0,0000	1,013	1		0	9,203E-14	56	2,24373E-06	3	2354,20841	1,29	39,06	0
	High	eLVAAIk	1	1	1	P73243	N-Term(iTRAQ4plex); K7(iTRAQ4plex)	0,0000	1,093	1		0	0,0009026	44	3,73224E-05	2	1031,67046	0,12	27,01	0
	High	aIPLVLmSGR	2	1	1	P73243	N-Term(iTRAQ4plex); M7(Oxidation)	0,0000	0,931	2	7,2	0	0,000008615	44	4,20698E-05	2	1216,72075	0,24	34,24	0
	High	kEEVLEk	3	1	1	P73243	N-Term(iTRAQ4plex); K1(iTRAQ4plex); K7(iTRAQ4plex)	0,0000	1,026	3	3,3	0	0,00018	42	6,32368E-05	3	1306,79468	0,32	15,30	1
	High	aIPLVLMSGR	2	1	1	P73243	N-Term(iTRAQ4plex)	0,0000	0,802	2	21,0	0	0,0000819	41	8,64908E-05	2	1200,72624	0,58	40,99	0
	High	qQVAQmHGEIDLLkk	2	1	1	P73243	N-Term(iTRAQ4plex); M6(Oxidation); K14(iTRAQ4plex); K15(iTRAQ4plex)	0,0000	0,820	1		0	0,000000456	33	0,000506991	4	2186,23354	0,34	24,64	1
	High	iPEPFEYFAFVEkPFEQR	1	1	1	P73243	N-Term(iTRAQ4plex); K13(iTRAQ4plex)	0,0000	1,269	1		0	7,524E-08	32	0,000651628	3	2561,33640	1,73	56,97	0
P73202	Phycobilisome 8.9 kDa linker polypeptide, phycocyanin-associated, rod OS=Synech	664,95	59,04	1	5	5	19	0,776	16	30,5	83	9,3	9,41							
	A3	Sequence	# PSMs	# Proteins	# Protein Groups	Protein Group Accessions	Modifications	ΔCn	114/115	114/115 Count	114/115 Variability [%]	q-Value	PEP	IonScore	Exp Value	Charge	MH+ [Da]	ΔM [ppm]	RT [min]	# Missed Cleavages
	High	mLGQSSLVGYSNTQAANR	4	1	1	P73202	N-Term(iTRAQ4plex); M1(Oxidation)	0,0000	1,090	4	12,2	0	4,551E-17	119	1,20495E-12	2	2057,02226	0,96	27,77	0
	High	qTDANENSAHDIR	3	1	1	P73202	N-Term(iTRAQ4plex)	0,0000	0,954	3	56,3	0	5,974E-10	55	3,17665E-06	3	1614,75913	0,14	10,28	0
	High	vFVYEVSGLR	5	1	1	P73202	N-Term(iTRAQ4plex)	0,0000	0,764	3	1,0	0	0,000001576	51	7,56781E-06	2	1312,73943	0,94	40,03	0
	High	qTDANENSAHDIRR	5	1	1	P73202	N-Term(iTRAQ4plex)	0,0000	0,620	4	6,0	0	3,705E-10	51	7,85181E-06	3	1770,86075	0,42	8,92	1
	High	sGSVFIk	2	1	1	P73202	N-Term(iTRAQ4plex); K7(iTRAQ4plex)	0,0000	0,851	2	5,0	0	0,0006736	39	0,000115337	2	1025,62126	-2,06	22,88	0
P52415	Glucose-1-phosphate adenylyltransferase OS=Synechocystis sp. (strain PCC 6803 /	656,46	24,37	1	11	11	31	0,743	29	21,5	439	49,3	6,47							
	A3	Sequence	# PSMs	# Proteins	# Protein Groups	Protein Group Accessions	Modifications	ΔCn	114/115	114/115 Count	114/115 Variability [%]	q-Value	PEP	IonScore	Exp Value	Charge	MH+ [Da]	ΔM [ppm]	RT [min]	# Missed Cleavages
	High	iYVLTQFNSASLNR	3	1	1	P52415	N-Term(iTRAQ4plex)	0,0000	0,850	3	24,6	0	1,251E-10	83	4,78597E-09	2	1769,96807	0,77	43,91	0
	High	vLAIILGGGAGTR	4	1	1	P52415	N-Term(iTRAQ4plex)	0,0000	0,557	4	16,4	0	3,999E-08	63	4,65554E-07	2	1341,83306	-0,35	48,10	0
	High	nVMIVNk	1	1	1	P52415	N-Term(iTRAQ4plex); K7(iTRAQ4plex)	0,0000	0,962	1		0	0,0000996	47	2,30245E-05	2	1105,66545	1,13	21,91	0
	High	kAPELGLmk	5	1	1	P52415	N-Term(iTRAQ4plex); K1(iTRAQ4plex); M8(Oxidation); K9(iTRAQ4plex)	0,0000	0,745	4	4,3	0	1,123E-07	42	0,000109439	3	1434,87329	1,26	20,40	1
	High	qYLWLFR	3	1	1	P52415	N-Term(iTRAQ4plex)	0,0000	0,573	3	1,6	0	0,0002663	41	7,24386E-05	2	1169,65886	0,05	47,86	0
	High	kAPELGLMk	1	1	1	P52415	N-Term(iTRAQ4plex); K1(iTRAQ4plex); K9(iTRAQ4plex)	0,0000	0,542	1		0	0,000003001	36	0,000223857	3	1418,87500	-1,10	25,04	1
	High	nVmIVNk	3	1	1	P52415	N-Term(iTRAQ4plex); M3(Oxidation); K7(iTRAQ4plex)	0,0000	0,917	3	0,6	0	0,0007738	36	0,000248869	2	1121,66533	5,54	18,90	0
	High	akPAVPLAGk	3	1	1	P52415	N-Term(iTRAQ4plex); K2(iTRAQ4plex); K10(iTRAQ4plex)	0,0000	0,781	2	1,7	0	0,000008626	30	0,001106547	3	1383,90561	0,57	18,91	0
	High	eVLHNLLEk	3	1	1	P52415	N-Term(iTRAQ4plex); K9(iTRAQ4plex)	0,0000	0,726	3	7,6	0	0,0002425	24	0,00400839	3	1382,82172	-2,09	30,99	0
	High	iTDFSEkPQGEALR	1	1	1	P52415	N-Term(iTRAQ4plex); K7(iTRAQ4plex)	0,0000	0,644	1		0	0,00002213	15	0,032613145	3	1879,01218	-2,25	28,51	0
	High	eVLHNLLEkYEGATDFGk	1	1	1	P52415	N-Term(iTRAQ4plex); K9(iTRAQ4plex); K18(iTRAQ4plex)	0,0000	1,291	1		0	0,00000706	10	0,094182454	4	2495,34902	-0,66	44,11	1
	High	iHHSVLGIR	1	1	1	P52415	N-Term(iTRAQ4plex)	0,0000	0,688	1		0,002	0,02167	11	0,113850192	3	1175,71231	-0,56	16,55	0
	High	lYPLTk	2	1	1	P52415	N-Term(iTRAQ4plex); K6(iTRAQ4plex)	0,0000	0,762	2	3,8	0,005	0,06912	25	0,003005869	2	1022,65190	2,99	28,09	0
P74720	Sll1106 protein OS=Synechocystis sp. (strain PCC 6803 / Kazusa) GN=sll1106 PE=4	655,01	25,73	1	5	5	26	1,015	20	14,0	171	18,0	4,82							
	A3	Sequence	# PSMs	# Proteins	# Protein Groups	Protein Group Accessions	Modifications	ΔCn	114/115	114/115 Count	114/115 Variability [%]	q-Value	PEP	IonScore	Exp Value	Charge	MH+ [Da]	ΔM [ppm]	RT [min]	# Missed Cleavages
	High	lQVEHLIEMEDAAVVVkDEk	8	1	1	P74720	N-Term(iTRAQ4plex); K17(iTRAQ4plex); K20(iTRAQ4plex)	0,0000	1,046	5	13,0	0	1,295E-16	66	3,38325E-07	3	2727,49985	1,27	43,80	1
	High	vLADVSk	3	1	1	P74720	N-Term(iTRAQ4plex); K7(iTRAQ4plex)	0,0000	0,883	3	22,2	0	0,0002146	57	2,78184E-06	2	1019,63353	-0,41	22,18	0
	High	lQVEHLIEmEDAAVVVkDEk	9	1	1	P74720	N-Term(iTRAQ4plex); M9(Oxidation); K17(iTRAQ4plex); K20(iTRAQ4plex)	0,0000	1,015	6	3,2	0	4,519E-11	51	8,22186E-06	3	2743,49302	0,62	41,34	1
	High	hGLLDGVPAAE	3	1	1	P74720	N-Term(iTRAQ4plex)	0,0000	0,764	3	24,1	0	0,00005916	35	0,000351536	2	1222,65483	-0,03	31,61	0
	High	kVTPDk	2	1	1	P74720	N-Term(iTRAQ4plex); K1(iTRAQ4plex); K6(iTRAQ4plex)	0,0000	1,184	2	5,0	0	0,005121	16	0,023986672	3	1119,70813	-1,48	8,97	1
	High	vTPDkVLADVSk	1	1	1	P74720	N-Term(iTRAQ4plex); K5(iTRAQ4plex); K12(iTRAQ4plex)	0,0000	1,127	1		0	0,0001465	15	0,034432615	3	1704,03128	2,63	31,81	1
P73297	DNA-directed RNA polymerase subunit alpha OS=Synechocystis sp. (strain PCC 680	647,77	21,97	1	9	9	24	1,044	18	7,3	314	35,0	4,79							
	A3	Sequence	# PSMs	# Proteins	# Protein Groups	Protein Group Accessions	Modifications	ΔCn	114/115	114/115 Count	114/115 Variability [%]	q-Value	PEP	IonScore	Exp Value	Charge	MH+ [Da]	ΔM [ppm]	RT [min]	# Missed Cleavages
	High	vLLSNLPGAAVTAIR	4	1	1	P73297	N-Term(iTRAQ4plex)	0,0000	1,036	4	3,6	0	2,992E-13	98	1,67869E-10	2	1639,00408	1,03	46,43	0
	High	iAGVNHEFATILGVR	6	1	1	P73297	N-Term(iTRAQ4plex)	0,0000	1,022	4	10,1	0	2,009E-10	78	1,44202E-08	2	1740,99016	1,36	44,01	0
	High	gQGTTVGNALR	4	1	1	P73297	N-Term(iTRAQ4plex)	0,0000	1,061	2	2,6	0	1,544E-07	72	6,16552E-08	2	1217,67278	0,71	16,53	0
	High	rVLLSNLPGAAVTAIR	2	1	1	P73297	N-Term(iTRAQ4plex)	0,0000	0,732	2	58,7	0	7,874E-07	48	1,6217E-05	3	1795,10227	-0,69	39,66	1
	High	kNQQQYSk	1	1	1	P73297	N-Term(iTRAQ4plex); K1(iTRAQ4plex); K8(iTRAQ4plex)	0,0000	1,330	1		0	0,0001688	25	0,003026704	3	1455,82807	0,05	8,30	1
	High	lGITLPHEk	4	1	1	P73297	N-Term(iTRAQ4plex); K9(iTRAQ4plex)	0,0000	0,980	2	2,0	0	0,002988	23	0,005332981	3	1295,79325	0,52	28,47	0
	High	nQQQYSk	1	1	1	P73297	N-Term(iTRAQ4plex); K7(iTRAQ4plex)	0,0000	1,311	1		0	0,001174	17	0,018922129	2	1183,63103	0,06	9,74	0
	High	lEmEFRVER	1	1	1	P73297	N-Term(iTRAQ4plex); M3(Oxidation)	0,0000	1,080	1		0,001	0,008315	18	0,0141244	3	1368,70612	-0,11	25,24	1
	High	rLGITLPHEk	1	1	1	P73297	N-Term(iTRAQ4plex); K10(iTRAQ4plex)	0,0000	0,930	1		0,006	0,07603	31	0,000805323	4	1451,89675	2,10	23,67	1
P73133	Acetylornithine aminotransferase OS=Synechocystis sp. (strain PCC 6803 / Kazusa)	647,50	35,20	1	12	12	22	0,978	20	8,3	429	46,5	5,24							
	A3	Sequence	# PSMs	# Proteins	# Protein Groups	Protein Group Accessions	Modifications	ΔCn	114/115	114/115 Count	114/115 Variability [%]	q-Value	PEP	IonScore	Exp Value	Charge	MH+ [Da]	ΔM [ppm]	RT [min]	# Missed Cleavages
	High	sYLDFVAGIATcTLGHAHPALVR	3	1	1	P73133	N-Term(iTRAQ4plex); C12(Carbamidomethyl)	0,0000	0,966	2	2,6	0	1,262E-15	96	2,36695E-10	4	2613,37636	1,33	54,86	0
	High	nQYPTLFTEVR	2	1	1	P73133	N-Term(iTRAQ4plex)	0,0000	0,956	2	3,5	0	0,000003565	62	5,87449E-07	2	1511,79888	0,91	37,92	0
	High	lWGYEHLGVEPDIFTSAk	1	1	1	P73133	N-Term(iTRAQ4plex); K18(iTRAQ4plex)	0,0000	0,997	1		0	4,978E-10	53	4,47682E-06	3	2350,23410	0,80	47,16	0
	High	tLATITATGQPk	2	1	1	P73133	N-Term(iTRAQ4plex); K12(iTRAQ4plex)	0,0000	1,108	2	18,8	0	0,000000108	53	5,48239E-06	2	1489,88237	-0,33	26,24	0
	High	yAHTVLDFLEQPVILTAk	2	1	1	P73133	N-Term(iTRAQ4plex); K18(iTRAQ4plex)	0,0000	0,962	2	10,7	0	0,000001431	50	1,06162E-05	3	2346,33597	2,03	54,26	0
	High	aVSDQIQk	2	1	1	P73133	N-Term(iTRAQ4plex); K8(iTRAQ4plex)	0,0000	1,108	2	6,5	0	0,00002171	49	1,75756E-05	2	1176,68633	3,09	17,58	0
	High	sGLAEIk	1	1	1	P73133	N-Term(iTRAQ4plex); K7(iTRAQ4plex)	0,0000	1,213	1		0	0,0007721	44	5,33265E-05	2	1005,62255	4,22	20,90	0
	High	gLAGGVPIGAmmck	1	1	1	P73133	N-Term(iTRAQ4plex); M11(Oxidation); M12(Oxidation); C13(Carbamidometh	0,0000	0,862	1		0	3,455E-08	33	0,00051047	3	1681,86765	-0,13	27,14	0
	High	lLDNVQAR	1	1	1	P73133	N-Term(iTRAQ4plex)	0,0000	0,785	1		0	0,0001358	31	0,000760274	2	1072,62297	-0,18	24,65	0
	High	wIVEHScADR	2	1	1	P73133	N-Term(iTRAQ4plex); C7(Carbamidomethyl)	0,0000	0,928	2	10,9	0	0,00000409	29	0,00112712	3	1416,68195	0,60	20,85	0
	High	aAmEQGLLLAPAGPk	1	1	1	P73133	N-Term(iTRAQ4plex); M3(Oxidation); K15(iTRAQ4plex)	0,0000	0,936	1		0	0,00004928	16	0,02883434	3	1771,00467	1,11	32,17	0
	High	fPIAIAR	4	1	1	P73133	N-Term(iTRAQ4plex)	0,0000	0,926	3	0,1	0,002	0,01982	29	0,00126174	2	931,58440	-0,21	34,30	0
P28371	Elongation factor G 1 OS=Synechocystis sp. (strain PCC 6803 / Kazusa) GN=fusA P	645,49	21,73	1	16	16	35	0,667	32	19,7	695	76,7	5,02							
	A3	Sequence	# PSMs	# Proteins	# Protein Groups	Protein Group Accessions	Modifications	ΔCn	114/115	114/115 Count	114/115 Variability [%]	q-Value	PEP	IonScore	Exp Value	Charge	MH+ [Da]	ΔM [ppm]	RT [min]	# Missed Cleavages
	High	gVLAGYPVVDLk	2	1	1	P28371	N-Term(iTRAQ4plex); K12(iTRAQ4plex)	0,0000	0,763	2	22,1	0	2,933E-07	58	1,7378E-06	2	1518,91423	0,52	41,43	0
	High	iYSGTLSk	3	1	1	P28371	N-Term(iTRAQ4plex); K8(iTRAQ4plex)	0,0000	0,557	2	11,6	0	0,0004065	56	2,57614E-06	2	1156,68816	5,66	24,27	0
	High	kLDFFPTYGGSSFk	3	1	1	P28371	N-Term(iTRAQ4plex); K1(iTRAQ4plex); K14(iTRAQ4plex)	0,0000	0,807	3	23,1	0	1,055E-09	55	3,34942E-06	3	2026,10135	0,16	40,27	1
	High	vTGGNVPR	3	1	1	P28371	N-Term(iTRAQ4plex)	0,0000	0,657	3	7,0	0	0,001334	48	1,43539E-05	2	943,54338	-0,85	13,62	0
	High	nIGIFAHVDAGk	3	1	1	P28371	N-Term(iTRAQ4plex); K12(iTRAQ4plex)	0,0000	0,615	3	30,5	0	5,969E-08	48	1,64048E-05	3	1529,86713	-0,49	31,63	0
	High	tHGVEVEmGkPQVAYR	3	1	1	P28371	N-Term(iTRAQ4plex); M8(Oxidation); K10(iTRAQ4plex)	0,0000	0,535	2	4,0	0	7,171E-08	40	0,000110146	3	2105,10477	-0,23	20,55	0
	High	sQETGPmGVR	2	1	1	P28371	N-Term(iTRAQ4plex); M7(Oxidation)	0,0000	0,702	2	6,0	0	0,0005144	38	0,000172572	2	1221,60295	1,22	11,49	0
	High	eSITQQVSDTYVHkk	1	1	1	P28371	N-Term(iTRAQ4plex); K14(iTRAQ4plex); K15(iTRAQ4plex)	0,0000	0,860	1		0	1,889E-10	34	0,000528666	4	2195,20131	-0,88	27,01	1
	High	gmGELHLDIk	1	1	1	P28371	N-Term(iTRAQ4plex); M2(Oxidation); K10(iTRAQ4plex)	0,0000	1,064	1		0	0,00005314	31	0,000803471	3	1416,77497	-0,70	26,58	0
	High	fGALTFTR	3	1	1	P28371	N-Term(iTRAQ4plex)	0,0000	0,572	3	3,3	0	0,004409	29	0,00129113	2	1056,59575	-0,13	36,25	0
	High	nVQTGHTLcDPk	1	1	1	P28371	N-Term(iTRAQ4plex); C9(Carbamidomethyl); K12(iTRAQ4plex)	0,0000	0,565	1		0	0,00003252	28	0,00141244	3	1657,85410	-1,78	16,41	0
	High	lIYINkLDR	2	1	1	P28371	N-Term(iTRAQ4plex); K6(iTRAQ4plex)	0,0000	0,564	2	12,0	0	0,0001668	23	0,004677028	3	1435,88642	-0,78	33,49	1
	High	tHGVEVEMGkPQVAYR	1	1	1	P28371	N-Term(iTRAQ4plex); K10(iTRAQ4plex)	0,0000	1,150	1		0	0,0004252	15	0,031839776	4	2089,10927	-0,51	25,18	0
	High	lIYINk	3	1	1	P28371	N-Term(iTRAQ4plex); K6(iTRAQ4plex)	0,0000	0,674	2	3,2	0,005	0,05746	30	0,001004546	2	1051,67510	-0,29	30,42	0
	High	lGmALSk	1	1	1	P28371	N-Term(iTRAQ4plex); M3(Oxidation); K7(iTRAQ4plex)	0,0000	0,761	1		0,009	0,1058	23	0,007483901	2	1023,61247	1,33	19,88	0
	Medium	qSLPk	2	1	1	P28371	N-Term(iTRAQ4plex); K5(iTRAQ4plex)	0,0000	0,739	2	0,5	0,022	0,2408	17	0,022282812	2	860,54588	1,74	12,42	0
	Medium	aLAFk	2	1	1	P28371	N-Term(iTRAQ4plex); K5(iTRAQ4plex)	0,0000	0,691	1		0,045	0,4715	22	0,006682978	2	837,54131	-2,82	22,18	0
P48957	50S ribosomal protein L20 OS=Synechocystis sp. (strain PCC 6803 / Kazusa) GN=rp	643,15	57,26	1	11	11	42	2,919	37	58,3	117	13,5	12,02							
	A3	Sequence	# PSMs	# Proteins	# Protein Groups	Protein Group Accessions	Modifications	ΔCn	114/115	114/115 Count	114/115 Variability [%]	q-Value	PEP	IonScore	Exp Value	Charge	MH+ [Da]	ΔM [ppm]	RT [min]	# Missed Cleavages
	High	kmLAQLAVLDPAAFSEVVk	5	1	1	P48957	N-Term(iTRAQ4plex); K1(iTRAQ4plex); M2(Oxidation); K19(iTRAQ4plex)	0,0000	1,690	5	104,3	0	3,804E-13	82	6,35287E-09	3	2478,44071	1,62	48,06	1
	High	tANQQVMk	3	1	1	P48957	N-Term(iTRAQ4plex); K8(iTRAQ4plex)	0,0000	3,667	2	31,9	0	0,00001773	48	1,76185E-05	2	1207,67693	5,12	13,93	0
	High	tANQQVmk	10	1	1	P48957	N-Term(iTRAQ4plex); M7(Oxidation); K8(iTRAQ4plex)	0,0000	3,539	8	51,6	0	0,000006681	39	0,000167846	2	1223,66680	0,93	9,38	0
	High	aNIEINRk	2	1	1	P48957	N-Term(iTRAQ4plex); K8(iTRAQ4plex)	0,0000	2,977	2	2,9	0	0,00007304	35	0,000349116	2	1245,75530	2,83	14,72	1
	High	kANIEINR	4	1	1	P48957	N-Term(iTRAQ4plex); K1(iTRAQ4plex)	0,0000	4,034	4	9,5	0	0,003878	34	0,000435482	2	1245,75224	0,38	15,00	1
	High	qEGmSYSk	3	1	1	P48957	N-Term(iTRAQ4plex); M4(Oxidation); K8(iTRAQ4plex)	0,0000	3,428	3	13,3	0	0,0008575	34	0,000437492	2	1233,60185	-0,43	9,97	0
	High	kANIEINRk	1	1	1	P48957	N-Term(iTRAQ4plex); K1(iTRAQ4plex); K9(iTRAQ4plex)	0,0000	0,391	1		0	0,001093	18	0,016142471	4	1517,94973	0,60	12,98	2
	High	vAATAk	7	1	1	P48957	N-Term(iTRAQ4plex); K6(iTRAQ4plex)	0,0000	1,988	5	2,8	0,001	0,01487	19	0,013000798	2	848,54357	-0,98	10,28	0
	High	lTGQLkk	1	1	1	P48957	N-Term(iTRAQ4plex); K6(iTRAQ4plex); K7(iTRAQ4plex)	0,0000	1,615	1		0,003	0,03897	12	0,062657059	3	1219,80933	-0,42	16,79	1
	High	rLWITR	2	1	1	P48957	N-Term(iTRAQ4plex)	0,0000	1,166	2	42,3	0,006	0,07686	23	0,004731186	3	988,61753	0,25	21,91	1
	Medium	iNAAAR	3	1	1	P48957	N-Term(iTRAQ4plex)	0,0000	1,919	3	11,1	0,028	0,3029	27	0,00196775	2	759,46129	2,51	13,53	0
	Medium	lWITR	1	1	1	P48957	N-Term(iTRAQ4plex)	0,0000	4,166	1		0,031	0,3317	24	0,004226394	2	832,51677	0,73	29,63	0
P72938	Extracellular nuclease OS=Synechocystis sp. (strain PCC 6803 / Kazusa) GN=nucH	627,92	3,67	1	5	5	18	0,510	16	31,8	1879	196,4	3,99							
	A3	Sequence	# PSMs	# Proteins	# Protein Groups	Protein Group Accessions	Modifications	ΔCn	114/115	114/115 Count	114/115 Variability [%]	q-Value	PEP	IonScore	Exp Value	Charge	MH+ [Da]	ΔM [ppm]	RT [min]	# Missed Cleavages
	High	vTGFNVLNYFk	6	1	1	P72938	N-Term(iTRAQ4plex); K11(iTRAQ4plex)	0,0000	0,481	6	22,8	0	2,177E-10	79	1,2473E-08	2	1589,89177	-0,78	51,26	0
	High	iIGGLSQNLGR	2	1	1	P72938	N-Term(iTRAQ4plex)	0,0000	0,465	2	2,7	0	5,486E-08	68	1,7578E-07	2	1271,75444	-0,64	33,34	0
	High	dLVTGETFTAVVNHFk	5	1	1	P72938	N-Term(iTRAQ4plex); K16(iTRAQ4plex)	0,0000	0,513	3	13,7	0	2,522E-14	67	2,11928E-07	3	2066,11594	-0,09	46,92	0
	High	qANLFTYAAGGTLSR	4	1	1	P72938	N-Term(iTRAQ4plex)	0,0000	0,971	4	23,2	0	1,623E-10	47	1,83641E-05	2	1713,90605	1,14	39,71	0
	High	vNDRPVTPENVGGSLk	1	1	1	P72938	N-Term(iTRAQ4plex); K16(iTRAQ4plex)	0,0000	0,441	1		0	2,617E-13	44	4,27533E-05	3	1970,09140	0,21	25,66	0
P73103	Slr1908 protein OS=Synechocystis sp. (strain PCC 6803 / Kazusa) GN=slr1908 PE=	622,65	11,34	1	3	6	15	0,777	7	12,6	591	64,5	5,26							
	A3	Sequence	# PSMs	# Proteins	# Protein Groups	Protein Group Accessions	Modifications	ΔCn	114/115	114/115 Count	114/115 Variability [%]	q-Value	PEP	IonScore	Exp Value	Charge	MH+ [Da]	ΔM [ppm]	RT [min]	# Missed Cleavages
	High	lmQEFEAELAALGAR	4	2	2	P73409;P73103	N-Term(iTRAQ4plex); M2(Oxidation)	0,0000				0	2,752E-11	80	1,11422E-08	2	1808,93474	0,74	54,13	0
	High	lQAGSVPNLSGPTGTNmAR	3	1	1	P73103	N-Term(iTRAQ4plex); M17(Oxidation)	0,0000	0,777	3	7,7	0	1,044E-08	72	6,28015E-08	2	2031,04228	0,61	30,69	0
	High	rLmQEFEAELAALGAR	1	2	2	P73409;P73103	N-Term(iTRAQ4plex); M3(Oxidation)	0,0000				0	9,957E-10	71	7,86991E-08	3	1965,03623	0,87	47,64	1
	High	lMQEFEAELAALGAR	2	2	2	P73409;P73103	N-Term(iTRAQ4plex)	0,0000				0	1,571E-09	69	1,38347E-07	2	1792,93596	-1,41	59,09	0
	High	lQAGSVPNLSGPTGTNMAR	1	1	1	P73103	N-Term(iTRAQ4plex)	0,0000	1,019	1		0	9,033E-07	68	1,66876E-07	2	2015,04875	1,30	33,84	0
	High	ySPFVNR	2	1	1	P73103	N-Term(iTRAQ4plex)	0,0000	0,722	2	1,5	0	0,005598	40	0,000106898	2	1026,54875	-0,16	24,49	0
	High	lIQENVAVLREDIDk	1	2	2	P73409;P73103	N-Term(iTRAQ4plex); K15(iTRAQ4plex)	0,0000				0	2,089E-09	29	0,001279293	3	2043,16916	0,12	41,73	1
	High	gPEGTGGALR	1	1	1	P73103	N-Term(iTRAQ4plex)	0,0000	1,034	1		0,003	0,03355	26	0,00232793	2	1058,57146	0,30	14,06	0
P37101	Phosphoribulokinase OS=Synechocystis sp. (strain PCC 6803 / Kazusa) GN=prk PE=	611,95	37,35	1	12	12	34	0,802	32	15,6	332	37,9	5,43							
	A3	Sequence	# PSMs	# Proteins	# Protein Groups	Protein Group Accessions	Modifications	ΔCn	114/115	114/115 Count	114/115 Variability [%]	q-Value	PEP	IonScore	Exp Value	Charge	MH+ [Da]	ΔM [ppm]	RT [min]	# Missed Cleavages
	High	gHTYEDILASINAR	5	1	1	P37101	N-Term(iTRAQ4plex)	0,0000	0,668	4	12,0	0	6,007E-10	77	2,52542E-08	2	1703,88469	0,78	38,48	0
	High	kAAGVTALDPR	4	1	1	P37101	N-Term(iTRAQ4plex); K1(iTRAQ4plex)	0,0000	0,805	4	2,4	0	0,00004506	50	9,01509E-06	2	1386,83086	0,06	18,30	1
	High	kPDFTAYIEPQk	7	1	1	P37101	N-Term(iTRAQ4plex); K1(iTRAQ4plex); K12(iTRAQ4plex)	0,0000	0,804	7	16,9	0	9,29E-12	45	3,76177E-05	3	1869,05124	1,60	33,52	0
	High	lIEDkESk	4	1	1	P37101	N-Term(iTRAQ4plex); K5(iTRAQ4plex); K8(iTRAQ4plex)	0,0000	0,838	4	5,0	0	0,00001378	44	4,11121E-05	2	1393,82573	-0,40	15,70	1
	High	kLTcTYPGIk	3	1	1	P37101	N-Term(iTRAQ4plex); K1(iTRAQ4plex); C4(Carbamidomethyl); K10(iTRAQ4pl	0,0000	0,799	3	2,9	0	0,00001463	41	8,12774E-05	3	1612,94547	-0,13	22,99	1
	High	vVVIEGLHPLYDER	4	1	1	P37101	N-Term(iTRAQ4plex)	0,0000	0,722	3	10,4	0	0,000006721	39	0,000130608	3	1782,98801	0,50	39,83	0
	High	vEPNkVVVIEGLHPLYDER	1	1	1	P37101	N-Term(iTRAQ4plex); K5(iTRAQ4plex)	0,0000	0,568	1		0	4,668E-09	34	0,000382798	4	2494,39223	0,55	39,91	1
	High	hkDYPGTDNGTGLFQVLVGLk	1	1	1	P37101	N-Term(iTRAQ4plex); K2(iTRAQ4plex); K21(iTRAQ4plex)	0,0000	0,840	1		0	1,097E-07	19	0,013962719	4	2691,48281	-0,10	46,53	1
	High	yYGEMTELLLk	1	1	1	P37101	N-Term(iTRAQ4plex); K11(iTRAQ4plex)	0,0000	1,125	1		0,001	0,01271	11	0,090864174	3	1647,89115	0,31	47,61	0
	High	qYADVVIQVLPTR	1	1	1	P37101	N-Term(iTRAQ4plex)	0,0000	1,038	1		0,002	0,03252	11	0,086039493	2	1645,94158	1,31	42,73	0
	High	aAGVTALDPR	2	1	1	P37101	N-Term(iTRAQ4plex)	0,0000	0,776	2	0,7	0,009	0,1153	20	0,01018521	2	1114,63469	0,85	22,95	0
	Medium	sTFLR	1	1	1	P37101	N-Term(iTRAQ4plex)	0,0000	0,919	1		0,049	0,5024	20	0,009907635	2	767,45317	-0,07	18,65	0
P73596	Sll1307 protein OS=Synechocystis sp. (strain PCC 6803 / Kazusa) GN=sll1307 PE=4	609,23	29,14	1	4	4	17	1,825	12	11,4	175	17,8	6,52							
	A3	Sequence	# PSMs	# Proteins	# Protein Groups	Protein Group Accessions	Modifications	ΔCn	114/115	114/115 Count	114/115 Variability [%]	q-Value	PEP	IonScore	Exp Value	Charge	MH+ [Da]	ΔM [ppm]	RT [min]	# Missed Cleavages
	High	aYSAVTLATNHNLDR	5	1	1	P73596	N-Term(iTRAQ4plex)	0,0000	1,868	4	5,2	0	4,736E-11	106	2,58803E-11	2	1789,94670	8,56	28,99	0
	High	sDGALVHTVQTSFNk	4	1	1	P73596	N-Term(iTRAQ4plex); K15(iTRAQ4plex)	0,0000	1,828	3	9,7	0	1,981E-10	77	2,89153E-08	2	1892,01616	2,39	31,03	0
	High	aAQSAIAAcR	4	1	1	P73596	N-Term(iTRAQ4plex); C9(Carbamidomethyl)	0,0000	1,713	2	9,3	0	0,000003663	68	1,48867E-07	2	1162,61321	1,07	15,41	0
	High	tSGILASmQAk	4	1	1	P73596	N-Term(iTRAQ4plex); M8(Oxidation); K11(iTRAQ4plex)	0,0000	1,442	3	41,9	0	0,00002156	51	8,18408E-06	2	1410,80022	9,71	19,25	0
Q55361	Sll0887 protein OS=Synechocystis sp. (strain PCC 6803 / Kazusa) GN=sll0887 PE=4	603,65	25,11	1	10	10	24	1,319	23	11,9	446	47,6	4,94							
	A3	Sequence	# PSMs	# Proteins	# Protein Groups	Protein Group Accessions	Modifications	ΔCn	114/115	114/115 Count	114/115 Variability [%]	q-Value	PEP	IonScore	Exp Value	Charge	MH+ [Da]	ΔM [ppm]	RT [min]	# Missed Cleavages
	High	fYLNSAGAk	3	1	1	Q55361	N-Term(iTRAQ4plex); K9(iTRAQ4plex)	0,0000	1,426	3	5,9	0	2,975E-07	71	7,67308E-08	2	1258,70415	0,58	26,83	0
	High	fDTQPTGHANIGSk	2	1	1	Q55361	N-Term(iTRAQ4plex); K14(iTRAQ4plex)	0,0000	1,315	2	8,1	0	1,328E-11	60	1,10655E-06	3	1760,91648	-0,31	20,09	0
	High	nGVLTGLLHSAGTAk	1	1	1	Q55361	N-Term(iTRAQ4plex); K15(iTRAQ4plex)	0,0000	1,192	1		0	2,609E-08	52	6,4859E-06	3	1727,00626	0,47	35,00	0
	High	rFDTQPTGHANIGSk	4	1	1	Q55361	N-Term(iTRAQ4plex); K15(iTRAQ4plex)	0,0000	1,218	3	23,2	0	2,059E-07	49	1,19116E-05	4	1917,01748	-0,34	17,55	1
	High	mLDIQAIADHAHQGAAALGIk	1	1	1	Q55361	N-Term(iTRAQ4plex); M1(Oxidation); K21(iTRAQ4plex)	0,0000	1,500	1		0	1,42E-09	48	1,62544E-05	4	2448,32900	0,72	45,04	0
	High	sYASIYLYSR	2	1	1	Q55361	N-Term(iTRAQ4plex)	0,0000	1,340	2	2,3	0	0,00008506	44	3,75811E-05	2	1366,71306	0,53	34,43	0
	High	yTVVFSAR	3	1	1	Q55361	N-Term(iTRAQ4plex)	0,0000	1,313	3	7,7	0	0,00009939	43	5,58432E-05	2	1086,60649	0,05	29,90	0
	High	iTVGAHFYHVYSEk	2	1	1	Q55361	N-Term(iTRAQ4plex); K14(iTRAQ4plex)	0,0000	1,702	2	5,9	0	1,657E-11	40	0,000102558	3	1939,03489	1,68	29,59	0
	High	mLDIQAIADHAHQGAAALGIk	1	1	1	Q55361	N-Term(iTRAQ4plex); K21(iTRAQ4plex)	0,0000	0,917	1		0	1,845E-11	39	0,000144607	4	2432,33388	0,64	47,07	0
	High	tLSHLDYRPIPTGk	3	1	1	Q55361	N-Term(iTRAQ4plex); K14(iTRAQ4plex)	0,0000	1,272	3	51,8	0	0,000271	25	0,003326366	3	1886,07602	1,15	27,60	0
	High	rVDIIk	2	1	1	Q55361	N-Term(iTRAQ4plex); K6(iTRAQ4plex)	0,0000	1,280	2	8,1	0,002	0,02074	22	0,006039069	3	1031,68064	-0,90	17,27	1
P0DJF8	S-methyl-5'-thioadenosine phosphorylase OS=Synechocystis sp. (strain PCC 6803 /	600,46	24,23	1	7	7	29	1,804	23	36,3	326	35,3	6,86							
	A3	Sequence	# PSMs	# Proteins	# Protein Groups	Protein Group Accessions	Modifications	ΔCn	114/115	114/115 Count	114/115 Variability [%]	q-Value	PEP	IonScore	Exp Value	Charge	MH+ [Da]	ΔM [ppm]	RT [min]	# Missed Cleavages
	High	nAVNAQQVILETVk	5	1	1	P0DJF8	N-Term(iTRAQ4plex); K14(iTRAQ4plex)	0,0000	1,092	5	82,1	0	4,528E-14	111	8,39402E-12	2	1815,06157	2,04	39,96	0
	High	qLAANPFESIAHR	2	1	1	P0DJF8	N-Term(iTRAQ4plex)	0,0000	1,680	1		0	5,399E-09	52	5,754E-06	3	1597,85703	0,17	34,29	0
	High	lSLLLGk	7	1	1	P0DJF8	N-Term(iTRAQ4plex); K7(iTRAQ4plex)	0,0000	2,287	5	5,2	0	0,0008163	50	9,88485E-06	2	1031,70769	0,94	39,65	0
	High	gHHLLPSEIPFR	4	1	1	P0DJF8	N-Term(iTRAQ4plex)	0,0000	1,804	3	0,2	0	0,000002382	40	9,88485E-05	3	1546,86124	0,08	29,76	0
	High	aLQYAVLTPPDkFPAATYEk	2	1	1	P0DJF8	N-Term(iTRAQ4plex); K12(iTRAQ4plex); K20(iTRAQ4plex)	0,0000	1,313	2	31,6	0	2,2E-09	37	0,000191412	4	2655,47793	0,77	42,44	1
	High	vAFLAR	5	1	1	P0DJF8	N-Term(iTRAQ4plex)	0,0000	2,172	3	1,0	0,001	0,01441	33	0,000885193	2	820,51714	1,17	27,60	0
	High	aNIHGMk	2	1	1	P0DJF8	N-Term(iTRAQ4plex); K7(iTRAQ4plex)	0,0000	1,187	2	12,1	0,001	0,01192	12	0,057938868	3	1058,60227	0,31	11,85	0
	High	aNIHGmk	2	1	1	P0DJF8	N-Term(iTRAQ4plex); M6(Oxidation); K7(iTRAQ4plex)	0,0000	2,832	2	5,8	0,001	0,0111	10	0,097044294	3	1074,59873	1,75	8,42	0
P74229	30S ribosomal protein S7 OS=Synechocystis sp. (strain PCC 6803 / Kazusa) GN=rps	594,29	53,85	1	10	10	23	1,308	22	22,2	156	17,4	10,45							
	A3	Sequence	# PSMs	# Proteins	# Protein Groups	Protein Group Accessions	Modifications	ΔCn	114/115	114/115 Count	114/115 Variability [%]	q-Value	PEP	IonScore	Exp Value	Charge	MH+ [Da]	ΔM [ppm]	RT [min]	# Missed Cleavages
	High	sLASSIVYNALASVGEk	2	1	1	P74229	N-Term(iTRAQ4plex); K17(iTRAQ4plex)	0,0000	1,222	2	6,4	0	1,489E-15	112	6,42688E-12	2	1997,11724	0,72	55,37	0
	High	rPVPPDPVYNSTLLSmTIR	1	1	1	P74229	N-Term(iTRAQ4plex); M16(Oxidation)	0,0000	0,972	1		0	2,36E-12	72	7,23424E-08	3	2316,25229	0,86	38,36	0
	High	nLTPLVEVk	5	1	1	P74229	N-Term(iTRAQ4plex); K9(iTRAQ4plex)	0,0000	1,341	5	15,0	0	0,00001385	59	1,22735E-06	2	1300,80828	0,29	33,06	0
	High	kSLASSIVYNALASVGEk	2	1	1	P74229	N-Term(iTRAQ4plex); K1(iTRAQ4plex); K18(iTRAQ4plex)	0,0000	0,799	2	92,2	0	1,604E-09	51	7,19399E-06	3	2269,31247	-0,15	49,51	1
	High	kRPVPPDPVYNSTLLSmTIR	2	1	1	P74229	N-Term(iTRAQ4plex); K1(iTRAQ4plex); M17(Oxidation)	0,0000	1,768	2	7,6	0	8,037E-10	47	2,02288E-05	3	2588,44937	0,79	35,34	1
	High	vGGATYQVPmEVRPAR	2	1	1	P74229	N-Term(iTRAQ4plex); M10(Oxidation)	0,0000	1,540	2	0,7	0	0,000002175	47	2,12799E-05	3	1890,99735	-0,20	26,03	0
	High	wLVHFSR	1	1	1	P74229	N-Term(iTRAQ4plex)	0,0000	1,404	1		0	0,0003205	44	4,33481E-05	3	1088,61225	0,05	31,69	0
	High	gTALALR	3	1	1	P74229	N-Term(iTRAQ4plex)	0,0000	1,293	2	10,3	0	0,003206	33	0,00051047	2	845,53215	-0,48	19,94	0
	High	rVGGATYQVPmEVRPAR	1	1	1	P74229	N-Term(iTRAQ4plex); M11(Oxidation)	0,0000	0,922	1		0	0,00003854	22	0,006652272	4	2047,09878	-0,03	21,83	1
	High	rVGGATYQVPMEVRPAR	1	1	1	P74229	N-Term(iTRAQ4plex)	0,0000	4,826	1		0	0,001608	16	0,023931505	4	2031,10854	2,27	25,11	1
	High	kRPVPPDPVYNSTLLSMTIR	1	1	1	P74229	N-Term(iTRAQ4plex); K1(iTRAQ4plex)	0,0000	0,960	1		0	8,749E-09	11	0,071116439	4	2572,45473	0,90	39,27	1
	Medium	aFAHYR	2	1	1	P74229	N-Term(iTRAQ4plex)	0,0000	1,158	2	2,3	0,013	0,1497	14	0,038545173	3	908,48709	1,30	13,88	0
Q59994	Triosephosphate isomerase OS=Synechocystis sp. (strain PCC 6803 / Kazusa) GN=t	593,06	19,83	1	6	6	25	1,645	22	20,4	242	26,1	5,16							
	A3	Sequence	# PSMs	# Proteins	# Protein Groups	Protein Group Accessions	Modifications	ΔCn	114/115	114/115 Count	114/115 Variability [%]	q-Value	PEP	IonScore	Exp Value	Charge	MH+ [Da]	ΔM [ppm]	RT [min]	# Missed Cleavages
	High	rQYFGETDETANLR	4	1	1	Q59994	N-Term(iTRAQ4plex)	0,0000	1,643	4	11,7	0	3,938E-12	71	7,83376E-08	3	1843,90647	0,49	25,12	1
	High	kIIIAGNWk	2	1	1	Q59994	N-Term(iTRAQ4plex); K1(iTRAQ4plex); K9(iTRAQ4plex)	0,0000	1,631	2	11,7	0	1,895E-07	52	6,03907E-06	3	1474,94849	1,00	29,78	1
	High	aGLIPILcVGESk	4	1	1	Q59994	N-Term(iTRAQ4plex); C8(Carbamidomethyl); K13(iTRAQ4plex)	0,0000	1,329	4	4,2	0	7,51E-09	48	1,44534E-05	2	1644,96111	0,82	45,67	0
	High	iIIAGNWk	5	1	1	Q59994	N-Term(iTRAQ4plex); K8(iTRAQ4plex)	0,0000	1,802	5	8,3	0	0,001746	45	3,53973E-05	2	1202,75115	0,96	36,42	0
	High	vLAAQk	3	1	1	Q59994	N-Term(iTRAQ4plex); K6(iTRAQ4plex)	0,0000	0,911	3	11,3	0,002	0,03007	25	0,002937447	2	917,60753	5,76	15,18	0
	High	vIGLIR	7	1	1	Q59994	N-Term(iTRAQ4plex)	0,0000	1,838	4	2,5	0,007	0,08738	16	0,027225133	2	814,56432	1,46	30,07	0
P73821	D-3-phosphoglycerate dehydrogenase OS=Synechocystis sp. (strain PCC 6803 / Kaz	591,22	20,04	1	13	13	31	0,747	28	23,4	554	59,2	5,69							
	A3	Sequence	# PSMs	# Proteins	# Protein Groups	Protein Group Accessions	Modifications	ΔCn	114/115	114/115 Count	114/115 Variability [%]	q-Value	PEP	IonScore	Exp Value	Charge	MH+ [Da]	ΔM [ppm]	RT [min]	# Missed Cleavages
	High	tLGVVGLGk	4	1	1	P73821	N-Term(iTRAQ4plex); K9(iTRAQ4plex)	0,0000	0,769	2	5,6	0	3,273E-07	77	2,08915E-08	2	1131,73271	-1,14	30,33	0
	High	gLLSQALR	3	1	1	P73821	N-Term(iTRAQ4plex)	0,0000	0,660	3	13,5	0	0,0005787	56	2,31191E-06	2	1001,62218	-0,25	31,47	0
	High	kQFIGTEVYk	3	1	1	P73821	N-Term(iTRAQ4plex); K1(iTRAQ4plex); K10(iTRAQ4plex)	0,0000	0,891	3	11,4	0	7,235E-07	47	1,91854E-05	3	1644,96476	-2,29	25,11	1
	High	qVAQVDVk	2	1	1	P73821	N-Term(iTRAQ4plex); K8(iTRAQ4plex)	0,0000	0,986	2	1,8	0	0,0005784	41	8,25463E-05	2	1174,70891	4,67	17,85	0

	High	iIQAGSQLk	3	1	1	P73821	N-Term(iTRAQ4plex); K9(iTRAQ4plex)	0,0000	0,772	3	3,6	0	0,00000629	39	0,000124443	2	1245,78264	4,59	25,85	0
	High	lAETLGTLVGQLAGGR	1	1	1	P73821	N-Term(iTRAQ4plex)	0,0000	0,797	1		0	0,000003703	36	0,00022751	3	1699,98172	-0,39	58,03	0
	High	lLAYDPFISQER	2	1	1	P73821	N-Term(iTRAQ4plex)	0,0000	0,723	2	22,1	0	0,002765	35	0,000304768	2	1595,85723	1,38	46,24	0
	High	kTLGVVGLGk	1	1	1	P73821	N-Term(iTRAQ4plex); K1(iTRAQ4plex); K10(iTRAQ4plex)	0,0000	0,990	1		0	0,00002058	34	0,000443578	3	1403,92810	-2,09	25,48	1
	High	iGSHVAGVAk	3	1	1	P73821	N-Term(iTRAQ4plex); K10(iTRAQ4plex)	0,0000	0,636	2	0,6	0	0,0007487	33	0,000523564	3	1226,74793	1,60	15,80	0
	High	hIPDANk	5	1	1	P73821	N-Term(iTRAQ4plex); K7(iTRAQ4plex)	0,0000	0,579	5	14,7	0	0,0007503	28	0,001482416	3	1082,62140	1,58	11,21	0
	High	lRPYLk	2	1	1	P73821	N-Term(iTRAQ4plex); K6(iTRAQ4plex)	0,0000	0,667	2	1,8	0	0,005422	13	0,045495664	3	1077,70163	-0,61	20,68	0
	High	dYSGSLHLk	1	1	1	P73821	N-Term(iTRAQ4plex); K9(iTRAQ4plex)	0,0000	0,970	1		0,002	0,02826	14	0,035724816	3	1307,71863	-0,88	22,73	0
	Medium	mkPTAR	1	1	1	P73821	N-Term(iTRAQ4plex); M1(Oxidation); K2(iTRAQ4plex)	0,0000	0,703	1		0,033	0,3563	13	0,046770284	3	1007,59064	-0,40	7,83	0
P73264	3-amino-5-hydroxybenzoic acid synthase OS=Synechocystis sp. (strain PCC 6803 /	590,79	36,24	1	10	10	19	1,065	18	18,1	378	41,5	5,64							
	A3	Sequence	# PSMs	# Proteins	# Protein Groups	Protein Group Accessions	Modifications	ΔCn	114/115	114/115 Count	114/115 Variability [%]	q-Value	PEP	IonScore	Exp Value	Charge	MH+ [Da]	ΔM [ppm]	RT [min]	# Missed Cleavages
	High	lkQAENTTVLVPTNTNFATVASVLR	1	1	1	P73264	N-Term(iTRAQ4plex); K2(iTRAQ4plex)	0,0000	0,872	1		0	4,352E-12	108	1,76999E-11	3	2975,68045	1,20	47,33	1
	High	aYELITTPLR	2	1	1	P73264	N-Term(iTRAQ4plex)	0,0000	0,885	2	26,8	0	5,123E-07	67	2,14274E-07	2	1320,76421	-0,15	38,67	0
	High	sGTLILGNHTQEFER	2	1	1	P73264	N-Term(iTRAQ4plex)	0,0000	1,161	2	17,7	0	1,667E-09	61	7,14447E-07	3	1845,95871	0,60	29,39	0
	High	mTEIHALLGQIHLAk	3	1	1	P73264	N-Term(iTRAQ4plex); K15(iTRAQ4plex)	0,0000	1,181	2	14,4	0	1,392E-13	57	1,95884E-06	3	1963,14164	0,74	44,20	0
	High	mTEIHALLGQIHLAk	4	1	1	P73264	N-Term(iTRAQ4plex); M1(Oxidation); K15(iTRAQ4plex)	0,0000	0,964	4	18,1	0	6,648E-13	55	3,08298E-06	3	1979,13846	1,70	40,69	0
	High	hIcPPLTSGTTPEEATR	2	1	1	P73264	N-Term(iTRAQ4plex); C3(Carbamidomethyl)	0,0000	1,230	2	13,7	0	3,43E-09	52	5,63599E-06	3	2011,00669	1,55	25,20	0
	High	lMVLLPEGk	1	1	1	P73264	N-Term(iTRAQ4plex); K9(iTRAQ4plex)	0,0000	1,122	1		0	0,00003677	46	2,902E-05	2	1287,79595	0,81	40,33	0
	High	tFAPSLAmVk	1	1	1	P73264	N-Term(iTRAQ4plex); M8(Oxidation); K10(iTRAQ4plex)	0,0000	0,416	1		0	0,0001026	31	0,000731089	3	1368,77918	-0,59	32,23	0
	High	rGmNFGGLHTDFGNSTR	1	1	1	P73264	N-Term(iTRAQ4plex); M3(Oxidation)	0,0000	1,033	1		0	0,00003422	24	0,003854517	4	2026,96389	0,21	24,07	1
	High	gLYVWEDTAHAHGSQLQGk	1	1	1	P73264	N-Term(iTRAQ4plex); K19(iTRAQ4plex)	0,0000	1,675	1		0	0,0000127	24	0,00413019	4	2385,22207	1,27	31,04	0
	High	gmNFGGLHTDFGNSTR	1	1	1	P73264	N-Term(iTRAQ4plex); M2(Oxidation)	0,0000	1,072	1		0,004	0,0556	14	0,043548179	3	1870,87003	4,11	27,75	0
P09193	Photosystem II 44 kDa reaction center protein OS=Synechocystis sp. (strain PCC 68	576,88	17,17	1	9	9	20	0,380	18	41,6	460	50,3	6,60							
	A3	Sequence	# PSMs	# Proteins	# Protein Groups	Protein Group Accessions	Modifications	ΔCn	114/115	114/115 Count	114/115 Variability [%]	q-Value	PEP	IonScore	Exp Value	Charge	MH+ [Da]	ΔM [ppm]	RT [min]	# Missed Cleavages
	High	lGANIASAQGPTGLGk	6	1	1	P09193	N-Term(iTRAQ4plex); K16(iTRAQ4plex)	0,0000	1,048	5	35,9	0	1,767E-16	120	1,03031E-12	2	1743,00005	-0,19	31,51	0
	High	aAAAGFEk	4	1	1	P09193	N-Term(iTRAQ4plex); K8(iTRAQ4plex)	0,0000	0,486	3	8,0	0	0,00007237	43	4,67703E-05	2	1052,59770	-0,19	17,52	0
	High	dLPSTGFAWWSGNAR	1	1	1	P09193	N-Term(iTRAQ4plex)	0,0000	0,312	1		0	0,000001359	42	5,96994E-05	2	1808,88469	0,53	45,97	0
	High	lINLSGk	2	1	1	P09193	N-Term(iTRAQ4plex); K7(iTRAQ4plex)	0,0000	0,325	2	20,5	0	0,0009155	42	0,000111499	2	1032,67143	5,67	29,06	0
	High	lRNDIQPWQVR	2	1	1	P09193	N-Term(iTRAQ4plex)	0,0000	0,363	2	9,7	0	9,873E-07	37	0,000181539	3	1568,87821	0,24	29,14	1
	High	gPWLEPLR	2	1	1	P09193	N-Term(iTRAQ4plex)	0,0000	0,452	2	38,8	0	0,0001044	37	0,000190972	2	1111,63725	-0,76	36,87	0
	High	nDIQPWQVR	1	1	1	P09193	N-Term(iTRAQ4plex)	0,0000	0,559	1		0	0,0001362	26	0,002546654	2	1299,69267	0,02	28,64	0
	High	gPNGLDLDkLR	1	1	1	P09193	N-Term(iTRAQ4plex); K9(iTRAQ4plex)	0,0000	0,638	1		0	0,001739	15	0,033263658	3	1485,85901	-2,55	29,22	1
	Medium	fWDFR	1	1	1	P09193	N-Term(iTRAQ4plex)	0,0000	0,277	1		0,039	0,4048	14	0,037236599	2	914,46404	-0,11	38,74	0
P73704	General secretion pathway protein G OS=Synechocystis sp. (strain PCC 6803 / Kazu	576,06	25,60	1	5	5	29	1,225	25	11,3	168	17,6	4,78							
	A3	Sequence	# PSMs	# Proteins	# Protein Groups	Protein Group Accessions	Modifications	ΔCn	114/115	114/115 Count	114/115 Variability [%]	q-Value	PEP	IonScore	Exp Value	Charge	MH+ [Da]	ΔM [ppm]	RT [min]	# Missed Cleavages
	High	smSGGTFYDSGTR	5	1	1	P73704	N-Term(iTRAQ4plex); M2(Oxidation)	0,0000	1,283	3	4,8	0	4,481E-09	78	1,61797E-08	2	1525,67912	5,33	17,40	0
	High	sMSGGTFYDSGTR	2	1	1	P73704	N-Term(iTRAQ4plex)	0,0000	1,206	2	3,7	0	2,995E-09	74	3,57248E-08	2	1509,67656	0,32	23,19	0
	High	aFSTVVcR	9	1	1	P73704	N-Term(iTRAQ4plex); C7(Carbamidomethyl)	0,0000	1,238	9	10,2	0	0,001038	40	9,3319E-05	2	1083,57463	0,80	23,44	0
	High	sTIGALNR	11	1	1	P73704	N-Term(iTRAQ4plex)	0,0000	1,158	9	8,3	0	0,0075	35	0,000286398	2	975,57451	4,22	17,86	0
	High	aQQGYFTEk	1	1	1	P73704	N-Term(iTRAQ4plex); K9(iTRAQ4plex)	0,0000	1,506	1		0,001	0,008274	22	0,005928844	2	1359,72356	6,51	19,97	0
	Medium	eSEAk	1	1	1	P73704	N-Term(iTRAQ4plex); K5(iTRAQ4plex)	0,0000	1,346	1		0,043	0,4517	10	0,099076351	2	851,46843	-3,35	7,87	0
P77965	DNA-directed RNA polymerase subunit beta OS=Synechocystis sp. (strain PCC 6803	568,52	19,51	1	20	20	26	0,888	24	8,5	1102	123,3	5,30							
	A3	Sequence	# PSMs	# Proteins	# Protein Groups	Protein Group Accessions	Modifications	ΔCn	114/115	114/115 Count	114/115 Variability [%]	q-Value	PEP	IonScore	Exp Value	Charge	MH+ [Da]	ΔM [ppm]	RT [min]	# Missed Cleavages
	High	lRPGEPPTVSGGQQLLESR	1	1	1	P77965	N-Term(iTRAQ4plex)	0,0000	0,924	1		0	1,433E-13	90	8,91189E-10	3	2165,18131	0,79	33,03	0
	High	eLQSLGLDIAAHk	1	1	1	P77965	N-Term(iTRAQ4plex); K13(iTRAQ4plex)	0,0000	0,793	1		0	3,707E-11	63	4,77496E-07	3	1682,96720	-0,48	37,23	0
	High	tHGIVTYVDATEIR	1	1	1	P77965	N-Term(iTRAQ4plex)	0,0000	0,827	1		0	1,574E-09	63	5,18764E-07	3	1718,92105	0,95	31,71	0
	High	sVGELLQNQIR	1	1	1	P77965	N-Term(iTRAQ4plex)	0,0000	0,855	1		0	3,639E-08	49	1,25017E-05	2	1400,79875	0,65	35,69	0
	High	sPGVYYk	2	1	1	P77965	N-Term(iTRAQ4plex); K7(iTRAQ4plex)	0,0000	0,883	2	4,9	0	0,006843	49	1,30608E-05	2	1101,62334	4,60	19,16	0
	High	dTVHGLLEEASQRPNk	1	1	1	P77965	N-Term(iTRAQ4plex); K16(iTRAQ4plex)	0,0000	0,878	1		0	2,986E-13	48	1,65566E-05	3	2082,11991	0,79	30,23	0
	High	lELHFLGk	3	1	1	P77965	N-Term(iTRAQ4plex); K8(iTRAQ4plex)	0,0000	0,859	2	12,5	0	0,0006825	40	0,000108136	2	1244,76091	0,29	37,94	0
	High	iSALGPGGLTR	1	1	1	P77965	N-Term(iTRAQ4plex)	0,0000	0,882	1		0	0,00006666	37	0,000209397	2	1185,70696	-0,24	30,62	0
	High	vQPHSPDNPAEkGEEIVYPIQk	1	1	1	P77965	N-Term(iTRAQ4plex); K12(iTRAQ4plex); K22(iTRAQ4plex)	0,0000	1,261	1		0	1,362E-09	35	0,00034512	4	2907,56020	0,85	31,07	1
	High	kLSAQVLLk	2	1	1	P77965	N-Term(iTRAQ4plex); K1(iTRAQ4plex); K9(iTRAQ4plex)	0,0000	0,922	2	9,9	0	0,00002501	33	0,000467703	3	1431,95981	-1,76	27,66	1
	High	lSAQVLLk	1	1	1	P77965	N-Term(iTRAQ4plex); K8(iTRAQ4plex)	0,0000	1,124	1		0	0,0001363	33	0,000492006	2	1159,76409	-1,04	33,29	0
	High	vIVNQIVR	2	1	1	P77965	N-Term(iTRAQ4plex)	0,0000	0,816	2	3,1	0	0,00006671	32	0,00059016	2	1084,69524	-0,64	29,51	0
	High	tYSASLIPNR	1	1	1	P77965	N-Term(iTRAQ4plex)	0,0000	1,186	1		0	0,00008235	30	0,001315613	2	1265,69805	0,79	27,31	0
	High	qAVPLLRPERPLVGTGLEAQAAR	1	1	1	P77965	N-Term(iTRAQ4plex)	0,0000	0,557	1		0	0,00000738	29	0,00120773	4	2586,49624	0,03	35,01	0
	High	kLRPGEPPTVSGGQQLLESR	1	1	1	P77965	N-Term(iTRAQ4plex); K1(iTRAQ4plex)	0,0000	1,235	1		0	8,488E-09	29	0,001336503	4	2437,37661	-0,01	29,01	1
	High	nLDEHGIIR	1	1	1	P77965	N-Term(iTRAQ4plex)	0,0000	0,899	1		0	0,003521	21	0,00790624	3	1210,66605	-0,04	19,77	0
	High	aLmGSNmQR	2	1	1	P77965	N-Term(iTRAQ4plex); M3(Oxidation); M7(Oxidation)	0,0000	0,906	1		0	0,001153	20	0,010938808	2	1183,56816	0,09	10,60	0
	High	sIPRPGTPESFk	1	1	1	P77965	N-Term(iTRAQ4plex); K12(iTRAQ4plex)	0,0000	0,871	1		0,001	0,01292	18	0,016404765	3	1603,90390	-0,48	25,46	0
	Medium	kIQVGDk	1	1	1	P77965	N-Term(iTRAQ4plex); K1(iTRAQ4plex); K7(iTRAQ4plex)	0,0000	0,893	1		0,012	0,1369	13	0,05116465	3	1219,77179	-1,37	13,63	1
	Medium	iYVAQk	1	1	1	P77965	N-Term(iTRAQ4plex); K6(iTRAQ4plex)	0,0000	1,094	1		0,049	0,5093	15	0,032060479	2	1009,63310	4,61	20,01	0
P73304	30S ribosomal protein S5 OS=Synechocystis sp. (strain PCC 6803 / Kazusa) GN=rps	565,62	50,29	1	10	10	22	1,276	19	15,8	173	18,2	10,59							
	A3	Sequence	# PSMs	# Proteins	# Protein Groups	Protein Group Accessions	Modifications	ΔCn	114/115	114/115 Count	114/115 Variability [%]	q-Value	PEP	IonScore	Exp Value	Charge	MH+ [Da]	ΔM [ppm]	RT [min]	# Missed Cleavages
	High	qLGSNNPLNNAR	3	1	1	P73304	N-Term(iTRAQ4plex)	0,0000	1,227	3	14,4	0	4,244E-10	85	3,20605E-09	2	1441,76348	0,43	23,39	0
	High	tVLELAGVk	4	1	1	P73304	N-Term(iTRAQ4plex); K9(iTRAQ4plex)	0,0000	1,260	3	20,6	0	0,00002602	62	6,93378E-07	2	1217,77080	0,01	36,50	0
	High	aAINALETLR	4	1	1	P73304	N-Term(iTRAQ4plex)	0,0000	1,059	3	5,8	0	3,574E-07	62	8,45632E-07	2	1215,71929	1,22	35,97	0
	High	vVVRPAAPGTGVIAGGAVR	2	1	1	P73304	N-Term(iTRAQ4plex)	0,0000	1,215	1		0	2,945E-09	55	3,49116E-06	3	1891,13633	0,23	29,88	0
	High	qLIEVPLTk	2	1	1	P73304	N-Term(iTRAQ4plex); K9(iTRAQ4plex)	0,0000	1,374	2	3,2	0	0,00001408	47	1,78636E-05	2	1328,84001	0,61	35,78	0
	High	kQLIEVPLTk	2	1	1	P73304	N-Term(iTRAQ4plex); K1(iTRAQ4plex); K10(iTRAQ4plex)	0,0000	1,136	2	46,4	0	0,00002673	21	0,007602738	3	1601,03739	0,72	30,51	1
	High	ekkEDTNWQER	1	1	1	P73304	N-Term(iTRAQ4plex); K2(iTRAQ4plex); K3(iTRAQ4plex)	0,0000	1,418	1		0	0,000004806	11	0,082029489	4	1894,99917	0,43	12,21	2
	High	kGVADGkk	1	1	1	P73304	N-Term(iTRAQ4plex); K1(iTRAQ4plex); K7(iTRAQ4plex); K8(iTRAQ4plex)	0,0000	0,564	1		0,002	0,02146	11	0,078157382	4	1378,88552	-0,66	8,05	2
	High	aGDVIGAVR	2	1	1	P73304	N-Term(iTRAQ4plex)	0,0000	1,345	2	7,8	0,006	0,08031	23	0,006293758	2	1001,58550	-0,57	22,90	0
	High	kGVADGk	1	1	1	P73304	N-Term(iTRAQ4plex); K1(iTRAQ4plex); K7(iTRAQ4plex)	0,0000	2,753	1		0,009	0,1128	20	0,010739152	3	1106,68906	-0,31	8,37	1
P74690	Probable phosphoketolase OS=Synechocystis sp. (strain PCC 6803 / Kazusa) GN=sl	557,45	12,55	1	9	9	25	0,579	24	13,9	805	90,8	6,21							
	A3	Sequence	# PSMs	# Proteins	# Protein Groups	Protein Group Accessions	Modifications	ΔCn	114/115	114/115 Count	114/115 Variability [%]	q-Value	PEP	IonScore	Exp Value	Charge	MH+ [Da]	ΔM [ppm]	RT [min]	# Missed Cleavages
	High	vTSPFSLSPFGQAR	3	1	1	P74690	N-Term(iTRAQ4plex)	0,0000	0,554	3	6,9	0	1,205E-10	83	4,61318E-09	2	1637,87737	0,33	45,36	0
	High	fVSVIDLLk	10	1	1	P74690	N-Term(iTRAQ4plex); K9(iTRAQ4plex)	0,0000	0,576	10	17,9	0	4,841E-07	58	1,6633E-06	2	1321,83330	-0,07	51,56	0
	High	aANYLAVGMIYLR	1	1	1	P74690	N-Term(iTRAQ4plex)	0,0000	0,518	1		0	1,043E-08	50	1,02322E-05	2	1598,88664	1,31	50,16	0
	High	sYkPEELFDEQGTLkPGFk	2	1	1	P74690	N-Term(iTRAQ4plex); K3(iTRAQ4plex); K15(iTRAQ4plex); K19(iTRAQ4plex)	0,0000	0,601	1		0	1,885E-08	46	2,89674E-05	4	2789,51528	-1,99	37,42	0
	High	iNNPSVLSR	2	1	1	P74690	N-Term(iTRAQ4plex)	0,0000	0,571	2	13,9	0	0,0003406	32	0,00064561	2	1143,66045	0,15	23,14	0
	High	qFFPNLR	2	1	1	P74690	N-Term(iTRAQ4plex)	0,0000	0,534	2	20,6	0	0,002465	25	0,003311083	2	1065,59526	-0,89	34,88	0
	High	fSLAIDVIDRLPQLR	1	1	1	P74690	N-Term(iTRAQ4plex)	0,0000	0,568	1		0	0,00006445	21	0,007833755	3	1900,11380	0,04	55,23	1
	High	aIAPEGDkR	3	1	1	P74690	N-Term(iTRAQ4plex); K8(iTRAQ4plex)	0,0000	0,701	3	5,5	0	0,00732	15	0,038850149	3	1244,71982	-0,27	10,92	1
	High	fINPIR	1	1	1	P74690	N-Term(iTRAQ4plex)	0,0000	0,689	1		0,003	0,04186	31	0,000851079	2	903,55266	-0,69	29,23	0
P74061	Ribulose-phosphate 3-epimerase OS=Synechocystis sp. (strain PCC 6803 / Kazusa)	556,95	42,17	1	9	9	25	0,945	23	22,9	230	25,0	5,43							
	A3	Sequence	# PSMs	# Proteins	# Protein Groups	Protein Group Accessions	Modifications	ΔCn	114/115	114/115 Count	114/115 Variability [%]	q-Value	PEP	IonScore	Exp Value	Charge	MH+ [Da]	ΔM [ppm]	RT [min]	# Missed Cleavages
	High	nIVVAPSILSADFSR	4	1	1	P74061	N-Term(iTRAQ4plex)	0,0000	0,818	4	25,5	0	7,199E-11	88	1,5775E-09	2	1732,97270	0,71	46,63	0
	High	aGADIISVHVEHNASPHLHR	4	1	1	P74061	N-Term(iTRAQ4plex)	0,0000	0,767	2	6,9	0	1,585E-12	51	8,07179E-06	4	2304,20913	0,55	23,38	0
	High	fVPNITIGPLIVDAIRPLTk	7	1	1	P74061	N-Term(iTRAQ4plex); K20(iTRAQ4plex)	0,0000	0,843	7	11,9	0	7,043E-09	44	4,22639E-05	4	2465,50966	-0,09	58,77	0
	High	rPEPQLATV	2	1	1	P74061	N-Term(iTRAQ4plex)	0,0000	0,829	2	5,6	0	0,00381	43	5,36995E-05	2	1154,66557	0,47	23,82	0
	High	kTLDVHLmIVEPEk	2	1	1	P74061	N-Term(iTRAQ4plex); K1(iTRAQ4plex); M8(Oxidation); K14(iTRAQ4plex)	0,0000	1,175	2	32,4	0	1,168E-07	34	0,000443578	4	2100,20913	-0,39	32,73	1
	High	tLDVHLmIVEPEkYVEDFAk	1	1	1	P74061	N-Term(iTRAQ4plex); M7(Oxidation); K13(iTRAQ4plex); K20(iTRAQ4plex)	0,0000	1,101	1		0	0,000001446	25	0,004348786	4	2824,51748	0,25	47,09	1
	High	kTLDVHLmIVEPEkYVEDFAk	1	1	1	P74061	K1(iTRAQ4plex); N-Term(iTRAQ4plex); M8(Oxidation); K14(iTRAQ4plex); K2	0,0000	1,870	1		0	1,645E-07	19	0,013595422	5	3096,71705	1,05	43,31	2
	High	lGEEIk	2	1	1	P74061	N-Term(iTRAQ4plex); K6(iTRAQ4plex)	0,0000	0,996	2	16,4	0,001	0,01592	18	0,014189595	2	976,59221	0,47	20,07	0
	High	tLcQIR	2	1	1	P74061	N-Term(iTRAQ4plex); C3(Carbamidomethyl)	0,0000	0,958	2	2,0	0,002	0,01979	33	0,000484139	2	934,52910	3,23	17,16	0
P73314	30S ribosomal protein S3 OS=Synechocystis sp. (strain PCC 6803 / Kazusa) GN=rps	553,61	36,67	1	9	9	28	1,089	25	20,9	240	27,1	9,98							
	A3	Sequence	# PSMs	# Proteins	# Protein Groups	Protein Group Accessions	Modifications	ΔCn	114/115	114/115 Count	114/115 Variability [%]	q-Value	PEP	IonScore	Exp Value	Charge	MH+ [Da]	ΔM [ppm]	RT [min]	# Missed Cleavages
	High	tALTTYGILGIk	6	1	1	P73314	N-Term(iTRAQ4plex); K12(iTRAQ4plex)	0,0000	1,023	6	22,0	0	7,537E-11	88	1,7457E-09	2	1538,94084	0,78	44,05	0
	High	tLNNAGISDIR	1	1	1	P73314	N-Term(iTRAQ4plex)	0,0000	0,646	1		0	0,00001203	53	5,61009E-06	2	1317,72466	0,24	26,07	0
	High	gGSGIEQLR	2	1	1	P73314	N-Term(iTRAQ4plex)	0,0000	1,156	2	1,4	0	0,000263	49	1,30608E-05	2	1060,59270	5,58	18,03	0
	High	kAEQIELGIHTARPGVVVGR	4	1	1	P73314	N-Term(iTRAQ4plex); K1(iTRAQ4plex)	0,0000	0,846	3	16,0	0	3,385E-08	40	9,48353E-05	5	2418,41962	0,49	27,70	1
	High	rYPELLQEDHk	3	1	1	P73314	N-Term(iTRAQ4plex); K11(iTRAQ4plex)	0,0000	1,039	3	5,5	0	4,942E-09	37	0,000311409	3	1715,93222	0,17	22,60	1
	High	vPLHTLR	6	1	1	P73314	N-Term(iTRAQ4plex)	0,0000	0,958	4	2,3	0	0,005332	30	0,001083852	3	979,61725	0,31	18,83	0
	High	vWIFk	3	1	1	P73314	N-Term(iTRAQ4plex); K5(iTRAQ4plex)	0,0000	1,274	3	5,0	0,004	0,05336	26	0,002666674	2	980,61730	0,13	38,88	0
	Medium	iHPVGFR	1	1	1	P73314	N-Term(iTRAQ4plex)	0,0000	1,272	1		0,016	0,1789	12	0,056489796	3	969,57639	1,35	19,58	0
	Medium	iQVSGR	2	1	1	P73314	N-Term(iTRAQ4plex)	0,0000	1,150	2	5,9	0,02	0,224	23	0,006370613	2	803,48937	4,69	14,31	0
P37277	Photosystem I reaction center subunit XI OS=Synechocystis sp. (strain PCC 6803 /	553,32	26,11	1	4	4	38	0,807	36	26,8	157	16,6	4,83							
	A3	Sequence	# PSMs	# Proteins	# Protein Groups	Protein Group Accessions	Modifications	ΔCn	114/115	114/115 Count	114/115 Variability [%]	q-Value	PEP	IonScore	Exp Value	Charge	MH+ [Da]	ΔM [ppm]	RT [min]	# Missed Cleavages
	High	tFIGNLPAYR	22	1	1	P37277	N-Term(iTRAQ4plex)	0,0000	0,730	21	16,9	0	0,000001617	58	1,76999E-06	2	1295,72331	0,34	37,20	0
	High	gLEVGmAHGYFLIGPWTLLGPLR	3	1	1	P37277	N-Term(iTRAQ4plex); M6(Oxidation)	0,0000	0,868	2	16,0	0	1,263E-11	57	1,86625E-06	3	2657,43216	-2,78	59,17	0
	High	gLSPILR	10	1	1	P37277	N-Term(iTRAQ4plex)	0,0000	0,971	10	17,3	0	0,0009328	46	2,4602E-05	2	899,57921	-0,33	28,49	0
	High	kGLSPILR	3	1	1	P37277	N-Term(iTRAQ4plex); K1(iTRAQ4plex)	0,0000	0,493	3	15,1	0	0,00006968	37	0,000208435	3	1171,77619	-0,30	21,74	1
Q55247	Nitrogen regulatory protein P-II OS=Synechocystis sp. (strain PCC 6803 / Kazusa) (	552,24	49,11	1	6	6	18	1,233	18	25,5	112	12,4	6,81							
	A3	Sequence	# PSMs	# Proteins	# Protein Groups	Protein Group Accessions	Modifications	ΔCn	114/115	114/115 Count	114/115 Variability [%]	q-Value	PEP	IonScore	Exp Value	Charge	MH+ [Da]	ΔM [ppm]	RT [min]	# Missed Cleavages
	High	iALVNAGIVGmTVSEVR	4	1	1	Q55247	N-Term(iTRAQ4plex); M11(Oxidation)	0,0000	1,159	4	20,5	0	6,669E-13	88	1,61135E-09	2	1889,06767	1,55	46,03	0
	High	gSEYTVEFLQk	2	1	1	Q55247	N-Term(iTRAQ4plex); K11(iTRAQ4plex)	0,0000	1,078	2	4,4	0	1,891E-07	64	3,9808E-07	2	1588,84722	0,68	36,00	0
	High	iFISPVDSVVR	3	1	1	Q55247	N-Term(iTRAQ4plex)	0,0000	1,156	3	0,2	0	0,0009205	50	9,37497E-06	2	1375,80766	0,76	41,95	0
	High	vEAIIRPFk	4	1	1	Q55247	N-Term(iTRAQ4plex); K9(iTRAQ4plex)	0,0000	1,554	4	10,1	0	0,00002332	45	3,4512E-05	2	1360,85686	0,98	34,01	0
	High	kVEAIIRPFk	3	1	1	Q55247	N-Term(iTRAQ4plex); K1(iTRAQ4plex); K10(iTRAQ4plex)	0,0000	1,567	3	8,9	0	0,0002031	40	9,7268E-05	3	1633,05657	2,45	27,98	1
	Medium	lVSAAR	2	1	1	Q55247	N-Term(iTRAQ4plex)	0,0000	1,387	2	30,4	0,017	0,1859	18	0,04366747	2	760,48461	6,34	14,15	0
P72939	Alkaline phosphatase OS=Synechocystis sp. (strain PCC 6803 / Kazusa) GN=sll0654	551,91	7,74	1	10	10	25	0,671	23	10,1	1409	149,3	4,15							
	A3	Sequence	# PSMs	# Proteins	# Protein Groups	Protein Group Accessions	Modifications	ΔCn	114/115	114/115 Count	114/115 Variability [%]	q-Value	PEP	IonScore	Exp Value	Charge	MH+ [Da]	ΔM [ppm]	RT [min]	# Missed Cleavages
	High	vVLLAHmQQIAIEQELAVR	1	1	1	P72939	N-Term(iTRAQ4plex); M7(Oxidation)	0,0000	0,649	1		0	3,818E-12	61	7,63783E-07	3	2321,31589	1,13	45,04	0
	High	aELQSAGVR	5	1	1	P72939	N-Term(iTRAQ4plex)	0,0000	0,696	3	3,8	0	0,00000286	59	1,49176E-06	2	1074,60869	5,82	16,04	0
	High	tYAFVGLER	3	1	1	P72939	N-Term(iTRAQ4plex)	0,0000	0,638	3	8,2	0	9,966E-07	58	1,75376E-06	2	1199,65337	-0,63	33,94	0
	High	hVDIIVAGGSNTR	2	1	1	P72939	N-Term(iTRAQ4plex)	0,0000	0,908	2	84,6	0	2,512E-07	54	3,72366E-06	2	1482,81511	0,37	20,80	0
	High	tINSGVSLISk	2	1	1	P72939	N-Term(iTRAQ4plex); K11(iTRAQ4plex)	0,0000	0,808	2	18,1	0	0,000002616	44	4,11121E-05	3	1406,84443	-0,93	31,10	0
	High	eSNVFGVSNVYLEGSRPAIR	2	1	1	P72939	N-Term(iTRAQ4plex)	0,0000	0,537	2	8,9	0	6,549E-10	36	0,000266054	3	2338,22898	0,73	40,24	0
	High	iQNLAFR	3	1	1	P72939	N-Term(iTRAQ4plex)	0,0000	0,611	3	3,7	0	0,001045	32	0,000812662	2	1005,59618	-0,04	29,62	0
	High	lLDENDRLR	3	1	1	P72939	N-Term(iTRAQ4plex)	0,0000	0,654	3	2,5	0	0,00793	26	0,002269708	3	1287,71463	0,66	23,53	1
	High	lTVTTk	3	1	1	P72939	N-Term(iTRAQ4plex); K6(iTRAQ4plex)	0,0000	0,675	3	0,7	0,006	0,07225	42	6,63697E-05	2	950,61241	-0,06	18,45	0
	Medium	eVIVAk	1	1	1	P72939	N-Term(iTRAQ4plex); K6(iTRAQ4plex)	0,0000	0,751	1		0,033	0,3548	26	0,002552525	2	946,61424	-3,51	18,76	0
P73488	Sll1130 protein OS=Synechocystis sp. (strain PCC 6803 / Kazusa) GN=sll1130 PE=4	547,80	46,96	1	5	5	38	0,813	32	26,6	115	12,9	8,81							
	A3	Sequence	# PSMs	# Proteins	# Protein Groups	Protein Group Accessions	Modifications	ΔCn	114/115	114/115 Count	114/115 Variability [%]	q-Value	PEP	IonScore	Exp Value	Charge	MH+ [Da]	ΔM [ppm]	RT [min]	# Missed Cleavages
	High	rPALILSDAPAFNNR	21	1	1	P73488	N-Term(iTRAQ4plex)	0,0000	0,860	19	26,2	0	1,008E-09	56	2,28544E-06	3	1799,00571	0,66	35,09	0
	High	sAGLFTPSVVR	4	1	1	P73488	N-Term(iTRAQ4plex)	0,0000	0,785	4	15,2	0	0,0003885	52	6,99794E-06	2	1277,73308	-0,29	33,60	0
	High	iGHSVmAmITSAk	4	1	1	P73488	N-Term(iTRAQ4plex); M6(Oxidation); M8(Oxidation); K13(iTRAQ4plex)	0,0000	0,690	2	7,7	0	4,037E-09	50	1,04225E-05	3	1665,89005	-0,38	23,06	0
	High	iGHSVMAMITSAk	1	1	1	P73488	N-Term(iTRAQ4plex); K13(iTRAQ4plex)	0,0000	1,524	1		0	3,022E-07	36	0,000273012	3	1633,90116	0,18	37,31	0
	High	lFTLEHk	3	1	1	P73488	N-Term(iTRAQ4plex); K7(iTRAQ4plex)	0,0000	0,757	3	10,3	0	0,0006443	32	0,000734362	2	1175,70183	-0,73	27,09	0
	High	sAFPHVFk	4	1	1	P73488	N-Term(iTRAQ4plex); K8(iTRAQ4plex)	0,0000	0,728	2	17,0	0	0,00001581	29	0,001129718	3	1220,70371	0,56	27,38	0
	High	iGHSVMAmITSAk	1	1	1	P73488	N-Term(iTRAQ4plex); M8(Oxidation); K13(iTRAQ4plex)	0,0000	0,749	1		0	0,000001579	19	0,013488697	3	1649,89304	-1,66	30,53	0
P73902	Sll0236 protein OS=Synechocystis sp. (strain PCC 6803 / Kazusa) GN=sll0236 PE=4	538,40	19,53	1	12	12	29	1,408	25	9,3	558	63,9	5,25							
	A3	Sequence	# PSMs	# Proteins	# Protein Groups	Protein Group Accessions	Modifications	ΔCn	114/115	114/115 Count	114/115 Variability [%]	q-Value	PEP	IonScore	Exp Value	Charge	MH+ [Da]	ΔM [ppm]	RT [min]	# Missed Cleavages
	High	fAITHALMAPR	3	1	1	P73902	N-Term(iTRAQ4plex)	0,0000	1,294	2	8,1	0	6,426E-08	69	1,27342E-07	3	1371,76911	0,22	33,59	0
	High	vEIGGWSFk	1	1	1	P73902	N-Term(iTRAQ4plex); K9(iTRAQ4plex)	0,0000	1,459	1		0	3,412E-07	53	5,59758E-06	2	1310,73613	1,06	40,87	0
	High	fAITHALmAPR	3	1	1	P73902	N-Term(iTRAQ4plex); M8(Oxidation)	0,0000	1,447	2	2,5	0	0,000001714	42	5,63599E-05	3	1387,76319	-0,38	30,65	0
	High	iHQELPHLEGR	2	1	1	P73902	N-Term(iTRAQ4plex)	0,0000	1,157	2	17,7	0	7,065E-07	39	0,000129113	3	1472,81018	0,75	22,86	0
	High	aEAEFWQQHR	3	1	1	P73902	N-Term(iTRAQ4plex)	0,0000	1,560	3	27,7	0	2,929E-07	38	0,000156664	3	1445,70389	-0,26	24,28	0
	High	kTDSVTLITGk	1	1	1	P73902	N-Term(iTRAQ4plex); K1(iTRAQ4plex); K11(iTRAQ4plex)	0,0000	1,495	1		0	0,00001419	38	0,000158844	3	1594,97257	-0,91	23,64	1
	High	wHPPEDDYk	2	1	1	P73902	N-Term(iTRAQ4plex); K9(iTRAQ4plex)	0,0000	1,374	2	5,6	0	0,0001498	34	0,000420698	3	1474,72071	0,12	23,71	0
	High	sSVRPYPYPEER	2	1	1	P73902	N-Term(iTRAQ4plex)	0,0000	1,358	1		0	0,00007876	31	0,000736156	3	1623,82468	-0,06	19,47	0
	High	yLTTPYLLR	4	1	1	P73902	N-Term(iTRAQ4plex)	0,0000	1,403	4	8,7	0	0,003469	31	0,000741259	2	1283,74870	0,55	41,50	0
	High	hLIWVIR	3	1	1	P73902	N-Term(iTRAQ4plex)	0,0000	1,412	2	8,9	0	0,0007144	28	0,001559445	2	1080,68083	0,88	32,83	0
	High	aVRPIQVTLk	2	1	1	P73902	N-Term(iTRAQ4plex); K10(iTRAQ4plex)	0,0000	2,151	2	12,9	0	0,001966	26	0,00232793	2	1412,92060	1,00	25,55	0
	High	kAVRPIQVTLk	2	1	1	P73902	N-Term(iTRAQ4plex); K1(iTRAQ4plex); K11(iTRAQ4plex)	0,0000	0,316	2	4,3	0	0,000004633	26	0,002773129	3	1685,11576	-0,28	23,13	1
	High	lTITAQAER	1	1	1	P73902	N-Term(iTRAQ4plex)	0,0000	1,408	1		0,001	0,01465	25	0,002818188	2	1146,66057	0,55	22,88	0
P73722	SOS function regulatory protein OS=Synechocystis sp. (strain PCC 6803 / Kazusa) (	536,96	29,56	1	11	11	30	1,088	26	13,7	203	22,7	6,11							
	A3	Sequence	# PSMs	# Proteins	# Protein Groups	Protein Group Accessions	Modifications	ΔCn	114/115	114/115 Count	114/115 Variability [%]	q-Value	PEP	IonScore	Exp Value	Charge	MH+ [Da]	ΔM [ppm]	RT [min]	# Missed Cleavages
	High	gVSVIGELk	9	1	1	P73722	N-Term(iTRAQ4plex); K9(iTRAQ4plex)	0,0000	1,226	6	12,8	0	0,00006475	60	1,02322E-06	2	1189,73857	-0,77	32,64	0
	High	sSVFALR	4	1	1	P73722	N-Term(iTRAQ4plex)	0,0000	0,995	3	0,9	0	0,0004522	45	2,83772E-05	2	923,54411	1,08	27,46	0
	High	kSSVFALR	3	1	1	P73722	N-Term(iTRAQ4plex); K1(iTRAQ4plex)	0,0000	0,871	3	15,6	0	0,00006162	43	5,48239E-05	2	1195,74085	0,59	22,79	1
	High	iLHQkPk	2	1	1	P73722	N-Term(iTRAQ4plex); K5(iTRAQ4plex); K7(iTRAQ4plex)	0,0000	0,993	2	5,0	0	0,005129	38	0,000164426	3	1295,85198	-0,30	10,31	0
	High	rYYQDGTk	1	1	1	P73722	N-Term(iTRAQ4plex); K8(iTRAQ4plex)	0,0000	1,047	1		0	0,00006493	33	0,000462349	3	1318,70349	3,12	10,53	1
	High	gPGQELk	2	1	1	P73722	N-Term(iTRAQ4plex); K7(iTRAQ4plex)	0,0000	1,039	2	22,4	0	0,007951	33	0,00052236	2	1016,59874	0,83	13,89	0
	High	aSNNkGPGQELk	3	1	1	P73722	N-Term(iTRAQ4plex); K5(iTRAQ4plex); K12(iTRAQ4plex)	0,0000	1,086	3	0,4	0	0,00002482	31	0,00084522	3	1674,95029	0,21	12,50	1
	High	iDFAPLmk	2	1	1	P73722	N-Term(iTRAQ4plex); M7(Oxidation); K8(iTRAQ4plex)	0,0000	1,220	2	7,2	0	0,001561	22	0,006901921	2	1238,70635	0,48	36,16	0
	High	iDFAPLmkk	1	1	1	P73722	N-Term(iTRAQ4plex); M7(Oxidation); K8(iTRAQ4plex); K9(iTRAQ4plex)	0,0000	1,196	1		0	0,0003524	21	0,008184083	3	1510,90464	1,23	30,63	1
	High	sPAPIQSR	2	1	1	P73722	N-Term(iTRAQ4plex)	0,0000	1,018	2	2,1	0,002	0,02906	18	0,014688248	2	999,57176	1,37	12,67	0
	High	yYQDGTk	1	1	1	P73722	N-Term(iTRAQ4plex); K7(iTRAQ4plex)	0,0000	1,148	1		0,005	0,0702	20	0,009930475	2	1162,60185	3,09	15,29	0
P73315	50S ribosomal protein L22 OS=Synechocystis sp. (strain PCC 6803 / Kazusa) GN=rp	534,83	43,80	1	6	6	26	2,161	17	124,9	121	13,5	9,99							
	A3	Sequence	# PSMs	# Proteins	# Protein Groups	Protein Group Accessions	Modifications	ΔCn	114/115	114/115 Count	114/115 Variability [%]	q-Value	PEP	IonScore	Exp Value	Charge	MH+ [Da]	ΔM [ppm]	RT [min]	# Missed Cleavages
	High	tkLDTTAEVk	10	1	1	P73315	N-Term(iTRAQ4plex); K2(iTRAQ4plex); K10(iTRAQ4plex)	0,0000	19,560	4	283,7	0	1,354E-07	63	4,97703E-07	2	1537,91496	-0,78	17,46	1
	High	kPTcHITVAVAPALADN	4	1	1	P73315	N-Term(iTRAQ4plex); K1(iTRAQ4plex); C4(Carbamidomethyl)	0,0000	1,180	3	146,3	0	1,155E-07	49	1,33045E-05	3	2066,13443	1,79	30,53	1
	High	rVLDQIR	3	1	1	P73315	N-Term(iTRAQ4plex)	0,0000	1,735	3	15,4	0	0,001011	37	0,000201823	3	1043,64383	-0,38	17,25	1
	High	acEPVLk	4	1	1	P73315	N-Term(iTRAQ4plex); C2(Carbamidomethyl); K7(iTRAQ4plex)	0,0000	11,421	2	50,9	0	0,0009817	37	0,000207477	2	1104,63970	6,45	18,73	0
	High	eALIILEFmPYk	1	1	1	P73315	N-Term(iTRAQ4plex); M9(Oxidation); K12(iTRAQ4plex)	0,0000	1,009	1		0	0,00006936	31	0,001064587	3	1770,99790	1,38	51,07	0
	High	eALIILEFMPYk	1	1	1	P73315	N-Term(iTRAQ4plex); K12(iTRAQ4plex)	0,0000	1,124	1		0	0,0007288	15	0,030336816	3	1755,00217	0,92	57,02	0
	High	vLDQIR	3	1	1	P73315	N-Term(iTRAQ4plex)	0,0000	1,137	3	62,1	0,001	0,01718	29	0,001965025	2	887,54863	6,22	23,81	0
P05429	Photosystem II CP47 chlorophyll apoprotein OS=Synechocystis sp. (strain PCC 680	529,94	21,10	1	10	10	31	0,423	26	60,1	507	55,9	5,85							
	A3	Sequence	# PSMs	# Proteins	# Protein Groups	Protein Group Accessions	Modifications	ΔCn	114/115	114/115 Count	114/115 Variability [%]	q-Value	PEP	IonScore	Exp Value	Charge	MH+ [Da]	ΔM [ppm]	RT [min]	# Missed Cleavages
	High	tGAmNSGDGIAQEWIGHPIFk	4	1	1	P05429	N-Term(iTRAQ4plex); M4(Oxidation); K21(iTRAQ4plex)	0,0000	0,456	3	132,3	0	8,177E-16	70	1,09388E-07	3	2533,27549	0,24	42,43	0
	High	rmPNFFETFPVImTDADGVVR	2	1	1	P05429	N-Term(iTRAQ4plex); M2(Oxidation); M13(Oxidation)	0,0000	0,415	2	260,5	0	5,99E-15	63	4,48714E-07	3	2618,28733	0,32	48,47	1
	High	gYFQEEIQR	3	1	1	P05429	N-Term(iTRAQ4plex)	0,0000	0,912	2	10,3	0	0,0001251	50	1,03746E-05	2	1313,66020	-0,35	27,71	0
	High	vHTVVLNDPGR	3	1	1	P05429	N-Term(iTRAQ4plex)	0,0000	0,294	2	64,0	0	0,00003746	47	1,96322E-05	3	1350,76055	-0,38	20,04	0
	High	rMPNFFETFPVIMTDADGVVR	1	1	1	P05429	N-Term(iTRAQ4plex)	0,0000	0,299	1		0	1,057E-09	40	9,18269E-05	3	2586,30356	2,67	55,80	1
	High	lAFYDYVGNSPAk	3	1	1	P05429	N-Term(iTRAQ4plex); K13(iTRAQ4plex)	0,0000	0,630	3	38,0	0	0,000001211	39	0,000120218	3	1732,91379	-0,63	39,72	0
	High	qGMFVLPFmAR	3	1	1	P05429	N-Term(iTRAQ4plex); M9(Oxidation)	0,0000	0,376	3	38,8	0	0,0006978	28	0,001516946	2	1456,75676	0,38	46,15	0
	High	aDIPFRR	3	1	1	P05429	N-Term(iTRAQ4plex)	0,0000	0,270	3	26,7	0	0,003804	26	0,002517503	2	1018,59099	-0,48	20,32	1
	High	qGmFVLPFmAR	2	1	1	P05429	N-Term(iTRAQ4plex); M3(Oxidation); M9(Oxidation)	0,0000	0,437	2	9,9	0	0,001713	22	0,006151344	2	1472,75163	0,35	40,87	0
	High	vGDLSTRk	3	1	1	P05429	N-Term(iTRAQ4plex); K8(iTRAQ4plex)	0,0000	0,342	2	4,5	0,001	0,01469	16	0,026422263	3	1163,69739	-1,11	14,66	1
	High	aDIPFR	4	1	1	P05429	N-Term(iTRAQ4plex)	0,0000	0,310	2	7,3	0,002	0,02957	13	0,04487144	2	862,48967	-0,79	25,16	0
	High	gLPWYR	1	1	1	P05429	N-Term(iTRAQ4plex)	0,0000	1,806	1		0,007	0,08425	20	0,010597155	2	935,52165	-0,34	31,96	0
P27724	NAD(P)H-quinone oxidoreductase subunit H OS=Synechocystis sp. (strain PCC 680	528,29	23,10	1	10	10	22	0,514	20	18,7	394	45,5	6,06							
	A3	Sequence	# PSMs	# Proteins	# Protein Groups	Protein Group Accessions	Modifications	ΔCn	114/115	114/115 Count	114/115 Variability [%]	q-Value	PEP	IonScore	Exp Value	Charge	MH+ [Da]	ΔM [ppm]	RT [min]	# Missed Cleavages
	High	iPAGEHYVR	3	1	1	P27724	N-Term(iTRAQ4plex)	0,0000	0,510	3	9,6	0	0,00006175	61	8,72911E-07	3	1185,65604	5,34	17,46	0
	High	tNImYVPYVSR	2	1	1	P27724	N-Term(iTRAQ4plex); M4(Oxidation)	0,0000	0,499	2	5,7	0	0,000000256	54	3,8104E-06	2	1502,78020	0,53	32,08	0
	High	sADFNNLQILPHILk	5	1	1	P27724	N-Term(iTRAQ4plex); K15(iTRAQ4plex)	0,0000	0,448	3	22,1	0	4,126E-11	48	1,68644E-05	3	2011,15903	0,55	45,68	0
	High	lITNNPIFR	2	1	1	P27724	N-Term(iTRAQ4plex)	0,0000	0,487	2	3,8	0	0,000001154	45	3,15479E-05	2	1231,72832	0,29	38,46	0
	High	vImLELNR	2	1	1	P27724	N-Term(iTRAQ4plex); M3(Oxidation)	0,0000	0,661	2	5,0	0	0,00007123	37	0,000351924	2	1147,66191	-0,60	33,45	0
	High	kSEWNDFQYQYIAk	1	1	1	P27724	N-Term(iTRAQ4plex); K1(iTRAQ4plex); K14(iTRAQ4plex)	0,0000	0,596	1		0	3,129E-09	37	0,00021576	3	2252,16959	-0,72	36,06	1
	High	kVAPTFk	1	1	1	P27724	N-Term(iTRAQ4plex); K1(iTRAQ4plex); K7(iTRAQ4plex)	0,0000	0,630	1		0	0,0008321	19	0,011748164	3	1222,78862	-1,44	17,10	1
	High	vAPTFk	2	1	1	P27724	N-Term(iTRAQ4plex); K6(iTRAQ4plex)	0,0000	0,725	2	10,1	0,001	0,01165	42	6,59128E-05	2	950,59532	4,18	21,10	0
	High	lINNNYFR	2	1	1	P27724	N-Term(iTRAQ4plex)	0,0000	0,558	2	18,1	0,002	0,02512	27	0,001999724	2	1197,64897	-0,60	33,84	0
	High	rVEGVGTVTR	1	1	1	P27724	N-Term(iTRAQ4plex)	0,0000	0,449	2	3,7	0,002	0,02028	25	0,003427441	2	1217,70769	-0,51	13,70	1
P16033	Photosystem Q(B) protein 2 OS=Synechocystis sp. (strain PCC 6803 / Kazusa) GN=	526,81	16,67	1	5	5	16	0,377	15	29,9	360	39,7	5,64							
	A3	Sequence	# PSMs	# Proteins	# Protein Groups	Protein Group Accessions	Modifications	ΔCn	114/115	114/115 Count	114/115 Variability [%]	q-Value	PEP	IonScore	Exp Value	Charge	MH+ [Da]	ΔM [ppm]	RT [min]	# Missed Cleavages
	High	fGQEEETYNIVAAHGYFGR	2	1	1	P16033	N-Term(iTRAQ4plex)	0,0000	0,419	2	16,8	0	1,877E-14	70	9,01509E-08	3	2332,11325	0,73	41,56	0
	High	lIFQYASFNNSR	4	1	1	P16033	N-Term(iTRAQ4plex)	0,0000	0,334	4	19,6	0	0,000000363	64	4,37492E-07	2	1603,83550	0,34	43,17	0
	High	vIGTWADVLNR	4	1	1	P16033	N-Term(iTRAQ4plex)	0,0000	0,435	4	22,9	0	0,00000844	62	5,62302E-07	2	1387,78203	0,40	45,03	0
	High	aNIGFEVmHER	3	1	1	P16033	N-Term(iTRAQ4plex); M8(Oxidation)	0,0000	0,310	2	1,2	0	5,59E-08	51	8,24081E-06	2	1462,72270	-0,19	22,66	0
	High	qWELSYR	2	1	1	P16033	N-Term(iTRAQ4plex)	0,0000	0,400	2	21,2	0	0,0004205	38	0,000155944	2	1125,58037	-0,51	29,69	0
	High	aNIGFEVMHER	1	1	1	P16033	N-Term(iTRAQ4plex)	0,0000	0,441	1		0	0,000001313	25	0,002818188	3	1446,72708	-0,68	28,43	0
P72760	Carbon dioxide-concentrating mechanism protein CcmK homolog 1 OS=Synechocys	526,67	73,87	1	4	7	36	1,651	6	9,0	111	12,1	6,55							
	A3	Sequence	# PSMs	# Proteins	# Protein Groups	Protein Group Accessions	Modifications	ΔCn	114/115	114/115 Count	114/115 Variability [%]	q-Value	PEP	IonScore	Exp Value	Charge	MH+ [Da]	ΔM [ppm]	RT [min]	# Missed Cleavages
	High	yTEAVEQFR	2	1	1	P72760	N-Term(iTRAQ4plex)	0,0000	1,731	2	1,2	0	0,000005549	51	8,14648E-06	2	1286,65105	1,00	30,90	0
	High	vTLVGYEk	19	2	2	P72760;P72761	N-Term(iTRAQ4plex); K8(iTRAQ4plex)	0,0000				0	0,0008475	49	1,13755E-05	2	1196,71440	1,24	27,93	0
	High	eIVNPSIIR	4	1	1	P72760	N-Term(iTRAQ4plex)	0,0000	1,673	2	7,8	0	0,000743	38	0,000144534	2	1184,71245	0,39	30,87	0
	High	gDVSEVQASVTAGIENIRR	1	1	1	P72760	N-Term(iTRAQ4plex)	0,0000	1,460	1		0	2,657E-07	33	0,000481049	3	2145,14042	1,07	42,04	1
	High	vNGGEVLSNHIIARPHENLEYVLPIR	1	1	1	P72760	N-Term(iTRAQ4plex)	0,0000	1,201	1		0	4,818E-07	18	0,014587135	4	3083,68911	0,62	41,30	0
	High	vTVIVR	7	2	2	P72760;P72761	N-Term(iTRAQ4plex)	0,0000				0,001	0,01412	37	0,000187055	2	830,55889	1,02	22,67	0
	Medium	iGSGR	2	2	2	P72760;P72761	N-Term(iTRAQ4plex)	0,0000				0,023	0,2444	16	0,0477829	2	633,38042	0,56	7,96	0
P73530	30S ribosomal protein S1 homolog A OS=Synechocystis sp. (strain PCC 6803 / Kazu	522,35	23,17	1	8	8	22	1,362	20	33,4	328	36,5	4,67							
	A3	Sequence	# PSMs	# Proteins	# Protein Groups	Protein Group Accessions	Modifications	ΔCn	114/115	114/115 Count	114/115 Variability [%]	q-Value	PEP	IonScore	Exp Value	Charge	MH+ [Da]	ΔM [ppm]	RT [min]	# Missed Cleavages
	High	kMNGLEVAQVVVGSVR	2	1	1	P73530	N-Term(iTRAQ4plex); K1(iTRAQ4plex)	0,0000	0,987	2	3,7	0	1,063E-10	89	1,47295E-09	3	1974,14188	0,48	42,81	1
	High	kmNGLEVAQVVVGSVR	2	1	1	P73530	N-Term(iTRAQ4plex); K1(iTRAQ4plex); M2(Oxidation)	0,0000	0,985	2	3,9	0	1,172E-09	63	5,39473E-07	3	1990,13761	0,89	36,56	1
	High	gALIDIGAk	3	1	1	P73530	N-Term(iTRAQ4plex); K9(iTRAQ4plex)	0,0000	2,010	2	8,9	0	0,000002666	50	1,34868E-05	2	1145,71440	0,98	29,86	0
	High	gFIPGSHISAR	1	1	1	P73530	N-Term(iTRAQ4plex)	0,0000	1,563	1		0	5,681E-08	45	3,52347E-05	3	1285,71320	-0,14	22,88	0
	High	tAAYIPIQEmSINR	2	1	1	P73530	N-Term(iTRAQ4plex); M10(Oxidation)	0,0000	1,041	2	46,2	0	5,718E-07	40	0,000109388	3	1766,92325	0,26	32,29	0
	High	sNVFATNR	5	1	1	P73530	N-Term(iTRAQ4plex)	0,0000	1,447	4	7,8	0	0,00008731	37	0,000214274	2	1052,56084	0,27	16,63	0
	High	iSLSTk	3	1	1	P73530	N-Term(iTRAQ4plex); K6(iTRAQ4plex)	0,0000	1,666	3	6,3	0,001	0,008932	41	7,2272E-05	2	936,59776	1,01	21,57	0
	High	rIEYMR	1	1	1	P73530	N-Term(iTRAQ4plex)	0,0000	1,075	1		0,007	0,08966	18	0,015274606	3	1011,55313	0,49	15,31	1
	Medium	lVLSHR	2	1	1	P73530	N-Term(iTRAQ4plex)	0,0000	1,324	2	0,1	0,014	0,1621	14	0,037065512	2	868,55162	3,57	15,70	0
	Medium	rIEYmR	1	1	1	P73530	N-Term(iTRAQ4plex); M5(Oxidation)	0,0000	1,309	1		0,035	0,3717	20	0,009246343	3	1027,54731	-0,24	11,35	1
P73608	Glycogen operon protein GlgX OS=Synechocystis sp. (strain PCC 6803 / Kazusa) GN	514,74	12,02	1	9	9	22	1,047	18	18,9	707	79,8	5,86							
	A3	Sequence	# PSMs	# Proteins	# Protein Groups	Protein Group Accessions	Modifications	ΔCn	114/115	114/115 Count	114/115 Variability [%]	q-Value	PEP	IonScore	Exp Value	Charge	MH+ [Da]	ΔM [ppm]	RT [min]	# Missed Cleavages
	High	sVVILLAk	3	1	1	P73608	N-Term(iTRAQ4plex); K8(iTRAQ4plex)	0,0000	1,193	2	2,3	0	0,000231	61	8,29793E-07	2	1130,77580	0,59	37,00	0
	High	gTFAGILSk	2	1	1	P73608	N-Term(iTRAQ4plex); K9(iTRAQ4plex)	0,0000	1,192	2	9,5	0	0,00003595	54	3,78416E-06	2	1181,71208	-1,00	34,33	0
	High	gPTISFR	3	1	1	P73608	N-Term(iTRAQ4plex)	0,0000	1,076	3	5,6	0	0,001648	48	1,93157E-05	2	921,52800	0,58	25,25	0
	High	yWTAEFHIDGFR	1	1	1	P73608	N-Term(iTRAQ4plex)	0,0000	1,083	1		0	1,967E-08	47	1,99054E-05	3	1685,81906	-0,14	43,60	0
	High	aGYAATGk	2	1	1	P73608	N-Term(iTRAQ4plex); K8(iTRAQ4plex)	0,0000	1,183	1		0	0,000895	46	2,29071E-05	2	1026,58293	0,68	13,51	0
	High	fDLASILGR	1	1	1	P73608	N-Term(iTRAQ4plex)	0,0000	1,059	1		0	0,00002502	46	2,42644E-05	2	1135,65972	0,43	49,57	0
	High	rAPQPFVEIPFPESFR	3	1	1	P73608	N-Term(iTRAQ4plex)	0,0000	0,855	3	8,6	0	8,612E-08	41	8,60934E-05	3	2061,10471	0,39	46,47	1
	High	vLAFMLcGR	2	1	1	P73608	N-Term(iTRAQ4plex); C7(Carbamidomethyl)	0,0000	0,932	2	6,8	0	0,0000179	38	0,000142223	2	1210,65557	-0,15	45,42	0
	High	vLAFmLcGR	1	1	1	P73608	N-Term(iTRAQ4plex); M5(Oxidation); C7(Carbamidomethyl)	0,0000	0,900	1		0	0,00001229	22	0,005767266	2	1226,65117	0,41	37,54	0
	High	lAHPVLR	4	1	1	P73608	N-Term(iTRAQ4plex)	0,0000	0,958	2	7,9	0,002	0,02583	22	0,006500848	3	949,60929	3,06	15,97	0
P52231	Thioredoxin OS=Synechocystis sp. (strain PCC 6803 / Kazusa) GN=trxA PE=1 SV=3	507,54	16,82	1	2	2	21	1,292	19	19,7	107	11,7	4,93							

	A3	Sequence	# PSMs	# Proteins	# Protein Groups	Protein Group Accessions	Modifications	ΔCn	114/115	114/115 Count	114/115 Variability [%]	q-Value	PEP	IonScore	Exp Value	Charge	MH+ [Da]	ΔM [ppm]	RT [min]	# Missed Cleavages
	High	tTLASTLEk	4	1	1	P52231	N-Term(iTRAQ4plex); K9(iTRAQ4plex)	0,0000	1,515	3	2,3	0	0,00001358	60	1,05189E-06	2	1251,74187	1,60	26,44	0
	High	sIPTLMIFk	9	1	1	P52231	N-Term(iTRAQ4plex); K9(iTRAQ4plex)	0,0000	1,207	9	14,5	0	0,00004216	59	1,38666E-06	2	1337,81389	2,50	47,28	0
	High	sIPTLmIFk	8	1	1	P52231	N-Term(iTRAQ4plex); M6(Oxidation); K9(iTRAQ4plex)	0,0000	1,292	7	6,9	0	0,0001016	51	7,81574E-06	2	1353,80669	0,91	43,40	0
Q55176	Slr0483 protein OS=Synechocystis sp. (strain PCC 6803 / Kazusa) GN=slr0483 PE=	503,54	25,50	1	4	4	22	1,095	18	11,2	149	16,9	7,24							
	A3	Sequence	# PSMs	# Proteins	# Protein Groups	Protein Group Accessions	Modifications	ΔCn	114/115	114/115 Count	114/115 Variability [%]	q-Value	PEP	IonScore	Exp Value	Charge	MH+ [Da]	ΔM [ppm]	RT [min]	# Missed Cleavages
	High	tDVGPITTPNPQk	3	1	1	Q55176	N-Term(iTRAQ4plex); K13(iTRAQ4plex)	0,0000	1,056	2	5,8	0	1,344E-10	102	6,59461E-11	2	1655,92168	0,59	24,79	0
	High	rQELASEFGALk	15	1	1	Q55176	N-Term(iTRAQ4plex); K12(iTRAQ4plex)	0,0000	1,091	13	14,5	0	2,28E-08	83	5,54587E-09	3	1636,92588	-0,16	29,96	1
	High	rQELASEFGALkEQIFGG	2	1	1	Q55176	N-Term(iTRAQ4plex); K12(iTRAQ4plex)	0,0000	0,918	1		0	7,627E-10	62	6,18016E-07	3	2268,22611	1,48	51,56	2
	High	tATAGIk	2	1	1	Q55176	N-Term(iTRAQ4plex); K7(iTRAQ4plex)	0,0000	1,115	2	1,9	0,005	0,05844	31	0,001829024	2	949,59416	2,20	14,02	0
P77973	Argininosuccinate synthase OS=Synechocystis sp. (strain PCC 6803 / Kazusa) GN=a	498,83	24,50	1	10	10	22	0,838	19	9,3	400	44,5	5,14							
	A3	Sequence	# PSMs	# Proteins	# Protein Groups	Protein Group Accessions	Modifications	ΔCn	114/115	114/115 Count	114/115 Variability [%]	q-Value	PEP	IonScore	Exp Value	Charge	MH+ [Da]	ΔM [ppm]	RT [min]	# Missed Cleavages
	High	sIQANALYENR	2	1	1	P77973	N-Term(iTRAQ4plex)	0,0000	0,786	2	6,1	0	7,386E-07	67	1,86196E-07	2	1422,74687	0,76	25,31	0
	High	lNEIAGNHGVGR	2	1	1	P77973	N-Term(iTRAQ4plex)	0,0000	0,687	2	8,5	0	2,004E-07	62	6,88605E-07	2	1380,74626	-0,16	20,60	0
	High	gLWYSPLk	2	1	1	P77973	N-Term(iTRAQ4plex); K8(iTRAQ4plex)	0,0000	0,731	1		0	0,000004872	47	2,11334E-05	2	1251,73406	0,06	38,53	0
	High	eIYEAPALLVLIDAHR	6	1	1	P77973	N-Term(iTRAQ4plex)	0,0000	0,878	6	10,8	0	1,591E-08	45	3,06175E-05	3	1967,11191	1,83	52,78	0
	High	sREIYEAPALLVLIDAHR	2	1	1	P77973	N-Term(iTRAQ4plex)	0,0000	0,834	1		0	9,399E-08	45	3,22827E-05	4	2210,24233	0,40	45,99	1
	High	yGADAVAHGcTGk	2	1	1	P77973	N-Term(iTRAQ4plex); C10(Carbamidomethyl); K13(iTRAQ4plex)	0,0000	0,844	1		0	8,004E-07	36	0,000229599	3	1594,78690	-1,09	17,25	0
	High	yGVESPVkk	2	1	1	P77973	N-Term(iTRAQ4plex); K8(iTRAQ4plex); K9(iTRAQ4plex)	0,0000	0,858	2	3,6	0	0,0007841	30	0,001023222	3	1438,85968	-2,30	18,45	1
	High	aAEGFIYIWGLPTk	1	1	1	P77973	N-Term(iTRAQ4plex); K14(iTRAQ4plex)	0,0000	1,101	1		0	0,0001237	26	0,002754038	3	1854,03916	-0,69	52,30	0
	Medium	sSPYSIDR	1	1	1	P77973	N-Term(iTRAQ4plex)	0,0000	1,087	1		0,017	0,1893	15	0,031693486	2	1068,54863	4,12	17,52	0
	Medium	vVGIk	2	1	1	P77973	N-Term(iTRAQ4plex); K5(iTRAQ4plex)	0,0000	0,834	2	6,8	0,029	0,3173	16	0,023603152	2	803,55950	0,22	18,41	0
P49433	Glyceraldehyde-3-phosphate dehydrogenase 1 OS=Synechocystis sp. (strain PCC 68	498,68	19,76	1	7	7	22	2,145	19	30,1	339	36,1	6,24							
	A3	Sequence	# PSMs	# Proteins	# Protein Groups	Protein Group Accessions	Modifications	ΔCn	114/115	114/115 Count	114/115 Variability [%]	q-Value	PEP	IonScore	Exp Value	Charge	MH+ [Da]	ΔM [ppm]	RT [min]	# Missed Cleavages
	High	gAAQNIIPSSTGAAk	6	1	1	P49433	N-Term(iTRAQ4plex); K15(iTRAQ4plex)	0,0000	1,889	4	63,1	0	1,531E-11	103	4,60225E-11	2	1673,94646	2,35	24,89	0
	High	aAALVLPQLk	7	1	1	P49433	N-Term(iTRAQ4plex); K10(iTRAQ4plex)	0,0000	2,303	6	8,5	0	0,00002585	67	2,09879E-07	2	1311,86308	2,14	39,36	0
	High	iGINGFGR	2	1	1	P49433	N-Term(iTRAQ4plex)	0,0000	1,502	2	10,5	0	0,0001206	39	0,000157464	2	977,56749	2,63	29,40	0
	High	lTGMAFR	2	1	1	P49433	N-Term(iTRAQ4plex)	0,0000	2,169	2	10,2	0	0,002628	32	0,000666761	2	939,52147	1,26	28,56	0
	High	vPTPNVSVVDLTFk	1	1	1	P49433	N-Term(iTRAQ4plex); K14(iTRAQ4plex)	0,0000	0,988	1		0	0,0000116	27	0,002123098	3	1804,04990	2,21	45,67	0
	High	rVIISAPSkDPEk	1	1	1	P49433	N-Term(iTRAQ4plex); K9(iTRAQ4plex); K13(iTRAQ4plex)	0,0000	2,145	1		0	0,0001255	22	0,006838644	4	1872,13454	3,54	20,35	2
	High	lTGmAFR	2	1	1	P49433	N-Term(iTRAQ4plex); M4(Oxidation)	0,0000	1,763	2	0,3	0,005	0,06285	22	0,006576124	2	955,51622	1,07	21,09	0
	High	vIISAPSkDPEk	1	1	1	P49433	N-Term(iTRAQ4plex); K8(iTRAQ4plex); K12(iTRAQ4plex)	0,0000	2,749	1		0,007	0,08593	17	0,019951245	3	1716,03495	4,75	23,71	1
P55038	Ferredoxin-dependent glutamate synthase 2 OS=Synechocystis sp. (strain PCC 680	495,20	12,15	1	18	18	25	0,518	23	41,7	1556	169,4	5,76							
	A3	Sequence	# PSMs	# Proteins	# Protein Groups	Protein Group Accessions	Modifications	ΔCn	114/115	114/115 Count	114/115 Variability [%]	q-Value	PEP	IonScore	Exp Value	Charge	MH+ [Da]	ΔM [ppm]	RT [min]	# Missed Cleavages
	High	aILANWSDYLGk	1	1	1	P55038	N-Term(iTRAQ4plex); K12(iTRAQ4plex)	0,0000	0,521	1		0	7,98E-08	62	6,03907E-07	2	1638,90984	0,26	46,76	0
	High	sLITAHVEHTGSPk	2	1	1	P55038	N-Term(iTRAQ4plex); K14(iTRAQ4plex)	0,0000	0,588	2	43,4	0	3,219E-11	52	8,20593E-06	3	1764,98362	-0,61	19,35	0
	High	hAGSPWELGVTEVHR	1	1	1	P55038	N-Term(iTRAQ4plex)	0,0000	0,521	1		0	1,686E-09	51	8,8914E-06	4	1818,93337	-1,89	29,14	0
	High	nPGYTSNFAVYHR	1	1	1	P55038	N-Term(iTRAQ4plex)	0,0000	0,719	1		0	9,866E-09	48	1,48926E-05	3	1669,81931	-0,62	25,82	0
	High	vcHTNNcPVGVATQQER	2	1	1	P55038	N-Term(iTRAQ4plex); C2(Carbamidomethyl); C7(Carbamidomethyl)	0,0000	0,237	1		0	5,937E-11	48	1,55228E-05	3	2113,99564	-1,51	17,50	0
	High	lTVLGWR	2	1	1	P55038	N-Term(iTRAQ4plex)	0,0000	0,320	1		0	0,001244	46	2,35489E-05	2	988,60521	-0,85	39,85	0
	High	fcTGGmSLGALSR	1	1	1	P55038	N-Term(iTRAQ4plex); C2(Carbamidomethyl); M6(Oxidation)	0,0000	0,518	1		0	0,000000281	44	4,2655E-05	2	1516,73674	-0,12	31,15	0
	High	gkPDHTLVEQALk	1	1	1	P55038	N-Term(iTRAQ4plex); K2(iTRAQ4plex); K13(iTRAQ4plex)	0,0000	0,426	1		0	9,393E-07	39	0,000114807	3	1868,09433	-1,22	23,12	0
	High	sLLAHLGYR	1	1	1	P55038	N-Term(iTRAQ4plex)	0,0000	0,321	1		0	0,000001698	39	0,000115337	3	1173,68598	-0,09	31,34	0
	High	lLYDYFk	3	1	1	P55038	N-Term(iTRAQ4plex); K7(iTRAQ4plex)	0,0000	0,364	3	0,3	0	0,0001844	39	0,000130674	2	1249,70586	-0,98	42,66	0
	High	eLLAQWFNTR	2	1	1	P55038	N-Term(iTRAQ4plex)	0,0000	0,523	2	5,5	0	0,0001741	36	0,000279235	2	1421,76482	-0,70	48,30	0
	High	ekELEVSGWTk	1	1	1	P55038	N-Term(iTRAQ4plex); K2(iTRAQ4plex); K11(iTRAQ4plex)	0,0000	0,418	1		0	0,000005524	32	0,000616595	3	1737,97153	-1,86	27,96	1
	High	eAHETLAIAmNR	1	1	1	P55038	N-Term(iTRAQ4plex); M10(Oxidation)	0,0000	0,539	1		0	0,000006259	25	0,003075884	3	1515,76892	-1,14	18,56	0
	High	kVSEYIAMLR	1	1	1	P55038	N-Term(iTRAQ4plex); K1(iTRAQ4plex)	0,0000	1,309	1		0	0,006108	13	0,053699471	3	1497,86725	-1,96	36,71	1
	Medium	fSTNTmPk	2	1	1	P55038	N-Term(iTRAQ4plex); M6(Oxidation); K8(iTRAQ4plex)	0,0000	0,504	2	10,5	0,016	0,177	18	0,016291835	2	1229,64141	-2,00	15,97	0
	Medium	lYIAR	1	1	1	P55038	N-Term(iTRAQ4plex)	0,0000	1,011	1		0,019	0,2042	15	0,032506485	2	779,48961	0,01	21,67	0
	Medium	dRPVTALR	1	1	1	P55038	N-Term(iTRAQ4plex)	0,0000	0,767	1		0,036	0,3842	11	0,084327651	3	1071,64405	4,57	12,78	0
	Medium	tIVYk	1	1	1	P55038	N-Term(iTRAQ4plex); K5(iTRAQ4plex)	0,0000	0,800	1		0,038	0,3983	13	0,051876421	2	911,58086	0,47	17,19	0
P74510	Dihydrolipoamide acetyltransferase component (E2) of pyruvate dehydrogenase cor	493,80	29,79	1	12	12	23	1,038	23	15,4	433	44,9	6,19							
	A3	Sequence	# PSMs	# Proteins	# Protein Groups	Protein Group Accessions	Modifications	ΔCn	114/115	114/115 Count	114/115 Variability [%]	q-Value	PEP	IonScore	Exp Value	Charge	MH+ [Da]	ΔM [ppm]	RT [min]	# Missed Cleavages
	High	aLVQNmVAAmAAPTFR	2	1	1	P74510	N-Term(iTRAQ4plex); M6(Oxidation); M10(Oxidation)	0,0000	1,083	2	6,3	0	1,606E-10	80	9,07758E-09	2	1866,97136	1,40	32,19	0
	High	vDLATIAGTGPHGR	2	1	1	P74510	N-Term(iTRAQ4plex)	0,0000	1,075	2	0,4	0	2,931E-07	71	8,74923E-08	2	1508,83013	-0,07	29,40	0
	High	aLVQNMVAAMAAPTFR	1	1	1	P74510	N-Term(iTRAQ4plex)	0,0000	0,747	1		0	1,298E-07	60	9,26766E-07	2	1834,98210	1,74	50,78	0
	High	vTSTPSVPVGQTVPLTTFQk	2	1	1	P74510	N-Term(iTRAQ4plex); K20(iTRAQ4plex)	0,0000	1,040	2	9,5	0	2,021E-08	41	8,22186E-05	3	2375,34189	-0,26	40,57	0
	High	vIYGAHAAAFLk	2	1	1	P74510	N-Term(iTRAQ4plex); K12(iTRAQ4plex)	0,0000	0,756	2	25,4	0	1,181E-08	38	0,000158855	3	1548,91513	0,67	31,92	0
	High	iVSWTk	2	1	1	P74510	N-Term(iTRAQ4plex); K6(iTRAQ4plex)	0,0000	1,061	2	12,0	0	0,002444	37	0,000178636	2	1021,62859	0,13	27,54	0
	High	gVTmTALLAk	1	1	1	P74510	N-Term(iTRAQ4plex); M4(Oxidation); K10(iTRAQ4plex)	0,0000	1,369	1		0	0,00007864	36	0,00022489	2	1308,78130	1,00	27,04	0
	High	qmAVNVTcDHR	1	1	1	P74510	N-Term(iTRAQ4plex); M2(Oxidation); C8(Carbamidomethyl)	0,0000	0,857	1		0	4,211E-08	36	0,000270377	3	1490,69593	-0,12	12,23	0
	High	sPGDkVEk	4	1	1	P74510	N-Term(iTRAQ4plex); K5(iTRAQ4plex); K8(iTRAQ4plex)	0,0000	1,173	4	3,4	0	0,005233	22	0,006236918	2	1291,76103	2,19	11,13	1
	High	kHPIVNASYTDQGIIYHk	1	1	1	P74510	N-Term(iTRAQ4plex); K1(iTRAQ4plex); K18(iTRAQ4plex)	0,0000	1,499	1		0	3,483E-07	20	0,010918959	4	2516,40224	1,45	25,00	1
	High	rQmAVNVTcDHR	1	1	1	P74510	N-Term(iTRAQ4plex); M3(Oxidation); C9(Carbamidomethyl)	0,0000	1,011	1		0	0,006279	13	0,054697818	4	1646,79799	0,47	9,85	1
	High	iIASPR	3	1	1	P74510	N-Term(iTRAQ4plex)	0,0000	0,934	3	6,4	0,001	0,01052	34	0,000412069	2	800,51134	0,32	16,86	0
	Medium	wkELVER	1	1	1	P74510	N-Term(iTRAQ4plex); K2(iTRAQ4plex)	0,0000	0,897	1		0,028	0,3012	19	0,013182567	3	1247,73401	-0,85	27,63	1
P74384	Adenylosuccinate lyase OS=Synechocystis sp. (strain PCC 6803 / Kazusa) GN=purB	490,25	18,10	1	7	7	18	1,420	13	20,5	431	48,4	5,92							
	A3	Sequence	# PSMs	# Proteins	# Protein Groups	Protein Group Accessions	Modifications	ΔCn	114/115	114/115 Count	114/115 Variability [%]	q-Value	PEP	IonScore	Exp Value	Charge	MH+ [Da]	ΔM [ppm]	RT [min]	# Missed Cleavages
	High	hAEYVQQLALLAASLER	3	1	1	P74384	N-Term(iTRAQ4plex)	0,0000	1,394	2	17,6	0	2,835E-12	84	4,03618E-09	2	2056,13042	-0,20	51,53	0
	High	vAQSIAVGk	4	1	1	P74384	N-Term(iTRAQ4plex); K9(iTRAQ4plex)	0,0000	1,448	2	2,8	0	7,119E-07	66	2,49442E-07	2	1160,72588	1,47	22,08	0
	High	vLLALVEk	4	1	1	P74384	N-Term(iTRAQ4plex); K8(iTRAQ4plex)	0,0000	1,570	3	11,8	0	0,00001293	55	2,90382E-06	2	1172,78691	1,03	43,13	0
	High	gHAVAALENVALWHER	1	1	1	P74384	N-Term(iTRAQ4plex)	0,0000	0,761	1		0	1,236E-10	48	1,61053E-05	4	1917,02199	0,40	34,42	0
	High	vAFPDccILTHFmLk	1	1	1	P74384	N-Term(iTRAQ4plex); C6(Carbamidomethyl); C7(Carbamidomethyl); M13(Ox	0,0000	1,599	1		0	9,506E-08	22	0,006011322	3	2156,09818	1,68	47,93	0
	High	yTVmVGR	2	1	1	P74384	N-Term(iTRAQ4plex); M4(Oxidation)	0,0000	0,523	2	129,0	0,004	0,05294	17	0,020557486	2	985,52593	0,18	20,75	0
	High	fAVEIR	3	1	1	P74384	N-Term(iTRAQ4plex)	0,0000	3,275	2	391,5	0,009	0,1111	36	0,000430646	2	878,52257	1,04	27,29	0
P73911	Catalase-peroxidase OS=Synechocystis sp. (strain PCC 6803 / Kazusa) GN=katG PE	490,14	22,94	1	17	17	39	2,385	36	47,0	754	84,4	5,74							
	A3	Sequence	# PSMs	# Proteins	# Protein Groups	Protein Group Accessions	Modifications	ΔCn	114/115	114/115 Count	114/115 Variability [%]	q-Value	PEP	IonScore	Exp Value	Charge	MH+ [Da]	ΔM [ppm]	RT [min]	# Missed Cleavages
	High	vLGTNHGGTk	5	1	1	P73911	N-Term(iTRAQ4plex); K10(iTRAQ4plex)	0,0000	3,304	3	0,2	0	0,000001349	76	2,72251E-08	2	1271,73540	3,43	10,37	0
	High	vDLVFGSNSILR	3	1	1	P73911	N-Term(iTRAQ4plex)	0,0000	1,296	3	53,7	0	0,00001151	49	2,06293E-05	2	1463,83525	0,92	45,74	0
	High	tQLmGLTAPEmTVLIGGmR	1	1	1	P73911	N-Term(iTRAQ4plex); M4(Oxidation); M11(Oxidation); M18(Oxidation)	0,0000	0,883	1		0	1,378E-07	43	4,92006E-05	3	2211,13364	1,42	40,24	0
	High	fYREPDYFAEVFAk	3	1	1	P73911	N-Term(iTRAQ4plex); K14(iTRAQ4plex)	0,0000	2,738	3	27,8	0	1,79E-11	43	5,30848E-05	3	2070,05912	0,76	44,51	1
	High	vYGFAGGR	2	1	1	P73911	N-Term(iTRAQ4plex)	0,0000	2,611	2	0,3	0	0,003431	39	0,000118569	2	970,52397	1,30	22,26	0
	High	sYAEVYAQDDNREk	1	1	1	P73911	N-Term(iTRAQ4plex); K14(iTRAQ4plex)	0,0000	2,614	1		0	1,031E-07	33	0,000515193	3	1975,96231	1,17	19,84	1
	High	vmNADRFDLPR	1	1	1	P73911	N-Term(iTRAQ4plex); M2(Oxidation)	0,0000	2,765	1		0	0,005544	25	0,003111501	3	1493,76606	0,58	25,99	1
	High	vMNADRFDLPR	3	1	1	P73911	N-Term(iTRAQ4plex)	0,0000	1,946	3	16,1	0	0,006266	25	0,003169349	3	1477,77060	0,22	30,97	1
	High	nWPGNEPTR	2	1	1	P73911	N-Term(iTRAQ4plex)	0,0000	1,354	2	50,9	0	0,000833	23	0,00478597	2	1214,60942	4,88	17,98	0
	High	dIYWGAEkEWLASSDHR	1	1	1	P73911	N-Term(iTRAQ4plex); K8(iTRAQ4plex)	0,0000	1,608	1		0	1,672E-07	23	0,004874948	4	2351,17031	1,86	38,18	1
	High	hNPImTDADmAmIkDPIYR	2	1	1	P73911	N-Term(iTRAQ4plex); M5(Oxidation); M10(Oxidation); M12(Oxidation); K14(i	0,0000	1,890	2	19,3	0	5,966E-07	18	0,015030382	4	2568,25088	0,35	28,40	1
	High	eDIWHPEkDIYWGAEk	1	1	1	P73911	N-Term(iTRAQ4plex); K8(iTRAQ4plex); K16(iTRAQ4plex)	0,0000	2,100	1		0	3,713E-08	12	0,084174636	4	2448,26455	3,47	37,02	1
	High	hGVFTDR	1	1	1	P73911	N-Term(iTRAQ4plex)	0,0000	1,393	1		0,001	0,01325	16	0,022697081	3	975,51368	0,82	13,41	0
	High	iRLEPQk	2	1	1	P73911	N-Term(iTRAQ4plex); K7(iTRAQ4plex)	0,0000	3,024	2	0,8	0,001	0,008719	16	0,027287893	3	1171,74131	0,99	18,64	1
	High	lLWPIk	6	1	1	P73911	N-Term(iTRAQ4plex); K6(iTRAQ4plex)	0,0000	3,675	5	29,6	0,002	0,02262	31	0,000746397	2	1057,70366	2,29	41,65	0
	Medium	rLLWPIk	2	1	1	P73911	N-Term(iTRAQ4plex); K7(iTRAQ4plex)	0,0000	2,715	2	21,4	0,013	0,1467	11	0,084327651	2	1213,80510	2,26	32,86	1
	Medium	qTGEVk	1	1	1	P73911	N-Term(iTRAQ4plex); K6(iTRAQ4plex)	0,0000	1,176	1		0,016	0,1742	20	0,013612671	2	949,55327	-2,55	10,30	0
	Medium	wTATk	2	1	1	P73911	N-Term(iTRAQ4plex); K5(iTRAQ4plex)	0,0000	2,863	2	14,4	0,035	0,3714	27	0,002455734	2	894,52825	-0,55	16,05	0
P73296	50S ribosomal protein L17 OS=Synechocystis sp. (strain PCC 6803 / Kazusa) GN=r	486,55	52,59	1	7	7	37	3,144	31	103,1	116	13,2	11,06							
	A3	Sequence	# PSMs	# Proteins	# Protein Groups	Protein Group Accessions	Modifications	ΔCn	114/115	114/115 Count	114/115 Variability [%]	q-Value	PEP	IonScore	Exp Value	Charge	MH+ [Da]	ΔM [ppm]	RT [min]	# Missed Cleavages
	High	aLGYmYDkPTVHALFADAPSR	8	1	1	P73296	N-Term(iTRAQ4plex); M5(Oxidation); K8(iTRAQ4plex)	0,0000	7,249	6	79,6	0	2,421E-11	50	9,97631E-06	3	2627,35489	0,68	37,65	0
	High	aLGYMYDkPTVHALFADAPSR	3	1	1	P73296	N-Term(iTRAQ4plex); K8(iTRAQ4plex)	0,0000	10,080	2	223,8	0	1,713E-10	50	1,08136E-05	3	2611,36209	1,50	40,88	0
	High	rGDNAEMAVIELV	2	1	1	P73296	N-Term(iTRAQ4plex)	0,0000	1,665	2	9,1	0	0,002497	45	3,05492E-05	2	1560,81926	1,26	45,68	1
	High	aLTTELIR	3	1	1	P73296	N-Term(iTRAQ4plex)	0,0000	6,884	3	66,5	0	0,0001449	39	0,000114807	2	1060,64897	0,62	30,86	0
	High	vPQLGkPADQR	4	1	1	P73296	N-Term(iTRAQ4plex); K6(iTRAQ4plex)	0,0000	3,335	4	123,3	0	0,00006462	30	0,000937497	2	1496,87944	0,44	20,25	0
	High	rRGDNAEMAVIELV	2	1	1	P73296	N-Term(iTRAQ4plex)	0,0000	2,208	2	11,8	0	0,001621	15	0,029442183	3	1716,91898	0,33	38,26	2
	High	rALGYMYDkPTVHALFADAPSR	1	1	1	P73296	N-Term(iTRAQ4plex); K9(iTRAQ4plex)	0,0000	1,352	1		0	0,000005176	11	0,083362361	5	2767,46259	1,19	35,76	1
	High	mITLAk	5	1	1	P73296	N-Term(iTRAQ4plex); K6(iTRAQ4plex)	0,0000	3,689	4	56,4	0,002	0,03197	20	0,008994355	2	964,61156	1,22	26,14	0
	High	rRGDNAEmAVIELV	1	1	1	P73296	N-Term(iTRAQ4plex); M8(Oxidation)	0,0000	1,048	1		0,003	0,03373	19	0,011802391	3	1732,91471	0,80	35,44	2
	High	rGDNAEmAVIELV	3	1	1	P73296	N-Term(iTRAQ4plex); M7(Oxidation)	0,0000	1,038	2	3,1	0,005	0,06196	31	0,000862919	2	1576,81328	0,68	40,98	1
	High	mITLAk	5	1	1	P73296	N-Term(iTRAQ4plex); M1(Oxidation); K6(iTRAQ4plex)	0,0000	3,148	4	12,4	0,005	0,06491	19	0,018236578	2	980,61089	5,71	21,01	0
P74421	Phosphoglycerate kinase OS=Synechocystis sp. (strain PCC 6803 / Kazusa) GN=pgk	484,45	26,68	1	10	10	23	0,708	19	12,6	401	41,8	5,19							
	A3	Sequence	# PSMs	# Proteins	# Protein Groups	Protein Group Accessions	Modifications	ΔCn	114/115	114/115 Count	114/115 Variability [%]	q-Value	PEP	IonScore	Exp Value	Charge	MH+ [Da]	ΔM [ppm]	RT [min]	# Missed Cleavages
	High	vILGSHFGRPk	3	1	1	P74421	N-Term(iTRAQ4plex); K11(iTRAQ4plex)	0,0000	0,572	3	13,4	0	8,336E-08	59	1,20218E-06	3	1498,90921	-0,32	22,15	0
	High	fHAGEEGNDAEFAk	2	1	1	P74421	N-Term(iTRAQ4plex); K14(iTRAQ4plex)	0,0000	0,660	2	0,8	0	6,457E-10	59	1,31513E-06	3	1809,86533	0,37	22,28	0
	High	rPLVAIVGGSk	6	1	1	P74421	N-Term(iTRAQ4plex); K11(iTRAQ4plex)	0,0000	0,664	4	0,7	0	0,000002744	56	2,29599E-06	2	1384,88811	0,15	24,05	0
	High	qSIANLTEADLAGkR	2	1	1	P74421	N-Term(iTRAQ4plex); K14(iTRAQ4plex)	0,0000	0,970	1		0	1,8E-11	47	1,89657E-05	3	1875,05417	0,16	31,43	1
	High	aQGLNTGk	3	1	1	P74421	N-Term(iTRAQ4plex); K8(iTRAQ4plex)	0,0000	0,784	2	15,2	0	0,00005372	45	3,38986E-05	2	1076,63262	2,19	13,83	0
	High	lGELLGQPVVk	2	1	1	P74421	N-Term(iTRAQ4plex); K11(iTRAQ4plex)	0,0000	0,954	2	36,8	0	0,00009588	33	0,000508124	3	1440,90287	-0,01	39,56	0
	High	sLVEEDkLDLAk	2	1	1	P74421	N-Term(iTRAQ4plex); K7(iTRAQ4plex); K12(iTRAQ4plex)	0,0000	0,726	2	2,2	0	0,00000205	30	0,001066522	3	1792,04203	-0,46	31,72	1
	High	eLQFLQGAIEAPk	1	1	1	P74421	N-Term(iTRAQ4plex); K13(iTRAQ4plex)	0,0000	0,639	1		0	0,0002892	21	0,008221859	3	1731,98819	-0,12	43,95	0
	High	aALPTIk	1	1	1	P74421	N-Term(iTRAQ4plex); K7(iTRAQ4plex)	0,0000	0,770	1		0,005	0,05852	31	0,000841337	2	1001,65807	-1,70	26,21	0
	Medium	sLmAk	1	1	1	P74421	N-Term(iTRAQ4plex); M3(Oxidation); K5(iTRAQ4plex)	0,0000	1,094	1		0,039	0,4075	14	0,043348094	2	853,50279	-3,25	11,80	0
P73597	Sll1306 protein OS=Synechocystis sp. (strain PCC 6803 / Kazusa) GN=sll1306 PE=4	482,04	32,84	1	9	9	20	2,611	17	14,2	335	38,2	8,18							
	A3	Sequence	# PSMs	# Proteins	# Protein Groups	Protein Group Accessions	Modifications	ΔCn	114/115	114/115 Count	114/115 Variability [%]	q-Value	PEP	IonScore	Exp Value	Charge	MH+ [Da]	ΔM [ppm]	RT [min]	# Missed Cleavages
	High	fFSSGQYLTELk	3	1	1	P73597	N-Term(iTRAQ4plex); K12(iTRAQ4plex)	0,0000	1,822	3	60,8	0	7,904E-11	71	7,31089E-08	2	1707,92571	3,56	44,63	0
	High	aVGYNAPGLR	4	1	1	P73597	N-Term(iTRAQ4plex)	0,0000	2,656	2	0,5	0	0,00001219	59	1,2473E-06	2	1161,65141	1,46	23,17	0
	High	lQRPEHVYVFEDFLk	2	1	1	P73597	N-Term(iTRAQ4plex); K15(iTRAQ4plex)	0,0000	2,771	2	3,2	0	1,772E-09	58	1,41244E-06	3	2208,21164	2,73	40,38	0
	High	iLDLLDkYk	2	1	1	P73597	N-Term(iTRAQ4plex); K7(iTRAQ4plex); K9(iTRAQ4plex)	0,0000	2,902	2	1,5	0	0,000002366	56	2,35489E-06	2	1552,97478	4,71	43,89	1
	High	kIDIANFALNDPNTIR	1	1	1	P73597	N-Term(iTRAQ4plex); K1(iTRAQ4plex)	0,0000	1,459	1		0	1,238E-08	51	7,72627E-06	3	2103,18491	2,27	40,54	1
	High	yGYTEGVPR	1	1	1	P73597	N-Term(iTRAQ4plex)	0,0000	2,611	1		0	0,000003663	50	9,79422E-06	2	1185,60271	0,54	22,80	0
	High	nVDIILk	2	1	1	P73597	N-Term(iTRAQ4plex); K7(iTRAQ4plex)	0,0000	2,028	2	8,4	0	0,0002576	47	2,15263E-05	2	1102,71367	5,64	29,89	0
	High	mmAVTmHDR	3	1	1	P73597	N-Term(iTRAQ4plex); M1(Oxidation); M2(Oxidation); M6(Oxidation)	0,0000	2,083	2	31,6	0	0,000005494	28	0,001644258	3	1283,56803	1,31	8,04	0
	High	gHEAAAHGWDWDNEFNmTAPQER	1	1	1	P73597	N-Term(iTRAQ4plex); M17(Oxidation)	0,0000	2,828	1		0	0,00002675	20	0,011142176	4	2829,22304	1,31	32,57	0
	High	mmAVTmHDR	1	1	1	P73597	N-Term(iTRAQ4plex); M2(Oxidation); M6(Oxidation)	0,0000	1,407	1		0	0,0000895	13	0,048749482	3	1267,57184	0,32	11,63	0
P72758	Carbon dioxide concentrating mechanism protein CcmM OS=Synechocystis sp. (stra	479,43	19,36	1	13	13	21	0,892	16	15,4	687	73,1	8,59							
	A3	Sequence	# PSMs	# Proteins	# Protein Groups	Protein Group Accessions	Modifications	ΔCn	114/115	114/115 Count	114/115 Variability [%]	q-Value	PEP	IonScore	Exp Value	Charge	MH+ [Da]	ΔM [ppm]	RT [min]	# Missed Cleavages
	High	sLLAQGYGIGAEHANER	2	1	1	P72758	N-Term(iTRAQ4plex)	0,0000	0,756	1		0	3,309E-11	84	3,86367E-09	3	1929,99387	2,02	30,74	0
	High	iQPGVSVAPGSSIR	1	1	1	P72758	N-Term(iTRAQ4plex)	0,0000	0,997	1		0	2,602E-09	63	4,56005E-07	2	1511,87053	2,82	28,19	0
	High	sAVAHQTGNLAGDSANQLR	2	1	1	P72758	N-Term(iTRAQ4plex)	0,0000	0,673	1		0	5,349E-12	62	5,95621E-07	3	2054,04856	-0,52	21,74	0
	High	rVLETIIQRP	1	1	1	P72758	N-Term(iTRAQ4plex)	0,0000	1,727	1		0	7,235E-09	48	1,61425E-05	3	1368,84574	0,97	29,89	1
	High	fLADHTNEYVR	2	1	1	P72758	N-Term(iTRAQ4plex)	0,0000	0,807	2	17,4	0	7,773E-08	48	1,69813E-05	3	1508,76212	0,45	25,94	0
	High	sLLAQGYk	4	1	1	P72758	N-Term(iTRAQ4plex); K8(iTRAQ4plex)	0,0000	0,999	2	4,8	0	0,0000555	45	3,17665E-05	2	1167,70183	3,62	25,59	0
	High	iGLEYASAR	1	1	1	P72758	N-Term(iTRAQ4plex)	0,0000	1,146	1		0	0,000119	44	3,90814E-05	2	1123,62175	-0,95	27,01	0
	High	vLETIIQRP	1	1	1	P72758	N-Term(iTRAQ4plex)	0,0000	0,957	1		0	0,002297	41	8,4522E-05	2	1212,74248	-0,66	36,84	0
	High	vLEQIIQRPGDNVVAGR	2	1	1	P72758	N-Term(iTRAQ4plex)	0,0000	0,878	1		0	1,303E-08	36	0,00026133	3	2008,14255	0,23	37,28	0
	High	rVLEQIIQRPGDNVVAGR	1	1	1	P72758	N-Term(iTRAQ4plex)	0,0000	0,836	1		0	0,00003456	30	0,000957128	3	2164,24399	0,36	31,52	1
	High	rVVEVIIQRPGDVPGSPSR	1	1	1	P72758	N-Term(iTRAQ4plex)	0,0000	0,816	1		0	0,0007835	26	0,003615164	3	2205,25449	-1,83	29,56	1
	High	vVEVIIQRPGDVPGSPSR	1	1	1	P72758	N-Term(iTRAQ4plex)	0,0000	0,811	1		0	0,00004813	22	0,006151344	3	2049,15763	0,11	34,51	0
	High	aLLHQGYk	2	1	1	P72758	N-Term(iTRAQ4plex); K8(iTRAQ4plex)	0,0000	0,837	2	12,5	0,001	0,009785	20	0,010350707	3	1217,72678	1,89	15,92	0
P10549	Photosystem II manganese-stabilizing polypeptide OS=Synechocystis sp. (strain PC	473,23	27,01	1	9	9	30	1,098	24	8,3	274	29,9	4,94							
	A3	Sequence	# PSMs	# Proteins	# Protein Groups	Protein Group Accessions	Modifications	ΔCn	114/115	114/115 Count	114/115 Variability [%]	q-Value	PEP	IonScore	Exp Value	Charge	MH+ [Da]	ΔM [ppm]	RT [min]	# Missed Cleavages
	High	gTIEVEPNTk	4	1	1	P10549	N-Term(iTRAQ4plex); K10(iTRAQ4plex)	0,0000	1,097	2	6,5	0	8,778E-08	72	8,0112E-08	2	1375,77678	7,00	19,03	0
	High	gLYTGYDNAVALPSAADkFR	3	1	1	P10549	N-Term(iTRAQ4plex); K18(iTRAQ4plex)	0,0000	1,112	2	4,6	0	7,785E-12	65	3,49945E-07	3	2417,27378	1,39	40,65	1
	High	qTTSLEQIR	3	1	1	P10549	N-Term(iTRAQ4plex)	0,0000	0,865	3	1,2	0	0,000001036	61	7,09529E-07	2	1219,67693	0,50	21,52	0
	High	qkAEYVk	4	1	1	P10549	N-Term(iTRAQ4plex); K2(iTRAQ4plex); K7(iTRAQ4plex)	0,0000	1,405	4	26,6	0	0,0005674	40	9,5273E-05	2	1297,78301	-0,77	11,13	1
	High	gAGFLDPk	5	1	1	P10549	N-Term(iTRAQ4plex); K8(iTRAQ4plex)	0,0000	1,044	3	1,4	0	0,003935	21	0,008510793	2	1092,62944	0,21	25,17	0
	High	kETPLGk	3	1	1	P10549	N-Term(iTRAQ4plex); K1(iTRAQ4plex); K7(iTRAQ4plex)	0,0000	1,085	2	12,3	0	0,007918	18	0,0141244	3	1204,76181	-0,62	12,85	1
	High	eEPVNkR	5	1	1	P10549	N-Term(iTRAQ4plex); K6(iTRAQ4plex)	0,0000	1,117	5	4,4	0	0,00298	18	0,015135612	3	1159,66834	0,83	8,52	1
	High	gIFYGR	2	1	1	P10549	N-Term(iTRAQ4plex)	0,0000	0,989	2	13,1	0,002	0,01896	23	0,004560054	2	856,48577	6,99	23,80	0
	Medium	aEYVk	1	1	1	P10549	N-Term(iTRAQ4plex); K5(iTRAQ4plex)	0,0000	1,111	1		0,012	0,137	26	0,002243727	2	897,53148	3,44	14,62	0
P80046	Isocitrate dehydrogenase [NADP] OS=Synechocystis sp. (strain PCC 6803 / Kazusa)	469,72	25,89	1	13	13	29	0,681	23	15,2	475	52,2	5,58							
	A3	Sequence	# PSMs	# Proteins	# Protein Groups	Protein Group Accessions	Modifications	ΔCn	114/115	114/115 Count	114/115 Variability [%]	q-Value	PEP	IonScore	Exp Value	Charge	MH+ [Da]	ΔM [ppm]	RT [min]	# Missed Cleavages
	High	gIGAAIANR	2	1	1	P80046	N-Term(iTRAQ4plex)	0,0000	0,771	2	10,0	0	0,00002838	72	5,80724E-08	2	986,58617	-0,23	21,85	0
	High	iTFVAGkPVVPNDPIIPYIR	1	1	1	P80046	N-Term(iTRAQ4plex); K7(iTRAQ4plex)	0,0000	0,702	1		0	1,89E-12	51	7,77983E-06	3	2497,48148	1,17	48,20	0
	High	kGIGAAIANR	3	1	1	P80046	N-Term(iTRAQ4plex); K1(iTRAQ4plex)	0,0000	0,621	2	12,6	0	1,022E-07	46	2,5408E-05	3	1258,78241	-0,81	16,92	1
	High	lQPPSVGSk	2	1	1	P80046	N-Term(iTRAQ4plex); K9(iTRAQ4plex)	0,0000	0,708	2	5,8	0	0,0002926	45	3,23571E-05	2	1200,71929	0,17	23,62	0
	High	gPLTTPVGGGIR	3	1	1	P80046	N-Term(iTRAQ4plex)	0,0000	0,675	2	1,7	0	0,00007047	44	4,1973E-05	2	1268,74419	-0,14	28,58	0
	High	lDSGIGIkPISk	5	1	1	P80046	N-Term(iTRAQ4plex); K8(iTRAQ4plex); K12(iTRAQ4plex)	0,0000	0,711	3	7,0	0	4,09E-08	35	0,000282468	3	1660,03617	-0,48	29,54	0
	High	tPEkLDIIVYR	2	1	1	P80046	N-Term(iTRAQ4plex); K4(iTRAQ4plex)	0,0000	0,583	2	5,3	0	8,156E-07	31	0,000758525	3	1634,97068	-0,81	33,64	1
	High	sLNVALR	3	1	1	P80046	N-Term(iTRAQ4plex)	0,0000	0,677	3	26,7	0	0,006799	22	0,006791567	2	916,56993	0,29	24,12	0
	High	qMVTLVHk	4	1	1	P80046	N-Term(iTRAQ4plex); K8(iTRAQ4plex)	0,0000	0,614	2	0,3	0	0,002779	21	0,008769403	3	1243,74341	-0,08	21,96	0
	High	qIFDLYTcVRPcR	1	1	1	P80046	N-Term(iTRAQ4plex); C8(Carbamidomethyl); C12(Carbamidomethyl)	0,0000	0,578	1		0	0,0002533	18	0,016748272	3	1871,93827	0,29	38,79	0
	High	yYPGTPSPHk	1	1	1	P80046	N-Term(iTRAQ4plex); K10(iTRAQ4plex)	0,0000	1,005	1		0	0,006441	15	0,033571443	3	1434,76105	-0,65	18,75	0
	High	vDkPLk	1	1	1	P80046	N-Term(iTRAQ4plex); K3(iTRAQ4plex); K6(iTRAQ4plex)	0,0000	0,709	1		0,002	0,03149	24	0,004285189	2	1131,74492	-1,11	14,15	0
	Medium	gNImk	1	1	1	P80046	N-Term(iTRAQ4plex); M4(Oxidation); K5(iTRAQ4plex)	0,0000	0,903	1		0,029	0,3129	11	0,076730849	2	866,49956	-1,46	12,21	0
P36239	50S ribosomal protein L19 OS=Synechocystis sp. (strain PCC 6803 / Kazusa) GN=r	463,08	39,34	1	7	7	17	1,904	15	36,7	122	13,8	10,64							
	A3	Sequence	# PSMs	# Proteins	# Protein Groups	Protein Group Accessions	Modifications	ΔCn	114/115	114/115 Count	114/115 Variability [%]	q-Value	PEP	IonScore	Exp Value	Charge	MH+ [Da]	ΔM [ppm]	RT [min]	# Missed Cleavages
	High	vFLLHSPR	4	1	1	P36239	N-Term(iTRAQ4plex)	0,0000	2,135	3	1,7	0	0,0000109	61	8,689E-07	2	1112,67058	0,78	27,43	0
	High	kIFQGVGVER	6	1	1	P36239	N-Term(iTRAQ4plex); K1(iTRAQ4plex)	0,0000	2,525	5	6,3	0	0,000002996	58	1,72175E-06	2	1420,85625	3,34	23,57	1
	High	iQPYEGTVIAk	2	1	1	P36239	N-Term(iTRAQ4plex); K11(iTRAQ4plex)	0,0000	2,101	2	48,9	0	5,044E-07	57	1,92296E-06	2	1506,87786	0,54	30,49	0
	High	nGGISETITVR	1	1	1	P36239	N-Term(iTRAQ4plex)	0,0000	1,528	1		0	0,000001734	50	1,03269E-05	2	1290,71428	0,65	25,00	0
	High	iFQGVGVER	2	1	1	P36239	N-Term(iTRAQ4plex)	0,0000	1,800	2	8,3	0	0,00008109	47	2,07477E-05	2	1148,65569	1,06	29,77	0
	High	iVEGGk	1	1	1	P36239	N-Term(iTRAQ4plex); K6(iTRAQ4plex)	0,0000	1,221	1		0,003	0,03607	24	0,009194755	2	890,55986	5,50	14,70	0
	Medium	iVEGGkER	1	1	1	P36239	N-Term(iTRAQ4plex); K6(iTRAQ4plex)	0,0000	1,427	1		0,017	0,189	15	0,040449207	3	1175,69785	-0,71	10,60	1
P36236	50S ribosomal protein L1 OS=Synechocystis sp. (strain PCC 6803 / Kazusa) GN=rpl	451,96	37,82	1	11	11	33	1,656	28	30,6	238	25,8	8,73							
	A3	Sequence	# PSMs	# Proteins	# Protein Groups	Protein Group Accessions	Modifications	ΔCn	114/115	114/115 Count	114/115 Variability [%]	q-Value	PEP	IonScore	Exp Value	Charge	MH+ [Da]	ΔM [ppm]	RT [min]	# Missed Cleavages
	High	tGIVHVmFGk	7	1	1	P36236	N-Term(iTRAQ4plex); M7(Oxidation); K10(iTRAQ4plex)	0,0000	2,081	4	10,6	0	0,000007603	52	7,03072E-06	3	1392,79227	0,76	26,95	0
	High	fDETAEAHIR	1	1	1	P36236	N-Term(iTRAQ4plex)	0,0000	1,677	1		0	9,616E-07	51	8,01623E-06	2	1332,66936	2,15	21,66	0
	High	aLQETIDR	1	1	1	P36236	N-Term(iTRAQ4plex)	0,0000	1,795	1		0	0,0000498	42	6,99794E-05	2	1089,60259	0,45	20,37	0
	High	mQAAIAk	3	1	1	P36236	N-Term(iTRAQ4plex); M1(Oxidation); K7(iTRAQ4plex)	0,0000	2,081	2	23,0	0	0,0003645	37	0,000178636	2	1036,60637	0,01	12,46	0
	High	gGTVTADLAAAVNEFk	1	1	1	P36236	N-Term(iTRAQ4plex); K16(iTRAQ4plex)	0,0000	1,806	1		0	0,00000285	36	0,000256431	3	1852,00650	0,53	49,85	0
	High	tGIVHVMFGk	1	1	1	P36236	N-Term(iTRAQ4plex); K10(iTRAQ4plex)	0,0000	1,078	1		0	0,00000312	33	0,000473119	3	1376,79554	-0,55	32,41	0
	High	lYSPLEAmELLk	2	1	1	P36236	N-Term(iTRAQ4plex); M8(Oxidation); K12(iTRAQ4plex)	0,0000	1,177	2	20,3	0	0,000006673	26	0,002238567	3	1710,96073	0,97	45,34	0
	High	lYSPLEAMELLk	5	1	1	P36236	N-Term(iTRAQ4plex); K12(iTRAQ4plex)	0,0000	1,428	5	7,3	0	6,882E-08	24	0,003999171	2	1694,96538	0,72	54,78	0
	High	nRPSGAk	2	1	1	P36236	N-Term(iTRAQ4plex); K7(iTRAQ4plex)	0,0000	2,245	1		0	0,001854	21	0,007160939	3	1017,60413	-0,24	7,52	1
	High	mQAAIAk	1	1	1	P36236	N-Term(iTRAQ4plex); K7(iTRAQ4plex)	0,0000	2,475	1		0	0,002324	20	0,009036495	2	1020,61797	6,39	18,88	0
	High	vAVLAR	4	1	1	P36236	N-Term(iTRAQ4plex)	0,0000	1,275	4	46,9	0,001	0,01434	36	0,000388447	2	772,52172	7,17	19,30	0
	High	tTVSLPk	1	1	1	P36236	N-Term(iTRAQ4plex); K7(iTRAQ4plex)	0,0000	1,940	1		0,002	0,0272	37	0,000346035	2	1033,65105	1,41	21,56	0
	High	rmQAAIAk	1	1	1	P36236	N-Term(iTRAQ4plex); M2(Oxidation); K8(iTRAQ4plex)	0,0000	1,022	1		0,002	0,02082	17	0,023844745	3	1192,70752	0,03	9,30	1
	High	lGIDPk	3	1	1	P36236	N-Term(iTRAQ4plex); K6(iTRAQ4plex)	0,0000	0,904	3	44,0	0,005	0,06572	22	0,006108998	2	930,58617	-0,11	21,16	0
P72854	Sulfite reductase [ferredoxin] OS=Synechocystis sp. (strain PCC 6803 / Kazusa) GN	445,10	17,80	1	11	11	22	1,228	19	17,5	635	71,4	8,47							
	A3	Sequence	# PSMs	# Proteins	# Protein Groups	Protein Group Accessions	Modifications	ΔCn	114/115	114/115 Count	114/115 Variability [%]	q-Value	PEP	IonScore	Exp Value	Charge	MH+ [Da]	ΔM [ppm]	RT [min]	# Missed Cleavages
	High	lTANHNILLYDINAQDk	1	1	1	P72854	N-Term(iTRAQ4plex); K17(iTRAQ4plex)	0,0000	1,017	1		0	1,505E-12	67	2,07E-07	3	2244,22446	0,78	38,00	0
	High	gFNVLAGGGLGR	4	1	1	P72854	N-Term(iTRAQ4plex)	0,0000	1,228	3	9,0	0	2,886E-08	65	3,19867E-07	2	1261,71404	0,50	37,13	0
	High	kVVDQFQLPLR	2	1	1	P72854	N-Term(iTRAQ4plex); K1(iTRAQ4plex)	0,0000	1,097	2	2,8	0	4,82E-10	52	6,91783E-06	3	1630,98978	0,88	34,98	1
	High	qGLQIHGIVk	5	1	1	P72854	N-Term(iTRAQ4plex); K10(iTRAQ4plex)	0,0000	1,248	3	5,2	0	0,000113	41	7,09529E-05	3	1380,86185	3,82	25,00	0
	High	lAAAYTEk	1	1	1	P72854	N-Term(iTRAQ4plex); K8(iTRAQ4plex)	0,0000	1,331	1		0	0,00004813	41	8,56979E-05	2	1154,66741	1,25	22,06	0
	High	nVmAPAAPFR	1	1	1	P72854	N-Term(iTRAQ4plex); M3(Oxidation)	0,0000	1,529	1		0	0,001198	26	0,002415294	2	1233,65337	0,21	26,12	0
	High	eFSHGYTAPAk	1	1	1	P72854	N-Term(iTRAQ4plex); K11(iTRAQ4plex)	0,0000	1,071	1		0	0,00005526	24	0,003681035	3	1495,77866	0,19	18,64	0
	High	vVDQFQLPLR	1	1	1	P72854	N-Term(iTRAQ4plex)	0,0000	1,048	1		0	0,0004537	17	0,018965749	2	1358,79411	2,07	41,60	0
	High	qVETYmGkPFQSFkPLPAWR	1	1	1	P72854	N-Term(iTRAQ4plex); M6(Oxidation); K8(iTRAQ4plex); K14(iTRAQ4plex)	0,0000	1,332	1		0	0,007472	12	0,074115666	4	2858,54433	1,92	39,61	0
	High	aIVATQR	2	1	1	P72854	N-Term(iTRAQ4plex)	0,0000	2,030	2	37,0	0,001	0,008377	39	0,000122172	2	902,55480	0,87	14,12	0
	High	vGFTALR	3	1	1	P72854	N-Term(iTRAQ4plex)	0,0000	1,097	3	22,8	0,004	0,04458	20	0,014267036	2	907,54839	0,20	28,34	0
P27183	ATP synthase subunit b' OS=Synechocystis sp. (strain PCC 6803 / Kazusa) GN=atp(	444,34	44,06	1	5	5	15	1,610	12	16,6	143	16,2	5,22							
	A3	Sequence	# PSMs	# Proteins	# Protein Groups	Protein Group Accessions	Modifications	ΔCn	114/115	114/115 Count	114/115 Variability [%]	q-Value	PEP	IonScore	Exp Value	Charge	MH+ [Da]	ΔM [ppm]	RT [min]	# Missed Cleavages
	High	qkETAAQEIEAQR	8	1	1	P27183	N-Term(iTRAQ4plex); K2(iTRAQ4plex)	0,0000	1,797	5	6,4	0	7,084E-09	70	1,20976E-07	3	1789,96744	1,53	17,80	1
	High	rQSQAVIADAQAEAR	1	1	1	P27183	N-Term(iTRAQ4plex)	0,0000	1,147	1		0	3,074E-09	63	4,94277E-07	3	1757,93735	-0,12	19,88	1
	High	lLGPELIk	3	1	1	P27183	N-Term(iTRAQ4plex); K8(iTRAQ4plex)	0,0000	1,571	3	3,1	0	0,000009337	56	2,25408E-06	2	1170,77190	1,57	37,79	0
	High	qSALSSLEQEVAALSNQILHk	1	1	1	P27183	N-Term(iTRAQ4plex); K21(iTRAQ4plex)	0,0000	1,460	1		0	1,016E-09	43	4,81915E-05	3	2554,41172	1,47	61,08	0
	High	rLAAEk	2	1	1	P27183	N-Term(iTRAQ4plex); K6(iTRAQ4plex)	0,0000	1,189	2	24,9	0,002	0,02562	16	0,022749403	3	975,61960	0,65	10,24	1
	>sp|K1C10_HUMAN|	437,68	18,21	1	12	12	22	1,547	21	19,8	593	59,5	5,21							
	A3	Sequence	# PSMs	# Proteins	# Protein Groups	Protein Group Accessions	Modifications	ΔCn	114/115	114/115 Count	114/115 Variability [%]	q-Value	PEP	IonScore	Exp Value	Charge	MH+ [Da]	ΔM [ppm]	RT [min]	# Missed Cleavages
	High	sQYEQLAEQNRk	3	1	1		N-Term(iTRAQ4plex); K12(iTRAQ4plex)	0,0000	1,792	2	2,4	0	3,027E-09	53	5,15193E-06	3	1781,94101	1,43	19,07	1
	High	iRLENEIQTYR	2	1	1		N-Term(iTRAQ4plex)	0,0000	1,537	2	12,1	0	0,000001486	50	9,74922E-06	3	1578,87210	0,03	29,19	1
	High	lkYENEVALR	4	1	1		N-Term(iTRAQ4plex); K2(iTRAQ4plex)	0,0000	1,726	4	8,5	0	8,376E-07	44	4,27533E-05	3	1522,88422	0,68	26,09	1
	High	lASYLDkVR	3	1	1		N-Term(iTRAQ4plex); K7(iTRAQ4plex)	0,0000	1,388	3	16,2	0	0,000005952	37	0,000187055	3	1352,81366	-0,28	32,70	1
	High	rVLDELTLTk	1	1	1		N-Term(iTRAQ4plex); K10(iTRAQ4plex)	0,0000	1,826	1		0	0,000007883	37	0,000222316	3	1475,90412	0,35	32,93	1
	High	ikEWYEk	2	1	1		N-Term(iTRAQ4plex); K2(iTRAQ4plex); K7(iTRAQ4plex)	0,0000	2,034	2	2,0	0	0,00009124	34	0,000405875	2	1427,82536	-0,36	24,84	1
	High	qSVEADINGLRR	1	1	1		N-Term(iTRAQ4plex)	0,0000	1,414	1		0	0,00007134	33	0,000509296	3	1501,82013	-0,17	26,14	1
	High	vTMQNLNDRLASYLDkVR	1	1	1		N-Term(iTRAQ4plex); K16(iTRAQ4plex)	0,0000	0,625	1		0	1,121E-09	30	0,00098168	4	2424,32851	0,53	44,88	2
	High	vTmQNLNDR	1	1	1		N-Term(iTRAQ4plex); M3(Oxidation)	0,0000	1,031	1		0	0,0008939	30	0,001066522	2	1250,63042	1,93	14,77	0
	High	kDAEAWFNEk	1	1	1		N-Term(iTRAQ4plex); K1(iTRAQ4plex); K10(iTRAQ4plex)	0,0000	1,281	1		0	0,00000147	20	0,009953367	3	1669,88980	-0,73	28,43	1
	High	aLEESNYELEGkIkEWYEk	1	1	1		N-Term(iTRAQ4plex); K12(iTRAQ4plex); K14(iTRAQ4plex); K19(iTRAQ4plex)	0,0000	1,011	1		0	2,535E-07	17	0,020782502	4	2934,56118	0,97	42,19	2
	High	sEITELRR	2	1	1		N-Term(iTRAQ4plex)	0,0000	1,460	2	3,9	0,001	0,009401	17	0,036684613	3	1147,65982	4,03	16,18	1
P73463	Slr1220 protein OS=Synechocystis sp. (strain PCC 6803 / Kazusa) GN=slr1220 PE=	435,57	18,93	1	6	6	18	1,338	13	7,3	317	36,0	4,32							
	A3	Sequence	# PSMs	# Proteins	# Protein Groups	Protein Group Accessions	Modifications	ΔCn	114/115	114/115 Count	114/115 Variability [%]	q-Value	PEP	IonScore	Exp Value	Charge	MH+ [Da]	ΔM [ppm]	RT [min]	# Missed Cleavages
	High	lRNEFINTEVITR	4	1	1	P73463	N-Term(iTRAQ4plex)	0,0000	1,261	2	3,7	0	3,532E-08	60	9,54927E-07	3	1748,97855	0,53	34,04	1
	High	vRDFQFDLATGk	5	1	1	P73463	N-Term(iTRAQ4plex); K12(iTRAQ4plex)	0,0000	1,351	3	4,2	0	1,317E-08	58	1,82469E-06	3	1684,92679	0,39	33,62	1
	High	lNQLSVGLLER	3	1	1	P73463	N-Term(iTRAQ4plex)	0,0000	1,342	3	0,4	0	0,00001382	58	2,11925E-06	2	1385,82427	0,67	41,97	0
	High	eIVALGLR	3	1	1	P73463	N-Term(iTRAQ4plex)	0,0000	1,274	3	11,7	0	0,0008635	50	1,10655E-05	2	1014,64324	0,39	34,60	0
	High	kLGVVk	1	1	1	P73463	N-Term(iTRAQ4plex); K1(iTRAQ4plex); K6(iTRAQ4plex)	0,0000	1,483	1		0	0,003691	30	0,001071445	3	1075,75412	-2,07	14,80	1
	High	lGIGRPSWER	2	1	1	P73463	N-Term(iTRAQ4plex)	0,0000	1,124	1		0	0,0003272	12	0,062946271	3	1314,74027	0,25	27,08	0
Q55141	Acetolactate synthase small subunit OS=Synechocystis sp. (strain PCC 6803 / Kazu	431,60	49,42	1	9	9	17	1,373	17	30,8	172	18,9	5,43							
	A3	Sequence	# PSMs	# Proteins	# Protein Groups	Protein Group Accessions	Modifications	ΔCn	114/115	114/115 Count	114/115 Variability [%]	q-Value	PEP	IonScore	Exp Value	Charge	MH+ [Da]	ΔM [ppm]	RT [min]	# Missed Cleavages
	High	rGFNIESLAVGSAEQGDVSR	1	1	1	Q55141	N-Term(iTRAQ4plex)	0,0000	1,032	1		0	5,637E-13	88	1,70596E-09	3	2236,14566	0,77	37,63	1
	High	aEVIELAQVFR	2	1	1	Q55141	N-Term(iTRAQ4plex)	0,0000	1,784	2	2,3	0	0,000005818	58	1,7457E-06	2	1418,81377	0,93	48,61	0
	High	vSANAPNRAEVIELAQVFR	1	1	1	Q55141	N-Term(iTRAQ4plex)	0,0000	1,655	1		0	4,104E-09	52	6,41165E-06	3	2228,22983	1,32	44,35	1
	High	mkHTLSVLVEDEAGVLTR	1	1	1	Q55141	N-Term(iTRAQ4plex); M1(Oxidation); K2(iTRAQ4plex)	0,0000	1,898	1		0	5,946E-11	48	1,66414E-05	3	2302,26944	0,63	37,21	1
	High	vSANAPNR	4	1	1	Q55141	N-Term(iTRAQ4plex)	0,0000	1,342	4	6,1	0	0,001734	39	0,000116942	2	972,53441	0,07	12,84	0

	High	iAGLFAR	2	1	1	Q55141	N-Term(iTRAQ4plex)	0,0000	1,722	2	3,5	0	0,002291	35	0,000337264	2	891,55358	0,32	34,17	0
	High	mVAILQmLAk	1	1	1	Q55141	N-Term(iTRAQ4plex); M7(Oxidation); K10(iTRAQ4plex)	0,0000	1,166	1		0	0,0002816	30	0,001044648	3	1421,84717	0,61	47,81	0
	High	mVAILQmLAk	1	1	1	Q55141	N-Term(iTRAQ4plex); M1(Oxidation); M7(Oxidation); K10(iTRAQ4plex)	0,0000	0,552	1		0	0,0002465	25	0,003019743	3	1437,84208	0,60	38,05	0
	High	lVNVIk	2	1	1	Q55141	N-Term(iTRAQ4plex); K6(iTRAQ4plex)	0,0000	1,272	2	46,8	0,005	0,05612	29	0,001129718	2	973,66509	0,25	27,82	0
	Medium	iALVR	2	1	1	Q55141	N-Term(iTRAQ4plex)	0,0000	1,228	2	13,6	0,028	0,3039	19	0,014027168	2	715,49437	-0,47	23,50	0
Q55585	Probable succinate-semialdehyde dehydrogenase [NADP(+)] OS=Synechocystis sp.	429,91	14,98	1	7	7	19	0,936	19	14,3	454	48,7	5,14							
	A3	Sequence	# PSMs	# Proteins	# Protein Groups	Protein Group Accessions	Modifications	ΔCn	114/115	114/115 Count	114/115 Variability [%]	q-Value	PEP	IonScore	Exp Value	Charge	MH+ [Da]	ΔM [ppm]	RT [min]	# Missed Cleavages
	High	fAAPALmAGNVGLLk	4	1	1	Q55585	N-Term(iTRAQ4plex); M7(Oxidation); K15(iTRAQ4plex)	0,0000	0,977	4	6,4	0	3,727E-10	90	1,03985E-09	3	1777,02701	-0,85	43,00	0
	High	fAAPALMAGNVGLLk	1	1	1	Q55585	N-Term(iTRAQ4plex); K15(iTRAQ4plex)	0,0000	1,288	1		0	6,823E-07	45	3,34942E-05	3	1761,03312	-0,28	48,33	0
	High	kAADILEQR	4	1	1	Q55585	N-Term(iTRAQ4plex); K1(iTRAQ4plex)	0,0000	0,954	4	7,4	0	0,00007782	44	4,05481E-05	3	1331,78821	-0,27	20,80	1
	High	rDEWAALMTLEMGk	1	1	1	Q55585	N-Term(iTRAQ4plex); K14(iTRAQ4plex)	0,0000	0,584	1		0	5,453E-11	39	0,000121051	3	1939,00138	-0,35	45,13	1
	High	hLPFAQR	2	1	1	Q55585	N-Term(iTRAQ4plex)	0,0000	0,892	2	7,1	0	0,0008346	28	0,001520443	3	1012,58091	0,01	19,38	0
	High	mGILEFVNAk	1	1	1	Q55585	N-Term(iTRAQ4plex); K10(iTRAQ4plex)	0,0000	0,735	1		0	0,00009647	24	0,005142582	3	1409,80716	0,44	45,08	0
	High	mGILEFVNAk	1	1	1	Q55585	N-Term(iTRAQ4plex); M1(Oxidation); K10(iTRAQ4plex)	0,0000	1,100	1		0	0,00013	19	0,012704864	3	1425,80078	-0,47	38,99	0
	High	iPFGGIk	3	1	1	Q55585	N-Term(iTRAQ4plex); K7(iTRAQ4plex)	0,0000	0,840	3	4,4	0,008	0,1008	29	0,001342672	2	1019,64910	-0,11	33,25	0
	Medium	aFQALk	2	1	1	Q55585	N-Term(iTRAQ4plex); K6(iTRAQ4plex)	0,0000	0,919	2	2,7	0,012	0,1383	31	0,000858954	2	965,60704	4,97	23,10	0
Q55727	Slr0645 protein OS=Synechocystis sp. (strain PCC 6803 / Kazusa) GN=slr0645 PE=:	429,51	36,89	1	6	6	17	0,831	15	13,1	206	22,8	4,74							
	A3	Sequence	# PSMs	# Proteins	# Protein Groups	Protein Group Accessions	Modifications	ΔCn	114/115	114/115 Count	114/115 Variability [%]	q-Value	PEP	IonScore	Exp Value	Charge	MH+ [Da]	ΔM [ppm]	RT [min]	# Missed Cleavages
	High	tDLAGALkDGLDNFFQR	3	1	1	Q55727	N-Term(iTRAQ4plex); K8(iTRAQ4plex)	0,0000	0,811	3	18,6	0	1,124E-10	56	2,76675E-06	4	2169,15517	0,39	53,90	1
	High	vAYIYANNEPmGR	3	1	1	Q55727	N-Term(iTRAQ4plex); M11(Oxidation)	0,0000	0,931	2	3,4	0	1,183E-08	55	3,48313E-06	2	1657,81450	1,20	26,60	0
	High	iDRDEELAISLIQVGR	2	1	1	Q55727	N-Term(iTRAQ4plex)	0,0000	0,752	2	10,1	0	0,000000523	47	1,92296E-05	3	1971,10086	0,84	46,74	1
	High	rYDNVTAEk	4	1	1	Q55727	N-Term(iTRAQ4plex); K9(iTRAQ4plex)	0,0000	0,818	4	6,5	0	0,000005701	42	5,68814E-05	3	1383,74787	0,57	14,18	1
	High	lILEASQk	4	1	1	Q55727	N-Term(iTRAQ4plex); K8(iTRAQ4plex)	0,0000	0,841	3	2,9	0	0,0007544	40	9,22508E-05	2	1189,74248	2,52	27,26	0
	High	wEIAQASTIALAk	1	1	1	Q55727	N-Term(iTRAQ4plex); K13(iTRAQ4plex)	0,0000	1,181	1		0	0,0005571	28	0,001774067	3	1689,98069	1,69	41,81	0
Q55660	Slr0001 protein OS=Synechocystis sp. (strain PCC 6803 / Kazusa) GN=slr0001 PE=:	428,75	33,75	1	5	5	24	1,810	23	24,4	160	17,4	5,15							
	A3	Sequence	# PSMs	# Proteins	# Protein Groups	Protein Group Accessions	Modifications	ΔCn	114/115	114/115 Count	114/115 Variability [%]	q-Value	PEP	IonScore	Exp Value	Charge	MH+ [Da]	ΔM [ppm]	RT [min]	# Missed Cleavages
	High	kIPHGLYVcGVk	5	1	1	Q55660	N-Term(iTRAQ4plex); K1(iTRAQ4plex); C9(Carbamidomethyl); K12(iTRAQ4pl	0,0000	1,669	4	28,1	0	9,109E-12	56	2,46587E-06	3	1803,06919	0,91	25,05	1
	High	dVAAQFFkPQR	9	1	1	Q55660	N-Term(iTRAQ4plex); K8(iTRAQ4plex)	0,0000	2,014	9	6,7	0	0,000001899	56	2,71E-06	2	1594,89629	1,16	29,64	0
	High	iPHGLYVcGVk	2	1	1	Q55660	N-Term(iTRAQ4plex); C8(Carbamidomethyl); K11(iTRAQ4plex)	0,0000	2,683	2	14,5	0	2,851E-07	41	8,72911E-05	3	1530,87284	1,52	29,71	0
	High	rVGDkFENFDVLDAPETGcPVLk	2	1	1	Q55660	N-Term(iTRAQ4plex); K5(iTRAQ4plex); C19(Carbamidomethyl); K23(iTRAQ4p	0,0000	2,105	2	10,3	0	4,804E-11	31	0,000751951	4	3038,60000	0,58	41,62	2
	High	mLDEQAkk	3	1	1	Q55660	N-Term(iTRAQ4plex); M1(Oxidation); K7(iTRAQ4plex); K8(iTRAQ4plex)	0,0000	1,658	3	3,0	0	0,0005227	20	0,011065474	3	1410,80024	1,08	11,20	1
	High	mLDEQAkk	3	1	1	Q55660	N-Term(iTRAQ4plex); K7(iTRAQ4plex); K8(iTRAQ4plex)	0,0000	1,617	3	10,9	0	0,00002314	19	0,012588385	3	1394,80341	-0,27	14,49	1
P54123	Ribonuclease J OS=Synechocystis sp. (strain PCC 6803 / Kazusa) GN=slr0551 PE=3	427,59	12,34	1	9	9	20	0,853	18	16,2	640	70,4	7,74							
	A3	Sequence	# PSMs	# Proteins	# Protein Groups	Protein Group Accessions	Modifications	ΔCn	114/115	114/115 Count	114/115 Variability [%]	q-Value	PEP	IonScore	Exp Value	Charge	MH+ [Da]	ΔM [ppm]	RT [min]	# Missed Cleavages
	High	iLPLGGLHEIGk	1	1	1	P54123	N-Term(iTRAQ4plex); K12(iTRAQ4plex)	0,0000	0,936	1		0	1,118E-09	53	4,5391E-06	3	1534,95499	-0,64	38,86	0
	High	lLEELIVR	5	1	1	P54123	N-Term(iTRAQ4plex)	0,0000	0,866	5	18,2	0	0,000006969	48	1,76592E-05	2	1128,71086	-0,05	46,35	0
	High	tNLQTVSPR	3	1	1	P54123	N-Term(iTRAQ4plex)	0,0000	0,781	2	3,8	0	0,00005294	47	2,14274E-05	2	1159,65544	0,22	18,03	0
	High	mLLALTRPk	2	1	1	P54123	N-Term(iTRAQ4plex); M1(Oxidation); K9(iTRAQ4plex)	0,0000	1,418	2	36,1	0	0,0003129	40	9,93047E-05	3	1346,84204	-0,90	25,29	0
	High	sFVVEFIR	3	1	1	P54123	N-Term(iTRAQ4plex)	0,0000	0,803	3	5,1	0	0,0002936	38	0,000162544	2	1140,65325	-0,14	43,66	0
	High	mLLALTRPk	1	1	1	P54123	N-Term(iTRAQ4plex); K9(iTRAQ4plex)	0,0000	0,901	1		0	1,874E-07	37	0,000184064	3	1330,84705	-0,97	32,48	0
	High	vNIILSLAQk	1	1	1	P54123	N-Term(iTRAQ4plex); K10(iTRAQ4plex)	0,0000	0,954	1		0	0,00000165	36	0,000271625	3	1386,89243	0,10	44,61	0
	High	sMLNVIAHAR	2	1	1	P54123	N-Term(iTRAQ4plex)	0,0000	0,625	1		0	0,0000236	28	0,001741687	3	1255,70642	0,18	29,72	0
	High	lAMALLR	1	1	1	P54123	N-Term(iTRAQ4plex)	0,0000	0,719	1		0,001	0,01086	35	0,00030267	2	931,58769	-0,30	38,31	0
	High	kLGYIk	1	1	1	P54123	N-Term(iTRAQ4plex); K1(iTRAQ4plex); K6(iTRAQ4plex)	0,0000	0,948	1		0,003	0,03736	25	0,003435342	3	1153,76456	-2,03	18,48	1
P73510	Sll1358 protein OS=Synechocystis sp. (strain PCC 6803 / Kazusa) GN=sll1358 PE=1	427,35	23,10	1	7	7	17	2,521	15	32,1	394	43,1	6,23							
	A3	Sequence	# PSMs	# Proteins	# Protein Groups	Protein Group Accessions	Modifications	ΔCn	114/115	114/115 Count	114/115 Variability [%]	q-Value	PEP	IonScore	Exp Value	Charge	MH+ [Da]	ΔM [ppm]	RT [min]	# Missed Cleavages
	High	qVGTYNFPVSk	4	1	1	P73510	N-Term(iTRAQ4plex); K11(iTRAQ4plex)	0,0000	1,701	3	12,3	0	3,877E-10	77	2,29823E-08	2	1527,84490	2,57	30,88	0
	High	iEVPHTHNLLGQQPLVSLGGNELR	3	1	1	P73510	N-Term(iTRAQ4plex)	0,0000	1,432	3	28,1	0	7,097E-13	64	3,90814E-07	3	2765,52481	2,47	42,26	0
	High	gWGHSIEGIGPDTAk	1	1	1	P73510	N-Term(iTRAQ4plex); K15(iTRAQ4plex)	0,0000	2,041	1		0	4,415E-09	55	3,44326E-06	3	1812,95249	2,29	29,77	0
	High	sLSNVVWGk	2	1	1	P73510	N-Term(iTRAQ4plex); K9(iTRAQ4plex)	0,0000	3,090	1		0	0,00009009	55	3,48313E-06	2	1277,75115	4,32	32,09	0
	High	iTLTSPEGk	1	1	1	P73510	N-Term(iTRAQ4plex); K9(iTRAQ4plex)	0,0000	3,067	1		0	0,00000443	48	1,56315E-05	2	1233,73381	3,65	24,87	0
	High	gGLWYFPR	3	1	1	P73510	N-Term(iTRAQ4plex)	0,0000	2,589	3	4,0	0	0,003106	29	0,001218905	2	1139,61467	2,46	41,39	0
	High	kLPVQDTIFSLPTQP	3	1	1	P73510	N-Term(iTRAQ4plex); K1(iTRAQ4plex)	0,0000	1,245	3	6,5	0	0,005738	24	0,004086888	3	1972,13999	2,12	47,25	1
P74233	Slr1160 protein OS=Synechocystis sp. (strain PCC 6803 / Kazusa) GN=slr1160 PE=:	421,39	40,20	1	6	6	13	1,427	12	11,6	204	22,2	5,55							
	A3	Sequence	# PSMs	# Proteins	# Protein Groups	Protein Group Accessions	Modifications	ΔCn	114/115	114/115 Count	114/115 Variability [%]	q-Value	PEP	IonScore	Exp Value	Charge	MH+ [Da]	ΔM [ppm]	RT [min]	# Missed Cleavages
	High	rLmNEANEAINLNQYTVALEk	2	1	1	P74233	N-Term(iTRAQ4plex); M3(Oxidation); K21(iTRAQ4plex)	0,0000	1,795	2	6,4	0	2,216E-16	94	3,74085E-10	3	2738,44461	2,18	40,29	1
	High	eFRPLNQEGSILSVGAGR	4	1	1	P74233	N-Term(iTRAQ4plex)	0,0000	1,456	3	12,4	0	1,998E-14	63	4,4978E-07	3	2074,11875	1,20	37,25	0
	High	aNNEPALAVPLLLQIIR	2	1	1	P74233	N-Term(iTRAQ4plex)	0,0000	1,386	2	3,4	0	0,000000623	52	6,54591E-06	2	1989,20610	4,17	61,51	0
	High	iAALETGQLR	2	1	1	P74233	N-Term(iTRAQ4plex)	0,0000	1,250	2	15,5	0	0,00001132	43	5,92513E-05	2	1215,71807	0,21	31,48	0
	High	sQNPTTDLGR	1	1	1	P74233	N-Term(iTRAQ4plex)	0,0000	1,079	1		0	0,00006771	40	0,000106898	2	1232,63530	0,10	15,84	0
	High	iAALETGQLRDEATYR	2	1	1	P74233	N-Term(iTRAQ4plex)	0,0000	1,454	2	1,9	0	0,0003323	30	0,000901509	3	1951,03763	0,53	33,67	1
P73867	Potassium-transporting ATPase B chain OS=Synechocystis sp. (strain PCC 6803 / Ka	417,79	12,90	1	9	9	15	0,118	15	61,3	690	72,9	6,14							
	A3	Sequence	# PSMs	# Proteins	# Protein Groups	Protein Group Accessions	Modifications	ΔCn	114/115	114/115 Count	114/115 Variability [%]	q-Value	PEP	IonScore	Exp Value	Charge	MH+ [Da]	ΔM [ppm]	RT [min]	# Missed Cleavages
	High	lIDLVTIGk	1	1	1	P73867	N-Term(iTRAQ4plex); K9(iTRAQ4plex)	0,0000	0,091	1		0	0,000001489	69	1,28816E-07	2	1259,81328	-3,54	44,04	0
	High	vAQFNVVATSGR	2	1	1	P73867	N-Term(iTRAQ4plex)	0,0000	0,062	2	39,5	0	0,0000755	55	3,38041E-06	2	1392,76848	-2,26	29,07	0
	High	fRPLSADQLLQR	3	1	1	P73867	N-Term(iTRAQ4plex)	0,0000	0,140	3	36,4	0	5,062E-08	51	8,8914E-06	3	1587,90671	-1,32	35,05	0
	High	sTQTQTYAQR	2	1	1	P73867	N-Term(iTRAQ4plex)	0,0000	0,082	2	10,7	0	0,000005851	45	3,10435E-05	2	1327,66985	-1,83	10,36	0
	High	tGTITLGNR	2	1	1	P73867	N-Term(iTRAQ4plex)	0,0000	0,125	2	7,8	0	0,0009852	45	3,96443E-05	2	1076,62297	4,55	19,16	0
	High	vSHLGGTPLAVcR	1	1	1	P73867	N-Term(iTRAQ4plex); C12(Carbamidomethyl)	0,0000	0,193	1		0	5,784E-08	36	0,000249442	3	1510,82544	-1,76	24,78	0
	High	dIIkPGIQER	1	1	1	P73867	N-Term(iTRAQ4plex); K4(iTRAQ4plex)	0,0000	0,103	1		0	0,00004841	20	0,009161572	3	1456,86774	-3,36	21,49	0
	High	qLLITR	2	1	1	P73867	N-Term(iTRAQ4plex)	0,0000	0,324	2	112,4	0,002	0,01892	24	0,004149254	2	887,57726	-2,52	23,29	0
	High	kGAVDAIR	1	1	1	P73867	N-Term(iTRAQ4plex); K1(iTRAQ4plex)	0,0000	0,230	1		0,002	0,03044	21	0,008169384	3	1117,68811	-4,56	13,03	1
P27320	Ferredoxin-1 OS=Synechocystis sp. (strain PCC 6803 / Kazusa) GN=petF PE=1 SV=	415,24	17,53	1	3	3	25	1,194	23	15,6	97	10,4	3,95							
	A3	Sequence	# PSMs	# Proteins	# Protein Groups	Protein Group Accessions	Modifications	ΔCn	114/115	114/115 Count	114/115 Variability [%]	q-Value	PEP	IonScore	Exp Value	Charge	MH+ [Da]	ΔM [ppm]	RT [min]	# Missed Cleavages
	High	aGAcSTcAGk	10	1	1	P27320	N-Term(iTRAQ4plex); C4(Carbamidomethyl); C7(Carbamidomethyl); K10(iTR	0,0000	1,231	9	15,6	0	2,263E-07	71	8,47169E-08	2	1270,61443	1,71	11,53	0
	High	mASYTVk	2	1	1	P27320	N-Term(iTRAQ4plex); K7(iTRAQ4plex)	0,0000	1,107	2	19,0	0	0,0004299	49	1,2473E-05	2	1087,61113	4,71	20,32	0
	High	mASYTVk	4	1	1	P27320	N-Term(iTRAQ4plex); M1(Oxidation); K7(iTRAQ4plex)	0,0000	1,196	3	0,2	0	0,0008641	37	0,000206524	2	1103,60759	6,04	15,34	0
	High	aSYTVk	9	1	1	P27320	N-Term(iTRAQ4plex); K6(iTRAQ4plex)	0,0000	1,162	9	10,5	0,002	0,03224	39	0,000134267	2	956,56670	1,24	13,92	0
P73954	Membrane-associated protein slr1513 OS=Synechocystis sp. (strain PCC 6803 / Kaz	412,78	32,73	1	5	5	18	0,484	12	36,6	110	12,0	7,08							
	A3	Sequence	# PSMs	# Proteins	# Protein Groups	Protein Group Accessions	Modifications	ΔCn	114/115	114/115 Count	114/115 Variability [%]	q-Value	PEP	IonScore	Exp Value	Charge	MH+ [Da]	ΔM [ppm]	RT [min]	# Missed Cleavages
	High	gYTVMNTGGk	4	1	1	P73954	N-Term(iTRAQ4plex); K10(iTRAQ4plex)	0,0000	0,715	2	23,1	0	1,928E-08	72	6,69838E-08	2	1315,69988	6,08	20,50	0
	High	gYTVmNTGGk	5	1	1	P73954	N-Term(iTRAQ4plex); M5(Oxidation); K10(iTRAQ4plex)	0,0000	0,386	3	21,9	0	7,726E-09	70	1,04947E-07	2	1331,68669	-0,07	15,43	0
	High	fEILTETR	2	1	1	P73954	N-Term(iTRAQ4plex)	0,0000	0,460	2	28,9	0	0,0000185	52	6,82292E-06	2	1152,63823	0,08	34,63	0
	High	lVIVTEk	3	1	1	P73954	N-Term(iTRAQ4plex); K7(iTRAQ4plex)	0,0000	0,628	2	17,9	0	0,0002579	43	5,4572E-05	2	1089,71404	1,69	28,44	0
	High	akPANk	3	1	1	P73954	N-Term(iTRAQ4plex); K2(iTRAQ4plex); K6(iTRAQ4plex)	0,0000	0,350	2	7,2	0,001	0,01723	11	0,082788498	3	1060,68436	0,42	7,80	0
	Medium	iLLkk	1	1	1	P73954	N-Term(iTRAQ4plex); K4(iTRAQ4plex); K5(iTRAQ4plex)	0,0000	0,436	1		0,029	0,311	12	0,064116529	3	1046,76596	-0,21	19,87	1
P72704	Probable peptidyl-prolyl cis-trans isomerase sll0227 OS=Synechocystis sp. (strain P(	412,25	26,42	1	7	7	18	1,367	18	24,6	246	26,6	5,14							
	A3	Sequence	# PSMs	# Proteins	# Protein Groups	Protein Group Accessions	Modifications	ΔCn	114/115	114/115 Count	114/115 Variability [%]	q-Value	PEP	IonScore	Exp Value	Charge	MH+ [Da]	ΔM [ppm]	RT [min]	# Missed Cleavages
	High	vITGQNNLEk	3	1	1	P72704	N-Term(iTRAQ4plex); K10(iTRAQ4plex)	0,0000	1,620	3	7,3	0	2,649E-09	79	1,37711E-08	2	1403,81047	0,56	21,85	0
	High	aLGNQAGFPVMLPHk	2	1	1	P72704	N-Term(iTRAQ4plex); K15(iTRAQ4plex)	0,0000	1,411	2	18,8	0	7,33E-11	58	1,88535E-06	3	1868,04581	0,14	37,65	0
	High	nIPLEIk	3	1	1	P72704	N-Term(iTRAQ4plex); K7(iTRAQ4plex)	0,0000	1,381	3	2,1	0	0,007473	49	1,13755E-05	2	1114,70708	-0,33	30,99	0
	High	aLGNQAGFPVmLPHk	2	1	1	P72704	N-Term(iTRAQ4plex); M11(Oxidation); K15(iTRAQ4plex)	0,0000	0,583	2	215,7	0	2,552E-10	44	5,06036E-05	3	1884,04227	0,95	35,24	0
	High	gDGTGGYVDkNTQRPR	2	1	1	P72704	N-Term(iTRAQ4plex); K10(iTRAQ4plex)	0,0000	1,026	2	6,3	0	6,636E-09	28	0,001485833	4	2009,04128	0,47	13,44	1
	High	qGDkIQSAk	1	1	1	P72704	N-Term(iTRAQ4plex); K4(iTRAQ4plex); K9(iTRAQ4plex)	0,0000	1,542	1		0	0,005567	13	0,048080614	3	1406,83316	0,28	10,21	1
	High	tGAVAMAR	3	1	1	P72704	N-Term(iTRAQ4plex)	0,0000	1,022	3	14,9	0,004	0,04855	20	0,009267658	2	920,51006	-0,43	13,76	0
	Medium	tGAVAmAR	1	1	1	P72704	N-Term(iTRAQ4plex); M6(Oxidation)	0,0000	0,905	1		0,012	0,1388	16	0,022540835	2	936,50621	0,90	10,06	0
	Medium	iQSAk	1	1	1	P72704	N-Term(iTRAQ4plex); K5(iTRAQ4plex)	0,0000	1,950	1		0,038	0,4002	20	0,010255811	2	834,52922	0,58	12,70	0
P72851	50S ribosomal protein L28 OS=Synechocystis sp. (strain PCC 6803 / Kazusa) GN=rp	412,06	61,54	1	9	9	25	1,342	22	30,8	78	9,0	11,81							
	A3	Sequence	# PSMs	# Proteins	# Protein Groups	Protein Group Accessions	Modifications	ΔCn	114/115	114/115 Count	114/115 Variability [%]	q-Value	PEP	IonScore	Exp Value	Charge	MH+ [Da]	ΔM [ppm]	RT [min]	# Missed Cleavages
	High	lSTTAIk	3	1	1	P72851	N-Term(iTRAQ4plex); K7(iTRAQ4plex)	0,0000	1,522	3	13,1	0	0,00003077	63	5,2236E-07	2	1021,64934	-0,24	21,88	0
	High	lQQANLQWk	4	1	1	P72851	N-Term(iTRAQ4plex); K9(iTRAQ4plex)	0,0000	1,433	4	16,0	0	0,00001233	44	4,23614E-05	2	1416,82134	0,81	31,20	0
	High	kLQQANLQWk	4	1	1	P72851	N-Term(iTRAQ4plex); K1(iTRAQ4plex); K10(iTRAQ4plex)	0,0000	1,259	4	11,1	0	7,075E-07	43	4,89745E-05	3	1689,01773	0,30	25,66	1
	High	eAGINLNkF	2	1	1	P72851	N-Term(iTRAQ4plex); K8(iTRAQ4plex)	0,0000	1,794	2	25,0	0	0,0009036	37	0,000211054	2	1293,74077	0,17	33,86	1
	High	gINAmAk	6	1	1	P72851	N-Term(iTRAQ4plex); M5(Oxidation); K7(iTRAQ4plex)	0,0000	1,087	3	3,2	0	0,001022	31	0,000812774	2	1008,57695	1,87	13,55	0
	High	rVWWPEGNR	2	1	1	P72851	N-Term(iTRAQ4plex)	0,0000	1,001	2	66,0	0	0,001885	18	0,017741895	3	1343,70884	-0,10	27,30	1
	High	eAGINLNk	1	1	1	P72851	N-Term(iTRAQ4plex); K8(iTRAQ4plex)	0,0000	1,631	1		0	0,0002098	16	0,030717982	2	1146,67339	1,10	20,54	0
	High	cQLTGk	1	1	1	P72851	N-Term(iTRAQ4plex); C1(Carbamidomethyl); K6(iTRAQ4plex)	0,0000	0,868	1		0,001	0,01731	23	0,004581102	2	994,56163	2,25	12,05	0
	High	vWWPEGNR	2	1	1	P72851	N-Term(iTRAQ4plex)	0,0000	1,122	2	26,4	0,002	0,02738	24	0,003819179	2	1187,60771	-0,12	34,60	0
P73307	30S ribosomal protein S8 OS=Synechocystis sp. (strain PCC 6803 / Kazusa) GN=rp:	408,45	51,88	1	8	8	22	1,292	19	21,1	133	14,7	9,99							
	A3	Sequence	# PSMs	# Proteins	# Protein Groups	Protein Group Accessions	Modifications	ΔCn	114/115	114/115 Count	114/115 Variability [%]	q-Value	PEP	IonScore	Exp Value	Charge	MH+ [Da]	ΔM [ppm]	RT [min]	# Missed Cleavages
	High	vLGGIGIAIVSTSHGImTDR	2	1	1	P73307	N-Term(iTRAQ4plex); M17(Oxidation)	0,0000	0,950	2	22,7	0	9,49E-13	69	1,52192E-07	3	2157,18290	0,46	45,41	0
	High	qPLINTLQR	2	1	1	P73307	N-Term(iTRAQ4plex)	0,0000	1,520	2	1,8	0	0,0001729	49	1,33036E-05	2	1226,73418	0,33	27,20	0
	High	mLVLTLk	2	1	1	P73307	N-Term(iTRAQ4plex); K7(iTRAQ4plex)	0,0000	1,327	2	4,0	0	0,0002377	46	2,61198E-05	2	1105,72563	-0,10	40,67	0
	High	tRQPLINTLQR	1	1	1	P73307	N-Term(iTRAQ4plex)	0,0000	1,068	1		0	0,00002242	44	4,2459E-05	3	1483,88340	0,55	22,27	1
	High	mLVLTLk	6	1	1	P73307	N-Term(iTRAQ4plex); M1(Oxidation); K7(iTRAQ4plex)	0,0000	1,438	3	4,7	0	0,0001949	41	8,31706E-05	2	1121,72063	-0,03	33,92	0
	High	mTLSIAk	2	1	1	P73307	N-Term(iTRAQ4plex); M1(Oxidation); K7(iTRAQ4plex)	0,0000	1,140	2	12,0	0	0,0006217	33	0,000463415	2	1067,63774	0,39	20,84	0
	High	hSTTQVPTTk	2	1	1	P73307	N-Term(iTRAQ4plex); K10(iTRAQ4plex)	0,0000	1,033	2	12,7	0,001	0,009917	19	0,011560324	3	1387,77948	0,80	10,86	0
	High	vSkPGLR	4	1	1	P73307	N-Term(iTRAQ4plex); K3(iTRAQ4plex)	0,0000	1,364	4	12,2	0,006	0,07385	22	0,006108998	2	1044,67644	-0,36	13,91	0
	Medium	vYSPSkk	1	1	1	P73307	N-Term(iTRAQ4plex); K6(iTRAQ4plex); K7(iTRAQ4plex)	0,0000	0,996	1		0,027	0,29	14	0,037928879	3	1240,76163	-0,73	12,14	1
P73244	Slr2025 protein OS=Synechocystis sp. (strain PCC 6803 / Kazusa) GN=slr2025 PE=:	406,24	17,65	1	2	2	17	1,196	9	6,3	153	16,9	4,44							
	A3	Sequence	# PSMs	# Proteins	# Protein Groups	Protein Group Accessions	Modifications	ΔCn	114/115	114/115 Count	114/115 Variability [%]	q-Value	PEP	IonScore	Exp Value	Charge	MH+ [Da]	ΔM [ppm]	RT [min]	# Missed Cleavages
	High	aITLVATLADDHDDRLk	12	1	1	P73244	N-Term(iTRAQ4plex); K17(iTRAQ4plex)	0,0000	1,222	6	6,9	0	8,414E-14	75	4,44973E-08	3	2155,19803	0,86	39,57	1
	High	eNHLVALHQR	5	1	1	P73244	N-Term(iTRAQ4plex)	0,0000	1,182	3	1,5	0	1,083E-07	66	2,53495E-07	2	1360,75676	0,09	14,14	0
Q55835	Iron uptake protein A2 OS=Synechocystis sp. (strain PCC 6803 / Kazusa) GN=futA2	405,13	31,50	1	11	11	18	0,884	16	27,3	346	38,1	6,18							
	A3	Sequence	# PSMs	# Proteins	# Protein Groups	Protein Group Accessions	Modifications	ΔCn	114/115	114/115 Count	114/115 Variability [%]	q-Value	PEP	IonScore	Exp Value	Charge	MH+ [Da]	ΔM [ppm]	RT [min]	# Missed Cleavages
	High	gTHVNVSGAGVLk	4	1	1	Q55835	N-Term(iTRAQ4plex); K13(iTRAQ4plex)	0,0000	1,077	3	0,6	0	1,372E-08	67	1,9542E-07	3	1526,89450	3,38	20,38	0
	High	iAIHGEPETR	1	1	1	Q55835	N-Term(iTRAQ4plex)	0,0000	0,952	1		0	0,0006632	51	8,14648E-06	2	1266,69036	-1,55	17,62	0
	High	hPDGLWYGFTQR	3	1	1	Q55835	N-Term(iTRAQ4plex)	0,0000	0,696	3	2,7	0	2,095E-09	50	9,35341E-06	2	1620,80490	0,57	35,60	0
	High	vSLFFPNTGSGER	1	1	1	Q55835	N-Term(iTRAQ4plex)	0,0000				0	4,756E-08	45	3,38041E-05	2	1554,80571	1,53	40,23	0
	High	iLVRPSSNVYNLSLTASR	2	1	1	Q55835	N-Term(iTRAQ4plex)	0,0000	0,744	2	1,9	0	6,296E-08	41	7,74408E-05	3	2134,20774	-1,12	38,71	0
	High	wLQGLVGNFAR	1	1	1	Q55835	N-Term(iTRAQ4plex)	0,0000	1,054	1		0	0,000002309	40	0,000111422	2	1404,78801	0,80	51,37	0
	High	tINLYSSR	1	1	1	Q55835	N-Term(iTRAQ4plex)	0,0000	0,895	1		0	0,0001658	30	0,001023222	2	1097,60564	-1,38	23,77	0
	High	iAIHGEPETRR	1	1	1	Q55835	N-Term(iTRAQ4plex)	0,0000	1,017	1		0	0,002525	19	0,011245273	4	1422,79287	-0,40	15,33	1
	High	aEQAGLFQPVR	1	1	1	Q55835	N-Term(iTRAQ4plex)	0,0000	0,781	1		0	0,0002077	15	0,030774379	2	1359,74907	-0,81	28,93	0
	High	vLYYSR	2	1	1	Q55835	N-Term(iTRAQ4plex)	0,0000	0,617	2	11,6	0,001	0,01058	29	0,001227354	2	944,53411	2,03	22,10	0
	Medium	iPENLR	1	1	1	Q55835	N-Term(iTRAQ4plex)	0,0000	0,873	1		0,034	0,3646	15	0,028573932	2	885,52764	0,19	21,98	0
P73458	Carboxyl-terminal protease OS=Synechocystis sp. (strain PCC 6803 / Kazusa) GN=p	403,19	16,31	1	8	8	21	2,513	19	36,9	423	46,8	5,21							
	A3	Sequence	# PSMs	# Proteins	# Protein Groups	Protein Group Accessions	Modifications	ΔCn	114/115	114/115 Count	114/115 Variability [%]	q-Value	PEP	IonScore	Exp Value	Charge	MH+ [Da]	ΔM [ppm]	RT [min]	# Missed Cleavages
	High	gkPGTSVVLTIER	4	1	1	P73458	N-Term(iTRAQ4plex); K2(iTRAQ4plex)	0,0000	3,128	3	3,0	0	1,654E-08	58	1,51695E-06	3	1644,99167	1,78	27,02	0
	High	gEVEQQSANkR	5	1	1	P73458	N-Term(iTRAQ4plex); K10(iTRAQ4plex)	0,0000	3,619	4	7,3	0	0,000002108	55	3,2561E-06	3	1533,82468	1,49	8,54	1
	High	rGEVEQQSANk	1	1	1	P73458	N-Term(iTRAQ4plex); K11(iTRAQ4plex)	0,0000	1,975	1		0	0,000001943	48	1,82263E-05	3	1533,82450	1,38	8,46	1
	High	tLIEIHPVR	4	1	1	P73458	N-Term(iTRAQ4plex)	0,0000	1,735	4	42,3	0	0,00001694	38	0,000148583	3	1221,74494	1,08	27,54	0
	High	lEkENVVGYIFDLR	2	1	1	P73458	N-Term(iTRAQ4plex); K3(iTRAQ4plex)	0,0000	1,646	2	97,8	0	0,00000611	36	0,000291199	4	1983,12307	3,87	48,46	1
	High	rGEVEQQSANkR	2	1	1	P73458	N-Term(iTRAQ4plex); K11(iTRAQ4plex)	0,0000	2,721	2	11,8	0	0,0001243	30	0,001086351	3	1689,92630	1,65	7,94	2
	High	kELQQNR	2	1	1	P73458	N-Term(iTRAQ4plex); K1(iTRAQ4plex)	0,0000	1,763	2	7,0	0	0,0002036	27	0,002618955	3	1203,70667	1,54	8,70	1
	Medium	iWLDEGGIVSTVDRR	1	1	1	P73458	N-Term(iTRAQ4plex)	0,0000	1,853	1		0,039	0,4063	15	0,029040227	3	1860,01224	1,39	41,34	1
P73311	30S ribosomal protein S17 OS=Synechocystis sp. (strain PCC 6803 / Kazusa) GN=r	403,17	66,67	1	6	6	20	1,406	15	11,7	81	9,3	9,94							
	A3	Sequence	# PSMs	# Proteins	# Protein Groups	Protein Group Accessions	Modifications	ΔCn	114/115	114/115 Count	114/115 Variability [%]	q-Value	PEP	IonScore	Exp Value	Charge	MH+ [Da]	ΔM [ppm]	RT [min]	# Missed Cleavages
	High	tVVVAVESR	2	1	1	P73311	N-Term(iTRAQ4plex)	0,0000	1,181	2	0,8	0	0,00005811	63	4,797E-07	2	1103,65325	-0,81	21,86	0
	High	iQETRPLSk	8	1	1	P73311	N-Term(iTRAQ4plex); K9(iTRAQ4plex)	0,0000	1,469	5	5,1	0	0,00000392	58	2,03194E-06	2	1359,81999	0,10	17,42	0
	High	vGIVVSNk	4	1	1	P73311	N-Term(iTRAQ4plex); K8(iTRAQ4plex)	0,0000	1,502	2	3,9	0	0,00006608	45	2,99206E-05	2	1103,70647	3,42	22,84	0
	High	aHDEENQcQEGDkVR	4	1	1	P73311	N-Term(iTRAQ4plex); C8(Carbamidomethyl); K13(iTRAQ4plex)	0,0000	1,334	4	12,8	0	7,201E-09	44	4,23614E-05	4	2102,97866	1,06	8,98	1
	High	wQVINImSHSS	1	1	1	P73311	N-Term(iTRAQ4plex); M7(Oxidation)	0,0000	0,985	1		0	0,00009058	42	6,54591E-05	2	1461,72869	0,67	35,40	0
	High	fkAHDEENQcQEGDkVR	1	1	1	P73311	N-Term(iTRAQ4plex); K2(iTRAQ4plex); C10(Carbamidomethyl); K15(iTRAQ4p	0,0000	1,406	1		0	0,000001582	12	0,057012489	5	2522,23829	-1,42	13,41	2
P73334	DNA-directed RNA polymerase subunit beta' OS=Synechocystis sp. (strain PCC 680:	394,04	8,81	1	11	11	18	0,963	17	32,2	1317	144,7	4,84							
	A3	Sequence	# PSMs	# Proteins	# Protein Groups	Protein Group Accessions	Modifications	ΔCn	114/115	114/115 Count	114/115 Variability [%]	q-Value	PEP	IonScore	Exp Value	Charge	MH+ [Da]	ΔM [ppm]	RT [min]	# Missed Cleavages
	High	tFHTGGVFTGEVAR	1	1	1	P73334	N-Term(iTRAQ4plex)	0,0000	0,845	1		0	2,41E-10	78	1,75792E-08	3	1622,84067	-0,07	27,18	0
	High	rLVDVSQDVIVR	3	1	1	P73334	N-Term(iTRAQ4plex)	0,0000	2,448	3	129,7	0	8,362E-11	71	8,53041E-08	3	1542,90836	-0,07	31,75	1
	High	vQNHSIVAELIDDRYR	2	1	1	P73334	N-Term(iTRAQ4plex)	0,0000	0,745	2	2,3	0	0,000006391	44	4,35482E-05	4	2072,10146	0,42	35,75	1
	High	yTPVLLGITk	2	1	1	P73334	N-Term(iTRAQ4plex); K10(iTRAQ4plex)	0,0000	0,926	2	28,1	0	0,00001113	43	5,40717E-05	2	1392,87139	0,64	40,86	0
	High	cSQLADELkELGFR	1	1	1	P73334	N-Term(iTRAQ4plex); C1(Carbamidomethyl); K9(iTRAQ4plex)	0,0000	0,983	1		0	2,017E-07	32	0,000677595	3	1954,03250	0,93	46,49	1
	High	kQGYEVTk	1	1	1	P73334	N-Term(iTRAQ4plex); K1(iTRAQ4plex); K8(iTRAQ4plex)	0,0000	1,048	1		0	0,0001722	29	0,001210515	3	1384,81556	-0,35	12,77	1
	High	gLkENVIIGR	3	1	1	P73334	N-Term(iTRAQ4plex); K3(iTRAQ4plex)	0,0000	1,165	2	27,6	0	0,0004716	28	0,001462076	3	1386,86606	-0,79	24,07	1
	High	kGLVDTALR	1	1	1	P73334	N-Term(iTRAQ4plex); K1(iTRAQ4plex)	0,0000	0,863	1		0	0,0005059	28	0,001545148	3	1260,78778	-0,04	21,68	1
	High	kLIALAYR	1	1	1	P73334	N-Term(iTRAQ4plex); K1(iTRAQ4plex)	0,0000	0,779	1		0	0,0009972	25	0,003213439	3	1235,80716	-0,54	28,74	1
	High	gLQAAQk	1	1	1	P73334	N-Term(iTRAQ4plex); K7(iTRAQ4plex)	0,0000	0,952	1		0,001	0,01654	34	0,000401229	2	1003,61766	3,77	12,20	0
	High	tTTGGmIR	2	1	1	P73334	N-Term(iTRAQ4plex); M6(Oxidation)	0,0000	1,106	2	20,8	0,002	0,02787	14	0,03638899	2	996,52709	0,60	11,23	0
P73817	Hemolysin OS=Synechocystis sp. (strain PCC 6803 / Kazusa) GN=sll1951 PE=4 SV=	390,97	3,73	1	6	6	12	0,968	12	136,2	1741	178,2	3,75							
	A3	Sequence	# PSMs	# Proteins	# Protein Groups	Protein Group Accessions	Modifications	ΔCn	114/115	114/115 Count	114/115 Variability [%]	q-Value	PEP	IonScore	Exp Value	Charge	MH+ [Da]	ΔM [ppm]	RT [min]	# Missed Cleavages
	High	aQAAQAAINLAQLR	2	1	1	P73817	N-Term(iTRAQ4plex)	0,0000	0,471	2	121,4	0	6,419E-12	102	6,17974E-11	2	1582,91496	0,21	37,84	0
	High	lEALTNk	2	1	1	P73817	N-Term(iTRAQ4plex); K7(iTRAQ4plex)	0,0000	1,862	2	0,2	0	0,0000725	51	1,13597E-05	2	1076,65801	2,41	24,30	0
	High	yPFFQAPQTADkR	4	1	1	P73817	N-Term(iTRAQ4plex); K12(iTRAQ4plex)	0,0000	1,695	4	17,9	0	6,218E-07	48	1,55955E-05	2	1856,99431	2,44	31,73	1
	High	nVAASGTk	2	1	1	P73817	N-Term(iTRAQ4plex); K8(iTRAQ4plex)	0,0000	0,225	2	18,8	0	0,005691	47	2,26148E-05	2	1035,59746	-6,03	11,30	0
	High	aLSPNVIAALQIMYTGR	1	1	1	P73817	N-Term(iTRAQ4plex)	0,0000	1,047	1		0	9,385E-07	37	0,000190546	3	1962,10508	4,44	55,58	0
	Medium	gFQLGk	1	1	1	P73817	N-Term(iTRAQ4plex); K6(iTRAQ4plex)	0,0000	0,315	1		0,014	0,1584	23	0,004863736	2	937,56981	-1,21	23,11	0
Q55118	Putative thylakoid lumen peptidyl-prolyl cis-trans isomerase sll0408 OS=Synechocys	389,03	15,57	1	8	8	18	1,397	18	17,3	379	41,4	4,84							
	A3	Sequence	# PSMs	# Proteins	# Protein Groups	Protein Group Accessions	Modifications	ΔCn	114/115	114/115 Count	114/115 Variability [%]	q-Value	PEP	IonScore	Exp Value	Charge	MH+ [Da]	ΔM [ppm]	RT [min]	# Missed Cleavages
	High	aIPLEILVk	2	1	1	Q55118	N-Term(iTRAQ4plex); K9(iTRAQ4plex)	0,0000	1,558	2	0,9	0	0,00000033	61	8,87095E-07	2	1283,85613	1,56	46,17	0
	High	rLQDSLEDISNHIR	2	1	1	Q55118	N-Term(iTRAQ4plex)	0,0000	1,269	2	19,1	0	1,226E-11	52	6,19398E-06	3	1839,97995	0,30	34,80	1
	High	aANLTITLk	1	1	1	Q55118	N-Term(iTRAQ4plex); K9(iTRAQ4plex)	0,0000	1,627	1		0	0,000005265	43	5,61009E-05	2	1232,78313	1,18	30,96	0
	High	kFYDGLPFIR	2	1	1	Q55118	N-Term(iTRAQ4plex); K1(iTRAQ4plex)	0,0000	1,601	2	2,1	0	0,0000059	38	0,000160694	3	1543,88907	0,99	38,33	1
	High	aANLTITLkEDk	2	1	1	Q55118	N-Term(iTRAQ4plex); K9(iTRAQ4plex); K12(iTRAQ4plex)	0,0000	1,546	2	4,7	0	2,114E-07	38	0,000165566	3	1749,04996	0,97	27,83	1
	High	fYDGLPFIR	3	1	1	Q55118	N-Term(iTRAQ4plex)	0,0000	1,262	3	4,2	0	0,001429	34	0,000410176	2	1271,69194	1,12	46,82	0
	High	dkEQVISFR	5	1	1	Q55118	N-Term(iTRAQ4plex); K2(iTRAQ4plex)	0,0000	1,267	5	5,5	0	0,0001342	32	0,000670177	3	1409,79984	0,50	22,94	1
	Medium	iVSAk	1	1	1	Q55118	N-Term(iTRAQ4plex); K5(iTRAQ4plex)	0,0000	1,280	1		0,022	0,2387	15	0,03040675	2	805,54149	3,62	15,08	0
P48939	30S ribosomal protein S4 OS=Synechocystis sp. (strain PCC 6803 / Kazusa) GN=rps	387,90	24,75	1	6	6	16	1,030	14	21,3	202	23,2	10,48							
	A3	Sequence	# PSMs	# Proteins	# Protein Groups	Protein Group Accessions	Modifications	ΔCn	114/115	114/115 Count	114/115 Variability [%]	q-Value	PEP	IonScore	Exp Value	Charge	MH+ [Da]	ΔM [ppm]	RT [min]	# Missed Cleavages
	High	lGmAGTIPGAR	3	1	1	P48939	N-Term(iTRAQ4plex); M3(Oxidation)	0,0000	1,183	2	1,5	0	0,00002374	78	1,72572E-08	2	1203,66423	0,46	23,91	0
	High	lGMAGTIPGAR	1	1	1	P48939	N-Term(iTRAQ4plex)	0,0000	0,902	1		0	0,000005081	48	1,94617E-05	2	1187,66875	-0,01	29,40	0
	High	rAYPPGQHGQAR	2	1	1	P48939	N-Term(iTRAQ4plex)	0,0000	0,703	2	24,9	0	5,394E-09	44	3,63053E-05	3	1481,78318	-0,73	7,86	1
	High	rATGSTGQALLELLEmR	4	1	1	P48939	N-Term(iTRAQ4plex); M16(Oxidation)	0,0000	0,858	4	9,8	0	3,504E-08	43	4,50786E-05	3	2006,08463	1,22	46,32	1
	High	aYPPGQHGQAR	1	1	1	P48939	N-Term(iTRAQ4plex)	0,0000	1,026	1		0	0,000004449	39	0,000127049	3	1325,68357	0,33	8,83	0
	High	rLGELPGLSR	3	1	1	P48939	N-Term(iTRAQ4plex)	0,0000	1,179	2	15,0	0	0,000005536	38	0,000148926	3	1241,74564	0,76	27,91	1
	High	lGELPGLSR	2	1	1	P48939	N-Term(iTRAQ4plex)	0,0000	1,193	2	21,4	0	0,002468	31	0,000887095	2	1085,64311	-0,42	35,03	0
Q55497	Ornithine carbamoyltransferase OS=Synechocystis sp. (strain PCC 6803 / Kazusa) G	383,04	8,77	1	4	4	12	0,822	12	30,7	308	33,6	5,64							
	A3	Sequence	# PSMs	# Proteins	# Protein Groups	Protein Group Accessions	Modifications	ΔCn	114/115	114/115 Count	114/115 Variability [%]	q-Value	PEP	IonScore	Exp Value	Charge	MH+ [Da]	ΔM [ppm]	RT [min]	# Missed Cleavages
	High	sLLQLAADLk	6	1	1	Q55497	N-Term(iTRAQ4plex); K10(iTRAQ4plex)	0,0000	0,976	6	24,3	0	6,481E-07	66	2,47155E-07	2	1359,84429	-0,54	49,13	0
	High	iLGLLFYk	2	1	1	Q55497	N-Term(iTRAQ4plex); K8(iTRAQ4plex)	0,0000	0,906	2	15,6	0	0,000001524	61	8,14648E-07	2	1254,80657	0,11	51,80	0
	High	yIDILAVR	3	1	1	Q55497	N-Term(iTRAQ4plex)	0,0000	0,739	3	2,6	0	0,0007134	45	3,07588E-05	2	1106,66911	0,06	42,96	0
	High	kILGLLFYk	1	1	1	Q55497	N-Term(iTRAQ4plex); K1(iTRAQ4plex); K9(iTRAQ4plex)	0,0000	0,667	1		0	4,925E-08	32	0,000659128	3	1527,00144	-1,32	42,74	1
	>sp|K22E_HUMAN|	381,82	13,02	1	7	8	13	1,600	11	13,3	645	65,8	8,00							
	A3	Sequence	# PSMs	# Proteins	# Protein Groups	Protein Group Accessions	Modifications	ΔCn	114/115	114/115 Count	114/115 Variability [%]	q-Value	PEP	IonScore	Exp Value	Charge	MH+ [Da]	ΔM [ppm]	RT [min]	# Missed Cleavages
	High	gFSSGSAVVSGGSR	3	1	1		N-Term(iTRAQ4plex)	0,0000	1,826	2	0,8	0	7,812E-12	84	3,81918E-09	2	1398,71086	1,02	20,16	0
	High	fLEQQNQVLQTk	1	2	2		N-Term(iTRAQ4plex); K12(iTRAQ4plex)	0,0000				0	2,846E-08	56	2,93561E-06	2	1763,99321	2,13	31,91	0
	High	sLVGLGGTk	2	1	1		N-Term(iTRAQ4plex); K9(iTRAQ4plex)	0,0000	1,627	2	8,3	0	0,00000769	47	1,98596E-05	2	1119,69902	1,25	25,37	0
	High	skEEAEALYHSk	2	1	1		N-Term(iTRAQ4plex); K2(iTRAQ4plex); K12(iTRAQ4plex)	0,0000	1,570	2	1,5	0	1,475E-10	37	0,000180289	3	1823,98917	1,53	21,22	1
	High	fASFIDkVR	1	1	1		N-Term(iTRAQ4plex); K7(iTRAQ4plex)	0,0000	1,600	1		0	0,00002226	34	0,000537145	3	1370,80039	-2,26	34,78	1
	High	skEEAEALYHSkYEELQVTVGR	1	1	1		N-Term(iTRAQ4plex); K2(iTRAQ4plex); K12(iTRAQ4plex)	0,0000	0,861	1		0	4,483E-08	33	0,000539135	5	2998,58900	1,45	35,86	2
	High	lQGEIAHVk	1	1	1		N-Term(iTRAQ4plex); K9(iTRAQ4plex)	0,0000	1,290	1		0	0,0000542	30	0,001111655	3	1282,77353	1,06	21,62	0
	High	kYEDEINkR	2	1	1		N-Term(iTRAQ4plex); K1(iTRAQ4plex); K8(iTRAQ4plex)	0,0000	1,797	2	4,0	0	0,00001782	22	0,006668068	3	1626,91971	1,32	14,72	2
P24602	Bacterioferritin OS=Synechocystis sp. (strain PCC 6803 / Kazusa) GN=bfr PE=1 SV=	377,69	37,82	1	6	6	29	1,166	23	16,2	156	18,3	4,82							
	A3	Sequence	# PSMs	# Proteins	# Protein Groups	Protein Group Accessions	Modifications	ΔCn	114/115	114/115 Count	114/115 Variability [%]	q-Value	PEP	IonScore	Exp Value	Charge	MH+ [Da]	ΔM [ppm]	RT [min]	# Missed Cleavages
	High	iGLQNYLQSQmS	1	1	1	P24602	N-Term(iTRAQ4plex); M11(Oxidation)	0,0000	1,060	1		0	1,505E-07	50	1,09388E-05	2	1541,77739	1,52	39,27	0
	High	gkPAVLAQLHk	10	1	1	P24602	N-Term(iTRAQ4plex); K2(iTRAQ4plex); K11(iTRAQ4plex)	0,0000	1,196	8	21,2	0	1,18E-08	46	2,34407E-05	4	1594,01699	0,31	24,21	0
	High	dQYFIHSR	12	1	1	P24602	N-Term(iTRAQ4plex)	0,0000	1,115	10	14,5	0	0,00001259	44	4,42558E-05	2	1209,61467	1,13	18,22	0
	High	iLADTEEDHAYWLEk	1	1	1	P24602	N-Term(iTRAQ4plex); K15(iTRAQ4plex)	0,0000	1,156	1		0	0,000005463	24	0,003556068	3	2121,07669	1,12	38,83	0
	High	qLGLIEk	3	1	1	P24602	N-Term(iTRAQ4plex); K7(iTRAQ4plex)	0,0000	1,421	2	3,1	0,001	0,01707	36	0,000415383	2	1088,69255	0,69	27,44	0
	High	gELAAR	2	1	1	P24602	N-Term(iTRAQ4plex)	0,0000	1,251	1		0,004	0,04865	12	0,088086634	2	760,44469	1,69	13,41	0
P74251	Sll1089 protein OS=Synechocystis sp. (strain PCC 6803 / Kazusa) GN=sll1089 PE=4	372,50	15,49	1	3	3	10	1,887	10	32,9	213	23,5	4,97							
	A3	Sequence	# PSMs	# Proteins	# Protein Groups	Protein Group Accessions	Modifications	ΔCn	114/115	114/115 Count	114/115 Variability [%]	q-Value	PEP	IonScore	Exp Value	Charge	MH+ [Da]	ΔM [ppm]	RT [min]	# Missed Cleavages
	High	iYAmGVIQPLTSTR	3	1	1	P74251	N-Term(iTRAQ4plex); M4(Oxidation)	0,0000	2,315	3	5,0	0	6,851E-12	90	1,02558E-09	2	1709,93987	1,26	37,48	0
	High	iYAMGVIQPLTSTR	4	1	1	P74251	N-Term(iTRAQ4plex)	0,0000	1,887	4	27,8	0	4,211E-10	74	4,42558E-08	2	1693,94426	0,86	43,94	0
	High	aYLVGNk	2	1	1	P74251	N-Term(iTRAQ4plex); K7(iTRAQ4plex)	0,0000	1,201	2	46,8	0	0,0001988	44	3,68952E-05	2	1052,63542	1,10	19,97	0
	High	fQRPPSGVPPIR	1	1	1	P74251	N-Term(iTRAQ4plex)	0,0000	2,292	1		0	0,00003135	31	0,000851079	3	1494,86698	0,51	26,21	0
P72839	Slr1301 protein OS=Synechocystis sp. (strain PCC 6803 / Kazusa) GN=slr1301 PE=:	370,32	15,51	1	9	9	17	1,028	15	10,9	535	61,7	4,84							
	A3	Sequence	# PSMs	# Proteins	# Protein Groups	Protein Group Accessions	Modifications	ΔCn	114/115	114/115 Count	114/115 Variability [%]	q-Value	PEP	IonScore	Exp Value	Charge	MH+ [Da]	ΔM [ppm]	RT [min]	# Missed Cleavages
	High	qTAALEAQGR	1	1	1	P72839	N-Term(iTRAQ4plex)	0,0000	1,040	1		0	6,955E-08	66	2,34407E-07	2	1188,64690	1,29	13,81	0
	High	eVLQGILHR	4	1	1	P72839	N-Term(iTRAQ4plex)	0,0000	0,869	2	2,1	0	1,249E-07	54	3,9443E-06	3	1208,72339	0,15	30,41	0
	High	nkLEQNLQSTR	3	1	1	P72839	N-Term(iTRAQ4plex); K2(iTRAQ4plex)	0,0000	0,990	3	2,0	0	4,592E-07	42	6,59128E-05	3	1618,91324	1,06	17,68	1
	High	nFLGGYk	1	1	1	P72839	N-Term(iTRAQ4plex); K7(iTRAQ4plex)	0,0000	1,165	1		0	0,0006609	37	0,000184064	2	1086,62163	2,77	27,72	0
	High	nQALIQQk	1	1	1	P72839	N-Term(iTRAQ4plex); K8(iTRAQ4plex)	0,0000	1,106	1		0	0,0001101	34	0,00042655	2	1230,74162	0,62	16,01	0
	High	aNQLAQVEQQR	1	1	1	P72839	N-Term(iTRAQ4plex)	0,0000	0,945	1		0	2,036E-07	32	0,000671382	2	1428,76958	1,38	21,90	0
	High	kTNFEQNR	1	1	1	P72839	N-Term(iTRAQ4plex); K1(iTRAQ4plex)	0,0000	1,521	1		0	0,00002025	30	0,000901509	3	1324,71997	-0,92	9,95	1
	High	lkEQQSEIER	3	1	1	P72839	N-Term(iTRAQ4plex); K2(iTRAQ4plex)	0,0000	1,100	3	4,0	0	5,912E-07	26	0,003502142	3	1547,86667	2,25	14,77	1
	High	qEVLLQHLk	2	1	1	P72839	N-Term(iTRAQ4plex); K9(iTRAQ4plex)	0,0000	0,952	2	9,6	0	0,0001508	21	0,007673085	3	1395,85535	-0,64	27,08	0
P72700	Slr0244 protein OS=Synechocystis sp. (strain PCC 6803 / Kazusa) GN=slr0244 PE=:	369,70	33,45	1	10	10	18	0,947	18	14,7	284	31,2	5,25							
	A3	Sequence	# PSMs	# Proteins	# Protein Groups	Protein Group Accessions	Modifications	ΔCn	114/115	114/115 Count	114/115 Variability [%]	q-Value	PEP	IonScore	Exp Value	Charge	MH+ [Da]	ΔM [ppm]	RT [min]	# Missed Cleavages
	High	vNAPcPVLLTR	2	1	1	P72700	N-Term(iTRAQ4plex); C5(Carbamidomethyl)	0,0000	0,948	2	3,2	0	0,000003827	67	2,06049E-07	2	1383,79070	0,56	33,20	0
	High	aSITILHVVPPQITTEAFTEk	3	1	1	P72700	N-Term(iTRAQ4plex); K21(iTRAQ4plex)	0,0000	0,865	3	32,3	0	1,105E-09	56	2,41529E-06	3	2583,46671	1,17	48,26	0
	High	lLGTSLSDYIR	2	1	1	P72700	N-Term(iTRAQ4plex)	0,0000	0,952	2	13,9	0	1,905E-07	54	4,44601E-06	2	1381,78105	0,19	45,89	0
	High	vNPDLkPDLLPLSR	4	1	1	P72700	N-Term(iTRAQ4plex); K6(iTRAQ4plex)	0,0000	0,855	4	15,5	0	2,061E-07	29	0,001121941	3	1865,11063	0,38	38,57	0
	High	cTVTGGkPGEk	2	1	1	P72700	N-Term(iTRAQ4plex); C1(Carbamidomethyl); K7(iTRAQ4plex); K11(iTRAQ4pl	0,0000	1,264	2	7,7	0	0,000972	24	0,0041973	3	1565,87015	1,26	11,53	0
	High	rVmVALDk	1	1	1	P72700	N-Term(iTRAQ4plex); M3(Oxidation); K8(iTRAQ4plex)	0,0000	0,857	1		0	0,002013	20	0,011116549	3	1235,73886	0,33	18,55	1
	High	rLGIAYR	1	1	1	P72700	N-Term(iTRAQ4plex)	0,0000	0,867	1		0,002	0,03217	25	0,003388208	3	992,61206	-0,13	18,28	1
	High	rPSIAk	1	1	1	P72700	N-Term(iTRAQ4plex); K6(iTRAQ4plex)	0,0000	1,327	1		0,008	0,09667	20	0,010255811	3	959,62140	-2,76	10,60	0
	Medium	vSTVLR	1	1	1	P72700	N-Term(iTRAQ4plex)	0,0000	0,965	1		0,025	0,2737	25	0,0035311	2	818,52623	5,60	18,94	0

	Medium	lGIAYR	1	1	1	P72700	N-Term(iTRAQ4plex)	0,0000	1,092	1		0,044	0,4622	20	0,014234223	2	836,51622	6,15	24,36	0
P12975	Photosystem I reaction center subunit IV OS=Synechocystis sp. (strain PCC 6803 /	368,30	17,57	1	2	2	26	0,917	25	15,8	74	8,1	8,50							
	A3	Sequence	# PSMs	# Proteins	# Protein Groups	Protein Group Accessions	Modifications	ΔCn	114/115	114/115 Count	114/115 Variability [%]	q-Value	PEP	IonScore	Exp Value	Charge	MH+ [Da]	ΔM [ppm]	RT [min]	# Missed Cleavages
	High	sGILYPVIVR	24	1	1	P12975	N-Term(iTRAQ4plex)	0,0000	0,928	23	13,9	0	0,0001054	52	6,96578E-06	2	1260,78032	0,52	38,90	0
	High	sGILYPVIVRFDR	2	1	1	P12975	N-Term(iTRAQ4plex)	0,0000	0,652	2	14,3	0	0,001612	16	0,025643069	3	1678,97458	-0,94	44,71	1
P17253	ATP synthase gamma chain OS=Synechocystis sp. (strain PCC 6803 / Kazusa) GN=a	364,78	23,89	1	9	9	17	0,964	13	12,3	314	34,6	6,89							
	A3	Sequence	# PSMs	# Proteins	# Protein Groups	Protein Group Accessions	Modifications	ΔCn	114/115	114/115 Count	114/115 Variability [%]	q-Value	PEP	IonScore	Exp Value	Charge	MH+ [Da]	ΔM [ppm]	RT [min]	# Missed Cleavages
	High	aLQESAASELAAR	1	1	1	P17253	N-Term(iTRAQ4plex)	0,0000	0,888	1		0	4,252E-08	72	5,79389E-08	2	1460,78447	1,28	27,55	0
	High	gLcGGYNVNAIk	3	1	1	P17253	N-Term(iTRAQ4plex); C3(Carbamidomethyl); K12(iTRAQ4plex)	0,0000	1,132	2	9,4	0	7,304E-10	68	1,66713E-07	2	1553,83623	0,88	29,07	0
	High	aVALLVVTGDR	2	1	1	P17253	N-Term(iTRAQ4plex)	0,0000	0,667	2	13,5	0	0,000003011	49	1,34267E-05	2	1257,76641	1,30	38,37	0
	High	lVLVGSk	2	1	1	P17253	N-Term(iTRAQ4plex); K7(iTRAQ4plex)	0,0000	0,957	1		0	0,001155	41	8,29793E-05	2	1003,67540	-0,02	27,52	0
	High	vELIYTR	1	1	1	P17253	N-Term(iTRAQ4plex)	0,0000	0,946	1		0	0,001886	36	0,00027477	2	1037,60893	-2,17	29,50	0
	High	nQGIAVk	1	1	1	P17253	N-Term(iTRAQ4plex); K7(iTRAQ4plex)	0,0000	1,277	1		0	0,0007356	30	0,000931044	2	1017,63579	6,15	14,30	0
	High	lVAAAk	4	1	1	P17253	N-Term(iTRAQ4plex); K6(iTRAQ4plex)	0,0000	0,962	2	1,6	0,002	0,03137	21	0,0080904	2	860,58202	1,43	16,96	0
	Medium	iQSVk	2	1	1	P17253	N-Term(iTRAQ4plex); K5(iTRAQ4plex)	0,0000	1,011	2	7,0	0,025	0,2626	17	0,021133431	2	862,56127	1,42	13,69	0
	Medium	kITEAmR	1	1	1	P17253	N-Term(iTRAQ4plex); K1(iTRAQ4plex); M6(Oxidation)	0,0000	1,042	1		0,046	0,4792	13	0,054572017	3	1152,66318	-1,53	9,28	1
P73853	IMP dehydrogenase subunit OS=Synechocystis sp. (strain PCC 6803 / Kazusa) GN=	364,31	21,71	1	7	7	13	0,785	10	11,2	387	40,2	5,34							
	A3	Sequence	# PSMs	# Proteins	# Protein Groups	Protein Group Accessions	Modifications	ΔCn	114/115	114/115 Count	114/115 Variability [%]	q-Value	PEP	IonScore	Exp Value	Charge	MH+ [Da]	ΔM [ppm]	RT [min]	# Missed Cleavages
	High	iNVGTTGTIR	2	1	1	P73853	N-Term(iTRAQ4plex)	0,0000	0,626	2	6,2	0	0,000000215	63	6,03105E-07	2	1175,68694	0,38	24,84	0
	High	gFHWGmATPSPVLPR	1	1	1	P73853	N-Term(iTRAQ4plex); M6(Oxidation)	0,0000	0,776	1		0	6,541E-12	57	2,14768E-06	3	1812,93308	-0,32	34,96	0
	High	eILVGPAk	2	1	1	P73853	N-Term(iTRAQ4plex); K8(iTRAQ4plex)	0,0000	0,830	1		0	0,00001679	46	2,63615E-05	2	1114,70683	-0,56	24,18	0
	High	rAYGIDEIALVPGVR	2	1	1	P73853	N-Term(iTRAQ4plex)	0,0000	0,698	2	3,1	0	1,376E-07	43	4,85255E-05	3	1773,01480	0,45	39,51	1
	High	tSmGTLGAk	3	1	1	P73853	N-Term(iTRAQ4plex); M3(Oxidation); K9(iTRAQ4plex)	0,0000	0,800	2	1,2	0	0,0008861	43	5,5077E-05	2	1169,64458	0,60	12,72	0
	High	aQQLGmGk	2	1	1	P73853	N-Term(iTRAQ4plex); M6(Oxidation); K8(iTRAQ4plex)	0,0000	0,863	1		0	0,002559	36	0,000306473	2	1136,63689	2,86	13,69	0
	High	aGAAAVLVGIGPGAAcTSR	1	1	1	P73853	N-Term(iTRAQ4plex); C16(Carbamidomethyl)	0,0000	0,798	1		0	0,000001257	34	0,000357273	3	1842,99808	0,19	36,42	0
P73546	Phosphorylase OS=Synechocystis sp. (strain PCC 6803 / Kazusa) GN=glgP PE=3 SV	364,24	10,18	1	9	9	17	0,620	15	28,6	855	98,2	6,14							
	A3	Sequence	# PSMs	# Proteins	# Protein Groups	Protein Group Accessions	Modifications	ΔCn	114/115	114/115 Count	114/115 Variability [%]	q-Value	PEP	IonScore	Exp Value	Charge	MH+ [Da]	ΔM [ppm]	RT [min]	# Missed Cleavages
	High	hLEIIYEINHR	2	1	1	P73546	N-Term(iTRAQ4plex)	0,0000	0,378	2	25,7	0	4,676E-11	65	2,8312E-07	3	1580,86686	0,18	29,72	0
	High	hLGNNLINLGLYDk	1	1	1	P73546	N-Term(iTRAQ4plex); K14(iTRAQ4plex)	0,0000	0,673	1		0	2,645E-10	56	2,45471E-06	3	1872,05692	-0,68	39,16	0
	High	qHLAALEIIHLYNR	1	1	1	P73546	N-Term(iTRAQ4plex)	0,0000	0,453	1		0	3,163E-10	54	4,43802E-06	4	1835,04079	-0,04	37,83	0
	High	nLAAYLLk	4	1	1	P73546	N-Term(iTRAQ4plex); K8(iTRAQ4plex)	0,0000	0,667	3	16,1	0	0,000009255	48	1,60313E-05	2	1193,74932	-0,27	41,73	0
	High	tFIFGGk	1	1	1	P73546	N-Term(iTRAQ4plex); K7(iTRAQ4plex)	0,0000	1,367	1		0	0,0009467	45	3,30347E-05	2	1057,62676	-1,62	32,21	0
	High	yEFGIFHQR	3	1	1	P73546	N-Term(iTRAQ4plex)	0,0000	0,586	2	8,5	0	0,00004595	44	3,96251E-05	2	1340,68401	-2,09	33,56	0
	High	qNPHAEIVPR	1	1	1	P73546	N-Term(iTRAQ4plex)	0,0000	0,713	1		0	0,000004577	35	0,000297831	3	1304,71799	-0,92	14,14	0
	High	lGGHTEIIHNEk	2	1	1	P73546	N-Term(iTRAQ4plex); K12(iTRAQ4plex)	0,0000	0,472	2	12,5	0	0,000005566	29	0,001667943	4	1635,90468	-0,63	17,69	0
	High	ikQNPHAEIVPR	2	1	1	P73546	N-Term(iTRAQ4plex); K2(iTRAQ4plex)	0,0000	0,757	2	21,9	0	0,001865	12	0,063236817	4	1689,99770	-1,54	17,77	1
P21697	Plastocyanin OS=Synechocystis sp. (strain PCC 6803 / Kazusa) GN=petE PE=1 SV=	357,48	30,16	1	4	4	14	1,287	12	9,9	126	13,1	6,77							
	A3	Sequence	# PSMs	# Proteins	# Protein Groups	Protein Group Accessions	Modifications	ΔCn	114/115	114/115 Count	114/115 Variability [%]	q-Value	PEP	IonScore	Exp Value	Charge	MH+ [Da]	ΔM [ppm]	RT [min]	# Missed Cleavages
	High	lSPHNIVFAADGVDADTAAk	3	1	1	P21697	N-Term(iTRAQ4plex); K20(iTRAQ4plex)	0,0000	1,209	3	12,6	0	2,077E-17	123	4,97703E-13	3	2300,21573	1,37	37,01	0
	High	gAGmVGk	7	1	1	P21697	N-Term(iTRAQ4plex); M4(Oxidation); K7(iTRAQ4plex)	0,0000	1,358	5	2,5	0	0,005286	20	0,011091748	2	923,52184	-0,50	9,90	0
	High	aGEEVk	1	1	1	P21697	N-Term(iTRAQ4plex); K6(iTRAQ4plex)	0,0000	1,246	1		0,001	0,01168	33	0,00049885	2	920,52910	-0,05	12,17	0
	High	gAGMVGk	2	1	1	P21697	N-Term(iTRAQ4plex); K7(iTRAQ4plex)	0,0000	1,176	2	7,4	0,002	0,02631	32	0,000629463	2	907,53234	5,45	14,30	0
	Medium	wVNNk	1	1	1	P21697	N-Term(iTRAQ4plex); K5(iTRAQ4plex)	0,0000	1,456	1		0,023	0,2483	27	0,001887861	2	948,55058	0,05	17,35	0
P72866	30S ribosomal protein S15 OS=Synechocystis sp. (strain PCC 6803 / Kazusa) GN=r	354,97	26,97	1	4	4	17	1,812	13	14,5	89	10,4	10,90							
	A3	Sequence	# PSMs	# Proteins	# Protein Groups	Protein Group Accessions	Modifications	ΔCn	114/115	114/115 Count	114/115 Variability [%]	q-Value	PEP	IonScore	Exp Value	Charge	MH+ [Da]	ΔM [ppm]	RT [min]	# Missed Cleavages
	High	iTQLTGHLk	6	1	1	P72866	N-Term(iTRAQ4plex); K9(iTRAQ4plex)	0,0000	1,794	4	7,4	0	1,175E-07	67	2,22316E-07	2	1298,80583	1,82	25,34	0
	High	rLLSFINAR	2	1	1	P72866	N-Term(iTRAQ4plex)	0,0000	1,404	1		0	1,016E-07	54	3,78416E-06	3	1233,75556	0,57	32,57	1
	High	lLSFINAR	5	1	1	P72866	N-Term(iTRAQ4plex)	0,0000	1,812	5	14,5	0	0,00002113	49	1,37625E-05	2	1077,65459	0,79	42,13	0
	High	yQALIk	4	1	1	P72866	N-Term(iTRAQ4plex); K6(iTRAQ4plex)	0,0000	2,033	3	4,7	0	0,00806	34	0,000414925	2	1023,64458	0,47	26,25	0
P73321	Uncharacterized protein slr1894 OS=Synechocystis sp. (strain PCC 6803 / Kazusa) (	353,98	46,79	1	8	8	16	0,898	13	8,8	156	17,7	5,57							
	A3	Sequence	# PSMs	# Proteins	# Protein Groups	Protein Group Accessions	Modifications	ΔCn	114/115	114/115 Count	114/115 Variability [%]	q-Value	PEP	IonScore	Exp Value	Charge	MH+ [Da]	ΔM [ppm]	RT [min]	# Missed Cleavages
	High	sLDVFAPGTYk	2	1	1	P73321	N-Term(iTRAQ4plex); K11(iTRAQ4plex)	0,0000	0,898	1		0	1,366E-07	59	1,23302E-06	2	1485,81975	0,38	37,59	0
	High	aTINIGIPEADR	2	1	1	P73321	N-Term(iTRAQ4plex)	0,0000	0,962	2	1,1	0	0,00006557	53	4,47682E-06	2	1413,78203	0,11	34,26	0
	High	lLADTYTLYLQTHNFHWNVTGPQFR	1	1	1	P73321	N-Term(iTRAQ4plex)	0,0000	1,511	1		0	3,667E-12	48	1,64048E-05	4	3179,62246	1,28	52,56	0
	High	iAESLkk	3	1	1	P73321	N-Term(iTRAQ4plex); K6(iTRAQ4plex); K7(iTRAQ4plex)	0,0000	0,876	2	2,7	0	0,001773	48	1,7457E-05	2	1220,79424	0,31	17,78	1
	High	aTINIGIPEADRIk	3	1	1	P73321	N-Term(iTRAQ4plex); K14(iTRAQ4plex)	0,0000	0,854	2	1,3	0	1,923E-07	40	0,000101384	3	1799,06412	0,64	36,03	1
	High	gHETIVQScR	2	1	1	P73321	N-Term(iTRAQ4plex); C9(Carbamidomethyl)	0,0000	1,207	2	53,8	0	0,000733	30	0,001104002	2	1330,66643	0,74	11,08	0
	High	tAWMLR	1	1	1	P73321	N-Term(iTRAQ4plex)	0,0000	0,737	1		0,001	0,01432	33	0,000484139	2	921,50847	-1,35	28,93	0
	Medium	iAESLk	1	1	1	P73321	N-Term(iTRAQ4plex); K6(iTRAQ4plex)	0,0000	1,089	1		0,023	0,2451	26	0,002624037	2	948,60057	3,95	20,96	0
	Medium	tAWmLR	1	1	1	P73321	N-Term(iTRAQ4plex); M4(Oxidation)	0,0000	0,928	1		0,026	0,2796	14	0,044052444	2	937,50389	-0,79	24,99	0
P54416	ATP-dependent Clp protease proteolytic subunit 1 OS=Synechocystis sp. (strain PC(	352,27	25,76	1	4	4	13	0,821	10	11,1	198	21,7	5,03							
	A3	Sequence	# PSMs	# Proteins	# Protein Groups	Protein Group Accessions	Modifications	ΔCn	114/115	114/115 Count	114/115 Variability [%]	q-Value	PEP	IonScore	Exp Value	Charge	MH+ [Da]	ΔM [ppm]	RT [min]	# Missed Cleavages
	High	imIHQPLGGAQGQATDIEIQAk	2	1	1	P54416	N-Term(iTRAQ4plex); M2(Oxidation); K22(iTRAQ4plex)	0,0000	0,860	2	9,0	0	3,74872E-20	96	2,38215E-10	3	2623,41373	0,77	35,72	0
	High	iVFLGQEVR	3	1	1	P54416	N-Term(iTRAQ4plex)	0,0000	0,879	3	7,8	0	0,00006385	50	1,15393E-05	2	1204,71782	0,62	38,72	0
	High	aLLNQHLANHTGk	7	1	1	P54416	N-Term(iTRAQ4plex); K13(iTRAQ4plex)	0,0000	0,743	4	14,8	0	1,34E-08	46	2,46144E-05	3	1704,97483	0,02	18,71	0
	High	aFDIYSR	1	1	1	P54416	N-Term(iTRAQ4plex)	0,0000	0,757	1		0	0,0004032	42	6,90192E-05	2	1015,53472	1,75	27,75	0
Q55087	Geranylgeranyl diphosphate reductase OS=Synechocystis sp. (strain PCC 6803 / Ka	350,87	21,87	1	9	9	24	0,838	21	16,2	407	44,9	8,00							
	A3	Sequence	# PSMs	# Proteins	# Protein Groups	Protein Group Accessions	Modifications	ΔCn	114/115	114/115 Count	114/115 Variability [%]	q-Value	PEP	IonScore	Exp Value	Charge	MH+ [Da]	ΔM [ppm]	RT [min]	# Missed Cleavages
	High	aGIETYLFER	2	1	1	Q55087	N-Term(iTRAQ4plex)	0,0000	0,896	2	6,3	0	5,269E-07	48	1,53098E-05	2	1342,71330	0,69	41,45	0
	High	tIGSLLR	3	1	1	Q55087	N-Term(iTRAQ4plex)	0,0000	0,814	3	3,7	0	0,003288	43	5,55866E-05	2	903,57280	-1,78	31,45	0
	High	kLEGGEIIk	3	1	1	Q55087	N-Term(iTRAQ4plex); K1(iTRAQ4plex); K9(iTRAQ4plex)	0,0000	0,986	3	4,1	0	0,00001692	41	7,7085E-05	3	1418,89276	-1,10	21,14	1
	High	rEVLDGFLR	2	1	1	Q55087	N-Term(iTRAQ4plex)	0,0000	0,764	1		0	0,00000849	39	0,000137711	3	1248,71738	-0,61	32,33	1
	High	yGATYLVLDILQR	1	1	1	Q55087	N-Term(iTRAQ4plex)	0,0000	0,895	1		0	0,0009334	36	0,000253495	2	1668,94707	1,74	56,41	0
	High	ikDLQAGIR	6	1	1	Q55087	N-Term(iTRAQ4plex); K2(iTRAQ4plex)	0,0000	0,815	4	4,9	0	0,002197	34	0,000369803	3	1301,81412	-0,20	23,35	1
	High	yDHVAVGTGTmk	1	1	1	Q55087	N-Term(iTRAQ4plex); M11(Oxidation); K12(iTRAQ4plex)	0,0000	1,106	1		0	9,592E-10	31	0,000748118	3	1582,81363	-0,10	18,45	0
	High	vEAHPIPEHPRPR	3	1	1	Q55087	N-Term(iTRAQ4plex)	0,0000	0,897	3	44,9	0	0,00001312	28	0,001823518	3	1678,92423	-0,97	13,69	0
	High	iRLPEDk	3	1	1	Q55087	N-Term(iTRAQ4plex); K7(iTRAQ4plex)	0,0000	0,739	3	15,4	0,001	0,01147	17	0,019062914	3	1158,70685	-1,44	21,53	1
Q55385	Probable 30S ribosomal protein PSRP-3 OS=Synechocystis sp. (strain PCC 6803 / Ka	348,80	56,25	1	5	5	11	1,812	11	4,2	112	12,6	5,12							
	A3	Sequence	# PSMs	# Proteins	# Protein Groups	Protein Group Accessions	Modifications	ΔCn	114/115	114/115 Count	114/115 Variability [%]	q-Value	PEP	IonScore	Exp Value	Charge	MH+ [Da]	ΔM [ppm]	RT [min]	# Missed Cleavages
	High	gTSPLTSYFFWPR	4	1	1	Q55385	N-Term(iTRAQ4plex)	0,0000	1,794	4	4,5	0	5,729E-10	79	1,36449E-08	2	1702,87261	0,94	52,09	0
	High	qISmAEAQGk	1	1	1	Q55385	N-Term(iTRAQ4plex); M4(Oxidation); K10(iTRAQ4plex)	0,0000	1,829	1		0	0,00001194	58	1,48583E-06	2	1366,73381	7,24	13,35	0
	High	iNVLNQATEVINFWQDLk	1	1	1	Q55385	N-Term(iTRAQ4plex); K18(iTRAQ4plex)	0,0000	1,812	1		0	4,209E-09	53	4,86374E-06	3	2433,34238	1,76	62,16	0
	High	aDAWQQLkDELEAk	3	1	1	Q55385	N-Term(iTRAQ4plex); K8(iTRAQ4plex); K14(iTRAQ4plex)	0,0000	1,818	3	0,8	0	2,394E-08	36	0,000224373	3	2077,13285	1,83	45,23	1
	High	hWIAEADR	2	1	1	Q55385	N-Term(iTRAQ4plex)	0,0000	1,312	2	4,1	0	0,0007775	21	0,008769403	3	1141,58677	-0,31	17,58	0
P73298	30S ribosomal protein S11 OS=Synechocystis sp. (strain PCC 6803 / Kazusa) GN=r	343,05	32,31	1	3	3	8	1,072	8	9,5	130	13,8	11,36							
	A3	Sequence	# PSMs	# Proteins	# Protein Groups	Protein Group Accessions	Modifications	ΔCn	114/115	114/115 Count	114/115 Variability [%]	q-Value	PEP	IonScore	Exp Value	Charge	MH+ [Da]	ΔM [ppm]	RT [min]	# Missed Cleavages
	High	aLQGAGLEITLIR	4	1	1	P73298	N-Term(iTRAQ4plex)	0,0000	0,974	4	3,5	0	6,094E-09	80	1,00224E-08	2	1498,90788	0,31	44,17	0
	High	kGTPYAAQTAADSAAR	3	1	1	P73298	N-Term(iTRAQ4plex); K1(iTRAQ4plex)	0,0000	1,094	3	3,5	0	4,049E-08	75	3,10456E-08	3	1866,99973	4,54	16,23	1
	High	qLEVmVSGPGAGR	1	1	1	P73298	N-Term(iTRAQ4plex); M5(Oxidation)	0,0000	1,075	1		0	2,71E-09	49	1,4378E-05	2	1460,76592	0,72	25,73	0
P36237	50S ribosomal protein L11 OS=Synechocystis sp. (strain PCC 6803 / Kazusa) GN=r	341,91	45,39	1	7	7	16	1,875	14	10,3	141	15,0	9,45							
	A3	Sequence	# PSMs	# Proteins	# Protein Groups	Protein Group Accessions	Modifications	ΔCn	114/115	114/115 Count	114/115 Variability [%]	q-Value	PEP	IonScore	Exp Value	Charge	MH+ [Da]	ΔM [ppm]	RT [min]	# Missed Cleavages
	High	sFTFILk	4	1	1	P36237	N-Term(iTRAQ4plex); K7(iTRAQ4plex)	0,0000	1,992	4	1,1	0	0,0001125	56	2,47725E-06	2	1143,70293	1,14	41,51	0
	High	tADkPGmIIPVEISVFEDR	1	1	1	P36237	N-Term(iTRAQ4plex); K4(iTRAQ4plex); M7(Oxidation)	0,0000	2,182	1		0	2,272E-09	43	4,48714E-05	3	2421,29844	1,88	45,24	0
	High	tPPASVLIR	3	1	1	P36237	N-Term(iTRAQ4plex)	0,0000	1,786	2	1,5	0	0,00128	43	4,97703E-05	2	1097,67986	-0,09	27,52	0
	High	kAAGVEk	2	1	1	P36237	N-Term(iTRAQ4plex); K1(iTRAQ4plex); K7(iTRAQ4plex)	0,0000	2,204	2	7,6	0	0,0008549	30	0,001107625	3	1134,72077	0,06	8,69	1
	High	gSSEPNkNk	1	1	1	P36237	N-Term(iTRAQ4plex); K7(iTRAQ4plex); K9(iTRAQ4plex)	0,0000	0,977	1		0	0,001582	15	0,034353422	3	1392,78019	-0,39	7,88	1
	High	lALPAGk	4	1	1	P36237	N-Term(iTRAQ4plex); K7(iTRAQ4plex)	0,0000	1,748	3	7,2	0,001	0,0165	37	0,000199054	2	957,63481	1,31	25,60	0
	High	eIAQTk	1	1	1	P36237	N-Term(iTRAQ4plex); K6(iTRAQ4plex)	0,0000	1,227	1		0,006	0,07738	23	0,00955592	2	977,59032	3,41	13,39	0
P73048	Sll1638 protein OS=Synechocystis sp. (strain PCC 6803 / Kazusa) GN=sll1638 PE=1	340,43	41,61	1	5	5	11	0,919	11	14,6	149	16,5	7,34							
	A3	Sequence	# PSMs	# Proteins	# Protein Groups	Protein Group Accessions	Modifications	ΔCn	114/115	114/115 Count	114/115 Variability [%]	q-Value	PEP	IonScore	Exp Value	Charge	MH+ [Da]	ΔM [ppm]	RT [min]	# Missed Cleavages
	High	iAQLQVYVNPIAVAR	4	1	1	P73048	N-Term(iTRAQ4plex)	0,0000	0,943	4	12,5	0	8,848E-12	77	1,89221E-08	2	1799,06755	0,84	46,13	0
	High	rLQGLIADQNWVDTQTYIHGPLGQLR	1	1	1	P73048	N-Term(iTRAQ4plex)	0,0000	0,955	1		0	3,462E-08	50	1,01384E-05	4	3136,69375	5,23	46,94	1
	High	rDmLGLASSLLPk	1	1	1	P73048	N-Term(iTRAQ4plex); M3(Oxidation); K13(iTRAQ4plex)	0,0000	1,014	1		0	0,00000229	37	0,000180717	3	1704,99222	0,06	37,30	1
	High	eVFGHLER	4	1	1	P73048	N-Term(iTRAQ4plex)	0,0000	0,836	4	5,8	0	0,0006254	37	0,000199512	2	1130,60661	-0,80	20,74	0
	High	dMLGLASSLLPk	1	1	1	P73048	N-Term(iTRAQ4plex); K12(iTRAQ4plex)	0,0000	1,066	1		0	0,005133	28	0,001733684	3	1532,89542	-0,44	51,51	0
P29107	Ketol-acid reductoisomerase OS=Synechocystis sp. (strain PCC 6803 / Kazusa) GN=	332,35	30,82	1	7	7	13	1,101	13	19,4	331	35,8	5,01							
	A3	Sequence	# PSMs	# Proteins	# Protein Groups	Protein Group Accessions	Modifications	ΔCn	114/115	114/115 Count	114/115 Variability [%]	q-Value	PEP	IonScore	Exp Value	Charge	MH+ [Da]	ΔM [ppm]	RT [min]	# Missed Cleavages
	High	rTYEQGQGVPALFAVYQDASGQAR	1	1	1	P29107	N-Term(iTRAQ4plex)	0,0000	1,028	1		0	1,174E-14	91	7,79776E-10	3	2756,39060	1,18	44,11	1
	High	aMFSWLk	3	1	1	P29107	N-Term(iTRAQ4plex); K7(iTRAQ4plex)	0,0000	1,080	3	0,1	0	0,0004844	56	2,64223E-06	2	1170,65874	0,29	43,09	0
	High	eFVLENQAGkPGFTAmR	4	1	1	P29107	N-Term(iTRAQ4plex); K10(iTRAQ4plex); M16(Oxidation)	0,0000	1,223	4	7,9	0	8,138E-08	43	5,30848E-05	3	2199,14884	0,77	31,96	0
	High	aGILETTFR	1	1	1	P29107	N-Term(iTRAQ4plex)	0,0000	0,880	1		0	0,00004033	26	0,002243727	2	1151,65447	0,29	33,17	0
	High	iVTEETkAEmR	1	1	1	P29107	N-Term(iTRAQ4plex); K7(iTRAQ4plex); M10(Oxidation)	0,0000	1,498	1		0	0,0004299	22	0,006667607	3	1610,86686	0,38	16,92	1
	High	tVAIIGYGSQGHAHALNLk	1	1	1	P29107	N-Term(iTRAQ4plex); K19(iTRAQ4plex)	0,0000	0,842	1		0	4,891E-07	22	0,006682978	4	2238,25918	-0,26	31,84	0
	High	rESEELIEEVGkDLR	1	1	1	P29107	N-Term(iTRAQ4plex); K12(iTRAQ4plex)	0,0000	1,338	1		0	0,00001419	19	0,014544468	4	2090,13711	1,84	35,58	2
	Medium	amFSWLk	1	1	1	P29107	N-Term(iTRAQ4plex); M2(Oxidation); K7(iTRAQ4plex)	0,0000	1,255	1		0,023	0,2451	27	0,002118215	2	1186,65410	0,66	35,59	0
P74002	Uncharacterized protein slr1322 OS=Synechocystis sp. (strain PCC 6803 / Kazusa) (	332,13	15,75	1	7	7	13	1,419	13	19,9	489	53,1	5,64							
	A3	Sequence	# PSMs	# Proteins	# Protein Groups	Protein Group Accessions	Modifications	ΔCn	114/115	114/115 Count	114/115 Variability [%]	q-Value	PEP	IonScore	Exp Value	Charge	MH+ [Da]	ΔM [ppm]	RT [min]	# Missed Cleavages
	High	qSYAYAAASR	2	1	1	P74002	N-Term(iTRAQ4plex)	0,0000	1,585	2	3,1	0	0,000001151	62	6,83864E-07	2	1231,61919	0,33	17,36	0
	High	eSLSTLLGLGR	3	1	1	P74002	N-Term(iTRAQ4plex)	0,0000	1,254	3	11,8	0	0,00001269	56	3,61688E-06	2	1289,75517	0,46	46,88	0
	High	iTQSVGFSLFcADGEHR	1	1	1	P74002	N-Term(iTRAQ4plex); C11(Carbamidomethyl)	0,0000	0,995	1		0	8,934E-09	47	2,07955E-05	3	2068,00614	1,07	41,77	0
	High	ekDQWLHR	2	1	1	P74002	N-Term(iTRAQ4plex); K2(iTRAQ4plex)	0,0000	1,301	2	27,0	0	0,003891	31	0,000709529	4	1399,76797	-0,37	16,46	1
	High	lDHYASHAQSR	2	1	1	P74002	N-Term(iTRAQ4plex)	0,0000	1,173	2	88,2	0	0,00008558	29	0,001199416	3	1428,71015	0,06	11,62	0
	High	gATLIGTATEImNk	1	1	1	P74002	N-Term(iTRAQ4plex); M12(Oxidation); K14(iTRAQ4plex)	0,0000	0,882	1		0	0,000006791	26	0,002387646	3	1723,94931	-0,58	34,59	0
	High	iTkPLk	2	1	1	P74002	N-Term(iTRAQ4plex); K3(iTRAQ4plex); K6(iTRAQ4plex)	0,0000	1,576	2	4,1	0,009	0,1082	25	0,00305471	2	1131,78179	-0,68	17,25	0
P74729	HrEpiB OS=Synechocystis sp. (strain PCC 6803 / Kazusa) GN=sll0576 PE=4 SV=1 -	330,04	34,94	1	11	11	18	0,624	17	23,9	312	34,9	7,62							
	A3	Sequence	# PSMs	# Proteins	# Protein Groups	Protein Group Accessions	Modifications	ΔCn	114/115	114/115 Count	114/115 Variability [%]	q-Value	PEP	IonScore	Exp Value	Charge	MH+ [Da]	ΔM [ppm]	RT [min]	# Missed Cleavages
	High	vFLHGLENFESmk	2	1	1	P74729	N-Term(iTRAQ4plex); M12(Oxidation); K13(iTRAQ4plex)	0,0000	0,572	2	8,5	0	4,761E-10	51	8,7694E-06	3	1854,95945	-3,69	38,71	0
	High	lATVFGmAPR	2	1	1	P74729	N-Term(iTRAQ4plex); M7(Oxidation)	0,0000	0,498	2	5,5	0	0,000001767	48	1,53805E-05	2	1222,67266	-0,70	33,96	0
	High	aVVIFEGHFk	3	1	1	P74729	N-Term(iTRAQ4plex); K10(iTRAQ4plex)	0,0000	0,509	2	8,2	0	0,000005076	47	2,04174E-05	3	1434,83393	-0,60	34,37	0
	High	fcTEESPLRPISLYGVTk	2	1	1	P74729	N-Term(iTRAQ4plex); C2(Carbamidomethyl); K18(iTRAQ4plex)	0,0000	0,621	2	0,8	0	2,721E-08	35	0,000321657	3	2385,27536	1,11	42,36	0
	High	mRVDLLVNDFVYR	2	1	1	P74729	N-Term(iTRAQ4plex); M1(Oxidation)	0,0000	0,892	2	39,3	0	4,234E-07	34	0,000382798	3	1799,95987	0,19	42,74	1
	High	iLmPVTNSGYGIGEk	1	1	1	P74729	N-Term(iTRAQ4plex); M3(Oxidation); K15(iTRAQ4plex)	0,0000	0,808	1		0	0,00001653	26	0,00227494	3	1883,01633	-1,27	35,01	0
	High	kEDLIkEQLk	1	1	1	P74729	N-Term(iTRAQ4plex); K1(iTRAQ4plex); K6(iTRAQ4plex); K10(iTRAQ4plex)	0,0000	0,753	1		0	2,909E-07	16	0,027100045	3	1820,13462	0,37	23,96	2
	High	nYIHIR	1	1	1	P74729	N-Term(iTRAQ4plex)	0,0000	0,809	1		0,002	0,02581	18	0,017059646	3	959,55429	-0,03	17,94	0
	High	gYTILR	2	1	1	P74729	N-Term(iTRAQ4plex)	0,0000	0,703	2	5,9	0,004	0,05291	22	0,006729302	2	866,52019	-1,67	23,03	0
	High	dYIVSNQR	1	1	1	P74729	N-Term(iTRAQ4plex)	0,0000	0,561	1		0,004	0,05435	11	0,071774472	2	1138,59612	-1,06	17,93	0
	High	kEDLIk	1	1	1	P74729	N-Term(iTRAQ4plex); K1(iTRAQ4plex); K6(iTRAQ4plex)	0,0000	0,748	1		0,006	0,07693	23	0,004507856	3	1177,74836	-2,81	16,06	1
Q05971	10 kDa chaperonin OS=Synechocystis sp. (strain PCC 6803 / Kazusa) GN=groS PE=	329,42	48,54	1	5	5	10	0,881	10	8,8	103	10,9	5,22							
	A3	Sequence	# PSMs	# Proteins	# Protein Groups	Protein Group Accessions	Modifications	ΔCn	114/115	114/115 Count	114/115 Variability [%]	q-Value	PEP	IonScore	Exp Value	Charge	MH+ [Da]	ΔM [ppm]	RT [min]	# Missed Cleavages
	High	ekPQIGEVVQVGPGk	4	1	1	Q05971	N-Term(iTRAQ4plex); K2(iTRAQ4plex); K15(iTRAQ4plex)	0,0000	0,950	4	4,0	0	1,314E-12	88	1,73768E-09	2	1997,18315	3,78	27,72	0
	High	aAISINVSTVkPLGDR	3	1	1	Q05971	N-Term(iTRAQ4plex); K11(iTRAQ4plex)	0,0000	0,892	3	7,2	0	3,255E-09	56	2,57022E-06	3	1929,13840	0,62	34,89	0
	High	vSPAEEk	1	1	1	Q05971	N-Term(iTRAQ4plex); K7(iTRAQ4plex)	0,0000	0,853	1		0,001	0,008567	37	0,000207679	2	1047,59441	1,84	12,29	0
	High	vLYSk	1	1	1	Q05971	N-Term(iTRAQ4plex); K5(iTRAQ4plex)	0,0000	0,870	1		0,008	0,0922	27	0,001844888	2	897,56713	2,62	18,51	0
	Medium	yAGTDIk	1	1	1	Q05971	N-Term(iTRAQ4plex); K7(iTRAQ4plex)	0,0000	0,754	1		0,013	0,1429	19	0,011856869	2	1055,60271	4,89	18,33	0
P73405	Pyruvate dehydrogenase E1 beta subunit OS=Synechocystis sp. (strain PCC 6803 /	329,36	11,11	1	3	3	10	0,685	9	9,0	324	35,7	5,54							
	A3	Sequence	# PSMs	# Proteins	# Protein Groups	Protein Group Accessions	Modifications	ΔCn	114/115	114/115 Count	114/115 Variability [%]	q-Value	PEP	IonScore	Exp Value	Charge	MH+ [Da]	ΔM [ppm]	RT [min]	# Missed Cleavages
	High	aETLLFAALR	6	1	1	P73405	N-Term(iTRAQ4plex)	0,0000	0,687	6	8,0	0	3,232E-08	68	1,54515E-07	2	1248,74297	-0,26	48,12	0
	High	lEAYFHAVPGLk	3	1	1	P73405	N-Term(iTRAQ4plex); K12(iTRAQ4plex)	0,0000	0,662	2	5,0	0	3,599E-08	63	5,39473E-07	2	1632,93706	1,12	41,62	0
	High	lTIVQPPQIVDAVk	1	1	1	P73405	N-Term(iTRAQ4plex); K14(iTRAQ4plex)	0,0000	1,104	1		0	9,939E-09	33	0,00049314	3	1809,11002	0,64	44,95	0
P74500	Slr1940 protein OS=Synechocystis sp. (strain PCC 6803 / Kazusa) GN=slr1940 PE=	326,90	8,30	1	4	4	8	1,136	8	2,7	482	51,2	4,89							
	A3	Sequence	# PSMs	# Proteins	# Protein Groups	Protein Group Accessions	Modifications	ΔCn	114/115	114/115 Count	114/115 Variability [%]	q-Value	PEP	IonScore	Exp Value	Charge	MH+ [Da]	ΔM [ppm]	RT [min]	# Missed Cleavages
	High	lTLSGSGNNLk	2	1	1	P74500	N-Term(iTRAQ4plex); K11(iTRAQ4plex)	0,0000	1,212	2	2,7	0	2,464E-07	76	2,52331E-08	2	1391,81072	0,74	26,73	0
	High	nVGSIGAPGAR	2	1	1	P74500	N-Term(iTRAQ4plex)	0,0000	1,117	2	0,8	0	0,00006519	58	1,75376E-06	2	1142,64775	6,88	18,30	0
	High	nQGFNLSk	2	1	1	P74500	N-Term(iTRAQ4plex); K8(iTRAQ4plex)	0,0000	1,140	2	1,6	0	0,000078	52	6,41165E-06	2	1195,67107	3,10	22,60	0
	High	fRPPSTYTLR	2	1	1	P74500	N-Term(iTRAQ4plex)	0,0000	1,024	2	16,7	0	0,00001365	40	0,000111679	3	1381,77033	-0,40	26,94	0
P73411	Glucose-6-phosphate 1-dehydrogenase OS=Synechocystis sp. (strain PCC 6803 / Ka	326,20	22,00	1	10	10	14	0,502	14	41,9	509	57,8	7,17							
	A3	Sequence	# PSMs	# Proteins	# Protein Groups	Protein Group Accessions	Modifications	ΔCn	114/115	114/115 Count	114/115 Variability [%]	q-Value	PEP	IonScore	Exp Value	Charge	MH+ [Da]	ΔM [ppm]	RT [min]	# Missed Cleavages
	High	rLPPELTVVGFAR	1	1	1	P73411	N-Term(iTRAQ4plex)	0,0000	0,527	1		0	4,461E-10	57	2,14768E-06	3	1598,95035	0,25	39,42	1
	High	kVSEIAIQFR	1	1	1	P73411	N-Term(iTRAQ4plex); K1(iTRAQ4plex)	0,0000	0,706	1		0	6,85E-08	55	3,50728E-06	3	1478,89121	-1,46	31,01	1
	High	qVPLLIFQSVAHQANPNVLSLR	1	1	1	P73411	N-Term(iTRAQ4plex)	0,0000	0,435	1		0	1,661E-08	54	3,79289E-06	3	2588,48087	0,57	54,28	0
	High	nFLGELDEkR	2	1	1	P73411	N-Term(iTRAQ4plex); K9(iTRAQ4plex)	0,0000	0,712	2	76,2	0	0,000000399	40	9,79422E-05	3	1508,83078	-0,25	31,29	1
	High	wQGVPFYLR	1	1	1	P73411	N-Term(iTRAQ4plex)	0,0000	0,350	1		0	0,0003025	40	0,000109136	2	1309,71709	-0,23	44,08	0
	High	iQPNEGISLR	1	1	1	P73411	N-Term(iTRAQ4plex)	0,0000	0,425	1		0	0,00001241	39	0,000134577	2	1270,72283	-0,62	28,02	0
	High	iDHYLGk	2	1	1	P73411	N-Term(iTRAQ4plex); K7(iTRAQ4plex)	0,0000	0,588	2	31,6	0	0,0011	37	0,000204974	2	1133,65325	-2,19	22,66	0
	High	fANAIFEPLWNR	1	1	1	P73411	N-Term(iTRAQ4plex)	0,0000	0,303	1		0	5,963E-07	36	0,000226971	2	1621,86003	-0,47	51,64	0
	High	iVIEkPFGR	3	1	1	P73411	N-Term(iTRAQ4plex); K5(iTRAQ4plex)	0,0000	0,562	3	13,0	0	0,00238	26	0,002243727	3	1346,83738	-1,86	30,77	0
	High	kLVPAIYQmk	1	1	1	P73411	N-Term(iTRAQ4plex); K1(iTRAQ4plex); M9(Oxidation); K10(iTRAQ4plex)	0,0000	0,804	1		0	0,00008531	23	0,004677028	3	1638,99497	-1,69	25,98	1
Q55196	Phosphate import ATP-binding protein PstB 1 OS=Synechocystis sp. (strain PCC 68(	324,73	18,22	1	6	6	11	0,397	11	24,0	269	30,2	6,02							
	A3	Sequence	# PSMs	# Proteins	# Protein Groups	Protein Group Accessions	Modifications	ΔCn	114/115	114/115 Count	114/115 Variability [%]	q-Value	PEP	IonScore	Exp Value	Charge	MH+ [Da]	ΔM [ppm]	RT [min]	# Missed Cleavages
	High	aSGFSLSGGQQQR	2	1	1	Q55196	N-Term(iTRAQ4plex)	0,0000	0,289	2	0,1	0	4,347E-11	86	2,32258E-09	2	1466,74602	-0,58	22,43	0
	High	gNLEELAEDSLRR	3	1	1	Q55196	N-Term(iTRAQ4plex)	0,0000	0,439	3	14,9	0	0,00001196	61	8,66902E-07	3	1645,86161	-0,64	36,88	1
	High	lNDLIESFR	2	1	1	Q55196	N-Term(iTRAQ4plex)	0,0000	0,428	2	1,9	0	0,000004315	36	0,000288524	2	1250,68755	1,11	46,07	0
	High	aALWDEVkDk	2	1	1	Q55196	N-Term(iTRAQ4plex); K8(iTRAQ4plex); K10(iTRAQ4plex)	0,0000	0,326	2	6,5	0	0,00001332	23	0,005533119	3	1606,90976	-4,21	31,60	1
	High	rAALWDEVkDk	1	1	1	Q55196	N-Term(iTRAQ4plex); K9(iTRAQ4plex); K11(iTRAQ4plex)	0,0000	0,078	1		0	0,0008185	20	0,0097949	4	1763,01137	-3,55	26,37	2
	Medium	lNDLIESFRLEGk	1	1	1	Q55196	N-Term(iTRAQ4plex); K13(iTRAQ4plex)	0,0000	0,466	1		0,011	0,1313	11	0,101554189	3	1822,02884	-1,37	47,54	1
P26290	Cytochrome b6-f complex iron-sulfur subunit 2 OS=Synechocystis sp. (strain PCC 6(	324,13	20,56	1	3	3	9	0,940	8	7,7	180	19,0	5,11							
	A3	Sequence	# PSMs	# Proteins	# Protein Groups	Protein Group Accessions	Modifications	ΔCn	114/115	114/115 Count	114/115 Variability [%]	q-Value	PEP	IonScore	Exp Value	Charge	MH+ [Da]	ΔM [ppm]	RT [min]	# Missed Cleavages
	High	vTEFLASHNAGDR	4	1	1	P26290	N-Term(iTRAQ4plex)	0,0000	0,900	4	7,7	0	4,113E-11	80	1,00224E-08	2	1560,78935	0,39	26,92	0
	High	vLAQGLk	4	1	1	P26290	N-Term(iTRAQ4plex); K7(iTRAQ4plex)	0,0000	0,987	3	2,0	0	0,00096	38	0,000206987	2	1016,67461	3,88	23,17	0
	High	yLIPPSSGGSGGGVTAk	1	1	1	P26290	N-Term(iTRAQ4plex); K17(iTRAQ4plex)	0,0000	0,766	1		0	3,192E-07	30	0,00090567	3	1836,01187	0,70	30,11	0
P74450	Slr0151 protein OS=Synechocystis sp. (strain PCC 6803 / Kazusa) GN=slr0151 PE=	322,17	25,62	1	7	7	14	1,621	12	30,1	320	34,9	5,07							
	A3	Sequence	# PSMs	# Proteins	# Protein Groups	Protein Group Accessions	Modifications	ΔCn	114/115	114/115 Count	114/115 Variability [%]	q-Value	PEP	IonScore	Exp Value	Charge	MH+ [Da]	ΔM [ppm]	RT [min]	# Missed Cleavages
	High	hQDLLAVVTELR	3	1	1	P74450	N-Term(iTRAQ4plex)	0,0000	1,762	3	9,5	0	0,000001653	66	3,20404E-07	2	1537,88396	1,33	43,68	0
	High	aTAAQIVQLVNQNQAHLQEYLQk	1	1	1	P74450	N-Term(iTRAQ4plex); K23(iTRAQ4plex)	0,0000	0,578	1		0	2,657E-12	60	1,10456E-06	4	2896,58510	-1,13	51,57	0
	High	lEAFQAAAAQTHAGLDIk	1	1	1	P74450	N-Term(iTRAQ4plex); K18(iTRAQ4plex)	0,0000	1,591	1		0	3,203E-12	53	5,66486E-06	3	2143,17679	0,81	39,69	0
	High	kQLEEHLLANQR	3	1	1	P74450	N-Term(iTRAQ4plex); K1(iTRAQ4plex)	0,0000	1,475	2	37,7	0	1,014E-09	42	6,83864E-05	3	1767,01389	1,30	20,63	1
	High	gDLAGAFHHR	2	1	1	P74450	N-Term(iTRAQ4plex)	0,0000	1,052	2	60,7	0	7,082E-07	35	0,000326565	3	1224,63709	1,33	15,86	0
	High	sLALQGk	3	1	1	P74450	N-Term(iTRAQ4plex); K7(iTRAQ4plex)	0,0000	1,930	2	10,7	0	0,001756	34	0,000483496	2	1004,64141	7,09	19,37	0
	High	qLEEHLLANQR	1	1	1	P74450	N-Term(iTRAQ4plex)	0,0000	0,896	1		0	0,0000197	28	0,001458714	3	1494,81598	0,96	23,45	0
Q55393	Diflavin flavoprotein A 1 OS=Synechocystis sp. (strain PCC 6803 / Kazusa) GN=dfa1	321,57	10,82	1	7	7	12	0,483	12	45,4	573	63,5	5,90							
	A3	Sequence	# PSMs	# Proteins	# Protein Groups	Protein Group Accessions	Modifications	ΔCn	114/115	114/115 Count	114/115 Variability [%]	q-Value	PEP	IonScore	Exp Value	Charge	MH+ [Da]	ΔM [ppm]	RT [min]	# Missed Cleavages
	High	vALQFLDNFVHQPFER	3	1	1	Q55393	N-Term(iTRAQ4plex)	0,0000	0,286	3	19,6	0	2E-14	64	4,31699E-07	3	2104,11087	0,56	54,29	0
	High	iTVVATk	1	1	1	Q55393	N-Term(iTRAQ4plex); K7(iTRAQ4plex)	0,0000	0,636	1		0	0,0006151	51	8,05323E-06	2	1019,66783	-2,45	21,75	0
	High	lSQAIAk	1	1	1	Q55393	N-Term(iTRAQ4plex); K7(iTRAQ4plex)	0,0000	0,454	1		0	0,001274	45	3,62401E-05	2	1018,65312	3,14	20,55	0
	High	lSTQTEAIAk	1	1	1	Q55393	N-Term(iTRAQ4plex); K10(iTRAQ4plex)	0,0000	0,634	1		0	3,073E-07	44	3,72366E-05	2	1349,78557	-1,72	22,61	0
	High	hNVGELLHR	2	1	1	Q55393	N-Term(iTRAQ4plex)	0,0000	0,442	2	33,6	0	0,000007796	43	5,27193E-05	4	1218,68032	-1,71	17,55	0
	High	fLDLGLR	3	1	1	Q55393	N-Term(iTRAQ4plex)	0,0000	0,658	3	46,6	0	0,001362	35	0,000544718	2	977,58934	-0,75	40,71	0
	Medium	nITAIR	1	1	1	Q55393	N-Term(iTRAQ4plex)	0,0000	0,513	1		0,01	0,1222	22	0,007919501	2	831,51525	-1,98	17,74	0
P73393	Sll1734 protein OS=Synechocystis sp. (strain PCC 6803 / Kazusa) GN=sll1734 PE=4	321,05	15,08	1	6	6	16	0,357	15	91,0	431	50,0	5,88							
	A3	Sequence	# PSMs	# Proteins	# Protein Groups	Protein Group Accessions	Modifications	ΔCn	114/115	114/115 Count	114/115 Variability [%]	q-Value	PEP	IonScore	Exp Value	Charge	MH+ [Da]	ΔM [ppm]	RT [min]	# Missed Cleavages
	High	sMYNVTSAVIQALR	2	1	1	P73393	N-Term(iTRAQ4plex)	0,0000	0,547	2	53,8	0	4,595E-09	68	1,46882E-07	2	1696,91692	-0,23	51,67	0
	High	lFPDLFLEQVR	1	1	1	P73393	N-Term(iTRAQ4plex)	0,0000	0,413	1		0	0,0005843	45	2,90382E-05	2	1520,85881	-0,38	54,46	0
	High	lkDIQSDSYR	3	1	1	P73393	N-Term(iTRAQ4plex); K2(iTRAQ4plex)	0,0000	0,602	3	5,3	0	0,0000176	45	3,05471E-05	3	1512,82541	-0,43	20,85	1
	High	smYNVTSAVIQALR	2	1	1	P73393	N-Term(iTRAQ4plex); M2(Oxidation)	0,0000	0,496	2	95,9	0	3,185E-09	44	3,91715E-05	3	1712,91153	-0,41	45,29	0
	High	sYNAQAk	4	1	1	P73393	N-Term(iTRAQ4plex); K7(iTRAQ4plex)	0,0000	0,202	3	4,9	0	0,0004676	37	0,000206524	2	1069,58708	-0,89	12,25	0
	High	aTLPPSTHPFADVIHR	1	1	1	P73393	N-Term(iTRAQ4plex)	0,0000	1,321	1		0	0,0001575	13	0,050115262	4	1903,03151	0,42	31,36	0
	High	vFLNPLR	3	1	1	P73393	N-Term(iTRAQ4plex)	0,0000	0,257	3	49,7	0,001	0,0138	23	0,005558659	2	1002,62297	1,26	35,35	0
P74410	30S ribosomal protein S16 OS=Synechocystis sp. (strain PCC 6803 / Kazusa) GN=r	315,08	70,73	1	7	7	16	1,242	15	17,1	82	9,6	10,52							
	A3	Sequence	# PSMs	# Proteins	# Protein Groups	Protein Group Accessions	Modifications	ΔCn	114/115	114/115 Count	114/115 Variability [%]	q-Value	PEP	IonScore	Exp Value	Charge	MH+ [Da]	ΔM [ppm]	RT [min]	# Missed Cleavages
	High	lkEGAQPTDTVR	5	1	1	P74410	N-Term(iTRAQ4plex); K2(iTRAQ4plex)	0,0000	1,341	4	11,2	0	0,00003145	47	1,8197E-05	2	1602,90862	2,01	15,97	1
	High	aQVFEQLkA	2	1	1	P74410	N-Term(iTRAQ4plex); K8(iTRAQ4plex)	0,0000	1,408	2	9,5	0	0,0001082	46	2,74138E-05	2	1321,77226	0,32	31,74	1
	High	dGRPLEELGFYNPR	3	1	1	P74410	N-Term(iTRAQ4plex)	0,0000	0,988	3	2,7	0	1,218E-10	38	0,000147901	3	1806,92594	0,20	36,09	0
	High	rDGRPLEELGFYNPR	2	1	1	P74410	N-Term(iTRAQ4plex)	0,0000	1,278	2	5,4	0	0,000002504	37	0,00021329	3	1963,02738	0,34	31,19	1
	High	iVAmHSTTR	2	1	1	P74410	N-Term(iTRAQ4plex); M4(Oxidation)	0,0000	0,977	2	3,6	0	0,0006457	32	0,000562302	2	1175,63286	0,43	9,89	0
	High	lDVPAIVk	1	1	1	P74410	N-Term(iTRAQ4plex); K8(iTRAQ4plex)	0,0000	1,282	1		0,005	0,07094	29	0,001276351	2	1142,73845	-0,27	35,11	0
	Medium	sILTk	1	1	1	P74410	N-Term(iTRAQ4plex); K5(iTRAQ4plex)	0,0000	1,042	1		0,013	0,1452	26	0,002382155	2	849,56902	4,98	18,23	0
P72699	UPF0045 protein sll0230 OS=Synechocystis sp. (strain PCC 6803 / Kazusa) GN=sll0	313,39	43,80	1	4	4	14	1,134	12	17,3	121	13,3	7,43							
	A3	Sequence	# PSMs	# Proteins	# Protein Groups	Protein Group Accessions	Modifications	ΔCn	114/115	114/115 Count	114/115 Variability [%]	q-Value	PEP	IonScore	Exp Value	Charge	MH+ [Da]	ΔM [ppm]	RT [min]	# Missed Cleavages
	High	acHEAVHALGAPR	8	1	1	P72699	N-Term(iTRAQ4plex); C2(Carbamidomethyl)	0,0000	1,073	6	12,1	0	7,32E-10	83	5,1047E-09	2	1532,79009	1,81	14,13	0
	High	hTmHAYGTNIEGDWDEVFAAVk	1	1	1	P72699	N-Term(iTRAQ4plex); M3(Oxidation); K22(iTRAQ4plex)	0,0000	2,042	1		0	1,269E-08	32	0,000594251	4	2795,33535	0,54	44,26	0
	High	vLAEAGLk	1	1	1	P72699	N-Term(iTRAQ4plex); K8(iTRAQ4plex)	0,0000	1,160	1		0	0,007852	29	0,002110809	2	1088,69231	0,46	27,15	0
	High	tDRPQTmDEk	2	1	1	P72699	N-Term(iTRAQ4plex); M7(Oxidation); K10(iTRAQ4plex)	0,0000	1,318	2	6,5	0	0,000252	19	0,011427994	3	1524,75812	0,94	8,45	0
	High	hTMHAYGTNIEGDWDEVFAAVk	1	1	1	P72699	N-Term(iTRAQ4plex); K22(iTRAQ4plex)	0,0000	0,979	1		0	0,0001338	14	0,043548179	4	2779,34047	0,56	45,53	0
	High	tDRPQTMDEk	1	1	1	P72699	N-Term(iTRAQ4plex); K10(iTRAQ4plex)	0,0000	1,041	1		0	0,004891	10	0,093749725	3	1508,76056	-0,80	12,09	0
Q55512	Aspartate-semialdehyde dehydrogenase OS=Synechocystis sp. (strain PCC 6803 / K	312,17	18,05	1	6	6	9	0,748	9	17,8	338	36,6	6,04							
	A3	Sequence	# PSMs	# Proteins	# Protein Groups	Protein Group Accessions	Modifications	ΔCn	114/115	114/115 Count	114/115 Variability [%]	q-Value	PEP	IonScore	Exp Value	Charge	MH+ [Da]	ΔM [ppm]	RT [min]	# Missed Cleavages
	High	gAALNAVQIAELLVER	2	1	1	Q55512	N-Term(iTRAQ4plex)	0,0000	0,825	2	33,0	0	3,066E-12	107	2,09879E-11	2	1811,05266	1,02	58,00	0
	High	kGAALNAVQIAELLVER	1	1	1	Q55512	N-Term(iTRAQ4plex); K1(iTRAQ4plex)	0,0000	0,520	1		0	2,143E-13	87	2,14274E-09	3	2083,24930	0,70	51,23	1
	High	aHSEAVNLEFATPFPVELAk	1	1	1	Q55512	N-Term(iTRAQ4plex); K20(iTRAQ4plex)	0,0000	0,748	1		0	3,15E-10	43	8,94301E-05	3	2458,32199	-0,05	46,93	0
	High	kIFAAEDIR	2	1	1	Q55512	N-Term(iTRAQ4plex); K1(iTRAQ4plex)	0,0000	0,924	2	19,7	0	0,000002663	39	0,000127635	3	1350,80051	1,56	24,40	1
	High	nFPLAELk	2	1	1	Q55512	N-Term(iTRAQ4plex); K8(iTRAQ4plex)	0,0000	0,778	2	17,3	0,002	0,02119	35	0,000315479	2	1219,72783	-0,89	36,54	0
	High	iTATcVR	1	1	1	Q55512	N-Term(iTRAQ4plex); C5(Carbamidomethyl)	0,0000	0,671	1		0,005	0,06267	12	0,067759471	2	964,53685	0,21	16,35	0
P74426	Sll0359 protein OS=Synechocystis sp. (strain PCC 6803 / Kazusa) GN=sll0359 PE=1	311,20	30,32	1	5	6	14	0,833	11	22,8	155	17,2	9,11							
	A3	Sequence	# PSMs	# Proteins	# Protein Groups	Protein Group Accessions	Modifications	ΔCn	114/115	114/115 Count	114/115 Variability [%]	q-Value	PEP	IonScore	Exp Value	Charge	MH+ [Da]	ΔM [ppm]	RT [min]	# Missed Cleavages
	High	acGYYSTSk	3	1	1	P74426	N-Term(iTRAQ4plex); C2(Carbamidomethyl); K9(iTRAQ4plex)	0,0000	1,212	3	3,6	0	3,723E-07	63	5,36995E-07	2	1324,65178	5,45	16,45	0
	High	gVPLDPSGTk	1	1	1	P74426	N-Term(iTRAQ4plex); K10(iTRAQ4plex)	0,0000	1,155	1		0	0,000001887	49	1,25305E-05	2	1258,72441	-0,12	22,03	0
	High	vkELSHLPR	2	1	1	P74426	N-Term(iTRAQ4plex); K2(iTRAQ4plex)	0,0000	0,786	2	8,7	0	0,000003992	36	0,000277778	3	1366,84156	0,46	19,60	1
	High	eLSHLPR	6	1	1	P74426	N-Term(iTRAQ4plex)	0,0000	0,770	4	1,9	0	0,0001913	31	0,000822186	3	995,57746	1,99	16,51	0
	High	vNLTDFYDAVLAAk	1	1	1	P74426	N-Term(iTRAQ4plex); K14(iTRAQ4plex)	0,0000	1,023	1		0	0,00108	13	0,049200555	3	1828,01364	2,25	55,10	0
	Medium	hIHLk	1	2	2	P74426;Q55432	N-Term(iTRAQ4plex); K5(iTRAQ4plex)	0,0000				0,03	0,329	12	0,068544088	2	935,60338	0,52	11,57	0
P74162	Sll1380 protein OS=Synechocystis sp. (strain PCC 6803 / Kazusa) GN=sll1380 PE=4	306,48	37,85	1	6	6	12	0,914	10	11,7	214	23,9	8,91							
	A3	Sequence	# PSMs	# Proteins	# Protein Groups	Protein Group Accessions	Modifications	ΔCn	114/115	114/115 Count	114/115 Variability [%]	q-Value	PEP	IonScore	Exp Value	Charge	MH+ [Da]	ΔM [ppm]	RT [min]	# Missed Cleavages
	High	fQVcQSGQVPTAPVR	3	1	1	P74162	N-Term(iTRAQ4plex); C4(Carbamidomethyl)	0,0000	0,923	3	1,8	0	4,374E-10	79	1,20495E-08	2	1817,94634	0,77	30,32	0
	High	nLDFTLNAPAGHLPSPR	1	1	1	P74162	N-Term(iTRAQ4plex)	0,0000	0,849	1		0	1,573E-10	64	4,11811E-07	3	1964,04673	-0,19	38,46	0
	High	lLLAATNR	2	1	1	P74162	N-Term(iTRAQ4plex)	0,0000	1,017	2	3,6	0	0,00005641	49	1,31817E-05	2	1015,63719	-0,88	27,77	0

	High	tIDQLWLQHSNNR	1	1	1	P74162	N-Term(iTRAQ4plex)	0,0000	0,846	1		0	3,754E-08	39	0,000117211	3	1768,92020	-0,53	33,37	0
	High	fGFTPQLEAFLATGNRPGR	4	1	1	P74162	N-Term(iTRAQ4plex)	0,0000	0,845	2	10,1	0	1,427E-09	33	0,000553312	3	2223,18015	0,42	50,26	0
	High	kENQWVIFk	1	1	1	P74162	N-Term(iTRAQ4plex); K1(iTRAQ4plex); K9(iTRAQ4plex)	0,0000	1,127	1		0	0,0002688	21	0,007888056	3	1623,95853	0,14	32,14	1
P72655	Uncharacterized protein slr1128 OS=Synechocystis sp. (strain PCC 6803 / Kazusa)	306,18	27,10	1	8	8	13	0,470	13	24,4	321	35,7	5,68							
	A3	Sequence	# PSMs	# Proteins	# Protein Groups	Protein Group Accessions	Modifications	ΔCn	114/115	114/115 Count	114/115 Variability [%]	q-Value	PEP	IonScore	Exp Value	Charge	MH+ [Da]	ΔM [ppm]	RT [min]	# Missed Cleavages
	High	nILSTLEGVR	3	1	1	P72655	N-Term(iTRAQ4plex)	0,0000	0,400	3	0,4	0	0,000002331	58	1,6633E-06	2	1245,72844	0,06	43,63	0
	High	aAILTSEGQR	2	1	1	P72655	N-Term(iTRAQ4plex)	0,0000	0,615	2	42,2	0	0,00005051	48	1,59945E-05	2	1189,66509	-0,57	19,61	0
	High	kLTPGLNFTVPILDR	2	1	1	P72655	N-Term(iTRAQ4plex); K1(iTRAQ4plex)	0,0000	0,469	2	19,3	0	1,813E-07	45	3,48313E-05	3	1972,18265	-0,39	46,29	1
	High	aAILNAEAEQQkk	1	1	1	P72655	N-Term(iTRAQ4plex); K12(iTRAQ4plex); K13(iTRAQ4plex)	0,0000	0,644	1		0	1,228E-08	40	9,74922E-05	3	1846,07517	-0,39	21,18	1
	High	tEINELLLR	1	1	1	P72655	N-Term(iTRAQ4plex)	0,0000	0,470	1		0	0,0003584	32	0,000601132	2	1244,73345	0,27	38,14	0
	High	iVNEkNEYLVER	2	1	1	P72655	N-Term(iTRAQ4plex); K5(iTRAQ4plex)	0,0000	0,551	2	19,9	0	0,00001007	28	0,001503038	3	1793,99942	-0,33	26,77	1
	High	aTAEALSILTEk	1	1	1	P72655	N-Term(iTRAQ4plex); K12(iTRAQ4plex)	0,0000	0,576	1		0,001	0,008786	17	0,023237064	2	1534,89164	-0,95	41,26	0
	High	lGSYNkk	1	1	1	P72655	N-Term(iTRAQ4plex); K6(iTRAQ4plex); K7(iTRAQ4plex)	0,0000	0,326	1		0,002	0,03235	16	0,027037716	3	1241,75379	-3,22	13,51	1
P73008	Methyl-accepting chemotaxis protein OS=Synechocystis sp. (strain PCC 6803 / Kazu	305,41	6,10	1	5	5	8	0,766	8	25,2	869	93,2	4,40							
	A3	Sequence	# PSMs	# Proteins	# Protein Groups	Protein Group Accessions	Modifications	ΔCn	114/115	114/115 Count	114/115 Variability [%]	q-Value	PEP	IonScore	Exp Value	Charge	MH+ [Da]	ΔM [ppm]	RT [min]	# Missed Cleavages
	High	iVAVISQIASR	4	1	1	P73008	N-Term(iTRAQ4plex)	0,0000	0,736	4	18,7	0	0,000006063	71	7,92447E-08	2	1300,80754	0,45	46,60	0
	High	aPSPTNPGmDHPFGR	1	1	1	P73008	N-Term(iTRAQ4plex); M9(Oxidation)	0,0000	1,033	1		0	2,196E-08	62	6,1372E-07	3	1740,82578	0,73	21,94	0
	High	tVGGILQIR	1	1	1	P73008	N-Term(iTRAQ4plex)	0,0000	0,668	1		0	0,0001187	53	5,02308E-06	2	1100,69036	-0,45	34,71	0
	High	rALEDIIEVSNR	1	1	1	P73008	N-Term(iTRAQ4plex)	0,0000	1,271	1		0	1,378E-07	27	0,001778157	3	1558,86637	-0,41	33,72	1
	High	iNTLVR	1	1	1	P73008	N-Term(iTRAQ4plex)	0,0000	0,735	1		0,005	0,06125	24	0,004102041	2	859,54784	-0,41	21,70	0
P74135	Sll1873 protein OS=Synechocystis sp. (strain PCC 6803 / Kazusa) GN=sll1873 PE=4	303,18	61,98	1	6	6	13	0,992	11	27,7	121	12,9	9,99							
	A3	Sequence	# PSMs	# Proteins	# Protein Groups	Protein Group Accessions	Modifications	ΔCn	114/115	114/115 Count	114/115 Variability [%]	q-Value	PEP	IonScore	Exp Value	Charge	MH+ [Da]	ΔM [ppm]	RT [min]	# Missed Cleavages
	High	aAAPVETPASAPVAPAPk	2	1	1	P74135	N-Term(iTRAQ4plex); K18(iTRAQ4plex)	0,0000	1,006	2	36,2	0	1,968E-09	64	3,89915E-07	3	1933,10172	1,00	26,57	0
	High	rTPGPSLNR	4	1	1	P74135	N-Term(iTRAQ4plex)	0,0000	0,845	4	12,6	0	0,00002528	44	4,28519E-05	3	1141,65470	-1,02	11,71	1
	High	sEVAPVEkPAPTTSk	4	1	1	P74135	N-Term(iTRAQ4plex); K8(iTRAQ4plex); K15(iTRAQ4plex)	0,0000	1,087	2	13,6	0	7,874E-08	42	5,662E-05	3	1973,14066	6,43	19,86	0
	High	akVDPSQVAFASGDPIPQNVAR	1	1	1	P74135	N-Term(iTRAQ4plex); K2(iTRAQ4plex)	0,0000	1,960	1		0	0,00001307	36	0,000257614	3	2555,39334	4,39	36,09	1
	Medium	lFGAk	1	1	1	P74135	N-Term(iTRAQ4plex); K5(iTRAQ4plex)	0,0000	1,168	1		0,045	0,4674	17	0,026802867	2	823,52587	-2,61	56,61	0
	Medium	fkEmAR	1	1	1	P74135	N-Term(iTRAQ4plex); K2(iTRAQ4plex); M4(Oxidation)	0,0000	0,835	1		0,046	0,4764	18	0,015957689	2	1085,59819	-3,15	10,31	1
P73302	Adenylate kinase 1 OS=Synechocystis sp. (strain PCC 6803 / Kazusa) GN=adk1 PE=	301,60	31,89	1	5	5	13	1,195	11	9,4	185	20,2	5,59							
	A3	Sequence	# PSMs	# Proteins	# Protein Groups	Protein Group Accessions	Modifications	ΔCn	114/115	114/115 Count	114/115 Variability [%]	q-Value	PEP	IonScore	Exp Value	Charge	MH+ [Da]	ΔM [ppm]	RT [min]	# Missed Cleavages
	High	gLIFLGAPGSGk	6	1	1	P73302	N-Term(iTRAQ4plex); K12(iTRAQ4plex)	0,0000	1,293	4	10,9	0	0,000000205	57	1,89657E-06	2	1404,84612	0,53	39,26	0
	High	nVNQAIFLDELLVNIGHR	2	1	1	P73302	N-Term(iTRAQ4plex)	0,0000	0,998	2	17,4	0	1,021E-10	54	3,90814E-06	3	2209,22550	2,01	55,67	0
	High	aGWILDGFPR	2	1	1	P73302	N-Term(iTRAQ4plex)	0,0000	1,222	2	8,6	0	0,000007046	38	0,000152746	2	1275,69805	1,07	46,38	0
	High	tHWVINLk	2	1	1	P73302	N-Term(iTRAQ4plex); K8(iTRAQ4plex)	0,0000	1,136	2	5,4	0	0,001216	37	0,00020416	2	1298,78313	0,61	31,64	0
	High	gRADDNETTIR	1	1	1	P73302	N-Term(iTRAQ4plex)	0,0000	1,269	1		0	0,005075	13	0,045078557	3	1391,69767	-1,39	10,22	1
P72673	Thylakoid-associated protein slr0729 OS=Synechocystis sp. (strain PCC 6803 / Kazu	301,54	30,69	1	3	3	10	1,465	10	5,4	101	10,9	4,70							
	A3	Sequence	# PSMs	# Proteins	# Protein Groups	Protein Group Accessions	Modifications	ΔCn	114/115	114/115 Count	114/115 Variability [%]	q-Value	PEP	IonScore	Exp Value	Charge	MH+ [Da]	ΔM [ppm]	RT [min]	# Missed Cleavages
	High	lLTLLEEYQSk	5	1	1	P72673	N-Term(iTRAQ4plex); K11(iTRAQ4plex)	0,0000	1,468	5	10,1	0	6,37E-08	71	7,11164E-08	2	1624,94243	1,48	49,44	0
	High	aGVEAALk	2	1	1	P72673	N-Term(iTRAQ4plex); K8(iTRAQ4plex)	0,0000	1,405	2	1,6	0	0,00006084	51	1,48064E-05	2	1046,64910	4,05	20,34	0
	High	vYPLLEkDELGR	3	1	1	P72673	N-Term(iTRAQ4plex); K7(iTRAQ4plex)	0,0000	1,479	3	1,8	0	3,308E-07	46	2,29615E-05	3	1719,98948	0,63	34,02	1
Q55119	Elongation factor P OS=Synechocystis sp. (strain PCC 6803 / Kazusa) GN=efp PE=3	297,05	21,93	1	4	4	10	1,456	9	17,5	187	20,4	4,89							
	A3	Sequence	# PSMs	# Proteins	# Protein Groups	Protein Group Accessions	Modifications	ΔCn	114/115	114/115 Count	114/115 Variability [%]	q-Value	PEP	IonScore	Exp Value	Charge	MH+ [Da]	ΔM [ppm]	RT [min]	# Missed Cleavages
	High	sVQTGNVVEk	4	1	1	Q55119	N-Term(iTRAQ4plex); K10(iTRAQ4plex)	0,0000	1,638	3	0,5	0	2,022E-08	80	9,57128E-09	2	1348,77483	5,44	17,30	0
	High	aGETVPQANIEk	2	1	1	Q55119	N-Term(iTRAQ4plex); K12(iTRAQ4plex)	0,0000	1,493	2	3,5	0	4,49E-09	52	5,80724E-06	2	1544,85491	1,69	22,70	0
	High	vVEFLHVkPGk	2	1	1	Q55119	N-Term(iTRAQ4plex); K8(iTRAQ4plex); K11(iTRAQ4plex)	0,0000	0,818	2	2,1	0	0,000001352	34	0,000439511	3	1685,04728	-0,12	28,62	0
	High	sVmQHTYk	2	1	1	Q55119	N-Term(iTRAQ4plex); M3(Oxidation); K8(iTRAQ4plex)	0,0000	1,422	2	3,5	0	0,001954	20	0,010989299	3	1297,68253	0,95	9,39	0
P72777	Ycf54-like protein OS=Synechocystis sp. (strain PCC 6803 / Kazusa) GN=slr1780 PE	295,66	23,31	1	3	3	16	1,880	11	18,3	133	15,4	4,91							
	A3	Sequence	# PSMs	# Proteins	# Protein Groups	Protein Group Accessions	Modifications	ΔCn	114/115	114/115 Count	114/115 Variability [%]	q-Value	PEP	IonScore	Exp Value	Charge	MH+ [Da]	ΔM [ppm]	RT [min]	# Missed Cleavages
	High	nVAIVSTNk	7	1	1	P72777	N-Term(iTRAQ4plex); K9(iTRAQ4plex)	0,0000	2,059	5	4,7	0	0,00000214	63	5,00253E-07	2	1233,74199	1,18	20,58	0
	High	fLLEEEPFEEVLkER	3	1	1	P72777	N-Term(iTRAQ4plex); K13(iTRAQ4plex)	0,0000	1,521	3	2,0	0	0,00007632	27	0,002164675	3	2195,18479	0,41	50,19	1
	High	sFIVWVk	6	1	1	P72777	N-Term(iTRAQ4plex); K7(iTRAQ4plex)	0,0000	1,880	3	1,3	0,001	0,01076	44	3,96251E-05	2	1166,71819	0,49	40,60	0
P73679	Isopenicillin N epimerase OS=Synechocystis sp. (strain PCC 6803 / Kazusa) GN=cef	295,21	26,68	1	8	8	15	1,480	13	10,7	401	44,0	5,87							
	A3	Sequence	# PSMs	# Proteins	# Protein Groups	Protein Group Accessions	Modifications	ΔCn	114/115	114/115 Count	114/115 Variability [%]	q-Value	PEP	IonScore	Exp Value	Charge	MH+ [Da]	ΔM [ppm]	RT [min]	# Missed Cleavages
	High	lEVDYYAFTGHk	5	1	1	P73679	N-Term(iTRAQ4plex); K12(iTRAQ4plex)	0,0000	1,442	3	1,1	0	2,387E-10	51	8,66902E-06	3	1730,90165	1,40	36,88	0
	High	accHYITNEEEIEHLLSR	1	1	1	P73679	N-Term(iTRAQ4plex); C2(Carbamidomethyl); C3(Carbamidomethyl)	0,0000	1,259	1		0	2,7E-14	45	3,21344E-05	4	2418,13076	0,35	39,66	0
	High	wFAGPAGVGGLYIHGDAk	2	1	1	P73679	N-Term(iTRAQ4plex); K18(iTRAQ4plex)	0,0000	1,071	2	3,4	0	2,419E-10	45	4,09431E-05	3	2104,13047	4,07	42,51	0
	High	yQAIcQR	2	1	1	P73679	N-Term(iTRAQ4plex); C5(Carbamidomethyl)	0,0000	1,522	2	0,2	0	0,0004679	42	5,87449E-05	2	1082,55290	-0,41	16,25	0
	High	sITYGSk	2	1	1	P73679	N-Term(iTRAQ4plex); K7(iTRAQ4plex)	0,0000	1,654	2	6,8	0	0,0008951	37	0,000192296	2	1043,60320	5,42	16,21	0
	High	qQFPGLANk	1	1	1	P73679	N-Term(iTRAQ4plex); K9(iTRAQ4plex)	0,0000	1,294	1		0	0,0001023	32	0,00058479	2	1290,74150	0,48	24,92	0
	High	lGEINPTYVGWR	1	1	1	P73679	N-Term(iTRAQ4plex)	0,0000	1,480	1		0	0,0005667	22	0,006775947	2	1548,83122	1,34	41,05	0
	High	fFPVADTLNQGDAAAVLVNHLGPk	1	1	1	P73679	N-Term(iTRAQ4plex); K24(iTRAQ4plex)	0,0000	2,166	1		0	0,00001817	21	0,007227199	3	2782,51517	0,73	51,22	0
P73196	DNA ligase OS=Synechocystis sp. (strain PCC 6803 / Kazusa) GN=lig PE=4 SV=1 -	294,11	13,17	1	7	7	13	1,608	12	7,9	562	61,4	5,08							
	A3	Sequence	# PSMs	# Proteins	# Protein Groups	Protein Group Accessions	Modifications	ΔCn	114/115	114/115 Count	114/115 Variability [%]	q-Value	PEP	IonScore	Exp Value	Charge	MH+ [Da]	ΔM [ppm]	RT [min]	# Missed Cleavages
	High	qAVNEVAETRPk	2	1	1	P73196	N-Term(iTRAQ4plex); K12(iTRAQ4plex)	0,0000	1,679	1		0	9,963E-10	60	9,423E-07	3	1629,91678	0,30	16,71	0
	High	lVQLNSLVTR	2	1	1	P73196	N-Term(iTRAQ4plex)	0,0000	1,606	2	0,7	0	5,553E-07	59	1,17482E-06	2	1286,79119	-0,08	38,99	0
	High	vVILGTLNAmNR	1	1	1	P73196	N-Term(iTRAQ4plex); M10(Oxidation)	0,0000	1,156	1		0	0,00000234	45	2,97146E-05	2	1460,83916	1,05	35,88	0
	High	vkLETTQAEGDRLk	1	1	1	P73196	N-Term(iTRAQ4plex); K2(iTRAQ4plex); K14(iTRAQ4plex)	0,0000	0,981	1		0	4,713E-08	33	0,000457057	4	2020,17739	0,52	21,88	2
	High	akGEPPSEVPEk	2	1	1	P73196	N-Term(iTRAQ4plex); K2(iTRAQ4plex); K12(iTRAQ4plex)	0,0000	1,324	2	12,4	0	0,000001675	33	0,000824311	3	1699,96598	4,03	16,03	1
	High	fVGLQk	2	1	1	P73196	N-Term(iTRAQ4plex); K6(iTRAQ4plex)	0,0000	1,671	2	5,1	0	0,006549	30	0,000931044	2	979,61839	0,51	25,29	0
	High	tEPPRENk	3	1	1	P73196	N-Term(iTRAQ4plex); K8(iTRAQ4plex)	0,0000	1,675	3	3,5	0	0,001837	25	0,003047684	3	1258,70166	1,79	8,32	1
P74344	Molybdopterin biosynthesis MoeB protein OS=Synechocystis sp. (strain PCC 6803 /	292,73	26,28	1	10	10	15	0,896	14	19,2	392	42,7	5,05							
	A3	Sequence	# PSMs	# Proteins	# Protein Groups	Protein Group Accessions	Modifications	ΔCn	114/115	114/115 Count	114/115 Variability [%]	q-Value	PEP	IonScore	Exp Value	Charge	MH+ [Da]	ΔM [ppm]	RT [min]	# Missed Cleavages
	High	iILGAPNTLSGR	2	1	1	P74344	N-Term(iTRAQ4plex)	0,0000	0,846	2	2,1	0	0,000002531	55	3,28073E-06	2	1355,81352	0,55	34,61	0
	High	hIILPEVGLDGQk	2	1	1	P74344	N-Term(iTRAQ4plex); K13(iTRAQ4plex)	0,0000	0,957	2	21,6	0	3,188E-08	48	1,7578E-05	3	1707,00418	-0,12	34,98	0
	High	lLLFNAWDmk	1	1	1	P74344	N-Term(iTRAQ4plex); M9(Oxidation); K10(iTRAQ4plex)	0,0000	0,974	1		0	3,292E-08	45	3,28829E-05	2	1554,85942	0,08	49,57	0
	High	aLSLLk	3	1	1	P74344	N-Term(iTRAQ4plex); K6(iTRAQ4plex)	0,0000	1,104	2	5,6	0	0,007807	42	6,02518E-05	2	932,63768	-0,66	30,25	0
	High	vkELLDGHEGILIAHck	1	1	1	P74344	N-Term(iTRAQ4plex); K2(iTRAQ4plex); C16(Carbamidomethyl); K17(iTRAQ4p	0,0000	0,823	1		0	0,000002375	36	0,000264241	4	2364,34585	1,02	33,20	1
	High	eLLDGHEGILIAHck	1	1	1	P74344	N-Term(iTRAQ4plex); C14(Carbamidomethyl); K15(iTRAQ4plex)	0,0000	0,636	1		0	2,373E-07	29	0,001313387	4	1993,07717	-0,40	32,22	0
	High	qIIHGTSWVGkPk	1	1	1	P74344	N-Term(iTRAQ4plex); K11(iTRAQ4plex); K13(iTRAQ4plex)	0,0000	0,546	1		0	0,00000101	25	0,002992058	4	1883,12136	-0,74	23,52	0
	High	lRPNPVRPVIEk	2	1	1	P74344	N-Term(iTRAQ4plex); K12(iTRAQ4plex)	0,0000	0,896	2	0,9	0	0,0002345	19	0,012415666	3	1706,06778	-0,13	22,84	0
	High	hIILPEVGLDGQkR	1	1	1	P74344	N-Term(iTRAQ4plex); K13(iTRAQ4plex)	0,0000	4,342	1		0	0,00008105	17	0,019497099	4	1863,10500	-0,27	30,83	1
	High	eLHALmESQEkDFILLDVR	1	1	1	P74344	N-Term(iTRAQ4plex); M6(Oxidation); K11(iTRAQ4plex)	0,0000	1,792	1		0	0,000001044	16	0,022907094	4	2590,37758	-0,55	42,16	1
P32422	Photosystem I iron-sulfur center OS=Synechocystis sp. (strain PCC 6803 / Kazusa)	290,85	65,43	1	5	5	18	0,672	16	17,6	81	8,8	5,95							
	A3	Sequence	# PSMs	# Proteins	# Protein Groups	Protein Group Accessions	Modifications	ΔCn	114/115	114/115 Count	114/115 Variability [%]	q-Value	PEP	IonScore	Exp Value	Charge	MH+ [Da]	ΔM [ppm]	RT [min]	# Missed Cleavages
	High	aAQIASSPR	5	1	1	P32422	N-Term(iTRAQ4plex)	0,0000	0,518	3	5,7	0	0,0002243	51	8,01623E-06	2	1044,59783	5,71	14,36	0
	High	vYLGAETTR	5	1	1	P32422	N-Term(iTRAQ4plex)	0,0000	0,598	5	37,4	0	0,00003907	40	9,01509E-05	2	1153,63432	0,82	22,30	0
	High	rcETAcPTDFLSIR	3	1	1	P32422	N-Term(iTRAQ4plex); C2(Carbamidomethyl); C6(Carbamidomethyl)	0,0000	0,733	3	1,8	0	3,619E-09	40	0,000102087	3	1869,90799	0,62	33,76	1
	High	iYDTcIGcTQcVR	1	1	1	P32422	N-Term(iTRAQ4plex); C5(Carbamidomethyl); C8(Carbamidomethyl); C11(Car	0,0000	0,923	1		0	0,00006447	29	0,001140171	2	1789,81670	0,82	28,94	0
	High	tEDcVGckR	4	1	1	P32422	N-Term(iTRAQ4plex); C4(Carbamidomethyl); C7(Carbamidomethyl); K8(iTRA	0,0000	0,700	4	6,1	0	0,00004937	19	0,01216102	3	1412,68622	-0,19	8,95	1
Q59987	Light-dependent protochlorophyllide reductase OS=Synechocystis sp. (strain PCC 6	287,42	24,84	1	10	10	25	0,880	23	26,6	322	36,0	8,38							
	A3	Sequence	# PSMs	# Proteins	# Protein Groups	Protein Group Accessions	Modifications	ΔCn	114/115	114/115 Count	114/115 Variability [%]	q-Value	PEP	IonScore	Exp Value	Charge	MH+ [Da]	ΔM [ppm]	RT [min]	# Missed Cleavages
	High	gYVSQELAGER	1	1	1	Q59987	N-Term(iTRAQ4plex)	0,0000	0,665	1		0	0,000009938	46	2,27494E-05	2	1352,69365	0,70	24,21	0
	High	kPIAmINNk	2	1	1	Q59987	N-Term(iTRAQ4plex); K1(iTRAQ4plex); M5(Oxidation); K9(iTRAQ4plex)	0,0000	1,022	2	2,4	0	4,891E-08	37	0,000179461	3	1476,89353	0,17	14,83	0
	High	tIFPWFQk	5	1	1	Q59987	N-Term(iTRAQ4plex); K8(iTRAQ4plex)	0,0000	0,776	5	20,0	0	0,00005395	35	0,000341956	2	1354,77605	-0,11	47,82	0
	High	rFVAQFR	1	1	1	Q59987	N-Term(iTRAQ4plex)	0,0000	1,247	1		0	0,00008382	34	0,000418765	3	1067,62314	0,04	20,81	1
	High	lDLGYLDSVRR	2	1	1	Q59987	N-Term(iTRAQ4plex)	0,0000	0,732	2	1,1	0	0,00006507	25	0,003531588	3	1450,81418	0,47	36,98	1
	High	lIILGTVTANSk	2	1	1	Q59987	N-Term(iTRAQ4plex); K12(iTRAQ4plex)	0,0000	1,227	2	68,7	0	0,00001918	22	0,005942511	3	1517,95237	1,20	39,43	0
	High	lcNmLTTR	2	1	1	Q59987	N-Term(iTRAQ4plex); C2(Carbamidomethyl); M4(Oxidation)	0,0000	0,995	2	6,3	0	0,003322	16	0,023279305	2	1168,59380	0,23	21,04	0
	High	acPDADkR	4	1	1	Q59987	N-Term(iTRAQ4plex); C2(Carbamidomethyl); K7(iTRAQ4plex)	0,0000	0,909	4	3,6	0	0,00135	14	0,043348094	3	1220,63019	0,47	8,33	1
	High	eLGRPLk	1	1	1	Q59987	N-Term(iTRAQ4plex); K7(iTRAQ4plex)	0,0000	0,724	1		0,007	0,08532	10	0,097716973	3	1100,70417	1,03	15,02	0
	Medium	fVAQFR	5	1	1	Q59987	N-Term(iTRAQ4plex)	0,0000	0,484	3	25,3	0,01	0,1227	31	0,000816526	2	911,52275	0,84	27,64	0
Q55776	Sll0180 protein OS=Synechocystis sp. (strain PCC 6803 / Kazusa) GN=sll0180 PE=4	282,97	11,98	1	6	6	10	0,472	9	23,4	501	53,9	5,78							
	A3	Sequence	# PSMs	# Proteins	# Protein Groups	Protein Group Accessions	Modifications	ΔCn	114/115	114/115 Count	114/115 Variability [%]	q-Value	PEP	IonScore	Exp Value	Charge	MH+ [Da]	ΔM [ppm]	RT [min]	# Missed Cleavages
	High	aTVQANQSSVSASR	2	1	1	Q55776	N-Term(iTRAQ4plex)	0,0000	0,383	2	31,7	0	9,999E-11	94	4,18765E-10	2	1549,80657	0,94	14,64	0
	High	aQANLNLAQINFk	2	1	1	Q55776	N-Term(iTRAQ4plex); K13(iTRAQ4plex)	0,0000	0,541	1		0	5,863E-08	47	2,19265E-05	3	1732,99472	-0,09	39,16	0
	High	aTINAQk	1	1	1	Q55776	N-Term(iTRAQ4plex); K7(iTRAQ4plex)	0,0000	0,552	1		0	0,0003215	40	0,000117596	2	1033,62834	3,78	12,44	0
	High	lGLPVEVVR	1	1	1	Q55776	N-Term(iTRAQ4plex)	0,0000	0,362	1		0	0,0005343	24	0,004158819	2	1125,71086	-0,36	37,94	0
	High	iSFISPTANR	1	1	1	Q55776	N-Term(iTRAQ4plex)	0,0000	0,311	1		0,001	0,01748	32	0,000635331	2	1249,70281	0,53	32,17	0
	High	sAVQQAk	3	1	1	Q55776	N-Term(iTRAQ4plex); K7(iTRAQ4plex)	0,0000	0,530	3	0,4	0,007	0,08492	28	0,002410571	2	1019,60631	-2,43	10,03	0
Q55113	Slr0431 protein OS=Synechocystis sp. (strain PCC 6803 / Kazusa) GN=slr0431 PE=	282,13	17,20	1	3	3	7	1,446	7	16,6	250	27,0	8,37							
	A3	Sequence	# PSMs	# Proteins	# Protein Groups	Protein Group Accessions	Modifications	ΔCn	114/115	114/115 Count	114/115 Variability [%]	q-Value	PEP	IonScore	Exp Value	Charge	MH+ [Da]	ΔM [ppm]	RT [min]	# Missed Cleavages
	High	vVLSSLNLTPQNIVR	4	1	1	Q55113	N-Term(iTRAQ4plex)	0,0000	1,278	4	11,2	0	3,598E-12	84	3,89915E-09	2	1797,07292	0,78	45,95	0
	High	rLEGYIGSNTVSFQYPSDQVGk	1	1	1	Q55113	N-Term(iTRAQ4plex); K22(iTRAQ4plex)	0,0000	1,650	1		0	4,405E-13	62	6,85441E-07	3	2733,41269	1,47	37,82	1
	High	lVAYLR	2	1	1	Q55113	N-Term(iTRAQ4plex)	0,0000	1,456	2	1,0	0,004	0,0497	34	0,000555789	2	878,55846	0,49	30,62	0
Q55387	Periplasmic binding protein of ABC transporter for natural amino acids OS=Synecho	280,72	9,25	1	4	4	11	1,899	9	36,9	454	46,6	4,56							
	A3	Sequence	# PSMs	# Proteins	# Protein Groups	Protein Group Accessions	Modifications	ΔCn	114/115	114/115 Count	114/115 Variability [%]	q-Value	PEP	IonScore	Exp Value	Charge	MH+ [Da]	ΔM [ppm]	RT [min]	# Missed Cleavages
	High	sNTGAGIQSk	6	1	1	Q55387	N-Term(iTRAQ4plex); K10(iTRAQ4plex)	0,0000	2,374	4	1,6	0	3,438E-08	66	2,74405E-07	2	1250,69682	2,00	10,66	0
	High	tAPPDTYQAQALAALAk	1	1	1	Q55387	N-Term(iTRAQ4plex); K17(iTRAQ4plex)	0,0000	1,032	1		0	2,418E-09	44	5,22328E-05	3	2018,12015	1,99	39,52	0
	High	sLEAFTAQWk	3	1	1	Q55387	N-Term(iTRAQ4plex); K10(iTRAQ4plex)	0,0000	1,024	3	41,8	0	0,0001119	26	0,002477251	3	1468,80622	1,59	42,07	0
	Medium	gFWAR	1	1	1	Q55387	N-Term(iTRAQ4plex)	0,0000	1,899	1		0,017	0,1898	16	0,023386757	2	780,42833	1,25	23,93	0
P77966	DNA gyrase subunit B OS=Synechocystis sp. (strain PCC 6803 / Kazusa) GN=gyrB	277,51	6,68	1	7	7	12	0,828	10	15,7	1078	122,7	6,64							
	A3	Sequence	# PSMs	# Proteins	# Protein Groups	Protein Group Accessions	Modifications	ΔCn	114/115	114/115 Count	114/115 Variability [%]	q-Value	PEP	IonScore	Exp Value	Charge	MH+ [Da]	ΔM [ppm]	RT [min]	# Missed Cleavages
	High	sALETVLTVLHAGGk	2	1	1	P77966	N-Term(iTRAQ4plex); K15(iTRAQ4plex)	0,0000	0,575	1		0	2,507E-11	55	2,85082E-06	3	1784,05149	-0,33	51,18	0
	High	fQAILPLR	3	1	1	P77966	N-Term(iTRAQ4plex)	0,0000	0,741	3	4,7	0	0,00001072	52	6,20826E-06	2	1101,68999	-0,12	41,70	0
	High	rFQAILPLR	2	1	1	P77966	N-Term(iTRAQ4plex)	0,0000	0,725	1		0	2,199E-07	41	7,58525E-05	3	1257,79090	-0,28	33,34	1
	High	rEFIETYGTk	1	1	1	P77966	N-Term(iTRAQ4plex); K10(iTRAQ4plex)	0,0000	0,818	1		0	0,0000097	39	0,000118842	3	1531,83496	-0,61	23,19	1
	High	lkELAYLNAGVR	1	1	1	P77966	N-Term(iTRAQ4plex); K2(iTRAQ4plex)	0,0000	0,908	1		0	9,737E-09	35	0,000286398	3	1634,98166	-0,97	36,27	1
	High	rADSLkEEQFYYEGGIR	2	1	1	P77966	N-Term(iTRAQ4plex); K6(iTRAQ4plex)	0,0000	0,901	2	2,2	0	0,00001426	22	0,005740768	4	2349,21425	2,75	28,88	2
	High	tLLLTFFYR	1	1	1	P77966	N-Term(iTRAQ4plex)	0,0000	1,057	1		0	0,005562	20	0,011720425	2	1317,77202	2,48	55,97	0
Q55557	Virginiamycin B hydrolase Vgb OS=Synechocystis sp. (strain PCC 6803 / Kazusa) G	273,49	21,88	1	6	6	14	2,985	14	40,1	352	38,5	6,80							
	A3	Sequence	# PSMs	# Proteins	# Protein Groups	Protein Group Accessions	Modifications	ΔCn	114/115	114/115 Count	114/115 Variability [%]	q-Value	PEP	IonScore	Exp Value	Charge	MH+ [Da]	ΔM [ppm]	RT [min]	# Missed Cleavages
	High	vTIYPTVPGIGTR	3	1	1	Q55557	N-Term(iTRAQ4plex)	0,0000	3,711	3	0,0	0	0,00009862	49	1,21051E-05	2	1517,88274	1,25	39,16	0
	High	iFPAPQGFGPYGmTATPk	2	1	1	Q55557	N-Term(iTRAQ4plex); M13(Oxidation); K18(iTRAQ4plex)	0,0000	1,960	2	78,4	0	5,499E-09	48	1,59945E-05	3	2184,14426	1,84	41,21	0
	High	iFPAPQGFGPYGMTATPk	1	1	1	Q55557	N-Term(iTRAQ4plex); K18(iTRAQ4plex)	0,0000	1,157	1		0	4,943E-09	48	1,61425E-05	3	2168,14927	1,81	44,49	0
	High	lYPLGTGSHPHGVVmGPDGGLWIcDESNAIR	1	1	1	Q55557	N-Term(iTRAQ4plex); M15(Oxidation); C24(Carbamidomethyl)	0,0000	4,131	1		0	5,845E-11	39	0,000123586	4	3465,68398	1,10	42,72	0
	High	ykVTIYPTVPGIGTR	1	1	1	Q55557	N-Term(iTRAQ4plex); K2(iTRAQ4plex)	0,0000	1,685	1		0	0,000004442	30	0,001078872	3	1953,14353	1,19	36,95	1
	Medium	fNPQNk	1	1	1	Q55557	N-Term(iTRAQ4plex); K6(iTRAQ4plex)	0,0000	1,871	1		0,011	0,1315	23	0,004666272	2	1035,58891	6,13	15,64	0
	Medium	lPGLGPR	5	1	1	Q55557	N-Term(iTRAQ4plex)	0,0000	3,345	5	22,7	0,013	0,1439	19	0,012472974	2	853,53801	0,43	25,81	0
P73654	Ssl3364 protein OS=Synechocystis sp. (strain PCC 6803 / Kazusa) GN=ssl3364 PE=	271,80	27,03	1	2	2	7	1,156	7	14,0	74	8,3	4,22							
	A3	Sequence	# PSMs	# Proteins	# Protein Groups	Protein Group Accessions	Modifications	ΔCn	114/115	114/115 Count	114/115 Variability [%]	q-Value	PEP	IonScore	Exp Value	Charge	MH+ [Da]	ΔM [ppm]	RT [min]	# Missed Cleavages
	High	qQHPTQTTLEk	4	1	1	P73654	N-Term(iTRAQ4plex); K11(iTRAQ4plex)	0,0000	1,106	4	5,7	0	4,683E-08	62	6,74481E-07	2	1598,88359	5,96	12,84	0
	High	iEQELANAR	3	1	1	P73654	N-Term(iTRAQ4plex)	0,0000	1,299	3	3,4	0	0,000001127	56	2,58208E-06	2	1187,65019	0,07	25,78	0
P74466	Putative ATP-dependent Clp protease proteolytic subunit-like OS=Synechocystis sp.	271,45	20,00	1	4	4	7	0,894	6	17,8	225	24,9	5,00							
	A3	Sequence	# PSMs	# Proteins	# Protein Groups	Protein Group Accessions	Modifications	ΔCn	114/115	114/115 Count	114/115 Variability [%]	q-Value	PEP	IonScore	Exp Value	Charge	MH+ [Da]	ΔM [ppm]	RT [min]	# Missed Cleavages
	High	tGAQGQATDIQIR	3	1	1	P74466	N-Term(iTRAQ4plex)	0,0000	0,773	2	4,2	0	2,621E-09	93	4,56005E-10	2	1502,80486	0,31	21,47	0
	High	tFYLTPAQAk	2	1	1	P74466	N-Term(iTRAQ4plex); K10(iTRAQ4plex)	0,0000	1,080	2	34,0	0	3,668E-07	66	2,48296E-07	2	1427,81511	0,99	30,32	0
	High	aSLPHATIVLNQNR	1	1	1	P74466	N-Term(iTRAQ4plex)	0,0000	1,010	1		0	6,028E-07	36	0,00025175	3	1677,95066	-0,64	28,90	0
	High	akEVISNk	1	1	1	P74466	N-Term(iTRAQ4plex); K2(iTRAQ4plex); K8(iTRAQ4plex)	0,0000	0,925	1		0	0,00117	23	0,005104697	3	1320,81791	-2,45	13,38	1
P52981	1,4-alpha-glucan branching enzyme GlgB OS=Synechocystis sp. (strain PCC 6803 /	268,56	11,69	1	9	9	13	0,668	13	22,8	770	89,5	5,62							
	A3	Sequence	# PSMs	# Proteins	# Protein Groups	Protein Group Accessions	Modifications	ΔCn	114/115	114/115 Count	114/115 Variability [%]	q-Value	PEP	IonScore	Exp Value	Charge	MH+ [Da]	ΔM [ppm]	RT [min]	# Missed Cleavages
	High	gVYFAVWAPNAR	2	1	1	P52981	N-Term(iTRAQ4plex)	0,0000	0,680	2	14,7	0	0,000007463	58	1,85949E-06	2	1494,80010	1,77	42,93	0
	High	lGAHLmTVDGVk	1	1	1	P52981	N-Term(iTRAQ4plex); M6(Oxidation); K12(iTRAQ4plex)	0,0000	0,574	1		0	0,000006764	42	7,04644E-05	3	1544,87015	-0,51	25,81	0
	High	vNQWVIR	1	1	1	P52981	N-Term(iTRAQ4plex)	0,0000	0,833	1		0	0,005491	38	0,000144202	2	1058,63201	8,73	30,36	0
	High	tDPYGFYQEVRPk	1	1	1	P52981	N-Term(iTRAQ4plex); K13(iTRAQ4plex)	0,0000	0,846	1		0	6,53E-08	36	0,000317932	3	1887,98325	-0,59	32,07	0
	High	aLFTYMFTHPGk	2	1	1	P52981	N-Term(iTRAQ4plex); K12(iTRAQ4plex)	0,0000	0,586	2	24,3	0	0,000001731	32	0,000568814	3	1700,90915	1,09	41,47	0
	High	aLFTYmFTHPGk	2	1	1	P52981	N-Term(iTRAQ4plex); M6(Oxidation); K12(iTRAQ4plex)	0,0000	0,656	2	2,8	0	0,000009455	28	0,001632939	3	1716,90085	-0,79	35,38	0
	High	qFFTELNHLYk	1	1	1	P52981	N-Term(iTRAQ4plex); K11(iTRAQ4plex)	0,0000	0,556	1		0	0,000001753	27	0,002218043	3	1727,93534	-0,35	41,16	0
	High	nWEGHIYEk	1	1	1	P52981	N-Term(iTRAQ4plex); K9(iTRAQ4plex)	0,0000	0,731	1		0	0,000002756	26	0,002564307	3	1463,75159	-0,39	21,92	0
	High	hSVVSFIR	1	1	1	P52981	N-Term(iTRAQ4plex)	0,0000	0,469	1		0	0,0001405	19	0,011884201	3	1088,63349	0,15	24,41	0
	High	lIPYVk	1	1	1	P52981	N-Term(iTRAQ4plex); K6(iTRAQ4plex)	0,0000	0,798	1		0,009	0,1126	26	0,002243727	2	1020,66808	-1,48	32,97	0
P73335	Uncharacterized deoxyribonuclease sll1786 OS=Synechocystis sp. (strain PCC 6803	268,45	18,77	1	4	4	12	1,014	11	25,6	261	29,2	5,87							
	A3	Sequence	# PSMs	# Proteins	# Protein Groups	Protein Group Accessions	Modifications	ΔCn	114/115	114/115 Count	114/115 Variability [%]	q-Value	PEP	IonScore	Exp Value	Charge	MH+ [Da]	ΔM [ppm]	RT [min]	# Missed Cleavages
	High	hVPLETLAQQTTTNAR	1	1	1	P73335	N-Term(iTRAQ4plex)	0,0000	0,661	1		0	1,334E-09	59	1,12712E-06	3	1924,03781	0,47	29,54	0
	High	hVAEAIAALR	4	1	1	P73335	N-Term(iTRAQ4plex)	0,0000	0,913	4	13,7	0	0,00000518	50	1,06216E-05	2	1194,70806	0,40	26,65	0
	High	aDNRDHQIEVFR	5	1	1	P73335	N-Term(iTRAQ4plex)	0,0000	1,104	4	16,5	0	1,676E-07	36	0,000253495	3	1643,83683	-0,16	19,68	1
	High	eLNkPVIIHcR	2	1	1	P73335	N-Term(iTRAQ4plex); K4(iTRAQ4plex); C10(Carbamidomethyl)	0,0000	1,640	2	6,9	0	0,00003596	36	0,000266054	4	1666,96804	0,89	21,86	0
P73312	50S ribosomal protein L29 OS=Synechocystis sp. (strain PCC 6803 / Kazusa) GN=rp	266,27	53,42	1	4	4	12	2,398	11	84,4	73	8,5	7,53							
	A3	Sequence	# PSMs	# Proteins	# Protein Groups	Protein Group Accessions	Modifications	ΔCn	114/115	114/115 Count	114/115 Variability [%]	q-Value	PEP	IonScore	Exp Value	Charge	MH+ [Da]	ΔM [ppm]	RT [min]	# Missed Cleavages
	High	kLGDEELATEILATk	3	1	1	P73312	N-Term(iTRAQ4plex); K1(iTRAQ4plex); K15(iTRAQ4plex)	0,0000	1,139	3	142,9	0	0,000000396	60	1,01384E-06	3	2063,19944	1,63	40,33	1
	High	lAQLLTVER	4	1	1	P73312	N-Term(iTRAQ4plex)	0,0000	2,456	3	3,5	0	0,00000054	57	1,77816E-06	2	1186,72783	0,17	39,19	0
	High	rPENPHEFk	3	1	1	P73312	N-Term(iTRAQ4plex); K9(iTRAQ4plex)	0,0000	3,933	3	15,2	0	0,0003215	38	0,000162918	2	1441,78166	1,81	11,31	0
	High	fQQATR	2	1	1	P73312	N-Term(iTRAQ4plex)	0,0000	1,197	2	8,4	0,005	0,06526	15	0,035397289	2	894,49181	0,45	12,58	0
Q55707	Uncharacterized protein sll0617 OS=Synechocystis sp. (strain PCC 6803 / Kazusa)	266,15	17,23	1	8	8	15	1,286	11	11,7	267	28,9	5,03							
	A3	Sequence	# PSMs	# Proteins	# Protein Groups	Protein Group Accessions	Modifications	ΔCn	114/115	114/115 Count	114/115 Variability [%]	q-Value	PEP	IonScore	Exp Value	Charge	MH+ [Da]	ΔM [ppm]	RT [min]	# Missed Cleavages
	High	aNLNDLVSk	1	1	1	Q55707	N-Term(iTRAQ4plex); K9(iTRAQ4plex)	0,0000	1,435	1		0	2,359E-07	62	6,4445E-07	2	1261,73601	0,44	29,38	0
	High	nLAALEAk	6	1	1	Q55707	N-Term(iTRAQ4plex); K8(iTRAQ4plex)	0,0000	1,273	3	1,1	0	0,00001286	55	3,99667E-06	2	1117,68254	0,51	26,12	0
	High	lNQDTQEAk	1	1	1	Q55707	N-Term(iTRAQ4plex); K9(iTRAQ4plex)	0,0000	1,417	1		0	0,000001772	41	7,74408E-05	2	1334,72002	3,42	13,53	0
	High	rNLAALEAk	2	1	1	Q55707	N-Term(iTRAQ4plex); K9(iTRAQ4plex)	0,0000	1,179	1		0	0,000005733	41	8,43277E-05	3	1273,78370	0,48	19,21	1
	High	lNQDTQEAkk	1	1	1	Q55707	N-Term(iTRAQ4plex); K9(iTRAQ4plex); K10(iTRAQ4plex)	0,0000	1,416	1		0	0,000001437	20	0,009615459	3	1606,91379	0,82	12,45	1
	High	tmSENLR	2	1	1	Q55707	N-Term(iTRAQ4plex); M2(Oxidation)	0,0000	1,228	2	4,8	0,005	0,0576	22	0,006698383	2	1010,50536	-0,39	11,59	0
	Medium	kNmLQAR	1	1	1	Q55707	N-Term(iTRAQ4plex); K1(iTRAQ4plex); M3(Oxidation)	0,0000	1,400	1		0,035	0,3719	12	0,062801498	3	1164,67737	1,03	8,63	1
	Medium	kWEDR	1	1	1	Q55707	N-Term(iTRAQ4plex); K1(iTRAQ4plex)	0,0000	1,286	1		0,046	0,4764	17	0,020557486	3	1021,56470	-2,18	12,87	1
P48946	30S ribosomal protein S18 OS=Synechocystis sp. (strain PCC 6803 / Kazusa) GN=rp	264,06	25,35	1	3	3	11	1,384	11	28,4	71	8,4	10,76							
	A3	Sequence	# PSMs	# Proteins	# Protein Groups	Protein Group Accessions	Modifications	ΔCn	114/115	114/115 Count	114/115 Variability [%]	q-Value	PEP	IonScore	Exp Value	Charge	MH+ [Da]	ΔM [ppm]	RT [min]	# Missed Cleavages
	High	lVALLPFVNk	9	1	1	P48946	N-Term(iTRAQ4plex); K10(iTRAQ4plex)	0,0000	1,384	9	24,3	0	0,00002781	56	2,69135E-06	2	1401,90788	0,47	50,37	0
	High	iTGLTAk	1	1	1	P48946	N-Term(iTRAQ4plex); K7(iTRAQ4plex)	0,0000	3,138	1		0	0,0007032	46	2,47725E-05	2	991,64299	4,00	22,74	0
	High	rITGLTAk	1	1	1	P48946	N-Term(iTRAQ4plex); K8(iTRAQ4plex)	0,0000	0,504	1		0,001	0,009235	15	0,031475309	3	1147,73984	-0,27	16,44	1
P73636	30S ribosomal protein S6 OS=Synechocystis sp. (strain PCC 6803 / Kazusa) GN=rps	258,94	30,09	1	6	6	15	2,071	15	26,8	113	13,2	8,37							
	A3	Sequence	# PSMs	# Proteins	# Protein Groups	Protein Group Accessions	Modifications	ΔCn	114/115	114/115 Count	114/115 Variability [%]	q-Value	PEP	IonScore	Exp Value	Charge	MH+ [Da]	ΔM [ppm]	RT [min]	# Missed Cleavages
	High	lTPEkPEk	5	1	1	P73636	N-Term(iTRAQ4plex); K5(iTRAQ4plex); K8(iTRAQ4plex)	0,0000	2,450	5	4,7	0	0,00002168	42	6,68949E-05	2	1373,83782	0,99	16,96	0
	High	vSQEVTk	2	1	1	P73636	N-Term(iTRAQ4plex); K7(iTRAQ4plex)	0,0000	1,690	2	2,4	0	0,0005523	38	0,00014756	2	1078,64275	7,49	14,82	0
	High	rLAYQIR	1	1	1	P73636	N-Term(iTRAQ4plex)	0,0000	2,324	1		0	0,0004041	22	0,005688529	3	1063,64966	0,34	18,56	1
	High	lNDNVmR	2	1	1	P73636	N-Term(iTRAQ4plex); M6(Oxidation)	0,0000	1,272	2	32,5	0,001	0,0154	27	0,001936288	2	1021,52184	0,10	13,83	0
	High	lAYQIR	1	1	1	P73636	N-Term(iTRAQ4plex)	0,0000	1,701	1		0,005	0,05859	29	0,001783749	2	907,54900	0,90	23,84	0
	High	fLSIk	4	1	1	P73636	N-Term(iTRAQ4plex); K5(iTRAQ4plex)	0,0000	1,907	4	13,2	0,005	0,05871	28	0,001698126	2	895,58537	-0,17	30,12	0
Q01950	Phycobilisome 7.8 kDa linker polypeptide, allophycocyanin-associated, core OS=Syn	258,27	50,75	1	3	3	6	0,840	6	9,1	67	7,7	10,93							
	A3	Sequence	# PSMs	# Proteins	# Protein Groups	Protein Group Accessions	Modifications	ΔCn	114/115	114/115 Count	114/115 Variability [%]	q-Value	PEP	IonScore	Exp Value	Charge	MH+ [Da]	ΔM [ppm]	RT [min]	# Missed Cleavages
	High	vELATGRPGTNAGLA	2	1	1	Q01950	N-Term(iTRAQ4plex)	0,0000	0,840	2	1,7	0	5,215E-08	66	2,43326E-07	2	1570,86809	0,68	29,39	0
	High	eLQNTYFTk	2	1	1	Q01950	N-Term(iTRAQ4plex); K9(iTRAQ4plex)	0,0000	0,984	2	9,0	0	0,00001527	60	1,02658E-06	2	1431,77153	-0,48	29,21	0
	High	iTAcVPSQTR	2	1	1	Q01950	N-Term(iTRAQ4plex); C4(Carbamidomethyl)	0,0000	0,790	2	0,3	0	0,000006966	43	4,87495E-05	2	1276,68059	0,44	21,21	0
Q59978	Protein GrpE OS=Synechocystis sp. (strain PCC 6803 / Kazusa) GN=grpE PE=3 SV=	256,88	20,88	1	6	6	16	1,412	15	15,8	249	27,6	4,68							
	A3	Sequence	# PSMs	# Proteins	# Protein Groups	Protein Group Accessions	Modifications	ΔCn	114/115	114/115 Count	114/115 Variability [%]	q-Value	PEP	IonScore	Exp Value	Charge	MH+ [Da]	ΔM [ppm]	RT [min]	# Missed Cleavages
	High	gYLLDDIVLR	3	1	1	Q59978	N-Term(iTRAQ4plex)	0,0000	1,271	3	3,7	0	3,911E-07	52	6,69838E-06	2	1320,76531	0,68	47,69	0
	High	yVALAAEFDNFR	1	1	1	Q59978	N-Term(iTRAQ4plex)	0,0000	1,200	1		0	3,895E-08	43	4,52866E-05	2	1559,79839	0,56	47,51	0
	High	yVALAAEFDNFRk	1	1	1	Q59978	N-Term(iTRAQ4plex); K13(iTRAQ4plex)	0,0000	1,355	1		0	0,000003196	35	0,000331872	3	1831,99674	1,20	43,11	1
	High	ekEEQAk	7	1	1	Q59978	N-Term(iTRAQ4plex); K2(iTRAQ4plex); K7(iTRAQ4plex)	0,0000	1,587	7	5,1	0	0,001037	34	0,00043451	2	1293,73796	0,38	7,89	1
	High	tQIkPNSDGENQIHk	2	1	1	Q59978	N-Term(iTRAQ4plex); K4(iTRAQ4plex); K15(iTRAQ4plex)	0,0000	1,270	1		0	0,000002207	26	0,00246827	4	2141,17202	2,09	16,57	0
	High	sYQGVYk	2	1	1	Q59978	N-Term(iTRAQ4plex); K7(iTRAQ4plex)	0,0000	1,469	2	10,7	0,003	0,03569	28	0,001530982	2	1132,62663	2,26	16,98	0
Q55734	Sll0395 protein OS=Synechocystis sp. (strain PCC 6803 / Kazusa) GN=sll0395 PE=4	256,77	25,00	1	5	5	8	0,503	7	7,1	212	23,7	6,52							
	A3	Sequence	# PSMs	# Proteins	# Protein Groups	Protein Group Accessions	Modifications	ΔCn	114/115	114/115 Count	114/115 Variability [%]	q-Value	PEP	IonScore	Exp Value	Charge	MH+ [Da]	ΔM [ppm]	RT [min]	# Missed Cleavages
	High	tmATATPLSk	1	1	1	Q55734	N-Term(iTRAQ4plex); M2(Oxidation); K10(iTRAQ4plex)	0,0000	0,503	1		0	1,577E-07	66	2,29071E-07	2	1324,74285	3,28	17,32	0
	High	tFTTGNVLVVSHk	2	1	1	Q55734	N-Term(iTRAQ4plex); K13(iTRAQ4plex)	0,0000	0,442	1		0	3,661E-11	58	2,17225E-06	3	1690,97239	-0,40	29,64	0
	High	tLNLYFLR	2	1	1	Q55734	N-Term(iTRAQ4plex)	0,0000	0,576	2	27,8	0	0,0001987	52	6,59128E-06	2	1183,69560	0,02	45,44	0
	High	lINLPIQk	1	1	1	Q55734	N-Term(iTRAQ4plex); K8(iTRAQ4plex)	0,0000	0,492	1		0	0,0008941	43	5,11646E-05	2	1226,80657	-0,76	38,67	0
	High	dVSWTAIFASPLHR	2	1	1	Q55734	N-Term(iTRAQ4plex)	0,0000	0,590	2	16,9	0	1,345E-08	42	5,74077E-05	3	1743,92997	0,02	47,65	0
Q6ZEQ0	Non-heme chloroperoxidase OS=Synechocystis sp. (strain PCC 6803 / Kazusa) GN=	256,70	24,82	1	6	6	12	0,946	10	9,7	274	30,1	5,60							
	A3	Sequence	# PSMs	# Proteins	# Protein Groups	Protein Group Accessions	Modifications	ΔCn	114/115	114/115 Count	114/115 Variability [%]	q-Value	PEP	IonScore	Exp Value	Charge	MH+ [Da]	ΔM [ppm]	RT [min]	# Missed Cleavages
	High	sQFFLDIASGPFFGFNRPGAk	3	1	1	Q6ZEQ0	N-Term(iTRAQ4plex); K21(iTRAQ4plex)	0,0000	0,904	2	3,3	0	1,353E-14	80	9,95337E-09	3	2591,36936	1,68	54,76	0
	High	iYPGGSHSLGDTSk	2	1	1	Q6ZEQ0	N-Term(iTRAQ4plex); K14(iTRAQ4plex)	0,0000	0,924	1		0	0,000007869	47	2,15263E-05	3	1706,89505	-0,09	21,11	0
	High	nAYDcIk	1	1	1	Q6ZEQ0	N-Term(iTRAQ4plex); C5(Carbamidomethyl); K7(iTRAQ4plex)	0,0000	1,008	1		0	0,0007832	44	4,40524E-05	2	1171,60381	1,56	17,92	0
	High	aVLMGAVPPLMLk	1	1	1	Q6ZEQ0	N-Term(iTRAQ4plex); K13(iTRAQ4plex)	0,0000	1,167	1		0	0,00005208	42	6,88605E-05	3	1627,98740	-0,51	48,70	0
	High	sTITTk	2	1	1	Q6ZEQ0	N-Term(iTRAQ4plex); K6(iTRAQ4plex)	0,0000	0,945	2	10,5	0,001	0,01231	41	7,96104E-05	2	938,57549	-0,63	13,23	0
	High	aVLmGAVPPLMLk	1	1	1	Q6ZEQ0	N-Term(iTRAQ4plex); M4(Oxidation); K13(iTRAQ4plex)	0,0000	0,967	1		0,001	0,01529	16	0,026422263	3	1643,98239	-0,46	44,31	0
	High	aVLmGAVPPLmLk	1	1	1	Q6ZEQ0	N-Term(iTRAQ4plex); M4(Oxidation); M11(Oxidation); K13(iTRAQ4plex)	0,0000	0,989	1		0,005	0,07038	10	0,090150887	3	1659,97940	0,81	40,78	0
	High	aAFLADR	1	1	1	Q6ZEQ0	N-Term(iTRAQ4plex)	0,0000	0,694	1		0,006	0,07622	25	0,00349921	2	907,51146	-0,40	22,20	0
Q55953	Sll0781 protein OS=Synechocystis sp. (strain PCC 6803 / Kazusa) GN=sll0781 PE=4	255,96	45,81	1	9	9	15	1,122	14	30,6	155	18,6	6,24							
	A3	Sequence	# PSMs	# Proteins	# Protein Groups	Protein Group Accessions	Modifications	ΔCn	114/115	114/115 Count	114/115 Variability [%]	q-Value	PEP	IonScore	Exp Value	Charge	MH+ [Da]	ΔM [ppm]	RT [min]	# Missed Cleavages
	High	tTPLVPPFTk	4	1	1	Q55953	N-Term(iTRAQ4plex); K10(iTRAQ4plex)	0,0000	1,411	4	11,2	0	0,00001563	53	4,80806E-06	2	1388,84001	0,59	35,06	0
	High	rEASINDkPIAESER	1	1	1	Q55953	N-Term(iTRAQ4plex); K8(iTRAQ4plex)	0,0000	1,086	1		0	3,705E-09	38	0,000148242	4	2003,07619	0,06	16,76	1
	High	eLWAFGENR	1	1	1	Q55953	N-Term(iTRAQ4plex)	0,0000	1,229	1		0	0,0008524	34	0,000417802	2	1265,63933	-0,18	38,08	0
	High	eASINDkPIAESER	2	1	1	Q55953	N-Term(iTRAQ4plex); K7(iTRAQ4plex)	0,0000	1,159	1		0	8,414E-09	31	0,000849122	3	1846,97458	-0,20	19,14	0
	High	lVkELWAFGENR	1	1	1	Q55953	N-Term(iTRAQ4plex); K3(iTRAQ4plex)	0,0000	0,994	1		0	0,000004869	28	0,001570254	3	1749,99222	1,80	43,10	1
	High	eASINDkPIAESERR	1	1	1	Q55953	N-Term(iTRAQ4plex); K7(iTRAQ4plex)	0,0000	0,886	1		0	0,000006264	20	0,010665225	4	2003,07509	-0,49	17,61	1
	High	rFFWDAPGDRPLDHPGLINTPE	1	1	1	Q55953	N-Term(iTRAQ4plex)	0,0000	0,768	1		0,001	0,015	15	0,028639802	4	2694,35586	0,46	42,90	1
	High	eIAIAk	2	1	1	Q55953	N-Term(iTRAQ4plex); K6(iTRAQ4plex)	0,0000	1,205	2	5,0	0,004	0,04493	42	0,000137414	2	932,60515	3,47	20,94	0
	Medium	iREFLR	2	1	1	Q55953	N-Term(iTRAQ4plex)	0,0000	0,912	2	3,1	0,011	0,131	19	0,013061709	3	977,60172	0,42	23,60	1
Q57038	Cytochrome b6 OS=Synechocystis sp. (strain PCC 6803 / Kazusa) GN=petB PE=3 S	253,87	16,22	1	4	4	8	0,971	7	14,1	222	25,0	9,01							
	A3	Sequence	# PSMs	# Proteins	# Protein Groups	Protein Group Accessions	Modifications	ΔCn	114/115	114/115 Count	114/115 Variability [%]	q-Value	PEP	IonScore	Exp Value	Charge	MH+ [Da]	ΔM [ppm]	RT [min]	# Missed Cleavages
	High	gSESVGQATLTR	2	1	1	Q57038	N-Term(iTRAQ4plex)	0,0000	0,914	2	9,0	0	0,000000448	84	3,58072E-09	2	1349,72209	5,87	17,75	0
	High	vYLTGGFk	2	1	1	Q57038	N-Term(iTRAQ4plex); K8(iTRAQ4plex)	0,0000	1,144	1		0	0,00003675	44	4,09232E-05	2	1172,69097	-0,70	32,09	0
	High	vFQWFNDR	3	1	1	Q57038	N-Term(iTRAQ4plex)	0,0000	0,939	3	8,9	0	0,00001701	35	0,000297831	2	1255,63506	0,79	42,85	0
	Medium	kQGIGGPL	1	1	1	Q57038	N-Term(iTRAQ4plex); K1(iTRAQ4plex)	0,0000	1,127	1		0,046	0,4851	22	0,006760363	2	1087,67656	4,75	21,34	1
P73672	4-hydroxy-3-methylbut-2-en-1-yl diphosphate synthase OS=Synechocystis sp. (strai	252,73	18,61	1	6	6	11	0,925	10	10,5	403	44,1	5,96							
	A3	Sequence	# PSMs	# Proteins	# Protein Groups	Protein Group Accessions	Modifications	ΔCn	114/115	114/115 Count	114/115 Variability [%]	q-Value	PEP	IonScore	Exp Value	Charge	MH+ [Da]	ΔM [ppm]	RT [min]	# Missed Cleavages
	High	tLFNLEDVLHEVR	4	1	1	P73672	N-Term(iTRAQ4plex)	0,0000	0,877	3	4,5	0	3,502E-11	59	1,16137E-06	3	1728,94095	0,46	50,21	0

	High	qAGYIALYR	1	1	1	P73672	N-Term(iTRAQ4plex)	0,0000	1,059	1		0	0,000001462	48	1,64048E-05	2	1198,66997	-0,10	29,23	0
	High	iRETLEPLVISLR	2	1	1	P73672	N-Term(iTRAQ4plex)	0,0000	0,848	2	8,6	0	0,000000376	38	0,000166713	3	1683,03000	0,84	43,60	1
	High	vPVmLAAYR	2	1	1	P73672	N-Term(iTRAQ4plex); M4(Oxidation)	0,0000	0,976	2	0,5	0	0,0006643	37	0,000208435	2	1179,66838	0,60	32,07	0
	High	rVPETDGVQELINLIk	1	1	1	P73672	N-Term(iTRAQ4plex); K16(iTRAQ4plex)	0,0000	0,813	1		0	4,264E-10	34	0,000385452	3	2112,22940	1,25	50,29	1
	High	iNPGLYVFEkPDAQR	1	1	1	P73672	N-Term(iTRAQ4plex); K10(iTRAQ4plex)	0,0000	1,131	1		0	0,00004403	16	0,025643069	3	2035,12333	0,87	37,82	0
Q55828	LL-diaminopimelate aminotransferase OS=Synechocystis sp. (strain PCC 6803 / Kaz	251,22	18,20	1	6	6	14	0,808	13	13,1	412	45,1	5,62							
	A3	Sequence	# PSMs	# Proteins	# Protein Groups	Protein Group Accessions	Modifications	ΔCn	114/115	114/115 Count	114/115 Variability [%]	q-Value	PEP	IonScore	Exp Value	Charge	MH+ [Da]	ΔM [ppm]	RT [min]	# Missed Cleavages
	High	kVDLIYLcFPNNPTGATATk	2	1	1	Q55828	N-Term(iTRAQ4plex); K1(iTRAQ4plex); C8(Carbamidomethyl); K20(iTRAQ4pl	0,0000	1,156	2	17,6	0	2,42E-11	59	1,66028E-06	3	2655,45133	-1,04	42,67	1
	High	rVNAFTTANPNAQVIk	3	1	1	Q55828	N-Term(iTRAQ4plex); K16(iTRAQ4plex)	0,0000	0,740	3	51,2	0	3,933E-10	54	3,67257E-06	3	2032,15335	-0,44	25,89	1
	High	tPHGLSSWDFFDk	1	1	1	Q55828	N-Term(iTRAQ4plex); K13(iTRAQ4plex)	0,0000	0,754	1		0	6,726E-11	46	2,79235E-05	3	1824,91495	-0,55	42,20	0
	High	iAQHDFQAR	4	1	1	Q55828	N-Term(iTRAQ4plex)	0,0000	0,833	3	4,4	0	0,0006559	26	0,002398667	3	1229,65198	0,98	15,96	0
	High	fNGVSYIIQR	1	1	1	Q55828	N-Term(iTRAQ4plex)	0,0000	0,843	1		0	0,002851	19	0,012273545	2	1340,74065	-2,75	37,16	0
	High	iSAFNSR	3	1	1	Q55828	N-Term(iTRAQ4plex)	0,0000	0,653	3	14,2	0,001	0,01718	18	0,016981264	2	938,52306	5,79	20,63	0
P74177	DNA-directed RNA polymerase subunit gamma OS=Synechocystis sp. (strain PCC 68	250,02	10,86	1	7	7	16	0,850	15	14,2	626	70,9	7,49							
	A3	Sequence	# PSMs	# Proteins	# Protein Groups	Protein Group Accessions	Modifications	ΔCn	114/115	114/115 Count	114/115 Variability [%]	q-Value	PEP	IonScore	Exp Value	Charge	MH+ [Da]	ΔM [ppm]	RT [min]	# Missed Cleavages
	High	lQEILAPEIIVR	1	1	1	P74177	N-Term(iTRAQ4plex)	0,0000	0,914	1		0	9,722E-07	47	1,98139E-05	2	1537,94597	1,63	47,42	0
	High	iYQcGLPR	2	1	1	P74177	N-Term(iTRAQ4plex); C4(Carbamidomethyl)	0,0000	0,782	2	19,1	0	0,0004472	44	4,37492E-05	2	1150,61565	-0,27	25,29	0
	High	eMAIELFQPFVIHR	3	1	1	P74177	N-Term(iTRAQ4plex)	0,0000	0,763	3	16,2	0	2,481E-10	41	9,17049E-05	3	1874,01157	0,00	51,51	0
	High	lAAPVTHVWYLk	2	1	1	P74177	N-Term(iTRAQ4plex); K12(iTRAQ4plex)	0,0000	0,692	1		0	0,000000248	39	0,00013551	3	1685,99887	0,43	39,52	0
	High	lREEILASk	2	1	1	P74177	N-Term(iTRAQ4plex); K9(iTRAQ4plex)	0,0000	0,798	2	1,4	0	0,0005167	35	0,000336488	2	1346,82524	0,46	25,44	1
	High	emAIELFQPFVIHR	3	1	1	P74177	N-Term(iTRAQ4plex); M2(Oxidation)	0,0000	0,927	3	24,7	0	0,000001474	28	0,00155228	3	1890,00730	0,43	47,88	0
	High	tVVGANNR	2	1	1	P74177	N-Term(iTRAQ4plex)	0,0000	1,334	2	8,4	0,003	0,03905	18	0,016864365	2	974,54991	-0,08	11,73	0
	High	kLILk	1	1	1	P74177	N-Term(iTRAQ4plex); K1(iTRAQ4plex); K5(iTRAQ4plex)	0,0000	0,878	1		0,003	0,03419	16	0,025820818	3	1046,76368	-2,39	19,20	1
Q55756	Carbamoyl-phosphate synthase large chain OS=Synechocystis sp. (strain PCC 6803	248,99	7,03	1	7	7	9	0,888	9	15,1	1081	119,0	4,98							
	A3	Sequence	# PSMs	# Proteins	# Protein Groups	Protein Group Accessions	Modifications	ΔCn	114/115	114/115 Count	114/115 Variability [%]	q-Value	PEP	IonScore	Exp Value	Charge	MH+ [Da]	ΔM [ppm]	RT [min]	# Missed Cleavages
	High	sLGITPVYk	1	1	1	Q55756	N-Term(iTRAQ4plex); K9(iTRAQ4plex)	0,0000	0,822	1		0	0,00002082	54	3,74947E-06	2	1265,77068	-0,07	29,62	0
	High	lRDYSIAIIR	2	1	1	Q55756	N-Term(iTRAQ4plex)	0,0000	0,934	2	7,5	0	4,274E-07	54	4,64718E-06	3	1363,81748	-0,27	34,11	1
	High	kTPASFEPTIDYVVTk	2	1	1	Q55756	N-Term(iTRAQ4plex); K1(iTRAQ4plex); K16(iTRAQ4plex)	0,0000	0,937	2	1,2	0	3,041E-12	47	2,33448E-05	3	2228,25766	1,69	37,67	1
	High	sVGEAmAIGR	1	1	1	Q55756	N-Term(iTRAQ4plex); M6(Oxidation)	0,0000	0,614	1		0	0,005521	35	0,000285739	2	1150,60063	-0,09	17,45	0
	High	vVATSGTQk	1	1	1	Q55756	N-Term(iTRAQ4plex); K9(iTRAQ4plex)	0,0000	1,126	1		0	0,0007788	33	0,000502308	2	1178,70024	1,62	12,31	0
	High	iSYPVVVRPSYVLGGR	1	1	1	Q55756	N-Term(iTRAQ4plex)	0,0000	0,771	1		0	0,002219	13	0,047859704	3	1906,10318	0,02	40,19	0
	Medium	sLLGWk	1	1	1	Q55756	N-Term(iTRAQ4plex); K6(iTRAQ4plex)	0,0000	0,776	1		0,033	0,3554	30	0,001169988	2	991,61632	-1,59	33,47	0
P74326	Ssr1600 protein OS=Synechocystis sp. (strain PCC 6803 / Kazusa) GN=ssr1600 PE=	248,57	57,14	1	4	4	12	1,553	10	15,4	70	7,5	7,42							
	A3	Sequence	# PSMs	# Proteins	# Protein Groups	Protein Group Accessions	Modifications	ΔCn	114/115	114/115 Count	114/115 Variability [%]	q-Value	PEP	IonScore	Exp Value	Charge	MH+ [Da]	ΔM [ppm]	RT [min]	# Missed Cleavages
	High	qGSVQIVTNPR	5	1	1	P74326	N-Term(iTRAQ4plex)	0,0000	1,406	4	14,8	0	5,198E-08	73	5,01153E-08	2	1342,75810	1,59	21,09	0
	High	fLHLHNSLAEAIAATTEG	1	1	1	P74326	N-Term(iTRAQ4plex)	0,0000	1,751	1		0	0,000007211	25	0,003243172	3	2039,06943	0,75	45,36	0
	Medium	vTQTVk	4	1	1	P74326	N-Term(iTRAQ4plex); K6(iTRAQ4plex)	0,0000	1,401	3	25,0	0,012	0,1388	10	0,095273036	2	963,61284	5,32	13,92	0
	Medium	lSQNk	2	1	1	P74326	N-Term(iTRAQ4plex); K5(iTRAQ4plex)	0,0000	1,653	2	6,3	0,035	0,3717	18	0,015666429	2	877,53514	0,68	10,46	0
Q55432	Sll0822 protein OS=Synechocystis sp. (strain PCC 6803 / Kazusa) GN=sll0822 PE=1	248,37	31,78	1	4	5	15	1,165	11	8,1	129	14,0	8,59							
	A3	Sequence	# PSMs	# Proteins	# Protein Groups	Protein Group Accessions	Modifications	ΔCn	114/115	114/115 Count	114/115 Variability [%]	q-Value	PEP	IonScore	Exp Value	Charge	MH+ [Da]	ΔM [ppm]	RT [min]	# Missed Cleavages
	High	icGYVTSTk	2	1	1	Q55432	N-Term(iTRAQ4plex); C2(Carbamidomethyl); K9(iTRAQ4plex)	0,0000	1,222	2	1,0	0	0,000005012	62	6,85441E-07	2	1316,71233	0,05	23,98	0
	High	sNATkPLTGEALLEk	7	1	1	Q55432	N-Term(iTRAQ4plex); K5(iTRAQ4plex); K15(iTRAQ4plex)	0,0000	1,194	4	25,0	0	7,264E-13	38	0,000174169	3	2004,17044	0,13	28,53	1
	High	vkELGDLPk	3	1	1	Q55432	N-Term(iTRAQ4plex); K2(iTRAQ4plex); K9(iTRAQ4plex)	0,0000	1,105	3	16,1	0	0,00001711	30	0,00101151	3	1430,90155	5,06	24,57	1
	High	vkELGDLPkEEk	2	1	1	Q55432	N-Term(iTRAQ4plex); K2(iTRAQ4plex); K9(iTRAQ4plex); K12(iTRAQ4plex)	0,0000	1,087	2	8,4	0	9,469E-08	22	0,006222573	3	1961,17673	0,09	23,89	2
	Medium	hIHLk	1	2	2	P74426;Q55432	N-Term(iTRAQ4plex); K5(iTRAQ4plex)	0,0000				0,03	0,329	12	0,068544088	2	935,60338	0,52	11,57	0
P74367	Photosystem II lipoprotein Psb27 OS=Synechocystis sp. (strain PCC 6803 / Kazusa)	247,78	20,90	1	3	3	10	1,483	8	19,0	134	14,8	9,33							
	A3	Sequence	# PSMs	# Proteins	# Protein Groups	Protein Group Accessions	Modifications	ΔCn	114/115	114/115 Count	114/115 Variability [%]	q-Value	PEP	IonScore	Exp Value	Charge	MH+ [Da]	ΔM [ppm]	RT [min]	# Missed Cleavages
	High	rLQLEFTQAER	3	1	1	P74367	N-Term(iTRAQ4plex)	0,0000	1,307	3	0,2	0	0,000002164	72	6,97257E-08	2	1534,84892	1,99	27,95	1
	High	kGDAGGLk	4	1	1	P74367	N-Term(iTRAQ4plex); K1(iTRAQ4plex); K8(iTRAQ4plex)	0,0000	1,915	3	11,7	0	0,0000832	45	3,04068E-05	3	1177,72913	2,22	9,84	1
	High	gQINDYISR	3	1	1	P74367	N-Term(iTRAQ4plex)	0,0000	1,478	2	4,7	0	0,00000219	42	6,82292E-05	2	1209,63530	0,71	24,31	0
Q55458	Uncharacterized protein slr0039 OS=Synechocystis sp. (strain PCC 6803 / Kazusa) (	247,76	11,20	1	4	4	8	0,763	7	2,4	357	38,4	4,91							
	A3	Sequence	# PSMs	# Proteins	# Protein Groups	Protein Group Accessions	Modifications	ΔCn	114/115	114/115 Count	114/115 Variability [%]	q-Value	PEP	IonScore	Exp Value	Charge	MH+ [Da]	ΔM [ppm]	RT [min]	# Missed Cleavages
	High	iNAAVLLVGR	4	1	1	Q55458	N-Term(iTRAQ4plex)	0,0000	0,753	3	0,4	0	4,416E-08	71	8,64908E-08	2	1169,74846	-0,22	40,36	0
	High	kPLVFLDEk	2	1	1	Q55458	N-Term(iTRAQ4plex); K1(iTRAQ4plex); K9(iTRAQ4plex)	0,0000	0,792	2	5,6	0	3,076E-08	47	1,88786E-05	3	1520,93955	-1,13	32,17	0
	High	yHSPLVVDGLLk	1	1	1	Q55458	N-Term(iTRAQ4plex); K12(iTRAQ4plex)	0,0000	0,830	1		0	9,743E-07	34	0,000404011	3	1628,96134	-0,06	36,83	0
	High	eLAHQLNAk	1	1	1	Q55458	N-Term(iTRAQ4plex); K9(iTRAQ4plex)	0,0000	1,008	1		0	0,002991	19	0,011560324	3	1311,76197	-0,28	15,97	0
P19050	NAD(P)H-quinone oxidoreductase subunit K 1 OS=Synechocystis sp. (strain PCC 68	245,87	20,16	1	7	7	15	0,592	15	27,5	248	27,3	8,13							
	A3	Sequence	# PSMs	# Proteins	# Protein Groups	Protein Group Accessions	Modifications	ΔCn	114/115	114/115 Count	114/115 Variability [%]	q-Value	PEP	IonScore	Exp Value	Charge	MH+ [Da]	ΔM [ppm]	RT [min]	# Missed Cleavages
	High	kVANESIQER	2	1	1	P19050	N-Term(iTRAQ4plex); K1(iTRAQ4plex)	0,0000	0,826	2	11,6	0	1,316E-07	48	2,01456E-05	3	1461,82761	0,82	12,38	1
	High	aITQQTHR	2	1	1	P19050	N-Term(iTRAQ4plex)	0,0000	0,674	2	26,6	0	0,001462	37	0,000187055	2	1098,61260	-0,95	10,02	0
	High	yLQQGTR	2	1	1	P19050	N-Term(iTRAQ4plex)	0,0000	0,680	2	6,4	0	0,004785	16	0,027126433	2	1009,55394	-0,78	16,41	0
	High	fGLVPR	3	1	1	P19050	N-Term(iTRAQ4plex)	0,0000	0,489	3	2,2	0,002	0,02502	33	0,000502308	2	832,51860	2,92	27,94	0
	High	iLNPASR	3	1	1	P19050	N-Term(iTRAQ4plex)	0,0000	0,592	3	49,2	0,009	0,1117	23	0,005081243	2	914,55937	5,86	18,89	0
	Medium	mAPALVR	1	1	1	P19050	N-Term(iTRAQ4plex); M1(Oxidation)	0,0000	0,569	1		0,029	0,3127	16	0,026240373	2	917,53508	-0,93	17,94	0
	Medium	vATAk	2	1	1	P19050	N-Term(iTRAQ4plex); K5(iTRAQ4plex)	0,0000	0,603	2	7,0	0,038	0,3946	32	0,001300684	2	777,50536	-2,47	11,06	0
P73293	30S ribosomal protein S9 OS=Synechocystis sp. (strain PCC 6803 / Kazusa) GN=rps	245,00	32,85	1	5	5	9	1,300	8	21,2	137	15,1	10,13							
	A3	Sequence	# PSMs	# Proteins	# Protein Groups	Protein Group Accessions	Modifications	ΔCn	114/115	114/115 Count	114/115 Variability [%]	q-Value	PEP	IonScore	Exp Value	Charge	MH+ [Da]	ΔM [ppm]	RT [min]	# Missed Cleavages
	High	iANYIQSLk	4	1	1	P73293	N-Term(iTRAQ4plex); K9(iTRAQ4plex)	0,0000	1,290	3	2,5	0	2,638E-07	69	1,25594E-07	2	1337,80437	0,93	39,84	0
	High	aLcQLSPENR	1	1	1	P73293	N-Term(iTRAQ4plex); C3(Carbamidomethyl)	0,0000	1,153	1		0	1,237E-07	62	6,68298E-07	2	1331,68718	1,00	23,12	0
	High	kAPQYSk	2	1	1	P73293	N-Term(iTRAQ4plex); K1(iTRAQ4plex); K7(iTRAQ4plex)	0,0000	1,822	2	0,6	0	0,003862	33	0,000529663	2	1253,75749	-0,24	10,26	1
	High	qPLkAEGYLTR	1	1	1	P73293	N-Term(iTRAQ4plex); K4(iTRAQ4plex)	0,0000	1,085	1		0	0,00006374	21	0,007260558	3	1563,90927	-0,30	23,94	1
	High	vVYWGTGR	1	1	1	P73293	N-Term(iTRAQ4plex)	0,0000	1,530	1		0,001	0,01216	29	0,001361351	2	1081,59111	-0,01	27,95	0
Q55900	Septum site-determining protein MinD OS=Synechocystis sp. (strain PCC 6803 / Ka	243,49	17,29	1	4	4	8	0,741	8	5,5	266	29,0	5,41							
	A3	Sequence	# PSMs	# Proteins	# Protein Groups	Protein Group Accessions	Modifications	ΔCn	114/115	114/115 Count	114/115 Variability [%]	q-Value	PEP	IonScore	Exp Value	Charge	MH+ [Da]	ΔM [ppm]	RT [min]	# Missed Cleavages
	High	tTTTANLGAALAR	1	1	1	Q55900	N-Term(iTRAQ4plex)	0,0000	0,678	1		0	4,419E-11	98	1,68267E-10	2	1404,79375	0,70	27,87	0
	High	lSVPGLAFQNIAR	3	1	1	Q55900	N-Term(iTRAQ4plex)	0,0000	0,741	3	0,2	0	0,000005199	49	1,1428E-05	2	1529,89263	0,35	45,87	0
	High	lPNLVLLPAAQNR	3	1	1	Q55900	N-Term(iTRAQ4plex)	0,0000	0,789	3	7,6	0	0,0009089	40	9,18269E-05	2	1562,94951	-0,28	46,12	0
	High	iSLIVNR	1	1	1	Q55900	N-Term(iTRAQ4plex)	0,0000	0,738	1		0,002	0,02505	32	0,000685441	2	958,61687	0,27	30,43	0
P73410	Cysteine synthase OS=Synechocystis sp. (strain PCC 6803 / Kazusa) GN=cysK PE=	240,23	9,94	1	3	3	6	1,367	6	6,5	312	33,2	5,80							
	A3	Sequence	# PSMs	# Proteins	# Protein Groups	Protein Group Accessions	Modifications	ΔCn	114/115	114/115 Count	114/115 Variability [%]	q-Value	PEP	IonScore	Exp Value	Charge	MH+ [Da]	ΔM [ppm]	RT [min]	# Missed Cleavages
	High	iASNITELIGR	3	1	1	P73410	N-Term(iTRAQ4plex)	0,0000	1,424	3	11,0	0	9,787E-07	69	1,17752E-07	2	1330,78166	0,40	42,39	0
	High	lIVMIQPSFGER	1	1	1	P73410	N-Term(iTRAQ4plex)	0,0000	1,393	1		0	9,128E-07	59	1,94701E-06	2	1533,86138	2,18	46,08	0
	High	lIVmIQPSFGER	1	1	1	P73410	N-Term(iTRAQ4plex); M4(Oxidation)	0,0000	1,341	1		0	0,000007541	35	0,000320605	2	1549,85307	0,09	41,49	0
	Medium	iGINmINR	1	1	1	P73410	N-Term(iTRAQ4plex); M5(Oxidation)	0,0000	1,110	1		0,037	0,3908	30	0,000931044	2	1090,61589	-0,08	24,50	0
P74769	Ssr1528 protein OS=Synechocystis sp. (strain PCC 6803 / Kazusa) GN=ssr1528 PE=	239,82	47,87	1	5	5	13	1,018	12	14,6	94	10,1	7,42							
	A3	Sequence	# PSMs	# Proteins	# Protein Groups	Protein Group Accessions	Modifications	ΔCn	114/115	114/115 Count	114/115 Variability [%]	q-Value	PEP	IonScore	Exp Value	Charge	MH+ [Da]	ΔM [ppm]	RT [min]	# Missed Cleavages
	High	hIVQAELDQk	3	1	1	P74769	N-Term(iTRAQ4plex); K10(iTRAQ4plex)	0,0000	0,983	3	0,7	0	3,678E-08	43	5,34527E-05	3	1468,83643	0,13	19,58	0
	High	lELYHQVmGLEAGR	1	1	1	P74769	N-Term(iTRAQ4plex); M8(Oxidation)	0,0000	0,911	1		0	6,195E-11	41	7,62026E-05	3	1775,92325	0,06	35,80	0
	High	lELYHQVMGLEAGR	2	1	1	P74769	N-Term(iTRAQ4plex)	0,0000	0,965	2	4,7	0	6,948E-09	38	0,000153451	3	1759,92899	0,43	40,19	0
	High	dkEIAFFYGAk	2	1	1	P74769	N-Term(iTRAQ4plex); K2(iTRAQ4plex); K11(iTRAQ4plex)	0,0000	1,083	2	8,2	0	1,728E-08	32	0,000651583	3	1720,96506	0,93	35,04	1
	High	lIDAGFAPLkDk	4	1	1	P74769	N-Term(iTRAQ4plex); K10(iTRAQ4plex); K12(iTRAQ4plex)	0,0000	1,178	3	4,4	0	0,000003449	30	0,000939658	3	1720,03727	0,17	36,77	1
	High	lIDAGFAPLkDkEIAFFYGAk	1	1	1	P74769	N-Term(iTRAQ4plex); K10(iTRAQ4plex); K12(iTRAQ4plex); K21(iTRAQ4plex)	0,0000	1,219	1		0	3,546E-10	23	0,00495416	4	2890,65688	0,11	49,79	2
P72799	Slr1794 protein OS=Synechocystis sp. (strain PCC 6803 / Kazusa) GN=slr1794 PE=	239,61	10,22	1	4	4	11	0,783	10	18,3	362	39,1	6,13							
	A3	Sequence	# PSMs	# Proteins	# Protein Groups	Protein Group Accessions	Modifications	ΔCn	114/115	114/115 Count	114/115 Variability [%]	q-Value	PEP	IonScore	Exp Value	Charge	MH+ [Da]	ΔM [ppm]	RT [min]	# Missed Cleavages
	High	tTVAIAAAk	2	1	1	P72799	N-Term(iTRAQ4plex); K9(iTRAQ4plex)	0,0000	0,821	1		0	2,675E-07	66	2,31723E-07	2	1133,71343	0,14	19,94	0
	High	aLVLTFLGk	6	1	1	P72799	N-Term(iTRAQ4plex); K9(iTRAQ4plex)	0,0000	0,736	6	30,9	0	0,0001073	46	2,76039E-05	2	1249,81194	-0,26	48,96	0
	High	lkEVEAQYLR	2	1	1	P72799	N-Term(iTRAQ4plex); K2(iTRAQ4plex)	0,0000	0,783	2	3,7	0	7,937E-07	35	0,000319867	3	1536,89902	0,12	29,29	1
	High	vFLPGFDkR	1	1	1	P72799	N-Term(iTRAQ4plex); K8(iTRAQ4plex)	0,0000	0,708	1		0	0,0002177	30	0,001051889	3	1366,80841	-0,12	33,86	1
Q55367	Cyanate hydratase OS=Synechocystis sp. (strain PCC 6803 / Kazusa) GN=cynS PE=	236,36	24,83	1	5	5	10	1,372	10	18,2	149	16,5	5,21							
	A3	Sequence	# PSMs	# Proteins	# Protein Groups	Protein Group Accessions	Modifications	ΔCn	114/115	114/115 Count	114/115 Variability [%]	q-Value	PEP	IonScore	Exp Value	Charge	MH+ [Da]	ΔM [ppm]	RT [min]	# Missed Cleavages
	High	aGTEISAITTk	2	1	1	Q55367	N-Term(iTRAQ4plex); K11(iTRAQ4plex)	0,0000	1,548	2	2,4	0	4,433E-07	73	4,80806E-08	2	1379,79997	1,10	25,69	0
	High	gLGPVVPTDPLIYR	2	1	1	Q55367	N-Term(iTRAQ4plex)	0,0000	1,181	2	8,0	0	0,000021	62	6,41165E-07	2	1640,95098	1,04	44,01	0
	High	eVIHEk	2	1	1	Q55367	N-Term(iTRAQ4plex); K6(iTRAQ4plex)	0,0000	1,277	2	17,6	0,003	0,03567	12	0,068544088	3	1042,61258	-0,92	10,81	0
	Medium	fLPYkk	2	1	1	Q55367	N-Term(iTRAQ4plex); K5(iTRAQ4plex); K6(iTRAQ4plex)	0,0000	1,445	2	14,6	0,014	0,1574	16	0,023712099	3	1227,78156	-0,81	23,61	1
	Medium	fLPYk	2	1	1	Q55367	N-Term(iTRAQ4plex); K5(iTRAQ4plex)	0,0000	1,390	2	18,2	0,025	0,2662	28	0,001530982	2	955,58586	0,36	28,31	0
P23350	50S ribosomal protein L10 OS=Synechocystis sp. (strain PCC 6803 / Kazusa) GN=rp	234,95	22,54	1	5	5	17	1,829	13	13,1	173	18,7	8,60							
	A3	Sequence	# PSMs	# Proteins	# Protein Groups	Protein Group Accessions	Modifications	ΔCn	114/115	114/115 Count	114/115 Variability [%]	q-Value	PEP	IonScore	Exp Value	Charge	MH+ [Da]	ΔM [ppm]	RT [min]	# Missed Cleavages
	High	gLQAHVDkE	9	1	1	P23350	N-Term(iTRAQ4plex); K8(iTRAQ4plex)	0,0000	1,783	5	3,8	0	0,0005516	49	1,23586E-05	2	1284,71465	-0,32	13,59	1
	High	eVPASVAR	2	1	1	P23350	N-Term(iTRAQ4plex)	0,0000	1,597	2	12,0	0	0,004947	35	0,00031115	2	972,56651	7,21	15,51	0
	High	lRPLGGTck	2	1	1	P23350	N-Term(iTRAQ4plex); C8(Carbamidomethyl); K9(iTRAQ4plex)	0,0000	2,400	2	6,5	0	0,0003738	23	0,007535777	3	1289,76505	3,73	16,74	0
	High	gGVLEGk	1	1	1	P23350	N-Term(iTRAQ4plex); K7(iTRAQ4plex)	0,0000	1,537	1		0	0,008042	14	0,063440705	2	947,57994	3,70	16,73	0
	High	iALGIk	3	1	1	P23350	N-Term(iTRAQ4plex); K6(iTRAQ4plex)	0,0000	1,991	3	10,2	0,008	0,09303	27	0,001866251	2	902,63341	6,28	28,66	0
P54224	Uroporphyrinogen decarboxylase OS=Synechocystis sp. (strain PCC 6803 / Kazusa)	233,11	20,57	1	7	7	14	0,811	14	8,6	350	39,1	5,96							
	A3	Sequence	# PSMs	# Proteins	# Protein Groups	Protein Group Accessions	Modifications	ΔCn	114/115	114/115 Count	114/115 Variability [%]	q-Value	PEP	IonScore	Exp Value	Charge	MH+ [Da]	ΔM [ppm]	RT [min]	# Missed Cleavages
	High	aGQGGHIFNLGHGVLVGTPEDNVR	2	1	1	P54224	N-Term(iTRAQ4plex)	0,0000	0,828	2	5,4	0	6,078E-10	51	7,26056E-06	4	2588,34658	0,57	35,29	0
	High	iAEAIAVYVR	1	1	1	P54224	N-Term(iTRAQ4plex)	0,0000	0,900	1		0	0,000046	51	8,72911E-06	2	1248,74468	1,11	42,99	0
	High	qmAFSEPAILHSFLDk	3	1	1	P54224	N-Term(iTRAQ4plex); M2(Oxidation); K16(iTRAQ4plex)	0,0000	0,824	3	26,7	0	3,328E-11	34	0,000392618	3	2138,11777	-0,81	47,29	0
	High	fFFETAk	2	1	1	P54224	N-Term(iTRAQ4plex); K7(iTRAQ4plex)	0,0000	0,827	2	0,4	0	0,0005538	29	0,001213305	2	1177,64824	-1,15	37,16	0
	High	kAGQGGHIFNLGHGVLVGTPEDNVR	1	1	1	P54224	N-Term(iTRAQ4plex); K1(iTRAQ4plex)	0,0000	1,412	1		0	0,00009214	13	0,052686317	4	2860,54262	0,17	32,60	1
	High	tILGTLR	4	1	1	P54224	N-Term(iTRAQ4plex)	0,0000	0,735	4	7,7	0,002	0,02914	26	0,002564307	2	917,58891	-1,25	30,50	0
	High	dkYPSFR	1	1	1	P54224	N-Term(iTRAQ4plex); K2(iTRAQ4plex)	0,0000	0,784	1		0,003	0,04165	12	0,060529907	3	1200,66043	-0,93	18,75	1
P74518	Light-repressed protein A homolog OS=Synechocystis sp. (strain PCC 6803 / Kazusa	232,59	36,13	1	5	5	9	1,103	9	10,4	191	21,9	6,54							
	A3	Sequence	# PSMs	# Proteins	# Protein Groups	Protein Group Accessions	Modifications	ΔCn	114/115	114/115 Count	114/115 Variability [%]	q-Value	PEP	IonScore	Exp Value	Charge	MH+ [Da]	ΔM [ppm]	RT [min]	# Missed Cleavages
	High	nkDTDEINVIYIR	4	1	1	P74518	N-Term(iTRAQ4plex); K2(iTRAQ4plex)	0,0000	1,240	4	16,0	0	4,55E-10	60	1,11422E-06	3	1881,03220	0,08	32,27	1
	High	lLIQGNNITVTEAIHDYVEEkVER	1	1	1	P74518	N-Term(iTRAQ4plex); K21(iTRAQ4plex)	0,0000	1,154	1		0	1,131E-13	47	1,96775E-05	4	3071,66616	1,47	50,11	1
	High	nHGGYGVIQPHQAS	2	1	1	P74518	N-Term(iTRAQ4plex)	0,0000	1,094	2	1,2	0	0,00000344	41	8,16526E-05	2	1608,80107	0,70	16,25	0
	High	hFQNLTTk	1	1	1	P74518	N-Term(iTRAQ4plex); K8(iTRAQ4plex)	0,0000	1,079	1		0	0,0004095	20	0,013711699	3	1276,72474	-0,37	16,76	0
	High	iQDkQHGNVk	1	1	1	P74518	N-Term(iTRAQ4plex); K4(iTRAQ4plex); K10(iTRAQ4plex)	0,0000	1,008	1		0	0,00006404	16	0,024432618	3	1598,93424	0,22	10,02	1
P74228	Elongation factor G 2 OS=Synechocystis sp. (strain PCC 6803 / Kazusa) GN=fusB PI	231,89	9,12	1	8	8	11	1,023	9	10,9	691	75,4	5,05							
	A3	Sequence	# PSMs	# Proteins	# Protein Groups	Protein Group Accessions	Modifications	ΔCn	114/115	114/115 Count	114/115 Variability [%]	q-Value	PEP	IonScore	Exp Value	Charge	MH+ [Da]	ΔM [ppm]	RT [min]	# Missed Cleavages
	High	gSYVLNSTk	1	1	1	P74228	N-Term(iTRAQ4plex); K9(iTRAQ4plex)	0,0000	1,231	1		0	6,317E-07	63	5,15193E-07	2	1256,71697	6,43	19,27	0
	High	rGHIEGQETEQGIAk	2	1	1	P74228	N-Term(iTRAQ4plex); K15(iTRAQ4plex)	0,0000	0,763	1		0	6,169E-10	59	1,3122E-06	3	1941,04007	0,41	14,07	1
	High	iVGGVIPk	2	1	1	P74228	N-Term(iTRAQ4plex); K8(iTRAQ4plex)	0,0000	0,950	1		0	0,0001565	44	3,99917E-05	2	1070,71697	-0,62	28,46	0
	High	iLFYSGVVHk	1	1	1	P74228	N-Term(iTRAQ4plex); K10(iTRAQ4plex)	0,0000	0,693	1		0	7,549E-07	34	0,000410176	3	1450,86573	-0,24	35,37	0
	High	kAVQAEGk	1	1	1	P74228	N-Term(iTRAQ4plex); K1(iTRAQ4plex); K8(iTRAQ4plex)	0,0000	1,063	1		0	0,000111	30	0,001076391	3	1262,77851	-0,61	8,76	1
	High	lIILk	2	1	1	P74228	N-Term(iTRAQ4plex); K5(iTRAQ4plex)	0,0000	1,001	2	3,1	0,002	0,03259	31	0,000753304	2	887,65282	-0,45	33,97	0
	High	aVQAEGk	1	1	1	P74228	N-Term(iTRAQ4plex); K7(iTRAQ4plex)	0,0000	1,134	1		0,003	0,0405	24	0,003917148	2	990,58220	-0,05	12,31	0
	High	vYSGVLEk	1	1	1	P74228	N-Term(iTRAQ4plex); K8(iTRAQ4plex)	0,0000	1,024	1		0,009	0,102	23	0,005432128	2	1182,69756	0,25	26,54	0
P74394	Sll0272 protein OS=Synechocystis sp. (strain PCC 6803 / Kazusa) GN=sll0272 PE=4	231,36	35,67	1	4	4	6	0,797	6	18,2	157	17,6	8,78							
	A3	Sequence	# PSMs	# Proteins	# Protein Groups	Protein Group Accessions	Modifications	ΔCn	114/115	114/115 Count	114/115 Variability [%]	q-Value	PEP	IonScore	Exp Value	Charge	MH+ [Da]	ΔM [ppm]	RT [min]	# Missed Cleavages
	High	kPIAIPGADNTPYWQVQGTWSGDR	1	1	1	P74394	N-Term(iTRAQ4plex); K1(iTRAQ4plex)	0,0000	0,733	1		0	9,127E-16	100	1,22572E-10	3	2945,51529	0,13	41,90	0
	High	iGLGPVALDFSk	2	1	1	P74394	N-Term(iTRAQ4plex); K12(iTRAQ4plex)	0,0000	1,038	2	69,4	0	2,418E-10	65	3,40385E-07	2	1504,89800	0,13	46,22	0
	High	qFNLYFFQEDIkGPk	2	1	1	P74394	N-Term(iTRAQ4plex); K12(iTRAQ4plex); K15(iTRAQ4plex)	0,0000	0,797	2	0,7	0	8,573E-10	45	3,06175E-05	3	2306,25620	0,71	45,70	1
	Medium	aPAEk	1	1	1	P74394	N-Term(iTRAQ4plex); K5(iTRAQ4plex)	0,0000	0,989	1		0,036	0,3777	16	0,023603152	2	803,48479	-2,19	9,66	0
P74494	Nucleoside diphosphate kinase OS=Synechocystis sp. (strain PCC 6803 / Kazusa) G	231,25	34,23	1	4	4	9	0,720	9	13,9	149	16,7	5,55							
	A3	Sequence	# PSMs	# Proteins	# Protein Groups	Protein Group Accessions	Modifications	ΔCn	114/115	114/115 Count	114/115 Variability [%]	q-Value	PEP	IonScore	Exp Value	Charge	MH+ [Da]	ΔM [ppm]	RT [min]	# Missed Cleavages
	High	qLIGEIVGR	2	1	1	P74494	N-Term(iTRAQ4plex)	0,0000	0,654	2	0,4	0	0,00001122	60	9,57128E-07	2	1128,68559	-0,16	34,69	0
	High	nIIHGSDAIETAk	1	1	1	P74494	N-Term(iTRAQ4plex); K13(iTRAQ4plex)	0,0000	0,702	1		0	1,248E-08	54	4,69009E-06	3	1656,91605	0,06	22,09	0
	High	qmIGATDPHAAAPGTIR	2	1	1	P74494	N-Term(iTRAQ4plex); M2(Oxidation)	0,0000	0,692	2	26,3	0	0,00002643	45	3,22827E-05	3	1866,97116	5,26	21,20	0
	High	tFIMIkPDGVQR	1	1	1	P74494	N-Term(iTRAQ4plex); K6(iTRAQ4plex)	0,0000	0,720	1		0	0,000003516	30	0,001157158	3	1692,97190	0,54	32,35	0
	High	tFImIkPDGVQR	2	1	1	P74494	N-Term(iTRAQ4plex); M4(Oxidation); K6(iTRAQ4plex)	0,0000	0,760	2	5,1	0	0,000009419	29	0,00132425	3	1708,96396	-1,14	29,06	0
	High	qMIGATDPHAAAPGTIR	1	1	1	P74494	N-Term(iTRAQ4plex)	0,0000	0,982	1		0	0,00001801	24	0,003706551	3	1850,96390	-1,36	23,98	0
Q55386	Slr0924 protein OS=Synechocystis sp. (strain PCC 6803 / Kazusa) GN=slr0924 PE=	231,13	14,34	1	3	3	10	1,551	8	23,7	286	31,3	5,72							
	A3	Sequence	# PSMs	# Proteins	# Protein Groups	Protein Group Accessions	Modifications	ΔCn	114/115	114/115 Count	114/115 Variability [%]	q-Value	PEP	IonScore	Exp Value	Charge	MH+ [Da]	ΔM [ppm]	RT [min]	# Missed Cleavages
	High	iVPTQEAIQFIR	4	1	1	Q55386	N-Term(iTRAQ4plex)	0,0000	1,696	4	28,9	0	6,577E-08	71	7,2272E-08	2	1558,90935	1,25	43,78	0
	High	sLSAQQPk	5	1	1	Q55386	N-Term(iTRAQ4plex); K8(iTRAQ4plex)	0,0000	1,652	3	1,4	0	0,00001618	38	0,000163453	2	1146,67314	0,89	13,26	0
	High	qkPDVGSQVSVQPVSLGEVVk	1	1	1	Q55386	N-Term(iTRAQ4plex); K2(iTRAQ4plex); K21(iTRAQ4plex)	0,0000	0,782	1		0	2,132E-07	25	0,003451199	4	2612,49941	0,38	35,24	0
P74361	Chaperone protein ClpB 2 OS=Synechocystis sp. (strain PCC 6803 / Kazusa) GN=cl	226,12	13,42	1	8	10	15	0,834	9	23,0	872	98,1	5,26							
	A3	Sequence	# PSMs	# Proteins	# Protein Groups	Protein Group Accessions	Modifications	ΔCn	114/115	114/115 Count	114/115 Variability [%]	q-Value	PEP	IonScore	Exp Value	Charge	MH+ [Da]	ΔM [ppm]	RT [min]	# Missed Cleavages
	High	sLLREEVLESDIAEIISk	1	1	1	P74361	N-Term(iTRAQ4plex); K18(iTRAQ4plex)	0,0000	0,834	1		0	1,076E-11	48	1,47901E-05	3	2332,32566	1,80	52,98	1
	High	tAIAEGLAQR	3	2	2	P74361;Q55662	N-Term(iTRAQ4plex)	0,0000				0	2,898E-07	45	3,07588E-05	2	1173,66985	-0,86	23,74	0
	High	aAELQYGk	1	1	1	P74361	N-Term(iTRAQ4plex); K8(iTRAQ4plex)	0,0000	0,920	1		0	0,00002322	39	0,000186693	2	1167,66533	3,52	18,42	0
	High	vDETIIFHGLQk	1	1	1	P74361	N-Term(iTRAQ4plex); K12(iTRAQ4plex)	0,0000	0,591	1		0	5,73E-08	30	0,000950539	3	1687,96067	-0,90	38,08	0
	High	aGLSDPNRPTASFIFLGPTGVGk	1	1	1	P74361	N-Term(iTRAQ4plex); K23(iTRAQ4plex)	0,0000	1,045	1		0	1,861E-07	27	0,0020854	4	2590,42568	0,94	43,09	0
	High	eNFRPEFLNR	2	1	1	P74361	N-Term(iTRAQ4plex)	0,0000	0,700	2	2,8	0	0,000008186	26	0,002269708	3	1465,76694	0,04	30,11	0
	High	yRGEFEER	3	2	2	P74361;Q55662	N-Term(iTRAQ4plex)	0,0000				0	0,003701	26	0,002306588	3	1229,60428	0,92	14,57	1
	High	iINHDVPESLRDR	1	1	1	P74361	N-Term(iTRAQ4plex)	0,0000	0,843	1		0	0,000321	18	0,015558582	3	1707,92649	0,34	23,91	1
	High	lSFTSLR	1	1	1	P74361	N-Term(iTRAQ4plex)	0,0000	0,730	1		0,005	0,06101	12	0,069337791	2	967,56963	0,31	33,48	0
	Medium	lVESEkEk	1	1	1	P74361	N-Term(iTRAQ4plex); K6(iTRAQ4plex); K8(iTRAQ4plex)	0,0000	0,973	1		0,024	0,2531	17	0,021328976	3	1393,82425	-1,46	14,07	1
P51634	Magnesium-chelatase subunit ChlI OS=Synechocystis sp. (strain PCC 6803 / Kazusa	225,63	13,17	1	4	4	10	1,092	10	9,4	357	39,4	5,08							
	A3	Sequence	# PSMs	# Proteins	# Protein Groups	Protein Group Accessions	Modifications	ΔCn	114/115	114/115 Count	114/115 Variability [%]	q-Value	PEP	IonScore	Exp Value	Charge	MH+ [Da]	ΔM [ppm]	RT [min]	# Missed Cleavages
	High	lALLLNVIDPk	7	1	1	P51634	N-Term(iTRAQ4plex); K11(iTRAQ4plex)	0,0000	1,095	7	10,0	0	4,598E-08	56	2,48296E-06	2	1496,96465	-0,55	54,35	0
	High	iRVDSQEPLSIVk	1	1	1	P51634	N-Term(iTRAQ4plex); K13(iTRAQ4plex)	0,0000	0,908	1		0	1,979E-08	34	0,000361385	3	1772,05167	-0,22	33,98	1
	High	rVVFPFTAIVGQDEmk	1	1	1	P51634	N-Term(iTRAQ4plex); M15(Oxidation); K16(iTRAQ4plex)	0,0000	1,162	1		0	1,861E-10	33	0,00058129	3	2141,17087	1,90	43,25	1
	High	vIVLcLR	1	1	1	P51634	N-Term(iTRAQ4plex); C5(Carbamidomethyl)	0,0000	1,089	1		0	0,003865	33	0,000548239	2	1016,64037	-0,35	37,45	0
Q55426	Slr0841 protein OS=Synechocystis sp. (strain PCC 6803 / Kazusa) GN=slr0841 PE=	223,89	11,34	1	3	3	7	1,088	7	16,6	291	31,5	4,92							
	A3	Sequence	# PSMs	# Proteins	# Protein Groups	Protein Group Accessions	Modifications	ΔCn	114/115	114/115 Count	114/115 Variability [%]	q-Value	PEP	IonScore	Exp Value	Charge	MH+ [Da]	ΔM [ppm]	RT [min]	# Missed Cleavages
	High	kAcTAEVmAQEQR	3	1	1	Q55426	K1(iTRAQ4plex); N-Term(iTRAQ4plex); C3(Carbamidomethyl); M8(Oxidation)	0,0000	0,750	3	22,6	0	9,192E-08	63	4,83025E-07	3	1825,91471	0,42	13,63	1
	High	kAcTAEVMAQEQR	1	1	1	Q55426	N-Term(iTRAQ4plex); K1(iTRAQ4plex); C3(Carbamidomethyl)	0,0000	1,505	1		0	5,098E-08	55	3,12586E-06	3	1809,92978	5,94	16,64	1
	High	aIPQLQFLGDR	1	1	1	Q55426	N-Term(iTRAQ4plex)	0,0000	1,145	1		0	5,497E-08	54	3,76678E-06	2	1401,79802	0,64	43,10	0
	High	lTGTGGcNR	2	1	1	Q55426	N-Term(iTRAQ4plex); C7(Carbamidomethyl)	0,0000	1,150	2	8,3	0,005	0,05978	17	0,021230978	2	1079,53911	0,61	12,11	0
P73886	Ribosome-binding ATPase YchF OS=Synechocystis sp. (strain PCC 6803 / Kazusa) G	223,15	19,83	1	6	6	7	0,860	7	21,2	363	39,3	4,84							
	A3	Sequence	# PSMs	# Proteins	# Protein Groups	Protein Group Accessions	Modifications	ΔCn	114/115	114/115 Count	114/115 Variability [%]	q-Value	PEP	IonScore	Exp Value	Charge	MH+ [Da]	ΔM [ppm]	RT [min]	# Missed Cleavages
	High	aPQAAGVIHTDFER	1	1	1	P73886	N-Term(iTRAQ4plex)	0,0000	0,991	1		0	5,806E-08	59	1,15337E-06	3	1655,86411	1,12	26,84	0
	High	eVDAIVHVVR	1	1	1	P73886	N-Term(iTRAQ4plex)	0,0000	0,757	1		0	2,174E-07	46	2,43204E-05	3	1280,74326	-0,86	29,22	0
	High	gEGLGNQFLANIR	1	1	1	P73886	N-Term(iTRAQ4plex)	0,0000	0,860	1		0	3,379E-08	45	3,40385E-05	2	1532,83220	1,29	40,30	0
	High	aTYELLGLR	1	1	1	P73886	N-Term(iTRAQ4plex)	0,0000	0,735	1		0	0,0001898	45	3,54813E-05	2	1179,68657	0,97	38,25	0
	High	gNkDLLEEVAILER	2	1	1	P73886	N-Term(iTRAQ4plex); K3(iTRAQ4plex)	0,0000	0,955	2	5,4	0	3,809E-10	43	5,20223E-05	3	1887,07907	0,03	44,54	1
	High	sTLFNALVANAk	1	1	1	P73886	N-Term(iTRAQ4plex); K12(iTRAQ4plex)	0,0000	0,612	1		0	0,000004367	39	0,000133036	3	1536,89829	-0,36	43,69	0
Q55436	Slr0848 protein OS=Synechocystis sp. (strain PCC 6803 / Kazusa) GN=slr0848 PE=	222,46	24,55	1	6	6	12	1,118	10	7,8	277	31,8	4,89							
	A3	Sequence	# PSMs	# Proteins	# Protein Groups	Protein Group Accessions	Modifications	ΔCn	114/115	114/115 Count	114/115 Variability [%]	q-Value	PEP	IonScore	Exp Value	Charge	MH+ [Da]	ΔM [ppm]	RT [min]	# Missed Cleavages
	High	qVTHQEIQQFR	1	1	1	Q55436	N-Term(iTRAQ4plex)	0,0000	1,079	1		0	1,068E-08	48	1,67098E-05	3	1557,82651	0,68	19,39	0
	High	iLDQIDVIAEFIPGAVHR	2	1	1	Q55436	N-Term(iTRAQ4plex)	0,0000	1,016	1		0	2,816E-10	40	9,35341E-05	3	2150,21152	1,12	59,01	0
	High	ikDESGIIQk	4	1	1	Q55436	N-Term(iTRAQ4plex); K2(iTRAQ4plex); K10(iTRAQ4plex)	0,0000	1,198	3	8,4	0	1,204E-07	37	0,00020463	3	1562,94980	1,27	21,90	1
	High	aIAILER	3	1	1	Q55436	N-Term(iTRAQ4plex)	0,0000	1,105	3	20,1	0	0,0004511	36	0,000232258	2	929,59020	0,13	30,63	0
	High	mIEVAHQEAQR	1	1	1	Q55436	N-Term(iTRAQ4plex); M1(Oxidation)	0,0000	1,142	1		0	0,0003606	31	0,000736156	3	1471,75266	5,58	12,87	0
	High	qTQQEcDTLRR	1	1	1	Q55436	N-Term(iTRAQ4plex); C6(Carbamidomethyl)	0,0000	1,088	1		0	0,0006851	18	0,016748272	3	1578,77781	0,18	11,43	1
P73437	ATP-dependent zinc metalloprotease FtsH 4 OS=Synechocystis sp. (strain PCC 6803	221,66	14,97	1	6	9	13	0,845	6	7,5	628	68,2	5,41							
	A3	Sequence	# PSMs	# Proteins	# Protein Groups	Protein Group Accessions	Modifications	ΔCn	114/115	114/115 Count	114/115 Variability [%]	q-Value	PEP	IonScore	Exp Value	Charge	MH+ [Da]	ΔM [ppm]	RT [min]	# Missed Cleavages
	High	gVLLVGPPGTGk	2	3	3	Q55700;P72991;P73437	N-Term(iTRAQ4plex); K12(iTRAQ4plex)	0,0000				0	1,209E-07	59	1,31513E-06	2	1382,86235	0,97	32,40	0
	High	vRDLFEQAk	3	2	2	P72991;P73437	N-Term(iTRAQ4plex); K9(iTRAQ4plex)	0,0000				0	0,00000137	46	2,69755E-05	3	1393,80437	0,11	27,68	1
	High	fLLNESELRDQIATLLGGR	1	1	1	P73437	N-Term(iTRAQ4plex)	0,0000	0,884	1		0	0,000001179	38	0,000142233	3	2289,27060	0,96	57,94	1
	High	nkQDSVTEADFR	1	1	1	P73437	N-Term(iTRAQ4plex); K2(iTRAQ4plex)	0,0000	0,883	1		0	0,0000134	30	0,001083852	3	1697,87015	0,24	18,82	1
	High	aIkPQPQWQR	1	1	1	P73437	N-Term(iTRAQ4plex); K3(iTRAQ4plex)	0,0000	1,087	1		0	6,077E-08	28	0,001516946	3	1539,90012	0,18	19,75	0
	High	yTALGAk	1	1	1	P73437	N-Term(iTRAQ4plex); K7(iTRAQ4plex)	0,0000	0,767	1		0	0,001434	25	0,002857393	2	1011,60893	1,20	19,47	0
	High	yQLkPEAEDEGkEk	1	1	1	P73437	N-Term(iTRAQ4plex); K4(iTRAQ4plex); K12(iTRAQ4plex); K14(iTRAQ4plex)	0,0000	0,799	1		0,001	0,009408	14	0,042361371	4	2240,24380	8,08	19,55	1
	High	iSIVPR	1	1	1	P73437	N-Term(iTRAQ4plex)	0,0000	0,809	1		0,006	0,07303	23	0,005545874	2	828,54210	-0,34	25,01	0
	Medium	tLLAk	2	3	3	Q55700;P72991;P73437	N-Term(iTRAQ4plex); K5(iTRAQ4plex)	0,0000				0,011	0,1328	30	0,001096402	2	833,57073	1,02	18,46	0
P73604	Slr1853 protein OS=Synechocystis sp. (strain PCC 6803 / Kazusa) GN=slr1853 PE=	220,50	20,35	1	2	2	8	1,208	6	25,6	113	12,1	4,84							
	A3	Sequence	# PSMs	# Proteins	# Protein Groups	Protein Group Accessions	Modifications	ΔCn	114/115	114/115 Count	114/115 Variability [%]	q-Value	PEP	IonScore	Exp Value	Charge	MH+ [Da]	ΔM [ppm]	RT [min]	# Missed Cleavages
	High	aAGAFAALk	7	1	1	P73604	N-Term(iTRAQ4plex); K9(iTRAQ4plex)	0,0000	1,255	5	12,9	0	0,00002368	65	3,45915E-07	2	1107,67705	0,50	28,94	0
	High	vAGEAWGLPLIDQk	1	1	1	P73604	N-Term(iTRAQ4plex); K14(iTRAQ4plex)	0,0000	0,944	1		0	0,00261	14	0,041397108	3	1785,01444	-0,30	46,43	0
Q55690	Glycine--tRNA ligase beta subunit OS=Synechocystis sp. (strain PCC 6803 / Kazusa)	219,30	8,73	1	7	7	11	0,754	11	28,0	722	80,0	4,86							
	A3	Sequence	# PSMs	# Proteins	# Protein Groups	Protein Group Accessions	Modifications	ΔCn	114/115	114/115 Count	114/115 Variability [%]	q-Value	PEP	IonScore	Exp Value	Charge	MH+ [Da]	ΔM [ppm]	RT [min]	# Missed Cleavages
	High	lALSWLk	2	1	1	Q55690	N-Term(iTRAQ4plex); K7(iTRAQ4plex)	0,0000	0,720	2	6,7	0	0,00005927	47	1,86625E-05	2	1118,71709	-0,47	44,07	0
	High	lNLLGLIR	3	1	1	Q55690	N-Term(iTRAQ4plex)	0,0000	0,684	3	13,1	0	0,00003803	47	2,08895E-05	2	1055,70525	-0,50	49,82	0
	High	gPPASAAFk	1	1	1	Q55690	N-Term(iTRAQ4plex); K9(iTRAQ4plex)	0,0000	0,954	1		0	0,003527	46	2,902E-05	2	1133,65288	-2,54	19,06	0
	High	eVTTTVmVTHQR	1	1	1	Q55690	N-Term(iTRAQ4plex); M7(Oxidation)	0,0000	0,406	1		0	1,802E-07	32	0,000571439	3	1561,81052	-1,27	17,22	0
	High	aVYQALLAIYPk	1	1	1	Q55690	N-Term(iTRAQ4plex); K12(iTRAQ4plex)	0,0000	1,288	1		0	0,0001161	23	0,004886186	3	1637,98606	-0,53	48,65	0
	High	qLQGQASR	2	1	1	Q55690	N-Term(iTRAQ4plex)	0,0000	0,915	2	10,8	0	0,006377	21	0,008629189	2	1031,57134	-0,11	10,53	0
	High	rLAVLIk	1	1	1	Q55690	N-Term(iTRAQ4plex); K7(iTRAQ4plex)	0,0000	0,490	1		0,002	0,02048	24	0,003944301	3	1100,77420	-1,46	25,29	1
P73826	3-oxoacyl-[acyl-carrier-protein] reductase 2 OS=Synechocystis sp. (strain PCC 6803	217,93	18,75	1	4	4	7	0,799	7	13,3	240	25,3	6,60							
	A3	Sequence	# PSMs	# Proteins	# Protein Groups	Protein Group Accessions	Modifications	ΔCn	114/115	114/115 Count	114/115 Variability [%]	q-Value	PEP	IonScore	Exp Value	Charge	MH+ [Da]	ΔM [ppm]	RT [min]	# Missed Cleavages
	High	gNVGQTNYSATk	2	1	1	P73826	N-Term(iTRAQ4plex); K12(iTRAQ4plex)	0,0000	0,783	2	2,9	0	2,245E-10	73	4,62349E-08	2	1527,80327	1,77	15,22	0
	High	vIVVTGGNR	2	1	1	P73826	N-Term(iTRAQ4plex)	0,0000	0,824	2	18,0	0	0,0002083	58	1,73768E-06	2	1058,64348	-0,40	21,26	0
	High	gVAYSIkPFIEGmYER	2	1	1	P73826	N-Term(iTRAQ4plex); K7(iTRAQ4plex); M13(Oxidation)	0,0000	0,745	2	12,6	0	0,0006792	21	0,009974934	3	2164,13706	0,88	41,87	0
	Medium	aGVIGmmk	1	1	1	P73826	N-Term(iTRAQ4plex); M6(Oxidation); M7(Oxidation); K8(iTRAQ4plex)	0,0000	0,984	1		0,034	0,3575	16	0,023440669	2	1126,62517	4,31	14,27	0
P72785	4-alpha-glucanotransferase OS=Synechocystis sp. (strain PCC 6803 / Kazusa) GN=r	216,47	5,74	1	3	3	8	1,014	8	29,3	505	57,1	4,88							
	A3	Sequence	# PSMs	# Proteins	# Protein Groups	Protein Group Accessions	Modifications	ΔCn	114/115	114/115 Count	114/115 Variability [%]	q-Value	PEP	IonScore	Exp Value	Charge	MH+ [Da]	ΔM [ppm]	RT [min]	# Missed Cleavages
	High	aTGFAWWIk	4	1	1	P72785	N-Term(iTRAQ4plex); K9(iTRAQ4plex)	0,0000	1,185	4	27,5	0	1,807E-07	53	5,32072E-06	2	1367,77178	0,23	45,39	0
	High	vLHFAFDSDR	3	1	1	P72785	N-Term(iTRAQ4plex)	0,0000	0,981	3	21,5	0	0,00000603	46	2,67898E-05	2	1350,69328	0,72	33,76	0
	High	aNLQYLDIVR	1	1	1	P72785	N-Term(iTRAQ4plex)	0,0000	0,877	1		0	0,000001506	42	6,98184E-05	2	1348,77092	0,27	39,81	0
P73479	Succinate dehydrogenase flavoprotein subunit OS=Synechocystis sp. (strain PCC 68	212,65	11,13	1	6	6	7	1,067	7	26,6	575	63,7	5,67							
	A3	Sequence	# PSMs	# Proteins	# Protein Groups	Protein Group Accessions	Modifications	ΔCn	114/115	114/115 Count	114/115 Variability [%]	q-Value	PEP	IonScore	Exp Value	Charge	MH+ [Da]	ΔM [ppm]	RT [min]	# Missed Cleavages
	High	lGSNSLLEcVVYGR	1	1	1	P73479	N-Term(iTRAQ4plex); C9(Carbamidomethyl)	0,0000	1,153	1		0	8,313E-09	70	9,43996E-08	2	1710,89641	-0,10	45,77	0
	High	aVmVATGGYGR	2	1	1	P73479	N-Term(iTRAQ4plex); M3(Oxidation)	0,0000	0,882	2	2,2	0	0,00001006	45	2,88383E-05	2	1241,64324	0,25	19,71	0
	High	iPFcWEEAHR	1	1	1	P73479	N-Term(iTRAQ4plex); C4(Carbamidomethyl)	0,0000	1,283	1		0	0,000002295	45	3,44326E-05	3	1488,71747	-0,01	34,76	0
	High	aITLEIR	1	1	1	P73479	N-Term(iTRAQ4plex)	0,0000	0,655	1		0	0,002607	34	0,000407352	2	959,60192	1,33	30,20	0
	High	rFmEDYAPSR	1	1	1	P73479	N-Term(iTRAQ4plex); M3(Oxidation)	0,0000	1,067	1		0	0,0000106	33	0,000477496	3	1431,68882	5,62	16,55	1
	High	hTLASFDGEQIk	1	1	1	P73479	N-Term(iTRAQ4plex); K12(iTRAQ4plex)	0,0000	1,203	1		0	0,0002361	31	0,000739554	3	1633,87955	0,44	26,56	0
P74266	50S ribosomal protein L21 OS=Synechocystis sp. (strain PCC 6803 / Kazusa) GN=rp	212,61	15,32	1	3	3	7	2,603	5	25,2	124	13,7	9,52							
	A3	Sequence	# PSMs	# Proteins	# Protein Groups	Protein Group Accessions	Modifications	ΔCn	114/115	114/115 Count	114/115 Variability [%]	q-Value	PEP	IonScore	Exp Value	Charge	MH+ [Da]	ΔM [ppm]	RT [min]	# Missed Cleavages
	High	sYAIIEIGGTQIR	2	1	1	P74266	N-Term(iTRAQ4plex)	0,0000				0	1,232E-10	86	2,31723E-09	2	1564,88481	2,07	40,15	0
	High	kVIVYk	2	1	1	P74266	N-Term(iTRAQ4plex); K1(iTRAQ4plex); K6(iTRAQ4plex)	0,0000	6,445	2	11,4	0	0,001	27	0,002172551	3	1181,79920	0,84	17,13	1

	Medium	vIVYk	3	1	1	P74266	N-Term(iTRAQ4plex); K5(iTRAQ4plex)	0,0000	2,386	3	11,9	0,011	0,1303	28	0,001527461	2	909,60112	-0,05	21,81	0
P20804	Acyl carrier protein OS=Synechocystis sp. (strain PCC 6803 / Kazusa) GN=acpP PE=	210,20	20,78	1	2	2	12	1,241	8	7,4	77	8,6	4,07							
	A3	Sequence	# PSMs	# Proteins	# Protein Groups	Protein Group Accessions	Modifications	ΔCn	114/115	114/115 Count	114/115 Variability [%]	q-Value	PEP	IonScore	Exp Value	Charge	MH+ [Da]	ΔM [ppm]	RT [min]	# Missed Cleavages
	High	aVEHIESk	11	1	1	P20804	N-Term(iTRAQ4plex); K8(iTRAQ4plex)	0,0000	1,227	7	4,5	0	0,0002853	58	1,68655E-06	2	1200,68205	-0,53	12,60	0
	High	mNQEIFEk	1	1	1	P20804	N-Term(iTRAQ4plex); M1(Oxidation); K8(iTRAQ4plex)	0,0000	1,567	1		0	0,0002944	32	0,0005754	2	1342,69280	0,93	21,40	0
P72968	Slr1590 protein OS=Synechocystis sp. (strain PCC 6803 / Kazusa) GN=slr1590 PE=	209,88	10,51	1	3	3	7	0,904	7	4,2	295	32,6	5,00							
	A3	Sequence	# PSMs	# Proteins	# Protein Groups	Protein Group Accessions	Modifications	ΔCn	114/115	114/115 Count	114/115 Variability [%]	q-Value	PEP	IonScore	Exp Value	Charge	MH+ [Da]	ΔM [ppm]	RT [min]	# Missed Cleavages
	High	vGESVNFLGFLNk	3	1	1	P72968	N-Term(iTRAQ4plex); K13(iTRAQ4plex)	0,0000	0,904	3	3,5	0	1,666E-07	64	3,90814E-07	2	1711,96489	1,58	50,54	0
	High	kVTGETDPALVk	3	1	1	P72968	N-Term(iTRAQ4plex); K1(iTRAQ4plex); K12(iTRAQ4plex)	0,0000	0,904	3	4,2	0	2,947E-09	55	3,49116E-06	3	1690,01181	0,39	20,13	1
	High	skPIFk	1	1	1	P72968	N-Term(iTRAQ4plex); K2(iTRAQ4plex); K6(iTRAQ4plex)	0,0000	1,103	1		0	0,001014	14	0,035478888	3	1151,75003	-1,06	15,86	0
Q55200	Protein SphX OS=Synechocystis sp. (strain PCC 6803 / Kazusa) GN=sphX PE=3 SV	209,55	10,42	1	5	5	10	0,621	10	12,6	336	36,8	4,77							
	A3	Sequence	# PSMs	# Proteins	# Protein Groups	Protein Group Accessions	Modifications	ΔCn	114/115	114/115 Count	114/115 Variability [%]	q-Value	PEP	IonScore	Exp Value	Charge	MH+ [Da]	ΔM [ppm]	RT [min]	# Missed Cleavages
	High	sLTVEELkR	5	1	1	Q55200	N-Term(iTRAQ4plex); K8(iTRAQ4plex)	0,0000	0,577	5	14,8	0	0,0007912	53	5,5077E-06	2	1362,81890	-0,46	24,21	1
	High	iWEPAAEk	1	1	1	Q55200	N-Term(iTRAQ4plex); K8(iTRAQ4plex)	0,0000	0,620	1		0	0,00001804	39	0,000117482	2	1231,69390	1,10	26,75	0
	High	rIWEPAAEk	1	1	1	Q55200	N-Term(iTRAQ4plex); K9(iTRAQ4plex)	0,0000	0,563	1		0	0,002186	29	0,00119116	3	1387,79111	-1,83	22,13	1
	High	tDIADASRPINk	2	1	1	Q55200	N-Term(iTRAQ4plex); K12(iTRAQ4plex)	0,0000	0,681	2	13,5	0	0,0001632	28	0,001698244	3	1588,88761	-1,35	19,51	0
	High	lFcEGk	1	1	1	Q55200	N-Term(iTRAQ4plex); C3(Carbamidomethyl); K6(iTRAQ4plex)	0,0000	0,728	1		0,005	0,05712	23	0,004753024	2	1041,55974	-4,24	21,62	0
P72991	ATP-dependent zinc metalloprotease FtsH 3 OS=Synechocystis sp. (strain PCC 6803	205,47	9,90	1	2	5	9	1,036	2	20,9	616	67,2	5,24							
	A3	Sequence	# PSMs	# Proteins	# Protein Groups	Protein Group Accessions	Modifications	ΔCn	114/115	114/115 Count	114/115 Variability [%]	q-Value	PEP	IonScore	Exp Value	Charge	MH+ [Da]	ΔM [ppm]	RT [min]	# Missed Cleavages
	High	rTPGFTGADLSNLLNEAAILAAR	1	1	1	P72991	N-Term(iTRAQ4plex)	0,0000	0,901	1		0	1,557E-09	76	4,48176E-08	3	2515,37876	1,48	56,76	1
	High	gVLLVGPPGTGk	2	3	3	Q55700;P72991;P73437	N-Term(iTRAQ4plex); K12(iTRAQ4plex)	0,0000				0	1,209E-07	59	1,31513E-06	2	1382,86235	0,97	32,40	0
	High	vRDLFEQAk	3	2	2	P72991;P73437	N-Term(iTRAQ4plex); K9(iTRAQ4plex)	0,0000				0	0,00000137	46	2,69755E-05	2	1393,80437	0,11	27,68	1
	High	qVVVDRPDYAGR	1	1	1	P72991	N-Term(iTRAQ4plex)	0,0000	1,191	1		0	0,0005774	16	0,024715534	3	1518,82193	4,85	20,38	0
	Medium	tLLAk	2	3	3	Q55700;P72991;P73437	N-Term(iTRAQ4plex); K5(iTRAQ4plex)	0,0000				0,011	0,1328	30	0,001096402	2	833,57073	1,02	18,46	0
P36265	Transcription termination/antitermination protein NusG OS=Synechocystis sp. (strai	204,06	24,39	1	5	5	13	1,170	10	7,3	205	23,4	6,35							
	A3	Sequence	# PSMs	# Proteins	# Protein Groups	Protein Group Accessions	Modifications	ΔCn	114/115	114/115 Count	114/115 Variability [%]	q-Value	PEP	IonScore	Exp Value	Charge	MH+ [Da]	ΔM [ppm]	RT [min]	# Missed Cleavages
	High	nTPHVINFVGSEQk	1	1	1	P36265	N-Term(iTRAQ4plex); K14(iTRAQ4plex)	0,0000	1,406	1		0	3,465E-09	47	2,10848E-05	3	1858,00815	1,07	27,36	0
	High	aLLSIFGR	6	1	1	P36265	N-Term(iTRAQ4plex)	0,0000	1,164	3	0,5	0	0,000863	44	3,75811E-05	2	1020,63310	0,81	44,60	0
	High	iHTLDVADR	1	1	1	P36265	N-Term(iTRAQ4plex)	0,0000	1,337	1		0	0,00000386	29	0,001235862	3	1183,65570	0,43	20,59	0
	High	iFPGYVLIR	4	1	1	P36265	N-Term(iTRAQ4plex)	0,0000	1,147	4	7,3	0	0,003652	26	0,002422085	2	1221,74809	0,38	44,86	0
	High	hVDEQEPVVk	1	1	1	P36265	N-Term(iTRAQ4plex); K10(iTRAQ4plex)	0,0000	0,939	1		0	0,00006321	20	0,009331898	3	1467,80555	0,64	15,50	0
P74360	Uncharacterized protein sll1526 OS=Synechocystis sp. (strain PCC 6803 / Kazusa) C	203,63	11,46	1	5	5	6	0,756	6	20,9	506	57,7	7,50							
	A3	Sequence	# PSMs	# Proteins	# Protein Groups	Protein Group Accessions	Modifications	ΔCn	114/115	114/115 Count	114/115 Variability [%]	q-Value	PEP	IonScore	Exp Value	Charge	MH+ [Da]	ΔM [ppm]	RT [min]	# Missed Cleavages
	High	lEQLLLLAk	2	1	1	P74360	N-Term(iTRAQ4plex); K9(iTRAQ4plex)	0,0000	0,685	2	1,7	0	5,241E-08	61	8,66902E-07	2	1328,87578	0,15	49,92	0
	High	fALVVPIk	1	1	1	P74360	N-Term(iTRAQ4plex); K8(iTRAQ4plex)	0,0000	0,894	1		0	0,0003997	51	8,7694E-06	2	1174,78044	0,18	42,94	0
	High	lALLPQEHLQALGNLR	1	1	1	P74360	N-Term(iTRAQ4plex)	0,0000	0,608	1		0	3,841E-10	51	8,83019E-06	3	1930,13614	0,32	44,29	0
	High	kQDGPQFVWQVLSSQGk	1	1	1	P74360	N-Term(iTRAQ4plex); K1(iTRAQ4plex); K17(iTRAQ4plex)	0,0000	1,085	1		0	4,635E-10	35	0,000385827	4	2364,30166	-0,85	44,54	1
	High	qAIVNFIR	1	1	1	P74360	N-Term(iTRAQ4plex)	0,0000	0,825	1		0	0,005929	26	0,002950598	2	1104,66448	-0,14	34,76	0
P73163	Putative carboxymethylenebutenolidase OS=Synechocystis sp. (strain PCC 6803 / K	203,39	13,82	1	3	3	6	1,113	6	20,9	246	26,9	4,96							
	A3	Sequence	# PSMs	# Proteins	# Protein Groups	Protein Group Accessions	Modifications	ΔCn	114/115	114/115 Count	114/115 Variability [%]	q-Value	PEP	IonScore	Exp Value	Charge	MH+ [Da]	ΔM [ppm]	RT [min]	# Missed Cleavages
	High	eGYVAIAPAIYQR	3	1	1	P73163	N-Term(iTRAQ4plex)	0,0000	1,108	3	1,3	0	0,000000591	59	1,22735E-06	2	1594,87224	0,77	36,19	0
	High	qLFQTELk	2	1	1	P73163	N-Term(iTRAQ4plex); K8(iTRAQ4plex)	0,0000	1,365	2	6,1	0	0,0001941	52	9,38278E-06	2	1294,76128	0,26	31,86	0
	High	yPGADHGFFcDQR	1	1	1	P73163	N-Term(iTRAQ4plex); C10(Carbamidomethyl)	0,0000	0,701	1		0	0,001624	32	0,000674481	3	1713,75345	-1,52	28,46	0
P27180	ATP synthase subunit delta OS=Synechocystis sp. (strain PCC 6803 / Kazusa) GN=a	203,33	29,73	1	6	6	11	1,512	10	24,0	185	20,1	6,79							
	A3	Sequence	# PSMs	# Proteins	# Protein Groups	Protein Group Accessions	Modifications	ΔCn	114/115	114/115 Count	114/115 Variability [%]	q-Value	PEP	IonScore	Exp Value	Charge	MH+ [Da]	ΔM [ppm]	RT [min]	# Missed Cleavages
	High	qFTNTVLAEVTSALk	3	1	1	P27180	N-Term(iTRAQ4plex); K15(iTRAQ4plex)	0,0000	1,076	3	20,1	0	1,48E-08	58	1,49968E-06	2	1910,08672	1,55	54,08	0
	High	vGSQVFDSSLR	2	1	1	P27180	N-Term(iTRAQ4plex)	0,0000	1,600	2	16,1	0	5,336E-08	52	5,95621E-06	2	1338,71404	0,44	31,55	0
	High	lTDAQkDQVk	2	1	1	P27180	N-Term(iTRAQ4plex); K6(iTRAQ4plex); K10(iTRAQ4plex)	0,0000	1,805	2	3,4	0,001	0,00844	15	0,036199106	3	1577,92710	3,03	16,03	1
	High	mkGSLYSSk	1	1	1	P27180	N-Term(iTRAQ4plex); M1(Oxidation); K2(iTRAQ4plex); K9(iTRAQ4plex)	0,0000	1,559	1		0,006	0,07345	14	0,037841645	3	1448,81467	0,23	12,71	1
	High	rVGLSLGTAL	2	1	1	P27180	N-Term(iTRAQ4plex)	0,0000	1,466	1		0,008	0,09472	17	0,018070493	2	1130,70195	0,47	36,78	1
	Medium	vGLSLGTAL	1	1	1	P27180	N-Term(iTRAQ4plex)	0,0000	1,409	1		0,025	0,2646	13	0,046341491	2	974,60271	2,47	43,87	0
P73534	Pyruvate kinase 2 OS=Synechocystis sp. (strain PCC 6803 / Kazusa) GN=pyk2 PE=	203,26	9,14	1	4	4	5	0,805	5	11,2	591	62,9	6,64							
	A3	Sequence	# PSMs	# Proteins	# Protein Groups	Protein Group Accessions	Modifications	ΔCn	114/115	114/115 Count	114/115 Variability [%]	q-Value	PEP	IonScore	Exp Value	Charge	MH+ [Da]	ΔM [ppm]	RT [min]	# Missed Cleavages
	High	aAGIITEEESLTSHAAIIGLR	1	1	1	P73534	N-Term(iTRAQ4plex)	0,0000	0,805	1		0	9,171E-13	88	2,02742E-09	3	2296,26468	0,74	47,71	0
	High	gVGIGQGAVSGR	1	1	1	P73534	N-Term(iTRAQ4plex)	0,0000	0,924	1		0	1,241E-08	51	7,79776E-06	2	1201,68474	6,43	18,96	0
	High	qLIQAGATTFR	2	1	1	P73534	N-Term(iTRAQ4plex)	0,0000	0,767	2	3,8	0	6,866E-07	47	2,10363E-05	2	1349,76653	0,53	30,40	0
	High	lGVPVIVGFk	1	1	1	P73534	N-Term(iTRAQ4plex); K10(iTRAQ4plex)	0,0000	0,911	1		0	0,0003521	30	0,000911948	2	1316,85430	-0,13	45,82	0
P73299	30S ribosomal protein S13 OS=Synechocystis sp. (strain PCC 6803 / Kazusa) GN=r	201,41	49,61	1	7	7	11	1,826	10	43,2	127	14,6	10,59							
	A3	Sequence	# PSMs	# Proteins	# Protein Groups	Protein Group Accessions	Modifications	ΔCn	114/115	114/115 Count	114/115 Variability [%]	q-Value	PEP	IonScore	Exp Value	Charge	MH+ [Da]	ΔM [ppm]	RT [min]	# Missed Cleavages
	High	vkDLSDEDALk	3	1	1	P73299	N-Term(iTRAQ4plex); K2(iTRAQ4plex); K11(iTRAQ4plex)	0,0000	2,878	2	7,3	0	2,297E-09	56	2,52912E-06	3	1664,94669	2,14	24,41	1
	High	sHEILDATGVSPDVR	1	1	1	P73299	N-Term(iTRAQ4plex)	0,0000	0,746	1		0	0,000003229	48	1,58478E-05	3	1739,91025	3,30	28,16	0
	High	lGDIGTYR	1	1	1	P73299	N-Term(iTRAQ4plex)	0,0000	1,785	1		0	0,0005405	22	0,005780561	2	1038,57073	0,65	27,38	0
	High	rLTVAGk	2	1	1	P73299	N-Term(iTRAQ4plex); K7(iTRAQ4plex)	0,0000	1,776	2	7,5	0	0,002058	21	0,010217244	3	1032,67801	1,16	13,47	1
	High	rWEAmNIk	1	1	1	P73299	N-Term(iTRAQ4plex); M5(Oxidation); K8(iTRAQ4plex)	0,0000	2,538	1		0	0,0007303	16	0,026422263	3	1351,73993	0,31	18,77	1
	High	iAGVDLPR	1	1	1	P73299	N-Term(iTRAQ4plex)	0,0000	2,026	1		0,002	0,02012	34	0,000413971	2	984,59642	0,54	29,37	0
	Medium	qGLPVR	2	1	1	P73299	N-Term(iTRAQ4plex)	0,0000	1,336	2	12,5	0,027	0,2851	21	0,008589542	2	813,51122	6,00	15,74	0
P73328	Slr1900 protein OS=Synechocystis sp. (strain PCC 6803 / Kazusa) GN=slr1900 PE=	200,62	19,43	1	7	7	10	1,398	10	36,5	247	27,4	4,87							
	A3	Sequence	# PSMs	# Proteins	# Protein Groups	Protein Group Accessions	Modifications	ΔCn	114/115	114/115 Count	114/115 Variability [%]	q-Value	PEP	IonScore	Exp Value	Charge	MH+ [Da]	ΔM [ppm]	RT [min]	# Missed Cleavages
	High	aHLTVTQGDER	1	1	1	P73328	N-Term(iTRAQ4plex)	0,0000	1,796	1		0	3,817E-07	61	7,11164E-07	3	1370,71451	0,01	14,15	0
	High	gTEIcQVSR	2	1	1	P73328	N-Term(iTRAQ4plex); C5(Carbamidomethyl)	0,0000	1,289	2	23,9	0	9,269E-07	56	2,57022E-06	2	1193,61016	3,04	15,58	0
	High	yNAIFk	2	1	1	P73328	N-Term(iTRAQ4plex); K6(iTRAQ4plex)	0,0000	1,216	2	20,8	0	0,004505	28	0,001499581	2	1043,61467	1,80	27,65	0
	High	kQGNFEk	2	1	1	P73328	N-Term(iTRAQ4plex); K1(iTRAQ4plex); K7(iTRAQ4plex)	0,0000	1,662	2	26,0	0	0,0005191	27	0,002417646	3	1282,74836	0,30	10,43	1
	Medium	yNAIFkDPk	1	1	1	P73328	N-Term(iTRAQ4plex); K6(iTRAQ4plex); K9(iTRAQ4plex)	0,0000	2,252	1		0,019	0,2065	11	0,088450001	3	1527,89078	0,81	26,23	1
	Medium	dIVTHR	1	1	1	P73328	N-Term(iTRAQ4plex)	0,0000	1,201	1		0,021	0,2336	26	0,002398667	2	884,50731	0,29	11,99	0
	Medium	aAYENR	1	1	1	P73328	N-Term(iTRAQ4plex)	0,0000	1,103	1		0,035	0,3689	17	0,020463033	2	867,44402	-0,11	11,35	0
P72929	Uncharacterized protein sll1021 OS=Synechocystis sp. (strain PCC 6803 / Kazusa) G	200,43	6,24	1	4	4	5	0,776	4	33,2	673	74,4	5,12							
	A3	Sequence	# PSMs	# Proteins	# Protein Groups	Protein Group Accessions	Modifications	ΔCn	114/115	114/115 Count	114/115 Variability [%]	q-Value	PEP	IonScore	Exp Value	Charge	MH+ [Da]	ΔM [ppm]	RT [min]	# Missed Cleavages
	High	lAVIVAQQDAQQHR	2	1	1	P72929	N-Term(iTRAQ4plex)	0,0000	0,492	1		0	9,702E-12	74	3,93523E-08	3	1720,95646	-0,63	26,88	0
	High	kELESQEAQQQR	1	1	1	P72929	N-Term(iTRAQ4plex); K1(iTRAQ4plex)	0,0000	0,991	1		0	4,307E-10	53	4,81915E-06	3	1761,93399	0,34	12,18	1
	High	nEILTAAAR	1	1	1	P72929	N-Term(iTRAQ4plex)	0,0000	0,939	1		0	0,00001212	53	5,09296E-06	2	1102,63298	-0,68	22,64	0
	High	vAQALQk	1	1	1	P72929	N-Term(iTRAQ4plex); K7(iTRAQ4plex)	0,0000	0,641	1		0	0,001521	44	3,82798E-05	2	1045,66374	2,79	18,79	0
P74456	Ribosome-recycling factor OS=Synechocystis sp. (strain PCC 6803 / Kazusa) GN=fr	200,17	27,47	1	6	6	15	1,180	13	18,0	182	20,2	5,81							
	A3	Sequence	# PSMs	# Proteins	# Protein Groups	Protein Group Accessions	Modifications	ΔCn	114/115	114/115 Count	114/115 Variability [%]	q-Value	PEP	IonScore	Exp Value	Charge	MH+ [Da]	ΔM [ppm]	RT [min]	# Missed Cleavages
	High	rIDELLAAk	6	1	1	P74456	N-Term(iTRAQ4plex); K9(iTRAQ4plex)	0,0000	1,104	4	9,0	0	0,000005146	44	3,64729E-05	2	1316,81548	1,08	30,93	1
	High	lNIPPLTAER	2	1	1	P74456	N-Term(iTRAQ4plex)	0,0000	1,183	2	3,5	0	0,00348	29	0,001121941	2	1267,75017	0,84	36,84	0
	High	aNASLLDR	1	1	1	P74456	N-Term(iTRAQ4plex)	0,0000	1,339	1		0	0,005713	27	0,002654056	2	1003,57207	6,73	20,62	0
	High	lAELkDHmQk	1	1	1	P74456	N-Term(iTRAQ4plex); K5(iTRAQ4plex); M8(Oxidation); K10(iTRAQ4plex)	0,0000	1,530	1		0	0,0006015	15	0,040661299	3	1660,94254	0,51	18,28	1
	High	lAELkDHMQk	1	1	1	P74456	N-Term(iTRAQ4plex); K5(iTRAQ4plex); K10(iTRAQ4plex)	0,0000	1,426	1		0	0,0006123	13	0,054663551	4	1644,94570	-0,65	24,63	1
	High	sFNTIR	3	1	1	P74456	N-Term(iTRAQ4plex)	0,0000	1,023	3	10,2	0,005	0,06071	27	0,002046445	2	881,49596	-0,22	18,30	0
	Medium	lTDQSTkR	1	1	1	P74456	N-Term(iTRAQ4plex); K7(iTRAQ4plex)	0,0000	1,318	1		0,021	0,2245	14	0,037322438	3	1236,71436	-0,56	9,32	1
P74507	2,3-bisphosphoglycerate-independent phosphoglycerate mutase OS=Synechocystis	199,36	9,40	1	3	3	6	1,044	6	6,4	532	57,9	5,83							
	A3	Sequence	# PSMs	# Proteins	# Protein Groups	Protein Group Accessions	Modifications	ΔCn	114/115	114/115 Count	114/115 Variability [%]	q-Value	PEP	IonScore	Exp Value	Charge	MH+ [Da]	ΔM [ppm]	RT [min]	# Missed Cleavages
	High	lkEAIQAIETVDLNLGR	2	1	1	P74507	N-Term(iTRAQ4plex); K2(iTRAQ4plex)	0,0000	1,091	2	11,4	0	1,583E-12	88	1,72175E-09	3	2171,26609	1,02	52,55	1
	High	kIPGHGGEVk	3	1	1	P74507	N-Term(iTRAQ4plex); K1(iTRAQ4plex); K10(iTRAQ4plex)	0,0000	1,038	3	1,9	0	6,465E-07	34	0,000422639	3	1453,88239	-1,90	10,99	1
	High	aEAPIAPVVLVILDGWGYRPDTR	1	1	1	P74507	N-Term(iTRAQ4plex)	0,0000	1,101	1		0	0,0002604	13	0,055586586	3	2652,46708	1,49	61,78	0
P72703	Agmatinase 1 OS=Synechocystis sp. (strain PCC 6803 / Kazusa) GN=speB1 PE=3 S	198,34	15,36	1	6	6	10	1,114	9	16,5	306	33,4	5,01							
	A3	Sequence	# PSMs	# Proteins	# Protein Groups	Protein Group Accessions	Modifications	ΔCn	114/115	114/115 Count	114/115 Variability [%]	q-Value	PEP	IonScore	Exp Value	Charge	MH+ [Da]	ΔM [ppm]	RT [min]	# Missed Cleavages
	High	fVVAIGGEHAITTGVVR	1	1	1	P72703	N-Term(iTRAQ4plex)	0,0000	1,109	1		0	2,817E-10	52	5,79389E-06	3	1870,06711	0,18	38,49	0
	High	vLELGLPTLPIAIR	3	1	1	P72703	N-Term(iTRAQ4plex)	0,0000	1,054	2	37,4	0	0,00005637	52	5,92884E-06	2	1649,05315	2,96	56,69	0
	High	nIPVFWAR	3	1	1	P72703	N-Term(iTRAQ4plex)	0,0000	1,221	3	2,9	0	0,002236	38	0,000160683	2	1146,65398	-0,08	41,10	0
	High	rVLELGLPTLPIAIR	1	1	1	P72703	N-Term(iTRAQ4plex)	0,0000	1,039	1		0	0,00006184	31	0,000820295	3	1805,15183	1,35	50,33	1
	High	ekNIPVFWAR	1	1	1	P72703	N-Term(iTRAQ4plex); K2(iTRAQ4plex)	0,0000	0,700	1		0	2,648E-07	24	0,004698328	3	1547,89286	-0,55	34,70	1
	Medium	lFQTk	1	1	1	P72703	N-Term(iTRAQ4plex); K5(iTRAQ4plex)	0,0000	1,441	1		0,036	0,3815	16	0,023931505	2	924,57952	4,14	21,43	0
P74582	Aconitate hydratase 2 OS=Synechocystis sp. (strain PCC 6803 / Kazusa) GN=acnB	198,18	7,49	1	7	7	9	0,916	8	9,1	868	93,5	5,31							
	A3	Sequence	# PSMs	# Proteins	# Protein Groups	Protein Group Accessions	Modifications	ΔCn	114/115	114/115 Count	114/115 Variability [%]	q-Value	PEP	IonScore	Exp Value	Charge	MH+ [Da]	ΔM [ppm]	RT [min]	# Missed Cleavages
	High	aGGYILGGk	2	1	1	P74582	N-Term(iTRAQ4plex); K9(iTRAQ4plex)	0,0000	0,998	1		0	7,702E-07	70	1,104E-07	2	1123,67070	-0,62	24,17	0
	High	aGFLTAIAk	1	1	1	P74582	N-Term(iTRAQ4plex); K9(iTRAQ4plex)	0,0000	0,928	1		0	0,000001119	54	3,97164E-06	2	1179,73381	-0,17	36,51	0
	High	gGVALRPGDGIIHSWLNR	1	1	1	P74582	N-Term(iTRAQ4plex)	0,0000	0,279	1		0	2,521E-08	41	0,000102137	4	2062,14174	-0,49	37,67	0
	High	scSGSTIk	1	1	1	P74582	N-Term(iTRAQ4plex); C2(Carbamidomethyl); K8(iTRAQ4plex)	0,0000	1,146	1		0	0,00476	27	0,002167554	2	1127,59685	-0,05	10,42	0
	High	gFTLAQk	1	1	1	P74582	N-Term(iTRAQ4plex); K7(iTRAQ4plex)	0,0000	0,953	1		0	0,003464	23	0,00512826	2	1052,63310	-1,11	23,26	0
	High	iTVTVFk	1	1	1	P74582	N-Term(iTRAQ4plex); K7(iTRAQ4plex)	0,0000	0,889	1		0,001	0,01004	41	7,42968E-05	2	1095,70134	-0,26	33,63	0
	High	iPLLIGR	2	1	1	P74582	N-Term(iTRAQ4plex)	0,0000	0,873	2	5,3	0,004	0,0506	12	0,070302376	2	925,63133	-0,23	38,48	0
P72585	GDP-L-fucose synthase OS=Synechocystis sp. (strain PCC 6803 / Kazusa) GN=fcl PE	194,88	8,33	1	3	3	7	0,760	6	22,2	312	34,7	5,90							
	A3	Sequence	# PSMs	# Proteins	# Protein Groups	Protein Group Accessions	Modifications	ΔCn	114/115	114/115 Count	114/115 Variability [%]	q-Value	PEP	IonScore	Exp Value	Charge	MH+ [Da]	ΔM [ppm]	RT [min]	# Missed Cleavages
	High	qVVAQLIAAGGDRPk	3	1	1	P72585	N-Term(iTRAQ4plex); K15(iTRAQ4plex)	0,0000	0,932	2	67,3	0	4,225E-15	81	7,60274E-09	3	1811,07383	-0,22	32,57	0
	High	kALLVQLESYR	1	1	1	P72585	N-Term(iTRAQ4plex); K1(iTRAQ4plex)	0,0000	0,669	1		0	6,026E-08	37	0,000190533	3	1607,97287	0,33	38,01	1
	High	aLLVQLESYR	3	1	1	P72585	N-Term(iTRAQ4plex)	0,0000	0,775	3	5,9	0	0,00001739	34	0,000375811	2	1335,77678	1,11	42,89	0
Q55522	Valine--tRNA ligase OS=Synechocystis sp. (strain PCC 6803 / Kazusa) GN=valS PE=	193,78	8,35	1	7	7	10	0,973	9	8,7	910	102,7	5,17							
	A3	Sequence	# PSMs	# Proteins	# Protein Groups	Protein Group Accessions	Modifications	ΔCn	114/115	114/115 Count	114/115 Variability [%]	q-Value	PEP	IonScore	Exp Value	Charge	MH+ [Da]	ΔM [ppm]	RT [min]	# Missed Cleavages
	High	lNQVIQQTR	2	1	1	Q55522	N-Term(iTRAQ4plex)	0,0000	0,883	2	14,9	0	0,00002739	53	5,15193E-06	2	1243,72331	-0,50	24,60	0
	High	qVLAHTLDSIIk	2	1	1	Q55522	N-Term(iTRAQ4plex); K12(iTRAQ4plex)	0,0000	0,714	1		0	9,467E-08	50	1,01151E-05	3	1625,98294	0,02	38,84	0
	High	gkVPVEPLLSTQWFVk	2	1	1	Q55522	N-Term(iTRAQ4plex); K2(iTRAQ4plex); K16(iTRAQ4plex)	0,0000	0,946	2	7,7	0	6,58E-11	50	1,06162E-05	3	2260,34232	-0,31	47,24	1
	High	hNLPFINLLNk	1	1	1	Q55522	N-Term(iTRAQ4plex); K11(iTRAQ4plex)	0,0000	1,609	1		0	0,000003842	26	0,002500173	3	1610,96079	-0,82	42,23	0
	High	kNVVQALEAQGFLVk	1	1	1	Q55522	N-Term(iTRAQ4plex); K1(iTRAQ4plex); K15(iTRAQ4plex)	0,0000	0,918	1		0	0,000002286	20	0,010326901	4	2076,25283	-0,66	45,33	1
	High	lkDWcISR	1	1	1	Q55522	N-Term(iTRAQ4plex); K2(iTRAQ4plex); C5(Carbamidomethyl)	0,0000	0,973	1		0	0,00655	18	0,017020409	3	1365,75400	-0,84	26,91	1
	Medium	wILSR	1	1	1	Q55522	N-Term(iTRAQ4plex)	0,0000	1,018	1		0,02	0,2222	19	0,011748164	2	818,50023	-0,35	29,17	0
Q55700	ATP-dependent zinc metalloprotease FtsH 2 OS=Synechocystis sp. (strain PCC 6803	193,47	11,32	1	5	7	10	1,076	6	20,1	627	68,5	5,41							
	A3	Sequence	# PSMs	# Proteins	# Protein Groups	Protein Group Accessions	Modifications	ΔCn	114/115	114/115 Count	114/115 Variability [%]	q-Value	PEP	IonScore	Exp Value	Charge	MH+ [Da]	ΔM [ppm]	RT [min]	# Missed Cleavages
	High	gVLLVGPPGTGk	2	3	3	Q55700;P72991;P73437	N-Term(iTRAQ4plex); K12(iTRAQ4plex)	0,0000				0	1,209E-07	59	1,31513E-06	2	1382,86235	0,97	32,40	0
	High	rTPGFSGADLANLLNEAAILTAR	1	1	1	Q55700	N-Term(iTRAQ4plex)	0,0000	1,340	1		0	9,089E-07	41	0,00014075	4	2515,38173	2,67	57,38	1
	High	qLAEQGHQmAR	1	1	1	Q55700	N-Term(iTRAQ4plex); M9(Oxidation)	0,0000	0,851	1		0	0,00001494	39	0,000139949	3	1428,71317	-0,21	8,67	0
	High	eILEVHAR	1	1	1	Q55700	N-Term(iTRAQ4plex)	0,0000	0,855	1		0	0,000252	25	0,002883832	3	1110,63864	-0,15	19,62	0
	High	vRDLFk	1	1	1	Q55700	N-Term(iTRAQ4plex); K6(iTRAQ4plex)	0,0000	1,071	1		0,003	0,0365	12	0,064416927	3	1065,66419	-1,62	23,13	1
	Medium	tLLAk	2	3	3	Q55700;P72991;P73437	N-Term(iTRAQ4plex); K5(iTRAQ4plex)	0,0000				0,011	0,1328	30	0,001096402	2	833,57073	1,02	18,46	0
	Medium	vTLIPR	2	1	1	Q55700	N-Term(iTRAQ4plex)	0,0000	1,105	2	3,3	0,017	0,1816	30	0,000986211	2	842,55876	0,87	25,91	0
P72940	Probable adenylyl-sulfate kinase OS=Synechocystis sp. (strain PCC 6803 / Kazusa) (	193,06	22,03	1	4	4	11	0,822	8	10,3	177	19,7	5,97							
	A3	Sequence	# PSMs	# Proteins	# Protein Groups	Protein Group Accessions	Modifications	ΔCn	114/115	114/115 Count	114/115 Variability [%]	q-Value	PEP	IonScore	Exp Value	Charge	MH+ [Da]	ΔM [ppm]	RT [min]	# Missed Cleavages
	High	iGFVSHLLTR	3	1	1	P72940	N-Term(iTRAQ4plex)	0,0000	0,728	2	2,9	0	0,000001329	55	3,53159E-06	3	1286,76981	-0,28	37,48	0
	High	gVTIWLTGLSGAGk	2	1	1	P72940	N-Term(iTRAQ4plex); K14(iTRAQ4plex)	0,0000	1,172	2	2,1	0	1,766E-07	50	9,0985E-06	2	1647,96659	-0,42	48,00	0
	High	tTITHALEk	4	1	1	P72940	N-Term(iTRAQ4plex); K9(iTRAQ4plex)	0,0000	0,828	2	4,2	0	0,006617	22	0,006972567	3	1301,76624	-0,39	18,88	0
	High	gLGFSk	2	1	1	P72940	N-Term(iTRAQ4plex); K6(iTRAQ4plex)	0,0000	0,822	2	0,5	0,003	0,03823	31	0,000862919	2	896,54167	-3,04	21,20	0
P74230	30S ribosomal protein S12 OS=Synechocystis sp. (strain PCC 6803 / Kazusa) GN=r	193,00	26,19	1	4	4	9	1,191	9	9,0	126	14,2	11,22							
	A3	Sequence	# PSMs	# Proteins	# Protein Groups	Protein Group Accessions	Modifications	ΔCn	114/115	114/115 Count	114/115 Variability [%]	q-Value	PEP	IonScore	Exp Value	Charge	MH+ [Da]	ΔM [ppm]	RT [min]	# Missed Cleavages
	High	pTIQQLIR	3	1	1	P74230	N-Term(iTRAQ4plex)	0,0000	1,157	3	13,3	0	0,00000468	54	3,74947E-06	2	1112,69036	-0,44	32,99	0
	High	vYTTTPk	3	1	1	P74230	N-Term(iTRAQ4plex); K7(iTRAQ4plex)	0,0000	1,142	3	0,1	0	0,0006568	37	0,000200433	2	1097,65117	6,09	15,69	0
	High	gTLDATGVkDR	1	1	1	P74230	N-Term(iTRAQ4plex); K9(iTRAQ4plex)	0,0000	1,343	1		0	0,0002314	25	0,003198674	3	1420,79999	0,09	15,78	1
	Medium	kPNSALR	2	1	1	P74230	N-Term(iTRAQ4plex); K1(iTRAQ4plex)	0,0000	1,243	2	6,4	0,034	0,3575	12	0,063969065	3	1073,66745	0,44	9,33	0
P73866	Potassium-transporting ATPase A chain OS=Synechocystis sp. (strain PCC 6803 / Ka	191,33	4,84	1	2	2	7	0,130	7	60,3	558	59,3	5,41							
	A3	Sequence	# PSMs	# Proteins	# Protein Groups	Protein Group Accessions	Modifications	ΔCn	114/115	114/115 Count	114/115 Variability [%]	q-Value	PEP	IonScore	Exp Value	Charge	MH+ [Da]	ΔM [ppm]	RT [min]	# Missed Cleavages
	High	gPVAHFEIIk	3	1	1	P73866	N-Term(iTRAQ4plex); K10(iTRAQ4plex)	0,0000	0,130	3	9,6	0	4,053E-07	58	1,50998E-06	2	1398,82976	-3,59	30,44	0
	High	iARPLESLIFAGSGITk	4	1	1	P73866	N-Term(iTRAQ4plex); K17(iTRAQ4plex)	0,0000	0,306	4	400,8	0	3,106E-08	51	9,84422E-06	3	2061,22867	-1,19	47,96	0
Q55131	Slr0049 protein OS=Synechocystis sp. (strain PCC 6803 / Kazusa) GN=slr0049 PE=	190,93	7,04	1	3	3	7	0,679	7	13,8	398	44,0	5,06							
	A3	Sequence	# PSMs	# Proteins	# Protein Groups	Protein Group Accessions	Modifications	ΔCn	114/115	114/115 Count	114/115 Variability [%]	q-Value	PEP	IonScore	Exp Value	Charge	MH+ [Da]	ΔM [ppm]	RT [min]	# Missed Cleavages
	High	iVPLQFLk	5	1	1	Q55131	N-Term(iTRAQ4plex); K8(iTRAQ4plex)	0,0000	0,745	5	12,9	0	0,0003257	63	5,58432E-07	2	1245,81755	0,17	46,15	0
	High	fWmTFSEAYINHLR	1	1	1	Q55131	N-Term(iTRAQ4plex); M3(Oxidation)	0,0000	0,679	1		0	0,000001193	33	0,000529627	3	1974,96567	0,17	47,00	0
	Medium	nIPTLk	1	1	1	Q55131	N-Term(iTRAQ4plex); K6(iTRAQ4plex)	0,0000	0,588	1		0,029	0,3081	20	0,009396584	2	973,63268	4,33	24,07	0
Q55848	Ribose-phosphate pyrophosphokinase OS=Synechocystis sp. (strain PCC 6803 / Kaz	190,23	11,71	1	4	4	7	0,852	7	25,8	333	36,4	7,20							
	A3	Sequence	# PSMs	# Proteins	# Protein Groups	Protein Group Accessions	Modifications	ΔCn	114/115	114/115 Count	114/115 Variability [%]	q-Value	PEP	IonScore	Exp Value	Charge	MH+ [Da]	ΔM [ppm]	RT [min]	# Missed Cleavages
	High	lVANLITGAGAQR	2	1	1	Q55848	N-Term(iTRAQ4plex)	0,0000	0,714	2	1,5	0	1,017E-09	61	8,50546E-07	2	1427,84563	0,34	38,60	0
	High	kLNDAPLAIIDkR	2	1	1	Q55848	N-Term(iTRAQ4plex); K1(iTRAQ4plex); K12(iTRAQ4plex)	0,0000	0,992	2	22,8	0	9,272E-08	44	3,56427E-05	4	1899,17629	0,57	30,19	2
	High	qITAVLPYYGYAR	2	1	1	Q55848	N-Term(iTRAQ4plex)	0,0000	0,893	2	18,6	0	0,0004198	40	9,28902E-05	2	1658,90361	0,80	41,73	0
	High	kLNDAPLAIIDk	1	1	1	Q55848	N-Term(iTRAQ4plex); K1(iTRAQ4plex); K12(iTRAQ4plex)	0,0000	1,117	1		0	0,000007114	24	0,003854517	3	1743,07248	-0,92	34,89	1
P72805	Sll1665 protein OS=Synechocystis sp. (strain PCC 6803 / Kazusa) GN=sll1665 PE=4	189,95	4,07	1	3	3	14	1,313	11	14,7	589	63,6	3,57							
	A3	Sequence	# PSMs	# Proteins	# Protein Groups	Protein Group Accessions	Modifications	ΔCn	114/115	114/115 Count	114/115 Variability [%]	q-Value	PEP	IonScore	Exp Value	Charge	MH+ [Da]	ΔM [ppm]	RT [min]	# Missed Cleavages
	High	aETQALQAR	2	1	1	P72805	N-Term(iTRAQ4plex)	0,0000	1,317	2	0,4	0	0,00009181	54	4,03618E-06	2	1131,62969	5,12	14,62	0
	High	lESLESSHR	8	1	1	P72805	N-Term(iTRAQ4plex)	0,0000	1,266	5	19,3	0	0,0001252	48	1,58114E-05	2	1201,62969	0,27	17,65	0
	High	kLDSTk	4	1	1	P72805	N-Term(iTRAQ4plex); K1(iTRAQ4plex); K6(iTRAQ4plex)	0,0000	1,365	4	26,0	0	0,008034	22	0,006011322	3	1123,70423	-0,42	9,60	1
P74535	Sll1336 protein OS=Synechocystis sp. (strain PCC 6803 / Kazusa) GN=sll1336 PE=4	189,94	9,65	1	6	6	8	0,797	8	14,4	705	78,3	5,62							
	A3	Sequence	# PSMs	# Proteins	# Protein Groups	Protein Group Accessions	Modifications	ΔCn	114/115	114/115 Count	114/115 Variability [%]	q-Value	PEP	IonScore	Exp Value	Charge	MH+ [Da]	ΔM [ppm]	RT [min]	# Missed Cleavages
	High	vLNFNLGVER	2	1	1	P74535	N-Term(iTRAQ4plex)	0,0000	0,742	2	8,8	0	0,000006169	57	2,15869E-06	2	1304,74492	0,43	39,78	0
	High	qLQQLTRPYSLAETL	2	1	1	P74535	N-Term(iTRAQ4plex)	0,0000	0,782	2	4,7	0	0,000001631	49	1,23018E-05	2	1905,05889	1,39	44,36	0
	High	rVELLVEQIAWEmR	1	1	1	P74535	N-Term(iTRAQ4plex); M13(Oxidation)	0,0000	1,048	1		0	9,622E-08	36	0,000365905	3	1932,04959	0,09	43,95	1
	High	vmVGVEGIR	1	1	1	P74535	N-Term(iTRAQ4plex); M2(Oxidation)	0,0000	1,116	1		0	0,00692	25	0,004017076	2	1119,63140	0,08	26,98	0
	High	sELDSHPYIAk	1	1	1	P74535	N-Term(iTRAQ4plex); K11(iTRAQ4plex)	0,0000	0,930	1		0	0,0001307	20	0,009374973	3	1547,83060	-0,13	21,76	0
	High	wLWAGYGFR	1	1	1	P74535	N-Term(iTRAQ4plex)	0,0000	0,749	1		0,001	0,01235	18	0,016179683	2	1299,67497	-0,43	48,84	0
P73316	30S ribosomal protein S19 OS=Synechocystis sp. (strain PCC 6803 / Kazusa) GN=r	189,81	28,26	1	3	3	7	1,427	7	5,5	92	10,3	11,14							
	A3	Sequence	# PSMs	# Proteins	# Protein Groups	Protein Group Accessions	Modifications	ΔCn	114/115	114/115 Count	114/115 Variability [%]	q-Value	PEP	IonScore	Exp Value	Charge	MH+ [Da]	ΔM [ppm]	RT [min]	# Missed Cleavages
	High	kGPFVAASLLR	2	1	1	P73316	N-Term(iTRAQ4plex); K1(iTRAQ4plex)	0,0000	1,625	2	17,7	0	0,000001235	50	1,00918E-05	3	1446,90378	0,16	33,37	1
	High	gPFVAASLLR	3	1	1	P73316	N-Term(iTRAQ4plex)	0,0000	1,427	3	4,1	0	0,00000593	47	2,28927E-05	2	1174,70769	1,00	41,08	0
	High	qHVPVFVSEQMVGHk	1	1	1	P73316	N-Term(iTRAQ4plex); K15(iTRAQ4plex)	0,0000	1,376	1		0	4,852E-07	39	0,00014002	4	2010,08022	-1,58	29,16	0
	High	qHVPVFVSEQmVGHk	1	1	1	P73316	N-Term(iTRAQ4plex); M11(Oxidation); K15(iTRAQ4plex)	0,0000	1,175	1		0	0,000000168	26	0,002540973	4	2026,07815	-0,08	24,34	0
Q55650	Slr0355 protein OS=Synechocystis sp. (strain PCC 6803 / Kazusa) GN=slr0355 PE=	189,33	9,67	1	2	2	4	1,109	4	20,7	331	35,4	5,52							
	A3	Sequence	# PSMs	# Proteins	# Protein Groups	Protein Group Accessions	Modifications	ΔCn	114/115	114/115 Count	114/115 Variability [%]	q-Value	PEP	IonScore	Exp Value	Charge	MH+ [Da]	ΔM [ppm]	RT [min]	# Missed Cleavages
	High	vGILGFGGLGQAAAR	3	1	1	Q55650	N-Term(iTRAQ4plex)	0,0000	1,134	3	35,3	0	2,161E-12	84	4,08291E-09	2	1530,88896	1,04	44,58	0
	High	aTIREDIAHmPGYNVDk	1	1	1	Q55650	N-Term(iTRAQ4plex); M10(Oxidation); K17(iTRAQ4plex)	0,0000	1,084	1		0	1,959E-07	27	0,002405366	4	2234,14980	0,87	24,54	1
P26525	NAD(P)H-quinone oxidoreductase subunit I OS=Synechocystis sp. (strain PCC 6803	189,07	45,08	1	10	10	15	0,688	14	18,7	193	22,1	6,76							
	A3	Sequence	# PSMs	# Proteins	# Protein Groups	Protein Group Accessions	Modifications	ΔCn	114/115	114/115 Count	114/115 Variability [%]	q-Value	PEP	IonScore	Exp Value	Charge	MH+ [Da]	ΔM [ppm]	RT [min]	# Missed Cleavages
	High	rPITVQYPYEk	3	1	1	P26525	N-Term(iTRAQ4plex); K11(iTRAQ4plex)	0,0000	0,660	3	19,4	0	1,685E-08	48	1,51346E-05	3	1681,95060	-0,59	24,70	0
	High	hDLNYDNVALGR	1	1	1	P26525	N-Term(iTRAQ4plex)	0,0000	0,819	1		0	7,337E-07	38	0,000176592	3	1530,77808	-0,06	25,44	0
	High	yIGQGLAVTFDHmSR	1	1	1	P26525	N-Term(iTRAQ4plex); M13(Oxidation)	0,0000	0,810	1		0	3,653E-09	34	0,000374085	3	1854,92905	0,05	35,99	0
	High	eLGYLPk	1	1	1	P26525	N-Term(iTRAQ4plex); K7(iTRAQ4plex)	0,0000	0,681	1		0	0,002216	32	0,000694976	2	1107,66911	3,50	28,20	0
	High	gVIEPHNLPk	2	1	1	P26525	N-Term(iTRAQ4plex); K10(iTRAQ4plex)	0,0000	0,551	1		0	0,00002016	31	0,00079794	3	1391,82401	-0,67	21,70	0
	High	aGQHPEDLVk	1	1	1	P26525	N-Term(iTRAQ4plex); K10(iTRAQ4plex)	0,0000	0,722	1		0	0,00204	27	0,002177559	3	1381,77069	2,08	16,85	0
	High	mFNNILk	2	1	1	P26525	N-Term(iTRAQ4plex); M1(Oxidation); K7(iTRAQ4plex)	0,0000	0,738	2	7,6	0	0,001127	24	0,003917148	2	1183,67473	-0,04	28,16	0
	High	iHFEFDk	2	1	1	P26525	N-Term(iTRAQ4plex); K7(iTRAQ4plex)	0,0000	0,587	2	9,6	0	0,0007563	24	0,00522288	2	1223,66680	0,40	31,17	0
	High	aGQHPEDLVkAE	1	1	1	P26525	N-Term(iTRAQ4plex); K10(iTRAQ4plex)	0,0000	0,605	1		0	0,0073	13	0,048861861	3	1581,84830	0,48	19,60	1
	Medium	lIPSER	1	1	1	P26525	N-Term(iTRAQ4plex)	0,0000	0,695	1		0,046	0,4845	15	0,032882892	2	858,51811	1,81	19,82	0
Q55602	NifS protein OS=Synechocystis sp. (strain PCC 6803 / Kazusa) GN=nifS PE=3 SV=1	188,98	15,80	1	5	5	8	0,839	8	32,3	386	41,7	6,46							
	A3	Sequence	# PSMs	# Proteins	# Protein Groups	Protein Group Accessions	Modifications	ΔCn	114/115	114/115 Count	114/115 Variability [%]	q-Value	PEP	IonScore	Exp Value	Charge	MH+ [Da]	ΔM [ppm]	RT [min]	# Missed Cleavages
	High	iPLDVHQLNIDLLSFTGHk	1	1	1	Q55602	N-Term(iTRAQ4plex); K19(iTRAQ4plex)	0,0000	0,655	1		0	3,473E-13	53	4,797E-06	4	2448,38442	-0,39	52,80	0
	High	aLLTTLQPR	2	1	1	Q55602	N-Term(iTRAQ4plex)	0,0000	0,704	2	0,3	0	0,00001061	40	0,000107887	2	1156,71721	0,13	32,41	0
	High	sGTLPVPLIVGLAk	2	1	1	Q55602	N-Term(iTRAQ4plex); K14(iTRAQ4plex)	0,0000	1,025	2	31,1	0	0,0001263	38	0,000173768	3	1653,05637	0,61	49,98	0
	High	tEASHVLYALGR	2	1	1	Q55602	N-Term(iTRAQ4plex)	0,0000	1,251	2	21,6	0	0,000001968	27	0,002055749	3	1460,79700	-0,57	29,62	0
	High	gIGGLYR	1	1	1	Q55602	N-Term(iTRAQ4plex)	0,0000	0,843	1		0,003	0,03824	35	0,000335714	2	879,51720	0,35	22,44	0
P74746	Slr0600 protein OS=Synechocystis sp. (strain PCC 6803 / Kazusa) GN=slr0600 PE=	188,81	10,12	1	4	4	7	0,693	7	8,1	326	36,0	6,05							
	A3	Sequence	# PSMs	# Proteins	# Protein Groups	Protein Group Accessions	Modifications	ΔCn	114/115	114/115 Count	114/115 Variability [%]	q-Value	PEP	IonScore	Exp Value	Charge	MH+ [Da]	ΔM [ppm]	RT [min]	# Missed Cleavages
	High	iFALGDLkk	3	1	1	P74746	N-Term(iTRAQ4plex); K8(iTRAQ4plex); K9(iTRAQ4plex)	0,0000	0,725	3	6,7	0	0,00000231	53	4,89745E-06	2	1436,91850	-1,14	35,59	1
	High	kDSLYPIFR	1	1	1	P74746	N-Term(iTRAQ4plex); K1(iTRAQ4plex)	0,0000	0,840	1		0	1,485E-09	43	4,64483E-05	3	1426,82920	-0,34	32,31	1
	High	lADHGYPLHEAAITk	2	1	1	P74746	N-Term(iTRAQ4plex); K15(iTRAQ4plex)	0,0000	0,648	2	2,1	0	9,315E-11	41	8,31706E-05	3	1924,05411	0,52	26,41	0
	High	iFALGDLk	1	1	1	P74746	N-Term(iTRAQ4plex); K8(iTRAQ4plex)	0,0000	0,675	1		0	0,001811	30	0,00095273	2	1164,72234	-0,66	41,17	0
P73061	Urease subunit alpha OS=Synechocystis sp. (strain PCC 6803 / Kazusa) GN=ureC P	188,01	13,36	1	7	7	15	0,797	12	12,1	569	61,0	6,21							
	A3	Sequence	# PSMs	# Proteins	# Protein Groups	Protein Group Accessions	Modifications	ΔCn	114/115	114/115 Count	114/115 Variability [%]	q-Value	PEP	IonScore	Exp Value	Charge	MH+ [Da]	ΔM [ppm]	RT [min]	# Missed Cleavages
	High	vIHTYHTEGAGGGHAPDIIk	3	1	1	P73061	N-Term(iTRAQ4plex); K20(iTRAQ4plex)	0,0000	1,109	2	9,2	0	8,934E-19	78	1,9204E-08	3	2361,25705	0,69	23,14	0
	High	aTTcTPGAWNIHR	1	1	1	P73061	N-Term(iTRAQ4plex); C4(Carbamidomethyl)	0,0000	0,737	1		0	0,00000323	48	1,49613E-05	3	1628,80771	-0,44	23,23	0
	High	lGLSkPLVAVk	5	1	1	P73061	N-Term(iTRAQ4plex); K5(iTRAQ4plex); K11(iTRAQ4plex)	0,0000	0,798	3	0,2	0	0,000001933	26	0,002460198	3	1557,04703	0,40	31,83	0
	High	tWQTAHk	2	1	1	P73061	N-Term(iTRAQ4plex); K7(iTRAQ4plex)	0,0000	0,848	2	3,5	0	0,002627	22	0,006266139	3	1159,64545	-0,67	12,30	0
	High	hSYAHTFGPTVGDk	1	1	1	P73061	N-Term(iTRAQ4plex); K14(iTRAQ4plex)	0,0000	0,721	1		0	0,00005413	13	0,050695569	4	1804,92238	0,16	19,49	0
	Medium	fGGGk	2	1	1	P73061	N-Term(iTRAQ4plex); K5(iTRAQ4plex)	0,0000	0,643	2	13,4	0,042	0,4363	11	0,075678062	2	753,45146	2,25	13,81	0
	Medium	iYAIGk	1	1	1	P73061	N-Term(iTRAQ4plex); K6(iTRAQ4plex)	0,0000	0,773	1		0,049	0,5066	23	0,005057897	2	952,61168	4,93	24,67	0
P73601	Sll1784 protein OS=Synechocystis sp. (strain PCC 6803 / Kazusa) GN=sll1784 PE=4	187,60	10,11	1	3	3	7	1,016	7	20,4	267	29,7	5,11							
	A3	Sequence	# PSMs	# Proteins	# Protein Groups	Protein Group Accessions	Modifications	ΔCn	114/115	114/115 Count	114/115 Variability [%]	q-Value	PEP	IonScore	Exp Value	Charge	MH+ [Da]	ΔM [ppm]	RT [min]	# Missed Cleavages
	High	gGLQLFQSVk	4	1	1	P73601	N-Term(iTRAQ4plex); K10(iTRAQ4plex)	0,0000	1,036	4	9,4	0	5,218E-08	59	1,20495E-06	2	1364,81474	0,51	37,74	0
	High	hLPAITEESk	2	1	1	P73601	N-Term(iTRAQ4plex); K10(iTRAQ4plex)	0,0000	0,848	2	6,7	0	0,0002402	40	9,0567E-05	3	1412,80286	2,88	20,31	0
	Medium	lPTSLTk	1	1	1	P73601	N-Term(iTRAQ4plex); K7(iTRAQ4plex)	0,0000	1,187	1		0,044	0,4597	30	0,000918269	2	1047,66435	-0,84	26,96	0
P72864	Carboxysome formation protein OS=Synechocystis sp. (strain PCC 6803 / Kazusa) G	187,26	24,50	1	8	8	13	0,739	13	13,8	351	37,6	6,37							
	A3	Sequence	# PSMs	# Proteins	# Protein Groups	Protein Group Accessions	Modifications	ΔCn	114/115	114/115 Count	114/115 Variability [%]	q-Value	PEP	IonScore	Exp Value	Charge	MH+ [Da]	ΔM [ppm]	RT [min]	# Missed Cleavages
	High	qLAVIGQTVGR	2	1	1	P72864	N-Term(iTRAQ4plex)	0,0000	0,603	2	26,5	0	3,012E-07	46	2,77313E-05	2	1285,77190	0,77	30,14	0
	High	nmQNFSmLk	2	1	1	P72864	N-Term(iTRAQ4plex); M2(Oxidation); M7(Oxidation); K9(iTRAQ4plex)	0,0000	0,760	2	5,3	0	0,00007727	40	9,18269E-05	2	1432,71758	0,59	20,45	0
	High	kVGAQDkPVLLk	1	1	1	P72864	N-Term(iTRAQ4plex); K1(iTRAQ4plex); K7(iTRAQ4plex); K12(iTRAQ4plex)	0,0000	0,420	1		0	4,703E-07	35	0,000298518	4	1872,20937	-1,87	22,08	1
	High	hGEYSEVIVSTPAGPVAIGk	1	1	1	P72864	N-Term(iTRAQ4plex); K20(iTRAQ4plex)	0,0000	0,962	1		0	9,816E-07	29	0,001397632	3	2299,25260	-0,48	41,23	0
	High	vGAQDkPVLLk	2	1	1	P72864	N-Term(iTRAQ4plex); K6(iTRAQ4plex); K11(iTRAQ4plex)	0,0000	0,792	2	10,2	0	0,002333	25	0,003061752	3	1600,01987	2,52	24,54	0
	High	tSPYAFQGHGESALGLLAAAk	1	1	1	P72864	N-Term(iTRAQ4plex); K21(iTRAQ4plex)	0,0000	0,584	1		0	0,001862	12	0,058880299	4	2377,27505	-0,19	42,96	0
	High	vEQPFkR	2	1	1	P72864	N-Term(iTRAQ4plex); K6(iTRAQ4plex)	0,0000	0,711	2	8,0	0,005	0,06622	16	0,031256716	3	1191,71106	1,86	15,58	1
	Medium	aSLTFR	2	1	1	P72864	N-Term(iTRAQ4plex)	0,0000	0,810	2	1,0	0,012	0,1414	13	0,050462644	2	838,48967	-0,80	22,33	0
P72937	Slr0670 protein OS=Synechocystis sp. (strain PCC 6803 / Kazusa) GN=slr0670 PE=	185,26	18,56	1	5	5	8	0,481	8	37,8	291	32,7	5,54							
	A3	Sequence	# PSMs	# Proteins	# Protein Groups	Protein Group Accessions	Modifications	ΔCn	114/115	114/115 Count	114/115 Variability [%]	q-Value	PEP	IonScore	Exp Value	Charge	MH+ [Da]	ΔM [ppm]	RT [min]	# Missed Cleavages
	High	lFGSHTLSLAk	2	1	1	P72937	N-Term(iTRAQ4plex); K11(iTRAQ4plex)	0,0000	0,444	2	10,7	0	1,495E-08	68	2,1974E-07	2	1461,86443	-1,63	34,04	0
	High	rATLLDWTVPSLTDSILNR	1	1	1	P72937	N-Term(iTRAQ4plex)	0,0000	0,343	1		0	1,221E-09	50	1,02322E-05	3	2315,28055	-1,51	53,82	1
	High	rPELLEIR	2	1	1	P72937	N-Term(iTRAQ4plex)	0,0000	0,527	2	45,4	0	0,000001531	43	4,90874E-05	3	1169,71259	0,23	26,62	0
	High	sWFPLLFFSPk	2	1	1	P72937	N-Term(iTRAQ4plex); K11(iTRAQ4plex)	0,0000	0,558	2	21,3	0	0,000003599	38	0,000186377	2	1656,93791	-0,81	59,24	0
	Medium	aPPGk	1	1	1	P72937	N-Term(iTRAQ4plex); K5(iTRAQ4plex)	0,0000	1,024	1		0,04	0,4188	12	0,063382593	2	757,47887	-2,90	9,61	0
P74338	NAD(P)H-quinone oxidoreductase subunit M OS=Synechocystis sp. (strain PCC 680	184,22	23,97	1	3	3	7	0,546	7	30,9	121	14,1	4,70							
	A3	Sequence	# PSMs	# Proteins	# Protein Groups	Protein Group Accessions	Modifications	ΔCn	114/115	114/115 Count	114/115 Variability [%]	q-Value	PEP	IonScore	Exp Value	Charge	MH+ [Da]	ΔM [ppm]	RT [min]	# Missed Cleavages

	High	vLNYSMGLPk	1	1	1	P74338	N-Term(iTRAQ4plex); K10(iTRAQ4plex)	0,0000	0,551	1		0	0,0002967	53	6,44438E-06	2	1409,80803	1,07	36,18	0
	High	vENQETAGk	1	1	1	P74338	N-Term(iTRAQ4plex); K9(iTRAQ4plex)	0,0000	0,999	1		0	0,00006627	46	2,44889E-05	2	1263,67705	-1,02	10,78	0
	High	vLNYSmGLPk	3	1	1	P74338	N-Term(iTRAQ4plex); M6(Oxidation); K10(iTRAQ4plex)	0,0000	0,546	3	30,9	0	0,0000614	45	3,52347E-05	2	1425,80046	-0,68	30,47	0
	High	iGSDLEHFIR	2	1	1	P74338	N-Term(iTRAQ4plex)	0,0000	0,448	2	2,3	0	0,00009921	36	0,000241529	2	1330,72441	0,60	36,54	0
P73862	Rubisco operon transcriptional regulator OS=Synechocystis sp. (strain PCC 6803 / K	183,75	11,08	1	5	5	9	0,274	8	156,2	316	35,7	8,50							
	A3	Sequence	# PSMs	# Proteins	# Protein Groups	Protein Group Accessions	Modifications	ΔCn	114/115	114/115 Count	114/115 Variability [%]	q-Value	PEP	IonScore	Exp Value	Charge	MH+ [Da]	ΔM [ppm]	RT [min]	# Missed Cleavages
	High	lAVQNLFHR	5	1	1	P73862	N-Term(iTRAQ4plex)	0,0000	0,205	4	45,2	0	1,806E-07	52	6,79157E-06	3	1241,72174	-1,45	35,50	0
	High	tVGLPLFEQIGkR	1	1	1	P73862	N-Term(iTRAQ4plex); K12(iTRAQ4plex)	0,0000	0,501	1		0	0,001003	32	0,00665227	3	1746,04923	-1,40	41,43	1
	High	tVGLPLFEQIGk	1	1	1	P73862	N-Term(iTRAQ4plex); K12(iTRAQ4plex)	0,0000	0,972	1		0	0,00004586	28	0,001705965	3	1589,94559	-3,12	46,45	0
	High	lAVITTAk	1	1	1	P73862	N-Term(iTRAQ4plex); K8(iTRAQ4plex)	0,0000	0,671	1		0,006	0,07858	22	0,005847497	2	1104,71831	-4,33	26,24	0
	Medium	yFIPR	1	1	1	P73862	N-Term(iTRAQ4plex)	0,0000	0,098	1		0,029	0,3192	23	0,005175711	2	839,48876	-1,01	27,77	0
P73407	Carbon dioxide-concentrating mechanism protein CcmK homolog 4 OS=Synechocys	181,24	23,21	1	3	3	7	1,197	7	8,1	112	11,9	6,06							
	A3	Sequence	# PSMs	# Proteins	# Protein Groups	Protein Group Accessions	Modifications	ΔCn	114/115	114/115 Count	114/115 Variability [%]	q-Value	PEP	IonScore	Exp Value	Charge	MH+ [Da]	ΔM [ppm]	RT [min]	# Missed Cleavages
	High	iTIVGYIR	3	1	1	P73407	N-Term(iTRAQ4plex)	0,0000	1,307	3	2,9	0	0,00001936	50	9,20386E-06	2	1078,67497	0,79	38,36	0
	High	tAmAAGIDAINR	1	1	1	P73407	N-Term(iTRAQ4plex); M3(Oxidation)	0,0000	1,197	1		0	0,000006734	42	6,69838E-05	2	1363,71306	0,71	22,52	0
	High	fTLNIR	3	1	1	P73407	N-Term(iTRAQ4plex)	0,0000	1,149	3	2,5	0,002	0,01854	29	0,001575193	2	907,54808	-0,12	32,63	0
P74607	UPF0296 protein ssl2874 OS=Synechocystis sp. (strain PCC 6803 / Kazusa) GN=ssl	179,14	35,96	1	3	3	6	0,691	5	8,9	89	9,9	9,60							
	A3	Sequence	# PSMs	# Proteins	# Protein Groups	Protein Group Accessions	Modifications	ΔCn	114/115	114/115 Count	114/115 Variability [%]	q-Value	PEP	IonScore	Exp Value	Charge	MH+ [Da]	ΔM [ppm]	RT [min]	# Missed Cleavages
	High	vVAIVSPESAPIkR	3	1	1	P74607	N-Term(iTRAQ4plex); K13(iTRAQ4plex)	0,0000	0,555	2	9,5	0	1,607E-10	77	1,99054E-08	2	1754,07756	-0,19	29,71	1
	High	sQLIDATYGR	1	1	1	P74607	N-Term(iTRAQ4plex)	0,0000	0,726	1		0	0,000002274	55	3,34172E-06	2	1267,67571	-0,48	25,83	0
	High	fVVDkDFk	2	1	1	P74607	N-Term(iTRAQ4plex); K5(iTRAQ4plex); K8(iTRAQ4plex)	0,0000	0,712	2	4,5	0	0,0001134	17	0,020275427	3	1429,83945	-1,47	28,86	1
P73120	Sll1830 protein OS=Synechocystis sp. (strain PCC 6803 / Kazusa) GN=sll1830 PE=4	179,00	10,06	1	3	3	11	0,613	10	8,5	358	39,6	4,87							
	A3	Sequence	# PSMs	# Proteins	# Protein Groups	Protein Group Accessions	Modifications	ΔCn	114/115	114/115 Count	114/115 Variability [%]	q-Value	PEP	IonScore	Exp Value	Charge	MH+ [Da]	ΔM [ppm]	RT [min]	# Missed Cleavages
	High	lYYLPFPVQAR	4	1	1	P73120	N-Term(iTRAQ4plex)	0,0000	0,631	4	8,5	0	0,00001124	47	2,1576E-05	2	1510,85478	0,60	48,74	0
	High	qLQSLTPVPALSILLPTHR	3	1	1	P73120	N-Term(iTRAQ4plex)	0,0000	0,599	3	13,4	0	1,091E-08	41	7,72627E-05	3	2228,32230	-1,11	52,69	0
	High	iALVLR	4	1	1	P73120	N-Term(iTRAQ4plex)	0,0000	0,610	3	5,2	0	0,007487	20	0,009289022	2	828,57805	-0,87	33,24	0
P73527	6,7-dimethyl-8-ribityllumazine synthase OS=Synechocystis sp. (strain PCC 6803 / K	178,52	22,56	1	3	3	7	0,441	6	8,7	164	17,6	6,29							
	A3	Sequence	# PSMs	# Proteins	# Protein Groups	Protein Group Accessions	Modifications	ΔCn	114/115	114/115 Count	114/115 Variability [%]	q-Value	PEP	IonScore	Exp Value	Charge	MH+ [Da]	ΔM [ppm]	RT [min]	# Missed Cleavages
	High	gQTPHFDFVAGEAAk	2	1	1	P73527	N-Term(iTRAQ4plex); K15(iTRAQ4plex)	0,0000	0,184	2	751,4	0	4,876E-12	55	3,29587E-06	3	1862,96408	0,05	30,67	0
	High	tVYEGSFTPPARPFR	1	1	1	P73527	N-Term(iTRAQ4plex)	0,0000	0,439	1		0	0,0004415	20	0,009620315	3	1868,97910	0,79	33,21	0
	High	fALVIAR	4	1	1	P73527	N-Term(iTRAQ4plex)	0,0000	0,442	3	1,0	0,001	0,01068	46	3,37573E-05	2	933,60002	-0,24	35,49	0
P73037	Peptidyl-prolyl cis-trans isomerase OS=Synechocystis sp. (strain PCC 6803 / Kazusa	177,33	17,91	1	3	3	8	1,452	8	32,3	201	21,5	8,94							
	A3	Sequence	# PSMs	# Proteins	# Protein Groups	Protein Group Accessions	Modifications	ΔCn	114/115	114/115 Count	114/115 Variability [%]	q-Value	PEP	IonScore	Exp Value	Charge	MH+ [Da]	ΔM [ppm]	RT [min]	# Missed Cleavages
	High	nkPFTFTIGVGQVIk	4	1	1	P73037	N-Term(iTRAQ4plex); K2(iTRAQ4plex); K15(iTRAQ4plex)	0,0000	1,432	4	45,7	0	8,085E-10	59	1,1587E-06	3	2081,25162	1,56	41,55	0
	High	vEVHYTGR	2	1	1	P73037	N-Term(iTRAQ4plex)	0,0000	1,452	2	13,6	0	0,00004627	33	0,000451825	3	1104,59406	2,00	14,63	0
	High	kLIIPPDLAYGSR	2	1	1	P73037	N-Term(iTRAQ4plex); K1(iTRAQ4plex)	0,0000	1,423	2	23,0	0	0,000004571	33	0,000457057	3	1731,04245	0,97	35,07	1
P54899	N-acetyl-gamma-glutamyl-phosphate reductase OS=Synechocystis sp. (strain PCC 6	176,64	14,81	1	4	4	6	0,597	6	17,2	351	38,2	6,71							
	A3	Sequence	# PSMs	# Proteins	# Protein Groups	Protein Group Accessions	Modifications	ΔCn	114/115	114/115 Count	114/115 Variability [%]	q-Value	PEP	IonScore	Exp Value	Charge	MH+ [Da]	ΔM [ppm]	RT [min]	# Missed Cleavages
	High	vIVLSAIDNLVk	2	1	1	P54899	N-Term(iTRAQ4plex); K12(iTRAQ4plex)	0,0000	0,861	2	56,2	0	3,737E-09	70	9,0985E-08	2	1571,99858	0,68	51,85	0
	High	gILATVYATLR	2	1	1	P54899	N-Term(iTRAQ4plex)	0,0000	0,630	2	9,8	0	1,275E-07	52	5,91521E-06	2	1321,79558	-0,35	51,18	0
	High	dPGLVRDDLITIYSAFYR	1	1	1	P54899	N-Term(iTRAQ4plex)	0,0000	0,535	1		0	5,923E-07	43	4,75302E-05	3	2258,19706	1,43	55,55	1
	High	iLPHGVYPQTk	1	1	1	P54899	N-Term(iTRAQ4plex); K11(iTRAQ4plex)	0,0000	0,516	1		0	0,00007578	25	0,002978311	3	1540,90720	-1,17	26,67	0
P72781	NarL subfamily OS=Synechocystis sp. (strain PCC 6803 / Kazusa) GN=slr1783 PE=4	176,44	13,48	1	3	3	8	1,286	8	17,4	282	31,4	5,53							
	A3	Sequence	# PSMs	# Proteins	# Protein Groups	Protein Group Accessions	Modifications	ΔCn	114/115	114/115 Count	114/115 Variability [%]	q-Value	PEP	IonScore	Exp Value	Charge	MH+ [Da]	ΔM [ppm]	RT [min]	# Missed Cleavages
	High	fQSLPVVFLTAR	6	1	1	P72781	N-Term(iTRAQ4plex)	0,0000	1,156	6	30,6	0	5,099E-08	54	4,4154E-06	2	1521,89263	1,05	51,52	0
	High	eQIGQPSGIHTTPSPIk	1	1	1	P72781	N-Term(iTRAQ4plex); K17(iTRAQ4plex)	0,0000	1,436	1		0	0,0000174	43	5,01153E-05	3	2078,14963	0,55	25,06	0
	High	fALQHGLTE	1	1	1	P72781	N-Term(iTRAQ4plex)	0,0000	1,282	1		0	0,002073	33	0,000527193	2	1159,62346	0,55	30,08	0
P74142	30S ribosomal protein S1 homolog B OS=Synechocystis sp. (strain PCC 6803 / Kazu	175,49	13,11	1	4	4	7	1,245	7	2,9	305	33,8	5,25							
	A3	Sequence	# PSMs	# Proteins	# Protein Groups	Protein Group Accessions	Modifications	ΔCn	114/115	114/115 Count	114/115 Variability [%]	q-Value	PEP	IonScore	Exp Value	Charge	MH+ [Da]	ΔM [ppm]	RT [min]	# Missed Cleavages
	High	aHILEANQDNNk	3	1	1	P74142	N-Term(iTRAQ4plex); K12(iTRAQ4plex)	0,0000	1,224	3	2,8	0	7,472E-09	50	1,11165E-05	3	1654,87754	1,44	14,67	0
	High	qNLGDkQEQLEk	1	1	1	P74142	N-Term(iTRAQ4plex); K6(iTRAQ4plex); K12(iTRAQ4plex)	0,0000	1,560	1		0	5,349E-08	31	0,000799779	3	1862,03464	0,13	18,14	1
	High	rIQQAESmGk	2	1	1	P74142	N-Term(iTRAQ4plex); M8(Oxidation); K10(iTRAQ4plex)	0,0000	1,207	2	7,4	0	9,269E-07	30	0,001083852	3	1451,78931	0,96	9,68	1
	High	lVLTQR	1	1	1	P74142	N-Term(iTRAQ4plex)	0,0000	1,245	1		0,005	0,06257	24	0,004101758	2	873,56413	0,33	21,39	0
Q6ZEI6	Slr7012 protein OS=Synechocystis sp. (strain PCC 6803 / Kazusa) GN=slr7012 PE=	174,48	17,63	1	6	6	7	1,000	7	14,2	329	36,5	6,35							
	A3	Sequence	# PSMs	# Proteins	# Protein Groups	Protein Group Accessions	Modifications	ΔCn	114/115	114/115 Count	114/115 Variability [%]	q-Value	PEP	IonScore	Exp Value	Charge	MH+ [Da]	ΔM [ppm]	RT [min]	# Missed Cleavages
	High	hSQSFPVFQTDGVLNTTR	1	1	1	Q6ZEI6	N-Term(iTRAQ4plex)	0,0000	0,891	1		0	2,662E-13	80	1,09893E-08	3	2178,10562	-0,20	37,51	0
	High	sQFQPSFPR	2	1	1	Q6ZEI6	N-Term(iTRAQ4plex)	0,0000	0,977	2	10,7	0	0,000167	39	0,000136135	2	1237,64482	0,16	28,74	0
	High	nLGLTSADk	1	1	1	Q6ZEI6	N-Term(iTRAQ4plex); K9(iTRAQ4plex)	0,0000	1,240	1		0	0,000002836	36	0,000255841	2	1206,69426	0,83	20,21	0
	High	tQAGLLEk	1	1	1	Q6ZEI6	N-Term(iTRAQ4plex); K8(iTRAQ4plex)	0,0000	1,582	1		0	0,0003985	22	0,005984116	2	1147,69548	2,57	20,25	0
	Medium	kQTTPER	1	1	1	Q6ZEI6	N-Term(iTRAQ4plex); K1(iTRAQ4plex)	0,0000	0,923	1		0,014	0,1627	29	0,00149686	3	1147,66834	0,85	7,73	1
	Medium	yGAQESR	1	1	1	Q6ZEI6	N-Term(iTRAQ4plex)	0,0000	1,000	1		0,045	0,4697	14	0,043448021	2	954,47667	0,55	10,93	0
P72720	Glutamine--fructose-6-phosphate aminotransferase [isomerizing] OS=Synechocystis	174,04	9,83	1	4	4	6	0,677	6	39,5	631	69,6	5,85							
	A3	Sequence	# PSMs	# Proteins	# Protein Groups	Protein Group Accessions	Modifications	ΔCn	114/115	114/115 Count	114/115 Variability [%]	q-Value	PEP	IonScore	Exp Value	Charge	MH+ [Da]	ΔM [ppm]	RT [min]	# Missed Cleavages
	High	aVHQLEGAFAIAVLDAHcPEQLIVAR	1	1	1	P72720	N-Term(iTRAQ4plex); C18(Carbamidomethyl)	0,0000	1,298	1		0	7,501E-14	62	6,4859E-07	4	2972,59487	1,71	52,59	0
	High	lTPLGVEVYDFNLkR	3	1	1	P72720	N-Term(iTRAQ4plex); K14(iTRAQ4plex)	0,0000	0,472	3	84,7	0	0,000000113	50	8,91189E-06	3	2052,17301	-0,12	44,67	1
	High	hGPIALLDAk	1	1	1	P72720	N-Term(iTRAQ4plex); K10(iTRAQ4plex)	0,0000	0,788	1		0	0,0002221	28	0,001485833	3	1322,80185	-1,24	30,21	0
	High	ekLENHSNFSR	1	1	1	P72720	N-Term(iTRAQ4plex); K2(iTRAQ4plex)	0,0000	0,592	1		0	0,00004437	26	0,002605974	3	1648,86179	-1,69	13,64	1
P73426	2-C-methyl-D-erythritol 2,4-cyclodiphosphate synthase OS=Synechocystis sp. (strai	173,34	13,04	1	2	2	8	1,009	6	12,1	161	17,4	6,52							
	A3	Sequence	# PSMs	# Proteins	# Protein Groups	Protein Group Accessions	Modifications	ΔCn	114/115	114/115 Count	114/115 Variability [%]	q-Value	PEP	IonScore	Exp Value	Charge	MH+ [Da]	ΔM [ppm]	RT [min]	# Missed Cleavages
	High	lLAQVHQLILER	5	1	1	P73426	N-Term(iTRAQ4plex)	0,0000	0,939	4	10,6	0	8,741E-07	58	1,4288E-06	2	1576,96758	1,26	43,81	0
	High	lkPHIQAMk	1	1	1	P73426	N-Term(iTRAQ4plex); K2(iTRAQ4plex); K9(iTRAQ4plex)	0,0000	1,082	1		0	0,002383	14	0,043951126	4	1497,93020	0,14	19,26	0
	High	lkPHIQAmk	2	1	1	P73426	N-Term(iTRAQ4plex); K2(iTRAQ4plex); M8(Oxidation); K9(iTRAQ4plex)	0,0000	1,673	1		0	0,006209	11	0,084912183	3	1513,92496	0,04	16,16	0
P73711	Threonine synthase OS=Synechocystis sp. (strain PCC 6803 / Kazusa) GN=thrC PE=	173,24	9,70	1	4	4	5	0,567	5	5,5	433	47,0	6,09							
	A3	Sequence	# PSMs	# Proteins	# Protein Groups	Protein Group Accessions	Modifications	ΔCn	114/115	114/115 Count	114/115 Variability [%]	q-Value	PEP	IonScore	Exp Value	Charge	MH+ [Da]	ΔM [ppm]	RT [min]	# Missed Cleavages
	High	lPDHIVAPLASGSLFTk	1	1	1	P73711	N-Term(iTRAQ4plex); K17(iTRAQ4plex)	0,0000	0,567	1		0	4,057E-13	81	7,99779E-09	3	2054,18698	-0,94	45,14	0
	High	gFQEFIk	1	1	1	P73711	N-Term(iTRAQ4plex); K7(iTRAQ4plex)	0,0000	0,547	1		0	0,0005008	47	2,22828E-05	2	1156,65642	-3,53	32,58	0
	High	vGLVDDkPVR	1	1	1	P73711	N-Term(iTRAQ4plex); K7(iTRAQ4plex)	0,0000	0,546	1		0	0,00011	32	0,000754037	3	1385,83298	-1,84	21,63	0
	High	aFLPVTGk	2	1	1	P73711	N-Term(iTRAQ4plex); K8(iTRAQ4plex)	0,0000	0,681	2	13,4	0,005	0,06361	37	0,00019542	2	1120,69426	-2,34	29,04	0
Q55390	Sll0553 protein OS=Synechocystis sp. (strain PCC 6803 / Kazusa) GN=sll0553 PE=4	172,40	17,10	1	7	7	8	0,571	8	12,3	345	38,2	5,38							
	A3	Sequence	# PSMs	# Proteins	# Protein Groups	Protein Group Accessions	Modifications	ΔCn	114/115	114/115 Count	114/115 Variability [%]	q-Value	PEP	IonScore	Exp Value	Charge	MH+ [Da]	ΔM [ppm]	RT [min]	# Missed Cleavages
	High	lAALNPNAIk	1	1	1	Q55390	N-Term(iTRAQ4plex); K10(iTRAQ4plex)	0,0000	0,610	1		0	1,246E-07	50	9,7717E-06	2	1312,81890	-0,18	32,03	0
	High	aEYAALSFVR	1	1	1	Q55390	N-Term(iTRAQ4plex)	0,0000	0,544	1		0	0,0001853	44	3,70655E-05	2	1270,69133	0,07	38,39	0
	High	vYPAFAADYR	1	1	1	Q55390	N-Term(iTRAQ4plex)	0,0000	0,566	1		0	0,001176	33	0,000477496	2	1316,67644	0,65	33,51	0
	High	sNFFAQLVSVPVLDR	1	1	1	Q55390	N-Term(iTRAQ4plex)	0,0000	0,685	1		0	0,00001856	31	0,000851079	2	1836,01079	-1,57	56,59	0
	High	aFVEIk	1	1	1	Q55390	N-Term(iTRAQ4plex); K6(iTRAQ4plex)	0,0000	0,514	1		0	0,005876	25	0,002870582	2	994,61748	-0,09	26,16	0
	High	rLAALNPNAIk	1	1	1	Q55390	N-Term(iTRAQ4plex); K11(iTRAQ4plex)	0,0000	0,575	1		0	0,002047	22	0,006515834	3	1468,91965	-0,41	26,41	1
	High	rFLALLQ	2	1	1	Q55390	N-Term(iTRAQ4plex)	0,0000	0,555	2	18,9	0,002	0,02115	35	0,000314335	2	1004,63677	-0,59	41,32	1
P74467	Probable ATP-dependent Clp protease proteolytic subunit 3 OS=Synechocystis sp. (	172,31	26,73	1	6	6	13	0,756	11	6,5	202	22,4	6,04							
	A3	Sequence	# PSMs	# Proteins	# Protein Groups	Protein Group Accessions	Modifications	ΔCn	114/115	114/115 Count	114/115 Variability [%]	q-Value	PEP	IonScore	Exp Value	Charge	MH+ [Da]	ΔM [ppm]	RT [min]	# Missed Cleavages
	High	lALPHAR	4	1	1	P74467	N-Term(iTRAQ4plex)	0,0000	0,629	2	0,1	0	0,001502	39	0,000138029	3	921,57511	0,03	19,14	0
	High	qQLNEImAQR	1	1	1	P74467	N-Term(iTRAQ4plex); M7(Oxidation)	0,0000	0,760	1		0	0,000003766	35	0,00030128	2	1390,72307	0,07	19,90	0
	High	wIDIYTR	3	1	1	P74467	N-Term(iTRAQ4plex)	0,0000	0,753	3	6,1	0	0,008025	29	0,001380289	2	1110,60747	0,93	38,47	0
	High	imIHQPmGGTGR	1	1	1	P74467	N-Term(iTRAQ4plex); M2(Oxidation); M7(Oxidation)	0,0000	0,801	1		0	0,001195	14	0,042753336	3	1473,74366	0,89	17,40	0
	High	pIGVPSVPFR	2	1	1	P74467	N-Term(iTRAQ4plex)	0,0000	0,723	2	8,0	0,001	0,01531	25	0,002883832	2	1212,72234	0,14	37,88	0
	High	lPGSQYER	2	1	1	P74467	N-Term(iTRAQ4plex)	0,0000	0,767	2	2,2	0,002	0,02835	18	0,014553586	2	1093,57561	-0,23	20,75	0
P73057	Nucleoid-associated protein slr1847 OS=Synechocystis sp. (strain PCC 6803 / Kazus	171,23	16,67	1	2	2	6	1,067	6	6,0	114	12,1	4,60							
	A3	Sequence	# PSMs	# Proteins	# Protein Groups	Protein Group Accessions	Modifications	ΔCn	114/115	114/115 Count	114/115 Variability [%]	q-Value	PEP	IonScore	Exp Value	Charge	MH+ [Da]	ΔM [ppm]	RT [min]	# Missed Cleavages
	High	aQQVQEGAk	4	1	1	P73057	N-Term(iTRAQ4plex); K9(iTRAQ4plex)	0,0000	1,036	4	1,7	0	2,671E-07	63	5,34797E-07	2	1246,69805	-1,09	9,71	0
	High	ikELQEAFQk	2	1	1	P73057	N-Term(iTRAQ4plex); K2(iTRAQ4plex); K10(iTRAQ4plex)	0,0000	1,252	2	20,8	0	6,724E-07	30	0,000999931	3	1665,99076	0,45	31,48	1
Q55758	Bifunctional protein PyrR OS=Synechocystis sp. (strain PCC 6803 / Kazusa) GN=pyr	170,68	29,21	1	4	4	6	0,736	6	15,8	178	19,9	9,17							
	A3	Sequence	# PSMs	# Proteins	# Protein Groups	Protein Group Accessions	Modifications	ΔCn	114/115	114/115 Count	114/115 Variability [%]	q-Value	PEP	IonScore	Exp Value	Charge	MH+ [Da]	ΔM [ppm]	RT [min]	# Missed Cleavages
	High	aAQIIEILSPEEIRR	2	1	1	Q55758	N-Term(iTRAQ4plex)	0,0000	0,668	2	4,2	0	2,981E-11	63	5,41963E-07	3	1882,08927	0,72	43,81	1
	High	tkIPLSLTGk	1	1	1	Q55758	N-Term(iTRAQ4plex); K2(iTRAQ4plex); K10(iTRAQ4plex)	0,0000	0,904	1		0	5,956E-07	50	1,01151E-05	3	1489,96625	-1,05	28,26	1
	High	aALNAVTEYGRPQVIR	2	1	1	Q55758	N-Term(iTRAQ4plex)	0,0000	0,730	2	11,5	0	4,174E-08	38	0,000186508	3	1902,06791	0,04	33,10	0
	High	rVVLVDDVIYk	1	1	1	Q55758	N-Term(iTRAQ4plex); K11(iTRAQ4plex)	0,0000	1,012	1		0	9,916E-07	34	0,0004385	3	1606,97641	-0,43	34,87	1
P74299	Phosphoenolpyruvate carboxylase OS=Synechocystis sp. (strain PCC 6803 / Kazusa	170,06	4,74	1	4	4	6	0,849	6	11,5	1034	118,9	5,78							
	A3	Sequence	# PSMs	# Proteins	# Protein Groups	Protein Group Accessions	Modifications	ΔCn	114/115	114/115 Count	114/115 Variability [%]	q-Value	PEP	IonScore	Exp Value	Charge	MH+ [Da]	ΔM [ppm]	RT [min]	# Missed Cleavages
	High	kLDQLQGSVTGR	3	1	1	P74299	N-Term(iTRAQ4plex); K1(iTRAQ4plex)	0,0000	0,887	3	8,1	0	3,795E-07	47	1,97229E-05	2	1589,92131	-0,04	23,54	1
	High	fLLPAWYGVGTALk	1	1	1	P74299	N-Term(iTRAQ4plex); K14(iTRAQ4plex)	0,0000	0,898	1		0	3,38E-09	39	0,000140919	3	1824,06589	-0,20	54,72	0
	High	sLQAVAQSHR	1	1	1	P74299	N-Term(iTRAQ4plex)	0,0000	0,812	1		0	0,004219	31	0,00072272	3	1240,68665	-1,00	15,81	0
	High	rPLIPQEmPFSER	1	1	1	P74299	N-Term(iTRAQ4plex); M8(Oxidation)	0,0000	0,717	1		0	0,000000178	30	0,000993047	3	1759,93009	1,05	29,26	0
Q55788	Sensory transduction histidine kinase OS=Synechocystis sp. (strain PCC 6803 / Kaz	169,26	17,46	1	6	6	10	1,103	9	12,1	315	35,0	5,86							
	A3	Sequence	# PSMs	# Proteins	# Protein Groups	Protein Group Accessions	Modifications	ΔCn	114/115	114/115 Count	114/115 Variability [%]	q-Value	PEP	IonScore	Exp Value	Charge	MH+ [Da]	ΔM [ppm]	RT [min]	# Missed Cleavages
	High	tLAPVVLk	2	1	1	Q55788	N-Term(iTRAQ4plex); K8(iTRAQ4plex)	0,0000	1,071	2	11,5	0	0,0002808	48	1,48242E-05	2	1128,75957	0,07	30,26	0
	High	qAIANPLHSYR	1	1	1	Q55788	N-Term(iTRAQ4plex)	0,0000	1,103	1		0	0,000001034	35	0,000316206	3	1413,77130	-0,45	22,94	0
	High	qENWQHLLSLSk	2	1	1	Q55788	N-Term(iTRAQ4plex); K12(iTRAQ4plex)	0,0000	1,017	1		0	2,76E-08	34	0,000358072	3	1770,97446	0,19	39,13	0
	High	ekLQEYcTR	2	1	1	Q55788	N-Term(iTRAQ4plex); K2(iTRAQ4plex); C7(Carbamidomethyl)	0,0000	1,198	2	1,1	0	0,0001358	24	0,004334809	3	1514,79279	3,45	15,24	1
	High	eHQLPVDQk	1	1	1	Q55788	N-Term(iTRAQ4plex); K9(iTRAQ4plex)	0,0000	1,148	1		0	0,0001323	23	0,004886186	3	1381,76676	-0,77	15,06	0
	High	mLQLFk	2	1	1	Q55788	N-Term(iTRAQ4plex); K6(iTRAQ4plex)	0,0000	0,767	2	1,5	0,006	0,078	27	0,002212942	2	1067,65251	-0,06	39,01	0
P73660	Porphobilinogen deaminase OS=Synechocystis sp. (strain PCC 6803 / Kazusa) GN=	168,47	9,38	1	4	4	7	1,031	5	11,6	320	34,9	5,73							
	A3	Sequence	# PSMs	# Proteins	# Protein Groups	Protein Group Accessions	Modifications	ΔCn	114/115	114/115 Count	114/115 Variability [%]	q-Value	PEP	IonScore	Exp Value	Charge	MH+ [Da]	ΔM [ppm]	RT [min]	# Missed Cleavages
	High	lREQGAGEILAEILAEAGR	1	1	1	P73660	N-Term(iTRAQ4plex)	0,0000	1,031	1		0	5,974E-13	91	8,24884E-10	3	2140,18595	0,74	54,66	1
	High	lREQGAGEILAEILAEAGRG	1	1	1	P73660	N-Term(iTRAQ4plex)	0,0000	1,295	1		0	6,717E-12	75	3,13307E-08	3	2197,20829	1,12	54,97	2
	High	rTDLAVHSLk	1	1	1	P73660	N-Term(iTRAQ4plex); K10(iTRAQ4plex)	0,0000	1,115	1		0	0,003939	22	0,006294627	4	1427,85647	-0,59	17,46	1
	High	tDLAVHSLk	4	1	1	P73660	N-Term(iTRAQ4plex); K9(iTRAQ4plex)	0,0000	0,916	2	9,5	0	0,00004269	17	0,018406449	3	1271,75525	-0,73	21,15	0
P74474	D-alanyl-D-alanine carboxypeptidase OS=Synechocystis sp. (strain PCC 6803 / Kazu	167,43	14,25	1	5	5	6	0,523	5	20,8	400	44,3	4,75							
	A3	Sequence	# PSMs	# Proteins	# Protein Groups	Protein Group Accessions	Modifications	ΔCn	114/115	114/115 Count	114/115 Variability [%]	q-Value	PEP	IonScore	Exp Value	Charge	MH+ [Da]	ΔM [ppm]	RT [min]	# Missed Cleavages
	High	sLFQIGSITk	1	1	1	P74474	N-Term(iTRAQ4plex); K10(iTRAQ4plex)	0,0000	0,523	1		0	1,943E-08	76	2,36592E-08	2	1381,82842	-0,68	41,02	0
	High	vYGQSYAELIDkk	1	1	1	P74474	N-Term(iTRAQ4plex); K12(iTRAQ4plex); K13(iTRAQ4plex)	0,0000	0,499	1		0	7,9E-09	46	3,14556E-05	3	1946,09909	1,63	32,60	1
	High	lVSLITGk	2	1	1	P74474	N-Term(iTRAQ4plex); K8(iTRAQ4plex)	0,0000	0,455	1		0	0,0004553	32	0,000628015	2	1118,73686	-1,68	38,28	0
	High	rEPEFFSAIQLSTk	1	1	1	P74474	N-Term(iTRAQ4plex); K14(iTRAQ4plex)	0,0000	1,519	1		0	2,374E-07	31	0,000739554	3	1941,06955	0,57	40,50	1
	High	sHAPNSPPIDAk	1	1	1	P74474	N-Term(iTRAQ4plex); K12(iTRAQ4plex)	0,0000	1,005	1		0	0,00001345	24	0,004120691	3	1521,82303	-2,22	14,92	0
P73736	N-acetylmuramoyl-L-alanine amidase OS=Synechocystis sp. (strain PCC 6803 / Kazu	167,11	4,47	1	3	3	5	1,877	5	23,4	649	70,0	9,83							
	A3	Sequence	# PSMs	# Proteins	# Protein Groups	Protein Group Accessions	Modifications	ΔCn	114/115	114/115 Count	114/115 Variability [%]	q-Value	PEP	IonScore	Exp Value	Charge	MH+ [Da]	ΔM [ppm]	RT [min]	# Missed Cleavages
	High	vQPTAQLIPNPTR	2	1	1	P73736	N-Term(iTRAQ4plex)	0,0000	2,029	2	11,6	0	0,000001054	61	1,21935E-06	2	1578,91069	1,41	32,74	0
	High	iVVDLPGTTLR	2	1	1	P73736	N-Term(iTRAQ4plex)	0,0000	1,979	2	28,7	0	0,002538	44	4,23614E-05	2	1327,80766	0,78	40,85	0
	Medium	fYVLR	1	1	1	P73736	N-Term(iTRAQ4plex)	0,0000	0,685	1		0,017	0,1846	15	0,029172255	2	841,50517	-0,09	29,97	0
Q55770	Sll0185 protein OS=Synechocystis sp. (strain PCC 6803 / Kazusa) GN=sll0185 PE=4	166,24	9,79	1	5	5	8	0,788	8	15,3	419	47,0	5,12							
	A3	Sequence	# PSMs	# Proteins	# Protein Groups	Protein Group Accessions	Modifications	ΔCn	114/115	114/115 Count	114/115 Variability [%]	q-Value	PEP	IonScore	Exp Value	Charge	MH+ [Da]	ΔM [ppm]	RT [min]	# Missed Cleavages
	High	sQGGQQLALR	2	1	1	Q55770	N-Term(iTRAQ4plex)	0,0000	0,753	2	3,0	0	1,172E-07	60	1,05674E-06	2	1201,67949	2,08	16,95	0
	High	kSQLLAEVEk	1	1	1	Q55770	N-Term(iTRAQ4plex); K1(iTRAQ4plex); K10(iTRAQ4plex)	0,0000	0,802	1		0	0,0002382	24	0,004045479	3	1576,96250	-0,61	27,02	1
	High	akEDRPSLk	1	1	1	Q55770	N-Term(iTRAQ4plex); K2(iTRAQ4plex); K9(iTRAQ4plex)	0,0000	1,065	1		0,001	0,01691	23	0,005259809	3	1475,88974	-0,60	10,85	1
	High	iTLMPR	1	1	1	Q55770	N-Term(iTRAQ4plex)	0,0000	0,950	1		0,005	0,06532	35	0,000314029	2	874,53166	1,77	27,45	0
	High	wLTLAR	1	1	1	Q55770	N-Term(iTRAQ4plex)	0,0000	0,773	1		0,005	0,05892	29	0,001330363	2	903,55345	0,19	35,59	0
	High	iTLmPR	2	1	1	Q55770	N-Term(iTRAQ4plex); M4(Oxidation)	0,0000	0,654	2	51,5	0,007	0,08418	15	0,030972054	2	890,52464	-0,43	22,08	0
P74186	Membrane protein OS=Synechocystis sp. (strain PCC 6803 / Kazusa) GN=pilM PE=4	165,77	13,62	1	5	5	9	0,765	7	33,4	367	40,9	4,60							
	A3	Sequence	# PSMs	# Proteins	# Protein Groups	Protein Group Accessions	Modifications	ΔCn	114/115	114/115 Count	114/115 Variability [%]	q-Value	PEP	IonScore	Exp Value	Charge	MH+ [Da]	ΔM [ppm]	RT [min]	# Missed Cleavages
	High	vNVAQLTSQGQSYk	1	1	1	P74186	N-Term(iTRAQ4plex); K14(iTRAQ4plex)	0,0000	1,298	1		0	5,205E-12	54	3,77572E-06	3	1810,99088	0,39	32,06	0
	High	vQVLLVATR	3	1	1	P74186	N-Term(iTRAQ4plex)	0,0000	0,606	2	1,8	0	0,0005806	44	4,04548E-05	2	1142,73723	-0,50	35,03	0
	High	fTSLFGR	2	1	1	P74186	N-Term(iTRAQ4plex)	0,0000	0,804	2	24,0	0,002	0,01856	32	0,000623692	2	971,54332	0,21	37,55	0
	High	vMGELTDELRR	1	1	1	P74186	N-Term(iTRAQ4plex)	0,0000				0,006	0,08123	19	0,011911597	3	1462,78159	0,74	35,04	1
	High	qVATAVPmR	2	1	1	P74186	N-Term(iTRAQ4plex); M8(Oxidation)	0,0000	0,869	2	19,1	0,008	0,09118	20	0,009704429	2	1132,62712	0,51	16,04	0
P73305	50S ribosomal protein L18 OS=Synechocystis sp. (strain PCC 6803 / Kazusa) GN=rp	165,19	11,67	1	2	2	6	2,185	4	8,8	120	13,2	10,92							
	A3	Sequence	# PSMs	# Proteins	# Protein Groups	Protein Group Accessions	Modifications	ΔCn	114/115	114/115 Count	114/115 Variability [%]	q-Value	PEP	IonScore	Exp Value	Charge	MH+ [Da]	ΔM [ppm]	RT [min]	# Missed Cleavages
	High	gINQVVFDR	5	1	1	P73305	N-Term(iTRAQ4plex)	0,0000	2,160	3	3,4	0	4,778E-07	67	1,84914E-07	2	1191,65886	-1,19	29,26	0
	Medium	lAVFR	1	1	1	P73305	N-Term(iTRAQ4plex)	0,0000	2,431	1		0,044	0,4557	23	0,005900788	2	749,47887	-0,25	26,99	0
P77962	Serine hydroxymethyltransferase OS=Synechocystis sp. (strain PCC 6803 / Kazusa)	164,35	15,46	1	7	7	11	1,222	11	9,9	427	46,2	6,32							
	A3	Sequence	# PSMs	# Proteins	# Protein Groups	Protein Group Accessions	Modifications	ΔCn	114/115	114/115 Count	114/115 Variability [%]	q-Value	PEP	IonScore	Exp Value	Charge	MH+ [Da]	ΔM [ppm]	RT [min]	# Missed Cleavages
	High	aVAFGEALkPEFk	2	1	1	P77962	N-Term(iTRAQ4plex); K9(iTRAQ4plex); K13(iTRAQ4plex)	0,0000	1,364	2	8,3	0	1,791E-07	58	1,61053E-06	3	1839,07596	1,01	35,56	0
	High	wFEVAHYGVEk	1	1	1	P77962	N-Term(iTRAQ4plex); K11(iTRAQ4plex)	0,0000	1,050	1		0	5,111E-09	33	0,000543213	3	1652,86832	0,47	37,17	0
	High	qIEFDkFR	2	1	1	P77962	N-Term(iTRAQ4plex); K6(iTRAQ4plex)	0,0000	1,194	2	2,5	0	0,00007021	28	0,001648049	3	1370,76862	1,11	28,78	1
	High	qQALEVkPk	2	1	1	P77962	N-Term(iTRAQ4plex); K7(iTRAQ4plex); K9(iTRAQ4plex)	0,0000	1,416	2	9,7	0	0,0001864	26	0,002243727	3	1472,91465	-0,99	15,92	0
	High	lDYDkIR	2	1	1	P77962	N-Term(iTRAQ4plex); K5(iTRAQ4plex)	0,0000	1,119	2	3,1	0	0,005756	16	0,026085111	3	1210,70176	-1,37	22,46	1
	High	yAEGLPGkR	1	1	1	P77962	N-Term(iTRAQ4plex); K8(iTRAQ4plex)	0,0000	1,300	1		0,005	0,07099	17	0,024344042	3	1278,74109	0,17	18,42	1
	Medium	lGSPAmTTR	1	1	1	P77962	N-Term(iTRAQ4plex); M6(Oxidation)	0,0000	1,222	1		0,046	0,4805	15	0,035234653	2	1093,58171	2,24	14,23	0
P74447	Ferredoxin OS=Synechocystis sp. (strain PCC 6803 / Kazusa) GN=slr0148 PE=4 SV=	164,14	10,75	1	2	2	6	1,284	5	2,9	186	20,6	5,06							
	A3	Sequence	# PSMs	# Proteins	# Protein Groups	Protein Group Accessions	Modifications	ΔCn	114/115	114/115 Count	114/115 Variability [%]	q-Value	PEP	IonScore	Exp Value	Charge	MH+ [Da]	ΔM [ppm]	RT [min]	# Missed Cleavages
	High	tLEVITTHNR	2	1	1	P74447	N-Term(iTRAQ4plex)	0,0000	1,269	2	1,1	0	0,000003716	50	9,03587E-06	2	1327,74614	0,81	20,14	0
	High	rAEENILNPR	4	1	1	P74447	N-Term(iTRAQ4plex)	0,0000	1,391	3	1,2	0	0,000004628	41	7,90624E-05	2	1355,75237	0,84	18,03	1
Q55466	Sll0518 protein OS=Synechocystis sp. (strain PCC 6803 / Kazusa) GN=sll0518 PE=4	162,81	9,87	1	4	4	6	0,952	6	25,3	385	43,1	8,38							
	A3	Sequence	# PSMs	# Proteins	# Protein Groups	Protein Group Accessions	Modifications	ΔCn	114/115	114/115 Count	114/115 Variability [%]	q-Value	PEP	IonScore	Exp Value	Charge	MH+ [Da]	ΔM [ppm]	RT [min]	# Missed Cleavages
	High	rPVFSVYR	2	1	1	Q55466	N-Term(iTRAQ4plex)	0,0000	0,952	2	1,6	0	0,000002336	40	9,18269E-05	3	1167,67554	0,01	23,51	0
	High	fYQILAR	2	1	1	Q55466	N-Term(iTRAQ4plex)	0,0000	0,793	2	2,1	0	0,0007502	40	0,000100918	2	1054,61699	0,37	36,24	0
	High	kTPFNPDSSEk	1	1	1	Q55466	N-Term(iTRAQ4plex); K1(iTRAQ4plex); K11(iTRAQ4plex)	0,0000	1,258	1		0	0,000001266	40	0,000102558	3	1681,91086	-0,77	17,23	1
	High	rAIAVAAALEGk	1	1	1	Q55466	N-Term(iTRAQ4plex); K12(iTRAQ4plex)	0,0000	1,124	1		0	0,0006113	30	0,000974922	3	1457,90531	0,70	28,77	1
P73607	Anti-sigma factor antagonist OS=Synechocystis sp. (strain PCC 6803 / Kazusa) GN=	162,70	19,27	1	2	2	6	1,136	6	13,5	109	11,9	4,41							
	A3	Sequence	# PSMs	# Proteins	# Protein Groups	Protein Group Accessions	Modifications	ΔCn	114/115	114/115 Count	114/115 Variability [%]	q-Value	PEP	IonScore	Exp Value	Charge	MH+ [Da]	ΔM [ppm]	RT [min]	# Missed Cleavages
	High	iHEVILPLAEPGSk	2	1	1	P73607	N-Term(iTRAQ4plex); K14(iTRAQ4plex)	0,0000	0,986	2	5,4	0	8,328E-11	47	1,87919E-05	3	1791,06205	0,08	35,95	0
	High	lLLYLYR	4	1	1	P73607	N-Term(iTRAQ4plex)	0,0000	1,187	4	6,6	0	0,0003857	46	2,72879E-05	2	1097,68474	0,72	45,27	0
P74468	Aminopeptidase P OS=Synechocystis sp. (strain PCC 6803 / Kazusa) GN=pepP PE=	162,07	18,59	1	6	6	7	1,353	7	25,0	441	49,5	5,63							
	A3	Sequence	# PSMs	# Proteins	# Protein Groups	Protein Group Accessions	Modifications	ΔCn	114/115	114/115 Count	114/115 Variability [%]	q-Value	PEP	IonScore	Exp Value	Charge	MH+ [Da]	ΔM [ppm]	RT [min]	# Missed Cleavages
	High	mHPVVSSAEYR	1	1	1	P74468	N-Term(iTRAQ4plex); M1(Oxidation)	0,0000	1,732	1		0	0,000001877	53	4,86374E-06	3	1435,71201	-0,03	14,80	0
	High	acDLSAIAHQR	1	1	1	P74468	N-Term(iTRAQ4plex); C2(Carbamidomethyl)	0,0000	1,146	1		0	0,000003747	49	1,2473E-05	3	1385,70856	0,66	22,12	0
	High	tGHWLGLDVHDAGNYk	1	1	1	P74468	N-Term(iTRAQ4plex); K16(iTRAQ4plex)	0,0000	1,353	1		0	0,000001709	36	0,000225408	4	2071,06093	0,45	32,11	0
	High	qGYGPQALVNSHPLVHPLR	2	1	1	P74468	N-Term(iTRAQ4plex)	0,0000	1,186	2	6,4	0	0,000001161	30	0,000928902	4	2227,22158	-0,06	32,90	0
	High	iHYHLGRDEALNQTILk	1	1	1	P74468	N-Term(iTRAQ4plex); K17(iTRAQ4plex)	0,0000	1,391	1		0	0,00002593	19	0,012472974	4	2309,29629	-0,26	29,73	1
	High	ykPFYmHR	1	1	1	P74468	N-Term(iTRAQ4plex); K2(iTRAQ4plex); M6(Oxidation)	0,0000	2,066	1		0,009	0,1097	22	0,005983703	4	1445,76247	1,56	16,69	0
P73066	Ycf23 protein OS=Synechocystis sp. (strain PCC 6803 / Kazusa) GN=ycf23 PE=4 SV	161,66	12,90	1	3	3	5	0,300	5	31,7	248	25,4	5,33							
	A3	Sequence	# PSMs	# Proteins	# Protein Groups	Protein Group Accessions	Modifications	ΔCn	114/115	114/115 Count	114/115 Variability [%]	q-Value	PEP	IonScore	Exp Value	Charge	MH+ [Da]	ΔM [ppm]	RT [min]	# Missed Cleavages
	High	aAPTLAAAHAISR	1	1	1	P73066	N-Term(iTRAQ4plex)	0,0000	0,300	1		0	4,935E-08	63	5,55866E-07	3	1393,80164	-1,17	22,81	0
	High	rFEGPEVLALTk	2	1	1	P73066	N-Term(iTRAQ4plex); K12(iTRAQ4plex)	0,0000	0,301	2	32,3	0	0,00005155	39	0,00011587	3	1647,96384	-2,09	35,17	1
	High	qLIQQVR	2	1	1	P73066	N-Term(iTRAQ4plex)	0,0000	0,383	2	53,2	0,001	0,01045	44	3,9808E-05	2	1028,63103	-2,23	19,64	0
P73598	Sll1305 protein OS=Synechocystis sp. (strain PCC 6803 / Kazusa) GN=sll1305 PE=4	154,73	8,47	1	3	3	5	0,416	5	6,0	295	32,9	4,97							
	A3	Sequence	# PSMs	# Proteins	# Protein Groups	Protein Group Accessions	Modifications	ΔCn	114/115	114/115 Count	114/115 Variability [%]	q-Value	PEP	IonScore	Exp Value	Charge	MH+ [Da]	ΔM [ppm]	RT [min]	# Missed Cleavages
	High	lLLEQLGEHFR	3	1	1	P73598	N-Term(iTRAQ4plex)	0,0000	0,401	3	0,5	0	7,725E-07	69	1,29178E-07	2	1498,84978	-0,07	44,84	0
	Medium	yYSRPGAmk	1	1	1	P73598	N-Term(iTRAQ4plex); M8(Oxidation); K9(iTRAQ4plex)	0,0000	0,631	1		0,039	0,4085	12	0,057540019	3	1376,72861	3,71	12,71	0
	Medium	aFLAk	1	1	1	P73598	N-Term(iTRAQ4plex); K5(iTRAQ4plex)	0,0000	0,985	1		0,04	0,4195	24	0,003664123	2	837,54173	-2,31	21,04	0
Q55514	Slr0552 protein OS=Synechocystis sp. (strain PCC 6803 / Kazusa) GN=slr0552 PE=	153,63	10,25	1	3	3	7	0,787	5	1,5	244	26,7	4,98							
	A3	Sequence	# PSMs	# Proteins	# Protein Groups	Protein Group Accessions	Modifications	ΔCn	114/115	114/115 Count	114/115 Variability [%]	q-Value	PEP	IonScore	Exp Value	Charge	MH+ [Da]	ΔM [ppm]	RT [min]	# Missed Cleavages
	High	aGSQLVFk	5	1	1	Q55514	N-Term(iTRAQ4plex); K8(iTRAQ4plex)	0,0000	0,793	3	0,5	0	0,00001531	51	7,58525E-06	2	1137,68645	-0,53	25,40	0
	High	vASAFVEk	1	1	1	Q55514	N-Term(iTRAQ4plex); K8(iTRAQ4plex)	0,0000	0,718	1		0	0,00001918	45	3,74712E-05	2	1138,67449	3,00	25,55	0
	High	tAEVVTVGk	1	1	1	Q55514	N-Term(iTRAQ4plex); K9(iTRAQ4plex)	0,0000	0,779	1		0	0,000001879	38	0,000181213	2	1191,71831	-0,37	20,22	0
P73913	Acetohydroxy acid synthase OS=Synechocystis sp. (strain PCC 6803 / Kazusa) GN=	152,72	3,54	1	2	2	5	0,789	4	25,7	621	67,7	6,18							
	A3	Sequence	# PSMs	# Proteins	# Protein Groups	Protein Group Accessions	Modifications	ΔCn	114/115	114/115 Count	114/115 Variability [%]	q-Value	PEP	IonScore	Exp Value	Charge	MH+ [Da]	ΔM [ppm]	RT [min]	# Missed Cleavages
	High	fEAAGEIEHILVR	3	1	1	P73913	N-Term(iTRAQ4plex)	0,0000	0,666	2	2,6	0	1,532E-09	66	2,64832E-07	3	1627,89255	0,03	44,94	0
	High	hVLEQLLQR	2	1	1	P73913	N-Term(iTRAQ4plex)	0,0000	0,941	2	3,4	0	0,000004315	43	5,58432E-05	3	1279,75989	-0,34	32,09	0
P74446	Slr0147 protein OS=Synechocystis sp. (strain PCC 6803 / Kazusa) GN=slr0147 PE=	150,86	8,90	1	3	3	4	1,816	4	18,4	292	32,3	8,44							
	A3	Sequence	# PSMs	# Proteins	# Protein Groups	Protein Group Accessions	Modifications	ΔCn	114/115	114/115 Count	114/115 Variability [%]	q-Value	PEP	IonScore	Exp Value	Charge	MH+ [Da]	ΔM [ppm]	RT [min]	# Missed Cleavages
	High	nLFILGLk	2	1	1	P74446	N-Term(iTRAQ4plex); K8(iTRAQ4plex)	0,0000	2,055	2	2,1	0	0,000004426	58	1,4756E-06	2	1205,78862	2,14	46,04	0
	High	lIALPDTLIk	1	1	1	P74446	N-Term(iTRAQ4plex); K10(iTRAQ4plex)	0,0000	1,492	1		0	0,0001515	49	1,22453E-05	2	1384,90398	1,57	47,13	0
	High	tWGSAFYk	1	1	1	P74446	N-Term(iTRAQ4plex); K8(iTRAQ4plex)	0,0000	1,629	1		0	0,003073	31	0,000799779	2	1247,67021	3,14	31,54	0
P74341	Sll1537 protein OS=Synechocystis sp. (strain PCC 6803 / Kazusa) GN=sll1537 PE=3	150,68	12,95	1	2	2	11	1,627	8	65,5	139	15,9	4,70							
	A3	Sequence	# PSMs	# Proteins	# Protein Groups	Protein Group Accessions	Modifications	ΔCn	114/115	114/115 Count	114/115 Variability [%]	q-Value	PEP	IonScore	Exp Value	Charge	MH+ [Da]	ΔM [ppm]	RT [min]	# Missed Cleavages
	High	vEWGETLEAALkR	2	1	1	P74341	N-Term(iTRAQ4plex); K12(iTRAQ4plex)	0,0000	1,179	2	8,4	0	1,147E-13	57	1,89657E-06	3	1790,00681	0,94	43,53	1
	High	vLIVk	9	1	1	P74341	N-Term(iTRAQ4plex); K5(iTRAQ4plex)	0,0000	2,592	6	149,7	0,007	0,08214	31	0,00072773	2	859,62230	0,45	26,52	0
P48944	30S ribosomal protein S14 OS=Synechocystis sp. (strain PCC 6803 / Kazusa) GN=rp	150,46	36,00	1	4	4	10	1,254	9	7,7	100	11,8	10,74							
	A3	Sequence	# PSMs	# Proteins	# Protein Groups	Protein Group Accessions	Modifications	ΔCn	114/115	114/115 Count	114/115 Variability [%]	q-Value	PEP	IonScore	Exp Value	Charge	MH+ [Da]	ΔM [ppm]	RT [min]	# Missed Cleavages
	High	eWAHQGLLPGVTk	5	1	1	P48944	N-Term(iTRAQ4plex); K13(iTRAQ4plex)	0,0000	1,212	4	3,5	0	4,988E-10	74	3,75811E-08	2	1723,97331	-0,06	32,29	0
	High	eALkEEFR	2	1	1	P48944	N-Term(iTRAQ4plex); K4(iTRAQ4plex)	0,0000	1,424	2	6,9	0	0,001352	25	0,003019743	2	1309,73894	2,65	19,41	1
	High	qAETLEDkLAVHQk	2	1	1	P48944	N-Term(iTRAQ4plex); K8(iTRAQ4plex); K14(iTRAQ4plex)	0,0000	1,275	2	5,2	0	0,000001293	23	0,004518247	3	2042,15805	-1,28	26,28	1
	High	rEALkEEFR	1	1	1	P48944	N-Term(iTRAQ4plex); K5(iTRAQ4plex)	0,0000	1,443	1		0	0,0001409	12	0,056489796	4	1465,83620	-0,26	16,64	2
P73600	Sll1785 protein OS=Synechocystis sp. (strain PCC 6803 / Kazusa) GN=sll1785 PE=1	149,31	13,81	1	4	4	12	1,512	12	15,4	268	30,0	5,30							
	A3	Sequence	# PSMs	# Proteins	# Protein Groups	Protein Group Accessions	Modifications	ΔCn	114/115	114/115 Count	114/115 Variability [%]	q-Value	PEP	IonScore	Exp Value	Charge	MH+ [Da]	ΔM [ppm]	RT [min]	# Missed Cleavages
	High	aAFASEAPk	2	1	1	P73600	N-Term(iTRAQ4plex); K9(iTRAQ4plex)	0,0000	1,638	2	3,2	0	0,00072	49	1,38347E-05	2	1179,67107	8,33	19,63	0
	High	eYFQAVGPR	2	1	1	P73600	N-Term(iTRAQ4plex)	0,0000	1,512	2	5,3	0	0,00003684	39	0,000119666	2	1210,63530	1,30	27,54	0
	High	tLPIVFVWmR	6	1	1	P73600	N-Term(iTRAQ4plex); M9(Oxidation)	0,0000	1,376	6	38,1	0	0,000007968	25	0,003357376	2	1421,81157	1,38	51,26	0
	High	lkNDkDLER	1	1	1	P73600	N-Term(iTRAQ4plex); K2(iTRAQ4plex); K5(iTRAQ4plex)	0,0000	2,185	1		0	0,003914	11	0,082788498	3	1562,92563	1,90	13,01	2
	Medium	tLPIVFVWMR	1	1	1	P73600	N-Term(iTRAQ4plex)	0,0000	1,444	1		0,01	0,1257	14	0,036472876	2	1405,81792	2,29	56,65	0
P72707	Sll0224 protein OS=Synechocystis sp. (strain PCC 6803 / Kazusa) GN=sll0224 PE=4	148,72	9,06	1	3	3	5	1,442	4	8,6	298	32,8	4,42							
	A3	Sequence	# PSMs	# Proteins	# Protein Groups	Protein Group Accessions	Modifications	ΔCn	114/115	114/115 Count	114/115 Variability [%]	q-Value	PEP	IonScore	Exp Value	Charge	MH+ [Da]	ΔM [ppm]	RT [min]	# Missed Cleavages
	High	iEQTGVINAGTR	1	1	1	P72707	N-Term(iTRAQ4plex)	0,0000	1,307	1		0	5,143E-09	68	1,44867E-07	2	1402,77727	0,11	23,44	0
	High	nLVNYSLVk	3	1	1	P72707	N-Term(iTRAQ4plex); K9(iTRAQ4plex)	0,0000	1,528	2	3,0	0	2,896E-07	54	4,18765E-06	2	1337,80474	1,20	33,00	0
	High	lGkPIk	1	1	1	P72707	N-Term(iTRAQ4plex); K3(iTRAQ4plex); K6(iTRAQ4plex)	0,0000	1,389	1		0	0,006549	11	0,074125904	3	1087,75586	-0,45	15,91	0
P74490	Pyruvate dehydrogenase E1 component, alpha subunit OS=Synechocystis sp. (strai	148,68	13,16	1	4	4	9	0,816	7	21,9	342	38,1	5,19							
	A3	Sequence	# PSMs	# Proteins	# Protein Groups	Protein Group Accessions	Modifications	ΔCn	114/115	114/115 Count	114/115 Variability [%]	q-Value	PEP	IonScore	Exp Value	Charge	MH+ [Da]	ΔM [ppm]	RT [min]	# Missed Cleavages
	High	dHVHALSAGVPAR	2	1	1	P74490	N-Term(iTRAQ4plex)	0,0000	0,862	2	13,6	0	3,76E-09	46	2,42644E-05	3	1473,80408	-0,17	16,67	0
	High	gHSLADPDELR	1	1	1	P74490	N-Term(iTRAQ4plex)	0,0000	0,548	1		0	0,000003841	33	0,000553312	3	1353,68808	0,09	19,79	0
	High	lPILFVVENNk	2	1	1	P74490	N-Term(iTRAQ4plex); K11(iTRAQ4plex)	0,0000	1,645	2	23,8	0	0,000009569	32	0,000639735	3	1573,95517	-0,29	50,61	0
	High	aTSQPEIYkk	4	1	1	P74490	N-Term(iTRAQ4plex); K9(iTRAQ4plex); K10(iTRAQ4plex)	0,0000	0,816	2	0,1	0	0,0005038	29	0,001790982	3	1596,93363	0,93	15,62	1
P74753	Slr0606 protein OS=Synechocystis sp. (strain PCC 6803 / Kazusa) GN=slr0606 PE=	148,39	6,62	1	2	2	6	0,643	5	5,5	317	34,7	7,40							
	A3	Sequence	# PSMs	# Proteins	# Protein Groups	Protein Group Accessions	Modifications	ΔCn	114/115	114/115 Count	114/115 Variability [%]	q-Value	PEP	IonScore	Exp Value	Charge	MH+ [Da]	ΔM [ppm]	RT [min]	# Missed Cleavages
	High	fIGLQVPSGk	4	1	1	P74753	N-Term(iTRAQ4plex); K10(iTRAQ4plex)	0,0000	0,636	3	1,4	0	8,231E-07	59	1,40272E-06	2	1333,80730	-0,70	35,66	0

	High	eADVkPALVLk	2	1	1	P74753	N-Term(iTRAQ4plex); K5(iTRAQ4plex); K11(iTRAQ4plex)	0,0000	0,694	2	6,0	0	0,000007635	28	0,001682558	3	1615,01511	-0,25	27,56	0
Q55356	Photosystem II reaction center Psb28 protein OS=Synechocystis sp. (strain PCC 68	148,23	16,07	1	2	2	5	1,440	5	3,1	112	12,6	4,94							
	A3	Sequence	# PSMs	# Proteins	# Protein Groups	Protein Group Accessions	Modifications	ΔCn	114/115	114/115 Count	114/115 Variability [%]	q-Value	PEP	IonScore	Exp Value	Charge	MH+ [Da]	ΔM [ppm]	RT [min]	# Missed Cleavages
	High	fYFLEPTILAk	4	1	1	Q55356	N-Term(iTRAQ4plex); K11(iTRAQ4plex)	0,0000	1,391	4	6,6	0	5,824E-10	60	9,22508E-07	2	1629,95110	0,99	52,85	0
	High	aEIQFSk	1	1	1	Q55356	N-Term(iTRAQ4plex); K7(iTRAQ4plex)	0,0000	1,441	1		0	0,0001919	39	0,00015388	2	1110,64006	0,27	24,93	0
P73609	Anti-sigma factor antagonist OS=Synechocystis sp. (strain PCC 6803 / Kazusa) GN=	148,05	17,59	1	2	2	5	1,130	5	10,2	108	12,0	4,74							
	A3	Sequence	# PSMs	# Proteins	# Protein Groups	Protein Group Accessions	Modifications	ΔCn	114/115	114/115 Count	114/115 Variability [%]	q-Value	PEP	IonScore	Exp Value	Charge	MH+ [Da]	ΔM [ppm]	RT [min]	# Missed Cleavages
	High	ekIEEVAQAEISk	1	1	1	P73609	N-Term(iTRAQ4plex); K2(iTRAQ4plex); K13(iTRAQ4plex)	0,0000	0,785	1		0	2,608E-07	43	5,25981E-05	3	1906,08835	1,35	31,20	1
	High	iLVFAk	4	1	1	P73609	N-Term(iTRAQ4plex); K6(iTRAQ4plex)	0,0000	1,156	4	6,3	0,002	0,0182	43	5,59719E-05	2	978,65917	0,13	35,35	0
P73851	Aspartate--tRNA ligase OS=Synechocystis sp. (strain PCC 6803 / Kazusa) GN=aspS	144,57	9,52	1	6	6	8	0,615	8	18,1	599	67,2	5,31							
	A3	Sequence	# PSMs	# Proteins	# Protein Groups	Protein Group Accessions	Modifications	ΔCn	114/115	114/115 Count	114/115 Variability [%]	q-Value	PEP	IonScore	Exp Value	Charge	MH+ [Da]	ΔM [ppm]	RT [min]	# Missed Cleavages
	High	lSQNLQLR	1	1	1	P73851	N-Term(iTRAQ4plex)	0,0000	0,569	1		0	0,00001449	59	1,18569E-06	2	1115,66460	-0,68	26,68	0
	High	dHGGVIFLDLR	1	1	1	P73851	N-Term(iTRAQ4plex)	0,0000	0,368	1		0	2,035E-07	44	3,99917E-05	3	1385,76542	-0,30	38,97	0
	High	lVmLLAk	2	1	1	P73851	N-Term(iTRAQ4plex); M3(Oxidation); K7(iTRAQ4plex)	0,0000	0,950	2	3,7	0	0,0006555	31	0,000820295	2	1091,70891	-1,09	35,86	0
	High	ikPGGDLFk	1	1	1	P73851	N-Term(iTRAQ4plex); K2(iTRAQ4plex); K9(iTRAQ4plex)	0,0000	0,624	1		0	0,000005449	26	0,002338676	3	1406,86979	-2,42	26,55	0
	High	nIDLPRPFPR	2	1	1	P73851	N-Term(iTRAQ4plex)	0,0000	0,584	2	5,6	0	0,0002165	20	0,008932438	3	1368,78641	-0,35	32,75	0
	Medium	vPGGNETISNVR	1	1	1	P73851	N-Term(iTRAQ4plex)	0,0000	0,717	1		0,028	0,3007	15	0,033882075	2	1386,74236	-2,49	22,78	0
Q55847	Sll0470 protein OS=Synechocystis sp. (strain PCC 6803 / Kazusa) GN=sll0470 PE=4	144,55	19,35	1	4	4	6	1,408	6	13,3	186	20,7	8,78							
	A3	Sequence	# PSMs	# Proteins	# Protein Groups	Protein Group Accessions	Modifications	ΔCn	114/115	114/115 Count	114/115 Variability [%]	q-Value	PEP	IonScore	Exp Value	Charge	MH+ [Da]	ΔM [ppm]	RT [min]	# Missed Cleavages
	High	vYLGTWIVSINR	2	1	1	Q55847	N-Term(iTRAQ4plex)	0,0000	1,770	2	14,5	0	1,246E-07	56	2,73508E-06	2	1564,89873	1,22	49,73	0
	High	rQGNIEIEDVVWDkESR	2	1	1	Q55847	N-Term(iTRAQ4plex); K14(iTRAQ4plex)	0,0000	1,408	2	7,0	0	1,419E-08	40	9,48353E-05	4	2361,24111	0,39	33,61	2
	High	lYIPAkk	1	1	1	Q55847	N-Term(iTRAQ4plex); K6(iTRAQ4plex); K7(iTRAQ4plex)	0,0000	1,343	1		0	0,0008159	13	0,048525498	3	1264,83524	-0,06	24,17	1
	High	lYIPAk	1	1	1	Q55847	N-Term(iTRAQ4plex); K6(iTRAQ4plex)	0,0000	1,144	1		0,002	0,025	31	0,000835545	2	992,63951	1,23	29,24	0
P73807	Histidinol-phosphate aminotransferase OS=Synechocystis sp. (strain PCC 6803 / Ka	143,90	24,64	1	7	7	9	0,935	9	17,5	349	38,7	5,12							
	A3	Sequence	# PSMs	# Proteins	# Protein Groups	Protein Group Accessions	Modifications	ΔCn	114/115	114/115 Count	114/115 Variability [%]	q-Value	PEP	IonScore	Exp Value	Charge	MH+ [Da]	ΔM [ppm]	RT [min]	# Missed Cleavages
	High	hTPAYVPGEQPQTNDFIk	1	1	1	P73807	N-Term(iTRAQ4plex); K18(iTRAQ4plex)	0,0000	0,540	1		0	1,778E-12	67	1,78636E-07	3	2330,20255	0,25	30,62	0
	High	lIFLASPNPPk	1	1	1	P73807	N-Term(iTRAQ4plex); K11(iTRAQ4plex)	0,0000	0,831	1		0	0,001814	40	0,000100925	3	1484,90705	-0,61	40,43	0
	High	vGLAIAAPALIEEMDkVR	2	1	1	P73807	N-Term(iTRAQ4plex); K16(iTRAQ4plex)	0,0000	0,703	2	45,8	0	0,0001065	36	0,000228544	3	2184,26731	0,35	51,49	1
	High	nQAEFVPLWEk	1	1	1	P73807	N-Term(iTRAQ4plex); K11(iTRAQ4plex)	0,0000	1,231	1		0	0,00004971	17	0,019362882	3	1648,89402	0,15	40,20	0
	High	wMAAADLYQALk	1	1	1	P73807	N-Term(iTRAQ4plex); K12(iTRAQ4plex)	0,0000	1,054	1		0,004	0,04822	12	0,066064782	3	1668,89774	-2,69	49,60	0
	High	sYSLAGmR	1	1	1	P73807	N-Term(iTRAQ4plex); M7(Oxidation)	0,0000	1,008	1		0,005	0,06555	16	0,02685159	2	1044,52556	-0,88	16,68	0
	High	vSIRPSVR	2	1	1	P73807	N-Term(iTRAQ4plex)	0,0000	0,899	2	5,8	0,006	0,07885	11	0,085895419	3	1057,66422	4,10	16,29	0
Q55766	Ribose-5-phosphate isomerase A OS=Synechocystis sp. (strain PCC 6803 / Kazusa)	143,55	16,60	1	3	3	7	1,035	7	21,2	235	24,7	5,03							
	A3	Sequence	# PSMs	# Proteins	# Protein Groups	Protein Group Accessions	Modifications	ΔCn	114/115	114/115 Count	114/115 Variability [%]	q-Value	PEP	IonScore	Exp Value	Charge	MH+ [Da]	ΔM [ppm]	RT [min]	# Missed Cleavages
	High	kAGPVVTDQGNLVIDVk	1	1	1	Q55766	N-Term(iTRAQ4plex); K1(iTRAQ4plex); K17(iTRAQ4plex)	0,0000	0,899	1		0	1,575E-10	62	6,90192E-07	3	2185,29270	0,45	32,51	1
	High	kYGIPLTTLDVADR	3	1	1	Q55766	N-Term(iTRAQ4plex); K1(iTRAQ4plex)	0,0000	1,167	3	6,2	0	2,878E-10	56	2,72251E-06	3	1850,06302	0,21	37,26	1
	High	lGGkPELR	3	1	1	Q55766	N-Term(iTRAQ4plex); K4(iTRAQ4plex)	0,0000	1,035	3	9,4	0	0,002509	26	0,002648317	2	1157,73039	5,08	17,37	0
P74561	1-(5-phosphoribosyl)-5-[(5-phosphoribosylamino)methylideneamino] imidazole-4-ca	142,68	8,98	1	2	2	4	1,014	4	23,2	256	27,3	5,02							
	A3	Sequence	# PSMs	# Proteins	# Protein Groups	Protein Group Accessions	Modifications	ΔCn	114/115	114/115 Count	114/115 Variability [%]	q-Value	PEP	IonScore	Exp Value	Charge	MH+ [Da]	ΔM [ppm]	RT [min]	# Missed Cleavages
	High	lGAAAIIYTDIHR	2	1	1	P74561	N-Term(iTRAQ4plex)	0,0000	1,203	2	58,6	0	1,332E-08	56	2,89533E-06	3	1557,88712	0,08	39,35	0
	High	lHLVDLDGAk	2	1	1	P74561	N-Term(iTRAQ4plex); K10(iTRAQ4plex)	0,0000	1,014	2	16,9	0	1,868E-08	50	9,83943E-06	3	1368,80957	0,44	31,16	0
P72688	Transcription termination/antitermination protein NusA OS=Synechocystis sp. (strai	142,30	17,03	1	8	8	9	0,935	9	15,1	458	51,3	4,61							
	A3	Sequence	# PSMs	# Proteins	# Protein Groups	Protein Group Accessions	Modifications	ΔCn	114/115	114/115 Count	114/115 Variability [%]	q-Value	PEP	IonScore	Exp Value	Charge	MH+ [Da]	ΔM [ppm]	RT [min]	# Missed Cleavages
	High	rEQLPNDNYR	1	1	1	P72688	N-Term(iTRAQ4plex)	0,0000	1,228	1		0	8,204E-08	43	4,78597E-05	3	1448,73722	0,64	15,45	1
	High	gPQLVVSR	2	1	1	P72688	N-Term(iTRAQ4plex)	0,0000	0,916	2	3,2	0	0,00133	40	0,000103031	2	999,60692	0,14	20,53	0
	High	rNNLPTTAVEEALR	1	1	1	P72688	N-Term(iTRAQ4plex)	0,0000	0,860	1		0	2,863E-07	39	0,000151842	3	1727,95273	0,35	32,54	1
	High	iHAVVNELRGEk	1	1	1	P72688	N-Term(iTRAQ4plex); K12(iTRAQ4plex)	0,0000	0,707	1		0	0,00001303	30	0,00091405	3	1652,96775	-0,56	25,35	1
	High	eAkPPSPSVGPR	1	1	1	P72688	N-Term(iTRAQ4plex); K3(iTRAQ4plex)	0,0000	1,035	1		0	0,00003154	19	0,01216102	3	1509,86249	-0,21	15,76	0
	High	qVLLQk	1	1	1	P72688	N-Term(iTRAQ4plex); K6(iTRAQ4plex)	0,0000	1,005	1		0,003	0,03507	16	0,02792351	2	1016,66966	-0,98	20,32	0
	Medium	iVAVAR	1	1	1	P72688	N-Term(iTRAQ4plex)	0,0000	0,414	1		0,023	0,2503	15	0,029105161	2	772,51641	0,30	17,90	0
	Medium	aNSTFRVYLk	1	1	1	P72688	N-Term(iTRAQ4plex); K10(iTRAQ4plex)	0,0000	1,528	1		0,046	0,4727	10	0,091194785	3	1486,85511	-4,67	50,26	1
P72950	Sll0647 protein OS=Synechocystis sp. (strain PCC 6803 / Kazusa) GN=sll0647 PE=4	141,88	9,77	1	3	3	5	1,847	4	24,8	256	28,7	5,52							
	A3	Sequence	# PSMs	# Proteins	# Protein Groups	Protein Group Accessions	Modifications	ΔCn	114/115	114/115 Count	114/115 Variability [%]	q-Value	PEP	IonScore	Exp Value	Charge	MH+ [Da]	ΔM [ppm]	RT [min]	# Missed Cleavages
	High	fSLYALGLSHR	3	1	1	P72950	N-Term(iTRAQ4plex)	0,0000	1,655	2	28,5	0	3,605E-09	61	1,16771E-06	3	1407,78641	-0,09	40,07	0
	High	kLPAAIk	1	1	1	P72950	N-Term(iTRAQ4plex); K1(iTRAQ4plex); K7(iTRAQ4plex)	0,0000	2,373	1		0	0,00005171	40	9,68211E-05	3	1172,80918	0,05	19,12	1
	High	lQEYFHR	1	1	1	P72950	N-Term(iTRAQ4plex)	0,0000	1,707	1		0	0,0004306	36	0,000239315	3	1136,59732	0,35	25,92	0
P73145	Thylakoid-associated single-stranded DNA-binding protein slr1034 OS=Synechocyst	141,84	35,66	1	5	5	8	1,342	7	25,5	129	14,4	4,74							
	A3	Sequence	# PSMs	# Proteins	# Protein Groups	Protein Group Accessions	Modifications	ΔCn	114/115	114/115 Count	114/115 Variability [%]	q-Value	PEP	IonScore	Exp Value	Charge	MH+ [Da]	ΔM [ppm]	RT [min]	# Missed Cleavages
	High	vVGWGNLANTIk	2	1	1	P73145	N-Term(iTRAQ4plex); K12(iTRAQ4plex)	0,0000	1,648	2	31,2	0	6,157E-08	66	3,01426E-07	2	1559,91680	1,26	41,76	0
	High	mNSFVLMATVIR	1	1	1	P73145	N-Term(iTRAQ4plex)	0,0000	1,134	1		0	0,006939	37	0,000183219	2	1525,84026	3,32	51,74	0
	High	eTYHPGDR	1	1	1	P73145	N-Term(iTRAQ4plex)	0,0000	1,453	1		0	0,003852	12	0,0608093	3	1118,53562	0,80	11,83	0
	High	rAELTASR	1	1	1	P73145	N-Term(iTRAQ4plex)	0,0000	2,181	1		0,002	0,02126	12	0,056620018	3	1047,60422	1,39	9,53	1
	High	lIIEGR	3	1	1	P73145	N-Term(iTRAQ4plex)	0,0000	0,918	2	41,5	0,002	0,02052	11	0,075852518	2	844,53801	0,84	23,71	0
P74371	Chromophore lyase CpcT/CpeT OS=Synechocystis sp. (strain PCC 6803 / Kazusa) G	141,02	21,94	1	4	4	10	1,251	9	11,8	196	22,6	5,33							
	A3	Sequence	# PSMs	# Proteins	# Protein Groups	Protein Group Accessions	Modifications	ΔCn	114/115	114/115 Count	114/115 Variability [%]	q-Value	PEP	IonScore	Exp Value	Charge	MH+ [Da]	ΔM [ppm]	RT [min]	# Missed Cleavages
	High	vWGSVAGAFHFVR	1	1	1	P74371	N-Term(iTRAQ4plex)	0,0000	1,077	1		0	1,836E-10	51	8,07179E-06	3	1576,85044	-0,08	43,31	0
	High	vAIRPLDQAk	5	1	1	P74371	N-Term(iTRAQ4plex); K10(iTRAQ4plex)	0,0000	1,227	4	2,0	0	0,00003205	46	2,72879E-05	2	1398,86931	1,54	22,44	0
	High	lkVVEDHIEIENFk	2	1	1	P74371	N-Term(iTRAQ4plex); K2(iTRAQ4plex); K14(iTRAQ4plex)	0,0000	1,370	2	1,8	0	2,503E-07	26	0,002365756	4	2145,22915	0,52	35,71	1
	Medium	vkDEEk	2	1	1	P74371	N-Term(iTRAQ4plex); K2(iTRAQ4plex); K6(iTRAQ4plex)	0,0000	1,408	2	4,8	0,017	0,185	14	0,035642652	3	1179,69367	-0,74	8,55	1
P72656	Ribonuclease E/G-like protein OS=Synechocystis sp. (strain PCC 6803 / Kazusa) GN	139,65	9,79	1	5	5	6	0,790	6	24,0	674	74,9	6,16							
	A3	Sequence	# PSMs	# Proteins	# Protein Groups	Protein Group Accessions	Modifications	ΔCn	114/115	114/115 Count	114/115 Variability [%]	q-Value	PEP	IonScore	Exp Value	Charge	MH+ [Da]	ΔM [ppm]	RT [min]	# Missed Cleavages
	High	aRPQIAQLTELGLVELTR	1	1	1	P72656	N-Term(iTRAQ4plex)	0,0000	0,684	1		0	1,364E-09	65	2,85739E-07	3	2152,25809	0,45	49,84	0
	High	lVEkPILSPPVAk	1	1	1	P72656	N-Term(iTRAQ4plex); K4(iTRAQ4plex); K13(iTRAQ4plex)	0,0000	0,822	1		0	4,019E-10	53	5,2236E-06	3	1823,17252	-0,31	32,92	0
	High	aLAVLIkPPGmGLLVR	2	1	1	P72656	N-Term(iTRAQ4plex); K7(iTRAQ4plex); M11(Oxidation)	0,0000	0,643	2	25,0	0	0,000006172	29	0,00123302	3	1952,23532	0,99	44,26	0
	High	hQVAAVFWk	1	1	1	P72656	N-Term(iTRAQ4plex); K9(iTRAQ4plex)	0,0000	1,066	1		0	0,00009819	23	0,004709448	3	1373,79187	-1,00	29,59	0
	High	lTGNISmPGR	1	1	1	P72656	N-Term(iTRAQ4plex); M7(Oxidation)	0,0000	0,941	1		0	0,005243	15	0,030127979	2	1205,64287	-0,05	20,97	0
P73643	Sll1762 protein OS=Synechocystis sp. (strain PCC 6803 / Kazusa) GN=sll1762 PE=4	139,54	14,40	1	5	5	5	1,300	5	11,2	382	42,0	4,94							
	A3	Sequence	# PSMs	# Proteins	# Protein Groups	Protein Group Accessions	Modifications	ΔCn	114/115	114/115 Count	114/115 Variability [%]	q-Value	PEP	IonScore	Exp Value	Charge	MH+ [Da]	ΔM [ppm]	RT [min]	# Missed Cleavages
	High	kGTVDAVAGDGIQLAGLR	1	1	1	P73643	N-Term(iTRAQ4plex); K1(iTRAQ4plex)	0,0000	1,206	1		0	9,216E-13	89	1,26174E-09	3	2029,16306	-0,71	36,71	1
	High	qFFNYLVITHEQVmEPk	1	1	1	P73643	N-Term(iTRAQ4plex); M14(Oxidation); K17(iTRAQ4plex)	0,0000	2,136	1		0	6,766E-14	36	0,000226464	3	2427,26401	0,77	43,38	0
	High	iREEVGkELGR	1	1	1	P73643	N-Term(iTRAQ4plex); K7(iTRAQ4plex)	0,0000	1,300	1		0	0,00009059	21	0,007924466	3	1573,92533	-0,73	21,54	2
	Medium	iREEVGk	1	1	1	P73643	N-Term(iTRAQ4plex); K7(iTRAQ4plex)	0,0000	1,346	1		0,011	0,1328	19	0,01570158	2	1118,67766	0,40	14,02	1
	Medium	vIPQPAETR	1	1	1	P73643	N-Term(iTRAQ4plex)	0,0000	1,180	1		0,029	0,3117	27	0,002055749	2	1154,66545	0,36	20,86	0
P73392	Uncharacterized protein sll1735 OS=Synechocystis sp. (strain PCC 6803 / Kazusa) G	138,90	21,05	1	2	2	6	0,595	6	20,1	133	14,1	4,48							
	A3	Sequence	# PSMs	# Proteins	# Protein Groups	Protein Group Accessions	Modifications	ΔCn	114/115	114/115 Count	114/115 Variability [%]	q-Value	PEP	IonScore	Exp Value	Charge	MH+ [Da]	ΔM [ppm]	RT [min]	# Missed Cleavages
	High	iLTYHVVAGk	5	1	1	P73392	N-Term(iTRAQ4plex); K10(iTRAQ4plex)	0,0000	0,573	5	10,8	0	2,348E-09	87	1,92739E-09	2	1388,85027	-0,11	27,10	0
	High	lPPGTITTLVQNIPQLAR	1	1	1	P73392	N-Term(iTRAQ4plex)	0,0000	0,765	1		0,002	0,01973	18	0,016671321	3	2076,23069	0,42	54,14	0
P73812	Slr2060 protein OS=Synechocystis sp. (strain PCC 6803 / Kazusa) GN=slr2060 PE=	138,86	4,09	1	2	2	7	1,026	4	3,8	465	53,0	6,02							
	A3	Sequence	# PSMs	# Proteins	# Protein Groups	Protein Group Accessions	Modifications	ΔCn	114/115	114/115 Count	114/115 Variability [%]	q-Value	PEP	IonScore	Exp Value	Charge	MH+ [Da]	ΔM [ppm]	RT [min]	# Missed Cleavages
	High	lVENFVAVTGHYVk	1	1	1	P73812	N-Term(iTRAQ4plex); K14(iTRAQ4plex)	0,0000	0,459	1		0	3,104E-10	47	1,92739E-05	3	1864,05649	-0,35	43,46	0
	High	lAIAk	6	1	1	P73812	N-Term(iTRAQ4plex); K5(iTRAQ4plex)	0,0000	1,050	3	0,6	0,002	0,02361	28	0,001523947	2	803,55895	-0,46	21,00	0
P49057	GMP synthase [glutamine-hydrolyzing] OS=Synechocystis sp. (strain PCC 6803 / Ka	138,81	16,61	1	7	7	8	0,750	8	31,6	542	61,0	6,24							
	A3	Sequence	# PSMs	# Proteins	# Protein Groups	Protein Group Accessions	Modifications	ΔCn	114/115	114/115 Count	114/115 Variability [%]	q-Value	PEP	IonScore	Exp Value	Charge	MH+ [Da]	ΔM [ppm]	RT [min]	# Missed Cleavages
	High	lIGHEFIQVFEEESNR	1	1	1	P49057	N-Term(iTRAQ4plex)	0,0000	0,557	1		0	4,847E-12	63	4,9885E-07	3	2091,06406	0,60	46,83	0
	High	aLFGVQFHPEVVHSVGGIALIR	1	1	1	P49057	N-Term(iTRAQ4plex)	0,0000	0,801	1		0	3,381E-09	39	0,000135198	4	2490,41030	0,01	47,53	0
	High	rHPFPGPGLAIR	2	1	1	P49057	N-Term(iTRAQ4plex)	0,0000	1,157	2	21,4	0	0,0008727	25	0,00309877	3	1461,85517	-0,56	23,98	1
	High	lVELFDHQFHIPVQYVNAR	1	1	1	P49057	N-Term(iTRAQ4plex)	0,0000	0,460	1		0	3,439E-07	16	0,023440669	4	2469,31508	-0,37	49,19	0
	Medium	lFkDEVR	1	1	1	P49057	N-Term(iTRAQ4plex); K3(iTRAQ4plex)	0,0000	0,661	1		0,024	0,2507	11	0,078699143	3	1194,70740	-0,93	21,49	1
	Medium	lNILR	1	1	1	P49057	N-Term(iTRAQ4plex)	0,0000	0,763	1		0,033	0,3505	32	0,00061372	2	772,51549	-0,87	25,93	0
	Medium	sVGVmGDkR	1	1	1	P49057	N-Term(iTRAQ4plex); M5(Oxidation); K8(iTRAQ4plex)	0,0000	0,737	1		0,044	0,4575	20	0,009659842	3	1252,69315	0,73	9,45	1
P77970	Glutamate--ammonia ligase OS=Synechocystis sp. (strain PCC 6803 / Kazusa) GN=	138,05	7,73	1	5	5	7	1,285	7	31,0	724	79,2	5,47							
	A3	Sequence	# PSMs	# Proteins	# Protein Groups	Protein Group Accessions	Modifications	ΔCn	114/115	114/115 Count	114/115 Variability [%]	q-Value	PEP	IonScore	Exp Value	Charge	MH+ [Da]	ΔM [ppm]	RT [min]	# Missed Cleavages
	High	tQSVHQIINRPLSSGk	2	1	1	P77970	N-Term(iTRAQ4plex); K16(iTRAQ4plex)	0,0000	1,423	2	15,2	0	2,199E-10	49	1,19942E-05	3	2053,17716	0,71	22,63	0
	High	tSPFAFTGNR	2	1	1	P77970	N-Term(iTRAQ4plex)	0,0000	1,481	2	23,2	0	0,00000555	37	0,000185768	2	1241,64043	0,71	29,43	0
	High	tVIYPAAVEYLSk	1	1	1	P77970	N-Term(iTRAQ4plex); K13(iTRAQ4plex)	0,0000	1,098	1		0	5,722E-07	31	0,000779776	3	1741,99656	-0,76	42,85	0
	High	aAIATASNDHR	1	1	1	P77970	N-Term(iTRAQ4plex)	0,0000	1,024	1		0	0,00008435	27	0,002147682	3	1270,66285	0,61	10,36	0
	High	kVPLLR	1	1	1	P77970	N-Term(iTRAQ4plex); K1(iTRAQ4plex)	0,0000	1,621	1		0,001	0,009561	21	0,007726272	3	1013,70682	-0,56	20,75	1
	>sp|K1C9_HUMAN|	137,95	5,94	1	3	4	5	1,677	4	5,7	623	62,1	5,30							
	A3	Sequence	# PSMs	# Proteins	# Protein Groups	Protein Group Accessions	Modifications	ΔCn	114/115	114/115 Count	114/115 Variability [%]	q-Value	PEP	IonScore	Exp Value	Charge	MH+ [Da]	ΔM [ppm]	RT [min]	# Missed Cleavages
	High	fSSSSGYGGGSSR	2	1	1		N-Term(iTRAQ4plex)	0,0000	1,692	2	5,7	0	8,753E-07	69	1,21891E-07	2	1379,63677	4,31	15,70	0
	High	kGPAAIQk	1	1	1		N-Term(iTRAQ4plex); K1(iTRAQ4plex); K8(iTRAQ4plex)	0,0000	1,600	1		0	0,00004789	26	0,002285441	3	1244,80460	-0,40	12,87	1
	High	ikFEMEQNLR	1	1	1		N-Term(iTRAQ4plex); K2(iTRAQ4plex)	0,0000	1,728	1		0	0,001858	15	0,03090082	3	1595,88315	0,83	31,77	1
	High	kAALEk	1	2	2	;P72675	N-Term(iTRAQ4plex); K1(iTRAQ4plex); K6(iTRAQ4plex)	0,0000				0,004	0,05468	21	0,008452205	3	1091,71195	-2,69	10,92	1
P26522	NAD(P)H-quinone oxidoreductase subunit 1 OS=Synechocystis sp. (strain PCC 6803	137,23	10,75	1	4	4	10	0,466	10	36,8	372	40,5	5,06							
	A3	Sequence	# PSMs	# Proteins	# Protein Groups	Protein Group Accessions	Modifications	ΔCn	114/115	114/115 Count	114/115 Variability [%]	q-Value	PEP	IonScore	Exp Value	Charge	MH+ [Da]	ΔM [ppm]	RT [min]	# Missed Cleavages
	High	kISAAAQQR	3	1	1	P26522	N-Term(iTRAQ4plex); K1(iTRAQ4plex)	0,0000	0,501	3	22,0	0	0,0000509	34	0,000400839	3	1260,76023	-1,94	9,58	1
	High	ySLLGGLR	2	1	1	P26522	N-Term(iTRAQ4plex)	0,0000	0,438	2	31,1	0	0,0004628	29	0,001321204	2	1022,61095	-0,56	36,83	0
	High	vRIDQLLNLGWk	1	1	1	P26522	N-Term(iTRAQ4plex); K12(iTRAQ4plex)	0,0000	0,574	1		0	0,00001909	25	0,003365116	3	1743,05088	-0,65	48,03	1
	High	lVFkEDVVPAk	4	1	1	P26522	N-Term(iTRAQ4plex); K4(iTRAQ4plex); K11(iTRAQ4plex)	0,0000	0,366	4	5,4	0	0,00000173	24	0,004120691	3	1677,02982	-0,80	32,28	1
Q55120	Biotin carboxyl carrier protein of acetyl-CoA carboxylase OS=Synechocystis sp. (stra	136,91	14,29	1	2	2	4	1,346	4	9,0	154	16,3	4,32							
	A3	Sequence	# PSMs	# Proteins	# Protein Groups	Protein Group Accessions	Modifications	ΔCn	114/115	114/115 Count	114/115 Variability [%]	q-Value	PEP	IonScore	Exp Value	Charge	MH+ [Da]	ΔM [ppm]	RT [min]	# Missed Cleavages
	High	wTAIVSPmVGTFYR	1	1	1	Q55120	N-Term(iTRAQ4plex); M8(Oxidation)	0,0000	1,440	1		0	0,000002878	47	2,14768E-05	2	1787,92876	0,89	45,22	0
	High	aINFTELR	3	1	1	Q55120	N-Term(iTRAQ4plex)	0,0000	1,276	3	3,1	0	0,00001861	45	3,61737E-05	2	1107,62920	1,16	34,33	0
Q55738	DNA gyrase subunit A OS=Synechocystis sp. (strain PCC 6803 / Kazusa) GN=gyrA F	134,87	7,21	1	5	5	5	0,717	5	13,0	860	95,0	5,26							
	A3	Sequence	# PSMs	# Proteins	# Protein Groups	Protein Group Accessions	Modifications	ΔCn	114/115	114/115 Count	114/115 Variability [%]	q-Value	PEP	IonScore	Exp Value	Charge	MH+ [Da]	ΔM [ppm]	RT [min]	# Missed Cleavages
	High	lRPLSTNSLLR	1	1	1	Q55738	N-Term(iTRAQ4plex)	0,0000	0,673	1		0	4,748E-07	63	5,1047E-07	3	1413,86551	-0,25	31,07	0
	High	yHPHGDTAVYDALVR	1	1	1	Q55738	N-Term(iTRAQ4plex)	0,0000	0,717	1		0	8,716E-12	50	9,95337E-06	3	1857,93564	-0,44	28,11	0
	High	vNAIIEEELEQIk	1	1	1	Q55738	N-Term(iTRAQ4plex); K13(iTRAQ4plex)	0,0000	1,365	1		0	0,0001547	33	0,000477496	3	1816,02719	-1,91	50,57	0
	High	gVASIETmEHPGRPDR	1	1	1	Q55738	N-Term(iTRAQ4plex); M8(Oxidation)	0,0000	0,865	1		0,001	0,01489	18	0,016594723	4	1911,94265	-1,97	17,88	0
	High	iVIELkR	1	1	1	Q55738	N-Term(iTRAQ4plex); K6(iTRAQ4plex)	0,0000	0,658	1		0,003	0,03817	23	0,005046264	3	1158,77854	-2,37	27,77	1
P72631	Sll1064 protein OS=Synechocystis sp. (strain PCC 6803 / Kazusa) GN=sll1064 PE=4	133,76	7,39	1	5	5	11	1,406	9	14,6	568	64,7	5,77							
	A3	Sequence	# PSMs	# Proteins	# Protein Groups	Protein Group Accessions	Modifications	ΔCn	114/115	114/115 Count	114/115 Variability [%]	q-Value	PEP	IonScore	Exp Value	Charge	MH+ [Da]	ΔM [ppm]	RT [min]	# Missed Cleavages
	High	sFFPVLQGR	3	1	1	P72631	N-Term(iTRAQ4plex)	0,0000	1,275	3	11,9	0	0,000007168	41	0,000107978	2	1194,67583	0,52	41,14	0
	High	hQAVITGLR	2	1	1	P72631	N-Term(iTRAQ4plex)	0,0000	1,098	2	9,9	0	0,0000169	38	0,00017099	3	1138,68564	3,77	16,05	0
	High	lSLRPIWR	2	1	1	P72631	N-Term(iTRAQ4plex)	0,0000	1,515	1		0	0,000004064	26	0,002301283	3	1184,73886	0,33	34,96	0
	High	aLHLQFk	3	1	1	P72631	N-Term(iTRAQ4plex); K7(iTRAQ4plex)	0,0000	1,530	2	1,9	0	0,00005452	22	0,005834048	3	1144,70798	-0,11	27,06	0
	High	aSEWFDDRR	1	1	1	P72631	N-Term(iTRAQ4plex)	0,0000	1,550	1		0,002	0,02383	15	0,03467129	3	1325,63578	0,18	23,51	1
P73833	Cyanophycin synthetase OS=Synechocystis sp. (strain PCC 6803 / Kazusa) GN=cph	133,25	7,90	1	6	6	9	0,840	9	24,3	873	94,6	5,58							
	A3	Sequence	# PSMs	# Proteins	# Protein Groups	Protein Group Accessions	Modifications	ΔCn	114/115	114/115 Count	114/115 Variability [%]	q-Value	PEP	IonScore	Exp Value	Charge	MH+ [Da]	ΔM [ppm]	RT [min]	# Missed Cleavages
	High	vAYFSmNPDNPVIQNHIR	1	1	1	P73833	N-Term(iTRAQ4plex); M6(Oxidation)	0,0000	0,830	1		0	1,397E-10	57	2,11334E-06	3	2275,14310	0,89	34,30	0
	High	amVQLGYGVHQQR	1	1	1	P73833	N-Term(iTRAQ4plex); M2(Oxidation)	0,0000	0,715	1		0	6,742E-11	46	2,72879E-05	3	1646,85550	0,08	20,85	0
	High	kIPWmLLSAR	1	1	1	P73833	N-Term(iTRAQ4plex); K1(iTRAQ4plex); M5(Oxidation)	0,0000	0,910	1		0	0,00000831	34	0,000485727	3	1518,90644	-0,32	38,30	1
	High	kIPWMLLSAR	1	1	1	P73833	N-Term(iTRAQ4plex); K1(iTRAQ4plex)	0,0000	1,143	1		0	0,0001344	22	0,00687021	3	1502,90915	-1,90	41,15	1
	High	iIVkEDDDkR	2	1	1	P73833	N-Term(iTRAQ4plex); K4(iTRAQ4plex); K9(iTRAQ4plex)	0,0000	0,954	2	19,1	0	0,005802	17	0,017822557	3	1662,97428	-0,49	15,88	2
	High	fGVVGGPGDRR	1	1	1	P73833	N-Term(iTRAQ4plex)	0,0000	0,941	1		0,001	0,01289	14	0,036980264	3	1260,69175	-1,00	19,50	1
	High	lLAHIYR	2	1	1	P73833	N-Term(iTRAQ4plex)	0,0000	0,659	2	11,7	0,002	0,03185	14	0,040176306	3	1029,63196	-0,60	26,81	0
Q55013	Cytochrome c-550 OS=Synechocystis sp. (strain PCC 6803 / Kazusa) GN=psbV PE=	133,20	24,38	1	3	3	5	1,053	5	8,3	160	17,9	4,93							
	A3	Sequence	# PSMs	# Proteins	# Protein Groups	Protein Group Accessions	Modifications	ΔCn	114/115	114/115 Count	114/115 Variability [%]	q-Value	PEP	IonScore	Exp Value	Charge	MH+ [Da]	ΔM [ppm]	RT [min]	# Missed Cleavages
	High	tNNNVSLGLADLAGAEPR	1	1	1	Q55013	N-Term(iTRAQ4plex)	0,0000	1,053	1		0	1,244E-09	58	1,63294E-06	3	1956,02744	0,34	40,50	0
	High	qFTNGQk	3	1	1	Q55013	N-Term(iTRAQ4plex); K7(iTRAQ4plex)	0,0000	0,986	3	7,8	0	0,0005358	42	5,88803E-05	2	1110,61370	-0,81	11,62	0
	High	iFVDTcTQcHLQGk	1	1	1	Q55013	N-Term(iTRAQ4plex); C6(Carbamidomethyl); C9(Carbamidomethyl); K14(iTR	0,0000	1,113	1		0	1,381E-09	31	0,000714447	3	1995,00382	0,37	27,81	0
P73104	NarL subfamily OS=Synechocystis sp. (strain PCC 6803 / Kazusa) GN=slr1909 PE=4	133,16	12,21	1	2	2	3	1,062	3	26,5	213	24,0	5,58							
	A3	Sequence	# PSMs	# Proteins	# Protein Groups	Protein Group Accessions	Modifications	ΔCn	114/115	114/115 Count	114/115 Variability [%]	q-Value	PEP	IonScore	Exp Value	Charge	MH+ [Da]	ΔM [ppm]	RT [min]	# Missed Cleavages
	High	vIQLVAQGLANR	1	1	1	P73104	N-Term(iTRAQ4plex)	0,0000	1,062	1		0	0,000000062	80	1,08885E-08	2	1425,86675	0,61	44,27	0
	High	lLQHNNHPLEDNAR	2	1	1	P73104	N-Term(iTRAQ4plex)	0,0000	1,115	2	34,3	0	1,223E-09	49	1,23302E-05	3	1814,93662	-0,69	17,65	0
P09192	Photosystem II D2 protein OS=Synechocystis sp. (strain PCC 6803 / Kazusa) GN=p	132,81	10,80	1	4	4	6	0,472	6	21,5	352	39,5	5,55							
	A3	Sequence	# PSMs	# Proteins	# Protein Groups	Protein Group Accessions	Modifications	ΔCn	114/115	114/115 Count	114/115 Variability [%]	q-Value	PEP	IonScore	Exp Value	Charge	MH+ [Da]	ΔM [ppm]	RT [min]	# Missed Cleavages
	High	aYDFVSQELR	1	1	1	P09192	N-Term(iTRAQ4plex)	0,0000	0,467	1		0	0,000004801	50	1,07639E-05	2	1371,69670	-4,25	37,05	0
	High	nILLNEGmR	2	1	1	P09192	N-Term(iTRAQ4plex); M8(Oxidation)	0,0000	0,408	2	23,6	0	0,0002587	49	1,17752E-05	2	1219,65813	-0,37	25,62	0
	High	fWSQIFGIAFSNk	1	1	1	P09192	N-Term(iTRAQ4plex); K13(iTRAQ4plex)	0,0000	0,490	1		0	1,921E-07	23	0,005284088	3	1832,99302	-0,44	57,12	0
	High	qFEISR	1	1	1	P09192	N-Term(iTRAQ4plex)	0,0000	0,606	1		0,002	0,02616	16	0,025643069	2	923,50652	-0,22	20,44	0
	High	nILLNEGMR	1	1	1	P09192	N-Term(iTRAQ4plex)	0,0000	0,321	1		0,008	0,09304	30	0,000990764	2	1203,66216	-1,25	30,29	0
Q55709	Protein translocase subunit SecA OS=Synechocystis sp. (strain PCC 6803 / Kazusa)	132,66	10,19	1	8	8	9	0,831	8	24,3	932	106,9	5,12							
	A3	Sequence	# PSMs	# Proteins	# Protein Groups	Protein Group Accessions	Modifications	ΔCn	114/115	114/115 Count	114/115 Variability [%]	q-Value	PEP	IonScore	Exp Value	Charge	MH+ [Da]	ΔM [ppm]	RT [min]	# Missed Cleavages
	High	vYNLQVTITPTNRPSSR	1	1	1	Q55709	N-Term(iTRAQ4plex)	0,0000	0,748	1		0	0,000005818	41	0,000102308	3	2090,14762	0,04	31,04	0
	High	tPLIISGQVERPTEk	2	1	1	Q55709	N-Term(iTRAQ4plex); K15(iTRAQ4plex)	0,0000	0,785	1		0	4,951E-07	40	0,000111679	3	1956,13639	-0,25	29,74	0
	High	gVHVVTVNDYLAR	1	1	1	Q55709	N-Term(iTRAQ4plex)	0,0000	1,001	1		0	0,00002741	35	0,000305471	3	1586,87748	0,20	31,77	0
	High	eHLQSmEALR	1	1	1	Q55709	N-Term(iTRAQ4plex); M6(Oxidation)	0,0000	1,131	1		0	0,000006833	30	0,001096402	3	1373,69556	-0,63	14,67	0
	High	hFDVQLLGGIILHk	1	1	1	Q55709	N-Term(iTRAQ4plex); K14(iTRAQ4plex)	0,0000	1,079	1		0	0,00001115	22	0,005767266	4	1878,11526	-2,75	44,33	0
	High	kVETYYYDIR	1	1	1	Q55709	N-Term(iTRAQ4plex); K1(iTRAQ4plex)	0,0000	0,881	1		0	0,000163	22	0,006606934	3	1637,87729	-0,27	27,88	1
	High	akELFLk	1	1	1	Q55709	N-Term(iTRAQ4plex); K2(iTRAQ4plex); K7(iTRAQ4plex)	0,0000	0,704	1		0	0,0004722	17	0,020987949	3	1280,82730	-2,30	25,15	1
	High	tFFHEEVRk	1	1	1	Q55709	N-Term(iTRAQ4plex); K9(iTRAQ4plex)	0,0000	0,711	1		0,001	0,008343	19	0,014687347	4	1480,81521	0,07	20,00	1
P72586	GDP-mannose 4,6-dehydratase OS=Synechocystis sp. (strain PCC 6803 / Kazusa) G	132,53	18,78	1	6	6	9	0,822	9	30,6	362	41,3	6,47							
	A3	Sequence	# PSMs	# Proteins	# Protein Groups	Protein Group Accessions	Modifications	ΔCn	114/115	114/115 Count	114/115 Variability [%]	q-Value	PEP	IonScore	Exp Value	Charge	MH+ [Da]	ΔM [ppm]	RT [min]	# Missed Cleavages
	High	yLRPAEVDLLIGDPAk	3	1	1	P72586	N-Term(iTRAQ4plex); K16(iTRAQ4plex)	0,0000	0,889	3	37,4	0	1,892E-10	40	0,000109388	3	2058,18363	-0,10	42,93	0
	High	iLEDVkPTEIYNLGAQSHVR	2	1	1	P72586	N-Term(iTRAQ4plex); K6(iTRAQ4plex)	0,0000	0,705	2	23,1	0	9,58E-10	38	0,000141244	4	2570,41836	0,08	40,05	0
	High	vYGHWQTVNYR	1	1	1	P72586	N-Term(iTRAQ4plex)	0,0000	0,763	1		0	0,0001877	13	0,051400815	3	1566,79337	-0,02	26,67	0
	High	rGETFVTR	1	1	1	P72586	N-Term(iTRAQ4plex)	0,0000	1,006	1		0,004	0,04663	20	0,009353411	2	1109,61882	0,37	12,60	1
	High	lLEAIR	1	1	1	P72586	N-Term(iTRAQ4plex)	0,0000	0,661	1		0,005	0,06266	23	0,004666272	2	858,55260	-0,41	29,40	0
	High	iVAGTQk	1	1	1	P72586	N-Term(iTRAQ4plex); K7(iTRAQ4plex)	0,0000	0,893	1		0,009	0,1079	25	0,003121711	2	1004,63890	4,60	14,94	0
Q55233	Protein DrgA OS=Synechocystis sp. (strain PCC 6803 / Kazusa) GN=drgA PE=1 SV=	132,27	31,90	1	6	6	11	0,519	11	39,5	210	23,7	9,19							
	A3	Sequence	# PSMs	# Proteins	# Protein Groups	Protein Group Accessions	Modifications	ΔCn	114/115	114/115 Count	114/115 Variability [%]	q-Value	PEP	IonScore	Exp Value	Charge	MH+ [Da]	ΔM [ppm]	RT [min]	# Missed Cleavages
	High	eVANYLVGAIASFYGGkPQLQR	1	1	1	Q55233	N-Term(iTRAQ4plex); K17(iTRAQ4plex)	0,0000	1,082	1		0	7,084E-08	57	2,10363E-06	4	2669,46767	0,84	55,10	0
	High	sIGmAmQNLmLAAk	1	1	1	Q55233	N-Term(iTRAQ4plex); M4(Oxidation); M6(Oxidation); M10(Oxidation); K14(iT	0,0000	0,686	1		0	6,113E-09	32	0,000628015	3	1814,93991	-1,02	28,76	0
	High	fLIIRDPQLR	2	1	1	Q55233	N-Term(iTRAQ4plex)	0,0000	0,340	2	5,7	0	0,0004088	21	0,007243859	3	1414,86463	-0,37	35,91	1
	High	lTAEEERk	3	1	1	Q55233	N-Term(iTRAQ4plex); K8(iTRAQ4plex)	0,0000	0,482	3	1,8	0	0,0004562	21	0,00839402	3	1263,71207	-2,10	12,80	1
	High	vAELVk	2	1	1	Q55233	N-Term(iTRAQ4plex); K6(iTRAQ4plex)	0,0000	0,548	2	18,5	0,001	0,01036	33	0,000820524	2	946,61968	2,23	22,87	0
	High	hFDPDHR	1	1	1	Q55233	N-Term(iTRAQ4plex)	0,0000	0,671	1		0,002	0,02438	12	0,058206301	3	1067,51300	-0,87	9,22	0
	High	sIGMAmQNLMLAAk	1	1	1	Q55233	N-Term(iTRAQ4plex); M6(Oxidation); K14(iTRAQ4plex)	0,0000	0,822	1		0,002	0,01854	10	0,097766327	3	1782,94645	-3,08	47,04	0
P73875	Ssl0467 protein OS=Synechocystis sp. (strain PCC 6803 / Kazusa) GN=ssl0467 PE=	132,03	30,99	1	2	2	5	0,862	4	4,2	71	8,2	4,64							
	A3	Sequence	# PSMs	# Proteins	# Protein Groups	Protein Group Accessions	Modifications	ΔCn	114/115	114/115 Count	114/115 Variability [%]	q-Value	PEP	IonScore	Exp Value	Charge	MH+ [Da]	ΔM [ppm]	RT [min]	# Missed Cleavages
	High	qNEQQLHENmAAR	4	1	1	P73875	N-Term(iTRAQ4plex); M10(Oxidation)	0,0000	0,867	3	6,6	0	8,777E-08	62	6,36752E-07	3	1728,82029	-0,10	9,31	0
	High	hVEETmLSR	1	1	1	P73875	N-Term(iTRAQ4plex); M6(Oxidation)	0,0000	0,857	1		0	0,00032	14	0,040454795	3	1261,63254	-0,16	12,64	0
Q55865	Sll0585 protein OS=Synechocystis sp. (strain PCC 6803 / Kazusa) GN=sll0585 PE=4	131,53	11,11	1	3	3	5	0,588	5	5,7	342	37,6	7,03							
	A3	Sequence	# PSMs	# Proteins	# Protein Groups	Protein Group Accessions	Modifications	ΔCn	114/115	114/115 Count	114/115 Variability [%]	q-Value	PEP	IonScore	Exp Value	Charge	MH+ [Da]	ΔM [ppm]	RT [min]	# Missed Cleavages
	High	sSLkPFQALAVTSTGTmER	1	1	1	Q55865	N-Term(iTRAQ4plex); K4(iTRAQ4plex); M17(Oxidation)	0,0000	1,107	1		0	9,899E-09	47	2,36017E-05	3	2328,25284	2,41	35,34	0
	High	qVFNILWR	3	1	1	Q55865	N-Term(iTRAQ4plex)	0,0000	0,572	3	1,8	0	0,00002309	45	2,90382E-05	2	1219,70793	0,90	45,94	0
	High	rLEVIGELAmL	1	1	1	Q55865	N-Term(iTRAQ4plex); M10(Oxidation)	0,0000	0,783	1		0	0,0007028	20	0,011065474	2	1403,80486	-0,05	48,70	1
P73942	Proline--tRNA ligase OS=Synechocystis sp. (strain PCC 6803 / Kazusa) GN=proS PE	130,93	8,79	1	4	4	10	0,782	8	6,0	603	66,9	5,47							
	A3	Sequence	# PSMs	# Proteins	# Protein Groups	Protein Group Accessions	Modifications	ΔCn	114/115	114/115 Count	114/115 Variability [%]	q-Value	PEP	IonScore	Exp Value	Charge	MH+ [Da]	ΔM [ppm]	RT [min]	# Missed Cleavages
	High	fkDSELIGIPYR	3	1	1	P73942	N-Term(iTRAQ4plex); K2(iTRAQ4plex)	0,0000	0,811	3	0,9	0	4,856E-07	37	0,000206524	3	1725,97812	0,17	37,08	1
	High	aLAGDRPVHDPTGELk	2	1	1	P73942	N-Term(iTRAQ4plex); K16(iTRAQ4plex)	0,0000	0,702	2	10,3	0	0,0002648	29	0,001258838	3	1964,08152	0,55	23,04	0
	High	gIEVGHIFQLGTk	4	1	1	P73942	N-Term(iTRAQ4plex); K13(iTRAQ4plex)	0,0000	0,751	2	8,0	0	0,000002891	25	0,003206048	3	1686,97831	0,08	39,06	0
	High	qLPVNLYQIQTk	1	1	1	P73942	N-Term(iTRAQ4plex); K12(iTRAQ4plex)	0,0000	0,816	1		0	0,000007016	16	0,023067472	3	1733,01926	-0,43	38,79	0
P74521	Glycogen synthase 1 OS=Synechocystis sp. (strain PCC 6803 / Kazusa) GN=glgA1 F	130,53	7,13	1	3	3	7	0,650	6	23,4	477	54,3	6,09							
	A3	Sequence	# PSMs	# Proteins	# Protein Groups	Protein Group Accessions	Modifications	ΔCn	114/115	114/115 Count	114/115 Variability [%]	q-Value	PEP	IonScore	Exp Value	Charge	MH+ [Da]	ΔM [ppm]	RT [min]	# Missed Cleavages
	High	nAmVVGIVAR	2	1	1	P74521	N-Term(iTRAQ4plex); M3(Oxidation)	0,0000	0,846	2	11,4	0	0,000009622	53	5,17571E-06	2	1189,68425	-0,13	24,45	0
	High	iPLYLFGHPAFDSR	2	1	1	P74521	N-Term(iTRAQ4plex)	0,0000	0,650	2	10,5	0	2,92E-08	35	0,000338041	3	1776,95652	0,62	47,61	0
	High	vLHQLGHDVR	3	1	1	P74521	N-Term(iTRAQ4plex)	0,0000	0,466	2	31,6	0	0,000482	16	0,026361493	3	1317,74997	-0,65	17,41	0
Q55383	Malate dehydrogenase OS=Synechocystis sp. (strain PCC 6803 / Kazusa) GN=mdh	130,49	5,56	1	2	2	3	0,605	3	3,7	324	34,3	5,03							
	A3	Sequence	# PSMs	# Proteins	# Protein Groups	Protein Group Accessions	Modifications	ΔCn	114/115	114/115 Count	114/115 Variability [%]	q-Value	PEP	IonScore	Exp Value	Charge	MH+ [Da]	ΔM [ppm]	RT [min]	# Missed Cleavages
	High	nANIVAQGAR	2	1	1	Q55383	N-Term(iTRAQ4plex)	0,0000	0,597	2	1,9	0	0,000002042	69	1,35198E-07	2	1157,65264	1,60	14,98	0
	High	vTGLPSQR	1	1	1	Q55383	N-Term(iTRAQ4plex)	0,0000	0,962	1		0,005	0,06912	28	0,002373384	2	1001,59276	6,71	18,32	0
Q55852	Sll0596 protein OS=Synechocystis sp. (strain PCC 6803 / Kazusa) GN=sll0596 PE=4	129,78	9,02	1	2	2	4	1,053	3	5,2	244	26,3	6,10							
	A3	Sequence	# PSMs	# Proteins	# Protein Groups	Protein Group Accessions	Modifications	ΔCn	114/115	114/115 Count	114/115 Variability [%]	q-Value	PEP	IonScore	Exp Value	Charge	MH+ [Da]	ΔM [ppm]	RT [min]	# Missed Cleavages
	High	mGSNPALATPEHGQR	3	1	1	Q55852	N-Term(iTRAQ4plex); M1(Oxidation)	0,0000	1,027	2	8,8	0	9,573E-09	62	6,561E-07	3	1725,85526	5,39	13,13	0
	High	gVYQLAk	1	1	1	Q55852	N-Term(iTRAQ4plex); K7(iTRAQ4plex)	0,0000	1,053	1		0	0,0002303	47	2,11821E-05	2	1066,65386	3,69	22,97	0
P74592	ATP phosphoribosyltransferase regulatory subunit OS=Synechocystis sp. (strain PCC	129,75	8,98	1	3	3	5	0,823	5	9,0	401	44,3	5,07							
	A3	Sequence	# PSMs	# Proteins	# Protein Groups	Protein Group Accessions	Modifications	ΔCn	114/115	114/115 Count	114/115 Variability [%]	q-Value	PEP	IonScore	Exp Value	Charge	MH+ [Da]	ΔM [ppm]	RT [min]	# Missed Cleavages
	High	nLALQLFDLR	2	1	1	P74592	N-Term(iTRAQ4plex)	0,0000	0,711	2	21,9	0	0,00001838	58	1,75376E-06	2	1346,79204	0,55	51,49	0
	High	lGLRPELTASIAR	2	1	1	P74592	N-Term(iTRAQ4plex)	0,0000	0,825	2	8,7	0	0,000002337	34	0,000363053	3	1540,92936	0,09	33,75	0
	High	lDLQGEcLGLVDR	1	1	1	P74592	N-Term(iTRAQ4plex); C7(Carbamidomethyl)	0,0000	1,032	1		0	0,003358	10	0,092463431	4	1631,85793	2,16	21,13	0
P73043	Glutamate decarboxylase OS=Synechocystis sp. (strain PCC 6803 / Kazusa) GN=ga	127,99	8,35	1	4	4	7	1,137	5	6,2	467	53,0	5,41							
	A3	Sequence	# PSMs	# Proteins	# Protein Groups	Protein Group Accessions	Modifications	ΔCn	114/115	114/115 Count	114/115 Variability [%]	q-Value	PEP	IonScore	Exp Value	Charge	MH+ [Da]	ΔM [ppm]	RT [min]	# Missed Cleavages
	High	aIAYFASQPDHkPk	3	1	1	P73043	N-Term(iTRAQ4plex); K12(iTRAQ4plex); K14(iTRAQ4plex)	0,0000	1,155	2	2,4	0	2,216E-07	40	0,000100686	4	2005,12356	0,20	24,48	0
	High	gWLVPAYR	1	1	1	P73043	N-Term(iTRAQ4plex)	0,0000	1,090	1		0	0,0005106	36	0,000239867	2	1105,62761	0,09	35,95	0
	High	nREDLVVQR	2	1	1	P73043	N-Term(iTRAQ4plex)	0,0000	0,984	1		0	0,0003096	36	0,000247725	3	1272,71308	-0,81	14,72	1
	High	gLANSVSk	1	1	1	P73043	N-Term(iTRAQ4plex); K8(iTRAQ4plex)	0,0000	1,204	1		0	0,00004305	35	0,000379658	2	1063,63640	1,31	15,43	0
Q55894	Phosphomethylpyrimidine synthase OS=Synechocystis sp. (strain PCC 6803 / Kazus	127,85	12,85	1	6	6	9	1,031	8	22,0	459	51,1	5,74							
	A3	Sequence	# PSMs	# Proteins	# Protein Groups	Protein Group Accessions	Modifications	ΔCn	114/115	114/115 Count	114/115 Variability [%]	q-Value	PEP	IonScore	Exp Value	Charge	MH+ [Da]	ΔM [ppm]	RT [min]	# Missed Cleavages
	High	iAAHAADIAR	2	1	1	Q55894	N-Term(iTRAQ4plex)	0,0000	0,884	2	15,9	0	0,000000208	49	1,19666E-05	2	1152,66338	2,39	18,08	0
	High	qGQTNVSQmHYAR	2	1	1	Q55894	N-Term(iTRAQ4plex); M9(Oxidation)	0,0000	1,010	1		0	0,00002075	35	0,000325065	3	1679,80387	-0,11	10,81	0
	High	rENLPVELIkDEVAR	1	1	1	Q55894	N-Term(iTRAQ4plex); K10(iTRAQ4plex)	0,0000	1,734	1		0	9,914E-08	26	0,002546654	4	2069,19570	-0,05	36,39	2
	High	tLGQLTR	1	1	1	Q55894	N-Term(iTRAQ4plex)	0,0000	1,386	1		0	0,006404	13	0,050929569	2	932,56536	0,84	20,77	0
	High	dkEmVSQR	2	1	1	Q55894	N-Term(iTRAQ4plex); K2(iTRAQ4plex); M4(Oxidation)	0,0000	1,156	2	14,0	0,004	0,05264	14	0,044562547	3	1296,68241	0,27	8,35	1
	Medium	lLLSVk	1	1	1	Q55894	N-Term(iTRAQ4plex); K6(iTRAQ4plex)	0,0000	0,947	1		0,02	0,2223	28	0,001761854	2	960,66820	-1,46	31,57	0
P74448	Slr0149 protein OS=Synechocystis sp. (strain PCC 6803 / Kazusa) GN=slr0149 PE=	127,73	12,82	1	2	2	4	1,711	4	19,3	156	18,4	5,26							
	A3	Sequence	# PSMs	# Proteins	# Protein Groups	Protein Group Accessions	Modifications	ΔCn	114/115	114/115 Count	114/115 Variability [%]	q-Value	PEP	IonScore	Exp Value	Charge	MH+ [Da]	ΔM [ppm]	RT [min]	# Missed Cleavages
	High	aYNYTEYAk	2	1	1	P74448	N-Term(iTRAQ4plex); K9(iTRAQ4plex)	0,0000	1,947	2	6,4	0	0,000001014	60	9,5273E-07	2	1410,71782	2,46	24,38	0
	High	iRDSEEQIIHR	2	1	1	P74448	N-Term(iTRAQ4plex)	0,0000	1,497	2	7,1	0	0,00002192	33	0,000503466	3	1539,83786	1,19	19,72	1
P52986	Homoserine dehydrogenase OS=Synechocystis sp. (strain PCC 6803 / Kazusa) GN=	127,43	5,08	1	2	2	3	0,727	3	1,0	433	45,5	5,01							
	A3	Sequence	# PSMs	# Proteins	# Protein Groups	Protein Group Accessions	Modifications	ΔCn	114/115	114/115 Count	114/115 Variability [%]	q-Value	PEP	IonScore	Exp Value	Charge	MH+ [Da]	ΔM [ppm]	RT [min]	# Missed Cleavages

	High	iAILASLGFGGR	1	1	1	P52986	N-Term(iTRAQ4plex)	0,0000	0,732	1		0	5,534E-07	64	3,96278E-07	2	1318,79607	-0,25	52,16	0
	High	sLILQAIAHk	2	1	1	P52986	N-Term(iTRAQ4plex); K10(iTRAQ4plex)	0,0000	0,637	2	19,9	0	9,758E-09	49	1,16942E-05	3	1381,87607	-0,66	35,80	0
Q55421	Elongation factor G-like protein OS=Synechocystis sp. (strain PCC 6803 / Kazusa) G	126,78	3,29	1	2	2	4	0,742	4	5,7	669	73,3	5,00							
	A3	Sequence	# PSMs	# Proteins	# Protein Groups	Protein Group Accessions	Modifications	ΔCn	114/115	114/115 Count	114/115 Variability [%]	q-Value	PEP	IonScore	Exp Value	Charge	MH+ [Da]	ΔM [ppm]	RT [min]	# Missed Cleavages
	High	vLTLAPLFk	3	1	1	Q55421	N-Term(iTRAQ4plex); K9(iTRAQ4plex)	0,0000	0,716	3	10,5	0	0,000008237	58	1,55944E-06	2	1289,84245	-0,85	47,46	0
	High	akQPFGEVLQALk	1	1	1	Q55421	N-Term(iTRAQ4plex); K2(iTRAQ4plex); K13(iTRAQ4plex)	0,0000	0,773	1		0	1,937E-07	45	3,41956E-05	3	1861,12699	-0,11	41,92	1
P73471	Phosphoribosylaminoimidazole-succinocarboxamide synthase OS=Synechocystis sp.	126,61	9,47	1	2	2	3	0,818	3	2,1	264	29,7	5,29							
	A3	Sequence	# PSMs	# Proteins	# Protein Groups	Protein Group Accessions	Modifications	ΔCn	114/115	114/115 Count	114/115 Variability [%]	q-Value	PEP	IonScore	Exp Value	Charge	MH+ [Da]	ΔM [ppm]	RT [min]	# Missed Cleavages
	High	gAINcAISAALFR	1	1	1	P73471	N-Term(iTRAQ4plex); C5(Carbamidomethyl)	0,0000	1,061	1		0	1,641E-08	85	3,28829E-09	2	1507,81865	0,95	44,96	0
	High	aVNIIPLEVVVR	2	1	1	P73471	N-Term(iTRAQ4plex)	0,0000	0,812	2	1,0	0	0,002432	35	0,00028312	2	1465,92497	1,80	47,86	0
P74188	Slr1276 protein OS=Synechocystis sp. (strain PCC 6803 / Kazusa) GN=slr1276 PE=	126,26	8,00	1	2	2	3	0,851	3	5,4	275	30,0	4,34							
	A3	Sequence	# PSMs	# Proteins	# Protein Groups	Protein Group Accessions	Modifications	ΔCn	114/115	114/115 Count	114/115 Variability [%]	q-Value	PEP	IonScore	Exp Value	Charge	MH+ [Da]	ΔM [ppm]	RT [min]	# Missed Cleavages
	High	lQPLILMk	1	1	1	P74188	N-Term(iTRAQ4plex); K8(iTRAQ4plex)	0,0000	0,805	1		0	0,00008571	54	3,68952E-06	2	1243,80486	-0,17	42,47	0
	High	tSLVNPEFPVNVVR	2	1	1	P74188	N-Term(iTRAQ4plex)	0,0000	0,867	2	2,7	0	0,000001003	54	3,95339E-06	2	1714,96343	1,47	42,09	0
P73274	Leucine--tRNA ligase OS=Synechocystis sp. (strain PCC 6803 / Kazusa) GN=leuS PE	125,76	3,45	1	3	3	6	0,666	5	13,3	869	98,2	5,29							
	A3	Sequence	# PSMs	# Proteins	# Protein Groups	Protein Group Accessions	Modifications	ΔCn	114/115	114/115 Count	114/115 Variability [%]	q-Value	PEP	IonScore	Exp Value	Charge	MH+ [Da]	ΔM [ppm]	RT [min]	# Missed Cleavages
	High	fAVRPETLVTNEPLTk	2	1	1	P73274	N-Term(iTRAQ4plex); K16(iTRAQ4plex)	0,0000	0,777	2	1,3	0	1,601E-11	57	1,88786E-06	3	2103,20597	0,32	37,05	0
	High	lRDWLISR	3	1	1	P73274	N-Term(iTRAQ4plex)	0,0000	0,651	2	3,3	0	0,000002786	40	0,000104277	3	1202,71222	-0,36	33,38	1
	Medium	mFILFk	1	1	1	P73274	N-Term(iTRAQ4plex); K6(iTRAQ4plex)	0,0000	0,609	1		0,014	0,1582	25	0,002857393	2	1086,66142	-0,91	46,19	0
P73762	Sll0837 protein OS=Synechocystis sp. (strain PCC 6803 / Kazusa) GN=sll0837 PE=	125,03	12,24	1	3	3	3	1,739	3	20,6	294	32,9	4,81							
	A3	Sequence	# PSMs	# Proteins	# Protein Groups	Protein Group Accessions	Modifications	ΔCn	114/115	114/115 Count	114/115 Variability [%]	q-Value	PEP	IonScore	Exp Value	Charge	MH+ [Da]	ΔM [ppm]	RT [min]	# Missed Cleavages
	High	kAEALAPEEAGIk	1	1	1	P73762	N-Term(iTRAQ4plex); K1(iTRAQ4plex); K13(iTRAQ4plex)	0,0000	1,739	1		0	6,628E-09	60	1,01151E-06	3	1759,03672	2,32	22,68	1
	High	fTLGNAYFQk	1	1	1	P73762	N-Term(iTRAQ4plex); K10(iTRAQ4plex)	0,0000	2,054	1		0	0,00001094	53	4,68781E-06	2	1476,81291	2,69	37,86	0
	High	aLFATPTMVEVLR	1	1	1	P73762	N-Term(iTRAQ4plex)	0,0000	1,516	1		0	0,003264	38	0,000165185	2	1591,90117	0,80	48,50	0
P73848	3-hydroxyacyl-[acyl-carrier-protein] dehydratase FabZ OS=Synechocystis sp. (strain	124,90	24,39	1	4	4	6	2,369	6	25,9	164	17,9	5,53							
	A3	Sequence	# PSMs	# Proteins	# Protein Groups	Protein Group Accessions	Modifications	ΔCn	114/115	114/115 Count	114/115 Variability [%]	q-Value	PEP	IonScore	Exp Value	Charge	MH+ [Da]	ΔM [ppm]	RT [min]	# Missed Cleavages
	High	fFAFAGIDGVR	2	1	1	P73848	N-Term(iTRAQ4plex)	0,0000	2,369	2	21,3	0	0,00001778	47	2,07E-05	2	1343,72441	1,12	47,57	0
	High	gkFFAFAGIDGVR	1	1	1	P73848	N-Term(iTRAQ4plex); K2(iTRAQ4plex)	0,0000	2,853	1		0	0,000006984	32	0,000588803	3	1672,94107	-0,20	41,32	1
	High	rPVVPGDQLImTVELQSFk	1	1	1	P73848	N-Term(iTRAQ4plex); M11(Oxidation); K19(iTRAQ4plex)	0,0000	1,089	1		0	6,074E-08	27	0,001972287	4	2461,37417	0,56	46,77	0
	High	yPFALVDR	2	1	1	P73848	N-Term(iTRAQ4plex)	0,0000	2,161	2	38,4	0	0,003544	25	0,0028772	2	1124,62261	0,46	38,81	0
P72623	Probable glycogen synthase 2 OS=Synechocystis sp. (strain PCC 6803 / Kazusa) GN	124,74	17,72	1	9	9	11	0,527	11	16,0	491	56,2	6,48							
	A3	Sequence	# PSMs	# Proteins	# Protein Groups	Protein Group Accessions	Modifications	ΔCn	114/115	114/115 Count	114/115 Variability [%]	q-Value	PEP	IonScore	Exp Value	Charge	MH+ [Da]	ΔM [ppm]	RT [min]	# Missed Cleavages
	High	yGAVPVVR	2	1	1	P72623	N-Term(iTRAQ4plex)	0,0000	0,495	2	5,5	0	0,001459	33	0,000929549	2	1004,60069	-0,27	24,82	0
	High	aAMEFLLR	1	1	1	P72623	N-Term(iTRAQ4plex)	0,0000	0,497	1		0	0,002095	32	0,000680722	2	1094,61455	-0,34	40,42	0
	High	yDHIWGLHDAYR	1	1	1	P72623	N-Term(iTRAQ4plex)	0,0000	0,501	1		0	6,633E-07	32	0,000696578	4	1689,82448	-0,56	31,36	0
	High	aIALYkDDPVAFk	2	1	1	P72623	N-Term(iTRAQ4plex); K6(iTRAQ4plex); K13(iTRAQ4plex)	0,0000	0,665	2	14,5	0	0,00001938	31	0,000717745	3	1883,09928	-0,54	35,42	1
	High	gHYYGALDDHmR	1	1	1	P72623	N-Term(iTRAQ4plex); M11(Oxidation)	0,0000	0,527	1		0	0,0000512	21	0,00799779	4	1594,71718	-1,09	15,98	0
	High	wFWHEk	1	1	1	P72623	N-Term(iTRAQ4plex); K6(iTRAQ4plex)	0,0000	0,411	1		0	0,002909	18	0,016556556	3	1220,64447	-0,85	30,23	0
	High	vcYTIHNFk	1	1	1	P72623	N-Term(iTRAQ4plex); C2(Carbamidomethyl); K9(iTRAQ4plex)	0,0000	0,709	1		0	0,001625	16	0,024601977	3	1469,77970	-1,12	26,17	0
	High	aGGLGDVIYGLSR	1	1	1	P72623	N-Term(iTRAQ4plex)	0,0000	1,609	1		0,004	0,04873	16	0,02940307	2	1421,78911	1,52	42,88	0
	High	fAFFSk	1	1	1	P72623	N-Term(iTRAQ4plex); K6(iTRAQ4plex)	0,0000	0,586	1		0,009	0,1151	25	0,002978516	2	1034,58977	-1,53	37,97	0
P73280	Sll1072 protein OS=Synechocystis sp. (strain PCC 6803 / Kazusa) GN=sll1072 PE=	124,49	10,76	1	3	3	7	1,010	4	4,5	223	25,2	5,20							
	A3	Sequence	# PSMs	# Proteins	# Protein Groups	Protein Group Accessions	Modifications	ΔCn	114/115	114/115 Count	114/115 Variability [%]	q-Value	PEP	IonScore	Exp Value	Charge	MH+ [Da]	ΔM [ppm]	RT [min]	# Missed Cleavages
	High	kAELIEGIVHVASPLR	1	1	1	P73280	N-Term(iTRAQ4plex); K1(iTRAQ4plex)	0,0000				0	2,726E-08	55	3,06881E-06	4	2020,21572	-0,04	40,73	1
	High	aELIEGIVHVASPLR	5	1	1	P73280	N-Term(iTRAQ4plex)	0,0000	1,012	3	9,0	0	1,459E-08	49	1,34887E-05	3	1748,01871	-0,03	46,06	0
	High	vYENHGVk	1	1	1	P73280	N-Term(iTRAQ4plex); K8(iTRAQ4plex)	0,0000	1,007	1		0,008	0,09839	13	0,057861899	3	1233,68406	0,85	11,69	0
P73390	Poly(3-hydroxyalkanoate) synthase OS=Synechocystis sp. (strain PCC 6803 / Kazus	124,03	7,14	1	3	3	6	0,513	6	6,2	378	42,9	4,77							
	A3	Sequence	# PSMs	# Proteins	# Protein Groups	Protein Group Accessions	Modifications	ΔCn	114/115	114/115 Count	114/115 Variability [%]	q-Value	PEP	IonScore	Exp Value	Charge	MH+ [Da]	ΔM [ppm]	RT [min]	# Missed Cleavages
	High	dLPPAIAHWLSER	2	1	1	P73390	N-Term(iTRAQ4plex)	0,0000	0,523	2	4,4	0	8,878E-07	54	3,8634E-06	3	1648,89438	0,94	46,76	0
	High	sLVANLLk	1	1	1	P73390	N-Term(iTRAQ4plex); K8(iTRAQ4plex)	0,0000	0,861	1		0	0,00005126	45	3,27318E-05	2	1145,74761	-1,78	38,86	0
	High	lLNFLR	3	1	1	P73390	N-Term(iTRAQ4plex)	0,0000	0,496	3	6,4	0,001	0,008637	30	0,001383386	2	919,58391	-0,73	43,62	0
P73914	Squalene-hopene-cyclase OS=Synechocystis sp. (strain PCC 6803 / Kazusa) GN=sh	123,97	7,26	1	5	5	8	0,610	8	10,0	647	72,0	4,96							
	A3	Sequence	# PSMs	# Proteins	# Protein Groups	Protein Group Accessions	Modifications	ΔCn	114/115	114/115 Count	114/115 Variability [%]	q-Value	PEP	IonScore	Exp Value	Charge	MH+ [Da]	ΔM [ppm]	RT [min]	# Missed Cleavages
	High	nYLLQQQR	2	1	1	P73914	N-Term(iTRAQ4plex)	0,0000	0,610	2	1,9	0	0,00001541	43	5,21666E-05	2	1206,66936	-1,49	22,32	0
	High	kQGAINk	1	1	1	P73914	N-Term(iTRAQ4plex); K1(iTRAQ4plex); K7(iTRAQ4plex)	0,0000	0,790	1		0	0,003651	17	0,018406449	3	1190,75672	-1,19	8,84	1
	High	aLAEADLGkDHPALVk	1	1	1	P73914	N-Term(iTRAQ4plex); K9(iTRAQ4plex); K16(iTRAQ4plex)	0,0000	0,547	1		0	0,000007686	16	0,025940002	4	2080,21157	-0,56	28,55	1
	High	qYFPLIALAR	3	1	1	P73914	N-Term(iTRAQ4plex)	0,0000	0,601	3	6,1	0,001	0,009853	27	0,002069998	2	1335,79045	-0,08	48,23	0
	Medium	fAPQIk	1	1	1	P73914	N-Term(iTRAQ4plex); K6(iTRAQ4plex)	0,0000	0,917	1		0,022	0,2348	24	0,004282505	2	991,62224	4,38	24,55	0
P73824	Hydroperoxy fatty acid reductase gpx2 OS=Synechocystis sp. (strain PCC 6803 / Ka	123,73	11,04	1	2	2	7	0,783	5	3,5	154	16,6	4,81							
	A3	Sequence	# PSMs	# Proteins	# Protein Groups	Protein Group Accessions	Modifications	ΔCn	114/115	114/115 Count	114/115 Variability [%]	q-Value	PEP	IonScore	Exp Value	Charge	MH+ [Da]	ΔM [ppm]	RT [min]	# Missed Cleavages
	High	tkYDVDFTLLEk	3	1	1	P73824	N-Term(iTRAQ4plex); K2(iTRAQ4plex); K12(iTRAQ4plex)	0,0000	0,870	3	1,0	0	2,106E-08	49	1,37148E-05	3	1904,07492	0,42	39,32	1
	High	fLIGR	4	1	1	P73824	N-Term(iTRAQ4plex)	0,0000	0,780	2	0,6	0,009	0,1016	12	0,084900454	2	749,47801	-1,39	27,32	0
P23353	Chorismate synthase OS=Synechocystis sp. (strain PCC 6803 / Kazusa) GN=aroC P	122,98	9,12	1	3	3	5	0,712	5	8,5	362	39,3	5,99							
	A3	Sequence	# PSMs	# Proteins	# Protein Groups	Protein Group Accessions	Modifications	ΔCn	114/115	114/115 Count	114/115 Variability [%]	q-Value	PEP	IonScore	Exp Value	Charge	MH+ [Da]	ΔM [ppm]	RT [min]	# Missed Cleavages
	High	vAAGAIAk	1	1	1	P23353	N-Term(iTRAQ4plex); K8(iTRAQ4plex)	0,0000	0,918	1		0	0,00288	45	3,9079E-05	2	988,64324	3,90	18,30	0
	High	tLGTPIAILVR	3	1	1	P23353	N-Term(iTRAQ4plex)	0,0000	0,707	3	1,0	0	0,00008826	41	7,58525E-05	2	1297,83257	0,09	44,97	0
	High	iAFkPTATIGQEQk	1	1	1	P23353	N-Term(iTRAQ4plex); K4(iTRAQ4plex); K14(iTRAQ4plex)	0,0000	0,835	1		0	2,393E-11	37	0,000205575	3	1964,15458	0,23	29,13	0
P74308	Aldehyde reductase OS=Synechocystis sp. (strain PCC 6803 / Kazusa) GN=slr0942	122,93	18,35	1	5	5	8	0,749	8	16,2	327	36,0	5,15							
	A3	Sequence	# PSMs	# Proteins	# Protein Groups	Protein Group Accessions	Modifications	ΔCn	114/115	114/115 Count	114/115 Variability [%]	q-Value	PEP	IonScore	Exp Value	Charge	MH+ [Da]	ΔM [ppm]	RT [min]	# Missed Cleavages
	High	rEELWITSk	2	1	1	P74308	N-Term(iTRAQ4plex); K9(iTRAQ4plex)	0,0000	0,704	2	1,6	0	0,00003182	40	0,00010964	3	1449,83054	0,08	27,36	1
	High	kLEmVLSmAR	1	1	1	P74308	K1(iTRAQ4plex); N-Term(iTRAQ4plex); M4(Oxidation); M8(Oxidation)	0,0000	0,845	1		0	0,0001167	37	0,000216755	3	1497,83603	-0,78	26,87	1
	High	kLEMVLSMAR	1	1	1	P74308	N-Term(iTRAQ4plex); K1(iTRAQ4plex)	0,0000	0,787	1		0	0,000000606	31	0,000839402	3	1465,84861	0,84	36,79	1
	High	lWSNAHHPDAVLPALEk	2	1	1	P74308	N-Term(iTRAQ4plex); K17(iTRAQ4plex)	0,0000	0,772	2	44,7	0	0,002604	21	0,008374714	4	2186,19741	0,59	36,37	0
	High	aVDLGLcHHIGVSNFSLk	1	1	1	P74308	N-Term(iTRAQ4plex); C7(Carbamidomethyl); K18(iTRAQ4plex)	0,0000	0,678	1		0	8,048E-07	11	0,096692477	4	2255,22133	0,17	39,30	0
	Medium	iALLDR	1	1	1	P74308	N-Term(iTRAQ4plex)	0,0000	0,842	1		0,041	0,4313	27	0,001995125	2	844,53655	-0,90	29,76	0
P73489	Ssl2245 protein OS=Synechocystis sp. (strain PCC 6803 / Kazusa) GN=ssl2245 PE=	121,40	15,91	1	2	2	7	1,152	7	4,0	88	9,2	4,35							
	A3	Sequence	# PSMs	# Proteins	# Protein Groups	Protein Group Accessions	Modifications	ΔCn	114/115	114/115 Count	114/115 Variability [%]	q-Value	PEP	IonScore	Exp Value	Charge	MH+ [Da]	ΔM [ppm]	RT [min]	# Missed Cleavages
	High	aQGPAPGk	6	1	1	P73489	N-Term(iTRAQ4plex); K8(iTRAQ4plex)	0,0000	1,139	6	2,1	0	0,001397	40	0,0001104	2	1013,59740	-0,83	9,18	0
	High	sINAYk	1	1	1	P73489	N-Term(iTRAQ4plex); K6(iTRAQ4plex)	0,0000	1,488	1		0	0,006505	29	0,001345767	2	983,58116	4,84	17,67	0
P74702	Heat shock protein OS=Synechocystis sp. (strain PCC 6803 / Kazusa) GN=htpG PE=	120,54	6,84	1	4	4	5	1,080	5	11,7	658	75,0	5,03							
	A3	Sequence	# PSMs	# Proteins	# Protein Groups	Protein Group Accessions	Modifications	ΔCn	114/115	114/115 Count	114/115 Variability [%]	q-Value	PEP	IonScore	Exp Value	Charge	MH+ [Da]	ΔM [ppm]	RT [min]	# Missed Cleavages
	High	aLNNPNINIk	1	1	1	P74702	N-Term(iTRAQ4plex); K10(iTRAQ4plex)	0,0000	1,168	1		0	7,945E-08	47	1,94971E-05	2	1398,83086	0,07	26,90	0
	High	gNITIHTENIFPIIk	2	1	1	P74702	N-Term(iTRAQ4plex); K15(iTRAQ4plex)	0,0000	1,141	2	15,5	0	8,979E-09	40	0,000107639	3	1998,16349	0,40	42,78	0
	High	fkQQVEDLLLYR	1	1	1	P74702	N-Term(iTRAQ4plex); K2(iTRAQ4plex)	0,0000	1,080	1		0	5,903E-10	37	0,000214274	3	1840,05667	-0,25	45,26	1
	High	rIADYIAk	1	1	1	P74702	N-Term(iTRAQ4plex); K8(iTRAQ4plex)	0,0000	0,686	1		0	0,00003772	22	0,006606478	3	1237,75138	0,54	24,28	1
P73370	Slr2070 protein OS=Synechocystis sp. (strain PCC 6803 / Kazusa) GN=slr2070 PE=	119,97	9,51	1	2	2	4	0,777	3	8,4	284	31,9	5,71							
	A3	Sequence	# PSMs	# Proteins	# Protein Groups	Protein Group Accessions	Modifications	ΔCn	114/115	114/115 Count	114/115 Variability [%]	q-Value	PEP	IonScore	Exp Value	Charge	MH+ [Da]	ΔM [ppm]	RT [min]	# Missed Cleavages
	High	sLGVVLVPQR	2	1	1	P73370	N-Term(iTRAQ4plex)	0,0000	0,799	2	4,2	0	0,000007553	53	5,43213E-06	2	1211,75932	0,04	33,81	0
	High	wIGHDPEILDIHSIELR	2	1	1	P73370	N-Term(iTRAQ4plex)	0,0000	0,616	1		0	3,336E-09	42	6,79157E-05	4	2187,16689	-0,49	44,01	0
Q55190	High-affinity Na(+)/H(+) antiporter NhaS3 OS=Synechocystis sp. (strain PCC 6803	118,17	6,94	1	3	3	5	0,485	5	74,5	461	47,8	4,61							
	A3	Sequence	# PSMs	# Proteins	# Protein Groups	Protein Group Accessions	Modifications	ΔCn	114/115	114/115 Count	114/115 Variability [%]	q-Value	PEP	IonScore	Exp Value	Charge	MH+ [Da]	ΔM [ppm]	RT [min]	# Missed Cleavages
	High	lLSPFYVSLVNR	3	1	1	Q55190	N-Term(iTRAQ4plex)	0,0000	0,310	3	24,8	0	1,647E-08	54	3,88124E-06	2	1551,90251	0,61	51,13	0
	High	aVFEGAk	1	1	1	Q55190	N-Term(iTRAQ4plex); K7(iTRAQ4plex)	0,0000	0,677	1		0	0,001664	29	0,001213305	2	1009,59520	3,08	18,94	0
	High	kEEAPEkPVPTPD	1	1	1	Q55190	N-Term(iTRAQ4plex); K1(iTRAQ4plex); K7(iTRAQ4plex)	0,0000	0,485	1		0	0,00161	15	0,028971435	3	1869,02945	-1,91	18,59	1
P74741	Bifunctional purine biosynthesis protein PurH OS=Synechocystis sp. (strain PCC 68	117,11	7,44	1	3	3	4	0,860	4	17,9	511	54,5	5,36							
	A3	Sequence	# PSMs	# Proteins	# Protein Groups	Protein Group Accessions	Modifications	ΔCn	114/115	114/115 Count	114/115 Variability [%]	q-Value	PEP	IonScore	Exp Value	Charge	MH+ [Da]	ΔM [ppm]	RT [min]	# Missed Cleavages
	High	tTLGVGAGQmNR	2	1	1	P74741	N-Term(iTRAQ4plex); M10(Oxidation)	0,0000	0,860	2	2,2	0	0,000003262	45	3,23571E-05	2	1364,70867	0,98	16,81	0
	High	iHGGILAR	1	1	1	P74741	N-Term(iTRAQ4plex)	0,0000	0,649	1		0	0,003509	40	0,000128216	3	980,61078	-1,45	18,04	0
	High	tAAAAGITTIIQPGGSmR	1	1	1	P74741	N-Term(iTRAQ4plex); M17(Oxidation)	0,0000	1,076	1		0	5,102E-08	34	0,000643179	3	1876,00724	-0,39	34,45	0
P74036	dTDP-glucose 4-6-dehydratase OS=Synechocystis sp. (strain PCC 6803 / Kazusa) G	116,64	7,93	1	2	2	4	0,618	3	1,9	328	36,9	5,81							
	A3	Sequence	# PSMs	# Proteins	# Protein Groups	Protein Group Accessions	Modifications	ΔCn	114/115	114/115 Count	114/115 Variability [%]	q-Value	PEP	IonScore	Exp Value	Charge	MH+ [Da]	ΔM [ppm]	RT [min]	# Missed Cleavages
	High	iLVTGGAGFIGSHLIDR	1	1	1	P74036	N-Term(iTRAQ4plex)	0,0000	0,618	1		0	3,447E-14	83	4,74209E-09	3	1870,06821	0,76	43,95	0
	High	gNVNTIGPR	3	1	1	P74036	N-Term(iTRAQ4plex)	0,0000	0,630	2	4,9	0	0,00002125	33	0,000477496	2	1071,60295	0,18	14,81	0
P54384	3-isopropylmalate dehydratase large subunit OS=Synechocystis sp. (strain PCC 68	116,57	2,99	1	2	2	7	1,056	5	28,0	468	50,4	5,69							
	A3	Sequence	# PSMs	# Proteins	# Protein Groups	Protein Group Accessions	Modifications	ΔCn	114/115	114/115 Count	114/115 Variability [%]	q-Value	PEP	IonScore	Exp Value	Charge	MH+ [Da]	ΔM [ppm]	RT [min]	# Missed Cleavages
	High	dVILHIIR	5	1	1	P54384	N-Term(iTRAQ4plex)	0,0000	0,869	3	0,1	0	0,00002141	45	3,17665E-05	2	1122,71111	-0,42	33,22	0
	High	aVQWWR	2	1	1	P54384	N-Term(iTRAQ4plex)	0,0000	1,270	2	0,1	0,001	0,01675	30	0,00101151	2	989,54460	0,83	31,28	0
P72662	Cysteine synthase OS=Synechocystis sp. (strain PCC 6803 / Kazusa) GN=cysM PE=	116,20	16,62	1	4	4	6	0,384	6	13,2	325	34,7	5,40							
	A3	Sequence	# PSMs	# Proteins	# Protein Groups	Protein Group Accessions	Modifications	ΔCn	114/115	114/115 Count	114/115 Variability [%]	q-Value	PEP	IonScore	Exp Value	Charge	MH+ [Da]	ΔM [ppm]	RT [min]	# Missed Cleavages
	High	vVYQLLR	3	1	1	P72662	N-Term(iTRAQ4plex)	0,0000	0,357	3	4,5	0	0,00687	45	3,38041E-05	2	1034,64702	-0,85	35,56	0
	High	tVPAVPYRDPNNYVk	1	1	1	P72662	N-Term(iTRAQ4plex); K15(iTRAQ4plex)	0,0000	0,387	1		0	0,0004609	23	0,005211587	3	2021,10587	0,00	28,63	1
	High	kLGPGHTIVTVLcDGGAR	1	1	1	P72662	N-Term(iTRAQ4plex); K1(iTRAQ4plex); C13(Carbamidomethyl)	0,0000	0,619	1		0	4,495E-07	21	0,008834649	4	2139,19619	0,67	31,83	1
	High	hGFVDSIGHTPLIR	1	1	1	P72662	N-Term(iTRAQ4plex)	0,0000	0,538	1		0	0,001969	15	0,031986742	4	1692,92983	-0,26	31,06	0
P74598	Uncharacterized WD repeat-containing protein sll1491 OS=Synechocystis sp. (strai	115,90	8,33	1	4	4	5	1,515	5	26,0	348	37,3	5,52							
	A3	Sequence	# PSMs	# Proteins	# Protein Groups	Protein Group Accessions	Modifications	ΔCn	114/115	114/115 Count	114/115 Variability [%]	q-Value	PEP	IonScore	Exp Value	Charge	MH+ [Da]	ΔM [ppm]	RT [min]	# Missed Cleavages
	High	iWDLSALAR	2	1	1	P74598	N-Term(iTRAQ4plex)	0,0000	1,805	2	24,2	0	0,000004907	51	7,46397E-06	2	1188,68645	0,57	45,71	0
	High	vETLVGHTDk	1	1	1	P74598	N-Term(iTRAQ4plex); K10(iTRAQ4plex)	0,0000	1,515	1		0	0,00003213	35	0,000282468	3	1386,78467	1,11	20,14	0
	High	eLFSVR	1	1	1	P74598	N-Term(iTRAQ4plex)	0,0000	1,275	1		0,001	0,01252	46	2,61452E-05	2	894,51958	3,37	27,01	0
	Medium	dGmRVETLVGHTDk	1	1	1	P74598	N-Term(iTRAQ4plex); M3(Oxidation); K14(iTRAQ4plex)	0,0000	1,142	1		0,013	0,1431	11	0,073615625	4	1861,96926	0,63	19,74	1
P73746	Sll0854 protein OS=Synechocystis sp. (strain PCC 6803 / Kazusa) GN=sll0854 PE=	115,09	13,64	1	4	4	5	0,877	5	10,6	308	34,5	5,12							
	A3	Sequence	# PSMs	# Proteins	# Protein Groups	Protein Group Accessions	Modifications	ΔCn	114/115	114/115 Count	114/115 Variability [%]	q-Value	PEP	IonScore	Exp Value	Charge	MH+ [Da]	ΔM [ppm]	RT [min]	# Missed Cleavages
	High	lLHSIIGGVAVSSTR	1	1	1	P73746	N-Term(iTRAQ4plex)	0,0000	0,877	1		0	3,183E-10	62	5,79389E-07	3	1653,97666	-0,14	38,39	0
	High	iYPASVVk	2	1	1	P73746	N-Term(iTRAQ4plex); K8(iTRAQ4plex)	0,0000	0,948	2	22,2	0	0,001218	48	1,55228E-05	2	1164,72209	-0,85	26,44	0
	High	sQQmmQLLk	1	1	1	P73746	N-Term(iTRAQ4plex); M4(Oxidation); M5(Oxidation); K9(iTRAQ4plex)	0,0000	0,942	1		0	0,003667	17	0,021977081	2	1426,76567	1,40	17,09	0
	High	nmLTTNAVAR	1	1	1	P73746	N-Term(iTRAQ4plex); M2(Oxidation)	0,0000	0,644	1		0,002	0,02693	14	0,042459024	2	1250,66423	-0,13	17,76	0
P73723	Succinate dehydrogenase iron-sulphur protein subunit OS=Synechocystis sp. (strai	115,03	6,65	1	2	2	6	0,513	4	9,5	331	36,8	7,03							
	A3	Sequence	# PSMs	# Proteins	# Protein Groups	Protein Group Accessions	Modifications	ΔCn	114/115	114/115 Count	114/115 Variability [%]	q-Value	PEP	IonScore	Exp Value	Charge	MH+ [Da]	ΔM [ppm]	RT [min]	# Missed Cleavages
	High	gIASILPLGLR	5	1	1	P73723	N-Term(iTRAQ4plex)	0,0000	0,502	3	12,0	0	5,833E-07	43	5,41963E-05	2	1253,80522	-0,81	50,15	0
	High	qkPQQSPYLEk	1	1	1	P73723	N-Term(iTRAQ4plex); K2(iTRAQ4plex); K11(iTRAQ4plex)	0,0000	0,525	1		0	0,00001037	29	0,001373947	3	1778,02549	4,62	17,05	0
P74395	N utilization substance protein B homolog OS=Synechocystis sp. (strain PCC 6803 /	114,74	14,93	1	3	3	5	1,310	4	18,4	221	24,7	6,02							
	A3	Sequence	# PSMs	# Proteins	# Protein Groups	Protein Group Accessions	Modifications	ΔCn	114/115	114/115 Count	114/115 Variability [%]	q-Value	PEP	IonScore	Exp Value	Charge	MH+ [Da]	ΔM [ppm]	RT [min]	# Missed Cleavages
	High	aSNLNSAk	3	1	1	P74395	N-Term(iTRAQ4plex); K8(iTRAQ4plex)	0,0000	1,482	2	6,8	0	0,0002792	49	1,35198E-05	2	1092,62529	0,11	13,02	0
	High	eLSLLSLSQLSR	1	1	1	P74395	N-Term(iTRAQ4plex)	0,0000	1,024	1		0	4,356E-07	44	4,23348E-05	2	1489,87151	0,56	47,55	0
	High	kTDGAPVTPGEPR	1	1	1	P74395	N-Term(iTRAQ4plex); K1(iTRAQ4plex)	0,0000	1,212	1		0	0,00001881	31	0,000855008	3	1612,89145	1,05	15,00	1
P74306	Zeta-carotene desaturase OS=Synechocystis sp. (strain PCC 6803 / Kazusa) GN=cr	114,17	7,36	1	3	3	4	1,164	4	40,8	489	54,3	5,80							
	A3	Sequence	# PSMs	# Proteins	# Protein Groups	Protein Group Accessions	Modifications	ΔCn	114/115	114/115 Count	114/115 Variability [%]	q-Value	PEP	IonScore	Exp Value	Charge	MH+ [Da]	ΔM [ppm]	RT [min]	# Missed Cleavages
	High	qAAQAILANQAR	1	1	1	P74306	N-Term(iTRAQ4plex)	0,0000	1,163	1		0	9,533E-08	66	2,26971E-07	2	1398,79631	2,06	22,90	0
	High	iSFAEWFLSk	2	1	1	P74306	N-Term(iTRAQ4plex); K10(iTRAQ4plex)	0,0000	0,893	2	40,8	0	0,000001109	53	5,40754E-06	2	1515,84282	-1,45	53,72	0
	High	lkEHTHTFVNQGGR	1	1	1	P74306	N-Term(iTRAQ4plex); K2(iTRAQ4plex)	0,0000	2,501	1		0	0,002604	15	0,028181883	4	1912,03798	-0,63	14,43	1
P74625	Phycobilisome rod-core linker polypeptide CpcG OS=Synechocystis sp. (strain PCC 6	114,11	17,67	1	5	5	7	0,248	6	41,3	249	28,5	9,10							
	A3	Sequence	# PSMs	# Proteins	# Protein Groups	Protein Group Accessions	Modifications	ΔCn	114/115	114/115 Count	114/115 Variability [%]	q-Value	PEP	IonScore	Exp Value	Charge	MH+ [Da]	ΔM [ppm]	RT [min]	# Missed Cleavages
	High	iSGELPFAR	1	1	1	P74625	N-Term(iTRAQ4plex)	0,0000	0,256	1		0	0,001652	42	7,13498E-05	2	1133,64189	-1,48	35,63	0
	High	ekLEAIGYFR	2	1	1	P74625	N-Term(iTRAQ4plex); K2(iTRAQ4plex)	0,0000	0,059	1		0	0,0003062	29	0,001734315	3	1513,85904	-1,77	33,00	1
	High	nFEVNNNYR	1	1	1	P74625	N-Term(iTRAQ4plex)	0,0000	0,380	1		0	0,002944	25	0,003380415	2	1313,63384	-1,27	21,23	0
	High	fVQmcIQR	1	1	1	P74625	N-Term(iTRAQ4plex); M4(Oxidation); C5(Carbamidomethyl)	0,0000	0,310	1		0	0,0005774	17	0,020557486	2	1241,62212	-2,45	22,44	0
	High	nQAPLTYR	2	1	1	P74625	N-Term(iTRAQ4plex)	0,0000	0,209	2	20,8	0,003	0,03327	33	0,000549503	2	1106,60649	-0,90	18,68	0
P74074	Slr1338 protein OS=Synechocystis sp. (strain PCC 6803 / Kazusa) GN=slr1338 PE=	112,76	12,00	1	2	2	3	1,059	3	2,6	200	22,4	4,51							
	A3	Sequence	# PSMs	# Proteins	# Protein Groups	Protein Group Accessions	Modifications	ΔCn	114/115	114/115 Count	114/115 Variability [%]	q-Value	PEP	IonScore	Exp Value	Charge	MH+ [Da]	ΔM [ppm]	RT [min]	# Missed Cleavages
	High	sRWELVQESTLALAR	2	1	1	P74074	N-Term(iTRAQ4plex)	0,0000	1,187	2	19,6	0	1,547E-10	77	1,78226E-08	3	1903,05173	-0,06	41,09	1
	High	rYDNVNASk	1	1	1	P74074	N-Term(iTRAQ4plex); K9(iTRAQ4plex)	0,0000	1,059	1		0	0,000881	27	0,001905329	3	1354,73252	0,56	11,37	1
P73599	Uncharacterized protein sll1304 OS=Synechocystis sp. (strain PCC 6803 / Kazusa) G	112,43	11,15	1	3	3	4	0,466	4	25,1	287	32,8	5,99							
	A3	Sequence	# PSMs	# Proteins	# Protein Groups	Protein Group Accessions	Modifications	ΔCn	114/115	114/115 Count	114/115 Variability [%]	q-Value	PEP	IonScore	Exp Value	Charge	MH+ [Da]	ΔM [ppm]	RT [min]	# Missed Cleavages
	High	lVAETLIk	1	1	1	P73599	N-Term(iTRAQ4plex); K8(iTRAQ4plex)	0,0000	0,456	1		0	0,00009421	46	2,45454E-05	2	1174,76213	-2,41	34,79	0
	High	gTPGSDSFAWGHWFk	1	1	1	P73599	N-Term(iTRAQ4plex); K15(iTRAQ4plex)	0,0000	0,476	1		0	4,971E-08	40	0,000109136	3	1967,96445	0,07	43,56	0
	High	fGVHTFIWk	2	1	1	P73599	N-Term(iTRAQ4plex); K9(iTRAQ4plex)	0,0000	0,423	2	66,6	0	0,0002407	39	0,000122797	2	1422,81072	-2,06	37,27	0
P74721	Zinc-containing alcohol dehydrogenase family OS=Synechocystis sp. (strain PCC 68	111,54	5,65	1	3	3	8	0,881	8	13,9	336	35,8	6,10							
	A3	Sequence	# PSMs	# Proteins	# Protein Groups	Protein Group Accessions	Modifications	ΔCn	114/115	114/115 Count	114/115 Variability [%]	q-Value	PEP	IonScore	Exp Value	Charge	MH+ [Da]	ΔM [ppm]	RT [min]	# Missed Cleavages
	High	vVLSHSkN	3	1	1	P74721	N-Term(iTRAQ4plex); K7(iTRAQ4plex)	0,0000	0,784	3	5,1	0	0,001692	33	0,000539473	2	1171,70281	-0,80	11,54	1
	High	fDYIISTVNLk	1	1	1	P74721	N-Term(iTRAQ4plex); K11(iTRAQ4plex)	0,0000	1,075	1		0	0,007409	30	0,001044648	3	1600,92057	1,04	45,93	0
	High	vVLSHSk	4	1	1	P74721	N-Term(iTRAQ4plex); K7(iTRAQ4plex)	0,0000	0,918	4	6,1	0,002	0,02635	34	0,000409232	2	1057,66423	3,23	12,31	0
P74527	Aminopeptidase OS=Synechocystis sp. (strain PCC 6803 / Kazusa) GN=ape2 PE=4	111,39	2,19	1	2	2	3	0,674	3	2,8	869	97,9	5,77							
	A3	Sequence	# PSMs	# Proteins	# Protein Groups	Protein Group Accessions	Modifications	ΔCn	114/115	114/115 Count	114/115 Variability [%]	q-Value	PEP	IonScore	Exp Value	Charge	MH+ [Da]	ΔM [ppm]	RT [min]	# Missed Cleavages
	High	iAWVLIk	2	1	1	P74527	N-Term(iTRAQ4plex); K7(iTRAQ4plex)	0,0000	0,668	2	1,4	0	0,00005275	56	2,52331E-06	2	1130,75334	-0,59	43,79	0
	High	eREQVFYFPLTR	1	1	1	P74527	N-Term(iTRAQ4plex)	0,0000	0,982	1		0	4,193E-08	25	0,002844265	3	1728,91916	0,08	39,71	1
Q55759	GTP cyclohydrolase 1 OS=Synechocystis sp. (strain PCC 6803 / Kazusa) GN=folE PE	111,07	11,11	1	3	3	6	0,903	5	1,5	234	26,6	6,62							
	A3	Sequence	# PSMs	# Proteins	# Protein Groups	Protein Group Accessions	Modifications	ΔCn	114/115	114/115 Count	114/115 Variability [%]	q-Value	PEP	IonScore	Exp Value	Charge	MH+ [Da]	ΔM [ppm]	RT [min]	# Missed Cleavages
	High	tREEFLNLIR	4	1	1	Q55759	N-Term(iTRAQ4plex)	0,0000	0,801	3	16,2	0	0,000008133	41	7,85236E-05	3	1434,81845	-0,09	36,57	1
	High	yHPENVELNk	1	1	1	Q55759	N-Term(iTRAQ4plex); K10(iTRAQ4plex)	0,0000	0,903	1		0	0,00009483	26	0,002360315	3	1530,81498	-0,33	21,65	0
	High	vVGLSk	1	1	1	Q55759	N-Term(iTRAQ4plex); K6(iTRAQ4plex)	0,0000	0,912	1		0,009	0,1086	22	0,007259555	2	890,58672	-5,20	19,27	0
P74241	Sulfate adenylyltransferase OS=Synechocystis sp. (strain PCC 6803 / Kazusa) GN=s	110,72	13,85	1	6	6	9	0,746	8	10,6	390	43,7	6,25							
	A3	Sequence	# PSMs	# Proteins	# Protein Groups	Protein Group Accessions	Modifications	ΔCn	114/115	114/115 Count	114/115 Variability [%]	q-Value	PEP	IonScore	Exp Value	Charge	MH+ [Da]	ΔM [ppm]	RT [min]	# Missed Cleavages
	High	nYGcTHFIVGR	2	1	1	P74241	N-Term(iTRAQ4plex); C4(Carbamidomethyl)	0,0000	0,751	1		0	0,000003278	36	0,00024042	3	1467,72910	0,50	24,27	0
	High	vILGINPSAmR	1	1	1	P74241	N-Term(iTRAQ4plex); M10(Oxidation)	0,0000	0,741	1		0	0,00005507	35	0,000336488	2	1330,76311	-0,21	33,12	0
	High	ePHPLFPk	2	1	1	P74241	N-Term(iTRAQ4plex); K8(iTRAQ4plex)	0,0000	0,699	2	2,3	0	0,000002764	28	0,001485833	3	1252,72922	-0,02	22,75	0
	High	tIVGFQTR	1	1	1	P74241	N-Term(iTRAQ4plex)	0,0000	1,569	1		0	0,003074	27	0,002182579	2	1065,61687	-0,45	25,67	0
	High	eAIFHALIR	2	1	1	P74241	N-Term(iTRAQ4plex)	0,0000	0,679	2	23,7	0	0,001329	22	0,007014069	3	1213,71695	-0,37	34,27	0
	High	aHEYIQk	1	1	1	P74241	N-Term(iTRAQ4plex); K7(iTRAQ4plex)	0,0000	0,918	1		0,002	0,02761	14	0,047719715	3	1176,65924	-1,95	13,13	0
P72967	Slr0695 protein OS=Synechocystis sp. (strain PCC 6803 / Kazusa) GN=slr0695 PE=	110,30	18,50	1	4	4	8	1,332	8	19,0	173	19,0	9,04							
	A3	Sequence	# PSMs	# Proteins	# Protein Groups	Protein Group Accessions	Modifications	ΔCn	114/115	114/115 Count	114/115 Variability [%]	q-Value	PEP	IonScore	Exp Value	Charge	MH+ [Da]	ΔM [ppm]	RT [min]	# Missed Cleavages
	High	gLENLSAR	2	1	1	P72967	N-Term(iTRAQ4plex)	0,0000	1,268	2	22,2	0	0,0001567	28	0,001599448	2	1003,56578	0,47	20,50	0
	High	nNPTAANk	4	1	1	P72967	N-Term(iTRAQ4plex); K8(iTRAQ4plex)	0,0000	1,332	4	12,3	0	0,004806	16	0,026666745	3	1117,61798	-2,18	10,36	0
	High	qGFAEIk	1	1	1	P72967	N-Term(iTRAQ4plex); K7(iTRAQ4plex)	0,0000	1,483	1		0,003	0,03473	17	0,017946097	2	1080,62969	0,45	21,55	0
	High	sPSFTPQDR	1	1	1	P72967	N-Term(iTRAQ4plex)	0,0000	1,150	1		0,008	0,09577	31	0,000741259	2	1178,59331	0,90	18,37	0
P55037	Ferredoxin-dependent glutamate synthase 1 OS=Synechocystis sp. (strain PCC 68	108,30	4,77	1	6	6	7	0,814	6	8,5	1550	169,0	5,92							
	A3	Sequence	# PSMs	# Proteins	# Protein Groups	Protein Group Accessions	Modifications	ΔCn	114/115	114/115 Count	114/115 Variability [%]	q-Value	PEP	IonScore	Exp Value	Charge	MH+ [Da]	ΔM [ppm]	RT [min]	# Missed Cleavages
	High	vSHDIVEQGLQmLVNLEHR	2	1	1	P55037	N-Term(iTRAQ4plex); M12(Oxidation)	0,0000	0,841	1		0	2,421E-11	51	8,35545E-06	4	2377,23940	-0,90	49,30	0
	High	vTATLPITNINR	1	1	1	P55037	N-Term(iTRAQ4plex)	0,0000	0,750	1		0	0,0004183	33	0,000478597	2	1456,86052	0,05	35,10	0
	High	gNVNWmQAR	1	1	1	P55037	N-Term(iTRAQ4plex); M6(Oxidation)	0,0000	0,798	1		0	0,001091	25	0,002944218	2	1235,60735	0,11	19,03	0
	High	hAGLPWELGLAETHQTLVLNNLR	1	1	1	P55037	N-Term(iTRAQ4plex)	0,0000	0,955	1		0	4,371E-10	24	0,003872309	4	2726,49038	1,62	46,73	0
	High	kLYVIR	1	1	1	P55037	N-Term(iTRAQ4plex); K1(iTRAQ4plex)	0,0000	0,717	1		0,002	0,02294	15	0,030972054	3	1079,71689	-0,97	21,19	1
	Medium	fFTLR	1	1	1	P55037	N-Term(iTRAQ4plex)	0,0000	0,829	1		0,023	0,249	25	0,003265653	2	827,48931	-0,37	34,36	0
P74226	30S ribosomal protein S10 OS=Synechocystis sp. (strain PCC 6803 / Kazusa) GN=r	107,88	37,14	1	6	6	11	1,328	11	40,7	105	12,0	9,77							
	A3	Sequence	# PSMs	# Proteins	# Protein Groups	Protein Group Accessions	Modifications	ΔCn	114/115	114/115 Count	114/115 Variability [%]	q-Value	PEP	IonScore	Exp Value	Charge	MH+ [Da]	ΔM [ppm]	RT [min]	# Missed Cleavages
	High	tNAAAVGPIPLPTkR	1	1	1	P74226	N-Term(iTRAQ4plex); K14(iTRAQ4plex)	0,0000	1,328	1		0	3,119E-09	42	6,79157E-05	3	1794,08695	1,62	29,51	1
	High	tNAAAVGPIPLPTk	1	1	1	P74226	N-Term(iTRAQ4plex); K14(iTRAQ4plex)	0,0000	0,983	1		0	0,00009225	30	0,001088855	3	1637,98423	0,79	34,77	0
	High	kIYcVLR	3	1	1	P74226	N-Term(iTRAQ4plex); K1(iTRAQ4plex); C4(Carbamidomethyl)	0,0000	1,618	3	10,0	0	0,0001276	26	0,002317235	3	1239,74842	-0,14	23,77	1
	High	rIIDIYQPSSk	2	1	1	P74226	N-Term(iTRAQ4plex); K11(iTRAQ4plex)	0,0000	0,974	2	10,3	0	0,00001802	25	0,003483133	3	1607,93570	-0,15	27,13	1
	High	eHFETR	2	1	1	P74226	N-Term(iTRAQ4plex)	0,0000	0,538	2	10,4	0,002	0,02506	13	0,049655802	3	962,48224	1,05	10,92	0
	High	iYcVLR	2	1	1	P74226	N-Term(iTRAQ4plex); C3(Carbamidomethyl)	0,0000	1,651	2	12,3	0,006	0,08015	18	0,017699867	2	967,55828	6,94	28,94	0
P74063	Photosystem I assembly protein Ycf3 OS=Synechocystis sp. (strain PCC 6803 / Kaz	107,28	14,45	1	2	2	3	1,493	3	3,8	173	19,9	4,82							
	A3	Sequence	# PSMs	# Proteins	# Protein Groups	Protein Group Accessions	Modifications	ΔCn	114/115	114/115 Count	114/115 Variability [%]	q-Value	PEP	IonScore	Exp Value	Charge	MH+ [Da]	ΔM [ppm]	RT [min]	# Missed Cleavages
	High	mPSALNNIAVIYHFQGEk	2	1	1	P74063	N-Term(iTRAQ4plex); M1(Oxidation); K18(iTRAQ4plex)	0,0000	1,487	2	4,5	0	8,133E-09	55	3,12586E-06	3	2336,23587	2,01	41,40	0
	High	eAFVYYR	1	1	1	P74063	N-Term(iTRAQ4plex)	0,0000	1,493	1		0	0,0008522	32	0,000578056	2	1091,56706	2,60	27,78	0
Q55366	Ferredoxin--nitrite reductase OS=Synechocystis sp. (strain PCC 6803 / Kazusa) GN=	106,93	7,37	1	4	4	4	1,112	4	8,4	502	55,9	6,61							
	A3	Sequence	# PSMs	# Proteins	# Protein Groups	Protein Group Accessions	Modifications	ΔCn	114/115	114/115 Count	114/115 Variability [%]	q-Value	PEP	IonScore	Exp Value	Charge	MH+ [Da]	ΔM [ppm]	RT [min]	# Missed Cleavages
	High	lPFPLLTAAPk	1	1	1	Q55366	N-Term(iTRAQ4plex); K11(iTRAQ4plex)	0,0000	1,161	1		0	0,00003477	64	3,88124E-07	2	1455,91826	0,32	49,42	0
	High	kFNIAIEGSR	1	1	1	Q55366	N-Term(iTRAQ4plex); K1(iTRAQ4plex)	0,0000	1,066	1		0	0,000001112	39	0,000150797	3	1422,83054	-0,16	26,05	1
	High	fREEVAAk	1	1	1	Q55366	N-Term(iTRAQ4plex); K8(iTRAQ4plex)	0,0000	0,972	1		0	0,00004894	30	0,001066522	3	1237,71512	0,63	15,77	1
	High	aNkFETVk	1	1	1	Q55366	N-Term(iTRAQ4plex); K3(iTRAQ4plex); K8(iTRAQ4plex)	0,0000	1,195	1		0	0,0004863	14	0,041492538	3	1368,81928	-1,36	17,57	1
P74565	RNA polymerase sigma factor SigA OS=Synechocystis sp. (strain PCC 6803 / Kazus	106,67	8,00	1	4	4	5	1,704	5	34,8	425	49,9	6,27							
	A3	Sequence	# PSMs	# Proteins	# Protein Groups	Protein Group Accessions	Modifications	ΔCn	114/115	114/115 Count	114/115 Variability [%]	q-Value	PEP	IonScore	Exp Value	Charge	MH+ [Da]	ΔM [ppm]	RT [min]	# Missed Cleavages
	High	lPVHLYETISR	2	1	1	P74565	N-Term(iTRAQ4plex)	0,0000	1,671	2	2,9	0	0,00002491	44	3,80163E-05	3	1471,84082	1,26	35,07	0
	High	lVVSIAk	1	1	1	P74565	N-Term(iTRAQ4plex); K7(iTRAQ4plex)	0,0000	2,140	1		0	0,0007316	42	5,87449E-05	2	1017,69200	0,92	29,33	0
	High	kkPYTEDSIR	1	1	1	P74565	N-Term(iTRAQ4plex); K1(iTRAQ4plex); K2(iTRAQ4plex)	0,0000	3,115	1		0	0,000004175	25	0,003097205	4	1668,96901	2,70	13,23	1
	High	nFAAFR	1	1	1	P74565	N-Term(iTRAQ4plex)	0,0000	0,649	1		0,003	0,03339	12	0,062657059	2	869,47521	0,20	23,90	0
P74220	Putative carboxypeptidase slr1534 OS=Synechocystis sp. (strain PCC 6803 / Kazusa	106,56	8,64	1	2	2	2	1,146	2	18,2	301	32,9	5,47							
	A3	Sequence	# PSMs	# Proteins	# Protein Groups	Protein Group Accessions	Modifications	ΔCn	114/115	114/115 Count	114/115 Variability [%]	q-Value	PEP	IonScore	Exp Value	Charge	MH+ [Da]	ΔM [ppm]	RT [min]	# Missed Cleavages
	High	aSGNLSQVAGIALGR	1	1	1	P74220	N-Term(iTRAQ4plex)	0,0000	1,015	1		0	6,872E-12	92	7,54037E-10	2	1557,88555	1,64	38,45	0
	High	lTVVSPSGSLR	1	1	1	P74220	N-Term(iTRAQ4plex)	0,0000	1,293	1		0,002	0,01979	28	0,001534511	2	1259,74150	-1,99	29,36	0
P73810	DNA topoisomerase 1 OS=Synechocystis sp. (strain PCC 6803 / Kazusa) GN=topA P	106,14	6,24	1	5	5	6	0,878	5	17,8	898	99,3	8,82							
	A3	Sequence	# PSMs	# Proteins	# Protein Groups	Protein Group Accessions	Modifications	ΔCn	114/115	114/115 Count	114/115 Variability [%]	q-Value	PEP	IonScore	Exp Value	Charge	MH+ [Da]	ΔM [ppm]	RT [min]	# Missed Cleavages
	High	fGPYVVHDQGk	1	1	1	P73810	N-Term(iTRAQ4plex); K11(iTRAQ4plex)	0,0000	0,989	1		0	0,000002448	44	3,73224E-05	3	1534,82865	1,94	23,19	0
	High	kASATGTAk	1	1	1	P73810	N-Term(iTRAQ4plex); K1(iTRAQ4plex); K9(iTRAQ4plex)	0,0000	0,353	1		0	0,0004271	33	0,000487495	3	1266,77121	-2,35	7,92	1
	High	rIDFPGYFR	2	1	1	P73810	N-Term(iTRAQ4plex)	0,0000	0,729	1		0	0,000004203	32	0,000594251	3	1314,70813	0,43	33,71	1
	High	iTQLSVLLk	1	1	1	P73810	N-Term(iTRAQ4plex); K9(iTRAQ4plex)	0,0000	0,878	1		0	0,000001545	32	0,000633826	2	1302,86003	0,08	43,79	0
	High	lTGkPWAVVNTEEkPGVR	1	1	1	P73810	N-Term(iTRAQ4plex); K4(iTRAQ4plex); K14(iTRAQ4plex)	0,0000	0,890	1		0	7,485E-10	17	0,022129419	4	2413,39297	0,06	32,25	0
Q55641	Ribonuclease D OS=Synechocystis sp. (strain PCC 6803 / Kazusa) GN=rnd PE=4 SV	105,73	13,36	1	4	4	7	0,748	6	20,1	217	24,4	6,21							
	A3	Sequence	# PSMs	# Proteins	# Protein Groups	Protein Group Accessions	Modifications	ΔCn	114/115	114/115 Count	114/115 Variability [%]	q-Value	PEP	IonScore	Exp Value	Charge	MH+ [Da]	ΔM [ppm]	RT [min]	# Missed Cleavages
	High	aQLAYAANDVR	1	1	1	Q55641	N-Term(iTRAQ4plex)	0,0000	0,680	1		0	5,07E-08	49	1,26465E-05	2	1335,71379	0,01	24,24	0
	High	iFHFAR	1	1	1	Q55641	N-Term(iTRAQ4plex)	0,0000	0,519	1		0,001	0,01759	32	0,000894063	3	934,53760	-0,39	24,57	0
	High	yLIPLR	4	1	1	Q55641	N-Term(iTRAQ4plex)	0,0000	0,890	3	11,5	0,002	0,02105	10	0,117745238	2	918,58898	-0,38	35,43	0
	Medium	hTFDIk	1	1	1	Q55641	N-Term(iTRAQ4plex); K6(iTRAQ4plex)	0,0000	0,737	1		0,02	0,2174	11	0,103593801	3	1048,60031	-2,53	18,07	0
Q55778	Glutamate--tRNA ligase OS=Synechocystis sp. (strain PCC 6803 / Kazusa) GN=gltX	105,45	7,04	1	3	3	5	0,807	5	37,5	483	54,0	5,48							
	A3	Sequence	# PSMs	# Proteins	# Protein Groups	Protein Group Accessions	Modifications	ΔCn	114/115	114/115 Count	114/115 Variability [%]	q-Value	PEP	IonScore	Exp Value	Charge	MH+ [Da]	ΔM [ppm]	RT [min]	# Missed Cleavages
	High	kAIQQLLDQGLAYR	1	1	1	Q55778	N-Term(iTRAQ4plex); K1(iTRAQ4plex)	0,0000	1,030	1		0	1,501E-10	70	9,5273E-08	3	1905,11716	0,58	42,18	1
	High	tAVFNWLFAR	3	1	1	Q55778	N-Term(iTRAQ4plex)	0,0000	0,676	3	15,3	0	0,0002676	25	0,002883832	2	1368,75688	1,72	52,72	0
	High	gEDHIANTAk	1	1	1	Q55778	N-Term(iTRAQ4plex); K10(iTRAQ4plex)	0,0000	1,036	1		0,002	0,0263	10	0,103846696	3	1343,71405	-1,29	10,71	0
P73270	Probable agmatinase 2 OS=Synechocystis sp. (strain PCC 6803 / Kazusa) GN=speB	105,38	17,18	1	6	6	8	1,858	7	22,5	390	42,9	5,24							
	A3	Sequence	# PSMs	# Proteins	# Protein Groups	Protein Group Accessions	Modifications	ΔCn	114/115	114/115 Count	114/115 Variability [%]	q-Value	PEP	IonScore	Exp Value	Charge	MH+ [Da]	ΔM [ppm]	RT [min]	# Missed Cleavages
	High	vIcDTMAHLVVSGQLPR	1	1	1	P73270	N-Term(iTRAQ4plex); C3(Carbamidomethyl)	0,0000	1,777	1		0	6,453E-09	41	8,22186E-05	3	2040,08622	0,52	44,66	0
	High	eALYLLk	1	1	1	P73270	N-Term(iTRAQ4plex); K7(iTRAQ4plex)	0,0000	2,159	1		0	0,0003121	40	0,000147006	2	1137,71269	0,45	35,43	0

	High	gELPHYAGINTFmk	1	1	1	P73270	N-Term(iTRAQ4plex); M13(Oxidation); K14(iTRAQ4plex)	0,0000	1,858	1		0	1,828E-09	36	0,000274138	3	1881,97959	1,28	32,80	0
	High	vGIIHFDR	3	1	1	P73270	N-Term(iTRAQ4plex)	0,0000	3,201	2	20,2	0	0,001442	24	0,004139711	3	1100,63349	0,14	28,29	0
	High	nLVQLGIGGWQVPR	1	1	1	P73270	N-Term(iTRAQ4plex)	0,0000	1,039	1		0	0,00007379	13	0,049087397	3	1680,96970	1,81	47,66	0
	Medium	sIPTFSR	1	1	1	P73270	N-Term(iTRAQ4plex)	0,0000	1,826	1		0,049	0,5016	15	0,031547867	2	951,53813	0,11	25,10	0
P74375	Slr0442 protein OS=Synechocystis sp. (strain PCC 6803 / Kazusa) GN=slr0442 PE=	103,48	9,00	1	5	5	6	2,260	6	56,8	611	62,9	5,21							
	A3	Sequence	# PSMs	# Proteins	# Protein Groups	Protein Group Accessions	Modifications	ΔCn	114/115	114/115 Count	114/115 Variability [%]	q-Value	PEP	IonScore	Exp Value	Charge	MH+ [Da]	ΔM [ppm]	RT [min]	# Missed Cleavages
	High	kFELNFDNTGGk	1	1	1	P74375	N-Term(iTRAQ4plex); K1(iTRAQ4plex); K12(iTRAQ4plex)	0,0000	3,053	1		0	2,007E-07	37	0,000207477	3	1801,98685	3,30	30,89	1
	High	aSTPmPSIPLSALASAR	1	1	1	P74375	N-Term(iTRAQ4plex); M5(Oxidation)	0,0000	1,392	1		0	0,0003157	36	0,00029268	2	1829,99504	2,08	40,28	0
	High	iPQGQVIGR	2	1	1	P74375	N-Term(iTRAQ4plex)	0,0000	2,610	2	41,4	0	0,001927	30	0,000924634	2	1111,67107	0,55	23,42	0
	High	vGVEEENkGk	1	1	1	P74375	N-Term(iTRAQ4plex); K8(iTRAQ4plex); K10(iTRAQ4plex)	0,0000	2,559	1		0	0,00002168	24	0,00522288	3	1520,86740	1,92	13,26	1
	Medium	tcTYIPR	1	1	1	P74375	N-Term(iTRAQ4plex); C2(Carbamidomethyl)	0,0000	1,382	1		0,012	0,1353	15	0,031547867	2	1054,55583	8,19	19,10	0
P73626	Sll1771 protein OS=Synechocystis sp. (strain PCC 6803 / Kazusa) GN=sll1771 PE=4	103,20	12,60	1	3	3	3	1,121	3	4,3	254	28,5	4,56							
	A3	Sequence	# PSMs	# Proteins	# Protein Groups	Protein Group Accessions	Modifications	ΔCn	114/115	114/115 Count	114/115 Variability [%]	q-Value	PEP	IonScore	Exp Value	Charge	MH+ [Da]	ΔM [ppm]	RT [min]	# Missed Cleavages
	High	hVLFQcLGR	1	1	1	P73626	N-Term(iTRAQ4plex); C6(Carbamidomethyl)	0,0000	0,970	1		0	0,000000103	65	3,39602E-07	3	1273,69754	1,51	27,93	0
	High	rDmGTTAVLIAFR	1	1	1	P73626	N-Term(iTRAQ4plex); M3(Oxidation)	0,0000	1,121	1		0	1,268E-07	52	7,38572E-06	3	1610,88187	0,81	32,88	1
	High	vTEDHTWVAR	1	1	1	P73626	N-Term(iTRAQ4plex)	0,0000	1,154	1		0	0,001218	10	0,097044294	3	1357,69864	0,38	19,74	0
P72952	Sll0645 protein OS=Synechocystis sp. (strain PCC 6803 / Kazusa) GN=sll0645 PE=4	103,09	9,02	1	2	2	3	3,883	3	5,5	255	27,1	5,07							
	A3	Sequence	# PSMs	# Proteins	# Protein Groups	Protein Group Accessions	Modifications	ΔCn	114/115	114/115 Count	114/115 Variability [%]	q-Value	PEP	IonScore	Exp Value	Charge	MH+ [Da]	ΔM [ppm]	RT [min]	# Missed Cleavages
	High	aQNQTTFAVSk	1	1	1	P72952	N-Term(iTRAQ4plex); K11(iTRAQ4plex)	0,0000	3,883	1		0	5,359E-10	76	2,46587E-08	2	1482,81792	1,63	20,27	0
	High	gQTATSGGGGVR	2	1	1	P72952	N-Term(iTRAQ4plex)	0,0000	3,702	2	12,7	0,001	0,01642	37	0,000179048	2	1191,62175	1,57	8,90	0
P74314	OmpR subfamily OS=Synechocystis sp. (strain PCC 6803 / Kazusa) GN=slr0947 PE=	102,66	18,80	1	3	3	4	0,851	4	11,8	234	26,2	4,91							
	A3	Sequence	# PSMs	# Proteins	# Protein Groups	Protein Group Accessions	Modifications	ΔCn	114/115	114/115 Count	114/115 Variability [%]	q-Value	PEP	IonScore	Exp Value	Charge	MH+ [Da]	ΔM [ppm]	RT [min]	# Missed Cleavages
	High	akLEEDPSNPELILTAR	1	1	1	P74314	N-Term(iTRAQ4plex); K2(iTRAQ4plex)	0,0000	0,845	1		0	2,953E-08	52	8,0112E-06	3	2184,21402	1,15	34,78	1
	High	gTGYLFQR	2	1	1	P74314	N-Term(iTRAQ4plex)	0,0000	0,921	2	10,8	0	0,0006598	43	5,16381E-05	2	1085,58586	-0,16	26,90	0
	High	iTGLELGADDYVVkPFSPk	1	1	1	P74314	N-Term(iTRAQ4plex); K14(iTRAQ4plex); K19(iTRAQ4plex)	0,0000	0,459	1		0	0,0004997	13	0,049770271	4	2481,39980	1,30	44,60	0
P72897	Slr1619 protein OS=Synechocystis sp. (strain PCC 6803 / Kazusa) GN=slr1619 PE=	101,56	7,23	1	2	2	5	0,785	4	10,5	249	27,7	5,14							
	A3	Sequence	# PSMs	# Proteins	# Protein Groups	Protein Group Accessions	Modifications	ΔCn	114/115	114/115 Count	114/115 Variability [%]	q-Value	PEP	IonScore	Exp Value	Charge	MH+ [Da]	ΔM [ppm]	RT [min]	# Missed Cleavages
	High	tAVWEYLk	3	1	1	P72897	N-Term(iTRAQ4plex); K8(iTRAQ4plex)	0,0000	0,785	2	9,3	0	0,000002215	55	3,30347E-06	2	1297,73943	-0,03	38,97	0
	High	eAIEAHPmVk	2	1	1	P72897	N-Term(iTRAQ4plex); M8(Oxidation); K10(iTRAQ4plex)	0,0000	0,886	2	30,3	0	0,0001523	28	0,001599448	3	1428,77323	-1,91	16,44	0
P74113	Slr1970 protein OS=Synechocystis sp. (strain PCC 6803 / Kazusa) GN=slr1970 PE=	100,58	20,23	1	3	3	5	1,144	5	10,7	173	19,4	4,58							
	A3	Sequence	# PSMs	# Proteins	# Protein Groups	Protein Group Accessions	Modifications	ΔCn	114/115	114/115 Count	114/115 Variability [%]	q-Value	PEP	IonScore	Exp Value	Charge	MH+ [Da]	ΔM [ppm]	RT [min]	# Missed Cleavages
	High	kPLEPLVk	3	1	1	P74113	N-Term(iTRAQ4plex); K1(iTRAQ4plex); K8(iTRAQ4plex)	0,0000	1,172	3	3,6	0	2,684E-07	40	9,65984E-05	3	1355,89753	-0,84	21,40	0
	High	lmAALLAGDVVAmPHkk	1	1	1	P74113	N-Term(iTRAQ4plex); M2(Oxidation); M13(Oxidation); K16(iTRAQ4plex); K17	0,0000	0,791	1		0	0,0001063	27	0,001918536	4	2229,28481	1,07	34,11	1
	High	yFDRPQIADR	1	1	1	P74113	N-Term(iTRAQ4plex)	0,0000	0,964	1		0	0,0004598	19	0,012792929	3	1424,74125	0,65	25,70	0
P74675	Sll1542 protein OS=Synechocystis sp. (strain PCC 6803 / Kazusa) GN=sll1542 PE=4	100,40	8,67	1	2	2	3	1,028	3	34,8	300	34,8	4,69							
	A3	Sequence	# PSMs	# Proteins	# Protein Groups	Protein Group Accessions	Modifications	ΔCn	114/115	114/115 Count	114/115 Variability [%]	q-Value	PEP	IonScore	Exp Value	Charge	MH+ [Da]	ΔM [ppm]	RT [min]	# Missed Cleavages
	High	rLGELSNEQIGQIR	2	1	1	P74675	N-Term(iTRAQ4plex)	0,0000	0,917	2	17,0	0	5,7E-10	49	1,1272E-05	3	1756,97953	0,48	28,55	1
	High	rLSLIETILANk	1	1	1	P74675	N-Term(iTRAQ4plex); K12(iTRAQ4plex)	0,0000	1,506	1		0	0,00009519	30	0,001035071	3	1659,04282	1,24	46,42	1
P74275	Delta-1-pyrroline-5-carboxylate dehydrogenase OS=Synechocystis sp. (strain PCC 6	99,73	4,65	1	5	5	6	0,649	6	14,2	990	110,0	5,67							
	A3	Sequence	# PSMs	# Proteins	# Protein Groups	Protein Group Accessions	Modifications	ΔCn	114/115	114/115 Count	114/115 Variability [%]	q-Value	PEP	IonScore	Exp Value	Charge	MH+ [Da]	ΔM [ppm]	RT [min]	# Missed Cleavages
	High	yFYQPR	1	1	1	P74275	N-Term(iTRAQ4plex)	0,0000	1,097	1		0	0,003271	45	3,21344E-05	2	1017,52654	-0,88	24,98	0
	High	qQAFQALTR	1	1	1	P74275	N-Term(iTRAQ4plex)	0,0000	0,645	1		0	0,000045	45	3,98747E-05	2	1206,67082	-0,29	25,12	0
	High	aQQNHWQIPVYVEk	1	1	1	P74275	N-Term(iTRAQ4plex); K14(iTRAQ4plex)	0,0000	0,667	1		0	0,0001094	11	0,075509223	3	2028,09171	0,57	34,81	0
	High	lLPGMAYLIR	2	1	1	P74275	N-Term(iTRAQ4plex)	0,0000	0,543	2	2,1	0,001	0,0155	32	0,0006109	2	1290,77214	-0,26	49,00	0
	Medium	vkQQLGk	1	1	1	P74275	N-Term(iTRAQ4plex); K2(iTRAQ4plex); K7(iTRAQ4plex)	0,0000	0,654	1		0,029	0,3131	12	0,057540019	3	1232,80405	-0,84	12,94	1
P74707	Peptide chain release factor 1 OS=Synechocystis sp. (strain PCC 6803 / Kazusa) GN	99,28	8,22	1	4	4	7	1,566	7	8,9	365	41,1	5,00							
	A3	Sequence	# PSMs	# Proteins	# Protein Groups	Protein Group Accessions	Modifications	ΔCn	114/115	114/115 Count	114/115 Variability [%]	q-Value	PEP	IonScore	Exp Value	Charge	MH+ [Da]	ΔM [ppm]	RT [min]	# Missed Cleavages
	High	lTILLLPk	3	1	1	P74707	N-Term(iTRAQ4plex); K8(iTRAQ4plex)	0,0000	1,566	3	8,4	0	0,001873	39	0,000121331	2	1198,83940	1,39	47,38	0
	High	vETAVDLFHkPTGIR	2	1	1	P74707	N-Term(iTRAQ4plex); K10(iTRAQ4plex)	0,0000	1,534	2	12,0	0	2,426E-07	29	0,001294106	4	1971,12771	0,54	34,77	0
	High	rLTILLLPk	1	1	1	P74707	N-Term(iTRAQ4plex); K9(iTRAQ4plex)	0,0000	0,864	1		0	0,001326	15	0,032506485	3	1354,93970	0,62	37,24	1
	Medium	vYSQLk	1	1	1	P74707	N-Term(iTRAQ4plex); K6(iTRAQ4plex)	0,0000	1,632	1		0,013	0,1463	22	0,005807243	2	1025,63005	6,53	20,07	0
P72941	Putative biopolymer transport protein ExbB-like 2 OS=Synechocystis sp. (strain PCC	98,93	7,93	1	2	2	4	0,744	4	11,6	227	25,1	5,24							
	A3	Sequence	# PSMs	# Proteins	# Protein Groups	Protein Group Accessions	Modifications	ΔCn	114/115	114/115 Count	114/115 Variability [%]	q-Value	PEP	IonScore	Exp Value	Charge	MH+ [Da]	ΔM [ppm]	RT [min]	# Missed Cleavages
	High	kVGSELEVLYR	1	1	1	P72941	N-Term(iTRAQ4plex); K1(iTRAQ4plex)	0,0000	0,694	1		0	9,564E-09	70	1,01618E-07	3	1580,92490	-0,10	32,29	1
	High	yLLAPLR	3	1	1	P72941	N-Term(iTRAQ4plex)	0,0000	0,799	3	3,7	0	0,002555	24	0,005494692	2	989,62566	-0,79	36,45	0
P74302	Slr0937 protein OS=Synechocystis sp. (strain PCC 6803 / Kazusa) GN=slr0937 PE=	98,65	7,67	1	2	2	2	1,809	2	30,8	417	46,1	4,37							
	A3	Sequence	# PSMs	# Proteins	# Protein Groups	Protein Group Accessions	Modifications	ΔCn	114/115	114/115 Count	114/115 Variability [%]	q-Value	PEP	IonScore	Exp Value	Charge	MH+ [Da]	ΔM [ppm]	RT [min]	# Missed Cleavages
	High	eVkPWASVGDVLASFTR	1	1	1	P74302	N-Term(iTRAQ4plex); K3(iTRAQ4plex)	0,0000	2,216	1		0	2,393E-13	63	5,1996E-07	3	2150,18863	1,74	53,15	0
	High	aHEALQEQVVSLGQR	1	1	1	P74302	N-Term(iTRAQ4plex)	0,0000	1,476	1		0	1,008E-09	49	1,30909E-05	3	1808,97214	-0,80	28,09	0
P27589	Cytochrome b6-f complex subunit 4 OS=Synechocystis sp. (strain PCC 6803 / Kazus	98,51	10,00	1	2	2	5	1,015	5	8,0	160	17,4	6,04							
	A3	Sequence	# PSMs	# Proteins	# Protein Groups	Protein Group Accessions	Modifications	ΔCn	114/115	114/115 Count	114/115 Variability [%]	q-Value	PEP	IonScore	Exp Value	Charge	MH+ [Da]	ΔM [ppm]	RT [min]	# Missed Cleavages
	High	kPDLSDPDLR	3	1	1	P27589	N-Term(iTRAQ4plex); K1(iTRAQ4plex)	0,0000	1,015	3	0,2	0	0,000001187	43	4,54957E-05	3	1443,80509	0,33	22,11	0
	High	fQNPFR	2	1	1	P27589	N-Term(iTRAQ4plex)	0,0000	1,090	2	18,7	0,002	0,02829	29	0,001218905	2	952,51286	0,76	30,67	0
P73449	Nitrate transport protein NrtD OS=Synechocystis sp. (strain PCC 6803 / Kazusa) GN	98,13	11,45	1	4	4	5	1,149	5	56,5	332	36,5	5,01							
	A3	Sequence	# PSMs	# Proteins	# Protein Groups	Protein Group Accessions	Modifications	ΔCn	114/115	114/115 Count	114/115 Variability [%]	q-Value	PEP	IonScore	Exp Value	Charge	MH+ [Da]	ΔM [ppm]	RT [min]	# Missed Cleavages
	High	nYALDFLFHR	1	1	1	P73449	N-Term(iTRAQ4plex)	0,0000	1,149	1		0	0,000001909	45	3,25814E-05	3	1439,75589	0,46	45,60	0
	High	iGEILDIPFDRPR	2	1	1	P73449	N-Term(iTRAQ4plex)	0,0000	2,062	2	1,7	0	0,0001476	37	0,000187581	3	1684,95169	0,80	45,92	0
	High	kPGQISGGmk	1	1	1	P73449	N-Term(iTRAQ4plex); K1(iTRAQ4plex); M9(Oxidation); K10(iTRAQ4plex)	0,0000	0,806	1		0	0,001155	19	0,014027168	3	1450,84842	4,95	10,62	0
	Medium	vAIAR	1	1	1	P73449	N-Term(iTRAQ4plex)	0,0000	0,866	1		0,038	0,3962	20	0,026434195	2	673,44664	-1,65	14,52	0
P73918	Acetolactate synthase OS=Synechocystis sp. (strain PCC 6803 / Kazusa) GN=ilvB PI	97,56	9,09	1	4	4	7	0,868	6	17,9	550	60,1	5,41							
	A3	Sequence	# PSMs	# Proteins	# Protein Groups	Protein Group Accessions	Modifications	ΔCn	114/115	114/115 Count	114/115 Variability [%]	q-Value	PEP	IonScore	Exp Value	Charge	MH+ [Da]	ΔM [ppm]	RT [min]	# Missed Cleavages
	High	qIVRPSITPEVVR	3	1	1	P73918	N-Term(iTRAQ4plex)	0,0000	0,901	2	17,9	0	1,723E-08	39	0,000130008	3	1637,98111	-0,53	29,33	0
	High	gTmPYTHPLSLWTVGLQQR	1	1	1	P73918	N-Term(iTRAQ4plex); M3(Oxidation)	0,0000	1,029	1		0	0,000000178	37	0,000200433	3	2345,21665	-1,14	43,06	0
	High	qGkPTPYGASLR	2	1	1	P73918	N-Term(iTRAQ4plex); K3(iTRAQ4plex)	0,0000	0,791	2	17,1	0	0,003821	29	0,001241567	3	1562,89798	5,53	17,13	0
	Medium	vYAAFR	1	1	1	P73918	N-Term(iTRAQ4plex)	0,0000	0,849	1		0,042	0,4378	18	0,015522798	2	870,49639	1,10	23,18	0
P74178	Uncharacterized protein sll1178 OS=Synechocystis sp. (strain PCC 6803 / Kazusa) G	97,49	5,20	1	3	3	4	0,846	4	11,4	615	69,1	5,68							
	A3	Sequence	# PSMs	# Proteins	# Protein Groups	Protein Group Accessions	Modifications	ΔCn	114/115	114/115 Count	114/115 Variability [%]	q-Value	PEP	IonScore	Exp Value	Charge	MH+ [Da]	ΔM [ppm]	RT [min]	# Missed Cleavages
	High	lLETYLAFAPR	1	1	1	P74178	N-Term(iTRAQ4plex)	0,0000	0,779	1		0	2,211E-09	80	9,1405E-09	2	1437,82329	0,71	50,22	0
	High	iQTVHPETNPR	2	1	1	P74178	N-Term(iTRAQ4plex)	0,0000	1,030	2	18,8	0	0,0005539	23	0,005610092	3	1435,77939	1,36	15,99	0
	High	kFADLFGAPR	1	1	1	P74178	N-Term(iTRAQ4plex); K1(iTRAQ4plex)	0,0000	0,787	1		0	5,685E-07	21	0,0095793	3	1409,81147	-2,08	34,47	1
P72817	Universal stress protein Sll1654 OS=Synechocystis sp. (strain PCC 6803 / Kazusa) G	96,88	14,01	1	2	2	3	1,072	3	4,6	157	16,8	5,52							
	A3	Sequence	# PSMs	# Proteins	# Protein Groups	Protein Group Accessions	Modifications	ΔCn	114/115	114/115 Count	114/115 Variability [%]	q-Value	PEP	IonScore	Exp Value	Charge	MH+ [Da]	ΔM [ppm]	RT [min]	# Missed Cleavages
	High	iHQSQLILLSVVEk	1	1	1	P72817	N-Term(iTRAQ4plex); K14(iTRAQ4plex)	0,0000	1,187	1		0	7,629E-08	48	1,72175E-05	3	1895,15915	1,21	43,55	0
	High	tILFPLDR	2	1	1	P72817	N-Term(iTRAQ4plex)	0,0000	1,055	2	2,3	0	0,00432	47	1,94536E-05	2	1118,66960	0,49	41,57	0
Q46363	Acetazolamide conferring resistance protein zam OS=Synechocystis sp. (strain PCC	96,32	6,65	1	5	5	7	0,777	6	30,8	782	87,5	4,93							
	A3	Sequence	# PSMs	# Proteins	# Protein Groups	Protein Group Accessions	Modifications	ΔCn	114/115	114/115 Count	114/115 Variability [%]	q-Value	PEP	IonScore	Exp Value	Charge	MH+ [Da]	ΔM [ppm]	RT [min]	# Missed Cleavages
	High	aVLNHLLANTLk	3	1	1	Q46363	N-Term(iTRAQ4plex); K12(iTRAQ4plex)	0,0000	0,777	2	1,4	0	8,143E-09	45	3,1115E-05	3	1594,98795	-0,24	38,38	0
	High	tGVELNAHTcR	1	1	1	Q46363	N-Term(iTRAQ4plex); C10(Carbamidomethyl)	0,0000	1,036	1		0	0,00001402	43	4,5811E-05	3	1401,70044	-1,51	14,50	0
	High	vIEPGELkk	1	1	1	Q46363	N-Term(iTRAQ4plex); K8(iTRAQ4plex); K9(iTRAQ4plex)	0,0000	0,613	1		0	0,0003578	18	0,016291835	3	1444,90812	-1,28	23,22	1
	Medium	yPIAQHPPLGEVTk	1	1	1	Q46363	N-Term(iTRAQ4plex); K14(iTRAQ4plex)	0,0000	0,657	1		0,024	0,2572	12	0,062946271	3	1838,05606	7,93	29,30	0
	Medium	tWITAk	1	1	1	Q46363	N-Term(iTRAQ4plex); K6(iTRAQ4plex)	0,0000	2,375	1		0,027	0,288	12	0,082448213	2	1007,61669	3,85	21,68	0
P73173	PilJ protein OS=Synechocystis sp. (strain PCC 6803 / Kazusa) GN=pilJ PE=4 SV=1	96,15	6,09	1	6	6	7	0,595	7	25,4	953	103,1	4,61							
	A3	Sequence	# PSMs	# Proteins	# Protein Groups	Protein Group Accessions	Modifications	ΔCn	114/115	114/115 Count	114/115 Variability [%]	q-Value	PEP	IonScore	Exp Value	Charge	MH+ [Da]	ΔM [ppm]	RT [min]	# Missed Cleavages
	High	sQLVSQSLQSLAk	1	1	1	P73173	N-Term(iTRAQ4plex); K13(iTRAQ4plex)	0,0000	1,129	1		0	0,000003015	34	0,000384565	3	1676,97837	-0,11	36,36	0
	High	nLQTTAQk	2	1	1	P73173	N-Term(iTRAQ4plex); K8(iTRAQ4plex)	0,0000	0,497	2	1,6	0	0,006088	29	0,001644857	2	1191,69707	2,93	12,57	0
	High	fSTIVSkEELEk	1	1	1	P73173	N-Term(iTRAQ4plex); K7(iTRAQ4plex); K12(iTRAQ4plex)	0,0000	0,503	1		0	0,00005891	19	0,012704864	3	1842,05802	-0,26	32,51	1
	High	rLAESSLEISk	1	1	1	P73173	N-Term(iTRAQ4plex); K11(iTRAQ4plex)	0,0000	0,691	1		0	0,002837	19	0,013433504	3	1520,88626	-1,59	23,87	1
	High	vAEISkk	1	1	1	P73173	N-Term(iTRAQ4plex); K6(iTRAQ4plex); K7(iTRAQ4plex)	0,0000	0,595	1		0,003	0,03965	21	0,007277295	2	1206,77300	-4,32	14,22	1
	High	sNLTLQk	1	1	1	P73173	N-Term(iTRAQ4plex); K7(iTRAQ4plex)	0,0000	0,693	1		0,004	0,0505	24	0,004265501	2	1091,66765	1,24	18,34	0
P73574	3-oxoacyl-[acyl-carrier-protein] reductase 1 OS=Synechocystis sp. (strain PCC 6803	95,14	9,31	1	2	2	2	0,956	2	6,0	247	25,7	6,77							
	A3	Sequence	# PSMs	# Proteins	# Protein Groups	Protein Group Accessions	Modifications	ΔCn	114/115	114/115 Count	114/115 Variability [%]	q-Value	PEP	IonScore	Exp Value	Charge	MH+ [Da]	ΔM [ppm]	RT [min]	# Missed Cleavages
	High	tALTAQVALVTGASR	1	1	1	P73574	N-Term(iTRAQ4plex)	0,0000	0,919	1		0	9,538E-10	64	4,23614E-07	2	1602,93083	0,77	37,80	0
	High	aGVIGFTk	1	1	1	P73574	N-Term(iTRAQ4plex); K8(iTRAQ4plex)	0,0000	0,995	1		0	0,001286	44	3,60554E-05	2	1080,66960	3,72	27,33	0
P74667	Diaminopimelate epimerase OS=Synechocystis sp. (strain PCC 6803 / Kazusa) GN=	94,88	10,04	1	2	2	3	0,861	3	16,9	279	30,1	4,91							
	A3	Sequence	# PSMs	# Proteins	# Protein Groups	Protein Group Accessions	Modifications	ΔCn	114/115	114/115 Count	114/115 Variability [%]	q-Value	PEP	IonScore	Exp Value	Charge	MH+ [Da]	ΔM [ppm]	RT [min]	# Missed Cleavages
	High	iHTLAGVITPQLLADGQVk	1	1	1	P74667	N-Term(iTRAQ4plex); K19(iTRAQ4plex)	0,0000	0,687	1		0	1,752E-11	53	4,81915E-06	3	2262,34494	1,10	44,23	0
	High	lYmTGPAQR	2	1	1	P74667	N-Term(iTRAQ4plex); M3(Oxidation)	0,0000	0,911	2	8,4	0	0,000005686	37	0,000221804	2	1196,62395	2,09	17,03	0
Q55621	Amidophosphoribosyltransferase OS=Synechocystis sp. (strain PCC 6803 / Kazusa)	94,51	10,10	1	3	3	4	1,140	3	19,2	495	53,6	5,76							
	A3	Sequence	# PSMs	# Proteins	# Protein Groups	Protein Group Accessions	Modifications	ΔCn	114/115	114/115 Count	114/115 Variability [%]	q-Value	PEP	IonScore	Exp Value	Charge	MH+ [Da]	ΔM [ppm]	RT [min]	# Missed Cleavages
	High	tVEAGELVHITESGLVSHR	2	1	1	Q55621	N-Term(iTRAQ4plex)	0,0000	1,002	1		0	1,241E-12	50	9,01571E-06	4	2178,16445	0,39	38,96	0
	High	lTYFGLYALQHR	1	1	1	Q55621	N-Term(iTRAQ4plex)	0,0000	1,140	1		0	0,000000124	40	0,000103507	3	1625,89231	0,16	43,56	0
	High	dPHGIRPLVIGVLEEETPR	1	1	1	Q55621	N-Term(iTRAQ4plex)	0,0000	1,296	1		0	7,854E-08	25	0,003676145	4	2271,25869	0,37	42,22	0
Q55128	Aspartate aminotransferase OS=Synechocystis sp. (strain PCC 6803 / Kazusa) GN=	93,54	6,17	1	3	3	5	0,939	5	7,9	389	42,3	5,26							
	A3	Sequence	# PSMs	# Proteins	# Protein Groups	Protein Group Accessions	Modifications	ΔCn	114/115	114/115 Count	114/115 Variability [%]	q-Value	PEP	IonScore	Exp Value	Charge	MH+ [Da]	ΔM [ppm]	RT [min]	# Missed Cleavages
	High	yGPAAGEPALR	2	1	1	Q55128	N-Term(iTRAQ4plex)	0,0000	0,886	2	16,8	0	0,00001387	25	0,003125863	2	1245,67070	-0,12	24,14	0
	High	iTPEQLR	1	1	1	Q55128	N-Term(iTRAQ4plex)	0,0000	1,076	1		0	0,006617	24	0,003689521	2	1000,59203	1,23	22,40	0
	High	qAITSk	2	1	1	Q55128	N-Term(iTRAQ4plex); K6(iTRAQ4plex)	0,0000	0,921	2	3,0	0,005	0,06216	34	0,000428519	2	935,57372	-2,89	13,11	0
Q55782	Slr0209 protein OS=Synechocystis sp. (strain PCC 6803 / Kazusa) GN=slr0209 PE=	93,49	7,18	1	2	2	4	0,996	4	3,2	348	40,7	5,63							
	A3	Sequence	# PSMs	# Proteins	# Protein Groups	Protein Group Accessions	Modifications	ΔCn	114/115	114/115 Count	114/115 Variability [%]	q-Value	PEP	IonScore	Exp Value	Charge	MH+ [Da]	ΔM [ppm]	RT [min]	# Missed Cleavages
	High	nVNDLAILTHLVNk	2	1	1	Q55782	N-Term(iTRAQ4plex); K14(iTRAQ4plex)	0,0000	0,974	2	0,5	0	7,031E-12	43	5,2236E-05	3	1852,08963	0,07	45,84	0
	High	gGLIEGIIHSk	2	1	1	Q55782	N-Term(iTRAQ4plex); K11(iTRAQ4plex)	0,0000	1,136	2	16,8	0	0,00000418	39	0,000121891	3	1411,85016	-0,71	35,43	0
P73424	Slr1540 protein OS=Synechocystis sp. (strain PCC 6803 / Kazusa) GN=slr1540 PE=	93,30	23,79	1	7	7	8	0,751	8	13,2	311	35,1	6,61							
	A3	Sequence	# PSMs	# Proteins	# Protein Groups	Protein Group Accessions	Modifications	ΔCn	114/115	114/115 Count	114/115 Variability [%]	q-Value	PEP	IonScore	Exp Value	Charge	MH+ [Da]	ΔM [ppm]	RT [min]	# Missed Cleavages
	High	qQHFFADIQk	2	1	1	P73424	N-Term(iTRAQ4plex); K10(iTRAQ4plex)	0,0000	0,680	2	3734,2	0	6,105E-09	47	2,03222E-05	3	1549,83542	-0,74	26,43	0
	High	fIGIHLcR	1	1	1	P73424	N-Term(iTRAQ4plex); C7(Carbamidomethyl)	0,0000	0,734	1		0	0,003019	26	0,002432036	3	1159,65232	-0,32	31,81	0
	High	lVHYDPk	1	1	1	P73424	N-Term(iTRAQ4plex); K7(iTRAQ4plex)	0,0000	0,687	1		0	0,005774	20	0,011186593	3	1159,67035	-0,88	18,41	0
	High	iLImGGTR	1	1	1	P73424	N-Term(iTRAQ4plex); M4(Oxidation)	0,0000	0,732	1		0	0,005819	18	0,018339719	2	1020,60002	0,74	22,00	0
	High	ekLEkEEFDVIFDNNGR	1	1	1	P73424	N-Term(iTRAQ4plex); K2(iTRAQ4plex); K5(iTRAQ4plex)	0,0000	0,769	1		0	2,177E-08	17	0,020426281	4	2514,32143	0,52	36,49	2
	High	gNRPDPVNGVAQIHGDRR	1	1	1	P73424	N-Term(iTRAQ4plex)	0,0000	0,927	1		0,001	0,01754	14	0,03801894	4	2102,11123	1,30	17,24	1
	Medium	kAFPLR	1	1	1	P73424	N-Term(iTRAQ4plex); K1(iTRAQ4plex)	0,0000	0,821	1		0,01	0,1242	14	0,044562547	3	1019,65995	-0,48	18,42	1
P73290	Adenylosuccinate synthetase OS=Synechocystis sp. (strain PCC 6803 / Kazusa) GN	92,99	4,50	1	3	3	3	1,241	3	12,6	444	48,8	5,45							
	A3	Sequence	# PSMs	# Proteins	# Protein Groups	Protein Group Accessions	Modifications	ΔCn	114/115	114/115 Count	114/115 Variability [%]	q-Value	PEP	IonScore	Exp Value	Charge	MH+ [Da]	ΔM [ppm]	RT [min]	# Missed Cleavages
	High	lLWTVEYk	1	1	1	P73290	N-Term(iTRAQ4plex); K8(iTRAQ4plex)	0,0000	1,350	1		0	0,00006555	49	1,39306E-05	2	1339,78642	0,01	41,98	0
	High	lRPHVVDSSLk	1	1	1	P73290	N-Term(iTRAQ4plex); K11(iTRAQ4plex)	0,0000	1,033	1		0	0,000001914	40	0,000104225	3	1538,92655	0,53	19,70	0
	High	kLLWTVEYk	1	1	1	P73290	N-Term(iTRAQ4plex); K1(iTRAQ4plex); K9(iTRAQ4plex)	0,0000	1,241	1		0	0,000007566	31	0,000862919	3	1611,98380	0,22	34,94	1
P74145	Sll1390 protein OS=Synechocystis sp. (strain PCC 6803 / Kazusa) GN=sll1390 PE=4	92,25	16,87	1	4	4	5	0,925	5	6,3	249	27,4	4,74							
	A3	Sequence	# PSMs	# Proteins	# Protein Groups	Protein Group Accessions	Modifications	ΔCn	114/115	114/115 Count	114/115 Variability [%]	q-Value	PEP	IonScore	Exp Value	Charge	MH+ [Da]	ΔM [ppm]	RT [min]	# Missed Cleavages
	High	rGETAESLLTDEMVDSLLR	1	1	1	P74145	N-Term(iTRAQ4plex)	0,0000	1,139	1		0	2,205E-10	44	3,60554E-05	3	2279,17197	2,16	50,95	1
	High	yNQALIEADkR	1	1	1	P74145	N-Term(iTRAQ4plex); K10(iTRAQ4plex)	0,0000	0,925	1		0	1,815E-08	44	4,00839E-05	3	1608,89408	-0,46	22,84	1
	High	lNSDLkk	1	1	1	P74145	N-Term(iTRAQ4plex); K6(iTRAQ4plex); K7(iTRAQ4plex)	0,0000	0,965	1		0,009	0,106	12	0,056620018	3	1249,78022	-3,04	16,11	1
	Medium	fVVIR	2	1	1	P74145	N-Term(iTRAQ4plex)	0,0000	0,753	2	25,6	0,026	0,2831	24	0,007641379	2	777,51036	0,01	27,51	0
P73652	Sll1757 protein OS=Synechocystis sp. (strain PCC 6803 / Kazusa) GN=sll1757 PE=4	91,80	10,27	1	3	3	4	1,019	4	3,2	292	31,7	5,47							
	A3	Sequence	# PSMs	# Proteins	# Protein Groups	Protein Group Accessions	Modifications	ΔCn	114/115	114/115 Count	114/115 Variability [%]	q-Value	PEP	IonScore	Exp Value	Charge	MH+ [Da]	ΔM [ppm]	RT [min]	# Missed Cleavages
	High	qkVESQASNQALk	2	1	1	P73652	N-Term(iTRAQ4plex); K2(iTRAQ4plex); K13(iTRAQ4plex)	0,0000	1,132	2	15,2	0	2,953E-08	57	2,03704E-06	3	1863,06662	0,32	13,66	1
	High	emSkDPAALk	1	1	1	P73652	N-Term(iTRAQ4plex); M2(Oxidation); K4(iTRAQ4plex); K10(iTRAQ4plex)	0,0000	0,980	1		0,003	0,03756	12	0,063737157	3	1537,86325	0,79	15,05	1
	Medium	skmEEIk	1	1	1	P73652	N-Term(iTRAQ4plex); K2(iTRAQ4plex); M3(Oxidation); K7(iTRAQ4plex)	0,0000	1,016	1		0,019	0,2031	11	0,07761935	3	1312,74726	-2,61	9,93	1
P73732	Extracellular solute-binding protein OS=Synechocystis sp. (strain PCC 6803 / Kazus	91,54	5,33	1	2	2	4	1,503	4	21,2	582	63,6	5,19							
	A3	Sequence	# PSMs	# Proteins	# Protein Groups	Protein Group Accessions	Modifications	ΔCn	114/115	114/115 Count	114/115 Variability [%]	q-Value	PEP	IonScore	Exp Value	Charge	MH+ [Da]	ΔM [ppm]	RT [min]	# Missed Cleavages
	High	vTSFkPGDVVLYEVNPHYR	1	1	1	P73732	N-Term(iTRAQ4plex); K5(iTRAQ4plex)	0,0000	1,772	1		0	7,678E-10	39	0,000124851	4	2508,35293	1,57	40,04	0
	High	fPHPFLQDPLVR	3	1	1	P73732	N-Term(iTRAQ4plex)	0,0000	1,335	3	8,2	0	0,000001302	35	0,000315479	3	1609,89804	0,54	41,80	0
P72807	Sll1663 protein OS=Synechocystis sp. (strain PCC 6803 / Kazusa) GN=sll1663 PE=4	91,00	9,09	1	2	2	4	0,900	3	5,1	220	24,1	4,65							
	A3	Sequence	# PSMs	# Proteins	# Protein Groups	Protein Group Accessions	Modifications	ΔCn	114/115	114/115 Count	114/115 Variability [%]	q-Value	PEP	IonScore	Exp Value	Charge	MH+ [Da]	ΔM [ppm]	RT [min]	# Missed Cleavages
	High	lALAVGHLEHPDR	3	1	1	P72807	N-Term(iTRAQ4plex)	0,0000	0,885	2	2,6	0	6,205E-08	47	2,20785E-05	3	1571,87693	-0,37	29,38	0
	Medium	rGFDLLk	1	1	1	P72807	N-Term(iTRAQ4plex); K7(iTRAQ4plex)	0,0000	1,148	1		0,027	0,2954	14	0,037928879	3	1136,70197	-0,94	26,29	1
P74122	Arginine biosynthesis bifunctional protein ArgJ OS=Synechocystis sp. (strain PCC 68	90,74	5,57	1	2	2	4	1,355	3	31,9	413	43,3	5,47							
	A3	Sequence	# PSMs	# Proteins	# Protein Groups	Protein Group Accessions	Modifications	ΔCn	114/115	114/115 Count	114/115 Variability [%]	q-Value	PEP	IonScore	Exp Value	Charge	MH+ [Da]	ΔM [ppm]	RT [min]	# Missed Cleavages
	High	tIAGSSLVk	2	1	1	P74122	N-Term(iTRAQ4plex); K9(iTRAQ4plex)	0,0000	1,355	1		0	0,00002236	55	3,01974E-06	2	1163,72405	0,19	22,87	0
	High	tIALETEIDGRPVR	2	1	1	P74122	N-Term(iTRAQ4plex)	0,0000	1,334	2	34,3	0	0,00001144	30	0,001172685	3	1713,96018	-0,86	31,43	0
Q55155	General secretion pathway protein E OS=Synechocystis sp. (strain PCC 6803 / Kazu	90,43	4,76	1	4	4	4	0,628	4	17,9	672	75,1	5,60							
	A3	Sequence	# PSMs	# Proteins	# Protein Groups	Protein Group Accessions	Modifications	ΔCn	114/115	114/115 Count	114/115 Variability [%]	q-Value	PEP	IonScore	Exp Value	Charge	MH+ [Da]	ΔM [ppm]	RT [min]	# Missed Cleavages
	High	kITPAVVAR	1	1	1	Q55155	N-Term(iTRAQ4plex); K1(iTRAQ4plex)	0,0000	0,583	1		0	0,00003233	48	1,47901E-05	3	1242,81049	-2,55	17,77	1
	High	nYFSPFGNk	1	1	1	Q55155	N-Term(iTRAQ4plex); K9(iTRAQ4plex)	0,0000	0,937	1		0	0,00009579	33	0,000557147	2	1361,70757	-1,22	34,55	0
	Medium	qALVQVk	1	1	1	Q55155	N-Term(iTRAQ4plex); K7(iTRAQ4plex)	0,0000	0,676	1		0,011	0,1331	23	0,004581102	2	1073,69011	-1,88	19,39	0
	Medium	vNTLPSR	1	1	1	Q55155	N-Term(iTRAQ4plex)	0,0000	0,532	1		0,035	0,3725	25	0,002992058	2	930,54759	-1,43	15,89	0
P74569	Aspartokinase OS=Synechocystis sp. (strain PCC 6803 / Kazusa) GN=lysC PE=1 SV	89,68	8,17	1	5	5	8	0,731	8	30,2	600	63,5	5,38							
	A3	Sequence	# PSMs	# Proteins	# Protein Groups	Protein Group Accessions	Modifications	ΔCn	114/115	114/115 Count	114/115 Variability [%]	q-Value	PEP	IonScore	Exp Value	Charge	MH+ [Da]	ΔM [ppm]	RT [min]	# Missed Cleavages
	High	aAHSAFNLAGTk	2	1	1	P74569	N-Term(iTRAQ4plex); K12(iTRAQ4plex)	0,0000	0,969	2	30,1	0	0,000004223	22	0,006123081	3	1475,82154	0,41	21,06	0
	High	vVAPPVQNR	2	1	1	P74569	N-Term(iTRAQ4plex)	0,0000	0,819	2	31,0	0,001	0,01007	30	0,001018521	2	1123,67790	6,63	18,65	0
	High	nYGIPLVVR	2	1	1	P74569	N-Term(iTRAQ4plex)	0,0000	0,752	2	30,2	0,001	0,008951	26	0,002643561	2	1174,70659	0,08	36,76	0
	High	fGGTSVGTVER	1	1	1	P74569	N-Term(iTRAQ4plex)	0,0000	0,466	1		0,001	0,009613	20	0,010989299	2	1253,66118	0,40	22,92	0
	Medium	iLEIRPDR	1	1	1	P74569	N-Term(iTRAQ4plex)	0,0000	0,672	1		0,023	0,2472	11	0,071774472	3	1155,69779	0,96	24,64	0
P73283	3-oxoacyl-[acyl-carrier-protein] synthase 2 OS=Synechocystis sp. (strain PCC 6803	89,27	5,53	1	2	2	3	0,673	2	14,1	416	44,0	5,73							
	A3	Sequence	# PSMs	# Proteins	# Protein Groups	Protein Group Accessions	Modifications	ΔCn	114/115	114/115 Count	114/115 Variability [%]	q-Value	PEP	IonScore	Exp Value	Charge	MH+ [Da]	ΔM [ppm]	RT [min]	# Missed Cleavages
	High	qALGNHAYNIAVSSTk	2	1	1	P73283	N-Term(iTRAQ4plex); K16(iTRAQ4plex)	0,0000	0,612	1		0	6,787E-10	56	2,618E-06	3	1962,06461	-0,07	25,84	0
	High	aIAWALk	1	1	1	P73283	N-Term(iTRAQ4plex); K7(iTRAQ4plex)	0,0000	0,739	1		0	0,0004861	43	6,53403E-05	2	1060,67497	-0,74	34,92	0
Q55625	Ribosome-binding factor A OS=Synechocystis sp. (strain PCC 6803 / Kazusa) GN=rl	89,05	18,80	1	2	2	4	1,232	4	29,2	133	14,7	5,91							
	A3	Sequence	# PSMs	# Proteins	# Protein Groups	Protein Group Accessions	Modifications	ΔCn	114/115	114/115 Count	114/115 Variability [%]	q-Value	PEP	IonScore	Exp Value	Charge	MH+ [Da]	ΔM [ppm]	RT [min]	# Missed Cleavages
	High	rTPEVSFLEDR	3	1	1	Q55625	N-Term(iTRAQ4plex)	0,0000	1,421	3	14,9	0	4,432E-07	51	8,41337E-06	3	1492,78690	-0,53	28,51	1
	High	eVSQmLLHEIkDDR	1	1	1	Q55625	N-Term(iTRAQ4plex); M5(Oxidation); K11(iTRAQ4plex)	0,0000	1,067	1		0	0,000001234	27	0,002892902	4	2017,06496	1,11	32,20	1
P73267	Sll1080 protein OS=Synechocystis sp. (strain PCC 6803 / Kazusa) GN=sll1080 PE=4	88,38	16,19	1	4	4	4	1,343	4	31,4	352	38,2	4,56							
	A3	Sequence	# PSMs	# Proteins	# Protein Groups	Protein Group Accessions	Modifications	ΔCn	114/115	114/115 Count	114/115 Variability [%]	q-Value	PEP	IonScore	Exp Value	Charge	MH+ [Da]	ΔM [ppm]	RT [min]	# Missed Cleavages
	High	lIQEkPEQVQALVNTWFDIR	1	1	1	P73267	N-Term(iTRAQ4plex); K5(iTRAQ4plex)	0,0000	1,470	1		0	1,08E-12	65	2,8312E-07	3	2715,51230	1,84	55,89	0
	High	rAGVSQEELQLFk	1	1	1	P73267	N-Term(iTRAQ4plex); K13(iTRAQ4plex)	0,0000	0,971	1		0	7,739E-07	27	0,001900947	3	1793,01725	0,69	32,81	1
	High	iLDAQFVk	1	1	1	P73267	N-Term(iTRAQ4plex); K8(iTRAQ4plex)	0,0000	1,228	1		0	0,0002544	22	0,006729302	2	1221,74492	0,29	33,92	0
	High	kFmEENPEkSDEImAk	1	1	1	P73267	N-Term(iTRAQ4plex); K1(iTRAQ4plex); M3(Oxidation); K9(iTRAQ4plex); M14	0,0000	1,881	1		0	0,0002548	12	0,073400301	4	2534,30117	1,83	21,07	2
Q59992	Tryptophan synthase beta chain OS=Synechocystis sp. (strain PCC 6803 / Kazusa) (	88,05	13,35	1	5	5	6	0,794	5	12,7	412	45,0	6,71							
	A3	Sequence	# PSMs	# Proteins	# Protein Groups	Protein Group Accessions	Modifications	ΔCn	114/115	114/115 Count	114/115 Variability [%]	q-Value	PEP	IonScore	Exp Value	Charge	MH+ [Da]	ΔM [ppm]	RT [min]	# Missed Cleavages
	High	iIAETGAGQHGVATATVcAR	2	1	1	Q59992	N-Term(iTRAQ4plex); C18(Carbamidomethyl)	0,0000	0,794	1		0	2,826E-11	56	2,69755E-06	3	2127,11057	0,36	25,67	0
	High	iNNALGQVLLAk	1	1	1	Q59992	N-Term(iTRAQ4plex); K12(iTRAQ4plex)	0,0000	0,864	1		0	0,00003683	32	0,00061372	3	1541,96207	0,18	42,18	0
	High	eDLNHTGAHk	1	1	1	Q59992	N-Term(iTRAQ4plex); K10(iTRAQ4plex)	0,0000	0,623	1		0,002	0,02182	18	0,014453399	4	1409,73745	-0,09	9,11	0
	High	iVINcSGR	1	1	1	Q59992	N-Term(iTRAQ4plex); C5(Carbamidomethyl)	0,0000	1,204	1		0,005	0,07063	16	0,024715534	2	1062,58525	0,55	20,39	0
	Medium	lNVFR	1	1	1	Q59992	N-Term(iTRAQ4plex)	0,0000	0,782	1		0,036	0,382	16	0,0332033	2	792,48442	-0,56	26,24	0
Q55573	Slr0184 protein OS=Synechocystis sp. (strain PCC 6803 / Kazusa) GN=slr0184 PE=	87,74	9,71	1	2	2	2	0,712	2	3,0	206	23,7	5,15							
	A3	Sequence	# PSMs	# Proteins	# Protein Groups	Protein Group Accessions	Modifications	ΔCn	114/115	114/115 Count	114/115 Variability [%]	q-Value	PEP	IonScore	Exp Value	Charge	MH+ [Da]	ΔM [ppm]	RT [min]	# Missed Cleavages
	High	vLVQILRPVS	1	1	1	Q55573	N-Term(iTRAQ4plex)	0,0000	0,698	1		0	0,00002607	51	7,60274E-06	2	1267,82292	0,83	42,48	0
	High	vPADLEHLLR	1	1	1	Q55573	N-Term(iTRAQ4plex)	0,0000	0,727	1		0	0,00004194	50	1,104E-05	3	1306,76163	1,24	39,87	0
Q55374	Slr0907 protein OS=Synechocystis sp. (strain PCC 6803 / Kazusa) GN=slr0907 PE=	87,66	4,14	1	3	3	3	0,723	3	30,0	1014	115,3	5,25							
	A3	Sequence	# PSMs	# Proteins	# Protein Groups	Protein Group Accessions	Modifications	ΔCn	114/115	114/115 Count	114/115 Variability [%]	q-Value	PEP	IonScore	Exp Value	Charge	MH+ [Da]	ΔM [ppm]	RT [min]	# Missed Cleavages
	High	fWILPAVVPHNLIk	1	1	1	Q55374	N-Term(iTRAQ4plex); K14(iTRAQ4plex)	0,0000	0,593	1		0	1,956E-08	44	4,385E-05	3	1935,18271	0,21	52,19	0
	High	kQVFTNITQIFADHEVSR	1	1	1	Q55374	N-Term(iTRAQ4plex); K1(iTRAQ4plex)	0,0000	0,882	1		0	4,985E-09	42	6,41165E-05	4	2421,30971	-1,33	41,41	1
	High	fILIDPLINR	1	1	1	Q55374	N-Term(iTRAQ4plex)	0,0000	0,723	1		0,001	0,008963	28	0,001520443	2	1357,83245	0,00	51,17	0
Q55187	Phenylalanine--tRNA ligase alpha subunit OS=Synechocystis sp. (strain PCC 6803 /	87,34	13,29	1	5	5	7	0,779	6	24,5	331	37,1	6,15							
	A3	Sequence	# PSMs	# Proteins	# Protein Groups	Protein Group Accessions	Modifications	ΔCn	114/115	114/115 Count	114/115 Variability [%]	q-Value	PEP	IonScore	Exp Value	Charge	MH+ [Da]	ΔM [ppm]	RT [min]	# Missed Cleavages
	High	kGELSLILk	1	1	1	Q55187	N-Term(iTRAQ4plex); K1(iTRAQ4plex); K9(iTRAQ4plex)	0,0000	1,103	1		0	0,0000093	36	0,000278593	3	1432,94272	-2,53	32,07	1
	High	gLAFTHLk	2	1	1	Q55187	N-Term(iTRAQ4plex); K8(iTRAQ4plex)	0,0000	0,595	1		0	0,001972	23	0,00508381	3	1174,71778	-0,77	26,95	0
	High	fAmVLHQIDDIRR	1	1	1	Q55187	N-Term(iTRAQ4plex); M3(Oxidation)	0,0000	0,918	1		0	0,000001379	22	0,006236918	3	1773,95469	-0,24	35,92	1
	High	lSAEERPk	2	1	1	Q55187	N-Term(iTRAQ4plex); K8(iTRAQ4plex)	0,0000	0,709	2	9,9	0,001	0,0117	24	0,004567703	3	1217,71515	4,85	12,46	0
	High	vQYLGk	1	1	1	Q55187	N-Term(iTRAQ4plex); K6(iTRAQ4plex)	0,0000	0,802	1		0,002	0,02919	22	0,006151344	2	995,61174	-1,06	20,15	0
P74250	Hydroperoxy fatty acid reductase gpx1 OS=Synechocystis sp. (strain PCC 6803 / Ka	86,89	8,88	1	2	2	5	0,415	5	14,7	169	18,4	7,09							
	A3	Sequence	# PSMs	# Proteins	# Protein Groups	Protein Group Accessions	Modifications	ΔCn	114/115	114/115 Count	114/115 Variability [%]	q-Value	PEP	IonScore	Exp Value	Charge	MH+ [Da]	ΔM [ppm]	RT [min]	# Missed Cleavages
	High	yGVTFPLFEk	4	1	1	P74250	N-Term(iTRAQ4plex); K10(iTRAQ4plex)	0,0000	0,515	4	33,3	0	0,000005323	53	6,68623E-06	2	1488,83171	-1,61	47,42	0
	Medium	wNFTk	1	1	1	P74250	N-Term(iTRAQ4plex); K5(iTRAQ4plex)	0,0000	0,376	1		0,032	0,3438	19	0,01216102	2	983,55199	-3,35	27,02	0
P74079	Slr1342 protein OS=Synechocystis sp. (strain PCC 6803 / Kazusa) GN=slr1342 PE=	86,51	11,69	1	3	3	4	0,801	4	13,5	445	48,5	5,30							
	A3	Sequence	# PSMs	# Proteins	# Protein Groups	Protein Group Accessions	Modifications	ΔCn	114/115	114/115 Count	114/115 Variability [%]	q-Value	PEP	IonScore	Exp Value	Charge	MH+ [Da]	ΔM [ppm]	RT [min]	# Missed Cleavages
	High	qGLRDESPIFFILSNTR	2	1	1	P74079	N-Term(iTRAQ4plex)	0,0000	0,918	2	38,3	0	1,308E-09	43	4,78597E-05	3	2137,15335	0,49	50,55	1
	High	eLGPFDAHFLVPAFFEGGR	1	1	1	P74079	N-Term(iTRAQ4plex)	0,0000	0,857	1		0	2,899E-07	31	0,000805323	3	2250,14902	1,11	55,66	0
	High	lALATEAVDHYLVVSR	1	1	1	P74079	N-Term(iTRAQ4plex)	0,0000	0,748	1		0	0,000001093	26	0,002553814	3	1901,06119	-0,08	46,99	0
Q55542	Slr0334 protein OS=Synechocystis sp. (strain PCC 6803 / Kazusa) GN=slr0334 PE=	86,00	13,64	1	3	3	5	18,900	4	19,0	110	11,6	7,85							
	A3	Sequence	# PSMs	# Proteins	# Protein Groups	Protein Group Accessions	Modifications	ΔCn	114/115	114/115 Count	114/115 Variability [%]	q-Value	PEP	IonScore	Exp Value	Charge	MH+ [Da]	ΔM [ppm]	RT [min]	# Missed Cleavages
	High	kPAYYEAk	2	1	1	Q55542	N-Term(iTRAQ4plex); K1(iTRAQ4plex); K8(iTRAQ4plex)	0,0000	23,530	2	39,1	0	0,00008589	46	2,44889E-05	2	1401,82182	8,28	15,48	0
	High	kAQLIVk	1	1	1	Q55542	N-Term(iTRAQ4plex); K1(iTRAQ4plex); K7(iTRAQ4plex)	0,0000	19,582	1		0	0,0003118	19	0,013930606	3	1231,85639	8,25	17,55	1
	High	aQLIVk	2	1	1	Q55542	N-Term(iTRAQ4plex); K6(iTRAQ4plex)	0,0000	15,188	1		0,002	0,02818	13	0,055843162	2	959,65642	7,53	21,79	0
P73804	Sll1961 protein OS=Synechocystis sp. (strain PCC 6803 / Kazusa) GN=sll1961 PE=4	85,94	3,61	1	2	2	3	0,889	3	6,5	388	42,9	5,95							
	A3	Sequence	# PSMs	# Proteins	# Protein Groups	Protein Group Accessions	Modifications	ΔCn	114/115	114/115 Count	114/115 Variability [%]	q-Value	PEP	IonScore	Exp Value	Charge	MH+ [Da]	ΔM [ppm]	RT [min]	# Missed Cleavages
	High	vLVTVPQR	2	1	1	P73804	N-Term(iTRAQ4plex)	0,0000	0,820	2	18,6	0	0,003925	39	0,000115072	2	1055,66850	-0,83	26,81	0
	High	sIAILk	1	1	1	P73804	N-Term(iTRAQ4plex); K6(iTRAQ4plex)	0,0000	0,889	1		0,001	0,01464	41	7,12804E-05	2	932,63811	-0,20	26,37	0
P74694	Slr0455 protein OS=Synechocystis sp. (strain PCC 6803 / Kazusa) GN=slr0455 PE=	85,67	19,15	1	3	3	4	1,094	4	21,5	141	15,8	5,10							
	A3	Sequence	# PSMs	# Proteins	# Protein Groups	Protein Group Accessions	Modifications	ΔCn	114/115	114/115 Count	114/115 Variability [%]	q-Value	PEP	IonScore	Exp Value	Charge	MH+ [Da]	ΔM [ppm]	RT [min]	# Missed Cleavages
	High	gEmNAEEARR	1	1	1	P74694	N-Term(iTRAQ4plex); M3(Oxidation)	0,0000	1,373	1		0	0,0001171	39	0,000130909	3	1322,62470	0,53	7,86	1
	High	rYVDELIR	2	1	1	P74694	N-Term(iTRAQ4plex)	0,0000	1,094	2	8,8	0	0,001134	27	0,002162569	3	1207,69031	-1,05	26,88	1
	High	iImLRDLVQk	1	1	1	P74694	N-Term(iTRAQ4plex); M3(Oxidation); K10(iTRAQ4plex)	0,0000	0,330	1		0,002	0,03127	41	8,22186E-05	2	1532,95159	5,14	23,79	1
P73067	1-deoxy-D-xylulose-5-phosphate synthase OS=Synechocystis sp. (strain PCC 6803 /	84,76	4,22	1	2	2	4	0,944	3	6,1	640	69,3	6,28							
	A3	Sequence	# PSMs	# Proteins	# Protein Groups	Protein Group Accessions	Modifications	ΔCn	114/115	114/115 Count	114/115 Variability [%]	q-Value	PEP	IonScore	Exp Value	Charge	MH+ [Da]	ΔM [ppm]	RT [min]	# Missed Cleavages
	High	fVkPLDTELILPLAER	3	1	1	P73067	N-Term(iTRAQ4plex); K3(iTRAQ4plex)	0,0000	0,944	3	6,1	0	3,173E-08	38	0,000173768	3	2142,28031	1,20	48,52	0
	High	vFAHTLTTLAk	1	1	1	P73067	N-Term(iTRAQ4plex); K11(iTRAQ4plex)	0,0000				0	0,007771	11	0,07128038	3	1489,89804	-0,04	32,75	0
P38382	Protein translocase subunit SecE OS=Synechocystis sp. (strain PCC 6803 / Kazusa)	84,74	23,46	1	2	2	4	1,516	4	17,6	81	9,1	5,31							
	A3	Sequence	# PSMs	# Proteins	# Protein Groups	Protein Group Accessions	Modifications	ΔCn	114/115	114/115 Count	114/115 Variability [%]	q-Value	PEP	IonScore	Exp Value	Charge	MH+ [Da]	ΔM [ppm]	RT [min]	# Missed Cleavages
	High	sNFIAATkDELAk	3	1	1	P38382	N-Term(iTRAQ4plex); K8(iTRAQ4plex); K13(iTRAQ4plex)	0,0000	1,634	3	12,8	0	0,000006919	37	0,000184489	3	1840,05631	1,21	33,80	1
	Medium	vVWPSR	1	1	1	P38382	N-Term(iTRAQ4plex)	0,0000	1,075	1		0,02	0,2242	18	0,014553586	2	887,52678	5,40	25,91	0
P72647	Sll1053 protein OS=Synechocystis sp. (strain PCC 6803 / Kazusa) GN=sll1053 PE=4	84,68	5,58	1	2	2	4	0,648	4	55,6	520	56,2	4,97							

	A3	Sequence	# PSMs	# Proteins	# Protein Groups	Protein Group Accessions	Modifications	ΔCn	114/115	114/115 Count	114/115 Variability [%]	q-Value	PEP	IonScore	Exp Value	Charge	MH+ [Da]	ΔM [ppm]	RT [min]	# Missed Cleavages
	High	IRPGLVVEILDGQEk	2	1	1	P72647	N-Term(iTRAQ4plex); K15(iTRAQ4plex)	0,0000	0,816	2	141,4	0	2,219E-10	43	5,48239E-05	3	1954,15921	0,82	44,44	0
	High	IQAGERPETIAQAR	2	1	1	P72647	N-Term(iTRAQ4plex)	0,0000	0,648	2	34,0	0	0,00001886	35	0,000289714	3	1683,92868	1,64	21,91	0
P73016	Enoyl-[acyl-carrier-protein] reductase [NADH] FabI OS=Synechocystis sp. (strain PC	84,39	9,30	1	2	2	3	1,100	3	8,9	258	27,6	6,43							
	A3	Sequence	# PSMs	# Proteins	# Protein Groups	Protein Group Accessions	Modifications	ΔCn	114/115	114/115 Count	114/115 Variability [%]	q-Value	PEP	IonScore	Exp Value	Charge	MH+ [Da]	ΔM [ppm]	RT [min]	# Missed Cleavages
	High	IDILIHcLAFADk	2	1	1	P73016	N-Term(iTRAQ4plex); C7(Carbamidomethyl); K13(iTRAQ4plex)	0,0000	0,969	2	19,0	0	0,000002067	45	3,32637E-05	3	1817,02494	0,84	51,19	0
	High	hAFVTGIANNR	1	1	1	P73016	N-Term(iTRAQ4plex)	0,0000	1,168	1		0	0,0000036	32	0,000639691	3	1343,73310	2,23	18,74	0
P72747	Slr1103 protein OS=Synechocystis sp. (strain PCC 6803 / Kazusa) GN=slr1103 PE=	84,02	2,69	1	2	2	3	0,743	3	0,1	706	79,8	5,39							
	A3	Sequence	# PSMs	# Proteins	# Protein Groups	Protein Group Accessions	Modifications	ΔCn	114/115	114/115 Count	114/115 Variability [%]	q-Value	PEP	IonScore	Exp Value	Charge	MH+ [Da]	ΔM [ppm]	RT [min]	# Missed Cleavages
	High	ikPTSQNEGYSYR	2	1	1	P72747	N-Term(iTRAQ4plex); K2(iTRAQ4plex)	0,0000	0,743	2	0,0	0	5,08E-09	44	3,59724E-05	3	1830,95963	0,42	18,73	0
	High	aNLFFR	1	1	1	P72747	N-Term(iTRAQ4plex)	0,0000	0,544	1		0,005	0,0699	31	0,000812774	2	911,52208	0,11	32,10	0
P74423	Alanine--tRNA ligase OS=Synechocystis sp. (strain PCC 6803 / Kazusa) GN=alaS PE	83,84	3,08	1	2	2	3	0,445	2	66,0	877	96,1	5,27							
	A3	Sequence	# PSMs	# Proteins	# Protein Groups	Protein Group Accessions	Modifications	ΔCn	114/115	114/115 Count	114/115 Variability [%]	q-Value	PEP	IonScore	Exp Value	Charge	MH+ [Da]	ΔM [ppm]	RT [min]	# Missed Cleavages
	High	rIEAVAGPSVLDYLNVR	1	1	1	P74423	N-Term(iTRAQ4plex)	0,0000	0,296	1		0	1,576E-11	61	7,63836E-07	3	2016,13785	0,96	44,92	1
	High	eSGIFIHFGR	2	1	1	P74423	N-Term(iTRAQ4plex)	0,0000	0,667	1		0	0,0008094	32	0,000603907	3	1306,70066	-1,40	34,67	0
Q55404	Acetyl-coenzyme A synthetase OS=Synechocystis sp. (strain PCC 6803 / Kazusa) GI	83,79	3,06	1	2	2	3	1,148	3	14,0	653	73,0	5,76							
	A3	Sequence	# PSMs	# Proteins	# Protein Groups	Protein Group Accessions	Modifications	ΔCn	114/115	114/115 Count	114/115 Variability [%]	q-Value	PEP	IonScore	Exp Value	Charge	MH+ [Da]	ΔM [ppm]	RT [min]	# Missed Cleavages
	High	yGVNIFYTAPTAIR	1	1	1	Q55404	N-Term(iTRAQ4plex)	0,0000	1,148	1		0	4,417E-09	52	6,96578E-06	2	1729,94011	0,40	44,67	0
	High	wIFDLk	2	1	1	Q55404	N-Term(iTRAQ4plex); K6(iTRAQ4plex)	0,0000	1,206	2	21,4	0	0,004135	30	0,000903587	2	1109,66045	0,61	45,59	0
P74100	Slr1962 protein OS=Synechocystis sp. (strain PCC 6803 / Kazusa) GN=slr1962 PE=	83,09	5,14	1	2	2	3	1,490	3	14,4	370	40,3	6,28							
	A3	Sequence	# PSMs	# Proteins	# Protein Groups	Protein Group Accessions	Modifications	ΔCn	114/115	114/115 Count	114/115 Variability [%]	q-Value	PEP	IonScore	Exp Value	Charge	MH+ [Da]	ΔM [ppm]	RT [min]	# Missed Cleavages
	High	kPVESLADLR	2	1	1	P74100	N-Term(iTRAQ4plex); K1(iTRAQ4plex)	0,0000	1,564	2	7,2	0	0,000004481	54	3,94457E-06	3	1415,84562	-0,33	26,71	0
	High	sANLLMLSR	1	1	1	P74100	N-Term(iTRAQ4plex)	0,0000	0,907	1		0,004	0,04966	16	0,025059362	2	1148,65959	1,52	32,86	0
P74094	Slr1958 protein OS=Synechocystis sp. (strain PCC 6803 / Kazusa) GN=slr1958 PE=	82,25	14,50	1	4	4	7	1,153	7	3,8	200	23,0	5,06							
	A3	Sequence	# PSMs	# Proteins	# Protein Groups	Protein Group Accessions	Modifications	ΔCn	114/115	114/115 Count	114/115 Variability [%]	q-Value	PEP	IonScore	Exp Value	Charge	MH+ [Da]	ΔM [ppm]	RT [min]	# Missed Cleavages
	High	iILETLNR	1	1	1	P74094	N-Term(iTRAQ4plex)	0,0000	1,153	1		0	0,00004674	36	0,000278593	2	1115,69219	1,50	35,08	0
	High	mVTFFFIR	2	1	1	P74094	N-Term(iTRAQ4plex); M1(Oxidation)	0,0000	1,224	2	5,1	0	0,0001287	35	0,000301974	2	1220,66277	0,74	47,41	0
	High	qLEAYQk	1	1	1	P74094	N-Term(iTRAQ4plex); K7(iTRAQ4plex)	0,0000	1,156	1		0,002	0,02711	28	0,002394021	2	1167,66484	3,11	17,64	0
	High	mVTFFFIR	2	1	1	P74094	N-Term(iTRAQ4plex)	0,0000	1,108	2	2,8	0,002	0,02093	17	0,017822557	2	1204,66838	1,19	51,35	0
	Medium	sPYGLk	1	1	1	P74094	N-Term(iTRAQ4plex); K6(iTRAQ4plex)	0,0000	0,866	1		0,048	0,496	14	0,046364037	2	952,57598	5,65	19,20	0
P74618	6-phosphogluconolactonase OS=Synechocystis sp. (strain PCC 6803 / Kazusa) GN=	81,29	10,83	1	3	3	4	0,867	4	9,1	240	26,5	5,27							
	A3	Sequence	# PSMs	# Proteins	# Protein Groups	Protein Group Accessions	Modifications	ΔCn	114/115	114/115 Count	114/115 Variability [%]	q-Value	PEP	IonScore	Exp Value	Charge	MH+ [Da]	ΔM [ppm]	RT [min]	# Missed Cleavages
	High	lITVGNk	1	1	1	P74618	N-Term(iTRAQ4plex); K7(iTRAQ4plex)	0,0000	1,165	1		0	0,001663	39	0,000207622	2	1032,67192	6,14	24,24	0
	High	lTFTIPLINR	2	1	1	P74618	N-Term(iTRAQ4plex)	0,0000	0,867	2	6,6	0	0,00001705	30	0,001091365	2	1331,81719	0,30	50,75	0
	High	iHVFWGDER	1	1	1	P74618	N-Term(iTRAQ4plex)	0,0000	0,802	1		0	0,00477	25	0,003515362	3	1302,67060	-0,45	32,54	0
P74771	NAD(P)H-quinone oxidoreductase subunit O OS=Synechocystis sp. (strain PCC 6803	81,03	29,17	1	3	3	6	0,435	6	30,4	72	8,3	7,25							
	A3	Sequence	# PSMs	# Proteins	# Protein Groups	Protein Group Accessions	Modifications	ΔCn	114/115	114/115 Count	114/115 Variability [%]	q-Value	PEP	IonScore	Exp Value	Charge	MH+ [Da]	ΔM [ppm]	RT [min]	# Missed Cleavages
	High	fYVPTPNVWLR	2	1	1	P74771	N-Term(iTRAQ4plex)	0,0000	0,396	2	18,4	0	0,005237	36	0,000248296	2	1535,84905	-0,06	50,42	0
	High	lPDYLFHSk	2	1	1	P74771	N-Term(iTRAQ4plex); K9(iTRAQ4plex)	0,0000	0,596	2	18,1	0	0,000005195	28	0,002019918	3	1407,78788	0,28	36,39	0
	High	rLPDYLFHSk	2	1	1	P74771	N-Term(iTRAQ4plex); K10(iTRAQ4plex)	0,0000	0,386	2	13,1	0	0,00006507	23	0,006716268	4	1563,88735	-0,80	29,22	1
P74463	Twitching motility protein OS=Synechocystis sp. (strain PCC 6803 / Kazusa) GN=pil	80,65	10,03	1	3	3	6	0,969	6	16,6	369	40,6	7,03							
	A3	Sequence	# PSMs	# Proteins	# Protein Groups	Protein Group Accessions	Modifications	ΔCn	114/115	114/115 Count	114/115 Variability [%]	q-Value	PEP	IonScore	Exp Value	Charge	MH+ [Da]	ΔM [ppm]	RT [min]	# Missed Cleavages
	High	lIFSmLNNSQR	1	1	1	P74463	N-Term(iTRAQ4plex); M5(Oxidation)	0,0000	0,790	1		0	0,00001458	50	1,09388E-05	2	1482,78655	0,66	35,46	0
	High	amLSNSLLAVFAQNLVk	2	1	1	P74463	N-Term(iTRAQ4plex); M2(Oxidation); K17(iTRAQ4plex)	0,0000	1,082	2	0,2	0	0,00585	25	0,003162059	3	2123,22013	3,00	58,26	0
	High	sGkPDELQR	3	1	1	P74463	N-Term(iTRAQ4plex); K3(iTRAQ4plex)	0,0000	0,900	3	10,0	0	0,007474	20	0,009549926	3	1317,73468	-1,40	12,40	0
Q55198	Phosphate transport system permease protein PstC OS=Synechocystis sp. (strain P	80,23	5,05	1	2	2	4	0,375	4	8,4	317	33,8	9,61							
	A3	Sequence	# PSMs	# Proteins	# Protein Groups	Protein Group Accessions	Modifications	ΔCn	114/115	114/115 Count	114/115 Variability [%]	q-Value	PEP	IonScore	Exp Value	Charge	MH+ [Da]	ΔM [ppm]	RT [min]	# Missed Cleavages
	High	qASLGLGATR	1	1	1	Q55198	N-Term(iTRAQ4plex)	0,0000	0,366	1		0	0,0005177	51	8,83019E-06	2	1117,64433	-0,27	19,89	0
	High	wETIFR	3	1	1	Q55198	N-Term(iTRAQ4plex)	0,0000	0,385	3	9,3	0,001	0,01611	32	0,000639691	2	995,54143	-1,69	37,98	0
Q55898	Polyphosphate kinase OS=Synechocystis sp. (strain PCC 6803 / Kazusa) GN=ppk PE	80,09	8,24	1	5	5	5	0,806	5	4,2	728	82,6	5,35							
	A3	Sequence	# PSMs	# Proteins	# Protein Groups	Protein Group Accessions	Modifications	ΔCn	114/115	114/115 Count	114/115 Variability [%]	q-Value	PEP	IonScore	Exp Value	Charge	MH+ [Da]	ΔM [ppm]	RT [min]	# Missed Cleavages
	High	eLSWLAFNQR	1	1	1	Q55898	N-Term(iTRAQ4plex)	0,0000	0,375	1		0	0,000001044	53	4,9885E-06	2	1407,75139	0,87	43,67	0
	High	vLHEGLDDRTPLLER	1	1	1	Q55898	N-Term(iTRAQ4plex)	0,0000	1,291	1		0	0,001797	25	0,00427689	4	1907,04213	-2,43	30,68	1
	High	yVHIGTGNYNPk	1	1	1	Q55898	N-Term(iTRAQ4plex); K12(iTRAQ4plex)	0,0000	0,806	1		0	0,00002729	21	0,008071793	3	1650,88272	-0,91	24,73	0
	High	rVEAVVPVEQPDLk	1	1	1	Q55898	N-Term(iTRAQ4plex); K14(iTRAQ4plex)	0,0000	0,810	1		0	0,00000663	16	0,027301675	3	1867,08872	-0,26	29,58	1
	Medium	lLVAPVNmR	1	1	1	Q55898	N-Term(iTRAQ4plex); M8(Oxidation)	0,0000	0,783	1		0,011	0,1283	19	0,014027168	2	1172,69109	-2,69	28,51	0
P19125	NAD(P)H-quinone oxidoreductase subunit J OS=Synechocystis sp. (strain PCC 6803	80,06	23,46	1	4	4	6	0,642	5	8,0	179	20,6	4,53							
	A3	Sequence	# PSMs	# Proteins	# Protein Groups	Protein Group Accessions	Modifications	ΔCn	114/115	114/115 Count	114/115 Variability [%]	q-Value	PEP	IonScore	Exp Value	Charge	MH+ [Da]	ΔM [ppm]	RT [min]	# Missed Cleavages
	High	iLmPEDWVGWPLRk	1	1	1	P19125	N-Term(iTRAQ4plex); M3(Oxidation); K14(iTRAQ4plex)	0,0000	0,624	1		0	5,391E-07	41	8,8914E-05	3	2044,13120	0,93	48,68	1
	High	eNPVVPSVYWIWk	1	1	1	P19125	N-Term(iTRAQ4plex); K13(iTRAQ4plex)	0,0000	1,560	1		0	0,00004917	35	0,000328829	3	1905,05252	0,63	51,64	0
	High	sLVSFYHLVk	2	1	1	P19125	N-Term(iTRAQ4plex); K10(iTRAQ4plex)	0,0000	0,608	1		0	0,000001019	29	0,001279293	3	1480,87616	-0,31	37,93	0
	Medium	vFLPR	2	1	1	P19125	N-Term(iTRAQ4plex)	0,0000	0,664	2	5,1	0,012	0,1373	11	0,073277392	2	775,49443	-0,35	25,64	0
P72895	Slr1617 protein OS=Synechocystis sp. (strain PCC 6803 / Kazusa) GN=slr1617 PE=	79,57	4,38	1	2	2	4	0,597	4	27,2	411	45,4	8,15							
	A3	Sequence	# PSMs	# Proteins	# Protein Groups	Protein Group Accessions	Modifications	ΔCn	114/115	114/115 Count	114/115 Variability [%]	q-Value	PEP	IonScore	Exp Value	Charge	MH+ [Da]	ΔM [ppm]	RT [min]	# Missed Cleavages
	High	fIFNFFk	2	1	1	P72895	N-Term(iTRAQ4plex); K7(iTRAQ4plex)	0,0000	0,530	2	35,8	0	0,00007426	33	0,000464483	2	1250,71868	0,85	52,02	0
	High	yLAEILIDHcR	2	1	1	P72895	N-Term(iTRAQ4plex); C10(Carbamidomethyl)	0,0000	0,637	2	27,2	0	0,00001194	31	0,000812774	3	1546,81583	-0,66	42,60	0
P72762	NADH-glutamate synthase small subunit OS=Synechocystis sp. (strain PCC 6803 / K	79,48	6,68	1	3	3	3	0,840	3	2,4	494	54,1	5,52							
	A3	Sequence	# PSMs	# Proteins	# Protein Groups	Protein Group Accessions	Modifications	ΔCn	114/115	114/115 Count	114/115 Variability [%]	q-Value	PEP	IonScore	Exp Value	Charge	MH+ [Da]	ΔM [ppm]	RT [min]	# Missed Cleavages
	High	tPGPNFISAk	1	1	1	P72762	N-Term(iTRAQ4plex); K10(iTRAQ4plex)	0,0000	1,262	1		0	0,00004677	52	7,62397E-06	2	1319,75432	-1,42	27,74	0
	High	gQSLVVWAFNEGR	1	1	1	P72762	N-Term(iTRAQ4plex)	0,0000	0,826	1		0	0,00002432	28	0,001733684	2	1606,84795	1,29	45,79	0
	High	aGHWVTVYER	1	1	1	P72762	N-Term(iTRAQ4plex)	0,0000	0,840	1		0	0,0001401	26	0,002264488	3	1361,70884	0,40	24,87	0
P73930	Signal recognition particle receptor FtsY OS=Synechocystis sp. (strain PCC 6803 / K	79,32	3,37	1	2	2	3	1,002	3	1,4	504	53,8	4,46							
	A3	Sequence	# PSMs	# Proteins	# Protein Groups	Protein Group Accessions	Modifications	ΔCn	114/115	114/115 Count	114/115 Variability [%]	q-Value	PEP	IonScore	Exp Value	Charge	MH+ [Da]	ΔM [ppm]	RT [min]	# Missed Cleavages
	High	vNLVNQLk	1	1	1	P73930	N-Term(iTRAQ4plex); K8(iTRAQ4plex)	0,0000	1,002	1		0	0,00003246	42	5,96994E-05	2	1215,76543	-0,76	30,90	0
	High	aAYQNIQAR	2	1	1	P73930	N-Term(iTRAQ4plex)	0,0000	0,958	2	8,1	0	0,0000868	39	0,00011885	2	1178,64067	0,69	16,56	0
P74756	47 kD protein OS=Synechocystis sp. (strain PCC 6803 / Kazusa) GN=slr0609 PE=4	79,32	12,79	1	3	3	4	0,975	4	2,4	383	43,2	5,91							
	A3	Sequence	# PSMs	# Proteins	# Protein Groups	Protein Group Accessions	Modifications	ΔCn	114/115	114/115 Count	114/115 Variability [%]	q-Value	PEP	IonScore	Exp Value	Charge	MH+ [Da]	ΔM [ppm]	RT [min]	# Missed Cleavages
	High	kFQYFLDNQLTPNIFR	1	1	1	P74756	N-Term(iTRAQ4plex); K1(iTRAQ4plex)	0,0000	0,966	1		0	2,28E-10	45	3,12586E-05	3	2332,27164	1,02	49,37	1
	High	qGLPVTIITGFLGSGk	1	1	1	P74756	N-Term(iTRAQ4plex); K16(iTRAQ4plex)	0,0000	0,983	1		0	0,000003753	29	0,001230184	3	1876,11545	0,41	56,16	0
	High	sNQLVLIGQDLDHDkIR	2	1	1	P74756	N-Term(iTRAQ4plex); K15(iTRAQ4plex)	0,0000	0,966	2	4,8	0	4,561E-07	22	0,005929253	4	2252,26357	1,51	37,39	1
P74175	HlyD family of secretion proteins OS=Synechocystis sp. (strain PCC 6803 / Kazusa)	79,27	5,15	1	3	3	4	1,016	4	0,6	583	64,7	5,45							
	A3	Sequence	# PSMs	# Proteins	# Protein Groups	Protein Group Accessions	Modifications	ΔCn	114/115	114/115 Count	114/115 Variability [%]	q-Value	PEP	IonScore	Exp Value	Charge	MH+ [Da]	ΔM [ppm]	RT [min]	# Missed Cleavages
	High	iLAGLLGR	2	1	1	P74175	N-Term(iTRAQ4plex)	0,0000	1,071	2	7,9	0	0,002267	43	5,24771E-05	2	956,63786	0,52	42,99	0
	High	kIESLETVR	1	1	1	P74175	N-Term(iTRAQ4plex); K1(iTRAQ4plex)	0,0000	1,016	1		0	0,00000963	28	0,001492794	3	1362,82157	1,50	21,87	1
	High	ikLDSQELVSHGR	1	1	1	P74175	N-Term(iTRAQ4plex); K2(iTRAQ4plex)	0,0000	1,008	1		0	0,000001284	26	0,002741385	3	1770,00785	-1,93	25,90	1
P74571	Oligopeptidase A OS=Synechocystis sp. (strain PCC 6803 / Kazusa) GN=prlC PE=3	79,07	4,21	1	2	2	3	1,511	3	18,3	713	80,2	5,11							
	A3	Sequence	# PSMs	# Proteins	# Protein Groups	Protein Group Accessions	Modifications	ΔCn	114/115	114/115 Count	114/115 Variability [%]	q-Value	PEP	IonScore	Exp Value	Charge	MH+ [Da]	ΔM [ppm]	RT [min]	# Missed Cleavages
	High	qINFSLLDLELHHR	2	1	1	P74571	N-Term(iTRAQ4plex)	0,0000	1,033	2	61,1	0	0,000001776	52	6,88605E-06	4	1879,02968	-0,54	41,89	0
	High	hGFEQVQPLVVGFISR	1	1	1	P74571	N-Term(iTRAQ4plex)	0,0000	1,708	1		0	0,000001473	30	0,001248343	3	1957,07755	-0,06	45,99	0
Q55746	Acyl-[acyl-carrier-protein]--UDP-N-acetylglucosamine O-acyltransferase OS=Synech	78,47	8,70	1	2	2	2	0,978	2	49,5	276	30,0	7,99							
	A3	Sequence	# PSMs	# Proteins	# Protein Groups	Protein Group Accessions	Modifications	ΔCn	114/115	114/115 Count	114/115 Variability [%]	q-Value	PEP	IonScore	Exp Value	Charge	MH+ [Da]	ΔM [ppm]	RT [min]	# Missed Cleavages
	High	iSGVLGVHQFVHIGR	1	1	1	Q55746	N-Term(iTRAQ4plex)	0,0000	1,341	1		0	4,116E-10	52	5,7544E-06	4	1763,02187	1,20	36,16	0
	High	sLNLIGLQR	1	1	1	Q55746	N-Term(iTRAQ4plex)	0,0000	0,713	1		0	0,0007858	39	0,000123302	2	1157,71208	-0,20	35,32	0
P73222	Slr2005 protein OS=Synechocystis sp. (strain PCC 6803 / Kazusa) GN=slr2005 PE=	78,30	6,13	1	2	2	2	0,927	2	27,5	261	28,0	9,64							
	A3	Sequence	# PSMs	# Proteins	# Protein Groups	Protein Group Accessions	Modifications	ΔCn	114/115	114/115 Count	114/115 Variability [%]	q-Value	PEP	IonScore	Exp Value	Charge	MH+ [Da]	ΔM [ppm]	RT [min]	# Missed Cleavages
	High	aAmAILNPR	1	1	1	P73222	N-Term(iTRAQ4plex); M3(Oxidation)	0,0000	0,773	1		0	0,000005241	50	1,104E-05	2	1116,63298	1,20	21,11	0
	High	iLANPFR	1	1	1	P73222	N-Term(iTRAQ4plex)	0,0000	1,112	1		0,001	0,01102	42	6,68298E-05	2	974,59136	0,98	34,15	0
P27319	Flavodoxin OS=Synechocystis sp. (strain PCC 6803 / Kazusa) GN=isiB PE=1 SV=2 -	77,61	11,18	1	2	2	3	0,416	3	53,5	170	18,8	4,30							
	A3	Sequence	# PSMs	# Proteins	# Protein Groups	Protein Group Accessions	Modifications	ΔCn	114/115	114/115 Count	114/115 Variability [%]	q-Value	PEP	IonScore	Exp Value	Charge	MH+ [Da]	ΔM [ppm]	RT [min]	# Missed Cleavages
	High	tWVSEIkPILQS	2	1	1	P27319	N-Term(iTRAQ4plex); K7(iTRAQ4plex)	0,0000	0,404	2	58,8	0	0,000001758	56	2,90523E-06	2	1688,98223	-0,21	46,10	0
	High	iSGLGGk	1	1	1	P27319	N-Term(iTRAQ4plex); K7(iTRAQ4plex)	0,0000	0,416	1		0	0,003265	35	0,000326565	2	919,58129	-0,25	19,74	0
P72683	Geranylgeranyl pyrophosphate synthase OS=Synechocystis sp. (strain PCC 6803 / K	77,33	7,62	1	2	2	3	1,335	3	42,7	302	32,4	5,11							
	A3	Sequence	# PSMs	# Proteins	# Protein Groups	Protein Group Accessions	Modifications	ΔCn	114/115	114/115 Count	114/115 Variability [%]	q-Value	PEP	IonScore	Exp Value	Charge	MH+ [Da]	ΔM [ppm]	RT [min]	# Missed Cleavages
	High	ySLLAGGk	1	1	1	P72683	N-Term(iTRAQ4plex); K8(iTRAQ4plex)	0,0000	1,335	1		0	0,00001081	55	3,14753E-06	2	1096,66033	-0,15	26,28	0
	High	tDITPETLTFIHTHk	2	1	1	P72683	N-Term(iTRAQ4plex); K15(iTRAQ4plex)	0,0000	1,421	2	53,6	0	0,000003327	31	0,000726056	4	2042,11513	-0,48	34,51	0
P74643	Phosphoglucomutase OS=Synechocystis sp. (strain PCC 6803 / Kazusa) GN=pgm P	77,28	4,76	1	2	2	2	0,620	2	20,8	567	61,1	6,11							
	A3	Sequence	# PSMs	# Proteins	# Protein Groups	Protein Group Accessions	Modifications	ΔCn	114/115	114/115 Count	114/115 Variability [%]	q-Value	PEP	IonScore	Exp Value	Charge	MH+ [Da]	ΔM [ppm]	RT [min]	# Missed Cleavages
	High	fTPTPAVSYAILTHNQGR	1	1	1	P74643	N-Term(iTRAQ4plex)	0,0000	0,539	1		0	2,96E-13	60	1,06407E-06	3	2117,12674	0,32	39,06	0
	High	rLDYEQALk	1	1	1	P74643	N-Term(iTRAQ4plex); K9(iTRAQ4plex)	0,0000	0,712	1		0	0,00001975	31	0,00087694	3	1423,81510	0,23	24,18	1
P74296	Phenylalanine--tRNA ligase beta subunit OS=Synechocystis sp. (strain PCC 6803 / K	77,10	6,05	1	6	6	7	0,725	6	12,7	810	87,8	4,97							
	A3	Sequence	# PSMs	# Proteins	# Protein Groups	Protein Group Accessions	Modifications	ΔCn	114/115	114/115 Count	114/115 Variability [%]	q-Value	PEP	IonScore	Exp Value	Charge	MH+ [Da]	ΔM [ppm]	RT [min]	# Missed Cleavages
	High	gEAIINLR	1	1	1	P74296	N-Term(iTRAQ4plex)	0,0000				0	0,0008954	39	0,000137395	2	1029,61772	0,36	28,12	0
	High	lQQLLGk	1	1	1	P74296	N-Term(iTRAQ4plex); K7(iTRAQ4plex)	0,0000	0,709	1		0	0,001344	21	0,007128038	2	1087,70647	-1,20	33,19	0
	High	gQSVPAGQR	2	1	1	P74296	N-Term(iTRAQ4plex)	0,0000	0,741	2	19,3	0	0,003525	19	0,012704864	2	1043,57048	-0,94	9,08	0
	High	gLLEAIFQR	1	1	1	P74296	N-Term(iTRAQ4plex)	0,0000	0,742	1		0	0,00005433	18	0,016710906	2	1190,70183	0,34	50,95	0
	High	fGQLHPQLR	1	1	1	P74296	N-Term(iTRAQ4plex)	0,0000	0,970	1		0	0,00103	16	0,026666745	3	1239,70676	-0,91	25,04	0
	Medium	qFAVSLR	1	1	1	P74296	N-Term(iTRAQ4plex)	0,0000	0,680	1		0,015	0,1672	25	0,002937447	2	964,57121	1,61	26,86	0
P72814	Slr1800 protein OS=Synechocystis sp. (strain PCC 6803 / Kazusa) GN=slr1800 PE=	76,40	4,51	1	2	2	4	1,224	3	2,3	355	39,4	4,84							
	A3	Sequence	# PSMs	# Proteins	# Protein Groups	Protein Group Accessions	Modifications	ΔCn	114/115	114/115 Count	114/115 Variability [%]	q-Value	PEP	IonScore	Exp Value	Charge	MH+ [Da]	ΔM [ppm]	RT [min]	# Missed Cleavages
	High	gTGFLNWLk	3	1	1	P72814	N-Term(iTRAQ4plex); K9(iTRAQ4plex)	0,0000	1,234	2	1,2	0	3,941E-07	50	1,22941E-05	2	1323,76714	0,58	46,52	0
	High	aFVETAk	1	1	1	P72814	N-Term(iTRAQ4plex); K7(iTRAQ4plex)	0,0000	0,589	1		0,002	0,02514	26	0,002602512	2	1053,61223	-5,76	17,79	0
P72749	GTP-binding protein TypA/BipA homolog OS=Synechocystis sp. (strain PCC 6803 / K	76,28	5,86	1	4	4	4	0,868	4	1,9	597	66,0	5,10							
	A3	Sequence	# PSMs	# Proteins	# Protein Groups	Protein Group Accessions	Modifications	ΔCn	114/115	114/115 Count	114/115 Variability [%]	q-Value	PEP	IonScore	Exp Value	Charge	MH+ [Da]	ΔM [ppm]	RT [min]	# Missed Cleavages
	High	tQLEFVIPAR	1	1	1	P72749	N-Term(iTRAQ4plex)	0,0000	0,856	1		0	0,000004648	36	0,000339581	2	1317,76543	0,51	38,17	0
	High	lLGFEGLNR	1	1	1	P72749	N-Term(iTRAQ4plex)	0,0000	0,957	1		0	0,0004879	34	0,000544617	2	1162,67009	-0,03	38,88	0
	High	gLRPLVVVNk	1	1	1	P72749	N-Term(iTRAQ4plex); K10(iTRAQ4plex)	0,0000	0,857	1		0	0,0001504	21	0,007277295	3	1382,90805	-0,41	26,83	0
	High	gLLGFR	1	1	1	P72749	N-Term(iTRAQ4plex)	0,0000	0,879	1		0,002	0,01866	28	0,00303217	2	806,50121	0,85	31,44	0
P74155	Membrane protein insertase YidC OS=Synechocystis sp. (strain PCC 6803 / Kazusa)	76,21	5,21	1	2	2	3	1,285	2	1,8	384	42,6	6,62							
	A3	Sequence	# PSMs	# Proteins	# Protein Groups	Protein Group Accessions	Modifications	ΔCn	114/115	114/115 Count	114/115 Variability [%]	q-Value	PEP	IonScore	Exp Value	Charge	MH+ [Da]	ΔM [ppm]	RT [min]	# Missed Cleavages
	High	nSQILTPTYSVTk	2	1	1	P74155	N-Term(iTRAQ4plex); K13(iTRAQ4plex)	0,0000	1,270	1		0	5,054E-09	57	2,08158E-06	2	1739,97966	0,84	30,46	0
	High	tGFLFIk	1	1	1	P74155	N-Term(iTRAQ4plex); K7(iTRAQ4plex)	0,0000	1,301	1		0,002	0,02622	32	0,000602518	2	1113,69182	0,68	39,46	0
P72802	Mitochondrial outer membrane 72K protein OS=Synechocystis sp. (strain PCC 6803	75,95	10,15	1	3	3	4	1,436	3	16,4	266	29,9	5,30							
	A3	Sequence	# PSMs	# Proteins	# Protein Groups	Protein Group Accessions	Modifications	ΔCn	114/115	114/115 Count	114/115 Variability [%]	q-Value	PEP	IonScore	Exp Value	Charge	MH+ [Da]	ΔM [ppm]	RT [min]	# Missed Cleavages
	High	aEAILTQIR	2	1	1	P72802	N-Term(iTRAQ4plex)	0,0000	1,436	1		0	0,000007826	45	3,64244E-05	2	1158,69695	0,53	33,15	0
	High	iNAQHANAYYNR	1	1	1	P72802	N-Term(iTRAQ4plex)	0,0000	1,286	1		0	8,035E-07	43	4,85255E-05	3	1578,79026	0,56	17,99	0
	High	kDmAQk	1	1	1	P72802	N-Term(iTRAQ4plex); K1(iTRAQ4plex); M3(Oxidation); K6(iTRAQ4plex)	0,0000	2,137	1		0,009	0,1052	13	0,052477121	3	1168,67328	1,07	7,75	1
P73503	Slr1437 protein OS=Synechocystis sp. (strain PCC 6803 / Kazusa) GN=slr1437 PE=	74,88	16,96	1	2	2	2	1,374	2	13,0	112	12,7	5,22							
	A3	Sequence	# PSMs	# Proteins	# Protein Groups	Protein Group Accessions	Modifications	ΔCn	114/115	114/115 Count	114/115 Variability [%]	q-Value	PEP	IonScore	Exp Value	Charge	MH+ [Da]	ΔM [ppm]	RT [min]	# Missed Cleavages
	High	cAYVAIGEFkPGR	1	1	1	P73503	N-Term(iTRAQ4plex); C1(Carbamidomethyl); K10(iTRAQ4plex)	0,0000	1,499	1		0	4,419E-11	67	2,14274E-07	3	1755,94846	1,69	32,02	0
	High	aISTYk	1	1	1	P73503	N-Term(iTRAQ4plex); K6(iTRAQ4plex)	0,0000	1,259	1		0,005	0,05966	21	0,007567806	2	970,58238	1,26	17,14	0
P74511	Thylakoid membrane protein slr1949 OS=Synechocystis sp. (strain PCC 6803 / Kazu	74,80	12,74	1	3	3	5	0,895	5	23,3	212	23,9	9,13							
	A3	Sequence	# PSMs	# Proteins	# Protein Groups	Protein Group Accessions	Modifications	ΔCn	114/115	114/115 Count	114/115 Variability [%]	q-Value	PEP	IonScore	Exp Value	Charge	MH+ [Da]	ΔM [ppm]	RT [min]	# Missed Cleavages
	High	nLLFWHEVAR	3	1	1	P74511	N-Term(iTRAQ4plex)	0,0000	0,777	3	12,3	0	0,00009736	40	0,0001104	2	1428,78874	1,30	38,46	0
	High	sLGSAFk	1	1	1	P74511	N-Term(iTRAQ4plex); K7(iTRAQ4plex)	0,0000	2,291	1		0,006	0,0784	18	0,017138389	2	997,59068	-1,40	22,13	0
	Medium	tLIEQTPNRR	1	1	1	P74511	N-Term(iTRAQ4plex)	0,0000	1,045	1		0,025	0,2642	11	0,085895419	3	1371,78293	0,30	15,96	1
P73632	D-alanine--D-alanine ligase OS=Synechocystis sp. (strain PCC 6803 / Kazusa) GN=d	74,68	9,32	1	2	2	2	0,828	2	14,0	354	38,7	5,05							
	A3	Sequence	# PSMs	# Proteins	# Protein Groups	Protein Group Accessions	Modifications	ΔCn	114/115	114/115 Count	114/115 Variability [%]	q-Value	PEP	IonScore	Exp Value	Charge	MH+ [Da]	ΔM [ppm]	RT [min]	# Missed Cleavages
	High	nRSELEAALDNAASYDRR	1	1	1	P73632	N-Term(iTRAQ4plex)	0,0000	0,754	1		0	0,00000113	72	6,60648E-08	4	2195,09560	1,54	34,71	2
	High	lLQLALDYHGRPGHQ	1	1	1	P73632	N-Term(iTRAQ4plex)	0,0000	0,910	1		0	7,735E-07	16	0,027001798	4	1862,01809	1,45	35,45	0
P73130	Sll0995 protein OS=Synechocystis sp. (strain PCC 6803 / Kazusa) GN=sll0995 PE=4	74,51	11,69	1	2	2	3	0,776	3	8,9	231	26,2	4,73							
	A3	Sequence	# PSMs	# Proteins	# Protein Groups	Protein Group Accessions	Modifications	ΔCn	114/115	114/115 Count	114/115 Variability [%]	q-Value	PEP	IonScore	Exp Value	Charge	MH+ [Da]	ΔM [ppm]	RT [min]	# Missed Cleavages
	High	vAPDVmVALGRPk	1	1	1	P73130	N-Term(iTRAQ4plex); M6(Oxidation); K13(iTRAQ4plex)	0,0000	0,776	1		0	0,000001071	43	4,73119E-05	3	1656,97062	-0,23	28,77	0
	High	lRAELAEAENDRLk	1	1	1	P73130	N-Term(iTRAQ4plex); K14(iTRAQ4plex)	0,0000	0,565	1		0	0,001697	29	0,001161368	4	1916,07937	-0,55	28,23	2
	High	vAPDVMVALGRPk	1	1	1	P73130	N-Term(iTRAQ4plex); K13(iTRAQ4plex)	0,0000	0,824	1		0	0,00006487	28	0,00160683	3	1640,97617	0,05	34,40	0
Q55629	Uncharacterized protein slr0782 OS=Synechocystis sp. (strain PCC 6803 / Kazusa) (	73,74	9,98	1	4	4	4	0,731	4	40,0	471	51,4	5,33							
	A3	Sequence	# PSMs	# Proteins	# Protein Groups	Protein Group Accessions	Modifications	ΔCn	114/115	114/115 Count	114/115 Variability [%]	q-Value	PEP	IonScore	Exp Value	Charge	MH+ [Da]	ΔM [ppm]	RT [min]	# Missed Cleavages
	High	fAIVATPPHLAGR	1	1	1	Q55629	N-Term(iTRAQ4plex)	0,0000	0,613	1		0	0,00002705	43	4,69862E-05	3	1493,86969	-0,85	34,05	0
	High	iHWAGTEIAPR	1	1	1	Q55629	N-Term(iTRAQ4plex)	0,0000	0,872	1		0	0,00001177	38	0,000237207	3	1394,76496	-0,86	28,84	0
	High	lLISYDRPFWR	1	1	1	Q55629	N-Term(iTRAQ4plex)	0,0000	0,313	1		0	0,00006185	20	0,010591806	3	1609,89548	-1,05	43,17	0
	Medium	yGkWIAMGEAER	1	1	1	Q55629	N-Term(iTRAQ4plex); K3(iTRAQ4plex)	0,0000	1,031	1		0,012	0,1415	10	0,096154586	3	1698,88767	0,01	40,77	1
Q55162	Sll0051 protein OS=Synechocystis sp. (strain PCC 6803 / Kazusa) GN=sll0051 PE=4	73,72	11,32	1	2	2	3	0,909	3	11,4	159	17,8	4,54							
	A3	Sequence	# PSMs	# Proteins	# Protein Groups	Protein Group Accessions	Modifications	ΔCn	114/115	114/115 Count	114/115 Variability [%]	q-Value	PEP	IonScore	Exp Value	Charge	MH+ [Da]	ΔM [ppm]	RT [min]	# Missed Cleavages
	High	tGGPFGVVIVk	2	1	1	Q55162	N-Term(iTRAQ4plex); K11(iTRAQ4plex)	0,0000	0,830	2	25,2	0	0,00001121	43	4,57057E-05	2	1361,83855	-0,73	37,99	0
	Medium	qAIAIMR	1	1	1	Q55162	N-Term(iTRAQ4plex)	0,0000	0,909	1		0,027	0,2965	25	0,003459155	2	946,56120	-1,35	23,06	0
P74737	Protein RecA OS=Synechocystis sp. (strain PCC 6803 / Kazusa) GN=recA PE=3 SV=	73,33	6,21	1	2	2	2	0,835	2	3,8	354	37,8	5,22							
	A3	Sequence	# PSMs	# Proteins	# Protein Groups	Protein Group Accessions	Modifications	ΔCn	114/115	114/115 Count	114/115 Variability [%]	q-Value	PEP	IonScore	Exp Value	Charge	MH+ [Da]	ΔM [ppm]	RT [min]	# Missed Cleavages
	High	sGcLVIFLNQLR	1	1	1	P74737	N-Term(iTRAQ4plex); C3(Carbamidomethyl)	0,0000	0,857	1		0	0,00004891	47	2,74101E-05	2	1563,88201	1,41	51,33	0
	High	kGTEGEYGIR	1	1	1	P74737	N-Term(iTRAQ4plex); K1(iTRAQ4plex)	0,0000	0,815	1		0	0,000006875	40	9,46172E-05	3	1397,76230	-0,31	14,36	1
Q55552	IMP dehydrogenase OS=Synechocystis sp. (strain PCC 6803 / Kazusa) GN=guaB PE	73,21	12,26	1	2	2	3	1,442	3	9,1	155	17,3	5,34							
	A3	Sequence	# PSMs	# Proteins	# Protein Groups	Protein Group Accessions	Modifications	ΔCn	114/115	114/115 Count	114/115 Variability [%]	q-Value	PEP	IonScore	Exp Value	Charge	MH+ [Da]	ΔM [ppm]	RT [min]	# Missed Cleavages
	High	rLPVLNVESR	2	1	1	Q55552	N-Term(iTRAQ4plex)	0,0000	1,508	2	6,7	0	1,303E-07	48	1,61053E-05	3	1326,79895	1,13	26,54	1
	High	eAAHLmNEk	1	1	1	Q55552	N-Term(iTRAQ4plex); M6(Oxidation); K9(iTRAQ4plex)	0,0000	1,357	1		0	0,0001564	17	0,020747702	3	1346,69822	0,38	11,52	0
P74309	Fructose-bisphosphate aldolase class 1 OS=Synechocystis sp. (strain PCC 6803 / Ka	73,04	5,00	1	2	2	2	1,204	2	8,5	300	33,1	5,83							
	A3	Sequence	# PSMs	# Proteins	# Protein Groups	Protein Group Accessions	Modifications	ΔCn	114/115	114/115 Count	114/115 Variability [%]	q-Value	PEP	IonScore	Exp Value	Charge	MH+ [Da]	ΔM [ppm]	RT [min]	# Missed Cleavages
	High	aQAEALLk	1	1	1	P74309	N-Term(iTRAQ4plex); K8(iTRAQ4plex)	0,0000	1,275	1		0	0,0001365	57	2,76906E-06	2	1131,69878	1,03	22,54	0
	High	qIVPILk	1	1	1	P74309	N-Term(iTRAQ4plex); K7(iTRAQ4plex)	0,0000	1,137	1		0	0,006434	30	0,001114218	2	1098,74858	-0,30	32,92	0
P28463	Glutamyl-tRNA reductase OS=Synechocystis sp. (strain PCC 6803 / Kazusa) GN=he	72,26	4,92	1	2	2	3	0,684	2	12,5	427	47,5	6,76							
	A3	Sequence	# PSMs	# Proteins	# Protein Groups	Protein Group Accessions	Modifications	ΔCn	114/115	114/115 Count	114/115 Variability [%]	q-Value	PEP	IonScore	Exp Value	Charge	MH+ [Da]	ΔM [ppm]	RT [min]	# Missed Cleavages
	High	lEEALTHLR	2	1	1	P28463	N-Term(iTRAQ4plex)	0,0000	0,629	1		0	0,00001246	49	1,15337E-05	3	1225,70084	-1,06	33,25	0
	High	yLFTLLHEDAVR	1	1	1	P28463	N-Term(iTRAQ4plex)	0,0000	0,744	1		0	3,456E-07	36	0,000302629	3	1620,88712	0,29	46,11	0
P72584	Magnesium-protoporphyrin IX monomethyl ester [oxidative] cyclase 1 OS=Synecho	72,24	14,53	1	4	4	5	0,834	5	8,0	358	42,1	7,12							
	A3	Sequence	# PSMs	# Proteins	# Protein Groups	Protein Group Accessions	Modifications	ΔCn	114/115	114/115 Count	114/115 Variability [%]	q-Value	PEP	IonScore	Exp Value	Charge	MH+ [Da]	ΔM [ppm]	RT [min]	# Missed Cleavages
	High	kLPIFASNGWQFIk	1	1	1	P72584	N-Term(iTRAQ4plex); K1(iTRAQ4plex); K14(iTRAQ4plex)	0,0000	0,791	1		0	1,336E-09	44	3,58897E-05	3	2081,22434	-1,39	46,65	1
	High	lYLmkPIAVDQLAGAVR	2	1	1	P72584	N-Term(iTRAQ4plex); M4(Oxidation); K5(iTRAQ4plex)	0,0000	0,850	2	9,1	0	0,00001597	26	0,002354887	3	2162,26169	0,30	43,69	1
	High	fIFYATYLSEk	1	1	1	P72584	N-Term(iTRAQ4plex); K11(iTRAQ4plex)	0,0000	1,126	1		0	0,0001723	21	0,008689004	3	1669,90757	-0,25	48,57	0
	High	aQPHTLNDWk	1	1	1	P72584	N-Term(iTRAQ4plex); K10(iTRAQ4plex)	0,0000	0,834	1		0	0,0001145	17	0,021487669	3	1497,80765	1,59	20,33	0
Q55505	Chaperone protein DnaJ 1 OS=Synechocystis sp. (strain PCC 6803 / Kazusa) GN=d	72,08	5,84	1	2	2	3	1,078	2	20,0	377	40,8	7,34							
	A3	Sequence	# PSMs	# Proteins	# Protein Groups	Protein Group Accessions	Modifications	ΔCn	114/115	114/115 Count	114/115 Variability [%]	q-Value	PEP	IonScore	Exp Value	Charge	MH+ [Da]	ΔM [ppm]	RT [min]	# Missed Cleavages
	High	gGLEGFLGGLFHk	2	1	1	Q55505	N-Term(iTRAQ4plex); K13(iTRAQ4plex)	0,0000	0,943	1		0	2,25E-08	47	2,06725E-05	3	1619,91391	-0,58	51,11	0
	High	dADkDEIkR	1	1	1	Q55505	N-Term(iTRAQ4plex); K4(iTRAQ4plex); K8(iTRAQ4plex)	0,0000	1,232	1		0	0,00002435	23	0,005023079	3	1521,86002	0,19	10,49	2
P73960	3-isopropylmalate dehydrogenase OS=Synechocystis sp. (strain PCC 6803 / Kazusa	71,83	10,77	1	3	3	4	0,872	4	21,0	362	38,6	4,84							
	A3	Sequence	# PSMs	# Proteins	# Protein Groups	Protein Group Accessions	Modifications	ΔCn	114/115	114/115 Count	114/115 Variability [%]	q-Value	PEP	IonScore	Exp Value	Charge	MH+ [Da]	ΔM [ppm]	RT [min]	# Missed Cleavages
	High	eLTGGIYFGkPk	2	1	1	P73960	N-Term(iTRAQ4plex); K10(iTRAQ4plex); K12(iTRAQ4plex)	0,0000	0,758	2	4,9	0	3,533E-08	39	0,00013365	3	1742,01999	-0,76	31,20	0
	High	sQRPETGLLAIR	1	1	1	P73960	N-Term(iTRAQ4plex)	0,0000	1,169	1		0	0,00009514	25	0,003250648	3	1484,86594	-0,45	26,53	0
	High	aNPLAQVLSAAmmLR	1	1	1	P73960	N-Term(iTRAQ4plex); M12(Oxidation); M13(Oxidation)	0,0000	0,971	1		0,004	0,05158	20	0,009267658	3	1761,94297	-2,45	56,59	0
P72644	Phosphoribosylformylglycinamidine synthase 2 OS=Synechocystis sp. (strain PCC 68	71,75	4,30	1	3	3	4	0,979	4	24,2	768	81,8	4,83							
	A3	Sequence	# PSMs	# Proteins	# Protein Groups	Protein Group Accessions	Modifications	ΔCn	114/115	114/115 Count	114/115 Variability [%]	q-Value	PEP	IonScore	Exp Value	Charge	MH+ [Da]	ΔM [ppm]	RT [min]	# Missed Cleavages
	High	eQELIDIFHR	1	1	1	P72644	N-Term(iTRAQ4plex)	0,0000	0,918	1		0	7,212E-08	49	1,38357E-05	3	1443,77076	-0,37	40,08	0
	High	aAVAEAAR	2	1	1	P72644	N-Term(iTRAQ4plex)	0,0000	1,197	2	20,5	0	0,006405	31	0,000814648	2	902,51793	0,32	11,67	0
	High	nSRPLLSNFPTEGER	1	1	1	P72644	N-Term(iTRAQ4plex)	0,0000	0,757	1		0	2,696E-07	18	0,014790062	3	1860,96585	-1,43	29,09	0
Q55589	Cell division cycle protein OS=Synechocystis sp. (strain PCC 6803 / Kazusa) GN=slr	71,47	6,19	1	3	3	5	0,567	5	27,1	501	56,4	6,27							
	A3	Sequence	# PSMs	# Proteins	# Protein Groups	Protein Group Accessions	Modifications	ΔCn	114/115	114/115 Count	114/115 Variability [%]	q-Value	PEP	IonScore	Exp Value	Charge	MH+ [Da]	ΔM [ppm]	RT [min]	# Missed Cleavages
	High	aRPASASVAEYQR	1	1	1	Q55589	N-Term(iTRAQ4plex)	0,0000	0,832	1		0	6,998E-09	47	1,99054E-05	3	1549,81968	-0,45	15,23	0
	High	aVGGLEELkk	2	1	1	Q55589	N-Term(iTRAQ4plex); K9(iTRAQ4plex); K10(iTRAQ4plex)	0,0000	0,655	2	21,5	0	0,00000031	28	0,001632939	3	1475,91510	-0,47	24,34	1
	High	lWGLPLLR	2	1	1	Q55589	N-Term(iTRAQ4plex)	0,0000	0,509	2	10,5	0,009	0,1057	16	0,024208618	2	1111,71025	-0,55	51,09	0
Q55410	Sll0540 protein OS=Synechocystis sp. (strain PCC 6803 / Kazusa) GN=sll0540 PE=4	69,66	5,21	1	2	2	2	0,746	2	4,7	307	32,5	4,93							
	A3	Sequence	# PSMs	# Proteins	# Protein Groups	Protein Group Accessions	Modifications	ΔCn	114/115	114/115 Count	114/115 Variability [%]	q-Value	PEP	IonScore	Exp Value	Charge	MH+ [Da]	ΔM [ppm]	RT [min]	# Missed Cleavages
	High	gVVNLILGk	1	1	1	Q55410	N-Term(iTRAQ4plex); K9(iTRAQ4plex)	0,0000	0,722	1		0	0,000001344	52	6,23692E-06	2	1200,79143	-0,36	42,22	0
	High	qLFYFYk	1	1	1	Q55410	N-Term(iTRAQ4plex); K7(iTRAQ4plex)	0,0000	0,769	1		0	0,0001611	31	0,000864908	2	1296,72331	0,19	41,69	0
P52276	Asparagine--tRNA ligase OS=Synechocystis sp. (strain PCC 6803 / Kazusa) GN=asn	69,48	6,49	1	3	3	4	0,747	4	40,7	462	52,7	5,43							
	A3	Sequence	# PSMs	# Proteins	# Protein Groups	Protein Group Accessions	Modifications	ΔCn	114/115	114/115 Count	114/115 Variability [%]	q-Value	PEP	IonScore	Exp Value	Charge	MH+ [Da]	ΔM [ppm]	RT [min]	# Missed Cleavages
	High	nAcATAIHQFFQER	1	1	1	P52276	N-Term(iTRAQ4plex); C3(Carbamidomethyl)	0,0000	1,088	1		0	6,209E-10	37	0,000206524	3	1836,89261	-0,34	34,68	0
	High	hSFEFLR	2	1	1	P52276	N-Term(iTRAQ4plex)	0,0000	0,687	2	40,7	0	0,00001841	32	0,000602518	3	1079,57499	-0,45	26,34	0
	Medium	tNTIGAVmR	1	1	1	P52276	N-Term(iTRAQ4plex); M8(Oxidation)	0,0000	0,624	1		0,036	0,3833	26	0,003820569	2	1122,60515	-0,59	15,92	0
Q55698	Slr0226 protein OS=Synechocystis sp. (strain PCC 6803 / Kazusa) GN=slr0226 PE=	68,47	2,90	1	2	2	5	0,902	5	3,4	482	50,8	4,98							
	A3	Sequence	# PSMs	# Proteins	# Protein Groups	Protein Group Accessions	Modifications	ΔCn	114/115	114/115 Count	114/115 Variability [%]	q-Value	PEP	IonScore	Exp Value	Charge	MH+ [Da]	ΔM [ppm]	RT [min]	# Missed Cleavages
	High	fLVLMHVk	1	1	1	Q55698	N-Term(iTRAQ4plex); K8(iTRAQ4plex)	0,0000	1,515	1		0,001	0,009928	15	0,032807263	3	1274,79068	0,73	37,09	0
	High	lIIFSR	4	1	1	Q55698	N-Term(iTRAQ4plex)	0,0000	0,892	4	2,4	0,003	0,03621	34	0,000416579	2	892,57372	0,04	39,57	0
P73735	NADH dehydrogenase OS=Synechocystis sp. (strain PCC 6803 / Kazusa) GN=ndh P	68,38	4,95	1	2	2	3	0,577	3	1,8	404	44,5	5,30							
	A3	Sequence	# PSMs	# Proteins	# Protein Groups	Protein Group Accessions	Modifications	ΔCn	114/115	114/115 Count	114/115 Variability [%]	q-Value	PEP	IonScore	Exp Value	Charge	MH+ [Da]	ΔM [ppm]	RT [min]	# Missed Cleavages
	High	gkEILAmSPEFNR	1	1	1	P73735	N-Term(iTRAQ4plex); K2(iTRAQ4plex); M7(Oxidation)	0,0000	0,502	1		0	2,686E-09	32	0,000650085	3	1795,96067	-0,49	26,30	1
	High	rLVYLYR	2	1	1	P73735	N-Term(iTRAQ4plex)	0,0000	0,580	2	0,9	0	0,0004788	31	0,000756781	3	1126,68399	-1,20	24,77	1
P74645	Circadian clock protein KaiB OS=Synechocystis sp. (strain PCC 6803 / Kazusa) GN=	67,83	21,90	1	2	2	2	0,945	2	2,1	105	11,9	6,79							
	A3	Sequence	# PSMs	# Proteins	# Protein Groups	Protein Group Accessions	Modifications	ΔCn	114/115	114/115 Count	114/115 Variability [%]	q-Value	PEP	IonScore	Exp Value	Charge	MH+ [Da]	ΔM [ppm]	RT [min]	# Missed Cleavages
	High	lYVAGNTPNSVR	1	1	1	P74645	N-Term(iTRAQ4plex)	0,0000	0,959	1		0	2,011E-07	65	3,12586E-07	2	1434,78240	0,15	25,17	0
	High	kIIGDLSDREk	1	1	1	P74645	N-Term(iTRAQ4plex); K1(iTRAQ4plex); K11(iTRAQ4plex)	0,0000	0,932	1		0	0,0003627	16	0,026300863	3	1706,01725	-0,03	20,25	2
P73326	Acetylglutamate kinase OS=Synechocystis sp. (strain PCC 6803 / Kazusa) GN=argB	67,65	8,75	1	2	2	2	0,828	2	31,6	297	31,5	6,32							
	A3	Sequence	# PSMs	# Proteins	# Protein Groups	Protein Group Accessions	Modifications	ΔCn	114/115	114/115 Count	114/115 Variability [%]	q-Value	PEP	IonScore	Exp Value	Charge	MH+ [Da]	ΔM [ppm]	RT [min]	# Missed Cleavages
	High	iLSEALPYIQHFAGR	1	1	1	P73326	N-Term(iTRAQ4plex)	0,0000	0,672	1		0	5,555E-13	68	1,71384E-07	3	1859,02860	-0,56	50,74	0
	Medium	dYkDPSTLIHk	1	1	1	P73326	N-Term(iTRAQ4plex); K3(iTRAQ4plex); K11(iTRAQ4plex)	0,0000	1,019	1		0,029	0,3147	11	0,077440831	4	1748,98574	-2,85	25,68	1
P73588	Sll1315 protein OS=Synechocystis sp. (strain PCC 6803 / Kazusa) GN=sll1315 PE=4	66,70	4,83	1	2	2	2	1,170	2	9,4	352	38,9	7,23							
	A3	Sequence	# PSMs	# Proteins	# Protein Groups	Protein Group Accessions	Modifications	ΔCn	114/115	114/115 Count	114/115 Variability [%]	q-Value	PEP	IonScore	Exp Value	Charge	MH+ [Da]	ΔM [ppm]	RT [min]	# Missed Cleavages
	High	lYQSIGVAR	1	1	1	P73588	N-Term(iTRAQ4plex)	0,0000	1,098	1		0	0,00000249	48	1,75376E-05	2	1150,66985	-0,22	27,80	0
	High	iISLTTGk	1	1	1	P73588	N-Term(iTRAQ4plex); K8(iTRAQ4plex)	0,0000	1,246	1		0,001	0,008505	32	0,000608093	2	1120,71794	-0,06	28,75	0
P74453	Sll0148 protein OS=Synechocystis sp. (strain PCC 6803 / Kazusa) GN=sll0148 PE=4	66,21	3,67	1	2	2	4	0,717	2	1,0	735	78,5	5,91							
	A3	Sequence	# PSMs	# Proteins	# Protein Groups	Protein Group Accessions	Modifications	ΔCn	114/115	114/115 Count	114/115 Variability [%]	q-Value	PEP	IonScore	Exp Value	Charge	MH+ [Da]	ΔM [ppm]	RT [min]	# Missed Cleavages
	High	gLSVAAILRPDLk	2	1	1	P74453	N-Term(iTRAQ4plex); K13(iTRAQ4plex)	0,0000	0,713	1		0	3,084E-11	40	9,90764E-05	3	1641,03092	0,43	42,40	0
	High	nLALTVALAHDLER	2	1	1	P74453	N-Term(iTRAQ4plex)	0,0000	0,722	1		0	0,000001008	35	0,000331872	3	1679,95365	-1,49	44,19	0
P72689	Translation initiation factor IF-2 OS=Synechocystis sp. (strain PCC 6803 / Kazusa) G	65,97	6,09	1	5	5	6	0,986	6	7,6	1001	108,1	5,95							

	A3	Sequence	# PSMs	# Proteins	# Protein Groups	Protein Group Accessions	Modifications	ΔCn	114/115	114/115 Count	114/115 Variability [%]	q-Value	PEP	IonScore	Exp Value	Charge	MH+ [Da]	ΔM [ppm]	RT [min]	# Missed Cleavages
	High	aAGVPLIVAINk	1	1	1	P72689	N-Term(iTRAQ4plex); K12(iTRAQ4plex)	0,0000	1,030	1		0	0,00003372	31	0,000814648	3	1453,93659	1,42	40,18	0
	High	rGDQVLFEGNIDSLkR	2	1	1	P72689	N-Term(iTRAQ4plex); K15(iTRAQ4plex)	0,0000	1,003	2	10,6	0	0,000003415	30	0,001570826	4	2135,18081	-0,19	31,77	2
	High	vNRPEIVSLk	1	1	1	P72689	N-Term(iTRAQ4plex); K10(iTRAQ4plex)	0,0000	1,035	1		0	0,000005213	22	0,005740768	3	1442,89258	-0,54	25,27	0
	High	lQQAmSSR	1	1	1	P72689	N-Term(iTRAQ4plex); M5(Oxidation)	0,0000	0,943	1		0	0,0002123	15	0,031766547	2	1080,56035	1,39	10,96	0
	High	rPPVVTImGHVDHGk	1	1	1	P72689	N-Term(iTRAQ4plex); M8(Oxidation); K15(iTRAQ4plex)	0,0000	0,870	1		0,001	0,008991	17	0,023506572	5	1947,08128	-1,26	17,94	0
P74506	Slr1944 protein OS=Synechocystis sp. (strain PCC 6803 / Kazusa) GN=slr1944 PE=	65,85	3,72	1	2	2	3	2,020	3	0,9	538	58,6	4,77							
	A3	Sequence	# PSMs	# Proteins	# Protein Groups	Protein Group Accessions	Modifications	ΔCn	114/115	114/115 Count	114/115 Variability [%]	q-Value	PEP	IonScore	Exp Value	Charge	MH+ [Da]	ΔM [ppm]	RT [min]	# Missed Cleavages
	High	iFTWLGk	2	1	1	P74506	N-Term(iTRAQ4plex); K7(iTRAQ4plex)	0,0000	1,812	2	17,1	0	0,0004912	43	4,75302E-05	2	1152,70671	4,12	44,87	0
	High	fYTQHLkPEELEk	1	1	1	P74506	N-Term(iTRAQ4plex); K7(iTRAQ4plex); K13(iTRAQ4plex)	0,0000	2,020	1		0	4,995E-08	23	0,005216662	4	2094,16372	1,97	28,91	0
P72741	Slr1097 protein OS=Synechocystis sp. (strain PCC 6803 / Kazusa) GN=slr1097 PE=	65,79	15,34	1	3	3	5	0,868	5	9,3	163	19,0	4,70							
	A3	Sequence	# PSMs	# Proteins	# Protein Groups	Protein Group Accessions	Modifications	ΔCn	114/115	114/115 Count	114/115 Variability [%]	q-Value	PEP	IonScore	Exp Value	Charge	MH+ [Da]	ΔM [ppm]	RT [min]	# Missed Cleavages
	High	yAAmLVPHEFHR	1	1	1	P72741	N-Term(iTRAQ4plex); M4(Oxidation)	0,0000	0,868	1		0	0,000006813	29	0,001124527	4	1630,82778	-0,20	28,95	0
	Medium	aISLNLNR	3	1	1	P72741	N-Term(iTRAQ4plex)	0,0000	0,896	3	14,0	0,011	0,132	21	0,008297935	2	1044,62749	-0,72	28,68	0
	Medium	lWFIR	1	1	1	P72741	N-Term(iTRAQ4plex)	0,0000	0,666	1		0,039	0,4022	21	0,008113146	2	878,53703	0,15	43,50	0
P73934	Slr2105 protein OS=Synechocystis sp. (strain PCC 6803 / Kazusa) GN=slr2105 PE=	65,63	3,87	1	2	2	2	0,688	2	0,0	595	65,4	4,79							
	A3	Sequence	# PSMs	# Proteins	# Protein Groups	Protein Group Accessions	Modifications	ΔCn	114/115	114/115 Count	114/115 Variability [%]	q-Value	PEP	IonScore	Exp Value	Charge	MH+ [Da]	ΔM [ppm]	RT [min]	# Missed Cleavages
	High	lQGAIAIFPLVRPVATk	1	1	1	P73934	N-Term(iTRAQ4plex); K17(iTRAQ4plex)	0,0000	0,688	1		0	6,018E-11	64	4,35482E-07	3	2082,30521	0,47	47,52	0
	Medium	lTALFR	1	1	1	P73934	N-Term(iTRAQ4plex)	0,0000	0,688	1		0,028	0,2984	15	0,031330693	2	864,54228	-0,12	35,74	0
P73757	Neopullulanase OS=Synechocystis sp. (strain PCC 6803 / Kazusa) GN=nplT PE=4 S	65,41	5,71	1	2	2	3	0,805	2	25,1	508	58,0	5,25							
	A3	Sequence	# PSMs	# Proteins	# Protein Groups	Protein Group Accessions	Modifications	ΔCn	114/115	114/115 Count	114/115 Variability [%]	q-Value	PEP	IonScore	Exp Value	Charge	MH+ [Da]	ΔM [ppm]	RT [min]	# Missed Cleavages
	High	fAIGQQQAIAVAQQPk	1	1	1	P73757	N-Term(iTRAQ4plex); K16(iTRAQ4plex)	0,0000	0,681	1		0	0,00001158	39	0,000126465	3	1986,14042	1,45	33,81	0
	High	aLPQFNHDHPDVR	2	1	1	P73757	N-Term(iTRAQ4plex)	0,0000	0,951	1		0	1,261E-07	38	0,000146545	4	1689,85854	0,42	23,36	0
P72675	Slr0731 protein OS=Synechocystis sp. (strain PCC 6803 / Kazusa) GN=slr0731 PE=	65,13	6,47	1	2	3	3	1,026	2	0,4	402	44,7	4,50							
	A3	Sequence	# PSMs	# Proteins	# Protein Groups	Protein Group Accessions	Modifications	ΔCn	114/115	114/115 Count	114/115 Variability [%]	q-Value	PEP	IonScore	Exp Value	Charge	MH+ [Da]	ΔM [ppm]	RT [min]	# Missed Cleavages
	High	iENWFFTLGGR	1	1	1	P72675	N-Term(iTRAQ4plex)	0,0000	1,024	1		0	0,00001743	36	0,000271	2	1483,78301	1,03	52,65	0
	High	tFEPVHAER	1	1	1	P72675	N-Term(iTRAQ4plex)	0,0000	1,029	1		0	0,0003009	35	0,000334172	3	1229,63962	0,06	16,52	0
	High	kAALEk	1	2	2	;P72675	N-Term(iTRAQ4plex); K1(iTRAQ4plex); K6(iTRAQ4plex)	0,0000				0,004	0,05468	21	0,008452205	3	1091,71195	-2,69	10,92	1
P73201	Serine--tRNA ligase OS=Synechocystis sp. (strain PCC 6803 / Kazusa) GN=serS PE=	64,75	5,81	1	2	2	2	0,866	2	21,5	430	48,0	5,59							
	A3	Sequence	# PSMs	# Proteins	# Protein Groups	Protein Group Accessions	Modifications	ΔCn	114/115	114/115 Count	114/115 Variability [%]	q-Value	PEP	IonScore	Exp Value	Charge	MH+ [Da]	ΔM [ppm]	RT [min]	# Missed Cleavages
	High	aETVGILPHWEIGEk	1	1	1	P73201	N-Term(iTRAQ4plex); K15(iTRAQ4plex)	0,0000	0,750	1		0	6,073E-13	49	1,1995E-05	3	1967,08518	0,54	40,24	0
	High	vPVVLQDFLk	1	1	1	P73201	N-Term(iTRAQ4plex); K10(iTRAQ4plex)	0,0000	0,999	1		0	6,062E-07	29	0,001393061	3	1445,89762	0,39	51,03	0
P73058	Histidinol dehydrogenase 1 OS=Synechocystis sp. (strain PCC 6803 / Kazusa) GN=h	64,51	9,68	1	3	3	3	1,172	3	36,2	434	46,5	5,53							
	A3	Sequence	# PSMs	# Proteins	# Protein Groups	Protein Group Accessions	Modifications	ΔCn	114/115	114/115 Count	114/115 Variability [%]	q-Value	PEP	IonScore	Exp Value	Charge	MH+ [Da]	ΔM [ppm]	RT [min]	# Missed Cleavages
	High	rSEQNIDQALAIAk	1	1	1	P73058	N-Term(iTRAQ4plex); K14(iTRAQ4plex)	0,0000	1,485	1		0	2,941E-08	39	0,000144938	3	1845,04300	-0,16	28,58	1
	High	lGGVQALAAIAYGTk	1	1	1	P73058	N-Term(iTRAQ4plex); K15(iTRAQ4plex)	0,0000	1,172	1		0	0,00005735	27	0,002032217	3	1721,01981	-0,13	48,19	0
	High	iTPLPTVGLYVPR	1	1	1	P73058	N-Term(iTRAQ4plex)	0,0000	0,858	1		0	0,00004332	25	0,003191317	3	1569,94699	-0,98	45,43	0
P73991	Short-chain alcohol dehydrogenase family OS=Synechocystis sp. (strain PCC 6803 /	64,33	6,02	1	2	2	2	1,352	2	66,5	249	26,5	5,50							
	A3	Sequence	# PSMs	# Proteins	# Protein Groups	Protein Group Accessions	Modifications	ΔCn	114/115	114/115 Count	114/115 Variability [%]	q-Value	PEP	IonScore	Exp Value	Charge	MH+ [Da]	ΔM [ppm]	RT [min]	# Missed Cleavages
	High	aAFLLAk	1	1	1	P73991	N-Term(iTRAQ4plex); K7(iTRAQ4plex)	0,0000	0,899	1		0	0,0002748	45	3,16935E-05	2	1021,66423	-0,61	32,45	0
	High	gGLIALTR	1	1	1	P73991	N-Term(iTRAQ4plex)	0,0000	2,034	1		0,001	0,01013	32	0,000580724	2	944,59972	-1,33	27,86	0
Q55716	Glycine--tRNA ligase alpha subunit OS=Synechocystis sp. (strain PCC 6803 / Kazusa	64,05	10,37	1	3	3	3	0,928	3	4,6	299	34,3	4,87							
	A3	Sequence	# PSMs	# Proteins	# Protein Groups	Protein Group Accessions	Modifications	ΔCn	114/115	114/115 Count	114/115 Variability [%]	q-Value	PEP	IonScore	Exp Value	Charge	MH+ [Da]	ΔM [ppm]	RT [min]	# Missed Cleavages
	High	gAGTmNPHTFLR	1	1	1	Q55716	N-Term(iTRAQ4plex); M5(Oxidation)	0,0000	0,957	1		0	0,000003344	48	1,66713E-05	3	1461,73954	0,39	18,41	0
	High	eVAHIYLQQR	1	1	1	Q55716	N-Term(iTRAQ4plex)	0,0000	0,778	1		0	0,00001621	23	0,00540717	3	1400,77759	0,64	22,38	0
	Medium	kVDkPISLV	1	1	1	Q55716	N-Term(iTRAQ4plex); K1(iTRAQ4plex); K4(iTRAQ4plex)	0,0000	0,928	1		0,013	0,1453	20	0,010888549	3	1430,92948	-0,85	27,18	1
P73355	Ferredoxin component OS=Synechocystis sp. (strain PCC 6803 / Kazusa) GN=slr12	63,04	15,24	1	2	2	5	1,106	4	4,8	164	17,5	8,31							
	A3	Sequence	# PSMs	# Proteins	# Protein Groups	Protein Group Accessions	Modifications	ΔCn	114/115	114/115 Count	114/115 Variability [%]	q-Value	PEP	IonScore	Exp Value	Charge	MH+ [Da]	ΔM [ppm]	RT [min]	# Missed Cleavages
	High	ikDGAIVcPFHR	2	1	1	P73355	N-Term(iTRAQ4plex); K2(iTRAQ4plex); C8(Carbamidomethyl)	0,0000	1,024	2	15,3	0	0,00002491	38	0,000164048	4	1700,95131	0,24	27,32	1
	High	mSAEkPLPVFPVR	3	1	1	P73355	N-Term(iTRAQ4plex); M1(Oxidation); K5(iTRAQ4plex)	0,0000	1,113	2	4,8	0	7,579E-07	28	0,001455459	3	1775,01358	0,41	31,60	0
P74416	Glycine dehydrogenase (decarboxylating) OS=Synechocystis sp. (strain PCC 6803 /	63,03	3,76	1	4	4	4	0,603	4	8,3	983	107,3	5,39							
	A3	Sequence	# PSMs	# Proteins	# Protein Groups	Protein Group Accessions	Modifications	ΔCn	114/115	114/115 Count	114/115 Variability [%]	q-Value	PEP	IonScore	Exp Value	Charge	MH+ [Da]	ΔM [ppm]	RT [min]	# Missed Cleavages
	High	gNNELVAHEcILDLRPLk	1	1	1	P74416	N-Term(iTRAQ4plex); C10(Carbamidomethyl); K18(iTRAQ4plex)	0,0000	0,619	1		0	1,626E-07	45	4,16815E-05	4	2379,30483	-0,39	39,04	0
	High	rLENYYPILFR	1	1	1	P74416	N-Term(iTRAQ4plex)	0,0000	0,588	1		0	0,00003355	21	0,009736272	3	1627,91013	1,48	40,22	1
	High	gNNELVAHEcILDLRPLkk	1	1	1	P74416	N-Term(iTRAQ4plex); C10(Carbamidomethyl); K18(iTRAQ4plex); K19(iTRAQ	0,0000	0,553	1		0	0,00002535	13	0,048525498	5	2651,50312	0,12	34,12	1
	High	fWPAVGR	1	1	1	P74416	N-Term(iTRAQ4plex)	0,0000	0,887	1		0,002	0,03195	25	0,003451199	2	976,54857	0,03	33,36	0
P73796	Urease subunit gamma OS=Synechocystis sp. (strain PCC 6803 / Kazusa) GN=ureA	62,60	31,00	1	3	3	4	0,810	3	0,5	100	11,0	5,21							
	A3	Sequence	# PSMs	# Proteins	# Protein Groups	Protein Group Accessions	Modifications	ΔCn	114/115	114/115 Count	114/115 Variability [%]	q-Value	PEP	IonScore	Exp Value	Charge	MH+ [Da]	ΔM [ppm]	RT [min]	# Missed Cleavages
	High	dkLLIFTASLVAER	1	1	1	P73796	N-Term(iTRAQ4plex); K2(iTRAQ4plex)	0,0000	0,810	1		0	6,276E-10	46	2,46587E-05	3	1864,11472	0,02	49,30	1
	High	lVTVHEPIR	2	1	1	P73796	N-Term(iTRAQ4plex)	0,0000	0,807	1		0	0,00002084	28	0,001541594	3	1207,72931	1,12	23,61	0
	High	mQLSPQEkDk	1	1	1	P73796	N-Term(iTRAQ4plex); M1(Oxidation); K8(iTRAQ4plex); K10(iTRAQ4plex)	0,0000	0,944	1		0,005	0,06463	14	0,036057864	3	1651,91306	4,90	12,77	1
P74060	Putative sulfur carrier protein slr0821 OS=Synechocystis sp. (strain PCC 6803 / Kaz	61,51	20,18	1	2	2	3	1,434	3	15,4	109	11,7	4,67							
	A3	Sequence	# PSMs	# Proteins	# Protein Groups	Protein Group Accessions	Modifications	ΔCn	114/115	114/115 Count	114/115 Variability [%]	q-Value	PEP	IonScore	Exp Value	Charge	MH+ [Da]	ΔM [ppm]	RT [min]	# Missed Cleavages
	High	rFLNFYVNEEDIR	2	1	1	P74060	N-Term(iTRAQ4plex)	0,0000	1,111	2	57,0	0	1,237E-08	40	0,000111165	3	1858,95957	1,45	40,07	1
	High	lcDEEGkPR	1	1	1	P74060	N-Term(iTRAQ4plex); C2(Carbamidomethyl); K7(iTRAQ4plex)	0,0000	1,434	1		0	0,002946	22	0,006652272	3	1391,71772	-1,05	13,65	0
P27182	ATP synthase subunit c OS=Synechocystis sp. (strain PCC 6803 / Kazusa) GN=atpE	61,46	11,11	1	2	2	5	1,121	5	1,9	81	8,0	4,91							
	A3	Sequence	# PSMs	# Proteins	# Protein Groups	Protein Group Accessions	Modifications	ΔCn	114/115	114/115 Count	114/115 Variability [%]	q-Value	PEP	IonScore	Exp Value	Charge	MH+ [Da]	ΔM [ppm]	RT [min]	# Missed Cleavages
	High	qPEAEGk	4	1	1	P27182	N-Term(iTRAQ4plex); K7(iTRAQ4plex)	0,0000	1,122	4	1,2	0	0,007605	27	0,001874865	2	1046,57207	-0,01	11,08	0
	High	qPEAEGkIR	1	1	1	P27182	N-Term(iTRAQ4plex); K7(iTRAQ4plex)	0,0000	0,913	1		0,002	0,02559	23	0,005175711	3	1315,75733	0,05	12,23	1
P74685	Sll0443 protein OS=Synechocystis sp. (strain PCC 6803 / Kazusa) GN=sll0443 PE=	61,31	7,25	1	2	2	2	0,973	2	30,0	469	51,0	4,77							
	A3	Sequence	# PSMs	# Proteins	# Protein Groups	Protein Group Accessions	Modifications	ΔCn	114/115	114/115 Count	114/115 Variability [%]	q-Value	PEP	IonScore	Exp Value	Charge	MH+ [Da]	ΔM [ppm]	RT [min]	# Missed Cleavages
	High	gRPFFILMTGPQETVDELVR	1	1	1	P74685	N-Term(iTRAQ4plex)	0,0000	1,186	1		0	1,131E-09	38	0,00017378	3	2449,30570	1,06	53,07	0
	High	kAEIAQNPGGENAR	1	1	1	P74685	N-Term(iTRAQ4plex); K1(iTRAQ4plex)	0,0000	0,798	1		0	3,334E-07	37	0,00021231	3	1742,93717	-0,96	13,48	1
P72874	Translation initiation factor IF-3 OS=Synechocystis sp. (strain PCC 6803 / Kazusa) G	61,07	19,21	1	4	4	5	0,939	5	10,9	177	20,5	9,33							
	A3	Sequence	# PSMs	# Proteins	# Protein Groups	Protein Group Accessions	Modifications	ΔCn	114/115	114/115 Count	114/115 Variability [%]	q-Value	PEP	IonScore	Exp Value	Charge	MH+ [Da]	ΔM [ppm]	RT [min]	# Missed Cleavages
	High	eIQHAHLAk	1	1	1	P72874	N-Term(iTRAQ4plex); K9(iTRAQ4plex)	0,0000	0,943	1		0	0,00006664	28	0,001523947	4	1334,77749	-0,62	12,71	0
	High	iRFPEIR	2	1	1	P72874	N-Term(iTRAQ4plex)	0,0000	0,870	2	0,4	0	0,000582	26	0,002605974	3	1074,65549	1,32	31,20	1
	High	ykIDEHDYQVR	1	1	1	P72874	N-Term(iTRAQ4plex); K2(iTRAQ4plex)	0,0000	0,939	1		0	0,00003852	25	0,003435342	4	1753,91311	1,10	22,24	1
	High	ykFEQEk	1	1	1	P72874	N-Term(iTRAQ4plex); K2(iTRAQ4plex); K7(iTRAQ4plex)	0,0000	1,429	1		0,001	0,01381	10	0,093104357	3	1403,78894	-0,39	18,11	1
Q55168	Phytochrome-like protein cph1 OS=Synechocystis sp. (strain PCC 6803 / Kazusa) Gl	60,41	3,74	1	3	3	3	1,550	3	6,2	748	84,2	5,12							
	A3	Sequence	# PSMs	# Proteins	# Protein Groups	Protein Group Accessions	Modifications	ΔCn	114/115	114/115 Count	114/115 Variability [%]	q-Value	PEP	IonScore	Exp Value	Charge	MH+ [Da]	ΔM [ppm]	RT [min]	# Missed Cleavages
	High	sVASGLLAIPIAR	1	1	1	Q55168	N-Term(iTRAQ4plex)	0,0000	1,550	1		0	0,00001049	35	0,000328073	2	1411,87676	0,97	45,26	0
	High	iFVIFQR	1	1	1	Q55168	N-Term(iTRAQ4plex)	0,0000	0,966	1		0	0,004491	24	0,004334809	2	1066,65422	1,14	43,89	0
	Medium	aIVNLILR	1	1	1	Q55168	N-Term(iTRAQ4plex)	0,0000	1,617	1		0,022	0,2356	28	0,001595769	2	1055,70781	1,93	42,64	0
P74673	Slr1668 protein OS=Synechocystis sp. (strain PCC 6803 / Kazusa) GN=slr1668 PE=	60,35	9,29	1	2	2	2	1,804	2	3,1	280	30,6	6,24							
	A3	Sequence	# PSMs	# Proteins	# Protein Groups	Protein Group Accessions	Modifications	ΔCn	114/115	114/115 Count	114/115 Variability [%]	q-Value	PEP	IonScore	Exp Value	Charge	MH+ [Da]	ΔM [ppm]	RT [min]	# Missed Cleavages
	High	lVLNFENQGTAHQLLR	1	1	1	P74673	N-Term(iTRAQ4plex)	0,0000	1,767	1		0	2,013E-10	45	3,88501E-05	3	1997,10935	2,21	40,00	0
	High	lSPVGGGASR	1	1	1	P74673	N-Term(iTRAQ4plex)	0,0000	1,842	1		0	0,006732	29	0,001244429	2	1044,59355	1,61	16,13	0
P73497	Slr1240 protein OS=Synechocystis sp. (strain PCC 6803 / Kazusa) GN=slr1240 PE=	60,34	14,53	1	3	3	4	1,187	4	20,9	179	21,0	5,52							
	A3	Sequence	# PSMs	# Proteins	# Protein Groups	Protein Group Accessions	Modifications	ΔCn	114/115	114/115 Count	114/115 Variability [%]	q-Value	PEP	IonScore	Exp Value	Charge	MH+ [Da]	ΔM [ppm]	RT [min]	# Missed Cleavages
	High	kLEVFPk	2	1	1	P73497	N-Term(iTRAQ4plex); K1(iTRAQ4plex); K7(iTRAQ4plex)	0,0000	1,454	2	31,5	0	0,0008928	39	0,000114807	3	1292,82712	-2,42	24,64	1
	High	lFHEETHAEk	1	1	1	P73497	N-Term(iTRAQ4plex); K10(iTRAQ4plex)	0,0000	0,902	1		0	0,00005043	18	0,015274606	4	1528,79995	0,07	16,14	0
	High	kLENDEHNk	1	1	1	P73497	N-Term(iTRAQ4plex); K1(iTRAQ4plex); K9(iTRAQ4plex)	0,0000	1,192	1		0,001	0,01154	17	0,019229589	4	1558,85366	-0,84	8,30	1
P74287	Isopentenyl-diphosphate delta-isomerase OS=Synechocystis sp. (strain PCC 6803 /	60,05	4,58	1	2	2	3	1,269	3	8,0	349	37,5	5,14							
	A3	Sequence	# PSMs	# Proteins	# Protein Groups	Protein Group Accessions	Modifications	ΔCn	114/115	114/115 Count	114/115 Variability [%]	q-Value	PEP	IonScore	Exp Value	Charge	MH+ [Da]	ΔM [ppm]	RT [min]	# Missed Cleavages
	High	lWSGLWSk	2	1	1	P74287	N-Term(iTRAQ4plex); K8(iTRAQ4plex)	0,0000	1,093	2	31,0	0	0,000698	36	0,000251995	2	1264,72905	-0,15	44,19	0
	Medium	gISTGFER	1	1	1	P74287	N-Term(iTRAQ4plex)	0,0000	1,269	1		0,043	0,4534	17	0,021775594	2	1010,53899	0,22	20,94	0
P73668	UDP-N-acetylmuramoylalanine--D-glutamate ligase OS=Synechocystis sp. (strain PC	59,96	6,19	1	2	2	2	0,682	2	21,7	452	49,0	5,76							
	A3	Sequence	# PSMs	# Proteins	# Protein Groups	Protein Group Accessions	Modifications	ΔCn	114/115	114/115 Count	114/115 Variability [%]	q-Value	PEP	IonScore	Exp Value	Charge	MH+ [Da]	ΔM [ppm]	RT [min]	# Missed Cleavages
	High	aITETLLTFTGVPHR	1	1	1	P73668	N-Term(iTRAQ4plex)	0,0000	0,590	1		0	5,178E-07	43	4,91122E-05	3	1800,01279	-0,49	46,22	0
	High	tTTTSLVQAIFQk	1	1	1	P73668	N-Term(iTRAQ4plex); K13(iTRAQ4plex)	0,0000	0,788	1		0	5,426E-07	30	0,001051889	3	1725,99808	-0,50	46,58	0
Q55658	Sll0023 protein OS=Synechocystis sp. (strain PCC 6803 / Kazusa) GN=sll0023 PE=	59,95	3,13	1	2	2	2	0,774	2	28,8	447	51,6	4,63							
	A3	Sequence	# PSMs	# Proteins	# Protein Groups	Protein Group Accessions	Modifications	ΔCn	114/115	114/115 Count	114/115 Variability [%]	q-Value	PEP	IonScore	Exp Value	Charge	MH+ [Da]	ΔM [ppm]	RT [min]	# Missed Cleavages
	High	lLLEGLHVR	1	1	1	Q55658	N-Term(iTRAQ4plex)	0,0000	0,640	1		0	3,624E-08	60	1,00918E-06	3	1193,74839	-0,27	36,47	0
	Medium	mQPIk	1	1	1	Q55658	N-Term(iTRAQ4plex); K5(iTRAQ4plex)	0,0000	0,936	1		0,038	0,3947	13	0,055203931	2	904,55882	6,60	16,61	0
Q55922	Slr0315 protein OS=Synechocystis sp. (strain PCC 6803 / Kazusa) GN=slr0315 PE=	59,86	7,79	1	2	2	3	0,564	2	3,0	244	25,9	5,47							
	A3	Sequence	# PSMs	# Proteins	# Protein Groups	Protein Group Accessions	Modifications	ΔCn	114/115	114/115 Count	114/115 Variability [%]	q-Value	PEP	IonScore	Exp Value	Charge	MH+ [Da]	ΔM [ppm]	RT [min]	# Missed Cleavages
	High	yAVWAISEGLR	1	1	1	Q55922	N-Term(iTRAQ4plex)	0,0000	0,576	1		0	8,084E-07	41	8,07179E-05	2	1408,77092	0,25	44,45	0
	High	gLLEELRk	2	1	1	Q55922	N-Term(iTRAQ4plex); K8(iTRAQ4plex)	0,0000	0,553	1		0	0,007123	32	0,000638219	3	1245,77597	-0,77	30,63	1
Q6YRQ8	Slr6096 protein OS=Synechocystis sp. (strain PCC 6803 / Kazusa) GN=slr6096 PE=	59,63	5,36	1	2	2	2	1,323	2	3,0	373	42,1	4,67							
	A3	Sequence	# PSMs	# Proteins	# Protein Groups	Protein Group Accessions	Modifications	ΔCn	114/115	114/115 Count	114/115 Variability [%]	q-Value	PEP	IonScore	Exp Value	Charge	MH+ [Da]	ΔM [ppm]	RT [min]	# Missed Cleavages
	High	gQNSIAAQR	1	1	1	Q6YRQ8	N-Term(iTRAQ4plex)	0,0000	1,350	1		0	0,002552	38	0,000150304	2	1088,59319	0,24	11,00	0
	High	kLGNDTTALER	1	1	1	Q6YRQ8	N-Term(iTRAQ4plex); K1(iTRAQ4plex)	0,0000	1,296	1		0	0,000001673	34	0,00036141	3	1505,85285	0,15	17,85	1
P73946	Slr1506 protein OS=Synechocystis sp. (strain PCC 6803 / Kazusa) GN=slr1506 PE=	59,55	4,34	1	3	3	5	0,954	5	8,4	622	68,8	5,30							
	A3	Sequence	# PSMs	# Proteins	# Protein Groups	Protein Group Accessions	Modifications	ΔCn	114/115	114/115 Count	114/115 Variability [%]	q-Value	PEP	IonScore	Exp Value	Charge	MH+ [Da]	ΔM [ppm]	RT [min]	# Missed Cleavages
	High	fYLISQk	2	1	1	P73946	N-Term(iTRAQ4plex); K7(iTRAQ4plex)	0,0000	0,986	2	7,1	0	0,001121	32	0,000570125	2	1186,70598	-1,22	34,39	0
	High	dISYVIDELERR	2	1	1	P73946	N-Term(iTRAQ4plex)	0,0000	0,928	2	4,2	0	0,000001935	20	0,011512808	3	1651,87754	0,19	47,55	1
	High	aSEHFIDk	1	1	1	P73946	N-Term(iTRAQ4plex); K8(iTRAQ4plex)	0,0000	1,082	1		0,004	0,04318	14	0,035972449	3	1234,67347	5,21	17,09	0
P73720	Putative OxPP cycle protein OpcA OS=Synechocystis sp. (strain PCC 6803 / Kazusa)	59,33	3,58	1	2	2	2	0,446	2	7,8	475	52,0	5,11							
	A3	Sequence	# PSMs	# Proteins	# Protein Groups	Protein Group Accessions	Modifications	ΔCn	114/115	114/115 Count	114/115 Variability [%]	q-Value	PEP	IonScore	Exp Value	Charge	MH+ [Da]	ΔM [ppm]	RT [min]	# Missed Cleavages
	High	qALQTAFETAHR	1	1	1	P73720	N-Term(iTRAQ4plex)	0,0000	0,470	1		0	1,979E-08	45	3,24317E-05	3	1516,79807	-0,55	25,40	0
	High	yVWWk	1	1	1	P73720	N-Term(iTRAQ4plex); K5(iTRAQ4plex)	0,0000	0,423	1		0,003	0,034	27	0,00179461	2	1069,60637	-0,88	37,75	0
P73921	Sll1979 protein OS=Synechocystis sp. (strain PCC 6803 / Kazusa) GN=sll1979 PE=	58,90	14,79	1	3	3	3	1,257	3	26,8	142	15,9	5,06							
	A3	Sequence	# PSMs	# Proteins	# Protein Groups	Protein Group Accessions	Modifications	ΔCn	114/115	114/115 Count	114/115 Variability [%]	q-Value	PEP	IonScore	Exp Value	Charge	MH+ [Da]	ΔM [ppm]	RT [min]	# Missed Cleavages
	High	iNYALAMLANkk	1	1	1	P73921	N-Term(iTRAQ4plex); K11(iTRAQ4plex); K12(iTRAQ4plex)	0,0000	1,500	1		0	1,131E-09	38	0,00014157	3	1782,06754	0,18	41,39	1
	High	iNYALAmLANk	1	1	1	P73921	N-Term(iTRAQ4plex); M7(Oxidation); K11(iTRAQ4plex)	0,0000	0,937	1		0	0,000005565	30	0,000965984	3	1525,86710	1,31	38,79	0
	High	iDGYHPQAR	1	1	1	P73921	N-Term(iTRAQ4plex)	0,0000	1,257	1		0	0,002634	16	0,023494705	3	1200,62464	0,35	14,91	0
P73389	Slr1829 protein OS=Synechocystis sp. (strain PCC 6803 / Kazusa) GN=slr1829 PE=	58,78	5,76	1	2	2	3	0,471	3	30,2	330	38,0	5,85							
	A3	Sequence	# PSMs	# Proteins	# Protein Groups	Protein Group Accessions	Modifications	ΔCn	114/115	114/115 Count	114/115 Variability [%]	q-Value	PEP	IonScore	Exp Value	Charge	MH+ [Da]	ΔM [ppm]	RT [min]	# Missed Cleavages
	High	gFAALMEDLLAR	1	1	1	P73389	N-Term(iTRAQ4plex)	0,0000	0,471	1		0	0,000003396	54	4,18765E-06	2	1450,78386	-0,46	58,85	0
	High	nLSSFmR	2	1	1	P73389	N-Term(iTRAQ4plex); M6(Oxidation)	0,0000	0,407	2	54,8	0,002	0,02891	18	0,015775023	2	1014,51628	0,36	20,46	0
P73123	Probable RuBisCO transcriptional regulator OS=Synechocystis sp. (strain PCC 6803	58,24	6,96	1	2	2	2	0,833	2	12,5	345	38,0	5,76							
	A3	Sequence	# PSMs	# Proteins	# Protein Groups	Protein Group Accessions	Modifications	ΔCn	114/115	114/115 Count	114/115 Variability [%]	q-Value	PEP	IonScore	Exp Value	Charge	MH+ [Da]	ΔM [ppm]	RT [min]	# Missed Cleavages
	High	vLSVIINPNR	1	1	1	P73123	N-Term(iTRAQ4plex)	0,0000	0,766	1		0	0,00008652	38	0,000168644	2	1268,78191	0,93	38,04	0
	High	lkIEmELNSIEAIk	1	1	1	P73123	N-Term(iTRAQ4plex); K2(iTRAQ4plex); M5(Oxidation); K14(iTRAQ4plex)	0,0000	0,906	1		0	0,000002042	34	0,000443578	3	2079,21000	0,18	44,05	1
Q55541	Slr0333 protein OS=Synechocystis sp. (strain PCC 6803 / Kazusa) GN=slr0333 PE=	57,34	21,70	1	2	2	3	3,246	3	17,5	106	11,4	8,48							
	A3	Sequence	# PSMs	# Proteins	# Protein Groups	Protein Group Accessions	Modifications	ΔCn	114/115	114/115 Count	114/115 Variability [%]	q-Value	PEP	IonScore	Exp Value	Charge	MH+ [Da]	ΔM [ppm]	RT [min]	# Missed Cleavages
	High	sSAPQYGLPGDkVEVIk	1	1	1	Q55541	N-Term(iTRAQ4plex); K12(iTRAQ4plex); K17(iTRAQ4plex)	0,0000	12,604	1		0	0,0001318	32	0,000642643	3	2220,27188	5,33	32,68	1
	High	aVGWIR	2	1	1	Q55541	N-Term(iTRAQ4plex)	0,0000	3,062	2	8,7	0,005	0,06612	27	0,002410911	2	845,51421	3,29	25,10	0
Q55972	Slr0708 protein OS=Synechocystis sp. (strain PCC 6803 / Kazusa) GN=slr0708 PE=	57,03	5,72	1	2	2	2	0,944	2	1,1	402	44,7	5,26							
	A3	Sequence	# PSMs	# Proteins	# Protein Groups	Protein Group Accessions	Modifications	ΔCn	114/115	114/115 Count	114/115 Variability [%]	q-Value	PEP	IonScore	Exp Value	Charge	MH+ [Da]	ΔM [ppm]	RT [min]	# Missed Cleavages
	High	tTPIQAPPVPVQYQVVk	1	1	1	Q55972	N-Term(iTRAQ4plex); K17(iTRAQ4plex)	0,0000	0,938	1		0	1,595E-11	44	4,2459E-05	3	2153,26187	2,12	38,42	0
	High	iNAWIk	1	1	1	Q55972	N-Term(iTRAQ4plex); K6(iTRAQ4plex)	0,0000	0,951	1		0,006	0,07505	27	0,002660378	2	1032,64116	-3,18	30,90	0
Q55449	Slr0031 protein OS=Synechocystis sp. (strain PCC 6803 / Kazusa) GN=slr0031 PE=	56,86	6,34	1	4	4	7	1,064	7	14,7	584	66,9	5,58							
	A3	Sequence	# PSMs	# Proteins	# Protein Groups	Protein Group Accessions	Modifications	ΔCn	114/115	114/115 Count	114/115 Variability [%]	q-Value	PEP	IonScore	Exp Value	Charge	MH+ [Da]	ΔM [ppm]	RT [min]	# Missed Cleavages
	High	iRPIALEk	4	1	1	Q55449	N-Term(iTRAQ4plex); K8(iTRAQ4plex)	0,0000	1,063	4	2,1	0	0,0007546	20	0,011090982	3	1227,80271	-0,04	25,27	0
	High	vkSEAGQEk	1	1	1	Q55449	N-Term(iTRAQ4plex); K2(iTRAQ4plex); K9(iTRAQ4plex)	0,0000	1,336	1		0	0,001567	19	0,012021814	3	1407,81845	1,18	8,97	1
	High	fPVWTPGSYLVR	1	1	1	Q55449	N-Term(iTRAQ4plex)	0,0000	1,358	1		0,002	0,01835	28	0,001555858	2	1565,86138	1,06	49,31	0
	Medium	fGFRDPEk	1	1	1	Q55449	N-Term(iTRAQ4plex); K8(iTRAQ4plex)	0,0000	0,964	1		0,015	0,165	16	0,029949714	3	1283,69812	-0,44	23,78	1
P73637	5-oxo-1,2,5-tricarboxilic-3-penten acid decarboxilase/isomer OS=Synechocystis sp.	56,59	11,96	1	2	2	2	0,960	2	6,2	276	30,0	4,49							
	A3	Sequence	# PSMs	# Proteins	# Protein Groups	Protein Group Accessions	Modifications	ΔCn	114/115	114/115 Count	114/115 Variability [%]	q-Value	PEP	IonScore	Exp Value	Charge	MH+ [Da]	ΔM [ppm]	RT [min]	# Missed Cleavages
	High	gFDGFcPLGPWVVR	1	1	1	P73637	N-Term(iTRAQ4plex); C6(Carbamidomethyl)	0,0000	0,921	1		0	0,00000112	39	0,000116673	2	1750,88408	-0,90	52,74	0
	High	hIDPEAHLETTVNDEETPR	1	1	1	P73637	N-Term(iTRAQ4plex)	0,0000	1,001	1		0	9,053E-09	30	0,000937497	4	2347,12832	-0,01	25,63	0
P33779	Global nitrogen regulator OS=Synechocystis sp. (strain PCC 6803 / Kazusa) GN=ntc	56,46	8,89	1	2	2	2	0,658	2	36,7	225	25,0	7,05							
	A3	Sequence	# PSMs	# Proteins	# Protein Groups	Protein Group Accessions	Modifications	ΔCn	114/115	114/115 Count	114/115 Variability [%]	q-Value	PEP	IonScore	Exp Value	Charge	MH+ [Da]	ΔM [ppm]	RT [min]	# Missed Cleavages
	High	iTVHNPVALSQQFT	1	1	1	P33779	N-Term(iTRAQ4plex)	0,0000	0,518	1		0	7,474E-07	43	5,29627E-05	2	1698,93083	0,74	39,61	0
	Medium	vYFLLk	1	1	1	P33779	N-Term(iTRAQ4plex); K6(iTRAQ4plex)	0,0000	0,837	1		0,027	0,2906	27	0,002127992	2	1070,68389	-1,26	40,86	0
Q55584	Cation or drug efflux system protein OS=Synechocystis sp. (strain PCC 6803 / Kazu	56,23	2,86	1	4	4	4	0,651	4	22,4	1083	117,5	4,88							
	A3	Sequence	# PSMs	# Proteins	# Protein Groups	Protein Group Accessions	Modifications	ΔCn	114/115	114/115 Count	114/115 Variability [%]	q-Value	PEP	IonScore	Exp Value	Charge	MH+ [Da]	ΔM [ppm]	RT [min]	# Missed Cleavages
	High	vLLDEIkR	1	1	1	Q55584	N-Term(iTRAQ4plex); K7(iTRAQ4plex)	0,0000	0,502	1		0	0,001317	23	0,004920056	3	1273,80494	-2,59	29,64	1
	High	lkNWDERPGQk	1	1	1	Q55584	N-Term(iTRAQ4plex); K2(iTRAQ4plex); K11(iTRAQ4plex)	0,0000	4,017	1		0	0,00005267	10	0,092676581	4	1803,02187	-1,06	18,80	1
	High	sLFFVk	1	1	1	Q55584	N-Term(iTRAQ4plex); K6(iTRAQ4plex)	0,0000	0,625	1		0,002	0,01887	45	3,19867E-05	2	1028,63713	-1,13	36,95	0
	Medium	fQINAR	1	1	1	Q55584	N-Term(iTRAQ4plex)	0,0000	0,677	1		0,01	0,12	14	0,037754611	2	892,51158	-0,63	20,69	0
Q55375	Slr0909 protein OS=Synechocystis sp. (strain PCC 6803 / Kazusa) GN=slr0909 PE=	56,22	5,33	1	2	2	2	0,743	2	12,7	450	52,5	5,36							
	A3	Sequence	# PSMs	# Proteins	# Protein Groups	Protein Group Accessions	Modifications	ΔCn	114/115	114/115 Count	114/115 Variability [%]	q-Value	PEP	IonScore	Exp Value	Charge	MH+ [Da]	ΔM [ppm]	RT [min]	# Missed Cleavages
	High	qIIVEQMINAIHQEFQR	1	1	1	Q55375	N-Term(iTRAQ4plex)	0,0000	0,682	1		0	1,416E-08	42	6,26571E-05	3	2241,19516	0,91	57,05	0
	High	wHIIIYR	1	1	1	Q55375	N-Term(iTRAQ4plex)	0,0000	0,809	1		0	0,0002565	27	0,001900947	3	1144,67456	-0,19	33,15	0
P72891	Slr1613 protein OS=Synechocystis sp. (strain PCC 6803 / Kazusa) GN=slr1613 PE=	55,99	12,76	1	2	2	2	0,510	2	104,8	196	22,3	4,86							
	A3	Sequence	# PSMs	# Proteins	# Protein Groups	Protein Group Accessions	Modifications	ΔCn	114/115	114/115 Count	114/115 Variability [%]	q-Value	PEP	IonScore	Exp Value	Charge	MH+ [Da]	ΔM [ppm]	RT [min]	# Missed Cleavages
	High	kEPDESYNIHIVk	1	1	1	P72891	N-Term(iTRAQ4plex); K1(iTRAQ4plex); K13(iTRAQ4plex)	0,0000	0,285	1		0	1,144E-08	42	7,52832E-05	4	2004,11049	-1,07	25,03	1
	High	sTIGLLLEAYmR	1	1	1	P72891	N-Term(iTRAQ4plex); M11(Oxidation)	0,0000	0,912	1		0	0,0000018	27	0,001790482	2	1526,83830	0,89	48,96	0
Q55673	UDP-N-acetylglucosamine 1-carboxyvinyltransferase OS=Synechocystis sp. (strain P	55,30	4,79	1	2	2	2	1,372	2	12,2	438	46,6	5,97							
	A3	Sequence	# PSMs	# Proteins	# Protein Groups	Protein Group Accessions	Modifications	ΔCn	114/115	114/115 Count	114/115 Variability [%]	q-Value	PEP	IonScore	Exp Value	Charge	MH+ [Da]	ΔM [ppm]	RT [min]	# Missed Cleavages
	High	aSFFVIGPLLSR	1	1	1	Q55673	N-Term(iTRAQ4plex)	0,0000	1,489	1		0	2,726E-07	55	2,94422E-06	2	1450,85588	1,36	53,40	0
	High	lQHVAELQR	1	1	1	Q55673	N-Term(iTRAQ4plex)	0,0000	1,264	1		0	0,001006	10	0,090150887	3	1237,71512	1,41	19,35	0
P72756	Sll1033 protein OS=Synechocystis sp. (strain PCC 6803 / Kazusa) GN=sll1033 PE=	54,57	2,54	1	2	2	3	0,766	3	12,0	668	73,7	4,46							
	A3	Sequence	# PSMs	# Proteins	# Protein Groups	Protein Group Accessions	Modifications	ΔCn	114/115	114/115 Count	114/115 Variability [%]	q-Value	PEP	IonScore	Exp Value	Charge	MH+ [Da]	ΔM [ppm]	RT [min]	# Missed Cleavages
	High	aIIPYFR	2	1	1	P72756	N-Term(iTRAQ4plex)	0,0000	0,726	2	8,0	0	0,005203	34	0,000487427	2	1023,61064	-0,15	37,95	0
	High	vRPQIPLDVW	1	1	1	P72756	N-Term(iTRAQ4plex)	0,0000	0,830	1		0,002	0,02072	23	0,005247712	2	1366,79729	0,65	45,53	0
P73288	Aklaviketone reductase OS=Synechocystis sp. (strain PCC 6803 / Kazusa) GN=sll18	54,51	8,57	1	2	2	3	0,868	3	7,8	210	22,0	5,85							
	A3	Sequence	# PSMs	# Proteins	# Protein Groups	Protein Group Accessions	Modifications	ΔCn	114/115	114/115 Count	114/115 Variability [%]	q-Value	PEP	IonScore	Exp Value	Charge	MH+ [Da]	ΔM [ppm]	RT [min]	# Missed Cleavages
	High	aIVTSLQGk	1	1	1	P73288	N-Term(iTRAQ4plex); K9(iTRAQ4plex)	0,0000	0,937	1		0	0,0004278	45	2,86398E-05	2	1204,75066	0,23	24,43	0
	High	lmGQVNLIR	2	1	1	P73288	N-Term(iTRAQ4plex); M2(Oxidation)	0,0000	0,845	2	3,9	0	0,0004756	23	0,007046012	2	1203,70012	0,06	29,54	0
P74795	Ssl0352 protein OS=Synechocystis sp. (strain PCC 6803 / Kazusa) GN=ssl0352 PE=	54,35	36,21	1	3	3	4	0,733	4	12,1	58	6,6	5,26							
	A3	Sequence	# PSMs	# Proteins	# Protein Groups	Protein Group Accessions	Modifications	ΔCn	114/115	114/115 Count	114/115 Variability [%]	q-Value	PEP	IonScore	Exp Value	Charge	MH+ [Da]	ΔM [ppm]	RT [min]	# Missed Cleavages
	High	fEGLVQR	1	1	1	P74795	N-Term(iTRAQ4plex)	0,0000	0,951	1		0,003	0,03292	24	0,0044154	2	992,56420	-0,39	26,94	0
	Medium	mIFPGATVR	1	1	1	P74795	N-Term(iTRAQ4plex)	0,0000	0,784	1		0,013	0,1512	29	0,001140171	2	1135,63909	-2,10	33,38	0
	Medium	lVTFR	2	1	1	P74795	N-Term(iTRAQ4plex)	0,0000	0,675	2	2,0	0,025	0,2671	21	0,00810905	2	779,48943	-0,24	25,19	0
P74067	Ssl1498 protein OS=Synechocystis sp. (strain PCC 6803 / Kazusa) GN=ssl1498 PE=	54,33	24,59	1	2	2	2	2,164	2	20,9	61	6,4	6,58							
	A3	Sequence	# PSMs	# Proteins	# Protein Groups	Protein Group Accessions	Modifications	ΔCn	114/115	114/115 Count	114/115 Variability [%]	q-Value	PEP	IonScore	Exp Value	Charge	MH+ [Da]	ΔM [ppm]	RT [min]	# Missed Cleavages
	High	lNNFAVEPk	1	1	1	P74067	N-Term(iTRAQ4plex); K9(iTRAQ4plex)	0,0000	1,882	1		0	0,00001029	36	0,000283733	2	1319,75749	0,98	28,41	0
	Medium	mNNYTk	1	1	1	P74067	N-Term(iTRAQ4plex); M1(Oxidation); K6(iTRAQ4plex)	0,0000	2,488	1		0,011	0,1344	31	0,000726056	2	1074,55168	2,30	11,97	0
Q55492	Sll0497 protein OS=Synechocystis sp. (strain PCC 6803 / Kazusa) GN=sll0497 PE=	54,33	8,00	1	2	2	3	0,848	3	5,0	275	31,5	5,74							
	A3	Sequence	# PSMs	# Proteins	# Protein Groups	Protein Group Accessions	Modifications	ΔCn	114/115	114/115 Count	114/115 Variability [%]	q-Value	PEP	IonScore	Exp Value	Charge	MH+ [Da]	ΔM [ppm]	RT [min]	# Missed Cleavages
	High	gLSLASLTFIR	2	1	1	Q55492	N-Term(iTRAQ4plex)	0,0000	0,834	2	2,5	0	0,0002119	36	0,000262404	2	1321,79766	1,21	50,33	0
	High	gSDAQERPALk	1	1	1	Q55492	N-Term(iTRAQ4plex); K11(iTRAQ4plex)	0,0000	0,936	1		0,002	0,02037	15	0,033573761	3	1459,80786	-1,99	13,24	0
P74328	Uncharacterized tRNA/rRNA methyltransferase slr0955 OS=Synechocystis sp. (strair	54,02	8,07	1	3	3	3	0,893	3	2,6	384	42,8	9,64							
	A3	Sequence	# PSMs	# Proteins	# Protein Groups	Protein Group Accessions	Modifications	ΔCn	114/115	114/115 Count	114/115 Variability [%]	q-Value	PEP	IonScore	Exp Value	Charge	MH+ [Da]	ΔM [ppm]	RT [min]	# Missed Cleavages
	High	iWITSHLR	1	1	1	P74328	N-Term(iTRAQ4plex)	0,0000	0,893	1		0	0,00000273	50	1,04947E-05	3	1169,69098	-0,16	31,59	0
	High	vAAGALEHFPVAR	1	1	1	P74328	N-Term(iTRAQ4plex)	0,0000	0,991	1		0	0,002172	17	0,01978758	3	1481,83545	0,59	32,64	0
	High	qTALHQADLR	1	1	1	P74328	N-Term(iTRAQ4plex)	0,0000	0,877	1		0,003	0,03683	12	0,062369176	3	1296,71509	0,76	14,89	0
P73069	Ycf48-like protein OS=Synechocystis sp. (strain PCC 6803 / Kazusa) GN=slr2034 PE	53,95	7,02	1	3	3	4	0,996	4	4,4	342	37,3	4,89							
	A3	Sequence	# PSMs	# Proteins	# Protein Groups	Protein Group Accessions	Modifications	ΔCn	114/115	114/115 Count	114/115 Variability [%]	q-Value	PEP	IonScore	Exp Value	Charge	MH+ [Da]	ΔM [ppm]	RT [min]	# Missed Cleavages
	High	yVAVSAR	2	1	1	P73069	N-Term(iTRAQ4plex)	0,0000	0,967	2	1,1	0	0,002221	28	0,001566643	2	909,52849	1,14	17,25	0
	High	vVFLSPEk	1	1	1	P73069	N-Term(iTRAQ4plex); K8(iTRAQ4plex)	0,0000	1,171	1		0	0,0002685	28	0,001614247	2	1206,73516	1,23	31,75	0
	High	rLQTmGYGk	1	1	1	P73069	N-Term(iTRAQ4plex); M5(Oxidation); K9(iTRAQ4plex)	0,0000	1,019	1		0	0,00267	20	0,009461718	3	1357,74744	-1,93	14,45	1
P09190	Cytochrome b559 subunit alpha OS=Synechocystis sp. (strain PCC 6803 / Kazusa) (	53,64	32,10	1	2	2	4	0,726	4	30,8	81	9,4	4,59							
	A3	Sequence	# PSMs	# Proteins	# Protein Groups	Protein Group Accessions	Modifications	ΔCn	114/115	114/115 Count	114/115 Variability [%]	q-Value	PEP	IonScore	Exp Value	Charge	MH+ [Da]	ΔM [ppm]	RT [min]	# Missed Cleavages
	High	qELPILQER	2	1	1	P09190	N-Term(iTRAQ4plex)	0,0000	0,557	2	9,5	0	0,002645	36	0,000234947	2	1269,72807	-0,23	32,95	0
	High	sGTTGERPFSDIVTSIR	2	1	1	P09190	N-Term(iTRAQ4plex)	0,0000	0,889	2	0,3	0	0,000003639	18	0,015169456	3	1967,03098	-0,27	36,34	0
Q55841	Arabinofuranosidase OS=Synechocystis sp. (strain PCC 6803 / Kazusa) GN=abfB PE	53,01	10,73	1	2	2	2	2,236	2	0,7	177	20,5	9,10							
	A3	Sequence	# PSMs	# Proteins	# Protein Groups	Protein Group Accessions	Modifications	ΔCn	114/115	114/115 Count	114/115 Variability [%]	q-Value	PEP	IonScore	Exp Value	Charge	MH+ [Da]	ΔM [ppm]	RT [min]	# Missed Cleavages
	High	fSGFTESTIFR	1	1	1	Q55841	N-Term(iTRAQ4plex)	0,0000	2,226	1		0	0,0001316	38	0,000169813	2	1435,73735	2,44	42,51	0
	High	nFPNYFLR	1	1	1	Q55841	N-Term(iTRAQ4plex)	0,0000	2,246	1		0,009	0,1104	28	0,001468825	2	1214,64568	1,48	39,86	0
P74379	Slr0443 protein OS=Synechocystis sp. (strain PCC 6803 / Kazusa) GN=slr0443 PE=	52,86	10,40	1	2	2	4	1,411	3	6,1	125	14,6	4,58							
	A3	Sequence	# PSMs	# Proteins	# Protein Groups	Protein Group Accessions	Modifications	ΔCn	114/115	114/115 Count	114/115 Variability [%]	q-Value	PEP	IonScore	Exp Value	Charge	MH+ [Da]	ΔM [ppm]	RT [min]	# Missed Cleavages
	High	lSHFIk	1	1	1	P74379	N-Term(iTRAQ4plex); K6(iTRAQ4plex)	0,0000	1,198	1		0	0,006894	12	0,070302376	3	1032,65238	7,69	22,53	0
	High	gLYGLLR	3	1	1	P74379	N-Term(iTRAQ4plex)	0,0000	1,440	2	3,1	0,002	0,0233	35	0,000328073	2	935,58037	0,94	37,06	0
P73719	Sll1628 protein OS=Synechocystis sp. (strain PCC 6803 / Kazusa) GN=sll1628 PE=	52,75	7,29	1	2	2	3	1,641	3	13,3	384	43,1	4,46							
	A3	Sequence	# PSMs	# Proteins	# Protein Groups	Protein Group Accessions	Modifications	ΔCn	114/115	114/115 Count	114/115 Variability [%]	q-Value	PEP	IonScore	Exp Value	Charge	MH+ [Da]	ΔM [ppm]	RT [min]	# Missed Cleavages
	High	fQTTVLASPSPNLVLAAR	1	1	1	P73719	N-Term(iTRAQ4plex)	0,0000	1,641	1		0	2,235E-08	46	2,47155E-05	3	2029,15702	0,34	44,69	0
	High	kEEAIPSYEk	2	1	1	P73719	N-Term(iTRAQ4plex); K1(iTRAQ4plex); K10(iTRAQ4plex)	0,0000	1,625	2	14,8	0	0,00001835	20	0,01099485	3	1625,91410	1,86	19,42	1
Q55484	Diaminopimelate decarboxylase OS=Synechocystis sp. (strain PCC 6803 / Kazusa) (	52,48	10,45	1	5	5	6	1,247	6	9,3	469	50,8	5,45							
	A3	Sequence	# PSMs	# Proteins	# Protein Groups	Protein Group Accessions	Modifications	ΔCn	114/115	114/115 Count	114/115 Variability [%]	q-Value	PEP	IonScore	Exp Value	Charge	MH+ [Da]	ΔM [ppm]	RT [min]	# Missed Cleavages
	High	tPASPSPNQNLLPLTAVINk	1	1	1	Q55484	N-Term(iTRAQ4plex); K20(iTRAQ4plex)	0,0000	1,160	1		0	0,00004513	33	0,000503466	3	2363,35947	2,42	45,22	0
	High	iYFHGNNk	2	1	1	Q55484	N-Term(iTRAQ4plex); K8(iTRAQ4plex)	0,0000	1,602	2	36,0	0	0,002437	13	0,047009897	3	1280,70060	1,26	17,49	0
	High	kVVPNIR	1	1	1	Q55484	N-Term(iTRAQ4plex); K1(iTRAQ4plex)	0,0000	1,183	1		0,005	0,06467	14	0,038634029	3	1113,73532	0,59	17,35	1
	High	lIAEPGR	1	1	1	Q55484	N-Term(iTRAQ4plex)	0,0000	1,229	1		0,008	0,09382	19	0,01166729	2	899,54369	0,63	20,02	0
	Medium	qNIPYPk	1	1	1	Q55484	N-Term(iTRAQ4plex); K7(iTRAQ4plex)	0,0000	2,218	1		0,03	0,328	25	0,003460838	2	1147,66936	-1,76	20,00	0
P72861	Uncharacterized protein sll0936 OS=Synechocystis sp. (strain PCC 6803 / Kazusa) (	52,34	3,19	1	2	2	2	0,555	2	29,6	501	55,2	5,35							
	A3	Sequence	# PSMs	# Proteins	# Protein Groups	Protein Group Accessions	Modifications	ΔCn	114/115	114/115 Count	114/115 Variability [%]	q-Value	PEP	IonScore	Exp Value	Charge	MH+ [Da]	ΔM [ppm]	RT [min]	# Missed Cleavages
	High	gFIQLLETR	1	1	1	P72861	N-Term(iTRAQ4plex)	0,0000	0,457	1		0	0,00003993	45	3,44494E-05	2	1220,71233	0,28	46,32	0
	Medium	iYTPILR	1	1	1	P72861	N-Term(iTRAQ4plex)	0,0000	0,675	1		0,029	0,3158	21	0,01209063	2	1019,63561	-1,37	33,28	0
P73557	RNA-binding protein OS=Synechocystis sp. (strain PCC 6803 / Kazusa) GN=ssr1480	52,11	14,46	2	4	4	10	1,168	9	32,2	83	9,4	5,01							
	A3	Sequence	# PSMs	# Proteins	# Protein Groups	Protein Group Accessions	Modifications	ΔCn	114/115	114/115 Count	114/115 Variability [%]	q-Value	PEP	IonScore	Exp Value	Charge	MH+ [Da]	ΔM [ppm]	RT [min]	# Missed Cleavages
	High	rVQLPTDRETGR	1	2	1	P73557	N-Term(iTRAQ4plex)	0,0000	1,115	1		0	0,00103	25	0,002857393	4	1571,87343	-0,03	16,66	2
	High	rVQLPTDR	4	2	1	P73557	N-Term(iTRAQ4plex)	0,0000	1,168	3	0,4	0	0,004845	21	0,007815738	3	1128,66327	2,36	16,52	1
	High	vQLPTDRETGR	3	2	1	P73557	N-Term(iTRAQ4plex)	0,0000	1,859	3	15,9	0,002	0,02408	13	0,052840875	3	1415,77457	1,56	19,64	1

	Medium	vQLPTDR	2	2	1	P73557	N-Term(iTRAQ4plex)	0,0000	1,189	2	62,7	0,028	0,3031	17	0,018195752	2	972,56078	1,32	22,06	0
P73257	Argininosuccinate lyase OS=Synechocystis sp. (strain PCC 6803 / Kazusa) GN=argH	51,80	2,60	1	2	2	2	0,847	2	11,1	461	50,9	5,54							
	A3	Sequence	# PSMs	# Proteins	# Protein Groups	Protein Group Accessions	Modifications	ΔCn	114/115	114/115 Count	114/115 Variability [%]	q-Value	PEP	IonScore	Exp Value	Charge	MH+ [Da]	ΔM [ppm]	RT [min]	# Missed Cleavages
	High	rLTELVGDVGk	1	1	1	P73257	N-Term(iTRAQ4plex); K11(iTRAQ4plex)	0,0000	0,913	1		0	3,662E-07	46	2,75231E-05	3	1474,88358	0,25	30,56	1
	High	rLTELVGDVGkk	1	1	1	P73257	N-Term(iTRAQ4plex); K11(iTRAQ4plex); K12(iTRAQ4plex)	0,0000	0,786	1		0	0,001357	19	0,012851979	4	1747,08327	1,74	27,00	2
P77964	Protein translocase subunit SecY OS=Synechocystis sp. (strain PCC 6803 / Kazusa)	51,77	3,39	1	2	2	2	1,132	2	14,0	442	48,0	9,70							
	A3	Sequence	# PSMs	# Proteins	# Protein Groups	Protein Group Accessions	Modifications	ΔCn	114/115	114/115 Count	114/115 Variability [%]	q-Value	PEP	IonScore	Exp Value	Charge	MH+ [Da]	ΔM [ppm]	RT [min]	# Missed Cleavages
	High	rIPIISAR	1	1	1	P77964	N-Term(iTRAQ4plex)	0,0000	1,031	1		0	0,00002037	42	6,99794E-05	3	1069,69672	0,42	24,08	1
	Medium	tSYLPLR	1	1	1	P77964	N-Term(iTRAQ4plex)	0,0000	1,244	1		0,017	0,1876	23	0,004742092	2	993,58714	2,19	26,98	0
P72845	Sll1188 protein OS=Synechocystis sp. (strain PCC 6803 / Kazusa) GN=sll1188 PE=4	51,59	21,34	1	3	3	4	0,893	4	22,9	164	18,8	5,00							
	A3	Sequence	# PSMs	# Proteins	# Protein Groups	Protein Group Accessions	Modifications	ΔCn	114/115	114/115 Count	114/115 Variability [%]	q-Value	PEP	IonScore	Exp Value	Charge	MH+ [Da]	ΔM [ppm]	RT [min]	# Missed Cleavages
	High	lELLPHPEGGFYk	1	1	1	P72845	N-Term(iTRAQ4plex); K13(iTRAQ4plex)	0,0000	0,732	1		0	0,000001391	27	0,002008954	3	1787,99393	0,25	39,12	0
	High	nVVTGIYFLmTk	2	1	1	P72845	N-Term(iTRAQ4plex); M10(Oxidation); K12(iTRAQ4plex)	0,0000	1,172	2	25,0	0	0,000002927	26	0,002333297	3	1689,94736	-0,87	43,49	0
	High	sAEIINQLTR	1	1	1	P72845	N-Term(iTRAQ4plex)	0,0000	0,803	1		0	0,0004553	22	0,005860977	2	1288,73308	-0,85	33,02	0
P48959	50S ribosomal protein L35 OS=Synechocystis sp. (strain PCC 6803 / Kazusa) GN=rp	51,02	32,84	1	2	2	3	1,260	3	19,7	67	7,9	11,36							
	A3	Sequence	# PSMs	# Proteins	# Protein Groups	Protein Group Accessions	Modifications	ΔCn	114/115	114/115 Count	114/115 Variability [%]	q-Value	PEP	IonScore	Exp Value	Charge	MH+ [Da]	ΔM [ppm]	RT [min]	# Missed Cleavages
	High	rLSNLALVHEADEk	2	1	1	P48959	N-Term(iTRAQ4plex); K14(iTRAQ4plex)	0,0000	1,139	2	15,0	0	3,028E-07	36	0,00025175	3	1883,06028	0,71	25,12	1
	High	fRPTGSGk	1	1	1	P48959	N-Term(iTRAQ4plex); K8(iTRAQ4plex)	0,0000	1,436	1		0	0,003987	11	0,08471689	3	1137,66217	0,24	10,94	0
Q55626	Bifunctional protein FolD OS=Synechocystis sp. (strain PCC 6803 / Kazusa) GN=folD	50,45	17,01	1	3	3	4	1,056	4	48,5	294	30,9	6,01							
	A3	Sequence	# PSMs	# Proteins	# Protein Groups	Protein Group Accessions	Modifications	ΔCn	114/115	114/115 Count	114/115 Variability [%]	q-Value	PEP	IonScore	Exp Value	Charge	MH+ [Da]	ΔM [ppm]	RT [min]	# Missed Cleavages
	High	dIAQLVAQGHR	1	1	1	Q55626	N-Term(iTRAQ4plex)	0,0000	0,728	1		0	0,00001407	31	0,000807179	3	1351,75568	-0,47	26,59	0
	High	sILVGkPLALmLLEk	2	1	1	Q55626	N-Term(iTRAQ4plex); K6(iTRAQ4plex); M11(Oxidation); K15(iTRAQ4plex)	0,0000	1,056	2	38,0	0	0,000000389	30	0,000903587	3	2073,30960	0,67	48,85	0
	High	lLHTIDPSkDADGLHPVNLGHLVR	1	1	1	Q55626	N-Term(iTRAQ4plex); K9(iTRAQ4plex)	0,0000	2,003	1		0	0,000001026	12	0,067915672	6	2905,61968	-1,88	36,90	1
	>sp|CAS1_BOVIN|	50,41	10,28	1	2	2	2	0,909	2	2,0	214	24,5	5,02							
	A3	Sequence	# PSMs	# Proteins	# Protein Groups	Protein Group Accessions	Modifications	ΔCn	114/115	114/115 Count	114/115 Variability [%]	q-Value	PEP	IonScore	Exp Value	Charge	MH+ [Da]	ΔM [ppm]	RT [min]	# Missed Cleavages
	High	yLGYLEQLLR	1	1	1		N-Term(iTRAQ4plex)	0,0000	0,897	1		0	5,158E-07	50	9,07758E-06	2	1411,80766	0,76	56,61	0
	High	fFVAPFPEVFGk	1	1	1		N-Term(iTRAQ4plex); K12(iTRAQ4plex)	0,0000	0,922	1		0	0,005542	13	0,052477121	2	1672,93804	2,30	54,24	0
P74572	Pyrroline-5-carboxylate reductase OS=Synechocystis sp. (strain PCC 6803 / Kazusa)	49,54	8,61	1	2	2	2	1,000	2	0,4	267	27,9	8,31							
	A3	Sequence	# PSMs	# Proteins	# Protein Groups	Protein Group Accessions	Modifications	ΔCn	114/115	114/115 Count	114/115 Variability [%]	q-Value	PEP	IonScore	Exp Value	Charge	MH+ [Da]	ΔM [ppm]	RT [min]	# Missed Cleavages
	High	lALQTVLGTAELIk	1	1	1	P74572	N-Term(iTRAQ4plex); K14(iTRAQ4plex)	0,0000	1,002	1		0	0,000001019	42	6,54591E-05	3	1758,10031	1,34	53,98	0
	High	gFPDHAIIR	1	1	1	P74572	N-Term(iTRAQ4plex)	0,0000	0,997	1		0	0,002634	21	0,008471689	3	1169,65445	-0,30	24,23	0
Q55139	Glutathione S-transferase OS=Synechocystis sp. (strain PCC 6803 / Kazusa) GN=gs	49,27	15,22	1	3	3	4	0,828	4	21,0	184	20,4	6,57							
	A3	Sequence	# PSMs	# Proteins	# Protein Groups	Protein Group Accessions	Modifications	ΔCn	114/115	114/115 Count	114/115 Variability [%]	q-Value	PEP	IonScore	Exp Value	Charge	MH+ [Da]	ΔM [ppm]	RT [min]	# Missed Cleavages
	High	sPFILGEk	1	1	1	Q55139	N-Term(iTRAQ4plex); K8(iTRAQ4plex)	0,0000	0,588	1		0	0,0009231	37	0,000193183	2	1178,70036	-1,70	32,32	0
	High	qAPYLAINPFGk	1	1	1	Q55139	N-Term(iTRAQ4plex); K12(iTRAQ4plex)	0,0000	0,882	1		0	0,000003884	16	0,023713737	3	1606,91947	-0,07	43,56	0
	Medium	lYGAPQSR	2	1	1	Q55139	N-Term(iTRAQ4plex)	0,0000	0,895	2	21,0	0,01	0,1233	19	0,011994165	2	1035,56902	-1,32	17,52	0
P22040	Uncharacterized ABC transporter ATP-binding protein sll0415 OS=Synechocystis sp.	47,66	6,78	1	3	3	3	0,791	3	2,8	354	39,5	8,10							
	A3	Sequence	# PSMs	# Proteins	# Protein Groups	Protein Group Accessions	Modifications	ΔCn	114/115	114/115 Count	114/115 Variability [%]	q-Value	PEP	IonScore	Exp Value	Charge	MH+ [Da]	ΔM [ppm]	RT [min]	# Missed Cleavages
	High	gTLLGkPIGDR	1	1	1	P22040	N-Term(iTRAQ4plex); K6(iTRAQ4plex)	0,0000	0,957	1		0,001	0,01484	12	0,065008479	3	1414,86353	1,03	23,05	0
	High	iLLGVIR	1	1	1	P22040	N-Term(iTRAQ4plex)	0,0000	0,791	1		0,002	0,03178	28	0,001458714	2	927,64678	-0,45	39,33	0
	High	iAILAR	1	1	1	P22040	N-Term(iTRAQ4plex)	0,0000	0,776	1		0,004	0,05535	32	0,000586098	2	800,54808	0,76	27,40	0
Q55171	Slr0476 protein OS=Synechocystis sp. (strain PCC 6803 / Kazusa) GN=slr0476 PE=4	46,75	17,65	1	2	2	5	1,085	4	32,4	136	14,4	7,59							
	A3	Sequence	# PSMs	# Proteins	# Protein Groups	Protein Group Accessions	Modifications	ΔCn	114/115	114/115 Count	114/115 Variability [%]	q-Value	PEP	IonScore	Exp Value	Charge	MH+ [Da]	ΔM [ppm]	RT [min]	# Missed Cleavages
	High	hIGGLDLLPTPk	3	1	1	Q55171	N-Term(iTRAQ4plex); K12(iTRAQ4plex)	0,0000	0,967	3	29,7	0	0,00002934	35	0,000309008	3	1548,93912	2,51	35,86	0
	High	gmPTNRPIEASk	2	1	1	Q55171	N-Term(iTRAQ4plex); M2(Oxidation); K12(iTRAQ4plex)	0,0000	1,488	1		0	0,00002734	19	0,017273009	3	1604,86667	-0,15	13,00	0
P73545	Uncharacterized thylakoid-associated protein ssl2501 OS=Synechocystis sp. (strain	46,30	25,84	1	2	2	2	1,087	2	0,1	89	10,6	4,67							
	A3	Sequence	# PSMs	# Proteins	# Protein Groups	Protein Group Accessions	Modifications	ΔCn	114/115	114/115 Count	114/115 Variability [%]	q-Value	PEP	IonScore	Exp Value	Charge	MH+ [Da]	ΔM [ppm]	RT [min]	# Missed Cleavages
	High	qFWDNYFQALR	1	1	1	P73545	N-Term(iTRAQ4plex)	0,0000	1,088	1		0	0,00005845	43	4,64483E-05	2	1631,81133	1,59	50,20	0
	High	kFFSTWmESLPk	1	1	1	P73545	N-Term(iTRAQ4plex); K1(iTRAQ4plex); M7(Oxidation); K12(iTRAQ4plex)	0,0000	1,087	1		0	0,000003864	17	0,032395445	3	1949,05753	0,41	38,76	1
Q6ZEQ5	Sll5075 protein OS=Synechocystis sp. (strain PCC 6803 / Kazusa) GN=sll5075 PE=4	45,82	16,55	1	2	2	3	1,073	3	1,9	139	15,6	5,15							
	A3	Sequence	# PSMs	# Proteins	# Protein Groups	Protein Group Accessions	Modifications	ΔCn	114/115	114/115 Count	114/115 Variability [%]	q-Value	PEP	IonScore	Exp Value	Charge	MH+ [Da]	ΔM [ppm]	RT [min]	# Missed Cleavages
	High	mkEDFGFTHIALFAk	1	1	1	Q6ZEQ5	N-Term(iTRAQ4plex); M1(Oxidation); K2(iTRAQ4plex); K15(iTRAQ4plex)	0,0000	1,073	1		0	0,000001191	32	0,00061372	4	2203,20131	3,03	39,64	1
	High	mkEDFGFTHIALFAk	1	1	1	Q6ZEQ5	N-Term(iTRAQ4plex); K2(iTRAQ4plex); K15(iTRAQ4plex)	0,0000	1,086	1		0	1,954E-07	28	0,002692278	4	2187,19863	-0,50	42,55	1
	High	lDkDTGkR	1	1	1	Q6ZEQ5	N-Term(iTRAQ4plex); K3(iTRAQ4plex); K7(iTRAQ4plex)	0,0000	0,787	1		0,001	0,01405	12	0,062657059	3	1364,82163	-0,43	9,31	2
P72622	Peptide methionine sulfoxide reductase MsrA 1 OS=Synechocystis sp. (strain PCC 6	45,40	9,46	1	2	2	4	1,295	4	5,5	222	24,2	5,26							
	A3	Sequence	# PSMs	# Proteins	# Protein Groups	Protein Group Accessions	Modifications	ΔCn	114/115	114/115 Count	114/115 Variability [%]	q-Value	PEP	IonScore	Exp Value	Charge	MH+ [Da]	ΔM [ppm]	RT [min]	# Missed Cleavages
	High	vFWESHNPTQGmR	1	1	1	P72622	N-Term(iTRAQ4plex); M12(Oxidation)	0,0000	0,658	1		0	0,00001047	26	0,002306588	3	1748,83023	0,38	26,22	0
	High	gFFDLFGk	3	1	1	P72622	N-Term(iTRAQ4plex); K8(iTRAQ4plex)	0,0000	1,296	3	0,3	0	0,00004047	22	0,005754002	2	1218,67742	1,03	50,33	0
P73707	Sll1692 protein OS=Synechocystis sp. (strain PCC 6803 / Kazusa) GN=sll1692 PE=4	44,90	11,72	1	2	2	2	0,860	2	8,5	145	17,2	5,00							
	A3	Sequence	# PSMs	# Proteins	# Protein Groups	Protein Group Accessions	Modifications	ΔCn	114/115	114/115 Count	114/115 Variability [%]	q-Value	PEP	IonScore	Exp Value	Charge	MH+ [Da]	ΔM [ppm]	RT [min]	# Missed Cleavages
	High	lmVLVAHLLk	1	1	1	P73707	N-Term(iTRAQ4plex); M2(Oxidation); K10(iTRAQ4plex)	0,0000	0,812	1		0	4,065E-07	45	3,22827E-05	3	1440,92008	-0,99	43,50	0
	High	wQVQHWk	1	1	1	P73707	N-Term(iTRAQ4plex); K7(iTRAQ4plex)	0,0000	0,911	1		0	0,002049	13	0,050346583	3	1299,71958	-0,38	26,27	0
Q55445	Putative methyl-accepting chemotaxis protein sll0041 OS=Synechocystis sp. (strain	44,62	1,60	1	2	2	3	0,625	3	12,6	1000	108,3	4,79							
	A3	Sequence	# PSMs	# Proteins	# Protein Groups	Protein Group Accessions	Modifications	ΔCn	114/115	114/115 Count	114/115 Variability [%]	q-Value	PEP	IonScore	Exp Value	Charge	MH+ [Da]	ΔM [ppm]	RT [min]	# Missed Cleavages
	High	lVFYYHPR	1	1	1	Q55445	N-Term(iTRAQ4plex)	0,0000	0,681	1		0	0,001094	31	0,000872911	3	1238,68000	-0,21	31,45	0
	High	qLAQQANR	2	1	1	Q55445	N-Term(iTRAQ4plex)	0,0000	0,597	2	6,9	0	0,0001556	25	0,003523465	2	1072,59734	-0,62	11,13	0
Q55791	Probable ATP-dependent transporter slr0075 OS=Synechocystis sp. (strain PCC 680	44,33	12,11	1	2	2	2	0,861	2	8,8	256	28,4	4,87							
	A3	Sequence	# PSMs	# Proteins	# Protein Groups	Protein Group Accessions	Modifications	ΔCn	114/115	114/115 Count	114/115 Variability [%]	q-Value	PEP	IonScore	Exp Value	Charge	MH+ [Da]	ΔM [ppm]	RT [min]	# Missed Cleavages
	High	nPDNATLVITHYQR	1	1	1	Q55791	N-Term(iTRAQ4plex)	0,0000	0,812	1		0	7,004E-09	36	0,000254665	3	1785,93546	-0,56	26,23	0
	High	lLNYIIPDHIHVmYDGk	1	1	1	Q55791	N-Term(iTRAQ4plex); M13(Oxidation); K17(iTRAQ4plex)	0,0000	0,914	1		0	0,000253	21	0,007227199	4	2345,25307	-1,53	43,14	0
Q55780	Slr0208 protein OS=Synechocystis sp. (strain PCC 6803 / Kazusa) GN=slr0208 PE=4	44,07	8,49	1	2	2	3	1,226	2	5,5	259	29,9	5,07							
	A3	Sequence	# PSMs	# Proteins	# Protein Groups	Protein Group Accessions	Modifications	ΔCn	114/115	114/115 Count	114/115 Variability [%]	q-Value	PEP	IonScore	Exp Value	Charge	MH+ [Da]	ΔM [ppm]	RT [min]	# Missed Cleavages
	High	dkLDQVIYSLIR	2	1	1	Q55780	N-Term(iTRAQ4plex); K2(iTRAQ4plex)	0,0000	1,272	1		0	4,766E-08	37	0,000205102	3	1751,03208	0,85	51,64	1
	High	iQFALPSIPk	1	1	1	Q55780	N-Term(iTRAQ4plex); K10(iTRAQ4plex)	0,0000	1,182	1		0	0,0009598	20	0,009527304	3	1401,87220	0,97	45,19	0
P74305	Protease OS=Synechocystis sp. (strain PCC 6803 / Kazusa) GN=pqqE PE=3 SV=1 -	43,90	4,96	1	2	2	2	1,299	2	3,2	524	59,6	5,15							
	A3	Sequence	# PSMs	# Proteins	# Protein Groups	Protein Group Accessions	Modifications	ΔCn	114/115	114/115 Count	114/115 Variability [%]	q-Value	PEP	IonScore	Exp Value	Charge	MH+ [Da]	ΔM [ppm]	RT [min]	# Missed Cleavages
	High	rPVIGYDEDIR	1	1	1	P74305	N-Term(iTRAQ4plex)	0,0000	1,271	1		0	0,000001228	39	0,000132425	3	1476,79282	0,04	24,16	0
	High	vAQETFRPENSTIGR	1	1	1	P74305	N-Term(iTRAQ4plex)	0,0000	1,327	1		0	0,0001185	18	0,016121026	3	1848,96842	-0,05	23,81	0
Q55799	General secretion pathway protein E OS=Synechocystis sp. (strain PCC 6803 / Kazu	43,89	3,20	1	4	4	4	0,872	4	13,9	813	90,1	5,52							
	A3	Sequence	# PSMs	# Proteins	# Protein Groups	Protein Group Accessions	Modifications	ΔCn	114/115	114/115 Count	114/115 Variability [%]	q-Value	PEP	IonScore	Exp Value	Charge	MH+ [Da]	ΔM [ppm]	RT [min]	# Missed Cleavages
	High	iLILGLR	1	1	1	Q55799	N-Term(iTRAQ4plex)	0,0000	0,713	1		0,001	0,0119	30	0,001044648	2	941,66271	-0,15	43,55	0
	Medium	kFNPFk	1	1	1	Q55799	N-Term(iTRAQ4plex); K1(iTRAQ4plex); K6(iTRAQ4plex)	0,0000	0,843	1		0,013	0,149	17	0,01874865	3	1212,74421	-1,90	23,05	1
	Medium	aLADLkk	1	1	1	Q55799	N-Term(iTRAQ4plex); K6(iTRAQ4plex); K7(iTRAQ4plex)	0,0000	1,015	1		0,016	0,177	18	0,016031347	3	1190,77814	-4,33	19,70	1
	Medium	vLVQIR	1	1	1	Q55799	N-Term(iTRAQ4plex)	0,0000	0,903	1		0,033	0,3513	18	0,016216981	2	871,58415	-0,49	25,84	0
P74373	Putative diflavin flavoprotein A 3 OS=Synechocystis sp. (strain PCC 6803 / Kazusa)	43,27	4,36	1	2	2	2	0,851	2	22,2	597	65,1	6,48							
	A3	Sequence	# PSMs	# Proteins	# Protein Groups	Protein Group Accessions	Modifications	ΔCn	114/115	114/115 Count	114/115 Variability [%]	q-Value	PEP	IonScore	Exp Value	Charge	MH+ [Da]	ΔM [ppm]	RT [min]	# Missed Cleavages
	High	lEAYPAQTYAPSHGPLVR	1	1	1	P74373	N-Term(iTRAQ4plex)	0,0000	0,987	1		0	1,123E-09	30	0,001061622	3	2114,11228	-1,37	32,41	0
	High	aIESLLHk	1	1	1	P74373	N-Term(iTRAQ4plex); K8(iTRAQ4plex)	0,0000	0,734	1		0	0,007938	27	0,002212942	3	1198,73865	-0,96	29,73	0
P74324	Fructose-1,6-bisphosphatase class 1 OS=Synechocystis sp. (strain PCC 6803 / Kazu	43,09	5,76	1	2	2	2	0,886	2	28,9	347	38,2	5,67							
	A3	Sequence	# PSMs	# Proteins	# Protein Groups	Protein Group Accessions	Modifications	ΔCn	114/115	114/115 Count	114/115 Variability [%]	q-Value	PEP	IonScore	Exp Value	Charge	MH+ [Da]	ΔM [ppm]	RT [min]	# Missed Cleavages
	High	ySGALVGDIHR	1	1	1	P74324	N-Term(iTRAQ4plex)	0,0000	0,732	1		0	0,0002545	42	5,88803E-05	3	1331,71964	0,59	26,03	0
	High	rLLDLIPSk	1	1	1	P74324	N-Term(iTRAQ4plex); K9(iTRAQ4plex)	0,0000	1,073	1		0,001	0,01343	14	0,041588189	3	1342,86481	-0,95	37,67	1
P72985	Slr1600 protein OS=Synechocystis sp. (strain PCC 6803 / Kazusa) GN=slr1600 PE=4	43,00	20,13	1	4	4	6	1,404	6	6,1	149	17,1	4,98							
	A3	Sequence	# PSMs	# Proteins	# Protein Groups	Protein Group Accessions	Modifications	ΔCn	114/115	114/115 Count	114/115 Variability [%]	q-Value	PEP	IonScore	Exp Value	Charge	MH+ [Da]	ΔM [ppm]	RT [min]	# Missed Cleavages
	High	mGVELVLR	2	1	1	P72985	N-Term(iTRAQ4plex); M1(Oxidation)	0,0000	1,457	2	9,6	0	0,003328	24	0,005396697	2	1076,62615	0,61	29,83	0
	High	iQkGDNLVQk	1	1	1	P72985	N-Term(iTRAQ4plex); K3(iTRAQ4plex); K10(iTRAQ4plex)	0,0000	1,484	1		0	0,0039	19	0,013273028	3	1574,96372	2,97	19,49	1
	High	vIVTPR	2	1	1	P72985	N-Term(iTRAQ4plex)	0,0000	1,228	2	16,5	0,005	0,06663	14	0,037065512	2	828,54387	1,79	19,55	0
	High	rPVALk	1	1	1	P72985	N-Term(iTRAQ4plex); K6(iTRAQ4plex)	0,0000	1,436	1		0,006	0,07639	13	0,0468781	3	971,66974	9,58	12,09	0
P73953	Slr1512 protein OS=Synechocystis sp. (strain PCC 6803 / Kazusa) GN=slr1512 PE=4	42,51	7,22	1	2	2	3	0,400	2	137,5	374	39,6	5,44							
	A3	Sequence	# PSMs	# Proteins	# Protein Groups	Protein Group Accessions	Modifications	ΔCn	114/115	114/115 Count	114/115 Variability [%]	q-Value	PEP	IonScore	Exp Value	Charge	MH+ [Da]	ΔM [ppm]	RT [min]	# Missed Cleavages
	High	tRQEYLSQQEPEDNRVk	2	1	1	P73953	N-Term(iTRAQ4plex); K17(iTRAQ4plex)	0,0000	0,199	1		0	0,000002775	34	0,000401763	4	2408,24184	0,40	18,63	2
	High	iGLTGGmAIR	1	1	1	P73953	N-Term(iTRAQ4plex); M7(Oxidation)	0,0000	0,800	1		0,004	0,0523	22	0,006965784	2	1148,65715	-0,62	26,53	0
P73793	Sll1150 protein OS=Synechocystis sp. (strain PCC 6803 / Kazusa) GN=sll1150 PE=4	41,93	7,14	1	2	2	2	0,844	2	80,9	252	26,7	8,69							
	A3	Sequence	# PSMs	# Proteins	# Protein Groups	Protein Group Accessions	Modifications	ΔCn	114/115	114/115 Count	114/115 Variability [%]	q-Value	PEP	IonScore	Exp Value	Charge	MH+ [Da]	ΔM [ppm]	RT [min]	# Missed Cleavages
	High	gQSLIGPNVIk	1	1	1	P73793	N-Term(iTRAQ4plex); K11(iTRAQ4plex)	0,0000	0,523	1		0	0,000003611	37	0,000214768	3	1413,86661	-0,15	30,93	0
	High	qImAEAk	1	1	1	P73793	N-Term(iTRAQ4plex); M3(Oxidation); K7(iTRAQ4plex)	0,0000	1,362	1		0,002	0,02488	18	0,014892582	2	1094,61479	2,70	12,24	0
Q55360	Sll0888 protein OS=Synechocystis sp. (strain PCC 6803 / Kazusa) GN=sll0888 PE=4	41,70	16,84	1	3	3	3	0,910	3	25,8	190	21,9	4,94							
	A3	Sequence	# PSMs	# Proteins	# Protein Groups	Protein Group Accessions	Modifications	ΔCn	114/115	114/115 Count	114/115 Variability [%]	q-Value	PEP	IonScore	Exp Value	Charge	MH+ [Da]	ΔM [ppm]	RT [min]	# Missed Cleavages
	High	gHGLELEVHGEPk	1	1	1	Q55360	N-Term(iTRAQ4plex); K13(iTRAQ4plex)	0,0000	0,910	1		0	0,000004944	31	0,000810905	4	1689,91469	-0,95	21,09	0
	High	nRFPDLTIIR	1	1	1	Q55360	N-Term(iTRAQ4plex)	0,0000	1,079	1		0	0,001833	24	0,004158819	3	1388,81174	-0,98	37,08	1
	High	fPDLTIIREEHIAQLAk	1	1	1	Q55360	N-Term(iTRAQ4plex); K17(iTRAQ4plex)	0,0000	0,500	1		0	0,00005071	11	0,076730849	4	2282,31386	1,19	42,26	1
P73832	Cyanophycinase OS=Synechocystis sp. (strain PCC 6803 / Kazusa) GN=cphB PE=1	40,87	8,49	1	2	2	2	0,798	2	11,0	271	29,4	6,52							
	A3	Sequence	# PSMs	# Proteins	# Protein Groups	Protein Group Accessions	Modifications	ΔCn	114/115	114/115 Count	114/115 Variability [%]	q-Value	PEP	IonScore	Exp Value	Charge	MH+ [Da]	ΔM [ppm]	RT [min]	# Missed Cleavages
	High	eILQTFWSR	1	1	1	P73832	N-Term(iTRAQ4plex)	0,0000	0,859	1		0	0,00005425	32	0,000628015	2	1323,71953	1,31	44,73	0
	High	lNILVHGEVYHQVk	1	1	1	P73832	N-Term(iTRAQ4plex); K14(iTRAQ4plex)	0,0000	0,741	1		0	1,958E-08	22	0,006500848	4	1937,12331	1,13	31,26	0
Q55891	Phycocyanobilin:ferredoxin oxidoreductase OS=Synechocystis sp. (strain PCC 6803	39,71	8,06	1	2	2	2	1,332	2	31,5	248	28,1	5,20							
	A3	Sequence	# PSMs	# Proteins	# Protein Groups	Protein Group Accessions	Modifications	ΔCn	114/115	114/115 Count	114/115 Variability [%]	q-Value	PEP	IonScore	Exp Value	Charge	MH+ [Da]	ΔM [ppm]	RT [min]	# Missed Cleavages
	High	qGQIHYcQQQQk	1	1	1	Q55891	N-Term(iTRAQ4plex); C7(Carbamidomethyl); K12(iTRAQ4plex)	0,0000	1,082	1		0	9,987E-07	40	0,000110655	3	1833,93344	3,60	12,87	0
	Medium	kmHLELAk	1	1	1	Q55891	N-Term(iTRAQ4plex); K1(iTRAQ4plex); M2(Oxidation); K8(iTRAQ4plex)	0,0000	1,639	1		0,011	0,1315	13	0,048191451	4	1417,85256	-2,54	14,84	1
P73803	Slr1263 protein OS=Synechocystis sp. (strain PCC 6803 / Kazusa) GN=slr1263 PE=4	39,59	5,53	1	2	2	2	1,506	2	23,4	470	51,7	9,14							
	A3	Sequence	# PSMs	# Proteins	# Protein Groups	Protein Group Accessions	Modifications	ΔCn	114/115	114/115 Count	114/115 Variability [%]	q-Value	PEP	IonScore	Exp Value	Charge	MH+ [Da]	ΔM [ppm]	RT [min]	# Missed Cleavages
	High	gRFDVFAHHVVQASPEGk	1	1	1	P73803	N-Term(iTRAQ4plex); K18(iTRAQ4plex)	0,0000	1,760	1		0	6,18E-10	26	0,002754038	4	2269,21181	1,64	27,76	1
	High	tANLmQPR	1	1	1	P73803	N-Term(iTRAQ4plex); M5(Oxidation)	0,0000	1,289	1		0,001	0,008346	27	0,001990536	2	1090,58183	2,06	13,39	0
P74219	Slr1533 protein OS=Synechocystis sp. (strain PCC 6803 / Kazusa) GN=slr1533 PE=4	39,24	17,39	1	2	2	2	1,654	2	15,5	115	12,8	5,12							
	A3	Sequence	# PSMs	# Proteins	# Protein Groups	Protein Group Accessions	Modifications	ΔCn	114/115	114/115 Count	114/115 Variability [%]	q-Value	PEP	IonScore	Exp Value	Charge	MH+ [Da]	ΔM [ppm]	RT [min]	# Missed Cleavages
	High	aIGAQVNQGGRPEk	1	1	1	P74219	N-Term(iTRAQ4plex); K14(iTRAQ4plex)	0,0000	1,835	1		0	6,896E-07	37	0,000218258	3	1712,96885	2,44	15,53	0
	High	vLLTLR	1	1	1	P74219	N-Term(iTRAQ4plex)	0,0000	1,491	1		0,006	0,07613	16	0,027225133	2	858,58983	0,58	32,59	0
P74357	Sll1530 protein OS=Synechocystis sp. (strain PCC 6803 / Kazusa) GN=sll1530 PE=4	38,82	9,59	1	3	3	3	0,612	3	12,2	386	43,7	6,18							
	A3	Sequence	# PSMs	# Proteins	# Protein Groups	Protein Group Accessions	Modifications	ΔCn	114/115	114/115 Count	114/115 Variability [%]	q-Value	PEP	IonScore	Exp Value	Charge	MH+ [Da]	ΔM [ppm]	RT [min]	# Missed Cleavages
	High	iANFDHLPAALYF	1	1	1	P74357	N-Term(iTRAQ4plex)	0,0000	0,452	1		0	0,001964	31	0,00086575	2	1635,87187	4,08	53,88	0
	High	tLVSAkPHLVLETQR	1	1	1	P74357	N-Term(iTRAQ4plex); K6(iTRAQ4plex)	0,0000	0,664	1		0	0,00000115	18	0,017537594	4	1980,18191	-1,30	29,02	0
	High	vLAPEIDkk	1	1	1	P74357	N-Term(iTRAQ4plex); K8(iTRAQ4plex); K9(iTRAQ4plex)	0,0000	0,612	1		0	0,0004454	17	0,022282812	3	1444,90766	-1,60	22,50	1
P74239	Ssl2148 protein OS=Synechocystis sp. (strain PCC 6803 / Kazusa) GN=ssl2148 PE=4	38,22	16,33	1	3	3	6	1,090	6	12,1	98	10,6	4,50							
	A3	Sequence	# PSMs	# Proteins	# Protein Groups	Protein Group Accessions	Modifications	ΔCn	114/115	114/115 Count	114/115 Variability [%]	q-Value	PEP	IonScore	Exp Value	Charge	MH+ [Da]	ΔM [ppm]	RT [min]	# Missed Cleavages
	High	rPDFIPk	3	1	1	P74239	N-Term(iTRAQ4plex); K7(iTRAQ4plex)	0,0000	1,141	3	9,6	0	0,003334	17	0,022459117	3	1160,70252	-0,45	21,78	0
	High	rPVGWER	1	1	1	P74239	N-Term(iTRAQ4plex)	0,0000	1,111	1		0,001	0,009105	23	0,005187642	3	1043,58808	1,30	13,96	0
	High	skRPDFIPk	2	1	1	P74239	N-Term(iTRAQ4plex); K2(iTRAQ4plex); K9(iTRAQ4plex)	0,0000	0,776	2	13,2	0,001	0,01392	16	0,024488941	4	1519,92922	-1,90	17,85	1
P74154	Slr1470 protein OS=Synechocystis sp. (strain PCC 6803 / Kazusa) GN=slr1470 PE=4	38,12	12,21	1	2	2	2	0,834	2	0,8	131	14,9	9,35							
	A3	Sequence	# PSMs	# Proteins	# Protein Groups	Protein Group Accessions	Modifications	ΔCn	114/115	114/115 Count	114/115 Variability [%]	q-Value	PEP	IonScore	Exp Value	Charge	MH+ [Da]	ΔM [ppm]	RT [min]	# Missed Cleavages
	High	tGRPLEAITQQ	1	1	1	P74154	N-Term(iTRAQ4plex)	0,0000	0,829	1		0	0,003317	35	0,000333403	2	1357,75578	0,10	24,64	0
	Medium	aIWLR	1	1	1	P74154	N-Term(iTRAQ4plex)	0,0000	0,839	1		0,027	0,2934	16	0,023065879	2	802,50536	-0,31	28,84	0
P74588	Slr1557 protein OS=Synechocystis sp. (strain PCC 6803 / Kazusa) GN=slr1557 PE=4	37,56	7,59	1	3	3	3	1,018	3	0,5	369	40,2	4,78							
	A3	Sequence	# PSMs	# Proteins	# Protein Groups	Protein Group Accessions	Modifications	ΔCn	114/115	114/115 Count	114/115 Variability [%]	q-Value	PEP	IonScore	Exp Value	Charge	MH+ [Da]	ΔM [ppm]	RT [min]	# Missed Cleavages
	High	rGELIVRPLk	1	1	1	P74588	N-Term(iTRAQ4plex); K10(iTRAQ4plex)	0,0000	0,816	1		0	0,0002043	25	0,003140292	4	1468,95888	1,52	20,70	1
	High	rPQIVSIk	1	1	1	P74588	N-Term(iTRAQ4plex); K8(iTRAQ4plex)	0,0000	1,018	1		0	0,007215	16	0,026544222	3	1228,79926	1,03	20,78	0
	High	fGVSISTVSR	1	1	1	P74588	N-Term(iTRAQ4plex)	0,0000	1,022	1		0,001	0,01451	23	0,005247712	2	1196,67363	-1,64	31,60	0
Q55376	Slr0912 protein OS=Synechocystis sp. (strain PCC 6803 / Kazusa) GN=slr0912 PE=4	37,12	8,14	1	2	2	2	0,908	2	11,3	258	29,3	5,21							
	A3	Sequence	# PSMs	# Proteins	# Protein Groups	Protein Group Accessions	Modifications	ΔCn	114/115	114/115 Count	114/115 Variability [%]	q-Value	PEP	IonScore	Exp Value	Charge	MH+ [Da]	ΔM [ppm]	RT [min]	# Missed Cleavages
	High	qALAAELHQR	1	1	1	Q55376	N-Term(iTRAQ4plex)	0,0000	0,980	1		0	0,0001485	28	0,001599448	3	1280,71872	-0,37	18,96	0
	High	wLHIDGEHTGR	1	1	1	Q55376	N-Term(iTRAQ4plex)	0,0000	0,842	1		0	0,00003113	22	0,006052991	4	1464,74599	-0,33	24,97	0
P73508	Slr1440 protein OS=Synechocystis sp. (strain PCC 6803 / Kazusa) GN=slr1440 PE=4	36,90	16,78	1	2	2	2	1,123	2	48,7	143	16,5	6,52							
	A3	Sequence	# PSMs	# Proteins	# Protein Groups	Protein Group Accessions	Modifications	ΔCn	114/115	114/115 Count	114/115 Variability [%]	q-Value	PEP	IonScore	Exp Value	Charge	MH+ [Da]	ΔM [ppm]	RT [min]	# Missed Cleavages
	High	tFFLADPFHNYLEFk	1	1	1	P73508	N-Term(iTRAQ4plex); K15(iTRAQ4plex)	0,0000	1,533	1		0	1,105E-07	32	0,000616552	3	2177,13736	2,91	53,17	0
	High	hQEAIFDLk	1	1	1	P73508	N-Term(iTRAQ4plex); K9(iTRAQ4plex)	0,0000	0,822	1		0	0,00004394	18	0,017729555	3	1388,77915	1,07	28,67	0
P73157	Probable signal peptidase I-2 OS=Synechocystis sp. (strain PCC 6803 / Kazusa) GN	36,62	12,39	1	2	2	2	1,361	2	28,7	218	24,7	5,52							
	A3	Sequence	# PSMs	# Proteins	# Protein Groups	Protein Group Accessions	Modifications	ΔCn	114/115	114/115 Count	114/115 Variability [%]	q-Value	PEP	IonScore	Exp Value	Charge	MH+ [Da]	ΔM [ppm]	RT [min]	# Missed Cleavages
	High	nFHDAFIk	1	1	1	P73157	N-Term(iTRAQ4plex); K8(iTRAQ4plex)	0,0000	1,646	1		0	0,001765	27	0,002051021	3	1279,70487	0,87	24,57	0
	High	lRDPERGEIVVFNPTDALk	1	1	1	P73157	N-Term(iTRAQ4plex); K19(iTRAQ4plex)	0,0000	1,126	1		0	0,000007463	23	0,005296269	4	2457,37197	0,60	38,95	2
Q55688	Aldehyde decarbonylase OS=Synechocystis sp. (strain PCC 6803 / Kazusa) GN=sll0	36,45	12,55	1	2	2	3	1,311	3	33,7	231	26,2	4,75							
	A3	Sequence	# PSMs	# Proteins	# Protein Groups	Protein Group Accessions	Modifications	ΔCn	114/115	114/115 Count	114/115 Variability [%]	q-Value	PEP	IonScore	Exp Value	Charge	MH+ [Da]	ΔM [ppm]	RT [min]	# Missed Cleavages
	High	aNFATAk	2	1	1	Q55688	N-Term(iTRAQ4plex); K7(iTRAQ4plex)	0,0000	1,222	2	45,2	0	0,0005348	35	0,000298518	2	1010,59215	4,77	15,31	0
	High	kITEGVVkDEYTHLNYGEEWLk	1	1	1	Q55688	N-Term(iTRAQ4plex); K1(iTRAQ4plex); K8(iTRAQ4plex); K22(iTRAQ4plex)	0,0000	1,311	1		0	0,000007034	13	0,044977985	4	3227,75063	2,21	38,03	2
P74176	HlyB family OS=Synechocystis sp. (strain PCC 6803 / Kazusa) GN=hlyB PE=4 SV=1	36,26	3,66	1	3	3	3	0,666	3	31,9	1011	112,0	5,68							
	A3	Sequence	# PSMs	# Proteins	# Protein Groups	Protein Group Accessions	Modifications	ΔCn	114/115	114/115 Count	114/115 Variability [%]	q-Value	PEP	IonScore	Exp Value	Charge	MH+ [Da]	ΔM [ppm]	RT [min]	# Missed Cleavages
	High	iDmSLGSEIIDHLLR	1	1	1	P74176	N-Term(iTRAQ4plex); M3(Oxidation)	0,0000	0,518	1		0	7,651E-08	36	0,000246587	3	1872,00290	0,59	54,00	0
	High	gVFGQmQVPFLIR	1	1	1	P74176	N-Term(iTRAQ4plex); M6(Oxidation)	0,0000	0,822	1		0	0,00171	12	0,066676072	2	1651,91472	2,17	47,08	0
	Medium	lIGLPPLPR	1	1	1	P74176	N-Term(iTRAQ4plex)	0,0000	0,666	1		0,035	0,3733	13	0,047859704	2	1119,73540	-1,50	42,63	0
Q55823	Ycf66-like protein OS=Synechocystis sp. (strain PCC 6803 / Kazusa) GN=slr0503 PE	36,14	8,01	1	2	2	3	1,192	3	4,6	337	37,6	5,29							
	A3	Sequence	# PSMs	# Proteins	# Protein Groups	Protein Group Accessions	Modifications	ΔCn	114/115	114/115 Count	114/115 Variability [%]	q-Value	PEP	IonScore	Exp Value	Charge	MH+ [Da]	ΔM [ppm]	RT [min]	# Missed Cleavages
	High	nAPPTNPNPRPTR	1	1	1	Q55823	N-Term(iTRAQ4plex)	0,0000	1,033	1		0	0,0008239	27	0,001954204	3	1575,84702	-0,16	11,73	0
	High	rPRPEAGNDPAPSR	2	1	1	Q55823	N-Term(iTRAQ4plex)	0,0000	1,211	2	2,3	0,001	0,01622	17	0,020322166	4	1663,87502	0,27	8,89	0
P72642	4-hydroxy-tetrahydrodipicolinate reductase OS=Synechocystis sp. (strain PCC 6803	35,78	7,27	1	2	2	2	1,145	1		275	29,1	5,55							
	A3	Sequence	# PSMs	# Proteins	# Protein Groups	Protein Group Accessions	Modifications	ΔCn	114/115	114/115 Count	114/115 Variability [%]	q-Value	PEP	IonScore	Exp Value	Charge	MH+ [Da]	ΔM [ppm]	RT [min]	# Missed Cleavages
	High	gGLGPGQIPIHSIR	1	1	1	P72642	N-Term(iTRAQ4plex)	0,0000				0	0,00009011	27	0,001931835	3	1545,89829	0,03	30,60	0
	High	kVVELk	1	1	1	P72642	N-Term(iTRAQ4plex); K1(iTRAQ4plex); K6(iTRAQ4plex)	0,0000	1,145	1		0,003	0,03902	22	0,006822916	3	1147,77161	-5,11	16,82	1
P74214	Signal recognition particle protein OS=Synechocystis sp. (strain PCC 6803 / Kazusa)	35,67	4,56	1	2	2	2	0,450	2	159,2	482	52,1	9,09							
	A3	Sequence	# PSMs	# Proteins	# Protein Groups	Protein Group Accessions	Modifications	ΔCn	114/115	114/115 Count	114/115 Variability [%]	q-Value	PEP	IonScore	Exp Value	Charge	MH+ [Da]	ΔM [ppm]	RT [min]	# Missed Cleavages
	High	iAQSGSHSETDVSk	1	1	1	P74214	N-Term(iTRAQ4plex); K14(iTRAQ4plex)	0,0000	0,960	1		0	0,00002155	30	0,001129562	3	1703,88260	1,35	13,99	0
	High	kNPDLLAk	1	1	1	P74214	N-Term(iTRAQ4plex); K1(iTRAQ4plex); K8(iTRAQ4plex)	0,0000	0,211	1		0	0,00001346	19	0,013395842	3	1330,84211	0,17	18,64	1
Q55508	Indole-3-glycerol phosphate synthase OS=Synechocystis sp. (strain PCC 6803 / Kaz	35,46	4,41	1	2	2	4	1,092	4	4,1	295	32,9	5,99							
	A3	Sequence	# PSMs	# Proteins	# Protein Groups	Protein Group Accessions	Modifications	ΔCn	114/115	114/115 Count	114/115 Variability [%]	q-Value	PEP	IonScore	Exp Value	Charge	MH+ [Da]	ΔM [ppm]	RT [min]	# Missed Cleavages
	High	lQQAGAR	3	1	1	Q55508	N-Term(iTRAQ4plex)	0,0000	1,080	3	3,4	0	0,003051	30	0,00090985	2	887,51885	0,99	12,00	0
	High	kASPSk	1	1	1	Q55508	N-Term(iTRAQ4plex); K1(iTRAQ4plex); K6(iTRAQ4plex)	0,0000	1,424	1		0,003	0,03868	16	0,023012829	3	1049,66590	-1,93	7,85	1
P72751	Sll1036 protein OS=Synechocystis sp. (strain PCC 6803 / Kazusa) GN=sll1036 PE=4	35,40	9,25	1	2	2	2	1,270	2	7,8	292	32,6	5,33							
	A3	Sequence	# PSMs	# Proteins	# Protein Groups	Protein Group Accessions	Modifications	ΔCn	114/115	114/115 Count	114/115 Variability [%]	q-Value	PEP	IonScore	Exp Value	Charge	MH+ [Da]	ΔM [ppm]	RT [min]	# Missed Cleavages
	High	qLILFHHDPGHDDSmLDVIWDR	1	1	1	P72751	N-Term(iTRAQ4plex); M15(Oxidation)	0,0000	1,338	1		0	6,622E-10	29	0,001137549	4	2819,36953	0,09	42,64	0
	Medium	fWGVR	1	1	1	P72751	N-Term(iTRAQ4plex)	0,0000	1,204	1		0,024	0,2578	19	0,01264649	2	808,45873	0,09	28,67	0
P48949	30S ribosomal protein S21 OS=Synechocystis sp. (strain PCC 6803 / Kazusa) GN=rp	35,17	31,67	1	2	2	2	1,041	2	10,0	60	7,3	11,60							
	A3	Sequence	# PSMs	# Proteins	# Protein Groups	Protein Group Accessions	Modifications	ΔCn	114/115	114/115 Count	114/115 Variability [%]	q-Value	PEP	IonScore	Exp Value	Charge	MH+ [Da]	ΔM [ppm]	RT [min]	# Missed Cleavages
	High	aGIYTDFkk	1	1	1	P48949	N-Term(iTRAQ4plex); K8(iTRAQ4plex); K9(iTRAQ4plex)	0,0000	1,113	1		0	0,00002743	25	0,003619911	3	1474,86243	-0,39	24,78	1
	High	hQFFETPQEk	1	1	1	P48949	N-Term(iTRAQ4plex); K10(iTRAQ4plex)	0,0000	0,974	1		0	0,00000389	23	0,004497488	3	1578,81992	2,80	21,84	0
Q6ZES9	Uncharacterized protein OS=Synechocystis sp. (strain PCC 6803 / Kazusa) GN=slr5	34,42	21,54	1	2	2	2	1,187	2	15,6	130	14,3	5,08							
	A3	Sequence	# PSMs	# Proteins	# Protein Groups	Protein Group Accessions	Modifications	ΔCn	114/115	114/115 Count	114/115 Variability [%]	q-Value	PEP	IonScore	Exp Value	Charge	MH+ [Da]	ΔM [ppm]	RT [min]	# Missed Cleavages
	High	rLENQEFTSR	1	1	1	Q6ZES9	N-Term(iTRAQ4plex)	0,0000	1,318	1		0	0,0007277	30	0,001051889	3	1423,74497	2,75	16,14	1
	Medium	lSGQIk	1	1	1	Q6ZES9	N-Term(iTRAQ4plex); K6(iTRAQ4plex)	0,0000	1,070	1		0,015	0,1717	18	0,017138389	2	933,59673	-0,46	16,50	0
P73697	Slr1812 protein OS=Synechocystis sp. (strain PCC 6803 / Kazusa) GN=slr1812 PE=4	34,05	12,03	1	2	2	2	0,800	2	15,2	158	19,1	5,07							
	A3	Sequence	# PSMs	# Proteins	# Protein Groups	Protein Group Accessions	Modifications	ΔCn	114/115	114/115 Count	114/115 Variability [%]	q-Value	PEP	IonScore	Exp Value	Charge	MH+ [Da]	ΔM [ppm]	RT [min]	# Missed Cleavages
	High	nHWEAEIINFR	1	1	1	P73697	N-Term(iTRAQ4plex)	0,0000	0,722	1		0	1,942E-07	34	0,000392618	3	1572,80344	-0,35	38,36	0
	High	lLFEHLLk	1	1	1	P73697	N-Term(iTRAQ4plex); K8(iTRAQ4plex)	0,0000	0,886	1		0	0,0007332	12	0,056230249	3	1300,82215	-0,77	39,72	0
P73716	Slr1732 protein OS=Synechocystis sp. (strain PCC 6803 / Kazusa) GN=slr1732 PE=4	33,50	12,75	1	2	2	2	1,620	2	13,5	149	16,8	6,10							
	A3	Sequence	# PSMs	# Proteins	# Protein Groups	Protein Group Accessions	Modifications	ΔCn	114/115	114/115 Count	114/115 Variability [%]	q-Value	PEP	IonScore	Exp Value	Charge	MH+ [Da]	ΔM [ppm]	RT [min]	# Missed Cleavages
	High	wDkFNFSLEk	1	1	1	P73716	N-Term(iTRAQ4plex); K3(iTRAQ4plex); K10(iTRAQ4plex)	0,0000	1,773	1		0	0,00002977	28	0,001545148	3	1745,96115	1,40	40,25	1
	High	tYQVRPQAk	1	1	1	P73716	N-Term(iTRAQ4plex); K9(iTRAQ4plex)	0,0000	1,480	1		0	0,001322	18	0,014420158	3	1378,80439	-0,10	13,59	0
P55175	Hydrolase sll0601 OS=Synechocystis sp. (strain PCC 6803 / Kazusa) GN=sll0601 PE	33,17	8,82	1	3	3	3	1,240	3	46,0	272	30,2	6,27							
	A3	Sequence	# PSMs	# Proteins	# Protein Groups	Protein Group Accessions	Modifications	ΔCn	114/115	114/115 Count	114/115 Variability [%]	q-Value	PEP	IonScore	Exp Value	Charge	MH+ [Da]	ΔM [ppm]	RT [min]	# Missed Cleavages
	High	dHWQVLLQAR	1	1	1	P55175	N-Term(iTRAQ4plex)	0,0000	2,850	1		0	0,00005424	27	0,002079553	3	1409,77668	-0,26	33,53	0
	High	fLQTmAQR	1	1	1	P55175	N-Term(iTRAQ4plex); M5(Oxidation)	0,0000	0,923	1		0	0,000701	15	0,031115014	2	1154,61162	0,63	20,20	0
	Medium	fPELYR	1	1	1	P55175	N-Term(iTRAQ4plex)	0,0000	1,240	1		0,019	0,2053	17	0,018576761	2	968,53270	0,51	31,52	0
P72705	Photosystem I assembly protein Ycf4 OS=Synechocystis sp. (strain PCC 6803 / Kazu	33,09	10,64	1	3	3	4	0,915	4	24,7	188	20,4	10,05							
	A3	Sequence	# PSMs	# Proteins	# Protein Groups	Protein Group Accessions	Modifications	ΔCn	114/115	114/115 Count	114/115 Variability [%]	q-Value	PEP	IonScore	Exp Value	Charge	MH+ [Da]	ΔM [ppm]	RT [min]	# Missed Cleavages
	High	rIELINk	2	1	1	P72705	N-Term(iTRAQ4plex); K7(iTRAQ4plex)	0,0000	0,777	2	8,3	0	0,0008737	12	0,065308544	3	1173,75464	-0,98	22,52	1
	Medium	sLYLk	1	1	1	P72705	N-Term(iTRAQ4plex); K5(iTRAQ4plex)	0,0000	1,253	1		0,016	0,1754	27	0,002172551	2	911,57988	-0,60	21,77	0
	Medium	sGQVTIFR	1	1	1	P72705	N-Term(iTRAQ4plex)	0,0000	1,019	1		0,043	0,4462	14	0,041397108	2	1051,60234	0,62	25,04	0
P72690	Slr0236 protein OS=Synechocystis sp. (strain PCC 6803 / Kazusa) GN=slr0236 PE=4	33,07	7,21	1	2	2	2	1,256	2	84,2	222	25,0	7,74							
	A3	Sequence	# PSMs	# Proteins	# Protein Groups	Protein Group Accessions	Modifications	ΔCn	114/115	114/115 Count	114/115 Variability [%]	q-Value	PEP	IonScore	Exp Value	Charge	MH+ [Da]	ΔM [ppm]	RT [min]	# Missed Cleavages
	High	fLEEFSPDRR	1	1	1	P72690	N-Term(iTRAQ4plex)	0,0000	2,058	1		0,001	0,01327	25	0,003183978	3	1439,73868	-0,92	34,70	1
	High	lALAFR	1	1	1	P72690	N-Term(iTRAQ4plex)	0,0000	0,767	1		0,005	0,06515	21	0,007726272	2	834,53124	-0,70	32,80	0
Q55444	Methyl-accepting chemotaxis protein II OS=Synechocystis sp. (strain PCC 6803 / Ka	32,99	11,04	1	2	2	3	1,022	3	23,0	163	17,7	5,26							
	A3	Sequence	# PSMs	# Proteins	# Protein Groups	Protein Group Accessions	Modifications	ΔCn	114/115	114/115 Count	114/115 Variability [%]	q-Value	PEP	IonScore	Exp Value	Charge	MH+ [Da]	ΔM [ppm]	RT [min]	# Missed Cleavages
	High	qFAAQTHLLALk	1	1	1	Q55444	N-Term(iTRAQ4plex); K12(iTRAQ4plex)	0,0000	0,877	1		0	0,0002372	17	0,022078522	3	1628,97269	0,01	33,80	0
	Medium	aINLIR	2	1	1	Q55444	N-Term(iTRAQ4plex)	0,0000	1,138	2	16,0	0,019	0,2102	21	0,007294071	2	843,55376	0,56	25,57	0
Q55354	Photosystem II reaction center protein L OS=Synechocystis sp. (strain PCC 6803 / I	32,66	33,33	1	2	2	3	0,906	3	6,2	39	4,5	8,84							
	A3	Sequence	# PSMs	# Proteins	# Protein Groups	Protein Group Accessions	Modifications	ΔCn	114/115	114/115 Count	114/115 Variability [%]	q-Value	PEP	IonScore	Exp Value	Charge	MH+ [Da]	ΔM [ppm]	RT [min]	# Missed Cleavages
	High	nSNPNRQPVELNR	1	1	1	Q55354	N-Term(iTRAQ4plex)	0,0000	0,869	1		0	0,00007923	17	0,017904822	3	1681,88431	-0,47	14,53	1
	High	qPVELNR	2	1	1	Q55354	N-Term(iTRAQ4plex)	0,0000	1,021	2	17,8	0,004	0,04363	20	0,00920386	2	999,57579	5,40	16,12	0
P72966	Photosystem I biogenesis protein BtpA OS=Synechocystis sp. (strain PCC 6803 / Ka	32,38	16,38	1	3	3	3	0,892	3	48,1	287	30,0	5,44							
	A3	Sequence	# PSMs	# Proteins	# Protein Groups	Protein Group Accessions	Modifications	ΔCn	114/115	114/115 Count	114/115 Variability [%]	q-Value	PEP	IonScore	Exp Value	Charge	MH+ [Da]	ΔM [ppm]	RT [min]	# Missed Cleavages
	High	mDLFQTFQTHNPVIGVVHLLPLPTSAR	1	1	1	P72966	N-Term(iTRAQ4plex); M1(Oxidation)	0,0000	0,892	1		0	0,001985	26	0,002594	4	3191,72280	2,22	57,44	0
	High	lQNLVVAPVGINVLR	1	1	1	P72966	N-Term(iTRAQ4plex)	0,0000	0,655	1		0	0,001164	20	0,011116549	2	1749,08757	0,45	49,81	0
	Medium	skPSk	1	1	1	P72966	N-Term(iTRAQ4plex); K2(iTRAQ4plex); K5(iTRAQ4plex)	0,0000	1,819	1		0,025	0,2682	12	0,064714262	3	978,62720	-3,68	7,90	0
Q55496	Sll0493 protein OS=Synechocystis sp. (strain PCC 6803 / Kazusa) GN=sll0493 PE=4	32,32	8,33	1	2	2	2	0,938	2	7,5	228	25,2	6,23							
	A3	Sequence	# PSMs	# Proteins	# Protein Groups	Protein Group Accessions	Modifications	ΔCn	114/115	114/115 Count	114/115 Variability [%]	q-Value	PEP	IonScore	Exp Value	Charge	MH+ [Da]	ΔM [ppm]	RT [min]	# Missed Cleavages
	High	rQFAVIGLGR	1	1	1	Q55496	N-Term(iTRAQ4plex)	0,0000	0,892	1		0	0,0009493	27	0,00207477	3	1260,76691	0,91	29,17	1
	High	tSHFLSLIR	1	1	1	Q55496	N-Term(iTRAQ4plex)	0,0000	0,987	1		0,001	0,01784	19	0,014092888	3	1217,71167	-0,52	32,90	0
P73789	Peptidyl-prolyl cis-trans isomerase slr1251 OS=Synechocystis sp. (strain PCC 6803 /	32,20	8,77	1	2	2	2	1,153	2	1,5	171	18,5	5,54							
	A3	Sequence	# PSMs	# Proteins	# Protein Groups	Protein Group Accessions	Modifications	ΔCn	114/115	114/115 Count	114/115 Variability [%]	q-Value	PEP	IonScore	Exp Value	Charge	MH+ [Da]	ΔM [ppm]	RT [min]	# Missed Cleavages
	High	aLcTGEk	1	1	1	P73789	N-Term(iTRAQ4plex); C3(Carbamidomethyl); K7(iTRAQ4plex)	0,0000	1,142	1		0	0,002097	32	0,000601132	2	1066,59014	9,00	12,74	0
	High	aGkPLHFk	1	1	1	P73789	N-Term(iTRAQ4plex); K3(iTRAQ4plex); K8(iTRAQ4plex)	0,0000	1,165	1		0	0,002631	11	0,070952876	4	1329,83559	-0,86	15,53	0

P73591	Sll1308 protein OS=Synechocystis sp. (strain PCC 6803 / Kazusa) GN=sll1308 PE=3	30,77	8,87	1	2	2	2	1,030	2	11,9	248	25,8	5,21							
	A3	Sequence	# PSMs	# Proteins	# Protein Groups	Protein Group Accessions	Modifications	ΔCn	114/115	114/115 Count	114/115 Variability [%]	q-Value	PEP	IonScore	Exp Value	Charge	MH+ [Da]	ΔM [ppm]	RT [min]	# Missed Cleavages
	High	kLESAAFLVDk	1	1	1	P73591	N-Term(iTRAQ4plex); K1(iTRAQ4plex); K11(iTRAQ4plex)	0,0000	1,116	1		0	0,00007705	25	0,00305471	3	1652,99430	-0,29	32,59	1
	High	aAAYSITQALR	1	1	1	P73591	N-Term(iTRAQ4plex)	0,0000	0,951	1		0	0,0004259	19	0,014679046	2	1308,73870	-0,43	33,41	0
Q55274	Iron stress-induced chlorophyll-binding protein OS=Synechocystis sp. (strain PCC 6	30,76	2,34	1	2	2	2	0,107	2	29,0	342	37,2	6,68							
	A3	Sequence	# PSMs	# Proteins	# Protein Groups	Protein Group Accessions	Modifications	ΔCn	114/115	114/115 Count	114/115 Variability [%]	q-Value	PEP	IonScore	Exp Value	Charge	MH+ [Da]	ΔM [ppm]	RT [min]	# Missed Cleavages
	High	aLGFDFk	1	1	1	Q55274	N-Term(iTRAQ4plex); K7(iTRAQ4plex)	0,0000	0,129	1		0,002	0,03132	27	0,002223156	2	1085,61980	-3,31	35,86	0
	Medium	aLGFDFkR	1	1	1	Q55274	N-Term(iTRAQ4plex); K7(iTRAQ4plex)	0,0000	0,088	1		0,011	0,1296	17	0,01883519	3	1241,72000	-3,63	29,63	1
P73997	3-dehydroquinate synthase OS=Synechocystis sp. (strain PCC 6803 / Kazusa) GN=a	30,15	13,85	1	3	3	4	0,885	3	15,1	361	38,8	5,80							
	A3	Sequence	# PSMs	# Proteins	# Protein Groups	Protein Group Accessions	Modifications	ΔCn	114/115	114/115 Count	114/115 Variability [%]	q-Value	PEP	IonScore	Exp Value	Charge	MH+ [Da]	ΔM [ppm]	RT [min]	# Missed Cleavages
	High	tkLPTEmPPTLAVENLLASLLHDk	1	1	1	P73997	N-Term(iTRAQ4plex); K2(iTRAQ4plex); M7(Oxidation); K24(iTRAQ4plex)	0,0000	0,885	1		0	0,0008719	24	0,003890183	4	3079,73379	-3,26	58,81	1
	High	aGYEVFQHLIPAGETHk	1	1	1	P73997	N-Term(iTRAQ4plex); K17(iTRAQ4plex)	0,0000	0,980	1		0	0,000005798	16	0,024041967	4	2185,16445	-0,01	38,47	0
	High	tGVNHPQGk	2	1	1	P73997	N-Term(iTRAQ4plex); K9(iTRAQ4plex)	0,0000	0,710	1		0,001	0,008658	14	0,041397108	3	1225,69013	0,78	8,15	0
Q6ZEU6	Sll5034 protein OS=Synechocystis sp. (strain PCC 6803 / Kazusa) GN=sll5034 PE=4	28,87	7,25	1	2	2	2	0,899	2	33,7	207	23,2	7,34							
	A3	Sequence	# PSMs	# Proteins	# Protein Groups	Protein Group Accessions	Modifications	ΔCn	114/115	114/115 Count	114/115 Variability [%]	q-Value	PEP	IonScore	Exp Value	Charge	MH+ [Da]	ΔM [ppm]	RT [min]	# Missed Cleavages
	High	tEAPTk	1	1	1	Q6ZEU6	N-Term(iTRAQ4plex); K6(iTRAQ4plex)	0,0000	1,122	1		0,005	0,06666	29	0,001816051	2	934,54186	-3,14	11,88	0
	Medium	eVcRPVGmR	1	1	1	Q6ZEU6	N-Term(iTRAQ4plex); C3(Carbamidomethyl); M8(Oxidation)	0,0000	0,721	1		0,017	0,1883	11	0,07924466	3	1263,64304	0,91	10,39	0
P37979	Protein IcfG OS=Synechocystis sp. (strain PCC 6803 / Kazusa) GN=icfG PE=4 SV=2	27,56	2,84	1	2	2	2	0,670	2	19,2	634	70,9	6,13							
	A3	Sequence	# PSMs	# Proteins	# Protein Groups	Protein Group Accessions	Modifications	ΔCn	114/115	114/115 Count	114/115 Variability [%]	q-Value	PEP	IonScore	Exp Value	Charge	MH+ [Da]	ΔM [ppm]	RT [min]	# Missed Cleavages
	High	vIVQFLNAVR	1	1	1	P37979	N-Term(iTRAQ4plex)	0,0000	0,762	1		0	0,00008153	26	0,002511713	2	1302,80156	0,07	49,63	0
	High	IIIQLISR	1	1	1	P37979	N-Term(iTRAQ4plex)	0,0000	0,589	1		0	0,0007524	15	0,034831326	2	1099,73100	-0,89	44,69	0
Q55501	Slr0929 protein OS=Synechocystis sp. (strain PCC 6803 / Kazusa) GN=slr0929 PE=	27,47	7,69	1	2	2	2	0,773	2	17,8	195	21,7	8,88							
	A3	Sequence	# PSMs	# Proteins	# Protein Groups	Protein Group Accessions	Modifications	ΔCn	114/115	114/115 Count	114/115 Variability [%]	q-Value	PEP	IonScore	Exp Value	Charge	MH+ [Da]	ΔM [ppm]	RT [min]	# Missed Cleavages
	High	kYPHIVVDTR	1	1	1	Q55501	N-Term(iTRAQ4plex); K1(iTRAQ4plex)	0,0000	0,686	1		0	0,004493	11	0,085895419	4	1515,88918	0,38	20,20	1
	Medium	iLLTk	1	1	1	Q55501	N-Term(iTRAQ4plex); K5(iTRAQ4plex)	0,0000	0,871	1		0,029	0,3174	27	0,001786364	2	875,61278	-4,62	25,03	0
P74232	Phosphoribosylamine--glycine ligase OS=Synechocystis sp. (strain PCC 6803 / Kazu	27,06	4,30	1	2	2	2	1,308	2	86,2	419	44,0	5,08							
	A3	Sequence	# PSMs	# Proteins	# Protein Groups	Protein Group Accessions	Modifications	ΔCn	114/115	114/115 Count	114/115 Variability [%]	q-Value	PEP	IonScore	Exp Value	Charge	MH+ [Da]	ΔM [ppm]	RT [min]	# Missed Cleavages
	High	vLGVTALGk	1	1	1	P74232	N-Term(iTRAQ4plex); K9(iTRAQ4plex)	0,0000	2,163	1		0	0,005268	21	0,007277295	2	1145,74870	-0,83	31,28	0
	High	vFHAGTELk	1	1	1	P74232	N-Term(iTRAQ4plex); K9(iTRAQ4plex)	0,0000	0,792	1		0	0,0002108	19	0,015963497	3	1289,74555	-0,06	20,94	0
P74751	Elongation factor 4 OS=Synechocystis sp. (strain PCC 6803 / Kazusa) GN=lepA PE=	26,83	4,64	1	3	3	3	0,998	3	26,1	603	66,9	5,22							
	A3	Sequence	# PSMs	# Proteins	# Protein Groups	Protein Group Accessions	Modifications	ΔCn	114/115	114/115 Count	114/115 Variability [%]	q-Value	PEP	IonScore	Exp Value	Charge	MH+ [Da]	ΔM [ppm]	RT [min]	# Missed Cleavages
	High	nFSIIAHIDHGk	1	1	1	P74751	N-Term(iTRAQ4plex); K12(iTRAQ4plex)	0,0000	0,642	1		0	0,00001123	25	0,00289048	4	1639,91555	-0,21	28,84	0
	High	lkVEEPFIk	1	1	1	P74751	N-Term(iTRAQ4plex); K2(iTRAQ4plex); K9(iTRAQ4plex)	0,0000	1,187	1		0	0,00004899	11	0,073955419	3	1534,95499	-1,26	32,31	1
	High	lkELIPR	1	1	1	P74751	N-Term(iTRAQ4plex); K2(iTRAQ4plex)	0,0000	0,998	1		0,003	0,04223	14	0,035807171	3	1156,76557	-0,06	28,76	1
P74001	Sll1239 protein OS=Synechocystis sp. (strain PCC 6803 / Kazusa) GN=sll1239 PE=4	26,57	12,36	1	3	3	3	1,425	3	30,2	178	20,6	5,35							
	A3	Sequence	# PSMs	# Proteins	# Protein Groups	Protein Group Accessions	Modifications	ΔCn	114/115	114/115 Count	114/115 Variability [%]	q-Value	PEP	IonScore	Exp Value	Charge	MH+ [Da]	ΔM [ppm]	RT [min]	# Missed Cleavages
	High	iELDSNkk	1	1	1	P74001	N-Term(iTRAQ4plex); K7(iTRAQ4plex); K8(iTRAQ4plex)	0,0000	2,516	1		0	0,002375	12	0,062805836	3	1378,82480	-1,32	17,79	1
	High	sQFFTYGLR	1	1	1	P74001	N-Term(iTRAQ4plex)	0,0000	1,168	1		0,002	0,02759	12	0,056881364	2	1262,66557	0,44	37,30	0
	Medium	qILLk	1	1	1	P74001	N-Term(iTRAQ4plex); K5(iTRAQ4plex)	0,0000	1,425	1		0,016	0,1753	27	0,002197708	2	902,63219	4,94	23,35	0
P73004	Long-chain-fatty-acid CoA ligase OS=Synechocystis sp. (strain PCC 6803 / Kazusa)	26,26	3,59	1	2	2	2	0,547	2	15,8	696	77,6	6,67							
	A3	Sequence	# PSMs	# Proteins	# Protein Groups	Protein Group Accessions	Modifications	ΔCn	114/115	114/115 Count	114/115 Variability [%]	q-Value	PEP	IonScore	Exp Value	Charge	MH+ [Da]	ΔM [ppm]	RT [min]	# Missed Cleavages
	High	dIkPHHIVGVPR	1	1	1	P73004	N-Term(iTRAQ4plex); K3(iTRAQ4plex)	0,0000	0,492	1		0	0,0002375	13	0,05163807	4	1655,99172	-1,87	17,73	0
	High	ySSGRPIPFTEIR	1	1	1	P73004	N-Term(iTRAQ4plex)	0,0000	0,608	1		0,003	0,03931	26	0,002484216	3	1666,90262	-0,45	31,90	0
Q55798	6-carboxy-5,6,7,8-tetrahydropterin synthase OS=Synechocystis sp. (strain PCC 680	26,19	17,05	1	2	2	2	0,914	2	8,0	129	14,5	7,01							
	A3	Sequence	# PSMs	# Proteins	# Protein Groups	Protein Group Accessions	Modifications	ΔCn	114/115	114/115 Count	114/115 Variability [%]	q-Value	PEP	IonScore	Exp Value	Charge	MH+ [Da]	ΔM [ppm]	RT [min]	# Missed Cleavages
	High	eFSFEAAHQLPHHEGk	1	1	1	Q55798	N-Term(iTRAQ4plex); K16(iTRAQ4plex)	0,0000	0,964	1		0	0,000001207	10	0,143670632	4	2152,08144	-0,01	24,25	0
	High	mWIIYk	1	1	1	Q55798	N-Term(iTRAQ4plex); K6(iTRAQ4plex)	0,0000	0,866	1		0,009	0,1026	26	0,002398667	2	1141,66765	-0,50	39,83	0
P73698	Slr1813 protein OS=Synechocystis sp. (strain PCC 6803 / Kazusa) GN=slr1813 PE=	26,16	9,15	1	2	2	3	0,834	2	87,0	153	18,4	5,62							
	A3	Sequence	# PSMs	# Proteins	# Protein Groups	Protein Group Accessions	Modifications	ΔCn	114/115	114/115 Count	114/115 Variability [%]	q-Value	PEP	IonScore	Exp Value	Charge	MH+ [Da]	ΔM [ppm]	RT [min]	# Missed Cleavages
	High	lFEHLLk	2	1	1	P73698	N-Term(iTRAQ4plex); K7(iTRAQ4plex)	0,0000	0,503	1		0	0,0008113	21	0,008729111	3	1187,73795	-0,95	33,87	0
	High	lASLLTR	1	1	1	P73698	N-Term(iTRAQ4plex)	0,0000	1,384	1		0	0,007082	19	0,013834708	2	917,58971	-0,39	33,04	0
P73114	Esterase OS=Synechocystis sp. (strain PCC 6803 / Kazusa) GN=slr1916 PE=4 SV=1	25,74	4,95	1	2	2	2	0,917	2	38,6	283	31,7	6,52							
	A3	Sequence	# PSMs	# Proteins	# Protein Groups	Protein Group Accessions	Modifications	ΔCn	114/115	114/115 Count	114/115 Variability [%]	q-Value	PEP	IonScore	Exp Value	Charge	MH+ [Da]	ΔM [ppm]	RT [min]	# Missed Cleavages
	High	lLDFLR	1	1	1	P73114	N-Term(iTRAQ4plex)	0,0000	0,713	1		0,002	0,02028	26	0,00546706	2	920,56835	-0,28	46,38	0
	Medium	vMELQYVk	1	1	1	P73114	N-Term(iTRAQ4plex); K8(iTRAQ4plex)	0,0000	1,180	1		0,025	0,2631	13	0,054950292	3	1297,74826	4,17	11,09	0
P74352	DNA-directed RNA polymerase subunit omega OS=Synechocystis sp. (strain PCC 68	25,61	22,37	1	2	2	2	1,511	2	46,2	76	8,7	5,48							
	A3	Sequence	# PSMs	# Proteins	# Protein Groups	Protein Group Accessions	Modifications	ΔCn	114/115	114/115 Count	114/115 Variability [%]	q-Value	PEP	IonScore	Exp Value	Charge	MH+ [Da]	ΔM [ppm]	RT [min]	# Missed Cleavages
	High	sNLDSNHIIYR	1	1	1	P74352	N-Term(iTRAQ4plex)	0,0000	1,123	1		0	0,0002683	22	0,006698383	3	1475,77274	0,27	21,03	0
	Medium	yNITVR	1	1	1	P74352	N-Term(iTRAQ4plex)	0,0000	2,033	1		0,013	0,153	17	0,020463033	2	909,52849	1,15	21,30	0
P48958	50S ribosomal protein L33 OS=Synechocystis sp. (strain PCC 6803 / Kazusa) GN=rp	24,35	26,15	1	3	3	3	1,305	3	4,8	65	7,5	10,24							
	A3	Sequence	# PSMs	# Proteins	# Protein Groups	Protein Group Accessions	Modifications	ΔCn	114/115	114/115 Count	114/115 Variability [%]	q-Value	PEP	IonScore	Exp Value	Charge	MH+ [Da]	ΔM [ppm]	RT [min]	# Missed Cleavages
	High	kFcTHcNk	1	1	1	P48958	N-Term(iTRAQ4plex); K1(iTRAQ4plex); C3(Carbamidomethyl); C6(Carbamido	0,0000	1,305	1		0	0,0007405	17	0,020322166	3	1526,79380	0,38	9,05	1
	Medium	lELkk	1	1	1	P48958	N-Term(iTRAQ4plex); K4(iTRAQ4plex); K5(iTRAQ4plex)	0,0000	2,625	1		0,024	0,2613	20	0,009015089	2	1062,72283	-1,77	17,86	1
	Medium	yTTmk	1	1	1	P48958	N-Term(iTRAQ4plex); M4(Oxidation); K5(iTRAQ4plex)	0,0000	1,264	1		0,032	0,3466	10	0,093534107	2	947,51055	-0,51	58,12	0
P74718	Slr1189 protein OS=Synechocystis sp. (strain PCC 6803 / Kazusa) GN=slr1189 PE=	23,79	8,55	1	2	2	2	0,638	2	29,0	351	38,4	5,76							
	A3	Sequence	# PSMs	# Proteins	# Protein Groups	Protein Group Accessions	Modifications	ΔCn	114/115	114/115 Count	114/115 Variability [%]	q-Value	PEP	IonScore	Exp Value	Charge	MH+ [Da]	ΔM [ppm]	RT [min]	# Missed Cleavages
	High	nYFHGFLDLAk	1	1	1	P74718	N-Term(iTRAQ4plex); K11(iTRAQ4plex)	0,0000	0,527	1		0	0,000000635	24	0,004168406	3	1612,87003	-1,60	41,05	0
	High	gFDLPAFAAHTLLSDPLGR	1	1	1	P74718	N-Term(iTRAQ4plex)	0,0000	0,773	1		0,001	0,01168	12	0,088289694	3	2142,14719	0,32	55,27	0
P73310	50S ribosomal protein L14 OS=Synechocystis sp. (strain PCC 6803 / Kazusa) GN=rp	23,68	17,21	1	3	3	3	1,452	3	3,1	122	13,3	10,08							
	A3	Sequence	# PSMs	# Proteins	# Protein Groups	Protein Group Accessions	Modifications	ΔCn	114/115	114/115 Count	114/115 Variability [%]	q-Value	PEP	IonScore	Exp Value	Charge	MH+ [Da]	ΔM [ppm]	RT [min]	# Missed Cleavages
	High	dkNFTk	1	1	1	P73310	N-Term(iTRAQ4plex); K2(iTRAQ4plex); K6(iTRAQ4plex)	0,0000	1,422	1		0	0,00549	20	0,009353411	2	1184,69988	-0,06	11,55	1
	High	dALPNmPIkk	1	1	1	P73310	N-Term(iTRAQ4plex); M6(Oxidation); K9(iTRAQ4plex); K10(iTRAQ4plex)	0,0000	1,452	1		0	0,0005516	16	0,022854409	3	1574,93131	0,79	19,87	1
	Medium	aVIVR	1	1	1	P73310	N-Term(iTRAQ4plex)	0,0000	1,648	1		0,043	0,454	13	0,052236011	2	701,48046	2,00	15,51	0
Q55811	Aldehyde dehydrogenase OS=Synechocystis sp. (strain PCC 6803 / Kazusa) GN=sl	22,90	3,29	1	2	2	2	1,029	2	34,3	456	51,2	7,97							
	A3	Sequence	# PSMs	# Proteins	# Protein Groups	Protein Group Accessions	Modifications	ΔCn	114/115	114/115 Count	114/115 Variability [%]	q-Value	PEP	IonScore	Exp Value	Charge	MH+ [Da]	ΔM [ppm]	RT [min]	# Missed Cleavages
	High	rPFWGETNLR	1	1	1	Q55811	N-Term(iTRAQ4plex)	0,0000	1,289	1		0	3,969E-07	23	0,005116465	3	1419,76096	-0,31	28,74	0
	Medium	mNLIk	1	1	1	Q55811	N-Term(iTRAQ4plex); K5(iTRAQ4plex)	0,0000	0,821	1		0,04	0,4198	12	0,070794578	2	906,56633	-2,40	20,70	0
Q55667	Slr0006 protein OS=Synechocystis sp. (strain PCC 6803 / Kazusa) GN=slr0006 PE=	22,44	8,76	1	2	2	3	1,384	3	4,3	217	23,4	8,46							
	A3	Sequence	# PSMs	# Proteins	# Protein Groups	Protein Group Accessions	Modifications	ΔCn	114/115	114/115 Count	114/115 Variability [%]	q-Value	PEP	IonScore	Exp Value	Charge	MH+ [Da]	ΔM [ppm]	RT [min]	# Missed Cleavages
	High	qFDkDQPLGILTR	2	1	1	Q55667	N-Term(iTRAQ4plex); K4(iTRAQ4plex)	0,0000	1,404	2	2,2	0	0,0002654	16	0,023548866	3	1819,03305	0,76	35,62	1
	Medium	aVAQLR	1	1	1	Q55667	N-Term(iTRAQ4plex)	0,0000	0,567	1		0,028	0,2989	19	0,012077304	2	801,50627	-0,08	15,55	0
Q55160	Biotin carboxylase OS=Synechocystis sp. (strain PCC 6803 / Kazusa) GN=accC PE=	21,12	3,13	1	2	2	2	0,916	2	7,2	448	49,1	7,28							
	A3	Sequence	# PSMs	# Proteins	# Protein Groups	Protein Group Accessions	Modifications	ΔCn	114/115	114/115 Count	114/115 Variability [%]	q-Value	PEP	IonScore	Exp Value	Charge	MH+ [Da]	ΔM [ppm]	RT [min]	# Missed Cleavages
	High	lIVWAGDRPSAIk	1	1	1	Q55160	N-Term(iTRAQ4plex); K13(iTRAQ4plex)	0,0000	0,872	1		0	0,00003615	21	0,007708502	3	1714,02646	0,58	35,98	0
	High	lIVWAGDRPSAIkR	1	1	1	Q55160	N-Term(iTRAQ4plex); K13(iTRAQ4plex)	0,0000	0,961	1		0	0,004188	12	0,062801498	4	1870,12722	0,34	30,48	1
P74236	Slr1161 protein OS=Synechocystis sp. (strain PCC 6803 / Kazusa) GN=slr1161 PE=	21,02	8,54	1	2	2	2	0,823	2	20,3	199	22,9	5,85							
	A3	Sequence	# PSMs	# Proteins	# Protein Groups	Protein Group Accessions	Modifications	ΔCn	114/115	114/115 Count	114/115 Variability [%]	q-Value	PEP	IonScore	Exp Value	Charge	MH+ [Da]	ΔM [ppm]	RT [min]	# Missed Cleavages
	High	sFLDRIENLk	1	1	1	P74236	N-Term(iTRAQ4plex); K10(iTRAQ4plex)	0,0000	0,718	1		0	0,0005533	21	0,007888056	3	1522,88340	0,13	40,17	1
	Medium	kLYELPR	1	1	1	P74236	N-Term(iTRAQ4plex); K1(iTRAQ4plex)	0,0000	0,942	1		0,022	0,243	13	0,066060728	3	1206,74366	-1,02	23,46	1

Supplementary Table S5

[NiFe]-hydrogenase is essential for cyanobacterium *Synechocystis* sp. PCC 6803 aerobic growth in the dark

Edith De Rosa, Vanessa Checchetto, Cinzia Franchin, Elisabetta Bergantino, Paola Berto, Ildikò Szabò, Giorgio M. Giacometti, Giorgio Arrigoni, and Paola Costantini

Accession	Description	ΣCoverage	Dark/Light Replicate 1	Dark/Light Replicate 2	Dark/Light Replicate 3	Average	p Value
Q55542	Slr0334 protein OS=Synechocystis sp. (strain PCC 6803 / Kazusa) GN=slr0334 PE=4 SV=1 - [Q55542_SYNY3]	13,64	4,43	7,59	18,92	10,31	3,88E-07
Q55168	Phytochrome-like protein cph1 OS=Synechocystis sp. (strain PCC 6803 / Kazusa) GN=cph1 PE=1 SV=1 - [PHY1_SYNY3]	4,81	23,48	3,31	1,55	9,45	4,84E-02
P77970	Glutamate--ammonia ligase OS=Synechocystis sp. (strain PCC 6803 / Kazusa) GN=glnN PE=3 SV=1 - [P77970_SYNY3]	9,94	7,05	2,58	1,29	3,64	3,35E-02
P73309	50S ribosomal protein L24 OS=Synechocystis sp. (strain PCC 6803 / Kazusa) GN=rplX PE=3 SV=1 - [RL24_SYNY3]	68,7	1,23	2,52	4,86	2,87	2,23E-02
P36239	50S ribosomal protein L19 OS=Synechocystis sp. (strain PCC 6803 / Kazusa) GN=rplS PE=3 SV=1 - [RL19_SYNY3]	44,26	3,87	2,26	1,77	2,63	8,10E-05
P74446	Slr0147 protein OS=Synechocystis sp. (strain PCC 6803 / Kazusa) GN=slr0147 PE=4 SV=1 - [P74446_SYNY3]	13,01	3,54	2,33	1,82	2,56	3,51E-06
Q55541	Slr0333 protein OS=Synechocystis sp. (strain PCC 6803 / Kazusa) GN=slr0333 PE=4 SV=1 - [Q55541_SYNY3]	37,74	1,92	2,29	3,25	2,48	1,05E-08
P42352	50S ribosomal protein L9 OS=Synechocystis sp. (strain PCC 6803 / Kazusa) GN=rplI PE=3 SV=2 - [RL9_SYNY3]	61,18	1,97	2,73	2,39	2,36	5,21E-19
P74565	RNA polymerase sigma factor SigA OS=Synechocystis sp. (strain PCC 6803 / Kazusa) GN=sigA PE=3 SV=1 - [SIGA_SYNY3]	8	3,33	1,94	1,71	2,33	9,17E-05
P73296	50S ribosomal protein L17 OS=Synechocystis sp. (strain PCC 6803 / Kazusa) GN=rplQ PE=3 SV=1 - [RL17_SYNY3]	52,59	1,73	2,09	3,15	2,32	4,22E-06
P73318	50S ribosomal protein L23 OS=Synechocystis sp. (strain PCC 6803 / Kazusa) GN=rplW PE=3 SV=1 - [RL23_SYNY3]	78,22	1,70	2,17	2,59	2,15	4,46E-10
P74390	Negative aliphatic amidase regulator OS=Synechocystis sp. (strain PCC 6803 / Kazusa) GN=amiC PE=4 SV=1 - [P74390_SYNY3	41,48	1,27	2,61	2,51	2,13	2,45E-03
Q55484	Diaminopimelate decarboxylase OS=Synechocystis sp. (strain PCC 6803 / Kazusa) GN=lysA PE=3 SV=1 - [DCDA_SYNY3]	22,39	2,58	2,56	1,25	2,13	3,48E-03
P74450	Slr0151 protein OS=Synechocystis sp. (strain PCC 6803 / Kazusa) GN=slr0151 PE=4 SV=1 - [P74450_SYNY3]	25,62	2,38	2,36	1,62	2,12	5,87E-09
P48957	50S ribosomal protein L20 OS=Synechocystis sp. (strain PCC 6803 / Kazusa) GN=rplT PE=3 SV=1 - [RL20_SYNY3]	57,26	1,58	1,83	2,92	2,11	1,29E-04
P73320	50S ribosomal protein L3 OS=Synechocystis sp. (strain PCC 6803 / Kazusa) GN=rplC PE=3 SV=1 - [RL3_SYNY3]	52,58	2,01	2,46	1,84	2,10	1,58E-17
P73848	3-hydroxyacyl-[acyl-carrier-protein] dehydratase FabZ OS=Synechocystis sp. (strain PCC 6803 / Kazusa) GN=fabZ PE=3 SV=2	24,39	1,56	2,30	2,37	2,08	9,04E-08
P72866	30S ribosomal protein S15 OS=Synechocystis sp. (strain PCC 6803 / Kazusa) GN=rpsO PE=3 SV=1 - [RS15_SYNY3]	33,71	2,25	2,08	1,78	2,04	6,46E-25
P36237	50S ribosomal protein L11 OS=Synechocystis sp. (strain PCC 6803 / Kazusa) GN=rplK PE=3 SV=1 - [RL11_SYNY3]	45,39	1,90	2,24	1,88	2,00	5,24E-35
P73306	50S ribosomal protein L6 OS=Synechocystis sp. (strain PCC 6803 / Kazusa) GN=rplF PE=3 SV=1 - [RL6_SYNY3]	77,65	1,75	1,66	2,52	1,98	5,11E-07
P73293	30S ribosomal protein S9 OS=Synechocystis sp. (strain PCC 6803 / Kazusa) GN=rpsI PE=3 SV=1 - [RS9_SYNY3]	37,23	2,82	1,76	1,30	1,96	5,78E-03
P73303	50S ribosomal protein L15 OS=Synechocystis sp. (strain PCC 6803 / Kazusa) GN=rplO PE=3 SV=1 - [RL15_SYNY3]	67,35	1,71	1,92	2,25	1,96	2,76E-17
P73315	50S ribosomal protein L22 OS=Synechocystis sp. (strain PCC 6803 / Kazusa) GN=rplV PE=3 SV=1 - [RL22_SYNY3]	43,8	1,40	2,28	2,16	1,95	2,67E-05
Q55698	Slr0226 protein OS=Synechocystis sp. (strain PCC 6803 / Kazusa) GN=slr0226 PE=4 SV=1 - [Q55698_SYNY3]	2,9	2,94	1,38	1,52	1,94	1,09E-02
P73305	50S ribosomal protein L18 OS=Synechocystis sp. (strain PCC 6803 / Kazusa) GN=rplR PE=3 SV=1 - [RL18_SYNY3]	20	1,72	1,89	2,19	1,93	8,96E-21
P74448	Slr0149 protein OS=Synechocystis sp. (strain PCC 6803 / Kazusa) GN=slr0149 PE=3 SV=1 - [P74448_SYNY3]	20,51	2,29	1,71	1,71	1,90	1,02E-10
P73319	50S ribosomal protein L4 OS=Synechocystis sp. (strain PCC 6803 / Kazusa) GN=rplD PE=3 SV=1 - [RL4_SYNY3]	55,71	1,89	2,10	1,69	1,89	5,09E-24
P73311	30S ribosomal protein S17 OS=Synechocystis sp. (strain PCC 6803 / Kazusa) GN=rpsQ PE=3 SV=1 - [RS17_SYNY3]	66,67	2,63	1,59	1,41	1,88	1,91E-03
P73317	50S ribosomal protein L2 OS=Synechocystis sp. (strain PCC 6803 / Kazusa) GN=rplB PE=3 SV=1 - [RL2_SYNY3]	61,59	1,87	1,58	2,15	1,87	5,29E-12
P73294	50S ribosomal protein L13 OS=Synechocystis sp. (strain PCC 6803 / Kazusa) GN=rplM PE=3 SV=1 - [RL13_SYNY3]	78,81	1,37	1,56	2,65	1,86	3,87E-03
P73307	30S ribosomal protein S8 OS=Synechocystis sp. (strain PCC 6803 / Kazusa) GN=rpsH PE=3 SV=1 - [RS8_SYNY3]	51,88	2,78	1,50	1,29	1,86	1,68E-02
P80505	Glyceraldehyde-3-phosphate dehydrogenase 2 OS=Synechocystis sp. (strain PCC 6803 / Kazusa) GN=gap2 PE=1 SV=3 - [G3P2	64,99	1,98	1,06	2,50	1,85	3,10E-02
P73308	50S ribosomal protein L5 OS=Synechocystis sp. (strain PCC 6803 / Kazusa) GN=rplE PE=3 SV=2 - [RL5_SYNY3]	65,56	2,47	1,66	1,37	1,83	8,35E-04
Q55662	ATP-dependent Clp protease regulatory subunit OS=Synechocystis sp. (strain PCC 6803 / Kazusa) GN=clpC PE=3 SV=1 - [Q55	24,36	2,69	1,61	1,12	1,81	3,92E-02
P77962	Serine hydroxymethyltransferase OS=Synechocystis sp. (strain PCC 6803 / Kazusa) GN=glyA PE=3 SV=1 - [GLYA_SYNY3]	19,67	2,57	1,61	1,22	1,80	1,26E-02
P73316	30S ribosomal protein S19 OS=Synechocystis sp. (strain PCC 6803 / Kazusa) GN=rpsS PE=3 SV=1 - [RS19_SYNY3]	36,96	2,27	1,69	1,43	1,80	2,65E-05
P74071	30S ribosomal protein S2 OS=Synechocystis sp. (strain PCC 6803 / Kazusa) GN=rpsB PE=3 SV=1 - [RS2_SYNY3]	35,32	1,93	1,77	1,66	1,79	8,38E-40
P73290	Adenylosuccinate synthetase OS=Synechocystis sp. (strain PCC 6803 / Kazusa) GN=purA PE=3 SV=2 - [PURA_SYNY3]	12,16	2,67	1,42	1,24	1,78	2,87E-02
P74267	50S ribosomal protein L27 OS=Synechocystis sp. (strain PCC 6803 / Kazusa) GN=rpmA PE=3 SV=1 - [RL27_SYNY3]	59,77	1,73	1,51	2,06	1,77	3,62E-10
P73409	Slr1841 protein OS=Synechocystis sp. (strain PCC 6803 / Kazusa) GN=slr1841 PE=4 SV=1 - [P73409_SYNY3]	17,78	1,58	1,92	1,72	1,74	7,79E-22
P73304	30S ribosomal protein S5 OS=Synechocystis sp. (strain PCC 6803 / Kazusa) GN=rpsE PE=3 SV=1 - [RS5_SYNY3]	61,27	2,29	1,64	1,28	1,74	2,05E-03
P73636	30S ribosomal protein S6 OS=Synechocystis sp. (strain PCC 6803 / Kazusa) GN=rpsF PE=3 SV=1 - [RS6_SYNY3]	44,25	1,51	1,80	1,87	1,73	9,28E-16
P74193	Threonine synthase OS=Synechocystis sp. (strain PCC 6803 / Kazusa) GN=thrC PE=3 SV=1 - [THRC_SYNY3]	26,7	1,86	1,59		1,73	2,16E-12
P73452	Nitrate transport protein NrtA OS=Synechocystis sp. (strain PCC 6803 / Kazusa) GN=nrtA PE=1 SV=1 - [NRTA_SYNY3]	38,12	1,12	1,76	2,16	1,68	1,25E-02
P48946	30S ribosomal protein S18 OS=Synechocystis sp. (strain PCC 6803 / Kazusa) GN=rpsR PE=3 SV=1 - [RS18_SYNY3]	64,79	1,99	1,65	1,38	1,68	1,53E-06
P73298	30S ribosomal protein S11 OS=Synechocystis sp. (strain PCC 6803 / Kazusa) GN=rpsK PE=3 SV=1 - [RS11_SYNY3]	32,31	2,25	1,53	1,07	1,62	4,17E-02
P23350	50S ribosomal protein L10 OS=Synechocystis sp. (strain PCC 6803 / Kazusa) GN=rplJ PE=3 SV=1 - [RL10_SYNY3]	22,54	1,50	1,50	1,81	1,61	1,68E-14
P74219	Slr1533 protein OS=Synechocystis sp. (strain PCC 6803 / Kazusa) GN=slr1533 PE=4 SV=1 - [P74219_SYNY3]	29,57	1,55	1,56	1,66	1,59	8,34E-102
P72851	50S ribosomal protein L28 OS=Synechocystis sp. (strain PCC 6803 / Kazusa) GN=rpmB PE=3 SV=1 - [RL28_SYNY3]	61,54	2,08	1,31	1,34	1,58	3,79E-03
P74395	N utilization substance protein B homolog OS=Synechocystis sp. (strain PCC 6803 / Kazusa) GN=nusB PE=3 SV=2 - [NUSB_SY	24,89	1,68	1,69	1,31	1,56	1,53E-07
P74226	30S ribosomal protein S10 OS=Synechocystis sp. (strain PCC 6803 / Kazusa) GN=rpsJ PE=3 SV=1 - [RS10_SYNY3]	45,71	1,82	1,49	1,33	1,54	3,12E-06
P48939	30S ribosomal protein S4 OS=Synechocystis sp. (strain PCC 6803 / Kazusa) GN=rpsD PE=3 SV=1 - [RS4_SYNY3]	28,22	2,01	1,54	1,03	1,53	4,58E-02
P36236	50S ribosomal protein L1 OS=Synechocystis sp. (strain PCC 6803 / Kazusa) GN=rplA PE=3 SV=1 - [RL1_SYNY3]	43,28	1,46	1,44	1,66	1,52	1,59E-20
P73289	50S ribosomal protein L25 OS=Synechocystis sp. (strain PCC 6803 / Kazusa) GN=rplY PE=3 SV=2 - [RL25_SYNY3]	65,31	1,04	1,93	1,56	1,51	3,52E-02
P73299	30S ribosomal protein S13 OS=Synechocystis sp. (strain PCC 6803 / Kazusa) GN=rpsM PE=3 SV=1 - [RS13_SYNY3]	54,33	1,15	1,54	1,83	1,51	3,91E-03
P48959	50S ribosomal protein L35 OS=Synechocystis sp. (strain PCC 6803 / Kazusa) GN=rpmI PE=3 SV=1 - [RL35_SYNY3]	32,84	1,20	2,02	1,26	1,49	2,43E-02
P73098	Chaperone protein dnaK3 OS=Synechocystis sp. (strain PCC 6803 / Kazusa) GN=dnaK3 PE=3 SV=2 - [DNAK3_SYNY3]	3,24	1,49	1,46		1,48	6,87E-221
P74229	30S ribosomal protein S7 OS=Synechocystis sp. (strain PCC 6803 / Kazusa) GN=rpsG PE=3 SV=1 - [RS7_SYNY3]	53,85	1,58	1,52	1,31	1,47	1,76E-11
P19050	NAD(P)H-quinone oxidoreductase subunit K 1 OS=Synechocystis sp. (strain PCC 6803 / Kazusa) GN=ndhK1 PE=1 SV=1 - [NDH	23,79	0,83	0,59	0,59	0,67	2,84E-04
P73061	Urease subunit alpha OS=Synechocystis sp. (strain PCC 6803 / Kazusa) GN=ureC PE=3 SV=1 - [URE1_SYNY3]	16,34	0,54	0,68	0,80	0,67	2,37E-04
P72977	16.6 kDa small heat shock protein, molecular chaperon OS=Synechocystis sp. (strain PCC 6803 / Kazusa) GN=hsp17 PE=3 SV	33,56	0,57	0,78		0,67	9,33E-03
P19569	Photosystem I reaction center subunit II OS=Synechocystis sp. (strain PCC 6803 / Kazusa) GN=psaD PE=1 SV=2 - [PSAD_SYNY	52,48	0,63	0,68	0,67	0,66	0,00E+00
Q55360	Sll0888 protein OS=Synechocystis sp. (strain PCC 6803 / Kazusa) GN=sll0888 PE=4 SV=1 - [Q55360_SYNY3]	16,84	0,44	0,62	0,91	0,66	2,59E-02
P73103	Slr1908 protein OS=Synechocystis sp. (strain PCC 6803 / Kazusa) GN=slr1908 PE=4 SV=1 - [P73103_SYNY3]	19,12	0,67	0,50	0,78	0,65	4,17E-04
P19125	NAD(P)H-quinone oxidoreductase subunit J OS=Synechocystis sp. (strain PCC 6803 / Kazusa) GN=ndhJ PE=1 SV=4 - [NDHJ_SY	23,46	0,63	0,62	0,64	0,63	0,00E+00
P73004	Long-chain-fatty-acid CoA ligase OS=Synechocystis sp. (strain PCC 6803 / Kazusa) GN=slr1609 PE=4 SV=1 - [P73004_SYNY3]	5,89		0,71	0,55	0,63	3,29E-04
P73807	Histidinol-phosphate aminotransferase OS=Synechocystis sp. (strain PCC 6803 / Kazusa) GN=hisC PE=3 SV=2 - [HIS8_SYNY3]	27,79	0,39	0,55	0,94	0,63	3,53E-02
P73321	Uncharacterized protein slr1894 OS=Synechocystis sp. (strain PCC 6803 / Kazusa) GN=slr1894 PE=3 SV=1 - [Y1894_SYNY3]	49,36	0,37	0,61	0,90	0,63	3,84E-02
Q55410	Sll0540 protein OS=Synechocystis sp. (strain PCC 6803 / Kazusa) GN=sll0540 PE=4 SV=1 - [Q55410_SYNY3]	5,21	0,40	0,70	0,75	0,62	7,09E-03
Q55233	Protein DrgA OS=Synechocystis sp. (strain PCC 6803 / Kazusa) GN=drgA PE=1 SV=1 - [DRGA_SYNY3]	39,05	0,87	0,46	0,52	0,61	7,02E-03
P72662	Cysteine synthase OS=Synechocystis sp. (strain PCC 6803 / Kazusa) GN=cysM PE=4 SV=1 - [P72662_SYNY3]	24,62	0,84	0,59	0,38	0,61	1,54E-02
P74591	Shikimate dehydrogenase OS=Synechocystis sp. (strain PCC 6803 / Kazusa) GN=aroE PE=3 SV=1 - [AROE_SYNY3]	16,21	0,69	0,52		0,61	2,81E-04
P09190	Cytochrome b559 subunit alpha OS=Synechocystis sp. (strain PCC 6803 / Kazusa) GN=psbE PE=1 SV=3 - [PSBE_SYNY3]	32,1	0,47		0,73	0,60	1,49E-02
P73720	Putative OxPP cycle protein OpcA OS=Synechocystis sp. (strain PCC 6803 / Kazusa) GN=opcA PE=4 SV=1 - [OPCA_SYNY3]	6,95	0,73	0,61	0,45	0,60	1,85E-04
Q55776	Sll0180 protein OS=Synechocystis sp. (strain PCC 6803 / Kazusa) GN=sll0180 PE=4 SV=1 - [Q55776_SYNY3]	11,98	0,71	0,56	0,47	0,58	2,18E-06
Q55199	Phosphate-binding protein OS=Synechocystis sp. (strain PCC 6803 / Kazusa) GN=pstS PE=3 SV=1 - [Q55199_SYNY3]	31,33	0,35	0,55	0,81	0,57	1,06E-02
Q55727	Slr0645 protein OS=Synechocystis sp. (strain PCC 6803 / Kazusa) GN=slr0645 PE=4 SV=1 - [Q55727_SYNY3]	57,77	0,27	0,61	0,83	0,57	4,73E-02
P32422	Photosystem I iron-sulfur center OS=Synechocystis sp. (strain PCC 6803 / Kazusa) GN=psaC PE=1 SV=2 - [PSAC_SYNY3]	65,43	0,44	0,59	0,67	0,57	4,10E-06
P74416	Glycine dehydrogenase (decarboxylating) OS=Synechocystis sp. (strain PCC 6803 / Kazusa) GN=gcvP PE=1 SV=1 - [GCSP_SYN	6,51	0,42	0,66	0,60	0,56	1,70E-05
Q55835	Iron uptake protein A2 OS=Synechocystis sp. (strain PCC 6803 / Kazusa) GN=futA2 PE=1 SV=1 - [FUTA2_SYNY3]	31,5	0,38	0,38	0,88	0,55	1,51E-02
Q55513	4-hydroxy-tetrahydrodipicolinate synthase OS=Synechocystis sp. (strain PCC 6803 / Kazusa) GN=dapA PE=3 SV=1 - [DAPA_S	8,31	0,60	0,47		0,53	1,45E-07
P09192	Photosystem II D2 protein OS=Synechocystis sp. (strain PCC 6803 / Kazusa) GN=psbD PE=3 SV=2 - [PSBD_SYNY3]	10,8	0,75	0,37	0,47	0,53	1,05E-03
Q55865	Sll0585 protein OS=Synechocystis sp. (strain PCC 6803 / Kazusa) GN=sll0585 PE=4 SV=1 - [Q55865_SYNY3]	11,11	0,32	0,67	0,59	0,53	2,09E-03
P73511	Glycogen phosphorylase OS=Synechocystis sp. (strain PCC 6803 / Kazusa) GN=glgP PE=3 SV=1 - [PHSG_SYNY3]	32,51	0,38	0,43	0,76	0,52	1,08E-03
P16033	Photosystem Q(B) protein 2 OS=Synechocystis sp. (strain PCC 6803 / Kazusa) GN=psbA2 PE=1 SV=1 - [PSBA2_SYNY3]	16,67	0,82	0,37	0,38	0,52	5,40E-03
P73527	6,7-dimethyl-8-ribityllumazine synthase OS=Synechocystis sp. (strain PCC 6803 / Kazusa) GN=ribH PE=3 SV=1 - [RISB_SYNY3	29,27	0,37	0,68	0,44	0,50	6,33E-05
Q55517	Sll0529 protein OS=Synechocystis sp. (strain PCC 6803 / Kazusa) GN=sll0529 PE=4 SV=1 - [Q55517_SYNY3]	36,25	0,49	0,47	0,51	0,49	0,00E+00
P72939	Alkaline phosphatase OS=Synechocystis sp. (strain PCC 6803 / Kazusa) GN=sll0654 PE=4 SV=1 - [P72939_SYNY3]	11,36	0,28	0,51	0,67	0,49	2,31E-03
P80046	Isocitrate dehydrogenase [NADP] OS=Synechocystis sp. (strain PCC 6803 / Kazusa) GN=icd PE=1 SV=2 - [IDH_SYNY3]	27,58	0,28	0,48	0,68	0,48	2,11E-03
Q55200	Protein SphX OS=Synechocystis sp. (strain PCC 6803 / Kazusa) GN=sphX PE=3 SV=1 - [SPHX_SYNY3]	17,26	0,37	0,45	0,62	0,48	5,01E-07
P74338	NAD(P)H-quinone oxidoreductase subunit M OS=Synechocystis sp. (strain PCC 6803 / Kazusa) GN=ndhM PE=1 SV=1 - [NDHM	23,97	0,28	0,56	0,55	0,46	2,57E-04
P73914	Squalene-hopene-cyclase OS=Synechocystis sp. (strain PCC 6803 / Kazusa) GN=shc PE=4 SV=1 - [P73914_SYNY3]	10,36	0,30	0,43	0,61	0,45	2,70E-05
P74771	NAD(P)H-quinone oxidoreductase subunit O OS=Synechocystis sp. (strain PCC 6803 / Kazusa) GN=ndhO PE=1 SV=1 - [NDHO_	40,28	0,45	0,45	0,44	0,44	0,00E+00
P73393	Sll1734 protein OS=Synechocystis sp. (strain PCC 6803 / Kazusa) GN=sll1734 PE=4 SV=1 - [P73393_SYNY3]	16,71	0,57	0,41	0,36	0,44	2,01E-09
P73599	Uncharacterized protein sll1304 OS=Synechocystis sp. (strain PCC 6803 / Kazusa) GN=sll1304 PE=3 SV=1 - [Y1304_SYNY3]	11,15	0,48	0,37	0,47	0,44	0,00E+00
Q55196	Phosphate import ATP-binding protein PstB 1 OS=Synechocystis sp. (strain PCC 6803 / Kazusa) GN=pstB1 PE=3 SV=1 - [PSTB	25,65	0,55	0,36	0,40	0,43	3,30E-11
P72646	Sll1054 protein OS=Synechocystis sp. (strain PCC 6803 / Kazusa) GN=sll1054 PE=3 SV=1 - [P72646_SYNY3]	9,09	0,26	0,61		0,43	2,94E-02
P72647	Sll1053 protein OS=Synechocystis sp. (strain PCC 6803 / Kazusa) GN=sll1053 PE=4 SV=1 - [P72647_SYNY3]	7,31	0,19	0,39	0,65	0,41	4,75E-03
P73618	Deoxyribose-phosphate aldolase OS=Synechocystis sp. (strain PCC 6803 / Kazusa) GN=deoC PE=3 SV=1 - [DEOC_SYNY3]	10,22	0,37	0,44		0,40	0,00E+00
P72891	Slr1613 protein OS=Synechocystis sp. (strain PCC 6803 / Kazusa) GN=slr1613 PE=4 SV=1 - [P72891_SYNY3]	12,76	0,27		0,51	0,39	1,84E-03
P74474	D-alanyl-D-alanine carboxypeptidase OS=Synechocystis sp. (strain PCC 6803 / Kazusa) GN=slr1924 PE=4 SV=1 - [P74474_SYN	14,25	0,30	0,34	0,52	0,39	6,32E-09
Q55198	Phosphate transport system permease protein PstC OS=Synechocystis sp. (strain PCC 6803 / Kazusa) GN=pstC PE=3 SV=1 - [	5,05		0,39	0,38	0,38	0,00E+00
P74250	Hydroperoxy fatty acid reductase gpx1 OS=Synechocystis sp. (strain PCC 6803 / Kazusa) GN=gpx1 PE=1 SV=1 - [GPX1_SYNY3	16,57	0,21	0,40	0,42	0,34	8,15E-07
P73954	Membrane-associated protein slr1513 OS=Synechocystis sp. (strain PCC 6803 / Kazusa) GN=slr1513 PE=1 SV=1 - [Y1513_SYN	48,18	0,16	0,35	0,48	0,33	2,10E-04
P72938	Extracellular nuclease OS=Synechocystis sp. (strain PCC 6803 / Kazusa) GN=nucH PE=4 SV=1 - [P72938_SYNY3]	6,55	0,16	0,33	0,51	0,33	4,25E-04
P73066	Ycf23 protein OS=Synechocystis sp. (strain PCC 6803 / Kazusa) GN=ycf23 PE=4 SV=1 - [P73066_SYNY3]	12,9	0,42	0,26	0,30	0,33	1,78E-14
P73862	Rubisco operon transcriptional regulator OS=Synechocystis sp. (strain PCC 6803 / Kazusa) GN=rbcR PE=4 SV=1 - [P73862_SY	14,24	0,48	0,15	0,27	0,30	9,28E-05
P72827	Iron uptake protein A1 OS=Synechocystis sp. (strain PCC 6803 / Kazusa) GN=futA1 PE=1 SV=1 - [FUTA1_SYNY3]	39,44	0,18	0,24	0,38	0,27	3,47E-10
P73866	Potassium-transporting ATPase A chain OS=Synechocystis sp. (strain PCC 6803 / Kazusa) GN=kdpA PE=3 SV=1 - [ATKA_SYNY	4,84	0,54	0,09	0,13	0,25	2,01E-03
P27319	Flavodoxin OS=Synechocystis sp. (strain PCC 6803 / Kazusa) GN=isiB PE=1 SV=2 - [FLAV_SYNY3]	11,18	0,15	0,16	0,42	0,24	2,86E-06

P73867	Potassium-transporting ATPase B chain OS=Synechocystis sp. (strain PCC 6803 / Kazusa) GN=kdpB PE=3 SV=1 - [ATKB_SYNY3	15,36	0,42	0,10	0,12	0,21	8,38E-05
P74625	Phycobilisome rod-core linker polypeptide CpcG OS=Synechocystis sp. (strain PCC 6803 / Kazusa) GN=cpcG PE=4 SV=1 - [P74	24,1	0,16	0,16	0,25	0,19	0,00E+00

Supplementary Table S6

[NiFe]-hydrogenase is essential for cyanobacterium *Synechocystis* sp. PCC 6803 aerobic growth in the dark

Edith De Rosa, Vanessa Checchetto, Cinzia Franchin, Elisabetta Bergantino, Paola Berto, Ildikò Szabò, Giorgio M. Giacometti, Giorgio Arrigoni, and Paola Costantini

Accession	Description	ΣCoverage	Mutant Dark/WT Dark Replicate 1 (114/116)	Mutant Dark/WT Dark Replicate 2 (115/117)	Mutant Dark/WT Dark Replicate 3 (116/114)	Average	Two-tailed Z Test
	>sp|CAS1_BOVIN|	17,29		0,2	0,1	0,2	1,6E-06
	>sp|K1C10_HUMAN|	23,1	1,8	0,7	0,8	1,1	9,6E-01
	>sp|K1C9_HUMAN|	14,29	1,2	1,0	1,0	1,1	3,3E-01
	>sp|K22E_HUMAN|	19,38	1,4	0,6	0,7	0,9	5,0E-01
	>sp|K2C1_HUMAN|	34,06	1,2	0,7	0,8	0,9	5,0E-01
	>sp|TRYP_PIG|	28,57	1,1	1,2	0,8	1,0	9,0E-01
P74561	1-(5-phosphoribosyl)-5-[(5-phosphoribosylamino)methylideneamino] imidazo	13,28	2,6		0,7	1,4	6,7E-01
Q55663	1-deoxy-D-xylulose 5-phosphate reductoisomerase OS=Synechocystis sp. (s	8,63	0,7	0,9		0,7	1,2E-02
P73067	1-deoxy-D-xylulose-5-phosphate synthase OS=Synechocystis sp. (strain PC	5	0,6	0,6	0,9	0,7	3,5E-03
P52981	1,4-alpha-glucan branching enzyme GlgB OS=Synechocystis sp. (strain PC	17,53	1,8	2,1	1,1	1,5	2,2E-02
Q05971	10 kDa chaperonin OS=Synechocystis sp. (strain PCC 6803 / Kazusa) GN=	72,82	0,7	0,7	0,4	0,6	9,1E-03
P72977	16.6 kDa small heat shock protein, molecular chaperon OS=Synechocystis s	33,56	2,2	2,5		2,1	1,1E-54
P73426	2-C-methyl-D-erythritol 2,4-cyclodiphosphate synthase OS=Synechocystis sp	13,04	2,2	1,5	1,0	2,1	6,4E-02
P48576	2-isopropylmalate synthase OS=Synechocystis sp. (strain PCC 6803 / Kazus	4,5	0,7			0,8	2,0E+00
P74507	2,3-bisphosphoglycerate-independent phosphoglycerate mutase OS=Synech	13,72	1,5	1,1	1,0	1,2	1,5E-01
P73264	3-amino-5-hydroxybenzoic acid synthase OS=Synechocystis sp. (strain PCC	42,06	2,3	1,8	1,1	1,7	3,0E-02
P73997	3-dehydroquinate synthase OS=Synechocystis sp. (strain PCC 6803 / Kazus	15,24		0,7	1,3	1,0	9,5E-01
P73848	3-hydroxyacyl-[acyl-carrier-protein] dehydratase FabZ OS=Synechocystis sp	24,39	1,0	1,0	0,7	0,9	2,8E-01
P54384	3-isopropylmalate dehydratase large subunit OS=Synechocystis sp. (strain F	4,06	1,8	0,9	0,8	1,2	7,1E-01
P74207	3-isopropylmalate dehydratase small subunit OS=Synechocystis sp. (strain F	10		1,1		1,3	2,0E+00
P73960	3-isopropylmalate dehydrogenase OS=Synechocystis sp. (strain PCC 6803 /	10,77	1,2		1,3	1,4	4,1E-25
Q55596	3-ketoacyl-acyl carrier protein reductase OS=Synechocystis sp. (strain PCC	21,24	1,0			1,1	2,0E+00
P73574	3-oxoacyl-[acyl-carrier-protein] reductase 1 OS=Synechocystis sp. (strain PC	18,62		0,8	0,9	0,8	8,3E-02
P73826	3-oxoacyl-[acyl-carrier-protein] reductase 2 OS=Synechocystis sp. (strain PC	34,58	0,8	0,7	0,8	0,8	0,0E+00
P73283	3-oxoacyl-[acyl-carrier-protein] synthase 2 OS=Synechocystis sp. (strain PC	5,53	1,2	0,8	0,8	0,9	5,9E-01
P73530	30S ribosomal protein S1 homolog A OS=Synechocystis sp. (strain PCC 680	33,54	1,1	1,2	1,2	1,2	2,1E-20
P74142	30S ribosomal protein S1 homolog B OS=Synechocystis sp. (strain PCC 680	16,72	0,5	0,8	0,6	0,6	5,0E-03
P74226	30S ribosomal protein S10 OS=Synechocystis sp. (strain PCC 6803 / Kazus	45,71	1,2	1,2	1,4	1,4	7,1E-10
P73298	30S ribosomal protein S11 OS=Synechocystis sp. (strain PCC 6803 / Kazus	32,31	1,4	1,3	1,7	1,9	5,6E-07
P74230	30S ribosomal protein S12 OS=Synechocystis sp. (strain PCC 6803 / Kazus	30,16	2,5	2,7	2,0	2,0	3,1E-25
P73299	30S ribosomal protein S13 OS=Synechocystis sp. (strain PCC 6803 / Kazus	54,33	1,3	0,8	0,7	0,9	5,0E-01
P48944	30S ribosomal protein S14 OS=Synechocystis sp. (strain PCC 6803 / Kazus	44	0,8	1,0	1,0	0,9	2,1E-01
P72866	30S ribosomal protein S15 OS=Synechocystis sp. (strain PCC 6803 / Kazus	33,71	1,4	1,1	1,3	2,4	7,0E-05
P74410	30S ribosomal protein S16 OS=Synechocystis sp. (strain PCC 6803 / Kazus	70,73	2,8	2,9	1,4	1,9	8,7E-04
P73311	30S ribosomal protein S17 OS=Synechocystis sp. (strain PCC 6803 / Kazus	66,67	3,3	3,3	2,0	2,4	4,1E-09
P48946	30S ribosomal protein S18 OS=Synechocystis sp. (strain PCC 6803 / Kazus	64,79	1,5	1,2	0,8	1,2	4,9E-01
P73316	30S ribosomal protein S19 OS=Synechocystis sp. (strain PCC 6803 / Kazus	36,96	1,3	1,3	1,0	1,2	9,8E-03
P74071	30S ribosomal protein S2 OS=Synechocystis sp. (strain PCC 6803 / Kazusa)	35,32	1,2	1,2	1,4	1,4	2,1E-05
P73336	30S ribosomal protein S20 OS=Synechocystis sp. (strain PCC 6803 / Kazus	16,49	1,3	1,3		1,7	3,0E-119
P48949	30S ribosomal protein S21 OS=Synechocystis sp. (strain PCC 6803 / Kazus	31,67			1,2	1,1	2,0E+00
P73314	30S ribosomal protein S3 OS=Synechocystis sp. (strain PCC 6803 / Kazusa)	36,67	1,3	1,5	1,7	1,5	2,6E-05
P48939	30S ribosomal protein S4 OS=Synechocystis sp. (strain PCC 6803 / Kazusa)	28,22	4,9	5,2	2,4	3,1	1,6E-08
P73304	30S ribosomal protein S5 OS=Synechocystis sp. (strain PCC 6803 / Kazusa)	61,27	1,5	1,6	1,7	1,5	2,2E-24
P73636	30S ribosomal protein S6 OS=Synechocystis sp. (strain PCC 6803 / Kazusa)	44,25	1,7	1,4	1,2	1,6	5,5E-04
P74229	30S ribosomal protein S7 OS=Synechocystis sp. (strain PCC 6803 / Kazusa)	53,85	2,2	2,1	1,4	2,5	7,5E-06
P73307	30S ribosomal protein S8 OS=Synechocystis sp. (strain PCC 6803 / Kazusa)	51,88	2,1	2,0	1,8	3,4	3,0E-41
P73293	30S ribosomal protein S9 OS=Synechocystis sp. (strain PCC 6803 / Kazusa)	37,23	1,4	1,3	1,4	1,3	1,9E-23
P72785	4-alpha-glucanotransferase OS=Synechocystis sp. (strain PCC 6803 / Kazus	8,51	9,0	7,5	1,9	4,8	1,0E-03
P73672	4-hydroxy-3-methylbut-2-en-1-yl diphosphate synthase OS=Synechocystis s	25,56	0,5	0,5	0,6	0,6	2,7E-13
P72642	4-hydroxy-tetrahydrodipicolinate reductase OS=Synechocystis sp. (strain PC	7,27			0,9	0,9	2,0E+00
Q55513	4-hydroxy-tetrahydrodipicolinate synthase OS=Synechocystis sp. (strain PC	8,31	2,1	3,5		2,0	6,7E-05
P74756	47 kD protein OS=Synechocystis sp. (strain PCC 6803 / Kazusa) GN=slr060	16,71	1,1	0,9	1,2	1,1	6,3E-01
P73637	5-oxo-1,2,5-tricarboxilic-3-penten acid decarboxilase/isomer OS=Synechocy	14,13	4,2		1,6	2,0	4,5E-02
P36236	50S ribosomal protein L1 OS=Synechocystis sp. (strain PCC 6803 / Kazusa)	43,28	1,4	1,4	1,2	1,4	4,4E-06
P23350	50S ribosomal protein L10 OS=Synechocystis sp. (strain PCC 6803 / Kazus	22,54	1,2	1,1	1,0	1,1	8,9E-03
P36237	50S ribosomal protein L11 OS=Synechocystis sp. (strain PCC 6803 / Kazus	45,39	0,8	0,9	1,0	0,9	5,9E-02
P73294	50S ribosomal protein L13 OS=Synechocystis sp. (strain PCC 6803 / Kazus	78,81	4,4	6,1	2,8	4,8	2,9E-10
P73310	50S ribosomal protein L14 OS=Synechocystis sp. (strain PCC 6803 / Kazus	27,87		1,8	1,4	1,6	2,2E-03
P73303	50S ribosomal protein L15 OS=Synechocystis sp. (strain PCC 6803 / Kazus	67,35	3,6	4,4	2,1	2,6	1,6E-07
P73313	50S ribosomal protein L16 OS=Synechocystis sp. (strain PCC 6803 / Kazus	35,25	3,8	8,5		4,3	1,8E-05
P73296	50S ribosomal protein L17 OS=Synechocystis sp. (strain PCC 6803 / Kazus	52,59	4,8	6,7	2,3	3,1	3,1E-06
P73305	50S ribosomal protein L18 OS=Synechocystis sp. (strain PCC 6803 / Kazus	20	2,0	1,8	1,5	1,8	1,1E-09
P36239	50S ribosomal protein L19 OS=Synechocystis sp. (strain PCC 6803 / Kazus	44,26	2,0	1,8	1,8	1,7	3,4E-195
P73317	50S ribosomal protein L2 OS=Synechocystis sp. (strain PCC 6803 / Kazusa)	61,59	8,8	7,9	3,7	5,4	7,5E-12
P48957	50S ribosomal protein L20 OS=Synechocystis sp. (strain PCC 6803 / Kazus	57,26	2,5	2,9	1,5	1,9	1,1E-04
P74266	50S ribosomal protein L21 OS=Synechocystis sp. (strain PCC 6803 / Kazus	69,35	5,3	5,7	2,0	3,4	9,0E-05
P73315	50S ribosomal protein L22 OS=Synechocystis sp. (strain PCC 6803 / Kazus	43,8	6,3	7,0	2,2	3,7	5,0E-05
P73318	50S ribosomal protein L23 OS=Synechocystis sp. (strain PCC 6803 / Kazus	78,22	4,2	4,2	1,8	2,8	4,7E-05
P73309	50S ribosomal protein L24 OS=Synechocystis sp. (strain PCC 6803 / Kazus	68,7	3,0	3,9	2,0	3,2	6,4E-08
P73289	50S ribosomal protein L25 OS=Synechocystis sp. (strain PCC 6803 / Kazus	65,31	7,8	8,0	2,4	3,9	3,8E-05
P74267	50S ribosomal protein L27 OS=Synechocystis sp. (strain PCC 6803 / Kazus	59,77	5,9	5,4	2,3	5,3	1,0E-06
P72851	50S ribosomal protein L28 OS=Synechocystis sp. (strain PCC 6803 / Kazus	61,54	4,9	3,7	1,8	3,3	1,6E-04
P73312	50S ribosomal protein L29 OS=Synechocystis sp. (strain PCC 6803 / Kazus	73,97	1,0	1,1	0,9	1,0	8,3E-01
P73320	50S ribosomal protein L3 OS=Synechocystis sp. (strain PCC 6803 / Kazusa)	52,58	7,6	8,9	2,8	4,2	1,1E-06
P48958	50S ribosomal protein L33 OS=Synechocystis sp. (strain PCC 6803 / Kazus	26,15			2,2	2,3	2,0E+00
P48959	50S ribosomal protein L35 OS=Synechocystis sp. (strain PCC 6803 / Kazus	32,84	3,2	2,1	1,7	1,8	1,1E-05
P73319	50S ribosomal protein L4 OS=Synechocystis sp. (strain PCC 6803 / Kazusa)	55,71	5,7	7,3	2,3	5,8	1,6E-05
P73308	50S ribosomal protein L5 OS=Synechocystis sp. (strain PCC 6803 / Kazusa)	65,56	2,9	3,5	1,4	2,5	1,2E-03
P73306	50S ribosomal protein L6 OS=Synechocystis sp. (strain PCC 6803 / Kazusa)	77,65	5,9	5,5	3,1	4,7	2,0E-14
P23349	50S ribosomal protein L7/L12 OS=Synechocystis sp. (strain PCC 6803 / Kaz	52,34	1,1	1,1	1,0	1,1	3,4E-01
P42352	50S ribosomal protein L9 OS=Synechocystis sp. (strain PCC 6803 / Kazusa)	61,18	4,1	4,0	2,3	2,4	6,1E-11
Q55798	6-carboxy-5,6,7,8-tetrahydropterin synthase OS=Synechocystis sp. (strain P	23,26	3,4	2,4	1,8	2,0	4,5E-07
Q55988	6-phosphofructokinase 2 OS=Synechocystis sp. (strain PCC 6803 / Kazusa)	5,99		0,9		1,0	2,0E+00
P52208	6-phosphogluconate dehydrogenase, decarboxylating OS=Synechocystis sp	24,9	0,6	0,6	1,1	0,8	9,0E-02
P74618	6-phosphogluconolactonase OS=Synechocystis sp. (strain PCC 6803 / Kazu	15,42	0,6	0,6	0,9	0,7	3,4E-03
P73527	6,7-dimethyl-8-ribityllumazine synthase OS=Synechocystis sp. (strain PCC 6	29,27	2,1	1,6	1,4	1,4	4,0E-06
Q05972	60 kDa chaperonin 1 OS=Synechocystis sp. (strain PCC 6803 / Kazusa) GN	58,96	0,8	0,9	0,9	0,8	3,6E-06
P22034	60 kDa chaperonin 2 OS=Synechocystis sp. (strain PCC 6803 / Kazusa) GN	62,5	1,2	1,5	1,2	1,7	2,9E-04
Q46363	Acetazolamide conferring resistance protein zam OS=Synechocystis sp. (stra	6,65		0,8	1,4	1,1	7,1E-01
P73913	Acetohydroxy acid synthase OS=Synechocystis sp. (strain PCC 6803 / Kazu	3,54	0,3		0,7	0,4	4,3E-02
P73918	Acetolactate synthase OS=Synechocystis sp. (strain PCC 6803 / Kazusa) GI	13,09	1,9	1,6	1,2	1,6	1,2E-03
Q55141	Acetolactate synthase small subunit OS=Synechocystis sp. (strain PCC 680	49,42	0,9	1,1	0,8	0,9	3,6E-01
P73825	Acetyl coenzyme A acetyltransferase (Thiolase) OS=Synechocystis sp. (strai	7,33		0,6		0,7	2,0E+00
Q55404	Acetyl-coenzyme A synthetase OS=Synechocystis sp. (strain PCC 6803 / Ka	10,41	0,8	1,1	1,4	1,2	6,7E-01
P73326	Acetylglutamate kinase OS=Synechocystis sp. (strain PCC 6803 / Kazusa) G	8,75			1,1	1,1	2,0E+00
P73133	Acetylornithine aminotransferase OS=Synechocystis sp. (strain PCC 6803 / I	41,03	0,7	0,5	0,4	0,5	8,2E-04
P74582	Aconitate hydratase 2 OS=Synechocystis sp. (strain PCC 6803 / Kazusa) GN	11,29	0,9	0,8	0,8	0,8	2,8E-11
P20804	Acyl carrier protein OS=Synechocystis sp. (strain PCC 6803 / Kazusa) GN=a	20,78	1,1	1,0	0,8	1,0	6,0E-01
Q55746	Acyl-[acyl-carrier-protein]--UDP-N-acetylglucosamine O-acyltransferase OS=	8,7			0,8	0,9	2,0E+00
P74008	Adenosylhomocysteinase OS=Synechocystis sp. (strain PCC 6803 / Kazusa	57,65	0,4	0,4	0,7	0,5	9,1E-06
P73302	Adenylate kinase 1 OS=Synechocystis sp. (strain PCC 6803 / Kazusa) GN=a	44,32	1,4	1,2	1,0	1,2	1,1E-01
P74384	Adenylosuccinate lyase OS=Synechocystis sp. (strain PCC 6803 / Kazusa) G	32,02	2,7	1,4	1,0	1,6	1,5E-01
P73290	Adenylosuccinate synthetase OS=Synechocystis sp. (strain PCC 6803 / Kaz	12,16	1,1	1,6	1,4	1,2	1,3E-03
P72703	Agmatinase 1 OS=Synechocystis sp. (strain PCC 6803 / Kazusa) GN=speB1	15,36	2,5	2,3	1,6	1,7	1,2E-08
P73288	Aklaviketone reductase OS=Synechocystis sp. (strain PCC 6803 / Kazusa) G	8,57		0,7	0,7	0,6	0,0E+00
P74423	Alanine--tRNA ligase OS=Synechocystis sp. (strain PCC 6803 / Kazusa) GN	4,56		0,6	1,8	1,2	9,8E-01
Q55688	Aldehyde decarbonylase OS=Synechocystis sp. (strain PCC 6803 / Kazusa)	12,55			0,9	0,9	2,0E+00
Q55811	Aldehyde dehydrogenase OS=Synechocystis sp. (strain PCC 6803 / Kazusa	3,29			1,3	1,2	2,0E+00
P74308	Aldehyde reductase OS=Synechocystis sp. (strain PCC 6803 / Kazusa) GN=	18,35	2,3	2,7	1,4	2,6	2,2E-04
P72939	Alkaline phosphatase OS=Synechocystis sp. (strain PCC 6803 / Kazusa) GN	11,36	0,4	0,3	0,3	0,3	0,0E+00
Q01951	Allophycocyanin alpha chain OS=Synechocystis sp. (strain PCC 6803 / Kazu	77,02	0,5	0,5	0,7	0,5	5,2E-05
Q01952	Allophycocyanin beta chain OS=Synechocystis sp. (strain PCC 6803 / Kazus	98,76	0,6	0,5	0,8	0,7	4,9E-04
P72870	Allophycocyanin subunit alpha-B OS=Synechocystis sp. (strain PCC 6803 / I	73,91	0,5	0,5	0,7	0,6	2,8E-05
P74551	Allophycocyanin subunit beta-18 OS=Synechocystis sp. (strain PCC 6803 / I	68,05	0,6	0,7	0,6	0,7	0,0E+00
Q55621	Amidophosphoribosyltransferase OS=Synechocystis sp. (strain PCC 6803 / I	12,12	0,7		1,2	0,9	7,0E-01
P74527	Aminopeptidase OS=Synechocystis sp. (strain PCC 6803 / Kazusa) GN=ape	3,34	0,6		1,2	0,9	6,7E-01
P74468	Aminopeptidase P OS=Synechocystis sp. (strain PCC 6803 / Kazusa) GN=p	18,82	1,6	1,2	0,9	1,3	2,4E-01
P73607	Anti-sigma factor antagonist OS=Synechocystis sp. (strain PCC 6803 / Kazu	19,27	0,7	0,6	0,4	0,5	2,0E-05
P73609	Anti-sigma factor antagonist OS=Synechocystis sp. (strain PCC 6803 / Kazu	45,37	0,4	0,3	0,2	0,3	1,6E-10
P26287	Apocytochrome f OS=Synechocystis sp. (strain PCC 6803 / Kazusa) GN=pe	44,21	1,2	1,3	0,9	1,2	1,8E-01
Q55841	Arabinofuranosidase OS=Synechocystis sp. (strain PCC 6803 / Kazusa) GN	10,73			0,7	0,8	2,0E+00
P74122	Arginine biosynthesis bifunctional protein ArgJ OS=Synechocystis sp. (strain	8,96	0,7		0,5	0,7	3,5E-03
P73257	Argininosuccinate lyase OS=Synechocystis sp. (strain PCC 6803 / Kazusa) (	2,6			0,8	0,7	2,0E+00
P77973	Argininosuccinate synthase OS=Synechocystis sp. (strain PCC 6803 / Kazus	26	0,7	0,7	1,1	0,8	1,3E-01
P52276	Asparagine--tRNA ligase OS=Synechocystis sp. (strain PCC 6803 / Kazusa)	9,74	0,8	0,9	1,3	1,0	8,3E-01
Q55128	Aspartate aminotransferase OS=Synechocystis sp. (strain PCC 6803 / Kazus	6,17			0,8	0,9	2,0E+00
P73851	Aspartate--tRNA ligase OS=Synechocystis sp. (strain PCC 6803 / Kazusa) G	11,02	0,5	0,8	1,0	0,8	1,0E-01
Q55512	Aspartate-semialdehyde dehydrogenase OS=Synechocystis sp. (strain PCC	19,82	0,9	1,1	1,1	1,1	7,8E-01
P74569	Aspartokinase OS=Synechocystis sp. (strain PCC 6803 / Kazusa) GN=lysC I	9,83	1,2	1,2	1,2	1,1	7,4E-33
P74215	Aspartyl/glutamyl-tRNA(Asn/Gln) amidotransferase subunit B OS=Synechoc	6,06		1,1		1,1	2,0E+00
P74592	ATP phosphoribosyltransferase regulatory subunit OS=Synechocystis sp. (st	12,47	0,7	0,7	1,1	0,8	1,5E-01
P26533	ATP synthase epsilon chain OS=Synechocystis sp. (strain PCC 6803 / Kazus	17,65	0,6			0,6	2,0E+00
P17253	ATP synthase gamma chain OS=Synechocystis sp. (strain PCC 6803 / Kazu	28,34	0,5	0,6	0,8	0,6	1,4E-04
P27179	ATP synthase subunit alpha OS=Synechocystis sp. (strain PCC 6803 / Kazu	33,4	0,4	0,5	0,8	0,6	5,3E-04
P27181	ATP synthase subunit b OS=Synechocystis sp. (strain PCC 6803 / Kazusa) (	51,96	0,6	0,6	0,5	0,4	4,0E-10
P27183	ATP synthase subunit b' OS=Synechocystis sp. (strain PCC 6803 / Kazusa)	53,85	0,4	0,4	0,5	0,5	0,0E+00
P26527	ATP synthase subunit beta OS=Synechocystis sp. (strain PCC 6803 / Kazus	53,83	0,4	0,5	0,7	0,5	7,2E-06
P27182	ATP synthase subunit c OS=Synechocystis sp. (strain PCC 6803 / Kazusa) (	11,11			0,7	0,6	2,0E+00
P27180	ATP synthase subunit delta OS=Synechocystis sp. (strain PCC 6803 / Kazus	36,22	0,5	0,5	0,5	0,5	0,0E+00
P54416	ATP-dependent Clp protease proteolytic subunit 1 OS=Synechocystis sp. (st	32,83	0,8	0,7	0,9	0,8	3,0E-04
Q55662	ATP-dependent Clp protease regulatory subunit OS=Synechocystis sp. (strai	24,36	0,8	0,9	1,2	0,9	6,2E-01
Q55700	ATP-dependent zinc metalloprotease FtsH 2 OS=Synechocystis sp. (strain P	11,48	0,9	0,7	0,9	0,9	1,2E-02
P72991	ATP-dependent zinc metalloprotease FtsH 3 OS=Synechocystis sp. (strain P	11,2	0,8		1,2	1,0	8,4E-01

P73437	ATP-dependent zinc metalloprotease FtsH 4 OS=Synechocystis sp. (strain P	17,83	0,8	1,1	1,8	1,3	4,2E-01
P72719	Bacterioferritin comigratory protein OS=Synechocystis sp. (strain PCC 6803	15,22	1,4	1,2		1,4	2,5E-04
P24602	Bacterioferritin OS=Synechocystis sp. (strain PCC 6803 / Kazusa) GN=bfr Pl	65,38	3,1	3,5	2,1	3,3	2,1E-11
P73287	Bacterioferritin OS=Synechocystis sp. (strain PCC 6803 / Kazusa) GN=bfr Pl	36,36	1,8	1,9	1,4	1,6	1,6E-09
Q55460	Bicarbonate-binding protein CmpA OS=Synechocystis sp. (strain PCC 6803	49,34	12,4	5,7	2,5	6,8	2,3E-04
Q55074	Bifunctional pantoate ligase/cytidylate kinase OS=Synechocystis sp. (strain F	4,48	0,8			0,7	2,0E+00
Q55626	Bifunctional protein FolD OS=Synechocystis sp. (strain PCC 6803 / Kazusa)	17,01	0,8		1,1	0,9	5,9E-01
Q55758	Bifunctional protein PyrR OS=Synechocystis sp. (strain PCC 6803 / Kazusa)	29,21	0,8	0,9	1,1	1,0	4,9E-01
P74741	Bifunctional purine biosynthesis protein PurH OS=Synechocystis sp. (strain F	11,15	1,0	0,9	1,0	0,9	4,1E-01
P74576	Biosynthetic arginine decarboxylase 1 OS=Synechocystis sp. (strain PCC 68	4,03	0,5	1,0		0,8	3,1E-01
P72587	Biosynthetic arginine decarboxylase 2 OS=Synechocystis sp. (strain PCC 68	2,43	0,5			0,5	2,0E+00
Q55120	Biotin carboxyl carrier protein of acetyl-CoA carboxylase OS=Synechocystis	14,29	1,2		0,4	0,8	5,0E-01
Q55160	Biotin carboxylase OS=Synechocystis sp. (strain PCC 6803 / Kazusa) GN=a	6,47	0,5		1,1	0,9	5,0E-01
Q54715	C-phycocyanin alpha chain OS=Synechocystis sp. (strain PCC 6803 / Kazus	79,01	0,5	0,5	0,7	0,6	1,1E-07
Q54714	C-phycocyanin beta chain OS=Synechocystis sp. (strain PCC 6803 / Kazusa	96,51	0,6	0,6	0,7	0,7	0,0E+00
P73589	C4-dicarboxylase binding protein OS=Synechocystis sp. (strain PCC 6803 / I	9,76	0,3	0,5		0,7	8,0E-05
Q55756	Carbamoyl-phosphate synthase large chain OS=Synechocystis sp. (strain P(	10,64	0,6	0,6	0,8	0,5	6,8E-04
P74587	Carbamoyl-phosphate synthase small chain OS=Synechocystis sp. (strain P(	4,17		0,9		0,9	2,0E+00
P72759	Carbon dioxide concentrating mechanism protein CcmL OS=Synechocystis s	18		0,9		0,9	2,0E+00
P72758	Carbon dioxide concentrating mechanism protein CcmM OS=Synechocystis	26,35	0,9	1,1	1,4	1,1	5,4E-01
P72760	Carbon dioxide-concentrating mechanism protein CcmK homolog 1 OS=Syn	73,87	3,1	2,7	0,9	2,0	6,5E-02
P72761	Carbon dioxide-concentrating mechanism protein CcmK homolog 2 OS=Syn	75,73	2,6	3,5	1,0	1,8	5,2E-02
P73407	Carbon dioxide-concentrating mechanism protein CcmK homolog 4 OS=Syn	23,21	0,7	0,7	0,7	0,7	0,0E+00
Q54735	Carbonic anhydrase OS=Synechocystis sp. (strain PCC 6803 / Kazusa) GN=	16,06	0,7	0,9		0,8	2,3E-02
P73458	Carboxyl-terminal protease OS=Synechocystis sp. (strain PCC 6803 / Kazus	22,7	1,1	0,7	0,6	0,8	1,5E-01
P72864	Carboxysome formation protein OS=Synechocystis sp. (strain PCC 6803 / K	37,32	1,4	1,3	0,9	1,2	1,9E-01
P73911	Catalase-peroxidase OS=Synechocystis sp. (strain PCC 6803 / Kazusa) GN	25,6	1,9	1,2	0,9	1,4	3,2E-01
Q55584	Cation or drug efflux system protein OS=Synechocystis sp. (strain PCC 680	3,79	0,9	1,0	1,1	1,0	8,9E-01
Q55589	Cell division cycle protein OS=Synechocystis sp. (strain PCC 6803 / Kazusa)	7,19	1,0	1,2	1,8	1,4	1,6E-01
P73456	Cell division protein FtsZ OS=Synechocystis sp. (strain PCC 6803 / Kazusa)	7,67	0,9	0,6		0,8	1,2E-01
P74459	Chaperone protein ClpB 1 OS=Synechocystis sp. (strain PCC 6803 / Kazusa	4,68	0,6			0,7	2,0E+00
P74361	Chaperone protein ClpB 2 OS=Synechocystis sp. (strain PCC 6803 / Kazusa	14,68	0,8		1,4	1,1	8,0E-01
Q55505	Chaperone protein DnaJ 1 OS=Synechocystis sp. (strain PCC 6803 / Kazusa	5,84			0,6	1,0	2,0E+00
P22358	Chaperone protein dnaK2 OS=Synechocystis sp. (strain PCC 6803 / Kazusa	41,04	1,2	1,0	0,8	1,0	8,3E-01
P73098	Chaperone protein dnaK3 OS=Synechocystis sp. (strain PCC 6803 / Kazusa	3,24	0,9	0,2		0,7	2,7E-01
P73243	CheY subfamily OS=Synechocystis sp. (strain PCC 6803 / Kazusa) GN=slr2	69,44	0,7	0,7	0,7	0,7	0,0E+00
P23353	Chorismate synthase OS=Synechocystis sp. (strain PCC 6803 / Kazusa) GN	12,71	1,0	0,9	1,2	1,1	5,5E-01
P73092	Chromophore lyase CpcS/CpeS 2 OS=Synechocystis sp. (strain PCC 6803 /	8,33	1,5			1,6	2,0E+00
P74371	Chromophore lyase CpcT/CpeT OS=Synechocystis sp. (strain PCC 6803 / K	21,94	1,6	0,9	0,9	1,1	7,2E-01
P74645	Circadian clock protein KaiB OS=Synechocystis sp. (strain PCC 6803 / Kazu	21,9	0,7		0,4	0,6	1,8E-03
Q59977	Citrate synthase OS=Synechocystis sp. (strain PCC 6803 / Kazusa) GN=gltA	10,33		1,1		1,0	2,0E+00
Q55822	CobW protein OS=Synechocystis sp. (strain PCC 6803 / Kazusa) GN=cobW	6,53		1,1		1,1	2,0E+00
Q55367	Cyanate hydratase OS=Synechocystis sp. (strain PCC 6803 / Kazusa) GN=c	36,91	2,1	1,6	1,0	1,4	7,4E-02
P73833	Cyanophycin synthetase OS=Synechocystis sp. (strain PCC 6803 / Kazusa)	13,75	1,1	1,1	1,7	1,3	9,3E-02
P73832	Cyanophycinase OS=Synechocystis sp. (strain PCC 6803 / Kazusa) GN=cph	8,49	2,2	1,8	1,2	1,5	4,4E-03
P73410	Cysteine synthase OS=Synechocystis sp. (strain PCC 6803 / Kazusa) GN=c	16,03	2,4	1,6	0,7	1,6	4,2E-01
P72662	Cysteine synthase OS=Synechocystis sp. (strain PCC 6803 / Kazusa) GN=c	24,62	1,1	1,5	1,1	1,3	1,2E-02
P09190	Cytochrome b559 subunit alpha OS=Synechocystis sp. (strain PCC 6803 / K	32,1	0,8		1,0	0,9	5,2E-01
Q57038	Cytochrome b6 OS=Synechocystis sp. (strain PCC 6803 / Kazusa) GN=petB	18,92	0,5	0,6	1,0	0,7	5,2E-02
P26290	Cytochrome b6-f complex iron-sulfur subunit 2 OS=Synechocystis sp. (strain	28,89	0,6	0,8	0,4	0,6	3,3E-04
P27589	Cytochrome b6-f complex subunit 4 OS=Synechocystis sp. (strain PCC 680	17,5	0,5		1,0	0,8	2,7E-01
Q55013	Cytochrome c-550 OS=Synechocystis sp. (strain PCC 6803 / Kazusa) GN=p	46,25	1,1	0,9	1,0	1,0	7,3E-01
P73468	Cytoplasmic membrane protein for maltose uptake OS=Synechocystis sp. (s	4,41		0,8		0,8	2,0E+00
P73821	D-3-phosphoglycerate dehydrogenase OS=Synechocystis sp. (strain PCC 68	20,04	0,8	0,8	1,1	1,0	1,8E-01
P73632	D-alanine--D-alanine ligase OS=Synechocystis sp. (strain PCC 6803 / Kazus	9,32			1,2	1,2	2,0E+00
P74474	D-alanyl-D-alanine carboxypeptidase OS=Synechocystis sp. (strain PCC 680	14,25	1,5	0,8	0,5	0,9	5,1E-01
P73922	D-fructose 1,6-bisphosphatase class 2/sedoheptulose 1,7-bisphosphatase O	43,77	0,7	0,7	0,9	0,8	7,3E-03
P74275	Delta-1-pyrroline-5-carboxylate dehydrogenase OS=Synechocystis sp. (strai	7,37	0,9	1,3	1,7	1,3	1,7E-01
P77969	Delta-aminolevulinic acid dehydratase OS=Synechocystis sp. (strain PCC 68	48,62	1,1	0,6	0,5	0,7	9,2E-02
P73618	Deoxyribose-phosphate aldolase OS=Synechocystis sp. (strain PCC 6803 / I	10,22	4,3	5,7		4,0	4,2E-33
Q55484	Diaminopimelate decarboxylase OS=Synechocystis sp. (strain PCC 6803 / K	22,39	1,1	1,0	0,7	0,9	4,7E-01
P74667	Diaminopimelate epimerase OS=Synechocystis sp. (strain PCC 6803 / Kazu	17,2	0,9	0,9	0,8	0,8	5,3E-05
Q55393	Diflavin flavoprotein A 1 OS=Synechocystis sp. (strain PCC 6803 / Kazusa) (	12,57	0,8	0,9	1,0	0,9	1,5E-01
P74510	Dihydrolipoamide acetyltransferase component (E2) of pyruvate dehydrogen	33,95	0,9	0,7	0,8	0,7	5,4E-04
P72740	Dihydrolipoyl dehydrogenase OS=Synechocystis sp. (strain PCC 6803 / Kazu	64,35	3,6	2,3	1,5	2,3	8,2E-04
P74438	Dihydroorotase OS=Synechocystis sp. (strain PCC 6803 / Kazusa) GN=pyrC	15,5	1,8	1,5		1,9	2,7E-11
P74689	Dihydroxy-acid dehydratase OS=Synechocystis sp. (strain PCC 6803 / Kazu	32,44	2,5	1,9	1,2	1,8	4,6E-03
Q55738	DNA gyrase subunit A OS=Synechocystis sp. (strain PCC 6803 / Kazusa) GN	15	1,2	1,0	1,5	1,2	1,5E-01
P77966	DNA gyrase subunit B OS=Synechocystis sp. (strain PCC 6803 / Kazusa) GN	6,68	0,8	0,9	1,5	0,9	9,9E-01
P73196	DNA ligase OS=Synechocystis sp. (strain PCC 6803 / Kazusa) GN=lig PE=4	26,33	1,3	1,0	0,7	1,0	7,6E-01
P72856	DNA polymerase III subunit beta OS=Synechocystis sp. (strain PCC 6803 / K	8,95		0,9		0,9	2,0E+00
P73810	DNA topoisomerase 1 OS=Synechocystis sp. (strain PCC 6803 / Kazusa) GN	10,36	1,1	0,8	1,3	1,1	8,1E-01
P73418	DNA-binding protein HU OS=Synechocystis sp. (strain PCC 6803 / Kazusa)	60	0,6	0,6	0,4	0,5	1,1E-04
P73297	DNA-directed RNA polymerase subunit alpha OS=Synechocystis sp. (strain I	24,52	0,8	0,8	1,1	0,9	1,8E-01
P77965	DNA-directed RNA polymerase subunit beta OS=Synechocystis sp. (strain P	19,51	0,7	0,9	1,3	1,0	7,1E-01
P73334	DNA-directed RNA polymerase subunit beta' OS=Synechocystis sp. (strain F	10,17	0,8	0,8	1,2	1,0	6,0E-01
P74177	DNA-directed RNA polymerase subunit gamma OS=Synechocystis sp. (strai	14,38	0,7	0,9	1,3	1,0	7,4E-01
P74352	DNA-directed RNA polymerase subunit omega OS=Synechocystis sp. (strair	22,37			0,9	1,0	2,0E+00
P74036	dTDP-glucose 4-6-dehydratase OS=Synechocystis sp. (strain PCC 6803 / K	7,93		0,6	1,0	0,8	2,9E-01
P74751	Elongation factor 4 OS=Synechocystis sp. (strain PCC 6803 / Kazusa) GN=l	4,64	0,6	0,6	1,0	0,8	3,0E-02
P28371	Elongation factor G 1 OS=Synechocystis sp. (strain PCC 6803 / Kazusa) GN	24,32	1,1	0,9	1,1	1,1	4,8E-01
P74228	Elongation factor G 2 OS=Synechocystis sp. (strain PCC 6803 / Kazusa) GN	10,56	1,1	1,1	1,4	1,2	3,0E-02
Q55421	Elongation factor G-like protein OS=Synechocystis sp. (strain PCC 6803 / K	3,29	1,2	1,2	1,5	1,6	1,7E-03
Q55119	Elongation factor P OS=Synechocystis sp. (strain PCC 6803 / Kazusa) GN=e	37,97	0,6	0,4	0,3	0,5	1,6E-05
P74070	Elongation factor Ts OS=Synechocystis sp. (strain PCC 6803 / Kazusa) GN=	59,63	1,1	1,2	1,0	1,2	6,9E-02
P74227	Elongation factor Tu OS=Synechocystis sp. (strain PCC 6803 / Kazusa) GN=	77,44	1,1	1,1	1,3	1,6	7,9E-04
P77972	Enolase OS=Synechocystis sp. (strain PCC 6803 / Kazusa) GN=eno PE=3 S	41,67	1,7	1,3	1,1	1,9	1,6E-02
P73016	Enoyl-[acyl-carrier-protein] reductase [NADH] FabI OS=Synechocystis sp. (s	13,95	1,0	1,0	1,6	1,4	2,5E-01
P73114	Esterase OS=Synechocystis sp. (strain PCC 6803 / Kazusa) GN=slr1916 PE	4,95			0,3	0,4	2,0E+00
P72938	Extracellular nuclease OS=Synechocystis sp. (strain PCC 6803 / Kazusa) GN	6,55	0,2	0,3	0,2	0,2	0,0E+00
P73732	Extracellular solute-binding protein OS=Synechocystis sp. (strain PCC 6803	10,48	1,3	0,9	0,8	1,0	8,5E-01
P73355	Ferredoxin component OS=Synechocystis sp. (strain PCC 6803 / Kazusa) G	31,1	0,9	0,7	0,8	0,8	6,3E-03
P74447	Ferredoxin OS=Synechocystis sp. (strain PCC 6803 / Kazusa) GN=slr0148 F	10,75	1,0	1,1	0,5	0,9	4,6E-01
Q55318	Ferredoxin--NADP reductase OS=Synechocystis sp. (strain PCC 6803 / Kazu	43,34	3,6	4,0	1,3	2,6	8,8E-03
Q55366	Ferredoxin--nitrite reductase OS=Synechocystis sp. (strain PCC 6803 / Kazu	14,14	0,8	1,0	0,7	0,7	4,5E-02
P27320	Ferredoxin-1 OS=Synechocystis sp. (strain PCC 6803 / Kazusa) GN=petF P	17,53	1,7	1,7	1,0	2,0	4,4E-02
P55037	Ferredoxin-dependent glutamate synthase 1 OS=Synechocystis sp. (strain P	7,29	1,1	0,7	1,1	1,0	6,4E-01
P55038	Ferredoxin-dependent glutamate synthase 2 OS=Synechocystis sp. (strain P	19,73	2,4	2,0	1,2	1,8	6,2E-03
Q55389	Ferredoxin-thioredoxin reductase, catalytic chain OS=Synechocystis sp. (stra	19,49		1,0		1,0	2,0E+00
P73050	Ferripyochelin binding protein OS=Synechocystis sp. (strain PCC 6803 / Kaz	13,16	2,4			7,4	2,0E+00
P27319	Flavodoxin OS=Synechocystis sp. (strain PCC 6803 / Kazusa) GN=isiB PE=	11,18	1,8	0,8	0,5	1,0	8,4E-01
P74324	Fructose-1,6-bisphosphatase class 1 OS=Synechocystis sp. (strain PCC 680	5,76			1,0	1,1	2,0E+00
P74309	Fructose-bisphosphate aldolase class 1 OS=Synechocystis sp. (strain PCC 6	7,33	2,0	1,9	1,2	2,6	9,6E-04
Q55664	Fructose-bisphosphate aldolase class 2 OS=Synechocystis sp. (strain PCC 6	38,44	0,8	0,8	0,7	0,7	1,3E-04
P72585	GDP-L-fucose synthase OS=Synechocystis sp. (strain PCC 6803 / Kazusa) (	15,71	1,1	1,1	1,5	1,1	9,1E-02
P72586	GDP-mannose 4,6-dehydratase OS=Synechocystis sp. (strain PCC 6803 / K	29,56	1,0	1,4	1,3	1,3	5,4E-02
P74189	General secretion pathway protein D OS=Synechocystis sp. (strain PCC 680	4,2		0,4		0,4	2,0E+00
Q55155	General secretion pathway protein E OS=Synechocystis sp. (strain PCC 680	4,76			1,7	1,4	2,0E+00
Q55799	General secretion pathway protein E OS=Synechocystis sp. (strain PCC 680	4,43	0,9	0,9	1,0	0,9	1,8E-01
P73704	General secretion pathway protein G OS=Synechocystis sp. (strain PCC 680	25,6	0,4	0,3	0,5	0,4	1,3E-09
Q55087	Geranylgeranyl diphosphate reductase OS=Synechocystis sp. (strain PCC 6	27,03	0,9	0,9	0,6	0,8	3,2E-02
P72683	Geranylgeranyl pyrophosphate synthase OS=Synechocystis sp. (strain PCC	7,62		0,5	0,8	0,6	2,0E-02
P33779	Global nitrogen regulator OS=Synechocystis sp. (strain PCC 6803 / Kazusa)	24,44	0,7	0,8	1,0	0,9	1,4E-01
P52415	Glucose-1-phosphate adenylyltransferase OS=Synechocystis sp. (strain PC(	29,84	0,5	0,7	1,1	0,7	1,6E-01
P73411	Glucose-6-phosphate 1-dehydrogenase OS=Synechocystis sp. (strain PCC (	22	0,9	1,2	1,4	1,2	3,0E-01
P52983	Glucose-6-phosphate isomerase OS=Synechocystis sp. (strain PCC 6803 / K	47,27	3,9	3,9	1,9	3,2	2,2E-06
P73043	Glutamate decarboxylase OS=Synechocystis sp. (strain PCC 6803 / Kazusa	8,35	0,8	0,9	1,0	0,8	1,2E-01
P77970	Glutamate--ammonia ligase OS=Synechocystis sp. (strain PCC 6803 / Kazus	9,94	0,8	0,8	1,0	0,8	9,7E-02
Q55778	Glutamate--tRNA ligase OS=Synechocystis sp. (strain PCC 6803 / Kazusa) (	10,14		1,3	1,7	2,3	1,1E-03
Q55665	Glutamate-1-semialdehyde 2,1-aminomutase OS=Synechocystis sp. (strain I	33,72	1,9	2,0	1,4	1,8	3,1E-08
P77961	Glutamine synthetase OS=Synechocystis sp. (strain PCC 6803 / Kazusa) GN	51,8	2,9	2,5	1,4	2,2	1,9E-04
P72720	Glutamine--fructose-6-phosphate aminotransferase [isomerizing] OS=Synecl	11,89	0,7	1,3	1,3	1,1	6,7E-01
P28463	Glutamyl-tRNA reductase OS=Synechocystis sp. (strain PCC 6803 / Kazusa)	4,92	0,5		1,3	0,9	5,8E-01
Q55139	Glutathione S-transferase OS=Synechocystis sp. (strain PCC 6803 / Kazusa	19,02	0,9	1,2	0,9	1,0	8,4E-01
P73493	Glutathione synthetase OS=Synechocystis sp. (strain PCC 6803 / Kazusa) G	9,69	0,9			0,9	2,0E+00
P49433	Glyceraldehyde-3-phosphate dehydrogenase 1 OS=Synechocystis sp. (strai	19,76	0,5	0,6	0,3	0,5	1,1E-03
P80505	Glyceraldehyde-3-phosphate dehydrogenase 2 OS=Synechocystis sp. (strai	64,99	1,6	2,3	0,8	1,6	2,5E-01
P74416	Glycine dehydrogenase (decarboxylating) OS=Synechocystis sp. (strain PC(	6,51	1,4	1,0	1,2	1,2	1,3E-01
Q55716	Glycine--tRNA ligase alpha subunit OS=Synechocystis sp. (strain PCC 6803	10,37	0,6	0,8	0,8	0,6	3,6E-04
Q55690	Glycine--tRNA ligase beta subunit OS=Synechocystis sp. (strain PCC 6803 /	10,39	0,5	0,6	1,0	0,7	8,0E-02
P72691	Glycogen operon protein GlgX OS=Synechocystis sp. (strain PCC 6803 / Ka	25,34	1,8	1,5	0,9	1,3	2,0E-01
P73608	Glycogen operon protein GlgX OS=Synechocystis sp. (strain PCC 6803 / Ka	14	1,6	1,5	0,3	1,1	8,5E-01
P73511	Glycogen phosphorylase OS=Synechocystis sp. (strain PCC 6803 / Kazusa)	32,51	5,6	4,9	0,9	2,7	8,4E-02
P74521	Glycogen synthase 1 OS=Synechocystis sp. (strain PCC 6803 / Kazusa) GN	8,6	0,5	0,7	1,2	0,8	2,8E-01
P49057	GMP synthase [glutamine-hydrolyzing] OS=Synechocystis sp. (strain PCC 6	18,27	1,0	1,2	1,1	1,3	9,4E-02
Q55759	GTP cyclohydrolase 1 OS=Synechocystis sp. (strain PCC 6803 / Kazusa) GN	16,24	0,9	0,7	0,9	0,7	4,3E-03
P72749	GTP-binding protein TypA/BipA homolog OS=Synechocystis sp. (strain PCC	11,89	0,9	0,8	1,0	0,8	8,6E-03
P74702	Heat shock protein OS=Synechocystis sp. (strain PCC 6803 / Kazusa) GN=h	6,84		6,0	1,6	5,2	7,9E-02
P72849	Heme oxygenase 1 OS=Synechocystis sp. (strain PCC 6803 / Kazusa) GN=	8,75		0,2		0,3	2,0E+00
P73817	Hemolysin OS=Synechocystis sp. (strain PCC 6803 / Kazusa) GN=sll1951 P	5,11	0,3	0,1	0,6	0,5	7,8E-03
Q55614	High-affinity branched-chain amino acid transport ATP-binding protein OS=S	6,43	1,2			1,1	2,0E+00
Q55190	High-affinity Na(+)/H(+) antiporter NhaS3 OS=Synechocystis sp. (strain PCC	6,94	1,0		1,5	1,2	3,6E-01
P73058	Histidinol dehydrogenase 1 OS=Synechocystis sp. (strain PCC 6803 / Kazus	9,68	0,9	1,2	1,2	1,1	3,4E-01
P73807	Histidinol-phosphate aminotransferase OS=Synechocystis sp. (strain PCC 6	27,79	5,4	4,5	1,2	4,2	2,1E-02
P74176	HlyB family OS=Synechocystis sp. (strain PCC 6803 / Kazusa) GN=hlyB PE	3,66			0,7	0,7	2,0E+00
P74175	HlyD family of secretion proteins OS=Synechocystis sp. (strain PCC 6803 / K	6,86	0,0	0,3	0,4	0,3	1,5E-02
P52986	Homoserine dehydrogenase OS=Synechocystis sp. (strain PCC 6803 / Kazu	5,08	1,0	0,7	0,9	0,8	5,5E-02
P74729	HrEpiB OS=Synechocystis sp. (strain PCC 6803 / Kazusa) GN=sll0576 PE=4	52,24	0,9	1,1	1,2	1,2	5,8E-01
P55175	Hydrolase sll0601 OS=Synechocystis sp. (strain PCC 6803 / Kazusa) GN=sl	8,82			1,1	1,0	2,0E+00

P74250	Hydroperoxy fatty acid reductase gpx1 OS=Synechocystis sp. (strain PCC 68	16,57	0,6	0,5	0,7	0,6	3,1E-07
P73824	Hydroperoxy fatty acid reductase gpx2 OS=Synechocystis sp. (strain PCC 68	31,82	1,4	1,2	0,8	1,2	4,9E-01
Q55552	IMP dehydrogenase OS=Synechocystis sp. (strain PCC 6803 / Kazusa) GN=	20,65	0,4		0,4	0,4	0,0E+00
P73853	IMP dehydrogenase subunit OS=Synechocystis sp. (strain PCC 6803 / Kazu	40,31	3,4	2,9	1,5	2,4	7,2E-04
Q55508	Indole-3-glycerol phosphate synthase OS=Synechocystis sp. (strain PCC 68	9,49	1,4		1,2	1,7	4,7E-05
P80507	Inorganic pyrophosphatase OS=Synechocystis sp. (strain PCC 6803 / Kazus	55,03	1,6	1,5	1,1	1,4	5,3E-03
Q55274	Iron stress-induced chlorophyll-binding protein OS=Synechocystis sp. (strain	2,34			0,4	0,4	2,0E+00
P72827	Iron uptake protein A1 OS=Synechocystis sp. (strain PCC 6803 / Kazusa) GI	39,44	1,9	1,2	0,9	1,4	3,1E-01
Q55835	Iron uptake protein A2 OS=Synechocystis sp. (strain PCC 6803 / Kazusa) GI	31,5	1,2	1,4	0,7	1,1	7,9E-01
P80046	Isocitrate dehydrogenase [NADP] OS=Synechocystis sp. (strain PCC 6803 /	27,58	2,8	1,6	1,2	1,8	2,2E-02
P73679	Isopenicillin N epimerase OS=Synechocystis sp. (strain PCC 6803 / Kazusa)	34,91	1,9	1,9	0,9	1,6	1,0E-01
P74287	Isopentenyl-diphosphate delta-isomerase OS=Synechocystis sp. (strain PCC	4,58			1,1	0,9	2,0E+00
P29107	Ketol-acid reductoisomerase OS=Synechocystis sp. (strain PCC 6803 / Kazu	34,74	0,5	0,6	0,9	0,7	4,3E-03
P73553	Large-conductance mechanosensitive channel OS=Synechocystis sp. (strair	14,48	1,2	1,8		1,3	3,9E-02
P73274	Leucine--tRNA ligase OS=Synechocystis sp. (strain PCC 6803 / Kazusa) GN	4,03	0,6	0,8	1,6	1,0	7,0E-01
Q59987	Light-dependent protochlorophyllide reductase OS=Synechocystis sp. (strain	30,12	0,7	0,8	1,1	0,9	2,5E-01
P74518	Light-repressed protein A homolog OS=Synechocystis sp. (strain PCC 6803	55,5	1,3	1,2	1,4	1,4	2,0E-07
Q57310	Lipid-A-disaccharide synthase OS=Synechocystis sp. (strain PCC 6803 / Ka:	8,63	0,6			0,7	2,0E+00
Q55828	LL-diaminopimelate aminotransferase OS=Synechocystis sp. (strain PCC 68	24,03	0,7	0,7	0,9	0,8	1,1E-02
Q55687	Long-chain acyl-[acyl-carrier-protein] reductase OS=Synechocystis sp. (strai	7,06		1,1		1,0	2,0E+00
P73004	Long-chain-fatty-acid CoA ligase OS=Synechocystis sp. (strain PCC 6803 / K	5,89		1,0	1,3	1,1	4,9E-01
P73443	Lysine--tRNA ligase OS=Synechocystis sp. (strain PCC 6803 / Kazusa) GN=	4,9	0,9			1,0	2,0E+00
P72772	Magnesium-chelatase subunit ChlD OS=Synechocystis sp. (strain PCC 6803	3,25	0,8			0,8	2,0E+00
P51634	Magnesium-chelatase subunit ChlI OS=Synechocystis sp. (strain PCC 6803	15,41	1,1	1,3	0,9	1,0	6,0E-01
P72584	Magnesium-protoporphyrin IX monomethyl ester [oxidative] cyclase 1 OS=Sy	18,99	0,4	0,4	0,7	0,5	2,5E-05
Q55383	Malate dehydrogenase OS=Synechocystis sp. (strain PCC 6803 / Kazusa) G	5,56	0,7	1,0	1,7	1,1	7,2E-01
P73377	Mannose-6-phosphate isomerase OS=Synechocystis sp. (strain PCC 6803 /	18,6	0,8			0,9	2,0E+00
P74155	Membrane protein insertase YidC OS=Synechocystis sp. (strain PCC 6803 /	5,21	0,6		0,9	0,8	1,1E-01
P74186	Membrane protein OS=Synechocystis sp. (strain PCC 6803 / Kazusa) GN=p	19,07	1,0	1,1	1,1	1,1	7,6E-02
P73954	Membrane-associated protein slr1513 OS=Synechocystis sp. (strain PCC 68	48,18	6,9	7,6	4,8	5,4	5,4E-39
Q55729	Methionine--tRNA ligase OS=Synechocystis sp. (strain PCC 6803 / Kazusa)	3,2		0,6		0,5	2,0E+00
Q55444	Methyl-accepting chemotaxis protein II OS=Synechocystis sp. (strain PCC 68	11,04	1,1		0,7	0,9	5,6E-01
P73008	Methyl-accepting chemotaxis protein OS=Synechocystis sp. (strain PCC 680	8,52	0,7	0,8	1,0	0,7	2,7E-02
P72802	Mitochondrial outer membrane 72K protein OS=Synechocystis sp. (strain PC	10,15	0,9		0,6	0,8	6,4E-02
P74344	Molybdopterin biosynthesis MoeB protein OS=Synechocystis sp. (strain PCC	28,32	1,1	0,9	1,2	1,1	5,5E-01
P74395	N utilization substance protein B homolog OS=Synechocystis sp. (strain PCC	24,89	0,7	0,8	0,8	0,8	0,0E+00
P54899	N-acetyl-gamma-glutamyl-phosphate reductase OS=Synechocystis sp. (strai	14,81	0,5	0,7	1,2	0,8	2,4E-01
P73736	N-acetylmuramoyl-L-alanine amidase OS=Synechocystis sp. (strain PCC 68(	5,86	0,6	0,6	0,4	0,6	1,1E-06
Q55498	N5-carboxyaminoimidazole ribonucleotide mutase OS=Synechocystis sp. (st	17,61	0,7			0,6	2,0E+00
P26522	NAD(P)H-quinone oxidoreductase subunit 1 OS=Synechocystis sp. (strain P	10,75	1,3	1,5	1,6	1,7	3,4E-10
P27724	NAD(P)H-quinone oxidoreductase subunit H OS=Synechocystis sp. (strain P	23,1	1,2	1,5	1,7	1,5	6,8E-05
P26525	NAD(P)H-quinone oxidoreductase subunit I OS=Synechocystis sp. (strain P(	50,78	1,4	1,2	1,2	1,6	4,5E-06
P19125	NAD(P)H-quinone oxidoreductase subunit J OS=Synechocystis sp. (strain P	23,46	1,8	1,7	1,3	1,7	3,8E-05
P19050	NAD(P)H-quinone oxidoreductase subunit K 1 OS=Synechocystis sp. (strain	23,79	1,5	1,5	1,3	2,2	6,3E-09
P74338	NAD(P)H-quinone oxidoreductase subunit M OS=Synechocystis sp. (strain F	23,97	1,1	1,2	1,0	1,8	1,0E-02
P74771	NAD(P)H-quinone oxidoreductase subunit O OS=Synechocystis sp. (strain F	40,28	1,2	1,3	0,9	1,1	3,2E-01
P73735	NADH dehydrogenase OS=Synechocystis sp. (strain PCC 6803 / Kazusa) G	4,95			1,9	2,4	2,0E+00
P72762	NADH-glutamate synthase small subunit OS=Synechocystis sp. (strain PCC	8,3		1,0	0,9	1,0	4,4E-01
P54386	NADP-specific glutamate dehydrogenase OS=Synechocystis sp. (strain PCC	11,21	1,4	1,8		1,6	3,9E-05
P72781	NarL subfamily OS=Synechocystis sp. (strain PCC 6803 / Kazusa) GN=slr17	13,48		0,9	0,7	0,9	1,0E-01
P73104	NarL subfamily OS=Synechocystis sp. (strain PCC 6803 / Kazusa) GN=slr19	17,84	1,0		0,9	0,9	2,5E-01
P74390	Negative aliphatic amidase regulator OS=Synechocystis sp. (strain PCC 680	41,48	1,5	1,6	1,3	1,9	2,8E-09
P73757	Neopullulanase OS=Synechocystis sp. (strain PCC 6803 / Kazusa) GN=nplT	11,42	2,0	1,2	1,6	1,6	2,3E-03
Q55602	NifS protein OS=Synechocystis sp. (strain PCC 6803 / Kazusa) GN=nifS PE:	26,68	2,7	4,2	1,4	2,7	2,9E-03
P73448	Nitrate reductase OS=Synechocystis sp. (strain PCC 6803 / Kazusa) GN=na	4,76	1,7	1,5		1,4	4,8E-20
P73450	Nitrate transport ATP-binding protein NrtC OS=Synechocystis sp. (strain PC(	2,69		0,5		0,7	2,0E+00
P73452	Nitrate transport protein NrtA OS=Synechocystis sp. (strain PCC 6803 / Kazu	38,12	1,1	0,8	0,7	0,9	2,1E-01
P73449	Nitrate transport protein NrtD OS=Synechocystis sp. (strain PCC 6803 / Kazu	11,45		0,3	0,7	0,6	4,1E-02
Q55247	Nitrogen regulatory protein P-II OS=Synechocystis sp. (strain PCC 6803 / Ka	70,54	0,7	0,6	0,6	0,5	8,6E-08
Q6ZEQ0	Non-heme chloroperoxidase OS=Synechocystis sp. (strain PCC 6803 / Kazu	31,02	4,0	3,8	2,0	2,4	2,2E-07
P73057	Nucleoid-associated protein slr1847 OS=Synechocystis sp. (strain PCC 6803	23,68	1,4	1,3	0,8	1,2	4,8E-01
P74494	Nucleoside diphosphate kinase OS=Synechocystis sp. (strain PCC 6803 / Ka	34,23	1,6	2,2	1,1	1,6	3,6E-02
P74571	Oligopeptidase A OS=Synechocystis sp. (strain PCC 6803 / Kazusa) GN=prl	6,45	0,7	0,5	1,3	0,7	3,4E-01
P74314	OmpR subfamily OS=Synechocystis sp. (strain PCC 6803 / Kazusa) GN=slr(	18,8			0,9	1,0	2,0E+00
P74102	Orange carotenoid-binding protein OS=Synechocystis sp. (strain PCC 6803	51,74	0,7	1,1	1,3	1,0	9,5E-01
Q55497	Ornithine carbamoyltransferase OS=Synechocystis sp. (strain PCC 6803 / Ka	8,77	0,9	1,0	1,5	1,1	5,7E-01
P72848	Oxygen-dependent coproporphyrinogen-III oxidase OS=Synechocystis sp. (s	40,88	1,2	0,9	0,6	0,9	4,7E-01
P74707	Peptide chain release factor 1 OS=Synechocystis sp. (strain PCC 6803 / Kaz	8,22	0,6	0,6	0,6	0,6	0,0E+00
P72622	Peptide methionine sulfoxide reductase MsrA 1 OS=Synechocystis sp. (strai	9,46	1,9		1,2	2,9	7,8E-02
P73037	Peptidyl-prolyl cis-trans isomerase OS=Synechocystis sp. (strain PCC 6803	17,91	1,1	0,9	0,8	0,9	3,5E-01
P73789	Peptidyl-prolyl cis-trans isomerase slr1251 OS=Synechocystis sp. (strain PC	8,77			0,9	0,8	2,0E+00
Q55387	Periplasmic binding protein of ABC transporter for natural amino acids OS=S	19,38	1,0	1,0	0,8	0,9	5,8E-01
Q55187	Phenylalanine--tRNA ligase alpha subunit OS=Synechocystis sp. (strain PC(	13,29	0,8	0,7	1,2	0,9	4,0E-01
P74296	Phenylalanine--tRNA ligase beta subunit OS=Synechocystis sp. (strain PCC	8,4	0,7	0,9	1,1	0,9	2,6E-01
Q55196	Phosphate import ATP-binding protein PstB 1 OS=Synechocystis sp. (strain I	25,65	0,1	0,1	0,6	0,4	5,5E-03
Q55198	Phosphate transport system permease protein PstC OS=Synechocystis sp. (	5,05		0,4	0,3	0,4	7,3E-15
P73785	Phosphate-binding protein OS=Synechocystis sp. (strain PCC 6803 / Kazusa	54,35	0,3	0,1	0,1	0,2	5,4E-09
Q55199	Phosphate-binding protein OS=Synechocystis sp. (strain PCC 6803 / Kazusa	31,33	0,7	0,5	0,8	0,7	9,6E-04
P74299	Phosphoenolpyruvate carboxylase OS=Synechocystis sp. (strain PCC 6803	9,96	0,9	1,2	1,1	1,1	5,4E-01
Q55905	Phosphoenolpyruvate synthase OS=Synechocystis sp. (strain PCC 6803 / K	3,42	0,6			0,8	2,0E+00
P74643	Phosphoglucomutase OS=Synechocystis sp. (strain PCC 6803 / Kazusa) GN	9,52	0,8	0,9	0,7	0,8	6,3E-04
P74421	Phosphoglycerate kinase OS=Synechocystis sp. (strain PCC 6803 / Kazusa)	40,65	0,6	0,6	0,7	0,8	1,4E-08
Q55894	Phosphomethylpyrimidine synthase OS=Synechocystis sp. (strain PCC 6803	12,85	1,2	0,8	0,8	0,9	5,2E-01
P74232	Phosphoribosylamine--glycine ligase OS=Synechocystis sp. (strain PCC 680	4,3			0,8	0,9	2,0E+00
P73471	Phosphoribosylaminoimidazole-succinocarboxamide synthase OS=Synecho	9,47	1,4	1,5	1,6	1,8	2,7E-20
Q55843	Phosphoribosylformylglycinamidine synthase 1 OS=Synechocystis sp. (strain	15,18	0,7			0,7	2,0E+00
P72644	Phosphoribosylformylglycinamidine synthase 2 OS=Synechocystis sp. (strair	4,3			1,0	1,0	2,0E+00
P37101	Phosphoribulokinase OS=Synechocystis sp. (strain PCC 6803 / Kazusa) GN	43,98	0,9	0,9	1,2	1,0	9,2E-01
P73546	Phosphorylase OS=Synechocystis sp. (strain PCC 6803 / Kazusa) GN=glgP	12,16	0,3	0,5	0,9	0,7	3,2E-02
P74063	Photosystem I assembly protein Ycf3 OS=Synechocystis sp. (strain PCC 68(	14,45			0,3	0,4	2,0E+00
P72705	Photosystem I assembly protein Ycf4 OS=Synechocystis sp. (strain PCC 68(	10,64			1,1	1,2	2,0E+00
P72966	Photosystem I biogenesis protein BtpA OS=Synechocystis sp. (strain PCC 6	16,38			0,9	0,8	2,0E+00
P32422	Photosystem I iron-sulfur center OS=Synechocystis sp. (strain PCC 6803 / K	65,43	1,1	1,0	1,4	1,2	8,9E-02
P29254	Photosystem I P700 chlorophyll a apoprotein A1 OS=Synechocystis sp. (stra	13,72	0,7	1,0	1,9	1,1	7,9E-01
P29255	Photosystem I P700 chlorophyll a apoprotein A2 OS=Synechocystis sp. (stra	17,65	0,7	1,0	2,3	1,3	7,0E-01
P19569	Photosystem I reaction center subunit II OS=Synechocystis sp. (strain PCC (	52,48	0,8	0,9	1,1	0,9	3,6E-01
P29256	Photosystem I reaction center subunit III OS=Synechocystis sp. (strain PCC	56,36	1,0	0,9	1,1	1,0	8,7E-01
P12975	Photosystem I reaction center subunit IV OS=Synechocystis sp. (strain PCC	41,89	0,2	0,1	0,5	0,3	5,9E-04
P37277	Photosystem I reaction center subunit XI OS=Synechocystis sp. (strain PCC	26,11	0,9	0,9	1,3	1,1	8,0E-01
Q55332	Photosystem II 12 kDa extrinsic protein OS=Synechocystis sp. (strain PCC 6	25,19	0,6			0,7	2,0E+00
P09193	Photosystem II 44 kDa reaction center protein OS=Synechocystis sp. (strain	17,17	1,0	1,1	1,8	1,2	3,0E-01
P05429	Photosystem II CP47 chlorophyll apoprotein OS=Synechocystis sp. (strain P	22,09	1,1	1,6	2,2	1,5	1,4E-02
P09192	Photosystem II D2 protein OS=Synechocystis sp. (strain PCC 6803 / Kazusa	10,8	1,0	1,2	2,0	1,2	1,7E-01
P74367	Photosystem II lipoprotein Psb27 OS=Synechocystis sp. (strain PCC 6803 / I	35,82	0,8	0,7	0,6	0,7	2,7E-04
P10549	Photosystem II manganese-stabilizing polypeptide OS=Synechocystis sp. (st	27,01	0,3	0,2	0,2	0,3	7,5E-11
Q55354	Photosystem II reaction center protein L OS=Synechocystis sp. (strain PCC (	33,33			1,0	1,0	2,0E+00
Q55356	Photosystem II reaction center Psb28 protein OS=Synechocystis sp. (strain I	16,07			0,6	0,5	2,0E+00
P16033	Photosystem Q(B) protein 2 OS=Synechocystis sp. (strain PCC 6803 / Kazus	16,67	1,1	1,1	1,9	1,4	1,1E-01
Q55544	Phycobiliprotein ApcE OS=Synechocystis sp. (strain PCC 6803 / Kazusa) GN	48,21	0,5	0,7	1,4	0,9	3,8E-01
P73203	Phycobilisome 32.1 kDa linker polypeptide, phycocyanin-associated, rod 1 O	66,32	0,8	1,1	1,3	1,0	8,3E-01
P73204	Phycobilisome 32.1 kDa linker polypeptide, phycocyanin-associated, rod 2 O	58,97	0,8	1,1	1,5	1,2	6,0E-01
Q01950	Phycobilisome 7.8 kDa linker polypeptide, allophycocyanin-associated, core	50,75	0,4	0,5	1,0	0,6	5,9E-02
P73202	Phycobilisome 8.9 kDa linker polypeptide, phycocyanin-associated, rod OS=	81,93	0,2	0,2	0,2	0,2	0,0E+00
P73093	Phycobilisome rod-core linker polypeptide CpcG OS=Synechocystis sp. (stra	59,57	0,5	0,6	1,0	0,7	6,9E-02
P74625	Phycobilisome rod-core linker polypeptide CpcG OS=Synechocystis sp. (stra	24,1	2,6	1,2	1,5	1,6	2,5E-02
Q55891	Phycocyanobilin:ferredoxin oxidoreductase OS=Synechocystis sp. (strain PC	8,06			0,9	0,9	2,0E+00
Q55168	Phytochrome-like protein cph1 OS=Synechocystis sp. (strain PCC 6803 / Ka	4,81	0,4	0,4	0,9	0,6	1,0E-02
P73173	PilJ protein OS=Synechocystis sp. (strain PCC 6803 / Kazusa) GN=pilJ PE=	9,55	1,3	0,7	1,0	0,9	7,8E-01
P21697	Plastocyanin OS=Synechocystis sp. (strain PCC 6803 / Kazusa) GN=petE P	44,44	1,5	1,6	0,9	1,3	1,6E-01
P73390	Poly(3-hydroxyalkanoate) synthase OS=Synechocystis sp. (strain PCC 6803	7,14	0,5	0,5	1,1	0,7	1,2E-01
Q55898	Polyphosphate kinase OS=Synechocystis sp. (strain PCC 6803 / Kazusa) GI	9,07		1,1	0,8	0,9	6,3E-01
P72659	Polyribonucleotide nucleotidyltransferase OS=Synechocystis sp. (strain PCC	21,73	0,8	0,9	1,3	1,0	9,9E-01
P73660	Porphobilinogen deaminase OS=Synechocystis sp. (strain PCC 6803 / Kazu	13,44	1,1	1,0	0,8	1,0	7,8E-01
P73866	Potassium-transporting ATPase A chain OS=Synechocystis sp. (strain PCC 6	4,84	0,6	0,4	0,6	0,6	2,9E-08
P73867	Potassium-transporting ATPase B chain OS=Synechocystis sp. (strain PCC (	15,36	0,5	0,3	0,7	0,6	2,4E-03
P73547	Precorrin decarboxylase OS=Synechocystis sp. (strain PCC 6803 / Kazusa)	11,01	0,8			0,8	2,0E+00
P73849	Probable 2-phosphosulfolactate phosphatase OS=Synechocystis sp. (strain I	9,54	1,5			1,5	2,0E+00
Q55385	Probable 30S ribosomal protein PSRP-3 OS=Synechocystis sp. (strain PCC	65,18	0,7	0,9	0,9	0,7	2,1E-02
P72940	Probable adenylyl-sulfate kinase OS=Synechocystis sp. (strain PCC 6803 / K	31,64	0,5	0,6	0,8	0,6	1,4E-03
P73270	Probable agmatinase 2 OS=Synechocystis sp. (strain PCC 6803 / Kazusa) G	17,18	4,8	5,0	2,2	4,6	1,0E-06
P74467	Probable ATP-dependent Clp protease proteolytic subunit 3 OS=Synechocys	39,11	0,6	0,7	1,0	0,8	5,2E-02
Q55791	Probable ATP-dependent transporter slr0075 OS=Synechocystis sp. (strain F	12,11			0,9	0,8	2,0E+00
P54691	Probable branched-chain-amino-acid aminotransferase OS=Synechocystis s	64,92	2,6	2,5	1,5	2,9	1,2E-05
P73971	Probable cytosol aminopeptidase OS=Synechocystis sp. (strain PCC 6803 /	9,96	2,5	3,6		2,2	9,2E-09
P54902	Probable gamma-glutamyl phosphate reductase OS=Synechocystis sp. (stra	6,7	1,0			1,1	2,0E+00
P72623	Probable glycogen synthase 2 OS=Synechocystis sp. (strain PCC 6803 / Ka:	17,72	1,0	1,0	1,5	1,2	3,6E-01
P72704	Probable peptidyl-prolyl cis-trans isomerase sll0227 OS=Synechocystis sp. (	26,42	1,7	1,5	1,0	1,4	4,4E-02
P74690	Probable phosphoketolase OS=Synechocystis sp. (strain PCC 6803 / Kazus	18,14	2,1	2,7	1,5	2,5	6,3E-05
P73123	Probable RuBisCO transcriptional regulator OS=Synechocystis sp. (strain PC	6,96			0,9	0,9	2,0E+00
P73157	Probable signal peptidase I-2 OS=Synechocystis sp. (strain PCC 6803 / Kaz	12,39			0,8	0,8	2,0E+00
Q55585	Probable succinate-semialdehyde dehydrogenase [NADP(+)] OS=Synechoc	17,62	1,0	1,3	0,9	1,1	5,9E-01
P73942	Proline--tRNA ligase OS=Synechocystis sp. (strain PCC 6803 / Kazusa) GN	10,12	0,6	1,1	1,3	1,0	9,5E-01
P74305	Protease OS=Synechocystis sp. (strain PCC 6803 / Kazusa) GN=pqqE PE=:	4,96	1,5		0,8	1,1	8,3E-01
Q55233	Protein DrgA OS=Synechocystis sp. (strain PCC 6803 / Kazusa) GN=drgA P	39,05	2,0	1,5	1,0	1,5	6,9E-02
Q59978	Protein GrpE OS=Synechocystis sp. (strain PCC 6803 / Kazusa) GN=grpE F	30,92	0,7	0,7	0,3	0,7	2,4E-02
P37979	Protein IcfG OS=Synechocystis sp. (strain PCC 6803 / Kazusa) GN=icfG PE	2,84			0,7	0,6	2,0E+00
P73176	Protein PatA OS=Synechocystis sp. (strain PCC 6803 / Kazusa) GN=sll1291	2,99		0,3		0,4	2,0E+00
P74737	Protein RecA OS=Synechocystis sp. (strain PCC 6803 / Kazusa) GN=recA P	7,91	1,5	1,7	1,5	2,1	1,6E-18
Q55200	Protein SphX OS=Synechocystis sp. (strain PCC 6803 / Kazusa) GN=sphX I	17,26	1,2	0,5	0,4	0,7	1,4E-01

Q55709	Protein translocase subunit SecA OS=Synechocystis sp. (strain PCC 6803 /	11,16	1,1	0,7	1,0	0,9	5,6E-01
P38382	Protein translocase subunit SecE OS=Synechocystis sp. (strain PCC 6803 /	23,46			0,9	1,3	2,0E+00
P77964	Protein translocase subunit SecY OS=Synechocystis sp. (strain PCC 6803 /	6,11	0,5		1,4	0,9	7,0E-01
P74466	Putative ATP-dependent Clp protease proteolytic subunit-like OS=Synechocy	20	0,8	0,8	0,7	0,8	0,0E+00
P72941	Putative biopolymer transport protein ExbB-like 2 OS=Synechocystis sp. (stra	10,57	0,6	0,6	0,9	0,7	4,1E-03
P73163	Putative carboxymethylenebutenolidase OS=Synechocystis sp. (strain PCC	34,55	1,4	1,5	1,5	1,5	0,0E+00
P74220	Putative carboxypeptidase slr1534 OS=Synechocystis sp. (strain PCC 6803	13,62		4,2	3,9	2,8	0,0E+00
P74373	Putative diflavin flavoprotein A 3 OS=Synechocystis sp. (strain PCC 6803 / K	5,19			1,0	1,1	2,0E+00
Q55445	Putative methyl-accepting chemotaxis protein sll0041 OS=Synechocystis sp.	6,2	0,9	0,9	1,8	1,2	5,9E-01
P73720	Putative OxPP cycle protein OpcA OS=Synechocystis sp. (strain PCC 6803 /	6,95	1,8	1,2	1,4	1,7	4,0E-03
P73728	Putative peroxiredoxin sll1621 OS=Synechocystis sp. (strain PCC 6803 / Ka	60,32	1,7	1,3	1,0	1,4	5,6E-02
P73790	Putative pterin-4-alpha-carbinolamine dehydratase OS=Synechocystis sp. (s	34,38	0,5			0,4	2,0E+00
P73623	Putative quercetin 2,3-dioxygenase sll1773 OS=Synechocystis sp. (strain PC	11,21	1,9			2,1	2,0E+00
P74060	Putative sulfur carrier protein slr0821 OS=Synechocystis sp. (strain PCC 680	20,18	1,0		1,1	1,6	4,8E-27
Q55118	Putative thylakoid lumen peptidyl-prolyl cis-trans isomerase sll0408 OS=Syn	19	1,1	1,0	0,7	0,9	4,9E-01
P72776	Pyridoxine 5'-phosphate synthase OS=Synechocystis sp. (strain PCC 6803 /	49,17	1,6	1,6	1,0	1,4	3,5E-02
P74211	Pyridoxine/pyridoxamine 5'-phosphate oxidase OS=Synechocystis sp. (strain	18,22	0,9	1,2		1,1	8,1E-01
P74572	Pyrroline-5-carboxylate reductase OS=Synechocystis sp. (strain PCC 6803 /	8,61			1,2	1,3	2,0E+00
P73405	Pyruvate dehydrogenase E1 beta subunit OS=Synechocystis sp. (strain PCC	17,9	0,7	0,9	1,2	0,9	5,5E-01
P74490	Pyruvate dehydrogenase E1 component, alpha subunit OS=Synechocystis s	19,88	1,1	1,2	1,3	1,3	8,3E-04
Q55863	Pyruvate kinase 1 OS=Synechocystis sp. (strain PCC 6803 / Kazusa) GN=py	5,59		0,8		0,8	2,0E+00
P73534	Pyruvate kinase 2 OS=Synechocystis sp. (strain PCC 6803 / Kazusa) GN=py	13,03		1,2	1,0	1,2	1,7E-01
P73348	Rehydrin OS=Synechocystis sp. (strain PCC 6803 / Kazusa) GN=slr1198 PE	48,82	1,1	0,9	0,7	0,9	2,6E-01
P74124	Renin-binding protein OS=Synechocystis sp. (strain PCC 6803 / Kazusa) GN	5,37	1,3			1,3	2,0E+00
Q55641	Ribonuclease D OS=Synechocystis sp. (strain PCC 6803 / Kazusa) GN=rnd	13,36	2,8		1,5	2,1	2,7E-02
P72656	Ribonuclease E/G-like protein OS=Synechocystis sp. (strain PCC 6803 / Kaz	11,28	1,1	1,0	1,2	1,1	5,3E-02
P54123	Ribonuclease J OS=Synechocystis sp. (strain PCC 6803 / Kazusa) GN=slr0	13,91	0,7	0,9	1,5	1,1	9,8E-01
Q55766	Ribose-5-phosphate isomerase A OS=Synechocystis sp. (strain PCC 6803 /	16,6	0,5	0,9	0,6	0,7	2,0E-02
Q55848	Ribose-phosphate pyrophosphokinase OS=Synechocystis sp. (strain PCC 6	11,71	0,7	0,7	0,8	0,6	0,0E+00
P73886	Ribosome-binding ATPase YchF OS=Synechocystis sp. (strain PCC 6803 /	19,83	1,0	0,9	1,1	1,0	7,5E-01
Q55625	Ribosome-binding factor A OS=Synechocystis sp. (strain PCC 6803 / Kazusa	30,08	1,2		1,5	1,8	1,3E-02
P74456	Ribosome-recycling factor OS=Synechocystis sp. (strain PCC 6803 / Kazusa	36,26	1,1	1,2	0,4	0,9	5,2E-01
P54205	Ribulose bisphosphate carboxylase large chain OS=Synechocystis sp. (strai	49,15	1,0	1,1	1,7	1,4	2,3E-01
P54206	Ribulose bisphosphate carboxylase small chain OS=Synechocystis sp. (strai	61,95	1,0	0,9	1,0	0,9	3,6E-01
P74061	Ribulose-phosphate 3-epimerase OS=Synechocystis sp. (strain PCC 6803 /	43,04	1,5	1,4	1,0	1,3	7,2E-02
P74565	RNA polymerase sigma factor SigA OS=Synechocystis sp. (strain PCC 6803	8	0,8	0,9	0,7	0,8	1,1E-02
P73557	RNA-binding protein OS=Synechocystis sp. (strain PCC 6803 / Kazusa) GN=	14,46	0,8	0,7	0,6	0,8	1,1E-03
P73862	Rubisco operon transcriptional regulator OS=Synechocystis sp. (strain PCC	14,24	0,7	0,8	1,4	1,0	7,7E-01
P73068	Rubredoxin OS=Synechocystis sp. (strain PCC 6803 / Kazusa) GN=rub PE=	20,87	0,7			0,8	2,0E+00
P72871	S-adenosylmethionine synthase OS=Synechocystis sp. (strain PCC 6803 / K	38,5	1,4	1,2	0,9	1,1	3,3E-01
P0DJF8	S-methyl-5'-thioadenosine phosphorylase OS=Synechocystis sp. (strain PCC	30,98	2,6	2,1	1,5	2,2	6,9E-06
P73010	Sec-independent protein translocase protein TatA OS=Synechocystis sp. (str	17,46		0,4		0,4	2,0E+00
Q55788	Sensory transduction histidine kinase OS=Synechocystis sp. (strain PCC 68	21,59	1,0	0,9	0,8	0,9	3,1E-02
Q55177	Sensory transduction histidine kinase OS=Synechocystis sp. (strain PCC 68	3,85	0,5			0,5	2,0E+00
Q55900	Septum site-determining protein MinD OS=Synechocystis sp. (strain PCC 68	25,94	0,6	1,1	1,0	0,9	4,6E-01
P77962	Serine hydroxymethyltransferase OS=Synechocystis sp. (strain PCC 6803 /	19,67	0,7	0,5	0,6	0,7	3,1E-11
P73201	Serine--tRNA ligase OS=Synechocystis sp. (strain PCC 6803 / Kazusa) GN=	7,91	1,0		1,3	1,1	4,4E-01
P74591	Shikimate dehydrogenase OS=Synechocystis sp. (strain PCC 6803 / Kazusa	16,21	2,7	2,1		1,6	3,2E-10
P73991	Short-chain alcohol dehydrogenase family OS=Synechocystis sp. (strain PC	6,02			0,6	0,8	2,0E+00
P74214	Signal recognition particle protein OS=Synechocystis sp. (strain PCC 6803 /	4,56			2,3	2,1	2,0E+00
P73930	Signal recognition particle receptor FtsY OS=Synechocystis sp. (strain PCC	3,37			1,3	1,1	2,0E+00
Q55499	Single-stranded DNA-binding protein 1 OS=Synechocystis sp. (strain PCC 6	69,42	1,9	2,2	1,5	1,6	6,1E-09
Q55658	Sll0023 protein OS=Synechocystis sp. (strain PCC 6803 / Kazusa) GN=sll00	5,37	1,1		1,7	1,3	1,9E-01
Q55162	Sll0051 protein OS=Synechocystis sp. (strain PCC 6803 / Kazusa) GN=sll00	11,32		2,8	1,2	2,0	1,4E-01
Q55140	Sll0066 protein OS=Synechocystis sp. (strain PCC 6803 / Kazusa) GN=sll00	2,54		1,1		1,0	2,0E+00
Q55138	Sll0068 protein OS=Synechocystis sp. (strain PCC 6803 / Kazusa) GN=sll00	1,53		1,7		2,1	2,0E+00
Q55134	Sll0071 protein OS=Synechocystis sp. (strain PCC 6803 / Kazusa) GN=sll00	6,31	0,4			0,4	2,0E+00
P74453	Sll0148 protein OS=Synechocystis sp. (strain PCC 6803 / Kazusa) GN=sll01	3,67	0,8	0,8	1,2	0,9	5,1E-01
Q55564	Sll0162 protein OS=Synechocystis sp. (strain PCC 6803 / Kazusa) GN=sll01	10,31	0,6			0,6	2,0E+00
Q55558	Sll0172 protein OS=Synechocystis sp. (strain PCC 6803 / Kazusa) GN=sll01	21,05	0,7	0,5		0,6	1,4E-04
Q55776	Sll0180 protein OS=Synechocystis sp. (strain PCC 6803 / Kazusa) GN=sll01	11,98	1,3	1,2	1,3	3,9	5,4E-14
Q55770	Sll0185 protein OS=Synechocystis sp. (strain PCC 6803 / Kazusa) GN=sll01	9,79	0,9	0,8	0,8	0,8	1,8E-08
Q55762	Sll0188 protein OS=Synechocystis sp. (strain PCC 6803 / Kazusa) GN=sll01	9,38		1,0		0,9	2,0E+00
P72707	Sll0224 protein OS=Synechocystis sp. (strain PCC 6803 / Kazusa) GN=sll02	17,45	1,5	1,0	0,7	1,1	8,8E-01
P73902	Sll0236 protein OS=Synechocystis sp. (strain PCC 6803 / Kazusa) GN=sll02	22,58	3,3	2,8	2,0	2,6	4,6E-11
P74394	Sll0272 protein OS=Synechocystis sp. (strain PCC 6803 / Kazusa) GN=sll02	35,67		1,3	1,0	1,1	4,1E-01
Q55648	Sll0314 protein OS=Synechocystis sp. (strain PCC 6803 / Kazusa) GN=sll03	32,8	1,6	1,2	1,0	1,3	1,1E-01
P74426	Sll0359 protein OS=Synechocystis sp. (strain PCC 6803 / Kazusa) GN=sll03	42,58	0,7	0,6	0,7	0,7	0,0E+00
Q55734	Sll0395 protein OS=Synechocystis sp. (strain PCC 6803 / Kazusa) GN=sll03	32,08	1,6	1,7	1,2	2,1	2,8E-05
P74685	Sll0443 protein OS=Synechocystis sp. (strain PCC 6803 / Kazusa) GN=sll04	13,01		5,6	1,4	3,0	1,5E-01
Q55184	Sll0456 protein OS=Synechocystis sp. (strain PCC 6803 / Kazusa) GN=sll04	5,41	1,1			1,2	2,0E+00
Q55847	Sll0470 protein OS=Synechocystis sp. (strain PCC 6803 / Kazusa) GN=sll04	19,35	1,0	0,7	0,6	0,7	6,2E-02
Q55496	Sll0493 protein OS=Synechocystis sp. (strain PCC 6803 / Kazusa) GN=sll04	8,33		0,7	1,3	1,0	9,1E-01
Q55492	Sll0497 protein OS=Synechocystis sp. (strain PCC 6803 / Kazusa) GN=sll04	8			1,6	1,6	2,0E+00
Q55466	Sll0518 protein OS=Synechocystis sp. (strain PCC 6803 / Kazusa) GN=sll05	16,36	0,7	0,8	1,1	0,9	2,8E-01
Q55517	Sll0529 protein OS=Synechocystis sp. (strain PCC 6803 / Kazusa) GN=sll05	36,25	1,2	1,1	1,0	1,2	2,4E-02
Q55410	Sll0540 protein OS=Synechocystis sp. (strain PCC 6803 / Kazusa) GN=sll05	5,21	0,9	0,6	0,5	0,7	1,2E-02
Q55390	Sll0553 protein OS=Synechocystis sp. (strain PCC 6803 / Kazusa) GN=sll05	25,51	1,0	1,8	2,6	1,7	5,0E-02
Q55865	Sll0585 protein OS=Synechocystis sp. (strain PCC 6803 / Kazusa) GN=sll05	11,11	1,5	1,3	1,0	1,2	5,1E-02
Q55862	Sll0588 protein OS=Synechocystis sp. (strain PCC 6803 / Kazusa) GN=sll05	11,11	0,8	0,7		0,7	1,8E-06
Q55852	Sll0596 protein OS=Synechocystis sp. (strain PCC 6803 / Kazusa) GN=sll05	22,54		1,3	1,5	1,2	4,7E-04
Q55726	Sll0602 protein OS=Synechocystis sp. (strain PCC 6803 / Kazusa) GN=sll06	4,92		0,9		0,9	2,0E+00
Q55723	Sll0606 protein OS=Synechocystis sp. (strain PCC 6803 / Kazusa) GN=sll06	4,41		1,7		1,3	2,0E+00
P72952	Sll0645 protein OS=Synechocystis sp. (strain PCC 6803 / Kazusa) GN=sll06	9,02			0,6	0,7	2,0E+00
P72950	Sll0647 protein OS=Synechocystis sp. (strain PCC 6803 / Kazusa) GN=sll06	9,77	2,5	2,1	1,6	2,5	3,3E-07
Q55984	Sll0659 protein OS=Synechocystis sp. (strain PCC 6803 / Kazusa) GN=sll06	3,26		0,8		0,8	2,0E+00
Q55966	Sll0676 protein OS=Synechocystis sp. (strain PCC 6803 / Kazusa) GN=sll06	12,22	0,5			0,5	2,0E+00
P74630	Sll0735 protein OS=Synechocystis sp. (strain PCC 6803 / Kazusa) GN=sll07	7,94	2,2			2,1	2,0E+00
Q55953	Sll0781 protein OS=Synechocystis sp. (strain PCC 6803 / Kazusa) GN=sll07	45,81	1,8	1,9	0,9	1,5	1,4E-01
Q55943	Sll0788 protein OS=Synechocystis sp. (strain PCC 6803 / Kazusa) GN=sll07	12,76		0,4		0,4	2,0E+00
Q55432	Sll0822 protein OS=Synechocystis sp. (strain PCC 6803 / Kazusa) GN=sll08	31,78	0,7	0,7	0,7	0,7	0,0E+00
P73762	Sll0837 protein OS=Synechocystis sp. (strain PCC 6803 / Kazusa) GN=sll08	15,99	0,6		0,6	0,6	0,0E+00
P73746	Sll0854 protein OS=Synechocystis sp. (strain PCC 6803 / Kazusa) GN=sll08	18,18	1,5	1,7	1,1	1,4	2,6E-02
Q55361	Sll0887 protein OS=Synechocystis sp. (strain PCC 6803 / Kazusa) GN=sll08	27,13	2,6	2,9	1,4	2,4	9,4E-04
Q55360	Sll0888 protein OS=Synechocystis sp. (strain PCC 6803 / Kazusa) GN=sll08	16,84	3,2	3,3	1,2	2,4	1,1E-02
P73130	Sll0995 protein OS=Synechocystis sp. (strain PCC 6803 / Kazusa) GN=sll09	11,69			1,2	1,1	2,0E+00
P72756	Sll1033 protein OS=Synechocystis sp. (strain PCC 6803 / Kazusa) GN=sll10	2,54	0,5	0,5	0,8	0,6	1,0E-03
P72751	Sll1036 protein OS=Synechocystis sp. (strain PCC 6803 / Kazusa) GN=sll10	17,81	2,2	2,1	1,4	1,8	2,0E-06
P72653	Sll1049 protein OS=Synechocystis sp. (strain PCC 6803 / Kazusa) GN=sll10	6,38	1,0	1,0		0,9	1,5E-01
P72647	Sll1053 protein OS=Synechocystis sp. (strain PCC 6803 / Kazusa) GN=sll10	7,31	2,5	2,4	1,1	1,7	1,4E-02
P72646	Sll1054 protein OS=Synechocystis sp. (strain PCC 6803 / Kazusa) GN=sll10	9,09	4,5	3,3		3,8	7,3E-20
P72631	Sll1064 protein OS=Synechocystis sp. (strain PCC 6803 / Kazusa) GN=sll10	16,37	2,7	2,3	1,3	2,1	8,8E-04
P73280	Sll1072 protein OS=Synechocystis sp. (strain PCC 6803 / Kazusa) GN=sll10	10,76	3,9	3,3	1,6	2,1	3,8E-04
P73267	Sll1080 protein OS=Synechocystis sp. (strain PCC 6803 / Kazusa) GN=sll10	16,19		1,5	1,2	1,4	4,0E-03
P74251	Sll1089 protein OS=Synechocystis sp. (strain PCC 6803 / Kazusa) GN=sll10	15,49	0,8	0,8	0,6	0,7	4,7E-05
P74720	Sll1106 protein OS=Synechocystis sp. (strain PCC 6803 / Kazusa) GN=sll11	49,12	1,4	1,7	1,4	1,6	4,0E-19
P74719	Sll1107 protein OS=Synechocystis sp. (strain PCC 6803 / Kazusa) GN=sll11	8,91	0,4			0,2	2,0E+00
P73488	Sll1130 protein OS=Synechocystis sp. (strain PCC 6803 / Kazusa) GN=sll11	46,96	0,2	0,2	0,8	0,4	1,4E-02
P73793	Sll1150 protein OS=Synechocystis sp. (strain PCC 6803 / Kazusa) GN=sll11	7,14			1,5	1,2	2,0E+00
P72845	Sll1188 protein OS=Synechocystis sp. (strain PCC 6803 / Kazusa) GN=sll11	21,34	1,4	2,1	1,0	1,7	6,2E-02
P74001	Sll1239 protein OS=Synechocystis sp. (strain PCC 6803 / Kazusa) GN=sll12	12,36			3,1	3,2	2,0E+00
P74073	Sll1258 protein OS=Synechocystis sp. (strain PCC 6803 / Kazusa) GN=sll12	14,51	2,0			2,7	2,0E+00
P73541	Sll1273 protein OS=Synechocystis sp. (strain PCC 6803 / Kazusa) GN=sll12	4,85		1,8		2,0	2,0E+00
P73598	Sll1305 protein OS=Synechocystis sp. (strain PCC 6803 / Kazusa) GN=sll13	8,47	0,3	0,2	0,5	0,3	2,9E-06
P73597	Sll1306 protein OS=Synechocystis sp. (strain PCC 6803 / Kazusa) GN=sll13	39,4	0,3	0,3	0,2	0,4	8,7E-10
P73596	Sll1307 protein OS=Synechocystis sp. (strain PCC 6803 / Kazusa) GN=sll13	41,71	0,4	0,2	0,2	0,3	2,5E-07
P73591	Sll1308 protein OS=Synechocystis sp. (strain PCC 6803 / Kazusa) GN=sll13	8,87			0,9	0,9	2,0E+00
P73588	Sll1315 protein OS=Synechocystis sp. (strain PCC 6803 / Kazusa) GN=sll13	4,83	0,9	0,5	0,5	0,7	1,3E-02
P74535	Sll1336 protein OS=Synechocystis sp. (strain PCC 6803 / Kazusa) GN=sll13	11,06	0,4		0,9	0,7	1,6E-01
P74534	Sll1338 protein OS=Synechocystis sp. (strain PCC 6803 / Kazusa) GN=sll13	17,11	0,9	0,8		0,8	1,2E-02
P73510	Sll1358 protein OS=Synechocystis sp. (strain PCC 6803 / Kazusa) GN=sll13	32,74	0,5	0,3	0,3	0,4	2,3E-11
P74162	Sll1380 protein OS=Synechocystis sp. (strain PCC 6803 / Kazusa) GN=sll13	38,79	1,0	0,8	0,7	0,9	1,3E-01
P74145	Sll1390 protein OS=Synechocystis sp. (strain PCC 6803 / Kazusa) GN=sll13	20,88	0,7	0,8	0,8	0,7	2,9E-12
P73439	Sll1461 protein OS=Synechocystis sp. (strain PCC 6803 / Kazusa) GN=sll14	9,09	2,1			1,6	2,0E+00
P74358	Sll1528 protein OS=Synechocystis sp. (strain PCC 6803 / Kazusa) GN=sll15	3,82	0,6	0,5		0,5	0,0E+00
P74357	Sll1530 protein OS=Synechocystis sp. (strain PCC 6803 / Kazusa) GN=sll15	11,4	0,6	0,5	1,0	0,8	6,1E-02
P74348	Sll1534 protein OS=Synechocystis sp. (strain PCC 6803 / Kazusa) GN=sll15	4,5	1,4			1,3	2,0E+00
P74341	Sll1537 protein OS=Synechocystis sp. (strain PCC 6803 / Kazusa) GN=sll15	12,95	1,3		0,5	0,9	6,5E-01
P74675	Sll1542 protein OS=Synechocystis sp. (strain PCC 6803 / Kazusa) GN=sll15	8,67		2,4	1,8	3,5	5,9E-07
P73719	Sll1628 protein OS=Synechocystis sp. (strain PCC 6803 / Kazusa) GN=sll16	9,38	0,8	0,9	0,7	0,9	1,8E-02
P73048	Sll1638 protein OS=Synechocystis sp. (strain PCC 6803 / Kazusa) GN=sll16	41,61	1,8	1,9	1,5	2,5	5,5E-11
P72807	Sll1663 protein OS=Synechocystis sp. (strain PCC 6803 / Kazusa) GN=sll16	14,09	0,7	0,7	0,6	0,7	4,4E-10
P72805	Sll1665 protein OS=Synechocystis sp. (strain PCC 6803 / Kazusa) GN=sll16	5,94	1,2	1,1	0,8	1,0	9,0E-01
P73707	Sll1692 protein OS=Synechocystis sp. (strain PCC 6803 / Kazusa) GN=sll16	11,72			0,8	0,9	2,0E+00
P73393	Sll1734 protein OS=Synechocystis sp. (strain PCC 6803 / Kazusa) GN=sll17	16,71	3,4	3,7	3,5	3,6	0,0E+00
P73652	Sll1757 protein OS=Synechocystis sp. (strain PCC 6803 / Kazusa) GN=sll17	10,27			1,0	1,0	2,0E+00
P73643	Sll1762 protein OS=Synechocystis sp. (strain PCC 6803 / Kazusa) GN=sll17	24,35	1,4	0,6	0,8	0,9	5,8E-01
P73626	Sll1771 protein OS=Synechocystis sp. (strain PCC 6803 / Kazusa) GN=sll17	12,6		1,1	1,2	1,3	1,6E-09
P73619	Sll1775 protein OS=Synechocystis sp. (strain PCC 6803 / Kazusa) GN=sll17	2,94		1,7		1,5	2,0E+00
P73601	Sll1784 protein OS=Synechocystis sp. (strain PCC 6803 / Kazusa) GN=sll17	10,11	1,5	0,8	0,7	1,0	7,1E-01
P73600	Sll1785 protein OS=Synechocystis sp. (strain PCC 6803 / Kazusa) GN=sll17	26,49	0,5	0,3	0,3	0,3	2,6E-13
P73120	Sll1830 protein OS=Synechocystis sp. (strain PCC 6803 / Kazusa) GN=sll18	14,53	1,1	1,2	1,0	1,1	2,9E-01
P73111	Sll1835 protein OS=Synechocystis sp. (strain PCC 6803 / Kazusa) GN=sll18	6,79	0,4			0,2	2,0E+00
P73107	Sll1837 protein OS=Synechocystis sp. (strain PCC 6803 / Kazusa) GN=sll18	15,17	2,1			1,8	2,0E+00
P74135	Sll1873 protein OS=Synechocystis sp. (strain PCC 6803 / Kazusa) GN=sll18	62,81	0,4	0,4	0,6	0,5	6,2E-10
P73804	Sll1961 protein OS=Synechocystis sp. (strain PCC 6803 / Kazusa) GN=sll19	3,61	1,9	1,3	0,9	1,4	2,4E-01
P73921	Sll1979 protein OS=Synechocystis sp. (strain PCC 6803 / Kazusa) GN=sll19	14,79	0,9	0,9	1,0	0,9	3,5E-03
Q6ZEU6	Sll5034 protein OS=Synechocystis sp. (strain PCC 6803 / Kazusa) GN=sll50	15,46	0,6		0,8	0,8	2,8E-02
Q6ZEQ5	Sll5075 protein OS=Synechocystis sp. (strain PCC 6803 / Kazusa) GN=sll50	21,58		1,5	1,3	3,3	1,6E-08

Q55660	Slr0001 protein OS=Synechocystis sp. (strain PCC 6803 / Kazusa) GN=slr00	39,38	2,7	2,3	1,3	2,1	1,7E-03
Q55667	Slr0006 protein OS=Synechocystis sp. (strain PCC 6803 / Kazusa) GN=slr00	8,76			2,2	1,7	2,0E+00
Q55449	Slr0031 protein OS=Synechocystis sp. (strain PCC 6803 / Kazusa) GN=slr00	6,34	4,1	3,2	1,7	2,5	1,4E-04
Q55131	Slr0049 protein OS=Synechocystis sp. (strain PCC 6803 / Kazusa) GN=slr00	11,06	0,5	0,6	0,9	0,6	4,1E-03
P74445	Slr0146 protein OS=Synechocystis sp. (strain PCC 6803 / Kazusa) GN=slr01	12,94	0,5			0,5	2,0E+00
P74446	Slr0147 protein OS=Synechocystis sp. (strain PCC 6803 / Kazusa) GN=slr01	13,01	0,5	0,5	0,5	0,5	0,0E+00
P74448	Slr0149 protein OS=Synechocystis sp. (strain PCC 6803 / Kazusa) GN=slr01	20,51	0,8	0,7	0,6	0,7	1,6E-08
P74450	Slr0151 protein OS=Synechocystis sp. (strain PCC 6803 / Kazusa) GN=slr01	25,62	0,5	0,4	0,7	0,6	4,4E-06
Q55573	Slr0184 protein OS=Synechocystis sp. (strain PCC 6803 / Kazusa) GN=slr01	9,71			1,6	1,5	2,0E+00
Q55780	Slr0208 protein OS=Synechocystis sp. (strain PCC 6803 / Kazusa) GN=slr02	8,49	0,7	0,5	0,7	0,6	1,3E-06
Q55782	Slr0209 protein OS=Synechocystis sp. (strain PCC 6803 / Kazusa) GN=slr02	7,18	0,4	0,8	0,9	0,7	1,4E-01
Q55698	Slr0226 protein OS=Synechocystis sp. (strain PCC 6803 / Kazusa) GN=slr02	2,9	1,1	1,3	0,9	1,1	3,6E-01
P72690	Slr0236 protein OS=Synechocystis sp. (strain PCC 6803 / Kazusa) GN=slr02	7,21			1,4	1,3	2,0E+00
P72700	Slr0244 protein OS=Synechocystis sp. (strain PCC 6803 / Kazusa) GN=slr02	33,45	0,8	1,0	1,1	1,0	8,7E-01
P72709	Slr0250 protein OS=Synechocystis sp. (strain PCC 6803 / Kazusa) GN=slr02	14,61	0,5			0,4	2,0E+00
P74396	Slr0280 protein OS=Synechocystis sp. (strain PCC 6803 / Kazusa) GN=slr02	7,87	0,8			0,8	2,0E+00
Q55922	Slr0315 protein OS=Synechocystis sp. (strain PCC 6803 / Kazusa) GN=slr03	7,79			1,9	1,6	2,0E+00
Q55541	Slr0333 protein OS=Synechocystis sp. (strain PCC 6803 / Kazusa) GN=slr03	37,74	0,6	0,3	0,3	0,5	5,5E-06
Q55542	Slr0334 protein OS=Synechocystis sp. (strain PCC 6803 / Kazusa) GN=slr03	13,64	0,3	0,2	0,2	0,3	0,0E+00
Q55650	Slr0355 protein OS=Synechocystis sp. (strain PCC 6803 / Kazusa) GN=slr03	9,67			2,0	2,3	2,0E+00
Q55737	Slr0416 protein OS=Synechocystis sp. (strain PCC 6803 / Kazusa) GN=slr04	14,38	1,6	1,2		2,3	7,1E-03
Q55113	Slr0431 protein OS=Synechocystis sp. (strain PCC 6803 / Kazusa) GN=slr04	20,8	0,8	0,5	0,5	0,6	7,2E-04
P74375	Slr0442 protein OS=Synechocystis sp. (strain PCC 6803 / Kazusa) GN=slr04	12,77	2,2	1,1	0,7	1,3	5,9E-01
P74379	Slr0443 protein OS=Synechocystis sp. (strain PCC 6803 / Kazusa) GN=slr04	21,6	0,4	0,4	0,3	0,5	0,0E+00
P74694	Slr0455 protein OS=Synechocystis sp. (strain PCC 6803 / Kazusa) GN=slr04	19,15			1,2	1,2	2,0E+00
Q55171	Slr0476 protein OS=Synechocystis sp. (strain PCC 6803 / Kazusa) GN=slr04	27,94	0,4	0,4	0,5	0,5	1,1E-12
Q55176	Slr0483 protein OS=Synechocystis sp. (strain PCC 6803 / Kazusa) GN=slr04	25,5	0,7	0,6	0,9	0,7	4,2E-03
Q55514	Slr0552 protein OS=Synechocystis sp. (strain PCC 6803 / Kazusa) GN=slr05	27,87	0,9	0,8	0,9	0,8	1,7E-02
Q55392	Slr0565 protein OS=Synechocystis sp. (strain PCC 6803 / Kazusa) GN=slr05	3,69		0,8		0,8	2,0E+00
P74746	Slr0600 protein OS=Synechocystis sp. (strain PCC 6803 / Kazusa) GN=slr06	10,12	1,6	1,1	0,6	1,1	9,7E-01
P74752	Slr0605 protein OS=Synechocystis sp. (strain PCC 6803 / Kazusa) GN=slr06	10,03	2,8	3,8		3,1	4,5E-14
P74753	Slr0606 protein OS=Synechocystis sp. (strain PCC 6803 / Kazusa) GN=slr06	6,62			1,1	1,1	2,0E+00
Q55727	Slr0645 protein OS=Synechocystis sp. (strain PCC 6803 / Kazusa) GN=slr06	57,77	2,8	2,2	1,0	1,8	5,7E-02
P74584	Slr0667 protein OS=Synechocystis sp. (strain PCC 6803 / Kazusa) GN=slr06	7,51			0,9	0,8	2,0E+00
P72937	Slr0670 protein OS=Synechocystis sp. (strain PCC 6803 / Kazusa) GN=slr06	31,27	0,9	2,0	1,9	1,6	1,1E-01
P72967	Slr0695 protein OS=Synechocystis sp. (strain PCC 6803 / Kazusa) GN=slr06	21,97	0,8		0,7	0,7	4,5E-12
Q55972	Slr0708 protein OS=Synechocystis sp. (strain PCC 6803 / Kazusa) GN=slr07	10,2		0,2	0,5	0,5	3,8E-03
P72675	Slr0731 protein OS=Synechocystis sp. (strain PCC 6803 / Kazusa) GN=slr07	12,19	0,5		1,0	0,7	3,0E-01
Q55426	Slr0841 protein OS=Synechocystis sp. (strain PCC 6803 / Kazusa) GN=slr08	14,43	2,0	1,8	1,2	1,5	1,8E-03
Q55436	Slr0848 protein OS=Synechocystis sp. (strain PCC 6803 / Kazusa) GN=slr08	24,55	0,9	0,8	0,6	0,7	1,1E-02
P73556	Slr0876 protein OS=Synechocystis sp. (strain PCC 6803 / Kazusa) GN=slr08	7,58		0,6		0,6	2,0E+00
Q55374	Slr0907 protein OS=Synechocystis sp. (strain PCC 6803 / Kazusa) GN=slr09	4,93	0,3	1,0	1,1	0,8	3,6E-01
Q55375	Slr0909 protein OS=Synechocystis sp. (strain PCC 6803 / Kazusa) GN=slr09	5,33			0,9	0,9	2,0E+00
Q55376	Slr0912 protein OS=Synechocystis sp. (strain PCC 6803 / Kazusa) GN=slr09	8,14		1,5	1,0	1,2	3,6E-01
Q55386	Slr0924 protein OS=Synechocystis sp. (strain PCC 6803 / Kazusa) GN=slr09	14,34	1,0	0,4	0,4	0,7	3,1E-02
Q55501	Slr0929 protein OS=Synechocystis sp. (strain PCC 6803 / Kazusa) GN=slr09	15,9	0,9	0,9	1,0	0,9	5,9E-02
P74302	Slr0937 protein OS=Synechocystis sp. (strain PCC 6803 / Kazusa) GN=slr09	11,99	0,5		0,4	0,5	0,0E+00
P72852	Slr0962 protein OS=Synechocystis sp. (strain PCC 6803 / Kazusa) GN=slr09	5,66	1,0			1,0	2,0E+00
P73007	Slr1043 protein OS=Synechocystis sp. (strain PCC 6803 / Kazusa) GN=slr10	14,44	0,9			1,0	2,0E+00
P72741	Slr1097 protein OS=Synechocystis sp. (strain PCC 6803 / Kazusa) GN=slr10	26,38	0,6	0,8	1,0	0,9	1,8E-01
P72747	Slr1103 protein OS=Synechocystis sp. (strain PCC 6803 / Kazusa) GN=slr11	3,97	1,4		1,4	1,3	0,0E+00
P74233	Slr1160 protein OS=Synechocystis sp. (strain PCC 6803 / Kazusa) GN=slr11	40,2	0,6	0,5	0,5	0,5	0,0E+00
P74236	Slr1161 protein OS=Synechocystis sp. (strain PCC 6803 / Kazusa) GN=slr11	8,54	1,1		0,8	0,9	6,3E-01
P74718	Slr1189 protein OS=Synechocystis sp. (strain PCC 6803 / Kazusa) GN=slr11	8,55			0,8	0,8	2,0E+00
P73342	Slr1194 protein OS=Synechocystis sp. (strain PCC 6803 / Kazusa) GN=slr11	9,46		1,4		1,6	2,0E+00
P73344	Slr1196 protein OS=Synechocystis sp. (strain PCC 6803 / Kazusa) GN=slr11	3,96	1,0			0,9	2,0E+00
P73463	Slr1220 protein OS=Synechocystis sp. (strain PCC 6803 / Kazusa) GN=slr12	18,93	1,4	1,0	0,8	1,1	7,7E-01
P73497	Slr1240 protein OS=Synechocystis sp. (strain PCC 6803 / Kazusa) GN=slr12	14,53			0,8	0,8	2,0E+00
P73803	Slr1263 protein OS=Synechocystis sp. (strain PCC 6803 / Kazusa) GN=slr12	8,94	1,1	0,8	0,7	0,9	2,9E-01
P74188	Slr1276 protein OS=Synechocystis sp. (strain PCC 6803 / Kazusa) GN=slr12	13,45	2,1	2,0	1,3	1,9	1,1E-04
P72839	Slr1301 protein OS=Synechocystis sp. (strain PCC 6803 / Kazusa) GN=slr13	19,63	1,5	1,2	0,8	1,2	4,7E-01
P74074	Slr1338 protein OS=Synechocystis sp. (strain PCC 6803 / Kazusa) GN=slr13	12	2,7	1,6	1,2	1,7	1,6E-02
P74079	Slr1342 protein OS=Synechocystis sp. (strain PCC 6803 / Kazusa) GN=slr13	18,43	0,7		1,4	1,0	9,6E-01
P73592	Slr1406 protein OS=Synechocystis sp. (strain PCC 6803 / Kazusa) GN=slr14	13,26	0,8			0,8	2,0E+00
P73503	Slr1437 protein OS=Synechocystis sp. (strain PCC 6803 / Kazusa) GN=slr14	32,14	1,0	1,0	0,8	1,0	3,8E-01
P73508	Slr1440 protein OS=Synechocystis sp. (strain PCC 6803 / Kazusa) GN=slr14	16,78			1,6	1,2	2,0E+00
P74154	Slr1470 protein OS=Synechocystis sp. (strain PCC 6803 / Kazusa) GN=slr14	12,21			1,0	1,0	2,0E+00
P73946	Slr1506 protein OS=Synechocystis sp. (strain PCC 6803 / Kazusa) GN=slr15	13,02	2,3	0,7	0,9	1,2	7,5E-01
P73953	Slr1512 protein OS=Synechocystis sp. (strain PCC 6803 / Kazusa) GN=slr15	7,22			3,6	7,0	2,0E+00
P74219	Slr1533 protein OS=Synechocystis sp. (strain PCC 6803 / Kazusa) GN=slr15	29,57	0,4	0,8	0,7	0,6	9,5E-03
P73424	Slr1540 protein OS=Synechocystis sp. (strain PCC 6803 / Kazusa) GN=slr15	28,3	1,1	1,2	1,6	1,4	4,0E-02
P74588	Slr1557 protein OS=Synechocystis sp. (strain PCC 6803 / Kazusa) GN=slr15	7,59	0,4	0,5	0,7	0,6	7,1E-06
P72968	Slr1590 protein OS=Synechocystis sp. (strain PCC 6803 / Kazusa) GN=slr15	10,51	1,2		1,1	1,2	1,4E-03
P72985	Slr1600 protein OS=Synechocystis sp. (strain PCC 6803 / Kazusa) GN=slr16	24,83		0,2	0,3	0,2	0,0E+00
P72891	Slr1613 protein OS=Synechocystis sp. (strain PCC 6803 / Kazusa) GN=slr16	12,76	2,6		1,4	1,6	3,2E-02
P72895	Slr1617 protein OS=Synechocystis sp. (strain PCC 6803 / Kazusa) GN=slr16	8,52	0,7		1,6	1,1	9,0E-01
P72897	Slr1619 protein OS=Synechocystis sp. (strain PCC 6803 / Kazusa) GN=slr16	7,23	1,5		1,0	1,5	2,6E-01
P74673	Slr1668 protein OS=Synechocystis sp. (strain PCC 6803 / Kazusa) GN=slr16	9,29			1,1	1,0	2,0E+00
P73716	Slr1732 protein OS=Synechocystis sp. (strain PCC 6803 / Kazusa) GN=slr17	12,75			0,4	0,6	2,0E+00
P73049	Slr1768 protein OS=Synechocystis sp. (strain PCC 6803 / Kazusa) GN=slr17	4,7		0,7		0,7	2,0E+00
P72765	Slr1772 protein OS=Synechocystis sp. (strain PCC 6803 / Kazusa) GN=slr17	7,16		2,2		2,6	2,0E+00
P72799	Slr1794 protein OS=Synechocystis sp. (strain PCC 6803 / Kazusa) GN=slr17	11,6	0,8	1,0	1,2	1,0	7,5E-01
P72814	Slr1800 protein OS=Synechocystis sp. (strain PCC 6803 / Kazusa) GN=slr18	8,17	0,9	1,1	1,2	1,1	3,9E-01
P73697	Slr1812 protein OS=Synechocystis sp. (strain PCC 6803 / Kazusa) GN=slr18	12,03	0,9		0,7	0,8	8,8E-02
P73698	Slr1813 protein OS=Synechocystis sp. (strain PCC 6803 / Kazusa) GN=slr18	9,15			1,9	1,6	2,0E+00
P73699	Slr1814 protein OS=Synechocystis sp. (strain PCC 6803 / Kazusa) GN=slr18	12,34	1,2			1,3	2,0E+00
P73706	Slr1818 protein OS=Synechocystis sp. (strain PCC 6803 / Kazusa) GN=slr18	8,46		0,8		0,8	2,0E+00
P73389	Slr1829 protein OS=Synechocystis sp. (strain PCC 6803 / Kazusa) GN=slr18	5,76			1,0	1,0	2,0E+00
P73409	Slr1841 protein OS=Synechocystis sp. (strain PCC 6803 / Kazusa) GN=slr18	17,78	0,5	0,3	1,2	0,7	1,5E-01
P73603	Slr1852 protein OS=Synechocystis sp. (strain PCC 6803 / Kazusa) GN=slr18	52,66	0,9	0,5	0,5	0,7	2,3E-02
P73604	Slr1853 protein OS=Synechocystis sp. (strain PCC 6803 / Kazusa) GN=slr18	20,35	0,4	0,3	0,2	0,3	1,8E-10
P73605	Slr1854 protein OS=Synechocystis sp. (strain PCC 6803 / Kazusa) GN=slr18	51,52	0,6	0,3	0,3	0,4	1,6E-06
P73606	Slr1855 protein OS=Synechocystis sp. (strain PCC 6803 / Kazusa) GN=slr18	48,93	1,0	0,7	0,5	0,7	8,3E-02
P73328	Slr1900 protein OS=Synechocystis sp. (strain PCC 6803 / Kazusa) GN=slr19	22,67	2,0	1,4	0,9	1,9	1,4E-01
P73103	Slr1908 protein OS=Synechocystis sp. (strain PCC 6803 / Kazusa) GN=slr19	19,12	0,9	0,5	0,9	0,8	1,4E-01
P74473	Slr1923 protein OS=Synechocystis sp. (strain PCC 6803 / Kazusa) GN=slr19	4,02	1,6			1,8	2,0E+00
P74478	Slr1926 protein OS=Synechocystis sp. (strain PCC 6803 / Kazusa) GN=slr19	14,35	0,8	0,7		0,7	1,3E-12
P74500	Slr1940 protein OS=Synechocystis sp. (strain PCC 6803 / Kazusa) GN=slr19	14,73	2,5	1,3	0,5	1,3	6,6E-01
P74506	Slr1944 protein OS=Synechocystis sp. (strain PCC 6803 / Kazusa) GN=slr19	6,69	0,5	0,4	0,3	0,4	0,0E+00
P74094	Slr1958 protein OS=Synechocystis sp. (strain PCC 6803 / Kazusa) GN=slr19	17		0,7	0,9	0,8	4,6E-03
P74100	Slr1962 protein OS=Synechocystis sp. (strain PCC 6803 / Kazusa) GN=slr19	5,14		1,2	0,7	1,0	7,5E-01
P74113	Slr1970 protein OS=Synechocystis sp. (strain PCC 6803 / Kazusa) GN=slr19	20,23	1,1		0,8	0,9	5,8E-01
P73222	Slr2005 protein OS=Synechocystis sp. (strain PCC 6803 / Kazusa) GN=slr20	13,03	0,5		0,6	0,5	0,0E+00
P73244	Slr2025 protein OS=Synechocystis sp. (strain PCC 6803 / Kazusa) GN=slr20	25,49	1,5	1,5	1,3	1,5	3,5E-10
P73812	Slr2060 protein OS=Synechocystis sp. (strain PCC 6803 / Kazusa) GN=slr20	4,09	0,8	0,9	1,4	1,0	9,4E-01
P73370	Slr2070 protein OS=Synechocystis sp. (strain PCC 6803 / Kazusa) GN=slr20	15,14	0,9	0,8	1,1	0,9	3,0E-01
P73934	Slr2105 protein OS=Synechocystis sp. (strain PCC 6803 / Kazusa) GN=slr21	3,87			1,9	3,3	2,0E+00
P73681	Slr2144 protein OS=Synechocystis sp. (strain PCC 6803 / Kazusa) GN=slr21	9,63	1,0	0,9		1,0	2,4E-01
Q6ZEP2	Slr5088 protein OS=Synechocystis sp. (strain PCC 6803 / Kazusa) GN=slr50	16,81	10,4			10,4	2,0E+00
Q6YRQ8	Slr6096 protein OS=Synechocystis sp. (strain PCC 6803 / Kazusa) GN=slr60	5,36			0,9	1,0	2,0E+00
Q6ZEI6	Slr7012 protein OS=Synechocystis sp. (strain PCC 6803 / Kazusa) GN=slr70	23,1	3,4	3,7	1,7	4,9	3,1E-05
Q6ZEC5	Slr7073 protein OS=Synechocystis sp. (strain PCC 6803 / Kazusa) GN=slr70	19,3		1,4		1,3	2,0E+00
P74281	Soluble hydrogenase 42 kD subunit OS=Synechocystis sp. (strain PCC 6803	44,79	2,9	2,2	1,0	2,2	3,7E-02
P73722	SOS function regulatory protein OS=Synechocystis sp. (strain PCC 6803 / Ka	50,25	0,6	0,4	0,4	0,5	0,0E+00
P73914	Squalene-hopene-cyclase OS=Synechocystis sp. (strain PCC 6803 / Kazusa	10,36	3,9	4,1	1,5	3,6	1,1E-03
P74795	Ssl0352 protein OS=Synechocystis sp. (strain PCC 6803 / Kazusa) GN=ssl0	36,21			1,2	1,3	2,0E+00
P73875	Ssl0467 protein OS=Synechocystis sp. (strain PCC 6803 / Kazusa) GN=ssl0	30,99			0,5	0,5	2,0E+00
P74067	Ssl1498 protein OS=Synechocystis sp. (strain PCC 6803 / Kazusa) GN=ssl1	24,59			0,7	0,7	2,0E+00
P73031	Ssl1918 protein OS=Synechocystis sp. (strain PCC 6803 / Kazusa) GN=ssl1	18,56	1,1			1,1	2,0E+00
P74239	Ssl2148 protein OS=Synechocystis sp. (strain PCC 6803 / Kazusa) GN=ssl2	16,33		1,1	1,0	1,0	9,7E-01
P73489	Ssl2245 protein OS=Synechocystis sp. (strain PCC 6803 / Kazusa) GN=ssl2	15,91	0,4	0,4	0,3	0,3	0,0E+00
P73654	Ssl3364 protein OS=Synechocystis sp. (strain PCC 6803 / Kazusa) GN=ssl3	27,03	0,5	0,5	0,5	0,4	0,0E+00
P73286	Ssl3451 protein OS=Synechocystis sp. (strain PCC 6803 / Kazusa) GN=ssl3	22,22	1,4			1,4	2,0E+00
P74769	Ssr1528 protein OS=Synechocystis sp. (strain PCC 6803 / Kazusa) GN=ssr1	47,87	1,6	1,1	0,8	1,1	6,7E-01
P74326	Ssr1600 protein OS=Synechocystis sp. (strain PCC 6803 / Kazusa) GN=ssr1	57,14	0,8	0,9	0,7	0,7	2,5E-06
P72914	Ssr1766 protein OS=Synechocystis sp. (strain PCC 6803 / Kazusa) GN=ssr1	10,53	1,7			1,7	2,0E+00
P73792	Ssr2087 protein OS=Synechocystis sp. (strain PCC 6803 / Kazusa) GN=ssr2	14,29		0,8		0,8	2,0E+00
P73479	Succinate dehydrogenase flavoprotein subunit OS=Synechocystis sp. (strain	13,74	3,7	3,1	1,2	4,1	1,8E-02
P73723	Succinate dehydrogenase iron-sulphur protein subunit OS=Synechocystis sp	6,65			1,4	1,5	2,0E+00
P74241	Sulfate adenylyltransferase OS=Synechocystis sp. (strain PCC 6803 / Kazus	13,85	0,7	0,6	1,0	0,7	8,0E-02
P72854	Sulfite reductase [ferredoxin] OS=Synechocystis sp. (strain PCC 6803 / Kazu	27,09	2,0	1,7	0,8	1,5	2,2E-01
P73128	Sulfolipid biosynthesis protein SqdB OS=Synechocystis sp. (strain PCC 6803	51,44	1,4	1,4	0,9	1,3	1,7E-01
P77968	Superoxide dismutase [Fe] OS=Synechocystis sp. (strain PCC 6803 / Kazus	39,2	1,2	1,0	0,9	1,1	6,7E-01
P52231	Thioredoxin OS=Synechocystis sp. (strain PCC 6803 / Kazusa) GN=trxA PE	46,73	0,8	0,7	0,7	0,6	0,0E+00
P73711	Threonine synthase OS=Synechocystis sp. (strain PCC 6803 / Kazusa) GN=	9,7			0,5	0,6	2,0E+00
P74193	Threonine synthase OS=Synechocystis sp. (strain PCC 6803 / Kazusa) GN=	26,7	1,7	1,3		1,6	2,4E-03
P72804	Thylakoid membrane protein slr1796 OS=Synechocystis sp. (strain PCC 680	8,96	1,1			1,1	2,0E+00
P74511	Thylakoid membrane protein slr1949 OS=Synechocystis sp. (strain PCC 680	16,04	0,6	0,5	0,8	0,7	6,6E-04
P73695	Thylakoid-associated protein sll1697 OS=Synechocystis sp. (strain PCC 680	8,72	1,2			1,4	2,0E+00
P72673	Thylakoid-associated protein slr0729 OS=Synechocystis sp. (strain PCC 680	46,53	0,9	1,0	0,8	0,9	1,3E-01
P73145	Thylakoid-associated single-stranded DNA-binding protein slr1034 OS=Syne	35,66	1,6	3,5	1,3	2,0	4,1E-02
P72797	Transaldolase OS=Synechocystis sp. (strain PCC 6803 / Kazusa) GN=tal PE	52,17	1,0	1,1	0,9	1,0	8,2E-01
P72688	Transcription termination/antitermination protein NusA OS=Synechocystis sp	19,65	1,0	1,1	1,1	1,1	6,0E-02
P36265	Transcription termination/antitermination protein NusG OS=Synechocystis sp	24,39	0,6	0,8	1,0	0,8	1,0E-01
P73282	Transketolase OS=Synechocystis sp. (strain PCC 6803 / Kazusa) GN=tktA P	43,58	1,7	1,2	0,8	1,3	4,4E-01
P72689	Translation initiation factor IF-2 OS=Synechocystis sp. (strain PCC 6803 / Ka	10,49	0,9	1,1	1,3	1,1	4,2E-01
P72874	Translation initiation factor IF-3 OS=Synechocystis sp. (strain PCC 6803 / Ka	19,21	0,7	0,6	0,5	0,5	2,7E-09
Q55511	Trigger factor OS=Synechocystis sp. (strain PCC 6803 / Kazusa) GN=tig PE	32,48	1,1	1,2	0,8	1,0	8,9E-01
Q59994	Triosephosphate isomerase OS=Synechocystis sp. (strain PCC 6803 / Kazus	37,19	2,4	2,1	1,3	1,7	1,6E-03

Q59992	Tryptophan synthase beta chain OS=Synechocystis sp. (strain PCC 6803 / K	17,48	1,3	1,8	1,7	1,5	1,3E-07
P73655	Tryptophan--tRNA ligase OS=Synechocystis sp. (strain PCC 6803 / Kazusa)	6,53	0,8			0,7	2,0E+00
P74463	Twitching motility protein OS=Synechocystis sp. (strain PCC 6803 / Kazusa)	21,95	0,5		0,8	0,8	7,7E-02
Q55673	UDP-N-acetylglucosamine 1-carboxyvinyltransferase OS=Synechocystis sp.	5,94	1,6	0,4	0,6	0,9	4,8E-01
Q55469	UDP-N-acetylmuramoyl-L-alanyl-D-glutamate--2,6-diaminopimelate ligase O	10,3	0,5	0,3		0,4	6,1E-11
P45450	UDP-N-acetylmuramoyl-tripeptide--D-alanyl-D-alanine ligase OS=Synechocy	7,93	1,4			1,4	2,0E+00
P73668	UDP-N-acetylmuramoylalanine--D-glutamate ligase OS=Synechocystis sp. (	6,19	0,4	0,5	1,1	0,8	1,2E-01
P22040	Uncharacterized ABC transporter ATP-binding protein sll0415 OS=Synechoc	6,78			1,2	1,2	2,0E+00
P73335	Uncharacterized deoxyribonuclease sll1786 OS=Synechocystis sp. (strain P	24,9	2,6	2,0	1,3	1,9	2,4E-03
Q6ZES9	Uncharacterized protein OS=Synechocystis sp. (strain PCC 6803 / Kazusa)	21,54			0,7	0,7	2,0E+00
Q6ZED7	Uncharacterized protein OS=Synechocystis sp. (strain PCC 6803 / Kazusa)	4,27	1,5			1,6	2,0E+00
Q55707	Uncharacterized protein sll0617 OS=Synechocystis sp. (strain PCC 6803 / K	34,46	0,6	0,5	0,3	0,4	2,5E-05
P72861	Uncharacterized protein sll0936 OS=Synechocystis sp. (strain PCC 6803 / K	4,59	1,0		2,0	1,2	2,7E-01
P72929	Uncharacterized protein sll1021 OS=Synechocystis sp. (strain PCC 6803 / K	12,33	0,9	0,8	0,7	0,7	1,9E-03
P74178	Uncharacterized protein sll1178 OS=Synechocystis sp. (strain PCC 6803 / K	5,2			0,9	1,0	2,0E+00
P73599	Uncharacterized protein sll1304 OS=Synechocystis sp. (strain PCC 6803 / K	11,15	2,1	1,3	0,6	1,4	6,0E-01
P74360	Uncharacterized protein sll1526 OS=Synechocystis sp. (strain PCC 6803 / K	14,03	0,5	0,6	1,0	0,7	6,4E-02
P73392	Uncharacterized protein sll1735 OS=Synechocystis sp. (strain PCC 6803 / K	27,07	6,6		2,0	4,2	3,7E-02
P73602	Uncharacterized protein sll1783 OS=Synechocystis sp. (strain PCC 6803 / K	23,13	0,8			0,8	2,0E+00
Q55458	Uncharacterized protein slr0039 OS=Synechocystis sp. (strain PCC 6803 / K	11,2	1,1	1,3	1,5	1,3	4,6E-04
Q55629	Uncharacterized protein slr0782 OS=Synechocystis sp. (strain PCC 6803 / K	9,98			1,0	1,0	2,0E+00
P72655	Uncharacterized protein slr1128 OS=Synechocystis sp. (strain PCC 6803 / K	27,1	0,8	1,4	1,8	1,3	2,5E-01
P74002	Uncharacterized protein slr1322 OS=Synechocystis sp. (strain PCC 6803 / K	26,58	2,0	2,4	1,5	3,1	2,5E-06
P73042	Uncharacterized protein slr1764 OS=Synechocystis sp. (strain PCC 6803 / K	23,46		12,4		8,0	2,0E+00
P73321	Uncharacterized protein slr1894 OS=Synechocystis sp. (strain PCC 6803 / K	49,36	1,5	1,8	1,5	1,8	1,4E-09
P73152	Uncharacterized thylakoid-associated protein sll0982 OS=Synechocystis sp.	17,97	1,1			1,1	2,0E+00
P73545	Uncharacterized thylakoid-associated protein ssl2501 OS=Synechocystis sp	37,08	1,1	1,6	1,2	1,5	3,2E-02
P74328	Uncharacterized tRNA/rRNA methyltransferase slr0955 OS=Synechocystis s	14,32	0,7	1,2	1,1	1,0	9,5E-01
Q55563	Uncharacterized WD repeat-containing protein sll0163 OS=Synechocystis sp	0,95		1,3		1,2	2,0E+00
P74598	Uncharacterized WD repeat-containing protein sll1491 OS=Synechocystis sp	16,67	0,9	1,0	0,6	0,8	1,2E-01
P72817	Universal stress protein Sll1654 OS=Synechocystis sp. (strain PCC 6803 / K	45,86	0,9	0,9	0,8	0,8	1,3E-05
P72699	UPF0045 protein sll0230 OS=Synechocystis sp. (strain PCC 6803 / Kazusa)	51,24	1,6	1,3	1,2	1,6	1,5E-03
P74607	UPF0296 protein ssl2874 OS=Synechocystis sp. (strain PCC 6803 / Kazusa	40,45	0,9	0,7	0,7	0,7	6,0E-04
P72753	Uracil phosphoribosyltransferase OS=Synechocystis sp. (strain PCC 6803 /	17,59	0,9			0,9	2,0E+00
P73061	Urease subunit alpha OS=Synechocystis sp. (strain PCC 6803 / Kazusa) GN	16,34	2,0	1,3	0,9	1,2	1,7E-01
P73796	Urease subunit gamma OS=Synechocystis sp. (strain PCC 6803 / Kazusa)	31	0,7		0,8	0,6	4,7E-06
P74457	Uridylate kinase OS=Synechocystis sp. (strain PCC 6803 / Kazusa) GN=pyr	6,15	0,7			0,7	2,0E+00
P54224	Uroporphyrinogen decarboxylase OS=Synechocystis sp. (strain PCC 6803 /	22,86	0,5	0,6	0,7	0,4	1,1E-08
Q55522	Valine--tRNA ligase OS=Synechocystis sp. (strain PCC 6803 / Kazusa) GN=	11,54	0,6	0,9	1,2	0,8	3,9E-01
Q55557	Virginiamycin B hydrolase Vgb OS=Synechocystis sp. (strain PCC 6803 / Ka	34,38	1,2	0,7	0,5	0,8	2,4E-01
P73066	Ycf23 protein OS=Synechocystis sp. (strain PCC 6803 / Kazusa) GN=ycf23	12,9	1,2	0,7	0,6	0,8	2,7E-01
P74429	Ycf39 gene product OS=Synechocystis sp. (strain PCC 6803 / Kazusa) GN=	3,99			1,3	1,3	2,0E+00
P73069	Ycf48-like protein OS=Synechocystis sp. (strain PCC 6803 / Kazusa) GN=sl	7,02	1,1		0,7	0,9	4,5E-01
P72777	Ycf54-like protein OS=Synechocystis sp. (strain PCC 6803 / Kazusa) GN=sl	27,82	1,1	0,9	0,9	1,0	5,6E-01
Q55823	Ycf66-like protein OS=Synechocystis sp. (strain PCC 6803 / Kazusa) GN=sl	8,01			0,8	0,8	2,0E+00
P74306	Zeta-carotene desaturase OS=Synechocystis sp. (strain PCC 6803 / Kazusa	7,36			1,0	1,0	2,0E+00
P74721	Zinc-containing alcohol dehydrogenase family OS=Synechocystis sp. (strain	5,65	1,6	1,2	0,7	1,2	7,0E-01

Supplementary Table S7

[NiFe]-hydrogenase is essential for cyanobacterium *Synechocystis* sp. PCC 6803 aerobic growth in the dark

Edith De Rosa, Vanessa Checchetto, Cinzia Franchin, Elisabetta Bergantino, Paola Berto, Ildikò Szabò, Giorgio M. Giacometti, Giorgio Arrigoni, and Paola Costantini

Accession	Description	ΣCoverage	Mutant Light/WT Light Replicate 1 (115/117)	Mutant Light/WT Light Replicate 2 (116/114)	Mutant Light/WT Light Replicate 3 (117/115)	Average	Two-Tailed Z Test
	>sp|CAS1_BOVIN|	17,29		0,1	0,2	0,2	6,4E-13
	>sp|K1C10_HUMAN|	23,1	0,9	1,2	1,2	1,1	4,9E-01
	>sp|K1C9_HUMAN|	14,29	1,0	1,1	1,0	1,1	3,8E-02
	>sp|K22E_HUMAN|	19,38	0,7	1,2	1,0	1,0	8,2E-01
	>sp|K2C1_HUMAN|	34,06	0,8	1,6	1,3	1,2	4,0E-01
	>sp|TRYP_PIG|	28,57	0,6	1,5	1,3	1,1	9,5E-01
P74561	1-(5-phosphoribosyl)-5-[(5-phosphoribosylamino)methylideneamino] imic	13,28	1,9		0,9	1,4	4,8E-01
Q55663	1-deoxy-D-xylulose 5-phosphate reductoisomerase OS=Synechocystis s	8,63	1,3	0,7		1,0	8,2E-01
P73067	1-deoxy-D-xylulose-5-phosphate synthase OS=Synechocystis sp. (strain	5	4,5	0,8	0,7	2,0	6,0E-01
P52981	1,4-alpha-glucan branching enzyme GlgB OS=Synechocystis sp. (strain	17,53	1,8	0,6	0,7	1,0	7,7E-01
Q05971	10 kDa chaperonin OS=Synechocystis sp. (strain PCC 6803 / Kazusa) G	72,82	0,5	0,7	0,6	0,6	2,6E-09
P72977	16.6 kDa small heat shock protein, molecular chaperon OS=Synechocys	33,56	0,8	0,6		0,7	3,4E-02
P73426	2-C-methyl-D-erythritol 2,4-cyclodiphosphate synthase OS=Synechocyst	13,04	0,5	2,3	2,0	1,6	6,5E-01
P48576	2-isopropylmalate synthase OS=Synechocystis sp. (strain PCC 6803 / K	4,5	1,5			1,5	2,0E+00
P74507	2,3-bisphosphoglycerate-independent phosphoglycerate mutase OS=Sy	13,72	0,6	0,8	1,2	0,9	3,9E-01
P73264	3-amino-5-hydroxybenzoic acid synthase OS=Synechocystis sp. (strain I	42,06	0,7	2,3	1,9	1,6	2,9E-01
P73997	3-dehydroquinate synthase OS=Synechocystis sp. (strain PCC 6803 / Ka	15,24		0,9	0,9	0,9	0,0E+00
P73848	3-hydroxyacyl-[acyl-carrier-protein] dehydratase FabZ OS=Synechocysti	24,39	1,1	2,7	1,9	1,9	2,3E-02
P54384	3-isopropylmalate dehydratase large subunit OS=Synechocystis sp. (stra	4,06	1,3	4,4	2,3	2,7	1,3E-02
P74207	3-isopropylmalate dehydratase small subunit OS=Synechocystis sp. (stra	10		2,6		2,6	2,0E+00
P73960	3-isopropylmalate dehydrogenase OS=Synechocystis sp. (strain PCC 68	10,77	1,1		0,7	0,9	6,1E-01
Q55596	3-ketoacyl-acyl carrier protein reductase OS=Synechocystis sp. (strain P	21,24	3,9			3,9	2,0E+00
P73574	3-oxoacyl-[acyl-carrier-protein] reductase 1 OS=Synechocystis sp. (strai	18,62		0,8	0,7	0,8	3,7E-04
P73826	3-oxoacyl-[acyl-carrier-protein] reductase 2 OS=Synechocystis sp. (strai	34,58	0,5	0,5	0,4	0,5	0,0E+00
P73283	3-oxoacyl-[acyl-carrier-protein] synthase 2 OS=Synechocystis sp. (strain	5,53	2,0	0,8	0,4	1,0	7,1E-01
P73530	30S ribosomal protein S1 homolog A OS=Synechocystis sp. (strain PCC	33,54	1,2	1,5	1,3	1,3	1,1E-07
P74142	30S ribosomal protein S1 homolog B OS=Synechocystis sp. (strain PCC	16,72	0,9	1,3	1,0	1,1	6,1E-01
P74226	30S ribosomal protein S10 OS=Synechocystis sp. (strain PCC 6803 / Ka	45,71	1,6	1,4	1,0	1,3	3,9E-02
P73298	30S ribosomal protein S11 OS=Synechocystis sp. (strain PCC 6803 / Ka	32,31	2,4	1,5	1,4	1,8	1,1E-03
P74230	30S ribosomal protein S12 OS=Synechocystis sp. (strain PCC 6803 / Ka	30,16	3,0	1,2	2,1	2,1	8,5E-03
P73299	30S ribosomal protein S13 OS=Synechocystis sp. (strain PCC 6803 / Ka	54,33	1,8	1,6	1,6	1,6	1,6E-38
P48944	30S ribosomal protein S14 OS=Synechocystis sp. (strain PCC 6803 / Ka	44	1,3	1,0	1,2	1,2	3,8E-02
P72866	30S ribosomal protein S15 OS=Synechocystis sp. (strain PCC 6803 / Ka	33,71	1,8	3,4	2,4	2,5	1,5E-06
P74410	30S ribosomal protein S16 OS=Synechocystis sp. (strain PCC 6803 / Ka	70,73	1,3	1,5	1,9	1,6	1,1E-05
P73311	30S ribosomal protein S17 OS=Synechocystis sp. (strain PCC 6803 / Ka	66,67	2,0	1,3	2,5	1,9	5,9E-04
P48946	30S ribosomal protein S18 OS=Synechocystis sp. (strain PCC 6803 / Ka	64,79	1,9	1,7	1,5	1,7	4,6E-19
P73316	30S ribosomal protein S19 OS=Synechocystis sp. (strain PCC 6803 / Ka	36,96	2,8	1,7	1,5	2,0	2,7E-04
P74071	30S ribosomal protein S2 OS=Synechocystis sp. (strain PCC 6803 / Kaz	35,32	2,5	2,0	1,7	2,1	1,5E-12
P73336	30S ribosomal protein S20 OS=Synechocystis sp. (strain PCC 6803 / Ka	16,49	1,0	1,2		1,1	8,7E-02
P48949	30S ribosomal protein S21 OS=Synechocystis sp. (strain PCC 6803 / Ka	31,67			0,9	0,9	2,0E+00
P73314	30S ribosomal protein S3 OS=Synechocystis sp. (strain PCC 6803 / Kaz	36,67	3,5	1,6	1,4	2,2	1,6E-02
P48939	30S ribosomal protein S4 OS=Synechocystis sp. (strain PCC 6803 / Kaz	28,22	1,8	1,5	2,6	2,0	1,1E-04
P73304	30S ribosomal protein S5 OS=Synechocystis sp. (strain PCC 6803 / Kaz	61,27	2,5	1,4	1,7	1,9	8,7E-04
P73636	30S ribosomal protein S6 OS=Synechocystis sp. (strain PCC 6803 / Kaz	44,25	1,5	1,9	1,8	1,7	1,8E-12
P74229	30S ribosomal protein S7 OS=Synechocystis sp. (strain PCC 6803 / Kaz	53,85	1,9	2,0	2,0	2,0	0,0E+00
P73307	30S ribosomal protein S8 OS=Synechocystis sp. (strain PCC 6803 / Kaz	51,88	2,1	1,3	2,0	1,8	3,8E-04
P73293	30S ribosomal protein S9 OS=Synechocystis sp. (strain PCC 6803 / Kaz	37,23	2,8	1,6	1,9	2,1	1,3E-05
P72785	4-alpha-glucanotransferase OS=Synechocystis sp. (strain PCC 6803 / K	8,51	0,9	1,0	1,6	1,2	4,5E-01
P73672	4-hydroxy-3-methylbut-2-en-1-yl diphosphate synthase OS=Synechocys	25,56	0,4	0,5	0,5	0,5	0,0E+00
P72642	4-hydroxy-tetrahydrodipicolinate reductase OS=Synechocystis sp. (strair	7,27			0,9	0,9	2,0E+00
Q55513	4-hydroxy-tetrahydrodipicolinate synthase OS=Synechocystis sp. (strain	8,31	0,6	0,7		0,7	8,5E-12
P74756	47 kD protein OS=Synechocystis sp. (strain PCC 6803 / Kazusa) GN=slı	16,71	2,4	1,1	1,4	1,6	5,3E-02
P73637	5-oxo-1,2,5-tricarboxilic-3-penten acid decarboxilase/isomer OS=Synech	14,13	0,8		1,7	1,2	7,1E-01
P36236	50S ribosomal protein L1 OS=Synechocystis sp. (strain PCC 6803 / Kaz	43,28	1,6	1,9	1,8	1,8	2,2E-30
P23350	50S ribosomal protein L10 OS=Synechocystis sp. (strain PCC 6803 / Ka	22,54	1,5	2,0	2,1	1,9	6,8E-11
P36237	50S ribosomal protein L11 OS=Synechocystis sp. (strain PCC 6803 / Ka	45,39	1,7	2,1	1,6	1,8	3,5E-13
P73294	50S ribosomal protein L13 OS=Synechocystis sp. (strain PCC 6803 / Ka	78,81	3,3	8,1	6,2	5,9	2,1E-10
P73310	50S ribosomal protein L14 OS=Synechocystis sp. (strain PCC 6803 / Ka	27,87		1,2	1,7	1,4	5,5E-02
P73303	50S ribosomal protein L15 OS=Synechocystis sp. (strain PCC 6803 / Ka	67,35	5,1	6,5	6,7	6,1	2,1E-93
P73313	50S ribosomal protein L16 OS=Synechocystis sp. (strain PCC 6803 / Ka	35,25	2,6	9,0		5,8	1,1E-02
P73296	50S ribosomal protein L17 OS=Synechocystis sp. (strain PCC 6803 / Ka	52,59	4,6	14,5	7,9	9,0	2,6E-10
P73305	50S ribosomal protein L18 OS=Synechocystis sp. (strain PCC 6803 / Ka	20	2,6	5,2	4,7	4,2	6,7E-11
P36239	50S ribosomal protein L19 OS=Synechocystis sp. (strain PCC 6803 / Ka	44,26	6,1	6,4	4,4	5,6	6,6E-49
P73317	50S ribosomal protein L2 OS=Synechocystis sp. (strain PCC 6803 / Kaz	61,59	7,0	11,9	7,1	8,7	6,9E-34
P48957	50S ribosomal protein L20 OS=Synechocystis sp. (strain PCC 6803 / Ka	57,26	2,2	5,5	4,1	3,9	1,1E-06
P74266	50S ribosomal protein L21 OS=Synechocystis sp. (strain PCC 6803 / Ka	69,35	2,8	4,3	3,5	3,5	6,6E-22
P73315	50S ribosomal protein L22 OS=Synechocystis sp. (strain PCC 6803 / Ka	43,8	3,2	19,2	5,1	9,1	4,1E-04
P73318	50S ribosomal protein L23 OS=Synechocystis sp. (strain PCC 6803 / Ka	78,22	3,8	11,7	5,4	7,0	3,3E-08
P73309	50S ribosomal protein L24 OS=Synechocystis sp. (strain PCC 6803 / Ka	68,7	2,2	11,2	11,3	8,2	6,5E-04
P73289	50S ribosomal protein L25 OS=Synechocystis sp. (strain PCC 6803 / Ka	65,31	3,2	12,7	3,7	6,5	1,3E-04
P74267	50S ribosomal protein L27 OS=Synechocystis sp. (strain PCC 6803 / Ka	59,77	4,7	12,2	8,1	8,3	2,0E-13
P72851	50S ribosomal protein L28 OS=Synechocystis sp. (strain PCC 6803 / Ka	61,54	4,3	4,2	4,3	4,3	0,0E+00
P73312	50S ribosomal protein L29 OS=Synechocystis sp. (strain PCC 6803 / Ka	73,97	0,9	3,4	2,8	2,4	6,7E-02
P73320	50S ribosomal protein L3 OS=Synechocystis sp. (strain PCC 6803 / Kaz	52,58	7,4	35,6	4,3	15,8	2,2E-04
P48958	50S ribosomal protein L33 OS=Synechocystis sp. (strain PCC 6803 / Ka	26,15			4,3	4,3	2,0E+00
P48959	50S ribosomal protein L35 OS=Synechocystis sp. (strain PCC 6803 / Ka	32,84	2,2	6,0	3,4	3,9	1,0E-05
P73319	50S ribosomal protein L4 OS=Synechocystis sp. (strain PCC 6803 / Kaz	55,71	4,5	19,4	4,0	9,3	1,2E-04
P73308	50S ribosomal protein L5 OS=Synechocystis sp. (strain PCC 6803 / Kaz	65,56	2,4	1,7	1,3	1,8	9,5E-04
P73306	50S ribosomal protein L6 OS=Synechocystis sp. (strain PCC 6803 / Kaz	77,65	5,4	8,4	9,5	7,8	6,2E-33
P23349	50S ribosomal protein L7/L12 OS=Synechocystis sp. (strain PCC 6803 /	52,34	0,9	2,5	1,7	1,7	1,6E-01
P42352	50S ribosomal protein L9 OS=Synechocystis sp. (strain PCC 6803 / Kaz	61,18	4,9	10,3	6,6	7,3	9,9E-20
Q55798	6-carboxy-5,6,7,8-tetrahydropterin synthase OS=Synechocystis sp. (stra	23,26	0,7	2,5	2,3	1,8	2,2E-01
Q55988	6-phosphofructokinase 2 OS=Synechocystis sp. (strain PCC 6803 / Kazu	5,99		0,7		0,7	2,0E+00
P52208	6-phosphogluconate dehydrogenase, decarboxylating OS=Synechocysti	24,9	1,1	0,5	0,3	0,6	8,8E-02
P74618	6-phosphogluconolactonase OS=Synechocystis sp. (strain PCC 6803 / K	15,42	0,5	0,5	0,6	0,5	0,0E+00
P73527	6,7-dimethyl-8-ribityllumazine synthase OS=Synechocystis sp. (strain PC	29,27	0,9	3,8	2,2	2,3	1,2E-01
Q05972	60 kDa chaperonin 1 OS=Synechocystis sp. (strain PCC 6803 / Kazusa)	58,96	0,8	0,9	0,8	0,8	2,6E-04
P22034	60 kDa chaperonin 2 OS=Synechocystis sp. (strain PCC 6803 / Kazusa)	62,5	1,0	1,0	1,0	1,0	5,4E-02
Q46363	Acetazolamide conferring resistance protein zam OS=Synechocystis sp.	6,65		0,8	1,0	0,9	4,4E-01
P73913	Acetohydroxy acid synthase OS=Synechocystis sp. (strain PCC 6803 / K	3,54	2,2		0,8	1,5	6,1E-01
P73918	Acetolactate synthase OS=Synechocystis sp. (strain PCC 6803 / Kazusa	13,09	1,0	1,0	1,0	1,0	8,7E-05
Q55141	Acetolactate synthase small subunit OS=Synechocystis sp. (strain PCC	49,42	0,9	1,6	1,3	1,3	2,3E-01
P73825	Acetyl coenzyme A acetyltransferase (Thiolase) OS=Synechocystis sp. (	7,33		0,4		0,4	2,0E+00
Q55404	Acetyl-coenzyme A synthetase OS=Synechocystis sp. (strain PCC 6803	10,41	3,2	1,0	1,2	1,8	2,1E-01
P73326	Acetylglutamate kinase OS=Synechocystis sp. (strain PCC 6803 / Kazus	8,75			1,0	1,0	2,0E+00
P73133	Acetylornithine aminotransferase OS=Synechocystis sp. (strain PCC 68(	41,03	0,4	0,7	0,6	0,5	2,4E-05
P74582	Aconitate hydratase 2 OS=Synechocystis sp. (strain PCC 6803 / Kazusa	11,29	0,7	1,1	0,8	0,8	1,7E-01
P20804	Acyl carrier protein OS=Synechocystis sp. (strain PCC 6803 / Kazusa) G	20,78	0,7	1,5	1,4	1,2	7,0E-01
Q55746	Acyl-[acyl-carrier-protein]--UDP-N-acetylglucosamine O-acyltransferase	8,7			0,8	0,8	2,0E+00
P74008	Adenosylhomocysteinase OS=Synechocystis sp. (strain PCC 6803 / Kaz	57,65	0,6	0,5	0,3	0,5	3,0E-04
P73302	Adenylate kinase 1 OS=Synechocystis sp. (strain PCC 6803 / Kazusa) G	44,32	0,8	1,8	1,6	1,4	3,2E-01
P74384	Adenylosuccinate lyase OS=Synechocystis sp. (strain PCC 6803 / Kazu	32,02	1,3	2,1	1,7	1,7	8,7E-04
P73290	Adenylosuccinate synthetase OS=Synechocystis sp. (strain PCC 6803 /	12,16	3,1	1,6	1,7	2,1	7,5E-04
P72703	Agmatinase 1 OS=Synechocystis sp. (strain PCC 6803 / Kazusa) GN=sp	15,36	0,8	3,0	1,9	1,9	1,9E-01
P73288	Aklaviketone reductase OS=Synechocystis sp. (strain PCC 6803 / Kazus	8,57		1,0	0,8	0,9	4,5E-01
P74423	Alanine--tRNA ligase OS=Synechocystis sp. (strain PCC 6803 / Kazusa)	4,56		0,6	1,1	0,8	4,0E-01
Q55688	Aldehyde decarbonylase OS=Synechocystis sp. (strain PCC 6803 / Kazu	12,55			1,8	1,8	2,0E+00
Q55811	Aldehyde dehydrogenase OS=Synechocystis sp. (strain PCC 6803 / Kaz	3,29			1,7	1,7	2,0E+00
P74308	Aldehyde reductase OS=Synechocystis sp. (strain PCC 6803 / Kazusa) (	18,35	1,4	0,9	1,3	1,2	2,4E-01
P72939	Alkaline phosphatase OS=Synechocystis sp. (strain PCC 6803 / Kazusa	11,36	0,1	0,2	0,3	0,2	6,1E-06
Q01951	Allophycocyanin alpha chain OS=Synechocystis sp. (strain PCC 6803 / K	77,02	0,5	0,9	1,0	0,8	2,0E-01
Q01952	Allophycocyanin beta chain OS=Synechocystis sp. (strain PCC 6803 / Ka	98,76	0,6	0,9	0,9	0,8	6,6E-02
P72870	Allophycocyanin subunit alpha-B OS=Synechocystis sp. (strain PCC 68(	73,91	0,6	1,0	1,0	0,8	2,7E-01
P74551	Allophycocyanin subunit beta-18 OS=Synechocystis sp. (strain PCC 68(	68,05	0,6	1,0	1,1	0,9	5,3E-01
Q55621	Amidophosphoribosyltransferase OS=Synechocystis sp. (strain PCC 68(	12,12	2,0		1,4	1,7	7,5E-03
P74527	Aminopeptidase OS=Synechocystis sp. (strain PCC 6803 / Kazusa) GN=	3,34	3,1		0,6	1,9	6,9E-01
P74468	Aminopeptidase P OS=Synechocystis sp. (strain PCC 6803 / Kazusa) G	18,82	0,8	2,1	1,5	1,5	2,9E-01
P73607	Anti-sigma factor antagonist OS=Synechocystis sp. (strain PCC 6803 / K	19,27	0,3	0,8	0,8	0,6	6,8E-02
P73609	Anti-sigma factor antagonist OS=Synechocystis sp. (strain PCC 6803 / K	45,37	0,3	0,6	0,5	0,5	1,3E-04
P26287	Apocytochrome f OS=Synechocystis sp. (strain PCC 6803 / Kazusa) GN	44,21	0,7	1,7	1,5	1,3	5,5E-01
Q55841	Arabinofuranosidase OS=Synechocystis sp. (strain PCC 6803 / Kazusa)	10,73			0,9	0,9	2,0E+00
P74122	Arginine biosynthesis bifunctional protein ArgJ OS=Synechocystis sp. (st	8,96	0,9		0,9	0,9	3,5E-02
P73257	Argininosuccinate lyase OS=Synechocystis sp. (strain PCC 6803 / Kazu	2,6			0,7	0,7	2,0E+00
P77973	Argininosuccinate synthase OS=Synechocystis sp. (strain PCC 6803 / K	26	0,9	0,6	0,7	0,7	1,7E-02
P52276	Asparagine--tRNA ligase OS=Synechocystis sp. (strain PCC 6803 / Kazu	9,74	1,1	0,4	0,8	0,8	2,5E-01
Q55128	Aspartate aminotransferase OS=Synechocystis sp. (strain PCC 6803 / K	6,17			1,0	1,0	2,0E+00
P73851	Aspartate--tRNA ligase OS=Synechocystis sp. (strain PCC 6803 / Kazus	11,02	1,7	0,5	0,5	0,9	4,8E-01
Q55512	Aspartate-semialdehyde dehydrogenase OS=Synechocystis sp. (strain P	19,82	1,5	1,1	0,8	1,1	7,3E-01
P74569	Aspartokinase OS=Synechocystis sp. (strain PCC 6803 / Kazusa) GN=ly	9,83	1,6	1,1	0,7	1,2	6,7E-01
P74215	Aspartyl/glutamyl-tRNA(Asn/Gln) amidotransferase subunit B OS=Synec	6,06		0,9		0,9	2,0E+00
P74592	ATP phosphoribosyltransferase regulatory subunit OS=Synechocystis sp	12,47	1,4	0,6	0,7	0,9	5,6E-01
P26533	ATP synthase epsilon chain OS=Synechocystis sp. (strain PCC 6803 / K	17,65	0,5			0,5	2,0E+00
P17253	ATP synthase gamma chain OS=Synechocystis sp. (strain PCC 6803 / K	28,34	1,3	0,6	0,5	0,8	2,6E-01
P27179	ATP synthase subunit alpha OS=Synechocystis sp. (strain PCC 6803 / K	33,4	1,1	0,5	0,5	0,7	8,7E-02
P27181	ATP synthase subunit b OS=Synechocystis sp. (strain PCC 6803 / Kazu	51,96	0,6	1,5	1,3	1,1	9,5E-01
P27183	ATP synthase subunit b' OS=Synechocystis sp. (strain PCC 6803 / Kazu	53,85	0,6	0,9	0,9	0,8	8,5E-02
P26527	ATP synthase subunit beta OS=Synechocystis sp. (strain PCC 6803 / Ka	53,83	0,7	0,6	0,7	0,7	0,0E+00
P27182	ATP synthase subunit c OS=Synechocystis sp. (strain PCC 6803 / Kazus	11,11			0,6	0,6	2,0E+00
P27180	ATP synthase subunit delta OS=Synechocystis sp. (strain PCC 6803 / Ka	36,22	0,6	0,9	0,7	0,7	2,6E-03
P54416	ATP-dependent Clp protease proteolytic subunit 1 OS=Synechocystis sp	32,83	0,7	1,0	0,9	0,9	1,9E-01
Q55662	ATP-dependent Clp protease regulatory subunit OS=Synechocystis sp. (	24,36	2,0	1,2	0,9	1,4	2,7E-01
Q55700	ATP-dependent zinc metalloprotease FtsH 2 OS=Synechocystis sp. (stra	11,48	1,5	1,1	0,9	1,1	4,8E-01
P72991	ATP-dependent zinc metalloprotease FtsH 3 OS=Synechocystis sp. (stra	11,2	1,5		1,3	1,4	4,6E-08

P73437	ATP-dependent zinc metalloprotease FtsH 4 OS=Synechocystis sp. (stra	17,83	2,2	1,1	1,2	1,5	9,1E-02
P72719	Bacterioferritin comigratory protein OS=Synechocystis sp. (strain PCC 6	15,22	1,1	1,1		1,1	1,8E-177
P24602	Bacterioferritin OS=Synechocystis sp. (strain PCC 6803 / Kazusa) GN=b	65,38	0,9	3,5	2,1	2,2	1,1E-01
P73287	Bacterioferritin OS=Synechocystis sp. (strain PCC 6803 / Kazusa) GN=b	36,36	0,7	3,2	2,6	2,1	2,6E-01
Q55460	Bicarbonate-binding protein CmpA OS=Synechocystis sp. (strain PCC 6	49,34	1,7	6,5	2,2	3,5	7,2E-03
Q55074	Bifunctional pantoate ligase/cytidylate kinase OS=Synechocystis sp. (str	4,48	1,3			1,3	2,0E+00
Q55626	Bifunctional protein FolD OS=Synechocystis sp. (strain PCC 6803 / Kazu	17,01	0,5		1,1	0,8	4,7E-01
Q55758	Bifunctional protein PyrR OS=Synechocystis sp. (strain PCC 6803 / Kazu	29,21	0,9	0,7	0,6	0,7	1,8E-03
P74741	Bifunctional purine biosynthesis protein PurH OS=Synechocystis sp. (str	11,15	1,8	0,6	0,7	1,1	8,4E-01
P74576	Biosynthetic arginine decarboxylase 1 OS=Synechocystis sp. (strain PC	4,03	1,9	0,4		1,1	8,3E-01
P72587	Biosynthetic arginine decarboxylase 2 OS=Synechocystis sp. (strain PC	2,43	3,2			3,2	2,0E+00
Q55120	Biotin carboxyl carrier protein of acetyl-CoA carboxylase OS=Synechocy	14,29	0,7		1,4	1,0	9,3E-01
Q55160	Biotin carboxylase OS=Synechocystis sp. (strain PCC 6803 / Kazusa) G	6,47	3,0		0,9	2,0	3,9E-01
Q54715	C-phycocyanin alpha chain OS=Synechocystis sp. (strain PCC 6803 / K	79,01	0,5	0,9	1,0	0,8	1,6E-01
Q54714	C-phycocyanin beta chain OS=Synechocystis sp. (strain PCC 6803 / Ka	96,51	0,4	0,8	0,9	0,7	8,4E-02
P73589	C4-dicarboxylase binding protein OS=Synechocystis sp. (strain PCC 68	9,76	0,6	0,3		0,5	1,7E-02
Q55756	Carbamoyl-phosphate synthase large chain OS=Synechocystis sp. (stra	10,64	1,6	0,7	0,5	0,9	5,6E-01
P74587	Carbamoyl-phosphate synthase small chain OS=Synechocystis sp. (stra	4,17		1,0		1,0	2,0E+00
P72759	Carbon dioxide concentrating mechanism protein CcmL OS=Synechocys	18		1,9		1,9	2,0E+00
P72758	Carbon dioxide concentrating mechanism protein CcmM OS=Synechocy	26,35	2,0	1,5	1,1	1,5	4,2E-02
P72760	Carbon dioxide-concentrating mechanism protein CcmK homolog 1 OS=	73,87	1,0	5,9	3,6	3,5	6,6E-02
P72761	Carbon dioxide-concentrating mechanism protein CcmK homolog 2 OS=	75,73	1,0	5,7	3,3	3,3	6,0E-02
P73407	Carbon dioxide-concentrating mechanism protein CcmK homolog 4 OS=	23,21	0,6	1,6	1,1	1,1	9,8E-01
Q54735	Carbonic anhydrase OS=Synechocystis sp. (strain PCC 6803 / Kazusa)	16,06	1,8	1,4		1,6	2,1E-04
P73458	Carboxyl-terminal protease OS=Synechocystis sp. (strain PCC 6803 / K	22,7	0,7	2,3	1,9	1,7	3,0E-01
P72864	Carboxysome formation protein OS=Synechocystis sp. (strain PCC 680	37,32	1,1	0,5	0,5	0,7	7,5E-02
P73911	Catalase-peroxidase OS=Synechocystis sp. (strain PCC 6803 / Kazusa)	25,6	0,6	1,7	2,5	1,6	4,3E-01
Q55584	Cation or drug efflux system protein OS=Synechocystis sp. (strain PCC	3,79	1,9	0,6	0,7	1,1	8,8E-01
Q55589	Cell division cycle protein OS=Synechocystis sp. (strain PCC 6803 / Kaz	7,19	2,0	0,9	1,0	1,3	5,2E-01
P73456	Cell division protein FtsZ OS=Synechocystis sp. (strain PCC 6803 / Kazu	7,67	1,0	0,9		0,9	4,5E-01
P74459	Chaperone protein ClpB 1 OS=Synechocystis sp. (strain PCC 6803 / Ka	4,68	1,5			1,5	2,0E+00
P74361	Chaperone protein ClpB 2 OS=Synechocystis sp. (strain PCC 6803 / Ka	14,68	1,7		0,7	1,2	8,2E-01
Q55505	Chaperone protein DnaJ 1 OS=Synechocystis sp. (strain PCC 6803 / Ka	5,84			0,9	0,9	2,0E+00
P22358	Chaperone protein dnaK2 OS=Synechocystis sp. (strain PCC 6803 / Ka	41,04	1,2	0,9	1,0	1,0	8,3E-01
P73098	Chaperone protein dnaK3 OS=Synechocystis sp. (strain PCC 6803 / Ka	3,24	1,4	0,5		0,9	7,0E-01
P73243	CheY subfamily OS=Synechocystis sp. (strain PCC 6803 / Kazusa) GN=	69,44	0,8	0,8	0,8	0,8	0,0E+00
P23353	Chorismate synthase OS=Synechocystis sp. (strain PCC 6803 / Kazusa	12,71	1,3	0,8	0,8	1,0	7,0E-01
P73092	Chromophore lyase CpcS/CpeS 2 OS=Synechocystis sp. (strain PCC 68	8,33	1,2			1,2	2,0E+00
P74371	Chromophore lyase CpcT/CpeT OS=Synechocystis sp. (strain PCC 680	21,94	0,9	1,6	1,3	1,3	1,8E-01
P74645	Circadian clock protein KaiB OS=Synechocystis sp. (strain PCC 6803 / K	21,9	0,7		0,5	0,6	8,4E-04
Q59977	Citrate synthase OS=Synechocystis sp. (strain PCC 6803 / Kazusa) GN	10,33		0,8		0,8	2,0E+00
Q55822	CobW protein OS=Synechocystis sp. (strain PCC 6803 / Kazusa) GN=c	6,53		1,4		1,4	2,0E+00
Q55367	Cyanate hydratase OS=Synechocystis sp. (strain PCC 6803 / Kazusa) G	36,91	0,6	1,3	1,6	1,2	7,8E-01
P73833	Cyanophycin synthetase OS=Synechocystis sp. (strain PCC 6803 / Kazu	13,75	2,0	0,9	1,0	1,3	4,8E-01
P73832	Cyanophycinase OS=Synechocystis sp. (strain PCC 6803 / Kazusa) GN	8,49	1,6	1,5	1,3	1,5	2,4E-09
P73410	Cysteine synthase OS=Synechocystis sp. (strain PCC 6803 / Kazusa) G	16,03	0,7	1,6	1,2	1,1	7,8E-01
P72662	Cysteine synthase OS=Synechocystis sp. (strain PCC 6803 / Kazusa) G	24,62	0,9	1,3	0,4	0,9	4,3E-01
P09190	Cytochrome b559 subunit alpha OS=Synechocystis sp. (strain PCC 680	32,1	0,9		1,0	0,9	4,4E-01
Q57038	Cytochrome b6 OS=Synechocystis sp. (strain PCC 6803 / Kazusa) GN=	18,92	1,7	0,8	0,9	1,1	8,5E-01
P26290	Cytochrome b6-f complex iron-sulfur subunit 2 OS=Synechocystis sp. (s	28,89	0,6	0,8	0,7	0,7	3,4E-06
P27589	Cytochrome b6-f complex subunit 4 OS=Synechocystis sp. (strain PCC	17,5	1,6		0,9	1,3	4,9E-01
Q55013	Cytochrome c-550 OS=Synechocystis sp. (strain PCC 6803 / Kazusa) G	46,25	0,7	1,7	1,6	1,3	5,2E-01
P73468	Cytoplasmic membrane protein for maltose uptake OS=Synechocystis s	4,41		0,7		0,7	2,0E+00
P73821	D-3-phosphoglycerate dehydrogenase OS=Synechocystis sp. (strain PC	20,04	2,1	0,7	0,6	1,1	9,5E-01
P73632	D-alanine--D-alanine ligase OS=Synechocystis sp. (strain PCC 6803 / K	9,32			1,5	1,5	2,0E+00
P74474	D-alanyl-D-alanine carboxypeptidase OS=Synechocystis sp. (strain PCC	14,25	0,4	0,7	0,9	0,7	5,2E-02
P73922	D-fructose 1,6-bisphosphatase class 2/sedoheptulose 1,7-bisphosphata	43,77	1,0	0,8	0,8	0,9	1,2E-01
P74275	Delta-1-pyrroline-5-carboxylate dehydrogenase OS=Synechocystis sp. (	7,37	3,4	0,6	0,7	1,6	7,7E-01
P77969	Delta-aminolevulinic acid dehydratase OS=Synechocystis sp. (strain PC	48,62	0,7	1,8	1,4	1,3	5,8E-01
P73618	Deoxyribose-phosphate aldolase OS=Synechocystis sp. (strain PCC 68	10,22	1,0	2,7		1,9	3,1E-01
Q55484	Diaminopimelate decarboxylase OS=Synechocystis sp. (strain PCC 680	22,39	2,1	1,7	1,2	1,6	6,9E-03
P74667	Diaminopimelate epimerase OS=Synechocystis sp. (strain PCC 6803 / K	17,2	1,0	0,7	0,7	0,8	6,5E-02
Q55393	Diflavin flavoprotein A 1 OS=Synechocystis sp. (strain PCC 6803 / Kazu	12,57	0,9	0,4	0,4	0,6	2,5E-02
P74510	Dihydrolipoamide acetyltransferase component (E2) of pyruvate dehydr	33,95	1,2	0,8	0,7	0,9	3,4E-01
P72740	Dihydrolipoyl dehydrogenase OS=Synechocystis sp. (strain PCC 6803 /	64,35	1,2	4,3	3,5	3,0	1,0E-02
P74438	Dihydroorotase OS=Synechocystis sp. (strain PCC 6803 / Kazusa) GN=	15,5	0,7	1,9		1,3	7,4E-01
P74689	Dihydroxy-acid dehydratase OS=Synechocystis sp. (strain PCC 6803 / K	32,44	0,9	3,9	2,1	2,3	1,1E-01
Q55738	DNA gyrase subunit A OS=Synechocystis sp. (strain PCC 6803 / Kazusa	15	1,3	1,4	1,1	1,2	2,6E-03
P77966	DNA gyrase subunit B OS=Synechocystis sp. (strain PCC 6803 / Kazusa	6,68	2,8	0,8	0,9	1,5	5,4E-01
P73196	DNA ligase OS=Synechocystis sp. (strain PCC 6803 / Kazusa) GN=lig P	26,33	1,6	1,5	2,2	1,8	1,5E-05
P72856	DNA polymerase III subunit beta OS=Synechocystis sp. (strain PCC 680	8,95		0,8		0,8	2,0E+00
P73810	DNA topoisomerase 1 OS=Synechocystis sp. (strain PCC 6803 / Kazusa	10,36	1,5	0,8	1,0	1,1	7,6E-01
P73418	DNA-binding protein HU OS=Synechocystis sp. (strain PCC 6803 / Kazu	60	0,6	1,1	1,2	1,0	7,9E-01
P73297	DNA-directed RNA polymerase subunit alpha OS=Synechocystis sp. (st	24,52	1,0	1,0	1,0	1,0	8,4E-01
P77965	DNA-directed RNA polymerase subunit beta OS=Synechocystis sp. (stra	19,51	2,5	0,9	0,9	1,4	5,5E-01
P73334	DNA-directed RNA polymerase subunit beta' OS=Synechocystis sp. (stra	10,17	2,0	0,9	0,8	1,3	6,3E-01
P74177	DNA-directed RNA polymerase subunit gamma OS=Synechocystis sp. (	14,38	2,3	0,9	1,1	1,4	3,4E-01
P74352	DNA-directed RNA polymerase subunit omega OS=Synechocystis sp. (s	22,37			1,3	1,3	2,0E+00
P74036	dTDP-glucose 4-6-dehydratase OS=Synechocystis sp. (strain PCC 680	7,93		0,5	0,6	0,5	0,0E+00
P74751	Elongation factor 4 OS=Synechocystis sp. (strain PCC 6803 / Kazusa) G	4,64	3,0	1,0	0,7	1,6	5,6E-01
P28371	Elongation factor G 1 OS=Synechocystis sp. (strain PCC 6803 / Kazusa	24,32	1,8	0,6	0,5	1,0	6,8E-01
P74228	Elongation factor G 2 OS=Synechocystis sp. (strain PCC 6803 / Kazusa	10,56	2,7	1,0	1,1	1,6	2,6E-01
Q55421	Elongation factor G-like protein OS=Synechocystis sp. (strain PCC 6803	3,29	2,3	0,7	1,1	1,4	5,8E-01
Q55119	Elongation factor P OS=Synechocystis sp. (strain PCC 6803 / Kazusa) G	37,97	0,5	0,7	1,0	0,8	1,1E-01
P74070	Elongation factor Ts OS=Synechocystis sp. (strain PCC 6803 / Kazusa)	59,63	1,4	1,6	1,6	1,5	2,5E-28
P74227	Elongation factor Tu OS=Synechocystis sp. (strain PCC 6803 / Kazusa)	77,44	1,7	1,4	1,2	1,4	1,8E-04
P77972	Enolase OS=Synechocystis sp. (strain PCC 6803 / Kazusa) GN=eno PE	41,67	0,5	2,0	1,3	1,3	8,3E-01
P73016	Enoyl-[acyl-carrier-protein] reductase [NADH] FabI OS=Synechocystis s	13,95	3,4	0,8	1,3	1,8	3,0E-01
P73114	Esterase OS=Synechocystis sp. (strain PCC 6803 / Kazusa) GN=slr191	4,95			0,5	0,5	2,0E+00
P72938	Extracellular nuclease OS=Synechocystis sp. (strain PCC 6803 / Kazusa	6,55	0,0	0,1	0,1	0,1	4,8E-11
P73732	Extracellular solute-binding protein OS=Synechocystis sp. (strain PCC 6	10,48	0,5	1,1	1,1	0,9	5,7E-01
P73355	Ferredoxin component OS=Synechocystis sp. (strain PCC 6803 / Kazus	31,1	0,9	1,0	1,4	1,1	7,1E-01
P74447	Ferredoxin OS=Synechocystis sp. (strain PCC 6803 / Kazusa) GN=slr01	10,75	0,8	1,4	1,5	1,2	4,1E-01
Q55318	Ferredoxin--NADP reductase OS=Synechocystis sp. (strain PCC 6803 /	43,34	1,1	1,2	1,5	1,3	7,6E-03
Q55366	Ferredoxin--nitrite reductase OS=Synechocystis sp. (strain PCC 6803 / K	14,14	2,3	0,7	0,8	1,3	7,5E-01
P27320	Ferredoxin-1 OS=Synechocystis sp. (strain PCC 6803 / Kazusa) GN=pe	17,53	0,6	1,4	1,1	1,1	9,7E-01
P55037	Ferredoxin-dependent glutamate synthase 1 OS=Synechocystis sp. (stra	7,29	0,7	0,7	0,7	0,7	0,0E+00
P55038	Ferredoxin-dependent glutamate synthase 2 OS=Synechocystis sp. (stra	19,73	1,6	0,8	0,6	1,0	7,7E-01
Q55389	Ferredoxin-thioredoxin reductase, catalytic chain OS=Synechocystis sp.	19,49		1,4		1,4	2,0E+00
P73050	Ferripyochelin binding protein OS=Synechocystis sp. (strain PCC 6803 /	13,16	0,7			0,7	2,0E+00
P27319	Flavodoxin OS=Synechocystis sp. (strain PCC 6803 / Kazusa) GN=isiB	11,18	0,2	0,1	0,4	0,2	8,5E-05
P74324	Fructose-1,6-bisphosphatase class 1 OS=Synechocystis sp. (strain PCC	5,76			0,9	0,9	2,0E+00
P74309	Fructose-bisphosphate aldolase class 1 OS=Synechocystis sp. (strain P	7,33	1,1	0,9	0,9	1,0	8,5E-01
Q55664	Fructose-bisphosphate aldolase class 2 OS=Synechocystis sp. (strain P	38,44	0,7	0,4	0,5	0,6	2,3E-04
P72585	GDP-L-fucose synthase OS=Synechocystis sp. (strain PCC 6803 / Kazu	15,71	1,5	1,0	0,9	1,1	4,9E-01
P72586	GDP-mannose 4,6-dehydratase OS=Synechocystis sp. (strain PCC 680	29,56	1,5	1,1	0,9	1,1	4,4E-01
P74189	General secretion pathway protein D OS=Synechocystis sp. (strain PCC	4,2		0,4		0,4	2,0E+00
Q55155	General secretion pathway protein E OS=Synechocystis sp. (strain PCC	4,76			0,9	0,9	2,0E+00
Q55799	General secretion pathway protein E OS=Synechocystis sp. (strain PCC	4,43	3,3	1,1	0,9	1,8	3,4E-01
P73704	General secretion pathway protein G OS=Synechocystis sp. (strain PCC	25,6	0,6	0,5	0,7	0,6	2,7E-08
Q55087	Geranylgeranyl diphosphate reductase OS=Synechocystis sp. (strain PC	27,03	1,7	1,6	0,9	1,4	1,1E-01
P72683	Geranylgeranyl pyrophosphate synthase OS=Synechocystis sp. (strain P	7,62		0,6	1,1	0,8	4,8E-01
P33779	Global nitrogen regulator OS=Synechocystis sp. (strain PCC 6803 / Kaz	24,44	1,3	0,6	0,4	0,8	3,1E-01
P52415	Glucose-1-phosphate adenylyltransferase OS=Synechocystis sp. (strain	29,84	1,9	0,5	0,5	1,0	6,2E-01
P73411	Glucose-6-phosphate 1-dehydrogenase OS=Synechocystis sp. (strain P	22	1,7	0,5	0,6	0,9	5,1E-01
P52983	Glucose-6-phosphate isomerase OS=Synechocystis sp. (strain PCC 68	47,27	0,8	2,7	2,0	1,9	1,5E-01
P73043	Glutamate decarboxylase OS=Synechocystis sp. (strain PCC 6803 / Kaz	8,35	2,8	0,9	0,9	1,5	5,0E-01
P77970	Glutamate--ammonia ligase OS=Synechocystis sp. (strain PCC 6803 / K	9,94	3,2	0,8	0,8	1,6	5,4E-01
Q55778	Glutamate--tRNA ligase OS=Synechocystis sp. (strain PCC 6803 / Kazu	10,14		0,8	0,9	0,9	6,6E-02
Q55665	Glutamate-1-semialdehyde 2,1-aminomutase OS=Synechocystis sp. (str	33,72	1,7	4,9	2,2	2,9	2,6E-03
P77961	Glutamine synthetase OS=Synechocystis sp. (strain PCC 6803 / Kazusa	51,8	0,9	3,9	2,3	2,4	1,1E-01
P72720	Glutamine--fructose-6-phosphate aminotransferase [isomerizing] OS=Sy	11,89	2,7	0,7	0,4	1,3	8,9E-01
P28463	Glutamyl-tRNA reductase OS=Synechocystis sp. (strain PCC 6803 / Kaz	4,92	2,5		0,7	1,6	6,6E-01
Q55139	Glutathione S-transferase OS=Synechocystis sp. (strain PCC 6803 / Kaz	19,02	1,0	0,6	0,5	0,7	7,4E-02
P73493	Glutathione synthetase OS=Synechocystis sp. (strain PCC 6803 / Kazus	9,69	1,3			1,3	2,0E+00
P49433	Glyceraldehyde-3-phosphate dehydrogenase 1 OS=Synechocystis sp. (	19,76	0,8	0,7	0,7	0,7	2,2E-06
P80505	Glyceraldehyde-3-phosphate dehydrogenase 2 OS=Synechocystis sp. (	64,99	2,5	1,7	1,8	2,0	5,6E-08
P74416	Glycine dehydrogenase (decarboxylating) OS=Synechocystis sp. (strain	6,51	0,4	0,5	0,7	0,5	5,4E-04
Q55716	Glycine--tRNA ligase alpha subunit OS=Synechocystis sp. (strain PCC 6	10,37	0,8	0,7	0,8	0,8	0,0E+00
Q55690	Glycine--tRNA ligase beta subunit OS=Synechocystis sp. (strain PCC 68	10,39	2,1	0,6	0,6	1,1	8,2E-01
P72691	Glycogen operon protein GlgX OS=Synechocystis sp. (strain PCC 6803	25,34	0,6	1,7	1,4	1,2	7,4E-01
P73608	Glycogen operon protein GlgX OS=Synechocystis sp. (strain PCC 6803	14	0,5	0,2	0,6	0,4	7,4E-04
P73511	Glycogen phosphorylase OS=Synechocystis sp. (strain PCC 6803 / Kaz	32,51	0,9	1,2	1,5	1,2	1,7E-01
P74521	Glycogen synthase 1 OS=Synechocystis sp. (strain PCC 6803 / Kazusa)	8,6	1,8	0,6	0,7	1,0	7,6E-01
P49057	GMP synthase [glutamine-hydrolyzing] OS=Synechocystis sp. (strain PC	18,27	1,2	0,8	0,9	0,9	5,6E-01
Q55759	GTP cyclohydrolase 1 OS=Synechocystis sp. (strain PCC 6803 / Kazusa	16,24	0,9	0,8	0,5	0,7	2,9E-02
P72749	GTP-binding protein TypA/BipA homolog OS=Synechocystis sp. (strain P	11,89	1,2	0,8	0,8	0,9	5,1E-01
P74702	Heat shock protein OS=Synechocystis sp. (strain PCC 6803 / Kazusa) G	6,84		4,2	1,9	3,0	8,3E-03
P72849	Heme oxygenase 1 OS=Synechocystis sp. (strain PCC 6803 / Kazusa)	8,75		0,9		0,9	2,0E+00
P73817	Hemolysin OS=Synechocystis sp. (strain PCC 6803 / Kazusa) GN=sll19	5,11	0,1	0,1	0,2	0,1	0,0E+00
Q55614	High-affinity branched-chain amino acid transport ATP-binding protein O	6,43	2,3			2,3	2,0E+00
Q55190	High-affinity Na(+)/H(+) antiporter NhaS3 OS=Synechocystis sp. (strain	6,94	1,1		0,6	0,8	4,2E-01
P73058	Histidinol dehydrogenase 1 OS=Synechocystis sp. (strain PCC 6803 / K	9,68	2,0	1,1	1,9	1,7	1,4E-02
P73807	Histidinol-phosphate aminotransferase OS=Synechocystis sp. (strain PC	27,79	1,6	3,6	1,6	2,3	7,2E-03
P74176	HlyB family OS=Synechocystis sp. (strain PCC 6803 / Kazusa) GN=hlyB	3,66			0,3	0,3	2,0E+00
P74175	HlyD family of secretion proteins OS=Synechocystis sp. (strain PCC 680	6,86	0,1	0,2	0,6	0,3	2,0E-03
P52986	Homoserine dehydrogenase OS=Synechocystis sp. (strain PCC 6803 / K	5,08	1,4	0,5	0,4	0,8	2,7E-01
P74729	HrEpiB OS=Synechocystis sp. (strain PCC 6803 / Kazusa) GN=sll0576	52,24	2,1	0,6	0,7	1,1	8,8E-01
P55175	Hydrolase sll0601 OS=Synechocystis sp. (strain PCC 6803 / Kazusa) G	8,82			1,3	1,3	2,0E+00

P74250	Hydroperoxy fatty acid reductase gpx1 OS=Synechocystis sp. (strain PC	16,57	0,3	0,5	0,5	0,4	5,5E-05
P73824	Hydroperoxy fatty acid reductase gpx2 OS=Synechocystis sp. (strain PC	31,82	0,4	1,3	1,0	0,9	5,0E-01
Q55552	IMP dehydrogenase OS=Synechocystis sp. (strain PCC 6803 / Kazusa)	20,65	0,7		1,0	0,8	2,1E-01
P73853	IMP dehydrogenase subunit OS=Synechocystis sp. (strain PCC 6803 / K	40,31	1,2	0,8	0,9	1,0	6,2E-01
Q55508	Indole-3-glycerol phosphate synthase OS=Synechocystis sp. (strain PCC	9,49	0,8		1,0	0,9	1,3E-01
P80507	Inorganic pyrophosphatase OS=Synechocystis sp. (strain PCC 6803 / K	55,03	0,5	1,8	1,5	1,3	7,9E-01
Q55274	Iron stress-induced chlorophyll-binding protein OS=Synechocystis sp. (s	2,34			0,1	0,1	2,0E+00
P72827	Iron uptake protein A1 OS=Synechocystis sp. (strain PCC 6803 / Kazusa	39,44	0,2	0,3	0,3	0,3	0,0E+00
Q55835	Iron uptake protein A2 OS=Synechocystis sp. (strain PCC 6803 / Kazusa	31,5	0,2	1,1	1,3	0,9	4,8E-01
P80046	Isocitrate dehydrogenase [NADP] OS=Synechocystis sp. (strain PCC 68	27,58	0,3	1,0	1,3	0,9	4,7E-01
P73679	Isopenicillin N epimerase OS=Synechocystis sp. (strain PCC 6803 / Kaz	34,91	0,6	2,2	1,6	1,4	5,8E-01
P74287	Isopentenyl-diphosphate delta-isomerase OS=Synechocystis sp. (strain	4,58			2,4	2,4	2,0E+00
P29107	Ketol-acid reductoisomerase OS=Synechocystis sp. (strain PCC 6803 / I	34,74	1,5	0,8	0,8	1,1	9,5E-01
P73553	Large-conductance mechanosensitive channel OS=Synechocystis sp. (s	14,48	1,2	0,8		1,0	9,5E-01
P73274	Leucine--tRNA ligase OS=Synechocystis sp. (strain PCC 6803 / Kazusa)	4,03	2,7	0,7	0,7	1,4	8,2E-01
Q59987	Light-dependent protochlorophyllide reductase OS=Synechocystis sp. (s	30,12	2,1	0,6	0,7	1,1	8,7E-01
P74518	Light-repressed protein A homolog OS=Synechocystis sp. (strain PCC 6	55,5	2,1	2,0	1,7	2,0	3,3E-32
Q57310	Lipid-A-disaccharide synthase OS=Synechocystis sp. (strain PCC 6803 /	8,63	1,9			1,9	2,0E+00
Q55828	LL-diaminopimelate aminotransferase OS=Synechocystis sp. (strain PCC	24,03	1,5	0,5	0,6	0,9	4,1E-01
Q55687	Long-chain acyl-[acyl-carrier-protein] reductase OS=Synechocystis sp. (s	7,06		1,2		1,2	2,0E+00
P73004	Long-chain-fatty-acid CoA ligase OS=Synechocystis sp. (strain PCC 680	5,89		0,7	0,6	0,6	1,8E-04
P73443	Lysine--tRNA ligase OS=Synechocystis sp. (strain PCC 6803 / Kazusa) (	4,9	2,2			2,2	2,0E+00
P72772	Magnesium-chelatase subunit ChlD OS=Synechocystis sp. (strain PCC	3,25	2,4			2,4	2,0E+00
P51634	Magnesium-chelatase subunit ChlI OS=Synechocystis sp. (strain PCC 6	15,41	1,7	1,2	1,4	1,4	1,4E-03
P72584	Magnesium-protoporphyrin IX monomethyl ester [oxidative] cyclase 1 OS	18,99	1,6	0,8	0,8	1,1	9,0E-01
Q55383	Malate dehydrogenase OS=Synechocystis sp. (strain PCC 6803 / Kazus	5,56	1,3	0,5	0,5	0,8	2,6E-01
P73377	Mannose-6-phosphate isomerase OS=Synechocystis sp. (strain PCC 68	18,6	0,7			0,7	2,0E+00
P74155	Membrane protein insertase YidC OS=Synechocystis sp. (strain PCC 68	5,21	1,7		0,6	1,1	9,5E-01
P74186	Membrane protein OS=Synechocystis sp. (strain PCC 6803 / Kazusa) G	19,07	0,7	0,7	0,8	0,7	3,2E-09
P73954	Membrane-associated protein slr1513 OS=Synechocystis sp. (strain PC	48,18	2,0	3,4	4,4	3,3	3,7E-07
Q55729	Methionine--tRNA ligase OS=Synechocystis sp. (strain PCC 6803 / Kazu	3,2		0,3		0,3	2,0E+00
Q55444	Methyl-accepting chemotaxis protein II OS=Synechocystis sp. (strain PC	11,04	0,9		1,7	1,3	5,4E-01
P73008	Methyl-accepting chemotaxis protein OS=Synechocystis sp. (strain PCC	8,52	1,5	0,6	0,7	0,9	5,7E-01
P72802	Mitochondrial outer membrane 72K protein OS=Synechocystis sp. (strai	10,15	0,7		1,7	1,2	8,6E-01
P74344	Molybdopterin biosynthesis MoeB protein OS=Synechocystis sp. (strain	28,32	2,2	1,5	1,2	1,6	1,3E-02
P74395	N utilization substance protein B homolog OS=Synechocystis sp. (strain	24,89	1,3	1,5	1,0	1,2	8,6E-02
P54899	N-acetyl-gamma-glutamyl-phosphate reductase OS=Synechocystis sp. (	14,81	1,1	0,4	0,6	0,7	1,2E-01
P73736	N-acetylmuramoyl-L-alanine amidase OS=Synechocystis sp. (strain PCC	5,86	0,7	1,3	1,9	1,3	4,4E-01
Q55498	N5-carboxyaminoimidazole ribonucleotide mutase OS=Synechocystis sp	17,61	0,6			0,6	2,0E+00
P26522	NAD(P)H-quinone oxidoreductase subunit 1 OS=Synechocystis sp. (stra	10,75	2,3	0,8	0,8	1,3	6,7E-01
P27724	NAD(P)H-quinone oxidoreductase subunit H OS=Synechocystis sp. (stra	23,1	2,5	1,2	0,8	1,5	3,8E-01
P26525	NAD(P)H-quinone oxidoreductase subunit I OS=Synechocystis sp. (strai	50,78	1,4	1,4	1,0	1,2	5,3E-02
P19125	NAD(P)H-quinone oxidoreductase subunit J OS=Synechocystis sp. (stra	23,46	0,9	1,4	1,0	1,1	5,7E-01
P19050	NAD(P)H-quinone oxidoreductase subunit K 1 OS=Synechocystis sp. (st	23,79	1,4	1,1	0,9	1,1	4,6E-01
P74338	NAD(P)H-quinone oxidoreductase subunit M OS=Synechocystis sp. (stra	23,97	0,4	1,4	1,0	0,9	6,1E-01
P74771	NAD(P)H-quinone oxidoreductase subunit O OS=Synechocystis sp. (stra	40,28	0,8	1,0	0,8	0,8	6,7E-03
P73735	NADH dehydrogenase OS=Synechocystis sp. (strain PCC 6803 / Kazus	4,95			0,7	0,7	2,0E+00
P72762	NADH-glutamate synthase small subunit OS=Synechocystis sp. (strain F	8,3		1,0	1,1	1,1	2,9E-01
P54386	NADP-specific glutamate dehydrogenase OS=Synechocystis sp. (strain	11,21	1,2	0,6		0,9	6,3E-01
P72781	NarL subfamily OS=Synechocystis sp. (strain PCC 6803 / Kazusa) GN=s	13,48		2,2	1,3	1,8	4,0E-02
P73104	NarL subfamily OS=Synechocystis sp. (strain PCC 6803 / Kazusa) GN=s	17,84	1,0		1,3	1,1	3,4E-01
P74390	Negative aliphatic amidase regulator OS=Synechocystis sp. (strain PCC	41,48	1,2	4,3	2,6	2,7	2,1E-02
P73757	Neopullulanase OS=Synechocystis sp. (strain PCC 6803 / Kazusa) GN=	11,42	0,4	1,4	1,2	1,0	6,8E-01
Q55602	NifS protein OS=Synechocystis sp. (strain PCC 6803 / Kazusa) GN=nifS	26,68	1,0	1,5	1,3	1,2	7,6E-02
P73448	Nitrate reductase OS=Synechocystis sp. (strain PCC 6803 / Kazusa) GN	4,76	1,0	1,9		1,4	3,6E-01
P73450	Nitrate transport ATP-binding protein NrtC OS=Synechocystis sp. (strain	2,69		1,1		1,1	2,0E+00
P73452	Nitrate transport protein NrtA OS=Synechocystis sp. (strain PCC 6803 / I	38,12	0,7	1,5	1,4	1,2	6,2E-01
P73449	Nitrate transport protein NrtD OS=Synechocystis sp. (strain PCC 6803 /	11,45		0,7	0,7	0,7	0,0E+00
Q55247	Nitrogen regulatory protein P-II OS=Synechocystis sp. (strain PCC 6803	70,54	0,5	0,8	0,8	0,7	2,0E-02
Q6ZEQ0	Non-heme chloroperoxidase OS=Synechocystis sp. (strain PCC 6803 / K	31,02	0,7	1,9	1,5	1,4	5,1E-01
P73057	Nucleoid-associated protein slr1847 OS=Synechocystis sp. (strain PCC	23,68	0,7	1,7	1,4	1,3	4,7E-01
P74494	Nucleoside diphosphate kinase OS=Synechocystis sp. (strain PCC 6803	34,23	0,6	0,6	0,5	0,6	3,6E-12
P74571	Oligopeptidase A OS=Synechocystis sp. (strain PCC 6803 / Kazusa) GN	6,45	1,2	0,7	2,3	1,4	5,6E-01
P74314	OmpR subfamily OS=Synechocystis sp. (strain PCC 6803 / Kazusa) GN	18,8			0,9	0,9	2,0E+00
P74102	Orange carotenoid-binding protein OS=Synechocystis sp. (strain PCC 6	51,74	1,5	0,7	0,5	0,9	5,0E-01
Q55497	Ornithine carbamoyltransferase OS=Synechocystis sp. (strain PCC 6803	8,77	1,7	0,7	0,6	1,0	7,6E-01
P72848	Oxygen-dependent coproporphyrinogen-III oxidase OS=Synechocystis s	40,88	0,6	2,6	2,3	1,8	4,0E-01
P74707	Peptide chain release factor 1 OS=Synechocystis sp. (strain PCC 6803 /	8,22	0,9	1,0	1,1	1,0	7,7E-01
P72622	Peptide methionine sulfoxide reductase MsrA 1 OS=Synechocystis sp. (s	9,46	1,4		2,1	1,7	1,3E-02
P73037	Peptidyl-prolyl cis-trans isomerase OS=Synechocystis sp. (strain PCC 68	17,91	0,6	1,9	1,8	1,4	5,4E-01
P73789	Peptidyl-prolyl cis-trans isomerase slr1251 OS=Synechocystis sp. (strain	8,77			1,0	1,0	2,0E+00
Q55387	Periplasmic binding protein of ABC transporter for natural amino acids O	19,38	0,8	1,3	1,3	1,1	6,0E-01
Q55187	Phenylalanine--tRNA ligase alpha subunit OS=Synechocystis sp. (strain	13,29	2,6	1,1	0,7	1,5	5,4E-01
P74296	Phenylalanine--tRNA ligase beta subunit OS=Synechocystis sp. (strain F	8,4	2,0	0,7	0,7	1,1	9,9E-01
Q55196	Phosphate import ATP-binding protein PstB 1 OS=Synechocystis sp. (str	25,65	0,7	0,3	0,4	0,5	4,2E-03
Q55198	Phosphate transport system permease protein PstC OS=Synechocystis	5,05		0,3	0,4	0,3	0,0E+00
P73785	Phosphate-binding protein OS=Synechocystis sp. (strain PCC 6803 / Ka	54,35	0,4	0,1	0,2	0,2	2,9E-04
Q55199	Phosphate-binding protein OS=Synechocystis sp. (strain PCC 6803 / Ka	31,33	0,5	0,5	0,8	0,6	1,2E-03
P74299	Phosphoenolpyruvate carboxylase OS=Synechocystis sp. (strain PCC 6	9,96	1,2	1,0	0,8	1,0	9,7E-01
Q55905	Phosphoenolpyruvate synthase OS=Synechocystis sp. (strain PCC 6803	3,42	2,1			2,1	2,0E+00
P74643	Phosphoglucomutase OS=Synechocystis sp. (strain PCC 6803 / Kazusa	9,52	1,0	0,4	0,3	0,6	6,5E-02
P74421	Phosphoglycerate kinase OS=Synechocystis sp. (strain PCC 6803 / Kaz	40,65	0,9	0,6	0,4	0,7	3,6E-02
Q55894	Phosphomethylpyrimidine synthase OS=Synechocystis sp. (strain PCC 6	12,85	1,0	1,1	1,1	1,1	3,9E-07
P74232	Phosphoribosylamine--glycine ligase OS=Synechocystis sp. (strain PCC	4,3			1,1	1,1	2,0E+00
P73471	Phosphoribosylaminoimidazole-succinocarboxamide synthase OS=Syne	9,47	2,3	2,3	1,2	1,9	5,2E-03
Q55843	Phosphoribosylformylglycinamidine synthase 1 OS=Synechocystis sp. (s	15,18	1,0			1,0	2,0E+00
P72644	Phosphoribosylformylglycinamidine synthase 2 OS=Synechocystis sp. (s	4,3			1,0	1,0	2,0E+00
P37101	Phosphoribulokinase OS=Synechocystis sp. (strain PCC 6803 / Kazusa)	43,98	1,2	0,7	0,7	0,8	2,6E-01
P73546	Phosphorylase OS=Synechocystis sp. (strain PCC 6803 / Kazusa) GN=g	12,16	1,7	0,4	0,7	0,9	5,0E-01
P74063	Photosystem I assembly protein Ycf3 OS=Synechocystis sp. (strain PCC	14,45			1,0	1,0	2,0E+00
P72705	Photosystem I assembly protein Ycf4 OS=Synechocystis sp. (strain PCC	10,64			0,8	0,8	2,0E+00
P72966	Photosystem I biogenesis protein BtpA OS=Synechocystis sp. (strain PC	16,38			1,3	1,3	2,0E+00
P32422	Photosystem I iron-sulfur center OS=Synechocystis sp. (strain PCC 680	65,43	0,8	0,9	1,0	0,9	1,6E-01
P29254	Photosystem I P700 chlorophyll a apoprotein A1 OS=Synechocystis sp. (	13,72	2,0	0,8	0,7	1,2	8,7E-01
P29255	Photosystem I P700 chlorophyll a apoprotein A2 OS=Synechocystis sp. (	17,65	2,1	0,8	0,8	1,2	8,3E-01
P19569	Photosystem I reaction center subunit II OS=Synechocystis sp. (strain P	52,48	0,8	0,8	1,0	0,9	4,0E-02
P29256	Photosystem I reaction center subunit III OS=Synechocystis sp. (strain F	56,36	0,9	1,2	0,9	1,0	8,7E-01
P12975	Photosystem I reaction center subunit IV OS=Synechocystis sp. (strain F	41,89	0,3	0,3	0,9	0,5	2,0E-02
P37277	Photosystem I reaction center subunit XI OS=Synechocystis sp. (strain F	26,11	1,1	0,9	1,0	1,0	7,5E-01
Q55332	Photosystem II 12 kDa extrinsic protein OS=Synechocystis sp. (strain PC	25,19	0,5			0,5	2,0E+00
P09193	Photosystem II 44 kDa reaction center protein OS=Synechocystis sp. (st	17,17	2,0	0,7	0,7	1,1	8,8E-01
P05429	Photosystem II CP47 chlorophyll apoprotein OS=Synechocystis sp. (stra	22,09	1,8	0,6	0,8	1,0	8,2E-01
P09192	Photosystem II D2 protein OS=Synechocystis sp. (strain PCC 6803 / Ka	10,8	1,4	0,5	0,7	0,9	4,4E-01
P74367	Photosystem II lipoprotein Psb27 OS=Synechocystis sp. (strain PCC 680	35,82	0,7	1,1	1,1	1,0	6,5E-01
P10549	Photosystem II manganese-stabilizing polypeptide OS=Synechocystis sp	27,01	0,6	0,5	1,2	0,8	1,7E-01
Q55354	Photosystem II reaction center protein L OS=Synechocystis sp. (strain P	33,33			1,2	1,2	2,0E+00
Q55356	Photosystem II reaction center Psb28 protein OS=Synechocystis sp. (str	16,07			1,4	1,4	2,0E+00
P16033	Photosystem Q(B) protein 2 OS=Synechocystis sp. (strain PCC 6803 / K	16,67	1,4	0,6	0,7	0,9	4,8E-01
Q55544	Phycobiliprotein ApcE OS=Synechocystis sp. (strain PCC 6803 / Kazusa	48,21	2,1	0,6	0,6	1,1	8,4E-01
P73203	Phycobilisome 32.1 kDa linker polypeptide, phycocyanin-associated, rod	66,32	1,4	0,5	0,6	0,8	3,2E-01
P73204	Phycobilisome 32.1 kDa linker polypeptide, phycocyanin-associated, rod	58,97	1,0	0,4	0,4	0,6	5,8E-02
Q01950	Phycobilisome 7.8 kDa linker polypeptide, allophycocyanin-associated, c	50,75	1,3	0,6	0,6	0,8	2,8E-01
P73202	Phycobilisome 8.9 kDa linker polypeptide, phycocyanin-associated, rod (	81,93	0,6	0,2	0,4	0,4	1,8E-04
P73093	Phycobilisome rod-core linker polypeptide CpcG OS=Synechocystis sp.	59,57	1,7	0,6	0,7	1,0	7,4E-01
P74625	Phycobilisome rod-core linker polypeptide CpcG OS=Synechocystis sp.	24,1	0,9	0,4	0,4	0,6	1,8E-02
Q55891	Phycocyanobilin:ferredoxin oxidoreductase OS=Synechocystis sp. (strai	8,06			0,7	0,7	2,0E+00
Q55168	Phytochrome-like protein cph1 OS=Synechocystis sp. (strain PCC 6803	4,81	2,5	0,5	0,8	1,3	9,9E-01
P73173	PilJ protein OS=Synechocystis sp. (strain PCC 6803 / Kazusa) GN=pilJ I	9,55	1,0	0,5	0,6	0,7	7,9E-02
P21697	Plastocyanin OS=Synechocystis sp. (strain PCC 6803 / Kazusa) GN=pe	44,44	0,7	1,3	1,4	1,1	7,0E-01
P73390	Poly(3-hydroxyalkanoate) synthase OS=Synechocystis sp. (strain PCC 6	7,14	0,5	0,2	0,5	0,4	1,5E-03
Q55898	Polyphosphate kinase OS=Synechocystis sp. (strain PCC 6803 / Kazusa	9,07		1,3	0,9	1,1	5,0E-01
P72659	Polyribonucleotide nucleotidyltransferase OS=Synechocystis sp. (strain I	21,73	2,3	0,7	0,6	1,2	9,7E-01
P73660	Porphobilinogen deaminase OS=Synechocystis sp. (strain PCC 6803 / K	13,44	1,4	1,4	1,1	1,3	4,7E-03
P73866	Potassium-transporting ATPase A chain OS=Synechocystis sp. (strain PC	4,84	0,2	0,0	0,1	0,1	1,7E-08
P73867	Potassium-transporting ATPase B chain OS=Synechocystis sp. (strain P	15,36	0,1	0,1	0,1	0,1	0,0E+00
P73547	Precorrin decarboxylase OS=Synechocystis sp. (strain PCC 6803 / Kazu	11,01	0,7			0,7	2,0E+00
P73849	Probable 2-phosphosulfolactate phosphatase OS=Synechocystis sp. (str	9,54	2,0			2,0	2,0E+00
Q55385	Probable 30S ribosomal protein PSRP-3 OS=Synechocystis sp. (strain F	65,18	0,9	2,5	1,8	1,8	1,1E-01
P72940	Probable adenylyl-sulfate kinase OS=Synechocystis sp. (strain PCC 680	31,64	0,8	0,8	0,8	0,8	7,4E-11
P73270	Probable agmatinase 2 OS=Synechocystis sp. (strain PCC 6803 / Kazus	17,18	0,4	2,2	2,4	1,7	6,2E-01
P74467	Probable ATP-dependent Clp protease proteolytic subunit 3 OS=Synech	39,11	1,0	0,6	0,5	0,7	5,2E-02
Q55791	Probable ATP-dependent transporter slr0075 OS=Synechocystis sp. (str	12,11			0,9	0,9	2,0E+00
P54691	Probable branched-chain-amino-acid aminotransferase OS=Synechocys	64,92	0,9	4,1	2,3	2,4	1,3E-01
P73971	Probable cytosol aminopeptidase OS=Synechocystis sp. (strain PCC 68	9,96	3,4	0,7		2,0	6,3E-01
P54902	Probable gamma-glutamyl phosphate reductase OS=Synechocystis sp. (	6,7	1,8			1,8	2,0E+00
P72623	Probable glycogen synthase 2 OS=Synechocystis sp. (strain PCC 6803	17,72	1,6	0,6	0,6	0,9	4,9E-01
P72704	Probable peptidyl-prolyl cis-trans isomerase sll0227 OS=Synechocystis s	26,42	1,0	2,2	1,7	1,7	4,3E-02
P74690	Probable phosphoketolase OS=Synechocystis sp. (strain PCC 6803 / Ka	18,14	1,8	0,4	0,6	0,9	5,3E-01
P73123	Probable RuBisCO transcriptional regulator OS=Synechocystis sp. (strai	6,96			0,8	0,8	2,0E+00
P73157	Probable signal peptidase I-2 OS=Synechocystis sp. (strain PCC 6803 /	12,39			1,9	1,9	2,0E+00
Q55585	Probable succinate-semialdehyde dehydrogenase [NADP(+)] OS=Synec	17,62	0,8	1,5	1,2	1,2	3,8E-01
P73942	Proline--tRNA ligase OS=Synechocystis sp. (strain PCC 6803 / Kazusa)	10,12	2,1	1,0	0,8	1,3	5,2E-01
P74305	Protease OS=Synechocystis sp. (strain PCC 6803 / Kazusa) GN=pqqE I	4,96	0,6		2,0	1,3	8,5E-01
Q55233	Protein DrgA OS=Synechocystis sp. (strain PCC 6803 / Kazusa) GN=drg	39,05	0,5	0,5	0,8	0,6	1,3E-03
Q59978	Protein GrpE OS=Synechocystis sp. (strain PCC 6803 / Kazusa) GN=grp	30,92	0,6	0,8	1,3	0,9	5,2E-01
P37979	Protein IcfG OS=Synechocystis sp. (strain PCC 6803 / Kazusa) GN=icfG	2,84			0,5	0,5	2,0E+00
P73176	Protein PatA OS=Synechocystis sp. (strain PCC 6803 / Kazusa) GN=sll1	2,99		0,3		0,3	2,0E+00
P74737	Protein RecA OS=Synechocystis sp. (strain PCC 6803 / Kazusa) GN=re	7,91	2,5	1,2	0,8	1,5	3,7E-01
Q55200	Protein SphX OS=Synechocystis sp. (strain PCC 6803 / Kazusa) GN=sp	17,26	0,6	0,4	0,4	0,5	2,9E-10

Q55709	Protein translocase subunit SecA OS=Synechocystis sp. (strain PCC 68	11,16	2,1	0,7	0,6	1,1	8,7E-01
P38382	Protein translocase subunit SecE OS=Synechocystis sp. (strain PCC 68	23,46			1,0	1,0	2,0E+00
P77964	Protein translocase subunit SecY OS=Synechocystis sp. (strain PCC 68	6,11	2,3		1,4	1,8	1,7E-02
P74466	Putative ATP-dependent Clp protease proteolytic subunit-like OS=Synec	20	1,0	0,6	0,6	0,7	4,4E-02
P72941	Putative biopolymer transport protein ExbB-like 2 OS=Synechocystis sp.	10,57	1,5	0,5	0,6	0,9	5,0E-01
P73163	Putative carboxymethylenebutenolidase OS=Synechocystis sp. (strain P	34,55	0,8	1,7	2,0	1,5	1,8E-01
P74220	Putative carboxypeptidase slr1534 OS=Synechocystis sp. (strain PCC 6	13,62		1,6	1,4	1,5	1,1E-09
P74373	Putative diflavin flavoprotein A 3 OS=Synechocystis sp. (strain PCC 680	5,19			0,8	0,8	2,0E+00
Q55445	Putative methyl-accepting chemotaxis protein sll0041 OS=Synechocystis	6,2	2,5	0,9	0,8	1,4	5,8E-01
P73720	Putative OxPP cycle protein OpcA OS=Synechocystis sp. (strain PCC 68	6,95	1,4	0,5	0,6	0,8	3,2E-01
P73728	Putative peroxiredoxin sll1621 OS=Synechocystis sp. (strain PCC 6803	60,32	0,6	1,1	1,1	0,9	6,5E-01
P73790	Putative pterin-4-alpha-carbinolamine dehydratase OS=Synechocystis s	34,38	0,6			0,6	2,0E+00
P73623	Putative quercetin 2,3-dioxygenase sll1773 OS=Synechocystis sp. (strai	11,21	1,0			1,0	2,0E+00
P74060	Putative sulfur carrier protein slr0821 OS=Synechocystis sp. (strain PCC	20,18	1,0		2,1	1,6	2,9E-01
Q55118	Putative thylakoid lumen peptidyl-prolyl cis-trans isomerase sll0408 OS=	19	0,7	1,2	1,3	1,1	7,6E-01
P72776	Pyridoxine 5'-phosphate synthase OS=Synechocystis sp. (strain PCC 68	49,17	0,8	2,5	1,6	1,6	2,8E-01
P74211	Pyridoxine/pyridoxamine 5'-phosphate oxidase OS=Synechocystis sp. (s	18,22	0,7	0,7		0,7	0,0E+00
P74572	Pyrroline-5-carboxylate reductase OS=Synechocystis sp. (strain PCC 68	8,61			1,1	1,1	2,0E+00
P73405	Pyruvate dehydrogenase E1 beta subunit OS=Synechocystis sp. (strain	17,9	2,1	0,7	0,6	1,1	9,6E-01
P74490	Pyruvate dehydrogenase E1 component, alpha subunit OS=Synechocys	19,88	1,7	0,9	1,2	1,3	2,5E-01
Q55863	Pyruvate kinase 1 OS=Synechocystis sp. (strain PCC 6803 / Kazusa) G	5,59		0,6		0,6	2,0E+00
P73534	Pyruvate kinase 2 OS=Synechocystis sp. (strain PCC 6803 / Kazusa) G	13,03		0,7	0,7	0,7	0,0E+00
P73348	Rehydrin OS=Synechocystis sp. (strain PCC 6803 / Kazusa) GN=slr119	48,82	0,7	1,6	1,2	1,2	6,6E-01
P74124	Renin-binding protein OS=Synechocystis sp. (strain PCC 6803 / Kazusa	5,37	2,0			2,0	2,0E+00
Q55641	Ribonuclease D OS=Synechocystis sp. (strain PCC 6803 / Kazusa) GN=	13,36	2,0		0,5	1,3	9,4E-01
P72656	Ribonuclease E/G-like protein OS=Synechocystis sp. (strain PCC 6803 /	11,28	2,5	1,0	1,0	1,5	3,2E-01
P54123	Ribonuclease J OS=Synechocystis sp. (strain PCC 6803 / Kazusa) GN=	13,91	3,3	0,9	0,7	1,6	5,7E-01
Q55766	Ribose-5-phosphate isomerase A OS=Synechocystis sp. (strain PCC 68	16,6	0,6	0,7	0,9	0,7	8,2E-03
Q55848	Ribose-phosphate pyrophosphokinase OS=Synechocystis sp. (strain PC	11,71	1,2	0,4	0,6	0,7	1,9E-01
P73886	Ribosome-binding ATPase YchF OS=Synechocystis sp. (strain PCC 680	19,83	1,8	0,6	0,8	1,0	8,2E-01
Q55625	Ribosome-binding factor A OS=Synechocystis sp. (strain PCC 6803 / Ka	30,08	1,1		2,2	1,7	1,9E-01
P74456	Ribosome-recycling factor OS=Synechocystis sp. (strain PCC 6803 / Ka	36,26	0,6	0,8	1,6	1,0	7,3E-01
P54205	Ribulose bisphosphate carboxylase large chain OS=Synechocystis sp. (	49,15	3,2	1,6	1,3	2,1	1,8E-02
P54206	Ribulose bisphosphate carboxylase small chain OS=Synechocystis sp. (	61,95	1,1	2,3	1,6	1,7	3,2E-02
P74061	Ribulose-phosphate 3-epimerase OS=Synechocystis sp. (strain PCC 68	43,04	0,4	2,1	1,4	1,3	8,5E-01
P74565	RNA polymerase sigma factor SigA OS=Synechocystis sp. (strain PCC 6	8	1,6	1,2	1,2	1,3	2,2E-03
P73557	RNA-binding protein OS=Synechocystis sp. (strain PCC 6803 / Kazusa)	14,46	1,0	1,9	1,5	1,4	8,1E-02
P73862	Rubisco operon transcriptional regulator OS=Synechocystis sp. (strain P	14,24	0,4	0,2	0,4	0,3	2,0E-05
P73068	Rubredoxin OS=Synechocystis sp. (strain PCC 6803 / Kazusa) GN=rub	20,87	1,4			1,4	2,0E+00
P72871	S-adenosylmethionine synthase OS=Synechocystis sp. (strain PCC 680	38,5	0,4	2,0	1,2	1,2	9,5E-01
P0DJF8	S-methyl-5'-thioadenosine phosphorylase OS=Synechocystis sp. (strain	30,98	0,9	4,1	2,4	2,5	1,0E-01
P73010	Sec-independent protein translocase protein TatA OS=Synechocystis sp	17,46		1,3		1,3	2,0E+00
Q55788	Sensory transduction histidine kinase OS=Synechocystis sp. (strain PC	21,59	0,8	1,0	1,0	0,9	2,7E-01
Q55177	Sensory transduction histidine kinase OS=Synechocystis sp. (strain PC	3,85	1,2			1,2	2,0E+00
Q55900	Septum site-determining protein MinD OS=Synechocystis sp. (strain PC	25,94	1,2	0,7	0,6	0,8	2,8E-01
P77962	Serine hydroxymethyltransferase OS=Synechocystis sp. (strain PCC 68	19,67	1,9	0,6	0,5	1,0	6,9E-01
P73201	Serine--tRNA ligase OS=Synechocystis sp. (strain PCC 6803 / Kazusa)	7,91	3,5		1,1	2,3	2,6E-01
P74591	Shikimate dehydrogenase OS=Synechocystis sp. (strain PCC 6803 / Ka	16,21	1,2	1,5		1,3	2,2E-02
P73991	Short-chain alcohol dehydrogenase family OS=Synechocystis sp. (strain	6,02			0,9	0,9	2,0E+00
P74214	Signal recognition particle protein OS=Synechocystis sp. (strain PCC 68	4,56			1,4	1,4	2,0E+00
P73930	Signal recognition particle receptor FtsY OS=Synechocystis sp. (strain P	3,37			1,3	1,3	2,0E+00
Q55499	Single-stranded DNA-binding protein 1 OS=Synechocystis sp. (strain PC	69,42	0,7	2,5	2,3	1,8	2,9E-01
Q55658	Sll0023 protein OS=Synechocystis sp. (strain PCC 6803 / Kazusa) GN=	5,37	1,5		1,0	1,2	3,5E-01
Q55162	Sll0051 protein OS=Synechocystis sp. (strain PCC 6803 / Kazusa) GN=	11,32		1,3	1,6	1,4	9,6E-04
Q55140	Sll0066 protein OS=Synechocystis sp. (strain PCC 6803 / Kazusa) GN=	2,54		0,8		0,8	2,0E+00
Q55138	Sll0068 protein OS=Synechocystis sp. (strain PCC 6803 / Kazusa) GN=	1,53		1,1		1,1	2,0E+00
Q55134	Sll0071 protein OS=Synechocystis sp. (strain PCC 6803 / Kazusa) GN=	6,31	1,7			1,7	2,0E+00
P74453	Sll0148 protein OS=Synechocystis sp. (strain PCC 6803 / Kazusa) GN=	3,67	1,8	0,8	0,9	1,2	7,5E-01
Q55564	Sll0162 protein OS=Synechocystis sp. (strain PCC 6803 / Kazusa) GN=	10,31	0,9			0,9	2,0E+00
Q55558	Sll0172 protein OS=Synechocystis sp. (strain PCC 6803 / Kazusa) GN=	21,05	0,3	1,3		0,8	4,9E-01
Q55776	Sll0180 protein OS=Synechocystis sp. (strain PCC 6803 / Kazusa) GN=	11,98	1,0	1,0	0,7	0,9	2,6E-01
Q55770	Sll0185 protein OS=Synechocystis sp. (strain PCC 6803 / Kazusa) GN=	9,79	1,1	0,8	0,7	0,9	2,1E-01
Q55762	Sll0188 protein OS=Synechocystis sp. (strain PCC 6803 / Kazusa) GN=	9,38		1,6		1,6	2,0E+00
P72707	Sll0224 protein OS=Synechocystis sp. (strain PCC 6803 / Kazusa) GN=	17,45	0,5	1,4	1,7	1,2	9,3E-01
P73902	Sll0236 protein OS=Synechocystis sp. (strain PCC 6803 / Kazusa) GN=	22,58	0,9	3,3	2,8	2,4	6,7E-02
P74394	Sll0272 protein OS=Synechocystis sp. (strain PCC 6803 / Kazusa) GN=	35,67		1,4	1,3	1,4	2,3E-06
Q55648	Sll0314 protein OS=Synechocystis sp. (strain PCC 6803 / Kazusa) GN=	32,8	1,2	3,7	3,2	2,7	1,8E-02
P74426	Sll0359 protein OS=Synechocystis sp. (strain PCC 6803 / Kazusa) GN=	42,58	1,0	0,7	0,6	0,8	6,0E-02
Q55734	Sll0395 protein OS=Synechocystis sp. (strain PCC 6803 / Kazusa) GN=	32,08	1,0	0,6	0,5	0,7	7,9E-02
P74685	Sll0443 protein OS=Synechocystis sp. (strain PCC 6803 / Kazusa) GN=	13,01		5,5	1,8	3,6	3,7E-02
Q55184	Sll0456 protein OS=Synechocystis sp. (strain PCC 6803 / Kazusa) GN=	5,41	2,1			2,1	2,0E+00
Q55847	Sll0470 protein OS=Synechocystis sp. (strain PCC 6803 / Kazusa) GN=	19,35	0,4	1,9	1,5	1,3	9,1E-01
Q55496	Sll0493 protein OS=Synechocystis sp. (strain PCC 6803 / Kazusa) GN=	8,33		0,9	1,4	1,2	5,4E-01
Q55492	Sll0497 protein OS=Synechocystis sp. (strain PCC 6803 / Kazusa) GN=	8			1,1	1,1	2,0E+00
Q55466	Sll0518 protein OS=Synechocystis sp. (strain PCC 6803 / Kazusa) GN=	16,36	2,2	1,0	1,0	1,4	3,3E-01
Q55517	Sll0529 protein OS=Synechocystis sp. (strain PCC 6803 / Kazusa) GN=	36,25	0,5	0,7	0,5	0,6	7,4E-09
Q55410	Sll0540 protein OS=Synechocystis sp. (strain PCC 6803 / Kazusa) GN=	5,21	0,5	0,8	0,5	0,6	1,1E-04
Q55390	Sll0553 protein OS=Synechocystis sp. (strain PCC 6803 / Kazusa) GN=	25,51	2,0	0,6	0,8	1,2	9,5E-01
Q55865	Sll0585 protein OS=Synechocystis sp. (strain PCC 6803 / Kazusa) GN=	11,11	0,5	2,1	1,5	1,4	7,2E-01
Q55862	Sll0588 protein OS=Synechocystis sp. (strain PCC 6803 / Kazusa) GN=	11,11	0,4	1,5		1,0	7,3E-01
Q55852	Sll0596 protein OS=Synechocystis sp. (strain PCC 6803 / Kazusa) GN=	22,54		1,8	1,6	1,7	3,7E-16
Q55726	Sll0602 protein OS=Synechocystis sp. (strain PCC 6803 / Kazusa) GN=	4,92		0,5		0,5	2,0E+00
Q55723	Sll0606 protein OS=Synechocystis sp. (strain PCC 6803 / Kazusa) GN=	4,41		0,8		0,8	2,0E+00
P72952	Sll0645 protein OS=Synechocystis sp. (strain PCC 6803 / Kazusa) GN=	9,02			2,2	2,2	2,0E+00
P72950	Sll0647 protein OS=Synechocystis sp. (strain PCC 6803 / Kazusa) GN=	9,77	0,6	3,1	1,7	1,8	4,1E-01
Q55984	Sll0659 protein OS=Synechocystis sp. (strain PCC 6803 / Kazusa) GN=	3,26		0,7		0,7	2,0E+00
Q55966	Sll0676 protein OS=Synechocystis sp. (strain PCC 6803 / Kazusa) GN=	12,22	0,8			0,8	2,0E+00
P74630	Sll0735 protein OS=Synechocystis sp. (strain PCC 6803 / Kazusa) GN=	7,94	1,1			1,1	2,0E+00
Q55953	Sll0781 protein OS=Synechocystis sp. (strain PCC 6803 / Kazusa) GN=	45,81	0,4	1,2	0,9	0,8	3,8E-01
Q55943	Sll0788 protein OS=Synechocystis sp. (strain PCC 6803 / Kazusa) GN=	12,76		0,9		0,9	2,0E+00
Q55432	Sll0822 protein OS=Synechocystis sp. (strain PCC 6803 / Kazusa) GN=	31,78	1,5	0,8	0,7	1,0	8,8E-01
P73762	Sll0837 protein OS=Synechocystis sp. (strain PCC 6803 / Kazusa) GN=	15,99	0,6		1,2	0,9	6,0E-01
P73746	Sll0854 protein OS=Synechocystis sp. (strain PCC 6803 / Kazusa) GN=	18,18	0,3	1,0	0,9	0,7	2,3E-01
Q55361	Sll0887 protein OS=Synechocystis sp. (strain PCC 6803 / Kazusa) GN=	27,13	0,7	2,2	1,4	1,4	4,1E-01
Q55360	Sll0888 protein OS=Synechocystis sp. (strain PCC 6803 / Kazusa) GN=	16,84	0,5	1,3	1,2	1,0	8,7E-01
P73130	Sll0995 protein OS=Synechocystis sp. (strain PCC 6803 / Kazusa) GN=	11,69			1,2	1,2	2,0E+00
P72756	Sll1033 protein OS=Synechocystis sp. (strain PCC 6803 / Kazusa) GN=	2,54	1,9	0,5	0,5	1,0	6,1E-01
P72751	Sll1036 protein OS=Synechocystis sp. (strain PCC 6803 / Kazusa) GN=	17,81	0,7	1,8	1,9	1,5	3,4E-01
P72653	Sll1049 protein OS=Synechocystis sp. (strain PCC 6803 / Kazusa) GN=	6,38	2,3	1,0		1,6	3,2E-01
P72647	Sll1053 protein OS=Synechocystis sp. (strain PCC 6803 / Kazusa) GN=	7,31	0,9	2,4	2,2	1,8	1,2E-01
P72646	Sll1054 protein OS=Synechocystis sp. (strain PCC 6803 / Kazusa) GN=	9,09	0,6	2,7		1,7	7,3E-01
P72631	Sll1064 protein OS=Synechocystis sp. (strain PCC 6803 / Kazusa) GN=	16,37	0,5	2,5	1,9	1,7	4,9E-01
P73280	Sll1072 protein OS=Synechocystis sp. (strain PCC 6803 / Kazusa) GN=	10,76	0,7	1,8	1,8	1,5	3,3E-01
P73267	Sll1080 protein OS=Synechocystis sp. (strain PCC 6803 / Kazusa) GN=	16,19		1,4	1,2	1,3	2,0E-02
P74251	Sll1089 protein OS=Synechocystis sp. (strain PCC 6803 / Kazusa) GN=	15,49	0,5	2,0	2,3	1,6	6,3E-01
P74720	Sll1106 protein OS=Synechocystis sp. (strain PCC 6803 / Kazusa) GN=	49,12	1,2	1,7	1,5	1,4	2,3E-04
P74719	Sll1107 protein OS=Synechocystis sp. (strain PCC 6803 / Kazusa) GN=	8,91	1,9			1,9	2,0E+00
P73488	Sll1130 protein OS=Synechocystis sp. (strain PCC 6803 / Kazusa) GN=	46,96	0,5	0,2	0,9	0,5	5,0E-02
P73793	Sll1150 protein OS=Synechocystis sp. (strain PCC 6803 / Kazusa) GN=	7,14			1,1	1,1	2,0E+00
P72845	Sll1188 protein OS=Synechocystis sp. (strain PCC 6803 / Kazusa) GN=	21,34	0,9	1,0	1,1	1,0	8,0E-01
P74001	Sll1239 protein OS=Synechocystis sp. (strain PCC 6803 / Kazusa) GN=	12,36			5,0	5,0	2,0E+00
P74073	Sll1258 protein OS=Synechocystis sp. (strain PCC 6803 / Kazusa) GN=	14,51	1,1			1,1	2,0E+00
P73541	Sll1273 protein OS=Synechocystis sp. (strain PCC 6803 / Kazusa) GN=	4,85		1,2		1,2	2,0E+00
P73598	Sll1305 protein OS=Synechocystis sp. (strain PCC 6803 / Kazusa) GN=	8,47	0,4	0,2	0,2	0,2	6,1E-11
P73597	Sll1306 protein OS=Synechocystis sp. (strain PCC 6803 / Kazusa) GN=	39,4	0,1	0,5	0,7	0,4	4,9E-02
P73596	Sll1307 protein OS=Synechocystis sp. (strain PCC 6803 / Kazusa) GN=	41,71	0,1	0,5	0,6	0,4	1,7E-02
P73591	Sll1308 protein OS=Synechocystis sp. (strain PCC 6803 / Kazusa) GN=	8,87			1,0	1,0	2,0E+00
P73588	Sll1315 protein OS=Synechocystis sp. (strain PCC 6803 / Kazusa) GN=	4,83	0,6	0,9	1,1	0,9	3,3E-01
P74535	Sll1336 protein OS=Synechocystis sp. (strain PCC 6803 / Kazusa) GN=	11,06	2,2		0,6	1,4	7,8E-01
P74534	Sll1338 protein OS=Synechocystis sp. (strain PCC 6803 / Kazusa) GN=	17,11	0,9	0,5		0,7	2,0E-01
P73510	Sll1358 protein OS=Synechocystis sp. (strain PCC 6803 / Kazusa) GN=	32,74	0,3	0,5	0,8	0,6	3,3E-02
P74162	Sll1380 protein OS=Synechocystis sp. (strain PCC 6803 / Kazusa) GN=	38,79	0,7	1,7	1,2	1,2	5,8E-01
P74145	Sll1390 protein OS=Synechocystis sp. (strain PCC 6803 / Kazusa) GN=	20,88	1,1	1,6	0,9	1,2	3,5E-01
P73439	Sll1461 protein OS=Synechocystis sp. (strain PCC 6803 / Kazusa) GN=	9,09	0,8			0,8	2,0E+00
P74358	Sll1528 protein OS=Synechocystis sp. (strain PCC 6803 / Kazusa) GN=	3,82	1,3	0,3		0,8	5,0E-01
P74357	Sll1530 protein OS=Synechocystis sp. (strain PCC 6803 / Kazusa) GN=	11,4	1,9	0,4	0,7	1,0	6,0E-01
P74348	Sll1534 protein OS=Synechocystis sp. (strain PCC 6803 / Kazusa) GN=	4,5	1,4			1,4	2,0E+00
P74341	Sll1537 protein OS=Synechocystis sp. (strain PCC 6803 / Kazusa) GN=	12,95	0,9		1,0	1,0	6,7E-01
P74675	Sll1542 protein OS=Synechocystis sp. (strain PCC 6803 / Kazusa) GN=	8,67		2,5	1,5	2,0	1,9E-02
P73719	Sll1628 protein OS=Synechocystis sp. (strain PCC 6803 / Kazusa) GN=	9,38	0,9	1,2	1,2	1,1	5,6E-01
P73048	Sll1638 protein OS=Synechocystis sp. (strain PCC 6803 / Kazusa) GN=	41,61	1,3	2,0	2,2	1,8	1,9E-04
P72807	Sll1663 protein OS=Synechocystis sp. (strain PCC 6803 / Kazusa) GN=	14,09	0,6	1,4	1,6	1,2	6,7E-01
P72805	Sll1665 protein OS=Synechocystis sp. (strain PCC 6803 / Kazusa) GN=	5,94	0,9	1,1	1,4	1,1	3,6E-01
P73707	Sll1692 protein OS=Synechocystis sp. (strain PCC 6803 / Kazusa) GN=	11,72			0,8	0,8	2,0E+00
P73393	Sll1734 protein OS=Synechocystis sp. (strain PCC 6803 / Kazusa) GN=	16,71	2,5	1,2	1,0	1,6	2,1E-01
P73652	Sll1757 protein OS=Synechocystis sp. (strain PCC 6803 / Kazusa) GN=	10,27			0,9	0,9	2,0E+00
P73643	Sll1762 protein OS=Synechocystis sp. (strain PCC 6803 / Kazusa) GN=	24,35	0,7	0,9	1,4	1,0	7,9E-01
P73626	Sll1771 protein OS=Synechocystis sp. (strain PCC 6803 / Kazusa) GN=	12,6		1,5	1,3	1,4	2,2E-07
P73619	Sll1775 protein OS=Synechocystis sp. (strain PCC 6803 / Kazusa) GN=	2,94		2,0		2,0	2,0E+00
P73601	Sll1784 protein OS=Synechocystis sp. (strain PCC 6803 / Kazusa) GN=	10,11	0,6	0,8	0,9	0,8	1,9E-02
P73600	Sll1785 protein OS=Synechocystis sp. (strain PCC 6803 / Kazusa) GN=	26,49	0,2	0,5	0,8	0,5	5,6E-02
P73120	Sll1830 protein OS=Synechocystis sp. (strain PCC 6803 / Kazusa) GN=	14,53	0,5	0,5	0,5	0,5	0,0E+00
P73111	Sll1835 protein OS=Synechocystis sp. (strain PCC 6803 / Kazusa) GN=	6,79	0,5			0,5	2,0E+00
P73107	Sll1837 protein OS=Synechocystis sp. (strain PCC 6803 / Kazusa) GN=	15,17	0,4			0,4	2,0E+00
P74135	Sll1873 protein OS=Synechocystis sp. (strain PCC 6803 / Kazusa) GN=	62,81	0,4	0,6	0,7	0,6	1,7E-03
P73804	Sll1961 protein OS=Synechocystis sp. (strain PCC 6803 / Kazusa) GN=	3,61	1,7	0,6	0,6	1,0	6,1E-01
P73921	Sll1979 protein OS=Synechocystis sp. (strain PCC 6803 / Kazusa) GN=	14,79	1,1	1,3	0,9	1,1	2,6E-01
Q6ZEU6	Sll5034 protein OS=Synechocystis sp. (strain PCC 6803 / Kazusa) GN=	15,46	0,4		0,8	0,6	1,4E-01
Q6ZEQ5	Sll5075 protein OS=Synechocystis sp. (strain PCC 6803 / Kazusa) GN=	21,58		3,0	2,5	2,8	3,6E-30

Q55660	Slr0001 protein OS=Synechocystis sp. (strain PCC 6803 / Kazusa) GN=	39,38	0,8	3,3	2,1	2,1	1,9E-01
Q55667	Slr0006 protein OS=Synechocystis sp. (strain PCC 6803 / Kazusa) GN=	8,76			2,8	2,8	2,0E+00
Q55449	Slr0031 protein OS=Synechocystis sp. (strain PCC 6803 / Kazusa) GN=	6,34	0,9	3,6	2,3	2,3	1,1E-01
Q55131	Slr0049 protein OS=Synechocystis sp. (strain PCC 6803 / Kazusa) GN=	11,06	0,8	0,3	0,3	0,5	9,8E-03
P74445	Slr0146 protein OS=Synechocystis sp. (strain PCC 6803 / Kazusa) GN=	12,94	1,2			1,2	2,0E+00
P74446	Slr0147 protein OS=Synechocystis sp. (strain PCC 6803 / Kazusa) GN=	13,01	1,0	0,7	0,7	0,8	7,5E-02
P74448	Slr0149 protein OS=Synechocystis sp. (strain PCC 6803 / Kazusa) GN=	20,51	0,9	1,3	1,1	1,1	3,8E-01
P74450	Slr0151 protein OS=Synechocystis sp. (strain PCC 6803 / Kazusa) GN=	25,62	0,9	1,1	0,9	1,0	4,9E-01
Q55573	Slr0184 protein OS=Synechocystis sp. (strain PCC 6803 / Kazusa) GN=	9,71			0,8	0,8	2,0E+00
Q55780	Slr0208 protein OS=Synechocystis sp. (strain PCC 6803 / Kazusa) GN=	8,49	0,5	0,6	0,9	0,7	2,0E-02
Q55782	Slr0209 protein OS=Synechocystis sp. (strain PCC 6803 / Kazusa) GN=	7,18	1,8	0,9	0,8	1,2	7,4E-01
Q55698	Slr0226 protein OS=Synechocystis sp. (strain PCC 6803 / Kazusa) GN=	2,9	0,4	0,9	0,9	0,7	1,4E-01
P72690	Slr0236 protein OS=Synechocystis sp. (strain PCC 6803 / Kazusa) GN=	7,21			1,4	1,4	2,0E+00
P72700	Slr0244 protein OS=Synechocystis sp. (strain PCC 6803 / Kazusa) GN=	33,45	2,0	1,1	0,9	1,3	2,7E-01
P72709	Slr0250 protein OS=Synechocystis sp. (strain PCC 6803 / Kazusa) GN=	14,61	0,4			0,4	2,0E+00
P74396	Slr0280 protein OS=Synechocystis sp. (strain PCC 6803 / Kazusa) GN=	7,87	0,8			0,8	2,0E+00
Q55922	Slr0315 protein OS=Synechocystis sp. (strain PCC 6803 / Kazusa) GN=	7,79			1,6	1,6	2,0E+00
Q55541	Slr0333 protein OS=Synechocystis sp. (strain PCC 6803 / Kazusa) GN=	37,74	0,7	0,8	2,0	1,2	9,4E-01
Q55542	Slr0334 protein OS=Synechocystis sp. (strain PCC 6803 / Kazusa) GN=	13,64	0,4	2,2	5,5	2,7	4,7E-01
Q55650	Slr0355 protein OS=Synechocystis sp. (strain PCC 6803 / Kazusa) GN=	9,67			3,4	3,4	2,0E+00
Q55737	Slr0416 protein OS=Synechocystis sp. (strain PCC 6803 / Kazusa) GN=	14,38	0,5	1,6		1,0	7,8E-01
Q55113	Slr0431 protein OS=Synechocystis sp. (strain PCC 6803 / Kazusa) GN=	20,8	0,3	0,8	0,8	0,6	1,0E-01
P74375	Slr0442 protein OS=Synechocystis sp. (strain PCC 6803 / Kazusa) GN=	12,77	0,8	0,8	1,1	0,9	3,6E-01
P74379	Slr0443 protein OS=Synechocystis sp. (strain PCC 6803 / Kazusa) GN=	21,6	0,2	0,6	0,5	0,5	2,5E-03
P74694	Slr0455 protein OS=Synechocystis sp. (strain PCC 6803 / Kazusa) GN=	19,15			2,0	2,0	2,0E+00
Q55171	Slr0476 protein OS=Synechocystis sp. (strain PCC 6803 / Kazusa) GN=	27,94	0,5	0,4	1,2	0,7	1,3E-01
Q55176	Slr0483 protein OS=Synechocystis sp. (strain PCC 6803 / Kazusa) GN=	25,5	0,8	0,8	0,9	0,8	3,8E-03
Q55514	Slr0552 protein OS=Synechocystis sp. (strain PCC 6803 / Kazusa) GN=	27,87	1,0	0,7	0,6	0,7	2,9E-02
Q55392	Slr0565 protein OS=Synechocystis sp. (strain PCC 6803 / Kazusa) GN=	3,69		1,0		1,0	2,0E+00
P74746	Slr0600 protein OS=Synechocystis sp. (strain PCC 6803 / Kazusa) GN=	10,12	0,4	1,4	0,7	0,8	3,5E-01
P74752	Slr0605 protein OS=Synechocystis sp. (strain PCC 6803 / Kazusa) GN=	10,03	2,3	0,6		1,5	7,5E-01
P74753	Slr0606 protein OS=Synechocystis sp. (strain PCC 6803 / Kazusa) GN=	6,62			0,4	0,4	2,0E+00
Q55727	Slr0645 protein OS=Synechocystis sp. (strain PCC 6803 / Kazusa) GN=	57,77	0,5	1,4	1,2	1,0	8,6E-01
P74584	Slr0667 protein OS=Synechocystis sp. (strain PCC 6803 / Kazusa) GN=	7,51			0,5	0,5	2,0E+00
P72937	Slr0670 protein OS=Synechocystis sp. (strain PCC 6803 / Kazusa) GN=	31,27	2,2	0,6	0,8	1,2	9,8E-01
P72967	Slr0695 protein OS=Synechocystis sp. (strain PCC 6803 / Kazusa) GN=	21,97	1,0		1,1	1,0	4,7E-01
Q55972	Slr0708 protein OS=Synechocystis sp. (strain PCC 6803 / Kazusa) GN=	10,2		0,4	0,4	0,4	0,0E+00
P72675	Slr0731 protein OS=Synechocystis sp. (strain PCC 6803 / Kazusa) GN=	12,19	1,0		1,1	1,0	5,7E-01
Q55426	Slr0841 protein OS=Synechocystis sp. (strain PCC 6803 / Kazusa) GN=	14,43	1,2	3,3	2,1	2,2	1,8E-02
Q55436	Slr0848 protein OS=Synechocystis sp. (strain PCC 6803 / Kazusa) GN=	24,55	0,9	1,0	1,1	1,0	6,6E-01
P73556	Slr0876 protein OS=Synechocystis sp. (strain PCC 6803 / Kazusa) GN=	7,58		0,6		0,6	2,0E+00
Q55374	Slr0907 protein OS=Synechocystis sp. (strain PCC 6803 / Kazusa) GN=	4,93	3,6	0,7	0,7	1,6	7,9E-01
Q55375	Slr0909 protein OS=Synechocystis sp. (strain PCC 6803 / Kazusa) GN=	5,33			0,5	0,5	2,0E+00
Q55376	Slr0912 protein OS=Synechocystis sp. (strain PCC 6803 / Kazusa) GN=	8,14		1,6	1,9	1,7	8,0E-12
Q55386	Slr0924 protein OS=Synechocystis sp. (strain PCC 6803 / Kazusa) GN=	14,34	0,9	2,0	1,4	1,4	2,5E-01
Q55501	Slr0929 protein OS=Synechocystis sp. (strain PCC 6803 / Kazusa) GN=	15,9	1,7	0,7	0,7	1,0	8,7E-01
P74302	Slr0937 protein OS=Synechocystis sp. (strain PCC 6803 / Kazusa) GN=	11,99	0,8		1,3	1,1	9,0E-01
P72852	Slr0962 protein OS=Synechocystis sp. (strain PCC 6803 / Kazusa) GN=	5,66	1,9			1,9	2,0E+00
P73007	Slr1043 protein OS=Synechocystis sp. (strain PCC 6803 / Kazusa) GN=	14,44	0,6			0,6	2,0E+00
P72741	Slr1097 protein OS=Synechocystis sp. (strain PCC 6803 / Kazusa) GN=	26,38	0,9	0,9	1,0	0,9	1,0E-03
P72747	Slr1103 protein OS=Synechocystis sp. (strain PCC 6803 / Kazusa) GN=	3,97	1,0		0,9	1,0	7,1E-01
P74233	Slr1160 protein OS=Synechocystis sp. (strain PCC 6803 / Kazusa) GN=	40,2	0,3	1,7	1,5	1,2	8,7E-01
P74236	Slr1161 protein OS=Synechocystis sp. (strain PCC 6803 / Kazusa) GN=	8,54	1,0		0,7	0,8	3,7E-01
P74718	Slr1189 protein OS=Synechocystis sp. (strain PCC 6803 / Kazusa) GN=	8,55			0,9	0,9	2,0E+00
P73342	Slr1194 protein OS=Synechocystis sp. (strain PCC 6803 / Kazusa) GN=	9,46		2,7		2,7	2,0E+00
P73344	Slr1196 protein OS=Synechocystis sp. (strain PCC 6803 / Kazusa) GN=	3,96	0,3			0,3	2,0E+00
P73463	Slr1220 protein OS=Synechocystis sp. (strain PCC 6803 / Kazusa) GN=	18,93	0,6	2,2	1,6	1,5	4,9E-01
P73497	Slr1240 protein OS=Synechocystis sp. (strain PCC 6803 / Kazusa) GN=	14,53			1,5	1,5	2,0E+00
P73803	Slr1263 protein OS=Synechocystis sp. (strain PCC 6803 / Kazusa) GN=	8,94	0,7	1,0	1,7	1,1	7,8E-01
P74188	Slr1276 protein OS=Synechocystis sp. (strain PCC 6803 / Kazusa) GN=	13,45	0,8	1,6	1,6	1,3	3,6E-01
P72839	Slr1301 protein OS=Synechocystis sp. (strain PCC 6803 / Kazusa) GN=	19,63	0,5	1,2	1,1	0,9	6,1E-01
P74074	Slr1338 protein OS=Synechocystis sp. (strain PCC 6803 / Kazusa) GN=	12	1,0	1,4	1,5	1,3	5,6E-02
P74079	Slr1342 protein OS=Synechocystis sp. (strain PCC 6803 / Kazusa) GN=	18,43	1,9		1,1	1,5	1,6E-01
P73592	Slr1406 protein OS=Synechocystis sp. (strain PCC 6803 / Kazusa) GN=	13,26	0,4			0,4	2,0E+00
P73503	Slr1437 protein OS=Synechocystis sp. (strain PCC 6803 / Kazusa) GN=	32,14	0,9	1,3	1,0	1,1	5,1E-01
P73508	Slr1440 protein OS=Synechocystis sp. (strain PCC 6803 / Kazusa) GN=	16,78			1,5	1,5	2,0E+00
P74154	Slr1470 protein OS=Synechocystis sp. (strain PCC 6803 / Kazusa) GN=	12,21			0,7	0,7	2,0E+00
P73946	Slr1506 protein OS=Synechocystis sp. (strain PCC 6803 / Kazusa) GN=	13,02	0,9	0,5	0,9	0,8	9,5E-02
P73953	Slr1512 protein OS=Synechocystis sp. (strain PCC 6803 / Kazusa) GN=	7,22			0,7	0,7	2,0E+00
P74219	Slr1533 protein OS=Synechocystis sp. (strain PCC 6803 / Kazusa) GN=	29,57	1,1	1,3	1,3	1,2	8,6E-03
P73424	Slr1540 protein OS=Synechocystis sp. (strain PCC 6803 / Kazusa) GN=	28,3	2,5	0,8	1,2	1,5	3,6E-01
P74588	Slr1557 protein OS=Synechocystis sp. (strain PCC 6803 / Kazusa) GN=	7,59	1,5	2,2	1,0	1,5	1,2E-01
P72968	Slr1590 protein OS=Synechocystis sp. (strain PCC 6803 / Kazusa) GN=	10,51	2,1		0,8	1,5	6,2E-01
P72985	Slr1600 protein OS=Synechocystis sp. (strain PCC 6803 / Kazusa) GN=	24,83		0,5	0,8	0,7	6,5E-02
P72891	Slr1613 protein OS=Synechocystis sp. (strain PCC 6803 / Kazusa) GN=	12,76	0,6		1,5	1,0	8,7E-01
P72895	Slr1617 protein OS=Synechocystis sp. (strain PCC 6803 / Kazusa) GN=	8,52	2,4		0,7	1,6	6,7E-01
P72897	Slr1619 protein OS=Synechocystis sp. (strain PCC 6803 / Kazusa) GN=	7,23	1,2		1,1	1,2	7,8E-02
P74673	Slr1668 protein OS=Synechocystis sp. (strain PCC 6803 / Kazusa) GN=	9,29			1,2	1,2	2,0E+00
P73716	Slr1732 protein OS=Synechocystis sp. (strain PCC 6803 / Kazusa) GN=	12,75			1,2	1,2	2,0E+00
P73049	Slr1768 protein OS=Synechocystis sp. (strain PCC 6803 / Kazusa) GN=	4,7		1,0		1,0	2,0E+00
P72765	Slr1772 protein OS=Synechocystis sp. (strain PCC 6803 / Kazusa) GN=	7,16		1,7		1,7	2,0E+00
P72799	Slr1794 protein OS=Synechocystis sp. (strain PCC 6803 / Kazusa) GN=	11,6	1,7	0,7	0,6	1,0	8,2E-01
P72814	Slr1800 protein OS=Synechocystis sp. (strain PCC 6803 / Kazusa) GN=	8,17	0,6	1,5	0,8	1,0	6,4E-01
P73697	Slr1812 protein OS=Synechocystis sp. (strain PCC 6803 / Kazusa) GN=	12,03	0,8		1,4	1,1	8,1E-01
P73698	Slr1813 protein OS=Synechocystis sp. (strain PCC 6803 / Kazusa) GN=	9,15			1,3	1,3	2,0E+00
P73699	Slr1814 protein OS=Synechocystis sp. (strain PCC 6803 / Kazusa) GN=	12,34	0,7			0,7	2,0E+00
P73706	Slr1818 protein OS=Synechocystis sp. (strain PCC 6803 / Kazusa) GN=	8,46		0,9		0,9	2,0E+00
P73389	Slr1829 protein OS=Synechocystis sp. (strain PCC 6803 / Kazusa) GN=	5,76			0,3	0,3	2,0E+00
P73409	Slr1841 protein OS=Synechocystis sp. (strain PCC 6803 / Kazusa) GN=	17,78	1,4	0,4	1,3	1,0	8,3E-01
P73603	Slr1852 protein OS=Synechocystis sp. (strain PCC 6803 / Kazusa) GN=	52,66	0,2	0,8	0,7	0,6	1,1E-01
P73604	Slr1853 protein OS=Synechocystis sp. (strain PCC 6803 / Kazusa) GN=	20,35	0,2	0,4	0,5	0,4	2,3E-03
P73605	Slr1854 protein OS=Synechocystis sp. (strain PCC 6803 / Kazusa) GN=	51,52	0,2	0,5	0,4	0,4	2,3E-05
P73606	Slr1855 protein OS=Synechocystis sp. (strain PCC 6803 / Kazusa) GN=	48,93	0,2	0,9	0,8	0,6	1,6E-01
P73328	Slr1900 protein OS=Synechocystis sp. (strain PCC 6803 / Kazusa) GN=	22,67	0,8	2,0	1,9	1,6	1,8E-01
P73103	Slr1908 protein OS=Synechocystis sp. (strain PCC 6803 / Kazusa) GN=	19,12	1,0	0,2	0,6	0,6	1,2E-01
P74473	Slr1923 protein OS=Synechocystis sp. (strain PCC 6803 / Kazusa) GN=	4,02	0,9			0,9	2,0E+00
P74478	Slr1926 protein OS=Synechocystis sp. (strain PCC 6803 / Kazusa) GN=	14,35	0,7	0,8		0,8	1,8E-04
P74500	Slr1940 protein OS=Synechocystis sp. (strain PCC 6803 / Kazusa) GN=	14,73	0,7	1,6	1,5	1,2	5,9E-01
P74506	Slr1944 protein OS=Synechocystis sp. (strain PCC 6803 / Kazusa) GN=	6,69	0,4	0,2	0,6	0,4	6,3E-04
P74094	Slr1958 protein OS=Synechocystis sp. (strain PCC 6803 / Kazusa) GN=	17		1,4	1,1	1,3	2,8E-02
P74100	Slr1962 protein OS=Synechocystis sp. (strain PCC 6803 / Kazusa) GN=	5,14		1,2	1,4	1,3	3,4E-04
P74113	Slr1970 protein OS=Synechocystis sp. (strain PCC 6803 / Kazusa) GN=	20,23	0,6		1,5	1,0	8,7E-01
P73222	Slr2005 protein OS=Synechocystis sp. (strain PCC 6803 / Kazusa) GN=	13,03	0,5		0,7	0,6	5,3E-04
P73244	Slr2025 protein OS=Synechocystis sp. (strain PCC 6803 / Kazusa) GN=	25,49	0,5	2,0	2,2	1,6	5,3E-01
P73812	Slr2060 protein OS=Synechocystis sp. (strain PCC 6803 / Kazusa) GN=	4,09	2,7	0,5	0,5	1,3	8,7E-01
P73370	Slr2070 protein OS=Synechocystis sp. (strain PCC 6803 / Kazusa) GN=	15,14	1,4	0,7	0,6	0,9	4,7E-01
P73934	Slr2105 protein OS=Synechocystis sp. (strain PCC 6803 / Kazusa) GN=	3,87			1,1	1,1	2,0E+00
P73681	Slr2144 protein OS=Synechocystis sp. (strain PCC 6803 / Kazusa) GN=	9,63	0,6	1,9		1,3	8,6E-01
Q6ZEP2	Slr5088 protein OS=Synechocystis sp. (strain PCC 6803 / Kazusa) GN=	16,81	0,5			0,5	2,0E+00
Q6YRQ8	Slr6096 protein OS=Synechocystis sp. (strain PCC 6803 / Kazusa) GN=	5,36			1,1	1,1	2,0E+00
Q6ZEI6	Slr7012 protein OS=Synechocystis sp. (strain PCC 6803 / Kazusa) GN=	23,1	0,8	2,1	1,8	1,5	2,6E-01
Q6ZEC5	Slr7073 protein OS=Synechocystis sp. (strain PCC 6803 / Kazusa) GN=	19,3		0,7		0,7	2,0E+00
P74281	Soluble hydrogenase 42 kD subunit OS=Synechocystis sp. (strain PCC (	44,79	0,9	3,9	1,6	2,2	1,6E-01
P73722	SOS function regulatory protein OS=Synechocystis sp. (strain PCC 6803	50,25	0,4	0,5	0,4	0,4	0,0E+00
P73914	Squalene-hopene-cyclase OS=Synechocystis sp. (strain PCC 6803 / Ka	10,36	0,5	0,5	1,0	0,6	2,8E-02
P74795	Ssl0352 protein OS=Synechocystis sp. (strain PCC 6803 / Kazusa) GN=	36,21			1,6	1,6	2,0E+00
P73875	Ssl0467 protein OS=Synechocystis sp. (strain PCC 6803 / Kazusa) GN=	30,99			0,7	0,7	2,0E+00
P74067	Ssl1498 protein OS=Synechocystis sp. (strain PCC 6803 / Kazusa) GN=	24,59			2,4	2,4	2,0E+00
P73031	Ssl1918 protein OS=Synechocystis sp. (strain PCC 6803 / Kazusa) GN=	18,56	0,5			0,5	2,0E+00
P74239	Ssl2148 protein OS=Synechocystis sp. (strain PCC 6803 / Kazusa) GN=	16,33		1,5	1,4	1,5	1,1E-20
P73489	Ssl2245 protein OS=Synechocystis sp. (strain PCC 6803 / Kazusa) GN=	15,91	0,3	0,6	0,4	0,4	2,8E-06
P73654	Ssl3364 protein OS=Synechocystis sp. (strain PCC 6803 / Kazusa) GN=	27,03	0,3	0,8	0,9	0,7	1,3E-01
P73286	Ssl3451 protein OS=Synechocystis sp. (strain PCC 6803 / Kazusa) GN=	22,22	1,3			1,3	2,0E+00
P74769	Ssr1528 protein OS=Synechocystis sp. (strain PCC 6803 / Kazusa) GN=	47,87	1,0	1,9	2,1	1,7	4,7E-02
P74326	Ssr1600 protein OS=Synechocystis sp. (strain PCC 6803 / Kazusa) GN=	57,14	0,7	1,4	1,3	1,2	6,6E-01
P72914	Ssr1766 protein OS=Synechocystis sp. (strain PCC 6803 / Kazusa) GN=	10,53	1,7			1,7	2,0E+00
P73792	Ssr2087 protein OS=Synechocystis sp. (strain PCC 6803 / Kazusa) GN=	14,29		0,9		0,9	2,0E+00
P73479	Succinate dehydrogenase flavoprotein subunit OS=Synechocystis sp. (s	13,74	1,3	2,4	1,6	1,8	1,2E-03
P73723	Succinate dehydrogenase iron-sulphur protein subunit OS=Synechocyst	6,65			0,7	0,7	2,0E+00
P74241	Sulfate adenylyltransferase OS=Synechocystis sp. (strain PCC 6803 / K	13,85	1,2	0,6	0,6	0,8	1,8E-01
P72854	Sulfite reductase [ferredoxin] OS=Synechocystis sp. (strain PCC 6803 / I	27,09	1,3	1,8	1,7	1,6	3,6E-07
P73128	Sulfolipid biosynthesis protein SqdB OS=Synechocystis sp. (strain PCC	51,44	0,7	1,2	1,3	1,0	9,9E-01
P77968	Superoxide dismutase [Fe] OS=Synechocystis sp. (strain PCC 6803 / Ka	39,2	0,5	1,9	1,4	1,3	7,4E-01
P52231	Thioredoxin OS=Synechocystis sp. (strain PCC 6803 / Kazusa) GN=trxA	46,73	0,5	1,2	1,2	1,0	7,4E-01
P73711	Threonine synthase OS=Synechocystis sp. (strain PCC 6803 / Kazusa) (	9,7			0,3	0,3	2,0E+00
P74193	Threonine synthase OS=Synechocystis sp. (strain PCC 6803 / Kazusa) (	26,7	1,9	2,0		2,0	1,4E-212
P72804	Thylakoid membrane protein slr1796 OS=Synechocystis sp. (strain PCC	8,96	0,8			0,8	2,0E+00
P74511	Thylakoid membrane protein slr1949 OS=Synechocystis sp. (strain PCC	16,04	0,9	0,6	0,9	0,8	1,4E-01
P73695	Thylakoid-associated protein sll1697 OS=Synechocystis sp. (strain PCC	8,72	0,8			0,8	2,0E+00
P72673	Thylakoid-associated protein slr0729 OS=Synechocystis sp. (strain PCC	46,53	0,6	1,7	1,6	1,3	5,8E-01
P73145	Thylakoid-associated single-stranded DNA-binding protein slr1034 OS=S	35,66	0,9	1,3	1,8	1,3	1,9E-01
P72797	Transaldolase OS=Synechocystis sp. (strain PCC 6803 / Kazusa) GN=ta	52,17	0,6	0,5	0,5	0,5	7,3E-15
P72688	Transcription termination/antitermination protein NusA OS=Synechocysti	19,65	1,2	1,1	0,8	1,1	7,4E-01
P36265	Transcription termination/antitermination protein NusG OS=Synechocyst	24,39	1,6	2,1	1,6	1,8	1,2E-09
P73282	Transketolase OS=Synechocystis sp. (strain PCC 6803 / Kazusa) GN=tk	43,58	0,6	2,0	1,3	1,3	6,9E-01
P72689	Translation initiation factor IF-2 OS=Synechocystis sp. (strain PCC 6803	10,49	1,5	1,1	1,1	1,2	9,7E-02
P72874	Translation initiation factor IF-3 OS=Synechocystis sp. (strain PCC 6803	19,21	1,0	0,7	0,7	0,8	7,3E-02
Q55511	Trigger factor OS=Synechocystis sp. (strain PCC 6803 / Kazusa) GN=tig	32,48	1,1	1,7	1,7	1,5	1,9E-02
Q59994	Triosephosphate isomerase OS=Synechocystis sp. (strain PCC 6803 / K	37,19	0,8	3,8	1,9	2,2	2,0E-01

Q59992	Tryptophan synthase beta chain OS=Synechocystis sp. (strain PCC 680	17,48	1,4	1,2	1,0	1,2	1,3E-01
P73655	Tryptophan--tRNA ligase OS=Synechocystis sp. (strain PCC 6803 / Kazu	6,53	0,6			0,6	2,0E+00
P74463	Twitching motility protein OS=Synechocystis sp. (strain PCC 6803 / Kazu	21,95	1,3		0,7	1,0	9,4E-01
Q55673	UDP-N-acetylglucosamine 1-carboxyvinyltransferase OS=Synechocystis	5,94	0,6	1,9	1,2	1,2	7,4E-01
Q55469	UDP-N-acetylmuramoyl-L-alanyl-D-glutamate--2,6-diaminopimelate ligas	10,3	1,1	0,6		0,8	4,4E-01
P45450	UDP-N-acetylmuramoyl-tripeptide--D-alanyl-D-alanine ligase OS=Synec	7,93	2,5			2,5	2,0E+00
P73668	UDP-N-acetylmuramoylalanine--D-glutamate ligase OS=Synechocystis s	6,19	2,3	0,5	0,8	1,2	9,6E-01
P22040	Uncharacterized ABC transporter ATP-binding protein sll0415 OS=Synec	6,78			0,8	0,8	2,0E+00
P73335	Uncharacterized deoxyribonuclease sll1786 OS=Synechocystis sp. (stra	24,9	1,0	3,9	2,3	2,4	5,1E-02
Q6ZES9	Uncharacterized protein OS=Synechocystis sp. (strain PCC 6803 / Kazu	21,54			0,9	0,9	2,0E+00
Q6ZED7	Uncharacterized protein OS=Synechocystis sp. (strain PCC 6803 / Kazu	4,27	2,6			2,6	2,0E+00
Q55707	Uncharacterized protein sll0617 OS=Synechocystis sp. (strain PCC 680	34,46	0,5	0,8	0,9	0,8	7,7E-02
P72861	Uncharacterized protein sll0936 OS=Synechocystis sp. (strain PCC 680	4,59	1,4		0,8	1,1	9,6E-01
P72929	Uncharacterized protein sll1021 OS=Synechocystis sp. (strain PCC 680	12,33	1,0	0,4	0,6	0,7	8,4E-02
P74178	Uncharacterized protein sll1178 OS=Synechocystis sp. (strain PCC 680	5,2			0,8	0,8	2,0E+00
P73599	Uncharacterized protein sll1304 OS=Synechocystis sp. (strain PCC 680	11,15	0,4	0,3	0,5	0,4	2,7E-14
P74360	Uncharacterized protein sll1526 OS=Synechocystis sp. (strain PCC 680	14,03	1,3	0,5	0,5	0,8	2,3E-01
P73392	Uncharacterized protein sll1735 OS=Synechocystis sp. (strain PCC 680	27,07	1,0		1,7	1,3	3,2E-01
P73602	Uncharacterized protein sll1783 OS=Synechocystis sp. (strain PCC 680	23,13	0,5			0,5	2,0E+00
Q55458	Uncharacterized protein slr0039 OS=Synechocystis sp. (strain PCC 680	11,2	1,4	0,9	0,9	1,1	7,5E-01
Q55629	Uncharacterized protein slr0782 OS=Synechocystis sp. (strain PCC 680	9,98			0,5	0,5	2,0E+00
P72655	Uncharacterized protein slr1128 OS=Synechocystis sp. (strain PCC 680	27,1	1,8	0,5	0,4	0,9	4,8E-01
P74002	Uncharacterized protein slr1322 OS=Synechocystis sp. (strain PCC 680	26,58	0,8	2,0	1,3	1,3	4,2E-01
P73042	Uncharacterized protein slr1764 OS=Synechocystis sp. (strain PCC 680	23,46		4,1		4,1	2,0E+00
P73321	Uncharacterized protein slr1894 OS=Synechocystis sp. (strain PCC 680	49,36	0,8	2,0	1,8	1,5	2,7E-01
P73152	Uncharacterized thylakoid-associated protein sll0982 OS=Synechocystis	17,97	0,5			0,5	2,0E+00
P73545	Uncharacterized thylakoid-associated protein ssl2501 OS=Synechocysti	37,08	0,4	0,1	0,3	0,3	3,2E-05
P74328	Uncharacterized tRNA/rRNA methyltransferase slr0955 OS=Synechocys	14,32	1,0	1,2	1,1	1,1	2,1E-03
Q55563	Uncharacterized WD repeat-containing protein sll0163 OS=Synechocyst	0,95		0,7		0,7	2,0E+00
P74598	Uncharacterized WD repeat-containing protein sll1491 OS=Synechocyst	16,67	0,6	2,0	1,8	1,5	4,6E-01
P72817	Universal stress protein Sll1654 OS=Synechocystis sp. (strain PCC 680	45,86	0,9	1,2	1,0	1,1	5,6E-01
P72699	UPF0045 protein sll0230 OS=Synechocystis sp. (strain PCC 6803 / Kazu	51,24	0,6	1,5	1,6	1,2	7,4E-01
P74607	UPF0296 protein ssl2874 OS=Synechocystis sp. (strain PCC 6803 / Kaz	40,45	0,7	0,9	0,5	0,7	3,0E-02
P72753	Uracil phosphoribosyltransferase OS=Synechocystis sp. (strain PCC 680	17,59	1,1			1,1	2,0E+00
P73061	Urease subunit alpha OS=Synechocystis sp. (strain PCC 6803 / Kazusa	16,34	0,8	1,8	1,4	1,3	3,3E-01
P73796	Urease subunit gamma OS=Synechocystis sp. (strain PCC 6803 / Kazus	31	0,4		1,1	0,7	3,7E-01
P74457	Uridylate kinase OS=Synechocystis sp. (strain PCC 6803 / Kazusa) GN=	6,15	1,1			1,1	2,0E+00
P54224	Uroporphyrinogen decarboxylase OS=Synechocystis sp. (strain PCC 68	22,86	0,7	0,9	0,8	0,8	7,9E-03
Q55522	Valine--tRNA ligase OS=Synechocystis sp. (strain PCC 6803 / Kazusa) (	11,54	2,5	0,7	0,8	1,3	7,6E-01
Q55557	Virginiamycin B hydrolase Vgb OS=Synechocystis sp. (strain PCC 6803	34,38	0,3	1,2	1,1	0,9	4,8E-01
P73066	Ycf23 protein OS=Synechocystis sp. (strain PCC 6803 / Kazusa) GN=yc	12,9	0,8	0,4	0,4	0,5	1,5E-03
P74429	Ycf39 gene product OS=Synechocystis sp. (strain PCC 6803 / Kazusa) (	3,99			0,8	0,8	2,0E+00
P73069	Ycf48-like protein OS=Synechocystis sp. (strain PCC 6803 / Kazusa) GN	7,02	1,4		0,7	1,1	9,7E-01
P72777	Ycf54-like protein OS=Synechocystis sp. (strain PCC 6803 / Kazusa) GN	27,82	0,7	1,8	1,8	1,4	3,7E-01
Q55823	Ycf66-like protein OS=Synechocystis sp. (strain PCC 6803 / Kazusa) GN	8,01			1,1	1,1	2,0E+00
P74306	Zeta-carotene desaturase OS=Synechocystis sp. (strain PCC 6803 / Kaz	7,36			1,2	1,2	2,0E+00
P74721	Zinc-containing alcohol dehydrogenase family OS=Synechocystis sp. (st	5,65	0,6	1,6	1,1	1,1	1,0E+00
